# First paleoproteome study of fossil fish otoliths and the pristine preservation of the biomineral crystal host

**DOI:** 10.1038/s41598-023-30537-8

**Published:** 2023-03-07

**Authors:** Jarosław Stolarski, Jeana Drake, Ismael Coronado, Ana R. Vieira, Urszula Radwańska, Elizabeth A. C. Heath-Heckman, Maciej Mazur, Jinming Guo, Anders Meibom

**Affiliations:** 1 Institute of Paleobiology, Polish Academy of Sciences, Twarda 51/55, 00818 Warsaw, Poland; 2 Department of Earth, Planetary, and Space Sciences, University of California, Los Angeles, CA USA; 3 Faculty of Biological and Environmental Sciences, Universidad de León, Campus of Vegazana S/N, 24171 Leon, Spain; 4 Department of Animal Biology, Faculty of Sciences, University of Lisbon, Campo Grande, 1749-016 Lisbon, Portugal; 5 Marine and Environmental Sciences Centre (MARE), University of Lisbon, Campo Grande, 1749-016 Lisbon, Portugal; 6 Department of Geology, University of Warsaw, Żwirki i Wigury 93, 02089 Warsaw, Poland; 7 Department of Integrative Biology, Michigan State University, East Lansing, MI USA; 8 Department of Chemistry, University of Warsaw, Pasteura 1, 02-093 Warsaw, Poland; 9 School of Materials Science and Engineering, Hubei University, Wuhan, 430062 Hubei China; 10 Laboratory for Biological Geochemistry, School of Architecture, Civil and Environmental Engineering, Ecole Polytechnique Fédérale de Lausanne, 1015 Lausanne, Switzerland; 11 Center for Advanced Surface Analysis, Institute of Earth Sciences, Université de Lausanne, 1015 Lausanne, Switzerland

**Keywords:** Palaeontology, Palaeontology

## Abstract

Otoliths are calcium carbonate components of the stato-acoustical organ responsible for hearing and maintenance of the body balance in teleost fish. During their formation, control over, e.g., morphology and carbonate polymorph is influenced by complex insoluble collagen-like protein and soluble non-collagenous protein assemblages; many of these proteins are incorporated into their aragonite crystal structure. However, in the fossil record these proteins are considered lost through diagenetic processes, hampering studies of past biomineralization mechanisms. Here we report the presence of 11 fish-specific proteins (and several isoforms) in Miocene (ca. 14.8–14.6 Ma) phycid hake otoliths. These fossil otoliths were preserved in water-impermeable clays and exhibit microscopic and crystallographic features indistinguishable from modern representatives, consistent with an exceptionally pristine state of preservation. Indeed, these fossil otoliths retain ca. 10% of the proteins sequenced from modern counterparts, including proteins specific to inner ear development, such as otolin-1-like proteins involved in the arrangement of the otoliths into the sensory epithelium and otogelin/otogelin-like proteins that are located in the acellular membranes of the inner ear in modern fish. The specificity of these proteins excludes the possibility of external contamination. Identification of a fraction of identical proteins in modern and fossil phycid hake otoliths implies a highly conserved inner ear biomineralization process through time.

## Introduction

Paleoproteomics is an accelerating research field providing new perspectives on, e.g., the evolution of biomineralization processes through time and refining our understanding of fossil remains^1^. While studies of ancient DNA are limited to few million years because DNA degrades relatively fast after cell death^2^, the study of more stable protein remains in the fossil record offers an opportunity to explore protein function and their evolution over geological time scales; several to hundreds millions of years^3^. Paleoproteomic studies of biominerals such as bones, teeth, and shells are particularly promising because such structures have high potential of preserving protein residues embedded within well-preserved fossil specimens. Here, we explore this potential in fossilized calcium carbonate structures of the inner ear of teleost fish (otoliths). Fish otoliths are, in contrast to osteological fish remains, frequently found in the fossil record during the Mesozoic, and with increasing abundance in Cenozoic strata^4^. Due to their taxon-specific morphology, these fossils are key to the interpretation of palaeobiodiversity of fish and to palaeoenvironmental reconstructions based on their isotope- and trace element compositions^5^. Although otolith calcium carbonate mineral structures can resemble inorganically precipitated aggregates of crystals, they are in fact complex organic-mineral composites, akin to many other biogenic carbonates^6^. Studies of the modern otolith biomineralization process have shown that the organic macromolecules, proteins in particular, control key aspects of otolith formation^7^, including regulation of calcium transport, nucleation, and saturation state at the crystallization site, thus actively modulating aragonite crystal growth^8^. Indeed, the strict selection of a specific calcium carbonate polymorph (aragonite as opposed to, e.g., calcite or vaterite) has also been shown to be controlled by proteins, such as aspartic acid and serine residues, that attract calcium cations to the growing crystal surface and favor denser (i.e., aragonitic) packing of ions^9^. To date, several hundred proteins have been identified in modern fish otoliths, many of which are thought to be directly involved in biomineralization^10^. Proteins known to be involved in otolith biomineralization can be divided into two main groups: (1) complexes of structural, insoluble collagen-like proteins and (2) soluble, non-collagenous proteins (NCPs). The collagen-like proteins, such as otolin-1, create a scaffold for the growing biomineral; otolin-1 has homology of sequences to collagen X, a protein also involved in endochondral ossification and bone fracture repair^11^. The soluble NCPs are usually highly acidic and intrinsically disordered proteins (IDPs) that directly regulate nucleation, orientation, and crystal growth. Such IDPs were identified in several fish taxa, e.g., Starmaker (Stm) in zebrafish^7^, Starmaker-like (Stm-l) in medaka^12^, and Otolith Matrix Macromolecule-64 (OMM-64) in rainbow trout^13^, and their role in the biomineralization has been thoroughly characterized^14–17^.

Finding evidence of proteins embedded into fossil otoliths would enhance our understanding of the evolution of an important aquatic biomineralization process, but such residues have not yet been identified and it is generally thought that these organic components are broken down and lost due to diagenetic alteration of the aragonite polymorph, which is metastable and normally transforms into a more stable calcite, e.g., via dissolution–precipitation processes in the presence of active solutions^18^. Such diagenetic processes also strongly affect the preservation of many inter/intra-crystalline proteins—in particular the highly labile NCPs^19^. In order to succeed in the detection and identification of remains of such proteins in fossil otoliths, we hypothesized that only specimens preserved in water-impermeable deposits and still composed entirely of aragonite with ultrastructural features similar to modern counterparts, have the potential to preserve organic components, most likely as inclusions embedded inside the aragonite crystals. Some easy-to-find sagittal Miocene otolith fossils from teleost fish fulfil these criteria. Here we report the results of a search for proteins embedded in fossil otoliths from phycid hake fish found in Miocene (about 14 million years old) water-impermeable clays exposed in Korytnica (Holy Cross Mountains, Central Poland; see Material)^20^.

## Results

### Mineral phase characteristics

**Figure 1:**
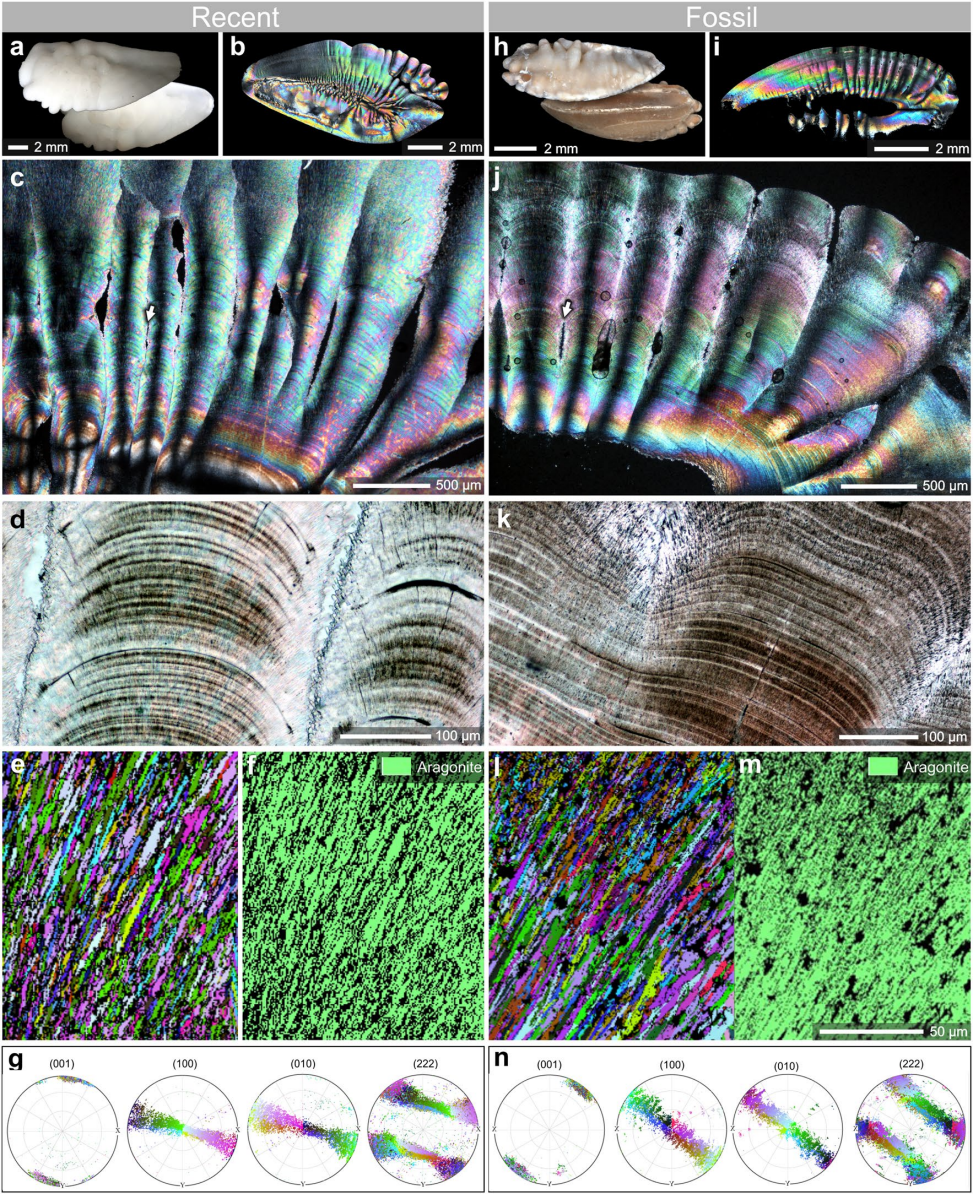
Morphological, microstructural and crystallographic similarity of modern *Phycis* *phycis* (**a–g**) and fossil *Phycis* *tenuis* (**h–n**) saggital otoliths. Modern (**a**) and fossil (**h**) sagittas are slim and elongated; the outer face (distal surface) most commonly is composed of irregular thickenings/protuberances and grooves. Thin-sections in polarized (**b,c,i,j**) and normal transmitted light (**d,k**) show numerous growth rings and point to ontogenetic continuity of protuberances (columns in longitudinal cut; occasionally separated by spindle-shaped voids, white arrows). Crystallographic orientation images (EBSD) show aragonite crystals of similar size in modern (**e**) and fossil (**l**) samples (phase maps (**f,m**) confirm aragonite mineralogy of both samples); pole figures are fully comparable between modern (**g**) and fossil samples (**n**): same crystal size, distribution, inclination, azimuthal dispersion and turbostratic distribution in the plane (222). ZPAL P.21/R-OTH-242/001 (**a–g**); ZPAL P.21/ C-OTH-07/006 (**h–n**). The BungeColorKey palette (more accessible for color-blind users) from MTEX was used to create orientation images and the pole figures.

**Figure 2:**
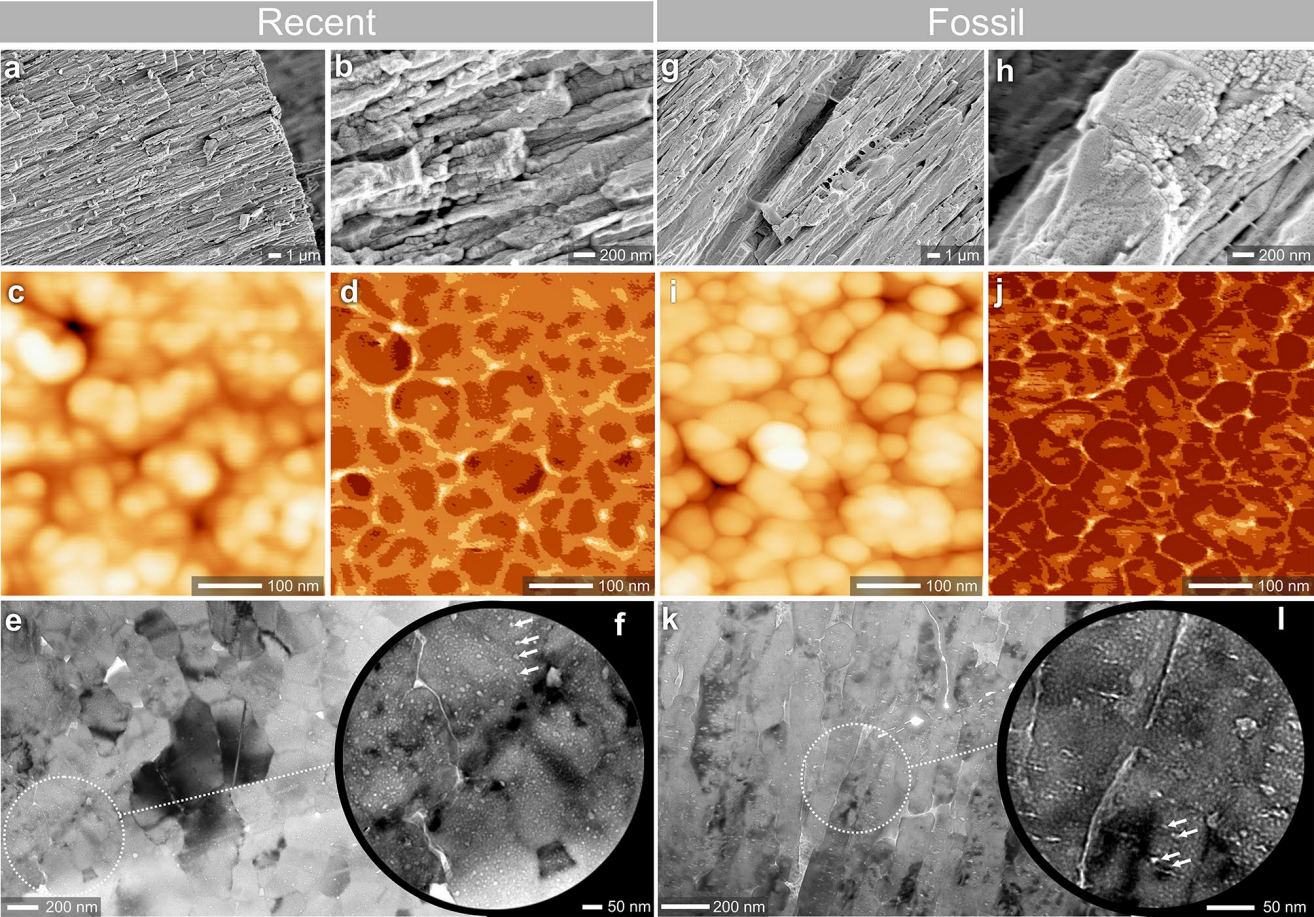
Composite, organic-mineral structure of *Phycis* *phycis* (**a–f**) and fossil *Phycis* *tenuis* (**g–l**) saggital otoliths. In FESEM the aragonite fibers of modern and fossil otoliths consist of slender units ca. 200–300 nm wide (**a,g**) that in higher magnification (**b,h**) show nanogranular organization; nanograins ca. 50–100 nm in diameter are visible in AFM height-mode (**c,i**) and peak force error-mode (**d,j**) images. TEM observations of skeletal lamellae (extracted by focused ion beam) show aragonite, fine-scale fibers (comparable in size with those observed in FESEM) that include numerous organic inclusions (arrows in **f,l**). Fibers in (**e**) cut obliquely or longitudinally (**k**) to the growth direction. ZPAL P.21/R-OTH-187/002 (**a–f**); ZPAL P.21/C-OTH-07/007 (**g–l**).

Modern (white) and fossil (brownish color) saggital otoliths of *Phycis* spp. are slim and elongated calcium carbonate biomineral structures; wider at the anterior and gradually narrowing towards the posterior parts (Fig. 1a,h). The inner face (proximal surface) is convex without distinct *sulcus* *acusticus* (the area where the sensory tissue comes into contact with the otolith). The outer face (distal surface) is most commonly composed of irregular thickenings/protuberances and grooves (Fig. 1a,h). Longitudinal thin-sections (i.e., in the sagittal plane) observed with both polarized and normal transmitted light exhibit ca. 500 µm thick columnar units that correspond in size to protuberances on the surface (Fig. 1c,j). Occasionally, spindle-shaped voids occur between columnar units that may correspond to the grooves between neighboring surface protuberances (white arrows in Fig. 1c,j). The columnar units and other parts (proximal) of modern and fossil otoliths in longitudinal sections show numerous layers (alternating dark-brown and colorless zones, ca. 5–7 µm thick; Fig. 1c,d,j,k. Layers are composed of crystals that in crystallographic orientation images (EBSD) are present with similar size in modern and fossil samples (Fig. 1e,l). Phase maps have confirmed the strictly aragonitic mineralogy of both modern and fossil samples (Fig. 1f,m) and the resulting pole figures are fully comparable between modern (Fig. 1g) and fossil specimens (Fig. 1n). A high degree of similarity between modern and fossil specimens was also observed for crystal size, inclination, azimuthal dispersion, and turbostratic distribution in the plane (222) (Fig. 1g,n). The aragonite fibres of modern and fossil otoliths consist of slender units ca. 200–300 nm wide that show nanogranular organization (Fig. 2a,b,g,h), with nanograins ca. 50–100 nm in diameter clearly visible in AFM height- and peak force error-mode images (Fig. 2c,d,i,j). TEM observations of skeletal lamellae (extracted by focused ion beam) show aragonite, fine-scale fibres comparable in size with those observed in FESEM cut either obliquely (Fig. 2e) or longitudinally (Fig. 2k) to the growth direction. The fibres include numerous intracrystalline low density defects (inclusions) ranging in size between 2 and 25 nm, which are clearly seen as bright spots in TEM images (white arrows in Fig. 2f,l). These inclusions are aligned perpendicularly with the crystal growth direction as seen in longitudinal cuts (Fig. 2f,l). Material of similar low density is also located at the grain boundaries (Fig. 2e,k). The modern and fossil samples show overall similar weight loss profiles, but the derivative curves suggest differences in decomposition. Modern otoliths decompose over broader temperature range in comparison to modern ones (ca. 200–430 °C vs. 250–400 °C) and show several weight-loss steps (at ca. 210 °C, 360 °C, and 410 °C) in comparison to the one relatively large weight-loss (at ca. 340 °C) in fossil otoliths (Supplementary Fig. S1).

### Protein preservation: amino acid racemization test

The amino acid racemization and their relative content in fossil otolith samples was used as a proxy of protein degradation. The analyses were performed using fossil *P*. *tenuis* otoliths compared directly with modern *P.* *phycis.* The measurements included free amino acids originally present within biomineral (FAAs) as well as those formed during hydrolysis of complete peptides into individual amino acids (so called total hydrolyzable amino acids, THAA). FAAs tend to result from the hydrolysis of highly-racemized N-terminal amino acids, so that the D/L ratio of FAA should be higher than that of the THAA for a given amino acid. As expected, the distribution of amino acids between pools of free amino acids (FAA) versus polymerized amino acids (THAA-FAA; as pmol amino acids per mg of starting otolith) shows that most amino acids in modern specimens are part of intact peptides whereas the majority of fossil peptides have broken down to individual amino acids. A dramatic loss of both Asx and Glx—which include the acidic amino acids aspartic and glutamic acid—as well as of Ser was observed in fossil specimens (Table 1).

**Table 1:** Amino acid racemization and relative abundance in modern *Phycis* *phycis* (bold fields) and fossil *P.* *tenuis* (italic fields) otoliths.

		DL Asx	DL Gx	Amino acid pmol/mg	% Asx	% Glx	% Ser	% Ala	% Val	% Phe	% Ile	% Leu
**Modern**	**FAA**	**0.252 (0.083)**	**0.167 (0.042)**	**90.7 (22.4)**	**20.4 (2.9**	**18.0 (2.0)**	**18.4 (3.8)**	**14.8 (2.1)**	**6.3 (2.2)**	**6.6 (1.8)**	**11.0 (3.7)**	**4.6 (1.8)**
	**THAA**	**0.070 (0.001)**	**0.026 (0.001)**	**6518.32 (1696.3)**	**23.7 (0.3)**	**21.5 (0.5)**	**13.4 (0.2)**	**14.4 (0.2)**	**11.2 (0.6)**	**3.4 (0.2)**	**3.1 (0.1)**	**9.2 (0.2)**
*Fossil*	*FAA*	*0.843* *(0.062)*	*1.006* *(0.003)*	*1489* *(430.7)*	*2.2* *(0)*	*10.8* *(0.6)*	*0.1* *(0)*	*48.3* *(0.2)*	*19.4* *(0.1)*	*2.0* *(0.2)*	*5.2* *(0.4)*	*12.0* *(0.2)*
	*THAA*	*0.808* *(0.052)*	*1.034* *(0.006)*	*1117.9* *(285.1)*	*1.6* *(0.1)*	*28.5* *(0.4)*	*0.4* *(0.1)*	*37.6* *(0.3)*	*15.5* *(0.3)*	*1.4* *(0.1)*	*4.0* *(0)*	*11.0* *(0.1)*

Note the decrease in % Asx and pmol THAA/mg starting otolith powder and increase in % Ala of fossil relative to modern otoliths. Average of replicates are shown with standard deviations in parentheses; technical duplicates of each from three modern and one fossil specimen were analyzed.

### Proteome analysis

In total, peptides for 132 fish-specific proteins and several isoforms were detected in modern *P*. *phycis* otoliths (Supplementary Table S2), while peptides for 11 fish-specific proteins were found in fossil *P.* *tenuis* otoliths (Table 2, Supplementary Tables S3–S5), which is a return ratio comparable to that of modern vs. fossil coral (aragonite) skeletons ^21,22^. Proteins were observed across both acid solubility fractions in all specimens and GluC improved protein detection in modern samples, but only tryptic digest peptides were detected in fossil samples (Supplementary Table S6). Additionally, deamidation of asparagine and glutamine and oxidation of methionine were detected (Supplementary Table S6)^23^. Five of 11 proteins sequenced from fossil otoliths represent proteins encoded by genes expressed in the inner ear (i.e., alpha-tectorin, beta-tectorin, otolin-1-like, otogelin/otogelin-like and otogelin-like). The other six proteins occur in modern fish otoliths, but are not specific to inner ear function. Proteins found in modern otoliths but not fossils include, e.g., usherin, a protein important to the development and homeostasis of the inner ear. Other modern otolith proteins include several types of collagens, contactin, low-density receptor-related lipoprotein, carbonic anhydrase that interconverts CO_2_ and bicarbonate (Supplementary Table S2).

**Table 2:** Proteins sequenced from a fossil *Phycis* *tenuis* otolith by LC–MS/MS.

Protein	Gene ID	Solubility fraction	Function
**Alpha-tectorin**	**g13180.t3**	**SOM, IOM**	**Noncollagenous components of the tectorial membrane (extracellular matrix) in the inner ear**
**Beta-tectorin**	**g51752.t1**	**IOM**	**The protein specifically expressed in the mammalian and avian inner ear; one of the major non-collagenous components of the tectorial membrane (extracellular matrix of the inner ear)**
**Otogelin/otogelin-like**	**g19658.t1, g45878.t1, g59712.t1, g40098.t2, g40101.t1**	**IOM**	**The protein encoded by gene that is expressed in the inner ear of vertebrates (essential for its normal function)**
**Otogelin-like (low quality protein)**	**g45228.t1, g67087.t1**	**IOM**	**As otogelin-like (above)**
**Otolin-1-like**	**g24402.t1**	**SOM, IOM**	**A short chain collagen-like protein providing a scaffold for otolith biomineralization and regulatory function by interacting with other matrix proteins**
*72* *kDa* *type* *IV* *collagenase*	*g30843.t1*	*IOM*	*The* *protein* *from* *the* *group* *of* *matrix* *metalloproteinases* *(MMPs)* *with* *ability* *to* *remodel* *the* *extracellular* *matrix,* *a* *function* *necessary* *for* *proper* *cellular* *migration* *and* *tissue* *morphogenesis.*
*Splicing* *factor,* *arginine/serine-rich* *19-like*	*g31972.t1*	*IOM*	*The* *protein* *belonging* *to* *serine* *arginine-rich* *protein* *family,* *involved* *in* *the* *splicing* *process* *of* *precursor* *RNA.*
*Protocadherin* *fat* *4-like*	*g45703.t1*	*IOM*	*The* *protein* *from* *a* *large* *family* *of* *proteins* *often* *involved* *in* *calcium-dependent* *cellular* *adhesion.*
*Transforming* *growth* *factor-beta-induced* *protein* *ig-h3* *isoform* *X2*	*g54590.t1*	*IOM*	*The* *extracellular* *matrix* *protein* *functionally* *associated* *with* *the* *adhesion,* *migration,* *proliferation,* *and* *differentiation* *of* *various* *cells.*
*Neuroserpin*	*g59144.t1*	*SOM,* *IOM*	*The* *protein* *from* *a* *large* *familiy* *of* *serine* *protease* *inhibitors* *that* *may* *regulate* *local* *protease* *activity* *during* *framework* *assembly.*
*Thrombospondin-1* *precursor*	*g6096.t1*	*IOM*	*The* *protein* *is* *an* *adhesive* *glycoprotein* *that* *mediates* *cell-to-cell* *and* *cell-to-matrix* *interactions.*

All proteins were also identified in modern otoliths of *Phycis* *phycis* (Supplementary Table S4). The bolded text highlights proteins specific to otolith/inner ear development; italicized text highlights proteins expressed in various tissues, including the inner ear.

## Discussion

One of the most distinct features of biominerals, carbonates included, is that they are invariably organic-mineral composites in which the organic components, such as polysaccharides, lipids, and proteins are incorporated/embedded into the inorganic mineral phase, forming meso- to nanoscale intra- and intercrystalline inclusions and networks ^24,25^. These organic components participate in the physiologically mediated process of biomineral formation. Because the proteomic profiles can be linked with transcriptomic resources/expression, studies of the proteome of modern biomineral structures provide insights into molecular mechanisms of biomineralization. Identification of proteins in fossil biomineral structures therefore raises hope to gain indirect access to genome-related information in the absence of preserved DNA in fossils much older than ca. 2 My, the age beyond which DNA is generally no longer surviving in the fossil record ^26,27^.

Increasingly, paleoproteomic data have been extracted from various fossil biomineral structures ^21,28–31^. However, to date no paleoproteomic information exists from fossil otoliths that represent the most abundant fish remains in Mesozoic and Cenozoic deposits. This study is the first report on protein identification in fossil otoliths conducted in direct comparison with proteome of congeneric modern otoliths (phycid hakes). The following discussion focuses on two key aspects: structural criteria of fossil biominerals that preserve pristine paleoproteomic information and comparative analysis of protein content in modern and fossil phycid hakes otoliths.

### Mineral phase vs. paleoproteome information preservation

The fossil material selected for this study came from Korytnica, a locality well known for exceptional preservation of aragonitic biominerals (see also “Material” section)^32,33^. Indeed, in all fossil otolith samples studied here, only the aragonite carbonate polymorph was detected; i.e., we observed no evidence of the presence of secondary calcite or other secondary phases. The exceptional preservation of these fossil otoliths is further supported by their crystallographic and ultrastructural features, which are indistinguishable from those characterizing the modern counterparts in terms of their distribution of crystal sizes, orientation/inclination, azimuthal dispersion, and turbostratic distribution (plane (222) (Figs. 1, 2). Further evidence of the extremely pristine preservation state of these fossil otoliths is provided by the occurrence of their nanogranular texture, typical of otoliths and most other biogenic minerals^6,34,35^. The nodular nanograins (ca. 100 nm in diameter), which are typically visualized with atomic force microscopy (Fig. 2c,d,i,j) are considered the product of a biomineralization process that involved amorphous precursors^36,37^. In this process, the organic molecules become incorporated into the crystallizing biomineral, e.g., as inclusions, and as organic-rich ‘envelopes’ around the resulting nanograins (Fig. 2e,f,k,l)^25^. Individual proteins involved in the carbonate biomineralization process have masses up to hundreds kDa and sizes from few to several nanometers in diameter/radius of random coil (structured/IDPs proteins). Their embedment into the crystal structure was interpreted as occurrence of intra- and intercrystalline inclusions^38,39^. Such inclusions are consistently present in modern and fossil otolith samples analysed herein, and we assume that they are the primary source of the proteinaceous material in this study. Prior (paleo)proteomic analyses examined amino acid racemization and their relative content measurements to assess the preservation potential of the samples^40^. Protein degradation is clearly suggested by the distribution of amino acids between pools of free amino acids (FAAs) versus polymerized amino acids (THAA-FAA, as pmol amino acids per mg of starting otolith material); most fossil peptides have broken down to individual amino acids. The observed bias toward acidic amino acids in fossil specimens suggests that highly acidic proteins (common to biominerals in general) are part of more soluble portions of the biomineral, which were preferentially degraded; this is reflected in the types of proteins that were sequenced by LC–MS/MS (Table 2). Protein degradation is also supported by the thermogravimetric data. The thermograms of Recent samples exhibit broader thermal decomposition range and a higher number of weight-loss steps in comparison to fossil ones. The Recent samples contain organic compounds that differ in their susceptibility to thermal decomposition, thus it is not surprising that the temperature range of their decay is relatively wide. It appears therefore that modern otoliths contain a greater diversity of organic (proteinaceous) components, which is manifested by higher thermal decomposition range (number of weight-loss steps) in comparison to fossil otoliths, for which the organic diversity is smaller.

### Comparative (paleo)proteome analysis of modern and fossil phycid hake otoliths

More than 130 proteins were identified in otoliths from modern adult *Phycis* *phycis*. These include otolins, otogelins, usherins, and a cochlin, which have been previously identified in otoliths^41,42^. Of the detected modern otolith proteins, 11 were also observed in the fossil *P*. *tenuis* otoliths. These include alpha and a beta tectorin, two otogelin-like proteins, otolin-1-like, and a neuroserpin, all of which have been suggested to be directly involved in calcium carbonate biomineralization. We also identified in the fossil otoliths additional proteins not previously detailed from fish otoliths, such as collagenase; transforming growth factor b-inducing protein, splicing factor; arginine/serine-rich 19-like protein; protocadherin FAT 4-like proteins; and thrombospondin. All of these additional proteins except the splicing factor have GO terms designating them as extracellular or associated with a membrane. Several are calcium binding and at least one, protocadherin, has been detected in biogenic carbonates from other organisms^43^.

There are several possible reasons why this particular suite of proteins previously established as otolith matrix proteins were preserved in the fossil otoliths studied here. Co-preservation of some proteins may result from their intimate interactions during biomineralization. Both otogelin and alpha tectorins are necessary for tethering of the otolith membrane to sensory structures in the ear^44^, and otogelin is crucial in early larval development of the initial seeding of the otolith^10^. Beta tectorins likely sequester calcium as biomineralization proceeds^10^, and may polymerize with otolin^45^, possibly enhancing their co-preservation. Similarly, alpha tectorins possess an N-terminal Nidogen domain that has been proposed to allow it to also interact with otolin^46^. The proteins detected in fossil otoliths are some of the highest scoring proteins in modern otoliths (higher scores indicate a more confident match between combined scores of all observed mass spectra and amino acid sequences within examined protein), which has been shown to be linearly related to relative protein abundance^47^. Lastly, some proteins with acidic residues that provide an organic scaffold for biomineral formation (e.g., otolin-1 like^48^) are strongly stabilized by calcium ions; such proteins may firmly adhere to the biomineral surface, which may enhance their preservation potential in fossil record^29^.

Our observations refine the structural criteria for exceptional preservation of carbonate biominerals in the fossil record, and the paleoprotein sequences indicate highly conserved inner ear biomineralization processes in fish through geological time.

## Material

### Modern samples

Forkbeard (*Phycis* *phycis*, Phycidae family) modern otoliths were collected from fish caught off by fishermen along the mainland Portuguese west coast between 2011 and 2012. Sagittal otoliths were removed with ventral cranium section through gills, rinsed with water, air dried and stored in labelled plastic tubes at Lisbon Sciences Faculty (Portugal) until analyses^49,50^. Two large specimens of *Phycis* *phycis* were selected for biomineral structure analyses (ZPAL P.21/R-OTH-242/001, ZPAL P.21/R-OTH-187/002) and three specimens (ca. 2.5 g in weight) were selected for proteome analysis (ZPAL P.21/R-OTH-196/003, ZPAL P.21/R-OTH-197/004, ZPAL P.21/R-OTH-198/005).

### Fossil samples

The fossil sagittal otolith samples of *P*. *tenuis* were collected from the Korytnica Clays [GPS position: 50°39′50′′ to 50°40′50′′ N and 20°31′20′′ to 20°33′00′′ E; three sites: Korytnica-Plebania, Korytnica-Forest, and Mt. Lysa^33^], a unique facies deposited in the terminal part of the bay (Korytnica Basin) developed in the Miocene along the rocky shore on the southern slopes of the Holy Cross Mountains, Central Poland^20^. The Korytnica Basin is filled by a shallowing-up sedimentary sequence composed of ca. 30–60 m thick sequence of clays, locally interfingering with oyster shellbeds. The absolute age of the Korytnica sequence is estimated as 14.8–14.6 Ma^51,52^ Korytnica Clays are renown from pristine preservation of Miocene fossils. Such exceptional preservation is supported by aragonite mineralogy (a metastable CaCO_3_ polymorph in normal conditions) of skeletons of e.g., scleractinian corals, gastropods, and fish otoliths and their distinct and fully comparable to modern counterparts micro- and nanostructural features^6^. The unusually favorable conditions of fossilization are further supported by exceptional, residual color patterns of some gastropod and barnacle shells^20,53,54^. Such preservation implies that fossils embedded in impermeable clays were virtually sealed off from extremal environment. After withdrawal of the Paratethys seas from the southern outskirts of the Holy Cross Mountains, these sediments were not covered by additional thick sedimentary cover that could cause any geothermal gradient heat effect on fossil material. The preset-day annual mean-temperature for Świętokrzyskie region in Poland ranges from 5.61 °C (in 1940) to 10.10 °C (in 2019); observations from 1901 till 2021^55^. Considering the very fine grained nature of Korytnica-clays and consequently, typical to such sediments, extremely low thermal conductivity^56^ it can be reliable suggested that the examined otolith samples (retrieved from sediments found today at 1–2 m depth) have not experienced any significant temperature fluctuations during their burial history.

The clay samples were washed and sieved through standard sieves (500/250/125 μm) and dried at 40 °C. Of ca. 300 otolith specimens of *P.* *tenuis*, 2 specimens were selected for biomineral structure analyses (ZPAL P.21/C-OTH-07/006, and ZPAL P.21/C-OTH-07/006); 20 specimens (ca. 1.6 g in weight) were selected for proteome analysis (collective number ZPAL P.21/C-OTH-07/008-027). Specimens selected for (paleo)proteome analyses were soaked in sodium hypochlorite (5%) for 3 h, rinsed and ultrasonicated with deionized water and dried at 40 °C overnight.

Material of modern and fossil otoliths is housed at the Institute of Paleobiology, Polish Academy of Sciences, Warsaw (abbreviation ZPAL). Detailed sample identification information is provided in Supplementary Table S1.

## Experimental

### Otolith biomineral structure

Structural features of the otolith were studied and photographed using a transmitted light microscope Nikon Eclipse 80i at Institute of Paleobiology, Polish Academy of Sciences, Field–Emission Scanning Electron Microscopy (FESEM, Zeiss Merlin) at the Department of Chemistry, University of Warsaw, Thermo Fisher Tecnai Osiris microscope at Central facility in electron microscopy (CIME) of Swiss Federal Institute of Technology in Lausanne (EPFL), and Atomic Force Microscopy (AFM) using Multimode 5 instrument (Veeco) upgraded to Multimode 8 version (Bruker), at the Department of Chemistry, University of Warsaw. Light microscope images (in polarized light) were taken from ultra-thin (2–12 μm thick) sections made in sagittal plane of the otolith. FE-SEM images were taken of transverse broken sections of otoliths mounted on stubs with double-sided adhesive tape and sputter coated with a conductive platinum film; the accelerating voltage was of 5 kV, working distance 4–6 mm. Atomic Force Microscopy imaging was acquired in ScanAsyst mode using dedicated silicone cantilevers. Two signals (height and peak force error) were simultaneously collected during each scan. Otolith polished sagittal sections (Buehler Topol 3 final polishing suspension with particle size 0.25 µm) were rinsed in Milli-Q water, washed in an ultrasonic cleaner for 10 s, and then etched with a Milli-Q water solution for 7 h. The images were processed with WSxM v5.0 Develop 10.2 software from Nanotec^57^. Samples for Transmitted Electron Microscopy (TEM) were prepared as cross-sectional TEM lamellae extracted and then milled using a dual-beam Gemini NVision 40 Focused Ion Beam machine. The initial chunks are milled with gallium ions at 30 kV, 6.5 nA and then thinned down with lower currents step by step until using 80 pA, and finally smoothed at 5 kV, 80 pA. TEM analyses were performed at 200 kV accelerating voltage. High angle annular dark field (HAADF) images in scanning transmission electron microscopy (STEM) mode were recorded with a spot size of 0.5 nm and camera length of 115 mm.

### Electron backscatter diffraction (EBSD)

The surface sample (of thick slides) was polished with alumina of 1 µm, 0.3 µm, and 0.05 µm and finally polished with colloidal silica (0.05 µm). Before analysis, samples were coated with a thin layer (ca*.* 2 nm) of carbon using a high vacuum coater. The EBSD study was carried out with Oxford NordlysMax detector mounted on a scanning electron microscope JEOL JSM-6610LV at the Institute of Materials Engineering, Łódź University of Technology. EBSD data were collected with AztecHKL software at high vacuum, 20 kV, large probe current, and 20 mm of working distance. EBSD patterns were collected at a resolution of 0.22 μm step size for crystallographic maps using the unit cell settings characteristic of aragonite and calcite as follows^58,59^: “Pmcn” symmetry and a = 4.96 Å, b = 7.97 Å, and c = 5.75 Å estimated for *Favia* coral using X-ray powder diffraction with synchrotron radiation (43) and a = b = 4.99 Å, and c = 17.06 Å, respectively. The EBSD data are represented in this study by crystallographic maps, phase images, and the pole figures, which represent the stereographic projection of crystallographic planes in reference to the (100), (010), (001) and (222) aragonite planes. Orientation images and the pole figures were created using MTEX open source plugin for Matlab program (https://mtex-toolbox.github.io/). To eliminate combination of red and green colors and create images more accessible for color-blind users we selected BungeColorKey palette from MTEX (the outcome was tested using Coblis, the Color Blindness Simulator at https://www.color-blindness.com/coblis-color-blindness-simulator/).

### Thermogravimetry

Thermogravimetric analysis was performed with a TGA Q50 apparatus (TA Instruments) at the Department of Chemistry, University of Warsaw. Otolith samples (37.48 and 37.16 mg for fossil and Recent samples, respectively) were heated in nitrogen environment under linear gradient (10 °C min^–1^) from ambient 20 °C to 550 °C.

### Amino acid racemization analysis

The first test of the assumption that original organic material remains in the otolith included amino acid racemization analysis (AAR)^60,61^. The AAR analysis provided information that the samples have been appropriately cleaned (D/L of fossil specimens approaching (1), that original protein material was still embedded (D/L of fossil specimens approaching (1), and gave an idea of the state of degradation (i.e., amino acid relative concentrations and abundance similar to those in modern otolith samples should suggest minimal degradation).

Amino acids for racemization analysis, both free and total hydrolysable amino acids, were extracted, hydrolyzed, and evaporated to dryness from cleaned skeleton powders (described below) by standard methods^61^. All samples were prepared in duplicate and analyzed at the Northern Arizona University Amino Acid Geochronology Laboratory using standard methods with modifications for microfossils^60,62^. Rehydrated samples were spiked with L-homo-arginine as an internal standard and then injected into an HPLC fitted with a reverse-phase C18-packed column. ‘Blank’ samples were included. We have previously shown that our ‘clean space’ set up and handling protocol are sufficient to prevent exogenous protein contamination in the laboratory^21^.

### Proteome analysis

The fossil and modern otoliths were powdered by mortar and pestle to 125 μm, oxidized in 50:50 concentrated bleach/H_2_O_2_ for 1 h while sonicating following modified methods of Stoll et al.^63^, rinsed five times with MilliQ, and dried. Oxidation and rinses were repeated two more times. Cleaned powders of approximately 0.5 g per sample were decalcified in 0.5 M acetic acid with all handling occurring in a laminar flow hood to minimize contamination. Soluble organic matrix (SOM) was concentrated by centrifugal filtration (Amicon, 3 kDa cutoff) and rinsed with filtered phosphate buffered saline. Insoluble organic matrix (IOM; material that pelleted at 43,000×*g* for 5 min) was three times washed washed with 80% acetone. Fossil proteins were prepared as single samples for each solubility fraction (sample 07); modern samples were extracted as biological triplicates (samples 196–198). Samples were solubilized in SDS buffer and then digested using the MED-FASP protocol on a 30 kDa Microcon Centrifugal Unit (Sigma Aldrich) after rinsing out the SDS buffer with 8 M urea ^64^ by sequential applications of trypsin and then Glu-C enzymes. Samples were sequenced by liquid chromatography tandem mass spectrometry (LC–MS/MS) at the UCLA Semel Institute Proteomics Facility. Each fraction was analyzed separately on a nano-liquid-chromatography system coupled to a benchtop high-resolution orbitrap mass spectrometer (QE-Plus; Thermo Fisher) and operated in positive ion mode with data-dependent acquisition. MS1 was performed at resolution of 70,000 (at 400 *m/z*) and MS2 at 17,500. Instrumental blanks were run between all samples to minimize carryover. Transformed mass spectra were analyzed in Mascot against the UniProt-Human database, a common contaminants database, and the *Phycis* *phycis* genome’s ^65^ predicted protein database. The *P.* *phycis* protein database (Supplementary Table S5) was generated using the BRAKER pipeline, which includes the use of the GeneMark-ES/ET and Augustus programs^66–69^, to predict protein-coding regions of the unannotated *P.* *phycis* genome. The Atlantic cod (*Gadus* *morhua*) predicted protein database (NCBI assembly GCA_902167405.1 gadMor3.0) (predicted protein file available) was used to input “hints” to guide CDS prediction. All samples allowed a fixed modification of carbamidomethylation on C and variable oxidation on MW, deamidation on NQ and protein N-terminal acetylation; fossil samples also allowed Phospho K & T and MOD. For each sample, a first decoy search was carried out to determine p-values for a 1% false discovery rate. Then an error tolerant search was conducted, with the p-value adjusted if necessary. Cutoff scores were applied at the value recommended by the Mascot algorithm. Returned sequences were annotated in Blast2GO software and further run in Blast2GO against the NCBI nr primates database to test for potential human contaminants not picked up by the UniProt-Human database in Mascot; likely human contaminant proteins were excluded from the final list if they were > 90% similar to primates with an e-value within 20 units of the original annotation, within 10 e-value units and 10% similarity for e-50 and lower, or within 5 e-value units and 10% similarity for e-50 and higher^22^. Duplicate sequences were checked in CD-HIT with > 90% similarity; duplicates are noted separately but counted together in total protein counts. Several proteins were predicted as separate peptides; those peptides were BLASTed against the Atlantic cod (*Gadus* *morhua*) predicted proteome (NCBI assembly GCA_902167405.1 gadMor3.0) and concatenated, with strings of XXs denoting regions of unknown sequence between known peptides.

## Supplementary Information

Supplementary Information.

Supplementary Table 1.

Supplementary Table 2.

Supplementary Table 3.

Supplementary Table 4.

Supplementary Table 5.

Supplementary Table 6.

## Data Availability

All data generated or analyzed during this study are included in this published article (and its Supplementary Information). The mass spectrometry proteomics data have been deposited to the Proteomic Xchange Consortium via the Pride partner repository^70^ (https://www.ebi.ac.uk/pride/login) under the dataset identifier PXD036742 and 10.6019/PXD036742. No ethical approval or guidance was required because this study only analyzed specimens were collected from commercial landings of fishing vessels and fossil material in museum collections.
