# Supplementary material for: First paleoproteome study of fossil fish otoliths and the pristine preservation of the biomineral crystal host
Source: Sci Rep. 2023 Mar 7;13:3822. doi: 10.1038/s41598-023-30537-8 (PMC9992438; doi:10.1038/s41598-023-30537-8)
Supplement: Supplementary file 1 — Supplementary Information. [file 41598_2023_30537_MOESM1_ESM.docx]

**Supplementary Information for:**

First paleoproteome study of fossil fish otoliths and the pristine preservation of the biomineral crystal host

Jarosław Stolarski^1^, Jeana Drake^2^, Ismael Coronado^3^, Ana R. Vieira^4,5^, Urszula Radwańska^6^, Elizabeth A. C. Heath-Heckman^7^, Maciej Mazur^8^, Jinming Guo^9^, and Anders Meibom^10,11^

^1^Institute of Paleobiology, Polish Academy of Sciences, Twarda 51/55, PL-00-818 Warsaw, Poland

^2^Department of Earth, Planetary, and Space Sciences, University of California, Los Angeles, CA, USA

^3^Faculty of Biological and Environmental Sciences, Universidad de León, Campus of Vegazana S/N 24171, León, Spain

^4^Department of Animal Biology, Faculty of Sciences, University of Lisbon, Campo Grande, 1749‑016 Lisbon, Portugal

^5^Marine and Environmental Sciences Centre (MARE), University of Lisbon, Campo Grande, 1749-016 Lisbon, Portugal.

^6^Department of Geology, University of Warsaw, Żwirki i Wigury 93, 02-089 Warsaw, Poland.

^7^Department of Integrative Biology, Michigan State University, East Lansing, MI, USA

^8^Department of Chemistry, University of Warsaw, Pasteura 1, 02-093 Warsaw, Poland,

^9^School of Materials Science and Engineering, Hubei University, Wuhan 430062, Hubei, China

^10^Laboratory for Biological Geochemistry, School of Architecture, Civil and Environmental Engineering, Ecole Polytechnique Fédérale de Lausanne (EPFL), CH-1015 Lausanne, Switzerland.

^11^Center for Advanced Surface Analysis, Institute of Earth Sciences, Université de Lausanne, CH-1015 Lausanne, Switzerland.


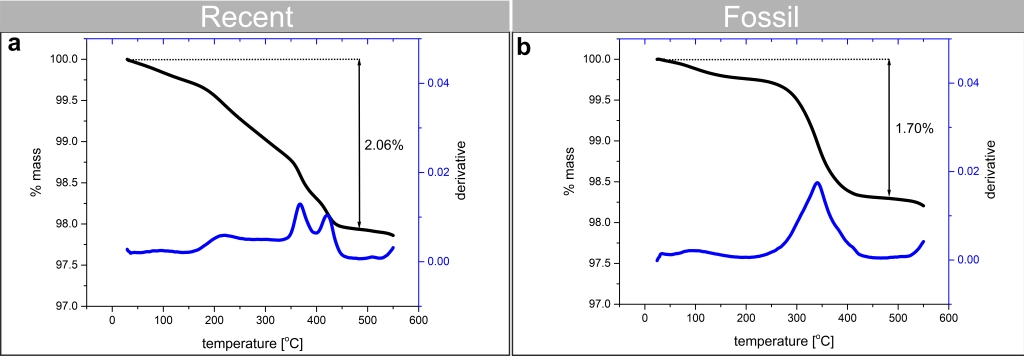


**Supplementary Figure S1**. Enlarged scale weight loss profiles and derivative w/t of Recent *P. phycis* (a) and fossil *P*. *tenuis* (b) otolith samples before the aragonite-calcite phase transition at ca. 500°C. Modern otoliths decompose over a broader temperature range in comparison to modern ones (ca. 200-430°C vs. 250-400°C), which exhibit several weight-loss steps (at. ca. 210°C, 360°C, and 410°C) in comparison to the one relatively large weight-loss (at ca. 340°C) in fossil otoliths.

**Captions of Supplementary Tables S1 to S6**

**Supplementary Table 1**. Sample identification information. Specimens housed at the Institute of Paleobiology, Polish Academy of Sciences, Warsaw (abbreviation ZPAL) received collection number ZPAL P.21, followed by prefix R-OTH (Recent otoliths) and C-OTH (fossil, Cenozoic otoliths). A. Samples used in otolith biomineral structural analyses. B. Samples used in proteome analysis. Sample numbers starting with F were the extracted and digested proteins run by LC-MS/MS. Sample numbers 19N are the modern collecting effort while sample number 07 is from the fossil collecting effort. All samples used in proteomic analysis were destructively sampled in their entirety.

**Supplementary Table 2**. Peptide detection information for 132 proteins sequenced from modern *P*. *phycis* otoliths by LC-MS/MS. Note that five peptide sequences were predicted with isoforms; in each case, the longest peptide was used for further analysis.

**Supplementary Table 3**. Peptide detection information for 11 proteins sequenced from fossil *P*. *tenuis* otoliths by LC-MS/MS.

**Supplementary Table 4**. FASTA sequences of 11 proteins sequenced from fossil *P*. *tenuis* otoliths by LC-MS/MS. ‘x’s’ represent where predicted peptides have been manually concatenated. There could be a few or very many amino acids between concatenated peptides.

**Supplementary Table 5**. Characterization information for all proteins detected in modern and fossil *Phycis* otoliths. Several proteins were predicted as separate peptides; those peptides were trained using the BRAKER pipeline against the *Gadus moria* predicted proteome (NCBI assembly GCA_902167405.1 gadMor3.0) and concatenated, with strings of XXs denoting regions of unknown sequence between known peptides.

**Supplementary Table 6**. Detected peptides with modifications commonly observed in degraded fossil specimens (deamidations of NQ and (di)oxidation of M). Peptides detected multiple times are only shown once with the longest detected peptide represented. Column names follow conventions of those found in Supplementary Tables 2 and 3.
