# Supplementary material for: First paleoproteome study of fossil fish otoliths and the pristine preservation of the biomineral crystal host
Source: Sci Rep. 2023 Mar 7;13:3822. doi: 10.1038/s41598-023-30537-8 (PMC9992438; doi:10.1038/s41598-023-30537-8)
Supplement: Supplementary file 5 — Supplementary Table 4. [file 41598_2023_30537_MOESM5_ESM.docx]

Supplementary Table 4. FASTA sequences of 11 proteins sequenced from fossil *P*. *tenuis* otoliths by LC-MS/MS. ‘x’s’ represent where predicted peptides have been manually concatenated. There could be a few or very many amino acids between concatenated peptides.

**>g13180.t3_g16293.t1_g49429.t1**

MVSRVNPVIALHLLIIFAQTKAGNQDIMYPYGPGHRDQETPKMDDGSSAEVVLLLPFVFFNAPYRSIYVNNNGVISFNVQVSQFTPEAFPLSDSRSFIAP

LWADVHNGIRGDVYYRETVDPEILARATQDIRKYFKNIPGFTATWAFIATWHQVTFYGGSQTTPCVLISDGATAFSMFNYGEILWSTGTASGGDPLTGLG

FNGGDIGHFFNLPGSRSNDVVNIEQTTNVNVPGRRFYRRGEIFWLSDQCSLRCRCLDMDNEVQCQEAPCGQLETCEQQEGAFYCQPVRTSTCVVFGDPHY

HTFDGFLYHFQGTCSYLLARPCWEVAGLPNFSVEAKNENRGMASVSWLRDVTVEVYGHRVTLPKGSLGIAQVDGLRKTLPVQLQLGAIKVYQSGVAIALE

TDFGLLVTYDGQHYASISLPSSYFNNTCGLCGNYNDDPADDPVLPDGSLAESVVELGGSWRADDTDWRCNDGCTQNCSLCDPVVEAFYFRPDYCGLINKT

DGPFRDCRSVVDPTAFVYSCVYDLCSNRDNITTLCHAIQAYSMACQALGVTIRPWRSRNFCALSCPEASHYQVCTSACPSSCSDLTSALYCAHPCTEGCQ

CDAGYVLSGSHCVHLDDCGCEHEGLYYPRNETFWAGASSKSSECTLRCVCGAAGEVSCFNDSCREAEVCTAEVGRLGCYPHREGTCSVAQNTVTSAFDGG

SLSFPDDSSYYLLKLCAAVPRNASSVEVKIGRKLVNKGPTWMRPVVVRVANLEAQIGGADFDVVKLNGEVVPLPYVHPLETMLIYKAPGNATVVECSGLL

RVYYNRQGFLNVSLSTIFYNVTCGLCGVFNRNASDDLRLPNGRLAETAEQFTEGWRAIADDLTCNGDCDDLYRMCTDLRIYQSPWMCGNINDPGNSSFLA

CHTAINPSPFFRNCLYNMCVKEGNRSALCSSLQAYASACQDAQVGLASWRSATNCPLPCPDHSHFDECVTACPLTCTNLDEPRLACPLPCVEGCQCEEGY

ALRDGHCVARSDCGCLSRGRQLATNQTFWRDRECLERCFCNGSDNSVYCQTGPCHAEEYCEESGGLYFCQPRTEALCAAAGYGHFLPFGGVPFELQSSCT

LQLATTYCRGNAEAGRLAGXXXXXXXVSPPFKLAARNEARDTSQAIWVRGFVLEVYGYEIEVSRSYKNTVTVNKDRLYLPLKLGAGKITVFTLGLLLVLE

SDFGLRVVFDWNTLLLLTLPHSIYNTTCGICQGVPPSTASTAALTSTTEWGMGWAERDTFCQVGCGDSCPRCGLGEKxxxxxxxxxxYVFVEPEAVRLCVLIVDRLGVFARCHSKVAPAFFYQSCLQDTCLDQGAQETICNWLQIYASTCQTQGVPVSGWRSNTPCALSCPANSHYSSCMSVCPPQC

APARGQRDCNQDCVEGCLCDQGYVLNGKSCLLPQNCGCYTDGKYYExxxxxxxxxxEICTNNTCEQENGGDLCGCPELPSSPSGDDDVIQAEVTCKHAQMEVSISKCKLFQLGFEREDVRINDQHCPGIEGEDFISFQINNTKAHCGSIVQSNGTH

IMYKNTVWIESVNNTGNVITRDRTINVEFSCAYELDLKISLETVLKPML

**>g19658.t1_g45878.t1_g59712.t1_g28107.t1_g36359.t2_g40098.t2_g40101.t1_g64937.t1_g59944.t1**

MSCRSWGQYNFETFDGLYYYFPGKCTYTLLRDCEDTTSSSIIVQVHNDPGCHASAYNCSRSVSLFLPWEGEVRLHSNSVTFNDQRVDLPAHIHDLELEQI

SHYILVTQQQGFTLAWDGHSGSIYIKLSPEFVGRTCGLCGNFNADVQDDLKTSYGVVTADIDMFGNSWMEAEPHEARCPIVASGFPSPCFGLDDHVLLRV

EELCSRLLEEPFLSCHAFVSPLPYMASCSNDLCLSSPGGEVVCQVFTEYARACAHADSPLKDWRKHIPQCAKQCPPGLQYRECISCCPASCTVERLCFDS

KLVCLDGCYCPPGESAVWGGVSPSRSAGGPHCATVTAVxxxLLEHSAQLPQLVDWYLRHYKEEKVGWRNLNSSPFWHRSGGTSSLPIPEKVEVVGPSQLIPCVLLSGLIYEDGRCVSPSDCPCEYHGMLYPTGQMVQEDCN

NCTCVGGMWNCTEYTCPGECSVTGDMYFQSFDGRIFTFPATCQYVLAKSRSSGKFTVTIQNTPCGPTLDGACIQSVSLVFDEDPRSQITLSHLGEVFRAG

QYRVSLPYSDGNSDLRHQDPRYXMFHIQELSSMFLQVSTTLGLQLHYSWREFRLYLRLDPSWKDDSLGLCGTFNGNTQDDFLxxxxxxxxxxSYASAMCDILNQDVFSPCHEYVSPSPFQQQCRADTCRCGTPCLCSALAHYARRCRKFSVIVEFRAHVPDCAVECPATMEYGTCVSSCQRRCSTLGSSHHC

GEECEEGCVCPDGTFYNLHTHTCVPRGPLSDNQWEATCPTEAAAVLPCLSPSTSQPSLPPAPRAPGVSESAEPLNLEARQVFxxxxxxxxxxMFSHVVNESGLVSVQTSPPLEPWSDLGPRRGAASPARILFLRHGDECFQPESCPCLWKGKEYYPGDKVSSPCHKCVCQHGAFQCVTRPCPAMCTAYGDRH

YKTFDGLLYDYVGACKVYLVKSSSAAGGLSVMTENLDCFSSGVICRKSLFINIGRSVVAFDDDSGKPNPASVVDRKQRMHIWTAGYFTVIHFPGEQVTVL

WDHKTTVHVQVGPQWQGKLSGLCGNFDLRTVNEMRTPDNIDSPTAQEFGNSWTAAExxxCVNSPDIRSPCSLSPLREPFAKRQCGVLLGEVFQSCHPVVDVSWFYVNCLADTCGCTHGGDCECFCSSVAAYAQRCCHQGVPVDWRTPALCLPGIExxxLGKGPFQLLTFLERGTVLAASRSSGRVYLQASPPSTSDVLTHFMMTPGLSRSRPHDSSLVSFEAVDRPNYFLDTSPEGSVRLTKWRESAAFWDGATFVLH

RDTSVPGFDALESFSKRGFFLHAAAPRLQLLKHRNWPAFRRGVLFKLTGVGPDTPAAGPRCQWRYDSCVSPCFRTCSDPSGTACVTIPQxxxxxxxxxxMSESSTLETTKHTVEITSPMVISSTRMCTPPYSEFIDECTKYICVNSQLVLFNKTQNCPYDSLPPNCGLLGFAILVNGDKCCPKWDCPCRCSVFPDLNVI

TFDGNSVAVYKAASYIISQLPNETVSVQVQECPADSDALYVNSRYAKPRFRKFGFEILDTGNMYLIRSPSGLRLQWFHSTGMMVMETESSSSRLPTMGLC

GYCDGDPANDLTLADGSVLVEGQDPAAFIDSWQVPNTTSYVSHSRRREVNCSTSDCSHCMAMLQDASFLPCHDFVGPCPLLYLSCGSEFQKEAFVPQVPP

STFCEMWVRDAEYVNNRCVALxxxxxxxxxxLPACTAQSCPNQDFESDPVQCSGLTEGCVCPEGTLLHRPYSALCIPPDKCACTDSGGVPRANGDLWKASKDGCCMYRCDNDAIKPVEYNCSAVALPTCRR

AGEEVVSLADDNSCCPRKACGKTGSSVPRGSRxxxxxxxxxxVSCPVGMSVSPVSSPDQCCQKSCGEYEPFTGTVENDQSGVFLREDLHPEMCPVRDAVCLDAERGVMRPGQTLVERSPDGLCHSTQCSGSLDPATGYHHLR

TSTTNCSAQCQP

**>g24402.t1**

EPGIPGEKGEPGLRGEPGLRGEPGPRGISGLEGPFGPPGAPGMRGQKGEKGECPPAPPGAPGQRGEPGLNGTDGLPGNKGDPGPKGDRGAVGPLGGRGMP

GMKGDKGIKGVRGPRGPKGAPGEDAPMVRSAFSVGLFPSKSFPPPGLPVKFDKVFYNEEGHWDPVLSKFNATHPGVYVFAYHLTVRNRPLRAALVVNGVR

KVRTRDSLYGQDIDQASNLVLLQLAPGDQVWLETLRDWNGVYSSSEDDSTF

**>g30842.t2_g30843.t3**

YSLFLVAAHEFGHALGLEHSQDPGALMAPVYTYTKDFRLSNDDIKGIQELYGVQTGGNPQNPTLGPVTPMDLCSEPVVFDAVAQIRGETFFFKDRxxxxxxxxxxMGLQGNGCVDVCSVFLFRSVNFRSKPTGPMLVATYWPDLPGKIDAAYENPLEEKTVFFAGNQMWIYRADQLERGYPKRISSLDLPTDLEQIDAAFSFRKN

QKTYLFSGDKFWRYNEEKKKMDPGFPRLIADSWNGIPDGMDAAFSLNNIGK

**>g31972.t1**

ALLDNLPSLVTFTARLRCVTGICPRDLEDYGCCCRYRGAGEPLDPLDSCCFQHRQCYQTVMDAPCWQEMPPLADNLTCSSLNTTCDPFSRLPVTSPMGGV

CPDVPRSEQPTQRPGRLLCPLSRVMTETWVPDVGDSCVRSFCECDTAAVECLALTAYNSSMKNLPESFCSGLEPTGGTLRNASENAGDVITNPGATGSEY

DNATALSSNQNATAFNSSYLDEVDRLLSLDWAPENSSDITGVGGDLVAPRPAPPAGSPDEEEGEREEELALPPTDDPIFVTKGSELEEEETEWDFITHNS

SAGDPESTSSLPAAASGRTGDRTASPTLLTHNPPGYEDHGPASPPGTQSVTRVSPPSTPPPERPSEEEEEEEEEEEEEMEEEEEEEEEAPIPPPASEAAA

PCCHAHSSPLRKRGGAMMRVRVNTSPFPRCSSGRPYGVLRHENPPVDHYRYQDNPANHRPLQGAGRDPDTSRLESPSYTHRPPDHHHGDRVPLISPSVVP

TNGHARRRITGGGGATSDHALR

**>g52865.t1_g67445.t1_g45228.t1_g67087.t1_g24538.t1**

TRTEEPVVFGNSWAVDLPHETACPSVEQDFNGPCQSESDMDDAIEKCSALLFFPFLSCHENIDPNPFVASCVSDLCVSDDEEIFCRALVEYTRACSHVGY

PVREWRDSFPSCNDGCEESFVHRDCISCCPPTCTFEKECLGTNLHCLDGCYCPDGLILQNGTCISVTHCPCVYHGTSYSQGHVLEQGCSVCVCMGGVWNC

TDSNCTAECTVVGDVFVATFDGRMFLQPGVCQYVLAKSHRSSRFTVTLQYTTCAEQKACVQSVTVVLDEDVNRQVTLTxxxxxxxxxxVVCLGGMLYHTCVSSCGRSCRALSSPESCNPDDCAEGCGCSEGSFYDEMRQRCVQQSQCHCFSMGGVSQPGEVSFSASGPCLCRNGQMECVPEEREPERT

DTQVCPEGKVYHSCGEQRGGVACEPTCRNLMLNLTCPPNAPCIPGCVCPPGLVLHHEECYYPESCPCVWLGLEYLPGESVDTACYKCVCQRGFFSCSYLP

CPAVCTVYSDRHFHTFDGLEYDYHSDCQVYLMKxxxxxxxxxxSSFVIPYRMMCEWRYRPCASPCVHTCNDPTATRCHFLPPVEGCFPRCPKNMVLDEVTRRCVYIEDCGCSGRAExxxxxxxxxxMSPRAPPPGPLATAGFGGMRFFDPEGQHEVSVNYRPARLPFSRQSLHMDDTGSMYLIQTPGGVNIQWYHSTGIMVLQYTAPGNQTGHTRGLCGCCDGNPA

DDLKLPNGTVVRELGDMMLFLQAWRVQTSDETEHMRRVGDNCTTGDCSMCLSMLKQRAFAPCHSKVSPEQFCDVMWAGDLHYKDHQCDFMAAYVAVCYTH

QVCISWRGHNFCxxxxxxxxxxLGPGQSLVQYFEGDLCYTVHCLHNKDPDTGFYTMEISSVNCSQRCGPHQVYVASSDPQVCCGSCRNVSCSYNNDNGTTELFTSGSSWVENCTRYDCMETA

VGAVVLASGVVCPPFNDTECVQNGGAVQSYVDGCCKTCKEDGKTCKRVAIRTTIRKDDCRSHAPVTVYSCDGKCPSATIFNFNINSHARFCKCCRESGLQ

TRTVTLYCSRNATLVDYNFQEPLDCSCQWN

**>g45703.t1**

MGLVGESSVTVRPNSRMITPPGTTAIPAGFRAVVGTVQAAYRESSRPITYSVLVDDGDKLFLLNRLSGDFVLSRGLDFETQRFYILTVGVQMEGGLVSGV

RVYFNVLDVNDHPPEFEPDTYSASLPEDAPVGTCFLLLNVSDEDTGENGDLDLTIFAGDDEHVFSVDPAGKLCLNDELDRERKPSYNLTVTARDRPQASS

QRFTSTAQVMISVRDINDNAPIFMSDRSVAIPEDAALQFLITTVRAVDADSGPNGEVFYYINDSVSSPWVGVHNTSGRVYLRRPLDREQADVFTVAVMAV

DQGSPRMASTMTLTVRVEDVNDHRPEFPQRSYSLAVREDVPRGSSVFRAQAWDADLGANGEVRYTLSRPSPFRVDAVRGVVVVTEKLDRERQANYTLTVT

AEDRGSPPRSSAAVIGITVEDVNDFTPVFSPATQTVHVMENEEDVSQFTQLVPLSETQKDSPAGTVFAIVTATDLDQGVNGKIRYSIVGPDSPFAIEETS

GELSSTDVLDRETAAIYNFTVIGRDTHPTHPLSSSVLVNVLVGDVNDHWPQFQNSPYVAHVPTVMDPEFHVNVSDVNDNVPQFIQSEFRCEIFENELPMW

VCDVLAIDADSGSYVDSVNGTITTADVFDYEREQIYDVTVQASNAMDAGTPPLTSTTVFHISVLDENDNPYMQRSIHIEVKYYGSSFQGGEATYNITIEA

TDPFLFLVNHSVYVNYKGFTNASMDSCILFYVSSSSVHDFLTHKYLRFVKALDSLFNLQASKIHVFGIKVLKTEILLLAAVKSYNGEYLSREVASSISTG

HRRVLEAQTNVTISLITSDPCLTGPCQNGATCGKNIHIGPNVAVLESATVIFVSPQMEIFNCTCLAGFTGTLCEADIDECESNPCRNEGTCVNNHGGFYC

HCQSGFFGPSCSTDADECPTQKCHNGGSCVKAQDGFHCLCDAGYKGVSGVHCEQDSIGFEELSFMEFPPLDPRSNLISLEVATVQQDSLLLYNPGGPTRQ

DFVALELVDGRVRFSFDLGAGPVRLETGKRVADGLFHSITAKRIDNVNSRALTPDMALGAHNILHRAVRAKMAVFATIIGLTLFVSANLHTQEVTVPQLK

DRKLQFMLDNPLLGFAVEAPIADGLWHILSVISDGQNAVVYLDGRAVLNMTRGIDLTPVTVDRIVLGGAVPQLGSRLNVPGFSGCVEYFKVAGYTLPGSG

HSDLVEVRPSPSLLQTGCPSSSCLPSPCPADNYTMPGEVSPQEDGQWQACGGPGQESTWACICLHNSSSRSCDVCSPMRDQGGGCSPDPQSTPLWVIAVL

LPLLLLCVTIAVILTLRHRRTKQDAWWCQNKSPPQRTATQGNENDAFASDDGASVRRGEAAEERRPPDIILNDNQSSGVGLYHEADSAGVQRAPHASELD

YYEIDSYSVAFHSDADSQKQRGKKPCSSPCCTRADPRRRGREIQNLLQSKKSPLNHERATKSERPARLSPSLHPELRPYVDTYHLLPCRPQEPKRSPGPG

PAPALSAEEVRQLNDGPQPKVRPRTTRAAPTNDISADGGTGSGSEFGRERHAVAGHTDVTDATAGSLASKHLSASTPSGAPAEWENTRNTCVQPTACALE

NIASLPSESKCDIQTDLEELI

**>g51752.t1**

MVSCFPNAIIANVPECPYGWEIGQLSLGGVCYTGVVSAGYFRFTIPDLTPKNHSYCGTHAEYLPGKDPKHLFFNSIVSNDTSLTVRNQPVNYTFSCTYRA

AYLVNNAVFSQRVATVYVNNGSLGTFKSQLSMNVFTDAPYVIDTSEIGSEVFIGIEAKGLSNSCSSDHTVTIFENAKDSRSMFKFNSFRFQRFEKVSTVW

LHCEINICDGERLVCQP

**>g54590.t1_g64615.t1_g25348.t1_g25000.t1**

LSVWSLKLYEGPHRNTVKAERLSGEQTPREQESRPPRARLAGAVVWVSWIPSPQVSPGERPPSIPGLPSADSSAVLEEMSLLRVAPDWLSSGDVXVISYE

CCPGYEKIPGEKGCPAALPLVDIYNTLGAVGASTTQMYTDRALLREEIDGPGSITFFAPSNKAWSALPHEILDALVSNVNIELLNALHYHMVDRRLTSDD

LKHGSSFTSMYQDFDVHIHHYPNGIVTVNCARLIKTDQHATNGIVHVVDRVITAISNNVHSFIDTDDDLETLRVSGPQPTSPSIPHTHALNESRGHSASI

SVPTHDPGQSxxxxxxxxxxMDGEMRTLMNNHVLKNQLNSKSLYHGQELETLGGLKLRVFVYRNVRVSLAVFRRLLIVSEFELVLTDALVCSALRLDLAIxxxxxxxxxxNLCIENACIAAHDKMGRFATMLTVDKVLTPPMGTIMDVLKADDRFSLLTGALQTAGMTERMNKQDLLTVFAPTNEAFNAMPRTELNRLLRKTSTQRRDAE

RMLKLxxxxxxxxxxRNYTVYVNRVPVVEADLMATNGVVHAVDSIIKPLPPKVDREQADGPAAVRSASSRVRKAPVRTAAGPGSTGPQGQP

**>g59144.t1**

EEFSLLHNLTAALSDDDGHYIVRLANSLFLQEGMAFNPEFLHLMRKYFRAEVDTVDFSESAAVAEQINSWVENHTESKIHKLLSAEDFSSMTRLTLVNAV

YFRGSWKNQFRPENTRTFSFSRDDGSEVQTLMMYQQGDFYYGSDMNLILPPLGHWKVIPPVFLSGPSIWAAPGRPTGEQTSAFTAARCQTALTSLCYVAL

LRRSATAPRVSVARRSERFRGA

**>g6096.t1**

MKLTGGVFLLLMLSGCESARVAESRDDNSVFDLFELVQVPKKNHGVTLVKGDDPYSPAYKILNPDLIPPVPDSAFGNLIDSIHAERGFLLLLNFKQFKRT

RGSLLTVEKRDGSGPVFEIVSNGKANTLDIVFSTENKQQVVSIEDVDLATSHWKNITLFVQEDRAQLFVGCEEVNTAELDASIQSILTQETPAVARLRLG

KGAVKDRFMGVLQNVRFVFGTTLDAILRNKGCQNSLPTDTMILENLNGSSAIRTEYTGHKTKDLQLLCGFSCEDLASMFKELRTLGVVVKELSNELRQLT

VENKLIQSRIDIHSGVCIHNGIVHKNRDEWTVDDCTECTCQ
