# Supplementary material for: First paleoproteome study of fossil fish otoliths and the pristine preservation of the biomineral crystal host
Source: Sci Rep. 2023 Mar 7;13:3822. doi: 10.1038/s41598-023-30537-8 (PMC9992438; doi:10.1038/s41598-023-30537-8)
Supplement: Supplementary file 6 — Supplementary Table 5. [file 41598_2023_30537_MOESM6_ESM.pdf]

>g1.t1
LMLDDDPTPCAFAGNGRFCMSNGTECRGKWEGPNGGITNFDNIFFAMLTVFQCITMEGWTDVLYWMNDAIGFEIPWIYFV
SLVIFGSFFIINLVLGVLSG
EFSKEREKAVARGELQKAQESKQMEEDMIGYMDWLIEAEDVDEEGNKRAAIAKKKMMKKFGWYKHSEEGESDSDDDIAYL
DDDSGFCASLMAKMMANSFC
DQLCQLNHTFRKNCRVAVKTTNFYWLVLLLVFLNTVASASEHYGQPKWLTEMQERANKILLLLFTLEMSMKMYAFGLQIY
FMALFNRFDCFVVCGGILET
LLVELEFIPPIGISVLRCIRLLRIFKMTRHWAALSDLVNSLLNSMKAICSLLLLLFLFLIIFALLGMQLFGGKFNFDETQ
MKRSTFDSFPQALLTCFQIL
TGEDWNAVMYDGIMAYGGPVFPNMVVCIYFVILFVCGNCILPKNTVAVVTEKKEEDDEWDEDEEKEDEDAGNEEDDWQEN
EELRAIEGLEGVAPMKPEFS
GPKEKIVPIPDGSSFFILGKKNCLRVACHNLIHHPYITNFILIFIILSSISLAAEDPIKSHSFRNIVLGYADYVFTSVFT
VEIILKMTVYGAFLHTGSFC
RNAFNLLDLLVVSVSLMSFFLHSSAISVVKILRVLRVLRPLRAINRAKGLKNVVQCVFVAIRTIGNILIVTSLLQFMFAC
IGVQLFKGRFYSCTDEAKHT
PDECKGTFVVYKDGDMNHPMVRERIWENSDFNFDNVLMGMLALFTVSTFEGWPQLLYRAVDANAINRGPIYNYRVEISIF
FIIYIIIIAFFMMNIFVGFV
IITFREQGEAEFKNCELNKNQRQCVYYALKAQPIKIYIPKNPSQLKFWRIINSSQFEYIMFVLILGNTLTLAVQHYEQSK
LFTSIMDILNMIFTVVFTVE
MVVKLLALRVHHYFIDPWNSFDALIVVGSVLDIAVSEFS
>g1.t2
MLDDDPTPCAFAGNGRFCMSNGTECRGKWEGPNGGITNFDNIFFAMLTVFQCITMEGWTDVLYWMNDAIGFEIPWIYFVS
LVIFGSFFIINLVLGVLSGE
FSKEREKAVARGELQKAQESKQMEEDMIGYMDWLIEAEDVDEEGNKRAAIAKKKMMKKFGWYKHSEEGESDSDDDIAYLD
DDSGFCASLMAKMMANSFCD
QLCQLNHTFRKNCRVAVKTTNFYWLVLLLVFLNTVASASEHYGQPKWLTEMQERANKILLLLFTLEMSMKMYAFGLQIYF
MALFNRFDCFVVCGGILETL
LVELEFIPPIGISVLRCIRLLRIFKMTRHWAALSDLVNSLLNSMKAICSLLLLLFLFLIIFALLGMQLFGGKFNFDETQM
KRSTFDSFPQALLTCFQILT
GEDWNAVMYDGIMAYGGPVFPNMVVCIYFVILFVCGNCILPKNTVAVVTEKKEEDDEWDEDEEKEDEDAGNEEDDWQENE
ELRAIEGLEGVAPMKPEFSG
PKEKIVPIPDGSSFFILGKKNCLRVACHNLIHHPYITNFILIFIILSSISLAAEDPIKSHSFRNIVLGYADYVFTSVFTV
EIILKMTVYGAFLHTGSFCR
NAFNLLDLLVVSVSLMSFFLHSSAISVVKILRVLRVLRPLRAINRAKGLKNVVQCVFVAIRTIGNILIVTSLLQFMFACI
GVQLFKGRFYSCTDEAKHTP
DECKGTFVVYKDGDMNHPMVRERIWENSDFNFDNVLMGMLALFTVSTFEGWPQLLYRAVDANAINRGPIYNYRVEISIFF
IIYIIIIAFFMMNIFVGFVI
ITFREQGEAEFKNCELNKNQRQCVYYALKAQPIKIYIPKNPSQLKFWRIINSSQFEYIMFVLILGNTLTLAVQHYEQSKL
FTSIMDILNMIFTVVFTVEM
VVKLLALRVHHYFIDPWNSFDALIVVGSVLDIAVSEFS
>g1.t3
LMLDDDPTPCAFAGNGRFCMSNGTECRGKWEGPNGGITNFDNIFFAMLTVFQCITMEGWTDVLYWMNDAIGFEIPWIYFV
SLVIFGSFFIINLVLGVLSG
EFSKEREKAVARGELQKAQESKQMEEDMIGYMDWLIEAEDVDEEGNKRAAIAKKKMMKKFGWYKHSEEGESDSDDDIAYL
DDDSGFCASLIDQLCQLNHT
FRKNCRVAVKTTNFYWLVLLLVFLNTVASASEHYGQPKWLTEMQERANKILLLLFTLEMSMKMYAFGLQIYFMALFNRFD
CFVVCGGILETLLVELEFIP
PIGISVLRCIRLLRIFKMTRHWAALSDLVNSLLNSMKAICSLLLLLFLFLIIFALLGMQLFGGKFNFDETQMKRSTFDSF
PQALLTCFQILTGEDWNAVM
YDGIMAYGGPVFPNMVVCIYFVILFVCGNCILPKNTVAVVTEKKEEDDEWDEDEEKEDEDAGNEEDDWQENEELRAIEGL
EGVAPMKPEFSGPKEKIVPI
PDGSSFFILGKKNCLRVACHNLIHHPYITNFILIFIILSSISLAAEDPIKSHSFRNIVLGYADYVFTSVFTVEIILKMTV
YGAFLHTGSFCRNAFNLLDL
LVVSVSLMSFFLHSSAISVVKILRVLRVLRPLRAINRAKGLKNVVQCVFVAIRTIGNILIVTSLLQFMFACIGVQLFKGR
FYSCTDEAKHTPDECKGTFV
VYKDGDMNHPMVRERIWENSDFNFDNVLMGMLALFTVSTFEGWPQLLYRAVDANAINRGPIYNYRVEISIFFIIYIIIIA

FFMMNIFVGFVIITFREQGE
AEFKNCELNKNQRQCVYYALKAQPIKIYIPKNPSQLKFWRIINSSQFEYIMFVLILGNTLTLAVQHYEQSKLFTSIMDIL
NMIFTVVFTVEMVVKLLALR
VHHYFIDPWNSFDALIVVGSVLDIAVSEFS
>g2.t1
LVHCSTARTESALFLLNLGFDNRPESPFQHPGVNFPGEAEQCDCTNVPVVKATWEKDAAFLEYYSDVTDSSIPTIHLGVP
NTEREEVPMTTAVRRIKLSL
LWCWIPAPQHLRCVHSTVPGVRGAIRQGTAFSRETCL
>g2.t2
MRTQLSRWLYKDRMARTNGPGTPYSLSTPHRIPRGTRSTESALFLLNLGFDNRPESPFQHPGVNFPGEAEQCDCTNVPVV
KATWEKDAAFLEYYSDVTDS
SIPTIHLGVPNTEREEVPMTTAVRRIKLSLLWCWIPAPQHLRCVHSTVPGVRGAIRQGTAFSRETCL
>g2.t3
LLCFPVPVVKATWEKDAAFLEYYSDVTDSSIPTIHLGVPNTEREEVPMTTAVRRIKLSLLWCWIPAPQHLRCVHSTVPGV
RGAIRQGTAFSRETCL
>g3.t1
MDDEEETYRLWKIRKTIMQLCHDRGYLVTQDELDQTLDEFKSQFGDKPSEGRPRRTDLTVLVAHNDDPTDQMFVFFPGRC
MWKS
>g3.t2
MDDEEETYRLWKIRKTIMQLCHDRGYLVTQDELDQTLDEFKSQFGDKPSEGRPRRTDLTVLVAHNDDPTDQMFVFFP
>g4.t1
LDRYCKTAMRPLGPSVLLSVLLLAAAQDPNTATSIYDFSATDIDGNVVSLERYRGDVVIITNVASK
>g5.t1
MESSADSGRSLPVGRRLCYAVGHFLNDLCASVWFTYLLVFYHSVLGLQSSSAGVLMLIGQVADGVCTPLIGYESDRTPGL
SGYGKRKTWHLLGTLCVLLS
FVFIFSPCLACDANTAQWAQVFYFSPLIIVFQFGWAASQISHLSLIPELVSSENAKVELTAYRYAFTVVANITVYAVTWL
LFQFQDREGPVMPDSLGEMD
VPLFRNLALTVLGIGALSSLMFHVGTKEPDPGDEEERQSPLACVPSSPPLLQWKHWLKQPSFYQVAFLYMCTRLIVNMSQ
TYISVYLTDSLLLPKNVIAI
IPLVMYVSGFVFSLVMKPISRLVGISVTYLLGLLLVLGFSLWVLVDPAVGEGRIYGAVALLGSGSATILVMSLSMTARLI
GEQTQSGAFVYGSMSLTDKL
ANGAGVMLIQSLRPCR
>g5.t2
MESSADSGRSLPVGRRLCYAVGHFLNDLCASVWFTYLLVFYHSVLGLQSSSAGVLMLIGQVADGVCTPLIGYESDRTPGL
SGYGKRKTWHLLGTLCVLLS
FVFIFSPCLACDANTAQWAQVFYFSPLIIVFQFGWAASQISHLSLIPELVSSENAKVELTAYRYAFTVVANITVYAVTWL
LFQFQDREGPVMPDSLGEMD
VPLFRNLALTVLGIGALSSLMFHVGTKEPDPGDEEERQSPLACVPSSPPLLQWKHWLKQPSFYQVAFLYMCTRLIVNMSQ
TYISVYLTDSLLLPKNVIAI
IPLVMYVSGFVFSLVMKPISRLVGISVTYLLGLLLVLGFSLWVLVDPAVGEGRIYGAVALLGSGSATILVMSLSMTARLI
GEQTQSGAFVYGSMSLTDKL
ANGAGVMLIQSLRPCRYRRSAASRTKRCFFGAVV
>g5.t3
MESSADSGRSLPVGRRLCYAVGHFLNDLCASVWFTYLLVFYHSVLGLQSSSAGVLMLIGQVADGVCTPLIGYESDRTPGL
SGYGKRKTWHLLGTLCVLLS
FVFIFSPCLACDANTAQWAQVFYFSPLIIVFQFGWAASQISHLSLIPELVSSENAKVELTAYRYAFTVVANITVYAVTWL
LFQFQDREGPVMPDSLGEMD
VPLFRNLALTVLGIGALSSLMFHVGTKEPDPGDEEERQSPLACVPSSPPLLQWKHWLKQPSFYQVAFLYMCTRLIVNMSQ
TYISVYLTDSLLLPKNVIAI
IPLVMYVSGFVFSLVMKPISRLVTYLLGLLLVLGFSLWVLVDPAVGEGRIYGAVALLGSGSATILVMSLSMTARLIGEQT
QSGAFVYGSMSLTDKLANGA
GVMLIQSLRPCR
>g6.t1
MFGQRYDSPPVYSPPYTSPSNNGYGPAGSFQDPRSEYDPYPPPPGSYYMEDNPQHFYKWLSPPGIVKAMMAVVVVLCVGI
FACVASTLMWDMQYGYGGGL
GSYGGGYGGGYGGGYG

>g7.t1
MYECLISLNERTKTDSSHGRFVSRVEEVLNDWKLIGSSAGRSSPEKGEYTSGTWEEKSQEINFADFKFTITHHYLKQESD
KSDLKEELEEDAFPLAMQDL
LCMNNDFPPRAHCLVRWYGMREFLVISPGANCEAIISESKCNLLLSSVSISLANSGW
>g7.t2
MTAQHDMNNGVVMVGEYTSGTWEEKSQEINFADFKFTITHHYLKQESDKSDLKEELEEDAFPLAMQDLLCMNNDFPPRAH
CLVRWYGMREFLVISPGANC
EAIISESKCNLLLSSVSISLANSGW
>g8.t1
GLRAAPGWRVEYPESFCVVEGSTTTIPCSFTYPKDYTVKRLVWCPNHEICQREMFSVYDSNVTASSRFVYLGDRAGNCTL
KIINTMKQDTATYRFRIETN
KNNFTERKGVHITVTDGDNVTVKSSISENKVNKSSEVTLTCTSGCSFHQLDVHWYRNGQALSEKGPALHLRHLTYHDTGD
YTCSLDSSGQNMSAPWSLVV
LGDGESTTDRHLYCLRLPQ
>g9.t1
GGAHPSLWVGQAPYGPPIATGPELEKCKANHLLILLRDGSCQYRGIYCYLTDTEEIFKFLGTGPRTITCKMIDKLFKYSC
DRKQFGVIPTKTLSITVDAL
TIHNQLWQVKRKGSLTVKK
>g9.t2
ELEKCKANHLLILLRDGSCQYRGIYCYLTDTEEIFKFLGTGPRTITCKMIDKLFKYSCDRKQFGVIPTKTLSITVDALTI
HNQLWQVKRKGSLTVKK
>g10.t1
MKSDGLEAQLHRYTMEKNLTKMEKLLRKGVDVDCVNHLGQTPLFCAALMGHEAAAELLLQYRADPNHRCKDWSTPVHAAV
FSCNPWLLSCLLDSGGDIRL
HDQEGRVPVEWLMADAQEHRVREVCIDKPCQSLGVLASIPVIKDSDLTQADDETLHSFNCGSLTSMTNQLFHPHLLQLMA
VSLSADLLKTSLVFERVHVD
SLHNLLHHKRAEFPALHIEWLLPVLLQVCEAVVYLHSRGLVIRALSSHSVVLTHPGLAKLTGFGFMVH
>g10.t2
LGVMKSDGLEAQLHRYTMEKNLTKMEKLLRKGVDVDCVNHLGQTPLFCAALMGHEAAAELLLQYRADPNHRCKDWSTPVH
AAVFSCNPWLLSCLLDSGGD
IRLHDQEGRVPVEWLMADAQEHRVREVCIDKPCQSLGVLASIPVIKDSDLTQADDETLHSFNCGSLTSMTNQLFHPHLLQ
LMAVSLSADLLKTSLVFERV
HVDSLHNLLHHKRAEFPALHIEWLLPVLLQVCEAVVYLHSRGLVIRALSSHSVVLTHPGLAKLTGFGFMVH
>g10.t3
MATLPFPCPVRLGVMKSDGLEAQLHRYTMEKNLTKMEKLLRKGVDVDCVNHLGQTPLFCAALMGHEAAAELLLQYRADPN
HRCKDWSTPVHAAVFSCNPW
LLSCLLDSGGDIRLHDQEGRVPVEWLMADAQEHRVREVCIDKPCQSLGVLASIPVIKDSDLTQADDETLHSFNCGSLTSM
TNQLFHPHLLQLMAVSLSAD
LLKTSLVFERVHVDSLHNLLHHKRAEFPALHIEWLLPVLLQVCEAVVYLHSRGLVIRALSSHSVVLTHPGLAKLTGFGFM
VH
>g11.t1
MDEGGPSSKSTLAKSRECLSESERQHQERMKATKLFKNAEKNTHAFLDKELKKLWRVHFSQYQQCSGSQREEEEEVADDK
KEEQRKRAIEGVLNIAQLCL
IEIKQEKLAEALRSSKRLVV
>g11.t2
MSQEMDEGGPSSKSTLAKSRECLSESERQHQERMKATKLFKNAEKNTHAFLDKELKKLWRVHFSQYQQCSGSQREEEEEV
ADDKKEEQRKRAIEGVLNIA
QLCLIEIKQEKLAEALRSSKRLVV
>g12.t1
MTTGVAGIGKTVSTRKFTLDWAECKTNQDIDFTFPFTFRELNLMEGKQFSLVELLHHFFIETKKAGICRFDQFHVVFIYD
GLDECRLPLDFQNNKIWTDI
TKPTSVDVLLTNLIKGNLLPSARIWITTRPAAANRIPADCVDMVTEVRGFTDLQKEEYFRKRFREETLASTIISHIKTSP
SLHVMCTSPVFCWIIATVLE
HILTTSQRGEELPKTLTEMYIHFLRVQSIQEDMKYDGRAETDCEFWNSARRKLIVSLGKLAFNQLEKDNLIFYEGDLEEC
GIDIRAALVYSGVFTQIFKE
ECGLYQDKVYCFVHLSVQEFMAALYVFYSFIDSGVNLLSEKQKKQKDLKRRISREAGIQLFYQSAVDKALQSVKGHLDLF

LRFLLGLSLENNQNLLRGLL
GWRGSSSPTNQETASYIKKKMNGGLEQEKSINLFHCLNELNDYSLVIDAQRYLTSGSLSSEALSPAQWSALVFILLSSEK
KLDIFELKKYSATEEGLLRM
LPVVKASKTSLRLSGCNLSERCCKALTSVLSSNSSSLGELALGINDLRDSGVKLLSAGLGSPHCRLESLSSKSSRLRELD
LSTNDLQDSGVQLLSAGLGS
PHCQLETLRLSGCLVTQEGCSSLASALSFNPTHLRVLDLSYNHPGDSGVTLLSAGLQNPRWRLDTLSVEHSGVRRLKPGV
RKYACELKLDPNTASNHLYL
SEDNRMVTTVKEKQLVSDHPERVDPYIQVLCGEGLTGRCYWEVEWKGKASIAVAYRGITGGGGVDGWLGGNDKSWSLYCS
EDRYGAWYNGRDTSICLPPS
GSNRVGVYVDWPAGTLSFYRVSPDVGGSSDTLTHIHTFQSTFTQDLHPGFGFLDCGSSVSLCSL
>g12.t2
MTTGVAGIGKTVSTRKFTLDWAECKTNQDIDFTFPFTFRELNLMEGKQFSLVELLHHFFIETKKAGICRFDQFHVVFIYD
GLDECRLPLDFQNNKIWTDI
TKPTSVDVLLTNLIKGNLLPSARIWITTRPAAANRIPADCVDMVTEVRGFTDLQKEEYFRKRFREETLASTIISHIKTSP
SLHVMCTSPVFCWIIATVLE
HILTTSQRGEELPKTLTEMYIHFLRVQSIQEDMKYDGRAETDCEFWNSARRKLIVSLGKLAFNQLEKDNLIFYEGDLEEC
GIDIRAALVYSGVFTQIFKE
ECGLYQDKVYCFVHLSVQEFMAALYVFYSFIDSGVNLLSEKQKKQKDLKRRISREAGIQLFYQSAVDKALQSVKGHLDLF
LRFLLGLSLENNQNLLRGLL
GWRGSSSPTNQETASYIKKKMNGGLEQEKSINLFHCLNELNDYSLVIDAQRYLTSGSLSSEALSPAQWSALVFILLSSEK
KLDIFELKKYSATEEGLLRM
LPVVKASKTSLRLSGCNLSERCCKALTSVLSSNSSSLGELALGINDLRDSGVKLLSAGLGSPHCRLESLSSKSSRLRELD
LSTNDLQDSGVQLLSAGLGS
PHCQLETLRLSGCLVTQEGCSSLASALSFNPTHLRVLDLSYNHPGDSGVTLLSAGLQNPRWRLDTLSVEHSGVRRLKPGV
RKXGQRCPGFPIDPMGSVGE
RRMMAKRLSLMENLFPSMCDTLSAPDSRDRLRYRRPFLLAAVTLHSQHCSKQITQTNTDSSPIL
>g12.t3
MTTGVAGIGKTVSTRKFTLDWAECKTNQDIDFTFPFTFRELNLMEGKQFSLVELLHHFFIETKKAGICRFDQFHVVFIYD
GLDECRLPLDFQNNKIWTDI
TKPTSVDVLLTNLIKGNLLPSARIWITTRPAAANRIPADCVDMVTEVRGFTDLQKEEYFRKRFREETLASTIISHIKTSP
SLHVMCTSPVFCWIIATVLE
HILTTSQRGEELPKTLTEMYIHFLRVQSIQEDMKYDGRAETDCEFWNSARRKLIVSLGKLAFNQLEKDNLIFYEGDLEEC
GIDIRAALVYSGVFTQIFKE
ECGLYQDKVYCFVHLSVQEFMAALYVFYSFIDSGVNLLSEKQKKQKDLKRRISREAGIQLFYQSAVDKALQSVKGHLDLF
LRFLLGLSLENNQNLLRGLL
GWRGSSSPTNQETASYIKKKMNGGLEQEKSINLFHCLNELNDYSLVIDAQRYLTSGSLSSEALSPAQWSALVFILLSSEK
KLDIFELKKYSATEEGLLRM
LPVVKASKTSLRLSGCNLSERCCKALTSVLSSNSSSLGELALGINDLRDSGVKLLSAGLGSPHCRLESLSSKSSRLRELD
LSTNDLQDSGVQLLSAGLGS
PHCQLETLRLSGCLVTQEGCSSLASALSFNPTHLRVLDLSYNHPGDSGVTLLSAGLQNPRWRLDTLSVEHSGVRRLKPGV
RKYACELKLDPNTASNHLYL
SEDNRMVTTVKEKQLVSDHPERVDPYIQVLCGEGGQRCPGFPIDPMGSVGERRMMAKRLSLMENLFPSMCDTLSAPDSRD
RLRYRRPFLLAAVTLHSQHC
SKQITQTNTDSSPIL
>g13.t1
MSITLDIRLKRANKVYHEGDLLAGVVVLASKEAVQHQGISLTMEGVVNLQLSSKSVGVFEAFYNSVKPIQLINSNIEVAK
AGKIPGGKTEIPFEFPLSTK
GNKVLYETYHGVFVNIQYTLRCDMKRSLLAKDLSRTFEFMVHCQPQKAKLVPTPVDFTITPETLQNVQEVGKSHRSLLPR
FLIRGHLNATNCVITEPLTG
ELMVENSQVAIKSIELQLVRVETCGCAEGYARDATEIQNIQIAEGDVCHGLSIPIYMVFPRLFTCPTLETTNFKVEFEVN
VVIVLHDDHLITENFPLKLC
RV
>g13.t2
LFTTQDLLAGVVVLASKEAVQHQGISLTMEGVVNLQLSSKSVGVFEAFYNSVKPIQLINSNIEVAKAGKIPGGKTEIPFE
FPLSTKGNKVLYETYHGVFV
NIQYTLRCDMKRSLLAKDLSRTFEFMVHCQPQKAKLVPTPVDFTITPETLQNVQEVGKSHRSLLPRFLIRGHLNATNCVI

TEPLTGELMVENSQVAIKSI
ELQLVRVETCGCAEGYARDATEIQNIQIAEGDVCHGLSIPIYMVFPRLFTCPTLETTNFKVEFEVNVVIVLHDDHLITEN
FPLKLCRV
>g13.t3
LLYLFTTQDLLAGVVVLASKEAVQHQGISLTMEGVVNLQLSSKSVGVFEAFYNSVKPIQLINSNIEVAKAGKIPGGKTEI
PFEFPLSTKGNKVLYETYHG
VFVNIQYTLRCDMKRSLLAKDLSRTFEFMVHCQPQKAKLVPTPVDFTITPETLQNVQEVGKSHRSLLPRFLIRGHLNATN
CVITEPLTGELMVENSQVAI
KSIELQLVRVETCGCAEGYARDATEIQNIQIAEGDVCHGLSIPIYMVFPRLFTCPTLETTNFKVEFEVNVVIVLHDDHLI
TENFPLKLCRV
>g14.t1
MRGRTLIRQPSTGQSFRATAWTGVHVTSPVSGPVSGRPCADDVVLPCVQCINSWLKEQSTCPTCRKHALIPEDFPMLVGR
RHVGRPAPSFS
>g14.t2
CINSWLKEQSTCPTCRKHALIPEDFPMLVGRRHVGRPAPSFS
>g15.t1
DSVEDLAESEEYAEARSILDSVKLEG
>g16.t1
ASGTVYTAIDVATGQEVAIKQINLQKQPKKELIINEILVMKEMKNPNIVNFLDSFLVGDELFVVMEYLAGGSLTDVVTET
CMDEAQIAAVCREALEFLHA
NQVIHRDIKSDNVLLGMDGSVKLTDFGFCAQITPEQSKRSTMVGTPYWMAPEVVTRKAYGPKVDIWSLGIMAIEMVEGEP
PYLNENPLRALYLIATNGTP
ELQSPEKLSPVFRSFLSRCLEMDVEKRGGGKELLQHPFLEMAKPLSTLTPLILAAKEAMKNNR
>g16.t2
ASGTVYTAIDVATGQEVAIKQINLQKQPKKELIINEILVMKEMKNPNIVNFLDSFLVGDELFVVMEYLAGGSLTDVVTET
CMDEAQIAAVCRECLQALEF
LHANQVIHRDIKSDNVLLGMDGSVKLTDFGFCAQITPEQSKRSTMVGTPYWMAPEVVTRKAYGPKVDIWSLGIMAIEMVE
GEPPYLNENPLRALYLIATN
GTPELQSPEKLSPVFRSFLSRCLEMDVEKRGGGKELLQHPFLEMAKPLSTLTPLILAAKEAMKNNR
>g17.t1
MSHPFIPLYHSFIILQLTSPLISKMSSGGGIESGRPAKVGSVVEVIGKGQRGTVAYVGATLFASGKWVGVILDEAKGKND
GTVQGKRYFTCEENHGIFVR
QSQIQLVEDGSAATSPETPDSVTSKIPRQKGVKASR
>g17.t2
MSSGGGIESGRPAKVGSVVEVIGKGQRGTVAYVGATLFASGKWVGVILDEAKGKNDGTVQGKRYFTCEENHGIFVRQSQI
QLVEDGSAATSPETPDSVTS
KIPRQKGVKASR
>g18.t1
MLGPDYLKFISTSPSIDIYQGSSWLCLTVSSSFLTHNFYHSSPFPFCCPILPADVVESIMVRAALVTATSLVLTGAVVAH
AYFLKHQFYPTVVYLTKSSP
SMAVLYIQAFVLVFLLGKFMRKVFFGQLRAAEMEHLIERSWYAVTETCLAFTVFRDDFSPRFVALFTLLLFLKCFHWLAE
DRVDFMERSPNISWVFHFRV
LSLMGLLGVLDFLFVNHACHSIITRGASVQLVFGFEYAILMTMVLTTIIKYLLHTIDLQSENPWDNKAVYMLYTELFTGF
IKVLLYMAFMTIMIKVHTFP
LFAIRPMYLAMRQFKKAVTDAIMSRRAIRNMNTLYPDATPADLQASDNVCIICREEMVTGAKKLPCNHIFHSSCLRSWFQ
RQQTCPTCRMDVLRASNAN
>g18.t2
MVRAALVTATSLVLTGAVVAHAYFLKHQFYPTVVYLTKSSPSMAVLYIQAFVLVFLLGKFMRKVFFGQLRAAEMEHLIER
SWYAVTETCLAFTVFRDDFS
PRFVALFTLLLFLKCFHWLAEDRVDFMERSPNISWVFHFRVLSLMGLLGVLDFLFVNHACHSIITRGASVQLVFGFEYAI
LMTMVLTTIIKYLLHTIDLQ
SENPWDNKAVYMLYTELFTGFIKVLLYMAFMTIMIKVHTFPLFAIRPMYLAMRQFKKAVTDAIMSRRAIRNMNTLYPDAT
PADLQASDNVCIICREEMVT
GAKKLPCNHIFHSSCLRSWFQRQQTCPTCRMDVLRASNAN
>g18.t3
MLGPDYLKFISTSPSIDIYQGSSWLCLTVSSSFLTHNFYHSSPFPFCCPILPADVVESIMVRAALVTATSLVLTGAVVAH

AYFLKHQFYPTVVYLTKSSP
SMAVLYIQAFVLVFLLGKFMRKVFFGQLRAAEMEHLIERSWYAVTETCLAFTVFRDDFSPRFVALFTLLLFLKCFHWLAE
DRVDFVSGTAHAVALMDVQM
ERSPNISWVFHFRVLSLMGLLGVLDFLFVNHACHSIITRGASVQLVFGFEYAILMTMVLTTIIKYLLHTIDLQSENPWDN
KAVYMLYTELFTGFIKVLLY
MAFMTIMIKVHTFPLFAIRPMYLAMRQFKKAVTDAIMSRRAIRNMNTLYPDATPADLQASDNVCIICREEMVTGAKKLPC
NHIFHSSCLRSWFQRQQTCP
TCRMDVLRASNAN
>g19.t1
MCVSADTPPPAYMPPDEQMGQDSQSMETSSSSVVPQNMPRGDVQPVEYEEPSHWCSIVYYELNNRVGEAYHASSTSVLVD
GFTDPSNNKNRFCLGLLSNV
NRNSTIENTRRHIGKGVHLYYVGGEVYAECLSDTSIFVQSRNCNYHHGFHTTTVCKIPSGCSLKIFNNQEFAQLLAQSVN
HGFEAVYELTKMCTIRMSFV
KGWGAEYHRQDVTSTPCWIEVHLHGPLQWLDKVLTQMGSPLNPISSVS
>g19.t2
MPPDEQMGQDSQSMETSSSSVVPQNMPRGDVQPVEYEEPSHWCSIVYYELNNRVGEAYHASSTSVLVDGFTDPSNNKNRF
CLGLLSNVNRNSTIENTRRH
IGKGVHLYYVGGEVYAECLSDTSIFVQSRNCNYHHGFHTTTVCKIPSGCSLKIFNNQEFAQLLAQSVNHGFEAVYELTKM
CTIRMSFVKGWGAEYHRQDV
TSTPCWIEVHLHGPLQWLDKVLTQMGSPLNPISSVS
>g19.t3
DTPPPAYMPPDEQMGQDSQSMETSSSSVVPQNMPRGDVQPVEYEEPSHWCSIVYYELNNRVGEAYHASSTSVLVDGFTDP
SNNKNRFCLGLLSNVNRNST
IENTRRHIGKGVHLYYVGGEVYAECLSDTSIFVQSRNCNYHHGFHTTTVCKIPSGCSLKIFNNQEFAQLLAQSVNHGFEA
VYELTKMCTIRMSFVKGWGA
EYHRQDVTSTPCWIEVHLHGPLQWLDKVLTQMGSPLNPISSVS
>g20.t1
MMSDMHFRSLRTKMSLMQRNEEASRQLEMMSDMHFRSLRTKMSLMQRNEEASRQLESSRQLASRFHEQFAVRDDLMGLAI
GTHGANIQQARKVPGVTTID
LDEETCTFHIYGEVGSTHT
>g20.t2
FYVIEYAACDATLNEIVTLERLRPVNPNTPATKNTFLKIHLDVPEDLRQMCSKESTHKDFKKAVGAFSITYDSDAKQLVI
LVSPRLIELD
>g20.t3
FYVIEYAACDATLNEIVTLERLRPVNPNTPATKNTFLKIHLDVPEDLRQMCSKESTHKDFKKAVGAFSITYDSDAKQLVI
LSVNEVSTKRAQMMSDMHFR
SLRTKMSLMQRNEEASRQLEMMSDMHFRSLRTKMSLMQRNEEASRQLESSRQLASRFHEQFAVRDDLMGLAIGTHGANIQ
QARKVPGVTTIDLDEETCTF
HIYGEVGSTHT
>g20.t4
FYVIEYAACDATLNEIVTLERLRPVNPNTPATKNTFLKIHLDVPEDLRQMCSKESTHKDFKKAVGAFSITYDSDAKQLVI
LVKSML
>g21.t1
MVLEINELNRRMSVVEGMLNRLERRVVSPTQRQLRQKLDQLTGDISDNGLTSDEEEGPEEEEEEEEISVPRWRSPAGLAK
ANDEAEPQVPASTSTSTSSL
EGQLIQPTDAHKVTAPTESKERGRRTPEDGSQGGFRGSNALLFELEDKVAQATASLQTTRTQVSYIENRIAALGAAGMSV
DKGRRKVRAAKTPSHHAS
>g21.t2
MSVVEGMLNRLERRVVSPTQRQLRQKLDQLTGDISDNGLTSDEEEGPEEEEEEEEISVPRWRSPAGLAKANDEAEPQVPA
STSTSTSSLEGQLIQPTDAH
KVTAPTESKERGRRTPEDGSQGGFRGSNALLFELEDKVAQATASLQTTRTQVSYIENRIAALGAAGMSVDKGRRKVRAAK
TPSHHAS
>g21.t3
LNRRMSVVEGMLNRLERRVVSPTQRQLRQKLDQLTGDISDNGLTSDEEEGPEEEEEEEEISVPRWRSPAGLAKANDEAEP
QVPASTSTSTSSLEGQLIQP
TDAHKVTAPTESKERGRRTPEDGSQGGFRGSNALLFELEDKVAQATASLQTTRTQVSYIENRIAALGAAGMSVDKGRRKV

RAAKTPSHHAS
>g22.t1
TPDAPGTPDATEVSGDSITLAWTAPTSDGGDAVQYYILEKREKKTTRFYKVITKKPITEGAHKVLHLTEGTEYEFRVMAV
NDAGVGTPSNVSIPIKAAEP
KDIPCAPSVVCVSDSSNTTISLEWSRPVDDGGMEVLGYIIEMVKGDEEEWKRVNKELATQTNYVVSGLETGAKYRFRVAA
VNHVGQGEVQETPEAAQAVD
RLAPPEVDIDASFKQTHIIKAGGSVCLGIHFRGKPIPTSSWTKEDGELGVLSEVSTTEGFSSLSMENCSRNESGKYSITL
ENPSGSKTITFTVKVMDTPG
APQTLALQEVSRGAVTLTWEPPINDGGARIHHYVIERREASRRTWQQSGGKCTKQILKIQDLLEGVPYFFRVSAENQHGL
GEALELTEPVIATAEPSSPK
RLDILDTTDSSVSLGWLKPEHDGGSRIQCYIVEVKAKGTDKWMVVGNTKSLTYSIEKLNKGDEYDFRVKAKNESGSSQPK
ETIASVLVKEPHIEPAADLS
EIINQLITCRSGNTFTIDVPFSGRPAPKVSWKLEEMRLKSSDRVSIKVSKDRTSISVKEAMRGDGGKYYLTLENVAGTKT
FAINVNVIGRPSPPAGPIEI
SSITSESCKLDWGAPDDDGGTDITNYIVEKRESGSTAWQLINSSVKRTSLHVGHLTKYMQYTFRVCAENRFGVSKSTESE
TIVAEHPFRPPTRPTVSGVT
ANSMHIKWEEPYNDGGSKVTGYWIEKKERNIILWVRENKIPCFECHYKVEGLVEGLEYQFRISAMNSAGLSKASEASKRA
FAQNPVGPPSKPEVTNVTRS
TVSLKWSSPLNDGGSPIVGYIIERKPYILTGEGRWLKCNYTNVVDTFFTVTALGEGEPYEFRVIAKNSNQVFSMPSESTG
SVQCKTDFEPPKAQLDSKLL
SDTVTVRAGSDLVLDAAVGGRPDPKACWSKGTRDLELCEKYHLQYTPTKAMAIIKFCDRDDTGRYILTVRNVSGTRTAEV
NVKVLDTPGVCQGSIAISRI
TEESCTLSWNPPLEDGGDDVSHYIVERRDTNRLNWVVMDDECKELSCNITRLFKNTEYLFRVRGVNKYGSGVYLQSEPMV
ARNTFTVPSPPGAPEILVSG
KDFATIEWLKPESDGGSEILHYLVERREKKSARWVKVNREGTLLDSMLKVSGLIEGNIYQFRVTAINKAGESEPSEVSLY
VICRVPTCTPAPPSIPRIVD
THADSISLAWSRPVDDGGADVIGYILEMQESGAEEWTKAHEKTLRTSEHVVSALSSSKKYCFRVAGININGTGDFSEACL
ETQPVERIETPDFELHDDLK
KTICLRAGGSLRMSVTVTGRPAPAITWSKPGVDLQSRGFIDVTNTSTTLIIDKVHRYDAGKYTLVAENSAGKQEVNILVK
IYDTPGPTGPLKIKELTKES
ATISWEASTVDGGAPVNNYIIERREASMRAYKTVTSKCSKTSYKIDGLMEGMLYYFRVLPENIYGVGEPCETPDVVLVCE
VPEPPHKLEMIDVTKTTVTL
GWEKPEHDGGSRLTGYTIEACKPGTDKWMKVATMKTTDFEHTIEKLNENEQYLFRIRATNSRGASEPKEMLTAVTVQEQR
VLPIVDLSSIPQKVVNVLAG
KTLELDLPIVGRPPPVCSWYFADNKLKVSERLKLKSTGKFSKLTMSDTTIDDSGDYTLEVKNITGVTTEVIKVIVLSKPD
EPKGPIRFEEVDATSVTCSW
DPPVRDGGAPTSGYMVEQRDAHRPGWVPVCDSVSRPTFKFENLTEGDEYVFRAAAVNRYGTGEFLQSDVAMCKSLKNVPG
PPGRPTVFDVSRDGMTVAWN
PPEEDGGLEVSGYIIERKEVRADRWVRANKNPVTMTRYRSTGLIEGLEYEHRITAINTRGMSKPSLLSKPAVATDPIDTT
KSSVSLAWSAPDDEGDARVD
GYLIEMQKVGSLAWIKCNTTPSLICEYTLTQMPQGEEFKFRVMACNAGGSGEPAEVPGTIKVIEMLESPDYDLELKYKDV
YVVRQGGVVRLSLPIKGKPH
PTCKWSKVDSNISEDAMIATTEDASELVIKGAQRSDSGVYDLLLENKVGKKKAQIKVKVIGRPSAPEGPLSFEEVQANSV
KVSWKAPSDDGGSEILGYIV
ERREATRNAWYTVDSRVTDSQLAVKGLREGIEYHFKVTAENSFGVSASLKSEYPLVPKTPLCVPEPSSIQPEIMDVTKSS
VALAWTRPKDDGGSPVTGYI
LEYKVVSSETWSRHQTKITSTMFTLPGLMPDAEYLLRIIAVNNIGESEAGPVSEPVTCKDPFDKPSQPGEIDTVNVAKNS
IAIRWQTPECDGGKEVLGYW
VEHRKSSESSWKKSNKQRLKEQEFTMLGLAEATEYEFRVFAENETGISRPRRTATCIKTKLSVETKPSLRKEMDEVTTKL
GQPAVMKCQIIGRPVPEIKW
YHAGKEMMECRKYEMSSDGRNHTLSIMTDQQEDEGEYTCKAINDAGDAETTGVLVLEAAPNFHPDYPLKDTYYAGLGTTL
RIHAVYIGRPEPKIMWLHGA
KTLENTEDISIETTEHYTHLVIKNVQRRVNGGKYRIRLHNHFGRADTAFNVEIYDKPDIPEGPIVLDALLKNSVIISWKA
PQDNGGSMITNYIVEKREDK
EGSDWELVSSSINGLSCRVPNLVDSAGYFFRVYAQNRYGNSQPLELAAPIVIKSQLEKPSPPLSPVVSGITRDSCVVSWK
PPSSDGGSKIKSYCLEKKDK

KGDWTPVTTDEIKQTVFSVKRLTEGTEYIFRVKCENLGGQSDYSEETAPMIPALAVDVRAPAFKEELRNLSVKHKSNATL
VCKITGQPKPVIKWFRRGKE
IHSDGKKIKIQEFKGGYHQLVISEADEEDSTIYQIRATNQGGSICATVSLDVEVPAKIHLPKNLKDKEAIPALRGEMVNI
KIPFGGKPDPVITWQKGQDL
IDNNGYYQVIVTRSFTSLVFPNGVEKKDAGFYIVCAKNRFGIDQQTVELDVADVPDPPRDIKASDVSRDSVTLNWVAPAN
DGGSKVISYIIEKCPTTAER
WERVAQARDTHYTVINLFGGTSYQFRTIAVNKFGQSTPSDTTGPIMTKEDKSRVLLYDREVDDTGCVPRGKAPHSGASNL
HNKFAIAEELGRGQFGIVHR
CIESSSEKTYMAKFVKVRGADQAIVKKEIATLNLAKHTNFLLLHESFDSPEELVLIYDFISGVDIFERLNMADFELNERE
IVNYIRQICLALEFLHDESY
GCFDIKPENIVYTTRTSSNIKIIELGQSRHLTPGDQIKIQYTTAEYAAPEIHQCDMVSSVTDMWSVGVLAYVLLSGLNPF
TAETTQQMIDNISNAAYSYD
DESFKQVSVESLDFTDRLMTKDRKHRMRAVEALEHPWLKRPAEELSNRMMQTSRHKRYYQAMVRKEWNTVVSAARVASGG
SIRSQRGVFVAKVKIAPFEH
GPVAGQIGHSVVNEGDNVKFVCNIENYDSTTEVTWYCGVRQLEAGEKYEIDYEDGIAVITINKVTRSDDGTYRCKIVNEY
GEDSAYAELFINSVRSYRDF
FTTRVVKRVKRRVDTARMLQKPPEFTLPLVNCTAYIGEDLRFGVTITVHPEPRVIWHKSGQKLIPGNDEKKYTFINDKGL
YQLIIHHLEMEDDAEYSVVA
RNRYGDDSCKARLTVVPRPKPADLTLRPMFKRLLANVDCREGQNVRFEIRVSGRPELKWEKDGTPLAFGPSIEVVHEGLD
YFILHVRDTLPEDSGVYRVT
ATNSAGSTSCQAILKVERVTHVKKEFELSEKDKKKLAVKEAMDKKVRLSQILSGTEVMPLPSVAMQAVREAASMFKPAVT
SKKGDKTEAEIQKEKEEKKK
RAAEKRLRMPYDVPAPRVINPAALEEDVEIKHFKPFSDMKWYKKLRDQYEFPEPMDKIQQKRLKRIRLSRWDQFYEVPLP
RIKDQYKPKWRIPSVSQDDL
ETVRPARHRTPSPEMEAYNRIRRRSLGDFSDEELLLPVNEYLTMKRTEEERLRLEEELELGCDCLPPRFSPIQFELSALR
PSSPRRVYSDDEEMEDVKRY
DSYRIPSKYEAGPSFIDLRQRHDKATFRPPRQKQRVYEEKEDQELLRPHKTNQRITSYKSELKRMEFEEKTRISRKETTV
SSSTVSEAVESEYISSVASA
FIPKPIKKLPPPEPERRSPTPERASKADVTFPKVDFLSRYEVRKQALRSERRSVERKVDVVSQAPFSLDHSPRVTIRMRS
HRVPYGSNTKFTLNIQAKPE
AEIKWLHDGNQIHQSGKYHMSNISGVLTLQIIECVTEDAGTYRVVCKNAKGETSDYATLDVSGAEYAAFSSFRKEEEPPS
VHLPEMTTTEVYHVSSSKTT
VQETVQETITVVEKKEEKPSFPAKILKKPQSLTVEEGDLAKFECDVDGSDAPCFLVQPSSQNAGQNIKLTCDIKGEPAPE
GKGLENNQMMSLASNRRLTH
SKNTWTTRTRVESCDECTAFKICAMKEDDVETYTLRLSNEFGFDSATVDVHIRSMDRHRGRVLCKPGGRQRRGPGRADPR
HRRLALWTWNVTSLAGKEPE
LVREVERYQLDIVGLTSTHSTGSGTKLLERGWTLSFSGVAKGERRRAGVGILTSPRLSGAVLEFSPVNERVASMRLQVVE
EKALTVVCAYAPNSSLESPA
FLECLDGVLEGAPSGDSVVLLGDFNAHVGNDGETWRGVIRRNGLPDLNPSGALLLDFCASHGLAITNTMFEHRVGHKCTW
YQNTLGQRSMIDLWSYHQIC
GRMSWTLG
>g22.t2
TPDAPGTPDATEVSGDSITLAWTAPTSDGGDAVQYYILEKREKKTTRFYKVITKKPITEGAHKVLHLTEGTEYEFRVMAV
NDAGVGTPSNVSIPIKAAEP
KDIPCAPSVVCVSDSSNTTISLEWSRPVDDGGMEVLGYIIEMVKGDEEEWKRVNKELATQTNYVVSGLETGAKYRFRVAA
VNHVGQGEVQETPEAAQAVD
RLAPPEVDIDASFKQTHIIKAGGSVCLGIHFRGKPIPTSSWTKEDGELGVLSEVSTTEGFSSLSMENCSRNESGKYSITL
ENPSGSKTITFTVKVMDTPG
APQTLALQEVSRGAVTLTWEPPINDGGARIHHYVIERREASRRTWQQSGGKCTKQILKIQDLLEGVPYFFRVSAENQHGL
GEALELTEPVIATAEPSSPK
RLDILDTTDSSVSLGWLKPEHDGGSRIQCYIVEVKAKGTDKWMVVGNTKSLTYSIEKLNKGDEYDFRVKAKNESGSSQPK
ETIASVLVKEPHIEPAADLS
EIINQLITCRSGNTFTIDVPFSGRPAPKVSWKLEEMRLKSSDRVSIKVSKDRTSISVKEAMRGDGGKYYLTLENVAGTKT
FAINVNVIGRPSPPAGPIEI
SSITSESCKLDWGAPDDDGGTDITNYIVEKRESGSTAWQLINSSVKRTSLHVGHLTKYMQYTFRVCAENRFGVSKSTESE
TIVAEHPFTPPGPPTRPTVS

GVTANSMHIKWEEPYNDGGSKVTGYWIEKKERNIILWVRENKIPCFECHYKVEGLVEGLEYQFRISAMNSAGLSKASEAS
KRAFAQNPVGPPSKPEVTNV
TRSTVSLKWSSPLNDGGSPIVGYIIERKPYILTGEGRWLKCNYTNVVDTFFTVTALGEGEPYEFRVIAKNSNQVFSMPSE
STGSVQCKTDFEPPKAQLDS
KLLSDTVTVRAGSDLVLDAAVGGRPDPKACWSKGTRDLELCEKYHLQYTPTKAMAIIKFCDRDDTGRYILTVRNVSGTRT
AEVNVKVLDTPGVCQGSIAI
SRITEESCTLSWNPPLEDGGDDVSHYIVERRDTNRLNWVVMDDECKELSCNITRLFKNTEYLFRVRGVNKYGSGVYLQSE
PMVARNTFTVPSPPGAPEIL
VSGKDFATIEWLKPESDGGSEILHYLVERREKKSARWVKVNREGTLLDSMLKVSGLIEGNIYQFRVTAINKAGESEPSEV
SLYVICRVPTCTPAPPSIPR
IVDTHADSISLAWSRPVDDGGADVIGYILEMQESGAEEWTKAHEKTLRTSEHVVSALSSSKKYCFRVAGININGTGDFSE
ACLETQPVERIETPDFELHD
DLKKTICLRAGGSLRMSVTVTGRPAPAITWSKPGVDLQSRGFIDVTNTSTTLIIDKVHRYDAGKYTLVAENSAGKQEVNI
LVKIYDTPGPTGPLKIKELT
KESATISWEASTVDGGAPVNNYIIERREASMRAYKTVTSKCSKTSYKIDGLMEGMLYYFRVLPENIYGVGEPCETPDVVL
VCEVPEPPHKLEMIDVTKTT
VTLGWEKPEHDGGSRLTGYTIEACKPGTDKWMKVATMKTTDFEHTIEKLNENEQYLFRIRATNSRGASEPKEMLTAVTVQ
EQRVLPIVDLSSIPQKVVNV
LAGKTLELDLPIVGRPPPVCSWYFADNKLKVSERLKLKSTGKFSKLTMSDTTIDDSGDYTLEVKNITGVTTEVIKVIVLS
KPDEPKGPIRFEEVDATSVT
CSWDPPVRDGGAPTSGYMVEQRDAHRPGWVPVCDSVSRPTFKFENLTEGDEYVFRAAAVNRYGTGEFLQSDVAMCKSLKN
VPGPPGRPTVFDVSRDGMTV
AWNPPEEDGGLEVSGYIIERKEVRADRWVRANKNPVTMTRYRSTGLIEGLEYEHRITAINTRGMSKPSLLSKPAVATDPI
DTTKSSVSLAWSAPDDEGDA
RVDGYLIEMQKVGSLAWIKCNTTPSLICEYTLTQMPQGEEFKFRVMACNAGGSGEPAEVPGTIKVIEMLESPDYDLELKY
KDVYVVRQGGVVRLSLPIKG
KPHPTCKWSKVDSNISEDAMIATTEDASELVIKGAQRSDSGVYDLLLENKVGKKKAQIKVKVIGRPSAPEGPLSFEEVQA
NSVKVSWKAPSDDGGSEILG
YIVERREATRNAWYTVDSRVTDSQLAVKGLREGIEYHFKVTAENSFGVSASLKSEYPLVPKTPLCVPEPSSIQPEIMDVT
KSSVALAWTRPKDDGGSPVT
GYILEYKVVSSETWSRHQTKITSTMFTLPGLMPDAEYLLRIIAVNNIGESEAGPVSEPVTCKDPFDKPSQPGEIDTVNVA
KNSIAIRWQTPECDGGKEVL
GYWVEHRKSSESSWKKSNKQRLKEQEFTMLGLAEATEYEFRVFAENETGISRPRRTATCIKTKLSVETKPSLRKEMDEVT
TKLGQPAVMKCQIIGRPVPE
IKWYHAGKEMMECRKYEMSSDGRNHTLSIMTDQQEDEGEYTCKAINDAGDAETTGVLVLEAAPNFHPDYPLKDTYYAGLG
TTLRIHAVYIGRPEPKIMWL
HGAKTLENTEDISIETTEHYTHLVIKNVQRRVNGGKYRIRLHNHFGRADTAFNVEIYDKPDIPEGPIVLDALLKNSVIIS
WKAPQDNGGSMITNYIVEKR
EDKEGSDWELVSSSINGLSCRVPNLVDSAGYFFRVYAQNRYGNSQPLELAAPIVIKSQLEKPSPPLSPVVSGITRDSCVV
SWKPPSSDGGSKIKSYCLEK
KDKKGDWTPVTTDEIKQTVFSVKRLTEGTEYIFRVKCENLGGQSDYSEETAPMIPALAVDVRAPAFKEELRNLSVKHKSN
ATLVCKITGQPKPVIKWFRR
GKEIHSDGKKIKIQEFKGGYHQLVISEADEEDSTIYQIRATNQGGSICATVSLDVEVPAKIHLPKNLKDKEAIPALRGEM
VNIKIPFGGKPDPVITWQKG
QDLIDNNGYYQVIVTRSFTSLVFPNGVEKKDAGFYIVCAKNRFGIDQQTVELDVADVPDPPRDIKASDVSRDSVTLNWVA
PANDGGSKVISYIIEKCPTT
AERWERVAQARDTHYTVINLFGGTSYQFRTIAVNKFGQSTPSDTTGPIMTKEDKSRVLLYDREVDDTGCVPRGKAPHSGA
SNLHNKFAIAEELGRGQFGI
VHRCIESSSEKTYMAKFVKVRGADQAIVKKEIATLNLAKHTNFLLLHESFDSPEELVLIYDFISGVDIFERLNMADFELN
EREIVNYIRQICLALEFLHD
ESYGCFDIKPENIVYTTRTSSNIKIIELGQSRHLTPGDQIKIQYTTAEYAAPEIHQCDMVSSVTDMWSVGVLAYVLLSGL
NPFTAETTQQMIDNISNAAY
SYDDESFKQVSVESLDFTDRLMTKDRKHRMRAVEALEHPWLKRPAEELSNRMMQTSRHKRYYQAMVRKEWNTVVSAARVA
SGGSIRSQRGVFVAKVKIAP
FEHGPVAGQIGHSVVNEGDNVKFVCNIENYDSTTEVTWYCGVRQLEAGEKYEIDYEDGIAVITINKVTRSDDGTYRCKIV
NEYGEDSAYAELFINSVRSY

RDFFTTRVVKRVKRRVDTARMLQKPPEFTLPLVNCTAYIGEDLRFGVTITVHPEPRVIWHKSGQKLIPGNDEKKYTFIND
KGLYQLIIHHLEMEDDAEYS
VVARNRYGDDSCKARLTVVPRPKPADLTLRPMFKRLLANVDCREGQNVRFEIRVSGRPELKWEKDGTPLAFGPSIEVVHE
GLDYFILHVRDTLPEDSGVY
RVTATNSAGSTSCQAILKVERVTHVKKEFELSEKDKKKLAVKEAMDKKVRLSQILSGTEVMPLPSVAMQAVREAASMFKP
AVTSKKGDKTEAEIQKEKEE
KKKRAAEKRLRMPYDVPAPRVINPAALEEDVEIKHFKPFSDMKWYKKLRDQYEFPEPMDKIQQKRLKRIRLSRWDQFYEV
PLPRIKDQYKPKWRIPSVSQ
DDLETVRPARHRTPSPEMEAYNRIRRRSLGDFSDEELLLPVNEYLTMKRTEEERLRLEEELELGCDCLPPRFSPIQFELS
ALRPSSPRRVYSDDEEMEDV
KRYDSYRIPSKYEAGPSFIDLRQRHDKATFRPPRQKQRVYEEKEDQELLRPHKTNQRITSYKSELKRMEFEEKTRISRKE
TTVSSSTVSEAVESEYISSV
ASAFIPKPIKKLPPPEPERRSPTPERASKADVTFPKVDFLSRYEVRKQALRSERRSVERKVDVVSQAPFSLDHSPRVTIR
MRSHRVPYGSNTKFTLNIQA
KPEAEIKWLHDGNQIHQSGKYHMSNISGVLTLQIIECVTEDAGTYRVVCKNAKGETSDYATLDVSGAEYAAFSSFRKEEE
PPSVHLPEMTTTEVYHVSSS
KTTVQETVQETITVVEKKEEKPSFPAKILKKPQSLTVEEGDLAKFECDVDGSDAPCFLVQPSSQNAGQNIKLTCDIKGEP
APEGKGLENNQMMSLASNRR
LTHSKNTWTTRTRVESCDECTAFKICAMKEDDVETYTLRLSNEFGFDSATVDVHIRSMDRHRGRVLCKPGGRQRRGPGRA
DPRHRRLALWTWNVTSLAGK
EPELVREVERYQLDIVGLTSTHSTGSGTKLLERGWTLSFSGVAKGERRRAGVGILTSPRLSGAVLEFSPVNERVASMRLQ
VVEEKALTVVCAYAPNSSLE
SPAFLECLDGVLEGAPSGDSVVLLGDFNAHVGNDGETWRGVIRRNGLPDLNPSGALLLDFCASHGLAITNTMFEHRVGHK
CTWYQNTLGQRSMIDLWSYH
QICGRMSWTLG
>g22.t3
TPDATEVSGDSITLAWTAPTSDGGDAVQYYILEKREKKTTRFYKVITKKPITEGAHKVLHLTEGTEYEFRVMAVNDAGVG
TPSNVSIPIKAAEPKDIPCA
PSVVCVSDSSNTTISLEWSRPVDDGGMEVLGYIIEMVKGDEEEWKRVNKELATQTNYVVSGLETGAKYRFRVAAVNHVGQ
GEVQETPEAAQAVDRLAPPE
VDIDASFKQTHIIKAGGSVCLGIHFRGKPIPTSSWTKEDGELGVLSEVSTTEGFSSLSMENCSRNESGKYSITLENPSGS
KTITFTVKVMDTPGAPQTLA
LQEVSRGAVTLTWEPPINDGGARIHHYVIERREASRRTWQQSGGKCTKQILKIQDLLEGVPYFFRVSAENQHGLGEALEL
TEPVIATAEPSSPKRLDILD
TTDSSVSLGWLKPEHDGGSRIQCYIVEVKAKGTDKWMVVGNTKSLTYSIEKLNKGDEYDFRVKAKNESGSSQPKETIASV
LVKEPHIEPAADLSEIINQL
ITCRSGNTFTIDVPFSGRPAPKVSWKLEEMRLKSSDRVSIKVSKDRTSISVKEAMRGDGGKYYLTLENVAGTKTFAINVN
VIGRPSPPAGPIEISSITSE
SCKLDWGAPDDDGGTDITNYIVEKRESGSTAWQLINSSVKRTSLHVGHLTKYMQYTFRVCAENRFGVSKSTESETIVAEH
PFTPPGPPTRPTVSGVTANS
MHIKWEEPYNDGGSKVTGYWIEKKERNIILWVRENKIPCFECHYKVEGLVEGLEYQFRISAMNSAGLSKASEASKRAFAQ
NPVGPPSKPEVTNVTRSTVS
LKWSSPLNDGGSPIVGYIIERKPYILTGEGRWLKCNYTNVVDTFFTVTALGEGEPYEFRVIAKNSNQVFSMPSESTGSVQ
CKTDFEPPKAQLDSKLLSDT
VTVRAGSDLVLDAAVGGRPDPKACWSKGTRDLELCEKYHLQYTPTKAMAIIKFCDRDDTGRYILTVRNVSGTRTAEVNVK
VLDTPGVCQGSIAISRITEE
SCTLSWNPPLEDGGDDVSHYIVERRDTNRLNWVVMDDECKELSCNITRLFKNTEYLFRVRGVNKYGSGVYLQSEPMVARN
TFTVPSPPGAPEILVSGKDF
ATIEWLKPESDGGSEILHYLVERREKKSARWVKVNREGTLLDSMLKVSGLIEGNIYQFRVTAINKAGESEPSEVSLYVIC
RVPTCTPAPPSIPRIVDTHA
DSISLAWSRPVDDGGADVIGYILEMQESGAEEWTKAHEKTLRTSEHVVSALSSSKKYCFRVAGININGTGDFSEACLETQ
PVERIETPDFELHDDLKKTI
CLRAGGSLRMSVTVTGRPAPAITWSKPGVDLQSRGFIDVTNTSTTLIIDKVHRYDAGKYTLVAENSAGKQEVNILVKIYD
TPGPTGPLKIKELTKESATI
SWEASTVDGGAPVNNYIIERREASMRAYKTVTSKCSKTSYKIDGLMEGMLYYFRVLPENIYGVGEPCETPDVVLVCEVPE
PPHKLEMIDVTKTTVTLGWE

KPEHDGGSRLTGYTIEACKPGTDKWMKVATMKTTDFEHTIEKLNENEQYLFRIRATNSRGASEPKEMLTAVTVQEQRVLP
IVDLSSIPQKVVNVLAGKTL
ELDLPIVGRPPPVCSWYFADNKLKVSERLKLKSTGKFSKLTMSDTTIDDSGDYTLEVKNITGVTTEVIKVIVLSKPDEPK
GPIRFEEVDATSVTCSWDPP
VRDGGAPTSGYMVEQRDAHRPGWVPVCDSVSRPTFKFENLTEGDEYVFRAAAVNRYGTGEFLQSDVAMCKSLKNVPGPPG
RPTVFDVSRDGMTVAWNPPE
EDGGLEVSGYIIERKEVRADRWVRANKNPVTMTRYRSTGLIEGLEYEHRITAINTRGMSKPSLLSKPAVATDPIDTTKSS
VSLAWSAPDDEGDARVDGYL
IEMQKVGSLAWIKCNTTPSLICEYTLTQMPQGEEFKFRVMACNAGGSGEPAEVPGTIKVIEMLESPDYDLELKYKDVYVV
RQGGVVRLSLPIKGKPHPTC
KWSKVDSNISEDAMIATTEDASELVIKGAQRSDSGVYDLLLENKVGKKKAQIKVKVIGRPSAPEGPLSFEEVQANSVKVS
WKAPSDDGGSEILGYIVERR
EATRNAWYTVDSRVTDSQLAVKGLREGIEYHFKVTAENSFGVSASLKSEYPLVPKTPLCVPEPSSIQPEIMDVTKSSVAL
AWTRPKDDGGSPVTGYILEY
KVVSSETWSRHQTKITSTMFTLPGLMPDAEYLLRIIAVNNIGESEAGPVSEPVTCKDPFDKPSQPGEIDTVNVAKNSIAI
RWQTPECDGGKEVLGYWVEH
RKSSESSWKKSNKQRLKEQEFTMLGLAEATEYEFRVFAENETGISRPRRTATCIKTKLSVETKPSLRKEMDEVTTKLGQP
AVMKCQIIGRPVPEIKWYHA
GKEMMECRKYEMSSDGRNHTLSIMTDQQEDEGEYTCKAINDAGDAETTGVLVLEAAPNFHPDYPLKDTYYAGLGTTLRIH
AVYIGRPEPKIMWLHGAKTL
ENTEDISIETTEHYTHLVIKNVQRRVNGGKYRIRLHNHFGRADTAFNVEIYDKPDIPEGPIVLDALLKNSVIISWKAPQD
NGGSMITNYIVEKREDKEGS
DWELVSSSINGLSCRVPNLVDSAGYFFRVYAQNRYGNSQPLELAAPIVIKSQLEKPSPPLSPVVSGITRDSCVVSWKPPS
SDGGSKIKSYCLEKKDKKGD
WTPVTTDEIKQTVFSVKRLTEGTEYIFRVKCENLGGQSDYSEETAPMIPALAVDVRAPAFKEELRNLSVKHKSNATLVCK
ITGQPKPVIKWFRRGKEIHS
DGKKIKIQEFKGGYHQLVISEADEEDSTIYQIRATNQGGSICATVSLDVEVPAKIHLPKNLKDKEAIPALRGEMVNIKIP
FGGKPDPVITWQKGQDLIDN
NGYYQVIVTRSFTSLVFPNGVEKKDAGFYIVCAKNRFGIDQQTVELDVADVPDPPRDIKASDVSRDSVTLNWVAPANDGG
SKVISYIIEKCPTTAERWER
VAQARDTHYTVINLFGGTSYQFRTIAVNKFGQSTPSDTTGPIMTKEDKSRVLLYDREVDDTGCVPRGKAPHSGASNLHNK
FAIAEELGRGQFGIVHRCIE
SSSEKTYMAKFVKVRGADQAIVKKEIATLNLAKHTNFLLLHESFDSPEELVLIYDFISGVDIFERLNMADFELNEREIVN
YIRQICLALEFLHDESYGCF
DIKPENIVYTTRTSSNIKIIELGQSRHLTPGDQIKIQYTTAEYAAPEIHQCDMVSSVTDMWSVGVLAYVLLSGLNPFTAE
TTQQMIDNISNAAYSYDDES
FKQVSVESLDFTDRLMTKDRKHRMRAVEALEHPWLKRPAEELSNRMMQTSRHKRYYQAMVRKEWNTVVSAARVASGGSIR
SQRGVFVAKVKIAPFEHGPV
AGQIGHSVVNEGDNVKFVCNIENYDSTTEVTWYCGVRQLEAGEKYEIDYEDGIAVITINKVTRSDDGTYRCKIVNEYGED
SAYAELFINSVRSYRDFFTT
RVVKRVKRRVDTARMLQKPPEFTLPLVNCTAYIGEDLRFGVTITVHPEPRVIWHKSGQKLIPGNDEKKYTFINDKGLYQL
IIHHLEMEDDAEYSVVARNR
YGDDSCKARLTVVPRPKPADLTLRPMFKRLLANVDCREGQNVRFEIRVSGRPELKWEKDGTPLAFGPSIEVVHEGLDYFI
LHVRDTLPEDSGVYRVTATN
SAGSTSCQAILKVERVTHVKKEFELSEKDKKKLAVKEAMDKKVRLSQILSGTEVMPLPSVAMQAVREAASMFKPAVTSKK
GDKTEAEIQKEKEEKKKRAA
EKRLRMPYDVPAPRVINPAALEEDVEIKHFKPFSDMKWYKKLRDQYEFPEPMDKIQQKRLKRIRLSRWDQFYEVPLPRIK
DQYKPKWRIPSVSQDDLETV
RPARHRTPSPEMEAYNRIRRRSLGDFSDEELLLPVNEYLTMKRTEEERLRLEEELELGCDCLPPRFSPIQFELSALRPSS
PRRVYSDDEEMEDVKRYDSY
RIPSKYEAGPSFIDLRQRHDKATFRPPRQKQRVYEEKEDQELLRPHKTNQRITSYKSELKRMEFEEKTRISRKETTVSSS
TVSEAVESEYISSVASAFIP
KPIKKLPPPEPERRSPTPERASKADVTFPKVDFLSRYEVRKQALRSERRSVERKVDVVSQAPFSLDHSPRVTIRMRSHRV
PYGSNTKFTLNIQAKPEAEI
KWLHDGNQIHQSGKYHMSNISGVLTLQIIECVTEDAGTYRVVCKNAKGETSDYATLDVSGAEYAAFSSFRKEEEPPSVHL
PEMTTTEVYHVSSSKTTVQE

TVQETITVVEKKEEKPSFPAKILKKPQSLTVEEGDLAKFECDVDGSDAPCFLVQPSSQNAGQNIKLTCDIKGEPAPEGKG
LENNQMMSLASNRRLTHSKN
TWTTRTRVESCDECTAFKICAMKEDDVETYTLRLSNEFGFDSATVDVHIRSMDRHRGRVLCKPGGRQRRGPGRADPRHRR
LALWTWNVTSLAGKEPELVR
EVERYQLDIVGLTSTHSTGSGTKLLERGWTLSFSGVAKGERRRAGVGILTSPRLSGAVLEFSPVNERVASMRLQVVEEKA
LTVVCAYAPNSSLESPAFLE
CLDGVLEGAPSGDSVVLLGDFNAHVGNDGETWRGVIRRNGLPDLNPSGALLLDFCASHGLAITNTMFEHRVGHKCTWYQN
TLGQRSMIDLWSYHQICGRM
SWTLG
>g23.t1
MALLWRRMKVMVTVVMVNLFIFILISRNNGQDKGGRHKVHIPKNPFWSKMVPNQAYWNLQQQVLDLQNNPILTANYSTED
LPGWLNDSTLISDPCHPDLK
VTTQVKDYNSLPHRFKDFLIYMRCRSYPLLVDQADLCADPPFLLLAIKSLAPHFDRRQAIRESWGRSGVLANRTVKTVFL
LGNTSEVDHHPDLSDMLHYE
SMRHHDIVQWDYRDSFFNLTVKEVLFLGWIETHCPGTQYIFKGDDDVFVNTYSILQFLGGLSGAKARDLFVGDVITNAGP
HRDKKVKYFIPESMYVGNYP
PYAGGGGYLYSGDLAARLHNASKRVALYPIDDVYTGMCLRKLGLAPEKHKGFRTFDIEEKYRANPCAYKSLMLVHPRTPQ
EMIQIWAWLSDPNLNCQ
>g24.t1
YRTFTVVKRELSRKAKLSIYQSIYVPTLTYGHELWVVTERTRSRIQAAKMSFLPRVAGLSLRDRARSFDIRRKLRVEPLL
LR
>g25.t1
MELHNGIPNSLEELIDEVKNACGLGGFTWLQYKDTDFVNLTSTTVIKDLTVLKINAEFMRVTTVPLEKKFLAQLDKHMSK
MLEVIRSKGGRVKEQTKATL
QVLDETVDITIRRECIFKSLMIYLGEPVHHLIKECQQRFPAPPGEPRGVPRPDEIYNPSSEIWVSPGVSYQLDVPGTPLT
GGAQEAY
>g25.t2
MELHNGIPNSLEELIDEVKNACGLGGFTWLQYKDTDFVNLTSTTINAEFMRVTTVPLEKKFLAQLDKHMSKMLEVIRSKG
GRVKEQTKATLQVLDETVDI
TIRRECIFKSLMIYLGEPVHHLIKECQQRFPAPPGEPRGVPRPDEIYNPSSEIWVSPGVSYQLDVPGTPLTGGAQEAY
>g25.t3
MEEINAEFMRVTTVPLEKKFLAQLDKHMSKMLEVIRSKGGRVKEQTKATLQVLDETVDITIRRECIFKSLMIYLGEPVHH
LIKECQQRFPAPPGEPRGVP
RPDEIYNPSSEIWVSPGVSYQLDVPGTPLTGGAQEAY
>g26.t1
MMEYMEDKFALKSQTMKGSEYYMGPGAALEQVMDGLEAGVQPSYYGKASPKCGQSFGPQGGDHHAGLERSSPCGDANYST
KPEEDPLNPELCRTMDSCCS
MRGGSPRGADKMGELDELGDPKCDSNVSSSKKRRHRTTFTSAQLEELEKVFQKTHYPDVYVREQLAMRTELTEARVQVWF
QNRRAKWRKRERYGQLQQAK
SHFATTYDLSVLPRADSYSQIPNNLWAGAGPGGSVVSSHCVLPRASPPCGVSSYSPHPPPRSSSSSSSSAGGGASVDHGY
VGFPNQQNQFGHVSLNNFFS
ADSLLAPAAGGHHVFETKPEYERRSSSIAVLRMKAKEHAANISWAM
>g26.t2
LDSGRAWLKTLRSLIGQTQSDAVLVFRAGEPVLFLIKRRPSQTPPKLSIGNSSWTMKGSEYYMGPGAALEQVMDGLEAGV
QPSYYGKASPKCGQSFGPQG
GDHHAGLERSSPCGDANYSTKPEEDPLNPELCRTMDSCCSMRGGSPRGADKMGELDELGDPKCDSNVSSSKKRRHRTTFT
SAQLEELEKVFQKTHYPDVY
VREQLAMRTELTEARVQVWFQNRRAKWRKRERYGQLQQAKSHFATTYDLSVLPRADSYSQIPNNLWAGAGPGGSVVSSHC
VLPRASPPCGVSSYSPHPPP
RSSSSSSSSAGGGASVDHGYVGFPNQQNQFGHVSLNNFFSADSLLAPAAGGHHVFETKPEYERRSSSIAVLRMKAKEHAA
NISWAM
>g27.t1
MYTSLRTMDDCPMRAWRGSLPADLSGSLRLPRRRFTTRTRPENKEHTPRAAIWCRPEDPGPGEALPHGPSVRDRAEPETN
RHRALVSPSCAVAACSLICS
GLPGHVVWLKVCEVTCQVSRQVTRQVIRQVTRSGPRSVGSELIPKGVMSSVVCALMRSNCPPPNHQSPRGPPDPKHPGRS
AASGMFYAE

>g27.t2
MDDCPMRAWRGSLPADLSGSLRLPRRRFTTRTRPENKEHTPRAAIWCRPEDPGPGEALPHGPSVRDRAEPETNRHRALVS
PSCAVAACSLICSGLPGHVV
WLKVCEVTCQVSRQVTRQVIRQVTRSGPRSVGSELIPKGVMSSVVCALMRSNCPPPNHQSPRGPPDPKHPGRSAASGMFY
AE
>g27.t3
MYTSLRTMDDCPMRAWRGSLPADLSGSLRLPRRRFTTRTRPENKEHTPRAAIWCRPEDPGPGEALPHGPSVRDRAEPETN
RHRALVSPSCAVAACSLICS
GLASRRGHGVTC
>g28.t1
MQSHLDTPAVREPGLLEPRVGRPSNIGQAQPIIDQLAEEARAFNRIYVASVHPDLSDEDIKSVFEAFGRIKSCTLARDPT
TGRHRSFGFIGELRGTPGRA
LAVVA
>g28.t2
MQSHLDTPAVREPGLLEPRVGRPSNIGQAQPIIDQLAEEARAFNRIYVASVHPDLSDEDIKSVFEAFGRIKSCTLARDPT
TGRHRSFGFI
>g29.t1
GCSVSAVNLTNQTDSVLSKRLSKSSATLWTSPSRNRSLALSPWESSVVDRLMTPTLAFLARSRSAASVLANGKDPQSPVC
PRSASASPLAQCSHRPAHRC
PQRWGVNASSPDIAPRRHSSTPVRHAVRSHSSSGGYLE
>g29.t2
GCSVSAVNLTNQTDSVLSKRLSKSSATLWTSPSRNRSLALSPWESSVVDRLMTPTLAFLARSRSAASVLANGKDPQSPVC
PRSASASPLAQCSHRPAHRC
PQRWGVNASSPDIAPRRHSSTP
>g30.t1
DMVTLSKPIIYISIEEIINTHSLLLEHLGAIAPEGADLLHELLQDLGEVPDVEALL
>g31.t1
LTKTKDLNHCQERIFKDIGLAYAEKCVQCEAQGKAMKGAVAFNYIMKPAATGALILEATATELIQFSPFNIMNGAAQMEA
KQFLSYAGISKTPVAPISAA
YIPRGSLQYEFATELLQTPIQLLRITNAEAQIVEVLNHLVTYSVAKVHEDAPLKFIELIQLLRVASFDRIDALWDQYKVK
PAYRHWILNAVPAIGTHEAL
RFIKEKFHIDDISVVEAAQALISSIHMVTADLEAIKLVEPIHDLLIQSVAKRDIEGAIIALKVLGNAGHPASLKPIMKIM
PGFSTAAAALPMNVHSEAIL
AMRNIAKKEPKLIQDVAVQVFMDKALDAEIRMAAALVLFETRLPMGLVTAMADAVLKESNLQVASFVYSYMKAMTKNTAP
DYASFATACAVAVKMLSPKF
DRLSFRFSRALYFNAYHNPWMVGAAASAFYVNDAATVMPKAVVAKARTYFAGAYADVLE
>g31.t2
LTKTKDLNHCQERIFKDIGLAYAEKCVQCEAGKAMKGAVAFNYIMKPAATGALILEATATELIQFSPFNIMNGAAQMEAK
QFLSYAGISKTPVAPISAAY
IPRGSLQYEFATELLQTPIQLLRITNAEAQIVEVLNHLVTYSVAKVHEDAPLKFIELIQLLRVASFDRIDALWDQYKVKP
AYRHWILNAVPAIGTHEALR
FIKEKFHIDDISVVEAAQALISSIHMVTADLEAIKLVEPIHDLLIQSVAKRDIEGAIIALKVLGNAGHPASLKPIMKIMP
GFSTAAAALPMNVHSEAILA
MRNIAKKEPKLIQDVAVQVFMDKALDAEIRMAAALVLFETRLPMGLVTAMADAVLKESNLQVASFVYSYMKAMTKNTAPD
YASFATACAVAVKMLSPKFD
RLSFRFSRALYFNAYHNPWMVGAAASAFYVNDAATVMPKAVVAKARTYFAGAYADVLE
>g31.t3
LTKTKDLNHCQERIFKDIGLAYAEKCVQCEAQGKAMKGAVAFNYIMKPAATGALILEATATELIQFSPFNIMNGAAQMEA
KQFLSYAGISKTPVAPISAA
YIPRGSLQYEFATELLQTPIQLLRITNAEAQIVEVLNHLVTYSVAKVHEDAPLKFIELIQLLRVASFDRIDALWDQYKVK
PAYRHWILNAVPAIGTHEAL
RFIKEKFHIDDISVVEAAQALISSIHMVTADLEAIKLVEPIHDLLIQSVAKRDIEGAIIALKVLGNAGHPASLKPIMKIM
PGFSTAAAALPMNVHSEAIL
AMRNIAKKEPKLVFMDKALDAEIRMAAALVLFETRLPMGLVTAMADAVLKESNLQVASFVYSYMKAMTKNTAPDYASFAT
ACAVAVKMLSPKFDRLSFRF
SRALYFNAYHNPWMVGAAASAFYVNDAATVMPKAVVAKARTYFAGAYADVLE

>g32.t1
MHRPSCRPSPSGGGQLFSDLRDDGSMFSRHLEKRESDQCPRSAVDGGKRMERGKRSLRISNLLLRQCLSEFLGSFIVIVS
KSSVVTMFGCAALAQMKTSR
GAKGQLLTVNLAFSAGIMVAMYLGRDISGAHLNPALSLSSCVVGSLPWSKLLPYCCSQVLGAYVASALVFLMYY
>g32.t2
LDSAVSSRGLDTILSLPPSESPCQPRSRSYFQSVTVTKVVKPDGSVEERHTVRDGQGNEETTVTRSGPTGNPQPPGTGDR
PLGPGGGQLFSDLRDDGSMF
SRHLEKRESDQCPRSAVDGGKRMERGKRSLRISNLLLRQCLSEFLGSFIVIVSKSSVVTMFGCAALAQMKTSRGAKGQLL
TVNLAFSAGIMVAMYLGRDI
SGAHLNPALSLSSCVVGSLPWSKLLPYCCSQVLGAYVASALVFLMYY
>g32.t3
MHRPSCRPSPSGGGQLFSDLRDDGSMFSRHLEKRESDQCPRSAVDGGKRMERGKRSLRISNLLLRQCLSEFLGSFIVIMF
GCAALAQMKTSRGAKGQLLT
VNLAFSAGIMVAMYLGRDISGAHLNPALSLSSCVVGSLPWSKLLPYCCSQVLGAYVASALVFLMYY
>g33.t1
MFKRRRRGKRADALVKLRQRGFQTALLSIHLANVRSLNNKMDELLLLNRRNSNFCRSAALCFTETWLSELTPDSGLYLPD
FQLLRADRACVTEALQHLAD
QITDEERKHPDSLLIVLGDFNRANLS
>g34.t1
TDVVKRELSRKAKLSIYQSIYVPTLTYGHELWVVTERTRLRIQAAEMSFLRRVAGLSLRDRVRSLDIRRELGVEPLLLRV
ERSQLRWFGHLIRMPPGRLP
LEVFRARPTGKTPRGRPRTRWRDYISHLAWECLRVPQEELESVAAQLHVARIDPWRLNRMTRLAGSQVKVQLSLLEKLQT
QVTYGRQELEVLLAHQAHPG
PSAGAPRQASSCRSFQSPVTFPLHSFPGVDKRALSGNLGLY
>g34.t2
TDVVKRELSRKAKLSIYQSIYVPTLTYGHELWVVTERTRLRIQAAEMSFLRRVAGLSLRDRVRSLDIRRELGVEPLLLRV
ERSQLRWFGHLIRMPPGRLP
LEVFRARPTGKTPRGRPRTRWRDYISHLAWECLRVPQEELESVAAQLHVARIDPWRLNRMTRLAGSQVKVQLSLLEKLQT
QVTYGRQELEVLLAHQAHPG
PSAGAPRQ
>g34.t3
TDVVKRELSRKAKLSIYQSIYVPTLTYGHELWVVTERTRLRIQAAEMSFLRRVAGLSLRDRVRSLDIRRELGVEPLLLRV
ERSQLRWFGHLIRMPPGRLP
LEVFRARPTAQLHVARIDPWRLNRMTRLAGSQVKVQLSLLEKLQTQVTYGRQELEVLLAHQAHPGPSAGAPRQASSCRSF
QSPVTFPLHSFPGVDKRALS
GNLGLY
>g35.t1
RNDILCHSGWAEVTSAGIVLSLTRRSPQTHALVTHVSLRLVRGAGMEFASGPRRTSTME
>g36.t1
MRRLCSRKVSPEEEHLWPLDHRDTCQRLSGTVAGAAGDLILSHGFNSCQEQCGNYSSSSMPAYVMDTLSSSCVGPYLRCS
SGTGLAVWYQHCGGSFMFTD
HVLDGQNTRLEVLISGPDWSVHTSPRPIRSDAAGSHLTHPVSAFTFHRVSCWTVSCLQVPSPRRETNSPDPQIGTAAGAL
FHTTVDSLVLGCLLLWDGPG
DSPTGQYTVHTKTQGKREDKAKTEAKMSDVATCRMKNHDESSATSGQL
>g36.t2
MPAYVMDTLSSSCVGPYLRCSSGTGLAVWYQHCGGSFMFTDHVLDGQNTRLEVLISGPDWSVHTSPRPIRSDAAGSHLTH
PVSAFTFHRVSCWTVSCLQV
PSPRRETNSPDPQIGTAAGALFHTTVDSLVLGCLLLWDGPGDSPTGQYTVHTKTQGKREDKAKTEAKMSDVATCRMKNHD
ESSATSGQL
>g36.t3
MRRLCSRKVSPEEEHLWPLDHRDTCQRLSGTVAGAAGDLILSHGFNSCQEQCGNYSSSSMPAYVMDTLSSSCVGPYLRCS
SGTGLAVWYQHCGGSFMFTD
HVLDGQNTRLEVLISGPDWSVHTSPRPIRSDAAGSHLTHPVSAFTFHRVSCWTVSCLQTNSPDPQIGTAAGALFHTTVDS
LVLGCLLLWDGPGDSPTGQY
TVHTKTQGKREDKAKTEAKMSDVATCRMKNHDESSATSGQL
>g37.t1

LRGGSRSRPKDKGAHSPFRGHRNPATATMGEWSFLGELFDSLQARSPMLGRFWLFIMLVFRILILGTVATDLFEDEQEEF
ACNTLQPGCRQVCYDMAFPI
SMYRFWVFHIVLISTPALLYLLYAMHQLTKNKSTNDGGGGSQDQHGERRLRQLYLVNVAIRLLAEVGVLLGQWWLYGFRV
EAQFPCSRFPCPYTVDCFTS
RPAEKTIFLVFYFSVGVVSGFSSLVELLYAAIKWFCPRATQHQMLERSYVSQNLSNLKMYDEQEENMKLGRGKAMSESAL
SSLKMKVGPVRSSGARKTSN
IGRKSHKPHGNRPYVV
>g38.t1
LQGGADGLQSRQGDSLRPSPSLSPSGVLSVQSQQPRLTVTRWSVSRHLGPLDPSLSGTEPDSGERRSSAVGFLMAGAARG
HVTCSGHVTQCSTQRPLFIV
GYMTPVTYWIY
>g39.t1
SLICHRRGPVCAWVSAEGPRDGGSMMCMQGNRSLRTTPWPVASGPSEGGACLGGPGPPPWYFGTATALVSMPTSTVQPPS
WTRPWTFFTDTDCQ
>g39.t2
SLICHRRGPVCAWVSAEGPRDGGSMMCMQGNRSLRTTPWPVASGPSTLDLLYRH
>g40.t1
FIKEKFHIDDISVVEAAQALISSIHMVTADLEAIKLVEPIHDLLIQSVAKRDIEGAIIALKVLGNAGHPASLKPIMKIMP
GFSTAAAALPMNVHSEAILA
MRNIAKKEPKLIQHVALQVFMDKALHAEIRMAAALVLFETRLPMGLVTAMAHAVLKESNLQVASFVYSYMKAMTRNTAPD
YRSSATACAVAVTMLSPKFD
RLSFRFSRASYFDAYYSPWMVGAAASAFYVNDVATVMPKAVVAKARTYFAGAYADVLEISKWRTSPVNQPLASVYVKFFG
QEIGFASIDKALMDQLIQVA
TGPSVVTYGRKALEALLSGFSFHYAKPMLVAEVRRIMPTTVGLPMELSFYTAAVAAATVD
>g40.t2
MFVFCRHWILSAVPAIGTHEALRFIKEKFHIDDISVVEAAQALISSIHMVTADLEAIKLVEPIHDLLIQSVAKRDIEGAI
IALKVLGNAGHPASLKPIMK
IMPGFSTAAAALPMNVHSEAILAMRNIAKKEPKLIQHVALQVFMDKALHAEIRMAAALVLFETRLPMGLVTAMAHAVLKE
SNLQVASFVYSYMKAMTRNT
APDYRSSATACAVAVTMLSPKFDRLSFRFSRASYFDAYYSPWMVGAAASAFYVNDVATVMPKAVVAKARTYFAGAYADVL
EISKWRTSPVNQPLASVYVK
FFGQEIGFASIDKALMDQLIQVATGPSVVTYGRKALEALLSGFSFHYAKPMLVAEVRRIMPTTVGLPMELSFYTAAVAAA
TVD
>g40.t3
LSAVPAIGTHEALRFIKEKFHIDDISVVEAAQALISSIHMVTADLEAIKLVEPIHDLLIQSVAKRDIEGAIIALKVLGNA
GHPASLKPIMKIMPGFSTAA
AALPMNVHSEAILAMRNIAKKEPKLIQHVALQVFMDKALHAEIRMAAALVLFETRLPMGLVTAMAHAVLKESNLQVASFV
YSYMKAMTRNTAPDYRSSAT
ACAVAVTMLSPKFDRLSFRFSRASYFDAYYSPWMVGAAASAFYVNDVATVMPKAVVAKARTYFAGAYADVLEISKWRTSP
VNQPLASVYVKFFGQEIGFA
SIDKALMDQLIQVATGPSVVTYGRKALEALLSGFSFHYAKPMLVAEVRRIMPTTVGLPMELSFYTAAVAAATVD
>g41.t1
MDPFPISWWKQRWIGTMFQGLEKITTWARMCFKPAKSRSLVLKRRKVTGKFCISLDNTQIPSVTEKPIRSLGKTFDCTLK
DTASIKASGQVGSASSLNEE
FMVTHAREVLQYWAWTRWGQAVNRKISWSELWQAEPCRIKFLISSIYDVLPSPSNLFCWGKVDTPSCPLCLRGGMLEHIL
SCCPKALGEGHRTWRHDQVL
KAIAETIFTSITQNKPFRPAPWNCTRLADEGQPGEAAQISRAHYEKPP
>g42.t1
MEEANERKRAKYGDLVEECRRRGWRAQCVPVEVGCRGFAARSLCKAYSLLGITGARQRNAIKTTPEAAENASRWLWIKRG
DPWVHAT
>g43.t1
DPAFLEKKERCEYLKNKLAHIKAKIQEYNKVMEWNDGYS
>g44.t1
LPLSFFFISQIQNADDVLISPLERFRKEQIGAVK
>g45.t1
MTSHDPNPKPRCWNPFRSIKGFLQLAQLMVSGCVKSSISTIERRYGFTSQKLGLLVSFNEVGNVMLIVFVSFFGSRIHRP

RFIGAGALLAGVSSLIIALP
HFLSQPHPYAGTHAGRSTNNMTTGLCQSQSSIATSLSNHSCGRNESPTHQGVFPVLLLGQLLLGISTVPIQPFGMSYVDD
FASRRNSPLYIGTLGWILLA
VTSLGPALGFMMGSFTLRFYVDFDKITADQVQIDSKDLRWVGAWWLGFLIASCLLFLSSLPYLFFPRSMPKESVALAEGS
KSVDGKELDTVQPVTLTQFL
RSFPKTALRTLRNPVFMLMCLGQVFCAGVIVGVVTFLAKFMERQFNLSASVATMLIGKSVPTASAKPGLSPXAVHIPVCV
VAIVVGGAVMRRLDLSRSGA
VKWCAAAIFVTLLCSVPLLFVGCPTQRVAGVFPADWNDSAILCSRGCACHRDAFNPVCASDGTENDSAILCSRGCACHRD
AFNPVCASDGTEFRSPCHAG
CIAMATDDINHRVQNYTSCGCVGSEGGGGGGGGYVLPGTCGSRCSHLLVLFMVLSALTCFVGPTCKAPCFSTILRTVPPE
DKSFAVGVCYMLFRVL
>g45.t2
MTSHDPNPKPRCWNPFRSIKGFLQLAQLMVSGCVKSSISTIERRYGFTSQKLGLLVSFNEVGNVMLIVFVSFFGSRIHRP
RFIGAGALLAGVSSLIIALP
HFLSQPHPYAGTHAGRSTNNMTTGLCQSQSSIATSLSNHSCGRNESPTHQGVFPVLLLGQLLLGISTVPIQPFGMSYVDD
FASRRNSPLYIGTLGWILLA
VTSLGPALGFMMGSFTLRFYVDFDKITADQVQIDSKDLRWVGAWWLGFLIASCLLFLSSLPYLFFPRSMPKESVALAEGS
KSVDGKELDTVQPVTLTQFL
RSFPKTALRTLRNPVFMLMCLGQVFCAGVIVGVVTFLAKFMERQFNLSASVATMLIGKSVPTASAKPGLSPXAVHIPVCV
VAIVVGGAVMRRLDLSRSGA
VKWCAAAIFVTLLCSVPLLFVGCPTQRVAGVFPADWNDSAILCSRGNDSAILCSRGCACHRDAFNPVCASDGTEFRSPCH
AGCIAMATDDINHRVQNYTS
CGCVGSEGGGGGGGGYVLPGTCGSRCSHLLVLFMVLSALTCFVGPTCKAPCFSTILRTVPPEDKSFAVGVCYMLFRVL
>g45.t3
MRYHGGKVKYCIQSTPGESSMIAFYVTQARPTGAHLWFLTPFSLKCAAQTSGYRYQPLDERLHVENQGFLQLAQLMVSGC
VKSSISTIERRYGFTSQKLG
LLVSFNEVGNVMLIVFVSFFGSRIHRPRFIGAGALLAGVSSLIIALPHFLSQPHPYAGTHAGRSTNNMTTGLCQSQSSIA
TSLSNHSCGRNESPTHQGVF
PVLLLGQLLLGISTVPIQPFGMSYVDDFASRRNSPLYIGTLGWILLAVTSLGPALGFMMGSFTLRFYVDFDKITADQVQI
DSKDLRWVGAWWLGFLIASC
LLFLSSLPYLFFPRSMPKESVALAEGSKSVDGKELDTVQPVTLTQFLRSFPKTALRTLRNPVFMLMCLGQVFCAGVIVGV
VTFLAKFMERQFNLSASVAT
MLIGKSVPTASAKPGLSPXAVHIPVCVVAIVVGGAVMRRLDLSRSGAVKWCAAAIFVTLLCSVPLLFVGCPTQRVAGVFP
ADWNDSAILCSRGCACHRDA
FNPVCASDGTENDSAILCSRGCACHRDAFNPVCASDGTEFRSPCHAGCIAMATDDINHRVQNYTSCGCVGSEGGGGGGGG
YVLPGTCGSRCSHLLVLFMV
LSALTCFVGPTCKAPCFSTILRTVPPEDKSFAVGVCYMLFRVL
>g46.t1
MEWYLCVLMLSFPWSIQTESSPRCQNCVRQTDSESKTGRPANILQTSASTGFSQHRTADSNAQQPRTSAEGSPKLPQLLY
NGVADKPNSQLTNAVAFPML
DNPAWNNEEQQSPETYEVDAAFNKLQLHDNAFHHFGPKSKRNDDAGKGSLEHDTLQKRYGGFMRRIRPKLSNLKWDNQKR
YGGFLRRHFKIAVRSEPHAI
DFSL
>g47.t1
MSGPPGSGMYRSLPQQQQSQAQQQQQQQQQQQQQQQQQQQQQQQQQQQQQQQQQQQQQQGLPYASASLPPTSQSSSLFSH
ASAGQPPPSSSLGFGSAQGS
LGVGLASALG
>g48.t1
MSVPCSILGMNDVAWQETRGGMLHANGAPETGVVRVHGGGPLATVGGAGQGPGGPHLQGMDRGSNPTPGTPQPPLSGRSQ
DDATVGYFFQRQPGEQLGGC
VPGKHRWPTGDGSHIDQVLDGSELNHVNQVRAVDEMNYDFQALALESRGMGELLPAKKLWDSDELAKDGRKGMLLGEEWR
DNAWGSSHHSVSQPIMVQQR
PGQSFHGNGDANSVLSPRSEGGGLGVSMVEYVLSSSPGDKMDSRYRNGGYGTGDADPDGREKSDVPDKTSPFEEDKNPEL
KSGEEGDSSKANGRGLLNGM
DKDFNPTPGSRQASPTEAVERMGPGQGGMEMMGQHPHQHQHQHPHHHQHPHQHPHHPHPLAHAHAMQQQNPVQNKGPN
EDFQGHDGQGMGGMEQQAGV

ESLQFDYAGNQIQVDSSGTPVGLFDYNSQQQLFQRPNHLTVQQLTAAQQQQYALAAAQQQHLAGLAPAFVPNPYIINTGP
PGTDPYTAAGLAAAATLAVV
PPQYYGVPWGVYPANLFQQQAAANANHSANQQASNQGPGPGQQVMRTGNNQRPLTPGQGQPSQQESLAAAAAAANPALAY
AGMSGYQVLAPAAYYDQNGA
LVMGPGARGGLGGPMRLVQTPLLINPAAAQAGEFWPPDGLLIGTRSPPGAQTGGSATRFDRVL
>g48.t2
MNDVAWQETRGGMLHANGAPETGVVRVHGGGPLATVGGAGQGPGGPHLQGMDRGSNPTPGTPQPPLSGRSQDDATVGYFF
QRQPGEQLGGCVPGKHRWPT
GDGSHIDQVLDGSELNHVNQVRAVDEMNYDFQALALESRGMGELLPAKKLWDSDELAKDGRKGMLLGEEWRDNAWGSSHH
SVSQPIMVQQRPGQSFHGNG
DANSVLSPRSEGGGLGVSMVEYVLSSSPGDKMDSRYRNGGYGTGDADPDGREKSDVPDKTSPFEEDKNPELKSGEEGDSS
KANGRGLLNGMDKDFNPTPG
SRQASPTEAVERMGPGQGGMEMMGQHPHQHQHQHQHPHHHQHPHQHPHHPHPLAHAHAMQQQNPVQNKGPNEDFQGHDGQ
GMGGMEQQAGVESLQFDYAG
NQIQVDSSGTPVGLFDYNSQQQLFQRPNHLTVQQLTAAQQQQYALAAAQQQHLAGLAPAFVPNPYIINTGPPGTDPYTAA
GLAAAATLAVVPPQYYGVPW
GVYPANLFQQQAAANANHSANQQASNQGPGPGQQVMRTGNNQRPLTPGQGQPSQQESLAAAAAAANPALAYAGMSGYQVL
APAAYYDQNGALVMGPGARG
GLGGPMRLVQTPLLINPAAAQAGEFWPPDGLLIGTRSPPGAQTGGSATRFDRVL
>g48.t3
MNDVAWQETRGGMLHANGAPETGVVRVHGGGPLATVGGAGQGPGGPHLQGMDRGSNPTPGTPQPPLSGRSQDDATVGYFF
QRQPGEQLGGCVPGKHRWPT
GDGSHIDQVRAVDEMNYDFQALALESRGMGELLPAKKLWDSDELAKDGRKGMLLGEEWRDNAWGSSHHSVSQPIMVQQRP
GQSFHGNGDANSVLSPRSEG
GGLGVSMVEYVLSSSPGDKMDSRYRNGGYGTGDADPDGREKSDVPDKTSPFEEDKNPELKSGEEGDSSKANGRGLLNGMD
KDFNPTPGSRQASPTEAVER
MGPGQGGMEMMGQHPHQHQHQHQHPHHHQHPHQHPHHPHPLAHAHAMQQQNPVQNKGPNEDFQGHDGQGMGGMEQQAGVE
SLQFDYAGNQIQVDSSGTPV
GLFDYNSQQQLFQRPNHLTVQQLTAAQQQQYALAAAQQQHLAGLAPAFVPNPYIINTGPPGTDPYTAAGLAAAATLAVVP
PQYYGVPWGVYPANLFQQQA
AANANHSANQQASNQGPGPGQQVMRTGNNQRPLTPGQGQPSQQESLAAAAAAANPALAYAGMSGYQVLAPAAYYDQNGAL
VMGPGARGGLGGPMRLVQTP
LLINPAAAQAGEFWPPDGLLIGTRSPPGAQTGGSATRFDRVL
>g49.t1
MADIRKKLVVVGDGACGKTCLLIVFSKDEFPEVYVPTVFETYVADIEVDSKQVQLALWDTAGQEDYDRLRPLSYPDTDVI
LMCFSVDSPDSLENIPEKWV
AEVKHFCPNVPIILVANKKDLRNDENVKYELSRLKQEPVKTDDGRAMATRIGAYDYLECSAKTKDGIWEVFETATKASLQ
KRPRQSGSCVNCCLLL
>g50.t1
STVHGECATDFTVNSGEAPDITVSRDLSKCKTYYPQREHTSPLALISGMPLFMDATEDKALIQTKDALLASLRELSTLAQ
GTEGQRRASIFHKLVTELRG
LNTEVLTTTTPEMMDVSSSLTWQALAQCGTPECTSAILTILRTFEPESIEVDAVVYAMGLLPSPSALLVKDMLLMAQYKQ
SKPIMFALSNVVRKHFEIDY
QVTPDITAVAKFMTSILGSDCPGEKDLTYLTLRVVGNMGEAMEAAEPAIKSILLKCMRQPATTLSVQLAAIQAFRRMELT
DEVRANLQRVSTYAKGAVQK
RLAAYLTLMRNPKTSDLEMVKKLLKQEQNKQVKAFVTSHIYNIVSSTDRHSQTLAKKIMDILQNTDVPTHTDYTTLSRNY
KLGMAHERMQANTKGNIIFD
PSSQLPREVMLETTMEVFGYTMDMWEFGMDGKGFEPSIEALFGKNGFFPDTISKALYWAVDKMPFTTLKDNGKKVPENLV
KEVVANFNKLVKELQAQESP
EAMAYLRIMGTELGYIKASELKGIAEYAAMYATILMKIIPQETMKKLISNTDNEFFAHYIFMDNKFGMATGAGFPLKVTM
AGIFAPGAKGGLSMTPNMEA
TFRPSMGIGFVTKMGVHVPEFVDTDVAMHTNIYHESALNARVTIDNNQIKLSIPAPQGSTQLFSVSNNVLLVNNGHVEVI
QGKDEGRINAVTCRPFFSGV
KYCTTARYYTASDTAAPYFPLNGETKFAVDIQPTGDVTEYTATIAYKLLSEGKGRQVDSLKMTLKAEGSASTEASVSMKY
NRNRNVFTTNVQIPDYDVET
GIRVGMVDSSSKGKSLTFDITNKNIPLLSLISRAKLQDQMDGMLQMQLMVPSMKTDASITATLKNSEDLVLEIKSDVKLL

ETNSVQAVVFKYGKDQAEVT
LMSNMNSDITMLKPYTEALQDWMELAMNEMMDKQVVKTDMKLRHIYTKALEAGNIWMDKIASDVPHVYTLKNQMAELEMP
TMPENLFMNLESKFRYQFNK
DRVTLTVAVPLGGKTSQEFRIPPMLTTPHISMPQMGLNLSPMNIRIPTFTIPSDYDLTLPLMGMVEVSGKVKVNYYNLEG
MISAGNNTLESPSYMAKFNV
MADSPMELLSFTSEGAAEISDTPQDAIKVTLDGSLSHKLIKTSFVVSEKIAITDKVLSTSNYNWMASSPLGLQTSLDLTS
QCSLSSNMLTGDANMDGSLK
VGSMSASTSYSQTFYAEPLKKEAKTEATLRVNSAFLEVFNKATATLANEQLLIESNTNVNIDPLKHTTKFTFSYKDAKLT
VQSNSATMANKRIIRNEVDF
TASRAGATIRVENQADDTVNNVYSLLSGSINPSSIEINTDASLNIFASRASHKATMSLNMNGLITSCTTTAQSSPLTFEN
VFTGGVDTSGATMAITTKGT
VKENTVEMSVDAKAGSSEVYLNTIFNGNVFDANTRNRLNLRVNKDGCSFTNNMVGSLGEMKTDSTQSLTLTLRSLALSSK
TENTLNTRNYYKHDIKVDVE
SFTASVNVKNDLKIQEVNILNEAKFTAKPYTLELTSTTTGSLSEENLRHTYELKFVDMILSAKCNTNGKLLGSQMTHTSE
MEIADWTMKFNNVANLNSQS
LRLDSSVKTIAEPFTLSIDAILNTNGEIQLYGQQSGDLYSKFLLKAKPLSVINSFEYRASTTHELQNGASFKTNMNNKFN
NVLSLKEQSVFVSIASKMNN
HELHHELTAFNKAEKMGMAMKGALATTFFNGASENHDYSISGSVNYDKNSHTNFIQIPFLEHLPETIEELKNLVVRLVDE
SMDILRNIDASYEITAKIRG
KVEELKDAIDTINPQLFIQDLKDFVQSMETFMYNMMAKIPTEELNNMLKSINAGIMEWIKKNKIDNKINLIYAKIEEFLS
NYEIEKIIEKIMDEAIVIMK
QYRIRERIHSSLAALKAIDVQPMFNKIMAPVKELINNMYAFDYKQMVEDISMYCIRMIEKIKSFDYETFTMELKDKVSDM
TRIPCFGKLSGEFKIVSPNY
HLQSTADLANTTTTSATPEFTMNLNSQATSTFNLLGYTLDASVHLAMPERSRLSFSENLKAVHTAFMIDHQGSMSLYGLS
AQASSKTVAKATTEPYDAVL
VSDTKLSIDSQMSATLDTTYKHGINMPLLGLLSEANAHQKMDAKLNDGILTVNVNNKGQGTYTLPGFSDDVAHKSDMNAV
MDIYTARVIYNDETKSDLFK
LMDNFDADIRFTRYIIVEGKVETESPFMKNSKTMMKFQAKAEDLKITLDASHNAELVGPVAGTLANSVLAEVAPYELTFD
TKNKANVKVTLPLKLIGKTD
FLNDVAITVKSDVQQASWTGLARFNQFKYTHYFTMDNGEGEIQVFGRVNGDANLDVLKQPITIPEINVPYFGLTYPKLVD
YSLWEDTGLSMLLATTKQTI
DWEATLRCTKAVDAIVIHLDMKPIMDIINANAEVLPSNMIVGKDKAVATLARMERDIIIPVLSDVTYEFSMKSALITLKT
DASVMNQGDITMKLDASSFS
TFEDLNGKIEGTSTLSLENGIRLDSLFSLKHTMVEGTHDSMIGFSKGGVDASLKNSAKVNLPVIKIDINQEISEKEGIII
SMSSPSAGLVGFHLQNKRIS
KEHQSYP
>g50.t2
STVHGECATDFTVNSGEAPDITVSRDLSKCKTYYPQREHTSPLALISGMPLFMDATEDKALIQTKDALLASLRELSTLAQ
GTEGQRRASIFHKLVTELRG
LNTEVLTTTTPEMMDVSSSLTWQALAQCGTPECTSAILTILRTFEPESIEVDAVVYAMGLLPSPSALLVKDMLLMAQYKQ
SKPIMFALSNVVRKHFEIDY
QVTPDITAVAKFMTSILGSDCPGEKDLTYLTLRVVGNMGEAMEAAEPAIKSILLKCMRQPATTLSVQLAAIQAFRRMELT
DEVRANLQRVSTYAKGAVQK
RLAAYLTLMRNPKTSDLEMVKKLLKQEQNKQVKAFVTSHIYNIVSSTDRHSQTLAKKIMDILQNTDVPTHTDYTTLSRNY
KLGMAHERMQANTKGNIIFD
PSSQLPREVMLETTMEVFGYTMDMWEFGMDGKGFEPSIEALFGKNGFFPDTISKALYWAVDKMPFTTLKDNGKKVPENLV
KEVVANFNKLVKELQAQESP
EAMAYLRIMGTELGYIKASELKGIAEYAAMYATILMKIIPQETMKKLISNTDNEFFAHYIFMDNKFGMATGAGFPLKVTM
AGIFAPGAKGGLSMTPNMEA
TFRPSMGIGFVTKMGVHVPEFVDTDVAMHTNIYHESALNARVTIDNNQIKLSIPAPQGSTQLFSVSNNVLLVNNGHVEVI
QGKDEGRINAVTCRPFFSGV
KYCTTARYYTASDTAAPYFPLNGETKFAVDIQPTGDVTEYTATIAYKLLSEGKGRQVDSLKMTLKAEGSASTEASVSMKY
NRNRNVFTTNVQIPDYDVET
GIRVGMVDSSSKGKSLTFDITNKNIPLLSLISRAKLQDQMDGMLQMQLMVPSMKTDASITATLKNSEDLVLEIKSDVKLL
ETNSVQAVVFKYGKDQAEVT
LMSNMNSDITMLKPYTEALQDWMELAMNEMMDKQVVKTDMKLRHIYTKALEAGNIWMDKIASDVPHVYTLKNQMAELEMP

TMPENLFMNLESKFRYQFNK
DRVTLTVAVPLGGKTSQEFRIPPMLTTPHISMPQMGLNLSPMNIRIPTFTIPSDYDLTLPLMGMVEVSGKVKVNYYNLEG
MISAGNNTLESPSYMAKFNV
MADSPMELLSFTSEGAAEISDTPQDAIKVTLDGSLSHKLIKTSFVVSEKIAITDKVLSTSNYNWMASSPLGLQTSLDLTS
QCSLSSNMLTGDANMDGSLK
VGSMSASTSYSQTFYAEPLKKEAKTEATLRVNSAFLEVFNKATATLANEQLLIESNTNVNIDPLKHTTKFTFSYKDAKLT
VQSNSATMANKRIIRNEVDF
TASRAGATIRVENQADDTVNNVYSLLSGSINPSSIEINTDASLNIFASRASHKATMSLNMNGLITSCTTTAQSSPLTFEN
VFTGGVDTSGATMAITTKGT
VKENTVEMSVDAKAGSSEVYLNTIFNGNVFDANTRNRLNLRVNKDGCSFTNNMVGSLGEMKTDSTQSLTLTLRSLALSSK
TENTLNTRNYYKHDIKAKPL
SVINSFEYRASTTHELQNGASFKTNMNNKFNNVLSLKEQSVFVSIASKMNNHELHHELTAFNKAEKMGMAMKGALATTFF
NGASENHDYSISGSVNYDKN
SHTNFIQIPFLEHLPETIEELKNLVVRLVDESMDILRNIDASYEITAKIRGKVEELKDAIDTINPQLFIQDLKDFVQSME
TFMYNMMAKIPTEELNNMLK
SINAGIMEWIKKNKIDNKINLIYAKIEEFLSNYEIEKIIEKIMDEAIVIMKQYRIRERIHSSLAALKAIDVQPMFNKIMA
PVKELINNMYAFDYKQMVED
ISMYCIRMIEKIKSFDYETFTMELKDKVSDMTRIPCFGKLSGEFKIVSPNYHLQSTADLANTTTTSATPEFTMNLNSQAT
STFNLLGYTLDASVHLAMPE
RSRLSFSENLKAVHTAFMIDHQGSMSLYGLSAQASSKTVAKATTEPYDAVLVSDTKLSIDSQMSATLDTTYKHGINMPLL
GLLSEANAHQKMDAKLNDGI
LTVNVNNKGQGTYTLPGFSDDVAHKSDMNAVMDIYTARVIYNDETKSDLFKLMDNFDADIRFTRYIIVEGKVETESPFMK
NSKTMMKFQAKAEDLKITLD
ASHNAELVGPVAGTLANSVLAEVAPYELTFDTKNKANVKVTLPLKLIGKTDFLNDVAITVKSDVQQASWTGLARFNQFKY
THYFTMDNGEGEIQVFGRVN
GDANLDVLKQPITIPEINVPYFGLTYPKLVDYSLWEDTGLSMLLATTKQTIDWEATLRCTKAVDAIVIHLDMKPIMDIIN
ANAEVLPSNMIVGKDKAVAT
LARMERDIIIPVLSDVTYEFSMKSALITLKTDASVMNQGDITMKLDASSFSTFEDLNGKIEGTSTLSLENGIRLDSLFSL
KHTMVEGTHDSMIGFSKGGV
DASLKNSAKVNLPVIKIDINQEISEKEGIIISMSSPSAGLVGFHLQNKRISKEHQSYP
>g50.t3
STVHGECATDFTVNSGEAPDITVSRDLSKCKTYYPQREHTSPLALISGMPLFMDATEDKALIQTKDALLASLRELSTLAQ
GTEGQRRASIFHKLVTELRG
LNTEVLTTTTPEMMDVSSSLTWQALAQCGTPECTSAILTILRTFEPESIEVDAVVYAMGLLPSPSALLVKDMLLMAQYKQ
SKPIMFALSNVVRKHFEIDY
QVTPDITAVAKFMTSILGSDCPGEKDLTYLTLRVVGNMGEAMEAAEPAIKSILLKCMRQPATTLSVQLAAIQAFRRMELT
DEVRANLQRVSTYAKGAVQK
RLAAYLTLMRNPKTSDLEMVKKLLKQEQNKQVKAFVTSHIYNIVSSTDRHSQTLAKKIMDILQNTDVPTHTDYTTLSRNY
KLGMAHERMQANTKGNIIFD
PSSQLPREVMLETTMEVFGYTMDMWEFGMDGKGFEPSIEALFGKNGFFPDTISKALYWAVDKMPFTTLKDNGKKVPENLV
KEVVANFNKLVKELQAQESP
EAMAYLRIMGTELGYIKASELKGIAEYAAMYATILMKIIPQETMKKLISNTDNEFFAHYIFMDNKFGMATGAGFPLKVTM
AGIFAPGAKGGLSMTPNMEA
TFRPSMGIGFVTKMGVHVPEFVDTDVAMHTNIYHESALNARVTIDNNQIKLSIPAPQGSTQLFSVSNNVLLVNNGHVEVI
QGKDEGRINAVTCRPFFSGV
KYCTTARYYTASDTAAPYFPLNGETKFAVDIQPTGDVTEYTATIAYKLLSEGKGRQVDSLKMTLKAEGSASTEASVSMKY
NRNRNVFTTNVQIPDYDVET
GIRVGMVDSSSKGKSLTFDITNKNIPLLSLISRAKLQDQMDGMLQMQLMVPSMKTDASITATLKNSEDLVLEIKSDVKLL
ETNSVQAVVFKYGKDQAEVT
LMSNMNSDITMLKPYTEALQDWMELAMNEMMDKQVVKTDMKLRHIYTKALEAGNIWMDKIASDVPHVYTLKNQMAELEMP
TMPENLFMNLESKFRYQFNK
DRVTLTVAVPLGGKTSQEFRIPPMLTTPHISMPQMGLNLSPMNIRIPTFTIPSDYDLTLPLMGMVEVSGKVKVNYYNLEG
MISAGNNTLESPSYMAKFNV
MADSPMELLSFTSEGAAEISDTPQDAIKVTLDGSLSHKLIKTSFVVSEKIAITDKVLSTSNYNWMASSPLGLQTSLDLTS
QCSLSSNMLTGDANMDGSLK
VGSMSASTSYSQTFYAEPLKKEAKTEATLRVNSAFLEVFNKATATLANEQLLIESNTNVNIDPLKHTTKFTFSYKDAKLT

VQSNSATMANKRIIRNEVDF
TASRAGATIRVENQADDTVNNVYSLLSGSINPSSIEINTDASLNIFASRASHKATMSLNMNGLITSCTTTAQSSPLTFEN
VFTGGVDTSGATMAITTKGT
VKENTVEMSVDAKAGSSEVYLNTIFNGNVFDANTRNRLNLRVNKDGCSFTNNMVGSLGEMKTDSTQSLTLTLRSLALSSK
TENTLNTRNYYKHDIKVDVE
SFTASVNVKNDLKIQEVNILNEAKFTAKPYTLELTSTTTGSLSEENLRHTYELKFVDMILSAKCNTNGKLLGSQMTHTSE
MEIADWTMKFNNVANLNSQS
LRLDSSVKTIAEPFTLSIDAILNTNGEIQLYGQQSGDLYSKFLLKAKPLSVINSFEYRASTTHELQNGASFKTNMNNKFN
NVLSLKEQSVFVSIASKMNN
HELHHELTAFNKAEKMGMAMKGALATTFFNGASENHDYSISGSVNYDKNSHTNFIQIPFLEHLPETIEELKNLVVRLVDE
SMDILRNIDASYEITAKIRG
KVEELKDAIDTINPQLFIQDLKDFVQSMETFMYNMMAKIPTEELNNMLKSINAGIMEWIKKNKIDNKINLIYAKIEEFLS
NYEIEKIIEKIMDEAIVIMK
QYRIRERIHSSLAALKAIDVQPMFNKIMAPVKELINNMYAFDYKQMVEDISMYCIRMIEKIKSFDYETFTMELKDKVSDM
TRIPCFGKLSGEFKIVSPNY
HLQSTADLANTTTTSATPEFTMNLNSQATSTFNLLGYTLDASVHLAMPERSRLSFSENLKAVHTAFMIDHQGSMSLYGLS
AQASSKTVAKATTEPYDAVL
VSDTKLSIDSQMSATLDTTYKHGINMPLLGLLSEANAHQKMDAKLNDGILTVNVNNKGQGTYTLPGFSDDVAHKSDMNAV
MDIYTARVIYNDETKSDLFK
LMDNFDADIRFTRYIIVEGKVETESPFMKNSKTMMKFQAKAEDLKITLDASHNAELVGPVAGTLANSVLAEVAPYELTFD
TKNKANVKVTLPLKLIGKTD
FLNDVAITVKSDVQQASWTGLARFNQFKYTHYFTMDNGEGEIQVFGRVNGDANLDVLKQPITIPEINVPYFGLTYPKLVD
YSLWEDTGLSMLLATTKQTI
DWEATLRCTKAVDAIVIHLDMKPIMDIINANAEVLPSNMIVGKDKAVATLARMERDIIIPVLSDVTYEFSMKSALITLKT
DASVMNQGDITMKLDASSFS
TFEDLNGKIEGTSTLSLENGIRLDSLFSLKHTMVEGTHDSMIGFSKGGVDASLKNSAKVNLPVIKIDINQEISEKEGIII
SMSSPSAGLVGFHLQNKCNV
>g51.t1
MMRRIQHRLCLSHVFCSLAVGRQDQLYRSELEKWSNQIYQEPEVAPEEAHMFDLDCFLSNTSDTLFTMTQKPCPWSSDRH
NTYSSNADMIQPALTPLQPN
LDDFMDIPDIFMNYRLESNDQSGLADFGYFDGPSGGAFTPVSTHQLLTDTSQHKLAEPGEPSGSHLSHVTSALPRPEQAR
ASGDYDPYHFPGVLPASYGH
QQLYPTPPVAVGDPGMPLPVSAGNHFLYPPPPPPPPPCHKYSYLGPPGGGATSTVITHTASS
>g51.t2
MMRRIQHRLCLSHVFCSLAVGRQDQLYRSELEKWSNQIYQEPEVAPEEAHMFDLDCFLSNTSDTLFTMTQKPCPWSSDRH
NTYSSNADMIQPALTPLQPN
LDDFMDIPDIFMNYRLESNDQSGLADFGYFDGPSGGAFTPVSTHQLLTDTSQHKLAEARASGDYDPYHFPGVLPASYGHQ
QLYPTPPVAVGDPGMPLPVS
AGNHFLYPPPPPPPPPCHKYSYLGPPGGGATSTVITHTASS
>g52.t1
MDIHCKGDPFSAMHWHGGVNQLGGVFVNGRPLPDVVRQRIVELAHQGVRPCDISRQLRVSHGCVSKILGRYYETGSIKPG
VIGGSKPKVATPKVVDKIAD
YKRQNPTMFAWEIRDRLLAEGICDNDTVPSVSSINRYRRHVTPAGR
>g52.t2
HGGVNQLGGVFVNGRPLPDVVRQRIVELAHQGVRPCDISRQLRVSHGCVSKILGRYYETGSIKPGVIGGSKPKVATPKVV
DKIADYKRQNPTMFAWEIRD
RLLAEGICDNDTVPSVSSINRYRRHVTPAGR
>g52.t3
MHWHGGVNQLGGVFVNGRPLPDVVRQRIVELAHQGVRPCDISRQLRVSHGCVSKILGRYYETGSIKPGVIGGSKPKVATP
KVVDKIADYKRQNPTMFAWE
IRDRLLAEGICDNDTVPSVSSINRYRRHVTPAGR
>g53.t1
KTARLERTLTDNLHHLHHHHYHHHHHHLHHHHHQHHHHQHHHHHYHHQHHQYHTTTTTTTTTTTTTTTTTTTYHHHHH
HRHHHQLPAPPNAVPS
>g54.t1
MAFPCGCSRDGCGNMAGRIEFNPLRVRTHYLHTIMKLGLEKRILGPAGGEQYPGDSNLLLSPSSTCPPSPELPCSSSSSS

SSSSSSSSSLSVAAGGLDFQL
EESEVEAVVAVHNLLAERDILEREN
>g54.t2
VDRMAFPCGCSRDGCGNMAGRIEFNPLRVRTHYLHTIMKLGLEKRILGPAGGEQYPGDSNLLLSPSSTCPPSPELPCSSS
SSSSSSSSSSSLSVAAGGLD
FQLEESEVEAVVAVHNLLAERDILEREN
>g55.t1
ARSYNRQVRPAVLDSSSRLKWTNTAQQPQYLVGDEAVYVSPSDCYDIHWPVVRGHLNVHPGPGGSLTAVLADLEAIWGHV
INKQLEIPLRDLKVGGASGR
GISQ
>g55.t2
SYNRQVRPAVLDSSSRLKWTNTAQQPQYLVGDEAVYVSPSDCYDIHWPVVRGHLNVHPGPGGSLTAVLADLEAIWGHVIN
KQLEIPLRDLKVGGASGRGI
SQ
>g55.t3
ARSYNRQVRPAVLDSSSRLKWTNTAQQPQYLVGDEAVYVSPSDCYDIHWPVVRGHLNVHPGPGGSLTAVLADLEAIWGHV
INKQLEIPLRDLK
>g56.t1
RHGDADRQRSSKLFDRHREDDSVQTLAKRRRASTLPPPCTLPPSSLYPVPYIHPSSTLKPASTLEPTSALSNCLCILPSG
FTCSSKYPSETLRNKKDVST
QKRERKAGSTLTCTHLLINSNVQTSPRGLYM
>g57.t1
DLTARVSCEDLTARLSCQETRLLRVGLRGHGRMSASFSSVNTENARDAQLRPTPQVSSGSRERLA
>g57.t2
LTVRVSCQDLTARVSCEDLTARLSCQETRLLRVGLRGHGRMSASFSSVNTENARDAQLRPTPQVSSGSRERLA
>g58.t1
PAYSYYPDDEFQHFYRWTSPPGIIKIMAVIVIVLCLATFACVASTLAWDTQGGLSGFGGGGFGGGSYGTGFGAGGYGAGY
PSGGSFGNTGYGAGNSYGYG
NIGGNYNDPRKGKGFLIAMAAITFIMALVVFIIIVSHQTLCQSRRFYLALVILCAILALMMLIATIVYLVAVNPMAQSSS
SIYAMQIQGLCAQYQQPAQS
GVFVNQYLYHYCVVEPQEVRGPKMSRGSEIPRGPLFVLLFGFLNPFWVYVLLWAIAIVCGFLVTAALIIMMVFALKTRQK
IRGYGKSNILWKKVKVVDDI
PPPGDVEAWVG
>g58.t2
PAYSYYPDDEFQHFYRWTSPPGIIKIMAVIVIVLCLATFACVASTLAWDTQGGLSGFGGGGFGGGSYGTGFGAGGYGAGY
PSGGSFGNTGYGAGNSYGYG
NIGGNYNDPRKGKGFLIAMAAITFIMALVVFIIIVSHQTLCQSRRFYLALVILCAILALMMLIATIVYLVAVNPMAQSSS
SIYAMQIQGLCAQYQQPAQS
GVFVNQYLYHYCVVEPQEVRGPKMSRGSEIPRGPLFVLLFGFLNPFWVYVLLWAIAIVCGFLVTAALIIMMVFALKTRQK
IRGYGKSNILWKKVKVVDDI
PPPGDVEAW
>g58.t3
PAYSYYPDDEFQHFYRWTSPPGIIKIMAVIVIVLCLATFACVASTLAWDTQGGLSGFGGGGFGGGSYGTGFGAGGYGAGY
PSGGSFGNTGYGAGNSYGYG
NIGGNYNDPRKGKGFLIAMAAITFIMALVVFIIIVSHQTLCQSRRFYLALVILCAILALMMLIATIVYLVAVNPMAQSSS
SIYAMQIQGLCAQYQQPAQS
GVFVNQYLYHYCVVEPQEAIAIVCGFLVTAALIIMMVFALKTRQKIRGYGKSNILWKKVKVVDDIPPPGDVEAWVG
>g59.t1
LALCERSLLAQMPALQLHGSQTVLEPVAQWDSPMQVKLSCWKGGLNTLLVELLPWALLANHSRWDLWLFEGETIVLQIPA
GKVIVPPNF
>g59.t2
LCERSLLAQMPALQLHGSQTVLEPVAQWDSPMQVKLSCWKGGLNTLLVELLPWALLANHSRWDLWLFEGETIVLQIPAGK
VIVPPNF
>g60.t1
MLDMPSRPVTNPRRRVVTQRTHLRELCEGQGSVLRVLMYAPGLGEGRRGAGERSPPGAVAGRTVALNVQPVSELFVYPWL
SGAQLCLGLSVDRCGHETAC

GSSPVDT
>g61.t1
LLRSSETCTSVPPCADFLTSRPQAVRIGNTTSSTLTLSTGAYQGCVLSPLLYSLFTHDCTSTHRSNTVIKFADDTPIIGC
ITGSDESAYRAERVVTTAQR
VTRTELPTIQDLYSQRCRRKAQWTISDHRHPSHSVFMLLPSGRRYRSHRMRTSRIRDSLIPQAIRLLNC
>g61.t2
LLRSSETCTSVPPCADFLTSRPQAVRIGNTTSSTLTLSTGAYQGCVLSPLLYSLFTHDCTSTHRSNTVIKFADDTPIIGC
ITGSDESAYRAERVVTTAQR
VTRTELPTIQDLYSQRCRRKAQWTISDHRHPSHSVFMLLPSGR
>g62.t1
MDRISRRSRGEENVQFGNLRIASLLFADDVVLLASSDCDLQQALGRFSAKCEAVGMRVSTSKSEAMVLCRKTVDCSRRVG
SELFPK
>g63.t1
MGTSGGIGLEPGTVVITKQSVDARFFLPKYEQVILGKTVVHSTDLDADLAEKLLQCSNRLNQFQAVIANTMCTMGFYEGQ
ARLDGAFCSYTDKDKQDYLA
KAYASGVRNIEMESSVFAAMCKQSGLK
>g63.t2
MGTSGGIGLEPGTVVITKQSVDARFFLPKYEQVILGKTVVHSTDLDADLAEKLLQCSNRLNQFQAVIANTMCTMGFYEGQ
ARLDGAFCSYTDKDKQDYLA
KAYASGVRNIEMESSVFAAMCKQSGLKGFS
>g63.t3
MGTSGGIGLEPGTVVITKQSVDARFFLPKYEQVILGKTVVHSTDLDADLAEKLLQCSNRLNQFQAVIANTMCTMGFYEGQ
ARLDGAFCSYTDKDKQDYLA
KAYASGVRNIEMESSVFAAMCKQSGLKAAVVCVTLLDRQKGDQLSSSHEVLLNLQQRPQILVGHFIKSQLGSSGKV
>g64.t1
ESILTSSITIWYAAATAKDKGRLQRVIRSAEKVIGCNLPSLQDLYTSRTLKRAGKIVADPSHPGHKLFETLPSGRRLRSI
RTKTSRHKNSFFPSATSLIN
KARNPPPHPDLHVTVT
>g65.t1
LKQEAHTFSFMTHISINFRTLSESSVPVSTNLTHLRSAASRRAAVPQGSVWPWCPSPVYFSQPLMKGLRKATYHRGTSQV
EHVWVNI
>g66.t1
MIPPDAKESMDRSKIGLKGPLKTPIAAGHPSMNLLLRKTFDLYANVRPCVSIEGYKTPYTDVNLVTIRENTEGEYSGIEH
VIVDGVVQSIKLITEDASRR
IAEYAFEYARNNQRSSVTAVHKANIMRMSDGLFLRKCREVAETYKDIKFTEMYLDTVCLNMVQDPTQFDVLVMPNLYGDI
LSDLCAGLIGGLGVTPSGNI
GANGVAIFESVHGTAPDIAGLDMANPTALLLSAVMMLRHMGLHDYGKKIEVACFDTIRDKKVLTKDLGGNSKCSEFTADI
CRRVQDMD
>g66.t2
MIPPDAKESMDRSKIGLKGPLKTPIAAGHPSMNLLLRKTFDLYANVRPCVSIEGYKTPYTDVNLVTIRENTEGEYSGIEH
VIVDGVVQSIKLITEDASRR
IAEYAFEYARNNQRSSVTAVHKANIMRMSDGLFLRKCREVAETYKDIKFTEMYLDTVCLNMVQDPTQFDVLVMPNLYGDI
LSDLCAGLIGGLGVTPSGNI
GANGVHGTAPDIAGLDMANPTALLLSAVMMLRHMGLHDYGKKIEVACFDTIRDKKVLTKDLGGNSKCSEFTADICRRVQD
MD
>g66.t3
MIPPDAKESMDRSKIGLKGPLKTPIAAGHPSMNLLLRKTFDLYANVRPCVSIEGYKTPYTDVNLVTIRENTEGEYSGIEH
VIVDGVVQSIKLITEDASRR
IAEYAFEYARNNQRSSVTAVHKANIMRMSDGLFLRKCREVAETYKDIKFTEMYLDTVCLNMVQDPTQFDVLVMPNLYGDI
LSDLCAGLIGGLGVTPSGNI
GANGVAIFESVHGTAPDIAGLDMANPTALLLSAVMMLRHMGLHDYGKKIEVACFDTIRDKKRLKPESMRELTWTGQQQAP
CRGLSEVLTKDLGGNSKCSE
FTADICRRVQDMD
>g67.t1
LRPPLRASIPLASDHQQHCARTLSGTAPRAPEWNYTRTSSRNTTSNSKMADCLSCGPGYKTPLDAMKGPREEIVYLPCIY
RNTEIMKPDYLATVDVDPKS

PTYCQVIHRLEMPNLRDELHHSGWNACSSCYGDASKSRSLLILPCLISSRVYVVDVATDARAPRIHKTVEPTELMWKCDL
ANPHTSHCLGSGQIMLSCIG
DPSGNGKGGFVLLDGESFEVLGNWEHPGDATAFGYDFWYQPRHNVMISTEWGAPKSLNKGFDLQHVKEGLYGTSIHVWDW
TSHKKLQSLDLGAEGAVPLE
VRFLHEPSAPEGYVGCALGSTVYRFFKTQKGDWATELVIKIPSKKVEGWALPEMPSLITDILISLDDRFLYFSNWLHGDI
RQYDITDRRAPRLVGQAFLG
GSIQNDGPVRVLEDPENQPQPPPCIIKGKRIQGAPQMLQLSLDGKRLYVTTSLYSGWDKQFYPNMLKEGSVMMQLDVDTV
NGGITLNKDFLVDFGKEPHG
PVLAHELRYPGGDCTSDIWL
>g67.t2
LSRDRTLSSTPIRQDWAPTTLRPPLRASIPLASDHQQHCARTLSGTAPRAPEWNYTRTSSRNTTSNSKMADCLSCGPGYK
TPLDAMKGPREEIVYLPCIY
RNTEIMKPDYLATVDVDPKSPTYCQVIHRLEMPNLRDELHHSGWNACSSCYGDASKSRSLLILPCLISSRVYVVDVATDA
RAPRIHKTVEPTELMWKCDL
ANPHTSHCLGSGQIMLSCIGDPSGNGKGGFVLLDGESFEVLGNWEHPGDATAFGYDFWYQPRHNVMISTEWGAPKSLNKG
FDLQHVKEGLYGTSIHVWDW
TSHKKLQSLDLGAEGAVPLEVRFLHEPSAPEGYVGCALGSTVYRFFKTQKGDWATELVIKIPSKKVEGWALPEMPSLITD
ILISLDDRFLYFSNWLHGDI
RQYDITDRRAPRLVGQAFLGGSIQNDGPVRVLEDPENQPQPPPCIIKGKRIQGAPQMLQLSLDGKRLYVTTSLYSGWDKQ
FYPNMLKEGSVMMQLDVDTV
NGGITLNKDFLVDFGKEPHGPVLAHELRYPGGDCTSDIWL
>g67.t3
LRPPLRASIPLASDHQQHCARTLSGTAPRAPEWNYTRTSSRNTTSNSKMADCLSCGPGYKTPLDAMKGPREEIVYLPCIY
RNTEIMKPDYLATVDVDPKS
PTYCQVIHRLEMPNLRDELHHSGWNACSSCYGDASKSRSLLILPCLISSRVYVVDVATDARAPRIHKTVEPTELMWKCDL
ANPHTSHCLGSGQIMLSCIG
DPSGNGKGGFVLLDGESFEVLGNWEHPGDATAFGYDFWYQPRHNVMISTEWGAPKSLNKGFDLQHVKEGLYGTSIHVWDW
TSHKKLQSLDLGAEGAVPLE
VRFLHEPSAPEGYVGCALGSTVYRFFKTQKGDWATELVIKIPSKKVEGWALPEMPSESLTIGYAKSMPCSLITDILISLD
DRFLYFSNWLHGDIRQYDIT
DRRAPRLVGQAFLGGSIQNDGPVRVLEDPENQPQPPPCIIKGKRIQGAPQMLQLSLDGKRLYVTTSLYSGWDKQFYPNML
KEGSVMMQLDVDTVNGGITL
NKDFLVDFGKEPHGPVLAHELRYPGGDCTSDIWL
>g68.t1
MEEGIQSFTEFTRKQVEFHRIPLSAVAGVVRLSIFLDADLLKKTSEGAWPTHALSLSGTGTPLMTAYLTVLADGTLLPTL
VLLWDPSRAEGPLGDGPPSA
PPSVLVETHPQGFSSAGGLELWVKRVWRPHASSPGQHRKRLLVLDRHRDHVAEPFLAALGQTRTLPVLIPPGCSLRLQPL
RVVTLPVLQRLLLTRWEEFT
SREGFLEGGASRRVMAAGLPHTLTGWLKDALVILQDRRVLLKNSFRLTGLVPEEGEKSQSRSQLISSLAEDMLGPGVLEP
EQPAETVPMEMEEPEEREGA
LETVKAIEKEGPNQGNAGGPSKEQPADTSLETREQVESEEEL
>g69.t1
MLVYKQTPRVAEQPHVTQVTVRDVIKGPADLQRSAELRQDADEGSTGAVQDRVDQPMRSPRSSEDRVDQPMRSPRSSEDR
VDQPMRSPRSSEDRVDQPMR
SPRSSEDRVDQPMRSPRSSEDRVDQPMRSPRSSEDRVDQPMRSPRSSEDRVDQPMRRRGGLAPDRASPRRASSSVVLGFC
RPEVCRRGAGDVVLRPSGAS
FLSASCSLLGNRLGTEHLLWACSGVLQLKKESVGRAAAAAQRTERPDGAGRPLTVGSAGSIPLPSSGCPHV
>g69.t2
MLVYKQTPRVAEQPHVTQVTVRDVIKGPADLQRSAELRQDADEGSTGAVQDRVDQPMRSPRSSEDRVDQPMRSPRSSEDR
VDQPMRSPRSSEDRVDQPMR
SPRSSEDRVDQPMRSPRSSEDRVDQPMRSPRSSEDRVDQPMRSPRSSEDRVDQPMRSSTFNSHCFASGGAMEGSVTHHTD
CIRRGGLAPDRASPRRASSS
VVLGFCRPEVCRRGAGDVVLRPSGASFLSASCSLLGNRLGTEHLLWACSGVLQLKKESVGRAAAAAQRTERPDGAGRPLT
VGSAGSIPLPSSGCPHV
>g69.t3
MLVYKQTPRVAEQPHVTQVTVRDVIKGPADLQRSAELRQDADEGSTGAVQDRVDQPMRSPRSSEDRVDQPMRSPRSSEDR

VDQPMRSPRSSEDRVDQPMR
SPRSSEDRVDQPMRSPRSSEDRVDQPMRSPRSSEDRVDQPMRSPRSSEDRHCFASGGAMEGSVTHHTDCIRRGGLAPDRA
SPRRASSSVVLGFCRPEVCR
RGAGDVVLRPSGASFLSASCSLLGNRLGTEHLLWACSGVLQLKKESVGRAAAAAQRTERPDGAGRPLTVGSAGSIPLPSS
GCPHV
>g70.t1
EDGGMPRGAFYPTLATITPQDNHDQRIVGGNEAIPGEIPWQVTLMSLSRIPFCGGSLLSEDWVITAAHCLTDQRFTVRDY
IVRV
>g70.t2
EDGGMPRGAFYPTLATITPQDNHDQRIVGGNEAIPGEIPWQVGTETTAEWADGCAAGWSEVTLMSLSRIPFCGGSLLSED
WVITAAHCLTDQRFTVRDYI
VRV
>g70.t3
MPRGAFYPTLATITPQDNHDQRIVGGNEAIPGEIPWQVTLMSLSRIPFCGGSLLSEDWVITAAHCLTDQRFTVRDYIVRV
>g71.t1
PNIHSFLALTHASTCSVCVLYVKSRGALRRHVFDANSCCRLLRFILSPQEAAPVKHIASLRSEEPALLCETVSNKAKTAM
GLRLAA
>g72.t1
RNVLSISTGIEDIGTLKWDLALCLLAVWVICFFCIWKGVKSTGKVVYITATFPFVMLIILLVRGVTLPGASEGIRFYLYP
DLTRLQDPEVWIDAGTQIFF
SYAICLGAMTSLGSYNKYKYNCYRDCLLLGGLNSGTSFVSGFAIFSVLGFMAQEQGVDIADVAESDAGWVWMVHVAERTV
LCSKPSKSCDKLRIC
>g72.t2
RNVLSISTGIEDIGTLKWDLALCLLAVWVICFFCIWKGVKSTGKVVYITATFPFVMLIILLVRGVTLPGASEGIRFYLYP
DLTRLQDPEVWIDAGTQIFF
SYAICLGAMTSLGSYNKYKYNCYRDCLLLGGLNSGTSFVSGFAIFSVLGFMAQEQGVDIADVAESVRETLPHVHMIIDAG
WVWMVHVAERTVLCSKPSKS
CDKLRIC
>g73.t1
PRDTGRRPDVDTQAGSAALRATEPQPAAGPEDTGLQEHSDDNCLYCSGLSVLGLKVSHGSRPDLVDGPAFSDWCSTSSHH
LEAVVQARLSGISGSTLSQG
SGGSGHSAELVL
>g73.t2
PRDTGRRPDVDTQAGSAALRATEPQPAAGPEDTGLQEHSDDNCLYCSGLSVLGLKVSHGSRPDLVDGPAFSDWCSTSSHH
LEAVVQARLSGISGSTLSQG
SGGSGHSAELVLGKGPS
>g74.t1
LLSAQSLGWFYVNVKCRFKRFGSAKVLKTPYRKHLAERHALAELSVLQDYCSETENYFHLKQKHDMM
>g75.t1
MWKAAVVTLGLLVMLVGRVRSNEGHPSFYGVKLCGREFIRAVIFTCGGSRWRRSVGDSGIIDEESFDPWNMPQLSGEQQP
SEAQVWKGETMDTASTGSGF
RRFARSPISEEVLEALRSADRKGRDVVVGLSNACCKWGCSKNEPTRSLSGLILKNNVKAHYQNFPPTVADFIKRECLNNI
GDPSPLIRATIGILITTIAS
KGELQMWPELLPQLCNLLNSEDYNTCEGSFGALQKICEDSSELLDSEALNRPLNIMIPKFLQFFKHCSPKIRSHAIACVN
QFIIGRAQALMDNIDTFIES
LFALAGDEDSEVRKNVCRALVMLLEVRIDRLIPHMHSIIQ
>g75.t2
MWKAAVVTLGLLVMLVGRVRSNEGHPSFYGVKLCGREFIRAVIFTCGGSRWRRSVGDSGIIDEESFDPWNMPQLSGEQQP
SEAQVWKGETMDTASTGSGF
RRFARSPISEEVLEALRSADRKGRDVVVGLSNACCKWGCSKNEPTRSLSGLILKNNVKAHYQNFPPTVADFIKRECLNNI
GDPSPLIRATIASKGELQMW
PELLPQLCNLLNSEDYNTCEGSFGALQKICEDSSELLDSEALNRPLNIMIPKFLQFFKHCSPKIRSHAIACVNQFIIGRA
QALMDNIDTFIESLFALAGD
EDSEVRKNVCRALVMLLEVRIDRLIPHMHSIIQ
>g76.t1
IIALTRPLDSWLEHVNFATLFSCLSNKEILLVFSAAVLERRIVFIAEELGTLSQVNHAISALLYPFTWQHTFISIVPSIL

IDVVMAPTPYLLGVQKKLLQ
RMDDTTDMLLVDLSENRKQTFIAAIGDEASILPPKLHNEILLALENKKEKATVEELNHVVSEAFLTFFVKTVGHYASFMT
PGGGAEACRTFQKRAFYKAI
KSKTTRHFVKKFIQTQMFDIFIQEVEQRQSSSQQGMSARRVHRFDSPKAAEIAERQSPGLTCSVSPYFFEGIFHQKIQEY
KKNVKAEKKKKKR
>g76.t2
IIALTRPLDSWLEHVNFATLFSCLSNKEILLVFSAAVLERRIVFIAEELGTLSQVNHAISALLYPFTWQHTFISIVPSIL
IDVVMAPTPYLLGVQKKLLQ
RMDDTTDMLLVDLSENRKQTFIAAIGDEASILPPKLHNEILLALENKKEKATVEELNHVVSEAFLTFFVKTVGHYASFMT
PGGGAEACRTFQKRAFYKAI
KSKTTRHFVKKFIQTQMFDIFIQEVEQRQSSSQQGIFHQKIQEYKKNVKAEKKKKKR
>g76.t3
IIALTRPLDSWLEHVNFATLFSCLSNKEILLVFSAAVLERRIVFIAEELGTLSQVNHAISALLYPFTWQHTFISIVPSIL
IDVVMAPTPYLLGVQKKLLQ
RMDDTTDMLLVDLSENRKQTFIAAIGDEASILPPKLHNEILLALENKKEKATVEELNHVVSEAFLTFFVKTVGHYASFMT
PGGGAEACRTFQKRAFYKAI
KSKTTRHFVKKFIQTQMFDIFIQEVEQRQSSSQQ
>g77.t1
MGDMKTPDFDDLLAAFDIPDIDAKEAIQSSPEEDEGQKANSGKSRTEVSQQPQAPISTHQKGSTLLQEEEYPEHVIDDRD
SPESPPPDQTGYAIPKRSTS
PVQTATFSQGTCLDKVEPMDSDLSQDSRTGDVNKTDGDKMEVDYDGKMDKESLTADSSTAAIVSTTTAATDRVSSPSRPL
KVKIKMVKTPTGTITRTVTR
PAAKGAGRAASKATAGSTPPAPPAPPASLTGKPKTDGSQQFLKKTGKGGESTATVSVLQDASTAMLVAASKVKSSKGSVD
ARAKVSAAAVSITKSAALPA
ISSSPRFRSAGANMRSLGHKTLTSGLAVSSPTALQPPQGSSRPASIVNNTGAIISKSQSNLVEAFNKILNSKNLLPSYKP
DLTSPPPAEWGLPLPAQGYR
CLECGDAFALERSLARHYDRRSLRIEVTCNHCAKRLAFFNKCSLLLHAREHKERGLPNSSSVNSSSAAGLPSSSAPGAST
FSAAAQPQTPATAGRKAAEA
VQYTDNKCPECLLQFCSKEEVAAHFQEVNPAFCTPCKDCTPPILLSNGCSTSAHQRIHKACAPHVCPECGGTSCSQSAFQ
THLEESCLHFTRRIGYRCSS
CQVVFGGLMSVKSHIQMAHCDMFHKCPSCPMAFKSSPSIQSHIAAQHPTLTGGQAKLIYKCVMCDTVFTQKPLLYVHFDT
HLANQTVHVFKCPECPKLYS
QRSSLVDHIAVTPCLG
>g77.t2
MGDMKTPDFDDLLAAFDIPDIDAKEAIQSSPEEDEGQKANSGKSRTEGGSPSSFPCSPVSQSNPPVVSGSISLKQHCSPE
NESGKLPGAPRLSSLLTSLK
PELHPDELTSTSSTPPLVSQQPQAPISTHQKGSTLLQEEEYPEHVIDDRDSPESPPPDQTGYAIPKRSTSPVQTATFSQG
TCLDKVEPMDSDLSQDSRTG
DVNKTDGDKMEVDYDGKMDKESLTADSSTAAIVSTTTAATDRVSSPSRPLKVKIKMVKTPTGTITRTVTRPAAKGAGRAA
SKATAGSTPPAPPAPPASLT
GKPKTDGSQQFLKKTGKGGESTATVSVLQDASTAMLVAASKVKSSKGSVDARAKVSAAAVSITKSAALPAISSSPRFRSA
GANMRSLGHKTLTSGLAVSS
PTALQPPQGSSRPASIVNNTGAIISKSQSNLVEAFNKILNSKNLLPSYKPDLTSPPPAEWGLPLPAQGYRCLECGDAFAL
ERSLARHYDRRSLRIEVTCN
HCAKRLAFFNKCSLLLHAREHKERGLPNSSSVNSSSAAGLPSSSAPGASTFSAAAQPQTPATAGRKAAEAVQYTDNKCPE
CLLQFCSKEEVAAHFQEVNP
AFCTPCKDCTPPILLSNGCSTSAHQRIHKACAPHVCPECGGTSCSQSAFQTHLEESCLHFTRRIGYRCSSCQVVFGGLMS
VKSHIQMAHCDMFHKCPSCP
MAFKSSPSIQSHIAAQHPTLTGGQAKLIYKCVMCDTVFTQKPLLYVHFDTHLANQTVHVFKCPECPKLYSQRSSLVDHIA
VTPCLG
>g77.t3
MGDMKTPDFDDLLAAFDIPDIDAKEAIQSSPEEDEGQKANSGKSRTEGGSPSSFPCSPVSQSNPPVVSGSISLKQHCSPE
NESVSQQPQAPISTHQKGST
LLQEEEYPEHVIDDRDSPESPPPDQTGYAIPKRSTSPVQTATFSQGTCLDKVEPMDSDLSQDSRTGDVNKTDGDKMEVDY
DGKMDKESLTADSSTAAIVS
TTTAATDRVSSPSRPLKVKIKMVKTPTGTITRTVTRPAAKGAGRAASKATAGSTPPAPPAPPASLTGKPKTDGSQQFLKK

TGKGGESTATVSVLQDASTA
MLVAASKVKSSKGSVDARAKVSAAAVSITKSAALPAISSSPRFRSAGANMRSLGHKTLTSGLAVSSPTALQPPQGSSRPA
SIVNNTGAIISKSQSNLVEA
FNKILNSKNLLPSYKPDLTSPPPAEWGLPLPAQGYRCLECGDAFALERSLARHYDRRSLRIEVTCNHCAKRLAFFNKCSL
LLHAREHKERGLPNSSSVNS
SSAAGLPSSSAPGASTFSAAAQPQTPATAGRKAAEAVQYTDNKCPECLLQFCSKEEVAAHFQEVNPAFCTPCKDCTPPIL
LSNGCSTSAHQRIHKACAPH
VCPECGGTSCSQSAFQTHLEESCLHFTRRIGYRCSSCQVVFGGLMSVKSHIQMAHCDMFHKCPSCPMAFKSSPSIQSHIA
AQHPTLTGGQAKLIYKCVMC
DTVFTQKPLLYVHFDTHLANQTVHVFKCPECPKLYSQRSSLVDHIAVTPCLG
>g78.t1
MTPYLAHSMKMLEQYSHNKYRQAYDLSKGTPPAHIADTPEMVRIRKAQEQLSEVKYRMEGNKARTGSLYDGEAKEVAHVK
HMSELLSKVLYRQKWDETKD
RYLLPPDAPELVLAVKNAANYSKKLYTEDWDLEKAGFLPYSDSPELRRVAKAQEALSDIKYKKGHDQRKLKFTSLADPPE
VELAKKVFTQRSDLKYKEDY
NKNVKGQWCETPYFDVATARVAMENLSNRRYTQEYEDTKDQIYFMQTDTPVYDTNKKAGTSASSVKYKKDYEKTKDKSGY
NTLPATENPLLRQLRYAGTI
LSDKVYKANYEKSRGFSINYCDTPKFQMDSVLKKFTDQNYKAEYEDIKTRCFFPQTVTPEYEADKKLQQCKDLNYKADYE
QIKVKNCLPADYPFFIQSRV
NAFNLSENCYKYNWEKDKAKQFEIKGDAIPILAARAHTNIASDVKYKKEYEKNRGHMVGALSIHDDPKILHSVHVAKIQS
DREYKKDYEKVKTKYHTSLD
MMSVTLAKKSQAIASMTGYKKISNRYLMPYDSVLLDLAKKANSIQSDNEYKADYNTYTKGTPWIPFGSMDVEKAKKAGEI
LNEKKYRQHPDTIPFTAIDD
HPVMLQARVNQLQRSDLFYKKDLEEIHQKYSLPADIPEFQQARCNAFNISKNYYKMAWEETIAKGYDLRPDAISIKAAKA
ARHAASDVQYKKAYEKARGH
HVGFRNVKDDPLLVHYMQVAKMQSEKNYKKDYHKAKLKYHSPVDMMSLVHAKQASKAQTNAGYRNIEHHYNLMPDAMNLV
L
>g78.t2
SGLPTRKVRKRVQVDTSKFMTPYLAHSMKMLEQYSHNKYRQAYDLSKGTPPAHIADTPEMVRIRKAQEQLSEVKYRMEGN
KARTGSLYDGEAKEVAHVKH
MSELLSKVLYRQKWDETKDRYLLPPDAPELVLAVKNAANYSKKLYTEDWDLEKAGFLPYSDSPELRRVAKAQEALSDIKY
KKGHDQRKLKFTSLADPPEV
ELAKKVFTQRSDLKYKEDYNKNVKGQWCETPYFDVATARVAMENLSNRRYTQEYEDTKDQIYFMQTDTPVYDTNKKAGTS
ASSVKYKKDYEKTKDKSGYN
TLPATENPLLRQLRYAGTILSDKVYKANYEKSRGFSINYCDTPKFQMDSVLKKFTDQNYKAEYEDIKTRCFFPQTVTPEY
EADKKLQQCKDLNYKADYEQ
IKVKNCLPADYPFFIQSRVNAFNLSENCYKYNWEKDKAKQFEIKGDAIPILAARAHTNIASDVKYKKEYEKNRGHMVGAL
SIHDDPKILHSVHVAKIQSD
REYKKDYEKVKTKYHTSLDMMSVTLAKKSQAIASMTGYKKISNRYLMPYDSVLLDLAKKANSIQSDNEYKADYNTYTKGT
PWIPFGSMDVEKAKKAGEIL
NEKKYRQHPDTIPFTAIDDHPVMLQARVNQLQRSDLFYKKDLEEIHQKYSLPADIPEFQQARCNAFNISKNYYKMAWEET
IAKGYDLRPDAISIKAAKAA
RHAASDVQYKKAYEKARGHHVGFRNVKDDPLLVHYMQVAKMQSEKNYKKDYHKAKLKYHSPVDMMSLVHAKQASKAQTNA
GYRNIEHHYNLMPDAMNLVL
>g78.t3
MTTTTVTHHTTETQSGLPTRKVRKRVQVDTSKFMTPYLAHSMKMLEQYSHNKYRQAYDLSKGTPPAHIADTPEMVRIRKA
QEQLSEVKYRMEGNKARTGS
LYDGEAKEVAHVKHMSELLSKVLYRQKWDETKDRYLLPPDAPELVLAVKNAANYSKKLYTEDWDLEKAGFLPYSDSPELR
RVAKAQEALSDIKYKKGHDQ
RKLKFTSLADPPEVELAKKVFTQRSDLKYKEDYNKNVKGQWCETPYFDVATARVAMENLSNRRYTQEYEDTKDQIYFMQT
DTPVYDTNKKAGTSASSVKY
KKDYEKTKDKSGYNTLPATENPLLRQLRYAGTILSDKVYKANYEKSRGFSINYCDTPKFQMDSVLKKFTDQNYKAEYEDI
KTRCFFPQTVTPEYEADKKL
QQCKDLNYKADYEQIKVKNCLPADYPFFIQSRVNAFNLSENCYKYNWEKDKAKQFEIKGDAIPILAARAHTNIASDVKYK
KEYEKNRGHMVGALSIHDDP
KILHSVHVAKIQSDREYKKDYEKVKTKYHTSLDMMSVTLAKKSQAIASMTGYKKISNRYLMPYDSVLLDLAKKANSIQSD

NEYKADYNTYTKGTPWIPFG
SMDVEKAKKAGEILNEKKYRQHPDTIPFTAIDDHPVMLQARVNQLQRSDLFYKKDLEEIHQKYSLPADIPEFQQARCNAF
NISKNYYKMAWEETIAKGYD
LRPDAISIKAAKAARHAASDVQYKKAYEKARGHHVGFRNVKDDPLLVHYMQVAKMQSEKNYKKDYHKAKLKYHSPVDMMS
LVHAKQASKAQTNAGYRNIE
HHYNLMPDAMNLVL
>g79.t1
MKLLHNCWSELLVLDHIFRQVHHGKDDSLLLVTGQEVELSCIVSQADGTLSGLVQRSQELAARLIALQVDRREVACLKFL
LLFNP
>g80.t1
YHIRRHSAQEVSGGVHEYSSFGDTKRGMVRTLSLCRLGSNHHCSLCIKCKPRRTTRATLTTGSCNLVSTNPIKVKQTIKS
LLVGGGEKDRDGRAQVGGSF
YCFPGALLEDWSPHCCCCVGGSGGGSVLLLWERERPRFLLVSREEPARIAPNPGLVLSGRQLVGEDLSEPRME
>g80.t2
MNGYHIRRHSAQEVSGGVHEYSSFGDTKRGMVRTLSLCRLGSNHHCSLCIKCKPRRTTRATLTTGSCNLVSTNPIKVKQT
IKSLLVGGGEKDRDGRAQVG
GSFYCFPGALLEDWSPHCCCCVGGSGGGSVLLLWERERPRFLLVSREEPARIAPNPGLVLSGRQLVGEDLSEPRME
>g80.t3
CDARTAGYHIRRHSAQEVSGGVHEYSSFGDTKRGMVRTLSLCRLGSNHHCSLCIKCKPRRTTRATLTTGSCNLVSTNPIK
VKQTIKSLLVGGGEKDRDGR
AQVGGSFYCFPGALLEDWSPHCCCCVGGSGGGSVLLLWERERPRFLLVSREEPARIAPNPGLVLSGRQLVGEDLSEPRME
>g81.t1
MPRFTRVQCYLTRAAEDLSSLKVPSTAAVNNVALLSALSAMTVGKESFSWGDVEQVARFSGAILQLCQPVAVTTGRQMVW
GNMGHGAM
>g82.t1
GRGGRTTAWSLINQTRPPPPSTTAGSCGCYPSQSNGVITATLVTHHHSVKLPEETARPASVFRPDTGLWLLIMAVFEL
>g83.t1
ESILTSSITIWYAAATAKDKGRLQRVIRSAEKVIGCNLPSLQDLYTSRTLKRAGKIVADPSHPGHKLFETLPSGRRLRSI
RTKTSRHKNSFFPSATSLIN
KARNPPPHPDLHVTVT
>g84.t1
RRTLSEDTIRVFLQQISRAMKILQSRGIIHRDLKPQNILLCSPPGRRCSPSNACLKIADFGFARHLQTNTMAATLCGSPM
YMAPEVIMSQNYDAKADLWS
IGTVVYQCLTGKAPFY
>g84.t2
LSEDTIRVFLQQISRAMKILQSRGIIHRDLKPQNILLCSPPGRRCSPSNACLKIADFGFARHLQTNTMAATLCGSPMYMA
PEVIMSQNYDAKADLWSIGT
VVYQCLTGKAPFY
>g85.t1
LKTLCWPVLMSSSPLSCLTWLQNFCKNKGQVGWELDTQPLQGYNFEVMVRLQSQSLHRLHTFHQAAHHPMSPARHPMDLS
LVVHTATRKVNWEGSTWDPD
GGCWVDILAPFPITDSGNCYVLVTMDYFRKWPEAPDQNVVTTGQRLVEYILP
>g86.t1
LWPRPRGSDYALLSAEGREWGSTEGTSTLLHPPSQRIHLPVRLWRLQPRTMKVSSAVLALLTRVFFLGGACQAINVTVLP
APFSAAQEGANVTLSCLVSQ
RHRNTGLPVVRWTFLPAGARRPEDEVLVARVNMRKARFYGNYTKSFPWPKLRLTVVKQGKIFDLLVLNISEGDRGLYMCH
AQEFRKHRERWKAFSNATAA
TELRDHNCMTWFI
>g86.t2
MKVSSAVLALLTRVFFLGGACQAINVTVLPAPFSAAQEGANVTLSCLVSQRHRNTGLPVVRWTFLPAGARRPEDEVLVAR
VNMRKARFYGNYTKSFPWPK
LRLTVVKQGKIFDLLVLNISEGDRGLYMCHAQEFRKHRERWKAFSNATAATELRDHNCMTWFI
>g86.t3
LLSAEGREWGSTEGTSTLLHPPSQRIHLPVRLWRLQPRTMKVSSAVLALLTRVFFLGGACQAINVTVLPAPFSAAQEGAN
VTLSCLVSQRHRNTGLPVVR
WTFLPAGARRPEDEVLVARVNMRKARFYGNYTKSFPWPKLRLTVVKQGKIFDLLVLNISEGDRGLYMCHAQEFRKHRERW

KAFSNATAATELRDHNCMTW
FI
>g87.t1
MGDWNLLGSILEEVHVHSTIVGKIWLTILFIFRMLVLGVAAEDVWEDEQTEFVCNTDQPGCKAVCYDRAFPISLIRFWVL
QVIFVSAPSLVYMGHALYRI
RFMDKERHRRRAQLREELGEPEVALDEHRRLERALRRLEDRRRVKKVPLRGSLLRTYVIHILTRSVVEVCFILGQYILYG
LRLEPLYKCERLPCPNSVDC
YISRPTEKTIFMAFMFVIAAVSLFLNVLEISHLGVRKIRQTIYGDKYSEEDSLIYKPKKKRTLQHLCLMRHLSPHNGPLT
QTIFRETPAGGTNAVHENTG
LRANQQTTTTTTSHNSLAPLGHYQTNCNTPGSRVPQNQYRTGQECATQGPQAHEGPEMNSRTLVDQHPLVWPAAPCNVEG
WQKEPTRAPEELLRPMEHPR
VLQTLGYSMAHRTSFAARDMQEEDERKDSIGSDLFHLNHRKTSFMAKPPSESMSTISGSSSPSIHSSEESDELGSLQGDM
PMMPPPGGRRMSMSVFLDIS
SIMKK
>g88.t1
MSLFSWTSPDTLSTERTMILGGNSLSRPNQPLPMGSGKVIQAIIPMRLQEVAVGEPSARGRCQRCLHTWEPVPKALTGTV
TQNRLTPNVASLLHGSFWVK
SCSA
>g88.t2
MILGGNSLSRPNQPLPMGSGKVIQAIIPMRLQEVAVGEPSARGRCQRCLHTWEPVPKALTGTVTQNRLTPNVASLLHGSF
WVKSCSA
>g88.t3
MSLFSWTSPDTLSTERTMILGGNSLSRPNQPLPMGSGKVIQAIIPMRLQEVAVGEPSARGRCQRCLHTWEPVPKALTGTV
TQNRLTPNVASLLHGSFWVK
SWYQQH
>g89.t1
MPFPFGKSQKSPAEIVKNLKENVAYMEKLDATESKKCEKVAEEVSKNLSSLKEVLCGTGDKEPQTEAVAQLAQELYNTNL
LIALIANLQRIDFEGKKDVV
HLFSNIVRRQIGTRTPTVEYISSHPQILFMLLKGYESAEVALNCGMMLRECLRHEPLARTVLFSEELYCFFRFVELSTFD
IASDAFASFKVFTEYEKLLH
SDNYVTKRQSLKLLGELLLDRHNFTVMTKYISRAENLKLMMNLLRDNSRNIQFEAFHVFKVFVANPNKTQPVLDILLKNQ
AKLVEFLSHFQTDRSEDEQF
CDEKNYLIKQIRDLKRAPAPEEA
>g89.t2
MPFPFGKSQKSPAEIVKNLKENVAYMEKLDATESKKCEKVAEEVSKNLSSLKEVLCGTGDKEPQTEAVAQLAQELYNTNL
LIALIANLQRIDFEGKKDVV
HLFSNIVRRQIGTRTPTVEYISSHPQILFMLLKGYESAEVALNCGMMLRECLRHEPLARTVLFSEELYCFFRFVELSTFD
IASDAFASFKDLLTRHKIMC
ADFLETNYDRVFTEYEKLLHSDNYVTKRQSLKLLGELLLDRHNFTVMTKYISRAENLKLMMNLLRDNSRNIQFEAFHVFK
VFVANPNKTQPVLDILLKNQ
AKLVEFLSHFQTDRSEDEQFCDEKNYLIKQIRDLKRAPAPEEA
>g90.t1
MSVLVQCPPGLSPMKDGTGCYDHHIGIDCSDGFNGGCEQLCLQQLAPLEDDPTLYNIQMFCGCIEDYRRGQDGRSCQPLS
EICTEGVDCGEPADIPANQT
VFGDLFYGYNNHSKETSSGQILKATFRFPEL
>g90.t2
MKDGTGCYDHHIGIDCSDGFNGGCEQLCLQQLAPLEDDPTLYNIQMFCGCIEDYRRGQDGRSCQPLSEICTEGVDCGEPA
DIPANQTVFGDLFYGYNNHS
KETSSGQILKATFRFPEL
>g90.t3
LSPMKDGTGCYDHHIGIDCSDGFNGGCEQLCLQQLAPLEDDPTLYNIQMFCGCIEDYRRGQDGRSCQPLSEICTEGVDCG
EPADIPANQTVFGDLFYGYN
NHSKETSSGQILKATFRFPEL
>g91.t1
SPAPPTVLPALLLLQPFYCEASLERTETQTSFR
>g92.t1

LLLSRLDVGMVLARDGSGPGWSWPGPGSMANLLTRPAVLRPLTFATEEKQALHSWSRVSSAGQSVVLETLKILGPKLGTP
HPSVPAQLTTRRPNSPQDFL
SPRPNGKQDLAIGSEKVLAGGARPRELPSTQELLIFLQELREEGHQATVLRSKDVYGYRSALSCPLTQDKLRALEKSQRP
TAKRRGRKPPARKREVHPSW
RTAQRSSPRIQGTYPPEAPLCPPSTMSRMAGKADPRSPQPCLRLTSLQGLPGAPTARLQIHTSWEPSSGPQLPQHL
>g92.t2
LSRLDVGMVLARDGSGPGWSWPGPGSMANLLTRPAVLRPLTFATEEKQALHSWSRVSSAGQSVVLETLKILGPKLGTPHP
SVPAQLTTRRPNSPQDFLSP
RPNGKQDLAIGSEKVLAGGARPRELPSTQELLIFLQELREEGHQATVLRSKDVYGYRSALSCPLTQDKLRALEKSQRPTA
KRRGRKPPARKREVHPSWRT
AQRSSPRIQGTYPPEAPLCPPSTMSRMAGKADPRSPQPCLRLTSLQGLPGAPTARLQIHTSWEPSSGPQLPQHL
>g92.t3
LDVGMVLARDGSGPGWSWPGPGSMANLLTRPAVLRPLTFATEEKQALHSWSRVSSAGQSVVLETLKILGPKLGTPHPSVP
AQLTTRRPNSPQDFLSPRPN
GKQDLAIGSEKVLAGGARPRELPSTQELLIFLQELREEGHQATVLRSKDVYGYRSALSCPLTQDKLRALEKSQRPTAKRR
GRKPPARKREVHPSWRTAQR
SSPRIQGTYPPEAPLCPPSTMSRMAGKADPRSPQPCLRLTSLQGLPGAPTARLQIHTSWEPSSGPQLPQHL
>g93.t1
VVLFPFPFPESQRQGADKLPDVLEKGVVLWMGADGLFAKRLCQGRVYWEGPLAPHKDKPNKLEKEHTCKLLDTQQFFSGT
YARALKRCSEMRSH
>g94.t1
MLRHDNLVKCLGGFVDDDRLFGIFELLDHSIVAELNDRCRGLDSDVLRKYTFQILQGARFLHGKNVSSRIFT
>g95.t1
DGAMLCGDESAWLPLKEALPRLERLQAESRQRSCAVQGAVPGSLLGADWKAALDVCGPLLSAAGGAVPDENVHKLRVADM
ALDACINLGIWTQALTYGMK
TLPAYSRESTEETQEGICTCDDIRILPLLVQRKR
>g95.t2
DGAMLCGDESAWLPLKEALPRLERLQAESRQRSCAVQGAVPGSLLGADWKAALDVCGPLLSAAGGAVPDENVHKLRVADM
ALDACINLGIWTQALTYGMK
TLPAYR
>g95.t3
MLDVCRVVTDGAMLCGDESAWLPLKEALPRLERLQAESRQRSCAVQGAVPGSLLGADWKAALDVCGPLLSAAGGAVPDEN
VHKLRVADMALDACINLGIW
TQALTYGMKTLPAYSRESTEETQEGICTCDDIRILPLLVQRKR
>g96.t1
GLPFFILTPQHSPFKRGFFCGDESIGYPVREETISYQLLGGVMIPFTLIVVKSLGVILDSTLSFQSHINSITRSAYFHLR
NINRLRPSLTPHTAAILVHS
LVTSRLDYCNSLLFGLPHKSLHILQLVQNSAARIITRTPSIHHITPVLQRLRWLPVQFRIRYKVLLVTFKAIHNLAPPYL
SDLLHVPTPSRSLRSSSSVH
LSVPSARLTTMGSRAFSHSA
>g97.t1
MQWWRSFDPHFWLSEQETRKQLLLKEAWEEKRRQAQAKKAAAEKTAMEMKERKEQERDVVVKRMEQVKKALEGRKEQREK
IIQETKKEQEKEKEDAQQEK
RAKEQDAEAERKKRQQMVKKAWEERKEKEVQLE
>g97.t2
MQWWRSFDPHFWLSEQETRKQLLLKEAWEEKRRQAQAKKAAAEKTAMEMKERKEQERDVVVKRMEQVKKALEGRKEQREK
IIQETKKEQEKEKEDAQQEK
RAKEQDAEAERKKRQQMVKKAWEERKEKEVQLECTGQAMGF
>g98.t1
FSVQTDNNSLKYVLSTARLDAMGQRFRLSAFDFDVQYRRGQSNANADALSRMSAQEVTQVLHSCPQQVTSSEPEDGAEQP
IKDPVGSTSAQSEPFRPNEP
YGDVGTESLPAMTKQEIRAGQKEGQ
>g99.t1
MFFKKIKGFFYRGCCYHGPGESRGSQGRLSFAKTGTLTVSSSPQAWSNSMTNHKATELGNLETLLVKWLGKDSAEHAKRI
RAIHVNLP
>g99.t2

MFFKKIKGFFYRGCCYHGPGESRGSQGRLSFAKTGTSGTLTVSSSPQAWSNSMTNHKATELGNLETLLVKWLGKDSAEHA
KRIRAIHVNLP
>g99.t3
MFFKKIKGFFYRGCCYHGPGESRGSQGRLSFAKTGTLTVSSSPQAWSNSMTNHKATELGNLETLLVKWLGKDSAEHAKRI
RAIHVNLPDKHKIRTDNSER
LCPIHKKPHPLQKCWAFREKPLEERKAFLK
>g100.t1
YGLQRILGDERSLLLLARAIDPKQTNMMTEIVKILSAVCIIGEENM
>g101.t1
MTRVCLLVLTTCFLLDNYGLEAATLDAQTGGVLLPRPAASAVLRRQRRYNTGRLEEIHKDNLERECIEERCNFEEAREVF
ENNEKTVGRLGPDTLLSRSG
FGPRADN
>g101.t2
MTRVCLLVLTTCFLLDNYGLEAATLDAQTGGVLLPRPAASAVLRRQRRYNTGRLEEIHKDNLERECIEERCNFEEAREVF
ENNEKTV
>g101.t3
MTRVCLLVLTTCFLLDNYGLEAATLDAQTGGVLLPRPAASAVLRRQRRYNTGRLEEIHKDNLERECIEERCNFEEAREVF
ENNEKTVDGILG
>g102.t1
MLNENTDYIKKRYMTYEANVRDTPSSQQDAVLGALRTNRALLLEQLQSDKSLQEAWALREAEWTGATWFCSGMEEDVPWA
FAQECFLLLLALARHLSTQM
ALFEQTPPSPVANRHTPEAAPPLPPDVLSVSQQKTLAAALQFTVSLGLCAHLAPGVGVSLEQRSAFGPALAGLMRRNGSE
PPAERRLLTATRVLLRLSEL
PSLATLVFTRHLGDLVAALCQLGHQPQCSPGAK
>g103.t1
MMHRKDMFAVCVLQILVLGVMFATAKPSSLPCFLEDELEVKAKKRLNHLFDQASHPEPGSTTAQCPVGLYAGLGRQLNDR
SISPWRYVSVTNTDRFPQTI
VVAQCLCSGCITIKREDNTPTENFTYNSVAVEQSKTFLKKELCSDGKRYRLVPYSETVAVGCTCAKPKN
>g103.t2
MMHRKDMFAVCVLQASHPEPGSTTAQCPVGLYAGLGRQLNDRSISPWRYVSVTNTDRFPQTIVVAQCLCSGCITIKREDN
TPTENFTYNSVAVEQSKTFL
KKELCSDGKRYRLVPYSETVAVGCTCAKPKN
>g103.t3
MFATAKPSSLPCFLEDELEVKAKKRLNHLFDQASHPEPGSTTAQCPVGLYAGLGRQLNDRSISPWRYVSVTNTDRFPQTI
VVAQCLCSGCITIKREDNTP
TENFTYNSVAVEQSKTFLKKELCSDGKRYRLVPYSETVAVGCTCAKPKN
>g104.t1
PPVTPQRRPSATELLLSPILQFVSQSQKAFEYLSPTPAADEDEGRLMAEIAED
>g105.t1
RNCPAARSLTILVQGTLTARAEPGRPYIGPSLGPGRRPDTARINRGGELMESEMARWRPLKRCPENYFTAV
>g106.t1
MSDLPSVRTGPAAPFEYTTLDLFGPYTVRDGVRRRTKKKVWGVVFSCMASRAVHADLVEDLSTEGFLKAYQRFTALRGHP
KKLWSDQGTNFVGTRPALED
LYSFLACIDKGEVQRRAVVAGTDWVWEFSPADSPHRNGAAEAAVRVLKRSLSSVGEGGDLTILEFQTLLYLAANLSNERP
VGARAQVQEETVDVVTPNSL
LLGRAGPRGDTQGFEFPTYPFSRLRAVQVEVDKFWRRWSQLAGPHLFVRQKWHTPSRNVSAGDLVWVADQNALRARFRLG
RVVEACPDDKGVVRDVKVKV
CHSLPICLAQARKDKEPCPSTILHRDVRRLVVLLPVEEQEVA
>g107.t1
MPNLKLISATDTQYSTAVLKSMNDQRNHGLFCDVTIIIQDKKFRAHKTILSASSTYFHQLFSVAGQVIELNFIKAEIFEQ
ILNYIYSSKIIRVRSDMLEE
LISAGQILGVKFIANLGQLLSQVKGLPGLSKDTESKSGASTKVMPIITESFSISAEEFNQTCKSGEDDGDSDNDVMFVSE
TETAKAAETPKSLPGVIDLD
GGAWEDAAAAAAASNKKLEPSAAGAAGPPPELEDA
>g107.t2
MLEELISAGQILGVKFIANLGQLLSQVKGLPGLSKDTESKSGASTKVMPIITESFSISAEEFNQTCKSGEDDGDSDNDVM

FVSETETAKAAETPKSLPGV
IDLDGGAWEDAAAAAAASNKKLEPSAAGAAGPPPELEDA
>g108.t1
MPIYDRSSDRGFDGKDHSPVRTRRRTRSRSRSRSGSHGGRSLSPDYFGPKLPKPRNRERERЕEHEHRLREARNIKRIRIG
SPRRSRSRSRERHNHWSDQR
GGFKDDYYDQQRDDAQRQRQEAFIARSKKSKKKKVKKTRKQSSGSSSEDSGGEEEEEEAGDEEEDGGVVWVEKTCMDENL
VGPEAPLTQMSQDDKPLDFG
HALLPGEGAAMAEYVKAGKRIPRRGEIGLTSDEIANFEKSGFVMSGSRHRRMEAVRLRKENQIYSADEKRALASFNQEER
RKRESKILSSFREMVYRKTK
GKDEK
>g108.t2
MPIYDRSSDRGFDGKDHSPVRTRRRTRSRSRSRSGSHGGRSLSPDYFGPKLPKPRNRERERЕEHEHRLREARNIKRIRIG
SPRRSRSRSRERHNHWSDQR
GGFKDDYYDQQRDDAQRQRQEAFIARRLQERERIGELGSPEVWGYSPRVKEPESKKSKKKKVKKTRKQSSGSSSEDSGGE
EEEEEAGDEEEDGGVVWVEK
TCMDENLVGPEAPLTQMSQDDKPLDFGHALLPGEGAAMAEYVKAGKRIPRRGEIGLTSDEIANFEKSGFVMSGSRHRRME
AVRLRKENQIYSADEKRALA
SFNQEERRKRESKILSSFREMVYRKTKGKDEK
>g109.t1
MKEDKENTRPKEKRMDVKCEDGDKIEKLKEKREAMTKEELEEQLQPLPELDYLEFFSNDTSLFPHLFARAYINFRKQEDI
VLFRDRFDGYVFIDGKGQEY
PAIVEFAPFQKIAKKRSKKRDAKCGTIDDARKTTPLLDFLKNKQRIREEKKEERRRRELERKRLRDEERRKWRDEERRRR
KDNEKMKKLDRPLEKDQLKD
EPKIKVATAEDGSVRKPDRGEEMDTEKLKDKPKRQERPTKDDKVMGGVDLKKRPNVEMKDDRARKVDEEGRKDFRERDAA
RDRERDRERRQKEKERVRRQ
DEDRRRRRERHDGENTCRKREEAGKRDKERDWEKKRPGSTAESAASERAAKESRKEENMKRDRMRNKDRPAIQLYQPGAR
SRNRGGGGGGGGGGGGGESQ
PAEGKPDNDSKRANDNADD
>g109.t2
MDVKCEDGDKIEKLKEKREAMTKEELEEQLQPLPELDYLEFFSNDTSLFPHLFARAYINFRKQEDIVLFRDRFDGYVFID
GKGQEYPAIVEFAPFQKIAK
KRSKKRDAKCGTIDDARKTTPLLDFLKNKQRIREEKKEERRRRELERKRLRDEERRKWRDEERRRRKDNEKMKKLDRPLE
KDQLKDEPKIKVATAEDGSV
RKPDRGEEMDTEKLKDKPKRQERPTKDDKVMGGVDLKKRPNVEMKDDRARKVDEEGRKDFRERDAARDRERDRERRQKEK
ERVRRQDEDRRRRRERHDGE
NTCRKREEAGKRDKERDWEKKRPGSTAESAASERAAKESRKEENMKRDRMRNKDRPAIQLYQPGARSRNRGGGGGGGGGG
GGGESQPAEGKPDNDSKRAN
DNADD
>g109.t3
MKEDKENTRPKEKRMDVKCEDGDKIEKLKEKREAMTKIVIRRLPPSLTKEELEEQLQPLPELDYLEFFSNDTSLFPHLFA
RAYINFRKQEDIVLFRDRFD
GYVFIDGKGQEYPAIVEFAPFQKIAKKRSKKRDAKCGTIDDARKTTPLLDFLKNKQRIREEKKEERRRRELERKRLRDEE
RRKWRDEERRRRKDNEKMKK
LDRPLEKDQLKDEPKIKVATAEDGSVRKPDRGEEMDTEKLKDKPKRQERPTKDDKVMGGVDLKKRPNVEMKDDRARKVDE
EGRKDFRERDAARDRERDRE
RRQKEKERVRRQDEDRRRRRERHDGENTCRKREEAGKRDKERDWEKKRPGSTAESAASERAAKESRKEENMKRDRMRNKD
RPAIQLYQPGARSRNRGGGG
GGGGGGGGGESQPAEGKPDNDSKRANDNADD
>g110.t1
MFHVVALWGQSEPSWGTLIGCENIHDYRFPSFFLFDRSHSGKSSEAIMSSHKTFKIKRFLAKKQKQNRPIPQWIRMKTGN
KISIDLVRPMVHHRGEGRCR
PTILRAISIVLLIPRAKELLHLKWGVSLQVLPGSFAIVVHHIDFRTKGTNKKFYYHILAVHTRDVERGETPAAF
>g110.t2
MFHVVALWGQSEPSWGTLIGCENIHDYRFPSFFLFDRSHSGKSSEAIMSSHKTFKIKRFLAKKQKQNRPIPQWIRMKTGN
KISIDLVRPMVHHRGEGRCR
PTILRAISIVLLIPRAKELLHLNCSERTSLRSVSGISVEESAGGRVGNF

>g111.t1
MLKTPVHAKKLLTPIQSGRKALGVVNKISATPAASRQGLKPQKVQEPKVKCVPQSKAEEYPDVERFVPYDPLEFEKHYIP
EDLVRLGCLAVPGLASIPRT
YCIPEEDFELIEPYTIQPSTVKAHRGSDDCATELNAFLQTISELTIDLPPESD
>g111.t2
MANIIFAERENLGLPGPTLKARQRLQSASESMLKTPVHAKKLLTPIQSGRKALGVVNKISATPAASRQGLKPQKVQEPKV
KCVPQSKAEEYPDVERFVPY
DPLEFEKHYIPEDLVRLGCLAVPGLASIPRTYCIPEEDFELIEPYTIQPSTVKAHRGSDDCATELNAFLQTISELTIDLP
PESD
>g111.t3
MLKTPVHAKKLLTPIQSGRKALGVVNKISATPAASRQGLKPQKVQEPKVKCVPQSKAEEYPDVERFVPYDPLAVPGLASI
PRTYCIPEEDFELIEPYTIQ
PSTVKAHRGSDDCATELNAFLQTISELTIDLPPESD
>g112.t1
MASTEIARQAGEGARAVPLAGHVGFDSMPDQLVNKSVNHGFCFNILCVGETGLGKSTLMDTLFNTKFEGEPTQHNQPGVQ
LKSNTYELQESNVRLKLTVV
NTVGFGDQINKEDSYKSIVEFIDAQFEAYLQEELKIKRTLHSYHDTRIHACLYFIAPTGHSLKSLDLVTMKKLDSK
>g112.t2
MASTEIARQAGEGARAVPLAGHVGFDSMPDQLVNKSVNHGFCFNILCVGETGLGKSTLMDTLFNTKFEGEPTQHNQPGVQ
LKSNTYELQESNVRLKLTVV
NTVGFGDQINKEDSYKSIVEFIDAQFEAYLQEELKIKRTLHSYHDTRIHACLYFIAPTGHSLKSLDLVTMKKLDSKLMCS
RCCIGSRSAVKCCCLGYCRL
ISS
>g112.t3
MASTEIARQAGEGARAVPLAGHVGFDSMPDQLVNKSVNHGFCFNILCVGETGLGKSTLMDTLFNTKFEGEPTQHNQPGVQ
LKSNTYELQESNVRLKLTVV
NTVGFGDQINKEDSYKSIVEFIDAQFEAYLQEELKIKRTLHSYHDTRIHACLYFIAPTGHSLKSLDLVTMKKLDSKKM
>g113.t1
MFYQTVVASVLFYAVVCWGGSAAKKNSTRLDRLIRRAGSVVGLKLDSVVTVAEKRTLKKLLAVMDDASNPLHTVISAQRS
QLSNRLLLPKCKTNRFKDYF
VPRAIKLYNSALGGRGRSSRRPH
>g114.t1
MVLIWTQFGVKNKNVNCKVRVMHRDDSSSSSSSSSGESSKSDQEVPCLSIKPAGKDFYKILGVPADSNEDDIKKAYRRMA
LKFHPDKNSDADAEDKFKEI
AEAYEILTDSKKRGIYDQYGEEGLKNGNCVSMAGHGNTYRGGFQGDARATFSTLYHGSDHFDIFFGSDVDSSSSSDDDIF
NPFSHFAFANGGGEGGRRGR
RRRLQGLAMVHDLLVSLEEVFSGCTKRIKISRSRLNPDGHSLRSEDKVLDVVVKKGWRAGTKITFPCEGDETPNNTPADV
TFVLRDKKHAQYKRDGSNIV
YTAKITLKEALCGCTVNVPTLDNRMMPLPSSDVIKPGSVRRLRGEGLPLPKTPAQRGDLVVEFQVLFPDRIPPQSREIIK
HSLAKC
>g114.t2
MRSSYLTTSMVGPGLDSDFGASAGVSLRIVSADSDGLTHGVVAAALEWDYYDPCYVQQNSVPVHKGSRPSIHAKQYWV
>g114.t3
MQQVGFQGQENLRFRVGAYSSSTYNLIKDCIPREDHRGARQALSETRWGQSSRVRIPDGAQHHTGEFCCV
>g114.t4
MVLIWTQFGVKNKNVNCKVRVMHRDDSSSSSSSSSGESSKSDQEVPCLSIKPAGKDFYKILGVPADSNEDDIKKAYRRMA
LKFHPDKNSDADAEDKFKEI
AEAYEILTDSKKRGIYDQYGEEGLKNGNCVSMAGHGNTYRGGFQGDARATFSTLYHGSDHFDIFFGSDVDSSSSSDDDIF
NPFSHFAFANGGGEGGRRGR
RRRLQGLAMVHDLLVSLEEVFSGCTKRIKISRSRLNPDGHSLRSEDKVLDVVVKKGWRAGTKITFPCEGDETPNNTPADV
TFVLRDKKHAQYKRDGSNIV
YTAKITLKEALCGCTVNVPTLDNRMMPLPSSDVIKPGSVRRLRGEGLPLPKTPAQRGDLVVEFQVLFPDRIPPQSRQENL
RFRVGAYSSSTYNLIKDCIP
REDHRGARQALSETRWGQSSRVRIPDGAQHHTGEFCCV
>g114.t5
MVLIWTQFGVKNKNVNCKVRVMHRDDSSSSSSSSSGESSKSDQEVPCLSIKPAGKDFYKILGVPADSNEDDIKKAYRRMA

LKFHPDKNSDADAEDKFKEI
AEAYEILTDSKKRGIYDQYGEEGLKNGNCVSMAGHGNTYRGGFQGDARATFSTLYHGSDHFDIFFGSDVDSSSSSDDDIF
NPFSHFAFANGGGEGGRRGR
RRRLQGLAMVHDLLVSLEEVFSGCTKRIKISRSRLNPDGHSLRSEDKVLDVVVKKGWRAGTKITFPCEGDETPNNTPADV
TFVLRDKKHAQYKRDGSNIV
YTAKITLKEALCGCTVNVPTLDNRMMPLPSSDVIKPGSVRRLRGEGLPLPKTPAQRGDLVVEFQVLFPDRIPPQSRQENL
RFRVGAYSSSTYNLIKDCIP
REDHRGARQALSETRWGQSSRLTKSLDLVRRSSTLESLVNINDGLGPPLHPLDRVELECEYPLHYNSARMRSSYLTTSMV
GPGLDSDFGASAGVSLRIVS
ADSDGLTHGVVAAALEWDYYDPCYVQQNSVPVHKGSRPSIHAKQYWV
>g115.t1
MSFLRRVAGLSLRDRVRSSDIRRELGVEPLLLRVERSQLRWFGHLIRMPPGRLPLEVFRARPTGKRPRGRPRTRWRDYIS
HLAWERLGVPQEELESVAGE
KDVWSSLLSLLPPRPGPG
>g116.t1
MDDCSSDGRLFENLIKDVRKETQKKPLLLIWTPPLGIKFDFNDCRKHFDITSCDLTYNRSLYQQAAGVLFYHKDIHSVGH
MPQGPRPPLQKWIWYHVESP
TNTWRIGGTDNIFNLTLSYRRDADITARYEVVIKKEPSDDFVLPKKDKLLCWIASNKATRGTGIRMKYYSELIKHIEVHV
YGAAFAGTLPPDRYYPTLAS
CKFYLAFENSIHKDYITEKTNGPLCSGTVPVVLGPPRDNYEEFIPGDAFIHVNDFPDAKALAEHLLALDRNDERYLEYFR
WRRFYSATPHLLTTENEFTQ
PTCLACDYISRDNRYSRQHPLYCPERCKAFQCDLTQDDLQGEVPQASVDVVTLIFVLSAIHPNKMARSLENIFRVLKPGG
SVLFRDYGLNDHAMQRFKAA
NRLGQNFYVRQDGTRSYFFSKEFLAKLFEGTGFRSVANGYVFRETVNKKEGLCVPRVFLQSKFTKPHRSQTSP
>g116.t2
MYFLYTRPVMDDCSSDGRLFENLIKDVRKETQKKPLLLIWTPPLGIKFDFNDCRKHFDITSCDLTYNRSLYQQAAGVLFY
HKDIHSVGHMPQGPRPPLQK
WIWYHVESPTNTWRIGGTDNIFNLTLSYRRDADITARYEVVIKKEPSDDFVLPKKDKLLCWIASNKATRGTGIRMKYYSE
LIKHIEVHVYGAAFAGTLPP
DRYYPTLASCKFYLAFENSIHKDYITEKTNGPLCSGTVPVVLGPPRDNYEEFIPGDAFIHVNDFPDAKALAEHLLALDRN
DERYLEYFRWRRFYSATPHL
LTTENEFTQPTCLACDYISRDNRYSRQHPLYCPERCKAFQCDLTQDDLQGEVPQASVDVVTLIFVLSAIHPNKMARSLEN
IFRVLKPGGSVLFRDYGLND
HAMQRFKAANRLGQNFYVRQDGTRSYFFSKEFLAKLFEGTGFRSVANGYVFRETVNKKEGLCVPRVFLQSKFTKPHRSQT
SP
>g116.t3
MDDCSSDGRLFENLIKDVRKETQKKPLLLIWTPPLGIKFDFNDCRKHFDITSCDLTYNRSLYQQAAGVLFYHKDIHSVGH
MPQGPRPPLQKWIWYHVESP
TNTWRIGGTDNIFNLTLSYRRDADITARYEVVIKKEPSDDFVLPKKDKLLCWIASNKATRGTGIRMKYYSELIKHIEVHV
YGAAFAGTLPPDRYYPTLAS
CKFYLAFENSIHKDYITEKTNGPLCSGTVPVVLGPPRDNYEEFIPGDAFIHVNDFPDAKALAEHLLALDRNDERYLEYFR
WRRFYSATPHLLTTENEFTQ
PTCLACDYISRDNRARLHCLPFRLCVYRQQHPLYCPERCKAFQCDLTQDDLQGEVPQASVDVVTLIFVLSAIHPNKMARS
LENIFRVLKPGGSVLFRDYG
LNDHAMQRFKAANRLGQNFYVRQDGTRSYFFSKEFLAKLFEGTGFRSVANGYVFRETVNKKEGLCVPRVFLQSKFTKPHR
SQTSP
>g117.t1
IRGYSKKIRGYSLECPGLTRLPQKHFSEDIQTCQYRSVEVLIGATYDTPADIWSTACMVSPAATAF
>g118.t1
LKTTLQKKLSSDTVDLSGIPLSSRDVRQVAFYLQNNGDAVVSVDISFTELHDDNLRLLLPLLGVMPKLGTLALNGNRLTL
AIVKDLTEMLKEPKQFSSLA
WIDLGNNVDIFTMPQPLLVALRRRCSLKSSLPTIYEYTEGQPYCYHLETSIEDPSLYEEEEQADGDGEVEGEEEELEEDE
EVGNKPELEPWGIGGKRLSK
DFTLHFCQR
>g119.t1
MSTFVGFHTWTVGGIKGIDLPTLRLVSCWGSQGCVEKHLFSNMSVLQPVEQLSLARLEVKFQWGALKAPGFFLGPRALDV

SHKSGPQGPDPEEELLPLQP
GNALPLQPAAASTPPPAVHAALVTVSLYPKQCHTRRRRSAVHLPVTPSNVCKPRRLYIDFKDVGWQDWIIAPQGYLANYC
HGECPFPLSESLNGTNHAIL
QTLVHSLDPRGTPQPCCVPIRLSPISMLYYDNNDNVVLRHYQDMVVDECGCR
>g119.t2
LVSCWGSQGCVEKHLFSNMSVLQPVEQLSLARLEVKFQWGALKAPGFFLGPRALDVSHKSGPQGPDPEEELLPLQPGNAL
PLQPAAASTPPPAVHAALVT
VSLYPKQCHTRRRRSAVHLPVTPSNVCKPRRLYIDFKDVGWQDWIIAPQGYLANYCHGECPFPLSESLNGTNHAILQTLV
HSLDPRGTPQPCCVPIRLSP
ISMLYYDNNDNVVLRHYQDMVVDECGCR
>g120.t1
MVEHLISSSLELVCIFKVLNQRCILRASYHPPLEGTRVVXGSGNMEKVGQIWGGEVVDGLECYREDFVFDTELNREPVEL
LEDRSDVVDGGGSCDDAGS
>g121.t1
LRPLVGTQLDPLQFAYQPGIGVDDAVIYLMHRSLLHLEDSRSTVRVMFFYFSSAFNTIQPPLLRVKMENVGVDHHLAAWT
TDYLTNRPQYVRLHHCVSDV
VLCSTGAPQGTVLSPFLFTLYTSDFTHNTTHCHIQTFSDDTAIVGCVSEEND
>g122.t1
MPPGRLPLEVFRARPTGKRPRGRPRTRWRDYISHLAWERLGVPQEELESVAGEKDVWSSLLSLLPPRPGPR
>g123.t1
LGHPNASRTHLWRFCWPTTQKIGLRVPASVDQSALEFEGVIFYHNGPFPWKQTRISPSSEVELRPPPDSAHEGPKMRPDW
SGTLGNWKTQRTPPDAPTPG
QRPHWGPSETAAG
>g124.t1
IFLNILPRLLRMQMRPWTPDPGTTGDWGHGGRGEDPRLAGNGASHLQHGRRRSSVGLMVKAEEYVLRTARTELMFSRLRE
RQGLIKTVFEKLHRDLDDVS
VEKLESSLAKLSPDLKQCVASCKHIAETAQGQNHFQSENEEWFLLARVIDRVCFITMALIFSIGTVGIFLMGHFNQPPTM
PFPGDPRRFLPPLGNATGSD
LQG
>g124.t2
IFLNILPRLLRMQMRPWTPDPGTTGDWGHGGRGEDPRLAGNGASHLQHGRRRSSVGLMVKAEEYVLRTARTELMFSRLRE
RQGLIKTVFEKLRGYL
>g124.t3
LVTVHLAVDRDLDDVSVEKLESSLAKLSPDLKQCVASCKHIAETAQGQNHFQSENEEWFLLARVIDRVCFITMALIFSIG
TVGIFLMGHFNQPPTMPFPG
DPRRFLPPLGNATGSDLQG
>g124.t4
LDDVSVEKLESSLAKLSPDLKQCVASCKHIAETAQGQNHFQSENEEWFLLARVIDRVCFITMALIFSIGTVGIFLMGHFN
QPPTMPFPGDPRRFLPPLGN
ATGSDLQG
>g125.t1
MDADKCQEDSNAMSEGLESAMEKMGVSETKEQEEPLAAVGEPEQVALNGHHEAEPSPVHDRQKDGFPEEVPESVQAPQTM
PEAAPPMETLVQDLPPHVSE
KVEEPMVTEAASDAEPAPPQRDGSPEKVSESPQRETAEEPAGAEEMSEEVPAQPQPDGSPVKIPEAPERDTLANASEGME
ESTGAEDSMGAEELSEAVLT
QHQPVESPAAVSPPDVLTTGTESETPQPELQLSAESDPEPKVELVPELADQTSPEPDSVAQIVDLAIHKEETPAAAGEVA
MDTEKVLTIDSPDEKAVEEL
SGNSVVSESVTAACPKAPMQTEEAQNVQSAEVLNAKDAEPVKEEEPKKAAEPDLPAPTAMDQVEQKEATADQKQPTKEEG
VGVPAPGSLAFALLEHEPTM
AALRASRTLVVLRGLPGSGKSFLARAIADSYQSLCTVVSADDHDPASAGGYKDLDEAVIARCGPATGASLVVVDDTNHTH
DRLVRLAEIAEQNRRLVLFL
EPRTEWCADLPELAKRTCRGLDEAQIRVMKVAYEEVCIPLFYGWFLLQDKIKCTAMDFLKTLDTLDAFKKHLADFPVETE
KEVDLEQYFKSDWALHCTTK
FCDYGRAKGAKEYAEQAAVQDLYGSAFELSLTALFVTPRTVGARVALTEAQLALWP
>g125.t2
RDSAEEIGDDKWLLNANVLVAQADLHLGDFQSSTSHFEEALKRAKALKDDSAAEAIQKALLLARKNLVK

>g125.t3
RDSAEEIGDDKWLLNANVLVAQADLHLGDFQSSTSHFEEALKRAKALKDDSAAEAIQKADRNQNMDADKCQEDSNAMSEG
LESAMEKMGVSETKEQEEPL
AAVGEPEQVALNGHHEAEPSPVHDRQKDGFPEEVPESVQAPQTMPEAAPPMETLVQDLPPHVSEKVEEPMVTEAASDAEP
APPQRDGSPEKVSESPQRET
AEEPAGAEEMSEEVPAQPQPDGSPVKIPEAPERDTLANASEGMEESTGAEDSMGAEELSEAVLTQHQPVESPAAVSPPDV
LTTGTESETPQPELQLSAES
DPEPKVELVPELADQTSPEPDSVAQIVDLAIHKEETPAAAGEVAMDTEKVLTIDSPDEKAVEELSGNSVVSESVTAACPK
APMQTEEAQNVQSAEVLNAK
DAEPVKEEEPKKAAEPDLPAPTAMDQVEQKEATADQKQPTKEEGVGVPAPGSLAFALLEHEPTMAALRASRTLVVLRGLP
GSGKSFLARAIADSYQSLCT
VVSADDHDPASAGGYKDLDEAVIARCGPATGASLVVVDDTNHTHDRLVRLAEIAEQNRRLVLFLEPRTEWCADLPELAKR
TCRGLDEAQIRVMKVAYEEV
CIPLFYGWFLLQDKIKCTAMDFLKTLDTLDAFKKHLADFPVETEKEVDLEQYFKSDWALHCTTKFCDYGRAKGAKEYAEQ
AAVQDLYGSAFELSLTALFV
TPRTVGARVALTEAQLALWP
>g125.t4
MSEGLESAMEKMGVSETKEQEEPLAAVGEPEQVALNGHHEAEPSPVHDRQKDGFPEEVPESVQAPQTMPEAAPPMETLVQ
DLPPHVSEKVEEPMVTEAAS
DAEPAPPQRDGSPEKVSESPQRETAEEPAGAEEMSEEVPAQPQPDGSPVKIPEAPERDTLANASEGMEESTGAEDSMGAE
ELSEAVLTQHQPVESPAAVS
PPDVLTTGTESETPQPELQLSAESDPEPKVELVPELADQTSPEPDSVAQIVDLAIHKEETPAAAGEVAMDTEKVLTIDSP
DEKAVEELSGNSVVSESVTA
ACPKAPMQTEEAQNVQSAEVLNAKDAEPVKEEEPKKAAEPDLPAPTAMDQVEQKEATADQKQPTKEEGVGVPAPGSLAFA
LLEHEPTMAALRASRTLVVL
RGLPGSGKSFLARAIADSYQSLCTVVSADDHDPASAGGYKDLDEAVIARCGPATGASLVVVDDTNHTHDRLVRLAEIAEQ
NRRLVLFLEPRTEWCADLPE
LAKRTCRGLDEAQIRVMKVAYEEVCIPLFYGWFLLQDKIKCTAMDFLKTLDTLDAFKKHLADFPVETEKEVDLEQYFKSD
WALHCTTKFCDYGRAKGAKE
YAEQAAVQDLYGSAFELSLTALFVTPRTVGARVALTEAQLALWP
>g126.t1
ESRLPQIQQNVRQAIGGNMSVFCLGFGDSVDYSFLDVMAKQNNGVARRIYEASDASLQLQGFYDEVASPLLSAVDLRYPD
NAVESLTTHHFSQLFNGSEI
VVAGRLSENDMDNFLVEVFANGEFQVQGQASVTDWGVTYPEKEYIFGDFTERLWAYLTIQQLLEASKSGPPEGKTNATAK
ALEMSLKYSFVTPLTSMVVT
KPETEEGPSGPLIADKLTEVLIEDYLEVQPSGMPMEDDLEVEEDFMMTSDLMAVDLKDLLTPSSS
>g126.t2
ESRLPQIQQNVRQAIGGNMSVFCLGFGDSVDYSFLDVMAKQNNGVARRIYEASDASLQLQGFYDEVASPLLSAVDLRYPD
NAVESLTTHHFSQLFNGSEI
VVAGRLSENDMDNFLVEVFANGYEQEFQVQGQASVTDWGVTYPEKEYIFGDFTERLWAYLTIQQLLEASKSGPPEGKTNA
TAKALEMSLKYSFVTPLTSM
VVTKPETEEGPSGPLIADKLTEVLIEDYLEVQPSGMPMEDDLEVEEDFMMTSDLMAVDLKDLLTPSSS
>g126.t3
ESRLPQIQQNVRQAIGGNMSVFCLGFGDSVDYSFLDVMAKQNNGVARRIYEASDASLQLQGFYDEVASPLLSAVDLRYPD
NAVESLTTHHFSQLFNGSEI
VVAGRLSENDMDNFLVEVFANGEFQVQGQASVTDWGVTYPEKEYIFGDFTERLWAYLTIQQLLEASKSGPPEGKTNATAK
ALEMSLKYSFVTPLTSMVVT
KPETEEGPSGPLIADKLTEGTITLGFTILPLPTISILPQ
>g127.t1
LAGAIVHHIARRSQSPAVRPRVVRGLPSPRRPLTNPGDADSRGPPGPAASGAAGQAGIDWLGLTGPDWRGVGSAVSGVEQ
VCLRRDSARR
>g128.t1
PGRGRVDQLFTLAALLRGSWEFAHPVYMCFVDLEKAYDRVPRGILWGVLREYGVLGPLIRAIRSLYNQRSRGAAEVRRVS
GLGTSELRLCFFQMMWFCWL
HQTVTSNRHEGGLQPLSGWEAAGMRVSTSKSEAMVLCRKTVDCSLRVGSELAPDKRTKNE
>g128.t2

LEKAYDRVPRGILWGVLREYGVLGPLIRAIRSLYNQRSRGAAEVRRVSGLGTSELRLCFFQMMWFCWLHQTVTSNRHEGG
LQPLSGWEAAGMRVSTSKSE
AMVLCRKTVDCSLRVGSELAPDKRTKNE
>g129.t1
MFKRRRRGKRASALVKLRQRGFLTALPSIHLANVRSLNNKMDELLLLNRRNSDFCRSAALCFTETWLSELTPDSGLYLPD
FQLLRADRVTELRGRAEGRN
LLLY
>g130.t1
MSRRTRNSRAWRYVWSGIRRDADARALVMASENEDWEYERLQRRLLVCSAAFKYSDSDSEADFPTVVVPPVPSAVPVTGE
SYCGCDSQAESSYNPRLRGF
HRVKDCRCGEDDQDFDWVWDVSSGSTATLLSCDNRKVNFHSEYSCGTAAVRGSKELADGQHFWEIKMTSPVYGTDMMVGI
GTSDINLDKYRHSFCSLLGK
DSDSWGLSYT
>g130.t2
MSRRTRNSRAWRYVWSGIRRDADARALVMASENEDWEYERLQRRLLVCSAAFKYSDSDSEADFPTVVVPPVPSAVPVTGE
SYCGCDSQAESSYNPRLRGF
HRVKDCRCGEDDQDFDWVWDVSSGSTATLLSCDNRKVNFHSEYSCGTAAVRGSKELADGQHFWEIKMTSPVYGTDMMVGI
GTSDINLDKYRHSFCSLLGK
DSDSWGLSYTGKEGRSCVGLP
>g130.t3
MASENEDWEYERLQRRLLVCSAAFKYSDSDSEADFPTVVVPPVPSAVPVTGESYCGCDSQAESSYNPRLRGFHRVKDCRC
GEDDQDFDWVWDVSSGSTAT
LLSCDNRKVNFHSEYSCGTAAVRGSKELADGQHFWEIKMTSPVYGTDMMVGIGTSDINLDKYRHSFCSLLGKDSDSWGLS
YT
>g131.t1
MEGTVHLSIATDMDLSRLGSHADMKAVATEPDEVHQLEEVLLQWYKQIKQDASVVIQKIKDCISLYQEYRSCFHKAKKQM
EQSGEKPLEVSELYIFSIFK
GFCERLEKV
>g131.t2
MEGTVHLSIATDMDLSRLGSHADMKAVATEPDEVHQLEEVLLQWYKQIKQDCISLYQEYRSCFHKAKKQMEQSGEKPLEV
SELYIFSIFKGFCERLEKV
>g131.t3
MSGATHPEGTEGLPGPDLSGVDQGAISGATGRTSRFCRPGCSAQTQESVKNVCTTEMLCGKVAFFAG
>g131.t4
MSGATHPEGTEGLPGPDLSGVDQGAISGATGRTSRFCRPGCSAQTQESVKNAGYKYAREARKKQLTSAHKYVFQVLACRL
SLEEKVVEEFILDSSSEIFF
SMFDCSAGLLQGIKDLYSKILIPSMNAKEDWGVLNQSKHGAGVIENFKDTLSHYVNFLEDAQLSMEGTVHLSIATDMDLS
RLGSHADMKAVATEPDEVHQ
LEEVLLQWYKQIKQDASVVIQKIKDCISLYQEYRSCFHKAKKQMEQSGEKPLEVSELYIFSIFKGFCERLEKV
>g132.t1
MSPKGKEVVTMYNKTAAVFMQYELLYHRAWMRQDFQLEHALHTTLLVRHPKTGKLLVNFDPKIVENIREAKCMLKMGLEV
PKHALRLVTLESTINANRLH
LEVLDFCESRIDHVLQDISVTMLIELQEDQPSSLQDLMDNNTVKQRHVAFPLDDKPAGVDHDTNSECQVGEKEDDFEEVI
IPSLEDIQRSIIRLLHLVVE
ISQGVACWGQNLPPAKGKGKSFYQSVAEHKDISKYAAILNSAMNSLKKPISNALEQFHSFKDIWAENRNLTVKKFMDGSP
YLTEIKAKLQHYTSAEQAIE
GIAPVIVMGCIELSTAPLKMSLTVEAKAWKMVLSKHLKEEYSKKMTDISGFTEKQLKQLSRPIVDLEDVRFAMEVLSQVR
DNEIHIDMTLGPIEEAYGML
SSSEVEVTQSEAKGLDTLRYSFLKLQSKARSVQDELVRLQPKFKQNLLESVTVFHTSVSNFIEQYETAVFDDLWRKYITY
SSGEKLFGLPVTEYEPLEKT
RKELSLLQKLYGLYDSVMSNLNGYYEIRWTDVDIEKINAELQEFQNRCRRLPKGLKDWQAFMDLKKKIDDFSESCPLLEM
MANKSMKLRHWSRITDLTGY
KFDVESESFALKSIMEAPLLQHKEDIEDICISAVKEKDIEAKLAQVVDIWSSEILSFMSFKCRGELMLKGTETAEVVTTM
EDSLMVLGSLMSNRYNAPFK
KDIQKWVSKLSSSSDIIEQWLIVQNLWVYLEAVFVGGDIAKQLPQEAKRFQNIDKSWIKIMVRAHEIANVVQCCVGDTTM
DQLLPYLQEQLELCQKSLTG

YLEKKRLRFPRFFFVSDPALLEILGQASDSHTIQLDVPVLAQGPVEEWLGELLVQQQTSLHSVIKAADFQINDPGFQLLS
FLNTFQAQIGLLGIQMLWTR
DSEEALKKASEERKIMSLTNHKFLELLNTLISRTTDDLSKFDRIKFETLVTIHVHQRDIFDDLVRLQIKSVTDFEWLKQS
RFYYKEDLDCVIVSITDVDF
IYQNEFLGCTDRLVITPLTDRCYITLSQALGMSMGGAPAGPAGTGKTETTKDMGRCLGKYVVVFNCSDQMDFRGLGRIYK
GLAQSGSWGCFDEFNRIDLP
VLSVAAQQIYIVLMARKERKTTFIFTDGDCVNLNPEFGLFITMIIMRVKLASCGFIENIILAQKFFVLYKLCEEQLTKQV
HYDFGLRNILSVLRTLGANK
RARPDDTESSIVMRVLRDMNLSKLVDEDEPLFLSLISDLFPGIQLDRSAYEDLQAAVAHQVELAGIVNHPPWNLKLVQLY
ETARVRHGLMTLGPSGAGKT
TVINILMRALTECGDPHREMRMNPKAITAPQMFGRLDASTNDWTDGIFSTLWRKTLKAKKGEFIWIVLDGPVDAIWIENL
NSVLDDNKTLTLANGDRIVM
SPRCKLVFEVHNMDNASPATVSRVGMVFMSSSALSWRPILQSVNLLEGLIPSKEAGGLASSMHLQRLFLFSLMWSLGALL
ELEDRDKLEAFVKDHSSKME
LPNTGPGQTMFEYMVNSQGEWDHWDTYVVEYLYPTDHVPDYASILVPNVDNTRTSFLLETIAKQRKAVLLIGEQGTAKTV
MIKGYTQKYDPETDLSKSLN
FSSATKPMMFQRTVESYIEKRMGSTYGPPGGRRMTIFVDDINMPIVNEWGDQITNEIVRQMMEMSGMYSVDKPGDFTTIV
DVQLLAAMIHPGVMGCGYFH
ECRGFQPSVTDMVMTLVSATRILWQWTKVKMLPTPSKFHYVFNLRDLSRIWQGMLKINAAECNDIATLLALFKCECIRVI
ADRFVCFEDKAWFETAISRL
IQEHVDPSLVSELHADPYFVDFLREPPEPTGDEGEDASFDAPKIYELVPGFEFLSEKLRTYQAQHNEAVRGTSLDLVFFT
DAMTHLIKISRIIRTDGGSA
MLVGVGGSGKQSLTRLASFIAGYSIFQITLTRTYNQVNLMDDLKLLYRTAGADGKGITFIFTDNEIKDEAFLEYLNNVLS
SGEVSNLFAKDEIDEITQGL
ISVMKKESPRVPPTFDNLYNYFISRARKNLHVVLCFSPVGEKFRSRSLKFPGLISGCTMDWFTPWPRDALVAVSNHFLSG
FPMVCSPDVKAAVVSTMGTY
HDKVSDACEAYFERFRRRTHVTPKSYLSFINGYKTLYAERHNNINTLAERMNVGLAKLVEASESVAQLSKVLVVKEKELA
AASIKADKVLTEVTVSAEAA
SVVKNAVQVVKDKAQKIVEGIVKEKALAEVKLEAAKPALAEAEAALNATDISTVRKLAKPPHLIMRIMDCVLLLFQKKLD
TITMDPERPCHKPSWGEALK
LLSAGGFMASLQQFPKDLINDETVELLDSYFDMVDYTLENAKKVCGSVAGLLSWTQAMATFFGINQEVLPLKCNLVIQEG
RLTVANAELASAQAQLDEKQ
AELDKVQAKFDAAMKEKQDLLDDAEMCRNKMTRASLLIDGLSGEKIRWTEQSKEFKAQINRQVQMKQRESLLVGDILQLT
GFLSYCGPFNQSFRNMLLND
IWAAELREHSIPFTEDLHLISALVDPPTVTSLNHKFFRTHLEDCLSLGHPLLIEDVHEELDPALDNVLEKNFIKSGSSYK
VKVGDKEVNVMEGFQLYITT
KLPNPLYTPEVSAKTSTIDFTVTMKGLENQLLGRVILTEKYELEEERLKLMEDVTANKRKMKELEDNLLYKLSSTQ
>g132.t2
MSPKGKEVVTMYNKTAAVFMQYELLYHRAWMRQDFQLEHALHTTLLVRHPKTGKLLVNFDPKIVENIREAKCMLKMGLEV
PKHALRLVTLESTINANRLH
LEVLDFCESRIDHVLQDISVTMLIELQEDQPSSLQDLMDNNTVKQRHVAFPLDDKPAGVDHDTNSECQVGEKEDDFEEVI
IPSLEDIQRSIIRLLHLVVE
ISQGVACWGQNLPPAKGKGKSFYQSVAEHKDISKYAAILNSAMNSLKKPISNALEQFHSFKDIWAENRNLTVKKFMDGSP
YLTEIKAKLQHYTSAEQAIE
GIAPVIVMGCIELSTAPLKMSLTVEAKAWKMVLSKHLKEEYSKKMTDISGFTEKQLKQLSRPIVDLEDVRFAMEVLSQVR
DNEIHIDMTLGPIEEAYGML
SSSEVEVTQSEAKGLDTLRYSFLKLQSKARSVQDELVRLQPKFKQNLLESVTVFHTSVSNFIEQYETAVFDDLWRKYITY
SSGEKLFGLPVTEYEPLEKT
RKELSLLQKLYGLYDSVMSNLNGYYEIRWTDVDIEKINAELQEFQNRCRRLPKGLKDWQAFMDLKKKIDDFSESCPLLEM
MANKSMKLRHWSRITDLTGY
KFDVESESFALKSIMEAPLLQHKEDIEDICISAVKEKDIEAKLAQVVDIWSSEILSFMSFKCRGELMLKGTETAEVVTTM
EDSLMVLGSLMSNRYNAPFK
KDIQKWVSKLSSSSDIIEQWLIVQNLWVYLEAVFVGGDIAKQLPQEAKRFQNIDKSWIKIMVRAHEIANVVQCCVGDTTM
DQLLPYLQEQLELCQKSLTG
YLEKKRLRFPRFFFVSDPALLEILGQASDSHTIQLDVPVLAQGPVEEWLGELLVQQQTSLHSVIKAADFQINDPGFQLLS
FLNTFQAQIGLLGIQMLWTR

DSEEALKKASEERKIMSLTNHKFLELLNTLISRTTDDLSKFDRIKFETLVTIHVHQRDIFDDLVRLQIKSVTDFEWLKQS
RFYYKEDLDCVIVSITDVDF
IYQNEFLGCTDRLVITPLTDRCYITLSQALGMSMGGAPAGPAGTGKTETTKDMGRCLGKYVVVFNCSDQMDFRGLGRIYK
GLAQSGSWGCFDEFNRIDLP
VLSVAAQQIYIVLMARKERKTTFIFTDGDCVNLNPEFGLFITMIIMRVKLASCGFIENIILAQKFFVLYKLCEEQLTKQV
HYDFGLRNILSVLRTLGANK
RARPDDTESSIVMRVLRDMNLSKLVDEDEPLFLSLISDLFPGIQLDRSAYEDLQAAVAHQVELAGIVNHPPWNLKLVQLY
ETARVRHGLMTLGPSGAGKT
TVINILMRALTECGDPHREMRMNPKAITAPQMFGRLDASTNDWTDGIFSTLWRKTLKAKKGEFIWIVLDGPVDAIWIENL
NSVLDDNKTLTLANGDRIVM
SPRCKLVFEVHNMDNASPATVSRVGMVFMSSSALSWRPILQSVNLLEGLIPSKEAGGLASSMHLQRLFLFSLMWSLGALL
ELEDRDKLEAFVKDHSSKME
LPNTGPGQTMFEYMVNSQGEWDHWDTYVVEYLYPTDHVPDYASILVPNVDNTRTSFLLETIAKQRKAVLLIGEQGTAKTV
MIKGYTQKYDPETDLSKSLN
FSSATKPMMFQRTVESYIEKRMGSTYGPPGGRRMTIFVDDINMPIVNEWGDQITNEIVRQMMEMSGMYSVDKPGDFTTIV
DVQLLAAMIHPGVMGCGYFH
ECRGFQPSVTDMVMTLVSATRILWQWTKVKMLPTPSKFHYVFNLRDLSRIWQGMLKINAAECNDIATLLALFKCECIRVI
ADRFVCFEDKAWFETAISRL
IQEHVDPSLVSELHADPYFVDFLREPPEPTGDEGEDASFDAPKIYELVPGFEFLSEKLRTYQAQHNEAVRGTSLDLVFFT
DAMTHLIKISRIIRTDGGSA
MLVGVGGSGKQSLTRLASFIAGYSIFQITLTRTYNQVNLMDDLKLLYRTAGADGKGITFIFTDNEIKDEAFLEYLNNVLS
SGEVSNLFAKDEIDEITQGL
ISVMKKESPRVPPTFDNLYNYFISRARKNLHVVLCFSPVGEKFRSRSLKFPGLISGCTMDWFTPWPRDALVAVSNHFLSG
FPMVCSPDVKAAVVSTMGTY
HDKVSDACEAYFERFRRRTHVTPKSYLSFINGYKTLYAERHNNINTLAERMNVGLAKLVEASESVAQLSKVLVVKEKELA
AASIKADKVLTEVTVSAEAA
SVVKNAVQVVKDKAQKIVEGIVKEKALAEVKLEAAKPALAEAEAALNASAVQHV
>g132.t3
MFTVGEFIWIVLDGPVDAIWIENLNSVLDDNKTLTLANGDRIVMSPRCKLVFEVHNMDNASPATVSRVGMVFMSSSALSW
RPILQGWTNKRNTTEAEQIL
SLYDKIFEDSYVFMKQNLKPKIELLECNYIMQSVNLLEGLIPSKEAGGLASSMHLQRLFLFSLMWSLGALLELEDRDKLE
AFVKDHSSKMELPNTGPGQT
MFEYMVNSQGEWDHWDTYVVEYLYPTDHVPDYASILVPNVDNTRTSFLLETIAKQRKAVLLIGEQGTAKTVMIKGYTQKY
DPETDLSKSLNFSSATKPMM
FQRTVESYIEKRMGSTYGPPGGRRMTIFVDDINMPIVNEWGDQITNEIVRQMMEMSGMYSVDKPGDFTTIVDVQLLAAMI
HPGVMGCGYFHECRGFQPSV
TDMVMTLVSATRILWQWTKVKMLPTPSKFHYVFNLRDLSRIWQGMLKINAAECNDIATLLALFKCECIRVIADRFVCFED
KAWFETAISRLIQEHVDPSL
VSELHADPYFVDFLREPPEPTGDEGEDASFDAPKIYELVPGFEFLSEKLRTYQAQHNEAVRGTSLDLVFFTDAMTHLIKI
SRIIRTDGGSAMLVGVGGSG
KQSLTRLASFIAGYSIFQITLTRTYNQVNLMDDLKLLYRTAGADGKGITFIFTDNEIKDEAFLEYLNNVLSSGEVSNLFA
KDEIDEITQGLISVMKKESP
RVPPTFDNLYNYFISRARKNLHVVLCFSPVGEKFRSRSLKFPGLISGCTMDWFTPWPRDALVAVSNHFLSGFPMVCSPDV
KAAVVSTMGTYHDKVSDACE
AYFERFRRRTHVTPKSYLSFINGYKTLYAERHNNINTLAERMNVGLAKLVEASESVAQLSKVLVVKEKELAAASIKADKV
LTEVTVSAEAASVVKNAVQV
VKDKAQKIVEGIVKEKALAEVKLEAAKPALAEAEAALNATDISTVRKLAKPPHLIMRIMDCVLLLFQKKLDTITMDPERP
CHKPSWGEALKLLSAGGFMA
SLQQFPKDLINDETVELLDSYFDMVDYTLENAKKVCGSVAGLLSWTQAMATFFGINQEVLPLKCNLVIQEGRLTVANAEL
ASAQAQLDEKQAELDKVQAK
FDAAMKEKQDLLDDAEMCRNKMTRASLLIDGLSGEKIRWTEQSKEFKAQINRQVQMKQRESLLVGDILQLTGFLSYCGPF
NQSFRNMLLNDIWAAELREH
SIPFTEDLHLISALVDPPTVTSLNHKFFRTHLEDCLSLGHPLLIEDVHEELDPALDNVLEKNFIKSGSSYKVKVGDKEVN
VMEGFQLYITTKLPNPLYTP
EVSAKTSTIDFTVTMKGLENQLLGRVILTEKYELEEERLKLMEDVTANKRKMKELEDNLLYKLSSTQ
>g132.t4

MSPKGKEVVTMYNKTAAVFMQYELLYHRAWMRQDFQLEHALHTTLLVRHPKTGKLLVNFDPKIVENIREAKCMLKMGLEV
PKHALRLVTLESTINANRLH
LEVLDFCESRIDHVLQDISVTMLIELQEDQPSSLQDLMDNNTVKQRHVAFPLDDKPAGVDHDTNSECQVGEKEDDFEEVI
IPSLEDIQRSIIRLLHLVVE
ISQGVACWGQNLPPAKGKGKSFYQSVAEHKDISKYAAILNSAMNSLKKPISNALEQFHSFKDIWAENRNLTVKKFMDGSP
YLTEIKAKLQHYTSAEQAIE
APLKMSLTVEAKAWKMVLSKHLKEEYSKKMTDISGFTEKQLKQLSRPIVDLEDVRFAMEVLSQVRDNEIHIDMTLGPIEE
AYGMLSSSEVEVTQSEAKGL
DTLRYSFLKLQSKAAVFDDLWRKYITYSSGEKLFGLPVTEYEPLEKTRKELSLLQKLYGLYDSVMSNLNGYYEIRWTDVD
IEKINAELQEFQNRCRRLPK
GLKDWQAFMDLKKKIDDFSESCPLLEMMANKSMKLRHWSRITDLTGYKFDVESESFALKSIMEAPLLQHKEDIEDICISA
VKEKDIEAKLAQVVDIWSSE
ILSFMSFKCRGELMLKGTETAEVVTTMEDSLMVLGSLMSNRYNAPFKKDIQKWVSKLSSSSDIIEQWLIVQNLWVYLEAV
FVGGDIAKQLPQEAKRFQNI
DKSWIKIMVRAHEIANVVQCCVGDTTMDQLLPYLQEQLELCQKSLTGYLEKKRLRFPRFFFVSDPALLEILGQASDSHTI
QLDVPVLAQGPVEEWLGELL
VQQQTSLHSVIKAADFQINDPGFQLLSFLNTFQAQIGLLGIQMLWTRDSEEALKKASEERKIMSLTNHKFLELLNTLISR
TTDDLSKFDRIKFETLVTIH
VHQRDIFDDLVRLQIKSVTDFEWLKQSRFYYKEDLDCVIVSITDVDFIYQNEFLGCTDRLVITPLTDRCYITLSQALGMS
MGGAPAGPAGTGKTETTKDM
GRCLGKYVVVFNCSDQMDFRGLGRIYKGLAQSGSWGCFDEFNRIDLPVLSVAAQQIYIVLMARKERKTTFIFTDGDCVNL
NPEFGLFITMIIMRVKLASC
GFIENIILAQKFFVLYKLCEEQLTKQVHYDFGLRNILSVLRTLGANKRARPDDTESSIVMRVLRDMNLSKLVDEDEPLFL
SLISDLFPGIQLDRSAYEDL
QAAVAHQVELAGIVNHPPWNLKLVQLYETARVRHGLMTLGPSGAGKTTVINILMRALTECGDPHREMRMNPKAITAPQMF
GRLDASTNDWTDGIFSTLWR
KTLKAKKGDAVYCYC
>g132.t5
LAKPPHLIMRIMDCVLLLFQKKLDTITMDPERPCHKPSWGEALKLLSAGGFMASLQQFPKDLINDETVELLDSYFDMVDY
TLENAKKVCGSVAGLLSWTQ
AMATFFGINQEVLPLKCNLVIQEGRLTVANAELASAQAQLDEKQAELDKVQAKFDAAMKEKQDLLDDAEMCRNKMTRASL
LIDGLSGEKIRWTEQSKEFK
AQINRQVQMKQRESLLVGDILQLTGFLSYCGPFNQSFRNMLLNDIWAAELREHSIPFTEDLHLISALVDPPTVTSLNHKF
FRTHLEDCLSLGHPLLIEDV
HEELDPALDNVLEKNFIKSGSSYKVKVGDKEVNVMEGFQLYITTKLPNPLYTPEVSAKTSTIDFTVTMKGLENQLLGRVI
LTEKYELEEERLKLMEDVTA
NKRKMKELEDNLLYKLSSTQ
>g133.t1
VGSGYDSEGLAGCPGRGGGSTDATAVKLIPRIDTERALLMAARSTPPTLSRSPEGEALHVLRAKR
>g134.t1
MSEPSFKFIAEMVHKILAYLDPPNKMLQSEDMDLLTGEIINIENLRKLRKKKEHTRTRFYKNPFKFVKDLFTKDKSGILK
TSKPELEKHLEKVHQDTKRH
EQLVIPHDIPPIQPPEFNIETGPPKWKEVENR
>g135.t1
TYAPVKQFLRVDKDKVQIFNPAFFKYIHDRWTKHHGRYPSTGMLVLFFALHVCDEVGRLSPPSSSSSSSLT
>g135.t2
MKQREHVVVYTNKAKAQVPAWFCSSVLSYTYAPVKQFLRVDKDKVQIFNPAFFKYIHDRWTKHHGRYPSTGMLVLFFALH
VCDEVGRLSPPSSSSSSSL
T
>g136.t1
MAAFKRTFWHITDQHWDPSYKLGGNPEAVCASSGQRPAVNAGPFGDYVCDSPWHLINSSVYFMKQVMPDPDFILWTGDDT
PHVPDQDLDVEGVLGIVRNL
THIIKEVFPNTKVYSALGNHDYQPKSQFPAASSYIYHQIAEMWSDWLEPSSQVTFKKGGYYSERLLNRTGYRIMVLNTNL
YYDQNKLTSKMSDPADQFHW
AEQVLKDAARNKERVYIIGHVPPGFFEKKRGKPWFRPEFNQRYVDMVRKHHPVIQGQFFGHHHTDSFRMFYSNDRPISTM
FLSPGVTPWKTTLPGVTDGA

NNPGIRSVEYDTQTLLVR
>g136.t2
LLSSIRLQDLPLESDMAAFKRTFWHITDQHWDPSYKLGGNPEAVCASSGQRPAVNAGPFGDYVCDSPWHLINSSVYFMKQ
VMPDPDFILWTGDDTPHVPD
QDLDVEGVLGIVRNLTHIIKEVFPNTKVYSALGNHDYQPKSQFPAASSYIYHQIAEMWSDWLEPSSQVTFKKGGYYSERL
LNRTGYRIMVLNTNLYYDQN
KLTSKMSDPADQFHWAEQVLKDAARNKERVYIIGHVPPGFFEKKRGKPWFRPEFNQRYVDMVRKHHPVIQGQFFGHHHTD
SFRMFYSNDRPISTMFLSPG
VTPWKTTLPGVTDGANNPGIRSVEYDTQTLLVR
>g136.t3
LPLESDMAAFKRTFWHITDQHWDPSYKLGGNPEAVCASSGQRPAVNAGPFGDYVCDSPWHLINSSVYFMKQVMPDPDFIL
WTGDDTPHVPDQDLDVEGVL
GIVRNLTHIIKEVFPNTKVYSALGNHDYQPKSQFPAASSYIYHQIAEMWSDWLEPSSQVTFKKGGYYSERLLNRTGYRIM
VLNTNLYYDQNKLTSKMSDP
ADQFHWAEQVLKDAARNKERVYIIGHVPPGFFEKKRGKPWFRPEFNQRYVDMVRKHHPVIQGQFFGHHHTDSFRMFYSND
RPISTMFLSPGVTPWKTTLP
GVTDGANNPGIRSVEYDTQTLLVR
>g137.t1
LHFYYYCTLGGISNSSVGSGYNQSPGGFASPSLSQGGEKKGRTRTNQIVPCTVSQLMSASQADEAFRVGEVEVAQITIVG
LIRSTDKSMTNIQYKVDDMT
AAPMDVKQWVDTEDPSMDSAVLPPGTYVKVSGNLRSFQNHRSVVAFSIRQVEDLNEITSHMLEVVQAHMALSKSPSTMGG
GPVGVNMPSARPGTMEEMGR
GYSGANEVSTNGLSPNQNQVLRLIRSSQDAQGISLQELERRLSGMSVVVIKQAVEFLSNEGHIFSTIDEDHFKSTDSDE
>g137.t2
LLSRDLVVRRRGGISNSSVGSGYNQSPGGFASPSLSQGGEKKGRTRTNQIVPCTVSQLMSASQADEAFRVGEVEVAQITI
VGLIRSTDKSMTNIQYKVDD
MTAAPMDVKQWVDTEDPSMDSAVLPPGTYVKVSGNLRSFQNHRSVVAFSIRQVEDLNEITSHMLEVVQAHMALSKSPSTM
GGGPVGVNMPSARPGTMEEM
GRGYSGANEVSTNGLSPNQNQVLRLIRSSQDAQGISLQELERRLSGMSVVVIKQAVEFLSNEGHIFSTIDEDHFKSTDSD
E
>g137.t3
MSASQADEAFRVGEVEVAQITIVGLIRSTDKSMTNIQYKVDDMTAAPMDVKQWVDTEDPSMDSAVLPPGTYVKVSGNLRS
FQNHRSVVAFSIRQVEDLNE
ITSHMLEVVQAHMALSKSPSTMGGGPVGVNMPSARPGTMEEMGRGYSGANEVSTNGLSPNQNQVLRLIRSSQDAQGISLQ
ELERRLSGMSVVVIKQAVEF
LSNEGHIFSTIDEDHFKSTDSDE
>g138.t1
SVYELSGNEVCFSTGDMVKVIGLTLESITCEDATSNQKSEMQISSKGLFKQAEKNRCFQTLKAMLDVRTQEKGDSCLPFS
FISHSELTLDALVVGPGTVM
VAISVEPCQRQGKERMLCQMRGSWGGSLGVALPLCCRGQFSECFTAQEIVSSSRLCRGRFCCVETADGVERPLVFWPVYN
VEARMHLRKDTVTFSSNLEV
DVVEVTDHDTSFIVPLTLDELLTMPDHIFPVVAQILAMPKLEHLVRSGWVEELRSQQTLVLHGTGAITMALCSSARRAKR
QYFLVSEGYAGMFRRRPREF
LSVFELYVAATQQPGLKVTVTRSGQEAADDMIGVVAGEVLEVVGFHVGGQGAQLQNAKQEGDVVICRRIEESSDDEQEEE
GVSSSSATVVLPLHWHGFFT
EVISNNKKYKIRDMCQKFSLPLNVKVVSRDSTLAFDPLYGISSFCLECITTETIIWASILTSPELCFTIPTRCLSMDIQF
TQQSLPWPKDEPPACLTDTV
IEVTESFYLEDCRLIYSQQPPPPRPPKTTNLSLAPKPCNPETLALSVPRKPAIPSPTPSHHSVDRLQHANQQGEVQSEDS
DHDYESGDLMDAIKKMQEFT
IS
>g138.t2
MLDVRTQEKGDSCLPFSFISHSELTLDALVVGPGTVMVAISVEPCQRQGKERMLCQMRGSWGGSLGVALPLCCRGQFSEC
FTAQEIVSSSRLCRGRFCCV
ETADGVERPLVFWPVYNVEARMHLRKDTVTFSSNLEVDVVEVTDHDTSFIVPLTLDELLTMPDHIFPVVAQILAMPKLEH
LVRSGWVEELRSQQTLVLHG
TGAITMALCSSARRAKRQYFLVSEGYAGMFRRRPREFLSVFELYVAATQQPGLKVTVTRSGQEAADDMIGVVAGEVLEVV

GFHVGGQGAQLQNAKQEGDV
VICRRIEESSDDEQEEEGVSSSSATVVLPLHWHGFFTEVISNNKKYKIRDMCQKFSLPLNVKVVSRDSTLAFDPLYGISS
FCLECITTETIIWASILTSP
ELCFTIPTRCLSMDIQFTQQSLPWPKDEPPACLTDTVIEVTESFYLEDCRLIYSQQPPPPRPPKTTNLSLAPKPCNPETL
ALSVPRKPAIPSPTPSHHSV
DRLQHANQQGEVQSEDSDHDYESGDLMDAIKKMQEFTIS
>g138.t3
MVSAGVAHSRSRLKVKARAVDGSVYELSGNEVCFSTGDMVKVIGLTLESITCEDATSNQKSEMQISSKGLFKQAEKNRCF
QTLKAMLDVRTQEKGDSCLP
FSFISHSELTLDALVVGPGTVMVAISVEPCQRQGKERMLCQMRGSWGGSLGVALPLCCRGQFSECFTAQEIVSSSRLCRG
RFCCVETADGVERPLVFWPV
YNVEARMHLRKDTVTFSSNLEVDVVEVTDHDTSFIVPLTLDELLTMPDHIFPVVAQILAMPKLEHLVRSGWVEELRSQQT
LVLHGTGAITMALCSSARRA
KRQYFLVSEGYAGMFRRRPREFLSVFELYVAATQQPGLKVTVTRSGQEAADDMIGVVAGEVLEVVGFHVGGQGAQLQNAK
QEGDVVICRRIEESSDDEQE
EEGVSSSSATVVLPLHWHGFFTEVISNNKKYKIRDMCQKFSLPLNVKVVSRDSTLAFDPLYGISSFCLECITTETIIWAS
ILTSPELCFTIPTRCLSMDI
QFTQQSLPWPKDEPPACLTDTVIEVTESFYLEDCRLIYSQQPPPPRPPKTTNLSLAPKPCNPETLALSVPRKPAIPSPTP
SHHSVDRLQHANQQGEVQSE
DSDHDYESGDLMDAIKKMQEFTIS
>g139.t1
SVSRIGQSKSVFSQSGNGREVLPIPFSQSQSSAPHSSTTPQVLSPTLVAPPNIQPRRSTRLFTSASSTAKVLVDRNTNGW
ARNNNMEPPPCGPTTCRDRH
WGRCYASRAAGKGGGLGMLISGIADWF
>g139.t2
SVSRIGQSKSVFSQSGNGREVLPIPFSQSQSSAPHSSTTPQVLSPTLVAPPNIQPRRSTRLFTSASSTAKVLVDRNTNAR
NNNMEPPPCGPTTCRDRHWG
RCYASRAAGKGGGLGMLISGIADWF
>g139.t3
SKSVFSQSGNGREVLPIPFSQSQSSAPHSSTTPQVLSPTLVAPPNIQPRRSTRLFTSASSTAKVLVDRNTNGWARNNNME
PPPCGPTTCRDRHWGRCYAS
RAAGKGGGLGMLISGIADWF
>g140.t1
SSPIPSISFSGHNIPPCSSFCKTSFFHLRNIAKLRPSLSLHAAEKLVHAFVSSRLDYCSALLIGIPGKSLQRLQHIQNSA
ARILMRMRKNDHITPVLHSL
HWLPVHLRIQYKILLHTHHCLHGEAPTYLTELLTPHTTSRTRSGQSHRVCPPRTKLKTMGDRAFQAAAPSLWNALPDHHR
APQTEDAFKKGLKTFLFRKA
YS
>g141.t1
MNPADSSPSSTQLPFRVRTSCLYPGEDFSSRSPATQPSYPALFPTCLALFLGLTRFHNSLVVQLNLQHSLEEHQV
>g142.t1
DGTVFCSALKGLSVSLLQRCKNKLYPDNLPRTSVVIVFHNEAWSTLLRTVHSVIDRSPHTLLEEIVLVDDASER
>g142.t2
LLQDGTVFCSALKGLSVSLLQRCKNKLYPDNLPRTSVVIVFHNEAWSTLLRTVHSVIDRSPHTLLEEIVLVDDASER
>g142.t3
CKNKLYPDNLPRTSVVIVFHNEAWSTLLRTVHSVIDRSPHTLLEEIVLVDDASER
>g143.t1
MTSLAHQLKRLALPQNDPNLFQRKDVASLLFDPIFAATMDRNTFYALGCTGLEELLGIEPAFAEFQDTLFNQASVTLERS
VQTKEINEKLDEGISLFLAR
VSPYFLLKPAQKCLEWLIHRFHIQLYNVDSLLACTLPYHETKTFTRVVQLLKISEPIHRWHWLHALQKSGVPLARGTLIT
HCYKNLSFMDFICTMVTKAV
KAYSSQSANCSQLRVVFSFYASTIVSALDAVDKVTDTIISKLLPFVQIGLTSSLADYKAATYMIVCQLAVKVVMEGSLVN
TLALHISKSLCKEPVLSKEG
LGCLIVLLQNQKEDSVGPKAFGHLCCMSSLVESLQAMAVLHHISPLLRYLLPHLVHSVVTARYDEMEQAAEAPSHGSLLE
TVIQSLPLSKGLDHMVA
>g143.t2

MTSLAHQLKRLALPQNDPNLFQRKDVASLLFDPIFAATMDRNTFYALGCTGLEELLGIEPAFAEFQDTLFNQASVTLERS
VQTKEINEKLDEGISLFLAR
VSPYFLLKPAQKCLEWLIHRFHIQLYNVDSLLACTLPYHETKTFTRVVQLLKISEPIHRWHWLHALQKSGVPLARGTLIT
HCYKNLSFMDFICTMVTKAV
KAYSSQSANCSQLRVVFSFYASTIVSALDAVDKVTDTIISKLLPFVQIGLTSSLADYKAATYMIVCQLAVKVVMEGSLVN
TLALHISKSLCKEPVLSKEG
LGCLIVLLQNQKEDSVGPKAFGHLCCMSSLVESLQAMAVLHHISPLLRYLLPHLVHSVVTARYDEMEQAAEAPSHGSLLE
TVIQSLPLSKGLDHMVARPS
H
>g143.t3
MTSLAHQLKRLALPQNDPNLFQRKDVASLLFDPIFAATMDRNTFYALEFQDTLFNQASVTLERSVQTKEINEKLDEGISL
FLARVSPYFLLKPAQKCLEW
LIHRFHIQLYNVDSLLACTLPYHETKTFTRVVQLLKISEPIHRWHWLHALQKSGVPLARGTLITHCYKNLSFMDFICTMV
TKAVKAYSSQSANCSQLRVV
FSFYASTIVSALDAVDKVTDTIISKLLPFVQIGLTSSLADYKAATYMIVCQLAVKVVMEGSLVNTLALHISKSLCKEPVL
SKEGLGCLIVLLQNQKEDSV
GPKAFGHLCCMSSLVESLQAMAVLHHISPLLRYLLPHLVHSVVTARYDEMEQAAEAPSHGSLLETVIQSLPLSKGLDHMV
A
>g144.t1
MSLQQPEVEAASDEEEGVFFIETDSECEWRDEDQVDHRELILQKADALASENRLKEAVDCFATAMRCGSVRPEQLGALVD
CVLRNFKRVVPEPEGGPGSG
EDTDMFDCPGCGCFLGEPVTLPCGHSYCRRCVHHGHLSRCQACGEPLRCAHAAGTPRKVNVVLGGLLGRFFPEELDTYKT
ICEIQELTTSRRFKAAVSLV
SDVIQPGSKVAASSSAMVARLCRAEAYTALGQYRLALEDAEFCLNTQPSAEGFFRKATVLHAMGQTDESLQVFLQCLVMD
GDFTRARRQVEMSCITASIL
RHSGGGTELNFIIIIFFIFFLGQILCDLLSPAGDALKEGLRATTQSNASPRLRGKALAAAAVSCGAHDDPEQTVPPDHHH
LTRAASLHGPPDAEK
>g144.t2
MSLQQPEVEAASDEEEGVFFIETDSECEWRDEDQVDHRELILQKADALASENRLKEAVDCFATAMRCGSVRPEQLGALVD
CVLRNFKRVVPEPEGGPGSG
EDTDMFDCPGCGCFLGEPVTLPCGHSYCRRCVHHGHLSRCQACGEPLRCAHAAGTPRKVNVVLGGLLGRFFPEELDTYKT
ICEIQELTTSRRFKAAVSLV
SDVIQPGSKVAASSSAMVARLCRAEAYTALGQYRLALEDAEFCLNTQPSAEGFFRKATVLHAMGQTDESLQVFLQCLVMD
GDFTRARRQVEMSCITASIL
RHSGGGTELNFIIIIFFIFFLGQILCDLLSPAGDALKEGLRATTQSNASPRLRGKALAAAAVSCGAHDDPEQTVPPDHHH
LTRAASLHGPPDAEKRLPFA
HLADSFIQSDSGGKLLEGLSRIHSSTAGPACRTAVWRSFECPFELPTPCLL
>g144.t3
MSLQQPEVEAASDEEEGVFFIETDSECEWRDEDQVDHRELILQKADALASENRLKEAVDCFATAMRCGSVRPEQLGALVD
CVLRNFKRVVPEPEGGPGSG
EDTDMFDCPGCGCFLGEPVTLPCGHSYCRRCVHHGHLSRCQACGEPLRCAHAAGTPRKVNVVLGGLLGRFFPEELDTYKT
ICEIQELTTSRRFKAAVSLV
SDVIQPGSKVAASSSAMVARLCRAEAYTALGQYRLALEDAEFCLNTQPSAEGFFRKATVLHAMGQTDESLQVFLQCLVMD
GDFTRARRQVEMVRDLDGRT
ICCSSH
>g145.t1
MDVGASEEERATLGRDQDCTGLPFPSHNGHWRVRE
>g146.t1
MCVDATDPMKGNWQRYVHRAHSTQEQNLFPLEINQAIYYKLLRPIRPGEELLVWYSAQDNPEITAALEEERVSSLSKKNS
PQAKRGRKEEEDERPSSSGP
GHAEMEPAPVESPAPTDVQDMSVVMIDWKKEEVETKVDQADDAGEPQQLPSMQTPQETSVTAEWREDEQQFPNCGKAGGE
SPQDLQPRSDSASLQGQDPE
SLDPDIDFDPDPDGDLEGDLQGESHPCLHCERHFSTKQGLERHIHIHTTANQQTHLFKCRYCGKSFGSQVGRRRHERRHE
SGAKKKPGSLAGTATLLSFA
GKAGASSPECTSPTSYIAIGSQFPVVHQPVPELAKKDLRPDADRPYTLEEYESKELHPCKYCNKAFGTHTNMRRHQRRIH
ERHLLPKGVRRKGMLPQEVS

AQRLPNESPSGSPPPVYVPSADTEDEADRDEYVVDISKNISENLSFYIDGKILSTSSVSNCEVIEVNSRSAALFGLDAVI
ISPNQISHALKVESKPNAGK
ALVSNLGQPTSKRRTTTPPHIPSLKMENEMGCSASSSSSSSSTSSSSNLLGGGLFSQSTESLAFQKEKTVYLSPKLKQLL
QSQDSQKSTLSQIGDSHRLA
SPLSVTSLPGASGRFKRRTSSPPSSPQLSPTQKPDNSKAEVGSLYTLKVPKLEGHRLSPVLNLTVHEDGDTVCSSEKDMS
SRSSSSCGGNSCNQQPLDLS
SSVNRRSDVLSKTLGDSALDLSVQRKSAMDPELKGTPPPQPPTKKRKPNTSMLEKVLMNEYAGLTSAVEEGPSALSSLGP
LQSLSPSMASESAHPSPPSL
TPVTMNPSSPAASSMTSPTPPPPVLPTIPPDMPISPRSEPSDCSVSRPLPVLSPKSSPRSNEDEALSDTEETSMAKEVQD
SVDENHEQLDSIPHTPVQDA
LESLSAPSPTEPTPGEPPSVETDVPQTPKDNGLTNGKPESEEPGSMTEAKSLSPDSAPLTSQQQQHSPALPTSPTISHNS
SPPPLSPSVALPVMKKDPVH
YSEEMSDTSHVHSDADDSTCPAANETPKEKGEDSETFCKTFVCNVCEEPFRSIKALNGHIVAHAVEWPYKCEFCVQLFVE
ARALLAHRTSLHGVGHIFVC
SECSKEFAFLCNLEQHQKDLHPSQTCTHTTVESGKLRPHNYTDPSRAKEESRPSTPVPEADVEAAPESDSIEKSPEVNGQ
SGEDEPEPEDSSEELYTTIK
IMASEGGKPKGPDVRLGLNQHYPSFKPPPFPYHSRSHAGSVASATNFTTHNIPQTFSTAIRCTKCGNSFDNMPELHKHIL
ACANASDKKRYTPKKNPIPL
KQIVKPQNGALSPAAAGAGQSAFRRMGQPKRLNFNQEPPGKAKMSSLNKKKNQLVQKAISQKNKTAISVKKASVKVEEQE
PEPHVCPHCSREFTYPASLN
KHIAISCPMKPEPKKGKKGDGKQDSPQMSLRRKATDADVPESEPKPLGKTRARSYGAAEPDPPQASRGRTGAPLGRPKRP
APFPASLAAPGKKRKKGQAQ
SSPAPPALSGSDPLNDAAQRPAVKAQRAAKEAAPKKAPEVTSPLQQQQQQQPKKEERFSLRTRERVGGPVTRSLQSVGAV
AAVEVKAEQPLAQEPKEPQE
SPLK
>g146.t2
MKGNWQRYVHRAHSTQEQNLFPLEINQAIYYKLLRPIRPGEELLVWYSAQDNPEITAALEEERVSSLSKKNSPQAKRGRK
EEEDERPSSSGPGHAEMEPA
PVESPAPTDVQDMSVVMIDWKKEEVETKVDQADDAGEPQQLPSMQTPQETSVTAEWREDEQQFPNCGKAGGESPQDLQPR
SDSASLQGQDPESLDPDIDF
DPDPDGDLEGDLQGESHPCLHCERHFSTKQGLERHIHIHTTANQQTHLFKCRYCGKSFGSQVGRRRHERRHESGAKKKPG
SLAGTATLLSFAGKAGASSP
ECTSPTSYIAIGSQFPVVHQPVPELAKKDLRPDADRPYTLEEYESKELHPCKYCNKAFGTHTNMRRHQRRIHERHLLPKG
VRRKGMLPQEVSAQRLPNES
PSGSPPPVYVPSADTEDEADRDEYVVDISKNISENLSFYIDGKILSTSSVSNCEVIEVNSRSAALFGLDAVIISPNQISH
ALKVESKPNAGKALVSNLGQ
PTSKRRTTTPPHIPSLKMENEMGCSASSSSSSSSTSSSSNLLGGGLFSQSTESLAFQKEKTVYLSPKLKQLLQSQDSQKS
TLSQIGDSHRLASPLSVTSL
PGASGRFKRRTSSPPSSPQLSPTQKPDNSKAEVGSLYTLKVPKLEGHRLSPVLNLTVHEDGDTVCSSEKDMSSRSSSSCG
GNSCNQQPLDLSSSVNRRSD
VLSKTLGDSALDLSVQRKSAMDPELKGTPPPQPPTKKRKPNTSMLEKVLMNEYAGLTSAVEEGPSALSSLGPLQSLSPSM
ASESAHPSPPSLTPVTMNPS
SPAASSMTSPTPPPPVLPTIPPDMPISPRSEPSDCSVSRPLPVLSPKSSPRSNEDEALSDTEETSMAKEVQDSVDENHEQ
LDSIPHTPVQDALESLSAPS
PTEPTPGEPPSVETDVPQTPKDNGLTNGKPESEEPGSMTEAKSLSPDSAPLTSQQQQHSPALPTSPTISHNSSPPPLSPS
VALPVMKKDPVHYSEEMSDT
SHVHSDADDSTCPAANETPKEKGEDSETFCKTFVCNVCEEPFRSIKALNGHIVAHAVEWPYKCEFCVQLFVEARALLAHR
TSLHGVGHIFVCSECSKEFA
FLCNLEQHQKDLHPSQTCTHTTVESGKLRPHNYTDPSRAKEESRPSTPVPEADVEAAPESDSIEKSPEVNGQSGEDEPEP
EDSSEELYTTIKIMASEGGK
PKGPDVRLGLNQHYPSFKPPPFPYHSRSHAGSVASATNFTTHNIPQTFSTAIRCTKCGNSFDNMPELHKHILACANASDK
KRYTPKKNPIPLKQIVKPQN
GALSPAAAGAGQSAFRRMGQPKRLNFNQEPPGKAKMSSLNKKKNQLVQKAISQKNKTAISVKKASVKVEEQEPEPHVCPH
CSREFTYPASLNKHIAISCP
MKPEPKKGKKGDGKQDSPQMSLRRKATDADVPESEPKPLGKTRARSYGAAEPDPPQASRGRTGAPLGRPKRPAPFPASLA
APGKKRKKGQAQSSPAPPAL

SGSDPLNDAAQRPAVKAQRAAKEAAPKKAPEVTSPLQQQQQQQPKKEERFSLRTRERVGGPVTRSLQSVGAVAAVEVKAE
QPLAQEPKEPQESPLK
>g146.t3
MCVDATDPMKGNWQRYVHRAHSTQEQNLFPLEINQAIYYKLLRPIRPGEELLVWYSAQDNPEITAALEEERVSSLSKKNS
PQAKRDVQDMSVVMIDWKKE
EVETKVDQADDAGEPQQLPSMQTPQETSVTAEWREDEQQFPNCGKAGGESPQDLQPRSDSASLQGQDPESLDPDIDFDPD
PDGDLEGDLQGESHPCLHCE
RHFSTKQGLERHIHIHTTANQQTHLFKCRYCGKSFGSQVGRRRHERRHESGAKKKPGSLAGTATLLSFAGKAGASSPECT
SPTSYIAIGSQFPVVHQPVP
ELAKKDLRPDADRPYTLEEYESKELHPCKYCNKAFGTHTNMRRHQRRIHERHLLPKGVRRKGMLPQEVSAQRLPNESPSG
SPPPVYVPSADTEDEADRDE
YVVDISKNISENLSFYIDGKILSTSSVSNCEVIEVNSRSAALFGLDAVIISPNQISHALKVESKPNAGKALVSNLGQPTS
KRRTTTPPHIPSLKMENEMG
CSASSSSSSSSTSSSSNLLGGGLFSQSTESLAFQKEKTVYLSPKLKQLLQSQDSQKSTLSQIGDSHRLASPLSVTSLPGA
SGRFKRRTSSPPSSPQLSPT
QKPDNSKAEVGSLYTLKVPKLEGHRLSPVLNLTVHEDGDTVCSSEKDMSSRSSSSCGGNSCNQQPLDLSSSVNRRSDVLS
KTLGDSALDLSVQRKSAMDP
ELKGTPPPQPPTKKRKPNTSMLEKVLMNEYAGLTSAVEEGPSALSSLGPLQSLSPSMASESAHPSPPSLTPVTMNPSSPA
ASSMTSPTPPPPVLPTIPPD
MPISPRSEPSDCSVSRPLPVLSPKSSPRSNEDEALSDTEETSMAKEVQDSVDENHEQLDSIPHTPVQDALESLSAPSPTE
PTPGEPPSVETDVPQTPKDN
GLTNGKPESEEPGSMTEAKSLSPDSAPLTSQQQQHSPALPTSPTISHNSSPPPLSPSVALPVMKKDPVHYSEEMSDTSHV
HSDADDSTCPAANETPKEKG
EDSETFCKTFVCNVCEEPFRSIKALNGHIVAHAVEWPYKCEFCVQLFVEARALLAHRTSLHGVGHIFVCSECSKEFAFLC
NLEQHQKDLHPSQTCHTTTV
ESGKLRPHNYTDPSRAKEESRPSTPVPEADVEAAPESDSIEKSPEVNGQSGEDEPEPEDSSEELYTTIKIMASEGGKPKG
PDVRLGLNQHYPSFKPPPFP
YHSRSHAGSVASATNFTTHNIPQTFSTAIRCTKCGNSFDNMPELHKHILACANASDKKRYTPKKNPIPLKQIVKPQNGAL
SPAAAGAGQSAFRRMGQPKR
LNFNQEPPGKAKMSSLNKKKNQLVQKAISQKNKTAISVKKASVKVEEQEPEPHVCPHCSREFTYPASLNKHIAISCPMKP
EPKKGKKGDGKQDSPQMSLR
RKATDADVPESEPKPLGKTRARSYGAAEPDPPQASRGRTGAPLGRPKRPAPFPASLAAPGKKRKKGQAQSSPAPPALSGS
DPLNDAAQRPAVKAQRAAKE
AAPKKAPEVTSPLQQQQQQQPKKEERFSLRTRERVGGPVTRSLQSVGAVAAVEVKAEQPLAQEPKEPQESPLK
>g147.t1
LVLRCQRSKSASPYAGARRRVPDSQLEAPPSRPSRRWLADRSTMMPGSALGKKLDLSKLTDEEAKHVWAVVQRDFDLRKK
EEDRLG
>g148.t1
MKLTDTGPPGDYKYGGSVFVNEAALAGSPAPNNHHKRDDPSAHNSLNALLVRSTRRQQAGPQNRAAAHQNRAVDTGFFYQ
PTGPAAELTSPSAGGKTGTR
PEVLVPMARFGGAEVQGLARVVKCPSQRGV
>g149.t1
MLTYNSGTSPAKHRHAITKGPIASVKNAVADLTVQNQRDNQGNQDQRHTWGNRDQQGPPRLNEDSNGQGGKSDVCFHCSR
GLLPRPLGKLPKERTTERTW
LPGGMEDTTGDVVFGTGIRDHHPTMVNNRRDGERGGQEERRRGQQHP
>g150.t1
MMMYPHYDPKSQQDGTTLNPAIRTFTRGQWSNKVEFLLAVAGQIIGLGNVWRFPYLCYKNGGGVFFIPYVLFLFTCGIPL
FLLETSLGQYTTQGSITCWR
KMCPLFEGMGYGSLVVVLYSSMYYIIILAWAFFYLFYSFSSELPWATCSNSWNTEQTVKSNINVDLPIAANWLKVVYFTA
TFPYVMLVVLLVRGLTLPGA
INGIKFYLYPVPSRLTDPMVWMDAGPGLAFIAYPRAVAMMPFPQLWAIFFFIMVILLGLDSQFVGLEAVTTAISDMNPAF
FHMGHRRKILLLIICIGCFF
VGLVMVTEGGLYIFQLFDYYACSGMTLLLFAILQSVCIGWVYGEWPLM
>g150.t2
MMYPHYDPKSQQDGTTLNPAIRTFTRGQWSNKVEFLLAVAGQIIGLGNVWRFPYLCYKNGGGVFFIPYVLFLFTCGIPLF
LLETSLGQYTTQGSITCWRK

MCPLFEGMGYGSLVVVLYSSMYYIIILAWAFFYLFYSFSSELPWATCSNSWNTEQTVKSNINVDLPIAANWLKVVYFTAT
FPYVMLVVLLVRGLTLPGAI
NGIKFYLYPVPSRLTDPMVWMDAGPGLAFIAYPRAVAMMPFPQLWAIFFFIMVILLGLDSQFVGLEAVTTAISDMNPAFF
HMGHRRKILLLIICIGCFFV
GLVMVTEGGLYIFQLFDYYACSGMTLLLFAILQSVCIGWVYGEWPLM
>g150.t3
MMMYPHYDPKSQQDGTTLNPAIRTFTRGQWSNKVEFLLAVAGQIIGLGNVWRFPYLCYKNGGGVFFIPYVLFLFTCGIPL
FLLETSLGQYTTQGSITCWR
KMCPLFEGMGYGSLVVVLYSSMYYIIILAWAFFYLFYSFSSELPWATCSNSWNTEQTVKSNINVDLPIAANWLKVVYFTA
TFPYVMLVVLLVRGLTLPGA
INGIKFYLYPVPSRLTDPMVWMDAGPGLAFIAYPRAVAMMPFPQLWAIFFFIMVILLGLDSQFVGLEAVTTAISDMNPAF
FHMGHRRKILLLIICIGCFF
VGLVMVTEGGLYIFQLFDYYACSGMTLLLFAILQSVCIGWVYGADRFYDIIEDMIGYRPLPLLKYCLKYVTPVICIATFV
FSLVKYTPLKFNNKIDYPCG
SLPFAPHQMTYR
>g151.t1
MIGDRVTVHYTGKLLNGQKVELLDFQGEMLTEDGGVMRRIKTKGEGFTNPNDGATVDVHLEGRCEGQLFDSRDVRFVVGE
AEDKGVPLGVDRAMEKMQKG
ECCLLQLQSKYGFGREGKPELQIGPNKDVLYEVTLRDFKKAGLLQQAVIQYQRIVSWLEMECGIGEEQQRKIQEFLLLSQ
LNLALCFLRLQEFSHVVDNC
NKVMELDAKNEKALYRRGEARLCRNEFSLAMADFRQVLQVNPLNRAARAQITICQRKVREHQEQDKKIYANMFQKFAEHD
KKVGKQKRKKDESVKNNGEV
GNKRRRRSQENS
>g151.t2
LQIVKGQGLDGDRPMIGDRVTVHYTGKLLNGQKVELLDFQGEMLTEDGGVMRRIKTKGEGFTNPNDGATVDVHLEGRCEG
QLFDSRDVRFVVGEAEDKGV
PLGVDRAMEKMQKGECCLLQLQSKYGFGREGKPELQIGPNKDVLYEVTLRDFKKAGLLQQAVIQYQRIVSWLEMECGIGE
EQQRKIQEFLLLSQLNLALC
FLRLQEFSHVVDNCNKVMELDAKNEKALYRRGEARLCRNEFSLAMADFRQVLQVNPLNRAARAQITICQRKVREHQEQDK
KIYANMFQKFAEHDKKVGKQ
KRKKDESVKNNGEVGNKRRRRSQENS
>g151.t3
MIGDRVTVHYTGKLLNGQKVELLDFQGEMLTEDGGVMRRIKTKGEGFTNPNDGATVDVHLEGRCEGQLFDSRDVRFVVGM
ALAEKAGLLQQAVIQYQRIV
SWLEMECGIGEEQQRKIQEFLLLSQLNLALCFLRLQEFSHVVDNCNKVMELDAKNEKALYRRGEARLCRNEFSLAMADFR
QVLQVNPLNRAARAQITICQ
RKVREHQEQDKKIYANMFQKFAEHDKKVGKQKRKKDESVKNNGEVGNKRRRRSQENS
>g152.t1
MNHKAPVAPHSRFLLPDTFTADLDILIHLPARREEETNVIPKTNPPSPRKAKVLMLVNCLEFNAAFHELFLKSDNWKFHN
DDGMEELIKESRFLSWFAVL
QTDFDDSRRSSTNIPWAKSSTLRKGSAVVACGSPFGSLCPDLFSNTLSTGIVSNTSGEDNAVILTDARCLPGTEGGGLFV
TESDRHAHLVGVIVASLCWK
GVEWIGLTLVCSVQVILRNISDITDRMNESPLRELSCRRGFEPEGLHLLNTASSSSSSSKLREYPTVCLIDSGQFWGSGV
MVAPHLALTCRHVVNGKKTV
TLKFHHKGRVHVIMGDVLFSTKPSSSFDFAVVQLRDSAPDVVVPVVAKSFQPGEEVFVVGYGALGKSCGPSLTGGILSKA
VSWDRRPVLLQTTCAVQAGA
SGGAVVRKHSGELLGIVSSNTRDCAANVTYPHLNFSIPGTVFQLPLERFSRSGDTEAFRDLDTTDAGVQRAWRLQNLPSK
L
>g152.t2
MIICNGFPFSRFMNHKAPVAPHSRFLLPDTFTADLDILIHLPARREEETNVIPKTNPPSPRKAKVLMLVNCLEFNAAFHE
LFLKSDNWKFHNDDGMEELI
KESRFLSWFAVLQTDFDDSRRSSTNIPWAKSSTLRKGSAVVACGSPFGSLCPDLFSNTLSTGIVSNTSGEDNAVILTDAR
CLPGTEGGGLFVTESDRHAH
LVGVIVASLCWKGVEWIGLTLVCSVQVILRNISDITDRMNESPLRELSCRRGFEPEGLHLLNTASSSSSSSKLREYPTVC
LIDSGQFWGSGVMVAPHLAL
TCRHVVNGKKTVTLKFHHKGRVHVIMGDVLFSTKPSSSFDFAVVQLRDSAPDVVVPVVAKSFQPGEEVFVVGYGALGKSC

GPSLTGGILSKAVSWDRRPV
LLQTTCAVQAGASGGAVVRKHSGELLGIVSSNTRDCAANVTYPHLNFSIPGTVFQLPLERFSRSGDTEAFRDLDTTDAGV
QRAWRLQNLPSKL
>g152.t3
LLPDTFTADLDILIHLPARREEETNVIPKTNPPSPRKAKVLMLVNCLEFNAAFHELFLKSDNWKFHNDDGMEELIKESRF
LSWFAVLQTDFDDSRRSSTN
IPWAKSSTLRKGSAVVACGSPFGSLCPDLFSNTLSTGIVSNTSGEDNAVILTDARCLPGTEGGGLFVTESDRHAHLVGVI
VASLCWKGVEWIGLTLVCSV
QVILRNISDITDRMNESPLRELSCRRGFEPEGLHLLNTASSSSSSSKLREYPTVCLIDSGQFWGSGVMVAPHLALTCRHV
VNGKKTVTLKFHHKGRVHVI
MGDVLFSTKPSSSFDFAVVQLRDSAPDVVVPVVAKSFQPGEEVFVVGYGALGKSCGPSLTGGILSKAVSWDRRPVLLQTT
CAVQAGASGGAVVRKHSGEL
LGIVSSNTRDCAANVTYPHLNFSIPGTVFQLPLERFSRSGDTEAFRDLDTTDAGVQRAWRLQNLPSKL
>g153.t1
GQVSLKELNLRPMEVFMCSVLKRQGYGDGFRWLSQYID
>g154.t1
MMINEAAARLGFLLSFCRMARCPILHRATPASTRLNLPGPVCTTLFQHRSAYAAVCHQFNLHHPPV
>g155.t1
MEYTSSPKPQLSSRANAFSIAALMSSGKPKDKETEENTIKPL
>g156.t1
ALEEYGGQESSEQNHSELEAKTEAARQNLRRAEWR
>g157.t1
MCPSFPHSFLRGFKARYGSGGPGAAGNPVHTVTRPEGHLWTKTGPKSLHHHRQSETTSPPPCGPADGQPGSRIPPAAATR
SRFIECSRSFGMSGRETDGG
VVLSAVTLPLAACEAAWLFDLCLSGGLPQHHAGQEAITVIPINTSLHVKLSSTLKHALARRRAAFRGGKTDSRRGAKKKP
VNPAEPGINELSVWCSLEPM
DVGDDLRAWGSEVQNAPLIS
>g157.t2
MCPSFPHSFLRGFKARYGSGGPGAAGNPVHTVTRPEGHLWTKTGPKSLHHHRQSETTSPPPCGPADGQPGSRIPPAAATR
SRFIECSRSFGMSGRETDGG
VVLSAVTLPLAACGGLPQHHAGQEAITVIPINTSLHVKLSSTLKHALARRRAAFRGGKTDSRRGAKKKPVNPAEPGINEL
SVWCSLEPMDVGDDLRAWGS
EVQNAPLIS
>g157.t3
MCPSFPHSFLRGFKARYGSGGPGAAGNPVHTVTRPEGHLWTKTGPKSLHHHRQSETTSPPPCGPADGQPGSRIPPAAATR
SRFIECSRSFGMSGRETDGG
VVLSAVTLPLAACEAAWLFDLCLSPQRDSKIMTVSHQCVSWGASDLL
>g158.t1
WTPFFVVHVTNVLCESCEIGPTLISVVTWLGYVNSAVNPIIYTAFNTEFRNVFHKLLCCRT
>g159.t1
MLAAMLRQSSGGSGGGEGMGGGGAATKLSLAIPRFSNSHFFPNSASTDTISMAGSVSSTGPSGSGPPVSGPPGRGSKTWC
PSTASHGSEGSEIPTTASPD
YIMQLVSDVRKFADVLLHLKEVFSSK
>g159.t2
LSQLEVLGFAQVKLWGQIRLWVMLAAMLRQSSGGSGGGEGMGGGGAATKLSLAIPRFSNSHFFPNSASTDTISMAGSVSS
TGPSGSGPPVSGPPGRGSKT
WCPSTASHGSEGSEIPTTASPDYIMQLVSDVRKFADVLLHLKEVFSSK
>g160.t1
MDVGLVSAIFTCLLTRALSQSNPGSTVTPVSKQNQSEPSVSGPAWKNALPPPPALGLLTIHWTHQSCEGEVQLMLPDHGP
SSCFPAGISQAMLEKFQQGQ
SEVCRGRLGCGGEPHWHKDNNTNCPSLRVTCT
>g160.t2
MDVGLVSAIFTCLLTRALSQSNPGSTVTPVSKQNQSEPSVSGPAWKNALPPPPALGLLTIHWTHQSCEGEVQLMLPDHGP
SSCFPAGISQAMLEKFQQGQ
SEVCRGRLGCGGEPHWHKDNNTNCPSLRVTCTGRSSTNRRVGGSIPLILLLLQLSTCQTPHMY
>g161.t1

MTSRQWVTSRRWILLMESSDPLAVTLGRCGQHQHLAWSSVILGRCGLHQHLACLQPLAAGLRKSCLVDP
>g161.t2
CLWRGSGTGETRHMTSRQWVTSRRWILLMESSDPLAVTLGRCGQHQHLAWSSVILGRCGLHQHLACLQPLAAGLRKSCLV
DP
>g162.t1
MVLGKVKSFTVSYDCLNDSNVPVFSSGDSVSDDDNSEEGLTTIHSGRHEYAFSLELPQTPLATSFEGKHGSVRYWVKAEL
HRPWLLPMKTKKEFTVFEHI
DINTPLLLSPQAGTKDKTLCCWFCTSGPISLSAKIERKGYTPGESIQIFAEIENCSSRMVVPKAALYQTQTFYAKGKMKE
VKQLVANLRGESLSSGKTET
WSGKMLKIPPVSPSILDCSIIRVEYSLM
>g162.t2
MVLGKVKSFTVSYDCLNDSNVPVFSSGDSVSGRSPPAEVYRLSDSSGVVYHTTVRLLKAVREGGTEDDDNSEEGLTTIHS
GRHEYAFSLELPQTPLATSF
EGKHGSVRYWVKAELHRPWLLPMKTKKEFTVFEHIDINTPLLLSPQAGTKDKTLCCWFCTSGPISLSAKIERKGYTPGES
IQIFAEIENCSSRMVVPKAA
LYQTQTFYAKGKMKEVKQLVANLRGESLSSGKTETWSGKMLKIPPVSPSILDCSIIRVEYSLM
>g162.t3
MVLGKVKSFTVSYDCLNDSNVPVFSSGDSVSDDDNSEEGLTTIHSGRHEPLATSFEGKHGSVRYWVKAELHRPWLLPMKT
KKEFTVFEHIDINTPLLLSP
QAGTKDKTLCCWFCTSGPISLSAKIERKGYTPGESIQIFAEIENCSSRMVVPKAALYQTQTFYAKGKMKEVKQLVANLRG
ESLSSGKTETWSGKMLKIPP
VSPSILDCSIIRVEYSLM
>g163.t1
MLGKLDYTSGSHSISNQVEAWTRGVKFLLSQEGRPVTELVGNASTVRVMTKDWETTDRAAEWIRDAAARSHQPFALYLGL
NLPHTYKTNSLGPTAGGSTF
RTSPYWLKKVSSELISIPKWLPMAAMHPVDYYSTFTKNCSGEFTQEEIRNIRAFYYAMCAEADSMLGQVLAALRSAGLLD
NTVVMFTADHGELAMEHRQY
YKMSMFEGSAHIPLLIMGPGLASGLKSNQTVSLVDIYPTVLELAGIPAPGNLSGHSLLPLLSKSGTRTAKTHPGWALSEF
HGCNANASTYMLRTGQWKYI
AYADDLTLDKAELHNVVTKFPQIGAYLEEQLRSIVAYPKVVLKLEKNNYNAWVFIGLAASELEQPDQSQTAYRKALEFEP
EQLLAWQGLANLYEKTDQWD
FKAELPDVYQKLVELYASSDKIKCYEVIRKLTDIYQSDKEHLKLAKLWLQFIHLKEEDAAEKAELLQLWQQMVQDLSGCI
EAQQPDIETQQHLVTAFEQA
MVLTCPAPGEEHRKLSADYISCLSKLPQEEARMKEACKSMLSLYPSQSYPLEVLCLHHLKTDVLSEEAGSCFSQLLEMSP
NNGLARVGMGTKALQEGKYQ
DAIRNLAQGLNQTGSAAGWCSLAQAQLKLHRYSDSALSCSQGLTLCASGDDDVRTTLLRMRLETLVRSGDAQAADQALET
LKQIPGGEMEMVLKALKGRA
YLNKGQAEQALQVSAELLASVPNMSQGLALRGLAQAAMGQLQLAEQSFLQAAAQCPDDWENDFLLGKLYWDMGEETRRDR
SKAHLLKAAKLDPHLGSAFR
HLGHYYRQVAKEAGRARGCYKKAFELDASDEESGAASVDLSMEQGDMVKRLHTERRAYQNTMAVAVYCQDTALAVLQSVI
DDSTPGSAKWAWMRRGLYHL
KVGQQPQAIADLQSALRADPEDWVCWECLGEAYLNRRSFTAALKAFGKAHKLQPSSIYSVYQMASIKQILGKFQEAAAEY
LQITSREDYVPALKGLGECL
LSLAKGLLEDYRDGSAIDLIQRAIKSLFRAVELRPDLSCLWKLLGDACTAVSAVSPNRARVLVPGPLTGLDPSTQNHLLN
QAQTLKVGERCYGHALKLRP
EAPSLWYDLGLNYHHQARLPCPAEGDNCTQALLLEKAQQ
>g163.t2
MLGKLDYTSGSHSISNQVEAWTRGVKFLLSQEGRPVTELVGNASTVRVMTKDWETTDRAAEWIRDAAARSHQPFALYLGL
NLPHTYKTNSLGPTAGGSTF
RTSPYWLKKVSSELISIPKWLPMAAMHPVDYYSTFTKNCSGEFTQEEIRNIRAFYYAMCAEADSMLGQVLAALRSAGLLD
NTVVMFTADHGELAMEHRQY
YKMSMFEGSAHIPLLIMGPGLASGLKSNQTVSLVDIYPTVLELAGIPAPGNLSGHSLLPLLSKSGTRTAKTHPGWALSEF
HGCNANASTYMLRTGQWKYI
AYADDLTLDKAELHNVVTKFPQIGAYLEEQLRSIVAYPKVVLKLEKNNYNAWVFIGLAASELEQPDQSQTAYRKALEFEP
EQLLAWQGLANLYEKTDQWD
FKAELPDVYQKLVELYASSDKIKCYEVIRKLTDIYQSDKEHLKLAKLWLQFIHLKEEDAAEKAELLQLWQQMVQDLSGCI

EAQQPDIETQQHLVTAFEQA
MVLTCPAPGEEHRKLSADYISCLSKLPQEEARMKEACKSMLSLYPSQSYPLEVLCLHHLKTDVLSEEAGSCFSQLLEMSP
NNGLARVGMGTKALQEGKYQ
DAIRNLAQGLNQTGSAAGWCSLAQAQLKLHRYSDSALSCSQGLTLCASGDDDVRTTLLRMRLETLVRSGDAQAADQALET
LKQVSAELLASVPNMSQGLA
LRGLAQAAMGQLQLAEQSFLQAAAQCPDDWENDFLLGKLYWDMGEETRRDRSKAHLLKAAKLDPHLGSAFRHLGHYYRQV
AKEAGRARGCYKKAFELDAS
DEESGAASVDLSMEQGDMVKRLHTERRAYQNTMAVAVYCQDTALAVLQSVIDDSTPGSAKWAWMRRGLYHLKVGQQPQAI
ADLQSALRADPEDWVCWECL
GEAYLNRRSFTAALKAFGKAHKLQPSSIYSVYQMASIKQILGKFQEAAAEYLQITSREDYVPALKGLGECLLSLAKGLLE
DYRDGSAIDLIQRAIKSLFR
AVELRPDLSCLWKLLGDACTAVSAVSPNRARVLVPGPLTGLDPSTQNHLLNQAQTLKVGERCYGHALKLRPEAPSLWYDL
GLNYHHQARLPCPAEGDNCT
QALLLEKAQQ
>g163.t3
MLGKLDYTSGSHSISNQVEAWTRGVKFLLSQEGRPVTELVGNASTVRVMTKDWETTDRAAEWIRDAAARSHQPFALYLGL
NLPHTYKTNSLGPTAGGSTF
RTSPYWLKKVSSELISIPKWLPMAAMHPVDYYSTFTKNCSGEFTQEEIRNIRAFYYAMCAEADSMLGQVLAALRSAGLLD
NTVVMFTADHGELAMEHRQY
YKMSMFEGSAHIPLLIMGPGLASGLKSNQTVSLVDIYPTVLELAGIPAPGNLSGHSLLPLLSKSGTRTAKTHPGWALSEF
HGCNANASTYMLRTGQWKYI
AYADDLTLDKAELHNVVTKFPQIGAYLEEQLRSIVAYPKVVLKLEKNNYNAWVFIGLAASELEQPDQSQTAYRKALEFEP
EQLLAWQGLANLYEKTDQWD
FKAELPDVYQKLVELYASSDKIKCYEVIRKLTDIYQSDKEHLKLAKLWLQFIHLKEEDAAEKAELLQLWQQMVQDLSGCI
EAQQPDIETQQHLVTAFEQA
MVLTCPAPGEEHRKLSADYISCLSKLPQEEARMKEACKSMLSLYPSQSYPLEVLCLHHLKTDVLSEEAGSCFSQLLEMSP
NNGLARVGMGTKALQEGKYQ
DAIRNLAQGLNQTGSAAGWCSLAQAQLKLHRYSDSALSCSQGLTLCASGDDDVRTTLLRMRLETLVRSGDAQAADQALET
LKQIPGGEMEMVLKALKGRA
YLNKGQAEQALQVSAELLASVPNMSQGLALRGLAQAAMGQLQLAEQSFLQAAAQCPDDWENDFLLGKLYWDMGEETRRDR
SKAHLLKAAKLDPHLGSAFR
HLGHYYRQVAKEAGRARGCYKKAFELDASDEESGAASVDLSMEQGDMDTALAVLQSVIDDSTPGSAKWAWMRRGLYHLKV
GQQPQAIADLQSALRADPED
WVCWECLGEAYLNRRSFTAALKAFGKAHKLQPSSIYSVYQMASIKQILGKFQEAAAEYLQITSREDYVPALKGLGECLLS
LAKGLLEDYRDGSAIDLIQR
AIKSLFRAVELRPDLSCLWKLLGDACTAVSAVSPNRARVLVPGPLTGLDPSTQNHLLNQAQTLKVGERCYGHALKLRPEA
PSLWYDLGLNYHHQARLPCP
AEGDNCTQALLLEKAQQ
>g164.t1
LDGPAGSAFCPSLVFKPTAPLCRKHGAGAAVMDMQIGLNVPARPSKGKRLPLSLTDDEPPGGGLAAVFTAAGLATGGQEW
VFIVKSRVYYPLLMSEALKE
VPSANG
>g165.t1
FSHVTEMNWDPAGIVGMVQSQNQETSSVRASMYTLKEDCRRDRT
>g166.t1
EQRVTIQREFNQWLSSCHEHLDKQVHFIGFNGTITRDDMSVRRMQHPWATFSSIEWDNKTYRAGQLVKSQRTQPIMYGSI
VQFLLYGDHDGDIYATGGQV
EVSLEPKEVHDERRIFLIAKIDRTATNASIQRVIDNELTKLPDKLEVEWPENNSWSQNAGIPAGTKLGPLKVVILNKKGD
AISRMPSPNQGAVRKLAMEL
RVVWHGPREANQDIHNYIAQHTVKWAFWFKPMGTAALLCRLIMHPGGHWEDVRLINITHGTVTMAASAVFLLPCTENLTK
LGKYTLELNTMLNESNAKEF
GGKQLPSYKLNFTITEGKAMRFEVDGFNSNLYVGMAFDMVLKLMDKFNHPVSPPPDLRPELTC
>g166.t2
EQRVTIQREFNQWLSSCHEHLDKQVHFIGFNGTITRDDMSVRRMQHPWATFSSIEWDNKTYRAGQLVKSQRTQPIMYGSI
VQFLLYGDHDGDIYATGGQV
EVSLEPKEVHDERRIFLIAKIDRTATNASIQRVIDNELTKLPDKLEVEWPENNSWSQNAGIPAGTKLGPLKVVILNKKGD

AISRMPSPNQGAVRKLAMEL
RVVWHGPREANQDIHNYIAQHTVKWAFWFKPMGTAALLCRLIMHPGGHWEDVRLINITHGTVTMAASAVFLLPCTENLTK
LGKYTLELNTMLNESNAKEF
GGKQLPSYKLNFTITEGKAMRFEVDGFNSNLYVGMAFDMVLKLMDKFNHPVSPPPDLRPELTCSDLDVSYEKTTSS
>g166.t3
EQRVTIQREFNQWLSSCHEHLDKQVHFIGFNGTITRDDMSVRRMQHPWATFSSIEWDNKTYRAGQLVKSQRTQPIMYGSI
VQFLLYGDHDGDIYATGGQV
EVSLEPKEVHDERRIFLIAKIDRTATNASIQRVIDNELTKLPDKLEVEWPENNSWSQNAGIPAGTKLGPLKVVILNKKGD
AISRMPSPNQGAVRKLAMEL
RVVWHGPREANQDIHNYIAQHTVKWAFWFKPMGTAALLCRLIMHPGGHWEDVRLINITHGTVTMAASAVFLLPCTENLTK
LGKYTLELNTMLNESNAKEF
GGKQLPSYKLNFTITGLFLLPTLPLGIHQHYASSKLKAVAWSAFM
>g167.t1
ITGSDYINANYIDGYRKQNAYIATQGPLPETFGDFWRMVWEQRAATVVMMTRLEEKSRIKCDQYWPSRGTETYGMTQVTL
LDTIELATFCVRTFSLHK
>g168.t1
MSQAPSFGQPAVGQSSSGFTTSSTPAFGQTSGSGQAWFGQTPAFGQSSTFGQTPGFTQQPPSFGLTSATGSTTTMVSTQP
AGFGQPSAFGQPSSTVTAST
FSTVQNVTQQEKGFGSTDFSFKPANEALFKPIFNASPEPTNPQATTGPSQAFGAMLSQTTPSTMESSSAATTTTGSTGFP
LLTGAKIGTLGFSFSQPTAA
PSISAHKDPMTTDGSGDSTSALKFTFSQPAAPSSTTSVTTTSTTTAQPTTPSSFSFSAKVLQPQAATLFGGALFGQTSAF
GDPKAPKQDPGADQRAAERG
SVPETNVFARLGKGTKRKEDPEVLSGSGSEKPPKEDQTSSSTTAADTAPRHPPKRALLRSRGPAGGLFGRAMSGLMKNPS
GASRREESQQQASDSGEVES
GESPAQVALQAAAPPRSQAPIREVLEKAGDSDSAKTPEPEAESGQATPTRRTRRSESSESGLGASPTDSTVLQCKNVPPA
LNKKEVMEKHFGRFGKVRRV
FCRPNKNLAIIHFHDHASAAKAKKKGKILHRHEILLFWQRKRQSSVDPEREGQAAREAPAAELKPESSPQRRPQIRASTL
GSSAGFSRSSPLKKPSIAKS
LQFDSDPQQEGAPDSQSLSSERPVPSSLLHLIGQTAETAEEKYRLLEQRDKIMRQGRPKRTDLVLSKVFVGTCPDMCPEK
ERYMRETRNQLSCFELVPDT
EMVDHRAAIKEYSRSSADQEEPLPHELRPLPVLSMTMDYLVTQVTDQGLDSYRDWYDFLWNRTRGIRK
>g168.t2
MVSTQPAGFGQPSAFGQPSSTVTASTFSTVQNVTQQEKGFGSTDFSFKPANEALFKPIFNASPEPTNPQATTGPSQAFGA
MLSQTTPSTMESSSAATTTT
GSTGFPLLTGAKIGTLGFSFSQPTAAPSISAHKDPMTTDGSGDSTSALKFTFSQPAAPSSTTSVTTTSTTTAQPTTPSSF
SFSAKVLQPQAATLFGGALF
GQTSAFGDPKAPKQDPGADQRAAERGSVPETNVFARLGKGTKRKEDPEVLSGSGSEKPPKEDQTSSSTTAADTAPRHPPK
RALLRSRGPAGGLFGRAMSG
LMKNPSGASRREESQQQASDSGEVESGESPAQVALQAAAPPRSQAPIREVLEKAGDSDSAKTPEPEAESGQATPTRRTRR
SESSESGLGASPTDSTVLQC
KNVPPALNKKEVMEKHFGRFGKVRRVFCRPNKNLAIIHFHDHASAAKAKKKGKILHRHEILLFWQRKRQSSVDPEREGQA
AREAPAAELKPESSPQRRPQ
IRASTLGSSAGFSRSSPLKKPSIAKSLQFDSDPQQEGAPDSQSLSSERPVPSSLLHLIGQTAETAEEKYRLLEQRDKIMR
QGRPKRTDLVLSKVFVGTCP
DMCPEKERYMRETRNQLSCFELVPDTEMVDHRAAIKEYSRSSADQEEPLPHELRPLPVLSMTMDYLVTQVTDQGLDSYRD
WYDFLWNRTRGIRK
>g168.t3
MSQAPSFGQPAVGQSSSGFTTSSTPAFGQTSGSGQAWFGQTPAFGQSSTFGQTPGFTQQPPSFGLTSATGSTTTMVSTQP
AGFGQPSAFGQPSSTVTAST
FSTVQNVTQQEKGFGSTDFSFKPANEALFKPIFNASPEPTNPQATTGPSQAFGAMLSQTTPSTMESSSAATTTTGSTGFP
LLTGAKIGTLGFSFSQPTAA
PSISAHKDPMTTDGSGDSTSALKFTFSQPAAPSSTTSVTTTSTTTAQPTTPSSFSFSAKVLQPQAATLFGGALFGQTSAF
GDPKAPKQDPGADQRAAERG
SVPETNVFARLGKGTKRKEDPEVLSGSGSEKPPKEDQTSSSTTAADTAPRHPPKRALLRSRGPAGGLFGRAMSGLMKNPS
GASRREESQQQASDSGEVES
GESPAQVALQAAAPPRSQAPIREVLEKAGDSAKTPEPEAESGQATPTRRTRRSESSESGLGASPTDSTVLQCKNVPPALN

KKEVMEKHFGRFGKVRRVFC
RPNKNLAIIHFHDHASAAKAKKKGKILHRHEILLFWQRKRQSSVDPEREGQAAREAPAAELKPESSPQRRPQIRASTLGS
SAGFSRSSPLKKPSIAKSLQ
FDSDPQQEGAPDSQSLSSERPVPSSLLHLIGQTAETAEEKYRLLEQRDKIMRQGRPKRTDLVLSKVFVGTCPDMCPEKER
YMRETRNQLSCFELVPDTEM
VDHRAAIKEYSRSSADQEEPLPHELRPLPVLSMTMDYLVTQVTDQGLDSYRDWYDFLWNRTRGIRK
>g169.t1
MGVVLRNLQKVVPLRRARLRRDVDTLRHILGVQKFDLGIICVDNRRIQQINRKYRKNNTPTDVLSFPFHEVVTAHGICHL
LGYRHETEEEWTEMFQRESY
ILGEYNRLTGQNLEPLTKRCGQDS
>g170.t1
MAATVTKLSSGVVKIRCISIELGTTKWKEGAFEIVEKDNKISLSLKFNSGGPPKTFQLNQNVKQIAQNMSRITVTLKDSS
IITLDKLPAALSQKTKEYLE
KVKQGKQSSKTSHGSANFSVLGNRAVKNETSPPGERQTTTRRQSVEGREETPRKPLGSPSRASSTPTRAGLSENRGEKRK
RLMNSDSDLNEDYPKENDSS
SNNKATSEPSRKFLLSCKEKLKQSEENRSSAPLGGPLQPSLFYGSRSVTKDFSQNHSFLERPSSTTQSPSAKRSLMLPNH
STPFKKVRPSVDYGGWNKQR
PSTLAQPQAPLQGFSNLGNTCYMNAILQSLFSLPSFSTDMLRQGIPWKKVPINALLRRFAHLMVKKDVGCPETKKDLLRK
VKSAISATAERFSGYMQNDA
HEFLSQCLDQLKEDVEKMNKSWKNETAAVSSSTSPAAWDDGQQGATSSSMTTATAAASAAAAAGKAEPGEETDTSRIYTC
PVAVNMEFEVQHTITCKGCG
EVVTKREQFNDLSIDLPRRKNTLPLRSIQDSLDLFFRMEEIEYSCEKCNGKAATVTHKFSKLPRVLILHLKRYSFNAQLS
LNSKLGQQVVIPRYLTLLSH
CTDATRAPLSLGWSAQSALRLGGKAAHPSCTSVLLDSDSEEELTRRGNVSRRRHLSDCLPEEEERTEERGVTMEQTDFND
DEMLAAVLEMSRQEAALSAP
PPMEDEPTSSPDTGFGDTDAHDLAYHTELLEADNKQPADVLDSLDLTMDENKENQTPDSAQQGELDWVQQYSLDQEREEQ
ELQQALAQSLQEHEAQEMRE
DDDLKRATELSLQEFNSSLPELLCSDEDSGNEDVLDMEYSEAETEDLKKNAEGGHLANSFRLISVVSHIGSSSSSGHYIS
DVFDMKKQSWLTYNDLDVSR
TQESAVQRDRDRSGYIFFYMHKDVFEELSEMDKSSANICVDAGRNVLQPL
>g170.t2
MAATVTKLSSGVVKIRCISIELGTTKWKEGAFEIVEKDNKISLSLKFNSGGPPKTFQLNQNVKQIAQNMSRITVTLKDSS
IITLDKLPAALSQKTKEYLE
KVKQGKQSSKTSHGSANFSVLGNRAVKNETSPPGERQTTTRRQSVEGREETPRKPLGSPSRASSTPTRAGLSENRGEKRK
RLMNSDSDLNEDYPKENDSS
SNNKATSEPSRKFLLSCKEKLKQSEENRSSAPLGGPLQPSLFYGSRSVTKDFSQNHSFLERPSSTTQSPSAKRSLMLPNH
STPFKKVRPSVDYGGWNKQR
PSTLAQPQAPLQGFSNLGNTCYMNAILQSLFSLPSFSTDMLRQGIPWKKVPINALLRRFAHLMVKKDVGCPETKKDLLRK
VKSAISATAERFSGYMQNDA
HEFLSQCLDQLKEDVEKMNKSWKNETAAVSSSTSPAAWDDGQQGATSSSMTTATAAASAAAAAGKAEPGEETDTSRIYTC
PVAVNMEFEVQHTITCKGCG
EVVTKREQFNDLSIDLPRRKNTLPLRSIQDSLDLFFRMEEIEYSCEKCNGKAATVTHKFSKLPRVLILHLKRYSFNAQLS
LNSKLGQQVVIPRYLTLLSH
CTDATRAPLSLGWSAQSALSRTLKTSQSVNSSSATPRRLGGKAAHPSCTSVLLDSDSEEELTRRGNVSRRRHLSDCLPEE
EERTEERGVTMEQTDFNDDE
MLAAVLEMSRQEAALSAPPPMEDEPTSSPDTGFGDTDAHDLAYHTELLEADNKQPADVLDSLDLTMDENKENQTPDSAQQ
GELDWVQQYSLDQEREEQEL
QQALAQSLQEHEAQEMREDDDLKRATELSLQEFNSSLPELLCSDEDSGNEDVLDMEYSEAETEDLKKNAEGGHLANSFRL
ISVVSHIGSSSSSGHYISDV
FDMKKQSWLTYNDLDVSRTQESAVQRDRDRSGYIFFYMHKDVFEELSEMDKSSANICVDAGRNVLQPL
>g170.t3
MAATVTKLSSGVVKIRCISIELGTTKWKEGAFEIVEKDNKISLSLKFNSGGPPKTFQLNQNVKQIAQNMSRITVTLKDSS
IITLDKLPAALSQKTKEYLE
KVKQGKQSSKTSHGSANFSVLGNRAVKNETSPPGERQTTTRRQSVEGREETPRKPLGSPSRASSTPTRAGLSENRGEKRK
RLMNSDSDLNEDYPKENDSS
SNNKATSEPSRKFLLSCKEKLKQSEENRSSAPLGGPLQPSLFYGSRSVTKDFSQNHSFLERPSSTTQSPSAKRSLMLPNH

STPFKKVRPSVDYGGWNKQR
PSTLAQPQAPLQGFSNLGNTCYMNAILQSLFSLPSFSTDMLRQGIPWKKVPINALLRRFAHLMVKKDVGCPETKKDLLRK
VKSAISATAERFSGYMQNDA
HEFLSQCLDQLKEDVEKMNKSWKNETAAVSSSTSPAAWDDGQQGATSSSMTTATAAASAAAAAGKAEPGEETDTSRIYTC
PVAVNMEFEVQHTITCKGCG
EVVTKREQFNDLSIDLPRRKNTLPLRSIQDSLDLFFRMEEIEYSCEKCNGKAATVTHKFSKLPRVLILHLKRYSFNAQLS
LNSKLGQQVVIPRYLTLLSH
CTDATRAPLSLGWSAQSALRLGGKAAHPSCTSVLLDSDSEEELTRRGNVSRRRHLSDCLPEEEERTEEVGTRLPGR
>g170.t4
MEQTDFNDDEMLAAVLEMSRQEAALSAPPPMEDEPTSSPDTGFGDTDAHDLAYHTELLEADNKQPADVLDSLDLTMDENK
ENQTPDSAQQGELDWVQQYS
LDQEREEQELQQALAQSLQEHEAQEMREDDDLKRATELSLQEFNSSLPELLCSDEDSGNEDVLDMEYSEAETEDLKKNAE
GGHLANSFRLISVVSHIGSS
SSSGHYISDVFDMKKQSWLTYNDLDVSRTQESAVQRDRDRSGYIFFYMHKDVFEELSEMDKSSANICVDAGRNVLQPL
>g171.t1
LLLLKLFEFLSGELCTVFLNQLLHIRIVYLEAHAFANSHNVLHYSHCHINLKALKAVTTALAQVDREKIYQWINELSSPE
TRENALLELSKKRESVPDLA
PMLWHSCGTIAALLQEIVNIYPSINPPTLTAHQSNRVCNALALLQCVASHPETRSAFLAAHIPLFLYPFLHTVSKTRPFE
YLRLTSLGVIGALVKTDEQE
VINFLLTTEIIPLCLRIMESGSELSKTVATFILQKILLDDTGLAYICQTYERFSHVAMILGKMVLQLSKEPSARLLKHVV
RCYLRLSDNLRAREALRQCL
PDQLKDNTFAQVLKDDTTTKRWLAQLVKNLQEGQVTDARGIPLSAQ
>g171.t2
MLATGAAVTTALAQVDREKIYQWINELSSPETRENALLELSKKRESVPDLAPMLWHSCGTIAALLQEIVNIYPSINPPTL
TAHQSNRVCNALALLQCVAS
HPETRSAFLAAHIPLFLYPFLHTVSKTRPFEYLRLTSLGVIGALVKTDEQEVINFLLTTEIIPLCLRIMESGSELSKTVA
TFILQKILLDDTGLAYICQT
YERFSHVAMILGKMVLQLSKEPSARLLKHVVRCYLRLSDNLRAREALRQCLPDQLKDNTFAQVLKDDTTTKRWLAQLVKN
LQEGQVTDARGIPLSAQ
>g171.t3
MMHRTKTVQARVSSAVTTALAQVDREKIYQWINELSSPETRENALLELSKKRESVPDLAPMLWHSCGTIAALLQEIVNIY
PSINPPTLTAHQSNRVCNAL
ALLQCVASHPETRSAFLAAHIPLFLYPFLHTVSKTRPFEYLRLTSLGVIGALVKTDEQEVINFLLTTEIIPLCLRIMESG
SELSKTVATFILQKILLDDT
GLAYICQTYERFSHVAMILGKMVLQLSKEPSARLLKHVVRCYLRLSDNLRAREALRQCLPDQLKDNTFAQVLKDDTTTKR
WLAQLVKNLQEGQVTDARGI
PLSAQ
>g172.t1
MKRRNADCSKLRRPLKRNRITEGIYGSTFLYLKFLVVWALVLLADFVLEFRFEYLWPFWLFIRSVYDSFRYQGLVMQWLF
FAASTYVWVQYVWHTERGVC
LPTVSLWILFVYIEAAIRFKDLKNFHVDLCRPFAAHCIGYPVVTLGFGFKSYVSYKMRLRKQKEVQKENEFYMQLLQQAL
PPEQQMLQRQEREAEE
>g172.t2
MKRRNADCSKLRRPLKRNRITEGIYGSTFLYLKFLVVWALVLLADFVLEFRFEYLWPFWLFIRSVYDSFRYQGLAFSVFF
VCVAFTSDIICLLFIPVQWL
FFAASTYVWVQYVWHTERGVCLPTVSLWILFVYIEAAIRFKDLKNFHVDLCRPFAAHCIGYPVVTLGFGFKSYVSYKMRL
RKQKEVQKENEFYMQLLQQA
LPPEQQMLQRQEREAEE
>g173.t1
MGTVIGKTNSVQLILIAILEVSGFIFNGWLLRTFLELHTFGAFFGLMLTWAMYWTRAKTPFEKEKGDRKSGLFSMFGTLF
LWMFWPSFNSVLEESGGHER
KLRVILSTYLALAVSAVATAAVSVFTSPRGKMNLV
>g173.t2
MAEICTASALISMGTVIGKTNSVQLILIAILEVSGFIFNGWLLRTFLELHTFGAFFGLMLTWAMYWTRAKTPFEKEKGDR
KSGLFSMFGTLFLWMFWPSF
NSVLEESGGHERKLRVILSTYLALAVSAVATAAVSVFTSPRGKMNLV

>g173.t3
MGTVIGKTNSVQLILIAILEVSGFIFNGWLLRTFLEVSPLNCAMQLHTFGAFFGLMLTWAMYWTRAKTPFEKEKGDRKSG
LFSMFGTLFLWMFWPSFNSV
LEESGGHERKLRVILSTYLALAVSAVATAAVSVFTSPRGKMNLV
>g174.t1
MAETHVRKSCATVRSSRRRSNRSRNRSNSRHRTSTGLVGRDRRRVSKSPSKSYHSRSRSSRRSAASPTLDEKKELLRAVK
ETATEALRVDLPVSMNPIVD
EPFEDCPTPEPQAWNSDEEPDVHRSKVSPIRRIISFSIHNSVAKPTVAPVMSAKVTPRVDSIESRKPYGHWVPVKRGVKR
PHKRTTSR
>g174.t2
MAETHVRKSCATVRSSRRRSNRSRNRSNSRHRTSTGLVGRDRRRVSKSPSKSYHSRSRSSRRSAASPTLDEKKELLRAVK
ETATEALRVDLPVSMNPIVD
EPFEDCPTPEPQAWVRPEDVPEKVTPQNSDEEPDVHRSKVSPIRRIISFSIHNSVAKPTVAPVMSAKVTPRVDSIESRKP
YGHWVPVKRGVKRPHKRTTS
R
>g174.t3
LVGRDRRRVSKSPSKSYHSRSRSSRRSAASPTLDEKKELLRAVKETATEALRVDLPVSMNPIVDEPFEDCPTPEPQAWNS
DEEPDVHRSKVSPIRRIISF
SIHNSVAKPTVAPVMSAKVTPRVDSIESRKPYGHWVPVKRGVKRPHKRTTSR
>g175.t1
IEKRAKYSRRRAYNDDADIDYINERNAKFNKKAERFYGKYTAEIKQNLERGTAV
>g176.t1
LVWPTGVTGTTVFSQLRTTQRTTVSTWIRDRLRAPGSGLRAPGRTGSSLRTVVPTHTGQHSASNNPTAVTSIPRTHRGTK
MAPGVWIRASRGSEETLLAL
RSLEQRAELSSQDHWRDRPFTPVGGGLS
>g177.t1
LTACHLTPRVSPRGRADDRTSRSPERGPASRNRREEDTAPGTRHPAGHGPGGVGVWSEPNRTRRRFKPGCSPLGAQPRWM
GIDSLGAILNNNQTPPPPCT
PTRDGQTGTVSTRVVNPVRFPTPDCPSPPRLSLLTPNLTQDYCDGKSNSLENQLFPDAVQTPADLLGLFVQRPHIPCPGC
RSDTHRDNEPSSSWSTGVGL
RDAHIWKGSD
>g178.t1
LFKRLNHHKAAGPDSVFPSTLKHCANQLSPVFTHIFNTSLETCHVPACFKASAIDPVPKKTRITGLNDYRPVALTSVVVN
TFERLVLTHLKDITDNLLDP
LQFAYRANRSVDDAVNMALHFILQHLDSPGSYARILFVDFSSAFNTIIPALLRDKLSQLNVP
>g179.t1
METSGTDILNDSDVRAPVSPLHLAAYHGHHQAMEVLVQSLLDLDVRNGQGRTPLDLAAFKGHVECVDVLINQGASILVKD
YTLKRTPIHAAATNGHSECL
RLLIGNADLPSAADIEDGNGQTPLMLSVLSGHTDCVYSMLNKGASVEAKDKWGRTALHRGAVTGHEECVEALLQHSANFL
VRDCKGRTPMHLAAACGHIG
VLGGLLHAAQSVETLPVLTDNKGYTPLHWACYNGHDTCVELLLEQEVFHKTEGNLFSPLHCAVIHDNEGAAEMLIDTIGL
GIVNAADTKKRTPLHAAAFT
DHVECLQLLLSNNAQVNSIDATGKTSLMMAAENGQTNAVGYTPALACAPNKDVADCLALILATMMPVFPCTRAPSLPFSA
INHYTTTSPSKSVTFDSLPA
LKDEHTSYCSFNNISHNEGSYKDEELNDSDSETY
>g179.t2
METSGTDILNDSDVRAPVSPLHLAAYHGHHQAMEVLVQSLLDLDVRNGQGRTPLDLAAFKGHVECVDVLINQGASILVKD
YTLKRTPIHAAATNGHSECL
RLLIGNADLPSAADIEDGNGQTPLMLSVLSGHTDCVYSMLNKGASVEAKDKWGRTALHRGAVTGHEECVEALLQHSANFL
VRDCKGRTPMHLAAACGHIG
VLGGLLHAAQSVETLPVLTDNKGYTPLHWACYNGHDTCVELLLEQEVFHKTEGNLFSPLHCAVTPLHAAAFTDHVECLQL
LLSNNAQVNSIDATGKTSLM
MAAENGQTNAVGYTPALACAPNKDVADCLALILATMMPVFPCTRAPSLPFSAINHYTTTSPSKSVTFDSLPALKDEHTSY
CSFNNISHNEGSYKDEELND
SDSETY
>g179.t3

METSGTDILNDSDVRAPVSPLHLAAYHGHHQAMEVLVQSLLDLDVRNGQGRTPLDLAAFKGHVECVDVLINQGASILVKD
YTLKRTPIHAAATNGHSECL
RLLIGNADLPSAADIEDGNGQTPLMLSVLSGHTDCVYSMLNKGASVEAKDKWGRTALHRGAVTGHEECVEALLQHSANFL
VRDCKGRTPMHLAAACGHIG
VLGGLLHAAQSVETLPVLTDNKGYTPLHWACYNGHDTCVELLLEQEVFHKTEGNLFSPLHCAVIHDNEGAAEMLIDTIGL
GIVNAADTKKRTPLHAAAFT
DHVECLQLLLSNNAQVNSIDATGKTSLMMAAENGQTNAVELLVSSAKADLTLQDAVKNTALHLACSYTPALACAPNKDVA
DCLALILATMMPVFPCTRAP
SLPFSAINHYTTTSPSKSVTFDSLPALKDEHTSYCSFNNISHNEGSYKDEELNDSDSETY
>g180.t1
LSHLKDITGPLLDPLQFAYRANRSADDAVNMGLHHILQHLDSPGTYARILFVDFSSAFNTIIPDILHSKLSQLTIPASTC
QWITNFLTDRRQQVRLGQIT
SSTRTISTGAPQGCVLSPLLFSLYTNDCTSSDPSVKLLKFADDTTVIGLIRDGDESAYRREVGQLALWCGQNHLELNTLK
TV
>g181.t1
PAPELGVLRQAVLSEPPTPQLTGHNASRPRGAQSLALRG
>g182.t1
MGMRPLCASYCNIIASAVNARLCLSSKGAHGKKKGPRTPSPPPPAPADGPLPGKKHKGKHKNKEKVAEEKAPKERGRDAE
KPKEKKKEKRRDRSDSSHKAK
RSATSEERSGSVSSTMRDPSPPSRKKSSSPTKASSHKALPPASPPR
>g182.t2
LRPTPWLWPLHPRLLRRPRVVTHHVPPARRSSKGAHGKKKGPRTPSPPPPAPADGPLPGKKHKGKHKNKEKVAEEKAPKE
RGRDAEKPKEKKKEKRRDRSD
SSHKAKRSATSEERSGSVSSTMRDPSPPSRKKSSSPTKASSHKALPPASPPR
>g183.t1
MSGRLSSSGVFSELLRLDIALKRFYGTIASSKIFPETLKADETLRGLKCQSSGLGPRQYFACWNPDPFDPPNHCLPCVLT
WFSPGSQQKDNQQIRAQVDG
SLARFIQTGLAPGQEYSATVTGEIDGKSEASTSVQFHTYQISSSSVSLVPVTSRPTNLRVVKTSTSSAVVQWEPAQGEID
RYRLTVSPNDGVGEAQEIAL
PPERDSAHIEHLEAGRFYDITLRAESGAGQSPEAAVQVTPGPMVPSSPLVTVTVPVPLATAQGTGDRPMNPASSTEAGRT
KISPFEQHQNWAWLETG
>g184.t1
MILKPRHGPPVGDDAMPESHSSRSSSEGDHRDERPARLVPVVNPSGADVRASSKETTIAPGSNMDPEVAMVTQGNQMENG
ELPELSVHDGTPINEKKCLN
KVKVTHIRLPHQEPDFSRTDACFIPCFIIIIIIVIVIITKDLLFITASITKFSVTIIIIVTITTNISIIILSSHLIKTNN
NKTIDSAPISFILR
>g185.t1
MKWLYQPSIPDPTVLGTQGGQLTDRLHQEEAGSPISGSDSDVHIESVDSGGSEHWAGTPPPRVLLSGSETLGRKVLTSTA
AQRRTGHSPTHRRNQNTVSQ
QSRKGHQSQSQSSKAAHSGMNGARHEFHVTPAGSPGDLPGEPLDYVGVRNRTSDGFTLVWDAPEGKYSNFVVTRREEHRS
TAEEERLEKEEEEEEEPPPP
PEEAMRHRGVGEENAIPESNVMHTPAARGAGLTGSDTLFTRKLPGSDRSFVFEKLAPRTPFTVTLRGTGTGLLSRLHKLV
ISTGPSPPSDLEFSQVSEDS
LTLTWTRPPDPVSSFKVTYTHTLE
>g185.t2
MKWLYQPSIPDPTVLGTQGGQLGSETLGRKVLTSTAAQRRTGHSPTHRRNQNTVSQQSRKGHQSQSQSSKAAHSGMNGAR
HEFHVTPAGSPGDLPGEPLD
YVGVRNRTSDGFTLVWDAPEGKYSNFVVTRREEHRSTAEEERLEKEEEEEEEPPPPPEEAMRHRGVGEENAIPESNVMHT
PAARGAGLTGSDTLFTRKLP
GSDRSFVFEKLAPRTPFTVTLRGTGTGLLSRLHKLVISTGPSPPSDLEFSQVSEDSLTLTWTRPPDPVSSFKVTYTHTLE
>g185.t3
MKWLYQPSIPDPTVLGTQGGQLTDRLHQEEAGSPISGSDSDVHIESVDSGGSEHWAGTPPPRVLLSGSETLGRKVLTSTA
AQRRTGHSPTHRRNQNTVSQ
QSRKGHQSQSQSSKAAHSGMNGARHEFHVTPAGSPGDLPGEPLDYVGVRNRTSDGFTLVWDAPEGKYSNFVVTRREEHRS
TAEEERLEKEEEEEEEPPPP
PEEAMRHRGVGEENAIPESNVMHTPAARGAGLTGSDTLFTRKLPGSDRSFVFEKLAPRTPFTVTLRGTGTGLLSRLHKLV

ISTGPSPPSDLEFSQVSEDS
LTLTWTRPPDPVSSFKVTYTHTLEGEPVSVVVGSQDSS
>g186.t1
MESLQGALEATHWDVFYKPHGEYIDGITDCISEYIGFCMDNTTSNADAVYKKGLSRLYFLRRLRSFNVCNRMLHMFYESV
VASTIFFAVVSWGAGIKAKD
ANRLNKLIKKTGSVVGSKLVTLEVVTVDRMLAKLLVIMDKITSHS
>g187.t1
LFPYTLVKYDTERDEPVRDARGLCIEALKGETGLLVSKITERAPFVGYVGNEAQTEKKRLRDVLRKGDLYFNSGDLLRVD
LEGFIYFQDRIGDTFRWKGE
NVATTEVADVLSLAGCVKESNVYGVEVPGHEGRAGMAVVSLKEDTPFDGSQIYRHVACLPSYARPRFIRIQMEVEVTATF
KQLKGRLVEQGFSPDSIGDP
LYILDDQAKCYSPLTLHMYCLISSGSVKL
>g187.t2
KLFPYTLVKYDTERDEPVRDARGLCIEALKGETGLLVSKITERAPFVGYVGNEAQTEKKRLRDVLRKGDLYFNSGDLLRV
DLEGFIYFQDRIGDTFRWKG
ENVATTEVADVLSLAGCVKESNVYGVEVPGHEGRAGMAVVSLKEDTPFDGSQIYRHVACLPSYARPRFIRIQMEVEVTAT
FKQLKGRLVEQGFSPDSIGD
PLYILDDQAKCYSPLTLHMYCLISSGSVKL
>g187.t3
MKLFPYTLVKYDTERDEPVRDARGLCIEALKGETGLLVSKITERAPFVGYVGNEAQTEKKRLRDVLRKGDLYFNSGDLLR
VDLEGFIYFQDRIGDTFRWK
GENVATTEVADVLSLAGCVKESNVYGVEVPGHEGRAGMAVVSLKEDTPFDGSQIYRHVACLPSYARPRFIRIQMEVEVTA
TFKQLKGRLVEQGFSPDSIG
DPLYILDDQAKCYSPLTLHMYCLISSGSVKL
>g188.t1
KIQLEAKLKETTERLEDEEEINAELTAKKRKLEDECSELKKDIDDLELTLAKVEKEKHATENKVKNLTEEMASQDESVAK
LSKEKKALQESHQQTLDDLQ
AEEDKVNTLTKAKTKLEQQVDDLEGSLEQEKKLRMDLERAKRKLEGDLKLAQESIMDLENDKQQSDEKIKKKDFETSQLL
SKIEDEQSLGAQLQKKIKEL
QARIEELEEEIEAERAARAKVEKQRADLSRELEEISERLEEAGGATAAQIEMNKKREAEFQKLRRDLEESTLQHEATAAA
LRKKQADSVAELGEQIDNLQ
RVKQKLEKEKSEYKMEIDDLSSNMEAVAKAKGNLEKICRTLEDQLSEVKAKSDENGRQINDISAQRARLLTENGEFGRQL
EEKEALVSQLTRGKQAFTQQ
IEELKRQIEEEVK
>g188.t2
LEDEEEINAELTAKKRKLEDECSELKKDIDDLELTLAKVEKEKHATENKVKNLTEEMASQDESVAKLSKEKKALQESHQQ
TLDDLQAEEDKVNTLTKAKT
KLEQQVDDLEGSLEQEKKLRMDLERAKRKLEGDLKLAQESIMDLENDKQQSDEKIKKKDFETSQLLSKIEDEQSLGAQLQ
KKIKELQARIEELEEEIEAE
RAARAKVEKQRADLSRELEEISERLEEAGGATAAQIEMNKKREAEFQKLRRDLEESTLQHEATAAALRKKQADSVAELGE
QIDNLQRVKQKLEKEKSEYK
MEIDDLSSNMEAVAKAKGNLEKICRTLEDQLSEVKAKSDENGRQINDISAQRARLLTENGEFGRQLEEKEALVSQLTRGK
QAFTQQIEELKRQIEEEVK
>g188.t3
KIQLEAKLKETTERLEDEEEINAELTAKKRKLEDECSELKKDIDDLELTLAKVEKEKHATENKVKNLTEEMASQDESVAK
LSKEKKALQESHQQTLDDLQ
AEEDKVNTLTKAKTKLEQQVDDLEGSLEQEKKLRMDLERAKRKLEGDLKLAQESIMDLENDKQQSDEKIKKKDFETSQLL
SKIEDEQSLGAQLQKKIKEL
QARIEELEEEIEAERAARAKVEKQRADLSRELEEISERLEEAGGATAAQIEMNKKREAEFQKLRRDLEESTLQHEATAAA
LRKKQADSVAELGEQIDNLQ
RVKQKLEKEKSEYKMEIDDLSSNMEAVAKAKAKSDENGRQINDISAQRARLLTENGEFGRQLEEKEALVSQLTRGKQAFT
QQIEELKRQIEEEVK
>g189.t1
KYYPPDFDPSKIPKLKLPKDRQYVVRLMAPFNMRCKTCGEYIYKGKKFNARKETVQNELYMGLPIFRFYIKCTRCLAEIT
FKTDPENTDYAMEHGATRNF
QAEKLLEQEETKIQRDREEEELNNPMKVLENRTRDSKLEMEVLENLQELKELNQRQAQVDFEGMIGQYRELEQREKEREK

EEDERETKEMLDRALIKRLR
DSDSDSDQEEKPPEPPRAGADRPTDILTASKLPEAQGPSAGGVKKAKLERWERSVGKLGGGGALGSLVVRKKPAGTPGPA
PPPGGCSAAP
>g190.t1
LTVTCYANNIPWITKDIKAILNDKKRAFREGDHAELRRVQGVLKIRIKVAKDRYRRKLEKKPQQNNTGDVWSGMRNITGF
QKTGSKGLEGGVARVNELNL
FFTTFDTAAPAPPPHDSAATAAGSPTTSATPPPPPSPPPYRPPLFTPSHFTPPPCHTPPPCSASCPPLPEDLIPPPTVTS
TVSFTPDLVRRQLIRLHSGK
AAGPDGVFPRVLKTCATQLCGVLNHVFNLSLHLQRVPLMWKTSCLVPVPKTLRPSSPQDYRPVALTSHIMKTLERLVLEQ
LRPMLNTQCHSASPSSSPSL
QTTSFHSQPLHTSTLPHPSLRIASCPPLPEDLIPPPTDTSTVSFTPGMWTWKSQPHHSPNPIPTSPFTQSLLPQSSLCVS
KSKV
>g191.t1
MFFDFSSAFNALPMGRLGEKMAAMQVEAALVSWIVDYLRPQFVRLQNCVSDRVVSNIGAPKGTVLSPFLFTLYTSD
>g192.t1
MSDEASVDRCVQRLNGKLVPGSNPPRKFKLNYATYGKRPEAGPEFSVFVGDLSSDVDDFQLHQVFKKYPSCKGAKVVTDQ
YGNSRGYGFVKFGEETEQKR
SIEECQGTVLGGKPLRLSIAVAKSQKVSNYHGGQGQGYHSNYNQPQSGYYGSHGNGGQGGYYSQWGGYDQYGGYGNSGYS
NAGYSPGYNYSYGAYGYPPP
GHALGPPPPTGAPPTSTEAPGPTEGCHDESGEAEDNGEGRTY
>g192.t2
GSAGYCFVEMSDEASVDRCVQRLNGKLVPGSNPPRKFKLNYATYGKRPEAGPEFSVFVGDLSSDVDDFQLHQVFKKYPSC
KGAKVVTDQYGNSRGYGFVK
FGEETEQKRSIEECQGTVLGGKPLRLSIAVAKSQKVSNYHGGQGQGYHSNYNQPQSGYYGSHGNGGQGGYYSQWGGYDQY
GGYGNSGYSNAGYSPGYNYS
YGAYGYPPPGHALGPPPPTGAPPTSTEAPGPTEGCHDESGEAEDNGEGRTY
>g192.t3
GSAGYCFVEMSDEASVDRCVQRLNGKLVPGSNPPRKFKLNYATYGKRPEAGPEFSVFVGDLSSDVDDFQLHQVFKKYPSC
KGAKVVTDQYGNSRGYGFVK
FGEETEQKRSIEECQGTVLGGKPLRLSIAVAKSQKVSNYHGGQGQGYHSNYNQPQSGYYGSHGNGGQGGYYSQWGGYDQY
GGYGNSGYSNAGYSPGYNYS
YGAYGYPPPGHALGPPPPTGAPPTSTEAPGPTEGCHDESGEAEDNGE
>g193.t1
MDESSVRRSLETLCQELNMDEQTAAEAMENFTVMWNTFTLEGEVVHWLACSLYAACRKSSTPTVGRGLMEGNCVSLTRIL
RTAKLSLIQFFSKMRKWADM
SNLQQDFRLRMDSLERNFEVSTVIFRKFEPIFMGVFQNPQGAEPPRPPRSRKH
>g193.t2
MGGDESPDSDSGRMDESSVRRSLETLCQELNMDEQTAAEAMENFTVMWNTFTLEGEVVHWLACSLYAACRKSSTPTVGRG
LMEGNCVSLTRILRTAKLSL
IQFFSKMRKWADMSNLQQDFRLRMDSLERNFEVSTVIFRKFEPIFMGVFQNPQGAEPPRPPRSRKH
>g194.t1
MEPSDSVTHKNGRQRKTTGANLHVENMKDGEMGGGSGPEVEEEAEGLLQGTDSEDRRRAKVRPGLRNELGNILLLLFLYV
LQGIPLGLAGSIPLIMQSKS
VSYKDQAFFSFVFWPFSLKLLWAPLVDALYFSRFGRRKSWLVPTQYLLGLFMLYLSVTVDSLLQSDGSHGPNVVTLTAVF
FLLAFLAATQDIAVDGWALT
MLSRENVGYASTCNSVGQTAGYFLGNVLFLALESANFCNQYLRSKPQDTGIVTLSDFLFYGGVVFLVSTTLVAIFKRESG
RGQGKRRVQEETQGVLETYK
LLFSIIKLPTVLSFCTLLLTAKVGFSAADAVTGLKLVEAGVPKEQLALLAVPMVPLQILLPLVISKYTAGPRPLDIFYKA
FPF
>g194.t2
MKDGEMGGGSGPEVEEEAEGLLQGTDSEDRRRAKVRPGLRNELGNILLLLFLYVLQGIPLGLAGSIPLIMQSKSVSYKDQ
AFFSFVFWPFSLKLLWAPLV
DALYFSRFGRRKSWLVPTQYLLGLFMLYLSVTVDSLLQSDGSHGPNVVTLTAVFFLLAFLAATQDIAVDGWALTMLSREN
VGYASTCNSVGQTAGYFLGN
VLFLALESANFCNQYLRSKPQDTGIVTLSDFLFYGGVVFLVSTTLVAIFKRESGRGQGKRRVQEETQGVLETYKLLFSII
KLPTVLSFCTLLLTAKVGFS

AADAVTGLKLVEAGVPKEQLALLAVPMVPLQILLPLVISKYTAGPRPLDIFYKAFPF
>g195.t1
MQHSPGSTQPCLLQQRPAGDVLLYVSALQTLDIGSTEQNGSCEGAVAILDAGAQYGKVIDRRVREMCVRSEILPLETPAF
HIREQGFRAIIISGGPASVY
AEDAPWFDRAIFTIGKPVLGICYGMQMMNKVFGGTVDRKSVREDGVFNIGLDNTCSLFRGLQKEELVLLTHGDSVDKVAE
GFKVVAQSGNIIAGIANEQK
KLYGTQFHPEVDLTENGIDILRNFLFEIAGCTSNFTVQDRQKACIDEIKEKVQKSKVLVLLSGGVDSTVCTALLNKALNQ
DQVIAVHIDNGFMRKRESQS
VEEALTKLGIKLQVVNASHTFYNGTTTLPISDEDRTPRKRISKTLNMTTNPEEKRKIIGDTFV
>g195.t2
MQHSPGSTQPCLLQQRPAGDVLLYVSALQTLDIGSTEQNGSCEGAVAILDAGAQYGKVIDRRVREMCVRSEILPLETPAF
HIREQGFRAIIISGGPASVY
AEDAPWFDRAIFTIGKPVLGICYGMQMMNKVFGGTVDRKSVREDGVFNIGLDNTCSLFRALREPDAWAFQVPCTSTERLT
NGGLQKEELVLLTHGDSVDK
VAEGFKVVAQSGNIIAGIANEQKKLYGTQFHPEVDLTENGIDILRNFLFEIAGCTSNFTVQDRQKACIDEIKEKVQKSKV
LVLLSGGVDSTVCTALLNKA
LNQDQVIAVHIDNGFMRKRESQSVEEALTKLGIKLQVVNASHTFYNGTTTLPISDEDRTPRKRISKTLNMTTNPEEKRKI
IGDTFV
>g195.t3
LDIGSTEQNGSCEGAVAILDAGAQYGKVIDRRVREMCVRSEILPLETPAFHIREQGFRAIIISGGPASVYAEDAPWFDRA
IFTIGKPVLGICYGMQMMNK
VFGGTVDRKSVREDGVFNIGLDNTCSLFRGLQKEELVLLTHGDSVDKVAEGFKVVAQSGNIIAGIANEQKKLYGTQFHPE
VDLTENGIDILRNFLFEIAG
CTSNFTVQDRQKACIDEIKEKVQKSKVLVLLSGGVDSTVCTALLNKALNQDQVIAVHIDNGFMRKRESQSVEEALTKLGI
KLQVVNASHTFYNGTTTLPI
SDEDRTPRKRISKTLNMTTNPEEKRKIIGDTFV
>g196.t1
MSNGPQRIMRPVNHQKPLPHTATVVKPAYAADQNMNPVTVTVNPASQQKPSVQADQPKPKTTKVNPEPAKNTTEPSKKEP
SQNETTKKESTNGNSKKRWS
LENFDIGRPLGKGKFGNVYLARERQTMFILALKVLFKKQLEKAGVEHQLRREVEIQSHLRHPNILRLYGYFHDASRVYLI
LEFAPKGELYGELQRCGSFN
EERSATYIMELADALNYCHSKKVIHRDIKPENLLLGANGELKIADFGWSVHTPSSRRSTLCGTLDYLPPEMIEGRTHDEK
VDLWSLGVLCYEFLVGSPPF
ETKSHEDTYRKISR
>g197.t1
LSCCLGVTSDLSSLQCVSGAQRRAGTLGERRPLCQWPCVCGPRAACAPGVSAVLDGCGCCKSCARQIGQTCNERDVCDPH
KGMYCDFSADRPRFQEGVCA
YMLAVGCDLNGLHYDNGHAFQPSPLYKCTCIAGAIGCTPAFTQKPAGLLSSAPKPAGILSKNLQDTSYRPGNKQEHITSP
PIALTSTSSDITSATSLTET
QGGYNVFLDPPLAWKKNCLVQTTPWSPCSKTCGMGISVRVNNDNSKCEMKKDRRLCLLRPCDKNLLRSIKAPKGKSCRAQ
VPARQAERLVLSGCTSTQRV
RPTYCGVCGGGRCCLPHTSRTRTLEFSCRGAPAVRWKMQWITSCVCQRKCSDPGDMFAELHFL
>g197.t2
MVKVRLWIDFMGPLCYHQLLPWSQTSSTPPNSSSPGLRPPLLPLAAPPLVSDVHQQMRDAVLALQITAAHPGPAGVSAVL
DGCGCCKSCARQIGQTCNER
DVCDPHKGMYCDFSADRPRFQEGVCAYMLAVGCDLNGLHYDNGHAFQPSPLYKCTCIAGAIGCTPAFTQKPAGLLSSAPK
PAGILSKNLQDTSYRPGNKQ
EHITSPPIALTSTSSDITSATSLTETQGGYNVFLDPPLAWKKNCLVQTTPWSPCSKTCGMGISVRVNNDNSKCEMKKDRR
LCLLRPCDKNLLRSIKAPKG
KSCRAQVPARQAERLVLSGCTSTQRVRPTYCGVCGGGRCCLPHTSRTRTLEFSCRGAPAVRWKMQWITSCVCQRKCSDPG
DMFAELHFL
>g197.t3
MVKVRLWIDFMGPLCYHQTSSTPPNSSSPGLRPPLLPLAAPPLVSDVHQQMRDAVLALQITAAHPGPAGVSAVLDGCGCC
KSCARQIGQTCNERDVCDPH
KGMYCDFSADRPRFQEGVCAYMLAVGCDLNGLHYDNGHAFQPSPLYKCTCIAGAIGCTPAFTQKPAGLLSSAPKPAGILS
KNLQDTSYRPGNKQEHITSP

PIALTSTSSDITSATSLTETQGGYNVFLDPPLAWKKNCLVQTTPWSPCSKTCGMGISVRVNNDNSKCEMKKDRRLCLLRP
CDKNLLRSIKAPKGKSCRAQ
VPARQAERLVLSGCTSTQRVRPTYCGVCGGGRCCLPHTSRTRTLEFSCRGAPAVRWKMQWITSCVCQRKCSDPGDMFAEL
HFL
>g198.t1
MNFEDILSLVGGFGKFQKLLYVWICLPRIFLAFHMLSSIFTGATPPHICRSTSLPGDRADPDLNASLLLGHGPACPNVQN
HSAGRAGSCPGGWEYQVVGS
TVVTEVCRQEQGLWDLVCEDRSRDWWVIVFLVLQWDLVCEDARLNSIISSIYMSGLLVGALLFGSLSDKYGRKPVLLLGL
SFLGVFGVGAAFAPNLYVYA
LLRFVVGTAVSAVLINTFVLGAEWTGPKHRMLAGVLTDYFFGLGYILLAGVAYLLRDWRHLQLAISAPCFLFFFYIWVLP
RSARWLMAHQRKGEALQLIC
RVAQLNGKPLQEDFTIQQMFGKDKANGRRHTMVDLVRTPHMRKRSLVLFYLWFVTILIYYGLAFNISGFGVNVYWSQLLF
GLVEIPARTMVLFTINRSRR
LSQAAYLGVGGLACLLTIFIPHDCTTLRMAMGMVGKFGVTASMSVIYIYSAEVFPTVVRQNGIGIGSTCARVGGVLAPVV
HLLQDYCLHAPMILFGLCAL
LGSSLTMLLPETANQPLPETIEDVNEDAGLR
>g198.t2
MNFEDILSLVGGFGKFQKLLYVWICLPRIFLAFHMLSSIFTGATPPHICRSTSLPGDRADPDLNASLLLGHGPACPNVQN
HSAGRAGSCPGGWEYQVVGS
TVVTEVCRQEQGLWDLVCEDRSRDWWVIVFLVLQWDLVCEDARLNSIISSIYMSGLLVGALLFGSLSDKYGRKPVLLLGL
SFLGVFGVGAAFAPNLYVYA
LLRFVVGTAVSAVLINTFVLGVLTDYFFGLGYILLAGVAYLLRDWRHLQLAISAPCFLFFFYIWVLPRSARWLMAHQRKG
EALQLICRVAQLNGKPLQED
FTIQQMFGKDKANGRRHTMVDLVRTPHMRKRSLVLFYLWFVTILIYYGLAFNISGFGVNVYWSQLLFGLVEIPARTMVLF
TINRSRRLSQAAYLGVGGLA
CLLTIFIPHDCTTLRMAMGMVGKFGVTASMSVIYIYSAEVFPTVVRQNGIGIGSTCARVGGVLAPVVHLLQDYCLHAPMI
LFGLCALLGSSLTMLLPETA
NQPLPETIEDVNEDAGLR
>g198.t3
MNFEDILSLVGGFGKFQKLLYVWICLPRIFLAFHMLSSIFTGATPPHICRSTSLPGDRADPDLNASLLLGHGPACPNVQN
HSAGRAGSCPGGWEYQVVGS
TVVTEVCRQEQGLWDLVCEDRSRDWWVIVFLVLQWDLVCEDARLNSIISSIYMSGLLVGALLFGSLSDKYGRKPVLLLGL
SFLGVFGVGAAFAPNLYVYA
LLRFVVGTAVSAVLINTFVLGAEWTGPKHRMLAGVLTDYFFGLGYILLAGVAYLLRDWRHLQLAISAPCFLFFFYIWVLP
RSARWLMAHQRKGEALQLIC
RVAQLNGKPLQEDFTIQQMFGKDKANGRRHTMVDLVRTPHMRKRSLVLFYLWFVTILIYYGLAFNISGFGVNVYWSQLLF
GLVEIPARTMVLFTINRSRR
LSQAAYLGVGGLACLLTIFIPHGEYCTTLRMAMGMVGKFGVTASMSVIYIYSAEVFPTVVRQNGIGIGSTCARVGGVLAP
VVHLLQDYCLHAPMILFGLC
ALLGSSLTMLLPETANQPLPETIEDVNEDAGLR
>g199.t1
LGGRPLVVRIDSPAPPSPPGRRAGYPPAAIAQWSSGASSRQQRDGLQRGGEDSGWGLDHSPPPNVGNSEENIVDQRQQGG
DVEVKRVSSFKVLVCTSQRT
PPENHPPQRTPLGQEEVWHNASIFTNFSGGHLQSY
>g199.t2
ASSRQQRDGLQRGGEDSGWGLDHSPPPNVGNSEENIVDQRQQGGDVEVKRVSSFKVLVCTSQRTPPENHPPQRTPLGQEE
VWHNASIFTNFSGGHLQSY
>g200.t1
CAKRLGYPTRRSCFFSLRHVVSGRQPRDERPSRAHLDEVISMGIFPAIKISSNSFFSSG
>g201.t1
MSNRPPEDHQAGLSPRNGAVVGTAVAGCRPPARGGTGTDRPGGPVLFRGTKGENERSVRFLSVERNEMRK
>g201.t2
LKAFCTGLFCTPPCVPGDESPSADPSERSDRVLMSNRPPEDHQAGLSPRNGAVVGTAVAGCRPPARGGTGTDRPGGPVLF
RGTKGENERSVRFLSVERNE
MRK
>g201.t3

ESPSADPSERSDRVLMSNRPPEDHQAGLSPRNGAVVGTAVAGCRPPARGGTGTDRPGGPVLFRGTKGENERSVRFLSVER
NEMRK
>g202.t1
DDVQYGVNLDVTSYTNGKRETHNPPGRALPFTVWDFYSSGCSLRLLNPQAFSSTVWNFLSVLQELFGSMAGANVYLTPPG
TQGFAPHYDDIEAFVVQLEG
KKHWRVYNPRSEEEVLPVNSSPNFSQSEIGKPVLEVVLEAGDLLYFPRGFIHQGDCLPDAHSLHITVSSYQKNSWGDLLA
KLVPAALGVAMEEDVEYRQG
LPLDYLTYMGVQNSDKAWHLAWSSASSPTCHVRHCNFAGVVSPRRVTTRDDDVIVAAQEDPRREKFLTRVGGLMRKLVGY
APVDAAVDEKAKEYLHDCLP
PALSPEEQASSVRGVSTRWGNGRPLNVGARLHSQTRVQLIRAGCARLCSDGEVIQLFYTTDNSRVYHKEELKSFEIKTEY
ADAIEFLIHSYPKFVSVQSV
PCDSAEEKIALAELLFEKGIIRTAEPL
>g202.t2
DDVQYGVNLDVTSYTNGKRETHNPPGRALPFTVWDFYSSGCSLRLLNPQAFSSTVWNFLSVLQELFGSMAGANVYLTPPG
TQGFAPHYDDIEAFVVQLEG
KKHWRVYNPRSEEEVLPVNSSPNFSQSEIGKPVLEVVLEAGDLLYFPRGFIHQGDCLPDAHSLHITVSSYQKNSWGDLLA
KLVPAALGVAMEEDVEYRQG
LPLDYLTYMGVQNSDKAWHLAWSSASRFHVLCVQRCSSAYLRPTCHVRHCNFAGVVSPRRVTTRDDDVIVAAQEDPRREK
FLTRVGGLMRKLVGYAPVDA
AVDEKAKEYLHDCLPPALSPEEQASSVRGVSTRWGNGRPLNVGARLHSQTRVQLIRAGCARLCSDGEVIQLFYTTDNSRV
YHKEELKSFEIKTEYADAIE
FLIHSYPKFVSVQSVPCDSAEEKIALAELLFEKGIIRTAEPL
>g202.t3
DDVQYGVNLDVTSYTNGKRETHNPPGRALPFTVWDFYSSGCSLRLLNPQAFSSTVWNFLSVLQELFGSMAGANVYLTPPG
TQGFAPHYDDIEAFVVQLEG
KKHWRVYNPRSEEEVLPVNSSPNFSQSEIGKPVLEVVLEAGDLLYFPRGFIHQGDCLPDAHSLHITVSSYQKNSWGDLLA
KLVPAALGVAMEEDVEYRQG
LPLDYLTYMGVQNSDKEDPRREKFLTRVGGLMRKLVGYAPVDAAVDEKAKEYLHDCLPPALSPEEQASSVRGVSTRWGNG
RPLNVGARLHSQTRVQLIRA
GCARLCSDGEVIQLFYTTDNSRVYHKEELKSFEIKTEYADAIEFLIHSYPKFVSVQSVPCDSAEEKIALAELLFEKGIIR
TAEPL
>g203.t1
MTSIIKLTAVSGVQEESALCYLLQVDEFRFLLDCGWDESFSMEIIDAMKRYVHQVDAVLLSHPDPLHLGALPYAVGKLGL
NCTIYATIPVYKMGQMFMYD
LFQSRNNSEDFNLFTLDDVDSAFDKIQQLKYSQIVNLKAGHMIGGTIWKIVKDGEEEIVYAVDFNHKREIHLNGCTLESV
SRPSLLITDSFNASYVQPRR
KQRDEQLLTNVMETLRGDGNVLIAADTAGRVLELAQLLDQIWRTKDAGLGVYSLALLNNVSYNVVEFSKSQVEWMSDKLM
RCFEDKRNNPFQFRHLSLCH
SLADLARVPSPKVVLCSQPDLESGFSRELFIQWCRDAKNSVILTYRTTPGTLARYLIDNPGERMLDLEVRKRVKLEGKEL
DEFLEKEKVKKEAAKKLEQA
KEVDVDSSDESDMEDDLDQPAVVKTKHHDLMMKGEGSRKGSFFKQAKKSYPMFPTHEERIKWDEYGEVIRPEDFLVPELQ
ATEEEKSKLEAGL
>g203.t2
MTSIIKLTAVSGVQEESALCYLLQVDEFRFLLDCGWDESFSMEIIDAMKRYVHQVDAVLLSHPDPLHLGALPYAVGKLGL
NCTIYATIPVYKMGQMFMYD
LFQSRNNSEDFNLFTLDDVDSAFDKIQQLKYSQIVNLKGKGHGLSITPLPAGHMIGGTIWKIVKDGEEEIVYAVDFNHKR
EIHLNGCTLESVSRPSLLIT
DSFNASYVQPRRKQRDEQLLTNVMETLRGDGNVLIAADTAGRVLELAQLLDQIWRTKDAGLGVYSLALLNNVSYNVVEFS
KSQVEWMSDKLMRCFEDKRN
NPFQFRHLSLCHSLADLARVPSPKVVLCSQPDLESGFSRELFIQWCRDAKNSVILTYRTTPGTLARYLIDNPGERMLDLE
VRKRVKLEGKELDEFLEKEK
VKKEAAKKLEQAKEVDVDSSDESDMEDDLDQPAVVKTKHHDLMMKGEGSRKGSFFKQAKKSYPMFPTHEERIKWDEYGEV
IRPEDFLVPELQATEEEKSK
LEAGL
>g204.t1
RGLHASCVSSSCLLQEGSERTACGADSLGSPVENIRMKDQQGRQWRQGARTAGDR

>g205.t1
RVWMAAGGASQASVGDVEEDASQLVFPKEFETAETLLNSEVHMLLEHRKQQNESAEDEQELSEVFMKTLNYTARFSRFKN
RETIASVRSLLLQKKLHKFE
LASLANLCPEAAEEAKALIPSLEGRFEDEELQQILDDIQTKRSFQY
>g205.t2
MAAGGASQASVGDVEEDASQLVFPKEFETAETLLNSEVHMLLEHRKQQNESAEDEQELSEVFMKTLNYTARFSRFKNRET
IASVRSLLLQKKLHKFELAS
LANLCPEAAEEAKALIPSLEGRFEDEELQQILDDIQTKRSFQY
>g206.t1
IWDPSNKRYEVPVPLSVPPSPETSEAKRSYRVLITKRPFGIQVVRKSTGTTIPGPGSRRHADTPAASRMLFFFFAEKTSG
WRGWDSSMPGFTFSDMFIQV
STRLSSHYVYGFGETEHLSYRHDLNYHTWGMFSKDQPPGPRGAGFGLQCSQPPCR
>g206.t2
IWDPSNKRYEVPVPLSVPPSPETSEAKRSYRVLITKRPFGIQVVRKSTGTTIWDSSMPGFTFSDMFIQVSTRLSSHYVYG
FGETEHLSYRHDLNYHTWGM
FSKDQPPGPRGAGFGLQCSQPPCR
>g206.t3
MPGFTFSDMFIQVSTRLSSHYVYGFGETEHLSYRHDLNYHTWGMFSKDQPPGPRGAGFGLQCSQPPCR
>g206.t4
IWDPSNKRYEVPVPLSVPPSPETSEAKRSYRVLITKRPFGIQVVRKSTGTTIPGPGSRRHADTPAASRMLFFFFAEKTSG
WRGLSQGGSSGSADGTNFPN
PCSYSSASGHAV
>g207.t1
MERWRRPRTDKEGWVGDRVSGAAGRQGGYWPSLLFYMFASCLRLLDNPPLPQPSLQLSEGLFSHSAPPWSLPSTPRQLKP
VNADGGRKTPTAERPGPTIN
IIVIVIVIIIIIIVIIVIIVIIIVIVVIILILILILIITIIILIIIITIIIITVIIIIVIIITVVIIIIIIIIN
>g208.t1
MIPDKAPKDDVFHAAGCLKEKAHVPSFEKYKELYEKSIENPD
>g209.t1
NRAPLLPVSYQHVQHSEPRGAPLFKRPEKAAPAPGSPPAVSACQCPADRPVVEHGLLGGGRSTVCGCCQRHGATAGGPPP
SGTTGLGGNFDIVSNLPAGI
AGAEPDWQPWSISWSPLRSTAVRGTGDGGRGTGDGGRGTGDGGPRCSGVIVYKPTTESGIHRP
>g210.t1
LADVIATQPTVLATIGKDAHLTCSHPRPSDPVIQVTWQKYQKKSEKWENVASYSKLFGTKVIQPFRKKVAYGGDIQLWNS
SIIIRNVSLEDESCYRCRFS
GNPDGVVDTNICLRVSNPALPTPVIGVHTQSSERVAGLSLRDRVRSSDIRRELGVEPLLLRVERSQLRWFGHLIRMPPGR
LPLEVFRARPTGKRPRGRPR
TRWRDYISHLAWERLGVPQEELESVAGEKDGWSSLLSLLPPRPGPG
>g210.t2
LQSTLADVIATQPTVLATIGKDAHLTCSHPRPSDPVIQVTWQKYQKKSEKWENVASYSKLFGTKVIQPFRKKVAYGGDIQ
LWNSSIIIRNVSLEDESCYR
CRFSGNPDGVVDTNICLRVSNPALPTPVIGVHTQSSERVAGLSLRDRVRSSDIRRELGVEPLLLRVERSQLRWFGHLIRM
PPGRLPLEVFRARPTGKRPR
GRPRTRWRDYISHLAWERLGVPQEELESVAGEKDGWSSLLSLLPPRPGPG
>g211.t1
MDGRKYNRAVRLHKLLYEALMRLTLNGFLSWLEETHRNDMVHLNETLKTIDSLGKEVSQHALKEVLENSSCTRIMDLFEV
YREFLRGGNGSLSNFWMSYL
DMVEILLGLIRASREGDWMLHLASIRAMIPWCFAYDRMNYARYLPYYYAQMSELPITHPDVYTEFMEGGFSVQLGSTNPF
GRIPVDQAIEETVNKDTQTA
GGTKGFSLKPGAVTKYYLTAEYRSMYLRQLRDLTGQGRCKWSHPDLQSPRIKRDEADVQSLMDLMENNWLNPMSPDEIDL
VSLSTGNMAPPDVTIDLLRA
LEKGEEAYQAFQQTRLDADPPPVKFHDKMTKQSLKTFSNVSTKQAHGKKAQDVVLKADRNLFSHMILVAESRKTSIKDAE
RLNRGGDITLQYKNLAGGHH
VQQWRKFLCSSSNKTSLIKFLVEEWKLPRYRAMLHGKVLYMTCEETCYKLTEDGCEEAAELHSTHEEADTRLLLHALHAA
NAGSKSVIITAEDTDVMVLC
LGFQKDITCPIYQKCGTQNRTRFVDITKLASSLGDSICDSLIGLHAFTGCDTVSAFAGRGKLNALKIMPQG

>g211.t2
MDGRKYNRAVRLHKLLYEALMRLTLNGFLSWLEETHRNDMVHLNETLKTIDSLGKEVSQHALKEVLENSSCTRIMDLFEV
YREFLRGGNGSLSNFWMSYL
DMVEILLGLIRASREGDWMLHLASIRAMIPWCFAYDRMNYARYLPYYYAQMSELPITHPDVYTEFMEGGFSVQLGSTNPF
GRIPVDQAIEETVNKDTQTA
GGTKGFSLKPGAVTKYYLTAEYRSMYLRQLRDLTGQGRCKWSHPDLQSPRIKRDEADVQSLMDLMENNWLNPMSPDEIDL
VSLSTGNMAPPDVTIDLLRA
LEKGEEAYQAFQQTRLDADPPPVKFHDKMTKQSLKTFSNVSTKQAHGKKAQDVVLKADRNLFSHMILVAESRKTSIKDAE
RLNRGGDITLQYKNLAGGHH
VQQWRKFLCSSSNKTSLIKFLVEEWKLPRYRAMLHGKVLYMTCEETCYKLTEDGCEEAAELHSTHEEADTRLLLHALHAA
NAGSKSVIITAEDTDVMVLC
LGFQKDITCPIYQKCGTQNRTRFVDITKLASSLGDSICDSLIGLHAFTGCDTVSAFAGRGKLNALKIVRKHTSCQETFSQ
LGQTWNVSDELFQKIEQFTC
RMYVANSSTAEVNKLRYQLFCTKRGEVESSQLPPCRDCLFMHVQRANYQAAIWKCCLQANPVVPSPTEYGWTDDEGKLAI
YWMRSPPAPDVVLEMLTCKC
VHSCKMPSCMCLSNGLPCTDMCRLQTCSNQKQQDGPELDFELGESDDERNEQFDE
>g211.t3
LLAIIGKRFQDAGLKDLCIESGMIAEGSIAGVMDGRKYNRAVRLHKLLYEALMRLTLNGFLSWLEETHRNDMVHLNETLK
TIDSLGKEVSQHALKEVLEN
SSCTRIMDLFEVYREFLRGGNGSLSNFWMSYLDMVEILLGLIRASREGDWMLHLASIRAMIPWCFAYDRMNYARYLPYYY
AQMSELPITHPDVYTEFMEG
GFSVQLGSTNPFGRIPVDQAIEETVNKDTQTAGGTKGFSLKPGAVTKYYLTAEYRSMYLRQLRDLTGQGRCKWSHPDLQS
PRIKRDEADVQSLMDLMENN
WLNPMSPDEIDLVSLSTGNMAPPDVTIDLLRALEKGEEAYQAFQQTRLDADPPPVKFHDKMTKQSLKTFSNVSTKQAHGK
KAQDVVLKADRNLFSHMILV
AESRKTSIKDAERLNRGGDITLQYKNLAGGHHVQQWRKFLCSSSNKTSLIKFLVEEWKLPRYRAMLHGKVLYMTCEETCY
KLTEDGCEEAAELHSTHEEA
DTRLLLHALHAANAGSKSVIITAEDTDVMVLCLGFQKDITCPIYQKCGTQNRTRFVDITKLASSLGDSICDSLIGLHAFT
GCDTVSAFAGRGKLNALKIM
PQG
>g212.t1
MDAAADFQAAIDKLRNENLESLANFQKECGAAISALQRTVDVHGKKIQDVEESLTDTCDQLAGLGETVARLTKDNEAMKK
QLDYLNNYTRRENIRIIGLP
ESVEMPKPADFVCNLLREVFGPNAFEMPIIIDRVHRTAAPKPPADAKPRPLLVRMHSYRVRELVLRLASRHEGPILYRDK
RIHFFPDVSPEVARLRSAFT
AVKRRLHQANITFRLYHPARLVFMWDTQKVSFTEPAEALSFIDQHVPATSSDSTSAADT
>g212.t2
MPETRHKTKDMLSTADSDSTAKLANQPSLCSPPPTVMDAAADFQAAIDKLRNENLESLANFQKECGAAISALQRTVDVHG
KKIQDVEESLTDTCDQLAGL
GETVARLTKDNEAMKKQLDYLNNYTRRENIRIIGLPESVEMPKPADFVCNLLREVFGPNAFEMPIIIDRVHRTAAPKPPA
DAKPRPLLVRMHSYRVRELV
LRLASRHEGPILYRDKRIHFFPDVSPEVARLRSAFTAVKRRLHQANITFRLYHPARLVFMWDTQKVSFTEPAEALSFIDQ
HVPATSSDSTSAADT
>g213.t1
LKHCANQLSPVFTHIFDTSLETCHVPACFKASAIVPVPKKTRITGLNDYRPVALTSVVMKTFERLVLTHLKDITDDLLDP
LQFAYRANRSVDDAVNMVLH
FILQHLDSPGSYGRILYVDLSSAFNMIIPALLRDKLSQINVPDSMCRWITDFLTDTRQFVRLGKHVSDTRTQGRRIGGKS
>g214.t1
MSLLTPEVEDGHPNKDASPCMSDTDSEDE
>g215.t1
MHSSDLKCNMTADDFYHLPLNLYVIILGIGLFVFMLSLMFCCYLFRYRGAEGRGPLHYDGKALENSMDIMRCLLKWLEIR
SVCPMCNKPISRLLPKAAVQ
GVQEPQDLLEV
>g215.t2
CLCGTGMHSSDLKCNMTADDFYHLPLNLYVIILGIGLFVFMLSLMFCCYLFRYRGAEGRGPLHYDGKALENSMDIMRCLL
KWLEIRSVCPMCNKPISRLL

PKAAVQGVQEPQDLLEV
>g216.t1
LTNDPSEDETKVVTAFPLCAGYREMFDNVDIWLVIDGEDNEMLSGLSSRITARSFSGLFCLTNQTSDNEKSLQPFCFLLL
PGSYQFRHFVLGQKLVKFFA
GEANCLTLKRLSVGVSRCTAVFHDIYSTNAFRVASIQADEK
>g217.t1
AVPLYLAGLLLSMVICLGSVLSFHVPPGTSGLQSAFSATRSNSVVGGSQKAVTFNKLLVNTGGDFNPDTGYFRCRVPGAY
YFSYSVGKFPKKMLSVILVK
NGDEVQAIAYDDYRKKGRKVQSQSLMITLKAMDTVWLLLQQSAQYAVYSNVGPYITFSGYLVYPDVTAASHYNNHNQLSQ
PSHPECPPQGGDPRYSWNHG
QASELQPRSAFSVARTSTSLGQSSTSGQLETEKKPLSFDVEYVNIGRHFNKTLGIFSCHYPGAYYFAFTVGKHPRRAVSV
KLMTGRGEVQAMVFDEDMSK
RREMQSQSLLLSLRQGDSVWLYSQQDERYAVYSNQGRYTTFSGFLVYPDAQAGSPDAHTPRTEQGI
>g218.t1
IPDEFITPMFNFYKSIGQLQMLQEEQALLTAITILTPERPYVKDQQAVERLQDPMLDVLRRICALHHPHEPQHFARLLGR
LTELRTLNHHHAEMLTSWRT
SDHKFTPLLCEIWDVQ
>g219.t1
LEPDLGGELRRASGGRALTHGARPGSARNSNMEEPLPCGLTTCRDRHRGRVQCKPGGRQRRGPGRADPRHRRLALGRWNV
TSLAGKEPKLVREVELY
>g220.t1
MLLIKAEGPSSPHYSIILLIKGGNMSETSSYQSLVHSAFGYPGFLLVSLLQFLYPFIAMVSYNVTAGDTLTKVFLRIPGV
GPGHMLAERHFVICITTLLV
TLPLSLPRHIGRLGKVSFLSMLLTLIILLVVMVRAVTLGPQMY
>g220.t2
MEMLLIKAEGPSSPHYSIILLIKGGNMSETSSYQSLVHSAFGYPGFLLVSLLQFLYPFIAMVSYNVTAGDTLTKVFLRIP
GVGPGHMLAERHFVICITTL
LVTLPLSLPRHIGRLGKVSFLSMLLTLIILLVVMVRAVTLGPQMY
>g220.t3
MSETSSYQSLVHSAFGYPGFLLVSLLQFLYPFIAMVSYNVTAGDTLTKVFLRIPGVGPGHMLAERHFVICITTLLVTLPL
SLPRHIGRLGKVSFLSMLLT
LIILLVVMVRAVTLGPQMY
>g221.t1
MKSFERLVLTHLKDITGPLLDPLQFAYRANRSVDDAVNMGLHYILQHLDSPGTYARVLFVDFSSAFNTIVPDILHSKLSQ
LTVPAPTYDTTDISLIRDGD
ESAYRREVEQLALWCGQNNLELNKLKTVEMTVDFRRSPPTLPPSPSSTALCLLWKPSGFWDLQSPRT
>g221.t2
LNDYRPVALTSVVMKSFERLVLTHLKDITGPLLDPLQFAYRANRSVDDAVNMGLHYILQHLDSPGTYARVLFVDFSSAFN
TIVPDILHSKLSQLTVPAPT
YDTTDISLIRDGDESAYRREVEQLALWCGQNNLELNKLKTVEMTVDFRRSPPTLPPSPSSTALCLLWKPSGFWDLQSPRT
>g222.t1
MYFLRQLRKFNLPQELLIQFYSAIIQSVLCTSITVWFGSATRQDRDRLPWTVSHHTLDTTCSNSSRLAKEEAHSGDRILY
NQARNILTKEIRVAKRSYTE
KLKNRFSANDPANDHLKNPLSHPSPAPTTSHIHLLPLTSVVMKSFERLVLTHLKDITGPLLDPLQFAYRANRSVDDAVNM
GLHYILQHLDSPGTYARVLF
VDFSSAFNTIVPDILHSKLSQLTVPAPTCQWITNFLTGRRQQETHLLNFADDTTVIGLIQDGDESAYRREVEQLALWCGQ
NNLELNKLKTVEMTVDFRRS
PPTLPPLTIINSTVSAVETFRFLGSTISQDLKWTPNIDTIIKKAQQRMYFLRQLRKFNLPQELMIQFYSAIIQSVLCTSI
TVWFGSATRQDRDRLQRTIR
TAEEIIGTKVKSDTGSL
>g222.t2
MYFLRQLRKFNLPQELLIQFYSAIIQSVLCTSITVWFGSATRQDRDRLPWTVSHHTLDTTCSNSSRLAKEEAHSGDRILY
NQARNILTKEIRVAKRSYTE
KLKNRFSANDPANDHLKNPLSHPSPAPTTSHIHLLPLTSVVMKSFERLVLTHLKDITGPLLDPLQFAYRANRSVDDAVNM
GLHYILQHLDSPGTYARVLF
VDFSSAFNTIVPDILHSKLSQLTVPAPTCQWITNFLTGRRQQETHLLNFADDTTVIGLIQDGDESAYRREVEQLALWCGQ

NNLELNKLKTVEMTVDFRRS
PPTLPPLTIINSTVSAVETFRFLGSTISQDLKWTPNIDTIIKKAQQRMYFLRQLRKFNLPQELMIQFYSAIIQETGIGKS
TLLNTLFNTNFENFESSHFE
PKGKLSAETYDLQEGNVQLKLTIVNMVGFGDQINKQQSYQPIMDYIVTQFESYLQEELKIKRSLHNYHDSCIHACIYFVA
PSGHWSP
>g222.t3
MYFLRQLRKFNLPQELLIQFYSAIIQSVLCTSITVWFGSATRQDRDRLPWTVSHHTLDTTCSNSSRLAKEEAHSGDRILY
NQARNILTKEIRVAKRSYTE
KLKNRFSANDPANDHLKNPLSHPSPAPTTSHIHLLPLTSVVMKSFERLVLTHLKDITGPLLDPLQFAYRANRSVDDAVNM
GLHYILQHLDSPGTYARVLF
VDFSSAFNTIVPDILHSKLSQLTVPAPTCQWITNFLTGRRQQETHLLNFADDTTVIGLIQDGDESAYRREVEQLALWCGQ
NNLELNKLKTVEMTVDFRRS
PPTLPPLTIINSTVSAVETFRFLGSTISQDLKWTPNIDTIIKKAQQRMYFLRQLRKFNLPQELMIQFYSAIIQETGIGKS
TLLNTLFNTNFENFESSHFE
PKGKLSAETYDLQEGNVQLKLTIVNMVGFGDQINKQQSYQPIMDYIVTQFESYLQEELKIKRSLHNYHDSYKPIKRSLHN
YHDSRIHACIYFIAPSGHSL
KSLDLVTMKKLDSKTNPGPRLHQFMDEVGAGSTWQGVQISRTNADDDTFNTLKLRLTNSFLLETCEDFESVEAARREWLD
LKILVACHYRHLDSQVLWQR
LIQGAIGRDGQFQHMQVVAEISLVLPMSSSCCERGFSSMKRIKSDSRACLSNCLLQISVHGPPVFLS
>g222.t4
LLNTLFNTNFENFESSHFEPKGKLSAETYDLQEGNVQLKLTIVNMVGFGDQINKQQSYQPIMDYIVTQFESYLQEELKIK
RSLHNYHDSYKPIKRSLHNY
HDSRIHACIYFIAPSGHSLKSLDLVTMKKLDSKTNPGPRLHQFMDEVGAGSTWQGVQISRTNADDDTFNTLKLRLTNSFL
LETCEDFESVEAARREWLDL
KILVACHYRHLDSQVLWQRLIQGAIGRDGQFQHMQVVAEISLVLPMSSSCCERGFSSMKRIKSDSRACLSNCLLQISVHG
PPVFLS
>g222.t5
LHNYHDSRIHACIYFIAPSGHSLKSLDLVTMKKLDSKTNPGPRLHQFMDEVGAGSTWQGVQISRTNADDDTFNTLKLRLT
NSFLLETCEDFESVEAARRE
WLDLKILVACHYRHLDSQVLWQRLIQGAIGRDGQFQHMQVVAEISLVLPMSSSCCERGFSSMKRIKSDSRACLSNCLLQI
SVHGPPVFLS
>g223.t1
IGCSQSAVSRHLSGKSVGRKKCGRKRCTTRRGDRTLRKIVEKGRFQTLGDLRKQWTESGVETSRATVHRRVQEMGYRSEV
KARQDVKPDIRQPPFTDYRQ
PPVDYRQPPVDYRQPPVDYRQPPVDYRQPPGLLEFRQHATATPFPLALPADFRPQDYDYFTVELEKSLKGFGFSIRGGRE
YKMDLFVLRLAEEGPAIRNG
RMRVGDQIIEINGDSTRDMTHARAIELIKAGGRRVRLLLKRGTGQVPDY
>g223.t2
GKSVGRKKCGRKRCTTRRGDRTLRKIVEKGRFQTLGDLRKQWTESGVETSRATVHRRVQEMGYRSEVKARQDVKPDIRQP
PFTDYRQPPVDYRQPPVDYR
QPPVDYRQPPVDYRQPPGLLEFRQHATATPFPLALPADFRPQDYDYFTVELEKSLKGFGFSIRGGREYKMDLFVLRLAEE
GPAIRNGRMRVGDQIIEING
DSTRDMTHARAIELIKAGGRRVRLLLKRGTGQVPDY
>g223.t3
IGCSQSAVSRHLSGKSVGRKKCGRKRCTTRRGDRTLRKIVEKGRFQTLGDLRKQWTESGVETSRATVHRRVQEMGYRSEV
KARQDVKPDIRQPPFTDYRQ
PPVDYRQPPVDYRQPPVDYRQPPVDYRQPPGLLEFRQHATATPFPLALPADFRPQDYDYFTVELEKSLKGFGFSIRGGRE
YKMDLFVLRLAEEGPAIRNG
RMRVGDQIIEINGDSTRDMTHARAIELIKAGGRRVRLLLKRGTGQVPDYGK
>g224.t1
VSSPVCRPQVTSASAFAQTLRRYTSLNHLAQAARAVLQNTAQINQMLSDLNRVDFANVQEQASWVCRCEDRVVQRLEQDF
KLTLQQQNSLEQWAAWLDGV
VSQVLKPYQHSAAFPKAAKLFLLKWSFYSSMVIRDLTLRSAASFGSFHLIRLLYDEYMYYLIEHRVAQAKGETPIAVMGE
FASLGRTLNPLDPDKGKTFL
DFSSRSVRRGQLLPAPLGAGCRARETHQRRLTFGLPYMSCVTEEEEEEEEEESDDEGQELSLQSDGGAGLGEESLEPPAK
LARTDQRVLFATGSADN

>g224.t2
VSSPVCRPQVTSASAFAQTLRRYTSLNHLAQAARAVLQNTAQINQMLSDLNRVDFANVQEQASWVCRCEDRVVQRLEQDF
KLTLQQQNSLEQWAAWLDGV
VSQVLKPYQHSAAFPKAAKLFLLKWSFYSSMVIRDLTLRSAASFGSFHLIRLLYDEYMYYLIEHRVAQAKGETPIAVMGE
RRLTFGLPYMSCVTEEEEEE
EEEESDDEGQELSLQSDGGAGLGEESLEPPAKLARTDQRVLFATGSADN
>g224.t3
VSSPVCRPQVTSASAFAQTLRRYTSLNHLAQAARAVLQNTAQINQMLSDLNRVDFANVQEQASWVCRCEDRVVQRLEQDF
KLTLQQQNSLEQWAAWLDGV
VSQVLKPYQHSAAFPKAAKLFLLKWSFYSSMVIRDLTLRSAASFGSFHLIRLLYDEYMYYLIEHRVAQAKGETPIAVMGE
FASLGRTLNPLDPDKEEEEE
EEEEESDDEGQELSLQSDGGAGLGEESLEPPAKLARTDQRVLFATGSADN
>g225.t1
DVTLDPNTAHPRLIISVDGKQVCCGDRHQPVQDTPERFDRVVCVLARQGFTSGRHYWEVSSVAASLAMAGQGLEWVAYRI
HMLNPIRHFSLLFSHRQSTY
VYL
>g225.t2
DVTLDPNTAHPRLIISVDGKQVCCGDRHQPVQDTPERFDRVVCVLARQGFTSGRHYWEQVSSVAASLAMAGQGLEWVAYR
IHMLNPIRHFSLLFSHRQST
YVYL
>g226.t1
PHHGTPMLQEGVKCLGTERDSQGKHSSSHKRK
>g227.t1
VHQVSVAVRASAAHAQRRTGSRPVTRVTRDTSARPTMAPLGKGPLHESSL
>g227.t2
MHHGSRSEPKMTHALTMLVDGTRQQPPVNTQAVHQVSVAVRASAAHAQRRTGSRPVTRVTRDTSARPTMAPLGKGPLHES
SL
>g228.t1
MGGVWNCQSAVKKADSISAFACLQTLHFLALTETWITPDNTATPAALSTAYSFSHTPRPSGQGGGTGLLISPKWSYRVLP
LEHLTRSVFELHAVAITIPI
KLYIAVIFRPPGPFHDFFDEMDALLSCFPEDSTLLVVLGDFNIQPAQSSPPPPWLSESLRAHRTELCTAERKWKKSGFAD
DLSHYLHLLSDFISTVSAAK
STYYWNKINSSASNPRKLFSMFSSLLNQTDRQTSKIPEGNCICYKYI
>g229.t1
LFQPGVTVVDCKGNGVYIAGSSWFSSLDLHSCSMLCWICEADVCQLFRKQKTKKAPGPDGVSPSCLKVCADQLTPIFTQI
FNRSLELCEVPSCFKRSTII
PVPKKPAISGLNDYRPVALTSVVMKTLRDWC
>g229.t2
LFQPGVTVVDCKGNGVYIAGSSWFSSLDLHSCSMICEADVCQLFRKQKTKKAPGPDGVSPSCLKVCADQLTPIFTQIFNR
SLELCEVPSCFKRSTIIPVP
KKPAISGLNDYRPVALTSVVMKTLRDWC
>g230.t1
MATRAMFPAGVFSVGGEMMEDEGPGLSELPGNNLPVPQLVMLANAATAVGGAESAAEDGASCDGLAEVKQMVELKTVGCS
YSDSEGESVRYSYDHQNRVE
FCIVEYVDSPAPRAW
>g231.t1
LDQSEEQQQCFLKELPKKRLFLDYEDPYRTGGDLQRPAPQCYRNWQGSATLIDRTFLMNEDCDEKSMRSFSDPVRRPKYK
AEDDEEEEEEEEEEGEEEAV
EVMGKLRVRV
>g231.t2
LDQSEEQQQCFLKELPKKRLFLDYEDPYRTGGDLQRPAPQCYRNWQGSATLIDRTFLMNEDCDEKSMRSFSDPVRRPKYK
AEDDEEEEEEEEEEGEEEAV
EVMGKLRVSLHLWLR
>g231.t3
LDQSEEQQQCFLKELPKKRLFLDYEDPYRTGGDLQRPAPQCYRNWQGSATLIDRTFLMNEDCDEKSMRSFSDPVRRPKYK
AEDDEEEEEEEEEEGEEEAV

EVMGKLR
>g232.t1
NTSLINQKKKLEADTSQLQSEVEEAVQECRNAEEKAKKAITDAAMMAEELKKEQDTSSHLERMKKNMEQTIKDLQHRLDE
AEQIAMKGGKKQIQKLENRV
KDLENEVESEQKKNSESIKGIRKYERRLKELTYQTEEDRKNVARLQDLVDKLQLKVKAYKRTSEEAEEQANTHLGKFRKL
QHELDEAEERADIAETQVNK
LRAKSRDVGSKKGFDEE
>g232.t2
NTSLINQKKKLEADTSQLQSEVEEAVQECRNAEEKAKKAITDAAMMAEELKKEQDTSSHLERMKKNMEQTIKDLQHRLDE
AEQIAMKGGKKQIQKLENRV
KDLENEVESEQKKNSESIKGIRKYERRLKELTYQTEEDRKNVARLQDLVDKLQLKVKAYKRTSEEAEEQANTHLGKFRKL
QHELDEAEERADIAETQVNK
LRAKSRDVGSKAISTLHARLPYRIHLAVRQVKPPSGSSCWV
>g233.t1
MGDATMREFGPAASYLRKSDKERLEAQTRPFDMKKECFVPDADEEYVKATVTSREGDSVTAQTERGKTVTVKECDVHPQN
PPKFDKIEDMAMFTFLHEPA
VLFNLKERYAAWMIYTYSGLFCVTVNPYKWLPVYNQEVVVAYRGKKRSEAPPHIFSISDNAYQYMLTDRENQSILITGES
GAGKTVNTKRVIQYFASIAA
GGGKKEGNSEKKGTLEDQIIQANPALEAFGNAKTIRNDNSSRFGKFIRIHFAASGKLASADIETXMLLITNNPYDYSFIS
QGETTVASINDAEELVATDD
AFDVLGFTQEDKNGIYKMIGAIMHYGNMRFKQKQREEQAEADGTEDADKVAYLMGLNSADLIKGLCHPRVKVGNEWVTKG
QSVQQVYYAIGALSKSVYEK
MFLWMVVRINHSLDTKQPRQYFIGVLDIAGFEIFDFNTFEQLCINFTNEKLQQFFNHHMFVLEQEEYKKEGIEWTFIDFG
MDLQACIDLIEKPMGIMSIL
EEECMFPKASDATFKAKLYDNHLGKSNNFQKPRIVKGKPEAHFSLVHYAGTVDYNICNWLVKNKDPLNETVVGLYQKSNL
KMLSNLFANYAGSDSDGKGG
KGGGSKKKGSSFQTVSALHRENLNKLMTNLRSTHPHFVRCIIPNESKTPGAMENPLVMHQLRCNGVLEGIRICRKGFPNR
ILYGDFKQRYRILNPAAIPD
GQFIDSRKGAEKLLGSLDIDHNQYKFGHTKVFFKAGLLGTLEEMRDDRLSLIITGIQSRARGLLARLEFQKIVERRDALL
VIQWNIRAFMGVKNWPWMKL
FFKIKPLLRSAEAEKEMANMKEEFLKLKEAYAKSEARRKELEEKMVSLLQEKNDLQLNVQAEQDNLADAEERCEGLIKNK
IQMEAKAKELSERLEDEEEM
NAELTAKKRKLEDECSELKKDIDDLELTLAKVEKEKHATENKVKNLVEEMASLDEIIAKLTKEKKALQESHQQTLDDLQS
EEDKVNTLTKAKAKLEQQVD
DLEGSLEQEKKVRMDLERAKRKLEGDLKLTQESLMDLENDKQQLEERLKKKDFEVSQLNNRIEDEQAISIQLQKKLKELQ
ARIEELEEELEAERAARAKV
EKQRADLARELEEISERLEEAGGATSAQIEMNKKREAEFQKLRRDLEEATLQHESTAATLRKKQADSVADLGEQIDNLQR
VKQKLEKEKSELRLELDDVV
SNMEHIVKTKV
>g233.t2
MGDATMREFGPAASYLRKSDKERLEAQTRPFDMKKECFVPDADEEYVKATVTSREGDSVTAQTERGKTVTVKECDVHPQN
PPKFDKIEDMAMFTFLHEPA
VLFNLKERYAAWMIYTYSGLFCVTVNPYKWLPVYNQEVVVAYRGKKRSEAPPHIFSISDNAYQYMLTDRENQSILITGES
GAGKTVNTKRVIQYFASIAA
GGGKKEGNSEKKGTLEDQIIQANPALEAFGNAKTIRNDNSSRFGKFIRIHFAASGKLASADIETXMLLITNNPYDYSFIS
QGETTVASINDAEELVATDD
AFDVLGFTQEDKNGIYKMIGAIMHYGNMRFKQKQREEQAEADGTEDADKVAYLMGLNSADLIKGLCHPRVKVGNEWVTKG
QSVQQVYYAIGALSKSVYEK
MFLWMVVRINHSLDTKQPRQYFIGVLDIAGFEIFDFNTFEQLCINFTNEKLQQFFNHHMFVLEQEEYKKEGIEWTFIDFG
MDLQACIDLIEKPMGIMSIL
EEECMFPKASDATFKAKLYDNHLGKSNNFQKPRIVKGKPEAHFSLVHYAGTVDYNICNWLVKNKDPLNETVVGLYQKSNL
KMLSNLFANYAGSDSVFVIN
HQMEKEERVVEENLNKLMTNLRSTHPHFVRCIIPNESKTPGAMENPLVMHQLRCNGVLEGIRICRKGFPNRILYGDFKQR
YRILNPAAIPDGQFIDSRKG
AEKLLGSLDIDHNQYKFGHTKVFFKAGLLGTLEEMRDDRLSLIITGIQSRARGLLARLEFQKIVERRDALLVIQWNIRAF
MGVKNWPWMKLFFKIKPLLR

SAEAEKEMANMKEEFLKLKEAYAKSEARRKELEEKMVSLLQEKNDLQLNVQAEQDNLADAEERCEGLIKNKIQMEAKAKE
LSERLEDEEEMNAELTAKKR
KLEDECSELKKDIDDLELTLAKVEKEKHATENKVKNLVEEMASLDEIIAKLTKEKKALQESHQQTLDDLQSEEDKVNTLT
KAKAKLEQQVDDLEGSLEQE
KKVRMDLERAKRKLEGDLKLTQESLMDLENDKQQLEERLKKKDFEVSQLNNRIEDEQAISIQLQKKLKELQARIEELEEE
LEAERAARAKVEKQRADLAR
ELEEISERLEEAGGATSAQIEMNKKREAEFQKLRRDLEEATLQHESTAATLRKKQADSVADLGEQIDNLQRVKQKLEKEK
SELRLELDDVVSNMEHIVKT
KV
>g233.t3
MREFGPAASYLRKSDKERLEAQTRPFDMKKECFVPDADEEYVKATVTSREGDSVTAQTERGKTVTVKECDVHPQNPPKFD
KIEDMAMFTFLHEPAVLFNL
KERYAAWMIYTYSGLFCVTVNPYKWLPVYNQEVVVAYRGKKRSEAPPHIFSISDNAYQYMLTDRENQSILITGESGAGKT
VNTKRVIQYFASIAAGGGKK
EGNSEKKGTLEDQIIQANPALEAFGNAKTIRNDNSSRFGKFIRIHFAASGKLASADIETXMLLITNNPYDYSFISQGETT
VASINDAEELVATDDAFDVL
GFTQEDKNGIYKMIGAIMHYGNMRFKQKQREEQAEADGTEDADKVAYLMGLNSADLIKGLCHPRVKVGNEWVTKGQSVQQ
VYYAIGALSKSVYEKMFLWM
VVRINHSLDTKQPRQYFIGVLDIAGFEIFDFNTFEQLCINFTNEKLQQFFNHHMFVLEQEEYKKEGIEWTFIDFGMDLQA
CIDLIEKPMGIMSILEEECM
FPKASDATFKAKLYDNHLGKSNNFQKPRIVKGKPEAHFSLVHYAGTVDYNICNWLVKNKDPLNETVVGLYQKSNLKMLSN
LFANYAGSDSDGKGGKGGGS
KKKGSSFQTVSALHRENLNKLMTNLRSTHPHFVRCIIPNESKTPGAMENPLVMHQLRCNGVLEGIRICRKGFPNRILYGD
FKQRYRILNPAAIPDGQFID
SRKGAEKLLGSLDIDHNQYKFGHTKVFFKAGLLGTLEEMRDDRLSLIITGIQSRARGLLARLEFQKIVERRDALLVIQWN
IRAFMGVKNWPWMKLFFKIK
PLLRSAEAEKEMANMKEEFLKLKEAYAKSEARRKELEEKMVSLLQEKNDLQLNVQAEQDNLADAEERCEGLIKNKIQMEA
KAKELSERLEDEEEMNAELT
AKKRKLEDECSELKKDIDDLELTLAKVEKEKHATENKVKNLVEEMASLDEIIAKLTKEKKALQESHQQTLDDLQSEEDKV
NTLTKAKAKLEQQVDDLEGS
LEQEKKVRMDLERAKRKLEGDLKLTQESLMDLENDKQQLEERLKKKDFEVSQLNNRIEDEQAISIQLQKKLKELQARIEE
LEEELEAERAARAKVEKQRA
DLARELEEISERLEEAGGATSAQIEMNKKREAEFQKLRRDLEEATLQHESTAATLRKKQADSVADLGEQIDNLQRVKQKL
EKEKSELRLELDDVVSNMEH
IVKTKV
>g234.t1
MEDKDSLVSQLTRGKMSYTQQIEDLKRQLEEETKAKNALAHAVQSSRHDCDLLREQYEEEQEAKAELQRSMSKANTEVAQ
WRTKYETDAIQRTEELEEAK
KKLAQRLQDAEEAVEAVNAKCSSLEKTKHRLQNEIEDLMVLSEWKQKYEESQCELESSQKEARCLSTELFKLKNSYEESL
DHLETMKRENKNLQEEISDL
TEQLGEGSKSIHELEKLRKQLEQEKSEIQSALEEAEASLEHEEGKILRAQLEFNQVKAEIERKLAEKDEEMEQSKRNLQR
NIDTLQSSLEAECRSRNEAL
RLKKKMEGDLNEMEIQLSQANRQASEAQKQLKTIHAHLKDSQIQLDESLRANDDLKENIAIVERRNNLMQAELEELRSAL
EQTERCRKLAEQELLDVSER
VQLLHSQNTSLINQKKKLEADTSQLQSEVEESVQECRNAEEKAKKAITDAAMMAEELKKEQDTSSHLERMKKNMEQTIKD
LQHRLDEAEQIAMKGGKKQV
QKLEARVRELENEVESEQKKSSEAVKGIRKYERRIKELTYQTEEDRKNVTRLQDLVDKLQLKVKAYKRTSEEAEEQANTH
LGKFRKLQHELDEAEERADI
AESQVNKLRAKSRDVGSKRGLDEE
>g234.t2
MEDKDSLVSQLTRGKMSYTQQIEDLKRQLEEETKAKNALAHAVQSSRHDCDLLREQYEEEQEAKAELQRSMSKANTEVAQ
WRTKYETDAIQRTEELEEAK
KKLAQRLQDAEEAVEAVNAKCSSLEKTKHRLQNEIEDLMVDVERSNAAAAALDKKQRNFDKARQYFKKRLCSIHKTAKII
KHYIVVLSEWKQKYEESQCE
LESSQKEARCLSTELFKLKNSYEESLDHLETMKRENKNLQEEISDLTEQLGEGSKSIHELEKLRKQLEQEKSEIQSALEE
AEASLEHEEGKILRAQLEFN

QVKAEIERKLAEKDEEMEQSKRNLQRNIDTLQSSLEAECRSRNEALRLKKKMEGDLNEMEIQLSQANRQASEAQKQLKTI
HAHLKDSQIQLDESLRANDD
LKENIAIVERRNNLMQAELEELRSALEQTERCRKLAEQELLDVSERVQLLHSQNTSLINQKKKLEADTSQLQSEVEESVQ
ECRNAEEKAKKAITDAAMMA
EELKKEQDTSSHLERMKKNMEQTIKDLQHRLDEAEQIAMKGGKKQVQKLEARVRELENEVESEQKKSSEAVKGIRKYERR
IKELTYQTEEDRKNVTRLQD
LVDKLQLKVKAYKRTSEEAEEQANTHLGKFRKLQHELDEAEERADIAESQVNKLRAKSRDVGSKRGLDEE
>g235.t1
MGDAVMAEFGIACTFLRKSDKERLEAQTRPFDMKKQCFVPDPEVEYVKASIVSRDGDKVTADTEFGKTVTVKECDVHPQN
PPKFDKIEDMAMFTFLHEPA
VLFNLKERYAAWMIYTYSGLFCVTVNPYKSLPVYNQEVVVAYRGKKRSEAPPHIFSISDNAYQYMLSDRENQSILITGES
GAGKTVNTKRVIQYFASIAA
VPNLLEKSRVTFQLKAERDYHIFYQILSQQKPELLEMLLITNNPYDYAFISQGETTVASINDADELVATDDAFDVLGFTQ
EEKNSIYKLTGAIMHHGNMK
FKQKQREEQAEADGTEDADKVAYLMGLNSADLIKGLCHPRVKVGNEWVTKGQNVAQVNYAIGALSKSVYEKMFLWMVVRI
NQSLETKQPRQYFIGVLDIA
GFEIFDYNTFEQLCINFTNEKLQQFFNHHMFVLEQEEYKKEGIEWTFIDFGMDLQACIDLIEKPMGIMSILEEECMFPKA
SDTTFKAKLYDNHLGKSNNF
QKPRVVKGKPEAHFSLVHYAGTVDYNISNWLVKNKDPLNETVENLNKLMTNLRSTHPHFVRCIIPNETKTPGAMENPLVM
HQLRCNGVLEGIRICRKGFP
NRILYGDFKQRYRILNPNAIPEGQFIDNKKAAEKLLGSLDLDHTQYRLGHTKVFFKAGLLGLLEEMRDDRLALIITGIQA
RSRGVLARIEFQKIVERRDA
LLVIQWNIRAFMGVKNWPWMKMYFKIKPLLKSAETEKEMANMKEEFLKLKEAYAKSEARRKELEEKMVSLLQEKNDLQIQ
VQAVC
>g235.t2
MGDAVMAEFGIACTFLRKSDKERLEAQTRPFDMKKQCFVPDPEVEYVKASIVSRDGDKVTADTEFGKTVTVKECDVHPQN
PPKFDKIEDMAMFTFLHEPA
VLFNLKERYAAWMIYTYSGLFCVTVNPYKSLPVYNQEVVVAYRGKKRSEAPPHIFSISDNAYQYMLSDRENQSILITGES
GAGKTVNTKRVIQYFASIAA
VPKMLLITNNPYDYAFISQGETTVASINDADELVATDDAFDVLGFTQEEKNSIYKLTGAIMHHGNMKFKQKQREEQAEAD
GTEDADKVAYLMGLNSADLI
KGLCHPRVKVGNEWVTKGQNVAQVNYAIGALSKSVYEKMFLWMVVRINQSLETKQPRQYFIGVLDIAGFEIFDYNTFEQL
CINFTNEKLQQFFNHHMFVL
EQEEYKKEGIEWTFIDFGMDLQACIDLIEKPMGIMSILEEECMFPKASDTTFKAKLYDNHLGKSNNFQKPRVVKGKPEAH
FSLVHYAGTVDYNISNWLVK
NKDPLNETVENLNKLMTNLRSTHPHFVRCIIPNETKTPGAMENPLVMHQLRCNGVLEGIRICRKGFPNRILYGDFKQRYR
ILNPNAIPEGQFIDNKKAAE
KLLGSLDLDHTQYRLGHTKVFFKAGLLGLLEEMRDDRLALIITGIQARSRGVLARIEFQKIVERRDALLVIQWNIRAFMG
VKNWPWMKMYFKIKPLLKSA
ETEKEMANMKEEFLKLKEAYAKSEARRKELEEKMVSLLQEKNDLQIQVQAVC
>g235.t3
MGDAVMAEFGIACTFLRKSDKERLEAQTRPFDMKKQCFVPDPEVEYVKASIVSRDGDKVTADTEFGKTVTVKECDVHPQN
PPKFDKIEDMAMFTFLHEPA
VLFNLKERYAAWMIYTYSGLFCVTVNPYKSLPVYNQEVVVAYRGKKRSEAPPHIFSISDNAYQYMLSDRENQSILITGES
GAGKTVNTKRVIQYFASIAA
VPNLLEKSRVTFQLKAERDYHIFYQILSQQKPELLEMLLITNNPYDYAFISQGETTVASINDADELVATDDAFDVLGFTQ
EEKNSIYKLTGAIMHHGNMK
FKQKQREEQAEADGTEDADKVAYLMGLNSADLIKGLCHPRVKVGNEWVTKGQNVAQVNYAIGALSKSVYEKMFLWMVVRI
NQSLETKQPRQYFIGVLDIA
GFEIFDYNTFEQLCINFTNEKLQQFFNHHMFVLEQEEYKKEGIEWTFIDFGMDLQACIDLIEKPMGIMSILEEECMFPKA
SDTTFKAKLYDNHLGKSNNF
QKPRVVKGKPEAHFSLVHYAGTVDYNISNWLVKNKDPLNETVENLNKLMTNLRSTHPHFVRCIIPNETKTPGAMENPLVM
HQLRCNGVLEGIRICRKGFP
NRILYGDFKQRYRILNPNAIPEGQFIDNKKAAEKLLGSLDLDHTQYRLGHTKVFFKAGLLGLLEEMRDDRLALIITGIQA
RSRGVLARIEFQKIVERRDA
LLVIQWNIRAFMGVKNWPWMKMYFKIKPLLKSAETEKEMANMKEEFLKLKEAYAKSEARRKELEEKMVSLLQEKNDLQIQ

VQAEQDNLCDAEERCEGLIK
SKIQLEAKSKELTERLEDEEEMNAELTAKKRKLEDECSELKKDIDDLELTLAKVEKEKHATENKVKNLTEEMAALDEIIA
KLTKEKKALQEAHQQTLDDL
QSEEDKVNTLTKAKAKLEQQVDDARIEELEEELEAERAARAKVEKQRADLSRELEEISERLEEAGGATSAQIEMNKKREA
EFLKLRRDLEEATLQHEATA
ATLRKKQADSVADLGEQIDNLQRVKQKLEKEKSELRLELDDVVSNMEHIVKSKAKNALAHAVQSSRHDCDLLREQYEEEQ
EAKAELQRSMSKANSEVAQW
RTKYETDAIQRTEELEEAKKKLAQRLQDAEEAVEAVNAKCSSLEKTKHRLQNEIEDLMVDVERSNAAAAALDKKQRNFDK
VLSEWKQKYEESQCELESSQ
KEARCLSTELFKLKNSYEESLDHLETMKRENKNLQEEVSDLTEQLGEGSKTIHELEKVRKQMDQEKCEIQAALEEAEASL
EHEEGKILRAQLEFNQIKAE
IERKMAEKDEEMEQCKKNMQRTIDTLHSSLESETRSRNEALRLKKKMEGDLNEMEIQLSQANRQASEAQKQLKSVHAHLK
DTQLQLDDSLRSNDDLKENI
AIVERRNNLMQAELEELRVLVEQTERARKLAEQELLDVTERVQLLHSQNTSLINQKKKLEADTSQLQSEVEEAVQECRNA
EEKAKKAITDAAMMAEELKK
EQDTSSHLERMKKNMEQTIKDLQHRLDEAEQIAMKGGKKQVQKLEARVSAL
>g235.t4
LIKSKIQLEAKSKELTERLEDEEEMNAELTAKKRKLEDECSELKKDIDDLELTLAKVEKEKHATENKVKNLTEEMAALDE
IIAKLTKEKKALQEAHQQTL
DDLQSEEDKVNTLTKAKAKLEQQVDDARIEELEEELEAERAARAKVEKQRADLSRELEEISERLEEAGGATSAQIEMNKK
REAEFLKLRRDLEEATLQHE
ATAATLRKKQADSVADLGEQIDNLQRVKQKLEKEKSELRLELDDVVSNMEHIVKSKAKNALAHAVQSSRHDCDLLREQYE
EEQEAKAELQRSMSKANSEV
AQWRTKYETDAIQRTEELEEAKKKLAQRLQDAEEAVEAVNAKCSSLEKTKHRLQNEIEDLMVDVERSNAAAAALDKKQRN
FDKVLSEWKQKYEESQCELE
SSQKEARCLSTELFKLKNSYEESLDHLETMKRENKNLQEEVSDLTEQLGEGSKTIHELEKVRKQMDQEKCEIQAALEEAE
ASLEHEEGKILRAQLEFNQI
KAEIERKMAEKDEEMEQCKKNMQRTIDTLHSSLESETRSRNEALRLKKKMEGDLNEMEIQLSQANRQASEAQKQLKSVHA
HLKDTQLQLDDSLRSNDDLK
ENIAIVERRNNLMQAELEELRVLVEQTERARKLAEQELLDVTERVQLLHSQNTSLINQKKKLEADTSQLQSEVEEAVQEC
RNAEEKAKKAITDAAMMAEE
LKKEQDTSSHLERMKKNMEQTIKDLQHRLDEAEQIAMKGGKKQVQKLEARVSAL
>g235.t5
LIKSKIQLEAKSKELTERLEDEEEMNAELTAKKRKLEDECSELKKDIDDLELTLAKVEKEKHATENKVKNLTEEMAALDE
IIAKLTKEKKALQEAHQQTL
DDLQSEEDKVNTLTKAKAKLEQQVDDARIEELEEELEAERAARAKVEKQRADLSRELEEISERLEEAGGATSAQIEMNKK
REAEFLKLRRDLEEATLQHE
ATAATLRKKQADSVADLGEQIDNLQRVKQKLEKEKSELRLELDDVVSNMEHIVKSKAKNALAHAVQSSRHDCDLLREQYE
EEQEAKAELQRSMSKANSEV
AQWRTKYETDAIQRTEELEEAKKKLAQRLQDAEEAVEAVNAKCSSLEKTKHRLQNEIEDLMVDVERSNAAAAALDKKQRN
FDKVLSEWKQKYEESQCELE
SSQKEARCLSTELFKLKNSYEESLDHLETMKRENKNLQEEVSDLTEQLGEGSKTIHELEKVRKQMDQEKCEIQAALEEAE
ASLEHEEGKILRAQLEFNQI
KAEIERKMAEKDEEMEQCKKNMQRTIDTLHSSLESETRSRNEALRLKKKMEGDLNEMEIQLSQANRQASEAQKQLKSVHA
HLKLQLDDSLRSNDDLKENI
AIVERRNNLMQAELEELRVLVEQTERARKLAEQELLDVTERVQLLHSQNTSLINQKKKLEADTSQLQSEVEEAVQECRNA
EEKAKKAITDAAMMAEELKK
EQDTSSHLERMKKNMEQTIKDLQHRLDEAEQIAMKGGKKQVQKLEARVSAL
>g236.t1
LIGTSYENRPMHVLKFSTGGTNRPAIWIDTGIHSREWVSPATGIWTANKIATDYGTDASLTSLLNTMDIYMLLLANPDGY
VYTQTNNRMWRKTRSRTPGS
NCRGADPNRNWDSGFGGPGTSKNPCSDLYHGPSAHSEVEVKNVVNLIKAHGNFKSFISVHAYSQLLMYPHGYSCKDVPDK
AELDSVGRVAVQKLASLYGT
TYKVGSICKIIYQASGGSIDWSYDVGIKYSFAFELRDTGRYGFLLPANQIIPTASETWLALRHLLEHVRDHPY
>g236.t2
KILIGTSYENRPMHVLKFSTGGTNRPAIWIDTGIHSREWVSPATGIWTANKIATDYGTDASLTSLLNTMDIYMLLLANPD

GYVYTQTNNRMWRKTRSRTP
GSNCRGADPNRNWDSGFGGPGTSKNPCSDLYHGPSAHSEVEVKNVVNLIKAHGNFKSFISVHAYSQLLMYPHGYSCKDVP
DKAELDSVGRVAVQKLASLY
GTTYKVGSICKIIYQASGGSIDWSYDVGIKYSFAFELRDTGRYGFLLPANQIIPTASETWLALRHLLEHVRDHPY
>g236.t3
LVSKILIGTSYENRPMHVLKFSTGGTNRPAIWIDTGIHSREWVSPATGIWTANKIATDYGTDASLTSLLNTMDIYMLLLA
NPDGYVYTQTNNRMWRKTRS
RTPGSNCRGADPNRNWDSGFGGPGTSKNPCSDLYHGPSAHSEVEVKNVVNLIKAHGNFKSFISVHAYSQLLMYPHGYSCK
DVPDKAELDSVGRVAVQKLA
SLYGTTYKVGSICKIIYQASGGSIDWSYDVGIKYSFAFELRDTGRYGFLLPANQIIPTASETWLALRHLLEHVRDHPY
>g237.t1
MDALGFVLGERVGALRVKHIRDLVHGAHAGHPLSGQAGVSMTYCGLDDQETVFYRKISGDSSEYFVNGVHVSLDKYKKEL
EKIGVVTKARNCLVFQGAVE
SIAMESPKERTKLFERISQSDEYAAEYELKRVALQKAKDDTQFHFHKKRTATVERKQISQEKLKARQCFPILGLGPHFLR
WPGGGLGLCMWGETAGKVLH
PCPPPTPQAQNYQALVDSLGQRRLRLSLFRLYHNQQDIQALASRLVEERQGAAAQRSSVEEGEGAVKALKKEHGGLTREQ
LAVEKDLRSEEQRLTQGQSQ
YIRAKVNTSHHMKKVEASKAALQKIHRLRAKREAAMEEVRQEMAELDTAWRRCERGMQEDGGPGGLAIDLEEGQVEEYKN
LKELARQQTALLCQQTEKLS
WEVQANLHKITLDHSKKKKVEAGIRNQQSQVEELTHRVEKLEEYTTGCRLSLEQYAQQERSLSEELEHGRQRSQVVEKEL
GQVLEELGNATLDHQETRQQ
QKRKEVLENLFRLYPDSVYGRLVDLCSPTHRKYQLAVVKVFGRYLNAIVVSSEKVARSCILYLKEQHAEPETFLPIDYLD
VGPLNERLRGLKGAQMVVDV
VQCNPGAISTLKKVLQFVCGNAVVCESIRDARSVAFDGPQRIKTVALDGTLFTKSGVISGGSKDLRTKARCWDEKDVRKL
KDRKDQLTAELRGLVKSRAK
ESELKGFQVQAQGVQIRLKYSNKEQENILRGNIPRCRAEISSLESELVNLDAEIQMQQENMEVKQAEMKRIKDRMEQMED
QVFADFCAQIGVDNIRQYEQ
EHLKQQSELDRKRCFYPSSIFFTXLEFEGQQGRLAAHLEYEQEQAEQQQRKLSQLEETMAKEELTVAQQAKEEKRLLDMV
EHSQNNILDIKNLLLAKKLQ
IRDSKAHLDQDTKTLQEANRQLARQQRALITVETSLEQQRHTKHNMLLDCKVQGLALSFLSGDLSQVQLDESESSALSMD
IYDRESQMVIDYSILGSELK
GVMTERDKQAKLDMLEEEVSSMEKELQTTNPPNLKALEQLAEEDRFHEVVRAFHSSSTATRKCSQEFEQLRAQRFRRFNQ
CFEHVAVAIDQIYKRLCRNA
SAQAILCVENLEDPYLGGINYNCVAPGKRFMPMDNLSGGEKAVAALALLFAIHSYRPAPFFVLDEVDAALDNTNIGKAGG
SVMSCWVLFGPDPMLLSQAF
SLHLCCNQVTSFIREESRENMQIIVISLKEEFYSRADALLGVYPKFNKCMFSSKLTLDLRPYPLNQEDLDEEVR
>g237.t2
MDALGFVLGERVGALRVKHIRDLVHGAHAGHPLSGQAGVSMTYCGLDDQETVFYRKISGDSSEYFVNGVHVSLDKYKKEL
EKIGVVTKARNCLVFQGAVE
SIAMESPKERTKLFERISQSDEYAAEYELKRVALQKAKDDTQFHFHKKRTATVERKQISQEKLKARQCFPILGLGPHFLR
WPGETAGKVLHPCPPPTPQA
QNYQALVDSLGQRRLRLSLFRLYHNQQDIQALASRLVEERQGAAAQRSSVEEGEGAVKALKKEHGGLTREQLAVEKDLRS
EEQRLTQGQSQYIRAKVNTS
HHMKKVEASKAALQKIHRLRAKREAAMEEVRQEMAELDTAWRRCERGMQEDGGPGGLAIDLEEGQVEEYKNLKELARQQT
ALLCQQTEKLSWEVQANLHK
ITLDHSKKKKVEAGIRNQQSQVEELTHRVEKLEEYTTGCRLSLEQYAQQERSLSEELEHGRQRSQVVEKELGQVLEELGN
ATLDHQETRQQQKRKEVLEN
LFRLYPDSVYGRLVDLCSPTHRKYQLAVVKVFGRYLNAIVVSSEKVARSCILYLKEQHAEPETFLPIDYLDVGPLNERLR
GLKGAQMVVDVVQCNPGAIS
TLKKVLQFVCGNAVVCESIRDARSVAFDGPQRIKTVALDGTLFTKSGVISGGSKDLRTKARCWDEKDVRKLKDRKDQLTA
ELRGLVKSRAKESELKGFQV
QAQGVQIRLKYSNKEQENILRGNIPRCRAEISSLESELVNLDAEIQMQQENMEVKQAEMKRIKDRMEQMEDQVFADFCAQ
IGVDNIRQYEQEHLKQQSEL
DRKRCFYPSSIFFTXLEFEGQQGRLAAHLEYEQEQAEQQQRKLSQLEETMAKEELTVAQQAKEEKRLLDMVEHSQNNILD
IKNLLLAKKLQIRDSKAHLD
QDTKTLQEANRQLARQQRALITVETSLEQQRHTKHNMLLDCKVQGLALSFLSGDLSQVQLDESESSALSMDIYDRESQMV

IDYSILGSELKGVMTERDKQ
AKLDMLEEEVSSMEKELQTTNPPNLKALEQLAEEDRFHEVVRAFHSSSTATRKCSQEFEQLRAQRFRRFNQCFEHVAVAI
DQIYKRLCRNASAQAILCVE
NLEDPYLGGINYNCVAPGKRFMPMDNLSGGEKAVAALALLFAIHSYRPAPFFVLDEVDAALDNTNIGKAGGSVMSCWVLF
GPDPMLLSQAFSLHLCCNQV
TSFIREESRENMQIIVISLKEEFYSRADALLGVYPKFNKCMFSSKLTLDLRPYPLNQEDLDEEVR
>g237.t3
MDALGFVLGERVGALRVKHIRDLVHGAHAGHPLSGQAGVSMTYCGLDDQETVFYRKISGDSSEYFVNGVHVSLDKYKKEL
EKIGVVTKARNCLVFQGAVE
SIAMESPKERTKLFERISQSDEYAAEYELKRVALQKAKDDTQFHFHKKRTATVERKQISQEKLKARQCFPILGLGPHFLR
WPGGGLGLCMWGETAGKVLH
PCPPPTPQAQNYQALVDSLGQRRLRLSLFRLYHNQQDIQALASRLVEERQGAAAQRSSVEEGEGAVKALKKEHGGLTREQ
LAVEKDLRSEEQRLTQGQSQ
YIRAKVNTSHHMKKVEASKAALQKIHRLRAKREAAMEEVRQEMAELDTAWRRCERGMQEDGGPGGLAIDLEEGQVEEYKN
LKELARQQTALLCQQTEKLS
WEVQANLHKITLDHSKKKKVEAGIRNQQSQVEELTHRVEKLEEYTTGCRLSLEQYAQQERSLSEELEHGRQRSQVVEKEL
GQVLEELGNATLDHQETRQQ
QKRKEVLENLFRLYPDSVVRSFRVLPFLVPMYGRLVDLCSPTHRKYQLAVVKVFGRYLNAIVVSSEKVARSCILYLKEQH
AEPETFLPIDYLDVGPLNER
LRGLKGAQMVVDVVQCNPGAISTLKKVLQFVCGNAVVCESIRDARSVAFDGPQRIKTVALDGTLFTKSGVISGGSKDLRT
KARCWDEKDVRKLKDRKDQL
TAELRGLVKSRAKESELKGFQVQAQGVQIRLKYSNKEQENILRGNIPRCRAEISSLESELVNLDAEIQMQQENMEVKQAE
MKRIKDRMEQMEDQVFADFC
AQIGVDNIRQYEQEHLKQQSELDRKRCFYPSSIFFTXLEFEGQQGRLAAHLEYEQEQAEQQQRKLSQLEETMAKEELTVA
QQAKEEKRLLDMVEHSQNNI
LDIKNLLLAKKLQIRDSKAHLDQDTKTLQEANRQLARQQRALITVETSLEQQRHTKHNMLLDCKVQGLALSFLSGDLSQV
QLDESESSALSMDIYDRESQ
MVIDYSILGSELKGVMTERDKQAKLDMLEEEVSSMEKELQTTNPPNLKALEQLAEEDRFHEVVRAFHSSSTATRKCSQEF
EQLRAQRFRRFNQCFEHVAV
AIDQIYKRLCRNASAQAILCVENLEDPYLGGINYNCVAPGKRFMPMDNLSGGEKAVAALALLFAIHSYRPAPFFVLDEVD
AALDNTNIGKAGGSVMSCWV
LFGPDPMLLSQAFSLHLCCNQVTSFIREESRENMQIIVISLKEEFYSRADALLGVYPKFNKCMFSSKLTLDLRPYPLNQE
DLDEEVR
>g238.t1
MLVALTALLLSGPGNPSQPTSLHYMSPYQLNAYAMALRAVGEIIQDYDSDKMFPALGFGAKLPPDGRVSHEFALNGNPQN
PYCSGIEGVMEAYYQSLKSV
RLYGPTHFSPVINHVARYASAVTDGSQYFILLIISDGVITDMAQTKESIVNVGPLQSYLHCLLCDLSLEQAHPQPGARLQ
LHQMTPRGGIQTVQSDRRVI
MSRPSGGGTLWPLISSAIIRDAPVMSIIKERRKTCSRVSPPAPTLPAALINNIARLQGPQGTVPLMGLIVVPPYTGPGGR
FCRLVDPGNKSAGLIEVGRE
SAGNTVQQCGWRMRAASLPMSIIIVGVGPAEFDEMIELDGDEERISSQGRLAERDIVQFVPFRDYVDRRGNHILSMARLA
KEVLAEIPDQFLSYMRTRGI
KPGTSPPPYTPAVHPLLTRRVSRI
>g238.t2
MLVALTALLLSGPGNPSQPTSLHYMSPYQLNAYAMALRAVGEIIQDYDSDKMFPALGFGAKLPPDGRVSHEFALNGNPQN
PYCSGIEGVMEAYYQSLKSV
RLYGPTHFSPVINHVARYASAVTDGSQYFILLIISDGVITDMAQTKESIVNVGPLQSYLHCLLCDLSLEQAHPQPGARLQ
LHQMTPRGGIQTVQSDRRVI
MSRPSGGGTLWPLISSAIIRDAPVMSIIKERRKTCSRVSPPAPTLPAALINNIARLQGPQGTVPLMGLIVVPPYTGPGGR
FCRLVDPGNKSAGLIEVGRE
SAGNTVQQCGWRMRAASLPMSIIIVGVGPAEFDEMIELDGDEERISSQGRLAERDIVQFVPFRDYVDRRGNHILSMARLA
KEVLAEIPDQFLSYMRTRGI
KPGTSPPPYTPAVHPLLTRRAPGSPWVPHPTWGSKDHPQAPPPASPTERVLGVDSFITRGLV
>g238.t3
MLVALTALLLSGPGNPSQPTSLHYMSPYQLNAYAMALRAVGEIIQDYDSDKMFPALGFGAKLPPDGRVSHEFALNGNPQN
PYCSGIEGVMEAYYQSLKSV

RLYGPTHFSPVINHVARYASAVTDGSQYFILLIISDGVITDMAQTKESIVNVGPLQSYLHCLLCDLSLEQAHPQPGARLQ
LHQMTPRGGIQTVQSDRRVI
MSRPSGGGTLWPLISSAIIRDAPVMSIIKERRKTCSRVSPPAPTLPAALINNIARLQGPQGTVPLMGLIVVPPYTGPGGR
FCRLVDPGNKSAGLIEVGRE
SAGNTVQQCGWRMRAASLPMSIIIVGVGPAEFDEMIELDGDEERISSQGRLAERDIVQFVPFRDYVDRRGNHILSMARLA
KEVLAEIPDQFLSYMRTRGI
KPGTSPPPYTPAVHPLLTRRAPGSPWVPHPTWGSKDHPQFKCDMMMLSKAQRVSLQLR
>g239.t1
MGKTADLTAVQKAIIDTLKQE
>g240.t1
MFYLALLLHLSSCDGYTNLTDPWRNRAFRRIDYPGFPTDDKQLFNKWWRFTGIGGDRANPLVPGQWILASLPIILCAVQT
PAVPLLDVTLKEAACVSLGM
KSLMSTFLLMVPMAVL
>g240.t2
MFYLALLLHLSSCDGYTNLTDPWRNRAFRRIDYPGFPTDDKQLFNKWWRFTGIGGDRASEKCGYNMGAFQYPXPLGAGSM
DTGFATYVTLKEAACVSLGM
KSLMSTFLLMVPMAVL
>g240.t3
MFYLALLLHLSSCDGYTNLTDPWRNRAFRRIDYPGFPTDDKQLFNKWWRFTGIGGDRASEKCGYNMGAFQYPWSVEIILC
AVQTPAVPLLDVTLKEAACV
SLGMKSLMSTFLLMVPMAVL
>g241.t1
MTTRAKLREQQERADSPASSCVSVKMEHSMEEPEVFKDGNQSLEKGRVQQERADYPGPSYVSMTSTSSMSKPFNFKDETL
EKRQHQERLKMQTEVIKRAE
ENAHTFLDKELKKLWKVIFLHDPECSESQREEDEVDGKKEEQRRRAIEGVVDITKLCLMELNHEELADTLCHGRVAFECQ
HKIKSHLKKKFRYGFEGIAK
AGQPVPLNDFYTELFITERGSGEVNKEHEVRLIETASRKPAKEETPIKCEDIFKTLPGQEIRTIMTTGVAGIGKTVLTHK
FTLDWAEGKTNYDIHFTFPL
TFRELNLLKGKEYSLVELLNYFFIETKGLCKYDLFQVVLILDGLDECRLPLDFQNNQICTDVTESTSVDVLLTNLIRGNL
LPSARIWITTRPAAADQIPA
ECVDMVTEVRGFTDSQKEEYFRKRFREENLANIIISHIKTSRSLRIMCHIPVFCWIIATVLEDFFKTSQIGKNMPKTMTQ
MYIHFLMVQSIQGDIKYQTH
WSSESREIIVSLGKLAFNQLEKGNLIFYEADLEECGIDIRAASLYSGVFTQIFKEVCALYQDKVFCFVHLSIQEFLAALY
VFLNFTDTGDNLLSEEKQSI
WSILLRNKSKVKLFQSAVDKALQSENGHLDLFLRFLLGLSLETNQILLQGLLGQRGNTSSTKEEIVSYIKDKISGDLSPE
RSINLFHCLNELNDRSLVEE
IQQHLTSGSLSKESLSPAQWSAIVFILLSTEKELDEFDLKKYSASEEGLLRLLPVVKSSKKSLSYYSSLRELDLSTNDLQ
DSGMKLLSAGLGSLNCTLET
LRLNGCNLSEGCCEALASALNSNSHLRELDLSTNDLQDSGVKLLSAGLGSAHCTLETLRLNGCNLSERCCEALASALSSN
SHLRELDLRTNDLQDSGVKL
LSAGLGSPQCALKTLRLNGCNLSERCCEALASALSSNSHLRELDLSTNDLQDSGVKLLSAGLGSPHCTLETIRKVDLSYN
HPGDSGFMHQSTGLDTFSVE
HGGVGRLKPALKKXVHLDRPAGTLSFYRVSPGVGGSSDTLTHIHTFQTTFTQEDLHPAFGQQILILNCLKGDISWQGSS
>g241.t2
MTTRAKLREQQERADSPASSCVSVKMEHSMEEPEVFKDGNQSLEKGRVQQERADYPGPSYVSMTSTSSMSKPFNFKDETL
EKRQHQERLKMQTEVIKRAE
ENAHTFLDKELKKLWKVIFLHDPECSESQREEDEVDGKKEEQRRRAIEGVVDITKLCLMELNHEELADTLCHGRVAFECQ
HKIKSHLKKKFRYGFEGIAK
AGQPVPLNDFYTELFITERGSGEVNKEHEVRLIETASRKPAKEETPIKCEDIFKTLPGQEIRTIMTTGVAGIGKTVLTHK
FTLDWAEGKTNYDIHFTFPL
TFRELNLLKGKEYSLVELLNYFFIETKGLCKYDLFQVVLILDGLDECRLPLDFQNNQICTDVTESTSVDVLLTNLIRGNL
LPSARIWITTRPAAADQIPA
ECVDMVTEVRGFTDSQKEEYFRKRFREENLANIIISHIKTSRSLRIMCHIPVFCWIIATVLEDFFKTSQIGKNMPKTMTQ
MYIHFLMVQSIQGDIKYQTH
WSSESREIIVSLGKLAFNQLEKGNLIFYEADLEECGIDIRAASLYSGVFTQIFKEVCALYQDKVFCFVHLSIQEFLAALY
VFLNFTDTGDNLLSEEKQSI

WSILLRNKSKVKLFQSAVDKALQSENGHLDLFLRFLLGLSLETNQILLQGLLGQRGNTSSTKEEIVSYIKDKISGDLSPE
RSINLFHCLNELNDRSLVEE
IQQHLTSGSLSKESLSPAQWSAIVFILLSTEKELDEFDLKKYSASEEGLLRLLPVVKSSKKSLSYYSSLRELDLSTNDLQ
DSGMKLLSAGLGSLNCTLET
LRLNGCNLSEGCCEALASALNSNSHLRELDLSTNDLQDSGVKLLSAGLGSAHCTLETLRLNGCNLSERCCEALASALSSN
SHLRELDLRTNDLQDSGVKL
LSAGLGSPQCALKTLSSNSHLRELDLSTNDLQDSGVKLLSAGLGSPHCTLETIRKVDLSYNHPGDSGFMHQSTGLDTFSV
EHGGVGRLKPALKKXVHLDR
PAGTLSFYRVSPGVGGSSDTLTHIHTFQTTFTQEDLHPAFGQQILILNCLKGDISWQGSS
>g241.t3
MTTRAKLREQQERADSPASSCVSVKMEHSMEEPEVFKDGNQSLEKGRVQQERADYPGPSYVSMTSTSSMSKPFNFKDETL
EKRQHQERLKMQTEVIKRAE
ENAHTFLDKELKKLWKVIFLHDPECSESQREEDEVDGKKEEQRRRAIEGVVDITKLCLMELNHEELADTLCHGRVAFECQ
HKIKSHLKKKFRYGFEGIAK
AGQPVPLNDFYTELFITERGSGEVNKEHEVRLIETASRKPAKEETPIKCEDIFKTLPGQEIRTIMTTGVAGIGKTVLTHK
FTLDWAEGKTNYDIHFTFPL
TFRELNLLKGKEYSLVELLNYFFIETKGLCKYDLFQVVLILDGLDECRLPLDFQNNQICTDVTESTSVDVLLTNLIRGNL
LPSARIWITTRPAAADQIPA
ECVDMVTEVRGFTDSQKEEYFRKRFREENLANIIISHIKTSRSLRIMCHIPVFCWIIATVLEDFFKTSQIGKNMPKTMTQ
MYIHFLMVQSIQGDIKYQTH
WSSESREIIVSLGKLAFNQLEKGNLIFYEADLEECGIDIRAASLYSGVFTQIFKEVCALYQDKVFCFVHLSIQEFLAALY
VFLNFTDTGDNLLSEEKQSI
WSILLRNKSKVKLFQSAVDKALQSENGHLDLFLRFLLGLSLETNQILLQGLLGQRGNTSSTKEEIVSYIKDKISGDLSPE
RSINLFHCLNELNDRSLVEE
IQQHLTSGSLSKESLSPAQWSAIVFILLSTEKELDEFDLKKYSASEEGLLRLLPVVKSSKKSLSYYSSLRELDLSTNDLQ
DSGMKLLSAGLGSLNCTLET
LRLNGCNLSEGCCEALASALNSNSHLRELDLSTNDLQDSGVKLLSAGLGSAHCTLETLSSNSHLRELDLRTNDLQDSGVK
LLSAGLGSPQCALKTLRLNG
CNLSERCCEALASALSSNSHLRELDLSTNDLQDSGVKLLSAGLGSPHCTLETIRKVDLSYNHPGDSGFMHQSTGLDTFSV
EHGGVGRLKPALKKXVHLDR
PAGTLSFYRVSPGVGGSSDTLTHIHTFQTTFTQEDLHPAFGQQILILNCLKGDISWQGSS
>g242.t1
MGGLWNCQSDSISAYACLQTLHFLALAETRITPDNKATPAALSTAYSSHTPGGVEEGEWTPHLTQVVLPVIYRPPGPFHD
FFDEMDALLSCFPEDGTPLV
VLGDFNIHPKQGYGQMISATTFTSTASTAKSTFYNKINCFPCSSLASNHPPPPPQLTADDFPDYFTKKVTDISISFTPTS
TAILPPPTSTSARLNRFTPL
SSEEVLRLVTSNHATTCPLDPIPSSLLQTISTDILPFITTLINSSLTSHLIPAPFKTASQTTPQETYPQHQWQLLKKI
>g242.t2
MGGLWNCQSDSISAYACLQTLHFLALAETRITPDNKATPAALSTAYSSHTPGGVEEGEWTPHLTQVVLPEQGYGQMISAT
TFTSTASTAKSTFYNKINCF
PCSSLASNHPPPPPQLTADDFPDYFTKKVTDISISFTPTSTAILPPPTSTSARLNRFTPLSSEEVLRLVTSNHATTCPLD
PIPSSLLQTISTDILPFITT
LINSSLTSHLIPAPFKTASQTTPQETYPQHQWQLLKKI
>g243.t1
MAQDKAPGLTLPYQYKLLAEMFRSADTIVGMLYNRSETVTFAKIQQGVRDMMHKHFEQSHLDQNEARPLLTASRLLERRR
IFYQNLVCFVKEHHKIRAKE
AQKLEAAMTRDPANEQRLLMMSRLTELSRILRTVFVAERKPTLIMEMVCNRMVASYRSALTIGEMEKHLQLLAELVPDWL
TIHPVRKDLYLKLCKNVDLH
IVQDKLSLRLQEEQRR
>g243.t2
MAQDKAPGLTLPYQYKLLAEMFRSADTIVGMLYNRSETVTFAKIQQGVRDMMHKHFEQSHLGQIKTVFPSAYTFRQERNI
PTFNSNIKKGSYQLTLEPVM
NSDQNEARPLLTASRLLERRRIFYQNLVCFVKEHHKIRAKEAQKLEAAMTRDPANEQRLLMMSRLTELSRILRTVFVAER
KPTLIMEMVCNRMVASYRSA
LTIGEMEKHLQLLAELVPDWLTIHPVRKDLYLKLCKNVDLHIVQDKLSLRLQEEQRR
>g243.t3

MAQDKAPGLTLPYQYKLLAEMFRSADTIVGMLYNRSETVTFAKIQQGVRDMMHKHFEQSHLDQNEARPLLTASRLLERRR
IFYQNLVCFVKEHHKIRAKE
AQKLEAAMTRDPANEQRLLMMSRLTELSRILRTVFVAERKPTLIMEMVCNRMVASYR
>g244.t1
VPFTRRLHPRRVCESPPSSDVGVQVRAPRATGLISRINTRRRLKQRLHGEEPYVSLVEPGPGSESSGPPLPHHHRIQRR
>g244.t2
VRAPRATGLISRINTRRRLKQRLHGEEPYVSLVEPGPGSESSGPPLPHHHRIQRR
>g244.t3
SPPSSDVGVQVRAPRATGLISRINTRRRLKQRLHGEEPYVSLVEPGPGSESSGPPLPHHHRIQRR
>g245.t1
MLTPSDNASGTVQDIGVLPPGSLLLDPEWPIHKECEKGGTIG
>g246.t1
MDFSGQESRVIEDPWEALCAAMEEGMSWRESAPPVSSVPPALHQTQPWFHSKLSRDEAQRVLSQHGLVDGMFLLRDSQSI
PNTFVLSLCHLQRVRHVQIR
PVDTERDAFYSLDSGQTCFTSLVQLVDFHQLNRGVLPCRLRQHCARLTL
>g246.t2
MDFSGQESRVIEDPWEALCAAMEEGMSWRRKSCHRLSPHGNLSASQGSMLSMALHQTQPWFHSKLSRDEAQRVLSQHGLV
DGMFLLRDSQSIPNTFVLSL
CHLQRVRHVQIRPVDTERDAFYSLDSGQTCFTSLVQLVDFHQLNRGVLPCRLRQHCARLTL
>g246.t3
MDFSGQESRVIEDPWEALCAAMEEGMSWRESAPPVSSVPPALHQTQPWFHSKLSRDEAQRVLSQHGLVDGMFLLRDSQSI
PNTFVDTERDAFYSLDSGQT
CFTSLVQLVDFHQLNRGVLPCRLRQHCARLTL
>g247.t1
DDKRSEWIYRGSTRLEPMFNLKLTTANTQEKKLAGQQRTRPNMGALRSKGPVVQYTSEGGSPSKAAPLMAATISPLSQFL
TPSQSARFDNKQQMARKSTS
PFIPGVGGTHASKMLQSLTSSASQAPSHSHCHSSTSTSTPTHTPTHTHTHSPS
>g247.t2
DDKRSEWIYRGSTRLEPMFNLKLTTANTQEKKLAGQQRTRPNMGALRSKGPVVQYTSEGGSPSKAAPLMAATISPLSQFL
TPSQSARFDNKQQMARKSTS
PFIPGVGGTHASKMLQSLTSSASQAPR
>g247.t3
DDKRSEWIYRGSTRLEPMFNLKLTTANTQEKKLAGQQRTRPNMGALRSKGPVVQYTSEGGSPSKAAPLMAATISPLSQFL
TPSQSARFDNKQQMARKSTS
PFIPGVGGTHASKMLQSLTSSASQAPSGELQALVPRPCSITRSFLHFIHTCTLTSARSSA
>g248.t1
MNPRYILFIHLVLNDMVQLTVSVLLFVISYVFFTINMCIC
>g249.t1
KGDGAGKQGKRKGKKCMEDAVQCAPSPVAAVPHIPTARSIHAEQRESVRSSLLL
>g250.t1
CDYINVPTTVFTPLEYGSCGLSEEKAIEMYGEENLEDRVIGFHYLGPNAGEVTQGYGVAIKCGVTKELLDNTIGIHPTCA
EVFTTLDVTKSSGGNITQAG
C
>g250.t2
CDYINVPTTVFTPLEYGSCGLSEEKAIEMYGEENLEIYHSLFWPLEFTVANRDNNKCYSKIICNKLDNDRVIGFHYLGPN
AGEVTQGYGVAIKCGVTKEL
LDNTIGIHPTCAEVFTTLDVTKSSGGNITQAGC
>g251.t1
MIDICHPVVKETGRVGFCNGRAKRHMSALGNGATPCQFDVETRHAFVGDQSGQVTILKVEQDSCSMVTTFKGHTVGGVEV
FRGAFSMLYHLS
>g251.t2
MIDICHPVVKETGRVGFCNGRAKRHMSALGNGATPCQFDVETRHAFVGDQSGQVTILKVEQDSCSMVTTFKGHT
>g252.t1
LTPSYGWQPKLSGGDLHYFGQLTTLNECVYRSMVRSRYVLLHDIDEIIMPYQHLDLVAFLQELQQQQPKAGVFLIENHIF
PKIHFEPSGRFHLPQWEGVP
GVNILEHVYREEPDRTGVTWHPHKIIAQPRSVEQTSVHSVLKSFGEVYNVPPDVCRIIHVRVALQGHLTLDQLHVDKRLW

DFSDRLIPMVDQALRRAGML
RSDVAGPS
>g253.t1
MTPIKALTMKDYENQIAALKKENFNLKLRIYFMEERMQQKCDDSTEDIFKTNIELKVELESMKREMVEKQELLVSASKAL
ESLASRDTGDSQRVREQARK
DMDALQHALDKKMADLEQDLLAAEKEVEKMAAIAEKAKLKNIDMEKQLVALGSGPKAFTPVPEPNNDLEQALQEKDGIIE
QLRISLREKEGLMKQKTNSD
HGADTPAHDHITQLTILISEKDHQLQELREQLGGERDKAQKDYKVSPAWRHSQ
>g253.t2
MDTMFGIDQTLPVDINGSCRLPDCIDDGDCSPDSMTALSFPDKMTPIKALTMKDYENQIAALKKENFNLKLRIYFMEERM
QQKCDDSTEDIFKTNIELKV
ELESMKREMVEKQELLVSASKALESLASRDTGDSQRVREQARKDMDALQHALDKKMADLEQDLLAAEKEVEKMAAIAEKA
KLKNIDMEKQLVALGSGPKA
FTPVPEPNNDLEQALQEKDGIIEQLRISLREKEGLMKQKTNSDHGADTPAHDHITQLTILISEKDHQLQELREQLGGERD
KAQKDYKVSPAWRHSQ
>g253.t3
MTALSFPDKMTPIKALTMKDYENQIAALKKENFNLKLRIYFMEERMQQKCDDSTEDIFKTNIELKVELESMKREMVEKQE
LLVSASKALESLASRDTGDS
QRVREQARKDMDALQHALDKKMADLEQDLLAAEKEVEKMAAIAEKAKLKNIDMEKQLVALGSGPKAFTPVPEPNNDLEQA
LQEKDGIIEQLRISLREKEG
LMKQKTNSDHGADTPAHDHITQLTILISEKDHQLQELREQLGGERDKAQKDYKVSPAWRHSQ
>g254.t1
MKDPCRVCGVRLVGSQCRWIFSATGQRKLQVILSHVLGRGVTRGDGCGEFLCGKCVFQLEKLVHCDIAINQLRDEHSAQV
QKLQAEKEHVIQCLNHVYSK
NNLSQAKGDGDSSRSKTTARSSGCSSPNEDDDEVMGQLASESYPQVKNQMRRCVSLDRLGGKAATPTRSGLRRLSPKLGS
GLTLEYSSGGLQAARHRSQS
MYLDLVSRKGSLPSPGFKGRSTSLQSLNEDFASNTFSDLEPKRHLREPKVFIARRSITDGSKGKAQARSLPCSSMVQSYL
ISDIIQLLRRISRRQILGPP
DSRIPVLQRPHTGHIPPNLLRRKRRRSEAQWKFIHDLTEEFNDEYTPITAEGFAKREHDVSQLEATNKLLNDELTQMKSS
NEHLTKELVEAQNQNKILEL
CHEIEDRDEALAKARETAHKAQLQKYQGAEEHQSLLMEKQAELAELQGEHYTKVLEAQKLQRSLARREQELADMQQAKGQ
LEVELEDFQQQKKKGDKALN
DLQNQLKKLSGEIVDRESALEQKYQGLLDQTTRKLQAHEVTIQHLTSSLADKEQQLQEYMNMVRDFEQSRSPGGSDVMLS
KLRERLKEKENALESFDSVI
KEKDVALQHLANTLKNLQRAKQDMEDNLNRALREKDSIISQLQLLLQGKTKDMEEMSQAMLSQAQSQARDLAEQMGQRLK
VTEAMLAEATKARESLVTDN
ESAVEGLLATISSKDQLLKESSEHYNRMLSESTREIQELKRQLVDGQQQLNTPEKPVATQDGYLETQELRVLLVEKDALI
KKLLEHGQDRDGFRAEPRIQ
ESDHVLELKHTVQILQGRLDETQAELSKINGEDNVENKTVTRNTMVILKKELVEKTAALNKALKRENELKISLVELQSVL
AELEAHVQGHKANVEALTST
LETKDQIISDLHQRLGQRGDRKNGNGTGTEVERSPSSLPQREKTIIGGDSQQRDLPSLSSLHDEHAALIRALKTEQQLFS
SLIRTIKEQDSSQRLHALQL
ELAAVQLLRQQLEEGIKTNEGLRTELENELHQAQLREGAVHPL
>g254.t2
MKDPCRVCGVRLVGSQCRWIFSATGQRKLQVILSHVLGRGVTRGDGCGEFLCGKCVFQLEKLVHCDIAINQLRDEHSAQV
QKLQAEKEHVIQCLNHVYSK
NNLSQAKGDGDSSRSKTTARSSGCSSPNEDDDEVMGQLASESYPQVKNQMRRCVSLDRLGGKAATPTRSGLRRLSPKLGS
GLTLEYSSGGLQAARHRSQS
MYLDLVSRKGSLPSPGFKGRSTSLQSLNEDFASNTFSDLEPKRHLREPKVFIARRSITDGSKGKAQARSLPCSSMVQSYL
ISDIIQLLRRISRRQILGPP
DSRIPVLQRPHTGHIPPNLLRRKRRRSEAQWKFIHDLTEEFNDEYTPITAEGFAKREHDVSQLEATNKLLNDELTQMKSS
NEHLTKELVEAQNQNKILEL
CHEIEDRDEALAKARETAHKAQLQKYQGAEEHQSLLMEKQAELAELQGEHYTKVLEAQKLQRSLARREQELADMQQAKGQ
LEVELEDFQQQKKKGDKALN
DLQNQLKKLSGEIVDRESALEQKYQGLLDQTTRKLQAHEVTIQHLTSSLADKEQQLQEYMNMVRDFEQSRSPGGSDVMLS
KLRERLKEKENALESFDSVI

KEKDVALQHLANTLKNLQRAKQDMEDNLNRALREKDSIISQLQLLLQGKTKDMEEMSQAMLSQAQSQARDLAEQMGQRLK
VTEAMLAEATKARESLVTDN
ESAVEGLLATISSKDQLLKESSEHYNRMLSESTREIQELKRQLVDGQQQLNTPEKPVATQDGYLETQELRVLLVEKDALI
KKLLEHGQDRDGFRAEPRIQ
ESDHVLELKHTVQILQGRLDETQAELSKINGEDNVENKTVTRNTMVILKKELVEKTAALNKALKRENELKISLVELQSVL
AELEAHVQGHKANVEALTST
LETKDQIISDLHQRLGQRGDRKNGNGTGTEVERSPSSLPQREKTIIGGDSQQRDLPSLSSLHDEHAALIRALKTEQQLFS
SLIRTIKEQDSSQRLHALQL
ELAAVQLLRQQLEEGIKTNEGLRTELENELHQAQLREGLT
>g254.t3
MKDPCRVCGVRLVGSQCRWIFSATGQRKLQVILSHVLGRGVTRGDGCGEFLCGKCVFQLEKLVHCDIAINQLRDEHSAQV
QKLQAEKEHVIQCLNHVYSK
NNLSQAKGDGDSSRSKTTARSSGCSSPNEDDDEVMGQLASESYPQVKNQMRRCVSLDRLGGKAATPTRSGLRRLSPKLGS
GLTLEYSSGGLQAARHRSQS
MYLDLVSRKGSLPSPGFKGRSTSLQSLNEDFASNTFSDLEPKRHLREPKVFIARRSITDGSKGKAQARSLPCSSMVQSYL
ISDIIQLLRRISRRQILGPP
DSRIPVLQRPHTGHIPPNLLRRKRRRSEAQWKFIHDLTEEFNDEYTPITAEGFAKREHDVSQLEATNKLLNDELTQMKSS
NEHLTKELVEAQNQNKILEL
CHEIEDRDEALAKARETAHKAQLQKYQGAEEHQSLLMEKQAELAELQGEHYTKVLEAQKLQRSLARREQELADMQQAKGQ
LEVELEDFQQQKKKGDKALN
DLQNQLKKLSGEIVDRESALEQKYQGLLDQTTRKLQAHEVTIQHLTSSLADKEQQLQEYMNMVRDFEQSRSPGGSDVMLS
KLRERLKEKENALESFDSVI
KEKDVALQHLANTLKNLQRAKQDMEDNLNRALREKDSIISQLQLLLQGKTKDMEEMSQAMLSQAQSQARDLAEQMGQRLK
VTEAMLAEATKARESLVTDN
ESAVEGLLATISSKDQLLKESSEHYNRMLSESTREIQELKRQLVDGQQQLNTPEKPVATQDGYLETQELRVLLVEKDALI
KKLLEHGQDRDGFRAEPRIQ
ESDHVLELKHTVQILQGRLDETQAELSKINGEDNVENKTVTRNTMVILKKELVEKTAALNKALKRENELKISLVELQSVL
AELEAHVQGHKANVEALTST
LETKDQIISDLHQRLGQRGDRKNGNGTGTEVERSPSSLPQREKTIIGGDSQQRDLPSLSSLHDEHAALIRALKTEQQLFS
SLIRTIKEQDSSQRLHALQL
ELAAVQLLRQQLEEGIKTNEGLRTELENELHQAQLREDTSSAHHLPLLYPQA
>g255.t1
KEQAATLYVNIRLTEPRLLPQYERVILNFVKEIKQHNSEMENLALAVKR
>g255.t2
KEQAATLYVNIRLTEPRLLPQYERVILNFVKEIKQHNSEMENLALAVK
>g256.t1
LTQQAYYVTAEPLTQQAYYVIAEPLTQQAYYVTAEPLTQQAYYVTAEPLTQQAYYVIAEPLTQQAYYVTAEPLIQLPYHV
MVETLIQQSYTQLSGVC
>g257.t1
MAMRQTPLTCSGHTRPVVDLAFSGITPFGYFLISACKDGKPMLRQGDTGDWIGTFLGHKGAVWGATLNTDATKAATAAAD
FTAKVWDAVSGAEVLTLAHK
HIVKSVNFTKDSNCLLTGGNDKVLRIYDLSKPDEAPQEIPGHTASIKKALWCHDDQHILSASEDRTVRLWDRNTPEAVKM
LSFDTSVSSMEYLADGDVVL
ITYGKTIAFYNAHSLELIKTIDTPANIHSASLHPEKDFIVAGGDDFKLYKYDYSTMEELESYKGHFGPVHCVRFSPDGEL
YASGSEDGTLRLWQTAVGKT
YGLWKCVLPVYKKNRLTMPYIFSKLLYELLWGG
>g257.t2
MRQTPLTCSGHTRPVVDLAFSGITPFGYFLISACKDGKPMLRQGDTGDWIGTFLGHKGAVWGATLNTDATKAATAAADFT
AKVWDAVSGAEVLTLAHKHI
VKSVNFTKDSNCLLTGGNDKVLRIYDLSKPDEAPQEIPGHTASIKKALWCHDDQHILSASEDRTVRLWDRNTPEAVKMLS
FDTSVSSMEYLADGDVVLIT
YGKTIAFYNAHSLELIKTIDTPANIHSASLHPEKDFIVAGGDDFKLYKYDYSTMEELESYKGHFGPVHCVRFSPDGELYA
SGSEDGTLRLWQTAVGKTYG
LWKCVLPVYKKNRLTMPYIFSKLLYELLWGG
>g258.t1
MAAGGGSGEAVDQYRAEVERLTRELAEANREKIRAAECGLVVLEENQTLKQQYAELEAEQEALRQELEQLQELNLAVMLW

LMMNDSGGMERWTRVPRKYY
ISDSV
>g259.t1
MQQRVAFGQAYSTQRKVAEDGESNEATLLQESATKEAYYMGRLLELQAELKHCRSSANSAQADSQHLGGLLQEIREVKGG
GNEEEDDDDDV
>g259.t2
MAFGQAYSTQRKVAEDGESNEATLLQESATKEAYYMGRLLELQAELKHCRSSANSAQADSQHLGGLLQEIREVKGGGNEE
EDDDDDV
>g260.t1
ESGRVLRISAGVRTPGPPPGAVAPALINTPL
>g261.t1
KNLTETQVVNEIYPVWTYSYLALLFPVFLATDYLRYKPVLVVQAASFVATYTALVQAQGVLAMQLLEFFFGLATATEVAY
YSYIYSVVPPARYQQVTGYC
RSVTLLGSAAGSLSGQLLVSMALVPLRYLVMATLASAVLAFMAPWFLPMPGRSLFFHKSGDKAGDKASDDASDEATEERC
CNGRPVAVTEEHCHGSAVER
LEDAESKVLLNVEVVLAAPGADARKHGGGGGSGGSSGLAEVLRVLWEDFLRCYSRRTLLAWSAWWALSTCGYFQVVNYAQ
VLWERVRPPHDLALYNGYVE
TLSTLLGALAAFAVGSVRVSWATWGELALCIFSTVMAACVFTMDLVHDIWVCYGSYVLFRATYMLLITIATFQIAASLSM
QRYALVFGVNTFVALLLQSV
LTLVVVDTAGLGLGIFPQFLIYGCYFAAIAAVFLLAAFCKLASRRRSKSPAVPGDSRGETDSDTGSLPN
>g261.t2
MGPDKNLTETQVVNEIYPVWTYSYLALLFPVFLATDYLRYKPVLVVQAASFVATYTALVQAQGVLAMQLLEFFFGLATAT
EVAYYSYIYSVVPPARYQQV
TGYCRSVTLLGSAAGSLSGQLLVSMALVPLRYLVMATLASAVLAFMAPWFLPMPGRSLFFHKSGDKAGDKASDDASDEAT
EERCCNGRPVAVTEEHCHGS
AVERLEDAESKVLLNVEVVLAAPGADARKHGGGGGSGGSSGLAEVLRVLWEDFLRCYSRRTLLAWSAWWALSTCGYFQVV
NYAQVLWERVRPPHDLALYN
GYVETLSTLLGALAAFAVGSVRVSWATWGELALCIFSTVMAACVFTMDLVHDIWVCYGSYVLFRATYMLLITIATFQIAA
SLSMQRYALVFGVNTFVALL
LQSVLTLVVVDTAGLGLGIFPQFLIYGCYFAAIAAVFLLAAFCKLASRRRSKSPAVPGDSRGETDSDTGSLPN
>g261.t3
VVNEIYPVWTYSYLALLFPVFLATDYLRYKPVLVVQAASFVATYTALVQAQGVLAMQLLEFFFGLATATEVAYYSYIYSV
VPPARYQQVTGYCRSVTLLG
SAAGSLSGQLLVSMALVPLRYLVMATLASAVLAFMAPWFLPMPGRSLFFHKSGDKAGDKASDDASDEATEERCCNGRPVA
VTEEHCHGSAVERLEDAESK
VLLNVEVVLAAPGADARKHGGGGGSGGSSGLAEVLRVLWEDFLRCYSRRTLLAWSAWWALSTCGYFQVVNYAQVLWERVR
PPHDLALYNGYVETLSTLLG
ALAAFAVGSVRVSWATWGELALCIFSTVMAACVFTMDLVHDIWVCYGSYVLFRATYMLLITIATFQIAASLSMQRYALVF
GVNTFVALLLQSVLTLVVVD
TAGLGLGIFPQFLIYGCYFAAIAAVFLLAAFCKLASRRRSKSPAVPGDSRGETDSDTGSLPN
>g262.t1
MLNNHRLVTLSEASILIGNGCRDRAELVKRVESSILLLLPTTSSRTNNASRSPSSYISSNDLERWEQMPKDWIYTVTCSG
LVEIPIVPPTPFPCLMHVCS
PKILYLHSLQLHHLSGGTNGFR
>g263.t1
MKFMTKGVKVNGKILRDLEALFVRLLVVGSKRRMELSTLFTSSARSLHPLPMSMDASERSAARTSMDAVPIYRCCFGKQQ
TAVIHQLWTSPSLGRDKKEE
EEVIMPVLDESPVAPPALFHIISCSCKAGTLVVYL
>g263.t2
MTKGVKVNGKILRDLEALFVRLLVVGSKRRMELSTLFTSSARSLHPLPMSMDASERSAARTSMDAVPIYRCCFGKQQTAV
IHQLWTSPSLGRDKKEEEEV
IMPVLDESPVAPPALFHIISCSCKAGTLVVYL
>g264.t1
PSAKRLRLRGDWSDTGPLARPESERERAGESRTEATLLQRISDMMSRWFEEASESQSPRLRSLPPAPSTATPRDNIPQGQ
VISVEAGGSMEGEPILSLHY
STQGTTASTIQLGWSAGPSQTHPLALGHEVGRVGASEAEEQEEEVRPLALQTRDAEAAPGQSRPMWGSGFNLRGSAIGER

VLRRSAAAHIQEIFRRRKER
RELEESESRRVGRPSACMVFKGHRNSRTVTKEACFWGEGFVLSGSDCGHVFVWDRRAGDLVMLLEADQHVVNCLQPHPYE
PILATSGIDSNIKMWYPMEE
SPAFDGQMAQELICRNELMLEETRNTITVPASFMLRMLASLNRAFRPGDPTTTTTSTTGTVRG
>g264.t2
PSAKRLRLRGDWSDTGPLARPESERERAGESRTEATLLQRISDMMSRWFEEASESQSPRLRSLPPAPSTATPRDNIPQGQ
VISVEAGGSMEGEPILSLHY
STQGTTASTIQLGWSAGPSQTHPLALGHEVGRVGASEAEEQEEEVRPLALQTRDAEAAPGQSRPMWGSGYVRFNLRGSAI
GERVLRRSAAAHIQEIFRRR
KERRELEESESRRVGRPSACMVFKGHRNSRTVTKEACFWGEGFVLSGSDCGHVFVWDRRAGDLVMLLEADQHVVNCLQPH
PYEPILATSGIDSNIKMWYP
MEESPAFDGQMAQELICRNELMLEETRNTITVPASFMLRMLASLNRAFRPGDPTTTTTSTTGTVRG
>g264.t3
PSAKRLRLRGDWSDTGPLARPESERERAGESRTEATLLQRISDMMSRWFEEASESQSPRLRSLPPAPSTATPRDNIPQGQ
VISVEAGGSMEGEPILSLHY
STQGTTASTIQLGWSAGPSQTHPLALGHEVGRVGASEAEEQEEEVRPLALQTRDAEAAPGQSRPMWGSGFNLRGSAIGER
VLRRSAAAHIQEIFRRRKER
RELEESESRRVGRPSACMVFKGHRNSRTVTKEACFWGEGFVLSGSDCGHVFVWDRRAGDLVMLLEADQHVVNCLQPHPYE
PILATSGIDSNIKMWYPMEE
SPAFDGQMAQELICRNELMLEETRNTITVPASFMLRMLASLNRAFRPDQSEERSEGSGPENDED
>g265.t1
MCLALRLLSPTASTRHVSLQPQRPHPGAQGPRGRWAEEEEEEEEEEEEEAVYIGINSLSTDFSSQKGVKGLPLNLQIDTYD
FSTGTNQLIHRAACQIKIFC
DKGAERKMRDEERKRTKRRGKTASDASANKGLVTSSMGIECTFFKSLDDHVTQPVLFIPETHLSTMQRGGL
>g265.t2
MHGACEKRPKVYIGINSLSTDFSSQKGVKGLPLNLQIDTYDFSTGTNQLIHRAACQIKIFCDKGAERKMRDEERKRTKRR
GKTASDASANKGLVTSSMGI
ECTFFKSLDDHVTQPVLFIPETHLSTMQRGGL
>g265.t3
MCLALRLLSPTASTRHVSLQPQRPHPGAQGPRGRWAEEEEEEEEEEEEEAVYIGINSLSTDFSSQKGVKGLPLNLQIDTYD
FSTGTNQLIHRAACQIKIFC
DKGAERKMRDEERKRTKRRGKTASDASANKGLVTSSMGIECTFFKSLDDHVTQPVLFIPETHLSTMQRGGLLQRW
>g266.t1
LKDSKGKEAKEKPCLSLKSQKAQKKKKPPDTTGAPFEL
>g266.t2
RLTAPGVTLIARLKDSKGKEAKEKPCLSLKSQKAQKKKKPPDTTGAPFEL
>g267.t1
LIPGYNITVTATDEGSPPLSSLRNIQVTIGDVNDNPPVFDEPSYSTYVPENVKSGHILCSVSARDPDWRQNGTVIYSLLP
GNSDGAQVSSYLSINGDTGV
IQTLRSFDYEQFRSFKVYVMARDNGSPPLSSNVTVSVFITDVNDNTPQILYPAPEGNSFMTELVPKAAQGGALVSKVIAV
DGDSGLNAWLSYHIIKSTDP
GLFKIDLHSGEVRTQRDISDSDNMKQNILVAVKDNGQPSLSATCAVHLLISDDLADVPELKHAQTYGDDDSRLTSYLIVA
LVSISTLFFTAIIITLGVRF
CRRRKPRLPLDRVAVPGAYLPTNYADVYATGTLCSTYNHDTYLTTGSTTNDFRFARSYNDSTLPTGQSPGKTPEDLLDVF
GEPKGLSAERYARRC
>g268.t1
MLLVSLSTNVEEDEGTPSAFPLHPHQQRQEAGRSGSVVIHVTVLDANDNAPVFSQAVYRASIPENAPLDTVVVVVRASDA
DEGVNGEVTYGLDHISDSSN
VFSLDPKSGEVRVIRSIDYEEESSYEIQVSAKDGLGLASYSTLMIDVTDINDNAPEIYLKSLTNPIPEDVRPGTEVGIIN
VHDEDSDSNRQVRCSIEQNT
PFKLVPSIKNYYSLVTTDKLDRELVTNYTVTILATDEGSPPLTSTKTFQLSVADVNDNPPVFTEQAYEAHVSENNKRGST
VCLVTAHDPDWRQNGTVFFS
LLPGAINGAPVSSFVSITGDTGVIQVVKSFDYELFRGFTFGVNARDNGSPPLSSNNYNAYVSENNKPGHSLGSVKAHDPD
WRQNGTVFFSLLPGAINGAP
VSSFVSITGDTGVIQVVKSFDYELFRGFTFGVNARDNGSPPLSSNVTVSVLITDLNDNPPQILYPASEGNSFMTELVPES
THGGALVSKVIAVDADSGQN

AWLSYHIVKSTDPGLFTIGLHSGEIRTQRDIAETDNMKSNLIVAVKDNGQPCLSVTCTIYLLISDSLSEVQERKELSYDD
NNSQLTLYLIIALVLVSMLL
LTSILIIILAVRFCHKTKPRMLFDGAVAIPGAYLPNYAEVDGTETLRSAYNYDGYLTTGSRTSDFKFVTSYNDVTLPADLT
LSKNEFGDMFGESEGSPETD
MAHGDLSYSIQEELKRGSVIGNIAADLGLGVGRQSSRNVRVDAEGNRKMYCDVNLATGDLIAAERIDRDGLCEDVSPGTE
VGIINVQDRDSESNGRVQCS
IQQDVPFKLVPSIKNYYSMVTTNQLDRETTSDYNITITATDEGYPPLTASKVIQLSIADMNDNAPVFEEHDYKAYVTENN
KPRSTLCSVKAHDPDWRQNG
TVIYSLLPGEVHGAPVSSYLSVNGDTGVIQSDRSFDYEQFRSFKVYVMARDNGSPPLSSNVTVSVFITDVNDNTPQILYP
APEGNSFMTELVPKAAQGGA
LVSKVIAVDGDSGQNAWLSYHIIKSTDRDFQNRPAQRGGQDPARHF
>g268.t2
MLLVSLSTNVEEDEGTPSAFPLHPHQQRQEAGRSGSVVIHVTVLDANDNAPVFSQAVYRASIPENAPLDTVVVVVRASDA
DEGVNGEVTYGLDHISDSSN
VFSLDPKSGEVRVIRSIDYEEESSYEIQVSAKDGLGLASYSTLMIDVTDINDNAPEIYLKSLTNPIPEDVRPGTEVGIIN
VHDEDSDSNRQVRCSIEQNT
PFKLVPSIKNYYSLVTTDKLDRELVTNYTVTILATDEGSPPLTSTKTFQLSVADVNDNPPVFTEQAYEAHVSENNKRGST
VCLVTAHDPDWRQNGTVFFS
LLPGAINGAPVSSFVSITGDTGVIQVVKSFDYELFRGFTFGNYNAYVSENNKPGHSLGSVKAHDPDWRQNGTVFFSLLPG
AINGAPVSSFVSITGDTGVI
QVVKSFDYELFRGFTFGVNARDNGSPPLSSNVTVSVLITDLNDNPPQILYPASEGNSFMTELVPESTHGGALVSKVIAVD
ADSGQNAWLSYHIVKSTDPG
LFTIGLHSGEIRTQRDIAETDNMKSNLIVAVKDNGQPCLSVTCTIYLLISDSLSEVQERKELSYDDNNSQLTLYLIIALV
LVSMLLLTSILIIILAVRFCH
KTKPRMLFDGAVAIPGAYLPNYAEVDGTETLRSAYNYDGYLTTGSRTSDFKFVTSYNDVTLPADLTLSKNEFGDMFGESE
GSPETDMAHGDLSYSIQEEL
KRGSVIGNIAADLGLGVGRQSSRNVRVDAEGNRKMYCDVNLATGDLIAAERIDRDGLCEDVSPGTEVGIINVQDRDSESN
GRVQCSIQQDVPFKLVPSIK
NYYSMVTTNQLDRETTSDYNITITATDEGYPPLTASKVIQLSIADMNDNAPVFEEHDYKAYVTENNKPRSTLCSVKAHDP
DWRQNGTVIYSLLPGEVHGA
PVSSYLSVNGDTGVIQSDRSFDYEQFRSFKVYVMARDNGSPPLSSNVTVSVFITDVNDNTPQILYPAPEGNSFMTELVPK
AAQGGALVSKVIAVDGDSGQ
NAWLSYHIIKSTDRDFQNRPAQRGGQDPARHF
>g268.t3
MLLVSLSTNVEEDEGTPSAFPLHPHQQRQEAGRSGSVVIHVTVLDANDNAPVFSQAVYRASIPENAPLDTVVVVVRASDA
DEGVNGEVTYGLDHISDSSN
VFSLDPKSGEVRVIRSIDYEEESSYEIQVSAKDGLGLASYSTLMIDVTDINDNAPEIYLKSLTNPIPEDVRPGTEVGIIN
VHDEDSDSNRQVRCSIEQNT
PFKLVPSIKNYYSLVTTDKLDRELVTNYTVTILATDEGSPPLTSTKTFQLSVADVNDNPPVFTEQAYEAHVSENNKRGST
VCLVTAHDPDWRQNGTVFFS
LLPGAINGAPVSSFVSITGDTGVIQVVKSFDYELFRGFTFGVNARDNGSPPLSSNVTAHDPDWRQNGTVFFSLLPGAING
APVSSFVSITGDTGVIQVVK
SFDYELFRGFTFGVNARDNGSPPLSSNVTVSVLITDLNDNPPQILYPASEGNSFMTELVPESTHGGALVSKVIAVDADSG
QNAWLSYHIVKSTDPGLFTI
GLHSGEIRTQRDIAETDNMKSNLIVAVKDNGQPCLSVTCTIYLLISDSLSEVQERKELSYDDNNSQLTLYLIIALVLVSM
LLLTSILIIILAVRFCHKTKP
RMLFDGAVAIPGAYLPNYAEVDGTETLRSAYNYDGYLTTGSRTSDFKFVTSYNDVTLPADLTLSKNEFGDMFGESEGSPE
TDMAHGDLSYSIQEELKRGS
VIGNIAADLGLGVGRQSSRNVRVDAEGNRKMYCDVNLATGDLIAAERIDRDGLCEDVSPGTEVGIINVQDRDSESNGRVQ
CSIQQDVPFKLVPSIKNYYS
MVTTNQLDRETTSDYNITITATDEGYPPLTASKVIQLSIADMNDNAPVFEEHDYKAYVTENNKPRSTLCSVKAHDPDWRQ
NGTVIYSLLPGEVHGAPVSS
YLSVNGDTGVIQSDRSFDYEQFRSFKVYVMARDNGSPPLSSNVTVSVFITDVNDNTPQILYPAPEGNSFMTELVPKAAQG
GALVSKVIAVDGDSGQNAWL
SYHIIKSTDRDFQNRPAQRGGQDPARHF
>g269.t1

YTVFVVGVADVDFTELQNIGSKPSERHIFVVDGFDSFDTIKENLITFICETATSSCPLIYLNGFTSPGFRMLEAFNLTEK
TYSSIKGVSMEPGSFNSYTS
YRLHKNAFLTQPSTAAPGMWLTDSSPRKTLRVPVLRKTAGVEVTPELRPSPGAEIHPEGLPPSYTILMMFRLLPDSPSKS
FDIWQVSSKDHKPETGVTID
PTSQTVSFYNKDERGEIQKVIFDSDPIKRIFHGSFHKAKLRPYHCEMPELW
>g269.t2
YTVFVVGVADVDFTELQNIGSKPSERHIFVVDGFDSFDTIKENLITFICETATSSCPLIYLNGFTSPGFRMLEAFNLTEK
TYSSIKGVSMEPGSFNSYTS
YRLHKNAFLTQPSTAAPGMWLTDSSPRKTLRVPVLRKTAGVEVTPELRPSPGAEIHPEGLPPSYTILMMFRLLPDSPSKS
FDIWQVSSKDHKPETGVTID
PTSQTVSFYNKDERGEIQKVIFDSDPIKRIFHGSFHKAKLRPYHCEMP
>g269.t3
YTVFVVGVADVDFTELQNIGSKPSERHIFVVDGFDSFDTIKENLITFICETATSSCPLIYLNGFTSPGFRMLEAFNLTEK
TYSSIKGVSMEPGSFNSYTS
YRLHKNAFLTQPSTAAPGMWLTDSSPRKTLRVPVLRKTAGVEVTPELRPSPGAEIHPEGLPPSYTILMMFRLLPDSPSKS
FDIWQVSSKDHKPETGVTID
PTSQTVSFYNKDERGEIQKVIFDSDPIKRIFHGSFHK
>g270.t1
MESSTIQRQRRELQLLITELKDRDRELTSMAASHHKQLHAWEQDRQRVLSLEERCARSEGELQKRAEVIRALSRRVRALE
AMEMDLQTELGTTQQQLQRL
GRERQDMDQTSQDLQ
>g270.t2
MESSTIQRQRRELQLLITELKDRDRELTSMAASHHKQLHAWEQDRQRVLSLEERCARSEGELQKRAEVIRALSRRVRALE
AMEMDLQTELGTTQQQLQRL
GRERQDMDQTSQDLQVPVC
>g271.t1
MYCEYMPEPAKCRHWLEKNFPDIFARVTVGPQGNIPKPEVVTGEEPPAVEEEEKKKQKRGGRGQIKQKKKTVPQKVTIAK
IPRAKKKYVTRVCGLATFDI
ELKDAQRFFAQKFSCGASVTAEDEIIIQGDFTDDIIDVIQDKWPEVDDDSIDDLGEVKK
>g271.t2
MPEPAKCRHWLEKNFPDIFARVTVGPQGNIPKPEVVTGEEPPAVEEEEKKKQKRGGRGQIKQKKKTVPQKVTIAKIPRAK
KKYVTRVCGLATFDIELKDA
QRFFAQKFSCGASVTAEDEIIIQGDFTDDIIDVIQDKWPEVDDDSIDDLGEVKK
>g271.t3
LVASVATTSVASVGDSLYCEYMPEPAKCRHWLEKNFPDIFARVTVGPQGNIPKPEVVTGEEPPAVEEEEKKKQKRGGRGQ
IKQKKKTVPQKVTIAKIPRA
KKKYVTRVCGLATFDIELKDAQRFFAQKFSCGASVTAEDEIIIQGDFTDDIIDVIQDKWPEVDDDSIDDLGEVKK
>g272.t1
MSQMFNLTPAEVCCAFESPILDQLLPPILVLEFIFGLLGNIVALWMFVFHMDNWKPNSVYLTHLAVADSIVLFCLPFRAD
YYRRGKDWLYGDIPCRILLF
LLAANRAAGIFFLTAVAVDRYLKIVHPLHPINRMGLAYALSVSLALWGLIIILATGYLLSDQHFFYRNNRTQCESFNICMG
YSPLSTWHNAFYVIQFFLPT
SIVAFCTVRILWQLRSKTIDSRGKIKRAVQFVLAVALIFVTCFFPSTISRVAVWILKTWYSECSHFREANLAFYTSVCFT
YFNSVLNPLVYYFSSPSFSG
IFQKVINKLLGRKEEEEQVATVAAVRM
>g273.t1
MTLRCDYTSHIKRHPSPSAKIPSTPAERPVSAVVLPGPPVGPPGPPVGPPDPHPRPARSGLVHDSVRLLDDALEVYLXGI
PAQPPRAPAHQQSALSQALC
SSALLSDPPTLLMDPVVLLSEPLPLPLCPLHFLSDPQAPLLDSLSSTPDLLYLAW
>g273.t2
MTLRCDYTSHIKRHPSPSAKIPSTPAERPVSAVVLPGPPVGPPGPPVGPPDPHPRPARSGLVTARVTFKVEHRPKCDVHD
SVRLLDDALEVYLXGIPAQP
PRAPAHQQSALSQALCSSALLSDPPTLLMDPVVLLSEPLPLPLCPLHFLSDPQAPLLDSLSSTPDLLYLAW
>g274.t1
MKSFKRLVFAHLKDITGLNTPCSFAYRANRDVRKGFVCGLQLATQCVIPEDLHSKLTQLILPEPNCQWITDLLTDQMLHV
RMEKITPGTQTISTRALTLL

SLLLFSLYTNDCTSGDPSVKLLKFADDDIMVIGLIQGSDESAYRREIHQPALWCSRKKPGVEATHNCGTEPSMESFKCPG
STISQDLQWTPNIGTIIGKA
QQKMNFLSLLRGSTFKLPQKLLIQFYTAIIQSLMTETG
>g275.t1
MPAKDTKPGAVQKAEQEKKPEPEKGKAPPSEQAKVEKAPSEPSKAAPSPAAPKAGQSTCPLCKVGLNMGSKDPPNYNTCT
DCKNTVCNQCGFNPQPNSTG
KAEEGKKSEQPSPKAAATQAAPNAGQSTCPLCNVGLNMGSKDTPNYNTCTECKNTVCNQCGFNPMPAKKEVEWLCLTCQM
QRALGESEAPGHLSIKPQAS
LKKASPSPEPQKKMSAAKDTKPPAVQKVEPEKKPEQPQQPKAAPSEQVKVEKAQSEPPKAAATQAAPKASQSTCPLCKVG
LNMGSKDPPNYNTCTDCKNT
VCNQCGFNLMPNVGEATETSSPDSFQVKETPDLAPSQVKETSSPAPSQAKDIPSPSPTQDTKPPPVQKAEQEKKPEQPQQ
SKAPPSEQAKVDKAPSEPPK
AAASQAAPKAGQVDTCPLCKVGLNMGSKEPPNYNTCTDSQNVKQESGGFFGFGGPKPEPASTKPAESVTGKMFGFGSSIF
NSASTLITTPPASPKMSAAK
DTKPPGAQKAEQEKKPEQPQQAKAPASEQVKVDEAPSEPPKAVASPAAPKAGQSTCPLCKVGLNMGSKEPPNYNTCTECK
NTVSKDTKSPPVQKAEQEKK
PEQPQKAKAPPSEQAKVDKAPSEPSKAAASPAAPKAGQSTCPLCKVGLNMGSKDPPNYNTCTDCKNSVCNQCGFSPMPNV
GGKEWLCLDCQVKRAAGAMD
AAGPPVTKPQASSVKASAQKETSMNESQKQSSPSPAQKTPQESQRQSGPQKPPDQTNQTGRKQSTQQESGGFFGFGGSKA
QPDAAKPAESVTGKMFGFGS
SIFNSASTLITTPPVSPKMTAAKDTKPPAVQKAEEGKKSEQPSEPPKAAATQAAPKAGQSTCPLCKVGLNMGSKDPPNYN
TCTECKNTVCNQCGFNTMQN
KSEVKEWLCLTCQMQRALGASEAPGQLSVKPQPSPKKVSPSPEPQKKVISESAESLQKKLSLPAGNPNKAEVVIGPESQK
QSSPSPGQKTPQESRKTSGP
QKPPDQTNQTGRKQSTQQESGGFFGFGGSKAQPDAAKPAESVTGKMFGFGSSIFNSASTLITTPPVSPKMSAAKDTKPPA
VQKAEQEKKPEQPQQAKAPP
SEQAKVDKAPSEPSKAAASPAAPKAGQSTCPLCKVGLNMGSKDPPNYNTCTECKNSVCNQCGFNPMPHSAALTG
>g275.t2
MPAKDTKPGAVQKAEQEKKPEPEKGKAPPSEQAKVEKAPSEPSKAAPSPAAPKAGQSTCPLCKVGLNMGSKDPPNYNTCT
DCKNTVCNQCGFNPQPNSTG
KAEEGKKSEQPSPKAAATQAAPNAGQSTCPLCNVGLNMGSKDTPNYNTCTECKNTVCNQCGFNPMPAKKEVEWLCLTCQM
QRALGESEAPGHLSIKPQAS
LKKASPSPEPQKKMSAAKDTKPPAVQKVEPEKKPEQPQQPKAAPSEQVKVEKAQSEPPKAAATQAAPKASQSTCPLCKVG
LNMGSKDPPNYNTCTDCKNT
VCNQCGFNLMPNVGEATETSSPDSFQVKETPDLAPSQVKETSSPAPSQAKDIPSPSPTQDTKPPPVQKAEQEKKPEQPQQ
SKAPPSEQAKVDKAPSEPPK
AAASQAAPKAGQVDTCPLCKVGLNMGSKEPPNYNTCTDSQNVKQESGGFFGFGGPKPEPASTKPAESVTGKMFGFGSSIF
NSASTLITTPPASPKMSAAK
DTKPPGAQKAEQEKKPEQPQQAKAPASEQVKVDEAPSEPPKAVASPAAPKAGQSTCPLCKVGLNMGSKEPPNYNTCTECK
NTVSKDTKSPPVQKAEQEKK
PEQPQKAKAPPSEQAKVDKAPSEPSKAAASPAAPKAGQSTCPLCKGKEWLCLDCQVKRAAGAMDAAGPPVTKPQASSVKA
SAQKETSMNESQKQSSPSPA
QKTPQESQRQSGPQKPPDQTNQTGRKQSTQQESGGFFGFGGSKAQPDAAKPAESVTGKMFGFGSSIFNSASTLITTPPVS
PKMTAAKDTKPPAVQKAEEG
KKSEQPSEPPKAAATQAAPKAGQSTCPLCKVGLNMGSKDPPNYNTCTECKNTVCNQCGFNTMQNKSEVKEWLCLTCQMQR
ALGASEAPGQLSVKPQPSPK
KVSPSPEPQKKVISESAESLQKKLSLPAGNPNKAEVVIGPESQKQSSPSPGQKTPQESRKTSGPQKPPDQTNQTGRKQST
QQESGGFFGFGGSKAQPDAA
KPAESVTGKMFGFGSSIFNSASTLITTPPVSPKMSAAKDTKPPAVQKAEQEKKPEQPQQAKAPPSEQAKVDKAPSEPSKA
AASPAAPKAGQSTCPLCKVG
LNMGSKDPPNYNTCTECKNSVCNQCGFNPMPHSAALTG
>g275.t3
MSAPAQGSRKMSITTAISPKSTPPVSPKLPPSKDTKPPPVQKAEQEKKPEQPQQSKAPPSEQAKVDKAPSEPPKAAASQA
APKAGQVDTCPLCKVGLNMG
SKEPPNYNTCTDSQNVKQESGGFFGFGGPKPEPASTKPAESVTGKMFGFGSSIFNSASTLITTPPASPKMSAAKDTKPPG
AQKAEQEKKPEQPQQAKAPA

SEQVKVDEAPSEPPKAVASPAAPKAGQSTCPLCKVGLNMGSKEPPNYNTCTECKNTVSKDTKSPPVQKAEQEKKPEQPQK
AKAPPSEQAKVDKAPSEPSK
AAASPAAPKAGQSTCPLCKVGLNMGSKDPPNYNTCTDCKNSVCNQCGFSPMPNVGGKEWLCLDCQVKRAAGAMDAAGPPV
TKPQASSVKASAQKETSMNE
SQKQSSPSPAQKTPQESQRQSGPQKPPDQTNQTGRKQSTQQESGGFFGFGGSKAQPDAAKPAESVTGKMFGFGSSIFNSA
STLITTPPVSPKMTAAKDTK
PPAVQKAEEGKKSEQPSEPPKAAATQAAPKAGQSTCPLCKVGLNMGSKDPPNYNTCTECKNTVCNQCGFNTMQNKSEVKE
WLCLTCQMQRALGASEAPGQ
LSVKPQPSPKKVSPSPEPQKKVISESAESLQKKLSLPAGNPNKAEVVIGPESQKQSSPSPGQKTPQESRKTSGPQKPPDQ
TNQTGRKQSTQQESGGFFGF
GGSKAQPDAAKPAESVTGKMFGFGSSIFNSASTLITTPPVSPKMSAAKDTKPPAVQKAEQEKKPEQPQQAKAPPSEQAKV
DKAPSEPSKAAASPAAPKAG
QSTCPLCKVGLNMGSKDPPNYNTCTECKNSVCNQCGFNPMPHSAALTG
>g276.t1
MSAAKDTKPPAVQKAEQEKKPEQSQQTKAPPSEQAKVDKAPSEPPKAAASQAASKAGQSTCPLCKVGLNMGSKDPPNYNT
CSDCSPKMSPSKEAKPPTVQ
TLEGEKKPEQPQQAKAPPSEQAKVDKAPSEPPKAAATQAAPKAGQSTCPLCKVGLNMGSKDPPNYNTCTDCKNTVCNQCG
FNPQPNVAEKEWLCLNCQMH
RALGASEPPGTLFMKPQPSPSKVQTASNIQNKDVPHEKKKEPSTPKSPLMKQSTPAAQPPMSEVDKDINTEKSVTGKMFG
FGSSIFNSASTLITTPPVSP
KIATKDAKLPAVQKAEEGKKSEQPPEPPKAAATQAAPKAGQSTCPLCKVGLNMGSKDPPNYNTCTDCKNIVCNQCGFNPM
PTKIEVKEWLCLTCQMQRAL
GASEAAGKPSVKPQASPKKVTPSPEPQKKVISESAQDSQKQSSPAPGQKTPQESRRLSSSPKPADQSKQTERKQSTQQES
GGFFGFGGSNAQPDAAKPAE
SVTGKMFGFGSSIFNSASTLITTPPVSPKMSAAKDTKPPAVQKAEQEKKPEKPQQAKAPPSEQAKVDKAPSEPSKDAVSQ
AAPKAGQSTCPLCKVGLNMG
SKDPPNYNTCTDCEGVAVSHMPDAESSGSI
>g276.t2
MSAAKDTKPPAVQKAEQEKKPEQSQQTKAPPSEQAKVDKAPSEPPKAAASQAASKAGQSTCPLCKVGLNMGSKDPPNYNT
CSDCSPKMSPSKEAKPPTVQ
TLEGEKKPEQPQQAKAPPSEQAKVDKAPSEPPKAAATQAAPKAGQSTCPLCKVGLNMGSKDPPNYNTCTDCKNTVCNQCG
FNPQPNVAEVGLNMGSKDPP
NYNTCTDCKNIVCNQCGFNPMPTKIEVKEWLCLTCQMQRALGASEAAGKPSVKPQASPKKVTPSPEPQKKVISESAQDSQ
KQSSPAPGQKTPQESRRLSS
SPKPADQSKQTERKQSTQQESGGFFGFGGSNAQPDAAKPAESVTGKMFGFGSSIFNSASTLITTPPVSPKMSAAKDTKPP
AVQKAEQEKKPEKPQQAKAP
PSEQAKVDKAPSEPSKDAVSQAAPKAGQSTCPLCKVGLNMGSKDPPNYNTCTDCEGVAVSHMPDAESSGSI
>g276.t3
MFGFGSSIFNSASTLITTPPVSPKMSAAKDTKPPAVQKAEQEKKPEQSQQTKAPPSEQAKVDKAPSEPPKAAASQAASKA
GQSTCPLCKVGLNMGSKDPP
NYNTCSDCSPKMSPSKEAKPPTVQTLEGEKKPEQPQQAKAPPSEQAKVDKAPSEPPKAAATQAAPKAGQSTCPLCKVGLN
MGSKDPPNYNTCTDCKNTVC
NQCGFNPQPNVAEKEWLCLNCQMHRALGASEPPGTLFMKPQPSPSKVQTASNIQNKDVPHEKKKEPSTPKSPLMKQSTPA
AQPPMSEVDKDINTEKSVTG
KMFGFGSSIFNSASTLITTPPVSPKIATKDAKLPAVQKAEEGKKSEQPPEPPKAAATQAAPKAGQSTCPLCKVGLNMGSK
DPPNYNTCTDCKNIVCNQCG
FNPMPTKIEVKEWLCLTCQMQRALGASEAAGKPSVKPQASPKKVTPSPEPQKKVISESAQDSQKQSSPAPGQKTPQESRR
LSSSPKPADQSKQTERKQST
QQESGGFFGFGGSNAQPDAAKPAESVTGKMFGFGSSIFNSASTLITTPPVSPKMSAAKDTKPPAVQKAEQEKKPEKPQQA
KAPPSEQAKVDKAPSEPSKD
AVSQAAPKAGQSTCPLCKVGLNMGSKDPPNYNTCTDCEGVAVSHMPDAESSGSI
>g277.t1
MSAGMSPMSAGHAPGMRMPGMPHNPVGYPRSMTNVPQYHQRPGMQPTRGGAPLGSMGSMGGQMPGPSYGGGNMAMRPSMG
PATMDPSRKRFLHQQQQQQQ
QPQGPMGGALRRG
>g278.t1

VGGTKAGVVRFLGETDFAKGDWCGVELDEPLGKNDGAVAGARAVTAGPNTVPPPMITSGREAGVSVMGQRTGVVNASDLH
EFSRASPGLGGHRSGKGLLC
LFALRPTSESHLSFSRLVARYFQCMPRYGLFAPVHKVTRIGFPCTTPTKAKSGRRRSTLKHSPSASSISSLSSVTTSSVG
GKPSRAGLLTETSARYARKI
SGTTALQEALKEKQQHIEQLLAERDMERCEVAKATSHAGEVQLELTSLRKGQDQ
>g278.t2
MQVGGTKAGVVRFLGETDFAKGDWCGVELDEPLGKNDGAVAGARAVTAGPNTVPPPMITSGREAGVSVMGQRTGVVNASD
LHEFSRASPGLGGHRSGKGL
LCLFALRPTSESHLSFSRLVARYFQCMPRYGLFAPVHKVTRIGFPCTTPTKAKSGRRRSTLKHSPSASSISSLSSVTTSS
VGGKPSRAGLLTETSARYAR
KISGTTALQEALKEKQQHIEQLLAERDMERCEVAKATSHAGEVQLELTSLRKGQDQ
>g278.t3
VGGTKAGVVRFLGETDFAKGDWCGVELDEPLGKNDGAVAGARTGVVNASDLHEFSRASPGLGGHRSGKGLLCLFALRPTS
ESHLSFSRLVARYFQCMPRY
GLFAPVHKVTRIGFPCTTPTKAKSGRRRSTLKHSPSASSISSLSSVTTSSVGGKPSRAGLLTETSARYARKISGTTALQE
ALKEKQQHIEQLLAERDMER
CEVAKATSHAGEVQLELTSLRKGQDQ
>g279.t1
WWNELPANVRTAESLPIFWKRLKTHLFRVHLNPA
>g280.t1
MVTGPSKTQSSTENFWKITCILRFREDKANYSLNTDDPLIFSSTLNLDYYTTHKYMGFTEEEFKRLNISSAKASFLPESE
KKDLLTKLHEAYGMLQSTAF
>g280.t2
MNGRVKVKNVYFLKKVVSSRVQVELHVHLDGAIRVQTILDVAKRRGISLPANTVEEMTQIIIVQKPATLTEFLGKFAEYM
HIIAGDREAIKRIAYEFVED
KAKEGVVYVEVRYSPHFLANTEVEPIPWNQEEGDVSPDEVVRLVGLVGFSHNLFPYMKSKDITGSSWSMNVVELCKKYRH
DGVVAIDLAGDESFDSEAYP
GHRKAYQGSQKLMFDWCDF
>g280.t3
MNGRVKVKNVYFLKKVVSSRVQVELHVHLDGAIRVQTILDVAKRRGISLPANTVEEMTQIIIVQKPATLTEFLGKFAEYM
HIIAGDREAIKRIAYEFVED
KAKEGVVYVEVRYSPHFLANTEVEPIPWNQEEALILSKLHQVGLVGFSHNLFPYMKSKDITGSSWSMNVVELCKKYRHDG
VVAIDLAGDESFDSEAYPGH
RKAYQGSQKLMFDWCDF
>g280.t4
MNGRVKVKNVYFLKKVVSSRVQVELHVHLDGAIRVQTILDVAKRRGISLPANTVEEMTQIIIVQKPATLTEFLGKFAEYM
HIIAGDREAIKRIAYEFVED
KAKEGVVYVEVRYSPHFLANTEVEPIPWNQEEGDVSPDEVVRLVGLVGFSHNLFPYMKSKDITGSSWSMNVVELCKKYRH
DGVVAIDLAGDESFDSEAYP
GHRKAYQNGFVKVCCFCVISGVPNFKFREDKANYSLNTDDPLIFSSTLNLDYYTTHKYMGFTEEEFKRLNISSAKASFLP
ESEKKDLLTKLHEAYGMLQS
TAF
>g281.t1
MDLKRGIKQAKHCYKLKVEEHFSNSNPRRMWQGIRVISDYKPANTSPLSTDTSFTNELNNFYARFEQDNHEPAIKMELTA
DHQPLSLSPIDVEAALSRIN
AHKAAGPDGIPGRALRACAGELAGVLTDLFNLSLARAVVPTCFKSTTIVPVPKHSTPTCPKDFR
>g282.t1
MDVVTFVVCLVLYFELFGVAEGNSFNHGSPFNHGSIVYTRDQLLALRPTPPLCVEWPVISEELQRRRRGCRAGVKRRERK
RRYKPCLPSVILGNVRSLPN
KMEELTALTRLQKEYRECSLKCFTETWLSELSPDSHASLDGFQFVRVDRKAAESGWGITVFVNNRWCHPGHISVKEQLCT
RDLELLAYVKYHTRDSKTLD
LLYANIKDAYTSSPLPLLGRSDHNLVHLVTDYTPVVNKQRPVKRAVKQWSEETSARLRDCFESTDWEALCSPHGSDLDSM
THCITDYVNFCVENTVPSKL
VWCFPNNKPWVTSESNLIPRKKRVFKSGDKEELRRVQKELRLRIRRGKDSYRRKLEKRLGCLERAAEHLRP
>g282.t2
LRPTPPLCVEWPVISEELQRRRRGCRAGVKRRERKRRYKPCLPSVILGNVRSLPNKMEELTALTRLQKEYRECSLKCFTE

TWLSELSPDSHASLDGFQFV
RVDRKAAESGWGITVFVNNRWCHPGHISVKEQLCTRDLELLAYVKYHTRDSKTLDLLYANIKDAYTSSPLPLLGRSDHNL
VHLVTDYTPVVNKQRPVKRA
VKQWSEETSARLRDCFESTDWEALCSPHGSDLDSMTHCITDYVNFCVENTVPSKLVWCFPNNKPWVTSESNLIPRKKRVF
KSGDKEELRRVQKELRLRIR
RGKDSYRRKLEKRLGCLERAAEHLRP
>g283.t1
MAASEEERVSFTPGKKQSRFETLMTKEEMEEEQR
>g284.t1
MKSWFSLNFLKLNSNKTEVLLIGTKSTLATLNTFSMSINNATVAPFPQ
>g285.t1
MDIYDPQTLGIIVFGGFMVISAIGIVLVSTFSMKETSYEEALAKQRRELGKIQPSRSDKKKKDKASEKKTKTKKKDDKPN
GKIPEPEEEPDTPETIPDPE
PAPVPLVAAAPEPKAPAQVPVDVQLKAAAPAEPSPKDKKKKKVAKVEPAPTPAPPASSAAPEPTKPTPTQAKSAPVPAKS
APLQAKAAPAEAKSAPATAA
PAKSAPAKPAAVPSKSAPTQSKPPQLTAKSAKAAPALETSTKEVPVMAVPPVGSQKNGAVAPAKAQESKKKAPKKKSEPG
KSVLPIRL
>g286.t1
MTSYELTTKVLVTTHGREWQKGIRHSEGGVVQFKTSIIIPVPKKPAPSCFNDYRPVALTPIVMKCFERLVMSHIKFILPP
NLDPSQFAYRAKHSTEDAIS
SALHPALTHLEKADVRMLFIDFSSAFNTIIPQQLICKLDQLGLNTSLCNWVLDFFSERPQAVRVGNNTSSSITLSTGAPQ
GCVLSPLLFTLLTHNCTPTY
SS
>g287.t1
LDVYLEVSANQVKGAYVVNKQLCCGPLGSETILCKEHKGSVCCGDDQYNPETQDCLGDPPKIKDLTVPCETYSDQELARD
PT
>g287.t2
LDVYLEVSANQVKGAYVVNKQLCCGPLGSETILCKEHKGSVCCGDDQYNPETQDCLGDPPKIKDLTVPCETYSDQELARD
PTDCCLSVVRLVTRVMWCLS
IAAGAEDTVVLLMDKVHF
>g288.t1
MKTPMSVLRLPRGPDPSSRGFDPNSARFIALCTTSISASAEAEADTASLTTEQRARSIMRERFLRCLIAMANKKIQFHMY
ERVKVDATFGASDIDVLNFQ
VSDLQTPIGVQREALIRCQDVISYSFDV
>g289.t1
MESAETTSAGLSMAAQVAIACNAFVGILLLLLFLVLYQACKVPSNPEMLPVLSLPSADSEAQPGPEHKYLLTS
>g290.t1
MVHVFDLSINKYEAICQQPVVAKKKTKLTHIEFNPMHPIIIVGDDRGYVTCFKLSPNLRKKPKEKKGQELPKGPEVEEAK
MEKLLSLLREPDYSSS
>g290.t2
MVHVFDLSINKYEAICQQPVVAKKKTKLTHIEFNPMHPIIIVGDDRGYVTCFKLSPNLRKKPKVGSIVTVGNHKQGSKEK
KGQELPKGPEVEEAKMEKLL
SLLREPDYSSS
>g291.t1
MFRAAVSEISRVHVVMALIARRTTDVHPRQASVSNQSDSRQEEALEQEKRQQMVLLEELEEQKSRLEAMLLDAQRQREDM
QAAVDQEVALREQDVPAHDQ
EVSPDAPTAASELEGVKLQDHSSRIRQHQQRLLEQN
>g291.t2
MFRAAVSEISRVHVVMALIARRTTDVHPRQASVSNQSDSRQEEALEQEKRQQMVLLEELEEQKSRLEAMLLDAQRQREDM
QAAVDQEVALREQDVPAHDQ
EVSPDAPTAASELEGVKLQDHSSRIRQHQQRLLEQNRCVCVIAGTDG
>g292.t1
LAGSSSSSPHAPLAPDNPTLVTHLSQGISWLQDEFLVTGGGFAPLCSSSNSSSNVPSIGSILQPCSTSNAGGDGGRHSLI
SPSLCRYCLSLSSPPLYPQK
NKHAFSRRARPRLPSCDGIPKRHANERALIKGEYSERVHHAASPSHRR
>g292.t2

LSQGISWLQDEFLVTGGGFAPLCSSSNSSSNVPSIGSILQPCSTSNAGGDGGRHSLISPSLCRYCLSLSSPPLYPQKNKH
AFSRRARPRLPSCDGIPKRH
ANERALIKGEYSERVHHAASPSHRR
>g293.t1
WTAYEGLRFSKNMYVLEEGEYPSPEAMGYLSSDTAIRSMQIAGH
>g294.t1
VPPLDLPVLGAEDVLELQLQQLSLAQFAVATMERAVPLLRTGV
>g295.t1
ANILRDSSGNVKLGDFGASKRIQTICMSGTGIKSITGTPYWMSPEVINGEGYGRKADVWSVACTVVEMLTQKPPWAEYEA
MAAIFKIATQPTRPLLPDSV
SDASRDFLRQVFVEEKWRPTAEVLLGHPFVQGVF
>g295.t2
LGDFGASKRIQTICMSGTGIKSITGTPYWMSPEVINGEGYGRKADVWSVACTVVEMLTQKPPWAEYEAMAAIFKIATQPT
RPLLPDSVSDASRDFLRQVF
VEEKWRPTAEVLLGHPFVQGVF
>g295.t3
MTEQIGPERHDRVEPSKGANILRDSSGNVKLGDFGASKRIQTICMSGTGIKSITGTPYWMSPEVINGEGYGRKADVWSVA
CTVVEMLTQKPPWAEYEAMA
AIFKIATQPTRPLLPDSVSDASRDFLRQVFVEEKWRPTAEVLLGHPFVQGVF
>g296.t1
TQPGIGECAAVERVPLTPPEEEEEPALGAPGVTAPKGTSDLCVAPDRFLMSQTKGVISA
>g296.t2
TQPGIGECAAVERVPLTPPEEEEEPALGAPGVTAPKVKRGTSDLCVAPDRFLMSQTKGVISA
>g297.t1
LNFGLDLKDTEAMKRALGLIDSKMAQAKNWLRDPHAQPGDAGEQAIRQILDEAGKVGELCAGKERRDVVGTARTLGQMTD
QVAEMRSSLLSCLVERRHLA
LNRLP
>g297.t2
LNFGLDLKDTEAMKRALGLIDSKMAQAKNWLRDPHAQPGDAGEQAIRQILDEAGKVGELCAGKERRDVVGTARTLGQMTD
QVAEMRSSCSVLTGERLALA
APKRCSLLSCLVERRHLALNRLP
>g297.t3
LNFGLDLKDTEAMKRALGLIDSKMAQAKNWLRDPHAQPGDAGEQAIRQILDEAGKVGELCAGKERRDVVGTARTLGQMTD
QVAEMRSR
>g298.t1
MTGSPGQGGLLPRSLDMIFNSIGPYQAKRFKVDPEFADMMNPEEACGAEGVDEDSSYGVFVSYIEIYNNYIFDLLEETPY
DPIRPKPPQSKILREDQNHN
MYVAGCTEVEVKTTEEAFEVFWRGQKRRRIANTQLNKESSRSHSVFIIKLAQAPLDADGDNILQDKSQVAVGQLCLVDLA
GSERTSRTRADGNRLREAGN
INQSLMTLRTCIEVLRENQMCGTNKMVPYRDSKVTHLFKNYFDGEGKVKMIVCINPKAEDYEETMLVMRFAEMTQEVEVA
RPADRPLCGFAAGRRQRNQA
VKDDLSRKLDERGGPADRDFTSTLGQLLQSIPPLSCCELVDPHDDITLPRVIQALEKRQRIRQMIIDEYKQAANKMRSSL
KELDGSLFSKEDLIHKQNGK
LAEKDRVIQNQKADMDRLEKKTKMQEYKIEVLQKTTNIYEVDKRSLQQELETREQRLKREHAEKKRMEQRMQGMVSDTQH
NNLQPRSSSRGSLRPGGLVG
PRRASPPVSRL
>g298.t2
MTGSPGQGGLLPRSLDMIFNSIGPYQAKRFKVDPEFADMMNPEEACGAEGVDEDSSYGVFVSYIEIYNNYIFDLLEETPY
DPIRPKPPQSKILREDQNHN
MYVAGCTEVEVKTTEEAFEVFWRGQKRRRIANTQLNKESSRSHSVFIIKLAQAPLDADGDNILQDKSQVAVGQLCLVDLA
GSERTSRTRADGNRLREAGN
INQSLMTLRTCIEVLRENQMCGTNKMVPYRDSKVTHLFKNYFDGEGKVKMIVCINPKAEDYEETMLVMRFAEMTQEVEVA
RPADRPLCGFAAGRRQRNQA
VKDDLSRKLDERGGPADRDFTSTLGQLLQSIPPLSCCELVDPHDDITLPRVIQALEKRQRIRQMIIDEYKQAANKMRSSL
KELDGSLFSKEDLIHKQNGK
LAEKDRVIQNQKADMDRLEKKTKMQEYKIEVLQKTTNIYEVDKRSLQQELETREQRLKREHAEKKRMEQRMQGMVSDTQH

NNLQPRSSSRGSLRPGGLER
RVNAKQLEMQNKLWVKNEKLKQLKAIVTETKGPSAPPPDTPQRQARPQRPPREDRTPAKRSASPSPMPPTHGLSRVEALQ
LRPVAGSAPAVRCQTRSSSP
TPSSTGSQSASSRVPACELCAAQVCSGPSSTATRYPATGPAATGPARLAVRCCAGEDAEEAGSRSPTSPYTGAVEGAYWT
ATPVRPLHRRSRSAGGEKWV
DHTPASSVDLGTMLQPNIPNSIKVSAPSEKVLAKCDKYVLTHQELASDGEIQTKLIKGEVIKTRGGGQSVQFTDIETLRQ
QLTTAHSRKRRSSEGSPPSS
DQAGEWTDVETRCAVAVEMRAGSNMGPGYEHRGYPK
>g298.t3
MTGSPGQGGLLPRSLDMIFNSIGPYQAKRFKVDPEFADMMNPEEACGAEGVDEDSSYGVFVSYIEIYNNYIFDLLEETPY
DPIRPKPPQSKILREDQNHN
MYVAGCTEVEVKTTEEAFEVFWRGQKRRRIANTQLNKESSRSHSVFIIKLAQAPLDADGDNILQDKSQVAVGQLCLVDLA
GSERTSRTRADGNRLREAGN
INQSLMTLRTCIEVLRENQMCGTNKMVPYRDSKVTHLFKNYFDGEGKVKMIVCINPKAEDYEETMLVMRFAEMTQEVEVA
RPADRPLCGFAAGRRQRNQA
VKDDLSRKLDERGGPADRDFTSTLGQLLQSIPPLSCCELVDPHDDITLPRVIQALEKRQRIRQMIIDEYKQAANKMRSSL
KELDGSLFSKEDLIHKQNGK
LAEKDRVIQNQKADMDRLEKKTKMQEYKIEVLQKTTNIYEVDKRSLQQELETREQRLKREHAEKKRMEQRMQGMVSDTQH
KWEKECERRVNAKQLEMQNK
LWVKNEKLKQLKAIVTETKGPSAPPPDTPQRQARPQRPPREDRTPAKRSASPSPMPPTHGLSRVEALQLRPVAGSAPAVR
CQTRSSSPTPSSTGSQSASS
RVPACELCAAQVCSGPSSTATRYPATGPAATGPARLAVRCCAGEDAEEAGSRSPTSPYTGAVEGAYWTATPVRPLHRRSR
SAGGEKWVDHTPASSVDLGT
MLQPNIPNSIKVSAPSEKVLAKCDKYVLTHQELASDGEIQTKLIKGEVIKTRGGGQSVQFTDIETLRQQLTTAHSRKRRS
SEGSPPSSDQAGEWTDVETR
CAVAVEMRAGSNMGPGYEHRGYPK
>g298.t4
LCDCRCVQERRVNAKQLEMQNKLWVKNEKLKQLKAIVTETKGPSAPPPDTPQRQARPQRPPREDRTPAKRSASPSPMPPT
HGLSRVEALQLRPVAGSAPA
VRCQTRSSSPTPSSTGSQSASSRVPACELCAAQVCSGPSSTATRYPATGPAATGPARLAVRCCAGEDAEEAGSRSPTSPY
TGAVEGAYWTATPVRPLHRR
SRSAGGEKWVDHTPASSVDLGTMLQPNIPNSIKVSAPSEKVLAKCDKYVLTHQELASDGEIQTKLIKGEVIKTRGGGQSV
QFTDIETLRQQLTTAHSRKR
RSSEGSPPSSDQAGEWTDVETRCAVAVEMRAGSNMGPGYEHRGYPK
>g299.t1
MAVGSKGGDIMLWNFDALSKTAFIQGKGAGDFIGGMKFCPTDLSKVYAASGDGTLSLRSFEGCTSTVLSTTQDCSHDYHD
VCHKLHKSKVTHAEFNPRCD
WLMVTASVDHTVKLWDLRNIKDKNSFLHEMPHDMAVNSAYFNPSDCSKLLTTDQGNQIQATWHPVYDLIVVGRYPNDKVC
PGDLRTIDLYDANTGELLRQ
LRDPNASGIIPVNKFNPRGDVIASGMGVNILVWKQHDSPVSERCKVTEGEASRVTSSRSQQGSRQQRSSRDGRSAAGDAR
LRKKLSSIEEAEAKTVAQTR
SKSTTKAKAPKGKKK
>g299.t2
MAVGSKGGDIMLWNFDALSKTAFIQGKGAGDFIGGMKFCPTDLSKVYAASGDGTLSLRSFEGCTSTVLSTTQDCSHDYHD
VXQMLVIGDNVGQVSLLSLD
GQKSFSHKLHKSKVTHAEFNPRCDWLMVTASVDHTVKLWDLRNIKDKNSFLHEMPHDMAVNSAYFNPSDCSKLLTTDQGN
QIQATWHPVYDLIVVGRYPN
DKVCPGDLRTIDLYDANTGELLRQLRDPNASGIIPVNKFNPRGDVIASGMGVNILVWKQHDSPVSERCKVTEGEASRVTS
SRSQQGSRQQRSSRDGRSAA
GDARLRKKLSSIEEAEAKTVAQTRSKSTTKAKAPKGKKK
>g299.t3
MAVGSKGGDIMLWNFDALSKTAFIQGKGAGDFIGGMKFCPTDLSKVYAASGDGTLSLRSFEGCTSTVLSTTQDCSHDYHD
VCRQMLVIGDNVGQVSLLSL
DGQKSFSHKLHKSKVTHAEFNPRCDWLMVTASVDHTVKLWDLRNIKDKNSFLHEMPHDMAVNSAYFNPSDCSKLLTTDQG
NQIQATWHPVYDLIVVGRYP
NDKVCPGDLRTIDLYDANTGELLRQLRDPNASGIIPVNKFNPRGDVIASGMGVNILVWKQHDSPVSERCKVTEGEASRVT

SSRSQQGSRQQRSSRDGRSA
AGDARLRKKLSSIEEAEAKTVAQTRSKSTTKAKAPKGKKK
>g300.t1
MASMRQGRAFLAQINDDIPKTLTSVEAMLSTVEDPRQASNHRIQFDSLVLSMVYSAHLVRTLRREEDRLAWSGILQRLAN
VTVYELRGNYLSFYDGYI
>g301.t1
MMESTEEGSLSVGGSVGEENYFLGYTFTDRSHSSRVVKSIMDLCLEDGLFADVTINVDGKEFQLHRLVLSAQSSFFRSMF
TSNLKESHNRTIELKDVSAT
VFQLLVDYIYHGTIKLRVEDLQDTYEMADMYQLTALFEECSRFLSRTVEVKNCLQVMWLADRHSDQELYTAAKHCAKIHL
VQLNQTEEFLNMPLCLLLDI
IKDGVPSSQNPTVAIESWINHNKVEREEFSCVLQENLKEIGEKVHIYLIGKEETRTHSLAVSLHCDEDDTISVSGQNSLC
HQITAACKHGGDLYVVGGSI
PRRMWKCNMHTMDWERCAPLPRDRLHHTMVSVPGEDAIYSLGGKTLQDTLSNAVIYYTVKDNMWTETSQLDTAVSGAAGV
NLGGTIYLLGGEENDMDFFT
KPSRLIQCFNTAAQKCQIKPYMLPFAGCMHAAAHMDVIFVVAEGDSLVCYNPLLESFTRLRFPEVWSCVPSLWKVASCNG
CIYVFRDKCKKGDANTLKFN
PATSVVSVIRGIKILLTNWQFVLA
>g301.t2
MESTEEGSLSVGGSVGEENYFLGYTFTDRSHSSRVVKSIMDLCLEDGLFADVTINVDGKEFQLHRLVLSAQSSFFRSMFT
SNLKESHNRTIELKDVSATV
FQLLVDYIYHGTIKLRVEDLQDTYEMADMYQLTALFEECSRFLSRTVEVKNCLQVMWLADRHSDQELYTAAKHCAKIHLV
QLNQTEEFLNMPLCLLLDII
KDGVPSSQNPTVAIESWINHNKVEREEFSCVLQENLKEIGEKVHIYLIGKEETRTHSLAVSLHCDEDDTISVSGQNSLCH
QITAACKHGGDLYVVGGSIP
RRMWKCNMHTMDWERCAPLPRDRLHHTMVSVPGEDAIYSLGGKTLQDTLSNAVIYYTVKDNMWTETSQLDTAVSGAAGVN
LGGTIYLLGGEENDMDFFTK
PSRLIQCFNTAAQKCQIKPYMLPFAGCMHAAAHMDVIFVVAEGDSLVCYNPLLESFTRLRFPEVWSCVPSLWKVASCNGC
IYVFRDKCKKGDANTLKFNP
ATSVVSVIRGIKILLTNWQFVLA
>g302.t1
MGQDQTKQQIEKGLKLYQSNQTEKALDVWTKVLERTSDPGGKFRVLGCLITAHSEMGKYKQMLKYALAQIDTAREMEDPD
YLTEGYLNLARSNEKLCDYQ
KTVSYCKTCLDMQGTTVSLQLNGQVCLSMGNAFLGLSVFQKALESYEKALRYAHNNDDKMLECRVCCSLGNFYVHLKDYE
KALFFPCKAAELVNDYGKGW
SLKYRAMSQYHMSVAYRKLDRLPDAMECCEESMKIALQHGDRPLQALCLLNFADIHRCRKDTDKAFPRYESAMCIMTEIG
NRLGQAHIYLGVAKCWLLQK
DFDKESLHRAEELAEELGNKLCTLKVCCLSEAMFRSREQGEQLRQQVVKLLQCVEELELYCGMCGESIGERDQQLQALPC
SHIFHLKCLQTNGTRGCPKC
RQSSMKPGFV
>g302.t2
MGQDQTKQQIEKGLKLYQSNQTEKALDVWTKVLERTSDPGGKFRVLGCLITAHSEMGKYKQMLKYALAQIDTAREMEDPD
YLTEGYLNLARSNEKLCDYQ
KTVSYCKTCLDMQGTTVSLQLNGQVCLSMGNAFLGLSVFQKALESYEKALRYAHNNDDKMLECRVCCSLGNFYVHLKDYE
KALFFPCKAAELVNDYGKGW
SLKYRAMSQYHMSVAYRKLDRLPDAMECCEESMKIALQHGDRPLQALCLLNFADIHRCRKDTDKAFPRYESAMCIMTEIG
NRLGQAHIYLGVAKCWLLQK
DFDKLCTLKVCCLSEAMFRSREQGEQLRQQVVKLLQCVEELELYCGMCGESIGERDQQLQALPCSHIFHLKCLQTNGTRG
CPKCRQSSMKPGFV
>g302.t3
MGQDQTKQQIEKGLKLYQSNQTEKALDVWTKVLERTSDPGGKFRVLGCLITAHSEMGKYKQMLKYALAQIDTAREMEDPD
YLTEGYLNLARSNEKLCDYQ
KTVSYCKTCLDMQGTTVSLQLNGQVCLSMGNAFLGLSVFQKALESYEKALRYAHNNDDKMLECRVCCSLGNFYVHLKDYE
KALFFPCKAAELVNDYGKGW
SLKYRAMSQYHMSVAYRKLDRLPDAMECCEESMKIALQHGDRPLQALCLLNFADIHRCRKDTDKAFPRYESAMCIMTEIG
NRLGQAHIYLGVAKCWLLQK
DFDKAQESLHRAEELAEELGNKLCTLKVCCLSEAMFRSREQGEQLRQQVVKLLQCVEELELYCGMCGESIGERDQQLQAL

PCSHIFHLKCLQTNGTRGCP
KCRQSSMKPGFV
>g303.t1
MGVPVLPSNSFGPDDLSFFNQDLDDSQREAVSFALSQRDVAVIHGPPGTGKTTTVVEVILQAVQQGNKVLCCASSNVAVD
NLVERLAQCRAKVLRLGHPA
RLLESIQKHSLDAILAQSDNTNIIADIRKDMDKALMGMKKVRDKGERFGFKRELGELRKELRTREATAIAQILKSADVVL
STNTGASKDGPLRHLPPDHF
DWVVIDECAQALESSCWIALLNARKCILAGDYKQLPPTIKSQTAASKGLSVSLMERLIQKFGDSVVRMLTVQYRMHSSIM
DWASREMYQGRLTAHSSVEG
HLLKDLPGIACVEETSTPLLLIDTAGCGLSEMEVTDEQSKGNQGEVDLVELHIKCLTDAGLKAKDIAVIAPYNLQVDLLR
QRLSVRYPALEIKSVDGFQG
REKEAVVLTLVRSNRKGEVGFLAEDRRINVAVTRARRHLAVICDSQTVQNHAFLESLVSHMTEHGEVRTAFEYLQDIVPQ
NYTRNHGNSKTKAPARSSPS
TKQKVKHQAADKATQKKCAGGKVSDDPANQHPKPTKPRTLTPEEEQKVKTRSAEIREQVENYLRDPSQDELQFPSSLNSH
DRMLVHQISEELGLGHESKG
EGKDRCITVSRPPPTSTATKGAVAPQLTHPEPAQEEQPISEPQAATTSQKPPEDLKSLHLERMKREQQRREENAQQKQKV
QVAIGQLHTQKKSAKGKKRV
KAGACDIAAAAGAEDDFEALISAVMKAESVCSFVKCKASVLVLGQLCPFCNRQYCLGHHVPEAKAHARMRISKEGVLYAG
SGKKDNSMDPNKKAYLQRKL
DSKLKDMESKRKLKSKEKSN
>g303.t2
MEIEQFVSKTLDLLQEEREAEIQETRLWQENSSLKELQSKGVCLLKLQIANDLSFFNQDLDDSQREAVSFALSQRDVAVI
HGPPGTGKTTTVVEVILQAV
QQGNKVLCCASSNVAVDNLVERLAQCRAKVLRLGHPARLLESIQKHSLDAILAQSDNTNIIADIRKDMDKALMGMKKVRD
KGERFGFKRELGELRKELRT
REATAIAQILKSADVVLSTNTGASKDGPLRHLPPDHFDWVVIDECAQALESSCWIALLNARKCILAGDYKQLPPTIKSQT
AASKGLSVSLMERLIQKFGD
SVVRMLTVQYRMHSSIMDWASREMYQGRLTAHSSVEGHLLKDLPGIACVEETSTPLLLIDTAGCGLSEMEVTDEQSKGNQ
GEVDLVELHIKCLTDAGLKA
KDIAVIAPYNLQVDLLRQRLSVRYPALEIKSVDGFQGREKEAVVLTLVRSNRKGEVGFLAEDRRINVAVTRARRHLAVIC
DSQTVQNHAFLESLVSHMTE
HGEVRTAFEYLQDIVPQNYTRNHGNSKTKAPARSSPSTKQKVKHQAADKATQKKCAGGKVSDDPANQHPKPTKPRTLTPE
EEQKVKTRSAEIREQVENYL
RDPSQDELQFPSSLNSHDRMLVHQISEELGLGHESKGEGKDRCITVSRPPPTSTATKGAVAPQLTHPEPAQEEQPISEPQ
AATTSQKPPEDLKSLHLERM
KREQQRREENAQQKQKVQVAIGQLHTQKKSAKGKKRVKAGACDIAAAAGAEDDFEALISAVMKAESVCSFVKCKASVLVL
GQLCPFCNRQYCLGHHVPEA
KAHARMRISKEGVLYAGSGKKDNSMDPNKKAYLQRKLDSKLKDMESKRKLKSKEKSN
>g303.t3
MEIEQFVSKTLDLLQEEREAEIQETRLWQENSSLKELQSKGVCLLKLQIASQSTGLYGRTLVVLEPRKHMGVPVLPSNSF
GPDDLSFFNQDLDDSQREAV
SFALSQRDVAVIHGPPGTGKTTTVVEVILQAVQQGNKVLCCASSNVAVDNLVERLAQCRAKVLRLGHPARLLESIQKHSL
DAILAQSDNTNIIADIRKDM
DKALMGMKKVRDKGERFGFKRELGELRKELRTREATAIAQILKSADVVLSTNTGASKDGPLRHLPPDHFDWVVIDECAQA
LESSCWIALLNARKCILAGD
YKQLPPTIKSQTAASKGLSVSLMERLIQKFGDSVVRMLTVQYRMHSSIMDWASREMYQGRLTAHSSVEGHLLKDLPGIAC
VEETSTPLLLIDTAGCGLSE
MEVTDEQSKGNQGEVDLVELHIKCLTDAGLKAKDIAVIAPYNLQVDLLRQRLSVRYPALEIKSVDGFQGREKEAVVLTLV
RSNRKGEVGFLAEDRRINVA
VTRARRHLAVICDSQTVQNHAFLESLVSHMTEHGEVRTAFEYLQDIVPQNYTRNHGNSKTKAPARSSPSTKQKVKHQAAD
KATQKKCAGGKVSDDPANQH
PKPTKPRTLTPEEEQKVKTRSAEIREQVENYLRDPSQDELQFPSSLNSHDRMLVHQISEELGLGHESKGEGKDRCITVSR
PPPTSTATKGAVAPQLTHPE
PAQEEQPISEPQAATTSQKPPEDLKSLHLERMKREQQRREENAQQKQKVQVAIGQLHTQKKSAKGKKRVKAGACDIAAAA
GAEDDFEALISAVMKAESVC
SFVKCKASVLVLGQLCPFCNRQYCLGHHVPEAKAHARMRISKEGVLYAGSGKKDNSMDPNKKAYLQRKLDSKLKDMESKR

KLKSKEKSN
>g304.t1
MVSSTLSKTDAKKSHKTEVDWNSHGYDIAKIFGSKLTSVSPVWLQVRRRGKNSYDVTGLHDHDPVPRLLFDGWSYQDYMS
VLESEDEIEELASELVDVAK
VMSYCGTFHSSLLCTTEKFDGFTLEMWSQLGGNRRRELVHLIKHVCETLKAKRLDCFLVIPPALTSTGQPGMFGREEFEQ
LAPVVDGFSLMTYDYSSAGS
QWRHTILLGLNMYGMDFSSQGADPILGGRNNGVKCKLYYPTLKGVMIF
>g304.t2
MVSSTLSKTDAKKSHKTEVDWNSHGYDIAKIFGSKLTSVSPVWLQVRRRGKNSYDVTGLHDHDPGWVKDVRRSNRKIRLV
PRLLFDGWSYQDYMSVLESE
DEIEELASELVDVAKVMSYCGTFHSSLLCTTEKFDGFTLEMWSQLGGNRRRELVHLIKHVCETLKAKRLDCFLVIPPALT
STGQPGMFGREEFEQLAPVV
DGFSLMTYDYSSAGSQWRHTILLGLNMYGMDFSSQGADPILGGRNNGVKCKLYYPTLKGVMIF
>g304.t3
LSVLDRGLVVSDPQWRDIWNSHGYDIAKIFGSKLTSVSPVWLQVRRRGKNSYDVTGLHDHDPVPRLLFDGWSYQDYMSVL
ESEDEIEELASELVDVAKVM
SYCGTFHSSLLCTTEKFDGFTLEMWSQLGGNRRRELVHLIKHVCETLKAKRLDCFLVIPPALTSTGQPGMFGREEFEQLA
PVVDGFSLMTYDYSSAGSQW
RHTILLGLNMYGMDFSSQGADPILGGRNNGVKCKLYYPTLKGVMIF
>g305.t1
LDHELKQNLRWTMQHKYKQPGEEGVTDAVDKMQQEFKCCGSNSSLDWSGSIWVLTSGKQGMVPDSCCKTPRDNCGSRDHP
SNIYKLEVXVTAKSFHQSFS
LCSSVFNLQTATPGGKSIDFAGVDESTARWVQDFNVKPYSTPAKLESIDGARYQALLIPDCPGALNDLAHSGSLHRILTH
FISQQSGHLI
>g305.t2
LAVGVWTVVGKSDYISLLNSTFYATSAYILIVTGTIVVVTGIIGCCATLREMKSLLIVLDHELKQNLRWTMQHKYKQPGE
EGVTDAVDKMQQEFKCCGSN
SSLDWSGSIWVLTSGKQGMVPDSCCKTPRDNCGSRDHPSNIYKLEVXVTAKSFHQSFSLCSSVFNLQTATPGGKSIDFAG
VDESTARWVQDFNVKPYSTP
AKLESIDGARYQALLIPDCPGALNDLAHSGSLHRILTHFISQQSGHLI
>g305.t3
LAVGVWTVVGKSDYISLLNSTFYATSAYILIVTGTIVVVTGIIGCCATLREMKSLLIVCFPYCYELDHELKQNLRWTMQH
KYKQPGEEGVTDAVDKMQQE
FKCCGSNSSLDWSGSIWVLTSGKQGMVPDSCCKTPRDNCGSRDHPSNIYKLEVXVTAKSFHQSFSLCSSVFNLQTATPGG
KSIDFAGVDESTARWVQDFN
VKPYSTPAKLESIDGARYQALLIPDCPGALNDLAHSGSLHRILTHFISQQSGHLI
>g306.t1
MGGGPEGTAQQMVSDPTLVHVLVRFFSGSSPHGTSQHSAQVGPTATQAMQEFLSRLQVHLSPTCPQMFSDFLLRLMHILS
TERGPFQSGQGPLDAQVKLL
EFTLEQNFEVVSVGTISSVIESSTFLVHHYITCSDKVVSRSGSDSSSGARACFGGLFANIIRPGDAKAVCGETTRDQLMF
DLLKLVNVLVQLPLSSDREF
SGRALA
>g307.t1
MHYFSPVDKMQLLEIITTDKTSKDTTAAAVAVGLKQGKVIIVVGDGPGFYTTRCLGPMLGEAVRVLQEGVGPKKLDQLTT
SFGFPVGTATLADEVGIDVA
AHVAEDLGKAFGPRFGGGNVEVLKTMVELGFKGRKSGKGCYVYQQGLKTKEVNTEIDEIM
>g307.t2
VIGMHYFSPVDKMQLLEIITTDKTSKDTTAAAVAVGLKQGKVIIVVGDGPGFYTTRCLGPMLGEAVRVLQEGVGPKKLDQ
LTTSFGFPVGTATLADEVGI
DVAAHVAEDLGKAFGPRFGGGNVEVLKTMVELGFKGRKSGKGCYVYQQGLKTKEVNTEIDEIM
>g308.t1
MHNDLSCSLSHSQGCLGDSSACLGEQMIRSLRDLKQAGRPR
>g309.t1
MEEKERAGSQAQYKLYEQARKKAEELSRENEALLQAHANVLQQNQEQAKEVEVLTNRLDQSRHKLEELHLKMNQLEEDKA
SISAEYEKIRSRFKTMLLAY
KAVNGTAPTYLQTLVKPHAPARALRSTTSAGRLVPPSLKGGKGRLAKSQLFSILAPQWWNELPANVRTAESLPIFRKRLK

THLFRVHLNPT
>g309.t2
MLNKLQAQLMEEKERAGSQAQYKLYEQARKKAEELSRENEALLQAHANVLQQNQEQAKEVEVLTNRLDQSRHKLEELHLK
MNQLEEDKASISAEYEKIRS
RFKTMLLAYKAVNGTAPTYLQTLVKPHAPARALRSTTSAGRLVPPSLKGGKGRLAKSQLFSILAPQWWNELPANVRTAES
LPIFRKRLKTHLFRVHLNPT
>g309.t3
SERDSLKEMLNKLQAQLMEEKERAGSQAQYKLYEQARKKAEELSRENEALLQAHANVLQQNQEQAKEVEVLTNRLDQSRH
KLEELHLKMNQLEEDKASIS
AEYEKIRSRFKTMLLAYKAVNGTAPTYLQTLVKPHAPARALRSTTSAGRLVPPSLKGGKGRLAKSQLFSILAPQWWNELP
ANVRTAESLPIFRKRLKTHL
FRVHLNPT
>g310.t1
MGKTADLTAVQKAIIDTLKQEENCTTRRGDRTLRKIVEKGRFQTLGDLRKQWTESGVETQSHRAQACAGNGLQVPHSP
>g310.t2
MGKTADLTAVQKAIIDTLKQEGKSVAENCTTRRGDRTLRKIVEKGRFQTLGDLRKQWTESGVETQSHRAQACAGNGLQVP
HSP
>g311.t1
MLSSKKSDAGSSSSTSSSSTGAAGVERPSGSDAQSGPSASVPGPADVKKKERASPSGEPGGPPLPHQAGPGGVDQDSAEV
RRTSRRKRAKVEYREMDESL
ANLSEDEYYSEEERNAKAEKERKQVVPPPAPPPEEENDSVEGAAFQSRLPHDRMTSQEAACFPDIISGPQQTQKVFLYIR
NRTLQLWLDNPKVQLTFEAT
AQQLEAPYNSDTVLVHRIHSYLERHGLVNFGIYKRVKPLPPKKTGKVIIIGGGVSGLAAARQLQSFGMDVTVLEARDRVG
GRVATFRKGNYVADLGAMVV
TGLGGNPMAVISKQVNMELAKIKQKCPLYEANGQAVPKEKDEMVEQEFNRLLEATSYLSHQLDFNFLNNKPVSLGQALEV
VI
>g311.t2
MLSSKKSDAGSSSSTSSSSTGAAGVERPSGSDAQSGPSASVPGPADVKKKERASPSGEPGGPPLPHQAGPGGVDQDSAEV
RRTSRRKRAKVEYREMDESL
ANLSEDEYYSEEERNAKAEKERKQVVPPPAPPPEEENDSVEGAAFQSRLPHDRMTSQEAACFPDIISGPQQTQKVFLYIR
NRTLQLWLDNPKVQLTFEAT
AQQLEAPYNSDTVLVHRIHSYLERHGLVNFGIYKRVKPLPPKKTGKVIIIGGGVSGLAAARQLQSFGMDVTVLEARDRVG
GRVATFRKGNYVADLGAMVV
TGLGGNPMAVISKQVNMELAKIKQKCPLYEANGQAVPKEKDEMVEQEFNRLLEATSYLSHQLDFNFLNNKPVSLGQALEV
VIQ
>g311.t3
LSSKKSDAGSSSSTSSSSTGAAGVERPSGSDAQSGPSASVPGPADVKKKERASPSGEPGGPPLPHQAGPGGVDQDSAEVR
RTSRRKRAKVEYREMDESLA
NLSEDEYYSEEERNAKAEKERKQVVPPPAPPPEEENDSVEGAAFQSRLPHDRMTSQEAACFPDIISGPQQTQKVFLYIRN
RTLQLWLDNPKVQLTFEATA
QQLEAPYNSDTVLVHRIHSYLERHGLVNFGIYKRVKPLPPKKTGKVIIIGGGVSGLAAARQLQSFGMDVTVLEARDRVGG
RVATFRKGNYVADLGAMVVT
GLGGNPMAVISKQVNMELAKIKQKCPLYEANGQAVPKEKDEMVEQEFNRLLEATSYLSHQLDFNFLNNKPVSLGQALEVV
I
>g312.t1
MVNGTKVAVPFDGKGISWWTDYNIKYRNPSVTPLENAFNGTVKPIFWPRPAYQLDTKNPSNNGFINQDFLVWMRRPAYQL
DTKNPSNNGFINQDFLVWMR
RSALPDFRKLYRRITEGNYAKGLPAGTYSLEIDYPVLAFNGRKKVVFSNVSWMGGKNQFLGIAYLVIGSMCVVMSIVMLI
VYAKFKFPDEE
>g312.t2
MSKEESTANRPDNTAFTQQRLPAWQPMLSAGIVIPGFVLIGVAFIGIGVALFVTSQNIQVLEGPVFFYYGLSNYFQNYRR
YGASKDANQLSGDVTYFKEP
SESCAPYQFDGKNPIVPCGSLPNSMFNGKVVV
>g312.t3
MVNGTKVAVPFDGKGISWWTDYNIKYRNPSVTPLENAFNGTVKPIFWPRPAYQLDTKNPSNNGFINQDFLVWMRRPAYQL
DTKNPSNNGFINQDFLVWMR

RSALPDFRKLYRRITEGNYAKGLPAGTYSLEIGYNYPVLAFNGRKKVVFSNVSWMGGKNQFLGIAYLVIGSMCVVMSIVM
LIVYAKFKFPDEE
>g312.t4
MMTSECLNPKDRKMGVPGRTSAMSKEESTANRPDNTAFTQQRLPAWQPMLSAGIVIPGFVLIGVAFIGIGVALFVTSQNI
QVLEGPVFFYYGLSNYFQNY
RRYGASKDANQLSGDVTYFKEPSESCAPYQFDGKNPIVPCGSLPNSMFNGKVVV
>g312.t5
MSKEESTANRPDNTAFTQQRLPAWQPMLSAGIVIPGFVLIGVAFIGIGVALFVTSQNIQVLEGPVFFYYGLSNYFQNYRR
YGASKDANQLSGDVTYFKEP
SESCAPYQFDGKNPIVPCGSLPNSMFNDTFKLFQMVNGTKVAVPFDGKGISWWTDYNIKYRNPSVTPLENAFNGTVKPIF
WPRPAYQLDTKNPSNNGFIN
QDFLVWMRRPAYQLDTKNPSNNGFINQDFLVWMRRSALPDFRKLYRRITEGNYAKGLPAGTYSLEIDYPVLAFNGRKKVV
FSNVSWMGGKNQFLGIAYLV
IGSMCVVMSIVMLIVYAKFKFPDEE
>g313.t1
MSDGGGGEENAGTMEVSAVQTVADTSVLQKHIRKLVPLLLEDGGEAPASLETALEEKSSVEQMKKFLSDPQINSILVERS
CLKEDVGDEGEEEKESIAYN
ISIDIHYGIKSNSLAFIKRAGVVDADKPISTQVRVLTLSEDSPYETLHSFISNAVAPFFKSYIRESGKADRDGDKMAPSV
EKKIAELEMGLLHLQQNIEI
PEISLLINPIITNISKQCYERSEKPNVTDFGDKVEEPTFLNQLQSGVNRWIREIQKVTKLDRDPASGTALQEISFWLNLE
RALNRIQEKRESPEVLLTLD
ILRHGKRFHATVSFDTDTGLKQAVETVNDYNPLMKDFPLNDLLSATELDKIRQALMAIFTHLKKIRNTKYPIQRALRLVE
AISRDLSSQLLKVLGTRKLM
HVAYEEFEKVMVACFEVFQTWEDEYEKLQVLLRDIVKRKREENLKMVWRLSPAHRKLQSRLDHMRRFRRQHEQLRAVIVR
VLRPQISAVPQHAPGETVEP
QDIKVAEVLFDAADANAIEEVNLAYENVKEVDGLDVSKEGMEAWEAAMKRYDERIDRVETRITARLRDQLGTAKNANEMF
RIFSRFNALFVRPHIRGAIR
EYQTQLIQRVKDDIESLHDKFKVQYPQSQACKMSHVRDLPPVSGSIIWAKQIDRQLTAYMKRVEDVLGKGWENHVEGLKL
KQDGDSFRAKLNTQEIFDDW
ARKVQQRNLGVSGRIFTIESSRARGRTGNMLKLKVNFLPEIITLSKEVRNLKWLSFRVPLAIVNKAHQANQLYPFAISLI
ESVRTYERTCEKVEERSSIS
LLVAGLKKEVQALVTEGIALVWESYKLDPYVQRLAETVFNFQEKVDDLLLIEEKIDLEVRSLDTCMFENKTFSEILNRIQ
KAVDDLNLHSYSNLPIWVNK
LDIEIERILGVRLQAGLRAWTQVLRGQMEDRADVDMDTEAPQAGHKPGGEPKIKNVVHELRITNQVIYLNPPIEDCRYKL
YQEMFSWKMVILSLPRIQSQ
RYQVGVHYELSEEEKFYRNALTRMPDGPAALEEAYNSVEDNVSEVEQYVKVWLQYQCLWDMQAENIYNRLGEDLTKWQAL
LVQIRKARGTFDNAETRKEF
GPVIIDYGKVQSKVNLKYDSWHKEVLSKFGQMLGQNMQDFHSQISKSRQELEQHSVDTASTSDAVTFITYVQTLKRKIKQ
FEKNVDLFRNGQRLLEKQRF
QFPPSWLYIDNIEGEWGAFSDIMKRKDTAIQQQVANLQMKIVQEDRAVENRTTDLLTDWEKTKPVGGSQRPEEALQSLTI
YEGKFGRLKDDREKCARAKE
ALELTDTGLLSGS
>g313.t2
MSDGGGGEENAGTMEVSAVQTVADTSVLQKHIRKLVPLLLEDGGEAPASLETALEEKSSVEQMKKFLSDPQINSILVERS
CLKEDVGDEGEEEKESIAYN
ISIDIHYGIKSNSLAFIKRAGVVDADKPISTQVRVLTLSEDSPYETLHSFISNAVAPFFKSYIRESGKADRDGDKMAPSV
EKKIAELEMGLLHLQQNIEI
PEISLLINPIITNISKQCYERSEKPNVTDFGDKVEEPTFLNQLQSGVNRWIREIQKVTKLDRDPASGTALQEISFWLNLE
RALNRIQEKRESPEVLLTLD
ILRHGKRFHATVSFDTDTGLKQAVETVNDYNPLMKDFPLNDLLSATELDKIRQALMAIFTHLKKIRNTKYPIQRALRLVE
AISRDLSSQLLKVLGTRKLM
HVAYEEFEKVMVACFEVFQTWEDEYEKLQVLLRDIVKRKREENLKMVWRLSPAHRKLQSRLDHMRRFRRQHEQLRAVIVR
VLRPQISAVPQHAPGETVEP
QDIKVAEVLFDAADANAIEEVNLAYENVKEVDGLDVSKEGMEAWEAAMKRYDERIDRVETRITARLRDQLGTAKNANEMF
RIFSRFNALFVRPHIRGAIR
EYQTQLIQRVKDDIESLHDKFKVQYPQSQACKMSHVRDLPPVSGSIIWAKQIDRQLTAYMKRVEDVLGKGWENHVEGLKL

KQDGDSFRAKLNTQEIFDDW
ARKVQQRNLGVSGRIFTIESSRARGRTGNMLKLKVNFLPEIITLSKEVRNLKWLSFRVPLAIVNKAHQANQLYPFAISLI
ESVRTYERTCEKVEERSSIS
LLVAGLKKEVQALVTEGIALVWESYKLDPYVQRLAETVFNFQEKVDDLLLIEEKIDLEVRSLDTCMFENKTFSEILNRIQ
KAVDDLNLHSYSNLPIWVNK
LDIEIERILGVRLQAGLRAWTQVLRGQMEDRADVDMDTEAPQAGHKPGGEPKIKNVVHELRITNQVIYLNPPIEDCRYKL
YQEMFSWKMVILSLPRIQSQ
RYQVGVHYELSEEEKFYRNALTRMPDGPAALEEAYNSVEDNVSEVEQYVKVWLQYQCLWDMQAENIYNRLGEDLTKWQAL
LVQIRKARGTFDNAETRKEF
GPVIIDYGKSKVNLKYDSWHKEVLSKFGQMLGQNMQDFHSQISKSRQELEQHSVDTASTSDAVTFITYVQTLKRKIKQFE
KNVDLFRNGQRLLEKQRFQF
PPSWLYIDNIEGEWGAFSDIMKRKDTAIQQQVANLQMKIVQEDRAVENRTTDLLTDWEKTKPVGGSQRPEEALQSLTIYE
GKFGRLKDDREKCARAKEAL
ELTDTGLLSGS
>g314.t1
MSSLEESGRFSSLRGLRSLSLANVEFYDAGLPDVCTLPRLESLDLSNTSVTNLKPLLGLRERLRSLTLHQLKRLEMSTAQ
VLGVIGQLDALQHLDISDDK
QFTSDVARQLLGQPGILPTLVSLDVSGRKQVTDAAIKTFVEERPGMAFLGLLATGAGFADFLNGEGSLKVTGEANETQIC
QALRRYSEREGFVREALFHL
FSLTHVMEKPRPDILKLVVLGMKNHPATLNVQLAASACVFNLTKQDLAADMPVRLLSSVTQLLLEAMRTFPNHQQVAALT
FHLGSPCLQLQKNCLLSLCS
DRILQEVPFNRFEAAKLVMQWLCNHEDQNMQRMAVAIISILAAKLSTEQTAQLGAELSIVMQLLNIVKQKAGQSVVDATL
KFTLSALWNLTDESPTTCSH
FIQNDGLNLFINVLESFPSESSIQQKVLGLLNNIAEVGDLHVQLMVQGFLDHICNLLHSPEVEVSYFAAGILAHLTSRGE
QAWTLSLSLRASLLEQLHSA
ILKWPTPECEMVAY
>g314.t2
MSSLEESGRFSSLRGLRSLSLANVEFYDAGLPDVCTLPRLESLDLSNTSVTNLKPLLGLRERLRSLTLHQLKRLEMSTAQ
VLGVIGQLDALQHLDISDDK
QFTSDVARQLLGQPGILPTLVSLDVSGRKQVTDAAIKTFVEERPGMAFLGLLATGAGFADFLNGEGSLKVTGEANETQIC
QALRRYSEREGFVREALFHL
FSLTHVMEKPRPDILKLVVLGMKNHPATLNVQLAASACVFNLTKQDLAADMPVRLLSSVTQLLLEAMRTFPNHQQLQKNC
LLSLCSDRILQEVPFNRFEA
AKLVMQWLCNHEDQNMQRMAVAIISILAAKLSTEQTAQLGAELSIVMQLLNIVKQKAGQSVVDATLKFTLSALWNLTDES
PTTCSHFIQNDGLNLFINVL
ESFPSESSIQQKVLGLLNNIAEVGDLHVQLMVQGFLDHICNLLHSPEVEVSYFAAGILAHLTSRGEQAWTLSLSLRASLL
EQLHSAILKWPTPECEMVAY
>g315.t1
LLTGYIFLPQPDTESVVNGNHQPCPTQSSPAPGVQSEHSAIVSSIEKDLQDIMDSLVMDDPQAPSSSDPREPTGVRHSIA
HSPLSPMLNGGGSYLLSPPT
SPGAMSVGSSYENTSPPFSPLSSPSAASSGSFTSPSPSGGPQQVQSSSSLPPVPVRSSSYNYSTQPTQQPRTGLSSYSSS
SIVVGPKVQESPRLQRKALL
DAPPLSPKPGRRAIGQGGHESPRQIHLLSSTESLTSHNIVPSSPRLTSKFPTCSSPSPTSPRTKVGLVLQEPPTIPSRQL
APQPMRAFQPPLDPIIHIIQ
GSPGLQPLPQSPRLLQPLESPRMARRNAELNGAICGSSMRELPPLSPSMARRGVPVLPGTLPGTMSSLKTPDSPSGLGKL
VPGSPRLRHKMASTSEDSFG
SRGIRTRSPSPTSMMTVDGVSGAGSGGSLGSRKASFGGSLTPAYSLGSLPGSSPVASPRSHRKTSGGGPREMGRPHPGMR
ERKNSITEISDNEDELLDYH
RRQREERLREQEMERLFKCASCAVPELGRVRGQSDRPSSRLQ
>g315.t2
MLCFGQSSFFRFNHPKEAYRMKSMMPQGGRGVAMGDYQLHKDTESVVNGNHQPCPTQSSPAPGVQSEHSAIVSSIEKDLQ
DIMDSLVMDDPQAPSSSDPR
EPTGVRHSIAHSPLSPMLNGGGSYLLSPPTSPGAMSVGSSYENTSPPFSPLSSPSAASSGSFTSPSPSGGPQQVQSSSSL
PPVPVRSSSYNYSTQPTQQP
RTGLSSYSSSSIVVGPKVQESPRLQRKALLDAPPLSPKPGRRAIGQGGHESPRQIHLLSSTESLTSHNIVPSSPRLTSKF
PTCSSPSPTSPRTKVGLVLQ

EPPTIPSRQLAPQPMRAFQPPLDPIIHIIQGSPGLQPLPQSPRLLQPLESPRMARRNAELNGAICGSSMRELPPLSPSMA
RRGVPVLPGTLPGTMSSLKT
PDSPSGLGKLVPGSPRLRHKMASTSEDSFGSRGIRTRSPSPTSMMTVDGVSGAGSGGSLGSRKASFGGSLTPAYSLGSLP
GSSPVASPRSHRKTSGGGPR
EMGRPHPGMRERKNSITEISDNEDELLDYHRRQREERLREQEMERLFKCASCAVPELGRVRGQSDRPSSRLQ
>g316.t1
LFPRDNPRNTRFAINFFTSIGLGGLTDELRDHLKNAPKMIMTQKQEVESSDSSDDSSSQSDSDSSSQSDSSSDSSSSSSS
SSSDSDSKHKKKKKKKEKTS
SSDRKKASNQDLKQKETTNKKASANSSSEEEEEGWGMARRRVSPPPHMKQRARELEERPLG
>g316.t2
LFPRDNPRNTRFAINFFTSIGLGGLTDELRDHLKNAPKMIMTQKQEVESSDSSDDSSSQSDSDSSSQSDSSSDSSSSSSS
SSSDSDSKHKKKKKKKEKTS
SSDRKKASNQDLKQKETTNKKASANSSSEEEEEGWGMARRRVSPPPHMKQRARELEERERARDRREDRSVGGRGRPGAGE
GEGAGERWRQREKDGERSGE
GRKERERSAEGKRERRKERERSVEGERRGRDEREKNKENRDRNRHREERR
>g317.t1
PAIRLLVRLAYPRSSIHPVPRAMEEKETTAPPAVPRGGNAQSGPHRSTDKTPDVTAATARSLLVNSPFAPSPDLVAISVE
VSENGKDVRRYVFDYSTGAD
MLSSVVERAPCQWAGLRSHSPSSAGDSRLTCSEFT
>g318.t1
MPLVKRNIEPRHLCQGALPEGIGGELEYVTNNSLSAIIRQLSCLSKHAENVFGELFDEANTFYVRANSLQDRVDRLAVKV
TQLDSNVEEVSLQDINMRKA
FRSSTLQSQQVVSKSSTPSSVNHLYSASEPAPPLGSLSAYREDRADAMKYYTDPSYFFDLWKEKMLQDTEEKKKERRRLR
EQKRLVDSTLQRQAKKVRKA
SNRRQEWNMMAFDKELRPDHRPPQSLRRGASSEGSLSPDGRQVSCILPDLPEYPAPPCPAHALHQPMGHGYVAAHPSPPT
EHEYQSIEAHYGEGRALRYA
NTGSHSAEAINGFPLPPVDYKSAECPTSSRHPPSGPSIPSTLTAFASPVGALPPT
>g318.t2
MPLVKRNIEPRHLCQGALPEGIGGELEYVTNNSLSAIIRQLSCLSKHAENVFGELFDEANTFYVRANSLQDRVDRLAVKV
TQLDSNVEEVSLQDINMRKA
FRSSTLQSQQVVSKSSTPSSVNHLYSASEPAPPLGSLSAYREDRADAMKYYTDPSYFFDLWKEKMLQDTEEKKKERRRLR
VGDIQDITALLLWDGQEQKR
LVDSTLQRQAKKVRKASNRRQEWNMMAFDKELRPDHRPPQSLRRGASSEGSLSPDGRQVSCILPDLPEYPAPPCPAHALH
QPMGHGYVAAHPSPPTEHEY
QSIEAHYGEGRALRYANTGSHSAEAINGFPLPPVDYKSAECPTSSRHPPSGPSIPSTLTAFASPVGALPPT
>g319.t1
NATVRCEPLVCLRPQCPPHSAAAYVPGSCCKECQ
>g319.t2
MIPREIAFVTILQCQNATVRCEPLVCLRPQCPPHSAAAYVPGSCCKECQ
>g320.t1
RTGSIRRPLDGSRSTANVTEERLMERLTATSGWLPQAHVGTATRDLQPWADLMISS
>g321.t1
NPPVFEEQAYSSYVSENNKPGHSLCSVKAHDPDWRQNEGNSFMTELVPKAAQGGALVSKVIAVDGDSGQNAWLSYHIIKS
TDRDFQNRPAQRGDVDGTGT
LRSTYNYDAYLTTGSRTSDFKFVTSYNDNTLPADQTLRKNPTDFEEVFGDLDSSPEVG
>g321.t2
NPPVFEEQAYSSYVSENNKPGHSLCSVKAHDPDWRQNEGNSFMTELVPKAAQGGALVSKVIAVDGDSGQNAWLSYHIIKS
TDRDFQNRPAQRGDQTLRKN
PTDFEEVFGDLDSSPEVG
>g321.t3
NPPVFEEQAYSSYVSENNKPGHSLCSVKAHDPDWRQNEGNSFMTELVPKAAQGGALVSKVIAVDGDSGQNAWLSYHIIKS
TDRDFQNRPAQRGDVDGTGT
LRSTYNYDAYLTTGSRTSDFKFVTSYNDNTLPADQTLRKNPTDFEEVFGDLDSSPEMKYLLNAVGC
>g322.t1
MTSWSFSLPRGVNGWWQGARQQKPNLGILSPRRCLGDKPQSPLSCCVNRAPGQQDFIQSPHPLSLDPPQTTSTTTTTTTS
TITTTTTTTTTSTTTTTTTH

TPTTLSTTTTTTHTPITLSTSTTTTRTSSTKTTTIPTSITTSNPTTSHNHHHHVHTAPLLTQSQPAPVPFIL
>g323.t1
GESGAGKTVNTKRVIQYFATISVGGGEKKKDQGAGKIQGSLEDQIIAANPLLEAYGNAKTVRNDNSSRFVNLLEKSRVTF
QLPDERGYHIFYQMMTNHKP
ELIEMTLITTNPYDFPMCSQGQITVASIDDKEELVATDTAIDILGFNNEEKMGIYKFTGAVLHHGNMKFKQKQREEQAEP
DGNEEADKISYLLGLNSADM
LKALCYPRVKVGNEFVTKGQTVPQVNNSVSALAKSIYERMFLWMVIRINQMLDTKQARQFFIGVLDIAGFEIFDYNSMEQ
LCINFTNEKLQQFFNHHMFV
LEQEEYKKEGIIWEFIDFGMDLAACIELIEKVHMT
>g323.t2
GESGAGKTVNTKRVIQYFATISVGGGEKKKDQGAGKIQGSLEDQIIAANPLLEAYGNAKTVRNDNSSRFVNLLEKSRVTF
QLPDERGYHIFYQMMTNHKP
ELIEMTLITTNPYDFPMCSQGQITVASIDDKEELVATDTAIDILGFNNEEKMGIYKFTGAVLHHGNMKFKQKQREEQAEP
DGNEVKVGNEFVTKGQTVPQ
VNNSVSALAKSIYERMFLWMVIRINQMLDTKQARQFFIGVLDIAGFEIFDYNSMEQLCINFTNEKLQQFFNHHMFVLEQE
EYKKEGIIWEFIDFGMDLAA
CIELIEKVHMT
>g323.t3
MTLITTNPYDFPMCSQGQITVASIDDKEELVATDTAIDILGFNNEEKMGIYKFTGAVLHHGNMKFKQKQREEQAEPDGNE
EADKISYLLGLNSADMLKAL
CYPRVKVGNEFVTKGQTVPQVNNSVSALAKSIYERMFLWMVIRINQMLDTKQARQFFIGVLDIAGFEIFDYNSMEQLCIN
FTNEKLQQFFNHHMFVLEQE
EYKKEGIIWEFIDFGMDLAACIELIEKVHMT
>g323.t4
GESGAGKTVNTKRVIQYFATISVGGGEKKKDQGAGKIQGSLEDQIIAANPLLEAYGNAKTVRNDNSSRFVNLLEKSRVTF
QLPDERGYHIFYQMMTNHKP
ELIGKMV
>g324.t1
MATEENAWVSSPSYSRYWQHYQQAMSWYQKHRRAYSKALIAAYGPPCYGQQYLGKSSSGDLNHRFPDWHGEDGGGHGRRG
EKRHRESPKPSDREKEDSGS
EEGDSEIECDVSNMEITEELRQYFAQTEKHREELKKQQELEDEEHGSYVPADQDLHRGAWLGTAAPPHERPGARRGAEMK
KLYGEDAAKIQAMEAALQLN
FDRNCDRKQPKYWPVIPLRL
>g324.t2
ATEENAWVSSPSYSRYWQHYQQAMSWYQKHRRAYSKALIAAYGPPCYGQQYLGKSSSGDLNHRFPDWHGEDGGGHGRRGE
KRHRESPKPSDREKEDSGSE
EGDSEIECDVSNMEITEELRQYFAQTEKHREELKKQQELEDEEHGSYVPADQDLHRGAWLGTAAPPHERPGARRGAEMKK
LYGEDAAKIQAMEAALQLNF
DRNCDRKQPKYWPVIPLRL
>g324.t3
PLLATTGVLMATEENAWVSSPSYSRYWQHYQQAMSWYQKHRRAYSKALIAAYGPPCYGQQYLGKSSSGDLNHRFPDWHGE
DGGGHGRRGEKRHRESPKPS
DREKEDSGSEEGDSEIECDVSNMEITEELRQYFAQTEKHREELKKQQELEDEEHGSYVPADQDLHRGAWLGTAAPPHERP
GARRGAEMKKLYGEDAAKIQ
AMEAALQLNFDRNCDRKQPKYWPVIPLRL
>g325.t1
MRVGDTNRLNRLIRRAGDVVGEELDTLTIVADRRTLSRLRSILDNASHPLFSTLAQRRSTFSRRLLSPRCTTERHRKSFL
PVAIKLYNTSL
>g326.t1
MTSYEVTTKVVVTTHRGEWQTGMADTQREVVMATGRNDFLCRSVVHLGERSLLLKVLLRWARVEKRGHRALWSTLRLLLQ
RGADPNTCRVPMPVLFLAIK
AAHPEAVLALLQCGARTDTPLPSEVKELHLQTPSMR
>g326.t2
MTSYEVTTKVVVTTHRGEWQTGMADTQREVVMATGRNDFLCRSVVHLGERSLLLKVLLRWARVEKRGHRALWSTLRLLLQ
RGADPNTCRVPMPVLFLAIK
AAHPEAVLALLQCGARTDTPLPSEVKELHLQTPSMRRNGIKE

>g326.t3
MADTQREVVMATGRNDFLCRSVVHLGERSLLLKVLLRWARVEKRGHRALWSTLRLLLQRGADPNTCRVPMPVLFLAIKAA
HPEAVLALLQCGARTDTPLP
SEVKELHLQTPSMR
>g327.t1
METQQAMASQPVTFSTDTSKTFVETWNEEHEELSDDQSLLDLGFLTDLMAKLNALNNKLQGKDRHLSNMISTVNGFKAKL
GVWNTQADTFPTWRKCHRPS
VKRTLFTLSSTVFTWTKWQQSSVSVSVN
>g327.t2
MDPADADKLLQAFSGQGTLLRQHQQHLQGLMETQQAMASQPVTFSTDTSKTFVETWNEEHEELSDDQSLLDLGFLTDLMA
KLNALNNKLQGKDRHLSNMI
STVNGFKAKLGVWNTQADTFPTWRKCHRPSVKRTLFTLSSTVFTWTKWQQSSVSVSVN
>g328.t1
GDSGSPLVCSGVLQGLVSWGRGCAEPKSPGVYVKVCQLLYWIEETMRTNR
>g329.t1
NPPVFEEQAYSSYVSENNKPGHSLCSVKAHDPDWRQNEGNSFMTELVPKAAQGGALVSKVIAVDGDSGQNAWLSYHIIKS
TDRDFQNRPAQRGDQTMRKN
PTDFEEVFGDLDVSPETSKALIATRKSIVRNDELEHKASLASTNTNEGKGVD
>g329.t2
NPPVFEEQAYSSYVSENNKPGHSLCSVKAHDPDWRQNEGNSFMTELVPKAAQGGALVSKVIAVDGDSGQNAWLSYHIIKS
TDRDFQNRPAQRGDVDGTGT
LRSTYNYDAYLTTGSRTSDFKFVTSYNDNTLPADQTMRKNPTDFEEVFGDLDVSPETSKALIATRKSIVRNDELEHKASL
ASTNTNEGKGVD
>g330.t1
MKGGVVPEGLVWNTLKKMQVDPGEKHPEFGDVKKLVTDEFVRQRYLEYTKTPHTEPIKHEFSWGQRAEVEVSKHKILEFL
GQLHDQDPQRWSQQYKEAVK
QLSGSAAQPCTSTPR
>g330.t2
MKGGVVPEGLVWNTLKKMQVDPGEKHPEFGDVKKLVTDEFVRQRYLEYTKTPHTEPIKHEFSWGQRAEVEVSKHKILEFL
GQDPQRWSQQYKEAVKQLSG
SAAQPCTSTPR
>g330.t3
MPQRKRLAKRSSGSVAEEDEDTRFTQPTTSQVQKGIEKLTKAQIDEKGGVVPEGLVWNTLKKMQVDPGEKHPEFGDVKKL
VTDEFVRQRYLEYTKTPHTE
PIKHEFSWGQRAEVEVSKHKILEFLGQLHDQDPQRWSQQYKEAVKQLSGSAAQPCTSTPR
>g331.t1
MYSLNLSYIKIFNVGSRYLVNRVQDHIQSRIVYYLMNIHVTPRSIYLSRHGESELNLLGRIGGDSGLSQRGARFASALGT
YMQGQNISDLKVWTSHMKRT
IQTAEALGVQYEQWKALNEIDAGVCEDMTYEEIQENYPEEFALRDQDKYRYRYPKGESYEDLVQRLEPVIMELERQENVL
VICHQAVMRCLLAYFLDKSA
GELPYLKCPLHTVLKLTPVAYGCKVESVFLNIEAVNTHRDRPENVDVDRDPEEALETVPDHI
>g331.t2
LSYIKIFNVGSRYLVNRVQDHIQSRIVYYLMNIHVTPRSIYLSRHGESELNLLGRIGGDSGLSQRGARFASALGTYMQGQ
NISDLKVWTSHMKRTIQTAE
ALGVQYEQWKALNEIDAGVCEDMTYEEIQENYPEEFALRDQDKYRYRYPKGESYEDLVQRLEPVIMELERQENVLVICHQ
AVMRCLLAYFLDKSAGELPY
LKCPLHTVLKLTPVAYGCKVESVFLNIEAVNTHRDRPENVDVDRDPEEALETVPDHI
>g332.t1
MQSSVAYVPQQAWIQNATLKDNIIFGQEWQESWYHRVVEACALLPDLDILPAGDNTEIGEKGVNLSGGQKQRVSMARAVY
CNCAVYLLDDSLSAVDAHVG
KHIFDNIIGPYGILKHRNVIKNMMDGHKPSNKAELLDFLRQEWHKVTQQQCERLVEACRGA
>g332.t2
MQSSVAYVPQQAWIQNATLKDNIIFGQEWQESWYHRVVEACALLPDLDILPAGDNTEIGEKGVNLSGGQKQRVSMARAVY
CNCAVYLLDDSLSAVDAHVG
KHIFDNIIGPYGILKHRVGSF
>g332.t3

MGEFVSVEHEAVKTLYNKSLFLEAKQCCVAYVPQQAWIQNATLKDNIIFGQEWQESWYHRVVEACALLPDLDILPAGDNT
EIGEKGVNLSGGQKQRVSMA
RAVYCNCAVYLLDDSLSAVDAHVGKHIFDNIIGPYGILKHRNVIKNMMDGHKPSNKAELLDFLRQEWHKVTQQQCERLVE
ACRGA
>g333.t1
MMVPQPRCHLGIPVTSCQQVCRQADGGQ
>g334.t1
MCLPEEDVYGEIMQQADVSALRLFEALVVTLPQTLLQTYVLICTDIGIKSPASVCFVVCLISLAWALVLYARACSLIRPG
HLPMTPAAILCRLLWR
>g334.t2
TSGLWLSPIRLWECMCLPEEDVYGEIMQQADVSALRLFEALVVTLPQTLLQTYVLICTDIGIKSPASVCFVVCLISLAWA
LVLYARACSLIRPGHLPMTP
AAILCRLLWR
>g335.t1
MEQFLQKLRARVRVGVVGGSDLNKIKEQLGEDVIQRVDYLFAENGLVAYKDGQLLSVQRGWSSLPLKEMSRMDDSLAIIL
LCPTSSMESREHPRTLALLT
SFKIGGFLLRKSTTISLVFLAFRWGR
>g335.t2
HVTPDMEQFLQKLRARVRVGVVGGSDLNKIKEQLGEDVIQRVDYLFAENGLVAYKDGQLLSVQRGWSSLPLKEMSRMDDS
LAIILLCPTSSMESREHPRT
LALLTSFKIGGFLLRKSTTISLVFLAFRWGR
>g335.t3
MEQFLQKLRARVRVGVVGGSDLNKIKEQLGEDVIQRVDYLFAENGLVAYKDGQLLSVQSIQTHMGEKLLQEFINFCLSYL
ATIKLPKKSKIGGFLLRKST
TISLVFLAFRWGR
>g336.t1
MKMPDPPEYVVYTDSELDMVRSQYFALLCNGKEKNYKMSKELCCRLLTIYTKMYKRLQNMRSPRKRQGSVPQRGAAKTAL
FSADNQDMETDWTDTSFSSD
NTILLESNDDISEDNSAEAKEWHQNITAAIRVSLMLCTHDCDCFTNHVAKATAAALTDFFPSLTSAAALSISVRGVLPQV
VFLVYCLGMMAFLQC
>g336.t2
MPDPPEYVVYTDSELDMVRSQYFALLCNGKEKNYKMSKELCCRLLTIYTKMYKRLQNMRSPRKRQGSVPQRGAAKTALFS
ADNQDMETDWTDTSFSSDNT
ILLESNDDISEDNSAEAKEWHQNITAAIRVSLMLCTHDCDCFTNHVAKATAAALTDFFPSLTSAAALSISVRGVLPQVVF
LVYCLGMMAFLQC
>g336.t3
MPSTSSMPPCQPQASAPISAEKTMKMPDPPEYVVYTDSELDMVRSQYFALLCNGKEKNYKMSKELCCRLLTIYTKMYKRL
QNMRSPRKRQGSVPQRGAAK
TALFSADNQDMETDWTDTSFSSDNTILLESNDDISEDNSAEAKEWHQNITAAIRVSLMLCTHDCDCFTNHVAKATAAALT
DFFPSLTSAAALSISVRGVL
PQVVFLVYCLGMMAFLQC
>g337.t1
MRATVPRRRPTPPQRALVSWRNQAIRLRPPARWRSQRVPAERAVSGEPVRGVAGRCPGSGNDNNFVTERRLQGAAARSLA
>g338.t1
GVGLLQGPGSASSNAAANSSPAGFLPPVRVSMVKLQPEDPGEESERVCFNIGRELYFYTYTNIKKAVDLSKPIDKRIYKG
TQPTCHDFNQYSATAESVAL
IVGFSAGQVQYLDPIKKETSKLFNEERLIDKSKVTCLKWLPKSENLFLASHASGHLYLYNVDHPCGTAAPQYSLLRQGEG
FAVYACKTKTPRNPLLRWAV
GEGGLNEFAFSPDGVHVACVGQDGCLRVFHFDSMELQGVMKSYFGGLLCVSWSPDGKYLATGGEDDLVTVWSFAESRVVA
RGHGHKSWVNVVAFDPFTTS
LEDEEPMELSGSEEDLHQGVAPPAPPPPPPAAAPAAAAAAATVPPASSMHFGRIRTSSTLSRLSRHSSKGGSPSVTYRFG
SVGQDTQFCLWDLTDDVLYP
RLPLSRALTSTLAPSLSHSGSGVVNSTTATVVGGVEGHHHPPNTTAASTGAGVVGAIPPTLPLPLP
>g339.t1
RSGVKSAGLATKPAGISAISCSPE
>g340.t1

MKVWPHSLAMHSYRSPTFCRHCGEMLWGLVHTNHQTLVLIYIMPASWVEPLVWLGVGYGDAVKTQVPLTFHIHSYTKPTV
CQYCFNGLFQCTDSAASASD
RGEREGDTESELVSVTSLYSGDEEMAADEDDDPLANGQEALVQQLPIR
>g340.t2
LSPPDLFEHRAGGPVCPLTRLSSPHLMKVWPHSLAMHSYRSPTFCRHCGEMLWGLVHTNHQTLVLIYIMPASWVEPLVWL
GVGYGDAVKTQVPLTFHIHS
YTKPTVCQYCFNGLFQCTDSAASASDRGEREGDTESELVSVTSLYSGDEEMAADEDDDPLANGQEALVQQLPIR
>g340.t3
MKVWPHSLAMHSYRSPTFCRHCGEMLWGLVHTNHQTLVLIYIMPASWVEPLVWLGVGYGDAVKTQVPLTFHIHSYTKPTV
CQYCFNGLFQCTDSAASASD
RGEREGDTESELVSVTSLYSGDEEMAADEDDDPLANGQEALVQQLPISCSAFEFCAHARELHAACYSTPTAEFAF
>g341.t1
MLTVTNRVKLTCITNTAAKITGLPTTNLSELNSPQNESWARTVLPPLALGGPSPVQSSGPLQLNEREKSQLFEMLGRTIF
APHCSVVGQGERVLLFRPRA
SSQQQGLRRMTQ
>g341.t2
MLTVTNRVKLTCITNTAAKITGLPTTNLSELNSPQNESWARTVLPPLALGGPSPVQSSGPLQLNEREKSQLFEMLGRTIF
WWNELPANVRTAESLPIFRK
RLKTHLFRAPHCSVVGQGERVLLFRPRASSQQQGLRRMTQ
>g342.t1
MGKTADLTAVQKAIIDTLKQEGKTQKEISERIGCSQSAVSRHLSGKSWKEKVWQKSCTTRRGDRTLRKIVEKGRFQTLGD
LRKQWTESGVETQSHRAQAC
AGNGLQVPHSP
>g343.t1
MAEVFPKGVHKHFKCKGFFCNINVTEYLATHKPDKPILGLSMVTVCLSSQLTILFYLCHLCEETCTRGNIMDHIFSAAHL
INYYVVHNGRRKRGCSEQVT
LQTLWFMLSDILYCKTYTDPDVLSFSWIPGGNMFRALKLAAVEHIKKNGNGVLQVLDLPRDLFNVTRRLPYAAASIPERM
SLQSYEADPGRKHTLLGQYT
QQSTPRAAPPTGG
>g343.t2
MAEVFPKGVHKHFKCKGFFCNINVTEYLATHKPDKPILGLSMVTVCLSSQLTILFYLCHLCEETCTRGNIMDHIFSAAHL
INYYVVHNGRRKRGCSEQVT
LQTLWFMLSDILYCKTYTDPDVLSFSWIPGGNMFRALKLAAVEHIKKNGNGVLQVLDLPRDLFNVTRRLPYAAGTSMLVL
LELSDPGP
>g343.t3
MAEVFPKGVHKHFKCKGLSMVTVCLSSQLTILFYLCHLCEETCTRGNIMDHIFSAAHLINYYVVHNGRRKRGCSEQVTLQ
TLWFMLSDILYCKTYTDPDV
LSFSWIPGGNMFRALKLAAVEHIKKNGNGVLQVLDLPRDLFNVTRRLPYAAASIPERMSLQSYEADPGRKHTLLGQYTQQ
STPRAAPPTGG
>g344.t1
MQVRHIDDKGRGVFATRGFAKGEFVVEYNGDLLDTACAKKREAEYALDPATGCYMYYFQYHSKAYWDIDAEEELLYDYGD
RSKASLSAHPWLKH
>g345.t1
LWVECGGDAGLLDAGVFQSEKLCPLYMTSSDRRPQGEGEEEEEEEEQYAATSSLGGCGGDEGADGWEDSDKRFTLQELRD
VLLEKNELKAQVFMLEEELA
YYKSEDLEDEGGYFADAMDPDPPHPAPAAQPESGIRRLATLPSSTSQTERLLAPAEESDPEFRIVRVLEMFEAVVKQYNL
SVEELRLERDNLKSELDRII
RESCTENGTPTAGPNQLVIDLKDPNRPRFTMQELKEVLQERNQLKGQLMVAQEELQMYKR
>g345.t2
LWVECGGDAGLLDAGVFQSEKLCPLYMTSSDRRPQGEGEEEEEEEEQYAATSSLGGCGGDEGADGWEDSDKRFTLQELRD
VLLEKNELKAQVFMLEEELA
YYKSEDLEDEGGYFADAMDPDPPHPAPAAQPESGIRRLATLPSSTSQTERLLAPAEESDPEMFEAVVKQYNLSVEELRLE
RDNLKSELDRIIRESCTENG
TPTAGPNQLVIDLKDPNRPRFTMQELKEVLQERNQLKGQLMVAQEELQMYKR
>g345.t3
AVVATPEPVRRNSLWVECGGDAGLLDAGVFQSEKLCPLYMTSSDRRPQGEGEEEEEEEEQYAATSSLGGCGGDEGADGWE

DSDKRFTLQELRDVLLEKNE
LKAQVFMLEEELAYYKSEDLEDEGGYFADAMDPDPPHPAPAAQPESGIRRLATLPSSTSQTERLLAPAEESDPEFRIVRV
LEMFEAVVKQYNLSVEELRL
ERDNLKSELDRIIRESCTENGTPTAGPNQLVIDLKDPNRPRFTMQELKEVLQERNQLKGQLMVAQEELQMYKR
>g346.t1
LYDLHHSFLVITFSGSSEEELLGHLENVREVAKRSGRHWARRRACFLLGRLCARRHKYSQARVYYEEALAVSVAGFADTP
LLTALYVNLTAVYLKQRMAD
KLPQTLEKACAALLCAPAHDFTSPDEMELLNLLLRRSVVQGDKHLEARLCYLAASLFLLLRKADDALPFAERLQFLMLNL
SDAEGRDAVAPLDLNWLLSQ
LYHRKYMPFLALASLSLDSRQDHTLPEAFRRVELFVRNSGRLNPRWREGATPLPAHIVVHLQRALAVAEQGKDWKTQRDL
CLGLAWVYQQYGVLDKAVRC
AQQAVETGGYINEEEGFQASVQLGWLLVLTGQTDRALDTLQPLLTSLQGTDSPAQRGVIHNLLALCLRRQARVREAGQHL
HSALAIARESGNERNQALAL
ANLGCLAIDTGASGLAERYLI
>g347.t1
MAPGMEAADIAVVGLYFVLVVSIGLVAMWKANRSTVSGYFLAGRSMTWVVIGASLFVSNIGSEHFIGLAGSGAASGYAVG
AWEFNSLFLLQLLGWVFIPV
YIHSGVYTMPEYLSRRYGGNRLKVYFAALSVLLYIFTKLSVDLYAGALFIQESLGWNIYLSIILLVSMTALLTVTGGLVA
VMYTDALQAVLMIGGALTLT
AISLVKVGGLDGVRTKYMNAVPNVSAIIASGNFSYNPSCRVEPNPNSLRIFRGPMDEDIPWPGFLLGQTPASIWYWCADQ
VIVQRVLAAKNIAHAKGATL
MAGVLKVLPMFLIVIPGMISRILFTDEIACIGPEHCMAVCGSKAGCSNIAYPRLVMSVMPVGLRGLMMAVMIAALMSDLD
SIFNSASTIFTLDIYQTARK
GASQKELLVVGRLFVLVMLVISIAWVPVIIEMQGGQMYLYIQEVAGYLTPPIAALFLLGVFWKRCNEKGAFWGGMTGLTL
GTLRLILAFVYREPRCDQPD
DRPALVARVHYMYVAAGLFWLSGLVAVVVSLCSCPPDPEQVRTTTVWGLRHIEKVPRKDPEEAHSLTDKHQCNGNLHKEL
AADVRNNGCLDGADVKLLVA
SADQDPATPSAETTPTTTPSEHSHRADQAEEGSNADGKRSTYLLLLDWFCGYKENQHNEKVTKEDERAIADMLYEPPWVK
VALNVGLVCVCSLGVVMFVY
FSL
>g348.t1
MISFLTEEDEVKFKSYCPKHSGLDEEDEEEEDQGCVADKRGKRRGRVRGEEEEQEEKKRGNLRKLKLQQLEEEFSMFVEV
EEVAEQLRLSSELVDFLFQY
WKLKRKANFNQPLLTPKNEEEESLARREQEVLLRRLQLFTHLRQDLERVRNLSYMVTRREKLKRSLCRLQEQMFHKQLLL
LEQLSGQSLPVTSL
>g348.t2
KNCRTAFHVTCGLQAKLKMISFLTEEDEVKFKSYCPKHSGLDEEDEEEEDQGCVADKRGKRRGRVRGEEEEQEEKKRGNL
RKLKLQQLEEEFSMFVEVEE
VAEQLRLSSELVDFLFQYWKLKRKANFNQPLLTPKNEEEESLARREQEVLLRRLQLFTHLRQDLERVRNLSYMVTRREKL
KRSLCRLQEQMFHKQLLLLE
QLSGQSLPVTSL
>g348.t3
CSAKNCRTAFHVTCGLQAKLKMISFLTEEDEVKFKSYCPKHSGLDEEDEEEEDQGCVADKRGKRRGRVRGEEEEQEEKKR
GNLRKLKLQQLEEEFSMFVE
VEEVAEQLRLSSELVDFLFQYWKLKRKANFNQPLLTPKNEEEESLARREQEVLLRRLQLFTHLRQDLERVRNLSYMVTRR
EKLKRSLCRLQEQMFHKQLL
LLEQLSGQSLPVTSL
>g349.t1
LVVRLPDPWGPLGGESVELPLLRLLSGFNLLPFEEEEEEEEEDDEEPSEKFLSRSVAEGSAEEHKGQIITLRSSREDHTV
FHTKYDSVRNTIDDRKVK
>g350.t1
LPPSASRSSTIWRPGGGSGVTIDIPPSTEEEHNLAPLLGHGSSSQPKEDDGESDKQRSHLHIRTRARFAGPPCL
>g351.t1
RVAQVLWGQLDTERREQHISCVELFYRLHCLAPSASICEDIICQALLHKDKAVRLEALHRFSILWHLTREIQNNRTMSLN
RSFDSLGGSDGSVSAAAQCW
LVRGLSLNDAMRVLEPILLLLLHPNTQRCSIQNVKQNLTAGNLKALSSRGRTSTKGSAGSEAGPGGAVLDLVNPVDREAL

WKLLESDPEAPTPKVPDAVP
ASRSESEATGEDSGEEDPEPESEHTESADTSGAHVSAENSSSSSGLFRSSLEEGAVYNGLRRAESEHTQVSDSLSSEEED
DQELEAMARSRLLKQEREKR
EAIDSLFRHVLLYSEVGGWQHLLQGLGLLASLLRSSAQRPLVDALSLTALDTSSAAHLNLVSNLLQRHQQAQDGNGFYGR
LLSPISSPSIPPSLLLELLM
SLCLRFLRSHYPSYLSLGPRDLQGNHEVQVQSVEVLVRLVNQLGGVARRQEGNGARLEPIRRLLSECRVQQYTLLSLSAS
MYVSQRGADRTALRGAELLG
EKGGLSEESLVHLGAAGSQEQYPLQVELLKLIQALITLENSIAQGGAVPPAGGPLGHPEESRDSPVAGAPLAREWQSAVL
FQQSIKAAQYVQNQPITAQG
MFVSAAARALQPQYGYAMHPHWVSLLCSSLPYLGRSLGIILAPIISQICVNLDDLVKVYEHDGGKVNQSLSARKENIAPD
YPLTLLEGLTTITHHCLLDH
QKSLTACDPTDLRNARSAVIEELPHMLNSMTLLWAVLKREENLKRCSEVSSSSKHATSSVYFRSTK
>g351.t2
RVAQVLWGQLDTERREQHISCVELFYRLHCLAPSASICEDIICQALLHKDKAVRLEALHRFSILWHLTREIQNNRTMSLN
RSFDRSLCVVLDSLGGSDGS
VSAAAQCWLVRGLSLNDAMRVLEPILLLLLHPNTQRCSIQNVKQNLTAGNLKALSSRGRTSTKGSAGSEAGPGGAVLDLV
NPVDREALWKLLESDPEAPT
PKVPDAVPASRSESEATGEDSGEEDPEPESEHTESADTSGAHVSAENSSSSSGLFRSSLEEGAVYNGLRRAESEHTQVSD
SLSSEEEDDQELEAMARSRL
LKQEREKREAIDSLFRHVLLYSEVGGWQHLLQGLGLLASLLRSSAQRPLVDALSLTALDTSSAAHLNLVSNLLQRHQQAQ
DGNGFYGRLLSPISSPSIPP
SLLLELLMSLCLRFLRSHYPSYLSLGPRDLQGNHEVQVQSVEVLVRLVNQLGGVARRQEGNGARLEPIRRLLSECRVQQY
TLLSLSASMYVSQRGADRTA
LRGAELLGEKGGLSEESLVHLGAAGSQEQYPLQVELLKLIQALITLENSIAQGGAVPPAGGPLGHPEESRDSPVAGAPLA
REWQSAVLFQQSIKAAQYVQ
NQPITAQGMFVSAAARALQPQYGYAMHPHWVSLLCSSLPYLGRSLGIILAPIISQICVNLDDLVKVYEHDGGKVNQSLSA
RKENIAPDYPLTLLEGLTTI
THHCLLDHQKSLTACDPTDLRNARSAVIEELPHMLNSMTLLWAVLKREENLKRCSEVSSSSKHATSSVYFRSTK
>g352.t1
MRQGDTVPSDSTLLTGSHPEEPHSSVRITLCHFSRALTSSGLFRPRGMSHVGAGQGGVTHDGFSMRATEGHDPLRRASEL
GL
>g353.t1
MSSAVRQNFHQDCEAAINRQINLELYASYVYLSMSYYFDRDDIALHNFAKFFRHQSHEEREHAEKLMKLQNERGGRIFLQ
DVRKPEKDEWASGVEALESA
LKLEKSVNQSLLDMHKVASDHTDPHMCDFIETHFLDEQVKSIKELSDWVSNLRRMGAPQNGMAEYLFDKHTLGKESS
>g353.t2
MRYVAAYLLAALGGNESPKASDIKKILESVGIEADDTRMQKVISELNGKSINEVIAQGNGKLASMPAGGAVVAAASSGGA
ASGGAAAPAAVEEKKEEKKE
ESEESDDDMGFGLFD
>g353.t3
MRYVAAYLLAALGGNESPKASDIKKILESVGIEADDTRMQKVISELNGKSINEVIAQGNGKLASMPAGGAVVAAASSGGA
ASGGAAAPAAVRQNFHQDCE
AAINRQINLELYASYVYLSMSYYFDRDDIALHNFAKFFRHQSHEEREHAEKLMKLQNERGGRIFLQDVRKPEKDEWASGV
EALESALKLEKSVNQSLLDM
HKVASDHTDPHMCDFIETHFLDEQVKSIKELSDWVSNLRRMGAPQNGMAEYLFDKHTLGKESS
>g353.t4
MFVFQKMSSAVRQNFHQDCEAAINRQINLELYASYVYLSMSYYFDRDDIALHNFAKFFRHQSHEEREHAEKLMKLQNERG
GRIFLQDVRKPEKDEWASGV
EALESALKLEKSVNQSLLDMHKVASDHTDPHMCDFIETHFLDEQVKSIKELSDWVSNLRRMGAPQNGMAEYLFDKHTLGK
ESS
>g354.t1
MSWTVRDPKSKKPCSCTKSQCLKRYCDCFANGEVCSNCNCLNCCNNVEHPVERHHAILNCLDRNPEAFKFNLVSRQSGEV
KGQPVKGCNCKQSSCLKNYC
ECYKAKMVCSSTCKCVGCLNCGGNSEVKAGVQGSSEISPSKKMHKETCHPSLITLAVVADTCNCLLAQAGQAEREGCDHT
VAQRMILQEFGHCLTSIVES
I

>g354.t2
MSWTVRDPKSKKPCSCTKSQCLKRYCDCFANGEVCSNCNCLNCCNNVEHPVERHHAILAKMVCSSTCKCVGCLNCGGNSE
VKAGVQGSSEISPSKKMHKE
TCHPSLITLAVVADTCNCLLAQAGQAEREGCDHTVAQRMILQEFGHCLTSIVESI
>g354.t3
MVYPLCSKTRVSPGEWESEDSGARMSWTVRDPKSKKPCSCTKSQCLKRYCDCFANGEVCSNCNCLNCCNNVEHPVERHHA
ILNCLDRNPEAFKFNLVSRQ
SGEVKGQPVKGCNCKQSSCLKNYCECYKAKMVCSSTCKCVGCLNCGGNSEVKAGVQGSSEISPSKKMHKETCHPSLITLA
VVADTCNCLLAQAGQAEREG
CDHTVAQRMILQEFGHCLTSIVESI
>g355.t1
PEGEHHWSKHYPFCGGAFQSPIDIQTDLLRFDPSLRPIELHDYDLSANEPLTLGNNGHSGGVTYRRSSTTTQWPLCASLP
SVIRCSAQTRRMLRMFLSSP
PHRYSAAQLHFHWGSTSTPAGSEHLVNGKRFAAEMHIVHFNSDKYANVSTAVDKSDGLAVLGVLVEVGEFNPAFDQFLKF
INGIKYRDQKVKVPGFNIRA
LLPARLDEYYRYDGSLTTPPCYPSVLWTIFRTPMQISQKQFLSLASAVYSSHAQESNSVPLNGNYRKPQFSDNRVVLVSF
QEGRGLHGIRTVTSPLVRKQ
IIQQLLVGDLADLADEGLYQLLPSSPERPLAADKKRKAFNNKHGPRTRHANPKKQQLHPPKSRNNDHYTGHLALTEEPLC
YVSLEHQVLHQAKQSHTEGQ
LVHTLRDLVFPDLNLRSYLGCKSELDFSTIRHIIRGRPMDEVVELDGSLRRASVGLMKKSPSLASKQGVPATKNEGFSNA
AGQVGSQPQGFPHPWLLPME
WED
>g355.t2
PEGEHHWSKHYPFCGGAFQSPIDIQTDLLRFDPSLRPIELHDYDLSANEPLTLGNNGHSGGVTYRRSSTTTQWPLCASLP
SVIRCSAQTRRMLRSVHYNV
TMEILPIRCLFCRSATVQLSLPSRMFLSSPPHRYSAAQLHFHWGSTSTPAGSEHLVNGKRFAAEMHIVHFNSDKYANVST
AVDKSDGLAVLGVLVEVGEF
NPAFDQFLKFINGIKYRDQKVKVPGFNIRALLPARLDEYYRYDGSLTTPPCYPSVLWTIFRTPMQISQKQFLSLASAVYS
SHAQESNSVPLNGNYRKPQF
SDNRVVLVSFQEGRGLHGIRTVTSPLVRKQIIQQLLVGDLADLADEGLYQLLPSSPERPLAADKKRKAFNNKHGPRTRHA
NPKKQQLHPPKSRNNDHYTG
HLALTEEPLCYVSLEHQVLHQAKQSHTEGQLVHTLRDLVFPDLNLRSYLGCKSELDFSTIRHIIRGRPMDEVVELDGSLR
RASVGLMKKSPSLASKQGVP
ATKNEGFSNAAGQVGSQPQGFPHPWLLPMEWED
>g355.t3
PEGEHHWSKHYPFCGGAFQSPIDIQTDLLRFDPSLRPIELHDYDLSANEPLTLGNNGHSGGVTYRRSSTTTQWPLCASLP
SVIRCSAQTRRMLRSVHYNV
TMEILPV
>g356.t1
LRIPETWSGKEVHLLWESDGEAMVWRDEQPVQGLTKEGEKTSYILSECLGDEDQHSVTLYVEMACNGLFGAGQGSMIAAP
DPNRKYSVQKAELVVLRRDV
REMLTDFEMLIDIVKQLGEGEQRGYQALFTVNEMVNLCDPAEPDSFSKARSLAHSFFTQRNGHSQHTVHAMGHCHIDSAW
LWPYEETIRKCGRSWVTVVR
LMEKNPDFVFTCSQAQQFQWVKSWYPGLYTQIQHYVKKGQFIPFWLPDTFGYSAQLPQIMQGSGISCFLTQKLSWNLVNT
FPHNTFFWEGLDGSQVLTHF
PPGNSYEMKGRVDELVNTVRNNKDKGRANHSAALFGFGDGGGGPTQLMLDRLTRVRDTDGLPKVRMSTPDQLFSELLADS
ALLCTWSGELFLELHNGTYT
TQAQIKRGNRQCESLLHDVEVASSLALWRDQAFHYPVDQLQELWRLLLLNQFHDVIPGSCIEMVVEDALRYYQALVNAPS
IGGAIVKDTQPVTPVSVTVQ
FWASYRKPVTEVVEPVKVVSSGGLRGSVSFTLRISPNSTVTQEMVLDAMCPYLKFNTQVEWAESHKFLKVEFPVRVRSAS
ATYEIQFGHLQRPTHRNTSW
DWARFEVTDGGLSSLQWLVGNKARQ
>g356.t2
MIAAPDPNRKYSVQKAELVVLRRDVREMLTDFEMLIDIVKQLGEGEQRGYQALFTVNEMVNLCDPAEPDSFSKARSLAHS
FFTQRNGHSQHTVHAMGHCH
IDSAWLWPYEETIRKCGRSWVTVVRLMEKNPDFVFTCSQAQQFQWVKSWYPGLYTQIQHYVKKGQFIPFWLPDTFGYSAQ

LPQIMQGSGISCFLTQKLSW
NLVNTFPHNTFFWEGLDGSQVLTHFPPGNSYEMKGRVDELVNTVRNNKDKGRANHSAALFGFGDGGGGPTQLMLDRLTRV
RDTDGLPKVRMSTPDQLFSE
LLADSALLCTWSGELFLELHNGTYTTQAQIKRGNRQCESLLHDVEVASSLALWRDQAFHYPVDQLQELWRLLLLNQFHDV
IPGSCIEMVVEDALRYYQAL
VNAPSIGGAIVKDTQPVTPVSVTVQFWASYRKPVTEVVEPVKVVSSGGLRGSVSFTLRISPNSTVTQEMVLDAMCPYLKF
NTQVEWAESHKFLKVEFPVR
VRSASATYEIQFGHLQRPTHRNTSWDWARFEVTDGGLSSLQWLVGNKARQ
>g356.t3
LRIPETWSGKEVHLLWESDGEAMVWRDEQPVQGLTKEGEKTSYILSECLGDEDQHSVTLYVEMACNGLFGAGQGSMIAAP
DPNRKYSVQKAELVVLRRDV
REMLTDFEMLIDIVKQLGEGEQRGYQALFTVNEMVNLCDPAEPDSFSKARSLAHSFFTQRNGHSQHTVHAMGHCHIDSAW
LWPYEETIRKCGRSWVTVVR
LMEKNPDFVFTCSQAQQFQWVKSWYPGLYTQIQHYVKKGQFIPFLEGQRFFHQEFGIYCKEFWLPDTFGYSAQLPQIMQG
SGISCFLTQKLSWNLVNTFP
HNTFFWEGLDGSQVLTHFPPGNSYEMKGRVDELVNTVRNNKDKGRANHSAALFGFGDGGGGPTQLMLDRLTRVRDTDGLP
KVRMSTPDQLFSELLADSAL
LCTWSGELFLELHNGTYTTQAQIKRGNRQCESLLHDVEVASSLALWRDQAFHYPVDQLQELWRLLLLNQFHDVIPGSCIE
MVVEDALRYYQALVNAPSIG
GAIVKDTQPVTPVSVTVQFWASYRKPVTEVVEPVKVVSSGGLRGSVSFTLRISPNSTVTQEMVLDAMCPYLKFNTQVEWA
ESHKFLKVEFPVRVRSASAT
YEIQFGHLQRPTHRNTSWDWARFEVTDGGLSSLQWLVGNKARQ
>g357.t1
MSFLDFEHQRPLMQTLIWALISLHTPLCHMQAAGVIQRAYNLNFPLRLFKTSADAAPWSAFSVDAKAIVLETIKQAEDSR
GTLLVRLYESHGSSVTAVLR
TVLPLQQAWQ
>g358.t1
MPEGPELHLASLFVNRVCEGVVFTGPVRKSVVSKSCDVAFTCQAYRIRAASRGKEVKLTLTPIKSEGKEGGSEQPMDIVF
RFGMSGYFRFTSEDELPKHS
HLRFFSDEKPCRVLSFVDTRRFGSWQPNGSWQPDRGPCVMFEYEGFRKNVLSNLSNRAFDRPICEALLNQKYFNGIGNYL
RAEILFRQV
>g358.t2
LASLFVNRVCEGVVFTGPVRKSVVSKSCDVAFTCQAYRIRAASRGKEVKLTLTPIKSEGKEGGSEQPMDIVFRFGMSGYF
RFTSEDELPKHSHLRFFSDE
KPCRVLSFVDTRRFGSWQPNGSWQPDRGPCVMFEYEGFRKNVLSNLSNRAFDRPICEALLNQKYFNGIGNYLRAEILFRQ
V
>g359.t1
LSSEHTTGLCKSYEDKHSALQDKLRETSLRLGRLNSVSWRVDCILSSSELKEVNEPAVHLKLQAHGAESGSTETTVVSIS
ADKFRVLLTELKQAQAMMNA
LQ
>g359.t2
LPGLGSCRDQHVGENXHTTGLCKSYEDKHSALQDKLRETSLRLGRLNSVSWRVDCILSSSELKEVNEPAVHLKLQAHGAE
SGSTETTVVSISADKFRVLL
TELKQAQAMMNALQ
>g359.t3
LPKEHTTGLCKSYEDKHSALQDKLRETSLRLGRLNSVSWRVDCILSSSELKEVNEPAVHLKLQAHGAESGSTETTVVSIS
ADKFRVLLTELKQAQAMMNA
LQ
>g360.t1
IHKFDYVFAENGTVQFKEGKLISKQAIQNQIGEELLQDLINFCLRYMGLMKLPKKR
>g360.t2
IHKFDYVFAENGTVQFKEGKLISKQAIQNQIGEELLQDLINFCLRYMGLMKLPKKRYGPLPQTSRCTHSEHTYVRY
>g360.t3
IHKFDYVFAENGTVQFKEGKLISKQAIQNQIGEELLQDLINFCLRYMGLMKLPKKRYGPLPQTSRCTHSEHT
>g361.t1
MNHNVVGLAQCSARDSMISTADGGVAGVQDVPFSGTVLVRPEAVDNTRDDFRGPEFRRSRVCRKDFPRRRGAGGQDYETV

SCTACGQQVNHFCCHPVLKV
LICEPGSLLKTAIMGKTADLTAVQKAIIDTLKQE
>g362.t1
PISDCIVGALSNREYGEGGTRRDKKEHEDDEV
>g363.t1
MNVIDHVRDMSVAGLHSNVRIMSSLLLTMSNNNPELFSPSQKYQLLVYHADAIFHDKEYRNAACKYSMALQQKKVISKTS
KVRTSTGGAASNVQAQSLPS
EIEVKYKIAECYTILKLDKDAIAVLDGIPSRQRTPKINMMLANLYRKAGQERSAVSSYKEVLRQCPLALDAIIGLLSLSV
KGAEVASMTMDVIQSIPNLD
WLSVWIKAYAFIHAGDNQRAINTICSLEKKSLLRDNVDLLVSLADVYFRAGDTKNSILKFEQAQMLDPYLIKGMDVYGYL
MAREGHLEDVEVLGGRLFNI
SDQHAEPWVISGCHSFYSKRYSRALYLGAKAIQLNSNSVQALLLKGAALRNMGRVQEAIIHFREAMRLAPCRLDCYEGLI
DCYLASNGIREAMGMANNIY
KTLGANAQTLTILATVCLEDPVTQEKAKALLDKALAQRPDYTKAVVKKAELLSREQKYEEGIALLRNALANQSDCVLHRM
LGDFLVAVNDYQEAMDQYSI
ALSLDPNDQKSLEGMQKMEKEESPTDATVELDGDDMEGSGEEGDLEGSDSEAAQWADQEQWFGMQ
>g363.t2
LLVYHADAIFHDKEYRNAACKYSMALQQKKVISKTSKVRTSTGGAASNVQAQSLPSEIEVKYKIAECYTILKLDKDAIAV
LDGIPSRQRTPKINMMLANL
YRKAGQERSAVSSYKEVLRQCPLALDAIIGLLSLSVKGAEVASMTMDVIQSIPNLDWLSVWIKAYAFIHAGDNQRAINTI
CSLEKKSLLRDNVDLLVSLA
DVYFRAGDTKNSILKFEQAQMLDPYLIKGMDVYGYLMAREGHLEDVEVLGGRLFNISDQHAEPWVISGCHSFYSKRYSRA
LYLGAKAIQLNSNSVQALLL
KGAALRNMGRVQEAIIHFREAMRLAPCRLDCYEGLIDCYLASNGIREAMGMANNIYKTLGANAQTLTILATVCLEDPVTQ
EKAKALLDKALAQRPDYTKA
VVKKAELLSREQKYEEGIALLRNALANQSDCVLHRMLGDFLVAVNDYQEAMDQYSIALSLDPNDQKSLEGMQKMEKEESP
TDATVELDGDDMEGSGEEGD
LEGSDSEAAQWADQEQWFGMQ
>g364.t1
TGREFDDLSPAAQRDAVTNARCFARVEPSHKSKIVEFLQGFDEITAMTGDGVNDAPALKKAEIGIAMGSGTAVAKSASEM
VLADDNFSSIVAAVEEGRAI
YNNMKQFIRYLISSNVGEVVCIFLTAALGFPEALIPVQLLWVNLVTDGLPATALGFNPPDLDIMDRPPRNAKEPLISGWL
FFRYLAIGGYVGAATVGAAA
WWFTVAEDGPQVTLYQLSHFLQCSSDNPDFDGLDCQVFESPYPMTMALSVLVTIEMCNALNSLSENQSLLRMPPWENVWL
LGAICLSMSLHFLILYVEPL
PVIFQITPLDGTQWVMVLKISLPVILLDEVLKFMARNYLESGKQPDEPAAKGCSLSACVQGISWPFVALCLPLVVWIYSI
DTNASAMFWP
>g365.t1
VNWVGEVNLNDLAMGRLPNIQVPMLVKNKSFYQISYKDQRASVDTSFYIVPCPQAPSQLKVTLDQASVKMGDVLPGDIHL
QLLDQFGNLTQKMTPQCVEH
IKVEGEELDSCLDVSWQESSKEVVVRGVRFRPGPPGPRELTFSLRTFVKQVKVMVTAGLPAKLQLVSGPRQPIQVLNLHG
ITSPFLIQLCDTWGNPSKDQ
RVVVVLAASSPSLKVMSSVMSKPLDEEGKASFMVDHVAGPKGQFSLEFSGLFNQKPIPGPSVPLTVVLDPNKPASLAVDY
DSNASITAGGTLPVTVVSEE
GGPVGDLSPAAIDMKLWKGETSTLTPPARDPHTSISTTSWPPSPLTTHLRLHLHHIITSTHNTPPSAPHHDLHHHSQHTS
FTTSTTSSPAPPTALHLRHP
YYTSFTTSSTPHHHHYPNNTPITISTTTITTTSFSTISSTPIHHHHTHHHSL
>g365.t2
VNWVGEVNLNDLAMGRLPNIQVPMLVKNKSFYQISYKDQRASVDTSFYIVPCPQAPSQLKVTLDQASVKMGDVLPGDIHL
QLLDQFGNLTQKMTPQCVEH
IKVEGEELDSCLDVSWQESSKEVVVRGVRFRPGPPGPRELTFSLRTFVKQVKVMVTAGLPAKLQLVSGPRQPIQVLNLHG
ITSPFLIQLCDTWGNPSKDQ
RVVVVLAASSPSLKVMSSVMSKPLDEEGKASFMVDHVAGPKGQFSLEFSGLFNQKPIPGPSVPLTVVLDPNKPASLAVDY
DSNASITAGGTLPVTVVSEE
GGPVGDLSPAAIDMKLWKGETSTLTPPARHHIMASITTHNTPPSPPPPHHHLHPQHTSISTTS
>g365.t3

VNWVGEVNLNDLAMGRLPNIQVPMLVKNKSFYQISYKDQRASVDTSFYIVPCPQAPSQLKVTLDQASVKMGDVLPGDIHL
QLLDQFGNLTQKMTPQCVEH
IKVEGEELDSCLDVSWQESSKEVVVRGVRFRPGPPGPRELTFSLRTFVKQVKVMVTAGLPAKLQLVSGPRQPIQVLNLHG
ITSPFLIQLCDTWGNPSKDQ
RVVVVLAASSPSLKVMSSVMSKPLDEEGKASFMVDHVAGPKGQFSLEFSGLFNQKPIPGPSVPLTVVLDPNKPASLAVDY
DSNASITAGGTLPVFSVTVV
SEEGGPVGDLSPAAIDMKLWKGETSTLTPPARDPHTSISTTSWPPSPLTTHLRLHLHHIITSTHNTPPSAPHHDLHHHSQ
HTSFTTSTTSSPAPPTALHL
RHPYYTSFTTSSTPHHHHYPNNTPITISTTTITTTSFSTISSTPIHHHHTHHHSL
>g366.t1
MEGQPDNDEDTEHHGHSPSGTRSPQSDAGNQRLQLSGHALHSERPVVKQEVPEREDYDLRLSRVQCFSMAAHFPQLKSEH
EDAVAVCREHHGMSGQALSA
DGPVRASQSRGPEGEDSSKSPVDPVSFKASSEACRSSLEVFLNSPSAASSPGLMMSVSPIPSSSAPISPSLPAAGSSSTT
NHSLEPGQHAVLLQSPKANR
STQRRNEKMANLNNIIHRLERAANREETLEWEF
>g367.t1
DAASFPFSSSPDSPCGRGAAARRGSLGVPRLPHDGEDKDEGAGAVRMPRGASGGVGSQHLTPSAILYRAAITRKQRRGVS
SEEEEGEVDSEVELPRRRRP
TSITKCPSVSTFSSENLSVSDGEEEGNTTDHSHSGTPDVVSTNTDERLDDKSDDLLSQGSEIPADPADPRQLASDGLSEK
EAALQHVKTQLTTDHNSYQV
GAQGSAGRTRR
>g368.t1
MTSQIRQNFHQDCEAAVNRQINLELYASYVYLSMSYYFERDDKSLPNLAKFFRSQSKEEREHAEKLMALQNQRGGRIFLQ
DIRKPDRDEWGSAVEALDCS
LQLEKSVNQSLLDVHKIASTHNDPHMCDFIETHYLDEQVKSIKELSDWVTNLRRMGAPENGMAEYLFDKHTLGGESA
>g369.t1
MMFSSGATASVGFDPPVLNVYGIGAVALSSVLTRLDRVSCGPPRRKRIEVFT
>g369.t2
MFSSGATASVGFDPPVLNVYGIGAVALSSVLTRLDRVSCGPPRRKRIEVFT
>g370.t1
MDLGMGSEKGSAEIQYGSSFQSNRSGQTVTYLGKFAFDTPPSGGIGGSGWCSDNNIISLVSAGILGVSPSPGSATTQTSS
SAANMGGQAADMEQVYGPPL
PAYSTCSDLYQEQVSFHHSPATSTALAYPGNEYHSASKPSMDGSLFSMIPDYNLFHHQGDVGVMEHKPFQTMDPIRVNPP
PITPLETIRAFKDKQQIHPG
FMGGQQHAPQHHGPPQTLTLKPIRPRKYPNRPSKTPVHERPHACPAENCDRRFSRSDELTRHLRIHTGHKPFQCRICMRS
FSRSDHLTTHIRTHTGEKPF
SCEFCGRKFARSDERKRHAKVHLKQKDKKQADKGSGAAGSHSSPPSSCGSGGPNSGNIMTVTTCA
>g371.t1
MFYESVVASAIFFAVSCWGSGMRVGDINRLNRLFRRAGDVVGEELDTLTIVAERRMLSRLRSILDNASHPLFSTLAQRRS
TFSRRLLSPRCTTERHRKSF
LPVAIKLYNHLPLSVCHSLPYVVTIALVVTS
>g372.t1
MHSRLQELKKEEETLLKIKVMLQDQLNRLKVQVNPDDASEINQTKLLLSAPMDYEEEEEEEDDDEEEDDDDYDEEDGDED
NDLEFVPEEDYEE
>g373.t1
MLLRCAVQVCHVLLLVLCVSAAEDFHWTKNERTSFYYGTFPAGFSWGAGSSAYQTEGAWDKDGKGLSIWDVFSHKKGRVQ
RNDTGDSSGEGYYKVKDDVS
LMNELKLNHYRFSISWPRLIPTGVKCLVGISLSGDWGEPVDINNQKDIEAAERYVQFYLGWFATPIFHGDYPQVMKDFIG
K
>g374.t1
LWFLDGGSHLIVDVHVSAYAGRKSTQQGMGTSRLPNFTSQEKGYIKGTCDFLGIGHFTTRYITQKNHPAGHSGASYFTDR
DLAELVDPRWPDPGSEWLYS
VPWGFRRLLNFVKAQYGNPMLYVTENGVSEKMTCTELCDDWRIKYYRDYINEMLKAIKDGVNVRGYTAWSLLDKFEWDEG
YSERFGLYYVDFRNKNKPRY
PKASVQYYKRIISSNGFPNQREVENWKRRATETCSSSNQLLAADPLTSHMEMVTEIVVPTVCTLCILLSAVFLMFLLRRR
N

>g375.t1
VPEEQVKEASDYCFETMEKINKSRMSGDYHEVVTMCRDCLKRAEPVLAETHIYLLRLWSTLSEVQAYLQFFDDAADLARK
MVDGYNKIYHPNNAALGLAT
MRAGVTHWQAGLIEVGHGMVCKAYAILMVTHGPTHPITKDLEAMRMQTEMEFRMFKQNEYVVYHSMREAALKNRPMTMMNE
PVSVQDGIKNLFHRRK
>g376.t1
MAFTGKYQLESQENFESFMKKIGLPDELIQKGKDIKSISEIEENGDTFKVTVTTGSKVLVNAFTVGQEADLETVTGEKVK
AVVKRDANKLKVSLKGIESV
TELVDANTIVNTMTIGDIVYKRISKRV
>g376.t2
MGSDSTFSPQGEGLGYLNWQQLWLIIVPRGRAVYKTGSWRSLSFTLAACHTPITMAFTGKYQLESQENFESFMKKIGLPD
ELIQKGKDIKSISEIEENGD
TFKVTVTTGSKVLVNAFTVGQEADLETVTGEKVKAVVKRDANKLKVSLKGIESVTELVDANTIVNTMTIGDIVYKRISKR
V
>g376.t3
ACHTPITMAFTGKYQLESQENFESFMKKIGLPDELIQKGKDIKSISEIEENGDTFKVTVTTGSKVLVNAFTVGQEADLET
VTGEKVKAVVKRDANKLKVS
LKGIESVTELVDANTIVNTMTIGDIVYKRISKRV
>g377.t1
AELSRREEVARRSLLVKVLYNEKEVSETQRRALNSDFRVHFGQIFNLKILNCPESIKLQVFEEVGSSSSLLAEVCVPVPE
PSVLTGRTPTEELEFSSNQR
VTFHHEGLGSVV
>g377.t2
AELSRREEVARRSLLVKVLYNEKEVSETQRRALNSDFRVHFGQIFNLKILNCPESIKLQVFEEVGSSSSLLAEVCVPVPE
PSVLTGRTPTEELEFSSNQR
VTFHHEGLGSALCQQCRTGPVDRTGTQRRKKTTC
>g377.t3
DQGLPDQVVYLTWGDALRPGVTFLIRGLLCVRAELSRREEVARRSLLVKVLYNEKEVSETQRRALNSDFRVHFGQIFNLK
ILNCPESIKLQVFEEVGSSS
SLLAEVCVPVPEPSVLTGRTPTEELEFSSNQRVTFHHEGLGSVV
>g378.t1
MGVQGFQEYIETHCPNAVVPVELQKLARGSMVGGGRQRPPQSPLRLLVDAENCLHRLYGGFYTDWVSGGQWNHMLGYLAA
LSKACFHGNIELLVCFNGAL
EKGRLHEWVKRQVNERQTAQQIVSHVQNKGTPPPKVWFLPPVCMAHCIRLALLRFRIGVSAVARSPEVVQSIEDHHLEVI
ALCRESGFHGLVAYDSDYAL
VNMPCYFSAHALKLSRNGKSLTTSQYLMHLVAKRLHLNPNRFVIFASLLGNHILPDEDLAAFHWSLLGPEHPLASLKVRA
HQLVLPPCDVVIKAVAEYVR
NVPDPSDLDTIAKDIFTHSQVGHVLIFTRSQIENN
>g378.t2
MGVQGFQEYIETHCPNAVVPVELQKLARGSMVGGGRQRPPQSPLRLLVDAENCLHRLYGGFYTDWVSGGQWNHMLGYLAA
LSKACFHGNIELLVCFNGAL
EKGRLHEWVKRQVNERQTAQQIVSHVQNKGTPPPKVWFLPPVCMAHCIRLALLRFRIGVSAVARSPEVVQSIEDHHLEVI
ALCRESGFHGLVAYDSDYAL
VNMPCYFSAHALKLSRNGKSLTTSQYLMHLVAKRLHLNPNRFVIFASLLGNHILPDEDLAAFHWSLLGPEHPLASLKVRA
HQLVLPPCDVVIKAVAEYVR
NVPDPSDLDTIAKDIFTHSQVGHVLIFTRSQCTSILHIARRTQWDPVVAHLSPDPQIENN
>g378.t3
MGVQGFQEYIETHCPNAVVPVELQKLARGSMVGGGRQRPPQSPLRLLVDAENCLHRLYGGFYTDWVSGGQWNHMLGYLAA
LSKACFHGNIELLVCFNGAL
EKGRLHEWVKRQVNERQTAQQIVSHVQNKGTPPPKVWFLPPVCMAHCIRLALLRFRIGVVQSIEDHHLEVIALCRESGFH
GLVAYDSDYALVNMPCYFSA
HALKLSRNGKSLTTSQYLMHLVAKRLHLNPNRFVIFASLLGNHILPDEDLAAFHWSLLGPEHPLASLKVRAHQLVLPPCD
VVIKAVAEYVRNVPDPSDLD
TIAKDIFTHSQVGHVLIFTRSQIENN
>g379.t1
MSLRADRVSHHAPDHINSFFVLCCSFRTFPRAAVEVKNEPILGFTEGSAERKDLLKALSALKGKTEEIPCVVGDEHVWTK

DVRYQLSPFDHSHKVAKFCY
ADKELLNKAILASVAARREWDLKPVRDRAQILFKAADVISGPKRAEILAKTMIGQAKTVVQAEIDAAPELIDFFRFNAKH
AIELEDQQPLDADGSTNTML
YRGLEGFVAAVSPFNFTAIGGNLAGTPTVMGNVVLWKPSDTAMSASYAVYKVLRECGLPPNIIQFVPSDGPVFGDTITSS
EHLAGINFTGSVPTFKRLWK
QVAQNLDIYRNFPRVAGECGGKNFHFVHASADVQSVVNGTIRSAFEYGGQKCSACSRMYVPDSLWPQIRDGLLAIHKNIK
PVEDFSSFFSAVIDDKSFAR
IKKWLDHARSSPDLTVIAGGNCDGQKGYFVEPTIIQVKDPQDPIMNEEIFGPVLSVYVYPDKDYKEVLTLVDNTSPYALT
GAVFAKDKSVVAEAAKALRN
AAGNYYVNDKSTGSIVAQQPFGGARASGTNDKPGGPHYVLRWTSPQVVKETFAPLASWEYPYMS
>g379.t2
MSLRADRVSHHAPDHINSFFVLCCSFRTFPRAAVEVKNEPILGFTEGSAERKDLLKALSALKGKTEEIPCVVGDEHVWTK
DVRYQLSELLNKAILASVAA
RREWDLKPVRDRAQILFKAADVISGPKRAEILAKTMIGQAKTVVQAEIDAAPELIDFFRFNAKHAIELEDQQPLDADGST
NTMLYRGLEGFVAAVSPFNF
TAIGGNLAGTPTVMGNVVLWKPSDTAMSASYAVYKVLRECGLPPNIIQFVPSDGPVFGDTITSSEHLAGINFTGSVPTFK
RLWKQVAQNLDIYRNFPRVA
GECGGKNFHFVHASADVQSVVNGTIRSAFEYGGQKCSACSRMYVPDSLWPQIRDGLLAIHKNIKPVEDFSSFFSAVIDDK
SFARIKKWLDHARSSPDLTV
IAGGNCDGQKGYFVEPTIIQVKDPQDPIMNEEIFGPVLSVYVYPDKDYKEVLTLVDNTSPYALTGAVFAKDKSVVAEAAK
ALRNAAGNYYVNDKSTGSIV
AQQPFGGARASGTNDKPGGPHYVLRWTSPQVVKETFAPLASWEYPYMS
>g379.t3
MSLRADRVSHHAPDHINSFFVLCCSFRTFPRAAVEVKNEPILGFTEGSAERKDLLKALSALKGKTEEIPCVVGDEHVWTK
DVRYQLSPFDHSHKVAKFCY
ADKELLNKAILASVAARREWDLKPVRDRAQILFKAADVISGPKRAEILAKTMIGQAKTVVQAEIDAAPELIDFFRFNAKH
AIELEDQQPLDADGSTNTML
YRGLEGFVAAVSPFNFTAIGGNLAGTPTVMGNVVLWKPSDTAMSASYAVYKVLRECGLPPNIIQFVPSDGPVFGDTITSS
EHLAGINFTGSVPTFKRLWK
QVAQNLDIYRNFPRVAGECGGKNFHFVHASADVQSVVNGTIRSAFEYGGQKCSACSRMYVPDSLWPQIRDGLLAIHKNIK
VGNPVEDFSSFFSAVIDDKS
FARIKKWLDHARSSPDLTVIAGGNCDGQKGYFVEPTIIQVKDPQDPIMNEEIFGPVLSVYVYPDKDYKEVLTLVDNTSPY
ALTGAVFAKDKSVVAEAAKA
LRNAAGNYYVNDKSTGSIVAQQPFGGARASGTNDKPGGPHYVLRWTSPQVVKETFAPLASWEYPYMS
>g380.t1
NPPVFEEQAYSSYVSENNKPGHSLCSVKAHDPDWRQNEGNSFMTELVPKAAQGGALVSKVIAVDGDSGQNAWLSYHIIKS
TDRDFQNRPAQRGDVDGTGT
LRSTYNYDAYLTTGSRTSDFKFVTSYNDNTLPADQTLRKNPTDFEEVFGDLDVSPESALVAPG
>g380.t2
NPPVFEEQAYSSYVSENNKPGHSLCSVKAHDPDWRQNEGNSFMTELVPKAAQGGALVSKVIAVDGDSGQNAWLSYHIIKS
TDRDFQNRPAQRGDQTLRKN
PTDFEEVFGDLDVSPESALVAPG
>g381.t1
MSHNAGCPSHYVYSSEPSVSSPAQSAGDTDPEAHSWNLEGAQEQDSTQHKCNSKAPCVSGVADESFHTLASYDVGLQAER
AASLDQPPAETVRNREQ
>g381.t2
MMHSSACKMCPSHYVYSSEPSVSSPAQSAGDTDPEAHSWNLEGAQEQDSTQHKCNSKAPCVSGVADESFHTLASYDVGLQ
AERAASLDQPPAETVRNREQ
>g381.t3
MKNILVFRCPSHYVYSSEPSVSSPAQSAGDTDPEAHSWNLEGAQEQDSTQHKCNSKAPCVSGVADESFHTLASYDVGLQA
ERAASLDQPPAETVRNREQ
>g382.t1
HLLHVFPSDIFTCAKSVRILRCPLGSMLSPVNTSKPHTLTPTLWVLRVHCADVQRVHAIGRQHESYRGQRVS
>g383.t1
PVLSKLLMADQEAPLDLTVKKPKAEPTEQDGVLDLSLRKNRYSSLPAVHSPYPSPHTPTVKGRSLRADGQVGLFMRSLQD
GRRENVGHSAYYKPFHRLAY

SLHIKEEVGLAQESEPESPPSQNHSSRTTDLSSDRSAPTLNSKTHFGALLKLRASRQASEHLKDLPRLLEATGLWPKSLA
VGKEALLQEAFGDQGLATSH
LSSFDLKIPQVRGLSTGKDQTWCSLTSEHSGTVSENGQGKALVSEQIRKDRSRVFNLPGTEKEYWPYESHQQGPGGHCAT
LDSESDLSSKQPRKKRGRYR
QYNTELLEEAIVVVMGGKMSVSKAQSVYGIPHSTLEYKVKERLGTLKHPPKKKLRLLSQADEQNALESPESKGDPQDSRD
TAFQQECPSTENGARLCNSG
LTPDEYSTMKPKCSDDDDDDDVDNEML
>g384.t1
MYVEEMWELKTVRTLAGRKKPYQRRNCPNPMPEIITLVTPKQALTKPDGVPPVDMIFAGSGGETCQQIPTEQSHKFAAEI
KHRSPNTDQSHSNTKSFCFR
KQSSVENYSS
>g385.t1
MHHPIQMKPADSEKNNAVEDRKLFIGMISKKCNENDIRLMFSPYGQIEECRILRGPDGLSRGCAFVTFTARQMALSAIKT
MHQSQTMEGCSSPIVVKFAD
TQKDKEQKRMAQQLQQQMQQLNAASMWGNLTGLNSLGPQYLAIAC
>g385.t2
MHHPIQMKPADSEKNNAVEDRKLFIGMISKKCNENDIRLMFSPYGQIEECRILRGPDGLSRGCAFVTFTARQMALSAIKT
MHQSQTMEDN
>g386.t1
MQNLAALAAAASATQATSSGSSAMTTSSSPLSMLTSSAGSSPTSSSNSSVNPMASLGALQSLAASAGAGLNMSSLAGMAA
LNGSLGSGSLSNGSGNTMEA
LSQAYSGIQQYAAAALPSLYNQSLLSQQSVSAAGSQKEGPEGANLFIYHLPQEFGDQDLLQMFMPFGNVISAKVFIDKQT
NLSKCFGFVSYDNPVSSQAA
IQSMNGFQIGMKRLKVQLKRSKNDSKPY
>g386.t2
MQNLAALAAAASATQATSSGSSAMTTSSSPLSMLTSSAGSSPTSSSNSSVNPMASLGALQSLAASAGAGLNMSSLAGCVT
GCLYHHHNSGMAALNGSLGS
GSLSNGSGNTMEALSQAYSGIQQYAAAALPSLYNQSLLSQQSVSAAGSQKEGPEGANLFIYHLPQEFGDQDLLQMFMPFG
NVISAKVFIDKQTNLSKCFG
FVSYDNPVSSQAAIQSMNGFQIGMKRLKVQLKRSKNDSKPY
>g386.t3
MQNLAALAAAASATQATSSGSSAMTTSSSPLSMLTSSAGSSPTSSSNSSVNPMASLGALQSLAASAGAGLNMSSLAGMAA
LNGSLGSGSLSNGSGNTMEA
LSQAYSGIQQYAAAALPSLYNQSLLSQQSVSAAGSQKEGANLFIYHLPQEFGDQDLLQMFMPFGNVISAKVFIDKQTNLS
KCFGFVSYDNPVSSQAAIQS
MNGFQIGMKRLKVQLKRSKNDSKPY
>g387.t1
MSLFLVHQNATSALVAARHASASTNLKDVLADLIPKEQTRIKNFKQQYGKTNIGQITVDMVYGGMRGMKGLVYETSVLDP
DEGIRFRGYSIPECQDLLPK
APGGQEPLPEGLFWLLVTGQVPTEEQVSWLSKEWAKRAALPSHVVTMLDNFPTNLHPMSQFSAAITALNSESGFARAYSE
GVHKSKYWEFVYEDSMDLIA
KLPCVAAKIYRNLYREGSSIGAIDSNLDWSHNFTNMLGYSDAQFTELMRLYLTIHSDHEGGNVSAHTSHLVGSALSDPYL
SFSAAMNGLAGPLHGLANQE
VLVWLTALQKELGGEVSDERMRDYIWNTLKSGRVVPGYGHAVLRKTDPRYTCQQEFAMKHLPNDPLFKMVAQLYKIVPNV
LLEQGKAKNPWPNVDAHSGV
LLQYYGMTEMNYYTVLFGVSRALGVLAQLVWSRALGFPLERPKSMSTDGLMGLVGAKSG
>g387.t2
MSLFLVHQNATSALVAARHASASTNLKDVLADLIPKEQTRIKNFKQQYGKTNIGQITVDMVYGGMRGMKGLVYETSVLDP
DEVRMLKGIRFRGYSIPECQ
DLLPKAPGGQEPLPEGLFWLLVTGQVPTEEQVSWLSKEWAKRAALPSHVVTMLDNFPTNLHPMSQFSAAITALNSESGFA
RAYSEGVHKSKYWEFVYEDS
MDLIAKLPCVAAKIYRNLYREGSSIGAIDSNLDWSHNFTNMLGYSDAQFTELMRLYLTIHSDHEGGNVSAHTSHLVGSAL
SDPYLSFSAAMNGLAGPLHG
LANQEVLVWLTALQKELGGEVSDERMRDYIWNTLKSGRVVPGYGHAVLRKTDPRYTCQQEFAMKHLPNDPLFKMVAQLYK
IVPNVLLEQGKAKNPWPNVD
AHSGVLLQYYGMTEMNYYTVLFGVSRALGVLAQLVWSRALGFPLERPKSMSTDGLMGLVGAKSG

>g388.t1
KEHTPHFPRNISAASLGSLIPHHHHHHHHHQATNHHHPHPAEPSVTDPLMGTTTVPGEAELRIDMEKNDAQFSQMKDSLG
TMHRSKSKHELKLLEKIPEN
AEATVVLVGSVDFLEQPTMAFVRLQEAVELDSVLEVPVPVRFLFVLLGPPSTSMDYHQIGRSISTLMSDKHFHEAAYLAD
DRQDLLNAINSFLDLSIVLP
PSEMGGDELLHSVARFQREMLRKREEQEVKLQEPKSMEEKEALLKPPRKEDDPLERTGRPFGGLVRDVLRRYPKYPSDFK
DALNPQCMAAVIFIYFAALS
PAITFGGLLEEKTGGLIGVSELIISTAFQGIVFTLLGAQPLLVVGFSGPLLVFEEAFYAFCKANEVEYLTGRVWIGFWLI
IIVTVMVAFEGSFLVRFVSR
FTQEIFSFLISLIFICETFIKLGKIFNDHPLKRCNSSDNSVNQTIANGTLRVLMPASGNFSAGPVRDEPNTALLSLVLMA
GTFLIAFYLRKFKNSAFFPG
RFRRMIGDFGVPIAILITVLVDYFIEDTYTQKLSVPSGFSVTSPKLRGWFINPLGSHKPFPIWMMFACVLPALLVFILIF
METQITTLIVSKKERMLVKG
SGFHLDLFLIVVLGGFSALFGLPWMAAATVRSVTHANALTVMSKAVAPGDKPRIQEVKEQRVTGLLVAILVGLSIVIGEL
LRKIPLAVLFGIFLYMGVMS
LNGIQLTERMMLLLMPPKYHPDHTYVRKVRTLRMHLFTCIQLVCLAALWVVMSTEASLAFPFVLILTVPVKMFLLPRIFT
PREIICLDADDAEPKFDERE
GHDEYDTMHMPV
>g388.t2
KEHTPHFPRNISAASLGSLIPHHHHHHHHHQATNHHHPHPAEPSVTDPLMGTTTVPGEAELRIDMEKNDAQFSQMKDSLG
TMHRSKSKHELKLLEKIPEN
AEATVVLVGSVDFLEQPTMAFVRLQEAVELDSVLEVPVPVRFLFVLLGPPSTSMDYHQIGRSISTLMSDKFHPDSPSLSP
LGPDQHFHEAAYLADDRQDL
LNAINSFLDLSIVLPPSEMGGDELLHSVARFQREMLRKREEQEVKLQEPKSMEEKEALLKPPRKEDDPLERTGRPFGGLV
RDVLRRYPKYPSDFKDALNP
QCMAAVIFIYFAALSPAITFGGLLEEKTGGLIGVSELIISTAFQGIVFTLLGAQPLLVVGFSGPLLVFEEAFYAFCKANE
VEYLTGRVWIGFWLIIIVTV
MVAFEGSFLVRFVSRFTQEIFSFLISLIFICETFIKLGKIFNDHPLKRCNSSDNSVNQTIANGTLRVLMPASGNFSAGPV
RDEPNTALLSLVLMAGTFLI
AFYLRKFKNSAFFPGRFRRMIGDFGVPIAILITVLVDYFIEDTYTQKLSVPSGFSVTSPKLRGWFINPLGSHKPFPIWMM
FACVLPALLVFILIFMETQI
TTLIVSKKERMLVKGSGFHLDLFLIVVLGGFSALFGLPWMAAATVRSVTHANALTVMSKAVAPGDKPRIQEVKEQRVTGL
LVAILVGLSIVIGELLRKIP
LAVLFGIFLYMGVMSLNGIQLTERMMLLLMPPKYHPDHTYVRKVRTLRMHLFTCIQLVCLAALWVVMSTEASLAFPFVLI
LTVPVKMFLLPRIFTPREII
CLDADDAEPKFDEREGHDEYDTMHMPV
>g388.t3
KEHTPHFPRNISAASLGSLIPHHHHHHHHHQATNHHHPHPAEPSVTDPLMGTTTVPGEAELRIDMEKNDAQFSQMKDSLG
TMHRSKSKHELKLLEKIPEN
AEATVVLVGSVDFLEQPTMAFVRLQEAVELDSVLEVPVPVRFLFVLLGPPSTSMDYHQIGRSISTLMSDKHFHEAAYLAD
DRQDLLNAINSFLDLSIVLP
PSEMGGDELLHSVARFQREMLRKREEQEVKLQEPKSMEEKEALLKPPRKEDDPLERTGRPFGGLVRDVLRRYPKYPSDFK
DALNPQCMAAVIFIYFAALS
PAITFGGLLAFDWLMVAEEKTGGLIGVSELIISTAFQGIVFTLLGAQPLLVVGFSGPLLVFEEAFYAFCKANEVEYLTGR
VWIGFWLIIIVTVMVAFEGS
FLVRFVSRFTQEIFSFLISLIFICETFIKLGKIFNDHPLKRCNSSDNSVNQTIANGTLRVLMPASGNFSAGPVRDEPNTA
LLSLVLMAGTFLIAFYLRKF
KNSAFFPGRFRRMIGDFGVPIAILITVLVDYFIEDTYTQKLSVPSGFSVTSPKLRGWFINPLGSHKPFPIWMMFACVLPA
LLVFILIFMETQITTLIVSK
KERMLVKGSGFHLDLFLIVVLGGFSALFGLPWMAAATVRSVTHANALTVMSKAVAPGDKPRIQEVKEQRVTGLLVAILVG
LSIVIGELLRKIPLAVLFGI
FLYMGVMSLNGIQLTERMMLLLMPPKYHPDHTYVRKVRTLRMHLFTCIQLVCLAALWVVMSTEASLAFPFVLILTVPVKM
FLLPRIFTPREIICLDADDA
EPKFDEREGHDEYDTMHMPV
>g389.t1
MARRAGILLWPAAAQKAYDRLPGEYKLGVDLVLEMRNSRAGVKHLSLFFKSMLQSDIQPGDGVIYIYRKFYLKETRCRNG

TVDTSNCLFRGKKPKIDCAI
CYQTFDGGIRPSPKPYVHCIRKAALTKAMTRVRENHCNYSSGAPVWLAGTGDIFSEGY
>g390.t1
MATVLLCLLAAGTLLVSAGAHEAYDELHETFKKGVDVAVDQVNSHVGISHHFLFLKTLAKYDTPAGFGVVYIYHNFYLKA
TRCPKGTVDNSNCAFRHDRP
VIDCAMCYKTYNGEIEATPKPYIHCIRRPSLTEDMTTARINHCNEMGYSSGAPTLLASRDP
>g391.t1
MCVLLFAVLAVGALLGPTQASDQYSQLAQNYRAGVDIVLEQLRAHVGIQTHFLFLRSIAKSDIEAGFGASYVFHHFHLKP
TSCAKGTVGTATAQTCPFRN
DREMTRTRVSSCRRMVYTSGSPTLLAVGTKHTDMFGVFGADID
>g391.t2
MCVLLFAVLAVGALLGPTQASDQYSQLAQNYRAGVDIVLEQLRAHVGIQTHFLFLRSIAKSDIEAGFGASYVFHHFHLKP
TSCAKGTVGTATAQTCPFRN
DRPLMDCQVCYKMFEGVIDDQPNPPYVHCIHKPKLTREMTRTRVSSCRRMVYTSGSPTLLAVGTKHTDMFGVFGADID
>g391.t3
LLVSAGAHEAYDELHKTVKKGVDVAVDQAGFGVVYIYHNFYLKATRCPKGTVDNSNCAFRHDRAGLGLSRPACGHAYAPL
HWPPEWKDMSEEFEIFNVLF
>g391.t4
LVSAGAHEAYDELHKTVKKGVDVAVDQAGFGVVYIYHNFYLKATRCPKGTVDNSNCAFRHDRAGLGLSRPACGHAYAPLH
WPPEWKDMSEEFEIFNVLF
>g391.t5
LLVSAGAHEAYDELHKTVKKGVDVAVDQAGFGVVYIYHNFYLKATRCPKGTVDNSNCAFRHDRASDQYSQLAQNYRAGVD
IVLEQLRAHVGIQTHFLFLR
SIAKSDIEAGFGASYVFHHFHLKPTSCAKGTVGTATAQTCPFRNDRPLMDCQVCYKMFEGVIDDQPNPPYVHCIHKPKLT
REMTRTRVSSCRRMVYTSGS
PTLLAVGTKHTDMFGVFGADID
>g392.t1
NTFRHGLLRSGLRTSLRTRLPRRPLLTQRDSPPGPPHPPPSLSVTSLSGGDSGQQPPRIHPGQPDGGPESSELCGFCRKT
VALAEPAIEALNRTYHDSCF
QCRQCQTPLAGQLYYNKAGIPLCEECYQASLELCWACGDTIKDLVIRALERTYHPACFTCATCHQQIGEQRFAQGEVGEV
YCIQDYYRKYAPQCSACKQL
IIPKEDGTDSYTVECQGSSFHEDCYRCEICAIQLSPDPNDHGCHPLDGRMLCKDCHLSLVSA
>g392.t2
RQADRHQDRRLDGNRELGALRGAGLRTSLRTRLPRRPLLTQRDSPPGPPHPPPSLSVTSLSGGDSGQQPPRIHPGQPDGG
PESSELCGFCRKTVALAEPA
IEALNRTYHDSCFQCRQCQTPLAGQLYYNKAGIPLCEECYQASLELCWACGDTIKDLVIRALERTYHPACFTCATCHQQI
GEQRFAQGEVGEVYCIQDYY
RKYAPQCSACKQLIIPKEDGTDSYTVECQGSSFHEDCYRCEICAIQLSPDPNDHGCHPLDGRMLCKDCHLSLVSA
>g392.t3
NTFRHGLLRSGLRTSLRTRLPRRPLLTQRDSPPGPPHPPPSLSVTSLRKGHHLVEGIQASSPPGSIPASRTAARRAAVRK
HRAMELCGFCRKTVALAEPA
IEALNRTYHDSCFQCRQCQTPLAGQLYYNKAGIPLCEECYQASLELCWACGDTIKDLVIRALERTYHPACFTCATCHQQI
GEQRFAQGEVGEVYCIQDYY
RKYAPQCSACKQLIIPKEDGTDSYTVECQGSSFHEDCYRCEICAIQLSPDPNDHGCHPLDGRMLCKDCHLSLVSA
>g393.t1
LFGLERSRSSETRERAYDHSPYGHHERSGTFDRQRHYNPDYYRDRSMFAAAGPGSSAMGPGFEASDPHFESRIRDPFTLA
SSARRDLYRDDRGRRVDRTY
HHRRSRSSHSSQSRHPSPQRSTGQAPKAPHSPKRAPLSPGRGPHSRSRSRSSSSDSVSSTSSTGSGSSDSNSSSSDGSRA
RSVQSSAAHAPPQSSMGLDS
EEPRRSFGIKVQNLPVRSTDTSLKDGLFHEFKKHGKVTSVQIHGATEDRYGLVFFRQQEDQEKALTVSKGKLFFGMLIEV
TAWNGPETESENEFRPLDGR
IDEFHPKATRTLFIGNLEKTTSYQQLLDIFQRFGEIVDIDIKKVNGVPQYAFVQYSDIASVCKAIKKMDGEYLGSNRLKV
LFDRLKGMALILYNNTDFAQ
AAVRETKGWKIGGNKIKVDFASQESQMAFYRSMQSSGQDIRDFYEIPPERREERRPPYHEFTAERAYYESVRTPGLYPED
ARREYSARSRERFPELEHFQ
GEPFDPRYHEDPREYRDFRDPFEQDIRKYTYIQRERERERERFEADRGRWSPSHPRRPITPTVSPSPSERAHRDSERRVY

SQSSERSGSASSLSPPHFEK
SDKTPLEHGSKGDKADKNSTADRVASAEKGKRARRKDKADKDKGEKIKSRKPKAQSPCDPPLDAEPEAGFDGASEKGLGS
VQDSTERQKFKVESDPLASN
QLPAVNPTETIKSERTEWAVGESDVDGKTRGKKHVKSENVNDVKESVDSDRLAARKRRFADASGRPIRQKRSRLDDEDGS
HPDFSASSACLKDVDGDKQQ
KDSERRDSRIKVEKNAFLGIPKEGQEEASGQREQSEVLVDVLDSKQHPGHNASRRFSHEGQGSTRDPELPGFKYQTADVE
KGTKDREDHVDIDLSQSYRK
QMEQNRRLHQQQQQQDFDKSDKPDSPQGGETEDLEHRSLVHDVGKPPEDVTDAFPSHKMCKKIDQFDNDASVKKERVYRI
FRQKTEEPEWANTASPGLHH
SPHHADEASVDQPQQKELSISDDKIHSDLELSLKQTHTTPIHKPNTPSLSIEEEQQKRWESRVKQDILPDLNFPRSLSKS
IHNRKCLGTPEDLNTWNVGT
RILNME
>g393.t2
LFGLERSRSSETRERAYDHSPYGHHERSGTFDRQRHYNPDYYRDRSMFAAAGPGSSAMGPGFEASDPHFESRIRDPFTLA
SSARRDLYRDDRGRRVDRTY
HHRRSRSSHSSQSRHPSPQRSTGQAPKAPHSPKRAPLSPGRGPHSRSRSRSSSSDSVSSTSSTGSGSSDSNSSSSDGSRA
RSVQSSAAHAPPQSSMGLDS
EEPRRSFGIKVQNLPVRSTDTSLKDGLFHEFKKHGKVTSVQIHGATEDRYGLVFFRQQEDQEKALTVSKGKLFFGMLIEV
TAWNGPETESENEFRPLDGR
IDEFHPKATRTLFIGNLEKTTSYQQLLDIFQRFGEIVDIDIKKVNGVPQYAFVQYSDIASVCKAIKKMDGEYLGSNRLKV
LFDRLKGMALILYNNTDFAQ
AAVRETKGWKIGGNKIKVDFASQESQMAFYRSMQSSGQDIRDFYEIPPERREERRPPYHEFTAERAYYESVRTPGLYPED
ARREYSARSRERFPELEHFQ
GEPFDPRYHEDPREYRDFRDPFEQDIRKYTYIQRERERERERFEADRGRWSPSHPRRPITPTVSPSPSERAHRDSERRVY
SQSSERSGSASSLSPPHFEK
SDKTPLEHGSKGDKADKNSTADRVASAEKGKRARRKDKADKDKGEKIKSRKPKAQSPCDPPLDAEPEAGFDGASEKGLGS
VQDSTERQKFKVESDPLASN
QLPAVNPTETIKSERTEWAVGESDVDGKTRGKKHVKSENVNDVKESVDSDRLAARKRRFADASGRPIRQKRSRLDDEDGS
HPDFSASSACLKDVDGDKQQ
KDSERRDSRIKVEKNAFLGIPKEGQEEASGQREQSEVLVDVLDSKQHPGHNASRRFSHEGQGSTRDPELPGFKYQTADVE
KGTKDREDHVDIDLSQSYRK
QMEQNRRLHQQQQQQDFDKSDKPDSPQGGETEDLEHRSLVHDVGKPPEDVTDAFPSHKMCKKIDQFDNDASVKKERVYRI
FRQKTEEPEWANTASPGLHH
SPHHADEASVDQPQQKELSISDDKIHSDLELSLKQTHTTPIHKPNTPSLSIEEEQQKRWESRVKQDILPDLNFPRSLSKS
IHNRKCLGYGVWNDLDSGEV
RTPEDLNTWNVGTRILNME
>g393.t3
MGDRDLRTDYNEPGSVPSAVRGLEDNPPSSSRDVTGFSRGTVGPVFGPPLSLHTREGRYERRIDGSSETRERAYDHSPYG
HHERSGTFDRQRHYNPDYYR
DRSMFAAAGPGSSAMGPGFEASDPHFESRIRDPFTLASSARRDLYRDDRGRRVDRTYHHRRSRSSHSSQSRHPSPQRSTG
QAPKAPHSPKRAPLSPGRGP
HSRSRSRSSSSDSVSSTSSTGSGSSDSNSSSSDGSRARSVQSSAAHAPPQSSMGLDSEEPRRSFGIKVQNLPVRSTDTSL
KDGLFHEFKKHGKVTSVQIH
GATEDRYGLVFFRQQEDQEKALTVSKGKLFFGMLIEVTAWNGPETESENEFRPLDGRIDEFHPKATRTLFIGNLEKTTSY
QQLLDIFQRFGEIVDIDIKK
VNGVPQYAFVQYSDIASVCKAIKKMDGEYLGSNRLKVLFDRLKGMALILYNNTDFAQAAVRETKGWKIGGNKIKVDFASQ
ESQMAFYRSMQSSGQDIRDF
YEIPPERREERRPPYHEFTAERAYYESVRTPGLYPEDARREYSARSRERFPELEHFQGEPFDPRYHEDPREYRDFRDPFE
QDIRKYTYIQRERERERERF
EADRGRWSPSHPRRPITPTVSPSPSERAHRDSERRVYSQSSERSGSASSLSPPHFEKSDKTPLEHGSKGDKADKNSTADR
VASAEKGKRARRKDKADKDK
GEKIKSRKPKAQSPCDPPLDAEPEAGFDGASEKGLGSVQDSTERQKFKVESDPLASNQLPAVNPTETIKSERTEWAVGES
DVDGKTRGKKHVKSENVNDV
KESVDSDRLAARKRRFADASGRPIRQKRSRLDDEDGSHPDFSASSACLKDVDGDKQQKDSERRDSRIKVEKNAFLGIPKE
GQEEASGQREQSEVLVDVLD
SKQHPGHNASRRFSHEGQGSTRDPELPGFKYQTADVEKGTKDREDHVDIDLSQSYRKQMEQNRRLHQQQQQQDFDKSDKP

DSPQGGETEDLEHRSLVHDV
GKPPEDVTDAFPSHKMCKKIDQFDNDASVKKERVYRIFRQKTEEPEWANTASPGLHHSPHHADEASVDQPQQKELSISDD
KIHSDLELSLKQTHTTPIHK
PNTPSLSIEEEQQKRWESRVKQDILPDLNFPRSLSKSIHNRKCLGTPEDLNTWNVGTRILNME
>g394.t1
MPTPAQPVVRQPASLLSPESKSVISVIASTATSVISRVCNPPEAEEKMNMTAGNPCADMAVSKQTYRPSIDDGGSHHGPS
VGEEGGSAGRFIVESTNLST
GSSPGLRVNTSEGVVVLSHSGQKIEGPQRISAKISQIPQATAVDIESQQLVPMPQIKQELYSHSQSGAQKGPPPLSDHGH
SGKTPSASIKQENTGLEKMD
SVYQSGPQGVVKRLPQAVGNLQAMSYHHPEYVLMKHQKKVEATDGHSSDGAKTSWPSAISPAISPHLPSPPGNHVAFVAA
ATPSHLGGAKPEPRSPRKSG
HPHSPFTKVSSPIGSSSPKGIPVMLPSGIAPMQQYVHHPEQSVIMPPHSSHSGLGRMSPHPSHVSQALPMSHLVQDVRVN
TPPLPVMNFGIHTEPLGSPW
SGSLQQRPTSPQAVGRDMVLKVNPGSVRGHEAEQEEARRFHQGTDPQAKQDGKMTQPVNMVQLLTKYPIVWQGLLALKND
TAAVQLHFVCGNKALAHRSL
PLQEGGVPVNTVEEYKYLGVYFNNKLDWTRNAEAIYKKGQSRLFFLRRLSGMRVGDTNRLNRLIRRAGDVVGEELDTLTI
VADRRMLSRLRSILYNASHP
LFSTLAQQRSTFSRRLLSPRCTTEGHRK
>g394.t2
MLNKCPLPDNRMPTPAQPVVRQPASLLSPESKSVISVIASTATSVISRVCNPPEAEEKMNMTAGNPCADMAVSKQTYRPS
IDDGGSHHGPSVGEEGGSAG
RFIVESTNLSTGSSPGLRVNTSEGVVVLSHSGQKIEGPQRISAKISQIPQATAVDIESQQLVPMPQIKQELYSHSQSGAQ
KGPPPLSDHGHSGKTPSASI
KQENTGLEKMDSVYQSGPQGVVKRLPQAVGNLQAMSYHHPEYVLMKHQKKVEATDGHSSDGAKTSWPSAISPAISPHLPS
PPGNHVAFVAAATPSHLGGA
KPEPRSPRKSGHPHSPFTKVSSPIGSSSPKGIPVMLPSGIAPMQQYVHHPEQSVIMPPHSSHSGLGRMSPHPSHVSQALP
MSHLVQDVRVNTPPLPVMNF
GIHTEPLGSPWSGSLQQRPTSPQAVGRDMVLKVNPGSVRGHEAEQEEARRFHQGTDPQAKQDGKMTQPVNMVQLLTKYPI
VWQGLLALKNDTAAVQLHFV
CGNKALAHRSLPLQEGGVPVNTVEEYKYLGVYFNNKLDWTRNAEAIYKKGQSRLFFLRRLSGMRVGDTNRLNRLIRRAGD
VVGEELDTLTIVADRRMLSR
LRSILYNASHPLFSTLAQQRSTFSRRLLSPRCTTEGHRK
>g394.t3
MDTDHSTSDLRQILMKPKNMPRQGSSTITSNLPTPRDKPSETTTQSSAMLNKCPLPDNRMPTPAQPVVRQPASLLSPESK
SVISVIASTATSVISRVCNP
PEAEEKMNMTAGNPCADMAVSKQTYRPSIDDGGSHHGPSVGEEGGSAGRFIVESTNLSTGSSPGLRVNTSEGVVVLSHSG
QKIEGPQRISAKISQIPQAT
AVDIESQQLVPMPQIKQELYSHSQSGAQKGPPPLSDHGHSGKTPSASIKQENTGLEKMDSVYQSGPQGVVKRLPQAVGNL
QAMSYHHPEYVLMKHQKKVE
ATDGHSSDGAKTSWPSAISPAISPHLPSPPGNHVAFVAAATPSHLGGAKPEPRSPRKSGHPHSPFTKVSSPIGSSSPKGI
PVMLPSGIAPMQQYVHHPEQ
SVIMPPHSSHSGLGRMSPHPSHVSQALPMSHLVQDVRVNTPPLPVMNFGIHTEPLGSPWSGSLQQRPTSPQAVGRDMVLK
VNPGSVRGHEAEQEEARRFH
QGTDPQAKQDGKMTQPVNMVQLLTKYPIVWQGLLALKNDTAAVQLHFVCGNKALAHRSLPLQEGGVPVNTVEEYKYLGVY
FNNKLDWTRNAEAIYKKGQS
RLFFLRRLSGMRVGDTNRLNRLIRRAGDVVGEELDTLTIVADRRMLSRLRSILYNASHPLFSTLAQQRSTFSRRLLSPRC
TTEGHRK
>g395.t1
MRSTENQIAFQEHKAKVVELEQRVAKVKTRYSVALRNLEQISEQIHAQRGRVHACHARPVACGARSSPVGAEAVEVRAAV
AQGLGSGAGGGGLGAGALES
DWADGEKTRQWVERHRKFGWGQQDAAGDSASVASLQTIASDLEKCDSVGHLGDLSDLGSLVSEEWDRERDQVLRGEEKSN
DRGGDAKVQAESVEMVSLAV
VAGADPATLLAPDRATEARGAMSRERHESFVKQHNRSVSL
>g396.t1
LFFSDPGEAPCITSAGFQFLLLDTASQLWYFTLQYLNTAQSRGMDLVEILSFLFQLSFSTLGRDYSVEGMSESLLTFLQH
LREFGLVFQRKRKSRRYYPT

RLAITLAAGVTTKASSSSNPMDVAPGTVDTSFIVVETNYRIYAYTNSELQIALVALFSEMLYRFPNVVVGQVTRESVQQA
IANGITAQQIIHFLRTRAHP
VMLKQTPVVPSTITDQIRLWELERDRLQFTEGVLYNQFLSQADFEVLRDRAQGELEHLNQASDEINKLELQLDDARSSYR
KILTDSARKLNAQGSKLGTC
IEKARPYYEARRLAKEAQQETQKAALRYERAVSMHTAAREMVYVAEQGLLADRNTLDPTWQEMLNHATAKVNEAEEERLR
SEREHQRVTQLCQDAEARVQ
NLQKALKKVIVKSKPYFELKAQFNHILEVRGMVANMT
>g396.t2
LFFSDPGEAPCITSAGFQFLLLDTASQLWYFTLQYLNTAQSRGMDLVEILSFLFQLSFSTLGRDYSVEGMSESLLTFLQH
LREFGLVFQRKRKSRRYYPT
RLAITLAAGVTTKASSSSNPMDVAPGTVDTSFIVVETNYRIYAYTNSELQIALVALFSEMLYRFPNVVVGQVTRESVQQA
IANGITAQQIIHFLRTRAHP
VMLKQTPVVPSTITDQIRLWELERDRLQFTEGVLYNQFLSQADFEVLRDRAQGDLREETPGEEAKDEESTLRERGGDGQG
KNTVNKMKTECGDEEETNTQ
EKADDDDGNKYEEELDPRIQA
>g396.t3
LFFSDPGEAPCITSAGFQFLLLDTASQLWYFTLQYLNTAQSRGMDLVEILSFLFQLSFSTLGRDYSVEGMSESLLTFLQH
LREFGLVFQRKRKSRRYYPT
RLAITLAAGVTTKASSSSNPMDVAPGTVDTSFIVVETNYRIYAYTNSELQIALVALFSEMLYRFPNVVVGQVTRESVQQA
IANGITAQQTPVVPSTITDQ
IRLWELERDRLQFTEGVLYNQFLSQADFEVLRDRAQGELEHLNQASDEINKLELQLDDARSSYRKILTDSARKLNAQGSK
LGTCIEKARPYYEARRLAKE
AQQETQKAALRYERAVSMHTAAREMVYVAEQGLLADRNTLDPTWQEMLNHATAKVNEAEEERLRSEREHQRVTQLCQDAE
ARVQNLQKALKKVIVKSKPY
FELKAQFNHILEVRGMVANMT
>g396.t4
LTLFQAQQETQKAALRYERAVSMHTAAREMVYVAEQGLLADRNTLDPTWQEMLNHATAKVNEAEEERLRSEREHQRVTQL
CQDAEARVQNLQKALKKVIV
KSKPYFELKAQFNHILEVRGMVANMT
>g397.t1
MSVCAALHGRLPGPPPYTLYVAEEGGRHWTDKEVRALLCVWADRHVRDQLKCTLRNKSIFQEMARQMQRNFGVVRNWKQC
RTKYKNLKYEYKTARNVQAA
ASGVGPGKYMKFFDEVESILTDRELDIGAWCNGDAEAGMLGSPVAQMPQTIRASENDNSDDFENDVEMEERLKGTDTCLS
SPVAASGSDQFHVVTVSDTG
RNWSDQEIRALIQVWSEEGMCKQLENSTRKKDIFVQISNRLLLQGVERDWKQCHTKYKNLKYLYRSLQKGKAEGADPRRL
MRFYDEVDALLTRTSTDSPC
VMGYAEPYLEPEGLSRLTGNAEIGTDGVGPTKPVTTKSSPGIGEDGAQNPDCFLSTTLEVTQNDAECKPHIVRERSLEQQ
QQRCILHTTVRLSKRLVISK
EEQDDIPIIEMDSVRSIWTPKPITDSKDNTSCKNAMATAPELKIGQKLNEGKTKEIFELVDQPGLVLVQSKNQITAGNAV
RKDQMEGKAAIANKTTSCVF
KLLQEA
>g397.t2
MPVGVCGRMSVCAALHGRLPGPPPYTLYVAEEGGRHWTDKEVRALLCVWADRHVRDQLKCTLRNKSIFQEMARQMQRNFG
VVRNWKQCRTKYKNLKYEYK
TARNVQAAASGVGPGKYMKFFDEVESILTDRELDIGAWCNGDAEAGMLGSPVAQMPQTIRASENDNSDDFENDVEMEERL
KGTDTCLSSPVAASGSDQFH
VVTVSDTGRNWSDQEIRALIQVWSEEGMCKQLENSTRKKDIFVQISNRLLLQGVERDWKQCHTKYKNLKYLYRSLQKGKA
EGADPRRLMRFYDEVDALLT
RTSTDSPCVMGYAEPYLEPEGLSRLTGNAEIGTDGVGPTKPVTTKSSPGIGEDGAQNPDCFLSTTLEVTQNDAECKPHIV
RERSLEQQQQRCILHTTVRL
SKRLVISKEEQDDIPIIEMDSVRSIWTPKPITDSKDNTSCKNAMATAPELKIGQKLNEGKTKEIFELVDQPGLVLVQSKN
QITAGNAVRKDQMEGKAAIA
NKTTSCVFKLLQEA
>g397.t3
MSVCAALHGRLPGPPPYTLYVAEEGGRHWTDKEVRALLCVWADRHVRDQLKCTLRNKSIFQEMARQMQRNFGVVRNWKQC
RTKYKNLKYEYKTARNVQAA

ASGVGPGKYMKFFDEVESILTDRELDIGAWCNGDAEAGMLGSPVAQMPQTIRASENDNSDDFENDVEMEERLKGTDTCLS
SPVAASGSDQFHVVTVSDTG
RNWSDQEIRALIQVWSEEGMCKQLENSTRKKDIFVQISNRLLLQGVERDWKQCHTKYKNLKYLYRSLQKGKAEGADPRRL
MRFYDEVDALLTRTSTDSPC
VMGYAEPYLEPEGLSRLTGNAEIGTDGVGPTKPVTTKSSPGIGEDGAQNPDCFLSTTLEVTQNDAECKPHIDNTSCKNAM
ATAPELKIGQKLNEGKTKEI
FELVDQPGLVLVQSKNQITAGNAVRKDQMEGKAAIANKTTSCVFKLLQEA
>g398.t1
MGLVSTAFPPEALQKLRNGNLGIGHTRYSTTGNSALQNCQPFVVDTLHGEIAVAHNGELINAAALRKKAGYNCYKVMNAS
KYKDVLKQRVHCCGVVGVAR
AVLQVMRHGVGLSTSSDSELITQLLALTPPMEEVDAPDWVARIKNLMTETPTSYSLLVMFRDVVYAIRDPYGNRPLCIGR
IVPISKLQSSGAGEGDTEGW
VVSSESCSFQSIGARYYREVQPGEIVQLSKHGVKSLSIVPRPEGDVPAFCIFEYVYFARPDSIFEGQMVYTVRQRCGRQL
AIEAPTDADLVSTVPESATP
AALGYAQQLGLPYVEVLCKNRYVGRTFIQPNTRLRQLGVAKKFGALTDNFAGKRIVLIDDSIVRGNTISPIIKLLKEAGA
KEVHIRVASPPIRHPCYMGI
NIPTKEELIANRPEFQDIAGHIGADSVQYLTVEGLVSAVQGGVASLQEKDRTISSNHKLSRKGHCTACLTGKYPVELEW
>g398.t2
LQGMGLVSTAFPPEALQKLRNGNLGIGHTRYSTTGNSALQNCQPFVVDTLHGEIAVAHNGELINAAALRKKAGYNCYKVM
NASKYKDVLKQRVHCCGVVG
VARAVLQVMRHGVGLSTSSDSELITQLLALTPPMEEVDAPDWVARIKNLMTETPTSYSLLVMFRDVVYAIRDPYGNRPLC
IGRIVPISKLQSSGAGEGDT
EGWVVSSESCSFQSIGARYYREVQPGEIVQLSKHGVKSLSIVPRPEGDVPAFCIFEYVYFARPDSIFEGQMVYTVRQRCG
RQLAIEAPTDADLVSTVPES
ATPAALGYAQQLGLPYVEVLCKNRYVGRTFIQPNTRLRQLGVAKKFGALTDNFAGKRIVLIDDSIVRGNTISPIIKLLKE
AGAKEVHIRVASPPIRHPCY
MGINIPTKEELIANRPEFQDIAGHIGADSVQYLTVEGLVSAVQGGVASLQEKDRTISSNHKLSRKGHCTACLTGKYPVEL
EW
>g399.t1
MEQTQASGPLDDTRGNLTDVLVQPEDQDEDLERTLSSHGSSLMELYPQMISQIGKARHRQHVSEAAGSVLRRYRRMRRTN
ANARNTADRKPTPRGDSQHH
HHHHLDRSCDEHNLTTAVESRSAAFHSLARRHSNRPEPLQMWQAPPEREADWERRDEKRPVLVMDFYSPKPSQASMNQTF
TVSRGSAPRSPLAGKPRRHS
YSPAVQSPTQTLRFDRSNGPRVMESSTVHNSPVRPQCSFETKWRHLEGYGGSPVAFARHSNTADMEGYAREAKQASYNPS
SPSSLHKHSPLKSRRRLYHQ
DSPSPSRLRLGSPRLPSETGRPSFQRHRSFDSALRPGGGALSPAKLEEEFTKLYHKFVCQGQSPLSDCFPCRCCAGRLEA
NRRSSSFSASSLSALALSPH
RSVLRKRVRELDQDKSPMSKRPRELTSSPGSLRHCKERLRNMCFGSRVPLSSTRLDFPSWRTPGVQQPAGEARGEGRGSR
IQPVFLDF
>g400.t1
MSAEDLVFLASPLTFDPSVVEIFLALSSGARLLIAPSVIKKAPNRLAKLLFRDHKTTVLQVTPTLLGRFGRLTLREEVLS
AGSPLRVLALGGEACPSPGL
LQSWRQEGNRTQMYNIYGITEVSCWACCHPVPESLLNSSHQAASSIPIGAPLLNTSVEVRDDRGGVIVEGQGQVFIGGRD
RVCLVDAESTLVPGTMRATG
DWVRVEGSHLHYLGRRDRLVKRHGQRVNLDTVQQVVMSLPQVESCAVGLSEGSRLVAFVVVSPSVHQVATTPRQAETSLS
VPGDREGGPADGGSAEAPDR
VLHRAVLQQLSQLVPSAGIPDSLVLVAALPLTAHG
>g400.t2
MSAEDLVFLASPLTFDPSVVEIFLALSSGARLLIAPSVIKKAPNRLAKLLFRDHKTTVLQVTPTLLGRFGRLTLREEVLS
AGSPLRVLALGGEACPSPGL
LQSWRQEGNRTQMYNIYGITEVSCWACCHPVPESLLNSSHQAASSIPIGAPLLNTSVEVRDDRGGVIVEGQGQVFIGGRD
RVCLVDAESTLVPGTMRATG
DWVRVEGSHLHYLGRRDRLVKRHGQRVNLDTVQQVESCAVGLSEGSRLVAFVVVSPSVHQVATTPRQAETSLSVPGDREG
GPADGGSAEAPDRVLHRAVL
QQLSQLVPSAGIPDSLVLVAALPLTAHG
>g400.t3

LCEFNRCHQLSCWTKRHSRKEPREPPVGHLPRPILLKTPQKEILHMCFTLLEPLGFQKLSLFQMSAEDLVFLASPLTFDP
SVVEIFLALSSGARLLIAPS
VIKKAPNRLAKLLFRDHKTTVLQVTPTLLGRFGRLTLREEVLSAGSPLRVLALGGEACPSPGLLQSWRQEGNRTQMYNIY
GITEVSCWACCHPVPESLLN
SSHQAASSIPIGAPLLNTSVEVRDDRGGVIVEGQGQVFIGGRDRVCLVDAESTLVPGTMRATGDWVRVEGSHLHYLGRRD
RLVKRHGQRVNLDTVQQVVM
SLPQVESCAVGLSEGSRLVAFVVVSPSVHQVATTPRQAETSLSVPGDREGGPADGGSAEAPDRVLHRAVLQQLSQLVPSA
GIPDSLVLVAALPLTAHG
>g401.t1
LGHPEDAAVEDDSHFLFSGGDSLKALRLCDDIAATVGSGVPGLLEAILEGSFTGLVSLVARATRLPTAAAADPPNVPAAR
KRSADRPASPAMSKRERAET
PSAPDYLHGSAGTTEFRRVKVLRRASQVLELNVRNPGAIEGSHRKTIAVNLESDSSAPGTSETGFRPASPRAPELEPTSP
ELRVRWSSDTGRCVDASPLL
LVEALDLSSGGLLWERVLGDRIESSAAVTRCGTQLAVGCYDGCVYFLCVASGATLWVFETGDAVKSSPTVDPATGLIFVG
SHDGHVYALDPQARQCVWRR
HCGGGAVFSSPYLQPAPRRLYAASLRGHLVCLHPDGGEVLWTHTRETPFFSSPCGAPGCVVIGSVDGHICAFTDTGQPRW
QFLAEGAVFSTPCFAADG
>g401.t2
LGHPEDAAVEDDSHFLFSGGDSLKALRLCDDIAATVGSGVPGLLEAILEGSFTGLVSLVARATRLPTAAAADPPNVPAAR
KRSADRPASPAMSKRERAET
PSAPDYLHGSAGTTEFRRVKVLRRASQVLELNVRNPGAIEGSHRKTIAVNLESDSSAPGTSETGFRPASPRAPELEPTSP
ELRVRWSSDTGRCVDASPLL
LVEALDLSSGGLLWERVLGDRIESSAAVTRCGTQLAVGCYDGCVYFLCVASGATLWVFETGDAVKSSPTVDPATGLIFVG
SHDGHVYALDPQARQCVWRR
HCGGGAVFSSPYLQPAPRRLYAASLRGHLVCLHPRSQWVSSPFIGVTRQDGGEVLWTHTRETPFFSSPCGAPGCVVIGSV
DGHICAFTDTGQPRWQFLAE
GAVFSTPCFAADG
>g401.t3
MYCLHGQDSLGHPEDAAVEDDSHFLFSGGDSLKALRLCDDIAATVGSGVPGLLEAILEGSFTGLVSLVARATRLPTAAAA
DPPNVPAARKRSADRPASPA
MSKRERAETPSAPDYLHGSAGTTEFRRVKVLRRASQVLELNVRNPGAIEGSHRKTIAVNLESDSSAPGTSETGFRPASPR
APELEPTSPELRVRWSSDTG
RCVDASPLLLVEALDLSSGGLLWERVLGDRIESSAAVTRCGTQLAVGCYDGCVYFLCVASGATLWVFETGDAVKSSPTVD
PATGLIFVGSHDGHVYALDP
QARQCVWRRHCGGGAVFSSPYLQPAPRRLYAASLRGHLVCLHPDGGEVLWTHTRETPFFSSPCGAPGCVVIGSVDGHICA
FTDTGQPRWQFLAEGAVFST
PCFAADG
>g402.t1
LKKVLEHLTENNYNWPSFNLILDLYYFLMAPTLCYQRDFPRSPKVRISKLLLRLLEMAPTHFLWLLLFFLCHSYLNLCAE
VLRFGDRHFYGDWWSERYVS
DNPLEELEHSLSEMVPQLLVTVVLGRYFQGNYGNGLVWLCLLLGPPLAVTMYFHDHYIGLLHVDVWI
>g402.t2
LKKVLEHLTENNYNWPSFNLILDLYYFLMAPTLCYQRDFPRSPKVRISKLLLRLLEMVILIQIMVGIVQQAPTHFLWLLL
FFLCHSYLNLCAEVLRFGDR
HFYGDWWSERYVSDNPLEELEHSLSEMVPQLLVTVVLGRYFQGNYGNGLVWLCLLLGPPLAVTMYFHDHYIGLLHVDVWI
>g402.t3
LKKVLEHLTENNYNWPSFNLILDLYYFLMAPTLCYQRDFPRSPKVRISKLLLRLLEMWIEPIFQRSENYFSAPTHFLWLL
LFFLCHSYLNLCAEVLRFGD
RHFYGDWWSERYVSDNPLEELEHSLSEMVPQLLVTVVLGRYFQGNYGNGLVWLCLLLGPPLAVTMYFHDHYIGLLHVDVW
I
>g403.t1
MATTARKRFLSVGTSFSDEEERAGLYQSVCGPDVAPPEQQHPYEAMLCCHRLHDSLLSSDSRFTAYRGILNWIIILLVAI
LAHPFAN
>g404.t1
MLIPYIESWPQAMSILLLPHNIPHSLKHLSSLVDDIWYHAGDRSTDMNWYTKRAALTGIYNTTELVMIQDSSPDFQDTWA
FLENRIQDVVDMAGTAKQVQ

STGEAVAQGLMGAALTDCTCDDFCPECSVELTLDVRCTEDQTRHVTSRDLLSNNPRVIPVTSRSRDNDPNDYVEQDDILL
VKLRKGQELRLRAYAKKGFG
KEHAKWNPTAGVSFEYDPDNALRHTVFPRPEEWPKSEYSEIEEDEVQAPYDPNGKPERFYYNVESCGSLRPETIVMSALA
VLKKKLSDLQTQLSHEIQSD
VLTIN
>g404.t2
MLIPYIESWPQAMSILLLPHNIPHSLKHLSSLVDDIWYHAGDRSTDMNWYTKRAALTGIYNTTELVMIQDSSPDFQDTWA
FLENRIQDVVDMAGTAKQVQ
STGEAVAQGLMGAALTDCTCDDFCPECSVELTLDVRCTEDQTRHVTSRDLLSNNPRVIPVTSRSRDNDPNDYVEQDDILL
VKLRKGQELRLRAYAKKGFG
KEHAKWNPTAGVSFEYDPDNALRHTVFPRPEEWFYYNVESCGSLRPETIVMSALAVLKKKLSDLQTQLSHEIQSDVLTIN
>g404.t3
MLIPYIESWPQAMSILLLPHNIPHSLKHLSSLVDDIWYHAGDRSTDMNWYTKRAALTGIYNTTELVMIQDSSPDFQDTWA
FLENRIQDVVDMAGTAKQDC
TCDDFCPECSVELTLDVRCTEDQTRHVTSRDLLSNNPRVIPVTSRSRDNDPNDYVEQDDILLVKLRKGQELRLRAYAKKG
FGKEHAKWNPTAGVSFEYDP
DNALRHTVFPRPEEWPKSEYSEIEEDEVQAPYDPNGKPERFYYNVESCGSLRPETIVMSALAVLKKKLSDLQTQLSHEIQ
SDVLTIN
>g405.t1
MDLGIKAGDWVLLVWNQPSSPLALKELAESVASHTSSTYDWILSSLLIDSSSIHTSETLAEMARVLKPGGKLVLDEPVTG
KIGHSVRTAEKLMTALKLSG
LTSVTEVSKEELTPGALSSLRSSTGYEGNTLSRARMSATKPDFEVGSSSQLKLSFGKKKAKP
>g405.t2
MDLGIKAGDWVLLVWNQPSSPLALKELAESVGTVTGSDGKVSVENMEMLLMSSHTSSTYDWILSSLLIDSSSIHTSETLA
EMARVLKPGGKLVLDEPVTG
KIGHSVRTAEKLMTALKLSGLTSVTEVSKEELTPGALSSLRSSTGYEGNTLSRARMSATKPDFEVGSSSQLKLSFGKKKA
KP
>g405.t3
MDLGIKAGDWVLLVWNQPSSPLALKELAESVASHTSSTYDWILSSLLIDSSSIHTSETLAEMARVLKPGGKLVLDEPVTG
KIGHSVRTAEKLMTALKLSG
LTSVTEVCVWSVSKEELTPGALSSLRSSTGYEGNTLSRARMSATKPDFEVGSSSQLKLSFGKKKAKP
>g406.t1
MYPAIALFYPRLARAVLEYRVRTIDGAKDNAQKQGYKGLKFPWESAVSGREVCPEDIYGEQEIHINGDVSLAFQHYLYLT
RDLSLFTEGQGCEVIWGVAD
YWVSRVTWSQKDQSYHLLGVMPPDEYYYNINDSVYTNTVAKSSLQFAVELANLLQCAAPKEWQDVADHMKIPFDQKHKYH
PEYDGYIKVGWLELGEAEKA
QKLLEKSFNNIQGPFQVWSESSDGSGAVNFLTGMGGFLQAVLFGYTGFRIQDDRLAFKPLLPNDISELCIRGVSYLGNKM
DWLLRKDEVCIILREQSTSA
GEATACALHVVLQGSGEKIPLIPELFAGKLQQFMSDIDDVHSVWLNEIQQEANRMLTRFSKGKRSKLRGSSVKSLVLIAE
DEGVPQVSTSGPAVEQPKRS
TRRNKQTPPEVPLEAAPISDCSAEQVPEEQPEVVDMEMETAVPNTEIMLGTDESQACFSNTAPSPPNIPVPKVSVRISSA
DRRSAELVAELEASPGRNAT
KIAIARTPTRARRSSVRHSLKLRYSKAGLRHSMTQDAVRRASRRSMLKKKSTRGGNSICKQKPVSSNERITRSMAVNSPS
VGSPALTGKFRGAPAVGSGV
LMSIVFLIYGSRQSKESCGKLEAVRPHMRAFLQTVQKNQMLMMTPMSVGRSSVVKSFIKHTTPIKIDPKSRERLKLEALK
KKQEQEEERIKKMEEEKRRK
QEEVKRKRDERLRRVVESRMKEDEEKKKRLEQKMSQSDEKNDKMRVERIAEEKAKKKVAAKRQEELDQKRRVEEEARLQK
IQQAEEERRQQELVAKRAAE
EEERVRKLAEARRALELRRERERERELERERQAAAERERAEREKALALQREVERAAREKERRELEEKRQLEERRMREEQQ
RLAAEKAAREREVAQQKMAA
ASVALNNAQSSVLKTPVGKGAALNVTMEVEKSPQSYSITPKGGNKPLIKSTNADDYGMDQKSDDSTDDESAPRKPIPSWA
E
>g406.t2
MYPAIALFYPRLARAVLEYRVRTIDGAKDNAQKQGYKGLKFPWESAVSGREVCPEDIYGEQEIHINGDVSLAFQHYLYLT
RDLSLFTEGQGCEVIWGVAD
YWVSRVTWSQKDQSYHLLGVMPPDEYYYNINDSVYTNTVAKSSLQFAVELANLLQCAAPKEWQDVADHMKIPFDQKHKYH

PEYDGYIKVGWLELGEAEKA
QKLLEKSFNNIQGPFQVWSESSDGSGAVNFLTGMGGFLQAVLFGYTGFRIQDDRLAFKPLLPNDISELCIRGVSYLGNKM
DWLLRKDEVCIILREQSTSA
GEATACALHVVLQGSGEKIPLIPELFAGKLQQFMSDIDDVHSVWLNEIQQEANRMLTRFSKGKRSKLRGSSVKSLVLIAE
DEGVPQVSTSGPAVEQPKRS
TRRNKQTPPEVPLEAAPISDCSAEQVPEEQPEVVDMEMETAVPNTEIMLGTDESQACFSNTAPSPPNIPVPKVSVRISSA
DRRSAELVAELEASPGRNAT
KIAIARTPTRARRSSVRHSLKLRYSKAGLRHSMTQDAVRRASRRSMLKKKSTRGGNSICKQKPVSSNERITRSMAVNSPS
VGSPALTGKFRGAPAVGSGV
LMSIVFLIYGSRQSKESCGKLEAVRPHMRAFLQTVQKNQMLMMTPMSVGRSSVVKSFIKHTTPIKIDPKSRERLKLEALK
KKQEQEEERIKKMEEEKRRK
QEEVKRKRDERLRRVVESRMKEDEEKKKRLEQKMSQSDEKNDKMRVERIAEEKAKKKVAAKRQEELDQKRRVEEEARLQK
IQQAEEERRQQELVAKRAAE
EEERVRKLAEARRALELRRERERERELERERQAAAERERAEREKALALQREVERAAREKERRELEEKRQLEERRMREEQQ
RLAAEKAAREREVAQQKMAA
ASVALNNAQSSVLKTPVGKGAALNVTMEVEKSPQSYSITPKGGNKPLIKSTNADDYGMDQKSDDSTDDESAPRKPIPSWA
EGHNLQQTIMKQYFNPPDLH
IYFGAIEPPRLENIFYKSKPRYFKRTSSAVWHSPPRLGHY
>g406.t3
MYPAIALFYPRLARAVLEYRVRTIDGAKDNAQKQGYKGLKFPWESAVSGREVCPEDIYGEQEIHINGDVSLAFQHYLYLT
RDLSLFTEGQGCEVIWGVAD
YWVSRVTWSQKDQSYHLLGVMPPDEYYYNINDSVYTNTVAKSSLQFAVELANLLQCAAPKEWQDVADHMKIPFDQKHKYH
PEYDGYIKVGWLELGEAEKA
QKLLEKSFNNIQGPFQVWSESSDGSGAVNFLTGMGGFLQAVLFGYTGFRIQDDRLAFKPLLPNDISELCIRGVSYLGNKM
DWLLRKDEVCIILREQSTSA
GEATACALHVVLQGSGEKIPLIPELFAGKLQQFMSDIDDVHSVWLNEIQQEANRMLTRFSKGKRSKLRGSSVKSLVLIAE
DEGVPQVSTSGPAVEQPKRS
TRRNKQTPPEVPLEAAPISDCSAEQVPEEQPEVVDMEMETAVPNTEIMLGTDESQACFSNTAPSPPNIPVPKVSVRISSA
DRRSAELVAELEASPGRNAT
KIAIARTPTRARRSSVRHSLKLRYSKAGLRHSMTQDAVRRASRRSMLKKKSTRGGNSICKQKPVSSNERITRSMAVNSPS
VGSPALTGKFRGAPAVGSGS
PKKAVGNWSGKRKSTESQEASPTKRPLPPKKSQSAVRPHMRAFLQTVQKNQMLMMTPMSVGRSSVVKSFIKHTTPIKIDP
KSRERLKLEALKKKQEQEEE
RIKKMEEEKRRKQEEVKRKRDERLRRVVESRMKEDEEKKKRLEQKMSQSDEKNDKMRVERIAEEKAKKKVAAKRQEELDQ
KRRVEEEARLQKIQQAEEER
RQQELVAKRAAEEEERVRKLAEARRALELRRERERERELERERQAAAERERAEREKALALQREVERAAREKERRELEEKR
QLEERRMREEQQRLAAEKAA
REREVAQQKMAAASVALNNAQSSVLKTPVGKGAALNVTMEVEKSPQSYSITPKGGNKPLIKSTNADDYGMDQKSDDSTDD
ESAPRKPIPSWAEGHNLQQT
IMKQYFNPPDLHIYFGAIEPPRLENIFYKSKPRYFKRTSSAVWHSPPRLGHY
>g407.t1
GQQNAVQYSSGGQQNAVQYSTVQEGSRMQYSTVQEGSRMQYSTVQFRRAAEYSTVQFRRAAECSTVQYSSGGQQQNTVHK
MVQNLQLLLSWL
>g408.t1
ASPVRLVNGFLNSPCSGRVEVYHNSQWGSVCDHDWDLNDAQVVCRQLGCGSAISAFMTANPGQDNAATEGEVRLVNGGNS
YCSGRVEIFLQGHWGTVCDD
DWELIDAQVCDHGRLGFHDCGHHEDAGVVCEAASPVRLVNGFLNSPCSGRVEVYHNSQWGSVCDHDWDLNDAQVVCRQLG
CGSAISAFMTANPGQDNNTA
TEGDVRLVNGGNSSCSGRVEIFLKGQWGTVCDDGWDLVNAQVVCKQLGCGRALSAPAASPVRLVNSLSHNPCSGRVEVFH
NSQWGSVCDHGWDLNDAQVV
CRQLGCGSAISTFTTVHSGQGTGPIWLDKNNQTHDIYSNILFIYAKNNGSLVTPVGIPFTCAYPLNTDASLDTIFSPYHP
FYGSNPSIGKKSKASMLLFS
SSKYTEPYPEGKVTLPLNSSVYVGVSLEKTDPRYVVVLDDCYATLSHNPDDQVRHFLIHNKCPSDQRLVTVDENGSSLQA
RFSAVMSHIFGEHHDMSLHC
SLSLCDPVASTCTPVCTRRTRRSVSVPGTVLSIGPISKRQSPQRCVSTTAYMQHYVLDEGALLLARAAWNDIFALDESQE
PGVEQRQFRPSTYRQFVLWQ

LGHLGVGRRVRQQLQALLGKHQGVFRSGQDGAANPSLIKHRIQTGDQSPIKRRAYRTSPKKRREIDRQVQRLLEDDIIEE
SCRPWSSPMVLEVHLARDHV
VFLGHVISAGGLRRDPRNTDKVKTWPIPRSAMERFKRYTGAAQIAVGAVLAQEVDGLEKVVAYASHSLTAAERRPNSHVE
QMDCDAPSAAHTLVNIGTQT
VMDSDPSAVCAIDTPTPTPATDLSSSPRSPPDFQAAADQALIYTLSHNGSNVGELQQQDADIGQVLVWLENGQRPPRWRL
KGARGSPRYYGTMPYENFAG
EETFTTCRS
>g408.t2
ASPVRLVNGFLNSPCSGRVEVYHNSQWGSVCDHDWDLNDAQVVCRQLGCGSAISAFMTANPGQDNAATEGEVRLVNGGNS
YCSGRVEIFLQGHWGTVCDD
DWELIDAQVCDHGRLGFHDCGHHEDAGVVCEAASPVRLVNGFLNSPCSGRVEVYHNSQWGSVCDHDWDLNDAQVVCRQLG
CGSAISAFMTANPGQDNNTA
TEGDVRLVNGGNSSCSGRVEIFLKGQWGTVCDDGWDLVNAQVVCKQLGCGRALSAPAASPVRLVNSLSHNPCSGRVEVFH
NSQWGSVCDHGWDLNDAQVV
CRQLGCGSAISTFTTVHSGQGTGPIWLDKNNQTHDIYSNILFIYAKNNGSLVTPVGIPFTCAYPLNTDASLDTIFSPYHP
FYGSNPSIGKKSKASMLLFS
SSKYTEPYPEGKVTLPLNSSVYVGVSLEKTDPRYVVVLDDCYATLSHNPDDQVRHFLIHNKCPSDQRLVTVDENGSSLQA
RFSAVMSHIFGEHHDMSLHC
SLSLCDPVASTCTPVCTRRTRRSVSVPGTVLSIGPISKRQSPQRCVSTTAYMQHYVLDEGALLLARAAWNDIFALDESQE
PGVEQRQFRPSTYRQFVLWQ
LGHLGVGRRVRQQLQALLGKHQGVFRSGQDGAANPSLIKHRIQTGDQSPIKRRAYRTSPKKRREIDRQVQRLLEDDIIEE
SCRPWSSPMVLEVHLARDHV
VFLGHVISAGGLRRDPRNTDKVKTWPIPRSAMEVRAFLGLCSYYRRFRFKRYTGAAQIAVGAVLAQEVDGLEKVVAYASH
SLTAAERRPNSHVEQMDCDA
PSAAHTLVNIGTQTVMDSDPSAVCAIDTPTPTPATDLSSSPRSPPDFQAAADQALIYTLSHNGSNVGELQQQDADIGQVL
VWLENGQRPPRWRLKGARGS
PRYYGTMPYENFAGEETFTTCRS
>g408.t3
ASPVRLVNGFLNSPCSGRVEVYHNSQWGSVCDHDWDLNDAQVVCRQLGCGSAISAFMTANPGQDNAATEGEVRLVNGGNS
YCSGRVEIFLQGHWGTVCDD
DWELIDAQVCDHGRLGFHDCGHHEDAGVVCEAASPVRLVNGFLNSPCSGRVEVYHNSQWGSVCDHDWDLNDAQVVCRQLG
CGSAISAFMTANPGQDNNTA
TEGDVRLVNGGNSSCSGRVEIFLKGQWGTVCDDGWDLVNAQVVCKQLGCGRALSAPAASPVRLVNSLSHNPCSGRVEVFH
NSQWGSVCDHGWDLNDAQVV
CRQLGCGSAISTFTTVHSGQGTGPIWLDKNNQTHDIYSNILFIYAKNNGSLVTPVGIPFTCAYPLNTDASLDTIFSPYHP
FYGSNPSIGKKSKASMLLFS
SSKYTEPYPEGKVTLPLNSSVYVGVSLEKTDPRYVVVLDDCYATLSHNPDDQVRHFLIHNKCPSDQRLVTVDENGSSLQA
RFSAVMSHIFGEHHDMSLHC
SLSLCDPVASTCTPVCTRRTRRSVSVPGTVLSIGPISSECNLY
>g408.t4
MIVPVKVESPRTAGPPIDSYLGYLEPDVCDNLGLVVTHTVAPAARLLNPTDHELKLHCWGVFYHECNLLTPGEVFESQQV
AASLPDVSECPLSAEQRQQL
QALLGKHQGVFRSGQDGAANPSLIKHRIQTGDQSPIKRRAYRTSPKKRREIDRQVQRLLEDDIIEESCRPWSSPMVLEVH
LARDHVVFLGHVISAGGLRR
DPRNTDKVKTWPIPRSAMERFKRYTGAAQIAVGAVLAQEVDGLEKVVAYASHSLTAAERRPNSHVEQMDCDAPSAAHTLV
NIGTQTVMDSDPSAVCAIDT
PTPTPATDLSSSPRSPPDFQAAADQALIYTLSHNGSNVGELQQQDADIGQVLVWLENGQRPPRWRLKGARGSPRYYGTMP
YENFAGEETFTTCRS
>g409.t1
SHQRSWFTPSEQSICRLDHLHRLVHLHRLDHLHRLVHLHRLDHLHRLVHGAT
>g410.t1
EATRGPGSHLQSRASADWTICTDWSIYTDWTICTDWSIYTDWTICTDWSTVRHE
>g411.t1
MGGYTFLETVCVYGYSLFIFIPTSLLWIIPLEWLHWLLILVSTVISGSVLVLTFWPVVRDHPKVMAVTTLVTIVILHSLL
AIGCKMYFFQTAVHMAATET
TTQLTMTTQVTKSH

>g411.t2
MGGYTFLETVCVYGYSLFIFIPTSLLWIIPLEWLHWLLILVSTVISGSVLVLTFWPVVRDHPKVMAVTTLVTIVILHSLL
AIGCKQCLV
>g412.t1
MAMSVFPGVRLLSIGDANGDIQRHSEQQPLRLEVKTTQDAALINLSNGEDTCVFKCSVSRETECSRVGKQSFIITLGCNS
VLLQFASPADFSSFYNLLKN
CRGHSGERSVFSDRTEESSAVQYFQFYGYLSQQQNMMQDYVRTGTYQRAILQNHTDFKDKVILDVGCGSGILSFFAAQAG
ARKVYAVEASTMAQHAEVLV
NSNRLGDRVVVIPGKVEEVTLPEQVDIIISEPMGYMLFNERMLESYLHAKKFLKPNGKMFPTIGDVHLAPFTDEQLYMEQ
FTKANFWYQPSFHGVDLSAL
RGAAVDEYFRQPIVDTFDIRILMAKSVKYTVNFLDTKEEDLYRIEIPFKFHMMHSGLVHGLAFWFDVAFIGSVYTGTSPT
PPPGSHYSSPSENMWNTGGA
YSMSQGMAVSGMPAAYDLSTVIGSGSAVSHNNLIPLVNSGIVNHTHSRMGSIMSTGIVQGATTGQAGPSSSSTHYPVTNQ
FTMGGAAISMASPMAIPSNT
MHYGS
>g412.t2
MAMSVFPGVRLLSIGDANGDIQRHSEQQPLRLEVKTTQDAALINLSNGEDTCVFKCSVSRETECSRVGKQSFIITLGCNS
VLLQFASPADFSSFYNLLKN
CRGHSGERSVFSDRTEESSAVQYFQFYGYLSQQQNMMQDYVRTGTYQRAILQNHTDFKDKVILDVGCGSGILSFFAAQAG
ARKVYAVEASTMAQHAEVLV
NSNRLGDRVVVIPGKVEEVTLPEQVDIIISEPMGYMLFNERMLESYLHAKKFLKPNGKMFPTIGDVHLAPFTDEQLYMEQ
FTKANFWYQPSFHGVDLSAL
RGAAVDEYFRQPIVDTFDIRILMAKSVKYTVNFLDTKEEDLYRIEIPFKFHMMHSGLVHGLAFWFDVAFIGSVQSYDISI
VAQVDQTGSKSSNLLDLKNP
FFRYTGTSPTPPPGSHYSSPSENMWNTGGAYSMSQGMAVSGMPAAYDLSTVIGSGSAVSHNNLIPLVNSGIVNHTHSRMG
SIMSTGIVQGATTGQAGPSS
SSTHYPVTNQFTMGGAAISMASPMAIPSNTMHYGS
>g412.t3
MAMSVFPGVRLLSIGDANGDIQRHSEQQPLRLEVKTTQDAALINLSNGEDTCVFKCSVSRETECSRVGKQSFIITLGCNS
VLLQFASPADFSSFYNLLKN
CRGHSGERSVFSDRTEESSAVQYFQFYGYLSQQQNMMQDYVRTGTYQRAILQNHTDFKDKVILDVGCGSGILSFFAAQAG
ARKVYAVEASTMAQHAEVLV
NSNRLGDRVVVIPGKVEEVTLPEQVDIIISEPMGYMLFNERMLESYLHAKKFLKPNDEQLYMEQFTKANFWYQPSFHGVD
LSALRGAAVDEYFRQPIVDT
FDIRILMAKSVKYTVNFLDTKEEDLYRIEIPFKFHMMHSGLVHGLAFWFDVAFIGSVYTGTSPTPPPGSHYSSPSENMWN
TGGAYSMSQGMAVSGMPAAY
DLSTVIGSGSAVSHNNLIPLVNSGIVNHTHSRMGSIMSTGIVQGATTGQAGPSSSSTHYPVTNQFTMGGAAISMASPMAI
PSNTMHYGS
>g413.t1
MHFSLLHPLSKSKLKAKKDFPETFGMQKGTKNRGQTSWKSAVKDRSQPESCQQRASPLEKAAAGRNMPRSAQDRDGALQH
GADLQRCPLNHRVPPTLVTG
V
>g414.t1
MLCVEVSPTISSWYRSTSRTSSGSFPAREVVSPQGSGSSMLLFRAEPGRAPWVKARPPDARRRAPLPGLAPVGGPGLPNL
GELWENLMELQLLAMAAMLI
LQSLDVLARCTYRPTTHPCV
>g415.t1
MKGAGTDAQVFLQIYGALGKSDEMKMENKSDSFEQGQVDKFMVTLQKALTKEKYSFDCGRWLDINEDDNEIIREIPATGD
VIPEPLPLIKYRVTICTGNI
SGSGTDASVFLTVIGDLGDTGERLMFMSKTNVNKFEKGNHDEFLIESVSLGTVRRVRVGHDGRGGGCGWFLDKVLVRVEG
QPESEAIEFPCNSLDNIFID
KDTVEAFD
>g415.t2
MKGAGTDAQVFLQIYGALGKSDEMKMENKSDSFEQGQVDKFMVTLQKALTKEKYSFDCGRWLDINEDDNEIIREIPATGD
VIPEPLPLIKYRVTICTGNI
SGSGTDASVFLTVIGDLGDTGERLMFMSKTNVNKFEKGNHDEFLIESVSLGTVRRVRVGHDGRGGGCGWFLDKVLVRVEG

QPESEAIEFPCNRCSAK
>g415.t3
MKGAGTDAQVFLQIYGALGKSDEMKMENKSDSFEQGQVDKFMVTLQKALTKEKYSFDCGRWLDINEDDNEIIREIPATGD
VIPEPLPLIKYRVTICTGNI
SGSGTDASVFLTVIGDLGDTGERLMFMSKTNVNKFEKGNHDEFLIESVSLGTVRRVRVGHDGRGGGCGWFLDKVLVRVEG
QPESEAIEFPCNR
>g416.t1
LTAPRAALTSHTPGTYPALRPGNVQRMHRPREQLWHIINPLIVTNKSRGGGRGSQPGDYGFVFDEKVGGAHFEDSEAKEV
TAKRRPTSADCGG
>g417.t1
LAVEENGLGNSVECCGEVQEDEDTDVTRVSSNEEVIGHFDGGGLCAVVSSEARLKGFIELMAGHVLVLELDGNCSFQDLA
EKRKIGDRLVVGEVIWVQTW
LFEDGNYCSLLKLEGTMPDSREELMMSTMMGHRNETKTVKMMFQKAEFPLAFIPEADCQ
>g418.t1
LEGTILPFVTSLVNSSLTSGLVPTPFKTARVKPLLKKPTLDTADILNYRPVSLLSFLSKTLERAVSNQLSSYLSENDLLD
PHQSGFKTAHSTETALLTVT
ESLRAAKASSLSSVLVLLDLSAAFDTVNHQTLLATLVKLGITDTALTWFTSYLMDRTYQ
>g419.t1
LGPGLALFTVVAFLSLTAGMGLSVHLMYVGECAPKKQRGFLTLTASIFIATGKVTGQIIGVRELLGSEGMWPYLLASSGI
PAILQFISLLFFPESPRYLY
IDKGDTEASKKGRLQTAQAAQGDGGGSPR
>g419.t2
MAKSFEMILLARFLFGYNVGMGLSVHLMYVGECAPKKQRGFLTLTASIFIATGKVTGQIIGVRELLGSEGMWPYLLASSG
IPAILQFISLLFFPESPRYL
YIDKGDTEASKKGRLQTAQAAQGDGGGSPR
>g420.t1
MLYNPCTFSALGDPAEPMQLGRTYLSPEERQRWVRGESSPELPDLSGMPPALKIRELFSKSRATALPPHRPFDCSGYEVL
RDMLNLFMFVYLDDRFQIFS
RSEQEHTQHIHFMFPSASLGTPYLGAVSEAGQPSPYACSPLSPRPKVRLHLPWTMRIHPTFHVFRVKHAIHSLLVPSPKL
PPPPRIIDGEPTFTIKWLMA
IHRRGRGFCT
>g420.t2
MEEYIEGTLKAGIIRPSSSPAGAGFFFVEKKDKTLHPCIDYRGSGYEVLRDMLNLFMFVYLDDRFQIFSRSEQEHTQHIH
FMFPSASLGTPYLGAVSEAG
QPSPYACSPLSPRPKVRLHLPWTMRIHPTFHVFRVKHAIHSLLVPSPKLPPPPRIIDGEPTFTIKWLMAIHRRGRGFCT
>g420.t3
MQLGRTYLSPEERQRWVRGESSPELPDLSGMPPALKIRELFSKSRATALPPHRPFDCSGYEVLRDMLNLFMFVYLDDRFQ
IFSRSEQEHTQHIHFMFPSA
SLGTPYLGAVSEAGQPSPYACSPLSPRPKVRLHLPWTMRIHPTFHVFRVKHAIHSLLVPSPKLPPPPRIIDGEPTFTIKW
LMAIHRRGRGFCT
>g421.t1
MRPIITSSRYTQNTEPISSYACRGSSTTGDIDERHTRENIKKRSPVLQPELRKDEVVDPRTEMTPANGADLDPHLLTAEQ
YPHSPEPLVLRSNPRSHLYT
HAYVSHQRGCQTIKPHNRSSALAQPPDGPRYGVRSRGRPGGWASSELLGRDDGMHGDVGPVMASPPSRTPPGPIQEVS
>g422.t1
MKGFKIAVMLSCLLVSLFCVAEAARSRKDSGSQNSFRRAANGIYQTLSNVFGDENIRGLYK
>g423.t1
MSSESMHFPGPGQYDCSQFKSKIQGGQSLQNRTKRFKEHPSEGPGPGAYDVLPASGNLLRAASANATQAKRAVNKMWLQA
VPNHVALGSQAGVPSIPSPG
QAYGYQQDSAGVLCRQPPPARDVTLGPAFYKPLLISLSKPSG
>g423.t2
MSSESMHFPGPGQYDCSQFKSKIQGGQSLQNRTKRFKEHPSEGPGPGAYDVLPASGNLLRAASANATQAKRAVNKMWLQA
VPNHVALGSQAGVPSIPSPG
QAYGYQQDSAGVLCRQPPPARDVTLGPAFYKPLLVR
>g423.t3
MSSESMHFPGPGQYDCSQFKSKIQGGQSLQNRTKRFKEHPSEGPGPGAYDVLPASGNLLRAASANATQAKRAVNKMWLQA

VPNHVALGSQAGVPSIPSPG
QAYGYQQDSAGVLCRQPPPARDVTLGPAFYKPLLSISGRQI
>g424.t1
RDHTGKMGFNEFKDLFTALNGWKQNFMMFDRDQSGTVEPQEMSQAITAMGEDGCHFELPFGA
>g425.t1
YDLLDVSTGRKVAEGHKASFCLEDTSCDAGGLSPGCYDTYHASIDCQWIDITDVAPGNYILKVTVNPSQLIQELDFSNNV
VRCDIRYTGSYVQAQNCRIS
S
>g425.t2
GLSPGCYDTYHASIDCQWIDITDVAPGNYILKVTVNPSQLIQELDFSNNVVRCDIRYTGSYVQAQNCRISS
>g426.t1
DKVISKEFMRKYIHFAKAVTPVLTQQAASHISEEYSRLRGQEQLAADVARTSPVTARTLETLIRLATAHAKARMSKAVEL
EDSEVAVELVQFAYFKKVLE
KERKRSRKDDDSASEEEEEVRSSQATPRSERKRVRRSSQGSEPYSPFDFSAEHDVPEIQAGTPKPTKTQEEEDPMDEVVP
PSQEAQPTLSADRLKSFKAR
LFSAFQEAHAQSLGMAASCXXXXXXXLLQAVGQQQEPPFSAAEVRGALSRMQDDNQVMVADDIVFLI
>g426.t2
DKVISKEFMRKYIHFAKAVTPVLTQQAASHISEEYSRLRGQEQLAADVARTSPVTARTLETLIRLATAHAKARMSKAVEL
EDSEVAVELVQFAYFKKVLE
KERKRSRKDDDSASEEEEEVRSSQATPRSERKRVRRSSQGSEPYSPFDFSAEHDVPESRTLLRTWSGPGTIQAGTPKPTK
TQEEEDPMDEVVPPSQEAQP
TLSADRLKSFKARLFSAFQEAHAQSLGMAASCXXXXXXXLLQAVGQQQEPPFSAAEVRGALSRMQDDNQVMVADDIVFLI
>g427.t1
LVSLFCSCINPQTLDSSPYKAEDAVDVGLGSMRDVEVIKLSQRASGQAFQVILRPPSFDGVPELNATLTLPPRRDPSLEE
IQRRLEAAEERRKCQEAELL
KHLAEKREHEREVVQKAMEENNNFIRNAKERLQQRMDTNRENRDALLAAMLERLQEKDKHAEEVRKNKEMKEEACR
>g427.t2
LFCSCINPQTLDSSPYKAEDAVDVGLGSMRDVEVIKLSQRASGQAFQVILRPPSFDGVPELNATLTLPPRRDPSLEEIQR
RLEAAEERRKCQEAELLKHL
AEKREHEREVVQKAMEENNNFIRNAKERLQQRMDTNRENRDALLAAMLERLQEKDKHAEEVRKNKEMKEEACR
>g427.t3
PAYREKIRELPLVSLFCSCINPQTLDSSPYKAEDAVDVGLGSMRDVEVIKLSQRASGQAFQVILRPPSFDGVPELNATLT
LPPRRDPSLEEIQRRLEAAE
ERRKCQEAELLKHLAEKREHEREVVQKAMEENNNFIRNAKERLQQRMDTNRENRDALLAAMLERLQEKDKHAEEVRKNKE
MKEEACR
>g428.t1
MVLSVCQWTGSPVSGGLQSVIHYAWPPSHLRPLAAEGSSDSDTGEPGCRLTQSQSGWRPFCKRGCEVHPQPPRLLGLLLR
PTAAELEVLVVQLLSGQPPF
VPDRGSERKTP
>g428.t2
MVLSVCQWTGSPVSGGLQSVIHYAWPPSHLRPLAAEGSSDSDTGEPGCRLTQSQSGWRPFCKRVLSASLRPEPTQRGCEV
HPQPPRLLGLLLRPTAAELE
VLVVQLLSGQPPFVPDRGSERKTP
>g428.t3
MVLSVCQWTGSPVSGGLQSVIHYAWPPSHLRPLAAEGSSDSDTGEPGCRLTQSQSGWRPFCKPPRLLGLLLRPTAAELEV
LVVQLLSGQPPFVPDRGSER
KTP
>g429.t1
MDGKGVGERSGYFEHVEEAEWAVQVLNATGMPVAASLCIGPEGDLNGVSPGDCAVRLVQAGAKIVGVNCHFDPTTAVKTV
KMMKEGVEKAGLEAYYMVQP
LAYHTPDCNRQGFIDLPEFPFALEPRVLTRWEMQKFAREAYDSGIRYIGGCCGFLPYHTRALAEELAEERGFLPAASEKH
GTWGSCLGMHTKPWVRARAR
RDYWEGLRPASGRPYCPALSSPDGWGVTKGHAQLMQKKESTTQDELKVLFERTTTNHTAGVFVVVPLSPAAPSLLELFWN
ESLFKSVEFGWKNTPWSRSW
MSFTMEESVESGWVSVRPNLFEEPERHKFVFIVAWNEIEGKFAITCHDRTVQKRSTFRDSLVDCPSTAPRVAQKHDPRPP
SRTDGSGVGSNSSTRSKEGA

KRSAALGGPITSEIVGKALGSWDVVSPKVMDIEVLDPEGFPGELSSHCDTEGKDGGAREEYSWAGLYSFQDLRAAHVQLC
AVNSDLEPCLPALPEEQSGM
WTVMFGAPEVSPRETDALCYQVQVYLGHALDTCGWKILSQVLFSEGDDTEEYYENLSELRQKGYEDALERAKTRLQERVS
FRREYDAQPHGCTVKLSVCC
SVRYCEPKPRVWYHAARVSSR
>g429.t2
MDGKGVGERSGYFEHVEEAEWAVQVLNATGMPVAASLCIGPEGDLNGVSPGDCAVRLVQAGAKIVGVNCHFDPTTAVKTV
KMMKEGVEKAGLEAYYMVQP
LAYHTPDCNRQGFIDLPEFPFALEPRVLTRWEMQKFAREAYDSGIRYIGGCCGFLPYHTRALAEELAEERGFLPAASEKH
GTWGSCLGMHTKPWVRARAR
RDYWEGLRPASGRPYCPALSSPDGWGVTKGHAQLMQKKESTTQDELKVLFERTTTNHTAGVFVVVPLSPAAPSLLELFWN
ESLFKSVEFGWKNTPWSRSW
MSFTMEESVESGWVSVRPNLFEEPERHKFVFIVAWNEIEGKFAITCHDRTVQKRSTFRDSLVDCPSTAPRVAQKHDPRPP
SRTDGSGVGSNSSTRSKEGA
KRSAALGGPITSEIVGKALGSWDVVSPKVMDIEVLDPEGFPGELSSHCDTEGKDGGAREEYSWAGLYSFQDLRAAHVQLC
AVNSDLEPCLPALPEEQSGM
WTVMFGAPEVSPRETDALCYQVQVYLGHALDTCGWKILSQVLFSEGDDTEEYYENLSELRQKGYEDALERAKTRLQESCP
LKEALPGWVVLSPGASVRGH
FLCLAAGSGVVVSVTMTTHISYARXNELLDRHRAADSMVELLQVYPEEDRAYEELLEETTQLYQYLLQPFRDMRELAMLR
RQQIKALQGCRLQPDRAGDQ
SERLCLLLPPSLTHPERLINQQKLYCRLCQLYYRLYCQLYCQLYCRLNCQLYCELCCRLYCRLYCRLCQLYYRLYCQLCT
PCPP
>g429.t3
MDGKGVGERSGYFEHVEEAEWAVQVLNATGMPVAASLCIGPEGDLNGVSPGDCAVRLVQAGAKIVGVNCHFDPTTAVKTV
KMMKEGVEKAGLEAYYMVQP
LAYHTPDCNRQGFIDLPEFPFALEPRVLTRWEMQKFAREAYDSGIRYIGGCCGFLPYHTRALAEELAEERGFLPAASEKH
GTWGSCLGMHTKPWVRARAR
RDYWEGLRPASGRPYCPALSSPDGWGVTKGHAQLMQKKESTTQDELKVLFERTTTNHTAGVFVVVPLSPAAPSLLELFWN
ESLFKSVEFGWKNTPWSRSW
MSFTMEESVESGWVSVRPNLFEEPERHKFVFIVAWNEIEGKFAITCHDRTVQKRSTFRDSLVDCPSTAPRVAQKHDPRPP
SRTDGSGVGSNSSTRSKEGA
KRSAALGGPITSEIVGKALGSWDVVSPKVMDIEVLDPEGFPGELSSHCDTEGKDGGAREEYSWAGLYSFQDLRAAHVQLC
AVNSDLEPCLPALPEEQSGM
WTVMFGAPEVSPRETDALCYQVQVYLGHALDTCGWKILSQVLFSEGDDTEEYYENLSELRQKGYEDALERAKTRLQESCP
LKEALPGWVVLSPGASVRGH
FLCLAAGSGVVVSVTMTTHISYARWCLHTALTNANTHTHPPLPGRPAIPRRLLEDRAFCRQTCSPWANSGPLELGVANEQ
LHALSTQSEVGQRLPWRPRT
KLLDRHRAADSMVELLQVYPEEDRAYEELLEETTQLYQYLLQPFRDMRELAMLRRQQIKALQGCRLQPDRAGDQSERLCL
LLPPSLTHPERLINQQKLYC
RLCQLYYRLYCQLYCQLYCRLNCQLYCELCCRLYCRLYCRLCQLYYRLYCQLCTPCPP
>g430.t1
LWPADNLKSSEDVQPKSIIQFELFKRGDQQAFQREKRNPAHDGTAGGPAGPSAAGPRGAFVCGKLFSSGRGVRE
>g430.t2
LKSSEDVQPKSIIQFELFKRGDQQAFQREKRNPAHDGTAGGPAGPSAAGPRGAFVCGKLFSSGRGVRE
>g431.t1
MEPSMNASFVDFVGGVQLLPGEGTRTALPAVLIGICVSGAVGNLLVMLIFYRDFRNARGSEVKALLAAVASTDLAILLLC
APVRAVTYYRRSWTLGGFVC
STTDWFQHACVVAKTLTLAATSRAKHDLVVPSGAARPPRLSPTWIHGALAFIWTVSMMFPIPQFLFASLQPRGADTSVCV
SEMPACAKDFLSLFYKIYPA
LTFALPTTFTLAYYIKRLHAAVNHAPDATRQQSKVTLVLLCVSGANTLLLLPEWSAFTWTRLGFNKPPVGLAIFAQVLVY
FGSCALPTILMTMYDDVRRG
VVGLWFVSTCRGAKHAPANRCPKSEENGAELGAHDAVGDEATSQELDKTFPDVEHFWTGRRNTHVEGDQDPVPWEREEKM
L
>g432.t1
MTLQPPHTPPREHSLLRPPRLVAMLLRRLAERSRISRGETMTTQWNESLGLPQALVPLSLHKGSRGSTPDPPAWLLAVRV
LGPGVLQECGEDLLHHMTKR

ASYGPRGLGLIPMQSNLYP
>g433.t1
MFYDYPPPTYLEYPILIAQGEIHWFVVGWRLLTVQKWIIDLAMSLCTFISAGSKFAQLQCLWRSKDSDQVSALSWSLAIY
TTLTRIYTTLVTTGDMQGKS
VIEYI
>g433.t2
MFYDYPPPTYLEYPILIAQGEIHWFVVGWRLLTVQKWIIDLAMSLCTFISAGSKFAQLQCLWRSKDSDQVSALSWSLAIY
TTLSEYSRNTTQPNRNVTCH
TAGIF
>g433.t3
MFYDYPPPTYLEYPILIAQDAILLLLLILHYNGDLRHSVGYALAFVVGWRLLTVQKWIIDLAMSLCTFISAGSKFAQLQCL
WRSKDSDQVSALSWSLAIYT
TLTRIYTTLVTTGDMQGKSVIEYI
>g434.t1
MSGPVLTTHRVWWCVGTVCLVRLSHSLRCGVEKQRRWAQITITEPRSRIGNPRGTEYKVAPAPHLPSSFSYPFSFYSFSF
YPFSSYFSSYPPSSSFSFFH
PAPSSSSFSSSFSSHFLFLFLLLLLLTAPLLFIPPTPPPPPPPPSPSPPPPCSPAPLLCSQSR
>g435.t1
MRDSLGETKTASTWPPPQVASQRSQTTELALLQLNVEGLTFIKSNVLEQIASKNNVTVIILQETHTENKNTLRVPGYTLA
GYIANKHHGIAIFVHKDM
>g436.t1
YCGADIKAVCSEAALCALRRRYPQIYSSSQKLVLDIDSISISSRDFLSAMTKMVPAAQRLAAAERCPCFTSVLTCAGLTR
RANPTGDCCACRAVVSPAKA
LIPALVPLLGAALHSLLDTLNRVFPHVELGLKKKAPTARRGRLT
>g436.t2
YCGADIKAVCSEAALCALRRRYPQIYSSSQKLVLDIDSISISSRDFLSAMTKMVPAAQRLAAAERCPCFTSVLTCAGLTR
RANPTGDCCACRAVVSPAKA
LIPALVPLLGAALHSLLDTLNRVFPHVELGLKKKAPTVTPLETRVLRSVY
>g436.t3
MITMASRLCQGQYKGYCGADIKAVCSEAALCALRRRYPQIYSSSQKLVLDIDSISISSRDFLSAMTKMVPAAQRLAAAER
CPCFTSVLTCAGLTRRANPT
GDCCACRAVVSPAKALIPALVPLLGAALHSLLDTLNRVFPHVELGLKKKAPTARRGRLT
>g437.t1
LEEEEEEEEEEEEDDAVLSEDEESLAPPSSRPQPNPLQGRLDFTRCVAFVPSSNLKNPPSSSSSPPASHPSAALSQPTSY
RPRLLLEGRPGSGQSSHLAP
AALHALERFTVYTLDVAVLFGASATAPEETCAQMFLEAKRTSPSILYIPRIGQWWETVGPALRATFLSLLHSIPAFSPIL
LLATCNLPYRLLSPELKELF
CLEYGEVFCVPLPSSRERRNFFEDLILNQAAKAPASKKKAMLQSLEVLPVAPPSAPRQLTKEETQRLEEQEEATLRELRL
FLRDVTNRLAQDKRFKAFTK
PVDLEEVEDYAEVIKEPMDLSTVLSKVDLHQYGTAKEFLYDVDLIWKNALEYNPDRVPSDRQIRHRACALKDTVHAIIKH
ELDEDFEKICEEIKESRSRR
DWSTSRSAPSFYHVLPKQPKACATEAKGGGGGDAAPAQEPVLPACAAPSSNPASVVTTSKNIGERTAH
>g437.t2
LEEEEEEEEEEEEDDAVLSEDEESLAPPSSRPQPNPLQGRLDFTRCVAFVPSSNLKNPPSSSSSPPASHPSAALSQPTSY
RPRLLLEGRPGSGQSSHLAP
AALHALERFTVYTLDVAVLFGASATAPEETCAQMFLEAKRTSPSILYIPRIGQWWETVGPALRATFLSLLHSIPAFSPIL
LLATCNLPYRLLSPELKELF
CLEYGEVFCVPLPSSRERRNFFEDLILNQAAKAPASKKKAMLQSLEVLPVAPPSAPRQLTKEETQRLEEQEEATLRELRL
FLRDVTNRLAQDKRFKAFTK
PVDLEEVEDYAEVIKEPMDLSTVLSKVDLHQYGTAKEFLYDVDLIWKNALEYNPDRVPSDRQIRHRACALKDTVHAIIKH
ELDEDFEKICEEIKESRSRR
DWSTSRSAPSFYHVLPKQPKACATEAKGGGGGDAAPAQEPVLP
>g438.t1
MSFLRRVAGLSLRDRVRSSDIRRELGVEPLLLRVERSQLRWFGHLIRMPPGRLPLEVFRAHPTGKRPRGRPRTRWRDYIS
HLAWERLGVPQEELESVAGE
KDVWSSLLSLLSPRPGRSAG

>g439.t1
MRQCWRDRPYERPPFSQISVQLNRMQEARKNKGIGFLPTKPVFQAYVNMALFENFTYAGIDATAEEA
>g440.t1
MSKTADLTAVQKAIIDTLKQEGKTQKEISERIGCSQSAVSRHLSGKSWKEKVWQKSCTTRRGDRTLRKIVEKGRFQTLGD
LRKQWTESGVETQSHRAQAC
AGNGLQVPHSP
>g441.t1
LRTNHQDKFLVYIYPLQRFVDEVGVPISSTGLSREYNDLLSAISDSDFYTDDVTRACLFVPSIDVLNQNSLRIRETAQAL
AMLPRWDRGMNHLLFNMLPG
GPPDYNTALDVPRDRALLAGGGFSTWTYRQGYDVSIPVYSPLSADVELPERQPG
>g441.t2
LLRTNHQDKFLVYIYPLQRFVDEVGVPISSTGLSREYNDLLSAISDSDFYTDDVTRACLFVPSIDVLNQNSLRIRETAQA
LAMLPRWDRGMNHLLFNMLP
GGPPDYNTALDVPRDRALLAGGGFSTWTYRQGYDVSIPVYSPLSADVELPERQPG
>g441.t3
MSYTLFFVYIYPLQRFVDEVGVPISSTGLSREYNDLLSAISDSDFYTDDVTRACLFVPSIDVLNQNSLRIRETAQALAML
PRWDRGMNHLLFNMLPGGPP
DYNTALDVPRDRALLAGGGFSTWTYRQGYDVSIPVYSPLSADVELPERQPG
>g442.t1
PIVRYAISMARKIGARVYALPDDLVEVKTKMVLTVFACLMGRGLKRAK
>g443.t1
MEVLEMSAMIPALQELTSASPSEYNNAVQKPRQILCQFIDRILTDVDVVALGLNKKSSSEPACVMLLDFVQHIIKSSSLM
FANPACHGAEYADTTQSCIE
FSWWVVGRLLRVAAAPDCGVLHPPVSDVLCSLLHTLRLRAPILFNRVAEELLAVCQDLCAVLYEGTCWPVTLERFRVPGE
RVSAHLAPSPPLLLVSHAAL
ESLVAVVMGIMTDSLRGVVSQRSLGVTWETACFMLAHSGCSTKLRPIAMAMLQRLVELGGYHEWQDGLFSAYLQLLETHF
HPDPEGAGLRGYGAALLSLT
HAVFQPTGGLWQHAPQLEPVHLAQLLDCVCALAVAGLRLGSEVSQSLCMLFNFLLSVAPSYEGATLIRRQRVTDVCRKLV
CTVGTESQAECAEGFLHIAL
EADVAMVTRELEGDGEVAAKRPCLPINPSASKRTRSCATAVDMRVRSVVWAETSGRIEHLLSSLTSDPDPESAVCALDGL
ALLLHLVALCTSPTSSSPLF
WVSAETLDRALQSCQALLESMAQSSSSHSHIQSAFQSTLMVLDSILYLTMSSQDEGEEGLHRQVCCLLSFPWVSELKALP
AYRACGFPSSQPALAHTLGP
GCNSQAECLSLLALLRRGVCDKWRVCVFCEALRSPEQGVRAAALRALPLLLHHLGPAHHQLLQTALPSKVEDSSEQVREA
LARVLGQLTCIQSRCSKLSI
IAAASPQATPPRILCSRLGLAAEHAGKATASLTASVIRLYLPLVGRQSPSSVKQVGGDSDSQAILSTLIGLIEDPDPVVR
TCFGQSVRFLLAEPAPSLQY
SLVSELLVARLKEAFTNAKLNRDDELRNTLILTTGEIGRAAHGSLVSFSLLRLLHCLLSKSSNVSVAAYTQIRALALTKG
LRLQTLFSQYKNPICQFLVE
SLHSRHASALRSTPDHSSESANQRELALDILAQIAHAFDFPDLHRFLTCTLQVLLPYLAAKASSTGSALIRTLANELKAN
RREILINNFKYIFSHLVCSC
TKEELEKAFHYLQSETEIELGSLLRQDFQGLHNELLLRLGEHYQQQAEQGTPDVLLTSNAFQLLLGDPEAFPGQMRYIIP
PASSGSPPGSLTSCLLTELP
SAVEVNVQRVDEGVRPAPPGYSANPCNA
>g443.t2
MEVLEMSAMIPALQELTSASPSEYNNAVQKPRQILCQFIDRILTDVDVVALGLNKKSSSEPACVMLLDFVQHIIKSSSLM
FANPACHGAEYADTTQSCIE
FSWWVVGRLLRVAAAPDCGVLHPPVSDVLCSLLHTLRLRAPILFNRVAEELLAVCQDLCAVLYEGTCWPVTLERFRVPGE
RVSAHLAPSPPLLLVSHAAL
ESLVAVVMGIMTDSLRGVVSQRSLGVTWETACFMLAHSGCSTKLRPIAMAMLQRLVELGGYHEWQDGLFSAYLQLLETHF
HPDPEGAGLRGYGAALLSLT
HAVFQPTGGLWQHAPQLEPVHLAQLLDCVCALAVAGLRLGSEVSQSLCMLFNFLLSVAPSYEGATLIRRQRVTDVCRKLV
CTVGTESQAECAEGFLHIAL
EADVAMVTRELEGDGEVAAKRPCLPINPSASKRTRSCATAVDMRVRSVVWAETSGRIEHLLSSLTSDPDPESAVCALDGL
ALLLHLVALCTSPTSSSPLF
WVSAETLDRALQSCQALLESMAQSSSSHSHIQSAFQSTLMVLDSILYLTMSSQDEGEEGLHRQVCCLLSFPWVSELKALP

AYRACGFPSSQPALAHTLGP
GCSESKVEDSSEQVREALARVLGQLTCIQSRCSKLSIIAAASPQATPPRILCSRLGLAAEHAGKATASLTASVIRLYLPL
VGRQSPSSVKQVGGDSDSQA
ILSTLIGLIEDPDPVVRTCFGQSVRFLLAEPAPSLQYSLVSELLVARLKEAFTNAKLNRDDELRNTLILTTGEIGRAAHG
SLVSFSLLRLLHCLLSKSSN
VSVAAYTQIRALALTKGLRLQTLFSQYKNPICQFLVESLHSRHASALRSTPDHSSESANQRELALDILAQIAHAFDFPDL
HRFLTCTLQVLLPYLAAKAS
STGSALIRTLANELKANRREILINNFKYIFSHLVCSCTKEELEKAFHYLQSETEIELGSLLRQDFQGLHNELLLRLGEHY
QQQAEQGTPDVLLTSNAFQL
LLGDPEAFPGQMRYIIPPASSGSPPGSLTSCLLTELPSAVEVNVQRVDEGVRPAPPGYSANPCNA
>g443.t3
MEVLEMSAMIPALQELTSASPSEYNNAVQKPRQILCQFIDRILTDVDVVALGLNKKSSSEPACVMLLDFVQHIIKSSSLM
FANPACHGAEYADTTQSCIE
FSWWVVGRLLRVAAAPDCGVLHPPVSDVLCSLLHTLRLRAPILFNRVAEELLAVCQDLCAVLYEGTCWPVTLERFRVPGE
RVSAHLAPSPPLLLVSHAAL
ESLVAVVMGIMTDSLRGVVSQRSLGVTWETACFMLAHSGCSTKLRPIAMAMLQRLVELGGYHEWQDGLFSAYLQLLETHF
HPDPEGAGLRGYGAALLSLT
HAVFQPTGGLWQHAPQLEPVHLAQLLDCVCALAVAGLRLGSEVSQSLCMLFNFLLSVAPSYEGATLIRRQRVTDVCRKLV
CTVGTESQAECAEGFLHIAL
EADVAMVTRELEGDGEVAAKRPCLPINPSASKRTRSCATAVDMRVRSVVWAETSGRIEHLLSSLTSDPDPESAVCALDGL
ALLLHLVALCTSPTSSSPLF
WVSAETLDRALQSCQALLESMAQSSSSHSHIQSAFQSTLMVLDSILYLTMSSQDEGEEGLHRQVCCLLSFPWVSELKALP
AYRACGFPSSQPALAHTLGP
GCKQGVRAAALRALPLLLHHLGPAHHQLLQTALPSKVEDSSEQVREALARVLGQLTCIQSRCSKLSIIAASPQATPPRI
LCSRLGLAAEHAGKATASLT
ASVIRLYLPLVGRQSPSSVKQVGGDSDSQAILSTLIGLIEDPDPVVRTCFGQSVRFLLAEPAPSLQYSLVSELLVARLKE
AFTNAKLNRDDELRNTLILT
TGEIGRAAHGSLVSFSLLRLLHCLLSKSSNVSVAAYTQIRALALTKGLRLQTLFSQYKNPICQFLVESLHSRHASALRST
PDHSSESANQRELALDILAQ
IAHAFDFPDLHRFLTCTLQVLLPYLAAKASSTGSALIRTLANELKANRREILINNFKYIFSHLVCSCTKEELEKAFHYLQ
SETEIELGSLLRQDFQGLHN
ELLLRLGEHYQQQAEQGTPDVLLTSNAFQLLLGDPEAFPGQMRYIIPPASSGSPPGSLTSCLLTELPSAVEVNVQRVDEG
VRPAPPGYSANPCNA
>g444.t1
MCRPCAADGCRYCERRDSRELCEEQQQQQVVRQAEVVVCRELQTVSAGEWRYQQEVDPDLQPVRQWVESQRRPPWEEVAR
LSQATKGLWSKFDSLCLSDG
VLQRTAMLQAVHGAPGSGHFGVTKTLRRLRQSCYWGRFRGDVVDFCRRCDLCTAQKGPIGSLKTPLLSPQALSMLEGSRT
GFIQLMHLPGCNRKEPHVPH
PTLLYHRHTPVNPHSKCKMVITLFP
>g444.t2
MCRPCAADGCRYCERRDSRELCEEQQQQQVVRQAEVVVCRELQTVSAGEWRYQQEVDPDLQPVRQWVESQRRPPWEEVAR
LSQATKGLWSKFDSLCLSDG
VLQRTAMLQAVHGAPGSGHFGVTKTLRRLRQSCYWGRFRGDVVDFCRRCDLCTAQKGPIGSLKTPLLSPQALSMLEGSRT
GFIQLMHLPGCNRKVRMHWK
NAIMMPGEGIFRL
>g445.t1
MSCLMISGMLFCLTNVQVGNLFDSHRSTVITMTSGFTDSSAGGFLVVKLLHEQGVSFRTTFLVCSXQESREKVTEKRNEG
EYMMEVKHETDKRVKAEVPS
FRSCVLSLFFLWHLLWTAILQFSHFLYLSTCNPTLSRLAGNDRDQVSQYTNVFAYIQTCGILFAPCNGLILDRHKHQPLA
QGESAQATNLSSFPLSLALT
SLQGFLFCVCFTVPVLPLQYVTFALQVVNCSFLYGGHQSFISHAFPGCHFGKLSGLIMSLSALTLMLQIPVVRYVKVVLH
GDPFYNGPSFDAALMSLRRV
LERIRAAGLKLHPDKCHFMLRQVTFLGHRVGQEGIITVQDKVQAVVDWPVPSTQKEVKSFLGLASYYRRFVRGFSCSPLF
QLLQKDRVCLDRSMPGGVRP
ASSGPCASPS
>g445.t2

MISGMLFCLTNVQVGNLFDSHRSTVITMTSGFTDSSAGGFLVVKLLHEQGVSFRTTFLVCSXQESREKVTEKRNEGEYMM
EVKHETDKRVKAEVPSFRSC
VLSLFFLWHLLWTAILQFSHFLYLSTCNPTLSRLAGNDRDQVSQYTNVFAYIQTCGILFAPCNGLILDRHKHQPLAQGES
AQATNLSSFPLSLALTSLQG
FLFCVCFTVPVLPLQYVTFALQVVNCSFLYGGHQSFISHAFPGCHFGKLSGLIMSLSALTLMLQIPVVRYVKVVLHGDPF
YNGPSFDAALMSLRRVLERI
RAAGLKLHPDKCHFMLRQVTFLGHRVGQEGIITVQDKVQAVVDWPVPSTQKEVKSFLGLASYYRRFVRGFSCSPLFQLLQ
KDRVCLDRSMPGGVRPASSG
PCASPS
>g445.t3
MSCLMISGMLFCLTNVQVGNLFDSHRSTVITMTSGFTDSSAGGFLVVKLLHEQGVSFRTTFLVCSCGCIWHLIRQESREK
VTEKRNEGEYMMEVKHETDK
RVKAEVPSFRSCVLSLFFLWHLLWTAILQFSHFLYLSTCNPTLSRLAGNDRDQVSQYTNVFAYIQTCGILFAPCNGLILD
RHKHQPLAQGESAQATNLSS
FPLSLALTSLQGFLFCVCFTVPVLPLQYVTFALQVVNCSFLYGGHQSFISHAFPGCHFGKLSGLIMSLSALTLMLQIPVV
RYVKVVLHGDPFYNGPSFDA
ALMSLRRVLERIRAAGLKLHPDKCHFMLRQVTFLGHRVGQEGIITVQDKVQAVVDWPVPSTQKEVKSFLGLASYYRRFVR
GFSCSPLFQLLQKDRVCLDR
SMPGGVRPASSGPCASPS
>g446.t1
FCQLRLDPNTAYRELHLSDGNKKVIRTRDLQPYNDSSERFDSFAQVLCREALSGGRFYWEIEWSGEFSIGVAYKGISRKG
KGSLCLLGYNDKSWSLLCSD
TGYSAWHNRVDKNVSGPHSPRIGVYLDHPAGILAFYSISGSMSLLHRFETRFGEPLYPGFGVGTSVKICQLK
>g447.t1
MNGAAVSDLTHAALTRMVRKCGDHITILVVDSETEKTYMRQKRPILPAMAVPCNLPHRARSFALAPGPQGYGFLLREEKT
SSGRTVHVVHEVRADGPAHA
AGLQEGQLLLEVNWVSAESLSHEDVVERVKQSRPQVTLTTISPKGLDFYRKLGLSPVLFCGDEASKDQRERDNLLPAVAE
ETPEQVVDSLHASLEECLDD
QSAGSEVVGASRSSERNDLEDVVVPAEAAGDPLSLGVTGQTAVTFSKPRLATFWHYARVELVPPSPAEIPKAIEGATNLL
KAFQSGRLAQTTVKDAVRNG
LVATEVLMWFYIGECIGKGGLVGYSV
>g447.t2
MNGAAVSDLTHAALTRMVRASSPDKNLCSAVYLCTNRHQCILLKQPPHETSRHPQVRKCGDHITILVVDSETEKTYMRQK
RPILPAMAVPCNLPHRARSF
ALAPGPQGYGFLLREEKTSSGRTVHVVHEVRADGPAHAAGLQEGQLLLEVNWVSAESLSHEDVVERVKQSRPQVTLTTIS
PKGLDFYRKLGLSPVLFCGD
EASKDQRERDNLLPAVAEETPEQVVDSLHASLEECLDDQSAGSEVVGASRSSERNDLEDVVVPAEAAGDPLSLGVTGQTA
VTFSKPRLATFWHYARVELV
PPSPAEIPKAIEGATNLLKAFQSGRLAQTTVKDAVRNGLVATEVLMWFYIGECIGKGGLVGYSV
>g447.t3
LVWMNGAAVSDLTHAALTRMVRKCGDHITILVVDSETEKTYMRQKRPILPAMAVPCNLPHRARSFALAPGPQGYGFLLRE
EKTSSGRTVHVVHEVRADGP
AHAAGLQEGQLLLEVNWVSAESLSHEDVVERVKQSRPQVTLTTISPKGLDFYRKLGLSPVLFCGDEASKDQRERDNLLPA
VAEETPEQVVDSLHASLEEC
LDDQSAGSEVVGASRSSERNDLEDVVVPAEAAGDPLSLGVTGQTAVTFSKPRLATFWHYARVELVPPSPAEIPKAIEGAT
NLLKAFQSGRLAQTTVKDAV
RNGLVATEVLMWFYIGECIGKGGLVGYSV
>g448.t1
YIEDVARCVDQSKRLIIVMTPNYVVRRGWSIFELETRLRNMLASGEIKVILIECAELRGIMNYQEKHRGCLQEGPKPTLF
LEEAKTLQHLQGDAEDVSVV
ASVIFFAVTCWGSGMRVADTNRLNRLIKRAGDVVGEELDTLTAVLCSLLGQEATAQRNSLAG
>g448.t2
YIEDVARCVDQSKRLIIVMTPNYVVRRGWSIFELETRLRNMLASGEIKVILIECAELRGIMNYQEGDAEDVSVVASVIFF
AVTCWGSGMRVADTNRLNRL
IKRAGDVVGEELDTLTAVLCSLLGQEATAQRNSLAG
>g448.t3

MAEQVYPILLNLAWNLTYIEDVARCVDQSKRLIIVMTPNYVVRRGWSIFELETRLRNMLASGEIKVILIECAELRGIMNY
QEKHRGCLQEGPKPTLFLEE
AKTLQHLQGDAEDVSVVASVIFFAVTCWGSGMRVADTNRLNRLIKRAGDVVGEELDTLTAVLCSLLGQEATAQRNSLAG
>g449.t1
RSPVFSHLSSSLQGLWTIRAFRAEERFQTIFDAHQDLHSEAWYLFLTTSRWFAMRLDLLCAVFVIIVTFGCVLLRDNLGA
GDVGLALSYAITLMGMFQWG
VRQSAEVETLMTSVERVIEYTELESEAPWETQKRPPPDWPSQGLVTFDKVNFSYSEDGPLVLRNMTATFLPREKVSC
>g450.t1
LTNSCAIESILTSSLSVWCGSCPVADQKAPQRVVRTAEKITRSAQPSIQDLHP
>g451.t1
MGKTADLTAVQKAIIDTLKQEGKTQKEISERIGCSQSAVSRHLSGKSWKEKVWQKSCTTRRGDRTLRKIVEKGRFQTLGD
LRKQWTESGVETQSHRAQAC
AGNGLQVPHSP
>g452.t1
MATAATGAGGTGSSGPATGPVGGGTAVGRKKDGGPSSKFWESSETVSQLETVRLWIGKHYKKYVQNDSPSSKSLAGLVVQ
LLQFQEDAFGRRVNNP
>g453.t1
QILAASFELHKFYHDAKEILGRILDKHKKLPEELGRDQNTVETLQRMHRTFQHDIQALGTQVRQLQEDAVRLQSAYAGDK
AEDIQRREGEVLEAWSSLLE
ACDGRRVRLLDTGDKFRFFSLVRDLLLWMEGVIRLIENQENPRDVSSVELLMNNHQGIKAEIDARNDSFTSCIELGKALL
ARKHYASEEIKEKLLQLTDK
RKDMIDKWEDRWEWLRLILEVHQFSRDAGVAEAWLLGQEPYLSGREVGQSVDEVEKLIKRHQAFEKSAATWEERFSALER
LTTMELLEVRRQQEEEERLR
RPPTPEVLVEPLQREGEQISHNGLPSDQESPRVSHHLCGPRNNLSCYLVTIIT
>g453.t2
QILAASFELHKFYHDAKEILGRILDKHKKLPEELGRDQNTVETLQRMHRTFQHDIQALGTQVRQLQEDAVRLQSAYAGDK
AEDIQRREGEVLEAWSSLLE
ACDGRRVRLLDTGDKFRFFSLVRDLLLWMEGVIRLIENQENPRDVSSVELLMNNHQGIKAEIDARNDSFTSCIELGKALL
ARKHYASEEIKEKLLQLTDK
RKDMIDKWEDRWEWLRLILEVHQFSRDAGVAEAWLLGQEPYLSGREVGQSVDEVEKLIKRHQAFEKSAATWEERFSALER
LTTMELLEVRRQQEEEERLR
RPPTPEVLVEPLQREGEQISHNGLPSDQESPR
>g454.t1
EGLAMEEELCRLRSHQELHAMQSRVLASLEGERATLERERDTLRSSVESLRTAQRKSDQLELTNQTLKAELERLGRSFEA
CRRREAELEAELREATLEAE
GLARGRDQALLEATRLEQEKEACVTELEGQKKEGRQREREAARLRQQLESTTLALEHSNQRARGLDTEH
>g455.t1
MLHCFLQGCPQILPGLDILVPAGLVRPITLRALNLPQPQSGQKNYECVFSIQGSTQRVPAVRFNSSCIQCQNTSYWYEGD
ETGNMAVDFSIVWDGDFFID
KPATMKALLYKCAAQRSSCGQCLKAPPTFDCGWCTESRKCLLRQHCPAPEANWMHHGRHNLRCSHPRITKIGPLTGPREG
GTRVTIEGENLGLQVREIAH
VQVAGVRCNPAPAEYISAERIVCEMAEALLPLSPGGPVELCIGVCSSEYRTLSVQTYLFVSPSVGRLSPEKGPVSGGTRL
TLWGRHLDAGSSVSVSIHQE
PCLFLRRSSRDIVCITPASQPGSGPAPIRLMIDRAEVTSETRFLYSEDPTISSIEPNWSILNGSTTITVTGSNLLTIQEP
KVRAKYAGIETTNVCKVLND
SVMTCLAPGLLYDKPSPPEGPLHPDEFGFILDQ
>g455.t2
LLGLQRLQPFTPLAHQMELGCYQLLPYWYEGDETGNMAVDFSIVWDGDFFIDKPATMKALLYKCAAQRSSCGQCLKAPPT
FDCGWCTESRKCLLRQHCPA
PEANWMHHGRHNLRCSHPRITKIGPLTGPREGGTRVTIEGENLGLQVREIAHVQVAGVRCNPAPAEYISAERIVCEMAEA
LLPLSPGGPVELCIGVCSSE
YRTLSVQTYLFVSPSVGRLSPEKGPVSGGTRLTLWGRHLDAGSSVSVSIHQEPCLFLRRSSRDIVCITPASQPGSGPAPI
RLMIDRAEVTSETRFLYSED
PTISSIEPNWSILNGSTTITVTGSNLLTIQEPKVRAKYAGIETTNVCKVLNDSVMTCLAPGLLYDKPSPPEGPLHPDEFG
FILDQ
>g455.t3

MCLCAADRLVVKLSLKSRETGHEFISTQFIYYNCSVLQSCTACVSSVFSCHWCKYRHICTNNLLDCSFQEGKVSKLEGCP
QILPGLDILVPAGLVRPITL
RALNLPQPQSGQKNYECVFSIQGSTQRVPAVRFNSSCIQCQNTSYWYEGDETGNMAVDFSIVWDGDFFIDKPATMKALLY
KCAAQRSSCGQCLKAPPTFD
CGWCTESRKCLLRQHCPAPEANWMHHGRHNLRCSHPRITKIGPLTGPREGGTRVTIEGENLGLQVREIAHVQVAGVRCNP
APAEYISAERIVCEMAEALL
PLSPGGPVELCIGVCSSEYRTLSVQTYLFVSPSVGRLSPEKGPVSGGTRLTLWGRHLDAGSSVSVSIHQEPCLFLRRSSR
DIVCITPASQPGSGPAPIRL
MIDRAEVTSETRFLYSEDPTISSIEPNWSILNGSTTITVTGSNLLTIQEPKVRAKYAGIETTNVCKVLNDSVMTCLAPGL
LYDKPSPPEGPLHPDEFGFI
LDQ
>g456.t1
MPSQPYTPRPAPSSRQHLDVRYTELASNLRTSPVPASVSPSRVIPISRSPRAQHQSPSSRRDMLSFNKARAEESTAAAAA
PSKPAAKSQNQNQNQNRHGQ
AAGTKAKEEHTVRIKTETRADDKASPAVVNTEAPKPARVESPPSVTVAVKTESEKKVRGEMEERARVSEGDQKGDGGKGP
ILDSSEKKILDSVTMEEIIS
KVMRPAAGLHTDGSAAADPPLTYHVEKTEQEDGSTKTQIILESKVEEELDISQDSALDALLSGGATTVSLHDIKDTPTGS
MIQELLSGLQGKEAMANKSV
NIEIMEQPSYRDDENDVEQDSDAKYLQPSSVTYCQIEEMESSPRDAKQLQQEVHHTSNISKPDTEHSKVEFVHDDELSRS
SKEPDSPYYGHDEDSQEYFV
STPDENLSELDEGVGIASYGHYGNLEDLSDERYYQDEGLPHQGMIAEELDEYELSKLMSEDDSYIKERFPECIIEEEVQV
SPTVQDEVLGFLKEDSMDPK
EQLEGALDSIQDSLSGPLREELAYFTRLSRESPQNMSVGVKRVQHTNDDGTMTIVAELNISQSLAESGLLDDDEGDLSEE
QILASLQSSNPGLQALLMQG
GAQGYNIKMSEEQEITHAGGLDDVFARGGSMTETTERFMELGPTERSFTFQKDQDQTSSGQGLQSHTMTSPIKISHEKKV
ATVYLESSTDD
>g457.t1
AHAIAVSQSPARPDRAHISHRLAMVHSSAAEPHHNESAAHYFEESLGVYRNTVGTQDPAFLTAQDDYCHFLLINGQKERC
VEIQKASLAPKRSVFGPWSG
EVADSLQLVGGVEMTQGLIRQAHRTLRKSSVSPNAKLPQIPRWTCSKINEFKTELGGTLSSLFCCSEKSCKWLQMFGERR
KIMDEISLCVC
>g457.t2
AHAIAVSQSPARPDRAHISHRLAMVHSSAAEPHHNESAAHYFEESLGVYRNTVGTQDPAFLTAQDDYCHFLLINGQKERC
VEIQKASLAPKRSVFGPWSG
EVADSLQLVGGVEMTQGLIRQAHRTLRKSSVSPNAKLPQIPR
>g457.t3
SQSPARPDRAHISHRLAMVHSSAAEPHHNESAAHYFEESLGVYRNTVGTQDPAFLTAQDDYCHFLLINGQKERCVEIQKA
SLAPKRSVFGPWSGEVADSL
QLVGGVEMTQGLIRQAHRTLRKSSVSPNAKLPQIPRWTCSKINEFKTELGGTLSSLFCCSEKSCKWLQMFGERRKIMDEI
SLCVC
>g458.t1
MDAWDPQTLAMLVFGGFMVVSAVGMALVSLVSMRETSYEDALDQQRREISRTQTQRSDRRRKEKTLDKRRTKKRELLEAA
DLEPAVASTSEPESDPEPAA
GPAVSPLAAEPGLLVAPESVETPLTASQVPPTTYVEAPPMPSVEAPPMTSVEAPPMTSVEAAPTSSPKEKKKKKRSGKSE
PVSAAETVTKETVVIAVEAA
VVAKETIAMGSAPSNPASDTGEKPKMDMPAKKAKAGPEPPPVQLRYKTLLSSITEAVLSQKEVQKLIQALQQKTSGEPDV
VAALKKKLEEKEKQLNNEKK
DTSAAKARLRDLTQEVSAERSKMRSLEAGLRAELLASGHTQATLTQQLQLKVCSLQEQLENGPSGQLTRLTQENSILRDA
LNQATSQAESRQNAELARLR
QEVLRGAREVGELSRRKDALQSSEERRRSLENKLSSMEQQLAQTQLTHVEKERDHQQRQKEMQEELGRCGDVIASLQESL
TQAQQHEVTIAELRGSLGSA
QEELQALKARLEQPEPQVGVSSKMEELNTRLQEREQQVTSLEEEVQHLKKNLENIPSASLSSNGVKESEGMTTPDSQTQE
DTTMLTSGRITELEAVLEER
ERRVVTLETELDKLKEEMETLKTKVQDSGSETESEQPFESLEKDSQLLALEEELTTTREDLEQMKQKNNELREKNWTAVE
ALAAAEKMNLQRLTEAQQAV
VVAEDSASHLQTEVQKCLHLLFPDVSVEPQQQGDWLELVTQKALETQRDAMQTSQEVLTKLKENERILQAECETNRNILA

KAKGELLSVQSRAEEQERVW
KEQWALADQQRQEAQDESLVLVNSESENKQLREQLMLLEAQLEKQLESSHFTQTNTEEVAQAQQPAGDLQLQVEKMEAEQ
ILSADLQEEQEQTSSKTPQE
QTSSQTPQDQLLPQDGTSV
>g458.t2
MLVFGGFMVVSAVGMALVSLVSMRETSYEDALDQQRREISRTQTQRSDRRRKEKTLDKRRTKKRELLEAADLEPAVASTS
EPESDPEPAAGPAVSPLAAE
PGLLVAPESVETPLTASQVPPTTYVEAPPMPSVEAPPMTSVEAPPMTSVEAAPTSSPKEKKKKKRSGKSEPVSAAETVTK
ETVVIAVEAAVVAKETIAMG
SAPSNPASDTGEKPKMDMPAKKAKAGPEPPPVQLRYKTLLSSITEAVLSQKEVQKLIQALQQKTSGEPDVVAALKKKLEE
KEKQLNNEKKDTSAAKARLR
DLTQEVSAERSKMRSLEAGLRAELLASGHTQATLTQQLQLKVCSLQEQLENGPSGQLTRLTQENSILRDALNQATSQAES
RQNAELARLRQEVLRGAREV
GELSRRKDALQSSEERRRSLENKLSSMEQQLAQTQLTHVEKERDHQQRQKEMQEELGRCGDVIASLQESLTQAQQHEVTI
AELRGSLGSAQEELQALKAR
LEQPEPQVGVSSKMEELNTRLQEREQQVTSLEEEVQHLKKNLENIPSASLSSNGVKESEGMTTPDSQTQEDTTMLTSGRI
TELEAVLEERERRVVTLETE
LDKLKEEMETLKTKVQDSGSETESEQPFESLEKDSQLLALEEELTTTREDLEQMKQKNNELREKNWTAVEALAAAEKMNL
QRLTEAQQAVVVAEDSASHL
QTEVQKCLHLLFPDVSVEPQQQGDWLELVTQKALETQRDAMQTSQEVLTKLKENERILQAECETNRNILAKAKGELLSVQ
SRAEEQERVWKEQWALADQQ
RQEAQDESLVLVNSESENKQLREQLMLLEAQLEKQLESSHFTQTNTEEVAQAQQPAGDLQLQVEKMEAEQILSADLQEEQ
EQTSSKTPQEQTSSQTPQDQ
LLPQDGTSV
>g458.t3
MLVFGGFMVVSAVGMALVSLVSMRETSYEDALDQQRREISRTQTQRSDRRRKEKTLDKRRTKKRELLEAADLEPAVASTS
EPESDPEPAAGPAVSPLAAE
PGLLVAPESVETPLTASQVPPTTYVEAPPMPSVEAPPMTSVEAPPMTSVEAAPTSSPKEKKKKKRSGKSEPVSAAETVTK
ETVVIAVEAAVVAKETIAMG
SAPSNPASDTGEKPKMDMPAKKAKAGPEPPPVQLRYKTLLSSITEAVLSQKEVQKLIQALQQKTSGEPDVVAALKKKLEE
KEKQLNNEKKDTSAAKARLR
DLTQEVSAERSKMRSLEAGLRAELLASGHTQATLTQQLQLKVRGHCKDVIPVVVSSLQEQLENGPSGQLTRLTQENSILR
DALNQATSQAESRQNAELAR
LRQEVLRGAREVGELSRRKDALQSSEERRRSLENKLSSMEQQLAQTQLTHVEKERDHQQRQKEMQEELGRCGDVIASLQE
SLTQAQQHEVTIAELRGSLG
SAQEELQALKARLEQPEPQVGVSSKMEELNTRLQEREQQVTSLEEEVQHLKKNLENIPSASLSSNGVKESEGMTTPDSQT
QEDTTMLTSGRITELEAVLE
ERERRVVTLETELDKLKEEMETLKTKVQDSGSETESEQPFESLEKDSQLLALEEELTTTREDLEQMKQKNNELREKNWTA
VEALAAAEKMNLQRLTEAQQ
AVVVAEDSASHLQTEVQKCLHLLFPDVSVEPQQQGDWLELVTQKALETQRDAMQTSQEVLTKLKENERILQAECETNRNI
LAKAKGELLSVQSRAEEQER
VWKEQWALADQQRQEAQDESLVLVNSESENKQLREQLMLLEAQLEKQLESSHFTQTNTEEVAQAQQPAGDLQLQVEKMEA
EQILSADLQEEQEQTSSKTP
QEQTSSQTPQDQLLPQDGTSV
>g459.t1
MSTTSKVVLGVSVLLTLSTVAGVHIKQRLDQERLHQGVVRDLERLERKRENLRLLDQQRVLTQQLEAQRNQREAQHNQHN
HH
>g460.t1
MESSSDQLVGENEELCTSASEGLEEGEVEGETVLMVESEDQGSVDLSHEQSGDSLTSDIGEEGEGGWACEDMAFYCDRCH
KWIPSGQLRGEQPSYLKGDN
FFKFICCSCSEDGKESFERMRLTWQQVVMLAMYNLSLEGTGRQGYFRWKEDICAFIGRHWTFLLGSRKKTITWWSTVAGC
LSVGSPAFFRSGAQEFGEPG
WWKLVQNRPPTLRPDLDKTTTKTKVSKPTLEPTITVEGLRKRGARNPVESAMQLKEKRSRTQEAKEIRRAQKEASYTDRS
SSSTPVKLGGRGGRRPDLLL
EKGEVVDFSSLSSSDRTPLTSPSPSPSPDFSAPGTPASHSATPSLLSEADLIPDLMPPQALFHDDEELEGEGMIDPGMEY
VPPPSAGHA

>g460.t2
MESSSDQLVGENEELCTSASEGLEEGEVEGETVLMVESEDQGSVDLSHEQSGDSLTSDIGEEGEGGWACEDMAFYCDRCH
KWIPSVYLSPQSACFTCKPV
YLCDPPGQLRGEQPSYLKGDNFFKFICCSCSEDGKESFERMRLTWQQVVMLAMYNLSLEGTGRQGYFRWKEDICAFIGRH
WTFLLGSRKKTITWWSTVAG
CLSVGSPAFFRSGAQEFGEPGWWKLVQNRPPTLRPDLDKTTTKTKVSKPTLEPTITVEGLRKRGARNPVESAMQLKEKRS
RTQEAKEIRRAQKEASYTDR
SSSSTPVKLGGRGGRRPDLLLEKGEVVDFSSLSSSDRTPLTSPSPSPSPDFSAPGTPASHSATPSLLSEADLIPDLMPPQ
ALFHDDEELEGEGMIDPGME
YVPPPSAGHA
>g461.t1
IDECQRVAGECGEGARCVNLEGDYECHCLLGYRAHNTTEPFHSLRDKGSCK
>g462.t1
MLTGLEKDVLSERSLAVMRNSAQIFNTNPRYILFIYMVINDTVQLILTILLFLLSYTLYRINVSLCCFIMLLALFTTENA
PIYL
>g462.t2
LCSQIFNTNPRYILFIYMVINDTVQLILTILLFLLSYTLYRINVSLCCFIMLLALFTTENAPIYL
>g463.t1
MTKGTSSFGKRRNKTHALCRRCGSKAYHLQKSTCGKCGYPEKRKRKYNWSAKAKRRNTTGTGRLRHLKVVYRRFRNGFRE
GTTPKPRRAAVAASSSS
>g463.t2
MTKGTSSFGKRRNKTHALCRRCGSKAYHLQKSTCGKCGYPEKRKRKYNWSAKAKRRNTTGTGRLRHLKVVYRRFRNGFRE
GTTPKPRRAAVAASSSS
>g464.t1
MTTLDDKLLGEKLQYYYSSSEDEGSENEDEEGEAKTIRNPDVLEPEIDYSPDGSAVNTGPKGVINDWRKYKQLEVEQRQE
QKKEMERLIKKLSMTCRSDL
DLEKDKEKQKELQDKINGKMTMQEYNMLQEQEDDEDFLQHYRMQRIEQMRRQLCRGKRFAQIFELASGEDFLEALDKEDK
STLVMIHIYEPEVVGCEAMR
GSLLCLAQEYPLVKFCSVRSAAISTSALFRGSALPALLVYKAGDLIGNFVRVTDQLGEDFFAVDVEALLQEYGLLPDKPA
TAIPKTIRNGNIVQHHRNDD
DDSDLDID
>g465.t1
MSSHLQWMVIRNCSSFLIKRNGQTYSTEPNNLKSKNSFRFNGLVHRKTVGVQPAADGKGIVVVLKKRAGQRKPVSTYEKI
TINKNSRATLSSVRHIIGKN
NYRRDLRMAALRRASAILKSQKPVVVKKKRTRAAKTA
>g466.t1
MEGEAALEALGEQLYNLIYPKHAEIAGKLTGMLLELPGPALSQLLQDEALLARALEKALSALNKQLEPRQNLETCMMSDS
SETEDIDRVGEKLFLLVERL
DPVHATDITELGNIVRAEGSFFQENFGCGLIHVFFNTNLAEAPPDHPPSEDMTLVQSSMVQSLWSFANLSLALLCFSLMK
GFT
>g466.t2
MEGEAALEALGEQLYNLIYPKHAEIAGKLTGPALSQLLQDEALLARALEKALSALNKQLEPRQNLETCMMSDSSETEDID
RVGEKLFLLVERLDPVHATD
ITELGNIVRAEGSFFQENFGCGLIHVFFNTNLAEAPPDHPPSEDMTLVQSSMVQSLWSFANLSLALLCFSLMKGFT
>g467.t1
SPPPPQQFKLISQAYEVLSDPKKRDLYDQGGEQAIKEGGMSGGGSPTDIFNMFFGGGGRTQRERRGLYEDVSRKNVVHQL
AVTLEEMYNGGTRKLGLQKN
VICEKCDGYGGKKGTLEKCSSCKGRGVQIKVQQIGPGMIQQIQSMCAECQGQGEKFSSKDRCKTCNGHKVERTKKILEVH
IDKGMKDGQRITFHGEGDQE
PGLEPGDVIIVLDQKDHPAFQRQDDNLIMKMDIKLVEALCGCKKSILTLDGRTLIISTQPGEVVKHNDLKCVHGEGMPVY
REPFEKGQLFVQFQVEFPEK
HWLPEHLMFQLERLLPPRDDVMVSDDMEEVTLSEVDSQSQQRSSSREAYEEDDEGPRQGVQCQTQ
>g467.t2
SPPPPQQFKLISQAYEVLSDPKKRDLYDQGGEQAIKEGGMSGGGSPTDIFNMFFGGGGRTQRERRGLYEDVSRKNVVHQL
AVTLEEMYNGGTRKLGLQKN
VICEKCDGYGGKKGTLEKCSSCKGRGVQIKVQQIGPGMIQQIQSMCAECQGQGEKFSSKDRCKTCNGHKVERTKKILEVH

IDKVLDQSVRTADMTGGMKD
GQRITFHGEGDQEPGLEPGDVIIVLDQKDHPAFQRQDDNLIMKMDIKLVEALCGCKKSILTLDGRTLIISTQPGEVVKHN
DLKCVHGEGMPVYREPFEKG
QLFVQFQVEFPEKHWLPEHLMFQLERLLPPRDDVMVSDDMEEVTLSEVDSQSQQRSSSREAYEEDDEGPRQGVQCQTQ
>g467.t3
SPPPPQQFKLISQAYEVLSDPKKRDLYDQGGEQAIKEGGMSGGGSPTDIFNMFFGGGGRTQRERRGKNVVHQLAVTLEEM
YNGGTRKLGLQKNVICEKCD
GYGGKKGTLEKCSSCKGRGVQIKVQQIGPGMIQQIQSMCAECQGQGEKFSSKDRCKTCNGHKVERTKKILEVHIDKVLDQ
SVRTADMTGGMKDGQRITFH
GEGDQEPGLEPGDVIIVLDQKDHPAFQRQDDNLIMKMDIKLVEALCGCKKSILTLDGRTLIISTQPGEVVKHNDLKCVHG
EGMPVYREPFEKGQLFVQFQ
VEFPEKHWLPEHLMFQLERLLPPRDDVMVSDDMEEVTLSEVDSQSQQRSSSREAYEEDDEGPRQGVQCQTQ
>g468.t1
MLVYDICNEKSFENIKNWIRNIEEHASSDVEKMILGNKCDISDRRQVSKDRGEKTDAPGAEGSVKITEKKSRRMKFFKCA
LL
>g468.t2
MLVYDICNEKSFENIKNWIRNIEEHASSDVEKMILGNKCDISDRRQVSKDRGEKAFYTIARDILQNLSTKTTDAPGAEGS
VKITEKKSRRMKFFKCALL
>g469.t1
MGQPAGAIANCAMVAQDKPKSPVSGPRNKARAKKGKGKGKAKPEPPEETDPRKLEIIRWVNSLEQAMMDALVLDRVDFVK
LLLENGVNIHHFLTIPRLEE
LYNTGNLPPDYQITLIDIGLVLEFLMGGAYRSNYTRKAFRNLYNTLYGLKRDDEPRKGKKKAKKKKEDEVEIDVDDPEVC
RFKFPFHELMVWAVLMKRQK
MALFLWQRGEEAMAKALVACKLYKAMAHESSESELVDDISQDLENNSKEFGQLAYELLDQSYKHDEQVAMKLLTYELINW
SNSTCLKLAVAAKQRDFIAH
TCSQMLLTDMWMGCLRIGKNPGLKVILGIIFPPLILLLDFRIGDDTSYDAPAAGEAGKEKDDDNKSSKEANTDTMSRKGD
EEEGSANVRKIPIAKRLFEF
YDAPFTKFWFNTIAYLVYLMLYNYIILVKMERWPSLQEWIVISYILTLGTEKVRQILMSEPGKLKQKINVWLEEYWNITD
LAAILTFLMGLMLRLQHEPY
MGYGRVIYCIDIIFWYIRVLDIFGVNKYLGPYVMMIGKMMIDMMYFVVIMLVVLMSFGVSRQAILHPDEEPTWRLARNIF
YMPYWMIYGEVFADSIDRKT
RIHTPCGEHLYDEDGKKLPPCIPGAWLTPALMAGYLLVANILLVNLLIAVFNNTFFEVKSISNQVWKFQRYQLIMTFHDR
PILPPPLIIFSHLYIVFNRL
FRRCAKKKQEGELDEKDRGLKLRLNPEELKNLYEFEEQCVEEYFREKEDEQQSSSDERIKVTSERVENMSMRLEEVNERE
NTMKATLQTVDLRLAQLEEI
HGRMAVALEKLAGVDRSELTRTYSRNSSVGDPSSLMRQGSINSADGYSLYRFHMDLEETAAKQKEVDEVGKTGLERQRSL
RQSSKEYGPTLDVSGPERVR
TSSCVDILISPCEQKSPQEGSQENVTNSSSDLTPDSGYPPAKSLQKVRPEMDKLKIISTNAKRAKSLRQYPSEELKTSPK
RAMSIVNIVPDVTDDVTVKD
EPTKKAQLWQPTPSSPKRTKSLRCCPTEGQQISTSTARRASSSVILSPDGEGLELLLPVDTKLQVERFTKSAHLLPVDLE
LAVNPLCVSDHMPLMAPTLV
QKLDVPEAFEEPSAGGPDQEQEHPGDGTPSDTAGVAPETNQRGEELAQTSVDEAFLSSHRSLSWNTSDMEARGLTVPLRS
RVSSSLSDIAGTSLVLPCAQ
QRGGEERGQESGGDQHRVKEGAEKVEASECPEVKSSDTGTQENKGPEGVELQQGPQ
>g469.t2
LDPGNPERDREHRGLSEFIHSALMHRPVIVVCPETRSLEPLAPGRAWRPASSHLHRRACDGALGFTSAGYGGTNASAPDQ
LSLALAWNRVDIARNQIFVY
GHNLPPAGAIANCAMVAQDKPKSPVSGPRNKARAKKGKGKGKAKPEPPEETDPRKLEIIRWVNSLEQAMMDALVLDRVDF
VKLLLENGVNIHHFLTIPRL
EELYNTGNLPPDYQITLIDIGLVLEFLMGGAYRSNYTRKAFRNLYNTLYGLKRDDEPRKGKKKAKKKKEDEVEIDVDDPE
VCRFKFPFHELMVWAVLMKR
QKMALFLWQRGEEAMAKALVACKLYKAMAHESSESELVDDISQDLENNSKEFGQLAYELLDQSYKHDEQVAMKLLTYELI
NWSNSTCLKLAVAAKQRDFI
AHTCSQMLLTDMWMGCLRIGKNPGLKVILGIIFPPLILLLDFRIGDDTSYDAPAAGEAGKEKDDDNKSSKEANTDTMSRK
GDEEEGSANVRKIPIAKRLF
EFYDAPFTKFWFNTIAYLVYLMLYNYIILVKMERWPSLQEWIVISYILTLGTEKVRQILMSEPGKLKQKINVWLEEYWNI

TDLAAILTFLMGLMLRLQHE
PYMGYGRVIYCIDIIFWYIRVLDIFGVNKYLGPYVMMIGKMMIDMMYFVVIMLVVLMSFGVSRQAILHPDEEPTWRLARN
IFYMPYWMIYGEVFADSIDR
KTRIHTPCGEHLYDEDGKKLPPCIPGAWLTPALMAGYLLVANILLVNLLIAVFNNTFFEVKSISNQVWKFQRYQLIMTFH
DRPILPPPLIIFSHLYIVFN
RLFRRCAKKKQEGELDEKDRGLKLRLNPEELKNLYEFEEQCVEEYFREKEDEQQSSSDERIKVTSERVENMSMRLEEVNE
RENTMKATLQTVDLRLAQLE
EIHGRMAVALEKLAGVDRSELTRTYSRNSSVGDPSSLMRQGSINSADGYSLYRFHMDLEETAAKQKEVDEVGKTGLERQR
SLRQSSKEYGPTLDVSGPER
VRTSSCVDILISPCEQKSPQEGSQENVTNSSSDLTPDSGYPPAKSLQKVRPEMDKLKIISTNAKRAKSLRQYPSEELKTS
PKRAMSIVNIVPDVTDDVTV
KDEPTKKAQLWQPTPSSPKRTKSLRCCPTEGQQISTSTARRASSSVILSPDGEGLELLLPVDTKLQVERFTKSAHLLPVD
LELAVNPLCVSDHMPLMAPT
LVQKLDVPEAFEEPSAGGPDQEQEHPGDGTPSDTAGVAPETNQRGEELAQTSVDEAFLSSHRSLSWNTSDMEARGLTVPL
RSRVSSSLSDIAGTSLVLPC
AQQRGGEERGQESGGDQHRVKEGAEKVEASECPEVKSSDTGTQENKGPEGVELQQGPQ
>g469.t3
LDPGNPERDREHRGLSEFIHSALMHRPVIVVCPETRSLEPLAPGRAWRPASSHLHRRACDGALGFTSAGYGGTNASAPDQ
LSLALAWNRVDIARNQIFVY
GHNLPPAGAIANCAMVAQDKPKSPVSGPRNKARAKKGKGKGKAKPEPPEETDPRKLEIIRWVNSLEQAMMDALVLDRVDF
VKLLLENGVNIHHFLTIPRL
EELYNTGNLPPDYQITLIDIGLVLEFLMGGAYRSNYTRKAFRNLYNTLYGLKRDDEPRKGKKKAKKKKEDEVEIDVDDPE
VCRFKFPFHELMVWAVLMKR
QKMALFLWQRGEEAMAKALVACKLYKAMAHESSESELVDDISQDLENNSKEFGQLAYELLDQSYKHDEQVAMKLLTYELI
NWSNSTCLKLAVAAKQRDFI
AHTCSQMLLTDMWMGCLRIGKNPGLKVILGIIFPPLILLLDFRIGDDTSYDAPAAGEAGKEKDDDNKSSKEANTDTMSRK
GDEEEGSANVRKIPIAKRLF
EFYDAPFTKFWFNTIAYLVYLMLYNYIILVKMERWPSLQEWIVISYILTLGTEKVRQILMSEPGKLKQKINVWLEEYWNI
TDLAAILTFLMGLMLRLQHE
PYMGYGRVIYCIDIIFWYIRVLDIFGVNKYLGPYVMMIGKMMIDMMYFVVIMLVVLMSFGVSRQAILHPDEEPTWRLARN
IFYMPYWMIYGEVFADSIDP
PCGEHLYDEDGKKLPPCIPGAWLTPALMAGYLLVANILLVNLLIAVFNNTFFEVKSISNQVWKFQRYQLIMTFHDRPILP
PPLIIFSHLYIVFNRLFRRC
AKKKQEGELDEKDRGLKLRLNPEELKNLYEFEEQCVEEYFREKEDEQQSSSDERIKVTSERVENMSMRLEEVNERENTMK
ATLQTVDLRLAQLEEIHGRM
AVALEKLAGVDRSELTRTYSRNSSVGDPSSLMRQGSINSADGYSLYRFHMDLEETAAKQKEVDEVGKTGLERQRSLRQSS
KEYGPTLDVSGPERVRTSSC
VDILISPCEQKSPQEGSQENVTNSSSDLTPDSGYPPAKSLQKVRPEMDKLKIISTNAKRAKSLRQYPSEELKTSPKRAMS
IVNIVPDVTDDVTVKDEPTK
KAQLWQPTPSSPKRTKSLRCCPTEGQQISTSTARRASSSVILSPDGEGLELLLPVDTKLQVERFTKSAHLLPVDLELAVN
PLCVSDHMPLMAPTLVQKLD
VPEAFEEPSAGGPDQEQEHPGDGTPSDTAGVAPETNQRGEELAQTSVDEAFLSSHRSLSWNTSDMEARGLTVPLRSRVSS
SLSDIAGTSLVLPCAQQRGG
EERGQESGGDQHRVKEGAEKVEASECPEVKSSDTGTQENKGPEGVELQQGPQ
>g470.t1
VTLNNAEVCSENITTLKRNLENDCSKLFMQGPASGDHAKIDSCLSDLVNTSSKFKDLLQVGGTPAGRRGDREPVLPGPRL
IPDTQAVLEGLSELNTTAIK
PQVKPWITAFLSISHNIEEEEFNEYEANDPWVQQLIVNLEHVMAEFKVLLQPRTNTSLNKCELSLWKGNVQ
>g470.t2
VTLNNAEVCSENITTLKRNLENDCSKLFMQGPASGDHAKIDSCLSDLVNTSSKFKDLLQVGGTPAGRRGDREPVLPGPRL
IPDTQAVLVCSHESAWLEGL
SELNTTAIKPQVKPWITAFLSISHNIEEEEFNEYEANDPWVQQLIVNLEHVMAEFKVLLQPRTNTSLNKCELSLWKGNVQ
>g470.t3
VTLNNAEVCSENITTLKRNLENDCSKLFMQGPASGDHAKIDSCLSDLVNTSSKFKDLLQVGGTPAGRRGDREPVLPGPRL
IPDTQAVLEGLSELNTTAIK
PQVKPWITAFLSISHNIEEVRMAVVYQFGSGTKSPLNEEFNEYEANDPWVQQLIVNLEHVMAEFKVLLQPRTNTSLNKCE

LSLWKGNVQ
>g471.t1
MHDQVPLPAPQSVPVPAPGISFSLELFTNTARCLTTRPLAEGQQESRWAPVQAAGSDYVTDTLAADGGLNSLSSFNAFLH
WLSGCFNFSTVAALPCVLTS
SKVKFFRSPQVDLAFWRLRESVTSAPVPALPKSRQHLIEEGDASDADVEAIVSQSESPTNWLPSSCGGGLSLRKGSDGPL
SIFSLCSGSHHQSNGLTEHQ
ELETHICCLVSQNPATSSKILIWCLSPLSRARQVLLKSSVRLKRMADCEVNSCPLLLARPEGLAFHSGPYPNVGHLH
>g471.t2
LAEGQQESRWAPVQAAGSDYVTDTLAADGGLNSLSSFNAFLHWLSGCFNFSTVAALPCVLTSSKVKFFRSPQVDLAFWRL
RESVTSAPVPALPKSRQHLI
EEGDASDADVEAIVSQSESPTNWLPSSCGGGLSLRKGSDGPLSIFSLCSGSHHQSNGLTEHQELETHICCLVSQNPATSS
KILIWCLSPLSRARQVLLKS
SVRLKRMADCEVNSCPLLLARPEGLAFHSGPYPNVGHLH
>g471.t3
MHDQVPLPAPQSVPVPAPGISFSLELFTNTARCLTTRPLAEGQQESRWAPVQAAGSDYVTDTLAADGGLNSLSSFNAFLH
WLSGCFNFSTVAALPCVLTS
SKVKFFRSPQVDLAFWRLRESVTSAPVPALPKSRQHLIEEGDASDADVEAIVSQXSESPTNWLPSSCGGGLSLRKGSDGP
LSIFSLCSGSHHQSNGLTEH
QELETHICCLVSQNPATSSKILIWCLSPLSRARQVLLKSSVRLKRMADCEVNSCPLLLARPEGLAFHSGPYPNVGHLH
>g472.t1
LQYEKRCREERSFDGHIPRSGSRETECLPASALVPPPHSRSASHRTSQGIEEWTTAATATGHGRRRIPDHLRRPATGRAR
HQIPDHPPVMGHAWHRIPDH
RTTVLPWAGPGIRYRTTAAALPRAMSGIGYRTTALPRATPGRRIPPPPSLLPNSS
>g473.t1
MGKTADLTAVQKAIIDTLKQE
>g474.t1
MDPPFGAEEEVADVQGLQAWAHLCCRNRRECKLCLAVELQIHIHPQLDTRDEENAKDNDSHEGGRRTNGHRVAHTEIGNN
VPINVGPAHISYGTMYRSFS
KN
>g475.t1
LLDLKVTVDGLEKERDFYFGKLRDIELICQEHESDNSPTLSKVMDVLYATEDRSLILPTVVRMDYVRTPLAEHTSSRPAS
GIHGDTWVTLAAWFSSKPHA
LHVHDGKRDC
>g475.t2
LLDLKVTVDGLEKERDFYFGKLRDIELICQEHESDNSPTLSKVMDVLYATEDGFAPPEDEENEGAQGDEDF
>g476.t1
AGTGRWPDRHQRTRRGRLPNATSEHGAGVGRASPTGDSSLRVTAAHPGAQLGSGAVGSVARLADCGPPHARRVPQNGLTS
AGLF
>g476.t2
AGTGRWPDRHQRTRRGRLPNATSEHGAGVGRASPTGDSSLRVTAAHPAGAQLGSGAVGSVARLADCGPPHARRVPQNGLT
SAGLF
>g477.t1
MDCKRLQRVVRTAEKITKTPLPSLQSIYHRRVHRRAASILRDPTHPQHGLFTLLPSGRRYRSVRSKTTRLKNSFFPTAIR
LLNR
>g478.t1
GPGFLLVHDNARPHVARVCRQFLEDEGIDTIDWPPRSPDLNPIEHLWDIMFRSIRWRQVAPQTVEELSDALVQIWEEIPQ
ETIRRLIRSMPRRCQACIQA
RGGNTKY
>g479.t1
LGVDGLMVSNTTISRPPNLQDPRCSETGGLSGRPLRELSTDTIREMFVLTRGKVPIVGIGGVASGKDALDKIRAGASLVQ
LYTAFSYQGPPLVTRIKREL
HQLLQ
>g480.t1
VKAFEKMDHLARAQRLLELQEAENARYVSLVGNTETAGSASGQILRPPGSLTTWFSDLSAQGQPG
>g481.t1
MHHHHHHHLHLTRDVVLQLVRDNADLRCELPKLEKRLRATAERVKALEGTLRAAKEGAMKDRKRYQQEVDRIKEALRSKN

VSRRGHSAQIAKPIRAGHHH
QPHPSSSSASAAPPSIRGSGGSGSSSSSSSSTSRGVSSPRHHHPSQHHPSQHRAQPQQPYPHPQHHRSK
>g481.t2
GSLAQKQRIVFLENNLEQLTKVHKQLVRDNADLRCELPKLEKRLRATAERVKALEGTLRAAKEGAMKDRKRYQQEVDRIK
EALRSKNVSRRGHSAQIAKP
IRAGHHHQPHPSSSSASAAPPSIRGSGGSGSSSSSSSSTSRGVSSPRHHHPSQHHPSQHRAQPQQPYPHPQHHRSK
>g482.t1
DLMEQLEKQDKTIRKLKKQLKVYSKRIGEMG
>g482.t2
MEQLEKQDKTIRKLKKQLKVYSKRIGEMG
>g483.t1
VDMLVPTKITGVITQGAKDFGHVQFVGSYKVAYSNDGERWTVYQDEKQGKDKP
>g484.t1
MSAVSRQVKLSSPDYVDCDKEEAVADFLKRIECYKVTYTPLDDDKDRIRLFLTREATQLLTQALVISRHDYCNSLLAGLP
ATAIKPLQRIQNAAACLVFN
RHKFSHVTPLFHDLHWLPVVARIKFKTMLLAYKAVNGIAPTYLQILWWNELPANVRTAESLPIFRKRLKTHLFRVHLNSA
>g484.t2
MSAVSRQVKLSSPDYVDCDKEEAVADFLKRIECYKVTYTPLDDDKDRFAVYNIRRIRLFLTREATQLLTQALVISRHDYC
NSLLAGLPATAIKPLQRIQN
AAACLVFNRHKFSHVTPLFHDLHWLPVVARIKFKTMLLAYKAVNGIAPTYLQILWWNELPANVRTAESLPIFRKRLKTHL
FRVHLNSA
>g484.t3
MSAVSRVFFVESICEDPEIIAENIKQVKLSSPDYVDCDKEEAVADFLKRIECYKVTYTPLDDDKDRIRLFLTREATQLLT
QALVISRHDYCNSLLAGLPA
TAIKPLQRIQNAAACLVFNRHKFSHVTPLFHDLHWLPVVARIKFKTMLLAYKAVNGIAPTYLQILWWNELPANVRTAESL
PIFRKRLKTHLFRVHLNSA
>g485.t1
MAGILPFGAMFIELFFIFSAIWENQFYYLFGFLFLVFIILVVSCSQISIVMVYFQLCAEDYRWWWRTFLVSGGSAFYVLV
YAVFYFINKPGSLLKTAIMG
KTADLTAVQKAIIDTLKQE
>g486.t1
MLISMRSMSRSSVVKSRPPKADLSPLGPLVTSPLRDRTPRHQVVMVIQLFFIFFDLHRTFFSCTCYSGVSVL
>g487.t1
TGLRYLAGSWLWVGDGDQEVPTGLPSCPDQHNVCGSVSQTLNVTQASNCMDRRNFFCQWTS
>g488.t1
MGKTADLTAVQKAIIDTLKQEGKTQKEISERIGCSQSAVSRHLSGKSWKEKVWQKSCTTRRGDRTLRKIVEKGRFQTLGD
LRKQWTESGVETQSHRAQAC
AGNGLQVPHSP
>g488.t2
MGKTADLTAVQKAIIDTLKQEGKTQKEISERIGCSQSAVSRHLSGKSWKEKVWQKSCTTRRGDRTLRKIVEKGRFQTLGD
LRKQWTESGVETQSHRAQAC
AGNGLQ
>g489.t1
MRGTQLELNGHKRDAGFHGDPVQQGPLPVPPRGRGGWRGAWRPLLSELREAEAPSQSYVSDADDSTMSGAREELLPVDAT
AAACNSSLNGSEAPGLQELH
RTEAALFEFQVFTITLAVLALCILAFTGLYCITVCFHRNRQSKRAHAYESAVTRGAQVDPVAVRAVKRSTSFINALTLFR
RAEGPRDKSRIYYIYSNPLP
IGLEDEEEQEEREAAAVAEEEEERGKERKKLRPREAPSRSLLIQKYAEDPNSGVTLDPPMF
>g490.t1
MAKRVKDAMKKELQQKEKNIMNRQKTYKEQEKESREAALHNTISDGNKGFALLQKMGYKAGQGLGKQDRGGIGMENVKKR
KAEQELEHYRQKAQARQQNE
TQSLEDFRVRVRSEREERKIEEDLKRSQRACEQLDSEKNISVPREDWYWPTPEAAEEAEDEAKQEEEKEDEEEEDETVEL
MPVNESKLTQPNYVCWLNEH
SQKNKVSLSAVENTAMELDQAKPGSLGNMEVSTGLTEPNYIGLLNEYCQKLDHHPDFPFVRKCGPAHCPQFFYRVVIGAK
EYPEVHGKTAKEAKRRAAKE
AWVAIQEQSDCDKTLKEWIEEKNKLQDDPLRGKECLDIMKKIMCGVKYIHSKNLVHRDLKPSNIMFGSNEVKIVDFGLVT

EDSDDVTTQMERTSSTGTKS
YMAPEQDSGRPQHCCGFYNFLCIYRKLPLLYILHTYSLSP
>g490.t2
MAKRVKDAMKKELQQKEKNIMNRQKTYKEQEKESREAALHNTISDGNKGFALLQKMGYKAGQGLGKQDRGGIGMENVKKR
KAEQELEHYRQKAQARQQNE
TQSLEDFRVRVRSEREERKIEEDLKRSQRACEQLDSEKNISVPREDWYWPTPEAAEEAEDEAKQEEEKEDEEEEDETVEL
MVFE
>g490.t3
MKHRICCTVWRIYNSIRQDVKPGLPMAKRVKDAMKKELQQKEKNIMNRQKTYKEQEKESREAALHNTISDGNKGFALLQK
MGYKAGQGLGKQDRGGIGME
NVKKRKAEQELEHYRQKAQARQQNETQSLEDFRVRVRSEREERKIEEDLKRSQRACEQLDSEKNISVPREDWYWPTPEAA
EEAEDEAKQEEEKEDEEEED
ETVELMPVNESKLTQPNYVCWLNEHSQKNKVSLSAVENTAMELDQAKPGSLGNMEVSTGLTEPNYIGLLNEYCQKLDHHP
DFPFVRKCGPAHCPQFFYRV
VIGAKEYPEVHGKTAKEAKRRAAKEAWVAIQEQSDCDKTLKEWIEEKNKLQDDPLRGKECLDIMKKIMCGVKYIHSKNLV
HRDLKPSNIMFGSNEVKIVD
FGLVTEDSDDVTTQMERTSSTGTKSYMAPEQDSGRPQHCCGFYNFLCIYRKLPLLYILHTYSLSP
>g490.t4
MASRIQRALEKPVNESKLTQPNYVCWLNEHSQKNKVSLSAVENTAMELDQAKPGSLGNMEVSTGLTEPNYIGLLNEYCQK
LDHHPDFPFVRKCGPAHCPQ
FFYRVVIGAKEYPEVHGKTAKEAKRRAAKEAWVAIQEQSDCDKTLKEWIEEKNKLQDDPLRGKECLDIMKKIMCGVKYIH
SKNLVHRDLKPSNIMFGSNE
VKIVDFGLVTEDSDDVTTQMERTSSTGTKSYMAPEQDSGRPQHCCGFYNFLCIYRKLPLLYILHTYSLSP
>g491.t1
MELAHSLLLNEDALAQITEAKRPVFIFEWLRFLDKVLVAANKVDVKEKQKKLVEQLTGLISSAPGPPTRKLLAKNLATLY
SIGDTFTVFQTLDKCNDIIK
SKDDTPAYLPTKLAAVACVGAFYEKMGRMLGSSFPDTINNMLKALKSAEGRGEILISLQKVLCGLGGAAASCHRDIYKNA
RSLLTDRSMAVRCAVAKCLL
ELQNEAVFMWTTELENLATLCFKALEGSNYGVRVAVSKLLGTVMATALMPKQAAVMRQNVKRASLEEVLELMATGFLRGG
SGFLKSGGEMLKGGGSVSRE
VRVGVTQASVHHSHIASRCDRVQTHVVSSPPQAYVVFVTTLGGQWLERNFATFLSHVLDLVSHPRATQTHVEAVYSRRCV
SFMLRATLGGLLGEKAQIAA
GMELCQAIGKQMRAVVTVLPAATVAWPQKGAALKLLMDWKPLSQCEFK
>g491.t2
MELAHSLLLNEDALAQITEAKRPVFIFEWLRFLDKVLVAANKVDVKEKQKKLVEQLTGLISSAPGPPTRKLLAKNLATLY
SIGDTFTVFQTLDKCNDIIK
SKDDTPAYLPTKLAAVACVGAFYEKMGRMLGSSFPDTINNMLKALKSAEGRGEILISLQKVLCGLGGAAASCHRDIYKNA
RSLLTDRSMAVRCAVAKCLL
ELQNEAVFMWTTELENLATLCFKALEGSNYGVRVAVSKLLGTVMATALMPKQAAVMRQNVKRASLEEVLELMATGFLRGG
SGFLKSGGEMLKGGGSVSRE
VRVGVTQAYVVFVTTLGGQWLERNFATFLSHVLDLVSHPRATQTHVEAVYSRRCVSFMLRATLGGLLGEKAQIAAGMELC
QAIGKQMRAVVTVLPAATVA
WPQKGAALKLLMDWKPLSQCEFK
>g491.t3
MELAHSLLLNEDALAQITEAKRPVFIFEWLRFLDKVLVAANKVDVKEKQKKLVEQLTGLISSAPGPPTRKLLAKNLATLY
SIGDTFTVFQTLDKCNDIIK
SKDDTPAYLPTKLAAVACVGAFYEKMGRMLGSSFPDTINNMLKALKSAEGRGEILISLQKVLCGLGGAAASCHRDIYKNA
RSLLTDRSMAVRCAVAKCLL
ELQNEAVFMWTTELENLATLCFKALEGSNYGVRVAVSKLLGTVMATALMPKQAADAPGVSLPLLCCPNVCALTASLSSRC
LSTVMRQNVKRASLEEVLEL
MATGFLRGGSGFLKSGGEMLKGGGSVSREVRVGVTQASVHHSHIASRCDRVQTHVVSSPPQAYVVFVTTLGGQWLERNFA
TFLSHVLDLVSHPRATQTHV
EAVYSRRCVSFMLRATLGGLLGEKAQIAAGMELCQAIGKQMRAVVTVLPAATVAWPQKGAALKLLMDWKPLSQCEFK
>g492.t1
MLMVNYRKCMEQGRTPMDICRAPVERVNSFRFPWVNITECLTWSHLTQSYLTRAHLTWAHLTWAHEGRVVKAVKCLFYLQ
QRKRLTWTPEYTDWLRHRQL

GQLHHPWPQGSPVMGSDSAVLTLAGSSFHHCGAKIEKS
>g493.t1
EKGHLFSGLLKKSPKAQRDIQEDLSGQRELSTSNDSLSEHKKTKEKGGAFSGMFKKPFSKHDEQPRDELSASNESLSENT
KDKLFGGMFRKPPRPVDAVQ
PHEDEQPLNSDLPPSNESSAETKEKGGLFGVILKKTPKATGDTAEDKDDDKTSLVSGDGLPEGNTGKEKSIFGGIFKKPQ
KQEDNSDKEESNQPEEELSA
SSEKLSETLTQKEKGGLFGGLLKKAPKPTVDNTEDKDADKEITASNDSLSEGAPAKEKSIFGGIFKKPQRPTQEDNSDKE
EKKMGLSGLFKRSASIDNLD
QEEKGGLFGGLLKKTPRATGDTTEDKDKEMTASNDSLSEGIAAKEKSIFGGIFKKPQKPTQEDSSEEKSIFSGMFKKSPK
ALEGSTADEDQVSGDELSET
AKERTGGISAMFKRPPKPSPRSQDPLHDGEDPGESDSETTGLKERGNEMSASNDSLNEMSAAKVVSFRVKRTLPRTAK
>g493.t2
EKGHLFSGLLKKSPKAQRDIQEDLSGQRELSTSNDSLSEHKKTKEKGGAFSGMFKKPFSKHDEQPRDELSASNESLSENT
KDKLFGGMFRKPPRPVDAVQ
PHEDEQPLNSDLPPSNESSAETKEKGGLFGVILKKTPKATGDTAEDKDDDKTSLVSGDGLPEGNTGKEKSIFGGIFKKPQ
KQEDNSDKEEKGGLFGGLLK
KAPKPTVDNTEDKDADKEITASNDSLSEGAPAKEKSIFGGIFKKPQRPTQEDNSDKEEKKMGLSGLFKRSASIDNLDQEE
KGGLFGGLLKKTPRATGDTT
EDKDKEMTASNDSLSEGIAAKEKSIFGGIFKKPQKPTQEDSSEDQVSGDELSETAKERTGGISAMFKRPPKPSPRSQDPL
HDGEDPGESDSETTGLKERG
NEMSASNDSLNEMSAAKVVSFRVKRTLPRTAK
>g493.t3
EKGHLFSGLLKKSPKAQRDIQEDLSGQRELSTSNDSLSEHKKTKEKGGAFSGMFKKPFSKHDEQPRDELSASNESLSENT
KDKLFGGMFRKPPRPVDAVQ
PHEDEQPLNSDLPPSNESSAETKEKGGLFGVILKKTPKATGDTAEDKDDDKTSLVSGDGLPEGNTGKEKSIFGGIFKKPQ
KQEDNSDKEEKGGLFGGLLK
KAPKPTVDNTEDKDADKEITASNDSLSEGAPAKEKSIFGGIFKKPQRPTQEDNSDKEEKKMGLSGLFKRSASIDNLDQEE
KGGLFGGLLKKTPRATGDTT
EDKDKEMTASNDSLSEGIAAKEKSIFGGIFKKPQKPTQEDSSEEKSIFSGMFKKSPKALEGSTADEDQVSGDELSETAKE
RTGGISAMFKRPPKPSPRSQ
DPLHDGEDPGESDSETTGLKERGNEMSASNDSLNEMSAAKVVSFRVKRTLPRTAK
>g494.t1
MAAITPILKKPGADPTDLNQYRPISNLPFISKTPERIVAAQLHSHLDSNNLHEPFQSGFRPKHSTETALVKITNDLLRAA
DSGLLTILILLDLTAAFDTM
SHPLRLGHLADIGITGMDIRSWMSRNFLRLNGSKTEALLIGSQNTLSKVQTTVATNIIIDGFPVPFSS
>g494.t2
MAAITPILKKPGADPTDLNQYRPISNLPFISKTPERIVAAQLHSHLDSNNLHEPFQSGFRPKHSTETALVKITNDLLRAA
DSGLLTILILLDLTAAFDTM
SHPLRLGHLADIGITGMVLAWFTSYLTDRQQFVQLKNHKSRSWMSRNFLRLNGSKTEALLIGSQNTLSKVQTTVATNIII
DGFPVPFSS
>g495.t1
ETWKCCRRRPRPRPNLCSSAHCKLHGTTVQCGCIIDRSFARLAVGISNCHGLQVGSSQGQGACNTRSSEGRPSRKEKFWM
ETPLRSNKLARGWYEGLEGG
WVVYASIQHLDTKAPPHYIHPSHPPLSGSFTAQEI
>g496.t1
LVLDCLLDLLLVLEKPPSSFTGPLEVPRRPCRHDDVVRLLLTNMEMEHKVALRRIYAAVLPKYIHRHLQRVERVLQGYLE
VSDPPQESSRSSALDALETL
LPVAWPRIDHRRAAVFLKCLLRLLVDVSSDGLL
>g496.t2
LVLDCLLDLLLVLEKPPSSFTGPLEVPRRPCRHDDVVRLLLTNMEMEHKVALRRIYAAVLPKYIHRHLQRVERVLQGYLE
VSDPPQESSRSSALDALETL
LPVAWPRIDHRRAAVFLKCLLRLLVDVSSDGLLCTSVQEKLTNQTSRCLTLLDHCSHGSVQVG
>g497.t1
LSDNNDESSADDRASVSGQDSWAPARYGPGPDGLAFPFGSVVSGFEFDCQHLKKKKKKKQKKKNQNQNQLYSL
>g498.t1
MGKTADLTAVQKAIIDTLKQE

>g499.t1
MGVGPGQEEEPIVAALPRGQRACRTLPSLDSAVESWDGSAADGYQSYSFNEWRSPKTNNSQSSTTARQRTNPLSDLGLSD
DDWDRPPLTGFNVGHDGTEP
DQEENFWSQALEDLETCGQSGILRELE
>g499.t2
MGVGPGQEEEPIVAALPRGQRACRTLPSLDSAVESWDGSAADGYQSYSFNEWRSPKTNNSQSSTTARQRTNPLSDLGLSD
DDWDRPPLTGFNVGHDGTEP
DQEENFWSQALEDLETCGQSGILRELEVPLSRC
>g500.t1
MQMCSVSSMVEAHDVGKIYKIRIGHDGSGIGDGWFLEMVDIKRLKMAMVQVEVKVEPKKDKKKDKKKKKKEEVEYIEELQ
EVVETLMFPCNRWLARDEED
GEIVVELLPEGTEDLEIISYEIHVFTGSLWGAGTDANVYITIYGEVGDTGERWLRKSNHLNKFEKSQEDIFNITAVDMGV
LKKLRIRHDNSNSNAAWFLE
RVEIIDNKDDTTYYFPCTRWLALDEDDGQIARELVPVDEAFMRKGDDDEEESSTTLGLEQKGEVIRYMNTKELKVAASLH
FKVEDVSEHLS
>g500.t2
VEAHDVGKIYKIRIGHDGSGIGDGWFLEMVDIKRLKMAMVQVEVKVEPKKDKKKDKKKKKKEEVEYIEELQEVVETLMFP
CNRWLARDEEDGEIVVELLP
EGTEDLEIISYEIHVFTGSLWGAGTDANVYITIYGEVGDTGERWLRKSNHLNKFEKSQEDIFNITAVDMGVLKKLRIRHD
NSNSNAAWFLERVEIIDNKD
DTTYYFPCTRWLALDEDDGQIARELVPVDEAFMRKGDDDEEESSTTLGLEQKGEVIRYMNTKELKVAASLHFKVEDVSEH
LS
>g500.t3
LRAGGTNANVFCQFYGEDGKTQVLALKNRSNNFERGTTEIFKVEAHDVGKIYKIRIGHDGSGIGDGWFLEMVDIKRLKMA
MVQVEVKVEPKKDKKKDKKK
KKKEEVEYIEELQEVVETLMFPCNRWLARDEEDGEIVVELLPEGTEDLEIISYEIHVFTGSLWGAGTDANVYITIYGEVG
DTGERWLRKSNHLNKFEKSQ
EDIFNITAVDMGVLKKLRIRHDNSNSNAAWFLERVEIIDNKDDTTYYFPCTRWLALDEDDGQIARELVPVDEAFMRKGDD
DEEESSTTLGLEQKGEVIRY
MNTKELKVAASLHFKVEDVSEHLS
>g501.t1
MGGLWNCQSAVKKADSISAYACLQTLHFLALTETWITPDNTATPAALSTAYSFSHTPRPSGRGGGTGLLISPKWSYRVLP
LAHLPRSAFELHAVAITAPI
KLYIAVIYRPPGPFHDFFDEMDALLSCFPEDGTPLVVLGDFNIQPEKLQ
>g502.t1
SLLDEPNPNSPANSQAAQLYQENKREYEKRVSAIVEQSWRDS
>g503.t1
SGAMDKSGGKRSRRQKNGENRDSYGGAEHGEDAGPGASREKRAKTSSKKKKRSKGKSEREVSPPDLPIDPDEPTYCLCEQ
VSYGEMIGCDNDECPIEWFH
FSCVGLHHKPKGKWFCPKCRGENEKTMDKALERAKKERSYNR
>g504.t1
EPDKVHHNNFLFYLGGVSALVVLLILIVGMLAAKRKSKHGVLWLPEGFMAKKDDKRREPVGQDDFGMKSVALASGF
>g505.t1
LSLNLLVLVQMLLYRLPDGRSGEQQGAEDTSLGGTGAEGSVLSVEEVALLILIACGLLVRMPRIQLHKVELMSLSFATSL
DSTMLLKTEL
>g505.t2
LTACGTKLSLNLLVLVQMLLYRLPDGRSGEQQGAEDTSLGGTGAEGSVLSVEEVALLILIACGLLVRMPRIQLHKVELMS
LSFATSLDSTMLLKTEL
>g506.t1
MMDHVIYWVRMDPTELGRTQLPGDEHINSMAVPMMLLCLVQQLTEERDNDLVAKYRETGNWCVKQILQHIQECKSGLCHK
ASAANFHRPYPGPPSLLLCV
RC
>g506.t2
MDPTELGRTQLPGDEHINSMAVPMMLLCLVQQLTEERDNDLVAKYRETGNWCVKQILQHIQECKSGLCHKASAANFHRPY
PGPPSLLLCVRC
>g507.t1

QPEGGGGGGDAGACYATRRRLTDASWETRGSLGPSLKDEDECCERIPGTAAEAG
>g508.t1
DSRRGEQGRGKERRAEEGRAEENRGEERRKGEEKEGERRGGEGRGEEKRGECLEKELGQGIDKSAAVPAPSALALSSLDK
DEPRKSFGIKVQNLPVRSTD
TSLKDGLFHEFKKHGKVTSVQIHGASEERYGLVFFRQQEDQEKAQVASKGKLFFGMQIEVIAWNGPETESENEFRPLDER
IDEFHPKATRTLFIGNLEKT
TSYHDLLNIFQRFGEIVDIDIKKVNGAPQYAFLQYCDIASVCKAIKKMDGEYLGSNRLKVVFDRMKGMALILYNNIEYAQ
AAVKEAKGWKIGGNKVKVDF
ANQESQMAFYRSMQASGQDIRDFYDLLTDRRDERRTQYEFSAERTYYDNVRTSATYSEDPRRKYPTRSREFYGEWDPYQG
DYYDPQYFEDPREYREYRAD
PYEQDIRKYSYLQRERERERERFETDRGRDHGRRTIECSQSPTHVPSHRPASPTASPSLSERIQSDSDRRLCCRSSERSG
SCSSVSPPRFEKPDKTHAER
YNKLDKLDKDRPFDVERGNASDKEKRVARKDKIDKQMLKKLKASSPNSQEIEPGVERDVSPVAAFQSKSIKTQKECSGKL
QGKDALKTLLGNPTDKKDPR
TRKTADRNLVSEETVLRS
>g508.t2
DSRRGEQGRGKERRAEEGRAEENRGEERRKGEEKEGERRGGEGRGEEKRGECLEKELGQGIDKSAAVPAPSALALSSLDK
DEPRKSFGIKVQNLPVRSTD
TSLKDGLFHEFKKHGKVTSVQIHGASEERYGLVFFRQQEDQEKAQVASKGKLFFGMQIEVIAWNGPETESENEFRPLDER
IDEFHPKATRTLFIGNLEKT
TSYHDLLNIFQRFGEIVDIDIKKVNGAPQYAFLQYCDIASVCKAIKKMDGEYLGSNRLKVVFDRMKGMALILYNNIEYAQ
AAVKEAKGWKIGGNKVDFAN
QESQMAFYRSMQASGQDIRDFYDLLTDRRDERRTQYEFSAERTYYDNVRTSATYSEDPRRKYPTRSREFYGEWDPYQGDY
YDPQYFEDPREYREYRADPY
EQDIRKYSYLQRERERERERFETDRGRDHGRRTIECSQSPTHVPSHRPASPTASPSLSERIQSDSDRRLCCRSSERSGSC
SSVSPPRFEKPDKTHAERYN
KLDKLDKDRPFDVERGNASDKEKRVARKDKIDKQMLKKLKASSPNSQEIEPGVERDVSPVAAFQSKSIKTQKECSGKLQG
KDALKTLLGNPTDKKDPRTR
KTADRNLVSEETVLRS
>g508.t3
IRGEESRGGERRGEQRRGEQRRTEEKRGEKGRRKKGRGEEGRGEERRKGESALKRSWARELTSDSSTSSSDDSPARSVQS
AAVPAPSALALSSLDKDEPR
KSFGIKVQNLPVRSTDTSLKDGLFHEFKKHGKVTSVQIHGASEERYGLVFFRQQEDQEKAQVASKGKLFFGMQIEVIAWN
GPETESENEFRPLDERIDEF
HPKATRTLFIGNLEKTTSYHDLLNIFQRFGEIVDIDIKKVNGAPQYAFLQYCDIASVCKAIKKMDGEYLGSNRLKVVFDR
MKGMALILYNNIEYAQAAVK
EAKGWKIGGNKVKVDFANQESQMAFYRSMQASGQDIRDFYDLLTDRRDERRTQYEFSAERTYYDNVRTSATYSEDPRRKY
PTRSREFYGEWDPYQGDYYD
PQYFEDPREYREYRADPYEQDIRKYSYLQRERERERERFETDRGRDHGRRTIECSQSPTHVPSHRPASPTASPSLSERIQ
SDSDRRLCCRSSERSGSCSS
VSPPRFEKPDKTHAERYNKLDKLDKDRPFDVERGNASDKEKRVARKDKIDKQMLKKLKASSPNSQEIEPGVERDVSPVAA
FQSKSIKTQKECSGKLQGKD
ALKTLLGNPTDKKDPRTRKTADRNLVSEETVLRS
>g509.t1
MNKTSEEVAEVEVSPSKLPESIPEDHQTINSKAVTAASTAGHHSSTPKSSVIRDIVLQSHLGVQRMHPGGGTSFPVYRDM
LGFTSQFPRRPSLGMNRANR
GIVSSQADSSLQAPPTFLPAPPPAVVSMGQQLRPDGKLEHSGHCSTDMVQLLKKYPIVWQGLLALKNDQAAVQLHFVSGN
EVLAQCSLPPPEGGSLLRIV
QRMRLEASQLDSVARRMTVENDYCLLLALPCGRDQEDVLSQTQALKTGFITYLQAKQAAGIINVPNPGSNQPAFVVQIFP
PCEFSEVHLSHLAPDLLNSI
SSISPHLMIVIASV
>g509.t2
MNKTSEEVAEVEVSPSKLPESIPEDHQTINSKAVTAASTAGHHSSTPKSSVIRDIVLQSHLGQQGVVSGGVNSSGTEEDP
RHFNQVLPRPPVPQADSSLQ
APPTFLPAPPPAVVSMGQQLRPDGKLEHSGHCSTDMVQLLKKYPIVWQGLLALKNDQAAVQLHFVSGNEVLAQCSLPPPE
GGSLLRIVQRMRLEASQLDS

VARRMTVENDYCLLLALPCGRDQEDVLSQTQALKTGFITYLQAKQAAGIINVPNPGSNQPAFVVQIFPPCEFSEVHLSHL
APDLLNSISSISPHLMIVIA
SV
>g509.t3
MNKTSEEVAEVEVSPSKLPESIPEDHQTINSKAVTAASTAGHHSSTPKSSVIRDIVLQSHLGQQGVVSGVQRMHPGGGTS
FPVYRDMLGFTSQFPRRPSL
GMNRANRGIVSSQADSSLQAPPTFLPAPPPAVVSMGQQLRPDGKLEHSGHCSTDMVQLLKKYPIVWQGLLALKNDQAAVQ
LHFVSGNEVLAQCSLPPPEG
GSLLRIVQRMRLEASQLDSVARRMTVENDYCLLLALPCGRDQEDVLSQTQALKTGFITYLQAKQAAGIINVPNPGSNQPA
FVVQIFPPCEFSEVHLSHLA
PDLLNSISSISPHLMIVIASV
>g510.t1
LTKLLKKAMTMLETDKENKRAERERILAERVPPLQMAGLTLQDLQNLCKELAQKIDVVDEERYDINSKVSKNDMEVQDLS
QKICDLKGKMKRPNLKRVKV
SAEAMLGALLGAKVKESVDFKANLKTVKKEEEKKEEVTDWRKNVESMSGMEGRKKLFDAA
>g510.t2
MLRLQAQGALLLQYHKTVMKKTRRRRRRKTKLLKKAMTMLETDKENKRAERERILAERVPPLQMAGLTLQDLQNLCKELA
QKIDVVDEERYDINSKVSKN
DMEVQDLSQKICDLKGKMKRPNLKRVKVSAEAMLGALLGAKVKESVDFKANLKTVKKEEEKKEEVTDWRKNVESMSGMEG
RKKLFDAA
>g510.t3
MWRQDVSTQQQHGEGRGSILVDHSHGAGFYISTHFVSFWAQKNCRSQTLSSKTKLLKKAMTMLETDKENKRAERERILAE
RVPPLQMAGLTLQDLQNLCK
ELAQKIDVVDEERYDINSKVSKNDMEVQDLSQKICDLKGKMKRPNLKRVKVSAEAMLGALLGAKVKESVDFKANLKTVKK
EEEKKEEVTDWRKNVESMSG
MEGRKKLFDAA
>g511.t1
MSKYKVILLRHGEGEWNKENRFCSWVDQKLSTDGVKEAQDCGRLLREQGYTFDVVFTSLLSRSIHTAWLVLEAMRQEWVP
VVKSWRLNERHYGALIGLNR
AEMALNHGEQQVKVWRRSYDVTPPPIDRSHPYFLEIYNDRRYSTCDVSQEELPRAESLKQVLDRLLPYWDGTVGPEVRRG
RSVLISAHGNSCRALLKHLE
GISDSEIANVTLPTGTPVLLELDENLRSVRPWQLLGDQVKIQAAIKKVEDQGKVQPSA
>g512.t1
LNARDADWNQNARVSYILEDSSLNGVPVSSYVSVSADSGVIHAVRSFDYEQIKDFQFRVKAQDGGSPPLSSNVSVKILIQ
DQNDNAPQVLYPVQTGGSLV
AEMVPRSADVGYLVTKVVAVDVDSGQNAWLSYKLQKATDRALFEVGLQNGEIRTIRQVTDKDAVKQRLTVIVEDNGQPSR
SATVIVNVAVADSFPEVLSE
FTDFTHDKEYNDNLTFYLVLALAVVSFLFITCLVVIVSVKIYRWRQSRILYHSNLPVIPYYPPRYSDTLGGTGTLQHVYN
YEVCRTTDSRKSDCKFGNPG
SQNVLIMDPSSTGTMQRMQSEKSILDEPDSPLE
>g513.t1
LIQDQNDNAPQVLYPVQTGGSLVAEMVPRSADVGYLVTKVVAVDVDSGQNAWLSYKLQKATDRALFEVGLQNGEIRTIRQ
VTDKDAVKQRLTVIVEDNGQ
PSRSATVIVNVAVADSFPEVLSEFTDFTHDKEYNDNLTFYLVLALAVVSFLFITCLVVIVSVKIYRWRQSRILYHSNLPV
IPYYPPRYSDTLGGTGTLQH
VYNYEVCRTTDSRKSDCKFGNPGSQNVLIMDPSSTGTMQRMQSEKSILDEPDSPLEVRTFP
>g514.t1
MVPRSADVGYLVTKVVAVDVDSGQNAWLSYKLQKATDRALFEVGLQNGEIRTIRQVTDKDAVKQRLTVIVEDNGQPSRSA
TVIVNVAVADSFPEVLSEFT
DFTHDKEYNDNLTFYLVLALAVVSFLFITCLVVIVSVKIYRWRQSRILYHSNLPVIPYYPPRYSDTLGGTGTLQHVYNYE
VCRTTDSRKSDCKFGNPGSQ
NVLIMDPSSTGTMQRMQSEKSILDEPDSPLEDQNDNAPQVLYPVQTGGSLVAEMVPRSADVGYLVTKVVAVDVDSGQNAW
LSYKLQKATDRALFEVGLQN
GEIRTIRQVTDKDAVKQRLTVIVEDNGQPSRSATVIVNVAVADSFPEVLSEFTDFTHDKEYNDNLTFYLVLALAVVSFLF
ITCLVVIVSVKIYRWRQSRI
LYHSNLPVIPYYPPRYSDTLGGTGTLQHVYNYEVCRTTDSRKSDCKFGNPGSQNVLIMDPSSTGTMQRMQSEKSILDEPD

SPLE
>g514.t2
LIQDQNDNAPQVLYPVQTGGSLVAEMVPRSADVGYLVTKVVAVDVDSGQNAWLSYKLQKATDRALFEVGLQNGEIRTIRQ
VTDKDAVKQRLTVIVEDNGQ
PSRSATVIVNVAVADSFPEVLSEFTDFTHDKEYNDNLTFYLVLALAVVSFLFITCLVVIVSVKIYRWRQSRILYHSNLPV
IPYYPPRYSDTLGGTGTLQH
VYNYEVCRTTDSRKSDCKFGNPGSQNVLIMDPSSTGTMQRMQSEKSILDEPDSPLEDQNDNAPQVLYPVQTGGSLVAEMV
PRSADVGYLVTKVVAVDVDS
GQNAWLSYKLQKATDRALFEVGLQNGEIRTIRQVTDKDAVKQRLTVIVEDNGQPSRSATVIVNVAVADSFPEVLSEFTDF
THDKEYNDNLTFYLVLALAV
VSFLFITCLVVIVSVKIYRWRQSRILYHSNLPVIPYYPPRYSDTLGGTGTLQHVYNYEVCRTTDSRKSDCKFGNPGSQNV
LIMDPSSTGTMQRMQSEKSI
LDEPDSPLE
>g515.t1
MEPELLTTARAVLEPTSEGEQGDMGAEEEEEAVEEELEAEDDRATAVQIAQLLRVNKY
>g516.t1
LSLSNAVDVDVGINSIKTYHISQSEFFDIEIQSGRDGSKFADLILKKTLDREQQPLHNLVLTALDGGVPTRSGTANIIIR
VLDTNDNAPRFDKDGYTINV
MENSPIGSLVSFYTIRVMENNAPGALLDSLTAYDPDLHENQYLVYFIIEREIANTSMSMLFSINPENGKLYALKTFDYEI
EKEFVFHIEARDSGVPPLSS
NVTVHIIIVDQNDNTPVIVSPWRAHGSVVEEKIPRSTDKGSLVSKVIAIDGDSVLNSRITYQFLQVTDATLFSLDQYNGE
IRTMRMFSYRDPRLQRLVVV
AKDNGEPALSATVTIKLSTVETAVKAYSDMTEVPLEYDIFSDLNLYLVIGLGSVSFLLLITILVTIVLKCQKPKSSKAAA
PCRNSVISERNSTIADSTLV
SNDAYWYSMFLAETRKGKLVVRQPAPKGSRYIVSSLPRSTGLTETSDSAPS
>g516.t2
MENNAPGALLDSLTAYDPDLHENQYLVYFIIEREIANTSMSMLFSINPENGKLYALKTFDYEIEKEFVFHIEARDSGVPP
LSSNVTVHIIIVDQNDNTPV
IVSPWRAHGSVVEEKIPRSTDKGSLVSKVIAIDGDSVLNSRITYQFLQVTDATLFSLDQYNGEIRTMRMFSYRDPRLQRL
VVVAKDNGEPALSATVTIKL
STVETAVKAYSDMTEVPLEYDIFSDLNLYLVIGLGSVSFLLLITILVTIVLKCQKPKSSKAAAPCRNSVISERNSTIADS
TLVSNDAYWYSMFLAETRKG
KLVVRQPAPKGSRYIVSSLPRSTGLTETSDSAPSTLQCQLVLNADFLVPVMWFLVEESMMSVLRLRRQGST
>g516.t3
MENNAPGALLDSLTAYDPDLHENQYLVYFIIEREIANTSMSMLFSINPENGKLYALKTFDYEIEKEFVFHIEARDSGVPP
LSSNVTVHIIIVDQNDNTPV
IVSPWRAHGSVVEEKIPRSTDKGSLVSKVIAIDGDSVLNSRITYQFLQVTDATLFSLDQYNGEIRTMRMFSYRDPRLQRL
VVVAKDNGEPALSATVTIKL
STVETAVKAYSDMTEVPLEYDIFSDLNLYLVIGLGSVSFLLLITILVTIVLKCQKPKSSKAAAPCRNSVISERNSTIADS
TLVSNDAYWYSMFLAETRKG
KLVVRQPAPKGSRYIVSSLPRSTGLTETSDSAPSTLQSFYTIRVMENNAPGALLDSLTAYDPDLHENQYLVYFIIEREIA
NTSMSMLFSINPENGKLYAL
KTFDYEIEKEFVFHIEARDSGVPPLSSNVTVHIIIVDQNDNTPVIVSPWRAHGSVVEEKIPRSTDKGSLVSKVIAIDGDS
VLNSRITYQFLQVTDATLFS
LDQYNGEIRTMRMFSYRDPRLQRLVVVAKDNGEPALSATVTIKLSTVETAVKAYSDMTEVPLEYDIFSDLNLYLVIGLGS
VSFLLLITILVTIVLKCQKP
KSSKAAAPCRNSVISERNSTIADSTLVSNDAYWYSMFLAETRKGKLVVRQPAPKGSRYIVSSLPRSTGLTETSDSAPS
>g517.t1
MASRLTSGSGISRSSPVMENADRRVQQQKLLSDSPAPATTPRPQLPSHQDAPPAPPQELGRTQQLIKRIEMANDEANGNQ
MQAGARGRPAVAPPRRAAQS
DVGKDTETQGRPEDGPAVKSPSGKQENLGVAREVSESNDSLSENHLKVKEKKGIFGGILRKSPKPTEAQDTQSLASELSA
SNDSLSETGKTTKDKGGIFS
GMFKKDPKPLSESSQPHEVTYRVVPRHRPNAVYNLYAVSNHSGTTIGGHYTAYCRNPASG
>g517.t2
LLSDSPAPATTPRPQLPSHQDAPPAPPQELGRTQQLIKRIEMANDEANGNQMQAGARGRPAVAPPRRAAQSDVGKDTETQ
GRPEDGPAVKSPSGKQENLG

VAREVSESNDSLSENHLKVKEKKGIFGGILRKSPKPTEAQDTQSLASELSASNDSLSETGKTTKDKGGIFSGMFKKDPKP
LSESSQPHEVTYRVVPRHRP
NAVYNLYAVSNHSGTTIGGHYTAYCRNPASG
>g517.t3
MASRLTSGSGISRSSPVMENADLPSHQDAPPAPPQELGRTQQLIKRIEMANDEANGNQMQAGARGRPAVAPPRRAAQSDV
GKDTETQGRPEDGPAVKSPS
GKQENLGVAREVSESNDSLSENHLKVKEKKGIFGGILRKSPKPTEAQDTQSLASELSASNDSLSETGKTTKDKGGIFSGM
FKKDPKPLSESSQPHEVTYR
VVPRHRPNAVYNLYAVSNHSGTTIGGHYTAYCRNPASG
>g518.t1
GAGISNQVNNSLDRKKVEKIVEDYQVKMADINKCMTFIKQGIENLRKFDLLKMRKHAYNRDFPALNNIYEDGAMMGKAIL
INANEIMRVVQIANVAGSTA
ARAVQIASMATGVLTGLFVGMDIYFVAKDSKELKKGAKSEFAGKIREVATQLHEGLVELNSIREELQSTTSTVDPEVTLD
LNKNKEHEEKKLEEDSDEDE
IDRIKKAIKKDYESREYV
>g519.t1
MITPGSSRKVAHLLACNRWELAPAPCSLLNGSSGGRITMMKRRESGAPKDKKKRSGVRQRLARHLLYDGGGGRLMHVVAG
RCRNNAPLGAITQATRRADT
SCIHLEAELGGKHRSLARAQPGPVNVTAQRQSASRGESL
>g520.t1
MPRSGDAPGIQERLDAAPLLMDIYPTQTPSMTQYSMLHPFQSALPGLWNCQSAVRKADSISTYACPQTLHFLHLTETWIT
PDNTATPAALSTAYSFSHTP
RPWGVSVFELHAVAITIPIKLYIAVIYRPLGPFHDFFDEMDALLSCFPEDSTPLLVLEPGTNLTLSSQGPVTPSVPSHPH
QRNLLLLLLGCPNICRLIEQ
SYGQLRGNGRNLAVLVIAPTTFNSSQISPPQCQQPNLHSTGTRSTPLPPTPENCFPCPCSSLLNPPFPPSSLIADDFANY
FTKKVTDISISLTPAIFPPP
TPTSVRLNYFTPLSSEEVLRLVTCIQATTCPLDPIPSSLLQAISTDILPFITTLINSSLTSGLIPAPFKTARGKPLLKKP
TLDSADIQSYKPSGFNTAHS
TETALLTVTESLRATKASSLSSVLVLLDLSAAYLMGRTYQWIQNAAARLTFNRHKFSHMTPSSMTSTGSL
>g520.t2
DIYPTQTPSMTQYSMLHPFQSALPGLWNCQSAVRKADSISTYACPQTLHFLHLTETWITPDNTATPAALSTAYSFSHTPR
PWGVSVFELHAVAITIPIKL
YIAVIYRPLGPFHDFFDEMDALLSCFPEDSTPLLVLEPGTNLTLSSQGPVTPSVPSHPHQRNLLLLLLGCPNICRLIEQS
YGQLRGNGRNLAVLVIAPTT
FNSSQISPPQCQQPNLHSTGTRSTPLPPTPENCFPCPCSSLLNPPFPPSSLIADDFANYFTKKVTDISISLTPAIFPPPT
PTSVRLNYFTPLSSEEVLRL
VTCIQATTCPLDPIPSSLLQAISTDILPFITTLINSSLTSGLIPAPFKTARGKPLLKKPTLDSADIQSYKPSGFNTAHST
ETALLTVTESLRATKASSLS
SVLVLLDLSAAYLMGRTYQWIQNAAARLTFNRHKFSHMTPSSMTSTGSL
>g520.t3
MPRSGDAPGIQERLDAAPLLMDIYPTQTPSMTQYSMLHPFQSALPGLWNCQSAVRKADSISTYACPQTLHFLHLTETWIT
PDNTATPAALSTAYSFSHTP
RPWGVSVFELHAVAITIPIKLYIAVIYRPLGPFHDFFDEMDALLSCFPEDSTPLLVLEPGTNLTLSSQGPVRNLLLLLLG
CPNICRLIEQSYGQLRGNGR
NLAVLVIAPTTFNSSQISPPQCQQPNLHSTGTRSTPLPPTPENCFPCPCSSLLNPPFPPSSLIADDFANYFTKKVTDISI
SLTPAIFPPPTPTSVRLNYF
TPLSSEEVLRLVTCIQATTCPLDPIPSSLLQAISTDILPFITTLINSSLTSGLIPAPFKTARGKPLLKKPTLDSADIQSY
KPSGFNTAHSTETALLTVTE
SLRATKASSLSSVLVLLDLSAAYLMGRTYQWIQNAAARLTFNRHKFSHMTPSSMTSTGSL
>g521.t1
MEVHKLFRRQNSHKAAGPDSVSPSTLQPCADKLSPVFMDIFNTSLETYRVPACFKVSTIIPVPKKPRTTGLNDYRLVALT
SVVMKSFLRLALSHLKAITN
PLLDPLQFPDRANRILFVDFSSAFNTIFPALLQDKLSQLHVPDSTCKWITDFLSDRKQCVKLGKHVSESQSISGSDHTQE
GMADTQREVLYSLMATGRND
FLCRSVVHLGERSLLLKVLLRWARVEKR
>g522.t1

MSTAKPSGIKIPTKITRPAPAKTATSAAGAKGTAAEKASAGGELQSSGESYQVGDRVWVNGNKPGVVQFLGEAQFAPGQW
AGIVLDEAIGKNDGSVAGVR
YFQCEALRGIFTRPSKLSRTEGEENGAQTAPPSRAA
>g523.t1
MCFNCGSPGHQIRDCPKPKDQAAINERRKEFSQNQSPSLGNQRYHAEVEERFAKYKAGLMSEELLSALGVGVNTLPPLIY
RMRQLGYPPGWLKEAEVENS
GLALYDGKESGTPHRYQGSGGFQFQSPLPPGSPSFGSPPPLPQGTPPATPTPPPLPKGTPPPTPTNGSPALPGRAWVVVD
ETGEGAEDGLTLEELEEQQR
LIWAALENADTATNSDCETQVTGTSVPSSPSVSTPARVDTETEMDTDDLVNLKTSTDDVKAGVEIAGPIRAQDESPLSPG
PIKAQDESPLSPGPIKAQDE
SPLSPGPIKAQEESPLSPGPIRAQEESPLSPGPSRAQDESPLSPASIGTLGASHPIPGWVRAPEDDPPSLGPVGAQTDTP
LSPKAGGTQDDNNLSPGPNR
VQGQDGLNPSLEASSVEKVPHRSRFAAGIVQFEDTPEYTEVAAATGTYLRIRDVLKSSPRNVSRSNK
>g523.t2
MCFNCGSPGHQIRDCPKPKDQAAINERRKEFSQNQSPSLGNQRYHAEVEERFAKYKAGLMSEELLSALGVGVNTLPPLIY
RMRQLGYPPGWLKEAEVENS
GLALYDGKGVCNSCVHCPEYMLYGSIPMQGNHLKPNFAAYLSNSFPSSGATSNKRRHDSDPAPHQTKKRRSSPSRSSDMD
LESESGTPHRYQGSGGFQFQ
SPLPPGSPSFGSPPPLPQGTPPATPTPPPLPKGTPPPTPTNGSPALPGRAWVVVDETGEGAEDGLTLEELEEQQRLIWAA
LENADTATNSDCETQVTGTS
VPSSPSVSTPARVDTETEMDTDDLVNLKTSTDDVKAGVEIAGPIRAQDESPLSPGPIKAQDESPLSPGPIKAQDESPLSP
GPIKAQEESPLSPGPIRAQE
ESPLSPGPSRAQDESPLSPASIGTLGASHPIPGWVRAPEDDPPSLGPVGAQTDTPLSPKAGGTQDDNNLSPGPNRVQGQD
GLNPSLEASSVEKVPHRSRF
AAGIVQFEDTPEYTEVAAATGTYLRIRDVLKSSPRNVSRSNK
>g523.t3
MCFNCGSPGHQIRDCPKPKDQAAINERRKEFSQNQSPSLGNQRYHAEVEERFAKYKAGLMSEELLSALGVGVNTLPPLIY
RMRQLGYPPGWLKEAEVENS
GLALYDGKGFNVAASPNMKDEYMLYGSIPMQGNHLKPNFAAYLSNSFPSSGATSNKRRHDSDPAPHQTKKRRSSPSRSSD
MDLESESGTPHRYQGSGGFQ
FQSPLPPGSPSFGSPPPLPQGTPPATPTPPPLPKGTPPPTPTNGSPALPGRAWVVVDETGEGAEDGLTLEELEEQQRLIW
AALENADTATNSDCETQVTG
TSVPSSPSVSTPARVDTETEMDTDDLVNLKTSTDDVKAGVEIAGPIRAQDESPLSPGPIKAQDESPLSPGPIKAQDESPL
SPGPIKAQEESPLSPGPIRA
QEESPLSPGPSRAQDESPLSPASIGTLGASHPIPGWVRAPEDDPPSLGPVGAQTDTPLSPKAGGTQDDNNLSPGPNRVQG
QDGLNPSLEASSVEKVPHRS
RFAAGIVQFEDTPEYTEMCHLIESARCLRGDTGLTYQGYKLVV
>g524.t1
MNYNISIRLDSEVCKSVPEIFQEPKKVETIDSQPGQAVKELVDASQKDTGSSCDTSADHNYCSLLEQSFQTQVESIQTTL
SSLELKLQSLEKKQRSENDA
DCTNKLKTALQLLDNIDCCLDGTLDERHLTQTVLTLLGAQCWGSVYSSHLINSVACWLGQQFHGANSCISQKVESFKVEH
IERITDLPPADQLATELFPE
AMRTLMLHWMGLSDEAHLGKRHSEYPILLLILEFANHNLITGAAHVLYSSLICR
>g525.t1
LLEKQQQQQQQPQQQQQEVPTVPAPLLCKAVTAAAPMATNPVLNVAALLASGTQVTPQIAMAAQMAALQARTLAETGIA
VPSYYNPSAVNPMKFAEQEK
KRKKLWQGKKEGDKSQTAELWEKLNFGNKDQNVKFRKLMGIKDEEEGEASKPINEEGLKTLQQQEEMFRNLDVQYEMARS
QTHTQRGMGLGFTTSFSRGM
DTI
>g526.t1
MLSSAGEVRTDLMSFTAEDYQHIEFGVDEVMESAPVGVKDPLFKVSSTLNP
>g527.t1
MEMTVAQITFXTSPSSIAAISKWACCCGEQTTRPPEHRPPDHLTTRTQTTRPPDHQTTDHQNTDHQNTDHQNTDHQNTTR
TQTTRTPPEHRPPEHKTTRP
PDQTTTRPPDQTTRPPDGSRGSQPLLSCDASVQRLDV
>g528.t1

MPEPSQLAPFDAKKLDPVGKHRCSLTERSLVIGRLTTSMEKQCVMFDLRRSALGPGYVKEVGVNRSEASGGGMLVEEVRE
EPVEVMEDWGDVIP
>g528.t2
MPEPSQLAPFDAKKLDPVGKHRCSLTERSLVIGRLTTSMEKQCVMFDLRRSALGPGYVKEVGVNRSEASGGGMLVEEEPV
EVMEDWGDVIP
>g528.t3
KLDPVGKHRCSLTERSLVIGRLTTSMEKQCVMFDLRRSALGPGYVKEVGVNRSEASGGGMLVEEVREEPVEVMEDWGDVI
P
>g529.t1
LREVCELHEGCDEMAETAGIVAAYTAYYGPVPF
>g530.t1
IGVISIERAYPLSLGANIGTTTTAILAAMASPGDTLGNSLQIALVHFLFNISGILLWYPIPFTRIPIRLAKGLGDITASY
RWFAAVYILCCFFVLPLSVF
ALSMAGWQVLVGVGVPLVAMLLVVMAINVLQRRRPGWLPAPLRSWDFLPLWAHSLEPWDGVVASLTARCCCCCKCCQVAP
GNGEEAGDAGCAVVAGEACA
AGTSG
>g530.t2
IGVISIERAYPLSLGANIGTTTTAILAAMASPGDTLGNSLQIALVHFLFNISGILLWYPIPFTRIPIRLAKGLGDITASY
RWFAAVYILCCFFVLPLSVF
ALSMAGWQVLVGVGVPLVAMLLVVMAINVLQRRRPGWLPAPLRSWDFLPLWAHSLEPWDGVVASLTARCCCCCKCCQVAP
GNGEEAGDAGCAVVAGEACA
AGTAERDAESQRKMELKILKSSRL
>g531.t1
VEDAEDQPKHLSSNNGQVSPGSRSATEWAGVIICPVPKKINPTGDKDHRSGEAELSEACSATDRGNYLDLNTSKTKDLLI
EFCWNSPPPYHYHLHEK
>g531.t2
VEDAEDQPKHLSSNNGQVSPGSRSATEWAGVIICPVPKKINPTGDKDHRSGEAELSEACSATDRGNYLDLNTSKTKDLLI
EFCWNSPPPYHYFNPMWTMY
HILEQNSGYFTLP
>g532.t1
MGKTADLTAVQKAIIDTLKQEGKTQKEISERIGCSQSAVSRHLSGKSWKEKVWQKSCTTRRGDRTLRKIVEKGRFQTLGD
LRKQWTESGVETQSHRAQAC
AGNGLQVPHSPGQATFEPETAAEAPDLGYREAALDCCSVVQSTFFG
>g533.t1
MPSLCLVRPAKVSPLVEEVSPSSTSSFEMMDMEAPPPPYQPVKPHWFYCRRADDSTSWLPFSREDSEKLESAYAS
>g533.t2
LAVMPSLCLVRPAKVSPLVEEVSPSSTSSFEMMDMEAPPPPYQPVKPHWFYCRRADDSTSWLPFSREDSEKLESAYAS
>g534.t1
MTMTVGQTVAGAKELSGLLTAQRSGSVSEVSSGLSPGPSSLSSVQSCMVSVSSTPAQGQAPATAQAPSASSLLQGLSFSL
QEIVTKAPSSPATSAGTGST
QVMSGRTQSQVLSSICSSTLLRAVPVTSAGGVSKTTAIHQLLTNGGLAKLASSLPGLAHIGHSQSAGQKAPTSIMVTLRG
SQPRVGPLNQPAAAALTLAK
TPDPKGLIWIND
>g534.t2
GASGRPMTMTVGQTVAGAKELSGLLTAQRSGSVSEVSSGLSPGPSSLSSVQSCMVSVSSTPAQGQAPATAQAPSASSLLQ
GLSFSLQEIVTKAPSSPATS
AGTGSTQVMSGRTQSQVLSSICSSTLLRAVPVTSAGGVSKTTAIHQLLTNGGLAKLASSLPGLAHIGHSQSAGQKAPTSI
MVTLRGSQPRVGPLNQPAAA
ALTLAKTPDPKGLIWIND
>g534.t3
MTPLFQDLHWLPVVARIKFKTMLLAYKAVNGTAPTYLQTLVKPHAPARALRSTTSAGRLVPPSLKGGKAGASGRPMTMTV
GQTVAGAKELSGLLTAQRSG
SVSEVSSGLSPGPSSLSSVQSCMVSVSSTPAQGQAPATAQAPSASSLLQGLSFSLQEIVTKAPSSPATSAGTGSTQVMSG
RTQSQVLSSICSSTLLRAVP
VTSAGGVSKTTAIHQLLTNGGLAKLASSLPGLAHIGHSQSAGQKAPTSIMVTLRGSQPRVGPLNQPAAAALTLAKTPDPK
GLIWIND

>g535.t1
WWNKLPVNVRTAESLPIFRERLKTHLFRVHLNPA
>g536.t1
MEVAGLYDEDSFAFHVRNSAVSPISNSSPYRPRRICDSMTELGIDEHEKAKDFCMYLDPAGHCQQLAAYGEAHHQGDVFS
DFLTESKIKRAAFQNLKSYA
FVNELESSQCGNLRNVREPAYVQGYPDLQETRVDTVYSPGLLENRCKAVESNEGKEDTKMDSGSSSFDMRSYLHYQSTPS
GSMGNISSASSTCSSPPGTP
APSGNNRSPSHNGKMSSGKSKKRLDKDSEEYKLRRERNNLAVRKSRDKAKMRNMETQHKVLELAAENDRLQKRVEQLSRE
LATLRNLLSATGQH
>g537.t1
MAISFLTLSPAEVYHNYPWYCYVYALAIFVTLTNQIHKWSHTYFGLPGWVVFLQDCHIILPRKHHRIHHVSPHETYFCIT
TGWLNYPLEKLGFWRYLEDI
IQSVTGEKPRADDLKWAHKVN
>g537.t2
MAISFLTLSPAEVYHNYPWYCYVYALAIFVTLTNQIHKWSHTYFGLPGWVVFLQDCHIILPRKHHRIHHVSPHETYFCIT
TGWLNYPLEKLGFWRYLEDI
IQSVTGEKPRADDLKWAHKFKHAELCRAAPEMNHLRSRPKAALPASKRGCRDVSPACCLKREVVSQAVVMQT
>g537.t3
MPCFALYFQGKRCQEWICVLLCFSLMAFNLIHLLANFHFGHLWFILLGVAEVYHNYPWYCYVYALAIFVTLTNQIHKWSH
TYFGLPGWVVFLQDCHIILP
RKHHRIHHVSPHETYFCITTGWLNYPLEKLGFWRYLEDIIQSVTGEKPRADDLKWAHKVN
>g537.t4
MAKFWGCQCIKQKQFKHAELCRAAPEMNHLRSRPKAALPASKRGCRDVSPACCLKREVVSQAVVMQT
>g538.t1
MSAAVRVKDCLTVPRNFRLLEELEEGQKGVGDGTVSWGLEDDEDMTLTRWRGMIIGPPRTIYENRMYSLKVECGPKYPES
PPCVRFVTKINLNGVDNSNG
VVDKQAVSALAKWQISYTIRTVLQDLRRLMLCKENIKLPQPPENQIYNN
>g538.t2
LLEELEEGQKGVGDGTVSWGLEDDEDMTLTRWRGMIIGPPRTIYENRMYSLKVECGPKYPESPPCVRFVTKINLNGVDNS
NGVVDKQAVSALAKWQISYT
IRTVLQDLRRLMLCKENIKLPQPPENQIYNN
>g539.t1
MKQANSTLLEALTAEVITAFQKWKEQKSWNAMFKAVMNYLHRLETILSFISESRNADLELHLQAGEALSKLFFAMDRIKY
KRLWQRYIADMHNLRTNHPQ
TWKELRTGNISVTKSVIPFVSVGADHACEHLNKLMKISSGIIGISNNANARQRFFMVAPELSRLAKEFKSQFDMEADRIT
EHHELGPSAVKRAHETINKI
RAAMLSHGNPFTTEGDKLHNLTTQAYIPDEYILNADVTGQKLFEDYASERINGDVSLWAPVKKENNKMFLSGNKRITVKL
RNNTVDLKETKDLFARLMVL
ARSNRDINYKEAI
>g540.t1
MSVQISDVNDNSPEFPQNPLELYLLENNTPGASIFSKSFDFETLKTFQFHVVATDSGAPPLSSNVTVNVFLLDQNDNPPV
ILHPLSSNGSAEGVEEVPRN
VLAGHLVTKVRAYDADIGYNGWLLFSLQEVSDHSLFGLDRYTGQIRTLRSFTETDEAQHKLVILVKDNGNVSLSATATVV
VKLVEPKEAISASDVKSAVT
>g541.t1
MSSQRSQTTELALLQLNVEGLTFIKINVLEQIASKNNVTVIILQETHTENKNTLRVPGYTLAGYIANKHHGIATFVHKDM
AWSAVGQSPEGAEVEWITTK
VQDTTIVNIYKPPPSRLVPSSLPDVPAPAVYAGDFNSHHTDWGYSSSNADGDFLVDWASTIVQHSLYDPKEPHTFYSARW
NSTTNPDLAFAKCHNNQPLP
VRRILDMFPRSHHRPSLITTPSLAQPVQGRDVKRWNFRKANWAGFTKQS
>g542.t1
LKNFYGSFSSPNYPDFYPPGSNCTWLIDTGDTRKVVLRFTDLKLDGTGYGDYVKVYDGLQENPRRLLRVLTAFDSRAPVA
VVSSSGRLRVHFYADKINAA
RGFNATYQVEGFCLPWEAPCGGNWGCYTEPQRCDGYWHCPNGRDELNCSSCQEEEFPCSRNGACYPRSDRCNYQNRCPNG
SDEKNCYFCQPGNFHCKDDC
GDGSDEQSCPVVVPTRVITAAVIGSLVCGLLLVIALGCTCKLYSLRMFERRSFETQLSRVEAELLRREAPPSYGQLIAQG

LIPPVEDFPVCSGTQASVLE
NLRLAVRSQLGFPSLRLPSSGRHGSLWRGLFWSSRS
>g542.t2
LKNFYGSFSSPNYPDFYPPGSNCTWLIDTGDTRKVVLRFTDLKLDGTGYGDYVKVYDGLQENPRRLLRVLTAFDSRAPVA
VVSSSGRLRVHFYADKINAA
RGFNATYQVEGFCLPWEAPCGGNWGCYTEPQRCDGYWHCPNGRDELNCSSCQEEEFPCSRNGACYPRSDRCNYQNRCPNG
SDEKNCYFCQPGNFHCKNNR
CVFESWVCDAQDDCGDGSDEQSCPVVVPTRVITAAVIGSLVCGLLLVIALGCTCKLYSLRMFERRSFETQLSRVEAELLR
REAPPSYGQLIAQGLIPPVE
DFPVCSGTQASVLENLRLAVRSQLGFPSLRLPSSGRHGSLWRGLFWSSRS
>g542.t3
LGDEIDCDVPTCGEHLKNFYGSFSSPNYPDFYPPGSNCTWLIDTGDTRKVVLRFTDLKLDGTGYGDYVKVYDGLQENPRR
LLRVLTAFDSRAPVAVVSSS
GRLRVHFYADKINAARGFNATYQVEGFCLPWEAPCGGNWGCYTEPQRCDGYWHCPNGRDELNCSSCQEEEFPCSRNGACY
PRSDRCNYQNRCPNGSDEKN
CYFCQPGNFHCKDDCGDGSDEQSCPVVVPTRVITAAVIGSLVCGLLLVIALGCTCKLYSLRMFERRSFETQLSRVEAELL
RREAPPSYGQLIAQGLIPPV
EDFPVCSGTQASVLENLRLAVRSQLGFPSLRLPSSGRHGSLWRGLFWSSRS
>g543.t1
MSRDMGRRRDMGHDIDRRRDTFSATGADTPSLMAKPRCSLPDAAGREDTLKKRRRRRK
>g544.t1
LGHEECEDADISTSAVDTVEPSSRLLASWTCTREPMVGADSEAARFLQVLSPAHGQRSMASPACPSGRPSTNRSRLSPFD
SDLESILEPGGQKRQQATQA
NMSINHHSEGGCVWLNPDASSSDTCLPRDRQVTALLEAPILPTHV
>g545.t1
GARPCSRLTQGDVAQGEDPEAGSGERKPPSRHFYPIALLLVSSHLLVVWLILSLVFLLAKYQ
>g546.t1
MNFLCEDDQFEHQASCQILEAETYKCVSCNRLGQHSCLRCKACYCDDHARSKVFKQEKGKGPPCPKCGHETQETKDLSMS
TRSVKFGRQSGAEEDSYGYG
ASGYAAYWKTAASSAGDQEEYDGAGDDEDDEDDDEEDEDDDEDEEDEEEGEEEHQDGKEGVVGPLAGLKLDETKT
>g546.t2
LFRCSFCMNFLCEDDQFEHQASCQILEAETYKCVSCNRLGQHSCLRCKACYCDDHARSKVFKQEKGKGPPCPKCGHETQE
TKDLSMSTRSVKFGRQSGAE
EDSYGYGASGYAAYWKTAASSAGDQEEYDGAGDDEDDEDDDEEDEDDDEDEEDEEEGEEEHQDGKEGVVGPLAGLKLDET
KT
>g546.t3
MHEIVRLRRKTSWDPQHWDGDGGVSCNRLGQHSCLRCKACYCDDHARSKVFKQEKGKGPPCPKCGHETQETKDLSMSTRS
VKFGRQSGAEEDSYGYGASG
YAAYWKTAASSAGDQEEYDGAGDDEDDEDDDEEDEDDDEDEEDEEEGEEEHQDGKEGVVGPLAGLKLDETKT
>g547.t1
MAVSLTRACTNIALSTCLLTVFFGHLLCMEFTVAEKEEWYTAFINTTYLDPVTLELKTDKSECGRYGEHSPKKEARGLVL
LPTFFRDRQGCHINVKFPMA
FPNLTWVALIAAGNCTFREKISNAASRNASAVVIYNVGSNNPNDTITMPHQGTGDIIAVMIPELKGQDIVALLEQHVTVM
MDITVGTRNLQKYVSRTSVV
FVSISFIVLMTISLAWLVFYYIQRFRYVNARDRNQRRLGDAAKKAICRLPVRTIRKGDKEIDSDFDSCAVCIESYKPNDV
VRILPCRHLFHKHCIDPWLQ
DQRTCPMCKTNILKALGIMPHADGSDEMPPEYEASVGAITGASEMTVNESSAVLDPGAPGSISLQRLSDHDDQVPPAAED
GPDYTGDHPALSSDLDPQST
GVYGGVPVEDQARGAPGDGPELPG
>g548.t1
TLFVPVGILSTAAPNNVTVLELEGSPCATGPCVVEFTRTPVLNATVAAARRRRPLFLRDG
>g549.t1
RADEEQDSATANTNCCVSANMDLWLTVMYAYKVPLLGLGCFIACSIRTRKDVNQEVTRECRRLALSVCAVAATSALGALG
SLLSSHQPQLHFLLPSLAIL
TGTASLLPWGVHAE
>g549.t2

RADEEQDSATANTNCCVSANMDLWLTVMYAYKVPLLGLGCFIACSIRTRKDVNQEVTRECRRLALSVCAVAATSALGALG
SLLSSHQPQLHFLLPSLAIL
TGTASLLPWGVHAEP
>g550.t1
MVPRSADVGYLVTKVVAVDVDSGQNAWLSYKLQKATDRALFEVGLQNGEIRTIRQVTDKDAVKQRLTVIVEDNGQPSRSA
TVIVNVAVADSFPEVLSEFT
DFTHDKEYNDNLTFYLVLALAVVSFLFITCLVVIVSVKIYRWRQSRILYHSNLPVIPYYPPRYSDTLGGTGTLQHVYNYE
VCRTTDSRKSDCKFGNPGSQ
NVLIMDPSSTGTMQRMQSEKSILDEPDSPLEVRYSIPEELATGSVVGNIAVDLGLDTSRLKSGRARIFTEDGSDSNVSVK
ILIQDQNDNAPQVLYPVQTG
GSLVAEMVPRSADVGYLVTKVVAVDVDSGQNAWLSYKLQKATDRALFEVGLQNGEIRTIRQVTDKDAVKQRLTVIVEDNG
QPSRSATVIVNVAVADSFPE
VLSEFTDFTHDKEYNDNLTFYLVLALAVVSFLFITCLVVIVSVKIYRWRQSRILYHSNLPVIPYYPPRYSDTLGGTGTLQ
HVYNYEVCRTTDSRKSDCKF
GNPGSQNVLIMDPSSTGTMQRMQSEKSILDEPDSPLE
>g551.t1
PPSKLGNPLRATSPMEFRSILTPESTVDCPLCGPYVWTGNPEAGQIGNHLTLKHGVWNPQERSLLCNLVYNRVDVQKEPC
PISGCGTVSYQLDHHLKTHR
ELTHSQVALLLKSLCLKVTKRKLASLRGSDPDLPLVSTLDLGGRDPAPAPPDRPSGPCPVAYCRWQAQQLDEARGKVVLL
SGLLSSLTARYYQLRLAKRQ
ATATLAEAGAQKDCQRPLTSLSCAPPGPPCEPWSLPHAPRSPLSPPRAPPSPLTVPSSPSSLPNAPRHTHSPPCDPGLSP
AGPACDPGPPAGPPCDPGLS
PATLRGWDLGHKCADSTSDVTALSTTKRLAPSRAIRSRAVIPREVLRRARRLAQEAMEEALCSMELTGTSSSLHRLYGYM
TVFLSTLYGHRGGVFKNLLV
EEVEAALPNETPGVYLINVENHPTNRAFETAQMVLTEEEYGWFTRLLFLKAVMGNHSPYLFFTFGPRPCRHLLNHLQGSW
RKMGLPGTPSFAQIRSSIAS
HAKSSHTAADWDRVARFMSHDASTADRFYVLNLNPEQAVLHRRLLEAALEDSEARGPGASCDPASAVTSHRGEAGGRGVS
LACCSGDEPATPSKEPGASS
DEPPSGREDEERRVNTVGLPSLHADDPPPRRCQLIRLMENAPMGNEGALPAKKAAEEEALMLWGSDLLLLVICKTSQPDG
MEEDQRLPGSEANPSSILIK
TEPDCDEVIKPSSILIKTEPDSDEISLYPGLRKLSVRLTDCSSWLGTHNHIRLQDLPGLARLKKRRVRGLSKGRKMTYCT
ECGKGFSKRSRMEAHRQADC
GRKEQYQCSHCDKTFVLKRDWMTHHRTHSSTMPFKCWFCHDGFSHQVTLNEHLDSHKDEKCYRCEVCDKRFVSYQGFKFH
RRSHQNQKLLSCPQCLKSFT
SPQSLKLHQVTHSGQKPHMCSVCGKGFKLQGGLQSHLRTHGDERAYRCTYCDKGFKRLQGLQQHHRVHLGIKRFQCAECS
KCFMDQSNLKAHQTTHMGER
PFLCTACGKRFARSCHLKKHTALYHCDDKPFTCKDCGKSFPVACYLAVHRRSHTKELCCSYCHKTYASKREWQEHERIHT
GEKPFPCSDCGENFRTPHAV
KRHQRVRH
>g551.t2
MLHQRLNKTYKGLLRCCLLFPDPPSKLGNPLRATSPMEFRSILTPESTVDCPLCGPYVWTGNPEAGQIGNHLTLKHGVWN
PQERSLLCNLVYNRVDVQKE
PCPISGCGTVSYQLDHHLKTHRELTHSQVALLLKSLCLKVTKRKLASLRGSDPDLPLVSTLDLGGRDPAPAPPDRPSGPC
PVAYCRWQAQQLDEARGKVV
LLSGLLSSLTARYYQLRLAKRQATATLAEAGAQKDCQRPLTSLSCAPPGPPCEPWSLPHAPRSPLSPPRAPPSPLTVPSS
PSSLPNAPRHTHSPPCDPGL
SPAGPACDPGPPAGPPCDPGLSPATLRGWDLGHKCADSTSDVTALSTTKRLAPSRAIRSRAVIPREVLRRARRLAQEAME
EALCSMELTGTSSSLHRLYG
YMTVFLSTLYGHRGGVFKNLLVEEVEAALPNETPGVYLINVENHPTNRAFETAQMVLTEEEYGWFTRLLFLKAVMGNHSP
YLFFTFGPRPCRHLLNHLQG
SWRKMGLPGTPSFAQIRSSIASHAKSSHTAADWDRVARFMSHDASTADRFYVLNLNPEQAVLHRRLLEAALEDSEARGPG
ASCDPASAVTSHRGEAGGRG
VSLACCSGDEPATPSKEPGASSDEPPSGREDEERRVNTVGLPSLHADDPPPRRCQLIRLMENAPMGNEGALPAKKAAEEE
ALMLWGSDLLLLVICKTSQP
DGMEEDQRLPGSEANPSSILIKTEPDCDEVIKPSSILIKTEPDSDEISLYPGLRKLSVRLTDCSSWLGTHNHIRLQDLPG
LARLKKRRVRGLSKGRKMTY

CTECGKGFSKRSRMEAHRQADCGRKEQYQCSHCDKTFVLKRDWMTHHRTHSSTMPFKCWFCHDGFSHQVTLNEHLDSHKD
EKCYRCEVCDKRFVSYQGFK
FHRRSHQNQKLLSCPQCLKSFTSPQSLKLHQVTHSGQKPHMCSVCGKGFKLQGGLQSHLRTHGDERAYRCTYCDKGFKRL
QGLQQHHRVHLGIKRFQCAE
CSKCFMDQSNLKAHQTTHMGERPFLCTACGKRFARSCHLKKHTALYHCDDKPFTCKDCGKSFPVACYLAVHRRSHTKELC
CSYCHKTYASKREWQEHERI
HTGEKPFPCSDCGENFRTPHAVKRHQRVRH
>g551.t3
MNMLHQRLNKTYKGLLRCCLLFPDPPSKLGNPLRATSPMEFRSILTPESTVDCPLCGPYVWTGNPEAGQIGNHLTLKHGV
WNPQERSLLCNLVYNRVDVQ
KEPCPISGCGTVSYQLDHHLKTHRELTHSQVALLLKSLCLKVTKRKLASLRGSDPDLPLVSTLDLGGRDPAPAPPDRPSG
PCPVAYCRWQAQQLDEARGK
VVLLSGLLSSLTARYYQLRLAKRQATATLAEAGAQKDCQRPLTSLSCAPPGPPCEPWSLPHAPRSPLSPPRAPPSPLTVP
SSPSSLPNAPRHTHSPPCDP
GLSPAGPACDPGPPAGPPCDPGLSPATLRGWDLGHKCADSTSDVTALSTTKRLAPSRAIRSRAVIPREVLRRARRLAQEA
MEEALCSMELTGTSSSLHRL
YGYMTVFLSTLYGHRGGVFKNLLVEEVEAALPNETPGVYLINVENHPTNRAFETAQMVLTEEEYGWFTRLLFLKAVMGNH
SPYLFFTFGPRPCRHLLNHL
QGSWRKMGLPGTPSFAQIRSSIASHAKSSHTAADWDRVARFMSHDASTADRFYVLNLNPEQAVLHRRLLEAALEDSEARG
PGASCDPASAVTSHRGEAGG
RGVSLACCSGDEPATPSKEPGASSDEPPSGREDEERRVNTVGLPSLHADDPPPRRCQLIRLMENAPMGNEGALPAKKAAE
EEALMLWGSDLLLLVICKTS
QPDGMEEDQRLPGSEANPSSILIKTEPDCDEVIKPSSILIKTEPDSDEISLYPGLRKLSVRLTDCSSWLGTHNHIRLQDL
PGLARLKKRRVRGLSKGRKM
TYCTECGKGFSKRSRMEAHRQADCGRKEQYQCSHCDKTFVLKRDWMTHHRTHSSTMPFKCWFCHDGFSHQVTLNEHLDSH
KDEKCYRCEVCDKRFVSYQG
FKFHRRSHQNQKLLSCPQCLKSFTSPQSLKLHQVTHSGQKPHMCSVCGKGFKLQGGLQSHLRTHGDERAYRCTYCDKGFK
RLQGLQQHHRVHLGIKRFQC
AECSKCFMDQSNLKAHQTTHMGERPFLCTACGKRFARSCHLKKHTALYHCDDKPFTCKDCGKSFPVACYLAVHRRSHTKE
LCCSYCHKTYASKREWQEHE
RIHTGEKPFPCSDCGENFRTPHAVKRHQRVRH
>g552.t1
GSKQLLGNYPLFITNKQWDEAVNSSKKDGRRLLRYLIRFVFTTDELKYSCGLGKRKRSVHSGEPGLERRPLNPVKVSCLR
>g553.t1
MGKTADLTAVQKAIIDTLKQEGDRTLRKIVEKGRFQTLGDLRKQWTESGVETQSHRAQACAGNGLQVPHSP
>g553.t2
MGKTADLTAVQKAIIDTLKQEGDRTLRKIVEKGRFQTLGDLRKQWTESGVETQSHRAQACAGNGLQ
>g554.t1
LCSPASEPQARSQRPCVCVSEPTISSSDHSTLPTDSYSPPITLQCNLSFAHGGHEESFWMKNGEEIEETRTANRNTEYKL
VKPRGDDAGLYMCVYTFEKS
PPANATIEVKAAPDITGHKRSENKNEGQRAVLYCKSVGFPHPNWTWRKLDSGAFM
>g554.t2
FELYCDVVGNPTPEIQWWYAEINRADSFRQLWGGARKRRVSINTAYGANGVSVLGVTRLALDDSGTYECRASNDPRRNDL
HQNPATTWVRAQATITVLQK
PTISSSDHSTLPTDSYSPPITLQCNLSFAHGGHEESFWMKNGEEIEETRTANRNTEYKLVKPRGDDAGLYMCVYTFEKSP
PANATIEVKAAPDITGHKRS
ENKNEGQRAVLYCKSVGFPHPNWTWRKLDSGAFM
>g554.t3
FELYCDVVGNPTPEIQWWYAEINRADSFRQLWGGARKRRVSINTAYGANGVSVLGVTRLALDDSGTYECRASNDPRRNDL
HQNPATTWVRAQATITVLQS
EWVRGETPSQDLPPYRLPQQHHVVFIHLPRTLRYRRVELQSSQKPXPTISSSDHSTLPTDSYSPPITLQCNLSFAHGGHE
ESFWMKNGEEIEETRTANRN
TEYKLVKPRGDDAGLYMCVYTFEKSPPANATIEVKAAPDITGHKRSENKNEGQRAVLYCKSVGFPHPNWTWRKLDSGAFM
>g554.t4
FELYCDVVGNPTPEIQWWYAEINRADSFRQLWGGARKRRVSINTAYGANGVSVLGVTRLALDDSGTYECRASNDPRRNDL
HQNPATTWVRAQATITVLQS

EWVRGETPSQDLPPYRLPQQHHVVFIHLPRTLRYRRVELQSSQKPCATTECSQGNIDP
>g555.t1
MSFLRRVAGLSLRDRVRSSDIRRELGVEPLLLRVERSQLRWFGHLIRMPPGRLPLEVFRARPTGKRPRGRPRTRWRDYIS
HLAWERLGVPQEELESVAGE
KDVWSSLLSLLPPRPGPG
>g556.t1
WVRKINYLGKGTYVDCALTNMTAEMMRARSDPQPLRFAVVITDGHVTGNPCGGIKVTAERARDAGIRMFVVAASKNVDET
GLREIANAPAAVYRRDFMAV
DLSQGRPIIHTETIDRIVNTMHHMAYVECYKVSCLETKGPAGPKGHRGQKGAKGDNGDPGLKGSRGRPGDPGIEGPIGQP
GIKGEYGLKGEKGVAGLSGR
NGTDGQKVRNCVSGTERCCDRANRPLYT
>g556.t2
WVRKINYLGKGTYVDCALTNMTAEMMRARSDPQPLRFAVVITDGHVTGNPCGGIKVTAERARDAGIRMFVVAASKNVDET
GLREIANAPAAVYRRDFMAV
DLSQGRPIIHTETIDRIVNTMHHMAYVECYKVSCLETKGPAGPKGHRGQKVSGAKGDNGDPGLKGSRGRPGDPGIEGPIG
QPGIKGEYGLKGEKGVAGLS
GRNGTDGQKVRNCVSGTERCCDRANRPLYT
>g556.t3
WVRKINYLGKGTYVDCALTNMTAEMMRARSDPQPLRFAVVITDGHVTGNPCGGIKVTAERARDAGIRMFVVAASKNVDET
GLREIANAPAAVYRRDFMAV
DLSQGRPIIHTETIDRIVNTMHHMAYVECYKVSCLETKGPAGPKGHRGQKGAKGDNGDPGLKGSRGRPGDPGIEGPIGQP
GIKGVAGLSGRNGTDGQKVR
NCVSGTERCCDRANRPLYT
>g557.t1
MFARLLEQRWVITAVLSDRSVTKPGNARNLELRDDYWKIMEEIEPVLKVLEVATTASCAEEAVSISDVYPVVCGLMNEHL
QPTDRRDVRVRAFVNTVRDS
LDKRFLPLDIETASQPAVMAAMLVPRHKKLRFLSSDVRAAAKMHMEGLFEAEKEKARARPGDEDGEREESQDEDPAPGAK
NIKKTENSGAMAFLLGASYS
VEEDEENTESELEQYRSLPAMPLEADPLVWWEAHELQYPITSKLARKFLSIPATSVASERVFSAAGQGVSKLRSRLLTDN
VNKLIFLNKNLPR
>g558.t1
MLYGNPLMAVVDLLLRGCMGETGGDRGRQGEGVLLGVSWLMIPPVDPLPSYSLGSGPEDFSNLPPEQRRKKLQGKLDELN
KDIQKEMDQRDALTKMKDVY
VKNPQMGDPASVDPRLGELAQSIEKLQYEVQKFEVR
>g558.t2
MAVVDLLLRGCMGETGGDRGRQGEGVLLGVSWLMIPPVDPLPSYSLGSGPEDFSNLPPEQRRKKLQGKLDELNKDIQKEM
DQRDALTKMKDVYVKNPQMG
DPASVDPRLGELAQSIEKLQYEVQKFEVR
>g559.t1
MVTANIFFTAAAAIVTDKWKRAESRYMVERAAIISHWNWLQAHISDLEYRIRQQTDIYRQIRTSKGSVELDGGSPSSPSV
GGAELKTELSISQDGSSERQ
EQPVTAPTSSSDPSPWRGQNGRPVNGVLNRMMESTETKLQQPAAASDSSCVAARTRPLVGCRRRRLLQPSSVPHLHGKAQ
RSGCVPSCCRLNPSCVMCGG
RSSPLEDPQYDLPTMERLSKLDPGVHPILSFSDDVCVGLRLQQVLKSQWQSKALDRSKPLKKLSLKHKLSSSREKHKFTS
SLMAVRLGHFKRRSEKARPG
EVGLGPARLDSQALCKTERLQPGPYDKSFSRKRPRERSLERGD
>g559.t2
MVTANIFFTAAAAIVTDKWKRAESRYMVERAAIISHWNWLQAHISDLEYRIRQQTDIYRQIRTSKGSVELDGGSPSSPSV
GGAELKTELSISQDGSSERQ
EQPVTAPTSSSDPSPWRGQNGRPVNGVLNRMMESTETKLQQPAAASDSSCVAARTRPLVGCRRRRLLQPSSVPHLHGKGL
SDLFSKKTIGGPFFSKCLHT
AQRSGCVPSCCRLNPSCVMCGGRSSPLEDPQYDLPTMERLSKLDPGVHPILSFSDDVCVGLRLQQVLKSQWQSKALDRSK
PLKKLSLKHKLSSSREKHKF
TSSLMAVRLGHFKRRSEKARPGEVGLGPARLDSQALCKTERLQPGPYDKSFSRKRPRERSLERGD
>g559.t3
MVTANIFFTAAAAIVTDKWKRAESRYMVERAAIISHWNWLQAHISDLEYRIRQQTDIYRQIRTSKGSVELDGGSPSSPSV

GGAELKTELSISQDGSSERQ
EQPVTAPTSSSDPSPWRGQNGRPVNGVLNRMMESTETKLQQPAAASDSSCVAARTRPLVGCRRRRLLQPSSVPHLHGKAQ
RSGCVPSCCRLNPSCVMCGG
RSSPLEDPQYDLPTMERLSKLDPGVHPILSFSDDVCVGLRLQQVLKSQWQSKALDRSKPLKKLSLKHKLSSSREKHKFTS
SLMAVRHFKRRSEKARPGEV
GLGPARLDSQALCKTERLQPGPYDKSFSRKRPRERSLERGD
>g560.t1
ARGRRVGHPCIRVLTNDIPNQYNEGHQHTALYASVSRGYENREGDNQASMSPEVSGDTRGAGAESDPRDTAAMKMKARL
>g561.t1
SNGSAEGVEEVPRNVLAGHLVTKVRAYDADIGYNGWLLFSLQEVSDHSLFGLDRYTGQIRTLRSFTETDEAQHKLVILVK
DNGNVSLSATATVVVKLVEP
KEAISASDVKSAVTDIGDGGVTFYLMITLASVSTLFLISIIVLIAMQCSKTTDVSSKYLPETNYDGTLCHSIQYRSGEKR
YMLVGPRMSVGSTIVPGSNG
NTLVVPDRRSTSTENAPIGTTVVQVNATDLDDGPNGDVVYSFGNSVNDKLFTLFDINQFTEKSVIIKIVDVNDNAPEIEV
TSFSSAIAEDSRPGTTVALI
SVTDFDSGLNGKSPYTFYLKENNAPGASIFTVKAFDRDENENAAIAFHIGRAGRDDDKLVSFLNINSQNGDVMALKRFDF
ETSKTFNFQVIATDSGNPPL
SSNVTVYVFILDQNDNAPVILYPPSTNGTSQGLEEIPRNVYGGHLVTKVRAYDADIGYNGWLLFSLQEVSDHSLFGLDRY
TGQIRTLRSFTETDEAQHKL
VILVKDNGNVSLSATATVVVKLVEPKEAISASDVKSAVTDIGDGGVTFYLMITLASVSTLFLISIIVLIAMQCSKTTDVS
SKYLPETNYDGTLCHSIQYR
SGEKRYMLVGPRMS
>g561.t2
LDQNDNPPVILHPLSSNGSAEGVEEVPRNVLAGHLVTKVRAYDADIGYNGWLLFSLQEVSDHSLFGLDRYTGQIRTLRSF
TETDEAQHKLVILVKDNGNV
SLSATATVVVKLVEPKEAISASDVKSAVTDIGDGGVTFYLMITLASVSTLFLISIIVLIAMQCSKTTDVSSKYLPETNYD
GTLCHSIQYRSGEKRYMLVG
PRMSVGSTIVPGSNGNTLVVPDRRSTSTENAPIGTTVVQVNATDLDDGPNGDVVYSFGNSVNDKLFTLFDINQFTEKSVI
IKIVDVNDNAPEIEVTSFSS
AIAEDSRPGTTVALISVTDFDSGLNGKSPYTFYLKENNAPGASIFTVKAFDRDENENAAIAFHIGRAGRDDDKLVSFLNI
NSQNGDVMALKRFDFETSKT
FNFQVIATDSGNPPLSSNVTVYVFILDQNDNAPVILYPPSTNGTSQGLEEIPRNVYGGHLVTKVRAYDADIGYNGWLLFS
LQEVSDHSLFGLDRYTGQIR
TLRSFTETDEAQHKLVILVKDNGNVSLSATATVVVKLVEPKEAISASDVKSAVTDIGDGGVTFYLMITLASVSTLFLISI
IVLIAMQCSKTTDVSSKYLP
ETNYDGTLCHSIQYRSGEKRYMLVGPRMS
>g561.t3
SNGSAEGVEEVPRNVLAGHLVTKVRAYDADIGYNGWLLFSLQEVSDHSLFGLDRYTGQIRTLRSFTETDEAQHKLVILVK
DNGNVSLSATATVVVKLVEP
KEAISASDVKSAVTDIGDGGVTFYLMITLASVSTLFLISIIVLIAMQCSKTTDVSSKYLPETNYDGTLCHSIQYRSGEKR
YMLVGPRMSVGSTIVPGSNG
NTLVVPDRRSTSTEVDREEVCERTPTCLIHLKTVLENPLEVHYVGVEVLDVNDHSPSFPEKDKTLEISESTSPGTRFQLR
PSRDPDGGPFSVQQYKLSSN
DHFRLEVKDKKDDGKIPILVLQKALDRETGRSHSLVLTALDGGKPPKSGEMNIRVDVLDVNDNTPVFSDDIYSVLLDENA
PIGTTVVQVNATDLDDGPNG
DVVYSFGNSVNDKLFTLFDINQFTEKSVIIKIVDVNDNAPEIEVTSFSSAIAEDSRPGTTVALISVTDFDSGLNGKSPYT
FYLKENNAPGASIFTVKAFD
RDENENAAIAFHIGRAGRDDDKLVSFLNINSQNGDVMALKRFDFETSKTFNFQVIATDSGNPPLSSNVTVYVFILDQNDN
APVILYPPSTNGTSQGLEEI
PRNVYGGHLVTKVRAYDADIGYNGWLLFSLQEVSDHSLFGLDRYTGQIRTLRSFTETDEAQHKLVILVKDNGNVSLSATA
TVVVKLVEPKEAISASDVKS
AVTDIGDGGVTFYLMITLASVSTLFLISIIVLIAMQCSKTTDVSSKYLPETNYDGTLCHSIQYRSGEKRYMLVGPRMS
>g562.t1
DDNSMFFQFGPSIEQQASVMLNIMEEYDWYIFSIVTTYYPGYQDFVTKVTRPGGEEA
>g562.t2
LGCTLHVLYPTPLDSEWMRIIQDVTSEDDNSMFFQFGPSIEQQASVMLNIMEEYDWYIFSIVTTYYPGYQDFVTKVTRPG

GEEA
>g563.t1
ASHSSTVPDPDCQLLSVLKHHDPPLIFDLESDPSENYPLTLDNEPALHSLLEKILEVKAKFEASMVFEQWIPTF
>g563.t2
LKHHDPPLIFDLESDPSENYPLTLDNEPALHSLLEKILEVKAKFEASMVFEQWIPTF
>g564.t1
MASTLLSRQLALSVHHSLRFPASGCRYVSKAAVNTQADYDFDRPLMKTQVPGPRSQNATSINFFCNYEESRGNYLVDADG
NRMLDVYTQISSIPIGDKLL
LAFQ
>g564.t2
MASTLLSRQLALSVHHSLRFPASGCRYVSKAAVNTQADYDFDRPLMKTQVPGPRSQELTKQMGEIQNATSINFFCNYEES
RGNYLVDADGNRMLDVYTQI
SSIPIGDKLLLAFQ
>g564.t3
MASTLLSRQLALSVHHSLRFPASGCRYVSKAAVNTQADYDFDRPLMKTQVPGPRSQNATSINFFCNYEESRGNYLVDADG
NRMLDVYTQISSIPIAFRLK
VCLD
>g565.t1
MPRLEDHCWTCSCTSSRGDSEDRVAAPKAVDMLSIITTVLRGAYGSLHSDRAANLLRRGLVLFSVGVFFALVLNLLQIQR
HVTLFPEEVMSTLFSSAWWI
PPCCGTGAAVIGLLYPCIDSHLGEPHKFKREWASVMRCIAVFVGINHASVVSFHPEAR
>g565.t2
MPRLEDHCWTCSCTSSRGDSEDRVAAPKAVDMLSIITTVLRGAYGSLHSDRAANLLRRGLVLFSVGVFFALVLNLLQIQR
HVTLFPEEVMSTLFSSAWWI
PPCCGTGAAVIGLLYPCIDSHLGEPHKFKREWASVMRCIAVFVGINHASVKLDCNNLQLSLTLAALSLGLWWTFDRSRSG
FGLGITTAFLATVITQLLVY
NGVY
>g565.t3
LVLNPRSPQKLDCNNLQLSLTLAALSLGLWWTFDRSRSGFGLGITTAFLATVITQLLVYNGVY
>g566.t1
LCVCRPTVDHGGGAPSRGSLASVALVASLLPLLLFRLEKEREPAVSLAVLYALPKLGTHKVLHVLQALAAPNLRAVVMRL
MTELWKKQPLPSDQNQLSVQ
RSVGPTFIDKDDTPMWSVSELRRALC
>g566.t2
LCVCRPTVDHGGGAPSRGSLASVALVSVVASLLPLLLFRLEKEREPAVSLAVLYALPKLGTHKVLHVLQALAAPNLRAVV
MRLMTELWKKQPLPSDQNQL
SVQRSVGPTFIDKDDTPMWSVSELRRALC
>g567.t1
GSLPTEYMRGKPLCMQLFPLLFSSCRIPGPKHDYVSHHALSRRSPTHITVVRNYQFFQLEVYNSDGSRMTESQIHGQLLR
IRSASWKTDKEPMGILTSEH
RHTWGLAYKRLLRDKVNKDSVRAIEKGLFTLCLDSPVMRISDEKYASRMAAQVLHGGGTFSNSGNRWFDKTLQ
>g567.t2
MRGKPLCMQLFPLLFSSCRIPGPKHDYVSHHALSRRSPTHITVVRNYQFFQLEVYNSDGSRMTESQIHGQLLRIRSASWK
TDKEPMGILTSEHRHTWGLA
YKRLLRDKVNKDSVRAIEKGLFTLCLDSPVMRISDEKYASRMAAQVLHGGGTFSNSGNRWFDKTLQ
>g568.t1
LFHHITRTHGTLIGVSTGLVLLLLIVSIFVQVKQPRKKVRPRPADAAYLPCVTHFPCRHADVVAVLLRKHNLFNRPEFHE
VFDPPHYELFTLRDKELSGD
LGELPEELQSLQALRRTSSERTRCVHEHH
>g568.t2
LFHHITRTHGTLIGVSTGLVLLLLIVSIFVQVKQPRKKVLLRKHNLFNRPEFHEVFDPPHYELFTLRDKELSGDLGELPE
ELQSLQALRRTSSERTRCVH
EHH
>g569.t1
RGCRPGKIVQMTEAEVRGLCIKSREIFLSQPILLELEAPLKICGQ
>g570.t1

LPELEQLLNGVRRYKRKQVTAGVRDRAPQLAVSHCTGDIHGQYTDLLRLFEYGGLPPEANYLFLGDYVDRGKQSLETICL
LLAYKIKYPENFFLLRGNHE
CASINRIYGFYDECECVTTMSSCISP
>g571.t1
MLLCTAPFERLKTFTEQSPGWFLILHKVDKKGERLPKPDDCPEDVYQTMRQCWARLPDHRPSFVVLREFLLENMPTDMSA
LHDFDQPDKLLIQTDDVIT
>g571.t2
MTGAWLSPMGRGNILHKVDKKGERLPKPDDCPEDVYQTMRQCWARLPDHRPSFVVLREFLLENMPTDMSALHDFDQPDKL
LIQTDDVIT
>g572.t1
GSTVSREASDKEKAKELPTFKDNDFLNEGHKLQIGDDNKKYFLEKLKHDVEFLATLKIMDYSLLVGIHDVERAEQEEMEV
EAAGDEEEYDDGLGGGALPG
SFGTPPDSPGNPLNFGGFFGPGEFDPSVDVYAIKSHDGGFVARPPRPSRCTFPPGCRSIASHV
>g573.t1
VEPFFVSLALFDVSKSCKISSDFHVDLNPPCVRDLLLPGDAPAPSPTASDSSDG
>g574.t1
MVPRSADVGYLVTKVVAVDVDSGQNAWLSYKLQKATDRALFEVGLQNGEIRTIRQVTDKDAVKQRLTVIVEDNGQPSRSA
TVIVNVAVADSFPEVLSEFT
DFTHDKEYNDNLTFYLVLALAVVSFLFITCLVVIVSVKIYRWRQSRILYHSNLPVIPYYPPRYSDTLGGTGTLQHVYNYE
VCRTTDSRKSDCKFGNPGSQ
NVLIMDPSSTGTMQRMQSEKSILDEPDSPLE
>g574.t2
LIQDQNDNAPQVLYPVQTGGSLVAEMVPRSADVGYLVTKVVAVDVDSGQNAWLSYKLQKATDRALFEVGLQNGEIRTIRQ
VTDKDAVKQRLTVIVEDNGQ
PSRSATVIVNVAVADSFPEVLSEFTDFTHDKEYNDNLTFYLVLALAVVSFLFITCLVVIVSVKIYRWRQSRILYHSNLPV
IPYYPPRYSDTLGGTGTLQH
VYNYEVCRTTDSRKSDCKFGNPGSQNVLIMDPSSTGTMQRMQSEKSILDEPDSPLE
>g575.t1
MADQCGLPAWATVGVDITQELTRKALESKMQCVNSAHDDGRPAWATVGMDITQEELRMELESHPDWEEPRTGQPEGLAYG
ALVLGLVILLAYLIHQGLKW
RRTQLLEEGVGIKGIEPSTLRLVDDPLCPPALPH
>g576.t1
MSNSDRVVLALGGAGTVGSGIVKALLDKGFKVAVISRDNSRLERLRSFVSASRKDNLTTIVGDVGTEEGVEAAKQALLKA
VGKVTDIISSLGFSWWQGGP
PHTQSLKDLQWVIETLLLSTFVSWKAFFPLVRDDPNSTYTFITGGAGEKLLMPGTGFLTVGAASTLSFCQVLREEYPEVA
CKLNQVKINTGVAPPERMAP
GFLSQLGLGEGVAALVERCTTSHAVYTVGCPADLKTVLIDGDL
>g577.t1
VVSPQGSGSSMLLFRAEPGRAPWVKARPPDARRRAPLPGLAPVGGPGLPNPGETLDSLRCYIVSVCDYRVCGDEICVLSN
RVPSIPRTVWLNRSTEFPRG
WIVPFIDEKIIPEQTGFRPGKSTTSQLVNLTQHIEDGVERGLITGAVFVDLSAAYDSQPQTTP
>g577.t2
VVSPQGSGSSMLLFRAEPGRAPWVKARPPDARRRAPLPGLAPVGGPGLPNPGETLDSLRCYIVSVCDYRVCGDEICVLSN
RVDSQDKINWQSGLPTKHXV
PSIPRTVWLNRSTEFPRGWIVPFIDEKIIPEQTGFRPGKSTTSQLVNLTQHIEDGVERGLITGAVFVDLSAAYDSQPQTT
P
>g577.t3
MPGISTPRPPPLPAARLALHPTPMSIPAGGLAPVGGPGLPNPGETLDSLRCYIVSVCDYRVCGDEICVLSNRVPSIPRTV
WLNRSTEFPRGWIVPFIDEK
IIPEQTGFRPGKSTTSQLVNLTQHIEDGVERGLITGAVFVDLSAAYDSQPQTTP
>g578.t1
MEGDMQPADEGPCAPKMCRQQQRGPCSSLKPFQSKRSAGKSRFDRSAMVEVPLFGGADDGGGGHHFSYNPGQPHPFTSSH
HQQRVQQQQYHQTTATISSS
QQHHATPQQQQQQQQQQLQHRFHCSENRPSSSRVASSTSAAVSSAAGCQQRRPGSGGAAPARLPEPRYSPGRAALVASPD
CSYGISS
>g578.t2

MQPADEGPCAPKMCRQQQRGPCSSLKPFQSKRSAGKSRFDRSAMVEVPLFGGADDGGGGHHFSYNPGQPHPFTSSHHQQR
VQQQQYHQTTATISSSQQHH
ATPQQQQQQQQQQLQHRFHCSENRPSSSRVASSTSAAVSSAAGCQQRRPGSGGAAPARLPEPRYSPGRAALVASPDCSYG
ISS
>g579.t1
LGTSVYNLPKTGPFRLRFLLDSIKDLRNSLVKKGRRGNPEDVIAELIKKLPSVSAVVFHEEVASEERAVEEAVKDVCARM
KVKVHTCWGSTLYHRDDLPF
PHLVRLPDVYTQFRKAVETQSTVRPLILSPDQVRPLPPGLEEGPVPTLEDLGQTDPEADPRSAFPCSGGESQALARLKHY
LWDT
>g579.t2
LGTSVYNLPKTGPFRLRFLLDSIKDLRNSLVKKGSTLVVRRGNPEDVIAELIKKLPSVSAVVFHEEVASEERAVEEAVKD
VCARMKVKVHTCWGSTLYHR
DDLPFPHLVRLPDVYTQFRKAVETQSTVRPLILSPDQVRPLPPGLEEGPVPTLEDLGQTDPEADPRSAFPCSGGESQALA
RLKHYLWDT
>g579.t3
LGTSVYNLPKTGPFRLRFLLDSIKDLRNSLVKKGSAVVFHEEVASEERAVEEAVKDVCARMKVKVHTCWGSTLYHRDDLP
FPHLVRLPDVYTQFRKAVET
QSTVRPLILSPDQVRPLPPGLEEGPVPTLEDLGQTDPEADPRSAFPCSGGESQALARLKHYLWDT
>g580.t1
LGKPDTAAGSAAFLAAEVSNPGSLYAGPGGGVALQYLVPRS
>g581.t1
SSPTIAQFGWTARSAKSSGRPKLLPFKDYGGHCGCSGHVQLVRDPGGDPELAGGIIYLIWPGNASGSPRRSWKALLGRRT
SGVPCSACCHRDPAPDKQTF
NNDDDDDDGGHYALRNLECCRTQLDSNEGVEPSQGGSEEMDSM
>g582.t1
LVVPVPIPITTSTMEPGAIKEELRLFQSTLLQDGLKELLNENKFVDCSLKVGDRSFPCHRLIMAACSPYFREIFFGEDGK
EVENTKEVVLEDINPTILDM
VIRYLYSAEIDLTDDNVQDIFAVANRFQIPSVFTVCVNYLQKKLSPGNCLAIFRMGLVLSCPRLAVAARNFVADRFELLH
KEEEFLQLAAHELFAIIGGD
SLNVEREELVYEAVMAWVRHDQDKRLPVLADAFDCIRFRLLPEKYFHEKVEANDIVKANPELQKKIQAVSDAFKGKLPEK
QPRQKKEGAEGAAAANGEEE
EEEEESLLPGYLNDNRRMGMYGRDFIMMINDTAAVAYDVVENECFLAAMSEQVPRNHVSTVTQRNQLYIIGGLFVDEEDK
DAPLQCYFYTLDPLISEWVA
LPPMPSPRCLFNIGESNDALFAIAGKDLQTNESLDTVMCFDIE
>g583.t1
LVEYFRTQMGKDPDMASAVAAIRTLLEFLKRDKGETLFGLRENLTWATDCLTGVDSSVAVSSAGELFLRFISLTSLEHQD
LSRCKKVMEERGELFLQKIS
MSRAKVAKLCHTFIKDGAKILTHSYSRVVLRVLEKAAAEKKRFSVYVTESQPDAA
>g583.t2
LVEYFRTQMGKDPDMASAVAAIRTLLEFLKRDKGETLFGLRENLTWATDCLTGVDSSVAVSSAGELFLRFISLTSLEHQD
LSRCKKVMEERGELFLQKIS
MSRAKVAKLCHTFIKDGAKILTHSYSRVVLRVLEKAAAEKKRFSVYVTESQPDAAG
>g583.t3
LSSLELVEYFRTQMGKDPDMASAVAAIRTLLEFLKRDKGETLFGLRENLTWATDCLTGVDSSVAVSSAGELFLRFISLTS
LEHQDLSRCKKVMEERGELF
LQKISMSRAKVAKLCHTFIKDGAKILTHSYSRVVLRVLEKAAAEKKRFSVYVTESQPDAA
>g584.t1
MAANEEEVRENQRGVVQRLKRVVLRCVSGGAADDGPPQKVRKDVAFHNYVAGVIGSRWFHAVIMLTIFLDALFSALETNH
HVKLRLSGFLRVADELFVAV
YAAEFLAKVYVEPVRFWKSNNNVFDAVLLAVSFPPMFADEASSGAVGSLGVIRCCRSLRVIKVSSMPMVKVLFAALYRTM
KSVLGVLCLIFLLVFLFAVI
GTQCFGDRDSGDPVHWGDLPSSLFTLFSMVTVDGWTDYQEELDRLVVGHTRVFTIVFIVLGNFILVNMYSAVVLTEIHHS
MDRFETRRQAARLRSQALKK
HAVGQRHKEEMRQLQQSRAGDAELLRXXXXXXXXELLRQLMPSLKRSLCPSDLVVVEDLCASLTFADLYLISLEDQDHTT
RKLRHLYGTIATTLGKMEEE
EQGKRRVIHVEEKGRE

>g585.t1
WWNELPANVRTAEALPIFRKRLKTHLFRVHLNPT
>g586.t1
EIKHKLPAMEAACKAAWRSAQLLTKDSPEEEVAQMLALMASVKDQLGKIRERCLPLLRESQSLLPPLEEMEKNITGFYQA
LEKASRITGSRESEGPGDFK
HKCQELVTYTHSCKKCLSVIERSHQSIQKTLDSSKTLQHMQTALLQKRVADLQSSSQAMIKESAEWRKHVEANSSLMKRF
EESRVELEKVLKTAQSCLTE
RGNPEELLRKHT
>g587.t1
MVFSTHCQYPTALGLTSPWTSSPVCHHPTAIPLLHHILTVVDWFSKLAHFVLLPKLPSAKEMAELVLQHVFRLHGLPIDV
VSDQGSQFTSIFWREFCKLV
GATVSLSSGFHPQSNGQTERKNQEMETAPRCMVSQNPASWSQQLMWVEYAHTLTSSATGLSPFQCAYGFQPPLFPALEKE
ASCPSVQAYELK
>g588.t1
MFRSIRRRQVAPQTVQELRDALVQIWEEIPQETIRRLIRSMPRHCTHFGGSEGDAQAYRKKSFTEKDESMATPNTICQFT
CLVAPLTWDVEERVRSALGG
HSGPSASPPDRLFVPTALRSDVLQWFHNSHLTCHPGIQCTKDVVQRRFW
>g588.t2
LVHDNARPHVARVCRQFLEDEGIDTIDWPPRSPDLNPIEHLWDIMFRSIRRRQVAPQTVQELRDALVQIWEEIPQETIRR
LIRSMPRHCTHFGGSEGDAQ
AYRKKSFTEKDESMATPNTICQFTCLVAPLTWDVEERVRSALGGHSGPSASPPDRLFVPTALRSDVLQWFHNSHLTCHPG
IQCTKDVVQRRFW
>g588.t3
MFRSIRRRQVAPQTVQELRDALVQIWEEIPQETIRRLIRSMPRHFSATGRNRSCCNRYFRDVTSVPQFTEKDESMATPNT
ICQFTCLVAPLTWDVEERVR
SALGGHSGPSASPPDRLFVPTALRSDVLQWFHNSHLTCHPGIQCTKDVVQRRFW
>g589.t1
MHLVEEPSVVDPTYIEDFLLTYRTFLSSPMEVGKKLLEWFMVDSHRDTVTRIVLLWVNNHFNDFEGAPVMTRFLEEFEKH
LEDTAKCRQITLQKVSREAP
LYFSLHGGSEKGFGIFVESVEPGSKAAEAGLKRGDQIMEINGQNFENICHSKAMDILKNNTHLSLTVKTNIFVFKELLSR
IVHEKQNGVPHIPKIQEKKG
NRFSIPNLPGDMEFPSDQKSTRKMKANTVSGGRNKIRKMLEKTRFSILPPKPFRGLFGDGSMGQSQDDSIVGTKQCRHSV
AIMPVPGSLSSSSPDLLQPA
SSVLDFSNPTEILFNQAHPFSLCPSRKGAYPGETSYSGNSENQSLAVEPPSSIVWKDCKDAGVFKLESFMSARQEHSDIP
DQVIRVFKVDQQSCYIIISK
DTTAKDVVSHTVNEFGLLSPPENYSLCEVSVSPEGFIKQRRLPDQLSKLADRIQLNGRYYLKNNMDTETLCSDEDAQDLL
RESLITLLQLSTVEVAAQLS
MRDFELFRNIESTEYVDDLFKLDSGVGSGHLKQFEEVINQETFWVATEILKESNALKRMKTIKHFIKIALHCRECKNFNS
MFAIISGLNLAPVARLRASW
EKLPSKYEKLFTDLQDVFDPSRNMAKYRNVLSSQSMQPPIIPLFPVVKKDLTFLHEGNDSSVDGLVNFEKLRMIAKEIRH
VVHMTSANMDPALMFRQ
>g589.t2
MHLVEEPSVVDPTYIEDFLLTYRTFLSSPMEVGKKLLEWFMVDSHRDTVTRIVLLWVNNHFNDFEGAPVMTRFLEEFEKH
LEDTKMNGHLRLLNIACAAK
AKCRQITLQKVSREAPLYFSLHGGSEKGFGIFVESVEPGSKAAEAGLKRGDQIMEINGQNFENICHSKAMDILKNNTHLS
LTVKTNIFVFKELLSRIVHE
KQNGVPHIPKIQEKKGNRFSIPNLPGDMEFPSDQKSTRKMKANTVSGGRNKIRKMLEKTRFSILPPKPFRGLFGDGSMGQ
SQDDSIVGTKQCRHSVAIMP
VPGSLSSSSPDLLQPASSVLDFSNPTEILFNQAHPFSLCPSRKGAYPGETSYSGNSENQSLAVEPPSSIVWKDCKDAGVF
KLESFMSARQEHSDIPDQVI
RVFKVDQQSCYIIISKDTTAKDVVSHTVNEFGLLSPPENYSLCEVSVSPEGFIKQRRLPDQLSKLADRIQLNGRYYLKNN
MDTETLCSDEDAQDLLRESL
ITLLQLSTVEVAAQLSMRDFELFRNIESTEYVDDLFKLDSGVGSGHLKQFEEVINQETFWVATEILKESNALKRMKTIKH
FIKIALHCRECKNFNSMFAI
ISGLNLAPVARLRASWEKLPSKYEKLFTDLQDVFDPSRNMAKYRNVLSSQSMQPPIIPLFPVVKKDLTFLHEGNDSSVDG
LVNFEKLRMIAKEIRHVVHM

TSANMDPALMFRQ
>g589.t3
MHLVEEPSVVDPTYIEDFLLTYRTFLSSPMEVGKKLLEWFMVDSHRDTVTRIVLLWVNNHFNDFEGAPVMTRFLEEFEKH
LEDTAKCRQITLQKVSREAP
LYFSLHGGSEKGFGIFVESVEPGSKAAEAGLKRGDQIMEINGQNFENICHSKAMDILKNNTHLSLTVKTNIFVFKELLSR
IVHEKQNGVPHIPKIQEKKG
NRFSIPNLPGDMEFPSDQKSTRKMKANTVSGGRNKIRKMLEKTRFSILPPKPFRGLFGDGSMGQSQDDSIVGTKQCRHSV
AIMPVPGSLSSSSPDLLQPA
SSVLDFSNPTEILFNQAHPFSLCPSRKGAYPGETSYSGNSENQSLAVEPPSSIVWKDCKDAGVFKLESFMSARQEHSDIP
DQVIRVFKVDQQSCYIIISK
DTTAKDVVSHTVNEFGLLSPPENYSLCEVSVSPEGFIKQRRLPDQLSKLADRIQLNGRYYLKNNMDTETLCSDEDAQDLL
RESLITLLQLSTVEVAAQLS
MRDFELFRNIESTEYVDDLFKLDSGVGSGHLKQFEEVINQETFWVATEILKESNALKRMKTIKHFIKIALHCRECKNFNS
MFAIISGLNLAPVARLRASW
EKLPSKYEKLFTDLQDVFDPSRNMAKYRNVLSSQSMQPPIIPLFPVVKKDLTFLHEGNDSSVDGLVNFEKLRMIAKEIRH
VVHMTSANMDPALMFRQR
>g590.t1
EDLSRMMDTIRSQYEGITEKNRKEMEAWYKVKFDELNKEVSTSADTLNSSRSQLTELKRTVQSLQIELQSQLSLKSALEG
QLAETEARYSNQLSQLQAMV
NALEMELSQVKMDIERQGLEYQRLLDIKTRLEMEIAEYRRLLDGEEVTTTVVTKVAKREPVVTKRVKMVVEQIVDGKVVS
RTEDVDEEVLSK
>g590.t2
MDTIRSQYEGITEKNRKEMEAWYKVKFDELNKEVSTSADTLNSSRSQLTELKRTVQSLQIELQSQLSLKSALEGQLAETE
ARYSNQLSQLQAMVNALEME
LSQVKMDIERQGLEYQRLLDIKTRLEMEIAEYRRLLDGEEVTTTVVTKVAKREPVVTKRVKMVVEQIVDGKVVSRTEDVD
EEVLSK
>g591.t1
AFEDIYIEQRKVIKVILEYADKIFTYIFILEMLLKWIAYGFKKYFTNYWCWLDFLIVD
>g592.t1
GPELEVPLMMLVEALCELRCPEAIQGLAAWSLCNTGKSLAWLSSVALQAEGRFERAAVEYQEHLCSVTGMDCSIPGAGRL
LKLSSSSVCSGAVSSPKHTG
NGDGRKTVLLKSTECSSEVLNFLANKACQCYVALCDWPSVQEWQASVQALKKNSSSSTPPGVHLKMDFNYIQALSHFEDG
DFEECRAQLELLPGEDCSLL
NATSAKDKLDLKRLLPSVLSLDPSELQKAIEVQLLRSAVSAMALANHQQEHKATQSSENLVRYLKQTGRISLGPLRLSTL
TLSSSLPSVSTLQLHCTTAL
DSTLTGQEDCVIPLDSEALSSCKQQDVQPWLQALRYTVFQRRLLLKLKAVDGHLMELCLVAVKFARKEGNVALATRLLNQ
CSNPEAKVGGDEEETLASSF
RCLNLECAAVEKWGPELEMEKAKVLYMAGQSVAAMMAMSSCALSYGHLGKCEPAACRSVLNLCKWLLVDWKDLTPQLKQA
RVVNNTCAVNSHTAGATGAS
SLLGHNITTSAASLSHNITALLLLPLGELGSPRITTESTVSVGVGEADFVLGQLYQLSTSLAPEMAKSWAALASWAYRWG
RKVVDNASQGEGVPLLSGEK
KEIEELLPDSTSEEDKNAIFSILGQAMCRPAGIQDEDLALQQDEDEEDDLVDVIWRQLLASCPWLADAGQVVTEGLIRVW
RRVVDRIFSLYRIACTAYFT
FLKLNAGKVPMNEEDPKLLLSSELCKQSNDDMIVMATLRLLRLLVKHASELREGLELGLASTPTAPWRGIIPQLFSRLNH
PEVYIRQSICSLLCRVAQDS
PHLILYPAIVGSLSLGGDAQNTVSKLPSALTPFLGALPAQRLAEEELSLPTSQESTGPGASGEDVAALCSSLDQAMMQDC
YSKIVEKLSSANPTMVVQVQ
LLVSELRRVTLLWDELWLGVLQQQHMHVLRRIQQLEEEVKRVQNNNTLRKDEKVAIMREKHSALMRPVVFALEHVCSITA
APAETPHETWFQQTYREAIS
SALERLRSPHNPASPASSWAPFKQIMLSLQQRAQKRASYLLHLNEISPRLASMAATEIAMPGEVSATDAVTIQSVGNTIT
ILPTKTKPKKLFFLGSDGRN
YPYLFKGLEDLHLDERIMQFLSIVNTMFTKVNQKEQPHFQARHYSVTPLGSRSGLIQWVDGAVPLFGLYKRWQQREAVLQ
AQKVQESLQAQPVPLVPRPS
ELYYSRIGPALKAMGLTLDVSRRDWPLSVMKEVLKELMEATPPNLLAKELWCSCTTPSEWWRVTQSYARSTAVMSMVGYI
IGLGDRHLDNVLIDMTTGEV
VHIDYNVCFEKGKSLRVPEKVPFRMTHNIETALGVTGVEGIFRLSCEQVVQMMRRGRETLLTLLEAFVYDPLVDWTAGGE

VGFAGAVYGGGGQQAENKQS
KREMERDITRSLFSSRVAEIKVNWFKNREEMLGVLPQVEEAVGEYLALQELLCQGEKLQSKLLEEATFLEGAENRTDHLI
VSLEQRYSEHTQLQSRQRTA
QEAIQSKLADLDQWISQYQAAFASLEATQLASLLQDISSPIDLGPPSYVPATAFLQNAGQAHLISQCEGLEAEVGAMLQQ
RRGALRGCLEQLHSYATVGL
LYPRATLLRHRAHQWKAWMEELLCSMTAEQCQNIYRHYEVLFAPQPPAASCQFLSACELALQHQAAELNGRVMRQAERLK
AEGTTTHTCEEQLQEIERCI
AVFQRENAEQGSLSLAAIITSALCALMSAGEQLVDLTSRDGAWFLEELCSMSSNIGVLATLLQRCQLLPHDLDLPAPAHT
LQVVYLANAVYTCLHELNTN
FRQIIFPEALRCMLRGEPTLESMLAELEQLVEQGSDGLDLQGLVDGMQASLHSTAIGLDRDAQAHCLHITRMLRSQYSEL
IQPRVMEPVGQEPPKMSAGQ
MLLVAFDGMFAQLETAFGQLTDKLSSLEVPPAWQKVDVIWEVRVSRGLLLDSPAQRRVMEEVFFLKRLQTIRDFFRLCTS
FAHTLSSEESSPSNGPVGYR
GPGAPVGSGVAVVSEEQMTRPIKCFTAEYVRQMLLGLPTQALGLALCSTLSALGLDLMAQVEAKDFGAENKVSLDDLCKK
AVEDGLASGRLSQMLLNRAT
VLASSYDTAWKKVDLLRRLDSSLDAAKVSLQRTQLHLAMFQWQHEDVLGPRSQQLSITPPPRSIILGSMKKKLYKLSQDE
AAIATVQVGPR
>g592.t2
GPELEVPLMMLVEALCELRCPEAIQGLAAWSLCNTGKSLAWLSSVALQAEGRFERAAVEYQEHLCSVTGMDCSIPGAGRL
LKLSSSSVCSGAVSSPKHTG
NGDGRKTVLLKSTECSSEVLNFLANKACQCYVALCDWPSVQEWQASVQALKKNSSSSTPPGVHLKMDFNYIQALSHFEDG
DFEECRAQLELLPGEDCSLL
NATSAKDKLDLKRLLPSVLSLDPSELQKAIEVQLLRSAVSAMALANHQQEHKATQSSENLVRYLKQTGRISLGPLRLSTL
TLSSSLPSVSTLQLHCTTAL
DSTLTGQEDCVIPLDSEALSSCKQQDVQPWLQALRYTVFQRRLLLKLKGSSAAVDGHLMELCLVAVKFARKEGNVALATR
LLNQCSNPEAKVGGDEEETL
ASSFRCLNLECAAVEKWGPELEMEKAKVLYMAGQSVAAMMAMSSCALSYGHLGKCEPAACRSVLNLCKWLLVDWKDLTPQ
LKQARVVNNTCAVNSHTAGA
TGASSLLGHNITTSAASLSHNITALLLLPLGELGSPRITTESTVSVGVGEADFVLGQLYQLSTSLAPEMAKSWAALASWA
YRWGRKVVDNASQGEGVPLL
SGEKKEIEELLPDSTSEEDKNAIFSILGQAMCRPAGIQDEDLALQQDEDEEDDLVDVIWRQLLASCPWLADAGQVVTEGL
IRVWRRVVDRIFSLYRIACT
AYFTFLKLNAGKVPMNEEDPKLLLSSELCKQSNDDMIVMATLRLLRLLVKHASELREGLELGLASTPTAPWRGIIPQLFS
RLNHPEVYIRQSICSLLCRV
AQDSPHLILYPAIVGSLSLGGDAQNTVSKLPSALTPFLGALPAQRLAEEELSLPTSQESTGPGASGEDVAALCSSLDQAM
MQDCYSKIVEKLSSANPTMV
VQVQLLVSELRRVTLLWDELWLGVLQQQHMHVLRRIQQLEEEVKRVQNNNTLRKDEKVAIMREKHSALMRPVVFALEHVC
SITAAPAETPHETWFQQTYR
EAISSALERLRSPHNPASPASSWAPFKQIMLSLQQRAQKRASYLLHLNEISPRLASMAATEIAMPGEVSATDAVTIQSVG
NTITILPTKTKPKKLFFLGS
DGRNYPYLFKGLEDLHLDERIMQFLSIVNTMFTKVNQKEQPHFQARHYSVTPLGSRSGLIQWVDGAVPLFGLYKRWQQRE
AVLQAQKVQESLQAQPVPLV
PRPSELYYSRIGPALKAMGLTLDVSRRDWPLSVMKEVLKELMEATPPNLLAKELWCSCTTPSEWWRVTQSYARSTAVMSM
VGYIIGLGDRHLDNVLIDMT
TGEVVHIDYNVCFEKGKSLRVPEKVPFRMTHNIETALGVTGVEGIFRLSCEQVVQMMRRGRETLLTLLEAFVYDPLVDWT
AGGEVGFAGAVYGGGGQQAE
NKQSKREMERDITRSLFSSRVAEIKVNWFKNREEMLGVLPQVEEAVGEYLALQELLCQGEKLQSKLLEEATFLEGAENRT
DHLIVSLEQRYSEHTQLQSR
QRTAQEAIQSKLADLDQWISQYQAAFASLEATQLASLLQDISSPIDLGPPSYVPATAFLQNAGQAHLISQCEGLEAEVGA
MLQQRRGALRGCLEQLHSYA
TVGLLYPRATLLRHRAHQWKAWMEELLCSMTAEQCQNIYRHYEVLFAPQPPAASCQFLSACELALQHQAAELNGRVMRQA
ERLKAEGTTTHTCEEQLQEI
ERCIAVFQRENAEQGSLSLAAIITSALCALMSAGEQLVDLTSRDGAWFLEELCSMSSNIGVLATLLQRCQLLPHDLDLPA
PAHTLQVVYLANAVYTCLHE
LNTNFRQIIFPEALRCMLRGEPTLESMLAELEQLVEQGSDGLDLQGLVDGMQASLHSTAIGLDRDAQAHCLHITRMLRSQ
YSELIQPRVMEPVGQEPPKM

SAGQMLLVAFDGMFAQLETAFGQLTDKLSSLEVPPAWQKVDVIWEVRVSRGLLLDSPAQRRVMEEVFFLKRLQTIRDFFR
LCTSFAHTLSSEESSPSNGP
VGYRGPGAPVGSGVAVVSEEQMTRPIKCFTAEYVRQMLLGLPTQALGLALCSTLSALGLDLMAQVEAKDFGAENKVSLDD
LCKKAVEDGLASGRLSQMLL
NRATVLASSYDTAWKKVDLLRRLDSSLDAAKVSLQRTQLHLAMFQWQHEDVLGPRSQQLSITPPPRSIILGSMKKKLYKL
SQDEAAIATVQVGPR
>g592.t3
GPELEVPLMMLVEALCELRCPEAIQGLAAWSLCNTGKSLAWLSSVALQAEGRFERAAVEYQEHLCSVTGMDCSIPGAGRL
LKLSSSSVCSGAVSSPKHTG
NGDGRKTVLLKSTECSSEVLNFLANKACQCYVALCDWPSVQEWQASVQALKKNSSSSTPPGVHLKMDFNYIQALSHFEDG
DFEECRAQLELLPGEDCSLL
NATSAKDKLDLKRLLPSVLSLDPSELQKAIEVQLLRSAVSAMALANHQQEHKATQSSENLVRYLKQTGRISLGPLRLSTL
TLSSSLPSVSTLQLHCTTAL
DSTLTGQEDCVIPLDSEALSSCKQQDVQPWLQALRYTVFQRRLLLKLKAVDGHLMELCLVAVKFARKEGNVALATRLLNQ
CSNPEAKVGGDEEETLASSF
RCLNLECAAVEKWGPELEMEKAKVLYMAGQSVAAMMAMSSCALSYGHLGKCEPAACRSVLNLCKWLLVDWKDLTPQLKQA
RVVNNTCAVNSHTAGATGAS
SLLGHNITTSAASLSHNITALLLLPLGELGSPRITTESTVSVGVGEADFVLGQLYQLSTSLAPEMAKSWAALASWAYRWG
RKVVDNASQGEGVPLLSGEK
KEIEELLPDSTSEEDKNAIFSILGQAMCRPAGIQDEDLALQQDEDEEDDLVDVIWRQLLASCPWLADAGQVVTEGLIRVW
RRVVDRIFSLYRIACTAYFT
FLKLNAGKVPMNEEDPKLLLSSELCKQSNDDMIVMATLRLLRLLVKHASELREGLELGLASTPTAPWRGIIPQLFSRLNH
PEVYIRQSICSLLCRVAQDS
PHLILYPAIVGSLSLGGDAQNTVSKLPSALTPFLGALPAQRLAEEELSLPTSQESTGPGASGEDVAALCSSLDQAMMQDC
YSKIVEKLSSANPTMVVQVQ
LLVSELRRVTLLWDELWLGVLQQQHMHVLRRIQQLEEEVKRVQNNNTLRKDEKVAIMREKHSALMRPVVFALEHVCSITA
APAETPHETWFQQTYREAIS
SALERLRSPHNPASPASSWAPFKQIMLSLQQRAQKRASYLLHLNEISPRLASMAATEIAMPGEVSATDAVTIQSVGNTIT
ILPTKTKPKKLFFLGSDGRN
YPYLFKGLEDLHLDERIMQFLSIVNTMFTKVNQKEQPHFQARHYSVTPLGSRSGLIQWVDGAVPLFGLYKRWQQREAVLQ
AQKVQESLQAQPVPLVPRPS
ELYYSRIGPALKAMGLTLDVSRRDWPLSVMKEVLKELMEATPPNLLAKELWCSCTTPSEWWRVTQSYARSTAVMSMVGYI
IGLGDRHLDNVLIDMTTGEV
VHIDYNVCFEKGKSLRVPEKVPFRMTHNIETALGVTGVEGIFRLSCEQVVQMMRRGRETLLTLLEAFVYDPLVDWTAGGE
VGFAGAVYGGGGQQAENKQS
KREMERDITRSLFSSRVAEIKVNWFKNREEMLGVLPQVEEAVGEYLALQELLCQGEKLQSKLLEEATFLEGAENRTDHLI
VSLEQRYSEHTQLQSRQRTA
QEAIQSKLADLDQWISQYQAAFASLEATQLASLLQDISSPIDLGPPSYVPATAFLQNAGQAHLISQCEGLEAEVGAMLQQ
RRGALRGCLEQLHSYATVGL
LYPRATLLRHRAHQWKAWMEELLCSMTAEQCQNIYRHYEVLFAPQPPAASCQFLSACELALQHQAAELNGRVMRQAERLK
AEGTTTHTCEEQLQEIERCI
AVFQRENAEQGSLSLAAIITSALCALMRWVCVLESEAAQLVTHCPLMGDAGLG
>g592.t4
MEGAAASAGEQLVDLTSRDGAWFLEELCSMSSNIGVLATLLQRCQLLPHDLDLPAPAHTLQVVYLANAVYTCLHELNTNF
RQIIFPEALRCMLRGEPTLE
SMLAELEQLVEQGSDGLDLQGLVDGMQASLHSTAIGLDRDAQAHCLHITRMLRSQYSELIQPRVMEPVGQEPPKMSAGQM
LLVAFDGMFAQLETAFGQLT
DKLSSLEVPPAWQKVDVIWEVRVSRGLLLDSPAQRRVMEEVFFLKRLQTIRDFFRLCTSFAHTLSSEESSPSNGPVGYRG
PGAPVGSGVAVVSEEQMTRP
IKCFTAEYVRQMLLGLPTQALGLALCSTLSALGLDLMAQVEAKDFGAENKVSLDDLCKKAVEDGLASGRLSQMLLNRATV
LASSYDTAWKKVDLLRRLDS
SLDAAKVSLQRTQLHLAMFQWQHEDVLGPRSQQLSITPPPRSIILGSMKKKLYKLSQDEAAIATVQVGPR
>g593.t1
APSPPNYRKRPNNEKATDDYHYEKFKKMNRRY
>g594.t1
MATQRVLPQSKETLLQNYNKRLKDDVRSIVDNFSEIIKTAKVRAGESLMKLVSDLKQFLILNDFPSVNDAITLQNQQLRS

LQDECDKKLTSLRDEIAVDL
YELEEEYYSSSLWDTTDLPLCEAYRRRDSWASPGPGSTQGDGDELHGPPSQEAKPQRHLNGHGTTSIEKA
>g594.t2
MATQRVLPQSKETLLQNYNKRLKDDVRSIVDNFSEIIKTAKVRAGESLMKLVSDLKQFLILNDFPSVNDAITLQNQQLRS
LQDECDKKLTSLRDEIAVDL
YELEEEYYSSSYSLWDTTDLPLCEAYRRRDSWASPGPGSTQGDGDELHGPPSQEAKPQRHLNGHGTTSIEKA
>g594.t3
LQNYNKRLKDDVRSIVDNFSEIIKTAKVRAGESLMKLVSDLKQFLILNDFPSVNDAITLQNQQLRSLQDECDKKLTSLRD
EIAVDLYELEEEYYSSSLWD
TTDLPLCEAYRRRDSWASPGPGSTQGDGDELHGPPSQEAKPQRHLNGHGTTSIEKA
>g595.t1
LQGESTGYTALREEQPPGPTEARSPRASDCDHSSDEDDEEEEEEEEAAGEKVMEAEERLEAEEAAEGDMAALDSSGSRPL
LLDSEEEEDLESGVQAQPTT
HSNASQTQQQKQQPQPPTFHTPLPVDTLAGNHSQPPPQSLRASEGAPDVFSKAPFQFAQDEVADVFANAPFPHANVHAAI
EPPPDVFSQAPFGKRKEAVG
VGGMASVTKTPYAQQAMAHAGTPDPQGVLGQVAPQPFRPQALAKYSRHFEGPVPQQPVAAQRVVSSVSRQAAVGSVPVGP
LHSWTSEPATTVDPFVSAPF
HLKAPHEKP
>g596.t1
MSLPPQDPGEVKPYLFCDEEIPVKSEEVVSHMWPTATPSFCAEHAYSSASKTCVQSLATPRKQPRKTPLVPRSLEPPVLE
LSPQAKAQLEELMMVGDLLE
VSLDETLHIWRILQATHPPSEERFLQVMEPGDSLLDKPLKIKLKESERKRRRKLERAEQHHMLMAAVTTGDLRHAKRQEL
KRAGLELALAGKAKKKKLKV
GTEKSRELKQLAKRLAKEEKERKRKEKAAAKVEGAREGPDKRKEKKILDIPSKYDWSGAEDSNDENAVCAAKNCQRPCKD
KVSSPSTGWSQGVRGSSRGH
RAPGGLSRSWTASVCSPKPNIGSPISMSVDI
>g596.t2
GHIQTFHQAGFSRVSAPRQQFVDYDDEETDSDEDIRETYGYDMKDPGEVKPYLFCDEEIPVKSEEVVSHMWPTATPSFCA
EHAYSSASKTCVQSLATPRK
QPRKTPLVPRSLEPPVLELSPQAKAQLEELMMVGDLLEVSLDETLHIWRILQATHPPSEERFLQVMEPGDSLLDKPLKIK
LKESERKRRRKLERAEQHHM
LMAAVTTGDLRHAKRQELKRAGLELALAGKAKKKKLKVGTEKSRELKQLAKRLAKEEKERKRKEKAAAKVEGAREGPDKR
KEKKILDIPSKYDWSGAEDS
NDENAVCAAKNCQRPCKDKVSSPSTGWSQGVRGSSRGHRAPGGLSRSWTASVCSPKPNIGSPISMSVDI
>g596.t3
MQVSRSNLTISGVCSNTVWGNDLRIFTGDLVLIGSGCEVDWVQCDGGCDGWFHQVCVGVSCEMAENEDYICMDCTCKTHV
VVSTAGVMGGGATAAV
>g596.t4
GHIQTFHQAGFSRVSAPRQQFVDYDDEETDSDEDIRETYGYDMKVLRPSVPAQRTDTWNLNSRGPPEVKFNG
>g596.t5
GHIQTFHQAGFSRVSAPRQQFVDYDDEETDSDEDIRETYGYDMKDPGEVKPYLFCDEEIPVKSEEVVSHMWPTATPSFCA
EHAYSSASKTCVQSLATPRK
QPRKTPLVPRSLEPPVLELSPQAKAQLEELMMVGDLLEVSLDETLHIWRILQATHPPSEERFLQVMEPGDSLLDKPLKIK
LKESERKRRRKLERAEQHHM
LMAAVTTGDLRHAKRQELKRAGLELALAGKAKKKKLKVGTEKSRELKQLAKRLAKEEKERKRKEKAAAKVEGAREGPDKR
KEKKILDIPSKYDWSGAEDS
NDENAVCAAKNCQRPCKDKVSSPSTGWSQGVRGSSRGHRAPGGLSRSWTASVCSPKPNIGSPISMSRSKWAWPVKFPVLQ
AEPGGCWAGQGLGPQTLTLV
LNPAGLSGARSRRVRAPLVRDYTLWMSS
>g596.t6
MPLLLQRSKWAWPVKFPVLQAEPGGCWAGQGLGPQTLTLVLNPAGLSGARSRRVRAPLVRDYTLWMSS
>g596.t7
LTISGVCSNTVWGNDLRIFTGDLVLIGSGCEVDWVQCDGGCDGWFHQVCVGVSCEMAENEDYICMDCTCKTHVVVSTAGV
MGGGATAAV
>g596.t8
MPLLLQRSKWAWPVKFPVLQAEPGGCWAGQGLGPQTLTLVLNPAGLSGARSRVCSNTVWGNDLRIFTGDLVLIGSGCEVD

WVQCDGGCDGWFHQVCVGVS
CEMAENEDYICMDCTCKTHVVVSTAGVMGGGATAAV
>g597.t1
LIGQEATAWTSVVHCLWYDCIAAVLIVSITALPLMAAEEAMITDLDENNPGTEWERVARLCDFNPKSSKQAKDVSRMRSV
LISLKQAPLVR
>g598.t1
MTSLLQEYQKEFPDIPIGYSGHESGVSITVAAVALGAKVVERHVTLDKTWKGSDHAASLEPAELAELVRAIRLVETALGS
GVKKMLPCEKPCHDKLGKSV
VARSAVSSGTVLTLDMLVVKVAEPRGVPPEDIFQLVGKTVLEDLEEDQSITPDLVDGYGKRAKC
>g598.t2
MVGGSQPCFIIAEIGQNHQGDLEIAKKMIKMAKDCGADCAKFQKSELEHKFNKLALARPYNSAHSWGKTYGDHKRHLEFS
HEQYRELQKYAEEVGIFFTA
SGMDEMAVEFLEVLNVPFFKVGSGDTNNFPYLEKTAKKGRPMVVSSGMQSMETMRRVYQTVREHNPNFCILQCTSAYPLD
PGDVNLRVISVRLTLSFQTP
PNLPEHPAVLSDQPCGPFA
>g598.t3
MVGGSQPCFIIAEIGQNHQGDLEIAKKMIKMAKDCGADCAKFQKSELEHKFNKLALARPYNSAHSWGKTYGDHKRHLEFS
HEQYRELQKYAEEVGIFFTA
SGMDEV
>g598.t4
MVGGSQPCFIIAEIGQNHQGDLEIAKKMIKMAKDCGADCAKFQKSELEHKFNKLALARPYNSAHSWGKTYGDHKRHLEFS
HEQYRELQKYAEEVGIFFTA
SGMDEMAVEFLEVLNVPFFKVGSGDTNNFPYLEKTAKKGRPMVVSSGMQSMETMRRVYQTVREHNPNFCILQCTSAYPLD
PGDVNLRVISEYQKEFPDIP
IGYSGHESGVSITVAAVALGAKVVERHVTLDKTWKGSDHAASLEPAELAELVRAIRLVETALGSGVKKMLPCEKPCHDKL
GKSVVARSAVSSGTVLTLDM
LVVKVAEPRGVPPEDIFQLVGKTVLEDLEEDQSITPDLVDGYGKRAKC
>g598.t5
MAVEFLEVLNVPFFKVGSGDTNNFPYLEKTAKKGRPMVVSSGMQSMETMRRVYQTVREHNPNFCILQCTSAYPLDPGDVN
LRVISVRLTLSFQTPPNLPE
HPAVLSDQPCGPFA
>g598.t6
LALMTSLLQEYQKEFPDIPIGYSGHESGVSITVAAVALGAKVVERHVTLDKTWKGSDHAASLEPAELAELVRAIRLVETA
LGSGVKKMLPCEKPCHDKLG
KSVVARSAVSSGTVLTLDMLVVKVAEPRGVPPEDIFQLVGKTVLEDLEEDQSITPDLVDGYGKRAKC
>g599.t1
MAVWQRSAVGRLTPSGQEVLLLDVNDVLISGSYLVLMAGQRVLFQKAGVEPKANYLHEEKAQRAAAQQDVLDSLRPLQRR
VEPQHCRPQHQHASPAVSLP
PRAQTRPTSPDPTAGGPAPPGAPWVSPVTSPPPDTMDLKFLDRSHRGDSAGADPAQAPLLGASRTPAPRPHVRRKLRPGR
LAGFMLSLVAVCAVFSWSAS
TLAGSGPGPEAERPAKPLGPQRTLLQHQNLSTQPPVAMRSSVEQMNHGDYPTDYFSLEERRQGYVALHMFGMLYMFIALA
IVCDEFFVPALTVITEKLAI
SDDVAGATFMAAGGSAPELFTSVIGVFVSHSNVGIGTIVGSAVFNILFVIGMCALFSREVLNLTWWPLFRDVSFYIVGLL
MLIYFFLDNQITVGESIGLL
SCYFFFYVTFMKFNATVERFIKGMLGGNQVEEVEQAPKLSVEVSLSKASAPDRLAVALKYK
>g599.t2
MAVWQRSAVGRLTPSGQEVLLLDVNDVLISGSYLVLMAGQRVLFQKAGVEPKANYLHEEKAQRAAAQQDVLDSLRPLQRR
VEPQHCRPQHQHASPAVSLP
PRAQTRPTSPDPTAGGPAPPGAPWVSPVTSPPPDTMDLKFLDRSHRGDSAGADPAQAPLLGASRTPAPRPHVRRKLRPGR
LAGFMLSLVAVCAVFSWSAS
TLAGSGPGPEAERPAKPLGPQRTLLQHQNLSTQPPVAMRSSVEQMNHGDYPTDYFSLEERRQGYVALHMFGMLYMFIALA
IVCDEFFVPALTVITEKLAI
SDDVAGATFMAAGGSAPELFTSVIGVFVSHSNVGIGTIVGSAVFNILFVIGMCALFSREVLNLTWWPLFRDVSFYIVGLL
MLIYFFLDNQITVGESIGLL
SCYFFFYVTFMKFNATVERFIKGMLGGNQVEEVEQAPKMSSSGGLVSVVLDQCSLSLQKRPGSRWRVPCQRFCLVLRMGAE
VLDPGKTLMSPGLLRVSSLS

PEGVPPYGLWRVVCEDLSFITHSLV
>g599.t3
MAVWQRSAVGRLTPSGQEVLLLDVNDVLISGSYLVLMAGQRVLFQKAGVEPKANYLHEEKAQRAAAQQDVLDSLRPLQRR
VEPQHCRPQHQVFGFGFAGT
GGTNSSSSAPPVTNMRRAQTRPTSPDPTAGGPAPPGAPWVSPVTSPPPDTMDLKFLDRSHRGDSAGADPAQAPLLGASRT
PAPRPHVRRKLRPGRLAGFM
LSLVAVCAVFSWSASTLAGSGPGPEAERPAKPLGPQRTLLQHQNLSTQPPVAMRSSVEQMNHGDYPTDYFSLEERRQGYV
ALHMFGMLYMFIALAIVCDE
FFVPALTVITEKLAISDDVAGATFMAAGGSAPELFTSVIGVFVSHSNVGIGTIVGSAVFNILFVIGMCALFSREVLNLTW
WPLFRDVSFYIVGLLMLIYF
FLDNQITVGESIGLLSCYFFYVTFMKFNATVERFIKGMLGGNQVEEVEQAPKLSVEVSLSKASAPDRLAVALKYK
>g600.t1
MRSAAMALLILGMCTTATWSLPMRSMMVTFPGDVIQDMTDIEMAEKYLKRFGYYQSVQRSGFQSMVSTDRALKALQRQMG
LEESGQLDKTTLDTMRRPRC
GVPDIRSYKTFDGDVKILNYSPDMEASLTDDTFARAFKVWSDVTPLTFTRLFDGNADIMISFGRANHGDPYPFDGKDGLL
AHAYPPGEGIQGDAHFDDDE
FWTLGKGTVVKTLYGNAEGALCHFPFTFNGKTYSSCTTDGRKDDLPWCSTTADFETDKKYGFCPSELLYTFGGNSEGSPC
VFPFVFLGETYDSCTAEGRS
DGYRWCSTTDNFDNDKKFGFCPSRDTAVSGGNAEGEPCHFPFQFQGQTYDSCTSDGRGDGKLWCATTADFDRDTKWGFCP
DKGYSLFLVAAHEFGHALGL
DHSNIRDALMYPMYSYVENFHLHADDVGGIQYLYGPKTGPEPSPPGPTDPTEPDTSTAVDPFPTNPTPVDPDSDVCQVTI
FDAITEIENVLHFFKDGNYW
KMSNKAGSKGPFAMSKRWPALPATIDSVFEDTPTKNIYFFAGTRFWVYSGKKVLGPRSIEKLGLPSSVQKVDGSLLRKNG
KVLLFSGDNYWRLDLKTLQM
DRGYPLATDSVFAGVPSDVHDVFQHKGRMYFCHDRFYWRMNSRRQVTRVGYIKYDLLRCSDNADQQK
>g600.t2
MRSAAMALLILGMCTTATWSLPMRSMMVTFPGDVIQDMTDIEMAEKYLKRFGYYQSVQRSGFQSMVSTDRALKALQRQMG
LEESGQLDKTTLDTMRRPRC
GVPDIRSYKTFDGDVKILNYSPDMEASLTDDTFARAFKVWSDVTPLTFTRLFDGNADIMISFGRANHGDPYPFDGKDGLL
AHAYPPGEGIQGDAHFDDDE
FWTLGKGTEGALCHFPFTFNGKTYSSCTTDGRKDDLPWCSTTADFETDKKYGFCPSELLYTFGGNSEGSPCVFPFVFLGE
TYDSCTAEGRSDGYRWCSTT
DNFDNDKKFGFCPSRDTAVSGGNAEGEPCHFPFQFQGQTYDSCTSDGRGDGKLWCATTADFDRDTKWGFCPDKGYSLFLV
AAHEFGHALGLDHSNIRDAL
MYPMYSYVENFHLHADDVGGIQYLYGPKTGPEPSPPGPTDPTEPDTSTAVDPFPTNPTPVDPDSDVCQVTIFDAITEIEN
VLHFFKDGNYWKMSNKAGSK
GPFAMSKRWPALPATIDSVFEDTPTKNIYFFAGTRFWVYSGKKVLGPRSIEKLGLPSSVQKVDGSLLRKNGKVLLFSGDN
YWRLDLKTLQMDRGYPLATD
SVFAGVPSDVHDVFQHKGRMYFCHDRFYWRMNSRRQVTRVGYIKYDLLRCSDNADQQK
>g600.t3
MRSAAMALLILGMCTTATWSLPMRSMMVTFPGDVIQDMTDIEMAEKYLKRFGYYQSVQRSGFQSMVSTDRALKALQRQMG
LEESGQLDKTTLDTMRRPRC
GVPDIRSYKTFDGDVKILNYSPDMEASLTDDTFARAFKVWSDVTPLTFTRLFDGNADIMISFGRANHGDPYPFDGKDGLL
AHAYPPGEGIQGDAHFDDDE
FWTLGKGTVVKTLYGNAEGALCHFPFTFNGKTYSSCTTDGRKDDLPWCSTTADFETDKKYGFCPSELLYTFGGNSEGSPC
VFPFVFLGETYDSCTAEGRS
DGYRWCSTTDNFDNDKKFGFCPSRDTAVSGGNAEGEPCHFPFQFQGQTYDSCTSDGRGDGKLWCATTADFDRDTKWGFCP
DKGYSLFLVAAHEFGHALGL
DHSNIRDALMYPMYSYVENFHLHADDVGGIQYLYGPKTGPEPSPPGPTDPTEPDTSTAVDPFPTNPTPVDPDSDVCQVTI
FDAITEIENVLHFFKDGNYW
KMSNKAGSKGPFAMSKRWPALPATIDSVFEDTPTKNIYFFAGMVLIVSKGTRFWVYSGKKVLGPRSIEKLGLPSSVQKVD
GSLLRKNGKVLLFSGDNYWR
LDLKTLQMDRGYPLATDSVFAGVPSDVHDVFQHKGRMYFCHDRFYWRMNSRRQVTRVGYIKYDLLRCSDNADQQK
>g601.t1
RLSGAPPPPTHAINRGIAAEGLGCILAGLWGTGNGTTSYSQNIAALGITKVGSRLVLQTTGLLMILLGIFGKFGAVFITI
PEPVIGGMFLVMFGMIAAVG

ISNLQVDLRVPLYYHQTPG
>g601.t2
RLSGAPPPPTHAINRGIAAEGLGCILAGLWGTGNGTTSYSQNIAALGITKVGSRLVLQTTGLLMILLGIFGKFGAVFITI
PEPVIGGMFLVMFGMIAAVG
ISNLQ
>g602.t1
MVLHFILQHLDSPGSYGRILYVDLSSAFNMIIPALLRDKLSQINVPDSMCRWITDFLTDTRQFVRLGKHVSDTRTQGRRI
GGKS
>g603.t1
MTLPFPKTLSASRPNPAMDIGGLETVVANNAYVSARGSIDGASAASMRDKKMRAKLNLPHIRNCEHTKSTVDAAFQSMCV
LQPIGKRLFQQYLDSEEMHK
NAAQLWKDMEDYNMSQEKDRMQKAQRIVNKYYESAAKTFCSFLEEKAVTRVKEDHKNIRSDLFKESEQLLLKHLEAKALE
GFKNSLFFLRYVQFKWLESQ
SVNEDWFMDFRVLGKGGFGEVHACQAKATGKMYANKKLDKKRLKKRKGYQGAIVEKRILAKVHSRFIVSLAYAFQTKSEL
CLVMTIMNGGDLRYHMYNVD
EKNPGFNEKRACFYTAQVICGLEHLHQHRIVYRDLKPENLLLDDAGFMAPELLQKKAYDYSVDYFTLGVTLYEMMAAKGP
FRVRGEKVENEEVTRRILND
AVAYPPTFSADSKAFCEGLMEKDPAKRLGFKNNECAELKNQPFFKELNWGRLEAGMLPPPFVPDPKMVYAKDIDDVGAFS
TIKGVVLDAKDTEFYDDFAS
GNIPIPWQEEMVETGVFGELNLWAEAGKLPNDLDPNFVESKGGGCVIL
>g603.t2
MTLPFPKTLSASRPNPAMDIGGLETVVANNAYVSARGSIDGASAASMRDKKMRAKLNLPHIRNCEHTKSTVDAAFQSMCV
LQPIGKRLFQQYLDSEEMHK
NAAQLWKDMEDYNMSQEKDRMQKAQRIVNKYYESAAKTFCSFLEEKAVTRVKEDHKNIRSDLFKESEQLLLKHLEAKALE
GFKNSLFFLRYVQFKWLESQ
SVNEDWFMDFRVLGKGGFGEVHACQAKATGKMYANKKLDKKRLKKRKGYQGAIVEKRILAKVHSRFIVSLAYAFQTKSEL
CLVMTIMNGGDLRYHMYNVD
EKNPGFNEKRACFYTAQVICGLEHLHQHRIVYRDLKPENLLLDDAGHVRLSDLGLAVELPPGKDTTTGYAGTPGFMAPEL
LQKKAYDYSVDYFTLGVTLY
EMMAAKGPFRVRGEKVENEEVTRRILNDAVAYPPTFSADSKAFCEGLMEKDPAKRLGFKNNECAELKNQPFFKELNWGRL
EAGMLPPPFVPDPKMVYAKD
IDDVGAFSTIKGVVLDAKDTEFYDDFASGNIPIPWQEEMVETGVFGELNLWAEAGKLPNDLDPNFVESKGGGCVIL
>g603.t3
MDIGGLETVVANNAYVSARGSIDGASAASMRDKKMRAKLNLPHIRNCEHTKSTVDAAFQSMCVLQPIGKRLFQQYLDSEE
MHKNAAQLWKDMEDYNMSQE
KDRMQKAQRIVNKYYESAAKTFCSFLEEKAVTRVKEDHKNIRSDLFKESEQLLLKHLEAKALEGFKNSLFFLRYVQFKWL
ESQSVNEDWFMDFRVLGKGG
FGEVHACQAKATGKMYANKKLDKKRLKKRKGYQGAIVEKRILAKVHSRFIVSLAYAFQTKSELCLVMTIMNGGDLRYHMY
NVDEKNPGFNEKRACFYTAQ
VICGLEHLHQHRIVYRDLKPENLLLDDAGFMAPELLQKKAYDYSVDYFTLGVTLYEMMAAKGPFRVRGEKVENEEVTRRI
LNDAVAYPPTFSADSKAFCE
GLMEKDPAKRLGFKNNECAELKNQPFFKELNWGRLEAGMLPPPFVPDPKMVYAKDIDDVGAFSTIKGVVLDAKDTEFYDD
FASGNIPIPWQEEMVETGVF
GELNLWAEAGKLPNDLDPNFVESKGGGCVIL
>g604.t1
GKKPPGRWLTFFSASKSGDAVFIRDSSAAHPLALLLLTDGDIAEI
>g604.t2
GKKPPGRWLTFFSASKSGDAVFIRDSSAAHPLALLLLTDGDIAEIGETLTMQTLVFSRR
>g605.t1
MRMFYESVVASAIFFAVSCWGSGMRVGDTNRLNRLIRRAGDAVGEELDTLTIVDAVQATWYCTSSDPSVKLLKFADDTTV
IGLIRDDDESAYRREVGQLA
LWCGQNHLELNTLKTVEMTVDFRSRPPTLPPITILNNTVSAVETFRFLGSIISQDLKWAHNVDTIIKKAHRGCTSCSAEE
VQPAPRTADPFYTAIVQSVL
CTSIIVWFGSATKQDKNRLTRTIRTAERIIGVKLPTIQDLHSSRVRKRAVNISADPSH
>g605.t2
MRMFYESVVASAIFFAVSCWGSGMRVGDTNRLNRLIRRAGDAVGEELDTLTIVDAVQATWYCTSSDPSVKLLKFADDTTV

IGLIRDDDESAYRREVGQLA
LWCGQNHLELNTLKTVEMTVDFRSRPPTLPPITILNNTVSAVETFRFLGSIISQDLKWAHNVDTIIKKAHRGCTSCSAEE
VQPAPRTADPFYTAIVQSVL
CTSIIVWFGSATKQDKNRLTRTIRTAERIIGVKLPTIQDLHSSR
>g606.t1
MRLDGPIWPVLSRGAEQSAARPTIDPLLFQASPRSQEITAFPNKKGPRSSDEEDEETNVLEKSCYYSFHSQGGNTSQMEL
QGEALLLAEAPALENKPLAT
LMTGRRTPEQECLPEGMVAWRGSGDGHCSDGAGLPRVGDKDRTVVPMTLYQHRVKGLVLALLVEPHLQRDMEAMEEVYHS
SLASLNGLEAHLRTGAPGPP
GPQGPYIFAHYDCIQNTLRTNVSGRVGEDQEHTFVRATALLHTHFCRSEALQEAIIRSSRTAVYATRSAAQETYFLQHGG
ALRNSGVPNHRDTAFSLPHK
ARHMLLKHGVSLL
>g606.t2
MRLDGPIWPVLSRGAEQSAARPTIDPLLFQASPRSQEITAFPNKKGPRSSDEEDEETNVLEKSCYYSFHSQGGNTSQMEL
QGEALLLAEAPALENKPLAT
LMTGRRTPEQECLPEGMVAWRGSGDGHCSDGAGLPRVGDKDRTVVPMTLYQHRVKGLVLALLVEPHLQRDMEAMEEVYHS
SLASLNGLEAHLRTGAPGPP
GPQGPYIFAHYDCIQNTLRTNVSGRVGEDQEHTFVRATALLHTHFCRSEALQEAIIRSSRTAVYATRSAAQETYFLQHGG
ALRNSGVPNHRDTAFSLPHK
ARHMLLKHGRGSRSVVFPGKSLFNFVRQAYRCCQEGFMCRNVKGIQGHLREGAQAEFLLTPDVLSVPITRAELHLQMFNP
RRLSIQPVLLFLAKRNLPTR
YDVWAQGDMVELKVDLLSVFRGLIETAGGLGGGGASSLVNMLLLGMRSRGGPPDAPGQQAPQDTNDDDDGGVWGDGLSHR
PPPPPLELGLALYCSHDGHG
LSCEHAGLRLSHMPFIALSYR
>g606.t3
MESTEQPAERATLLRLRIRSFAQRGSRSVVFPGKSLFNFVRQAYRCCQEGFMCRNVKGIQGHLREGAQAEFLLTPDVLSV
PITRAELHLQMFNPRRLSIQ
PVLLFLAKRNLPTRYDVWAQGDMVELKVDLLSVFRGLIETAGGLGGGGASSLVNMLLLGMRSRGGPPDAPGQQAPQDTND
DDDGGVWGDGLSHRPPPPPL
ELGLALYCSHDGHGLSCEHAGLRLSHMPFIALSYR
>g606.t4
MGSDSAVRSTTAVPKWRRAEISPADLFLLLVMEVPVDLLRSHSTPGKGSRPRRTRLSRTLSDIPSTDSNTDLETDSPHTP
MRLDGPIWPVLSRGAEQSAA
RPTIDPLLFQASPRSQEITAFPNKKGPRSSDEEDEETNVLEKSCYYSFHSQGGNTSQMELQGEALLLAEAPALENKPLAT
LMTGRRTPEQECLPEGMVAW
RGSGDGHCSDGAGLPRVGDKDRTVVPMTLYQHRVKGLVLALLVEPHLQRDMEAMEEVYHSSLASLNGLEAHLRTGAPGPP
GPQGPYIFAHYDCIQNTLRT
NVSGRVGEDQEHTFVRATALLHTHFCRSEALQEAIIRSSRTAVYATRSAAQETYFLQHGGALRNSGVPNHRDTAFSLPHK
ARHMLLKHGRGSRSVVFPGK
SLFNFVRQAYRCCQEGFMCRNVKGIQGHLREGAQAEFLLTPDVLSVPITRAELHLQMFNPRRLSIQPVLLFLAKRNLPTR
YDVWAQGDMVELKVDLLSVF
RGLIETAGGLGGGGASSLVNMLLLGMRSRGGPPDAPGQQAPQDTNDDDDGGVWGDGLSHRPPPPPLELGLALYCSHDGHG
LSCEHAGLRLSHMPFIALSY
R
>g607.t1
MFCVSDVESPACCPTYVAWHPHQRSTIAFGDELGRVAVKDLQGSQPTVMKTIHKRRVSGLAFSTHSAPLLASISDDCSVA
VMNSELAEQLRDRRHQDFVK
GVCWLHGGSDSLTTVGWDHQVLHHTVGAAAEATSGAP
>g607.t2
MFCVSDVESPACCPTYVAWHPHQRSTIAFGDELGRVAVKDLQGSQPTVMKTIHKRRVSGLAFSTHRLRDRRHQDFVKGVC
WLHGGSDSLTTVGWDHQVLH
HTVGAAAEATSGAP
>g608.t1
MSSSDSVHGFAVPTPTVPLFHMFNGLKSDSATGNASSKTSFTAARITGSHLAEAATHDSSQASVNLLQCLNPNFYITGKH
GSRSPRVPNICTTQTPFRKA
TEIREGSPTKTHSVVFNLRHRWLHPTKFCSCLPPDDHTLQFQSPEHC

>g608.t2
MSSSDSVHGFAVPTPTVPLFHMFNGLKSDSATGNASSKTSFTAARITGSHLAEAATHDSSQASVNLLQCLNPNFYITGKH
DGFIPPNSAPASHQMTTPSS
FNPQNTAKLNYAPKTSVLSGHGDSDTRPKQILLLQDSNPAVQLDGKASPTTPSKPLRGTAPGTQLDREPKGNLQANVPSK
AAQLQPKSESKPSPKPPALP
VLQEKSPPPPLPQVSPKFKRETFSPRLSEVEKSPRQKGQFSPLIDRKLQSMKGPETSRDGPSASPLALLKAAKEREKHRS
SGFHENGPKSYVKHINSPPH
SFVITPRSNSISSRSSTQGREEDSQPAVNASRPTSTRQTEALHLAPPSVRSPLAPSTPAQSTAATSASLSRLAAEKQDVR
QAPPSLDHGKSTASPPDPPM
TLLPPPPEFANFGYDNSIDAPPDFPPPDPPAKKAPLPTLKPVSSAAVPAPPPKPKTATTPPSQAPVQVVKPQVSFQTLPK
VPVPKPLPVSESQATLLSIL
QKKMQEMDSKISPKREMDSSSDEWGPHMSDEDTKIPFAPRTTPQHYKSNTLPAKTATLDMRELETKVAKIQQDGSSASSN
GTHSKHGMTFTVRPGTKQPI
TIVSKGEPS
>g608.t3
MKGPETSRDGPSASPLALLKAAKEREKHRSSGFHENGPKSYVKHINSPPHSFVITPRSNSISSRSSTQGREEDSQPAVNA
SRPTSTRQTEALHLAPPSVR
SPLAPSTPAQSTAATSASLSRLAAEKQDVRQAPPSLDHGKSTASPPDPPMTLLPPPPEFANFGYDNSIDAPPDFPPPDPP
AKKAPLPTLKPVSSAAVPAP
PPKPKTATTPPSQAPVQVVKPQVSFQTLPKVPVPKPLPVSESQATLLSILQKKMQEMDSKISPKREMDSSSDEWGPHMSD
EDTKIPFAPRTTPQHYKSNT
LPAKTATLDMRELETKVAKIQQDGSSASSNGTHSKHGMTFTVRPGTKQPITIVSKGEPS
>g608.t4
MSHTSCQLYFMSSSDSVHGFAVPTPTVPLFHMFNGLKSDSATGNASSKTSFTAARITGSHLAEAATHDSSQASVNLLQCL
NPNFYITGKHDGFIPPNSAP
ASHQMTTPSSFNPQNTAKLNYAPKTSVLSGHGDSDTRPKQILLLQDSNPAVQLDGKASPTTPSKPLRGTAPGTQLDREPK
GNLQANVPSKAAQLQPKSES
KPSPKPPALPVLQEKSPPPPLPQVSPKFKRETFSPRLSEVEKSPRQKGQFSPLIDRKLQSMKGPETSRDGPSASPLALLK
AAKEREKHRSSGFHENGPKS
YVKHINSPPHSFVITPRSNSISSRSSTQGREEDSQPAVNASRPTSTRQTEALHLAPPSVRSPLAPSTPAQSTAATSASLS
RLAAEKQDVRQAPPSLDHGK
STASPPDPPMTLLPPPPEFANFGYDNSIDAPPDFPPPDPPAKKAPLPTLKPVSSAAVPAPPPKPKTATTPPSQAPVQVVK
PQVSFQTLPKVPVPKPLPVS
ESQATLLSILQKKMQEMDSKISPKREMDSSSDEWGPHMSDEDTKIPFAPRTTPQHYKSNTLPAKTATLDMRELETKVAKI
QQDGSSASSNGTHSKHGMTF
TVRPGTKQPITIVSKGEPS
>g609.t1
VVACFRHLASCVGGCTDTLQLRDELRQTREKAQGLAGLCCQRLTARLRDKSLPDGDRREMELLWVAFSSGLELLHADLCK
VFGMANIFSVANGGAFVQTG
IQGGGSELAARALSLPDLNSAGPGPELPDSLEPQEASAMEQEIHRVDRMIEDMEAKVNVLRWTVEPRGGAEAAD
>g610.t1
LINNSGRSQRSLCLETSDDVYHALMELNFLGTISLTKEVLPHMMRRRGFFNCLRTELTDYPKIVISTACPGPVQSKIVHN
CFTEELDKAMDKVEDQQHKM
STSRCVRWILVGMANGAKEMWIAEQPVLLFCYLWQYAPTGEPGRHGLELCVNRLTQLSVTSPQSVAWGFIRVARPGARLK
HVKSAPVGTASQSLSNWNFE
NKSLQCVTSLGTWPPLTEIAMLRSP
>g610.t2
MLTSRYFGHVCLGANQIDILINNSGRSQRSLCLETSDDVYHALMELNFLGTISLTKEVLPHMMRRRGFFNCLRTELTDYP
KIVISTACPGPVQSKIVHNC
FTEELDKAMDKVEDQQHKMSTSRCVRWILVGMANGAKEMWIAEQPVLLFCYLWQYAPTGEPGRHGLELCVNRLTQLSVTS
PQSVAWGFIRVARPGARLKH
VKSAPVGTASQSLSNWNFENKSLQCVTSLGTWPPLTEIAMLRSP
>g610.t3
MGWWYGLLEHQIDILINNSGRSQRSLCLETSDDVYHALMELNFLGTISLTKEVLPHMMRRRGFFNCLRTELTDYPKIVIS
TACPGPVQSKIVHNCFTEEL
DKAMDKVEDQQHKMSTSRCVRWILVGMANGAKEMWIAEQPVLLFCYLWQYAPTGEPGRHGLELCVNRLTQLSVTSPQSVA

WGFIRVARPGARLKHVKSAP
VGTASQSLSNWNFENKSLQCVTSLGTWPPLTEIAMLRSP
>g611.t1
MGAFLDKPKMEKHNAHGDGNSLSYGLSSMQGWRVEMEDAHTAVVGLANGLDLWSFFAVYDGHAGSQVAKYCCEHLLEHIT
SNPDFQSALEEDPCVESVKN
GIRTGFLQIDEHMRTISEKKNGADRSGSTAVGVMISPGHIYFINCGDSRGLLSRGGAVHFFTQDHKPSNPLEKERIQNAG
GSVMIQRVNGSLAVSRALGD
FDYKCVHGKGPTEQLVSPEPEVYAIERCEGDDEFIVLACDGIWDVMANEELCEFVRSRLEVTDDLERVCNEIVDTCLYKG
SRDNMSVVLICFPGAPKVSP
EAVKREMELDKYLENRVEGKCFV
>g611.t2
MEKHNAHGDGNSLSYGLSSMQGWRVEMEDAHTAVVGLANGLDLWSFFAVYDGHAGSQVAKYCCEHLLEHITSNPDFQSAL
EEDPCVESVKNGIRTGFLQI
DEHMRTISEKKNGADRSGSTAVGVMISPGHIYFINCGDSRGLLSRGGAVHFFTQDHKPSNPLEKERIQNAGGSVMIQRVN
GSLAVSRALGDFDYKCVHGK
GPTEQLVSPEPEVYAIERCEGDDEFIVLACDGIWDVMANEELCEFVRSRLEVTDDLERVCNEIVDTCLYKGSRDNMSVVL
ICFPGAPKVSPEAVKREMEL
DKYLENRVEGKCFV
>g612.t1
MSKRASLASCIAKMYDGSKSENSGAASGAVRKPCIIPGAGIVSTLGNMEVKLVQLESHVQQIASGHADVLHRLDMVCKGL
GSLEKGLAQIQRTWGREADF
RAVNLSGFSTSAEEGLPTVSSDVRGLCWENMKLLQNLTQEGKSQRERLAGIEESVSAVDTMLEYIAEVFQSSKGTEQEFS
EEQKPKPSGAVPKEKTQAEN
EAFKLTTVNTKVETDAGDNAIVNIEEAPREPGSVQQTAQLQTGENEELPKPPAPEMDPSVGSCTAATPLPNAVIDDCPPA
PAPFEHRIVSAKQVPMGSYY
SIMPNEVLGGGRFGQVHKCAELSSGLTLAAKIIKVRDEVKNEIGVMNQLNHVNLIQLYDAFESRTNLTLIMEYVEGGELF
DRIVDENYKLTELDAIVFTK
QICEGVQYLHQQYILHLDLKPENILCVSATGNQIKIIDFGLARKYRPREKLKVNFGTPEFLAPEVVNYDFVSFPTDMWSV
GVITYMLLSGLSPFLGDNDA
ETMNNILHGSWEFDSDGFENVTSEAKDFVSELLIPTKSGRLSASGCTKHRWLNKLEEKAKQHHVTLKSQIKLQRYLAAHR
QWKV
>g612.t2
MSKRASLASCIAKMYDGSKSENSGAASGAVRKPCIIPGAGIVSTLGNMEVKLVQLESHVQQIASGHADVLHRLDMVCKGL
GSLEKGLAQIQRTWGREADF
RAVNLSGFSTSAEEGLPTVSSDVRGLCWENMKLLQNLTQEGKSQRERLAGIEESVSAVDTMLEYIAEVFQSSKVVEFTLK
RDVPWRIKSLLGSTVSKRRQ
GTEQEFSEEQKPKPSGAVPKEKTQAENEAFKLTTVNTKVETDAGDNAIVNIEEAPREPGSVQQTAQLQTGENEELPKPPA
PEMDPSVGSCTAATPLPNAV
IDDCPPAPAPFEHRIVSAKQVPMGSYYSIMPNEVLGGGRFGQVHKCAELSSGLTLAAKIIKVRDEVKNEIGVMNQLNHVN
LIQLYDAFESRTNLTLIMEY
VEGGELFDRIVDENYKLTELDAIVFTKQICEGVQYLHQQYILHLDLKPENILCVSATGNQIKIIDFGLARKYRPREKLKV
NFGTPEFLAPEVVNYDFVSF
PTDMWSVGVITYMLLSGLSPFLGDNDAETMNNILHGSWEFDSDGFENVTSEAKDFVSELLIPTKSGRLSASGCTKHRWLN
KLEEKAKQHHVTLKSQIKLQ
RYLAAHRQWKV
>g612.t3
MSKRASLASCIAKMYDGSKSENSGAASGAVRKPCIIPGAGIVSTLGNMEVKLVQLESHVQQIASGHADVLHRLDMVCKGL
GSLEKGLAQIQRTWGREADF
RAVNLSGFSTSAEEGLPTVSSDVRGLCWENMKLLQNLTQEGKSQRERLAGIEESVSAVDTMLEYIAEVFQSSKVVEFTLK
RDVPWRIKSLLGSTVSKRRQ
GTEQEFSEEQKPKPSGAVPKEKTQAENEAFKLTTVNTKVETDAGDNAIVNIEEAPREPGSVQQTAQLQTGENEELPKPPA
PEMDPSVGSCTAATPLPNAV
IDDCPPAPAPFEHRIVSAKQVPMGSYYSIMPNEVLGGAPTAVSLVCISLSAPFGQKALDNIETLLLALLFPTLSEEIDSR
GRFGQVHKCAELSSGLTLAA
KIIKVRDEVKNEIGVMNQLNHVNLIQLYDAFESRTNLTLIMEYVEGGELFDRIVDENYKLTELDAIVFTKQICEGVQYLH
QQYILHLDLKPENILCVSAT

GNQIKIIDFGLARKYRPREKLKVNFGTPEFLAPEVVNYDFVSFPTDMWSVGVITYMLLSGLSPFLGDNDAETMNNILHGS
WEFDSDGFENVTSEAKDFVS
ELLIPTKSGRLSASGCTKHRWLNKLEEKAKQHHVTLKSQIKLQRYLAAHRQWKV
>g613.t1
MEENGAHFFEGTEKLLEVWFTRQDEANGTGDLRTIPSLFTTNQLKYRTLNSASCRPPCGPHRVLSDMSTSEANWYPLVLH
WYDTAWALIGCLRGAVSGEL
>g614.t1
MGIQGLLQFLKDASEPTHVKKYRGQTVAVDTYCWLHKGAFSCAEKLAKGEPTDQYVTYCMKFVDMLLSFNVRPILVFDGC
NLPSKQEVEKSRRERRQANL
QKGKQLLREGKLSEARDCFTRSVNITPSMAHDVIKAARARGVDCVVAPYEADAQLAFLSKAGLAQAIITEDSDLLAFGCK
KVLLKMDKEGNGLEIDQAHL
GRCRSLGGVFTEEKFRYMCILSGCDYLASLYGIGLGKACKLLKVANNPDILTVIRKMGQYLKMSVTVPEEYLQGFVKADN
TFLYQLVFDPLRRKVVPLNP
YPEHLDPSTLGYAGLNKGDEKGYQMALGNLDINTMERID
>g614.t2
MGIQGLLQFLKDASEPTHVKKYRGQTVAVDTYCWLHKGAFSCAEKLAKGEPTDQYVTYCMKFVDMLLSFNVRPILVFDGC
NLPSKQEVEKSRREQANLQK
GKQLLREGKLSEARDCFTRSVNITPSMAHDVIKAARARGVDCVVAPYEADAQLAFLSKAGLAQAIITEDSDLLAFGCKKV
LLKMDKEGNGLEIDQAHLGR
CRSLGGVFTEEKFRYMCILSGCDYLASLYGIGLGKACKLLKVANNPDILTVIRKMGQYLKMSVTVPEEYLQGFVKADNTF
LYQLVFDPLRRKVVPLNPYP
EHLDPSTLGYAGLNKGDEKGYQMALGNLDINTMERID
>g615.t1
MQFSPTNGDFTFVSSTEAEELSGTIDAPDVKLNLGSDAGKDPYATTFLRQRGYGWLLEVEEEENEDSKPLLEELDIDLKD
IFYKIRCVLMPMPSLGFNRQ
VVRDNPDFWGPLAVVLLFSMISIYGQFRVVSWIITIWIFGSLTVFLLARVLGGEVSYGQVLGVIGYSLLPLIVISPLLLV
IRSFDVVATVIKLFGVFWAA
YSAASLLVGDEFKTKKPLLIYPIFLLYIYFLSLYTGV
>g615.t2
MQFSPTNGDFTFVSSTEAEELSGTIDAPDVKLNLGSDAGKDPYATTFLRQRGYGWLLEVEEEENEDSKPLLEELDIDLKD
IFYKIRCVLMPMPSLGFNRQ
VVRDNPDFWGPLAVVLLFSMISIYGQFRCCSYNPCSLVSLKVVSWIITIWIFGSLTVFLLARVLGGEVSYGQVLGVIGYS
LLPLIVISPLLLVIRSFDVV
ATVIKLFGVFWAAYSAASLLVGDEFKTKKPLLIYPIFLLYIYFLSLYTGV
>g616.t1
MAAAASPGSGSSAASDWAVLRDGCLRCDEQGLRSLSYHPALNAILAVTSRGSIKVIDGTSGAILQASALHGQYPADE
>g616.t2
MFALLSLLLAVSRAAKPGGRVRCQYFPAVDKVLFVDDYVVGCRKDLNGILLLDTALQPPVSKPEDMVQLELPVTEAQQML
CACQEKIDVSNTEGYDLFIT
QLKEGVKNTSHETAANHKVAKNPQPPRGNGE
>g616.t3
MAAAASPGSGSSAASDWAVLRDGCLRCDEQGLRSLSYHPALNAILAVTSRGSIKVIDGTSGAILQASALHAKPGGRVRCQ
YFPAVDKVLFVDDYVVGCRK
DLNGILLLDTALQPPVSKPEDMVQLELPVTEAQQMLCACQEKIDVSNTEGYDLFITQLKEGVKNTSHETAANHKVAKNPQ
PPRGNGE
>g616.t4
MFALLSLLLAVSRAAKPGGRVRCQYFPAVDKVLFVDDYVVGCRKDLNGILLLDTALQPPVSKPEDMVQLELPVTEAQQML
CACQEKIDVSNTEGYDLFIT
QLKEGVKNTSHETAANHKVAK
>g617.t1
MSSSSMPDPPQEDPVEAQANPAPELNEKHGEGTIVLNHHVPSDKPSGSKTSPTKDEPHKREKPQKKLGLKMGDPDNAFSP
YRPYRCVHEGCSAAFTIQQN
LILHYRAMHQASLPLTRVEGDAEKPSITSGQESVVKDDEVRCQVKDCSRVFMGITKLVQHYLLLHKFTRDKATAMMSSMN
VGTFKCDREECSLPFESVDK
YIEHIKNFHKEIAISESGTVDTTFKCEYDGCDCVYTTKSNLLRHLIKKHDYVYDPKSSDGRRTKSVGLFGPGNNTKEIFE
SKYKVKKKNIKKKEVNSIDH

WSSFGKPALKSHEEASALCTKKSSLQYPCMIIGCDTVERAERSIFKHYTTHGLTERYIEDHRSQFIFCKKYSRSRFKDAN
KSEGMSTSSSSEVSETEEKI
IQILVLPHLKRNSVMRVFCLDHLSL
>g618.t1
MAQQGGVWSNATLSSILSNGLPHAEQVHEFLELEGPMLLNMRLKHLIKMDSIEKAAVLAKICSEYPGYEGKGNFKQIYLV
CICMSKTQEQLMEEITSIDC
KDALDMICNLESEGDEKGALCLCTAFLKRQLLQEEVYCAWELTLFWSKLLIRLESSADVFLEQSKKMALLCRSVCHILFF
IKVIQNEVGEVGLPVCVEMC
IQAVKMTTSDHKDSKSTICKTIYCLLPNDLEVKRACQLTEFLLQPTVDSYYSVESLYNEPDQKPEEEGNLPVPNSLRCEL
LLALKTQWPFDPEFWDWRTL
KRNCLALMGEEAAIVSSIDTLNDAEEQEEEGALAKVPEYKDLEDFLLNTTNELNEITDEREKNREAKKLREQGFVSARFR
NWRAYMQYCVLCDKEFLGHR
IVRHAQKHFKDGHP
>g618.t2
MAQQGGVWSNATLSSILSNGLPHAEQVHEFLELEGPMLLNMRLKHLIKMDSIEKAAVLAKICSEYPGYEGKGNFKQIYLV
CICMSKTQEQLMEEITSIDC
KDALDMICNLESEGDEKGALCLCTAFLKRQLLQEEVYCAWELTLFWSKLLIRLESSADVFLEQSKKMALLCRSVCHILFF
IKVIQNEVGEVGLPVCVEMC
IQAVKMTTSDHKDSKSTICKTIYCLLPNDLEVKRACQLTEFLLQPTVDSYYSVESLYNEPDQKPEEEGNLPVPNSLRCEL
LLALKTQWPFDPEFWDWRTL
KRNCLALMGEEAAIVSSIDTLNDAEEQEEEVLKAEKF
>g618.t3
MAQQGGVWSNATLSSILSNGLPHAEQVHEFLELEGPMLLNMRLKHLIKMDSIEKAAVLAKICSEYPGYEGKGNFKQIYLV
CICMSKTQEQLMEEITSIDC
KDALDMICNLESEGDEKGALCLCTAFLKRQLLQEEVYCAWELTLFWSKLLIRLESSADVFLEQSKKMALLCRSVCHILFF
IKVIQNEVGEVGLPVCVEMC
IQAVKMTTSDHKDSKSTICKTIYCLLPNDLEVKRACQLTEFLLQPTVDSYYSVESLYNEPDQKPEEEGNLPVPNSLRCEL
LLALKTQWPFDPEFWDWRTL
KRNCLALMGEEAAIVSSIDTLNDAEEQEEEGALAKVPEYKDLEDFLLNTTNELNEITDEREKNREAKKLREQGFVSARFR
NWRAYMQYCVLCDKEFLGHR
IVRHAQKHFKDVQNCSKAFKFFRNLMAHGTPKMEDKGSAFVRLERIDTEAYMKDVAMASTSPSKSPLILKEPIPHGEARE
ILLG
>g619.t1
LVSHSDLDDRAIEALKEFNEDGALQVLLQFKESDLSHVQALLQRTGYTLDVTTGQRKYGGPPPESAHSGLQPTIGTEIFV
GKIPRDLFEDELVPLFEKAG
PIWDLRLMMDPLSGLNRGYAFVTFCTKDAAQEAVKLCNNNEIRPGKHIGVCISVANNRLFVGSIPKSKTKEQIVEEFAKV
TEGLNDVILYHQPDDKKKNR
GFCFLEYEDHKTAAQARRRLMSGKVKVWGNVVTVEWADPIEDPDPEVMAKVKVLFVRNLASTVTEEILENTFSQFGKLER
VKKLKDYAFIHFEERDGAVK
ALADLNGKDLEGEHIEIVFAKPPDQKRKERKALRHATKTQMYDEYYFYGPPHMPPPTRGRGRGGRGGYSYPPDYYGYEDY
YDYYGYDYHNYRGGYEDPYY
GYEDYQVSGRGRGGRGGGVRGGTSQIRGRGGATPRGRASFSQRGGPGTSRGTLSEMG
>g619.t2
MDSSAAVTHSEHFQTLLDAGLPQKVAEKLDDIYIAGLVSHSDLDDRAIEALKEFNEDGALQVLLQFKESDLSHVQALLQR
TGYTLDVTTGQRKYGGPPPE
SAHSGLQPTIGTEIFVGKIPRDLFEDELVPLFEKAGPIWDLRLMMDPLSGLNRGYAFVTFCTKDAAQEAVKLCNNNEIRP
GKHIGVCISVANNRLFVGSI
PKSKTKEQIVEEFAKVTEGLNDVILYHQPDDKKKNRGFCFLEYEDHKTAAQARRRLMSGKVKVWGNVVTVEWADPIEDPD
PEVMAKVKVLFVRNLASTVT
EEILENTFSQFGKLERVKKLKDYAFIHFEERDGAVKALADLNGKDLEGEHIEIVFAKPPDQKRKERKALRHATKTQMYDE
YYFYGPPHMPPPTRGRGRGG
RGGYSYPPDYYGYEDYYDYYGYDYHNYRGGYEDPYYGYEDYQVSGRGRGGRGGGVRGGTSQIRGRGGATPRGRASFSQRG
GPGTSRGTLSEMG
>g620.t1
DNNFIKDFPQLADGLMVIPLPVEEQCRGVLSEPLPNLQLLTGDAQFNEAMGYPMVQQWRVRSNLYRVKLSSITLSAGATY
PSALCVRCRPCP

>g620.t2
DNNFIKDFPQLADGLMVIPLPVEEQCRGVLSEPLPNLQLLTGDAQFNEAMGYPMVQQWRVRSNLYRVKLSSITLSA
>g621.t1
MAYHLAQAQRCSCPSPPSSPGHGCHCNPMGGPCEDCPAGEAGANGGQSSGSANRASGHRPLGCHWPHGSHGRGAHKAAQR
HVVTVGLSQVMLDYPLAVLV
GCGLFLLGCSLAGLLIGPLPDFSDPLLGFEPRGTNIGVRQSSLDKLQENTGPGKVLSYVPWQVQDGPLR
>g621.t2
VPREAPGMAYHLAQAQRCSCPSPPSSPGHGCHCNPMGGPCEDCPAGEAGANGGQSSGSANRASGHRPLGCHWPHGSHGRG
AHKAAQRHVVTVGLSQVMLD
YPLAVLVGCGLFLLGCSLAGLLIGPLPDFSDPLLGFEPRGTNIGVRQSSLDKLQENTGPGKVLSYVPWQVQDGPLR
>g621.t3
MAYHLAQAQRCSCPSPPSSPGHGCHCNPMGGPCEDCPAGEAGANGGQSSGSANRASGHRPLGCHWPHGSHGRGAHKAAQR
HVVTVGLSQVMLDYPLAVLV
GCGLFLLGCSLAGLLIGPLPDFSDPLLGFEPRGTNIGVRQSSLDKLQENTGPGK
>g622.t1
NACFGFAISLLMLTISAMMVYGAITHRDGWLIPFFCYQLFDFALSCLVAISPLTYLPRIKDYLDQLPDFPYKDNLLSMDS
SCLLLFVLLFFAFLIILKVK
PCPHLSAAHVFLCLSTKDLALHVKQQNLFVLVRLMGSTPSEVVT
>g622.t2
NACFGFAISLLMLTISAMMVYGAITHRDGWLIPFFCYQLFDFALSCLVAISPLTYLPRIKDYLDQLPDFPYKDNLLSMDS
SCLLLFVLLFFAFLIILKVK
PCPHLSAAHVLT
>g623.t1
GTYRFKNGGSYVGDYHNNIKHGQGSFYYPDGSKYEGSWVEDLRQGHGIYTYSNGDKYDGAWLHHVRHGQGTYWHHHTGSS
YRGAWTNGQIDGSGEIIHST
HHYKGRFLNNYVSL
>g623.t2
NEAGERHGSGKATLPNGDKYQGMYENGRRSGQGTYRFKNGGSYVGDYHNNIKHGQGSFYYPDGSKYEGSWVEDLRQGHGI
YTYSNGDKYDGAWLHHVRHG
QGTYWHHHTGSSYRGAWTNGQIDGSGEIIHSTHHYKGRFLNNYVSL
>g624.t1
LLIDGSENVGAANFPRVRDLALRIIEGLDVGRDTIRIAVALYSADPEIQFYLNSYDSKAAVLDAVKGLSFTGGEEANLGA
ALETVSESLLSQASGGRAEE
GVPQAVVVISAGPSTDDISTGDRALRTANVITFGVAVGGASSADLEPIATDKSFVLAAPDFRTAASMGDQLQPYIVGVAQ
RTIVIQNEFTEAIAVGRRDI
IFLLDSTMGTNVINSVRDLIKRFIDMLPIGPEDVQIGVAQFTNVARLEIDLNSYGSREDLTAALSRIRPKQGQTVNIGAA
LDFVRTNMFRADKGSRISQG
VPQLLLVMTSKKSSDSVDAPAQALQQMGVLTIAAGSKTADEAELKRIAFDDKLVFMMKDFRFLLRNPKAIISPLSTLSGI
MVTEEPELEITTVQTAKIVR
DIVFLVDGSSYVGNQNLPYVRQFITNLVNKLDVRPDRVQIGLMQFSERPKIEFYLNTHQSNQEVLNTISQMRVTGGSVLN
TGAAMSYALSNMFRSSTGSR
RRENVPQALVLITGGPAQDEVKKVADSVALAGVLTYTVSTGQADPQLLQSVAFVPELAFYTEDFADMPQLVEQIMQPLIT
VVGDTDVTGYVDSVITGAER
DVAFLIDGSDNVRGDFPYIQDFILKVIQPLDIGSDRVRISVVQHSETPTSVFYLNTYQTKDEVIRAVNDMRLAGGRSLNT
GSALKFMKETILSESNGSRA
PQNVPQFLIVLTGGRSRDNVKESAVALKTGGVVPFGVGVKDADPRQIEEISHNPSFAFKVKEFSELGSVQQKLNNYVSLP
KEQLQVVLEQVLKTPEMDCR
PFESSSEEWQKD
>g624.t2
LALRIIEGLDVGRDTIRIAVALYSADPEIQFYLNSYDSKAAVLDAVKGLSFTGGEEANLGAALETVSESLLSQASGGRAE
EGVPQAVVVISAGPSTDDIS
TGDRALRTANVITFGVAVGGASSADLEPIATDKSFVLAAPDFRTAASMGDQLQPYIVGVAQRTIVIQNEFTEAIAVGRRD
IIFLLDSTMGTNVINSVRDL
IKRFIDMLPIGPEDVQIGVAQFTNVARLEIDLNSYGSREDLTAALSRIRPKQGQTVNIGAALDFVRTNMFRADKGSRISQ
GVPQLLLVMTSKKSSDSVDA
PAQALQQMGVLTIAAGSKTADEAELKRIAFDDKLVFMMKDFRFLLRNPKAIISPLSTLSGIMVTEEPELEITTVQTAKIV

RDIVFLVDGSSYVGNQNLPY
VRQFITNLVNKLDVRPDRVQIGLMQFSERPKIEFYLNTHQSNQEVLNTISQMRVTGGSVLNTGAAMSYALSNMFRSSTGS
RRRENVPQALVLITGGPAQD
EVKKVADSVALAGVLTYTVSTGQADPQLLQSVAFVPELAFYTEDFADMPQLVEQIMQPLITVVGDTDVTGYVDSVITGAE
RDVAFLIDGSDNVRGDFPYI
QDFILKVIQPLDIGSDRVRISVVQHSETPTSVFYLNTYQTKDEVIRAVNDMRLAGGRSLNTGSALKFMKETILSESNGSR
APQNVPQFLIVLTGGRSRDN
VKESAVALKTGGVVPFGVGVKDADPRQIEEISHNPSFAFKVKEFSELGSVQQKLNNYVSLPKEQLQVVLEQVLKTPEMDC
RPFESSSEEWQKD
>g624.t3
LLIDGSENVGAANFPRVRDLALRIIEGLDVGRDTIRIAVALYSADPEIQFYLNSYDSKAAVLDAVKGLSFTGGEEANLGA
ALETVSESLLSQASGGRAEE
GVPQAVVVISAGPSTDDISTGDRALRTANVITFGVAVGGASSADLEPIATDKSFVLAAPDFRTAASMGDQLQPYIVGVAQ
RTIVIQNEFTEAIAVGRRDI
IFLLDSTMGTNVINSVRDLIKRFIDMLPIGPEDVQIGVAQFTNVARLEIDLNSYGSREDLTAALSRIRPKQGQTVNIGAA
LDFVRTNMFRADKGSRISQG
VPQLLLVMTSKKSSDSVDAPAQALQQMGVLTIAAGSKTADEAELKRIAFDDKLVFMMKDFRFLLRNPKAIISPLSTLSGI
MVTEEPELEITTVQTAKIVR
DIVFLVDGSSYVGNQNLPYVRQFITNLVNKLDVRPDRVQIGLMQFSERPKIEFYLNTHQSNQEVLNTISQMRVTGGSVLN
TGAAMSYALSNMFRSSTGSR
RRENVPQALVLITGGPAQDEVKKVADSVALAGVLTYTVSTGQADPQLLQSVAFVPELAFYTEDFADMPQLVEQIMQPLIT
VVGDTDVTGYVDSVITGAER
DVAFLIDGSDNVRGDFPYIQDFILKVIQPLDIGSDRVRISVVQHSETPTSVFYLNTYQTKDEVIRAVNDMRLAGGRSLNT
GSALKFMKETILSESNGSRA
PQNVPQFLIVLTGGRSRDNVKESAVALKTGGVVPFGVGVKDADPRQIEEISHNPSFAFKVKEFSELGSVQQKLNNYVSLP
KEQLQVVLEQVCR
>g625.t1
MRPSNCFALEAGLERAVPWGCDLFTFVTSAAGHMMRTLQKPRKNRPSKRQVNHRRFLHNMIQRKFTNIEAANQRLASALL
LTKEDNTSPSSPSQKPTAPE
PSIKPATDDCEPAKDPITNTSRDIDRVSKFGSDSVYGSSEIEVNNKRQPELRHIWGHLPNSQTNTCTWRGKSQGHRAPRP
ARQSRIGNENQTALISFLNS
PEHTKVKHKPGALPTACYSVEKTSQRTSIHLSEDSVYTASLKETDKNNNNPQFKSGSNSDVIIHLDDDLDLSLSPSFSPD
LSPLSLDSCDFSVQSMLDVS
TCLQKDHKDDTVENQWRDMMELLQNEVPHRDDVEDCVNMEEYFENACLCQEGSSELEGAFLCI
>g625.t2
MRPSNCFALEAGLERAVPWGCDLFTFVTSAAGHMMRTLQKPRKNRPSKRQVNHRRFLHNMIQRKFTNIEAANQRLASALL
LTKEDNTSPSSPSQKPTAPE
PSIKPATDDCEPAKDPITNTSRDIDRVSKFGSDSVYGSSEIEVNNKRQPELRHIWGHLPNSQTNTCTWRGKSQGHRAPRP
ARQSRIGNENQTALISFLNS
PEHTKVKHKPGALPTACYSVEKTSQRTSIHLSEDSVYTASLKETDKNNNNPQFKSGSNSDVIIHLDDDLDLSLSPSFSPD
LSPLSLDSCDFSVQSMLDVS
TCLQKDHKDDTVENQWRDMMELLQNEVPHRDDVEDCVNMEEYFENACLCQEGSSELEGVAQSFSAPTESPQYHPILTPPF
ENDWLFTEIV
>g626.t1
MSRAWALFKAHPYFGNVVGYTTLFASADVIQQSVLGGSRPATADSKSGDSPQPDSRTVGIDWSQTAKVALVGFCFHANFN
YHWLRGLERLLPGGGFRKIT
LKVGLDQLIAAPATISAFFIGLSILERKEDPLEDWRGKFWTSYKGVDIRHNKDRKVHRKEPRSQDIYLRLLVKLYRFLAR
RSNAPFNKVVLRRLFMSKSN
RPPLSVSSLVCALRVTVGARRRILKAGGEIMTFDQLAMSAPKGQGTVLLSGRRKAREVYRHFGKAPGTPHSHSKPYIRSK
GRKFERARGRRASRGYKN
>g626.t2
MSRAWALFKAHPYFGNVVGYTTLFASADVIQQSVLGGSRPATADSKSGDSPQPDSRTVGIDWSQTAKVALVGFCFHANFN
YHWLRGLERLLPGGGFRKIT
LKVGLDQLIAAPATISAFFIGLSILERKEDPLEDWRGKFWTSYKTGVVYWSTMQGVDIRHNKDRKVHRKEPRSQDIYLRL
LVKLYRFLARRSNAPFNKVV
LRRLFMSKSNRPPLSVSSLVCALRVTVGARRRILKAGGEIMTFDQLAMSAPKGQGTVLLSGRRKAREVYRHFGKAPGTPH

SHSKPYIRSKGRKFERARGR
RASRGYKN
>g626.t3
MSRAWALFKAHPYFGNVVGYTTLFASADVIQQSVLGGSRPATADSKSGDSPQPDSRTVGIDWSQTAKVALVGFCFHANFN
YHWLRGLERLLPGGGFRKIT
LKVGLDQLIAAPATISAFFIGLSILERKEDPLEDWRGKFWTSYKGVDIRHNKDRKVHRKEPRSQDIYLRLLVKLYRFLAR
RSNAPFNKVVLRRLFMSKSN
RPPLSVSSLIRKMRLAGRENRTAVVVGTITDDVRIQDVPKLKVCALRVTVGARRRILKAGGEIMTFDQLAMSAPKGQGTV
LLSGRRKAREVYRHFGKAPG
TPHSHSKPYIRSKGRKFERARGRRASRGYKN
>g627.t1
MEYLLQVKVGALVGLLLLTLFFGFIPVRLKWFRNTSGSETHRVVLSLISCFAGGVFLAACLLDIIPDYLEDINTELNARK
LEISFPLPEFIIALGFFVVL
ILERIVLTFQDAHGDHGERAPLVHAGHGHAPGPDLEASGPHVHVDFQAHSPFRSFVLFLSLSLHSVFEGLAIGLQRTDSK
VLEICIAILVHKSIIVFSLS
VKLVQSAVRPAWVAAYVGGLAAGTFVYITFMEILPHELNSPERQLPKVLFILVGFSVMAALAFLD
>g627.t2
MEYLLQVKVGALVGLLLLTLFFGFIPVRLKWFRNTSGSETHRVVLSLISCFAGGVFLAACLLDIIPDYLEDINTELNARK
LEISFPLPEFIIALGFFVVL
ILERIVLTFQDAHGDHGERAPLVHAGHGHAPGPDLEASGPHVHVDFQAHSPFRSFVLFLSLSLHSVFEGLAIGLQRTDSK
VLEICIAILVHKSIIVFSLS
VKLVQSAVRPAWGLAAGTFVYITFMEILPHELNSPERQLPKVLFILVGFSVMAALAFLD
>g627.t3
MEYLLQVKVGALVGLLLLTLFFGFIPVRLKWFRNTSGSETHRVVLSLISCFAGGVFLAACLLDIIPDYLEDINTELNARK
LEISFPLPEFIIALGFFVVL
ILERIVLTFQDAHGDHGERAPLVHAGHGHAPGPDLEASGPHVHVDFQAHSPFRSFVLFLSLSLHSVFEGLAIGLQRTDSK
VLEICIAILVHKSIIVFSLS
VKLVQSAVRPAWVAAYGLAAGTFVYITFMEILPHELNSPERQLPKVLFILVGFSVMAALAFLD
>g628.t1
MPSDCGAGVAAEPADQGRRPSTWSSTMRCEIRQGYGIQEWPDGSKYEGEYLNGLKHGAGRFTWKNGEYYEGMFHRDYRHG
EGLYSWPSGYRFTGKFYLNK
KEGYGKKEYPDGSTFQ
>g628.t2
MRCEIRQGYGIQEWPDGSKYEGEYLNGLKHGAGRFTWKNGEYYEGMFHRDYRHGEGLYSWPSGYRFTGKFYLNKKEGYGK
KEYPDGSTFQ
>g628.t3
MTVNQLSGSLVPKTSPVLNKSPDCFFLSLRDGSHLKCRQTAVLVSQQNPRTREDGRYYEGMFHRDYRHGEGLYSWPSGYR
FTGKFYLNKKEGYGKKEYPD
GSTFQ
>g629.t1
DLAKVQRAVCMLSNTTAIAEAWARLDHKFDLMYAKRAFVHWYVGEGMEEGEFSEAREDMAALEKDYEERECISIHVGQAG
VQIGNACWELYCLEHGIQPD
GQMPSDKAIGGGDDSFNTFFSETGAGKHVPRAVFVDLEPTVIDEVRTGTYRQLFHPEQLITGKEDAANNYARGHYTIGKE
IIDLVLDRVRKL
>g629.t2
DLAKVQRAVCMLSNTTAIAEAWARLDHKFDLMYAKRAFVHWYVGEGMEEGEFSEAREDMAALEKDYEERECISIHVGQAG
VQIGNACWELYCLEHGIQPD
GQMPSDKAIGGGDDSFNTFFSETGAGKHVPRAVFVDLEPTVIDEVRTGTYRQLFHPEQLITGKEDAANNYARGHYTIGKE
IIDLVLDRVRKLVSIL
>g629.t3
DLAKVQRAVCMLSNTTAIAEAWARLDHKFDLMYAKRAFVHWYVGEGMEEGEFSEAREDMAALEKDYEERECISIHVGQAG
VQIGNACWELYCLEHGIQPD
GQMPSDKAIGGGDDSFNTFFNEVRTGTYRQLFHPEQLITGKEDAANNYARGHYTIGKEIIDLVLDRVRKL
>g630.t1
MQVLSPTGSPARYLLARLADRSCSLAFLLSCLKKMDHQHAVQYLTAMVMEQIHITVQPQSQQALEGGRVVLTCRAAGPVG
LSYQWFRGKEQILGGTGSAA

DLVLCPLGPVHQGHYICRVNHGDNCTFSQWAQIQLAHSEYSSPGCSSTLFPGSKSGLCITRQPQSQTVTEGDTLILECLA
EANPPAQFHWYHNMRAMPQQ
KSCVLRLCAPMADVHELANLLRQLDFKVVSLLDLNWQEMHSAVTEFLLLLDKGVYGLLYFAGHGYENYGNSFMVPIDAPA
SYTSDHCLCVQSVLQRMQER
ETGLNVFLLDMCRKRCIDAEAYEVKTDDPHNGVFISFLKPRLCEDEKVTVMLDRVAEGMYLDISMKKYMGRCELMRGRQA
LELRSNLSERRALTDRIRST
ESSPDHSARNLQWAIAHVLPKSRKLHFECGVEVQIGFAAEFSNIMIIYTRILEKPKEIVSCFAELIDFPEKGVDLDLKKS
NQESIIEAGSLLPIMESLPS
QQSPSLYTRLKSLQRLKTDLTFAVCVHYRYSNMDEEVQEMQSVTVGKPLVSKLNLHEARLSRSSSADLQSFNAPEASLYG
EDLGPDFPASETSSIGSAST
TWSYYSQAIEDEGGNPEEIFVAAESTMKKTPKRAVKADARLDSEENMIGEDSHDGQDDHENMQDMCIFDMSVVDEAENDN
GIPAEVEDDKMQDFTEDLCD
EDELLRDSEEQYHEKMGSEEESAREEKSESELLDIDMPCTSPDVVSHTKTQPKRDDSILRLNAMLDMKAIGLELKPVNAK
WTSSNTELFSPLDQVLLFMH
VESKNDKVAGSCLSEPLNEDPQESREATAEDEQAGSPTGGQDEARRDTARTAEAVPGQVARDTEVKEVTDVDEGGDGQTV
DAQGESTQAVSVNEQGAAGE
TVESQMGSKEGEEEETKTEENASADASSAAKESSTVEGDDQKKSSEEKDANAESKDEKAAAGGTVAAGAGRNLWVSGLSS
TTRATDLKTLFSKYGKVVGA
KVVTNAKSPGARCYGFVTMSSGDEATTCISHLHRTELHGKMISVERVGLPAARGRPARGRMVRSVGGSSSSGSVLLNLSI
WLQAKNEPAGKKPADTKTPV
AERRNSTDSR
>g630.t2
MQVLSPTGSPARYLLARLADRSCSLAFLLSCLKKMDHQHAVQYLTAMVMEQIHITVQPQSQQALEGGRVVLTCRAAGPVG
LSYQWFRGKEQILGGTGSAA
DLVLCPLGPVHQGHYICRVNHGDNCTFSQWAQIQLAHSEYSSPGCSSTLFPGSKSGLCITRQPQSQTVTEGDTLILECLA
EANPPAQFHWYHNMRAMPQQ
KSCVLRLCAPMADVHELANLLRQLDFKVVSLLDLNWQEMHSAVTEFLLLLDKGVYGLLYFAGHGYENYGNSFMVPIDAPA
SYTSDHCLCVQSVLQRMQER
ETGLNVFLLDMCRKRCIDAEAYEVKTDDPHNGVFISFLKPRLCEDEKVTVMLDRVAEGMYLDISMKKYMGRCELMRGRQA
LELRSNLSERRALTDRIRST
ESSPDHSARNLQWAIAHVLPKSRKLHFECGVEVQIGFAAEFSNIMIIYTRILEKPKEIVSCFAELIDFPEKGVDLDLKKS
NQESIIEAGSLLPIMESLPS
QQSPSLYTRLKSLQRLKTDLTFAVCVHYRYSNMDEEVQEMQSVTVGKPLVSKLNLHEARLSRSSSADLQSFNAPEASLYG
EDLGPDFPASETSSIGSAST
TCFSTMADSSSEIDIPEGCEVRKLSELRVIDLKAELKKRNLDTTGIKSVLSERLKKAIEDEGGNPEEIFVAAESTMKKTP
KRAVKADARLDSEENMIGED
SHDGQDDHENMQDMCIFDMSVVDEAENDNGIPAEVEDDKMQDFTEDLCDEDELLRDSEEQYHEKMGSEEESAREEKSESE
LLDIDMPCTSPDVVSHTKTQ
PKRDDSILRLNAMLDMKAIGLELKPVNAKWTSSNTELFSPLDQVLLFMHVESKNDKVAGSCLSEPLNEDPQESREATAED
EQAGSPTGGQDEARRDTART
AEAVPGQVARDTEVKEVTDVDEGGDGQTVDAQGESTQAVSVNEQGAAGETVESQMGSKEGEEEETKTEENASADASSAAK
ESSTVEGDDQKKSSEEKDAN
AESKDEKAAAGGTVAAGAGRNLWVSGLSSTTRATDLKTLFSKYGKVVGAKVVTNAKSPGARCYGFVTMSSGDEATTCISH
LHRTELHGKMISVERVGLPA
ARGRPARGRMVRSVGGSSSSGSVLLNLSIWLQAKNEPAGKKPADTKTPVAERRNSTDSR
>g630.t3
MQVLSPTGSPARYLLARLADRSCSLAFLLSCLKKMDHQHAVQYLTAMVMEQIHITVQPQSQQALEGGRVVLTCRAAGPVG
LSYQWFRGKEQILGGTGSAA
DLVLCPLGPVHQGHYICRVNHGDNCTFSQWAQIQLAHSEYSSPGCSSTLFPGSKSGLCITRQPQSQTVTEGDTLILECLA
EANPPAQFHWYHNMRAMPQQ
KSCVLRLCAPMADVHELANLLRQLDFKVVSLLDLNWQEMHSAVTEFLLLLDKGVYGLLYFAGHGYENYGNSFMVPIDAPA
SYTSDHCLCVQSVLQRMQER
ETGLNVFLLDMCRKRCIDAEAYEVKTDDPHNGVFISFLKPRLCEDEKVTVMLDRVAEGMYLDISMKKYMGRCELMRGRQA
LELRSNLSERRALTDRIRST
ESSPDHSARNLQWAIAHVLPKSRKLHFECGVEVQIGFAAEFSNIMIIYTRILEKPKEIVSCFAELIDFPEKGVDLDLKKS
NQESIIEAGSLLPIMESLPS

QQSPSLYTRLKSLQRLKTDLTFAVCVHYRYSNMDEEVQEMQSVTVGKPLVSKLNLHEARLSRSSSADLHFSTMADSSSEI
DIPEGCEVRKLSELRVIDLK
AELKKRNLDTTGIKSVLSERLKKAIEDEGGNPEEIFVAAESTMKKTPKRAVKADARLDSEENMIGEDSHDGQDDHENMQD
MCIFDMSVVDEAENDNGIPA
EVEDDKMQDFTEDLCDEDELLRDSEEQYHEKMGSEEESAREEKSESELLDIDMPCTSPDVVSHTKTQPKRDDSILRLNAM
LDMKAIGLELKPVNAKWTSS
NTELFSPLDQVLLFMHVESKNDKVAGSCLSEPLNEDPQESREATAEDEQAGSPTGGQDEARRDTARTAEAVPGQVARDTE
VKEVTDVDEGGDGQTVDAQG
ESTQAVSVNEQGAAGETVESQMGSKEGEEEETKTEENASADASSAAKESSTVEGDDQKKSSEEKDANAESKDEKAAAGGT
VAAGAGRNLWVSGLSSTTRA
TDLKTLFSKYGKVVGAKVVTNAKSPGARCYGFVTMSSGDEATTCISHLHRTELHGKMISVERVGLPAARGRPARGRMVRS
VGGSSSSGSVLLNLSIWLQA
KNEPAGKKPADTKTPVAERRNSTDSR
>g631.t1
MAGEAQHSHAVKELDIESKLQDAGTSLEKLSDKTKKHSLAHFPSPGADAGPDSTGAETDPLEKERDCTQQREAVIRPQKA
GKIDFRSLQNRPKCSSDRTW
PSGKSSPQSPSGKGRSRDKARRSGRSERGNPQQLYRLSITNPRANPTIGIAYPQQKVPPPKKLEAGRRPLSGSYRFHVPS
VPEREAELQQEELSYSRCFQ
EASSNLTPTSYTSQTLTSAGAAAAHQHSAMAQQPSAAMENISPQPSSQLLLTDFQLSCSNAWHSPERIFNGANYGISSQK
STTLSEVNKAGVFASAPFQY
GYQFLEDSTTSPFSCEQNSQSQDFTDSAHVSAHVTHTSFSFTMGGEPTSSNTQFCKEPQQEERTCYPTPLSSQYTPAPPG
APSSLLCPRSQSEDSTSSDS
SGSSTQPSEQGKNALQKSTEDFEKANIGDLALTSGSKRNGHPKDAVHNPTSSSTCSSLSPASTSPVNHSSEDSQMTKTGG
PPPFYHQHQTKTPEKSRERE
IKETDGSKLFTSEDFRAPRLVLSSKFAERCGLKGFQDSPVSRASTHSQPSSATQTSAAIAKAIKENKRKRSSGGTWSKEL
IHKIVQQKNKLHKLHVKGTK
NLQFSLVMDKVTPSVQNPAFGEYDYVSDADDECEPVKIASQGRLSQSSRCKYTYTKESKWRARGEREQAAWRHESKECFE
VKKSEVVSLTSEKYAPHQKA
RRRDSRCSTSSDLSTSVSASSDGIGSPKSTDRTDSDCERKAEMQTKEYPEQRTAARPSPHKMYDETRTSVALSFTKSAKK
VNTHQSGHSSIKDSPKTHKS
CHSSPDIPDPVSLSPKANDALKNHDKCKNLLRISKSESHSRETGTFSYDLNTSQRTDEALHKEDSTAQTSHAHIEQLQLD
SPHSVTVNEEDFNQEKERSS
EGTVPCKRSDSNAATQDKLSDSDFFDKHSEVVCRGEEAATLAKDFAPKPPSLCSSFMDDVCLSPPESHDPLLQKDTSHLM
PYPLDQDHGLMKSPLSFDTS
SVFGDLTVAGFDNSLYSDIPLQKEGFSSIENTLDKKEAFDSSFSPFLEQREWSLMPYVSPVLPEEIAHYKGDTVKSSENK
SDFNHVPLSLPEKIMDYSAN
LNSCASEDELEIKRIVSELENQLQTTKIDSPPLLPENTQKHLQMSKFSPLRLSDDSDNDGTSLEVHCPVQSLSVPVNNMS
SEHFTDQVLPWSSPFQFDLI
EEDHDAQAATHNEPGPLEHFSERGENNNTGAKADDASLQKDIALESDTLEAAYCSADMAVEGSHVIPGICENSNALDNLN
KLFKSNMDNDKHSTTDSQEE
EEEAVKGIAFDSDAQSPSADNPADAAVELSEIKENEKETGLTQRASEVHADATHLYSPFADEQGVESSENLQEESDIQTT
ITVQDYGRPEIYSAGLAVDM
SMEIVTTEKPYSPILMETNAEXQFQG
>g631.t2
MAGEAQHSHAVKELDIESKLQDAGTSLEKLSDKTKKHSLAHFPSPGADAGPDSTGAETDPLEKERDCTQQREAVIRPQKA
GKIDFRSLQNRPKCSSDRTW
PSGKSSPQSPSGKGRSRDKARRSGRSERGNPQQLYRLSITNPRANPTIGIAYPQQKVPPPKKLEAGRRPLSGSYRFHVPS
VPEREAELQQEELSYSRCFQ
EASSNLTPTSYTSQTLTSAGAAAAHQHSAMAQQPSAAMENISPQPSSQLLLTDFQLSCSNAWHSPERIFNGANYGISSQK
STTLSEVNKAGVFASAPFQY
GYQFLEDSTTSPFSCEQNSQSQDFTDSAHVSAHVTHTSFSFTMGGEPTSSNTQFCKEPQQEERTCYPTPLSSQYTPAPPG
APSSLLCPRSQSEDSTSSDS
SGSSTQPSEQGKNALQKSTEDFEKANIGDLALTSGSKRNGHPKDAVHNPTSSSTCSSLSPASTSPVNHSSEDSQMTKTGG
PPPFYHQHQTKTPEKSRERE
IKETDGSKLFTSEDFRAPRLVLSSKFAERCGLKGFQDSPVSRASTHSQPSSATQTSAAIAKAIKENKRKRSSGGTWSKEL
IHKIVQQKNKLHKLHVKGTK

NLQFSLVMDKVTPSVQNPAFGEYDYVSDADDECEPVKIASQGRLSQSSRCKYTYTKESKWRARGEREQAAWRHESKECFE
VKKSEVVSLTSEKYAPHQKA
RRRDSRCSTSSDLSTSVSASSDGIGSPKSTDRTDSDCERKAEMQTKEYPEQRTAARPSPHKMYDETRTSVALSFTKSAKK
DHGLMKSPLSFDTSSVFGDL
TVAGFDNSLYSDIPLQKEGFKKIMDYSANLNSCASEDELEIKRIVSELENQLQTTKIDSPPLLPENTQKHLQMSKFSPLR
LSDDSDNDGTSLEVHCPVQS
LSVPVNNMSSEHFTDQVLPWSSPFQFDLIEEDHDAQAATHNEPGPLEHFSERGENNNTGAKADDASLQKDIALESDTLEA
AYCSADMAVEGSHVIPGICE
NSNALDNLNKLFKSNMDNDKHSTTDSQEEEEEAVKGIAFDSDAQSPSADNPADAAVELSEIKENEKETGLTQRASEVHAD
ATHLYSPFADEQGVESSENL
QEESDIQTTITVQDYGRPEIYSAGLAVDMSMEIVTTEKPYSPILMETNAEXQFQG
>g631.t3
MAGEAQHSHAVKELDIESKLQDAGTSLEKLSDKTKKHSLAHFPSPGADAGPDSTGAETDPLEKERDCTQQREAVIRPQKA
GKIDFRSLQNRPKCSSDRTW
PSGKSSPQSPSGKGRSRDKARRSGRSERGNPQQLYRLSITNPRANPTIGIAYPQQKVPPPKKLEAGRRPLSGSYRFHVPS
VPEREAELQQEELSYSRCFQ
EASSNLTPTSYTSQTLTSAGAAAAHQHSAMAQQPSAAMENISPQPSSQLLLTDFQLSCSNAWHSPERIFNGANYGISSQK
STTLSEVNKAGVFASAPFQY
GYQFLEDSTTSPFSCEQNSQSQDFTDSAHVSAHVTHTSFSFTMGGEPTSSNTQFCKEPQQEERTCYPTPLSSQYTPAPPG
APSSLLCPRSQSEDSTSSDS
SGSSTQPSEQGKNALQKSTEDFEKANIGDLALTSGSKRNGHPKDAVHNPTSSSTCSSLSPASTSPVNHSSEDSQMTKTGG
PPPFYHQHQTKTPSDPLGAN
SQHFHAEVPRNMPYGSDRGKDDMLSYLQNGVNLKNTMDGNKGYIDSFGLDHHQPPPPYSAHQLATSLATANLDQEKSRER
EIKETDGSKLFTSEDFRAPR
LVLSSKFAERCGLKGFQDSPVSRASTHSQPSSATQTSAAIAKAIKENKRKRSSGGTWSKELIHKIVQQKNKLHKLHVKGT
KNLQFSLVMDKVTPSVQNPA
FGEYDYVSDADDECEPVKIASQGRLSQSSRCKYTYTKESKWRARGEREQAAWRHESKECFEVKKSEVVSLTSEKYAPHQK
ARRRDSRCSTSSDLSTSVSA
SSDGIGSPKSTDRTDSDCERKAEMQTKEYPEQRTAARPSPHKMYDETRTSVALSFTKSAKKVNTHQSGHSSIKDSPKTHK
SCHSSPDIPDPVSLSPKAND
ALKNHDKCKNLLRISKSESHSRETGTFSYDLNTSQRTDEALHKEDSTAQTSHAHIEQLQLDSPHSVTVNEEDFNQEKERS
SEGTVPCKRSDSNAATQDKL
SDSDFFDKHSEVVCRGEEAATLAKDFAPKPPSLCSSFMDDVCLSPPESHDPLLQKDTSHLMPYPLDQDHGLMKSPLSFDT
SSVFGDLTVAGFDNSLYSDI
PLQKEGFSSIENTLDKKEAFDSSFSPFLEQREWSLMPYVSPVLPEEIAHYKGDTVKSSENKSDFNHVPLSLPEKIMDYSA
NLNSCASEDELEIKRIVSEL
ENQLQTTKIDSPPLLPENTQKHLQMSKFSPLRLSDDSDNDGTSLEVHCPVQSLSVPVNNMSSEHFTDQVLPWSSPFQFDL
IEEDHDAQAATHNEPGPLEH
FSERGENNNTGAKADDASLQKDIALESDTLEAAYCSADMAVEGSHVIPGICENSNALDNLNKLFKSNMDNDKHSTTDSQE
EEEEAVKGIAFDSDAQSPSA
DNPADAAVELSEIKENEKETGLTQRASEVHADATHLYSPFADEQGVESSENLQEESDIQTTITVQDYGRPEIYSAGLAVD
MSMEIVTTEKPYSPILMETN
AEXQFQG
>g632.t1
MVSVKMESEKATPPAGEDPLSAEGFLDDETTFSQLFPRDEDAKRKKPPRVYSKKNKRQKLSPDSYTLQDCPPSQDQHADT
QTDKTLDNSQSNHCEYETIS
IDDAIMLNMCHNSSLKGDAKTVSEINQCVCDHENTPDNQEKPGSNHLNPIESTIDKSSMEWSGLSEQNSLPAEPKVCSDQ
ISSEEDGPTPQSSVKPAPVP
HALEPSVTDNVPNFHSIDIQNIKTTFQLPEIQFFESSKDITVGPPIAAADIENKDVEKSKKPTERRGRKRQDGSIKVKDK
QYKCKVCFTWFLTLGELNFH
KLSHNPSPPPTCYMCVQRKFSSREQLRDHLREKHAKNKAGIWTCGMCLKEISDVWMYNEHLREHATQFARKGQTQGSMLG
IPGTFMQETAVKNFITSIMQ
HRPSKANKEPTKASKAQEKPVGQEMAVGEVKTAAAESVEPKVQKAKNNSGTGEKPLEVLHKTDTSKSVEMHPNCKDPSRD
CHHCGKQFPKPFKLQRHLVV
HNLEKIFLCHKCPVSYQEAQELKDHLKREHEEMEDLDSKHTTLYTCELCADVMHVIKKSFICSTCNYTFSKKEQFDRHME
KHLLGGNKIFKFKGVLRPVK

VSTSKDDLTVDIPASKKRKVVNDCVQENSSDSGIASVGSLHLNPSSETSSSKSNVSVADDSTQTSANEYRNDPQGTSVKT
EEDAELYSDLFGELDQCSFH
NESSESESPKKEEPCPSTLPMLPAKEGPERPASELCDVKEEDEVVGIPAETTPRWSKENSDVGQERVKENPDSAEGGTDG
AREKSESRCSVQPSEDLITV
ANTQTSEKSIDQQRDDSQGKESNKPNSSSDGKQDTLSTNAEPPAGNPTKDKAPLTKTNEGCRTSTRPSDTQMIKGSSNGS
DLIEDRESLRHQKKRKEMKS
PYCSQRICSPATRENLDVDAKAKKKFRLNKSESPSSQRKPDGPSDYSVLSSVRDDVVSNKIISKSKMSNLGLQAKRGLLD
NCLPKKAEIVAHVNGDCKAK
KGPLGRCLQSPVSKGSTAPKNNSLNKPRPKMGIRSMESHSYRTAESQNHLLSQLFGQKLTSFKIPLRKDTSESMN
>g633.t1
MSCLGLPAIQRPQVDRAAQAISGAPYDFEFDLRLTNEQTRLRWAGTTVFQTILDLSLDFSLDLSLDPNQDLSLDLSLDPS
HTV
>g634.t1
LAEQAERYDDMASAMKSVTEGGAELSNEERNLLSVAYKNVVGARRSAWRVISSIEQKTDGADKKQKMITEYREKVESELK
NICNDVLGLLDKFLIGNSKN
AESKVFYLKMKGDYYRYLAEVDRGEQSKPGEVIRYMNTKELKVVDSLQFSSIDVYWMVVSSPNLMMAFELEVAAQSCMYR
EYRRGLRGASARSGLSWGLL
AGTSSEDTAWDAIWSWSLAGLTYLKDLLTFATDSISGQSSLSSDAVANSCNAYENAFQISREEMDSTHPIRLGLALNFSV
FYYEILNSPEKACELAKSAF
DDAIAELDKLNEESYKDSTLIMQLLRDNLTLWTSDNAAEEGDAGEAGEAGENEN
>g634.t2
LAEQAERYDDMASAMKSVTEGGAELSNEERNLLSVAYKNVVGARRSAWRVISSIEQKTDGADKKQKMITEYREKVESELK
NICNDVLGLLDKFLIGNSKN
AESKVFYLKMKGDYYRYLAEVDRGEQSKPDDSRVNSECAGWLGSLMFLCLPQATGEVIRYMNTKELKVVDSLQFSSIDVY
WMVVSSPNLMMAFELEVAAQ
SCMYREYRRGLRGASARSGLSWGLLAGTSSEDTAWDAIWSWSLAGLTYLKDLLTFATDSISGQSSLSSDAVANSCNAYEN
AFQISREEMDSTHPIRLGLA
LNFSVFYYEILNSPEKACELAKSAFDDAIAELDKLNEESYKDSTLIMQLLRDNLTLWTSDNAAEEGDAGEAGEAGENEN
>g634.t3
LAEQAERYDDMASAMKSVTEGGAELSNEERNLLSVAYKNVVGARRSAWRVISSIEQKTDGADKKQKMITEYREKVESELK
NICNDVLGLLDKFLIGNSKN
AESKVFYLKMKGDYYRYLAEVDRGEQSKPVSPGEVIRYMNTKELKVVDSLQFSSIDVYWMVVSSPNLMMAFELEVAAQSC
MYREYRRGLRGASARSGLSW
GLLAGTSSEDTAWDAIWSWSLAGLTYLKDLLTFATDSISGQSSLSSDAVANSCNAYENAFQISREEMDSTHPIRLGLALN
FSVFYYEILNSPEKACELAK
SAFDDAIAELDKLNEESYKDSTLIMQLLRDNLTLWTSDNAAEEGDAGEAGEAGENEN
>g635.t1
CVISSSLYKAPWARVMVYGLGHKVRRNGQLNLMEAVCYPLDASPTNTGLTPPPTTNQYPSVIIPTDKVHIKLGE
>g636.t1
MTHSLLTRHLSHSHFFISCSNVLLTSPKRSSSPSSKNWESQALHSPCSHPPSRITLSRDTVKLGSMVLPHYEETPGATQL
NLDCLLSWRQDDGTRPPEDS
FCSSTQDLGSLGLEGNGPGTNPQGGPGEDCLLADLQGCS
>g636.t2
LSHSHFFISCSNVLLTSPKRSSSPSSKNWESQALHSPCSHPPSRITLSRDTVKLGSMVLPHYEETPGATQLNLDCLLSWR
QDDGTRPPEDSFCSSTQDLG
SLGLEGNGPGTNPQGGPGEDCLLADLQGCS
>g636.t3
LMTHSLLTRHLSHSHFFISCSNVLLTSPKRSSSPSSKNWESQALHSPCSHPPSRITLSRDTVKLGSMVLPHYEETPGATQ
LNLDCLLSWRQDDGTRPPED
SFCSSTQDLGSLGLEGNGPGTNPQGGPGEDCLLADLQGCS
>g637.t1
PPPGRRGSAQSGCREEADTKVNAKFVQDSSKFWYKAGISRDQAIAVLKDKEPGAFLIRDSNSFQGAYGLALKVTTPPLNA
NLNASKGDPQELLVRHFLIE
KGPRGVKIKGCQNESYFGSLSALVYQHSITPISLPCALLLPDKDLVGELQEIQSVTNTSTAANLLKQGAACNVLYLNSVE
TESLTGPQAISKATKCTLAL
TPRPSAAVVHFKVSMQGITLTDSKRRWTNSNSTSSKIFGFVARRTGSTTENVCHLFAEIDPEQPAVAIVNFINKVMLGPQ

LRR
>g637.t2
PPPGRRGSAQSGCREEADTKVNAKFVQDSSKFWYKAGISRDQAIAVLKDKEPGAFLIRDSNSFQGAYGLALKVTTPPLNA
NLNASKGDPQELLVRHFLIE
KGPRGVKIKGCQNESYFGSLSALVYQHSITPISLPCALLLPDKDLVGELQEIQSVTNTSTAANLLKQGAACNVLYLNSVE
TESLTGPQAISKATKCTLAL
TPRPSAAVVHFKVSMQGITLTDSKRRIFGFVARRTGSTTENVCHLFAEIDPEQPAVAIVNFINKVMLGPQLRR
>g637.t3
PPPGRRGSAQSGCREEADTKVNAKFVQDSSKFWYKAGISRDQAIAVLKDKEPGAFLIRDSNSFQGAYGLALKVTTPPLNA
NLNASKGDPQELLVRHFLIE
KGPRGVKIKGCQNESYFGSLSALVYQHSITPISLPCALLLPDKDLVGELQEIQSVTNTSTAANLLKQGAAVVHFKVSMQG
ITLTDSKRRWTNSNSTSSKI
FGFVARRTGSTTENVCHLFAEIDPEQPAVAIVNFINKVMLGPQLRR
>g638.t1
MAQRVPVADSGARKTSRVRVAVRLRPYMEKEDDKSEGPCVRGLDSQNLEIINWRNATETVKYHFDAFHDEQTTQQGVFLS
SVKPILPHILSGQNASVFAY
GPTGAGKTHTMLGSPEQPGVIPRAVREVFNLVRTRGEGQDGWDCSVTMSYLEIYNEKVLDLLSPNAQDLPIREDKDRNIL
IPGLTQVPLTSFSDFDHHFI
PASLNRTTASTKLNQRSSRSHAILLIKVVRTQRSRPHRQQTGKLYLVDLAGSEDNRRTGNQGIRLKESGAINLSLFTLSK
VVDSLNTGTGGRVPYRDSKL
TRLLQDSLGGSAHSVMITNIAPEYSYYFDTFSALNFAAKSKLIVNKPFVRETLTVPVPQVKRAREEQEVERQRKRPKDDR
KPNSTSPSAPPHSSTSEPSV
LERLIALEKLMMGCQEKDRQTMQQSRKEIEPGSLLKTAIMGKTADLTAVQKAIIDTLKQEECRIKAPQWEVWEGKSGRKR
CTTRRGDRTLRKIVEKGRFQ
TLGDLRKQWTESGVETQSHRAQACAGNGLQVPHSP
>g638.t2
MAQRVPVADSGARKTSRVRVAVRLRPYMEKEDDKSEGPCVRGLDSQNLEIINWRNATETVKYHFDAFHDEQTTQQGVFLS
SVKPILPHILSGQNASVFAY
GPTGAGKTHTMLGSPEQPGVIPRAVREVFNLVRTRGEGQDGWDCSVTMSYLEIYNEKVLDLLSPNAQDLPIREDKDRNIL
IPGLTQVPLTSFSDFDHHFI
PASLNRTTASTKLNQRSSRSHAILLIKVVRTQRSRPHRQQTGKLYLVDLAGSEDNRRTGNQGIRLKESGAINLSLFTLSK
VVDSLNTGTGGRVPYRDSKL
TRLLQDSLGGSAHSVMITNIAPEYSYYFDTFSALNFAAKSKLIVNKPFVRETLTVPVPQVKRAREEQEVERQRKRPKDDR
KPNSTSPSAPPHSSTSEPSV
LERLIALEKLMMGCQEKDRQTMQQSRKEIEPGSLLKTAIMGKTADLTAVQKAIIDTLKQEECRIKAPQWEVWEGKSGRKR
CTTRRGDRTLRKIVEKGRFQ
TLGDLRKQWTESGVETQSHRAQACAGNGLQ
>g638.t3
MAQRVPVADSGARKTSRVRVAVRLRPYMEKEDDKSEGPCVRGLDSQNLEIINWRNATETVKYHFDAFHDEQTTQQGVFLS
SVKPILPHILSGQNASVFAY
GPTGAGKTHTMLGSPEQPGVIPRAVREVFNLVRTRGEGQDGWDCSVTMSYLEIYNEKVLDLLSPNAQDLPIREDKDRNIL
IPGLTQVPLTSFSDFDHHFI
PASLNRTTASTKLNQRSSRSHAILLIKVVRTQRSRPHRQQTGKLYLVDLAGSEDNRRTGNQGIRLKESGAINLSLFTLSK
VVDSLNTGTGGRVPYRDSKL
TRLLQDSLGGSAHSVMITNIAPEYSYYFDTFSALNFAAKSKLIVNKPFVRETLTVPVPQVKRAREEQEVERQRKRPKDDR
KPNSTSPSAPPHSSTSEPSV
LERLIALEKLMMGCQEKDRQTMQQSRKEIEPGSLLKTAIMGKTADLTAVQKAIIDTLKQEGDRTLRKIVEKGRFQTLGDL
RKQWTESGVETQSHRAQACA
GNGLQVPHSP
>g639.t1
MQAEAMEPSLREGIEALAVGNQTAGEKDEKSGDLGMNDVKMGDATGEETTSPDEKTNNGGEEGGGEGQETQRGDSLLESD
GGEKTGEVEEEEEWEVPYSD
EELEQPGTWMPPPVEIKRLYEILAKGEALELSFEPLPRRPPTPERTPSPERDGDDDDAERDRADRESKAPSPTEFDFDEE
QTQATPKHSFINRRRTPGSS
ARSSMKPKARLDKVLSDMKRHRRMEEQILRTGRDLFKSDKRVEEPLSPNSQKEREKERERDSNPDTIFSPRQRRY
>g640.t1

MSPIADVTIGAFMADSVVLL
>g641.t1
MDAMDSPATRRRKAKGMFGLKVKKKKKVNTGLVLVNKGAE
>g642.t1
MGYVSPNNPYPTHITQAVLGMWPTRTPPQAVPKSFHRPGKWRGVRIGETVWTQPHPPPPCRHLQEGPSSTDVEVGTGVTG
PNTIAPLPAAPGSISRGHST
PALLPQAEEGGGAACHTENLKTEFLRTEACQKAFIQLRRALSNALVLAPADPSLPFMLDTDASGVGVGGVLSQVGADGET
VVAYFSWVFNKAERRYCVTR
LGGVTAEATVARGGKTLTGHQGAEAYALPDQEAETVAGALLGGIFRQFGLPDTLHSDQPGELVRVHNPQRRKGRFPKLAS
QWVGPCRVLERMGEVVYRVQ
LPPRGRKVVLHRDRLAPYQGSASPQVPGTLRSPHSPLIYHPHHTPINPHSKCKMGVTLFP
>g642.t2
MGYVSPNNPYPTHITQAVLGMWPTRTPPQAVPKSFHRPGKWRGVRIGETVWTQPHPPPPCRHLQEGPSSTDVEVGTGVTG
PNTIAPLPAAPGSISRGHST
PALLPQAEEGGGAACHTENLCCRKTEFLRTEACQKAFIQLRRALSNALVLAPADPSLPFMLDTDASGVGVGGVLSQVGAD
GETVVAYFSWVFNKAERRYC
VTRLGGVTAEATVARGGKTLTGHQGAEAYALPDQEAETVAGALLGGIFRQFGLPDTLHSDQPGELVRVHNPQRRKGRFPK
LASQWVGPCRVLERMGEVVY
RVQLPPRGRKVVLHRDRLAPYQGSASPQVPGTLRSPHSPLIYHPHHTPINPHSKCKMGVTLFP
>g642.t3
MGYVSPNNPYPTHITQAVLGMWPTRTPPQAVPKSFHRPGKWRGVRIGETVWTQPHPPPPCRHLQEGPSSTDVEVGTGVTG
PNTIAPLPAAPGSISRGHST
PALLPQAEEGGGAACHTENLCPSSHLPNQRLPFALDVGSGSSRKTEFLRTEACQKAFIQLRRALSNALVLAPADPSLPFM
LDTDASGVGVGGVLSQVGAD
GETVVAYFSWVFNKAERRYCVTRLGGVTAEATVARGGKTLTGHQGAEAYALPDQEAETVAGALLGGIFRQFGLPDTLHSD
QPGELVRVHNPQRRKGRFPK
LASQWVGPCRVLERMGEVVYRVQLPPRGRKVVLHRDRLAPYQGSASPQVPGTLRSPHSPLIYHPHHTPINPHSKCKMGVT
LFP
>g643.t1
DKVASESLPLQGLTVKLPTRSEEGGGSSMFQLYHKKTLFYSFRAEDQHSAHRWVNAIEEATIL
>g644.t1
ALYRTVVVKRELSRKAKLSIYQSIYVPTLTYGHELWVVTERTRSRIQAAEMSFLRRVAGLSLRDRERSSDIRRELGVEPL
LLRIERSQLRWFGHLIRMPP
GRLPLEVFRARPTGKRPRGRPRTRWRDYISHLAWERLGVPQEELESVAGEKDVWSSLLSLLPPRPGPG
>g645.t1
EPAKGEGPQGVAKEPQKEGEPEDKELRKQMCPYAAVGECRYGVNCAYLHGDVCDMCGLQVLHPTDDSQRSDHTKVTAAMP
RGGVLMRLTLRD
>g646.t1
MPFASLSHNQQTMGNCPLKSRTKQSSPVTPVAPGLRGCVADLERSLVVSLHDFPCHGVAEFSLRVGERLSVVSDDGDFLM
VKSARTGHEIYVPTECTASV
LNRWFFKGLSRYKAEELLMQPNNCTGAFIVRESQSHQDHYSLSILHRPDSYLGSVKHYHISRLDNGWFYIARGLTFSSLK
HLVEHYSEFSDGLCCALSEP
CFILGSDNTTTRMTGPGPLGVRRPTIIWKDVTRSMLFRGNRTESEHSLVSDGLREAISSYLYMTNGNEQDIR
>g646.t2
MGNCPLKSRTKQSSPVTPVAPGLRGCVADLERSLVVSLHDFPCHGVAEFSLRVGERLSVVSDDGDFLMVKSARTGHEIYV
PTECTASVLNRWFFKGLSRY
KAEELLMQPNNCTGAFIVRESQSHQDHYSLSILHRPDSYLGSVKHYHISRLDNGWFYIARGLTFSSLKHLVEHYSEFSDG
LCCALSEPCFILGSDNTTTR
MTGPGPLGVRRPTIIWKDVTRSMLFRGNRTESEHSLVSDGLREAISSYLYMTNGNEQDIR
>g646.t3
MPFASLSHNQQTMGNCPLKSRTKQSSPVTPVAPGLRGCVADLERSLVVSLHDFPCHGVAEFSLRVGERLSVVSEWFFKGL
SRYKAEELLMQPNNCTGAFI
VRESQSHQDHYSLSILHRPDSYLGSVKHYHISRLDNGWFYIARGLTFSSLKHLVEHYSEFSDGLCCALSEPCFILGSDNT
TTRMTGPGPLGVRRPTIIWK
DVTRSMLFRGNRTESEHSLVSDGLREAISSYLYMTNGNEQDIR
>g647.t1

MLQKLHDSSHPNSDLSHAHSLLKVLLSQLSMEAMSFVTEDRKTAQESTFPNTYTFELFGGVDTEGVTRSLAARDDFVLFL
FTLMNNKKTFLQTATLIEDI
LGAKKEMIQLEGIPNLSGLVQSFDQQQLANFCRILSVTISEPDIGNDDKHTLLAKNAQQKKNSNPSRAEVNQVALMNIPG
FIERLCKLATRKVSEATGAS
SFLQELEDWYTWLDNALVLDALMQMASEETEQSSTESSDESSLTTSPLRHHLPQSMKIVHEIMYKVEVLYVLCMLLMGRQ
RNQVHKMLAEFRLIPGLNNL
FDKLIWRKYTASNHVVHGQNENCDCSPEISFKIQFLRLLQSFSDHHENKYLLLNSQELNELSAISMKANIPEVEALVNTD
RSLVCDGKKGLLTRLLTVMK
REPPDSSFRFWQARAVESFLRGATSYADQMFLLKRGLLEHILFCIIDSGCTSRDVLQSYFDLLGELMKFNIDAFNRFNKY
VNTEEKFQTFLTQINSSLVD
SNMLVRCIVLSLDRFENQTEDVKVVEVLSECCLLSYMARVENRLTFLFRLVNIINVQTLTQENVSCLNTSLVILMLARRR
SKLPFYLDALRQKEYAEKYP
GCLLNNFHNLLRFWQRHYLNKDKDSTCLENVSEGLLCAGPASLLWQEHVVSSCIPFSYWKETVSVLLGSDRTSSCAIASY
IDEPYMDLDTDPLEE
>g647.t2
MLQKLHDSSHPNSDLSHAHSLLKVLLSQLSMEAMSFVTEDRKTAQESTFPNTYTFELFGGVDVMNDDLVKDCLSVLYNCC
MCTEGVTRSLAARDDFVLFL
FTLMNNKKTFLQTATLIEDILGAKKEMIQLEGIPNLSGLVQSFDQQQLANFCRILSVTISEPDIGNDDKHTLLAKNAQQK
KNSNPSRAEVNQVALMNIPG
FIERLCKLATRKVSEATGASSFLQELEDWYTWLDNALVLDALMQMASEETEQSSTESSDESSLTTSPLRHHLPQSMKIVH
EIMYKVEVLYVLCMLLMGRQ
RNQVHKMLAEFRLIPGLNNLFDKLIWRKYTASNHVVHGQNENCDCSPEISFKIQFLRLLQSFSDHHENKYLLLNSQELNE
LSAISMKANIPEVEALVNTD
RSLVCDGKKGLLTRLLTVMKREPPDSSFRFWQARAVESFLRGATSYADQMFLLKRGLLEHILFCIIDSGCTSRDVLQSYF
DLLGELMKFNIDAFNRFNKY
VNTEEKFQTFLTQINSSLVDSNMLVRCIVLSLDRFENQTEDVKVVEVLSECCLLSYMARVENRLTFLFRLVNIINVQTLT
QENVSCLNTSLVILMLARRR
SKLPFYLDALRQKEYAEKYPGCLLNNFHNLLRFWQRHYLNKDKDSTCLENVSEGLLCAGPASLLWQEHVVSSCIPFSYWK
ETVSVLLGSDRTSSCAIASY
IDEPYMDLDTDPLEE
>g647.t3
LPGILLEERDRRVKWHGIPTMLQKLHDSSHPNSDLSHAHSLLKVLLSQLSMEAMSFVTEDRKTAQESTFPNTYTFELFGG
VDVMNDDLVKDCLSVLYNCC
MCTEGVTRSLAARDDFVLFLFTLMNNKKTFLQTATLIEDILGAKKEMIQLEGIPNLSGLVQSFDQQQLANFCRILSVTIS
EPDIGNDDKHTLLAKNAQQK
KNSNPSRAEVNQVALMNIPGFIERLCKLATRKVSEATGASSFLQELEDWYTWLDNALVLDALMQMASEETEQSSTESSDE
SSLTTSPLRHHLPQSMKIVH
EIMYKVEVLYVLCMLLMGRQRNQVHKMLAEFRLIPGLNNLFDKLIWRKYTASNHVVHGQNENCDCSPEISFKIQFLRLLQ
SFSDHHENKYLLLNSQELNE
LSAISMKANIPEVEALVNTDRSLVCDGKKGLLTRLLTVMKREPPDSSFRFWQARAVESFLRGATSYADQMFLLKRGLLEH
ILFCIIDSGCTSRDVLQSYF
DLLGELMKFNIDAFNRFNKYVNTEEKFQTFLTQINSSLVDSNMLVRCIVLSLDRFENQTEDVKVVEVLSECCLLSYMARV
ENRLTFLFRLVNIINVQTLT
QENVSCLNTSLVILMLARRRSKLPFYLDALRQKEYAEKYPGCLLNNFHNLLRFWQRHYLNKDKDSTCLENVSEGLLCAGP
ASLLWQEHVVSSCIPFSYWK
ETVSVLLGSDRTSSCAIASYIDEPYMDLDTDPLEE
>g648.t1
LKTSSLANHREPRSAAKAGLRVHFRLPEDEEEEEQEEQCEPSGWPYEDAGLAANRGPPPVLTKPKLDPAQLQLLHKQVLL
EQQQDGDSHPHSKARPQSPP
WTQALPQSQSPYQPPPPAQSLTQSQPPLPVQSLTQSQPPLPVQSLTQSQPAPYSQSLTQSLPQSQPQYQPPPPAQSLSQS
QPAQSQPQISASDSVVWSPR
LHREPTQQHFQLYQNIAVTTQSHTPVSSLTMAPAMPTLTSSLAPVLRTTPSAPAPATQTNTIHASHLNLSLAALSKAMPP
GQFSSPAAPQFHATPVAAAA
TSQGTPPSLLEVTSPAPMLRSTHASLMNLAATSQSFSYARPREFMAAQTLSPVRSPSPTESPVPLLNELAAELNSSAASS
PTIPPFSPPPRVFPTRVLQS
PTSPLSMLSSPTLISAPFLNSVFAGRAQSPPQAPSPASSSSTPSPIQNPVAFLSSVLPSLHLAQATNSMGLPKGAPAGQL

KRLPKSRTPSSEDIRVSKEV
ILNDIERRLRFNNDSSHFGFQQEYKVSNFEQRLMSEIEFRLERTPVEESDDEVHHDEVPTGRCIAPIFDKKLRNFRAMEG
VPVTFSCKLVGIPIPKMGSD
SAVLTLAGSSFHHCGAKTEKS
>g648.t2
LKTSSLANHREPRSAAKAGLRVHFRLPEDEEEEEQEEQCEPSGWPYEDAGLAANRGPPPVLTKPKLDPAQLQLLHKQVLL
EQQQDGDSHPHSKARPQSPP
WTQALPQSQSPYQPPPPAQSLTQSQPPLPVQSLTQSQPPLPVQSLTQSQPAPYSQSLTQSLPQSQPQYQPPPPAQSLSQS
QPAQSQPQISASDSVVWSPR
LHREPTQQHFQLYQNIAVTTQSHTPVSSLTMAPAMPTLTSSLAPVLRTTPSAQLTAIPAPATGRPSAPAPATQTNTIHAS
HLNLSLAALSKAMPPGQFSS
PAAPQFHATPVAAAATSQGTPPSLLEVTSPAPMLRSTHASLMNLAATSQSFSYARPREFMAAQTLSPVRSPSPTESPVPL
LNELAAELNSSAASSPTIPP
FSPPPRVFPTRVLQSPTSPLSMLSSPTLISAPFLNSVFAGRAQSPPQAPSPASSSSTPSPIQNPVAFLSSVLPSLHLAQA
TNSMGLPKGAPAGQLKRLPK
SRTPSSEDIRVSKEVILNDIERRLRFNNDSSHFGFQQEYKVSNFEQRLMSEIEFRLERTPVEESDDEVHHDEVPTGRCIA
PIFDKKLRNFRAMEGVPVTF
SCKLVGIPIPKMGSDSAVLTLAGSSFHHCGAKTEKS
>g648.t3
LANHREPRSAAKAGLRVHFRLPEDEEEEEQEEQCEPSGWPYEDAGLAANRGPPPVLTKPKLDPAQLQLLHKQVLLEQQQD
GDSHPHSKARPQSPPWTQAL
PQSQSPYQPPPPAQSLTQSQPPLPVQSLTQSQPPLPVQSLTQSQPAPYSQSLTQSLPQSQPQYQPPPPAQSLSQSQPAQS
QPQISASDSVVWSPRLHREP
TQQHFQLYQNIAVTTQSHTPVSSLTMAPAMPTLTSSLAPVLRTTPSAPAPATQTNTIHASHLNLSLAALSKAMPPGQFSS
PAAPQFHATPVAAAATSQGT
PPSLLEVTSPAPMLRSTHASLMNLAATSQSFSYARPREFMAAQTLSPVRSPSPTESPVPLLNELAAELNSSAASSPTIPP
FSPPPRVFPTRVLQSPTSPL
SMLSSPTLISAPFLNSVFAGRAQSPPQAPSPASSSSTPSPIQNPVAFLSSVLPSLHLAQATNSMGLPKGAPAGQLKRLPK
SRTPSSEDIRVSKEVILNDI
ERRLRFNNDSSHFGFQQEYKVSNFEQRLMSEIEFRLERTPVEESDDEVHHDEVPTGRCIAPIFDKKLRNFRAMEGVPVTF
SCKLVGIPIPKMGSDSAVLT
LAGSSFHHCGAKTEKS
>g649.t1
VAFHIQPYRGRNDQSVHDNVKYIIDRYGDHGAFYKFPSTTGKNLPMFYIYDSYLTPPDAWADLLTPAGSHSLRGSAYDAV
FIALIVEERHKHDIVAAGFD
GMYTYFASNGFSFGSSHQNWKSVKAFCDGNSLLFVPSVGPGYIDTAVRPWNNHNTRNRVNGRYYETALQAALNARPDIVS
ITSFNEWHEGTQIERAAPKK
TVTRLYLDYLPHGPDHYLELTRRWAEQFNKEKEQWLI
>g650.t1
MFYGVMSAFAPVYGWILVLRALIFWALGTVFEVLLAILVMPTLGWRWLLALSTIPLFIFASLCFWLPESARYDVLTGNQE
KALATLKRIAAENGVPMPLG
KLVAARQEGRGKFQDLFSPHFRCTTVLLWFIWFSNAFSYYGLVLLTPELFQESANCGVSQDRRAEPRCSLECKYLNSDDY
KDLLWTTLSEFPGLLVTLWA
IDRLGRRKTMALCFFIYSLCLIPLYACVGRTSMTVLIFIARAFIAGGFQAAYVVYTPEVYPTATRALGLGTSSGMARVGAL
ITPFVAQVMMESSVHLTLSV
YCCCCLLAAVASCALPIETTGRGLQESSQREWGQEMMGRPHSPGGVPHSSSGSQN
>g650.t2
GLKMSVLWTMFYGVMSAFAPVYGWILVLRALIFWALGTVFEVLLAILVMPTLGWRWLLALSTIPLFIFASLCFWLPESAR
YDVLTGNQEKALATLKRIAA
ENGVPMPLGKLVAARQEGRGKFQDLFSPHFRCTTVLLWFIWFSNAFSYYGLVLLTPELFQESANCGVSQDRRAEPRCSLE
CKYLNSDDYKDLLWTTLSEF
PGLLVTLWAIDRLGRRKTMALCFFIYSLCLIPLYACVGRTSMTVLIFIARAFIAGGFQAAYVVYTPEVYPTATRALGLGTS
SGMARVGALITPFVAQVMME
SSVHLTLSVYCCCCLLAAVASCALPIETTGRGLQESSQREWGQEMMGRPHSPGGVPHSSSGSQN
>g650.t3
MFYGVMSAFAPVYGWILVLRALVGFGIGGAPQSATCILLIEIFWALGTVFEVLLAILVMPTLGWRWLLALSTIPLFIFAS

LCFWLPESARYDVLTGNQEK
ALATLKRIAAENGVPMPLGKLVAARQEGRGKFQDLFSPHFRCTTVLLWFIWFSNAFSYYGLVLLTPELFQESANCGVSQD
RRAEPRCSLECKYLNSDDYK
DLLWTTLSEFPGLLVTLWAIDRLGRRKTMALCFFIYSLCLIPLYACVGRTSMTVLIFIARAFIAGGFQAAYVYTPEVYPT
ATRALGLGTSSGMARVGALI
TPFVAQVMMESSVHLTLSVYCCCCLLAAVASCALPIETTGRGLQESSQREWGQEMMGRPHSPGGVPHSSSGSQN
>g651.t1
MRVAVIGAGVIGLSTAQCIYEQYHSTKEDLTIEVYADLFTPLTTSDGAAGFWQPYLYDTGRTGETKWNKETFDYLLSFLS
SPESVKMGLFLQSGYNLCTD
YAPEPAFKDMVLGFRHLTPRELDLFPGYKYVLGGLRRLADAGVDVIINCTGMRSGDLQPDPDLIPGRGQIIKVDAPWLKH
WVLTHKKNSTVYNSPYIIPG
SRLVTVGGVFQFGNWSEANNSVDHKKIWEEACHLEPSLKHARIVEDWAGLRPARTTVRLERERISCGQKDIEVIHNYGHG
GFGLTIHRGCAREATRLLGQ
IMEHNAKAPKARL
>g651.t2
MVLGFRHLTPRELDLFPGYKYVLGGLRRLADAGVDVIINCTGMRSGDLQPDPDLIPGRGQIIKVDAPWLKHWVLTHKKNS
TVYNSPYIIPGSRLVTVGGV
FQFGNWSEANNSVDHKKIWEEACHLEPSLKHARIVEDWAGLRPARTTVRLERERISCGQKDIEVIHNYGHGGFGLTIHRG
CAREATRLLGQIMEHNAKAP
KARL
>g651.t3
MRVAVIGAGVIGLSTAQCIYEQYHSTKEDLTIEVYADLFTPLTTSDGAAGFWQPYLYDTGRTGETKWNKETFDYLLSFLS
SPESVKMGLFLQSGYNLCTD
YAPWWNEVPANVRTAESLPIFRKRLKTHLFREPAFKDMVLGFRHLTPRELDLFPGYKYVLGGLRRLADAGVDVIINCTGM
RSGDLQPDPDLIPGRGQIIK
VDAPWLKHWVLTHKKNSTVYNSPYIIPGSRLVTVGGVFQFGNWSEANNSVDHKKIWEEACHLEPSLKHARIVEDWAGLRP
ARTTVRLERERISCGQKDIE
VIHNYGHGGFGLTIHRGCAREATRLLGQIMEHNAKAPKARL
>g651.t4
MRVAVIGAGVIGLSTAQCIYEQYHSTKEDLTIEVYADLFTPLTTSDGAAGFWQPYLYDTGRTGETKWNKETFDYLLSFLS
SPESVKMGLFLQSGYNLCTD
YAPWWNEVPANVRTAESLPIFRKRLKTHLFRVHLNPA
>g652.t1
GEVLVEQGGHTGHVEDPSSISSISSISSTLFPWLVRLRLRGGVGGPDRYGLTAPLEDHLLHTEKEAGLLQERPHPAEEEE
EDRNEEQEEELI
>g653.t1
QGEVKLGNPLLVLLQSPSQLVSHLLFDRQVPPERYPSWDGLACPQLCDGGWRSGDDRVQDPNMSSLQHSAALLLTA
>g654.t1
LNDVSPLVSVEQRAAASGGPAASSLLSSADTASHNAK
>g655.t1
MVILICQVLLGESNHASLIPEEGKKKVPGLQGRSAAQSHDLLRDFEATLLHMFGLQRRPRPSRLATVPRYLLDLYRLQSG
EAEDGSPQDIAFEYPERLAS
RANTVRGFHHEETLQCDSKLGSVEVWQEVIGSVGSQGSVGRRRHIHPHQEEGGWTDQDESALMAPV
>g655.t2
MIPGNRMLMVILICQVLLGESNHASLIPEEGKKKVPGLQGRSAAQSHDLLRDFEATLLHMFGLQRRPRPSRLATVPRYLL
DLYRLQSGEAEDGSPQDIAF
EYPERLASRANTVRGFHHEETLQCDSKLGSVEVWQEVIGSVGSQGSVGRRRHIHPHQEEGGWTDQDESALMAPV
>g656.t1
ENILERIHLHIVSDTAADGCSSKSCITHQKFAMTLYEQFVCRSCGASSDPHPFTELVHYVSTTALCQQVDVLLGKSERPR
ADMFGELLQAANTSGDMRSC
PGAGSGSPLSRSPASPCVPSQQVDVLLGKSERPRADMFGELLQAANTSGDMRSCP
>g656.t2
ENILERIHLHIVSDTAADGCSSKSCITHQKFAMTLYEQFVCRSCGASSDPHPFTELVHYVSTTALCQQVDVLLGKSERPR
ADMFGELLQAANTSGDMRSC
PGAGSGSPLSRSPASPCVPSQQVDVLLGKSERPRADMFGELLQAANTSGDMRSCPSRRRGVIHAVTQIYFQTLTSPLRGI
GLVITRRSAVGAL

>g656.t3
ENILERIHLHIVSDTAADGCSSKSCITHQKFAMTLYEQFVCRSCGASSDPHPFTELVHYVSTTALCQQVDVLLGKSERPR
ADMFGELLQAANTSGDMRSC
PGAGSGSPLSRSPASPCVPSQQVDVLLGKSERPRADMFGELLQAANTSGDMRSCPSSQRDRACNHTPQRRRRPVTAASC
>g657.t1
VYGKTIDGQQTIIACIEGHQFQPKNFWNGRCRSEWKFSISQSTAQVVGVLKIQVHYYEDGNVQLVSHKDIQEALTVSNEV
QTAKEFVKIIEDAENEYQTA
ISENYQTMSDTTFKALRRQLPVTRTKIDWNKILSYKIGKEMQNA
>g657.t2
VYGKTIDGQQTIIACIEGHQFQPKNFWSVDMNGRCRSEWKFSISQSTAQVVGVLKIQVHYYEDGNVQLVSHKDIQEALTV
SNEVQTAKEFVKIIEDAENE
YQTAISENYQTMSDTTFKALRRQLPVTRTKIDWNKILSYKIGKEMQNA
>g658.t1
NLSVTTAFSMRWLSFKPFCRRAPSRPDYAGDGYHGFTEPRECVNAVVPPSPSSSVLRPRGRVLPPDRLRAAVGETAATRL
MEGQGRTARGQAAAVLSLPE
RWLSSSSGVCP
>g658.t2
NLSVTTAFSMRWLSFKPFCRRAPSRPDYAGDGYHGFTEPRECVNAVVPPSPSSSVLRPRGRVLPPDRLRAAVGETAATRL
MEGQGRTARGQAAAVLSLPE
RWLSSSSGFLGVCGKAAPGLEAHALWCLCNGLVEEMGFTTLAVRPGHCSLVTAAWSLQPGATWRRDLLGVHSASLTRYPA
AQRRPLT
>g658.t3
NLSVTTAFSMRWLSFKPFCRRAPSRPDYAGDGYHGFTEPRECVNAVVPPSPSSSVLRPRGRVLPPDRLRAAVGETAATRL
MEGQGRTARGQAAAVLSLPE
RWLSSSSGTVKGCPLLSGQVLAKDIRGILNVMVKPGWFC
>g659.t1
MRLKSRRRFATHAQELLRTTPTDISKASWVKARWRDQWKSAEPSRLHHYIQDPTDVLGQHLPRKQWTTLNRLRTGVGHTL
QQCRNGAW
>g660.t1
LHRAGPPAASRLPRHATTQPALTDSNACLSLGLGDTAPPGSMSSSSCPTAPPPPAPPPPVPLLLLLLHCSSTISSYCSSS
PHSLLPLAHFTPLLRSSLSY
QQVL
>g661.t1
TAMQRKPTVTEVQTPTDTWSGLGFSKSMPAEAVKELRSVSRRSYKPYLSDGAAGQPPRVSSAPPPPPTCRYLAPARRIGE
FLPSVAICTLSYLGLDSISA
RSETKEAVRCVHRPVPSPPPPPGASPRSLCGASVCAGSTRRGGAMDDATRESRGPSQSAASSVTAGTSSEEDQNTVITNR
TFGVQ
>g661.t2
TAMQRKPTVTEVQTPTDTWSGLGFSKSMPAEAVKELRSVSRRSYKPYLSDGAAGQPPRDFVCAAKAFSHSDIHFKGMAFC
RIFRSAFRSALCRLAPTQRV
SSAPPPPPTCRYLAPARRIGEFLPSVAICTLSYLGLDSISARSETKEAVRCVHRPVPSPPPPPGASPRSLCGASVCAGST
RRGGAMDDATRESRGPSQSA
ASSVTAGTSSEEDQNTVITNRTFGVQ
>g662.t1
CYLATASPAKFQSAVQRAGLTLDLPESLSSLDKMATRYQDLEKSQNWTKDWEDKLREKIQSISSARERNMTYYT
>g663.t1
LDSLSVSSLFSDDDLEEDEEDGERGEEEDDDDEEGLGDLEVNPYDGLPFSSRYYVLLEERRRLPVWGLKYRLLELLESHS
MVLLSSPSGVGKSTQWCVEY
ALSHEFSQGLVCCSQPYSVAAASLALRVADEMDLSLGLEVGYRVPHDDSCTPDTLLR
>g663.t2
LDSLSVSSLFSDDDLEEDEEDGERGEEEDDDDEEGLGDLEVNPYDGLPFSSRYYVLLEERRRLPVWGLKYRLLELLESHS
MVLLSSPSGVGKSTQWCVEY
ALSHEFSQGLVCCSQPYSVAAASLALRVADEMDLSLGLEVGYRVPHDDSCTPDTLLRPFSEGNVVLLFFHPDPAQAKRAI
P
>g663.t3
LDSLSVSSLFSDDDLEEDEEDGERGEEEDDDDEEGLGDLEVNPYDGLPFSSRYYVLLEERRRLPVWGLKYRLLELLESHS

MVLLSSPSGVGKSTQWCVEY
ALSHEFSQGLVCCSQPYSVAAASLALRVADEMDLSLGLEVGYRVPHDDSCTPDTLLRRHALTSH
>g664.t1
ASRTTRSDEDSDLGWDAWGPWSDCSRTCGGGASYSLRRCLNGG
>g665.t1
MTVLLRYARPRFSRAGRGTVQPVQRYLVPHWSLSWYLTGASPGTSLEPLLVPHWSLSWYLTGAPPGTSLEPLLVPHWSLS
WYLTGASPGTSPEPLLVPHR
SLSWYLTGASPGTSPEPLLVPHWSLSWYAERGTEERRPAPELLTRPPGVCSAGDGPQKYGSV
>g666.t1
DVGATETPPPDLRVGLMTYDSRIHLYDLSPALSRPHMLVVTETEDLQLPVRDGLLVSLRDCMDSID
>g666.t2
MTYDSRIHLYDLSPALSRPHMLVVTETEDLQLPVRDGLLVSLRDCMDSID
>g667.t1
TITNKPPPCLAPPAPPLNLDHIEEVKRKVTQQANIHTIKELTEKCKMIANSKEEMAIAKPHVSDDES
>g668.t1
IIRARRLHVHDVSTNSAKLQWRPVLAGRGGYYEIRFGPVPTGGGAGGGGVGSGTSPSTGGGQYQRLVRPADISSAELTGL
RPGTTYTATLTPEFNEDILT
PLSVTFTTKP
>g668.t2
MEPRWLPEIIRARRLHVHDVSTNSAKLQWRPVLAGRGGYYEIRFGPVPTGGGAGGGGVGSGTSPSTGGGQYQRLVRPADI
SSAELTGLRPGTTYTATLTP
EFNEDILTPLSVTFTTKP
>g669.t1
ISPPSPGVDHRHVQKELGDVMVRLHNPVVMSPTTIQVTWTVSARQRYISPPSPGVDHRHVQKELGDVMVRLHNPVVMSPT
TIQVTWTVSARQRCVTRRRD
AVSTSVRL
>g670.t1
MKVCLIALRLGCVVKPCRPAFGPQVRLRCQKGIPPALRGRAWLYLSGGKVKREQSQGKFQELDNQAGDPKWLEVIEKDLH
RQFPFHEMFVSRGGHGQQDL
FRVLKAYTLYRPEEGYCQAQAPIAAVLLMHMPAEDAFWGLVQICEKYLPGYYSPGLEAIQLDGEILFALLRRVCPLAYRH
LEKHKMDPILYMTEWFMCAF
SRTLPWASVLRVWDMFLCD
>g671.t1
MTTLRAFTCDDLFKFNNMRVLGTGQQGARVLGTGRQGGTGRQGADYTLLCPDSNLDPLTETYGIPFYLQYLAHWPEYFIV
AQAPGGELMGYIMGKAEGSV
AREDWHGHVTALSVAPEFRRLGLAARLMDTLEEISERKGGFFVDLFVRVSNQVAVSMYKRLGYSVYRT
>g671.t2
LFVHSVLSGVSEEDPGASGAADMTTLRAFTCDDLFKFNNMRVLGTGQQGARVLGTGRQGGTGRQGADYTLLCPDSNLDPL
TETYGIPFYLQYLAHWPEYF
IVAQAPGGELMGYIMGKAEGSVAREDWHGHVTALSVAPEFRRLGLAARLMDTLEEISERKGGFFVDLFVRVSNQVAVSMY
KRLGYSVYRT
>g671.t3
MTTLRAFTCDDLFKFNNMRVLGTGQQGARVLGTGRQGGTGRQGADYTLLCPDSNLDPLTETDQSWSPDSPVQPRPWYGIP
FYLQYLAHWPEYFIVAQAPG
GELMGYIMGKAEGSVAREDWHGHVTALSVAPEFRRLGLAARLMDTLEEISERKGGFFVDLFVRVSNQVAVSMYKRLGYSV
YRT
>g672.t1
MDWLRVWGLFGLCVLSRAQRQNMTNTLSPGALPVPSASYLLLAPSQLCVGVPFTLSISVLTSDPKVLVTCQLHGTYFNTT
ATTSLSGGSSALVELFVPGP
VADRTLRLLVRGIAGKKLLFTNSTVLRVRRQDVSTFIQTDKHTYRPGETVHIRAVSIGASRRPHKSPVDMIVKDPRGNII
RQWLSLNSTLGVATQELQLS
EYPPLGSWTIQTTVEDVESVNKFTVSHYVLPKFAVVVTAPSVVYYRDILFGSVTAKYTYGRPVRGQMVVSYFDGWNIWTL
EPTKIDGSANFELDLSKHPE
DSMLDNDHEAVTVVVNVTESLTGLTYSSEAMVYVVRSSYKISFECSPFILKPSLNFTVKIRISSYNGQPLTLSDQAKTVF
VSVIQQPAFHWGGHDFMGLD
MEPLDFRRSVAPGQQQELQFPVPADGVIPLSIDIIDGIGSLEIEASLEDSYKTFSLSSNYHSPSKSYLQIQKPHTPAQVG

LPLLLPVLSNFEATLFFYTV
KSKDRIVASGRGFSPLVVTPEASWAPLACVVVYCIRADNEVVNDILLLPIPLELQNKVSLSWSEYQRKPGEQVSLSLSVS
EPGSLVGLLVVDEATHRRGS
RNDITTETVLEELYGGMSELPRPDNRRGDPESLFQACDLIVLTDATLNRNPLHVPMRLEVEKDEEEAEYHEPREPRKRQN
FPETWLWMNINTGEATASEV
SVMVPDSITTWRATAFVVSENLGLGILEEPARLTVFQDFFISLNLPVCVIRGEELVLEVNLYNYLSMDLQVLLTVAQSSS
FEFVFPDSQVLSMASSRQVL
VRSDSGASILFPIRPLALGPMPISVKAMSSAASDAIQRTVLVKAEGLEQSYSTTLFVELSSGTPSYVRDLNFVFPADMVA
GSQRAALMVVGDLLGPSING
LDSLIKMPYGCGEQNMINFAPNIYIIGYLEASGQSAPEVVAKATLYMER
>g672.t2
MDWLRVWGLFGLCVLSRAQRQNMTNTLSPGALPVPSASYLLLAPSQLCVGVPFTLSISVLTSDPKVLVTCQLHGTYFNTT
ATTSLSGGSSALVELFVPGP
VADRTLRLLVRGIAGKKLLFTNSTVLRVRRQDVSTFIQTDKHTYRPGETVHIRAVSIGASRRPHKSPVDMIVKDPRGNII
RQWLSLNSTLGVATQELQLS
EYPPLGSWTIQTTVEDVESVNKFTVSHYVLPKFAVVVTAPSVVYYRDILFGSVTAKYTYGRPVRGQMVVSYFDGWNIWTL
EPTKIDGSANFELDLSKHPE
DSMLDNDHEAVTVVVNVTESLTGLTYSSEAMVYVVRSSYKISFECSPFILKPSLNFTVKIRISSYNGQPLTLSDQAKTVF
VSVIQQPAFHWGGHDFMGLD
MEPLDFRRSVAPGQQQELQFPVPADGVIPLSIDIIDGIGSLEIEASLEDSYKTFSLSSNYHSPSKSYLQIQKPHTPAQVG
LPLLLPVLSNFEATLFFYTV
KSKDRIVASGRGFSPLVVTPEASWAPLACVVVYCIRADNEVVNDILLLPIPLELQNKVSLSWSEYQRKPGEQVSLSLSVS
EPGSLVGLLVVDEATHRRGS
RNDITTETVLEELYGGMSELPRPDNRRGDPESLFQACDLIVLTDATLNRNPLHVPMHLMMDVGLEVEKDEEEAEYHEPRE
PRKRQNFPETWLWMNINTGE
ATASEVSVMVPDSITTWRATAFVVSENLGLGILEEPARLTVFQDFFISLNLPVCVIRGEELVLEVNLYNYLSMDLQVLLT
VAQSSSFEFVFPDSQVLSMA
SSRQVLVRSDSGASILFPIRPLALGPMPISVKAMSSAASDAIQRTVLVKAEGLEQSYSTTLFVELSSGTPSYVRDLNFVF
PADMVAGSQRAALMVVGDLL
GPSINGLDSLIKMPYGCGEQNMINFAPNIYIIGYLEASGQSAPEVVAKATLYMER
>g672.t3
MTNTLSPGALPVPSASYLLLAPSQLCVGVPFTLSISVLTSDPKVLVTCQLHGTYFNTTATTSLSGGSSALVELFVPGPVA
DRTLRLLVRGIAGKKLLFTN
STVLRVRRQDVSTFIQTDKHTYRPGETVHIRAVSIGASRRPHKSPVDMIVKDPRGNIIRQWLSLNSTLGVATQELQLSEY
PPLGSWTIQTTVEDVESVNK
FTVSHYVLPKFAVVVTAPSVVYYRDILFGSVTAKYTYGRPVRGQMVVSYFDGWNIWTLEPTKIDGSANFELDLSKHPEDS
MLDNDHEAVTVVVNVTESLT
GLTYSSEAMVYVVRSSYKISFECSPFILKPSLNFTVKIRISSYNGQPLTLSDQAKTVFVSVIQQPAFHWGGHDFMGLDME
PLDFRRSVAPGQQQELQFPV
PADGVIPLSIDIIDGIGSLEIEASLEDSYKTFSLSSNYHSPSKSYLQIQKPHTPAQVGLPLLLPVLSNFEATLFFYTVKS
KDRIVASGRGFSPLVVTPEA
SWAPLACVVVYCIRADNEVVNDILLLPIPLELQNKVSLSWSEYQRKPGEQVSLSLSVSEPGSLVGLLVVDEATHRRGSRN
DITTETVLEELYGGMSELPR
PDNRRGDPESLFQACDLIVLTDATLNRNPLHVPMRLEVEKDEEEAEYHEPREPRKRQNFPETWLWMNINTGEATASEVSV
MVPDSITTWRATAFVVSENL
GLGILEEPARLTVFQDFFISLNLPVCVIRGEELVLEVNLYNYLSMDLQVLLTVAQSSSFEFVFPDSQVLSMASSRQVLVR
SDSGASILFPIRPLALGPMP
ISVKAMSSAASDAIQRTVLVKAEGLEQSYSTTLFVELSSGTPSYVRDLNFVFPADMVAGSQRAALMVVGDLLGPSINGLD
SLIKMPYGCGEQNMINFAPN
IYIIGYLEASGQSAPEVVAKATLYMER
>g673.t1
MPTALKMKIKEVKKEKGDRKIVAAQKKKKVLKQGVLRKKDLKKLTLYIKNGANCPCAQLDSLGSNFLIMGRKVDQQLLLM
SIHKWDKKSKELKFAIKYMK
SHQCPTYHTVFQ
>g674.t1
GSRAAQRVPQIAVVITDGDSSDDVLVPAQQLRRHGVIVFAIGVGSANMAELQGIANRPHDRFLFSIDSYQALQRLSDGLL

QTVCVSVEDQRIALVQKFSD
IYFLVDSGLNPVEFQAFWTMLLRLINQLNVGASAHRIGLAQYGKDTQVEFFLNQHKTKDDLLASLRRFRARHGQTNEQRN
LGSALTYAAANFFSTEAGSR
EDQGFRQFLVVASGTNSSDSVYQPARLVKSQGVTVMGIGIGSGMTDQMKTVATAPYVFQATNIVPMLKTAFETEEEPAEV
IEDCAAARVADVVFIVDESG
SIGSANFQLVRSFLHKMVERLDVNPNRVRVGIVMYNDNPTGRIYLNTFDDKNEILQFIRILPYHGGGTNTGRALKFARQN
FFVKERGSRKSQGVQQVAVV
ITDGESQDEVDKQAADLRRDGVTVYAIGIKDANKTQLVQMASHPSTKHVFNVDSFRKLKTLQKSLEKIVCHNIFHQAITV
KTRRTNIKEGNIWPNDFHDM
KKFIIEFLHTFHIGLEHVRVGVVKFADTPTLEFDLTTYNDSKALENAVINIQQIGNGTKTGTALSFMAPHFERAMTTRGH
TVHEYLIVITDKKSMDEVKL
PAEKLRAQGVIIYAIGVKSASETELKAIAGSPQKTFFVHNFDSLKPIKDDIITDICSRDACKDTPADLLFLIDSSGSIEV
KDYQMMKDFMKAMIGKSRVG
KDKVHIGVMQFSTIQTLEFPLNHFYDRSDMWEAIDKMEQVRGGTHTGQALTNISPYFDRVSGGRPGMRQNLIVITDGEAQ
DQVKGPAEVLRNKGVHIYAI
GVVDANTTQLLEISGSQDRVFTERNFDALKDLESQLALDLCKKECKKTQMADIIFLVDGSTSISDSKFLSMLKFMESIVN
ETTVSEKMTRFGVILYSTNP
KNVFSLNKYFSKRQVLKAIQALKSPSGDTYTGKALDYTRQYFDAQHGGRGAINVPQILMVITDSEATDTNNLMAPSVALR
KSGIGVFSI
>g674.t2
GSRAAQRVPQIAVVITDGDSSDDVLVPAQQLRRHGVIVFAIGVGSANMAELQGIANRPHDRFLFSIDSYQALQRLSDGLL
QTVCVSVEDQRIALVQKFSD
IYFLVDSGLNPVEFQAFWTMLLRLINQLNVGASAHRIGLAQYGKDTQVEFFLNQHKTKDDLLASLRRFRARHGQTNEQRN
LGSALTYAAANFFSTEAGSR
EDQGFRQFLVVASGTNSSDSVYQPARLVKSQGVTVMGIGIGSGMTDQMKTVATAPYVFQATNIVPMLKTAFETEEEPAEV
IEDCAAARVADVVFIVDESG
SIGSANFQLVRSFLHKMVERLDVNPNRVRVGIVMYNDNPTGRIYLNTFDDKNEILQFIRILPYHGGGTNTGRALKFARQN
FFVKERGSRKSQGVQQVAVV
ITDGESQDEVDKQAADLRRDGVTVYAIGIKDANKTQLVQMASHPSTKHVFNVDSFRKLKTLQKSLEKIVCHNIFHQAITV
KTRRTNIKEGCAQTEEADIF
FLIGSGNIWPNDFHDMKKFIIEFLHTFHIGLEHVRVGVVKFADTPTLEFDLTTYNDSKALENAVINIQQIGNGTKTGTAL
SFMAPHFERAMTTRGHTVHE
YLIVITDKKSMDEVKLPAEKLRAQGVIIYAIGVKSASETELKAIAGSPQKTFFVHNFDSLKPIKDDIITDICSRDACKDT
PADLLFLIDSSGSIEVKDYQ
MMKDFMKAMIGKSRVGKDKVHIGVMQFSTIQTLEFPLNHFYDRSDMWEAIDKMEQVRGGTHTGQALTNISPYFDRVSGGR
PGMRQNLIVITDGEAQDQVK
GPAEVLRNKGVHIYAIGVVDANTTQLLEISGSQDRVFTERNFDALKDLESQLALDLCKKECKKTQMADIIFLVDGSTSIS
DSKFLSMLKFMESIVNETTV
SEKMTRFGVILYSTNPKNVFSLNKYFSKRQVLKAIQALKSPSGDTYTGKALDYTRQYFDAQHGGRGAINVPQILMVITDS
EATDTNNLMAPSVALRKSGI
GVFSI
>g675.t1
PGWSSEEDDKKGKGCMLQFQHAVVRVLTHFMADQEDPRDPKAYFLGSDWQVDVTRLVRTFIKVEDPRVARLQDGRVLSGR
DLGTTSLQVFSPLSEAILAK
QTITVVDDKVSITELGVQMVTGLSMTLQLSQASNRAILATTTSQEVLQNPKQEALVSTWLQFSDGSQTPLDIYDPAYYRL
TITSLNAGVVSVQGSPSAVV
AEGEGQGVLVKVEMSICEACQKSKRKSTIAVGNGSLKVKFQSNGLLPDSTGTGSNTSNVSSSGGEDGGEDGGSDYGSDGE
ELNSDWKQRQPAQDPVASST
ISGQKGRAKQKVTTIKPVERTIITSGSLGGVGKTNGSKSLSNPTSAVSNPDDKAYNLDNILAEDMGAIALGSTVKAPGNL
VNYNSFPPQVPGQGPGVEIG
GEDVLVNRPLTDLEIGMYALLGVFCLAILVFLVNCISYVVKFRHKKPPSHGQEPMGHRHDWVWLGTDAELVMSMPGSPLQ
QDNHTATTVMDIGPDKTASL
SRRPSCLASLTDSPPPGCVGPLRSKHLQADSLHSPTSKRKRVQFTTFSTLERQHSLHLPPGRENGHQGIHWVGKESSHNK
EPQVPVTEPEDQL
>g676.t1
LSGSHVDQYRVFLLVCGLRRVKDPLYLAEVFTEWHREDPLNVLVIIGPEIDPVLTEEVEAVAQRRAGVFLAQGRSQRELH

AAMRRCFAVVNSSVSEGMSA
AILEAMHLEVPVVARDIPGNAAVVQHGVTGLLYSSPQEFVCQSQRLLSDEELRESVVRKAKLYVEQQHSVARERETYHSL
VQSLQ
>g677.t1
MVTLCGDQMAFPVLSPDCLNTNSTPPSHVNCTSRTPYCRPVIYCGLILIAVGVGTVKSNITPFGADQVKDRGPEATRRFF
NWFYWCINLGAIVSLGGVAY
IQQNLNFEVGYIIPTTCLGVSFLVFLLGRTVFITKPADGSAFTEMFQILGFACCSSQIPKEPNLLRPKPTLLDGAKASYG
GKFPEEKVEEVKALLKVLPV
FFALIPYWTVYFQMQTTYILQSLHLRIPGYFNSTSSSQQV
>g677.t2
MAFPVLSPDCLNTNSTPPSHVNCTSRTPYCRPVIYCGLILIAVGVGTVKSNITPFGADQVKDRGPEATRRFFNWFYWCIN
LGAIVSLGGVAYIQQNLNFE
VGYIIPTTCLGVSFLVFLLGRTVFITKPADGSAFTEMFQILGFACCSSQIPKEPNLLRPKPTLLDGAKASYGGKFPEEKV
EEVKALLKVLPVFFALIPYW
TVYFQMQTTYILQSLHLRIPGYFNSTSSSQQV
>g677.t3
MVTLCGDQMAFPVLSPDCLNTNSTPPSHVNCTSRTPYCRPVIYCGLILIAVGVGTVKSNITPFGADQVKDRGPEATRRFF
NWFYWCINLGAIVSLGGVAY
IQQNLNFEVGYIIPTTCLGVSFLVFLLGRTVFITKPADGSAFTEMFQILGFACCSSQIPKEPNLLRPKPTLLDGAKASYG
GKFPEEKVEEVKALLKVLPV
FFALIPYWTVYFQMQTTYILQSLHLRIPGYFNSTSSSQQVSV
>g678.t1
MAQVNACLKRTFTFFNVLFAIVGGLIIGLALLVQTLSRNNGGQE
>g678.t2
MELLMFQIVGGLIIGLALLVQTLSRNNGGQE
>g679.t1
MASKSLGKGYSKRRQRSVQPPIDDSTQILHESCNSREDKEEGDDDGPAVFFCGKCNLPIGDSLSWAGSDNESQIHLKRVT
DNVLIGKEHRIYEPRPKHRC
LIVDLSCKGCQSVLGMVYLSTPKALDYKRLTFSFKVKQLESYVLGSANQEVAGESPLAPLMVEDRGVVEQQLLE
>g679.t2
MSYNNTNMASKSLGKGYSKRRQRSVQPPIDDSTQILHESCNSREDKEEGDDDGPAVFFCGKCNLPIGDSLSWAGSDNESQ
IHLKRVTDNVLIGKEHRIYE
PRPKHRCLIVDLSCKGCQSVLGMVYLSTPKALDYKRLTFSFKVKQLESYVLGSANQEVAGESPLAPLMVEDRGVVEQQLL
E
>g679.t3
MASKSLGKGYSKRRQRSVQPPIDDSTQILHESCNSREDKEEGDDDGPAVFFCGKCNLPIGDSLSWAGSDNESVTDNVLIG
KEHRIYEPRPKHRCLIVDLS
CKGCQSVLGMVYLSTPKALDYKRLTFSFKVKQLESYVLGSANQEVAGESPLAPLMVEDRGVVEQQLLE
>g680.t1
MKWKRIHGLEWSGLICFTLLLWTKRTEGLADFSNYLSRIIVSQHALACDGQPLRLHCPRHSTVSIHSAFYGSGDDGAHPC
GSGSAGGSTDSPVRAQNGSC
SAFTALQ
>g680.t2
MCEKIKKVHAWLSSVLGEQPVPQFEVNSRTVDVLLQLAEASEVRCKDTSLLIDDFKQKASEYHADSIHLQDVLLQGVGLS
CACLSKPAADYTSALVANAM
VLGVRDTSLGSFIPAVNRLTNDVMEAEKSDRRLDRELKALRRKLGATLVLRKNLQEDINKTVKDQEDKISSRNMNKKLSH
QALMEIFEEVNTLRQEIIPL
TKKLEPYLDLSPIDRFGFPGAELTVDRHAHHHHHHSVLGWTWKRVSFHASPFSVSPLKSFHAGFWSISVVFVSPVSSNHL
LRSFYFQSSPVPCILK
>g680.t3
MCEKIKKVHAWLSSVLGEQPVPQFEVNSRTVDVLLQLAEASEVRCKDTSLLIDDFKQKASEYHADSIHLQDVLLQGVGLS
CACLSKPAADYTSALVANAM
VLGVRDTSLGSFIPAVNRLTNDVMEAEKSDRRLDRELKALRRKLGATLVLRKNLQEDINKTVKDQEVKGAQAEERLLNMD
FVKTKSRELINRRKIAEDKI
SSRNMNKKLSHQALMEIFEEVNTLRQEIIPLTKKLEPYLDLSPIDRFGFPGAELTVDRHAHHHHHHSVLGWTWKRVSFHA
SPFSVSPLKSFHAGFWSISV

VFVSPVSSNHLLRSFYFQSSPVPCILK
>g680.t4
MCEKIKKVHAWLSSVLGEQPVPQFEVNSRTVDVLLQLAEASEVRCKDTSLLIDDFKQKASEYHADSIHLQDVLLQGVGLS
CACLSKPAADYTSALVANAM
VLGVRDTSLGSFIPAVNRLTNDVMEAEKSDRRLDRELKALRRKLGATLVLRKNLQEDINKTVKDQEDKISSRNMNKKLSH
QALMEIFEEVNTLRQEIIPL
TKKLEPYLDLSPIDRFGFPGAELTVDRHAHHHHHHSVLGWTWKRVSFHASPFSVSPLKSFHAGFWSISVVFVSPVSSNHL
LRSFYFQSSPVPCILKEWEC
KTMKWKRIHGLEWSGLICFTLLLWTKRTEGLADFSNYLSRIIVSQHALACDGQPLRLHCPRHSTVSIHSAFYGSGDDGAH
PCGSGSAGGSTDSPVRAQNG
SCSAFTALQ
>g681.t1
LDARFSRKAASKGLPDASPPARSEHAGSTRPLNVSLEYQFILLSSPFLPLTDFSLPSLLEVFLSREARLPPLRAGQKAGP
DAQNSNRRSHAGSTAWKSRR
SRRTAQRYSVKEARKSQLSTSDSDANAENKAAGSGQGGSRSRRTHSAALRTSGQHHRPEAPQTHPTGAAETMSVPYPHPR
ALGPQTLDAETLTDPAAMSI
FNRMENSPFDLCHVLLSLLEKVCKFDMSINHNPGLAVSVVPTLTEFLTEFGDCCGPGGGGGGGVGPEELAGGWTEEPIAL
VQRMLLRTILHLMSVDVGQS
ETLPDSLRRSLTDLLRATLKIRSCLERQADPFAPRPKKTLQEVQDDFSFSRYRHRALLLPELLEGVLQVLLGCLQASTSN
PFFFSQALELIHEFIQHNGL
ELFEVTVLRLEGLARARDSEVGGEAAERLRGLIAGVLKIISAVKKAKSEQLHQSVCARRRHRRCEYSHFLHHHRDLSGLP
VSAFKQAARRNPFEEEEEGC
REGSEGAAVRYPERCCCLAACAHQCLRLLQRLSPNGPAVLQVLAGVQAVGICCCMDPRSVVGPLLHAFQAPGLRSYQSHV
LNVLGRLILDQLGGGQPSEK
AKLASCNICTLDSSQLPSLEETLQQGEPFAASLGLSYRSQGILPSGGGAEDMLWKWDALEAYQELVFGEDRQLSQQIAGH
VCQLTLRGNAVVQWQLYTHI
FNPVMQRGVELVHHAQQLGVSTMCAQMCNYHTQCLPVQVLLVYLQTLPALLKSRVIRDLFVSCNGLTQITELIHLDHTRS
WALKVFQKLILGAGEQPADG
PQELEEKEAGPGPAED
>g682.t1
STDSTIIRGGASESNVFFCTCGQIRPKGCVPTAPVWYDHVSHVQHRDAMSGGCGRSHHQPSTLAALQSPGRPNQPRLHRH
CDADAPPPVMEPQLRRLENE
PSRCCVVSCSISTSIMNWLTLSY
>g683.t1
GTSARAARRQEPEDTPADLHEETRSRGASPAPTFSGNGSTDNQGADENRIERGRGTPRRCEPLRGLRLT
>g684.t1
MDACARIRGVPLRTRASIRKETVRDSGAQCVKSLLRTKKELCRVSVELQRGDALRLTEALSSLPAELCDLLRSLHVHSLR
NDEVLLLKDSRRPAEGRDPA
TQCWLRVVCVLRHSPNSGPCPQAVRVSLVGLLRRYAAGIRYALELQALHRGSAESHQAEEDDTNQSVSSIEDDFVTALEH
LEEEDTRESSSAAAAPFHHG
TKRDVASQTVPAHKRRKDLTGSRVIISSSAKRPATKHKCGPDVSITVQRPSAPESQWTHYNPGTRLPAPSTRFSESDDSD
GSSPSPIIFLDEVGYQKSLL
AKLDIPQVPGGPREPVEDSDSEVSEFFDSFDQFDDLDELSSDSCTLTLPLDLVDPSSQGQKKKPSETSVGGSKYVSRGCS
TKGRNLQRFDQPPLPANVKK
PTPLKPGSPYSPNSEVPDSPRPVRSSCEENGGPLFSPVSASAFSPLGDSGGPLEYFWKSEGDVSELRKPQDLCSLYKTYS
DFASSLSREILGSVCGYQSP
VDISDNKNLSCVCHKEFQNSSGHLMKLSEIQEAVTMDKLQLRPQSLKDGIQRFATDLVEMSLGSALRDLQKGVSQCTTTL
CHLAARLTSSVFQMAFHEIG
MRRAYVLKERAISGLAGFLVGEAVSGALKEFLTVKKQIFNSTVSQFAADLAEELVFEGIMEVCQFSHPSTPLTPSDWSFG
QGREEARDDEDDDEEEEVVS
SYASDLSESVIQEAFIELSQADVAFTSQAAISVSLDNVCYVSSDDTSTHTCSTFIGTQVCHANPASDGTPGPSGENGVCT
VKKALFTVSGMASCIPVPQA
GQVLSLSQPSEETCQYKSALAHTSQTSPKRTVVTTASSDTTAATQTEQYPQGPHTHCPQGDASEGKSPFHHFSGNMVDMI
VSEACELMTATKMKKGFGDC
AEFFTKTIGSRRGSSSRPGSVSDDVPDSPLVQRGGQESFTYNNKDCGGVSQTGPPELTAGTQIEPSQSKCQSRSSAECIP
GTSGGTGEILPVTMDSLDLP

GARTGIQRKISIPRDESAPNCGQKSTRTPGTPPSSPQQPSEVSKEKQIRQFSKKLKGKLAKEFSPATPPPTPHYQPEPGP
GPTDPSPEADKADFMVKLMR
SLSEEAEGHGQEEEEEDEEQEEHGEGGAGGTPRGSGIGGTSRATARRMSNKEAFRYAEKLACHIVSMATEMDTLIGAEKK
EEEEEDGSRNPGGEARRDSV
AQFSEETLNTLWVYAGEVAGEVIGDVKRLVNSGQQCPHHRALRRRSLDRPVPECHQHQHHHHHPMASGPHHPMSTATSSP
GLSSQYPSCESVTDEYAGYL
IRVLKKEGGSRELVLDQYASRLAYRSIKQGLTHAARKMKQKSSTANRLHSSRSLPEDWKAAGDDASPPGDEAGAGESSVC
TCPRDPEEQHQQEYADLVNF
AESLAYSITCDVTRKLCLPSVLLPKSLTDSCLYRKSKLEDMAENLIRGAFSCPLLAKEGKGRQYHSTGSLSEGGLCTGVM
QVIEHYARKIVEDTLEMGLS
AAGHVSGEPPRHHTRDRHAHTDRLSEGATPGDIACRYCQVRACPRCTKPRVHHHHCRQAGPRLRPKRGQEYEGGAEHLSS
LEIPKIQIDLDLRVAFAEEM
VSVAMETAKRELSNTSLNADSGIGHDGASYAESLTAEIMTSALSNLCQAANLSSPGRDATESTMPQQLSVGDDSLGSWSN
LSFEDEHPDENSSFLHLSDS
SNGNSSSWSSLGLEGEVCEERLSFSPSDRHLIATALLSAAIMAELLYYRMPLSRPHTLSPSLPVPSALRCPVACSLRS
>g684.t2
MDACARIRGVPLRTRASIRKETVRDSGAQCVKSLLRTKKELCRVSVELQRGDALRLTEINFVCLPGLCEGKDGFPQALSS
LPAELCDLLRSLHVHSLRND
EVLLLKDSRRPAEGRDPATQCWLRVVCVLRHSPNSGPCPQAVRVSLVGLLRRYAAGIRYALELQALHRGSAESHQAEEDD
TNQSVSSIEDDFVTALEHLE
EEDTRESSSAAAAPFHHGTKRDVASQTVPAHKRRKDLTGSRVIISSSAKRPATKHKCGPDVSITVQRPSAPESQWTHYNP
GTRLPAPSTRFSESDDSDGS
SPSPIIFLDEVGYQKSLLAKLDIPQVPGGPREPVEDSDSEVSEFFDSFDQFDDLDELSSDSCTLTLPLDLVDPSSQGQKK
KPSETSVGGSKYVSRGCSTK
GRNLQRFDQPPLPANVKKPTPLKPGSPYSPNSEVPDSPRPVRSSCEENGGPLFSPVSASAFSPLGDSGGPLEYFWKSEGD
VSELRKPQDLCSLYKTYSDF
ASSLSREILGSVCGYQSPVDISDNKNLSCVCHKEFQNSSGHLMKLSEIQEAVTMDKLQLRPQSLKDGIQRFATDLVEMSL
GSALRDLQKGVSQCTTTLCH
LAARLTSSVFQMAFHEIGMRRAYVLKERAISGLAGFLVGEAVSGALKEFLTVKKQIFNSTVSQFAADLAEELVFEGIMEV
CQFSHPSTPLTPSDWSFGQG
REEARDDEDDDEEEEVVSSYASDLSESVIQEAFIELSQADVAFTSQAAISVSLDNVCYVSSDDTSTHTCSTFIGTQVCHA
NPASDGTPGPSGENGVCTVK
KALFTVSGMASCIPVPQAGQVLSLSQPSEETCQYKSALAHTSQTSPKRTVVTTASSDTTAATQTEQYPQGPHTHCPQGDA
SEGKSPFHHFSGNMVDMIVS
EACELMTATKMKKGFGDCAEFFTKTIGSRRGSSSRPGSVSDDVPDSPLVQRGGQESFTYNNKDCGGVSQTGPPELTAGTQ
IEPSQSKCQSRSSAECIPGT
SGGTGEILPVTMDSLDLPGARTGIQRKISIPRDESAPNCGQKSTRTPGTPPSSPQQPSEVSKEKQIRQFSKKLKGKLAKE
FSPATPPPTPHYQPEPGPGP
TDPSPEADKADFMVKLMRSLSEEAEGHGQEEEEEDEEQEEHGEGGAGGTPRGSGIGGTSRATARRMSNKEAFRYAEKLAC
HIVSMATEMDTLIGAEKKEE
EEEDGSRNPGGEARRDSVAQFSEETLNTLWVYAGEVAGEVIGDVKRLVNSGQQCPHHRALRRRSLDRPVPECHQHQHHHH
HPMASGPHHPMSTATSSPGL
SSQYPSCESVTDEYAGYLIRVLKKEGGSRELVLDQYASRLAYRSIKQGLTHAARKMKQKSSTANRLHSSRSLPEDWKAAG
DDASPPGDEAGAGESSVCTC
PRDPEEQHQQEYADLVNFAESLAYSITCDVTRKLCLPSVLLPKSLTDSCLYRKSKLEDMAENLIRGAFSCPLLAKEGKGR
QYHSTGSLSEGGLCTGVMQV
IEHYARKIVEDTLEMGLSAAGHVSGEPPRHHTRDRHAHTDRLSEGATPGDIACRYCQVRACPRCTKPRVHHHHCRQAGPR
LRPKRGQEYEGGAEHLSSLE
IPKIQIDLDLRVAFAEEMVSVAMETAKRELSNTSLNADSGIGHDGASYAESLTAEIMTSALSNLCQAANLSSPGRDATES
TMPQQLSVGDDSLGSWSNLS
FEDEHPDENSSFLHLSDSSNGNSSSWSSLGLEGEVCEERLSFSPSDRHLIATALLSAAIMAELLYYRMPLSRPHTLSPSL
PVPSALRCPVACSLRS
>g684.t3
MDACARIRGVPLRTRASIRKETVRDSGAQCVKSLLRTKKELCRVSVELQRGDALRLTEALSSLPAELCDLLRSLHVHSLR
NDEVLLLKDSRRPAEGRDPA
TQCWLRVVCVLRHSPNSGPCPQAVRVSLVGLLRRYAAGIRYALELQALHRGSAESHQAEEDDTNQSVSSIEDDFVTALEH

LEEEDTRESSSAAAAPFHHG
TKRDVASQTVPAHKRRKDLTGSRVIISSSAKRPATKHKCGPDVSITVQRPSAPESQWTHYNPGTRLPAPSTRFSESDDSD
GSSPSPIIFLDEVGYQKSLL
AKLDIPQVPGGPREPVEDSDSEVSEFFDSFDQFDDLDELSSDSCTLTLPLDLVDPSSQGQKKKPSETSVGGSKYVSRGCS
TKGRNLQRFDQPPLPANVKK
PTPLKPGSPYSPNSEVPDSPRPVRSSCEENGGPLFSPVSASAFSPLGDSGGPLEYFWKSEGDVSELRKPQDLCSLYKTYS
DFASSLSREILGSVCGYQSP
VDISDNKNLSCVCHKEFQNSSGHLMKLSEIQEAVTMDKLQLRPQSLKDGIQRFATDLVEMSLGSALRDLQKGVSQCTTTL
CHLAARLTSSVFQMAFHEIG
MRRAYVLKERAISGLAGFLVGEAVSGALKEFLTVKKQIFNSTVSQFAADLAEELVFEGIMEVCQFSHPSTPLTPSDWSFG
QGREEARDDEDDDEEEEVVS
SYASDLSESVIQEAFIELSQADVAFTSQAAISVSLDNVCYVSSDDTSTHTCSTFIGTQVCHANPASDGTPGPSGENGVCT
VKKALFTVSGMASCIPVPQA
GQVLSLSQPSEETCQYKSALAHTSQTSPKRTVVTTASSDTTAATQTEQYPQGPHTHCPQGDASEGKSPFHHFSGNMVDMI
VSEACELMTATKMKKGFGDC
AEFFTKTIGSRRGSSSRPGSVSDDVPDSPLVQRGGQESFTYNNKDCGGVSQTGPPELTAGTQIEPSQSKCQSRSSAECIP
GTSGGTGEILPVTMDSLDLP
GARTGIQRKISIPRDESAPNCGQKSTRTPGTPPSSPQQPSEVSKEKQIRQFSKKLKGKLAKEFSPATPPPTPHYQPEPGP
GPTDPSPEADKADFMVKLMR
SLSEEAEGHGQEEEEEDEEQEEHGEGGAGGTPRGSGIGGTSRATARRMSNKEAFRYAEKLACHIVSMATEMDTLIGAEKK
EEEEEDGSRNPGGEARRDSV
AQFSEETLNTLWVYAGEVAGEVIGDVKRLVNSGQQCPHHRALRRRSLDRPVPECHQHQHHHHHPMASGPHHPMSTATSSP
GLSSQYPSCESVTDEYAGYL
IRVLKKEGGSRELVLDQYASRLAYRSIKQGLTHAARKMKQKSSTANRLHSSRSLPEDWKAAGDDASPPGDEAGAGESSVC
TCPRDPEEQHQQEYADLVNF
AESLAYSITCDVTRKLCLPSVLLPKSLTDSCLYRKSKLEDMAENLIRGAFSCPLLAKEGKGRQYHSTGSLSEGGLCTGVM
QVIEHYARKIVEDTLEMGLS
AAGHVSGEPPRHHTRDRHAHTDRLSEGATPGDIACRYCQVRACPRCTKPRVHHHHCRQAGPRLRPKRGQEYEGGAEHLSS
LEIPKIQIDLDLRVAFAEEM
VSVAMETAKRELSNTSLNADSGIGHDGASYAESLTAEIMTSALSNLCQAANLRDATESTMPQQLSVGDDSLGSWSNLSFE
DEHPDENSSFLHLSDSSNGN
SSSWSSLGLEGEVCEERLSFSPSDRHLIATALLSAAIMAELLYYRMPLSRPHTLSPSLPVPSALRCPVACSLRS
>g685.t1
MYSTPHSALTASGNDNSITELLGALCDCSIYGVSWKRRAQGGLSKEGARKALKKRVYGALLSKLFQPGTSKGSASGPSTT
PPRNRVLMISFDLRVAGCQE
EAERLEELLGALPEGDPADPVLELLSAELQRLEARAWSLVCGEEWGSLGGLCGTLGLMDALPGTGLPALGMKLDAEERFE
RETRLSLFGALQHARTSDLH
IRLDLPPVPSDADVTGLAIKVPQCFDQSEDEGFQSASNLTPDSQSEPSPCPEFDIWEALRTFEPGKRRCWESIGCPPGNK
ELPYVSEGGREAFDQLYRLW
EGQMRVVSTVATPSPVLPLPLDSQTQLVSDLLNVLIGVASTTFPLSQ
>g685.t2
MYSTPHSALTASGNDNSITELLGALCDCSIYGVSWKRRAQGGLSKEGARKALKKRVYGALLSKLFQPGTSKGSASGPSTT
PPRNRVLMISFDLRVAGCQE
EAERLEELLGALPEGDPADPVLELLSAELQRLEARAWSLVCGEEWGSLGGLCGTLGLMDALPGTGLPALGMKLDAEERFE
RETRLSLFGALQHARTSDLH
IRLDLPPVPSDADVTGLAIKVPQCFDQSEDEGFQSASNLTPDSQSEPSPCPEFDIWEALRTFEPGKRRCWESIGCPGLMI
SLQDVYIGIQTQNLCPPGNK
ELPYVSEGGREAFDQLYRLWEGQMRVVSTVATPSPVLPLPLDSQTQLVSDLLNVLIGVASTTFPLSQ
>g686.t1
PIVRPYAGAVGPGFLLVHDNARPHVARVCRQFLEDEGIDTIDWPPRSPDLNPIEHLWDIMFRAIRRRQVAPQTVQELSDA
LVQIWEEIPQETIRRLIRSM
PQRCQACIQARGGNTNY
>g687.t1
MGIFIIVTMATGHVEGTEWVQIGEQRSNCPGRLYLRLRMRRPTRETQEPTSGIGTLRSLTNHFSELQGEGLEGGTG
>g687.t2
MGIFIIVTMATGHVEGTEWVQIGEQRSNCPGRLYLRLRMRRPTREVSLMSTSSDTQEPTSGIGTLRSLTNHFSELQGEGL

EGGTG
>g687.t3
MATGHVEGTEWVQIGEQRSNCPGRLYLRLRMRRPTRETQEPTSGIGTLRSLTNHFSELQGEGLEGGTG
>g688.t1
MLRPASHLSVLLRDLLTYWHQTPGALLYITTVAFPDITSSSSSMKPCSPSPPLRVVPDGNPRVLHWRPGRLQSLPHKLSI
LFALLPHTFASENGGLHHHQ
PSDGQSSSLGNG
>g688.t2
MLRPASHLSVLLRDLLTYWHQTPGALLYITTVAFPDITSSSSSMKPCSPSPPLSSDTSSSCFHSTRVVPDGNPRVLHWRP
GRLQSLPHKLSILFALLPHT
FASENGGLHHHQPSDGQSSSLGNG
>g689.t1
LLTRELSFQGKEGLCLSLETRRCMSWKLGCFPTSSWNTIXLALGTWNVTSLAGKEPELVREVERYQLDIVGLTSTHSIGS
GTKLLERGWTLSFSGVAQGE
RRRAGVGILTSPRLSAAVLEFSPVNERVASLRLQVVEGKALTVVCAYAPNSSSEYPAFLSVWVVSWKGSHLGIL
>g689.t2
MSWKLGCFPTSSWNTIXLALGTWNVTSLAGKEPELVREVERYQLDIVGLTSTHSIGSGTKLLERGWTLSFSGVAQGERRR
AGVGILTSPRLSAAVLEFSP
VNERVASLRLQVVEGKALTVVCAYAPNSSSEYPAFLSVWVVSWKGSHLGIL
>g689.t3
LETRRCMSWKLGCFPTSSWNTIXLALGTWNVTSLAGKEPELVREVERYQLDIVGLTSTHSIGSGTKLLERGWTLSFSGVA
QGERRRAGVGILTSPRLSAA
VLEFSPVNERVASLRLQVVEGKALTVVCAYAPNSSSEYPAFLSVWVVSWKGSHLGIL
>g690.t1
MGFNEFKELTGVLNAWKQNFMSMDRDRSGTMEAHEIQQALMSMGYKLSPQALGAIIRRYSSHGRIAFDDYVACCVRLRSL
TGVAQYRTLMATMTPRVLLL
LSLFAAAANGRRCRPLTIDFCQDVSYNTTCNGARSAAKGGGAEEIKRVVETRCSPYVAELMCRVVAPQGDPVTGMSTTPC
RAFCARPVALGAEQQTCEAI
NIPLCTRESYNHTVFPNLLGHASQEDAGLELHSYYPLVKVECSPHLKSFLCSVYTPECVEGRP
>g690.t2
MGFNEFKELTGVLNAWKQNFMSMDRDRSGTMEAHEIQQALMSMGYKLSPQALGAIIRRYSSHGRIAFDDYVACCVRLRSL
TGVAQYRTLMATMTPRVLLL
LSLFAAAANGRRCRPLTIDFCQDVSYNTTCNGARSAAKGGGAEEIKRVVETRCSPYVAELMCRVVAPQGDPVTGMSTTPC
RAFCARVQRDCAQPVALGAE
QQTCEAINIPLCTRESYNHTVFPNLLGHASQEDAGLELHSYYPLVKVECSPHLKSFLCSVYTPECVEGRP
>g690.t3
MGFNEFKELTGVLNAWKQNFMSMDRDRSGTMEAHEIQQALMSMGYKLSPQALGAIIRRYSSHGRIAFDDYVACCVRLRSL
TGVAQYRTLMATMTPRVLLL
LSLFAAAANGRRCRPLTIDFCQDVSYNTTCNGARSAAKGGGAEEIKRVVETRCSPYVAELMCRVVAPQGDPVTGMSTTPC
RAFCARVQRDCAQVLRERRI
PWPTRVRCSTLPTTHQQPDCVHPVALGAEQQTCEAINIPLCTRESYNHTVFPNLLGHASQEDAGLELHSYYPLVKVECSP
HLKSFLCSVYTPECVEGRP
>g691.t1
MVVEEVRHQEDAARCAKAVSQAQQGRWMKWDGVERRKITWNELWSMETSRLSFTIRATYDVLLSPTNLHLWYGEDPACLQ
CAAPANLKHILVGCKTSLTQ
GRYTWRHNQVLKCLAAELENNAMPPNYQTTHPKSQRPTCVQTGWSECEGVACGGGLQRLCSQFHHKAPEGSRDQSLTQHH
RPISLPRARGSMQSARARRH
ARGASGARPSAACHLTDDQAVAQHFGHCGCL
>g692.t1
LVVPLVGAPEEEEKEEEEEEEEEEEEEGCPRKEERRLGRGMLWYFYSSTVSTATPLDERKGHRCFCPPQVHYELLCLCSIQ
LKIVLLLFSVSHQKDRSSRR
PH
>g693.t1
LGTRVSRRALGISSVFLQGMRSSAVAVAPRPGEHAHTGPSQGLNGCLMEYRDKVRALEQLNQQLEEQIRLGLDRKASSAG
AWGALRRDWEDVYRQVSEAI
LDNARLMLQTENVQASAEDFKERYENEQPFRKAVEEEISSLYKVMEDARQTQADLEQQMENMRAEIQDLEQNHEEDVRVL

YHQIAGLDVDDLEAPIQTSL
DQILAYIRSHWEKVIQRNRAETDNYLEYKQDEGLGGKMSRGQEELEGLKTQCSESGSKIQSLQAETESIRALKRGLENSL
SDARHWHDIELQNLGSVVGR
LEAELGEVRGDIEQQRRDYDTLMGNKTRLEQDISAYHGVLDWEEKRYQPRHATTTAIACETQEATDTSRTSGASPAQ
>g693.t2
LGTRVSRRALGISSVFLQGMRSSAVAVAPRPGEHAHTGPSQGLNGCLMEYRDKVRALEQLNQQLEEQIRLGLDRKASSAG
AWGALRRDWEDVYRQVSEAI
LDNARLMLQTENVQASAEDFKERYENEQPFRKAVEEEISSLYKVMEDARQTQADLEQQMENMRAEIQDLEQNHEEDVRVL
YHQIAGLDVDDLEAPIQTSL
DQILAYIRSHWEKVIQRNRAETDNYLEYKDEGLGGKMSRGQEELEGLKTQCSESGSKIQSLQAETESIRALKRGLENSLS
DARHWHDIELQNLGSVVGRL
EAELGEVRGDIEQQRRDYDTLMGNKTRLEQDISAYHGVLDWEEKRYQPRHATTTAIACETQEATDTSRTSGASPAQ
>g694.t1
LQTRSNTLLLRAAKETQNSHRLHACLSPCLSAYFPEYLPTSFFASLSACVLVSLIVQYTFHPPEEEKKKMTKKKKKKKKK
KKKKKKKEEEKKKEEEEKGK
KKKKKKKKKKKKKKKKKS
>g694.t2
LKSHQRGWSTALLQTRSNTLLLRAAKETQNSHRLHACLSPCLSAYFPEYLPTSFFASLSACVLVSLIVQYTFHPPEEEKK
KMTKKKKKKKKKKKKKKKE
EEKKKEEEEKGKKKKKKKKKKKKKKKKKKS
>g695.t1
MADKHSILRCTYSAPTHSAVLLQGLSALRSRGQLLDVVLSVNEDRFQVHKAVLASCSDYF
>g696.t1
MTWAVFVGSTTAPPFENAQTWTGLRTNWITVNDDCLIKKRPVYIVVTVHTGNASCSSTSRAVVLNTIVKFPAPQNMAASW
TDHKLNLTWRGQGQSASADV
RVQSLENATQTWRHETNVETTANSMYWLLVKDLKRHMAYQVHVRQRATNFNHSLWSEWSSTLTVPAAARAGGVKYTMTYP
HHCCPRRGKKKSCGQTTVSS
THHSFFVTHSSVNFSIRATNAVGSSSPRYVMVPAEPAKDLNVAEMKDYVCYVYHGYQCVDRIPRLLDMCLSYSKEGVPVD
KPLGFMSLNETASSVALSWL
PIPPAGRRGVITHSILCHTKEHGTEECRNLTGPQTQFRLENLEPESRYNISLAMVTSKGSGPKAFLTVNTFPSDHPARDT
HRVWISLGLLVIFFVASIMC
SILVKR
>g696.t2
MAASWTDHKLNLTWRGQGQSASADVRVQSLENATQTWRHETNVETTANSMYWLLVKDLKRHMAYQVHVRQRATNFNHSLW
SEWSSTLTVPAAARAGGVKY
TMTYPHHCCPRRGKKKSCGQTTVSSTHHSFFVTHSSVNFSIRATNAVGSSSPRYVMVPAEPAKDLNVAEMKDYVCYVYHG
YQCVDRIPRLLDMCLSYSKE
GVPVDKPLGFMSLNETASSVALSWLPIPPAGRRGVITHSILCHTKEHGTEECRNLTGPQTQFRLENLEPESRYNISLAMV
TSKGSGPKAFLTVNTFPSDH
PARDTHRVWISLGLLVIFFVASIMCSILVKR
>g696.t3
MTWAVFVGSTTAPPFENAQTWTGLRTNWITVNDDCLIKKRPVYIVVTVHTGNASCSSTSRAVVLNTIDWLLVKDLKRHMA
YQVHVRQRATNFNHSLWSEW
SSTLTVPAAARAGGVKYTMTYPHHCCPRRGKKKSCGQTTVSSTHHSFFVTHSSVNFSIRATNAVGSSSPRYVMVPAEPAK
DLNVAEMKDYVCYVYHGYQC
VDRIPRLLDMCLSYSKEGVPVDKPLGFMSLNETASSVALSWLPIPPAGRRGVITHSILCHTKEHGTEECRNLTGPQTQFR
LENLEPESRYNISLAMVTSK
GSGPKAFLTVNTFPSDHPARDTHRVWISLGLLVIFFVASIMCSILVKR
>g697.t1
MVAEILTSSTTRSTQSIPGGTGCPEGIIASALILAIITCQCCDVRHTIRLRRKDTHTTLFSADCSHQPGPGLDSVPVRSA
QKATHW
>g698.t1
MSRRRAHSSADAGLSPDLHTAFTEPVGPDRIHGDFLDLDTDEELLCSVSEITEHLGRNISVVLETALTEIRKMVSVRIRV
LKME
>g699.t1
MGSAGGLPGAEQGTSGRTLPEAGSEGGNLPEAGGGGATLWTQGTQLGAESLERLQTRGAESLERLQTREAESLERLQTRG

GLQEDYQGRSRETQVEPFRR
QGRNPVDSGNAAGTRASGEAEATDSGAESLERLQTRGAGVSRTSPTGDSCGPAAETGGCCGPSSWAAELRAWWPVSHTAV
LPVPDGFPRIGWPQMACSEA
WWPVWLTEVPPVPNGCTSLGYSLLGPFVGSSLVTASSTCCQCSFVIIIIIIIIIIIIIITIIIIIITSSSSTMSAA
>g699.t2
MGSAGGLPGAEQGTSGRTLPEAGSEGGNLPEAGGGGATLWTQGTQLGAESLERLQTRGAESLERLQTREAESLERLQTRG
GLQEDYQGRSRETQVEPFRR
QGRNPVDSGNAAGTRASGEAEATDSGAESLERLQTRGAGVSRTSPTGDSCGPAAETGGCCGPSRIGWPQMACSEAWWPVW
LTEVPPVPNGCTSLGYSLLG
PFVGSSLVTASSTCCQCSFVIIIIIIIIIIIIIITIIIIIITSSSSTMSAA
>g700.t1
LPTPPKNRHLNVVEGFVCPCDPGSCVVGGKSPWETGAPYWSQTWEGRSPASVWWPGFDPWGLAGHSPKQLHGGAAALWAH
HLQG
>g700.t2
MLANIITSWKADLNQQRFFKLSSAPVENKALHLLLFLGVVANSQRLNRHLNVVEGFVCPCDPGSCVVGGKSPWETGAPYW
SQTWEGRSPASVWWPGFDPW
GLAGHSPKQLHGGAAALWAHHLQG
>g700.t3
MLANIITSWKADLNQQRFFKLSSAPVENKALHLLLFLGVNRHLNVVEGFVCPCDPGSCVVGGKSPWETGAPYWSQTWEGR
SPASVWWPGFDPWGLAGHSP
KQLHGGAAALWAHHLQG
>g701.t1
MFSLLLNPPSPPPPSSLTADDFADHFTKKVTDISVSLTPAATFPNPTSTAILPPPAPASARLNSFTPLSSEEVLKLVTSN
HATTCPLDPIPSSLLQAIST
DILPFVTSLVNSSLTSGLVRTPFKTVRVKPLLKKPTLDTADILNYRPVSLLSFLPKTLEHTLIATRISECLADISSWTTA
HQLKLNLAKTELFIPGKDCP
HRDLLVSVEGITVSASLTARNLGKMLLLFYHAVVESILRYGISAWFGNLSVQLKAQITRLTQRAMKIMGVRQHPTLQAIF
DKTIIRQAQKIISEPTHVLH
PEYQLLPSGRRFRVPQCRLNHFKESFVPLSIKALNGKLARA
>g701.t2
MFSLLLNPPSPPPPSSLTADDFADHFTKKVTDISVSLTPAATFPNPTSTAILPPPAPASARLNSFTPLSSEEVLKLVTSN
HATTCPLDPIPSSLLQAIST
DILPFVTSLVNSSLTSGLVRTPFKTVRVKPLLKKPTLDTADILNYRPVSLLSFLPKTLEHTLIATRISECLADISSWTTA
HQLKLNLAKTELFIPGKDCP
HRDLLVSVEGITVSASLTARNLGVIVDNQLSCTANITAVTRYCRFALYNFDRSL
>g701.t3
MFSLLLNPPSPPPPSSLTADDFADHFTKKVTDISVSLTPAATFPNPTSTAILPPPAPASARLNSFTPLSSEEVLKLVTSN
HATTCPLDPIPSSLLQAIST
DILPFVTSLVNSSLTSGLVRTPFKTVRVKPLLKKPTLDTADILNYRPVSLLSFLPKTLEHTLIATRISECLADISSWTTA
HQLKLNLAKTELFIPGKDCP
HRDLLVSVEGITVSASLTARNLGVIKMLLLFYHAVVESILRYGISAWFGNLSVQLKAQITRLTQRAMKIMGVRQHPTLQA
IFDKTIIRQAQKIISEPTHV
LHPEYQLLPSGRRFRVPQCRLNHFKESFVPLSIKALNGKLARA
>g702.t1
REEGHSSSDEREREREERESPAKEQHVTTMQVSKSIYFSICEEPESTLAREVHPHSEGECSPSECVFYSRSSSPKSDSEL
VVKPQESSGPQMQWNWGGFP
KVCPSERSPVESQRISTSDYSHFRTIERQDSFDDNDDFQTTVRCSRGGRVTVVRPLPRTMSLDLDLLSLEASTPTLEITP
HLTHSTPNGLPDTPASLATV
STDSCDGQKTLAGGFRSIPEQEDRRTSPNCTESKACTAITDTLASDKMISISPGGSVFVTAQHSRVETLGGADISEQYSA
TLEPSEMGAMNTIPVGDGDF
TEANEAGKRSQHLGSTGIYLDDLTKLDPEVAALYFPKSDSEAASPSRADQGSNSGTQSPQSVGSGAMDSGTEYLSDSTAD
PMDISMSLCGCVGDRSRISQ
EKFKEHIVTYKDFIKNPGIIEDPNLVICINSQYYNWAVAAPMILSKQAFQKNLPKSAVDRLVKDKMLKKSGRWWFSWGRR
EMDINQPEPSTQEQEVSLFA
STTLKATLDDMYSDEEAGLGRQAVMPSDSPSTDTMSTCQCISQMYRKSLRLTSEQIERLKLREGANQVVFSVTTQYQGTC
RCEAAIYLWNWDDRVIISDI

DGTITK
>g702.t2
REEGHSSSDEREREREEERESPAKEQHVTTMQVSKSIYFSICEEPESTLAREVHPHSEGECSPSECVFYSRSSSPKSDSEL
VVKPQESSGPQMQWNWGGFP
KVCPSERSPVESQRISTSDYSHFRTIERQDSFDDNDDFQTTVRCSRGGRVTVVRPLPRTMSLDLDLLSLEASTPTLEITP
HLTHSTPNGLPDTPASLATV
STDSCDGQKTLAGGFRSIPEQEDRRTSPVRATDLSEFARPEDSQNKDLTTTIVESSVIDNVVSLSQNCTESKACTAITDT
LASDKMISISPGGSVFVTAQ
HSRVETLGGADISEQYSATLEPSEMGAMNTIPVGDGDFTEANEAGKRSQHLGSTGIYLDDLTKLDPEVAALYFPKSDSEA
ASPSRADQGSNSGTQSPQSV
GSGAMDSGTEYLSDSTADPMDISMSLCGCVGDRSRISQEKFKEHIVTYKDFIKNPGIIEDPNLVICINSQYYNWAVAAPM
ILSKQAFQKNLPKSAVDRLV
KDKMLKKSGRWWFSWGRREMDINQPEPSTQEQEVSLFASTTLKATLDDMYSDEEAGLGRQAVMPSDSPSTDTMSTCQCIS
QMYRKSLRLTSEQIERLKLR
EGANQVVFSVTTQYQGTCRCEAAIYLWNWDDRVIISDIDGTITK
>g703.t1
MEFKSKQWFGASVRSDGEHILLKFLQKGRDSAKLALVQISS
>g703.t2
NRKTDKGVQMEFKSKQWFGASVRSDGEHILLKFLQKGRDSAKLALVQISS
>g704.t1
MPFGGGNKCGRCQKTVYFAEEVQCEGQSWHKSCFQCMVCHKSLDSTTVAVHLDEIFCKSCYGKKFGPKGYGFGGGAGTLS
MDRGEGLGIKHDAPAAHQPT
NNPNASKFAKTAGGSDACPRCGQAVYAAEKVIGGGNVRPFT
>g704.t2
MPFGGGNKCGRCQKTVYFAEEVQCEGQSWHKSCFQCMVCHKSLDSTTVAVHLDEIFCKSCYGKKFGPKGYGFGGGAGTLS
MDRGEGLGIKHDAPAAHQPT
NNPNASKFAKTAGGSDACPRCGQAVYAAEKVIGGGNVRPFT
>g704.t3
MPFGGGNKCGRCQKTVYFAEEVQCEGQSWHKSCFQCMVCHKSLDSTTVAVHLDEIFCKSCYGKKFGPKGYGFGGGAGTLS
MDRGEGLGIKHDAPAAHQPT
NNPNASKFAKTAGGSDACPRCGQAVYAAEKVIGGGN
>g705.t1
LSIRTLHHHLQYCSPLRSLHPPHLNPYHHHHHLHHCSPLRSLHPPHLHLPHSCTSSHQSVKLLKFADDTTLIGLISDGDE
SAH
>g706.t1
MHRLPASTHASVFHFTRAASTQQRASRTQRAPRVSQFPGARRGHVDRQTKKPGRFDPVFDELHRGCRASPRRSGEASKTK
REEREPERSERRQRGSKQEG
KLFPRLSSFILNVFW
>g707.t1
MADTQREMLYSLMATGSNDFLCRSVVHLGERSLLLKVLLRWARVEKRGGGRNEPCPEQPPPRKVCVKRITSLTELDPDVL
ESMHSLGCFRDRGKLTRDLQ
CEELSVPRHQDNDGGKKCGSRAETRMRLVVVVIRLPGARSDPQCQQFPAHITLCQFVSTENQEKMIYYLLLDRKERYPSC
EDEDLPPRNDLDPPRKRVDS
PMLTRHGRCRPERKSLEVLSVTEQGSPTPPRRALETSAHSQRYAHPPQQGRT
>g707.t2
MATGSNDFLCRSVVHLGERSLLLKVLLRWARVEKRGGGRNEPCPEQPPPRKVCVKRITSLTELDPDVLESMHSLGCFRDR
GKLTRDLQCEELSVPRHQDN
DGGKKCGSRAETRMRLVVVVIRLPGARSDPQCQQFPAHITLCQFVSTENQEKMIYYLLLDRKERYPSCEDEDLPPRNDLD
PPRKRVDSPMLTRHGRCRPE
RKSLEVLSVTEQGSPTPPRRALETSAHSQRYAHPPQQGRT
>g707.t3
MLYSLMATGSNDFLCRSVVHLGERSLLLKVLLRWARVEKRGGGRNEPCPEQPPPRKVCVKRITSLTELDPDVLESMHSLG
CFRDRGKLTRDLQCEELSVP
RHQDNDGGKKCGSRAETRMRLVVVVIRLPGARSDPQCQQFPAHITLCQFVSTENQEKMIYYLLLDRKERYPSCEDEDLPP
RNDLDPPRKRVDSPMLTRHG
RCRPERKSLEVLSVTEQGSPTPPRRALETSAHSQRYAHPPQQGRT

>g708.t1
MIPGSLSNVSKDDRELGKAVLVATYSFNNQSNDAYLFKTSEVERAQRQIVKGVRYILEVRLSRTMCLKRQGWDLLDCDVQ
PEGHLHQVAHPGGLHPSGKC
VWKTDSKECCFHRELYRSFSKD
>g709.t1
MNLQVFQEASLLVAELLKLPLKVLGPALSSEPSVLLVLTATAVSSPSERTEEWRREDRGRRSGEERGKKTEGADGGGRAG
RLPLMFRRLRLLAGTQHRGH
RATGPPAGARSGERSGQSGTYPAYQTDEYPQNPGMPSLLIKGTDIAC
>g710.t1
MELSDLSFPLGSAEDFYDDPCFNTSDMHFFDDLDPRLASVHVGLAKPDDSRHHHHHLHHHLHEEEEDEEEEDGDGHVRAP
GGHHGAGRCLLWACKACKRK
TTSADRRKAATMRERRRLGKVNDAFETLKRCTSSNPTQRLPKVEILRNAISYIESLQSLLRGPGEEGYYYPAQEPYSGED
SDLGSSPRSNCSDGMMDFSG
PSYQVQSARRAPYDRSFTDSPAGKTLRDPH
>g711.t1
MDQAVKYIEDMQDEFDLGYKTLQSQDPAEKTTDIMKEAVTRLQEMLNRLDYKRKEILSKMNDVIKEIDNMVTCQLHPELL
EWRRKQQVSIIGGPLLTGID
LLQNWFTGTALSLFQIKRQLDKLGELVVKVTYEGDPIPQQTPHMQESVKHLVTLVIKSSFVVETQPCMPTHLQRSLIIKT
GVQFTTKV
>g711.t2
MDQAVKYIEDMQDEFDLGYKTLQSQDPAEKTTDIMKEAVTRLQEMLNRLDYKRKEILSKMNDVIKEIDNMVTCQLHPELL
EWRRKQQVSIIGGPLLTGID
LLQNWFTGTALSLFQIKRQLDKLGELVVKVTYEGDPIPQQTPHMQESVKHLVTLVIKSSFVVETQPCMPTHLQRSLIIKT
GVQFTTKVRYVSHNS
>g712.t1
MHETHYREEVDRLTKWCKQNNLLLNVSKTKEIVVDFRRGHTEHPPLTIDGAAVERVSSTKFLGVHVSEDLSWTTNTASLA
KKAQRCLYFLRKLKRAKAPI
PVLKSFYLDIILKASCPAASLCGAVAAPNTAEKPCNA
>g713.t1
MDRSDGTVKYVLSGDGAGTVFVIDENNGDLHATRRLDREEKSYYTLRATVVNKQTGYKLEPETDFVIKLHDINDNEPQFS
KEVYIGRVPERSDIGTSVIQ
VTATDADDSMYGNSAKLVYSISQGHPYFSVDPNTGTSVIQVTATDADDSMYGNSAKLVYSISQGHPYFSVDPNTGVIRTA
LHAEDMDREQREHYQVVIEA
KDMAGQRGGLTGVALVNITLTDVNDNPPLFTQTIFQFSVLESSKAGTPVGRIQATDKDIGQNAEMFFTIAKGDGMDMFEI
STDKATQEGVITISKPLDYE
RKRSYTVEIRVQNTVLDTRFHSTDSNNTATVRISVDDVDEAPLFNMASYLMEVKEDAALGAAVGTVSALDPDAAHSPIR
>g713.t2
MDRSDGTVKYVLSGDGAGTVFVIDENNGDLHATRRLDREEKSYYTLRATVVNKQTGYKLEPETDFVIKLHDINDNEPQFS
KEVYIGRVPERSDIGTSVIQ
VTATDADDSMYGNSAKLVYSISQGHPYFSVDPNTGTSVIQVTATDADDSMYGNSAKLVYSISQGHPYFSVDPNTGVIRTA
LHAEDMDREQREHYQVVIEA
KDMAGQRGGLTGVALVNITLTDVNDNPPLFTQTIFQFSVLESSKAGTPVGRIQATDKDIGQNAEMFFTIAKGDGMDMFEI
STDKATQEGVITISKPLDYE
RKRSYTVEIRVQNTVLDTRFHSTDSNNTATVRISVDDVDEAPLFNMASYLMEVKEDAALGAAVGTVSALDPDAAHSPIRC
HAKVLIP
>g713.t3
MDRSDGTVKYVLSGDGAGTVFVIDENNGDLHATRRLDREEKSYYTLRATVVNKQTGYKLEPETDFVIKLHDINDNEPQFS
KEVYIGRVPERSDIGTSVIQ
VTATDADDSMYGNSAKLVYSISQGHPYFSVDPNTGTSVIQVTATDADDSMYGNSAKLVYSISQGHPYFSVDPNTGVIRTA
LHAEDMDREQREHYQVVIEA
KDMAGQRGGLTGVALVNITLTDVNDNPPLFTQTIFQFSVLESSKAGTPVGRIQATDKDIGQNAEMFFTIAKGDGMDMFEI
STDKATQEGVITISKPLDYE
RKRSYTVEIRVQNTVLDTRFHSTDSNNTATVRISVDDVDEAPLFNMASYLMEVKEDAALGAAVGTVSALDPDAAHSPIRL
QLRLCCTCQAAVEIDQVQGW
APQVQGLPA
>g714.t1

LVTSNHATTCPLYPIPSSLLQAISTDILPVITTLINSSLTSGLVLAPFKKPTLDNADSLNYRPVSLLSFLSKTLECAVSN
QLSSYLSENDLIDPHQSDFK
TAHSTETALLMVTESLRAAKASSLLLVLVLLDLSAAFDTVNHQTLLATLAELGITDTALAWFTSYLMDRTYQVTWNGSLA
KPRSLNTGVPQGSVWWNALP
ANVRTAESLPIFRKRLKTHLFRVHLNPA
>g715.t1
MSPQRFGQPRKRRRSECAGTAGPCEDKVKLVPSSVDVEVAQDNQRSMSSGKQLRRASISSKKSWKGPGGQ
>g716.t1
LSTALRRAVASNASTIALTILSHHA
>g717.t1
VEAHDPTSKSITVSWKAPDYDGGCAIQGYILEKMVKDGDRYERINQNLVAGLSYVVKDLQEDMEYQFRVRAENAAGVSEP
SRSTQLIKAADPVEKPKVTL
HARVQSGVCVKKGQEVRIDAYITGCPYPKITWLRNDENVTKEQTKKITPLVKKKKSKAKVPEPEEEFVTPMRERLGLDET
QKGQAALMIRDTVRADHGVF
TIQVENQHGASTASCVVNILDKPAAPINFTYDEIRNTSMVCQWEAPGDDGGSEILNYILEKKDNNNDEIGWITITSTLKG
TRYPVTKLIEGKEYIFRVTA
ENRFGVGPPCISEPVVAKNQFDPPDAPNTPRVHEVTACSARISWHEPKDNGSPILGYWIERKEVNSKHWTRVNRALLNSL
EVKVTGLMEGLTYIFRVCAE
NLAGPGPFSEPTDRTVAMDAILPPGPPIPWVVDTGKDSIMIAWKPPLYNGGGDILGYHVEKVVAGEKEWSRSTEFRCKEL
SYTVSGLTEGTDYYIRVLAV
NDAGPGAPGVTEPVTVKEPQVKNGVIIRAGEALRLPAVVSGRPQPEVKWSKDEGEVDQERGLVETEGRNSVLFIRKAVRA
DHGKYQVTGTNSSGTKSAET
RVDVMDVPGPVVDLKTVAVTKKNITLTWADPLDNGGSDIIGYEVLKKDAKMKLFRQPVETASCKCDIQDLLVGEEYDFRV
IARNKYGDGAPADLGPILAV
DPKGTPSAPEKLHYTDRGKTSISLAWQLPYTDGGSPIMGYYVEKMRSDSNEFDVAGRMFPECCGTIENLSENQEYQFRVF
AVNEVGNGLKSKPISAKIQD
DEIPPAISIMKYFKSNSITVKKGAAVDIPAEVKGLPLPTIYWMKDDVVIDKPDPEKMTLETEEVNRTTLQTKIVLPETIR
QDKGNYKLTAENCYGKAQHV
IRVEILDRPMPPRNIVVSDIKSESCYLTWDAPEDNGGSDITNYIVEQRDASKKKSDFEVLTINLIDRRYGVYKLFMNGAY
QFRIKAENKFGVSDGCDSEK
VQLRDPFGLPGPPRNPKIISATASTMTLTWEPPLDNGGSSIQGYWLEKRERGAVYWGKVNRAPVTKPAVKALQYTVLRLI
EGTEYQFRIAACNAAGIGPH
SEYSECRFAVDPCNPPSAPGCPEVKDKTKSSITLSWTPPDKDGGSPIKGYIIEVHEDGSPDWRRVNPADKLHPSTEITVP
DLKEGKKCRFRIYAVNAAGN
SEPSAKTADIVVQDILIEPNISLDVNAYDLLTCRAGTSIRIPVSISGRPAPTITWTFDGPAETEKKNELHTLPIDSEVKN
NSLV
>g717.t2
VEAHDPTSKSITVSWKAPDYDGGCAIQGYILEKMVKDGDRYERINQNLVAGLSYVVKDLQEDMEYQFRVRAENAAGVSEP
SRSTQLIKAADPVEKPKVTL
HARVQSGVCVKKGQEVRIDAYITGCPYPKITWLRNDENVTKEQTKKITPLVKKKKSKAKVPEPEEEFVTPMRERLGLDET
QKGQAALMIRDTVRADHGVF
TIQVENQHGASTASCVVNILDKPAAPINFTYDEIRNTSMVCQWEAPGDDGGSEILNYILEKKDNNNDEIGWITITSTLKG
TRYPVTKLIEGKEYIFRVTA
ENRFGVGPPCISEPVVAKNQFDPPDAPNTPRVHEVTACSARISWHEPKDNGSPILGYWIERKEVNSKHWTRVNRALLNSL
EVKVTGLMEGLTYIFRVCAE
NLAGPGPFSEPTDRTVAMDAILPPGPPIPWVVDTGKDSIMIAWKPPLYNGGGDILGYHVEKVVAGEKEWSRSTEFRCKEL
SYTVSGLTEGTDYYIRVLAV
NDAGPGAPGVTEPVTVKEPQVKNGVIIRAGEALRLPAVVSGRPQPEVKWSKDEGEVDQERGLVETEGRNSVLFIRKAVRA
DHGKYQVTGTNSSGTKSAET
RVDVMDVPGPVVDLKTVAVTKKNITLTWADPLDNGGSDIIGYEVLKKDAKMKLFRQPVETASCKCDIQDLLVGEEYDFRV
IARNKYGDGAPADLGPILAV
DPKGTPSAPEKLHYTDRGKTSISLAWQLPYTDGGSPIMGYYVEKMRSDSNEFDVAGRMFPECCGTIENLSENQEYQFRVF
AVNEVGNGLKSKPISAKIQD
DEIPPAISIMKYFKSNSITVKKGAAVDIPAEVKGLPLPTIYWMKDDVVIDKPDPEKMTLETEEVNRTTLQTKIVLPETIR
QDKGNYKLTAENCYGKAQHV
IRVEILDIKSESCYLTWDAPEDNGGSDITNYIVEQRDASKKKSDFEVLTINLIDRRYGVYKLFMNGAYQFRIKAENKFGV

SDGCDSEKVQLRDPFGLPGP
PRNPKIISATASTMTLTWEPPLDNGGSSIQGYWLEKRERGAVYWGKVNRAPVTKPAVKALQYTVLRLIEGTEYQFRIAAC
NAAGIGPHSEYSECRFAVDP
CNPPSAPGCPEVKDKTKSSITLSWTPPDKDGGSPIKGYIIEVHEDGSPDWRRVNPADKLHPSTEITVPDLKEGKKCRFRI
YAVNAAGNSEPSAKTADIVV
QDILIEPNISLDVNAYDLLTCRAGTSIRIPVSISGRPAPTITWTFDGPAETEKKNELHTLPIDSEVKNNSLV
>g717.t3
VEAHDPTSKSITVSWKAPDYDGGCAIQGYILEKMVKDGDRYERINQNLVAGLSYVVKDLQEDMEYQFRVRAENAAGVSEP
SRSTQLIKAADPVEKPKVTL
HARVQSGVCVKKGQEVRIDAYITGCPYPKITWLRNDENVTKEQTKKITPLVKKKKSKAKVPEPEEEFVTPMRERLGLDET
QKGQAALMIRDTVRADHGVF
TIQVENQHGASTASCVVNILDKPAAPINFTYDEIRNTSMVCQWEAPGDDGGSEILNYILEKKDNNNDEIGWITITSTLKG
TRYPVTKLIEGKEYIFRVTA
ENRFGVGPPCISEPVVAKNQFDPPDAPNTPRVHEVTACSARISWHEPKDNGSPILGYWIERKEVNSKHWTRVNRALLNSL
EVKVTGLMEGLTYIFRVCAE
NLAGPGPFSEPTDRTVAMDAILPPGPPIPWVVDTGKDSIMIAWKPPLYNGGGDILGYHVEKVVAGEKEWSRSTEFRCKEL
SYTVSGLTEGTDYYIRVLAV
NDAGPGAPGVTEPVTVKEPQVKNGVIIRAGEALRLPAVVSGRPQPEVKWSKDEGEVDQERGLVETEGRNSVLFIRKAVRA
DHGKYQVTGTNSSGTKSAET
RVDVMDVPGPVVDLKTVAVTKKNITLTWADPLDNGGSDIIGYEVLKKDAKMKLFRQPVETASCKCDIQDLLVGEEYDFRV
IARNKYGDGAPADLGPILAV
DPKGTPSAPEKLHYTDRGKTSISLAWQLPYTDGGSPIMGYYVEKMRSDSNEFDVAGRMFPECCGTIENLSENQEYQFRVF
AVNEVGNGLKSKPISAKIQD
DEIPPAISIMKYFKSNSITVKKGAAVDIPAEVKGLPLPTIYWMKDDVVIDKPDPEKMTLETEEVNRTTLQTKIVLPETIR
QDKGNYKLTAENCYGKAQHV
IRVEILDRPMPPRNIVVSDIKSESCYLTWDAPEDNGGSDITNYIVEQRDASKKKSDFEVLTINLIDRRYGVYKLFMNGAY
QFRIKAENKFGVSDGCDSEK
VQLRDPFGLPGPPRNPKIISATASTMTLTWEPPLDNGGSSIQGYWLEKRERGAVYWGKVNRAPVTKPAVKALQYTVLRLI
EGTEYQFRIAACNAAGIGPH
SEYSECRFAVDPCNPPSAPGCPEVKDKTKSSITLSWTPPDKDGGSPIKGYIIEVHEDGSPDWRRVNPADKLHPSTEITVP
DLKEGKKCRFRIYAVNAAGN
SEPSAKTADIVVQDILIEPNISLDVNAYDLLTCRAGTSIRIPVSISGRPAPTITWTFDGPAETEKKNELHTLPIDSEVVS
TDTSSVVMIPDSKLNHTGRY
ILNAESLAGHKVLKVRVNVLDLPGAPQDLKVSDVTRGTCRLTWKPPQSDGGERVKSYFIERKTVQGKAWTKVNPACSAQS
LVVTDLIGGQEYLFRVRAEN
RFGFGPYAETVDSSMARDPIHPPDPPTRLKIQAVKKDTITLTWKAPKNDGGSPVTHYSMDLLCWDASGTQKESWRRL
>g717.t4
MSMFSDLPGAPQDLKVSDVTRGTCRLTWKPPQSDGGERVKSYFIERKTVQGKAWTKVNPACSAQSLVVTDLIGGQEYLFR
VRAENRFGFGPYAETVDSSM
ARDPIHPPDPPTRLKIQAVKKDTITLTWKAPKNDGGSPVTHYSMDLLCWDASGTQKESWRRL
>g717.t5
MSMFSDLPGAPQDLKVSDVTRGTCRLTWKPPQSDGGERVKSYFIERKTVQGKAWTKVNPACSAQSLVVTDLIGGQEYLFR
VRAENRFGFGPYAETVDSSM
ARDPIHPPDPPTRLKIQAVKKDTITLTWKAPKNDGGSPVTHYSMDLLCWDASGTQKESWRRY
>g718.t1
LVIGYCIVLLDTQRDLKKGIKDAKMAYKQRIEDHFTDADPWRVWQGIQHIYRNCNAIPREGNPTLAEGLNYFFAHFEVKG
PAAEMIPPASNNHTLTVQPQ
DVMHLLRAVNARKAAGPEGIPGRILKVCAVLNSQSFLPTYSTVPVTVHYPEVNHHYSGHQAVRHQHPQ
>g719.t1
MVRWCLMSHSSEHLCVSGVKNISANLLEKVTVGQTGADGSAWVEQEMELLKFIVLLLVAVFGQVYVALGSPLSSEDGEMW
NTESWQGFPPEGALNLRLAD
LMKRSRAQQFHGLMGRSTGAGNRGEMFVGLMGRRSLRGDVEEELLPKYY
>g719.t2
MELLKFIVLLLVAVFGQVYVALGSPLSSEDGEMWNTESWQGFPPEGALNLRLADLMKRSRAQQFHGLMGRSTGAGNRGEM
FVGLMGRRSLRGDVEEELLP
KYY

>g719.t3
MVRWCLMSHSSEHLCVSGVKNISANLLEKVTVGQTGADGSAWVEQVAVFGQVYVALGSPLSSEDGEMWNTESWQGFPPEG
ALNLRLADLMKRSRAQQFHG
LMGRSTGAGNRGEMFVGLMGRRSLRGDVEEELLPKYY
>g720.t1
MHSRPNELAESENDQTFYGFSANEMGDQNSCFEDGPEDIPDPLLSEKKTSSKRPGGAQRFTLRVSLRLRSNTPHSNDKDG
EATKAEEKEVKEEPVMKKKG
MANPRKKRRVKFEEEEMAVAPLPVKRGSDSEDDSKETFLSKRDQNIRDNKAMLAQLMAALQEMPGEVIFDGVTLLQQKLP
RSAGESRRNPERATRRQTRS
MGGCEDPSGPPDKELELSLEDELLEVRHAPRRGTPRPSQSKPHAVRPVEDITEEEIQLVADNMTEKVYNKATGSTCHQCR
QKTIDTKTCCRSEECRGVQG
QFCGPCLRNRYGEDVKKALLDPGWQCPPCRGICNCSFCRQREGRCPTGILFPLAQYHGFSDVHSYLSR
>g720.t2
MHSRPNELAESENDQTFYGFSANEMGDQNSCFEDGPEDIPDPLLSEKKTSSKRPGGAQRFTLRVSLRLRSNTPHSNDKDG
EATKAEEKEVKEEPVMKKKG
MANPRKKRRVKFEEEEMAVAPLPVKRGSDSEDDSKETFLSKRDQNIRDNKAMLAQLMAALQEMPGEVIFDGVTLLQQKLP
RSAGESRRNPERATRRQTRS
MGGCEDPSGPPDKELELSLEDELLEVRHAPRRGTPRPSQSKPHAVRPVEDITEEEIQLVADNMTEKVYNKATGSTCHQCR
QKTIDTKTCCRSEECRGVQG
QFCGPCLRNRYGEDVKKALLDPGWQCPPCRGICNCSFCRQREGRCPTGILFPLAQYHGFSDVHSYLSSLRNKLKSGEEDE
DL
>g720.t3
MHSRPNELAESENDQTFYGFSANEMGDQNSCFEDGPEDIPDPLLSEKKTSSKRPGGAQRFTLRVSLRLRSNTPHSNDKDG
EATKAEEKEVKEEPVMKKKG
MANPRKKRRVKFEEEEMAVAPLPVKRGSDSEDDSKETFLSKRDQNIRDNKAMLAQLMAALQEMPGEVIFDGVTLLQQKLP
RSAGESRRNPERATRRQTRS
MGGCEDPSGPPDKELELSLEDELLEVRHAPRRGTPRPSQSKPHAVRPVEDITEEEIQLVADNMTEKVYNKATCRQKTIDT
KTCCRSEECRGVQGQFCGPC
LRNRYGEDVKKALLDPGWQCPPCRGICNCSFCRQREGRCPTGILFPLAQYHGFSDVHSYLSR
>g721.t1
MLVAFKWRHLFKEHDEKTLTSVRGDALSPLCSCQTATTRPINSADVKLQQAVGRGHFATVWQGEYQGSAVAVKVFPAGHK
HEFSSERDIYELPLMEHSGI
AHFLGAGQSRDGGQWLMVLELASSVRMHKPVVAHRDLSSSNVLVRADGTCALSDFGCSTVLRSCWGRPGWQCPRSMEEPG
QVGSLRYMSPEILEGSVDLS
SGRCLVQADVYSLGLLLWEIWTRCPDLVHDGSVPEHCLPYETELGSSVTLDRLLFYVSQMNKRPSIPEQWEWDTRVGDLQ
YS
>g721.t2
MLVAFKWRHLFKEHDEKTLTSVRGDALSPLCSCQTATTRPINSADVKLQQAVGRGHFATVWQGEYQGSAVAVKVFPAGHK
HEFSSERDIYELPLMEHSGI
AHFLGAGQSRDGGQWLMVLELASSVRMHKPVVAHRDLSSSNVLVRADGTCALSDFGCSTVLRSCWGRPGWQCPRSMEVGS
LRYMSPEILEGSVDLSSGRC
LVQADVYSLGLLLWEIWTRCPDLVHDGSVPEHCLPYETELGSSVTLDRLLFYVSQMNKRPSIPEQWEWDTRVGDLQYS
>g722.t1
TPAEEQQQQQEEEPRGRRGAMGKATPTPDEAREACSSVASVAPSTSDGAGPPGAGQEGSRHAPSQVLSNGGPPQDMLDTL
YRTVEATIAVVTKLSGTMTS
PSSS
>g723.t1
MSEPKGTNLFRIGTSTGEIRTKRRMSDNDLKTHPLVVLVSDNGEPSLTATVSIDVVVVENTGDIQTQFRHVPLKEDSFSA
LNLYLLIAIASVSVIFLLSL
ISLIAVKCHRTDNSFSRYSAPMITTHPDGSWSYSKSTQQYDVCFSSDTLKSDVVVFPAQFPPVDAELISINGGDTLNRTR
TLPNTDTVGHML
>g724.t1
LSNSAPTSPPPEHSARAPLRGACWSANTKAVVKKAQQRLHFLRVLRKSNINQKLLLTFYRSSVESILTYCTSVWFSLCTE
ADRKKLQRVTKTAQKIVGCP
LPSLKDLYTSRCLSRAQTIIRDITHPAHHLFDLLPSGRRYRCIRARTNRLKDSFFPKAVTILNAHMH
>g725.t1

MPDTMSLEYTIDIQLTELGFPDILQTYPAAVRESPRLSASQYVSLSGAPTKPGTMCSDDAFTFSVLLGTKKRRRGKHVIS
EIPGGLAAGEKADDEPHQGV
NDEQVANKNTQDVEDESADDSEVSEDYDEESLDEDQDCFDDESGDTGDYRCGVCGLQLASNMKLLDHMNLHTGQRPHCCS
ECGKRFCQLGNYRAHLKTHA
KGEPELVHSLWCPLCTKPFGSKEDLRGHLTTKHAERDFYECDSCKLLFTERKECKKHIEWHKSTHRRFACDTCGRRFSTQ
RSLKHHRNRKCLRSFACTDC
DRTFSRKNALLKHSFSHVGLLPYTCMLCCCHFRLARLYRQHKCNPQSVHCVACLRAFSSMEDFRQHKKDTGCWGHQEPKG
GDIRCPECGESFATREELKK
HGGAHQRVLTCAECGKGFRSALLLMSHMGGHAVQTPCLCQSCGRGFLHQLDYDRHLERCGLAPLSPVVTKKRAQKSWAPP
DQPTPPPDQPTPPPDQPTPP
PDQPTPPPVTDLPVASAASASGSDPASAAVPVPVPVPAPSPETPATGPWRLTLDKPPPVGHQLILFFPVNPRVGAGWKGA
VQPPTQAILDASVELHRRTA
GQRTSSAAGSRREVADDNMPLDLSMKSTRLVHANGQCSWFEMQQFIGDLITSGNTSSQSLKEQHDVLHVSWGLAGGEYLG
MKGAAQDKSDLDPNRLMLSG
TPERRAEFRDRKEVENELHACTCSGCPFSTSPPSYETLKPRKSLYSQTQPDKACLSKNHTSNPAMNLSLCLPEENPAEPS
STTTSDLKPRGSRRDDRERG
ADCSSSRGMTGTFPHFPMFACLCCHGLQTCAQLLRKQDGSDSALPHGNAHGHHFHHHHHCPFSSCLPCPQLAHSHPHAAQ
LRGHFPYLGCQHSFGGCPQP
SHKQGAQKSGQHPEDGARAAMFPSSLHPCMHCPAAFQRPSQLLQHQRSEHSQKPAGFLCTECGKAFNSHSNLRIHQNVHT
GARPYVCPDCGKSFSQSGAL
KIHKRIHTGERPYSCAFCGRGFPHLAGVRAHQRTHTGEKPYRCGLCGKCFTQSGALKIHTRMHTGERPFTCGVCEKGFTN
LAAIRFHYRTVHGLTYEAAG
RPAAGGQYRDQANPGCP
>g725.t2
MPDTMSLEYTIDIQLTELGFPDILQTYPAAVRESPRLSASQYVSLSGGDGHTHLPTKPTTRKLLEKDPSVSQSSQPSRDT
RGSSPAPTKPGTMCSDDAFT
FSVLLGTKKRRRGKHVISEIPGGLAAGEKADDEPHQGVNDEQVANKNTQDVEDESADDSEVSEDYDEESLDEDQDCFDDE
SGDTGDYRCGVCGLQLASNM
KLLDHMNLHTGQRPHCCSECGKRFCQLGNYRAHLKTHAKGEPELVHSLWCPLCTKPFGSKEDLRGHLTTKHAERDFYECD
SCKLLFTERKECKKHIEWHK
STHRRFACDTCGRRFSTQRSLKHHRNRKCLRSFACTDCDRTFSRKNALLKHSFSHVGLLPYTCMLCCCHFRLARLYRQHK
CNPQSVHCVACLRAFSSMED
FRQHKKDTGCWGHQEPKGGDIRCPECGESFATREELKKHGGAHQRVLTCAECGKGFRSALLLMSHMGGHAVQTPCLCQSC
GRGFLHQLDYDRHLERCGLA
PLSPVVTKKRAQKSWAPPDQPTPPPDQPTPPPDQPTPPPDQPTPPPVTDLPVASAASASGSDPASAAVPVPVPVPAPSPE
TPATGPWRLTLDKPPPVGHQ
LILFFPVNPRVGAGWKGAVQPPTQAILDASVELHRRTAGQRTSSAAGSRREVADDNMPLDLSMKSTRLVHANGQCSWFEM
QQFIGDLITSGNTSSQSLKE
QHDVLHVSWGLAGGEYLGMKGAAQDKSDLDPNRLMLSGTPERRAEFRDRKEVENELHACTCSGCPFSTSPPSYETLKPRK
SLYSQTQPDKACLSKNHTSN
PAMNLSLCLPEENPAEPSSTTTSDLKPRGSRRDDRERGADCSSSRGMTGTFPHFPMFACLCCHGLQTCAQLLRKQDGSDS
ALPHGNAHGHHFHHHHHCPF
SSCLPCPQLAHSHPHAAQLRGHFPYLGCQHSFGGCPQPSHKQGAQKSGQHPEDGARAAMFPSSLHPCMHCPAAFQRPSQL
LQHQRSEHSQKPAGFLCTEC
GKAFNSHSNLRIHQNVHTGARPYVCPDCGKSFSQSGALKIHKRIHTGERPYSCAFCGRGFPHLAGVRAHQRTHTGEKPYR
CGLCGKCFTQSGALKIHTRM
HTGERPFTCGVCEKGFTNLAAIRFHYRTVHGLTYEAAGRPAAGGQYRDQANPGCP
>g725.t3
MSLEYTIDIQLTELGFPDILQTYPAAVRESPRLSASQYVSLSGAPTKPGTMCSDDAFTFSVLLGTKKRRRGKHVISEIPG
GLAAGEKADDEPHQGVNDEQ
VANKNTQDVEDESADDSEVSEDYDEESLDEDQDCFDDESGDTGDYRCGVCGLQLASNMKLLDHMNLHTGQRPHCCSECGK
RFCQLGNYRAHLKTHAKGEP
ELVHSLWCPLCTKPFGSKEDLRGHLTTKHAERDFYECDSCKLLFTERKECKKHIEWHKSTHRRFACDTCGRRFSTQRSLK
HHRNRKCLRSFACTDCDRTF
SRKNALLKHSFSHVGLLPYTCMLCCCHFRLARLYRQHKCNPQSVHCVACLRAFSSMEDFRQHKKDTGCWGHQEPKGGDIR
CPECGESFATREELKKHGGA

HQRVLTCAECGKGFRSALLLMSHMGGHAVQTPCLCQSCGRGFLHQLDYDRHLERCGLAPLSPVVTKKRAQKSWAPPDQPT
PPPDQPTPPPDQPTPPPDQP
TPPPVTDLPVASAASASGSDPASAAVPVPVPVPAPSPETPATGPWRLTLDKPPPVGHQLILFFPVNPRVGAGWKGAVQPP
TQAILDASVELHRRTAGQRT
SSAAGSRREVADDNMPLDLSMKSTRLVHANGQCSWFEMQQFIGDLITSGNTSSQSLKEQHDVLHVSWGLAGGEYLGMKGA
AQDKSDLDPNRLMLSGTPER
RAEFRDRKEVENELHACTCSGCPFSTSPPSYETLKPRKSLYSQTQPDKACLSKNHTSNPAMNLSLCLPEENPAEPSSTTT
SDLKPRGSRRDDRERGADCS
SSRGMTGTFPHFPMFACLCCHGLQTCAQLLRKQDGSDSALPHGNAHGHHFHHHHHCPFSSCLPCPQLAHSHPHAAQLRGH
FPYLGCQHSFGGCPQPSHKQ
GAQKSGQHPEDGARAAMFPSSLHPCMHCPAAFQRPSQLLQHQRSEHSQKPAGFLCTECGKAFNSHSNLRIHQNVHTGARP
YVCPDCGKSFSQSGALKIHK
RIHTGERPYSCAFCGRGFPHLAGVRAHQRTHTGEKPYRCGLCGKCFTQSGALKIHTRMHTGERPFTCGVCEKGFTNLAAI
RFHYRTVHGLTYEAAGRPAA
GGQYRDQANPGCP
>g726.t1
AIMGFVNKQQAQDMLLSKPNGTFLLRFSDSEIGGITIAWVAENPNKA
>g726.t2
MCGALPLRAIMGFVNKQQAQDMLLSKPNGTFLLRFSDSEIGGITIAWVAENPNKA
>g727.t1
MGPAHEALRKADGSGVMLEPFVHQVAGHSCIMRFGEQAICKPLIPREHQFYKSLPPEIRKFTPQYKGVVSVRFEETKEGK
LCLIAYPSRGEAGDPENQDL
CVDSEPKTKMLKRGGEKQPGEKEDELVSLEEVQQAGGLFYSQEKIKHNPWRLKCHQQLLQKLRDTTKLRNQHKFILLENL
TWRYSVPCVLDLKMGTRQHG
DDASDEKKANHTRKCQQSTSASIGVRLCGMQVYRPDTGQLIFMNKYHGRKLTLAGFKEALYRFFHNGRRLRHELLAPVLR
RLRDMRATLEACESYRFFSS
SLLINYDGDPQRARARPRPRGGEEGDEDEPSDEEEYDEEDEGAFGFPSSSAGGAAG
>g728.t1
LQPADCRTLIEKLKACSDEQLLVELQHIKTWNIGKCELYHWVDLLDRFDGILCDAGQTVESMSWLLVCDRPDNGHLKALL
LAVLNFTALLIEYSFSRHLY
SSIEHLTTLLASCDMQVVLSVLNLLYVFSKRSNYITRLGSDKRTPLLARLQHLAESWGGKENGFGLAECCRDLPMSKYPP
SATTLHFEFYAEPGPEVKVE
RKISESPSEIMESLTVMYNIPKDKQTLLFTHIRLAHGFSNHKKRLQAVQARLHAISILVYSNALQESANSILYNGLIEEL
VDVLQITDKQLVDIKAASLR
TLTSIVHLERTPKLSNIIDCTGTASYHGFLPVLVRNCIQAMIDPLMEPYPHQFATALFSFLYHLASYDAGGEALVSCGMM
EALLKVIKFLGDEQDQITFV
TRAVRVVDLITNLDMAAFQSHTGLTIFICRLEVGVGTATQRPPQPTALHRGPERRIHSVPLPSVFNAQHEVDLSRKECPF
VIKPKIQRPSTAVESEDMDT
DMDSESHSSFS
>g728.t2
MKVDRSKLKKTPTEAPADCRTLIEKLKACSDEQLLVELQHIKTWNIGKCELYHWVDLLDRFDGILCDAGQTVESMSWLLV
CDRPDNGHLKALLLAVLNFT
ALLIEYSFSRHLYSSIEHLTTLLASCDMQVVLSVLNLLYVFSKRSNYITRLGSDKRTPLLARLQHLAESWGGKENGFGLA
ECCRDLPMSKYPPSATTLHF
EFYAEPGPEVKVERKISESPSEIMESLTVMYNIPKDKQTLLFTHIRLAHGFSNHKKRLQAVQARLHAISILVYSNALQES
ANSILYNGLIEELVDVLQIT
DKQLVDIKAASLRTLTSIVHLERTPKLSNIIDCTGTASYHGFLPVLVRNCIQAMIDPLMEPYPHQFATALFSFLYHLASY
DAGGEALVSCGMMEALLKVI
KFLGDEQDQITFVTRAVRVVDLITNLDMAAFQSHTGLTIFICRLEVGVGTATQRPPQPTALHRGPERRIHSVPLPSVFNA
QHEVDLSRKECPFVIKPKIQ
RPSTAVESEDMDTDMDSESHSSFS
>g728.t3
MKVDRSKLKKTPTEACELYHWVDLLDRFDGILCDAGQTVESMSWLLVCDRPDNGHLKALLLAVLNFTALLIEYSFSRHLY
SSIEHLTTLLASCDMQVVLS
VLNLLYVFSKRSNYITRLGSDKRTPLLARLQHLAESWGGKENGFGLAECCRDLPMSKYPPSATTLHFEFYAEPGPEVKVE
RKISESPSEIMESLTVMYNI

PKDKQTLLFTHIRLAHGFSNHKKRLQAVQARLHAISILVYSNALQESANSILYNGLIEELVDVLQITDKQLVDIKAASLR
TLTSIVHLERTPKLSNIIDC
TGTASYHGFLPVLVRNCIQAMIDPLMEPYPHQFATALFSFLYHLASYDAGGEALVSCGMMEALLKVIKFLGDEQDQITFV
TRAVRVVDLITNLDMAAFQS
HTGLTIFICRLEVGVGTATQRPPQPTALHRGPERRIHSVPLPSVFNAQHEVDLSRKECPFVIKPKIQRPSTAVESEDMDT
DMDSESHSSFS
>g729.t1
LGCDIPTLPFPRDGLCQSEFPPRALVAIWEERKRKLGAAWARTAVMHHLSGECSQGVVLVGNCLSEVVVSDAMITGNKQD
RESGTGGRQTEIRKDKAEIG
VGDRQDRETGTGGRRIAE
>g730.t1
LVFLGLRQGGRTVSDYIIDFRTRACQSEWNPAVQCDAFLLGLGSYLRDELVSYELPKSLDGLIELATPVGSHTLAFFIDS
GSDINIIDKELARQLDIQQV
PLPRPAPACTLNCHLMGTVTHQMTLLHLLLSGNHHDTVQFHILRSHLPFILGFP
>g731.t1
MEEYIDDSLVAGNIRPSSSPAGAGFFFVEKKDKTLRPCIDYRGLNKNRYLLPLISSAFELREGVTILSKLDLRNTYHLVR
IREGDEWKTAFNTPTGHYEY
LVMPFGLTNAPAVFQALVNDVLRDMLDRYVFMYLDDILIFS
>g732.t1
MLGGHAPEGRLHLLVFPLRLAIGPQMKTKRQADSGPDQSAKLPPKNGGKLRSPVGHNIDRKALWGLGIVDPLEDIRAESS
RDKQAVGRAGSGIWRMGAEV
NLALRGRKAVSGAQEGDLGGXGRQT
>g733.t1
MPEPSQLAPFDAKEQRLYSELPPDVRAPHPISKAEPSHPAEETHFGRLYPRSRSFGHYPELVTI
>g734.t1
MSWVSLLLTVWSSRALSWLLVVMVSRDVGLSVGLEGVQTSTCSRWGSRVEEFNQGQDGDIVIGGLFNLHYQPPVIDQEFI
KPPFYNPCTGLQVEMLKAAY
AMEFAVEEINRNPIILPGVKLNY
>g734.t2
MVSRDVGLSVGLEGVQTSTCSRWGSRVEEFNQGQDGDIVIGGLFNLHYQPPVIDQEFIKPPFYNPCTGLQVEMLKAAYAM
EFAVEEINRNPIILPGVKLN
Y
>g735.t1
KLPDNFNEAKFITFSMLIFCTVWVAFVPAYISSPGKYSVAVEIFAILASSYGLLLCIFAPKCFIILLRPERNTKKHLMAK
>g736.t1
IIKVGRWNPLPVHFLTDAPDATVADMLMEVYHMVTLRIQLQ
>g736.t2
IIKVGRWNPLPVHFLTDAPDATVADMLMEVYHMVTLRIQLQR
>g737.t1
GRAHLCGRSGSMSPDHHADADQSMEGHPPVTENPHAEYGLTDNIQRIVENEKGSTAEKSSKQKVDLQALPTRAYLDQTVV
PILLQGLSVLAKDRPPNPIE
YLAAFLLKNKGQFEERN
>g737.t2
GRAHLCGRSGSMSPDHHADADQSMEGHPPRIVENEKGSTAEKSSKQKVDLQALPTRAYLDQTVVPILLQGLSVLAKDRPP
NPIEYLAAFLLKNKGQFEER
N
>g737.t3
PAELISAAAPGPCRQHFRCTKQYKDHHADADQSMEGHPPVTENPHAEYGLTDNIQRIVENEKGSTAEKSSKQKVDLQALP
TRAYLDQTVVPILLQGLSVL
AKDRPPNPIEYLAAFLLKNKGQFEERN
>g738.t1
MNSSDSDEDSYDERSALVSSETPTVPSYQPDRADPEPRAQGPMLRREKGVGPERGAGPGRGAGPEVDLEEEDELTLKYGA
KHVIMLFIPVTLCMVVVVAS
IKSVSFYTEKSDQRLFYTPFSEDQVSVGRRLLNSFLNSIIMISVIVVMTILLVVLYKQRCYKFIHGWLILSSLMLLFWFS
YLYLGEVFKTYNLAVDYPTV
GVLIWNFGAVGMICIHWKGPLQLQQAYLILISALMALVFIKYLPEWSAWVILGAISIYDLVAVLSPRGPLRMLVETAQER

NEPIFPALIYSSGMAWMVGM
AKPAEGSRQHSDGGSLEAGDSPDPPAGAQEPRLEEEDQEEEERGVKLGLGDFIFYSVLVGKAAATGGDWNTTLACFVAIL
IGLCLTLLLLAIFKKALPAL
PISITFGLVFYFSTDYLVRPFMDNLAANQYYI
>g738.t2
LNRSERQVTVQVNCVGRRSVGRRSAGSSRHPDADAEQVEVGFTHGPSTMNSSDSDEDSYDERSALVSSETPTVPSYQPDR
ADPEPRAQGPMLRREKGVGP
ERGAGPGRGAGPEVDLEEEDELTLKYGAKHVIMLFIPVTLCMVVVVASIKSVSFYTEKSDQRLFYTPFSEDQVSVGRRLL
NSFLNSIIMISVIVVMTILL
VVLYKQRCYKFIHGWLILSSLMLLFWFSYLYLGEVFKTYNLAVDYPTVGVLIWNFGAVGMICIHWKGPLQLQQAYLILIS
ALMALVFIKYLPEWSAWVIL
GAISIYDLVAVLSPRGPLRMLVETAQERNEPIFPALIYSSGMAWMVGMAKPAEGSRQHSDGGSLEAGDSPDPPAGAQEPR
LEEEDQEEEERGVKLGLGDF
IFYSVLVGKAAATGGDWNTTLACFVAILIGLCLTLLLLAIFKKALPALPISITFGLVFYFSTDYLVRPFMDNLAANQYYI
>g739.t1
MDVRSSETCPVHPGQRTPGCSSGSGGKVSRKTRSRKGKRGRRRKSRRSRSNKSPPSCFPPELKLVSPTPQRFEHLATQLK
WRLQEGGGEAVYQIGVEDNG
MLVGLSQDDMKASLHTLQRMAEKVGADITVLRQRDVDNDSGRMARKITEVLVRKVPDDQQFLDLRVAVLGNVDSGKSTLL
GVLTQGELDNGRGRARLNLF
RHLHEIQTGRTSSISFEILGFNSKGE
>g739.t2
MDVRSSETCPVHPGQRTPGCSSGSGGKVSRKTRSRKGKRGRRRKSRRSRSNKSPPSCFPPELKLVSPTPQRFEHLATQLK
WRLQEGGGEAVYQIGVEDNG
MLVGLSQDDMKASLHTLQRMAEKVGADITVLRQRDVDNDSGRMARKITEVLVRKVPDDQQFLDLRVAVLGNVDSGKSTLL
GVLTQGELDNGRGRARLNLF
RHLHEIQTGRTSSISFEILGFNSKGEKLLVHD
>g740.t1
LDLFDRTQRMGFVVVGWTGGGVQSLQEVSRPDLCDSRGVCHGRLRDHSKTEATGGVITGCQSITWFIHPDPRTGSREPGQ
RLGLGATCPVQASLVPCPLG
VEQHHLVLTKLNTETT
>g740.t2
LDLFDRTQRMGFVVVGWTGGGVQSLQEVSRPDLCDSRGVCHGRLRDHSKTEATGIRRMWPISRSYIGMASLALAPVQASL
VPCPLGVEQHHLVLTKLNTE
TT
>g741.t1
MVILNSPPLQVIVCSHNHHRHFQEGPEAPSSSEVADPRTALRRRRRKRGKRGGLHARLKARASQPPLPSLLLANVRSLEN
KLDELRTRITTQRELRECCA
LIFTETWLSDKVPEHAIQLETHSMHMRRPDSNV
>g742.t1
SALWQSLASEIDPVASTKELVDGENRSRSSSSSAWEEDRSGKHVNLLSREPPRGPARHPARHPASPPLSLAGRQAALGRR
SKRSRAAHG
>g742.t2
VELMPLSGYLSFTFKTNTLPGSALWQSLASEIDPVASTKELVDGENRSRSSSSSAWEEDRSGKHVNLLSREPPRGPARHP
ARHPASPPLSLAGRQAALGR
RSKRSRAAHG
>g742.t3
LPQCLEALFLLLHTGVELMPLSGYLSFTFKTNTLPGSALWQSLASEIDPVASTKELVDGENRSRSSSSSAWEEDRSGKHV
NLLSREPPRGPARHPARHPA
SPPLSLAGRQAALGRRSKRSRAAHG
>g743.t1
EVSIFPEGFHNGSVGPSTTFLEGLWQIPVVEGGNRLYARFTQRIDQFVVVKSSFLRMSPLVAMREYTVPGDGKLLMIPMM
NPGMSLECILEFDILQLIQI
FWYSSIMKDYWRLETDLRQLVQQGLITGVKLNLEGQCPCMF
>g743.t2
MIPMMNPGMSLECILEFDILQLIQIFWYSSIMKDYWRLETDLRQLVQQGLITGVKLNLEGQCPCMF
>g744.t1

MRYMLWVVSMYLWILSLFLNGCQTYVLNGKEDFMLLAGPLINQQSRKDGVDSAFDCTTKSIPDGSRGYFEYLQGNGATHF
KVPLSWAQLLPTGLPSQPQL
TVVRCYRALLNQLVEVGLQPLVILHGSTVPEDLRWRYGGWESPELVEMFQQYAEFAFLEFGELVQSWVTLSHLDELKNVE
LHNALQAHGSAYNRYYELFP
EKDLACDSYHKIEYDVYLLRGLNVNTYQFSISWARVFPHGHQGRHSEKGALYYDKLINALIESGIQPVATLHYWDLPQAL
QECGGWTNASIVEAFREYAD
FCFSRFGDRVKAWNTFSSPWVVSHAGIPVFTADESRRIRGTADFFGLNHYTSRLVNNRVGGCTSGPQGVGDFQAHVDPSW
SSTASDWIYSAPWGLRRLLN
FISAEYLSETKVPIYITGNGMPTEYSGDTLNDTSRIDYIRSYVNEALKAINVDGVDVQRFTVQSLMDGFEGPQGYSERFG
LHHVNFDLPDRPRTPKQSAY
FYAKLIENNGFVWARQSFNTHALKNYDSSKIPSLPPSKVPSEAMIVWRNFSSQTIFQRKLYHYGTFSQGFNWGVGSSAYQ
VEGGWNADGKGPSVWDTFSQ
RPGNIPGNANGNVACDSYHRLEEDLYMLKALKVKSYKFSLSWSRILPNGRRASLNQIGVDYYNRLINGLVAHNITPMIEG
GWRADGKGLSIWDKFAHTPL
RVFNDDTGDIACDSYNKVEEDVAMLSKLKVTHYRFSISWSRVLPDGTNTHINEPGLNYYHRLVDALLAANIQPHITLYHW
DLPQALQDVGGWENDTIIYR
FRDYADIIFSRLGPKVKYWITINEPYNIAMVGHGYGAAAPGISFRPGTLPYLVGHNLIKAHAEAWHLYNDKYRAKQRGII
SITINSDWSEPRNPHKQEDI
DAARRVVQFYIGWFAHPIFNGDYSNVMKTIIRDRSLAAGLSKSRGAGTIADRTWLDSGSSWLKVFPPGFRRILNFIKKEY
GDPPIIIITENGMSERGPIDL
NDAHRSHYYEKYINQALKARLLDDVNIRGYTAWSLMDNLEWATGFSERFGLFYVNHSDPDLPRVAKRSVEYYSTVISCNG
FPDPASGPHACLNPEPEGTS
TPTLPTQPEHMVRFLGMDLSPSDAETGLYVTFGLVVVATLGVASVSLCLFRSSKRLASTA
>g744.t2
MRYMLWVVSMYLWILSLFLNGCQTYVLNGKEDFMLLAGPLINQQSRKDGVDSAFDCTTKSIPDGSRGYFEYLQGNGATHF
KVPLSWAQLLPTGLPSQPQL
TVVRCYRALLNQLVEVGLQPLVILHGSTVPEDLRWRYGGWESPELVEMFQQYAEFAFLEFGELVQSWVTLSHLDELKNVE
LHNALQAHGSAYNRYYELFP
EKDLACDSYHKIEYDVYLLRGLNVNTYQFSISWARVFPHGHQGRHSEKGALYYDKLINALIESGIQPVATLHYWDLPQAL
QECGGWTNASIVEAFREYAD
FCFSRFGDRVKAWNTFSSPWVVSHAGYGTASDWIYSAPWGLRRLLNFISAEYLSETKVPIYITGNGMPTEYSGDTLNDTS
RIDYIRSYVNEALKAINVDG
VDVQRFTVQSLMDGFEGPQGYSERFGLHHVNFDLPDRPRTPKQSAYFYAKLIENNGFVWARQSFNTHALKNYDSSKIPSL
PPSKVPSEAMIVWRNFSSQT
IFQRKLYHYGTFSQGFNWGVGSSAYQVEGGWNADGKGPSVWDTFSQRPGNIPGNANGNVACDSYHRLEEDLYMLKALKVK
SYKFSLSWSRILPNGRRASL
NQIGVDYYNRLINGLVAHNITPMIEGGWRADGKGLSIWDKFAHTPLRVFNDDTGDIACDSYNKVEEDVAMLSKLKVTHYR
FSISWSRVLPDGTNTHINEP
GLNYYHRLVDALLAANIQPHITLYHWDLPQALQDVGGWENDTIIYRFRDYADIIFSRLGPKVKYWITINEPYNIAMVGHG
YGAAAPGISFRPGTLPYLVG
HNLIKAHAEAWHLYNDKYRAKQRGIISITINSDWSEPRNPHKQEDIDAARRVVQFYIGWFAHPIFNGDYSNVMKTIIRDR
SLAAGLSKSRGAGTIADRTW
LDSGSSWLKVFPPGFRRILNFIKKEYGDPPIIIITENGMSERGPIDLNDAHRSHYYEKYINQALKARLLDDVNIRGYTAWS
LMDNLEWATGFSERFGLFYV
NHSDPDLPRVAKRSVEYYSTVISCNGFPDPASGPHACLNPEPEGTSTPTLPTQPEHMVRFLGMDLSPSDAETGLYVTFGL
VVVATLGVASVSLCLFRSSK
RLASTA
>g744.t3
MRYMLWVVSMYLWILSLFLNGCQTYVLNGKEDFMLLAGPLINQQSRKDGVDSAFDCTTKSIPDGSRGYFEYLQGNGATHF
KVPLSWAQLLPTGLPSQPQL
TVVRCYRALLNQLVEVGLQPLVILHGSTVPEDLRWRYGGWESPELVEMFQQYAEFAFLEFGELVQSWVTLSHLDELKNVE
LHNALQAHGSAYNRYYELFP
EKDLACDSYHKIEYDVYLLRGLNVNTYQFSISWARVFPHGHQGRHSEKGALYYDKLINALIESGIQPVATLHYWDLPQAL
QECGGWTNASIVEAFREYAD
FCFSRIPVFTADESRRIRGTADFFGLNHYTSRLVNNRVGGCTSGPQGVGDFQAHVDPSWSSTASDWIYSAPWGLRRLLNF
ISAEYLSETKVPIYITGNGM

PTEYSGDTLNDTSRIDYIRSYVNEALKAINVDGVDVQRFTVQSLMDGFEGPQGYSERFGLHHVNFDLPDRPRTPKQSAYF
YAKLIENNGFVWARQSFNTH
ALKNYDSSKIPSLPPSKVPSEAMIVWRNFSSQTIFQRKLYHYGTFSQGFNWGVGSSAYQVEGGWNADGKGPSVWDTFSQR
PGNIPGNANGNVACDSYHRL
EEDLYMLKALKVKSYKFSLSWSRILPNGRRASLNQIGVDYYNRLINGLVAHNITPMIEGGWRADGKGLSIWDKFAHTPLR
VFNDDTGDIACDSYNKVEED
VAMLSKLKVTHYRFSISWSRVLPDGTNTHINEPGLNYYHRLVDALLAANIQPHITLYHWDLPQALQDVGGWENDTIIYRF
RDYADIIFSRLGPKVKYWIT
INEPYNIAMVGHGYGAAAPGISFRPGTLPYLVGHNLIKAHAEAWHLYNDKYRAKQRGIISITINSDWSEPRNPHKQEDID
AARRVVQFYIGWFAHPIFNG
DYSNVMKTIIRDRSLAAGLSKSRGAGTIADRTWLDSGSSWLKVFPPGFRRILNFIKKEYGDPPIIITENGMSERGPIDLN
DAHRSHYYEKYINQALKARL
LDDVNIRGYTAWSLMDNLEWATGFSERFGLFYVNHSDPDLPRVAKRSVEYYSTVISCNGFPDPASGPHACLNPEPEGTST
PTLPTQPEHMVRFLGMDLSP
SDAETGLYVTFGLVVVATLGVASVSLCLFRSSKRLASTA
>g745.t1
MLTLYTNTVHLHEPYILQRRSDFLSVRRCGLSGPP
>g746.t1
MDTAALTRQWRRPWTPRPSHTVETSLDTAALTHSGDVPGHRGPHQTVETSLDTAVLTHSGDVPGHRGPHQTVETSLDTAV
PTRQWRRPWTPQSSPDSGDV
PGHRGPHQTVETSLDTAVLTRQWRRHWTLRPSPDSRDVTRPFLTQHVVSRSMSCRDLFVPRAGLCSAPPEDTWYLN
>g747.t1
MNHELRFGLAADGAFEGTKSERHGYPWLFREPVTSHGDHAEKDAAQTPTPKRQEIPKFECLRGRAFAQTLTRTSPPIPFV
VSMLSLCSVCLMRSGVSRSP
VAMHTLPGLDHSAPGQGFSDAAKGARSSSSSLPAARLHCVSAKPQSLKQKWSTGPSATGPSANRVGSHWLALRRRRIPLV
PGGLSGKQRGQERISGLGFA
PT
>g747.t2
MNHELRFGLAADGAFEGTKSERHGYPWLFREPVTSHGDHAEKDAAQTPTPKRQEIPKFECLRGRAFAQTLTREAGLDHSA
PGQGFSDAAKGARSSSSSLP
AARLHCVSAKPQSLKQKWSTGPSATGPSANRVGSHWLALRRRRIPLVPGGLSGKQRGQERISGLGFAPT
>g748.t1
MNIVIIGAHWLDGIQYPYHLYISPSARTGANGPDRPFQCPTCGVRFTRIQNLKQHMLIHSGIKPFQCDRCGKKFTRAYSL
KMHRLKHE
>g748.t2
LTWDSRLDGIQYPYHLYISPSARTGANGPDRPFQCPTCGVRFTRIQNLKQHMLIHSGIKPFQCDRCGKKFTRAYSLKMHR
LKHE
>g748.t3
MNIVIIGAHWLDGIQYPYHLYISPSARTGANGPDRPFQCPTCGVRFTRIQNLKQHMLIHSGIKPFQCDRCGKKFTRAYSL
KMHRLKHEGKRCFRCQICSA
TFTSFGEYKHHMRVS
>g749.t1
MLRVVIPLAAGSCLGRGESFPHKEMGICQWLRLIHVIVDKGRQALSTSDSDTGSPQSGHEWVSHSTVRLLTGITEHMESG
CRAALLIIFSIPA
>g749.t2
MGICQWLRLIHVIVDKGRQALSTSDSDTGSPQSGHEWVSHSTVRLLTGITEHMESGCRAALLIIFSIPA
>g750.t1
MTVAVESVKSTKFLEVHITDDLTWPTNTTSQVKTAQQRLHFLRGMRADLPPSTLTTFYRGAIESIFTSSLSVWYGSCPAD
QKALQRVVRTAENITRSAQP
FIQDLYPSCCLKRATNITKDPTHPAHELFTLLPSSKHYHSMRCRSTRLSKSFFPQAIRLLNPVKKSP
>g751.t1
MGVKTFTHSSPSHSQEMLNKLNALRSEGHLCDVTIRVQDKLFLAHKVVLACCSDFFRSKLVGRSEEEDKFVLDLHHVTVS
GFTPLLEYAYTSTLSISTEN
IIDVLAAASYMQMFAVASTCSEFMKSSILWSPGNNANNTLAADKLHESAPESASSNCVLTPLDGSLSPVSSDCSLMERNV
PVCRESRRKRKSYGVAMASP
ESLLKCPTQLVATSPQIPNPSPSFSDTAAQPVESSLAFPWTFPFGMDRRFHSDKAKLPESPRGLDHTAAAGTSSVEAVVV

GRRLSDFLTCEGAKAGSSLT
VAEEEDVRVKVERLSDEEAQEGSSQPVSASLSDQQTVPGSEQVQEELLISPQSSSIGEGCFST
>g752.t1
MLTCRKITGSYSKAKYGYSDIVTIPVGATNIDIKQRSHRGIKHDGHYLAIKRAKGSYVLNGNFSVSTLEQDIPVQGATLK
YSGSSTTLERIQSFRRLREA
ITIQLLVLGGDTNPPKVKYTFFIPRDTTFSSNAAKERPGAALSPHMLHPFGEPQWVLGEWSECSKSCGSGWSRRNVDCMD
SAGFHSGQCGRDLKPIDIRP
CGDMPCPMWQMGPWSACSRTCGQGVRRRSVFCSDYTGNIVEAVKCDPKKMPEPVSGECLNRECV
>g753.t1
MQSIQYRNDNTFPTKVALIKNNVKKNRYKDIVPFDHTRVKLSLNTSKNDEDYINASFIKGVSGSRAYIATQGPLPHTVLD
FLRMIWEYKVQVVVMACREF
EMGRCGRALHQLHYYNWPDHGVPDSIPPILEMLQEMRICQKHDDIPICIHCSAGCGRTGALCVIDFTWNLVKSQEQYELV
YKTVKLLFEKYLQSFDSLAC
QNKVQPTASPSPTASVDDKGERESEGSQSDSRPPSLPALDSESQAPRLTPTEEQGRGHIQPGPAQEKAAAHSKRDVQAQP
SHHHQYPPHHLPLDLPQRPS
EAATAARPQDPQSRDPGLTSATREEESNEPEDSEPPPKPAMPGLATACLTVEDPYFGSPETVLFAEEPFDCLMDELVSLH
TPTLALNDQSLELNGTCCEH
PESSQRAEVIIPPNDAALAIREMGERTPESFELAHNGAARPILSSSHLPPPPPPPPALAQQPQVELLLQEDLNPPLPHRT
PESFILATEEVDESHGAHHQ
APDPALRIGTSSEWAGSPQPNKLLDFVMNRSKSVRAKSSRQEPVVQQLSPPAVVFSVAGSEFEVLQIQR
>g753.t2
MQSIQYRNDNTFPTKVALIKNNVKKNRYKDIVPFDHTRVKLSLNTSKNDEDYINASFIKGVSGSRAYIATQGPLPHTVLD
FLRMIWEYKVQVVVMACREF
EMGRKKCERYWPETQADQFVCGPFTVGCCGRALHQLHYYNWPDHGVPDSIPPILEMLQEMRICQKHDDIPICIHCSAGCG
RTGALCVIDFTWNLVKSQEQ
YELVYKTVKLLFEKYLQSFDSLACQNKVQPTASPSPTASVDDKGERESEGSQSDSRPPSLPALDSESQAPRLTPTEEQGR
GHIQPGPAQEKAAAHSKRDV
QAQPSHHHQYPPHHLPLDLPQRPSEAATAARPQDPQSRDPGLTSATREEESNEPEDSEPPPKPAMPGLATACLTVEDPYF
GSPETVLFAEEPFDCLMDEL
VSLHTPTLALNDQSLELNGTCCEHPESSQRAEVIIPPNDAALAIREMGERTPESFELAHNGAARPILSSSHLPPPPPPPP
ALAQQPQVELLLQEDLNPPL
PHRTPESFILATEEVDESHGAHHQAPDPALRIGTSSEWAGSPQPNKLLDFVMNRSKSVRAKSSRQEPVVQQLSPPAVVFS
VAGSEFEVLQIQR
>g753.t3
MQSIQYRNDNTFPTKVALIKNNVKKNRYKDIVPFDHTRVKLSLNTSKNDEDYINASFIKGVSGSRAYIATQGPLPHTVLD
FLRMIWEYKVQVVVMACREF
EMGRCGRALHQLHYYNWPDHGVPDSIPPILEMLQEMRICQKHDDIPICIHCSAGCGRTGALCVIDFTWNLVKSQEQYELV
YKTVKLLFEKYLQSFDSLAC
QNKVQPTASPSPTASVDDKGERESEGSQSDSRPPSLPALDSESQAPRLTPTEEQGRGHIQPGPAQEKAAAHSKRDVQAQP
SHHHQYPPHHLPLDLPQRPS
EAATAARPQDPQSRDPGLTSATREEESNEPEDSEPPPKPAMPGLATACLTVEDPYFGSPETVLFAEEPFDCLMDELVSLH
TPTLALNDQSLELNGTCCEH
PESSQRAEVIIPPNDAALAIREMGERTPESFELAHNGAARPILSSSHLPPPPPPPPALAQQPQVELLLQEDLNPPLPHRT
PESFILATEEVDESHGAHHQ
APDPALRIGTSSEWAGSPQPNKLLDFVMNRSKNLSFSSCLLRLWCSAWREVVSRPSD
>g754.t1
MSGLTNLQSIAIPGGIELFNHSPSYSNPDVCNGRDRRPAERQTGRQAGRQEGRQTGTPTGGELEVGGXVAFGGGNKCGCC
QKTVYFAEEVQCDGRSFHKS
CFLCSEYPPPRGGAGKCVILVLKNLDSTTIAVHMDEIYCKACYGKKYGPKGYGFGQGAGTLSMDGGESLGIQPATAAPHR
PTTNTNTSKLAQKSGGSDKC
PRCGKAVYAAEKVIGGGSAWHKNGCFRCALCGKGLESTTLADKDGEIYCKGCYAKNFGPKGFGYGQGAGALAHT
>g754.t2
MSGLTNLQSIAIPGGIELFNHSPSYSNPDVCNGRDRRPAERQTGRQAGRQEGRQTGTPTGGELEVGGXVAFGGGNKCGCC
QKTVYFAEEVQCDGRSFHKS
CFLCSEYPPPRGGAGKCVILVLKNLDSTTIAVHMDEIYCKACYGKKYGPKGYGFGQGAGTLSMDGGESLGIQPATAAPHR
PTTNTNTSKLAQKSGGSDKC

PRCGKAVYAAEKVIGGGSAWHKNGCFRCALCGKGLESTTLADKDGEIYCKGAT
>g754.t3
MSGLTNLQSIAIPGGIELFNHSPSYSNPDVCNGRDRRPAERQTGRQAGRQEGRQTGTPTGGELEVGGVAFGGGNKCGCCQ
KTVYFAEEVQCDGRSFHKSC
FLCSEYPPPRGGAGKCVILVLKNLDSTTIAVHMDEIYCKACYGKKYGPKGYGFGQGAGTLSMDGGESLGIQPATAAPHRP
TTNTNTSKLAQKSGGSDKCP
RCGKAVYAAEKVIGGGSAWHKNGCFRCALCGKGLESTTLADKDGEIYCKGCYAKNFGPKGFGYGQGAGALAHT
>g755.t1
VCISRHPGEPSLKDPVYSSPGLYCQGRKATGAQSLSKDHTMRKKRGGWAPTRSGTELSPARPRRSAVLQGHLGQASEAGG
PGRGAGGCTRTGSWEASCPS
PNEPRWRAAGQQPVAFLDIRKHSSTTNYYYYYYHHYQYYYYYHYHHYYYYYYYYYHHYQYY
>g755.t2
LYCQGRKATGAQSLSKDHTMRKKRGGWAPTRSGTELSPARPRRSAVLQGHLGQASEAGGPGRGAGGCTRTGSWEASCPSP
NEPRWRAAGQQPVAFLDIRK
HSSTTNYYYYYYHHYQYYYYYHYHHYYYYYYYYYHHYQYY
>g756.t1
MSNGEIRSNGEIRSNVTRERGQARPPRTRREPVDFQAWKRKLCEVNAAFLTV
>g757.t1
MGCDGGTIPKRHELVKGPKKVEKVDRNAELAAKWKYCALSQEKLKRPIITCELGRLYNKDAVIEYLLDKTAERPNTEVVS
HIRGMKDIKELNLTDNPEWE
GEGRTTKGDRYEDMHCAMFICPVVGLEMSGKHR
>g757.t2
LVKGPKKVEKVDRNAELAAKWKYCALSQEKLKRPIITCELGRLYNKDAVIEYLLDKTAERPNTEVVSHIRGMKDIKELNL
TDNPEWEGEGRTTKGDRYED
MHCAMFICPVVGLEMSGKHR
>g757.t3
MGCDGGTIPKRHELVKGPKKVEKVDRNAELAAKWKYCALSQEKLKRPIITCELGRLYNKDAVIEYLLDKTAERPNTEVVS
HIRGMKDIKELNLTDNPEWE
GEGRTTKGDRYEDMHCAMFICPVVGLEMSGKHRY
>g758.t1
MIPLEGAKCSFLVCLGISTLICSLIYLRAWTQTELRPQGGANGCRSFADKCEAFLPRAEEGLQWQRRDCQVDSYILKNNV
TCSHLIEDLHFITRPLSQEE
QDYPLAFILTVHKELQLLVRLLRAIYTPQNVYCIHVDAKAPRDYQETVQRLAGCFPNVFLSSHRETVTYAGFSRLQADIN
CMMDLEHSKVGWKKVINLCG
QDFPVKSNLELVRYMQSKKWKDQNMTPGVKQPASMRHRTQVQHTEVTGSHVSPKGWGMRKGPPPNQMQIYFGTAYYALTR
QFVNFVLKNQVARDLLEWSK
DTFSPDEHYWVTLNHLKEAPGNHIDGGWDGAIRAIKWQDQEGTAHSGCKGRYVRDICIYGMEDLPWIIKKNSMFANKFES
RIFPEALDCLEQWHRGKVLR
QATVPIEPSWLLAVPYLIPISKPTAVVDNEIH
>g758.t2
MIPLEGAKCSFLVCLGISTLICSLIYLRAWTQTELRPQGGANGCRSFADKCEAFLPRAEEGLQWQRRDCQVDSYILKNNV
TCSHLIEDLHFITRPLSQEE
QDYPLAFILTVHKELQLLVRLLRAIYTPQNVYCIHVDAKAPRDYQETVQRLAGCFPNVFLSSHRETVTYAGFSRLQADIN
CMMDLEHSKVGWKKVINLCG
QDFPVKSNLELVRYMQSKKWKDQNMTPGVKQPASMRHRTQVQHTEVTGSHVSPKGWGMRKGPPPNQMQIYFGTAYYALTR
QFVNFVLKNQVARDLLEWSK
DTFSPDEHYWVTLNHLKGRYVRDICIYGMEDLPWIIKKNSMFANKFESRIFPEALDCLEQWHRGKVLRQATVPIEPSWLL
AVPYLIPISKPTAVVDNEIH
>g758.t3
LRPRAVAEVNEKEALANQGPAEGAKCSFLVCLGISTLICSLIYLRAWTQTELRPQGGANGCRSFADKCEAFLPRAEEGLQ
WQRRDCQVDSYILKNNVTCS
HLIEDLHFITRPLSQEEQDYPLAFILTVHKELQLLVRLLRAIYTPQNVYCIHVDAKAPRDYQETVQRLAGCFPNVFLSSH
RETVTYAGFSRLQADINCMM
DLEHSKVGWKKVINLCGQDFPVKSNLELVRYMQSKKWKDQNMTPGVKQPASMRHRTQVQHTEVTGSHVSPKGWGMRKGPP
PNQMQIYFGTAYYALTRQFV
NFVLKNQVARDLLEWSKDTFSPDEHYWVTLNHLKEAPGNHIDGGWDGAIRAIKWQDQEGTAHSGCKGRYVRDICIYGMED

LPWIIKKNSMFANKFESRIF
PEALDCLEQWHRGKVLRQATVPIEPSWLLAVPYLIPISKPTAVVDNEIH
>g759.t1
MTTVAPTHHEGTALTPPPVRHLTNRTSSPSRPLPSPGSAPPAELSELLRDKERRAQQQLGRCVEERERRLEEQRRKEQRR
RAAAEEKRRLQQEEEKVCGK
HQKTPRQPGRVTKPRPSRYSHAPLVTEPGQNKLNKAASGEGVQNAPVSSCLLTLRL
>g759.t2
MTTVAPTHHEGTVRHLTNRTSSPSRPLPSPGSAPPAELSELLRDKERRAQQQLGRCVEERERRLEEQRRKEQRRRAAAEE
KRRLQQEEEKVCGKHQKTPR
QPGRVTKPRPSRYSHAPLVTEPGQNKLNKAASGEGVQNAPVSSCLLTLRL
>g759.t3
MTTVAPTHHEGTALTPPPVRHLTNRTSSPSRPLPSPGSAPPAELSELLRDKERRAQQQLGRCVEERERRLEEQRRKEQRR
RAAAEEKRRLQQEEEKVGGQ
TLSNANCEMSLKVVTGNRKISD
>g760.t1
PPLPDPKPSPGAVPKDPPQGFLVTGEQRFLEGLRETFDLQLPDNPGDHGLRMIVIDGSNVAMSHGLGHFFSCRGIALAVQ
HFWNRGHRRISALLPHWRQK
RDPKITGRCFVLRDAQH
>g761.t1
TLSFLGMSLCLSLMFISSWYYALVAMVIAGCMYKYIEYRGAVKEWGDGIRGLSLNAARYALIRLEEAPPHTKNWRPQLLV
LCKLDSELEVKHPRLLSLTT
QMKAGKGLTIVSSVLEGAYMTRGSEAKTGEQRLKAAMSEEKTKGFSHVVVTSNLRDGFSLLIQSAGLGGMKHNTVLLAWP
ATWRQAQDSYPWKNFIETVR
ETTSAHQALLVAKNIDRFPGNQQRLEGTVDVWWIVHDGGLLMLLPFLLRQHKVWKKCTMRIFTVAQMDDNSIQMKKDLQM
FLYHLRLDAQVEVVEMHESD
ISAFTYEKTLVMEQRSQMLKQMQLSRTERQREVRVVLGAAGWEDARARLTQSSLQAQLIHDRNTASHGSSGGRPVPEQVH
MTWTKEKLVTERNRVREGGM
GVKDMFNMRPYVQLTCLNHLSVWLTCLTKQFVLLTCLFI
>g761.t2
MCYLFVNLACAVQTLLRTPNWRPRFKFYHWTLSFLGMSLCLSLMFISSWYYALVAMVIAGCMYKYIEYRGAVKEWGDGIR
GLSLNAARYALIRLEEAPPH
TKNWRPQLLVLCKLDSELEVKHPRLLSLTTQMKAGKGLTIVSSVLEGAYMTRGSEAKTGEQRLKAAMSEEKTKGFSHVVV
TSNLRDGFSLLIQSAGLGGM
KHNTVLLAWPATWRQAQDSYPWKNFIETVRETTSAHQALLVAKNIDRFPGNQQRLEGTVDVWWIVHDGGLLMLLPFLLRQ
HKVWKKCTMRIFTVAQMDDN
SIQMKKDLQMFLYHLRLDAQVEVVEMHESDISAFTYEKTLVMEQRSQMLKQMQLSRTERQREVRVVLGAAGWEDARARLT
QSSLQAQLIHDRNTASHGSS
GGRPVPEQVHMTWTKEKLVTERNRVREGGMGVKDMFNMRPYVQLTCLNHLSVWLTCLTKQFVLLTCLFI
>g761.t3
TLLRTPNWRPRFKFYHWTLSFLGMSLCLSLMFISSWYYALVAMVIAGCMYKYIEYRGAVKEWGDGIRGLSLNAARYALIR
LEEAPPHTKNWRPQLLVLCK
LDSELEVKHPRLLSLTTQMKAGKGLTIVSSVLEGAYMTRGSEAKTGEQRLKAAMSEEKTKGFSHVVVTSNLRDGFSLLIQ
SAGLGGMKHNTVLLAWPATW
RQAQDSYPWKNFIETVRETTSAHQALLVAKNIDRFPGNQQRLEGTVDVWWIVHDGGLLMLLPFLLRQHKVWKKCTMRIFT
VAQMDDNSIQMKKDLQMFLY
HLRLDAQVEVVEMHESDISAFTYEKTLVMEQRSQMLKQMQLSRTERQREVRVVLGAAGWEDARARLTQSSLQAQLIHDRN
TASHGSSGGRPVPEQVHMTW
TKEKLVTERNRVREGGMGVKDMFNMRPYVQLTCLNHLSVWLTCLTKQFVLLTCLFI
>g762.t1
PPVVQPPAEQPPQGGGVRAYPSNSRSPRSPPLFPGGPRPGRSGSGEPPPPPAEARGEAAATVKVTIESSSEEEEEEEAEE
EEQLVVVKVQKSPSPVDPRG
GRGQQQYQRVQG
>g762.t2
PPVVQPPAEQPPQGGGVRAYPSNSRSPRSPPLFPGGPRPGRSGSGEPPPPPAEARGEAAATVKVTIESSSEEEEEEEAEE
EEQLVVVKVQKSPSPVDPRG
GRGQQQYQRVQLEEEGDQGDRTADPTVSGRPALSTRSTH

>g762.t3
PPVVQPPAEQPPQGGGVRAYPSNSRSPRSPPLFPGGPRPGRSGSGEPPPPPAEARGEAAATVKVTIESSSEEEEEEEEAEE
EEQLVVVKVQKSPSPVDPRG
GRGQQQYQRVQSSEDMRSSIVDPSAFPFSN
>g763.t1
MMNWVIKKPPALRLLVYQVKWTKRIQMNGNKAHCPQWKTARKQLKSFQTNQLRYRREAARQTTDSLHRGPGALHAGVPAW
HLQPPHPVEVDAFSQWDRSQ
APVGGASLERQESVATTEARLRMEGVELKEEWQDEDFPRPLPEEEDLDEELFGGTSADGDPGYPVNQGKKPKKKLAAPDI
SLTLDRSEGSQLSDELEDST
ELDLDDMDTPSENSNEFEWEDDLPKPKATELLQKGVESVQEYSAAEEREEGRRWRVFRIGDQEHRVDMKAIEPYKKVISH
GGTLELLVAENYMIVYLNGA
TSRKKMPNVGWLRKCYQQIDRRLRKNLKSLIIVHPSWFIRTLLALTKPFISSKFSQKIKFVYNLTDLAELVPMEYVSIPD
CIK
>g763.t2
MMNWVIKKPPALRLLVYQVKWTKRIQMNGNKAHCPQWKTARKQLKSFQTNQLRYRREAARQTTDSLHRGPGALHAGVPAW
HLQPPHPVEVRCPVDAFSQW
DRSQAPVGGASLERQESVATTEARLRMEGVELKEEWQDEDFPRPLPEEEDLDEELFGGTSADGDPGYPVNQGKKPKKKLA
APDISLTLDRSEGSQLSDEL
EDSTELDLDDMDTPSENSNEFEWEDDLPKPKATELLQKGVESVQEYSAAEEREEGRRWRVFRIGDQEHRVDMKAIEPYKK
VISHGGTLELLVAENYMIVY
LNGATSRKKMPNVGWLRKCYQQIDRRLRKNLKSLIIVHPSWFIRTLLALTKPFISSKFSQKIKFVYNLTDLAELVPMEYV
SIPDCIK
>g763.t3
MMNWVIKKPPALRLLVYQVKWTKRIQMNGNKAHCPQWKTARKQLKSFQTNQLRYRREAARQTTDSLHRGPGALHAGVPAW
HLQPPHPVEVDAFSQWDRSQ
APVGGASLERQESVATTEARLRMEGVELKEEWQDEDFPRPLPEEEDLDEELFGGTSADGDPGYPVNQGKKPKKKLAAPDI
SLTLDRSEGSQLSDELEDST
ELDLDDMDTPSENSNEFEWEDDLPKPKATELLQKGVESVQEYSAAEEREEGRRWRVFRIGDQEHRVDMKAIEPYKKVISH
GGTLELLVAENYMIVYLNGA
TSRKKMPNVGWLRKCYQQIDRSSKFSQKIKFVYNLTDLAELVPMEYVSIPDCIK
>g764.t1
MDPANTEDLRQVVSRQGALLQELTAGRYGGNLGGCPSFLVQCFIAFEQQPRTYPAKRSRIGYLIRLRTGDALSWASAPWE
NDNVICFSCAAFTQEMRKTT
FHGTLNDSLKDELAFREDLENLDHLIQLAIRIDRQALVETLQRLWWGHYPGFGGDYPDFGGDTTQALVGTLPRHWRGHYP
GFGGDYPGFGGDTTQALLKA
TICWRNQSFPFTALIDSGAEESFLDGEVVQQLGLETRPVVPPQSCEGPERGRDRSDRTQDHSLGTSAVRHSFCLNPGAAP
EEVRRWFVSAPILVQPDPAW
QFIVEVDASDTGVRAV
>g764.t2
MDPANTEDLRQVVSRQGALLQELTAGRYGGNLGGCPSFLVQCFIAFEQQPRTYPAKRSRIGYLIRLRTGDALSWASAPWE
NDNVICFSCAAFTQEMRKTT
FHGTLNDSLKDELAFREDLENLDHLIQLAIRIDRQALVETLQRLWWGHYPGFGGDYPDFGGDTTQALVGTLPRHWRGHYP
GFGGDYPGFGGDTTQALLKA
TICWRNQSFPFTALIDSGAEESFLDGEVVQQLGLETRPVVPPQSCEGPERGRDRSDRTQDHSLGTSAVRHSFCLNPGAAP
EEVRRWFVSAPILVQPDPAW
QFIVEVDASDTGVLQWGHSNRLTCHPGVHHTLAFLRHRSWWPTMIPDTRLFVVACSVCARNKTLYPF
>g764.t3
MDPANTEDLRQVVSRQGALLQELTAGRYGGNLGGCPSFLVQCFIAFEQQPRTYPAKRSRIGYLIRLRTGDALSWASAPWE
NDNVICFSCAAFTQEMRKTT
FHGTLNDSLKDELAFREDLENLDHLIQLAIRIDRQALVETLQRLWWGHYPGFGGDYPDFGGDTTQALVGTLPRHWRGHYP
GFGGDYPGFGGDTTQALVGT
LPRLWRGSAICIIC
>g765.t1
MTGLDFDALLIKPKEVQEGGKRPLIVIPHGGPHSVIVAEWLLSPSALCRMGFAVLLVNYRGSIGYGQDSILSLPGNVGSQ
DVQDVHFAVESVLNGGEFDR
QKVALSGGSHGGFLVCHLIGQYPGFYKACVARNPVINMASMIGSTDIPDW

>g765.t2
LDFDALLIKPKEVQEGGKRPLIVIPHGGPHSVIVAEWLLSPSALCRMGFAVLLVNYRGSIGYGQDSILSLPGNVGSQDVQ
DVHFAVESVLNGGEFDRQKV
ALSGGSHGGFLVCHLIGQYPGFYKACVARNPVINMASMIGSTDIPDW
>g765.t3
LTAGPTHCGDGLLGRGVESGKQRKTRFTGTQEQGLGQAGLDFDALLIKPKEVQEGGKRPLIVIPHGGPHSVIVAEWLLSP
SALCRMGFAVLLVNYRGSIG
YGQDSILSLPGNVGSQDVQDVHFAVESVLNGGEFDRQKVALSGGSHGGFLVCHLIGQYPGFYKACVARNPVINMASMIGS
TDIPDW
>g766.t1
LTHGAWLGTTRNSYMEEPLPCGPTTCRDRHGGRVLCKPGGRQRRGPGRADPRHRRLALGTWNVTSLAGKEPELVREVERY
QLDIVGLTSTHSTGSGTKLL
ERGWTLSFSGGAQGERRRAGVGILTNPRLSRCVGVLPGERVASIRQVVEGKALTVVCAYAPNSSSEYPAFLKCLGGVLEG
APSGD
>g767.t1
MHRVVSEQEEPVAACQQFRHSVSWSATAGLLYKAIDSNLEDTGPVYNNRVAISIYFIIYIILIAFFMMNIFVGFVIVTFQ
EQGEQEYKNCELDKNQNPYQ
YQVWYIVTSCYFEYLMFLLIMLNTMCLGTQA
>g767.t2
MHRVVSEQEEPVAACQQFRHSVSWSATAGLLYKAIDSNLEDTGPVYNNRVAISIYFIIYIILIAFFMMNIFVGFVIVTFQ
EQGEQEYKNCELDKNQNPYQ
YQVWYIVTSCYFEYLMFLLIMLNTMCLGTQFNKAEKIILMKHEKKLNMTLNKL
>g767.t3
MHRVVSEQEEPVAACQQFRHSVSWSATAGLLYKAIDSNLEDTGPVYNNRVAISIYFIIYIILIAFFMMNIFVGFVIVTFQ
EQGEQEYKNCELDKNQVGYD
GEG
>g768.t1
DSIHHCYHALGSMVVLSLPSGSQARPVSGPGPPSQPVGSNPH
>g768.t2
LVGCCGPLFNVFQLRVMAYDVSPGICSVPEDSIHHCYHALGSMVVLSLPSGSQARPVSGPGPPSQPVGSNPH
>g769.t1
MIYMEPHMLGWQPLLVSWLSTLASTVSSMHRDLISGMFHRVLPVCLQLVRKATKELSPTSDTNLVRSLMNLMDCMMDEFQ
DEARIKSMDDRDICSWLEHP
SPQHITPETHTCNKFCSDL
>g769.t2
MIYMEPHMLGWQPLLVSWLSTLASTVSSMHRDLISGMFHRVLPVCLQLVRKATKSSAPRRPSFPVDGVLELNLPSALLRS
KHQAWRCPFLPRIELSPTSD
TNLVRSLMNLMDCMMDEFQDEARIKSMDDRDICSWLEHPSPQHITPETHTCNKFCSDL
>g769.t3
MIYMEPHMLGWQPLLVSWLSTLASTVSSMHRDLISGMFHRVLPVCLQLVRKATKSSAPRRPSFPVDGVLELNLPSALLRS
KHQAWRCPFLPRIELSPTSD
TNLVRSLMNLMDCMMDEFQDEARIKSMDDRDICSWLEHITPETHTCNKFCSDL
>g770.t1
MSYGTDVCCLFVKVQHTQQCSFCDSVSMSSHSAVREPTADVLELGRASTVRRKRILKPIPGEFRRRKCRGHRSPPGHARV
LLPGTSLLRSPLCPPKAAHP
NPPRLAWRTPSSARYNGDNCRGVTEGYDPECALNGVELAEIIVFFPPFVPEDRPGFEKMEQGYNAACTSYALHAKEAVGI
VFLSFPNVSRLICKRTADAG
PLLLVVSATINRARSVAI
>g771.t1
MTACPPMERRQHVLLHKPLLLNNPLPPTILISTNPHQVDLKIFPLTMDDISGGQTLRLKINHEESNMNYYFLRSYSGRVQ
GRTAWYQLNIVPKKETQNSR
RGCEQSLPLSRPPSTATELGGREDWLHGAWPNKVSPRGRLSWAAGTRTRGPPRAHEP
>g772.t1
DEGSGSSSGNGCAADGCTPPPTEPIPPAGSEAPVVRADRSDERAAAPGGSSAALCSSPLLLLLTTGLLLIIACSPPAARD
RQKPLGAYGNHVGSPGTSRN
LWEHHRTSRNL

>g773.t1
DKKYSCQECEAAFSRSDHLKIHLKTHSSSKPFKCSVCKRGFSSTSSLQSHMQAHRKNREHLALRAGRDGSRKGGEGDLDQ
DLYMCDYCEETFSQTDELEK
HVVTRHPQLSDRADLQCIHCPEIFLDEASLLTHIETQHGNRKHK
>g774.t1
MLHHHLLTVAQKVIKLAFEEFDLERGYDTLTVGDGGKIGDTRRVLYVLTGSSVPDLIVSLSNQMWLHLQSDDTIGSQGFK
AVYEELTVDALPGNRPHSLE
VVTGRAVYCWPTGTPGSGRG
>g775.t1
MALADTESGISNCNEFSPFPEIIELNVGGQVYVTRHTTLVAVPDSLLWNMFSKKTPKELARDNKGRFFLDRDGFLFRYIL
DYLRDLNLVLPDYFPERSRL
QREADFFQLRELSKRLLHPKMSKDDSISEEICQSDTDEGAPQCSAMDTLRMDTLRTITAVGSSPRSPSLDSRKSGYITIG
YRGSYTIGRDIQTDAKFRRV
ARITVCGKTSLAKEVFGDTLNESRDPDRPPERYTSRYYLKYNFLEQAFDKLTEVGFHMVACSSTGTCAYTSNDPNEDKIW
TSYTEYVFCRE
>g775.t2
MFSKKTPKELARDNKGRFFLDRDGFLFRYILDYLRDLNLVLPDYFPERSRLQREADFFQLRELSKRLLHPKMSKDDSISE
EICQSDTDEGAPQCSAMDTL
RMDTLRTITAVGSSPRSPSLDSRKSGYITIGYRGSYTIGRDIQTDAKFRRVARITVCGKTSLAKEVFGDTLNESRDPDRP
PERYTSRYYLKYNFLEQAFD
KLTEVGFHMVACSSTGTCAYTSNDPNEDKIWTSYTEYVFCRE
>g776.t1
MLRKSVTGSFGAAISGLGRSHLTEGVISRSKRMMLDTLGVGLIGTRTDVYDKALQYSQSFASRDQSQVWGRAETALPPSY
AAFVNGVAVHSMDFDDTWHP
ATHPSGAILPTVLALAQVLPRQPTGLDLLLAFNVGIEVQGRLLSFSREAVDIPQKFHPPTVVGVMGSAAASAKLLGLSPA
QCTHALAIAASFAGSPLANA
ATQTKPLHMGNAAGRGLEAAWLACLGLEGNPDVLDMESGFGVFFTEYMPSAMADVGGFQWALEKQNVAVKRFPAHLGMHW
VAEAALAAKAELERGGGSRG
HPDHIRRVTLRVPPSKYIDCPYPSTEHQARHSFQFNACSALLDGRVTVESFSDARRNRPALGELLSKVTVESPKDNHPSF
DRMYCDVVIETHRGDRYTGR
CDTFYGHWRRPLSQEDLERKFRDNASTVLSPEGVEGVVDAVGNMEQKTDSSTLSLYLQMTRNQ
>g776.t2
MLRKSVTGSFGAAISGLGRSHLTEGVISRSKRMMLDTLGVGLIGTRTDVYDKALQYSQSRRAFHLQSFASRDQSQVWGRA
ETALPPSYAAFVNGVAVHSM
DFDDTWHPATHPSGAILPTVLALAQVLPRQPTGLDLLLAFNVGIEVQGRLLSFSREAVDIPQKFHPPTVVGVMGSAAASA
KLLGLSPAQCTHALAIAASF
AGSPLANAATQTKPLHMGNAAGRGLEAAWLACLGLEGNPDVLDMESGFGVFFTEYMPSAMADVGGFQWALEKQNVAVKRF
PAHLGMHWVAEAALAAKAEL
ERGGGSRGHPDHIRRVTLRVPPSKYIDCPYPSTEHQARHSFQFNACSALLDGRVTVESFSDARRNRPALGELLSKVTVES
PKDNHPSFDRMYCDVVIETH
RGDRYTGRCDTFYGHWRRPLSQEDLERKFRDNASTVLSPEGVEGVVDAVGNMEQKTDSSTLSLYLQMTRNQ
>g777.t1
MYAPNVDQMHVVDHLKGGPTEEKRNVLVESARLARGDIQDLSELLAKDHDAIIFPGGFGAAKNLCTWATEGKDCSVNEQV
KAVLQAFHGAGKAIGLCCIS
PVLAARVLPGCEVTVGRENDDKYPNTTGTAAAIGQLGCTHVSKSVAESHVDEKNKVVTTCAFMCDAPLHEIFDGIGTMVQ
AVLKLA
>g778.t1
MEWYELFQLGNCTFPHLRDNVSAPFWCNQGAACFFEGIDDVHWSQNGTLEKVAVITGDMFNDMARWVQEDNQTGIYYETW
TVRSDPGPNATVWFESFDCS
QFVHRAYRKLADLGAKLSSRSRTDYTKVYLYSGEPTYLGNESTIFGQPSMENLAADIQKFYYTFRPHQSYVDFAVSLLDI
YEKVVVHKNFYLYYNSEYWH
LPMKPPYMKIVYEEVPLP
>g778.t2
MDQHSGGFVDFGAIKHSTAGKNYSNALSHGDVDSYCEALYPFCPAGDRDGRIPYMRDTDRISVFRLQAPVWEFKYGDLMG
KFHIMHDAIGFSSAESERNY
TMEWYELFQLGNCTFPHLRDNVSAPFWCNQGAACFFEGIDDVHWSQNGTLEKVAVITGDMFNDMARWVQEDNQTGIYYET

WTVRSDPGPNATVWFESFDC
SQFVHRAYRKLADLGAKLSSRSRTDYTKVYLYSGEPTYLGNESTIFGQPSMENLAADIQKFYYTFRPHQSYVDFAVSLLD
IYEKVVVHKNFYLYYNSEYW
HLPMKPPYMKIVYEEVPLP
>g778.t3
MDQHSGGFVDFGAIKHSTAGKNYSNALSHGGVARPIQHIMHDAIGFSSAESERNYTMEWYELFQLGNCTFPHLRDNVSAP
FWCNQGAACFFEGIDDVHWS
QNGTLEKVAVITGDMFNDMARWVQEDNQTGIYYETWTVRSDPGPNATVWFESFDCSQFVHRAYRKLADLGAKLSSRSRTD
YTKVYLYSGEPTYLGNESTI
FGQPSMENLAADIQKFYYTFRPHQSYVDFAVSLLDIYEKVVVHKNFYLYYNSEYWHLPMKPPYMKIVYEEVPLP
>g779.t1
MKRVKGDEEESNSPSHESPPEVCKKVCKRLSEESHEDVACDFGFDWARLPQEILLHIFQYLPLLDRAFASQVCRGWNEAF
HMPELWRCFEFELNQPASSY
LKATHPDLIKQIIKRHSNHLQYVSFKVDSSTESAEAACDILSQLVNCSLKTLGLISTARPSFMELPKSHFISALTVVFVN
SKSLSSLKIDDTPVDDPSLK
VLVANNSDTLKLLKMSSCPHVSPAGILCVADQCHGLRELALNYHLLSDELLLALSSEKHVHLEHLRIDVVSENPGQHFHT
IRKSSWDAMVRHSPKFNLVM
YFFLYEDEFGPFFCEEIPVTHLYFGRSVSKDVLGRVGLHCPRLVELVVCANGLRPLDEELIRIAKRCTQLSAIGLGECEV
SCSAFVEFVKMCGRRLSQLS
IMEEVLIPDHKYSLDDIHWEVSKHLGRVWFPDMMPTW
>g780.t1
MRLRNLPVDPPGGASPLFEDTSRVGAGAQPRGCRTQPAGPQAGPYPGQSILSDPMSNLAMAYGSSLASQGKEMVDKNLDR
FIPISRLKYYFAVDTMYVGK
KLGLLVFPYMHENWEVSYQQDTPVAPRFDVNAPDLYIPFMGFITYVLVAGLALGTQDRFSPELLGVQASSAFVWLIMEVV
AVLLSLYLVTVNTDLTTVDL
LAFSGYKYVGMIVGVIGGLLFGRLGYYLSLLWCCAAIFVFMIRTLRLKLLSEAAAEGKLVRGARNQLRMYLTMSIAAAQP
VFMYWLTYHLVR
>g780.t2
PAMRLRNLPVDPPGGASPLFEDTSRVGAGAQPRGCRTQPAGPQAGPYPGQSILSDPMSNLAMAYGSSLASQGKEMVDKNL
DRFIPISRLKYYFAVDTMYV
GKKLGLLVFPYMHENWEVSYQQDTPVAPRFDVNAPDLYIPFMGFITYVLVAGLALGTQDRFSPELLGVQASSAFVWLIME
VVAVLLSLYLVTVNTDLTTV
DLLAFSGYKYVGMIVGVIGGLLFGRLGYYLSLLWCCAAIFVFMIRTLRLKLLSEAAAEGKLVRGARNQLRMYLTMSIAAA
QPVFMYWLTYHLVR
>g780.t3
APHQPDDPLTTDQADRQGQGGKPAMRLRNLPVDPPGGASPLFEDTSRVGAGAQPRGCRTQPAGPQAGPYPGQSILSDPMS
NLAMAYGSSLASQGKEMVDK
NLDRFIPISRLKYYFAVDTMYVGKKLGLLVFPYMHENWEVSYQQDTPVAPRFDVNAPDLYIPFMGFITYVLVAGLALGTQ
DRFSPELLGVQASSAFVWLI
MEVVAVLLSLYLVTVNTDLTTVDLLAFSGYKYVGMIVGVIGGLLFGRLGYYLSLLWCCAAIFVFMIRTLRLKLLSEAAAE
GKLVRGARNQLRMYLTMSIA
AAQPVFMYWLTYHLVR
>g781.t1
MAEAQSDYASGEREEGGPSPGSSNKDAIHLPCSRLLRSTVIFRLKDCSCQRMMNTMYTDKSGVRSGISRGKAQ
>g782.t1
LGHLDSVPEEELCEASDITRPLDVGTLQAKLLQVLQNMPESHDPSVLKEVIESLGIGLGSDSQADRRHTLESIKEESSEE
EETDPGPGLHLSTKLRHPPS
DQTASEVTSDGDDARHDTPYCTQDYLECVGGLQDHADFLDDVKNELLSQCSFSSNMEELLAQLRDVQEMESNLQKGAHLV
LEDVAFDIQSLVSENADFLS
SAQSKHILQLLSTTQRAFREQTETLATQRHTLEGLLESRERDMQQKVVTEKQKEFSSKLDELCDSLTLTENRLIGHQHET
GHPDSVSNLQQYQQEHQALQ
KDMLTNAGALSDVISSTKKFLEENRAKMTPEQVAAVEKKLEEAKSKAKLINQRAEESRKDLEKVVTTAIKQESEKTAAVE
QLEESKHKIEGLLGWISNIG
NEKDGVGSPGHMSKENGNLPEDTSAAGVIGEEEEEKEDDANGNGPDATETDSVSRTNEKNSSLDIDKQYDRVKARHQEIL
SQQQELIMATQSAQALLDKQ
AQVLSPEDQEKLQRDIQELRGRYDASLGRAELEMKQVQCVQEELRKFRGDSQEFEVWLGQAEAEMEKLSAPGDCLAALGQ

KLLRQKSFSEDVISHKGDLR
FVTISGQKVLDAARACGSADAAGGEGSQLPVDTAGTCASVKEKLDSAAGRYKTLYTQCNNLASNLKDVVDKYTKYEDSSA
DLVKWLNSSEEEARRQQSEA
IAADPQTLQRQLEDTKARRTARHQYPPARMGPA
>g782.t2
LGSDSQADRRHTLESIKEESSEEEETDPGPGLHLSTKLRHPPSDQTASEVTSDGDDARHDTPYCTQDYLECVGGLQDHAD
FLDDVKNELLSQCSFSSNME
ELLAQLRDVQEMESNLQKGAHLVLEDVAFDIQSLVSENADFLSSAQSKHILQLLSTTQRAFREQTETLATQRHTLEGLLE
SRERDMQQKVVTEKQKEFSS
KLDELCDSLTLTENRLIGHQHETGHPDSVSNLQQYQQEHQALQKDMLTNAGALSDVISSTKKFLEENRAKMTPEQVAAVE
KKLEEAKSKAKLINQRAEES
RKDLEKVVTTAIKQESEKTAAVEQLEESKHKIEGLLGWISNIGNEKDGVGSPGHMSKENGNLPEDTSAAGVIGEEEEEKE
DDANGNGPDATETDSVSRTN
EKNSSLDIDKQYDRVKARHQEILSQQQELIMATQSAQALLDKQAQVLSPEDQEKLQRDIQELRGRYDASLGRAELEMKQV
QCVQEELRKFRGDSQEFEVW
LGQAEAEMEKLSAPGDCLAALGQKLLRQKSFSEDVISHKGDLRFVTISGQKVLDAARACGSADAAGGEGSQLPVDTAGTC
ASVKEKLDSAAGRYKTLYTQ
CNNLASNLKDVVDKYTKYEDSSADLVKWLNSSEEEARRQQSEAIAADPQTLQRQLEDTKARRTARHQYPPARMGPA
>g782.t3
LDSVPEEELCEASDITRPLDVGTLQAKLLQVLQNMPESHDPSVLKEVIESLGIGLGSDSQADRRHTLESIKEESSEEEET
DPGPGLHLSTKLRHPPSDQT
ASEVTSDGDDARHDTPYCTQDYLECVGGLQDHADFLDDVKNELLSQCSFSSNMEELLAQLRDVQEMESNLQKGAHLVLED
VAFDIQSLVSENADFLSSAQ
SKHILQLLSTTQRAFREQTETLATQRHTLEGLLESRERDMQQKVVTEKQKEFSSKLDELCDSLTLTENRLIGHQHETGHP
DSVSNLQQYQQEHQALQKDM
LTNAGALSDVISSTKKFLEENRAKMTPEQVAAVEKKLEEAKSKAKLINQRAEESRKDLEKVVTTAIKQESEKTAAVEQLE
ESKHKIEGLLGWISNIGNEK
DGVGSPGHMSKENGNLPEDTSAAGVIGEEEEEKEDDANGNGPDATETDSVSRTNEKNSSLDIDKQYDRVKARHQEILSQQ
QELIMATQSAQALLDKQAQV
LSPEDQEKLQRDIQELRGRYDASLGRAELEMKQVQCVQEELRKFRGDSQEFEVWLGQAEAEMEKLSAPGDCLAALGQKLL
RQKSFSEDVISHKGDLRFVT
ISGQKVLDAARACGSADAAGGEGSQLPVDTAGTCASVKEKLDSAAGRYKTLYTQCNNLASNLKDVVDKYTKYEDSSADLV
KWLNSSEEEARRQQSEAIAA
DPQTLQRQLEDTKARRTARHQYPPARMGPA
>g783.t1
MSEEERQKAQEQLMALSQAYCDLSQQCADQMPSTDQAVKRMLGITHPTTTKALTRTMKARLPRVLHLSPLFP
>g784.t1
MGLTTVVEVQATIAQFLSKPKYIAGLYLESSKKRISFLEAAEKGILAKANAIEFLEAQAATGSSINLAAGKPQSFEDALE
KENLDTILKDKLRQAHLAFS
GYLHGNKKLSVFEAIEARLVDRCKGNSLLEVQIATGGLVKPELGVRVPADVAVDRGMLNKATLQSLYEPVSNPKVFYNPS
TKRTAHYRDLLNMCVYDVHD
GIYLLPFGDAHQSGTSLTKAREVSVISSHRGTEMSAYEAYKGKHISQGTYLFLSRGGGEWVEESGIDVNGHTRHIIADSQ
SGRQLCIETALSQRLLEVTE
LASYRSGDLSIYELVDLIYSRMVVVEDVNGPIAGLWDATQKKRLSVFQALQQNLIDHVTALRLLEAQVCTGGILDPASGE
KATLKEAACRGLLDQSLVHQ
LPLYEQAYCGIIQPKTGKRISVAEAIQGNYFPKDIGLRCLEFQLLTGGLINPETQSRISLEEVTQSGLLDKHMLAVLKDK
SYSKILICPKTRRGISFKEA
LERSVHDQYTGLRLLEAVRANWNYRYM
>g785.t1
MNHNLAYLSGVSSVSSSTLTSLGSAESMWVGVEPPFCSSPFYLNNSSYGRLEMLQQIANRVQRDCVNGEDKLSLARTALQ
SDAKRLEAGIQFQNEAEIAG
YLLECENTLRQQVVDIQMLLDGKFPFADQLVQRVSKLRDALLSLRTECSSVYNQGRTLTAEQTKRMISGISQSLNFPQSP
ALAPGGAGAGAGGFTSGLGP
ALTPGPQAQSVPGGGGGMESESLKHQIRKPMGKSSLLDANMTEEEVNMNFVQDLLDWVEEMQVQVDRGDWGTDLPSVETQ
IEDFKSVHKAIEEFQMSLKE
AKHSEFQMTLPLKLTYSEKVDKLQSNYEKLLNCSKKRRNHLENLHDFVSRATQELIWLNEKEEEVAFDWSDRNVNITTK

RDYHADLMRELEEKEEVIKS
VQDMAEGLMLEKHPARLTIEAYRAAMQTQWNWILQLCSCVEQHLKENAVYFEFFSEAKESLDCLKNLQDAIQRKYSCDRT
SSLHKLEDLVQESMEEKEQL
LQYCSTVAGLVGRSKTIVQLRPRNPDSPIRSSIPVRAICDYRQIEITIFKDDECVLANNSHRAKWKVINPGGNEAMVPSV
CFTVPPPNKEAVEMASRIEQ
TYQEVLSLWHHSHVNMKSVVSWHFLMADIRAIRNWNVASIKTMLPGAHQQEVLGNLDSHFEEFLEDSEESEVFTVADRAH
SEREVVACKQYYEDLLRSAE
KGRLSSEEREESEYNNYISEVHNFRMHLEAHEEHLIRKIRTPLDRDDLEQSLLRITEQEKKMCELEQLKDDLETMKEKCE
VFLKEASTSPSLPTLSSELH
VLVQNMGQVYSMASIFMDKLKTVSLVVKNSQRAEALVKVYESKLYEEDAVNCDLKSIETVISTLKHWRSEIDEKQEVFHD
MEDELKKARAISERMFKVHN
ERDFDLDWHREKADQLGERWQSVHSQIDNRLRELESISKSLKYYKDSYGSLDEWVKDMEAAQLKAQENKPEDSKALAELL
NTQKVLVAEIEQKQSRITEC
QKHSEQYSSAVKDYELQLMTYRAMVDSQHKSPLKKRRMQNSSDTIQQEFMDLRTRYTALVTLMTQCVKFASETLRRAEDE
EVRLVLIMR
>g785.t2
MNHNLAYLSGVSSVSSSTLTSLGSAESMWVGVEPPFCSSPFYLNNSSYGRLEMLQQIANRVQRDCVNGEDKLSLARTALQ
SDAKRLEAGIQFQNEAEIAG
YLLECENTLRQQVVDIQMLLDGKFPFADQLVQRVSKLRDALLSLRTECSSVYNQGRTLTAEQTKRMISGISQSLNFPQSP
ALAPGGAGAGAGGFTSGLGP
ALTPGPQAQSVPGGGGGMESESLKHQIRKPMGKSSLLDANMTEEEVNMNFVQDLLDWVEEMQVQVDRGDWGTDLPSVETQ
IEDFKSVHKAIEEFQMSLKE
AKHSEFQMTLPLKLTYSEKVDKLQSNYEKLLNCSKKRRNHLENLHDFVSRATQELIWLNEKEEEEVAFDWSDRNVNITTK
RDYHADLMRELEEKEEVIKS
VQDMAEGLMLEKHPARLTIEAYRAAMQTQWNWILQLCSCVEQHLKENAVYFEFFSEAKESLDCLKNLQDAIQRKYSCDRT
SSLHKLEDLVQESMEEKEQL
LQYCSTVAGLVGRSKTIVQLRPRNPDSPIRSSIPVRAICDYRQIEITIFKDDECVLANNSHRAKWKVINPGGNEAMVPSV
CFTVPPPNKEAVEMASRIEQ
TYQEVLSLWHHSHVNMKSVVSWHFLMADIRAIRNWNVASIKTMLPGAHQQEVLGNLDSHFEEFLEDSEESEVFTVADRAH
SEREVVACKQYYEDLLRSAE
KGRLSSEEREESEYNNYISEVHNFRMHLEAHEEHLIRKIRTPLDRDDLEQSLLRITEQEKKMCELEQLKDDLETMKEKCE
VFLKEASTSPSLPTLSSELH
VLVQNMGQVYSMASIFMDKLKTVSLVVKNSQRAEALVKVYESKLYEEDAVNCDLKSIETVISTLKHWRSEIDEKQEVFHD
MEDELKKARAISERMFKVHN
ERDFDLDWHREKADQLGERWQSVHSQIDNRLRELESISKSLKYYKDSYGSLDEWVKDMEAAQLKAQENKPEDSKALAELL
NTQKVLVAEIEQKQSRITEC
QKHSEQYSSAVKDYELQLMTYRAMVDSQHKSPLKKRRMQNSSDTIQQEFMDLRTRYTALVTLMTQCVKFASETLRRAEDE
ESKKAVERVESTKWTENIEL
QKAAGEQEISQLQQRVQELEASLTERQAQLGHLEGEVESQRRTIEDLTLERSMLEDKTFQYFAELEQTANSKAAMEEELR
HMQQLIKESEAKRISLGEAL
ANMKKITDECTSQVLEDDCKKKENEKLENGQNLLKSGKVTRSECHSQDPDIRAVNNGVLESTTARKYNTEAFTISEVGSD
DLRHEIEGLTNDKNRAEMEV
KKLMSELNVAVEQRNGAEKDAQRCKGLLDDSNAELKELRADMELERNRMKVQSDELRQETVDMKTSIHLLQDQIKVLQRE
KSSLEQKIVFHTTEVEGLKE
QLKINQGKICRLNSVEQEYDNKMQNLEEELSAKQAEIDQLKIKLSGYNEEAKAIAQLQEHITNYQKEIKKLKLELNSSST
ERNVSQQKSXSLRQEMIVLQ
RDRAEAQEKSRSMGSEVRELKEKLDKAQDEAKEKHNECALHKVRCKQMEEQLNNYEQEVQIQMDKLQQEKAILGCNLLKA
QYQITLLEDEKVKLKSKISQ
LESHRCPRSEDMEPFKQRLLDSEQNVRIKEEEARSLREQIACFVEEVETLEEKLRELELLIKMEGRHKDEHTKRAVDQPC
AKDNVASTRLSKLSIQTTVT
TSYEDGSHEEEAVKKRVSSEV
>g785.t3
MNHNLAYLSGVSSVSSSTLTSLGSAESMWVGVEPPFCSSPFYLNNSSYGRLEMLQQIANRVQRDCVNGEDKLSLARTALQ
SDAKRLEAGIQFQNEAEIAG
YLLECENTLRQQVVDIQMLLDGKFPFADQLVQRVSKLRDALLSLRTECSSVYNQGRTLTAEQTKRMISGISQSLNFPQSP
ALAPGGAGAGAGGFTSGLGP

ALTPGPQAQSVPGGGGGMESESLKHQIRKPMGKSSLLDANMTEEEVNMNFVQDLLDWVEEMQVQVDRGDWGTDLPSVETQ
IEDFKSVHKAIEEFQMSLKE
AKHSEFQMTLPLKLTYSEKVDKLQSNYEKLLNCSKKRRNHLENLHDFVSRATQELIWLNEKEEEEVAFDWSDRNVNITTK
RDYHADLMRELEEKEEVIKS
VQDMAEGLMLEKHPARLTIEAYRAAMQTQWNWILQLCSCVEQHLKENAVYFEFFSEAKESLDCLKNLQDAIQRKYSCDRT
SSLHKLEDLVQESMEEKEQL
LQYCSTVAGLVGRSKTIVQLRPRNPDSPIRSSIPVRAICDYRQIEITIFKDDECVLANNSHRAKWKVINPGGNEAMVPSV
CFTVPPPNKEAVEMASRIEQ
TYQEVLSLWHHSHVNMKSVVSWHFLMADIRAIRNWNVASIKTMLPGAHQQEVLGNLDSHFEEFLEDSEESEVFTVADRAH
SEREVVACKQYYEDLLRSAE
KGRLSSEEREESEYNNYISEVHNFRMHLEAHEEHLIRKIRTPLDRDDLEQSLLRITEQEKKMCELEQLKDDLETMKEKCE
VFLKEASTSPSLPTLSSELH
VLVQNMGQVYSMASIFMDKLKTVSLVVKNSQRAEALVKVYESKLYEEDAVNCDLKSIETVISTLKHWRSEIDEKQEVFHD
MEDELKKARAISERMFKVHN
ERDFDLDWHREKADQLGERWQSVHSQIDNRLRELESISKSLKYYKDSYGSLDEWVKDMEAAQLKAQENKPEDSKALAELL
NTQKVLVAEIEQKQSRITEC
QKHSEQYSSAVKDYELQLMTYRAMVDSQHKSPLKKRRMQNSSDTIQQEFMDLRTRYTALVTLMTQCVKFASETLRRAEDE
ESKKAVERVESTKWTENIEL
QKAAGEQEISQLQQRVQELEASLTERQAQLGHLEGEVESQRRTIEDLTLERSMLEDKTFQYFAELEQTANSKAAMEEELR
HMQQLIKESEAKRISLGEAL
ANMKKITDECTSQVLEDDCKKKENEKLENGQNLLKSGKVTRSECHSQDPDIRAVNNGVLESTTARKYNTEAFTISEVGSD
DLRHEIEGLTNDKNRAEMEV
KKLMSELNVAVEQRNGAEKDAQRCKGLLDDSNAELKELRADMELERNRMKVQSDELRQETVDMKTSIHLLQDQIKVLQRE
KSSLEQKIVFHTTEVEGLKE
QLKINQGKICRLNSVEQEYDNKMQNLEEELSAKQAEIDQLKIKLSGYNEEAKAIAQLQEHITNYQKEIKKLKLELNSSST
ERNVSQQKSCELKSEVNELN
AMLKKTTAELQKESTQKSLRQEMIVLQRDRAEAQEKSRSMGSEVRELKEKLDKAQDEAKEKHNECALHKVRCKQMEEQLN
NYEQEVQIQMDKLQQEKAIL
GCNLLKAQYQITLLEDEKVKLKSKISQLESHRCPRSEDMEPFKQRLLDSEQNVRIKEEEARSLREQIACFVEEVETLEEK
LRELELLIKMEGRHKDEHTK
RAVDQPCAKDNVASTRLSKLSIQTTVTTSYEDGSHEEEAVKKRVSSEV
>g785.t4
MLEDKTFQYFAELEQTANSKAAMEEELRHMQQLIKESEAKRISLGEALANMKKITDECTSQVLEDDCKKKENEKLENGQN
LLKSGKVTRSECHSQDPDIR
AVNNGVLESTTARKYNTEAFTISEVGSDDLRHEIEGLTNDKNRAEMEVKKLMSELNVAVEQRNGAEKDAQRCKGLLDDSN
AELKELRADMELERNRMKVQ
SDELRQETVDMKTSIHLLQDQIKVLQREKSSLEQKIVFHTTEVEGLKEQLKINQGKICRLNSVEQEYDNKMQNLEEELSA
KQAEIDQLKIKLSGYNEEAK
AIAQLQEHITNYQKEIKKLKLELNSSSTERNVSQQKSXSLRQEMIVLQRDRAEAQEKSRSMGSEVRELKEKLDKAQDEAK
EKHNECALHKVRCKQMEEQL
NNYEQEVQIQMDKLQQEKAILGCNLLKAQYQITLLEDEKVKLKSKISQLESHRCPRSEDMEPFKQRLLDSEQNVRIKEEE
ARSLREQIACFVEEVETLEE
KLRELELLIKMEGRHKDEHTKRAVDQPCAKDNVASTRLSKLSIQTTVTTSYEDGSHEEEAVKKRVSSEV
>g786.t1
MAAKSIALHVLVSDDGEPPPSSTLRTSEKSTMEHLVERACFRDYQRLGLGTITVSTSRSNNDEQFRITVANRLYSLCRSY
PGLLVVPQGVQDSSLHKVAR
CYRHNRLPVVCWKHPRSRALLLRSGGFHGKSVVGLFKSPNPSTTTTSSSSESSSSLEQEKYLQAILDSIPIHFKMNSSNT
LSGRSQTGVSPGCPHPVGVG
RLGACS
>g786.t2
MAAKSIALHVLVSDDGEPPPSSTLRTSEKSTMEHLVERACFRDYQRLGLGTITVSTSRSNNDEQFRITVANRLYSLCRSR
LSSSYPGLLVVPQGVQDSSL
HKVARCYRHNRLPVVCWKHPRSRALLLRSGGFHGKSVVGLFKSPNPSTTTTSSSSESSSSLEQEKYLQAILDSIPIHFKM
NSSNTLSGRSQTGVSPGCPH
PVGVGRLGACS
>g786.t3

SIALHVLVSDDGEPPPSSTLRTSEKSTMEHLVERACFRDYQRLGLGTITVSTSRSNNDEQFRITVANRLYSLCRSYPGLL
VVPQGVQDSSLHKVARCYRH
NRLPVVCWKHPRSRALLLRSGGFHGKSVVGLFKSPNPSTTTTSSSSESSSSLEQEKYLQAILDSIPIHFKMNSSNTLSGR
SQTGVSPGCPHPVGVGRLGA
CS
>g787.t1
LKPDVVKHTLQVNTLAIATLRTADYHPVLSGLRVTPLPVQLQTLKKSLDLEKDTEDDETVLQNTYRPEQGSPRFTKNRRR
EREAGEKVINVSHGADEEEA
PQPAKGKRKSRRELPLPP
>g788.t1
MPMLGVRAPSRSSTPPTPADREGAESPLFQSRCSSPLNLLQLEESPSNRLEVATAMAASQQTTPSSVQGGVAPGQSSTNE
RSSDESKETENGETNESNQD
AMSTSSDLLDLLLQEDSRSGTGSAASGSGSSGSGSSGSGSSGSGSSGSGSNGCSSSGTSGTRSSHTSKYFGSVDSSENDH
SRKQPAAGGGGGSISGGGDG
GEEQFIKCVLQDPIWLLMANTDDKVMMTYQLPVRDMETVLREDREALRAMQKHQPRFSEEQKRELSQVHPWIRTGRLPRA
INVSACTGCKSPAAASAPYD
VEIHEMELCADRAAPRRADAPMEEGSQPLEEEEDQQMQAGAGTKFVGGGDQEMTTETQEVTSGQVGEGKPGAIATEADMT
L
>g788.t2
MLGVRAPSRSSTPPTPADREGAESPLFQSRCSSPLNLLQLEESPSNRLEVATAMAASQQTTPSSVQGGVAPGQSSTNERS
SDESKETENGETNESNQDAM
STSSDLLDLLLQEDSRSGTGSAASGSGSSGSGSSGSGSSGSGSSGSGSNGCSSSGTSGTRSSHTSKYFGSVDSSENDHSR
KQPAAGGGGGSISGGGDGGE
EQFIKCVLQDPIWLLMANTDDKVMMTYQLPVRDMETVLREDREALRAMQKHQPRFSEEQKRELSQVHPWIRTGRLPRAIN
VSACTGCKSPAAASAPYDVE
IHEMELCADRAAPRRADAPMEEGSQPLEEEEDQQMQAGAGTKFVGGGDQEMTTETQEVTSGQVGEGKPGAIATEADMTL
>g789.t1
LCLWTGGVAGWSYFYSNSTMDWTSARGWCQDHYTDMVAIQNQE
>g790.t1
MKDHHLNPVLLRFTDGELETRYSSEKEGRSSVAFGSCCVVLLFITAMEVFIDPRLTVNYVTLAIGEVLLLILTICSVAAI
FPR
>g791.t1
KDDPKKNCRWCNCHVCGIKQDPDKQLLCDECDMAFHMYCLDPPLTSIPDDEDWYCPGCRNDASEVVLAGEKLKESKKKSK
MASASSSSQRDWGKGMACVG
RTKQCTIVPSNHYGPVPGVPVGSQWKFRVQVSESGAHRPHVAGIHGRSNDGAYSLVLAGGYEDDVDDGNEFTYTGSGGRD
LSGNKRTAEQSCDQKLTNMN
RALALNCNTPVNDKDGGEAKNWKAGKSVRVIRSCKGRKHSKFSPEDGNRYDGIYKVVQYWPEKGKSGFLVWRYLLKRDDD
EPAPWTRDGKERIKKLGLTM
QYPEGYLETQAAKEKEKEKENKSEPEEETPTKGKRKRKSQGTAKGTPKKMKVEVYKLTVEQKSLIKEDEANKKIWMEAKQ
SLALGPKFLNKVEEMFLCIC
CQEVVYQPITTECQHNVCKECLQRSFRADVYTCPACRHDLGKNYAMTINNPLQVILNQFFPGYSNGR
>g791.t2
KDDPKKNCRWCNCHVCGIKQDPDKQLLCDECDMAFHMYCLDPPLTSIPDDEDWYCPGCRNDASEVVLAGEKLKESKKKSK
MASASSSSQRDWGKGMACVG
RTKQCTIVPSNHYGPVPGVPVGSQWKFRVQVSESGAHRPHVAGIHGRSNDGAYSLVLAGGYEDDVDDGNEFTYTGSGGRD
LSGNKRTAEQSCDQKLTNMN
RALALNCNTPVNDKDGGEAKNWKAGKSVRVIRSCKGRKHSKFSPEDGNRYDGIYKVVQYWPEKGKSGFLVWRYLLKRDDD
EPAPWTRDGKERIKKLGLTM
QYPEGYLETQAAKEKEKEKENKSEPEEETPTKGKRKRKSQGTDSAKSSPAKGTPKKMKVEVYKLTVEQKSLIKEDEANKK
IWMEAKQSLALGPKFLNKVE
EMFLCICCQEVVYQPITTECQHNVCKECLQRSFRADVYTCPACRHDLGKNYAMTINNPLQVILNQFFPGYSNGR
>g792.t1
VPGLYNQGVVPMAMPAAVPVVASQVAECSEEDLKALQDMFPNLDREVVRTVLEAQQGNKDAAINSLLQMAEEL
>g793.t1
MSVQCTPVSCPVEPSPNCSDPGYKAVNKTDGCCFKETCGFETNITKASGDCCKVYTCVPKEVCVSSNVEYSGTETTDSKG
CCKTCQLRSVCEVKTEATVI

EIDGCKNLEPVQMSFCSGTCGSSTKFSVAANMMVHRCACCKELSTSVKEVQLVCADGSTRTQKYTTVESCSCEDSVCRAK
EKRRRRR
>g793.t2
MSVQCTPVSCPVEPSPNCSDPGYKAVNKTDGCCFKETCGFETNITKASGDCCKVYTCVPKEVCVSSNVEYSGYEYQTVQN
KCCGICVQTSCIITYNNIQY
ITQGTETTDSKGCCKTCQLRSVCEVKTEATVIEIDGCKNLEPVQMSFCSGTCGSSTKFSVAANMMVHRCACCKELSTSVK
EVQLVCADGSTRTQKYTTVE
SCSCEDSVCRAKEKRRRRR
>g793.t3
MSVQCTPVSCPVEPSPNCSDPGYKAVNKTDGCCFKETCGFETNITKASGDCCKVYTCVPKEVCVSSNVEYSGTETTDSKG
CCKTCQLRSVCEVKTEATVI
EIDGCKNLEPVQMSFCSGTCGSSTNVKEVQLVCADGSTRTQKYTTVESCSCEDSVCRAKEKRRRRR
>g794.t1
MITLRIPALDTDIIFGASSGGSYFRINLSPTLFSGITEGLCGTCDNSRDNDCHGPNGQFEECTDSAGKWLVPDTPCVTST
APPTVIPSYTTSSTSPAPCQ
PTACDILNSSVFSPCHLHVSPTPFLKSCQSDVCNGDNNTCAIFAVYATECAQAGVCIDWRNATNGQCDHDCPSDKVYNAC
GPPVQPTCNSK
>g794.t2
MITLRIPALDTDIIFGASSGGSYFRINLSPTLFSGITEGLCGTCDNSRDNDCHGPNGQFEECTDSADHDCPSDKVYNACG
PPVQPTCNSK
>g795.t1
GAFSYVKRVTEKTKNSGKPVEFAAKFISSRGKRKAAALAEMALLSELDHEKIAFFHDAFEKKSTLVLVLELYPLNGVE
>g796.t1
SSIYSIALKSVISVSTVLLVGLIAAYHCCEVQLYMHDNSAEDWRIAMTMERVTLMALELGAVAIHPCPVGLLAYIRRAPA
SSETELEIVLALPMFLRLYL
LGRAMMLHSRLFTDTASRSIGALNK
>g796.t2
SSIYSIALKSVISVSTVLLVGLIAAYHCCEVQLYMHDNSAEDWRIAMTMERVTLMALELGAVAIHPCPVGLLAYIRRAPA
SSETELEIVLALPMFLRLYL
LGRAMMLHSRLFTDTASRSIGALNKFYARGTAWEFLVEKLEAPPSIIW
>g796.t3
SSIYSIALKSVISVSTVLLVGLIAAYHCCEVQLYMHDNSAEDWRIAMTMERVTLMALELGAVAIHPCPVGLLAYIRRAPA
SSETELEIVLALPMFLRLYL
LGRAMMLHSRLFTDTASRSIGALNKFYARGTAWEFLVEKLEAPPSII
>g797.t1
MPCCCHSSESWVASEVERPAVTTNTRRPLNQAAAHELSHGHRLHPSKVRRVFLTMYYGYCGLHVDHNYNPWISARRPLY
>g798.t1
MSAPPTADAHGPSAPPSYDSVPGYEGTVAGGGGEPVDAFSQPPPGPWAIAAQAPTFFQDDQQVIKVPYTSSMKVHPVMGF
PDLNVVGAAGRLIREHQATY
AQTARVLQQRQTIELIPITKLVNSYVMISRELSLNTGACRLLQNIMPGLETAVVFQEKEGIVEKLFKWAQEAEQPLRIYA
TGLLAGAMENQDIAANYREE
NSVLVPLMLQRLRELQDAESQREFKRPRKGLSQPLLPLDEEAVDGGFGVAAFGSSRTRVSQRGEAEEDRNEVAENDAPFF
LGSPHRAGSRALSAVKTMMK
PMSAPVSLTHQDMLDGSGYLKRKAEVESGKKAKQKMHYSLPDPDRNFNELSNSSWSEMSPWVIGNNYHLYPLTPEIEQRL
ILQYLTPLGEYQEVRPRFHH
VL
>g798.t2
MNDRMESQAMSAPPTADAHGPSAPPSYDSVPGYEGTVAGGGGEPVDAFSQPPPGPWAIAAQAPTFFQDDQQVIKVPYTSS
MKVHPVMGFPDLNVVGAAGR
LIREHQATYAQTARVLQQRQTIELIPITKLVNSYVMISRELSLNTGACRLLQNIMPGLETAVVFQEKEGIVEKLFKWAQE
AEQPLRIYATGLLAGAMENQ
DIAANYREENSVLVPLMLQRLRELQDAESQREFKRPRKGLSQPLLPLDEEAVDGGFGVAAFGSSRTRVSQRGEAEEDRNE
VAENDAPFFLGSPHRAGSRA
LSAVKTMMKPMSAPVSLTHQDMLDGSGYLKRKAEVESGKKAKQKMHYSLPDPDRNFNELSNSSWSEMSPWVIGNNYHLYP
LTPEIEQRLILQYLTPLGEY
QEVRPRFHHVL

>g798.t3
MSAPPTADAHGPSAPPSYDSVPGYEGTVAGGGGEPVDAFSQPPPGPWAIAAQAPTFFQDDQQVIKVPYTSSMKVHPVMGF
PDLNVVGAAGRLIREHQATY
AQTARVLQQRQTIELIPITKNIMPGLETAVVFQEKEGIVEKLFKWAQEAEQPLRIYATGLLAGAMENQDIAANYREENSV
LVPLMLQRLRELQDAESQRE
FKRPRKGLSQPLLPLDEEAVDGGFGVAAFGSSRTRVSQRGEAEEDRNEVAENDAPFFLGSPHRAGSRALSAVKTMMKPMS
APVSLTHQDMLDGSGYLKRK
AEVESGKKAKQKMHYSLPDPDRNFNELSNSSWSEMSPWVIGNNYHLYPLTPEIEQRLILQYLTPLGEYQEVRPRFHHVL
>g799.t1
MAALRPLTKPKIVKKRTKKFIRHQSDRYVKIAKNWRKPRGIDNRVRRRFKGQMLMPNIGYGSNNKTKYMLPTGFKKFLVH
NIKELEVLMMSNKTHCAEIA
HNVSSKNRKLIVERAAQLAIKITNPNARLRSEENE
>g800.t1
DFVESCRFLGTIIAQDLKWDLNIRSLTKKGQQRTYFLRQLKRFNLPKTMMVHFYIAIIESILIPPSPSGMLQPLLRTRAD
CSVSFALLRR
>g801.t1
MTENAVNLALHYILQCLDSARTYARILFVDFSSAFNTVILALVQDKLSQLSVPDFTCRHNSVLKITDNTDLLWLISGEDE
SAYKWEIKHLVTWCRLNNFS
PHLML
>g801.t2
MTENAVNLALHYILQCLDSARTYARILFVDFSSAFNTVILALVQDKLSQLSVPDFTCRHNSVLKITDNTDLLWLISGEDE
SAYKWEIKHLVTWCRLNNFS
PHLMLGERVKESVTGMGGVGHNCTPQSPRGVQVL
>g802.t1
MSLRTEQAQAQAELQSQAEAQRLAFREQVRHLQDEHRSTVETLHAQLSRLEEQLFALQSQSGSPSVQSSRRPRLADLQRR
NPDVGHGGPGSIDLQTTARE
EGEGMETAEMESPLPPHPPLPSLELLLGSPDPKQEPFVWTVEPTKQELSQQLTTATRSMEHMNSLLHETEATNAVLMEQV
TLLKSELRRLERNQEREKSV
SNMEYLKNVLLQFIFLNSGNERQALLPVIHTMLQLSPEEKSRLAAIAQGEQDASGSRGSGWSSYLHSWSGIR
>g802.t2
LLSAQSENMSLRTEQAQAQAELQSQAEAQRLAFREQVRHLQDEHRSTVETLHAQLSRLEEQLFALQSQSGSPSVQSSRRP
RLADLQRRNPDVGHGGPGSI
DLQTTAREEGEGMETAEMESPLPPHPPLPSLELLLGSPDPKQEPFVWTVEPTKQELSQQLTTATRSMEHMNSLLHETEAT
NAVLMEQVTLLKSELRRLER
NQEREKSVSNMEYLKNVLLQFIFLNSGNERQALLPVIHTMLQLSPEEKSRLAAIAQGEQDASGSRGSGWSSYLHSWSGIR
>g802.t3
LRCRHILIKTVNEIIIPFRLLSAQSENMSLRTEQAQAQAELQSQAEAQRLAFREQVRHLQDEHRSTVETLHAQLSRLEEQ
LFALQSQSGSPSVQSSRRPR
LADLQRRNPDVGHGGPGSIDLQTTAREEGEGMETAEMESPLPPHPPLPSLELLLGSPDPKQEPFVWTVEPTKQELSQQLT
TATRSMEHMNSLLHETEATN
AVLMEQVTLLKSELRRLERNQEREKSVSNMEYLKNVLLQFIFLNSGNERQALLPVIHTMLQLSPEEKSRLAAIAQGEQDA
SGSRGSGWSSYLHSWSGIR
>g803.t1
MQRVEDAFYTLVREIRLHRLSKLCKEEKTPRCVKLKKLREIRKHKEKMSKDGKKKKKKSKTKCTLM
>g804.t1
MNQKHLLKFIQTTFKTDADRVVLERAGEKLTLGQVFQGLAMDPYDLTVDSLDVHAGRQTFHRFDKFNSKYNPVGASELRE
IYLKTDNYIEGEYFARLIKE
VAHELEESKYQHAEPRLSIYGRSPDEWLTLASWFAQHQLYSPNLRWMIQVPRIYDIFKSRKIIPNFSKMLENIFLPLFDA
TVNPQKNKEVHLFLKYLTGF
DSVDDESKHSDHMFSFSSPKPESWTTDDNPPYSYYLFHMYANVMVLNNLRKERGLNTFQFRPHCGEAGSITHLVSAFLTA
DNISHGLNLKKSPVLQYLYY
LAQVPIAMSPLSNNSLFLEYSKNPLREFLHKGLCVSLSTDDPMQFHYTKEALMEEYAIAAQLWKLSTCDVCEIARNSVLQ
SGLSHQEKQHFLGPNYLQDG
PEGNDIRRTNVAQIRLAYRQETLCNELSFLVDAMKMEASATPGQ
>g805.t1
MTGILMCAAGLPVCLTRAPKPVLHPPPINKSDMKPVPGVSGTRRKTKKKHLRRVSLSKTLNPNLLPGR

>g805.t2
LSRPARSLSCPVGQCCPGLPGLCPVLWVSAIQACQVSVLSCGVPALPVKNLTGSGPVHPALAGMTGILMCAAGLPVCLTR
APKPVLHPPPINKSDMKPVP
GVSGTRRKTKKKHLRRVSLSKTLNPNLLPGR
>g806.t1
LTRLSHCSTAHSTIPPIANYFGTKGRYEEVNPHLLNDITAVDTSLLIPHSPDGCTPVHVTAIIRHGTRYPTVKNIRRMQR
LYELVVHEASGDRAWLDEIK
SSWKMWYTDDMDGKLVEKGRDDHRHLAVRLSKLLPSLLNVENLRRHRVRFQTSSKHRCVESIEAFQQGLHNHWGVE
>g806.t2
LSHCSTAHSTIPPIANYFGTKGRYEEVNPHLLNDITAVDTSLLIPHSPDGCTPVHVTAIIRHGTRYPTVKNIRRMQRLYE
LVVHEASGDRAWLDEIKSSW
KMWYTDDMDGKLVEKGRDDHRHLAVRLSKLLPSLLNVENLRRHRVRFQTSSKHRCVESIEAFQQGLHNHWGVE
>g806.t3
MASTLSKQSLILTTFSLALTRLSHCSTAHSTIPPIANYFGTKGRYEEVNPHLLNDITAVDTSLLIPHSPDGCTPVHVTAI
IRHGTRYPTVKNIRRMQRLY
ELVVHEASGDRAWLDEIKSSWKMWYTDDMDGKLVEKGRDDHRHLAVRLSKLLPSLLNVENLRRHRVRFQTSSKHRCVESI
EAFQQGLHNHWGVE
>g807.t1
MQMDPVDVLKVLEFHNSPEGVSKTTGFCTNRRASNPDTAYRIAKQAQISAPTKQFFPGKGGVFPEDFSILATVRAKAGLQ
SFLLSIYNEQGVQQLGMELG
RSPVFLYEDPTGKPAPEDYPLFRNLNLADGKWHRIAISVEKKSITIIVDCNNKITKNLARSDHTTIDTNGITVFGTRLLD
EDVFQSFQ
>g807.t2
MLIFHTYVKATMTMMLRSISRLDPVDVLKVLEFHNSPEGVSKTTGFCTNRRASNPDTAYRIAKQAQISAPTKQFFPGKGG
VFPEDFSILATVRAKAGLQS
FLLSIYNEQGVQQLGMELGRSPVFLYEDPTGKPAPEDYPLFRNLNLADGKWHRIAISVEKKSITIIVDCNNKITKNLARS
DHTTIDTNGITVFGTRLLDE
DVFQSFQ
>g807.t3
MQMDPVDVLKVLEFHNSPEGVSKTTGFCTNRRASNPDTAYRIAKQAQISAPTKQFFPGGVFPEDFSILATVRAKAGLQSF
LLSIYNEQGVQQLGMELGRS
PVFLYEDPTGKPAPEDYPLFRNLNLADGKWHRIAISVEKKSITIIVDCNNKITKNLARSDHTTIDTNGITVFGTRLLDED
VFQSFQ
>g808.t1
MVVIIISVSLALLVCALIPVIFYGRWRSTKGPDTEKGERAEAGYKENEEEQEMGSQPGGQHVDLGPAAMTQQASI
>g808.t2
LLIQTWADVTHGGKYVVSRSGVDGGFAVTVMRLENRDAGRYHCASTIMSKVLYHDTYHLKVLDGPPIVTELVTRYDHPST
ARYPATAVTSPPHGDHKLNQ
DATTRITGTMVVIIISVSLALLVCALIPVIFYGRWRSTKGPDTEKGERAEAGYKENEEEQEMGSQPGGQHVDLGPAAMTQ
QASI
>g808.t3
LCSSEDLLIQTWADVTHGGKYVVSRSGVDGGFAVTVMRLENRDAGRYHCASTIMSKVLYHDTYHLKVLDGPPIVTELVTR
YDHPSTARYPATAVTSPPHG
DHKLNQDATTRITGTMVVIIISVSLALLVCALIPVIFYGRWRSTKGPDTEKGERAEAGYKENEEEQEMGSQPGGQHVDLG
PAAMTQQASI
>g808.t4
LCSSEDLLIQTWADVTHGGKYVVSRSGVDGGFAVTVMRLENRDAGRYHCASTIMSKVLYHDTYHLKVLDGPPIVTELVTR
YDHPSTARYPATAVTSPPHG
DHKLNQDATTRITG
>g809.t1
MGKDYYDVLGITKSATEDDIKKAYRKQALRYHPDKNKSPGAEDKFKEIAEAYDILSDPKKKDIYDRFGEEGLKGGGGPAD
AGPGTFSYTFQGDPHAMFAE
FFGGRNPFDQLFGSRNNGGGFHQEMNPDDPFACFGMAGGGPGGLPHPFSGGMAPMGGACGTCLTKRQDPPVLHDLQVTLE
EVLSGCVKKMKISRHRLNAD
RRTHRTEDKILEVEIKKGWKEGTRITFAKEGDETATNIPADVVFVLKDKPHPVFCRHGADLVYPARVSLRDALCGCDVRV
PTLDGRTVTVATRDTVVRPG

TRRRLPGEGLPLPKAPGRRGDLVVEFEVTFPETLSAAAREAISRALPPSSSS
>g809.t2
MPRPPVESSGGCGRGVPTHPDRSEAVRDEWRTFSGAPCLGTKERPRLVGSHSQRHPGSQWDRLLILFRLFALPPHLSAVK
MGKDYYDVLGITKSATEDDI
KKAYRKQALRYHPDKNKSPGAEDKFKEIAEAYDILSDPKKKDIYDRFGEEGLKGGGGPADAGPGTFSYTFQGDPHAMFAE
FFGGRNPFDQLFGSRNNGGG
FHQEMNPDDPFACFGMAGGGPGGLPHPFSGGMAPMGGACGTCLTKRQDPPVLHDLQVTLEEVLSGCVKKMKISRHRLNAD
RRTHRTEDKILEVEIKKGWK
EGTRITFAKEGDETATNIPADVVFVLKDKPHPVFCRHGADLVYPARVSLRDALCGCDVRVPTLDGRTVTVATRDTVVRPG
TRRRLPGEGLPLPKAPGRRG
DLVVEFEVTFPETLSAAAREAISRALPPSSSS
>g810.t1
DSVESCRFLGTIITQDLKWDLYIRSLIKKAQQRMYFLRQLKKFNLPKTMMVQFYTAIIESILTSSINVWYTAATAATKDK
SRLQRIIRSVEKEAVSIRTR
TSRHMNSFFQQQLCS
>g810.t2
HQSPFDSVESCRFLGTIITQDLKWDLYIRSLIKKAQQRMYFLRQLKKFNLPKTMMVQFYTAIIESILTSSINVWYTAATA
ATKDKSRLQRIIRSVEKEAV
SIRTRTSRHMNSFFQQQLCS
>g811.t1
PAPGSQILCCQNVEQKLQNEFSAPAQWSAVSLAPPTQPVWYTWYLVKNQYHFSTVSLL
>g812.t1
MKFCLRSGTVVHVDRHGDARPENDGEVDPVTRLASPQLVVNSDRIVPHAVRTTQPAFLTQTVCLDDTTVKFEIWDTAGQE
RYHSLAPMYYRGAQAAIVVY
DITNTDTFTRAKNWVKELQRQASPNIVIALAGNKADLANKRAVDFQ
>g812.t2
MKFCLRSGTVVHVDRHGDARPESCQNDGEVDPVTRLASPQLVVNSDRIVPHAVRTTQPAFLTQTVCLDDTTVKFEIWDTA
GQERYHSLAPMYYRGAQAAI
VVYDITNTDTFTRAKNWVKELQRQASPNIVIALAGNKADLANKRAVDFQ
>g813.t1
SVNVFILDENDNSPGILPPYSEHGSVNSENIPYSAEAGYFVAKIRAVDADSGYNALLSYHMSEPKGTNLFRIGTSTGEIR
TKRRMSDNDLKTHPLVVLVS
DNGEPSLTATVSIDVVVVENTGDIQTQFRHVPLKEDSFSALNLYLLIAIASVSVIFLLSLISLIAVKCHRTDNSFSRYSA
PMITTHPDGSWSYSKSTQQY
DVCFSSDTLKSDVVVFPAQFPPVDAELISINGGDTLNRTQTLPTTVKVRITSISR
>g814.t1
MLVTNKQPSLGRSHSWDTLGGNEGQWDGAGSVYEQQARVAARRNSLSYGEGGGWYEPQPGVRPPDLDLKRDPYSHQDSLY
GQGYPERQDPRALRKGSVPD
LNHYDRPAMAHRGSIGHQDYYPHDPAAAPRQADGFFRGEHQPVPPPPHPLSRSASHFGMSPGTRAPSSPLPLPPPPPTAH
EINRLYRDPGAVANAAAKMM
PDGQRITSRAPSPAHYGAEHGSPRYASEPPPMSQPGYTDINGHPVDPQQPGAATCLVVDPACQGIIMRQEAASPFTIQQQ
QQLQQQQLQQQQQQLQQQQQ
LQQQQHLQQQQQLQQQQLQQQQIQQQQLQQQQLQQPQPLPPPPMSVYDPNLQMAAPLPPPPAPAQVAAPVPVNIPAPPPP
ASQIHSPLPPLPPTAAQTPA
PVDPKKTDPEFVALLRNEGLSERTISSLIQQGFDSTGMLAVMEENDVRTVASNLGQARVLSRLALNCKRPTDSLPAPSQP
QTPMRGRSNSFSHRSDIYHH
QQYQHQPHPPQALVIDPQLMPPATPGGMQTISPRMGEMMARRPSSAPSQQLMEANGGYQGQASRALGAYTGAMVPVQPRP
MSAYSHHSGMSMPMQMMPQQ
MTGSMSAIPTAFHQLPQQMPLPMPALQQPQQVPKAYSTNYTVPMELMKRDRNLPLSPMHSPHPSPQMRKTGVPNADGSMV
AVSGPIQSQGSLAPHQKMSR
RTGPPVIVSSMASPDLSKTISFFF
>g815.t1
MSVKLNALHSDSYVDISEYRDQYFKGNRYDQEKLLTQSNTLYVGNLSFYTTEEQVDLLFLSRYYTRVDAENAMRFINGTR
LDDRIIRTDWDAGFKEGRQY
GRGKSGGQVRDEYRQDYDPARGGYGKIAQQRAPEARSSF
>g816.t1

MSMYAKDIYAMAKTASFKIAAGESKADGLGATYYAVAVVKKKNSAINIFNLADKKSCHTGKDRTVGWNMPLGYLIDQGLM
SVMGCNVLQGVADFFNASCV
PGAQSGPPALCQLCAGDINGNNKCDKSTSERYFSYDGAFRCLAEDAGDVAFIKHTTVSDYTDGKGPSWAQSFRSPDFELL
CRDGTRAPISDWRRCHLVRV
PSRGIVVRNDISSSEVYNMLNTGLAEYENPKEWMGKVYYNALLAMDCKSEELPASLRWCVLSNGEQRKCVAMADAFNIKG
ITPSIQCIYGDSIPDCMKKI
QNNEADAITLDGGYIYTAGKDYGLVPAAGESYTEVGSFFKQSCVPGANQPGFPESLCGLCVGDTAGQNKCEKGKDLYDGY
NGAFRCLAKGDGDVAFIKHS
TVLQNTDGHSGESWASNLQSKDFQLLCSQNSRAEVTQYQTCHLARVPSHAVMVRPGTNTHAIYGLLDYAQRYFGSDLGTG
FKMFDSSLYEGSDLIFKDST
LRLVAVADKKNYREWLGQGYMQSLMDMECNSSAAASVSGSEKKKLQIGIKKRVDNCPIKSRKGDVLNMHYTGKLEDGTEF
DSSIPRDSPFTFTLGTGQVI
KGWDQGLLGMCKGEKRKLVIPSELGYGDRGAPPKIPGGATLIFEVELLSIERRSDL
>g816.t2
MSMYAKDIYAMAKTASFKIAAGESKADGLGATYYAVAVVKKKNSAINIFNLADKKSCHTGKDRTVGWNMPLGYLIDQGLM
SVMGCNVLQGVADFFNASCV
PGAQSGPPALCQLCAGDINGNNKCDKSTSERYFSYDGAFRCLAEDAGDVAFIKHTTVSDYTDGKGPSWAQSFRSPDFELL
CRDGTRAPISDWRRCHLVRV
PSRGIVVRNDISSSEVYNMLNTGLAEYENPKEWMGKVYYNALLAMDCKSEELPASLRWCVLSNGEQRKCVAMADAFNIKG
ITPSIQCIYGDSIPDCMKKI
QNNEADAITLDGGYIYTAGKDYGLVPAAGESYTEERDGSMYYAVAVVKTSRVDIHSLDDLRNLRSCHTGYGRTAGWNIPV
STLIARGLIKQQCQIPQEVG
SFFKQSCVPGANQPGFPESLCGLCVGDTAGQNKCEKGKDLYDGYNGAFRCLAKGDGDVAFIKHSTVLQNTDGHSGESWAS
NLQSKDFQLLCSQNSRAEVT
QYQTCHLARVPSHAVMVRPGTNTHAIYGLLDYAQRYFGSDLGTGFKMFDSSLYEGSDLIFKDSTLRLVAVADKKNYREWL
GQGYMQSLMDMECNSSAAAS
VSGSEKKKLQIGIKKRVDNCPIKSRKGDVLNMHYTGKLEDGTEFDSSIPRDSPFTFTLGTGQVIKGWDQGLLGMCKGEKR
KLVIPSELGYGDRGAPPKIP
GGATLIFEVELLSIERRSDL
>g816.t3
MPPLGLGATYYAVAVVKKKNSAINIFNLADKKSCHTGKDRTVGWNMPLGYLIDQGLMSVMGCNVLQGVADFFNASCVPGA
QSGPPALCQLCAGDINGNNK
CDKSTSERYFSYDGAFRCLAEDAGDVAFIKHTTVSDYTDGKGPSWAQSFRSPDFELLCRDGTRAPISDWRRCHLVRVPSR
GIVVRNDISSSEVYNMLNTG
LAEYENPKEWMGKVYYNALLAMDCKSEELPASLRWCVLSNGEQRKCVAMADAFNIKGITPSIQCIYGDSIPDCMKKIQNN
EADAITLDGGYIYTAGKDYG
LVPAAGESYTEVGSFFKQSCVPGANQPGFPESLCGLCVGDTAGQNKCEKGKDLYDGYNGAFRCLAKGDGDVAFIKHSTVL
QNTDGHSGESWASNLQSKDF
QLLCSQNSRAEVTQYQTCHLARVPSHAVMVRPGTNTHAIYGLLDYAQRYFGSDLGTGFKMFDSSLYEGSDLIFKDSTLRL
VAVADKKNYREWLGQGYMQS
LMDMECNSSAAASVSGSEKKKLQIGIKKRVDNCPIKSRKGDVLNMHYTGKLEDGTEFDSSIPRDSPFTFTLGTGQVIKGW
DQGLLGMCKGEKRKLVIPSE
LGYGDRGAPPKIPGGATLIFEVELLSIERRSDL
>g817.t1
MNVTSFYASRKKVELALPVDVSEDELELSSDEADELQANLQDESESDEKPKNEDENEDQDEDENEDEDSEGESAATAIPM
DERLCVDEQIVPFKGKSGLK
QYNPKKPKRWGYKIYDSNGIAYNFEVHTGPISPMDGMPNIGASGNIVLRLASIIPCNQSHKLFFDNWFTSVDLQTIEEKA
TMKRKPQAMMESNSGQ
>g817.t2
MNVTSFYASRKKVELALPVDVSEDELELSSDEADELQANLQDESESDEKPKNEDENEDQDEDENEDEDSEGESAATAIPM
DERLCVDEQIVPFKGKSGLK
QYNPKKPKRWGYKIYDSNGIAYNFEVHTGPISPMDGMPNIGASGNIVLRLASIIPCNQSHKLFFDNWFTSVDLQTIEEKA
TMKRKPQAMMESNSGQDCKD
CGVPKKDVYSLLKFKAEVASCLCNERKVLKKRGRPSQNVEGDLAEKKRRGPASSVPSTPVRQDNTDHWPVWVEKKGRCKY
PGCKGIVKVQCSKCVTYLCF
TAEKNRFMNFHKQ

>g818.t1
FQAEKGQLEQCLAQKESFSLGESTNKHQAGLEQQRTVLLNKHAQELHVITTRHKEQLDALSSRHQDQLATVAMELDTKHT
AVLVAMEASLDSKWRAELEH
LAAVFQETNQVQLEALDSELTARHQKERDELERRMLSNMDMLEATYLKEVQALRDDMERLLEDHAQEVEQLKSAHRLEVE
CVQTELVSNREELKMELAQM
HMAKFSAMATELSNAHKADLSAQKEALDAEHCKALDTLKMQVLELEEQHCSALQELSHTYNTDKLQLCDQQQLQIQELKS
VSPQELEACRREVEEESSRQ
RLQFLEQMELLKAELEQLKEAELEEQRRSFRTEREQKERGYTEKMSQLTAQLQRLDVVVAQLQAEVGCLQGELEGKCVEM
ETLDSLLQRRERESQEGANL
LSMLGQDLQAAKEDRLQLHQANEKLRKVLLHVIRSTVATEELIGQKINVGPRKSPQPDKADRSSTGNSDVQESGVSVGED
SELTQLLCESLLVCGTQLHP
AGDEAAVGACGRLHLAVDTLLELLNQANAQLEQAHSVHVSLEQRFSQGREDSCHLLKQHTVLLKQLDQEAGLKSHLHLEL
HKAEGLLDGYLTEKVLLEEA
LQQKESREEKLAEELEGLRVQLLQTEGLSAELEALQLQLRQTEGLPAALDHLRAQHQELTEEHAVLLRQREHLSAGLGER
ERALLEEAERLAQERLDLQR
QAEKDRNTLSLRLRALETELEERETKGLETELHHKAHSEDLHMHVQALEKQIKHNRQFIDEQAVEREHERDEFQQEINRL
EAQLSQTSRGQASGENRGQR
VESLQALIKDRTEDYDSLIAANQQAQHDVIERNDEIDKLAGRIRELEQALLNSTESIRSVSQLDQELHRTKLREQELMQD
KQGLEQQQLSNKLQIAALQS
KLDESRHYHDNTPDSDQLLRDALDTAQLSLQSREQEVEVLMSQLEQVKANLSVKEVELKHLTLQLQLMTNQNADQLHQLQ
EKIVILEENVSALSILQEER
EETREEEESLPSALLQEKNLEIDQLNQEIQRLEAELESSESDVALGAELEELRSQVEHLRCDISRLRQDKQDEEERLHEV
ISTLQAELATLGPALHEVSD
SQEGDSVNPSPSPSPELCNYSLHGDAGRKGAPGSLRQELSLTHASSSSALSSHARLHALQAQLETAAAEKEALQQLLLTQ
EEEYRGHGEELRRRLKEERE
RAKELQGLLAHKEAELEEVNVHAHWRAQALEISALRGERDQLSSVIVELRQSEEELGETQKKKENELEQRVESLREDNLA
LQRQIQEAGAQVAAMEKLVA
DGKAQVLTLEMVKGELFVEQEALRRTEGRLQEEMECLRQEVVTQRALIQQLQLRDNEELPERDQKEMLTHAESTLAKADL
ALREKEAELARLSAEQQALR
AELAAVKEGLNSSTERAEKLQEEGHTKDRALADLETYNHRLKAELRRLQEDLAVQEEELAYQQRELHHLRQRCHEQDSEH
HNQCFPQKGCYAFMHVYCLY
VLRFG
>g818.t2
FQAEKGQLEQCLAQKESFSLGESTNKHQAGLEQQRTVLLNKHAQELHVITTRHKEQLDALSSRHQDQLATVAMELDTKHT
AVLVAMEASLDSKWRAELEH
LAAVFQETNQVQLEALDSELTARHQKERDELERRMLSNMDMLEATYLKEVQALRDDMERLLEDHAQEVEQLKSAHRLEVE
CVQTELVSNREELKMELAQM
HMAKFSAMATELSNAHKADLSAQKEALDAEHCKALDTLKMQVLELEEQHCSALQELSHTYNTDKLQLCDQQQLQIQELKS
VSPQELEACRREVEEESSRQ
RLQFLEQMELLKAELEQLKEAELEEQRRSFRTEREQKERGYTEKMSQLTAQLQRLDVVVAQLQAEVGCLQGELEGKCVEM
ETLDSLLQRRERESQEGANL
LSMLGQDLQAAKEDRLQLHQANEKLRKVLLHVIRSTVATEELIGQKINVGPRKSPQPDKADRSSTGNSDVQESGVSVGED
SELTQLLCESLLVCGTQLHP
AGDEAAVGACGRLHLAVDTLLELLNQANAQLEQAHSVHVSLEQRFSQGREDSCHLLKQHTVLLKQLDQEAGLKSHLHLEL
HKAEGLLDGYLTEKVLLEEA
LQQKESREEKLAEELEGLRVQLLQTEGLSAELEALQLQLRQTEGLPAALDHLRAQHQELTEEHAVLLRQREHLSAGLGER
ERALLEEAERLAQERLDLQR
QAEKDRNTLSLRLRALETELEERETKGLETELHHKAHSEDLHMHVQALEKQIKHNRQFIDEQAVEREHERDEFQQEINRL
EAQLSQTSRGQASGENRGQR
VESLQALIKDRTEDYDSLIAANQQAQHDVIERNDEIDKLAGRIRELEQALLNSTESIRSVSQLDQELHRTKLREQELMQD
KQGLEQQQLSNKLQIAALQS
KLDESRHYHDNTPDSDQLLRDALDTAQLSLQSREQEVEVLMSQLEQVKANLSVKEVELKHLTLQLQLMTNQNADQLHQLQ
EKIVILEENVSALSILQEER
EETREEEESLPSALLQEKNLEIDQLNQEIQRLEAELESSESDVALGAELEELRSQVEHLRCDISRLRQDKQDEEERLHEV
ISTLQAELATLGPALHEVSD
SQEGDSVNPSPSPSPELCNYSLHGDAGRKGAPGSLRQELSLTHASSSSALSSHARLHALQAQLETAAAEKEALQQLLLTQ

EEEYRGHGEELRRRLKEERE
RAKELQGLLAHKEAELEEVNVHAHWRAQALEISALRGERDQLSSVIVELRQSEEELGETQKKKENELEQRVESLREDNLA
LQRQIQEAGAQVAAMEKLVA
DGKAQVLTLEMVKGELFVEQEALRRTEGRLQEEMECLRQEVVTQRALIQQLQLRDNEELPERDQKEMLTHAESTLAKADL
ALREKEAELARLSAEQQALR
AELAAVKEGLNSSTERAEKLQEEGHTKDRALADLETYNHRLKAELRRLQEDLAVQEEELAYQQRELHHLRQRCHEQDSEH
HNQCFPQKANLALTQMRDLK
G
>g818.t3
FQAEKGQLEQCLAQKESFSLGESTNKHQAGLEQQRTVLLNKHAQELHVITTRHKEQLDALSSRHQDQLATVAMELDTKHT
AVLVAMEASLDSKWRAELEH
LAAVFQETNQVQLEALDSELTARHQKERDELERRMLSNMDMLEATYLKEVQALRDDMERLLEDHAQEVEQLKSAHRLEVE
CVQTELVSNREELKMELAQM
HMAKFSAMATELSNAHKADLSAQKEALDAEHCKALDTLKMQVLELEEQHCSALQELSHTYNTDKLQLCDQQQLQIQELKS
VSPQELEACRREVEEESSRQ
RLQFLEQMELLKAELEQLKEAELEEQRRSFRTEREQKERGYTEKMSQLTAQLQRLDVVVAQLQAEVGCLQGELEGKCVEM
ETLDSLLQRRERESQEGANL
LSMLGQDLQAAKEDRLQLHQANEKLRKVLLHVIRSTVATEELIGQKINVGPRKSPQPDKADRSSTGNSDVQESGVSVGED
SELTQLLCESLLVCGTQLHP
AGDEAAVGACGRLHLAVDTLLELLNQANAQLEQAHSVHVSLEQRFSQGREDSCHLLKQHTVLLKQLDQEAGLKSHLHLEL
HKAEGLLDGYLTEKVLLEEA
LQQKESREEKLAEELEGLRVQLLQTEGLSAELEALQLQLRQTEGLPAALDHLRAQHQELTEEHAVLLRQREHLSAGLGER
ERALLEEAERLAQERLDLQR
QAEKDRNTLSLRLRALETELEERETKGLETELHHKAHSEDLHMHVQALEKQIKHNRQFIDEQAVEREHERDEFQQEINRL
EAQLSQTSRGQASGENRGQR
VESLQALIKDRTEDYDSLIAANQQAQHDVIERNDEIDKLAGRIRELEQALLNSTESIRSVSQLDQELHRTKLREQELMQD
KQGLEQQQLSNKLQIAALQS
KLDESRHYHDNTPDSDQLLRDALDTAQLSLQSREQEVEVLMSQLEQVKANLSVKEVELKHLTLQLQLMTNQNADQLHQLQ
EKIVILEENVSALSILQEER
EETREEEESLPSALLQEKNLEIDQLNQEIQRLEAELESSESDVALGAELEELRSQVEHLRCDISRLRQDKQDEEERLHEV
ISTLQAELATLGPALHEVSD
SQEGDSVNPSPSPSPELCNYSLHGDAGRKGAPGSLRQELSLTHASSSSALSSHARLHALQAQLETAAAEKEALQQLLLTQ
EEEYRGHGEELRRRLKEERE
RAKELQGLLAHKEAELEEVNVHAHWRAQALEISALRGERDQLSSVIVELRQSEEELGETQKKKENELEQRVESLREDNLA
LQRQIQEAGAQVAAMEKLVA
DGKAQVLTLEMVKGELFVEQEALRRTEGRLQEEMECLRQEVVTQRALIQQLQLRDNEELPERDQKEMLTHAESTLAKADL
ALREKEAELARLSAEQQALR
AELAAVKEGLNSSTERAEKLQEEGHTKDRALADLETYNHRLKAELRRLQEDLAVQEEELAYQQRELHHLRQRCHEQDSEH
HNQCFPQKAHSHGGFEDHLG
VSRVEASLSSPEVLRRLECSEDRTQEPFHASVLQGSCLSELSALNSTAGLDLPQGKASPRARTREQPCSRTITPYSLSTR
SASASDNFSMACSLDVDKMD
TIDLTAPPSPVGSTSSLSAPEWASDGYGSNVSSELGVRLRAELEHTERLDAQFVEYLRCRGMNPAANTDSAAGSMSLSEE
LLSPELQEILKRVHQESCRV
LTLSQRRATSSSQHHVSTVLSLDQTLGQPQDQASPLDRPDRFPSAPPMEWQQEKRALQETVVALRELLCRMAQRDTQTDL
GGDGDRQREMTSESDGQVEA
QLRLELEEAQRQMRADLGDTQSQLSTSQTALQEHLNVVQSLRLALKVGEEALQTEQSHVQDLQTLLEQERALTLRREREE
EDRREVLQGSLEQKGTEVVA
LRGQLEQEKVAFSNLRRELQIEQSRSGLLEKRLGDAHSDLQEECKCSAQQQDLHLQERNRLERLLVEAESRSDDSHRKLE
EERERCASTLDQLDRRQEAN
IELDRKVIKDLQAQLEQERRQREELASVTDRLRAELLQVKRRSEEKEEQRRHDEVQMEKETAARLQHCLESLKKQKWEAS
CALAAEKELCLRQATEMAEL
KEQVQAAVDKEREREKQRERERRKDSQKQLERERQLERTNNKLCELELLRKQDQQRMQELQHTLSELEKEEREMAAQRLS
CSATIQPAHHTRPGLAVNRC
ARTDDTIHGHQPGVSSGLVETLLGENSALTERVASLSQETLVLKQTAARLERRLRCAENDLTKVTVETENRPVISDAASS
GKDYGGLYWPVLCQVQRLYE
RYLRAESFRKSLVYQKRYLVLLLGGFQECEQATLCLIANMGARPSPTGPLQRRPLTRFRAAVRAVIAVSRMKFLTKKWQR

AIRRMSISGSVNGHFPGGRP
EILKQQRSNSECSPPTRDGKALHREPVSTWGRPVKSPFRLHNRDNSGSTAVLVQSRTSQDPEHSLTEYIHHLEKVQRRLG
GSKPGGIFDAGKDRVQTLPS
RSALGVGLGAAAANAELSHLCFGHNRFYKPSLTVEEAIRQNDKEHYRRLLEMVVTEKYSKSQPLPFNRTKPQDVSLLQGE
CRQGHFARAFDSMSRKTGSI
SGKPLCSLLLWTQPSDLDLSTEVATRLNLVDRENPVLSRLSNQPPTHGSLTRHCEEDLPRLTKDMAAEVSAALTQADPNL
VLSTAFKLRITQRDLATLRE
GSWLNDEEPELVWEVERYQLDIVGLTSTHSIGSGTKLLERGWTLSFSGVAQSRLVRCSSVAAAMLEPRWAMEGCGTTVRT
ARVPAAPSSGAPATGDLKET
ITYLLGRRNSGWVLLGSSGAWLVSLHRRALSFSPRLWLGGGERRRHVRGGA
>g818.t4
LGVSRVEASLSSPEVLRRLECSEDRTQEPFHASVLQGSCLSELSALNSTAGLDLPQGKASPRARTREQPCSRTITPYSLS
TRSASASDNFSMACSLDVDK
MDTIDLTAPPSPVGSTSSLSAPEWASDGYGSSECQYVSSELGVRLRAELEHTERLDAQFVEYLRCRGMNPAANTDSAAGS
MSLSEELLSPELQEILKRVH
QESCRVLTLSQRRATSSSQHHVSTVLSLDQTLGQPQDQASPLDRPDRFPSAPPMEWQQEKRALQETVVALRELLCRMAQR
DTQTDLGGDGDRQREMTSES
DGQVEAQLRLELEEAQRQMRADLGDTQSQLSTSQTALQEHLNVVQSLRLALKVGEEALQTEQSHVQDLQTLLEQERALTL
RREREEEDRREVLQGSLEQK
GTEVVALRGQLEQEKVAFSNLRRELQIEQSRSGLLEKRLGDAHSDLQEECKCSAQQQDLHLQERNRLERLLVEAESRSDD
SHRKLEEERERCASTLDQLD
RRQEANIELDRKVIKDLQAQLEQERRQREELASVTDRLRAELLQVKRRSEEKEEQRRHDEVQMEKETAARLQHCLESLKK
QKWEASCALAAEKELCLRQA
TEMAELKEQVQAAVDKEREREKQRERERRKDSQKQLERERQLERTNNKLCELELLRKQDQQRMQELQHTLSELEKEEREM
AAQRLSCSATIQPAHHTRPG
LAVNRCARTDDTIHGHQPGVSSGLVETLLGENSALTERVASLSQETLVLKQTAARLERRLRCAENDLTKVTVETENRPVI
SDAASSGKDYGGLYWPVLCQ
VQRLYERYLRAESFRKSLVYQKRYLVLLLGGFQECEQATLCLIANMGARPSPTGPLQRRPLTRFRAAVRAVIAVSRMKFL
TKKWQRAIRRMSISGSVNGH
FPGGRPEILKQQRSNSECSPPTRDGKALHREPVSTWGRPVKSPFRLHNRDNSGSTAVLVQSRTSQDPEHSLTEYIHHLEK
VQRRLGGSKPGPSGGIFDAG
KDRVQTLPSRSALGVGLGAAAANAELSHLCFGHNRFYKPSLTVEEAIRQNDKEHYRRLLEMVVTEKYSKSQPLPFNRTKP
QDVSLLQGECRQGHFARAFD
SMSRKTGSISGKPLCSLLLWTQPSDLDLSTEVATRLNLVDRENPVLSRLSNQPPTHGSLTRHCEEDLPRLTKDMAAEVSA
ALTQADPNLVLSTAFKLRIT
QRDLATLREGSWLNDEEPELVWEVERYQLDIVGLTSTHSIGSGTKLLERGWTLSFSGVAQSRLVRCSSVAAAMLEPRWAM
EGCGTTVRTARVPAAPSSGA
PATGDLKETITYLLGRRNSGWVLLGSSGAWLVSLHRRALSFSPRLWLGGGERRRHVRGGA
>g818.t5
LGVSRVEASLSSPEVLRRLECSEDRTQEPFHASVLQGSCLSELSALNSTAGLDLPQGKASPRARTREQPCSRTITPYSLS
TRSASASDNFSMACSLDVDK
MDTIDLTAPPSPVGSTSSLSAPEWASDGYGSNVSSELGVRLRAELEHTERLDAQFVEYLRCRGMNPAANTDSAAGSMSLS
EELLSPELQEILKRVHQESC
RVLTLSQRRATSSSQHHVSTVLSLDQTLGQPQDQASPLDRPDRFPSAPPMEWQQEKRALQETVVALRELLCRMAQRDTQT
DLGGDGDRQREMTSESDGQV
EAQLRLELEEAQRQMRADLGDTQSQLSTSQTALQEHLNVVQSLRLALKVGEEALQTEQSHVQDLQTLLEQERALTLRRER
EEEDRREVLQGSLEQKGTEV
VALRGQLEQEKVAFSNLRRELQIEQSRSGLLEKRLGDAHSDLQEECKCSAQQQDLHLQERNRLERLLVEAESRSDDSHRK
LEEERERCASTLDQLDRRQE
ANIELDRKVIKDLQAQLEQERRQREELASVTDRLRAELLQVKRRSEEKEEQRRHDEVQMEKETAARLQHCLESLKKQKWE
ASCALAAEKELCLRQATEMA
ELKEQVQAAVDKEREREKQRERERRKDSQKQLERERQLERTNNKLCELELLRKQDQQRMQELQHTLSELEKEEREMAAQR
LSCSATIQPAHHTRPGLAVN
RCARTDDTIHGHQPGVSSGLVETLLGENSALTERVASLSQETLVLKQTAARLERRLRCAENDLTKVTVETENRPVISDAA
SSGKDYGGLYWPVLCQVQRL
YERYLRAESFRKSLVYQKRYLVLLLGGFQECEQATLCLIANMGARPSPTGPLQRRPLTRFRAAVRAVIAVSRMKFLTKKW

QRAIRRMSISGSVNGHFPGG
RPEILKQQRSNSECSPPTRDGKALHREPVSTWGRPVKSPFRLHNRDNSGSTAVLVQSRTSQDPEHSLTEYIHHLEKVQRR
LGGSKPGGIFDAGKDRVQTL
PSRSALGVGLGAAAANAELSHLCFGHNRFYKPSLTVEEAIRQNDKEHYRRLLEMVVTEKYSKSQPLPFNRTKPQDVSLLQ
GECRQGHFARAFDSMSRKTG
SISGKPLCSLLLWTQPSDLDLSTEVATRLNLVDRENPVLSRLSNQPPTHGSLTRHCEEDLPRLTKDMAAEVSAALTQADP
NLVLSTAFKLRITQRDLATL
REGSWLNDEPCGPTTCRDRHRGRVQCKPGGRQRRHRRLALGTWNVTSLAGKEPELVWEVERYQLDIVGLTSTHSIGSGTK
LLERGWTLSFSGVAQSRLVR
CSSVAAAMLEPRWAMEGCGTTVRTARVPAAPSSGAPATGDLKETITYLLGRRNSGHTDRGPSVCLSQSCRSSIRWVLLGS
SGAWLVSLHRRALSFSPRLW
LGGGERRRHVRGGA
>g819.t1
VSAQVPIGQDLDGMNILGLVVFAIVFGVALRKLGEEGEILIKFFNSFNEATMVLVSWIMWYAPLGIMFLVAGKIVEMEDV
ATLFASLGKYIACCMVGHAI
HGFLVLPAIYAVITRKNPYTFLWGIFTALATAFGTSSSSATLPLMMKCVEENNGVSKHISRFILPIGATVNMDGAALFQC
VAAVFIAQLNDVSLNFIQVI
TIL
>g819.t2
VSAQVPIGQDLDGMNILGLVVFAIVFGVALRKLGEEGEILIKFFNSFNEATMVLVSWIMWCVALFLPFPISPALLHCRRY
APLGIMFLVAGKIVEMEDVA
TLFASLGKYIACCMVGHAIHGFLVLPAIYAVITRKNPYTFLWGIFTALATAFGTSSSSATLPLMMKCVEENNGVSKHISR
FILPIGATVNMDGAALFQCV
AAVFIAQLNDVSLNFIQVITIL
>g820.t1
PPESGSRRERITPGVKRKSECDRRRRNFAGKRALPYVPLLKELFGFVL
>g821.t1
LSSRVSDNISAARQQHPGDGQCSCGCCDPLPTEEDMRVSLWDLLPPDVGDLAAPGDNTSDAALSLINSAAL
>g822.t1
LGAGSYLAGKNFSLADVLVFPSIAYVFRFGLSVKNYPRLAEYYALLKERPSIKASWPPNWLGSPQAPDKLKDV
>g823.t1
SGPMLVSYNKREVSFVVTVKNNMENAYNTQVLVRHSSNLYYASITYPASYDTGDGASVQCASTAQSLLVACQVGYPAVKT
GQELTFNIHFDFNLDELHKQ
AEVHFEAKRQSNINFYVVEETAAVKTEVRTLDDIGPEFNFSVKVTTDNFPVEILYLTIAIPMTTEAGNPLLYITGLDTQV
GGSTSCDSRSSVNPLKIGGK
PRKVTFAEESLRGLKVLVSRPPDTSVLVPMTPPGGWWRKPE
>g823.t2
SGPMLVSYNKREVSFVVTVKNNMENAYNTQVLVRHSSNLYYASITYPASYDTGDGASVQCASTAQSLLVACQVGYPAVKT
GQELTFNIHFDFNLDELHKQ
AEVHFEAKRQSNINFYVVEETAAVKTEVRTLDDIGPEFNFSVKVTTDNFPVEILYLTIAIPMTTEAGNPLLYITGLDTQV
GGSTSCDSRSSVNPLKIGGK
PRKVTFAEESLRGLKVLGCKEAKCHVVKCILKDIETKKDYFVNATFQAVDLSSMVEIETSQPDLLIINHKNLPVVVTVSK
PGVAADVPVGVIVGSIIGGL
LLLALAVGLLWK
>g823.t3
SGPMLVSYNKREVSFVVTVKNNMENAYNTQVLVRHSSNLYYASITYPASYDTGDGASVQCASTAQSLLVACQVGYPAVKT
GQELTFNIHFDFNLDELHKQ
AEVHFEAKRQSNINFYVVEETAAVKTEVRTLDDIGPEFNFSVKGGSTSCDSRSSVNPLKIGGKPRKVTFAEESLRGLKVL
VSRPPDTSVLVPMTPPGGWW
RKPE
>g824.t1
MEWPAQSPDLNPIELLWEQLDRMVHKKCPSSQSNLWEGLLEAWDPAPHLFSCSSSSCSSSSTCSCSSSCSSPLSSAGRRS
TSSCVDDLLEEDERDRAKRA
SRNKSEKKRRDQFNVLIKELCTMLQGQGLPRKMDKSTILQRTIGFLQKQKDMSAQNDSCDVRQDWKPSFLSNEEFTQLML
EALDGFLVALTTDGNIIYVS
DSVSSLIGHLPSDMVDQNILNFLPEREHSDVYKLLSCHSLGSDPAGPAFLDNESHVEFCCHLARGNMDPKEPPVYEYVKF

VGDFKFHNNVPVSSCNGLEL
PLPRTLQSSLEEQVCLISTVRLVTPQFLKDLCNVEDPCNEFTSRHSLEWKFLFLDH
>g824.t2
MEWPAQSPDLNPIELLWEQLDRMVHKKCPSSQSNLWEGLLEAWDPAPHLFSCSSSSCSSSSTCSCSSSCSSPLSSAGRRR
PASKATPLNTWLQSARPEED
SGHDEDREQGADTSSCVDDLLEEDERDRAKRASRNKSEKKRRDQFNVLIKELCTMLQGQGLPRKMDKSTILQRTIGFLQK
QKDMSAQNDSCDVRQDWKPS
FLSNEEFTQLMLEALDGFLVALTTDGNIIYVSDSVSSLIGHLPSDMVDQNILNFLPEREHSDVYKLLSCHSLGSDPAGPA
FLDNESHVEFCCHLARGNMD
PKEPPVYEYVKFVGDFKFHNNVPVSSCNGLELPLPRTLQSSLEEQVCLISTVRLVTPQFLKDLCNVEDPCNEFTSRHSLE
WKFLFLDH
>g824.t3
MEWPAQSPDLNPIELLWEQLDHPAPHLFSCSSSSCSSSSTCSCSSSCSSPLSSAGRRRPASKATPLNTWLQSARPEEDSG
HDEDREQGADTSSCVDDLLE
EDERDRAKRASRNKSEKKRRDQFNVLIKELCTMLQGQGLPRKMDKSTILQRTIGFLQKQKDMSAQNDSCDVRQDWKPSFL
SNEEFTQLMLEALDGFLVAL
TTDGNIIYVSDSVSSLIGHLPSDMVDQNILNFLPEREHSDVYKLLSCHSLGSDPAGPAFLDNESHVEFCCHLARGNMDPK
EPPVYEYVKFVGDFKFHNNV
PVSSCNGLELPLPRTLQSSLEEQVCLISTVRLVTPQFLKDLCNVEDPCNEFTSRHSLEWKFLFLDH
>g825.t1
MLTFHRDQLGLLVHLVSQDRPEQRERLAPLEPVALRALRGPAERLAPLDLPDPPEPLAILVPMVFPEAKDLLVVPVLLEP
QVSLAPVAHPDPRERQDPLG
PRASLETPVSLASKEKLDPKESLAPWDPKVPLDLQERRANEEQEESPALLGHSDPPEREALLVTVVSLVKMVLLVLREPL
ETAVWLVPLDPREAPVIRAA
QESPVFPAPGVLLAVLVMLVPKAKLAPLVLLARTAGPAHLDPRVPVDSLESWDSQDPREPMVSLEKMARLEHLDLPALRD
PLEREESKDNLDPQVSRVFL
DLQAPQESRANLESRVFPERAELLALLDPEASVVSLVRGVVLEPRVFRDPADFPVPPDPMDPRELLGQLVLSEAKDPPAC
RECLAREELVASPERRETEV
TMERRDLRDLLARMVQEVKPDPLDQLVLLVHVVLLVTAVRLDPPGPLASLGLLVQMVSPVPRERWVRVDRRERPVPLAPR
DPLEPPDLMDLLVFLDLKEP
VVPRERPVPLASPVPRAELDLLAPTVTLGLPVPPALLVKTDPRVFAEMPAPRGDRETPGSEEPLAPRERRESLARMEFSV
SLDFAVWLVCPVNVEREASL
DSLGLLGSLVSRDLLAALETEDPLDPWAPPG
>g825.t2
MLTFHRDQLGLLVHLVSQDRPEQRERLAPLEPVALRALRGPAERLAPLDLPDPPEPLVVPVLLEPQVSLAPVAHPDPRER
QDPLGPRASLETPVSLASKE
KLDPKESLAPWDPKVPLDLQERRANEEQEESPALLGHSDPPEREALLVTVVSLVKMVLLVLREPLETAVWLVPLDPREAP
VIRAAQESPVFPAPGVLLAV
LVMLVPKAKLAPLVLLARTAGPAHLDPRVPVDSLESWDSQDPREPMVSLEKMARLEHLDLPALRDPLEREESKDNLDPQV
SRVFLDLQAPQESRANLESR
VFPERAELLALLDPEASVVSLVRGVVLEPRVFRDPADFPVPPDPMDPRELLGQLVLSEAKDPPACRECLAREELVASPER
RETEVTMERRDLRDLLARMV
QEVKPDPLDQLVLLVHVVLLVTAVRLDPPGPLASLGLLVQMVSPVPRERWVRVDRRERPVPLAPRDPLEPPDLMDLLVFL
DLKEPVVPRERPVPLASPVP
RAELDLLAPTVTLGLPVPPALLVKTDPRVFAEMPAPRGDRETPGSEEPLAPRERRESLARMEFSVSLDFAVWLVCPVNVE
REASLDSLGLLGSLVSRDLL
AALETEDPLDPWAPPG
>g825.t3
LMLTFHRDQLGLLVHLVSQDRPEQRERLAPLEPVALRALRGPAERLAPLDLPDPPEPLAILVPMVFPEAKDLLVVPVLLE
PQVSLAPVAHPDPRERQDPL
GPRASLETPVSLASKEKLDPKESLAPWDPKVPLDLQERRANEEQEESPALLGHSDPPEREALLVTVVSLVKMVLLVLREP
LETAVWLVPLDPREAPVIRA
AQESPVFPAPGVLLAVLVMLVPKAKLAPLVLLARTAGPAHLDPRVPVDSLESWDSQDPREPMVSLEKMARLEHLDLPALR
DPLEREESKDNLDPQVSRVF
LDLQAPQESRANLESRVFPERAELLALLDPEASVVSLVRGVVLEPRVFRDPADFPVPPDPMDPRELLGQLVLSEAKDPPA
CRECLAREELVASPERRETE

VTMERRDLRDLLARMVQEVKPDPLDQLVLLVHVVLLVTAVRLDPPGPLASLGLLVQMVSPVPRERWVRVDRRERPVPLAP
RDPLEPPDLMDLLVFLDLKE
PVVPRERPVPLASPVPRAELDLLAPTVTLGLPVPPALLVKTDPRVFAEMPAPRGDRETPGSEEPLAPRERRESLARMEFS
VSLDFAVWLVCPVNVEREAS
LDSLGLLGSLVSRDLLAALETEDPLDPWAPPG
>g826.t1
LVPPSFRDPQESREHVDQLELKAIRETLDPEAETESLALPETLDQPDHPDPLDWEGARWVPVGHLAPPELLARKVSKVTP
ERLESLDQLDLPAPVGPLDP
LENQEVTVKQVNLERLGSADLLDLRELVDSLELLDFLELKDTEVTLVWMEPRERVVLPEPRVRVDLLERTAPPDQWDPAA
CPAREDGPEPLDLLVPVVTM
VCLVLLVPLYVSPPLTVA
>g826.t2
MAPWDPQESREHVDQLELKAIRETLDPEAETESLALPETLDQPDHPDPLDWEGARWVPVGHLAPPELLARKVSKVTPERL
ESLDQLDLPAPVGPLDPLEN
QEVTVKQVNLERLGSADLLDLRELVDSLELLDFLELKDTEVTLVWMEPRERVVLPEPRVRVDLLERTAPPDQWDPAACPA
REDGPEPLDLLVPVVTMVCL
VLLVPLYVSPPLTVA
>g826.t3
LVPPSFRDPQESREHVDQLELKAIRETLDPEAETESLALPETLDQPDHPDPLDWEGARWVPVGHLAPPELLARKVSKVTP
ERLESLDQLDLPAPVGPLDP
LENQEVTVKQVNLERLGSADLLDLRVTLVWMEPRERVVLPEPRVRVDLLERTAPPDQWDPAACPAREDGPEPLDLLVPVV
TMVCLVLLVPLYVSPPLTVA
>g827.t1
MGEAEEEEEEEEEEASAGQMGQHSPGGTAGWAHSLISSRTMSPGRSQRRTHTDATRLGPPRVLVIVTVAILDAATHLLWQ
PKRHHRLGCGVVEEEVVVVV
VVVVCVAGGQQGAVLGAVGVVVVCGGGGGGMST
>g828.t1
DGWADRDEVVEGYELEAEGGITMKLQSAEVTSFDDYFLKLRLDTNTRNPWFAEFWQYRFQCRL
>g829.t1
MPAGYLAQGCPQTRHALTAALTDRCQTDSYRSAGDVTHDTALITKLIFDPPIGH
>g829.t2
MTRHALTAALTDRCQTDSYRSAGDVTHDTALITKLIFDPPIGH
>g830.t1
MAALTHVLACLMGMGAWVSINGLWVELPLLVPHIPEGWYLPSYLSVLIQMANVGPLFVTLMHRFQPGKLSETAVIYFIIG
VGMLASLLMALFWKETTVVA
GAPRSLALLVLTFFLSTVDCTSSVTFLPFMMRLKTQYLTTYYIGEGLSGLLPALVALIQGVGVVECVNGTTAAWGTNQTE
VYDLVAEYQPANFSAEVFFY
LLSGMMLVCLVAFHILNQRSSSARERQGGEYSNGVQAPHSGRTRTSEQGPVRTDSFRAAKPRSSFGTGTYSWPEVIYIFG
ILMFVNAMTNAVMPGVQSYS
CLPYGNKAYHMSATLAAVANPLACLIAVFLPIRSLLLMGVLSALGTAIAMYIMAMAILSPCPVLVHGISGAVIIVLAWII
LVLALSYVKVIIALIMRDEG
HSPLVWCGAIMQLGSFLGAVIMFPLVSVYSYFSSGDPCNTVCPMAMLDHTVTINMRQKTLHATVFVDMRGKVERCLGRAF
SRAAGTATSHTDSAQTSLHL
YFLNSG
>g830.t2
MAALTHVLACLMGMGAWVSINGLWVELPLLVPHIPEGWYLPSYLSVLIQMANVGPLFVTLMHRFQPGKLSETAVIYFIIG
VGMLASLLMALFWKETTVVA
GAPRSLALLVLTFFLSTVDCTSSVTFLPFMMRLKTQYLTTYYIGEGLSGLLPALVALIQGVGVVECVNGTTAAWGTNQTE
VYDLVAEYQPANFSAEVFFY
LLSGMMLVCLVAFHILNQRSSSARERQGGEYSNGVQAPHSGRTRTSEQGPVRTDSFRAAKPRSSFGTGTYSWPEVIYIFG
ILMFVNAMTNAVMPGVQSYS
CLPYGNKAYHMSATLAAVANPLACLIAVFLPIRSLLLMGVLSALGTAIAMYIMAMAILSPCPVLVHGISGAVIIVLAWII
LVLALSYVKVIIALIMRDEG
HSPLVXMAMLDHTVTINMRQKTLHATVFVDMRGKVERCLGRAFSRAAGTATSHTDSAQTSLHLYFLNSG
>g830.t3
MAALTHVLACLMGMGAWVSINGLWVELPLLVPHIPEGWYLPSYLSVLIQMANVGPLFVTLMHRFQPGKLSETAVIYFIIG

VGMLASLLMALFWKETTVVA
GAPRSLALLVLTFFLSTVDCTSSVTFLPFMMRLKTQYLTTYYIGEGLSGLLPALVALIQGVGVVECVNGTTAAWGTNQTE
VYDLVAEYQPANFSAEVFFY
LLSGMMLVCLVAFHILNQRSSSARERQGGEYSNGVQAPHSGRTRTSEQGPVRTDSFRAAKPRSSFGTGTYSWPEVIYIFG
ILMFVNAMTNAVMPGVQSYS
CLPYGNKAYHMSATLAAVANPLACLIAVFLPIRSLLLMGVLSALGTAIAMYIMAMAILSPCPVLVHGISGAVIIALADEK
REEKVKVKLNQVYTDVTLMV
TAAQSSVHSDIEEPAEVGQMAMLDHTVTINMRQKTLHATVFVDMRGKVERCLGRAFSRAAGTATSHTDSAQTSLHLYFLN
SG
>g831.t1
MFLQVLAWFEEGEETVTAFVEPFVILLILIANAVVGVWQERNAEDAIEALKEYEPEMGKVYRSDRKSVQMIKAREIVPGD
IVEVSVGDKVPADIRIVTIK
STTLRVDQSILTGESVSVIKHTDAVPDPRAVNQDKKNMLFSGTNIAAGKAIGVAVATGVSTEIGKIRDQMAATEQEKTPL
QAKLDEFGEQLSKVITLICI
AVWAINIGHFNDPVHGGSWIRGAVYYFKIAVALAVAAIPEGLPAVITTCLALGTRRMAKKNAIVRSLPSVETLGCTSVIC
SDKTGTLTTNQMCVTKMFIV
KNVDGENVNLDAFDISGSKYTPEGEVSQAGARTNCSAFDGLVELSTICALCNDSSLDYNESKKIFEKVGEATETALCCLV
EKMNVFNSNVKNLSKIERAN
ACCTVIKQLMKKNFTLEFSRDRKSMSVYCTPGKGDGGAKMFVKGAPEGVIERCTYVRVGTTRVPLTNAIKDKIMTVIKDW
GTGRDTLRCLALATCDNPLK
PDEMNLEDSTKFADYECDLTFVGCVGMLDPPRKEVCGSIQLCRDAGIRVIMITGDNKGTAIAICRRIGIFGEDEDVTGRA
YTGREFDDLPNAEQAEAVVR
ASCFARVEPSHKSKIVEFLQGHDDITAMTGDGVNDAPALKKAEIGIAMGSGTAVAKSASEMVLADDNFSSIVAAVEEGRA
IYNNMKQFIRYLISSNVGEV
VCIFLTAALGLPEALIPVQLLWVNLVTDGLPATALGFNPPDLDIMGRAPRSPKEPLISGWLFFRYMAIGAYVGAATVGAA
GWWFIYDPTGPGLTYYQLSH
FMQCHDENPDFEDVNCHIFEAATPMTMALSVLVTIEMCNALNSLSENQSLVRMPPWSNFWLLAAMTLSMSLHFLIIYVDP
LP
>g831.t2
MFLQVLAWFEEGEETVTAFVEPFVILLILIANAVVGVWQERNAEDAIEALKEYEPEMGKVYRSDRKSVQMIKAREIVPGD
IVEVSVGDKVPADIRIVTIK
STTLRVDQSILTGESVSVIKHTDAVPDPRAVNQDKKNMLFSGTNIAAGKAIGVAVATGVSTEIGKIRDQMAATEQEKTPL
QAKLDEFGEQLSKVITLICI
AVWAINIGHFNDPVHGGSWIRGAVYYFKIAVALAVAAIPEGLPAVITTCLALGTRRMAKKNAIVRSLPSVETLGCTSVIC
SDKTGTLTTNQMCVTKMFIV
KNVDGENVNLDAFDISGSKYTPEGEVSQAGARTNCSAFDGLVELSTICALCNDSSLDYNESKKIFEKVGEATETALCCLV
EKMNVFNSNVKNLSKIERAN
ACCTVIKQLMKKNFTLEFSRDRKSMSVYCTPGKGDGGAKMFVKGAPEGVIERCTYVRVGTTRVPLTNAIKDKIMTVIKDW
GTGRDTLRCLALATCDNPLK
PDEMNLEDSTKFADYECDLTFVGCVGMLDPPRKEVCGSIQLCRDAGIRVIMITGDNKGTAIAICRRIGIFGEDEDVTGRA
YTGREFDDLPNAEQAEAVVR
ASCFARVEPSHKSKIVEFLQGHDDITAMTGDGVNDAPALKKAEIGIAMGSGTAVAKSASEMVLADDNFSSIVAAVEEGRA
IYNNMKQFIRYLISSNVGEV
VCIFLTAALGLPEALIPVQLLWVNLVTDGLPATALGFNPPDLDIMGRAPRSPKEPLISGWLFFRYMAIGAYVGAATVGAA
GWWFIYDPTGPGLTYYQLSH
FMQCHDENPDFEDVNCHIFEAATPMTMALSVLVTIEMCNALNSLSENQSLVRMPPWSNFWLLAAMTLSMSLHFLIIYVDP
LPVSHMTSPHPFTLLSCHPL
P
>g831.t3
VLAWFEEGEETVTAFVEPFVILLILIANAVVGVWQERNAEDAIEALKEYEPEMGKVYRSDRKSVQMIKAREIVPGDIVEV
SVGDKVPADIRIVTIKSTTL
RVDQSILTGESVSVIKHTDAVPDPRAVNQDKKNMLFSGTNIAAGKAIGVAVATGVSTEIGKIRDQMAATEQEKTPLQAKL
DEFGEQLSKVITLICIAVWA
INIGHFNDPVHGGSWIRGAVYYFKIAVALAVAAIPEGLPAVITTCLALGTRRMAKKNAIVRSLPSVETLGCTSVICSDKT
GTLTTNQMCVTKMFIVKNVD
GENVNLDAFDISGSKYTPEGEVSQAGARTNCSAFDGLVELSTICALCNDSSLDYNESKKIFEKVGEATETALCCLVEKMN

VFNSNVKNLSKIERANACCT
VIKQLMKKNFTLEFSRDRKSMSVYCTPGKGDGGAKMFVKGAPEGVIERCTYVRVGTTRVPLTNAIKDKIMTVIKDWGTGR
DTLRCLALATCDNPLKPDEM
NLEDSTKFADYECDLTFVGCVGMLDPPRKEVCGSIQLCRDAGIRVIMITGDNKGTAIAICRRIGIFGEDEDVTGRAYTGR
EFDDLPNAEQAEAVVRASCF
ARVEPSHKSKIVEFLQGHDDITAMTGDGVNDAPALKKAEIGIAMGSGTAVAKSASEMVLADDNFSSIVAAVEEGRAIYNN
MKQFIRYLISSNVGEVVCIF
LTAALGLPEALIPVQLLWVNLVTDGLPATALGFNPPDLDIMGRAPRSPKEPLISGWLFFRYMAIGAYVGAATVGAAGWWF
IYDPTGPGLTYYQLSHFMQC
HDENPDFEDVNCHIFEAATPMTMALSVLVTIEMCNALNSLSENQSLVRMPPWSNFWLLAAMTLSMSLHFLIIYVDPLP
>g832.t1
LRRQRRGTWKRGGQNTVLYGGLAPGLNKNKPYKAAGLNGLRVKTLSFSMRFPKTGPPDRSRSTRLSGERWRGVGPSVPAM
PRCRMAGWREGGVAEAL
>g833.t1
MLPKKVAVYPGGGSLASLSAVYDLLDEQQYFYTTLIRQQERNYRAFLQMLANRMDGRMDGLVKELHDLRNSLQGVRSELA
EVRTRGAENTKQTACLAEGM
ATARKALDGPSAGAGLPKRATGKGGGTGPELRGGAGATVLRLDGGAARAREPKAMKLVADLTDICFDNFKADQLTEEQIA
EFKEAFSLFDKDGDGTITTK
ELGTVMRSLGQNPTEAELQDMINEVDADGNGTIDFPEFLTMMARKMKDTDSEEEIREAFRVFDKDGNGYISAAELRHVMT
NLGEKLTDEEVDEMIREADI
DGDGQVNYE
>g833.t2
LSFCFAQTPVLCGPVMLPKKVAVYPGGGSLASLSAVYDLLDEQQYFYTTLIRQQERNYRAFLQMLANRMDGRMDGLVKEL
HDLRNSLQGVRSELAEVRTR
GAENTKQTACLAEGMATARKALDGPSAGAGLPKRATGKGGGTGPELRGGAGATVLRLDGGAARAREPKAMKLVADLTDIC
FDNFKADQLTEEQIAEFKEA
FSLFDKDGDGTITTKELGTVMRSLGQNPTEAELQDMINEVDADGNGTIDFPEFLTMMARKMKDTDSEEEIREAFRVFDKD
GNGYISAAELRHVMTNLGEK
LTDEEVDEMIREADIDGDGQVNYE
>g833.t3
MLPKKVAVYPGGGSLASLSAVYDLLDEQQYFYTTLIRQQERNYRAFLQMLANRMDGRMDGLVKELHDLRNSLQGVRSELA
EVRTRGAENTKQTACLAEGM
ATARKALDGPSAGAGLPKRATGKGGGTGPELRGGAGATVLRLDGGAARAREPKAMKLVADLTDICFDNFKADQLTEEQIA
EFKEAFSLFDKDGDGTITTK
ELGTVMRSLGQNPTEAELQDMINEVDADGNGTIDFPEFLTMMARKMKDTDSEEEIREAFRVFDKDGNGYISAAELRHVMT
NLGEKLTDEEVDEMIREADI
D
>g834.t1
EALAVQRTALLRYHLGTSVFPLATLRGALTSSLAAWPRSAPLWTSYIQVENRYHSAGRSRRFFDSVTRGCKDIVPRLFAI
VAEQQRKQLVDAAQRSESHD
AALPILPENGLGNRIRGLFGNATLSETGTHCPLLWRMYMHFLVSEGKVDSAKAIFYKALQNVPWSKGLYMDAVQLFPGHL
QEFVDLMTEKELRLRLPLEE
LDILLED
>g834.t2
LLRYHLGTSVFPLATLRGALTSSLAAWPRSAPLWTSYIQVENRYHSAGRSRRFFDSVTRGCKDIVPRLFAIVAEQQRKQL
VDAAQRSESHDAALPILPEN
GLGNRIRGLFGNATLSETGTHCPLLWRMYMHFLVSEGKVDSAKAIFYKALQNVPWSKGLYMDAVQLFPGHLQEFVDLMTE
KELRLRLPLEELDILLED
>g835.t1
MEISSKVDLVRGYQVEVVAQFPHPLTVKALQEFLGMVNVYHRFIPRAAQLMRPLHEALKEGRHLTVFVDHKPLTFAMAKV
AQPWSTRQQRHLSYISEFTT
DLQHVAAVLGQSFPRDGDQGTDIEMVTSYWYLGVQLNNTGLTTLLHFTRRARADSIC
>g835.t2
MTSAISYGVVCWSSSISSTDRKRLVKLIKKASSILSCPLNSVQVVGERRVMAKLSSLLVEDSHPLQVIRTSLGSSFSGRP
QVCEGEMVQFLLHKRPNGAG
DRACLVEFCLNTFFDCNSPLDVLTFADSIFKHQCRFIQYLVQLSLS

>g835.t3
MEISSKVDLVRGYQVEVVAQFPHPLTVKALQEFLGMVNVYHRFIPRAAQLMRPLHEALKGRHLTVFVDHKPLTFAMAKVA
QPWSTRQQRHLSYISEFTTD
LQHVAAVLGQSFPRDGDQGTDIEMVTSYWYLGVQLNNTGLTTLLHFTRRARADSIC
>g835.t4
MEISSKVDLVRGYQVEVVAQFPHPLTVKALQEFLGMVNVYHRFIPRAAQLMRPLHEALKEGRHLTVFVDHKPLTFAMAKV
AQPWSTRQQRHLSYISEFTT
DLQHVAAVLGQSFPRDGDQGTDIEMVTSYWKRLVKLIKKASSILSCPLNSVQVVGERRVMAKLSSLLVEDSHPLQVIRTS
LGSSFSGRPQVCEGEMVQFL
LHKRPNGAGDRACLVEFCLNTFFDCNSPLDVLTFADSIFKHQCRFIQYLVQLSLS
>g836.t1
MGLVSAIDCRALKRSLSSGVDNLHHSSPPSLSLTKSPSSITLTSFDLPVDCNTASQDKITDVAPKPFDGLSPTTPPVFRR
KHTRGHANLSRIKMRELRAL
SMPELDKLCTEDFSSDPSAVYLKTDPGIQPTDLKESHMDNSSDQTATGVVVTEGNTMTVKESIQEHSRPDVHNPHLSSWS
VSLKELANSPLDQSRLQAVL
SLLKSKSDVLSLLQETKALSEVKVDTFFVVLVKEEGSGLGFSIAGGVDLEQKSITVHRVFTKGAAGLEGTVRRGDSVLSV
NGTSLEGTTHGEALFRLHQA
RVSTQTIVVIRRGQESERGGVSPCHEVSHQPRQLSLCTKKNSLEAGAELHKTSAGLGFSLEGGRASAQGDRPLHVKRIFK
GGAAELSRAIDVGDEVLAVN
SHPLVGLMHYDAWNLIKATSDGPVYLVIRKPRTSV
>g836.t2
MTVKESIQEHSRPDVHNPHLSSWSVSLKELANSPLDQSRLQAVLSLLKSKSDVLSLLQETKALSEVKVDTFFVVLVKEEG
SGLGFSIAGGVDLEQKSITV
HRVFTKGAAGLEGTVRRGDSVLSVNGTSLEGTTHGEALFRLHQARVSTQTIVVIRRGQESERGGVSPCHEVSHQPRQLSL
CTKKNSLEAGAELHKTSAGL
GFSLEGGRASAQGDRPLHVKRIFKGGAAELSRAIDVGDEVLAVNSHPLVGLMHYDAWNLIKATSDGPVYLVIRKPRTSV
>g837.t1
MRVKSMMSIEVPKNGQPPRLKGGEDPPQAPASSPSQDAALGGALARTPSGPLPQHCRRAEAGELAGVCTIDRVVLRKSKE
ETFGLDLEIKSSPLKVVVTA
LRPGGAAERESNGKMSVGDEIVVIGDLQVCTSSYQDICDLMRNLPEMLSLEVKRPISGPLLFRHQHSPLCTFPPRK
>g837.t2
KKDAPAGRDAGQVESKRSPKLEHKAVMRVKSMMSIEVPKNGQPPRLKGGEDPPQAPASSPSQDAALGGALARTPSGPLPQ
HCRRAEAGELAGVCTIDRVV
LRKSKEETFGLDLEIKSSPLKVVVTALRPGGAAERESNGKMSVGDEIVVIGDLQVCTSSYQDICDLMRNLPEMLSLEVKR
PISGPLLFRHQHSPLCTFPP
RK
>g837.t3
MRVKSMMSIEVPKNGQPPRLKGGEDPPQAPASSPSQDAALGGALARTPSGPLPQHCRRAEAGELAGVCTIDRVVLRKSKE
ETFGLDLEIKSSPLKVVVTA
LRPGGAAERESNGKMSVGDEIVVIGDLQVCTSSYQDICDLMRNLPEMLSLEVKRPISGNCEQLPLYPW
>g838.t1
LQSIPPQGGKLEIAGMVVGQWAGNKPIRGRGGFNMQVVSVGAGKGRGKEVPAKLRGGKKATANRPQTSSSPPSGSLLKPS
EEADFAPEVATQQLNGSSSA
SAIGQPLDLAPLAQPIPCALASRDNQSEGVEVEAPCCLDNCPSDGALQKEE
>g839.t1
MVGLVTGGASGLGRATVERLVRNGASAVILDLPSSDGHALAASLGDRCAFAPADVTSETDVQSAVSLARERFGKLDLAVN
CAGIAVAVKTYNIKKDVPHN
LEDFQRVITVNIGGTFNVIRLAVGAMAKNEPDADGHRGCIINTASVAAFDGQVGQAAYSASKGGIVGMTLPIARDLAPMG
IRVVTIAPGLFSTPLLAGLP
EKVRSFLARQVPFPSRLGDPAEFAHLVTSLAENPMINGEVIRLDGAIRMQP
>g839.t2
MFGYWCPHTRFGRKDGEHDTGNHLGWXSHVICVLYCVKMANIRSVKGMVGLVTGGASGLGRATVERLVRNGASAVILDLP
SSDGHALAASLGDRCAFAPA
DVTSETDVQSAVSLARERFGKLDLAVNCAGIAVAVKTYNIKKDVPHNLEDFQRVITVNIGGTFNVIRLAVGAMAKNEPDA
DGHRGCIINTASVAAFDGQV
GQAAYSASKGGIVGMTLPIARDLAPMGIRVVTIAPGLFSTPLLAGLPEKVRSFLARQVPFPSRLGDPAEFAHLVTSLAEN

PMINGEVIRLDGAIRMQP
>g839.t3
MANIRSVKGMVGLVTGGASGLGRATVERLVRNGASAVILDLPSSDGHALAASLGDRCAFAPADVTSETDVQSAVSLARER
FGKLDLAVNCAGIAVAVKTY
NIKKDVPHNLEDFQRVITVNIGGTFNVIRLAVGAMAKNEPDADGHRGCIINTASVAAFDGQVGQAAYSASKGGIVGMTLP
IARDLAPMGIRVVTIAPGLF
STPLLAGLPEKVRSFLARQVPFPSRLGDPAEFAHLVTSLAENPMINGEVIRLDGAIRMQP
>g840.t1
VFNGLAILASFASNDDPYQGPRDITTPEHMADYLQPKLLGILAFFNMQLLSSSAGEKDRKKLALTSVMALMRLIGSRHIS
SVRVKMMTTLRTGLRYRDDF
PLLCCQTWECFVKSVEPTHLGPLLSHVIVALLPLIPLQPGETAGIIRFLILDNREEVRDYLHEIYFLPDHPELKDIHTVL
QDYKKLTSSSSDLAAALQLS
MRAVQHENVDVRIHALTSLRDMMHSKQCWVAFTLAGSSQLQVALQGFPGSQWPLGEWLLRQVCASEAVEPVISSLVGVLL
KGCQDSSPEARLLCGECLGE
LGAVDPGRLDLSHSQTHGDRNTFVSGVDDPNFAYELLTELTRTFLAYADDVRAQDSAAYGMQELLSVFECREGRTDSPGR
RLWRRFPEQVQEILEPHLNS
RYKSSQKVVNWSKLKKPVYLSSRGNKFSDWSANWAGYLISKVRHELAGKVFNCCSFIIKHDYKVTIYLLPHILLHMLLGC
TPAEQQEVTDEMLAVLTECE
GRGGARVQENASSLSQLSTQTVFSMLAHLTQWSRHILSSKPKHSGSGEYQRVVAFLKGVPQDVLARASLRSKAYTRALMH
FEMHILENKESVQDHLTFLQ
TLYAAMHEPDGVRGVNALRREEPSLREQILEHESIGLLRDASACYDRAIELEPEQIGHYHGMMSSMLGLGQLSSVITQLN
GVLTNRPQWKLELNSYRVEA
AWRLNKWDLLHEYLSS
>g840.t2
MAPWQCCMPRAMSGRASSGPKVRHELAGKVFNCCSFIIKHDYKVTIYLLPHILLHMLLGCTPAEQQEVTDEMLAVLTECE
GRGGARVQENASSLSQLSTQ
TVFSMLAHLTQWSRHILSSKPKHSGSGEYQRVVAFLKGVPQDVLARASLRSKAYTRALMHFEMHILENKESVQDHLTFLQ
TLYAAMHEPDGVRGVNALRR
EEPSLREQILEHESIGLLRDASACYDRAIELEPEQIGHYHGMMSSMLGLGQLSSVITQLNGVLTNRPQWKLELNSYRVEA
AWRLNKWDLLHEYLSS
>g840.t3
VFNGLAILASFASNDDPYQGPRDITTPEHMADYLQPKLLGILAFFNMQLLSSSAGEKDRKKLALTSVMALMRLIGSRHIS
SVRVKMMTTLRTGLRYRDDF
PLLCCQTWECFVKSVEPTHLGPLLSHVIVALLPLIPLQPGETAGIIRFLILDNREEVRDYLHEIYFLPDHPELKDIHTVL
QDYKKLTSSSSDLAAALQLS
MRAVQHENVDVRIHALTSLRDMMHSKQCWVAFTLAGSSQLQVALQGFPGSQWPLGEWLLRQVCASEAVEPVISSLVGVLL
KGCQDSSPEARLLCGECLGE
LGAVDPGRLDLSHSQTHGDRNTFVSGVDDPNFAYELLTELTRTFLAYADDVRAQDSAAYGMQELLSVFECREGRTDSPGR
RLWRRFPEQVQEILEPHLNS
RYKSSQKVVNWSKLKKPVYLSSRGNKFSDWSANWAGYLISKVSGMQLLT
>g840.t4
VFNGLAILASFASNDDPYQGPRDITTPEHMADYLQPKLLGILAFFNMQLLSSSAGEKDRKKLALTSVMALMRLIGSRHIS
SVRVKMMTTLRTGLRYRDDF
PLLCCQTWECFVKSVEPTHLGPLLSHVIVALLPLIPLQPGETAGIIRFLILDNREEVRDYLHEIYFLPDHPELKDIHTVL
QDYKKLTSSSSDLAAALQLS
MRAVQHENVDVRIHALTSLRDMMHSKQCWVAFTLAGSSQLQVALQGFPGSQWPLGYLRCIVVVQKHPSYNSSLAQYIMQT
TLIKISNDLPTAANSGHTSI
FILLDFDTFDTFDTFSHTNLLNRLSQCLRLTACSPLASNHGLCFHCYLKICGPQTLEWLLRQVCASEAVEPVISSLVGVL
LKGCQDSSPEARLLCGECLG
ELGAVDPGRLDLSHSQTHGDRNTFVSGVDDPNFAYELLTELTRTFLAYADDVRAQDSAAYGMQELLSVFECREGRTDSPG
RRLWRRFPEQVQEILEPHLN
SRYKSSQKVVNWSKLKKPVYLSSRGNKFSDWSANWAGYLISKVRHELAGKVFNCCSFIIKHDYKVTIYLLPHILLHMLLG
CTPAEQQEVTDEMLAVLTEC
EGRGGARVQENASSLSQLSTQTVFSMLAHLTQWSRHILSSKPKHSGSGEYQRVVAFLKGVPQDVLARASLRSKAYTRALM
HFEMHILENKESVQDHLTFL
QTLYAAMHEPDGVRGVNALRREEPSLREQILEHESIGLLRDASACYDRAIELEPEQIGHYHGMMSSMLGLGQLSSVITQL

NGVLTNRPQWKLELNSYRVE
AAWRLNKWDLLHEYLSS
>g841.t1
MAASMARTCARLFRFASCIRRRLSPHYLVFNDNFHSSPAVHGGLLLSLNKRGVLKESFPEDAAHDQLARLLESGGQTVYC
GFDPTADSLHVGNLLTIIGL
LHFRSAGHHVLAVLGGATAQIGDPSGKTSERVPISADALDENTRSIRESIQRIFTNHELNFHDSARKKMGTMAVLNNMSW
YKDWNVVSFLSETGRRFRMG
TMLSRHSVQSRLKSEEGMSLTEFTYQVFQAHDFYHLNQVYGCRIQLGGSDQMGNLMSGHEYIRKVCGEEVFGLTLPLVTC
SMGDKLGKTAGNAVWLNKER
TSAFELYQFFLRQPDAIIERYVGW
>g841.t2
LSNMAASMARTCARLFRFASCIRRRLSPHYLVFNDNFHSSPAVHGGLLLSLNKRGVLKESFPEDAAHDQLARLLESGGQT
VYCGFDPTADSLHVGNLLTI
IGLLHFRSAGHHVLAVLGGATAQIGDPSGKTSERVPISADALDENTRSIRESIQRIFTNHELNFHDSARKKMGTMAVLNN
MSWYKDWNVVSFLSETGRRF
RMGTMLSRHSVQSRLKSEEGMSLTEFTYQVFQAHDFYHLNQVYGCRIQLGGSDQMGNLMSGHEYIRKVCGEEVFGLTLPL
VTCSMGDKLGKTAGNAVWLN
KERTSAFELYQFFLRQPDAIIERYVGW
>g842.t1
VNVGCGPAEERVLLTGLHAVADIYCENCKTTLGWKYVRCV
>g843.t1
MNEVEGLRFRRLNRLQVITEDLQTLQSKGSSYSGKVFCVVLVSLGVFLLFPLLDMGLLLESPIQPDVLRLSEPPVMSGFS
LETNQIVLRGLLGQTGSSSQ
TNQNTVSYIKEKISGDLSPERSINLFHCLNELNDCRGDPMVPDIRKSLPKASLPCSVVSAGLRWSGIRVISDYKPAKASP
PSTDTSFINELNNFYARFEK
DNHEPALKMELTADHQPLSLSPIDVEAALSRINAHKAVGHDGIPGRALRACAGEL
>g843.t2
MNEVEGLRFRRLNRLQVITEDLQTLQSKGSSYSGKVFCVVLVSLGVFLLFPLLDMGLLLESPIQPDVLRLSEPPVMSGFS
LETNQIVLRVVSAGLRWSGI
RVISDYKPAKASPPSTDTSFINELNNFYARFEKDNHEPALKMELTADHQPLSLSPIDVEAALSRINAHKAVGHDGIPGRA
LRACAGEL
>g843.t3
MNEVEGLRFRRLNRLQVITEDLQTLQSKGSSYSGKVFCVVLVSLGVFLLFPLLDMGLLLESPIQPDVLRLSEPPVMMVSA
GLRWSGIRVISDYKPAKASP
PSTDTSFINELNNFYARFEKDNHEPALKMELTADHQPLSLSPIDVEAALSRINAHKAVGHDGIPGRALRACAGEL
>g844.t1
MDIQSHAEAFFDDCRYCCVMMSRGTEQGRGGMIKGTREGMKRQERISKTNNGTKMATASATGRAIAPRPPGRREEPPTPG
CPVRRSRADKTATTRRNRRA
VLSRRHHRRRRRRRRANM
>g845.t1
MSADNDKKRSKRRYAAGRGNKRFKGSKELEIQDCDGSNSEEEDDAEAALKKEVDQLKKTGKGEKRFQALDSGANNVVFIR
TNNVEPDKLVHHILSDLHTT
KKKKSRVILRMLPVSGTCKAFQEDMDKYLATFLEPWFKTPNHATYQVAFKARNSSHNKRDDIIKSIAGLVGKMNPKNKVD
LTNPELTIIAEVIKTVCCLS
VVPDYALYRKYNLQEVVKEDTPKADAADGKEDQKATDQSDGADVGKTEEQTDKQSEDIKVPETDKQVEEDTEEGK
>g845.t2
MSADNDKKRSKRRYAAGRGNKRFKGSKELEIQDCDGSNSEEEDDAEAALKKEVDQLKKTGKGEKRFQALDSGANNVSGTC
KAFQEDMDKYLATFLEPWFK
TPNHATYQVAFKARNSSHNKRDDIIKSIAGLVGKMNPKNKVDLTNPELTIIAEVIKTVCCLSVVPDYALYRKYNLQEVVK
EDTPKADAADGKEDQKATDQ
SDGADVGKTEEQTDKQSEDIKVPETDKQVEEDTEEGK
>g846.t1
MKSKPPFGKAPDGDTEKKENVSGDEKTISGEDTSSKSPERSLRIKARPETKLPLSDTKKNRNSIPEPFGDYKSLVSPRTL
LNGTTTPLLGKATAAGKKTS
VDLFSPVLVNAPSLRDNGHGLVLCSTTVGGVSLVPQGHLTFEPQAVVRGHTKDDAETDIAVEAEEWYGCYDHVREGSSKG
FGFQRDPKAHAGQSAHRNQN

MLKETKWKGDGLAYLLRQRRNSSHPSEYHPLSLEQHHVQSILQFDYQAFIPCTAIGWAARAAQDHCSSVRMWAPSEAADR
V
>g847.t1
LFPFLEAYTLSLLSIVEFPAVLLNTSTGEIDSEFHTYVQPQEHPFLSEFCTELTGITQVQVEAGVPLHICLSRFSRWLRA
LQLQKGLAFPGERSLSTSAA
PQKPCAFLTWSDWDLAVCLYYECKRKQLHKPDVLNSWIDLRATYRLFYNRKPKGLNGALQDLGIQFSGREHSGLDDSRNT
AQLALRMMRDGCVMKLTRTE
ERVSGCLTSPPVLTPLLPTSLPFSPKNYCR
>g847.t2
LSLLSIVEFPAVLLNTSTGEIDSEFHTYVQPQEHPFLSEFCTELTGITQVQVEAGVPLHICLSRFSRWLRALQLQKGLAF
PGERSLSTSAAPQKPCAFLT
WSDWDLAVCLYYECKRKQLHKPDVLNSWIDLRATYRLFYNRKPKGLNGALQDLGIQFSGREHSGLDDSRNTAQLALRMMR
DGCVMKLTRTEERVSGCLTS
PPVLTPLLPTSLPFSPKNYCR
>g847.t3
LFPFLEAYTLSLLSIVEFPAVLLNTSTGEIDSEFHTYVQPQEHPFLSEFCTELTGITQVQVEAGVPLHICLSRFSRWLRA
LQLQKGLAFPGERSLSTSAA
PQKPCAFLTWSGLDDSRNTAQLALRMMRDGCVMKLTRTEERVSGCLTSPPVLTPLLPTSLPFSPKNYCR
>g848.t1
MELLHFAALGKNNGAKQPRDQESNQLRDVLRLHPVIRKFGTEVKGLTAYTLTKEEMIKRRFPVRGVQGFEDFLSTESADY
VTDSSPLYGLDCEMCLTTAG
YELARISLVDSSGKCVLDDLVQPPNRILNYLTRFSGITHAMLRPITTVVSDVQVKLLSLLPRDAVLVGHSLENDLRAIKL
IHPHVIDTSLLYRKEFGQRF
KLKVLAETVLGKQIQTEERLGHDPMEDASAALELAQYFIRTGPRQVVERHMEELWGDSLTAAKPTVAQPAPSTRFADVLQ
RSGRSLTFIGKHPFLNGTIN
PSNQQWHSSDKEVLASFRRQTAWLSFSVVQFSSFSEHMKYSGLQQERHHQRVRAHLRRMCVVFAGPFPAGFGQRDVRRLC
RRCGAVKRIRMLPTPHRMHA
EIEFMLLEGAVLAVQTLSGHSVRGQHIRVQRPVHESTLDLDLTLGALMEDPLNVNHVHVVRLKPNPRGTPDTSAHTPAPG
TPGSRRTDHNANGTTTTLSE
EELNETFGHFGAVERILLPIKANGRRERHASIKFQSREAVGAALGSSEELRGQRYLICPSVTPGHLCSWAAGTNTEMEEV
DAQEFSGSSLMENNPSDEKI
ERVMKKLDQRLRKLFKSLPDQTLSVVVLPGHTSFSGHFPGLCFMEVKNRFL
>g848.t2
MKRNVTDIERVIHHPPRISVPFTHLQQQVTLDMLMELLHFAALGKNNGAKQPRDQESNQLRDVLRLHPVIRKFGTEVKGL
TAYTLTKEEMIKRRFPVRGV
QGFEDFLSTESADYVTDSSPLYGLDCEMCLTTAGYELARISLVDSSGKCVLDDLVQPPNRILNYLTRFSGITHAMLRPIT
TVVSDVQVKLLSLLPRDAVL
VGHSLENDLRAIKLIHPHVIDTSLLYRKEFGQRFKLKVLAETVLGKQIQTEERLGHDPMEDASAALELAQYFIRTGPRQV
VERHMEELWGDSLTAAKPTV
AQPAPSTRFADVLQRSGRSLTFIGKHPFLNGTINPSNQQWHSSDKEVLASFRRQTAWLSFSVVQFSSFSEHMKYSGLQQE
RHHQRVRAHLRRMCVVFAGP
FPAGFGQRDVRRLCRRCGAVKRIRMLPTPHRMHAEIEFMLLEGAVLAVQTLSGHSVRGQHIRVQRPVHESTLDLDLTLGA
LMEDPLNVNHVHVVRLKPNP
RGTPDTSAHTPAPGTPGSRRTDHNANGTTTTLSEEELNETFGHFGAVERILLPIKANGRRERHASIKFQSREAVGAALGS
SEELRGQRYLICPSVTPGHL
CSWAAGTNTEMEEVDAQEFSGSSLMENNPSDEKIERVMKKLDQRLRKLFKSLPDQTLSVVVLPGHTSFSGHFPGLCFMEV
KNRFL
>g848.t3
MLMELLHFAALGKNNGAKQPRDQESNQLRDVLRLHPVIRKFGTEVKGLTAYTLTKEEMIKRRFPVRGVQGFEDFLSTESA
DYVTDSSPLYGLDCEMCLTT
AGYELARISLVDSSGKCVLDDLVQPPNRILNYLTRFSGITHAMLRPITTVVSDVQVKLLSLLPRDAVLVGHSLENDLRAI
KLIHPHVIDTSLLYRKEFGQ
RFKLKVLAETVLGKQIQTEERLGHDPMEDASAALELAQYFIRTGPRQVVERHMEELWGDSLTAAKPTVAQPAPSTRFADV
LQRSGRSLTFIGKHPFLNGT
INPSNQQWHSSDKEVLASFRRQTAWLSFSVVQFSSFSEHMKYSGLQQERHHQRVRAHLRRMCVVFAGPFPAGFGQRDVRR
LCRRCGAVKRIRMLPTPHRM

HAEIEFMLLEGAVLAVQTLSGHSVRGQHIRVQRPVHESTLDLDLTLGALMEDPLNVNHVHVVRLKPNPRGTPDTSAHTPA
PGTPGSRRTDHNANGTTTTL
SEEELNETFGHFGAVERILLPIKANGRRERHASIKFQSREAVGAALGSSEELRGQRYLICPSVTPGHLCSWAAGTNTEME
EVDAQEFSGSSLMENNPSDE
KIERVMKKLDQRLRKLFKSLPDQTLSVVVLPGHTSFSGHFPGLCFMEVKNRFL
>g849.t1
MQVIATGKGGGLGVLIPGIADLLSGRGMSPLWRGRSQSWRRPRTPFPGTFLSYVRSLSKKLDELQLLVGKNRDCSSFSFL
CFTET
>g850.t1
SNKWFCGSSSPRVLPFPGSSSLEVRGHCPSQFVLASFPSSSHAFHVTKQLLDRRSEVDNGSEEKPCGAGRSAGLDGELDP
QESRKKPHTSTDSKRRRQEV
RQEEDPAWPRYTCALQKDILYQGKMFVSDNWICFHSKVFGRDTKIAIPVLSVALIKKTKTAILVPNAIVIATNSERHVFV
SFLSRDNTFKLLKSVSVHLE
VRGL
>g850.t2
SNKWFCGSSSPRVLPFPGSSSLEVRGHCPSQFVLASFPSEVDNGSEEKPCGAGRSAGLDGELDPQESRKKPHTSTDSKRR
RQEVRQEEDPAWPRYTCALQ
KDILYQGKMFVSDNWICFHSKVFGRDTKIAIPVLSVALIKKTKTAILVPNAIVIATNSERHVFVSFLSRDNTFKLLKSVS
VHLEVRGL
>g850.t3
SNKWFCGSSSPRVLPFPGSSSLEVRGHCPSQFVLASFPSSSHAFHVTKQLLDRRYRWSNHRTDSSLQYRHLTNFYPPANX
SEVDNGSEEKPCGAGRSAGL
DGELDPQESRKKPHTSTDSKRRRQEVRQEEDPAWPRYTCALQKDILYQGKMFVSDNWICFHSKVFGRDTKIAIPVLSVAL
IKKTKTAILVPNAIVIATNS
ERHVFVSFLSRDNTFKLLKSVSVHLEVRGL
>g851.t1
SRLSSLFKDFGIAEDFATKALELKPWSYEAYYARARAKRSSRLTLLLQSVGCPHSSKGLVVLVFWLNADAGFVRHFAAAL
EDLKEAARLAPSNREIRRLL
TRVEEESLLHQKTSASSCPLQENSPSHHPLPAEPEAGGLERRSPTERLLQEREEQGQQEEQEERQTQSCGKRSTEYWPQN
PYGPRRPGGPGEQQTNQTQP
SHSLQTGAGGRVGGRPLS
>g851.t2
DFGIAEDFATKALELKPWSYEAYYARARAKRSSRLTLLLQSVGCPHSSKGLVVLVFWLNADAGFVRHFAAALEDLKEAAR
LAPSNREIRRLLTRVEEESL
LHQKTSASSCPLQENSPSHHPLPAEPEAGGLERRSPTERLLQEREEQGQQEEQEERQTQSCGKRSTEYWPQNPYGPRRPG
GPGEQQTNQTQPSHSLQTGA
GGRVGGRPLS
>g851.t3
SRLSSLFKDFGIAEDFATKALELKPWSYEAYYARARAKRSSRHFAAALEDLKEAARLAPSNREIRRLLTRVEEESLLHQK
TSASSCPLQENSPSHHPLPA
EPEAGGLERRSPTERLLQEREEQGQQEEQEERQTQSCGKRSTEYWPQNPYGPRRPGGPGEQQTNQTQPSHSLQTGAGGRV
GGRPLS
>g852.t1
MNQSPPPHLRTKVKVLQEKGEQGLVARGVEPLTQPQPIRAYQEGEPKIQTLPVHELARHRRQLLQELVLAQPHLVHDMSR
PEVQETARSTHVKTPSSPQA
NATQTTPRQGIASARTYQGIARAPSTAKHTLDVVEVMERMVHLHPPQETQETPEPRGAPHSPDPVMKPQAGPGTADSSLE
ASGLYGGDTEDRERAEDGED
RERRGGAEREEEDWEKRAGGEEGGEADGTLRDVQLWEDLEEVNYTQPDLDAFIGYSPSFHQAGPRQGDLSSPGRQHHAGL
EEEIPAALVSEALPVGRGEG
EGQASGNGIFRHSVTVATPASSSSSSSSSWRPAATEPMETETGTDFGLVGFAGSDTPREEGEEEEEGEEEEDDDDDDDEE
ETGLTQLNVFTQNPTVPEVG
VAPSRGPLQPAVETEEESEDLMVSPQTRFKLFRYRPLPRTTDFSRDLLVQTTPPMTPGSEVRGGGVTELHLSETLLDDLM
GPQQVVCVDWSELASRGDVF
LNISRNMTCEEFRVYQGVRLLDVLERVFARRLSSPAESWILYLSKPNYHQPQLLMNLASEHGVIAAKDVLQMLGEVGVLE
FGAASSCAVQPAASRGDYSK
LLVVLLIIGSVCLLIISSGLGYICWQRRLVAAKTMSRAEELHFVENGCHDNPTLDVTNDSQPEMHEKKPSTNGLGGGREG

GGADGSRWQVQISAY
>g852.t2
MNQSPPPHLRTKVKVLQEKGEQGLVARGVEPLTQPQPIRAYQEGEPKIQTLPVHELARHRRQLLQELVLAQPHLVHDMSR
PEVQETARSTHVKTPSSPQA
NATQTTPRQGIASARTYQGIARAPSTAKHTLDVVEVMERMVHLHPPQETQETPEPRGAPHSPDPVMKPQAGPGTADSSLE
ASGLYGGDTEDRERAEDGED
RERRGGAEREEEDWEKRAGGEEGGEADGTLRDVQLWEDLEEVNYTQPDLDAFIGYSPSFHQAGPRQGDLSSPGRQHHAGL
EEEIPAALVSEALPVGRGEG
EGQASGNGIFRHSVTVATPASSSSSSSSSWRPAATEPMETETGTDFGLVGFAGSDTPREEGEEEEEGEEEEDDDDDDDEE
ETGLTQLNVFTQNPTVPEVG
VAPSRGPLQPAVETEEESEDLMVSPQTRFKLFRYRPLPRTTDFSRDLLVQTTPPMTPGSEVRGGGVTELHLSETLLDDLM
GPQQVVCVDWSELASRGDVF
LNISRNMTCEEFRVYQGVRLLDVLERVFARRLSSPAESWILYLSKPNYHQPQLLMNLASEHGVGVLEFGAASSCAVQPAA
SRGDYSKLLVVLLIIGSVCL
LIISSGLGYICWQRRLVAAKTMSRAEELHFVENGCHDNPTLDVTNDSQPEMHEKKPSTNGLGGGREGGGADGSRWQVQIS
AY
>g852.t3
MNQSPPPHLRTKVKVLQEKGEQGLVARGVEPLTQPQPIRAYQEGEPKIQTLPVHELARHRRQLLQELVLAQPHLVHDMSR
PEVQETARSTHVKTPSSPQA
NATQTTPRQGIASARTYQGIARAPSTAKHTLDVVEVMERMVHLHPPQETQETPEPRGAPHSPDPVMKPQAGPGTADSSLE
ASGLYGGDTEDRERAEDGED
RERRGGAEREEEDWEKRAGGEEGGEADGTLRDVQLWEDLEEVNYTQPDLDAFIGYSPSFHQAGPRQGDLSSPGRQHHAGL
EEEIPAALVSEALPVGRGEG
EGQASGNGIFRHSVTVATPASSSSSSSSSWRPAATEPMETETGTDFGLVGFAGSDTPREEGEEEEEGEEEEDDDDDDDEE
ETGLTQLNVFTQNPTVPEVG
VAPSRGPLQPAVETEEESEDLMVSPQTRFKLFRYRPLPRTTDFSRDLLVQTTPPMTPGSEVRGGGVTELHLSETLLDDLM
GPQQVVCVDWSELASRGDVF
LNISRNMTCEEFRVYQGVRLLDVLERVFARRLSSPAESWILYLSKPNYHQPQLLMNLASEHGVIAAKDVLQMLGEVGVLE
FGAASSCAVQPAASRGDYSK
LLVVLLIIGSVCLLIISSGLGYICWQRRLVAAKTMSRAEELHFVENGCHDNPTLDVTNDSQPEMHEKKPSTNGLGGGREG
GGADGSRWQVFVNQAGCEEE
EEEQDTHL
>g853.t1
MAPREGDNVTLTAREGDNVTLTCRLDTDMNLQQSVFDWKKGTEEVFFYDKGKTYNNGREGQNPQFNGRVGLPPGGLQAGD
ASVILSNVREEDGGMYTCFF
PLLPLRKWIHLTVVVKPRLRLQMEEAPLSSGWVLLSCVSRGGRPQPSGLEWRNQDQAVIPAEPPATSPDIGDPSVIARYV
KVTTPGRYTCTALQDGVHPA
VSDDIQVFGNWQLTNPMMMPLLLFLEFCICCFFFFLFFLRRFKRNIESQNIKRNQGKIKKEPHYFTCSTRRQVLMLWTAR
DSTLEVTSRYSCHANTIKEA
SKGLQRVIQRSESVSEMEGGSCRTPPASVVPGPQSRSVGRAPNDGSEMSGGGVAVTTGGRRGAGSELV
>g853.t2
MAPREGDNVTLTAREGDNVTLTCRLDTDMNLQQSVFDWKKGTEEVFFYDKGKTYNNGREGQNPQFNGRVGLPPGGLQAGD
ASVILSNVREEDGGMYTCFF
PLLPLRKWIHLTVVVKPRLRLQMEEAPLSSGWVLLSCVSRGGRPQPSGLEWRNQDQAVIPAEPPATSPDIGDPSVIARYV
KVTTPGRYTCTALQDGVHPA
VSDDIQVFGNWQLTNPMMMPLLLFLEFCICCFFFFLFFLRRFKRNIESQATVLFTAGSKERLEDGEEKTESSASS
>g853.t3
MAPREGDNVTLTAREGDNVTLTCRLDTDMNLQQSVFDWKKGTEEVFFYDKGKTYNNGREGQNPQFNGRVGLPPGGLQAGD
ASVILSNVREEDGGMYTCFF
PLLPLRKWIHLTVVVKPRLRLQMEEAPLSSGWVLLSCVSRGGRPQPSGLEWRNQDQAVIPAEPPATSPDIGDPSVIARYV
KVTTPGRYTCTALQDGVHPA
VSDDIQVFGNWQLTNPMMMPLLLFLEFCICCFFFFLFFLRRFKRNIESQVLMLWTARDSTLEVTSRYSCHANTIKEASKG
LQRVIQRSESVSEMEGGSCR
TPPASVVPGPQSRSVGRAPNDGSEMSGGGVAVTTGGRRGAGSELV
>g854.t1
MSETNARLRDCFETTDWEALCSPHGSDLDGMTDCVTDYINFCVENTVPAKLVRCFPNNKPWVTPDIKALLNEKKRVFKSG

DKEELRRVQKELRLRIRRGK
DSYRRKLEKRLEDNNARDVWRGLQNISGHGKGVERNQAN
>g855.t1
MESEEEQQELEDVELEPEQARQSQARNAFLSFQTAVKQHFTLKKEKGERNITVQCNLCLSVTNLLSVSEDSTSNLKKHLK
CIAPMHRPALRLMLGFREA
>g856.t1
MLFIDYSSAFNTIAPSKLVIERRVLGLNSSLCKWLQDLLTGRPQVVRIGTNTSTTLTQHWRTAGVPEDESAFREEVLALA
E
>g857.t1
MSTEELAQKTDAAVHILNAAFAVDTVGLVHIDISEQLVGLDSTVQIVCHLSQKEPSVQEWSQSTPKEVSPITNGTESVVT
SDGVQTTTLCLRHVSAIWKG
LITCTFRPVELTIRTPQSQILHRASVTLDVALLPQIDIISEPQFPQCNAGSTVVAVEVKCEIQNSSEAYTTAWNFQQNKS
EIVTEKNFESKLISYRVKSH
VGCLEQDTEELATVICVFTNRKNQTRNASIPINVIYESTLFCKGVDGWPNTKVHFSARLRCEGQAGLRLRRCNDQEQWEE
VESWCVNHDLSIIIQDTLII
NKGLGSVSINSEKLFIRLANATTHTENINTVSNLNTSINILDNMQRLNTIFNESTIDNIIKSSSNILNVPLEDVWKPTRN
AESKMSMAELYLASVEKLIR
QTNILGTLTRESPNVQFQACVSSDCDSRVFNVSVSVGGRRGLVKMAGFKNLVNFLPPIQPDADPNSIVVTTSLDDKLLRS
KPRVWIKFQLSRPRPRNHAM
RCVAMDAGGHQWLLDRCEWGGPSDEDSCYCQSSSSVFAALLSKRGVDLPALHEITYVGLTCSIVSLVALLVIEGLVWSAV
VKTNSLYLRHVTLVNISLSV
LVGDCSSLASTVPEVSEYWCQACTILQHFFYLAQFCWMFCLSTLLLYQTMFVFSDLSKGRYQGVCYFMGYGAPFMIVTTA
FVTNNGGAEGVYYDDKNCWL
THHGLLKGSFYSFIIPVGIIVFFNIFSMALVIIKLLNHPRGGGHSGRHVALRILRSIIILTPVFGITWIFGFATLVLDLA
EGLVAHSVYYIFSISNSFQD
PHSSTKDTSLTLQSVMDK
>g857.t2
MSTEELAQKTDAAVHILNAAFAVDTVGLVHIDISEQLVGLDSTVQIVCHLSQKEPSVQEWSQSTPKEVSPITNGTESVVT
SDGVQTTTLCLRHVSAIWKG
LITCTFRPVELTIRTPQSQILHRASVTLDVALLPQIDIISEPQFPQCNAGSTVVAVEVKCEIQNSSEAYTTAWNFQQNKS
EIVTEKNFESKLISYRVKSH
VGCLEQDTEELATVICVFTNRKNQTRNASIPINVIYESTLFCKGVDGWPNTKVHFSARLRCEGQAGLRLRRCNDQEQWEE
VESWCVNHDLSIIIQDTLII
NKGLGSVSINSEKLFIRLANATTHTENINTVSNLNTSINILDNMQRLNTIFNESTIDVSTSLSSWSRCVSLRCAWVLLTL
TPSSQNIIKSSSNILNVPLE
DVWKPTRNAESKMSMAELYLASVEKLIRQTNILGTLTRESPNVQFQACVSSDCDSRVFNVSVSVGGRRGLVKMAGFKNLV
NFLPPIQPDADPNSIVVTTS
LDDKLLRSKPRVWIKFQLSRPRPRNHAMRCVAMDAGGHQWLLDRCEWGGPSDEDSCYCQSSSSVFAALLSKRGVDLPALH
EITYVGLTCSIVSLVALLVI
EGLVWSAVVKTNSLYLRHVTLVNISLSVLVGDCSSLASTVPEVSEYWCQACTILQHFFYLAQFCWMFCLSTLLLYQTMFV
FSDLSKGRYQGVCYFMGYGA
PFMIVTTAFVTNNGGAEGVYYDDKNCWLTHHGLLKGSFYSFIIPVGIIVFFNIFSMALVIIKLLNHPRGGGHSGRHVALR
ILRSIIILTPVFGITWIFGF
ATLVLDLAEGLVAHSVYYIFSISNSFQDPHSSTKDTSLTLQSVMDK
>g857.t3
LVHIDISEQLVGLDSTVQIVCHLSQKEPSVQEWSQSTPKEVSPITNGTESVVTSDGVQTTTLCLRHVSAIWKGLITCTFR
PVELTIRTPQSQILHRASVT
LDVALLPQIDIISEPQFPQCNAGSTVVAVEVKCEIQNSSEAYTTAWNFQQNKSEIVTEKNFESKLISYRVKSHVGCLEQD
TEELATVICVFTNRKNQTRN
ASIPINVIYESTLFCKGVDGWPNTKVHFSARLRCEGQAGLRLRRCNDQEQWEEVESWCVNHDLSIIIQDTLIINKGLGSV
SINSEKLFIRLANATTHTEN
INTVSNLNTSINILDNMQRLNTIFNESTIDNIIKSSSNILNVPLEDVWKPTRNAESKMSMAELYLASVEKLIRQTNILGT
LTRESPNVQFQACVSSDCDS
RVFNVSVSVGGRRGLVKMAGFKNLVNFLPPIQPDADPNSIVVTTSLDDKLLRSKPRVWIKFQLSRPRPRNHAMRCVAMDA
GGHQWLLDRCEWGGPSDEDS
CYCQSSSSVFAALLSKRGVDLPALHEITYVGLTCSIVSLVALLVIEGLVWSAVVKTNSLYLRHVTLVNISLSVLVGDCSS

LASTVPEVSEYWCQACTILQ
HFFYLAQFCWMFCLSTLLLYQTMFVFSDLSKGRYQGVCYFMGYGAPFMIVTTAFVTNNGGAEGVYYDDKNCWLTHHGLLK
GSFYSFIIPVGIIVFFNIFS
MALVIIKLLNHPRGGGHSGRHVALRILRSIIILTPVFGITWIFGFATLVLDLAEGLVAHSVYYIFSISNSFQDPHSSTKD
TSLTLQSVMDK
>g858.t1
MNGFVEDVLMPDRIILIDIPLIATFLSRGRMAKKIQAGPFKSLVISSLDSPKGQPSTPCSISPVLTGGCAQEATGQYLYL
ELQPGLFENIEVTRVNDPLS
LFCKLSIFSKELKNLSKQMQLHYNGRSALGLGRPWARGSPCATRSADGTWQRSQLTQDTAHNTGCVDVLHIDHGETQRIS
VSDVRHLHESFVRIPVVTYH
CAIDGVCGTTWSKDQMAVLKSLMHNAVVARFECYDAVKDMYDVTLFGVEAECINAYFTEESTVLADSRHARKDFAAEDCF
STMGDLTNTFLRCRLSPKSA
NPPAMRRTDNDNCVDKKCWPNPSPLRRENACSVAEVKNPLQSVCNLLTEIMVSQSVNPLEAAPGTLVTPENKLNGSHPPH
PSPSSSANWSTLEIPVDNCK
MQKQIVTEPTVRTVQQNEACSRNGQRAGVEQRASGASIRGASVAAGKPVNPPAEPRNVFQHATLADLPPRILERGSTSAV
HVSHFSSPTSFFVQLQQDET
EIYAIAERLNAPEALFSGPLEFDSLNVGDLVGAEFPDDGSWYRAVVGKKLGNGTLTVQFIDFGNEATIPCVKTCSLDKEF
LAQPRFSIPCEFGEIGNNQE
WWDQEAISAFRSSTVENPEKNYPCTFMKETLSVWDVVLEDQGIALAQTLKQKSQTGSNLKQTDCTEETSQYTKPDIIPNH
PYQVVYATCIVGPHFFWCQHD
DSAALGKVSKLVEEAGRSGGQDPAWVDALSLGSPCLALFPDDRRWYRAQVLRRTGSTLSILFVDYGNEAEVDVSSVRAVP
RFLFATAPQAFLCALEGFDE
SKGTWEECASDEFFELLVDKALSVTPRHVGENRHTAIPQYTVKVEVGETMVNKAMDNHWKMFGENDGPPSAAAAGDRDAV
EELLKNNTCDPNQRDLDWNH
KTPLHWAAAKGHADIVRVLVENGARPCMRTENGWTPAHFAAESGRLGALRLLHALHACVDKEDVSGDRPIRIAEMYGHDD
CVRFLKTAETESKAFRLAAA
QRGLSLDDTDHEWEERQRENGMDPRE
>g858.t2
MTELHQAAAAGDRDAVEELLKNNTCDPNQRDLDWNHKTPLHWAAAKGHADIVRVLVENGARPCMRTENGWTPAHFAAESG
RLGALRLLHALHACVDKEDV
SGDRPIRIAEMYGHDDCVRFLKTAETESKAFRLAAAQRGLSLDDTDHEWEERQRENGMDPRE
>g859.t1
LNHNELLVPASGASAGTAASRLRGKPLKTERSVPLLFLGDTSSYLADEAFGTTRKTTVLSRVITTVGSVSKSVKLHHSSR
NHQNCDSSRAFVADEGKMFG
PVVYPPSFCAFERNKIREGMNK
>g860.t1
MYFSSLFPYVVLICFLVRALLLKGSWDGIRHMFTPKLEIMLEAKVWREAATQVFFALGLGFGGVIAFSSYNKRDNNCHFD
AVLVSFINFFTSVLATLVVF
AVLGFKANIMNAKCVFMNTKKIMALVGTQLSHDLIPHHINLSQVSAEDYQQMTEIIRGVKEGEYAGLGLDNCSIEEELNK
AVQGTGLAFIAFTEAMTHFP
ASPFWSVMFFLMLINLGLGSMFGTIEGILTPLVDTFKVRKEFLTVGCCLLAFLIGLLFVQRSGNYFVAMFDDYSATLPLL
IVVILENVAVAWVYGVDKFF
EDMKDMLGFTPYRFYYYMWKYVTPILLLVLLGSSLIQLCMTPPSYSAWIQDLAMEKTVAFPPWGVFVCISLVVTAILPVP
VVFGLRYFNILEDSAGGTSA
VSYKKGRIIKETSRP
>g860.t2
MYFSSLFPYVVLICFLVRALLLKGSWDGIRHMFTPKLEIMLEAKVWREAATQVFFALGLGFGGVIAFSSYNKRDNNCHFD
AVLVSFINFFTSVLATLVVF
AVLGFKANIMNAKCVFMNTKKIMALVGTQLSHDLIPHHINLSQVSAEDYQQMTEIIRGVKEGEYAGLGLDNCSIEEELNK
AVQGTGLAFIAFTEAMTHFP
ASPFWSVMFFLMLINLGLGSMFGTIEGILTPLVDTFKVRKEFLTVGCCLLAFLIGLLFVQRSGNYFVAMFDDYSATLPLL
IVVILENVAVAWVYGVDKFF
EDMKDMLGFTPYRFYYYMWKYVTPILLLVLLGSSLIQLCMTPPSYSAWIQDLVSVRLPPPTTTSLILCSSEATRSYSQEP
K
>g860.t3
MYFSSLFPYVVLICFLVRALLLKGSWDGIRHMFTPKPWCETTLQFGSDEHSLLEIMLEAKVWREAATQVFFALGLGFGGV

IAFSSYNKRDNNCHFDAVLV
SFINFFTSVLATLVVFAVLGFKANIMNAKCVFMNTKKIMALVGTQLSHDLIPHHINLSQVSAEDYQQMTEIIRGVKEGEY
AGLGLDNCSIEEELNKAVQG
TGLAFIAFTEAMTHFPASPFWSVMFFLMLINLGLGSMFGTIEGILTPLVDTFKVRKEFLTVGCCLLAFLIGLLFVQRSGN
YFVAMFDDYSATLPLLIVVI
LENVAVAWVYGVDKFFEDMKDMLGFTPYRFYYYMWKYVTPILLLVLLGSSLIQLCMTPPSYSAWIQDLAMEKTVAFPPWG
VFVCISLVVTAILPVPVVFG
LRYFNILEDSAGGTSAVSYKKGRIIKETSRP
>g861.t1
MACQSIAAIMDEQALLGLNPNADARYRHRAMAYFEQLKESQDAWEVCAEALAKGIYNDDHVKFFCFQVLEHQIKYRHNGL
SGPQQQLIRETLMKWLQCQL
MNSQPEKPFIRNKAAQVFALTFVMEYLTLWPKFFFDILSLVGLNPHGVDVYLRTLMAIDAEVVDRDTLHSPEARNLIYNS
TFQDTRRNTLIKDTMREQCI
PNLVESWFQILQAYQQTQPEITCQCLEVVGSYVSWIDLNLIANDRFVNLLLSQMSVEELREEACDCLFEIVNKGMDPVDK
TKLVESLCQVLQSAGFFNVD
QEEDVDFLAKFSRLVNGMGQSLVLSWTKLAKAGNVKAATDTLQALEGKVPLLLQLLVHADDDISANIVAFCYDYLHVLKQ
AIMLAVMKKLTYDDEYNFDN
EGEDEAMFVEYRKQLKMLLDRLAQVSPDLLLEAVRRVFNNTMQTWQTAAFMEVEVAIRLVYMLGEALPASHGAHFTGDTT
KASVLQEMMRTLVSCGVSGY
QHTSVSLEFFETVVRYEKFFLVEPHHIPLVLVAFLDQRGLRHSSPKVRSRVAYLFSRFVKTL
>g861.t2
MACQSIAAIMDEQALLGLNPNADARYRHRAMAYFEQLKESQDAWEVCAEALAKGIYNDDHVKFFCFQVLEHQIKYRHNGL
SGPQQQLIRETLMKWLQCQL
MNSQPEKPFIRNKAAQVFALTFVMEYLTLWPKFFFDILSLVGLNPHGVDVYLRTLMAIDAEVVDRDTLHSPEARNLIYNS
TFQDTRRNTLIKDTMREQCI
PNLVESWFQILQAYQQTQPEITCQCLEVVGSYVSWIDLNLIANDRFVNLLLSQMSVEELREEACDCLFEIVNKGMDPVDK
TKLVESLCQVLQSAGFFNVD
QEEDVDFLAKFSRLVNGMGQSLVLSWTKLAKAGNVKAATDTLQALEGKVPLLLQLLVHADDDISANIVAFCYDYLHVLKQ
AIMLAVMKKLTYDDEYNFDN
EGEDEAMFVEYRKQLKMLLDRLAQVSPDLLLEAVRRVFNNTMHHSHKVPNSTLRYSHFSLLQSMSKNAALLSLLNGVCWC
YSRTWQTAAFMEVEVAIRLV
YMLGEALPASHGAHFTGDTTKASVLQEMMRTLVSCGVSGYQHTSVSLEFFETVVRYEKFFLVEPHHIPLVLVAFLDQRGL
RHSSPKVRSRVAYLFSRFVK
TL
>g861.t3
MACQSIAAIMDEQALLGLNPNADARYRHRAMAYFEQLKESQDAWEVCAEALAKGIYNDDHVKFFCFQVLEHQIKYRHNGL
SGPQQQLIRETLMKWLQCQL
MNSQPEKPFIRNKAAQVFALTFVMEYLTLWPKFFFDILSLVGLNPHGVDVYLRTLMAIDAEVVDRDTLHSPEARNLIYNS
TFQDTRRNTLIKDTMREQCI
PNLVESWFQILQAYQQTQPEITCQCLEVVGSYVSWIDLNLIANDRFVNLLLSQMSVEELREEACDCLFEIVNKGMDPVDK
TKLVESLCQVLQSAGFFNVD
QEEDVDFLAKFSRLVNGMGQSLVLSWTKLAKAGNVKAATDTLQALEGKVPLLLQLLVHADDDISANIVAFCYDYLHVLKQ
AIMLAVMKKLTYDDEYNFDN
EVSRSAETEQPFYLSTRVVAPSGWLMTALCGPAFQGEDEAMFVEYRKQLKMLLDRLAQVSPDLLLEAVRRVFNNTMQTWQ
TAAFMEVEVAIRLVYMLGEA
LPASHGAHFTGDTTKASVLQEMMRTLVSCGVSGYQHTSVSLEFFETVVRYEKFFLVEPHHIPLVLVAFLDQRGLRHSSPK
VRSRVAYLFSRFVKTL
>g862.t1
MRIFAPNHVVAKSRFWYFVSQLRKMKKASGETVYCGLVHEKTPQKVKNFGIWLRYDSRSGTHNMYREYRDLTTSGAVTQC
YRDMGARHRARAHAIQIMKV
QEIAANKCRRPAIKQFHDSKIKFPLPHRVLRRQHKPRFTTKRPNTFF
>g863.t1
MPSSFKGNHGKILYTLDTKLSRFMRMPSKASTKINFVSAVDPQAVPILMMPQYATKEKKMKVFTRGSVSMDVDLEKTGYF
PGEGIKVVAVIQNNSSREVK
PKYCVYSKHSFFANGKRKVDTKDLFKEIGEPIPPSTIQTITRILSIPHDVEPAILNSHIIKVEYRLRHSVLPPNGQWHLS
LLNPHLLMTCMKCSHL

>g863.t2
MPQYATKEKKMKVFTRGSVSMDVDLEKTGYFPGEGIKVVAVIQNNSSREVKPKYCVYSKHSFFANGKRKVDTKDLFKEIG
EPIPPSTIQTITRILSIPHD
VEPAILNSHIIKVEYRLRHSVLPPNGQWHLSLLNPHLLMTCMKCSHL
>g864.t1
LLKLLAGLEPDRSASAVEAGDQVSEFDQQPPGPRLGIGMDSCVIPLRHGGLSLVQTTDFFYPLVEDPYMMGRIACANVLS
DLYAMGITECDNMLMLLSVS
QKMNE
>g864.t2
LAGLEPDRSASAVEAGDQVSEFDQQPPGPRLGIGMDSCVIPLRHGGLSLVQTTDFFYPLVEDPYMMGRIACANVLSDLYA
MGITECDNMLMLLSVSQKMN
E
>g864.t3
MVRRINCPCRKVPQEALLKLLAGLEPDRSASAVEAGDQVSEFDQQPPGPRLGIGMDSCVIPLRHGGLSLVQTTDFFYPLV
EDPYMMGRIACANVLSDLYA
MGITECDNMLMLLSVSQKMNE
>g865.t1
MSSKKNKSKPKRSSEGESSNNSSILQSGNGNPRNVHSFSVIDFLEKGESGFYEGSQHVHREASSAQQLSGTAGAAKVGLQ
RCVQTSLGVKPGDGLAVHPL
TGPVLQAQEVVLCPRQKDDILETEEFKNYFVRSLEGRVLLPGSQLSLSYYGRAYSLRVEEVHGEDGPLLRGSEDRIPGDP
SLLGCTSSDLSLQLGMLTVE
EQ
>g865.t2
MSSKKNKSKPKRSSEGESSNNSSILQSGNGNPRNVHSFSVIDFLEKADDRTPRRCRWSLAQVSLGSMKAASMCIGRPVLL
SSSAGRQEVCLAWPVASFPA
AKVGLQRCVQTSLGVKPGDGLAVHPLTGPVLQAQEVVLCPRQKDDILETEEFKNYFVRSLEGRVLLPGSQLSLSYYGRAY
SLRVEEVHGEDGPLLRGSED
RIPGDPSLLGCTSSDLSLQLGMLTVEEQ
>g865.t3
MSSKKNKSKPKRSSEGESSNNSSILQSGNGNPRNVHSFSVIDFLEKASMCIGRPVLLSSSAGRQEVCLAWPVASFPAAKV
GLQRCVQTSLGVKPGDGLAV
HPLTGPVLQAQEVVLCPRQKDDILETEEFKNYFVRSLEGRVLLPGSQLSLSYYGRAYSLRVEEVHGEDGPLLRGSEDRIP
GDPSLLGCTSSDLSLQLGML
TVEEQ
>g866.t1
MVAFGCKLFSVLSSRLCKVHCPSAHSARALWVSAGRLQQPSGQDADVLTGKVDRFGGVTVNLADSNLPADISETAFSRLL
QDGVARWRGEGRVAVWLHLP
ISLSRCAAAASTLGFTFHHAQHHQAVLALWLGPGTSRLPAYATHQMGVAAIRNVLYFHLEVQQQTSRPVEEPGSNMSGRR
PCSSPLLQSSAPVLCSSPLL
QSSPSQSSLSPVSAQSQASLRPVSVQSQSSLSPVSEQSLVRSQMHHVQGDMQ
>g866.t2
MVAFGCKLFSVLSSRLCKVHCPSAHSARALWVSAGRLQQPSGQDADVLTGKVDRFGGVTVNLADSNLPADISETAFSRLL
QDGVARWRGEGRVAVWLHLP
ISLSRCAAAASTLGFTFHHAQHHQAVLALWLGPGTSRLPAYATHQMGVAGGRVIIFLLWKLLSAIRNVLYFHLEVQQQTS
RPVEEPGSNMSGRRPCSSPL
LQSSAPVLCSSPLLQSSPSQSSLSPVSAQSQASLRPVSVQSQSSLSPVSEQSLVRSQMHHVQGDMQ
>g867.t1
VSQYPDDGAGSMLQETLHGAQTEEMEQMQRYNQRLVEEMKNRQTQERGRMPKIQRSEAKTRMAMFKKSLRITISTGTPEQ
ERERIKQFAAQEEKRQKNEK
LAQHQKHENQMRDLQLQCDSNIRELQQLQNEKCHLLIEHETQKLKELDEEHVQEIREWRERLRPRKKALEEEFTRKLQEQ
EVFFRMSGESECLNPTTQSR
VSKFYPIPNMHNSGL
>g868.t1
MTSGGQPGPPGPRGPPGPPGPGLGSTTAIIDYSALTRNSDFRAMLSQAARQPGQPGPPGHPGATGAPGPQGPPGAPGHSS
STTYGSSGHTSYRMEDIQQY
LQSSGFRGLQGPPGPPGPQGPPGPSQDRGTYSGTYSGSHQPRETIRLELQEYLSTDSVRRAMRGPPGPPGEQGPRGYQGE

RGEPGLVQGFLQNQGFSQGT
SGRDSEGLVTIDVRTLTENLDYSRVASRVSDYIRNQGLLQEYTVDGPVKTTVRAVQGLPGPPGPPGPPGYSRVIGAYGNV
TADLMDFFRSKELGF
>g868.t2
MTSGGQPGPPGPRGPPGPPGPGLGSTTAIIDYSALTRNSDFRAMLSQAARQPGQPGPPGHPGATGAPGPQGPPGAPGHSS
STTYGSSGHTSYRMEDIQQY
LQSSGFRGLQGPPGPPGPQGPPGPSQDRGTYSGTYSGSHQPRETIRLELQEYLSTDSVRRAMRGPPGPPGEQGPRGYQGE
RGEPGLVQGFLQNQGFSQGT
SGRDSEGLVTIDVRTLTENLDYSRVASRVSDYIRNQGLLQEYTVDGPVKTTVRAVQGLPGPPGPPGPPGYSRVIGAYGNV
TADLMDFFRIMYFLQHMVPS
RAPWGHLVLRETEDTQDPEEKELQLAVPCPGELEYRMEDRGVYLVCLSFTGEVGTPGAPGLPGLHTIHTTHRVEKRDADE
AGHRRKRLRRSSGV
>g868.t3
MYFLQHMVPSRAPWGHLVLRETEDTQDPEEKELQLAVPCPGELEYRMEDRGVYLVCLSFTGEVGTPGAPGLPGLHTIHTT
HRVEKRDADEAGHRRKRLRR
SSGV
>g868.t4
MVPSRAPWGHLVLRETEDTQDPEEKELQLAVPCPGELEYRMEDRGVYLVCLSFTGEVGTPGAPGLPGLHTIHTTHRVEKR
DADEAGHRRKRLRRSSGV
>g868.t5
MTSGGQPGPPGPRGPPGPPGPGLGSTTAIIDYSALTRNSDFRAMLSQAARQPGQPGPPGHPGATGAPGPQGPPGAPGHSS
STTYGSSGHTSYRMEDIQQY
LQSECDSWEQLGPPGPSQDRGTYSGTYSGSHQPRETIRLELQEYLSTDSVRRAMRGPPGPPGEQGPRGYQGERGEPGLVQ
GFLQNQGFSQGTSGRDSEGL
VTIDVRTLTENLDYSRVASRVSDYIRNQGLLQEYTVDGPVKTTVRAVQGLPGPPGPPGPPGYSRVIGAYGNVTADLMDFF
RSKELGF
>g869.t1
MYDQAEEIMSRLMGKARDVVKIALRVDPVLDVKKKPELIYNVLLHYFSDAPSCLPLADFYATLPRHKENPVDYWIRLNKA
ADLALEGLHRQGKRTDNMND
EVALMFVKHCPDPELSCTLKSVAQNLQQSGERLLTRMVNMFQEMMEKMQGRNTSHPTRGQHDELILGTNVIKHILRQSKL
RDSYWETVSGPCPTGDPETV
QFLSMLSGLNPWRGSAVMTEPTSSHSAPRGVLVARVVTALWGDRWVPLKLINTSDKPVLVRRNAKLADVYPCAALEDVDV
AESSEAPLVSCAQSAEPPSA
GANSTKDELRSVGLSAVDVESCEVAENCKRRMADLVLQYEDVFSRHHLDCGEAKGFVHRIHLSDSRPFRLPYRRVPPAQY
QKLRQVLSEMEEKEIIRKST
SEYASPLVLVWKKNGDLRICTDFRWLNKRTLKDAHPLPHQADCLAALGGNCLFSTMDLTSGFYNMPLHEEDRKYSAFTTP
MGLYEYNRLPQGLCNSPGSF
MRMMTSIFGDQNYLSLLCYLDDLLVFAPDEETALLRLRMVFERLRGHNLKLAPKKCFFLRRSVKFLGHVIDENGVSTDTS
KVENIANMSSTDLMEPDGVT
PSQKRIRSFLGMINYYQHFVPRYSAIAKPLFDLLKGERRKGKQHKNKLSSRRLCASDWTLEREQAFEDLKTSLVHSVVLA
HPDFSRPFMLSTDASLDGIG
AVLSQIQEGEKRARPIAFASKSLSQSQKNYPAHRLEFLALKWSVCDKFSHWLKGHKFTVWTDNNPLTHILTKPKLDCCEQ
CWVAKLASYDFDIKYVPGQQ
NVVADALSRVPFVKESVGHRLLAEPYAGLLADVKDMSSASVQNAFRSSSGHKEISAVSDVAQSSYSPLHMHTQSVAMEDV
SAVLQSHVEWEAGPRARAVE
MLQYLPQLVPPGQEALPAYTEKDLRDKQLEDRTLSRVLSYVERRRRPSWRERFKESVSVTRYLKHWDRLAVSNGVLYRIS
RDQKTKAKRFQYVVPDSLRV
EVLQGVHDGAGHQAQYRSLSLTRQRFFWPNLDRDVRDYVRHCKRCIVSKTVEPEGRAPLESITSSRPLELVCIDFWSAED
SRNKSVDVLVITDHFTRMAQ
AFSCKDQTAKQVAKVLWDRYFCVFGFPERIHSDQGANFESQLISELLRVSGVKKSHTTPYHPMGNGSVERFNRTLGGMIR
ELSPEEKADWPRRLQTLTFM
YNCTAHETTGYPPFYLMFGRVPRLPVDVLFRSVLHDSAVTSYDKYVTCLAGALKEAMVIAQEHAAKEQNRHAQLYNRKVK
GSRIEIGDRVLLANRKERGK
KKLADRWESTVYTVVDMNTETHTYRIRDTVTGQEKVIHRNLLMLVNFFPVGDTCEMSDAASSMPVTGSSAPGTDDEGEAG
VTLFEGERETLGGASGRLDA
DGDGHSLVTVGEGQGPLTDSEPVDSERRTMEWITKLSAPSLSEADATAINSVTCDSQDTSVSPEDDMTDQSVTGVSGPVT

ADDVPAHVRLPDPTLDTMTQ
TDDASNTLHSGDHTSLMPWGRSDAQTQVRSRFGRLVKPVNRFIQTMYRQDVV
>g870.t1
MGRESRSKSKEKEIDVSEKKKTKEEKEDEKVEDQDFDQNKLEEEMRKRKERVEKWREEQRKKAIENIGEIKRELEEMKQG
KKWSLEDDEDDDEEGGPAPM
EGDEEEEEGEGKEEKKESEIKHEKPEEGEEEEEEGEKVTPMEQEEAAEEEDDDDVDPLDAYMEGVKEEVKKFNMGGMRGN
DKGNITSFIFVQKGGMMVTN
VTVVKTKKGPHTHKKKGELMINDQDAMEYSSEEEEVDLQTALTGYQTKQRKVLEPVDHQKIQYEPYRKNFYVEVPELARM
TPEDVHSYRLELEGITVKGK
GCPKPIKTWVQCGTSMKILNALKRHSYEKPTPIQTQAIPAIMSGRDLIGIAKTGSGKTIAFLLPMFRHIMDQRPLEESEG
PIAVIMTPTRELALQITKEC
KKFSRSLNLRVVCVYGGTGISEQIAELKRGAEIIVCTPGRMIDMLGANSGRVTNLRRATYVVVDEADRMFDMGFEPQ
>g870.t2
MVTLHAEIALCRLDSGDNGLSKSKEKEIDVSEKKKTKEEKEDEKVEDQDFDQNKLEEEMRKRKERVEKWREEQRKKAIEN
IGEIKRELEEMKQGKKWSLE
DDEDDDEEGGPAPMEGDEEEEEGEGKEEKKESEIKHEKPEEGEEEEEEGEKVTPMEQEEAAEEEDDDDVDPLDAYMEGVK
EEVKKFNMGGMRGNDKGNIT
SFIFVQKGGMMVTNVTVVKTKKGPHTHKKKGELMINDQDAMEYSSEEEEVDLQTALTGYQTKQRKVLEPVDHQKIQYEPY
RKNFYVEVPELARMTPEDVH
SYRLELEGITVKGKGCPKPIKTWVQCGTSMKILNALKRHSYEKPTPIQTQAIPAIMSGRDLIGIAKTGSGKTIAFLLPMF
RHIMDQRPLEESEGPIAVIM
TPTRELALQITKECKKFSRSLNLRVVCVYGGTGISEQIAELKRGAEIIVCTPGRMIDMLGANSGRVTNLRRATYVVVDEA
DRMFDMGFEPQ
>g870.t3
MGRESRRSRSRERRRSRSRERKRSRSRSRARRSRSNSPSKSRKTDEKSKSKEKEIDVSEKKKTKEEKEDEKVEDQDFDQN
KLEEEMRKRKERVEKWREEQ
RKKAIENIGEIKRELEEMKQGKKWSLEDDEDDDEEGGPAPMEGDEEEEEGEGKEEKKESEIKHEKPEEGEEEEEEGEKVT
PMEQEEAAEEEDDDDVDPLD
AYMEGVKEEVKKFNMGGMRGNDKGNITSFIFVQKGGMMVTNVTVVKTKKGPHTHKKKGELMINDQDAMEYSSEEEEVDLQ
TALTGYQTKQRKVLEPVDHQ
KIQYEPYRKNFYVEVPELARMTPEDVHSYRLELEGITVKGKGCPKPIKTWVQCGTSMKILNALKRHSYEKPTPIQTQAIP
AIMSGRDLIGIAKTGSGKTI
AFLLPMFRHIMDQRPLEESEGPIAVIMTPTRELALQITKECKKFSRSLNLRVVCVYGGTGISEQIAELKRGAEIIVCTPG
RMIDMLGANSGRVTNLRRAT
YVVVDEADRMFDMGFEPQ
>g871.t1
LDLNRTEPWPSSTSSSSPRPSPFLQEAIHSRSSTPERRGSPLPDLGDPVGGSLEDDMRGVAAGLRQRPRGERPLDDPSGS
PRRGLQKYLSRFDDAMKLRG
QLAVANEKSAEEGDSEPEEFDAFRMFRLIGSLLLAVFVRVSVCQYLSIFAPFLTLELAYMGLYKYFPKVERKPQTTVLTA
ALLLSGIPAEVINRSMDTYR
RMGDVFADLCLYFFTFVLSHEILLIVGSETP
>g871.t2
MDIYSFTCNTRCSHHVVYAAGTPRRPAEPRFHLDLNRTEPWPSSTSSSSPRPSPFLQEAIHSRSSTPERRGSPLPDLGDP
VGGSLEDDMRGVAAGLRQRP
RGERPLDDPSGSPRRGLQKYLSRFDDAMKLRGQLAVANEKSAEEGDSEPEEFDAFRMFRLIGSLLLAVFVRVSVCQYLSI
FAPFLTLELAYMGLYKYFPK
VERKPQTTVLTAALLLSGIPAEVINRSMDTYRRMGDVFADLCLYFFTFVLSHEILLIVGSETP
>g871.t3
LDLNRTEPWPSSTSSSSPRPSPFLQEAIHSRSSTPERRGSPLPDLGDPVGGSLEDDMRGVAAGLRQRPRGERPLDDPSGS
PRRGLQKYLSRFDDAMKLRG
QLAVANEKSAEEGDSEPEEFDAFRMFRLIGSLLLAVFVRVSVCQYLVSLDRRRRLVYKSIFAPFLTLELAYMGLYKYFPK
VERKPQTTVLTAALLLSGIP
AEVINRSMDTYRRMGDVFADLCLYFFTFVLSHEILLIVGSETP
>g872.t1
PYKVKVLPTHDASKVRASGPGLNTTGVPASLPVEFTIDAKDAGEGLLAVQITDPEGKPKKANIRDNHDGTYLVSYVPDMT
GRYTILIKYGGDEIPYSPYR

IRALPTGDASKCTVT
>g872.t2
PYKVKVLPTHDASKVRASGPGLNTTGVPASLPVEFTIDAKDAGEGLLAVQITDPEGKPKKANIRDNHDGTYLVSYVPDMT
GRYTILIKYGGDEIPYSPYR
IRALPTGDASKCTVTGCWGQ
>g873.t1
LGGVNKHSTSLGKIWLSVLFIFRITILVLAAESVWGDEQSDFTCNTQQPGCKNVCYDHFFPVSHIRLWCLQLIFVSTPAL
LVAMHVAYRKRGDKRSMLAS
NGAEKTTDLELETLKRRRLPITGPLWWTYTCSLFFRLLFEGGFMYALYYLYDGFQMPRLVKCEQWPCPNKVDCFISRPTE
KTIFTIFMVSSSTICMVLNV
AELAYLVAKALLRCSNRASRRQLPYVHHDGGVARDRALLENKKNEMMMMPPCTESSNKTA
>g874.t1
MFNGDETKYELWETKVLGHLHLLGLKDTVLREPNGEDTVAADGKKNTDAYAELIQLLDDKSLSLIMRDAPDNARKAQKIL
REYYAGKGKPRIINLYTMLT
SLQKTSSETVTDYIIRAETAITALRNADGARKVADKSGNGSGAAADYAFRIKDTDIGVQQQPAHNIKEKGLMVDTWASSH
IVTDIAKFKSFNDTFKSETH
CVELSDGTQCRGVAQRKGNAEVFLIDSTGQQHRAMLRDALFIPSTKTSFQ
>g874.t2
LPRLMFNGDETKYELWETKVLGHLHLLGLKDTVLREPNGEDTVAADGKKNTDAYAELIQLLDDKSLSLIMRDAPDNARKA
QKILREYYAGKGKPRIINLY
TMLTSLQKTSSETVTDYIIRAETAITALRNADGARKVADKSGNGSGAAADYAFRIKDTDIGVQQQPAHNIKEKGLMVDTW
ASSHIVTDIAKFKSFNDTFK
SETHCVELSDGTQCRGVAQRKGNAEVFLIDSTGQQHRAMLRDALFIPSTKTSFQ
>g875.t1
WSAGAVEAQRGTGRDSTSSSGSVLDCTLESIKVVGERRMLAKLTSIMDNSSHPLHETVGHLSSSFSGRLLHPRCKKDRYR
RSFIPAAVRLYNTSLTL
>g876.t1
GHLIRMPPGRLPLEVFRARPTGKRPRGRPRTRWRDYISHLAWERLGVPQEELESVAGEKDVWSSLLSLLPPRPGRR
>g877.t1
MQSEECMISLWRNKILSQWEAWRRDQRVRSEWQPYGEMIGTMVQGWYRGSQGKGCSSFGVRVMGGAIHPT
>g878.t1
MGKAEPTRHRFRRKAKGQGSSSGASEQPCDSASSSQSPSPLCDQSATENLLCSSPLDSASSPADSAPLLPDDLTANHGPC
RSQEALATDRNASASVTEVN
GVDPVDPASRGDPHDAELCPLSRHLPEELCSLPRSEITPLCSHGSLALSACRVQTDHSSLLVVFVCNTSDSDAPQFALKM
TSGDLE
>g879.t1
MLGYEASFSYIHAIKLAQQGTALDKRVGYLAVSLFLNESHELLLLLVNTVLKDLQSTNLMEVCMALTVVSQVFPRDMIPA
VLPLVEEKLNHPKEIIRRKA
VLALYKFYLIAPNQVQHIHNKFRKALCDKDPGVMSASLHIYLEMVKEKPESYKDLAVSFVTILKQVVGGKLPIDFNYHSV
PAPWLQIQLLRILSLLGNND
RSTSELMYEVLDESLRRAEMNHNITYGLKALTYVVQQDANLALQHQMTIIECLDHSDLIIKRETLELLFRITNGQNVKVI
VEKLLEFLRRSKDDYVTIDL
GGKTMNTVFLLVGDQMQPDIPNSFLRLLSEGSDSVEEDRQLRLLAVESYLSVLQDRPARLSQRFLQVISWVLGEYACLKE
GLEPATVLRLLAQLLDLRHS
SGETKSWVLTAMTKLCEGPACAAVAQQLADRHCGSLDTVLRQKLHELQHLASHAELRARVLPRRGAPEPLEVDSSLSFLD
GFVSEALAAGAAPYKPPHQR
QEELAQDKALSVEPYGLSLPPSISSCSVAERLSPTVLSVSSGLSGNSTELSPKGGSSALKLEGVMRVWGKEGYLAQTEAK
VEEAAQVEVPCSLGLSALQE
GGPASQTPLAALPTPVSTPTPEADREKQQLASSLFVGLGSQSSSGPCLVRSKSDAPHSAPVSDGTAS
>g879.t2
MKELMVRAIYCEMLGYEASFSYIHAIKLAQQGTALDKRVGYLAVSLFLNESHELLLLLVNTVLKDLQSTNLMEVCMALTV
VSQVFPRDMIPAVLPLVEEK
LNHPKEIIRRKAVLALYKFYLIAPNQVQHIHNKFRKALCDKDPGVMSASLHIYLEMVKEKPESYKDLAVSFVTILKQVVG
GKLPIDFNYHSVPAPWLQIQ
LLRILSLLGNNDRSTSELMYEVLDESLRRAEMNHNITYGLKALTYVVQQDANLALQHQMTIIECLDHSDLIIKRETLELL
FRITNGQNVKVIVEKLLEFL

RRSKDDYVTIDLGGKTMNTVFLLVGDQMQPDIPNSFLRLLSEGSDSVEEDRQLRLLAVESYLSVLQDRPARLSQRFLQVI
SWVLGEYACLKEGLEPATVL
RLLAQLLDLRHSSGETKSWVLTAMTKLCEGPACAAVAQQLADRHCGSLDTVLRQKLHELQHLASHAELRARVLPRRGAPE
PLEVDSSLSFLDGFVSEALA
AGAAPYKPPHQRQEELAQDKALSVEPYGLSLPPSISSCSVAERLSPTVLSVSSGLSGNSTELSPKGGSSALKLEGVMRVW
GKEGYLAQTEAKVEEAAQVE
VPCSLGLSALQEGGPASQTPLAALPTPVSTPTPEADREKQQLASSLFVGLGSQSSSGPCLVRSKSDAPHSAPVSDGTAS
>g879.t3
MATQEVQPNETLANLKTESETLKSKLEEERAKLHDVELHQVAEKVEGLGQFIMKTRRTLKGHGNKVLCMDWCKDKRRIVS
SSQEHAVTMPCTWVMACAYA
PSGCAVACGGLDNKCSVYPLSLDKNENLAAKKKSVAMHTNYLSACSFTNSDMQIGKLENQQPDREGQHCSIAWCQWPGLA
MYYPSGDAFASGSDDATCRL
YDLRADREVAIYSKESIIFGVSSVDFSLSGRLLFGGYNDYTINVWDVLKGTRVSILFGHENRVSTLRVSPDGTAFCTGSW
DHTLRMKELMVRAIYCEMLG
YEASFSYIHAIKLAQQGTALDKRVGYLAVSLFLNESHELLLLLVNTVLKDLQSTNLMEVCMALTVVSQVFPRDMIPAVLP
LVEEKLNHPKEIIRRKAVLA
LYKFYLIAPNQVQHIHNKFRKALCDKDPGVMSASLHIYLEMVKEKPESYKDLAVSFVTILKQVVGGKLPIDFNYHSVPAP
WLQIQLLRILSLLGNNDRST
SELMYEVLDESLRRAEMNHNITYGLKALTYVVQQDANLALQHQMTIIECLDHSDLIIKRETLELLFRITNGQNVKVIVEK
LLEFLRRSKDDYVTIDLGGK
TMNTVFLLVGDQMQPDIPNSFLRLLSEGSDSVEEDRQLRLLAVESYLSVLQDRPARLSQRFLQVISWVLGEYACLKEGLE
PATVLRLLAQLLDLRHSSGE
TKSWVLTAMTKLCEGPACAAVAQQLADRHCGSLDTVLRQKLHELQHLASHAELRARVLPRRGAPEPLEVDSSLSFLDGFV
SEALAAGAAPYKPPHQRQEE
LAQDKALSVEPYGLSLPPSISSCSVAERLSPTVLSVSSGLSGNSTELSPKGGSSALKLEGVMRVWGKEGYLAQTEAKVEE
AAQVEVPCSLGLSALQEGGP
ASQTPLAALPTPVSTPTPEADREKQQLASSLFVGLGSQSSSGPCLVRSKSDAPHSAPVSDGTAS
>g879.t4
MATQEVQPNETLANLKTESETLKSKLEEERAKLHDVELHQVAEKVEGLGQFIMKTRRTLKGHGNKVLCMDWCKDKRRIVS
SSQEHAVTMPCTWVMACAYA
PSGCAVACGGLDNKCSVYPLSLDKNENLAAKKKSVAMHTNYLSACSFTNSDMQIGKLENQQPDREGQHCSIAWCQWPGLA
M
>g879.t5
MATQEVQPNETLANLKTESETLKSKLEEERAKLHDVELHQVAEKVEGLGQFIMKTRRTLKGHGNKVLCMDWCKDKRRIVS
SSQEHAVTMPCTWVMACAYA
PSGCAVACGGLDNKCSVYPLSLDKNENLAAKKKSVAMHTNYLSACSFTNSDMQIGKLENQQPDREGQHCSIAWCQWPGLA
MYYPSGDAFASGSDDATCRL
YDLRADREVAIYSKESIIFGVSSVDFSLSGRLLFGGYNDYTINVWDVLKGTRVSILFGHENRVSTLRVSPDGTAFCTGSW
DHTLRVWA
>g879.t6
MEHVHYGMLRVGSCYRVSMAMQQTCSVLIWPRPRLETHLSQGYYPSGDAFASGSDDATCRLYDLRADREVAIYSKESIIF
GVSSVDFSLSGRLLFGGYND
YTINVWDVLKGTRVSILFGHENRVSTLRVSPDGTAFCTGSWDHTLRVWA
>g880.t1
MNLSSAAEVVKTQATPRSRIAKNLGIARPKSGSSLPSVFRPTEHTLGNMRADIGAASLSVYGDPYDLDPFVDVGVEEEEP
HTRVTSLLEAKRRGISHSAL
RSHQPVARPIASGLSRRGGALPLSSQCMAEAAPVPDLLGSILSGQSMLLMDSSAVVINRDGTLKAAQP
>g880.t2
MTNMNHEVVVKTQATPRSRIAKNLGIARPKSGSSLPSVFRPTEHTLGNMRADIGAASLSVYGDPYDLDPFVDVGVEEEEP
HTRVTSLLEAKRRGISHSAL
RSHQPVARPIASGLSRRGGALPLSSQCMAEAAPVPDLLGSILSGQSMLLMDSSAVVINRDGTLKAAQP
>g880.t3
MNLSSAAEVVKTQATPRSRIAKNLGIARPKSGSSLPSVFRPTEHTLGNMRADIGAASLSVYGDPYDLDPFVDVGVEEEEP
HTRVTSLLEAKRRGISHSAL
RSHQPVARPIASGLSRRGGALPLSSQCMAEAAPVPDLLGSILSGQSMLLMDSSAVVINRDGTLKAAQPERPSSL
>g881.t1

QTFPTCQGLGSGGGLSYAPYLAPMSHTMGLVPADILPSAPVLVPGSPPISMTTASSGCSSSSSSSAQKLLRTDKLEVTPV
>g882.t1
SLVDDESMVGILSTTQQTAAEVSEKLRVAAETELKINTAQEEYRPAASRGSILYFLITEMSMVNIMYQTSLSQFLKVFDL
SMARSPKSPVTQKRIGNIID
YLTFEVFRYTVRGLYENHKFIFTLLLALKIDLQKTQIKHHEFQILIKGGAALDLKACPSKPFRWILDMTWLNLVELSKLP
QFSEIEIQLSRNEKEWKVWF
DMDAPEEGVIPDGYNALLDVFHRLLLIRSWCPDRTLSQARKYVGDSMGARFAEPVILNLEMTWEESDPRTPLICFLSMGS
DPTNQIDALARKLHLECRAI
SMGQGQEVHARKLLQTSIAQNCHLGLDFMEELLDTVTMVESMHDSFRVWITTEPHNRFSITLLQSSIKFTNDPPQGVRAG
LKRTFVGISQDQLEVSNLPM
WKPLLYGVAFLHTVVQERRKFGPLGWNIPYEFNSADFTASVQFVQNHLDECSAKTGVSWVTVRYMLGEVQYGGRVTDDYD
KRLLNCFSRVWFGDKMFDPT
FCYYTGYKIPVCNAVEEYMEYIQTLPALDSPQVFGLHPNADITYQTNTSADVLDTITNIQPKESGGGAGETRESVVYQLA
RDMLDKLPPNYVPHEVKARL
VKMGALSSMNIFLRQEVDRMQRIISVVRISLVDLKLAIDGTIIMSENLRDVLDNMFDARVPSLWKKISWESSTLGFWFTE
LLERNSQFHSWVFDGRPKTF
WMTGFFNPQSFLTAMRQEVTRAHQGWALDTVTLHNDVLKQSKEDITAAPAEGVYVYGLHLDGAGWDRRNSRLVESTPKVL
FAALPVIHMMAINSTAPKDP
KLYLCPVYKKPRRTDLTYITGVVLRTAQSPDHWILRGVALLCDIK
>g882.t2
SLVDDESMVGILSTTQQTAAEVSEKLRVAAETELKINTAQEEYRPAASRGSILYFLITEMSMVNIMYQTSLSQFLKVFDL
SMARSPKSPVTQKRIGNIID
YLTFEVFRYTVRGLYENHKFIFTLLLALKIDLQKTQIKHHEFQILIKGGAALDLKACPSKPFRWILDMTWLNLVELSKLP
QFSEIEIQLSRNEKEWKVWF
DMDAPEEGVIPDGYNALLDVFHRLLLIRSWCPDRTLSQARKYVGDSMGARFAEPVILNLEMTWEESDPRTPLICFLSMGS
DPTNQIDALARKLHLECRAI
SMGQGQEVHARKLLQTSIAQGGWVLLQNCHLGLDFMEELLDTVTMVESMHDSFRVWITTEPHNRFSITLLQSSIKFTNDP
PQGVRAGLKRTFVGISQDQL
EVSNLPMWKPLLYGVAFLHTVVQERRKFGPLGWNIPYEFNSADFTASVQFVQNHLDECSAKTGVSWVTVRYMLGEVQYGG
RVTDDYDKRLLNCFSRVWFG
DKMFDPTFCYYTGYKIPVCNAVEEYMEYIQTLPALDSPQVFGLHPNADITYQTNTSADVLDTITNIQPKESGGGAGETRE
SVVYQLARDMLDKLPPNYVP
HEVKARLVKMGALSSMNIFLRQEVDRMQRIISVVRISLVDLKLAIDGTIIMSENLRDVLDNMFDARVPSLWKKISWESST
LGFWFTELLERNSQFHSWVF
DGRPKTFWMTGFFNPQSFLTAMRQEVTRAHQGWALDTVTLHNDVLKQSKEDITAAPAEGVYVYGLHLDGAGWDRRNSRLV
ESTPKVLFAALPVIHMMAIN
STAPKDPKLYLCPVYKKPRRTDLTYITGVVLRTAQSPDHWILRGVALLCDIK
>g882.t3
SLVDDESMVGILSTTQQTAAEVSEKLRVAAETELKINTAQEEYRPAASRGSILYFLITEMSMVNIMYQTSLSQFLKVFDL
SMARSPKSPVTQKRIGNIID
YLTFEVFRYTVRGLYENHKFIFTLLLALKIDLQKTQIKHHEFQILIKALDLKACPSKPFRWILDMTWLNLVELSKLPQFS
EIEIQLSRNEKEWKVWFDMD
APEEGVIPDGYNALLDVFHRLLLIRSWCPDRTLSQARKYVGDSMGARFAEPVILNLEMTWEESDPRTPLICFLSMGSDPT
NQIDALARKLHLECRAISMG
QGQEVHARKLLQTSIAQNCHLGLDFMEELLDTVTMVESMHDSFRVWITTEPHNRFSITLLQSSIKFTNDPPQGVRAGLKR
TFVGISQDQLEVSNLPMWKP
LLYGVAFLHTVVQERRKFGPLGWNIPYEFNSADFTASVQFVQNHLDECSAKTGVSWVTVRYMLGEVQYGGRVTDDYDKRL
LNCFSRVWFGDKMFDPTFCY
YTGYKIPVCNAVEEYMEYIQTLPALDSPQVFGLHPNADITYQTNTSADVLDTITNIQPKESGGGAGETRESVVYQLARDM
LDKLPPNYVPHEVKARLVKM
GALSSMNIFLRQEVDRMQRIISVVRISLVDLKLAIDGTIIMSENLRDVLDNMFDARVPSLWKKISWESSTLGFWFTELLE
RNSQFHSWVFDGRPKTFWMT
GFFNPQSFLTAMRQEVTRAHQGWALDTVTLHNDVLKQSKEDITAAPAEGVYVYGLHLDGAGWDRRNSRLVESTPKVLFAA
LPVIHMMAINSTAPKDPKLY
LCPVYKKPRRTDLTYITGVVLRTAQSPDHWILRGVALLCDIK
>g883.t1

MSASPEYSSFFAVMGASSAMVFSGAAYGTAKSGTGIAAMSVMRPELIMKSIIPVVMAGIIAIYGLVVAVLIANNISENVT
LYKSFLHLGAGLSVGLSGLA
AGFAIGIVGDAGVRGTAQQPRLFVGMILILIFAEVLGLYGLIVALILSTK
>g883.t2
MADFKAVQCQARFWLGAAYGTAKSGTGIAAMSVMRPELIMKSIIPVVMAGIIAIYGLVVAVLIANNISENVTLYKSFLHL
GAGLSVGLSGLAAGFAIGIV
GDAGVRGTAQQPRLFVGMILILIFAEVLGLYGLIVALILSTK
>g884.t1
MSRYNSTYASIKDGGNVHCDFLKVKDKENTAAASPRIVTSSTLAQRHAMYSGPRPHQRAPGGLEGVRVILACRGH
>g885.t1
MSHLLSFLEQYSHFQQLQQQVDQYRVQLKRHRVHHRRQMKVLRASYRERLRNKDSIICSLEEAVSQPHTPSPLTPSEDAG
SSSSGGLAGVHSLVESLYGL
QGERSRLRGELRLLHSQLEDKEQDRHSRILDFQQQIDDLKGCIEEREEELFRLRAASGATDSEKRVLCLSVENDSLKQSL
SMTQGLLEQLSAIPSQSSSL
LMKENESLRSRVQQLEMSLQERAEQLSHLERKSENSEWRRGEELRKREERVRELQLELDRERGKEPVVKYVTQTVEVESP
STLKQLSKARQKNELLSEKL
SAQNQRCKHLEEQIRKSDEHSCNLQHKIAAYEREISKLREELLKEISHLEEKKEEAVKAASSCSAEHFQSLQEQFLGLQQ
RLAALPPALRSMKTDYASLR
SQVRNFSDFYGAAVTDAKKQITAAIHEMSAANKDLMEKYRKEVALRRKYHEQLVELKGNIRVLCRVKPVLKEDQHDDGQA
VVVTTDPNNESALSVMNKGK
SRAFELDKVFHPQATQEEVFQEIEPLVTSCIDGYHVCIFAYGQTGSGKTYTMEGSVENPGINQRALKHLFNEIEDRKDMW
SYTVNVSSVEIYNEVLRDLL
SKEGDKLDIKINPDGTGQLHVPGLRVTEVKGFQHIKKILAKARRNRITFGTQMNQHSSRSHALLCITVYGTDLANGSKTT
GETTGKLNLVDLAGSERVWK
SGAEGERLKEAQNINRSLLALGDVIQALRGNQTHVPFRNSRLTYLLQDSLGKGNKTAMVVQVSALESNVGETLCSLKFAQ
KVCKVELGPAARKIESGAGL
CD
>g885.t2
LAESPLVAVMSHLLSFLEQYSHFQQLQQQVDQYRVQLKRHRVHHRRQMKVLRASYRERLRNKDSIICSLEEAVSQPHTPS
PLTPSEDAGSSSSGGLAGVH
SLVESLYGLQGERSRLRGELRLLHSQLEDKEQDRHSRILDFQQQIDDLKGCIEEREEELFRLRAASGATDSEKRVLCLSV
ENDSLKQSLSMTQGLLEQLS
AIPSQSSSLLMKENESLRSRVQQLEMSLQERAEQLSHLERKSENSEWRRGEELRKREERVRELQLELDRERGKEPVVKYV
TQTVEVESPSTLKQLSKARQ
KNELLSEKLSAQNQRCKHLEEQIRKSDEHSCNLQHKIAAYEREISKLREELLKEISHLEEKKEEAVKAASSCSAEHFQSL
QEQFLGLQQRLAALPPALRS
MKTDYASLRSQVRNFSDFYGAAVTDAKKQITAAIHEMSAANKDLMEKYRKEVALRRKYHEQLVELKGNIRVLCRVKPVLK
EDQHDDGQAVVVTTDPNNES
ALSVMNKGKSRAFELDKVFHPQATQEEVFQEIEPLVTSCIDGYHVCIFAYGQTGSGKTYTMEGSVENPGINQRALKHLFN
EIEDRKDMWSYTVNVSSVEI
YNEVLRDLLSKEGDKLDIKINPDGTGQLHVPGLRVTEVKGFQHIKKILAKARRNRITFGTQMNQHSSRSHALLCITVYGT
DLANGSKTTGETTGKLNLVD
LAGSERVWKSGAEGERLKEAQNINRSLLALGDVIQALRGNQTHVPFRNSRLTYLLQDSLGKGNKTAMVVQVSALESNVGE
TLCSLKFAQKVCKVELGPAA
RKIESGAGLCD
>g885.t3
MSHLLSFLEQYSHFQQLQQQVDQYRVQLKRHRVHHRRQMKVLRASYRERLRNKDSIICSLEEAVSQPHTPSPLTPSEDAG
SSSSGGLAGVHSLVESLYGL
QGERSRLRGELRLLHSQLEDKEQDRHSRILDFQQQIDDLKGCIEEREEELFRLRAASGATDSEKRVLCLSVENDSLKQSL
SMTQGLLEQLSAIPSQSSSL
LMKENESLRSRVQQLEMSLQERAEQLSHLERKSENSEWRRGEELRKREERVRELQLELDRERGKEPVVKTVEVESPSTLK
QLSKARQKNELLSEKLSAQN
QRCKHLEEQIRKSDEHSCNLQHKIAAYEREISKLREELLKEISHLEEKKEEAVKAASSCSAEHFQSLQEQFLGLQQRLAA
LPPALRSMKTDYASLRSQVR
NFSDFYGAAVTDAKKQITAAIHEMSAANKDLMEKYRKEVALRRKYHEQLVELKGNIRVLCRVKPVLKEDQHDDGQAVVVT
TDPNNESALSVMNKGKSRAF

ELDKVFHPQATQEEVFQEIEPLVTSCIDGYHVCIFAYGQTGSGKTYTMEGSVENPGINQRALKHLFNEIEDRKDMWSYTV
NVSSVEIYNEVLRDLLSKEG
DKLDIKINPDGTGQLHVPGLRVTEVKGFQHIKKILAKARRNRITFGTQMNQHSSRSHALLCITVYGTDLANGSKTTGETT
GKLNLVDLAGSERVWKSGAE
GERLKEAQNINRSLLALGDVIQALRGNQTHVPFRNSRLTYLLQDSLGKGNKTAMVVQVSALESNVGETLCSLKFAQKVCK
VELGPAARKIESGAGLCD
>g886.t1
MAPLYLIIFKIGVYNSESRHVRRALQEFPLLRAVLKDDGTTGKELLASWLHSFNSNCHGREIKLRNYTPEDEELKDRQVP
KAKPASVEDKVKEQLEAANP
EPIIEEVDLANLAPRKPDWDLKRDVAKKLEKLERRTQRAIAELISMDHLSRFSSHDLNSLMCPGA
>g886.t2
LRNYTPEDEELKDRQVPKAKPASVEDKVKEQLEAANPEPIIEEVDLANLAPRKPDWDLKRDVAKKLEKLERRTQRAIAEL
ISMDHLSRFSSHDLNSLMCP
GA
>g886.t3
EIKLRNYTPEDEELKDRQVPKAKPASVEDKVKEQLEAANPEPIIEEVDLANLAPRKPDWDLKRDVAKKLEKLERRTQRAI
AELISMDHLSRFSSHDLNSL
MCPGA
>g887.t1
AVVASPPAALASLEEAVTWIGYFCFTSNPFFYGCLNRQIREELGKHMPCLFRRARAEEEDRLPSREGSIEENFLQFLQGT
GCNLEPPDSRSASSPQGEAP
PQGPPPPQPVPVDFRIPGQVAEETSELADGEHVRRNHVTKDS
>g888.t1
MDCFGPFLTKQGRKESKSSSTACTSPTQLQQVEDDWAASRTPHTCHHFHQINPSAYKKPSSAVTPCQIFPRNHLMNPLWI
TWFKLLQNLLLRQNLSSLLP
SGLFSPSPKYNKTPSRANHSSAASVVYLSPEFTEP
>g889.t1
MVQFTDLEKAISTLVEEFHKAGSGATLKADEFKGLVSSQLPTFAKTVGTDAGWTDILQKMGVSGDGEGVSFERFWGLIQS
LATKQHSLLNIDKVSKCSCI
VL
>g889.t2
MGLCKYIQRFTDLEKAISTLVEEFHKAGSGATLKADEFKGLVSSQLPTFAKTVGTDAGWTDILQKMGVSGDGEGVSFERF
WGLIQSLATKQHSLLNIDKV
SKCSCIVL
>g890.t1
FNQCFGVLGVLDRLHGTDSKFRQTKQYERHSLLTNLTPLSKSIPDQPKKLQ
>g891.t1
DSFGHKHVVKVLTEEVEDGSVELVDEVEESREWRDTVQLHNEEKSLLRYYVFEGLRFVWLPRKGAFCKVSVLNEDWTCND
LHRFQSGLNPAEQSS
>g891.t2
DSFGHKHVVKVLTEEVEDGSVELVDEVEESREWRDTVQLHNEEKSLLRYYVFEGLRFVWLPRKGAFCKVSAGITPLSICS
VLNEDWTCNDLHRFQSGLNP
AEQSS
>g891.t3
DSFGHKHVVKVLTEEVEDGSVELVDEVEESREWRDTVQLHNEEVRKSLLRYYVFEGLRFVWLPRKGAFCKVSVLNEDWTC
NDLHRFQSGLNPAEQSS
>g892.t1
PTSDPLRPCTCGALSDRGHGHQVHLYATPRSVARDGDQPRPPTATATPMQTSDGVPTSPPCENAGGVAGSLSPPSPPTHP
GHVQAALIQSGDSRKSVVAP
PGDTGQSYTSKTGFTVCSTCGGYKVGDVWF
>g892.t2
PTSDPLRPCTCGALSDRGHGHQVHLYATPRSVARDGDQPRPPTATATPMQTSDGVPTSPPCENAGGVAGSLSPPSPPTHP
GHVQAALIQSGDSRKSVVAP
PGDTGQSYTSKTGFTVCSTCGGYKPAVPHFCLLVKRPRLARPSGFCLKQV
>g893.t1
DLYFVLKSCFKYYSSDDISKDGDGMDEQCRWCAEGGNLICCDYCSNAFCKKCILRNLGRKELSGILESKWYCYVCSPEPL

EGLVVACQGALQNLDYLSQP
RHQRRHRVEPEKELYAMLPALPQILYQKWDHSGMDGNVVFNYNTLDVPKELMKKAKHLVDSTNTLNRTFVEFIKSARNDK
RTLGARPLYLQSFLLVLKGL
RKSHALLEESLKEEFCDGDPLSCSESVLQERPGTEPEPPREAESEVDVTDKCTLTALQNLAAEHLEEDDLDFKGLTDEVK
SFYAQTSGVQDAAPLPERPT
IKPPKADLNLSHGLLKKLVVKLTPVPVGQQVASSAVGEGVKVSPEAQETLGAEAEPQDVGVCLKKDQETRTEAPAEAGEG
PQPEEELDNRRSPRVKTTPL
RRPSDVKPKPSVTTSGSDSDPEGGGDANLASGASPRRRDESEESDEVPAALLQXXXXXXXTRTPRCPR
>g893.t2
LYFVLKSCFKYYSSDDISKDGDGMDEQCRWCAEGGNLICCDYCSNAFCKKCILRNLGRKELSGILESKWYCYVCSPEPLE
GLVVACQGALQNLDYLSQPR
HQRRHRVEPEKELYAMLPALPQILYQKWDHSGMDGNVVFNYNTLDVPKELMKKAKHLVDSTNTLNRTFVEFIKSARNDKR
TLGARPLYLQSFLLVLKGLR
KSHALLEESLKEEFCDGDPLSCSESVLQERPGTEPEPPREAESEVDVTDKCTLTALQNLAAEHLEEDDLDFKGLTDEVKS
FYAQTSGVQDAAPLPERPTI
KPPKADLNLSHGLLKKLVVKLTPVPVGQQVASSAVGEGVKVSPEAQETLGAEAEPQDVGVCLKKDQETRTEAPAEAGEGP
QPEEELDNRRSPRVKTTPLR
RPSDVKPKPSVTTSGSDSDPEGGGDANLASGASPRRRDESEESDEVPAALLQXXXXXXXTRTPRCPR
>g894.t1
LSTATPLSPKRVCRKRKMLYSEASERRAKAQRVGSDSSNDDLKEDKTGTAGSQSSAVAGSRTDQRGPPDYQHATSTPVPS
DNRTKRLRTTWDPSSPSSSS
TSRSEGEEGDPALGSGSDSDDQRMKPITENVSILGASGFQQSSGDEDQSHSGPSWAAEDDDDAENRCNRKPDKPCPNSRK
KSNLQTAQSLNPTPKSDLQT
AQSLSQKPKSGQSPRPKTLPQPFHQTAQTRSSQARPRRSCSQAKGVKYSQ
>g894.t2
MLYSEASERRAKAQRVGSDSSNDDLKEDKTGTAGSQSSAVAGSRTDQRGPPDYQHATSTPVPSDNRTKRLRTTWDPSSPS
SSSTSRSEGEEGDPALGSGS
DSDDQRMKPITENVSILGASGFQQSSGDEDQSHSGPSWAAEDDDDAENRCNRKPDKPCPNSRKKSNLQTAQSLNPTPKSD
LQTAQSLSQKPKSGQSPRPK
TLPQPFHQTAQTRSSQARPRRSCSQAKGVKYSQ
>g894.t3
LSTATPLSPKRVCRKRKMLYSEASERRAKAQRVGSDSSNDDLKEDKTGTAGSQSSAVAGSRTDQRGPPDYQHATSTPVPS
DNRTKRLRTTWDPSSPSSSS
TSRSEGEEGDPALGSGSDSDDQRMKPITENVSILGASGFQQSSGDEDQSHSGPSWAAEDDDDAENRYGSKTLDIVPHSSA
RCNRKPDKPCPNSRKKSNLQ
TAQSLNPTPKSDLQTAQSLSQKPKSGQSPRPKTLPQPFHQTAQTRSSQARPRRSCSQAKGVKYSQ
>g895.t1
MQNVANDRAVPLPWSPFSPDVVVQMLXIAKKILLAQIKAEYSSGDDLSSDDESQDEEDSEDEDEGVKEGRHDDDDE
>g895.t2
MLHLTLLVLHHRTHAKALKASCCLVRPEDCFICVLWNNTMSHAAGPQHGIAKKILLAQIKAEYSSGDDLSSDDESQDEED
SEDEDEGVKEGRHDDDDE
>g895.t3
MQNVANDRAVPLPWSPFSPDVVVQMLXIAKKILLAQIKAEYSSGDDLSSDDESQDEEDSEDEDEGVKEGRHDDDDEGK
>g896.t1
PLHREKRQAHNHKKNTCPLLLVADYRFFQHMGRREESITLNYLIELIDRVDDIYRNTTWNDEFKGYGVQIQQIIINKKPT
EQPSDHKGRPWVHYNMKDSP
VEEKDVWDVKKLLEQFSSDIADNASTVCLAHLFTYQDFDEGTLGLAYVAPSKAMALGGLCPRPYLPSRSATKASYLNTGL
TSTMNFGKTILTKEADLVTT
HELGHNFGAEHDPDNLPACAPTDDRGGKFIMYPIAVSGDQVNNKHFSNCSKISIGKTLRFKAPECFKKRNSKVCGNSRVE
EGEDCDPGLLHLNDDPCCTA
ECKFKPSAQCSDRNSPCCKSCKFEQAGKRCQEAINATCKGVSNCTGDSSECPAPGNASDNTTCVDMGLCLAGDCNPFCEA
TQSLQSCACNGESRSTPHSP
LRLSGDAAVGRGALQ
>g896.t2
PLHREKRQAHNHKKNTCPLLLVADYRFFQHMGRREESITLNYLIELIDRVDDIYRNTTWNDEFKGYGVQIQQIIINKKPT
EQPSDHKGRPWVHYNMKDSP

VEEKDVWDVKKLLEVRARGDRQFSSDIADNASTVCLAHLFTYQDFDEGTLGLAYVAPSKAMALGGLCPRPYLPSRSATKA
SYLNTGLTSTMNFGKTILTK
EADLVTTHELGHNFGAEHDPDNLPACAPTDDRGGKFIMYPIAVSGDQVNNKHFSNCSKISIGKTLRFKAPECFKKRNSKV
CGNSRVEEGEDCDPGLLHLN
DDPCCTAECKFKPSAQCSDRNSPCCKSCKFEQAGKRCQEAINATCKGVSNCTGDSSECPAPGNASDNTTCVDMGLCLAGD
CNPFCEATQSLQSCACNGES
RSTPHSPLRLSGDAAVGRGALQ
>g896.t3
PLHREKRQAHNHKKNTCPLLLVADYRFFQHMGRREESITLNYLIELIDRVDDIYRNTTWNDEFKGYGVQIQQIIINKKPT
EQPSDHKGRPWVHYNMKDSP
VEEKDVWDVKKLLEQFSSDIADNASTVCLAHLFTYQDFDEGTLGLAYVAPSKAMALGGLCPRPYLPSRSATKASYLNTGL
TSTMNFGKTILTKEADLVTT
HELGHNFGAEHDPDNLPACAPTDDRGGKFIMYPIAVSGDQVNNKHFSNCSKISIGKTLRFKAPECFKKRNSKVCGNSRVE
EGEDCDPGLLHLNDDPCCTA
ECKFKPSAQCSDRNSPCCKSCKFEQAGKRCQEAINATCKGVSNCTGDSSECPAPGNASDNTTCVDMGLCLAGDCNPFCEA
TQSLQSCACN
>g897.t1
YLKIPVSSAETPDALRVFSFYLSSDSKDKPQASFDCIHQYVSGAGRELLEGKGSIQDKITVCATDDSYQMTRERVSQVEK
DIWSRSAIEIKPGPSKCVKV
QRKLGNAALGVDGSNRQSPSNKRSAAPSSVAHRPLRDRIIHLLALKPYRKPELLLWLERERASLKDKADLTSVLEEIAKL
NSKDLSYTLKEELYRHVQKD
WPGFLEEERQLIHRLLARKLQSIPGSQKRPLPSDSQVCQTPKKQRLLDHVLPLRSPSNGEAGPVKSRGSAAPGNTSVQTR
TEFETTNSHVQGSPTGLYPR
HTLSQPAADPKPRKAELEPPAPSPKPPQANISICTEQRLANGLHRKKKSKKHKDKERERLKPNWIETSPDQKQNQENINA
TNQEGDRTPATHASDEELPD
YLIKYRAIAAVEQRQQYTEDFCAEYDEYRALHERIGTVTEMFVQLGSKINTLPPGTQEHKVT
>g897.t2
YLKIPVSSAETPDALRVFSFYLSSDSKDKPQASFDCIHQYVSGAGRELLEGKGSIQDKITVCATDDSYQMTRERVSQVEK
DIWSRSAIEIKPGPSKCVKV
QRKLGNAALGVDGSNRQSPSNKRSAAPSSVAHRPLRDRIIHLLALKPYRKPELLLWLERERASLKDKADLTSVLEEIAKL
NSKDLSYTLKEELYRHVQKD
WPGFLEEERQLIHRLLARKLQSIPGSQVKSLQSNPSFHKAPGDSPPQLSPAKTLCLKRPLPSDSQVCQTPKKQRLLDHVL
PLRSPSNGEAGPVKSRGSAA
PGNTSVQTRTEFETTNSHVQGSPTGLYPRHTLSQPAADPKPRKAELEPPAPSPKPPQANISICTEQRLANGLHRKKKSKK
HKDKERERLKPNWIETSPDQ
KQNQENINATNQEGDRTPATHASDEELPDYLIKYRAIAAVEQRQQYTEDFCAEYDEYRALHERIGTVTEMFVQLGSKINT
LPPGTQEHKVT
>g897.t3
MVSRTSRRVHGLLGCRALCGAHIKEAWQPMGYLKIPVSSAETPDALRVFSFYLSSDSKDKPQASFDCIHQYVSGAGRELL
EGKGSIQDKITVCATDDSYQ
MTRERVSQVEKDIWSRSAIEIKPGPSKCVKVQRKLGNAALGVDGSNRQSPSNKRSAAPSSVAHRPLRDRIIHLLALKPYR
KPELLLWLERERASLKDKAD
LTSVLEEIAKLNSKDLSYTLKEELYRHVQKDWPGFLEEERQLIHRLLARKLQSIPGSQVKSLQSNPSFHKAPGDSPPQLS
PAKTLCLKRPLPSDSQVCQT
PKKQRLLDHVLPLRSPSNGEAGPVKSRGSAAPGNTSVQTRTEFETTNSHVQGSPTGLYPRHTLSQPAADPKPRKAELEPP
APSPKPPQANISICTEQRLA
NGLHRKKKSKKHKDKERERLKPNWIETSPDQKQNQENINATNQEGDRTPATHASDEELPDYLIKYRAIAAVEQRQQYTED
FCAEYDEYRALHERIGTVTE
MFVQLGSKINTLPPGTQEHKVT
>g898.t1
MLEQFSTKTAEADTSSELAKKSKETFRKEISQFIVQCLNPYRKPDCKLGRISNTDDFKHLARKLTHGVMNKELKACKNPE
DLECNEEVKHKAKEYIKKYM
HRFGSVYRPKEDTELE
>g898.t2
MLEQFSTKTAEADTSSELAKKSKETFRKEISQFIVQCLNPYRKPDCKLGRISNTDDFKHLARKLTHGVMNKELKACKNPE
DLECNEEVKHKAKEYIKKYM

HRFGSVYRPKEDTEL
>g899.t1
MNPSNPVSVHSTETALVKITNDLLHAADSGLLTILILLYLSAAFDTISHPLVLDRLAGIGISGTALSWFTSYLTDRQPFV
QLRNHKSGCSGVSLGVPQGS
VLGPLLFTIYLLPLGSLLRHHGVHFHCYADDTQVYISSKPTAAIPPTSLITCLDDKLNGNKTEALLIGFKSIITKSQHTP
APPIIIDGFPVPFSPQVKSL
GVILDSTLSFAPHIQNITRTAFFHLRNISRLRPSLTQSSTEILDHSFVTSRINYCNALLTGLPSKLNSTAARIITCTKSS
DHITPVLIQLHWLPVQYRID
YKNLLLTIKLSTIKPPPTFATSFMNTLPPTPSAQPPLDC
>g900.t1
FDSQEEERVLLACDRFEKEAGLSFPGRPCLTTVIDISGKTVFITRYIRPLNPPQELLDAFPNSPQEATVNSISFSGVCDL
WSTCDQFLTLLAGDEEEHAV
LLCNYFLSMGKKAWLIIGTAIPEVGNRSVLWFQLQGQSLVLQGPTAYVLTHEQNRYLIWNPSSGHYYGQYDTFCPLQAVG
CLVNADNVWFNIQDHSSPMR
ISLDTGRAHLWKPFFSRSFPHPGLASVQPQELVYRASDRAAAAELQERMEKILKEKIMEWRPRQPTRWNRYCTSTLRHFL
PRLELSGGRDMADEHRLELQ
SLLGDYRVSGFPLHLPFSELRPVIEAVYSTGVHSVETSNVEFALAVYVHPYPNNVLSVWVYLASLLRSR
>g900.t2
FDSQEEERVLLACDRFEKEAGLSFPGRPCLTTVIDISGKTVFITRYIRPLNPPQELLDAFPNSPQEATVNSISFSGVCDL
WSTCDQFLTLLAGDEEEHAV
LLCNYFLSMGKKAWLIIGTAIPESLVLQGPTAYVLTHEQNRYLIWNPSSGHYYGQYDTFCPLQAVGCLVNADNVWFNIQD
HSSPMRISLDTGRAHLWKPF
FSRSFPHPGLASVQPQELVYRASDRAAAAELQERMEKILKEKIMEWRPRQPTRWNRYCTSTLRHFLPRLELSGGRDMADE
HRLELQSLLGDYRVSGFPLH
LPFSELRPVIEAVYSTGVHSVETSNVEFALAVYVHPYPNNVLSVWVYLASLLRSR
>g900.t3
FDSQEEERVLLACDRFEKEAGLSFPGRPCLTTVIDISGKTVFITRYIRPLNPPQELLDAFPNSPQEATVNSISFSGVCDL
WSTCDQFLTLLAGDEEEHAV
LLCNYFLSMGKKAWLIIGTAIPEVGNRSVLWFQLQGQGPTAYVLTHEQNRYLIWNPSSGHYYGQYDTFCPLQAVGCLVNA
DNVWFNIQDHSSPMRISLDT
GRAHLWKPFFSRSFPHPGLASVQPQELVYRASDRAAAAELQERMEKILKEKIMEWRPRQPTRWNRYCTSTLRHFLPRLEL
SGGRDMADEHRLELQSLLGD
YRVSGFPLHLPFSELRPVIEAVYSTGVHSVETSNVEFALAVYVHPYPNNVLSVWVYLASLLRSR
>g901.t1
ALSQRGGLPSLEHLNLSGCLLVTEVGLQELVSACPAINDEHFYYCDNIDGKAAGTLRRPGDASGLRGSEPRGAAVVRWKL
RGLGPHADTASGCQNLQCGF
RACCRSGE
>g902.t1
SKQILLSKHIDLKGVEPVKGALLSYPIPDLRIPLSYEVRLAPITTYSSGDYVSRTIQYSE
>g902.t2
SKQILLSKHIDLKGVEPVKGALLSYPIPDLRIPLSYEVRLAPITTYSSGDYVSRTIQYSERKPLPCDGSDLDRKEKGFAD
R
>g903.t1
MNRHHYSLYVHNCRLILLLRQEPTETENYKPAEFHWKLDQVCDKEWHHYVVNVEFPAVSLFVDGTTYEPFLVTEDYPLRS
SKIDTQLTIGACWQGNAAGP
RPPPREFRGSSVASHLFGRRRGRVTKPTRRWLERLFGKRVLLTAVHNEDPLIGIGTAQLPTVSPVNATRYTPWPSASPVS
AVICAGLPGKIEGDE
>g904.t1
MVILVDVSGSVSGLTLKLIKASVMEMLDTLSDDDYVNVARFNEKAEAVVPCFKHLVQANIRNKKIFKDAVQQMQAKGTTD
YKSGFHFAFNQLLNVRVFTF
SVGQHNYDVTPLQWIACTNK
>g905.t1
MLTLSCMSGGRSKNIKAYAQSRQFDSAKGNAELEPILNRVHIVAPVQAASASLSSGFHPQSNGQTGRVNQDLEMPLRCLV
DSSLITRSSQLMWAEYTHNP
ADGKPIYMVRQILNCPRVHPAPGGLEGL
>g905.t2

MLTLSCMSGGRSKNIKAYAQSRQFDSAKGNAELEPILNRVHIVAPVQVTIELKAASASLSSGFHPQSNGQTGRVNQDLEM
PLRCLVDSSLITRSSQLMWA
EYTHNPADGKPIYMVRQILNCPRVHPAPGGLEGL
>g906.t1
LIDMLSDSGLSVIEATSFVSPKWVPQMADQMEVMKGIHRKPGVSYPVLTPNLKGFEAAVEAGALEVAIFGAASELFSKKN
INCSVEESLQRFEQVTTAAK
EAGVPVRGYVSCVLGCPYEGKVAPAKVAHVAKRLYSMGCYEISLGDTIGVGTPGAMSEMLEAVSKEVPVGALAVHCHDTY
GQALANILVALQ
>g906.t2
MLSDSGLSVIEATSFVSPKWVPQMADQMEVMKGIHRKPGVSYPVLTPNLKGFEAAVEAGALEVAIFGAASELFSKKNINC
SVEESLQRFEQVTTAAKEAG
VPVRGYVSCVLGCPYEGKVAPAKVAHVAKRLYSMGCYEISLGDTIGVGTPGAMSEMLEAVSKEVPVGALAVHCHDTYGQA
LANILVALQ
>g906.t3
MAALIRIINRSSFISRMGHPCLALGCSTAVAKSVGGSSSKRLPDRVKIVEVGPRDGLQNEKTIVPAETKIRLIDMLSDSG
LSVIEATSFVSPKWVPQMAD
QMEVMKGIHRKPGVSYPVLTPNLKGFEAAVEAGALEVAIFGAASELFSKKNINCSVEESLQRFEQVTTAAKEAGVPVRGY
VSCVLGCPYEGKVAPAKVAH
VAKRLYSMGCYEISLGDTIGVGTPGAMSEMLEAVSKEVPVGALAVHCHDTYGQALANILVALQ
>g907.t1
MEGKVLVTGGGGYIGSHCVVELIEAGYRPVVIDNFSNSVRGEGDVPESLRRIEKLMKTPIEFHELDLLDLAGLEELFKQH
SFIAVMHFAGLKAVGESVQK
PLLYYQVNLTASMNLIQVMQNHGVHNLVFSSSATVYGDPQRLPMDERHPVGGCTNPYGKTKFFIEEIITDQCKANKDWNA
ILLRYFNPIGAHVSGDIGED
PLGIPNNLLPYVAQVAIGRREHLNVFGNDYDTPDGTGVRDYIHVVDLAKGHIAALKKLQDNCGCKAYNLGTGTGYSVLQM
VQAMEKASGKKIACQIAPRR
TGDVASCYADPRLAADELGWKAEFDLERMXPQTQAQRWFNAADITVAHQRHLLKQLDQQRCQELFCDCSVQVEGQLFHAH
RNVLFASSGYFRMLLSPQGS
DRGRHPGLSDTASATFDAFSPDTFALILDFIYSGQLDLSSHNVIEVMSAASYLQMNSVINYCKTFIKSSLEISVKEEEEE
LEVEGGGDGGGEHCLDLMDQ
QGSLSCGDGDENLSDQEVSPPHRHLRLHQKDAQTPVGSVSPPPALWTHDGAHLTQQVFMMKDPDQAAPALATRADNDGGG
ISPAHAGLEEANTGAEDPLY
TVSGPERRRRKRGVRRTAPYCTRSSTTNDPPEVQEARSQKAEKAEELYATLPTIVGVIDSNPTMRFKCPFCTHTVKRKAD
LKRHLRCHTGERPYPCQACN
KRFTRLEHLRSHFETIHQARKLVCKKCKCPVTEEASHVVCEGTRRYRICTTCIQEEVGFDNLPMEDTLEGLDQEPALLLG
VDGEEEEGDTQRSWMMTMDQ
EDDEDEDNLAEDSGTDLIIHEVDDSDEEVQ
>g908.t1
TVCQTVAAKDLAADTVEVKSERSHYNNYNSNSYNSCYHYNSYNKSNRYNSCYHSNNNSNSNNSSNSNNSSKRTRSSLTTL
PSLASSGTGMTEHTEAYMDW
CLWNSLLINAEKTKELVVHHPGNGHRDGDILQSRLYLLWKLRSFGVQGPLLKSFDDSVVASAIFYGVDCWSSSISVIDRK
RLDKLIKKASSILGCPLNSC
RWWQRGG
>g908.t2
TVAAKDLAADTVEVKSERSHYNNYNSNSYNSCYHYNSYNKSNRYNSCYHSNNNSNSNNSSNSNNSSKRTRSSLTTLPSLA
SSGTGMTEHTEAYMDWCLWN
SLLINAEKTKELVVHHPGNGHRDGDILQSRLYLLWKLRSFGVQGPLLKSFDDSVVASAIFYGVDCWSSSISVIDRKRLDK
LIKKASSILGCPLNSCRWWQ
RGG
>g908.t3
HYNNYNSNSYNSCYHYNSYNKSNRYNSCYHSNNNSNSNNSSNSNNSSKRTRSSLTTLPSLASSGTGMTEHTEAYMDWCLW
NSLLINAEKTKELVVHHPGN
GHRDGDILQSRLYLLWKLRSFGVQGPLLKSFDDSVVASAIFYGVDCWSSSISVIDRKRLDKLIKKASSILGCPLNSCRWW
QRGG
>g909.t1
LEVNFPGEAEQCDTPIIGAHPLVPLFENGNHHPGLPLHRYCPRPPRDTEEACQPRHPNNVQSLQHLRANLIHTWRLATEE

LLDCLRDLCQPPDSVMQLEI
RMFTRDLI
>g910.t1
MAVHVFCSVLCSGGTRNTFSTLVFAGVMNHQTSLPVARSASTSHAVATATISRELGAQRDQRDHLAGNEFKTAVRPEQCL
TLV
>g911.t1
MELCAGRRPGTQRYITPGASVSAQSHSLLVALGGGIMTGRLPEPQPRRPNSSNICAPLFIACWRRGERPTDTAGGPETPS
KVGNIQLVTFYRMSTQGRQG
FSCPTRTTYRLLVGAL
>g912.t1
EMNDHQNTLSYVLINPPPDTLLELNDIVYIIRSDPLAHMPDETPVGLAECTKNLQEFCTETRDETHL
>g913.t1
MLHGAGRLYGVAVLLLWEQLGMGHACFADGQTLICQDVPTFFSPGYLSLVVLIPDLGQISSTMFLSGNLVNVTRLTVKSN
VTEMVPGALSSLLNLQTLIL
DENALTQIDPAWFSPRSAPALKDLSLVGNHIEVLSESTLGGLVGLRSLNLSRNRIRAIEPGSFGLQTELGSLDLSRNRLT
RLSPLVLGSVRSTLLRLSEN
PWDCSCAAEDFVELLKDLKNRALLEQENEVTCGTPSHLHGRPVWNVLVCETSPPSSSSSSSSPPSLSTRPLMTSLRGTSM
TTDLQPAGLSASAVLCGVLV
PLCALLGLLGAMGGLYGRKRRNRAVKGGPSAGEDPDVGQASQLQPPGSIADPVKRDAAETIRWHAERMLSVNSTTTRAKS
ADAALSTSPFSVGVKVSPGA
PQSETARVQPGEPGENPAEPRGGLGTARGKVEGRHGGTGHPEDGQRHADKRVPSVPVDTEEVPYLSIGALPPKQEANIAA
GAPGQRSGTGMSRVSSWPPS
AAQWQDKGMTLVQGERANILTKHPAAHQRVNECEHGTHTVWASRLLRQKTPDGGICHSGFLSVTSDPEHCSVAVTRSTTE
MLPLGGANGSQTEDPCVLSP
TAPRQCTADTPGGRAESRRPVPRAAPPGALCDEHLTVDYNICPNLSIGTSSLLSDNESRWRSERGPGDQVEQRPTGLTTG
AVGPGGPTEGGRQRAPVRYE
DLLHEVVQNHGRWTRDRWRQTQRNKPHAAGGR
>g913.t2
MCNGEADVRMLHGAGRLYGVAVLLLWEQLGMGHACFADGQTLICQDVPTFFSPGYLSLVVLIPDLGQISSTMFLSGNLVN
VTRLTVKSNVTEMVPGALSS
LLNLQTLILDENALTQIDPAWFSPRSAPALKDLSLVGNHIEVLSESTLGGLVGLRSLNLSRNRIRAIEPGSFGLQTELGS
LDLSRNRLTRLSPLVLGSVR
STLLRLSENPWDCSCAAEDFVELLKDLKNRALLEQENEVTCGTPSHLHGRPVWNVLVCETSPPSSSSSSSSPPSLSTRPL
MTSLRGTSMTTDLQPAGLSA
SAVLCGVLVPLCALLGLLGAMGGLYGRKRRNRAVKGGPSAGEDPDVGQASQLQPPGSIADPVKRDAAETIRWHAERMLSV
NSTTTRAKSADAALSTSPFS
VGVKVSPGAPQSETARVQPGEPGENPAEPRGGLGTARGKVEGRHGGTGHPEDGQRHADKRVPSVPVDTEEVPYLSIGALP
PKQEANIAAGAPGQRSGTGM
SRVSSWPPSAAQWQDKGMTLVQGERANILTKHPAAHQRVNECEHGTHTVWASRLLRQKTPDGGICHSGFLSVTSDPEHCS
VAVTRSTTEMLPLGGANGSQ
TEDPCVLSPTAPRQCTADTPGGRAESRRPVPRAAPPGALCDEHLTVDYNICPNLSIGTSSLLSDNESRWRSERGPGDQVE
QRPTGLTTGAVGPGGPTEGG
RQRAPVRYEDLLHEVVQNHGRWTRDRWRQTQRNKPHAAGGR
>g913.t3
MLHGAGRLYGVAVLLLWEQLGMGHACFADGQTLICQDVPTFFSPGYLSLVVLIPDLGQISSTMFLSGNLVNVTRLTVKSN
VTEMVPGALSSLLNLQTLIL
DENALTQIDPAWFSPRSAPALKDLSLVGNHIEVLSESTLGGLVGLRSLNLSRNRIRAIEPGSFGLQTELGSLDLSRNRLT
RLSPLVLGSVRSTLLRLSEN
PWDCSCAAEDFVELLKDLKNRALLEQENEVTCGTPSHLHGRPVWNVLVCETSPPSSSSSSSSPPSLSTRPLMTSLRGTSM
TTDLQPAGLSASAVLCGVLD
PRVPFGEKLLLLKVTVCIFSFPVPLCALLGLLGAMGGLYGRKRRNRAVKGGPSAGEDPDVGQASQLQPPGSIADPVKRDA
AETIRWHAERMLSVNSTTTR
AKSADAALSTSPFSVGVKVSPGAPQSETARVQPGEPGENPAEPRGGLGTARGKVEGRHGGTGHPEDGQRHADKRVPSVPV
DTEEVPYLSIGALPPKQEAN
IAAGAPGQRSGTGMSRVSSWPPSAAQWQDKGMTLVQGERANILTKHPAAHQRVNECEHGTHTVWASRLLRQKTPDGGICH
SGFLSVTSDPEHCSVAVTRS

TTEMLPLGGANGSQTEDPCVLSPTAPRQCTADTPGGRAESRRPVPRAAPPGALCDEHLTVDYNICPNLSIGTSSLLSDNE
SRWRSERGPGDQVEQRPTGL
TTGAVGPGGPTEGGRQRAPVRYEDLLHEVVQNHGRWTRDRWRQTQRNKPHAAGGR
>g914.t1
VDPAPPATADASADEGLPGAEPDSSDGRGKESEAAEELRRSRGHLIEVDLSGLRDPEEEEAQDASVDVVDGEQKSVLGFF
FKDEQKAEDELAKKRAAFLL
KQQKKAEEARLRKFQLEAESELKRDEARRKADEDQRKKEDEKMRREIIKQEYLRRKQQEVLEEQGLARPRLPKTPKTPKP
KQKARPKSIFREESSSDKCC
SSMPDNLSTAQSGSSLSLASAATNEADSVNSGGGGSQRCDSVESFPGSRNASRMAERDWDNGSTASSITSMAEYTGQSSI
ASMAEYTGPKLFKEPSAKSN
KPIIHNAISHCCLAGKVNEPQKNQILEELERSDSNHLMILFRDGGCQFRSLYAYLPDTEEIHKLTGTGPKSISKKMIDKL
YKYSSDRKQFTVIPAKTVSV
SVDALTIHNHLWQAKRGTAVPKKSAK
>g915.t1
PTKEEQQEAKPRLSDTKKPSITPAGPLRPSTGSTEARGGTSPGVEAPSWSSRAPLGEEDLGSQMEEMRSQMKDLVMSVDL
IKNQQTKEMALLRAELDEER
LKRLSLQ
>g915.t2
EEQQEAKPRLSDTKKPSITPAGPLRPSTGSTEARGGTSPGVEAPSWSSRAPLGEEDLGSQMEEMRSQMKDLVMSVDLIKN
QQTKEMALLRAELDEERLKR
LSLQ
>g915.t3
PTKEEQQEAKPRLSDTKKPSITPAGPLRPSTGSTEARGGTSPGVEAPSWSSRAPLGEEDLGSQMEEMRSQMKDLVMSVDL
IKNQQTKEMALLRAELDEER
LKRLSLQVKTNTPISM
>g916.t1
MPTSRKDSEPLGKAIQKIAALKEDIRRLSAELQRKDSLLSSFMEVAAGQSKRITSLSAGRPRWEPASGLRC
>g917.t1
MNRLVTFRLAAMDKAPPNLYPSSQSPSAGLRASRTNRLRRSSRTQQSQQPPSPATQAPESFETGKRGKSRMTGAVIGISE
IVRRIAPMHGRPAVVEPTLQ
QWIGDSAAIPCTPEVHHPKKKSPRPNGVDDLLKLAKQFDFNMFQQDEEQAEDMHQQTLELLSEDILEYENGFQEGPQLLP
AAPPAPAPEPASRDPSPGPQ
GHSGEDELELLFDEPTQRISGNLSQVGFLGSMETSSLNSRTMRGAPCCPTPTVSTQNTEDGPAGSRLDEDWGDDDWGDDD
FLNDSLVIEMTQNPQCFAPP
KHCSTQNGSTPHDRTRPCRGTPGRLEGCGGGTKNEPRIQKNSTTFKLEANPVPMHVTTNQSNVDTHTRFSTAQSEALSRR
NHAIPGSHLKRQSQPPGGAA
SESPAGPQPQTPLTNPRTPCNPATAPIMTNKMKENQPKRSSNVLAMANVNPLAAHSTRQTPPAAPAGEDLLDLDEVFSLE
SAWDDDGDDDLLCKMCEDPE
APAEVPERPAAPKQRLPLGGKPGLGGKPGLGGQRPAPAAASSRAGDDWEGHLDAHVWK
>g917.t2
MNRLVTFRLAAMDKAPPNLYPSSQSPSAGLRASRTNRLRRSSRTQQSQQPPSPATQAPESFETGKRGKSRMTGAVIGISE
IVRRIAPMHGRPAVVEPTLQ
QWIGDSAAIPCTPEVHHPKKKSPRFFSPAWAHISSQSRTLRGSGVKQGLKVSKRHSVQAYVQMESLGAFLMKPNGVDDLL
KLAKQFDFNMFQQDEEQAED
MHQQTLELLSEDILEYENGFQEGPQLLPAAPPAPAPEPASRDPSPGPQGHSGEDELELLFDEPTQRISGNLSQVGFLGSM
ETSSLNSRTMRGAPCCPTPT
VSTQNTEDGPAGSRLDEDWGDDDWGDDDFLNDSLVIEMTQNPQCFAPPKHCSTQNGSTPHDRTRPCRGTPGRLEGCGGGT
KNEPRIQKNSTTFKLEANPV
PMHVTTNQSNVDTHTRFSTAQSEALSRRNHAIPGSHLKRQSQPPGGAASESPAGPQPQTPLTNPRTPCNPATAPIMTNKM
KENQPKRSSNVLAMANVNPL
AAHSTRQTPPAAPAGEDLLDLDEVFSLESAWDDDGDDDLLCKMCEDPEAPAEVPERPAAPKQRLPLGGKPGLGGKPGLGG
QRPAPAAASSRAGDDWEGHL
DAHVWK
>g917.t3
MAPCPWVEQPPSPSAGLRASRTNRLRRSSRTQQSQQPPSPATQAPESFETGKRGKSRMTGAVIGISEIVRRIAPMHGRPA
VVEPTLQQWIGDSAAIPCTP

EVHHPKKKSPRPNGVDDLLKLAKQFDFNMFQQDEEQAEDMHQQTLELLSEDILEYENGFQEGPQLLPAAPPAPAPEPASR
DPSPGPQGHSGEDELELLFD
EPTQRISGNLSQVGFLGSMETSSLNSRTMRGAPCCPTPTVSTQNTEDGPAGSRLDEDWGDDDWGDDDFLNDSLVIEMTQN
PQCFAPPKHCSTQNGSTPHD
RTRPCRGTPGRLEGCGGGTKNEPRIQKNSTTFKLEANPVPMHVTTNQSNVDTHTRFSTAQSEALSRRNHAIPGSHLKRQS
QPPGGAASESPAGPQPQTPL
TNPRTPCNPATAPIMTNKMKENQPKRSSNVLAMANVNPLAAHSTRQTPPAAPAGEDLLDLDEVFSLESAWDDDGDDDLLC
KMCEDPEAPAEVPERPAAPK
QRLPLGGKPGLGGKPGLGGQRPAPAAASSRAGDDWEGHLDAHVWK
>g918.t1
LTADELHVLYEGIKLNNVHHYDYVLTGTLYTSYHNITNVHHYDNVITGYTRDTSFLEMVVDIIQELKRANPSLVYDEGCS
CGWDSREVRCLIPSVLSRPV
SIRQQDATNPTVSPYCISCVTQCWEIMDLWLLTGKKISTEKEAVEVMDLLHAMGPDTVVITSSDLPSRLGDQFLVSLGSQ
CTVHPDGSRSTQRVRMDVPK
VDAVFVGTGDLFAAMLLAWTHHYPNDLKTACEKTFSVMHHVIQRTISYAHELAGPGRRPSPPQLELRMVQSKADIEDPAI
VMEATVIS
>g918.t2
MFCYADWLKEFVCQGKWSRPMKCSQRSTLICVAKRNVKSLGYTRDTSFLEMVVDIIQELKRANPSLVYDEGCSCGWDSRE
VRCLIPSVLSRPVSIRQQDA
TNPTVSPYCISCVTQCWEIMDLWLLTGKKISTEKEAVEVMDLLHAMGPDTVVITSSDLPSRLGDQFLVSLGSQCTVHPDG
SRSTQRVRMDVPKVDAVFVG
TGDLFAAMLLAWTHHYPNDLKTACEKTFSVMHHVIQRTISYAHELAGPGRRPSPPQLELRMVQSKADIEDPAIVMEATVI
S
>g918.t3
LTADELHVLYEGIKLNNVHHYDYVLTGTLYTSYHNITNVHHYDNVITEHRDHMFCYADWLKEFVCQGKWSRPMKCSQRST
LICVAKRNVKSLGYTRDTSF
LEMVVDIIQELKRANPSLVYDEGCSCGWDSREVRCLIPSVLSRPVSIRQQDATNPTVSPYCISCVTQCWEIMDLWLLTGK
KISTEKEAVEVMDLLHAMGP
DTVVITSSDLPSRLGDQFLVSLGSQCTVHPDGSRSTQRVRMDVPKVDAVFVGTGDLFAAMLLAWTHHYPNDLKTACEKTF
SVMHHVIQRTISYAHELAGP
GRRPSPPQLELRMVQSKADIEDPAIVMEATVIS
>g919.t1
MDITFSQVTSSQPVVTLDDTKRDSLTPTNEAEDLDEQDRPPSTAKELQVVYHLELHVAVNHIPDRFMTSSLLFFLRNTKE
TVVEPVDMSEANELLPCVME
IGMLNGHSLLMLRDVLSHLYLPMLSVSQLNGPAGSHQTEEASPRPQDEGYRPPESKGTMVMRDELLNSAHKFLGHINTTL
QQLDGGSLVGL
>g919.t2
LLNRDDGEDEQKIITFLNNLTTEESASALFFFKEVREEEIEVQFTADDTKRDSLTPTNEAEDLDEQDRPPSTAKELQVVY
HLELHVAVNHIPDRFMTSSL
LFFLRNTKETVVEPVDMSEANELLPCVMEIGMLNGHSLLMLRDVLSHLYLPMLSVSQLNGPAGSHQTEEASPRPQDEGYR
PPESKGTMVMRDELLNSAHK
FLGHINTTLQQLDGGSLVGL
>g919.t3
MDITFSQVTSSQPVVTLDDTKRDSLTPTNEAEDLDEQDRPPSTAKELQVVYHLELHVAVNHIPDRFMTSSLLFFLRNTKE
TVVEPVDMSEANELLPCVME
IGMLNGHSLLMLRDVLSHVRLYLPMLSVSQLNGPAGSHQTEEASPRPQDEGYRPPESKGTMVMRDELLNSAHKFLGHINT
TLQQLDGGSLVGL
>g920.t1
MDLGEDVDVLVSSPEVVDMLEQCVMNWHAQITIIIEEQLKKKPQAPGPIAEIDFWRERSAILSALSEQLNLPVAKRILEV
LAKVDPVTVQNLELSVAELS
SHHEEAVQNVRFLNTLERHFKNLATGADFSVILNTTPQIMNSLRLVWIISSHYNKDERMVPLMERIAWELSERVKRVVNV
RVLFKDKREVAKAKVLDGKM
VLDQWRECYLETRARIEASARDPRWEFDRKRLFEKTDYMASICQDLHNVLQSLEEFSNIFGPELKAVTGDPKRIDDVLQR
LDRLVVPIQELSFDPFSIQK
RGRWNIIMRDFNSKVMDFEEEAISFIDQSFKKLRSATAAFDMLLKFKHIRSREALNKQMMKKFNDILAQYCKEVDMISMN
FDAHKEKPPLKNQPLVAGAI

SWERLLFRRIKSPILRFQEMGELLESEEGKEARAKYLEVGKRMRDYELQKYEHWRVETEKILPLLMNRNILVDPSTRGLR
LDRNVTYHMNFAPEIKEIIS
ETMNLEQLGFAVPELARNVALQGEKFLLTYMDGLNDLLSRYNSLMDSLNDAQFLLLQEHIQELRKVMHFGSKMLNWNSLG
ISDFIIGGIQATSKFESLVV
PIQRNEREMDSKLQAMEMANLFKSPLPDKSTELPGVKMFFELIEHERTKDLNVLTRKYSAIGPILIKVESLILHTSTGKS
KRMAQYYAYWEHKVFDSLVQ
MVLRNMQLFNKVLMGNTPLFQIETILSAPEILLQPKSSEVYKLIMNCVRNCVDGTKRFVRWMHGTCMECPPQGGAGEDQL
LTFSFYSDVCQHPQISELAV
ALSQNTQRLLSSLGRYLSSWKRYCPLWKLDKAVAMEKFASKKPPCVLYDDKLQFYAKVHQEVLMEPLVKNEHVICLNLAP
LAHTVQESAQAWVSALGRLL
NQSAREDLLGLRDEFTVPNLELKDTGSRSGALVLIYHHLSENLKRNPNSLEDLKFVLGTISNIRAMSLTVEMRLADIQEK
YRTLAMYKVEVPHEDLELVA
RIGPMWSGLLTESKLVDRSLGRVKKTFTEITKEQIEEFTQELSIFAESFNMHGPGAVGDDLEKGLAVLGTYEAELAKMES
GRQDLANAEKLFDLPFTMYP
ELLQVQKDMKGLRLIYDTYNAQKIAKAEWSQTLWVNLNLQLLQEGMEGFIRTLRKLPKEVRALPVAFFLEGRMKEFKESL
PLLLDLKNEALRERSIQRLA
NTSKVI
>g920.t2
MDLGEDVDVLVSSPEVVDMLEQCVMNWHAQITIIIEEQLKKKPQAPGPIAEIDFWRERSAILSALSEQLNLPVAKRILEV
LAKVDPVTVQNLELSVAELS
SHHEEAVQNVRFLNTLERHFKNLATGADFSVILNTTPQIMNSLRLVWIISSHYNKDERMVPLMERIAWELSERVKRVVNV
RVLFKDKREVAKAKVLDGKM
VLDQWRECYLETRARIEASARDPRWEFDRKRLFEKTDYMASICQDLHNVLQSLEEFSNIFGPELKAVTGDPKRIDDVLQR
LDRLVVPIQELSFDPFSIQK
RGRWNIIMRDFNSKVMEEAISFIDQSFKKLRSATAAFDMLLKFKHIRSREALNKQMMKKFNDILAQYCKEVDMISMNFDA
HKEKPPLKNQPLVAGAISWE
RLLFRRIKSPILRFQEMGELLESEEGKEARAKYLEVGKRMRDYELQKYEHWRVETEKILPLLMNRNILVDPSTRGLRLDR
NVTYHMNFAPEIKEIISETM
NLEQLGFAVPELARNVALQGEKFLLTYMDGLNDLLSRYNSLMDSLNDAQFLLLQEHIQELRKVMHFGSKMLNWNSLGISD
FIIGGIQATSKFESLVVPIQ
RNEREMDSKLQAMEMANLFKSPLPDKSTELPGVKMFFELIEHERTKDLNVLTRKYSAIGPILIKVESLILHTSTGKSKRM
AQYYAYWEHKVFDSLVQMVL
RNMQLFNKVLMGNTPLFQIETILSAPEILLQPKSSEVYKLIMNCVRNCVDGTKRFVRWMHGTCMECPPQGGAGEDQLLTF
SFYSDVCQHPQISELAVALS
QNTQRLLSSLGRYLSSWKRYCPLWKLDKAVAMEKFASKKPPCVLYDDKLQFYAKVHQEVLMEPLVKNEHVICLNLAPLAH
TVQESAQAWVSALGRLLNQS
AREDLLGLRDEFTVPNLELKDTGSRSGALVLIYHHLSENLKRNPNSLEDLKFVLGTISNIRAMSLTVEMRLADIQEKYRT
LAMYKVEVPHEDLELVARIG
PMWSGLLTESKLVDRSLGRVKKTFTEITKEQIEEFTQELSIFAESFNMHGPGAVGDDLEKGLAVLGTYEAELAKMESGRQ
DLANAEKLFDLPFTMYPELL
QVQKDMKGLRLIYDTYNAQKIAKAEWSQTLWVNLNLQLLQEGMEGFIRTLRKLPKEVRALPVAFFLEGRMKEFKESLPLL
LDLKNEALRERSIQRLANTS
KVI
>g921.t1
NFQGRSYECMSDCSDMSSYMSRCQSCRVDSGCFMLYERNNYMGQQFFMRRGEYNDMQRMQSMGMMFDNIRSCRMIPFHRG
QFRMKIFERENFGGQSYELM
DDCENIMDRYRMSDCQSCNVMDGHWLMYEQPQYRGRMMYMRPGEYRSFREMMSGQRFMSMRRITDMC
>g922.t1
MGKITFYEERNFQGRSYECMSDCSDMSSYMSRCQSCRVDSGCFMLYERNNYMGQQFFMRRGEYNDMQRMQSMGMMFDNIR
SCRMIPFHRGQFRMKIFERE
NFGGQSYELMDDCENIMDRYRMSDCQSCNVMDGHWLMYEQPQYRGRMMYMRPGEYRSFREMMSGQRFMSMRRITDMC
>g923.t1
SVNVFILDENDNSPGILPPYSEHGSVNSENIPYSAEAGYFVAKIRAVDADSGYNALLSYHMSEPKGTNLFRIGTSTGEIR
TKRRMSDNDLKTHPLVVLVS
DNGEPSLTATVSIDVVVVENTGDIQTQFRHVPLKEDSFSALNLYLLIAIASVSVIFLLSLISLIAVKCHRTDNSFSRYSA
PMITTHPDGSWSYSKSTQQY

DVCFSSDTLKSDVVVFPAQFPPGDAELISINGGDTLNRTQTLPNKDKWLWAPLYESLSVVLRR
>g924.t1
MSAHCKVLVEVVDLNDNAPEISVTSLLATVKEDAKIGTAIALVSVLDKDEGENSVVRVVIPNDVPFKLETNYKNYYSLVV
NGLIDRETNAHHKVIIVATD
EGTPSLSSSAVVTVHVSDVNDNQPRFTETRMSVYVKENSPVGVVLKTASAIDVDAGQNGQISYSLMADSVSSLPLSTMVN
INSETGDILSLKSFNYEDLK
TFQFKVKATDSGVPPLSSNVTVNVFILDENDNSPGILPPYSEHGSVNSENIPYSAEAGYFVAKIRAVDADSGYNALLSYH
MSEPKGTNLFRIGTSTGEIR
TKRRMSDNDLKTHPLVVLVSDNGEPSLTATVSIDVVVVENTGDIQTQFRHVPLKEDSFSALNLYLLIAIASVSVIFLLSL
ISLIAVKCHRTDNSFS
>g924.t2
MSAHCKVLVEVVDLNDNAPEISVTSLLATVKEDAKIGTAIALVSVLDKDEGENSVVRVVIPNDVPFKLETNYKNYYSLVV
NGLIDRETNAHHKVIIVATD
EGTPSLSSSAVVTVHVSDVNDNQPRFTETRMSVYVKENSPVGVVLKTASAIDVDAGQNVKATDSGVPPLSSNVTVNVFIL
DENDNSPGILPPYSEHGSVN
SENIPYSAEAGYFVAKIRAVDADSGYNALLSYHMSEPKGTNLFRIGTSTGEIRTKRRMSDNDLKTHPLVVLVSDNGEPSL
TATVSIDVVVVENTGDIQTQ
FRHVPLKEDSFSALNLYLLIAIASVSVIFLLSLISLIAVKCHRTDNSFS
>g925.t1
MCGQKSGLVGRMREKMREKNCA
>g926.t1
MENLTFDANIIFLEGLKVTPQSSFPAFILFLLIYIFIMVSNIGLMALICMERSLHQPMYLLFCNMSVNDVFGASAIIPRL
LSDIFILTKNRHITYTECVI
QAFCAHFHAGTSHSVLMVMAFDRYVAISNPLRYANIMTNKMVLKLTLFAWFLALGSVVILIGLNVRLSRCRRVILNPFCD
NASLFKLSCDNIIINHIYGL
GSAVVILGSSIGSITITYLKIAMVCLTNNGKLASIMFHVVPPALNAVIYGLQIKE
>g927.t1
DEESREENGRIEPIGSADSFLDHAPVRNIDSVETLSWQHSGDDSASVVSGGLVLQGSSA
>g928.t1
PAACGNGRLDHCCGHPDHSHGGDLHVLPYQLTSWLTPYSTSWAVRTSPAGKYGNFREPAVAHWSPAALHSQSICLTDSGA
PQPGRGQVGDTAMPRECMTA
LTDKNRYYT
>g929.t1
MLKTIGQALFSKIQTPKFTPGEKQGQDYEVRTYQAAKWVSTSTTGLQWDPAISTGFRRLFKYIQGANKNKVNVEMTAPVA
CLVEPGAGPACESTFTVSFY
IPEELQENPPEPSDETVFLESKKEFTAYVRTYGGFSNEQTKRENLLKLVESLQRDGVPFQDQPYYVAGYDSPFKLTNRRN
EVWILKKTEEVQQ
>g929.t2
MLKTIGQALFSKIQTPKFTPGEKQGQDYEVRTYQAAKWVSTKVNVEMTAPVACLVEPGAGPACESTFTVSFYIPEELQEN
PPEPSDETVFLESKKEFTAY
VRTYGGFSNEQTKRENLLKLVESLQRDGVPFQDQPYYVAGYDSPFKLTNRRNEVWILKKTEEVQQ
>g929.t3
LPPAATAAWTIAAAIRTTATGGICMCFLTSSPLGSPHTAPVGLSGPPLLVSTGILGNRPLPTGLQQRCTPRAYASPTQER
HNLVEGRRDMLKTIGQALFS
KIQTPKFTPGEKQGQDYEVRTYQAAKWVSTSTTGLQWDPAISTGFRRLFKYIQGANKNKVNVEMTAPVACLVEPGAGPAC
ESTFTVSFYIPEELQENPPE
PSDETVFLESKKEFTAYVRTYGGFSNEQTKRENLLKLVESLQRDGVPFQDQPYYVAGYDSPFKLTNRRNEVWILKKTEEV
QQ
>g930.t1
LYQQFSSLMACGMKLSLKLPALVRRYILGYT
>g931.t1
MPWRPLHPPPTSVTKAAVVRPHPSPVSLGLRVCPRDTQGL
>g932.t1
MSSSCKAPRVSQDVAKAQAGLFSGPQEEPSSGGSGRPFPRVNPEPTLPKPKLPIHKHSNSTTLPEARPTFTKPPLVIPPA
AKPSWVREGAGGGTLPNQGS
TPPKAPPTQQKPSSAITRMRLQGEEAKAEPESKNGHCSASRIPAGFTVPSSPFKADAREKGEQVPFRVNSVGKLKPNPLN

ARTTTDARTTTDARTTTDAR
TATDARTTTDTRTATDARTATNGRNATDATDTRNATDTRNVTDARNATDTRNVTDTRNVTDAKNSTPPPKPLANKKSSFV
RRQVDFQAAGRDKAVKPASV
SASAGQDTAPRKNTLPNVLLLGSAPSKPGRPPRVNLEPFRRDAKAPGQY
>g933.t1
MLPWSEALVKRTDANSNSHICEICPDYNVAKSAAYQNGSSQGKNHHAAPQQELPDPGCPAPGEPPSRFLGWRHVSLLQPA
WRQLSSVGSRIQLVASSDMW
DLFLVRLLMAIGIMLYYSNFTLAMEERFFIKPKVTGYLISYSSTLGALAGCLVGPVTHLYNKDMPALLLHSTVLTCGLIF
LYAAAPNVWQVVLSSTFFAI
STTIGRTCITDLELQRGGLQASGTLIGAGQSVTAVGRILAPLLSGLAQEFSPCGPPSLGVLLALSAAILLLFRIPKWNGR
GKTKMAKL
>g934.t1
GEWSRWLGLAAFVPQLALLLLVSWAYHSDLGLCCFLHTAIFVSFNKVCTSQYFLWYLCLLPLVIPQLSLSAWQGVGLLAL
WFAAQGVWLGPAYFLEFQGS
NTFLAIWLAGLLFLVINCFILVQIVLHYTPTARATQRLKDK
>g934.t2
GEWSRWLGLAAFVPQLALLLLVSWAYHSDLGLCCFLHTAIFVSFNKVCTSQYFLCGYRSFETYFINPCTGCVCLALFLQY
FLWYLCLLPLVIPQLSLSAW
QGVGLLALWFAAQGVWLGPAYFLEFQGSNTFLAIWLAGLLFLVINCFILVQIVLHYTPTARATQRLKDK
>g934.t3
GEWSRWLGLAAFVPQLALLLLVSWAYHSDLGLCCFLHTAIFVSFNKVCTSQYFLWYLCLLPLVIPQLSLSAWQGGVWLGP
AYFLEFQGSNTFLAIWLAGL
LFLVINCFILVQIVLHYTPTARATQRLKDK
>g935.t1
GSPEVGGALVDVHMIDFAHTTCRHYGEDSVLHEGQDSGFIFGLQNLITLISQLEEHSGD
>g936.t1
MEGRGLAVTLWIRIVALLCSFNTSSAQLSYSVSEEVDKGTVVGNLAKDLHINVRELEYRETRITSGNAKTYFDVDLKTGI
ITVKGDLDFEENAAYDIRVQ
AKDRGAPSRSVHGKVLVEVIDVNDNIPEIIITSLMSPVKEDSQ
>g937.t1
MSFLRRVAGLSLRDRVRSSDIRRELGVEPLLLHVERSQLRWFGHLIRMPPGRLPLEVFRARPTGKRPRGRPRTRWRDYIS
HLAWERLGVPEEELESVAGE
KDVWSSLLSLLPPRPGPG
>g938.t1
RRSDSGSNSPSSSSSSSSSCSSSSGSSCSPSPERKRFCPRRSDSSPCSSSSSGSCSPPRRYHFTYSRSRSKSWSQSGSRS
RSRSPLNHRKHWRGEVYSPN
GERGPPISSRWSGALTPVVKRASELMLTAASLQDERRVVYVGRIRRTMTHDELRERFSLIGEVDSVSLHFRDRRDSYGFV
TFHHMEDAFAAIENGSKLRR
PDELPFDICFGGRRQFCKSDYADLGEGPQDAESRTEIPDPSPS
>g938.t2
RRSDSGSNSPSSSSSSSSSCSSSSGSSCSPSPERKRFCPRRSDSSPCSSSSSGSCSPPRRYHFTYSRSRSKSWSQSGSRS
RSRSPLNHRKHWRGEVYSPN
GERGPPISSRWSGALTPVVKRASELMLTAASLQDERRVVYVGRIRRTMTHDELRERFSLIGEVDSVSLHFRDRRDSYGFV
TFHHMEDAFAAIENGSKLRR
PDELPFDICFGGRRQFCKSDYADLGEGPQDAESRTEIPDPSPSGEGRVRTNI
>g939.t1
SGRTSKLQSGKALQAAMASMTVKRPSADAADEAALQYLQWNIEHSILKHVGYYWTSWLRVKQPGHYYVYAKVSFSRGGGP
DQSLVSVVRLKHNETAKTQD
VMKAYCSMGMVTTSQCTASQGQVMHLQAGNLLGVWVEHHKWVDYERGATTFGIYKL
>g940.t1
LCVSQVDIEMSFVEQPGVMALVEGLLQYSWPPQLGGLSCPFPCITYAEAMRDYGSDKPDTRFHMKLVDISEVFVNTEIEF
LKSALGRSEGSVQAICVPSG
VNLFKSKDLETLKETARTLFGQELSVVAVREGGTLKSPLSKHLSPSDTEALLG
>g940.t2
MSFVEQPGVMALVEGLLQYSWPPQLGGLSCPFPCITYAEAMRDYGSDKPDTRFHMKLVDISEVFVNTEIEFLKSALGRSE
GSVQAICVPSGVNLFKSKDL

ETLKETARTLFGQELSVVAVREGGTLKSPLSKHLSPSDTEALLG
>g940.t3
LCVSQVDIEMSFVEQPGVMALVEGLLQYSWPPQLGGLSCPFPCITYAEAMRDYGSDKPDTRFHMKLVDISEVFVNTEIEF
LKSALGRSEGSVQAICVPSG
VNLFKSKDLETLKETARTLFGQVIALIIS
>g941.t1
MEALTGKPKVELTYDKMYTVTSRLAFTVSKEDDGVPVACIVDHPAVKDFQAQKYLEHSSVHMKQRGFLVGMRPKAQPKAS
KYGSGNYF
>g941.t2
MEALTGKPKVELTYDKMYTVTSRLAFTVSKEDDGVPVACIVDHPAVKDFQAQKYLEAQPKASKYGSGNYF
>g942.t1
MTCTAMGSKPAATIKWMKGDKELPASPHQQCKQHDHDNNSGGGGGGGSKTARPIEWSAVKLKGGVLGGGQRDWFDTKLLT
SRNTATLKAAAGVRAASPPM
FTPAPLQISVYCKQQNVYALGFQLFTSK
>g942.t2
CVCARLPVVPPGNPIIEARDEVLREGNETEMTCTAMGSKPAATIKWMKGDKELPASPHQQCKQHDHDNNSGGGGGGGSKT
ARPIEWSAVKLKGGVLGGGQ
RDWFDTKLLTSRNTATLKAAAGVRAASPPMFTPAPLQISVYCKQQNVYALGFQLFTSK
>g943.t1
SANEQDLRNNRVLDELVTGFRPA
>g943.t2
SANEQDLRNNRVLDELVTGFRPARVGSGSAVLK
>g944.t1
MGTVMQSLGLFRYKCTGLTQITFYEERNFQGRSYECMSDCSDMSSYMSRCQSCRVDSGCFMLYERNNYMGQQFFMRRGEY
NDMQRMQSMGMMFDNIRSCR
MIPFHRGQFRMKIFERENFGGQSYELMDDCDNIMDRYRMSDCQSCNVMDGHWLMYEQPQYRGRMMYMRPGEYRSFREMMS
GQRFMSMRRITDMC
>g944.t2
MGKITFYEERNFQGRSYECMSDCSDMSSYMSRCQSCRVDSGCFMLYERNNYMGQQFFMRRGEYNDMQRMQSMGMMFDNIR
SCRMIPFHRGQFRMKIFERE
NFGGQSYELMDDCDNIMDRYRMSDCQSCNVMDGHWLMYEQPQYRGRMMYMRPGEYRSFREMMSGQRFMSMRRITDMC
>g945.t1
MSDCSDMSSYMSRCQSCRVDSGCFMLYERNNYMGQQFFMRRGEYNDMQRMQSMGMMFDNIRSCRMIPFHRGQFRMKIFER
ENFGGQSYELMDDCDNIMDR
YRMSDCQSCNVMDGHWLMYEQPQYRGRMMYMRPGEYRSFREMMSGQRFMSMRRITDMC
>g946.t1
QWQRGVPEYHNQNGIVRPL
>g947.t1
MQTQWGSCPNADPVVIMSTCRPGLDTMLDLVRFAQLNDSTTPSPLENISTFPTWQPDSEANITKPHKCLQVLYEDIGNSR
VRFWDLLLLVPNVAFFIFLM
WKLPSARAKIRLTSSPIFITFYLLVFVVAAVGITRAVVSMTVSASSAATIIDKVLWEITRFFLLAIELSVIILGLAFGHL
ESKSSIKRVLAITAVLALAY
SITQGTLEIRYPDHHLSAEDFNIYGHGGRHFWLASSCFFFLVSRHMLTCNGKGKTLVQGLGSALLCAEIIEGLCCVDVTT
FLYFSIFAPLIYITFLRRFF
GYVTAALHPVVSLYLVHYRSTAPPPLT
>g947.t2
MSTCSPGLDTMLDLVRFAQLNDSTTPSPLENISTFPTWQPDSEANITKPHKCLQVLYEDIGNSRVRFWDLLLLVPNVAFF
IFLMWKLPSARAKIRLTSSP
IFITFYLLVFVVAAVGITRAVVSMTVSASSAATIIDKVLWEITRFFLLAIELSVIILGLAFGHLESKSSIKRVLAITAVL
ALAYSITQGTLEIRYPDHHL
SAEDFNIYGHGGRHFWLASSCFFFLVSRHMLTCNGKGKTLVQGLGSALLCAEIIEGLCCVDVTTFLYFSIFAPLIYITFL
RRFFGYVTAALHPVVSLYLV
HYRSTAPPPLT
>g947.t3
MQTQWGSCPNADPVVIMSTCRPGLDTMLDLVRFAQLNDSTTPSPLENISTFPTWQPDSEANITKPHKCLQVLYEDIGNSR
VRFWDLLLLVPNVAFFIFLM

WKLPSARAKIRLTSSPIFITFYLLVFVVAAVGITRAVVSMTVSASSAATIIDKVLWEITRFFLLAIELSVIILGLAFGHL
ESKSSIKRVLAITAVLALAY
SITQGTLEIRYPDHHLSAEDFNIYGHGGRHFWLASSCFFFLVSRHMLTCNGKGKTLVQGLGSALLCAEIIEGLCCVDVTT
FLYFSIFAPLIYITFLRRFF
>g948.t1
MSFTGESGQTVTEHNEEELICPICMMLFTEPMSLPCGHTYCLPCLLTMSAGLDQNNCPECQQEYGGTEALVKNMKMCSIV
EAYKATLERPRPTQPEQQSI
TTKKTEMDEPKFRLASQVTELTVRLDVAECILRKEQEREEEASATNEELRADTAQLLHAFRALSVNYSQAVTQLIEGGLS
PGEEALRARVSRATWLTESL
RHTVLSAESLLTEQDEARFTEDLNLLQPRIAQLLGQPPAEEGEPGDGSKMDASLACAKLEEMNADFKAGVGEIHRSLRNV
FNPSEVTFDPDTAHPNLVLS
ADMKTVTYSATKRACPASPLRFTSFLQVLSIQSFYGGEHCWRVELEGAPWAIGVCYSRQLARSGLPSALESSRCSWCLMW
FENRLRAFEQGHDVPLQLTT
MSRTIEVRLSFKTHRLSFYNVGFSGGKTHVYTFKAHLTEPVHLAYRMMSGNPTARITISEG
>g949.t1
MAPEDKILQRFSMERQRLHDKKDLFNLNEDEELTHYGQSLAEMEKFNDLMDSDSEPEEKGLLSAELTASHFGGGGGLLRR
KTGEDKGEGPRAKSRQELIE
ELIHKSKQEKRERQVQKEETFELTEKLDKDWKSIQALLANKAPKAQRDDGAQQEKPKLDEYDVMVRELGFEMKAQPSEKL
KTPEEVAREERERLQKLEVC
SYARRPGFSVAADRLRRMMGDVVERAPKAPVHLSADDLNDGFILEKDDKKTLTYKDGKWLSGEGEDGDNEEDDEEDGEEG
ESGEEEEEEGEQEDGEGADA
DGDDDDDDDDDEEEAEEVDSHSDLDSERGSEGEEEEVEEEEEEEGGAAPGVSLSEEEVKAQQEAAKAELPYTFAAPENYA
EFQALLKGHSPDNQGVILAR
TQKCNHPSLAVGNKLKLQVCAQCVGAQIFKLFGFLLEYMGDLATSSPPQLAIIDKLMPQLYDLCQMFPDAASKAMRGMLA
DGCHVMEEEMEGTGRASFPA
LDMVGAAILGLSPWKRARGRCRRADRPGSVKSHRVEPFH
>g949.t2
MGKPQKKRSVADKVRKSKTSAAIKNNPFEVKINRKKFEVLGRKTKHDVGLPGVSRSKAINKRKETLLKEHKNKHKVNKFV
DRRFGEYDTNMAPEDKILQR
FSMERQRLHDKKDLFNLNEDEELTHYGQSLAEMEKFNDLMDSDSEPEEKGLLSAELTASHFGGGGGLLRRKTGEDKGEGP
RAKSRQELIEELIHKSKQEK
RERQVQKEETFELTEKLDKDWKSIQALLANKAPKAQRDDGAQQEKPKLDEYDVMVRELGFEMKAQPSEKLKTPEEVAREE
RERLQKLEVCSYARRPGFSV
AADRLRRMMGDVVERAPKAPVHLSADDLNDGFILEKDDKKTLTYKDGKWLSGEGEDGDNEEDDEEDGEEGESGEEEEEEG
EQEDGEGADADGDDDDDDDD
DEEEAEEVDSHSDLDSERGSEGEEEEVEEEEEEEGGAAPGVSLSEEEVKAQQEAAKAELPYTFAAPENYAEFQALLKGHS
PDNQGVILARTQKCNHPSLA
VGNKLKLQVCAQCVGAQIFKLFGFLLEYMGDLATSSPPQLAIIDKLMPQLYDLCQMFPDAASKAMRGMLADGCHVMEEEM
EGTGRASFPALDMVGAAILG
LSPWKRARGRCRRADRPGSVKSHRVEPFH
>g949.t3
MAPEDKILQRFSMERQRLHDKKDLFNLNEDEELTHYGQSLAEMEKFNDLMDSDSEPEEKGLLSAELTASHFGGGGGLLRR
KTGEDKGEGPRAKSRQELIE
ELIHKSKQEKRERQVQKEETFELTEKLDKDWKSIQALLANKAPKAQRDDGAQQEKPKLDEYDVMVRELGFEMKAQPSEKL
KTPEEVAREERERLQKLEVC
SYARRPGFSVAADRLRRMMGDVVERAPKAPVHLSADDLNDGFILEKDDKKTLTYKDGKWLSGEGEDGDNEEDDEEDGEEG
ESGEEEEEEGEQEDGEGADA
DGDDDDDDDDDEEEAEEVDSHSDLDSERGSEGEEEEVEEEEEEEGGAAPGVSLSEEEVKAQQEAAKAELPYTFAAPENYA
EFQALLKGHSPDNQGVILAR
TQKCNHPSLAVGNKLKLQVCAQCVGAQIFKLFGFLLEYMGDLATSSPPQLAIIDKLMPYVGAGFCHSIKGLLFQLYDLCQ
MFPDAASKAMRGMLADGCHV
MEEEMEGTGRASFPALDMVGAAILGLSPWKRARGRCRRADRPGSVKSHRVEPFH
>g950.t1
MLARRWCSITLFQCEWETHGPETLGLVFTQRSGREYGSDDVSVGFFRRNLWVGYQYPGYRGYQYLFECGDYRHYNEFSAF
QPQIQSMRRVRDMQFHQRGC
FTLTSASK

>g950.t2
MHQMMRQTCRDYADKELAPIAATLDKNHTYPAKQVNRSARPAAEWTLVCTGLCSWPLLLASAPGLCSWPLLLATTASLGS
VWIQELGAMGAMAVEVPEEL
GGTGMDYLAYSLAMEEISRGCASTGVVVSVNN
>g950.t3
MMRQTCRDYADKELAPIAATLDKNHTYPAKQVNRSARPAAEWTLVCTGLCSWPLLLASAPGLCSWPLLLATTASLGSVWI
QELGAMGAMAVEVPEELGGT
GMDYLAYSLAMEEISRGCASTGVVVSVNN
>g950.t4
MLARRWCSITLFQCEWETHGPETLGLVFTQRSGREYGSDDVSVGFFRRNLWVGYQYPGYRGYQYLFECGDYRHYNEFSAF
QPQIQSMRRVRDMQFHQRGC
FTLTSASKPLSQLAELPEMHQMMRQTCRDYADKELAPIAATLDKNHTYPAKQVNRSARPAAEWTLVCTGLCSWPLLLASA
PGLCSWPLLLATTASLGSVW
IQELGAMGAMAVEVPEELGGTGMDYLAYSLAMEEISRGCASTGVVVSVNN
>g951.t1
MANQSFAIDGPGSLLAVLASQSGLAKGGSSTSSGGSSGGGISASDVSAYFKLIFLGLIICVSLAGNLLVSLLVLRDRALH
KAPYFFLLDLCLADAVRSAA
CFPFVLVSVHHSSAWTYSALSCKVVAFMAVLFCFHAAFMLFCVAVTRYLAIAHHRFYAKRMTIWTCAAIICMVWTLAIAM
AFPPVFDVGTYKFIQDEDQC
IFEHRYLKTNDTLGFMLMLAVVVLATHGFYAKLLLFEYRHRKMKPVQLVPAISQNWTFHGPGATGQAAANWIAGFGRGPM
PPTLLGIRQNLHNQHRRLLG
MEEVRSERRLGRMFYTITLLFLVLWAPYIVACFWRVFVTSWAIPHRYLSITVWMSFAQAGVNPIFCILLNEDLRKVLRAH
LPTYWRTKQNLPQDEAYCIM
>g952.t1
MMYVLLVCLTLWLLVEMVYCYRKISRADDQTQDP
>g953.t1
MSKERSKRSERAQDGDDEDDLDALLATLTADEVKELESEFMVIDPDPGVPVGLRQRNQTEKTPSISYDRKAMLDFCQARV
IEALSC
>g954.t1
MREYKLVVLGSGGVGKSALTVQFVQGIFVEKYDPTIEDSYRKQVEVDGQQCMLEILDTAGTEQFTAMRDLYMKNGQGFAL
VYSITAQSTFNDLQDLREQI
LRVKDTEDVSVAVSV
>g955.t1
MNLHQVLTGAVNPGDNCFSVGSVSNVPFTGPEVTGSPTAGPLRGAAGCASAQLSSSSLPECPPPDFPLCPAVRQAYASGC
DVVILGSDFERLQIIPGAKH
GNIQVGCVDCSLDGGRVRELLAATTGQLLRTDIDLQHACCYFESTF
>g955.t2
MNLHQVLTGAVNPGDNCFSVGSVSNVPFTGPEVTGSPTAGPLRGAAGCASAQLSSSSLPECPPPDFPLCPAVRQAYASGC
DVVILGSDFERLQIIPGAKH
GNIQVGCVDCSLDGGR
>g956.t1
MLVVLLGFPGLFLASTVITIAFPTTPSVKMIKNTSSVMKLSVPIPNKSSWSSSSAVLMFRCSSEHTTNPDPFIFQSVWLK
SVKTRLSKRQNGSEFITAST
FLPSVEGLGSECNVSLYHRRT
>g957.t1
LRPVERFRDLLPEEVADLFTTTQRVANLVERHFNGTSLTVAIQDGPEAGQTVKGPGARTKVVMHVMELMELMELK
>g957.t2
LVCPLRPVERFRDLLPEEVADLFTTTQRVANLVERHFNGTSLTVAIQDGPEAGQTVKGPGARTKVVMHVMELMELMELK
>g957.t3
LRPVERFRDLLPEEVADLFTTTQRVANLVERHFNGTSLTVAIQDGPEAGQTVKGPGARTKVVMHVMELMELMELNFKVFL
KHVHVHVLPRRAGDFEQNDS
VYDQEKTAQHNTCASQ
>g958.t1
SFLVGSVEKSRLITCFKSRHTSLTFDSVQLYCAEADQFSSRQYKDFHVIKRP
>g958.t2
LVGSVEKSRLITCFKSRHTSLTFDSVQLYCAEADQFSSRQYKDFHVIKRP

>g959.t1
MKRLKNYATSPRIGKPGPPTGARPGRGVRRRASGGRALTHGARPGTARNSNMEEPLPCGPTTCRDRYWGQVLCKVGGRQR
RGPGRADPRHRRLALGTWNI
TSLAGKEPELVREVERYQLDIVGTAQHWLWNQTPGKGLDSFSGVAQGEL
>g960.t1
LCAEEKRRRRRGEEGGGVGVLRRAAQQQQQQQQPTLGNWIPRTHVADGGDFCGFRGFCSIASRNAALSEDAQPLRILSSP
RPEDVRPRGGRLHRAAQHRG
SQ
>g960.t2
MQMSPESCAGLSRRDWDGVSAPLCAEEKRRRRRGEEGGGVGVLRRAAQQQQQQQQPTLGNWIPRTHVADGGDFCGFRGFC
SIASRNAALSEDAQPLRILS
SPRPEDVRPRGGRLHRAAQHRGSQ
>g961.t1
LVFRPRGVRRDAKSILPLDGERCVDPQAVTTTSINTSFLPLPSVSCGRHTGSHHKRWLRLKEEPTGETGGIVKPIFKS
>g962.t1
MSQGELPTTKPPKRVNEETISAENEQIAKKVKLEEENTDHEKKYPKKKVVLLLAYSGKGYYGMQGVSAAGQVVSLKLRLI
EDIIDNINKHLPAQIRVLGF
KRVTQGFNSKNNCDARTYSYMLPTVAFAPKDYDTQKSAAFRLDAETLARVNGLFSLYKGTHNFHNFTSQKSPQDPSARRY
ITEMSCEAPFLCGEAEFAVI
TVRGQSFMLHQIRKMIGLVIAVIKGYAKEEVLQRSWGHEKVDVPRAPGLGLVLERVHFDRYNQRFGGDGLHQRLEWEAEE
SSIQGFKKAHIYPSIVDTES
EERSMISWMASLPIHDYEASAADRREKDQKQV
>g962.t2
MSQGELPTTKPPKRVNEETISAENEQIAKKVKLEEENTDHEKKYPKKKVVLLLAYSGKGYYGMQGVSAAGQVVSLKLRLI
EDIIDNINKHLPAQIRVLGF
KRVTQGFNSKNNCDARTYSYMLPTVAFAPKDYDTQKSAAFRLDAETLARVNGLFSLYKGTHNFHNFTSQKSPQDPSARRY
ITEMSCEAPFLCGEAEFAVI
TVRGQSFMLHQIRKMIGLVIAVIKGYAKEEVLQRSWGHEKVDVPRAPGLGLVLERVHFDRYNQRFGGDGLHQRLEWEAEE
SSIQGFKKAHIYPSIVDTES
EERSMISWMASLPIHDYEASAADRREKDQKQDVDVGNESD
>g963.t1
MYKKILVILHDSILPHMSNPTLMIDFLTAAYEVGGAISLLALNGLFVLIHHHNLDYPDFYQKLYNLLEPSVFHVKYRARF
FHLANLFLSSTHLPVYLVAA
FAKRLARLALTAPPESLLMVLPFIYNLIRRHPACRVLVHRPTSSDESCEDPYMMDEADPILSHALESSLWEIKTLQTHYH
PDVAKVAMKMNKPLSVQEDD
IDELLEITNFELMERDLKATKSVPLEFEPARKLLQGGGGILGQHFCLE
>g963.t2
LPNNMYKKILVILHDSILPHMSNPTLMIDFLTAAYEVGGAISLLALNGLFVLIHHHNLDYPDFYQKLYNLLEPSVFHVKY
RARFFHLANLFLSSTHLPVY
LVAAFAKRLARLALTAPPESLLMVLPFIYNLIRRHPACRVLVHRPTSSDESCEDPYMMDEADPILSHALESSLWEIKTLQ
THYHPDVAKVAMKMNKPLSV
QEDDIDELLEITNFELMERDLKATKSVPLEFEPARKLLQGGGGILGQHFCLE
>g963.t3
LTCSQANILALRVGQLPNNMYKKILVILHDSILPHMSNPTLMIDFLTAAYEVGGAISLLALNGLFVLIHHHNLDYPDFYQ
KLYNLLEPSVFHVKYRARFF
HLANLFLSSTHLPVYLVAAFAKRLARLALTAPPESLLMVLPFIYNLIRRHPACRVLVHRPTSSDESCEDPYMMDEADPIL
SHALESSLWEIKTLQTHYHP
DVAKVAMKMNKPLSVQEDDIDELLEITNFELMERDLKATKSVPLEFEPARKLLQGGGGILGQHFCLE
>g964.t1
IWDIRSLECVHVLQTSGGSVYSIAVTNHHIVCGTYENLIHVWDIDTKEQVRTLTGHVGTVYALAVISTPDQTKVFSASYD
RSLR
>g964.t2
MAAVTERKIWDIRSLECVHVLQTSGGSVYSIAVTNHHIVCGTYENLIHVWDIDTKEQVRTLTGHVGTVYALAVISTPDQT
KVFSASYDRSLR
>g964.t3
MIWDIRSLECVHVLQTSGGSVYSIAVTNHHIVCGTYENLIHVWDIDTKEQVRTLTGHVGTVYALAVISTPDQTKVFSASY

DRSLR
>g965.t1
LAGCFGFTARKENGENRSVCGVPPRPPSAARKGKSRERVGFFPANFVQRVRPGERVWKVTAGFHGNRDKGQMTVKEGQ
>g966.t1
LGFPSAVLILEKPHDTVALEGETVTLACTISDPKATVTWTRDSVAIKAGLKYDLRKSGSSEQLRIHNLVPEDSGTYSCDT
GDACCDVSLTVEGAPVFFKK
ELKSQQAEEGEDLTLSVEVSKRGACVEWRKGGMVLQPSAKYDMRQEGCVRELHIRGLETEDGGFYTCDAGDQLTTASLAV
QVKEAYIVSGLMSTDVFVGE
WATFSCQLSGRAAGKVQWWLDGTQLEHSPFSEIGLCPGHIHTLTFKNLAPDDSGTVTFSAGSLVSAAKLLVKASTREVLL
HPSTQAGHILILTHPSTSAM
PSLTLACKH
>g966.t2
DMGHSPRTGPCGTRKSENSCYMSVKASAVVSTNEDSWAQGSRGAAEDVMVASTTTVKTYAETQLHHRDEQDAGVRAVKAS
AVVSTNQDSRTQGSRGLAEA
VKASALASTNQDFRTQGSSGAVEVHFTRELESVEAVEEGSVTLRCELSQPGAVAEWRKGTQVLKDGEKYHTTHDRCVHEL
HILDLKHVDTGSYSCCSGDA
ESFASLRVNALPVVFTKELESQEVDEGHHVALHCVLSKPGVPLEWRKGELCLCACAKYDISQTGHSATLVIRDVDPEDSG
NYTCDSGDRQSTSELVVKAR
PILFKSQLVDLETEAGQTAVLRCETTKPGSGVVWRHGDAVLETNSKHQLRREGTVAELLIYKLRAEDAGEYTCDTGSHRT
RAVLTVLAVEVTIAKFLESC
VVREGDDVRFECVVSHREAPPAQWRLQDVPLQHNEMNVIRCEDRTHSLTLRGVTKADSGTVTFSVGNHTSTASLTVKGKG
TTAGRREAGGRWEELCPPLC
PTAAPVVFQKELQSVEAVEGDRAVLSCETSGAGGRVTWRKGSLLLTDGHKYSMERQGSACTLVVSRLRAEDSGEYTCDTG
DRQTTATLTVKECVRVTREL
CDVTVTTGRDAVFEVELSHAGVTHGEWWLADNQLQNNDLNEMTSQGRVHRLALKMVTTDESGDVAFVVGEARSVACLLVE
EKAKVLILEKPHDTVALEGE
TVTLACTISDPKATVTWTRDSVAIKAGLKYDLRKSGSSEQLRIHNLVPEDSGTYSCDTGDACCDVSLTVEGAPVFFKKEL
KSQQAEEGEDLTLSVEVSKR
GACVEWRKGGMVLQPSAKYDMRQEGCVRELHIRGLETEDGGFYTCDAGDQLTTASLAVQVKEAYIVSGLMSTDVFVGEWA
TFSCQLSGRAAGKVQWWLDG
TQLEHSPFSEIGLCPGHIHTLTFKNLAPDDSGTVTFSAGSLVSAAKLLVKASTREVLLHPSTQAGHILILTHPSTSAMPS
LTLACKH
>g966.t3
MRSVPGHGPQSKDGALWDPTTTVKTYAETQLHHRDEQDAGVRAVKASAVVSTNQDSRTQGSRGLAEAVKASALASTNQDF
RTQGSSGAVEVHFTRELESV
EAVEEGSVTLRCELSQPGAVAEWRKGTQVLKDGEKYHTTHDRCVHELHILDLKHVDTGSYSCCSGDAESFASLRVNALPV
VFTKELESQEVDEGHHVALH
CVLSKPGVPLEWRKGELCLCACAKYDISQTGHSATLVIRDVDPEDSGNYTCDSGDRQSTSELVVKARPILFKSQLVDLET
EAGQTAVLRCETTKPGSGVV
WRHGDAVLETNSKHQLRREGTVAELLIYKLRAEDAGEYTCDTGSHRTRAVLTVLAVEVTIAKFLESCVVREGDDVRFECV
VSHREAPPAQWRLQDVPLQH
NEMNVIRCEDRTHSLTLRGVTKADSGTVTFSVGNHTSTASLTVKGKGTTAGRREAGGRWEELCPPLCPTAAPVVFQKELQ
SVEAVEGDRAVLSCETSGAG
GRVTWRKGSLLLTDGHKYSMERQGSACTLVVSRLRAEDSGEYTCDTGDRQTTATLTVKECVRVTRELCDVTVTTGRDAVF
EVELSHAGVTHGEWWLADNQ
LQNNDLNEMTSQGRVHRLALKMVTTDESGDVAFVVGEARSVACLLVEEKAKVLILEKPHDTVALEGETVTLACTISDPKA
TVTWTRDSVAIKAGLKYDLR
KSGSSEQLRIHNLVPEDSGTYSCDTGDACCDVSLTVEGAPVFFKKELKSQQAEEGEDLTLSVEVSKRGACVEWRKGGMVL
QPSAKYDMRQEGCVRELHIR
GLETEDGGFYTCDAGDQLTTASLAVQVKEAYIVSGLMSTDVFVGEWATFSCQLSGRAAGKVQWWLDGTQLEHSPFSEIGL
CPGHIHTLTFKNLAPDDSGT
VTFSAGSLVSAAKLLVKASTREVLLHPSTQAGHILILTHPSTSAMPSLTLACKH
>g966.t4
DMGHSPRTGPCGTRKSENSCYMSVKASAVVSTNEDSWAQGSRGAAEDVMVASTTTVKTYAETQLHHRDEQDAGVRAVKAS
AVVSTNQDSRTQGSRGLAEA
VKASALASTNQDFRTQGSSGAVEVHFTRELESVEAVEEGSVTLRCELSQPGAVAEWRKGTQVLKDGEKYHTTHDRCVHEL

HILDLKHVDTGSYSCCSGDA
ESFASLRVNVFTKELESQEVDEGHHVALHCVLSKPGVPLEWRKGELCLCACAKYDISQTGHSATLVIRDVDPEDSGNYTC
DSGDRQSTSELVVKARPILF
KSQLVDLETEAGQTAVLRCETTKPGSGVVWRHGDAVLETNSKHQLRREGTVAELLIYKLRAEDAGEYTCDTGSHRTRAVL
TVLAVEVTIAKFLESCVVRE
GDDVRFECVVSHREAPPAQWRLQDVPLQHNEMNVIRCEDRTHSLTLRGVTKADSGTVTFSVGNHTSTASLTVKGKGTTAG
RREAGGRWEELCPPLCPTAA
PVVFQKELQSVEAVEGDRAVLSCETSGAGGRVTWRKGSLLLTDGHKYSMERQGSACTLVVSRLRAEDSGEYTCDTGDRQT
TATLTVKECVRVTRELCDVT
VTTGRDAVFEVELSHAGVTHGEWWLADNQLQNNDLNEMTSQGRVHRLALKMVTTDESGDVAFVVGEARSVACLLVEEKAK
GKEMGWLLSRFEPALVSHRR
PTRLARCRPPPPS
>g967.t1
MTRVSHSEQLGLTSARISGWKAATADVVAILDAHIEVHDLWAEPLLTQIKGDRTVIVSPVFDRVNYYDLEVVTYLPASHA
FDWALWCMYERFRPEWYKLN
DPSMPGKSPSIMGILVADRKYLGEIGVLDGGMKVYGGENVELGIRVWTCGGSIEVVPCSKIAHIERNHKPYMLDLSITMK
RNALRVAEVWMDDYKHNINL
AWNLPFKDHGIDIGDVSERKELRKRLNCKPFKWYLDNVYPLLDAWDNILGYGGVSWLEL
>g967.t2
MTRVSHSEQLGLTSARISGWKAATADVVAILDAHIEVHDLWAEPLLTQIKGDRTVIVSPVFDRVNYYDLEVVTYLPASHA
FDWALWCMYERFRPEWYKLN
DPSMPGKSPSIMGILVADRKYLGEIGVLDGGMKVYGGENVELGIRVWTCGGSIEVVPCSKIAHIERNHKPYMLDLSITMK
RNALRVAEVWMDDYKHNINL
AWNLPFKDHGIDIGDVSERKELRKRLNCKPFKWYLDNVYPLLDAWDNILGYGG
>g967.t3
MTRVSHSEQLGLTSARISGWKAATADVVAILDAHIEVHDLWAEPLLTQIKGDRTVIVSPVFDRVNYYDLEVVTYLPASHA
FDWALWCMYERFRPEWYKLN
DPSMPGKSPSIMGILVADRKYLGEIGVLDGGMKVYGGENVELGIRVWTCGGSIEVVPCSKIAHIERNHKPYMLDLSITMK
RNALRVAEVWMDDYKHNINL
AWNLPFKDHGIDIGDVSERKELRKRLNCKPFKWYLDNVYPLLDAWDNILGYGGYTYFRETGELYIGGIKSHKYNPNRCLT
DDGNKDNVPGLNDCHESLQK
RIGIYWDFTQGKEFKNKQTSRCLEIIKGKLVVQGCTGQRWEIQNIIKTF
>g968.t1
MLRRILQMTPGKGGSQNTESTEQPGANLNHEQTSEGFICPQCMKSHNSAEELFKHYDLFHDTGDLPSNVAPTREDLTMLR
QEVQDLHASLK
>g969.t1
MFGSPIVLTLSGLGLQNLKNRISLQVLLVSSSRHPDQWIVPGGGVEPEEEPGGAAVREVYEEHNQDRKHRTYVYVLIVTE
TVEDWEDSVTIGRKRKWFKI
EDALQVLQNHKPVHAEYLIRLTTSRCGAPCSPSCADTNGNSPVSALTDNNGPHYGRPTQGSSLVNR
>g969.t2
MFGSPIVLTLSGLGLQNLKNRISLQVLLVSSSRHPDQWIVPGGGVEPEEEPGGAAVREVYEEAGVKGKLGRLLGVFEHNQ
DRKHRTYVYVLIVTETVEDW
EDSVTIGRKRKWFKIEDALQVLQNHKPVHAEYLIRLTTSRCGAPCSPSCADTNGNSPVSALTDNNGPHYGRPTQGSSLVN
R
>g970.t1
NDTVYWTDMGLNRISRAKRDQTWREDIITTGISRVEGIAVDWIAGNLYWTDHGFNLIEISRLSGAYRSVVISEGLDQPRA
IAVHPHKGYLFWSEWGQNPC
IGRSRLDGSDQVTLVNSGITWPNGISIDYEPRRSARQLISRRSHPAPKGRGAANVHLGNDFQENRLYWCDARTDKIERIN
LETGESREIVLSAANVDLFS
VAVFGPYIYWSDRAHSNGSIRRGFKNDAADAVTMRSGLGVNLKDVTVFNRGREKGTNPCARSNGGCQQLCFHLGSGRRTC
SCAHGHLAADGFACERYDGY
LLYSERTILKSIHLSDESDLNSPVQPFEKPAFFKNVIALAFDYSQPAAGTNRIFYSDVHFGNIQMISDDWTGRVIIAENV
GSVEGLAYHRAWDTLYWTSS
TSSTISRKTVDANRAGAFARQAVVTLSEEDHPHVLSLDECQNLMFWTNWNEQRPSIMRSTLAGNNLRVIASTDILTPNGL
TIDHKAEKLYFSDGSLGRIE
RCDYDGSNRYACNEPSNHFLRPE

>g970.t2
NDTVYWTDMGLNRISRAKRDQTWREDIITTGISRVEGIAVDWIAGNLYWTDHGFNLIEISRLSGAYRSVVISEGLDQPRA
IAVHPHKGYLFWSEWGQNPC
IGRSRLDGSDQVTLVNSGITWPNGISIDYEPRRSARQLISRRSHPAPKGRGAANVHLGNDFQENRLYWCDARTDKIERIN
LETGESREIVLSAANVDLFS
VAVFGPYIYWSDRAHSNGSIRRGFKNDAADAVTMRSGLGVNLKDVTVFNRGREKGTNPCARSNGGCQQLCFHLGSGRRTC
SCAHGHLAADGFACERYDGY
LLYSERTILKSIHLSDESDLNSPVQPFEKPAFFKNVIALAFDYSQPAAGTNRIFYSDVHFGNIQMISDDWTGRVIIAENV
GSVEGLAYHRAWDTLYWTSS
TSSTISRKTVDANRAGAFARQAVVTLSEEDHPHVLSLDECQNLMFWTNWNEQRPSIMRSTLAGNNLRVIASTDILTPNGL
TIDHKAEKLYFSDGSLGRIE
RCDYDGSNRY
>g970.t3
NDTVYWTDMGLNRISRAKRDQTWREDIITTGISRVEGIAVDWIAGNLYWTDHGFNLIEISRLSGAYRSVVISEGLDQPRA
IAVHPHKGYLFWSEWGQNPC
IGRSRLDGSDQVTLVNSGITWPNGISIDYEPRRSARQLISRRSHPAPKGRGAANVHLGNDFQENRLYWCDARTDKIERIN
LETGESREIVLSAANVDLFS
VAVFGPYIYWSDRAHSNGSIRRGFKNDAADAVTMRSGLGVNLKDVTVFNRGREKGTNPCARSNGGCQQLCFHLGSGRRTC
SCAHGHLAADGFACERYDGY
LLYSERTILKSIHLSDESDLNSPVQPFEKPAFFKNVIALAFDYSQPAAGTNRIFYSDVHFGNIQMISDDWTGRVIIAENV
GSVEGLAYHRAWDTLYWTSS
TSSTISRKTVDANRAGAFARQAVVTLSEEDHPHVLSLDECQNLMFWTNWNEQRPSIMRSTLAGNNLRVIASTDILTPNGL
TIDHKAEKLYFSDGSLGRIE
RCDYDGSNRYSVPQNYC
>g971.t1
MWSAMLMLSRFCSPVVPVLLTCCPGSPHLLSRFCSPVVPVLLTCCPGSPHLLSRFCSPVELQLCTLVWTHSLPPGLDLLV
WTHSPPPGLDLLVWFSPVQR
QMTSLTGKD
>g972.t1
MPFHYCKPEPCWQVYVEFDDQEWEKREWVKVYEDFQLFLLQHQLVWAKRKEGGGERERERGGGGGGGLMQGTKAKHIQWP
ALVSSF
>g973.t1
AIEACLGHMLKRRAAGFLRSDKIAALFTKIGKVNDTTSDVCCKVQEQLQQQSDGSRGSLTSTAGLRPGGCDLLRQACCRA
VQSE
>g974.t1
VQTGNACWELFCLEHGIGPDGVSLDVPLQPNSRQDPFNTFFNTGSSGRHVPRALYVDLEPTVIDEVRVGTYKDLFHPEQL
ISGKEDAANNYARGHYTVGR
EMIDTVLERIRKMRLLFQTDQCTGLQGFLVFHSFGGGTGSGFTSLLMERLSVDYGKKSKLEFAIYPAPQVSTAVVEPYNS
ILTTHTTLEHTDCAFMVDNE
AIYDICHRNMDIEHPSYINLNRLIGQIVSSITASLRFDGALNVDLTEFQTNLVPFPRIHFPLVTYSPIISAEKAYHEQLT
VLEITSSCFEPANQMVKCDP
RHGMYMACCMLYRGDVVPKDVNAAIANIKTRRSIQFVDWCPTGFKVGINYQPPTAVPGGDLAKIQRSVCMLSNTTAIAEA
WARLDHKFDLMYSKRAFVHW
YVGEGMEEGEFSEAREDMACLEKDYEELGKMSIDLDDDELSEE
>g974.t2
VQTGNACWELFCLEHGIGPDGVSLDVPLQPNSRQDPFNTFFNTGSSGRHVPRALYVDLEPTVIGTCLPDEVRVGTYKDLF
HPEQLISGKEDAANNYARGH
YTVGREMIDTVLERIRKMRLLFQTDQCTGLQGFLVFHSFGGGTGSGFTSLLMERLSVDYGKKSKLEFAIYPAPQVSTAVV
EPYNSILTTHTTLEHTDCAF
MVDNEAIYDICHRNMDIEHPSYINLNRLIGQIVSSITASLRFDGALNVDLTEFQTNLVPFPRIHFPLVTYSPIISAEKAY
HEQLTVLEITSSCFEPANQM
VKCDPRHGMYMACCMLYRGDVVPKDVNAAIANIKTRRSIQFVDWCPTGFKVGINYQPPTAVPGGDLAKIQRSVCMLSNTT
AIAEAWARLDHKFDLMYSKR
AFVHWYVGEGMEEGEFSEAREDMACLEKDYEELGKMSIDLDDDELSEE
>g974.t3
MWYECVQTGNACWELFCLEHGIGPDGVSLDVPLQPNSRQDPFNTFFNTGSSGRHVPRALYVDLEPTVIDEVRVGTYKDLF

HPEQLISGKEDAANNYARGH
YTVGREMIDTVLERIRKMRLLFQTDQCTGLQGFLVFHSFGGGTGSGFTSLLMERLSVDYGKKSKLEFAIYPAPQVSTAVV
EPYNSILTTHTTLEHTDCAF
MVDNEAIYDICHRNMDIEHPSYINLNRLIGQIVSSITASLRFDGALNVDLTEFQTNLVPFPRIHFPLVTYSPIISAEKAY
HEQLTVLEITSSCFEPANQM
VKCDPRHGMYMACCMLYRGDVVPKDVNAAIANIKTRRSIQFVDWCPTGFKVGINYQPPTAVPGGDLAKIQRSVCMLSNTT
AIAEAWARLDHKFDLMYSKR
AFVHWYVGEGMEEGEFSEAREDMACLEKDYEELGKMSIDLDDDELSEE
>g975.t1
MSRPRTDEFKPPPECPVFEPSWEEFRDPFAFINKIRPIAEKTGICKVRPPPGWQPPFACDVDRLHFVPRIQRLNELEAQT
RVKLNFLDQIAKFWDLQGCA
LKIPHVERKILDLYKLNKLVADEGGFDLVCRDRRWTKIALQMGFAPGKAIGSHLRAHYEKVLYPYNLFQSGANLLAPEPA
SKVARLEAEPEYEKKPSLLA
DSTDSKDFVAHDTPQRAPQQPEMVCPNAYRAKHLKSEVKTEPGEGCENRPNLRRRMGSFVAKPEPEKEIAITVKQEPVEV
KEPGIEADKIKSRYKKNLPP
VPLVPPSPVDLVVCLVCGSGADEDRLLLCDGCDDSYHTFCLIPSLHEVPKGDWRCPKCLAQVSAPPAPSSPGQRALPRCT
QPPRRSERASGREEEETRFT
AECSKPQEAFGFEQAYRDYSLRAFGQMADAFKSDYFNMPVHMVPTELVEKEFWRLVGAIEEDVTVEYGADIASKEFGSGF
PIPNGRFKVSPADEKYLKCG
WNLNNMAMMNPSVLTHVTADICGMTLPWLYVGMCFSSFCWHIEDHWSYSINYLHWGEPKTWYGAPGSAAEQLESVMKKLA
PELFDSQPDLLHQLVTIMNP
NTLMAHGVPIFRTNQCAGEFVVTFPRAYHSGFNQGFNFAEAVNFCTVDWMPLGRQCVDHYRLLHRYNVFSHDEMVCNMAS
KADTLDVVLASAVHRDMALM
IREEKELREKVKKMGVSQCHQAKYDHLQDDERQCIKCRTTCYLSAITCPCSPGALVCLHHIRDLCPCPVSNYTLKFTLDD
LYPMMTAVKHRAELYDDWAS
RVTQTLDAKLEKKKGLAVFRSLVSESEVNIFPDNDLLFRLRLVTKDAEKCSSVAQQLLGGKRQTRYRCGGKSQSLLTVEE
LRSFVRQLYNLPCSLTQAPL
LKKLLNDIEDFQQHSEKVLSEDVSSVVEIQGLLDVSFDFDVELPELPCLRVKLEHSRWLEGVQQATSRPSTLTLEAMRRL
IDQGLGLAPHSPVEKAMARL
QELLSVSEHWEDQASSLLNVSPPHHIETLSAAAEKASTIPAHLPNCLLLKDSIREAREWLQEVEKLQAGGCVPMADVVSD
VVLRGQAIQVHLEPLERLKS
LSAEVQEWKESAASMLLKKNSPFTLLEVLCPRSDVGSVGSPKRKPKKAKESLQLGKKKAVPLDSPADVEKALSESEDSTS
AAAVLEELRLREMDTLTVLR
AHNESKLLPTADCMDLTVCVCHKMPMGAMLQCELCRDAFHSVCVRDLCESPTERPWLCPLCRRSEAPPMSKVLSLLASLK
RTQVRLPEADALHFLVERAV
SWQDRARQTSDTLRLQEMQEGPGTPPTLTRWASGSTTADSNAQAPYLTPEWNKTSHSQTVFYTEQKCIPLQGLSRELEEL
MVEGLLLPVSLPETRDLYHV
LMHRPQSQPPAGCLSPPQDDSSDCNEHIPFNAQGTNLSWNEDEDGGALAAAEKNGKRHLEREGVQTERRPGKDKKPHYKR
QKMSKEKSLERRAASTSSSP
RSDFSQSDDSDEEMAVCPAERCQQPEGDEVDWVQCDGRCNQWFHQACVGVTAELAEKEDYVCVSCAIDDGHLRQ
>g975.t2
MSRPRTDEFKPPPECPVFEPSWEEFRDPFAFINKIRPIAEKTGICKVRPPPGWQPPFACDVDRLHFVPRIQRLNELEAQT
RVKLNFLDQIAKFWDLQGCA
LKIPHVERKILDLYKLNKLVADEGGFDLVCRDRRWTKIALQMGFAPGKAIGSHLRAHYEKVLYPYNLFQSGANLLAPEPA
SKVARLEAEPEYEKKPSLLA
DSTDSKDFVAHDTPQRAPQQPEMVCPNAYRAKHLKSEVKTEPGEGCENRPNLRRRMGSFVAKPEPEKEIAITVKQEPVEV
KEPGIEADKIKSRYKKNLPP
VPLVPPSPVDLVVCLVCGSGADEDRLLLCDGCDDSYHTFCLIPSLHEVPKGDWRCPKCLAQECSKPQEAFGFEQAYRDYS
LRAFGQMADAFKSDYFNMPV
HMVPTELVEKEFWRLVGAIEEDVTVEYGADIASKEFGSGFPIPNGRFKVSPADEKYLKCGWNLNNMAMMNPSVLTHVTAD
ICGMTLPWLYVGMCFSSFCW
HIEDHWSYSINYLHWGEPKTWYGAPGSAAEQLESVMKKLAPELFDSQPDLLHQLVTIMNPNTLMAHGVPIFRTNQCAGEF
VVTFPRAYHSGFNQGFNFAE
AVNFCTVDWMPLGRQCVDHYRLLHRYNVFSHDEMVCNMASKADTLDVVLASAVHRDMALMIREEKELREKVKKMGVSQCH
QAKYDHLQDDERQCIKCRTT
CYLSAITCPCSPGALVCLHHIRDLCPCPVSNYTLKFTLDDLYPMMTAVKHRAELYDDWASRVTQTLDAKLEKKKGLAVFR

SLVSESEVNIFPDNDLLFRL
RLVTKDAEKCSSVAQQLLGGKRQTRYRCGGKSQSLLTVEELRSFVRQLYNLPCSLTQAPLLKKLLNDIEDFQQHSEKVLS
EDVSSVVEIQGLLDVSFDFD
VELPELPCLRVKLEHSRWLEGVQQATSRPSTLTLEAMRRLIDQGLGLAPHSPVEKAMARLQELLSVSEHWEDQASSLLNV
SPPHHIETLSAAAEKASTIP
AHLPNCLLLKDSIREAREWLQEVEKLQAGGCVPMADVVSDVVLRGQAIQVHLEPLERLKSLSAEVQEWKESAASMLLKKN
SPFTLLEVLCPRSDVGSVGS
PKRKPKKAKESLQLGKKKAVPLDSPADVEKALSESEDSTSAAAVLEELRLREMDTLTVLRAHNESKLLPTADCMDLTVCV
CHKMPMGAMLQCELCRDAFH
SVCVRDLCESPTERPWLCPLCRRSEAPPMSKVLSLLASLKRTQVRLPEADALHFLVERAVSWQDRARQTSDTLRLQEMQE
GPGTPPTLTRWASGSTTADS
NAQAPYLTPEWNKTSHSQTVFYTEQKCIPLQGLSRELEELMVEGLLLPVSLPETRDLYHVLMHRPQSQPPAGCLSPPQDD
SSDCNEHIPFNAQGTNLSWN
EDEDGGALAAAEKNGKRHLEREGVQTERRPGKDKKPHYKRQKMSKEKSLERRAASTSSSPRSDFSQSDDSDEEMAVCPAE
RCQQPEGDEVDWVQCDGRCN
QWFHQACVGVTAELAEKEDYVCVSCAIDDGHLRQ
>g975.t3
MSRPRTDEFKPPPECPVFEPSWEEFRDPFAFINKIRPIAEKTGICKVRPPPAQTRVKLNFLDQIAKFWDLQGCALKIPHV
ERKILDLYKLNKLVADEGGF
DLVCRDRRWTKIALQMGFAPGKAIGSHLRAHYEKVLYPYNLFQSGANLLAPEPASKVARLEAEPEYEKKPSLLADSTDSK
DFVAHDTPQRAPQQPEMVCP
NAYRAKHLKSEVKTEPGEGCENRPNLRRRMGSFVAKPEPEKEIAITVKQEPVEVKEPGIEADKIKSRYKKNLPPVPLVPP
SPVDLVVCLVCGSGADEDRL
LLCDGCDDSYHTFCLIPSLHEVPKGDWRCPKCLAQVSAPPAPSSPGQRALPRCTQPPRRSERASGREEEETRFTAECSKP
QEAFGFEQAYRDYSLRAFGQ
MADAFKSDYFNMPVHMVPTELVEKEFWRLVGAIEEDVTVEYGADIASKEFGSGFPIPNGRFKVSPADEKYLKCGWNLNNM
AMMNPSVLTHVTADICGMTL
PWLYVGMCFSSFCWHIEDHWSYSINYLHWGEPKTWYGAPGSAAEQLESVMKKLAPELFDSQPDLLHQLVTIMNPNTLMAH
GVPIFRTNQCAGEFVVTFPR
AYHSGFNQGFNFAEAVNFCTVDWMPLGRQCVDHYRLLHRYNVFSHDEMVCNMASKADTLDVVLASAVHRDMALMIREEKE
LREKVKKMGVSQCHQAKYDH
LQDDERQCIKCRTTCYLSAITCPCSPGALVCLHHIRDLCPCPVSNYTLKFTLDDLYPMMTAVKHRAELYDDWASRVTQTL
DAKLEKKKGLAVFRSLVSES
EVNIFPDNDLLFRLRLVTKDAEKCSSVAQQLLGGKRQTRYRCGGKSQSLLTVEELRSFVRQLYNLPCSLTQAPLLKKLLN
DIEDFQQHSEKVLSEDVSSV
VEIQGLLDVSFDFDVELPELPCLRVKLEHSRWLEGVQQATSRPSTLTLEAMRRLIDQGLGLAPHSPVEKAMARLQELLSV
SEHWEDQASSLLNVSPPHHI
ETLSAAAEKASTIPAHLPNCLLLKDSIREAREWLQEVEKLQAGGCVPMADVVSDVVLRGQAIQVHLEPLERLKSLSAEVQ
EWKESAASMLLKKNSPFTLL
EVLCPRSDVGSVGSPKRKPKKAKESLQLGKKKAVPLDSPADVEKALSESEDSTSAAAVLEELRLREMDTLTVLRAHNESK
LLPTADCMDLTVCVCHKMPM
GAMLQCELCRDAFHSVCVRDLCESPTERPWLCPLCRRSEAPPMSKVLSLLASLKRTQVRLPEADALHFLVERAVSWQDRA
RQTSDTLRLQEMQEGPGTPP
TLTRWASGSTTADSNAQAPYLTPEWNKTSHSQTVFYTEQKCIPLQGLSRELEELMVEGLLLPVSLPETRDLYHVLMHRPQ
SQPPAGCLSPPQDDSSDCNE
HIPFNAQGTNLSWNEDEDGGALAAAEKNGKRHLEREGVQTERRPGKDKKPHYKRQKMSKEKSLERRAASTSSSPRSDFSQ
SDDSDEEMAVCPAERCQQPE
GDEVDWVQCDGRCNQWFHQACVGVTAELAEKEDYVCVSCAIDDGHLRQ
>g976.t1
LKGSVSFGYGLFHLCISMVPPHLLKIVNLLGFPGDRLQGLSALAYASESKDMKAPLATLALLWYHTVVQPFFALDGADTQ
AGLLEAKAILQRREAVYPNS
SLFMFFKGRVQRLEGQISGALTSFNGALELATDQREIQHVCLYEIGWCSMIELSFSDAYRAFERLKSESRWSQCYYAYLT
GVCQGASGDLEGAASVLKDV
QRLFKRKNNQIELFSLKRAEKLRKPSPSQELCSLAVIEVLYLWKALANCSVPKLQAMIQILQGIDDASCAGLKHLLLGAI
HKTLHNTKDAIQHFQLAERD
EVGCLSNSYVQPYSCYEVACVLLDSPEEEYAGYDFENRLHVRIHSALASTRAEAPQ

>g976.t2
LKGSVSFGYGLFHLCISMVPPHLLKIVNLLGFPGDRLQGLSALAYASESKDMKAPLATLALLWYHTVVQPFFALDGADTQ
AGLLEAKAILQRREAVYPNS
SLFMFFKGRVQRLEGQISGALTSFNGALELATDQREIQHVCLYEIGWCSMIELSFSDAYRAFERLKSESRWSQCYYAYLT
GVCQGASGDLEGAASVLKDV
QRLFKRKNNQIELFSLKRAEKLRKPSPSQELCSLAVIEVLYLWKALANCSVPKLQAMIQILQGIDDASCAGLKHLLLGAI
HKTLHNTKDAIQHFQLAERD
EVGCLSNSYVQPYSCYEVACVLLDSPEAAERGRMLMLQAKEEYAGYDFENRLHVRIHSALASTRAEAPQ
>g976.t3
LKGSVSFGYGLFHLCISMVPPHLLKIVNLLGFPGDRLQGLSALAYASESKDMKAPLATLALLWYHTVVQPFFALDGADTQ
AGLLEAKAILQRREAVYPNS
SLFMFFKGRVQRLEGQISGALTSFNGALELATDQREIQHVCLYEIGWCSMIELSFSDAYRAFERLKSESRWSQCYYAYLT
GVCQGASGDLEGAASVLKDV
QRLFKRKNNQIELFSLKRAEKLRKPSPSQELCSLAVIEVLYLWKALANCSVPKLQAMIQILQGIDDASCAGLKHLLLGAI
HKTLHNTKDAIQHFQLAERD
EVGCLSNSYVQPYSCYEVACVLLDSPEAAERGRMLMLQAKVMYCRSCFTWTCFFCLYTYLMNDLVFICLCLWFLQEEYAG
YDFENRLHVRIHSALASTRA
EAPQ
>g977.t1
MWCPTSLVVNGRETEYPVPEPYLPLNFLNSTGMRYEAEEVRQCLLKGLKESPGMTHADSLLLAGLEDEARRQLGVVYSQD
HQVTSQ
>g977.t2
MWCPTSLVVNGRETEYPVPEPYLPLNFLNSTGMRYEAEEVRQCLLKGKVTWLHVNSRPPQGLKESPGMTHADSLLLAGLE
DEARRQLGVVYSQDHQVTSQ
>g978.t1
IIKVGSWNPLPINLLTDAPTATVADILLDVYHMVTLRILLH
>g978.t2
LQSSSFWVKLQATLSGSTGIIKVGSWNPLPINLLTDAPTATVADILLDVYHMVTLRILLH
>g979.t1
LVLSQTEQQSSTKWSARSDCTRSTRGTPRELLSSDYSETYYLDDGSPVTTVPPNIDPCYYHGHIVNDSQSAVSISTCDGL
RGYFRTAAQRYVIEPLAAAG
EGQDEGDHGVRVLSERPGPQQGSGPMVCGVTNTTWETDFEPPTGRSRSRAAGPSLLQRQKYVELFLVADNREAGNRKWAR
LTAACDVFQYLNMKKDQDAL
RKRIFEVVNFINMVYKPMNTFIALVGLEVWSNQDLINVTPPAGDTLNAFNNWRNTELVKRKKHDNAHLISAIDFEGATVG
LAYVGTLCSGHSSGVVQ
>g979.t2
LVLSQTEQQSSTKWSARSDCTRSTRGTPRELLSSDYSETYYLDDGSPVTTVPPNIDPCYYHGHIVNDSQSAVSISTCDGL
RGYFRTAAQRYVIEPLAAAG
EGQDEGDHGVRVLSERPGPQQGSGPMVCGVTNTTWETDFEPPTGRSRSRAAGPSLLQRQKYVELFLVADNREAGNRKWAR
LTAACDVFQYLNMKKDQDAL
RKRIFEVVNFINMVYKPMNTFIALVGLEVWSNQDLINVTPPAGDTLNAFNNWRNTELVKRKKHDNAHLISAIDFEGATVG
LAYVGTLCSGHSSGVVQVSL
QDSLGFRVLERQGPVSL
>g979.t3
LVLSQTEQQSSTKWSARSDCTRSTRGTPRELLSSDYSETYYLDDGSPVTTVPPNIDPCYYHGHIVNDSQSAVSISTCDGL
RGYFRTAAQRYVIEPLAAAG
EGQDEGDHGVRVLSERPGPQQGSGPMVCGVTNTTWETDFEPPTGRSRSRAAGPSLLQRQKYVELFLVADNREYLNMKKDQ
DALRKRIFEVVNFINMVYKP
MNTFIALVGLEVWSNQDLINVTPPAGDTLNAFNNWRNTELVKRKKHDNAHLISAIDFEGATVGLAYVGTLCSGHSSGVVQ
>g980.t1
MEPLEQSGPDAGAQDVNPIYLQQLRELDIPEEAAKQALLHTRNVSAEEAAMYYFNKLENEEEGDDDLTFKMVFVVNMDLA
MGVGKVAAQVGHAAVGLYQA
LQGKNSWREMAWKWDHSGAKKVVVQGTNMAHLLELQALAMSLSLPTYLVQDAGLTQVESGSRTVLAIMGEDEMVNNVTGS
LKLL
>g980.t2
MEPLEQSGPDAGAQDVNPIYLQQLRELDIPEEAAKQALLHTRNVSAEEAAMYYFNKLENEEEGDDDLTFKMVFVVNMDLA

MGVGKVHSRFIPGIAESTLI
LMDTKFNYKNMIFEEVAAQVGHAAVGLYQALQGKNSWREMAWKWDHSGAKKVVVQGTNMAHLLELQALAMSLSLPTYLVQ
DAGLTQVESGSRTVLAIMGE
DEMVNNVTGSLKLL
>g981.t1
MLRRYPALRLWLPLVYARLPLRHVWLPSGLPWCVPLLVLPGVCPSWSCLVCAPPGPAASYCLAGPGIFK
>g982.t1
MEKFFTPVYRPPTQEEMKLRKPKSHHHYQNDHPQHHPHPHVAYHSQDNELHSYAKLDGTDDNTDDSHAHHHQNHHRRHRQ
GHHVHENLPHDFHQPGHPQR
HQHPSSESETQNPQQTTVTLSRASSSSSSLSSSSSSSLLSSYSWHTETSAKDLFSTGPTQRPHHSLSCSNILEVRTAFRQ
DGGSEPIVFATVKRGVPEAS
ACDRTGGRPRQNTHAHASSLVRGQSHSNEGLLLTSEDEAVAGAQRRAARPHHGGLYKTASLGRSLAFSEENLVLGASGRP
GRGVSTNQIPGKGILKNREP
QPDIRKAKSMEVLSPRVANRQGKGEDNGKGSTPAEIQIAKENFVQGKLQFSAFLDEITKQVISPSNLNILGVNQVKSPGE
TPDSVRGAAKPQLPIKKHRE
SCSGEEREQHHKPPATQEKREPRGHSLSYHSSHPDQSDPNRLTSYGATWHRGSPPLHPHPQTSSHHPHPGGNPQSQSCPN
NKTTASTGTHPPTVHRHYIP
STRRTFKPATGVQSLGIKRKDMAPTRPWAQHWVLNQSLAPVSQTHQNRDHLEPETQPPPVTARTQTGVSDIALTQHARDT
PIHIRALQEENTDLHQNLLQ
TVVCIESLEAELTRTREELSCLKDKHRRLSTETPTGTHQHNPRLGEHLHMMSESPSSERKYLINSIAELSSELEEAQRTI
AALETINVPSLIKELLEKHF
DSTEAVQKFLKTATTVNHSKTPSPPDPSQSGPSNVVEVPYDWLTQSEAGPHRVTAFMPWKQEASAAAPSPVAPSAAAPSA
DGGLSGAHNASQSQPFSIAD
ISTAIYMKIASEIAANHMSSQPKSVQGCPKGTNHTSTAGTPAHGQQVPETAELAPGHEDVTYLSAQQVLDEFIHQLHPQN
QASDGEEQQPHMSTTYKQE
>g982.t2
MEKFFTPVYRPPTQEEMKLRKPKSHHHYQNDHPQHHPHPHVAYHSQDNELHSYAKLDGTDDNTDDSHAHHHQNHHRRHRQ
GHHVHENLPHDFHQPGHPQR
HQHPSSESETQNPQQTTVTLSRASSSSSSLSSSSSSSLLSSYSWHTETSAKDLFSTGPTQRPHHSLSCSNILEVRTAFRQ
DGGSEPIVFATVKRGVPEAS
ACDRTGGRPRQNTHAHASSLVRGQSHSNEGLLLTSEDEAVAGAQRRAARPHHGGLYKTASLGRSLAFSEENLVLGASGRP
GRGVSTNQIPGKGILKNREP
QPDIRKAKSMEVLSPRVANRQGKGEDNGKGSTPAEIQIAKENFVQGKLQFSAFLDEITKQVISPSNLNILGVNQVKSPGE
TPDSVRGAAKPQLPIKKHRE
SCSGEEREQHHKPPATQEKREPRGHSLSYHSSHPDQSDPNRLTSYGATWHRGSPPLHPHPQTSSHHPHPGGNLHRHYIPS
TRRTFKPATGVQSLGIKRKD
MAPTRPWAQHWVLNQSLAPVSQTHQNRDHLEPETQPPPVTARTQTGVSDIALTQHARDTPIHIRALQEENTDLHQNLLQT
VVCIESLEAELTRTREELSC
LKDKHRRLSTETPTGTHQHNPRLGEHLHMMSESPSSERKYLINSIAELSSELEEAQRTIAALETINVPSLIKELLEKHFD
STEAVQKFLKTATTVNHSKT
PSPPDPSQSGPSNVVEVPYDWLTQSEAGPHRVTAFMPWKQEASAAAPSPVAPSAAAPSADGGLSGAHNASQSQPFSIADI
STAIYMKIASEIAANHMSSQ
PKSVQGCPKGTNHTSTAGTPAHGQQVPETAELAPGHEDVTYLSAQQVLDEFIHQLHPQNQASDGEEQQPHMSTTYKQE
>g982.t3
MKLRKPKSHHHYQNDHPQHHPHPHVAYHSQDNELHSYAKLDGTDDNTDDSHAHHHQNHHRRHRQGHHVHENLPHDFHQPG
HPQRHQHPSSESETQNPQQT
TVTLSRASSSSSSLSSSSSSSLLSSYSWHTETSAKDLFSTGPTQRPHHSLSCSNILEVRTAFRQDGGSEPIVFATVKRGV
PEASACDRTGGRPRQNTHAH
ASSLVRGQSHSNEGLLLTSEDEAVAGAQRRAARPHHGGLYKTASLGRSLAFSEENLVLGASGRPGRGVSTNQIPGKGILK
NREPQPDIRKAKSMEVLSPR
VANRQGKGEDNGKGSTPAEIQIAKENFVQGKLQFSAFLDEITKQVISPSNLNILGVNQVKSPGETPDSVRGAAKPQLPIK
KHRESCSGEEREQHHKPPAT
QEKREPRGHSLSYHSSHPDQSDPNRLTSYGATWHRGSPPLHPHPQTSSHHPHPGGNPQSQSCPNNKTTASTGTHPPTVHR
HYIPSTRRTFKPATGVQSLG
IKRKDMAPTRPWAQHWVLNQSLAPVSQTHQNRDHLEPETQPPPVTARTQTGVSDIALTQHARDTPIHIRALQEENTDLHQ
NLLQTVVCIESLEAELTRTR

EELSCLKDKHRRLSTETPTGTHQHNPRLGEHLHMMSESPSSERKYLINSIAELSSELEEAQRTIAALETINVPSLIKELL
EKHFDSTEAVQKFLKTATTV
NHSKTPSPPDPSQSGPSNVVEVPYDWLTQSEAGPHRVTAFMPWKQEASAAAPSPVAPSAAAPSADGGLSGAHNASQSQPF
SIADISTAIYMKIASEIAAN
HMSSQPKSVQGCPKGTNHTSTAGTPAHGQQVPETAELAPGHEDVTYLSAQQVLDEFIHQLHPQNQASDGEEQQPHMSTTY
KQE
>g983.t1
MALLKMKFPLQRRVRLAQGLWLLSWLAVLSGAFTFCLGVYLKTELLRRAEVMDNSEIHVVPNILMLVGLAAIGTNWFAGR
VCQDSLDPERFPRWKNLLLV
WFAVAVLLCSLLISAVALSYALQGHLEESLKVGLRNGIRFYRDTDVPGRCFQKETIDRLQMELRCCGNNNFRDWFEVQWI
SNRYLDFTSKEVKDNVDGRY
LVDGVPFSCCNPASPRPCLQYHLLDNNAHYNYEHQSEELNLYSRGCRQALVVYYMEIMNAIGPGVLSIIFIQVSVIASMR
YLQTAVEGALALENPEEESE
GYLLEKSLKDSLVDFQVAILSLLKMGQVGPSTQGAATDEEGGAEAGEKPAN
>g983.t2
MKFPLQRRVRLAQGLWLLSWLAVLSGAFTFCLGVYLKTELLRRAEVMDNSEIHVVPNILMLVGLAAIGTNWFAGRVCQDS
LDPERFPRWKNLLLVWFAVA
VLLCSLLISAVALSYALQGHLEESLKVGLRNGIRFYRDTDVPGRCFQKETIDRLQMELRCCGNNNFRDWFEVQWISNRYL
DFTSKEVKDNVDGRYLVDGV
PFSCCNPASPRPCLQYHLLDNNAHYNYEHQSEELNLYSRGCRQALVVYYMEIMNAIGPGVLSIIFIQVSVIASMRYLQTA
VEGALALENPEEESEGYLLE
KSLKDSLVDFQVAILSLLKMGQVGPSTQGAATDEEGGAEAGEKPAN
>g983.t3
MALLKMKFPLQRRVRLAQGLWLLSWLAVLSGAFTFCLGVYLKTELLRRAEVMDNSEIHVVPNILMLVGLAAIGTNWFAGR
VCQDSLDPERFPRWKNLLLV
WFAVAVLLCSLLISAVALSYALQGHLEESLKVGLRNGIRFYRDTDVPGRCFQKETIDRLQMELRCCGNNNFRDWFEVQWI
SNRYLDFTSKEVKDRIRSNV
DGRYLVDGVPFSCCNPASPRPCLQYHLLDNNAHYNYEHQSEELNLYSRGCRQALVVYYMEIMNAIGPGVLSIIFIQVSVI
ASMRYLQTAVEGALALENPE
EESEGYLLEKSLKDSLVDFQVAILSLLKMGQVGPSTQGAATDEEGGAEAGEKPAN
>g984.t1
MGSVWRESTHSGTWESTDLSWTDNITAVTKKAQQHLHFLRVLRKNNLDKKLLLAFYHSSIESLLTYCISTWFGSCTEADR
ARLQKTVRAAQRIIGCPLPS
PSDIYTTRCLSRALKISKDSFHPGSQVFELLPSGRRYRC
>g985.t1
MKRLLLQSQIHHALDPLQFAYQEKVGVDDAIIYLLHWTHSHLDKGKSAEGEYRGLVEDFVQWCRLNHLQLNTAKTKEMVV
DFRRSKPALLPVSIEGVNVE
VVSTYKYLAVHLDNKLDCLVASVLFYAVVCWGGSTKKRDAGRLERLVRKAGSVAGTELECITAVADKGTLSKLLTIMDHE
VVRSKCPSYVGTTGILVQEF
KHVFKIITKEDRLKVIPKKNSVFAVEVNGFVSHIYGSKFEYRASERSVKKFKVKGSIDL
>g985.t2
MAGMRKSSSCVEGGLDRANELNQFYNRFDCPAAAAAAVYPPPPPRNSFWRGVFFRRAGPTRTSPPLTHTHPPPPTIKGVT
YLYRQPSDTVHRRTQGQRVT
PLPTITADQVRAGLKRLKPRKAAGPDRSQIHHALDPLQFAYQEKVGVDDAIIYLLHWTHSHLDKGKSAEGEYRGLVEDFV
QWCRLNHLQLNTAKTKEMVV
DFRRSKPALLPVSIEGVNVEVVSTYKYLAVHLDNKLDCLVASVLFYAVVCWGGSTKKRDAGRLERLVRKAGSVAGTELEC
ITAVADKGTLSKLLTIMDHE
VVRSKCPSYVGTTGILVQEFKHVFKIITKEDRLKVIPKKNSVFAVEVNGFVSHIYGSKFEYRASERSVKKFKVKGSIDL
>g985.t3
MAGMRKSSSCVEGGLDRANELNQFYNRFDCPAAAAAAVYPPPPPRNSFWRGVFFRRAGPTRTSPPLTHTHPPPPTIKGVT
YLYRQPSDTVHRRTQGQRVT
PLPTITADQVRAGLKRLKPRKAAGPDRIHHALDPLQFAYQEKVGVDDAIIYLLHWTHSHLDKGKSAEGEYRGLVEDFVQW
CRLNHLQLNTAKTKEMVVDF
RRSKPALLPVSIEGVNVEVVSTYKYLAVHLDNKLDCLVASVLFYAVVCWGGSTKKRDAGRLERLVRKAGSVAGTELECIT
AVADKGTLSKLLTIMDHEVV
RSKCPSYVGTTGILVQEFKHVFKIITKEDRLKVIPKKNSVFAVEVNGFVSHIYGSKFEYRASERSVKKFKVKGSIDL

>g985.t4
MAGMRKSSSCVEGGLDRANELNQFYNRFDCPAAAAAAVYPPPPPRNSFWRGVFFRRAGPTRTSPPLTHTHPPPPTIKGVT
YLYRQPSDTVHRRTQGQRVT
PLPTITADQVRAGLKRLKPRKAAGPDRVSPRVLRTCAAKLGEPLKYIFDLSLHLGRAPTL
>g986.t1
MSWCDLAFTQVNQARILAVESSFGQRGAPLRKPGRVLVGEGRLSKLCRRRIQPKVFFLFSDVLVYGSITGSGHHYTNQKI
ISLEHIQLEDVEDSTRMENH
WLLRTPRKSFYMAAGCPEEKRAWM
>g987.t1
IKITTSCYPVPSICCHDLVYDWFESLAQCLHWNVRKRQAGLAQPSDSSDTEN
>g988.t1
MKTAQGSGHRTLLYGHAVLLRHSYSGMYLCCLSTSRSSTDKLAFDVGLQEDTTGEACWWTIHPASKQRSEGEKVRVGDDL
ILQGGRQQHQDKPNVKPGLK
AAVVGTVHAMFKHLSYGYEDLHVDAAFQQTLWSVVPICSGSEVAQGYLIGGDVMRLLHGHMDECLTVPSGDHGEEHRRSV
NPRHVSENAARRSEVNAWEN
PAVLQQNFWGETAKSPGTRKTEGRTVNYEGGAVSIHARSLWRLEMLRVVWSGSHTRWGQPFRLRHVTTGKYLSLTDDRSL
LLMDKDKADVKSTAFCFKSS
KEKLDSGMKKEMDGMGVADIKYGDSVCYVQHMDTSLWLTYQAMDTKGARMGGVQRKAIMHHEGHMDDGLTLSRSQHEESR
TARVIRSTVFLFNRFIRGLD
MLSKKGNTSALDLPIESVSLSLRDLIGYFQPPDEHLEHEEKQNRLRALKNRQNLFQEEGFQPSCNS
>g988.t2
MKTAQGSGHRTLLYGHAVLLRHSYSGMYLCCLSTSRSSTDKLAFDVGLQEDTTGEACWWTIHPASKQRSEGEKVRVGDDL
ILHLSYGYEDLHVDAAFQQT
LWSVVPICSGSEVAQGYLIGGDVMRLLHGHMDECLTVPSGDHGEEHRRSVNPRHVSENAARRSEVNAWENPAVLQQNFWG
ETAKSPGTRKTEGRTVNYEG
GAVSIHARSLWRLEMLRVVWSGSHTRWGQPFRLRHVTTGKYLSLTDDRSLLLMDKDKADVKSTAFCFKSSKEKLDSGMKK
EMDGMGVADIKYGDSVCYVQ
HMDTSLWLTYQAMDTKGARMGGVQRKAIMHHEGHMDDGLTLSRSQHEESRTARVIRSTVFLFNRFIRGLDMLSKKGNTSA
LDLPIESVSLSLRDLIGYFQ
PPDEHLEHEEKQNRLRALKNRQNLFQEEGFQPSCNS
>g988.t3
MKTAQGSGHRTLLYGHAVLLRHSYSGMYLCCLSTSRSSTDKLAFDVGLQEDTTGEACWWTIHPASKQRSEGEKVRVGDDL
ILQGGRQQHQDKPNVKPGLK
AAVVGTVHAMFKHLSYGYEDLHVDAAFQQTLWSVVPICSGSEVAQGYLIGGDVMRLLHGHMDECLTVPSGDHGEEHRRSV
NPRHVSENAARRSEVNAWEN
PAVLQQNFWGETAKSPGTRKTEGRWSGSHTRWGQPFRLRHVTTGKYLSLTDDRSLLLMDKDKADVKSTAFCFKSSKEKLD
SGMKKEMDGMGVADIKYGDS
VCYVQHMDTSLWLTYQAMDTKGARMGGVQRKAIMHHEGHMDDGLTLSRSQHEESRTARVIRSTVFLFNRFIRGLDMLSKK
GNTSALDLPIESVSLSLRDL
IGYFQPPDEHLEHEEKQNRLRALKNRQNLFQEEGFQPSCNS
>g989.t1
MPCYDQNLPQVGSSLHLIHGSQCWIQATKGATKLLPLHENSIFWESFLDQLDFSGAEGQKCPQLKCCFALASGEISHKLT
DIASCADCNLHLSQLRKL
>g990.t1
MFKARLSIWVPDSYLGEKPEDDEVVLQCSATVHKEQQKLCLAAEGFGNRLCFLESTSNSKNVPPDLSICAFVLEQSLSVR
ALQEMLANTEEKAEGVSRTL
PSWSRGFMLTHTLTKKIYK
>g990.t2
MFKARLSIWVPDSYLGEKPEDDEVVLQCSATVHKEQQKLCLAAEGFGNRLCFLESTSNSKNVPPDLSICAFVLEQSLSVR
ALQEMLANTEEKAEGVPEPC
>g991.t1
SALSRSFNSQHAPLGWSPRHHHGRRPMHASQGSLLCSPRDGLSPGAAGFCVSTA
>g992.t1
LVEPGMPSDNITVKRQVGKLPVTPSNYPGARPLQYNHNPGNTAPDAFPGTTVTAGDGRHERGEVGEGM
>g993.t1
AASSSGPSCIKDAVRHPGLPTALQTACCSLNTREPLQPFTEISMVCLSASTTVERAPHQTACPRQCCGQVASVNAMAIRG

LSTRLKPATMTLTLQ
>g994.t1
MQQKRNIQIIEWEDLDKRKFYSFGVFMTMSIRATVYPATLIRTRLQVQKGKSLYSGTFDAFFKILRRDGVRGLYRGFMVN
SFTLISGQAYITTYELVRKY
VSNYSHNNTVKSLVAGGSASLVAQSITVPIDVVSQQLMMQGQGGHPTRFQLQAKVQEGMSKRMFGQTRDIMAQIFAADGF
RGFYRGYVASLLTYIPNSAV
WWPFYHFYAEQLSKWAPSDCPHLLLQAMAGPLAAATASTVTNPMDVVRARVQVEGRSSIVETFKQLMAEEGAWGMTKGLS
ARIISSTPTTIVMVVGYETL
KKVSLRPELVDSRHW
>g994.t2
MTMSIRATVYPATLIRTRLQVQKGKSLYSGTFDAFFKILRRDGVRGLYRGFMVNSFTLISGQAYITTYELVRKYVSNYSH
NNTVKSLVAGGSASLVAQSI
TVPIDVVSQQLMMQGQGGHPTRFQLQAKVQEGMSKRMFGQTRDIMAQIFAADGFRGFYRGYVASLLTYIPNSAVWWPFYH
FYAEQLSKWAPSDCPHLLLQ
AMAGPLAAATASTVTNPMDVVRARVQVEGRSSIVETFKQLMAEEGAWGMTKGLSARIISSTPTTIVMVVGYETLKKVSLR
PELVDSRHW
>g995.t1
MEAIGHAGTCLGILANDGVLLAAERRNIHKLLDEVFFSEKIYKLNEYLLQYQEPMPCEQLVTALCDIKQAYTQFGGKRPF
GVSLLYMGWDKHYGFQLYQS
DPSGNYGGWKATCIGNNSA
>g995.t2
LYQVEYAMEAIGHAGTCLGILANDGVLLAAERRNIHKLLDEVFFSEKIYKLNEYLLQYQEPMPCEQLVTALCDIKQAYTQ
FGGKRPFGVSLLYMGWDKHY
GFQLYQSDPSGNYGGWKATCIGNNSA
>g995.t3
MIPGQQSFLPKAGRLYQVEYAMEAIGHAGTCLGILANDGVLLAAERRNIHKLLDEVFFSEKIYKLNEYLLQYQEPMPCEQ
LVTALCDIKQAYTQFGGKRP
FGVSLLYMGWDKHYGFQLYQSDPSGNYGGWKATCIGNNSA
>g996.t1
MRPLYRTVVVKRELSRKAKLSIYQSIYVPTLTYGHELWVVTERTRSRIQAAEMSFLHRVGEELGHPEGARSRAAAPSRRK
EPVEMVRASDQDASWAPPEE
LESVAGEKDVWSSLLSLLPPRPGPGEADV
>g997.t1
MHWRKTAELKLVSKKEEGADTERLYTVSFQEICDRFGKQYTWEVKSKVMGKKALDAAQMIRDALDLPMTAQELLHETRLV
QERIFPSAQLMPGVEKLIRH
LLKHGVPIAVATSSAGATYELKTSSHEAFFSQFSHVVLGDDPDVKNSKPQPDSFLVCAGRFQPPAAPDKAWLWKKRVWVV
AYLPADLAPLCTKTGTK
>g998.t1
VGDREIRESRLLKARVPLASSLGRPVTGAIQDGAARPMTAVRAAGYTSALTRGTTFDPLGQSKGPAPPLEARNEDTPEEK
IKILEKRVNELIAESCIAHS
QGSLQLALEKAKEAGRKERALVRQREQSGNADHINPDLTYAVLFNLANQYANNEMYPEALNTYQVIVKNKMFTNAGRLKV
NMANIYFKQKNYPKAIKFYR
MALDQISNAHKETRIKILQNIGVVFVRMGQYSDAVTSFEHIMSESPNVRTGFNLILCYYATGDRERMKKAFQKLIAVPLG
IGEEDKYIPTNVSHTALFPL
TASPQQDSLPGGGPCWTTRKSLAEKFIMTSAKLIAPAIESSFAAGFDWFVVPSTATRWGVVALTVXCVDMVKTSQYVELA
NDLEINKAITYLRQKDFKQV
RAAVLSGRGSIWRKQMGVV
>g998.t2
VGDREIRESRLLKARVPLASSLGRPVTGAIQDGAARPMTAVRAAGYTSALTRGTTFDPLGQSKGPAPPLEARNEDTPEEK
IKILEKRVNELIAESCIAHS
QGSLQLALEKAKEAGRKERALVRQREQSGNADHINPDLTYAVLFNLANQYANNEMYPEALNTYQVIVKNKMFTNAGRLKV
NMANIYFKQKNYPKAIKFYR
MALDQISNAHKETRIKILQNIGVVFVRMGQYSDAVTSFEHIMSESPNVRTGFNLILCYYATGDRERMKKAFQKLIAVPLG
IGEEDKYIPTNVSHTALFPL
TASPQQDSLPGGGPCWTTRKSLAEKFIMTSAKLIAPAIESSFAAGFDWCVDMVKTSQYVELANDLEINKAITYLRQKDFK
QVRAAVLSGRGSIWRKQMGV

V
>g998.t3
VGDREIRESRLLKDGAARPMTAVRAAGYTSALTRGTTFDPLGQSKGPAPPLEARNEDTPEEKIKILEKRVNELIAESCIA
HSQGSLQLALEKAKEAGRKE
RALVRQREQSGNADHINPDLTYAVLFNLANQYANNEMYPEALNTYQVIVKNKMFTNAGRLKVNMANIYFKQKNYPKAIKF
YRMALDQISNAHKETRIKIL
QNIGVVFVRMGQYSDAVTSFEHIMSESPNVRTGFNLILCYYATGDRERMKKAFQKLIAVPLGIGEEDKYIPTNVSHTALF
PLTASPQQDSLPGGGPCWTT
RKSLAEKFIMTSAKLIAPAIESSFAAGFDWFVVPSTATRWGVVALTVXCVDMVKTSQYVELANDLEINKAITYLRQKDFK
QVRAAVLSGRGSIWRKQMGV
V
>g999.t1
GSSSVRSSMGRAGVAPCSSVDSDCSDSPGLLSPIGNGRAPSLSNTFSAFGSNNLFNLSGVFSGMTSARSVAEPQSSWSDF
SHMSPPPTCPPASSSAWPSE
ALEPWPSSTSTTGSSSSVRHAHAHTHPAAA
>g999.t2
GSSSVRSSMGRAGVAPCSSVDSDCSDSPGLLSPIGNGRAPSLSNTFSAFGSNNLFNLSGVFSGMTSARSVAEPQSSWSDF
SHMSPPPTCPPASSSAWPSE
ALEPWPSSTSTTGSSSSQSILSSSLWSSSPPFSPSIWSTSADSSPLHTFSSPPPPNRHLGDLMMGSE
>g999.t3
GSSSVRSSMGRAGVAPCSSVDSDCSDSPGLLSPIGNGRAPSLSNTFSAFGSNNLFNLSGVFSGMTSARSVAEPQSSWSDF
SHMSPPPTCPPASSSAWPSE
ALEPWPSSTSTTGSSSSCFSLFLNNNVK
>g1000.t1
MRAAHYRVNSNRRKMIGLSVFPWLHHMPARALLFPPTVTVICPSGRHFCRRPDTSYRMLCRSRDTSYRMLCRSRDTSYRM
LCRSRDTSYRMLCRSRDTSY
RMLCRSRDTSYRMLCRSRDTSYRMLCRSRDTSYRMLCRSRDTSYRMLCRSRDTSYRMLCRSRDTSYRMLCRSRDTSYRML
WFSC
>g1001.t1
DRGVTVGTWRNETFCPLPCGPNSHYVPCAPPCQESCGGRPSGCGGPCSEACVCDPGYVLSAGKCVMKSSCGCNYTLNGQY
YEPGDEFYVDNCQLKCKCNA
PFITCQASVCPPQQECKLQGGELGCYPTSSQDCVVSGDPHYNTFDNKFYTFMGTCTYTLARNCGNKTGPWFSVEGKNEER
GLAGVSYLKKLYVTVGGVTV
TLMKSRRTLVNGLRVALPSSPSPLVMVSLAGQYVTLQTTFGLRVRWDGNHHAQISVP
>g1001.t2
LPCGPNSHYVPCAPPCQESCGGRPSGCGGPCSEACVCDPGYVLSAGKCVMKSSCGCNYTLNGQYYEPGDEFYVDNCQLKC
KCNAPFITCQASVCPPQQEC
KLQGGELGCYPTSSQDCVVSGDPHYNTFDNKFYTFMGTCTYTLARNCGNKTGPWFSVEGKNEERGLAGVSYLKKLYVTVG
GVTVTLMKSRRTLVNGLRVA
LPSSPSPLVMVSLAGQYVTLQTTFGLRVRWDGNHHAQISVP
>g1002.t1
MKKAFRMLLGVGRAVGSPSSPRAGCRAAQHVLYSSSSQEDRTTHTLMLRHLLTKIKATGPISVAEYMREALTNPVAGYYV
NNDMLGPDGDFITSPEISQV
FGELLGIWCVSEWMGAGRPSSLQLVELGPGRGSLMNDILR
>g1002.t2
MKKAFRMLLGVGRAVGSPSSPRGCRAAQHVLYSSSSQEDRTTHTLMLRHLLTKIKATGPISVAEYMREALTNPVAGYYVN
NDMLGPDGDFITSPEISQVF
GELLGIWCVSEWMGAGRPSSLQLVELGPGRGSLMNDILR
>g1002.t3
MRSVCAEQPIRTTVVRCERVTWSEPPSMKKAFRMLLGVGRAVGSPSSPRAGCRAAQHVLYSSSSQEDRTTHTLMLRHLLT
KIKATGPISVAEYMREALTN
PVAGYYVNNDMLGPDGDFITSPEISQVFGELLGIWCVSEWMGAGRPSSLQLVELGPGRGSLMNDILR
>g1003.t1
MAPRNRYRKAGVQKDNTDAEEKHTPPAECVDEDEGEDQDHAEEDEELTLEDVLRLGGKKADFLLVSNVNDSHDLIDGGKK
GAIDDLEAGELERFITKLGV
RAYASQQIIADDAEEAEDGSSSQKKKGKKDPSGGDQPAAPAETSRVADRKPAKKTKEEVTHAGTKKSRQNVDVLEFHQRL

VLLIKPGGKWFDQEYTAEGC
GEPQDEAQVAGYRALALRLYEAEVALYRSKKSLQKGANSAWMKTVVSKGTLVDRMAAMTVLLQDAPVHGLEHVESLIAMV
KKKGGRRMGLMALDTLRELL
LSDLLPPNRKLRPFPQHPFHQLEERASGNRDSRDRRLVLWFFEDRLKHLVADFVTALDEVSRDNVAATKAKALTTAHQLL
SGRPEQEKALLVQVVNKLGD
PEYKMAARASHLLETLLHRHPNMKAVVCGEVERLMFRPNISAKAQYYGVCFLSQVPLSHEEPQLAGKLISVYFTFFRACV
KKKDVESKMLGALLTGVSRA
YPYAAAADDQVKEQLDTLFRVVHAVKFNTAVQALMLLFQVMDSQQSVSDRYYVALYKKLLDPELTFCSKQSMFLNLIYKS
LKADVVLRRVKAFVKRLLQV
SCEQNATFACGALFLVSEVMRAKPGLKLLLQDNQVSGRRFQTHCTTRCHRSTGAVLGPAGEEENFQDLAEEDDDEEERFV
DADKVEERQTTEEPEKPKPK
ASWVHHQNLEGGKTTVTYDPLHRNPMFCGADRTTLWELQQLSLHFHPSVSLFAKTILKGESVQYSGDPLQDFTLIRFLDR
FVFRNPKQLKGKQNTEATVM
RPQQKLTFDKTRSLAVNCKEFLNKEESQIPVDQVFFYRYFKKRAQEKFLRRPPGGSDTESLEDVEDDEFEKILDSVEGDS
YYTDLPDNDLDFAGNMKSRT
GPEDSDSDSEDLEDLDDDEVSLGSMNEEDFGDQLEEDGGTFMDEDDDDEEDSEEEAGPPETTSHSRKRRKTSAGLDFSSS
KASRSSRTGSTPYWGTCVCL
TPGSAGVKKRRKKDCALFTSAEEFGDLLDENAGSQVDSIGMNAMANKDKAGLRQLKWEAQRDAWVHGRDDQTMRRRKSTF
SRRKDYGKAKVAKKVFRKKK
>g1003.t2
MAPRNRYRKAGVQKDNTDAEEKHTPPAECVDEDEGEDQDHAEEDEELTLEDVLRLGGKKADFLLVSNVNDSHDLIDGGKK
GAIDDLEAGELERFITKLGV
RAYASQQIIADDAEEAEDGSSSQKKKGKKDPSGGDQPAAPAETSRVADRKPAKKTKEEVTHAGTKKSRQNVDVLEFHQRL
VLLIKPGGKWFDQEYTAEGC
GEPQDEAQVAGYRALALRLYEAEVALYRSKKSLQKGANSAWMKTVVSKGTLVDRMAAMTVLLQDAPVHGLEHVESLIAMV
KKKGGRRMGLMALDTLRELL
LSDLLPPNRKLRPFPQHPFHQLEERASGNRDSRDRRLVLWFFEDRLKHLVADFVTALDEVSRDNVAATKAKALTTAHQLL
SGRPEQEKALLVQVVNKLGD
PEYKMAARASHLLETLLHRHPNMKAVVCGEVERLMFRPNISAKAQYYGVCFLSQVPLSHEEPQLAGKLISVYFTFFRACV
KKKDVESKMLGALLTGVSRA
YPYAAAADDQVKEQLDTLFRVVHAVKFNTAVQALMLLFQVMDSQQSVSDRYYVALYKKLLDPELTFCSKQSMFLNLIYKS
LKADVVLRRVKAFVKRLLQV
SCEQNATFACGALFLVSEVMRAKPGLKLLLQDNQVSGRRFQTHCTTRCHRSTGAVLGPAGEEENFQDLAEEDDDEEERFV
DADKVEERQTTEEPEKPKPK
ASWVHHQNLEGGKTTVTYDPLHRNPMFCGADRTTLWELQQLSLHFHPSVSLFAKTILKGESVQYSGDPLQDFTLIRFLDR
FVFRNPKQLKGKQNTEATVM
RPQQKLTFDKTRSLAVNCKEFLNKEESQIPVDQVFFYRYFKKRAQEKFLRRPPGGSDTESLEDVEDDEFEKILDSVEGDS
YYTDLPDNDLDFAGNMKSRT
GPEDSDSDSEDLEDLDDDEVSLGSMNEEDFGDQLEEDGGTFMDEDDDDEEDSEEEAGPPETTSHSRKRRKTSAGLDFSSS
KGSAGVKKRRKKDCALFTSA
EEFGDLLDENAGSQVDSIGMNAMANKDKAGLRQLKWEAQRDAWVHGRDDQTMRRRKSTFSRRKDYGKAKVAKKVFRKKK
>g1003.t3
MAPRNRYRKAGVQKDNTDAEEKHTPPAECVDEDEGEDQDHAEEDEELTLEDVLRLGGKKADFLLVSNVNDSHDLIDGGKK
GAIDDLEAGELERFITKLGV
RAYASQQIIADDAEEAEDGSSSQKKKGKKDPSGGDQPAAPAETSRVADRKPAKKTKEEVTHAGTKKSRQNVDVLEFHQRL
VLLIKPGGKWFDQEYTAEGC
GEPQDEAQVAGYRALALRLYEAEVALYRSKKSLQKGANSAWMKTVVSKGTLVDRMAAMTVLLQDAPVHGLEHVESLIAMV
KKKGGRRMGLMALDTLRELL
LSDLLPPNRKLRPFPQHPFHQLEERASGNRDSRDRRLVLWFFEDRLKHLVADFVTALDEVSRDNVAATKAKALTTAHQLL
SGRPEQEKALLVQVVNKLGD
PEYKMAARASHLLETLLHRHPNMKAVVCGEVERLMFRPNISAKAQYYGVCFLSQVPLSHEEPQLAGKLISVYFTFFRACV
KKKDVESKMLGALLTGVSRA
YPYAAAADDQVKEQLDTLFRVVHAVKFNTAVQALMLLFQVMDSQQSVSDRYYVALYKKLLDPELTFCSKQSMFLNLIYKS
LKADVVLRRVKAFVKRLLQV
SCEQNATFACGALFLVSEVMRAKPGLKLLLQDNQVSGRRFQTHCTTRCHRSTGAVLGPAGEEENFQDLAEEDDDEEERFV
DADKVEERQTTEEPEKPKPK

ASWVHHQNLEGGKTTVTYDPLHRNPMFCGADRTTLWELQQSVQALVVKHIYMVVVVLSLHFHPSVSLFAKTILKGESVQY
SGDPLQDFTLIRFLDRFVFR
NPKQLKGKQNTEATVMRPQQKLTFDKTRSLAVNCKEFLNKEESQIPVDQVFFYRYFKKRAQEKFLRRPPGGSDTESLEDV
EDDEFEKILDSVEGDSYYTD
LPDNDLDFAGNMKSRTGPEDSDSDSEDLEDLDDDEVSLGSMNEEDFGDQLEEDGGTFMDEDDDDEEDSEEEAGPPETTSH
SRKRRKTSAGLDFSSSKASR
SSRTGSTPYWGTCVCLTPGSAGVKKRRKKDCALFTSAEEFGDLLDENAGSQVDSIGMNAMANKDKAGLRQLKWEAQRDAW
VHGRDDQTMRRRKSTFSRRK
DYGKAKVAKKVFRKKK
>g1004.t1
MFGFGSYRQQMFGSCNGLTSRYTKADVDATQTQGGGQETPCSLRLSSLGLQTPVTGSIPGMTTSHSCGEHMGRVLMSRIL
VFHILVRCPTLDECEQSQFQ
GDHQMAPRPLEPLAKRLFKGLIVLELAGLFGAYGLFYKMKTSQDFRSTMNRRFPSILEVYYQSNEWAGVYGAREKDHQTW
STKTD
>g1004.t2
MAPRPLEPLAKRLFKGLIVLELAGLFGAYGLFYKMKTSQDFRSTMNRRFPSILEVYYQSNEWAGVYGAREKDHQTWSTKT
D
>g1004.t3
MFGFGSYRQQMFGSCNGLTSRYTKADVDATQTQGGGQETPCSLRLSSLGLQTPVTGSIPGMTTSHSCGEHMGRVLMSRIL
VFHILVPLFRRCPTLDECEQ
SQFQGDHQMAPRPLEPLAKRLFKGLIVLELAGLFGAYGLFYKMKTSQDFRSTMNRRFPSILEVYYQSNEWAGVYGAREKD
HQTWSTKTD
>g1005.t1
VIFELLWRDYFKFVGVKYGNRLFHIKEGRTGVPFVDANMRELAMTGFMSNRGRQNVASFLTKDLGLDWRMGAEWFEYLLV
DHDVCSNYGNWLYSAGLGND
PRENRKFNMIKQGLDYDAKGDYVRRWLPELQGIGGGDVHTPWTLSTSALARAQVTLGSSYPTPVVMAHEWSRHAGRKPVD
MGEIPLVALNMSVRKRLGLY
LNPQNTVAADWSNVAESMGFAYMEIRNFGSLNNPTYEDVEKYLKKKRDPPVQVPVVDSCVPRTPDTLNDVEGSPELFDAF
ICYCQSDFEFVHEMIERLEQ
TEYNLKLCVFDRDVLPGSCVWTITAELIEKRCKRMVVVISDEYLKSDACDFQTKFALSLCPGARSKRLIPVVYKSMTEPF
PSILRFLTICDYTRPCTQSW
FWVRLAKALALS
>g1005.t2
VIFELLWRDYFKFVGVKYGNRLFHIKEGRTGVPFVDANMRELAMTGFMSNRGRQNVASFLTKDLGLDWRMGAEWFEYLLV
DHDVCSNYGNWLYSAGLGND
PRENRKFNMIKQGLDYDAKGDYVRRWLPELQGIGGGDVHTPWTLSTSALARAQVTLGSSYPTPVVMAHEWSRHAGRKPVD
MGEIPLVALNMSVRKRLGLY
LNPQNTVAADWSNVAESMGFAYMEIRNFGSLNNPTCSPELFDAFICYCQSDFEFVHEMIERLEQTEYNLKLCVFDRDVLP
GSCVWTITAELIEKRCKRMV
VVISDEYLKSDACDFQTKFALSLCPGARSKRLIPVVYKSMTEPFPSILRFLTICDYTRPCTQSWFWVRLAKALALS
>g1005.t3
MIMGVIFELLWRDYFKFVGVKYGNRLFHIKEGRTGVPFVDANMRELAMTGFMSNRGRQNVASFLTKDLGLDWRMGAEWFE
YLLVDHDVCSNYGNWLYSAG
LGNDPRENRKFNMIKQGLDYDAKGDYVRRWLPELQGIGGGDVHTPWTLSTSALARAQVTLGSSYPTPVVMAHEWSRHAGR
KPVDMGEIPLVALNMSVRKR
LGLYLNPQNTVAADWSNVAESMGFAYMEIRNFGSLNNPTYEDVEKYLKKKRDPPVQVPVVDSCVPRTPDTLNDVEGSPEL
FDAFICYCQSDFEFVHEMIE
RLEQTEYNLKLCVFDRDVLPGSCVWTITAELIEKRCKRMVVVISDEYLKSDACDFQTKFALSLCPGARSKRLIPVVYKSM
TEPFPSILRFLTICDYTRPC
TQSWFWVRLAKALALS
>g1006.t1
TLSVSSNDALQPGSSHTLGPHSSPGPKRPGNTLRKWLTSPVRRLSSGKADGHVKKLANKHKKNRGEVVRKTAADLPLGAH
KDPDDTAATPQDETLEE
>g1007.t1
HGEQSQNQSQNQRQNQSQNQSQNQRQNQRQNQRQGGEHEG
>g1008.t1

MKNMKTNTHTLQKVSRLEEQEKDVVSALRYFKTIVDKMVVEKKVLEMLPGSASKVLEAILPLVQLEARIQHR
>g1009.t1
RSPSLDEDSIGSALSLQERNRQELPWEDDDLELEDEAEPENSPLETFLAAQSLGEFVPVFRREKIDLQALLLCTDRDLTS
IHVPLGPRKKLLDACKRRLD
ALSEPEALGDTEL
>g1010.t1
LIPVGLGSGSVTVPSDAGQAQTENSSPIRTRQVYGRAPSALTPILPWRCVSHGLLVSCRLAKEKLMYISEATQQEEKIER
LKAEAEDEYVIKKQVDYRDH
VVYEGQVDYRDQIVYEGQVDYGEQVEVLQEAKMMIPDCHRRLAKAHADLLELMVRTPSRLHHPPPPSPPSPHHLHHVTIS
ITSPSPSPHHLHHPTTSITH
HLHHPPPPSPHHLSSPS
>g1010.t2
LIPVGLGSGSVTVPSDAGQAQTENSSPIRTRQVYGRAPSALTPILPWRCVSHGLLVSCRLAKEKLMYISEATQQEEKIER
LKAEAEDEYVIKKQVDYRDH
VVYEGQVDYRDQIVYEGQVDYGEQVEVLQEAKMMIPDCHRRLAKAHADLLELM
>g1011.t1
MSGLKMALQPGPYPAAARAPSPSPGSGMRDATLPESQNGAGEEETEREEEHPPRLHQEEDGLQRLGGPGRREAAHLKGCE
MIRVHDMFFTQRAWRSARHS
MDVG
>g1012.t1
MCFSRADIRCFKRRLFGAXFPRCLHIDANIPALVRVLLRQDQAWPLQSRLPPGHRPADHGSPLMMGSNACGVAIMELRLK
EQWGFSPSG
>g1013.t1
MSINRNLVTGPPLPISSLRLLFSPVRLVSGAIWHTVQKNVVSDYGMLEEFVAMVTDIVPELLTSEQKTQLLLGLRARVAL
MLADIPSALEECLKSVSHRS
ELQSLLGCQKRLLDVRLDKNALHLCAGGDIKTEEGRAHSQGHALPGVHASGAPIEGQYVSTAVTASEAIETAVENAAKEG
TRHQEFSDVFEKDCRVLLKR
LDDVPPVRRRPPQLHKHKLAHSHLYCEVCRRVMRDAKTLARHKVSHTPFQCTRCEKNFGIFKHLSSHCHNVHHLRKPYKC
HYCFKVFSMFHVFIGHEWKH
TGQLPFQCSQCAMRFEHDSGLVIHQRVHTHEKPYLCMDCGKYFASYSNRSRHRRLVHNESRKVQRFSSSQRVKDGAHQKQ
HQEKPQKKQLNGPCKYCGTV
LHSSRLCPQMSLSANESNPLTSEMAAMEGAHVEILDSDFVSLLQDPSHVAPDGSSSKDDFFPSYSNDHSSLPLNEDCSAA
LQERDSPLGKHAAANKTEND
SSSQKGQCFICKKHVQMDIKEHMKTHFPDGVLTCPRCDTLFKSMSSLRLHFKRICIEQLQVEPGEARVGQGLFKCDECVK
SYRYKLSLDGHKRTHHQLYC
EVCRRVVRDAETLARHKVSHMPFQCTLCEKHFGIFKHLSRHYENIHKLSKPYKCHHCPKVFSKLRFFIRHEWQHTGQLPF
RCSQCSMRFKQDTDLVVHQR
VHTHEKPYLCADCGKTFSCNSNLRRHVKFRHSEARDMRHFPCTQCSKTFKEKGALQKHEQRQHQNQKKARHPCIYCGKMF
SQSGMTRHKLIHTGEKPLKC
HIPECDQRFRSASEVKNHVLRHHSDDRPFECKTCGKGFIRASILNDHMKIHSGEKDHVCVLCGKAFVKLYGMTRHIQLVH
TPGRQ
>g1013.t2
MSINRNLVTGPPLPISSLRLLFSPVRLVSGAIWHTVQKNVVSDYGMLEEFVAMVTDIVPELLTSEQKTQLLLGLRARVAL
MLADIPSALEECLKSVSHRS
ELQSLLGCQKRLLDVRLDKNALHLCAGGDIKTEEGRAHSQGHALPGVHASGAPIEGQYVSTAVTASEAIETAVENAAKEG
TRHQEFSDVFEKDCRVLLKR
LDDVPPVRRRPPQLHKHKLAHSHLYCEVCRRVMRDAKTLARHKVSHTPFQCTRCEKNFGIFKHLSSHCHNVHHLRKPYKC
HYCFKVFSMFHVFIGHEWKH
TGQLPFQCSQCAMRRVVRDAETLARHKVSHMPFQCTLCEKHFGIFKHLSRHYENIHKLSKPYKCHHCPKVFSKLRFFIRH
EWQHTGQLPFRCSQCSMRFK
QDTDLVVHQRVHTHEKPYLCADCGKTFSCNSNLRRHVKFRHSEARDMRHFPCTQCSKTFKEKGALQKHEQRQHQNQKKAR
HPCIYCGKMFSQSGMTRHKL
IHTGEKPLKCHIPECDQRFRSASEVKNHVLRHHSDDRPFECKTCGKGFIRASILNDHMKIHSGEKDHVCVLCGKAFVKLY
GMTRHIQLVHTPGRQ
>g1013.t3
MSINRNLVTGPPLPISSLRLLFSPVRLVSGAIWHTVQKNVVSDYGMLEEFVAMVTDIVPELLTSEQKTQLLLGLRARVAL

MLADIPSALEECLKSVSHRS
ELQSLLGCQKRLLDVRLDKNALHLCAGGDIKTEEGRAHSQGHALPGVHASGAPIEGQYVSTAVTASEAIETAVENAAKEG
TRHQEFSDVFEKDCRVLLKR
LDDVPPVRRRPPQLHKHKLAHSHLYCEVCRRVMRDAKTLARHKVSHTPFQCTRYGHKRTHHQLYCEVCRRVVRDAETLAR
HKVSHMPFQCTLCEKHFGIF
KHLSRHYENIHKLSKPYKCHHCPKVFSKLRFFIRHEWQHTGQLPFRCSQCSMRFKQDTDLVVHQRVHTHEKPYLCADCGK
TFSCNSNLRRHVKFRHSEAR
DMRHFPCTQCSKTFKEKGALQKHEQRQHQNQKKARHPCIYCGKMFSQSGMTRHKLIHTGEKPLKCHIPECDQRFRSASEV
KNHVLRHHSDDRPFECKTCG
KGFIRASILNDHMKIHSGEKDHVCVLCGKAFVKLYGMTRHIQLVHTPGRQ
>g1014.t1
LISAAIWQTVKQKVVLDYGMLEEFVYMVTDMVPQLLKARQRAELIFGLRARLILELCRSEETSESKIIQPHLDRMQVLLS
MWDVEVASLLTDSSSVLQEN
MESVFHLQELKPLLEGQRDLNIFEDTDSSADTCILPALFLPPAGRVVIASELTASIAEGDLVYTVDTGLGVEVVETESGI
FIGTSANEEGAADAQQWLAL
>g1014.t2
LLVPPIRLISAAIWQTVKQKVVLDYGMLEEFVYMVTDMVPQLLKARQRAELIFGLRARLILELCRSEETSESKIIQPHLD
RMQVLLSMWDVEVASLLTDS
SSVLQENMESVFHLQELKPLLEGQRDLNIFEDTDSSADTCILPALFLPPAGRVVIASELTASIAEGDLVYTVDTGLGVEV
VETESGIFIGTSANEEGAAD
AQQWLAL
>g1014.t3
LVPPIRLISAAIWQTVKQKVVLDYGMLEEFVYMVTDMVPQLLKARQRAELIFGLRARLILELCRSEETSESKIIQPHLDR
MQVLLSMWDVEVASLLTDSS
SVLQENMESVFHLQELKPLLEGQRDLNIFEDTDSSADTCILPALFLPPAGRVVIASELTASIAEGDLVYTVDTGLGVEVV
ETESGIFIGTSANEEGAADA
QQWLAL
>g1015.t1
EENSRHVVVNCGEALLKNNTYWPLVSDFINILSHQSVAKRFLEDHSLLMLWMSFVSFFQGKGPSRALLRVGGMNLNKREL
NEHVEFESQTYYAAFAAELE
ACAQPMWGLLTHCKVKVSIEKIEI
>g1016.t1
SRGKYEVIAGSPKSPDELVDVYQALLLKYPAVVALIQPLRKEDVDQWERLSSVIGASCSLLCDISGLPPSQALPPALPGV
KGQVLQQVNGTTVSDLIRIT
TENPGM
>g1017.t1
MSELSRKKEALTEDLNRNDNYSRDLMDLINELECKYQRALGKRTELKTELFSRMDEIGQLESVEAVINEKLAFCQRILTE
PRRTCSIPDPFRGPQDVLGK
>g1017.t2
MSELSRKKEALTEDLNRNDNYSRDLMDLINELECKYQRALGKRTELKTELFSRMDEIGQLESVEAVINEKLAFCQRILTE
PRRTCSIPDPFRGPQDVLGK
VKSN
>g1017.t3
ADTLRQMSELSRKKEALTEDLNRNDNYSRDLMDLINELECKYQRALGKRTELKTELFSRMDEIGQLESVEAVINEKLAFC
QRILTEPRRTCSIPDPFRGP
QDVLGK
>g1018.t1
LSLQRQSILTSTIWYTAATAKVKERWQCIIHSTEKVIGSSLPSLQNLHASRTLRHAASYHKTPSAEVGPPQADQTRVCVA
WWHHVVIWKPMSCPYVSATA
SDCSVLPQWKVL
>g1019.t1
MVLIQGTVPSDADRPALCSRFQVDFQCGSSVKPRADVAFHFNPRFKKRAAAIVCNSMQKESWGKEEVHQQMPFTPGEMFE
IIILVQKEVFKVAVNGHHLL
EYGQRVALDQVDT
>g1019.t2
MVLIQGTVPSDADRPALCSRFQVDFQCGSSVKPRADVAFHFNPRFKKRAAAIVCNSMQKESWGKEEVHQQMPFTPGEMFE

IIILVQKEVFKVAVNGHHLL
EYGQRVALDQ
>g1019.t3
MVLIQGTVPSDADRFQVDFQCGSSVKPRADVAFHFNPRFKKRAAAIVCNSMQKESWGKEEVHQQMPFTPGEMFEIIILVQ
KEVFKVAVNGHHLLEYGQRV
ALDQVDT
>g1020.t1
LNSTRVLASRTARKIFEDAEPKKSKKSTCEDEPLLKNNPRRFVVFPIQYHDIWQMYKKAEASFWTAEEVDLSKDLQHWET
LKDEEKYFVSHVLAFFAASD
GIVNENLVERFTQEVQVTEARCFYGFQIAMENIHSEMYSLLIDTYIKNSAEREYLFNAIETLPCVKKKADWALNWIGNQN
AGFGERVVAFAAVEGIFFSG
SFAAIFWLKKRGLMPGLTFSNELISRDEGLHCDFACLMFKHLVNKPSKETVTTLIMDAVAIEQEFLTQALPVKLIGMNCD
MMKQYIEFVADRLMVELGFS
KVYKVENPFDFMENISLEGKTNFFEKRVGEYQRMGVMSGSTDNTFRLDADF
>g1020.t2
LASRTARKIFEDAEPKKSKKSTCEDEPLLKNNPRRFVVFPIQYHDIWQMYKKAEASFWTAEEVDLSKDLQHWETLKDEEK
YFVSHVLAFFAASDGIVNEN
LVERFTQEVQVTEARCFYGFQIAMENIHSEMYSLLIDTYIKNSAEREYLFNAIETLPCVKKKADWALNWIGNQNAGFGER
VVAFAAVEGIFFSGSFAAIF
WLKKRGLMPGLTFSNELISRDEGLHCDFACLMFKHLVNKPSKETVTTLIMDAVAIEQEFLTQALPVKLIGMNCDMMKQYI
EFVADRLMVELGFSKVYKVE
NPFDFMENISLEGKTNFFEKRVGEYQRMGVMSGSTDNTFRLDADF
>g1020.t3
LNSTRVLASRTARKIFEDAEPKKSKKSTCEDEPLLKNNPRRFVVFPIQYHDIWQMYKKAEASFWTAEEVDLSKDLQHWET
LKDEEKYFVSHVLAFFAASD
GIVNENLVERFTQEVQVTEARCFYGFQIAMENIHSEMYSLLIDTYIKNSAEREYLFNAIETLPCVKKKADWALNWIGNQN
AGFGERVVAFAAVEGIFFSG
SFAAIFWLKKRGLMPGLTFSNELISRDEVSTVSCVRHLVGKAFVGLHCDFACLMFKHLVNKPSKETVTTLIMDAVAIEQE
FLTQALPVKLIGMNCDMMKQ
YIEFVADRLMVELGFSKVYKVENPFDFMENISLEGKTNFFEKRVGEYQRMGVMSGSTDNTFRLDADF
>g1021.t1
MHQRAVLTMEQARSVARPAGCSGTVEYHDLEAAEALVSMSFWGQGSHKPRPLTPTSDSCDSIHLQPEAADTPKDLVALST
LCMTPPHSPSFAETSTTTTT
TVLNTTTTSCSSVSSTVTRPALPRPRVCSGPLLRTSLPQPALVRGPGVSGALTPEPLESGSVEGPAVTPGRAMVTSVIRH
TADRSLPSHSVAPAAALAPS
PCQQPGAPAQRSLCSMGPASQQQLQIIQTKEGTAGGPCAMDTSDGSLPSSPESTSSSVVSLVHSPPTVSSYPPCCSSSSS
SPPSSSPATLPSSPVLCQVY
PVRGRSGMISAFVQAPVQVQAPGGPKPILPQSPPSFSQQLLVGSSVTQGTVMFVVPQTSVSQAPQGAQQVMTLGNTKLLP
LAPAPVYMATGQNGGPSQAD
FSRRRNYVCNFPGCKKTYFKSSHLKAHLRTHTGEKPFSCHWEGCDKKFARSDELSRHRRTHTGEKKFVCTVCERRFMRSD
HLTKHARRHITTKRASSWPA
ETRDLNKMALPKTQTQARASPHPGGLLVPSPN
>g1021.t2
MSCPLQADLMHQRAVLTMEQARSVARPAGCSGTVEYHDLEAAEALVSMSFWGQGSHKPRPLTPTSDSCDSIHLQPEAADT
PKDLVALSTLCMTPPHSPSF
AETSTTTTTTVLNTTTTSCSSVSSTVTRPALPRPRVCSGPLLRTSLPQPALVRGPGVSGALTPEPLESGSVEGPAVTPGR
AMVTSVIRHTADRSLPSHSV
APAAALAPSPCQQPGAPAQRSLCSMGPASQQQLQIIQTKEGTAGGPCAMDTSDGSLPSSPESTSSSVVSLVHSPPTVSSY
PPCCSSSSSSPPSSSPATLP
SSPVLCQVYPVRGRSGMISAFVQAPVQVQAPGGPKPILPQSPPSFSQQLLVGSSVTQGTVMFVVPQTSVSQAPQGAQQVM
TLGNTKLLPLAPAPVYMATG
QNGGPSQADFSRRRNYVCNFPGCKKTYFKSSHLKAHLRTHTGEKPFSCHWEGCDKKFARSDELSRHRRTHTGEKKFVCTV
CERRFMRSDHLTKHARRHIT
TKRASSWPAETRDLNKMALPKTQTQARASPHPGGLLVPSPN
>g1021.t3
ADLMHQRAVLTMEQARSVARPAGCSGTVEYHDLEAAEALVSMSFWGQGSHKPRPLTPTSDSCDSIHLQPEAADTPKDLVA

LSTLCMTPPHSPSFAETSTT
TTTTVLNTTTTSCSSVSSTVTRPALPRPRVCSGPLLRTSLPQPALVRGPGVSGALTPEPLESGSVEGPAVTPGRAMVTSV
IRHTADRSLPSHSVAPAAAL
APSPCQQPGAPAQRSLCSMGPASQQQLQIIQTKEGTAGGPCAMDTSDGSLPSSPESTSSSVVSLVHSPPTVSSYPPCCSS
SSSSPPSSSPATLPSSPVLC
QVYPVRGRSGMISAFVQAPVQVQAPGGPKPILPQSPPSFSQQLLVGSSVTQGTVMFVVPQTSVSQAPQGAQQVMTLGNTK
LLPLAPAPVYMATGQNGGPS
QADFSRRRNYVCNFPGCKKTYFKSSHLKAHLRTHTGEKPFSCHWEGCDKKFARSDELSRHRRTHTGEKKFVCTVCERRFM
RSDHLTKHARRHITTKRASS
WPAETRDLNKMALPKTQTQARASPHPGGLLVPSPN
>g1022.t1
LCNHHTQILTSFVSLSKQLLRAPVATRELYRSGPHNPSQPRARPPASCRSAAQWPLLEDPTTRPQKDDCERMGTLFGMLN
KCLRRVGFSQMYFGEKIVEP
VVIVFFWLLLWFLGLQALGLVGTLCIIIIYIQK
>g1023.t1
MSITLIAPSLTDMCCQWVKAGTASPTEPSTLKEPRTSNTSSPLSQIQRAYLSSRDGTEPRASPGLGHTIPKWPH
>g1024.t1
MQYIPTNAPADTVRLRLENTLISRVPRAAFYNLSELRFLWLTYNSITSIHPSSFLNLKSLHELRLDGNLLSAFPWEGLRD
TPHLCTLGLHNNRLSSLPAH
AAVFLPNVTYLDLSSNRSVCM
>g1025.t1
LNLVMAESMDCDEQAKRYCIRGRHVAFICLAVVFSAVAVGLGVGLTHSECSVANPTAEPTPLPPPVDRGPCRPSNDSSGD
WRNFRLPDYVNPLHYELHLE
PDLNTDVYEGTVAIHLKVSRPTRHLWLHIRETFVRAVPRLQRVTRGVQEEVSVKHCFEYKPQQYVVVEATEDLAMTGPDE
VYILSLDFRGWLNGSVVGFY
RVIYTEDGITK
>g1025.t2
MAESMDCDEQAKRYCIRGRHVAFICLAVVFSAVAVGLGVGLTHSECSVANPTAEPTPLPPPVDRGPCRPSNDSSGDWRNF
RLPDYVNPLHYELHLEPDLN
TDVYEGTVAIHLKVSRPTRHLWLHIRETFVRAVPRLQRVTRGVQEEVSVKHCFEYKPQQYVVVEATEDLAMTGPDEVYIL
SLDFRGWLNGSVVGFYRVIY
TEDGITK
>g1026.t1
MENWGLITYRENNLLYDENESSAYNKQRVASVIAHELVHQWFGNIVTMDWWDDLWLNEGFASFFEYIGVEEREPTWGMRD
IMLISDVLPVMVDDGLLSSH
PIIVNVSTPAEITSVFDGISYSKGASILRMLEDWMGKDKFRDGCRKYLKDYQYKNAKTEDFWASLANVSGLPVAETMDTW
TKQMGYPFLHLSAQGPLRTL
TQKRFLLDPKADPSQPPSPLRADNVDYGTAFNLTKYLESEEGYIVWDRVASSIAYIRDMLSDTDLYPKFQMDDPEAITKA
SQIFDQWIAKNISRVGVNLR
LLVYRYGMRNSGTEAKWNVMFQKYKDSPLAQEKDKLLYGLASVKNVHLLSKLLEATKDESVMRSQELFTVVRYVSLNPLG
KSMAWDWTTLNWDYLVQRWS
TFSSKHPMLVLEKCPGSKLLKL
>g1026.t2
MENWGLITYRENNLLYDENESSAYNKQRVASVIAHELVHQWFGNIVTMDWWDDLWLNEGFASFFEYIGVEEREPTWGMRD
IMLISDVLPVMVDDGLLSSH
PIIVNVSTPAEITSVFDGISYSKGASILRMLEDWMGKDKFRDGCRVSGLPVAETMDTWTKQMGYPFLHLSAQGPLRTLTQ
KRFLLDPKADPSQPPSPLRA
DNVDYGTAFNLTKYLESEEGYIVWDRVASSIAYIRDMLSDTDLYPKFQMDDPEAITKASQIFDQWIAKNISRVGVNLRLL
VYRYGMRNSGTEAKWNVMFQ
KYKDSPLAQEKDKLLYGLASVKNVHLLSKLLEATKDESVMRSQELFTVVRYVSLNPLGKSMAWDWTTLNWDYLVQRWSTF
SSKHPMLVLEKCPGSKLLKL
>g1026.t3
MENWGLITYRENNLLYDENESSAYNKQRVASVIAHELVHQWFGNIVTMDWWDDLWLNEGFASFFEYIGVEEREPTWGMRD
IMLISDVLPVMVDDGLLSSH
PIIVNVSTPAEITSVFDGISYSKGASILRMLEDWMGKDKFRDGCRKYLKDYQYKNAKTEDFWASLANVSGLPVAETMDTW
TKQMGYPFLHLSAQGPLRTL

TQKRFLLDPKADPSQPPSPLRADNVDYGTAFNLTKYLESEEGYIVWDRVASSIAYIRDMLSDTDLYPKFQMDDPEAITKA
SQIFDQWIAKNISRVGVNLR
LLVYRYGMRNSGTEAKWNVMFQKYKDSPLAQEKDKLLYGLASVKNVHLLSKLLEATKDESVMRSQELFTVVRYVSLNPLG
KSMAWDWTTLNWDYLVQRSV
QCNASGKVEGGKNFTYLNLSNGLYQYQK
>g1027.t1
MGLDSLVNASGEVVPYGGKSAFTQLEHNIVASYLITAGIISLASNIVVLLMFVKFKELRTATNAIIINLAFTDIGVAGIG
YPLSAASDLHGSWKFGYTGC
QIYAALNIFFGMASIGLLTVVAIDRYLSICRPDIGQKMTVRSYTLLIVAAWLNAVFWSSMPVVGWSGYAPDPTGATCTIN
WRQNDPSFVSYTMAVIAVNF
VAPLSVMFYCYYNVSVTMKRYKASNCLENLAMDWSDQMDVTKVKRQLPAWL
>g1027.t2
MGHSSGPPGEVVPYGGKSAFTQLEHNIVASYLITAGIISLASNIVVLLMFVKFKELRTATNAIIINLAFTDIGVAGIGYP
LSAASDLHGSWKFGYTGCQI
YAALNIFFGMASIGLLTVVAIDRYLSICRPDIGQKMTVRSYTLLIVAAWLNAVFWSSMPVVGWSGYAPDPTGATCTINWR
QNDPSFVSYTMAVIAVNFVA
PLSVMFYCYYNVSVTMKRYKASNCLENLAMDWSDQMDVTKVKRQLPAWL
>g1027.t3
MGHSSGPPGEVVPYGGKSAFTQLEHNIVASYLITAGIISLASNIVVLLMFVKFKELRTATNAIIINLAFTDIGVAGIGYP
LSAASDLHGSWKFGYTGCQI
YAALNIFFGMASIGLLTVVAIDRYLSICQKMTVRSYTLLIVAAWLNAVFWSSMPVVGWSGYAPDPTGATCTINWRQNDPS
FVSYTMAVIAVNFVAPLSVM
FYCYYNVSVTMKRYKASNCLENLAMDWSDQMDVTKVKRQLPAWL
>g1028.t1
MSGLLKRAATRVAPALRATMITQRATINSQPAQEKIGFTETAIGLGMLSLGILGPSGWILVHLEDYKKRE
>g1029.t1
MLLLCLLPPTSCTTCPQKCRCEDLQFYCDTQGLQAPPDGVDKGALGLSLRHNSISELSPDQFYGFSQLTWLHLDHNQITT
VQEDAFQGLYKLKDLNLSSN
RITKLPNTTFIHLINLQILDLSFNQMTALEPELFHGLRKLQILHLRSNSLRTTPVRAFWDCRSLEYLGLSNNRLRSLARN
GFAGLIKLRELHLEHNQLTK
INLAHFPRLVALQSLHLQWNKINNLTCGMEWTWTTLEKLDLTGNEIRVLTPDVFETLPNLKVLLLDNNKLSSLDPLVMDM
WQSLGTIGLSSNIWECTKRI
CSLATWLSTFKGRWEHSILCHSPEYAQGEEILDAVYGFQLCQNFSAPVVQTISTTTEATTTPEVTSSLFGIMQQTATQDY
AEDFGSFTTFTTTTTTTKTQR
TALATTTTTMEEAAVTDDFETDNTVLTQRVIIGTIALLFSFFLIIFIVYISRKCCPPTLRRIRHCSAIQNRRQMRNQQRQ
PMADLATQVPYNEYEPSHEE
GALVIINGYGQCKCQQLPYKECEV
>g1030.t1
AGQERFRAVTRSYYRGAAGALMVYDITRRSTYNHLSSWLTDARNLTNPNTVIILIGNKADLEAQRDVTYEEAKQFAEENG
ENVEEAFLEAAKRIYQNIQD
GSLDLNAAEGENVEEAFLEAAKRIYQNIQDGSLDLNAAESGVQHKPSVPQGGRLNSDTQPAKEGCSC
>g1030.t2
AGQERFRAVTRSYYRGAAGALMVYDITRRSTYNHLSSWLTDARNLTNPNTVIILIGNKADLEAQRDVTYEEAKQFAEENG
LLFLEASAKTGENVEEAFLE
AAKRIYQNIQDGSLDLNAAEGENVEEAFLEAAKRIYQNIQDGSLDLNAAESGVQHKPSVPQGGRLNSDTQPAKEGCSC
>g1031.t1
MSVYMTLCADVRALNIPLCSFTRAPSRLLRSKLSQSGASLENRVPTPASAASADLHSQHAVADLPVQENKAEIRHIKQEF
EPVSIKIEPLKTEMDDSGAL
LIKKEKVTEKQEPMEVEKKPEMKVDPKDEEDRGAAANTSSSSSSSSPSQPRRKIFKPEELRQALMPTLESLYRQDPESLP
FRQPVDPMQLGIPDYFDIVK
TPMDLSTVKRKLDTGQYQEPWQYVDDVWLMFNNAWLYNRKTSRVYKYCSKLAEVFEADI
>g1031.t2
MSGVWCPAPPCMSVYMTLCADVRALNIPLCSFTRAPSRLLRSKLSQSGASLENRVPTPASAASADLHSQHAVADLPVQEN
KAEIRHIKQEFEPVSIKIEP
LKTEMDDSGALLIKKEKVTEKQEPMEVEKKPEMKVDPKDEEDRGAAANTSSSSSSSSPSQPRRKIFKPEELRQALMPTLE
SLYRQDPESLPFRQPVDPMQ

LGIPDYFDIVKTPMDLSTVKRKLDTGQYQEPWQYVDDVWLMFNNAWLYNRKTSRVYKYCSKLAEVFEADI
>g1031.t3
MSVYMTLCADVRALNIPLCSFTRAPSRLLRSKLSQSGASLENRVPTPASAASADLHSQHAVADLPVQENKAEIRHIKQEF
EPVSIKIEPLKTEECVSKSN
KDAHRTKQMDDSGALLIKKEKVTEKQEPMEVEKKPEMKVDPKDEEDRGAAANTSSSSSSSSPSQPRRKIFKPEELRQALM
PTLESLYRQDPESLPFRQPV
DPMQLGIPDYFDIVKTPMDLSTVKRKLDTGQYQEPWQYVDDVWLMFNNAWLYNRKTSRVYKYCSKLAEVFEADI
>g1032.t1
VNECETGEHQCTESQMCVNIHGRYQCVDTNRCQEPYVQLSDNRCICPGTKPVCRDLPYSIVNRYMSITSERSVPSDIFQI
QATSLSSGAYNTFRIRSGDD
NSDFYIR
>g1033.t1
MLLDHLSTDSSDRGLVQFNTSDIHNTTTLLLSNDGSTLYVGARNAILSLDVSDPDVINLKEKRDWSPSPEDTRACRDKQK
NEKVDCPNFIHVLQPKNSTH
LYACGSYAYNPQDAYLPSRCSSDSSSLPPQDQQTLALTPISMAKGRCPFNPFERNTAITVDGELFTGTTEDFKGTQPVIS
RHFTRGGGPSVRQEASNTLL
NEPTFISSSLDQSEGKLYFFFTEVLEEFPYIVEHKGARVAQVCKNDVGGQRTLQKKWTSFTKSLLLCQPPKQQPFDILLD
MFTLEPPEGDSSKETLFYGV
FTSQWSLGSESSAVCAFKMQNIRDVFSGYYRTFDPGSHQWSTPGTKRTAQGKCGLGNATDVDLEVVKKSFLSHANVQAEG
EAPLMVSRGPNGVRYSRLVA
VTTQAANQKEYTVLFLLTEKGFLHKVVLLDGGPWVIEEVQVFKEPQLVKSLLLSSFKGVVYVGWSGGVTAVPLAHCSSYQ
SCGQCVLARDPFCSWDPSQQ
ACTKHHTLQTAQDVENGNVTKQCPPPSSSLTPHTVQIALNQVLTLPCARPSDLATLSWTSGRHRLPLPQNLFLQRPDGGL
TFPAVATTLGDYTCTSHEAG
YHEVQAVFAVTTPSPSIEWVGTGSDLLDQVRHTTVRHTTAKPLSTGAAPTDHPADPPADHPTDHPAGRDPDQTQPGCTGR
LTVPTEASRPEKEVFRQKSY
YTEMVVVSLLLALCVSVMLLSLAYRWSQRGPALPPKAQGSLGEGDQESLENDSILKQEPAAPEVKMVV
>g1033.t2
MLLDHLSTDSSDRGLVQFNTSDIHNTTTLLLSNDGSTLYVGARNAILSLDVSDPDVINLKEKRDWSPSPEDTRACRDKQK
NEKVDCPNFIHVLQPKNSTH
LYACGSYAYNPQDAYLPSRCSSDSSSLPPQDQQTLALTPISMAKGRCPFNPFERNTAITVDGELFTGTTEDFKGTQPVIS
RHFTRGGGPSVRQEASNTLL
NEPTFISSSLDQSEGKLYFFFTEVLEEFPYIVEHKGARVAQVCKNDVGGQRTLQKKWTSFTKSLLLCQPPKQQPFDILLD
MFTLEPPEGDSSKETLFYGV
FTSQWSLGSESSAVCAFKMQNIRDVFSGYYRTFDPGSHQWSTPGTKRTAQGKCGLGNATDVDLEVVKKSFLSHANVQAEG
EAPLMVSRGPNGVRYSRLVA
VTTQAANQKEYTVLFLLTEKGFLHKVVLLDGGPWVIEEVQVFKEPQLVKSLLLSSFKGVVYVGWSGGVTAVPLAHCSSYQ
SCGQCVLARDPFCSWDPSQQ
ACTKHHTLQTAQDVENGNVTKQCPPPSSSLTPHTGSHLLKHTPL
>g1033.t3
MLLDHLSTDSSDRGLVQFNTSDIHNTTTLLLSNDGSTLYVGARNAILSLDVSDPDVINLKEKRDWSPSPEDTRACRDKQK
NEKVDCPNFIHVLQPKNSTH
LYACGSYAYNPQDAYLPSRCSSDSSSLPPQDQQTLALTPISMAKGRCPFNPFERNTAITVDGELFTGTTEDFKGTQPVIS
RHFTRGGGPSVRQEASNTLL
NEPTFISSSLDQSEGKLYFFFTEVLEEFPYIVEHKGARVAQVCKNDVGGQRTLQKKWTSFTKSLLLCQPPKQQPFDILLD
MFTLEPPEGDSSKETLFYGV
FTSQWSLGSESSAVCAFKMQNIRDVFSGYYRTFDPGSHQWSTPGTKRTAQGKCGLGNATDVDLEVVKKSFLSHANVQAEG
EAPLMVSRGPNGVRYSRLVA
VTTQAANQKEYTVLFLLTGGSMVWISEKGFLHKVVLLDGGPWVIEEVQVFKEPQLVKSLLLSSFKGVVYVGWSGGVTAVP
LAHCSSYQSCGQCVLARDPF
CSWDPSQQACTKHHTLQTAQDVENGNVTKQCPPPSSSLTPHTVQIALNQVLTLPCARPSDLATLSWTSGRHRLPLPQNLF
LQRPDGGLTFPAVATTLGDY
TCTSHEAGYHEVQAVFAVTTPSPSIEWVGTGSDLLDQVRHTTVRHTTAKPLSTGAAPTDHPADPPADHPTDHPAGRDPDQ
TQPGCTGRLTVPTEASRPEK
EVFRQKSYYTEMVVVSLLLALCVSVMLLSLAYRWSQRGPALPPKAQGSLGEGDQESLENDSILKQEPAAPEVKMVV
>g1034.t1

MGLTKQYLRYVSSAVFGVIGSQKANIAYVTLRGGEKGRHVAVAACEHVFIWDVRKGEK
>g1035.t1
LSSVTMDPLGARSRFVDIQTLPTWEQQKPEPELQKQEEYDDNMVSQQDFSSPFPFRPDINRKIVLL
>g1036.t1
VDNSEYMRNGDFLPTRLQAQQDAVNIVCHSKTRSNPENNVGLITMANNCEVLTTLTPDTGRILSKLHAVQPRGNICFCTG
IRVAHLALKHRQGKNHKMRI
IAFVGSPVEDNEKDVSLESCV
>g1036.t2
VDNSEYMRNGDFLPTRLQAQQDAVNIVCHSKTRSNPENNVGLITMANNCEVLTTLTPDTGRILSKLHAVQPRGNICFCTG
IRVAHLALKHRQGKNHKMRI
IAFVGSPVEDNEKD
>g1037.t1
TFYEERNFQGRSYECMSDCSDMSSYMSRCQSCRVDSGCFMLYERNNYMGQQFFMRRGEYNDMQRMQSMGMMFDNIRSCRM
IPFHRGQFRMKIFERENFGG
QSYELMDDCDNIMDRYRMSDCQSCNVMDGHWLMYEQPQYRGRMMYMRPGEYRSFREMMSGQRFMSMRRITDMC
>g1038.t1
MGTVMQSLGLFRYKCTGLTQITFYEERNFQGRSYECMSDCSDMSSYMSRCQSCRVDSGCFMLYERNNYMGQQFFMRRGEY
NDMQRMQSMGMMFDNIRSCR
MIPFHRGQFRMKIFERENFGGQSYELMDDCDNIMDRYRMSDCQSCNVMDGHWLMYEQPQYRGRMMYMRPGEYRSFREMMS
GQRFMSMRRITDMC
>g1038.t2
MGKITFYEERNFQGRSYECMSDCSDMSSYMSRCQSCRVDSGCFMLYERNNYMGQQFFMRRGEYNDMQRMQSMGMMFDNIR
SCRMIPFHRGQFRMKIFERE
NFGGQSYELMDDCDNIMDRYRMSDCQSCNVMDGHWLMYEQPQYRGRMMYMRPGEYRSFREMMSGQRFMSMRRITDMC
>g1039.t1
VSVHHGAAVLLFLRLSLRSLEPPAVTRVRVYLHLSSHDHDDPGSLDDAAACRCLTLMQMRPRARRAHEYNVASFRPRKTC
CRFNWLPERRALHQPTHSQL
TQKGSLFSLRSPSPFPEMLTAGGVALITGGDDELPATVVLGDAQVGAGCRGRSGND
>g1039.t2
VSVHHGAAVLLFLRLSLRSLEPPAVTRVRVYLHLSSHDHDDPGSLDDAAACRCLTLMQMRPRARRAHEYNVASFRPRKTC
CRFNWLPERRALHQPTHSQL
TQKGSLFSLRSPSPFPEMLTAGGVALITGGDDELPATVVLGDAQGMTE
>g1039.t3
VSVHHGAAVLLFLRLSLRSLEPPAVTRVRVYLHLSSHDHDDPGSLDDAAACRCLTLMQMRPRARRAHEYNVASFRPRKTC
CRFNWLPERRALHQPTHSQL
TQKGSLFSLRSPSPFPEMLTAGGVALITGGDDELPATVVLGDAQASGSSSPG
>g1040.t1
TVLVCSLDSKETVVRSMGASALWALLHNSQKAKTNLKSPSVLLRLERAHALAKKVGLNVEYHPPPTVLESLRFYPDNAGD
SKQEVKCSVTRYNLSSMLLS
WRLYPRTTGTSWYSASWRPCSK
>g1040.t2
TVLVCSLDSKETVVRSMGASALWALLHNSQKAKTNLKSPSVLLRLERAHALAKKGSEVLSHPLQPLINAAFMASVPKDHW
YFVVLCIVAPLLK
>g1041.t1
MSAAVARSGGRAGSLFPNCKIVFLALQSFVFLPCRPDTPTLLPLSADAARQGTRSPLRSSAWCPLKPIVSNHSTDVADCD
NYYYYYYYYYYYYYYY
>g1042.t1
MARTKQTARKSTGGKAPRKQLATKAARKSAPATGGVKKPHRYRPGTVALREIRRYQKSTELLIRKLPFQRLVREIAQDFK
TDLRFQSSAVMALQEASEAY
LVGLFEDTNLCAIHAKRVTIMPKDIQLARRIRGERA
>g1043.t1
MSGRGKGGKGLGKGGAKRHRKVLRDNIQGITKPAIRRLARRGGVKRISGLIYEETRGVLKVFLENVIRDAVTYTEHAKRK
TVTAMDVVYALKRQGRTLYG
FGG
>g1044.t1
MLVVNERIDREQLCGDNTPCSFSFEIILENPMELHRIVVEVLDVNDHSPVFPAKDINLDINGGSPPLSSNVSVKILIQDQ

NDNAPQVLYPVQTGGSLVAE
MVPRSADVGYLVTKVVAVDVDSGQNAWLSYKLQKATDRALFEVGLQNGEIRTIRQVTDKDAVKQRLTVIVEDNGQPSRSA
TVIVNVAVADSFPEVLSEFT
DFTHDKEYNDNLTFYLVLALAVVSFSSSRV
>g1045.t1
MNVRLSSVLLFPSGTPLSSPQPADFLLHEQAGGRPDRPLASRIYRHNCPAESCYCCSLRAETSSQLVAGVMQRLSVHCHN
ARRRALTPGANGPSRKCVLP
RRRRRCTGHGWVEWCNGRPLESHAPLQSPNPSLPAELRGIFWPPPNTEAKHAHSLMSLAAVLFLTGQRHGSI
>g1046.t1
MDSAGLRNTDRGPARRADARRAGEQKEMEEQQSGQQNTKEPRAQREAARPFIEKKREESRRAHRTARRPRPSGVSSSSSS
CSFSPSSSSCSSPSPSPSSS
STPGPWCSGTTRPELFRMDCADQSRSLLCAVLYGP
>g1047.t1
MGQQVGRVGESTSTALQPPQPTHAPQPHQAKGNRGRRPREVGGGTAGTPPGRTAAVNVPDPGVNIFTKHSDLLKSHSGIT
RDAEDRVPAPCPWGGSL
>g1048.t1
SELTAPSRLDVPPVYSAHPRRSACVRAAPARPMAAPPLAGRSSEYQSSFSRAPPHVATTTGPPVSQTRPSDVRLAVFAVP
RMYATESDNYGSGKPRVV
>g1049.t1
MFRLCDARVVKDMTTGKSKGYGFVSFYNKLDAENAIGQMGGQWLGGRQIRTNWATRKPPAPKSIPDNGSKQLRFEDVVTQ
SSPQNCTVYCGGIQSGLTGN
YP
>g1049.t2
MRQTFSPFGQIMEIRVFPEKGYSFIRFSSHESAAHAIVSVNCTAIEGHLVKCYWGKESPDMAKSPQQ
>g1049.t3
MFRLCDARVVKDMTTGKSKGYGFVSFYNKLDAENAIGQMGGQWLGGRQIRTNWATRKPPAPKSIPDNGSKQLRFEDVVTQ
SSPQNCTVYCGGIQSGLTGN
YPXFSSHESAAHAIVSVNCTAIEGHLVKCYWGKESPDMAKSPQQ
>g1049.t4
MFRLCDARVVKDMTTGKSKGYGFVSFYNKLDAENAIGQMGGQWLGGRQIRTNWATRKPPAPKSIPDKHLMRQTFSPFGQI
MEIRVFPEKGYSFIRFSSHE
SAAHAIVSVNCTAIEGHLVKCYWGKESPDMAKSPQQ
>g1050.t1
MMRRALWWMRRSRRWPRRSVKMEEDERPVALEVSQQTLKGPNVPPDTSPQEAQKTFVREMKHPTLRQGYIPIPIFHEGAE
LRQLQQQHQHPQQQQQPPFS
YTQPSAAQNQSVCADGRTPSPTPGLHCRPRSPLHGPPDCCSSPEPTKPCSPTSHTTEVYSAPFQQPQRRSSAGLQAGYIP
IPVIHEGSGSQAPSQSAQLN
PTVYSQRLPYSEHQQPFHRPQPDERTSHCGSMQHPRHMDAAPIHHPQHLRSQSPAVTQVMGERPQVQQHISREVPVKIEQ
ETFASQQKPDKPQSPQSFHM
EIDNEPFPPPPPPVHFHEPTPQQWQPAQQAQYAPPFPQQQSPPPQEFPK
>g1050.t2
MMRRALWWMRRSRRWPRRSVKMEEDERPVALEVSQQTLKGPNVPPDTSPQEAQKTFVREMKHPTLRQGYIPIPIFHEGAE
LRQLQQQHQHPQQQQQPPFS
YTQPSAAQNQSVCADGRTPSPTPGLHCRPRSPLHGPPDCCSSPEPTKPCSPTSHTTEVYSAPFQQPQRRSSAGLQAGYIP
IPVIHEGSGSQAPSQSAQLN
PTVYSQRLPYSEHQQPFHRPQPDERTSHCGSMQHPRHMDAAPIHHPQHLRSQSPAVTQVMGERPQVQQHISREVPVKIEQ
ETFASQQKPDKPQSPQSFHM
EIDNEPFPPPPPPVHFHEPTPQQWQPAQQA
>g1050.t3
MRRSRRWPRRSVKMEEDERPVALEVSQQTLKGPNVPPDTSPQEAQKTFVREMKHPTLRQGYIPIPIFHEGAELRQLQQQH
QHPQQQQQPPFSYTQPSAAQ
NQSVCADGRTPSPTPGLHCRPRSPLHGPPDCCSSPEPTKPCSPTSHTTEVYSAPFQQPQRRSSAGLQAGYIPIPVIHEGS
GSQAPSQSAQLNPTVYSQRL
PYSEHQQPFHRPQPDERTSHCGSMQHPRHMDAAPIHHPQHLRSQSPAVTQVMGERPQVQQHISREVPVKIEQETFASQQK
PDKPQSPQSFHMEIDNEPFP
PPPPPVHFHEPTPQQWQPAQQAQYAPPFPQQQSPPPQEFPK

>g1051.t1
MGKGFYLSKNLALAGLLLAAVALVTIIVLSVVYDRERRNSSCFQDGLPSSGASSTSFSATSSSSSSTPSPGPLEPWQRYR
LPGDLAPVSYNITLWPRLEP
DGQGLFIFTGNSTVRFVCVKDTDLILIHTNKLNFTLFQGHEARLLALGGTPPAPAIRNAWVEVLTQYLVIQLEGRLEAGR
SYLLHTEFQGELADDLGGFY
RSTYKENGQEKIVATTQMQPTDARKAFPCFDEPAMKAVFHLTMIHPPKTVALSNAMNYDPVNVTVSGQALLETRFQPTEL
MSTYLLAFVVCEFGFIRSTV
DSPVLIRIWARMQAIEEGQGDYALEKTGPILNYFQNYYSSPYPLQKSDQIALPDFSAGAMENWGLITYRETALLYNPAFS
SNEDKEWVVTVISHELAHMW
FGNLVTMRWWNDLWLNEGFATYVSYLGAAAVEPTWNIKDLMVVNEVHGVMGVDALTISHPLSFAEDDIQQPGHIRRLFDS
ITYSKGAAVLRMLSDFITEG
VFTQGLR
>g1051.t2
MGKGFYLSKNLALAGLLLAAVALVTIIVLSVVYDRERRNSSCFQDGLPSSGASSTSFSATSSSSSSTPSPGPLEPWQRYR
LPGDLAPVSYNITLWPRLEP
DGQGLFIFTGNSTVRFVCVKDTDLILIHTNKLNFTLFQGHEARLLALGGTPPAPAIRNAWVEVLTQYLVIQLEGRLEAGR
SYLLHTEFQGELADDLGGFY
RSTYKENGQEKIVATTQMQPTDARKAFPCFDEPAMKAVFHLTMIHPPKTVALSNAMNYDPVNVTVSGQALLETRFQPTEL
MSTYLLAFVVCEFGFIRSTV
DSPVLIRIWARMQAIEEGQGDYALEKTGPILNYFQNYYSSPYPLQKSDQIALPDFSAGAMENWGLITYRETALLYNPAFS
SNEDKEWVVTVISHELAHMW
FGNLVTMRWWNDLWLNEGFATYVSYLGAAAVEPTWNIKDLMVVNEVHGVMGVDALTISHPLSFAEDDIQQPGHIRRLFDS
ITYSKVA
>g1052.t1
MAQAVFSRGGELLTWTGDIVGRWKEHFEELLNPTNMSSVEEAESEDSGEASSISLAEVSEAVKKLLSGKAPGVEEIRPEM
LKALDIVGLSWLARLFSVAW
RSGT
>g1053.t1
LVLGTWNVTSLAGKEPELVQEERYQLDIVVHSTGSGTKLLERGWTISFSLFRSCPGREAPGGCGDTHKPRAERR
>g1054.t1
LNLFYLANDVIQNCKRKNAIAFRSAFAEVLPNAAYLIKDGKVRKSVERIFTIWEERSVYSEELIAQFKAGLHRKDRERER
EKERLKEKEKEKEKEKEKEK
EKEKVKEKKEKEAQAPPALANPKAALKSKIVAEFTPSSFIDQLSVHKRAIEEEALREKQLEALRVDVCSTEALKRLKDKA
GGNKFATDFEEGSLKLQEFV
SFLEEELKTGPPLQDALGNADIFYEMQYKEVKIVANAYNAFASRVASLKRKLDALKSSLPGPEDSPIPSPSEDAPSPTGS
DSPFLAVGASRAPLDPELDG
KAMDEGDLVRDNVDPEDMDMSDEEGVSGMSVDEKTEKASAAVSKTTKASVPSKLNLPDATTKSPKTTPPATAPAPPTPLT
PTTTAGQSTPTSPLGVNLAN
VDLGKISSILSSITSAMKNTTSPSSRPSPGTPTTPSGQSTASKAAPPSPALASILSRVDITPEGILNALSKTNTP
>g1054.t2
MTACQEHSDIESETDTNHRLNLFYLANDVIQNCKRKNAIAFRSAFAEVLPNAAYLIKDGKVRKSVERIFTIWEERSVYSE
ELIAQFKAGLHRKDRERERE
KERLKEKEKEKEKEKEKEKEKEKVKEKKEKEAQAPPALANPKAALKSKIVAEFTPSSFIDQLSVHKRAIEEEALREKQLE
ALRVDVCSTEALKRLKDKAG
GNKFATDFEEGSLKLQEFVSFLEEELKTGPPLQDALGNADIFYEMQYKEVKIVANAYNAFASRVASLKRKLDALKSSLPG
PEDSPIPSPSEDAPSPTGSD
SPFLAVGASRAPLDPELDGKAMDEGDLVRDNVDPEDMDMSDEEGVSGMSVDEKTEKASAAVSKTTKASVPSKLNLPDATT
KSPKTTPPATAPAPPTPLTP
TTTAGQSTPTSPLGVNLANVDLGKISSILSSITSAMKNTTSPSSRPSPGTPTTPSGQSTASKAAPPSPALASILSRVDIT
PEGILNALSKTNTP
>g1054.t3
MTACQEHSDIETDTNHRLNLFYLANDVIQNCKRKNAIAFRSAFAEVLPNAAYLIKDGKVRKSVERIFTIWEERSVYSEEL
IAQFKAGLHRKDRERERERKE
RLKEKEKEKEKEKEKEKEKEKVKEKKEKEAQAPPALANPKAALKSKIVAEFTPSSFIDQLSVHKRAIEEEALREKQLEAL
RVDVCSTEALKRLKDKAGGN
KFATDFEEGSLKLQEFVSFLEEELKTGPPLQDALGNADIFYEMQYKEVKIVANAYNAFASRVASLKRKLDALKSSLPGPE

DSPIPSPSEDAPSPTGSDSP
FLAVGASRAPLDPELDGKAMDEGDLVRDNVDPEDMDMSDEEGVSGMSVDEKTEKASAAVSKTTKASVPSKLNLPDATTKS
PKTTPPATAPAPPTPLTPTT
TAGQSTPTSPLGVNLANVDLGKISSILSSITSAMKNTTSPSSRPSPGTPTTPSGQSTASKAAPPSPALASILSRVDITPE
GILNALSKTNTP
>g1055.t1
YLMKSYRQMQAGRQLSGAVSYLNRVQHGMVQPQAVSSQLDLLHLNTLVDLYELRAGSLVELAAQSIQQEFERGKSQEDSL
VELAAQSIQQEFERGKSQED
SWNASTIDLVRASDVSSRLLRREGVSLQHPSKAHCHYMVLKMFRDKVDQIEDPGVHSVLCTLARLYAIHGIGNHAGDFLQ
AGVLSGAQVLLVSGHLKDLL
AEVRPSAVLLVDAFDHHDKMLNSVLGRYDGNVYENMFEWARSSPLNRTEVHESFHKHLKPLQSKL
>g1055.t2
YLMKSYRQMQAGRQLSGAVSYLNRVQHGMVQPQAVSSQLDLLHLNTLVDLYELRAGSLVELAAQSIQQEFERGKSQEDSW
NASTIDLVRASDVSSRLLRR
EGVSLQHPSKAHCHYMVLKMFRDKVDQIEDPGVHSVLCTLARLYAIHGIGNHAGDFLQAGVLSGAQVLLVSGHLKDLLAE
VRPSAVLLVDAFDHHDKMLN
SVLGRYDGNVYENMFEWARSSPLNRTEVHESFHKHLKPLQSKL
>g1055.t3
YLMKSYRQMQAGRQLSGAVSYLNRVQHGMVQPQAVSSQLDLLHLNTLVDLYELRAGSLVELAAQSIQQEFERGKSQEDSL
VELAAQSIQQEFERGKSQED
SWNASTIDLVRASDVSSRLLRREGVSLQHPSKAHCHYMVLKMFRDKVDQIEDPGVHSVLCTLARLYAIHGIGNHAGDFLQ
AGVLSGAQVLLVSGHLKDLL
AEVRPSAVLLVDAFDHHDKMLNSVLGRYDGNVYENMFEWARSSPLNRTEVHESFHKHLKPLQSKLSRGSWEEDASSERSA
GYVCLEVFEAETTGCACPGV
HGRGDGVAGSPMFEKVHGNLKAFCLEVV
>g1056.t1
MAKMGFEKCPQEHQRILDGGLSSEKVTQFPVDPDCRGMMSFISCCLTAPAKTSEAPVGAQ
>g1056.t2
MWMKQHGHSNGRPPVLAGSTFWECSRMAKMGFEKCPQEHQRILDGGLSSEKVTQFPVDPDCRGMMSFISCCLTAPAKTSE
APVGAQ
>g1057.t1
MRQVCHHHHYRLVALTAIMRKCFEMLIKESITFRLPPTFDPFQFAYRPNRSTRDTISSTLYLSLAHLEQNNTHVQMLFLH
FSSAHNTIIPQHLVDKLGPL
GFSIPLCNWLLDFLTERPQSVRVRQNTSCVITLSTGAPQGCVLSPLLFTLMTHNCIPRAATKGREGGAAVKVISSTKFQG
VHTTDDLSWTQSEHKVTGHE
GTSCAE
>g1057.t2
MRKCFEMLIKESITFRLPPTFDPFQFAYRPNRSTRDTISSTLYLSLAHLEQNNTHVQMLFLHFSSAHNTIIPQHLVDKLG
PLGFSIPLCNWLLDFLTERP
QSVRVRQNTSCVITLSTGAPQGCVLSPLLFTLMTHNCIPRAATKGREGGAAVKVISSTKFQGVHTTDDLSWTQSEHKVTG
HEGTSCAE
>g1058.t1
MEESAIVKYLCANFGSVNKEELVANCVFGDATTVSVVISNRERFAVGTYNGEKMLIAKTSLRLCRKRECPGCSCLHLCKT
YLYGECQFVPGRHGACSFSH
DLHSEHNARLLQTHDLAHLDKKELCVLLLQNDAPLLPPVCHDYNNGEGAHGRCNESCTRIHICENNLHRPCSCFRAHDFH
QPQPLQALQKSRIPAALMGA
MKPLYINKKVLASLEYRAKNKGGGAPPAGDAXXXXXXXXRRGPQSSWAKREERSVSRALCDPPLDVFFFFLDKTEICLFF
LKGHCKHETGNCYKAHLKLP
YSWEVQEGHQWTALASSEQVEKDFCDPKKTY
>g1058.t2
MEESAIVKYLCANFGSVNKEELVANCVFGDATTVSVVISNRERFAVGTYNGEKMLIAKTSLRLCRKRECPGCSCLHLCKT
YLYGECQFVPGRHGACSFSH
DLHSEHNARLLQTHDLAHLDKKELCVLLLQNDAPLLPPVCHDYNNGEGAHGRCNESCTRIHICENNLHRPCSCFRAHDFH
QPQPLQALQKSRIPAALMGA
MKPLYINKKVLASLEYRAKNKGGGAPPAGDAXXXXXXXXRRGPQSSWAKREERSVSRALCDPPLDVFFFFLDKTEICLFF
LKGHCKHETGNCYKAHLKLP

YSWEVQEGHQWTALASSEQVEKDFCDPKKTYRWAEGADRSRAPLPVLSPLLNLWSQPS
>g1058.t3
MEESAIVKYLCANFGSVNKEELVANCVFGDATTVSVVISNRERFAVGTYNGEKMLIAKTSLRLCRKRECPGCSCLHLCKT
YLYGECQFVPGRHGACSFSH
DLHSEHNARLLQTHDLAHLDKKELCVLLLQNDAPLLPPVCHDYNNGEGAHGRCNESCTRIHICENNLHRPCSCFRAHDFH
QPQPLQALQKSRIPAALMGA
MKPLYINKKVLASLEYRAKNKGGGAPPAGDAXXXXXXXXRRGPQSSWAKREERSVSRALCDPPLDVFFFFLDKTEICLFF
LKGHCKHETGNCYKAHLKLP
YSWEVQEGHQWTALASSEQVEKDFCDPKKTYRGSEAD
>g1059.t1
MSMNDECQHSSLSSAEKLHRLQRRCCRSARRVGQGALAEPRGDTRPRRSREPGSPQNVHGPRCRREGFEPSTMSFCCPVV
QERSSFSPSASPQPNSTSSP
VHAPAARPASRMEEDAARLPTHLRK
>g1059.t2
MNDECQHSSLSSAEKLHRLQRRCCRSARRVGQGALAEPRGDTRPRRSREPGSPQNVHGPRCRREGFEPSTMSFCCPVVQE
RSSFSPSASPQPNSTSSPVH
APAARPASRMEEDAARLPTHLRK
>g1060.t1
MLLLNMMVDPDRSPPIGVLTPVPSQDAGNATLEAMGATAVPYYLDGQQGWRPQIEELRRALRSAGEACNPAALYVINPGD
PTGMVQLSVALFYEPAEIRT
AKEVEKIAQ
>g1061.t1
MALSSGGWAASPVPASSQAMHGNEALRLQLSMDPSRAGEFRLALRDTSGNRSVFITEFDLRSVQYEVKSPRSHEMRLATP
PHDCIRFNFRCDREAEEWAT
VVMSSLREAHRVANITAHELDDRRLHTAAAIEQWSTASLPLTEELCLELTRAIEAGDTQSASQHASSLARQKASLSIQLS
EKSYANGEISLSVAVEDMTS
SCCVTVKVLPHMTVAALKQQVFVEYGFHPRVQRWVIGQCLCTEPRTLASYGVQKDGDTAYLYLVSARQARTTRQMLQQDQ
ESAALPG
>g1061.t2
MDPSRAGEFRLALRDTSGNRSVFITEFDLRSVQYEVKSPRSHEMRLATPPHDCIRFNFRCDREAEEWATVVMSSLREAHR
VANITAHELDDRRLHTAAAI
EQWSTASLPLTEELCLELTRAIEAGDTQSASQHASSLARQKASLSIQLSEKSYANGEISLSVAVEDMTSSCCVTVKVLPH
MTVAALKQQVFVEYGFHPRV
QRWVIGQCLCTEPRTLASYGVQKDGDTAYLYLVSARQARTTRQMLQQDQESAALPG
>g1062.t1
FWKATSASCSSPLLVLVNSKSGDNQGVKFLRKFKQLLNPAQVFDLMNGGPQLGLRLFQKFVTFRILVCGGDGSVGWVLSE
LDKLNLHKQCQLGVLPLGTG
NDLARVLGWGALCDDDAQLLQILEKLERATTKMLDRWSVLTYEVPGKSSPVVKDEDSPDFPLQVHITQYADSVASHLTKI
LDSDKHSDVISSAK
>g1062.t2
HNSCKDQMGKVCPLGQCRVSIISPTTLNSIDSDGFWKATSASCSSPLLVLVNSKSGDNQGVKFLRKFKQLLNPAQVFDLM
NGGPQLGLRLFQKFVTFRIL
VCGGDGSVGWVLSELDKLNLHKQCQLGVLPLGTGNDLARVLGWGALCDDDAQLLQILEKLERATTKMLDRWSVLTYEVPG
KSSPVVKDEDSPDFPLQVHI
TQYADSVASHLTKILDSDKHSDVISSAK
>g1062.t3
FWKATSASCSSPLLVLVNSKSGDNQGVKFLRKFKQLLNPAQVFDLMNGGPQLGLRLFQKFVTFRILVCGGDGSVGWVLSE
LDKLNLHKQVGAGQVWTGNH
RTRQVCGQCQLGVLPLGTGNDLARVLGWGALCDDDAQLLQILEKLERATTKMLDRWSVLTYEVPGKSSPVVKDEDSPDFP
LQVHITQYADSVASHLTKIL
DSDKHSDVISSAK
>g1063.t1
PTAQPGNPTSRHRVTTHTRVLIRNGTGSRAVAGLRGPARSQYPGPLGVTAQH
>g1064.t1
LIVVRAFRVAKLARITPNPMAFFLSPETTAPETTLVGLDLAATCGAFRNEVAGVDRATRFVRRIGSDDIACRFLLLRQAL
SFLLLGWKVGRGTALCASGV

VTLAGGSSRCACND
>g1065.t1
MALPCLLSMLQSFGLQTLLVVVVGLWWSSSSLEGSISFSVDGWSPTPEVEEGWRGGGGPQRVTMTPTATGAGDVSDGSGS
TRVIGAHLYPRSVRGPDWLT
DDGNLRHFRRWQVRQVEAMRCRRAWHRQLGVENAGSLDCRFKLNTHKMVFVMNSEDYMYRRGALVKARMSQHKVAVTQHE
RFDKWHQGWLANHVSPSAER
SVQRWLMGEDEEDMIPCKTMCVATELKGQAVNRYQVHYSGSKAPASP
>g1065.t2
MALPCLLSMLQSFGLQTLLVVVVGLWWSSSSLEGSISFSVDGWSPTPEVEEGWRGGGGPQRVTMTPTATGAGDVSDGSGS
TRVIGAHLYPRSVRGPDWLT
DDGNLRHFRRWQVRQVEAMRCRRAWHRQLGVENAGSLDCRFKLNTHKMVFVMNSEDYMYRRGALVKARMMHVTREQVNPY
LLSSDMQLSLRLACLQSQHK
VAVTQHERFDKWHQGWLANHVSPSAERSVQRWLMGEDEEDMIPCKTMCVATELKGQAVNRYQVHYSGSKAPASP
>g1066.t1
EELERAQRLEERERIIREIWRNGQPDILGTGTGTIGRVHYY
>g1067.t1
KSNGDTLPADQFDAVAPSPEKLSHPTAGRAKPPARRPPSGLVSPGAVSSEESELLSPSRKSPSVPKVSETVVPPAKPHPV
AKSLPRDPSLESLQADVKEL
RMALELLKTRQERKYSVCG
>g1067.t2
KSNGDTLPADQFDAVAPSPEKLSHPTAGRAKPPARRPPSGLVSPGAVSETVVPPAKPHPVAKSLPRDPSLESLQADVKEL
RMALELLKTRQERKYSVCG
>g1067.t3
KSNGDTLPADQFDAVAPSPEKLSHPTAGRAKPPARRPPSGLVSPGAVSSEESELLSPSRKSPSVPKVSETVVPPAKPHPV
AKSLPRDPSLESLQADVKEL
RMALELLKTRQEQDMKEVKEELNQERQKRMMLQEEVLSLRMKN
>g1068.t1
MDGEGTENREPGDAFVVVENEAESIPAQEKPSAINTLNLLDTCPSCHLNFQSREPKLLPCLHSFCKKCLPPPSRNLAVAD
PPTVQVEGPANQP
>g1069.t1
MDLTAESHTVLLSDGNRIPLLGLGTYADPRTTAKGTALECVKHAIDVGYRHLDGALVYFNEHEVGQAIREKIADGTVKRE
DIFYCGKLWSTFQPPELVRP
ALENTLKALKLDYVDLYIVELPMAFKPGKDFYPKDENGKYLYHPTDLCATWEALEACKDAGLVKSLGVSNFNYRQLELLF
NKPGLKHKPVSNQIECHPYF
TQPKMLEYCRQKGVVVVGYSPIGTSRDPSWVNLKSPPLLEDDVLVSIGRKYAKSSAQVALRFNVQRGVVVIPKSFSPQRI
HHNFQVRCVTRSVGPVADT
>g1069.t2
MDLTAESHTVLLSDGNRIPLLGLGTYADPRTTAKGTALECVKHAIDVGYRHLDGALVYFNEHEVGQAIREKIADGTVKRE
DIFYCGKLWSTFQPPELVRP
ALENTLKALKLDYVDLYIVELPMAFKPGKDFYPKDENGKYLYHPTDLCATWEALEACKDAGLVKSLGVSNFNYRQLELLF
NKPGLKHKPVSNQVKLTFNP
RLDTNHSLIECHPYFTQPKMLEYCRQKGVVVVGYSPIGTSRDPSWVNLKSPPLLEDDVLVSIGRKYAKSSAQVALRFNVQ
RGVVVIPKSFSPQRIHHNFQ
VRCVTRSVGPVADT
>g1069.t3
MDLTAESHTVLLSDGNRIPLLGLGTYADPRTTAKGTALECVKHAIDVGYRHLDGALVYFNEHEVGQAIREKIADGTVKRE
DIFYCGKLWSTFQPPELVRP
ALENTLKALKLDYVDLYIVELPMAFKPGKDFYPKDENGKYLYHPTDLCATWEALEACKDAGLVKSLGVSNFNYRQLELLF
NKPGLKHKPVSNQVKLTFNP
RLDTNHSLIECHPYFTQPKMLEYCRQKGVVVVGYSPIGTSRDPSWVNLKSPPLLEDDVLVSIGRKYAKSSAQVALRFNVQ
RGVVVIPKSFSPQRIHHNFQ
IFDFSLTEEEMNAIEMLNKNVRFVELLM
>g1070.t1
MPGSTASSKARVYTDVNTQKSREYWDYDAHVPSWSNQDNYQLVRKLGRGKYSEVFEAINVSNNEKVVVKILKPVKKKKIK
REIKILENLRGGTNIIRLAD
TVKDPVELYQKLTDFDIRYYMYELLKALDYCHSMGIMHRDVKPHNVMIDHQLRKLRLIDWGLAEFYHPAQEYNVRVASRY

FKGPELLVDYQMYDYSLDMW
SLGCMLASMIFLKEPFFHGQDNYDQLVRIAKVLGTDELFGYLHKYHIELDTRFKDLLGQQTRKRWEQFVQTENQHLVSPE
ALDLLDKLLRYDHQQRLTAA
EAMQHPYFCE
>g1070.t2
MPGSTASSKARVYTDVNTQKSREYWDYDAHVPSWSNQDNYQLVRKLGRGKYSEVFEAINVSNNEKVVVKILKPVKKKKIK
REIKILENLRGGTNIIRLAD
TVKDPVSRTPALVFEFINNTDFKELYQKLTDFDIRYYMYELLKALDYCHSMGIMHRDVKPHNVMIDHQLRKLRLIDWGLA
EFYHPAQEYNVRVASRYFKG
PELLVDYQMYDYSLDMWSLGCMLASMIFLKEPFFHGQDNYDQLVRIAKVLGTDELFGYLHKYHIELDTRFKDLLGQQTRK
RWEQFVQTENQHLVSPEALD
LLDKLLRYDHQQRLTAAEAMQHPYFCE
>g1070.t3
MPGSTASSKARVYTDVNTQKSREYWDYDAHVPSWSNQDNYQLVRKLGRGKYSEVFEAINVSNNEKVVVKILKPVKKKKIK
REIKILENLRGGTNIIRLAD
TVKDPVELYQKLTDFDIRYYMYELLKALDYCHSMGIMHRDVKPHNVMIDHQLRKLRLIDWGLAEFYHPAQEYNVRVASRY
FKGPELLVDYQLVRIAKVLG
TDELFGYLHKYHIELDTRFKDLLGQQTRKRWEQFVQTENQHLVSPEALDLLDKLLRYDHQQRLTAAEAMQHPYFCE
>g1071.t1
MRSGRMTEAWQQQHAVAPPSVVHTLPQASDNPLGCAVYGVVLQPDSSLQQPQHSQHGQQHSVPAQQPSLQVGGERGHKCG
ACGHDISHLANPHEHQCMVS
QDRSFQCTQCMKIFSQATDLLEHQCVQVEQKPFVCGVCKMGFSLLTSLAQHHNSHGNGNNPMKCSICEKTYRPGSGNVTP
TSSAANPQQPSTGETSASGA
AISASSPPAFEASAPDRPYKCSVCHKAFRHLSELTRHERVHTGEKPYKCDTCDKSFSQSSHLAHHQRTHSSERPYKCAVC
EKSFKHRSHLVRHMYAHSGE
HLFKCNLCEMHFKESSELLHHQCQPIGERPFRCGSCGKSFKRPSDLRQHERTHSEERPFQCEECQMSFKQQYALVRHRRT
HKNPADRPFKCNLCDKGFLQ
PSHLLYHQQVHGMESLFKCASCQKSFSQSGELLRHKCGGEVEKPYKCDVCGKGYKKNSTLQRHQNSHCTEKPLKCSLCDR
RFVSSSEFVQHRCDPTREKP
LKCPDCEKRFKYSSELQRHRRVHTGEKPFKCASCDKSFKQREHLAKHQSVHSRETQFKCVWCGERFVDLTALQEHTVQHT
AEGDSFPAPPCIP
>g1072.t1
YYDILEDRVPAGLVAEYHSLLARCSRLFQEFSSLRNNLTCDSDSELDNVSMVEGVKLYDQLEILKRKLCIIENPLLRYVL
GYKGNARQHCVQAKGARTSG
GRVVHVVMASTTAIQLQSLLNDKLLLLCSAEDTEIQFHSDPVLAVDACFEGDAVVVLPGHYSVTSSMLLPDSITIEGVRQ
GKLEMENCVLQCETTGVIVR
TSAHLTMNLCDLYGSKGAGIEIYPGSVCSLTGNGIHHCKEGVLIKDFADELDVMPRITLENNVIHNNEGYGVILVKPTSQ
TQDSNGSGPLESAMDDLVEK
DEGSEMADEKLQQQQQQEEGTSPPHPEFPSTDGLLSPTAAASTTPKTTSAVDDTGLQRHSQVETMELATAAAAAAAATAA
TGSTRKWQLCRQLSRNKDGG
ATQADAGLLEQKMFVSIQGNQFRRNGMGDFGTFLY
>g1072.t2
PPLNDVPLIPDRSYYDILEDRVPAGLVAEYHSLLARCSRLFQEFSSLRNNLTCDSDSELDNVSMVEGVKLYDQLEILKRK
LCIIENPLLRYVLGYKGNAR
QHCVQAKGARTSGGRVVHVVMASTTAIQLQSLLNDKLLLLCSAEDTEIQFHSDPVLAVDACFEGDAVVVLPGHYSVTSSM
LLPDSITIEGVRQGKLEMEN
CVLQCETTGVIVRTSAHLTMNLCDLYGSKGAGIEIYPGSVCSLTGNGIHHCKEGVLIKDFADELDVMPRITLENNVIHNN
EGYGVILVKPTSQTQDSNGS
GPLESAMDDLVEKDEGSEMADEKLQQQQQQEEGTSPPHPEFPSTDGLLSPTAAASTTPKTTSAVDDTGLQRHSQVETMEL
ATAAAAAAAATAATGSTRKW
QLCRQLSRNKDGGATQADAGLLEQKMFVSIQGNQFRRNGMGDFGTFLY
>g1072.t3
YYDILEDRVPAGLVAEYHSLLARCSRLFQEFSSLRNNLTCDSDSELDNVSMVEGVKLYDQLEILKRKLCIIENPLLRYVL
GYKGNARQHCVQAKGARTSG
GRVVHVVMASTTAIQLQSLLNDKLLLLCSAEDTEIQVGAQNGEQGSEYWSPSGCGRDFRVAEFHSDPVLAVDACFEGDAV
VVLPGHYSVTSSMLLPDSIT

IEGVRQGKLEMENCVLQCETTGVIVRTSAHLTMNLCDLYGSKGAGIEIYPGSVCSLTGNGIHHCKEGVLIKDFADELDVM
PRITLENNVIHNNEGYGVIL
VKPTSQTQDSNGSGPLESAMDDLVEKDEGSEMADEKLQQQQQQEEGTSPPHPEFPSTDGLLSPTAAASTTPKTTSAVDDT
GLQRHSQVETMELATAAAAA
AAATAATGSTRKWQLCRQLSRNKDGGATQADAGLLEQKMFVSIQGNQFRRNGMGDFGTFLY
>g1073.t1
MVSLIGAMVVYWVLMSNFLYNTGKFVYNYNHNLSNPDFGSNTSVICPFQVHNSTLDGHLGSSNDTSDSLFHFWWNKTNTI
PFYLIILLLPLLNLRSASFF
ARFTFLGTISVMYLIFLVTLKAFQLGFHLEFHWFHSTQFFVAEFRTLFPQLTGVLTLAFFIHNCIITLMKNNRHQENNIR
DLSVAYLLVGLTYMYVGVLI
FASFPSPPFTKDCIQPNFLDNFPSSDVLVFVARACLLFQMTSVYPLLGYLARVQIMGYLFGNNYPSFFHVIALNVVIVAA
GVLMARFYPDIGSIIRYSGA
TCGLALVFVFPSLVHMISLKRQGELKWPSAIFHSFLILLGVANLIAQFFM
>g1073.t2
LSSLVFSMVSLIGAMVVYWVLMSNFLYNTGKFVYNYNHNLSNPDFGSNTSVICPFQVHNSTLDGHLGSSNDTSDSLFHFW
WNKTNTIPFYLIILLLPLLN
LRSASFFARFTFLGTISVMYLIFLVTLKAFQLGFHLEFHWFHSTQFFVAEFRTLFPQLTGVLTLAFFIHNCIITLMKNNR
HQENNIRDLSVAYLLVGLTY
MYVGVLIFASFPSPPFTKDCIQPNFLDNFPSSDVLVFVARACLLFQMTSVYPLLGYLARVQIMGYLFGNNYPSFFHVIAL
NVVIVAAGVLMARFYPDIGS
IIRYSGATCGLALVFVFPSLVHMISLKRQGELKWPSAIFHSFLILLGVANLIAQFFM
>g1073.t3
LVFSMVSLIGAMVVYWVLMSNFLYNTGKFVYNYNHNLSNPDFGSNTSVICPFQVHNSTLDGHLGSSNDTSDSLFHFWWNK
TNTIPFYLIILLLPLLNLRS
ASFFARFTFLGTISVMYLIFLVTLKAFQLGFHLEFHWFHSTQFFVAEFRTLFPQLTGVLTLAFFIHNCIITLMKNNRHQE
NNIRDLSVAYLLVGLTYMYV
GVLIFASFPSPPFTKDCIQPNFLDNFPSSDVLVFVARACLLFQMTSVYPLLGYLARVQIMGYLFGNNYPSFFHVIALNVV
IVAAGVLMARFYPDIGSIIR
YSGATCGLALVFVFPSLVHMISLKRQGELKWPSAIFHSFLILLGVANLIAQFFM
>g1074.t1
MAAIYTAINIVVFYSSSTFWTWLLLAFALAVYIGSYRSMSAMAKPVFGEDGSLLDGGIDLNMEQGMAEHLKDVILLTAIV
QVLSAISSYFWYLWLLAPAR
ALHLLWVNILGPWFSADAPSAPEEVNEKKQRRQERRQMKRF
>g1074.t2
MALLLAFALAVYIGSYRSMSAMAKPVFGEDGSLLDGGIDLNMEQGMAEHLKDVILLTAIVQVLSAISSYFWYLWLLAPAR
ALHLLWVNILGPWFSADAPS
APEEVNEKKQRRQERRQMKRF
>g1075.t1
MMPSVLRQVCPLGTPLDVPRTGTPSAAVVPAPAGSQRQPAWRTGQHRGRAVVSVALTEMVRCMQYGNPSKQDLWDVYGTV
TCKWWNELPANVRTAESLPI
FRKRLKTHLLRVHLNPA
>g1075.t2
LHKFLCGSGNKTDSNGLASRLAMMPSVLRQVCPLGTPLDVPRTGTPSAAVVPAPAGSQRQPAWRTGQHRGRAVVSVALTE
MVRCMQYGNPSKQDLWDVYG
TVTCKWWNELPANVRTAESLPIFRKRLKTHLLRVHLNPA
>g1076.t1
EEAQELIGQACRHSQHATELEGLLEGVTGQVQALEDRCLAQAVQQRSHAHRLQQDALEAQVFSLMQPQSLSRCQALELQL
TNEREARSDLQRKLCFAVRE
EEWRVARQTRAFQRIHELSAQQDAPADQQVLDVIDFYETQIAQLQEDVSRGPQDSQASNTSNGERNSGDATASLKAALKS
SQALLEETKVQREELKAEVQ
RLKKELDTRPTAKDLKTLKHQHLLLERSIQRSRRPATGADAAGSRSDAAAQPAAVPGEGRDRRLLIELGAILMGPRAPLP
LLRQRPALGQSEPAEFGGLR
PTLESWANQLAMLADLHIALSRLSQRLMPWRPVDNGGVTVETVRVEDLMLLVDTLLEDTATNDDNVLRSPTHHSLQCMVG
HFQKLFDVGSLRGVYPRMND
LYTRLGEMTNAMTNLRDILDLDDKASPSEVVSRVATVVASRTDSPAVQFHSLLAEGDIERMGPGPA
>g1076.t2

EEAQELIGQACRHSQHATELEGLLEGVTGQVQALEDRCLAQAVQQRSHAHRLQQDALEAQSRCQALELQLTNEREARSDL
QRKLCFAVREEEWRVARQTR
AFQRIHELSAQQDAPADQQVLDVIDFYETQIAQLQEDVSRGPQDSQASNTSNGERNSGDATASLKAALKSSQALLEETKV
QREELKAEVQRLKKELDTRP
TAKDLKTLKHQHLLLERSIQRSRRPATGADAAGSRSDAAAQPAAVPGEGRDRRLLIELGAILMGPRAPLPLLRQRPALGQ
SEPAEFGGLRPTLESWANQL
AMLADLHIALSRLSQRLMPWRPVDNGGVTVETVRVEDLMLLVDTLLEDTATNDDNVLRSPTHHSLQCMVGHFQKLFDVGS
LRGVYPRMNDLYTRLGEMTN
AMTNLRDILDLDDKASPSEVVSRVATVVASRTDSPAVQFHSLLAEGDIERMGPGPA
>g1076.t3
EEAQELIGQACRHSQHATELEGLLEGVTGQVQALEDRCLAQAVQQRSHAHRLQQDALEAQVFSLMQPQSLSRCQALELQL
TNEREARSDLQRKLCFAVRE
EEWRVARQTRAFQRIHELSAQQDAPADQQVLDVIDFYETQIAQLQEDVSRGPQDSQASNTSNGERNSGDATASLKAALKA
LLEETKVQREELKAEVQRLK
KELDTRPTAKDLKTLKHQHLLLERSIQRSRRPATGADAAGSRSDAAAQPAAVPGEGRDRRLLIELGAILMGPRAPLPLLR
QRPALGQSEPAEFGGLRPTL
ESWANQLAMLADLHIALSRLSQRLMPWRPVDNGGVTVETVRVEDLMLLVDTLLEDTATNDDNVLRSPTHHSLQCMVGHFQ
KLFDVGSLRGVYPRMNDLYT
RLGEMTNAMTNLRDILDLDDKASPSEVVSRVATVVASRTDSPAVQFHSLLAEGDIERMGPGPA
>g1077.t1
TYESKRQEFQGELQRREEEMRQSFVQRVKEKEMELKEKERELQGRFEQLKRIHQEEKAKLEEKRRSLEDEINVFSNKKAA
AELLQGQTFNTNSSNLKKDK
DRKK
>g1077.t2
TYESKRQEFQGELQRREEEMRQSFVQRVKEKEMELKEKERELQGRFEQLKRIHQEEKAKLEEKRRSLEDEINVFSNKKAA
AELLQGQTFNTNSSNLKKDK
DRKNVQGEKSGCLQLGCAAASPSGGQPPPRVPEGKLREHER
>g1078.t1
TGGRLAIGFAVLLRALWKGTHHAFQPSKLKAIVASKASQFTGYAQHDAQEFMAFLLDGLHEDLNRIQNKPYTESVDSDGR
LDEVPAARRDRDRGGVGVVA
EEAWQRHKMRNDSFIVDLFQGQFKSKLVCPMCSKVSITFDPFLYLPVPLPQKQKVLTVFYFAKEPHKKPVKFLVSVSKEN
SRAAEVLESIAKSVRVKPEN
LTLTAVEKSRFSRVFPPTHSLDTLSSADMLLCFQLLSKEVAKERVVLLRVQQRLQVPSLPISKCAACLKPPVADDDKLKR
CMRCYRVGYCNQACQKSHWA
IHKGQCGSSCENVGLPFLVSVPQSRLTYTRLTQLLEGYSRYSVNVFQPPFQSGRGSPEXXXXXXXXXXETTRAADGPPAG
RPDGSPHHEEDEEEDQEAAG
GGDTESGHATSSPLPGESCDSLSSAPSSLTRTADSGFSEPPSLASSCGAPADPQAEKETTCEKVTRPEGEVTPLETPGAH
LGTPVLKP
>g1078.t2
MTAVGSQSLGQYPAVGQDPVLAVVTGYQQPSGEVPGHACQFYISLLDANNKEQRLDENEDLLADLPDDCTLALVWKNNER
LKEYVLVRSKDLEFQEDPGS
LSETSRAGHFTLEQCLKLFTKPEVLAPEEAWYCPKCQQHREASKQLLLWRLPNVLIIQLKRFSFRSFIWRDKINDMVDFP
VRYPPTAPPVLPRRSPNRGP
YSRAGVSPSPGYSHGGGEWSHLVAS
>g1078.t3
TGGRLAIGFAVLLRALWKGTHHAFQPSKLKAIVASKASQFTGYAQHDAQEFMAFLLDGLHEDLNRIQNKPYTESVDSDGR
LDEVPAARRDRDRGGVGVVA
EEAWQRHKMRNDSFIVDLFQGQFKSKLVCPMCSKVSITFDPFLYLPVPLPQKQKVLTVFYFAKEPHKKPVKFLVSVSKEN
SRAAEVLESIAKSVRVKPEN
LTLTAVEKSRFSRVFPPTHSLDTLSSADMLLCFQLLSKEVAKERVVLLRVQQRLQVPSLPISKCAACLKPPVADDDKLKR
CMRCYRVGYCNQACQKSHWA
IHKGQCGSSCENVGLPFLVSVPQSRLTYTRLTQLLEGYSRYSVNVFQPPFQSGRGSPEXXXXXXXXXXETTRAADGPPAG
RPDGSPHHEEDEEEDQEAAG
GGDTESGHATSSPLPGESCDSLSSAPSSLTRTADSGFSEPPSLASSCGAPADPQAEKETTCEKVTRPEAVVTGYQQPSGE
VPGHACQFYISLLDANNKEQ
RLDENEDLLADLPDDCTLALVWKNNERLKEYVLVRSKDLEFQEDPGSLSETSRAGHFTLEQCLKLFTKPEVLAPEEAWYC

PKCQQHREASKQLLLWRLPN
VLIIQLKRFSFRSFIWRDKINDMVDFPVRYPPTAPPVLPRRSPNRGPYSRAGVSPSPGYSHGGGEWSHLVAS
>g1078.t4
TGGRLAIGFAVLLRALWKGTHHAFQPSKLKAIVASKASQFTGYAQHDAQEFMAFLLDGLHEDLNRIQNKPYTESVDSDGR
LDEVPAARRDRDRGGVGVVA
EEAWQRHKMRNDSFIVDLFQGQFKSKLVCPMCSKVSITFDPFLYLPVPLPQKQKVLTVFYFAKEPHKKPVKFLVSVSKEN
SRAAEVLESIAKSVRVKPEN
LTLTAVEKSRFSRVFPPTHSLDTLSSADMLLCFQLLSKEVAKERVVLLRVQQRLQVPSLPISKCAACLKPPVADDDKLKR
CMRCYRVGYCNQACQKSHWA
IHKGQCGSSCENVGLPFLVSVPQSRLTYTRLTQLLEGYSSLPKYLLHFDQWKPHVFPPRYSVNVFQPPFQSGRGSPEXXX
XXXXXXXETTRAADGPPAGR
PDGSPHHEEDEEEDQEAAGGGDTESGHATSSPLPGESCDSLSSAPSSLTRTADSGFSEPPSLASSCGAPADPQAEKETTC
EKVTRPEGEVTPLETPGAHL
GTPVLKP
>g1079.t1
CDFDDTAQYRVSAMNAKGEQSAYASVVVKMGLVSEYGVTFKTHIVDTFGVSFGREGETMSLGCTVIIYPALERYQPGVEW
YRDDVLLAPSKWHQMHWSGD
RATLTLTHLNKEDEGLYTLRITTKSGYETYSAYVFVRDADAEVEGAPGAPLDVRCLDANKDYVIVSWKQPAVDGGTSILG
YFVDSKAGYPRVRSDSMYPG
PVPRCE
>g1079.t2
CDFDDTAQYRVSAMNAKGEQSAYASVVVKTLTPVLFLLLRPGRPRVLSTSSVSMKTTQQGWTAECLRPPVGLVSEYGVTF
KTHIVDTFGVSFGREGETMS
LGCTVIIYPALERYQPGVEWYRDDVLLAPSKWHQMHWSGDRATLTLTHLNKEDEGLYTLRITTKSGYETYSAYVFVRDAD
AEVEGAPGAPLDVRCLDANK
DYVIVSWKQPAVDGGTSILGYFVDSKAGYPRVRSDSMYPGPVPRCE
>g1079.t3
CDFDDTAQYRVSAMNAKGEQSAYASVVVKMGLVSEYGVTFKTHIVDTFGVSFGREGETMSLGCTVIIYPALERYQPGVEW
YRDDVLLAPSKWHQMHWSGD
RATLTLTHLNKEDEGLYTLRITTKSGYETYSAYVFVRDADAEVEGAPGAPLDVRCLDANKDYVIVSWKQPAVDGGTSILG
YFVDSKAGYPRVRSDSMYPG
PVP
>g1080.t1
NVKEGMLVLACVKEVGDFDVTMRLPCGLQGFLQIKNVCDAYTKLLSEQLNAQGTEEIYSLDRIFRPGMLVRCVVLKLEVT
KGGSLSIKLSINPKLLNKAL
QPGMLKSGMTLSGCVESVEDHGYLVDIGVGETKAFLPKQAAKDQHNNPDELKVGQNVTCRVDEVKGSGRVVRLSVNPTTL
AKAFADASQGWTLTNLQPGL
LVKAKIKTVTKHGLFLSFLSFSGQVDFLHMEPGEVSTYSVGQEVQACVLYIEPITRLVALSLRSHLLQPGTLIDVTPAAG
DRVGEVVEGCKVTSMHNTSG
AVLELPDSTSVFVHKNHLKEMAQNSEISKLLSIPELSCRILSFSPMEQIHFASLRKSVRDRAHFRYEDIQVGEVVAGTVS
ELLEHGMMVLLSGNLKGLVP
KTHLSDILLRNPEKKYSEGMKIKCRVLAVEADRKRVTLTRKKTMMESTLPIIRTFQEARPGLVSHGFIVSIKNFGCIVRL
YGDVKGLVPLRELNSEPVGN
PEDLFYIGQVVKVKVLECEPEKERLLLSFKAVAEGDRVLPQIDLEVGQKCEAKVVSKSAACLEVAILPEEVPALLPTMHL
SDHVSNCPLMWEALQAGDVI
TNLVCFSRVRETIVSFFLNGVHLLVKTLTKKPLVKWGLEAGMFAKGFSDILVGMDLVGWIKRVMPYGVFVEFPCGIMGLA
PKSALFDRFITDTAVVYQVG
QTVVAKVTTFDEEKQRFLVTLKVSEVTAPEGGAQALLLRGLRERRTAEEVLADRETSGVHQELLALTVGQKLTLTLDTVE
KGGATFKSDQLTGVTIAASK
HHIIGVSLTSGQKVNAVVLHVDFPSAHVHVSVLPKLLAKKKPLAAGSVHGVMVQHVAGDFAVVA
>g1080.t2
NVKEGMLVLACVKEVGDFDVTMRLPCGLQGFLQIKNVCDAYTKLLSEQLNAQGTEEIYSLDRIFRPGMLVRCVVLKLEVT
KGGSLSIKLSINPKLLNKAL
QPGMLKSGMTLSGCVESVEDHGYLVDIGVGETKAFLPKQAAKDQHNNPDELKVGQNVTCRVDEVKGSGRVVRLSVNPTTL
AKAFADASQGWTLTNLQPGL
LVKAKIKTVTKHGLFLSFLSFSGQVDFLHMEPGEVSTYSVGQEVQACVLYIEPITRLVALSLRSHLLQPGTLIDVTPAAG

DRVGEVVEGCKVTSMHNTSG
AVLELPDSTSVFVHKNHLKEMAQNSEISKLLSIPELSCRILSFSPMEQIHFASLRKSVRDRAHFRYEDIQVGEVVAGTVS
ELLEHGMMVLLSGNLKGLVP
KTHLSDILLRNPEKKYSEGMKIKCRVLAVEADRKRVTLTRKKTMMESTLPIIRTFQEARPGLVSHGFIVSIKNFGCIVRL
YGDVKGLVPLRELNSEPVGN
PEDLFYIGQVVKVKVLECEPEKERLLLSFKAVAEGDRVLPQIDLEVGQKCEAKVVSKSAACLEVAILPEEVPALLPTMHL
SDHVSNCPLMWEALQAGDVI
TNLVCFSRTLTKKPLVKWGLEAGMFAKGFSDILVGMDLVGWIKRVMPYGVFVEFPCGIMGLAPKSALFDRFITDTAVVYQ
VGQTVVAKVTTFDEEKQRFL
VTLKVSEVTAPEGGAQALLLRGLRERRTAEEVLADRETSGVHQELLALTVGQKLTLTLDTVEKGGATFKSDQLTGVTIAA
SKHHIIGVSLTSGQKVNAVV
LHVDFPSAHVHVSVLPKLLAKKKPLAAGSVHGVMVQHVAGDFAVVA
>g1081.t1
DRFLVQNHQGPGVTEPWIRESEWDRRLSSLSNMSPGSQPSVPQELHMSV
>g1082.t1
MHIYFCGSIRGGRQDVAIYQKIVQKLQTYGDVLTEHVSHSALTEKGEYAALKGEKAIHQRDIDWLSTSDALSAMIRGADN
GDLVQVQDYAEEEVEAILED
YFAKLNKV
>g1082.t2
MHIYFCGSIRGGRQDVAIYQKIVQKLQTYGDVLTEHVSHSALTEKGEYAALKGEKAIHQRDIDWLSTSDVVVAEVTQPSL
GVGYELGRAVAMEKRIFCLF
RPSSERSLSAMIRGADNGDLVQVQDYAEEEVEAILEDYFAKLNKV
>g1083.t1
LEVANLNQLNHRFLRFSPIRPPWPWMEVAVTGDMGPERILPNSEDDLGQERPSDGAAHDVVGVGGEWSGEDIEEEEGGDG
EVDGEEEENAGYYYQPLNQD
PEGLNGLDPGDQGDPHAQQLQEVQDRIEAMGLHLPPPPDSDEEVELEGEVAERSHASIPMDEDHVELVKRTMATIALPTL
GVPAWARDISEDQWTDMVQH
TLQTHQSSSALRMPRGGDLD
>g1084.t1
MLNKPNAHMIHDISSQAFNNRWRPCLPLTQPPPHTASLTHQRRSARTAPLRAALLPSPSARCVLHHHATLSSRGPVNRDG
DHYARSPVSQRGGAQADTQT
AADVSPVTEACGPLAGQTRGLVPGPLAPWEQAGSRAGLMNGSPPYDLLPSCSAMTFLLVQAGVWGWAQCVWAWS
>g1084.t2
MLNKPNAHMIHDISSQAFNNRWRPCLPLTQPPPHTASLTHQRRSARTAPLRAALLPSPSARCVLHHHATLSSRGPVNRDG
DHYARSPVSQRGGAQADTQT
AADVSPVTEACGPLGGPVTAGQTRGLVPGPLAPWEQAGSRAGLMNGSPPYDLLPSCSAMTFLLVQAGVWGWAQCVWAWS
>g1085.t1
MVNDGKDTDLLAECAPKEAAEEKEAAVEKEAAEEREAAVEKEPSEPPSETIDPLNVYKWQTGSKGTAGEGRADGGATAAA
SQTERFQKSANTKWSRIQNW
RKALSDELGERAFTLSSSGKGAAGAKPEKAPGAHKHPFRRAVSEPAGSLISALTSSSTPGHAAPSTATTDASGASTADAS
QKGVGGIHFRKYLKTVSQKL
KRPKLQSRHSTQTLLSDAGKNVSVPTEQPRLQWAPPLQVPLWDISNCILEDGQILISPEDEPVMWTRNRVSSCVSVLSMQ
NLAEINTASFLPSPECSPDA
VGQGSGARFKAQDGGVRALIKRRLEGRAKRSSTTQINALGLSSRLYGSRESVSVPVSVAESLDLSADTSTVVRPVHSSIL
GEKYCFEVINSQNSHCFGCS
SAAERDRWIEDLRRAAQPNKDNIERTENSLSLWVNEAKDLPPKRRYFWEVHLDGTLFARTSSRAVDKCAQRSNGAADGCT
GTSGGPGGSGGQVGGFQLFW
GELFELDNLPSVSEVTLYLFRDEDTKKKRLSRDESCLHPLGSVAISLAEIRGRTHQEKWHAIVPFKAVGSGSGKELLGPQ
ASLRIKARFQNLKVLPMERY
KEFAEFVTVDYVEMCQSLEPLLSVKEKEELAGALVHVLQSIGKAKEFLIDLGDAEVERLGEKEALIFRENTLATKAIDEY
MKLVGQKYLIDTLGHFIFGL
YASAESCEVDPLKCPPADLLNNQRHLTEACEEVVKKITEMHGSFPEELNKVFSSWVTQCEDRGRPEIGHRLISASLFLRF
LCPAILSPSLFGLTQPYPEP
LTLRTLTLTAKVIQNLANFRLFGEKEEYMLFMNDFLQQHWEAMRGFLQAVSNPDSELPMASFDGYVDLPLRLAVLHGLLV
DIISKKEQNTIEQLHPLPSI
LNNITESLGPGAPRIPVSRRPGHSRYAPRRQISTEDLQTEGPEEEPEAGAAHPLDISSPQTGVSVKPAPEPVPWIKHNHK

EISEKQKREYEQFNSLDRHA
AELSELRLGVDQVTERELEMAKRLEDFIGQSLEQNDLLQAEVQELRNTVATCEEQLASATFRSANSLCTLNCVSTVR
>g1085.t2
MVNDGKDTDLLAECAPKEAAEEKEAAVEKEAAEEREAAVEKEPSEPPSETIDPLNVYKWQTGSKGTAGEGRADGGATAAA
SQTERFQKSANTKWSRIQNW
RKALSDELGERAFTLSSSGKGAAGAKPEKAPGAHKHPFRRAVSEPAGSLISALTSSSTPGHAAPSTATTDASGASTADAS
QKGVGGIHFRKYLKTVSQKL
KRPKLQSRHSTQTLLSDAGKNVSVPTEQPRLQWAPPLQVPLWDISNCILEDGQILISPEDEPVMWTRNRVSSCVSVLSMQ
NLAEINTASFLPSPECSPDA
VGQGSGARFKAQDGGVRALIKRRLEGRAKRSSTTQINALGLSSRLYGSRESVSVPVSVAESLDLSADTSTVVRPVHSSIL
GEKYCFEVINSQNSHCFGCS
SAAERDRWIEDLRRAAQPNKDNIERTENSLSLWVNEAKDLPPKRRYFWEVHLDGTLFARTSSRAVDKCAQRSNGAADGCT
GTSGGPGGSGGQVGGFQLFW
GELFELDNLPSVSEVTLYLFRDEDTKKKRLSRDESCLHPLGSVAISLAEIRGRTHQEKWHAIVPFKAVGSGSGKELLGPQ
ASLRIKARFQNLKVLPMERY
KEFAEFVTVDYVEMCQSLEPLLSVKEKEELAGALVHVLQSIGKAKEFLIDLGDAEVERLGEKEALIFRENTLATKAIDEY
MKLVGQKYLIDTLGHFIFGL
YASAESCEVDPLKCPPADLLNNQRHLTEACEEVVKKITEMHGSFPEELNKVFSSWVTQCEDRGRPEIGHRLISASLFLRF
LCPAILSPSLFGLTQPYPEP
LTLRTLTLTAKVIQNLANFRLFGEKEEYMLFMNDFLQQHWEAMRGFLQAVSNPDSELPMASFDGYVDLPLRLAVLHGLLV
DIISKKEQNTIEQLHPLPSI
LNNITESLGPGAPRIPVSRRPGHSRYAPRRQISTEDLQTEGPEEEPEAGAAHPLDISSPQTGVSVKPAPEPVPWIKHNHK
EISEKQKREYEQFNSLDRHA
AELSELRLGVDQVTERELEMAKRLEDFIGQSLEQNDLLQAEVQELRNTVAT
>g1085.t3
MVNDGKDTDLLAECAPKEAAEEKEAAVEKEAAEEREAAVEKEPSEPPSETIDPLNVYKWQTGSKGTAGEGRADGGATAAA
SQTERFQKSANTKWSRIQNW
RKALSDELGERAFTLSSSGKGAAGAKPEKAPGAHKHPFRRAVSEPAGSLISALTSSSTPGHAAPSTATTDASGASTADAS
QKGVGGIHFRKYLKTVSQKL
KRPKLQSRHSTQTLLSDAGKNVSVPTEQPRLQWAPPLQVPLWDISNCILEDGQILISPEDEPVMWTRNRVSSCVSVLSMQ
NLAEINTECSPDAVGQGSGA
RFKAQDGGVRALIKRRLEGRAKRSSTTQINALGLSSRLYGSRESVSVPVSVAESLDLSADTSTVVRPVHSSILGEKYCFE
VINSQNSHCFGCSSAAERDR
WIEDLRRAAQPNKDNIERTENSLSLWVNEAKDLPPKRRYFWEVHLDGTLFARTSSRAVDKCAQRSNGAADGCTGTSGGPG
GSGGQVGGFQLFWGELFELD
NLPSVSEVTLYLFRDEDTKKKRLSRDESCLHPLGSVAISLAEIRGRTHQEKWHAIVPFKAVGSGSGKELLGPQASLRIKA
RFQNLKVLPMERYKEFAEFV
TVDYVEMCQSLEPLLSVKEKEELAGALVHVLQSIGKAKEFLIDLGDAEVERLGEKEALIFRENTLATKAIDEYMKLVGQK
YLIDTLGHFIFGLYASAESC
EVDPLKCPPADLLNNQRHLTEACEEVVKKITEMHGSFPEELNKVFSSWVTQCEDRGRPEIGHRLISASLFLRFLCPAILS
PSLFGLTQPYPEPLTLRTLT
LTAKVIQNLANFRLFGEKEEYMLFMNDFLQQHWEAMRGFLQAVSNPDSELPMASFDGYVDLPLRLAVLHGLLVDIISKKE
QNTIEQLHPLPSILNNITES
LGPGAPRIPVSRRPGHSRYAPRRQISTEDLQTEGPEEEPEAGAAHPLDISSPQTGVSVKPAPEPVPWIKHNHKEISEKQK
REYEQFNSLDRHAAELSELR
LGVDQVTERELEMAKRLEDFIGQSLEQNDLLQAEVQELRNTVATCEEQLASATFRSANSLCTLNCVSTVR
>g1086.t1
VSILDSKKSMNIAIFLKTFKRPVGEMVEDILQGNWLRFGTGKLKELCKLLPETCEVKKLCAFRGNVLLLAEADQFMVQLV
KVPGYEERLKTMVLREEFFP
LMEEMKNSVDVMTKAANELLDCEDLHSVIRLVLKAGNYMNAGGHNGNAIGFRMNSLLRLADTKANKPAMNLMHYVAKQAE
DIDAELLTFPSQLEHIGMAS
RICQEEVKADFEREVKKIEEVQLYANRQPGLLQQMETFLLRASMKLSDLSSSLGELRILSQAVAEYFCEDPNTFKLEDCC
SVFHSFCQRFLTAIQENRNR
EAVELRRMDPARCSIKRRSTWSGREPRPPGKHNTSSEVLDTSTGDQETSGLESALHSFLLAAPARRCRRNIVPVEMGPLV
ELSSTPTKTPSPDSEELDSS
LDATPRHSSEKKQAELNEVQDQDLALTVEHQEEAEKMRAVNRKLICFQNRLGKGTNAACLPTLELTPASLAVPGTPPSTT

RDYFLRSNEHSSSPWTIVSP
FTVPDGKARPRTRRIPHLRRAFSISNTGDLNDSIWQMSPTPSSCSSTTVTPSTPCNSFALEDPAENRGGGGGGGGGRGAG
DFQSCYPDHLASRGLIPDYL
GQRPLSLGPLLRSCSLDETRRSPGASSWLGELIHKSISHRTKSCSSRSRMNSSTEEVFPAKEGSGSGLISFFRRFGNKSK
PPDSRELKSGDAET
>g1087.t1
MSDINIMAEDPEETTATEEIQVDEKQKYFDSGDYNMAKAKMKNKQLLTAAPEKTDMQHNMPTPKDLPQRKASLVASKLAG
>g1087.t2
MSDINIMAEDPEETTATEEIQVDEKDEKVVSPEKAEEAKLKSRYPNLGHKPGGSDLLRKRLQKGQKYFDSGDYNMAKAKM
KNKQLLTAAPEKTDMQHNMP
TPKDLPQRKASLVASKLAG
>g1088.t1
MSTSGDPHMNQASTSIADRDSSSPTPVALGIKRKRDDLDGLPSLEAAGIDLDCRATGYEWTGQRVAARTGTGHHEYEDTA
GLQWGRRRREQEAEELLSEP
KRMERLPESSMAPVETVGSDHHVAELSEKPPSDFCSSQNIQERIDLTHDSQSHSDLSHQEGPAPTGSRDTGMDFTESLIQ
SQSDFEAGLRPGEGTSTQKA
PEPTGGPRLNEDNGERRLKCSQPRREHVHDVSTAPRPLDEPTQPKTKPSLCRESDQIRPKAEKPMFVPERASATPNHSPR
DIGSRHRLQGDGADLLFTQD
SEGFRVIAHHGPWAPRTPLKDHSNLGNLAADLLFTQDSEGFRVIAHRGPRAPRTPLKDHSNLGNLARGKGPPSRKHFVFE
AVGEAMLFTQDSQGNTVIKH
>g1089.t1
MAELFAVVKTLQALEKAYIQDCVTPNEYTAFCSRLLVQYKAAFKQVQGSDVGSIDDFCRKYRLDCPLAMERIKEDRPITI
KDDMGNLNRCIADIVSLFIT
VMDKLRLEIRAMDEIQPDLRELMETMNRMSNMPPDSEAKDKVSLWLTTLSSMSASDELDDSQVRQMLFDLESAYNAFNRF
LHSS
>g1089.t2
MAELFAVVKTLQALEKAYIQDCVTPNEYTAFCSRLLVQYKAAFKQVQGSDVGSIDDFCRKYRLDCPLAMERIKEDRPITI
KDDMGNLNRCIADIVSIQPD
LRELMETMNRMSNMPPDSEAKDKVSLWLTTLSSMSASDELDDSQVRQMLFDLESAYNAFNRFLHSS
>g1089.t3
MAELFAVVKTLQALEKAYIQDCVTPNELLVQYKAAFKQVQGSDVGSIDDFCRKYRLDCPLAMERIKEDRPITIKDDMGNL
NRCIADIVSLFITVMDKLRL
EIRAMDEIQPDLRELMETMNRMSNMPPDSEAKDKVSLWLTTLSSMSASDELDDSQVRQMLFDLESAYNAFNRFLHSS
>g1090.t1
MLTPPEWDKLHRLETLLEPCRYVTEILGGETYVSCSVVLPALCHVRHVMEVSDEDPAYVILNQRKRYSLTEPYTGHVVNK
KRSALLSENVDKVVCLSNWL
KDE
>g1090.t2
MLTPPEWDKLHRLETLLEPCRYVTEILGGETYVSCSVVLPALCHVRHVMEVSDEDPAYVMGHPRRKLAFSCRWTQILNQR
KRYSLTEPYTGHVVNKKRSA
LLSENVDKVVCLSNWLKDE
>g1090.t3
MLTPPEWDKLHRLETLLEPCRYVTEILGGETYVSCSVVLPALCHVRHVMEVSDEDPAYVNPPEDLHTLLKMGHPRRKLAF
SCRWTQILNQRKRYSLTEPY
TGHVVNKKRSALLSENVDKVVCLSNWLKDE
>g1091.t1
MLLFVCRTELQGAHAWTGLENLMERFNNTWASYTLGVFICQSCSGLHRNITYISKVKSVLLDPWSDSEVEFLASMGNTKA
RGRYEQRLPPFYYRPTHTDC
TLLREQWIRARYERKEFVSVERQKPYSSENSSSVHPVTLSSLSSLSTLSSLSTLSSLSTLSTLSSLSSLSSLSTLSSLSS
LSSLFTLSSLSTLSTLSSLS
SLSSLSTLSSLSTLSSLSSLSTLFTPFAGLI
>g1092.t1
MVHVFPTACSVNVKLQYITTDIPQQDLTLTPCCGPAPWGPSAALVQQLSSDSHARLEAGPAVETMGRLDDNAKLKVVELR
KAGLSFRKIKAVLELENIKV
SAQAIYLYLREFQGRPAGRARA
>g1093.t1

METIWLYQFRLIVIGDSTVGKSCLIRRFTEGRFAQVSDPTVGVDFFSRLVEIEPGKRIKLQIWDTAGQERFRSITRAYYR
NSVGGLLLFDITNRRSFQNI
HDWLEEAKSHVQPHNIVFLLVGHKCDLEAQRQVTRQEAEKLAGVYGMHYVETSARDAINVEKAFVDLTRDIFELVGRGDI
QIQEGWEGVKSGLVPNTVHS
SEEVTKGDR
>g1094.t1
LSRRRIIPLPQWKANPETDSEALFSKDQLVLALYPQTTCFYRALIHSPPHRTTCFYRALIHSPPHRDDYSVLFEDTSYAD
GYSPPLNVAQRYVVACKENK
KK
>g1094.t2
LSRRRIIPLPQWKANPETDSEALFSKDQLVLALYPQTTCFYRALIHSPPHRDDYSVLFEDTSYADGYSPPLNVAQRYVVA
CKENKKK
>g1095.t1
MSDSESELGAPVKPPPKRRRIIFDPSTISSVPIYSNKVNKCLQLKPAPAALAHNNNTCDLEATLCSDSAPPEAEVDEFCF
TDSEEEEEHKLLYQLIQTRT
RESVGLCSPSPPLTPSKNQDQVSTRVWRVMDEVNSRLGAVSSLLSPSEAVGGMRTRGHRARPPATAGPSLTDAHEVNSRL
GAVSSLLSPSEAVGGMRTRG
HRARPPATAGPSLTDAQYDNDAVHHHPGHLSHPVETEDPPVAREIQLKFRYRTEIHRVLAVSSRPLGEAVAVLAYKLKVP
PSRILLLRNETELPAHSSAN
QLGLSIADIIDCVVMAEDAKQETQESSDIITVRLQGKDRGSTRQYSLHKEAPLGTVLSKYTASCSAGRRSKLQFRFDGSK
IKASHTPAQLDMEDGDVIEV
WS
>g1095.t2
MSDSESELGAPVKPPPKRRRIIFDPSTISSVPIYSNKVNKCLQLKPAPAALAHNNNTCDLEATLCSDSAPPEAEVDEFCF
TDSEEEEEHKLLYQLIQTRT
RESVGLCSPSPPLTPSKNQDQVSTRVWRVMDEVNSRLGAVSSLLSPSEAVGGMRTRGHRARPPATAGPSLTDAHEVNSRL
GAVSSLLSPSEAVGGMRTRG
HRARPPATAGPSLTDAQYDNDAVHHHPGHLSHPVETEDPPVAREIQLKFRYRTEIHRVLAVSSRPLGEAVAVLAYKLKVP
PSRILLLRNETELPAHSSAN
QLGLSIADIIDCVVMAEDAKQETQESSDIITVRLQGKDRGSTRQYSLHKEAPLGTVLSKYTASCSAGRRSKLQFRFDGSK
IKASHTPAQLDMEDGDVIES
TSFTSSLFSSFLASLSFLVVSVVCLQSAAAAEAAAMSLSDPIRDTTHFLSDDSEGEEPGARPSREEEEEPVSGVSVTRAR
LTVAILCYINLLNYMDRFTV
AVFVCSYMLLAPCFGYLGDRYNRKWIMCVGITFWSLVTLASSYTPEHYFWALLLTRGLVGVGEASYSTIAPTIIADLYVG
PTRTVMLSVFYFAIPVGR
>g1095.t3
MSDSESELGAPVKPPPKRRRIIFDPSTISSVPIYSNKVNKCLQLKPAPAALAHNNNTCDLEATLCSDSAPPEAEVDEFCF
TDSEEEEEHKLLYQLIQTRT
RESVGLCSPSPPLTPSKNQDQVSTRVWRVMDEVNSRLGAVSSLLSPSEAVGGMRTRGHRARPPATAGPSLTDAHEVNSRL
GAVSSLLSPSEAVGGMRTRG
HRARPPATAGPSLTDAQYDNDAVHHHPGHLSHPVETEDPPVAREIQLKFRYRTEIHRVLAVSSRPLGEAVAVLAYKLKVP
PSRILLLRNETELPAHSSAN
QLGLSIADIIDCVVMAEDAKQETQESSDIITVRLQGKDRGSTRQYSLHKEAPLGTVLSKYTASCSAGRRSKLQFRFDGSK
IKASHTPAQLDMEDGDVIES
TSFTSSLFSSFLASLSFLVVSVVCLQSAAAAEAAAMSLSDPIRDTTHFLSDDSEGEEPGARPSREEEEEPVSGVSVTRAR
LTVAILCYINLLNYMDRFTV
AVFVCSYMLLAPCFGYLGDRYNRKWIMCVGITFWSLVTLASSYTPEHYFWALLLTRGLVGVGEASYSTIAPTIIADLYVG
PTRTVMLSVFYFAIPVG
>g1096.t1
MDDLSVGGATRATLAGMAAAFGWRMRGHEPFAVTLYQTQTTVTRSTAAMTDAVIGGSSDRWVSNGGQRSMHTKGNWTTQP
GPGAAPDLTDEEKEIINNVI
ARAEKMEAMEQERIGRLVNRLDDMKKTACGDGVSRCLLCGEQLGSPGVSSVVCEDCKKVWKRSGAWFFKGFPKHFLPSPM
PISKPKDPNPGGQQPAAQQA
QGPPASAAREKPAQPQQQSQPQGSYAEQTAGK
>g1096.t2
MDDLSVGGATRATLAGMAAAFGWRMRGHEPFAVTLYQTQTTVTRSTAAMTDAVIGGSSDRWVSNGGQRSMHTKGNWTTQP

GPGAAPDLTDEEKEIINNVI
ARAEKMEAMEQERIGRLVNRLDDMKKTACGDGVSRCLLCGEQLGSPGVSSVVCEDCKKHMCTKCGTQADSRPRSVWKRSG
AWFFKGFPKHFLPSPMPISK
PKDPNPGGQQPAAQQAQGPPASAAREKPAQPQQQSQPQGSYAEQTAGK
>g1096.t3
MDDLSVGGATRATLAGMAAAFGWRMRGHEPFAQTTVTRSTAAMTDAVIGGSSDRWVSNGGQRSMHTKGNWTTQPGPGAAP
DLTDEEKEIINNVIARAEKM
EAMEQERIGRLVNRLDDMKKTACGDGVSRCLLCGEQLGSPGVSSVVCEDCKKVWKRSGAWFFKGFPKHFLPSPMPISKPK
DPNPGGQQPAAQQAQGPPAS
AAREKPAQPQQQSQPQGSYAEQTAGK
>g1097.t1
MQKNMRQEKEAAAAASNAAPGWPGGDLAIQQSHKAPPPYPQAAIARKMPMGVGGMDEKLICPQPLGTSAVMDPNAMRQPG
PGNPQGMYGRPPFPPQWQGP
APRRFPQPGMESMVPRHPLNPAVNMQGMVNPRGMMAGPGGEAMQSMGQGPPPQFIELRHNSQRLPLGPHFMSRGQQARPR
LCPPQQDLAAPYAQQHPMQV
DGGSDSQMGLQQGGLSMLMSQQPTGPATQQGQLQQQTITPSSNHPGSQQQQQQQQHHHHHPPPPPQPSIVPGPQVDPSVD
LPEPDLEGLTDAPGEGGVED
DDLALDLDPDKGDDDLGNLDNLETNDPHLDDLLNSDEFDLLAYTDPELDQGDPKDVFSDQLRLVEAESEAPSASGSADIK
VEEKPKMEPGQSSDTSATSA
LHASSSETSSTSKIKLEDRGLTSQLQDGQTVIKDEMGEAVSMLLGGAGASGKQTQPQTQTASLSSVRLGGVSYPLPGQGD
PLSFPPSTPHPDLGDDPLGL
PDVGGQTSPAVDLAKVESSLDGELPLLIQDLLEHEKKEQQKQQQQQQQQQQQQQQQQQLSSMHQGGMPSHMQGMPGQQP
NPQAPPGALMLHQHHHRPPP
QVMMGQQGMGPRPTHPMQPQQQQRFMGPGMAPPPHMAQQQAMMRMGQPGAMHPGLNHQPQQQPQMVKQPPMANNFFPNKD
DAEQHQYEEWLLHTQQLLQM
QLKFLEEQIGAHRKSRKALCAKQRTAKKAGREFAEADAEKLKLVTEEQSKIQKQLDQVRKQQKEHTNLIAEYRSKQQQHQ
QGSGLLKPGPSTLAPPHMLS
KMPGQMMGQPPGMMPQGGMPVRMPQGQPFMAGGQPQNPGVLGPRPPGPPGNPAGYFPQGPGMQGTDPRLLQERQLQHRMQ
MAKLQQQQAMMMPHPGPQPG
LMPHPGQQPGMMPQAQPGMMGNPLMTQQQPSIQQGMPVDPSNQQGMVQVPQGMVGGQPVPQLPQNMVGQPMNQPQGMMSA
QPGIMGNQPVGPSQQPQLMQ
RPQLMMGPQGMVVAPGHPGIRGPQAQLTPQQQNILAQRMISQQQMQQQQMQQQQMAQKRQQSQGLINQPNQDQRTSQPST
PQMGSSPSAGSNTSQQQGST
EGLQQAFPPDTQNPSLKERAILSPAPRTPTQQSGPSTPSPMAQQGSTGEPHIELAQQQQQQNPAYTVQNQPPNAQQPPGL
VGNQQTGMASHVQPQSVTIK
QERQQTDVSTPQQQQQQQQQQHAVQNVPQQSQDPSTLQHEGMGQVQNVMGQNPGQMQPQPAMMGHPSPQQQALMVQKQQA
MMGMLRPQQPNMMRPGVPTG
QIRMPINIQAILAQNPQLRNLPPNQQIQHIQAMIAQRQQGQMRMSMAQGQPGQMRPQMAPGQVPQVGQRMPGMEGQQMQF
AAVGKPGPVGPQQPIMGQQP
GMPPQMLQGMMVQGQQGMMVQGQQGMMVQGQQQQQQVGQMMQQQMIRGHQSLPNSPGDPQRHAMAQAMGMRPPTPNQQQQ
AHMVATAGRMPGSPSHAGSP
RGTSLQRMDNSPATPGTPHSTHVPSPSLAEGGACRGSPYNQIRASPLRSPGAKSPHHYTGLKAEPHASGNEMSQTPLVPL
NGPQQDERMQQHLQQKASGG
HATQLGSREGDLCRMTLQNIKQEPREVQCDIESVAGTHSGVIKREAPGELINCGNSSGFINAENVGGDPATQARSETGQQ
LLQKLLRTKNLQLAAQRPSE
GIHNEINGHINSKLAMLEQKLQGTPRNMEDLQSITKKAPMAKAKRTNKPTGERGPNPRKKNKKEEVGKSAEALMKQLKQG
LSLLPLMEPSITASLDLFAP
FGSSSANGKAPLKGSFGNAVLDNIPDYYSQLLTKSNLSNPPTPPSSLPPTPPPSVQHKLPNGVTAVEELSDAQKQTETAE
DTMDPVSQEVKSVDILAALP
TPPHNQNEDIRMESDDEDASESIIQASSPESNMGDAMARFPCLREPKEEETERAISPIIPLIPRTAIPVFPENKPFEAAD
DKAVATSNHWDSSKNNEVSV
TFMLSSAAAKNLNHMMVAMAQLLHIRMPGSYEVTFPPSPGTPGATGPGNAPEEPGDGSLNDGPSASQDEWLRQFDVTLPG
CTLKKQVDVLSLIKQEFSQQ
EDKPAQHCYTTNVNDLDVRHLPVIPVEESPPPSPSPPPPPPDPAPVEEATVETKPPCTPPALQIKTEPEQEAVAPPVDAQ
EKQSECAEAAEVSSAPQESA
DPADGAAPDPAAPSEDTSPAKEEPVAATTDTSAQTPGDSPDAMEVDTTKPAKQRRPFSPDETAVQPRQKKWKGVRWKRLQ

IVITIRKGGSKKENHREVSE
LMDRLRVALRPDRLPRDKRKCCFCHEEGDGATDGPARLLNIDVDLWVHLNCALWSTEVYETQGGALINVEVALRRGLRTL
CAYCQKTGATSSCNRLRCPN
VYHFACAMRARCMFFKDKTMLCTQHKLKGPSEEELGSFSVYRRVYIERDEVKQIASILQRGDRIHLFRVGGLIFHSVGQL
LPSQMANFHSPSAIFPVGYE
ATRIYWSTRVPNKRCRYRCRINEQDSRPFFEVRVLEHGMEDLHYSDTTPEGIWDRVVQQVAKLRDESAMLKLFSDRVKGE
EMYGLTIHAVMRITESLPGV
ENCQNYQFRYGRHPLMELPLMINPSGCARSEAKVPTHCKR
>g1097.t2
MQKNMRQEKEAAAAASNAAPGWPGGDLAIQQSHKAPPPYPQDRVAATNPGPQAAIARKMPMGVGGMDEKLICPQPLGTSA
VMDPNAMRQPGPGNPQGMYG
RPPFPPQWQGPAPRRFPQPGMESMVPRHPLNPAVNMQGMVNPRGMMAGPGGEAMQSMGQGPPPQFIELRHNSQRLPLGPH
FMSRGQQARPRLCPPQQDLA
APYAQQHPMQVDGGSDSQMGLQQGGLSMLMSQQPTGPATQQGQLQQQTITPSSNHPGSQQQQQQQHHHHHPPPPPQPSI
VPGPQVDPSVDLPEPDLEGL
TDAPGEGGVEDDDLALDLDPDKGDDDLGNLDNLETNDPHLDDLLNSDEFDLLAYTDPELDQGDPKDVFSDQLRLVEAESE
APSASGSADIKVEEKPKMEP
GQSSDTSATSALHASSSETSSTSKIKLEDRGLTSQLQDGQTVIKDEMGEAVSMLLGGAGASGKQTQPQTQTASLSSVRLG
GVSYPLPGQGDPLSFPPSTP
HPDLGDDPLGLPDVGGQTSPAVDLAKVESSLDGELPLLIQDLLEHEKKEQQKQQQQQQQQQQQQQQQQQLSSMHQGGMP
SHMQGMPGQQPNPQAPPGAL
MLHQHHHRPPPQVMMGQQGMGPRPTHPMQPQQQQRFMGPGMAPPPHMAQQQAMMRMGQPGAMHPGLNHQPQQQPQMVKQP
PMANNFFPNKDDAEQHQYEE
WLLHTQQLLQMQLKFLEEQIGAHRKSRKALCAKQRTAKKAGREFAEADAEKLKLVTEEQSKIQKQLDQVRKQQKEHTNLI
AEYRSKQQQHQQGSGLLKPG
PSTLAPPHMLSKMPGQMMGQPPGMMPQGGMPVRMPQGQPFMAGGQPQNPGVLGPRPPGPPGNPAGYFPQGPGMQGTDPRL
LQERQLQHRMQMAKLQQQQA
MMMPHPGPQPGLMPHPGQQPGMMPQAQPGMMGNPLMTQQQPSIQQGMPVDPSNQQGMVQVPQGMVGGQPVPQLPQNMVGQ
PMNQPQGMMSAQPGIMGNQP
VGPSQQPQLMQRPQLMMGPQGMVVAPGHPGIRGPQAQLTPQQQNILAQRMISQQQMQQQQMQQQQMAQKRQQSQGLINQP
NQDQRTSQPSTPQMGSSPSA
GSNTSQQQGSTEGLQQAFPPDTQNPSLKERAILSPAPRTPTQQSGPSTPSPMAQQGSTGEPHIELAQQQQQQNPAYTVQN
QPPNAQQPPGLVGNQQTGMA
SHVQPQSVTIKQERQQTDVSTPQQQQQQQQQQHAVQNVPQQSQDPSTLQHEGMGQVQNVMGQNPGQMQPQPAMMGHPSPQ
QQALMVQKQQAMMGMLRPQQ
PNMMRPGVPTGQIRMPINIQAILAQNPQLRNLPPNQQIQHIQAMIAQRQQGQMRMSMAQGQPGQMRPQMAPGQVPQVGQR
MPGMEGQQMQFAAVGKPGPV
GPQQPIMGQQPGMPPQMLQGMMVQGQQGMMVQGQQGMMVQGQQQQQQVGQMMQQQMIRGHQSLPNSPGDPQRHAMAQAMG
MRPPTPNQQQQAHMVATAGR
MPGSPSHAGSPRGTSLQRMDNSPATPGTPHSTHVPSPSLAEGGACRGSPYNQIRASPLRSPGAKSPHHYTGLKAEPHASG
NEMSQTPLVPLNGPQQDERM
QQHLQQKASGGHATQLGSREGDLCRMTLQNIKQEPREVQCDIESVAGTHSGVIKREAPGELINCGNSSGFINAENVGGDP
ATQARSETGQQLLQKLLRTK
NLQLAAQRPSEGIHNEINGHINSKLAMLEQKLQGTPRNMEDLQSITKKAPMAKAKRTNKPTGERGPNPRKKNKKEEVGKS
AEALMKQLKQGLSLLPLMEP
SITASLDLFAPFGSSSANGKAPLKGSFGNAVLDNIPDYYSQLLTKSNLSNPPTPPSSLPPTPPPSVQHKLPNGVTAVEEL
SDAQKQTETAEDTMDPVSQE
VKSVDILAALPTPPHNQNEDIRMESDDEDASESIIQASSPESNMGDAMARFPCLREPKEEETERAISPIIPLIPRTAIPV
FPENKPFEAADDKAVATSNH
WDSSKNNEVSVTFMLSSAAAKNLNHMMVAMAQLLHIRMPGSYEVTFPPSPGTPGATGPGNAPEEPGDGSLNDGPSASQDE
WLRQFDVTLPGCTLKKQVDV
LSLIKQEFSQQEDKPAQHCYTTNVNDLDVRHLPVIPVEESPPPSPSPPPPPPDPAPVEEATVETKPPCTPPALQIKTEPE
QEAVAPPVDAQEKQSECAEA
AEVSSAPQESADPADGAAPDPAAPSEDTSPAKEEPVAATTDTSAQTPGDSPDAMEVDTTKPAKQRRPFSPDETAVQPRQK
KWKGVRWKRLQIVITIRKGG
SKKENHREVSELMDRLRVALRPDRLPRDKRKCCFCHEEGDGATDGPARLLNIDVDLWVHLNCALWSTEVYETQGGALINV

EVALRRGLRTLCAYCQKTGA
TSSCNRLRCPNVYHFACAMRARCMFFKDKTMLCTQHKLKGPSEEELGSFSVYRRVYIERDEVKQIASILQRGDRIHLFRV
GGLIFHSVGQLLPSQMANFH
SPSAIFPVGYEATRIYWSTRVPNKRCRYRCRINEQDSRPFFEVRVLEHGMEDLHYSDTTPEGIWDRVVQQVAKLRDESAM
LKLFSDRVKGEEMYGLTIHA
VMRITESLPGVENCQNYQFRYGRHPLMELPLMINPSGCARSEAKVPTHCKR
>g1097.t3
MQKNMRQEKEAAAAASNAAPGWPGGDLAIQQSHKAPPPYPQAAIARKMPMGVGGMDEKLICPQPLGTSAVMDPNAMRQPG
PGNPQGMYGRPPFPPQWQGP
APRRFPQPGMESMVPRHPLNPAVNMQGMVNPRGMMAGPGGEAMQSMGQGPPPQFIELRHNSQRLPLGPHFMSRGQQARPR
LCPPQQDLAAPYAQQHPMQV
DGGSDSQMGLQQGGLSMLMSQQPTGPATQQGQLQQTITPSSNHPGSQQQQQQQQHHHHHPPPPPQPSIVPGPQVDPSVD
LPEPDLEGLTDAPGEGGVED
DDLALDLDPDKGDDDLGNLDNLETNDPHLDDLLNSDEFDLLAYTDPELDQGDPKDVFSDQLRLVEAESEAPSASGSADIK
VEEKPKMEPGQSSDTSATSA
LHASSSETSSTSKIKLEDRGLTSQLQDGQTVIKDEMGEAVSMLLGGAGASGKQTQPQTQTASLSSVRLGGVSYPLPGQGD
PLSFPPSTPHPDLGDDPLGL
PDVGGQTSPAVDLAKVESSLDGELPLLIQDLLEHEKKEQQKQQQQQQQQQQQQQQQQQQLSSMHQGGMPSHMQGMPGQQP
NPQAPPGALMLHQHHHRPPP
QVMMGQQGMGPRPTHPMQPQQQQRFMGPGMAPPPHMAQQQAMMRMDDAEQHQYEEWLLHTQQLLQMQLKFLEEQIGAHRK
SRKALCAKQRTAKKAGREFA
EADAEKLKLVTEEQSKIQKQLDQVRKQQKEHTNLIAEYRSKQQQHQQGSGLLKPGPSTLAPPHMLSKMPGQMMGQPPGMM
PQGGMPVRMPQGQPFMAGGQ
PQNPGVLGPRPPGPPGNPAGYFPQGPGMQGTDPRLLQERQLQHRMQMAKLQQQQAMMMPHPGPQPGLMPHPGQQPGMMPQ
AQPGMMGNPLMTQQQPSIQQ
GMPVDPSNQQGMVQVPQGMVGGQPVPQLPQNMVGQPMNQPQGMMSAQPGIMGNQPVGPSQQPQLMQRPQLMMGPQGMVVA
PGHPGIRGPQAQLTPQQQNI
LAQRMISQQQMQQQQMQQQQMAQKRQQSQGLINQPNQDQRTSQPSTPQMGSSPSAGSNTSQQQGSTEGLQQAFPPDTQNP
SLKERAILSPAPRTPTQQSG
PSTPSPMAQQGSTGEPHIELAQQQQQQNPAYTVQNQPPNAQQPPGLVGNQQTGMASHVQPQSVTIKQERQQTDVSTPQQQ
QQQQQQQHAVQNVPQQSQDP
STLQHEGMGQVQNVMGQNPGQMQPQPAMMGHPSPQQQALMVQKQQAMMGMLRPQQPNMMRPGVPTGQIRMPINIQAILAQ
NPQLRNLPPNQQIQHIQAMI
AQRQQGQMRMSMAQGQPGQMRPQMAPGQVPQVGQRMPGMEGQQMQFAAVGKPGPVGPQQPIMGQQPGMPPQMLQGMMVQG
QQGMMVQGQQGMMVQGQQQQ
QQVGQMMQQQMIRGHQSLPNSPGDPQRHAMAQAMGMRPPTPNQQQQAHMVATAGRMPGSPSHAGSPRGTSLQRMDNSPAT
PGTPHSTHVPSPSLAEGGAC
RGSPYNQIRASPLRSPGAKSPHHYTGLKAEPHASGNEMSQTPLVPLNGPQQDERMQQHLQQKASGGHATQLGSREGDLCR
MTLQNIKQEPREVQCDIESV
AGTHSGVIKREAPGELINCGNSSGFINAENVGGDPATQARSETGQQLLQKLLRTKNLQLAAQRPSEGIHNEINGHINSKL
AMLEQKLQGTPRNMEDLQSI
TKKAPMAKAKRTNKPTGERGPNPRKKNKKEEVGKSAEALMKQLKQGLSLLPLMEPSITASLDLFAPFGSSSANGKAPLKG
SFGNAVLDNIPDYYSQLLTK
SNLSNPPTPPSSLPPTPPPSVQHKLPNGVTAVEELSDAQKQTETAEDTMDPVSQEVKSVDILAALPTPPHNQNEDIRMES
DDEDASESIIQASSPESNMG
DAMARFPCLREPKEEETERAISPIIPLIPRTAIPVFPENKPFEAADDKAVATSNHWDSSKNNEVSVTFMLSSAAAKNLNH
MMVAMAQLLHIRMPGSYEVT
FPPSPGTPGATGPGNAPEEPGDGSLNDGPSASQDEWLRQFDVTLPGCTLKKQVDVLSLIKQEFSQQEDKPAQHCYTTNVN
DLDVRHLPVIPVEESPPPSP
SPPPPPPDPAPVEEATVETKPPCTPPALQIKTEPEQEAVAPPVDAQEKQSECAEAAEVSSAPQESADPADGAAPDPAAPS
EDTSPAKEEPVAATTDTSAQ
TPGDSPDAMEVDTTKPAKQRRPFSPDETAVQPRQKKWKGVRWKRLQIVITIRKGGSKKENHREVSELMDRLRVALRPDRL
PRDKRKCCFCHEEGDGATDG
PARLLNIDVDLWVHLNCALWSTEVYETQGGALINVEVALRRGLRTLCAYCQKTGATSSCNRLRCPNVYHFACAMRARCMF
FKDKTMLCTQHKLKGPSEEE
LGSFSVYRRVYIERDEVKQIASILQRGDRIHLFRVGGLIFHSVGQLLPSQMANFHSPSAIFPVGYEATRIYWSTRVPNKR

CRYRCRINEQDSRPFFEVRV
LEHGMEDLHYSDTTPEGIWDRVVQQVAKLRDESAMLKLFSDRVKGEEMYGLTIHAVMRITESLPGVENCQNYQFRYGRHP
LMELPLMINPSGCARSEAKV
PTHCKR
>g1098.t1
MKIWKDPGKNLSTEDEAVLEVGRLCRKSPKLVRGRPNDSQLVRGRPNDSQPGDATHTHGLTFGGPEKVWSTPHTNSSSNS
NSESNQKLEGNFDPSRTNSD
ASHRVLKVGSLRMYPGMFWNMHETSLEPEPFPELPQEIPGERETWKSRRSASVPDILEFHGNSTSEVPRTDMELSPWYTR
RQRAHLSPVEGLLERAKVQ
>g1099.t1
DAQQTPTYFALTSLAGITGVIITLCLILIITSSMEVIRRSYFEVFWYTHHLFVIFFIGLVIHGAGRIVRSQQNVEEHNFT
ICAGRINDWGRVPECPLPTF
AGGFPQTWMWVIGPIFLYLCERILRFIRYMQAVRYRKIVIRPSKVLELQLVKNGFKMEVGQYVFLNCPAISHLEWHPFTM
TSAPEEDFFSVHIRSVGDWT
QKLISMVEELPEGGQGPKMGVDGPFGTASEDVFDYEVSMLVGAGIGVTPFASILKSIWYKFKDSDPKLRTRKIYFYWLCR
ETHAFEWFADLLQVLEKEME
ERGMGDFLTYKLFLTGWDQSHTAHVMVRFDEDTDVVTGLKQKTNYGRPNWEKEFEQVRKENPSSTVGTFLCGPQALAKVL
GKKCAKFSDVDPRKTRFYFN
KENF
>g1099.t2
DAQQTPTYFALTSLAGITGVIITLCLILIITSSMEVIRRSYFEVFWYTHHLFVIFFIGLVIHGAGRIVRSQQNVEEHNFT
ICAGRINDWGRVPECPLPTF
AGGFPQTWMWVIGPIFLYLCERILRFIRYMQAVRYRKIVIRPSKVLELQLVKNGFKMEVGQYVFLNCPAISHLEWHPFTM
TSAPEEDFFSVHIRSVGDWT
QKLISMVEELPEGGQGPKMGVDGPFGTASEDVFDYEVSMLVGAGIGVTPFASILKSIWYKFKDSDPKLRTRKIYFYWLCR
ETHAFEWFADLLQVLEKEME
ERGMGDFLTYKLFLTGWDQSHTAHVMVRFDEDTDVVTGLKQKTNYGRPNWEKEFEQALAKVLGKKCAKFSDVDPRKTRFY
FNKENF
>g1100.t1
LEQEERTSPSIRPPGLPHDSGSSIPTLHSASVLPWMRGKRGAECDVFWMSGSRDGCERSHARGPQVVSPSPTGTLQRNSA
LVKNYQCRELRLKG
>g1101.t1
MQPGMHVNGAPQMMQPPPMGGGPGPAPGPGPVGPPGNLQHSPTGASVQAGSERSQGTGLEQQGPATEHNVSPDWPGGLPP
RGLLGDGPNDPRGGTLLSVT
GEVIEPAGYMGPPPPHQAPPMHMGQMGSGPPPDMRGPPHDMRGPPMGEPRNMMGDPRGPPMMEPRGPPIETRGRDPRAMD
ARGPVAGQRVPMAGGGMPGP
GPHNMAANAPPPARPSGGGFSPGQSQVSSQDQEKAALIMQVLQLTPEQIAMLPPEQRQSILILKEQIQKTAGAP
>g1101.t2
MVMPRSSSSSAPPVDDQTAQPGMHVNGAPQMMQPPPMGGGPGPAPGPGPVGPPGNLQHSPTGASVQAGSERSQGTGLEQQ
GPATEHNVSPDWPGGLPPRG
LLGDGPNDPRGGTLLSVTGEVIEPAGYMGPPPPHQAPPMHMGQMGSGPPPDMRGPPHDMRGPPMGEPRNMMGDPRGPPMM
EPRGPPIETRGRDPRAMDAR
GPVAGQRVPMAGGGMPGPGPHNMAANAPPPARPSGGGFSPGQSQVSSQDQEKAALIMQVLQLTPEQIAMLPPEQRQSILI
LKEQIQKTAGAP
>g1101.t3
MVMPRSSSSSAPPVDDQTAVSQPGMHVNGAPQMMQPPPMGGGPGPAPGPGPVGPPGNLQHSPTGASVQAGSERSQGTGLE
QQGPATEHNVSPDWPGGLPP
RGLLGDGPNDPRGGTLLSVTGEVIEPAGYMGPPPPHQAPPMHMGQMGSGPPPDMRGPPHDMRGPPMGEPRNMMGDPRGPP
MMEPRGPPIETRGRDPRAMD
ARGPVAGQRVPMAGGGMPGPGPHNMAANAPPPARPSGGGFSPGQSQVSSQDQEKAALIMQVLQLTPEQIAMLPPEQRQSI
LILKEQIQKTAGAP
>g1102.t1
MGALRTVLAWVGCVGTLGVAVGLWAIVSPSEERRKEMIKHLPEANPLRLEETRKRNALMMQVLKEAAETDANLARGFGGT
GSCLIPDSSRAAPSSFCCSS
YSYSCSSSSSSSSSTERLPDYGDTMPKSKEVLSSTSGSDSDSEVETKVKRKKPSAPEKAAKKPKGGEPSRPGGSARGSST
TDDHMFQIGKMRYVSVRDFK

GKVLVDIREYWMNQDGEMKPGKKGISLNPEQWSQLKDQMSEIDEAMKKL
>g1103.t1
APMFLDNLSGSLQSVAAAASSSALVTSSSTRYDPSVHAGPSAHETCVITGGGGGPGSISDIISLD
>g1104.t1
ERSSSGLLEWESINDAMEALALMNHYQMKNPTHHAN
>g1105.t1
MGESGRRQAVSLGVGPSASTLVGLDAGVWRNGEVMAELAESARSKSEGWPNRPKAVRDVLVTLNGCVPFNVRFRKDHNLI
GRVLAFDANTNPDFELKVFA
EYPCGSGIVYSQIPDLVRQMFRDSAKDAGKAVNSGLRYVEYEKRQGTGDWRGLTELLNDGVRLFKEPPIPEVLPQPYPEA
GMPMASAGRPVPPKGAARRR
KASPGSENGEGEAARPDHPSRETWQRSAYQGMEPLQGMAAPPEGAPPRLHSQPSPISALMGVAESLSTNQMARESPGMSA
THSSSSAGRPTSGSPSTASG
SQASLGQGGSQGGPSSSSSGSAAGEPGIGAQGALLCCTLCRERLEDTHFVQCPSVPHHKFCFPCTRASIRSQGPGGEVYC
PSGERCPLSGSTVPWAFMQG
EISTILAGDGDVTVKKESDP
>g1106.t1
MASWGRVRNILRNFAKAPRVLTALGQSRAVGTGVLASLVGTGVIVHHYGTSGSFPAVLYAQGPREDAVPPRSTRRIRFDQ
FASLTYDQEVYMTPRDFLFS
VMLENVDRLISYTEYLFLLTILTKPGTGFHIAFQMLDDDGNEQVDKKEFLKKPAAAVEDVNTTLQAFFFGRRGQNKLQYK
EFRRFMEDLQAEVQEMEFLQ
FSKGMDTMRREDFAEWLLHYTNEEDNDTYWENVRKRIPAGQSITFAEFKAFCLFTNNLEDFAFTMKLITEANRPIGMAQF
KRAVRIATGHDLSENVLDTV
FKIFDLDGDNCLSHKEFLCVMKDRMLRGLKVQPQYGLAGYWKCVKRETLKGAQEALRDSGSPF
>g1106.t2
MASWGRVRNILRNFAKAPRVLTALGQSRAVGTGVLASLVGTGVIVHHYGTSGSFPAVLYAQGPREDAVPPRSTRRIRFDQ
FASLTYDQEVYMTPRDFLFS
VMLENVDRKLRKKDLKKEEVDIMLTTAARARAGNTLFRNVGDNGLISYTEYLFLLTILTKPGTGFHIAFQMLDDDGNEQV
DKKEFLKKPAAAVEDVNTTL
QAFFFGRRGQNKLQYKEFRRFMEDLQAEVQEMEFLQFSKGMDTMRREDFAEWLLHYTNEEDNDTYWENVRKRIPAGQSIT
FAEFKAFCLFTNNLEDFAFT
MKLITEANRPIGMAQFKRAVRIATGHDLSENVLDTVFKIFDLDGDNCLSHKEFLCVMKDRMLRGLKVQPQYGLAGYWKCV
KRETLKGAQEALRDSGSPF
>g1107.t1
MNGGKMEREMDRQIGAASAVMRALYRTVVVKRELSQKAKLSIYQSIYVPTLTYGHELWVVTERTRSRIEAAEMSFLRRVA
GLSLRDKGEELGHPEGARSR
AAAPLRRKEPVEMVRASDQDASWAPPVRVVPGTSNWQETPGETQNSLEGLYISSGLGTPRGPPGGAGKHCWGEGRLEFLA
QPAATATRPRISGRLMMMMM
MMMMMMMIVRPELNWA
>g1108.t1
MMGGLWNCQSAVKKADSISAYACLQTLYFLALTETWITPENTATPAALSTAYSFSHTPRPSHLNSMLLLSKLYIAVIYPP
PGPFFDEMDALLSCFPEDGT
PLVVLGNFNIQPEVAII
>g1109.t1
MRPPVLLDPIPSSLLQAISTDILPFITTLINSSLIPAPFKTARVKPLLKKPTLDTADIQNYRLVLVLLDLFAAFDTVKHQ
TLLTTLADLGITDTALSQFT
SSLLNRTHQTL
>g1110.t1
MSAARGHAAVLPQEVQSGEEEGEEEEEEVFSKLCGHFFSSVVTSSALWQFPQLCGHFLSSVAISSALWPLPQLCGHFLSS
SSLSRAAIGSVFSSIPNSPG
GSMVNQYQLEDDQLRAGVTPRDMFITVDNPESHVTAIETFIVYRVMTRTTRGDFDSSEYEVQRRYQDFLWLKGRLEEAHP
TLIIHPLPEKFVMKGVVERF
NETFIETRRRALQLFLSKTSEHPVLSRSPQLKVFLTEQLALFGMACTHKPCSCFVFPIMGGIVELGAHKKQGPSLLSRIG
ESVRAVAFSVRGVKARPEDF
AAMQEYADDFSSKMVSLDKITLRIIKELQEYLDELRQYSPLYTHWANLETRLAEPLKGVASCLELCGSETEEQIYHMQEV
LEPALHQYLLCADSLKTVLR
RRDHMQAELEAKTEALTPSSSSKKVDIETGPCPCPHINPDLNPNLNPDLNHDPDLNPDLNPDLNPDLDPDLNPDLNPDRN

PDPDLNPNLDPVTLPL
>g1110.t2
MSAARGHAAVLPQEVQSGEEEGEEEEEEVFSKLCGHFFSSVVTSSALWQFPQLCGHFLSSVAISSALWPLPQLCGHFLSS
SSLSRAAIGSVFSSIPNSPG
GSMVNQYQLEDDQLRAGVTPRDMFITVDNPESHVTAIETFIVYRVMTRTTRGDFDSSEYEVQRRYQDFLWLKGRLEEAHP
TLIIHPLPEKFVMKGVVERF
NETFIETRRRALQLFLSKTSEHPVLSRSPQLKVFLTEQVVTITVPSSLPTSGPVRYGLHAQAVLLLCLSNNGRHCGANTQ
DKHRLVLLLTELSLQELGAH
KKQGPSLLSRIGESVRAVAFSVRGVKARPEDFAAMQEYADDFSSKMVSLDKITLRIIKELQEYLDELRQYSPLYTHWANL
ETRLAEPLKGVASCLELCGS
ETEEQIYHMQEVLEPALHQYLLCADSLKTVLRRRDHMQAELEAKTEALTPSSSSKKVDIETGPCPCPHINPDLNPNLNPD
LNHDPDLNPDLNPDLNPDLD
PDLNPDLNPDRNPDPDLNPNLDPVTLPL
>g1110.t3
MSAARGHAAVLPQEVQSGEEEGEEEEEEVFSKLCGHFFSSVVTSSALWQFPQLCGHFLSSVAISSALWPLPQLCGHFLSS
SSLSRAAIGSVFSSIPNSPG
GSMVNQYQLEDDQLRAGVTPRDMFITVDNPESHVTAIETFIVYRVMTRTTRGDFDSSEYEVQRRYQDFLWLKGRLEEAHP
TLIIHPLPEKFVMKGVVERF
NETFIETRRRALQLFLSKTSEHPVLSRSPQLKVFLTEQLALFGMACTHKPCSCFVFPIMGGIVVQTLKTNTEGSTTPPDL
LTSGSHVALVLLLTELSLQE
LGAHKKQGPSLLSRIGESVRAVAFSVRGVKARPEDFAAMQEYADDFSSKMVSLDKITLRIIKELQEYLDELRQYSPLYTH
WANLETRLAEPLKGVASCLE
LCGSETEEQIYHMQEVLEPALHQYLLCADSLKTVLRRRDHMQAELEAKTEALTPSSSSKKVDIETGPCPCPHINPDLNPN
LNPDLNHDPDLNPDLNPDLN
PDLDPDLNPDLNPDRNPDPDLNPNLDPVTLPL
>g1111.t1
MCFIASNQDPDGVIAQREAHKAQGGGGGHGPGGHGGMGGMDRMERMDRMGGMNMDRMGGMNMDRMERMDRMGGMNMDRMG
GMNMDRMDRMGGMNMDRMGG
MERMGGMDRMNMDRMGPGPGGPPAVNIPPSLMNNPNIPNEIIHGLQAGKIGSTVFVANLDYKVGWKKLKEVFGMAGVVVR
TDILEDKDGKSRGMGTVTFD
MPIEAVQAVCGLSGIGLGLGPGGQPIDATQLNRGEGRGTGMGNMGPGGMDGMNFGGMGGRAGGMDNFGSGGGGGGGGGG
GGGGGNNMDRFGPSGMGRMN
EMDRGIAAGFDRDFGRNEMGMTRNNFGDSFERGVGNSLGGDRMNSGLERLGTGMDRLAGLDRLGMDRMDRGSDLDRLGTG
FDRMGSGMDRLGPSMDRLGS
GLDRMSSSMDRLGPAGFDRLGPSSLDRISTSLDFSSPMGMDRMGNTGLDRMGQNNFDRMGSSGGLDRFPTGGLDRIGSNM
DRMGAGNVGGQFDRPADMDR
GSFGGNSFVGAGAGGPGAGGVNARKGCQIFVRNLPFDFTWKMLKDTFSTCGIVQYADIKMENGKSKGCGVVRFDNPETAE
RACRSMNGYRLNGREIDVRH
SSKGGGRKGFIGEFLDNLKQELSKNKEMTENIKKFRDEAKKLEESDALQQARRKYKTIESETAKTSEVLRKKLGTISETV
KEGLEEVSRTDIGKKIKEGV
EEAAKTAKSSAETVSKSGEKLGKTGAFRAISQGMESVKKEIDDLGHTGPYRPPSRLRKRTEFSSKGAGDQGKVFEANEDA
MGVVLHKDSKWFQQWKDFKD
NNVVVN
>g1111.t2
TAAEKPGQPQPQPPQQQQQQQQQPPPQQPLQGEVNGKPKHESGPSRKERPQKRGGGGRFEPYGNVNKRYRVFVSNIPYDV
KWQALKDLMKEKADFANTMC
FIASNQDPDGVIAQREAHKAQGGGGGHGPGGHGGMGGMDRMERMDRMGGMNMDRMGGMNMDRMERMDRMGGMNMDRMGGM
NMDRMDRMGGMNMDRMGGME
RMGGMDRMNMDRMGPGPGGPPAVNIPPSLMNNPNIPNEIIHGLQAGKIGSTVFVANLDYKVGWKKLKEVFGMAGVVVRTD
ILEDKDGKSRGMGTVTFDMP
IEAVQAVCGLSGIGLGLGPGGQPIDATQLNRGEGRGTGMGNMGPGGMDGMNFGGMGGRAGGMDNFGSGGGGGGGGGGGGG
GGGNNMDRFGPSGMGRMNEM
DRGIAAGFDRDFGRNEMGMTRNNFGDSFERGVGNSLGGDRMNSGLERLGTGMDRLAGLDRLGMDRMDRGSDLDRLGTGFD
RMGSGMDRLGPSMDRLGSGL
DRMSSSMDRLGPAGFDRLGPSSLDRISTSLDFSSPMGMDRMGNTGLDRMGQNNFDRMGSSGGLDRFPTGGLDRIGSNMDR
MGAGNVGGQFDRPADMDRGS

FGGNSFVGAGAGGPGAGGVNARKGCQIFVRNLPFDFTWKMLKDTFSTCGIVQYADIKMENGKSKGCGVVRFDNPETAERA
CRSMNGYRLNGREIDVRHSS
KGGGRKGFIGEFLDNLKQELSKNKEMTENIKKFRDEAKKLEESDALQQARRKYKTIESETAKTSEVLRKKLGTISETVKE
GLEEVSRTDIGKKIKEGVEE
AAKTAKSSAETVSKSGEKLGKTGAFRAISQGMESVKKEIDDLGHTGPYRPPSRLRKRTEFSSKGAGDQGKVFEANEDAMG
VVLHKDSKWFQQWKDFKDNN
VVVN
>g1111.t3
MCFIASNQDPDGVIAQREAHKAQGGGGGHGPGGHGGMGGMDRMERMDRMGGMNMDRMGGMNMDRMERMDRMGGMNMDRMG
GMNMDRMDRMGGMNMDRMGG
MERMGGMDRMNMDRMGPGPGGPPAVNIPPSLMNNPNIPNEIIHGLQAGKIGSTVFVANLDYKVGWKKLKEVFGMAGVVVR
TDILEDKDGKSRGMGTVTFD
MPIEAVQAVCGLSGIGLGLGPGGQPIDATQLNRGEGRGTGMGNMGPGGMDGMNFGGMGGRAGGMDNFGSGGGGGGGGGG
GGGGGNNMDRFGPSGMGRMN
EMDRGIAAGFDRDFGRNEMGMTRNNFGDSFERGVGNSLGGDRMNSGLERLGTGMDRLAGLDRLGMDRMDRGSDLDRLGTG
FDRMGSGMDRLGPSMDRLGS
GLDRMSSSMDRLGPAGFDRLGPSSLDRISTSLDFSSPMGMDRMGNTGLDRMGQNNFDRMGSSGGLDRFPTGGLDRIGSNM
DRMGAGNVGGQFDRPADMDR
GSFGGNSFVGAGAGGPGAGGVNARKGCQIFVRNLPFDFTWKMLKDTFSTCGIVQYADIKMENGKSKGCGVVRFDNPETAE
RACRSMNGYRLNGREIDVRH
SSKGGGRKGFIGEFLDNLKQELSKNKEMTENIKKFRDEAKKLEESDALQQARRKYKTIESETAKTSEVLRKKLGTISETV
KEGLEEVSRTDIGKKIKEGV
EEAAKTAKSSAETVSKSGEKLGKTGAFRAISQGMESVKKEIDDLGHTGPYRPPSRLRKRTEFSSKGAGDQGKVFEANEDA
MGVVLHKDSKWFQQWKDFKD
NNVVVNSKDDVTFVVEHVC
>g1112.t1
LSVQALHTAPKKPVILNGQKTDEVAADSRKTRGAAPAPSIVIVWRRGCRGEVSPAIPPRALQTSTLIWKEQQRLYILRKP
RTARLPQTFCCTRKGRIALW
GVVKPAPHNIFGTQLPAVEDIDPGAASPAGPSAASVTRPTLPTAY
>g1112.t2
LSVQALHTAPKKPVILNGQKTDEVAADSRKTRGAAPAPASERYPSCTRKGRIALWGVVKPAPHNIFGTQLPAVEDIDPGA
ASPAGPSAASVTRPTLPTAY
>g1113.t1
LSCQFNVTGEKLLIIHCNITESYPSTPPIWFVDSDDPSLTQVLERLEDVRRGNTLLLQQLKKLICDLCRLYNLPQHPDVE
MLDQPLPAGPHGTTDEVTSE
EEEEEEMGEDIEDLDHYDMKEEEPVDGKKSEDDGIEKENLAILEKIRKNQRQDHLNVSAHSGAVSGSVQASDRLMKELRE
IYRSQSYKTGIYSVELVNDS
LYEWHVKLRTVDPDSPLHSDLQVLKEKEGMDYILLNFSYKDHFPFDPPFVRVVSPVLSGGYVLGGGALCMELLTKQAGLG
SPGWSSAYSIESVIMQINAT
LVKGKARVQFGANKNQYNLARAQQSYKSLVQIHEKN
>g1113.t2
LSCQFNVTGEKLLIIHCNITESYPSTPPIWFVDSDDPSLTQVLERLEDVRRGNTLLLQQLKKLICDLCRLYNLPQHPDVE
MLDQPLPAGPHGTTDEVTSE
EEEEEEMGEDIEDLDHYDMKEEEPVDGKKSEDDGIEKENLAILEKIRKNQRQDHLNVSAHSGAVSGSVQASDRLMKELRE
IYRSQSYKTGIYSVELVNDS
LYEWHVKLRTVDPDSPLHSDLQVLKEKEGMDYILLNFSYKDHFPFDPPFVRVVSPVLSGGYVLGGGALCMELLTKQAGLG
SPGWSSAYSIESVIMQINAT
LVKGKARVQFGANKNQYNLARAQQSYKSLVQIHEKNVESMLPLT
>g1113.t3
LSCQFNVTGEKLLIIHCNITESYPSTPPIWFVDSDDPSLTQVLERLEDVRRGNTLLLQQLKKLICDLCRLYNLPQHPDVE
MLDQPLPAGPHGTTDEVTSE
EEEEEEMGEDIEDLDHYDMKEEEPVDGKKSEDDGIEKENLAILEKIRKNQRQDHLNVSAHSGAVSGSVQASDRLMKELRE
IYRSQSYKTGIYSVELVNDS
LYEWHVKLRTVDPDSPLHSDLQVLKEKEGMDYILLNFSYKDHFPFDPPFVRVVSPVLSGGYVLGGGALCMELLTKQAGLG
SPGWSSAYSIESVIMQINAT
LVKGKARVQFGANKNQYNLARAQQSYKSLVQIHEKN

>g1114.t1
MDSRNLSSAFSPDTDMFHRASPAGPPGGTGLSGRAAAVERVSADRPYLSSLVTDHGHVPAGPPCHTTLNTVTPVRRSQPM
VCHGKLSRLTPLSSQEVLRP
VTSNHATTCPLDPILSSLLQAISTDILPFITTLINSSLTSGWLQDCTLYRDGPPDESLRAAKASSLSSVLVLLDLSAAFD
TVNHQTLLTTLADLGITDTA
LAWFTSYLMNRTYQWIQNAAAHVFNRHKFSHVTPLFHDLHWLPVVASIKFKMMELPLPTSKH
>g1114.t2
MDSRNLSSAFSPDTDMFHRASPAGPPGGTGLSGRAAAVERVSADRPYLSSLVTDHGHVPAGPPCHTTLNTVTPVRRSQPM
VCHGKLSRLTPLSSQEVLRP
VTSNHATTCPLDPILSSLLQAISTDILPFITTLINSSLTSGWLQDCTLYRDGPPDESLRAAKASSLSSVLVLLDLSAAFD
TVNHQTLLTTLADLGITDTA
LAWFTSYLMNRTYQTL
>g1114.t3
MDSRNLSSAFSPDTDMFHRASPAGPPGGTGLSGRAAAVERVSDRPYLSSLVTDHGHVPAGPPCHTTLNTVTPVRRSQPMV
CHGKLSRLTPLSSQEVLRPV
TSNHATTCPLDPILSSLLQAISTDILPFITTLINSSLTSGWLQDCTLYRDGPPDESLRAAKASSLSSVLVLLDLSAAFDT
VNHQTLLTTLADLGITDTAL
AWFTSYLMNRTYQWIQNAAAHVFNRHKFSHVTPLFHDLHWLPVVASIKFKMMELPLPTSKH
>g1115.t1
LESIEQALQHYVVRLTVTHDKLRLTIANQDHSLAVLATQMGHLTAVLTKAAPFVPAPTPTPQASASSPPESLREPQAFAS
ELRRVFGEPSRGPDAASCLL
GLRQGGRTVSDYAIDFHTRAFQSEWNPAVQCDAFLLGLGSYTGLLGPSWAGVPLAIRGHIFSLSPLLRSAEHYSAAANRH
HTTAPSYQVERIFNPTAVQL
KLPQSMRLIDGGPTYTVHRLLQSRRCGWGLQYLVDWEDYGTERSWVPARHVLDKDLIRDFHRRHPDQLSRTTASTRDPGR
RSPSPQLLKPPGSDDKENEA
SSGEDIPVDANISQEY
>g1115.t2
LESIEQALQHYVVRLTVTHDKLRLTIANQDHSLAVLATQMGHLTAVLTKAAPFVPAPTPTPQASASSPPESLREPQAFAS
ELRRVFGEPSRGPDAASCLL
GLRQGGRTVSDYAIDFHTRAFQSEWNPAVQCDAFLLGLGSYTGLLGPSWAGVPLAIRGHIFSLSPLLRSAEHYSAAANRH
HTTAPSYQVERIFNPTAVQL
KLPQSMRCHPTFHVSRVKPVRVSPHVPAAPPPPLPRLIDGGPTYTVHRLLQSRRCGWGLQYLVDWEDYGTERSWVPARHV
LDKDLIRDFHRRHPDQLSRT
TASTRDPGRRSPSPQLLKPPGSDDKENEASSGEDIPVDANISQEY
>g1115.t3
MLGQHISRLESIEQALQHYVVRLTVTHDKLRLTIANQDHSLAVLATQMGHLTAVLTKAAPFVPAPTPTPQASASSPPESL
REPQAFASELRRVFGEPSRG
PDAASCLLGLRQGGRTVSDYAIDFHTRAFQSEWNPAVQCDAFLLGLGSYTGLLGPSWAGVPLAIRGHIFSLSPLLRSAEH
YSAAANRHHTTAPSYQVERI
FNPTAVQLKLPQSMRLIDGGPTYTVHRLLQSRRCGWGLQYLVDWEDYGTERSWVPARHVLDKDLIRDFHRRHPDQLSRTT
ASTRDPGRRSPSPQLLKPPG
SDDKENEASSGEDIPVDANISQEY
>g1116.t1
MNWLLVNQRYNKLIRPAMNRSERVTVKLQVSLAQLISVNEREQIMTTNVWLTQNWVDYRLSWDPSKYEGIEKLCIPSKHI
WLPDIVLYNNADGTYEVTVF
TNAIVQFNGSIFWLPPAIYKSACKIEVKHFPFDQQNCTLKFRSWTYDHTELDLVLKSEVASIDDFTPSGEWDILALPGRR
TTTPLDPAYVDLTYDFIIKR
KPLFYTINLIIPCVLITSLAILVFYLPSDCGEKMTLCISVLLALTVFLLLISKIVPPTSLDVPLIGKYLMFTMVLVTFSI
ITSVCVLNVHHRSPSTHTMP
SWVKMVFLIKLPTLLFMRRPQDNIVQQRLKKQRCERPSRSMLAVGHDLRRAGATMSCSSSALLASDSVFSIPGHFYNRDA
VPPLAHRHMNRKVDVEYLPI
GSVCSQDHRPRPDQDWATELQDAVNKVRFVADHTMEDHDDQSVVEDWKYVAMVVDRLFLWIFVIVCVLGTLGLFLQPVFQ
KPVVPAQGPGSETPRI
>g1116.t2
MNWLLVNQRYNKLIRPAMNRSERVTVKLQVSLAQLISVNWVDYRLSWDPSKYEGIEKLCIPSKHIWLPDIVLYNNADGTY
EVTVFTNAIVQFNGSIFWLP

PAIYKSACKIEVKHFPFDQQNCTLKFRSWTYDHTELDLVLKSEVASIDDFTPSGEWDILALPGRRTTTPLDPAYVDLTYD
FIIKRKPLFYTINLIIPCVL
ITSLAILVFYLPSDCGEKMTLCISVLLALTVFLLLISKIVPPTSLDVPLIGKYLMFTMVLVTFSIITSVCVLNVHHRSPS
THTMPSWVKMVFLIKLPTLL
FMRRPQDNIVQQRLKKQRCERPSRSMLAVGHDLRRAGATMSCSSSALLASDSVFSIPGHFYNRDAVPPLAHRHMNRKVDV
EYLPIGSVCSQDHRPRPDQD
WATELQDAVNKVRFVADHTMEDHDDQSVVEDWKYVAMVVDRLFLWIFVIVCVLGTLGLFLQPVFQKPVVPAQGPGSETPR
I
>g1116.t3
MSVPRPGSRGADSEERLMNWLLVNQRYNKLIRPAMNRSERVTVKLQVSLAQLISVNEREQIMTTNVWLTQNWVDYRLSWD
PSKYEGIEKLCIPSKHIWLP
DIVLYNNADGTYEVTVFTNAIVQFNGSIFWLPPAIYKSACKIEVKHFPFDQQNCTLKFRSWTYDHTELDLVLKSEVASID
DFTPSGEWDILALPGRRTTT
PLDPAYVDLTYDFIIKRKPLFYTINLIIPCVLITSLAILVFYLPSDCGEKMTLCISVLLALTVFLLLISKIVPPTSLDVP
LIGKYLMFTMVLVTFSIITS
VCVLNVHHRSPSTHTMPSWVKMVFLIKLPTLLFMRRPQDNIVQQRLKKQRCERPSRSMLAVGHDLRRAGATMSCSSSALL
ASDSVFSIPGHFYNRDAVPP
LAHRHMNRKVDVEYLPIGSVCSQDHRPRPDQDWATELQDAVNKVRFVADHTMEDHDDQSVVEDWKYVAMVVDRLFLWIFV
IVCVLGTLGLFLQPVFQKPV
VPAQGPGSETPRI
>g1117.t1
MPLNQTLPSQVGTRAAPSDQLERGSPQSPGPAEDDEGGCSEAEHRLFSVVFSHYNQFIRPVENVSDPVVVQFEVSMSQLV
KVPVVGDEWVFVMAGSQSRS
KRPSCLVAPSSQDQSSPRSLNNCKHR
>g1117.t2
MPLNQTLPSQVGTRAAPSDQLERGSPQSPGPAEDDEGGCSEAEHRLFSVVFSHYNQFIRPVENVSDPVVVQFEVSMSQLV
KVSRSKRPSCLVAPSSQDQS
SPRSLNNCKHR
>g1118.t1
LVTRSVRVLSDLLAGNTFAMAASKRLQRELEDIINSDAKNAQNVQVDESNIFNWQWLIIPENPPYDKGAFRVDIVFPVEY
PFKPPKITFKTKIYHPNIDE
KGQVCLPIITSENWKPATKTHQVFGR
>g1118.t2
LSDLLAGNTFAMAASKRLQRELEDIINSDAKNAQNVQVDESNIFNWQWLIIPENPPYDKGAFRVDIVFPVEYPFKPPKIT
FKTKIYHPNIDEKGQVCLPI
ITSENWKPATKTHQVFGR
>g1118.t3
DLLAGNTFAMAASKRLQRELEDIINSDAKNAQNVQVDESNIFNWQWLIIPENPPYDKGAFRVDIVFPVEYPFKPPKITFK
TKIYHPNIDEKGQVCLPIIT
SENWKPATKTHQVFGR
>g1119.t1
MSSEHVGASYLFFSGFPAGQTIEYFVLSYHGEFAFVTETEEGSIESFSGVRQGSRPEAFSLSLQGKLSFP
>g1120.t1
ITPQILNADNKKCTLPGTPTKVSCFKVKYCLKASGKGAPVTLSFRVELVLDRMKHKEATKRVLFLHSRTFQYAKNMSVFN
GQGPACEEQEAYLKNGLNLT
HYRLKAFIIQMLLAGDRLEYYFDHVNEPLPYSSMMGSMEWIKGPFYSLLE
>g1120.t2
ITPQILNADNKKCTLPGTPTKVSCFKVKYCLKASGKGAPVTLSFRVELVLDRMKHKEATKRVLFLHSRTFQYAKNMSVFN
GQGPACEEQEAYLKVSSC
>g1121.t1
IDLISNLLQVKMRKRFSVDKTLSHPWLQDYQMWSDLRSLELRVDERYITHESDDPRWSGHAPQAGPDHAPYPPCGPHGNM
IQGAGRYAEEEEELGTLSER
VSEL
>g1121.t2
IDLISNLLQVKMRKRFSVDKTLSHPWLQSRPVQTSPDQTRPVQSRPDQNLQCVVLLQDYQMWSDLRSLELRVDERYITHE
SDDPRWSGHAPQAGPDHAPY

PPCGPHGNMIQGAGRYAEEEEELGTLSERVSEL
>g1122.t1
MSRDRFNRNRGGGMNNFQPRRGGGPGGPMRGGLMGNPNFRNHPFGNQNRGGHMGNNFNKPPNGNQGPQTPQQKPPQTQST
PIIPPPAPGPALTMKGPIQQ
QKEQQQQQQQQQQQQQQQQQQQQQKLPTTPVPQPKISSPPPKPNIVSPPPKPNISSPPPKPNLSSPPPNQANKNPNQHQKP
PMNNVNGQQQRQMGHQGHKP
GQQHMNKPRPQQQLHKPDSGPEGGHPTELKATMSLLLKPGEKTYTQRCRLFIGNLPNDISDDEFKKLFAKYGEPSEVFIN
KNKGFGFIRLESRALAEIAK
AELDDTPMKGRPLRVRFATHSAALSIKNLSPFVSNELLEEAFSQFGMVERAVVIVDDRGHSTGKGLVEFASKHAARKALD
RCNEGVFLLTTSPRPVVVEP
LEQYDDEDGLPEKLAQKNPRYQKEREEPPRFARPGTFEFEYSKRWKSLDEMEKQQRQQVEVNMREAREKLESEMEDAFHE
HQASLLRQDLLRRQEELRRM
EEMHSQEMLKRKEMQIRQEEERRRREEEMIHKREMEEQMRRQREEKYRMGNFMDNRDRDMRMNPGGAMGMADMPFGSPNQ
KFPMGGLGFEGQQGMGGPSP
GGMMPNEMVRYPTPGNIRSYH
>g1122.t2
MGRADGTCEVSKGDGERRLQWMGTVCTEEGGLKRVEEELVRDGYQHFKAIIDQETKNGIPATRIMSAFSQVVSGSGNKNT
PILQCHGEMDPMILVQFGAM
AAEKLKNVVSHDEICFRSPIHRFNRNRGGGMNNFQPRRGGGPGGPMRGGLMGNPNFRNHPFGNQNRGGHMGNNFNKPPNG
NQGPQTPQQKPPQTQSTPII
PPPAPGPALTMKGPIQQQKEQQQQQQQQQQQQQQQQQQQQQKLPTTPVPQPKISSPPPKPNIVSPPPKPNISSPPPKPNLS
SPPPNQANKNPNQHQKPPMN
NVNGQQQRQMGHQGHKPGQQHMNKPRPQQQLHKPDSGPEGGHPTELKATMSLLLKPGEKTYTQRCRLFIGNLPNDISDDE
FKKLFAKYGEPSEVFINKNK
GFGFIRLESRALAEIAKAELDDTPMKGRPLRVRFATHSAALSIKNLSPFVSNELLEEAFSQFGMVERAVVIVDDRGHSTG
KGLVEFASKHAARKALDRCN
EGVFLLTTSPRPVVVEPLEQYDDEDGLPEKLAQKNPRYQKEREEPPRFARPGTFEFEYSKRWKSLDEMEKQQRQQVEVNM
REAREKLESEMEDAFHEHQA
SLLRQDLLRRQEELRRMEEMHSQEMLKRKEMQIRQEEERRRREEEMIHKREMEEQMRRQREEKYRMGNFMDNRDRDMRMN
PGGAMGMADMPFGSPNQKFP
MGGLGFEGQQGMGGPSPGGMMPNEMVRYPTPGNIRSYH
>g1122.t3
MRRSQAMGRADGTCEVSKGDGERRLQWMGTVCTEEGGLKRVEEELVRDGYQHFKAIIDQETKNGIPATRIMSAFSQVVSG
SGNKNTPILQCHGEMDPMIL
VQFGAMAAEKLKNVVSHDEICFRSPIHRFNRNRGGGMNNFQPRRGGGPGGPMRGGLMGNPNFRNHPFGNQNRGGHMGNNF
NKPPNGNQGPQTPQQKPPQT
QSTPIIPPPAPGPALTMKGPIQQQKEQQQQQQQQQQQQQQQQQQQQQKLPTTPVPQPKISSPPPKPNIVSPPPKPNISSPP
PKPNLSSPPPNQANKNPNQH
QKPPMNNVNGQQQRQMGHQGHKPGQQHMNKPRPQQQLHKPDSGPEGGHPTELKATMSLLLKPGEKTYTQRCRLFIGNLPN
DISDDEFKKLFAKYGEPSEV
FINKNKGFGFIRLESRALAEIAKAELDDTPMKGRPLRVRFATHSAALSIKNLSPFVSNELLEEAFSQFGMVERAVVIVDD
RGHSTGKGLVEFASKHAARK
ALDRCNEGVFLLTTSPRPVVVEPLEQYDDEDGLPEKLAQKNPRYQKEREEPPRFARPGTFEFEYSKRWKSLDEMEKQQRQ
QVEVNMREAREKLESEMEDA
FHEHQASLLRQDLLRRQEELRRMEEMHSQEMLKRKEMQIRQEEERRRREEEMIHKREMEEQMRRQREEKYRMGNFMDNRD
RDMRMNPGGAMGMADMPFGS
PNQKFPMGGLGFEGQQGMGGPSPGGMMPNEMVRYPTPGNIRSYH
>g1122.t4
MTRAWTKTCAAFWVSCEPDNLEAVVASWTFRWVREQRRGQSRERNESDGMRRSQAMGRADGTCEVSKGDGERRLQWMGTV
CTEEGGLKRVEEELVRDGYQ
HFKAIIDQETKNGIPATRIMSAFSQVVSGSGNKNTPILQCHGEMDPMILVQFGAMAAEKLKNVVSHDEICFRSPIHRGWR
L
>g1123.t1
LLELHSPDGLHTVVLRCKDAPTANSWFTAIHTNIAALLPHVLAHINAYLGATVASSSASHPHLKHIGWMAEQVTLPPSPP
PPPAPSPFGSCF
>g1123.t2

LLELHSPDGLHTVVLRCKDAPTANSWFTAIHTNIAALLPHVLAHINAYLGATVASSSASHPHLKHIGWMAEQ
>g1124.t1
MTDMWPSASSSTPPCPSFSSSSSSSIVLPLSPPLPPNTELLSVLTDTKLTLDVYRAGATALPVLWSFVPEQLKELQYLRL
GSEDKHGLEEALTTLPHLTR
LRSLAFRGLLTTLPASFSSLNLLVHLDLSFNQLSLLPPCLLTLPLLSSLLLGHNRLSLLPPDLGRLSSLRYLSLLGNGLL
ALPPSLGQLRALHTLDVSHN
RLADLPDEVGSLGELVTLELSHNQLRQLPETMASLRSLTELVVHSNQLHHVPASLHRLPLLKMDTRNNPLGRPPTPPPLP
PAAGGAELLFSPGCLSTTTR
LKWFEQKANRKWVWLDEHDILLSQPLELRPHGITFLKPVEVCVPYHRKRGRQVVVRRFSGELWSSLPTDLRRGSPSNSCH
PGGSPVRLACSSVDHFSWFV
AVARLTRDSCRVTPEGALLVSSRDPGIKLRFPPNSTVQIRTITLEVLQVPLAEIQAVCRDPQASASPLLCLSQTPSLNFL
QPVKVQIPLPSGVT
>g1124.t2
MTDMWPSASSSTPPCPSFSSSSSSSIVLPLSPPLPPNTELLSVLTDTKLTLDVYRAGATALPVLWSFVPEQLKELQYLRL
GSEDKHGLEEALTTLPHLTR
LRSLAFRGLLTTLPASFSSLNLLVHLDLSFNQLSLLPPCLLTLPLLSSLLLGHNRLSLLPPDLGRLSSLRYLSLLGNGLL
ALPPSLGQLRALHTLDVSHN
RLADLPDEVGSLGELVTLELSHNQLRQLPETMASLRSLTELVVHSNQLHHVPASLHRLPLLKMDTRNNPLGRPPTPPPLP
PAAGGAELLFSPGCLSTTTR
LKWFEQKANRKWVWLDEHDILLSQPLELRPHGITFLKPVEVCVPYHRKRGRQVVVRRFSGELWSSLPTDLRRGSPSNSCH
PGGSPVRLACSSVDHFSWFV
AVARLTRDSCRVTPEGALLVSSRDPGIKLRFPPNSTVQIRTITLEVPLAEIQAVCRDPQASASPLLCLSQTPSLNFLQPV
KVQIPLPSGVT
>g1124.t3
MTDMWPSASSSTPPCPSFSSSSSSSIVLPLSPPLPPNTELLSVLTDTKLTLDVYRAGATALPVLWSFVPEQLKELQYLRL
GSEDKHGLEEALTTLPHLTR
LRSLAFRGLLTTLPASFSSLNLLVHLDLSFNQLSLLPPCLLTLPLLSSLLLGHNRLSLLPPDLGRLSSLRYLSLLGNGLL
ALPPSLGQLRALHTLDVSHN
RLADLPDEVGSLGELVTLELSHNQLRQLPETMASLRSLTELVVHSNQLHHVPASLHRLPLLKMDTRNNPLGRPPTPPPLP
PAAGGAELLFSPGCLSTTTR
LKWFEQKANRKWVWLDEHDILLSQPLELRPHGITFLKPVEVCVPYHRKRGRQVVVRRFSGELWSSLPTDLRRGSPSNSCH
PGGSPVRLACSSVDHFSWFV
AVARLTRDSCRVTPEGALLVSSRDPGIKLRFPPNSTVQIRTITLEVLQVPLAEIQAVCRDPQASASPLLCLSQTPSLNFL
QPVKVQIPLPSGVTGS
>g1125.t1
MHPSFNLVKIMRHMLERTLPPDAHHRATGRLGISLTRVADGENVLVSVFNSKEEVAQACVCSAYIPVYCGLIPPTLRGVR
YVDGGISDNLPQYELKNTIT
VSPFSGESDICPRDSSTNLHELRFTNTSIQFTLKNLYRVSRALFPPDPMVMKAMCKQGYKDALQFLKRNGLLNLNGPYRD
RPLLLWNREEKEALVEACKE
RRSLSQSLSNLLPIRLASAMMLPYTLPLESAMSMTV
>g1125.t2
MHPSFNLVKIMRHMLERTLPPDAHHRATGRLGISLTRVADGENVLVSVFNSKEEVAQACVCSAYIPVYCGLIPPTLRGVR
YVDGGISDNLPQYELKNTIT
VSPFSGESDICPRDSSTNLHELRFTNTSIQFTLKNLYRVSRALFPPDPMVMKAMCKQGYKDALQFLKRNGLLNLNGPYRD
RPLLLWNREEKEALVEACKE
RRSLSQSLSNLLPIRLASAMMLPYTLPLESAMSMTVRIAATLIF
>g1125.t3
MHPSFNLVKIMRHMLERTLPPDAHHRATGRLGISLTRVADGENVLVSVFNSKEEVAQACVCSAYIPVYCGLIPPTLRGVR
YVDGGISDNLPQYELKNTIT
VSPFSGESDICPRDSSTNLHELRFTNTSIQFTLKNLYRVSRALFPPDPMVMKAMCKQGYKDALQFLKRNGLLNLNGPYRD
RPLLLWNREEKEGDVSSSGD
EEDEDIANADNIEERPHVEDSAEVHHSSSIEAHIFEHLPPNLHRALVEACKERRSLSQSLSNLLPIRLASAMMLPYTLPL
ESAMSMTV
>g1126.t1
PIVRPYAGAVGPGFLLVHDNARPHVARVCRQFLEDEGIDTIDWPPRSPDLNPIEHLWDIMFRSIRRRQVAPQTVQELSDA
LVQIWEEILQETIRRLIRSM

PRRCQACIQASGGNTKY
>g1127.t1
LARRLSGAAGRPLTGGLEVGSLPHGSSENITMVLEETQCLFCSSFPGLGANVRLCLVGSIFQVANSILAELEQVLVSRGQ
LERALSALKERLNEALPSVA
LLACSLIAAVVLHTLEPSVSFGEEVKPGDDTPYCVNIPVRREVEEERPPTQLEERLRRERQASREEFPPPPPLRWLIILE
PRLGTRPTCHLWLVWFY
>g1127.t2
LARRLSGAAGRPLTGGLEVGSLPHGSSENITMVLEETQCLFCSSFPGLGANVRLCLVGSIFQVANSILAELEQVLVSRGQ
LERALSALKERLNEALPSVA
LLACSLIAAVVLHTLEPSVSFGEEVKPGDDTPYCVNIPVRREVEEERPPTQLEERLRRERQASREEFPPPPPLRWLIILE
PRLGTRPTCHLWLESTEVKH
IKKRKKWFSWLRRLIPCMKTANDNETQHGTKHHRTSPKRTSTVTWATNMDI
>g1127.t3
LARRLSGAAGRPLTGGLEVGSLPHGSSENITMVLEETQCLFCSSFPGWPTPSWLSWSRCWSAEGSWRGLCRPSRRG
>g1128.t1
LFVVSLVIYSNYCKSARPGCDNASLNCSTALLNIRSLTSKGHLIQDLITDRKLDFLCLTETWQQPQDYSQLNKSTPSGFV
YICQPRGSGRGGGLAIIYRE
NWKVSQ
>g1128.t2
LVIYSNYCKSARPGCDNASLNCSTALLNIRSLTSKGHLIQDLITDRKLDFLCLTETWQQPQDYSQLNKSTPSGFVYICQP
RGSGRGGGLAIIYRENWKVS
Q
>g1129.t1
TFGAIEGAEPIGFPVGGKGQDADLETTVMPYLKVLSDFREGVRRVAREQKVTELLQLCDEVRDDTLPELGVRLEDHEGTG
LPTVVKLVDTETLLKERDEK
KRMEEEKRKKKEEAARKKQEQEGFPTHDPEGKEISKGQAKKLRKLYEAQEKLHQDYLQTSQNGS
>g1129.t2
TFGAIEGAEPIGFPVGGKGQDADLETTVMPYLKVLSDFREGVRRVAREQKVTELLQLCDEVRDDTLPELGVRLEDHEGLP
TVVKLVDTETLLKERDEKKR
MEEEKRKKKEEAARKKQEQEGFPTHDPEGKEISKGQAKKLRKLYEAQEKLHQDYLQTSQNGS
>g1129.t3
TFGAIEGAEPIGFPVGGKGQDADLETTVMPYLKVLSDFREGVRRVAREQKVTELLQLCDEVRDDTLPELGVRLEDHEGTG
LPTVVKLVDTETLLKERDEK
KRMEEEKRKKKEEAARKKQEQEMAKLAKMKIPACEMFLKEADKYSKFDETGFPTHDPEGKEISKGQAKKLRKLYEAQEKL
HQDYLQTSQNGS
>g1130.t1
LNQKARQKVVAVVPPVKASEAPLPSIPEESADVSMKEEELCQAFSDALLTVEDIDDQDGDLPHLCAEYVKDIYTYLMALE
VQPVSCRKLQLAGVTAMLVA
SKYEEIYSPEVADFAYITDDAFSKAQILQMEQLILRRLNFDLGRPLPLHFLRRASKAASSNVEKHTLAKYLMELTLVDYD
MVHYRPSEIAAASLCLSQLL
LEDLPWSPTQHHYSTYDESHLKPIMQHMAKNVVTVNEGKTKFQAVKNKYSSSKLMKISLIPHLKSPVVVDLAAPLLFH
>g1130.t2
LPYQKTLAKGTTKQALNQKARQKVVAVVPPVVPPVKAPEAPLPSMPEESADVSMKEEELCQAFSDALLTVEDIDDQDGDM
PQLCAEYVKDIYAYLLALEQ
KQSVRPKYLQGYEINERMRALLIDWLIQVHSRFQLLQETLYLTVAILDRFLQVQPVSRRKLQLAGVTAMLVASKYEEMYS
PEVADFAYITDNAFTKAQIL
QMEQLILRCLNFDLGRPLPLHFLRRASKAASSNVEKHTLAKYLMELTLVDYDMVHYRPSEIAAASLCLSQLLLEDLPWSP
TQHHYSTYDESHLKPIMQHM
AKNVVTVNEGKTKFQAVKNKYSSSKLMKISLIPQLKSAVVTSMAAPLIQQ
>g1130.t3
LNQKARQKVVAVVPPVKASEAPLPSIPEESADVSMKEEELCQAFSDALLTVEDIDDQDGDLPHLCAEYVKDIYTYLMALE
VQPVSCRKLQLAGVTAMLVA
SKYEEIYSPEVADFAYITDDAFSKAQILQMEQLILRRLNFDLGRPLPLHFLRRASKAASSNVEKHTLAKYLMELTLVDYD
MVHYRPSEIAAASLCLSQLL
LEDLPWSPTQHHYSTYDESHLKPIMQHMAKNVVTVNEGKTKFQAVKNKYSSSKLMKISLIPHLKSPVVKTLAKGTTKQAL
NQKARQKVVAVVPPVVPPVK

APEAPLPSMPEESADVSMKEEELCQAFSDALLTVEDIDDQDGDMPQLCAEYVKDIYAYLLALEQKQSVRPKYLQGYEINE
RMRALLIDWLIQVHSRFQLL
QETLYLTVAILDRFLQVQPVSRRKLQLAGVTAMLVASKYEEMYSPEVADFAYITDNAFTKAQILQMEQLILRCLNFDLGR
PLPLHFLRRASKAASSNVEK
HTLAKYLMELTLVDYDMVHYRPSEIAAASLCLSQLLLEDLPWSPTQHHYSTYDESHLKPIMQHMAKNVVTVNEGKTKFQA
VKNKYSSSKLMKISLIPQLK
SAVVTSMAAPLIQQ
>g1130.t4
LNQKARQKVVAVVPPVKASEAPLPSIPEESADVSMKEEELCQAFSDALLTVEDIDDQDGDLPHLCAEYVKDIYTYLMALE
QKQSVQPKYMQGYEINGRMR
ALLIDWLIQVHSRFQLLQETLYLTVAILDRFLQVQPVSCRKLQLAGVTAMLVASKYEEIYSPEVADFAYITDDAFSKAQI
LQMEQLILRRLNFDLGRPLP
LHFLRRASKAASSNVEKHTLAKYLMELTLVDYDMVHYRPSEIAAASLCLSQLLLEDLPWSPTQHHYSTYDESHLKPIMQH
MAKNVVTVNEGKTKFQAVKN
KYSSSKLMKISLIPHLKSPVVKTLAKGTTKQALNQKARQKVVAVVPPVVPPVKAPEAPLPSMPEESADVSMKEEELCQAF
SDALLTVEDIDDQDGDMPQL
CAEYVKDIYAYLLALEQKQSVRPKYLQGYEINERMRALLIDWLIQVHSRFQLLQETLYLTVAILDRFLQVQPVSRRKLQL
AGVTAMLVASKYEEMYSPEV
ADFAYITDNAFTKAQILQMEQLILRCLNFDLGRPLPLHFLRRASKAASSNVEKHTLAKYLMELTLVDYDMVHYRPSEIAA
ASLCLSQLLLEDLPWSPTQH
HYSTYDESHLKPIMQHMAKNVVTVNEGKTKFQAVKNKYSSSKLMKISLIPQLKSAVVTSMAAPLIQQ
>g1131.t1
MNIFTKVTGLAQQTSKLGSVLQRAFYGSSGKVTLFEQRNFAGRRLDLSSDCQKLSDKNFPERCNSVQVESGAWIGYEHDN
FRGRQYLWDMSERGEYNCHD
KWCAQVDHISSVRGIKQDNNPARAQLFERAGFSGKKTEIQDDVPNLMTRHSLNRAASIRVLGGAWVVYQEPNYRGPHYVL
EKRDFNNSSDWGSQNSTVGS
MRRVRFN
>g1131.t2
MNIFTKVTGLAQQTSKLGSVLQRAFYGSSGKVTLFEQRNFAGRRLDLSSDCQKLSDKNFPERCNSVQVESGAWIGYEHDN
FRGRQYLWDMSERGEYNCHD
KWCAQVDHISSVRGIKQDNNPARAQLFERAGFSGKKTEIQDDVPNLMTRHSLNRAASIREPNYRGPHYVLEKRDFNNSSD
WGSQNSTVGSMRRVRFN
>g1132.t1
MLLQSVLRTCRVLVLVLCVSAIEDFDWTKNDRGSFYYGVFPTGFSWGAGGSAYQTEGAWNVDGKGMSIWDAFAHKKGKIF
LNDTGDFSCEGYHKVKMLED
KYGGWQNSSMVNLFNDFANLCFERFGNRVKHWITFNNPWAHAKVWHTYDTQWRSKQKGLVGISLSGDWGEPVDITNQRDI
EAAERYVQFYMGWFATPLFN
GDYPQVMKDYVGRKSAQQGLGASRLPVFTAQEKSYIKGTCDFLGIGHFTTRYITQKNYPSGRGNSYFTDRDLAELVDPRW
PDPGSEWLYSVPWGFRRLLN
FVKTQYANPMIYVTENGVSEKMMCTDLCDDWRMQYFKDYINEMLKAIKDGVNIKAYTAWSLLDKFEWDEGYSERFGLYYV
DFRDKNKPRYPKASVQYYKS
IISSNGFPNQRELEESSGKIRDMQESQRLGQHMMKFRYTFRITSHPLISHMGMVTEIVVPTMCTLCILLSAVFLMFLLRK
RF
>g1132.t2
MLLQSVLRTCRVLVLVLCVSAIEDFDWTKNDRGSFYYGVFPTGFSWGAGGSAYQTEGAWNVDGKGMSIWDAFAHKKGKIF
LNDTGDFSCEGYHKVKDDIS
LMKDMNLNHYRFSISWPRILPTGIKSNPPSQPSHRKTSASCIFEDDCDVIMLNPDWFGFTYLSDENINEKGIKYYDDLIN
KLLENKITPIVTLYHWDLPQ
MLEDKYGGWQNSSMVNLFNDFANLCFERFGNRVKHWITFNNPWAHAKVWHTYDTQWRSKQKGLVGISLSGDWGEPVDITN
QRDIEAAERYVQFYMGWFAT
PLFNGDYPQVMKDYVGRKSAQQGLGASRLPVFTAQEKSYIKGTCDFLGIGHFTTRYITQKNYPSGRGNSYFTDRDLAELV
DPRWPDPGSEWLYSVPWGFR
RLLNFVKTQYANPMIYVTENGVSEKMMCTDLCDDWRMQYFKDYINEMLKAIKDGVNIKAYTAWSLLDKFEWDEGYSERFG
LYYVDFRDKNKPRYPKASVQ
YYKSIISSNGFPNQRELEESSGKIRDMQESQRLGQHMMKFRYTFRITSHPLISHMGMVTEIVVPTMCTLCILLSAVFLMF
LLRKRF

>g1132.t3
LPNIAMLLQSVLRTCRVLVLVLCVSAIEDFDWTKNDRGSFYYGVFPTGFSWGAGGSAYQTEGAWNVDGKGMSIWDAFAHK
KGKIFLNDTGDFSCEGYHKV
KMLEDKYGGWQNSSMVNLFNDFANLCFERFGNRVKHWITFNNPWAHAKVWHTYDTQWRSKQKGLVGISLSGDWGEPVDIT
NQRDIEAAERYVQFYMGWFA
TPLFNGDYPQVMKDYVGRKSAQQGLGASRLPVFTAQEKSYIKGTCDFLGIGHFTTRYITQKNYPSGRGNSYFTDRDLAEL
VDPRWPDPGSEWLYSVPWGF
RRLLNFVKTQYANPMIYVTENGVSEKMMCTDLCDDWRMQYFKDYINEMLKAIKDGVNIKAYTAWSLLDKFEWDEGYSERF
GLYYVDFRDKNKPRYPKASV
QYYKSIISSNGFPNQRELEESSGKIRDMQESQRLGQHMMKFRYTFRITSHPLISHMGMVTEIVVPTMCTLCILLSAVFLM
FLLRKRF
>g1133.t1
MSDSLGNGRFDIPDFDQLEDESFPPLPPPHSPGLGGDNPFGDEDGDLSKLEDVPAAKRKIIRKPQPKLNSQRYPADEMSG
SQDESCRSIRMLLLNISLQ
>g1133.t2
MSDSLGNGRFDIPDFDQLEDESFPPLPPPHSPGLGGDNPFGDEDGDLSKLEDVPAAKRKIIRKPQPKLNSQ
>g1134.t1
FLTWVNCSSVRWATRIQDVFTVGKLLALGLIIVVGLVQICNGNYESLRPQVAFAMETAPSIGQIALAFLQASFAFSGWNF
LNYVTEEVVDPRRNLPRAIY
ISVPVVTLVYTLTNIAYFCSMSPEELLASNAVAVTFGEKLLGMFSVIMPISVALSTFGGINGYLFTSSRLCFSGAREGHL
PSLLAMIHLKHCTPIPALLV
CCAATMVILCVGETHNLINYVSFINYLSYGVTVAALLYYRWKKPDLYRPIKVSLLVPVCYLCFWALLLGFSLLSEPLVCG
VGLLIILTGVPIYLLAVYWP
HKPHTVQQLLEWVTNVGQCLCYVVFPQVDAIETTPPSDWTDTSNMAAHS
>g1134.t2
MTSAYREKDVAAIDVNMGCPKEYSTKGGMGAALLSDPDKIEAILTKLVQGVTKPITCKIRILPSIEETVRLVRRIEKTGV
AAIAVHGRMRQERPRDPVHC
DFIQAVSRAVDVPVIANGGSLDMVKSFCDVEAFRSATGASGVMLARAAMWNPSVFRRQGLLPVQEVMEDYLRYAVRYDNH
AFNTKYCLCQMLRDKVESVM
GKQVQTAQTLAEICAAYGMQAFYEEVQALLRSRRERCPPETPLTQGGVTTMALRFQRREYPPEITPKMFLLEWSRKEKLE
QPHYHTVERPQDRLFQSTVS
VGNKQYRSSLWEKSKKGAEQAAAVVCLRVLRLPEGRLGEEDSGLVGKRKRAATALLNGSTAAAAAPQEEQEVEQEANKKI
HLSNREETAVSNGNHHHQPH
TP
>g1134.t3
FLTWVNCSSVRWATRIQDVFTVGKLLALGLIIVVGLVQICNGNYESLRPQVAFAMETAPSIGQIALAFLQASFAFSGWNF
LNYVTEEVVDPRRNLPRAIY
ISVPVVTLVYTLTNIAYFCSMSPEELLASNAVAVTFGEKLLGMFSVIMPISVALSTFGGINGYLFTSSREGHLPSLLAMI
HLKHCTPIPALLVCCAATMV
ILCVGETHNLINYVSFINYLSYGVTVAALLYYRWKKPDLYRPIKVSLLVPVCYLCFWALLLGFSLLSEPLVCGVGLLIIL
TGVPIYLLAVYWPHKPHTVQ
QLLEWVTNVGQCLCYVVFPQVDAIETTPPSDWTDTSNMAAHS
>g1134.t4
MTSAYREKDVAAIDVNMGCPKEYSTKILTKLVQGVTKPITCKIRILPSIEETVRLVRRIEKTGVAAIAVHGRMRQERPRD
PVHCDFIQAVSRAVDVPVIA
NGGSLDMVKSFCDVEAFRSATGASGVMLARAAMWNPSVFRRQGLLPVQEVMEDYLRYAVRYDNHAFNTKYCLCQMLRDKV
ESVMGKQVQTAQTLAEICAA
YGMQAFYEEVQALLRSRRERCPPETPLTQGGVTTMALRFQRREYPPEITPKMFLLEWSRKEKLEQPHYHTVERPQDRLFQ
STVSVGNKQYRSSLWEKSKK
GAEQAAAVVCLRVLRLPEGRLGEEDSGLVGKRKRAATALLNGSTAAAAAPQEEQEVEQEANKKIHLSNREETAVSNGNHH
HQPHTP
>g1134.t5
MKLFQDRWGARCRQVTLEVIXLSTMAAAGGLGFRGITALAPMVRVGTLPMRLLALDYGADMVYCEVGMDTRLTQPVYPEL
IDIKMVQXELDTVDFVAPDE
RVMFRTCEKERDRVVFQMGTADPERALAVARLVEKDVAAIDVNMGCPKEYSTKGGMGAALLSDPDKIEAILTKLVQGVTK
PITCKIRILPSIEETVRLVR

RIEKTGVAAIAVHGRMRQERPRDPVHCDFIQAVSRAVDVPVIANGGSLDMVKSFCDVEAFRSATGASGVMLARAAMWNPS
VFRRQGLLPVQEVMEDYLRY
AVRYDNHAFNTKYCLCQMLRDKVESVMGKQVQTAQTLAEICAAYGMQAFYEEVQALLRSRRERCPPETPLTQGGVTTMAL
RFQRREYPPEITPKMFLLEW
SRKEKLEQPHYHTVERPQDRLFQSTVSVGNKQYRSSLWEKSKKGAEQAAAVVCLRVLRLPEGRLGEEDSGLVGKRKRAAT
ALLNGSTAAAAAPQEEQEVE
QEANKKIHLSNREETAVSNGNHHHQPHTP
>g1134.t6
FLTWVNCSSVRWATRIQDVFTVGKLLALGLIIVVGLVQICNGNYESLRPQVAFAMETAPSIGQIALAFLQASFAFSGWNF
LNYVTEEVVDPRRNLPRAIY
ISVPVVTLVYTLTNIAYFCSMSPEELLASNAVAVTFGEKLLGMFSVIMPISVALSTFGGINGYLFTSSREGHLPSLLAMI
HLKHCTPIPALLVCCAATMV
ILCVGETHNLINYVSFINYLSYGVTVAALLYYRWKKPDLYRPIKVSLLVPVCYLCFWALLLGFSLLSEPLVCGVGLLIIL
TGVPIYLLAVYWPHKPHTVQ
QLLGLSTMAAAGGLGFRGITALAPMVRVGTLPMRLLALDYGADMVYCEVGMDTRLTQPVYPELIDIKMVQXELDTVDFVA
PDERVMFRTCEKERDRVVFQ
MGTADPERALAVARLV
>g1134.t7
MAAAGGLGFRGITALAPMVRVGTLPMRLLALDYGADMVYCEVGMDTRLTQPVYPELIDIKMVQXELDTVDFVAPDERVMF
RTCEKERDRVVFQMGTADPE
RALAVARLV
>g1135.t1
MVAGMSHLTVAHVVSYTQTLRHYPVFVCFHRGQGYRSELCYGTPNFLDRADPPLLRTGTFRAERFFTSIQ
>g1136.t1
LDLRTLWSPGYTQDLISPPCLPAPPHLLATSQHPHQTQGTQYGSLGHPRALPCPSIHITSIAPHSHMEPSSSTQGTEDTL
GSGVPEHARLSLEPRYRDTA
LTPSLCSSLSSRSWLSDLSSCESFSHVYDDVEGELEEAAARFAMGSPRA
>g1137.t1
MMSVFYENAHVKRAELWDVVGTDKWQINNILDDQYEPRPAHVVVREARALVGTKMSYCVFQRNCEHFATELRYGKARSRQ
>g1138.t1
MSVVCDGNPPKAMQTSHTHTPQNALSAQELSQEIKSFISGVDSVQGRKLSVREHARCAVRLLRSVPACRGAVLEHLRGVY
DEHVSAFLHHLETEGDASAG
VSSNLEDIIQEVHGVLSEFIRLNPRAWAPLVSTWAVDLLGQMSSKHAGRRATPHSSSLNELLQLWMSCAATRSLMEAYSQ
CLAAMLAWCPDACVDALLDT
SVQHSPHFDWVVAHIGSAFPGTIISRVLACGLKDFCSHGSKDPLLAPAGGDKGSRVPKIGSVVGILGHLAAHHSESIRKE
LLRMFQESLSPSGPLSPTSS
ASSWESSPQLRRSAVPFLLQLAALSPTLLGAVSAELVELLRPPVLLQLQALLLGLPREELENMLGLAVHLISQSPTGGAR
VLRFLADTATPASVIISGPT
PSPLDGIREACDRLLQMLLLHLHKLVYNRSDGTEGMGPSHHSAQPQRVIPFLEELQAHVGELCAETLRLERKRHLWLHQL
LCLLSVYGGPSVATEALCQL
LTQARNPEELALAWQLHTPLSACLPGLIPAAVGRCVAQIHTHTLGPRQLRQLLLNLAAAVQSQDEERRGAGPAAQASMAI
QVGGAVSGHLHDFCPLLLHG
DPAVSHAAARLLSTSPLPTR
>g1138.t2
MQTSHTHTPQNALSAQELSQEIKSFISGVDSVQGRKLSVREHARCAVRLLRSVPACRGAVLEHLRGVYDEHVSAFLHHLE
TEGDASAGVSSNLEDIIQEV
HGVLSEFIRLNPRAWAPLVSTWAVDLLGQMSSKHAGRRATPHSSSLNELLQLWMSCAATRSLMEAYSQCLAAMLAWCPDA
CVDALLDTSVQHSPHFDWVV
AHIGSAFPGTIISRVLACGLKDFCSHGSKDPLLAPAGGDKGSRVPKIGSVVGILGHLAAHHSESIRKELLRMFQESLSPS
GPLSPTSSASSWESSPQLRR
SAVPFLLQLAALSPTLLGAVSAELVELLRPPVLLQLQALLLGLPREELENMLGLAVHLISQSPTGGARVLRFLADTATPA
SVIISGPTPSPLDGIREACD
RLLQMLLLHLHKLVYNRSDGTEGMGPSHHSAQPQRVIPFLEELQAHVGELCAETLRLERKRHLWLHQLLCLLSVYGGPSV
ATEALCQLLTQARNPEELAL
AWQLHTPLSACLPGLIPAAVGRCVAQIHTHTLGPRQLRQLLLNLAAAVQSQDEERRGAGPAAQASMAIQVGGAVSGHLHD
FCPLLLHGDPAVSHAAARLL

STSPLPTR
>g1139.t1
LPCHIWFAVSAEPRPRGAVPPRRGAPPQGKARHSITQNPAGRPSPAFPLATRRRRDGTVTQGQRGPR
>g1139.t2
LPCHIWFAVSAEPRPRGAVPPRRGAPPQGKARHSITQNPAGRPSPAFPLATRRRRDGTVTQGQRGPRVA
>g1139.t3
MTRDPGRRLPCHIWFAVSAEPRPRGAVPPRRGAPPQGKARHSITQNPAGRPSPAFPLATRRRRDGTVTQGQRGPR
>g1140.t1
QRLFENLRMLPHAPGVQMQAIPEDAVQEDSGDEEE
>g1141.t1
MPCFLSPSQTSCIMADDLDIEAMLEAPFRKKLEGSKAHRVLLYCGPHGNTTVLWSPWQHDCTVVPTVT
>g1142.t1
MTIDQLREQHPLLDMVDHNRRPKEPEKRQIEAVILFGEPIRWETNLQLLVDVLLTNGSPASPHCLNMSAQLPILACNTDL
LWLADAPSPRLGHGVFMLCL
ETVFWKLAGQRLRYEAVMGKPSLLTYQHAELLIRQQTANQGWNTPVTTLYAIGDNLMTDVYGANSYNRYLKQQLMAPAAK
VLTQGRGTEWSAAPLQVEDQ
LDTPSATQCHSILVCTGVFNPGAALPIGQGNEDAVLMEPDYIVDDVEAAIDLIAQKEGFDL
>g1142.t2
MTIDQLREQHPLLDMVDHNRRPKEPVSPQEKRQIEAVILFGEPIRWETNLQLLVDVLLTNGSPASPHCLNMSAQLPILAC
NTDLLWLADAPSPRLGHGVF
MLCLETVFWKLAGQRLRYEAVMGKPSLLTYQHAELLIRQQTANQGWNTPVTTLYAIGDNLMTDVYGANSYNRYLKQQLMA
PAAKVLTQGRGTEWSAAPLQ
VEDQLDTPSATQCHSILVCTGVFNPGAALPIGQGNEDAVLMEPDYIVDDVEAAIDLIAQKEGFDL
>g1142.t3
MTIDQLREQHPLLDMVDHNRRPKEPVAVILFGEPIRWETNLQLLVDVLLTNGSPASPHCLNMSAQLPILACNTDLLWLAD
APSPRLGHGVFMLCLETVFW
KLAGQRLRYEAVMGKPSLLTYQHAELLIRQQTANQGWNTPVTTLYAIGDNLMTDVYGANSYNRYLKQQLMAPAAKVLTQG
RGTEWSAAPLQVEDQLDTPS
ATQCHSILVCTGVFNPGAALPIGQGNEDAVLMEPDYIVDDVEAAIDLIAQKEGFDL
>g1143.t1
MQVLRRGVLDQGLFSQIRGLCWRSRSSPTKPGLLLDVDGVLLRGRSVIPAARRVFQRLQDAAGHFLLPVVFVTNAGSCLR
HSKAQQLSDLLGVQVSPDQV
ILSHGPLQMFQSFHEKCVLVSGQGPVLDIAHEYPLAFDTSTNTITTTKATTSSNTITTATTTSNTITTTTSITIIIIIVL
ITLT
>g1143.t2
LLGSTPEQLTEQKNRDGSMQVLRRGVLDQGLFSQIRGLCWRSRSSPTKPGLLLDVDGVLLRGRSVIPAARRVFQRLQDAA
GHFLLPVVFVTNAGSCLRHS
KAQQLSDLLGVQVSPDQVILSHGPLQMFQSFHEKCVLVSGQGPVLDIAHEYPLAFDTSTNTITTTKATTSSNTITTATTT
SNTITTTTSITIIIIIVLIT
LT
>g1144.t1
MAEDPQIQVDELMSEQQEEEEGAESFIAHVPVPSQKEVEEALVRRKKMELLQRYASETLLAQSQEARTLLGL
>g1144.t2
VDELMSEQQEEEEGAESFIAHVPVPSQKEVEEALVRRKKMELLQRYASETLLAQSQEARTLLGL
>g1145.t1
LLRQRRLNYLGHYIDEDGIRPDETKVQGITQLQPPNNVHKLRRILGMIHYLGRYLPNLSEVTKPLTDLLKTEPEMTQDIA
ELTSELEAYEEAVCEAWPIS
PSKLHMVKQHTLQDEEL
>g1145.t2
LLRQRRLNYLGHYIDEDGIRPDETKVQGITQLQPPNNVHKLRRILGMIHYLGRYLPNLSEVTKPLTDLLKTEQLVIADTL
FRHPQPEMTQDIAELTSELE
AYEEAVCEAWPISPSKLHMVKQHTLQDEEL
>g1145.t3
RRLNYLGHYIDEDGIRPDETKVQGITQLQPPNNVHKLRRILGMIHYLGRYLPNLSEVTKPLTDLLKTEPEMTQDIAELTS
ELEAYEEAVCEAWPISPSKL
HMVKQHTLQDEEL

>g1146.t1
CMMAADMGLLMRGLAKLSQAVIETQATALRSGTGAAGVEAAAQNLQATAEQGIAAAMMKMQEFSGQQQTSSSSSSAFDSE
FDFPPAGEGAENHSFGQDEE
GFKFGEGSAEDAGQDAGAGGAPHAKGKQSLFEGYQDPSRQFTGQTRSYRRDPRHSVGHPTFYHQHLNRHGLRQLHRLDPA
SLSQFAGQPRGYHQDPTTVG
GLTAEDLEKARQGKRAEMKPHKQMLSERARERKVPVTRLGRLANFG
>g1147.t1
MAVCFCRWRNVFICLCSPCILLALLFVYITVMMCMTMAGSSELARVTSLHDVQHFIAQGAYRNEGWAPLPKLFWDQHLSL
GIWNLLQLSVDRSLNPILRP
HHHSGLPYRGTNVSYESLLAEGSPGAQDTAHRPDRLAGLPPGIQEFARHMHKRDYPVVMEPRGACGAGANDSGETLLLLA
IKTTKQNIRNRQTIRNTWGQ
TGWVGGQRGSGGGYVRRVFLLGTAPGRRPXXXXXXXRPGTEPSPLLQSEGLLHGDILWWDFKDSFFNLTLKDVLFWEWFS
GECRRTAFVFKGDDDVFVNT
PALVDYLRRQAGVPGAAGAMGDFVVGDVIEAAQPDRRMDSKYFVPEAFYSGAYPPYAGGGGLVYSGLLTRHLHLVSKRVH
LYPIDDVYVGMCLQRLNAHP
SHHAAFLTFDFSSDEEMEPCAYHKTIMVHKRTPRRVAQLWMQLKERRPQCWNVTLRDDRKN
>g1147.t2
LMAVCFCRWRNVFICLCSPCILLALLFVYITVMMCMTMAGSSELARVTSLHDVQHFIAQGAYRNEGWAPLPKLFWDQHLS
LGIWNLLQLSVDRSLNPILR
PHHHSGLPYRGTNVSYESLLAEGSPGAQDTAHRPDRLAGLPPGIQEFARHMHKRDYPVVMEPRGACGAGANDSGETLLLL
AIKTTKQNIRNRQTIRNTWG
QTGWVGGQRGSGGGYVRRVFLLGTAPGRRPXXXXXXXRPGTEPSPLLQSEGLLHGDILWWDFKDSFFNLTLKDVLFWEWF
SGECRRTAFVFKGDDDVFVN
TPALVDYLRRQAGVPGAAGAMGDFVVGDVIEAAQPDRRMDSKYFVPEAFYSGAYPPYAGGGGLVYSGLLTRHLHLVSKRV
HLYPIDDVYVGMCLQRLNAH
PSHHAAFLTFDFSSDEEMEPCAYHKTIMVHKRTPRRVAQLWMQLKERRPQCWNVTLRDDRKN
>g1148.t1
MSQATKRKHVVQEVLGEFVNPKENQQIVKVIGSRGNNLHEVVTAQGEAFLVSMPTKFRKNIWIKRGDYVIVDPINEGEKV
KAEISVILYKDHIQHLQRLQ
LWPDGFVEQPASQEKTDRRPERRAREEGAEEGKDEEEDEGNGSSSSDSEDDDSDLFVNTNRPSFHHSESEEEEEEEDEDE
DKEESQEDTEERTHAGS
>g1148.t2
MSQATKRKHVVQEVLGEFVNPKENQQIVKGEAFLVSMPTKFRKNIWIKRGDYVIVDPINEGEKVKAEISVILYKDHIQHL
QRLQLWPDGFVEQPASQEKT
DRRPERRAREEGAEEGKDEEEDEGNGSSSSDSEDDDSDLFVNTNRPSFHHSESEEEEEEEDEDEDKEESQEDTEERTHAG
S
>g1149.t1
ESTTEETNSVDDLVAPTGHPMQENVELLGMFKEFMGKYNKVYRSQEEADRRLQIFHQNLKTAEKLQSLDQGSAEYGVTKF
SDLTEEEFRSIYLNPLLSQW
TLHRPMKPAAPARGPAPDSWDWRDHGAVTSVKNQGMCGSCWAFSVTGNIEGQWFLQNGKLLSLSEQELLDCDKLDQACRG
GLPSNAYESIETLGGLETES
DYSYTGHKDRCDFTSKKVVAYINSSLELSKDEKEIAAWLAENGPVSVALNAFAMQFYRKGVSHPLRIFCSPWMIDHAVLL
VGYGQRKGIPFWAIKNSWGE
DYGEQGYYYLYRGAKLCGINQMGSSAVVN
>g1149.t2
MLLRPPDKHAEQRQRGSPAHSRCLSGGCQINALHAVVCTSDVVPKGTVKRSHRTSVLSESTTEETNSVDDLVAPTGHPMQ
ENVELLGMFKEFMGKYNKVY
RSQEEADRRLQIFHQNLKTAEKLQSLDQGSAEYGVTKFSDLTEEEFRSIYLNPLLSQWTLHRPMKPAAPARGPAPDSWDW
RDHGAVTSVKNQGMCGSCWA
FSVTGNIEGQWFLQNGKLLSLSEQELLDCDKLDQACRGGLPSNAYESIETLGGLETESDYSYTGHKDRCDFTSKKVVAYI
NSSLELSKDEKEIAAWLAEN
GPVSVALNAFAMQFYRKGVSHPLRIFCSPWMIDHAVLLVGYGQRKGIPFWAIKNSWGEDYGEQGYYYLYRGAKLCGINQM
GSSAVVN
>g1150.t1
MDTTQPSADTSVTTREANMNAEPQFTASSAPSSERLDPSTDTEKLSHAELLPGETVVKEGKAAILFPSANEVFYNPVQEF
NRDLTCAVITEFAREWMAQK

GVKLLVPGEKDRVVVSLSEEEKESTSGVALEEEEEEEEKIGKEELTATASVGESCE
>g1150.t2
MNAEPQFTASSAPSSERLDPSTDTEKLSHAELLPGETVVKEGKAAILFPSANEVFYNPVQEFNRDLTCAVITEFAREWMA
QKGVKLLVPGEKDRVVVSLS
EEEKESTSGVALEEEEEEEEKIGKEELTATASVGESCE
>g1151.t1
MSRMTGPQRERIKRSSISVRVAGQILLGHAP
>g1151.t2
LRDCAEELKDISMDIFNTSLSQAAVPSHFKTSIIIPVPKKPAPSCFNDYRPVALTPIVMKCFERLVMSHIKSILPPTLDP
SQFAYRPKRSTEDAISSALH
PALTHLEKADSYVRMLFIDFSSAFNTIIPQQLICKLDQLGLNTSLCNWVLDFLSERPQAVRVGNNTSSSITLSTGAPQGC
VLSPLLFTLLTQHTSHPAHS
LFSLLPFRQKV
>g1151.t3
LRDCAEELKDISMDIFNTSLSQAAVPSHFKTSIIIPVPKKPAPSCFNDYRPVALTPIVMKCFERLVMSHIKSILPPTLDP
SQFAYRPKRSTEDAISSALH
PALTHLEKADSYVRMLFIDFSSAFNTIIPQQLICKLDQLGLNTSLCNWVLDFLSERPQAVRVGNNTSSSITLSTGAPQGC
VLSPLLFTLLTQHTSHPAHS
LFSLLPFRQKKKKKSQNRTTWQDEGSWVSPMSRMTGPQRERIKRSSISVRVAGQILLGHAP
>g1152.t1
RQLLQRGPTTITTTITNATTITTHHHHHHHHHSPAASPPPPSLTSSVTATTTITTTITTTTTTTITTTSTTHQQRHHLER
DPVHAALDAVLLQAAVHPSR
GVPGRQLERNETHMGDRRRRRRDA
>g1153.t1
MDTDVLGAHAVMESAPDASAKGKTKGNSGMRRSEKPPYSYIALIVMAIQSSPTKRLTLSEIYQFLQARFPFFRGSYQGWK
NSVRHNLSLNECFMKLPKGL
GRPGKGHYWTIDPGSEFMFEEGSFRRRPRGFRRKCQALKPMYRMMNSFGFGPPMLQQNFDFQSTSASLPCHANAYNIDFI
GNTVPSNFEALGGGHHVTHM
SPSPGSGVTLFY
>g1154.t1
MQARYSVSSPNSLGVVPYISSDPSYYRAAAGGGYTGMPAPMNMYSHAAHDQYPASMARAYGPYTPQPQPKDMVKPPYSYI
ALITMAIQNSPDKKVTLNGI
YQFIMERFPFYRDNKQGWQNSIRHNLSLNECFVKVPRDDKKPGKGSYWTLDPDSYNMFENGSFLRRRRRFKKKDAMKEKE
DRLQKDTPARQQGRDQEQPV
QGSQPVRIQDIKTENGNVTPPQADSPSLSAVPKIESPDSSSSMSSGSPQSIPSNRSIVMENTDHQHHHHHGPASTQGFSV
DNIMTSLRASPQGATELPPG
LLASSRTPGITPSLSLNYSPNQTSVYNSQCNQNSISTTSNATYHCNMQAMSLYAGDRSSHLAPSTTVEDTLPDYSITTSS
SSLGHGNLNSGQDGGHHPHQ
GRLTSWYLNQAGDLGHLGAAYPTQQQNFHSVREIFESQRIGLNNSPVNGNNSCQMSFPPSQSIYRTSGAFVYDCSKF
>g1155.t1
MSGSLASVGLLGFEEDFGQAQWELLWSLILLFTFCLLLVWCYFWWEAHNDYNEFNXALPVMASRSSACSGVRWVIVLLQA
VLWLPSDSVLPQESLIRL
>g1156.t1
MAEVSQRLSSGAHGLSALLLTQCRPVRPVQGVNLSSSCQSTGASASGAAPVSAEVEPMASKGLPGDKFRPQQVKPHLHIL
DLRSIARAYSSNHTKEVGPL
YRSRTAYYDLLKVSPHATQSQIKTAYYKQSFIFHPDKNPGNKDATLRFSEVSEAYTVLGNIGLRRKYDSGILSPSDVHSA
GRPSSKEARSRAPGPQRHHP
QHPRPSASQQQYSRVGGRPMFDFDAFYQAHYGEQLEREKRARARKELLEARRKHNIEKWRRDKMWDLTIGVLLAAGGLIL
ISIR
>g1157.t1
MIEIQTQMSERAVELLNLPEGQPCFLLDVGCGSGLSGDYLSEEGHVWVGVDISTAMLGSFNISCCCNGVRFPNVSKFCMG
SVLNVAGLCCADVALDREVE
GDLVLGDMGQGMPFKAGTFDGCISISALQWLCNADKKSHSPPRRLHAFFSSLYSSLLELITSQAMRAGFSGGMVVDYPNS
SKAKKNLKGKSLKKGKEWIL
DKKERRRRQGR
>g1157.t2

MIEIQTQMSERAVELLNLPEGQPCFLLDVGCGSGLSGDYLSEEGHVWVGVDISTAMLGSFNISCCCNGVRFPNVSKFCMG
SVLNVAGLCCADVALDREVE
GDLVLGDMGQGMPFKAGTFDGCISLITFQSRGGRAVFQLYPENSEQLELITSQAMRAGFSGGMVVDYPNSSKAKKNLKGK
SLKKGKEWILDKKERRRRQG
R
>g1157.t3
LDGSDGSTLGQQEGRQPVSPAAQSLTDLCHFAGAARAGGKNKEAQLLHPFYNQEEAKKYSQNSRMIEIQTQMSERAVELL
NLPEGQPCFLLDVGCGSGLS
GDYLSEEGHVWVGVDISTAMLGSFNISCCCNGVRFPNVSKFCMGSVLNVAGLCCADVALDREVEGDLVLGDMGQGMPFKA
GTFDGCISISALQWLCNADK
KSHSPPRRLHAFFSSLYSSLLELITSQAMRAGFSGGMVVDYPNSSKAKKNLKGKSLKKGKEWILDKKERRRRQGR
>g1158.t1
MEMEGVEAAVKRGLMKYLYLAGAAEGGRAWNARAGVRPAVPTLSHRLSHRRATLGRLAPALALSRSVFVLHTRCFDFD
>g1159.t1
MFRGADRRPYSETDADPQKENRTPVFNGYRHRTQSESSDDGFDQYLVERATPKGKAPLQKHCTAKKNSMKHFIVDDFSSD
DDFVQKKPSRVPKSSRTPVS
LASVRTPLVRCDSPVFLSDSDDDNGIVVKSTWRTRHKKPQAPAETRTPSGMLARDDDEGSPPVFQSPLPLPSFCLPPVQV
PAHRPTSRRAVPPAPPTRVH
SAPTNFDNSSSSEEEFASLLERLKRKNTVGGNSPAIRNTGCFLQSLSVPGSSYERNFKQMKESLTSKLYQLYNTSVFDSK
LPTDMSVTWNKKMRKTAGYC
ISGQERVGGKRYTRIELSEKVCDSPDRLRDTLVHEMCHAATWLINEVRDGHGSFWKLYARKATLAHPELPMVTRCHSYDI
KYKFQYQCTRCKNTIGRHSK
SLDTQKFVCALCTGALVLLTPASTRAPTPFANFVKENYGNVRQELVGKSHADVMRKLSADFATKTKLGQS
>g1159.t2
MFRGADRRPYSETDADPQKENRTPVFNGYRHRTQSESSDDGFDQYLVERATPKGKAPLQKHCTAKKNSMKHFIVDDFSSD
DDFVQKKPSRVPKSSRTPVS
LASVRTPLVRCDSPVFLSDSDDDNGIVVKSTWRTRHKKPQAPAETRTPSGMLARDDDEGSPPVFQSPLPLPSFCLPPVQV
PAHRPTSRRAVPPAPPTRVH
SAPTNFDNSSSSEEEFASLLERLKRKNTVGGCFLQSLSVPGSSYERNFKQMKESLTSKLYQLYNTSVFDSKLPTDMSVTW
NKKMRKTAGYCISGQERVGG
KRYTRIELSEKVCDSPDRLRDTLVHEMCHAATWLINEVRDGHGSFWKLYARKATLAHPELPMVTRCHSYDIKYKFQYQCT
RCKNTIGRHSKSLDTQKFVC
ALCTGALVLLTPASTRAPTPFANFVKENYGNVRQELVGKSHADVMRKLSADFATKTKLGQS
>g1159.t3
MTSETHGMFRGADRRPYSETDADPQKENRTPVFNGYRHRTQSESSDDGFDQYLVERATPKGKAPLQKHCTAKKNSMKHFI
VDDFSSDDDFVQKKPSRVPK
SSRTPVSLASVRTPLVRCDSPVFLSDSDDDNGIVVKSTWRTRHKKPQAPAETRTPSGMLARDDDEGSPPVFQSPLPLPSF
CLPPVQVPAHRPTSRRAVPP
APPTRVHSAPTNFDNSSSSEEEFASLLERLKRKNTVGGNSPAIRNTGCFLQSLSVPGSSYERNFKQMKESLTSKLYQLYN
TSVFDSKLPTDMSVTWNKKM
RKTAGYCISGQERVGGKRYTRIELSEKVCDSPDRLRDTLVHEMCHAATWLINEVRDGHGSFWKLYARKATLAHPELPMVT
RCHSYDIKYKFQYQCTRCKN
TIGRHSKSLDTQKFVCALCTGALVLLTPASTRAPTPFANFVKENYGNVRQELVGKSHADVMRKLSADFATKTKLGQS
>g1160.t1
KRALRDLEAGVSHADGLRARQQDEERKQQQELERRAAALKREAELQRAAVRQLRAQSQQQQQLLTAREQEHRKELASRLT
PGGPEFREAVASEVASAQQR
HAQREAELEEKVAAGRSQYGALEDEFRMALTIEAARFSEVRQRADGVPFNSASGLLPGGVPAAASRRRRPVKEACDHLSA
ELSESRASLAQTQQRERRSS
ALVQELTAMVKEQKRRISELAHAKKEAVHELKGRLHSLEAEAEQARRLSVQLELLKKDKSRLLSQLTAQESVIDGLRAER
KIWGQELAQQGASLAQDRGR
LEARVEVLATELETQKKHGEQDGDALRIKTKIIDDQTGTIRKLKEGTQERDEQIRRLREEAAQAQKSFQKRLQEEAAPLA
ELRDRVEQLTLRKEQLKQQL
EDKELEMEELKDVYRQGVDCSSKKKWQEKAELLTRLEGQVKRMKDNFDSKERLLLEDRDRAADAHKAAVDKLHSVDDAFR
RQLESLQASHHAELLRLASD
KQKQLERANQKVIQVEEEMRMLLEETESSKRAMGEKMSRLSSVLKDF
>g1160.t2

KRALRDLEAGVSHADGLRARQQDEERKQQQELERRAAALKREAELQRAAVRQLRAQSQQQQQLLTAREQEHRKELASRLT
PGGPEFREAVASEVASAQQR
HAQREAELEEKVAAGRSQYGALEDEFRMALTIEAARFSEVKEACDHLSAELSESRASLAQTQQRERRSSALVQELTAMVK
EQKRRISELAHAKKEAVHEL
KGRLHSLEAEAEQARRLSVQLELLKKDKSRLLSQLTAQESVIDGLRAERKIWGQELAQQGASLAQDRGRLEARVEVLATE
LETQKKHGEQDGDALRIKTK
IIDDQTGTIRKLKEGTQERDEQIRRLREEAAQAQKSFQKRLQEEAAPLAELRDRVEQLTLRKEQLKQQLEDKELEMEELK
DVYRQGVDCSSKKKWQEKAE
LLTRLEGQVKRMKDNFDSKERLLLEDRDRAADAHKAAVDKLHSVDDAFRRQLESLQASHHAELLRLASDKQKQLERANQK
VIQVEEEMRMLLEETESSKR
AMGEKMSRLSSVLKDF
>g1160.t3
KRALRDLEAGVSHADGLRARQQDEEVAPPPPPQFGEEAAGTPVWSLPVLRFQRKQQQELERRAAALKREAELQRAAVRQL
RAQSQQQQQLLTAREQEHRK
ELASRLTPGGPEFREAVASEVASAQQRHAQREAELEEKVAAGRSQYGALEDEFRMALTIEAARFSEVRQRADGVPFNSAS
GLLPGGVPAAASRRRRPVKE
ACDHLSAELSESRASLAQTQQRERRSSALVQELTAMVKEQKRRISELAHAKKEAVHELKGRLHSLEAEAEQARRLSVQLE
LLKKDKSRLLSQLTAQESVI
DGLRAERKIWGQELAQQGASLAQDRGRLEARVEVLATELETQKKHGEQDGDALRIKTKIIDDQTGTIRKLKEGTQERDEQ
IRRLREEAAQAQKSFQKRLQ
EEAAPLAELRDRVEQLTLRKEQLKQQLEDKELEMEELKDVYRQGVDCSSKKKWQEKAELLTRLEGQVKRMKDNFDSKERL
LLEDRDRAADAHKAAVDKLH
SVDDAFRRQLESLQASHHAELLRLASDKQKQLERANQKVIQVEEEMRMLLEETESSKRAMGEKMSRLSSVLKDF
>g1161.t1
MSLSKTCPELLGDVDVLLERQRGLAFGSKRNLQMAVKHRVKVLTRSMCYRVVETWMRAGLRRAVDTSF
>g1162.t1
VMQVVREQITRTLSSKPTSLELFKNKVNALNYSEILKLRQTERLHQEETLAPPVVELKERLKPELMELIRQQRLNRLCQG
TLFRKISSRRRQDKLWYCRL
SPNHKVLHYGDVEEDTENPQIELLQEKIPVADIKALLTGKDCPHMKENKGKQTKEGLDLAFSITYDVEDYNLNFIASSRA
DFCLWTDGLNVLLGRDMSSE
SMRSELEILLSMEIKLRLLDLENVQIPDHAPPIPLPPSSYHFCYDFSQAEQ
>g1162.t2
LRASPNSREANILATIRFHSQVMQVVREQITRTLSSKPTSLELFKNKVNALNYSEILKLRQTERLHQEETLAPPVVELKE
RLKPELMELIRQQRLNRLCQ
GTLFRKISSRRRQDKLWYCRLSPNHKVLHYGDVEEDTENPQIELLQEKIPVADIKALLTGKDCPHMKENKGKQTKEGLDL
AFSITYDVEDYNLNFIASSR
ADFCLWTDGLNVLLGRDMSSESMRSELEILLSMEIKLRLLDLENVQIPDHAPPIPLPPSSYHFCYDFSQAEQ
>g1163.t1
MNSSSPPQIFTCCSPVAPEGLIQTKSGAAAQIASFSHSWHTDEDFYLVHLDLPPCHNPASIFLFPFVLNPIIIIIIIIII
IIIIIIIIIIHYIYIYIKG
RRFCPKRQTFVRR
>g1164.t1
MLNQIKEKVDTKRADLSTRLLCQAKEEAHRSGDRILYNQARNTLTKEIKVAKRSYTEKLKNRFSANNPANDPASVWRGLQ
ELTSYRKPPPQTVENLQLA
>g1165.t1
MENHPTFLYFAYGSNLLKERLQLQNPSAAVHCVARLKDYKLAFGNYKGRASDRWCGGVATMEQSLLQEVWGVVWRMNMSD
LESLDSQENVGLGAYSPVEV
VVQARDQELSCRTYIMNSCVRAAPSPQYLKSGQHVDVNNGPRSGSPAARFPHGSVPVVAAVGCQTVPMHREHKTTAGIYS
QVLWVVVMGAVQNGLPQQYQ
DMLRAIETNGYDGPLLVMTDLQLALEKAGETSEDNSEPKHTQNYTV
>g1165.t2
MNDFNPCATSPPYKQVVCLGARQRGLPLDYIEKLEAVPTNGAGEDSTFESVSPSASTSASTLHDRIDFTAFAQPDVLRTR
SASTRCVHGRIITRHRELLV
SRAKGVQCILDNLLQSDFFCVEDVEIVHRTPTKSDQQEEARLDDLYTLTQMEFLNDCNESLGFLQNLDQLLDESGVFNPQ
ADVVYIMGDAGMGKSLLLQK
LQNVWSKRELQTDAIFFFRFRCRMFTAFRETAEISLRDLLFKHSCYPDNDPDDEVFDYIVQFPERTIFTFDGYDEIEGDV

DLLEVTQVVSPEDKAHPLVL
LINLLCGKLLGGSQKVLTARTGTDVLKRVVKKKVTLRGFSPANLQSYVALHFKKPEHRRLVSVHLDASPHLCGLCSTPLF
CWIVFKSFKHLRSVHKDFEI
PEKCFTLTDVFLLFAEVFLSHWALPAKHLRKKSARCTSETFKLGLRPLGAHAKLALQGMESGRFIFNQEEVSACDLTEED
LLMGFLRPVHCYDACGSEAT
YEFLHMTLQSFLAALALVLDNSIDVRSILKFFVECKRKRERFNFPWVPCVTKSQEPRRKELFKSNEHLQFTNLFLCGLLA
KDNGSLVEHMVAPQLLKKKR
ALLKTYLSTSIKSHLRGLPTYNSNEGKRVHVLPNFVWMLRCIFETGSNDVAQLTAKGITANFIKLGYCNMYSGDCTALNF
VLQHRQKLLGVDMDNNNIND
YGVQQLRPSFSKMTIVRLCVNHISDSSVKVLAEELCKHKIVKVLGLGKNEITSVGAKCLANAIKKSTSIFDVGMWGNTIG
DEGAKVFAEALRNHPSLNSL
SLSANGITSEGGKILAEALLENTVMTIFWLVENKLTDEVAPHLAKLVQANTGLLHLWLISNQLTTAGLGLLQEALTHNTA
LKEIW
>g1165.t3
LYRPIQVNVTTDQGEILCRSYQMNDFNPCATSPPYKQVVCLGARQRGLPLDYIEKLEAVPTNGAGEDSTFESVSPSASTS
ASTLHDRIDFTAFAQPDVLR
TRSASTRCVHGRIITRHRELLVSRAKGVQCILDNLLQSDFFCVEDVEIVHRTPTKSDQQEEARLDDLYTLTQMEFLNDCN
ESLGFLQNLDQLLDESGVFN
PQADVVYIMGDAGMGKSLLLQKLQNVWSKRELQTDAIFFFRFRCRMFTAFRETAEISLRDLLFKHSCYPDNDPDDEVFDY
IVQFPERTIFTFDGYDEIEG
DVDLLEVTQVVSPEDKAHPLVLLINLLCGKLLGGSQKVLTARTGTDVLKRVVKKKVTLRGFSPANLQSYVALHFKKPEHR
RLVSVHLDASPHLCGLCSTP
LFCWIVFKSFKHLRSVHKDFEIPEKCFTLTDVFLLFAEVFLSHWALPAKHLRKKSARCTSETFKLGLRPLGAHAKLALQG
MESGRFIFNQEEVSACDLTE
EDLLMGFLRPVHCYDACGSEATYEFLHMTLQSFLAALALVLDNSIDVRSILKFFVECKRKRERFNFPWVPCVTKSQEPRR
KELFKSNEHLQFTNLFLCGL
LAKDNGSLVEHMVAPQLLKKKRALLKTYLSTSIKSHLRGLPTYNSNEGKRVHVLPNFVWMLRCIFETGSNDVAQLTAKGI
TANFIKLGYCNMYSGDCTAL
NFVLQHRQKLLGVDMDNNNINDYGVQQLRPSFSKMTIVRLCVNHISDSSVKVLAEELCKHKIVKVLGLGKNEITSVGAKC
LANAIKKSTSIFDVGMWGNT
IGDEGAKVFAEALRNHPSLNSLSLSANGITSEGGKILAEALLENTVMTIFWLVENKLTDEVAPHLAKLVQANTGLLHLWL
ISNQLTTAGLGLLQEALTHN
TALKEIW
>g1165.t4
MAACDPDGLSVTTESSNSSDYFLYFAYGSNLLKERLQKKNPSATVVSNYALRFGWQQNRTQNNWHGGVATVERSDGAHVW
GCVWRMSSEDLQTLDELQSG
LYRPIQVNVTTDQGEILCRSYQMNDFNPCATSPPYKQVVCLGARQRGLPLDYIEKLEAVPTNGAGEDSTFESVSPSASTS
ASTLHDRIDFTAFAQPDVLR
TRSASTRCVHGRIITRHRELLVSRAKGVQCILDNLLQSDFFCVEDVEIVHRTPTKSDQQEEARLDDLYTLTQMEFLNDCN
ESLGFLQNLDQLLDESGVFN
PQADVVYIMGDAGMGKSLLLQKLQNVWSKRELQTDAIFFFRFRCRMFTAFRETAEISLRDLLFKHSCYPDNDPDDEVFDY
IVQFPERTIFTFDGYDEIEG
DVDLLEVTQVVSPEDKAHPLVLLINLLCGKLLGGSQKVLTARTGTDVLKRVVKKKVTLRGFSPANLQSYVALHFKKPEHR
RLVSVHLDASPHLCGLCSTP
LFCWIVFKSFKHLRSVHKDFEIPEKCFTLTDVFLLFAEVFLSHWALPAKHLRKKSARCTSETFKLGLRPLGAHAKLALQG
MESGRFIFNQEEVSACDLTE
EDLLMGFLRPVHCYDACGSEATYEFLHMTLQSFLAALALVLDNSIDVRSILKFFVECKRKRERFNFPWVPCVTKSQEPRR
KELFKSNEHLQFTNLFLCGL
LAKDNGSLVEHMVAPQLLKKKRALLKTYLSTSIKSHLRGLPTYNSNEGKRVHVLPNFVWMLRCIFETGSNDVAQLTAKGI
TANFIKLGYCNMYSGDCTAL
NFVLQHRQKLLGVDMDNNNINDYGVQQLRPSFSKMTIVRLCVNHISDSSVKVLAEELCKHKIVKVLGLGKNEITSVGAKC
LANAIKKSTSIFDVGMWGNT
IGDEGAKVFAEALRNHPSLNSLSLSANGITSEGGKILAEALLENTVMTIFWLVENKLTDEVAPHLAKLVQANTGLLHLWL
ISNQLTTAGLGLLQEALTHN
TALKEIW
>g1165.t5

MENHPTFLYFAYGSNLLKERLQLQNPSAAVHCVARLKDYKLAFGNYKGRASDRWCGGVATMEQSLLQEVWGVVWRMNMSD
LESLDSQENVGLGAYSPVEV
VVQARDQELSCRTYIMNSCVRAAPSPQYLKVNAVP
>g1165.t6
MENHPTFLYFAYGSNLLKERLQLQNPSAAVHCVARLKDYKLAFGNYKGRASDRWCGGVATMEQSLLQEVWGVVWRMNMSD
LESLDSQENVGLGAYSPVEV
VVQARDQELSCRTYIMNSCVRAAPSPQYLKSGQHVDVNNGPRSGSPAARFPHGSVPVVAAVGCQTVPMHREHKTTAGIYS
QVLWVVVMGAVQNGLPQQYQ
DMLRAIETNGYDGPLLVMTDLQLALEKAGETSEDNSEPKHTQNYTTRGGRGRNTVLQEAESTQDSQFWVCSVHITVRHKA
TGSQPGIPCTRNYALRFGWQ
QNRTQNNWHGGVATVERSDGAHVWGCVWRMSSEDLQTLDECVKQHHYY
>g1165.t7
MAACDPDGLSVTTESSNSSDYFLYFAYGSNLLKERLQKKNPSATVVSNYALRFGWQQNRTQNNWHGGVATVERSDGAHVW
GCVWRMSSEDLQTLDECVKQ
HHYY
>g1165.t8
MRLSAHPQSGQHVDVNNGPRSGSPAARFPHGSVPVVAAVGCQTVPMHREHKTTAGIYSQVLWVVVMGAVQNGLPQQYQDM
LRAIETNGYDGPLLVMTDLQ
LALEKAGETSEDNSEPKHTQNYTV
>g1166.t1
MSLLRNFSVEAALLFLSVAQLSQDVQEDSSHSEVPFSRRAGECGCAGPCVCHLCTNHTCETEPAGACWNSVTLVSGKEER
VQSCLTPAQYSGQMFCYGSH
NRSKRHCCSTDFCNNETLRLYS
>g1166.t2
MSLLRNFSVEAALLFLSVAQLSQDVQEDSSHSEVPFSRRAGECVCHLCTNHTCETEPAGACWNSVTLVSGKEERVQSCLT
PAQYSGQMFCYGSHNRSKRH
CCSTDFCNNETLRLYS
>g1166.t3
MSLLRNFSVEAALLFLSVAQLSQDVQEDSSHSEVPFSRRAGECGCAGPSLPVARAVFHAPVNAASAAARHGNADKMEGGL
ADCVPPGGPPALRCVCHLCT
NHTCETEPAGACWNSVTLVSGKEERVQSCLTPAQYSGQMFCYGSHNRSKRHCCSTDFCNNETLRLYS
>g1167.t1
MVAFTSLSLILLVLGPLSAVQAQLRLFNIRAKDLPSDIFGIADGYVEVSCGPAYLGQTSVQSNNANPWWEEEFVYFKAQP
NDVLRLTVHDSDILLDDLLG
TCQRPIKVGTHTHDCYLKKGTLNYSYTLDGDIGQADV
>g1168.t1
MYEKLAEETLDALVDFFEDLSDEPFTGAQYDVQFSSGVLTVTLGEQGTYVINKQTPNKQIWLSSPTSGPKRYDWTGTHWA
YSRDGVTLHQLLSKEFSVIF
KMDMDFSKLLYS
>g1168.t2
MYEKLAEETLDALVDFFEDLSDEPFTGAQYDVQFSGTYVINKQTPNKQIWLSSPTSGPKRYDWTGTHWAYSRDGVTLHQL
LSKEFSVIFKMDMDFSKLLY
S
>g1169.t1
MLTRLFRMHGMLVASHPWEVIVGTVTLTICMMSMNMFTGNDQICGWNFDCPKTEEQILSSDIIILTITRCIAIIYIYFQF
QNLRQLGSKYILGIAGLFTI
FSSFVFSTVVIHFLDKELTGLNEALPFFLLLIDLSKACALAKFALSSNSQDEVRDNIARGMAVLGPAFTLDAVVECLVIG
VGTMSGVRQLEIMCCFGCMS
VLANYFVFMTFFPACVSLVLELSRENQEGHPIWQLSHFSKVMEEEDNKPNPVTQRVKMIMSLGLVMVHAHSRWIAEPLNI
NTTMDVQVGMGFGHDSPRRI
HPETALWQFYLARMITMDVEQVICLGLALLLAVKYIFFEQVEMESTLSLKNPMSMSSPAGAQRHPAESCCRMEPLARPST
SPASPAQDHRQKLAPTQATL
SAIKEEKGTRIVQPQTQSVFFLGDGKTEGGPLPQTPNVETSTPTEPRHLDECLAILKNPEMGAGFLSDGEVMQLVNSKHI
PAYKLEALMESPERGVAIRR
QMISAKLPAPAALSSLPYADYDYSKVIGTCCENVIGYMPVPVGVAGPLHLDGKQFQVPMATTEGCLVASTNRGCRAIALG
GGASSRILADGMTRGPVVRL

PSACHAAQVKAWLESEEGFQAIKEVFDSTSRFARLQKLQICLAGRNLYIRFQSMTGDAMGMNMISKGTEQALSRLQEEFP
EVQVLAVSGNYCTDKKPAAI
NWIEGRGKSAVCEATIPAKVVREILKTTTASLVDVNINKNLVGSAMAGSIGGYNAHAANLVAAIYIACGQDPAQSVGSSN
CITLMEPSGPMGEDLYISCT
MPSIELGTVGGGTNLPPQQACLKMLGVQGACQECPGDNARQLARVVCATVLAGELSLMAALSAGHLVKSHMTHNRSKVNL
QQTPGTCTKNAS
>g1169.t2
MLTRLFRMHGMLVASHPWEVIVGTVTLTICMMSMNMFTGNDQICGWNFDCPKTEEQILSSDIIILTITRCIAIIYIYFQF
QNLRQLGSKYILGIAGLFTI
FSSFVFSTVVIHFLDKELTGLNEALPFFLLLIDLSKACALAKFALSSNSQDEVRDNIARGMAVLGPAFTLDAVVECLVIG
VGTMSGVRQLEIMCCFGCMS
VLANYFVFMTFFPACVSLVLELSRENQEGHPIWQLSHFSKVMEEEDNKPNPVTQRVKMIMSLGLVMVHAHSRWIAEPLNI
NTTMDVQVGMGFGHDSPRRI
HPETALWQFYLARMITMDVEQVICLGLALLLAVKYIFFEQVEMESTLSLKNPMSMSSPAGAQRHPAESCCRMEPLARPST
SPASPAQDHRQKLAPTQATL
SAIKEEKDEMIRPLPAPQVQPXXXXXXXPQVQPQTQSVFFLGDGKTEGGPLPQTPNVETSTPTEPRHLDECLAILKNPEM
GAGFLSDGEVMQLVNSKHIP
AYKLEALMESPERGVAIRRQMISAKLPAPAALSSLPYADYDYSKVIGTCCENVIGYMPVPVGVAGPLHLDGKQFQVPMAT
TEGCLVASTNRGCRAIALGG
GASSRILADGMTRGPVVRLPSACHAAQVKAWLESEEGFQAIKEVFDSTSRFARLQKLQICLAGRNLYIRFQSMTGDAMGM
NMISKGTEQALSRLQEEFPE
VQVLAVSGNYCTDKKPAAINWIEGRGKSAVCEATIPAKVVREILKTTTASLVDVNINKNLVGSAMAGSIGGYNAHAANLV
AAIYIACGQDPAQSVGSSNC
ITLMEPSGPMGEDLYISCTMPSIELGTVGGGTNLPPQQACLKMLGVQGACQECPGDNARQLARVVCATVLAGELSLMAAL
SAGHLVKSHMTHNRSKVNLQ
QTPGTCTKNAS
>g1170.t1
MQSLDQSLWPENFLFLSHVMCVLRGALRDHRTKENEPLIFMIHSMIENSAPRPQLYQQ
>g1170.t2
MCVLRGALRDHRTKENEPLIFMIHSMIENSAPRPQLYQQ
>g1170.t3
LISGLGMQSLDQSLWPENFLFLSHVMCVLRGALRDHRTKENEPLIFMIHSMIENSAPRPQLYQQ
>g1171.t1
MVFLKLAKGAGGSLALQAEGGVRLGHFLVFQYYLHSPTALSTLALAEPYDPASLYRQKVGLNKDDIIVANHGIQVPLGRS
VFLDPFNDLVTEVQPGDRCQ
ITVLDNDPLAQTPGTLSPKKFPCEFGTDEVKYTHFGARSPSTDRVKLQLRYDSQTETVIIPFMLEVTVVFRQLEIITRNM
PLSVDKLNGNSNPIDKKGLE
FSFEDGVTQCKIATLVGAGGLPRYGILLNNFTNGQMIDCDEFVKQGIRYKHTASTKSPNRDYIPMMVEMQDIDGNTIMQE
HFQMMVRIKEGVENTAPKPS
FVAMMMMEIDQFVMTAITSDMLAAEDVESDPDDLIFNITSPLGYEQGYVISTDDQNLPITSFYQRDIRDLKIAYKPPSED
SEVERIFQLEFEIVDTDGAV
SDTFAFMIVVKPMNTLAPVATKNTGQLLFEGQSRALSSAQNLEISDEDNLEDVRITVIDGLKHGALTVLGSRRKFFTPAD
LDAGIVVYQHDGSDTYSDNI
IFRMSDGSNDVEFLFPITIAPTDDEPPIINANTGLVLFENEVMQISPFILSATDIDSEDSTIKFIIEAPYSSIGQLLLRQ
VEIPTDPSVWLFSETDEMFE
QVVTEWFQQDILDGKLFYRHIGPHSTTTVIDHLVFRVQDDNDPPNQSGEHTFTIKIHAVDDLTPELSAGATLHMTVREYH
LTPLRSTYLSFSDLDSDDRD
IKYTITKSPTDTDENNPSHLGHIVLTDSPDTVITEFTQAQVNHHKVAYKPPDQELGITPKVVQFTFTVEDTSGNTADGVF
TIFLQPVDNKPPVITNTGFT
VLERSMYIIGKKDLHATDKDTEDENIIFTVTQVPHYGKLQNLGIDMSNGETFRLQDIANSHLSYVHSGKESLSDVIKIDV
SDQFHVVPIIIKITVEPVDD
ESPTITLPDGMLGASIDVLENGVTEITSNVIQGRDEDTDDLALTFIVEEPPRLGEILVNGIPAERFTQQDIINGVVVYAH
TSGEIGPVRAQDSFNLTLTD
MSEQWVVGGNKIQGLTVYVTILPVDSIPPVVSVGEQFVVVEGEKNVITLEHIQTEDIDTENDDILCTIIVQPTSGYVENI
SPAPGSEKSRAGTAITAFSV
KDIGLGHIYYVQSIHKGVEPVEDRFTFRCSDGINFSERHFFPIVIIPANDEKPEIFIREFVVMEGMSLVIDTPILNGADS

DIPGDELEFEIIKNPKYGTI
VQQLSTGSIPVTKFNLEQIKEGSNIVYEHNDSETKEDSFEIRLSDGKNTVVKKVIIMVISVDDETPRMAINDGLEVEIGE
TKIISNKVLKATDLDSEDKT
LTYVIRYGPTQGYLQRITKFGDVIGNITLGKNFTQDEVDRQLIQYVHTGQEGIRDLLKFDVTDGINPLIDRYFYINIGSI
DMVFPDVINKGVTLKEGGRV
TLTTDLLSTTDINSPDEHLSFSITRAPSRGHLESTDHPGVPISTFTQLQLAGNKVYYIHTSDDEMKMDSFEFEVTDGYNP
VFRTFRVSITDVDNKKPVLT
INKLVVGEGETKLITPFELTAEDRDTPDKHLRFIITQVPVHGQLLFNNTQPITSFTKQDLNQNLITYKHDGTETNEDSFS
FTVTDGTHADFYVFPDTVYE
TRKPQTMTIQINTVDNGVPQIVVNRAAASLRLLTTGHVGFLVTSKALRSEDRDSPRNVLKYVVTQAPLHGLIINTAVGND
SIKTFTQADIDDMKISYVLL
DGSNATSDVFYFSVEDHGRSHDVLASELTLVDTIDGTHLRAAVPLRGTHILL
>g1171.t2
MVFLKLAKGAGGSLALQAEGGVRLGHFLVFQYYLHSPTALSTLALVGLNKDDIIVANHGIQVPLGRSVFLDPFNDLVTEV
QPGDRCQITVLDNDPLAQTP
GTLSPKKFPCEFGTDEVKYTHFGARSPSTDRVKLQLRYDSQTETVIIPFMLEVTVVFRQLEIITRNMPLSVDKLNGNSNP
IDKKGLEFSFEDGVTQCKIA
TLVGAGGLPRYGILLNNFTNGQMIDCDEFVKQGIRYKHTASTKSPNRDYIPMMVEMQDIDGNTIMQEHFQMMVRIKEGVE
NTAPKPSFVAMMMMEIDQFV
MTAITSDMLAAEDVESDPDDLIFNITSPLGYEQGYVISTDDQNLPITSFYQRDIRDLKIAYKPPSEDSEVERIFQLEFEI
VDTDGAVSDTFAFMIVVKPM
NTLAPVATKNTGQLLFEGQSRALSSAQNLEISDEDNLEDVRITVIDGLKHGALTVLGSRRKFFTPADLDAGIVVYQHDGS
DTYSDNIIFRMSDGSNDVEF
LFPITIAPTDDEPPIINANTGLVLFENEVMQISPFILSATDIDSEDSTIKFIIEAPYSSIGQLLLRQVEIPTDPSVWLFS
ETDEMFEQVVTEWFQQDILD
GKLFYRHIGPHSTTTVIDHLVFRVQDDNDPPNQSGEHTFTIKIHAVDDLTPELSAGATLHMTVREYHLTPLRSTYLSFSD
LDSDDRDIKYTITKSPTDTD
ENNPSHLGHIVLTDSPDTVITEFTQAQVNHHKVAYKPPDQELGITPKVVQFTFTVEDTSGNTADGVFTIFLQPVDNKPPV
ITNTGFTVLERSMYIIGKKD
LHATDKDTEDENIIFTVTQVPHYGKLQNLGIDMSNGETFRLQDIANSHLSYVHSGKESLSDVIKIDVSDQFHVVPIIIKI
TVEPVDDESPTITLPDGMLG
ASIDVLENGVTEITSNVIQGRDEDTDDLALTFIVEEPPRLGEILVNGIPAERFTQQDIINGVVVYAHTSGEIGPVRAQDS
FNLTLTDMSEQWVVGGNKIQ
GLTVYVTILPVDSIPPVVSVGEQFVVVEGEKNVITLEHIQTEDIDTENDDILCTIIVQPTSGYVENISPAPGSEKSRAGT
AITAFSVKDIGLGHIYYVQS
IHKGVEPVEDRFTFRCSDGINFSERHFFPIVIIPANDEKPEIFIREFVVMEGMSLVIDTPILNGADSDIPGDELEFEIIK
NPKYGTIVQQLSTGSIPVTK
FNLEQIKEGSNIVYEHNDSETKEDSFEIRLSDGKNTVVKKVIIMVISVDDETPRMAINDGLEVEIGETKIISNKVLKATD
LDSEDKTLTYVIRYGPTQGY
LQRITKFGDVIGNITLGKNFTQDEVDRQLIQYVHTGQEGIRDLLKFDVTDGINPLIDRYFYINIGSIDMVFPDVINKGVT
LKEGGRVTLTTDLLSTTDIN
SPDEHLSFSITRAPSRGHLESTDHPGVPISTFTQLQLAGNKVYYIHTSDDEMKMDSFEFEVTDGYNPVFRTFRVSITDVD
NKKPVLTINKLVVGEGETKL
ITPFELTAEDRDTPDKHLRFIITQVPVHGQLLFNNTQPITSFTKQDLNQNLITYKHDGTETNEDSFSFTVTDGTHADFYV
FPDTVYETRKPQTMTIQINT
VDNGVPQIVVNRAAASLRLLTTGHVGFLVTSKALRSEDRDSPRNVLKYVVTQAPLHGLIINTAVGNDSIKTFTQADIDDM
KISYVLLDGSNATSDVFYFS
VEDHGRSHDVLASELTLVDTIDGTHLRAAVPLRGTHILL
>g1171.t3
MVFLKLAKGAGGSLALQAEGGVRLGHFLVFQYYLHSPTALSTLALASLYRQKVGLNKDDIIVANHGIQVPLGRSVFLDPF
NDLVTEVQPGDRCQITVLDN
DPLAQTPGTLSPKKFPCEFGTDEVKYTHFGARSPSTDRVKLQLRYDSQTETVIIPFMLEVTVVFRQLEIITRNMPLSVDK
LNGNSNPIDKKGLEFSFEDG
VTQCKIATLVGAGGLPRYGILLNNFTNGQMIDCDEFVKQGIRYKHTASTKSPNRDYIPMMVEMQDIDGNTIMQEHFQMMV
RIKEGVENTAPKPSFVAMMM
MEIDQFVMTAITSDMLAAEDVESDPDDLIFNITSPLGYEQGYVISTDDQNLPITSFYQRDIRDLKIAYKPPSEDSEVERI

FQLEFEIVDTDGAVSDTFAF
MIVVKPMNTLAPVATKNTGQLLFEGQSRALSSAQNLEISDEDNLEDVRITVIDGLKHGALTVLGSRRKFFTPADLDAGIV
VYQHDGSDTYSDNIIFRMSD
GSNDVEFLFPITIAPTDDEPPIINANTGLVLFENEVMQISPFILSATDIDSEDSTIKFIIEAPYSSIGQLLLRQVEIPTD
PSVWLFSETDEMFEQVVTEW
FQQDILDGKLFYRHIGPHSTTTVIDHLVFRVQDDNDPPNQSGEHTFTIKIHAVDDLTPELSAGATLHMTVREYHLTPLRS
TYLSFSDLDSDDRDIKYTIT
KSPTDTDENNPSHLGHIVLTDSPDTVITEFTQAQVNHHKVAYKPPDQELGITPKVVQFTFTVEDTSGNTADGVFTIFLQP
VDNKPPVITNTGFTVLERSM
YIIGKKDLHATDKDTEDENIIFTVTQVPHYGKLQNLGIDMSNGETFRLQDIANSHLSYVHSGKESLSDVIKIDVSDQFHV
VPIIIKITVEPVDDESPTIT
LPDGMLGASIDVLENGVTEITSNVIQGRDEDTDDLALTFIVEEPPRLGEILVNGIPAERFTQQDIINGVVVYAHTSGEIG
PVRAQDSFNLTLTDMSEQWV
VGGNKIQGLTVYVTILPVDSIPPVVSVGEQFVVVEGEKNVITLEHIQTEDIDTENDDILCTIIVQPTSGYVENISPAPGS
EKSRAGTAITAFSVKDIGLG
HIYYVQSIHKGVEPVEDRFTFRCSDGINFSERHFFPIVIIPANDEKPEIFIREFVVMEGMSLVIDTPILNGADSDIPGDE
LEFEIIKNPKYGTIVQQLST
GSIPVTKFNLEQIKEGSNIVYEHNDSETKEDSFEIRLSDGKNTVVKKVIIMVISVDDETPRMAINDGLEVEIGETKIISN
KVLKATDLDSEDKTLTYVIR
YGPTQGYLQRITKFGDVIGNITLGKNFTQDEVDRQLIQYVHTGQEGIRDLLKFDVTDGINPLIDRYFYINIGSIDMVFPD
VINKGVTLKEGGRVTLTTDL
LSTTDINSPDEHLSFSITRAPSRGHLESTDHPGVPISTFTQLQLAGNKVYYIHTSDDEMKMDSFEFEVTDGYNPVFRTFR
VSITDVDNKKPVLTINKLVV
GEGETKLITPFELTAEDRDTPDKHLRFIITQVPVHGQLLFNNTQPITSFTKQDLNQNLITYKHDGTETNEDSFSFTVTDG
THADFYVFPDTVYETRKPQT
MTIQINTVDNGVPQIVVNRAAASLRLLTTGHVGFLVTSKALRSEDRDSPRNVLKYVVTQAPLHGLIINTAVGNDSIKTFT
QADIDDMKISYVLLDGSNAT
SDVFYFSVEDHGRSHDVLASELTLVDTIDGTHLRAAVPLRGTHILL
>g1172.t1
VTIWEALTNSKPGTHPLSCDPSRLDRTPQHKPRPPTSLCSTPTKKQSNTCSYNEQLRIAMEISAREQDAAEVRRRQEEEE
LQRIIQLSLMEK
>g1173.t1
MPGPCQSNNVCSFDDLETELEDLLREFDNVVEELKAPLRSSSCGNRRPLEEGTHTASRQCGVSGDSGVEDSDY
>g1173.t2
MPGPCQSNNVCSFDDLETELEDLLREFDNVVEELKAPLRSSSCGNRRPLEEGTHTASRQCGVSGDSGVEDSDYSK
>g1174.t1
MVHLRQTVCGTSRFHILEEQEAGIRVGTLRRDFPPPFKLLNLNYLWVDKNTGDLYTAEQKMDRESLCPEETMAGECIIPH
NAIVGPSGDLVQFHVVVEDI
NDNAPHFENTEIHMSLLEDLEVGSSVPLDDRATDSDSGVNGELLYDLQDPSGAFCLSVEKGALVSLVVQSALDRELRDTY
RMSLVATDGGAEPLSATATL
IIDVMDVNDNCPRFVSDSPQHVTMQGDYGKGSVVTQVVATDPDLGPNAAITYSLSPKVSESAKKLFSLDSLTGHIRLILN
LQTDSAEELVLHVLASGPYC
PPADTQVTVSVLRSTSHSPEIKIGFIAEHENQTIVLPENQPPSVVAVLEVRGSSDLGDSTLIIRGEVPFRLSPQNGKYLL
STSTPLDYEMKGEYLISVGV
SGPSTGGHTNTGSGLEIRVLVLDVNDNIPQFPLPHYHLDVEENNQPTSPLLTVEATDADSGPNGQVTYRLGKHTAAIFRI
NSQTGQLSVTVPLDREQQDV
HTISVLARDNGSPPLESLTTVSIHVHDQNDNAPVFVTPHFIFFIPENLAPLAHVGRIEVTDADKEKNGNVEVRVVNTTAP
FVMDNAQGVLRSTTDVDRET
VDRYELHLLATDHGHPFPLTSVAKVTIFVEDVNDNQPRVILPDSNLSCLTVSPATAAGTTVTKIYATDEDSGLNSEITYT
VAAQEPAPHGGPFRLDPRSG
NITLAQRLLETDLGMHHLFIVVSDGGKPTPLHTTVWVNLLVNETVEPCHLDSPPRSLPYRLAQTPSEASICDAKYAHLML
LVGLGMMPTSICLFCATAVL
YLKRRRGLQASRTGKEDENMIPLSHTKRHYDE
>g1175.t1
MKEILLIGFYQPNEELTRYLQEYAALGTGGGLYHFRDQILSGGPQAFFVMNADVCSEFPLSEMLEFHKEHGDPSSLVILG
TTANRKQSLNYGCIVENQDT

NEVLHYVEKPSTFVSDIINCGIYLFTPDIFQHIAGVFQKNQQDILL
>g1175.t2
MKEILLIGFYQPNEELTRYLQEYAALGTGGGLYHFRDQILSGGPQAFFVMNADVCSEFPLSEMLEFHKEHGDPSSLVILG
TTPSELSHWRWKEREPGFET
LCQRQSLSASSGTPANRKQSLNYGCIVENQDTNEVLHYVEKPSTFVSDIINCGIYLFTPDIFQHIAGVFQKNQQDILL
>g1175.t3
MKEILLIGFYQPNEELTRYLQEYAALGTGGGLYHFRDQILSGGPQAFFVMNADVCSEFPLSEMLEFHKEHGDPSSLVILG
TTANRKQSLNYGCIVENQDT
NEVLHYVEKPSTFVSDIINCGIYLFTPDIFQHIAGVFQKNQQDIL
>g1176.t1
MLCPSVFQPTQVNMGLMPFMDPHWPIRSLWPETRPIFYQMEQEMIRHMQEMRHNMEFMERLHQKIFDELEQSASSLASSS
ASLPLPSPSIYKPIAFQVGK
DGGSFALSLDTKDFSPEELSVKQVGRKLRVSGKTEQKQDDGKGSFCYRCQEFRQEFDVPEGVNPDSITCSLAGGQLQIQA
PRERTGDGKERVVPIQFTHP
AIGSSRAVEGATTAEEAPAKEAPAKEDSAKED
>g1177.t1
DGKGGRSPELSVESQPSESNKALQNPPIVSIRRGAHHHTRSPSALKVNAKVKENGRWANCQQLSCNTPELHKYDASSAQS
IF
>g1177.t2
DGKGGRSPELSVESQPSESNKALQNPPIVSIRRGAHHHTRSPSALKVNAKVKENGRWANCQQLSCNTPELHNLHEVTGLP
WERSGLPAEALGEGPETGGK
TILPKQHSA
>g1178.t1
MEAFLDLDRIVNHAPGQRGQVSRGSQGLVGVGGRSRRWGTGGRAGRRRGDLHLVVEKCNRSGGSTKLMVNKCTPALVRLQ
SEVSTLDVASDK
>g1179.t1
MTAYIPCDIVIASESQWGAMNKQPSKESLKDKVKGIFGIGPPRPPIKQNESKPSEFIINNDILKDLHPECGIGNRIKVIN
HVCDLAKAKKFEEGERLGPL
RAYYFKVIKDYQPPNEELADRLEVFKALTENGKDITYLEEDIACFVLLWMDNGLTSDFLHVLVNLVKFNSCYLDQNVSIM
VQKICVLCNSTTSSMDIEVA
LQVLDAVVCYNCLPSDSLTVFIITLCRTVNVKEFCETCWKLMRKVLGTHLGHSAIYTMCRIMEERVNVEDAALLRGAVFF
VGMALWGAHRLPALKNTPTL
VLPSFYKAMSCANEVVSYEIVLSITRLIKKYGKDLQVVTWDILLGIIERLLQQIQARTLTQLPHTIGSAELKAIVYELLT
TVEELYEQNGYHGSTEKFFS
LVEKCADKRPDSSVLTLISYRAQSIQPAKDGWIQNLHRLMDTFFKNESRSGIRIKVLHILSFVLSTNRQLYEVWTANTGS
GSISKALSPLPHRALSVLSA
FCLPFVFVSRPSPLS
>g1179.t2
MTAYIPCDIVIASESQWGAMNKQPSKESLKDKVKGIFGIGPPRPPIKQNESKPSEFIINNDILKDLHPECGIGNRIKVIN
HVCDLAKAKKFEEGERLGPL
RAYYFKVIKDYQPPNEELADRLEVFKALTENGKDITYLEEDIACFVLLWMDNGLTSDFLHVLVNLVKFNSCYLDQNVSIM
VQKICVLCNSTTSSMDIEVA
LQVLDAVVCYNCLPSDSLTVFIITLCRTVNVKEFCETCWKLMRKVLGTHLGHSAIYTMCRIMEERVNVEDAALLRGAVFF
VGMALWGAHRLPALKNTPTL
VLPSFYKAMSCANEVVSYEIVLSITRLIKKYGKDLQVVTWDILLGIIERLLQQIQARTLTQLPHSHPPRSVVVVVVPTQT
IGSAELKAIVYELLTTVEEL
YEQNGYHGSTEKFFSLVEKCADKRPDSSVLTLISYRAQSIQPAKDGWIQNLHRLMDTFFKNESRSGIRIKVLHILSFVLS
TNRQLYEVWTANTGSGSISK
ALSPLPHRALSVLSAFCLPFVFVSRPSPLS
>g1179.t3
MTAYIPCDIVIASESQWGAMNKQPSKESLKDKVKGIFGIGPPRPPIKQNESKPSEFIINNDILKDLHPECGIGNRIKVIN
HVCDLAKAKKFEEYAVEAVW
KAVEDMLTPEQPPEARHAVLLLLRAIIQGQACSCRPSIAHPSSYPLHWLIPVRVVWDGGERLGPLRAYYFKVIKDYQPPN
EELADRLEVFKALTENGKDI
TYLEEDIACFVLLWMDNGLTSDFLHVLVNLVKFNSCYLDQNVSIMVQKICVLCNSTTSSMDIEVALQVLDAVVCYNCLPS
DSLTVFIITLCRTVNVKEFC

ETCWKLMRKVLGTHLGHSAIYTMCRIMEERVNVEDAALLRGAVFFVGMALWGAHRLPALKNTPTLVLPSFYKAMSCANEV
VSYEIVLSITRLIKKYGKDL
QVVTWDILLGIIERLLQQIQARTLTQLPHSHPPRSVVVVVVPTQTIGSAELKAIVYELLTTVEELYEQNGYHGSTEKFFS
LVEKCADKRPDSSVLTLISY
RAQSIQPAKDGWIQNLHRLMDTFFKNESRSGIRIKVLHILSFVLSTNRQLYEVWTANTGSGSISKALSPLPHRALSVLSA
FCLPFVFVSRPSPLS
>g1180.t1
LASSSRRRRQLKIEYNENDEEPRPKTERWEPPNWRTQLDHIREMRSSRDAPVDKMGAEKCYDAGAPAPVRRFQVLVSLML
SSQTKDQVTAAAMQRLRARG
CTIETILNTDDETLGKLIYPVGFWRTKVKYLKKTSLMLQQEFAGDIPDSVEGLVRLPGVGPKMAHLAMDIAWDQVSGIGV
DTHVHRISNRLGWLRKSTKT
PEQTRKALEDWLPRELWSEINWLLVGFGQQVCLPVNPLCSMCLNQHCCPSAHKTSPAKRTKAERTKAEPPQPPDPRPPPA
SLKGKQGYGFHLHGARNRSG
QLIRKIEPGSCAELSGLRPGDRVVEVNGENVEQDSHHQVVDRIQEVGHRTRLLVIDRETDDYLRSRNLPCTEDLAVEMGT
FSPRSTPSTSPLRRPNGPIS
PKELLKHWAKPPELTPSPADSFADRSSSLARSARTDRASVAASSSGTDAEVSTVWRNVS
>g1180.t2
LASSSRRRRQLKIEYNENDEEPRPKTERWEPPNWRTQLDHIREMRSSRDAPVDKMGAEKCYDAGAPAPVRRFQVLVSLML
SSQTKDQVTAAAMQRLRARG
CTIETILNTDDETLGKLIYPVGFWRTKVKYLKKTSLMLQQEFAGDIPDSVEGLVRLPGVGPKMAHLAMDIAWDQVSGIGV
DTHVHRISNRLGWLRKSTKT
PEQTRKALEDWLPRELWSEINWLLVGFGQQVCLPVNPLCSMCLNQHCCPSAHKTSPAKRTKAERTKAEPPQPPDPRPPPA
SLKVEPGPGEQAGGTPARPP
TSDPLRRQRRRKV
>g1180.t3
LASSSRRRRQLKIEYNENDEEPRPKTERWEPPNWRTQLDHIREMRSSRDAPVDKMGAEKCYDAGAPAPVRRFQVLVSLML
SSQTKDQVTAAAMQRLRARG
CTIETILNTDDETLGKLIYPVGFWRTKVKYLKKTSLMLQQEFAGDIPDSVEGLVRLPGVGPKMAHLAMDIAWDQVSGIGV
DTHVHRISNRLGWLRKSTKT
PEQTRKALEDWLPRELWSEINWLLVGFGQQVCLPVNPLCSMCLNQHCCPSAHKTSPAKRTKAERTKAEPPQPPDPRPPPA
SLKGKQGYGFHLHGARNRSG
QLIRKIEPGSCAELSGLRPGDRVVEVNGENVEQDSHHQVVDRIQEVGHRTRLLVIDRETDDYLRSRNLPCTEDLAVEMGT
FSPRSTPSTSPLRRPNGPIS
PKELLKHWAKPPELTPSPADSFADRSSSLARSARTDRASVAASSSGTDAEVFQYATGCSLAMEIHRDAGPGRSRRRAAVL
PVDT
>g1180.t4
MGSELRPRVCFLTKGKQGYGFHLHGARNRSGQLIRKIEPGSCAELSGLRPGDRVVEVNGENVEQDSHHQVVDRIQEVGHR
TRLLVIDRETDDYLRSRNLP
CTEDLAVEMGTFSPRSTPSTSPLRRPNGPISPKELLKHWAKPPELTPSPADSFADRSSSLARSARTDRASVAASSSGTDA
EVSTVWRNVS
>g1181.t1
WLVDQMKDYSAAFYLSGFCFLCSTVFVFAVDLLVQRKLGVHGKEVASVT
>g1182.t1
MVVCGSHVAVVVVMVVVMVAAAAAAAAAAVVV
>g1182.t2
MWRWCGVGVMVVCGSHVAVVVVMVVVMVAAAAAAAAAAVVV
>g1183.t1
MQPFSPCCAQGPQQVRCQGLGQVHKQEHQHVLSCFFSRACLSVPSSKGTKAFFIGDNVPRPDTSVLYNRGQLNGQKDKEM
CLSDMM
>g1184.t1
MEVPARYLDLRRTCPGSHQREEEQEEEEQEEAEQRSSLFQHQQPFTENTESKVLNSLPIPRRPTFKAPSWPGICSSLLLA
AGSAVHLWREAKGTQAHTET
PVIAVSDSHSGRPPHTPVQNRTAELKWQLVWRQLPTGRRPGGREEPEGTPMSRCQGCELQRTASSRSSRS
>g1185.t1
MIEYGVVLNRRSMKWSKLLQGKAHIEKNLTVKRYVRKGIPNVHRGRIWMLSSGAQEQLESHPGFYQALLAAEHDSKLQEV
IRIDMHRTFPDNVLFQSTSE

PSQRTALYNVLLAYGHHNKAVGYCQGMNFIAGYLLIITKDEEKSFWLMDALLGKILP
>g1185.t2
LAPTATCLARHIDPQDENKIGIDGIQQFCDDLSLDPASITVLVVAWKFRAATQCEFSKKEFLDGMTELGCDSPEKLKALL
PRLEQELKDCGKFKDFYQFT
FNFAKNPGQKGLDLEMAVAYWNLVLTGRFKFLDLWNRFLLAWESYEEAMIEYGVVLNRRSMKWSKLLQGKAHIEKNLTVK
RYVRKGIPNVHRGRIWMLSS
GAQEQLESHPGFYQALLAAEHDSKLQEVIRIDMHRTFPDNVLFQSTSEPSQRTALYNVLLAYGHHNKAVGYCQGMNFIAG
YLLIITKDEEKSFWLMDALL
GKILP
>g1185.t3
MHPNDGLPVAHLGRSDRPRLLVLQQRVDAYGFSIPQAWESYEEAMIEYGVVLNRRSMKWSKLLQGKAHIEKNLTVKRYVR
KGIPNVHRGRIWMLSSGAQE
QLESHPGFYQALLAAEHDSKLQEVIRIDMHRTFPDNVLFQSTSEPSQRTALYNVLLAYGHHNKAVGYCQGMNFIAGYLLI
ITKDEEKSFWLMDALLGKIL
P
>g1185.t4
LAPTATCLARHIDPQDENKIGIDGIQQFCDDLSLDPASITVLVVAWKFRAATQCEFSKKEFLDGMTELGCDSPEKLKALL
PRLEQELKDCGKFKDFYQFT
FNFAKNPGQKGLDLEMAVAYWNLVLTGRFKFLDLWNRFLLHLFPGQLPALGRPPRGAPSRQARPRPQRGWGLTEEEEEEE
EEAQQHL
>g1185.t5
LAPTATCLARHIDPQDENKIGIDGIQQFCDDLSLDPASITVLVVAWKFRAATQCEFSKKEFLDGMTELGCDSPEKLKALL
PRLEQELKDCGKFKDFYQFT
FNFAKNPGQKGLDLEMAVAYWNLVLTGRFKFLDLWNRFLLPSVALQEERRPARHGLDLRGGGG
>g1186.t1
MNLGLQILLPRLGSLQHRPDKRRLIGLWLCNGATVFSAGLRDNLPLQCAVWTGLGPEPPLKECSATGLNESGPSFDLRPM
TCFYGR
>g1187.t1
PHQRQDRIRALETGPESEKQPGSFVMFETQTHSPVSLSTDTPCCLVTGGSWMGRDDGGVAWRKQTRRTNKTRNKHR
>g1188.t1
MQNSTVTMNGGGLLDSLDEIKKEIIRQKHLHDDLIKEQTYLSKISTDREHMAEEFRDRFLKTTKALEQDKRKHMAAMDSK
KAEVDSLHREQAQLQELLEM
AESALKEQEAKHAEVEKAANVVALLPKREVVFKGTTGHSGVTPHNFDMKTHIRYPMEGGTALITFEKDIVAQKVRSLQRH
KVTLEGECYIMVEAHPIQLL
VPTSVELDTRVCPNSVLVSQLPNLDSEAILDKLELHFSKSLNGGGEVVSRDFLEDAGNAVLVFKDDHCVAGLAEKQFHEV
QFQGKKKKHKVRVTPFINGA
ISRLETKSLSCDCSVLLTGIPPVMDADNLQDMLEIYFQKGSNGGGEISNMVYNPLGQTTVALFERDL
>g1188.t2
MQNSTVTMNGGGLLDSLDEIKKEIIRQKHLHDDLIKEQTYLSKISTDREHMAEEFRDRFLKTTKALEQDKRKHMAAMDSK
KVVALLPKREVVFKGTTGHS
GVTPHNFDMKTHIRYPMEGGTALITFEKDIVAQKVRSLQRHKVTLEGECYIMVEAHPIQLLVPTSVELDTRVCPNSVLVS
QLPNLDSEAILDKLELHFSK
SLNGGGEVVSRDFLEDAGNAVLVFKDDHCVAGLAEKQFHEVQFQGKKKKHKVRVTPFINGAISRLETKSLSCDCSVLLTG
IPPVMDADNLQDMLEIYFQK
GSNGGGEISNMVYNPLGQTTVALFERDL
>g1188.t3
MNGGGLLDSLDEIKKEIIRQKHLHDDLIKEQTYLSKISTDREHMAEEFRDRFLKTTKALEQDKRKHMAAMDSKKAEVDSL
HREQAQLQELLEMAESALKE
QEAKHAEVEKAANVVALLPKREVVFKGTTGHSGVTPHNFDMKTHIRYPMEGGTALITFEKDIVAQKVRSLQRHKVTLEGE
CYIMVEAHPIQLLVPTSVEL
DTRVCPNSVLVSQLPNLDSEAILDKLELHFSKSLNGGGEVVSRDFLEDAGNAVLVFKDDHCVAGLAEKQFHEVQFQGKKK
KHKVRVTPFINGAISRLETK
SLSCDCSVLLTGIPPVMDADNLQDMLEIYFQKGSNGGGEISNMVYNPLGQTTVALFERDL
>g1189.t1
MFMWQEQALHAVHSILMLVDKDTVPRPERQPGLQMDVVTSLKALNKIVEASQLTSDLGGTFAYSHADWLQLHQRLVSFTA
DLQGADSLLQKAIKKVDETK

TMDTAQ
>g1189.t2
MWQEQALHAVHSILMLVDKDTVPRPERQPGLQMDVVTSLKALNKIVEASQLTSDLGGTFAYSHADWLQLHQRLVSFTADL
QGADSLLQKAIKKVDETKTM
DTAQ
>g1189.t3
MFMWQEQALHAVHSILMLVDKDTVPRPERQPGLQMDVVTSLKALNKIVEASQLTSDLGGTFAYSHADWLQLHQ
>g1190.t1
RALGPVPLWRRGQNRRSYTEGETRFLCDAGAEVLSENYTWGRVKDLVRRTKLRNLSSSRLGLSSTSLKMSCPQLYRPDLG
PPEPPRSRNRNSMITLGHQS
KLDLSWLQ
>g1190.t2
RALGPVPLWRRGQNRRSYTEGETRFLCDAGAELSENYTWGRVKDLVRRTKLRNLSSSRLGLSSTSLKMSCPQLYRPDLGP
PEPPRSRNRNSMITLGHQSK
LDLSWLQ
>g1191.t1
DSEISAFELKKILNKVVTKRSDIKTSGFSLDTCRNMVNLLDKDGSGHLGLVEFKILWTKIEKLLTIYREKDVDKSGCMSS
TEMRGAVEEAAKRHQHTNHI
FLRTQKQQQQHLCVRGPLDHSHFDVFPPDLEEPPEELSGWDRNF
>g1191.t2
DSEISAFELKKILNKVVTKRSDIKTSGFSLDTCRNMVNLLDKDGSGHLGLVEFKILWTKIEKLLTIYREKDVDKSGCMSS
TEMRGAVEEAAKRHQHTNHI
FLRTQKQQQQHLCVSCV
>g1191.t3
DSEISAFELKKILNKVVTKRSDIKTSGFSLDTCRNMVNLLDKDGSGHLGLVEFKILWTKIEKLLTIYREKDVDKSGCMSS
TEMRGAVEEAACCFEAQMIL
RWVRGPLDHSHFDVFPPDLEEPPEELSGWDRNF
>g1192.t1
AQAPQYAPPKLPAPQKPRYTPPQLPRYQPQPQPEPGKTPPGKTPPGKTPPGETPPGKTPPGPEPVQSCDVAAAYKIQCGP
QDISGAACEDINCCFDGHMC
YYGKAVTVQCTKDAQFIVVVARDATLPNFDLESVSLLGNDPGCEPVGSTSAFAIYQFPVTACGTIMTCRYIGTSVEALVI
EVMLSPPHPPLAALGPIRVQ
LQLGNGVCTNKGCIEEDVAYSSFYTEGDYPVVKVLRDPVYVDIHLLERTDPNLVLTLGRCWTTTNSNPYSLPQWDLLIDG
CPYRDDRYQTTLVGVSDVAQ
PSHHKRFIFKMFTFVDQSSLTPLREKVYIHCNTAVCVPSSGNNCEPICYRKKRNAGKATPRDEATALVTTSPKYTVHPAP
RLCHSRQSVRRSFSRSFGKL
TMQTEDKPDMPDRLLPPTVTYLRAPSPFGGSGDDDSEAESYRDGHTDEVDRETRISAFAWCRDFLAGSWKTIPERDFQIS
IVSGGLSNLLYMCNLPDNVA
CVAEEPRQVLLRVYGAILQGVDSLVLESVMFAILAERTLGPKLYGIFPEGRLEEYLPNDRMHTEELSDPSISAEIASKVA
RFHKMEMPFNKEPKWLLGTI
DRYMDQVMELKFTRDAHLKKYQKLMKFNLPAELESLRQLLAATPSPVVFCHNDVQEGNILKLEGRDPTSTERLMLIDFEY
SSYNYRGFDFGNHFCEWMYD
YTYKQWPFYKATMGNYPTREQQLHFIRSYLAEFKALSEDPTEEDQTQVEEDMLTEANRYALSSHFLWGLWSIIQAKISKI
EFGYMDYALCRFDAYFKQKK
LCS
>g1192.t2
AQAPQYAPPKLPAPQKPRYTPPQLPRYQPQPQPEPGKTPPGKTPPGKTPPGETPPGKTPPGPEPVQSCDVAAAYKIQCGP
QDISGAACEDINCCFDGHMC
YYGKAVTVQCTKDAQFIVVVARDATLPNFDLESVSLLGNDPGCEPVGSTSAFAIYQFPVTACGTIMTCRYIGTSVEALVI
EVMLSPPHPPLAALGPIRVQ
LQLGNGVCTNKGCIEEDVAYSSFYTEGDYPVVKVLRDPVYVDIHLLERTDPNLVLTLGRCWTTTNSNPYSLPQWDLLIDG
CPYRDDRYQTTLVGVSDVAQ
PSHHKRFIFKMFTFVDQSSLTPLREKVYIHCNTAVCVPSSGNNCEPICYRKKRNAGKATPRDEATALVTTSPKYTVHPAP
RLCHSRQSVRRSFSRSFGKL
TMQTEDKPDMPDRLLPPTVTYLRAPSPFGGSGDDDSEAESYRDGHTDEVDRETRISAFAWCRDFLAGSWKTIPERDFQIS
IVSGGLSNLLYMCNLPDNVA

CVAEEPRQVLLRVYGAILQNDRMHTEELSDPSISAEIASKVARFHKMEMPFNKEPKWLLGTIDRYMDQVMELKFTRDAHL
KKYQKLMKFNLPAELESLRQ
LLAATPSPVVFCHNDVQEGNILKLEGRDPTSTERLMLIDFEYSSYNYRGFDFGNHFCEWMYDYTYKQWPFYKATMGNYPT
REQQLHFIRSYLAEFKALSE
DPTEEDQTQVEEDMLTEANRYALSSHFLWGLWSIIQAKISKIEFGYMDYALCRFDAYFKQKKLCS
>g1192.t3
AQAPQYAPPKLPAPQKPRYTPPQLPRYQPQPQPEPGKTPPGKTPPGKTPPGETPPGKTPPGPEPVQSCDVAAAYKIQCGP
QDISGAACEDINCCFDGHMC
YYGKAVTVQCTKDAQFIVVVARDATLPNFDLESVSLLGNDPGCEPVGSTSAFAIYQFPVTACGTIMTCRYIGTSVEALVI
EVMLSPPHPPLAALGPIRVQ
LQLGNGVCTNKGCIEEDVAYSSFYTEGDYPVVKVLRDPVYYVDIHLLERTDPNLVLTLGRCWTTTNSNPYSLPQWDLLIDG
CPYRDDRYQTTLVGVSDVAQ
PSHHKRFIFKMFTFVDQSSLTPLREKVYIHCNTAVCVPSSGNNCEPICYRKKRNAGKATPRDEATALVTTSPKYTVHPAP
RLCHSRQSVRRSFSRSFGKL
TMQTEDKPDMPDRLLPPTVTYLRAPSPFGGSGDDDSEAESYRDGHTDEVDRETRISAFAWCRDFLAGSWKTIPERDFQIS
IVSGGLSNLLYMCNLPDNVA
CVAEEPRQVLLRVYGAILQGVDSLVLESVMFAILAERTLGPKLYGIFPEGRLEEYLPNDRMHTEELSDPSISAEIASKVA
RFHKMEMPFNKEPKWLLGTI
DRYMDQVMELKFTRDAHLKKYQKLMKFNLPAELESLRQLLAATPSPVVFCHNDVQEGNILKLEGRDPTSTERLMLIDFEY
SSYNYRGFDFGNHFCEWMYD
YTYKQWPFYKATMGNYPTREQQLHFIRSYLAEFKALSEDPTEEDQTQVEEDMLTEANRYALSSHFLWGLWSIIQAKISKI
EFGYMVDLERLSECSEYFRG
MSQCRMREMSEGLIRLGHVPSSTFHNLLEFSFHQHFRVPEDELAENIQVASYLLAEPFLSECLSVLADVLTPDNCLSYLG
LAQDIYCAKLRMTVLTYLSR
NLLEQSHLTRYGCSCSMRGFSPEEMEEMISLRTRGDQRLCVLRKENLKSWADPETAPARHLFRLRGAEDGGDPSWHPIAE
LPFRADKWCFTAVVLLNYLY
ILGGYRQPIRRGWEFKMASFRYNPLTHTWVTTAPLIKHRRHFSAVACEGGIYAIGGWYLESLVSPDSNTALYTAVERYDP
WDDTWRFVSPPMLVSSLPLT
DFQFTMSLSHDVPLATSLGHCVYVLGNIQRTGEKLLLRTDADLPTLYFLGATDTLIVIGGNTSENVVTSFCPRTRHPSIA
CTSPTQLQQVEDDWAASRTP
HTCHHFHLINPSAYKKPSSAVTPGQIFPRNHLMNPLWITWLKLLPDLLLHQNLSSLWPSGLLSPSPNTETALVKITNDLL
HAADSGLLTILILLDLTAAF
DTISHPLLLERLADIGITGVVYISSKPTTALPLSSLTTCLQDIRSWISRNFLKLNGSKTEALLIGSKNTLSIYIYI
>g1192.t4
MSQCRMREMSEGLIRLGHVPSSTFHNLLEFSFHQHFRVPEDELAENIQVASYLLAEPFLSECLSVLADVLTPDNCLSYLG
LAQDIYCAKLRMTVLTYLSR
NLLEQSHLTRYGCSCSMRGFSPEEMEEMISLRTRGDQRLCVLRKENLKSWADPETAPARHLFRLRGAEDGGDPSWHPIAE
LPFRADKWCFTAVVLLNYLY
ILGGYRQPIRRGWEFKMASFRYNPLTHTWVTTAPLIKHRRHFSAVACEGGIYAIGGWYLESLVSPDSNTALYTAVERYDP
WDDTWRFVSPPMLVSSLPLT
DFQFTMSLSHDVPLATSLGHCVYVLGNIQRTGEKLLLRTDADLPTLYFLGATDTLIVIGGNTSENVVTSFCPRTRQWGEI
HAATKVAFAGQGTVSEDHVL
MPATEHRGVVRLDLLDLSLSLLPPLPIPAHYDAVFHLYF
>g1192.t5
MSQCRMREMSEGLIRLGHVPSSTFHNLLEFSFHQHFRVPEDELAENIQVASYLLAEPFLSECLSVLADVLTPDNCLSYLG
LAQDIYCAKLRMTVLTYLSR
NLLEQSHLTRYGCSCSMRGFSPEEMEEMISLRTRGDQRLCVLRKENLKSWADPETAPARHLFRLRGAEDGGDPSWHPIAE
LPFRADKWCFTAVVLLNYLY
ILGGYRQPIRRGWEFKMASFRYNPLTHTWVTTAPLIKHRRHFSAVACEGGIYAIGGWYLESLVSPDSNTALYTAVERYDP
WDDTWRFVSPPMLVSSLPLT
DFQFTMSLSHDVPLATSLGHCVYVLGNIQRTGEKLLLRTDADLPTLYFLGATDTLIVIGGNTSENVVTSFCPRTRHPSIA
CTSPTQLQQVEDDWAASRTP
HTCHHFHLINPSAYKKPSSAVTPGQIFPRNHLMNPLWITWLKLLPDLLLHQNLSSLWPSGLLSPSPNTETALVKITNDLL
HAADSGLLTILILLDLTAAF
DTISHPLLLERLADIGITGVDIRSWISRNFLKLNGSKTEALLIGSKNTLSIYIYI
>g1192.t6

LQQVEDDWAASRTPHTCHHFHLINPSAYKKPSSAVTPGQIFPRNHLMNPLWITWLKLLPDLLLHQNLSSLWPSGLLSPSP
NTETALVKITNDLLHAADSG
LLTILILLDLTAAFDTISHPLLLERLADIGITGVVYISSKPTTALPLSSLTTCLQDIRSWISRNFLKLNGSKTEALLIGS
KNTLSIYIYI
>g1193.t1
MGSDPSSRFALSKVQTTIAANIIIDGFPVPFSSQVRSLGVTLDVPLHPTKLLNRLQMIQNSAARLVTRTKSSDHITPILI
RLHWLPVQYRVDPKNLMLTF
KALHNLAPTYLSDLLQDYTPSRSLRSSSAGLLFTPTSRPAP
>g1194.t1
LSSFLCILSGTSTKDHRPPPPKTTDHLHQTLQFHSHYRIHFPGGGTLTIMYDSLLCSLTYRRRPLYSLSKHSPRETHLPA
RVRVRSFSESAVCYAAKDGT
TKDGPSDGGKKSVSEGKRLSGSGGSGKGGSQLRCPKCGDPCTHVETFVSSTRFVKCEKCHHFFVVLSETDSKKGLNKEPE
SAAEAVKLAFAQKPPPPPKK
IYAYLDKYVVGQCYAKKVLAVAVYNHYKRIYNNIPAGSRQQVEVEKQASLTPRELEMRRREDEYRFT
>g1194.t2
LSSFLCILSGTSTKDHRPPPPKTTDHLHQTLQFHSHYRIHFPGGGTLTIMYDSLLCSLTYRRRPLYSLSKHSPRETHLPA
RVRVRSFSESAVCYAAKDGT
TKDGPSDGGKKSVSEGKRLSGSGGSGKGGSQLRCPKCGDPCTHVETFVSSTRFVKCEKCHHFFVVLSETDSKKGLNKEPE
SAAEAVKLAFAQKPPPPPKK
IYAYLDKYVVGQCYAKKVLAVAVYNHYKRIYNNIPAGSRQQVEVEKQASLTPRGQYHYVHAFTLLVAVCVASTSTLELGI
PYGRSQVFAPQMPRASMHFF
MPHYMQ
>g1194.t3
LSSFLCILSGTSTKDHRPPPPKTTDHLHQTLQFHSHYRIHFPGGGTLTIMYDSLLCSLTYRRRPVQSQQQRDVLSVCVGG
EAILTFCASRVRVRSFSESA
VCYAAKDGTTKDGPSDGGKKSVSEGKRLSGSGGSGKGGSQLRCPKCGDPCTHVETFVSSTRFVKCEKCHHFFVVLSETDS
KKGLNKEPESAAEAVKLAFA
QKPPPPPKKIYAYLDKYVVGQCYAKKVLAVAVYNHYKRIYNNIPAGSRQQVEVEKQASLTPRELEMRRREDEYRFT
>g1195.t1
MQYIIPPFCVGCESRGSEFKECFSLYDKKQKGKIEAVDLITVMRSLGTSPTFTKKGELDFSTFLTMMHRQMQQEDPKTEI
LEALKMADKHNKGYIQAAEL
RAKLTKLGEKLTNKEVDDLFKEANVKSNGTVNYEEFTRMMTLPPVDY
>g1196.t1
MVLAYTGELDLRLWKAYYHEWFPLNKPSAGDYFHMAYNVITPILLLKLIERSPKVLSRSTVYLGIITLVMGASIHLVGDS
INHRLLLSGYQLHLSVRENP
IMKDLKPASLIDSFELLYFYDEHLGHLMWYLVTEGQIFELFLLTFVAMVAVVILQQRRGLRPDCNGLFLFGTFAVTMALV
VVWVAYLWNDTDLRRKYPGL
IYVPEPWSYYSLHVRDGH
>g1196.t2
MQGAGQLIGHCRYEIFQRKCIIKESPVPFGPMVLAYTGELDLRLWKAYYHEWFPLNKPSAGDYFHMAYNVITPILLLKLI
ERSPKVLSRSTVYLGIITLV
MGASIHLVGDSINHRLLLSGYQLHLSVRENPIMKDLKPASLIDSFELLYFYDEHLGHLMWYLVTEGQIFELFLLTFVAMV
AVVILQQRRGLRPDCNGLFL
FGTFAVTMALVVVWVAYLWNDTDLRRKYPGLIYVPEPWSYYSLHVRDGH
>g1196.t3
MVLAYTGELDLRLWKAYYHEWFPLNKPSAGDYFHMAYNVITPILLLKLIERSPKVLSRSTVYLGIITLVMGASIHLVGDS
INHRLLLSGYQLHLSVRENP
IMKDLKPASLGTSLARLKSRLESRRYLVTEGQIFELFLLTFVAMVAVVILQQRRGLRPDCNGLFLFGTFAVTMALVVVWV
AYLWNDTDLRRKYPGLIYVP
EPWSYYSLHVRDGH
>g1197.t1
MTVDPSSKMMTSVAHSTFIPMPYGHKHHNRSVRSADAAALGLGATGPFFSSRTVPLETPRADHVRQLPALVRTGPQSPPS
QAWAHLLLFLYLLRPSLRVM
SLLG
>g1197.t2
MMTSVAHSTFIPMPYGHKHHNRSVRSADAAALGLGATGPFFSSRTVPLETPRADHVRQLPALVRTGPQSPPSQAWAHLLL

FLYLLRPSLRVMSLLG
>g1197.t3
LMTVDPSSKMMTSVAHSTFIPMPYGHKHHNRSVRSADAAALGLGATGPFFSSRTVPLETPRADHVRQLPALVRTGPQSPP
SQAWAHLLLFLYLLRPSLRV
MSLLG
>g1198.t1
MQRFRRTFEDEKHQLQELNDRLAQYLSRTKQLEEENSSLITEIHALRQAKTTALDQGYKAEVRELRRMVGRLSSEKTQAE
MEREKLWREVQMVQSLRREE
TEVCQDIGGELRGCEKDLRHANESHASLEQRLFQLENEYVNLEEQHRQETAHLRRQLDSRVVSIMTPSHQGHPMVSPEEV
QQFARCVSEGWMDTFEMYQR
KVEELEQSIQSDRAMLDDLQKEKMLYAAEFDKLRVEAHKQSQIQVHLEEQLGHMQDTFRMELSQYQGRRPHANHAHTGLL
NRSVQRAIEELEHERNMLAD
NIAAKLWDHQQLMQVKMDLSMEVAAYSMYINK
>g1198.t2
MQRFRRTFEDEKHQLQELNDRLAQYLSRTKQLEEENSSLITEIHALRQAKTTALDQGYKAEVRELRRMVGRLSSEKTQAE
MEREKLWREVQMVQSLRREE
TEVCQDIGGELRGCEKDLRHANESHASLEQRLFQLENEYVNLEEQHRQETAHLRRQLDSRVVSIMTPSHQGHPMVSPEEV
QQFARCVSEGWMDTFEMYQR
KVEELEQSIQSDRAMLDDLQKEKMLYAAEFDKLRVEAHKQSQIQVHLEEQLGHMQDTFRMELSQYQRAIEELEHERNMLA
DNIAAKLWDHQQLMQVKMDL
SMEVAAYSMYINK
>g1198.t3
MQRFRRTFEDEKHQLQELNDRLAQYLSRTKQLEEENSSLITEIHALRQAKTTALDQGYKAEVRELRRMVGRLSSEKTQAE
MEREKLWREVQMVQSLRREE
TEVCQDIGGELRGCEKDLRHANESHASLEQRLFQLENEYVNLEEQHRQETAHLRRQLDSRVVSIMTPSHQGHPMVSPEEV
QQFARCVSEGWMDTFEMYQR
KVEELEQSIQSDRAMLDDLQKEKMLYAAEFDKLRVEAHKQSQIQVHLEEQLGHMQDTFRMELSQYQGRRPHANHAHTGLL
NRSVQRAIEELEHERNMLAD
NIAAKLWDHQQLMQVKMDLSMEVAAYR
>g1199.t1
LGPPRWELSLCLVAVVFILYFSLWKGVKSSGKVVYITATMPYIVLFVLLIRGITLPGAMDGIRAYLHIDLKMLNNPKVWI
DAATQIFYSLGAGFGVLIAF
ASYNKFDNNCYRDALLTSTINCVTSFVSGFAIFSVLGYMAQKHGVNIEDVATEGPGLVFIIYPEAISTLPGSTFWAIVFF
IMLLTLGIDSSMGGMEAVIT
GLADDFKILKRHRKLFTFATAFGTFLFALL
>g1199.t2
MPLIVPTERGVLHLHESRGIGDLGPPRWELSLCLVAVVFILYFSLWKGVKSSGKVVYITATMPYIVLFVLLIRGITLPGA
MDGIRAYLHIDLKMLNNPKV
WIDAATQIFYSLGAGFGVLIAFASYNKFDNNCYRDALLTSTINCVTSFVSGFAIFSVLGYMAQKHGVNIEDVATEGPGLV
FIIYPEAISTLPGSTFWAIV
FFIMLLTLGIDSSMGGMEAVITGLADDFKILKRHRKLFTFATAFGTFLFALL
>g1199.t3
MTNKCPPQLPFPFVGQGNGFWYPLFIYKPDEVRGVLHLHESRGIGDLGPPRWELSLCLVAVVFILYFSLWKGVKSSGKVV
YITATMPYIVLFVLLIRGIT
LPGAMDGIRAYLHIDLKMLNNPKVWIDAATQIFYSLGAGFGVLIAFASYNKFDNNCYRDALLTSTINCVTSFVSGFAIFS
VLGYMAQKHGVNIEDVATEG
PGLVFIIYPEAISTLPGSTFWAIVFFIMLLTLGIDSSMGGMEAVITGLADDFKILKRHRKLFTFATAFGTFLFALL
>g1200.t1
LALYKLKLKQEGARRPASSAHSANQAPGSSRRGGGAPGGLAPDDQGDPKEGDPPRKDEGITELLDSLRCNLAPSDGPKHN
VPEVFYWI
>g1200.t2
LALYKLKLKQEGARRPASSAHSANQAPGSSRRGGGAPGGLAPDDQGDPKEGDPPRKDETTDGFLGYLLPALDQGITELLD
SLRCNLAPSDGPKHNVPEVF
YWI
>g1200.t3
LALYKLKLKQEGARRPASSAHSANQGTPPASCVRARGARSGQPLIVMIESQRWLGLLDTSRVKLQGSWTPRVTLQTYWTP

RAPGSSRRGGGAPGGLAPDD
QGDPKEGDPPRKDEGITELLDSLRCNLAPSDGPKHNVPEVFYWI
>g1201.t1
MTPCPRHWAAAPPCSRWSTATFYPQHWVTADPSWGRAADGSPTFAGGSPADAVSGVCCIEAPPPVTAASPSPESTGFRLH
RRPPEGSCLRRSPRKGSGFA
SGLRKGSAFAAGLLLVSAFAANLLRDFVLAADLLRDFTFAAGLQRSFACAQPMVSPQAVRLRFPVPPLVSPQAVRLRFPA
LPLVPSSGPSRPSLVRTFWT
WAVWDLSLDRVHSTFMIRVLS
>g1202.t1
LRTAEKITRSAQPSIQDLYPPRCLKRAINITKDPHPPSTQTVHPPTIWQALPYHADHQTQKKLFSLRPSDY
>g1203.t1
MKDLRNLSLHRGFISPLLKELLRDPTVSCRGATVEYRVLINHLITYLVLEELFLLFSTVFSEAADNGIPLFVNALGLKGD
AF
>g1204.t1
MSAQRGYNEARKFFTFHYGNEQKIAAAYVDKAVKWPQIKAEDAKSLHSFSIFLTGCNNVMKDMEYLEEVNSPVGTSPRSL
RLYCKEESLGSSTRLLGRTT
VVSGSNR
>g1205.t1
ATLYRKLKAYLMTEEQLQEHGYPRPTPESSGRAVIHNQPEKKNPADPFTKICCRCGAEYKINVHGSCVRKEECHHHWGRL
RKHK
>g1205.t2
ATLYRKLKAYLMTEEQLQEHGYPRPTPESSGRAVIHNQPEKKNPADPFTKICCRCGAEYKINVHGSCVRKEECHHHWGRL
RKHKGGRV
>g1205.t3
LYRKLKAYLMTEEQLQEHGYPRPTPESSGRAVIHNQPEKKNPADPFTKICCRCGAEYKINVHGSCVRKEECHHHWGRLRK
HK
>g1206.t1
MPNFCAAPNCTRKSTQSDLAFFRFPRDPERCRMWVENCRRADLEAKTSDQLNKHYRLCAKHFDPAMVCKTTEEDIRKIKE
RRLASSIKQLISKKDSVAED
SPANDGEPPLSPEEKEFRDYLHSLFKVIVLLGKQSIPLTTDRPPEASEPDCNSNFQMLLHYHINAGDVALRKRFEGMAVN
AQYLSAAQQSQLLEVCENTV
REEILMEVRDSRFFSLVTGDLVEFDGEKFLPLFLRFVNQRNLLREEFLDLLPFEDSEAALVEKLEDQLSDRWGLSMEDCR
GHAHKATGTSTTKMKAVAVM
LTEKYPLALHMPCSTTALNIHLANSLPFPNVQNAMEALRKIDVFFRQPSTQGELEKAITACFRGNEEKESMLKQVCHTCW
TDQHNVFDLMVDMLESLVLC
MDNIRNNDGAKFPDVVVANAYSISEILADFELIVTIVILKNTLTYTRAFGRNLQGETLEVFFAANSLTAVLHSLNEVIDN
IDVYHEFWFEEAVCLAAGMV
IPVTMPRLFLRKQRSADTGAMQAEAYFKEYVTIPVIRGVIQEVEEMFSETNLKALKCLSLVPAVMGQMKFNTSEENNADV
YRDDLPSPDTLPAELHCWRI
KWKHRGKEVRLPTTIHETLQLPDVKFFPNVNSFLKVLSALPVLRFDYDGETAPASQRLRAYLDATPVEQRNKSMAVLHIN
AHVKHDLDVMVDQYCRLYPE
DEAEVEVEPAVEVEPTMEVEPAVEAEPVVEAEPAVEAVPAVEEEPMEEDTPKTT
>g1207.t1
MAWPCITRACCINRFWSELDKADIAVPLVFTKYSDVADVQHLQHHPQPKQKRTGPIAIETQPHLSEAESKAPPAATCAAE
GKDGSASVTRQDFKAWKVRP
EPSCKPKNAYHPSVGPFDTETQYQKDYKPWPIPKKGDHPWIPKPSPTTSGVRKGLAGSKKEHAAAEAESGVEKSEIEEKV
QEKEFKEVAKISTKREKSVD
RKPVEKQEEKQPSDPSAERKGRAAADALNRQIKEVVSTGSCYRNEYKAYKDVKPAKSFKASTHFQPPGVEACRETSYSAT
FKGEQGKTLTADNKLMERRR
IRSLYNEPSKEAAKVSASQRMDSIVTEPCV
>g1208.t1
MPVFIYSMPLNMCTIRERMLYSSCKNPLVDMLENELQMEIEKKLELDNGDDLTPDFLYDEVHPKQHAHKQAFAKPKGPPG
KRGARRITRPATEGEDDD
>g1208.t2
MCIFTSEMFIYSMPLNMCTIRERMLYSSCKNPLVDMLENELQMEIEKKLELDNGDDLTPDFLYDEVHPKQHAHKQAFAKP
KGPPGKRGARRITRPATEGE

DDD
>g1208.t3
MGFGKDLRNSHEGLLKLQDWELKVGGLVEGKVGELKLLETVKRFMTLRVKSDKEYAAMLLNLSRQTENQNPDYSSTVSQC
SSLFNLPLGTELQGVVIFPY
VGQPYKTQRHHE
>g1208.t4
MGFGKDLRNSHEGLLKLQDWELKLLETVKRFMTLRVKSDKEYAAMLLNLSRQTENQNPDYSSTVSQCSSLFNLPLGTELQ
GVVIFPYVGQPYKTQRHHE
>g1208.t5
MGFGKDLRNSHEGLLKLQDWELKVGGLVEGKVGELKLLETVKRFMTLRVKSDKEYAAMLLNLSRQTENQNPDYSSTVSQQ
TSRRISTRVSEFQMPVFIYS
MPLNMCTIRERMLYSSCKNPLVDMLENELQMEIEKKLELDNGDDLTPDFLYDEVHPKQHAHKQAFAKPKGPPGKRGARRI
TRPATEGEDDD
>g1209.t1
EEDILAEELRLQFQQEIQTLKTLKHPNLVDMVGFSSDGQYPCLVYVFMANGSVLDRLAXRQPSSVVEQQMRDSHRKCQSG
NILLDENFVAKISDFGLTRA
SATRSAATMLTDRIVGTRAYMAPEALRGEITPISDIFSFGVMELRYDIDDEDEDLTLVDFLDKKMTDTDMAQVERTYSLA
CHCLQDRKVKRPNIKQVLAE
LEDVSRGHSAEASGTGS
>g1209.t2
EGGFGTVYKGFINHKPVAVKKLSPEEDILAEELRLQFQQEIQTLKTLKHPNLVDMVGFSSDGQYPCLVYVFMANGSVLDR
LAXRQPSSVVEQQMRDSHRK
CQSGNILLDENFVAKISDFGLTRASATRSAATMLTDRIVGTRAYMAPEALRGEITPISDIFSFGVMELRYDIDDEDEDLT
LVDFLDKKMTDTDMAQVERT
YSLACHCLQDRKVKRPNIKQVLAELEDVSRGHSAEASGTGS
>g1209.t3
EEDILAEELRLQFQQEIQTLKTLKHPNLVDMVGFSSDGQYPCLVYVFMANGSVLDRLACMPSSVVEQQMRDSHRKCQSGN
ILLDENFVAKISDFGLTRAS
ATRSAATMLTDRIVGTRAYMAPEALRGEITPISDIFSFGVMELRYDIDDEDEDLTLVDFLDKKMTDTDMAQVERTYSLAC
HCLQDRKVKRPNIKQVLAEL
EDVSRGHSAEASGTGS
>g1210.t1
DKCYKCTLKQGQTLFIPSGWINAILTPVDCLAFSGHFVHNLSVEMQMRAYEVEKRLKVKTLTPFPNFETACWYVGRHFLE
RFKGLHKANKQPAPYLVHGA
KILNAAFRVWTKKQALLEHEDELPENMKPTQLIKDLAREIRVAENATKAIKSEPSSAKVPVEEPPSTHSEPEEPDSPARI
PSPGREKTPRKKATKPSKPP
KPPKMPKAPKPPKVPKVKEGGKKKGKKVKELTPPPPKPSSFAALESHAKDILSKMDQPKKGKAVKNVLSMSEKEISKQNN
VDKFEIREQNKNKTEAKWKY
KIHVQSLKSLISSPSSHIPTSHPLACSSSRLLCGPQNSKPDSLLKMEEESKMDRSSLLGNKDRFSFTMSHKKLLGYVGHR
AGDHPANSGQQLLSGRGAFN
LWCDLNRRATGVAAHHV
>g1210.t2
DKCYKCTLKQGQTLFIPSGWINAILTPVDCLAFSGHFVHNLSVEMQMRAYEVEKRLKVKTLTPFPNFETACWYVGRHFLE
RFKGLHKANKQPAPYLVHGA
KILNAAFRVWTKKQALLEHEDELPENMKPTQLIKDLAREIRVAEVTLSFCFSCTGIEITFFVPLSGSNATKAIKSEPSSA
KVPVEEPPSTHSEPEEPDSP
ARIPSPGREKTPRKKATKPSKPPKPPKMPKAPKPPKVPKVKEGGKKKGKKVKELTPPPPKPSSFAALESHAKDILSKMDQ
PKKGKAVKNVLSMSEKEISK
QNNVDKFEIREQNKNKTEAKWKYKIHVQSLKSLISSPSSHIPTSHPLACSSSRLLCGPQNSKPDSLLKMEEESKMDRSSL
LGNKDRFSFTMSHKKLLGYV
GHRAGDHPANSGQQLLSGRGAFNLWCDLNRRATGVAAHHV
>g1210.t3
DKCYKCTLKQGQTLFIPSGWINAILTPVDCLAFSGHFVHNLSVEMQMRAYEVEKRLKVKTLTPFPNFETACWYVGRHFLE
RFKGLHKANKQPAPYLVHGA
KILNAAFRVWTKKQALLEHEDELPENMKPTQLIKDLAREIRVAENATKAIKSEPSSAKVPVEEPPSTHSEPEEPDSPARI
PSPGREKTPRKKATKPSKPP

KPPKMPKAPKPPKVPKVKEGGKKKGKKVKELTPPPPKPSSFAALESHAKDILSKMDQPKKGKAVKNVLSMSEKEISKQNN
VDKFEIREQNKNKTEAKWKY
KNSKPDSLLKMEEESKMDRSSLLGNKDRFSFTMSHKKLLGYVGHRAGDHPANSGQQLLSGRGAFNLWCDLNRRATGVAAH
HV
>g1211.t1
PQAADGHLSISYGTESGELSLVGQEFYTQNSSAASSSSSSLAPPQGLGEEAEELLFLPHGVQMFFVTPDGQVSAPSYPGV
LRLVRFTAERPRGAPARPPA
FLQVCDWLYPLMASETPVLLCNTGVYMFPDMMAPAPGSYVGVVLSSELPAADRELFQDLLSQMTELRVQVLPGPVLRCLA
VALCLTSNSPVSVRGFEMSQ
RGDGNDP
>g1212.t1
MWKVIAVVAIATFATGKTLKYQIYEEQKVGTVIARLKDDVADVLAKLPSTVSLRFRAMQRGSMSLLAVREQDGEISIRTK
IDREKLCEKNLNCSIQFDVL
TLPTEHLQLFHIEVEVLDINDNTPQFARSIIPIEISESAAVGARIPLDSATDPDVGENSLYSYALEPNNFFKIDIQSRTD
GAKYAELVVLKELDREVRAG
YELQYTASDKGVPARTGSTLLKISVADSNDNSPIFDKSSYIINLPENAPVGTLLINLNATDADEGTNGKIVYSFSSHVSG
KILDTFKINPDSGHLTLIRR
VDYETASSYDIDVQAQDMGPNSMPAHCKILVKVGDVNDNKPDISINLMSSQGKEDAAYLSEASPLDTFVALVRVEDMDSG
LNGEVVCKLHGQGYFKLQKT
YENNYLILTNVTLDREKRSEFSLTVVAEDRGTPSLSTVKHFTVHVTDENDNPPRFEKGRYEIFKSENNAPGAYLYSLVAT
DPDLNANGQVSYSIVENVIH
GSSISTYVTIDPSNGAIYALRTFDREDVSHISFVVQAKDAGAPPLLSNATVSLNILDENDNPPAIVVPQLWNFTADVPVS
KFTEAGHLVTALRATDRDTG
VNAELICSIAGGNEEGFFIMDPRTCEVHANASLEGFPRERVELTVVVRDQGSESLSAKAVLKITLYENMENHVQVMDPGE
SSFDASLIIIISLGAICALL
LVIMVAFAARCSREKKETRHSYNCRVAESNHQHHPKKPSRQIHKGDITLVPTVNGTLPIRAHHRSPLATPPLDRAPMGSR
QSHHSRQSLNSLVTISSNHI
PESFALELTHTTPPVE
>g1213.t1
QVRRELERLKQRKAAGPDRISPHVSKAFSSQLCKVFQYLFNLSLHFQRVPVLWKTSCLVPVPKKRSCSSLDCPNTSVRVM
FFDFSSAFNTIQPRLLKAKL
ENMQLNVAKTREMVVDFRRNKPLPSPVGTGGTIFLAMVSWGADIRTKDANRLNKLIKKAESVVGSKLVTVEEERMLAITD
NHSHPLHTTLDKLKSSFSHR
LIQPRFKKEQYRKSFLPTAIRLYNSSASVRSTITELD
>g1213.t2
VRRELERLKQRKAAGPDRISPHVSKAFSSQLCKVFQYLFNLSLHFQRVPVLWKTSCLVPVPKKRSCSSLDCPNTSVRVMF
FDFSSAFNTIQPRLLKAKLE
NMQLNVAKTREMVVDFRRNKPLPSPVGTGGTIFLAMVSWGADIRTKDANRLNKLIKKAESVVGSKLVTVEEERMLAITDN
HSHPLHTTLDKLKSSFSHRL
IQPRFKKEQYRKSFLPTAIRLYNSSASVRSTITELD
>g1213.t3
LLTPTTDPVTPPTPQSGLHITSSQVRRELERLKQRKAAGPDRISPHVSKAFSSQLCKVFQYLFNLSLHFQRVPVLWKTSC
LVPVPKKRSCSSLDCPNTSV
RVMFFDFSSAFNTIQPRLLKAKLENMQLNVAKTREMVVDFRRNKPLPSPVGTGGTIFLAMVSWGADIRTKDANRLNKLIK
KAESVVGSKLVTVEEERMLA
ITDNHSHPLHTTLDKLKSSFSHRLIQPRFKKEQYRKSFLPTAIRLYNSSASVRSTITELD
>g1214.t1
LATLQSYRPLVNDYGPPSLGFSQGSSGNQVPQSKYAELLAIIEELGKEIRPTYAGSKSAMERLKRGIIHARGLVRECLAE
TERNARS
>g1214.t2
MSISTFRELLETRGSSGNQVPQSKYAELLAIIEELGKEIRPTYAGSKSAMERLKRGIIHARGLVRECLAETERNARS
>g1214.t3
MTPRAAIGPTQPFFVSTNGQETDSILGNIQNIQSPSAANLATLQSYRPLVNDYGPPSLGFSQGSSGNQVPQSKYAELLAI
IEELGKEIRPTYAGSKSAME
RLKRGIIHARGLVRECLAETERNARS
>g1215.t1

MSTDDSISEDVSTSGALSHCHASADGDGGKESEISCVSSEGTSASCLAGLEDGHTRPPKTSEGGAVGSRPMDMTPACDKI
RSLCLSTEEAFDQGKSLPFI
NPSSLETLRALVQEIQNSGETDPEMWKDCEGRWLHLFQLVEKQYQEQILAQQEQYQCQIQLIQDEIKALVQLQNRQPHAE
LSPTSATNTTLTTATTITAN
TTTAPPP
>g1215.t2
MSTDDSISEDVSTSGALSHCHASADGDGGKESEISCVSSEGTSASCLAGLEDGHTRPPKTSEGGAVGSRPMDMTPACDKI
RWSLCLSTEEAFDQGKSLPF
INPSSLETLRALVQEIQNSGETDPEMWKDCEGRWLHLFQLVEKQYQEQILAQQEQYQCQIQLIQDEIKALVQLQNRQPHA
ELSPTSATNTTLTTATTITA
NTTTAPPP
>g1216.t1
VFSHSHITLWEISTGLLRGRFIHLTFLVVNTTAPRVALSAPAATRGNLHMHFQRRVTKRTRMRARKGSRGRGPCSCGNEL
LSLITTIIIITIIIIAIIST
IISTIISTITHHR
>g1216.t2
LWEISTGLLRGRFIHLTFLVVNTTAPRVALSAPAATRGNLHMHFQRRVTKRTRMRARKGSRGRGPCSCGNELLSLITTII
IITIIIIAIISTIISTIIST
ITHHR
>g1216.t3
VVRRQVFSHSHITLWEISTGLLRGRFIHLTFLVVNTTAPRVALSAPAATRGNLHMHFQRRVTKRTRMRARKGSRGRGPCS
CGNELLSLITTIIIITIIII
AIISTIISTIISTITHHR
>g1217.t1
MARFEDDLSNRYGGGCPPGPGRGGGRQPGPPPPGPRVYKQTMAQRARTMAIYNPIPVKQSCLTVNRSLFIF
>g1218.t1
MHVIHEAGACGLLLVNECGPGSRFDYGDHYWRIKRRFFGCQCGSPRCRHSSSSSSSSSSASPAGAVQRGRGLETTTRRL
FVDANQMEN
>g1219.t1
DDRLSLSEILDKVEYIRSSTITDYGGMAVDEHDELMSMNASALLSPPPQQLHLLHLLLLHHFTTEPSHLHHRPGLFGLQR
HPRPREASSSLREDEDDDGT
NKQQSGQ
>g1219.t2
MIIFSPKLVGIVKDDRLSLSEILDKVEYIRSSTITDYGGMAVDEHDELMSMNASALLSPPPQQLHLLHLLLLHHFTTEPS
HLHHRPGLFGLQRHPRPREA
SSSLREDEDDDGTNKQQSGQ
>g1219.t3
DDRLSLSEILDKVEYIRSSTITDYGGMAVDEHDEL
>g1220.t1
MTRHGKNCTAGAVYTQDGYLYEKQAILEYILHQKTDIAKKMKAYEKQKKAQKSDSNLESKSGEREQVERFKTRESSIVSK
PLNPFSSGQCQGKEAGPSTK
ADNQSGASTSAGTSSKADSAQSLPSFWIPSLTPEAKPTLLKKPSKTVLCPMSGRPIKMNELITVRFTPLDPSLDRVALLT
RQDRYVCAVTKDTLGNSLPC
SVLRPSGAVVTQECVEKLIRKDMLDPVTGDKLAEKDIILLQRVSKITMRASLEANSVILESL
>g1220.t2
MTRHGKNCTAGAVYTYHEKRKDTGKQDGYLYEKQAILEYILHQKTDIAKKMKAYEKQKKAQKSDSNLESKSGEREQVERF
KTRESSIVSKPLNPFSSGQC
QGKEAGPSTKADNQSGASTSAGTSSKADSAQSLPSFWIPSLTPEAKPTLLKKPSKTVLCPMSGRPIKMNELITVRFTPLD
PSLDRVALLTRQDRYVCAVT
KDTLGNSLPCSVLRPSGAVVTQECVEKLIRKDMLDPVTGDKLAEKDIILLQRVSKITMRASLEANSVILESL
>g1220.t3
MTRHGKNCTAGAVYTQDGYLYEKQAILEYILHQKTDIAKKMKAYEKQKKAQKSDSNLESKSGEREQVERFKTRESSIVSK
PLNPFSSGQGKEAGPSTKAD
NQSGASTSAGTSSKADSAQSLPSFWIPSLTPEAKPTLLKKPSKTVLCPMSGRPIKMNELITVRFTPLDPSLDRVALLTRQ
DRYVCAVTKDTLGNSLPCSV
LRPSGAVVTQECVEKLIRKDMLDPVTGDKLAEKDIILLQRVSKITMRASLEANSVILESL

>g1221.t1
MVIIIIIIIVVVIVVVVIIIIIIIVVIVVVIIIVVIIVVIIVVIIIIVIIIVIIVIIVVIVIIVVVIIIVVIIIIIIVVI
IVIVVIIIIVVIVIIIIIIVIV
VIIIIVVIIVVIIVVIIIVIIIVVVIIIIIIVIVVVVVIIII
>g1222.t1
AEAVTMNSNHQAFQGRSQNGHPYLARQRLALQTMQGCSGRGNLEMKLEGVDREEMMHTNHSSDSQRSDEEGDHKLHDDNS
TPASLHEDEDLSPEQKAERE
RERRMANNARERLRVRDINEAFKELGHMCQLHLNSEKPQTKLLVLHQAVAVILSLEQQRNLNPKAACLRRREEEKASAGV
AEQQSMHLAFHPSLSDSTAP
MGRL
>g1222.t2
AEAVTMNSNHQAFQGRSQNGHPYLARQRLALQTMQGCSGRGNLEMKLEGVDREEMMHTNHSSDSQRSDEEGDHKLHDDNS
TPASLHEDEDLSPEQKAERE
RERRMANNARERLRVRDINEAFKELGHMCQLHLNSEKPQTKLLVLHQAVAVILSLEQQVRERNLNPKAACLRRREEEKAS
AGVAEQQSMHLAFHPSLSDS
TAPMGRL
>g1223.t1
MNGSPDIRGYSLPHRRPLSSPDNMFGVRVQIQGIKGRPYVVMNNNDQESHKDFPAHHSENQGQRRGASLDRPSDEEPAGR
RGQLTDFSSSEFRDLGRTVA
SNALHYQRHPELLRPYDPEKNNLNLLIPPQTIADARAAKPHSGPPPAQTTQATSLPVKARMQLPAAGLVPDHNENQHREA
VPPGKAAPARSPNSVETDNI
SSVGKLIDRFNSTQQKRGRFGPRNRIDLDDRRRSRSVDSRQTSDSSTSSSTSSSFSSSRASSLKGPRAETLNQDGGPPEE
PGSRFLSGSRDTLVDSHRQQ
SSSSMQLYPAHQGKESATTSPQTAKPLNGLEKCTPRSQAASNHVDEPQERDAQVTPDLLKGQQEVSADSNEDTAKQILFC
YLKDGTTDNESTTEKKVNLV
LDKLNTLKWKTAESVEKEERDHTADVKDLQEKQTALEYEVDGLKKQLTVEIKNEKTLAKACEKARNDRKSITEELNKSEF
ELSKLRDRLAEVEAELQSTK
QELAQLTAERDRSKTEMKDLQHQLSEMHDELDHAKSTEEINTEKEVLLQDMAQLRDGFQELLLVQEEQEEMLQLREGELA
VLKGALKEEVESHERSVTAL
KEEHQHKVQKLLRAVDVAKESNTLLGQEKAEAEKVREATQDKFRAMSQERGHLKEQVRQLESNVDELHQAVREAKALEKQ
LEQRANQLEVILVKMLKYLS
VLLSVQKEKQQVERTLMEVRQKEDQMSQSNQSLITRLEDVQIELTKLNHEHRELKDKLREEKSQVLELWRTRQELEEERK
MQDRTVEQLQKKMSSIMGEC
EVSTDKLQQQVTEARDRSQRELAELRRQLEEKGVELDKSRLTAKKLQEEDSQLNWINTFVFCVLWSKLLSLEKDMQQSRR
EQQEAQGRSRQLEQKVEELE
KRDLATLENHEQQVKLMERRIGQLEEDLTEERSSADRLMDRVEKSKDQAS
>g1223.t2
MEPHKMNGSPDIRGYSLPHRRPLSSPDNMFGVRVQIQGIKGRPYVVMNNNDQESHKDFPAHHSENQGQRRGASLDRPSDE
EPAGRRGQLTDFSSSEFRDL
GRTVASNALHYQRHPELLRPYDPEKNNLNLLIPPQTIADARAAKPHSGPPPAQTTQATSLPVKARMQLPAAGLVPDHNEN
QHREAVPPGKAAPARSPNSV
ETDNISSVGKLIDRFNSTQQKRGRFGPRNRIDLDDRRRSRSVDSRQTSDSSTSSSTSSSFSSSRASSLKGPRAETLNQDG
GPPEEPGSRFLSGSRDTLVD
SHRQQSSSSMQLYPAHQGKESATTSPQTAKPLNGLEKCTPRSQAASNHVDEPQERDAQVTPDLLKGQQEVSADSNEDTAK
QILFCYLKDGTTDNESTTEK
KVNLVLDKLNTLKWKTAESVEKEERDHTADVKDLQEKQTALEYEVDGLKKQLTVEIKNEKTLAKACEKARNDRKSITEEL
NKSEFELSKLRDRLAEVEAE
LQSTKQELAQLTAERDRSKTEMKDLQHQLSEMHDELDHAKSTEEINTEKEVLLQDMAQLRDGFQELLLVQEEQEEMLQLR
EGELAVLKGALKEEVESHER
SVTALKEEHQHKVQKLLRAVDVAKESNTLLGQEKAEAEKVREATQDKFRAMSQERGHLKEQVRQLESNVDELHQAVREAK
ALEKQLEQRANQLEVILVKM
LKYLSVLLSVQKEKQQVERTLMEVRQKEDQMSQSNQSLITRLEDVQIELTKLNHEHRELKDKLREEKSQVLELWRTRQEL
EEERKMQDRTVEQLQKKMSS
IMGECEVSTDKLQQQVTEARDRSQRELAELRRQLEEKGVELDKSRLTAKKLQEEDSQLNWINTFVFCVLWSKLLSLEKDM
QQSRREQQEAQGRSRQLEQK
VEELEKRDLATLENHEQQVKLMERRIGQLEEDLTEERSSADRLMDRVEKSKDQAS

>g1223.t3
MNGSPDIRGYSLPHRRPLSSPDNMFGVRVQIQGIKGRPYVVMNNNDQESHKDFPAHHSENQGQRRGASLDRPSDEEPAGR
RGQLTDFSSSEFRDLGRTVA
SNALHYQRHPELLRPYDPEKNNLNLLIPPQTIADARAAKPHSGPPPAQTTQATSLPVKARMQLPAAGLVPDHNENQHREA
VPPGKAAPARSPNSVETDNI
SSVGKLIDRFNSTQQKRGRFGPRNRIDLDDRRRSRSVDSRQTSDSSTSSSTSSSFSSSRASSLKGPRAETLNQDGGPPEE
PGSRFLSGSRDTLVDSHRQQ
SSSSMQLYPAHQGKESATTSPQTAKPLNGLEKCTPRSQAASNHVDEPQERDAQVTPDLLKGQQEVSADSNEDTAKQILFC
YLKDGTTDNESTTEKKVNLV
LDKLNTLKWKTAESVEKEERDHTADVKDLQEKQTALEYEVDGLKKQLTVEIKNEKTLAKACEKARNDRKSITEELNKSEF
ELSKLRDRLAEVEAELQSTK
QELAQLTAERDRSKTEMKDLQHQLSEMHDELDHAKSTEEINTEKEVLLQDMAQLRDGFQELLLVQEEQEEMLQLREGELA
VLKGALKEEVESHERSVTAL
KEEHQHKVQKLLRAVDVAKESNTLLGQEKAEAEKVREATQDKFRAMSQERGHLKEQVRQLESNVDELHQAVREAKALEKQ
LEQRANQLEVILVKMLKYLS
VLLSVQKEKQQVERTLMEVRQKEDQMSQSNQSLITRLEDVQIELTKLNHEHRELKDKLREEKSQVLELWRTRQELEEERK
MQDRTVEQLQKKMSSIMGEC
EVSTDKLQQQVTEARDRSQRELAELRRQLEEKGVELDKSRLTAKKLQEELLSLEKDMQQSRREQQEAQGRSRQLEQKVEE
LEKRDLATLENHEQQVKLME
RRIGQLEEDLTEERSSADRLMDRVEKSKDQAS
>g1224.t1
MDQMRGEVQQERAARQDLECDKMSLERQNKDLKSRVNHLEGVRGVNQQDSVVSRLTTRIQDLEDRLQGEK
>g1224.t2
MRGEVQQERAARQDLECDKMSLERQNKDLKSRVNHLEGVRGVNQQDSVVSRLTTRIQDLEDRLQGEK
>g1225.t1
ELLMSRLAGNFDATHRVNFDLGSESDVAPARFDPLVQFDHVGKEKLEQVRP
>g1225.t2
MSRLAGNFDATHRVNFDLGSESDVAPARFDPLVQFDHVGKEKLEQVRP
>g1226.t1
LFDNEWPTALHRSAACVGRRSGTDRIFLTGIFVFPFRSPNQLPSEVIKVGHPRIIPAASRPIYKTTTTPTTITTTTTTTT
TTTTTHQAAWRTPGGS
>g1226.t2
LFDNEWPTALHRSAACVGRRSGTDRIFLTGIFVFPFRSPNQLPSEVIKDIDLTICQIWQRLALSSRALLRDVYHTSLQVG
HPRIIPAASRPIYKTTTTPT
TITTTTTTTTTTTTTHQAAWRTPGGS
>g1227.t1
AADASEASDSGSEQERDWFDRATEVDKRRKNNHGRRVNLSRDEK
>g1228.t1
MVNNHTAKHNSDLGPRERIEKVIIIIIIIILLIRVLEDGCKMGQSHLSGQRWPALASREQMSEEEVEHQIGFILEEPRRR
SGACSLNHQAFLPQEPDQWR
NVDATGPADEPRAVHLPCVGRGRGRFTTWARFPRGPSHRWAVAWLQPVYVHH
>g1229.t1
AISTDILPFITTLINSSLTSSTLIATCISECLADISSWTTAHQLKLNLAKTELLFIPGTDCPHRDLLVSVKGITVSASPT
ARNLGVIVDDQLSCTANINA
MTRSCRIALYNIRRIRHFLTRDTTHLPPAIKPLQRIQNAAAHLVFNRHKFSHVTPLFQDLHWLPVVARIKFKMMLLAYKA
EMLYTSNG
>g1230.t1
MEGRLLHPWRMKLPRLILTKPHTGASARRRANALTAHSGPDGVHEVTVLTASPSLTLQGKRGRRLSFGGALRWGRGSTGG
YGAIPSTRGGTRAGSRRCWK
DWDLALVRKKSCSPKKNE
>g1231.t1
MAANEPHFTEKYFSQIVDHFNFNSMGNATFNQRYLITEKFWNRKRGPIFFYTGNEGNIWDFALNSGFITELAEQQGALVI
FAEHRYYGKSLPFGQESFGI
PQVGLLTVEQALADFALIISQLKQELGASDCPVIAFGGSYGGMLSAYMRQRYPNLVAGALAASAPVRSTAGMGDPGQFFR
DVTS
>g1231.t2

MGNATFNQRYLITEKFWNRKRGPIFFYTGNEGNIWDFALNSGFITELAEQQGALVIFAEHRYYGKSLPFGQESFGIPQVG
LLTVEQALADFALIISQLKQ
ELGASDCPVIAFGGSYGGMLSAYMRQRYPNLVAGALAASAPVRSTAGMGDPGQFFRDVTS
>g1231.t3
MAANEPHFTEKYFSQIVDHFNFNSMGNATFNQRYLITEKFWNRKRGPIFFYTGNEGNIWDFALNSGFITELAEQQGALVI
FAEHRYYGKSLPFGQESFGI
PQVGLLTVEQALADFALIISQLKQELGASDCPVIAFGGSYGGMLSAYMRQRYPNLVAGALAASAPVRSTAGMGDPGQFFR
DVTSVS
>g1232.t1
MLYTELLRLWDEAVKAMDARDWRRALEMAGTVTEPNSRTLFIEASAHLALQELEPALQALNQVIVKDGRLAVAFFQRSVV
LMQLDRLDDALSDCIWAQKH
MRGNTVIDYKQLGLHHKLFSWQVLYNAAAVYCRMGQWERATEVLMSASQERGAGRSSAITLGLETIS
>g1233.t1
WWNELPANVRTAESLPIFRKRLKTHLFRVHLNPT
>g1234.t1
GTFEVIKNTDIDLDKKIPEDYCPLDVQIPSDLEGSAYLKVSIQKQAPDIGDLGTVNLFRRQPKTKAGTQPWHVKLEAAQN
VLLCKEIFAQLSREAVQIKS
QIPHIVVKNQIISQPFPGLQLSISLCHSTGEKKSQKGSPERPKPDDHLYVLEHNLHQLMREFHKQRLSSVVMPHPASAPF
GHKRMRLAGPLAYDKAEISS
LQQTQGLLEKIIKQAKHIFLRSRTARTIDSLASHIEDPQIQAHWSNINDVYESSVKVLITSQGYEQICKSIQLQLNIGVE
QIRVVHRDGRVVTLSHQEQE
LQDFLLSQMSQHQVHAVQQLAKVMGWHVLSFSNHVGLGPVESIGNASAITVASANGEYAISVRNGPESGCKVLVQFPRSQ
NKDQPKSEVVQDAKWTHLRG
AYKEVHWSKMEGRNFVYKMELLMAALTPCP
>g1234.t2
MVHAGSLFPHQGTFEVIKNTDIDLDKKIPEDYCPLDVQIPSDLEGSAYLKVSIQKQAPDIGDLGTVNLFRRQPKTKAGTQ
PWHVKLEAAQNVLLCKEIFA
QLSREAVQIKSQIPHIVVKNQIISQPFPGLQLSISLCHSTGEKKSQKGSPERPKPDDHLYVLEHNLHQLMREFHKQRLSS
VVMPHPASAPFGHKRMRLAG
PLAYDKAEISSLQQTQGLLEKIIKQAKHIFLRSRTARTIDSLASHIEDPQIQAHWSNINDVYESSVKVLITSQGYEQICK
SIQLQLNIGVEQIRVVHRDG
RVVTLSHQEQELQDFLLSQMSQHQVHAVQQLAKVMGWHVLSFSNHVGLGPVESIGNASAITVASANGEYAISVRNGPESG
CKVLVQFPRSQNKDQPKSEV
VQDAKWTHLRGAYKEVHWSKMEGRNFVYKMELLMAALTPCP
>g1234.t3
GTFEVIKNTDIDLDKKIPEDYCPLDVQIPSDLEGSAYLKVSIQKQAPDIGDLGTVNLFRRQPKTKAGTQPWHVKLEAAQN
VLLCKEIFAQLSREAVQIKS
QIPHIVVKNQIISQPFPGLQLSISLCHSTGEKKSQKGSPERPKPDDHLYVLEHNLHQLMREFHKQRLSSVVMPHPASAPF
GHKRMRLAGPLAYDKAEISS
LQQTQGLLEKIIKQAKHIFLRSRTARTIDSLASHIEDPQIQAHWSNINDVYESSVKVLITSQGYEQICKSIQLQLNIGVE
QIRVVHRDGRVVTLSHQEQE
LQDFLLSQMSQHQVHAVQQLAKVMGWHVLSFSNHVGLGPVESIGNASAITVASANGEYAISGKTGQTRPATTSDRHLFSS
EFENQLPHVRNGPESGCKVL
VQFPRSQNKDQPKSEVVQDAKWTHLRGAYKEVHWSKMEGRNFVYKMELLMAALTPCP
>g1235.t1
LTRSVQEDVVFSVHVTCRGVPTLHWTFMSGAVSRSIGSWRPDVGGGGGDGNGSYSNITVDYRDRVEAHSNGSLGLARLRL
QDAGYYVLTVTEDSGSSKDC
GFVLKVNEVIYEDLQYLSVSALALAGLAGLLMLSMWLLHRAYKKLKACRRHRKQRTGTTMFNATEVGALDEWMD
>g1235.t2
GAISVHSPQRSLTRSVQEDVVFSVHVTCRGVPTLHWTFMSGAVSRSIGSWRPDVGGGGGDGNGSYSNITVDYRDRVEAHS
NGSLGLARLRLQDAGYYVLT
VTEDSGSSKDCGFVLKVNEVIYEDLQYLSVSALALAGLAGLLMLSMWLLHRAYKKLKACRRHRKQRTGTTMFNATEVGAL
DEWMD
>g1235.t3
LTRSVQEDVVFSVHVTCRGVPTLHWTFMSGAVSRSIGSWRPDVGGGGGDGNGSYSNITVDYRDRVEAHSNGSLGLARLRL
QDAGYYVLTVTEDSGSSKDC

GFVLKVNEVIYEDLQYLSVSALALAGLAGLLMLSMWLLHRAYKKLKACRRHRKQRTDNETELQPL
>g1236.t1
MEEANERKQAKYADLVEECRRRGWRAQCVPIEVGCRGFVARSLCKAYSLLGITGARQRKAIKTITEAAEKASRWLWIKRG
NPKEIKLLMNELDEEKRVRL
TLQ
>g1236.t2
MEEANERKQAKYADLVEECRRRGWRAQCVPIEAGSPGKEIKLLMNELDEEKRVRLTLQ
>g1237.t1
VNLLLREYLLSGDSREAERCLRELEVPHFHHEFVYEALVMVLESKGEKTFKMVLQLLKRLCVSTVITVDQMRRGYERVYM
DIPEINIDVPRAYFILELFV
DKSFSAGVIDKHLRDLCPCRGRKRFVSEGDGGCLKLESF
>g1237.t2
DTLIDEVNVEAEVIMLTVVILCWQVNLLLREYLLSGDSREAERCLRELEVPHFHHEFVYEALVMVLESKGEKTFKMVLQL
LKRLCVSTVITVDQMRRGYE
RVYMDIPEINIDVPRAYFILELFVDKSFSAGVIDKHLRDLCPCRGRKRFVSEGDGGCLKLESF
>g1238.t1
MAEVIQQRIEDRIPELEHLERVGLFTSKEVKSLIKRATALEYKLQRLALTKEDFITYATKGYLSKVFSELIAIHPDKPAL
WIMAAKSELEDMDSSESARH
LFLRALRFHPEDKKVFQEYFRMELLHAEKLRKEKSMLETAEVDLGEYEFSPEIMSGKLAEIVYKDATEKIQGAEFIISLL
NIAAIFDFTKELQDTILKDI
QSKYTEDCVAWDFLARRELEAPGAGEELTTAKGKASDMARREDRVSMVYEEGLKVLHTEPIFTCYASFCLERHKRKSNVQ
ELKDKREQRLLEVFQRAHDS
SMLKETFYKNWLKTLLTSGDAEGVARVAMAAKQRYSQSVSTWILSLQTLMQLESRDMDALFKEALECISPKNQK
>g1238.t2
MAEVIQQRIEDRIPELEHLERVGLFTSKEVKSLIKRATALEYKLQRLALTKEDFITYATKGYLSKVFSELIAIHPDKPAL
WIMAAKSELEDMDSSESARH
LFLRALRFHPEDKKVFQEYFRMELLHAEKLRKEKSMLETAEVDLGEYEFSPEIMSGKLAEIVYKDATEKIQGAEFIISLL
NIAAIFDFTKELQDTILKDI
QSKYTEDCVAWDFLARRELEAPGAGEELTTAKGKASDMARREDRVSMVYEEGLKVLHTEPIFTCYASFCLERHKRKSNVQ
ELKDKREQRLLEVFQRAHDS
SMLKETFYKNWLKTLLTSGDAEGVARVAMAAKQRYSQSVSTWILSLQTLMQLESRDMDALFKEALECISPK
>g1238.t3
MAEVIQQRIEDRIPELEHLERVGLFTSKEVKSLIKRATALEYKLQRLALTKEDFITYHIKYHFKKEQIEFPIIYRIQGVF
KRATNKWKATKGYLSKVFSE
LIAIHPDKPALWIMAAKSELEDMDSSESARHLFLRALRFHPEDKKVFQEYFRMELLHAEKLRKEKSMLETAEVDLGEYEF
SPEIMSGKLAEIVYKDATEK
IQGAEFIISLLNIAAIFDFTKELQDTILKDIQSKYTEDCVAWDFLARRELEAPGAGEELTTAKGKASDMARREDRVSMVY
EEGLKVLHTEPIFTCYASFC
LERHKRKSNVQELKDKREQRLLEVFQRAHDSSMLKETFYKNWLKTLLTSGDAEGVARVAMAAKQRYSQSVSTWILSLQTL
MQLESRDMDALFKEALECIS
PKNQK
>g1239.t1
MSGGSCKANGAVYPASSAGMNSVKKPKMEQIQADHELFLQAFEKKPSQNSENEQNSVSLEVLLVKVCHKKRKDVSCPVKQ
VPTGKKQVPLNPDSNQTKLG
SYPSLLVPSSEFEPSNSHMVKSYSLLFRVSRAGRREANGLVNGEANENIDVTETPSRKKRSSSHREEDGTTETYIAQMTV
FDKNRRLQLLDGEYEVSMQG
MEDSPVNKKRATWETILDGK
>g1239.t2
MSGGSCKANGAVYPASSAGMNSVKKPKMEQIQADHELFLQAFEKPTQIYRYLRTRNLIADVSCPVKQVPTGKKQVPLNPD
SNQTKLGSYPSLLVPSSEFE
PSNSHMVKSYSLLFRVSRAGRREANGLVNGEANENIDVTETPSRKKRSSSHREEDGTTETYIAQMTVFDKNRRLQLLDGE
YEVSMQGMEDSPVNKKRATW
ETILDGK
>g1239.t3
MSGGSCKANGAVYPASSAGMNSVKKPKMEQIQADHELFLQAFEKKPSQNSENEQNSVSLEVLLVKVCHKKRKDVSCPVKQ
VPTGKKQVPLNPDSNQTKLG

SYPSLLVPSSEFEPSNSHMVKSYSLLFRVSRAGRREANGLVNGEANENIGKGNYVTETPSRKKRSSSHREEDGTTETYIA
QMTVFDKNRRLQLLDGEYEV
SMQGMEDSPVNKKRATWETILDGK
>g1240.t1
MDHLPGKIQIPEKEPFEKVYQVGSVLGSGGFGTVYAGSRITDGAPVAVKHVAKERVNEWGTLNGSLVPLEILLLKKVSSS
FRGVIKLLDYYERSDGWLLV
MERPELVKDLFDFITEKGALDEDTARGFFRQVLEAVRHCYSCGVVHRDIKDENLLVDLRTGELKLIDFGSGALLKDTVYT
DFDGTRVYSPPEWVRFHRYH
GRSATVWSLGVLLFDMVCGDIPFEQDDEILRGKLCFRRRVSAGLFPWKLVSPAEVVGGAARAGQEGRWSAL
>g1240.t2
MQQDIFNGAVYFAVKSEKEPFEKVYQVGSVLGSGGFGTVYAGSRITDGAPVAVKHVAKERVNEWGTLNGSLVPLEILLLK
KVSSSFRGVIKLLDYYERSD
GWLLVMERPELVKDLFDFITEKGALDEDTARGFFRQVLEAVRHCYSCGVVHRDIKDENLLVDLRTGELKLIDFGSGALLK
DTVYTDFDGTRVYSPPEWVR
FHRYHGRSATVWSLGVLLFDMVCGDIPFEQDDEILRGKLCFRRRVSAGLFPWKLVSPAEVVGGAARAGQEGRWSAL
>g1240.t3
KEPFEKVYQVGSVLGSGGFGTVYAGSRITDGAPVAVKHVAKERVNEWGTLNGSLVPLEILLLKKVSSSFRGVIKLLDYYE
RSDGWLLVMERPELVKDLFD
FITEKGALDEDTARGFFRQVLEAVRHCYSCGVVHRDIKDENLLVDLRTGELKLIDFGSGALLKDTVYTDFDGTRVYSPPE
WVRFHRYHGRSATVWSLGVL
LFDMVCGDIPFEQDDEILRGKLCFRRRVSAGLFPWKLVSPAEVVGGAARAGQEGRWSAL
>g1241.t1
LLELVLEEQSVSRVLRQMSTGPEGPLGASGDGVGALNQEWSQICSRVGRSDGRRARHHLMSCWHSETEEEVRRRCGGGKE
EVLSGRSSQWTGGQGV
>g1242.t1
DPEAAQRKQGTYAAPTMKGKQGVPGGRSTDLLLLLCFIRVDHSHAERPRWTFVCTVSALTDPSCLHISITSGC
>g1243.t1
DGDGKDGGDDKKKKKKGRTGPLVPETAKDPGVQFVCGLTDTAANVGDRAELSCKLSSDTAKGCWYKNGKLLSDGDGVKII
QDGACHRLVIDCCTNDDGAV
YRFEAEGRKSEATLNVEDPPKMDLDALGKFSKPVIVRAGETADWKLPFSGGSPMTVQWYKDDEELLPGLTVRLETSSTDS
QLRLTKCQRKDGGDVKIKLK
NAFGTIEATSKLIVLDKPTPPQGPVEVLESSVGSVEFKWRPPKDDGGCPLTAYTLERQQVGRNKWAGLGEVPGGNPSYRD
ADVDPGRRYCYRIRAKNEEG
LSDYLETQDMSAGVLRYPGSPSAPKVVSAFKDCINLTWSAPCDTGGSAIVGYNLEKHKTGSNYWSLVNQEGPIKGGITPI
>g1243.t2
DGDGKDGGDDKKKKKKGRTGPLVPETAKGVQFVCGLTDTAANVGDRAELSCKLSSDTAKGCWYKNGKLLSDGDGVKIIQD
GACHRLVIDCCTNDDGAVYR
FEAEGRKSEATLNVEDPPKMDLDALGKFSKPVIVRAGETADWKLPFSGGSPMTVQWYKDDEELLPGLTVRLETSSTDSQL
RLTKCQRKDGGDVKIKLKNA
FGTIEATSKLIVLDKPTPPQGPVEVLESSVGSVEFKWRPPKDDGGCPLTAYTLERQQVGRNKWAGLGEVPGGNPSYRDAD
VDPGRRYCYRIRAKNEEGLS
DYLETQDMSAGVLRYPGSPSAPKVVSAFKDCINLTWSAPCDTGGSAIVGYNLEKHKTGSNYWSLVNQEGPIKGGITPI
>g1244.t1
MSFLRRVAGLSLRDRVRSSDIRRELGVEPLLLRVERSQFRWFRHLIRMPPGRLPLEVFQARPTGKRPRGRPRTRWRDYIS
HLAWERLRVPQEELESVAGE
KDVWISLLSLLPRDPAPDKRTFDDDDDAR
>g1245.t1
LHLVSSDSDFTVDSNGTIVVAVVSMVTKTRSFWVWVNTGRGQPRRVDVQLIPDSKQLQQYALHAQSAPWNQAHKRFKRRW
SPLPFNIVENDTPPFPKEVE
LIGSDSSVNYTVYYTISGPGVDLPPLNLFSVVANTGMVKVNGAVDREQYPKLVVRKCTRDPELASLLGRVVYTVVNTNGK
LFLARVSDILTGKETDRPLD
ITVLVMDVNDNAPTFSGPLEFRVDEQSSPDTDVGVVTATDRDQEDTPHTLIKFSLLTGNQLFNIHPKSGMITTKSGTLDR
EVQATIPVTIEIRDMNGAWN
GLFNTATATVRLLDINDNPPTFTQASLTAKIDENKVNVLVLRIPVEDRDEVNTKNWKAKFEVIQGNENNNFRVDTDPDTN
EGLLYLVKPLDYEKGKAVTL
TVSAQNEVPLVKTLTSWNTIPVELSVGNVDEGPEFFPLNKVIQVKENTPIGTLIGSYTALDPETKSNAGI

>g1245.t2
LHLVSSDSDFTVDSNGTIVVAVVSMVTKTRSFWVWVNTGRGQPRRVDVQLIPDSKQLQQYALHAQSAPWNQAHKRFKRRW
SPLPFNIVENDTPPFPKEVE
LIGSDSSVNYTVYYTISGPGVDLPPLNLFSVVANTGMVKVNGAVDREQYPKLVFLARVSDILTGKETDRPLDITVLVMDV
NDNAPTFSGPLEFRVDEQSS
PDTDVGVVTATDRDQEDTPHTLIKFSLLTGNQLFNIHPKSGMITTKSGTLDREVQATIPVTIEIRDMNGAWNGLFNTATA
TVRLLDINDNPPTFTQASLT
AKIDENKVNVLVLRIPVEDRDEVNTKNWKAKFEVIQGNENNNFRVDTDPDTNEGLLYLVKPLDYEKGKAVTLTVSAQNEV
PLVKTLTSWNTIPVELSVGN
VDEGPEFFPLNKVIQVKENTPIGTLIGSYTALDPETKSNAGI
>g1245.t3
LHLVSSDSDFTVDSNGTIVVAVVSMVTKTRSFWVWVNTGRGQPRRVDVQLIPDSKQLQQYALHAQSAPWNQAHKRFKRRW
SPLPFNIVENDTPPFPKEVE
LIGSDSSVNYTVYYTISGPGVDLPPLNLFSVVANTGMVKVNGAVDREQYPKLVVRKCTRDPELASLLGRVVYTVVNTNGK
LFLARVSDILTGKETDRPLD
ITVLVMDVNDNAPTFSGPLEFRVDEQSSPDTDVGVVTATDRDQEDTPHTLIKFSLLTGNQLFNIHPKSGMITTKSGTLDR
EVQATIPVTIEIRDMNGAWN
GLFNTATATVRLLDINDNPPTFTQASLTAKIDENKVNVLVLRIPVEDRDEVNTKNWKAKFEVIQGNENNNFRVDTDPDTN
EGLLYLVKPLDYEKGKAVTL
TVSAQNEVPLVKTLTSWNTIPVELSVGNVDEGPEFFPLNKVIQVKENTPIGTLIGSYTALDPETKSNAGIR
>g1246.t1
LSGWFQTHASYVLTVVARDLKGAAGGNTGTGTLTVQIKDINDNAPTLEKDAYEGSIEENTQGVEVMRIKTQDLDIEHTDN
WDVVFDIVSGNEAGYFSITT
DPKTNEGILMLDKAGQPLESIYLEVVVEVEQEQEQEQEQEQEQEQEQEQEQEQEQEQEQEQEQEQEAGTGTGAGAGAGTG
TGTGTGTAGTLTGTGSGTGT
GTAGAAPSKGKSYPIKINVKNMPEGPHFDPQVKAIPISEGGNTFSVNDVLTKYPAIDGDTGEMAENVRYAKGSDPDNWFT
VDPETAEIRLNKMPDRESPF
LQNGTYYAKVLCISEDMPSETATGTIAIQVEDFNDHCPTLTSTVQSMCTTANAVTVSALDEDDFPNSAPFSFHIVEEGTE
GKWDVEYLNETAAILRCHET
PWPGSYEVTVEILDQQGVMCPEPQKVKVEVCTCEDGKICGRASKRSELGPAAIGLIFLGLLALLRESYPRHLNGPAALRS
ARHS
>g1246.t2
LSGWFQTHASYVLTVVARDLKGAAGGNTGTGTLTVQIKDINDNAPTLEKDAYEGSIEENTQGVEVMRIKTQDLDIEHTDN
WDVVFDIVSGNEAGYFSITT
DPKTNEGILMLDKAGQPLESIYLEVVVEVEQEQEQEQEQEQEQEQEQEQEQEQEQEQEQEQEQEQEAGTGTGAGAGAEGP
HFDPQVKAIPISEGGNTFSV
NDVLTKYPAIDGDTGEMAENVRYAKGSDPDNWFTVDPETAEIRLNKMPDRESPFLQNGTYYAKVLCISEDMPSETATGTI
AIQVEDFNDHCPTLTSTVQS
MCTTANAVTVSALDEDDFPNSAPFSFHIVEEGTEGKWDVEYLNETAAILRCHETPWPGSYEVTVEILDQQGVMCPEPQKV
KVEVCTCEDGKICGRASKRS
ELGPAAIGLIFLGLLALLRESYPRHLNGPAALRSARHS
>g1246.t3
MSRDFSLVSVLSVLAALAILADVVKGNLVRKKREWIIPPTKLEENVDYTGKDYIAKIRSDNQAYVGAVMYSLHGAGADKF
PYNVFYVDEDTGFVKVTRAL
DRETHASYVLTVVARDLKGAAGGNTGTGTLTVQIKDINDNAPTLEKDAYEGSIEENTQGVEVMRIKTQDLDIEHTDNWDV
VFDIVSGNEAGYFSITTDPK
TNEGILMLDKAGQPLESIYLEVVVEVEQEQEQEQEQEQEQEQEQEQEQEQEQEQEQEQEQEQEQEAGTGTGAGAGAGTGTGT
GTGTAGTLTGTGSGTGTGTA
GAAPSKGKSYPIKINVKNMPEGPHFDPQVKAIPISEGGNTFSVNDVLTKYPAIDGDTGEMAENVRYAKGSDPDNWFTVDP
ETAEIRLNKMPDRESPFLQN
GTYYAKVLCISEDMPSETATGTIAIQVEDFNDHCPTLTSTVQSMCTTANAVTVSALDEDDFPNSAPFSFHIVEEGTEGKW
DVEYLNETAAILRCHETPWP
GSYEVTVEILDQQGVMCPEPQKVKVEVCTCEDGKICGRASKRSELGPAAIGLIFLGLLALLRESYPRHLNGPAALRSARH
S
>g1247.t1
MFCQCGGPGAFPGGFAEIPFDTKPNLMTYHTEGQGENTDVPLLNMPIQMDSEISKMAMPGHHLPPLGPIGPKFQQSTSSM

GGVFYQDSVAGGYGTGNWRR
PQEANGYYSEFDGRESVVVDKFSGFALPESFLGNYYAQKATFAPESMAVKDNLLLYDYEGQESPVGSVGCCSLLEADNDL
QFLDNLGPKFKTLAEVCGGK
METPEVKPKHHAPVNTMTTPRTTDTMTTVRSVVSTQQQPPPRPQPQPLPPPQPQTITQHTEHRVIGNSSTNSKTITEGST
LVRGGAAGQSQKLVMQQPPV
YYTTAPVMQPMTYIMQPQIPNTMYIANSPATTNMQGMMVVNGSTQGLMMPGQSVGQGGGGVIHTGSLPQGMMVVEGQVPA
GSQQMLPGQTYLVQGGTLKG
GLSGSRGTLLVEGQNATPVGRGIHIEGGGGVIRTGSLPQGMVVVEGQVPSGAQQMLHGGGQTYVVQGGLSAVGQSRMVVA
GQNIGEGSLIQNGGMASESL
VQMGSVRLPGRTEQGGAKRVTTSTTSDVIAPTKTVVHKKRVVTTEKITR
>g1247.t2
MFCQCGGPGAFPGGFAEIPFDTKPNLMTYHTEGQGENTVILPLDVPLLNMPIQMDSEISKMAMPGHHLPPLGPIGPKFQQ
STSSMGGVFYQDSVAGGYGT
GNWRRPQEANGYYSEFDGRESVVVDKFSGFALPESFLGNYYAQKATFAPESMAVKDNLLLYDYEGQESPVGSVGCCSLLE
ADNDLQFLDNLGPKFKTLAE
VCGGKMETPEVKPKHHAPVNTMTTPRTTDTMTTVRSVVSTQQQPPPRPQPQPLPPPQPQTITQHTEHRVIGNSSTNSKTI
TEGSTLVRGGAAGQSQKLVM
QQPPVYYTTAPVMQPMTYIMQPQIPNTMYIANSPATTNMQGMMVVNGSTQGLMMPGQSVGQGGGGVIHTGSLPQGMMVVE
GQVPAGSQQMLPGQTYLVQG
GTLKGGLSGSRGTLLVEGQNATPVGRGIHIEGGGGVIRTGSLPQGMVVVEGQVPSGAQQMLHGGGQTYVVQGGLSAVGQS
RMVVAGQNIGEGSLIQNGGM
ASESLVQMGSVRLPGRTEQGGAKRVTTSTTSDVIAPTKTVVHKKRVVTTEKITR
>g1248.t1
MDTKETLYYTLKGHGASLEPYNLFVVDETTGLVRIFDILDREQREYYI
>g1248.t2
MDTKETLYYTLKGHGASLEPYNLFVVDETTGLVRIFDILDREQREYYIGLFSVDSLLEWPNSAMGQWLRKRST
>g1249.t1
MSGGDLIDRLIACSSKSELGIHMGEEGKHGEEQEEEEEEEEEEVAADMTTEAPEQQLDPPPPPLRSSPSSGVLHPTMLS
PQDATESSSTAVKGKKASSG
LRRPEKPPYSYIALIVMAIQSSPTKRLTLSEIYQFLQARFPFFRGSYQGWKNSVRHNLSLNECFIKLPKGLGRPGKGHYW
TIDPASEFMFEEGSFRRRPR
GFRRKCQALKPMYRMMNGIGFGTSIIPQSFDFQAPSATFACHSNGYNLDMMSNSMAGGYDGLSGGHHVPHMSPSPGSTYV
ASCPVPSNGEYGPDSSSSPV
PSSPAMASALDGHSQYAGTSAHWASGGGAPYIKQQPLASSSSASSGLHSGMSPYSLEQSYIHQNARDTHASDLSVGIPRY
QSHSSVCDRKDFLLNFNGIS
SFHPSTSGSYYHHHHHHHHQSVCQDIKPCVM
>g1249.t2
MTTEAPEQQLDPPPPPLRSSPSSGVLHPTMLSPQDATESSSTAVKGKKASSGLRRPEKPPYSYIALIVMAIQSSPTKRLT
LSEIYQFLQARFPFFRGSYQ
GWKNSVRHNLSLNECFIKLPKGLGRPGKGHYWTIDPASEFMFEEGSFRRRPRGFRRKCQALKPMYRMMNGIGFGTSIIPQ
SFDFQAPSATFACHSNGYNL
DMMSNSMAGGYDGLSGGHHVPHMSPSPGSTYVASCPVPSNGEYGPDSSSSPVPSSPAMASALDGHSQYAGTSAHWASGGG
APYIKQQPLASSSSASSGLH
SGMSPYSLEQSYIHQNARDTHASDLSVGIPRYQSHSSVCDRKDFLLNFNGISSFHPSTSGSYYHHHHHHHHQSVCQDIKP
CVM
>g1249.t3
MSGGDLIDRLIACSSKSELGIHMGEEGKHGEEQEEEEEEEEEEGLRADMTTEAPEQQLDPPPPPLRSSPSSGVLHPTML
SPQDATESSSTAVKGKKASS
GLRRPEKPPYSYIALIVMAIQSSPTKRLTLSEIYQFLQARFPFFRGSYQGWKNSVRHNLSLNECFIKLPKGLGRPGKGHY
WTIDPASEFMFEEGSFRRRP
RGFRRKCQALKPMYRMMNGIGFGTSIIPQSFDFQAPSATFACHSNGYNLDMMSNSMAGGYDGLSGGHHVPHMSPSPGSTY
VASCPVPSNGEYGPDSSSSP
VPSSPAMASALDGHSQYAGTSAHWASGGGAPYIKQQPLASSSSASSGLHSGMSPYSLEQSYIHQNARDTHASDLSVGIPR
YQSHSSVCDRKDFLLNFNGI
SSFHPSTSGSYYHHHHHHHHQSVCQDIKPCVM
>g1250.t1

MELCMDAEGNLPSPLSGDELGSDGDCVANSPAPVAQSGDAKGKPYTRRPKPPYSYIALIAMAIRDSNSGRLTLAEINDYL
MKKFPFFRGSYTGWRNSVRH
NLSLNDCFLKVLRDPSRPWGKDNYWMLNPHSEYTFADGVFRRRRKRISKRTPKEHESPEVHTSSDDMRVPVPVGSPPARE
EKAGAKFSSSFAIDSILSRP
FKRREDSRDMVGSVSADLRLYWPIEAHALPCAAHYQTVGGPTQHPYRRYSLAEAAYSAERSRDALTYYHLTSQFMPNSGT
FSSLKMPKARGGSFLIDSLL
S
>g1251.t1
HSIGSASSLYPSFPPHSLRPCTLTSNPHGSHVHIKEEPIDELDETESPPPPASSPLQEPTVVNVASHASQSARFIKHWDR
GYNSCARTDLFFTPLASSKL
AKKREEAMEKSRREAELNTRQERERDKDRERERERbusEVDKNARVSSSSHDSRISDAQMSAHGHMRSSFEQLPTTVAAVPPY
IGPDTPALRTLSEYARPHVM
SPTNRNHPFFMSLSTGEPLLAYHMPGLYGAEPGLRERELRNLRERELRERMKPGFEMKPPELEAMHPSANPMEHFARHGA
LVLPHIAGPPHHFAPFHPGL
NHLERERMMMANQQLRPELSYAERLTAERLHAERMASVASDPAARLQMLNVTPHHHQHSHIHSHLHLHQQDPLNQGPVPN
PLLNDLPHDHDMLRHPLFGV
AYPREMQGQIPQMSAAHQLQAMHAQSAELQRMAMEQQWLHGHHHLHGAPLPGQEDFYRCVHGTT
>g1251.t2
IGPAPIPNIGPAPSHSIGSASSLYPSFPPHSLRPCTLTSNPHGSHVHIKEEPIDELDETESPPPPASSPLQEPTVVNVAS
HASQSARFIKHWDRGYNSCA
RTDLFFTPLASSKLAKKREEAMEKSRREAELNTRQERERDKDREREREREVDKNARVSSSSHDSRISDAQMSAHGHMRSS
FEQLPTTVAAVPPYIGPDTP
ALRTLSEYARPHVMSPTNRNHPFFMSLSTGEPLLAYHMPGLYGAEPGLRERELRNLRERELRERMKPGFEMKPPELEAMH
PSANPMEHFARHGALVLPHI
AGPPHHFAPFHPGLNHLERERMMMANQQLRPELSYAERLTAERLHAERMASVASDPAARLQMLNVTPHHHQHSHIHSHLH
LHQQDPLNQGPVPNPLLNDL
PHDHDMLRHPLFGVAYPREMQGQIPQMSAAHQLQAMHAQSAELQRMAMEQQWLHGHHHLHGAPLPGQEDFYRCVHGTT
>g1251.t3
HSIGSASSLYPSFPPHSLRPCTLTSNPHGSHVHIKEEPIDELDETESPPPPASSPLQEPTVVNVASHASQSARFIKHWDR
GYNSCARTDLFFTPLASSKL
AKKREEAMEKSRREAELNTRQERERDKDREREREREVDKNARVSSSSHDSRISDAQMSAHGHMRSSFEQLPTTVAAVPPY
IGPDTPALRTLSEYARPHVM
SPTNRNHPFFMSLSTGEPLLAYHMPGLYGAEPGLRERELRNLRERELRERMKPGFEMKPPELEAMHPSANPMEHFARHGA
LVLPHIAGPPHHFAPFHPGL
NHLERERMMMANQQLRPELSYAERLTAERLHAERMASVASDPAARLQMLNVTPHHHQHSHIHSHLHLHQQDPLNQGSSPH
PLDPMATGPRLARFPFPTGP
VPNPLLNDLPHDHDMLRHPLFGVAYPREMQGQIPQMSAAHQLQAMHAQSAELQRMAMEQQWLHGHHHLHGAPLPGQEDFY
RCVHGTT
>g1252.t1
GLWGAVTVGYPSSSSSSMWGAPHMEERHQMDNPAALLPGDLLGGGADSI
>g1253.t1
VVSPQGSGSSMLLFRAEPGRAPWVKDRPPDACRRAPLPGLAPVGGPGFPSPGETPCLPLTTSIHSHLLHTSTLLLPHPPA
LHPVLHYLRTLSLPPTDTST
VSFTPDRVRRQLIRLHSGKVAGPDGVFPRVLKACAPQLCGALNHVFSLSLHL
>g1253.t2
VVSPQGSGSSMLLFRAEPGRAPWVKDRPPDACRRAPLPGLAPVGGPGFPSPGEVAQMFFGLFIRVFESLFVWSLPWDHFA
MGDPTRGFCPRQHSSQTPCL
PLTTSIHSHLLHTSTLLLPHPPALHPVLHYLRTLSLPPTDTSTVSFTPDRVRRQLIRLHSGKVAGPDGVFPRVLKACAPQ
LCGALNHVFSLSLHL
>g1254.t1
MTSLRFPVQSGARQGCIISPIFLLVAMDWITTNTTADRPRGIQWTLFSQLEDLNFADDLALLSTNQYKIVFNLATRVCFN
FEE
>g1254.t2
MKTLERLVLGQLRPMVRPFTDQLQFAYQPHLGVDDGISYLLNKVCNTGALQGTVLSPFLYTSDFNYCTESCHLQKFCDDS
AIVGCIEQGDESEYRTVVGN
FVCWCELNHVQINGTKTKELIVDLRRAKTPVCPVFIQWVPVDTVELIVDLRRAKTPVCPVFIQWVPVDTVEEYKYLGSVL

>g1254.t3
MVRPFTDQLQFAYQPHLGVDDGISYLLNKVCNTGALQGTVLSPFLYTSDFNYCTESCHLQKFCDDSAIVGCIEQGDESEY
RTVVGNFVCWCELNHVQING
TKTKELIVDLRRAKTPVCPVFIQWVPVDTVELIVDLRRAKTPVCPVFIQWVPVDTVEEYKYLGSVL
>g1254.t4
MKTLERLVLGQLRPMVRPFTDQLQFAYQPHLGVDDGISYLLNKVCNTGALQGTVLSPFLYTSDFNYCTESCHLQKFCDDS
AIVGCIEQGDESEYRTVVGN
FVCWCELNHVQINGTKTKELIVDLRRAKTPVCPVFIQWVPVDTVELIVDLRRAKTPVCPVFIQWVPVDTVEEYKYLGSAT
VYLGQCLTPSPCHTGPAEEH
LQQKTPFTEPHHRAIQGIIPACGHQTIQHLPLRVSYPYKVTTKSGARQGCIISPIFLLVAMDWITTNTTADRPRGIQWTL
FSQLEDLNFADDLALLSTNQ
YKIVFNLATRVCFNFEE
>g1255.t1
MRVADTNRLNRLIKRAGDVVGEELDTLMTVAKRMMLSRLRSILDNASHPLHATRAQRRSTFSRRLLSPSRTTEPYRGSFL
PVVIKLYNTSL
>g1256.t1
FYSAEIICGLQFLHSKGIIYRDLKLDNVMLDQDGHIKIADFGMCKENVFGDNRATTFCGTPDYIAPEILLGQKYSFSVDW
WSFGVLLYEMLIGQSPFHGD
DEDELFESIRMDTPHYPRWITKEAKDLMEKLFERDPTRRLGIVGNICLHPFFKTINWPALERREIDPPFKPKVKAANDCS
NFDREFLSEKPRLSYSEKNF
IDSMDQAAFQGFSFINPNMEHLLEK
>g1257.t1
MSSHDVLKNASSLASYNVLLQVMFRVLTFLMNAFTLRFVSKEVVGVVNVRLMLLYSTLVFLSREAFRRACLSGSPGPGHN
WRQTINLLWLALPLGVVWAS
ILVCVWLWLLEVPDPQDIPHYAPAVWLFALAALQELLAEPLWVLAQAHMFVRLKVVAESLAMVAKCILTVALVVLAPGWG
LYIFSAAQLVYTGFLVLCYL
IYFVHFLGSEEADKKRFPIHTVGDLLPHTARGEPAIDWTLARLTWSFFKQSFLKQILTEGERYVMTFLNVLSFGEQGVYD
IVNNLGSMVARFIFLPIEES
FYVFFAKVLERGRDVKSQKQEDVSIAAEVLECLLRLVVVVGLVITVFGYAYSHLALDIYGGSLLSSGAGPVLLRCYSCYV
LMLAVNGVTECFVFAAMSQE
EVDQYNVVMLALSVSFLLLSYGLTWWAGAVGFIMANCLNMGLRILHSLLYIHRYFRPGPWEPLRGLLPSPALLLALGLSA
AVTALSEGVLCCDRGWPLRL
LHVAVGALSLLCVLLTALRAETRLVHFQGEDEGQEQRPEVRDEDLVEAKDKLGTRGPMKSKTFEVMEECEKVGKAAPSVF
SSVRSGGETALSKPSARPVR
K
>g1257.t2
MSSHDVLKNASSLASYNVLLQVMFRVLTFLMNAFTLRFVSKEVVGVVNVRLMLLYSTLVFLSREAFRRACLSGSPGPGHN
WRQTINLLWLALPLGVVWAS
ILVCVWLWLLEVPDPQDIPHYAPAVWLFALAALQELLAEPLWVLAQAHMFVRLKVVAESLAMVAKCILTVALVVLAPGWG
LYIFSAAQLVYTGFLVLCYL
IYFVHFLGSEEADKKRFPIHTVGDLLPHTARGEPAIDWTLARLTWSFFKQSFLKQILTEGERYVMTFLNVLSFGEQGVYD
IVNNLGSMVARFIFLPIEES
FYVFFAKVLERGRDVKSQKQEDVSIAAEVLECLLRLVVVVGLVITVFGYAYSHLALDIYGGSLLSSGAGPVLLRCYSCYV
LMLAVNGVTECFVFAAMSQE
EVDQYNVVMLALSVSFLLLSYGLTWWAGAVGFIMANCLNMGLRILHSLLYIHRYFRPGPWEPLRGLLPSPALLLALGLSA
AVTALSEGVLCCDRGWPLRL
LHVAVGALSLLCVLLTALRAETRLVHFVRTQLWPRQRKRSV
>g1257.t3
MSSHDVLKNASSLASYNVLLQVMFRVLTFLMNAFTLRFVSKEVVGVVNVRLMLLYSTLVFLSREAFRRACLSGSPGPGHN
WRQTINLLWLALPLGVVWAS
ILVCVWLWLLEVPDPQDIPHYAPAVWLFALAALQELLAEPLWVLAQAHMFVRLKVVAESLAMVAKCILTVALVVLAPGWG
LYIFSAAQLVYTGFLVLCYL
IYFVHFLGSEEADKKRFPIHTVGDLLPHTARGEPAIDWTLARLTWSFFKQSFLKQILTEGERYVMTFLNVLSFGEQGVYD
IVNNLGSMVARFIFLPIEES
FYVFFAKVLERGRDVKSQKQEDVSIAAEVLECLLRLVVVVGLVITVFGYAYSHLALDIYGGSLLSSGAGPVLLRCYSCYV
LMLAVNGVTECFVFAAMSQE

EVDQYNVVMLALSVSFLLLSYGLTWWAGAVGFIMANCLNMGLRILHSLLYIHRYFRPGPWEPLRGLLPSPALLLALGLSA
AVTALSEGVLCCDRGWPLRL
LHVAVGALSLLCVLLTALRAETRLVHFVRTQLWPRQRKRSQGEDEGQEQRPEVRDEDLVEAKDKLGTRGPMKSKTFEVME
ECEKVGKAAPSVFSSVRSGG
ETALSKPSARPVRK
>g1257.t4
MSQQGEDEGQEQRPEVRDEDLVEAKDKLGTRGPMKSKTFEVMEECEKVGKAAPSVFSSVRSGGETALSKPSARPVRK
>g1258.t1
LAHLGEIGTKTTLRYAVQLLTPASLLGRVQGKEGVEREQVEEINELFYDAKSSAKILQDQHHKFMK
>g1259.t1
LVGYAVSPALQEALEACQTRISKMELQQQQQQAVQLEGLENATARTLLGKLINVLLAVMAVLLVFVSTVANCVVPLMRTR
TRSLTTLLLVVLLAFLWRN
>g1260.t1
VTKRDEDSSLMQLKEEFRSYDALRREHDAQIVHIAMEAGLRISPEQWSSLLYGDLVHKSHMQSIIDKLQSPESFAKSVQE
LTIVLQRTGDPANLNSLRAP
LELLANIDHNPDAPAPSWEDLESVMLAVKLVIQGLVEFIQNFSKKSHDSPQPQANSKYKTSMCRDLRQQGGCPRGTSCTF
AHTQDELEKFRLRNKKIASA
GRPFPLSQGIMGKTVSGGEGEELSAGEGQAFKRTLEKAGPAASEAVATAMCQAQLLLRSSSGTEEAVRATANGAGGDSAG
PSGSSRSSAGSMEQTGVSPP
RTPARNSSSPSSMATVGHQECAASMPKQGHFLLRAPQPSQASELYYQENHPYQAT
>g1260.t2
VTKRDEDSSLMQLKEEFRSYDALRREHDAQIVHIAMEAGLRISPEQWSSLLYGDLVHKSHMQSIIDKLQSPESFAKSVQE
LTIVLQRTGDPANLNSLRAP
LELLANIDHNPDAPAPSWEDLESVMLAVKLVIQGLVEFIQNFSKKSHDSPQANSKYKTSMCRDLRQQGGCPRGTSCTFAH
TQDELEKFRLRNKKIASAGR
PFPLSQGIMGKTVSGGEGEELSAGEGQAFKRTLEKAGPAASEAVATAMCQAQLLLRSSSGTEEAVRATANGAGGDSAGPS
GSSRSSAGSMEQTGVSPPRT
PARNSSSPSSMATVGHQECAASMPKQGHFLLRAPQPSQASELYYQENHPYQAT
>g1261.t1
LLHLASPALSPAAGQPAAMYGSSYTHVPRYTLEAQKKKQLQRSKGKSCGYYMRIIFFFSSLIQSLIIVSLVLFLVYGKKQ
DSADSGRIADLEQSFSRLSL
ENVALNLQRKNLTRSLNVSSTEKLQLEQDVLKMRNLTNISAHYIQFFRLELDQCNADKLKMAPPPGGVSCPGLRRGMEPP
AECRSQLEHQKAKEQLLQSQ
LNQTVLENRIMRDKLTKERDDISLEAIDLRNHKQRLEWEIEKYQLQCKRDFVASLSGIFNVSKAFLGKIDSLFSHHMPFQ
LSCNKQREHLGQLCSNCTTL
SRQVEYRFQQYLDDLSIKVNDCVGKTSRLQADNDMKTKKYDSCNHNRTAMAHAHQQDLQRVQLRCDQNTKKLLLEKDRVN
QDKTLLDKKLHVRETEIVTL
KEENKQLNASCVTRVSGPHVLAALSIPFSD
>g1261.t2
LPLLHLASPALSPAAGQPAAMYGSSYTHVPRYTLEAQKKKQLQRSKGKSCGYYMRIIFFFSSLIQSLIIVSLVLFLVYGK
KQDSADSGRIADLEQSFSRL
SLENVALNLQRKNLTRSLNVSSTEKLQLEQDVLKMRNLTNISAHYIQFFRLELDQCNADKLKMAPPPGGVSCPGLRRGME
PPAECRSQLEHQKAKEQLLQ
SQLNQTVLENRIMRDKLTKERDDISLEAIDLRNHKQRLEWEIEKYQLQCKRDFVASLSGIFNVSKAFLGKIDSLFSHHMP
FQLSCNKQREHLGQLCSNCT
TLSRQVEYRFQQYLDDLSIKVNDCVGKTSRLQADNDMKTKKYDSCNHNRTAMAHAHQQDLQRVQLRCDQNTKKLLLEKDR
VNQDKTLLDKKLHVRETEIV
TLKEENKQLNASCVTRVSGPHVLAALSIPFSD
>g1261.t3
MGGWATGRFVGTSPVQGGGASNTVPGNPGSGNPGSGNPGLRNPGSGNPGSGNPGSGNPGSGNPGSGSPFFGGSNLGRGTG
TAGSYGGLGGRRTAGLEANI
EQHLQELQRFLLKPSSTE
>g1261.t4
LLHLASPALSPAAGQPAAMYGSSYTHVPRYTLEAQKKKQLQRSKGKSCGYYMRIIFFFSSLIQSLIIVSLVLFLVYGKKQ
DSADSGRIADLEQSFSRLSL
ENVALNLQRKNLTRSLNVSSTEKLQLEQDVLKMRNLTNISAHYIQFFRLELDQCNADKLKMAPPPGGVSCPGLRRGMEPP

AECRSQLEHQKAKEQLLQSQ
LNQTVLENRIMRDKLTKERDDISLEAIDLRNHKQRLEWEIEKYQLQCKRDFVASLSGIFNVSKAFLGKIDSLFSHHMPFQ
LSCNKQREHLGQLCSNCTTL
SRQVEYRFQQYLDDLSIKVNDCVGKTSRLQADNDMKTKKYDSCNHNRTAMAHAHQQDLQRVQLRCDQNTKKLLLEKDRVN
QDKTLLDKKLHVRETEIVTL
KEENKQLNASCVTRGGGASNTVPGNPGSGNPGSGNPGLRNPGSGNPGSGNPGSGNPGSGNPGSGSPFFGGSNLGRGTGTA
GSYGGLGGRRTAGLEANIEQ
HLQELQRFLLKPSSTE
>g1262.t1
MGTPGWRVTRVKVSSQSADLWFSLLCIRGCQAKVNFTKRLDCRCRHHGLVVPLKGHARKCPFSGCTCDMCGVYAQRAKIM
AVHRKMVASRKRGIETNKAA
RSTPDLRPGPPAPPPAPEDAKAARGVGRQKDVLPPAPPWALPTLCDAYTPTLLKTTFRGPFSE
>g1263.t1
MATVIAQSSYIFGLRKGVTNNLCFLDDQTVVFPSGNNCVRYHIDQKWQKFIPGTEKSQGMQALALSADRRYLAVSERGER
PSVTVYDLTHEQGRKRKVLT
AGELPVGEFLCVAFSPDSKYLIGQSGAPTWTLVLWLWERQKVMATVSSSDGDVVNQVSFSPLDNTQVCVSGLGVLRLYTY
GEGVLKRAAQAKVEGVAVQC
HAWVSGKRVAAGTDTGRLLMIESGELRWELDAGAR
>g1264.t1
MLRLMSPKEKSSFVAHCRVLSGLCDIVLARVYSHSTLEELEKEASIPIINGLSDLYHPIQILADFLTLQEHYGKLDGLTV
SWIGDGNNVLHSFMMTAAKL
GVHLKIATPKHGTGLVLTSDPMEAAKGSDVLVTDTWVSMGQEDEKKRRLKDFQGYQITNKTGGVANPDWTFLHCLPRKQE
EVDDEVFYSSRSLVFPEAEN
RKWTIMLFKLLSEEMHLRYLVDEYGMTIKDDHIPPLRLANWGVTSICGKLWSP
>g1264.t2
MLRLMSPKEKSSFVAHCRVLSGLCDIVLARVYSHSTLEELEKEASIPIINGLSDLYHPIQILADFLTLQEHYGKLDGLTV
SWIGDGNNVLHSFMMTAAKL
GVHLKIATPKHGTGLVLTSDPMEAAKGSDVLVTDTWVSMGQEDEKKRRLKDFQGYQITNKTGGVANPDWTFLHCLPRKQE
EVDDEVFYSSRSLVFPEAEN
RKWTIMQLFKLLSEEMHLRYLVDEYGMTIKDDHIPPLRLANWGVTSICGKLWSP
>g1264.t3
MSPKEKSSFVAHCRVLSGLCDIVLARVYSHSTLEELEKEASIPIINGLSDLYHPIQILADFLTLQEHYGKLDGLTVSWIG
DGNNVLHSFMMTAAKLGVHL
KIATPKHGTGLVLTSDPMEAAKGSDVLVTDTWVSMGQEDEKKRRLKDFQGYQITNKTGGVANPDWTFLHCLPRKQEEVDD
EVFYSSRSLVFPEAENRKWT
IMLFKLLSEEMHLRYLVDEYGMTIKDDHIPPLRLANWGVTSICGKLWSP
>g1265.t1
LGSHEGPAVLETVTIVTITLLAFLGNLLIVGTLYRRPYLLTPSNKFVFSLTLSNLLLSVFVLPFVAVSSAKREWVFGVVW
CNFTALLHLLISSASMLTLG
AIAIDRYYAVLYPMIYPMKITGNRAVVVIAYVWLHSLVACLPPLFGWSSFEFDCSKRTCVASWHRDRGYTAFWVTWCSVP
SFLAMLGCYGVIFRVARMKA
RKVHCGTVVVAEDESGGGGAGGSQRNGRKNSSTSTCSNGSRRSLVYAGSQCKALVTILVVIGTFLVTWGPYVVAVCSEAA
WGQDAASQTVGTLVAWLSFC
SAVCHPLIYGLWNKTVRKELLGMCFGDRYYREPFATRQRTSRLFSISNRITDLGMSPHLTAMLAGGQLVAPGSSTGDTGF
SFTQDSSTRLFFIPQGTDVM
LLDNFSTDGSSHPHSHGNTSGKRRSSVTFEDQVEQSKADGTLPSVVQVRAEIHKSLDSFAAGLARAIESDARLVLFGDDA
ALGAGMFAARPAPARPRYLD
GQRLRLESIDEGIVKDDRDQEQEDEGLVGLNAASVM
>g1265.t2
MNTSRNCSALGSHEGPAVLETVTIVTITLLAFLGNLLIVGTLYRRPYLLTPSNKFVFSLTLSNLLLSVFVLPFVAVSSAK
REWVFGVVWCNFTALLHLLI
SSASMLTLGAIAIDRYYAVLYPMIYPMKITGNRAVVVIAYVWLHSLVACLPPLFGWSSFEFDCSKRTCVASWHRDRGYTA
FWVTWCSVPSFLAMLGCYGV
IFRVARMKARKVHCGTVVVAEDESGGGGAGGSQRNGRKNSSTSTCSNGSRRSLVYAGSQCKALVTILVVIGTFLVTWGPY
VVAVCSEAAWGQDAASQTVG
TLVAWLSFCSAVCHPLIYGLWNKTVRKELLGMCFGDRYYREPFATRQRTSRLFSISNRITDLGMSPHLTAMLAGGQLVAP

GSSTGDTGFSFTQDSSTRLF
FIPQGTDVMLLDNFSTDGSSHPHSHGNTSGKRRSSVTFEDQVEQSKADGTLPSVVQVRAEIHKSLDSFAAGLARAIESDA
RLVLFGDDAALGAGMFAARP
APARPRYLDGQRLRLESIDEGIVKDDRDQEQEDEGLVGLNAASVM
>g1266.t1
RLEGDRSEGSGQENED
>g1267.t1
MTAGYWKARVLCAIMMITFIILAVLVLRNLGQNKDVFEKVRIPSKRFWRCWKWSESFWNREQQQLDLIYSPILNVAPTND
SLVARMPWWLDNTQALKPCE
PDNRVSRQVGDYNTLPQRFQDFLLYMRCRAYPILINQPHLCDDGPFLLLAIKSLAPHFDRRQAIRQSWGQAGKVANRTVV
AVFLLGTATTSDHFPDLSAL
LSHEAERHRDLLQWDYRDSFFNLTLKETHFLEWFSQHCSNAHFVLKGDDDVFVNTWQIIHYLEHLPEVMTKDLFVGDVIQ
NASPHRDHRLKYFIPESVFV
GGYPPYAGGGGYLYSGPLALRLFKVSQQVALFPIDDVFTGMCLEKLGLVPEKHNGFRTFDVDKKYRENPCMYKSLILVHS
RTPQEMIQIWSWLVDPKLDQ
CQ
>g1268.t1
MWTQESGEEQKRSSGSSENQRGSGEEEQEVVVRTKEVAEKRSRGAGERSLYQPGAGWPFHAVTQPIDMQKAFITPPNHQS
AGWRASSH
>g1269.t1
GVLGLAVAVAEMDRPYLVKNKSDEARETNIDRDDINAPEEEWN
>g1270.t1
MEMFSLSGVGFNWGNLTCPLCKAVFAILDIALLTDANEERVARVVGEACTRLHLADEEVCRQIAELFRDDFIRALEISLL
WPSEACALLVGTSCGKFDLY
APWNVTLPKVPKPPVRPPSPPQPGSPQSRVLFLTDIHWDQAYAAGSNADCKEPLCCRNESKDPEWQHHRRRRQAGYWGTY
SKCDLPLRTVQNLLEDAART
GPWDWVYWTGDIPAHNVWAQTRKQQLSELTIISKLIHKHLGPNVTVYPAIGNHESTPVNSFPPPFVHGNRSAAWLYDVMA
EEWAPWLPAQALKTLRYGGF
YSVLVQPGLRVVSLNMNFCARENFWLMVNSTDPANQLQWLVHILQASEDLGEKVHIIGHIPPGLCLSSWSWNYYHILNRY
ESTITGQFFGHTHVDEFQMF
YDEETMTRPVGVAFIAPSATTYINLNPGYRVYFVDGNYKGSSRLVLDHETYILNLTEANHGQPGSGSGGGVSLPDPKWTR
LYRATEAYGLPSLYPSDWDG
LLRVFQNDDRVFQRFWYLRHKGHVSAPCKDTCKTSQLCFLRSGKYDELQHCALLNGFGGNMARAMVKTLC
>g1270.t2
MEMFSLSGVGFNWGNLTCPLCKAVFAILDIALLTDANEERVARVVGEACTRLHLADEEVCRQIAELFRDDFIRALEISLL
WPSEACALLVGTSCGKFDLY
APWNVTLPKVPKPPVRPPSPPQPGSPQSRVLFLTDIHWDQAYAAGSNADCKEPLCCRNESKDPEWQHHRRRRQAGYWGTY
SKCDLPLRTVQNLLEDAART
GPWDWVYWTGDIPAHNVWAQTRKQQLSELTIISKLIHKHLGPNVTVYPAIGNHESTPVNSFPPPFVHGNRSAAWLYDVMA
EEWAPWLPAQALKTLRYGGF
YSVLVQPGLRVVSLNMNFCARENFWLMVNSTDPANQLQWLVHILQASEDLGEKVHIIGHIPPGLCLSSWSWNYYHILNR
>g1270.t3
MEMFSLSGVGFNWGNLTCPLCKAVFAILDIALLTDANEERVARVVGEACTRLHLADEEVCRQIAELFRDDFIRALEISLL
WPSEACALLVGTSCGKFDLY
APWNVTLPKVPKPPVRPPSPPQPGSPQSRVLFLTDIHWDQAYAAGSNADCKEPLCCRNESKDPEWQHHRRRRQAGYWGTY
SKCDLPLRTVQNLLEDAART
GPWDWVYWTGDIPAHNVWAQTRKQQLSELTIISKLIHKCVTHLGPNVTVYPAIGNHESTPVNSFPPPFVHGNRSAAWLYD
VMAEEWAPWLPAQALKTLRY
GGFYSVLVQPGLRVVSLNMNFCARENFWLMVNSTDPANQLQWLVHILQASEDLGEKVHIIGHIPPGLCLSSWSWNYYHIL
NRYESTITGQFFGHTHVDEF
QMFYDEETMTRPVGVAFIAPSATTYINLNPGYRVYFVDGNYKGSSRLVLDHETYILNLTEANHGQPGSGSGGGVSLPDPK
WTRLYRATEAYGLPSLYPSD
WDGLLRVFQNDDRVFQRFWYLRHKGHVSAPCKDTCKTSQLCFLRSGKYDELQHCALLNGFGGNMARAMVKTLC
>g1270.t4
MTRPVGVAFIAPSATTYINLNPGYRVYFVDGNYKGSSRLVLDHETYILNLTEANHGQPGSGSGGGVSLPDPKWTRLYRAT
EAYGLPSLYPSDWDGLLRVF

QNDDRVFQRFWYLRHKGHVSAPCKDTCKTSQLCFLRSGKYDELQHCALLNGFGGNMARAMVKTLC
>g1271.t1
RTGTLVCTWLIDSDQFENAQESLDYFGERRTDRSQSSKFQGVETPSQSRYVGYYEVMKNKFDRQLPPPRRLKMKSIRIHS
IAGVGRGDGSDLRVKVILKK
EQVFKCVCAKQEGCAVFPDVGSNAVVISLHEGPLVEGDVKVMFESSAGLPKGYEDCPFYFWFNTSFIEDHKFVTSTSVFA
SHVDCFTVTCQYTIRLYLSR
EELDNPHKPKTWDLYREDFGVTLLFSDPE
>g1271.t2
RTGTLVCTWLIDSDQFENAQESLDYFGERRTDRSQSSKFQGVETPSQSRYVGYYEVMKNKFDRQLPPPRRLKMKSIRIHS
IAGVGRGDGSDLRVKVILKK
EQVFKCVCAKQEGCAVFPDVGSNAVVISLHEGPLVEGDVKVMFESSAGLPKGYEDCPFYFWFNTSFIEDHKLYLSREELD
NPHKPKTWDLYREDFGVTLL
FSDPE
>g1272.t1
MASEELARKLQRRLATEQVSAPAPDPTEAEVPAKRGEPETPETERAATAASDDAASSELTAKLTRRLDINDGIAAPKRSK
VFNPYTEFKEFSRKQIKEME
AMFKR
>g1272.t2
MASEELARKLQRRLATEQVSAPAPDPTEAEVPAKRGEPETPETERAATAASDDAASSELTAKLTRRLDINDGIAAPKRSK
VFNPYTEFKEFSRKQIKEME
AMFK
>g1273.t1
MANDPLAEAGFHFDELNKLRVLDPEVGQKTSELKEECKEFVDKIGQFQKIVGGLIELVDELAKEAETEKMKAIGARNLLK
SVAKQRDAQQQQLQALIAEK
KMQLERYRIEYEALSKVEAEQNEFIDQFILQK
>g1273.t2
MANDPLAEAGFHFDELNKLRVLDPEVGQKTSELKEECKEFVDKIGQFQKIVGGLIELVDELAKEAETEKMKAIGARNLLK
SVAKQRDAQQQQLQALIAEK
KMQLERKKPLVLPGIVTLDRYRIEYEALSKVEAEQNEFIDQFILQK
>g1273.t3
MANDPLAEAGFHFDELNKLRVLDPEVGQKTSELKEECKEFVDKIGQFQKIVGGLIELVDELAKEAETEKMKAIGARNLLK
SVAKQRDAQQQQLQALIAEK
KMQLERLFNACPNAIKIKPMVFSQARKKPLVLPGIVTLDRYRIEYEALSKVEAEQNEFIDQFILQK
>g1274.t1
MSGASALHTPQSLDDPPLACPKTKTRTNSSAVGLGNKYVRLNVGGTLFYTTLQVLTRQDSMLKAMFSGKQEVFTDREGWI
LIDRSGKHFGSILGYLREGT
ATLPKGHQAVLELLAEAKFYLLEGLVELCQNSLQENEEQAQCVIPVITSVKEEERLIEACTKPVVKLVYNRSNNKYSYTS
NSDDNLLKNIELFDRLSMRY
NGRILFIKDVIGDEICCWSFYGQSRKLAEVCCTSILYATEKKQTKVSCRLPCWNRVK
>g1274.t2
LRTSVCRVVIYLRKDMSGASALHTPQSLDDPPLACPKTKTRTNSSAVGLGNKYVRLNVGGTLFYTTLQVLTRQDSMLKAM
FSGKQEVFTDREGWILIDRS
GKHFGSILGYLREGTATLPKGHQAVLELLAEAKFYLLEGLVELCQNSLQENEEQAQCVIPVITSVKEEERLIEACTKPVV
KLVYNRSNNKYSYTSNSDDN
LLKNIELFDRLSMRYNGRILFIKDVIGDEICCWSFYGQSRKLAEVCCTSILYATEKKQTKVSCRLPCWNRVK
>g1275.t1
MADSEVTPQSGGYDVELFVDPPDYDLICTICQGVLRCPVRAACHHIFCKRCILQWLKRQETCPCCRKAVNQSVIFVMFKL
SRSIGRMKIK
>g1275.t2
LVPFRRYNDTLSQADSEVTPQSGGYDVELFVDPPDYDLICTICQGVLRCPVRAACHHIFCKRCILQWLKRQETCPCCRKA
VNQSVIFVMFKLSRSIGRMK
IK
>g1276.t1
MRGVTFAINLLQVLVTASRALLLKQWDWKQAQCTRSWRAIHTIPIASMAFDPTSTLVATGGCDGTIKLWDIVKQYCTHNL
KGSSRVVHLVQFHPDASLLQ
LFSSSGDCGIRLWDLRSSQCLCVLQSHFSAVTSLAFTPSGDTMVSSGRDKICTVWDLKTRTAKRTVPVYETVEGVVLLPE

DQDLSQIGVQNSDLHFITAG
SK
>g1276.t2
MRGVTFAINLLQVLVTASRALLLKQWDWKQAQCTRSWRAIHTIPIASMAFDPTSTLVATGGCDGTIKLWDIVKQYCTHNL
KGSSRVVHLVQFHPDASLLQ
LFSSSGDCGIRLWDLRSSQCLCVLQSHFSAVTSLAFTPSGDTMVSSGRDKICTVWDLKTRTAKRTVPVYETVEGVVLLPE
DQDLSQIGVQNSDLHFITAG
SKGQGSVLNTMTTQLNGLTLKHQVIR
>g1276.t3
MISKDEKYIFCTCGSRVNILEISTGKIVHSVEQVLVTASRALLLKQWDWKQAQCTRSWRAIHTIPIASMAFDPTSTLVAT
GGCDGTIKLWDIVKQYCTHN
LKGSSRVVHLVQFHPDASLLQLFSSSGDCGIRLWDLRSSQCLCVLQSHFSAVTSLAFTPSGDTMVSSGRDKICTVWDLKT
RTAKRTVPVYETVEGVVLLP
EDQDLSQIGVQNSDLHFITAGSK
>g1277.t1
MKCFEKVVLAHIQNSIPDTLDPLQFAYRSNRSTSDAIAAALHYSLSHLETKDSYIRMLFVGYSSAFNTVIPHKLTQKLST
LGLDPTLCDW
>g1278.t1
MLFSTINRLLRPLDSPHPSGAPDLCSKFLDFFQAKVDSIHQQLLAPASPPPPHTPQPQGVPPSAVRLPQCSLSSFSLVDA
LQVTKLVTTAKASTCSLHPM
PTALVKVCIPTLCPIMVDIINSSLESGMVPSTFKMTTVIPILKKPGLNPDDLTNYRPISNLPFLRKILERAVAVQLHQHM
SNHELYEPLQSGFRAHHSTE
TALIKITLITHLIKITALINDFLIAADSGHISIVILLDLSAAFDTVSHTILLNRLSEYLGLTGAALSWFHSYLSDRKQFV
TIRDFSSTPASVNQGMPQGS
VLGPLLFTIYMLPLGQIIRHHGLS
>g1279.t1
CSKDMYQRCFPDSTRCLREGRATASSAGVINSLPNMRRAGVHEVESWIAVRHAIRTRVAGSSHARWRTPSNSSKEENLAH
SPFVHGTNSRSLASLQSQYR
QPSAWHGRQDSAILLHVHTPVHQSGFEGEPVP
>g1280.t1
MLDTTGVGLLKSVEQLLSEIFIPTLRKMNHGWGELASPQAQAIKRDFMASLDNFVSVLAGAQDNLQEKVNLTECEGFDLR
SLRGPSDYMAAANSSETLEK
MEACLKVWIKQIEQVLAESDQLRKEADDLGPRAELDHWKKRMSRFNYLLDQLKSPDVKAVLGVLIVAKSKLIKSWKELDT
RITDSANEAKDNVKYLYTLE
KFCDPLYNSDPVSMVDAIPGLVNAIRMIHSISRYYNTSEKITSLFVKVTNQMITACKAYITTSGSVTIWDQPPQQVTERI
TAAIHLNQEYQRYFHKTKEN
LEQTPSERQFDFSEMYIFGKFDTFQRRLTKILELFDTISTYSALQDSKIEGLETMATRFQVVMVVVMVMVVVMVMVMVVV
VVVVVVMVVVVVVMIVVVVV
VKEVEEVVVVVGVMGGVVVGGMVVVVV
>g1280.t2
MGVGGFSPKVNLTECEGFDLRSLRGPSDYMAAANSSETLEKMEACLKVWIKQIEQVLAESDQLRKEADDLGPRAELDHWK
KRMSRFNYLLDQLKSPDVKA
VLGVLIVAKSKLIKSWKELDTRITDSANEAKDNVKYLYTLEKFCDPLYNSDPVSMVDAIPGLVNAIRMIHSISRYYNTSE
KITSLFVKVTNQMITACKAY
ITTSGSVTIWDQPPQQVTERITAAIHLNQEYQRYFHKTKENLEQTPSERQFDFSEMYIFGKFDTFQRRLTKILELFDTIS
TYSALQDSKIEGLETMATRF
QVVMVVVMVMVVVMVMVMVVVVVVVVVVMVVVVVVMIVVVVVVKEVEEVVVVVGVMGGVVVGGMVVVVV
>g1280.t3
MLDTTGVGLLKSVEQLLSEIFIPTLRKMNHGWGELASPQAQAIKRDFMASLDNFVSVLAGAQDNLQEKVNLTECEGFDLR
SLRGPSDYMAAANSSETLEK
MEACLKVWIKQIEQVLAESDQLRKEADDLGPRAELDHWKKRMSRFNYLLDQLKSPDVKAVLGVLIVAKSKLIKELDTRIT
DSANEAKDNVKYLYTLEKFC
DPLYNSDPVSMVDAIPGLVNAIRMIHSISRYYNTSEKITSLFVKVTNQMITACKAYITTSGSVTIWDQPPQQVTERITAA
IHLNQEYQRYFHKTKENLEQ
TPSERQFDFSEMYIFGKFDTFQRRLTKILELFDTISTYSALQDSKIEGLETMATRFQVVMVVVMVMVVVMVMVMVVVVVV
VVVMVVVVVVMIVVVVVVKE

VEEVVVVVGVMGGVVVGGMVVVVV
>g1281.t1
METWQNADILSCLETLQSSKKKALQLVSRDAEKVTEYFDNLVGAFELKKQEILSDFETLRLVVMQAHDPEINRLSAALDE
QSQALSIAESFRNVSDPLCF
LQQMQEFREKLRVVRETPLPSRTEIDVGHHVRGFDVRDWDSLRLKDVDKISVPHELGSHRATKSRLRMRSCIGERGSLRA
PVYWAHVNITP
>g1282.t1
MSRTVHSSPFNVDARTRPHVSPPPHRACALTRDPAGSGR
>g1283.t1
MMSSLYNFIDAKDKFAVEQKATCDEYLALIYISIEAATEGICSATTPFLLLGDVLDCLPLDQCDNIFSFVEENVSTWKSS
TFYAAGKNYLLRMCNDLLRR
LSKSQNTVFCGRIQLFLARLFPLSEKSGLNLQSQFNLENVTVFNKNEQESTLGQKHTEEKEDGMEVEEGEMGEEEAPAPC
SIPIDYNLYRKFWTLQDYFR
NPLQCYDKFSWMTFLKYSDETLAVFKSFKLDDMQASKRKLEELRTSAGEHVYFAKFLTSEKLMDLQLSDSNFRRHILLQY
LILFQYLKGQVKFKSSSCVL
NDDQTAWIEETTKLVHQLLKEIPPEGEKFSAMVEHILNTEENWNGWKNEGCPSFVKERVADDKPKRPTRKRQAPEDFLGK
GPDRKLFMGNDELTRLWNLN
ADNMEACKSDDREFMPSLDEFFAEAIEQADPANMVEDEYKVVRNSNYGWRALRLLSRRSPHFFQPTNQKFKSLADYLDSM
VSKLAKELPKDIPSEEIKTG
EEDDDDNGDNLLKESNDSPSMQNKIVSNQQMDDVAAKLGAKWKTLAVQLEMKASELREIETDSEDVDMQAKLLLVAWQDR
EGAQATVENLVSALNAASFS
QIADNLSEA
>g1283.t2
MMSSLYNFIDAKDKFAASALSALTGRTSKPLINAFNQITGNETEKKNTLDQSLRGVLGDQIVEQKATCDEYLALIYISIE
AATEGICSATTPFLLLGDVL
DCLPLDQCDNIFSFVEENVSTWKSSTFYAAGKNYLLRMCNDLLRRLSKSQNTVFCGRIQLFLARLFPLSEKSGLNLQSQF
NLENVTVFNKNEQESTLGQK
HTEEKEDGMEVEEGEMGEEEAPAPCSIPIDYNLYRKFWTLQDYFRNPLQCYDKFSWMTFLKYSDETLAVFKSFKLDDMQA
SKRKLEELRTSAGEHVYFAK
FLTSEKLMDLQLSDSNFRRHILLQYLILFQYLKGQVKFKSSSCVLNDDQTAWIEETTKLVHQLLKEIPPEGEKFSAMVEH
ILNTEENWNGWKNEGCPSFV
KERVADDKPKRPTRKRQAPEDFLGKGPDRKLFMGNDELTRLWNLNADNMEACKSDDREFMPSLDEFFAEAIEQADPANMV
EDEYKVVRNSNYGWRALRLL
SRRSPHFFQPTNQKFKSLADYLDSMVSKLAKELPKDIPSEEIKTGEEDDDDNGDNLLKESNDSPSMQNKIVSNQQMDDVA
AKLGAKWKTLAVQLEMKASE
LREIETDSEDVDMQAKLLLVAWQDREGAQATVENLVSALNAASFSQIADNLSEA
>g1283.t3
MMSSLYNFIDAKDKFAVEQKATCDEYLALIYISIEAATEGICSATTPFLLLGDVLDCLPLDQCDNIFSFVEENVSTWKSS
TFYAAGKNYLLRMCNDLLRR
LSKSQNTVFCGRIQLFLARLFPLSEKSGLNLQSQFNLENVTVFNKNEQESTLGQKHTEEKEDGMEVEEGEMGEEEAPAPC
SIPIDYNLYRKFWTLQDYFR
NPLQCYDKFSWMTFLKYSDETLAVFKSFKLDDMQASKRKLEELRTSAGEHVYFAKFLTSEKLMDLQLSDSNFRRHILLQY
LILFQYLKGQVKFKSSSCVL
NDDQTAWIEETTKLVHQLLKEIPPEGEKFSAMVEHILNTEENWNGWKNEGCPSFVKERVADDKPKRPTRKRQAPEDFLGK
GPDRKLFMGNDELTRLWNLN
ADNMEACKSDDREFMPSLDEFFAEAIEQADPANMVEDEYKVVRNSNYGWRALRLLSRRSPHFFQPTNQKFKSLADYLDSM
VSKLAKELPKDIPSEEIKTG
EEDDDDNGDNLLKESNDSMFQEPSPRPSMQNKIVSNQQMDDVAAKLGAKWKTLAVQLEMKASELREIETDSEDVDMQAKL
LLVAWQDREGAQATVENLVS
ALNAASFSQIADNLSEA
>g1284.t1
NAPHTLNSRRAARRRSPTHNQCNRSADEWVKFDDDKVSVVSPEDILRLSGGGDWHIAYVLLYGPRRLEVLDEDD
>g1285.t1
MTLSDCGDTYRRPEYNDISVDCGTYSIRLSILLCPVVFTGYNESLLILNKIYDDPQCKGSLDESVTPPVIRFEFPLNNSN
ACGSVFK
>g1285.t2

MVLESSIAQMTLSDCGDTYRRPEYNDISVDCGTYSIRLSILLCPVVFTGYNESLLILNKIYDDPQCKGSLDESVTPPVIR
FEFPLNNSNACGSVFK
>g1286.t1
SAAAISVKDSNGTFVSTLNLMLYNDSTYVQQLSIPMAGLTLKTRVFAAVRASNLDRRWNVLMDYCYTTPSGNPNDDLRYD
LFFSCDKDPQTTVFENGKSQ
MGRFAFEVFRFVKHKNQKMSTVFLHCVTKLCRSDDCPMLMPICGSRKKRDIPDGKETNSASGNAVITAGPIITRSDETPT
NNSQLGQPNPPAFQMNAVTS
ALLSGVVILGVVSVGFFVLSLTLLRGKNVPAGIASSGVRNPAFT
>g1286.t2
TSALLSQRSAAAISVKDSNGTFVSTLNLMLYNDSTYVQQLSIPMAGLTLKTRVFAAVRASNLDRRWNVLMDYCYTTPSGN
PNDDLRYDLFFSCDKDPQTT
VFENGKSQMGRFAFEVFRFVKHKNQKMSTVFLHCVTKLCRSDDCPMLMPICGSRKKRDIPDGKETNSASGNAVITAGPII
TRSDETPTNNSQLGQPNPPA
FQMNAVTSALLSGVVILGVVSVGFFVLSLTLLRGKNVPAGIASSGVRNPAFT
>g1286.t3
SAAAISVKDSNGTFVSTLNLMLYNDSTYVQQLSIPMAGLTLKTRVFAAVRASNLDRRWNVLMDYCYTTPSGNPNDDLRYD
LFFSCDKDPQTTVFENGKSQ
MGRFAFEVFRFVKHKNQKMSTVFLHCVTKLCRSDDCPMLMPICGSRKKRDIPDGKETNSASGNAVITAGPIITRSGTIRT
CSSANIAIAPL
>g1287.t1
MREFGYSAATICSRTRRKIKVNIVRNDLQQFTGGFYFPMAKCFQIAPHAIALVLSRVRGDPEDAASNNPSEKLEHLSGYE
IFANFKAANMQHFWNKSMIH
AATEIFFLGWIDQHVLLVQGKEEHLEVLRNGWARRALTPPQGFHIKCIGPA
>g1288.t1
MESMDVDSKPNAHSFRYSLKYPCIGQCIIINNKNFDRRTDVDAGNAMQVFKKLGYIVKVFNDQSVAEIQHLLSAGLIPPR
SAAYLYTSVSKMPLTPYLLL
RDLHLIAPSVPKEPLCLAAAGSDHGGFASFVCVLLSHGDEGVFFGTDGSVELKSLTALFRGDRCRSLAGKPKLFFIQACR
GTQLDGGIETDSGPDYGTTK
IPVEADFLYAYSTAPGYYSWRNTSSGSWFMQSLCEMIGKYASELELHHILTRVNHKVAVAFESVSNDPGFDAKKQIPCVV
SMLTKEMYFTP
>g1288.t2
MESMDVDSKPNAHSFRYSLKYPCIGQCIIINNKNFDRRTDVDAGNAMQVFKKLGYIVKVFNDQSVAEIQHLLSAGLIPPR
SAAYLYTSVSKMPLTPYLLL
RDLHLIAPSVPKEPLCLAAAGSDHGGFASFVCVLLSHGDEGVFFGTDGSVELKSLTALFRGDRCRSLAGKPKLFFIQACR
GTQLDGGIETDSGPDYGTTK
IPVEADFLYAYSTAPGRVLVLVRCSLDCWL
>g1288.t3
MESMDVDSKPNAHSFRYSLKYPCIGQCIIINNKNFDRRTDVDAGNAMQVFKKLGYIVKVFNDQSVAEIQHLLSAAYLYTS
VSKMPLTPYLLLRDLHLIAP
SVPKEPLCLAAAGSDHGGFASFVCVLLSHGDEGVFFGTDGSVELKSLTALFRGDRCRSLAGKPKLFFIQACRGTQLDGGI
ETDSGPDYGTTKIPVEADFL
YAYSTAPGYYSWRNTSSGSWFMQSLCEMIGKYASELELHHILTRVNHKVAVAFESVSNDPGFDAKKQIPCVVSMLTKEMY
FTP
>g1289.t1
NRQDHAPVLLPRDPGVPACVAPRVMPGGTAGQFESRPP
>g1290.t1
LAFLVKEGYEDRIVVAHDIHTKNRLTKYGGHGFSHILKNIVPKMLTRGFTQSQVDKVLIENPKRWLTFK
>g1291.t1
LAFPPGGSQFVAQPNCQQLLASRWYDEFPGWRRRHWAGKFLTCVFIGLLFPIFALCYLCAPKSRYGLFIRKPFIKFICHT
ASYLAFLFLLLLASQHVVST
EPDRQGPAPTPVEWMILPWVLGFIWTEIKQMWDGGFQDYIHDWWNLMDFVMNSLYLATISLKIVAYTKYSGCKPRHLWEM
WHPTLVAEAVFAIANIFSSL
RLISLFTANSHLGPLQISLGRMLLDILKFLFIYCLVRPEHCLSGRSLQALSLEDY
>g1291.t2
LAFPPGGSQFVAQPNCQQLLASRWYDEFPGWRRRHWAGKFLTCVFIGLLFPIFALCYLCAPKSRYGLFIRKPFIKFICHT
ASYLAFLFLLLLASQHVVST

EPDRQGPAPTPVEWMILPWVLGFIWTEIKQMWDGGFQDYIHDWWNLMDFVMNSLYLATISLKIVAYTKYSGCKPRHLWEM
WHPTLVAEAVFAIANIFSSL
RLISLFTANSHLGPLQISLGRMLLDILKFLFIYCL
>g1292.t1
LVDDRCVVQPDTGDLNNPPKKFRVFETGWVCGAGSPWPCLLPHGK
>g1292.t2
LVDDRCVVQPDTGDLNNPPKKFRGEAKGGRPFFLCAASITLRDWMGLWRWESLAVPSASWQ
>g1293.t1
MESTSASGAGFLTRMALNDNKAGMEGLDRDKINKIIIESSKGSRFYENEVKKELQVNQRIEKMMIQKAQITEQQLQKAHA
QVERITHELDTSRDLSRVIV
HVDMDAFYAAVEMRDCPELKDKPMAVGSMSMLSTSNYHARKFGVRAAMPGFIAKKLCPNLVIVPTNFDKYRAVSSEIREI
FAEYDPHFQPMSLDEAYLDF
TDHLEQRISWPESLRTHCLRTDITETGELHSNVPQESDPCAENLSPVLFEDSPSVSPSLPASGDKVEPGGGSEVFGTCAE
EAVREMRFRIEQKTTLTASA
GIAPNTLLAKVCSDKNKPNGQYRLPSNREAVMDFIQSLPVRKVFGIGKVSEKMLSSLGITSCAHLGQQMALLSLLFSETA
WHHFARISSGLGSTHLERTF
KEMSAADEQLSLCRELCEDLAADMKKEGLRGKTVTLKLKNVKFEVKTRAWTLPCAVATSDDIFAAAKDLLKTEMDNVSVR
ISAFESTDDKKPQQKSIVGF
LKQGASGPSPGV
>g1293.t2
MESTSASGAGFLTRMALNDNKAGMEGLDRDKINKIIIESSKGSRFYENEVKKELQVNQRIEKMMIQKAQITEQQLQKAHA
QVERITHELDTSRDLSRVIV
HVDMDAFYAAVEMRDCPELKDKPMAVGSMSMLSTSNYHARKFGVRAAMPGFIAKKLCPNLVIVPTNFDKYRAVSSEIREI
FAEYDPHFQPMSLDEAYLDF
TDHLEQRISWPESLRTHCLRTDITETGELHSNVPQESDPCAENLSPVLFEDSPSVSPSLPASGDKVEPGGGSEVFGTCAE
EAVREMRFRIEQKTTLTASA
GIAPNTLLAKVCSDKNKPNGQYRLPSNREAVMDFIQSLPVRKVFGIGKVSEKMLSSLGITSCAHLGQQMALLSLLFSETA
WHHFARISSGLGSTHLERTF
KEMSAADEQLSLCRELCEDLAADMKKEGLRGKTVTLKLKNVKFEVKTRAWTLPCAVATSDDIFAAAKDLLKTEMDNVSPQ
RLRLRLMGVRISAFESTDDK
KPQQKSIVGFLKQGASGPSPGV
>g1293.t3
MESTSASGAGFLTRMALNDNKAGMEGLDRDKINKIIIESSKGSRFYENEVKKELQVNQRIEKMMIQKAQITEQQLQKAHA
QVERITHELDTSRDLSRVIV
HVDMDAFYAAVEMRDCPELKDKPMAVGSMSMLSTSNYHARKFGVRAAMPGFIAKKLCPNLVIVPTNFDKYRAVSSEIREI
FAEYDPHFQPMSLDEAYLDF
TDHLEQRISWPESLRTHCLRTDITETGELHSNVPQESDPCAENLSPVLFEDSPSVSPSLPASGDKVEPGGGSEVFGTCAE
EAVREMRFRIEQKTTLTASA
GIAPNTLLAKVCSDKNKPNGQYRLPSNREAVMDFIQSLPVFGIGKVSEKMLSSLGITSCAHLGQQMALLSLLFSETAWHH
FARISSGLGSTHLERTFKEM
SAADEQLSLCRELCEDLAADMKKEGLRGKTVTLKLKNVKFEVKTRAWTLPCAVATSDDIFAAAKDLLKTEMDNVSVRISA
FESTDDKKPQQKSIVGFLKQ
GASGPSPGV
>g1294.t1
VNHSDVTVVAFFIMPARTNNFNVETLKGQAVRKQLWDTAQTVKERFGKKLYESLLVGQLPDVSKMLDKEDFTMMKRAIFS
TQRQCQPPICTHNMLEDSSD
PILNCIRRIGLFNCSADRVKAKFLSSTSPLLPMDYEEFVRGCHLGVFPSYYEPWGYTPAECTVMGIPSISTNLSGFGCFM
EEHIADPSAYGIYILDRRFR
GVDESCNQLTSFLFQFCKQSRRQRIIQRNRTERLSDLLDWRYLGRYYISARHMALAKAFPETYMYEAQDPTSSSGFRYPR
PASVPPSPSLSRHSSPHHSD
DEDERYDEDLEAEKDRVNIRQPFAARAKAGDGVPSESD
>g1294.t2
VNHSDVTVVAFFIMPARTNNFNVETLKGQAVRKQLWDTAQTVKERFGKKLYESLLVGQLPDVSKMLDKEDFTMMKRAIFS
TQRQCQPPICTHNMLEDSSD
PILNCIRRIGLFNCSADRVKASPRREFLSSTSPLLPMDYEEFVRGCHLGVFPSYYEPWGYTPAECTVMGIPSISTNLSGF
GCFMEEHIADPSAYGIYILD

RRFRGVDESCNQLTSFLFQFCKQSRRQRIIQRNRTERLSDLLDWRYLGRYYISARHMALAKAFPETYMYEAQDPTSSSGF
RYPRPASVPPSPSLSRHSSP
HHSDDEDERYDEDLEAEKDRVNIRQPFAARAKAGDGVPSESD
>g1294.t3
MDYEEFVRGCHLGVFPSYYEPWGYTPAECTVMGIPSISTNLSGFGCFMEEHIADPSAYGIYILDRRFRGVDESCNQLTSF
LFQFCKQSRRQRIIQRNRTE
RLSDLLDWRYLGRYYISARHMALAKAFPETYMYEAQDPTSSSGFRYPRPASVPPSPSLSRHSSPHHSDDEDERYDEDLEA
EKDRVNIRQPFAARAKAGDG
VPSESD
>g1294.t4
VNHSDVTVVAFFIMPARTNNFNVETLKGQAVRKQLWDTAQTVKERFGKKLYESLLVGQLPDVSKMLDKEDFTMMKRAIFS
TQRQCQPPICTHNMLEDSSD
PILNCIRRIGLFNCSADRVKASPRRG
>g1295.t1
MVPGPVQIVTVCKEDHSFALDTEALGKVLLAPEVRDKHVVVLSVAGAFRKGKSFLLDFMLRYMYRKSDENWLGGDDEPLT
GFSWRGGSEPETTGIQLWSE
VFLVQKSDGTEVAVLLMDTQGAFDNQSTVKDCATIFALSTMTSSMQLFTEYGRLALDEIFQKPFQSLMFLIRDWSFPYEY
TYGVKGGQEFLEKRLQVKDA
QHEELQTVREHIHSCFTDISCFLLPHPGLKVATSPSFEGQLNDVAPEFKEQLRSLIPSLLHPDKLAEKEINSNKVTCRGL
LEFFKAYIKIYQGEDLPHPK
SMLQATAEANNLAAVATAKDQYYKNMEKVCGGEVPYVSPESLDEKHHFFFNDALHSFSSTKKMGGAEFCDRYRHQLEIEL
QDMWQSFSKHNE
>g1295.t2
MVPGPVQIVTVCKEDHSFALDTEALGKVLLAPEVRDKHVVVLSVAGAFRKGKSFLLDFMLRYMYRKSDENWLGGDDEPLT
GFSWRGGSEPETTGIQLWSE
VFLVQKSDGTEVAVLLMDTQGAFDNQSTVKDCATIFALSTMTSSMQIYNLSQNIQEDDLQQLQLFTEYGRLALDEIFQKP
FQSLMFLIRDWSFPYEYTYG
VKGGQEFLEKRLQVKDAQHEELQTVREHIHSCFTDISCFLLPHPGLKVATSPSFEGQLNDVAPEFKEQLRSLIPSLLHPD
KLAEKEINSNKVTCRGLLEF
FKAYIKIYQGEDLPHPKSMLQATAEANNLAAVATAKDQYYKNMEKVCGGEVPYVSPESLDEKHHFFFNDALHSFSSTKKM
GGAEFCDRYRHQLEIELQDM
WQSFSKHNE
>g1295.t3
MVPGPVQIVTVCKEDHSFALDTEALGKVLLAPEVRDKHVVVLSVAGAFRKGKSFLLDFMLRYMYRKSDENWLGGDDEPLT
GFSWRGGSEPETTGIQLWSE
VFLVQKSDGTEVAVLLMDTQGAFDNQSTVKDCATIFALSTMTSSMQNIQEDDLQQLQLFTEYGRLALDEIFQKPFQSLMF
LIRDWSFPYEYTYGVKGGQE
FLEKRLQVKDAQHEELQTVREHIHSCFTDISCFLLPHPGLKVATSPSFEGQLNDVAPEFKEQLRSLIPSLLHPDKLAEKE
INSNKVTCRGLLEFFKAYIK
IYQGEDLPHPKSMLQATAEANNLAAVATAKDQYYKNMEKVCGGEVPYVSPESLDEKHHFFFNDALHSFSSTKKMGGAEFC
DRYRHQLEIELQDMWQSFSK
HNE
>g1296.t1
SIQSSPLSNASSHCLEEHNVHCRSTANTSDQDDLTCLSWLHQRGSNLLPLQPLPKINLLSQQPETPPAFAPAESLSPSSN
KPPYSFSSLIFMAIEDSPNK
LLPVKGIYEWIINSFPYYRTASGGWRNSVRHNLSLSKSFRRIQRDKSQSVGKGSLWCVCPEYRAALLEVLRKTHHYHSTN
SNLVNMPALLEGVDYGTSMV
CDSVDISDSLSQSLLMSTPSSQILTSDNPTLFENSSGALTPDHEELVTMETVEEEEAGEEMEKDPLSDSGYIELHYYQCH
QYQYLVLPGEAELDLESVEI
LQLDAEAQEAAGSLLDLAGGWHQCL
>g1296.t2
LTCLSWLHQRGSNLLPLQPLPKINLLSQQPETPPAFAPAESLSPSSNKPPYSFSSLIFMAIEDSPNKLLPVKGIYEWIIN
SFPYYRTASGGWRNSVRHNL
SLSKSFRRIQRDKSQSVGKGSLWCVCPEYRAALLEVLRKTHHYHSTNSNLVNMPALLEGVDYGTSMVCDSVDISDSLSQS
LLMSTPSSQILTSDNPTLFE
NSSGALTPDHEELVTMETVEEEEAGEEMEKDPLSDSGYIELHYYQCHQYQYLVLPGEAELDLESVEILQLDAEAQEAAGS

LLDLAGGWHQCL
>g1296.t3
LEEHNVHCRSTANTSDQDDLTCLSWLHQRGSNLLPLQPLPKINLLSQQPETPPAFAPAESLSPSSNKPPYSFSSLIFMAI
EDSPNKLLPVKGIYEWIINS
FPYYRTASGGWRNSVRHNLSLSKSFRRIQRDKSQSVGKGSLWCVCPEYRAALLEVLRKTHHYHSTNSNLVNMPALLEGVD
YGTSMVCDSVDISDSLSQSL
LMSTPSSQILTSDNPTLFENSSGALTPDHEELVTMETVEEEEAGEEMEKDPLSDSGYIELHYYQCHQYQYLVLPGEAELD
LESVEILQLDAEAQEAAGSL
LDLAGGWHQCL
>g1297.t1
LGLMWWRLRKTCYQIVEHSWFESFIIFMILLSSGALHLLTAAVLSHQSYGLHKHPGASGGHGGGSFPSTGALLVTLRVSL
VAGEQSQTHAGVIPKTEDVF
TDLWSRKLAFQQAVDENRRMMSLQTETGCVILSLTERGVR
>g1297.t2
MWWRLRKTCYQIVEHSWFESFIIFMILLSSGALHLLTAAVLSHQSYGLHKHPGASGGHGGGSFPSTGALLVTLRVSLVAG
EQSQTHAGVIPKTEDVFTDL
WSRKLAFQQAVDENRRMMSLQTETGCVILSLTERGVR
>g1298.t1
DGGLFTFGEGSRGQLGHDSTHNEPLPRRVLELMGSEVSQIACGRRHTLAYVPSSGMVYAFGCNVHGQLGTGTQQDARSPA
PLQRSLLIGRSHPTESEHCS
ITKINCGGDHNFILYSDEQTAGRSQDFRAPGVPRGVARISGDSLNTWRVQLVDNTDSTLTK
>g1298.t2
LGHDSTHNEPLPRRVLELMGSEVSQIACGRRHTLAYVPSSGMVYAFGCNVHGQLGTGTQQDARSPAPLQRSLLIGRSHPT
ESEHCSITKINCGGDHNFIL
YSDEQTAGRSQDFRAPGVPRGVARISGDSLNTWRVQLVDNTDSTLTK
>g1299.t1
LEFAVYPAPQVSTAVVEPYNSILTTHTTLEHSDCAFMVDNEAIYDICRRNLDIERPSYTNLNRLIGQIVSSITASLRFDG
ALNVDLTEFQTNLVPYPRIH
FPLATYAPVISAEKAYHEQLSVADITNACFEPANQMVKCDPRHGKYMACCLLYRGDVVPKDVNSAIAAIKTKRTIQFVDW
CPTGFKVGINYQPPTVVPGG
DLAKVQRAVCMLSNTTAIAEAWARLDHKFDLMYAKRAFVHWYVGEGMEEGEFSEAREDMAALEKDYEEVGIDSFEEDEEG
EEY
>g1300.t1
GQEDKKERVWDRKHLSALRPPLLREDMRRRAVWMFLSCRKGL
>g1301.t1
LFAAVAWGIKKVTCGAQQQQPGPGGPTNRLLLPDTLCSQVLRDPTTWSSQLVWVEYAQNTLKSSTPSLSSLVSSPLCFQT
RRKRSTCLKRMLRIRPQGSA
ITSGLGVLRP
>g1302.t1
VKKLLTEALNKNEYLKRLEVQQVRDMVECMYERSFQQGEYVLQQGQPGHHLFVLADGRLDVVQHSKLLTSIAGWTTFGEL
AILYNCTRTASVIAMSSVLT
WALDREVFQNIMRRTAETRHQQYRNFMRSVSLLANLPEDMLSKIVDCLEVEYYDKGEYVIREGEEGSTFYIIAQGKVKVT
QSTQLHKEPQVINSLQKGDY
FGEKALISDDVRSANIIAEDNGLECLVIDRETFNQTVGTFNELQKYLQGYVATLDRDDKKRNAKINFMILLTTYKALNNL
ALSYLSDLLHQHVPSRCLRR
TVSRHLSRSISPDALQLEEKLSAFSSTSPFDHLEVITTLGVGGFGRVELVRIKGEDAAFALKVMKKQHIVDNRQEEHIHS
ERRILAESRSPFIVKLYRTF
KDNKYVYMLLEACLGGEIWSLLRDRGSFDETTAKFCVGCVTEAFHYLHRAGVIYRDLKPENLMLDAEGYVKLVDFGFAKK
IKSGQRTWTFCGTPEYVAPE
VILNKGHSFSVDFWSLGILVFELLIGSPPFSGSDQMMTYTFILKGVEKMDFPKKISKRPEDLIRKLCRRNPVERLGNLKN
GIIDIKKH
>g1302.t2
VKKLLTEALNKNEYLKRLEVQQVRDMVECMYERSFQQGEYVLQQGQPGHHLFVLADGRLDVVQHSKLLTSIAGWTTFGEL
AILYNCTRTASVIAMSSVLT
WALDREVFQNIMRRTAETRHQQYRNFMRSVSLLANLPEDMLSKIVDCLEVEYYDKGEYVIREGEEGSTFYIIAQGKVKVT
QSTQLHKEPQVINSLQKGDY

FGEKALISDDVRSANIIAEDNGLECLVIDRETFNQTVGTFNELQKYLQGYVATLDRDDKKRNAKINFMILLTTYKALNNL
ALSYLSDLLHQHVPSRCLRR
TVSRHLSRSISPDALQLEEKLSAFSSTSPFDHLEVITTLGVGGFGRVELVRIKGEDAAFALKVMKKQHIVDNRQEEHIHS
ERRILAESRSPFIVKLYRTF
KDNKYVYMLLEACLGGEIWSLLRDRGSFDETTAKFCVGCVTEAFHYLHRAGVIYRDLKPENLMLDAEGYVKLVDFGFAKK
IKSGQRTWTFCGTPEYVAPE
VILNKGHSFSVDFWSLGILVFELLIGSPPFSGSDQMMTYTFILKGVEKMDFPKKISKRPEDLIRKLCRRNPVERLGNLKN
GIIDIKKHRWFTGFNWEGLK
GRTLPSPLRRE
>g1302.t3
MRISLDRIGVKWGWVVYTTSVKKLLTEALNKNEYLKRLEVQQVRDMVECMYERSFQQGEYVLQQGQPGHHLFVLADGRLD
VVQHSKLLTSIAGWTTFGEL
AILYNCTRTASVIAMSSVLTWALDREVFQNIMRRTAETRHQQYRNFMRSVSLLANLPEDMLSKIVDCLEVEYYDKGEYVI
REGEEGSTFYIIAQGKVKVT
QSTQLHKEPQVINSLQKGDYFGEKALISDDVRSANIIAEDNGLECLVIDRETFNQTVGTFNELQKYLQGYVATLDRDDKK
RNAKINFMILLTTYKALNNL
ALSYLSDLLHQHVPSRCLRRTVSRHLSRSISPDALQLEEKLSAFSSTSPFDHLEVITTLGVGGFGRVELVRIKGEDAAFA
LKVMKKQHIVDNRQEEHIHS
ERRILAESRSPFIVKLYRTFKDNKYVYMLLEACLGGEIWSLLRDRGSFDETTAKFCVGCVTEAFHYLHRAGVIYRDLKPE
NLMLDAEGYVKLVDFGFAKK
IKSGQRTWTFCGTPEYVAPEVILNKGHSFSVDFWSLGILVFELLIGSPPFSGSDQMMTYTFILKGVEKMDFPKKISKRPE
DLIRKLCRRNPVERLGNLKN
GIIDIKKHRWFTGFNWEGLKGRTLPSPLRRE
>g1303.t1
NGTGSSSQISTPVSKHSPTSTPTSPGLGRKQKDLYLPLSQEDEDSLGESM
>g1303.t2
VNGTGSSSQISTPVSKHSPTSTPTSPGLGRKQKDLYLPLSQEDEDSLGESM
>g1304.t1
MDTGRDLQAGCTMFSSSTPYKNQHYDTLKRSCLTDRTLFEDPEFPTTNKSLFFRKPPPGRVEWKRPGEISGEPHLFVEGI
SAHDLHQGLVGNCWFVAACS
CLALKPQLWKKV
>g1304.t2
MDTGRDLQAGCTMFSSSTPYKNQHYDTLKRSCLTDRTLFEDPEFPTTNKSLFFRKPPPGRVEWKRPGVSAVTEVVEVVEE
ISGEPHLFVEGISAHDLHQG
LVGNCWFVAACSCLALKPQLWKKV
>g1305.t1
MIDDIEDMVSASDPTPQERQSARKSIIPRARKRKDQLVTEELQADSFIPGTQKIWMRTWGCSHNNSDGEYMAGQLAASGY
KMTGKT
>g1305.t2
MPAACDSMIDDIEDMVSASDPTPQERQSARKSIIPRARKRKDQLVTEELQADSFIPGTQKIWMRTWGCSHNNSDGEYMAG
QLAASGYKMTGKT
>g1306.t1
MAGSGDVDYPLNGPATPKMAEGGGTPCTRTSIPECAICLQSCVHPVCLPCHHVFCFLCMKGASWQSKRCALCRQEVPEDF
LERPKLLSPEELRAVAAGSR
GGGGDHAWYYEGRGGWWQYDERTSGELEDAFAKGTASTEMLIAGILYVADLENMVQYRRNE
>g1307.t1
MGLGLLLVILMVNFVEFAKSHGRNHVLHRRWCREDCGSCFSRHFCTRCRSGQFLFRGRCEVSCPEGLMASSVLRECTST
>g1308.t1
MKLVGQLKRVPRTGWVYRDVKQPESVSDHMYRMALMAMTITDPTVDKERCMKLALVHDMAECIVGDIAPMDNVSKAEKHR
RENEYETQSSPEARLVKQFD
LVEMILQAHEYEELEKTPGRLQEFFDSTEGRFHHPDVIQLVDCLKEERRGHVSDVAGATHSRTSEDPAAGPAEGSESQPP
APTS
>g1308.t2
MRVPRTGWVYRDVKQPESVSDHMYRMALMAMTITDPTVDKERCMKLALVHDMAECIVGDIAPMDNVSKAEKHRRENEYET
QSSPEARLVKQFDLVEMILQ
AHEYEELEKTPGRLQEFFDSTEGRFHHPDVIQLVDCLKEERRGHVSDVAGATHSRTSEDPAAGPAEGSESQPPAPTS

>g1309.t1
MSMPTMRNSPSFKSFEEKVETTVTNIKTKVGGTEPGGSFEEVLSSTAQASSQDRPSPPLTDTPERPC
>g1310.t1
QHQGELLLVAQRDFTSSSSSFSTCKPTSSNKSLVWRPW
>g1311.t1
MAPCFSNSSVHHGRFPRKDNLRVSSDIQLVYLLLKPGLHLLMYVQPPAHPRLVSQQLRKPRVEKMRRDRINTCIDQLKLL
LEKELHQQEPNAKLEKADVL
EMTVSFLRQQLLHTSGGPREPTLLGSSQGPRECVQDLLCSVGPSRETSIHAARPPPSLHVLQQHQGELLLVAQRDFTSSS
SSFSTCTPTSSNKSLVWRPW
>g1312.t1
LLPWLKKVNFTVGNAPMYFDENGDPPTGYDIVAWIWRGSRWTVRKVGSYTPVPSTLTIDASRVEWSHAGDATSVPLSICS
PDCDVGHRRLQTGQHKCCFD
CLACPATTFLNKSGATSCQPCPEKEWAPPKSEKCLKRTVLFLAWDAPLSIALLLLLAATLLLTTGSSVILLLHLNTPVVK
SAGGRTCLVMLAALTAAAAS
ALCHFGPPTRPGCLLKQPLFVVSFAVCLACVTVRAFQVVCIFKLASRLPRVYDTWAKNHGPEYTILLLSGAVLCVSVLRV
AVNPPEPSLDLRFYPDSIVL
ECSNTMSPGSWVELACASLLSGLCFSLSYMGKDLPANYNEAKCVAFSLVVYMLSWICYFTLYLISRDPFTMAVNVFAILS
SVLGVFGGYFLPKVCIILFR
PQMNTSAHFQNCIQMYTMNKQ
>g1312.t2
LPWLKKVNFTVGNAPMYFDENGDPPTGYDIVAWIWRGSRWTVRKVGSYTPVPSTLTIDASRVEWSHAGDATSVPLSICSP
DCDVGHRRLQTGQHKCCFDC
LACPATTFLNKSGATSCQPCPEKEWAPPKSEKCLKRTVLFLAWDAPLSIALLLLLAATLLLTTGSSVILLLHLNTPVVKS
AGGRTCLVMLAALTAAAASA
LCHFGPPTRPGCLLKQPLFVVSFAVCLACVTVRAFQVVCIFKLASRLPRVYDTWAKNHGPEYTILLLSGAVLCVSVLRVA
VNPPEPSLDLRFYPDSIVLE
CSNTMSPGSWVELACASLLSGLCFSLSYMGKDLPANYNEAKCVAFSLVVYMLSWICYFTLYLISRDPFTMAVNVFAILSS
VLGVFGGYFLPKVCIILFRP
QMNTSAHFQNCIQMYTMNKQ
>g1312.t3
MYFDENGDPPTGYDIVAWIWRGSRWTVRKVGSYTPVPSTLTIDASRVEWSHAGDATSVPLSICSPDCDVGHRRLQTGQHK
CCFDCLACPATTFLNKSGAT
SCQPCPEKEWAPPKSEKCLKRTVLFLAWDAPLSIALLLLLAATLLLTTGSSVILLLHLNTPVVKSAGGRTCLVMLAALTA
AAASALCHFGPPTRPGCLLK
QPLFVVSFAVCLACVTVRAFQVVCIFKLASRLPRVYDTWAKNHGPEYTILLLSGAVLCVSVLRVAVNPPEPSLDLRFYPD
SIVLECSNTMSPGSWVELAC
ASLLSGLCFSLSYMGKDLPANYNEAKCVAFSLVVYMLSWICYFTLYLISRDPFTMAVNVFAILSSVLGVFGGYFLPKVCI
ILFRPQMNTSAHFQNCIQMY
TMNKQ
>g1313.t1
GQRFGVQTVPTLQEALQECIKHSLTVFLNVKGYPDKVTHPRPTSP
>g1314.t1
RKELETKLKEREEFLLPIYHQVAVQFADLHDTPGRMQEKGVITVRRTPRRLSRVGWGASRLGRDTTSCRGAVVRADDVRA
QSRRFW
>g1315.t1
YRAIKNQPPPIMSIELDHNPFKCPAEGCTKSFRKGSLLHYHIKYYHSEQKLEGQASQHGPPRRKRSSSEGRDFLSERRKE
NQSNNNSHHHDNAEHITTAA
EEVKPEEGREEKEKRRDFMRMKTKKRKKKGKKRRHSADEGSDWSTLTAESGEDSPPWPVAMEMEDCEVVRCVCEVEEEND
FMIQCELCLCWQHGTCMGLY
EDNVPHSYICYSCRDATGWKRGRYFLSDSDWLRSGHMFGLSCVRENYSELNASKITHTTQLLAHTLGLTQVLRGLQLKLC
LLHCPTHPDLQLWRLPWRLG
EGPTAVANQRSDTDGSNSVLAGSYVSSEHCYQKTDASWENEEKHENITASGTSQNYKDEGCVDVKVEESLCASAAECQLH
LLEHMEALHNQTSTRMDLIE
RELD
>g1315.t2
AIKNQPPPIMSIELDHNPFKCPAEGCTKSFRKGSLLHYHIKYYHSEQKLEGQASQHGPPRRKRSSSEGRDFLSERRKENQ

SNNNSHHHDNAEHITTAAEE
VKPEEGREEKEKRRDFMRMKTKKRKKKGKKRRHSADEGSDWSTLTAESGEDSPPWPVAMEMEDCEVVRCVCEVEEENDFM
IQCELCLCWQHGTCMGLYED
NVPHSYICYSCRDATGWKRGRYFLSDSDWLRSGHMFGLSCVRENYSELNASKITHTTQLLAHTLGLTQVLRGLQLKLCLL
HCPTHPDLQLWRLPWRLGEG
PTAVANQRSDTDGSNSVLAGSYVSSEHCYQKTDASWENEEKHENITASGTSQNYKDEGCVDVKVEESLCASAAECQLHLL
EHMEALHNQTSTRMDLIERE
LD
>g1315.t3
YRAIKNQPPPIMSIELDHNPFKCPAEGCTKSFRKGSLLHYHIKYYHSEQKLEGQASQHGPPRRKRSSSEGRDFLSERRKE
NQSNNNSHHHDNAEHITTAA
EVKPEEGREEKEKRRDFMRMKTKKRKKKGKKRRHSADEGSDWSTLTAESGEDSPPWPVAMEMEDCEVVRCVCEVEEENDF
MIQCELCLCWQHGTCMGLYE
DNVPHSYICYSCRDATGWKRGRYFLSDSDWLRSGHMFGLSCVRENYSELNASKITHTTQLLAHTLGLTQVLRGLQLKLCL
LHCPTHPDLQLWRLPWRLGE
GPTAVANQRSDTDGSNSVLAGSYVSSEHCYQKTDASWENEEKHENITASGTSQNYKDEGCVDVKVEESLCASAAECQLHL
LEHMEALHNQTSTRMDLIER
ELD
>g1316.t1
MWRSLKLGTRLTLYKKLKPFYSILDRFLDTARKHPTKTFLHFEGRAYSYSEVDRRSNQVANALRTHAGLQAGDAVALFVG
NEPWFVWTWLGLAKLGCAAA
LLNFNIRSKSLLHCFSCCGAKVLIASS
>g1317.t1
MLSLCLHLVLGQAMGHHRTNCIGVDDLILHLLHHAYTHLDEPRGPVTIMLSPGHSARPSVSCWEETDKAAGSCDGVLDFL
TRSGLQGNKCLENMPDWTTN
WNAVSKKAQTRLYFPGEAPVLQCLLHCAATVVRVCRGQQTLPTAVG
>g1318.t1
SEPGGRPARWGEGFGELTPDASDVTTYNEFAKRPANVPSAKRTCREPTPGSEVPGGRVFSALRRCQQDSGFHSPFSQHQ
>g1319.t1
MLVLHASAPRPRVSPPGEDVHVGLHGVSEELDVRLQPDSVALEKTFISLCSVRTVSLANPGNVPLRYCWKSWPSQQEEDA
YAM
>g1319.t2
MQYGKPNTHAVNKGEDVHVGLHGVSEELDVRLQPDSVALEKTFISLCSVRTVSLANPGNVPLRYCWKSWPSQQEEDAYAM
>g1320.t1
MCVFVCLQIAGGVGPPGRAVSALSPSEESAARQQVEEQQWYVETLRKEIQVEQRREESELEREQARLRQQHAEVQQWILQ
EKQRLLSISPKGTQESGVQT
DLLPAPLLERLCGATGGSEGGAAAWLPDPPPPGLVRVRKKVVQDELLRNHALRRAESRVRRKRLHLQLERIARKRHLLEA
KRELQRLEQTLPPGLDSPGS
PELGSPYGSGGRPPCNPRRHSFSVDVLSRLYPQHPPIFSHYLKRNTSTELMLNPLRTGDHFSRKWVSDECLPRERTRSCS
SGVSPGQNQAPGSRAGSSEN
LKRPIEESPPARSYQERPERKPLRPNRSLTFKTNQKPLGSLKSPQRTALQPISNEREKNPSVENPKAQKIACVDVRTPQG
SNKGLGTIRKVFSRSVGPGL
KTALSKVFRKPPPGVNGRRNPKPVARMHRLHWRKTNRDRSLKDGTTHRHQCALKTTVSCEELDQMTFPEDVKHRRWHSME
ALMTQTGSWVERLQLGLAGW
VEDEGGERDEGSSDCDSLLSVDSLSSAYATALAEQLRREEALGSESDSVDSQMSRDSLALPESSTLQSRVNRTVVPKYTR
VTGSSHLLVRHVRIPDSTVP
EHTDSQSTQGSPAEAYWSQNGGLSTIASGETGAAMEPPVHKPREEPVLTQTSFTGSPRSLSSCSVREPDLQLALTDAWSS
TDAADSPRIPRECEAGALQK
ETPFRARDSNSGSSSSPSPISIDLSDCRNEVKSCNSTTSSSSEGIHITMEDQNLEASTELKRNKELTESLVGGLKILKRS
DEGALETPCATQIAGQDVGW
SDGNSDPEEKVPGLDEDVLCPDSDHANANARQPLCRKQQQAGKNVADCGPETHNMPSLSNIPVGGTNETETDGKSDKATS
LEKRFRMSVGIVDQERKPEE
RRPLRQWDAICSAIDLRIAEVVKGRLAAAPLMSGGDNAGRGRSHSLTALPQRTTEKGPAGGRATRGAAGPTGEGDARTRD
EGGDQMKAEVVRAECPSAPR
GELQREEGSGTNDRIYALMDEDEMRLPVQGMSAAERAANVAGTMSGIPLNDYRALSDNADTDNSSRENPVRLTFPLDRIS
PRHGPRKSPGKCPPSSLNDG

DEVTCTETCHSTPASPNVCSKPSDRQACLAAPVWKPDAPRPQNVFPDIHTTQSSSEEEDEMPSRVHRSRPDSMQLKSSIH
EGPAREHCPKSHSSQPQEMA
RNPNKERTTPCLKVSQSHCLEVPQEPQMHFASSDINPFVHQWQEDPSLRKCPWNPTFGSAADLSSKSPLWSSGEKRMTRC
CSADNGLNGQNSPFNSHLST
FANNKGLSSTLSSSEDYKEQAGGATSRDTSQASRPQSSGLDSRLAGLTLNTGSSCNEVPGADGSGRADEIMSLYSSEQES
SLADARDDSCRAQRRRTFEE
HGTQTECHAAETLGGPRGSRPTAAPGRDLTTNQRHRRSRTHVPTTLRTKENFRESPTWASMENMSTHLTQLIHSTSDLLG
DVQGMRTGKTPVKPGLRRNV
NISKDTVPWCPLRDSTGTHTSFDIGIQTEASSTTFVEEQWTSPTEKSTSHEINVIVKVIGSDILSVSHENGFPGVVMEKA
GSDERKWSKSDLKINTVASK
ESPDLLNASHCLTSVENPTESKVCHTRSDLPSNSKQSRAVCTKGCGLSQSSHSLSKSSVSVETGRSLHCVEKTRYTDRAT
SPIVTLGVSESIESHGQRRP
PCLERRKGSAFARDDASSNRSAESASLESVREISLKDTGASSVGLRSSVDGNVSRKSIPNGENNNDPISANADSAKIPQQ
RTSTSSNGINRRSRPSPGGR
HGDVGGQEDVDRHDDMNMRETQRTERHSGPPTDDSAGRGPAPSSLSDAPIREDDAAST
>g1320.t2
MCVFVCLQIAGGVGPPGRAVSALSPSEESAARQQVEEQQWYVETLRKEIQVEQRREESELEREQARLRQQHAEVQQWILQ
EKQRLLSISPKGTQESGVQT
DLLPAPLLERLCGATGGSEGGAAAWLPDPPPPGLVRVRKKVVQDELLRNHALRRAESRVRRKRLHLQLERIARKRHLLEA
KRELQRLEQTLPPGLDSPGS
PELGSPYGSGGRPPCNPRRHSFSVDVLSRLYPQHPPIFSHYLKRNTSTELMLNPLRTGDHFSRKWVSDECLPRERTRSCS
SGVSPGQNQAPGSRAGSSEN
LKRPIEESPPARSYQERPERKPLRPNRSLTFKTNQKPLGSLKSPQRTALQPISNEREKNPSVENPKAQKIACVDVRTPQG
SNKGLGTIRKVFSRSVGPGL
KTALSKVFRKPPPGVNGRRNPKPVARMHRLHWRKTNRDRSLKDGTTHRHQCALKTTVSCEELDQMTFPEDVKHRRWHSME
ALMTQTGSWVERLQLGLAGW
VEDEGGERDEGSSDCDSLLSVDSLSSAYATALAEQLRREEALGSESDSVDSQMSRDSLALPESSTLQSRVNRTVVPKYTR
VTGSSHLLVRHVRIPDSTVP
EHTDSQSTQGSPAEAYWSQNGGLSTIASGETGAAMEPPVHKPREEPVLTQTSFTGSPRSLSSCSVREPDLQLALTDAWSS
TDAADSPRIPRECEAGALQK
ETPFRARDSNSGSSSSPSPISIDLSDCRNEVKSCNSTTSSSSEGIHITMEDQNLEASTELSDTHFPDTEAPQEPGKVIGL
IGSRKRNKELTESLVGGLKI
LKRSDEGALETPCATQIAGQDVGWSDGNSDPEEKVPGLDEDVLCPDSDHANANARQPLCRKQQQAGKNVADCGPETHNMP
SLSNIPVGGTNETETDGKSD
KATSLEKRFRMSVGIVDQERKPEERRPLRQWDAICSAIDLRIAEVVKGRLAAAPLMSGGDNAGRGRSHSLTALPQRTTEK
GPAGGRATRGAAGPTGEGDA
RTRDEGGDQMKAEVVRAECPSAPRGELQREEGSGTNDRIYALMDEDEMRLPVQGMSAAERAANVAGTMSGIPLNDYRALS
DNADTDNSSRENPVRLTFPL
DRISPRHGPRKSPGKCPPSSLNDGDEVTCTETCHSTPASPNVCSKPSDRQACLAAPVWKPDAPRPQNVFPDIHTTQSSSE
EEDEMPSRVHRSRPDSMQLK
SSIHEGPAREHCPKSHSSQPQEMARNPNKERTTPCLKVSQSHCLEVPQEPQMHFASSDINPFVHQWQEDPSLRKCPWNPT
FGSAADLSSKSPLWSSGEKR
MTRCCSADNGLNGQNSPFNSHLSTFANNKGLSSTLSSSEDYKEQAGGATSRDTSQASRPQSSGLDSRLAGLTLNTGSSCN
EVPGADGSGRADEIMSLYSS
EQESSLADARDDSCRAQRRRTFEEHGTQTECHAAETLGGPRGSRPTAAPGRDLTTNQRHRRSRTHVPTTLRTKENFRESP
TWASMENMSTHLTQLIHSTS
DLLGDVQGMRTGKTPVKPGLRRNVNISKDTVPWCPLRDSTGTHTSFDIGIQTEASSTTFVEEQWTSPTEKSTSHEINVIV
KVIGSDILSVSHENGFPGVV
MEKAGSDERKWSKSDLKINTVASKESPDLLNASHCLTSVENPTESKVCHTRSDLPSNSKQSRAVCTKGCGLSQSSHSLSK
SSVSVETGRSLHCVEKTRYT
DRATSPIVTLGVSESIESHGQRRPPCLERRKGSAFARDDASSNRSAESASLESVREISLKDTGASSVGLRSSVDGNVSRK
SIPNGENNNDPISANADSAK
IPQQRTSTSSNGINRRSRPSPGGRHGDVGGQEDVDRHDDMNMRETQRTERHSGPPTDDSAGRGPAPSSLSDAPIREDDAA
ST
>g1321.t1
LLCRGGTCRNTEGSYDCHCPPGHSLSTDGSVCEDVNECQLSENLCRNGHCVNMVGTYQCSCDTGYQATPDRQGCVDIDEC

TIMNGGCDTHCTNSEGSYEC
SCREGYALMPDLRTCAVFSNNRYYDDHAVLDIDECEESPDICDGGQCTNIPGEYRCLCYDGFMASMDMRTCIDVNECDLN
PNICLHGDCENTKGSFICHC
QLGYFVKKGSTGCTDYKCLMILYCKKCRYT
>g1321.t2
LCRGGTCRNTEGSYDCHCPPGHSLSTDGSVCEDVNECQLSENLCRNGHCVNMVGTYQCSCDTGYQATPDRQGCVDIDECT
IMNGGCDTHCTNSEGSYECS
CREGYALMPDLRTCAVFSNNRYYDDHAVLDIDECEESPDICDGGQCTNIPGEYRCLCYDGFMASMDMRTCIDVNECDLNP
NICLHGDCENTKGSFICHCQ
LGYFVKKGSTGCTDYKCLMILYCKKCRYT
>g1321.t3
LLCRGGTCRNTEGSYDCHCPPGHSLSTDGSVCEDVNECQLSENLCRNGHCVNMVGTYQCSCDTGYQATPDRQGCVDIDEC
TIMNGGCDTHCTNSEGSYEC
SCREGYALMPDLRTCAVFSNNRYYDDHAVLDIDECEESPDICDGGQCTNIPGEYRCLCYDGFMASMDMRTCIDVNECDLN
PNICLHGDCENTKGSFICHC
QLGYFVKKGSTGCT
>g1322.t1
MSATDWYAHKSLGDGLYWIQERFYESENRANIWLLRGSHQDVVIDTGLGLKSLPDYITSKGLLGEDPQRKNPLLAIATHA
HFDHSGGLHQFQQVGVHSAE
VEALVNGDNFETATWLSDREIVQAPTPGWRARHYKVHAVQPTHILQEGDVINLGDRQLTVLHMPGHSRGSICLHDGDHKL
LFSGDVVYDGAMIDWLPYSR
VSDYISSCERIVGLVDSEQVDQVLPGHYNTFGAKRLHRIATSYISRAGTCPAKFSTFAWKTLAGVVLRASNPRSAC
>g1322.t2
LYWIQERFYESENRANIWLLRGSHQDVVIDTGLGLKSLPDYITSKGLLGEDPQRKNPLLAIATHAHFDHSGGLHQFQQVG
VHSAEVEALVNGDNFETATW
LSDREIVQAPTPGWRARHYKVHAVQPTHILQEGDVINLGDRQLTVLHMPGHSRGSICLHDGDHKLLFSGDVVYDGAMIDW
LPYSRVSDYISSCERIVGLV
DSEQVDQVLPGHYNTFGAKRLHRIATSYISRAGTCPAKFSTFAWKTLAGVVLRASNPRSAC
>g1323.t1
LKDDEEEAYMLALKQEMRGTMQRLPDNIKPLSTKADVERYKERYNKQNQLDDGDWTPEFFLWYSHQTCIQCSIEGFLQEL
EKKDGAEEEKSDDEKPKTKK
TAEGEEEELEEEYEEEELEEENDYIDSYFDNGEDFGALSDDNIDGEATY
>g1323.t2
LKDDEEEAYMLALKQEMRGTMQRLPDNIKPLSTKADVERYKERYNKQNQLDDGDWTPGRKKPVKPSAKDTKDELEKKDGA
EEEKSDDEKPKTKKTAEGEE
EELEEEYEEEELEEENDYIDSYFDNGEDFGALSDDNIDGEATY
>g1323.t3
LKDDEEEAYMLALKQEMRGTMQRLPDNIKPLSTKADVERYKERYNKQNQLDDGDWTPGRKKPELEKKDGAEEEKSDDEKP
KTKKTAEGEEEELEEEYEEE
ELEEENDYIDSYFDNGEDFGALSDDNIDGEATY
>g1324.t1
MASRSQHYGFQTATTVQPANGGHAYLFGNNGSENDLSEDDGEIFELRSRGRERLRRSASRERMDDIVYLARDVQEGDTLN
SLSLHYHCSVADLKRANNLL
TEQDFFALRSVKIPVRRFSVLTETYSAAAAALKPSSSSPSTTWSLSQISPGTSSVPTECSTDSSSSADSVEGFLLEKDKD
IERLVKSTGPSRSSLNEVVS
SLTQLEQPLLGEVAAEYKPAQRKDPYYGADWGMRWWTAVAIMLVVGIVTPVFYLLYYEVLVKADVSHHSAPANPNLAAPQ
GNKLELAGQEDSHAGAGLED
GKGNVAVGDPKAPGAENNKDGSDGKQAKT
>g1325.t1
EKFGSQSAHQMSAERGARDGRRRRPLRALVS
>g1326.t1
EATGFNVLLFSCLIFIPPPPHQPVSSSLLISLFVLLMSLSSFSSTPPLILSSPHCPRPPFPHVSLSPPYSATCLLSPSSS
LLINLSPLLISLSPPPPPY
>g1327.t1
MSLQDHKLKMPPPGSGMRTSPSEKDLSKLAESYQAADTTLESPEDLSHEQDKSPLSDSGFETRSEKTPSAPQSAESTGPK
PLFADSPIPPCVTETRTEVV

HIRSYEQPDEPCEPSLVEEAATAYPSIESDIASPSSLTDKVKALQVKMSEDDSMMSKSMCLKEETHITTTTRMVYHKPQI
AEGADMMEETMSVRDIMKAF
QSGRDPSKELAGLFEHKASQDSVKGDELTPRFLDKDTKSKPKVERIIEVHIGKGHSTSEPTEVIIRETKKHPELYAYKGD
RGIKELSEYDEAQQEEEELT
AEESLPSFLETSRVNTPVSHEEDSRPSSAQLIADDSYKALKLLSQHSIEYCDDELSEIRGESYRFAEKMLLSEKLDTSSQ
SHSDTEDSAMMADQTHPHSS
TEGTSQQQGSPKKLFSSRTPKEGSPKSGKFSSNKDDQAQFDKVTVLHYSTDQGSPKHAVWMRFTEDKHDRSRDKLLYEDR
VDKTVKEAEEKLSEVVRKVN
IEGSCASSGTETEMMEVNLQEEHDRYLLTEPIIRIQPPTPVLTDADDSQSNPEDDGDDESVFQPIPYKNATFPEEEKDKK
GSKSKKHDKNGNNKEPPGGT
NGSNGKEAEDVDCEQNGDQSITDCSIATTAEFSHDTDATEIDSLDGYELQDEDDGLCEQADSRLFGLPDSRRDVWATESF
RSTDRSFPPTKLEVIEEEKS
PEECSKNDCKNGSPSGGENIEDVCKDEDKKPEQKPSDKEGFSDTYFSYKLEEEFNSTFKTVATKGLDFDPWPSKGEEDMV
VDMTEPRNCHDEPKPFGLAA
EDHSQNTTPDTTPARTPTDDSTPTSEPNPFPFHEGKMFEMTRSGAIDMSKRDMVEERLQFFQIGEHPYSAGRYRKFQIFS
YLHHHCLQHDHHHHHHHHHH
HTTTPH
>g1327.t2
MSLQDHKLKMPPPGSGMRTSPSEKDLSKLAESYQAADTTLESPEDLSHEQDKSPLSDSGFETRSEKTPSAPQSAESTGPK
PLFADSPIPPCVTETRTEVV
HIRSYEQPDEPCEPSLVEEAATAYPSIESDIASPSSLTDKVKALQVKMSEDDSMMSKSMCLKEETHITTTTRMVYHKPQI
AEGADMMEETMSVRDIMKAF
QSGRDPSKELAGLFEHKASQDSVKGDELTPRFLDKDTKSKPKVERIIEVHIGKGHSTSEPTEVIIRETKKHPELYAYKGD
RGIKELSEYDEAQQEEEELT
AEESLPSFLETSRVNTPVSHEEDSRPSSAQLIADDSYKALKLLSQHSIEYCDDELSEIRGESYRFAEKMLLSEKLDTSSQ
SHSDTEDSAMMADQTHPHSS
TEGTSQQQGSPKKLFSSRTPKEGSPKSGKFSSNKDDQAQFDKVTVLHYSTDQGSPKHAVWMRFTEDKHDRSRDKLLYEDR
VDKTVKEAEEKLSEVVRKVN
IEGQFCSCASSGTETEMMEVNLQEEHDRYLLTEPIIRIQPPTPVLTDADDSQSNPEDDGDDESVFQPIPYKNATFPEEEK
DKKGSKSKKHDKNGNNKEPP
GGTNGSNGKEAEDVDCEQNGDQSITDCSIATTAEFSHDTDATEIDSLDGYELQDEDDGLCEQADSRLFGLPDSRRDVWAT
ESFRSTDRSFPPTKLEVIEE
EKSPEECSKNDCKNGSPSGGENIEDVCKDEDKKPEQKPSDKEGFSDTYFSYKLEEEFNSTFKTVATKGLDFDPWPSKGEE
DMVVDMTEPRNCHDEPKPFG
LAAEDHSQNTTPDTTPARTPTDDSTPTSEPNPFPFHEGKMFEMTRSGAIDMSKRDMVEERLQFFQIGEHPYSAGRYRKFQ
IFSYLHHHCLQHDHHHHHHH
HHHHTTTPH
>g1327.t3
MMEVNLQEEHDRYLLTEPIIRIQPPTPVLTDADDSQSNPEDDGDDESVFQPIPYKNATFPEEEKDKKGSKSKKHDKNGNN
KEPPGGTNGSNGKEAEDVDC
EQNGDQSITDCSIATTAEFSHDTDATEIDSLDGYELQDEDDGLCEQADSRLFGLPDSRRDVWATESFRSTDRSFPPTKLE
VIEEEKSPEECSKNDCKNGS
PSGGENIEDVCKDEDKKPEQKPSDKEGFSDTYFSYKLEEEFNSTFKTVATKGLDFDPWPSKGEEDMVVDMTEPRNCHDEP
KPFGLAAEDHSQNTTPDTTP
ARTPTDDSTPTSEPNPFPFHEGKMFEMTRSGAIDMSKRDMVEERLQFFQIGEHPYSAGRYRKFQIFSYLHHHCLQHDHHH
HHHHHHHHTTTPH
>g1327.t4
MSLQDHKLKMPPPGSGMRTSPSEKDLSKLAESYQAADTTLESPEDLSHEQDKSPLSDSGFETRSEKTPSAPQSAESTGPK
PLFADSPIPPCVTETRTEVV
HIRSYEQPDEPCEPSLVEEAATAYPSIESDIASPSSLTDKVKALQVKMSEDDSMMSKSMCLKEETHITTTTRMVYHKPQI
AEGADMMEETMSVRDIMKAF
QSGRDPSKELAGLFEHKASQDSVKGDELTPRFLDKDTKSKPKVERIIEVHIGKGHSTSEPTEVIIRETKKHPELYAYKGD
RGIKELSEYDEAQQEEEELT
AEESLPSFLETSRVNTPVSHEEDSRPSSAQLIADDSYKALKLLSQHSIEYCDDELSEIRGESYRFAEKMLLSEKLDTSSQ
SHSDTEDSAMMADQTHPHSS
TEGTSQQQGSPKKLFSSRTPKEGSPKSGKFSSNKDDQAQFDKVTVLHYSTDQGSPKHAVWMRFTEDKHDRSRDKLLYEDR

VDKTVKEAEEKLSEVVRKVN
IEGQFCENEV
>g1328.t1
YYKVSDPGSWVSVGETTGELKVTNTIDLESDLVTDAMYNITIKAVDLSSKSGMGMVTLLIEDTNDNVPKLVDQDLVLCEE
EGKRGSVTVAAHDPDRLPYS
SPYNFQLSGESQNKWRLRDAQNVSVVLEQAVDMPRGSYKVEMRIADLQDKGDVQVATVTVCRCVGGQCVADRTSVGMGAW
GVLAVLFALAMLLLLCLLFV
FVCNTRGEKMYIDDSSGGMLLHSNTEAPGEEVKAHNLLMIPTTGGGDTIDCSAKGTGVLDQNQNMGGTSVMNQQTMGQNS
MYQSSHREFMSTSVQHQNSF
YTSAGQYRGGASYSDSPYQKQANLSLFNTWDTNRLYLDEKLDYFGGEMEVCHADDLPRTYQYEGEGSAAGSVGCCSEMGD
QDGLDFLESLGPKFKTLANI
CLNKEARE
>g1329.t1
LFLSPLLLPLSSSALLLELPYGWEKIDDPIYGSYYVDHINRRTQFENPVLEAKRRLDQQQQIQSQGLSSLPLPTIYREKP
LFTRDPTQLKGSFLSTALQK
SNMGFGFTIIGGDEPDEFLQVKSVIPEGPAAQDAKMDTGNTCNIACHSFSEHALNDM
>g1329.t2
LFLSPLLLPLSSSALLLELPYGWEKIDDPIYGSYYVDHINRRTQFENPVLEAKRRLDQQQQIQSQGLSSLPLPTIYREKP
LFTRDPTQLKGSFLSTALQK
SNMGFGFTIIGGDEPDEFLQVKSVIPEGPAAQDAKMDT
>g1329.t3
MVEASMLWLSRLGSPYPTKLPYGWEKIDDPIYGSYYVDHINRRTQFENPVLEAKRRLDQQQQIQSQGLSSLPLPTIYREK
PLFTRDPTQLKGSFLSTALQ
KSNMGFGFTIIGGDEPDEFLQVKSVIPEGPAAQDAKMDTGNTCNIACHSFSEHALNDM
>g1330.t1
LTVDAQLQAHCLSTIWKAPDDRCVFPKMLLDPVGHKHVLVAFRNELKRVAVSQCEQHSSLESCWNAQDPYCGWCASQRRC
SFEDECPSSLWLSVPDDPSQ
RRMLSHSFLEDGDGQWWNELPANVRTAESLPIFRKRLKTHLFRVHLNPA
>g1330.t2
LTVDAQLQAHCLSTIWKAPDDRCVFPKMLLDPVGHKHVLVAFRNELKRVAVSQCEQHSSLESCWNAQDPYCGXCSFEDEC
PSSLWLSVPDDPSQRRMLSH
SFLEDGDGQWWNELPANVRTAESLPIFRKRLKTHLFRVHLNPA
>g1331.t1
MCCFLSDRKSVIHLQVEYGTCSWESLSCSRAGMMVLKAELKSTDRILGGDFCLPYLAHVSDILYFWTLQDNKLDEQRSRL
AFQRDIKNCNIYFSEPEAVG
LRSGLQGAESTDSSLPLWTLISTPEAYRPNRSTDDAIAFTLHTARSHLDQRNTYVWMLFIDYSSAFNSIVPSRFVVKATL
LSLPGKVYSRVLERRLRPIV
EPQIQKEQFRPGRGTVDQPSPESPILFVIFMDRISRRSRGEERVRFGNLRIAFLFFADDVVLLASSDCDLQHALGQFAAE
CEAVGMRVSTSKSEAMVLCR
KMVDCSLRVGGELLPHVKVFKYLWILFTSEGKMEREMDRRIGAASAVMRALYRTVVVKRELSRKAKLSIYQSIYVPTLTY
GHELWVVTKRTRSWIQAVEM
SFLRRVGRDRVRSLDIRRELGVEPLLLCVERSQLRWFGHLIRMPPEHLSLEVFRARPTDTLDSGERLPSQGPAGLGRGHH
SICLTDHKEPGRNS
>g1331.t2
MCCFLSDRKSVIHLQVEYGTCSWESLSCSRAGMMVLKAELKSTDRILGGDFCLPYLAHVSDILYFWTLQDNKLDEQRSRL
AFQRDIKNCNIXAVGLRSGL
QGAESTDSSLPLWTLISTPEAYRPNRSTDDAIAFTLHTARSHLDQRNTYVWMLFIDYSSAFNSIVPSRFVVKATLLSLPG
KVYSRVLERRLRPIVEPQIQ
KEQFRPGRGTVDQPSPESPILFVIFMDRISRRSRGEERVRFGNLRIAFLFFADDVVLLASSDCDLQHALGQFAAECEAVG
MRVSTSKSEAMVLCRKMVDC
SLRVGGELLPHVKVFKYLWILFTSEGKMEREMDRRIGAASAVMRALYRTVVVKRELSRKAKLSIYQSIYVPTLTYGHELW
VVTKRTRSWIQAVEMSFLRR
VGRDRVRSLDIRRELGVEPLLLCVERSQLRWFGHLIRMPPEHLSLEVFRARPTDTLDSGERLPSQGPAGLGRGHHSICLT
DHKEPGRNS
>g1331.t3
MCCFLSDRKSVIHLQVEYGTCSWESLSCSRAGMMVLKAELKSTDRILGGDFCLPYLAHVSDILYFWTLQDNKLDEQRSRL

AFQRDIKNCNIYFSEPEAVG
LRSGLQGAESTDSSLPLWTLISTPEAYRPNRSTDDAIAFTLHTARSHLDQRNTYVWMLFIDYSSAFNSIVPSRFVVKWWL
LKKISGGAIFLIMIKATLLS
LPGKVYSRVLERRLRPIVEPQIQKEQFRPGRGTVDQPSPESPILFVIFMDRISRRSRGEERVRFGNLRIAFLFFADDVVL
LASSDCDLQHALGQFAAECE
AVGMRVSTSKSEAMVLCRKMVDCSLRVGGELLPHVKVFKYLWILFTSEGKMEREMDRRIGAASAVMRALYRTVVVKRELS
RKAKLSIYQSIYVPTLTYGH
ELWVVTKRTRSWIQAVEMSFLRRVGRDRVRSLDIRRELGVEPLLLCVERSQLRWFGHLIRMPPEHLSLEVFRARPTDTLD
SGERLPSQGPAGLGRGHHSI
CLTDHKEPGRNS
>g1332.t1
LELLDKLRNPEKLRSCLSSEEEDMEVAVPGHMRRIQSMNRHSHAMRTDSGSSGECRKQSPELGLS
>g1332.t2
LELLDKLRNPEKLRSCLSSEEEDMEVAVPGHMRRIQSMNRHSHAMRTDSGSSDTVAAIASSMWRDENMESDDDYDDVDM
>g1332.t3
LELLDKLRNPEKLRSCLSSEEEDMEVAVPGHMRRIQSMNRHSHAMRTDSGSSAENRIYTSRPVPKDSQDRTVRLHRIEPV
>g1333.t1
YSPVCGGGPRELSHSLGLSNCAYPLSCTPSRKHRDRLRHRPAVDDVRGILTLCGGRTIPSKYMWMRSTQVEPRGLQPLRE
GPSVDAAQLHAACDVPVTGV
AYILAGTV
>g1334.t1
MRSTVFFQTKLGDIRQVLGRGGGGPLGQNVNKMICGFPGSVAGQQVARARVGILLLPPPHYLLSHNFSLPPPSSALAALD
EVGRFPSSLFCLFLSILCFT
PSPPFSKPLAATQTPSSSNTKKAKGS
>g1335.t1
MKAIPXKLVHAFVSSRLDYCNALLIGIPGKSLQRLQHIQNSAARILMR
>g1335.t2
MLQSHAPLAKPSWSPPPCGSMLKVWPWMESIKGTVHTVLVEKLVHAFVSSRLDYCNALLIGIPGKSLQRLQHIQNSAARI
LMR
>g1335.t3
MLQSHAPLAKPSWSPPPCGSMLKVWPWMESIKGTVHTVLVEQTPIPCSSSQTVFISTLLGLHSVLITVNIIYQRHCTYSP
TEKLVHAFVSSRLDYCNALL
IGIPGKSLQRLQHIQNSAARILMR
>g1336.t1
MALDPSFDADILARALGVPHDKHMLRRHLYQEMMLLSAAHSSAEQERETLAGISLSPGAANRYATNRMAMRRSVMSPSRL
HPKGHSVDRGLGFHGSCGSL
PREARVHAVPRTKESPVHTYQTVEITNV
>g1336.t2
EYADNLQGKGIHGALMALDPSFDADILARALGVPHDKHMLRRHLYQEMMLLSAAHSSAEQERETLAGISLSPGAANRYAT
NRMAMRRSVMSPSRLHPKGH
SVDRGLGFHGSCGSLPREARVHAVPRTKESPVHTYQTVEITNV
>g1337.t1
VSWSLTQQHDGKLAAWHNRRKTRLACQMRGNRVGVALDYDAGTITFCEVGPSGGLAHLHTFSTTFSQPVCLGFGLYKAEL
NSRVSVLKV
>g1338.t1
ELDAPFRLYGLTMNPLLYNITQVVILSAVSGVISDLLGFNLKVRARSLGPPPSWFTSSSSALEFRTQFPPHV
>g1339.t1
FPTLVNGAIALPLPALDPSPHLLLSHSPHTS
>g1340.t1
AHSGTETGAAETDPSVAQTPGRAQHALLTCCLPSLPRVHCVRPPLPTQGALRQPSPPYPGCTASGLPSLPRVHCVRPPLP
TQGALRQPSPPYPGCTASAL
PSLPRVHCVSPPLPTQGALLQASAPHHCRKPGLRSCSEGVPLGLERCLTSGRRRPPRCSALFSPLGFERPARLPQSVRPR
RNCDGAAVEGRLLSP
>g1341.t1
MRFDASVDDAVLSVLVLLRKGFVTWFVTFVKASSCPFSYVRTSQPPRVLFPAERQDTYIEVTPGKRTAAAGIPTLFLLKI
FLSSIVSFLPFVPLPRPLCP

RVSLWNIQPLADLPAGGCL
>g1342.t1
MQYALLMIRSACVHLGLCSREANRQLALKIQLNKPNQPNKLRKKKNLIHWFIGMGCIGVGAAAATFRHLQTPSDTFHASD
AGLESVFGGVPVGFSTVLLP
GKVKAQRC
>g1343.t1
NSIRHNLSLHSRFVRVQNEGTGKSSWWMLNPEGGKSGKSPRRRAASMDNSSKFAKCRGRAAKKKAQWPLFCSLAAMLPIA
KHVPLQEHSDGGVTSPAPPY
PKWMESSNSHSNDDFETWSTFRTRTSSDASTLSGRHSPFLSEQDDVGDGDVIHIGYPGGSGAKLSASPLPSVSELPGPMS
QCRSEGVMEGLLDNMNLCLS
PKIPQLPPDIPHSTAGPLLQKGPYGTPAAMPPRLQQDYLKRMAYGQQQQMTSLSPVHVNPVPDGQPGFGSFDSRYNCHAG
LLKDLLTSNAERGREQNMPL
MEMGLTLPGHGGCSLPSYSSQEYAGVHEGVKMIQALVAHPGPRRHAPVMHRQGPSGAQDLNGCNMIPLTSVSLTRGPPRM
TNPRTCMQMPISHSGHTGAI
PANYGSSIGGYGGPVPGHLRGQYPERLPSDLDNMSLERLECDMESVLHETLMDGDALDFNFDPTGGPQLFAQRVKNSSHS
WVSG
>g1343.t2
NSIRHNLSLHSRFVRVQNEGTGKSSWWMLNPEGGKSGKSPRRRAASMDNSSKFAKCRGRAAKKKAQWPLFCSLAAMLPIA
KHVS
>g1344.t1
MYPCITYLEKNLRVDCEFPPTYQLPGPYCEYRQESYLVGSTYPNMVNLVSVENRRRANVSLINPTLCRLTWAPMGEEQPY
TFTCRVYQGLDWKENSMAIH
QRLLPICSALSVTCGPWLLLVALSLHMVLGSVWL
>g1345.t1
GGGGDSPGGDSPLPPPPDGTPTKQGEEVGEEQEEEQEEEEVGEEQEEEGAEPNGEGTLGSPSGSGVLVLRAEREGPEEEQ
AAEFRSCDSF
>g1346.t1
HTLGVIFPDRASVMEEPRVIQLHVDKPLAGELHVKVVDRILRTFCQCLLDASSPPNKQSQWAAQYVSVMLGHTELVSVLM
FRLWDLFHNQGPSLASSHVL
GLAALVVHLQASSTHSPLVQVMPCVALQPVPLTEALSAALLCNTQDNMLFCVR
>g1346.t2
HTLGVIFPDRASVMEEPRVIQLHVDKPLAGELHVKVVDRILRTFCQCLLDASSPPNKQSQWAAQYVSVMLGHTELVSVLM
FRLWDLFHNQGPSLASSHVL
GLAALVVHLQASSTHSPLVQVMPCVALQPVPLTEALSAALLCNTQDNMLFCV
>g1346.t3
HTLGVIFPDRASVMEEPRVIQLHVDKPLAGELHVKVVDRILRTFCQCLLDASSPPNKLWDLFHNQGPSLASSHVLGLAAL
VVHLQASSTHSPLVQVMPCV
ALQPVPLTEALSAALLCNTQDNMLFCVR
>g1347.t1
MLIHESKSSFTELRTENDGLLAKMKMLEQDKIRQQRIEEELTRIKLSLETELRNKQRLVDENASVRKDLAYFKSQYELRD
SEKRQCDSDRDRADRERSTL
KSEVERLKRELRSIEERYKSQLLISEKEAQELSFRRDQLEREILMLQQKPSSLNRHTQTDEKVSTIDPSKLLFDGVRRKV
TAHQLCDCGIISKSTLEKLL
MGRKTVDEVTVDIQLNLKGTGIIAGMSSGSQGTMPFTKAKNAGLLSSESALMLLEAQAATGYILDPAFDERMPVDTACSR
GIVETEDRDVLLTAEAASAG
FKDPYSGKLLSVGQVLKQGRIDKQTAIRLLQAQESVGGILDPVLSVFLPKDLALDRNLIDEELYMALNKKPACYLDPTSE
GKISYSDLKLKCKIDSLSGL
LLLPGKQEKSMTVKGLRGEVTISSLVSAELLTQADLQEVNQGTLSCKDIEDRLKSYLHGSTCIAGIYDEAKDRIMPFYMA
MKEGLLMRGTTLELLEAQAA
SGFMIDPVNNVFLTVEEAVKRGLVGNDFKNKLMSAEKAVVGYTDHATGKTVSLFEAIDKELIEKGHGIRLLEAQIASGGI
IDPKGSHRIDVEMAYKRGYF
NKEMNEILSYEGDDTKGFFDPNTKENLTYLQLKERCKTDPKTGLVLLPLKDKSKKSQQSHESRSNVLRKRRVVIVDPDTG
LEMSVREAYHRELIDYNTFL
DLSEQECEWEEITVKGANGAVRVVLVDKKTGTQYDINDCLERGIIEQSSFDQYRSGSITLTELADLIAHKTSSTEMTITA
SNVDDVATCTSPTQATPSSP
TVRKRFNSISITLSPPEQFDDLSPVGAIFDTETLEKITILEGCRRGIVDTITAQRLLEAQACTGGIINPSTSQRLSLQDA

VHQTIIDDDMAGRLKASQKA
FTGFEDVKTKRKMSAAEAMKEKWLPYDAGQRFLEFQYLTGGLLEPDTGRRISIEEAIRRKWLDGREAQKLQDTRNHQKNL
TCPKTKLKISYKQAMDGCMV
EESNGMKMLQATSVSSKGISSPYNVSNPGSRSGSRQGSRAGSRSGSRRGSVDYSSSYSMSFSTTTLNKSFSSNSLS
>g1348.t1
MVSKDLEALHDEYIRLRERILKQLDGIPADSEQAKYLRSQLEYINLRIGDLKGLNNAYFNRLSALKGVLQNMTQAEDVVK
VHEALLTEKDTTSLDTNEVA
EYHSILKQMKQDMDHKRELFSAMDSELASAVLYNSQISQNFHKCDVDLSKYSEQVGQLTDRWRRIQAQIDSRMWDLEKQE
KQLKHYQTTSSNLETWINNS
RMRQDGLQTVKFTDIQALMDHLHQQKARKQEKVEDVQKDAETCASSIKDYELQLASYSSGLETLLNIPIKRTVLKSPATV
VRQEASDLHSNYIELLTRSG
DYSKFLGEMLKSMEEMKIRNTKIELLEEELRRLKLSFQDRSQKNQALEDALARYKLELNQSQEKLLSLEEVKMTAAIQCN
ATKESLDSTQSQLKDLNDRL
SRLQYQLDDEMRKRRLAEERYTSQQEEYESAVRRRQKELEELNWSKLDLEKVVKDRERELERLKLQMEEEAARRRGAESE
ISKVRSQHNQELSQLKQTFE
TEIHVTKTSVQKSSQQKEEETAGVRMMCDRLGNEKRDLEDELRRLRLSMAQRDEQKGRAEQEIHQQKELMTEEINRRSEL
EVQLRTLVQKRGEDDLRLKE
ANNTNQEMTRKISLLKQNLDEEERKRRSFEMEISQLRKVQTELQMKNKAHLEAMNKLEASEKERSVIHLQLEKQTSSKSK
AEQSAARLQSHTQELQCSLD
AMESQLEKQKKAFQEEFTRRKRLDAELERMKNACREHTTTIASLRSIQEEASTCERRYDMDVKALQEALDKSVRDLRLTT
DKLNQMAEELKKVKQQLLQE
QTRYRELNQRCEGLYKTIEEKSRLLNENTAEVDRLKSQTQSITKERLRLEEELRTSKQEKDDLRANRESVDEESRTQIRA
LHLQLQSSNKKSMELEAFIN
DLTKEREKLKLEIDKIQKQSIEVYLLRGIILVY
>g1348.t2
KSILKDNEGILKDNSQRSKWLVTGPGGLDMLIPSVCLLIPPPNPLGIGLANKNEQYYEAIMGIWNQLYINIKSLISWQYC
VKDINYINSLTLSMLSKMRP
DEYRSVIKRLETHYQEFLRTSQGSDMFGDEEKKTIKGQFAFAQNHYDGLIVQLPTYKPPAVKXXXXXXXXEPAKPATVKP
DTIKPQKITKVSAQPPASTT
STLSLTLLNDLNALRRRLEVTESSLTYHLHIPLGENGAHECTQHLQTLQMVSKDLEALHDEYIRLRERILKQLDGIPADS
EQAKYLRSQLEYINLRIGDL
KGLNNAYFNRLSALKGVLQNMTQAEDVVKVHEALLTEKDTTSLDTNEVAEYHSILKQMKQDMDHKRELFSAMDSELASAV
LYNSQISQNFHKCDVDLSKY
SEQVGQLTDRWRRIQAQIDSRMWDLEKQEKQLKHYQTTSSNLETWINNSRMRQDGLQTVKFTDIQALMDHLHQQKARKQE
KVEDVQKDAETCASSIKDYE
LQLASYSSGLETLLNIPIKRTVLKSPATVVRQEASDLHSNYIELLTRSGDYSKFLGEMLKSMEEMKIRNTKIELLEEELR
RLKLSFQDRSQKNQALEDAL
ARYKLELNQSQEKLLSLEEVKMTAAIQCNATKESLDSTQSQLKDLNDRLSRLQYQLDDEMRKRRLAEERYTSQQEEYESA
VRRRQKELEELNWSKLDLEK
VVKDRERELERLKLQMEEEAARRRGAESEISKVRSQHNQELSQLKQTFETEIHVTKTSVQKSSQQKEEETAGVRMMCDRL
GNEKRDLEDELRRLRLSMAQ
RDEQKGRAEQEIHQQKELMTEEINRRSELEVQLRTLVQKRGEDDLRLKEANNTNQEMTRKISLLKQNLDEEERKRRSFEM
EISQLRKVQTELQMKNKAHL
EAMNKLEASEKERSVIHLQLEKQTSSKSKAEQSAARLQSHTQELQCSLDAMESQLEKQKKAFQEEFTRRKRLDAELERMK
NACREHTTTIASLRSIQEEA
STCERRYDMDVKALQEALDKSVRDLRLTTDKLNQMAEELKKVKQQLLQEQTRYRELNQRCEGLYKTIEEKSRLLNENTAE
VDRLKSQTQSITKERLRLEE
ELRTSKQEKDDLRANRESVDEESRTQIRALHLQLQSSNKKSMELEAFINDLTKEREKLKLEIDKIQKQSIEVYLLRGIIL
VY
>g1348.t3
KSILKDNEGILKDNSQRSKWLVTGPGGLDMLIPSVCLLIPPPNPLGIGLANKNEQYYEAIMGIWNQLYINIKSLISWQYC
VKDINYINSLTLSMLSKMRP
DEYRSVIKRLETHYQEFLRTSQGSDMFGDEEKKTIKGQFAFAQNHYDGLIVQLPTYTPTQVVTVVTQEPPAVKXXXXXXX
XEPAKPATVKPDTIKPQKIT
KVSAQPPASTTSTLSLTLLNDLNALRRRLEVTESSLTYHLHIPLGENGAHECTQHLQTLQMVSKDLEALHDEYIRLRERI
LKQLDGIPADSEQAKYLRSQ

LEYINLRIGDLKGLNNAYFNRLSALKGVLQNMTQAEDVVKVHEALLTEKDTTSLDTNEVAEYHSILKQMKQDMDHKRELF
SAMDSELASAVLYNSQISQN
FHKCDVDLSKYSEQVGQLTDRWRRIQAQIDSRMWDLEKQEKQLKHYQTTSSNLETWINNSRMRQDGLQTVKFTDIQALMD
HLHQQKARKQEKVEDVQKDA
ETCASSIKDYELQLASYSSGLETLLNIPIKRTVLKSPATVVRQEASDLHSNYIELLTRSGDYSKFLGEMLKSMEEMKIRN
TKIELLEEELRRLKLSFQDR
SQKNQALEDALARYKLELNQSQEKLLSLEEVKMTAAIQCNATKESLDSTQSQLKDLNDRLSRLQYQLDDEMRKRRLAEER
YTSQQEEYESAVRRRQKELE
ELNWSKLDLEKVVKDRERELERLKLQMEEEAARRRGAESEISKVRSQHNQELSQLKQTFETEIHVTKTSVQKSSQQKEEE
TAGVRMMCDRLGNEKRDLED
ELRRLRLSMAQRDEQKGRAEQEIHQQKELMTEEINRRSELEVQLRTLVQKRGEDDLRLKEANNTNQEMTRKISLLKQNLD
EEERKRRSFEMEISQLRKVQ
TELQMKNKAHLEAMNKLEASEKERSVIHLQLEKQTSSKSKAEQSAARLQSHTQELQCSLDAMESQLEKQKKAFQEEFTRR
KRLDAELERMKNACREHTTT
IASLRSIQEEASTCERRYDMDVKALQEALDKSVRDLRLTTDKLNQMAEELKKVKQQLLQEQTRYRELNQRCEGLYKTIEE
KSRLLNENTAEVDRLKSQTQ
SITKERLRLEEELRTSKQEKDDLRANRESVDEESRTQIRALHLQLQSSNKKSMELEAFINDLTKEREKLKLEIDKIQKQS
IEVYLLRGIILVY
>g1348.t4
KSILKDNEGILKDNSQRSKWLVTGPGGLDMLIPSVCLLIPPPNPLGIGLANKNEQYYEAIMGIWNQLYINIKSLISWQYC
VKDINYINSLTLSMLSKMRP
DEYRSVIKRLETHYQEFLRTSQGSDMFGDEEKKTIKGQFAFAQNHYDGLIVQLPTYTPTQVVTVVTQEPPAVKXXXXXXX
XEPAKPATVKPDTIKPQKIT
KVSAQPPASTTSTLSLTLLNDLNALRRRLEVTESSLTYHLHIPLGENGAHECTQHLQTLQVCRTALSVFPVGSLVAFRPR
RR
>g1349.t1
MGSDSAVLTLAGSSFHHCGAKTEKSLDFASRPFPPFSAPPPRQRSHFSACDTRITTANPPLTRRCSIDRSPPDYESMVTP
TTDYDYLEFYCDRSSARAFR
AQYEPPLYWTITVVGGLGNLMVLWTFLHVRQRLKTMTDVYLVNLAVADLLFLVTLPLWATEAAKGWSFGLGLCKVNSALY
KINLFSSTMLLTCISADRYV
VIVETTRAQNSKTERLCYSKLICGAVWLLALLLALPELIFSSNKQSGDMEYCRLVYPGGNGNRTKILVLALQVSMGFCLP
FLIMVFCYSMIIYTLLKTRN
FGKHKAMRVILVVVAVFVLSQLPYTCSLVLEATLATNVTMANCEQTKRIDIAGQVLKSLAYAHACINPFLYAFVGVRFRN
DMVRVLQAYRCWPSKRPQSN
LSFLSRPSITSDPDNSRTLSL
>g1350.t1
MYRFAKAAVNVTGVAARQVRNGSTVRQDFHAKYGTGLLAGSLVFCTSVWGYVITQTGITWNLSPVGKLMPKQWREKEE
>g1350.t2
MSVPWSVVSGWCTCSRHLESPLFKGILVLSRLAIMYRFAKAAVNVTGVAARQVRNGSTVRQDFHAKYGTGLLAGSLVFCT
SVWGYVITQTGITWNLSPVG
KLMPKQWREKEE
>g1351.t1
MDDVEASEKTSPGILQEIFTSPLNLCLLGLCLFLLYKIFKGDTPVDLGEVEQPLPKLKRRDFTLSDLKPYDGLQNPRILM
AVNNKVFDVTRGKKFYGPEG
PYGVFAGRDASRGLATFCLDKEALKDEHDDLSDLNSMQQESLSEWETQFTQKYDYIGKLLKPGDEPTEYTDDEETKDKKT
D
>g1352.t1
MSAMRVDTKVVMLGKESVGKTSLVERYVHHRFLVGPYQNTIGAAFVAKPIQVGEKVVTLGIWDTAGSERYEAMSRIYYRG
ARAAIVCYGSK
>g1352.t2
MRVDTKVVMLGKESVGKTSLVERYVHHRFLVGPYQNTIGAAFVAKPIQVGEKVVTLGIWDTAGSERYEAMSRIYYRGARA
AIVCYGSK
>g1352.t3
MSAMRVDTKVVMLGKESVGKTSLVERYVHHRFLVGPYQNTIGAAFVAKPIQVGEKVVTLGIWDTAGSERYEAMSRIYYRG
ARAAIVCYAVGSASASVSST
QSNVVFFYMFYYPDGSSF

>g1353.t1
SLPLNPKPFLNGLTGKPVMVKLKWGMEYKGYLVSVDGYMNMQLANTEEYVDGALAGHLGEVLIRCNNVLYIRGVEEEEED
GEMRE
>g1353.t2
MSLPLNPKPFLNGLTGKPVMVKLKWGMEYKGYLVSVDGYMNMQLANTEEYVDGALAGHLGEVLIRCNNVLYIRGVEEEEE
DGEMRE
>g1354.t1
MHTEYRLLVKNASQVVVICSNGEKYLQKNAMQNLCVVENGSVVIGCDGLIKEVGPADTIQAQYSEELFEKVIDATGMCVI
PGLVDAHTHPVWAGDRVHEF
AMKLAGATYMDVHRAGGGIHFTVEHTRAAPASELLASLTARLGRMLRAGSTLVECKSGYGLEVETEIKMLEVIEKARRSL
PINVSSTYCGAHAVPRGKTM
AEATEDVLRVQLPALRQRLSSGDLRVDNIDVFCERGVFDLEATRAILRAGQDMGLHINFHGDELHPMNSAQLGAELGALA
ISHLEEVTDEGIAAMATAKT
AAVLLPTTAYILRLPQPRARDMLEAGVIVALGSDFNPNAYCCSMPLVMHLACVNMRLTMPEALAGATINAAYALGRSDTH
GSLEAGKHGDLVVLNCRRWE
HLIYQLGGHQEVIRHVIIKGSVVHDNDKTLGF
>g1354.t2
MHTEYRLLVKNASQVVVICSNGEKYLQKNAMQNLCVVENGSVVIGCDGLIKELAGATYMDVHRAGGGIHFTVEHTRAAPA
SELLASLTARLGRMLRAGST
LVECKSGYGLEVETEIKMLEVIEKARRSLPINVSSTYCGAHAVPRGKTMAEATEDVLRVQLPALRQRLSSGDLRVDNIDV
FCERGVFDLEATRAILRAGQ
DMGLHINFHGDELHPMNSAQLGAELGALAISHLEEVTDEGIAAMATAKTAAVLLPTTAYILRLPQPRARDMLEAGVIVAL
GSDFNPNAYCCSMPLVMHLA
CVNMRLTMPEALAGATINAAYALGRSDTHGSLEAGKHGDLVVLNCRRWEHLIYQLGGHQEVIRHVIIKGSVVHDNDKTLG
F
>g1355.t1
MARGCICCVKYMLFLFNLLFWLGGCGLLGVGVWLSVSQGSFATLSPSFPSLSAANLVITLGTVVMVTGFLGCLGAIKENK
CLLLSEADICLQDEGCRVCV
WLMKLRPHSSLIQFFIVLLVILLAELILLILFFVYTDKVSENARRDLKEGLVLYTTENNAGLRNAWNTIQTEWRCCGVDA
HTDWHAALQDKVVPDRCCQD
IQPGCGRNVSSTFWTRGCYDKVEEWLDDNKHLIGTIAMCVLVIQLLGMAFSMTLYQQIHRAGKKYEA
>g1355.t2
MARGCICCVKYMLFLFNLLFWLGGCGLLGVGVWLSVSQGSFATLSPSFPSLSAANLVITLGTVVMVTGFLGCLGAIKENK
CLLLSFFIVLLVILLAELIL
LILFFVYTDKVSENARRDLKEGLVLYTTENNAGLRNAWNTIQTEWRCCGVDAHTDWHAALQDKVVPDRCCQDIQPGCGRN
VSSTFWTRGCYDKVEEWLDD
NKHLIGTIAMCVLVIQLLGMAFSMTLYQQIHRAGKKYEA
>g1356.t1
VAALLVSLTCYFWKKNKRLEYKYSRLVMSANREGELPAADSCALAEGEEPEDDVVYAQKPTLLGKLRAIANKVVTDT
>g1357.t1
MVAKMSGHDQLVLCLCEFTRLLWKLLLPLLLFTVFLVAVLILLVAGLRLWLQSNRIARRARDGRPTVAFFHPYCHAGGGG
ERVLWCAIRALQNRYPEVNV
VVYTGDQEV
>g1358.t1
LWDPAAPELIREEADVHLFVPGNSSYAVLELLETHAVAHKVLLTNPKELIEMQTRNDTTDPRSGSSYYDKYHSLEDIYYW
LNKTAMDYPDRIKVILIGSS
FEKRPLYVIKQSRDITQILDTMDVYVLPVMNPDGYKYTWTTDRMWRKNRSVREDSSCTGVDLNRNFDANWCTEGASHNPC
SEIYCGNFPESEPETGAVAA
FLRRQKETVKLYVSIHSYSQMLLFPYSCTTALAENHNTLVGPVIRVVASACDGRASSVGRM
>g1358.t2
MQTRNDTTDPRSGSSYYDKYHSLEDIYYWLNKTAMDYPDRIKVILIGSSFEKRPLYVIKQSRDITQILDTMDVYVLPVMN
PDGYKYTWTTDRMWRKNRSV
REDSSCTGVDLNRNFDANWCTEGASHNPCSEIYCGNFPESEPETGAVAAFLRRQKETVKLYVSIHSYSQMLLFPYSCTTA
LAENHNTLVGPVIRVVASAC
DGRASSVGRM
>g1358.t3

LWDPAAPELIREEADVHLFVPGNSSYAVLELLETHAVAHKVLLTNPKELIEMQTRNDTTDPRSGSSYYDKYHSLEDIYYW
LNKTAMDYPDRIKVILIGSS
FEKRPLYVIKILDTMDVYVLPVMNPDGYKYTWTTDRMWRKNRSVREDSSCTGVDLNRNFDANWCTEGASHNPCSEIYCGN
FPESEPETGAVAAFLRRQKE
TVKLYVSIHSYSQMLLFPYSCTTALAENHNTLVGPVIRVVASACDGRASSVGRM
>g1359.t1
MAQEAAQKIRRYYKNTYKYGAGAKTIYLSPGGSDDWAYDLGIKYSFTFELQDRGQYGFLLPPSHIPKACNEALIALKTIA
LRVIEQLQPPV
>g1360.t1
MSKIRRKVTVENSKTISDSSSSTATSTASSSTTTSANPAAPSRRPSVFERLGPSTGSNAVDSQCRNWLKTGNCSYGTTCR
YTHGPQPRGKGFSFSRSAER
PPGDLRERMKNKRQDVDSEKRDLEEPASPTVKRDSSRGRHREKEDIKITKERTPASEEETAEWEASREDSDVGDYDYELS
LEMKRQKIQRELLKLEQENQ
EKREEIVIKKKKEEVAASTTSTPTTTKTRSSSTTTTTTK
>g1360.t2
MSKIRRKVTVENSKTISDSSSSTATSTASSSTTTSANPAAPSRRPSVFERLGPSTGSNAVDSQCRNWLKTGNCSYGTTCR
SAERPPGDLRERMKNKRQDV
DSEKRDLEEPASPTVKRDSSRGRHREKEDIKITKERTPASEEETAEWEASREDSDVGDYDYELSLEMKRQKIQRELLKLE
QENQEKREEIVIKKKKEEVA
ASTTSTPTTTKTRSSSTTTTTTK
>g1360.t3
MSKIRRKVTVENSKTISDSSSSTATSTASSSTTTSANPAAPSRRPSVFERLGPSTGSNAVDSQCRNWLKTGNCSYGTTCR
YTHGPQPRGKGFSFSRSAER
PPGDLRERMKNKRQDVDSEKRDLEEPASPTVKRDSSRGRHREKEDIKITKERTPASEEETAEWEASREDSDVGDYDYELS
LEMKRQKIQRELLKLEQENQ
EKREEIVIKKKKEEVAASTTSTPTTTKTRSSSTTTTTTKFGNILER
>g1361.t1
LTDFKKFIRKVNYLASQISPSRGQPLYELLKYKVGVVVGKPTAGRIPQTKGKRAALATAAVLASYDAGKSTEVSAKAGGY
GAWRSEDLEEPEQCRDKYGR
RVGTGDDYSRRRYSLLKANMGISLPMRMRGWPKYADKTSSPITDNQDRIHNGHQELAKCRERANQSVGWPKIYSGDHS
>g1362.t1
RDNVVRSFRSGDIWVLICTALLARGIDFKGVNLVLNYDFPTSAVEYIHRIGRTGRAGHTGKAVTFFTENDKPLLRSIANV
IKQAGCPVPDYMMGLKKVHG
YESSFSSSSSSSVFHIQRHAVVKQRRVLVPLYQQVLELALLQQENEAVAQTETTPESHDIHNTTLHDAQTCQRTKEAAAA
AAAAAAEEEIPAGSRKARVC
SPKKKGKKNKGGGKA
>g1362.t2
RDNVVRSFRSGDIWVLICTALLARGIDFKGVNLVLNYDFPTSAVEYIHRIGRTGRAGHTGKAVTFFTENDKPLLRSIANV
IKQAGCPVPDYMMGLKKVHG
YESSFSSSSSSSVFHIQRHAVVKQRRVLVPLYQQVLELALLQQENEAVAQTETTPESHDIHNTTLHDAQTCQRTKEAAAA
AAAAAAEEEIPAGSRKARV
>g1362.t3
RDNVVRSFRSGDIWVLICTALLARGIDFKGVNLVLNYDFPTSAVEYIHRIGRTGRAGHTGKAVTFFTENDKPLLRSIANV
IKQAGCPVPDYMMGLKKVHG
YESSFSSSSSSSVFHIQRHAVVKQRRVLVPLYQQVLELALLQQENEAVAQTETTPESHDIHNTTLHDAQTCQRTKEAAAA
AAAAAAEEEIPAGSRKARVW
>g1363.t1
MGVGTQSLSAGKPNPAVLPYHVLSSCLLMVSSCPLIISSCPLIVSTCSLILSPHPLILSPHGLILSPHGLTLSPHRLNLF
PHPVPSWSHPVPSWSHPLIL
SPHRLILSSCPLNGSSCPSSSHPVPSSSHPAWCW
>g1364.t1
MCFTSQFPSKTEVFITAEGLELRLQLEKN
>g1365.t1
MAKSNFEYVRQFETDDTCLPNCYIVVRLDGRNFHKFSEKHNFAKPNDIRALGLMTRSAQSVMEDLDDVIIAYGQSDEFSF
VFKRTSTWFKRRASKFMTHV
ASQFSSSYVFYWKEYFAEQPLLYPPGFDGRVVLYPSNRNLRDYLSWRQADCHVNNLYNTVFWTLVQKGGLTTVQAEQRLK

GTLAADKNEILFSEFTINYN
NEPLMLRKGTALIWEKRDEPVTKQVKLPNEEVAQEMTVTRSRKRVCPHYCDIIRDQFWEEHPDILESD
>g1365.t2
LSHSTTAMAKSNFEYVRQFETDDTCLPNCYIVVRLDGRNFHKFSEKHNFAKPNDIRALGLMTRSAQSVMEDLDDVIIAYG
QSDEFSFVFKRTSTWFKRRA
SKFMTHVASQFSSSYVFYWKEYFAEQPLLYPPGFDGRVVLYPSNRNLRDYLSWRQADCHVNNLYNTVFWTLVQKGGLTTV
QAEQRLKGTLAADKNEILFS
EFTINYNNEPLMLRKGTALIWEKRDEPVTKQVKLPNEEVAQEMTVTRSRKRVCPHYCDIIRDQFWEEHPDILESD
>g1365.t3
MAKSNFEYVRQFETDDTCLPNCYIVVRLDGRNFHKFSEKHNFAKPNDIRALGLMTRSAQSVMEDLDDVIIAYGQSDEFSF
VFKRTSTWFKRRASKFMTHV
ASQFSSSYVFYWKEYFAEQPLLYPPGFDGRVVLYPSNRNLRDYLSWRQADSIANGTTLPQPVTLSRITHGNTVQGTLAAD
KNEILFSEFTINYNNEPLML
RKGTALIWEKRDEPVTKQVKLPNEEVAQEMTVTRSRKRVCPHYCDIIRDQFWEEHPDILESD
>g1366.t1
MEERERRKPNTTEHDDRGAASASNPSTQPARKDESSPLHGDTEEDGDSVKLDICSDTFDPLLALYSPTVPLPFPNIKCFN
NVSEYESFLKGGRGRAKPEN
VEKRLRKAMKGVVDPERIERLKKLMVCNVPEEGEASRPPRRRQKKTHKNVVTRMQRRFCDTPYVFILMSHSCVARVNKGS
PLGELYRCVEERIRVKVHIR
TFKGLRGVCSGFVVAFDKFWNLLFEKLRLQHSSAETSTTGKGQGKKAQPPRPDPGRADRRQLPGSAGQPSEAERGAKEAQ
AGGSEGGGARGKTAHPYGKV
HTRHLNQLFIRGENVLLINPQPL
>g1366.t2
LHGDTEEDGDSVKLDICSDTFDPLLALYSPTVPLPFPNIKCFNNVSEYESFLKGGRGRAKPENVEKRLRKAMKGVVDPER
IERLKKLMVCNVPEEGEASR
PPRRRQKKTHKNVVTRMQLNKGSPLGELYRCVEERIRVKVHIRTFKGLRGVCSGFVVAFDKFWNLLFEKLRLQHSSAETS
TTGKGQGKKAQPPRPDPGRA
DRRQLPGSAGQPSEAERGAKEAQAGGSEGGGARGKTAHPYGKVHTRHLNQLFIRGENVLLINPQPL
>g1366.t3
MEERERRKPNTTEHDDRGAASASNPSTQPARKDESSPLHGDTEEDGDSVKLDICSDTFDPLLALYSPTVPLPFPNIKCFN
NVSEYESFLKGGRGRAKPEN
VEKRLRKAMKGVVDPERIERLKKLMVCNVPEEGEASRPPRRRQKKTHKNVVTRMQLNKGSPLGELYRCVEERIRVKVHIR
TFKGLRGVCSGFVVAFDKFW
NLLFEKLRLQHSSAETSTTGKGQGKKAQPPRPDPGRADRRQLPGSAGQPSEAERGAKEAQAGGSEGGGARGKTAHPYGKV
HTRHLNQLFIRGENVLLINP
QPL
>g1367.t1
LFLFIVKEFLAKAKEDFLRKWECPPQSTTCLDDFDRLKTLGTGSFGRVMLVKHKGTDQFYAMKILDKQKVVKLKQIEHTL
NEKRILQAVSFPFLVRLEYS
FKDNSNLYMVMEYVPGGEMFSHLRRIGRFSENHARFYAAQIVLTFEYLHSLDLIYRDLKPENLLIDQHGYIQVTDFGFAK
RVKGRTWTLCGTPEYLAPEI
ILSKGYNKAVDWWALGVLIYEMAAGYPPFFADQPIQIYEKIVSGKVRFPSHFSSDLKDLLRNLLQVDLTKRFGNLKNGVN
DIKNHKWFSTTDWIAIYERK
VEAPFMPKCRGPGDTSNFDDYEEEDVRVSETEKCAKEFAEF
>g1367.t2
LDELANRLFHKYRKGSQESDGPQVSEFTVIWDKLMKEFLAKAKEDFLRKWECPPQSTTCLDDFDRLKTLGTGSFGRVMLV
KHKGTDQFYAMKILDKQKVV
KLKQIEHTLNEKRILQAVSFPFLVRLEYSFKDNSNLYMVMEYVPGGEMFSHLRRIGRFSENHARFYAAQIVLTFEYLHSL
DLIYRDLKPENLLIDQHGYI
QVTDFGFAKRVKGRTWTLCGTPEYLAPEIILSKGYNKAVDWWALGVLIYEMAAGYPPFFADQPIQIYEKIVSGKVRFPSH
FSSDLKDLLRNLLQVDLTKR
FGNLKNGVNDIKNHKWFSTTDWIAIYERKVEAPFMPKCRGPGDTSNFDDYEEEDVRVSETEKCAKEFAEF
>g1367.t3
LDDFDRLKTLGTGSFGRVMLVKHKGTDQFYAMKILDKQKVVKLKQIEHTLNEKRILQAVSFPFLVRLEYSFKDNSNLYMV
MEYVPGGEMFSHLRRIGRFS
ENHARFYAAQIVLTFEYLHSLDLIYRDLKPENLLIDQHGYIQVTDFGFAKRVKGRTWTLCGTPEYLAPEIILSKGYNKAV

DWWALGVLIYEMAAGYPPFF
ADQPIQIYEKIVSGKVRFPSHFSSDLKDLLRNLLQVDLTKRFGNLKNGVNDIKNHKWFSTTDWIAIYERKVEAPFMPKCR
GPGDTSNFDDYEEEDVRVSE
TEKCAKEFAEF
>g1368.t1
MVQMMESQCEYFMYFPAVPISDLSDPARYRTLPRRSHLYLGETVRFLLVLRCKDVSTGGTPTDVLG
>g1369.t1
TRQCHLLLDVFNSTEHEVTVSAKNNQDLVLHASECQR
>g1370.t1
MGCFYSKKSKRKSKEGQTPTAGESTAASNNVPLGDGSDEAPKQYSWDTREKVDPKDYMLNGLKDSTVGRLPGTLNGQQLV
IQDCENCNIFVFDHSATITV
DDCVNCRVFLGPVKGSVFFRDCKDVRCVVACQQFRTRDCKKMEVYLCCATQPIIESSTGMRFACYQYYYPELAFHFKDAG
LSIFNNNWSNVHDFTPVSGE
INWTLLAEGAAVLDAVPAPDPESEFKAVRVSADASRSIVPLTRGGRRKDSEESCLFVFFAGDYTTANARKLIDEVGGVAW
HGGAGGRGGVVVWCGVVWIG
FAGNL
>g1371.t1
MLPPDGRKMTEKGFVLIQTKEVPMRPEDVKRVFQNDTEDLLEWSTKGPVTALELNGDGVVDACKTMANEVFKGSKVFVSE
SKGTSSRDVDNFFNFADMQM
GL
>g1371.t2
MLTLMLPPDGRKMTEKGFVLIQTKEVPMRPEDVKRVFQNDTEDLLEWSTKGPVTALELNGDGVVDACKTMANEVFKGSKV
FVSESKGTSSRDVDNFFNFA
DMQMGL
>g1371.t3
MTEKGFVLIQTKEVPMRPEDVKRVFQNDTEDLLEWSTKGPVTALELNGDGVVDACKTMANEVFKGSKVFVSESKGTSSRD
VDNFFNFADMQMGL
>g1372.t1
MLVCSECQENCPGWLMQATGPCCLSGRYRMKRIRTSSSSDSSDNESKSTWSRRKRPHPGPEWALNPTWRAGH
>g1373.t1
MLADVWTKFRLFQKPANFDTRLADCERTLAGVKGQLGVLDVRSVELDVVQSQLEQCMGRRDARKACPVNAVRPSVPLSVC
PSGAEQKLYKVLSEVKGEVE
TVIKTGRQVVQRQQTEQPKELDDRLTALKLLYNQLGAQ
>g1373.t2
MLADVWTKFRLFQKPANFDTRLADCERTLAGVKGQLGVLDVRSVELDVVQSQLEQCMQKLYKVLSEVKGEVETVIKTGRQ
VVQRQQTEQPKELDDRLTAL
KLLYNQLGAQ
>g1374.t1
VTSDSHYERVFLLDPQYQNPAALRNKLPELSQSAETSGCGGGYAEPDITQCTPHQCFHSNAPHYAETDVVSLQGVTGSNM
YAVPALTVDSLTHQDISAAE
FPRQRLVFREKLGEGQFGEVHLCEGEGLPGGAVLVAVKQLRADATSQARNDFLKEIKIMSRLSDPNIIQLLCVCVASDPL
CMVTEYMENGDLNMFLSQRE
IESTLTHANNIPSVSLSDLLHMSVQISSGMKYLASLNFVHRDLATRNCLLDRRLTIKIADFGMSRNLYSSDYYRIQGRAV
LPIRWMAWESILLGKFTTAS
DVWAFGVTLWEVFTLCKEQPYSLLSDQQVIQNTGEFFRNQGRQVLLYAPPLCPPSLFKLMLCCWGRDAAQRPSFHGLYQA
LRPHVNQ
>g1374.t2
CVAVTSDSHYERVFLLDPQYQNPAALRNKLPELSQSAETSGCGGGYAEPDITQCTPHQCFHSNAPHYAETDVVSLQGVTG
SNMYAVPALTVDSLTHQDIS
AAEFPRQRLVFREKLGEGQFGEVHLCEGEGLPGGAVLVAVKQLRADATSQARNDFLKEIKIMSRLSDPNIIQLLCVCVAS
DPLCMVTEYMENGDLNMFLS
QREIESTLTHANNIPSVSLSDLLHMSVQISSGMKYLASLNFVHRDLATRNCLLDRRLTIKIADFGMSRNLYSSDYYRIQG
RAVLPIRWMAWESILLGKFT
TASDVWAFGVTLWEVFTLCKEQPYSLLSDQQVIQNTGEFFRNQGRQVLLYAPPLCPPSLFKLMLCCWGRDAAQRPSFHGL
YQALRPHVNQ
>g1374.t3

VTSDSHYERVFLLDPQYQNPAALRNKLPELSQSAETSGCGGGYAEPDITQCTPHQCFHSNAPHYAETDVVSLQGVTGSNM
YAVPALTVDSLTHQDISAAE
FPRQRLVFREKLGEGQFGEVHLCEGEGLPGGAVLVAVKQLRADATSQARNDFLKEIKIMSRLSDPNIIQLLCVCVASDPL
CMVTEYMENGDLNMFLSQRE
IESTLTHANNIPSVSLSDLLHMSVQISSGMKYLASLNFVHRDLATRNCLLDRRLTIKIADFGMSRNLYSSDYYRIQGRAV
LPIRWMAWESILLVGTLGKF
TTASDVWAFGVTLWEVFTLCKEQPYSLLSDQQVIQNTGEFFRNQGRQVLLYAPPLCPPSLFKLMLCCWGRDAAQRPSFHG
LYQALRPHVNQ
>g1375.t1
LRRCLQPTEVAQVLQLIQDGTSMRTLARRFAVSPSTVSRAWRRYQETGQNTRRHGGGRRRTTAQQQDRYLVLCARRNRRS
TARALQNDHQQATNVHVSGL
GVWGGIPLEGCTDLHVLARSTVTAIRYRDEILRPIVRPYAGAVGPEFLLMHDNARPHVAGVLGSPDLNPTEHIWDIMSRS
IHRCHVAPQTVQELTDALIQ
AWEEIPQENIRRLIRSMPRRCRECIQARRGHTHY
>g1376.t1
LGGRVRRVTAFRTCLATLKGFSESEIKNCLNIELKMSLGFLPANMSFSNKCNDLLKGNVSMGFYQGFMTHKIEVIGGERY
FPDILYDQDPSESYHSWMNS
LHDNPDVVSYAIFPLHQLVEDPEVRENLKSLVTDYIKENRLQEEHMGSKNCSPSPNLDHHCCPLRAGRGTLKLEIHRAAG
LRADTFTKTDAYVKIFYNEV
YEETGTVMDDNDPVWNATFNFGSVEIGNLMRLEVWDRDVIYNDVAGRCAVFPERGTHSLSCQLKRGVLYFTYTSTCDPHL
TGYRCGRYSPSAE
>g1377.t1
LQRIPNVFPHFPNELSIPVENFEDHSAPPSPDEKDSGFFMLRKDSERRATLHHILTEDRDTVVGNLMEALSQ
>g1378.t1
MSGFDSNPFVESVDVNPFQVSRTVHPLDARNGLHVVDAFSRGFHIVPWRLRELPAWPXXXXXXXELEKKAAELQRKEQDF
QNLATGRGLPMGERPNNWPP
LPRICPIKPCFHQDFEEDIPEQYRKICKRVYYLWIFHCATLFLNVWACLAYFIADTQYGVDFGLSILWFVLFSPVSFVCW
YRPVYKAFRSDSSFSFFFFF
FVFFFQVAVYIIQTVGIPKWGNSGWISSISMIRTNIGVAVVMMVVAGFFTVNAILSVILLKMVHSKYRNTGASFSKAQVE
FSQEVMANRSFQEATASAAT
SAAQGMFRRN
>g1378.t2
LEKKAAELQRKEQDFQNLATGRGLPMGERPNNWPPLPRICPIKPCFHQDFEEDIPEQYRKICKRVYYLWIFHCATLFLNV
WACLAYFIADTQYGVDFGLS
ILWFVLFSPVSFVCWYRPVYKAFRSDSSFSFFFFFFVFFFQVAVYIIQTVGIPKWGNSGWISSISMIRTNIGVAVVMMVV
AGFFTVNAILSVILLKMVHS
KYRNTGASFSKAQVEFSQEVMANRSFQEATASAATSAAQGMFRRN
>g1378.t3
MSGFDSNPFVESVDVNPFQVSRTVHPLDARNGLHVVDAFSRGFHIVPWRLRELPAWPXXXXXXXELEKKAAELQRKEQDF
QNLATGRGLPMGERPNNWPP
LPRICPIKPCFHQDFEEDIPEQYRKICKRVYYLWIFHCATLFLNVWACLAYFIADTQYGVDFGLSILWFVLFSPVSFVCW
YRPVYKAFRSQSVLVEFLQS
GRIEFSLQVRPIEPRQSSVPEVVSDRLRSGSTAVFSSASRAVPGVRPDQGFSPGTVGPNCVMAASDSSFSFFFFFFVFFF
QVAVYIIQTVGIPKWGNSGW
ISSISMIRTNIGVAVVMMVVAGFFTVNAILSVILLKMVHSKYRNTGASFSKAQVEFSQEVMANRSFQEATASAATSAAQG
MFRRN
>g1379.t1
LKRRQVKPLAASLLDALDYDSSDDSDFEVGDASGSDGTGNGSDEEGSKGSVAGSDSDSDNANASANDDAGAEEEEAAKEA
AASDCVIVDEEKGRPQPSSD
GSGK
>g1380.t1
MLFSSCLRSLSWRWLPPSSGSKVTSSQRLGAARTLL
>g1381.t1
RGEPPTSKVSDHQMF
>g1382.t1
MRQAGHALLLCPILTWPDPPGCRCSVGPACRDPCVTCHPTSCLPPYFLSATLGTSVCCSISQPSVQCGVCQISRLSLPTA

AAAVRGRGRRSEEPELPSSN
LDQTVTSPITSVAGRGAHVTHLPAVAA
>g1383.t1
GPGFLLVHDNARPHVARVCRQFLEDEGIDTIDWPPRSPDLNPIEHLWDIMFRSIRRRQVVPQTVQELSDALVQIWEEIPQ
ETIRRLIRSMPQRCQASIQA
HGGNTKY
>g1384.t1
MAAVDSFSLLYREMARSCNTYGETLALVGALYMACKTCMVLRSCCSLVRIHFLPRLMPTRKTLAQRFGGWAVIHGATDDT
ARAYAEELARQGMNIVFVNH
GSTVNTAALSQHYGVEVTLVEADLALGDVAIKSLKDALWDKDIGILVNCVSGSLASPSHLTEMSERGLLELINRSVVSTT
LMTRLVLPRMVQRRRGAVVH
LCSGAGGRSSAGRAALVASEVSSSGRVASSVFVMRGWLAPKAEVYARHAISTLGVAHRTTGYWPHTLQVGSAERTPGI
>g1384.t2
MARSCNTYGETLALVGALYMACKTCMVLRSCCSLVRIHFLPRLMPTRKTLAQRFGGWAVIHGATDDTARAYAEELARQGM
NIVFVNHGSTVNTAALSQHY
GVEVTLVEADLALGDVAIKSLKDALWDKDIGILVNCVSGSLASPSHLTEMSERGLLELINRSVVSTTLMTRLVLPRMVQR
RRGAVVHLCSGAGGRSSAGR
AALVASEVSSSGRVASSVFVMRGWLAPKAEVYARHAISTLGVAHRTTGYWPHTLQVGSAERTPGI
>g1384.t3
MVFLILLLLSGGSAPYRLSIFPLAGDSLAVQLAPAMGEWDLLSRLLDQVQTHSTVIGKVWLTVLFVFRILVLSTATEK
>g1384.t4
MAAVDSFSLLYREMARSCNTYGETLALVGALYMACKTCMVLRSCCSLVRIHFLPRLMPTRKTLAQRFGGWAVIHGATDDT
ARAYAEELARQGMNIVFVNH
GSTVNTAALSQHYGVEVTLVEADLALGDVAIKSLKDALWDKDIGILVNCVSGSLASPSHLTEMSERGLLELINRSVVSTT
LMTRLVLPRMVQRRRGAVVH
LCSGAGGRSSAGRAALVASEVSSSGRVASSVFVMRGWLAPKAEVYARHAISTLGVAHRTTGYWPHTLQARGLQMVFLILL
LLSGGSAPYRLSIFPLAGDS
LAVQLAPAMGEWDLLSRLLDQVQTHSTVIGKVWLTVLFVFRILVLSTATEK
>g1384.t5
MVFLILLLLSGGSAPYRLSIFPLAGDSLAVQLAPAMGEWDLLSRLLDQVQTHSTVIGKVWLTVLFVFRILVLSTATEKVC
TYMH
>g1385.t1
MQRGLKGTRPPDQHQTMSHFSSEDEAELQYLLRQLLKSTKDRISGAPSIECAEEILLHLEETDKKFHNYEFVRYLRQHVD
RSLGAVVEAETERGAGGDTG
GSTHDIHAVTRRTRDSAEYKQMMQTLKNTMSMVVESLINKFEEDQLRKEEIDRENLHGQSESHFNDNCSDSDSSLNNGIH
LACQSLNGRET
>g1385.t2
MLLTTAPSGTRPPDQHQTMSHFSSEDEAELQYLLRQLLKSTKDRISGAPSIECAEEILLHLEETDKKFHNYEFVRYLRQH
VDRSLGAVVEAETERGAGGD
TGGSTHDIHAVTRRTRDSAEYKQMMQTLKNTMSMVVESLINKFEEDQLRKEEIDRENLHGQSESHFNDNCSDSDSSLNNG
IHLACQSLNGRET
>g1385.t3
MLQHVTNYGPVRPPDQHQTMSHFSSEDEAELQYLLRQLLKSTKDRISGAPSIECAEEILLHLEETDKKFHNYEFVRYLRQ
HVDRSLGAVVEAETERGAGG
DTGGSTHDIHAVTRRTRDSAEYKQMMQTLKNTMSMVVESLINKFEEDQLRKEEIDRENLHGQSESHFNDNCSDSDSSLNN
GIHLACQSLNGRET
>g1386.t1
LPGDIIKQAKDIKPVTEIQQSGNDFTITSKTPGKSVTNSFTIGKEANITTMDGKSLKCTVTMEGGKMVCDTGKFRHVQEL
KGGEMIETLTMASTTLVRKS
KKM
>g1386.t2
MARYLRPPNTSLFVRNISDESSTFEDVRDAEDALHNLDRKWVCGRQIEIQFAQGDRKTPNQMKTKESSPRSYSRYDD
>g1386.t3
LPGDIIKQAKDIKPVTEIQQSGNDFTITSKTPGKSVTNSFTIGKEANITTMDGKSLKCTVTMEGGKMVCDTGKFRHVQEL
KGGEMIEAYTCDVSTFEDVR
DAEDALHNLDRKWVCGRQIEIQFAQGDRKTPNQMKTKESSPRSYSRYDD

>g1386.t4
LPGDIIKQAKDIKPVTEIQQSGNDFTITSKTPGKSVTNSFTIGKEANITTMDGKSLKCTVTMEGGKMVCDTGKFRHVQEL
KGGEMIETLTMASTTLVRKR
RLKGYARDVVPDCDLMLCLSGSTAAQPVCPLCSVPLLYPSALSLCSIPLLYPSALSLCSIPLLCPLWSPEIGDR
>g1387.t1
YRTVVVKRELSRKAKLSIYQSIYVPTLTYGHELWVVTERTRLWIQAAEMSFLRRVAGLSLRDRVSSDIRRELGVEPLLLH
VERSQLRWFRHLIRMPPGRL
PLEVFRARPTGKRARGRPRTRWRDYISHLAWERLGVPQEELESVAGEKDVWSSLLSLLPPRAGPG
>g1388.t1
LLYVVLVSVLSLIARPLCTAKKVLCTHLLCVKKHKEESGCSGLRDKTAFVALSKFDEMNLLNDYRFLEDTTRLAGSSSRD
NLIRTPRITFKAKKLTACAR
KMNITLRFLPVSFTKSRHNSTMFLTK
>g1388.t2
MMDHRNLFLFVSHSLLCVKKHKEESGCSGLRDKTAFVALSKFDEMNLLNDYRFLEDTTRLAGSSSRDNLIRTPRITFKAK
KLTACARKMNITLRFLPVSF
TKSRHNSTMFLTK
>g1388.t3
MDHRNLFLFVSHSLLCVKKHKEESGCSGLRDKTAFVALSKFDEMNLLNDYRFLEDTTRLAGSSSRDNLIRTPRITFKAKK
LTACARKMNITLRFLPVSFT
KSRHNSTMFLTK
>g1389.t1
MKNGEFQKELWLRIRRGKDSYRRKLEKRLEDNNARGVWRGLQNISGHGKGVERNQANGDKDWADELNLFFNRFDSTPSPS
LPLPAP
>g1390.t1
MEVFIVLKDRELCTPPWLCLSERPPAGPGWAGVELALVVVVPCCLLGVLVLLGVLSLHTQRCSYRRANKQDPEEPLEDLV
LMSPDKCLKELIYDMSTSGS
GSGLPLLVQRTIARTILLQECVGKGRFGEVWRGKWRGEDVAVKIFSSREERSWFREAEIYQTVMLRHENILGFIAADNKD
NGSWTQLWLVSEYHEHGSLF
DYLNRYTVSVEAMVLLASSLASGLAHLHMEIIGTQGKPAIAHRDIKSKNVLVKKSGTAAIADLGLAVKHDSNASTLDIPA
NHRVGTKRYMSPELLDDSIN
MSSFESFKRADIYSLGLVFWELARRCSAQGLNEDFQLPYYDLVPSDPSVEDMRRVVCDQKLRPSIPNQWQSCEALRVMGK
LMRECCYGNAAARLTALRVK
KTISQ
>g1390.t2
MEVFIVLKDRELCTPPWLCLSERPPAGPGWAGVELALVVVVPCCLLGVLVLLGVLSLHTQRCSYRRANKQDPEEPLEDLV
LMSPDKCLKELIYDMSTSGS
GSGLPLLVQRTIARTILLQECVGKGRFGEVWRGKWRGEDVAVKIFSSREERSWFREAEIYQTVMLRHENILGFIAADNKD
NGSWTQLWLVSEYHEHGSLF
DYLNRYTVSVEAMVLLASSLASGLAHLHMEIIGTQGKPAIAHRDIKSKNVLVKKSGTAAIADLGLAVKHDSNASTLDIPA
NHRVGTKRYMSPELLDDSIN
MSSFESFKRADIYSLGLVFWELARRCSAQGLNEDFQLPYYDLVPSDPSVEDMRRVVCDQKLRPSIPNQWQSCEALRVMGK
LMRECCYGNAAARLTALRVK
KTISQVTVFKDV
>g1391.t1
MSGHETIRVLEVEVDSSTASSVSDDKVDSRVEGRDDQPPKQPDVSGIQDTPTAPQIAGGADVKEQQAITVDATGPALQTL
ASTIPISAALHQTQAALPIN
VQACPQESLTTLMTGMMAQAGSLGQPLLIPLSMAGSLGGQGGLAVLTLPTTNVATLPGLAAATTAGNLLKLPLAGLQAAT
VLNSLQPQLQTTAATLFQPQ
AAALQQVTSPPTQVTSTQAAAGQPTAAQMTSAVATAAAATTTTAITQPSISVAALQTAGLSLNPAIFSAASLGAQPQFLS
SLTSTPIFTTAMSNMAGLTS
QVITNAQGQEHNDHVVTSGEGEIGGDRERGGGVCGGGRAGEDGAGGRQAEIRKGEAEVIGTLPLLLNPASLAGGAATHTL
PLQGLQLQTVTPQLLFNAQG
QIVATLGGG
>g1391.t2
MDAQDIPAKDAPLTVNEQVIVMSGHETIRVLEVEVDSSTASSVSDDKVDSRVEGRDDQPPKQPDVSGIQDTPTAPQIAGG
ADVKEQQAITVDATGPALQT

LASTIPISAALHQTQAALPINVQACPQESLTTLMTGMMAQAGSLGQPLLIPLSMAGSLGGQGGLAVLTLPTTNVATLPGL
AAATTAGNLLKLPLAGLQAA
TVLNSLQPQLQTTAATLFQPQAAALQQVTSPPTQVTSTQAAAGQPTAAQMTSAVATAAAATTTTAITQPSISVAALQTAG
LSLNPAIFSAASLGAQPQFL
SSLTSTPIFTTAMSNMAGLTSQVITNAQGQEHNDHVVTSGEGEIGGDRERGGGVCGGGRAGEDGAGGRQAEIRKGEAEVI
GTLPLLLNPASLAGGAATHT
LPLQGLQLQTVTPQLLFNAQGQIVATLGGG
>g1391.t3
MSGHETIRVLEVEVDSSTASSVSDDKVDSRVEGRDDQPPKQPDVSGIQDTPTAPQIAGGADVKEQQAITVDATGPALQTL
ASTIPISAALHQTQAALPIN
VQACPQESLTTLMTGMMAQAGSLGQPLLIPLSMAGSLGGQGGLAVLTLPTTNVATLPGLAAATTAGNLLKLPLAGLQAAT
VLNSLQPQLQTTAATLFQPQ
AAALQQVTSPPTQVTSTQAAAGQPTAAQMTSAVATAAAATTTTAITQPSISVAALQTAGLSLNPAIFSAASLGAQPQFLS
SLTSTPIFTTAMSNMAGLTS
QVITNAQGQEHNDHVVTSGEGEIGGDRERGGGVCGGGRAGEDGAGGRQAEIRKGEAEVRSRGQEAGEFAGETCMW
>g1392.t1
MSSPYPGAQVFMPMQQHIPVGAVGQNVPMAYYPMGAMYPPGSTVMVDGGYDAGARFGATSSVSIPPPPPGHPPNAAQLAA
MQGANVVMAQRKNNFFLGGS
SGGCSVTP
>g1393.t1
MESRDDVFDSWLQRIHDQQSDGTRIHKDLRLYLAAARDMRSASRRLSQSLFDAYETDWAGDDDLGAIVEAEDLLWNDYEV
KLSDQALRTMESYMGQFPDI
RAEEEHNAAKYVYEAINTELKEDFPAFHESRIGCYASVFSAVSSLRDIFYKEMSVLNNDLQNVMKDLQAQHPDNVFAVKG
LQRYGSLKRRSLMSPKSWKS
SFSDFHQTYSPKMGTQRFSFRSPDRLRYGGSLTREGSVGRAAFAKSPPSETPSSESRDEEAHSDADDSPAEAQQPGSGDH
SGATGATEEERKAEESQNGG
GGEDEPAKEDGAEAGPAPQARQEDKGEGEAKALSESSSELNNSFDSVSLDLQLSAVANGTLYIEEKEDSPQSNGLAADSL
GSKPGSQKVSRQGQESPFIW
MVFAAVLFPVVITLLLVTLAVVGFLLYRYLCHNKGAYRTTGEPAPGEDFAEDSKPNQATNDKKEYFI
>g1394.t1
MNMGSTLVKCAVTDLGIQWVGWALAAAFKTEKFYDLAGTFIALAHLSHLWGGAGHVRQNVQTGLVTAWGIRLGTFLFMRI
LKDGQDRRFNGVRDSPGTFF
VYWSIQAVWVFMTLLPTLMLNSERRDVPLGVRDYIGWGIWGLGFVTQAVADQQKWYFKRDPHNAGKFIQHGLWSFSRHPN
YLGEILQWSGLWLAASSVMR
GQQYLSAVSPLFVWLLLRHVSGVPILEKQAMKRWGSDPAFQNYVKNTPLLWPVPRF
>g1394.t2
MGSTLVKCAVTDLGIQWVGWALAAAFKTEKFYDLAGTFIALAHLSHLWGGAGHVRQNVQTGLVTAWGIRLGTFLFMRILK
DGQDRRFNGVRDSPGTFFVY
WSIQAVWVFMTLLPTLMLNSERRDVPLGVRDYIGWGIWGLGFVTQAVADQQKWYFKRDPHNAGKFIQHGLWSFSRHPNYL
GEILQWSGLWLAASSVMRGQ
QYLSAVSPLFVWLLLRHVSGVPILEKQAMKRWGSDPAFQNYVKNTPLLWPVPRF
>g1394.t3
MNMGSTLVKCAVTDLGIQWVGWALAAAFKTEKFYDLAGTFIALAHLSHLWGGAGHVRQNVQTGLVTAWGIRLGTFLFMRI
LKDGQDRRFNAVWVFMTLLP
TLMLNSERRDVPLGVRDYIGWGIWGLGFVTQAVADQQKWYFKRDPHNAGKFIQHGLWSFSRHPNYLGEILQWSGLWLAAS
SVMRGQQYLSAVSPLFVWLL
LRHVSGVPILEKQAMKRWGSDPAFQNYVKNTPLLWPVPRF
>g1395.t1
MTPLGPHGAKEGVCSPLGLTGVLLQLQENESLVAKTTLTVPAGGGPVEAISTIETVPAPYWTRSKRITETLEWDSESVQS
VDIFKQPGVPDRVTRAAPST
PQSTGSVRLHEFVSKTVRKPCLKVIKPESCVPCGKRIKFGKLSLKCRDCRVVSHPECRERCPLPCTPNVAGTPVKIGEGT
LADYVSATSPMIPPLVVHCV
NEIEQRGLSEMDRGKTNSEHVLQTGLYRLSGAERTIRDLKDKFLRSRSVPLLSKVEDIHAITGLLKDFLRNLKEPLLTFR
LNRSFMEAAEITDDDNSVAQ
MYHIISQLPQPNRDTLAFLAVHFQRVAQSVATRMDASNLSRVLGPTLVGHAVPNPEPMAILQDTKRQPKVVERLLALPLE
YWSQFLMVENDHPAPNIKMS

ILGPLTTPEHQLNKTPSSSSLKQRVKSSLTPSFGSKSKSAVGFTRSANFFASPLLQ
>g1395.t2
MTPLGPHGAKEGVCSPLGLTGVLLQLQENESLVAKTTLTVPAGGGPVEAISTIETVPAPYWTRSKRITETLEWDSESVQS
VDIFKQPGVPDRVTRAAPST
PQSTGSVRLHEFVSKTVIKPESCVPCGKRIKFGKLSLKCRDCRVVSHPECRERCPLPCTPNVAGTPVKIGEGTLADYVSA
TSPMIPPLVVHCVNEIEQRG
LSEMDRGKTNSEHVLQTGLYRLSGAERTIRDLKDKFLRSRSVPLLSKVEDIHAITGLLKDFLRNLKEPLLTFRLNRSFME
AAEITDDDNSVAQMYHIISQ
LPQPNRDTLAFLAVHFQRVAQSVATRMDASNLSRVLGPTLVGHAVPNPEPMAILQDTKRQPKVVERLLALPLEYWSQFLM
VENDHPAPNIKMSILGPLTT
PEHQLNKTPSSSSLKQRVKSSLTPSFGSKSKSAVGFTRSANFFASPLLQ
>g1395.t3
MTPLGPHGAKEGVCSPLGLTGVLLQLQENESLVAKTTLTVPAGGGPVEAISTIETVPAPYWTRSKRITETLEWDSESVQS
VDIFKQPGVPDRVTRAAPST
PQSTGSVRLHEFVSKTVRKPCLKVIKPESCVPCGKRIKFGKLSLKCRDCRVVSHPECRERCPLPCTPNVAGTPVKIGEGT
LADYVSATSPMIPPLVVHCV
NEIEQRGLSEMDRGKTNSEHVLQTGLYRLSGAERTIRDLKDKFLRSRSVPLLSKVEDIHAITGLLKDFLRNLKEPLLTFR
LNRSFMEAAEITDDDNSVAQ
MYHIISQLPQPNRDTLAFLAVHFQRVAQSVATRMDASNLSRVLGPTLVGHAVPNPEPMAILQDTKRQPKVVERLLALPLE
YWSQFLMVENDHPAPNIKMS
ILGPLTTPEHQLNKTPSSSSLKQRVKSSLTPRYMTSDTLCCRCLLVN
>g1396.t1
MALNFDDCRRKWLRLEQDLGSCKEVLAKTQTERGSLEVKLKHARNQVDVEIRRRQKAEAECEKLVCRLLTIDESASILSD
ISYDKSEDSLVSVISPS
>g1397.t1
VIHEDSLRELLTTMGDRFSDEDVDQLFREAPIDKTGNFNYVAFTRILKHGAKDKDD
>g1398.t1
LKHASSRTEAGSVNSKHVLTAHSTHYQLIDGSAVSDTSHSDVSSFIPAAASFKGKVAVRHLSKGCRQQGNTGDTGAQILW
RPGSGNDDPDEPLMCRPGRL
MVCVLLPPPELDALGDELLLDDDSSYLDEASASPAIPEGMPSDSKTNKNSPEEEAELKKLKDIEKSVEQAAKKLEKVDGE
LTGLKNGFLAKDLQAEALGK
LDHRVKVASEQFMKILEQIDSLSLPENFNDCRMKKKGLVKTVQDFLAQCDKVEAGISDHLAKIQSKNLALAE
>g1398.t2
MHGGVTGKSLKDMEENLSSFGVKQGCKLMMIGKRNSPEEEAELKKLKDIEKSVEQAAKKLEKVDGELTGLKNGFLAKDLQ
AEALGKLDHRVKVASEQFMK
ILEQIDSLSLPENFNDCRMKKKGLVKTVQDFLAQCDKVEAGISDHLAKIQSKNLALAE
>g1398.t3
MEENLSSFGVKQGCKLMMIGKRNSPEEEAELKKLKDIEKSVEQAAKKLEKVDGELTGLKNGFLAKDLQAEALGKLDHRVK
VASEQFMKILEQIDSLSLPE
NFNDCRMKKKGLVKTVQDFLAQCDKVEAGISDHLAKIQSKNLALAE
>g1398.t4
LKHASSRTEAGSVNSKHVLTAHSTHYQLIDGSAVSDTSHSDVSSFIPAAASFKGKVAVRHLSKGCRQQGNTGDTGAQILW
RPGSGNDDPDEPLMCRPGRL
MVCVLLPPPELDALGDELLLDDDSSYLDEASASPAIPEGMPSDSKTNKDGVLVDEFGLPQIPAT
>g1399.t1
PGAEREMNEETWARIQRQNEGALRETPDDTMFRWLAMAYAHSHLTMTESYRGSCHGDDVTGGQGIVNRAAWKPVVGSMND
FSYLHTNCFELTIFLGCDKF
PHESELSKEWENNREALLSFIEQ
>g1399.t2
PGAEREMNEETWARIQRQNEGALRETPDDTMFRWLAMAYAHSHLTMTESYRGSCHGDDVTGGQGIVNRAAWKPVVGSMND
FSYLHTNCFELTIFLGCDKF
PHESELSKEWENNREALLSFIEQILYGVQVRRVGRPIEHSNTMVSKPFTSGFGTVSRCQVVLKNEIFISIKLFSRWKHEV
LQNLL
>g1399.t3
PGAEREMNEETWARIQRQNEGALRETPDDTMFRWLAMAYAHSHLTMTESYRGSCHGDDVTGGQGIVNRAAWKPVVGSMND
FSYLHTNCFELTIFLGCDKF

PHESELSKEWENNREALLSFIEQVRRVGRPIEHSNTMVSKPFTSGFGTVSRCQVVLKNEIFISIKLFSRWKHEVLQNLL
>g1400.t1
DDGEFQDKITPISVAMEYSLDYKLAADSTGLLPILDMKAPSNLTKQAHILLDCGDDNICTPDLRLSVKSDRQQLYIGDDN
ELTLEINAENRGEGAYEAEL
HVYPPPQAEYTSVVRSQALPRLSCNSKIENQTRVVVCELGNPMKGGAKVLAELRFSVHQLSEQDTSVKFDLQIV
>g1400.t2
MNLSSAAEDDGEFQDKITPISVAMEYSLDYKLAADSTGLLPILDMKAPSNLTKQAHILLDCGDDNICTPDLRLSVKSDRQ
QLYIGDDNELTLEINAENRG
EGAYEAELHVYPPPQAEYTSVVRSQALPRLSCNSKIENQTRVVVCELGNPMKGGAKVLAELRFSVHQLSEQDTSVKFDLQ
IV
>g1400.t3
DDGEFQDKITPISVAMEYSLDYKLAADSTGLLPILDMKAPSNLTKQAHILLDCGDDNICTPDLRLSVKSDRQQLYIGDDN
ELTLEINAENRGEGAYEAEL
HVYPPPQAEYTSVVRSQALPRLSCNSKIENQTRVVVCELGNPMKGGAKVLAELRFSVHQLSEQDTSVKFDLQIVRYTPLW
LLCASVPSCLGEKLW
>g1401.t1
MNSCLSMSPSASVSKPHLFLMQASSDPQPSLLLDVLTIAGVQRLVKRRGPWEMTPGFLDYVSMDVYSFPAAHASRAAMVS
KFLLSHLVLAVPLRVLLVLW
AFLVGVSRVMLGKHHLTDMACGFVLGLLHFSLMENVWLSSGTCQTLISISTFSWSPFS
>g1402.t1
LVASLCKVKRRTRHANMPSSSHTRSRTRDRNNVLGRPEFMSLNQPMRSGAPAAENRASSRRLSQIKPQTSQSSEEPADSA
TTTTTNTNNNKDKDKKEPGK
MPEEDCMQLNPSFKGIAMNALLAIDISLSKRMGVCAYTSSSWGGCRSMVSLLELTGYGITWIIGTIVCLTRSNTLAGQEV
LVNLLLVSLTSQNNHRSVQ
>g1403.t1
DPTSPAYGADVLRWWVAEANVFSEVQIGPTALQSARDSISKVLRSAAHRSSGQCVSLGPFRLRNTIRFLLGNLQSFDPRT
QAVDPKEMHYVDQYMLHLLR
DYSIKVTDAYSEFDAGRAIRVLQAFINRDLSSFYFSIVKDRLYCEPEGSVGRRSCQTVLEEVLDGLTRSIAPILPHLAEE
VYLHAPGHEGVCVLC
>g1403.t2
DPTSPAYGADVLRWWVAEANVFSEVQIGPTALQSARDSISKVLRSAAHRSSGQCVSLGPFRVKSPELRNTIRFLLGNLQS
FDPRTQAVDPKEMHYVDQYM
LHLLRDYSIKVTDAYSEFDAGRAIRVLQAFINRDLSSFYFSIVKDRLYCEPEGSVGRRSCQTVLEEVLDGLTRSIAPILP
HLAEEVYLHAPGHEGVCVLC
>g1403.t3
DPTSPAYGADVLRWWVAEANVFSEVQIGPTALQSARDSISKLRNTIRFLLGNLQSFDPRTQAVDPKEMHYVDQYMLHLLR
DYSIKVTDAYSEFDAGRAIR
VLQAFINRDLSSFYFSIVKDRLYCEPEGSVGRRSCQTVLEEVLDGLTRSIAPILPHLAEEVYLHAPGHEGVCVLC
>g1404.t1
MKRRRDFYAENRVLEPGEVVETFNIAEDEIISLSIQFYERNKSVERPRSDTDGDKSNGKRFLQCPAAMSVMHLAKFLRSK
MDIPSSYRVEVLYGDEPLKD
YYTLMDIAYFYEWRRVS
>g1404.t2
MQPDCVKITDLNPHLTCPLCAGYFIDATTIVECLHSYEMKRRRDFYAENRVLEPGEVVETFNIAEDEIISLSIQFYERNK
SVERPRSDTDGDKSNGKRFL
QCPAAMSVMHLAKFLRSKMDIPSSYRVEVLYGDEPLKDYYTLMDIAYFYEWRRVS
>g1404.t3
MTRFHRADKTLQDIVYKLVPGLFKDEMKRRRDFYAENRVLEPGEVVETFNIAEDEIISLSIQFYERNKSVERPRSDTDGD
KSNGKRFLQCPAAMSVMHLA
KFLRSKMDIPSSYRVEVLYGDEPLKDYYTLMDIAYFYEWRRVS
>g1405.t1
LTLPTVTSALFSRPARENLPSRFKFKEYCPMVFRNLRERFCIDDQDYQNSLTRSAPLNSDSQGRFGNRFLSSYDHRFVIK
TVSSEDIAEMHNILKKYHQ
>g1405.t2
LTLPTVTSALFSRPARENLPSRFKFKEYCPMVFRNLRERFCIDDQDYQNSLTRSAPLNSDSQGRFGNRFLSSYDHRFVIK
TVSSEDIAEMHNILKKYHQV

GASAGRRASGGGRVTPPDPRVPFHQIDNRSSRFYYRLFIRVLRGVAMGYCYTKRTPRLPDC
>g1406.t1
LLLNQGNPSPEEEEARRIAEMGKPILGEHRRLEVVIEENCNFKSTVDKLLKDTNLAAVIGTHSWKEQFIEAVTVSG
>g1406.t2
LLLNQGNPSPEEEEARRIAEMGKPILGEHRRLEVVIEENCNFKSTVDKLLKDTNLAAVIGTHSWKEQFIEAVTVSGGF
>g1407.t1
MMDVEEDGTVVDLGDPVPLASPAEDEGRQPSHPSAEGPQDEPAPPQIPPTDISPAQPPADQAAQSRWVSALDQFKSKRFF
VLRPGTLSQAIEDINALVDK
EQDGAVQGLWLMAEVDHWNNEKERMVVLTEKSLLVCKYDFVMLNCERVHRIPLNFVDRISHGAFSFPQHSILT
>g1407.t2
MMDVEEDGTVVDLGDPVPLASPAEDEGRQPSHPSAEGPQDEPAPPQIPPTDISPAQPPADQAAQSRWVSALDQFKSKRFF
VLRPGTLSQAIEDINALVDK
EQDGAVQGLWLMAEVDHWNNEKERMVVLTEKSLLVCKYDFVMLNCERVHRIPLNFVDRISHGAFSFPQHSIL
>g1408.t1
MAHRSQSSSVGDNPLDPNYLHPHYCEDYRLAIDALIEDDVQGYYEFLQTAETVPFLAQSEIDHIKSTIHIPSQSQSVNVP
ELTYHEGVMEDDGSSDTYWP
VHSDLDAPGLDLGWPLQQHSFIGPTEVTTLINPSDPEMPSIKEQARRLIKNAHHVIAVVMDIFTDVDIFSDLLAAAARHV
PVYILLDEQNCHHFVTMVNN
CRVNLDMIHMMRVRTVAGITYYCRTGKSFKGQVKDRFLLADCRAVISGNYSFMWSYEKIHRCIAHLFLGELVASFDEEFR
ILFAQSEPLVIDKSIVPHGN
SETSGSGYLSNQFGLKRSQSLRSNRSFLRQNELPSAFPYGDSDRNPLAFRRNDPYRHSMEPTAGITIGKYAQQQFHLQQS
YLEQGRSIVSRQMELSSSAF
KRHSYAEGTQESYSSSKQYMKHRVMNDLDETDHQREQSQRHYYMEGSGPESGLGQLDRLRRHASHLAIDQRSDSSFTSEP
PAAGGYGRDYFSSDDLKGPD
GFQGLHETGRYEGGSSHKRPTVGQAYACLSSPTQPHLAEKQQFLKQSEQEREQDPSARQGMRSWRINSYLNTCEDGGEEG
LPQAIGSDAFDDPPSAQHVA
DPEGSASRFGIKEMPSKPRPDIQRPRFGKPILPDSDKDSGPKMPRDLLSSSTSFNPYVAEKDRDKDFDMERDWDRTGTRD
RAADMDVKAGPEVTLSKHES
FRSRVNPMLQRSSRLRSSLIFASSKVEKHSGGLGLKPATEEDEDLDHIRTSSIVAQILEKRTSMSREPFEWRRKTEEKDR
DDETQRQLDKEKGQTSVKEL
KDKFQNASQDIKMSPDEVPSSVITGSLKTQQESTLNMNDPASRLQYFKDLAAKRKASKMGMESSLSAIEPADKKPDLLDK
PLASTATQNIPSVSVNPPEP
EPRKLDISSRLAELTRRSSFSGNKPPVVSPRSPVKQLKPPEPEPTRKEANAPEAQKKDSFKSLKPFPSPKIFRRDPLKLK
GLNPRRVSAGEETLTADATD
AEKSELKKTRSHSSSSTMRLDSKDVLNKACGSNTSINTIGSDGKSEGKPLDFLKKQTQRLKGFLGPKDKDKKGASGGDEK
TMRTVMEITEDASKTSRPSA
HTGSSNTEQSTTNHKVSAGTSGPSRYQSSGGSVLFSSNLRDDTKVILGQISANSQKNRQDQDKAADAKEVDSGDKGMERQ
NSMRKGLFPRLQPNPQEREG
LLKRIESMRKEKKVYSRFEVCVPILHNK
>g1408.t2
MAHRSQSSSVGDNPLDPNYLHPHYCEDYRLAIDALIEDDVQGYYEFLQTAETVPFLAQSEIDHIKSTIHIPSQSQSVNVP
ELTYHEGVMEDDGSSDTYWP
VHSDLDAPGLDLGWPLQQHSFIGPTEVTTLINPSDPEMPSIKEQARRLIKNAHHVIAVVMDIFTDVDIFSDLLAAAARHV
PVYILLDEQNCHHFVTMVNN
CRVNLDMIHMMRVRTVAGITYYCRTGKSFKGQVKDRFLLADCRAVISGNYSFMWSYEKIHRCIAHLFLGELVASFDEEFR
ILFAQSEPLVIDKSIVPHGN
SETSGSGYLSNQFGLKRSQSLRSNRSFLRQNELPSAFPYGDSDRNPLAFRRNDPYRHSMEPTAGITIGKYAQQQFHLQQS
YLEQGRSIVSRQMELSSSAF
KRHSYAEGTQESYSSSKQYMKHRVMNDLDETDHQREQSQRHYYMEGSGPESGLGQLDRLRRHASHLAIDQRSDSSFTSEP
PAAGGYGRDYFSSDDLKGPD
GFQGLHETGRYEGGSSHKRPTVGQAYACLSSPTQPHLAEKQQFLKQSEQEREQDPSARQGMRSWRINSYLNTCEDGGEEG
LPQAIGSDAFDDPPSAQHVA
DPEGSASRFGIKEMPSKPRPDIQRPRFGKPILPDSDKDSGPKMPRDLLSSSTSFNPYVAEKDRDKDFDMERDWDRTGTRD
RAADMDVKAGPEVTLSKHES
FRSRVNPMLQRSSRLRSSLIFASSKVEKHSGGLGLKPATEEDEDLDHIRTSSIVAQILEKRTSMSREPFEWRRKTEEKDR
DDETQRQLDKEKGQTSVKEL

KDKFQNASQDIKMSPDEVPSSVITGSLKTQQESTLNMNDPASRLQYFKDLAAKRKASKMGMESSLSAIEPADKKPDLLDK
PLASTATQNIPSVSVNPPEP
EPRKLDISSRLAELTRRSSFSGNKPPVVSPRSPVKQLKPPEPEPTRKEANAPEAQKKDSFKSLKPFPSPKIFRRDPLKLK
GLNPRRVSAGEETLTADATD
AEKSELKKTRSHSSSSTMRLDSKDVLNKACGSNTSINTIGSDGKSEGKPLDFLKKQTQRLKGFLGPKDKDKKGASGGDEK
TMRTVMEITEDASKTSRPSA
HTGSSNTEQSTTNHKVSAGTSGPSRYQSSGGSVLFSSNLRDDTKVILGQISANSQKNRQDQDKAADAKEVDSGDKGMERQ
NSMRKGLFPRLQPNPQEREG
LLKRIESMRKEKKVYSRFEMGNSLG
>g1408.t3
MAHRSQSSSVGDNPLDPNYLHPHYCEDYRLAIDALIEDDVQGYYEFLQTAETVPFLAQSEIDHIKSTIHIPSQSQSVNVP
ELTYHEGVMEDDGSSDTYWP
VHSDLDAPGLDLGWPLQQHSFIGPTEVTTLINPSDPEMPSIKEQARRLIKNAHHVIAVVMDIFTDVDIFSDLLAAAARHV
PVYILLDEQNCHHFVTMVNN
CRVNLDMIHVKDRFLLADCRAVISGNYSFMWSYEKIHRCIAHLFLGELVASFDEEFRILFAQSEPLVIDKSIVPHGNSET
SGSGYLSNQFGLKRSQSLRS
NRSFLRQNELPSAFPYGDSDRNPLAFRRNDPYRHSMEPTAGITIGKYAQQQFHLQQSYLEQGRSIVSRQMELSSSAFKRH
SYAEGTQESYSSSKQYMKHR
VMNDLDETDHQREQSQRHYYMEGSGPESGLGQLDRLRRHASHLAIDQRSDSSFTSEPPAAGGYGRDYFSSDDLKGPDGFQ
GLHETGRYEGGSSHKRPTVG
QAYACLSSPTQPHLAEKQQFLKQSEQEREQDPSARQGMRSWRINSYLNTCEDGGEEGLPQAIGSDAFDDPPSAQHVADPE
GSASRFGIKEMPSKPRPDIQ
RPRFGKPILPDSDKDSGPKMPRDLLSSSTSFNPYVAEKDRDKDFDMERDWDRTGTRDRAADMDVKAGPEVTLSKHESFRS
RVNPMLQRSSRLRSSLIFAS
SKVEKHSGGLGLKPATEEDEDLDHIRTSSIVAQILEKRTSMSREPFEWRRKTEEKDRDDETQRQLDKEKGQTSVKELKDK
FQNASQDIKMSPDEVPSSVI
TGSLKTQQESTLNMNDPASRLQYFKDLAAKRKASKMGMESSLSAIEPADKKPDLLDKPLASTATQNIPSVSVNPPEPEPR
KLDISSRLAELTRRSSFSGN
KPPVVSPRSPVKQLKPPEPEPTRKEANAPEAQKKDSFKSLKPFPSPKIFRRDPLKLKGLNPRRVSAGEETLTADATDAEK
SELKKTRSHSSSSTMRLDSK
DVLNKACGSNTSINTIGSDGKSEGKPLDFLKKQTQRLKGFLGPKDKDKKGASGGDEKTMRTVMEITEDASKTSRPSAHTG
SSNTEQSTTNHKVSAGTSGP
SRYQSSGGSVLFSSNLRDDTKVILGQISANSQKNRQDQDKAADAKEVDSGDKGMERQNSMRKGLFPRLQPNPQEREGLLK
RIESMRKEKKVYSRFEVCVP
ILHNK
>g1409.t1
MAVVNDGALKKMLKQYRYKEMTVREITNVISQYKDLKPVMDSYVFNDGSTRELMSLTGTVPVSYRGNVYNIPVCLWLLDT
YPYNPPICFVKPTSAMMIKT
GKHIDANGKIYLPYLHEWKHPQSDLYGLIQVMIVVFGDEPPVFSRPTTQAPHQAFQAAGPPNSSYMPGLPAVSPYGPNAN
PGGYPGYPYPGGASYPATAV
QGHYPSQTPGSAVGPSRDGTIGEDTIRASLISAVSDKLRWRMKEEMDRAQAELDALKRTEEDLKKGHQKLEEMVSRLDQE
V
>g1409.t2
MAVVNDGALKKMLKQYRYKEMTVREITNVISQYKDLKPVMDSYVFNDGSTRELMSLTGTVPVSYRGNVYNIPVCLWLLDT
YPYNPPICFVKPTSAMMIKT
GKHIDANGKIYLPYLHEWKHPQSDLYGLIQVMIVVFGDEPPVFSRPTTQAPHQAFQAAGPPNSSYMPGLPAVSPYGPNAN
PGGYPGYPYPGGASYPATAV
QGHYPSQTPGSAVGPSRDGTIGEDTIRASLISAVSDKLRWRMKEEMDRAQAELDALKRTEEDLKKGHQKLEEMVSRLDQE
VVSGSRQFL
>g1409.t3
MTVREITNVISQYKDLKPVMDSYVFNDGSTRELMSLTGTVPVSYRGNVYNIPVCLWLLDTYPYNPPICFVKPTSAMMIKT
GKHIDANGKIYLPYLHEWKH
PQSDLYGLIQVMIVVFGDEPPVFSRPTTQAPHQAFQAAGPPNSSYMPGLPAVSPYGPNANPGGYPGYPYPGGASYPATAV
QGHYPSQTPGSAVGPSRDGT
IGEDTIRASLISAVSDKLRWRMKEEMDRAQAELDALKRTEEDLKKGHQKLEEMVSRLDQEV
>g1410.t1

YYRACVLSTPPTLSLLTIGFHFRKVGRQLSTVVLPLELANRTGNNDRRTEGEVLARFPSLARGAPLMCAREKGSLFTKPR
YLSELCCVDGPSGVGRATFP
VVSRAV
>g1410.t2
YYRACVLSTPPTLSLLTIGFHFRKVGRQLRYLSELCCVDGPSGVGRATFPVVSRAV
>g1410.t3
YYRACVLSTPPTLSLLTIGFHFRKVGRQLSVRQFRFQIAPGSCLYNQGISSMIGTVVLPLELANRTGNNDRRTEGEVLAR
FPSLARGAPLMCAREKGSLF
TKPRYLSELCCVDGPSGVGRATFPVVSRAV
>g1411.t1
MAGTWDPHPLHQATPVSSKGHGASSTLAPALSQPRLSCAKRLTFFKSGDNKFGGVKMAIHRRRFKCFDALLDDLSQKVPL
PFGVRTVTTPKGTHTINHLD
QLQDGGCYLCSDHRPAKPVDMGLAGRRPVVWQPHHHHHRDTAQAQRPNVSALLGPSAPFRSRRLLLVKNTEPGRRRSVVL
GRWTARSLRAFLEEASDAMQ
FHVRRLYTAEGRTIDTVQSLMACHGLLVCAGREPFNPMLAEFIRKSSEERLPGLGTRSPAQGHRTSGNGARSPATQGARS
PAHGARSRGSECSEGRDSRK
NVNFGLETKKSIIHPRSDSSTRSTRFSLSSEKSYPNGLTPCSNSHSRPAIMNDDIEKRVVVNTDGSLSVEMKVRFRLHSN
ETLQWSTQMKKSHFLNNDCS
QSREARPSYLQQGHSETCSDPDSGSCDPKATNIYTDAAVRHPPEANHCPCCHQRREQQQYDLWENPAHTNNHAPKPPHNA
TRPAHTRVRQTHSSSSSSSS
HSRRVVCRRSRLSNSRGDSSLGQARVEVQEELCVTEEVCTVNRCCSNSGATVGNGGTGAAEEDQCTQADGEPTFSEGDDE
HALSVVSDSSHVLQALREDQ
DEEEGELPQSVSQCCQCESLPTSVSRTLQKGEDRGSRASSCHCGVTTPHSLAQGGEIDRDDSSVFKTSRTKTPCQNDAKD
YQEDVQDRAISSLSSHHGLP
GDDVRSRQSSACTNCRGCQGTTMPVLPASQRSSRSHTATPKPDPRLPDQGENHGDDEDKRTPSQMSESSEGSAVSLRSNK
SNLTHQDSTSEMSNIPEERG
TSAMSEASNQRKDSGAPGKTLTEPAADSQERVASVASATSQNSGRSHKSGCSRGIADVEERSASARSAKSNVSSQSCISH
KAVKEAEKRADSALSCQLAT
SKHNGTVEVEDEGEGVSERSASPLSAESEYSAKSRISHGSRTSTGSALPTNNTPDNTGNKEAEDKILRSISVLSAKTGVS
AKSDKSAKSHCSRCSKAVAP
DLEDAEDAEELQLENGGKGAEHYVCAIHGLEVKVSLQRFFENPDKRGGRTPSRLRPNSAASTCSDAKPTPASTEKSTTPG
PNGPRVKSDVGSNSGSVTLL
RTSRLNRESGNVCISQTTACVEKPTEGALSHSLSAADLLRDTPASAGPVSRPTSGSTKSRCQRNGMCAEEEEGELTPASL
PNQSANEVVSNWLQNIPANG
CLFADGDETSDTEGGEEVKATGTFAREGSPEGQNANEVAETPKKGVEGKEEDDEKKEQEVARDKDEEDSAKEVEVSELGL
SPPVEEVTSSRDHKTMFQHS
ESMSRNCNSSVAVMKVLLNPSLGRSNSLPEVSPVYGRKLSTSAKGLLDCLAQLQLIQPLMTNPGCERYAEIMSILHSLWL
NEGRDAPDAAGDIPKDKITP
PRSSSGVDHDQGGGADSLVMQGEEEAEAAAGEGEPECEEPPGADTDTKGDGADVTPSSSDKLSSAAGSSKSPTDN
>g1411.t2
MAGTWDPHPLHQATPVSSKGHGASSTLAPALSQPRLSCAKRLTFFKSGDNKFGGVKMAIHRRRFKCFDALLDDLSQKVPL
PFGVRTVTTPKGTHTINHLD
QLQDGGCYLCSDHRPAKPVDMGLAGRRPVVWQPHHHHHRDTAQAQRPNVSALLGPSAPFRSRRLLLVKNTEPGRRRSVVL
GRWTARSLRAFLEEASDAMQ
FHVRRLYTAEGRTIDTVQSLMACHGLLVCAGREPFNPMLAEFIRKSSEERLPGLGTRSPAQGHRTSGNGARSPATQGARS
PAHGARSRGSECSEGRDSRK
NVNFGLETKKSIIHPRSDSSTRSTRFSLSSEKSYPNGLTPCSNSHSRPAIMNDDIEKRVVVNTDGSLSVEMKVRFRLHSN
ETLQWSTQMKKSHFLNNDCS
QSREARPSYLQQGHSETCSDPDSGSCDPKATNIYTDAAVRHPPEANHCPCCHQRREQQQYDLWENPAHTNNHAPKPPHNA
TRPAHTRVRQTHSSSSSSSS
HSRRVVCRRSRLSNSRGDSSLGQARVEVQEELCVTEEVCTVNRCCSNSGATVGNGGTGAAEEDQCTQADGEPTFSEGDDE
HALSVVSDSSHVLQALREDQ
DEEEGELPQSVSQCCQCESLPTSVSRTLQKGEDRGSRASSCHCGVTTPHSLAQGGEIDRDDSSVFKTSRTKTPCQNDAKD
YQEDVQDRAISSLSSHHGLP
GDDVRSRQSSACTNCRGCQGTTMPVLPASQRSSRSHTATPKPDPRLPDQGENHGDDEDKRTPSQMSESSEGSAVSLRSNK
SNLTHQDSTSEMSNIPEERG

TSAMSEASNQRKDSGAPGKTLTEPAADSQERVASVASATSQNSGRSHKSGCSRGIADVEERSASARSAKSNVSSQSCISH
KAVKEAEKRADSALSCQLAT
SKHNGTVEVEDEGEGVSERSASPLSAESEYSAKSRISHGSRTSTGSALPTNNTPDNTGNKEAEDKILRSISVLSAKTGVS
AKSDKSAKSHCSRCSKAVAP
DLEDAEDAEELQLENGGKGAEHYVCAIHGLEVKVSLQRFFENPDKRGGRESGNVCISQTTACVEKPTEGALSHSLSAADL
LRDTPASAGPVSRPTSGSTK
SRCQRNGMCAEEEEGELTPASLPNQSANEVVSNWLQNIPANGCLFADGDETSDTEGGEEVKATGTFAREGSPEGQNANEV
AETPKKGVEGKEEDDEKKEQ
EVARDKDEEDSAKEVEVSELGLSPPVEEVTSSRDHKTMFQHSESMSRNCNSSVAVMKVLLNPSLGRSNSLPEVSPVYGRK
LSTSAKGLLDCLAQLQLIQP
LMTNPGCERYAEIMSILHSLWLNEGRDAPDAAGDIPKDKITPPRSSSGVDHDQGGGADSLVMQGEEEAEAAAGEGEPECE
EPPGADTDTKGDGADVTPSS
SDKLSSAAGSSKSPTDN
>g1411.t3
MAGTWDPHPLHQATPVSSKGHGASSTLAPALSQPRLSCAKRLTFFKSGDNKFGGVKMAIHRRRFKCFDALLDDLSQKVPL
PFGVRTVTTPKGTHTINHLD
QLQDGGCYLCSDHRPAKPVDMGLAGRRPVVWQPHHHHHRDTAQAQRPNVSALLGPSAPFRSRRLLLVKNTEPGRRRSVVL
GRWTARSLRAFLEEASDAMQ
FHVRRLYTAEGRTIDTVQSLMACHGLLVCAGREPFNPMLAEFIRKSSEERLPGLGTRSPAQGHRTSGNGARSPATQGARS
PAHGARSRGSECSEGRDSRK
NVNFGLETKKSIIHPRSDSSTRSTRFSLSSEKSYPNGLTPCSNSHSRPAIMNDDIEKRVVVNTDGSLSVEMKVRFRLHSN
ETLQWSTQMKKSHFLNNDCS
QSREARPSYLQQGHSETCSDPDSGSCDPKATNIYTDAAVRHPPEANHCPCCHQRREQQQYDLWENPAHTNNHAPKPPHNA
TRPAHTRVRQTHSSSSSSSS
HSRRVVCRRSRLSNSRGDSSLGQARVEVQEELCVTEEVCTVNRCCSNSGATVGNGGTGAAEEDQCTQADGEPTFSEGDDE
HALSVVSDSSHVLQALREDQ
DEEEGELPQSVSQCCQCESLPTSVSRTLQKGEDRGSRASSCHCGVTTPHSLAQGGEIDRDDSSVFKTSRTKTPCQNDAKD
YQEDVQDRAISSLSSHHGLP
GDDVRSRQSSACTNCRGCQGTTMPVLPASQRSSRSHTATPKPDPRLPDQGENHGDDEDKRTPSQMSESSEGSAVSLRSNK
SNLTHQDSTSEMSNIPEERG
TSAMSEASNQRKDSGAPGKTLTEPAADSQERVASVASATSQNSGRSHKSGCSRGIADVEERSASARSAKSNVSSQSCISH
KAVKEAEKRADSALSCQLAT
SKHNGTVEVEDEGEGVSERSASPLSAESEYSAKSRISHGSRTSTGSALPTNNTPDNTGNKEAEDKILRSISVLSAKTGVS
AKSDKSAKSHCSRCSKAVAP
DLEDAEDAEELQLENGGKGAEHYVCAIHGLEVKVSLQRFFENPDKRGGRPNSAASTCSDAKPTPASTEKSTTPGPNGPRV
KSDVGSNSGSVTLLRTSRLN
RESGNVCISQTTACVEKPTEGALSHSLSAADLLRDTPASAGPVSRPTSGSTKSRCQRNGMCAEEEEGELTPASLPNQSAN
EVVSNWLQNIPANGCLFADG
DETSDTEGGEEVKATGTFAREGSPEGQNANEVAETPKKGVEGKEEDDEKKEQEVARDKDEEDSAKEVEVSELGLSPPVEE
VTSSRDHKTMFQHSESMSRN
CNSSVAVMKVLLNPSLGRSNSLPEVSPVYGRKLSTSAKGLLDCLAQLQLIQPLMTNPGCERYAEIMSILHSLWLNEGRDA
PDAAGDIPKDKITPPRSSSG
VDHDQGGGADSLVMQGEEEAEAAAGEGEPECEEPPGADTDTKGDGADVTPSSSDKLSSAAGSSKSPTDN
>g1412.t1
MGLIFHDVSMIPPTASKVDLRTYMKGSSPDDLECEIRRYSTMGIQVRWPSQGAQAYDRWFKCTIKREEDFIFTAYLRHTP
AQAPSGMDDYQGVVPIGDRE
IITTSASMVLHTRSVALKVGLQSQQNLHCQFAVDHRAPKFRLTWSAKRRGDWAELYSHNSHSGDTRGSGVSRTALVSMGD
ATLSLPSTSVSSEGKYICSV
SVTPLDASLDMTLHIFAKVRSLTQPCSLLRLTWASLLLKELLRDPTVSCRGVSINVGPQLELVAGAEQKVVCEAEGYYPL
DVEIEWYQEPPGEPGAYRAG
APLPTKLLNVLLSSHRQNREGVFSLSSFFYYQAVLRDSGRRFTCRVMHSSLRTPVRKSFTLLVHGECVGGPGCCVAK
>g1412.t2
MKGSSPDDLECEIRRYSTMGIQVRWPSQGAQAYDRWFKCTIKREEDFIFTAYLRHTPAQAPSGMDDYQGVVPIGDREIIT
TSASMVLHTRSVALKVGLQS
QQNLHCQFAVDHRAPKFRLTWSAKRRGDWAELYSHNSHSGDTRGSGVSRTALVSMGDATLSLPSTSVSSEGKYICSVSVT
PLDASLDMTLHIFAKVRSLT

QPCSLLRLTWASLLLKELLRDPTVSCRGVSINVGPQLELVAGAEQKVVCEAEGYYPLDVEIEWYQEPPGEPGAYRAGAPL
PTKLLNVLLSSHRQNREGVF
SLSSFFYYQAVLRDSGRRFTCRVMHSSLRTPVRKSFTLLVHGECVGGPGCCVAK
>g1412.t3
LNADGHHETEFHPRDAVLQFGQADDAPVNPLATTFMVTASKVDLRTYMKGSSPDDLECEIRRYSTMGIQVRWPSQGAQAY
DRWFKCTIKREEDFIFTAYL
RHTPAQAPSGMDDYQGVVPIGDREIITTSASMVLHTRSVALKVGLQSQQNLHCQFAVDHRAPKFRLTWSAKRRGDWAELY
SHNSHSGDTRGSGVSRTALV
SMGDATLSLPSTSVSSEGKYICSVSVTPLDASLDMTLHIFAKVRSLTQPCSLLRLTWASLLLKELLRDPTVSCRGVSINV
GPQLELVAGAEQKVVCEAEG
YYPLDVEIEWYQEPPGEPGAYRAGAPLPTKLLNVLLSSHRQNREGVFSLSSFFYYQAVLRDSGRRFTCRVMHSSLRTPVR
KSFTLLVHGECVGGPGCCVA
K
>g1413.t1
LCHPSSLSAIQKGDWERVGQPILDAVGELCKQAECNGGPASSAIHCKTNIVAVLWCKLLSNESGEDMETAWRENPFFAVQ
NGLPEINRIVLLELVKATAA
ADIYGQLLLRLSKSQICSELSWVAEYVVCRPTSDGDVGLLLDVWWELWRGGGRGEAGGTGSPEDIFANEFARLTNQPSSE
NSGVSHQAAKRFKLDTSVPL
KPPPSLPMSLSPPPTDFLYILLHTLWDVRDCIATADLSQQALAVALDAIYTSFLLDRVVILTTEEKLHFLTKVVSLKEKE
DRTSSLAQLREAQLDLHTCG
RPAKFRPTITTLPHALVIIAELTQCLQQKGLLKGSDSNNPSYSTFRLKQSLQRSLVVLENSTVYQSMSGAEDQQTLKDIV
TLIRGVQESLTSPTIHCSPE
AYARLTMTVIDHRLEEHLDFAALFARKTNWATSGSEWTDCLERNQATFQKKDTVVALATTLMSISDREKVDVSHCKKLSK
IIANIFLELPLGDKNEVLAS
MLSLSSRGLFGGEPPLALAEGFNQELNMAFNCIIQGGGGDMAGTRAGSPGNLSTATSLVARVAFQNPEATLQRCCSAAIF
NKGVFTLMAEILLQLPGLRG
RGITEESLLCCCLQKAIGKKALLANEQDQFRRFLALLMQPVTEDCCGVEGDQGQWCFLSPADVVRTFVLPHISSLGAGPI
SMELAVQILHSALRVEVQET
ASSPHWVLQCSPFPLLYILAQHLNQASRCWEPPAVGTTSCLSLDTKELLVWVLGTLGQAVGQVVASEPSSWSRALSWLYS
KVEELDWTVRFHLKPVWGEH
FKNEVPSSLLAVCDLAEQEWSGLELPQYGQGSGLLAWMECCCISDSLRSTMLSHLCLDRHQPNHVTMFSKGLLVALAQTL
PWCSLSEWRRLLGALRELLD
SGLLHTPYSLEYVDYLPLLDLRRFSSQLRLSVLLLRALQLLCGSSCDGWLPAQGWAHVGRLYAYAIRETLEVLRTKLAPS
SSSSSSSADPTSGSPKPPKN
KPRTQSGEVQQRKETSPEKSPFSPHPKSTKSPQPPPAAPTEEEPGGVGDLSAEVLFVLGQLFCHVQHVQVMMPGGRCEAL
FLCALEILSLHESIMATYPD
SSTHLESANTRHFFTTITDNLESLEMKAVLQQKIAKLSSSMA
>g1413.t2
MSVNVYYSPMEKRLKGDWERVGQPILDAVGELCKQAECNGGPASSAIHCKTNIVAVLWCKLLSNESGEDMETAWRENPFF
AVQNGLPEINRIVLLELVKA
TAAADIYGQLLLRLSKSQICSELSWVAEYVVCRPTSDGDVGLLLDVWWELWRGGGRGEAGGTGSPEDIFANEFARLTNQP
SSENSGVSHQAAKRFKLDTS
VPLKPPPSLPMSLSPPPTDFLYILLHTLWDVRDCIATADLSQQALAVALDAIYTSFLLDRVVILTTEEKLHFLTKVVSLK
EKEDRTSSLAQLREAQLDLH
TCGRPAKFRPTITTLPHALVIIAELTQCLQQKGLLKGSDSNNPSYSTFRLKQSLQRSLVVLENSTVYQSMSGAEDQQTLK
DIVTLIRGVQESLTSPTIHC
SPEAYARLTMTVIDHRLEEHLDFAALFARKTNWATSGSEWTDCLERNQATFQKKDTVVALATTLMSISDREKVDVSHCKK
LSKIIANIFLELPLGDKNEV
LASMLSLSSRGLFGGEPPLALAEGFNQELNMAFNCIIQGGGGDMAGTRAGSPGNLSTATSLVARVAFQNPEATLQRCCSA
AIFNKGVFTLMAEILLQLPG
LRGRGITEESLLCCCLQKAIGKKALLANEQDQFRRFLALLMQPVTEDCCGVEGDQGQWCFLSPADVVRTFVLPHISSLGA
GPISMELAVQILHSALRVEV
QETASSPHWVLQCSPFPLLYILAQHLNQASRCWEPPAVGTTSCLSLDTKELLVWVLGTLGQAVGQVVASEPSSWSRALSW
LYSKVEELDWTVRFHLKPVW
GEHFKNEVPSSLLAVCDLAEQEWSGLELPQYGQGSGLLAWMECCCISDSLRSTMLSHLCLDRHQPNHVTMFSKGLLVALA
QTLPWCSLSEWRRLLGALRE

LLDSGLLHTPYSLEYVDYLPLLDLRRFSSQLRLSVLLLRALQLLCGSSCDGWLPAQGWAHVGRLYAYAIRETLEVLRTKL
APSSSSSSSSADPTSGSPKP
PKNKPRTQSGEVQQRKETSPEKSPFSPHPKSTKSPQPPPAAPTEEEPGGVGDLSAEVLFVLGQLFCHVQHVQVMMPGGRC
EALFLCALEILSLHESIMAT
YPDSSTHLESANTRHFFTTITDNLESLEMKAVLQQKIAKLSSSMA
>g1414.t1
MAYDIYAMYLTHYHQQRLRNYSSALQKHSLQTVKAFLSKERLLVLHHMALLLVFMPIVLLGLENSKLHWINGVMVLASFF
TCRILLFPYMYWMYGRQFDI
PLHSVPFHLPLHCNVGNLSILAPQVYWFILLVKKAHRLYLKKGKAWGTKTE
>g1414.t2
MAYDIYAMYLTHYHQQRLRNYSSALQKHSLQTVKAFLSKERLLVLHHMALLLVFMPIVLFFRRGLGDFFIACFWMTEFST
PFVSLGKILIQLGLENSKLH
WINGVMVLASFFTCRILLFPYMYWMYGRQFDIPLHSVPFHLPLHCNVGNLSILAPQVYWFILLVKKAHRLYLKKGKAWGT
KTE
>g1414.t3
LHRRHWLVNSFVLFGAPYMAYDIYAMYLTHYHQQRLRNYSSALQKHSLQTVKAFLSKERLLVLHHMALLLVFMPIVLLGL
ENSKLHWINGVMVLASFFTC
RILLFPYMYWMYGRQFDIPLHSVPFHLPLHCNVGNLSILAPQVYWFILLVKKAHRLYLKKGKAWGTKTE
>g1415.t1
MIISWTIDLQPSSLAACSSLFSSMVELRSLFPSSPSGAQVQRSSCRTVEDQSDGVKIVVDAFGLGNAQYLGQ
>g1415.t2
MIISWTIDLQPSSLAACSSLFSSMVELRSLFPSSPSGAQVQRSSCRTVEDQSDGVKIVVDAFGLGLLHGGMDHHLTVLLN
LYSPGPLVPRSPGLEGLEGL
EEPSRSHLAECSTMFYGSIPTSHTKHAAAPFSSFGSRKSARPPRRRRRLSRVPTFDTFLGCWISVALVKPALRPGRIAGK
RH
>g1416.t1
MRPGRRAGFTRATLIQQPRKVSNVGTRDSRRRRRGGLADFLLPNELNGAAACFVCDVASAGLLQTLQTLQTRGSGHQGTR
GVQVE
>g1417.t1
MYLRSLRPRQCAKFLDNVSLLPDILDRRQRDTCLRDADLTFQRLDAASAKPGSGSSDFAIGWRQRPIRDKDTVPDKLLHV
RFLSYNASMCPLPGSATLKA
AALEMHGMPTLSYITSNNQAHLKLAVGQFPQIAGPPAA
>g1417.t2
MYLRSLRPRQCAKFLDNVSLLPDILDRRQRDTCLRDADLTFQRLDAASAKPGSGSSDFAIGWRQRPIRDKDTVLYLNLQR
YPGRRGVSELRGIIRTSVKA
LQRICDIPRFPPRSAANRRAFEIPQASFCPCVHTYTGSATLKAAALEMHGMPTLSYITSNNQAHLKLAVGQFPQIAGPPA
A
>g1418.t1
MLHYGFRQPSLSKLKIIPQLLQRTLASLVEQVHKSLPSQQGLHCANKSKWTCTTTTMLYWDVMLTVVCT
>g1419.t1
MCKRSYSEIERAFGQAYSNHRKVAADGESREESLIQESACKEAHYKQRVLELQTELRQSRNIMTNTQSENERLASINLEM
REHQQANGYSTPLHIRASGS
SHLHCLSSVLDGL
>g1419.t2
MVEIRKSSSSLSRALNARVDQWVRERPGQLQTLLGFPRGLGLFHAGVCTHAAFGQAYSNHRKVAADGESREESLIQESAC
KEAHYKQRVLELQTELRQSR
NIMTNTQSENERLASINLEMREHQQANGYSTPLHIRASGSSHLHCLSSVLDGL
>g1419.t3
MCKRSYSEIERAFGQAYSNHRKVAADGESREESLIQESACKEAHYKQRVLELQTELRQSRNIMTNTQSENERLASINLEM
REV
>g1420.t1
MTACRHLRYDPAAPPMVFPELAGRTASTKTNRDTSLPNGGQDPLVGLEFCGGCEDTSSVDYRARMQTNRPLVSGPSRQGK
HFSTAGPPGARIKQGTLLRT
DANMDQWTLKALKRRTLLWPAVTDTGNNKGSREELAAQGSSGKLLPRQVDYFYLAWRGDKCGALRQRNPPQETRVISYMS
VSPSKRGKPAQSLCSSQEGV
PCQVFALTPIPLRRPVTHISAKMWTHARSGQRALMPL

>g1420.t2
MTACRHLRYDPAAPPMVFPELAGRTASTKTNRDTSLPNGGQGLEFCGGCEDTSSVDYRARMQTNRPLVSGPSRQGKHFST
AGPPGARIKQGTLLRTDANM
DQWTLKALKRRTLLWPAVTDTGNNKGSREELAAQGSSGKLLPRQVDYFYLAWRGDKCGALRQRNPPQETRVISYMSVSPS
KRGKPAQSLCSSQEGVPCQV
FALTPIPLRRPVTHISAKMWTHARSGQRALMPL
>g1420.t3
LFLIHLCITMQAIRPAVTDTGNNKGSREELAAQGSSGKLLPRQVDYFYLAWRGDKCGALRQRNPPQETRVISYMSVSPSK
RGKPAQSLCSSQEGVPCQVF
ALTPIPLRRPVTHISAKMWTHARSGQRALMPL
>g1421.t1
MTYGPALTLRDFRVRSAWLGAVNCGQGNRGKEGRKGIPRADAGWTHKPCSLDQTSWLHLYLWSAAWLWQAVEQSAFSQAP
DHWSCSKLPSGSHLSHGYQP
YSYGFTSTSEAP
>g1421.t2
MATAFQMTYGPALTLRDFRVRSAWLGAVNCGQGNRGKEGRKGIPRADAGWTHKPCSLDQTSWLHLYLWSAAWLWQAVEQS
AFSQAPDHWSCSKLPSGSHL
SHGYQPYSYGFTSTSEAP
>g1422.t1
MALSSLHLLQDRRTRGPRTSQHYTAGHRDHRDAKDIKPIIHPATGQEQDVLQYDPKLVIFGSPCRLVKGLKKGECGARRE
EGDEEKALETTTMDPECIIQ
SYYTGEHLVPPPRETGEHREEEEEEEEEGGSSVGTAHRLRDHAHAACPPPPPLSRPQSPQTPSTMPC
>g1422.t2
MALSSLHLLQDRRTRGPRTSQHYTAGHRDHRDAKDIKPIIHPATGQEQDVLQYDPKLVIFGSPCRLVKGLKECIIQSYYT
GEHLVPPPRETGEHREEEEE
EEEEGGSSVGTAHRLRDHAHAACPPPPPLSRPQSPQTPSTMPC
>g1422.t3
MALSSLHLLQDRRTRGPRTSQHYTAGHRDHRDAKDIKPIIHPATGQEQDVLQYDPKLVIFGSPCRLVKGLK
>g1423.t1
DLLDSDWSSVRSVLEQADILVIKYSVNDKLAFQQVRNGYAPRLRPLLRHWGVPVILVAVGARLHGSEKPTISYESHESSL
GAEAQLSGTGFDLPAVLTHC
LSLFSS
>g1423.t2
DLLDSDWSSVRSVLEQADILVIKYSVNDKLAFQQVRNGYAPRLRPLLRHWGVPVILVAVGARLH
>g1423.t3
MPPPQPPPLLIQDLLDSDWSSVRSVLEQADILVIKYSVNDKLAFQQVRNGYAPRLRPLLRHWGVPVILVAVGARLHGSEK
PTISYESHESSLGAEAQLSG
TGFDLPAVLTHCLSLFSS
>g1424.t1
MLTGLRAEVTAGGDSPGCVSCGLPALDKDAEERLMIEVAKQQILSKLHLKERPNITQTVPRVALLTALRKLHAGRVRQDG
TLVLDNNLPTKDQSYEIVSF
AETEDRGSGYNTSLGLTFQFLQEHGQNMQVLQSSLWVYARSSGQSPRLRAQVFLSGTSGDSNRTLLMEKMLDVQESNWHT
FTITHPLQAFLDRGQSHLHL
EVSCREDGGENMCSPQGPSGSFRTSDNPHHQPFLVAKVRYRDDQSRHSLRKRSLRCGDDVTVCCKRDFYIKFKDIQWQDW
IIAPEGYHMNYCMGQCPQHL
SGSPGIASSFHATVFSQLKINGINTAVSSCCVPTERRPLSMMYFNSQHNIVKMDVPDMIVDSCGCT
>g1425.t1
MHSKETPSDSSDDGLDLHLKKIPPSPGRKEELSLYKKTKRAIISKKYGVSKHEAEATTPIELISRGPDGRFVMDPSDGEM
SVKPRRIEGFPFVEESDLYP
EFRQSDEENDDPRPLPPVMATLRPHQISPISSQESYLRPPAYSPRFQRPLEGMTIMESSRLQATGQIRSSLHHRAFYGYL
GSHRDHDPPPFYMPETSPLS
SVMSSPPYLAEGPFGHPMIPEDMVDGDYQQYAVSGFPLPLTLASSSRSPEIWQGVDFTFASLEGPHFIFPPQRPFHIHHH
DSPYPPPPPPPHVLPLSSFQ
PSPLPMPTYPAVLPLEASKGRPGKSPSKARPRGSPARHIAMQEAHLGQLRHTGHGMGVPVLPYAEQMARIVPSTFSSLDT
RWFETAPRFSPRQVRRMDPG
IHQVVLQPSRLSPLTQSPLSSHQGSPEILVRPRPRPSIVQPPIPPEMSEITLLPPSTASFSRRSSPSSSPAKGQASRRAS

PSFRPHMAFATSATSYPSQS
PSPLMESSNIFGQMPSQRRTEEGILPSEPSPTQLSASG
>g1426.t1
MNHRGAFSVPLAVLLSTATLWLLPKQASSDVGPDAERQNGGPHPLAAHAALAAGGLPVGCRPPRERWSALRGVKWEPDDV
EEETLCLRLALRCAYTSGTP
PPPP
>g1426.t2
MNHRGAFSVPLAVLLSTATLWLLPKQASSDVGPDAERHSSPRRQNGGPHPLAAHAALAAGGLPVGCRPPRERWSALRGVK
WEPDDVEEETLCLRLALRCA
YTSGTPPPPP
>g1427.t1
MSADDAGFETEVYNNSVVRTPNLRALAQRSLIFKNAFTSVSSCSPSRSTILTGLPQ
>g1428.t1
MEQPLLGGSVAGRWVGSWLPIVSWLPRYNLKWLQMDLIAGLTVGLTAVPQALAYAEVARLPVQTKICDPLKTGL
>g1429.t1
TYIFTDTTDEDISSEGFNLVVTDCLSDHSQQALSCKMSAEYDHFMASDKRWGYLICPCGAVG
>g1429.t2
TYIFTDTTDEDISSEGFNLVVTDCLSDHSQQALSCKMSAEYDHFMASDKR
>g1429.t3
TYIFTDTTDEDISSEGFNLVVTDCLSDHSQQALSCKMSAEYDHFMASDK
>g1430.t1
EEVLEVRSLQRNLALCNTCTKSSPHRPALCCPTERRDSLVSSVQ
>g1431.t1
RRSWRSAACRGTLHFVTRVQRAVLIDRLSAVPLRGGTPWCPRYNEEHKALLPLPPPGGPAGQSEAAGQPSDHQDDNTFPR
SEKRPEC
>g1432.t1
MASLEMPPQSKKQTALSRPDLVKVPLDIPVPDPAKEEAKRKKKEEKEAAEKGKKDKDSDKEDKEEDEGPPCGPIYSNQHV
EQALKEVKPQLNALKEKLNT
VSMWMQLQIPKIEDGNNFGVAVQEKVFELLTNTRTKIEAFQTQISKYYSERGDAVAKASKQPHVGDYRELVHQLDEYQYS
ELRLVVLEIRNTYAVLFDII
NKNYDKIMKPRGHSKALIY
>g1432.t2
MASLEMPPQSKKQVDDFCQQLTKETALSRPDLVKVPLDIPVPDPAKEEAKRKKKEEKEAAEKGKKDKDSDKEDKEEDEGP
PCGPIYSNQHVEQALKEVKP
QLNALKEKLNTVSMWMQLQIPKIEDGNNFGVAVQEKVFELLTNTRTKIEAFQTQISKYYSERGDAVAKASKQPHVGDYRE
LVHQLDEYQYSELRLVVLEI
RNTYAVLFDIINKNYDKIMKPRGHSKALIY
>g1433.t1
LRRHRNNIFTDLYRKCFLLARMLKLDTQKLNTGPCRAAAQRCGQEGGEEHQATSSHPHQESSPQCHS
>g1434.t1
YDVLTGAVVARLCGHDACVRDVSWHPYQDSIISSSWDGAVRMWEHRQTHPLEELRHQE
>g1435.t1
GLPTNFKDLIQAKAEENNIVFMPLVAKRYEGKQLYTFGRIVIYIDRGVVFVQGEKTWVPTSLQSLIDMAK
>g1436.t1
MSSEALKKRKGKVLRNEGGTPEVKRGRGEGDPQ
>g1437.t1
ICQDVTMSSNSTDPGIFLTLITSRPGGAAYPESNALHQRGESGDPWPLLRLSALDDNASLCGGDLYNLTPDADNSTTAVA
GSYPAWQVAFIVLLTSLLSL
VTIIGNVLVVVSFKVNRQLKTVNNYFLLSLAVADLIIGVISMNLYTAYIVMGQWSMGNWACDLWLAIDYVASNASVMNLL
VISFDRYFSVTRPLTYRAKR
TTKRAVLMIGLAWFVSLVLWAPAILLWQYFVGERTVPSNECYIQFLSEPTITFCTAVAAFYLPVTIMSVLYWRIYQETQN
>g1438.t1
LTRCFVFHQLDDELMDLQKKMKQTEDELDKYSEGLKDAQEKLELADKKATDFTARGLHASARRELPLITPGLASNAGISR
FLGSQS
>g1439.t1
PLGQRRPDAAAMSQDEEHNAKQKDSVERLEKERIEKEVQARVEKERIEREKREEIERVEKEKEKLSKEKAAREMAEREQR

ERHEKVRIEQERVEQERMEQ
ERIQERVEQARRAEELQKMEGEINAPAMDKDKDKEEEEEAQLGRVKAAQEQEENKLDEPLKKGGRDLKERLEEREDEAIA
AQRSEEKDTDLRRRRRALEP
LRDAPRPGGVPGLEPLLDLGGADLHAALEGQLLGGAMVRSRQIKQTTETEKDV
>g1439.t2
MSQDEEHNAKQKDSVERLEKERIEKEVQARVEKERIEREKREEIERVEKEKEKLSKEKAAREMAEREQRERHEKVRIEQE
RVEQERMEQERIQERVEQAR
RAEELQKMEGEINAPAMDKDKDKEEEEEAQLGRVKAAQEQEENKLDEPLKKGGRDLKERLEEREDEAIAAQRSEEKDTDL
RRRRRALEPLRDAPRPGGVP
GLEPLLDLGGADLHAALEGQLLGGAMVRSRQIKQTTETEKDV
>g1440.t1
MSGPNVWTRSRDRFRRFPELVAQCPAEASAYGACVVATTGKQELKKDLCVKEFEALKTCFLNAVSMMQSQTQS
>g1440.t2
MSGPNVWTRSRDRFRRFPELVAQCPAEASAYGACVVATTGKQELKKDLCVKEFEALKTCFLNAVMMRVRGRCVEHPITK
ASSSARNVSVERVVL
>g1441.t1
MTVEISHESAGCGQFLLEYLLERLQVDSCHVKIKVLKIFVHLCAHGSHHFLTELRRNSTFIQQASVYSGPADPIHGTALF
QKVRNTAQEVGRLLFTDTLL
EKSPGSHSCVAAQATGMGSEATHRTAMQGFGCSPGKQGAASSSSSLLDKIQHAAGVVASAVLPPHEQQGIRLHDNHYRAV
AAPPTPIEVAVQACAYELPP
PRGASGTQRCPGQVGGGWEEMDSSHCSSHNSSQGAAANSRASVGRSSKSGGTGSQSGGSRESSGDLSDRVEAMQSGDCGQ
EMALISRLTEGSRVFLTREE
SQYFIKECSTLNCEVVVELLSRQLQEPSHTVQMRALCAVACFLTSDLLSLEQIYGAMHHRLSNLSEGPPGPLTNKATKIL
RQLEAMLGGAGRIPRQDVAA
SDHLASEEQLFNSAFPVAPPPPAAGLPTGLSRLNGSQETPLTVKGPSTDVPEQPSPSGPARDQSPPSARDHGETPQSPAE
ESGGSQAEENSAGRSVRPGG
GGSGSSSASEPPCVGRLSLFRGMELVARPGAPSFSPGTDLRDAAGLCHAGGNSDTDDYSQTLPNSAGSCFSKTSDVASQS
SQTVSAFSFLNC
>g1441.t2
MTVEISHESAGCGQFLLEYLLERLQVDSCHVKIKVLKIFVHLCAHGSHHFLTELRRNSTFIQQASVYSGPADPIHGTALF
QKVRNTAQEVGRLLFTDTLL
EKSPGSHSCVAAQATGMGSEATHRTAMQGFGCSPGKQGAASSSSSLLDKIQHAAGVVASAVLPPHEQQGIRLHDNHYRAV
AAPPTPIEVAVQACAYELPP
PRGASGQCVEVLRCPGQVGGGWEEMDSSHCSSHNSSQGAAANSRASVGRSSKSGGTGSQSGGSRESSGDLSDRVEAMQSG
DCGQEMALISRLTEGSRVFL
TREESQYFIKECSTLNCEVVVELLSRQLQEPSHTVQMRALCAVACFLTSDLLSLEQIYGAMHHRLSNLSEGPPGPLTNKA
TKILRQLEAMLGGAGRIPRQ
DVAASDHLASEEQLFNSAFPVAPPPPAAGLPTGLSRLNGSQETPLTVKGPSTDVPEQPSPSGPARDQSPPSARDHGETPQ
SPAEESGGSQAEENSAGRSV
RPGGGGSGSSSASEPPCVGRLSLFRGMELVARPGAPSFSPGTDLRDAAGLCHAGGNSDTDDYSQTLPNSAGSCFSKTSDV
ASQSSQTVSAFSFLNC
>g1442.t1
RGQSNCISQSSFSPQPEDSASASLQGFHGNGGSSSSSSSSSAGVSYHHINRVLREAHFSSLQIRALPGSTLRVMEKICRH
QDTTSHKTLAIYRLLGEFLE
AGGCAREVHKTTLCSTRHETFLFVKLGFFCT
>g1442.t2
RGQSNCISQSSFSPQPEDSASASLQGFHGNGGSSSSSSSSSAGVSYHHINRVLREAHFSSLQIRALPGSTTRHGRSRLAT
IKHRDLRVMEKICRHQDTTS
HKTLAIYRLLGEFLEAGGCAREVHKTTLCSTRHETFLFVKLGFFCT
>g1443.t1
SRDRERPRERERDKERGGASSRYRRAPEFWWSSGGEPRLLDPLVENNVHLTVLKDVLMENIVHLTVLKDILMENIIYWTV
QKTNF
>g1444.t1
MDAAFYHDTFKPPPTGHSVPPSSCLSANSMSADTLLPFLYTDHAPQPRTTQEQEGFADGFVRALASLHQQGGGVSGGIGA
NGGSSVSSAGGVICASVTSN
LCSAIGGLSSSRGDISFSSASGGSVDIGPSNLSGGTLVYTNLAANCHAPQHHKVTTAYQPVPGGISMTTAYQANADVSER

KKLRNRIAASRCRHRKLERI
ARLEERVRVLRSLNGRLVAKATRLHGQVGALRSRAAKHLTGGCQVNYHYR
>g1445.t1
LPCALCVCLTSPVDTAVSRPVWPGHCAVVDNMSRLLCLLFRRALTPRRPGFESVSATAASPRGDSHRRLI
>g1446.t1
MAETDVDNELLDYEEDEEPQGAPESVAPAGKKEVKGSYVSIHSSGFRDFLLKPELLRAIVDCGFEHPSEVQHECIPQAIL
GMDILCQAKSGMGKTAVFVL
ATLQQIEPVDGQVSVLVMCHTRELAFQISKEYERFSKYMPTVKAAVFFGGMAIKKDEDVLKKNCPHIVVGTPGRILALIR
NKTLNLKNVKHFVLDECDKM
LEQLDMRRDVQDIFRLTPHEKQCMMFSATLSKEIRPVCRKFMQDPMEVFVDDETKLTLHGLQQYYCKLKDSEKNRKLFDL
LDVLEFNQVVIFVKSVQRCV
ALSQLLVEQNFPAIAIHRGMAQEER
>g1446.t2
MAETDVDNELLDYEEDEEPQGAPESVAPAGKKEVKGSYVSIHSSGFRDFLLKPELLRAIVDCGFEHPSEVQHECIPQAIL
GMDILCQAKSGMGKTAVFVL
ATLQQIEPVDGQVSVLVMCHTRELAFQISKEYERFSKYMPTVKAAVFFGGMAIKKDEDVLKKNCPHIVVGTPGRILALIR
NKTLNLKNVKHFVLDECDKM
LEQLDMRRDVQDIFRLTPHEKQCMMFSATLSKEIRPVCRKFMQDPMEVFVDDETKLTLHGLQQYYCKLKDSEKNRKLFDL
LDVLEFNQVVIFVKSVQRCV
ALSQLLVEQNFPAIAIHRGMAQEERSAGVWQKPRPSSDGSQPTELPQVTNGPQFGHQGETFTKLPDQSHSVVFSSSGHVD
TPCGAP
>g1447.t1
MKTSEHFEELLKALGVNVFVRKIAVAAASSPSVEISQEGESLHIQTSTSVRTTQVSFTVGKSFNETTVDGRPCTSFPKWE
TENKISCEQTLQKGEGPKTA
WTRELTGHGELILTMRAGDVVCTRVYERA
>g1447.t2
MVPPCSGVNVFVRKIAVAAASSPSVEISQEGESLHIQTSTSVRTTQVSFTVGKSFNETTVDGRPCTSFPKWETENKISCE
QTLQKGEGPKTAWTRELTGH
GELILTMRAGDVVCTRVYERA
>g1447.t3
MKTSEHFEELLKALGVNVFVRKIAVAAASSPSVEISQEGESLHIQTSTSVRTTQVSFTVGKSFNETTVDGRPCTFMSSAS
LQSFPKWETENKISCEQTLQ
KGEGPKTAWTRELTGHGELILTMRAGDVVCTRVYERA
>g1448.t1
MTTMALHYSSRPEEGSGDPARLTEALQKRVKRQENLLLKCKELLHTHKERSAQLGSENETLQEQLQERLQELEKMKELHT
TEKTKLITQLRDAKNLIEQL
EQDKGMVIAETKRQMHETLEMKEDEIAQLRTRIQQITSQKEELQEQKEKNEKSELERALGMAQRAEEARKQLQVQLEEQV
KEVEREGEEERKSLQQELSR
VKQEVVSIMKEQCREEFSQAAKEREQQSSLALEDAELQKTALKTEAENKVKEAQLELEAARTRILELESMVEKSSEDGSV
QSNDFSNQMKELKAKHKEQM
STLKQKQRAQLEKHTDTLTQQHNAALETLKQNHSLEMETILKDKELQFQAHIEDMNQKTLEKLDMKQTELEVLASELTEA
LRVKRLLEEKLAALDGSVSL
TQKAMEQRLQDEVAKHNAEMANIMQQHEQSLGGMEKTLKEELNNLKMVLEEKQRELEEHILVEKTLKEKSEAALQDLNVK
SSEMEELSRSLSQAQSAKNH
LEDSNAQLSNITKDLLQCKKQLKQLEHQLEMTQADFKEKDELLQQKLKELEEKEQQLQQAREEAKLRLEEVTTRQEEEQR
LRKELDEEKAAHENKIASTV
KEMDGKLKTQETKMDKIKQKAKEMQEKFKKRLQENEESMKKELAKKEDELQQKEQQVRDKILDMAQKSSHGLSSAMSELQ
TNHQEELEKLRKAHKNEVED
LEHHWREKLRQQEEDVAEKHLHVLQEKALELEDTSQQLSTSREEKDQALLEINKFKEELAIRETTVQKLQVELKEAAAKV
DGLSQSEALLKEQIESAERN
LNQALNERNGLQDQLSRAEEESRANLKALSEKFDATEGTLKALESSKSKHGEDLERKLEESALQLQAKEGDFQKQFMVIT
NQMEHYCQEVQSKVESGTTD
LYERVEFRVTGLKDRVMCNQKRIEKLNTFILTKLDTICTLEEKLHKQTEENMNLCSSIEQLTAQLGVQAEHIEALTNERD
GLLKEAESHSQSISENVLRV
QQLSEENKTISENMSANVLHISNLESIIDTLKSQLAGSTMEKEEAINLLNQRCKEESQQIVANMRESIERLEQEKMSAVE
QADALRNSLSEFKNKAESRF

TQNHHTVVSLQDRLQDLEREILEKNEALQRMTANIDNQSISKSEMDQALSEKEQRVSALASELESCNGRLCELQDQLALK
TKECEQLSADLKQQLSIRES
EKREFSEQLQQTQEQFTQNGHLFQETEDKLRLLEKENHTCKLELEAQRVEFEKMSSEILKSKEESLKDTEERLSAESAKK
MAELKKKAEQKIGQIKKQLT
SQIEEKEQIVQSLKSSLEKINEKEAGNKRHIETLEEQGRTLEEKLGKLQEDHEKRLEQTQSDQRLEKEKSLEELRRDYEE
KMLLLQRDETAALDAKADET
HAKLEELGGKLSEAEEQNRNLVAEMGHLKEELLEREARLDRQQVSTASMEEQTLVEQEDVVRMEQSSTVHAPRPEKVDQR
DEDGESLESLKEKLTEVKNE
KLKIQKDFTRLQKDFRSLRREHEQDLDHLKKELAEENEKNLKLELEDLEMKQNSALKQLMRDFNTQLALKEREIELTVKE
TIGKAQSVEAEIISSHREEV
SQLHQLMAQKDDDLQRTVQKYEQVLQSREEEMGGRVWQVQKELEDLQARAGGDSE
>g1448.t2
MALHYSSRPEEGSGDPARLTEALQKRVKRQENLLLKCKELLHTHKERSAQLGSENETLQEQLQERLQELEKMKELHTTEK
TKLITQLRDAKNLIEQLEQD
KGMVIAETKRQMHETLEMKEDEIAQLRTRIQQITSQKEELQEQKEKNEKSELERALGMAQRAEEARKQLQVQLEEQVKEV
EREGEEERKSLQQELSRVKQ
EVVSIMKEQCREEFSQAAKEREQQSSLALEDAELQKTALKTEAENKVKEAQLELEAARTRILELESMVEKSSEDGSVQSN
DFSNQMKELKAKHKEQMSTL
KQKQRAQLEKHTDTLTQQHNAALETLKQNHSLEMETILKDKELQFQAHIEDMNQKTLEKLDMKQTELEVLASELTEALRV
KRLLEEKLAALDGSVSLTQK
AMEQRLQDEVAKHNAEMANIMQQHEQSLGGMEKTLKEELNNLKMVLEEKQRELEEHILVEKTLKEKSEAALQDLNVKSSE
MEELSRSLSQAQSAKNHLED
SNAQLSNITKDLLQCKKQLKQLEHQLEMTQADFKEKDELLQQKLKELEEKEQQLQQAREEAKLRLEEVTTRQEEEQRLRK
ELDEEKAAHENKIASTVKEM
DGKLKTQETKMDKIKQKAKEMQEKFKKRLQENEESMKKELAKKEDELQQKEQQVRDKILDMAQKSSHGLSSAMSELQTNH
QEELEKLRKAHKNEVEDLEH
HWREKLRQQEEDVAEKHLHVLQEKALELEDTSQQLSTSREEKDQALLEINKFKEELAIRETTVQKLQVELKEAAAKVDGL
SQSEALLKEQIESAERNLNQ
ALNERNGLQDQLSRAEEESRANLKALSEKFDATEGTLKALESSKSKHGEDLERKLEESALQLQAKEGDFQKQFMVITNQM
EHYCQEVQSKVESGTTDLYE
RVEFRVTGLKDRVMCNQKRIEKLNTFILTKLDTICTLEEKLHKQTEENMNLCSSIEQLTAQLGVQAEHIEALTNERDGLL
KEAESHSQSISENVLRVQQL
SEENKTISENMSANVLHISNLESIIDTLKSQLAGSTMEKEEAINLLNQRCKEESQQIVANMRESIERLEQEKMSAVEQAD
ALRNSLSEFKNKAESRFTQN
HHTVVSLQDRLQDLEREILEKNEALQRMTANIDNQSISKSEMDQALSEKEQRVSALASELESCNGRLCELQDQLALKTKE
CEQLSADLKQQLSIRESEKR
EFSEQLQQTQEQFTQNGHLFQETEDKLRLLEKENHTCKLELEAQRVEFEKMSSEILKSKEESLKDTEERLSAESAKKMAE
LKKKAEQKIGQIKKQLTSQI
EEKEQIVQSLKSSLEKINEKEAGNKRHIETLEEQGRTLEEKLGKLQEDHEKRLEQTQSDQRLEKEKSLEELRRDYEEKML
LLQRDETAALDAKADETHAK
LEELGGKLSEAEEQNRNLVAEMGHLKEELLEREARLDRQQVSTASMEEQTLVEQEDVVRMEQSSTVHAPRPEKVDQRDED
GESLESLKEKLTEVKNEKLK
IQKDFTRLQKDFRSLRREHEQDLDHLKKELAEENEKNLKLELEDLEMKQNSALKQLMRDFNTQLALKEREIELTVKETIG
KAQSVEAEIISSHREEVSQL
HQLMAQKDDDLQRTVQKYEQVLQSREEEMGGRVWQVQKELEDLQARAGGDSE
>g1448.t3
MDQLAKKHLQEEFDATLEEKDQIITVLQTQVALMKKRLKGVDELTVPSEDEVSVPEDAANTASGAPQTPSKDKDAEGVPT
EEEGSGDPARLTEALQKRVK
RQENLLLKCKELLHTHKERSAQLGSENETLQEQLQERLQELEKMKELHTTEKTKLITQLRDAKNLIEQLEQDKGMVIAET
KRQMHETLEMKEDEIAQLRT
RIQQITSQKEELQEQKEKNEKSELERALGMAQRAEEARKQLQVQLEEQVKEVEREGEEERKSLQQELSRVKQEVVSIMKE
QCREEFSQAAKEREQQSSLA
LEDAELQKTALKTEAENKVKEAQLELEAARTRILELESMVEKSSEDGSVQSNDFSNQMKELKAKHKEQMSTLKQKQRAQL
EKHTDTLTQQHNAALETLKQ
NHSLEMETILKDKELQFQAHIEDMNQKTLEKLDMKQTELEVLASELTEALRVKRLLEEKLAALDGSVSLTQKAMEQRLQD
EVAKHNAEMANIMQQHEQSL

GGMEKTLKEELNNLKMVLEEKQRELEEHILVEKTLKEKSEAALQDLNVKSSEMEELSRSLSQAQSAKNHLEDSNAQLSNI
TKDLLQCKKQLKQLEHQLEM
TQADFKEKDELLQQKLKELEEKEQQLQQAREEAKLRLEEVTTRQEEEQRLRKELDEEKAAHENKIASTVKEMDGKLKTQE
TKMDKIKQKAKEMQEKFKKR
LQENEESMKKELAKKEDELQQKEQQVRDKILDMAQKSSHGLSSAMSELQTNHQEELEKLRKAHKNEVEDLEHHWREKLRQ
QEEDVAEKHLHVLQEKALEL
EDTSQQLSTSREEKDQALLEINKFKEELAIRETTVQKLQVELKEAAAKVDGLSQSEALLKEQIESAERNLNQALNERNGL
QDQLSRAEEESRANLKALSE
KFDATEGTLKALESSKSKHGEDLERKLEESALQLQAKEGDFQKQFMVITNQMEHYCQEVQSKVESGTTDLYERVEFRVTG
LKDRVMCNQKRIEKLNTFIL
TKLDTICTLEEKLHKQTEENMNLCSSIEQLTAQLGVQAEHIEALTNERDGLLKEAESHSQSISENVLRVQQLSEENKTIS
ENMSANVLHISNLESIIDTL
KSQLAGSTMEKEEAINLLNQRCKEESQQIVANMRESIERLEQEKMSAVEQADALRNSLSEFKNKAESRFTQNHHTVVSLQ
DRLQDLEREILEKNEALQRM
TANIDNQSISKSEMDQALSEKEQRVSALASELESCNGRLCELQDQLALKTKECEQLSADLKQQLSIRESEKREFSEQLQQ
TQEQFTQNGHLFQETEDKLR
LLEKENHTCKLELEAQRVEFEKMSSEILKSKEESLKDTEERLSAESAKKMAELKKKAEQKIGQIKKQLTSQIEEKEQIVQ
SLKSSLEKINEKEAGNKRHI
ETLEEQGRTLEEKLGKLQEDHEKRLEQTQSDQRLEKEKSLEELRRDYEEKMLLLQRDETAALDAKADETHAKLEELGGKL
SEAEEQNRNLVAEMGHLKEE
LLEREARLDRQQVSTASMEEQTLVEQEDVVRMEQSSTVHAPRPEKVDQRDEDGESLESLKEKLTEVKNEKLKIQKDFTRL
QKDFRSLRREHEQDLDHLKK
ELAEENEKNLKLELEDLEMKQNSALKQLMRDFNTQLALKEREIELTVKETIGKAQSVEAEIISSHREEVSQLHQLMAQKD
DDLQRTVQKYEQVLQSREEE
MGGRVWQVQKELEDLQARAGGDSE
>g1448.t4
MDQLAKKHLQEEFDATLEEKDQIITVLQTQVALMKKRLKGVDELTVPSEDEVSVPEDAANTASGAPQTPSKDKDAEGVPT
EGQRAAE
>g1448.t5
MDQLAKKHLQEEFDATLEEKDQIITVLQTQSSVSFSLTPPLAKKDMTQLRAQVALMKKRLKGVDELTVPSEDEVSVPEDA
ANTASGAPQTPSKDKDAEGV
PTEGQRAAE
>g1449.t1
PPTQPLRDSQFYSDLPSPPPLTLPSAEGVKLEHHTKFRPKPPNRTKPSRVRLGPSSSEPQGAEQSLLGKLDNGLDAFFHR
TVNRLSFKQGSPGTQEVVDR
KRESRRSGFLQLLKPRTSRSSITAPPPTTVVMVTEETGGELQLKDPPAESQPPSPKNSPPGPTREDEEEKEEEKEEEEKE
EHHPALHHFGVPLLGLDLLA
EMKARQERKAATQKAPCPADGHAAGPPPTDLSDQPPETAPHSRPQPFTPRPPKPPLGPRDSGPPSTSSRPSSTTGPEERS
TREPLPAPLPAPRLKKPSEQ
EIEDASLGGPESPPEAELLGDSSAFDSPDEGSRGGGRQWSSLKNTPPPTENLNRQRATSLPSHAVLRWWDGGAWRPAFGS
IVTGNSELNTAHLLLLEQEK
LTVISREMEEVMERECSALGGLFQTLMGDMKSSSPVWEDLISKATKLQSHLRTTVVMVAAFLDAFQKVADLATNSRGGTR
EIGGALTRMCMRQRSIEAKL
RHFSMVFLDCLILPLQDQMEDWKRNTHTLDKDHAK
>g1449.t2
PPTQPLRDSQFYSDLPSPPPLTLPSAEGVKLEHHTKFRPKPPNRTKPSRVRLGPSSSEPQGAEQSLLGKLDNGLDAFFHR
TVNRLSFKQGSPGTQEVVDR
KRESRRSGFLQLLKPRTSRSSITAPPPTTVVMVTEETGGELQLKDPPAESQPPSPKNSPPGPTREDEEEKEEEKEEEEKE
EHHPALHHFGVPLLGLDLLA
EMKARQERKAATQKAPCPADGHAGPPPTDLSDQPPETAPHSRPQPFTPRPPKPPLGPRDSGPPSTSSRPSSTTGPEERST
REPLPAPLPAPRLKKPSEQE
IEDASLGGPESPPEAELLGDSSAFDSPDEGSRGGGRQWSSLKNTPPPTENLNRQRATSLPSHAVLRWWDGGAWRPAFGSI
VTGNSELNTAHLLLLEQEKL
TVISREMEEVMERECSALGGLFQTLMGDMKSSSPVWEDLISKATKLQSHLRTTVVMVAAFLDAFQKVADLATNSRGGTRE
IGGALTRMCMRQRSIEAKLR
HFSMVFLDCLILPLQDQMEDWKRNTHTLDKDHAK

>g1449.t3
PPTQPLRDSQFYSDLPSPPPLTLPSAEGVKLEHHTKFRPKPPNRTKPSRVRLGPSSSEPQGAEQSLLGKLDNGLDAFFHR
TVNRLSFKQGSPGTQEVVDR
KRESRRSGFLQLLKPRTSRSSITAPPPTTVVMVTEETGGELQLKDPPAESQPPSPKNSPPGPTREDEEEKEEEKEEEEKE
EHHPALHHFGVPLLGLDLLA
EMKARQERKAATQKAPCPADGHAAGPPPTDLSDQPPETAPHSRPQPFTPRPPKPPLGPRDSGPPSTSSRPSSTTGPEERS
TREPLPAPLPAPRLKKPSEQ
EIEDASLGGPESPPEAELLGDSSAFDSPDEGSRGGGRQWSSLKNTPPPTENLNRQRATSLPSHAVLRWWDGGAWRPAFGS
IVTGNSELNTAHLLLLEQEK
LTVISREMEEVMERECSALGGLFQTLMGDMKSSSPVWEDLISKATKLQSHLRTTVVMVAAFLDAFQKVADLATNSRGGTR
EIGGALTRMCMRQRSIEAKL
RHFSM
>g1450.t1
MNLDSLSLALSQISYLVDNLTKKNYRASQQEIQHFLIQECVSLISKPNFISSLCYAIDNPLHYQKSLKPSAHLFTQLSKV
LKLSKVQEVIFGLALLNSCN
TDLRGFAAVFVKQKLPDLLRSYVDADLGGHQEGGFQDIAIEVLHLLLSHLLFGQKGASGVGQEQTEAFLNTLCRDFPQDR
CPVVLAPLLYPEKRDILMDR
ILPDSGELAKTMMESSLADFMQEVGYGFCASVDECRNIILQYGVREVTASQVARVLGMMARTHSGLADGIPLQSISTPGS
SIWSDGKDKSDGSQTHTWNV
DVFIDVVKEVNPNLNFKEVTYELDHHGFMIRDSKGLKIVVCGIVRGLAMELFPVDLIYRPWKHAEGQLSFIQHSLMSPEV
FCFADYPCHTVAIDILKAPP
EDDNKEIATWKSLDLVESLLRLSEVGQYEPVKQLFSFPIKHCPDMLVLALLQISTSWHTLRHELISTLMPIFLGNHPNSA
IILHYAWHGQGQSPSIRQLI
MHSMAEWYMRGEHYDQAKLSRILDVAQDLKSLSMLLNATPFAFVIDLAALASRREYLKLDKWLTDKIREHGEPFIQACVT
FLKRRCPSIMGGLAPDKEQP
KSAQLPPETLATMLACLQSCAGYDTSVVHHK
>g1450.t2
MNLDSLSLALSQISYLVDNLTKKNYRASQQEIQHIVNCHGPEADRHLLRCLFSHVDFSGDGKSSGKDFHQFLIQECVSLI
SKPNFISSLCYAIDNPLHYQ
KSLKPSAHLFTQLSKVLKLSKVQEVIFGLALLNSCNTDLRGFAAVFVKQKLPDLLRSYVDADLGGHQEGGFQDIAIEVLH
LLLSHLLFGQKGASGVGQEQ
TEAFLNTLCRDFPQDRCPVVLAPLLYPEKRDILMDRILPDSGELAKTMMESSLADFMQEVGYGFCASVDECRNIILQYGV
REVTASQVARVLGMMARTHS
GLADGIPLQSISTPGSSIWSDGKDKSDGSQTHTWNVDVFIDVVKEVNPNLNFKEVTYELDHHGFMIRDSKGLKIVVCGIV
RGLAMELFPVDLIYRPWKHA
EGQLSFIQHSLMSPEVFCFADYPCHTVAIDILKAPPEDDNKEIATWKSLDLVESLLRLSEVGQYEPVKQLFSFPIKHCPD
MLVLALLQISTSWHTLRHEL
ISTLMPIFLGNHPNSAIILHYAWHGQGQSPSIRQLIMHSMAEWYMRGEHYDQAKLSRILDVAQDLKSLSMLLNATPFAFV
IDLAALASRREYLKLDKWLT
DKIREHGEPFIQACVTFLKRRCPSIMGGLAPDKEQPKSAQLPPETLATMLACLQSCAGYDTSVVHHK
>g1450.t3
MNLDSLSLALSQISYLVDNLTKKNYRASQQEIQHTQFLIQECVSLISKPNFISSLCYAIDNPLHYQKSLKPSAHLFTQLS
KVLKLSKVQEVIFGLALLNS
CNTDLRGFAAVFVKQKLPDLLRSYVDADLGGHQEGGFQDIAIEVLHLLLSHLLFGQKGASGVGQEQTEAFLNTLCRDFPQ
DRCPVVLAPLLYPEKRDILM
DRILPDSGELAKTMMESSLADFMQEVGYGFCASVDECRNIILQYGVREVTASQVARVLGMMARTHSGLADGIPLQSISTP
GSSIWSDGKDKSDGSQTHTW
NVDVFIDVVKEVNPNLNFKEVTYELDHHGFMIRDSKGLKIVVCGIVRGLAMELFPVDLIYRPWKHAEGQLSFIQHSLMSP
EVFCFADYPCHTVAIDILKA
PPEDDNKEIATWKSLDLVESLLRLSEVGQYEPVKQLFSFPIKHCPDMLVLALLQISTSWHTLRHELISTLMPIFLGNHPN
SAIILHYAWHGQGQSPSIRQ
LIMHSMAEWYMRGEHYDQAKLSRILDVAQDLKSLSMLLNATPFAFVIDLAALASRREYLKLDKWLTDKIREHGEPFIQAC
VTFLKRRCPSIMGGLAPDKE
QPKSAQLPPETLATMLACLQSCAGYDTSVVHHK
>g1451.t1
MVANCSNVMTKARQPPPGVMPKGRAPSTSSLDAISPVQIDPLSAMGSLNLGGSTTSHTQNMQGFPSSLSSAFSNPQSPAK

AFPPLTNPNPSTPFGGIGLS
SQLPGMDSGPLGSGIGSSIGSGLGMPAVTSDPFGTRKMSTPGLNPPTFQQTDLSQVWPEANQHFSKEIDDEANSYFQRIY
NHPPHPTMSVDEVLEMLQRF
KDSTIKREREVFNCMLRNLFEEYRFFPQYPDKELHITACLFGGIIEKGLVTYMALGLALRYVLEALRKPFGSKMYYFGIA
ALDRFKNRLKDYPQYCQHLA
SIGHFLQFPHHLQEYIEYGQQSRDPPVKMQGSITTPGSLALAQVQAQSQQPGVPKAPQPGQASTLGTSTTTTTTVAKTTT
IARPTPSSFKKDVPPSINTT
NIDTLLVATDQTERIVEPPENVQEKIAFIFNNLSQSNMTQKVEELKETVKEEFMPWVSQYLVMKRVSIEPNFHSLYSNFL
DTLKNPEFGKMVLSETYRNI
KVLLTSDKAAANFSDRSLLKNLGHWLGMITLAKNKPILYTDLEVKSLLLEAYVKGQQELLYVVPFVAKVLESSLRSVVFR
PQNPWTMAIMNVLAELHQEH
DLKLNLKFEIEVLCKNLSLDINDLKPGNLLKDKDKLKSLEEQLSAPKKEAKPPEEMLPIVTTVAPSTPAATTTTCTTTGP
PTPQFSYHDINVYALAGLAP
HINININIPLLQAHPQLKQCVRQSVERAVQELVHPVVDRSIKIAMTTCEQIVRKDFALDSEESRMRVAAHHMMRNLTAGM
AMITCREPLLMSIATNLKNS
FAAALRAPTPQQREMMEDAAARIAQDNCELACCFIQKTAVEKAGPEMDKRLATEFELRKHARQEGRRYCDPVVLTYQAER
MPEQIRLKVGGVDPKQLAVY
EEFARNVPGFLPSNDLSQPTGFLAQPMKQQAWATDDVAQIYDKCMADLEQHLHAVPPTLALNPQTQALRSLLEAVALARN
SRDGIAALGLLQKAVEGLLD
ATSGADADLLLRYRECHLLVLKALQDGRAYGPQWCNKQITRCLIECRDEYKYNVEAVELLIRNHLVNMQQYDLHLAQSME
NGLHYMAVAFAMQLVKLLLV
DERSVGHITEADLFHTIETLMRTSAHSRANAPEGLPQLMDVVRSNYEAMIDRAHGGPNFMMHSGISQASEYDDPPGLREK
AEYLLREWVNLYHSAAAGRD
STKAFSAFVGQMHQQGILKTDDLITRFFRLCTEMCVEISYRAQAEQQHNPAASAAIIRAKCYHNLDAFVRLIALLVKHSG
EATNTVTKINLLNKVRPGHG
LMLKVLGIVVGVLIQDHDVRQTEFQQLPYHRIFIMLLLELNAPEHVLETINFQTLTAFCNTFHILRPTKAPGFVYAWLEL
ISHRIFIARMLAHTPQQKGW
PMYAQLLIDLFKYLAPFLRNVELNKPMQILYKGTLRVLLVLLHDFPEFLCDYHYGFCDVIPPNCIQLRNLILSAFPRNMR
LPDPFTPNLKVDMLSEINIA
PRILTNFTGVMPSQFKKDLDSYLKTRSPVTFLSELRSNLQVSNEPGNRYNIQLINALVLYVGTQAIAHIHNKGSTPSMST
ITHSAHMDIFQNLAVDLDTE
GRYLFLNAIANQLRYPNSHTHYFSCTMLYLFAEANTEAIQEQITRVLLERLIVNRPHPWGLLITFIELIKNPAFKFWSHD
FVHCAPEIEKLFQSVAQCCM
GQKQAQQVMEGTGAS
>g1451.t2
MVANCSNVMTKARQPPPGVMPKGRAPSTSSLDAISPVQIDPLSAMGSLNLGGSTTSHTQNMQGFPSSLSSAFSNPQSPAK
AFPPLTNPNPSTPFGGIGLS
SQLPGMDSGPLGSGIGSSIGSGLGMPAVTSDPFGTRKMSTPGLNPPTFQQTDLSQVWPEANQHFSKEIDDEANSYFQRIY
NHPPHPTMSVDEVLEMLQRF
KDSTIKREREVFNCMLRNLFEEYRFFPQYPDKELHITACLFGGIIEKGLVTYMALGLALRYVLEALRKPFGSKMYYFGIA
ALDRFKNRLKDYPQYCQHLA
SIGHFLQFPHHLQEYIEYGQQSRDPPVKMQGSITTPGSLALAQVQAQSQQPGVPKAPQPGQASTLGTSTTTTTTVAKTTT
IARPTPSSFKKDVPPSINTT
NIDTLLVATDQTERIVEPPENVQEKIAFIFNNLSQSNMTQKVEELKETVKEEFMPWVSQYLVMKRVSIEPNFHSLYSNFL
DTLKNPEFGKMVLSETYRNI
KVLLTSDKAAANFSDRSLLKNLGHWLGMITLAKNKPILYTDLEVKSLLLEAYVKGQQELLYVVPFVAKVLESSLRSVVFR
PQNPWTMAIMNVLAELHQEH
DLKLNLKFEIEVLCKNLSLDINDLKPGNLLKDKDKLKSLEEQLSAPKKEAKPPEEMLPIVTTGDFLPFAVAPSTPAATTT
TCTTTGPPTPQFSYHDINVY
ALAGLAPHINININIPLLQAHPQLKQCVRQSVERAVQELVHPVVDRSIKIAMTTCEQIVRKDFALDSEESRMRVAAHHMM
RNLTAGMAMITCREPLLMSI
ATNLKNSFAAALRAPTPQQREMMEDAAARIAQDNCELACCFIQKTAVEKAGPEMDKRLATEFELRKHARQEGRRYCDPVV
LTYQAERMPEQIRLKVGGVD
PKQLAVYEEFARNVPGFLPSNDLSQPTGFLAQPMKQQAWATDDVAQIYDKCMADLEQHLHAVPPTLALNPQTQALRSLLE
AVALARNSRDGIAALGLLQK
AVEGLLDATSGADADLLLRYRECHLLVLKALQDGRAYGPQWCNKQITRCLIECRDEYKYNVEAVELLIRNHLVNMQQYDL

HLAQSMENGLHYMAVAFAMQ
LVKLLLVDERSVGHITEADLFHTIETLMRTSAHSRANAPEGLPQLMDVVRSNYEAMIDRAHGGPNFMMHSGISQASEYDD
PPGLREKAEYLLREWVNLYH
SAAAGRDSTKAFSAFVGQMHQQGILKTDDLITRFFRLCTEMCVEISYRAQAEQQHNPAASAAIIRAKCYHNLDAFVRLIA
LLVKHSGEATNTVTKINLLN
KVRPGHGLMLKVLGIVVGVLIQDHDVRQTEFQQLPYHRIFIMLLLELNAPEHVLETINFQTLTAFCNTFHILRPTKAPGF
VYAWLELISHRIFIARMLAH
TPQQKGWPMYAQLLIDLFKYLAPFLRNVELNKPMQILYKGTLRVLLVLLHDFPEFLCDYHYGFCDVIPPNCIQLRNLILS
AFPRNMRLPDPFTPNLKVDM
LSEINIAPRILTNFTGVMPSQFKKDLDSYLKTRSPVTFLSELRSNLQVSNEPGNRYNIQLINALVLYVGTQAIAHIHNKG
STPSMSTITHSAHMDIFQNL
AVDLDTEGRYLFLNAIANQLRYPNSHTHYFSCTMLYLFAEANTEAIQEQITRVLLERLIVNRPHPWGLLITFIELIKNPA
FKFWSHDFVHCAPEIEKLFQ
SVAQCCMGQKQAQQVMEGTGAS
>g1451.t3
MVANCSNVMTKARQPPPGVMPKGRAPSTSSLDAISPVQIDPLSAMGSLNLGGSTTSHTQNMQGFPSSLSSAFSNPQSPAK
AFPPLTNPNPSTPFGGIGLS
SQLPGMDSGPLGSGIGSSIGSGLGMPAVTSDPFGTRKMSTPGLNPPTFQQTDLSQVWPEANQHFSKEIDDEANSYFQRIY
NHPPHPTMSVDEVLEMLQRF
KDSTIKREREVFNCMLRNLFEEYRFFPQYPDKELHITACLFGGIIEKGLVTYMALGLALRYVLEALRKPFGSKMYYFGIA
ALDRFKNRLKDYPQYCQHLA
SIGHFLQFPHHLQEYIEYGQQSRDPPVKMQGSITTPGSLALAQVQAQSQQPGVPKAPQPGQASTLGTSTTTTTTVAKTTT
IARPTPSSFKKDVPPSINTT
NIDTLLVATDQTERIVEPPENVQEKIAFIFNNLSQSNMTQKVEELKETVKEEFMPWVSQYLVMKRVSIEPNFHSLYSNFL
DTLKNPEFGKMVLSETYRNI
KVLLTSDKAAANFSDRSLLKNLGHWLGMITLAKNKPILYTDLEVKSLLLEAYVKGQQELLYVVPFVAKVLESSLRSVVFR
PQNPWTMAIMNVLAELHQEH
DLKLNLKFEIEVLCKNLSLDINDLKPGNLLKDKDKLKSLEEQLSAPKKEAKPPEEMLPIVTTVAPSTPAATTTTCTTTGP
PTPQFSYHDINVYALAGLAP
HINININIPLLQAHPQLKQCVRQSVERAVQELVHPVVDRSIKIAMTTCEQIVRKDFALDSEESRMRVAAHHMMRNLTAGM
AMITCREPLLMSIATNLKNS
FAAALRAPTPQQREMMEDAAARIAQDNCELACCFIQKTAVEKAGPEMDKRLATEFELRKHARQEGRRYCDPVVLTYQAER
MPEQIRLKVGGVDPKQLAVY
EEFARNVPGFLPSNDLSQPTGFLAQPMKQQAWATDDVAQIYDKCMADLEQHLHAVPPTLALNPQTQALRSLLEAVALARN
SRDGIAALGLLQKAVEGLLD
ATSGADADLLLRYRECHLLVLKALQDGRAYGPQWCNKQITRCLIECRDEYKYNVEAVELLIRNHLVNMQQYDLHLAQSME
NGLHYMAVAFAMQLVKLLLV
DERSVGHITEADLFHTIETLMRTSAHSRANAPEGLPQLMDVVRSNYEAMIDRAHGGPNFMMHSGISQASEYDDPPGLREK
AEYLLREWVNLYHSAAAGRD
STKAFSAFVGQMHQQGILKTDDLITRFFRLCTEMCVEISYRAQAEQQHNPAASAAIIRAKCYHNLDAFVRLIALLVKHSG
EATNTVTKINLLNKVLGIVV
GVLIQDHDVRQTEFQQLPYHRIFIMLLLELNAPEHVLETINFQTLTAFCNTFHILRPTKAPGFVYAWLELISHRIFIARM
LAHTPQQKGWPMYAQLLIDL
FKYLAPFLRNVELNKPMQILYKGTLRVLLVLLHDFPEFLCDYHYGFCDVIPPNCIQLRNLILSAFPRNMRLPDPFTPNLK
VDMLSEINIAPRILTNFTGV
MPSQFKKDLDSYLKTRSPVTFLSELRSNLQVSNEPGNRYNIQLINALVLYVGTQAIAHIHNKGSTPSMSTITHSAHMDIF
QNLAVDLDTEGRYLFLNAIA
NQLRYPNSHTHYFSCTMLYLFAEANTEAIQEQITRVLLERLIVNRPHPWGLLITFIELIKNPAFKFWSHDFVHCAPEIEK
LFQSVAQCCMGQKQAQQVME
GTGAS
>g1452.t1
MLAKEDIEEGEVLFTIPRSLLLHQGTSKVSAVLEQDKGSLESASGWVPLLMALLYEYTCPESHWAPYLSLWTDFSSLDQP
LFWSTAERENLLRGTGIPEA
VERDLANIQREYSTVVLPFISKHADLWSPSTHTLQLYTRLVAFVMAYSFQEPQEEEEEDDDDDEDEKAPNLPLMVPMADM
LNHVSNHNANLEYTPDCLKM
VCVRPIRKGQEVFNTYGQMANWQLLHMYGFTEPYPGNGNDTADIPVANAYAAAAQGIQSDLDRQLVEEKKALTLEMMGEK

AAFIFGKQGCLTDMELHTTL
KVLCMSREEFQEFQDNEGWEEDEEANEEKVSLAFSNEGLPGLKAPWQGLIHAAARLTLEGYGSVEGSEEGACVDSDRMLI
EDEAKLAVLDSRQQRALQVR
HGQKSILNRVVALTKT
>g1452.t2
MLAKEDIEEGEVLFTIPRSLLLHQGTSKVSAVLEQDKGSLESASGWVPLLMALLYEYTCPESHWAPYLSLWTDFSSLDQP
LFWSTAERENLLRGTGIPEA
VERDLANIQREYSTVVLPFISKHADLWSPSTHTLQLYTRLVAFVMAYSFQEPQEEEEEDDDDDEDEKAPNLPLMVPMADM
LNHVSNHNANLEYTPVRTSP
PHCRATPRKPTTM
>g1452.t3
MLAKEDIEEGEVLFTIPRSLLLHQGTSKVSAVLEQDKGSLESASGWVPLLMALLYEYTCPESHWAPYLSLWTDFSSLDQP
LFWSTAERENLLRGTGIPEA
VERDLANIQREYSTVVLPFISKHADLWSPSTHTLQLYTRLVAFVMAYSFQEPQEEEEEDDDDDEDEKAPNLPLMVPMADM
LNHVSNHNANLEYTPDCLKM
VCVRPIRKGQEVFNTYGQMANWQLLHMYGFTEPYPGNGNDTADIPVANAYAAAAQGDQPRGIQSDLDRQLVEEKKALTLE
MMGEKAAFIFGKQGCLTDME
LHTTLKVLCMSREEFQEFQDNEGWEEDEEANEEKVSLAFSNEGLPGLKAPWQGLIHAAARLTLEGYGSVEGSEEGACVDS
DRMLIEDEAKLAVLDSRQQR
ALQVRHGQKSILNRVVALTKT
>g1453.t1
MDPCDCAKSGTCKCGTSCTCANCSCTKCKKSCCECCPAGCTKCASGCVCKDKTCDTSCCHWNLQLRHIMHLRKLHLHHVQ
EK
>g1453.t2
MDPCDCAKSGTCKCGTSCTCANCSCTKCKKSCCECCPAGCTKCASGCVCKDKTCDTSCCHWNLQLRHIMHLRKLHLHHVQ
EKLLRVLSIRLQQVRLRVRV
QGQCLRRKLLPVRSGAL
>g1453.t3
MDPCDCAKSGTCKCGTSCTCANCSCTKCKKSCCECCPAGCTKCASGCVCKDKTCDTSCCHWNLQLRHIMHLRKLHLHHVQ
ENKCASGCVCKDNACDANCC
Q
>g1454.t1
MRPKDKARVIDAAKHAHKFCNVEDDENVYIKRPDGIERQYRKKCGKCGLVLFYQHQMKNTPVTFIVDEAVVKFGQGFGNT
SVYSQKPPEAPKKVRVMMTK
RTKDMGKFSSVTVSTIDEEEEEIEAREVADSYAQNAKVIEKQLERKGMSKKRLQELAELEAKKAKMKGTLIDNQFKXAMD
TEGFGELLQQAEQLAAETEA
VSELPHVERNLQEIQQAGERLRSRTLTRTSQDAADVKASILLGSRGLDIFHISQRLESLSAATSFEPLEPVKDTDIQVKQ
ESLSLFMGLSVVPVVLKEFE
NDPDRVGKSEEGNECLFVVTGH
>g1454.t2
MDTEGFGELLQQAEQLAAETEAVSELPHVERNLQEIQQAGERLRSRTLTRTSQDAADVKASILLGSRGLDIFHISQRLES
LSAATSFEPLEPVKDTDIQV
KQESLSLFMGLSVVPVVLKEFENDPDRVGKSEEGNECLFVVTGH
>g1455.t1
MALCGRRNQSKSIIFLFGAFLFVGYVLFVRNSSDDVSSARRRDAPGAQDQELHDEQLAKPVYDKPPFDASGLGELGRAVR
LGLHGEEKKKEQESIDKHQI
NIYVSDKISLHRTLSERRNPLCKEEKYDYRSLPTTSVVIAFYNEAWSTLLRTVHSVLETSPDLLLKEVILVDDYSDR
>g1455.t2
LFGCLIIASESQTAPPVWADGAREERTNSDDVSSARRRDAPGAQDQELHDEQLAKPVYDKPPFDASGLGELGRAVRLGLH
GEEKKKEQESIDKHQINIYV
SDKISLHRTLSERRNPLCKEEKYDYRSLPTTSVVIAFYNEAWSTLLRTVHSVLETSPDLLLKEVILVDDYSDR
>g1456.t1
LLHQLHHYPQLHRYHRHHHCHHFPHCSNHHHHSFPTATTSTTTTSTPTXXXXXXXHPNHHHKHHLHHCYHHYDCHQLHPL
CHFYHPHQLHYHYLHHHLHQ
HYHLHYLHPHYRRNHTMTSNPLPPPPPLPPPLPPEPHHDLQPSTTCTNTTTPTTAGTTP
>g1457.t1

LSVCAAACEPGFYKAYAGNIKCSKCPPHSSSHDQAASICHCDKGFYRAAKDPPSSPCSSHILNISTRMSFESRVGPPSAP
RNLVSFINDTALFLQWAAPS
DAGGRKDLSYNVLCQRCLGNRGDGARCEPCEADLRFIPRPLGLTGTSVAVLDFALHANYTFHVEAINGVSGLAVVARSLA
NVTVTTHQSASLLQNMPAQH
GRDQPMQKNLTVLSTRAADGCLKPTNTNDLYILAGIAPPDIRRAVASRVERCRQTTDERHPLYGHVPAPSRLKSLRSFLT
SSAPLETTPTEARLNIWQEK
LFNHPHIPTIPIPPAETLPPGDNNWAKWKCLNRLRSGVGRSRETLSSWGYLSGPTTCDCGTEPQTMEHLLRCPLLGGPCT
AEDLTLYNTKGQQCTKHWLG
IL
>g1457.t2
LSVCAAACEPGFYKAYAGNIKCSKCPPHSSSHDQAASICHCDKGFYRAAKDPPSSPCSSHILNISTRMSFESRVGPPSAP
RNLVSFINDTALFLQWAAPS
DAGGRKDLSYNVLCQRCLGNRGDGARCEPCEADLRFIPRPLGLTGTSVAVLDFALHANYTFHVEAINGVSGLAVVARSLA
NVTVTTHQSDGVHVPRHCVL
QPLPSASLLQNMPAQHGRDQPMQKNLTVLSTRAADGCLKPTNTNDLYILAGIAPPDIRRAVASRVERCRQTTDERHPLYG
HVPAPSRLKSLRSFLTSSAP
LETTPTEARLNIWQEKLFNHPHIPTIPIPPAETLPPGDNNWAKWKCLNRLRSGVGRSRETLSSWGYLSGPTTCDCGTEPQ
TMEHLLRCPLLGGPCTAEDL
TLYNTKGQQCTKHWLGIL
>g1457.t3
MSFESRVGPPSAPRNLVSFINDTALFLQWAAPSDAGGRKDLSYNVLCQRCLGNRGDGARCEPCEADLRFIPRPLGLTGTS
VAVLDFALHANYTFHVEAIN
GVSGLAVVARSLANVTVTTHQSASLLQNMPAQHGRDQPMQKNLTVLSTRAADGCLKPTNTNDLYILAGIAPPDIRRAVAS
RVERCRQTTDERHPLYGHVP
APSRLKSLRSFLTSSAPLETTPTEARLNIWQEKLFNHPHIPTIPIPPAETLPPGDNNWAKWKCLNRLRSGVGRSRETLSS
WGYLSGPTTCDCGTEPQTME
HLLRCPLLGGPCTAEDLTLYNTKGQQCTKHWLGIL
>g1458.t1
MWNGLRRISDYKGKIRDVVQPSASMLDELDTFLCLVQAGRQLSSDVVITFKDNVTILGLITMRRHPGRRCTIAGLLAGCG
SVWNGNCSAHGR
>g1459.t1
MWSEPLDLNVSSMCPRVHPHLYFKLSTGVNELDQVILVLFTTHMFIGGFLGFVLDNTIPGSEKERGINGWRQMVQDKGPG
NAASRACYDIPFCDGVFQRF
ACFQYLPFLPSYRDSARETLQ
>g1459.t2
MCPRVHPHLYFKLSTGVNELDQVILVLFTTHMFIGGFLGFVLDNTIPGSEKERGINGWRQMVQDKGPGNAASRACYDIPF
CDGVFQRFACFQYLPFLPSY
RDSARETLQ
>g1460.t1
MTRLGSRSPPTGLTRSARTAITHSACGPSGVVGPEARSGTVTDGAFSPPCPSAASSGGVTAVALSRVCRGAVLENTIPGK
NLWKSNPRIEPSGSC
>g1461.t1
DGSGELIFYERCDTEGPKPSNYSISPTSDPQALRDIKAILNDKKRAFREGNYAELRRVQGVLKVTIKEAKDRYRRKLEKK
LQLNKTRDVWSGMRNITGFQ
KT
>g1461.t2
DGSGELIFYERCDTEGPKPSNYSISPTSDPQALRDIKAILNDKKRAFREGNYAELRRVQGVLKVTIKEAKDRYRRKLEKK
LQLNKTRDVWSGMRNITGFQ
KTGSTGLEGGVARANELNLFFN
>g1462.t1
VRWTKTAGSASDKFQETSIYNETLRIESILRVQGGRYYCKADNGVGVAAIKSIRVDVQCDTPPSDRGSGYGAAPSADGAR
LRGAAVVQPVRRRLGDGVVL
>g1462.t2
MICTSRDTRVFPPLQVRWTKTAGSASDKFQETSIYNETLRIESILRVQGGRYYCKADNGVGVAAIKSIRVDVQCDTPPSD
RGSGYGAAPSADGARLRGAA
VVQPVRRRLGDGVVL

>g1463.t1
MHAASLRMLIPGLLLLGLQGLHGVPLLVFLIDGFRYDYMDDLSALPGFRELGENGVKVDYVTPDFPSLSYPNYYSLMTGR
NCDIHQMIGNYMWDEVSMKE
FLIGTNADSRLPLWWDGSEPIWVTMQKKKKDVYMYNWPGCEVEILGVRPSFCQEYVYKPSVSDLTNSTEHALDTLQSGKA
EMAAIYYEKIDVEGHHFGPD
SHQLKMAVRELDTAMQSLNKRIK
>g1463.t2
MHAASLRMLIPGLLLLGLQGLHGVPLLVFLIDGFRYDYMDDLSALPGFRELGENGVKVDYVTPDFPSLSYPNYYSLMTGR
NCDIHQMIGNYMWDEVSMKE
FLIGTNADSRLPLWWDGSEPIWVTMQKKKKDVYMYNWPGSYEKRMKAFRCEVEILGVRPSFCQEYVYKPSVSDLTNSTEH
ALDTLQSGKAEMAAIYYEKI
DVEGHHFGPDSHQLKMAVRELDTAMQSLNKRIK
>g1463.t3
MHAASLRMLIPGLLLLGLQGLHGVPLLVFLIDGFRYDYMDDLSALPGFRELGENGVKVDYVTPDFPSLSYPNYYSLMTGL
YHRGVEGRNCDIHQMIGNYM
WDEVSMKEFLIGTNADSRLPLWWDGSEPIWVTMQKKKKDVYMYNWPGCEVEILGVRPSFCQEYVYKPSVSDLTNSTEHAL
DTLQSGKAEMAAIYYEKIDV
EGHHFGPDSHQLKMAVRELDTAMQSLNKRIK
>g1464.t1
MACTLQKVLGDARTLLERLKEHDNAAEGLIDQSGALSHRVRSMREVGHALPDKHIENSDVQELSKYKPHVLLTQENTQIK
ELQQENKELWLSLEEHQYAL
ELIMGRYRKQMLQMMMGEKELDTKPVLTLHQDHAKEVQGRVERICEMGQVMRRAVQVDDQHYCSVRERLAQLEMENKELR
DLLVISKSSVKIQREHGNQP
AAVAPLDPPSDTEANQ
>g1465.t1
MVRWAALTKLPASSSSPETGSRGTKCRCFPDRRHRRRGKLPPKLQLERQGGAVLHDPITWGEPGQRAAVCTFIGFCPSEK
LGKDRNPRAALRRV
>g1466.t1
MALHTALSHLDKRNTCPNAVFDYSSAFNTIVPGKLITKLKDLGLSPALCKQSPGLTDTTARTARQHLFFLRRQDAAHPR
>g1467.t1
PDNFNEAKFITFSMLIFCAVWVAFVPAYISSPGKYSVAVEIFAILASSYGLLLCIFAPKCFIILLRPERNTKKHLMAR
>g1468.t1
DASMMAMRRRIHGLSPEEIRALSKEELLMPVTMEDFTQTLKKISKSVSAADLEKYEAWMAEFGSV
>g1469.t1
MALNLWPVPHGLEDLLNPIGVTGSNPNKETPCLELEFDHFSSLVKYPDMNSVEEHANWSVSREMGFSYNLSGQSNRMARD
IALTEGDIEQLRQLSNRDPL
SEITEQEKDFLWRHRHYCMNIPEILPKILLAVKWNSRDEVAQMYCLLKEWPSIRPEQAMELLDCNYPDPMVRCFAVRCLD
KYLTDDKLSQYLIQLVQVLK
YEQYLDNPLVRFLLKKALTNQRIGHFFFWHLKSEMHNKTVSQRFGLLLESYCRACGMYLKHLSRQVEAMEKLIHLTDLLK
QEKKDETQKVQMRFLVDQMK
RPDYMDALQSFTSPLNPAHQLGSIRLDECRIMSSAKRPLWLNWENPDIMSELLFQNNEIIFKNGD
>g1469.t2
LAVNDASVTIQAKLQEHCPLAWGNVNLFDYTHTLVANKMALNLWPVPHGLEDLLNPIGVTGSNPNKETPCLELEFDHFSS
LVKYPDMNSVEEHANWSVSR
EMGFSYNLSGQSNRMARDIALTEGDIEQLRQLSNRDPLSEITEQEKDFLWRHRHYCMNIPEILPKILLAVKWNSRDEVAQ
MYCLLKEWPSIRPEQAMELL
DCNYPDPMVRCFAVRCLDKYLTDDKLSQYLIQLVQVLKYEQYLDNPLVRFLLKKALTNQRIGHFFFWHLKSEMHNKTVSQ
RFGLLLESYCRACGMYLKHL
SRQVEAMEKLIHLTDLLKQEKKDETQKVQMRFLVDQMKRPDYMDALQSFTSPLNPAHQLGSIRLDECRIMSSAKRPLWLN
WENPDIMSELLFQNNEIIFK
NGD
>g1469.t3
LPLSEHNEHCPLAWGNVNLFDYTHTLVANKMALNLWPVPHGLEDLLNPIGVTGSNPNKETPCLELEFDHFSSLVKYPDMN
SVEEHANWSVSREMGFSYNL
SGQSNRMARDIALTEGDIEQLRQLSNRDPLSEITEQEKDFLWRHRHYCMNIPEILPKILLAVKWNSRDEVAQMYCLLKEW
PSIRPEQAMELLDCNYPDPM

VRCFAVRCLDKYLTDDKLSQYLIQLVQVLKYEQYLDNPLVRFLLKKALTNQRIGHFFFWHLKSEMHNKTVSQRFGLLLES
YCRACGMYLKHLSRQVEAME
KLIHLTDLLKQEKKDETQKVQMRFLVDQMKRPDYMDALQSFTSPLNPAHQLGSIRLDECRIMSSAKRPLWLNWENPDIMS
ELLFQNNEIIFKNGD
>g1470.t1
QVAQLETLEHRGDLALDPKEIEDRQDRLDLQGPPGLIGPPGGAGPEGIGLPGPKGDRGYPGLTGPAGPQGIGIAGVKEPQ
APKEYLEKAFKVPRDRIQRN
PRTQRASRCGSPGGEGCGVTCRQTPLELVFVIDSSESVGPENFEAIKDFVNSLVDRASVGPNTTRVGVVLYSHINTVVLD
LGPQVSRDQVKLAVRSMPYM
GEGTFTGSAIHRSNRVFLSARRGVRKVAIVITDGQADERDSVSLEGAVKEAHGSGIEMFVIGVVNQSDALYEQFRRELHL
MASDPDQEHVYLIRDFALLS
ALESKLLSKICEEQDTGLFSSIPSSRTSPGTGEGAGNVREPPYRTDTDTPTFTGDARRIQLGVRGLRPDPRPSSPSDTGG
HPYDREPYRHPTDPEVEPPT
AGRRPPTATLPSGPTVHDKGSCRQTLDPGPCRQYLVRWYYDPDANACAKFWYGGCLGNSNRFLSEAICRNTCVTA
>g1470.t2
QVAQLETLEHRGDLALDPKEIEDRQDRLDLQGPPGLIGPPGGAGPEGIGLPGPKGDRGYPGLTGPAGPQGIGIAGVKEPQ
APKEYLEKAFKVPRYCFTMV
KYHCLGETGYRGTPGLRGPPGCGVTCRQTPLELVFVIDSSESVGPENFEAIKDFVNSLVDRASVGPNTTRVGVVLYSHIN
TVVLDLGPQVSRDQVKLAVR
SMPYMGEGTFTGSAIHRSNRVFLSARRGVRKVAIVITDGQADERDSVSLEGAVKEAHGSGIEMFVIGVVNQSDALYEQFR
RELHLMASDPDQEHVYLIRD
FALLSALESKLLSKICEEQDTGLFSSIPSSRTSPGTGEGAGNVREPPYRTDTDTPTFTGDARRIQLGVRGLRPDPRPSSP
SDTGGHPYDREPYRHPTDPE
VEPPTAGRRPPTATLPSGPTVHDKGSCRQTLDPGPCRQYLVRWYYDPDANACAKFWYGGCLGNSNRFLSEAICRNTCVTA
>g1470.t3
QVAQLETLEHRGDLALDPKEIEDRQDRLDLQGPPGLIGPPGGAGPEGIGLPGPKGDRGYPGLTGPAGPQGIGIAGVKEPQ
APKEYLEKAFKVPRDRIQRN
PRTQRASRCGSPGGEGCGVTCRQTPLELVFVIDSSESVGPENFEAIKDFVNSLVDRASVGPNTTRVGVVLYSHINTVVLD
LGPQVSRDQVKLAVRSMPYM
GEGTFTGSAIHRSNRVFLSARRGVRKVAIVITDGQADERDSVSLEGAVKEAHGSGIEMFVIGVVNQSDALYEQFRRELHL
MASDPDQEHVYLIRDFALLS
ALESKLLSKICEEQDTGLFSSIPSSRTSPGTGEGAGNVREPPYRTDTDTPTFTGDARRIQLGPPQPDPRPSSPSDTGGHP
YDREPYRHPTDPEVEPPTAG
RRPPTATLPSGPTVHDKGSCRQTLDPGPCRQYLVRWYYDPDANACAKFWYGGCLGNSNRFLSEAICRNTCVTA
>g1471.t1
MTTHVILDDKSDPDLDNNPADVNSKTQHWGIEPGDTVTAFRVPTLDGEFSYSPGALRGALIIHAFTHKSGFLECMWNNES
SLADLVRELPESTHVLFLSR
DDSAFEDAIWMRKQVYRAVTQHGREEVMARLHFSPVPVFALGNWIPGVLNSWGCTRHRCGLAQASFTSEGWNIPVIVKRQ
DARYDWLARWGQRSRHLVDA
GDGCEPSVGVAGHVAWVADGNCSYFTKVQSMSKSNASGVLVVAQDGQPLQDMNCQGEECSTKLLGIAAAMVHHEGSVAEA
LRLGQAVNVSLQNTPSPSIF
IAVNKQGALSEMGGFLYPSFSFLVWQAQWFEFSEGLRLKLLAPAQVVPVFNKVVMHGHKGAVATVEVSPGLLVSDTLELD
VSLSCPGRRDGSCAHWDHTV
QLFVCCDRLGPYCNMELGRWITPFRRVLSGSGRVSILYRRGTGQWLTDVSPLMPLLTNGSCTFTMKHAWWAMPWVVSLNL
RFGADQAGARXXXXXXXXMT
AMRQVVLLYAVITGHGSDDNSCGEFCVTSHIFPINGINNSLVFADAGSPLGCAGRVKEGALPNEYGTWLYGRGGWCDGLQ
VEPWVTDVTRQVSCPALRSQ
QGCVAGLDLSGQLTNRLLYYGLFEDRDPDPAKDPGYIIMSSFLVFYKWGQRTENSTEFL
>g1471.t2
MTTHVILDDKSDPDLDNNPADVNSKTQHWGIEPGDTVTAFRVPTLDGEFSYSPGALRGALIIHAFTHKSGFLECMWNNES
SLADLVRELPESTHVLFLSR
DDSAFEDAIWMRKQVYRAVTQHGREEVMARLHFSPVPVFALGNWIPGVLNSWGCTRHRCGLAQASFTSEGWNIPVIVKRQ
DARYDWLARWGQRSRHLVDA
GDGCEPSVGVAGHVAWVADGNCSYFTKVQSMSKSNASGVLVVAQDGQPLQDMNCQGEECSTKLLGIAAAMVHHEGSVAEA
LRLGQAVNVSLQNTPSPSIF
IAVNKQGALSEMGGFLYPSFSFLVWQAQWFEFSEGLRLKLLAPAQVVPVFNKVVMHGHKGAVATVEVSPGLLVSDTLELD

VSLSCPGRRDGSCAHWDHTV
QLFVCCDRLGPYCNMELGRWITPFRRGTGQWLTDVSPLMPLLTNGSCTFTMKHAWWAMPWVVSLNLRFGADQAGARXXXX
XXXXMTAMRQVVLLYAVITG
HGSDDNSCGEFCVTSHIFPINGINNSLVFADAGSPLGCAGRVKEGALPNEYGTWLYGRGGWCDGLQVEPWVTDVTRQVSC
PALRSQQGCVAGLDLSGQLT
NRLLYYGLFEDRDPDPAKDPGYIIMSSFLVFYKWGQRTENSTEFL
>g1471.t3
MTTHVILDDKSDPDLDNNPADVNSKTQHWGIEPGDTVTAFRVPTLDGEFSYSPGALRGALIIHAFTHKSGFLECMWNNES
SLADLVRELPESTHVLFLSR
DDSAFEDAIWMRKQVYRAVTQHGREEVMARLHFSPVPVFALGNWIPGVLNSWGCTRHRCGLAQASFTSEGWNIPVIVKRQ
DARYDWLARWGQRSRHLVDA
GDGCEPSVGVAGHVAWVADGNCSYFTKVQSMSKSNASGVLVVAQDGQPLQDMNCQGEECSTKLLGIAAAMVHHEGSVAEA
LRLGQAVNVSLQNTPSPSIF
IAVNKQGALSEMGGFLYPSFSFLVWQAQWFEFSEGLRLKLLAPAQVVPVFNKVVMHGHKGAVATVEVSPGLLVSDTLELD
VSLSCPGRRDGSCAHWDHTV
QLFVCCDRLGPYCNMELGRWITPFRRVLSGSGRVSILYRRGTGQWLTDVSPLMPLLTNGSCTFTMKHAXTFDQNYNKGRQ
PIEIPIPASTKKVLLYAVIT
GHGSDDNSCGEFCVTSHIFPINGINNSLVFADAGSPLGCAGRVKEGALPNEYGTWLYGRGGWCDGLQVEPWVTDVTRQVS
CPALRSQQGCVAGLDLSGQL
TNRLLYYGLFEDRDPDPAKDPGYIIMSSFLVFYKWGQRTENSTEFL
>g1472.t1
LAPKSPLNAQTGCSSRGQLVVVKMMIYLILQLPRHLPQSPPAMAAIGPAVMDEAQHSLTTLTTLTTVTTVTD
>g1473.t1
MARLSARIFGDVVRPTDSKSMKVVQLFKDQPLAQRKEVYDWYPQHKIYFSMTQRLRFMGLF
>g1474.t1
LTSPEGLAVDPTRRLMFWVDSAPDKIERANLDGGGRRTLFQSDLVNPRAIIVVPSTG
>g1475.t1
LTFDLHVFALQHWIVFTEVTEVFILVPALLGLKGNLEMTLASRLSTA
>g1476.t1
ESSQPLSSSGNGSEHGWVVATQFSQSANARSDMFMFGEAHYDAGDGPVARRPSQTSLRTEPFCGIETPPATAYAVVYASA
PVSSLTSADAQTGSQVVVQG
SLPESIQVRKLLDALHLSA
>g1476.t2
ESSQPLSSSGNGSEHGWVVATQFSQSANARSDMFMFGEAHYDAGDGPVARRPSQTSLRTEPFCGIETPPATAYAVVYASA
PVSSLTSADAQTGSQVVVQG
SLPESIQVRMS
>g1477.t1
MSLGFLPANMSFSNKRNDLLKGKVRMGFYQGFMTHKSEVIGGERYFPDILYDQDPSESYHSWMNSLHGV
>g1478.t1
VDEQQLKKQLDEITAIIKVRESCNTKVP
>g1479.t1
MQPLPLSTALGIKDREVYKVLVRKSQDESWVVFRRYADFSRLNNKNSALMILNSPGNTPCSPNAHRIVSTALISYMISAA
LSAGIQCAY
>g1479.t2
VYKVLVRKSQDESWVVFRRYADFSRLNNKNSALMILNSPGNTPCSPNAHRIVSTALISYMISAALSAGIQCAY
>g1479.t3
MAFDLEYNAMETGINVKRASDSWGIFVYKVLVRKSQDESWVVFRRYADFSRLNNKNSALMILNSPGNTPCSPNAHRIVST
ALISYMISAALSAGIQCAY
>g1480.t1
LSGSIGGGNTERLVKNGKLSEPLTSSKSQQLCIQSGSHSIKTSGYLNLLANCIDNFTHGLAVAGSFLVSKKVGFLTTFAI
LLHEIPHE
>g1480.t2
MKATGRGKPSWALAAVFVSVSLLVLTSRCASSNQKMTQTAMGYSTDAAAGPGNSLLEELLGFRALADLFASEQGEVWLYS
LVGSVVVGLSGIFPLLVIPI
EAGAALKTDAGGQKLKQLLSFAIGGLLGDVFLHLLPEAWTLCSQSDGSHHHHYMVQGLWVIVGLLSFLLLEKMFPDANTE
PSPTSGLETTSIALSGSIGG

GNTERLVKNGKLSEPLTSSKSQQLCIQSGSHSIKTSGYLNLLANCIDNFTHGLAVAGSFLVSKKVGFLTTFAILLHEIPH
E
>g1480.t3
MKATGRGKPSWALAAVFVSVSLLVLTSRCASSNQKMTQTAMGYSTDAAAGPGNSLLEELLGFRALADLFASEQGEVWLYS
LVGSVVVGLSGIFPLLVIPI
EAGAALKTDAGGQKLKQLLSFAIGGLLGDVFLHLLPEAWTLCSQSDGSHHHHYMVQGLWVIVGLLSFLLLEKMFPDANTE
PSPTSGLETTSIVSLIPSIA
FSTSLC
>g1480.t4
MKATGRGKPSWALAAVFVSVSLLVLTSRCASSNQKMTQTAMGYSTDAAAGPGNSLLEELLGFRALADLFASEQGEVWLYS
LVGSVVVGLSGIFPLLVIPI
EAGAALKTDAGGQKLKQLLSFAIGGLLGDVFLHLLPEAWTLCSQSDGSHHHHYMVQGLWVIVGLLSFLLLEKMFPDANTE
PSPTSGLETTSIVEYRPVLH
GLDVWQQCEPALSGSIGGGNTERLVKNGKLSEPLTSSKSQQLCIQSGSHSIKTSGYLNLLANCIDNFTHGLAVAGSFLVS
KKVGFLTTFAILLHEIPHE
>g1481.t1
LGFVWGPGDILVYDTIFKASGAEGTCSPFIHEVRKDEDIYSPILRKLFNESHHIFVGLQTIREDLPNKNKKTQFVSVSKN
YRSVIRACMEELQQVAVLTQ
DTELATQYGNQVSILLAIELIWNLCEVLFIDAAPGGSLLLHLLDWVRLHKADVDEKAQEVLQSESPTEHHGYWDVVLSYM
LQGRMDEARQMLVKWSVLKP
AARSMYKQMDNLLSKMPVYNPGGNQTLTEFDVKWRHWHDEVDHCLQDNSFASDPNLELICKILVGDEDALLEQKDLLGTW
YHFLVTRLLFSHPSVKPTEL
HYYAQSCLAMFDSRSAPEPLDSILLAAFEFDIHQVIKDCSFGSNLREFLLLEYASGLFTHHSLWQLAVDYFDYCPEFGRV
YLEQQIEHVPLETERKALKV
LRICEQRQMSEQVRSICKIMAKRALRNNRLGSALSWSIRAKDAAFATLISE
>g1481.t2
LGFVWGPGDILVYDTIFKASGAEGTCSPFIHEVRKDEDIYSPILRKLFNESHHIFVGLQTIREDLPNKNKKTQFVSVSKN
YRSVIRACMEELQQVAVLTQ
DTELATQYGNQVSILLAIELIWNLCEVLFIDAAPGGSLLLHLLDWVRLHKADVDEKAQEVLQSESPTEHHGYWDVVLSYM
LQGRMDEARQMLVKWSVLKP
AARSMYKQMDNLLSKMPVYNPGGNQTLTEFDVKWRHWHDEVDHCLQDNSFASDPNLELICKILVGDEDALLEQKDLLGTW
YHFLVTRLLFSHPSVKPTEL
HYYAQRVLQSCLAMFDSRSAPEPLDSILLAAFEFDIHQVIKDCSFGSNLREFLLLEYASGLFTHHSLWQLAVDYFDYCPE
FGRVYLEQQIEHVPLETERK
ALKVLRICEQRQMSEQVRSICKIMAKRALRNNRLGSALSWSIRAKDAAFATLISE
>g1481.t3
LGFVWGPGDILVYDTIFKASGAEGTCSPFIHEVRKDEDIYSPILRKLFNESHHIFVGLQTIREDLPNKNKKTQFVSVSKN
YRSVIRACMEELQQVAVLTQ
DTELATQYGNQVSILLAIELIWNLCEVLFIDAAPGGSLLLHLLDWVRLHKADVDEKAQEVLQSESPTEHHGYWDVVLSYM
LQGRMDEARQMLVKWSVLKP
AARSMYKQMDNLLSKMPVYNPGGNQTLTEFDVKWRHWHDEVDHCLQDNSFASDPNLELICKILVGDEDALLEQKDLLGTW
YHFLVTRLLFSHPSVKPTEL
HYYAQRVLQSCLAMFDSRSAPEPLDSILLAAFEFDIHQVIKDCSIALNNWWFVAHLTDLLDHCNLLQSHNLHFGSNLREF
LLLEYASGLFTHHSLWQLAV
DYFDYCPEFGRVYLEQQIEHVPLETERKALKVLRICEQRQMSEQVRSICKIMAKRALRNNRLGSALSWSIRAKDAAFATL
ISE
>g1482.t1
MDDLTRALSASFAVSKVPNSTACAHPRLAQYKSKFSVLEQSERRRRFLDLQK
>g1483.t1
MILEKAVENQNMSVKAKAIYTFQSENKEEISIQENEELVIFDENSVDGWLQGENSRGERGLFPASYVEILRQRSNSNLTD
SSISPAGSLGNDSPHFPSTP
NTTTSFLPHQGYYDDDDDDWDDWDDSSTVVEDDDPNHHHHRRSGGGGPNGHAHHSPNSNNPNVHYRAKPRMERQDSISSS
RKGSMVGRNLNRFSSFVRSG
VEAFVLGDVPMMAKIAESYTIEIGPKGPQWKESPQPFSCSIEDPTKQTKFKGIKTYISYRVTPSHTGSAVYRRYKHFDWL
YNRLLHKFTVISVPHLPEKQ
ATGRFEEDFIDKRKRRLILWMNHMTSHPVLSQYEGFEHFLMCADDKQWKLGKRRAEKDEMVGAHFMLTFQIPNEHQDLQD

VEERVDSFKTFTKKMDDSVM
QLTHGASEMVRRHLGEFRKEFHRLGSAFHSLSLAFMLEPPPSSEPLNNAIAHTGRTYENIGEMFAEQPKYDLFQMLDKLS
LYQGLLANFPDIIHLQKGNE
HSGGL
>g1484.t1
FRSFILFGSPRREHPDSLLVWFYQRVLDWFYHQILVWCTSTKDGSAQQLSSMEMAETAGWKDLLPTPSSIISSSITSFSL
TSFISPNTSFTPSLNSFMSS
FIPSNKLVDFVIYSSMQHDMALCHSPFTP
>g1484.t2
FILFGSPRREHPDSLLVWFYQRVLDWFYHQILVWCTSTKDGSAQQLSSMEMAETAGWKDLLPTPSSIISSSITSFSLTSF
ISPNTSFTPSLNSFMSSFIP
SNKLVDFVIYSSMQHDMALCHSPFTP
>g1485.t1
MQTGVDVLCTNGNEGEAGPSHSPDVVGIPQRARKRNLEDMVLIGDSGVGKSNLLSRFTRNEFNLESKSTIGVEFATRSLQ
VEGKTVKAQIWDTAGQERYR
AITSAYYRGAVGALLVYDIAKHLTYENAERWLKELQDHADSNIVIMLVGNKSDLRHLRAVPTDEAKGFSEKHGLSFLETS
ALDSSNVELAFQTILTAIYN
MVSQRQISGRSDSDFSPGSNVVPITVQPTQNPPRQNSCCQNN
>g1485.t2
LDRVKVLTNRQLVRTRGGTMSSKEDEYDYLFKVVLIGDSGVGKSNLLSRFTRNEFNLESKSTIGVEFATRSLQVEGKTVK
AQIWDTAGQERYRAITSAYY
RGAVGALLVYDIAKHLTYENAERWLKELQDHADSNIVIMLVGNKSDLRHLRAVPTDEAKGFSEKHGLSFLETSALDSSNV
ELAFQTILTAIYNMVSQRQI
SGRSDSDFSPGSNVVPITVQPTQNPPRQNSCCQNN
>g1486.t1
LIGLISDGDESAYRSETDRLVSWCSMYNLELNPLKTVEMIVGFRKDPPALPPVILCDSTVTLVESFRFLGTTISQELKWE
QNISSLTKKAQQRIYFLRQL
KKFHLPVKMLANFYTATIESIHTSSMPVWCAAATTRDKAKLQRVIHSAERVIGCSRPSLQELYISRTRRRAARIVADPSH
PGHKLFQSLPSGKRLRTRTS
RHKNSFFPSAAY
>g1487.t1
MIRPLLMCFALCVVYATSKPTEKKERVHHEEPLSSREHNDAEGFEYDHEAFLGREEAKTFDDLTPQESKRRLGVIVDKID
GDKDGFVTEEELRAWIKKAQ
KRYIYDNVDRQWKDFDINTDGKITWEEYKNVTYGTYLDDPEPDAGYNYQQMMARDERRFKIADKNGDLIADKEEFTAFLH
PEDYDHMKEIVVQETLEDID
KDGDGFIDLKEYIGDMYTRDEEMEEPDWVASEREQFAEFRDKNKDGKMDREETMDWILPSDYDHAEAEAKHLVYESDTDK
DGKLSKEEVLNKYDLFVGSQ
ATDFGEALVRHDEF
>g1487.t2
MIRPLLMCFALCVVYATSKPTEKKERVHHEEPLSSREHNDAEGFEYDHEAFLGREEAKTFDDLTPQESKRRLGVIVDKID
GDKDGFVTEEELRAWIKKAQ
KRYIYDNVDRQWKDFDINTDGKITWEEYKNVTYGTYLDDPEPDAGYNYQQMMARDERRFKIADKNGDLIADKEEFTAFLH
PEDYDHMKEIVVQETLEDID
KDGDGFIDLKEYIGDMYTRDEEMEEPDWVASEREQFAEFRDKNKDGKMDREETMDWILPSDYDHAEAEAKHLVYESDTDK
AFGVRYDSSVLAVFIPSCVL
PIVITADCCVFLCTSLFLQDGKLSKEEVLNKYDLFVGSQATDFGEALVRHDEF
>g1488.t1
MILVLHYFAGPLDQLVAVPWTGTPAGNQEHMSPIVSHHPSRPETAEMRTETHFLAEAWRPNNKATQPDKKGKTVVPDRRY
AQLHRPRADRDAPSPSTSVP
FSSRVLRLCDLHPRSLCLSPMWWRLPYVGLLLFFFPVTPRALSRPGGGAAQGGMGATVLLQHHYRG
>g1488.t2
MILVLHYFAGPLDQLVAVPWTGTPAGNQEHMSPIVSHHPSRPETAEMRTETHFLAEAWRPNNKATQPDKKGKTVVPDRRY
AQLHRSRVLRLCDLHPRSLC
LSPMWWRLPYVGLLLFFFPVTPRALSRPGGGAAQGGMGATVLLQHHYRG
>g1489.t1
MTGTLDTDSSSSGDSQAMEAAGNGFLPDLPVLPPSAEWSLEQAPPEPVSQNTVLPVDSHLSQDSFGPQQQQEQMSPGLAS

VEKAVEQFQLANAQLFQEEQ
PPLPTEAAPQSPAAESTAAPEDTAQQGQEILVASEAAYQDGSQGGAETTLMAEDGMELEEVPQEPPLPPEPEVPSEFDKL
FKECQENPEDFNSWVYLLQY
VEQEVYRRGLQAIPLSVDLWLHYLTFIKENADPTDPETEGRIRAAYEHAVLAAGTDFRSDRLWEAYINWESEQQKLVHVT
NIYDRILGIPTQLYSQHFQR
FKEHVQNNNPKHFLSEEEFIQLRVELSKANITTVAGEDGDTPAVGEELPPGMEDLPDPAKRVTEIENMRHKVIETRQEYF
NHNEHEVSKRWAFEEGIKRP
YFHVKSLEKTQLNNWKEYLDFEMENGTPERVVVLFERCLIACALYEEFWTK
>g1489.t2
MTGTLDTDSSSSGDSQAMEAAGNGFLPDLPVLPPSAEWSLEQAPPEPVSQNTVLPVDSHLSQDSFGPQQQQEQMSPGLAS
VEKAVEQFQLANAQLFQEEQ
PPLPTEAAPQSPAAESTAAPEDTAQQGQEILVASEAAYQDGSQETTLMAEDGMELEEVPQEPPLPPEPEVPSEFDKLFKE
CQENPEDFNSWVYLLQYVEQ
EVYRRGLQAIPLSVDLWLHYLTFIKENADPTDPETEGRIRAAYEHAVLAAGTDFRSDRLWEAYINWESEQQKLVHVTNIY
DRILGIPTQLYSQHFQRFKE
HVQNNNPKHFLSEEEFIQLRVELSKANITTVAGEDGDTPAVGEELPPGMEDLPDPAKRVTEIENMRHKVIETRQEYFNHN
EHEVSKRWAFEEGIKRPYFH
VKSLEKTQLNNWKEYLDFEMENGTPERVVVLFERCLIACALYEEFWTK
>g1490.t1
MGKILSKIFGNKEMRILMLGLDAAGKTTILYKLKLGQSVTTIPTVGFNVETVTYKNVKFNVWDVGGQDKIRPLWRHYYTG
TQGLIFVVDCADRDRIDEAR
QELHRIINDREMRDAIILIFANKQDLPDAMKPHEIQEKLGLTRIRDRNWYVQPSCATTGDGLYEGLTWLTSNHKS
>g1491.t1
MKKTKDKRAVEPTSHDIDHVPQQPWMKVLKIKTVRVEGAEADGLGEGVDAVAITGKCFDYVSRTPHTAQCSGTPPCTCQW
NLVLTAVPSINSVRWPRQRR
GDSSAERRLDRRFGA
>g1492.t1
MSCSFSCSVMLGFTSAEKALCGWPDGRACERSIIRSQRAAIPRCSPDVIELPQAGGHPHLDPTPPPSVLGG
>g1493.t1
ASPGTSITVLSVKPTDPREPWTSSVGIPTPSTPRSANADSDRAELQIRSSCRGDGGFCWSVARC
>g1494.t1
MSGVRWPGLKRSWMGQTSAHMAPHGVWEEPAKRQGLQTEVGRSPACQRAPRQNQQRVKAKRALMQNQRKAVRWRRPTRAT
QIAVGSATVRLGLSPRRF
>g1495.t1
MAKRFLCSPTHRLDFGTASSDLLSDVLINLNNRNKHELNKLEVCSGCFKHKNVIECVFLMTQELRLDPLVGYCAIEILER
FMVKHLKDMFTANTPCSAVA
GPASSYQDLVFEKLKVKLTLTVFSCVQIASKLWLHSDVVEDLSHITGISQRSIWDFSQVTLELITKNSSPVLST
>g1495.t2
MTQELRLDPLVGYCAIEILERFMVKHLKDMFTANTPCSAVAGPASSYQDLVFEKLKVKLTLTVFSCVQIASKLWLHSDVV
EDLSHITGISQRSIWDFSQV
TLELITKNSSPVLST
>g1495.t3
MAKRFLCSPTHRLDFGTASSDLLSDVLINLNNRNKHELNKLEVCSGCFKHKNVIECVFLMTQELRLDPLVGYCAIEILER
FMVKHLKDMFTANTPCSAVA
GPASSYQDLVFEKLKVKLTLTVFSCVQIASKLWLHSDRTAIYESLLKVVEDLSHITGISQRSIWDFSQVTLELITKNSSP
VLST
>g1496.t1
MADKTPKESSAPFATRIDPSKEGLSNEQIQFIRQVELQQWKKKTQTLSTRNVVTGLAIGAIVLGIYGYTFYSVSQERIMD
DLDKEAKIARVQEPKTGAK
>g1497.t1
MAVADVDVVRAFFLQLLVLDGQMLKTEPASIMDKIQKFLSLTNVVNYHKILAFDPKKGFWCQLLEGGKTKCLGKSKGRRY
PDMSPESQAFLREYYRDHNI
ELSKLLYRMGQPLPSWLREELVHSR
>g1497.t2
FVLPRSRGQTMAVADVDVVRAFFLQLLVLDGQMLKTEPASIMDKIQKFLSLTNVVNYHKILAFDPKKGFWCQLLEGGKTK
CLGKSKGRRYPDMSPESQAF

LREYYRDHNIELSKLLYRMGQPLPSWLREELVHSR
>g1497.t3
LLVLDGQMLKTEPASIMDKIQKFLSLTNVVNYHKILAFDPKKGFWCQLLEGGKTKCLGKSKGRRYPDMSPESQAFLREYY
RDHNIELSKLLYRMGQPLPS
WLREELVHSR
>g1498.t1
LPKTTRSSRGQTNGVSPQCHGAVVRHQIAISAITTVTTISAAGLPSAADCFRGVALTGKLSAWQQSAVGSASPSIAPLSC
DITPSSTSSITTTSSTTNAL
IHHHLHQHHHHLHQYHHRGLHHLNGS
>g1499.t1
VFRGTITDADDFDPTADAEALYNAMKGMGSDKEAILELVTSRSNAQRQEVITAYKSSYGKDLIGDLKYELTGNFERLIVS
LMRNPAQHDAKEIHDALKGA
GTNERCLIEVLASRNNHQMHAMVEAYKEAYGKDMEEDIVRDTSGHFKKMLVVLLQGTRDESGVVDADQVEQDAQDLFAAG
EEQWGTDEAKFIMILGSRSV
THLKMVFDEYEKIAEKSIEDSIKSELSGDFERLMLAVVQCIRSVSMFFARRLYKSMKGLGTADNTLIRIMICRSEIDMLD
IRECFRLRYEKSLYNMIK
>g1500.t1
MSEPRQSPTSSVQTRKQEIIKVTEQLIESINNGDFEAYAKICDPGLTSFEPEALGNLVEGHDFHRFYFDNGESVGS
>g1500.t2
MSEPRQSPTSSVQTRKQEIIKVTEQLIESINNGDFEAYAKICDPGLTSFEPEALGNLVEGHDFHRFYFDNGES
>g1500.t3
MFSAQNALKQVTLSCCRPHTQPSPQRPRLWAASTENIYHGFEGIGSRGHLKHMTTSRKQEIIKVTEQLIESINNGDFEAY
AKICDPGLTSFEPEALGNLV
EGHDFHRFYFDNGESVGS
>g1501.t1
RLPDENKAYFLALQQMIEEMAEKAGGPVVLIAHSMGNMYTLYFLNHQPQAWKDRYIKAFVALGAPWGGVAKTLRVVTSGD
NNHIAVISPLKIRTQQRTAI
STTWLFPYASTWPKNQVLVQTPTSNYTVMDYQRFFSDVGFPEGWQIRQDTEHLVSDLTPPGVAVHCLYGSGVATPETYHY
SDKFPNVEPAAIEMGDGDGT
VNLRSATQCSRWVGRQPQPVKLEELPGNEHVQMLLNYTTVAYIKRVLFSP
>g1502.t1
MLLHTDLVTTDPPDEDFAELLEDLLEAARAHGVLLTQQREFHLTVSQTVVLRHHWIQPFTQSLRAGLTTCKRFVCSAGLL
RVYSNAERTSLIDHHEEGPF
RLALDCQELRCTAGNKTFSFPLES
>g1502.t2
MLVGYSSSSDEEIENAREITVSTDPPDEDFAELLEDLLEAARAHGVLLTQQREFHLTVSQTVVLRHHWIQPFTQSLRAGL
TTCKRFVCSAGLLRVYSNAE
RTSLIDHHEEGPFRLALDCQELRCTAGNKTFSFPLES
>g1502.t3
MLLHTDLVTTDPPDEDFAELLEDLLEAARAHGVLLTQQREFHLTVSQTVVLRHHWIQPFTQSLRAGLTTCKSLIDHHEEG
PFRLALDCQELRCTAGNKTF
SFPLES
>g1503.t1
LNPYTRARMTEAWQQHAVAPPPVVHAIPPGAENALGCAVYGIVLQPDPPSLQHGQHGQQHGGHGGGQHSQQQHPAQAQQP
TLQVGNEGGHKCGACGHDIS
HLANPHEHQCMVSQDRSFQCTQCMKIFHQATDLLEHQCVQVEQKPFVCGVCKMGFSLLTSLAQHHTSHNSTNPMKCSICE
KTYRPGTSGNPTPTTSGSSH
HSHGNRASASSSSSILPFPSARDRPYKCSVCQKGFKHLSELTRHERVHTGEKPFKCDTCDKAFSQSSHLAHHQRTHSNER
PFKCAVCEKSFKHRSHLVRH
MYAHSGEHLFKCNLCELHFKESSELLHHPCHPQGARPFRCATCGKGFKRPSDLRQHERAHSEERPFHCDECQMSFKQQYA
LVRHRRTHKDPTVRPFKCNL
CDKGFMQPSHLLYHQHVHGMDNLFKCASCQKEFSQSGELLRHKCGEPSSASPDKPYKCDVCGKGYKKSSTLQRHQNSHCQ
EKPLKCSLCDRRFLSSSDFV
QHRCDPSREKPLKCPECERRFKYVSDLNRHRRIHTGEKPYKCANCNKGFKQREHMTKHQATHSREGQFKCVWCGERYSDL
GSLQDHTVQHTAEGGGYPVS
SCI

>g1503.t2
MTEAWQQHAVAPPPVVHAIPPGAENALGCAVYGIVLQPDPPSLQHGQHGQQHGGHGGGQHSQQQHPAQAQQPTLQVGNEG
GHKCGACGHDISHLANPHEH
QCMVSQDRSFQCTQCMKIFHQATDLLEHQCVQVEQKPFVCGVCKMGFSLLTSLAQHHTSHNSTNPMKCSICEKTYRPGTS
GNPTPTTSGSSHHSHGNRAS
ASSSSSILPFPSARDRPYKCSVCQKGFKHLSELTRHERVHTGEKPFKCDTCDKAFSQSSHLAHHQRTHSNERPFKCAVCE
KSFKHRSHLVRHMYAHSGEH
LFKCNLCELHFKESSELLHHPCHPQGARPFRCATCGKGFKRPSDLRQHERAHSEERPFHCDECQMSFKQQYALVRHRRTH
KDPTVRPFKCNLCDKGFMQP
SHLLYHQHVHGMDNLFKCASCQKEFSQSGELLRHKCGEPSSASPDKPYKCDVCGKGYKKSSTLQRHQNSHCQEKPLKCSL
CDRRFLSSSDFVQHRCDPSR
EKPLKCPECERRFKYVSDLNRHRRIHTGEKPYKCANCNKGFKQREHMTKHQATHSREGQFKCVWCGERYSDLGSLQDHTV
QHTAEGGGYPVSSCI
>g1504.t1
LLRPLALRRDKVCAPGRLGRGRASSSSLGLTGRLPLHSAYHHGLRRGVLLSPARSGARPRLSAHGGAGVFGWLCDADGSA
VQIRPQQLASVTRLPVATDT
>g1505.t1
MQIVEELSMLPAMLQTTNVGNAAGPTVAAVGAGGGVPTKQGAGVGGAGATGGASGGDGDYQLVKHEVLCSMKYTYEVLDF
LGRGTFGQVVKCWKRGTGEV
VAVKILKNHPSYARQGQIEVGILARLSGENADEHNLVRAFECFQHRSHTCLVFEMLEQNLYDFLKQNKFSPLPLKVIRPV
LQQVATALRKLKSMGLIHAD
LKPENIMLVDPVRQPYRVKVIDFGSASHVSKAVCSTYLQSRYYR
>g1506.t1
MDNDAAGGGRPLPIKLLSAQLRDGTSKRLNDSMECSVNVGNGRSISVSMVWMQGTVMEAKLDRNMVLLLDETGTFVVQGV
NNIPKGKPCLSQGKYVMVMG
VIQATSPEPVIRAVKMADLTKDAAVHRKMWKLEVEDLQQVST
>g1506.t2
MEAEVKPEDTEPLCEIAEKADSDSASQTEEGNATTQDSSIINGGKADGEQEMVDLKIIWNKIKYDLKIPLDSSGAKLKEA
IHSLTSLPPAMQKVMYKGLL
PEDKTLQRTEKVPMGSIKNVVTENIEGHDGYHMMVTAALASSQF
>g1506.t3
MEAEVKPEDTEPLCEIAEKADSDSASQTEEGNATTQDSSIINGGKADGEQEMVDLKIIWNKIKYDLKIPLDSSGAKLKEA
IHSLTSLPPAMQKVMYKGLL
PEDKTLQRTEKVPMGSIKNVVTENIEGHDGYHMMLRDGTSKRLNDSMECSVNVGNGRSISVSMVWMQGTVMEAKLDRNMV
LLLDETGTFVVQGVNNIPKG
KPCLSQGKYVMVMGVIQATSPEPVIRAVKMADLTKDAAVHRKMWKLEVEDLQQVST
>g1506.t4
MVWMQGTVMEAKLDRNMVLLLDETGTFVVQGVNNIPKGKPCLSQGKYVMVMGVIQATSPEPVIRAVKMADLTKDAAVHRK
MWKLEVEDLQQVST
>g1507.t1
MDQLYEMWLMSQKPSWSEENTNGAVRENGKQIVMPTDFAEEASNHCWLCGKNCNSDKQWQQHITSEKHKDKVFNSEDDQN
CWQYRFPRGTFQVCERYLKG
TCPERGSCQLAHGKQELDEWRERREFLLMKLAKARKDHLIAPKDNDFGKYSFLLDIQ
>g1507.t2
MWLMSQKPSWSEENTNGAVRENGKQIVMPTDFAEEASNHCWLCGKNCNSDKQWQQHITSEKHKDKVFNSEDDQNCWQYRF
PRGTFQVCERYLKGTCPERG
SCQLAHGKQELDEWRERREFLLMKLAKARKDHLIAPKDNDFGKYSFLLDIQ
>g1508.t1
RTGPSLRPPGRNTDLQGLSWDASTWRKESGGNLRSEVLLVKEPRWAGAERSGVRAVITVDPRGPPAHSRRRESLDGIQLG
>g1509.t1
EDRAVSPAARQEHRPPGALLGRFHLAEGKTENKLDYHGSTFIGPSSSLFGSQEKNNPAPRSSVQPRKARI
>g1510.t1
MTNTWTQTHYKHLDPLLTPRPMTNTWTQTHYKHLDPLQTPRPMTNTWTQIHYKHLGPLQTPRPMTNTWTQTHYKHLDPLL
TPRPTTNTQTHYQHLDPLLT
PRPTTNTQTHDQHLDPLLTPRPMTNTWTQTNYKHLDPLQTPRPMTNTWTQIHYKHLDPLQTPRPMTNTWTHY
>g1511.t1

MTNTWTQTHYKHLDPLLTPRPTTNTQTHDQHLDPLLTPRPMTNTWTQTHYKHLDPGPLPTPRPRRAANTWTQTHYKHLDP
DPLPTPRP
>g1512.t1
MKGHRIKGSGHQNPAFTEASRFGSRRALNDETDVFCCAGSPEASLSDPRATGPPGRRCRGANLRTAGSLRILPLFLFHAK
RSISFEQKKHELQASVGPNP
TAVPLEVPYPPDLDQVPGPGQYPWRCRIHQTWISYLSLNRLWEEGAHPCLPLEVPYPPDLDQVPGPGQYPWRCRIHQTWI
SLN
>g1512.t2
MKGHRIKGSGHQNPAFTEASRFGSRRALNDETDVFCCAGSPEASLSDPRATGPPGRRCRGANLRTAGSLRILPLFLFHAK
RSISFEQKKHELQASVGPNP
TAGPRPGSVPLEVPYPPDLDQVPGPGQYPWRCRIHQTWISYLSLNRLWEEGAHPCLPLEVPYPPDLDQVPGPGQYPWRCR
IHQTWISLN
>g1512.t3
MKGHRIKGSGHQNPAFTEASRFGSRRALNDETDVFCCAGSPEASLSDPRATGPPGRRCRGANLRTAGSLRILPLFLFHAK
RSISFEQKKHELQASVGPNP
TAGAVSTRPGSGPRPGSVPLEVPYPPDLDQVPGPGQYPWRCRIHQTWISYLSLNRLWEEGAHPCLPLEVPYPPDLDQVPG
PGQYPWRCRIHQTWISLN
>g1513.t1
QKVLASRKELLEYDLQQREAATKSSRSSSGGSQPTFTASQYRALSVLGCGHTSSNKCYGCASAVTGHCITLLRALATNPA
LRQILVVQGLIRELFEYNLR
RGTAAMREEVRQLVCLLTRDHPEATQQMNDLIIAKVSSALKGHWANPDLVSNLQYEMLLLTDSISKEGGCWELRLRCGRL
FNLYSPFTSPALSLFLMSVN
IKTPVVVENITLMCLRILQKLIKPPAPTSKKNKDAAVESLTTVKPYSNEIHAQAQLWLKKDPKASYEAWKKCLPARCQET
VSKPQTKVELRRQYLLEKYV
WKWKQFMRARTEGLFPRLLKLSHDNWLRQVLFTPATQAARQAACTIVEALTTIPSRKQQVLDLLTR
>g1513.t2
QKVLASRKELLEYDLQQREAATKSSRSSSGGSQPTFTASQYRALSVLGCGHTSSNKCYGCASAVTGHCITLLRALATNPA
LRQILVVQGLIRELFEYNLR
RGTAAMREEVRQLVCLLTRDHPEATQQMNDLIIAKVSSALKGHWANPDLVSNLQYEMLLLTDSISKEGGCWELRLRCGRL
FNLYSPFTSPALSLFLMSVN
IKTPVVVENITLMCLRILQKLIKPPAPTSKKNKDAAVESLTTVKPYSNEIHAQAQLWLKKDPKASYEAWKKCLPARCQET
VSKPQTKVELRRQYLLEKYV
WKWKQFMRARTEGLFPRLLKLSHDNWLRQVLFTPATQAARQAACTIVEALTTIPSRKQQVLDLLT
>g1514.t1
MMLRKGWRCLECIVCEVCGKASDPSRLLLCDDCDVSYHTYCLDPPLHTVPKGGWKCKWCVSCVQCGSNTPGFHCEWQNNY
THCGPCASLVTCPVCRENFM
EEDLLLQCQHCDRWVHAVCESLYTEDEVEQASDEGFACTSCTPYVPKPVVVESAIMASIKIKEPEPQFYRLEGVWLTESG
MSLLRSISMSPLHKRRQRRS
RLGGLCGEGADGLELKEGEGDGDEGKGCGEPMDCETKMEPSGSPDRELGADGSGEGPGYGDGVKGGAEETEDSKKRKRKP
YRPGIGGFMVRQRKCHTRIK
KGFFAQLAGETTLDGQPLERTIEEGPHPDTDNIMDPKPAEGEEQKKRRGRKKSKLEDMFPAYLQEAFFGKTLIDMSKKAV
MIPPVQRPGMCPSRPPEPAL
PGVKPPTQEAEAQDRVMEATLPLKKEVVDTPQTQGEGARVSTPSSSVKDQASMDPQGSDAEKSDGLPQGMESQDSEQFFR
KVLGVSDGSTLEGMKPILEG
SKGELNRNALPQRPLLSGSLPSAGVMDTFPGLAQSPFFDMRDRGGLFSPDGGEESPWATPSTPATPSTPPTPTEVEGDGL
SYNQRSLQRWEKDEELGELS
TISPVLYANTNFPTLKQDYPDWASRCKQIMKIWRKVSAADKVPFLQKAKDNRAAQRINKAQKAESQVCRPIKTEPGRVKG
ERPSLHLQIPPPSGSASTPS
QPSSAESPFPFPPNHGSSSAFFPDGPLKTPGSAEIRTDPFAKLPPQSPHAHSQPSTPFSQPGTSPLQANSSGYPPPGPQG
PPQGRPAPGPFDMQPGTPGT
PRRAQQVDPYFRSQLQQQQQQQQQQQQQQQQKQQSQQLQMQQGSLESLGPPQSPHSRAAGPGEPLFSPPHTPHYGDPFRC
QQGMGRPEYGSSSSPSAMAS
SPAGPGQYRADMNAPSPRSSSMGRPDMATGSPAGMLESTDGLFKAPMMPRMHQGEGGPLHPGASPNHPSEAYRQSPSNPF
SDPYGQPPLTPRPQSGDGCS
PLPQRHPVAQQESCPRVPSSPQSQGSAPSPLTPGGLSNDAFSVQSPVTNRFQSPDPYSRPPSRPQSRDPFATLHKPPRPG
SAAPEGAPSYRGSPHHNQPP

PASPASNPTGDPLSGKPSAPPPFSRSPSTSTYQMIQQQAQMTTQQAQMTQQQAQMTQHQAQMTQQQAQMNQPQLQLQQQV
QQQAMATDGFNPRLSSGSHD
PTAVRPPDSSQQPAMPGSQEMADMSVVQDPALVGLSPSELEKHRQ
>g1514.t2
MLRKGWRCLECIVCEVCGKASDPSRLLLCDDCDVSYHTYCLDPPLHTVPKGGWKCKWCVSCVQCGSNTPGFHCEWQNNYT
HCGPCASLVTCPVCRENFME
EDLLLQCQHCDRWVHAVCESLYTEDEVEQASDEGFACTSCTPYVPKPVVVESAIMASIKIKEPEPQFYRLEGVWLTESGM
SLLRSISMSPLHKRRQRRSR
LGGLCGEGADGLELKEGEGDGDEGKGCGEPMDCETKMEPSGSPDRELGADGSGEGPGYGDGVKGGAEETEDSKKRKRKPY
RPGIGGFMVRQRKCHTRIKK
GFFAQLAGETTLDGQPLERTIEEGPHPDTDNIMDPKPAEGEEQKKRRGRKKSKLEDMFPAYLQEAFFGKTLIDMSKKAVM
IPPVQRPGMCPSRPPEPALP
GVKPPTQEAEAQDRVMEATLPLKKEVVDTPQTQGEGARVSTPSSSVKDQASMDPQGSDAEKSDGLPQGMESQDSEQFFRK
VLGVSDGSTLEGMKPILEGS
KGELNRNALPQRPLLSGSLPSAGVMDTFPGLAQSPFFDMRDRGGLFSPDGGEESPWATPSTPATPSTPPTPTEVEGDGLS
YNQRSLQRWEKDEELGELST
ISPVLYANTNFPTLKQDYPDWASRCKQIMKIWRKVSAADKVPFLQKAKDNRAAQRINKAQKAESQVCRPIKTEPGRVKGE
RPSLHLQIPPPSGSASTPSQ
PSSAESPFPFPPNHGSSSAFFPDGPLKTPGSAEIRTDPFAKLPPQSPHAHSQPSTPFSQPGTSPLQANSSGYPPPGPQGP
PQGRPAPGPFDMQPGTPGTP
RRAQQVDPYFRSQLQQQQQQQQQQQQQQQQKQQSQQLQMQQGSLESLGPPQSPHSRAAGPGEPLFSPPHTPHYGDPFRCQ
QGMGRPEYGSSSSPSAMASS
PAGPGQYRADMNAPSPRSSSMGRPDMATGSPAGMLESTDGLFKAPMMPRMHQGEGGPLHPGASPNHPSEAYRQSPSNPFS
DPYGQPPLTPRPQSGDGCSP
LPQRHPVAQQESCPRVPSSPQSQGSAPSPLTPGGLSNDAFSVQSPVTNRFQSPDPYSRPPSRPQSRDPFATLHKPPRPGS
AAPEGAPSYRGSPHHNQPPP
ASPASNPTGDPLSGKPSAPPPFSRSPSTSTYQMIQQQAQMTTQQAQMTQQQAQMTQHQAQMTQQQAQMNQPQLQLQQQVQ
QQAMATDGFNPRLSSGSHDP
TAVRPPDSSQQPAMPGSQEMADMSVVQDPALVGLSPSELEKHRQ
>g1514.t3
MVILLSQDMCVVCGSFGKGAEGQLLACAQCAQCYHPYCVNSKITKMMLRKGWRCLECIVCEVCGKASDPSRLLLCDDCDV
SYHTYCLDPPLHTVPKGGWK
CKWCVSCVQCGSNTPGFHCEWQNNYTHCGPCASLVTCPVCRENFMEEDLLLQCQHCDRWVHAVCESLYTEDEVEQASDEG
FACTSCTPYVPKPVVVESAI
MASIKIKEPEPQFYRLEGVWLTESGMSLLRSISMSPLHKRRQRRSRLGGLCGEGADGLELKEGEGDGDEGKGCGEPMDCE
TKMEPSGSPDRELGADGSGE
GPGYGDGVKGGAEETEDSKKRKRKPYRPGIGGFMVRQRKCHTRIKKGFFAQLAGETTLDGQPLERTIEEGPHPDTDNIMD
PKPAEGEEQKKRRGRKKSKL
EDMFPAYLQEAFFGKTLIDMSKKAVMIPPVQRPGMCPSRPPEPALPGVKPPTQEAEAQDRVMEATLPLKKEVVDTPQTQG
EGARVSTPSSSVKDQASMDP
QGSDAEKSDGLPQGMESQDSEQFFRKVLGVSDGSTLEGMKPILEGSKGELNRNALPQRPLLSGSLPSAGVMDTFPGLAQS
PFFDMRDRGGLFSPDGGEES
PWATPSTPATPSTPPTPTEVEGDGLSYNQRSLQRWEKDEELGELSTISPVLYANTNFPTLKQDYPDWASRCKQIMKIWRK
VSAADKVPFLQKAKDNRAAQ
RINKAQKAESQVCRPIKTEPGRVKGERPSLHLQIPPPSGSASTPSQPSSAESPFPFPPNHGSSSAFFPDGPLKTPGSAEI
RTDPFAKLPPQSPHAHSQPS
TPFSQPGTSPLQANSSGYPPPGPQGPPQGRPAPGPFDMQPGTPGTPRRAQQVDPYFRSQLQQQQQQQQQQQQQQQQKQQS
QQLQMQQGSLESLGPPQSPH
SRAAGPGEPLFSPPHTPHYGDPFRCQQGMGRPEYGSSSSPSAMASSPAGPGQYRADMNAPSPRSSSMGRPDMATGSPAGM
LESTDGLFKAPMMPRMHQGE
GGPLHPGASPNHPSEAYRQSPSNPFSDPYGQPPLTPRPQSGDGCSPLPQRHPVAQQESCPRVPSSPQSQGSAPSPLTPGG
LSNDAFSVQSPVTNRFQSPD
PYSRPPSRPQSRDPFATLHKPPRPGSAAPEGAPSYRGSPHHNQPPPASPASNPTGDPLSGKPSAPPPFSRSPSTSTYQMI
QQQAQMTTQQAQMTQQQAQM
TQHQAQMTQQQAQMNQPQLQLQQQVQQQAMATDGFNPRLSSGSHDPTAVRPPDSSQQPAMPGSQEMADMSVVQDPALVGL
SPSELEKHRQ

>g1515.t1
MERMSEEALRLNLLKRGLETSAEREEALAKRLKMEGHEAMERLKMLALLKRKDLADLAALEGAAAAAAVAAGSLGDGKGP
GISSQHHHHQHQSIIGSTTA
YEEKLNGSLRVAGHGELNKNGKENILDEPVDMSASRRCETACERRSLSPDVIVLSDNEATSPRSTPHPEERLHHANREIF
RGKTGLERQQMIKVLREELR
LEEARLVLLKKLRQSQMQKENVVQKVPVVQNATSLQQTPIHSSQGMGKLPIRPGLNTTEPQNLRTVQEARSTTAVPKRRR
VEISPADLFLLLMMAVPVDQ
LR
>g1515.t2
MERMSEEALRLNLLKRGLETSAEREEALAKRLKMEGHEAMERLKMLALLKRKDLADLAALEGAAAAAAVAAGSLGDGKGP
GISSQHHHHQHQSIIGSTTA
YEEKLNGSLRVAGHGELNKNGKENILDEPVDMSASRRCETACERRSLSPDVIVLSDNEATSPRSTPHPEERLHHANREIF
RGKTGLERQQMIKVLREELR
LEEARLVLLKKLRQSQMQKENVVQKVPVVQNATSLQQTPIHSSQGMGKLPIRPGLNTTEPQNLRTVQG
>g1515.t3
MERMSEEALRLNLLKRGLETSAEREEALAKRLKMEGHEAMERLKMLALLKRKDLADLAALEGAAAAAAVAAGSLGDGKGP
GISSQHHHHQHQSIIGSTTA
YEEKLNGSLRVAGHGELNKNGKENILDEPVDMSASRRCETACERRSLSPDVIVLSDNEATSPRSTPHPEERLHHANREIF
RGKTGLERQQMIKVLREELR
LEEARLVLLKKLRQSQMQKENVVQKVPVVQNATSLQQTPIHSSQGMGKLPIRPGLNTTEPQNLRTVQGSLKADEMHINPL
VV
>g1516.t1
MWPIAAKDKGEKKLFGYSFVPLMQEDGRTLPDGTHELIIHK
>g1516.t2
KDKGEKKLFGYSFVPLMQEDGRTLPDGTHELIIHK
>g1517.t1
MESAMGQYTQEGQVTCWRKLCPLAQGIGYGQLVIKLYDFAYIVIQAWALLYLIFSFSALLPWAHCGNSWNTGIDELGGVR
WDLALCLLACWTMCYFSIWK
GVRSSGKVVYFTAVFPYVMLLVLLIRGVTLPGAWEGIKYYLYPGVERLAALEVWVEAGSQIFFSFSLASGTLVVLGSYNG
ANNNCYKDTLLLCLLNSGTS
FVAGFVVFSVLGFMAEQKGVPVDMVVDSVCDGRVFHHFGQRPVSQTLSPTGKE
>g1517.t2
MESAMGQYTQEGQVTCWRKLCPLAQGIGYGQLVIKLYDFAYIVIQAWALLYLIFSFSALLPWAHCGNSWNTDNCVDLQHS
DTNITMGHFNNTTSAATEFW
EWDLALCLLACWTMCYFSIWKGVRSSGKVVYFTAVFPYVMLLVLLIRGVTLPGAWEGIKYYLYPGVERLAALEVWVEAGS
QIFFSFSLASGTLVVLGSYN
GANNNCYKDTLLLCLLNSGTSFVAGFVVFSVLGFMAEQKGVPVDMVVDSVCDGRVFHHFGQRPVSQTLSPTGKE
>g1517.t3
MESAMGQYTQEGQVTCWRKLCPLAQGIGYGQLVIKLYDFAYIVIQAWALLYLIFSFSALLPWAHCGNSWNTGIDELGGVR
WDLALCLLACWTMCYFSIWK
GVRSSGKVVYFTAVFPYVMLLVLLIRGVTLPGAWEGIKYYLYPGVERLAALEVEDTLLLCLLNSGTSFVAGFVVFSVLGF
MAEQKGVPVDMVVDSVCDGR
VFHHFGQRPVSQTLSPTGKE
>g1518.t1
MADPAECTIKVMCRFRPLNSSEVTRGDRYIPKFQGDETVVIALWYRRDATRYDFNACCQKFHIQVALVDEF
>g1519.t1
MSAWCAGLPSLLFSAVKPRHAPLGGAWLLRWKSTGAGSLQDRQRQLMAKGLPKQKPISGVKHVVVVASGKGGVGKSTTAV
NLALGLMANDP
>g1519.t2
MSAWCAGLPSLLFSAVKPRHAPLGGAWLLRWKSTGAGSLQDRQRQLMAKGLPKQKPISGVKHVVVVASGKGGVGKSTTAV
NLALGLMANDPVRSNRMTGS
FH
>g1520.t1
MAEKAAPAPLARTVVQQCLHARLQVSPPGPDSEEQWVQVGRGMVIYVCFFKGATEDLLPKMGLSAPVVDVMLLTYGNNVT
LSSLLQQRLCERPSGGLGSV
LEVQGSVLVVPQATLGGRAKGRAMQYHGNIGKEAGLALYEGFVSLCRAQLQAAGGGGEVKHGTYGNRQVLQLDTNGPYTH

LIEF
>g1521.t1
MADGSSRPFAGLSDVSISEDIPVEGDISMPAASSSQDDGFSTLDEPVKDTVLRDLKAVGNKFVHVLYPKKSQALLRDWDL
WGPLLLCVSLSLLLQGGAGD
HMQDGGPQFAEVFVIIWFGSVIITMNSKLLGGTISFFQSLCVLGYCILPLTVAMVVCRLVLLGGSGPVSFAVRMIVVSVS
FGWSTFASTAFLADSQPPNR
KALVVYPVFLFYFVIGWMVLTFAPAQ
>g1522.t1
MADCRKQLTDDCEDLLARFQKTESVRFEIFANIWGEMKFSEIFLGTTEGTEGKVFSRQALAVACRYFLPPYTFQIRVGAL
YLLYGLYSHQLARPKVQIQL
ALKDWEHVQRFEKDAAEAQHFDGVYVLRKLFSQHAFCFAAMPCHLSYSHKKTQNKHSMCEGFIERPSRPQELLNSELLEE
LSNVHEHYERYRLSQDRVRP
DQVRPDLVRPDLVSLLRQTVMEFSSWQSKMETPAQDGEDSGEGSSQKESSRRAELLAAIKSRSYGQVVEASRGRRHRQVE
LGRHITPRAPKKPTLKSRAD
TILIQSDVKKEMSKYTKVWRLTTPETGADSEKTAWNSQEPTPPDLERPSGTSELGVKR
>g1522.t2
MADCRKQLTDDCEDLLARFQKTESVRFEIFANIWGEMKFSEIFLGTTEGTEGKVFSRQALAVACRYFLPPYTFQIRVGAL
YLLYGLYSHQLARPKVQLAL
KDWEHVQRFEKDAAEAQHFDGVYVLRKLFSQHAFCFAAMPCHLSYSHKKTQNKHSMCEGFIERPSRPQELLNSELLEELS
NVHEHYERYRLSQDRVRPDQ
VRPDLVRPDLVSLLRQTVMEFSSWQSKMETPAQDGEDSGEGSSQKESSRRAELLAAIKSRSYGQVVEASRGRRHRQVELG
RHITPRAPKKPTLKSRADTI
LIQSDVKKEMSKYTKVWRLTTPETGADSEKTAWNSQEPTPPDLERPSGTSELGVKR
>g1523.t1
GDSGGPLIYQGVAVGITSNGGKKCGLVKKPGIYTIISHYTTWISTTMAL
>g1524.t1
MMFGNTYVVCVCVALRAAEAHLSECEAEMEDQVNTASDPTTPPFSPLVMALPQHDRQVTQERQGRGRRDQQLWIDRSQKE
IQDLQRRLKEMETDRNLLLA
SVQERGHVTEVRGARMASSLPRTTTNTS
>g1524.t2
MFGNTYVVCVCVALRAAEAHLSECEAEMEDQVNTASDPTTPPFSPLVMALPQHDRQVTQERQGRGRRDQQLWIDRSQKEI
QDLQRRLKEMETDRNLLLAS
VQERGHVTEVRGARMASSLPRTTTNTS
>g1525.t1
MHFVGPQLEILEKESEEEIRRLRNKEARSREEVQMLLREAERNSREEEEEATRRNLELTTQKENLQKSHQIEVSAIFLFL
QLQQLAASHTAVLEAATHAR
DQHQNSLDAMATEMLQLQGHLEKVVSERDVLRNQLSQASQVLEGQATELQNLRGYIGELEPQRGQEERLAEIVQKLCREK
EALEKTADLLTIRLKCVNEI
LTLQEQKLLHKSLSDPLLSSGGCEGLRVLRLWRERVFSLCLCLRTQELELKTERERNRSGVRRTQNQGPLVKECSHTSTV
AHCLRSDLVPRLFCKEKTLQ
QEVKQEQLRNTLLQHSLDNRLAQLHLEEVAVQSLKEELATLGRENTELTTWKHQASTDLATVKDELQRFSAAFEARVADV
NKSQEQLHVFSKRLTFAKRR
VQTIQGLLARRSALQKVQHCSRQAAQEDHRVRRLQAELQLLGEEREELCEELRRTPELIDKALAQLRETCRTPPPPPCPS
SSTSISTTILTILTNSTSIT
TISSSMTSRPPPPLSPPPLVGCQLKEQQVELDQSRGALAEALSSQKEAQQREQEALARLEETEARLEEAQEQSQQGEEMV
VVVGPFMLANLG
>g1525.t2
MHFVGPQLEILEKESEEEIRRLRNKEARSREEVQMLLREAERNSREEEEEATRRNLELTTQKENLQKSHQIEVCVFHRTS
WLTAIFLFLQLQQLAASHTA
VLEAATHARDQHQNSLDAMATEMLQLQGHLEKVVSERDVLRNQLSQASQVLEGQATELQNLRGYIGELEPQRGQEERLAE
IVQKLCREKEALEKTADLLT
IRLKCVNEILTLQEQKLLHKSLSDPLLSSGGCEGLRVLRLWRERVFSLCLCLRTQELELKTERERNRSGVRRTQNQGPLV
KECSHTSTVAHCLRSDLVPR
LFCKEKTLQQEVKQEQLRNTLLQHSLDNRLAQLHLEEVAVQSLKEELATLGRENTELTTWKHQASTDLATVKDELQRFSA
AFEARVADVNKSQEQLHVFS
KRLTFAKRRVQTIQGLLARRSALQKVQHCSRQAAQEDHRVRRLQAELQLLGEEREELCEELRRTPELIDKALAQLRETCR

TPPPPPCPSSSTSISTTILT
ILTNSTSITTISSSMTSRPPPPLSPPPLVGCQLKEQQVELDQSRGALAEALSSQKEAQQREQEALARLEETEARLEEAQE
QSQQGEEMVVVVGPFMLANL
G
>g1525.t3
MHFVGPQLEILEKESEEEIRRLRNKEARSREEVQMLLREAERNSREEEEEATRRNLELTTQKENLQKSHQIEVSAIFLFL
QLQQLAASHTAVLEAATHAR
DQHQNSLDAMATEMLQLQGHLEKVVSERDVLRNQLSQASQVLEGQATELQNLRGYIGELEPQRGQEERLAEIVQSLVVQV
ICSTLTRIVCYGDPSRSLQK
LCREKEALEKTADLLTIRLKCVNEILTLQEQKLLHKSLSDPLLSSGGCEGLRVLRLWRERVFSLCLCLRTQELELKTERE
RNRSGVRRTQNQGPLVKECS
HTSTVAHCLRSDLVPRLFCKEKTLQQEVKQEQLRNTLLQHSLDNRLAQLHLEEVAVQSLKEELATLGRENTELTTWKHQA
STDLATVKDELQRFSAAFEA
RVADVNKSQEQLHVFSKRLTFAKRRVQTIQGLLARRSALQKVQHCSRQAAQEDHRVRRLQAELQLLGEEREELCEELRRT
PELIDKALAQLRETCRTPPP
PPCPSSSTSISTTILTILTNSTSITTISSSMTSRPPPPLSPPPLVGCQLKEQQVELDQSRGALAEALSSQKEAQQREQEA
LARLEETEARLEEAQEQSQQ
GEEMVVVVGPFMLANLG
>g1526.t1
GSGKHRTKEPKKKNTKQKLRFRAQNQHSDHLASVRSGHRD
>g1527.t1
WAVPFMILIVCIESNLLISYMVSVAAGISVAAAYLLPWSMLPDVVDDFKLKNPDIRGYEAIFFSFYVFFTKFASGVSLGI
STLSLDFAGYETRGCSQPQS
VILTLKVLVSVVPVALIALGLLIFKTYPIDEAKRKSNRKLLQKLL
>g1527.t2
WAVPFMILIVCIESNLLISYMVSVAAGISVAAAYLLPWSMLPDVVDDFKLKNPDIRGYEAIFFSFYVFFTKFASGVSLGI
STLSLDFAGYETRGCSQPQS
VILTLKVLVSVVPVALIALGLLIFKTYPIDEAKRKSNRKLLQKLLDAEDSEQDSPELGSVV
>g1528.t1
LGGTCTSNGVLRPRGDLLFTFPVTECDVQREMHPDYIAYKYVLAYTPSPNRSKGGGHSFALNIECRYPRYHHVYQLAVRP
TWQTAILRKNLRGRPNKFQI
QLMDDTWNMPAKSQIYVLGQTVNLQVSASHLPSGGKVYIRSCFATPYNGSASPLKYSLIDNFGCMSASQKDPVGSRFVPS
QDDQTVRFSLSAFQFTSHPD
TQLPASGDHSPSATPPQITTTVEGEPGDILALTNKTIRPITEQGSPRNPANTSHSSKTSGREDRQSSVDSVEEDKMEEVE
EELEGDGIVWVDGAKAIGRL
NKPGFGSGLITKEWEGEMSGMGGDSDEFRDDGEPRYGATGGPLETEENDELGSADSRGTKETAPNDEARGFSGGTPGPHL
MREEEAETQVTVAQRKTIAS
GMKGMGRT
>g1528.t2
MHPDYIAYKYVLAYTPSPNRSKGGGHSFALNIECRYPRYHHVYQLAVRPTWQTAILRKNLRGRPNKFQIQLMDDTWNMPA
KSQIYVLGQTVNLQVSASHL
PSGGKVYIRSCFATPYNGSASPLKYSLIDNFGCMSASQKDPVGSRFVPSQDDQTVRFSLSAFQFTSHPDTQLPASGDHSP
SATPPQITTTVEGEPGDILA
LTNKTIRPITEQGSPRNPANTSHSSKTSGREDRQSSVDSVEEDKMEEVEEELEGDGIVWVDGAKAIGRLNKPGFGSGLIT
KEWEGEMSGMGGDSDEFRDD
GEPRYGATGGPLETEENDELGSADSRGTKETAPNDEARGFSGGTPGPHLMREEEAETQVTVAQRKTIASGMKGMGRT
>g1528.t3
LEREPGSLGPRSQPSFAYLPDVSVTCSTSDFVVRVKPAFYGLGANADELRLGGTCTSNGVLRPRGDLLFTFPVTECDVQR
EMHPDYIAYKYVLAYTPSPN
RSKGGGHSFALNIECRYPRYHHVYQLAVRPTWQTAILRKNLRGRPNKFQIQLMDDTWNMPAKSQIYVLGQTVNLQVSASH
LPSGGKVYIRSCFATPYNGS
ASPLKYSLIDNFGCMSASQKDPVGSRFVPSQDDQTVRFSLSAFQFTSHPDTQLPASGDHSPSATPPQITTTVEGEPGDIL
ALTNKTIRPITEQGSPRNPA
NTSHSSKTSGREDRQSSVDSVEEDKMEEVEEELEGDGIVWVDGAKAIGRLNKPGFGSGLITKEWEGEMSGMGGDSDEFRD
DGEPRYGATGGPLETEENDE
LGSADSRGTKETAPNDEARGFSGGTPGPHLMREEEAETQVTVAQRKTIASGMKGMGRT

>g1529.t1
GRQKFISDVLNSMPVVRQNEDEDATEKLHFYKPTLKRDIPRVRANDTAEDSLKPPSLHARKRLRQTEGSESPPGKRFSSS
TLTAEDRDLLDSY
>g1530.t1
MIWSFRVHRRKNFMHANEAAACSLSVSLLATLMDMVPRIHMLYPLALVRGFWDDFMNHAHSLITSSWTFYLQADDGKVVI
FQTEPQVEIIPHFELEKDVK
EEPDKSFPSLSLPVYKDYHRSFIQERDRDMFEDRSPSAVGDENNFFTCLSRNPWLPGWILTTTLVLSVLVLIWICCATVA
TAVDQYVPAEKLSISGDMDY
MKEQKLTPYPASSLLIVTSTGQEEEEEQAGPLPAKVSLEQSNI
>g1530.t2
MIWSFRVHRRKNFMHANEAAACSLSVSLLATVRISIRPKLNVNQLMDMVPRIHMLYPLALVRGFWDDFMNHAHSLITSSW
TFYLQADDGKVVIFQTEPQV
EIIPHFELEKDVKEEPDKSFPSLSLPVYKDYHRSFIQERDRDMFEDRSPSAVGDENNFFTCLSRNPWLPGWILTTTLVLS
VLVLIWICCATVATAVDQYV
PAEKLSISGDMDYMKEQKLTPYPASSLLIVTSTGQEEEEEQAGPLPAKVSLEQSNI
>g1530.t3
MIWSFRVHRRKNFMHANEAAACSLSVSLLATSDEQYACNLGCQNQLPIAAQRQEQLMDMVPRIHMLYPLALVRGFWDDFM
NHAHSLITSSWTFYLQADDG
KVVIFQTEPQVEIIPHFELEKDVKEEPDKSFPSLSLPVYKDYHRSFIQERDRDMFEDRSPSAVGDENNFFTCLSRNPWLP
GWILTTTLVLSVLVLIWICC
ATVATAVDQYVPAEKLSISGDMDYMKEQKLTPYPASSLLIVTSTGQEEEEEQAGPLPAKVSLEQSNI
>g1531.t1
MLEFPGQTKDNLLHALAELKKKMPSREVLKSTKIGHTVNKMRKNPDPEVSDMATQVYTEWRTFIEENSNKPSIEVHCDKQ
SEALRNNARRLMADALELKT
DCSLIDNIEREVFHQSSRLVSSSYRRTVRALVFAFKHKPEVRSQLKDSTISVTQLVSSYKK
>g1531.t2
MPSREVLKSTKIGHTVNKMRKNPDPEVSDMATQVYTEWRTFIEENSNKPSIEVHCDKQSEALRNNARRLMADALELKTDC
SLIDNIEREVFHQSSRLVSS
SYRRTVRALVFAFKHKPEVRSQLKDSTISVTQLVSSYKK
>g1531.t3
MLEFPGQTKDNLLHALAELKKKMPSREVLKSTKIGHTVNKMRKNPDPEVSDMATQVYTEWRTFIEENSNKPSIEVHCDKQ
SEALRNNARRLMADALELKT
DCSLIDNIEREVFHQSSRLVSSSYRRTVRALVFAFKHKPE
>g1532.t1
GEPRAVAVDGRLPETRHDRQTDTRTRIAFDTGAIECEDCVGTDDDSQRQ
>g1533.t1
MGQAHQTGRNDEEIDVKALQDMYKRFVVECPSGVLFLHEFKRFFGVDPTGEVSDYAEAMFRAFDGNGDNTIDFLEFVAAL
NLVFRGDLEHKLRWSFKVYD
KDGNGYVDRSEVRSIIDSIYRINKSTKKDWSDGQLSVDEVVDRILEAVDTDRDGHINIDEFIKGAQQDPWVLNMLKLDMN
PYGWVLEQRRKSAHF
>g1533.t2
MGQAHQTGRNDEEIDVKALQDMYKRFVVECPSGVLFLHEFKRFFGVDPTGEVSDYAEAMFRAFDGNGDNTIDFLEFVAAL
NLVFRGDLEHKLRWSFKVYD
KDGNGYVDRSEVRSIIDSIYRINKSTKKDWSDGQLSVDEVVDRILEAVDTDRDGEELFRSVEQAQNKSSRERGTDGEGLV
LTRLNCFATGHINIDEFIKG
AQQDPWVLNMLKLDMNPYGWVLEQRRKSAHF
>g1534.t1
MGQEESHNEEMDKARIQELCIIFMKECPSGALHLHEFKRIFGVPSSSEEESSYIETVFRSFDTNKDNALDFIEYVAALHL
VLCGNLEDRLKWSFKIYDKD
GNGKLDRHEVKQIIRIIYKIKLKR
>g1534.t2
MGQEESHNEEMDKARIQELCIIFMKECPSGALHLHEFKRIFGVPSSSEEESSYIETVFRSFDTNKDNALDFIEYVAALHL
VLCGNLEDRLKWSFKIYDKD
GNGKLDRHEVKQIIR
>g1534.t3
MGQEESHNEEMDKARIQELCIIFMKECPSGALHLHEFKRIFGVPSSSEEESSYIETVFRSFDTNKDNALDFIEYVAALHL

VLCGNLEDRLKWSFKIYDKD
GNGKLDRHEVKQIIRIIYKIKLKRGDIKMTPNEICDRIFELVDLNHD
>g1535.t1
MLNQAFLWSRFDLLANGGASLTLSFERAPFLTQYRTVWVPWNVFYVMDTLVMKKEENDIPSCDLSGFVRPSPIIVASPLS
TCYRSSPEDGPIVPETQ
>g1535.t2
MLNQAFLWSRFDLLANGGASLTLSFERAPFLTQYRTVWVPWNVFYVMDTLVMKKEENDIPSCDLSGFVRPSPIIVASPLS
TCYRSSPEDGPIVPETQSAV
LQDPFDPLCSSHFLFQATPV
>g1536.t1
MEMLCTDGKDNEGDGLADCMDPDCCLQPSCQSQLYCRGSPDPSEVLSQSPAVLVPQQVARSFYQRIHFLLGPESTHVITA
DSPFNKR
>g1536.t2
LCNNNGRCTLESSGWHCVCQAGWRGAGCHVAMEMLCTDGKDNEGDGLADCMDPDCCLQPSCQSQLYCRGSPDPSEVLSQS
PAVLVPQQVARSFYQRIHFL
LGPESTHVITADSPFNKR
>g1536.t3
MEMLCTDGKDNEGDGLADCMDPDCCLQPSCQSQLYCRGSPDPSEVLSQSPAVLVPQQRIHFLLGPESTHVITADSPFNKR
>g1537.t1
LLCAFQSRALIGSRVAPILTSRPGKSRAGRWFQTDPAAVTPTSVSTAPSLLRSAPTR
>g1538.t1
MRDLNAKPCCGYLSVGRPLVAVRCSQKTRRVLNAYNDHMKHDVTAAPRRQVWILGAWPAVMMRKSLQNNHAERVDFLTNQ
EKISSKKTTTGVVIGVLLGL
LLLAALAAFLIWFFVSGLRCRPAVTVQTLCCCEYSLQLAGSLQLALCACGWCLQWKADFGLPLLQVTDRKTEGSISHQRT
AATLVFSGHLKMEDFKYNQT
LEDTKSTEFSVLADDLQEIMANSYNEEKFLAGYYSKSVVTAFSEGVLAYFWSEFKIPADDLEVLPEFTEELIQETLWIGV
KRYSRANRDNYRISEVTASI
TDPRKARNPRA
>g1538.t2
LQLFMRDLNAKPCCGYLSVGRPLVAVRCSQKTRRVLNAYNDHMKHDVTAAPRRQVWILGAWPAVMMRKSLQNNHAERVDF
LTNQEKISSKKTTTGVVIGV
LLGLLLLAALAAFLIWFFVSGLRCRPAVTVQTLCCCEYSLQLAGSLQLALCACGWCLQWKADFGLPLLQVTDRKTEGSIS
HQRTAATLVFSGHLKMEDFK
YNQTLEDTKSTEFSVLADDLQEIMANSYNEEKFLAGYYSKSVVTAFSEGVLAYFWSEFKIPADDLEVLPEFTEELIQETL
WIGVKRYSRANRDNYRISEV
TASITDPRKARNPRA
>g1538.t3
MRDLNAKPCCGYLSVGRPLVAVRCSQKTRRVLNAYNDHMKHDVTAAPRRQVWILGAWPAVMMRKSLQNNHAERVDFLTNQ
EKISSKKTTTGVVIGVLLGL
LLLAALAAFLIWFFVSGLRCRPAVTVQTLCCCEYSLQLAGSLQLALCACGWCLQWKADFGLPLLQVTDRKTEGSISHQRT
AATLVFSGHLKMEDFKYNQT
LEDTKSTEFSVLADDLQEIEYQLSNLFHSRSFHLQADLLMADVVATCLSSPPVPLDSQRPRPTMANSYNEEKFLAGYYSK
SVVTAFSEGVLAYFWSEFKI
PADDLEVLPEFTEELIQETLWIGVKRYSRANRDNYRISEVTASITDPRKARNPRA
>g1539.t1
LLDVLMQESRLYLIFEFLSMDLKKYLDSIPSGEYMDTMLVKSYLYQILEGIQFCHCRRVLHRDLKPQNLLIDNKGVIKLA
DFGLARAFGVPIRVYTHEVV
TLWYRAPEVLLGSARYATPVDVWSIGTIFAELATKKPLFHGDSEIDQLFRIFRTLGTPNNDTWPEVESLPDYKNTFPKWK
SGNLASMVKNLDTNGLDLLA
KTLIYNPPKRISARQAMAHPYFDDLDKSTLPASSLQI
>g1539.t2
WTPQFCLGLVTVRVLEMQGMFCLKSYSSNTFYDGGVKWLLDVLMQESRLYLIFEFLSMDLKKYLDSIPSGEYMDTMLVKS
YLYQILEGIQFCHCRRVLHR
DLKPQNLLIDNKGVIKLADFGLARAFGVPIRVYTHEVVTLWYRAPEVLLGSARYATPVDVWSIGTIFAELATKKPLFHGD
SEIDQLFRIFRTLGTPNNDT
WPEVESLPDYKNTFPKWKSGNLASMVKNLDTNGLDLLAKTLIYNPPKRISARQAMAHPYFDDLDKSTLPASSLQI

>g1540.t1
MCGRAALVMLGLWVLSGRVRGFYLPGLAPVSFCEPAKEGEGNPVPDCKSTIELFVNRLDSVESVLPYEYTAFDFCAVATE
KRPSENLGQVLFGERIEPSP
YKFQFKKSEKCKMVCEKKYTNAPAHKASLDFLKKGMLLNYQHHWIVDNMPVTWCYDVEEGQKFCNPGFPIGCYVTEGGQA
KDACVVNLDFKERDTFYIFN
HVDITIYYHMVENEQLEARLVAAKMEPKSYKHTNPDTPDCLGGPMFIQNKFSGDIRIPYTYSVEFLVCDGLLQGACTHAF
TRTEDKKIRWASRWDYILES
MPHTNIQWFSIMNSLVIVLFLSGMVAMIMLRTLHKDIARYNQMDSVEDAQEEFGWKLVHGDVFRPPRKGMLLSVFLGSGT
QIFIMTFVTLFFACLGFLSP
ANRGALMTCAVVLWVLLGTPAGYVAARFYKSFGGEKWKTNVLLTAFLCPGVVFADFFLMNLILWGEGSSAAMPFGTLVAI
LALWFCISVPLTFIGAYFGF
KKTGIEHPVRTNQIPRQIPEQSFYTKPLPGIVMGGILPFGCIFIQLFFILNSIWSHQMYYMFGFLFLVFIILVITCSEAT
ILLCYFHLCAEDYHWQWRSF
LTSGFTAVYFLVYAIHYFLSKLQITGLASTILYFGYTMIMALIFFLFTGTIGFFACFWFVTKIYSVVKVD
>g1540.t2
MCGRAALVMLGLWVLSGRVRGFYLPGLAPVSFCEPAKEGEGNPVPDCKSTIELFVNRLDSVESVLPYEYTAFDFCAVATE
KRPSENLGQVLFGERIEPSP
YKFQFKKSEKCKMVCEKKYTNAPAHKASLDFLKKGMLLNYQHHWIVDNMPVTWCYDVEEGQKFCNPGFPIGCYVTEGGQA
KDACVVNLDFKERDTFYIFN
HVDITIYYHMVENEQLEARLVAAKMEPKSYKHTNPDTPDCLGGPMFIQNKFSGDIRIPYTYSVEFLEDKKIRWASRWDYI
LESMPHTNIQWFSIMNSLVI
VLFLSGMVAMIMLRTLHKDIARYNQMDSVEDAQEEFGWKLVHGDVFRPPRKGMLLSVFLGSGTQIFIMTFVTLFFACLGF
LSPANRGALMTCAVVLWVLL
GTPAGYVAARFYKSFGGEKWKTNVLLTAFLCPGVVFADFFLMNLILWGEGSSAAMPFGTLVAILALWFCISVPLTFIGAY
FGFKKTGIEHPVRTNQIPRQ
IPEQSFYTKPLPGIVMGGILPFGCIFIQLFFILNSIWSHQMYYMFGFLFLVFIILVITCSEATILLCYFHLCAEDYHWQW
RSFLTSGFTAVYFLVYAIHY
FLSKLQITGLASTILYFGYTMIMALIFFLFTGTIGFFACFWFVTKIYSVVKVD
>g1540.t3
MCGRAALVMLGLWVLSGRVRGFYLPGLAPVSFCEPAKEGEGNPVPDCKVGTRKDHHHWVRWVRGGGPVESTIELFVNRLD
SVESVLPYEYTAFDFCAVAT
EKRPSENLGQVLFGERIEPSPYKFQFKKSEKCKMVCEKKYTNAPAHKASLDFLKKGMLLNYQHHWIVDNMPVTWCYDVEE
GQKFCNPGFPIGCYVTEGGQ
AKDACVVNLDFKERDTFYIFNHVDITIYYHMVENEQLEARLVAAKMEPKSYKHTNPDTPDCLGGPMFIQNKFSGDIRIPY
TYSVEFLVCDGLLQGACTHA
FTRTEDKKIRWASRWDYILESMPHTNIQWFSIMNSLVIVLFLSGMVAMIMLRTLHKDIARYNQMDSVEDAQEEFGWKLVH
GDVFRPPRKGMLLSVFLGSG
TQIFIMTFVTLFFACLGFLSPANRGALMTCAVVLWVLLGTPAGYVAARFYKSFGGEKWKTNVLLTAFLCPGVVFADFFLM
NLILWGEGSSAAMPFGTLVA
ILALWFCISVPLTFIGAYFGFKKTGIEHPVRTNQIPRQIPEQSFYTKPLPGIVMGGILPFGCIFIQLFFILNSIWSHQMY
YMFGFLFLVFIILVITCSEA
TILLCYFHLCAEDYHWQWRSFLTSGFTAVYFLVYAIHYFLSKLQITGLASTILYFGYTMIMALIFFLFTGTIGFFACFWF
VTKIYSVVKVD
>g1541.t1
DVYPMSAIIQLCSPASASVGLYVLAVHIKSRLGDQHYAVGSFVLLCNPWIKEDSVYMPLDLHLQEYVQSDCGLVFMGTPL
NVCSRPWAFGQYEPQVLDAC
LSMLQVSPQHLHDEQMDYLRRSDPVYLSRVVCAMVNCNDDRGVLQGKWAGLYHGGVHPTEWSGSADILTRWARSNFSPVR
YGQCWVFACVLCTGGWCWCC
RL
>g1541.t2
MSAIIQLCSPASASVGLYVLAVHIKSRLGDQHYAVGSFVLLCNPWIKEDSVYMPLDLHLQEYVQSDCGLVFMGTPLNVCS
RPWAFGQYEPQVLDACLSML
QVSPQHLHDEQMDYLRRSDPVYLSRVVCAMVNCNDDRGVLQGKWAGLYHGGVHPTEWSGSADILTRWARSNFSPVRYGQC
WVFACVLCTGGWCWCCRL
>g1541.t3
DVYPMSAIIQLCSPASASVGLYVLAVHIKSRLGDQHYAVGSFVLLCNPWIKEDSVYMPLDLHLQEYVQSDCGLVFMGTPL

NVCSRPWAFGQYEPQVLDAC
LSMLQVSPQHLHDEQMDYLRRSDPVYLSRVVCAMVNCNDDRGVLQGKWAGLYHGGVHPTEWSGSADILTRWARSNFSPVR
YGQCWVFACVLCTGGWCWCW
WGRL
>g1542.t1
LKTVTTREGTPSALITVEGRPQTPWSLWRGDPRHPGHWRGDPPRHHGNCGGETPDTLVTGGQTPDTEDTVEGRPQTPWSL
WRGDPRHPGHCGGETPPDTM
VTVEGRPQTPWSLEDRPQTPRTLWRGDPPQTPWSLWRGDPRHPGHCGGETPDTLVTGGQTPDTEDTGRETPDTKSLEERH
HRSLRRGDPQVTVAVAICSQ
YLSHAPSCHGHAVNT
>g1543.t1
LSPSLTSQFSPTSPRPQRASRRIKAGLSPSPGGQGSRKQSHHVLTPEVSPPRPTVKLWPVGECVTQAGGATWEAGRHGPP
DPQGDVRPEPHAHRTLRGTS
GQSPTPTGPSGGRQARAPRPPDPQGDAHRTLRGTSGQSPMPTGPS
>g1544.t1
MSEMATGFSALLWRSPGRGGERQPLLDRNRANSLRDNHRNDFSDDPEFREIVRKAEQAIDEGIFPERICQGSSGSYFVKD
PQGKIIGVFKPKNEEPYGQL
NPKWTKWLQKLCCPCCFGRDCLVLNQGYLSEAGASLVDQKLDLNVVPKTKVVYLGSDTFNYNAIDRVKSRGKKLALEKVP
KVGQRFHRIGLPPKVGSFQL
FVEGYKDAEHWLERFKAEPLPENVNRQLQIHFERLVVLDYIIRNTDRGNDNWLIKYDYPMDTGSIRDTDWVVVKDPIVRL
AAIDNGLAFPQKHPDSWRAY
PFYWAWLPQAKVPFSQEIKDLVLPKLNDPNFIKDLEEDLYELFK
>g1544.t2
MSEMATGFSALLWRSPGRGGERQPLLDRNRANSLRDNHRNDFSDDPEFREIVRKAEQAIDEGIFPERICQGSSGSYFVKD
PQGKIIGVFKPKNEEPYGQL
NPKWTKWLQKLCCPCCFGRDCLVLNQGYLSEAGASLVDQKLDLNVVPKTKVVYLGSDTFNYNAIDRVKSRGKKLALEKVP
KVGSFQLFVEGYKDAEHWLE
RFKAEPLPENVNRQLQIHFERLVVLDYIIRNTDRGNDNWLIKYDYPMDTGSIRDTDWVVVKDPIVRLAAIDNGLAFPQKH
PDSWRAYPFYWAWLPQAKVP
FSQEIKDLVLPKLNDPNFIKDLEEDLYELFK
>g1545.t1
TPWANARNTYFARGKNKQSLDAIEKAAFFVTLDDTQQRFDASSPVKSLTSYAKSLLHGQCFDRWFDKSFNLIVFKNGTMG
LNAEHSWADAPIIGHLWEHV
LSMDPKLSYTEEGHCCGEPHPNLPGPQRMLWDIPAECQEVIQSSLSVARMLAEDVDSHIIPFSDFGKGLIKKCRTSPDAF
IQLALQLAHFRDKGKFCLTY
EASMTRLFREGRTETVRSCTNETCAFVRAMLRNETTVECLKLLKEAAEKHQSLYRLAMTGMGIDRHLFCLYVVSKYLGED
SPFLKEVLSEPWRLSTSQTP
LQQVELFDLVRHPEYVSSGGGFGPVADDGYGVSYIIVGENLINFHISSKHSSPETDSHRFGENIRQAMLDILALFQLDKK
GAK
>g1545.t2
TPWANARNTYFARGKNKQSLDAIEKAAFFVTLDDTQQRFDASSPVKSLTSYAKSLLHGQCFDRWFDKSFNLIVFKNGTMG
LNAEHSWADAPIIGHLWEHV
LSMDPKLSYTEEGHCCGEPHPNLPGPQRMLWDIPAECQEVIQSSLSVARMLAEDVDSHIIPFSDFGKGLIKKCRTSPDAF
IQLALQLAHFRDKGKFCLTY
EASMTRLFREGRTETVRSCTNETCAFVRAMLRNETTVECLKLLKEAAEKHQSLYRLAMTGMGIDRHLFCLYVVSKYLGED
SPFLKEVLSEPWRLSTSQTP
LQQVELFDLVRHPEYVSSGGGFGPVSTARVADDGYGVSYIIVGENLINFHISSKHSSPETDSHRFGENIRQAMLDILALF
QLDKKGAK
>g1545.t3
TPWANARNTYFARGKNKQSLDAIEKAAFFVTLDDTQQRFDASSPVKSLTSYAKSLLHGQCFDRWFDKSFNLIVFKNGTMG
LNAEHSWADAPIIGHLWEHV
LSMDPKLSYTEEGHCCGEPHPNLPGPQRMLWDIPAEQVVLIPLANASSPHEARAEHPLSDETLNPGPTSLVSIKITLIHT
YTMCQEVIQSSLSVARMLAE
DVDSHIIPFSDFGKGLIKKCRTSPDAFIQLALQLAHFRDKGKFCLTYEASMTRLFREGRTETVRSCTNETCAFVRAMLRN
ETTVECLKLLKEAAEKHQSL
YRLAMTGMGIDRHLFCLYVVSKYLGEDSPFLKEVLSEPWRLSTSQTPLQQVELFDLVRHPEYVSSGGGFGPVADDGYGVS

YIIVGENLINFHISSKHSSP
ETDSHRFGENIRQAMLDILALFQLDKKGAK
>g1546.t1
MPRGPPTGPLRTPVLSPRVLSSETPVTRPGRRPGAKCASVKATVEHLTAGGADWVSCVCRTFSSPVVLVSGLSKAPRTQA
SREQLIHIPAVH
>g1546.t2
MPRGPPTGPLRTPVLSPRVLSSETPVTRPGRRPGAKCASVKRKRTATTMSHKASNASACAGIQLVKGHLKYCTNLTSYDS
PAEPLWNI
>g1547.t1
MRRCLQPTEVASGSAAHPGWHNARCGEKVCCVSQHSVKSMEEIPWAVGGQQPSSRTAIWSFVQGGTGGALPEPYKTTSSR
PLMCMLLLKLRETDSMRVSG
ASAHSSTPCSLIGICHRTPGLADLPLAPVLFTDESRFTLSTCDRRESRDAVENVSSMIGLAVDRNPIEHIWEIMSCSIHR
CHVAPQTDTLVQVWEEIPQE
NIHRLIRNMPRRCRECIQARGGHTQY
>g1548.t1
MEGEEFVPPPECPVFEPSWEEFQDPLGYIAKIRPIAEKSGICKIRPPAAETRVKLNYLDRIAKFWEIQGSSLKIPNIERR
ILDLFSLSKIVTDEGGFDLV
CKERRWARVAQRLGYPPGKNIGSLLRSHYERIVYPFEMFHSGASLPHCKPKHYDGEEVDREYKPHSIPLRQSVQPSKMNS
YGRRANRCQPD
>g1548.t2
GWGSRGQRSERAEEAGSRSRARSRSTRTDSSGASEKRRRSLLRLCYFHLRFPHMVMEGEEFVPPPECPVFEPSWEEFQDP
LGYIAKIRPIAEKSGICKIR
PPAAETRVKLNYLDRIAKFWEIQGSSLKIPNIERRILDLFSLSKIVTDEGGFDLVCKERRWARVAQRLGYPPGKNIGSLL
RSHYERIVYPFEMFHSGASL
PHCKPKHYDGEEVDREYKPHSIPLRQSVQPSKMNSYGRRANRCQPD
>g1548.t3
MEGEEFVPPPECPVFEPSWEEFQDPLGYIAKIRPIAEKSGICKIRPPAAETRVKLNYLDRIAKFWEIQGSSLKIPNIERR
ILDLFSLSKIVTDEGGFDLV
CKERRWARVAQRLGYPPGKNIGSLLRSHYERIVYPFEMFHSGASLPHCKPKHYDGEEVDREYKPHSIPLRQSVQPSKMNS
YGRRANRCQPDVSAKTQWSN
VSLRPSLALHPCLGWRLHKSSHIVVKR
>g1549.t1
MSERKGTAKLDYLRKIEKEIQDKWEKEKTFDCDAPTTIGQSTNVLAELYLSIPSQFGVGYQRLRGKQCLFPFGLHCTGMP
IKACADKLKREMELYGNPPQ
FPDEEKDEEEKADVSDEIIIKDKAKGKKSKAAAKSGTAKFQWDIMKSLGLSDCEIVKFANAEHWLEYFPPLAIKDLKQMG
VKVDWRRSFITTDVNPFYDS
FVRWQFVTLKERKKIKFGKRYTIYSPKDGQPCMDHDRQTGEGVGPQEYTLIKMKVVEPYPAKLKSKVFYSSGLKGKNIFL
VAATMRPETMFGQTNCWVRP
DMKYVAFETANGDVFISTGRAARNMSYQGFTKENGVVPVVMEILGQDILGCALSAPLTSYKTIYALPMLTIKEDK
>g1549.t2
MSERKGTAKLDYLRKIEKEIQDKWEKEKTFDCDAPTTIGQSTKGFSSVLAELYLSIPSQFGVGYQRLRGKQCLFPFGLHC
TGMPIKACADKLKREMELYG
NPPQFPDEEKDEEEKADVSDEIIIKDKAKGKKSKAAAKSGTAKFQWDIMKSLGLSDCEIVKFANAEHWLEYFPPLAIKDL
KQMGVKVDWRRSFITTDVNP
FYDSFVRWQFVTLKERKKIKFGKRYTIYSPKDGQPCMDHDRQTGEGVGPQEYTLIKMKVVEPYPAKLKSKVFYSSGLKGK
NIFLVAATMRPETMFGQTNC
WVRPDMKYVAFETANGDVFISTGRAARNMSYQGFTKENGVVPVVMEILGQDILGCALSAPLTSYKTIYALPMLTIKEDK
>g1550.t1
MMIRRCEADPSALANYVVALVKKDKPEKELKALCADQLDVFLQKETTGFVDKLFECLTTKNYLVNNPAKEPPKEELKPLV
VKPDLVEAEPAEEERESRRR
RSPPRNHESRSRDDRRREERKRRDLERHGKGGGGGGGGGGGGSDSHRERERPERRRGSPRGRSYSRSRSRSGSRGKSRDR
EHRGGGRGKDREQRRRR
>g1550.t2
MMIRRCEADPSALANYVVALVKKDKPEKELKALCADQLDVFLQKETTGFVDKLFECLTTKNYLVNNPAKEPPKEELKPLA
EPAEEERESRRRRSPPRNHE
SRSRDDRRREERKRRDLERHGKGGGGGGGGGGGGSDSHRERERPERRRGSPRGRSYSRSRSRSGSRGKSRDREHRGGGRG

KDREQRRRR
>g1550.t3
MMIRRCEADPSALANYVVALVKKDKPEKELKALCADQLDVFLQKETTGFVDKLFECLTTKNYLVNNPAKEPPKEELKPLV
VKPDLVEAEPAEEERESRRR
RSPPRNHESRSRDDRRREERKRRDLERHGKGGGGGGGGGGGGSDSHRERERPERRRGSPRGRSYSRSRSRSGSRGKSRDR
EHRGGGRGSSSSSSQCSRSV
RCPQNDTMTPGGEDSGGSDAEDRMVSGIEVPVTCVT
>g1551.t1
LDWTDNTHTLYKKGQSCLHLLRRLRSFGVCRTLLRTFYDSVVASAIFYTVVCWRGGSTDRYRSRLNRLIRRASSVLDCLL
DSIEEVGERRMLTKLTSIMD
NTSHPLQDTVGSLSSSFSSRLIHPR
>g1552.t1
LVSVEGITVSASPTARNLGIIVDNQLSCTANITAVTRSCRFALYNIRRIRPFLTREATQLLTQALVISRLDYCNSLLAGL
PATAIKPLQRIQNAAARLVF
NRNKFSNVTPLFQDLHWLPVAARIKFKTMLLAYKAVKGTAPTYLQTLVKPHAPARALRSTTSAGRLWWNELPANVRTAES
LPIFRKRLKTHLFRVHLNPA
>g1553.t1
MIPGSKCEANTFDGERCVYSSLKDSIRRLLKEHKCITAKTMQRDYFDYFLHMILEQGHHSDVKFMIHGQMFTAHRCVLST
RSEYFTTMFETKWKGKNLIH
LKHPLVLSFGPHNDLLQDNMMQLAVGAMPAELRIGCGQLPFIRTDDYPTYPDIVFRVDGYSFSCHKVFLL
>g1553.t2
MIPGSKCEANTFDGERCVYSSLKDSIRRLLKEHKCITAKTMQRDYFDYFLHMILEQGHHSDVKFMIHGQMFTAHRCVLST
RSEYFTTMFETKWKGKNLIH
LKHPLVRL
>g1553.t3
MIPGSKCEANTFDGERCVYSSLKDSIRRLLKEHKCITAKTMQRDYFDYFLHMILEQGHHSDVKFMIHGQMFTAHRCVLST
RSEYFTTMFETKWKGKNLIH
LKHPLCKMEYLTEELQHKCKQVSEYVTHKPGICVKVLSFGPHNDLLQDNMMQLAVGAMPAELRIGCGQLPFIRTDDYPTY
PDIVFRVDGYSFSCHKVFLL
>g1554.t1
EEVVRALQAACDRILSLADEELETAGYQQFQKSTGASGPWSNSAASPVNTGEKLGLAVLSPVRRLSLTNLHRGTTFQALA
GK
>g1555.t1
TSGAAQGTSYTPSMIQISIGSIHQGICGFLCDVTMTHQHQHSAWKAVM
>g1556.t1
MYCKSVGYPHPVWTWRKLEGTFYTDIDNSSGRYLITSLDNYTELNVLDLDINMDPGEYQCNATNIIGTTGSTTILRVRSL
LAPLWPFLGVLAEVIILVII
IVVYEKRKKPDEIMDGKSR
>g1557.t1
MANSVTWVLKRYGRFVPGSEGTDGDKWKVFEADGSHGELVLTVLDSGHMLVSRGTDLVGESRIMRMQFAGGSKAEALERC
SSAVAKLTKYLPVTTQDDMA
SPPPCAPAPPNQPPDASPTHTEQTARCTAGRGEPEAIHGSLSIQRLAQHFIGEQGLSLPLVYRHSPLPVGNLEAFLRACL
LDSSFPAFVEEVEAELMKLL
QA
>g1557.t2
MANSVTWVLKRYGRFVPGSEGTDGDKWKVFEADGSHGELVLTVLDSGHMLVSRGTDLVDGISLIGATEFLKVQRKTDNLL
FRCSIKGESRIMRMQFAGGS
KAEALERCSSAVAKLTKYLPVTTQDDMASPPPCAPAPPNQPPDASPTHTEQTARCTAGRGEPEAIHGSLSIQRLAQHFIG
EQGLSLPLVYRHSPLPVGNL
EAFLRACLLDSSFPAFVEEVEAELMKLLQA
>g1557.t3
MANSVTWVLKRYGRFVPGSEGTDGDKWKVFEADGSHGELVLTVLDSGHMLVSRGTDLVCIPTHWLQLFNSPCRTPPGHSQ
NQRCWSILSVTFESVFSDQG
QMLLLVGESRIMRMQFAGGSKAEALERCSSAVAKLTKYLPVTTQDDMASPPPCAPAPPNQPPDASPTHTEQTARCTAGRG
EPEAIHGSLSIQRLAQHFIG
EQGLSLPLVYRHSPLPVGNLEAFLRACLLDSSFPAFVEEVEAELMKLLQA

>g1558.t1
MGEGEDGDKDTRRKSIRMKPLPSPSPTSGGSCKGIGETKSGDSVIMETDIGRQLKTSSNGDCRRFRGSLSSITSRHVHDS
ESAEERRLITEAGDVTPSEE
SPPGAGGGGPDQVAQGAGDGGGSPDGSPDQQPGFIKLDGSDQISPDDEGLYQAGFMHRQFGAMLQPGVNKFSLRMFGSEK
AVEREQERVKSAGFWIIHPY
SDFR
>g1559.t1
MVVKGLSLLLEGNSQIYQDICFRDRKLLVDGNNLLYHLYFKSNLDQNHGGEYLALHVQ
>g1560.t1
SVLTALMPVLDWLVVQSGQEPTFLRSEAQLSQLVLGKYNEEAAPLLAKDQACLDLFVQALKSPVQPYPEIPSIQIMALQQ
ITKSFFSALGEEGVQQQLLG
VMFDLLVESQDPLVSNTICSVFKGLAVDAELVARELVPPEKPKGSVTVQQTRRSKMLQRKAEESGGAKPEEEVVSWQRVT
LILELLQHKKKLRRAQALVP
ALFTLLSRSLQLGPGAQGNVDYPRQLLLSCLLNICQKLSPDGGPVASDVLEEDKFNVELVVQCVRVSDVPQTHHHALLLL
GTVAGIFPEKVLHNIMPIFA
FMGANIMRLDDSYSFQVINKTVQTVIPALLKAHEGGEDQQTGSTDLVVVKIVHVFVDALPHVPDHRRLPILSQLVTTLGP
ARFLWVLMLLLFKQHAAKAS
GSAADKARPKG
>g1561.t1
MLALAMRTQDVALDMDMEFWTSVCCTFEVSEQLSSIIKILQYLIGLPQDREDEVPVKHTPAPRRGARRKEQQQQEEEEEE
KMEDLLFNVEAHSSKELRHF
KFLSVNFAAQLLASSSFIGKLADTTDDGQDEGLLQLQQSLLEEALRYIHSVARCVEDNVDKPTAKFWRALLNKAYDVLDK
VNSLLPTDTFITVMKGLMAN
RLPSIRRKAMELLNNKLQHKTKWEKHQVTVLLQLSDDLLSIVGAGYSAAAEEEAMKGEQGEEQAINRQTALYSLKLLCRN
FGAGHPQVFVPVLRRAVEVA
TATHEEKNVAGSALLCIAEVTGALKTLAIPQLPRLMPAVLQTMADRKDLLSNEIYLLSAVTALQRVAETLPHFISPYLHG
TVSQVCRISRLAEASATSPQ
LSARLASLSATLATKVPPRVLLPTLAKCYSDMVPAKQRHLGPLMSILKGHISHMEKDELNSHQSELTSFFLTALDFRAKH
CQGDLQKTEEVEGFTIDCLL
AMVMKLSEITFRPLFFKLFDWSKTETETEGKQRLLTFYRLADRIADRLKGLFVLFAGNLVKPFSDLLQQTNVTKTGGFQS
QGEALFDSENGVEKSSMLLR
YVLDCMLKIFLYDTHRFLSKDRAEALMGPLVDQLENTLGGEESYQLRVTKHLVPCLGQFSVAMGDDTQWKTLNYQVLLKT
RHSSSKVRFSALLMVMELAS
KLRENYMVLLPETIPFLAELMEDDTEEVEHQVQKVIQEMENILGEPLQSYF
>g1561.t2
MLALAMRTQDVALDMDMEFWTSVCCTFEVSEQLSSIIKILQYLIGLPQDREDEVPVKHTPAPRRGARRKEQQQQEEEEEE
KMEDLLFNVEAHSSKELRHF
KFLSVNFAAQLLASSSFIGKLADTTDDGQDEGLLQLQQSLLEEALRYIHSVARCVEDNVDKPTAKFWRALLNKAYDVLDK
VNSLLPTDTFITVMKGLMAN
RLPSIRRKAMELLNNKLQHKTKWEKHQVTVLLQLSDDLLSIVGAGYSAAAEEEAMKGEQGEEQAINRQTALYSLKLLCRN
FGAGHPQVFVPVLRRAVEVA
TATHEEKNVAGSALLCIAEVTGALKTLAIPQLPRLMPAVLQTMADRKDLLSNEIYLLSAVTALQRVAETLPHFISPYLHG
TVSQVRACPVSPSMLVCRIS
RLAEASATSPQLSARLASLSATLATKVPPRVLLPTLAKCYSDMVPAKQRHLGPLMSILKGHISHMEKDELNSHQSELTSF
FLTALDFRAKHCQGDLQKTE
EVEGFTIDCLLAMVMKLSEITFRPLFFKLFDWSKTETETEGKQRLLTFYRLADRIADRLKGLFVLFAGNLVKPFSDLLQQ
TNVTKTGGFQSQGEALFDSE
NGVEKSSMLLRYVLDCMLKIFLYDTHRFLSKDRAEALMGPLVDQLENTLGGEESYQLRVTKHLVPCLGQFSVAMGDDTQW
KTLNYQVLLKTRHSSSKVRF
SALLMVMELASKLRENYMVLLPETIPFLAELMEDDTEEVEHQVQKVIQEMENILGEPLQSYF
>g1561.t3
MLALAMRTQDVALDMDMEFWTSVCCTFEVSEQLSSIIKILQYLIGLPQDREDEVPVKHTPAPRRGARRKEQQQQEEEEEE
KMEDLLFNVEAHSSKELRHF
KFLSVNFAAQLLASSSFIGKLADTTDDGQDEGLLQLQQSLLEEALRYIHSVARCVEDNVDKPTAKFWRALLNKAYDVLDK
VNSLLPTDTFITVMKGLMAN
RLPSIRRKAMELLNNKLQHKTKWEKHQVTVLLQLSDDLLSIVGAGYSAAAEEEAMKGEQGEEQAINRQTALYSLKLLCRN

FGAGHPQVFVPVLRRAVEVA
TATHEEKNVAGSALLCIAEVTGALKTLAIPQLPRLMPAVLQTMADRKDLLSNEIYLLSAVTALQRVAETLPHFISPYLHG
TVSQVCRISRLAEASATSPQ
LSARLASLSATLATKVPPRVLLPTLAKCYSDMVPAKQRHLGPLMSILKGHISHMEKDELNSHQSELTSFFLTALDFRAKH
CQGDLQKTEEVEGFTIDCLL
AMVMKLSEITFRPLFFKLFDWSKTETETEGKQRLLTFYRLADRIADRLKGLFVLFAGNLVKPFSDLLQQTNVTKTGEALF
DSENGVEKSSMLLRYVLDCM
LKIFLYDTHRFLSKDRAEALMGPLVDQLENTLGGEESYQLRVTKHLVPCLGQFSVAMGDDTQWKTLNYQVLLKTRHSSSK
VRFSALLMVMELASKLRENY
MVLLPETIPFLAELMEDDTEEVEHQVQKVIQEMENILGEPLQSYF
>g1562.t1
MEGKQVGRQGRQGRQVNSKQILRSECIVLGRLPGLCDTEKYDRFVNSQLTVHDCQRVMREFKNHMLVILGKLMNNDLFYF
TPSPPHGGEGRAEGGPVGAL
AKANDGAQRRTRQVESEQDVTASTHMALPTLCGENIPLTNV
>g1562.t2
MEGKQVGRQGRQGRQVNSKQCIVLGRLPGLCDTEKYDRFVNSQLTVHDCQRVMREFKNHMLVILGKLMNNDLFYFTPSPP
HGGEGRAEGGPVGALAKAND
GAQRRTRQVESEQDVTASTHMALPTLCGENIPLTNV
>g1562.t3
MEGKQVGRQGRQGRQVNSKQILRSECIVLGRLPGLCDTEKYDRFVNSQLTVHDCQRVMREFKNHMLVILGKLMNNEYVEE
EQNRQNTFCLLKELQDCCCC
LFYFTPSPPHGGEGRAEGGPVGALAKANDGAQRRTRQVESEQDVTASTHMALPTLCGENIPLTNV
>g1563.t1
MLQVLRGAGKVGTALVNTQGEHLRLMACNSSLGAVVKAAQDVVEGLVGTAMGGSASSIKEDVFPDAEGWEMDPEEAAQWV
VGSEFSSGESRQSHAGPETT
GAPLHQGTGQGGEGWPRHPARFLHTPPGLYHRYQTRFIHDTVVARLTPEDIKKAREAKQALVKPVRQKLSDRARERKVPA
TRISRLANFGGLAVGLGLGA
IAEVAKQSFGSKRSGDMGAVLDSPLLSEANAERIVNTLCKVRGAALKIGQMLSIQDNSFINPQLQKIFERVRQSADFMPS
WQVNKVLEEDLGSDWRDQLL
SFEDKPFAAASIGQVHHGVLKDGREIAMKIQYPGVADSIHSDINNLMSVLKLSVVLPE
>g1563.t2
MLQVLRGAGKVGTALVNTQGEHLRLMACNSSLGAVVKAAQDVVEGLVGTAMGGSASSIKEDVFPDAEGWEMDPEEAAQWV
VGSEFSSGESRQSHAGPETT
GAPLHQGTGQGGEGWPRHPARFLHTPPGLYHRYQTRFIHDTVVARLTPEDIKKAREAKQALVKPVRQKLSDRARERKVPA
TRISRLANFGGLAVGLGLGA
IAEVAKQSFGSKRSGDMGAVLDSPLLSEANAERIVNTLCKVRGAALKIGQMLSIQDNSFINPQLQKIFERVRQSADFMPS
WQVNKVLEEDLGSDWRDQLL
SFEDKPFAAASIGQVHHGVLKDGREIAMKIQYPGVADSIHSDINNLMSVLKLSVVLPEGAHSHAAC
>g1563.t3
MLQVLRGAGKVGTALVNTQGEHLRLMACNSSLGAVVKAAQDVVEGLVGTAMGGSASSIKEDVFPDAEGWEMDPEEAAQWV
VGSEFSSGESRQSHAGPETT
GAPLHQGTGQGGEGWPRHPARFLHTPPGLYHRYQTRFIHDTVVARLTPEDIKKAREAKQALVKPVRQKLSDRARERKVPA
TRISRLANFGGLAVGLGLGA
IAEVAKQSFGSKRSGDMGAVLDSPLLSEANAERIVNTLCKVRGAALKIGQMLSIQDNSFINPQLQKIFERVRQSADFMPS
WQVNKVLEEDLGSDWRDQLL
SFEDKPFAAASIGQVHHGVLKDGREIAMKIQYPGVADSIHSDINNLMSVLKLSVVLPEDVRPLFLPG
>g1564.t1
MLSLQLLVDMVTGQLGDQGEQSGAATISRVLLAGNLLSQSTQDKEASTKVLLWSWLPPVAKYLTKKTQAGSVEAMRLLDE
LLLQLAASVPVDVMPGQFDP
TNYTLPQQALHRCMFPLSSVYPTLQLVTNPYQANVDGVRFLGTSGQNVMDIHKYTSMTNHLDILENTLRLGHLAPTAPDT
LGCYPFYQKDPFILEECPHV
YFSGNAPSFDSRMVTGQYEYPTLSSTVVLASQCNTNSYLWHCHEGPDGQQVLLVTIPEFVTTQTACLVNLRTLECEPVSF
SAFSTADEDECEMNISH
>g1564.t2
MFSDLSAQRESSTLLCRPGSEDQGPVFDRVLPAYTPCSERFKLGDRSFNRQFVLLASGLGLGSTRADSMLSLQLLVDMVT
GQLGDQGEQSGAATISRVLL

AGNLLSQSTQDKEASTKVLLWSWLPPVAKYLTKKTQAGSVEAMRLLDELLLQLAASVPVDVMPGQFDPTNYTLPQQALHR
CMFPLSSVYPTLQLVTNPYQ
ANVDGVRFLGTSGQNVMDIHKYTSMTNHLDILENTLRLGHLAPTAPDTLGCYPFYQKDPFILEECPHVYFSGNAPSFDSR
MVTGQYEYPTLSSTVVLASQ
CNTNSYLWHCHEGPDGQQVLLVTIPEFVTTQTACLVNLRTLECEPVSFSAFSTADEDECEMNISH
>g1564.t3
MLSLQLLVDMVTGQLGDQGEQSGAATISRVLLAGNLLSQSTQDKEASTKVLLWSWLPPVAKYLTKKTQAGSVEAMRLLDE
LLLQLAASVPVDVMPGQFDP
TNYTLPQQALHRCMFPLSSVYPTLQLVTNPYQANVDGVRFLGTSGQNVMDIHKYTSMTNHLDILENTLRLGHLAPTAPDT
LGCYPFYQKDPFILEECPHV
YFSGNAPSFDSRMVTGQYEYPTLSSTVVLASQCNTNSYLWHCHEGPDGQQVLLVTIPEFVTTQTACLVNLRTLECEPVSF
SAFSTADEDE
>g1565.t1
MQGNRGPPQNHGPRQPGPAEQKKPGGMNTNGQHPDHNVKPEQNEALTLDLQNFRKPGEKAYTQRSRLFVGNLPTGVSEQE
VEKLFSKYGKPSEIFINKDR
GFGFIRLESRIIAEIARAELDDTPFRGRQIRVRFATHGAALTVKNLPDFVSNELLEEAFAVFGQIERAIVIVDDRGRPTG
KGIVEYTSKPAARKALDKCG
EGAFLLTAFPRPVTVEPMDQFDDDEGLSEKLVNKNQQYHKEREQPPRFAQPGTFEYEYAMRWKALMEMEKQQYEMVDRNI
KEAHEKLEAEMEAARHEHQV
ILMRQDLLRRQEELRRMEELHSQEMQKRKQAELRQEEDRRRREEEVRMRGEEMMKRQQEGFRGNFPEGREQDMRMHMAGG
HGMPMNRNSMGGNAGPAGNP
PMSAENTSLMGPGPGSNNNNIMPGGGQQGGFPRGLPGPAKEFYFPTDIIRSKRLQIRNSSSPELYVGLDVVVITLVTFRA
QVSNPILEGRCPCMF
>g1565.t2
MNTNGQHPDHNVKPEQNEALTLDLQNFRKPGEKAYTQRSRLFVGNLPTGVSEQEVEKLFSKYGKPSEIFINKDRGFGFIR
LESRIIAEIARAELDDTPFR
GRQIRVRFATHGAALTVKNLPDFVSNELLEEAFAVFGQIERAIVIVDDRGRPTGKGIVEYTSKPAARKALDKCGEGAFLL
TAFPRPVTVEPMDQFDDDEG
LSEKLVNKNQQYHKEREQPPRFAQPGTFEYEYAMRWKALMEMEKQQYEMVDRNIKEAHEKLEAEMEAARHEHQVILMRQD
LLRRQEELRRMEELHSQEMQ
KRKQAELRQEEDRRRREEEVRMRGEEMMKRQQEGFRGNFPEGREQDMRMHMAGGHGMPMNRNSMGGNAGPAGNPPMSAEN
TSLMGPGPGSNNNNIMPGGG
QQGGFPRGLPGPAKEFYFPTDIIRSKRLQIRNSSSPELYVGLDVVVITLVTFRAQVSNPILEGRCPCMF
>g1566.t1
MLVEWMMNGHAILYTGVTLAFWSTILVVGICYTIFDLGFRFDVAWGIYITGSSIIGGGVKAPRIKTKNLVSIIFCEAVAI
YGIIMAIVISNMAENFSGTT
PETIGARNYQAGYSMFGAGLTVGFSNLFCGICVGIVGSGAALADAQNASLFVKILIVEIFGSAIGLFGVIVAILQ
>g1566.t2
MLVEWMMNGHAILYTGVTLAFWSTILVVGICYTIFDLGFRFDVAWFLTETSPFMWANLGIGLAISLSVVGAAWGIYITGS
SIIGGGVKAPRIKTKNLVSI
IFCEAVAIYGIIMAIVISNMAENFSGTTPETIGARNYQAGYSMFGAGLTVGFSNLFCGICVGIVGSGAALADAQNASLFV
KILIVEIFGSAIGLFGVIVA
ILQ
>g1566.t3
MLVEWMMNGHAILYTGVTLAFWSTILVVGICYTIFDLGFRFDVAWFLTETSPFMWANLGIGLAISLSVVGAAWGIYITGS
SIIGGGVKAPRIKTKNLVSI
IFCEAVAIYGIIMAIVISNMAENFSGTTPETIGARNYQAGYSMFGAGLTVGFSNLFCGICVGIVGSGAALADAQNASLFV
KILIVEIFGSAIGLFGVIVA
ILQPLVA
>g1567.t1
MAEADKLNIDSIIQRLLEVKGSRPGKNVQLTENEIRGLCLKSREIFLSQPILLELEAPLKICGDVHGQYYDLLRLFEYGG
FPPESNYLFLGDYVDRGKQS
LETICLLLAYKVKYPENFFLLRGNHECASINRIYGFYDECKRRYNIKLWKTFTDCFNCLPVAAIVDEKIFCCHG
>g1568.t1
MPEEAEDMWHTYNLLQVGDSLRASTIRKVQTESATGSVGSSRVRTTLTVCVETIDFDSQACQLRVKGTNLEENQYVKMGA
YHTIELEPNRKFTLAKKVWD

SVVLDRIEQACDAAQKADVAAVVMQEGLANVVLVTPAMTLLRAKVEVTIPRKRKGSALERFYEAVMQAILRHINFDVVKC
VLVASPGFVKDQFISYLYRE
AVRQDNKVLLENRPKFMLVHSSSGHKYSLKEILSDPTVTSRLSDTKAAAEVRALEEFYKMLQHEPDRAFYG
>g1568.t2
MPEEAEDMWHTYNLLQVGDSLRASTIRKVQTESATGSVGSSRVRTTLTVCVETIDFDSQACQLRVKGTNLEENQYVKMGA
YHTIELEPNRKFTLAKKVWD
SVVLDRIEQACDAAQKADVAAVVMQEGLANVVLVTPAMTLLRAKVEVTIPRKRKGSALERFYEAVMQAILRHINFDVVKC
VLVASPGFVKDQFISYLYRE
AVRQDNKVLLENRPKFMLVHSSSGHKYSLKEILSDPTVTSRLSDTKAAAEVRALEEFYKMLQHEPDRAFY
>g1569.t1
CCVRFSQEPSDQSVVLGERVVLSCVVFNYTGIVQWTKDGLALGVGEGLRGERPAPRLKMSHRFGQRSPKM
>g1570.t1
MCLFWSSIGPLVLCQIPQKDVDVSMLRGLIITMVILGAGQACPSGQENCSVHNDAKDYCYSARIRSTVLQGLPFGGVPTV
LALDFMCFLVLLFVFSILRK
VAWDYGRLALVTDADSVASAMHSETPDRYERLTSVSSSVDFDQRDN
>g1570.t2
MCLFWSSIGPLVLCQIPQKDVDVSMLRGLIITMVILGAGQACPSGQENCSVHNDAKDYCYSARIRSTVLQGLPFGGVPTV
LALDFMCFLVLLFVFSILRK
VAWDYGRLALVTDADSRRRDRDDYEPVKSVASAMHSETPDRYERLTSVSSSVDFDQRDN
>g1570.t3
LLPAPCSHPLPLAFTFPSQTPTQPVALVSGVVYWSFCPLVLCQIPQKDVDVSMLRGLIITMVILGAGQACPSGQENCSVH
NDAKDYCYSARIRSTVLQGL
PFGGVPTVLALDFMCFLVLLFVFSILRKVAWDYGRLALVTDADSRRRDRDDYEPVKSVASAMHSETPDRYERLTSVSSSV
DFDQRDN
>g1571.t1
MGIPTPNGDVFQHSFLASYLIYNLHTREVRELNPPEVSNSVLQFASWGVRDQQLVYIFENNIYYQADVQSSSWRLTSSGQ
EGAVFNGISDWLYEEQVLHT
QVTHWWSPDGSHLAYLSINDTLVPSMLLPRFIGALYPQGKKYPYPKVPALPPYFIIHCWLKVNDVTNGGLRCMGQINPTV
KLYVIALDGGAQTTELRPPD
SFEDREHYITMVTWVTWQQLAVRWVNRAQNMSILTLCEVTTAACVKNEEPVFSQDGSVFFITLPLENGGGGAFNHIAMIT
DQPEEQDVSVRHLTSGGWEV
SRLLAFDENTDSVVSTVSPFERSCLSCSLSKVQCTHFHARLSPGCQHVLLDCRGPGIPSTSVHQLSDMI
>g1571.t2
MGIPTPNGDVFQHSFLASYLIYNLHTREVRELNPPEVSNSVLQFASWGVRDQQLVYIFENNIYYQADVQSSSWRLTSSGQ
EGAVFNGISDWLYEEQVLHT
QVTHWWSPDGSHLAYLSINDTLVPSMLLPRFIGALYPQGKKYPYPKMGQINPTVKLYVIALDGGAQTTELRPPDSFEDRE
HYITMVTWVTWQQLAVRWVN
RAQNMSILTLCEVTTAACVKNEEPVFSQDGSVFFITLPLENGGGGAFNHIAMITDQPEEQDVSVRHLTSGGWEVSRLLAF
DENTDSVVSTVSPFERSCLS
CSLSKVQCTHFHARLSPGCQHVLLDCRGPGIPSTSVHQLSDMI
>g1571.t3
VFQHSFLASYLIYNLHTREVRELNPPEVSNSVLQFASWGVRDQQLVYIFENNIYYQADVQSSSWRLTSSGQEGAVFNGIS
DWLYEEQVLHTQVTHWWSPD
GSHLAYLSINDTLVPSMLLPRFIGALYPQGKKYPYPKVPALPPYFIIHCWLKVNDVTNGGLRCMGQINPTVKLYVIALDG
GAQTTELRPPDSFEDREHYI
TMVTWVTWQQLAVRWVNRAQNMSILTLCEVTTAACVKNEEPVFSQDGSVFFITLPLENGGGGAFNHIAMITDQPEEQDVS
VRHLTSGGWEVSRLLAFDEN
TDSVVSTVSPFERSCLSCSLSKVQCTHFHARLSPGCQHVLLDCRGPGIPSTSVHQLSDMI
>g1572.t1
IAIMSDLPMKILRKKIQKRNEKTKERKVLAQKTKNEDIDKSNETSTETIVAPETAIKSKIKQNKQKLPEKQEPTQAISTK
KKKRKLAKDSETPEVKKAKT
KKQAVEAEDEEDTEPKVDEQNGDQELGGEEDACSNEEGGDDKEEDENDDEADDDNAKLPMGLSGAFEDTTFESLAALVSE
HTLKAVKEMGFEHMTEIQHK
SIRPLLEGRDILAAARTGSGKTLAFLIPGIELIYKLKFMPRNGAGVLILSPTRELAMQTYGVLKELMAHHVHTFGLLMGG
SNRSAEAQRLANGVNILVAT
PGRLLDHLQNTAGFMYKNLQCLVIDEADRILEVGFEEELKQIIRLLPKRRQTMLFSATQTRKVEDLARISLKKEPLYVGV

DDDKDKATVDGLEQGYVVCP
SEKRFLLLFTFLKKNRKKKMMVFFSSCMSVKFHYELLNYIDLPVMAIHGKQKQTKRTTTFFQFCNADTGILLCTDVAARG
LDIPEVDWIVQYDPPDDPKE
YIHRVGRTARGIDGKGHALLILRPEELGFLRFLKQARVPLSEFEFSWSKISDIQSQLDKLIEKNYYLHKSAQEAYKSYVR
AYDSHSLKQIYNVSTLNLPM
VALSFGFKVPPFVDLSILPCTR
>g1572.t2
MSDLPMKILRKKIQKRNEKTKERKVLAQKTKNEDIDKSNETSTETIVAPETAIKSKIKQNKQKLPEKQEPTQAISTKKKK
RKLAKDSETPEVKKAKTKKQ
AVEAEDEEDTEPKVDEQNGDQELGGEEDACSNEEGGDDKEEDENDDEADDDNAKLPMGLSGAFEDTTFESLAALVSEHTL
KAVKEMGFEHMTEIQHKSIR
PLLEGRDILAAARTGSGKTLAFLIPGIELIYKLKFMPRNGAGVLILSPTRELAMQTYGVLKELMAHHVHTFGLLMGGSNR
SAEAQRLANGVNILVATPGR
LLDHLQNTAGFMYKNLQCLVIDEADRILEVGFEEELKQIIRLLPKRRQTMLFSATQTRKVEDLARISLKKEPLYVGVDDD
KDKATVDGLEQGYVVCPSEK
RFLLLFTFLKKNRKKKMMVFFSSCMSVKFHYELLNYIDLPVMAIHGKQKQTKRTTTFFQFCNADTGILLCTDVAARGLDI
PEVDWIVQYDPPDDPKEYIH
RVGRTARGIDGKGHALLILRPEELGFLRFLKQARVPLSEFEFSWSKISDIQSQLDKLIEKNYYLHKSAQEAYKSYVRAYD
SHSLKQIYNVSTLNLPMVAL
SFGFKVPPFVDLSILPCTR
>g1572.t3
IAIMSDLPMKILRKKIQKRNEKTKERKVLAQKTKNEDIEVKKAKTKKQAVEAEDEEDTEPKVDEQNGDQELGGEEDACSN
EEGGDDKEEDENDDEADDDN
AKLPMGLSGAFEDTTFESLAALVSEHTLKAVKEMGFEHMTEIQHKSIRPLLEGRDILAAARTGSGKTLAFLIPGIELIYK
LKFMPRNGAGVLILSPTREL
AMQTYGVLKELMAHHVHTFGLLMGGSNRSAEAQRLANGVNILVATPGRLLDHLQNTAGFMYKNLQCLVIDEADRILEVGF
EEELKQIIRLLPKRRQTMLF
SATQTRKVEDLARISLKKEPLYVGVDDDKDKATVDGLEQGYVVCPSEKRFLLLFTFLKKNRKKKMMVFFSSCMSVKFHYE
LLNYIDLPVMAIHGKQKQTK
RTTTFFQFCNADTGILLCTDVAARGLDIPEVDWIVQYDPPDDPKEYIHRVGRTARGIDGKGHALLILRPEELGFLRFLKQ
ARVPLSEFEFSWSKISDIQS
QLDKLIEKNYYLHKSAQEAYKSYVRAYDSHSLKQIYNVSTLNLPMVALSFGFKVPPFVDLSILPCTR
>g1573.t1
MLVKREAHHLGCTQHGLLSYQGRKNLNLFYGRFDIEGDGHQPFTLAPADVVYREEVQYLTVWCANNNLSLNATKTKDYRK
SKGGTHTPHHINGAEVERVT
SFKFLGVHSEHLSWYLNTSALIKKANITPLH
>g1574.t1
DILVRGRFSTASPPTSAPSPHGNHDKQLVVICNQHAFTSSPIL
>g1575.t1
MVVSEGAAARFWCAVAASPQATITWELNQSTLPLETDRITILPNGVLQIQKVQMEDAGKYRCVATNIGNRMKSREAALTV
SPGTAPKSRQSPRIVAGPQN
VTASMHQTVVLECIATGNPQPIISWSRADSKPIDVYNARVLGSGNLVLSDVKSQHSGIYLCRATTPGTRNHTLAAANLTV
LVPPSFVEKPESQTRPRAGT
ARFMCQAEGVPQPHISWLKNGQKVHLNGRIKIKLVITQIIPEDDAIYQCVAESEQGSVLSVARLIVVMSEDRPSAPRNVH
AETISSSAILLAWERPLYNA
NKVIAYSIHYMKAEGLNNEEYQAVIGNDTTSYIIEDLEPARNYSFYIVAYMPMGASRMSDQVSQHTLEDVPLRTPELSLI
SRSTSDIQVSWQPLPAKLSR
GRVSAYQLSYRTAADPAVTSVEVPRNSTQHLLENLEPDTIYLLRIAVATRVGWCEPSAWTSHRTPSVSSSKVPSAPLLQL
EPLNCTSIIARWQTVPGSVV
VQGYRLFYHEEGRPEQPTVQLPAPHSDYTISGLASLSSLPDALWELWLSEIGGHCQMSPPTTVFPSLALHNDPLAIVLGP
GATPALRPSPPSPRSRGPEI
SAAERWL
>g1575.t2
MVVSEGAAARFWCAVAASPQATITWELNQSTLPLETDRITILPNGVLQIQKVQMEDAGKYRCVATNIGNRMKSREAALTV
SPGTAPKSRQSPRIVAGPQN
VTASMHQTVVLECIATGNPQPIISWSRADSKPIDVYNARVLGSGNLVLSDVKSQHSGIYLCRATTPGTRNHTLAAANLTV

LVPPSFVEKPESQTRPRAGT
ARFMCQAEGVPQPHISWLKNGQKVHLNGRIKIKLVITQIIPEDDAIYQCVAESEQGSVLSVARLIVVMSEDRPSAPRNVH
AETISSSAILLAWERPLYNA
NKVIAYSIHYMKAEGLNNEEYQAVIGNDTTSYIIEDLEPARNYSFYIVAYMPMGASRMSDQVSQHTLEDVPLRTPELSLI
SRSTSDIQVSWQPLPAKLSR
GRVSAYQLSYRTAADPAVTSVEVPRNSTQHLLENLEPDTIYLLRIAVATRVGWCEPSAWTSHRTPSVSSSKVPSAPLLQL
EPLNCTSIIARWQTVPGSVV
VQGYRLFYHEEGRPEQPTVQLPAPHSDYTISGLASLSSLPDALWELWLSEIGGHCQMSPPTTVFPSLALHNDPLAIVLGP
GATPAL
>g1575.t3
MVVSEGAAARFWCAVAASPQATITWELNQSTLPLETDRITILPNGVLQIQKVQMEDAGKYRCVATNIGNRMKSREAALTV
SPGTAPKSRQSPRIVAGPQN
VTASMHQTVVLECIATGNPQPIISWSRADSKPIDVYNARVLGSGNLVLSDVKSQHSGIYLCRATTPGTRNHTLAAANLTV
LVPPSFVEKPESQTRPRAGT
ARFMCQAEGVPQPHISWLKNGQKVHLNGRIKIKLVITQIIPEDDAIYQCVAESEQGSVLSVARLIVVMSEDRPSAPRNVH
AETISSSAILLAWERPLYNA
NKVIAYSIHYMKAEGLNNEEYQAVIGNDTTSYIIEDLEPARNYSFYIVAYMPMGASRMSDQVSQHTLEDVPLRTPELSLI
SRSTSDIQVSWQPLPAKLSR
GRVSAYQLSYRTAADPAVTSVEVPRNSTQHLLENLEPDTIYLLRIAVATRVGWCEPSAWTSHRTPSVSSSKVPSAPLLQL
EPLNCTSIIARWQTVPGSVV
VQGYRLFYHEEGRPEQPTVQLPAPHSDYTISGLASLSSLPDALWELWLSEIGGHCQMSPPTTVFPSLALHNDPLAIVLGP
GATPA
>g1576.t1
MGRVIRGQRKGAGSVFKAHVHHRKGAAKLRHIDFAERHGYIKGIVKDIIHDPGRGAPLAKVAFRDPYRFKKRTELFIAAE
GIHTGQFIYCGKKAQLNIGN
VLPVGTMPEGTIICCVEEKPGDRGKLARASGNYATVISHNPETKKSRVKLPSGSKKVIASANRAVVGVVAGGGRIDKPIL
KAGRAYHKYKAKRNCWPRVR
GVAMNPVEHPFGGGNHQHIGKPSTIRRDAPAGRKVGLIAARRTGRLRGTKTVQEKEN
>g1577.t1
MFCHEDSPGWDGGHSNGFVNGYHDNRMNGGFGGRGTPRNDRGFGYDNKDGGGWAPPVRDARDGAYNSFGGRSDRGKSAYF
NDRGSGSRGRYDRGSYSSGG
NSRWGEDSREEEDWSKPTPANERLEHELFSGGNTGINFEKYDDIPVDATGNNCPPHIESFHDVDMGEIIMCNINLSRYTR
PTPVQKYAIPIIKSKRDLMA
CAQTGSGKTAAFLLPVLSQIFTEGPGEALQAAKNSGHENGKYSRRKQYPIALVLAPTRELALQIYEEARKQIRELERGCH
LLVATPGRLVDMMERGKIGL
DYCNYLVLDEADRMLDMGFEPQIRRIVEQDTMPPKGSRQTMMFSATFPKEIQILARDFLEDYIFLAVGRVGSTSENITQK
VVWVEDNDKRSFLLDLLNAT
GTAWSRGTHSSAIAMEMARRRPAARSRGQPFVTGGVAITLRSVLNGFSSLPLLCVGKDSLTLVFVETKKGADALEDFLYR
EGYACTSIHGDRSQRDREEA
LNQFRSGRCPILVATAVRHTDTPRTPPGAFIHLAP
>g1577.t2
MNGGFGGRGTPRNDRGFGYDNKDGGGWAPPVRDARDGAYNSFGGRSDRGKSAYFNDRGSGSRGRYDRGSYSSGGNSRWGE
DSREEEDWSKPTPANERLEH
ELFSGGNTGINFEKYDDIPVDATGNNCPPHIESFHDVDMGEIIMCNINLSRYTRPTPVQKYAIPIIKSKRDLMACAQTGS
GKTAAFLLPVLSQIFTEGPG
EALQAAKNSGHENGKYSRRKQYPIALVLAPTRELALQIYEEARKQIRELERGCHLLVATPGRLVDMMERGKIGLDYCNYL
VLDEADRMLDMGFEPQIRRI
VEQDTMPPKGSRQTMMFSATFPKEIQILARDFLEDYIFLAVGRVGSTSENITQKVVWVEDNDKRSFLLDLLNATGTAWSR
GTHSSAIAMEMARRRPAARS
RGQPFVTGGVAITLRSVLNGFSSLPLLCVGKDSLTLVFVETKKGADALEDFLYREGYACTSIHGDRSQRDREEALNQFRS
GRCPILVATAVRHTDTPRTP
PGAFIHLAP
>g1577.t3
MFCHEDSPGWDGGHSNGFVNGYHDNRMNGGFGGRGTPRNDRGFGYDNKDGGGWAPPVRDARDGAYNSFGGRSDRGKSAYF
NDRGSGSRGRYDRGSYSSGG
NSRWGEDSREEEDWSKPTPANERLEHELFSGGNTGINFEKYDDIPVDATGNNCPPHIESFHDVDMGEIIMCNINLSRYTR

PTPVQKYAIPIIKSKRDLMA
CAQTGSGKTAAFLLPVLSQIFTEGPGEALQAAKNSGHENGKYSRRKQYPIALVLAPTRELALQIYEEARKQIRELERGCH
LLVATPGRLVDMMERGKIGL
DYCNYLVLDEADRMLDMGFEPQIRRIVEQDTMPPKGSRQTMMFSATFPKEIQILARDFLEDYIFLAVGRVGSTSENITQK
VVWVEDNDKRSFLLDLLNAT
GTAWSRGTHSSAIAMEMARRRPAARSRGQPFVTGGTLRSVLNGFSSLPLLCVGKDSLTLVFVETKKGADALEDFLYREGY
ACTSIHGDRSQRDREEALNQ
FRSGRCPILVATAVRHTDTPRTPPGAFIHLAP
>g1578.t1
MEEKLYGSLPVPSEYLQQWIQSTQQFQALQAQYSGFSPHHHPHYPEVPAAEQATAHMAAHPEPMMAQQEPANLTKPPAKK
RGRPAQPKEPKPPKIPKEPK
PPKPPKAPKAPKAPKAPKVPKAPKAPKDPNAPKAKPGPKSKKAKEGEAEGKQEKIDESFKKVKRKVDRFKGMSEEEVMTK
TLPDLLDYNLDYVIIGINPG
LMAAFIGRWFPGPGNHFWKCLFLSGFTEELLNHMHDTILPAKYKIGFTNMVARATPGSKDLSREMFGKKPKTLTFGLQPH
KIPDCDVALYLMPSSSARCA
QFPRAQDKVHFYIKLRELRDELKGIQKSKEIEEVNYSFDLGLAKEDAKRMAIKEEAYDPGYEDAYGGAYVEGGPEGGQQP
PGEAKANGHCNFSAAENTEA
AEKATTSQAAGGAEGQLPDGQWMTQSFADQIPDIGGAAAGVAAAGAAPAAAMTEATA
>g1578.t2
MEEKLYGSLPVPSEYLQQWIQSTQQFQALQAQYSGFSPHHHPHYPEVPAAEQATAHMAAHPEPMMAQQEPANLTKPPAKK
RGRPAQPKEPKPPKIPKEPK
PPKPPKAPKAPKAPKAPKVPKAPKAPKDPNAPKAKPGPKSKKAKEGEAEGKQEKIDESFKKVKRKVDRFKGMSEEEVMTK
TLPDLLDYNLDYVIIGINPG
LMAAFIGRWFPGPGNHFWKCLFLSGFTEELLNHMHDTILPAKYKIGFTNMVARATPGSKDLSSKELREGGKILTEKLIKY
KPLIAVFNGKCIYEMFCREM
FGKKPKTLTFGLQPHKIPDCDVALYLMPSSSARCAQFPRAQDKVHFYIKLRELRDELKGIQKSKEIEEVNYSFDLGLAKE
DAKRMAIKEEAYDPGYEDAY
GGAYVEGGPEGGQQPPGEAKANGHCNFSAAENTEAAEKATTSQAAGGAEGQLPDGQWMTQSFADQIPDIGGAAAGVAAAG
AAPAAAMTEATA
>g1578.t3
MEEKLYGSLPVPSEYLQQWIQSTQQFQALQAQYSGFSPHHHPHYPEVPAAEQATAHMAAHPEPMMAQQEPANLTKPPAKK
RGRPAQPKEPKPPKIPKEPK
PPKPPKAPKAPKAPKAPKVPKAPKAPKDPNAPKAKPGPKSKKAKEGEAEGKQEKIDESFKKVKRKVDRFKGMSEEEVMTK
TLPDLLDYNLDYVIIGINPG
LMAAFIGRWFPGPGNHFWKCLFLSGFTEELLNHMHDTILPAKYKIGFTNMVARATPGSKDLSSARCAQFPRAQDKVHFYI
KLRELRDELKGIQKSKEIEE
VNYSFDLGLAKEDAKRMAIKEEAYDPGYEDAYGGAYVEGGPEGGQQPPGEAKANGHCNFSAAENTEAAEKATTSQAAGGA
EGQLPDGQWMTQSFADQIPD
IGGAAAGVAAAGAAPAAAMTEATA
>g1579.t1
MVRRFQPDVALPPNVKVEVVLLEAILNFVNQNMSRMGLEVADLDTQRFCVLDPQRAHSPWAPQSKTTSMILKQQHLHSLR
HLGGSRLL
>g1579.t2
MVRRFQPDVALPPNVKVEVVLLEVNKTGIKSNIEYEYLTEESHPVTDSLDSSSGEDPMDKLLRLDAHKILTAKTAILNFV
NQNMSRMGLEVADLDTQRFC
VLDPQRAHSPWAPQSKTTSMILKQQHLHSLRHLGGSRLL
>g1579.t3
MVRRFQPDVALPPNVKVEVVLLEVSVPRHPVTDSLDSSSGEDPMDKLLRLDAHKILTAKTAILNFVNQNMSRMGLEVADL
DTQRFCVLDPQRAHSPWAPQ
SKTTSMILKQQHLHSLRHLGGSRLL
>g1580.t1
LLWSEGPHGCCPADPPLRGRAGGERCPARPSPRLITDASSSSSTTGCSVLQGLLSRTSGSPRRPRSAELQSLLWFWPPRR
DGVRIQELRGAGGQRPGVPG
RVGKWRETRGGGDMRPTRREALSRVLLMLMPAVVCPLGTAPGVRGEERGYLQEALGALGLSDRAEGPPRLQKAQLGVLSG
RALRIVHCKERIGATQDICD
QCLEPGVVLSALEDDGDVYVSEENYRRVATVILYYIIHMGHLCVSNTASSYSPLSSTPPPDFSFYLLALADLHPEEEDPD

LLSAAEVQSILQVINQNYQP
ASPHTQCVDATHLMTEAGGAGEEVLGLPSSSSSPSSSSSSVSRLAAAVLTHLLLGDCFNRADLPPPAFFTDYIFHTLNCT
AGLHLTDLEELLHHLGVGGG
GGGGGGANHIHSRKSRGLTGPSQEEAGPRQDDFSHQTLSPATDWTALCFSANQLVDIFALDSHSLISKDRFSQMCPAIIQ
QLLGNACQSSERVSRGDPPT
ALESKTFDLLPWLYLTNQLTN
>g1580.t2
MLMPAVVCPLGTAPGVRGEERGYLQEALGALGLSDRAEGPPRLQKAQLGVLSGRALRIVHCKERIGATQDICDQCLEPGV
VLSALEDDGDVYVSEENYRR
VATVILYYIIHMGHLCVSNTASSYSPLSSTPPPDFSFYLLALADLHPEEEDPDLLSAAEVQSILQVINQNYQPASPHTQC
VDATHLMTEAGGAGEEVLGL
PSSSSSPSSSSSSVSRLAAAVLTHLLLGDCFNRADLPPPAFFTDYIFHTLNCTAGLHLTDLEELLHHLGVGGGGGGGGGA
NHIHSRKSRGLTGPSQEEAG
PRQDDFSHQTLSPATDWTALCFSANQLVDIFALDSHSLISKDRFSQMCPAIIQQLLGNACQSSERVSRGDPPTALESKTF
DLLPWLYLTNQLTN
>g1580.t3
GLLSRTSGSPRRPRSAELQSLLWFWPPRRDGVRIQELRGAGGQRPGVPGRVGKWRETRGGGDMRPTRREALSRVLLMLMP
AVVCPLGTAPGVRGEERGYL
QEALGALGLSDRAEGPPRLQKAQLGVLSGRALRIVHCKERIGATQDICDQCLEPGVVLSALEDDGDVYVSEENYRRVATV
ILYYIIHMGHLCVSNTASSY
SPLSSTPPPDFSFYLLALADLHPEEEDPDLLSAAEVQSILQVINQNYQPASPHTQCVDATHLMTEAGGAGEEVLGLPSSS
SSPSSSSSSVSRLAAAVLTH
LLLGDCFNRADLPPPAFFTDYIFHTLNCTAGLHLTDLEELLHHLGVGGGGGGGGGANHIHSRKSRGLTGPSQEEAGPRQD
DFSHQTLSPATDWTALCFSA
NQLVDIFALDSHSLISKDRFSQMCPAIIQQLLGNACQSSERVSRGDPPTALESKTFDLLPWLYLTNQLTN
>g1581.t1
MSAWIRNCARTQGIVGRGVMQQPPVLHHLLLGGALLLLLATPALCCPARCDCSAHSKSVSCHRKRLPTAPEGIPIETRAL
DLSKNKLRSITPDNFSSFQQ
LEDLDLSDNLISTVEPGSFRFQIALRFLTFRNNLLQLIPFGVLSGLTNLTHLDLSHNRLVVLLDHGFHDLRRLASLEVGD
NELVFISQRAFTGLQALRTL
TLERSNLTVVPTEALTHLHSLVNLHMLHLSIGFLKPYSFKRLGNLRHLEIDYWPWLETVPPLSLHGLNLTTLSITNTNLS
TFPGVALRNLPYLTHVNLSY
CRIQHIQQGELGQHPHLLELRLQGAQLMSIEPMAFTGLKALQLLDVSQNHLDSLESSVFASPHSLSKLCLGGNPLVCDCR
LLWLLNDRKVPFLQLLDAQP
ECTAPERLIGKLLRDLKEPLVSRYVTCTRPRIGPNITQLLMADEGQPAHLSCIAEGAPRPSVVWVTPHRRQITAKSSGRV
EVQANGTLEIKAAELHDSGV
YLCIASNAAGNASLSASLAVKSSGISDRWLYNNRSSNYLTDSNTTWGNETVLYNMTFPIDLKTIIISTAMGCVSFLGVVI
FCFLLLFAWSRGKGRHKSNF
DIEYVPRKSNGNSTEVIETSGPRRVNMKMI
>g1582.t1
TEPLPPELQPLVATDDEGAAVNATDPVVQLFLKRQRRMNEVKKRREANVLERLNRFTVKKYYLLRRGFLMTAIAMRNLAW
GLPPVDQLTREVAFANMKEP
PQEPNQNDPLKEEVGHGEL
>g1583.t1
LLSQGSQYQPPRQRDAAKDQAQSRPVTIIRDGDAAARAARNRDQLDQPSSRPSVPDSGNPVRARGADGRRWLPPQVQRRV
TPDNDEHPPQHQQQQPAGDL
ARTHSHNATKNDSRDASPPRREDMMVGDDPYDPYKSTDRDNPYYNYYDAYEQSRPRPRGGGGYGTRYHQHGLPDLVPDPY
YIQASAYVQRVPMYNLRCAG
EENCLSSSAYRGDVRDYDTRMLLRFPQRVKNQGTSDFLPSRPRYSWEWHSCHQHYHSMDEFSHYELLDSTSQRSVAEGHK
ASFCLEDTSCDYGYYRRFAC
TAHTQGLSPGCYDTYNADIDCQWIDITDVKPGNFVLKVSVNPSYQVPESDYSNNVVRCDVRYTGNYAYVSGCHISPSVAS
SRLRHVKVQFNSSACFTFTF
SVSCCRVSIPSFRSEISSLEKGYTVFKIEVLMNGRQHAIEKRYSEFHALHKMLKKSMKPPEVPSKHVRNWVPKVLEQRRH
GLELYLQTIIMENEVLPKIF
LDFLNIRHFPSVPKTESCGSFETDSDELSKLTHQPVMLYLRDPYLFPTAHDTFSNVVSEGVIHGVFYPDLQPR
>g1583.t2

LSQGSQYQPPRQRDAAKDQAQSRPVTIIRDGDAAARAARNRDQLDQPSSRPSVPDSGNPVRARGADGRRWLPPQVQRRVT
PDNDEHPPQHQQQQPAGDLA
RTHSHNATKNDSRDASPPRREDMMVGDDPYDPYKSTDRDNPYYNYYDAYEQSRPRPRGGGGYGTRYHQHGLPDLVPDPYY
IQASAYVQRVPMYNLRCAGE
ENCLSSSAYRGDVRDYDTRMLLRFPQRVKNQGTSDFLPSRPRYSWEWHSCHQHYHSMDEFSHYELLDSTSQRSVAEGHKA
SFCLEDTSCDYGYYRRFACT
AHTQGLSPGCYDTYNADIDCQWIDITDVKPGNFVLKVSVNPSYQVPESDYSNNVVRCDVRYTGNYAYVSGCHISPSVASS
RLRHVKVQFNSSACFTFTFS
VSCCRVSIPSFRSEISSLEKGYTVFKIEVLMNGRQHAIEKRYSEFHALHKMLKKSMKPPEVPSKHVRNWVPKVLEQRRHG
LELYLQTIIMENEVLPKIFL
DFLNIRHFPSVPKTESCGSFETDSDELSKLTHQPVMLYLRDPYLFPTAHDTFSNVVSEGVIHGVFYPDLQPR
>g1583.t3
LLSQGSQYQPPRQRDAAKDQAQSRPVTIIRDGDAAARAARNRDQLDQPSSRPSVPDSGNPVRARGADGRRWLPPQVQRRV
TPDNDEHPPQHQQQQPAGDL
ARTHSHNATKNDSRDASPPRREDMMVGDDPYDPYKSTDRDNPYYNYYDAYEQSRPRPRGGGGYGTRYHQHGLPDLVPDPY
YIQASAYVQRVPMYNLRCAG
EENCLSSSAYRGDVRDYDTRMLLRFPQRVKNQGTSDFLPSRPRYSWEWHSCHQHYHSMDEFSHYELLDSTSQRSVAEGHK
ASFCLEDTSCDYGYYRRFAC
TAHTQGLSPGCYDTYNADIDCQWIDITDVKPGNFVLKVSVNPSYQVPESDYSNNVVRCDVRYTGNYAYVSGCHISPSVAS
SRLRHVKVQFNSSACFTFTF
SVSCCRVSIPSFRSEISSLEKGYTVFKIEVLMNGRQHAIEKRYSEFHALHKMLKKSMKPPEVPSKHVRNWVPKVLEQRRH
GLELYLQTIIMENEVLPKIF
LDFLNIRHFPSVPKTESCGKLTHQPVMLYLRDPYLFPTAHDTFSNVVSEGVIHGVFYPDLQPR
>g1584.t1
MGVIDLLVSYMTEGLKDIFDYQFGGSIPTVISRYCLPPTTFPTLPFLISACLHPAPSPQPGLSPITNFTSPLLVPAELPP
TQHDGRSEHHCIPEHAAVKV
VVLNHQCLCETVHV
>g1585.t1
MRRMKANARERNRMHGLNDALEGLRRVVPCYSKTQKLSKIETLRLAKNYIWALSEVLHSGKSPDLLSFVQALCKGLSQPT
TNLVAGCLQLNPRTFLPEQS
CEPHPHLQGVGSYSFQGPGLTPGLPSPPYGHLDFSHAKPLPYVYDGPLLTDCSSPAFDGAPLSPPLSVNGNFSSFKQEVV
TGDFPF
>g1585.t2
MKANARERNRMHGLNDALEGLRRVVPCYSKTQKLSKIETLRLAKNYIWALSEVLHSGKSPDLLSFVQALCKGLSQPTTNL
VAGCLQLNPRTFLPEQSCEP
HPHLQGVGSYSFQGPGLTPGLPSPPYGHLDFSHAKPLPYVYDGPLLTDCSSPAFDGAPLSPPLSVNGNFSSFKQEVVTGD
FPF
>g1586.t1
MTKCCTEERVLEEGGALEPCEEEEEEEEDNQRLEEEDEEEEEEEEDEEEQDGDNKPKRRGPKKKKMTKARMQR
>g1587.t1
VLSLEADASVEEITHIYRELAKTWHPDHNPSKEAEAMFIKINDAYKASGGRMNADDHNDSYLPANSIKVELYSDDEPAVG
PQPRESVREDRDSTTEEQST
EHGAGAGGAGGGSAGERGHIYSELSSPKPASPGPLRLPNGKLQCDICGMICIGPNVLMVHKRSHTGERPFQCSQCGASFT
QKGNLLRHIKLHSGEKPFKC
PICNYACRRRDALTGHLRTHAVSSPKAGKTLKCEHCGRSYKQQSTLEEHMERCHSCLQSLDHQTTTGTQAAQGEDSPNPE
LMTKPVIQPSTEKLPFVERL
ANSITKRKRSTPQKFLGEKHMRLNMPEAPYELSSGSEKEGDRLNSQSAGETVGLLGTSSKYLQGGRVKSETYDPPKLSQP
LPGHLSELPMAMTSVYSRMA
SQSTCVLGGSGMAVVAMVRAGPDMGEGHDEPPSAHSANNSPNDCPDSTDTESTVEEQSTGAAAPTSTSNHRNLQHLHYPP
APALARSRPNSIPSQAKDSD
PEWDRACPVRLSAVKKSPGSPQSSRETLQVVDREGRAMRCFHCPHCRILFLDHVMFTIHMGCHGFRQPFECNICGHRSQD
RYEFSSHISRGEHQVG
>g1587.t2
VLSLEADASVEEITHIYRELAKTWHPDHNPSKEAEAMFIKINDAYKASGGRMNADDHNDSYLPANSIKVELYSDDEPAVG
PQPRESVREDRDSTTEEQST
EHGAGAGGAGGGSAGERGHIYSELSSPKPASPGPLRLPNGKLQCDICGMICIGPNVLMVHKRSHTGERPFQCSQCGASFT

QKGNLLRHIKLHSGEKPFKC
PICNYACRRRDALTGHLRTHAVSSPKAGKTLKCEHCGRSYKQQSTLEEHMERCHSCLQSLDHQTTTGEDSPNPELMTKPV
IQPSTEKLPFVERLANSITK
RKRSTPQKFLGEKHMRLNMPEAPYELSSGSEKEGDRLNSQSAGETVGLLGTSSKYLQGGRVKSETYDPPKLSQPLPGHLS
ELPMAMTSVYSRMASQSTCV
LGGSGMAVVAMVRAGPDMGEGHDEPPSAHSANNSPNDCPDSTDTESTVEEQSTGAAAPTSTSNHRNLQHLHYPPAPALAR
SRPNSIPSQAKDSDPEWDRA
CPVRLSAVKKSPGSPQSSRETLQVVDREGRAMRCFHCPHCRILFLDHVMFTIHMGCHGFRQPFECNICGHRSQDRYEFSS
HISRGEHQVG
>g1587.t3
MNADDHNDSYLPANSIKVELYSDDEPAVGPQPRESVREDRDSTTEEQSTEHGAGAGGAGGGSAGERGHIYSELSSPKPAS
PGPLRLPNGKLQCDICGMIC
IGPNVLMVHKRSHTGERPFQCSQCGASFTQKGNLLRHIKLHSGEKPFKCPICNYACRRRDALTGHLRTHAVSSPKAGKTL
KCEHCGRSYKQQSTLEEHME
RCHSCLQSLDHQTTTGTQAAQGEDSPNPELMTKPVIQPSTEKLPFVERLANSITKRKRSTPQKFLGEKHMRLNMPEAPYE
LSSGSEKEGDRLNSQSAGET
VGLLGTSSKYLQGGRVKSETYDPPKLSQPLPGHLSELPMAMTSVYSRMASQSTCVLGGSGMAVVAMVRAGPDMGEGHDEP
PSAHSANNSPNDCPDSTDTE
STVEEQSTGAAAPTSTSNHRNLQHLHYPPAPALARSRPNSIPSQAKDSDPEWDRACPVRLSAVKKSPGSPQSSRETLQVV
DREGRAMRCFHCPHCRILFL
DHVMFTIHMGCHGFRQPFECNICGHRSQDRYEFSSHISRGEHQVG
>g1587.t4
VLSLEADASVEEITHIYRELAKTWHPDHNPSKEAEAMFIKINDAYKVLLHRHKPKRL
>g1588.t1
MTKKRRNNGRAKKGRGHVQPIRCTNCSRCVPKDKAIKKFVIRNIVEAAAVRDISEASVFDSYVLPKLYVKLHYCISCAIH
SKVVRNRSTEARKDRTPPPR
FRPAGGPPRAPPKPM
>g1588.t2
TKKRRNNGRAKKGRGHVQPIRCTNCSRCVPKDKAIKKFVIRNIVEAAAVRDISEASVFDSYVLPKLYVKLHYCISCAIHS
KVVRNRSTEARKDRTPPPRF
RPAGGPPRAPPKPM
>g1588.t3
MTKKRRNNGRAKKGRGHVQPIRCTNCSRCVPKDKAIKKFVIRNIVEAAAVRDISEASVFDSYVLPKLYVKLHYCISCAIH
SKVVRNRSTEARKDRTPPPR
FRPAVSRSSQNFVGP
>g1589.t1
SLSSQSYHLQARGDMHPGLVLDPSTNYNSPKFRSRNQSYMRAVSTLSQASCVSQVSETEINGQFESVCESVFSEVESQAM
EALDLPGCFRTRSHSYLRAI
QAGYSQDDDCIPSMGSSTVTSTIRSTTGKSLPLGDPTPVVPYFVVKRSPVMVVNG
>g1589.t2
SLSSQSYHLQARGDMHPGLVLDPSTNYNSPKFRSRNQSYMRAVSTLSQASCVSQVSQVSETEINGQFESVCESVFSEVES
QAMEALDLPGCFRTRSHSYL
RAIQAGYSQDDDCIPSMGSSTVTSTIRSTTGKSLPLGDPTPVVPYFVVKRSPVMVVNG
>g1590.t1
MGAEAKPVSGPPVSPIYFRSPRSSIPERPSPKAIQASIKESATLAAAISMQWKEEVSAMRRELADLRRDLCTELRAFNSN
FNTFTQHYNTWSPQGGVVAT
ASGAGVGGGVAGAGLRASGAERGATGGAREKTPQVSKVSVGTQAKSKVLMTPHKMKHKKNHIFKTQ
>g1591.t1
MESSLSHMAQAVDDRPKDPLPQVIVTISPPSPVFPDPEESIDVDSGPVHPASLTDSDSHPLDSDPLLSDPEDIQVPSIDP
PPVEPHNTETPPSMDPSKNL
AEEAPGSPERSSCEQGFLWYRWQKRSREPKSIHRSASVELWSGRYEYNSAEITYMSLSL
>g1592.t1
MTYGDNVTFGIPLEQAWTDLISLATENVDVASFYWTLTGEDVNVNSSFEVPGVTILESLKKLPSRNVSVRVVSSVPSVPT
NSTDLKVLRENAKDLHKIFQ
SFWAMGHPNSSLPEPWPSKYDTNINKDHPLVLIDGNASSRIYLSDLDAILSAISEAEDFIHVGVMDYSPTTWFGHNRRYW
PDIDEAIREAAFERQVKIRL

LVSCGRDSNPAMLIFLQSLASLRDPHLNISIQVAPDPLQEARTLHHQLKAVFNRDWHSQFSVDLDDLGHNPDCALSVHEK
T
>g1592.t2
MTYGDNVTFGIPLEQAWTDLISLATENVDVASFYWTLTGEDVNVNSSFEVPGVTILESLKKLPSRNVSVRVVSSVPSVPT
NSTDLKVLRENAKDLHKIFQ
SFWAMGHPNSSLPEPWPSKYDTNINKDHPLVLIDGNASSRIYLSDLDAILSAISEAEDFIHVGVMDYSPTTWFGHNRRYW
PDIDEAIREAAFERQVKIRL
LVSCGRDSNPAMLIFLQSLASLRDPHLNISIQVAYSNLLRAHMDGLKKKDKKSKSKKSKATQ
>g1592.t3
MTYGDNVTFGIPLEQAWTDLISLATENVDVASFYWTLTGEDVNVNSSFEVPGVTILESLKKLPSRNVSVRVVSSVPSVPT
NSTDLKVLRENGENVLRRD
>g1592.t4
MGHPNSSLPEPWPSKYDTNINKDHPLVLIDGNASSRIYLSDLDAILSAISEAEDFIHVGVMDYSPTTWFGHNRRYWPDID
EAIREAAFERQVKIRLLVSC
GRDSNPAMLIFLQSLASLRDPHLNISIQVAPDPLQEARTLHHQLKAVFNRDWHSQFSVDLDDLGHNPDCALSVHEKT
>g1593.t1
ELQPMASSASVASLVIAVETGMSDEEGSPDPSLLAEKS
>g1594.t1
MLRTTGAFPFRHQQRYQRYELTSQPVLARYIPDAYLRMWLHTGPRRVNGWNPGLESAPTPPSPLSPRRRDNPGQRR
>g1595.t1
MAEDYTAMQQASLHRGHPHPSQASHMMAYSGRARGVGESPAPQANLHSSAGGSGGNPYRKEAMDYYFSMGGKERHRRGGV
MGYGAGFGYPGVDGHIPQQY
RHAASGSAQSPGMMSPYPVDYGATAGSGGEAGAFSPSHQYSLSQNPAAMQLRQQGQGYQSAHHGQQHRNYPQAAVRMTPQ
YPHYSPQGGTPSGSSGMYSP
PPQRYHDGTGTAGFDSKVNSSPGVASSSNTAGSASAAPNNVAHMENVQQSYHASPYPGYSPQSHSLHKQAALQHRSSQHG
LAAGYDPPVKMQPHAPSTAS
LYAKHHQASNPNIPHTVSQEIAKSPMHSNAQQNPINQNFSPVSNPSPAASAVQSPNCSSSPSPLMGVSEAHANSAGHPPA
HPIAPPRGGHGQGRVLQTVP
QLSPTPNSNSSISSCGSSGGHKAQGMNAAMASGHSSTTRSRMGAGMGDRSREDGSAMYSTPLEKMQDAGLNSLNALTSQV
ANLPNTVQHMLLTDTVFSQK
KGKDGGQMQQQAAHAIPQMQQRSRNASAASSTSTVREGSGGGGGDGSEAGVDEDSLVMSERVASVAKVEREEQYMEGEHR
RARQMSGASSGSEPAGFCAP
SHSLNQSQAGQASHDKIMVNSEPMTKQAGFSEMKPNDSRIPSASPSVCPPPAEPGQHYSTAPIPRTHSSLCDDPPPKPNC
RPEPPHGDNRYGHRKMMEVK
KEVIKSESGGVVKTRDKGDNQMQGKGDGNLLQDGRDKENRLNSGSSGHSNEREGPQKMSEEQQNASSVGVIVSSRPEAGH
PDKTKGLQDPSTEEKGPRPY
VRDSSSRNGEEGVDLTVYSSHHQLHQKPNFGRSQNHPQLAPHKYSYSEPQYGPEVSLKSRGRSGPVGAMEAVSRYQGHQQ
PQSAYAAAHSKDLVEASLKR
TEGAGPRVHEDNAQLQQYPSLLQEVLQGYNLDRRYGRAEQAFPPHLQLQQQFQTRHGFSMTGMTEAALHSSQMATSGKPP
QLNHKQGPASADAHPKMPPN
TDRAKGGVSQGNFPQALESAPLPTKHINLADYSLPQKKTLSTMTAPSSAVQELLLQETEPLTGSSNQSETQKSSTSLLPL
SSERRSVICDVSPNRRSTPE
RDEREREKATGGSSVIQEPFSLAPGASDLSKKDPGVKPVVKMEMALKDRFPGAANMHPNRHTSGSTKEAGSKPGHPADPY
RRGTVDMSPMPPHPTATTNP
LSPPSRHQAYVHGFDLTSGHASGFPGYRYGDTREGGMIPRGNPHFPSHHPYHNLSPQGQLSNPQATNKLQMYPHPCGPSH
PPQDISEWVAAMNRPSKEMM
MMQSGSSPGRHKLPSQSEQRQRMVHQTDVMTNEHLTKQPLPHQSPYYDIKMWEASHGGREGAVLMEGDPYYRTPPSAPPP
PSAPVAHHGVLPMASASNLV
DPDVTRGATEETKPARSVSASTSIKPFTEANSAPLQGPRPAKIGGSGDTNPLMLRRRVRSFISPIPAKRQLQEARASLNS
HHSPGAQSESSHNNEDDSSS
SDIVCPRLSSPISGENAYSQSMSPSGGKSKPLTPRKGRGLKLEAIVQKITPNVQKPMGLDGETNHYPGYSHSELASFSDS
QGQDMPHYPRVVGGDDGYLD
EGHSLNDLTFRGVDDAGPLPPIAYPCEPHQTSQVLKQTDFDFGLGAAVVSASGDKEDFTLLGPLPPPPPLPRQVQGSPPP
SSSALSDIQHFTNTYQQLET
RRGEQSAANLLRQKLQESGIGFEDYPGRDYYATTPPHHSQTPGLLLSRPQHHMSSGRSSLSPQDSKMTDSSVPKGYFPSG
KKKGRPVGSVNKQKRAQGQG

QNTSPGHNTMQGGPLTPSAPSLAALPSLP
>g1596.t1
LLSRAKGPLTAADRRTGAVQQTAAGRTWYRSGRAAAGYTPHLVPLRWGSSGLHATLGTAQVGQQPIEHRDVTCPGRT
>g1597.t1
MGQGRATHDSAIFFTVSCWGSGMRVGDTNRLNRLIRRAGDVVGEELDTLTTAAERRMLSRLRSILDNASHPLCSTLAQQR
STFSRRLLSPRRTTERHRKS
FLPVAIKLYNISL
>g1598.t1
MKRLNNICATSPGIGKPHRAAAPPCSCFRLCPAEPHGSKPGHQMLAGELPSQFWLQLALGTWNVTSLAGKEPELVREVEQ
YQLDIVGLTSTHSTGSGTKL
LERGRTLSFSGVAQGERRRVGVGILTSPRMSAAVLEFSPVNERVASMRLQVVEGKALTVVCAYAPNSSSEYPAFLECLGG
VLEGAPSGDSVVLLGDFNAH
VGNDGETWKGVIGRNGLPDLNPSGALLLDFCASHGLA
>g1599.t1
MLPSALYLKRSSSQDLFFTVSTGRHCRYFECWKAPGREEAGSRDELVIDRTMSEGSSGSSITQASASLPADGWLREDVPL
PLSQVAG
>g1600.t1
MTQINNPKIAFAYLRPACVLLTREPTVANVETLGGQLRALDDATLQQLQEYVLFPLRFVLKLPAAPPRREQLVQSVVEAM
THVLETTCVQSWETLRELLS
ELCLCLSSPCDPGRPSGDASEELKGAVLRCLSALLHAAYGDTLLRLYEPAALPGLGAAVSLLLALGEQEGSREVRLAALS
VLQTLGGRCDCDGREEEHVV
LGEGERRVVGGAMASFLPGITVALTRVVAGDPRHGHAITVGAIKAWYRTVGLVMDDSQLRGSVPGEAGPAGLGRAGELVV
LRTPDWANSTAGRLAILLKR
LIS
>g1601.t1
MDEESLEAAIAAYRAQLQQVETALSSGLDPTHQADLLQLREDLTQLVELTEASLVSVKKSQLLASLEESGGGAVKASGEA
ASGGFDHEFAAFYSELEGPS
GTSTVARVNGRVSLGAEEEENGGDDDDGGGGGDDDDGDEDTLSGTKVRAPYRTTWGTLEYHNAMVVGREAPDGEEAQVRV
LYVYPTQKSLKPCPFFLEDK
CRFEQNCRFSHGEVVYVSELRPFLELDLSNLLEGSTCLARHEDGIWYPARVLDIDHGFYTVKFDSLLLKDAVVEGDGIIP
PMREDDLSSSDSSDQDDDED
AVYAKVFQSSAGDSTTTTTSSSDFGGWEAHTRGIGSRLMLKMGYEYGKGLGKQQEGRVEPVMAVVLPKGASLDQSAELSQ
PRAQRRGPR
>g1601.t2
MDEESLEAAIAAYRAQLQQVETALSSGLDPTHQADLLQLREDLTQLVELTEASLVSVKKSQLLASLEESGGGAVKASGEA
ASGGFDHEFAAFYSELEGPS
GTSTVARVNGRVSLGAEEEENGGDDDDGGGGGDDDDGDEDTLSGTKVRAPYRTTWGTLEYHNAMVVGREAPDGEEAQVRV
LYVYPTQKSLKPCPFFLEDK
CRFEQNCRFSHGEVVYVSELRPFLELDLSNLLEGSTCLARHEDGIWYPARVLDIDHGFYTVKFDSLLLKDAVVEGDGIIP
PMREDDLSSSDSSDQDDDED
AVYAKVFQSSAGDSTTTTTSSSDFGGWEAHTRGIGSRLMLKMGYEYGKGEGRHPARMLASRPSTSC
>g1602.t1
EASPIHNHSINIENSVVSAAWTARRLGMATYDQLSLAANIAVTTHSCRFMLRNVRK
>g1603.t1
MAQLSASIEVTVKTLDSQSRSYKVKGELTVKEFKEHIATSVEIPVDKQRLIYQGRVLQDERTLTDYNVDGKVIHLVERAP
PQPSSSGSGGTGSSTGGADA
GPSTSHSSTTTQGIPHDRNGNSYVMLGTFNLPVNIMDPQQIQSNGSVDVHINMDQSVQSEPRMRLQLAENLLRDAQALID
RLEGQSRDAASAPEPVVSQP
SSGVGASTLQPMDTTTPSAPAPPSSSSSSSTQTEGPPHPGSNHPSPAEFVEVLSEVRRVEERLRPFIERTHCILGTATLA
DYNNNMREREEDQRTLNVVG
DTLRLLGNSLVALGDLRCNLTSNPPRHLYVVRPMAHYASPVLLQAGLPHMPIPINLGTTVTMTSNGRPTTEGQAQPSHTT
GQSEQQGPGQAPPPPPNAAP
TANQMPGGPGQGGPRVIRITHQTMEPVVMMQMNIDGESASGPGQPNVAAPGQPGATPIHIPGLPPEFMHAIVHQISHQAA
AMAAAAAAGHPTHMPPGAMP
GAPPPPTATPGTEGTTPPQQPPLPPQARVVITRPAFSPRVPQPMGTRGATINLRPTVPQATGPQPGQQTTPLAPTPLSQM
ISGLVGQFLLPGQQTDSSFP

STMAPPPPPSVNLSGHTSTSTSSSSSSSSSSSSSSTTSSSTQQPADGVPEGHLAQLLGSLLGGPAGGVGGAPAITVTLPG
MPGSFFQGMSDFIQVSY
>g1603.t2
MAQLSASIEVTVKTLDSQSRSYKVKGELTVKEFKEHIATSVEIPVDKQRLIYQGRVLQDERTLTDYNVDGKVIHLVERAP
PQPSSSGSGGTGSSTGGADA
GPSTSHSSTTTQGIPHDRNGNSYVMLGTFNLPVNIMDPQQIQSNGSVDVHINMDQSVQSEPRMRLQLAENLLRDAQALID
RLEGQSRDAASAPEPVVSQP
SSGVGASTLQPMDTTTPSAPAPPSSSSSSSTQTEGPPHPGSNHPSPAEFVEVLSEVRRVEERLRPFIERTHCILGTATLA
DYNNNMREREEDQRTLNVVG
DTLRLLGNSLVALGDLRCNLTSNPPRHLYVVRPMAHYASPVLLQAGLPHMPIPINLGTTVTMTSNGRPTTEGQAQPSHTT
GQSEQQGPGQAPPPPPNAAP
TANQMPGGPGQGGPRVIRITHQTMEPVVMMQMNIDGESASGPGQPNVAAPGQPGATPIHIPGLPPEFMHAIVHQISHQAA
AMAAAAAAGHPTHMPPGAMP
GAPPPPTATPGTEGTTPPQQPPLPPQARVVITRPAFSPRVPQPMGTRGATINLRPTVPQATGPQPGQQTTPLAPTPLSQM
ISGLVGQFLLPGQQTDSSFP
STMAPPPPPSVNLSGHTSTSTSSSSSSSSSSSSSSTTSSSTQQPADGVPEGHLAQLLGSLLGGPAGGVGGAPAITVTLPG
MPGSFFQGMSDFIQ
>g1603.t3
MAQLSASIEVTVKTLDSQSRSYKVKGELTVKEFKEHIATSVEIPVDKQRLIYQGRVLQDERTLTDYNVDGKVIHLVERAP
PQPSSSGSGGTGSSTGGADA
GPSTSHSSTTTQGIPHDRNGNSYVMLGTFNLPVNIMDPQQIQSNGSVDVHINMDQSVQSEPRMRLQLAENLLRDAQALID
RLEGQSRDAASAPEPVVSQP
SSGVGASTLQPMDTTTPSAPAPPSSSSSSSTQTEGPPHPGSNHPSPAEFVEVLSEVRRVEERLRPFIERTHCILGTATLA
DYNNNMREREEDQRTLNVVG
DTLRLLGNSLVALGDLRCNLTSNPPRHLYVVRPMAHYASPVLLQAGLPHMPIPINLGTTVTMTSNGRPTTEGQAQPSHTT
GQSEQQGPGQAPPPPPNAAP
TANQMPGGPGQGGPRVIRITHQTMEPVVMMQMNIDGESASGPGQPNVAAPGQPGATPIHIPGLPPEFMHAIVHQISHQAA
AMAAAAAAGHPTHMPPGAMP
GAPPPPTATPGTEGTTPPQQPPLPPQARVVITRPAFSPRVPQPMGTRGATINLRPTVPQATGPQPGQMISGLVGQFLLPG
QQTDSSFPSTMAPPPPPSVN
LSGHTSTSTSSSSSSSSSSSSSSTTSSSTQQPADGVPEGHLAQLLGSLLGGPAGGVGGAPAITVTLPGMPGSFFQGMSDF
IQVSY
>g1604.t1
LASAQKVTRLSYCAIDESHLRMDCKYEPAADSPEVMCKYTQNERIVDSTDPEETKDGVYKARTKVRIIDGNICQLRFNNL
WEGKQNFTCHIKQTEIATKT
AVVEKKLLATCSAGLLRFTGALLGLAALPLLLQISGLL
>g1604.t2
MFLLATVLLIGLASAQKVTRLSYCAIDESHLRMDCKYEPAADSPEVMCKYTQNERIVDSTDPEETKDGVYKARTKVRIID
GNICQLRFNNLWEGKQNFTC
HIKQTEIATKTAVVEKKLLATCSAGLLRFTGALLGLAALPLLLQISGLL
>g1605.t1
LLTGVDLSCIPSSDFGTNPRVEWKFNDMKGSQTYVVFDGSPTGHYKNRVTVLKNGLRLNKVTRMDNGEFDCEVSANDNFR
EVKVKLTVLVPAAAPVCKIP
TSATNAGKALLTCTDKVGSPPPTYTWYKGDTLLPVNPKQFPAFQNSTYKIHPMNGNLEFSPISKADTGDYYCEAVNEAGP
AQRCKAVRMEV
>g1605.t2
LLTGVDLSCIPSSDFGTNPRVEWKFNDMKGSQTYVVFDGSPTGHYKNRVTVLKNGLRLNKVTRMDNGEFDCEVSANDNFR
EVKVKLTVLVPAAAPVCKIP
TSATNAGKALLTCTDKVGSPPPTYTWYKGDTLLPVNPKQFPAFQNSTYKIHPMNGNLEFSPISKADTGDYYCEAVNEAGP
AQRCKAIQLQVVGVENTSLY
SLEEIFKLSKLQQLRFFE
>g1605.t3
LLTGVDLSCIPSSDFGTNPRVEWKFNDMKGSQTYVVFDGSPTGHYKNRVTVLKNGLRLNKVTRMDNGEFDCEVSANDNFR
EVKVKLTVLVPAAAPVCKIP
TSATNAGKALLTCTDKVGSPPPTYTWYKGDTLLPVNPKQFPAFQNSTYKIHPMNGNLEFSPISKADTGDYYCEAVNEAGP
AQRCKAVRMEVRDVNTGGIV

AGVIVALLLVALLICGLWYANKKGYLP
>g1606.t1
MAATIDDSNAAQATKKKHLGIPEAIFVEDVDSFMKLPENESADAVLRKLDEQYQKYKYMEVNLAQKKLRLKSQIPQIKQT
LEILRHMQKKKETTEPMQTH
FLLADNVYCKASVPPTDKVCLWLGANVMLEYDIDEAQALLEKNLSTASRNLDSLEEDLDFLRDQFTTTEVSILPPICLKA
GRQLVQ
>g1606.t2
MAATIDDSNAAQATKKKHLGIPEAIFVEDVDSFMKLPENESADAVLRKLDEQYQKYKYMEVNLAQKKLRLKSQIPQIKQT
LEILRHMQKKKETTEPMQTH
FLLADNVYCKASVPPTDKVCLWLGVSANVMLEYDIDEAQALLEKNLSTASRNLDSLEEDLDFLRDQFTTTEVSILPPICL
KAGRQLVQ
>g1607.t1
MRQSLRIKLSSAAVDPNDPEVSQAILKQCTHEVWISARRRSESRTGRIEKPGQVAGELLERLACRNSGEQSGPRLKGWFV
ASTNHLMQHSCESS
>g1608.t1
ESSTEPLSFIIFVPEWRDPATPALIRLESSRFLRHQLSISAYEHEYRSGSQHICKRYD
>g1609.t1
MAQSMRRSNTLGTMDHKLQRDKSRTGYFGNVKNRLGSKLSSSGSQPQELSKSPLEAEPRARCAAAEARDAGRHPHHAAAS
GPLHHVHHIRNPKLRQYYLQ
GAHLLDKVVRTSSELSTAQQEGTSCKTPPGDTVFSITSQFDVHHAGDSPTIASSTFAVIHETVGDPF
>g1609.t2
MDHKLQRDKSRTGYFGNVKNRLGSKLSSSGSQPQELSKSPLEAEPRARCAAAEARDAGRHPHHAAASGPLHHVHHIRNPK
LRQYYLQGAHLLDKVVRTSS
ELSTAQQEGTSCKTPPGDTVFSITSQFDVHHAGDSPTIASSTFAVIHETVGDPF
>g1609.t3
MAQSMRRSNTLGTMDHKLQRDKSRTGYFGNVKNRLGSKLSSSGSQPQELSKSPLEAEPRARCAAAEARDAGRHPHHAAAS
GPLHHVHHIRNPKLRQYYLQ
GAHLLDKVVRTSSELSTAQQEGTSCKTPPGDTVFSITSQFDVHHAGDSPTIASSTFAVIHET
>g1610.t1
MEVIPIWQNKPHGSTRSVELPGLPSLKPLAGPMVPTLADIAWIAADEEETYARVRSDARPLRHEWRPTPLLVLHRNSSVP
NFRREGKKVEGLRKPGVTAL
NRTTALQDELSRLRAQIAKIVSGDGGSNPLTPDLLSPDDTSMSFSMAPFESVSYQPPAATAAASFVISDVTEEEEEEEEE
DEEDVASVVSELVPDPLPPI
SMTASATFDLDRPSMDFREAEEDTVSLSKSTSFADVMDILKDMNRMKMSKERYNRGCASLREEDSATLISEALRKKFTLK
EEDVAAMPRK
>g1610.t2
MELPGLPSLKPLAGPMVPTLADIAWIAADEEETYARVRSDARPLRHEWRPTPLLVLHRNSSVPNFRREGKKVEGLRKPGV
TALNRTTALQDELSRLRAQI
AKIVSGDGGSNPLTPDLLSPDDTSMSFSMAPFESVSYQPPAATAAASFVISDVTEEEEEEEEEDEEDVASVVSELVPDPL
PPISMTASATFDLDRPSMDF
REAEEDTVSLSKSTSFADVMDILKDMNRMKMSKERYNRGCASLREEDSATLISEALRKKFTLKEEDVAAMPRK
>g1610.t3
MVPTLADIAWIAADEEETYARVRSDARPLRHEWRPTPLLVLHRNSSVPNFRREGKKVEGLRKPGVTALNRTTALQDELSR
LRAQIAKIVSGDGGSNPLTP
DLLSPDDTSMSFSMAPFESVSYQPPAATAAASFVISDVTEEEEEEEEEDEEDVASVVSELVPDPLPPISMTASATFDLDR
PSMDFREAEEDTVSLSKSTS
FADVMDILKDMNRMKMSKERYNRGCASLREEDSATLISEALRKKFTLKEEDVAAMPRK
>g1611.t1
QQAQIPISTSAGVVYPGAITMATTTPSPQMTSECSSASASPEPPLPVIQSTYNMKLEAGPQLVNSSSERPNGEDEMDLYE
DFEDEPKSDYSSENEAQEPV
SAT
>g1612.t1
MRAQYPDLPFPEITKRLGAEWSRLVPIDKQRYLDEAEREKAQYARDLKEYHQTEAYQITSAKIQDKKVKKAREAELRRLR
KANVEFEEQNAVLQRHIKDM
C
>g1612.t2

LQLLFLLGREVHDRPRKRGWPKGKKRKKVLPNGPKAPVTGYVRFLNERREQMRAQYPDLPFPEITKRLGAEWSRLVPIDK
QRYLDEAEREKAQYARDLKE
YHQTEAYQITSAKIQDKKVKKAREAELRRLRKANVEFEEQNAVLQRHIKDMC
>g1612.t3
MIGKRGWPKGKKRKKVLPNGPKAPVTGYVRFLNERREQMRAQYPDLPFPEITKRLGAEWSRLVPIDKQRYLDEAEREKAQ
YARDLKEYHQTEAYQITSAK
IQDKKVKKAREAELRRLRKANVEFEEQNAVLQRHIKDMC
>g1613.t1
LMMGRYTEPIEDVPCGNIVGLVGVDQYIVKTGTITTFEQAHNMRVMKFSVSPVVRVAVEAKNPADLPKLVEGLKRLAKSD
PMVQCIIEESGEHIVAGAGE
LHLEICLKDLEEDHAGIPLKKSDPVVSYRETVSEESSQMCLSKSPNKHNRLFMKARPFPDGLSEDIEKGDVTARQELKAR
ARYLVEKYEWDVGEARKIWC
FGPDGTGPNLLVDVTKGVQYLNEIKDSVVAGFQWASKEGVLCEENMRAIRYDVHDVSLHTDAIHRGGGQIIPTARRVLYA
CQLTAEPRIMEPVYLVEIQC
PEAVVGGIYGVLNRRRGHVFEECQVMGTPMFIVKAYLPVNESFGFTADLRSNTGGQAFPQCVFDHWQIMPGDPKDATSKP
YTIVTEIRKRKGIKEGIPLL
ENFLDKM
>g1613.t2
MRVMKFSVSPVVRVAVEAKNPADLPKLVEGLKRLAKSDPMVQCIIEESGEHIVAGAGELHLEICLKDLEEDHAGIPLKKS
DPVVSYRETVSEESSQMCLS
KSPNKHNRLFMKARPFPDGLSEDIEKGDVTARQELKARARYLVEKYEWDVGEARKIWCFGPDGTGPNLLVDVTKGVQYLN
EIKDSVVAGFQWASKEGVLC
EENMRAIRYDVHDVSLHTDAIHRGGGQIIPTARRVLYACQLTAEPRIMEPVYLVEIQCPEAVVGGIYGVLNRRRGHVFEE
CQVMGTPMFIVKAYLPVNES
FGFTADLRSNTGGQAFPQCVFDHWQIMPGDPKDATSKPYTIVTEIRKRKGIKEGIPLLENFLDKM
>g1613.t3
LVGVDQYIVKTGTITTFEQAHNMRVMKFSVSPVVRVAVEAKNPADLPKLVEGLKRLAKSDPMVQCIIEESGEHIVAGAGE
LHLEICLKDLEEDHAGIPLK
KSDPVVSYRETVSEESSQMCLSKSPNKHNRLFMKARPFPDGLSEDIEKGDVTARQELKARARYLVEKYEWDVGEARKIWC
FGPDGTGPNLLVDVTKGVQY
LNEIKDSVVAGFQWASKEGVLCEENMRAIRYDVHDVSLHTDAIHRGGGQIIPTARRVLYACQLTAEPRIMEPVYLVEIQC
PEAVVGGIYGVLNRRRGHVF
EECQVMGTPMFIVKAYLPVNESFGFTADLRSNTGGQAFPQCVFDHWQIMPGDPKDATSKPYTIVTEIRKRKGIKEGIPLL
ENFLDKM
>g1614.t1
LSGASGGQPNDWSQEDHGCPGSTDPSGRELQQQMARRRRRSHIPQKPNYSLNLWSIMKNCIGKELSKIPMP
>g1614.t2
RSQSNLSGASGGQPNDWSQEDHGCPGSTDPSGRELQQQMARRRRRSHIPQKPNYSLNLWSIMKNCIGKELSKIPMP
>g1615.t1
PEYGQRAEASLARRSAPGPAPLPATTLGVSLSPDDPDPAVAALAQPHAPRPSATDSQ
>g1616.t1
RKAKLSIYQSIYVPTLTYGHELWVVTERTRSRIQAAEMSFLRGVAGLSLRYRARSLDIRRELGVEPLLLRVERSQLRWFG
HLIRMPPGRLPLEVFRARPT
GKRPRGRPRTRWRDYISHLAWECLGVLQEELESVAGEKEVWSSLLSLLPL
>g1617.t1
LSLECGVMTGAFYGGVWAAGSRQQQQQQQKLHSFSWCRVDERADDAERGTESSNGEIRGTGGGV
>g1618.t1
MLGDLSVPPHSSVSSHSSVCLTPLYRLTLLYHLTPLYHFTSLCSHSSVPSHSSVPSHSSVRLTALYRLTTLSCLTLWQLK
GSVWQQAGPLSLLKSSYFTT
RCGLDRTRPPSSYTANGVSTQIWLWRTPAQLSSSSAFPPCVRDMTAERPAASRPAQLQPDNLQHASS
>g1618.t2
VPVSQHQQSTAELGLNASKTNKEQQKFYRAAEPPHHQLKGSVWQQAGPLSLLKSSYFTTRCGLDRTRPPSSYTANGVSTQ
IWLWRTPAQLSSSSAFPPCV
RDMTAERPAASRPAQLQPDNLQHASS
>g1618.t3
VPVSQHQQSTAELGLNASKTNKEQQKFYRAAEPPHHDCWCCLSGVMLGDLSVPPHSSVSSHSSVCLTPLYRLTLLYHLTP

LYHFTSLCSHSSVPSHSSVP
SHSSVRLTALYRLTTLSCLTLWQLKGSVWQQAGPLSLLKSSYFTTRCGLDRTRPPSSYTANGVSTQIWLWRTPAQLSSSS
AFPPCVRDMTAERPAASRPA
QLQPDNLQHASS
>g1619.t1
PVEKSVVVERHYIFLKRLCQVLCALGSQVTSLVGSEVAVETPANLNKYMEAMLSFTTHHSQFLKSATQTMWGTLFRHEIL
SKVPVVVEMSLKYLKTTRTN
LVKTGFPSRNDSPSCEYSRVDFDSDEEFSSFFNLFRSQQGEVVRTACRLVPMEALSDAAEWLRYQLSCPILTGNT
>g1619.t2
EKSVVVERHYIFLKRLCQVLCALGSQVTSLVGSEVAVETPANLNKYMEAMLSFTTHHSQFLKSATQTMWGTLFRHEILSK
VPVVVEMSLKYLKTTRTNLV
KTGFPSRNDSPSCEYSRVDFDSDEEFSSFFNLFRSQQGEVVRTACRLVPMEALSDAAEWLRYQLSCPILTGNT
>g1619.t3
MDAITGTNSLCQAVSACQETGLPMEKSVVVERHYIFLKRLCQVLCALGSQVTSLVGSEVAVETPANLNKYMEAMLSFTTH
HSQFLKSATQTMWGTLFRHE
ILSKVPVVVEMSLKYLKTTRTNLVKTGFPSRNDSPSCEYSRVDFDSDEEFSSFFNLFRSQQGEVVRTACRLVPMEALSDA
AEWLRYQLSCPILTGNT
>g1620.t1
MCVWLERALETPDQVRSPETHGDAQYNVLRRVLLSHTENAIAVFGLSALKRLALKDAPSPSGRCGDVIGCLPPTGAASAT
RASEAEST
>g1621.t1
MKRALEQGWMGEVDEYSLHGGCPVTRGEKWVANSWVNVDPDHQRQARYQRLVANGQKAKAGREEHYHPLSHSELHQDL
>g1622.t1
LGAVLSGAKAPGRVSQGKVVSGEGPDKERFKNPDGKAKQDQRYLARIGETGAPYWSQTWEGSSPASVWWPGFDPWGSAGL
SPKQQHGG
>g1623.t1
MDRSKWSLLLLTLALDSVCASPGFFISAPKVFHVNVKEKVFVQSSSVTTLYLEDERTNKVVSDIQTAQAGVQTVELMIDM
MKMSSLEKFKTDPYLLLVAE
GTTAGNTRSMTRVLVSMHRGYLFIQTNQPIYKPSDQVLPSFEVTLNTDEPYILLERETFHFTVKAKYTYGEPVNGSYHSW
FRAEGLQGSEQCLKVTQMIR
GLEQTGSATELKRTLQNSGCNLSSWDQATARLVMSVSVTEQQSRCELKVFLRRPDGSPAADVDIDIHVPALQPKERVVTN
QEGAAFALLSVPASPPADLV
VKVTAEDVKKEKALRRVLSLNQHYLYSSVPDRKIKAGQHLPVTFHIINGVPKNDVLYYLILSHGVLVDQGSLSTLGQTAS
KSIKITAQMVPSLRLVSYYY
NQQGDIISDSLWVDVDDECETKVKIQSSQSRPVPGGTIKLDVEVKGKARVALAVVDTAMYALNRHNKLTAKKVFSAMESY
DQGCSYGGGSDSASTFTNAG
LSFVSSGSPSPTFHEFGCEARSSQRARRSVDLQHELLAMKWNLSAELQQCCLEGFSPIPMRRSCKDRMKRVSEMKVEQQC
AEVFLNCCLKGEALRQSAED
DGLGRRFCVVEPLELTAFKDIFVSVRLPYSVRIYEQMSIVAVIYNYGFEAIQLAAHMEQTPGLCSPGSARVAAFVNVSLE
AGSSAPITFSAVPMMSGPVP
IKIRLFDVNNNREVDAIEKSLNVWTEGQLKTKEMTMEHYLDGAGFGHSSARALLSAKGVNALLELPSGCAEQTMQKMAPT
VLALRYLDMSQQWVQLPAGT
RDQALRHTEAGLMKILTYKKKDGSYGVWQTSKSSHWLTALIVKVLSLLAERQAXXXXXXXAAGVGQRGQAGRDVTNKDLS
ESVQYLLSMQKPDGSFSDPH
PVMGGVTGEGKDAAMTAFITVALKHGLSFVPTGTRSHVEAGIIRATTYLLSHVKMLDTPFAVAITAYCLAFCLPNKQDAK
PAWQQLQLLATLDNSCRVWK
ANPKSSSQNDAITVETTAYALLTAVAHQDLEWAHTAACWLVAQENYGGGFKSTQDTIVALEALAEYDVMKPPETFNRVEA
EFFVEGRSDKVQTSLTNPQD
RVEVELKVLRCIMDTLENNEQKLSSLSVQVMKHFYLMEPMESCASLNIEVKVEGKALYTSKLLNSYQYFDENYEYDEDVQ
QQTHTRRRRDSDQRSETENS
VMYHVCVSLRSNLSGMAIADITLLSGFEAKTDDLDRINEENRCITFEAKQTVPISVLQPAPASFYDYYEPAKDRTVATNS
IRVFANRLQCREKLQVGKKF
FIMGKDSSTVDSKGKCESPRYKQRCKTYHDFTSDFNDCRL
>g1623.t2
MDRSKWSLLLLTLALDSVCASPGFFISAPKVFHVNVKEKVFVQSSSVTTLYLEDERTNKVVSDIQTAQAGVQTVELMIDM
MKMSSLEKFKTDPYLLLVAE

GTTAGNTRSMTRVLVSMHRGYLFIQTNQPIYKPSDQVLPSFEVTLNTDEPYILLERETFHFTVKAKYTYGEPVNGSYHSW
FRAEGLQGSEQCLKVTQMIR
GLEQTGSATELKRTLQNSGCNLSSWDQATARLVMSVSVTEQQSRCELKVFLRRPDGSPAADVDIDIHVPALQPKERVVTN
QEGAAFALLSVPASPPADLV
VKVTAEDVKKEKALRRVLSLNQHYLYSSVPDRKIKAGQHLPVTFHIINGVPKNDVLYYLILSHGVLVDQGSLSTLGQTAS
KSIKITAQMVPSLRLVSYYY
NQQGDIISDSLWVDVDDECETKVKIQSSQSRPVPGGTIKLDVEVKGKARVALAVVDTAMYALNRHNKLTAKKVFSAMESY
DQGCSYGGGSDSASTFTNAG
LSFVSSGSPSPTFHEFGCEARSSQRARRSVDLQHELLAMKLQQCCLEGFSPIPMRRSCKDRMKRVSEMKVEQQCAEVFLN
CCLKGEALRQSAEDDGLGRR
FCVVEPLELTAFKDIFVSVRLPYSVRIYEQMSIVAVIYNYGFEAIQLAAHMEQTPGLCSPGSARVAAFVNVSLEAGSSAP
ITFSAVPMMSGPVPIKIRLF
DVNNNREVDAIEKSLNVWTEGQLKTKEMTMEHYLDGAGFGHSSARALLSAKGVNALLELPSGCAEQTMQKMAPTVLALRY
LDMSQQWVQLPAGTRDQALR
HTEAGLMKILTYKKKDGSYGVWQTSKSSHWLTALIVKVLSLLAERQAXXXXXXXAAGVGQRGQAGRDVTNKDLSESVQYL
LSMQKPDGSFSDPHPVMGGV
TGEGKDAAMTAFITVALKHGLSFVPTGTRSHVEAGIIRATTYLLSHVKMLDTPFAVAITAYCLAFCLPNKQDAKPAWQQL
QLLATLDNSCRVWKANPKSS
SQNDAITVETTAYALLTAVAHQDLEWAHTAACWLVAQENYGGGFKSTQDTIVALEALAEYDVMKPPETFNRVEAEFFVEG
RSDKVQTSLTNPQDRVEVEL
KVLRCIMDTLENNEQKLSSLSVQVMKHFYLMEPMESCASLNIEVKVEGKALYTSKLLNSYQYFDENYEYDEDVQQQTHTR
RRRDSDQRSETENSVMYHVC
VSLRSNLSGMAIADITLLSGFEAKTDDLDRINEENRCITFEAKQTVPISVLQPAPASFYDYYEPDKKCTIFYSAPKRNTE
VSKLCSDDVCQCAETKDRTV
ATNSIRVFANRLQCREKLQVGKKFFIMGKDSSTVDSKGKCESPRYKQRCKTYHDFTSDFNDCRL
>g1623.t3
MDRSKWSLLLLTLALDSVCASPGFFISAPKVFHVNVKEKVFVQSSSVTTLYLEDERTNKVVSDIQTAQAGVQTVELMIDM
MKMSSLEKFKTDPYLLLVAE
GTTAGNTRSMTRVLVSMHRGYLFIQTNQPIYKPSDQVLPSFEVTLNTDEPYILLERETFHFTVKAKYTYGEPVNGSYHSW
FRAEGLQGSEQCLKVTQMIR
GLEQTGSATELKRTLQNSGCNLSSWDQATARLVMSVSVTEQQSRCELKVFLRRPDGSPAADVDIDIHVPALQPKERVVTN
QEGAAFALLSVPASPPADLV
VKVTAEDVKKEKALRRVLSLNQHYLYSSVPDRKIKAGQHLPVTFHIINGVPKNDVLYYLILSHGVLVDQGSLSTLGQTAS
KSIKITAQMVPSLRLVSYYY
NQQGDIISDSLWVDVDDECETKVKIQSSQSRPVPGGTIKLDVEVKGKARVALAVVDTAMYALNRHNKLTAKKVFSAMESY
DQGCSYGGGSDSASTFTNAG
LSFVSSGSPSPTFHEFGCEARSSQRARRSVDLQHELLAMKWNLSAELQQCCLEGFSPIPMRRSCKDRMKRVSEMKVEQQC
AEVFLNCCLKGEALRQSAED
DGLGRRFCVVEPLELTAFKDIFVSVRLPYSVRIYEQMSIVAVIYNYGFEAIQLAAHMEQTPGLCSPGSARVAAFVNVSLE
AGSSAPITFSAVPMMSGPVP
IKIRLFDVNNNREVDAIEKSLNVWTEGQLKTKEMTMEHYLDGAGFGHSSARALLSAKGVNALLELPSGCAEQTMQKMAPT
VLALRYLDMSQQWVQLPAGT
RDQALRHTEAGLMKILTYKKKDGSYGVWQTSKSSHWLTALIVKVLSLLAERQAXXXXXXXAAGVGQRGQAGRDVTNKDLS
ESVQYLLSMQKPDGSFSDPH
PVMGGVTGEGKDAAMTAFITVALKHGLSFVPTGTRSHVEAGIIRATTYLLSHVKMLDTPFAVAITAYCLAFCLPNKQDAK
PAWQQLQLLATLDNSCRVWK
ANPKSSSQNDAITVETTAYALLTAVAHQDLEWAHTAACWLVAQENYGGGFKSTQDTIVALEALAEYDVMKPPETFNRVEA
EFFVEGRSDKVQTSLTNPQD
RVEVELKVLRCIMDTLENNEQKLSSLSVQVMKHFYLMEPMESCASLNIEVKVEGKALYTSKLLNSYQYFDENYEYDEDVQ
QQTHTRRRRDSDQRSETENS
VMYHVCVSLRSNLSGMAIADITLLSGFEAKTDDLDRIKGPVEQYISHYEISYGRVILYFNEAIVFAFLADKKCTIFYSAP
KRNTEVSKLCSDDVCQCAET
KDRTVATNSIRVFANRLQCREKLQVGKKFFIMGKDSSTVDSKGKCESPRYKQRCKTYHDFTSDFNDCRL
>g1624.t1
MADGGGGDDDVIHLASFNVHRSQGRRSRRKELITISDDSDEEPITLVPGSPVLVPDDTDDDVRILESLTPSRRHVIRPAA
TWCGTARNLVHVPIKRDAGE

VVVPSTSR
>g1624.t2
MADGGGGDDDVIHLASFNVHRSQGRRSRRKELITISDDSDEEPITLVPGSPVLVPDDTDDDVRILEVGSPLPRWLWNPAS
Q
>g1625.t1
WGNELPANVRTAESLPIFRKRLKTHLFRVHLNPA
>g1626.t1
GQDDLRQDAVMQQVFAMCSTLLQRNAETRRRKLLIRRYKVCLASCLAVSLVVCLVVCLVVSLASCLMVCLASCLEVCPEV
CLVVCLEVSLASCLEVCLEV
CLASCLLVVPFSQRSGVLEWCSGTVPIGEFLVDPNKGAHVRFRPQDWTNLACRRRMMASGDLPVLPLGSSQEVQRQGLEG
KMTAYSDVCRNFRPVFRYFC
MERFLDPAVWMEKRLAYTRSVATSSIGTLSPRVESRRKAPRASVAR
>g1626.t2
GQDDLRQDAVMQQVFAMCSTLLQRNAETRRRKLLIRRYKVCLASCLAVSLVVCLVVCLVVSLASCLMVCLASCLEVCPEV
CLVVCLEVSLASCLEVCLEV
CLASCLLVVPFSQRSGVLEWCSGTVPIGEFLVDPNKGAHVRFRPQDWTNLACRRRMMASGDLPVLPLGSSEVQRQGLEGK
MTAYSDVCRNFRPVFRYFCM
ERFLDPAVWMEKRLAYTRSVATSSIGTLSPRVESRRKAPRASVAR
>g1627.t1
VYSLCVLAPHGRQADPGNEYENQPRIGDIKPRLLIKPDRNRMLGYIWQYVYASFLRYWLKWVVRQVLRGSLQTSRDNLEQ
CVDHIVQAKNVKPQKDPLFR
KSLHTCLLQISGYASLYTCVEELRKETFSSDNMEHEDMLLTLWKLLMPTVKLESRVTKQWGDIGFQGDDPKTDFRGMGML
GLTNLVFFSEHYTEEARQVL
SHANHPKLGYSYAIVGINLTEMAYSLLKSGDLKPHFYNTVAGNPELHHFHHLYCYLAYEFDKFWVASKPQSIMEFNQYRE
KFHSQVKLMLQEPGVALTLT
WPAGTGVSCFTRGVRDMPACGLTTG
>g1627.t2
MLGYIWQYVYASFLRYWLKWVVRQVLRGSLQTSRDNLEQCVDHIVQAKNVKPQKDPLFRKSLHTCLLQISGYASLYTCVE
ELRKETFSSDNMEHEDMLLT
LWKLLMPTVKLESRVTKQWGDIGFQGDDPKTDFRGMGMLGLTNLVFFSEHYTEEARQVLSHANHPKLGYSYAIVGINLTE
MAYSLLKSGDLKPHFYNTVA
GNPELHHFHHLYCYLAYEFDKFWVASKPQSIMEFNQYREKFHSQVKLMLQEPGVALTLTWPAGTGVSCFTRGVRDMPACG
LTTG
>g1627.t3
VYSLCVLAPHGRQADPGNEYENQPRIGDIKPRLLIKPDRNRMLGYIWQYVYASFLRYWLKWVVRQVLRGSLQTSRDNLEQ
CVDHIVQAKNVKPQKDPLFR
KSLHTCLLQISGYASLYTCVEELRKETFSSDNMEHEDMLLTLWKLLMPTVKLESRVTKQWGDIGFQGDDPKTDFRGMGML
GLTNLVFFSEHYTEEARQVL
SHANHPKLGYSYAIVGINLTEMAYSLLKSGDLKPHFYNTVAGNPELHHFHHLYCYLAYEFDKFWVASKPQSIMEFNQYRE
KFHSQVKLMLQEPGVALTLT
WPAGTGVSCFTRGVRDMPACGLTT
>g1628.t1
LPWTKTFDADSERDRPSDQPIKKNERRRRKRKKDRKREEMTKLLPAPETEDEDDPRPPTPPSPAPSQPREEKKKKRAADS
EKEEEEEPAPSKKHRRGEGK
EKQKAESAEDEAAAPPEQDLSEGQAKQKQGQRSEPKLQGIKDLLHDKKARKTEAANCKAMGRVLGRLAVHLPPITTETSS
KLIGKLEKVNGDSPAQKPHA
DEMTTKQTEVKMRRIRKLFFSASVVRTIIDNSNTNAEKRLKSGLKFPWKTLTVEDFYIFLTIVLFSGIVSVHEVKEYWRK
DWPYNFRFPTDTMTQGRFEA
IMWSLHLSHPEEDEVHDAKKTTAEYDCLFKIKPLYTEMINACKSNFQPYQNISIDERMVTSNAKMSVTQQCMKSRRTKWG
YKLFVLADSATAYTWNFFVH
EGKTQSLKDSKGKFRPGQALSHTAVMNLTSPLLDMGYTLFVDNFYTSPALFEDLRTRNTGA
>g1628.t2
LPWTKTFDADSERDRPSDQPIKKNERRRRKRKKDRKREEMTKLLPAPETEDEDDPRPPTPPSPAPSQPREEKKKKRAADS
EKEEEEEPAPSKKHRRGEGK
EKQKAESAEDEAAAPPEQDLSEGQAKQKQGQRSEPKLQGIKDLLHDKKARKTEAANCKAMGRVLGRLAVHLPPITTETSS
KLIGKLEKVNGDSPAQKPHA

DEMTTKQTEVKMRRIRKVMTHQPPVDNSLLPFLSYSFRFTQAYL
>g1628.t3
LPWTKTFDADSERDRPSDQPIKKNERRRRKRKKDRKREEMTKLLPAPETEDEDDPRPPTPPSPAPSQPREEKKKKRAADS
EKEEEEEPAPSKKHRRGEGK
EKQKAESAEDEAAAPPEQDLSEGQAKQKQGQRSEPKLQGIKDLLHDKKARKTEAANCKAMGRVLGRLAVHLPPITTETSS
KLIGKLEKVNGDSPAQKPHA
DEMTTKQTEVKMRRIRKLFFSASVVRTIIDNSNTNAEKRLKSGLKFPWKTLTVEDFYIFLTIVLFSGIVSVHEVKEYWRK
DWPYNFRFPTDTMTQGRFEA
IMWSLHLSHPEEDEVHDAKKTTAEYDCLFKIKPLYTEMINACKSNFQPYQNISIDERMVTSNAKMSVTQQCMKSRRTKWG
YKLFVLADSATAYTWNFFVH
EGKTQSLKDSKGKFRPGQALSHTAVMNLTSPLLDMGYTLFVDNFYTSPALFEDLRTRNTGACGTIRSGLRDFPRTQQNDL
PQKPDRGDLRWIRKNNMLFV
KWM
>g1629.t1
MDLGTRLHTMDLGTRLFNNLKEVPWSSTSLPLDQLVSAYRLPQIVQLEHGESVEGLRDQDYLLLHSCRQWTTITAHSLEE
GHYVIGPKIEIPVHYEGQFK
LLEQDRDIREPVQYFSSVEEVAKAFPERVYVMEDITFNVKMASGECNEDTEVYNITLSTGDELTLMGQAEILYTKTSKEK
SRFNTIFRKIGKLHSMGRIG
RGKMPCLICMNHRTNESVSLPFQCKGRFSTCSPLELQMQEGEHTIRHIVEKTRLPVNVAVPGGPPRNQHDLHHIRQGHRY
KLVNVQTKTVVVCCVLRSDK
IVPIHFPLHAAVPRCSLPEGLLQGELWLDTMVHRWFTFCQEQFHIDDYSRAVRDVRADWGDEGKSP
>g1629.t2
MDLGTRLFNNLKEVPWSSTSLPLDQLVSAYRLPQIVQLEHGESVEGLRDQDYLLLHSCRQWTTITAHSLEEGHYVIGPKI
EIPVHYEGQFKLLEQDRDIR
EPVQYFSSVEEVAKAFPERVYVMEDITFNVKMASGECNEDTEVYNITLSTGDELTLMGQAEILYTKTSKEKSRFNTIFRK
IGKLHSMGRIGRGKMPCLIC
MNHRTNESVSLPFQCKGRFSTCSPLELQMQEGEHTIRHIVEKTRLPVNVAVPGGPPRNQHDLHHIRQGHRYKLVNVQTKT
VVVCCVLRSDKIVPIHFPLH
AAVPRCSLPEGLLQGELWLDTMVHRWFTFCQEQFHIDDYSRAVRDVRADWGDEGKSP
>g1630.t1
LSSVSSASVTSSVKLWITTMSFVDTLRQWDEAVVCVDKANLPQALQILVSIQEPNSKIYFNIGCLHLINQDLDAAEKAFD
RSIGKDEHLAIAFFQRGITF
HRKERYSTTVLSIIATNSSHVFQSSRVMDDMEVSELFLFKVLHNMALSEAHLGAWEKAQAHLVAALNYKTESKLGLIDRA
LQSILKQKLFQPAVVPSTML
FRPNKNYVAELEKRDYLGKAKPRKVTQAVRSRSPPAPRAFEGEPHVVLFEFVPETSGELAVVPGNVVFVLQKGADNWASV
VFNERRGLVPYNYLERMEIN
LTPKPNQAADGGETSACIVKVHYTFNLVLDVDWGSPYAALVDRISRKLQLPAESITLSCTSPAAGQTAMDGSTDMQAVWS
TANCGRITLWCAVKEEVGGF
CGQAG
>g1630.t2
LSSVSSASVTSSVKLWITTMSFVDTLRQWDEAVVCVDKANLPQALQILVSIQEPNSKIYFNIGCLHLINQDLDAAEKAFD
RSIGKDEHLAIAFFQRGITF
HRKERYSTTVLSIIATNSSHVFQSSRVMDDMEVSELFLFKVLHNMALSEAHLGAWEKAQAHLVAALNYKTESKLGLIDRA
LQSILKQKLFQPAVVPSTML
FRPNKNYVAELEKRDYLGKAKAVRSRSPPAPRAFEGEPHVVLFEFVPETSGELAVVPGNVVFVLQKGADNWASVVFNERR
GLVPYNYLERMEINLTPKPN
QAADGGETSACIVKVHYTFNLVLDVDWGSPYAALVDRISRKLQLPAESITLSCTSPAAGQTAMDGSTDMQAVWSTANCGR
ITLWCAVKEEVGGFCGQAG
>g1630.t3
LSSVSSASVTSSVKLWITTMSFVDTLRQWDEAVVCVDKANLPQALQILVSIQEPNSKIYFNIGCLHLINQDLDAAEKAFD
RSIGKDEHLAIAFFQRGITF
HRKERYSTTVLSIIATNSSHVFQSSRVMDDMEVSELFLFKVLHNMALSEAHLGAWEKAQAHLVAALNYKTESKLGLIDRA
LQSILKQKLFQPAVVPSTML
FRPNKNYVAELEKRDYLGKAKVRDGPTSPKAPEVLRAFEGEPHVVLFEFVPETSGELAVVPGNVVFVLQKGADNWASVVF
NERRGLVPYNYLERMEINLT
PKPNQAADGGETSACIVKVHYTFNLVLDVDWGSPYAALVDRISRKLQLPAESITLSCTSPAAGQTAMDGSTDMQAVWSTA

NCGRITLWCAVKEEVGGFCG
QAG
>g1631.t1
MRSDLLLVLTAAVVTVAGKPTTGRTTGHNKLLMISFDGFRWDYDQDVDTPNLDQLVLDGVKAKYITPPAITMTSPSHFTT
ITESSSLTRGLWPLLVGRWI
EDHGVVHNMMYNSTTNLKVPHKTTMTKSEWWDNGVLPLWITAQNQGLKAASFYYPGGGANYSGQAVNRALVETGIRPDDN
ETEWQENIDQVMGWFSQENF
DFVTLYYGEPDNVGHAKGPDHPKRKEIIRQIDRTLGYLRQAIASHGLADRLNVIITSDHGMTTVKKRPAVDEIILNKYLN
LLKLTSFEILDYGGYGIVTP
RPGRDQEVFELLSKAPNLTVYKKDELPESFHLQKSARITPIMVVADLGFNLNSRFIVYVNKGDHGFHNGEMDMKMIFRAF
GPDFKKNFVAEPFDSIHIYA
LMCRLLKIQSEPNNGSLVFTEDMLVRAGAVPVPSQTSLAVLASLAAYLLTVS
>g1632.t1
MVRVSRGAVHEGAAWAAGQGDHGGRQLAFYLMEWPAQSPDLNPIELLWEQLDRMWWNELRANVRTAESLPIFRKRLKTHL
FRVRLNPA
>g1632.t2
MVRVSRGAVHEGAAWAAGQGDHGGRQLAFYLMEWPAQSPDLNPIELLWEQLDRMDSTQRGGQDEGHTDHFRWWNELRANV
RTAESLPIFRKRLKTHLFRV
RLNPA
>g1632.t3
MVRVSRGAVHEGAAWAAGQGDHGGSQSIRLQWKGSVALLHLDLNHILHRKDSTQRGGQDEGHTDHFRWWNELRANVRTAE
SLPIFRKRLKTHLFRVRLNP
A
>g1633.t1
MIRKPKKPNSANRKCARVRLSNGKEAVVFIPGEGHNLQEHNVVLVQGGRTQDLPGVKLTVVRGKYDCAHVVKKKHVLSPR
GFPCPQAGSAGRRRVLDLRA
QEQEEDGEDSGVVLFSDEPGQSPVQPDETAAVVRKRSPRLLELSRALSSESIFCCSLCHKAF
>g1633.t2
MIRKPKKPNSANRKCARVRLSNGKEAVVFIPGEGHNLQEHNVVLVQGGRTQDLPGVKLTVVRGKYDCAHVVKKKHSVLSP
RGFPCPQAGSAGRRRVLDLR
AQEQEEDGEDSGVVLFSDEPGQSPVQPDETAAVVRKRSPRLLELSRALSSESIFCCSLCHKAF
>g1634.t1
LLTEADAGHTEFTDEVFQNETRFPGGEWAPASEPYTDVNGEQAQSPEDMECPPGWSWEDVWSSDVNRAVDEKGWEYGVTI
PPDDKPRSWVAAEKMYHIHR
RKRLVRPRRRTSDTAPMLERRTSGDDWEYSSLIGWKFHRKERSSDMFRRRRWRRKMAPSDRMGASAIFRLEGALGVDVDE
KAGKADATKRFGANTPNVSC
DFSRCYRYHLRVYVYQAKSLCAMDKDSFSDPYVHVSFLNVSKTTEVQRATLNPTWDQTLIFDNVELYGDPKTIARSPPDV
VMELYDSDQVGRDEPLGRCV
CPPVVNLNPAVAVTPKLLWFPVTKKGRAAGQMLVAAELLLKDGVTLHGSVGVGGENMWMWGG
>g1634.t2
LLTEADAGHTEFTDEVFQNETRFPGGEWAPASEPYTDVNGEQAQSPEDMECPPGWSWEDVWSSDVNRAVDEKGWEYGVTI
PPDDKPRSWVAAEKMYHIHR
RKRLVRPRRRTSDTAPMLERRTSGDDWEYSSLIGWKFHRKERSSDMFRRRRWRRKMAPSDRMGASAIFRLEGALGVDVDE
KAGKADATKRFGANTPNVSC
DFSRCYRYHLRVYVYQAKSLCAMDKDSFSDPYVHVSFLNVSKTTEVQRATLNPTWDQTLIFDNVELYGDPKTIARSPPDV
VMELYDSDQVVRPHCPPSSH
PSHESVETLSAMSGCVFSSTVVYRDSLVGRDEPLGRCVCPPVVNLNPAVAVTPKLLWFPVTKKGRAAGQMLVAAELLLKD
GVTLHGSVGVGGENMWMWGG
>g1635.t1
MTSWVHSAYVQRHPSCASDHISPFISATNRLVQQRAVGCTFLKKTFRFLLGTKRHVAVTDLHRELSVPRQGGQLSLGVTE
AALTPADEWAA
>g1635.t2
MTSWVHSAYVQRHPSCASDHISPFISATNRLVQQRAVGCTFLKKTFRFLLGTKRHVAVTDLHRELSVPRQGGQLSLGVTE
AALTPADEWAAVST
>g1635.t3
MTSWVHSAYVQRHPSCASDHISPFISATNRLVQQRAVGCTCTIHGQWCLTTEIQRCENWSQTFRFLLGTKRHVAVTDLHR

ELSVPRQGGQLSLGVTEAAL
TPADEWAA
>g1636.t1
MPQQKTLDMGSAFIQTQQLNAAMADTFLEHLCLLDIDSEPTTARNTGIICTIGPASRTVDMLKEMIKSGMNVARLNFSHG
SHEYHAQTIKNVREACESFE
PGSIHYRPVAIALDTKGPEIRTGLIRGSGEAEVELKKGNMIKLTLDDSYKENCDEEVLWLDYQNITNVVEIGSKIYIDDG
LMSLQVKEIGSNFLMCEIEN
GGTLGSKKGVNLPGAAVDLPAVSEKDIQDLQFGLEQDVDMVFASFIRKAADVNAVRAVLGEKGKNIKIISKLENHEGVRR
FDEIMEASDGIMVARGDLGI
EIPTEKVFLAQKMMIGRCNRAGKPITCATQMLESMIKKPRPTRAEGSDVANAVLDGADCIMLSGETAKGDYPLEAVRTQH
MVSRLGASWEPPAGLSRAEQ
G
>g1636.t2
MPQQKTLDMGSAFIQTQQLNAAMADTFLEHLCLLDIDSEPTTARNTGIICTIGPASRTVDMLKEMIKSGMNVARLNFSHG
SHEYHAQTIKNVREACESFE
PGSIHYRPVAIALDTKGPEIRTGLIRGSGEAEVELKKGNMIKLTLDDSYKENCDEEVLWLDYQNITNVVEIGSKIYIDDG
LMSLQVKEIGGYNQLVPETP
LRQEGLSDTWAPLGVMEVLMM
>g1637.t1
MCPKGNVSVLCASLSKQTIYLSTSLFILICLMMGAGERDLRGERLFFDLLVYDLTIFDLTVNDIVLFDLLVCDLTIFDLT
VYDIVVFDLLVCDLTIFDLT
VYHIVVFDLLVCDLTIFDPNFSDIVVF
>g1638.t1
MLITHDDLVKISDFGTSRELSDKSTKMSFAGTVAWMAPEVIRNEPVSEKVDIWSFGVVLWEMLTGEIPYRDVDSSAIIWG
VGNNSLQLPIPDSCPDGFKI
LLRQCWNVKPRNRPSFRQILLHLDIASADVLSTPQETYFKSQAEWREEVKQHFEKIKSEGTCIHRLDEELVNRRREELRH
ALDIREHYERKLERANNLYM
ELNAVMLQLELKEKELQRREQSLDKKYPGLFKHSSRQSSSSNSMDKLIKKRNVPQKLPSGKRPDILKSEVIIPKMDSSVM
QVTLPSCPNRSSTSPSRSRR
VKTRHRKPGKGSSGDLAGLKATQSAPSRGVAAQDGSGSAPDPSKQ
>g1638.t2
MLITHDDLVKISDFGTSRELSDKSTKMSFAGTVAWMAPEVIRNEPVSEKVDIWSFGVVLWEMLTGEIPYRDVDSSAIIWG
VGNNSLQLPIPDSCPDGFKI
LLRQCWNVKPRNRPSFRQILLHLDIASADVLSTPQETYFKSQAEWREEVKQHFEKIKSEGTCIHRLDEELVNRRREELRH
ALDIREHYERKLERANNLYM
ELNAVMLQLELKEKELQRREQSLDKKYPGLFKHSSRQSSSSNSMDKLIKKRNVPQKLPSGKRPDILKSEVIIPKMDSSVM
QVTLPSCPNRSSTSPSRSRR
VKTRHRKPGKGSSGDLAGLKATQSAPSRGVAAQDGSGSAPDPSKQ
>g1639.t1
FIVGEDGSWGLLYEQATAEGPPIATLLDHVLDYCQKPDPKRAPLVPLPMPRKLYFYIDQDIKRDIEHAKQNLDILINDLD
VNVFNFRRFGKELPKQNGLS
PNAFVQVALQLAYYRYELQG
>g1639.t2
MLFFPGQFIVGEDGSWGLLYEQATAEGPPIATLLDHVLDYCQKPDPKRAPLVPLPMPRKLYFYIDQDIKRDIEHAKQNLD
ILINDLDVNVFNFRRFGKEL
PKQNGLSPNAFVQVALQLAYYRYELQG
>g1640.t1
LFTTGKAARTLNALAGGVFNGNLCLQRFPAVCTRPLCQSEPQLPRDLLNRPAGLELSCPFWRGELLNLENNMMELRDLFT
DIYLLVEEQGAHIDHIQTNV
ERTSDYVAASNEKFKLAARYKKKNPLRQLCCCCCPPWRCCF
>g1640.t2
LPSGVNTSRCPHKAIHKTACRLLFTTGKAARTLNALAGGVFNGNLCLQRFPAVCTRPLCQSEPQLPRDLLNRPAGLELSC
PFWRGELLNLENNMMELRDL
FTDIYLLVEEQGAHIDHIQTNVERTSDYVAASNEKFKLAARYKKKNPLRQLCCCCCPPWRCCF
>g1640.t3
AIHKTACRLLFTTGKAARTLNALAGGVFNGNLCLQRFPAVCTRPLCQSEPQLPRDLLNRPAGLELSCPFWRGELLNLENN

MMELRDLFTDIYLLVEEQGA
HIDHIQTNVERTSDYVAASNEKFKLAARYKKKNPLRQLCCCCCPPWRCCF
>g1641.t1
MILATLTSVVQCSQSAAALVSKSYPRGNHWAVGHLMGRKSVEMLPDPQLDEQDNSDYRLPSLERRDATLGQQYPSRLERA
SLGSSPNRQEPDDADRGFLR
KSRWSEEIRFKYLREVK
>g1642.t1
MWVLGSSAAVMMDDRLSPSARFAQSPGNPARVHSIDAILGFKDDSVFNKSPTYTAREKSCKKDAELKGNVIITPMTKKEQ
NSETFESVCCTTTSDSPTHS
PADSPGGDGKYSDDEDNPKKKHRRNRTTFTTFQLHELERAFEKSHYPDVYSREELALKVNLPEVRVQVWFQNRRAKWRRQ
EKLEVSAIRLQDTPSSLFPF
GCSSTNPLHGSGGLHLDPWLTTPITTTTATSSLPTFPGFIGGAQGPSGGSYTSPQPPPSMASSMPATFLHSAPLAGTNHH
QLPHIGSICPPAQAYPCSGF
MDKLAPEETDPRNSSIASLRMKAKEHIQSFGKTW
>g1642.t2
MMDDRLSPSARFAQSPGNPARVHSIDAILGFKDDSVFNKSPTYTAREKSCKKDAELKGNVIITPMTKKEQNSETFESVCC
TTTSDSPTHSPADSPGGDGK
YSDDEDNPKKKHRRNRTTFTTFQLHELERAFEKSHYPDVYSREELALKVNLPEVRVQVWFQNRRAKWRRQEKLEVSAIRL
QDTPSSLFPFGCSSTNPLHG
SGGLHLDPWLTTPITTTTATSSLPTFPGFIGGAQGPSGGSYTSPQPPPSMASSMPATFLHSAPLAGTNHHQLPHIGSICP
PAQAYPCSGFMDKLAPEETD
PRNSSIASLRMKAKEHIQSFGKTW
>g1643.t1
MESITTEAQRTKPAQQPRQTSSAGQEEDVRTRTKRVLVLAQGVRQVRVSSSRKTKVFQVAPTVYIQHHPVSLKHPPCLEL
VIPEDHHHHDA
>g1644.t1
LDNAGKTTILKKFNGEDISTISPTLGFNIKTLEHRGFKLNIWDVGGQKSLRSYWRNYFESTDGLVWVVDSADRLRLEDCK
QELSSLLLEERLAGATLLVF
ANKQDLPGALSKEAIGEAMGLDNIKTHHWCIVGCSAVTGDNLLSGLDWLLDDIAARVFTAH
>g1645.t1
MPPLYMDAKSTSSGFTSGEELDSSNSSLHPGEASGPAGRKPRPGPGQRKDRNRDAARKSRMKQTEKADELHKEQQCLERS
NLALEKEIAALRADVRHYTE
MLEHHEPVCLLPPPPPPPSVR
>g1645.t2
LDRSVTLRVAVPSSPQHPGEASGPAGRKPRPGPGQRKDRNRDAARKSRMKQTEKADELHKEQQCLERSNLALEKEIAALR
ADVRHYTEMLEHHEPVCLLP
PPPPPPSVR
>g1646.t1
MNLQSCTRICSPGALPPKITARESLRFESPLLTTSSAPPQHLLTTSSAPPQHLLTTSSPPPHHLLSTSSPPPHHLP
>g1647.t1
MRAAARPERRGGSAVEICGDAPRPLPPAGSPENKTTCGPGVSVLAAPRPPNTPLRHPSVARVHSVAAGEADDMSFVEYSE
RVQEGDVVIIYLGHESMMPV
KVKGGDKTQTRYGAIRHSADLIGQRYGSKVTCSKGWVLVLHPTPELWTVNLPHRTQILYTTDIATITMMLELKPGSVVCE
SGKTARLRPAEPHPQNHTVL
TEPPATRQAGRGPQCLLGL
>g1648.t1
MKLKYSSEKEYPDLSQHNNHMAKVLTPAMYELLRKRETPSGFTLDDVIQTGIDNPAQVSISRRSAEEDFPDLSGHHNHMA
SVLTLDLYKQLRQRATPNGF
TVDDVIQTGVDNPGHPFIMTVGCVAGDEETYEVFKELLDPVIQDRHGGYKPTDKHKTDLNPAHLKGGDDLDPNYVLSSRV
RTGRSIRGFCLPPHCSRGER
RAVETLSIEALDTLSGDLKGKYYALKNMSDAEQQQLIDDHFLFDKPVSPLLLASGMARDWPDGRGIWHNVNKTFLVWVNE
EDHLRVISMQKGGNMKEVFN
RFCTGLTKIESLFKERKHAFMWNEHLGYVLTCPSNLGTGLRAGVHVKLPNLSKNAKFEDVLKRLRLQKRGTGGVDTAAVG
GTFDISNADRLGFSEVELVQ
MVVDGVKLLVEMEKKLEKGQSIDDLVPAQK
>g1648.t2

MPFGNTHNAMKLKYSSEKEYPDLSQHNNHMAKVLTPAMYELLRKRETPSGFTLDDVIQTGIDNPAQVSISRRSAEEDFPD
LSGHHNHMASVLTLDLYKQL
RQRATPNGFTVDDVIQTGVDNPGHPFIMTVGCVAGDEETYEVFKELLDPVIQDRHGGYKPTDKHKTDLNPAHLKGGDDLD
PNYVLSSRVRTGRSIRGFCL
PPHCSRGERRAVETLSIEALDTLSGDLKGKYYALKNMSDAEQQQLIDDHFLFDKPVSPLLLASGMARDWPDGRGIWHNVN
KTFLVWVNEEDHLRVISMQK
GGNMKEVFNRFCTGLTKIESLFKERKHAFMWNEHLGYVLTCPSNLGTGLRAGVHVKLPNLSKNAKFEDVLKRLRLQKRGT
GGVDTAAVGGTFDISNADRL
GFSEVELVQMVVDGVKLLVEMEKKLEKGQSIDDLVPAQK
>g1648.t3
MKLKYSSEKEYPDLSQHNNHMAKVLTPAMYELLRKRETPSGFTLDDVIQTGIDNPAQVSISRRSAEEDFPDLSGHHNHMA
SVLTLDLYKQLRQRATPNGF
TVDDVIQTGVDNPGHPFIMTVGCVAGDEETYEVFKELLDPVIQDRHGGYKPTDKHKTDLNPAHLKSGSGDPLVGPDTAAC
FGASCQALTRRVSPQGGDDL
DPNYVLSSRVRTGRSIRGFCLPPHCSRGERRAVETLSIEALDTLSGDLKGKYYALKNMSDAEQQQLIDDHFLFDKPVSPL
LLASGMARDWPDGRGIWHNV
NKTFLVWVNEEDHLRVISMQKGGNMKEVFNRFCTGLTKIESLFKERKHAFMWNEHLGYVLTCPSNLGTGLRAGVHVKLPN
LSKNAKFEDVLKRLRLQKRG
TGGVDTAAVGGTFDISNADRLGFSEVELVQMVVDGVKLLVEMEKKLEKGQSIDDLVPAQK
>g1649.t1
MRTTSSLEPADIMREIRKVLDANNCDYEQPESFLLLCVHGEGRAEDLVQWEMEVCKLPRLSLNGVRFKRISGSSIAFKNI
ASKVSNELKL
>g1650.t1
MNNHSFPLSPVMPLTSFANVLVFLFSILLATTIIFLNFSVLVSILINKSLRNENRFMFMLSTCLSDICTGVSYYYAGVFD
RVHCAQGNSVHPRLEIPRPE
RVTRTLWHTAIPYPDCGLFLECPCSTMDRFPLCFK
>g1651.t1
LTSGFVAFSSSLSPLHSSTPDVSLKPRAAEASEQHNGAPYLPLSRAPFPAVTVDQSITNYSVFGIRPSLCVNSFLLSRKL
LEPRCSQ
>g1652.t1
MPGFTCCVPGCYNNSHRDRELRFYTFPKDITQRELWLKNISRAGVSGCFSTFQPTTGHRVCSVHFSGGRKTYNIRVPTLF
PLRGVNERKNRRGRGRGKKV
SAAVPAAARPRPASHPGIVIANVFSTAADDADGSATAETGIDNLTVVQIDQNGEYVGATSVAVQPYRIGVPADVSSTTDP
TGEERSPDDPLEYISVISNP
LDHSYSLTTGTTSTELLRKLNEQRDIIALMEVKMKEMKATIRQLRVSEAKLQEEVRDRDRVLSCGPAVSFTVRKKI
>g1653.t1
MLPGVGVFGTGSTARVLVPLLQAEGFEVHALWGQSEEEAGCLAKELGIPFHTSRSDDVLLHPDVHLVCIYIPPPMTRQIA
VNALGIGKNVVCEKAATALD
SFKMVTAARYYPQLLSIVGNTLRFLPTFVAMRRLLMEGYVGDVQVCDVRVYGPSLLDRAYGWTCEELMGGGGLHTVGSTI
IDLLSHLIGSRAHRVHGLLR
TFLQQSGSICGIRRVTSDDFCFFQMLMGESGSGHGPGAGSGGVCCTVTLNFNMPGAFTQEVMVVGSTGRLVARGTQLYGQ
RNGGDGEELL
>g1653.t2
LAVRRAAMLPGVGVFGTGSTARVLVPLLQAEGFEVHALWGQSEEEAGCLAKELGIPFHTSRSDDVLLHPDVHLVCIYIPP
PMTRQIAVNALGIGKNVVCE
KAATALDSFKMVTAARYYPQLLSIVGNTLRFLPTFVAMRRLLMEGYVGDVQVCDVRVYGPSLLDRAYGWTCEELMGGGGL
HTVGSTIIDLLSHLIGSRAH
RVHGLLRTFLQQSGSICGIRRVTSDDFCFFQMLMGESGSGHGPGAGSGGVCCTVTLNFNMPGAFTQEVMVVGSTGRLVAR
GTQLYGQRNGGDGEELL
>g1654.t1
MALRLLQLMALWTTLFGVARACPGACTCMDKYGNLQFADCAYKDLLLVPVGLPPNVTTLSLSANKIKLLKAKSFVNVTQV
TSLWLAHNEIVTIETNTLAP
LVQLRNLDVSHNKIVHFPWGDLHNLTGLQLLKMNNNEMTFLPKDAFSTLKELRSLRINHNQLTTILEDTFSALTSLSHLQ
IFDNPFSCSCSLEWVRDWVA
TTKIAIPDQNLILCAAPEDLKGIMVTKMPKLGCKPPTALITFQPNLKTSEIYEGFMVVLNCETKGIPKPEVTWEVNAGTQ
KFQFTIPSAMEESDDPINDQ

ATNSRFHVFQNGTLIVPRMSKKDDGNYSCMATNDLGKAESIVKLALAGTQKHTTNSMQDTTGTKSVTVDKPSHPKTSGNN
VINGPKSDGKTNTSPTSSSK
KPGAAGQVDGSGRDLTFSGKCGMSDGSEYISNHAFNLSLDDLKQYTFDFGVIALEVSETEAKVQLNPLQLPSSKSNLHLS
QTQNLETVNKEQFSLYQMAA
QKAPLDMLYLCIKTGNGHSMVQWSKIEEGVNAYRFHGLHPGTNYTLCLTYGGQDCQVQVVFTTRKKIPSLLIIVVVSTFL
LALATVPLLGATCCHLLYKY
QGKTYKLIMKAQNPDQMEKQIAGDFDPRASFVESEKNPEPSELGEGEGEGEADGEDVDGEGELEGSVATESIPGSSSKTN
QEEFEMGSEYSDRLPLGAEA
VNISEEINGNYKQPSR
>g1655.t1
MCLNSYCDTHLEPHKTISALKKHQLVDPMDHLEDRRCKKHNQLLELFCKTEQVCVCQICTETDHESHPIVPLKEEFEMKT
AQLGDIESEVQQMVQDRQHK
IQEIKDTLEFSKIDADREIADGDQVIERCKDHYNQMVKAKLKSVEKQAKGHIKELEQEVQDLTNRISQLKELSHTEDHLH
LLQTFRYLKNFPHTRDWTGV
EVCPLSYVETLRRSLGQLENTLNMEMKKLLDLKPALKKCKCS
>g1656.t1
MSRNTERVGVQPLTRQLTGGGGCVFFASVGGASCSPKLTGNIGFGSEEVKVMGSDGHRMFSVMKKGKPPGRDLSVVDLQR
LEKKEVVVVVVMGGGVKLQS
>g1657.t1
MSSGAVSPPAGCAASVDPLYFNLCDLGAVWGVAVEAVAVAGAVTSFILLVFHVGIRPFVTNGQRKHMLPLQATLTVCTLG
LFGLALAFITGRDFTTCAAR
RFVFGVLFAGCLVCLVMHGWWVTLLGSGKREVRCGVVYLGALALWLVEVVINTEWLTISLARIPPDALPPDLGVSCGVTN
QDFVMALIYVMVLLLAAVLM
ATLSLAQKDRQWRRDGVLLLVTGVLTAAIWGAWISMYLYGNQAVGSGAWDDATLAVALVSNGWVFLVSYVIPETCILNES
GAGPEDERPDGDQVVYPARS
SLDYGPAQSQVHQNLYLDNAAFSMDEPSAEPEQPGAADGSYVGQLRRGVYQPTEIALISKGLTYSDFHQNTGLQMSPDSL
SDDPLPSLAW
>g1657.t2
MSSGAVSPPAGCAASVDPLYFNLCDLGAVWGVAVEAVAVAGAVTSFILLVFHVGIRPFVTNGQRKHMLPLQATLTVCTLG
LFGLALAFITGRDFTTCAAR
RFVFGVLFAGCLVCLVMHGWWVTLLGSGKREVRCGVVYLGALALWLVEVVINTEWLTISLARIPPDALPPDLGVSCGVTN
QDFVMALIYVMVLLLAAVLM
ATLSLAQKDRQWRRDGVLLLVTGVLTAAIWGAWISMYLYGNQAVGSGAWDDATLAVALVSNGWVFLVSYVIPETCILNES
GAGPEDERPDGDQVVYPARS
SLDYGPAQSQVHQNLYLDNAAFSMDEPSAEPEQPGAADGSYVGQLRRGVYQPTEIALISKGLTY
>g1658.t1
MKVGCPLNDTRVYFAHLPGLVTHLLPSILAAALKQEVALETGGTTVLNHSMALLQRMESLMAAGNGSDVSLRVQTASTEE
VKVIQAHALVLTLQSDVFQE
LLLATGSEPGPLVVTETAECAAVFDKFVRYLYCGDISVRLDQAIPLHKLASKYHVAGLQQGLTQFMGQHLSSESPAGHVV
GWYQYAVQIGDESLRDSCLQ
YLSWNLSSVLQSAEWTSVGPDLLLSLLRRSDLVVQSELELYDALETWAARNGPSGATVERALEAIRYGMIPPQQLFRLQR
NSPLIRRHYESVRDLLYLAF
QFHSAPPVQLAKYFEVNCSIFTPRNYLSAPWGAPWVISSPTRDDRSFSFQTQLGPSGHDASKRVTWNALFSPRWFPLGTR
TTYAEPGAMQPSSTDGGRPR
IIVTPATSSPDFAGVSFQKTVMVMAKQQGKVVVRHVYNFHQSTEEAGDFLAEADLQRRVSEYLIDSSLYLHVVVKPVYQT
LIVAKK
>g1659.t1
MSARSSAAGGGDRATGERADELSVDSMSPSVAAALSSSMEMPRKRKGSMDNQDSKSASIPDADMEDEEEGSDGDDQQVKI
QCFREPHSQIEKRRRDKMNS
LIDELSAMIPTCNPMSRKLDKLTVLRMAVQHLKSLKGSSTSFSETSYKPSFLPDEELKQLVLKAADGFLFVVGCDRGKIV
FVSESITKILNYSRAELIGQ
SLFDYIHPKDMGKVKEQLSASELYPRERLIDAKTGLQVQADLPVGATRLCSGARRSFFCRMKYNKICVKVEKELQAATSK
KKETQKYCTVHCTGYMRSWP
TSHLGVEGECEADKESSHFSCLVAMGRVHSHAAPQTNGAVQVKPTEFITRYAMDGKFTFVDQRHEQSWTSQRGNKADQSS
YSKTSEGMSCNPGTMIYAGS
IGTQIANELLDYNRINSSPSSGNASPFSLLQDKSPQGHSQVNT

>g1659.t2
MSARSSAAGGGDRATGERADELSVDSMSPSVAAALSSSMEMPRKRKGSMDNQDSKSASIPDADMEDEEEGSDGDDQQVKIQCFREPHSQIEKRRRDKMNS
LIDELSAMIPTCNPMSRKLDKLTVLRMAVQHLKSLKGSSTSFSETSYKPSFLPDEELKQLVLKAADGFLFVVGCDRGKIVFVSESITKILNYSRAELIGQ
SLFDYIHPKDMGKVKEQLSASELYPRERLIDAKTGLQVQADLPVGATRLCSGARRSFFCRMKYNKICVKVEKELQAATSKKKETQKYCTVHCTGYMRSWP
TSHLGVEGECEADKESSHFSCLVAMGRVHSHAAPQTNGAVQVKPTEFITRYAMDGKFTFVDQRHEQSWTSQRGNKADQSSYSKTSEGMSCNPGTMIYAGS
IGTQIANELLDYNSGNASPFSLLQDKSPQGHSQVNT
>g1659.t3
MSPSVAAALSSSMEMPRKRKGSMDNQDSKSASIPDADMEDEEEGSDGDDQQVKIQCFREPHSQIEKRRRDKMNSLIDELSAMIPTCNPMSRKLDKLTVLR
MAVQHLKSLKGSSTSFSETSYKPSFLPDEELKQLVLKAADGFLFVVGCDRGKIVFVSESITKILNYSRAELIGQSLFDYIHPKDMGKVKEQLSASELYPR
ERLIDAKTGLQVQADLPVGATRLCSGARRSFFCRMKYNKICVKVEKELQAATSKKKETQKYCTVHCTGYMRSWPTSHLGVEGECEADKESSHFSCLVAMG
RVHSHAAPQTNGAVQVKPTEFITRYAMDGKFTFVDQRHEQSWTSQRGNKADQSSYSKTSEGMSCNPGTMIYAGSIGTQIANELLDYNRINSSPSSGNASP
FSLLQDKSPQGHSQVNT
>g1660.t1
SCSWAQGVSRPPSVLLPRALYDIITASDGSGLPRCTSFLPHMSVAWASSFRPLLSKMMTCTEQSLYYRQWTAPRAHHMDRTNRTEGRSDNFHPRRLLLSG
PPQVGKTGAYLHFLGILSRMLIRLMEVDIYDEDDIHC
>g1660.t2
SCSWAQGVSRPPSVLLPRALYDIITASDGSGLPRCTSFLPHMSVAWASSFRPLLSKMMTCTEQSLYYRQWTAPRAHHMDRTNRTEGRSDNFHPRRLLLSG
PPQLAVRSSTFMHSLLFVVQVGKTGAYLHFLGILSRMLIRLMEVDIYDEDDIHC
>g1660.t3
SCSWAQGVSRPPSVLLPRALYDIITASDGSGLPRCTSFLPHMSVAWASSFRPLLSKMMTCTEQSLYYRQWTAPRAHHMDRTNRTEGRSDNFHPRRLLLSG
PPQVGKTGAYLHFLGILSRMLIRLMEVDIYDEDDIHCSQYTHTHTRYQIEEI
>g1661.t1
MMPNRAVLGGGSSNNKVKYSRLAADEDGYIDLQFKRSPPKVPYKAIALATFLFVVGALLIIVGSLLLAEYIKVEHPDRTIPVLIIGVLVFLPGFYHLRIA
YYASKGYRGYSYDDIPDFGD
>g1662.t1
MEKHARYHAMHATSLHYTAGSDSSRGSTPTSRAVHKSTYCLLRHGIPLFLKGGPQVIEVLGYRVTSLYTVTQLNPIGFLWDSGLEKVRATPFERTHTPWL
TRDGDDDDDDDDDAWWCGQPSGDTL
>g1662.t2
MEKHARYHAMHATSLHYTAGSDSSRGSTPTSRAVHKSTYCLLRHGIPLFLKGGPQVIEVLGYRVTSLYTVTQLNPIGFLWDSGLEKVRATPFERTHTPWL
TRDGDDDDDDDDDAWWCGQVPLQVAYQADHANVSGFECGHFPLRTPRLQPRNGEVLLMNR
>g1662.t3
MEKHARYHAMHATSLHYTAGSDSSRGSTPTSRAVHKSTYCLLRHGIPLFLKGGPQVIEVLGYRVTSLYTVTQLNPIGFLWDSGLEKVRATPFERTHTPWL
TRDGDDDDDDDDDAWWCGQCP
>g1663.t1
MREMSPQQRLYCSRGTSPFTRSAGGGAPAAPRRPGIIAGVFQHGKVEIIANDQGNRTTPSYVAFTDSERLIGDAAKNQVAMNPTNTVFDAKRLIGRRFED
TVVQSDMKHWPFNVISDSGRPKVEVDYKGETKSFYPEEISSMVLVKMKEIAEAYLGKSVSNAVVTVPAYFNDSQRQATKDAGTISGLNVLRIINEPTAAA
IAYGLDKKVGSERNVLIFDLGGGTFDVSILTIEDGIFEVKSTAGDTHLGGEDFDNRMVNHFIGEFKRKYKKDISDNKRAVRRLRTACERAKRTLSSSTQA
SIEIDSLYEGVDFYTSIT

>g1663.t2
MSPQQRLYCSRGTSPFTRSAGGGAPAAPRRPGIIAGVFQHGKVEIIANDQGNRTTPSYVAFTDSERLIGDAAKNQVAMNP
TNTVFDAKRLIGRRFEDTVV
QSDMKHWPFNVISDSGRPKVEVDYKGETKSFYPEEISSMVLVKMKEIAEAYLGKSVSNAVVTVPAYFNDSQRQATKDAGT
ISGLNVLRIINEPTAAAIAY
GLDKKVGSERNVLIFDLGGGTFDVSILTIEDGIFEVKSTAGDTHLGGEDFDNRMVNHFIGEFKRKYKKDISDNKRAVRRL
RTACERAKRTLSSSTQASIE
IDSLYEGVDFYTSIT
>g1663.t3
MREMSPQQRLYCSRGTSPFTRSAGGGAPAAPRRPGIIAGVFQHGKVEIIANDQGNRTTPSYVAFTDSERLIGDAAKNQVA
MNPTNTVFDAKRLIGRRFED
TVVQSDMKHWPFNVISDSGRPKVEVDYKGETKSFYPEEISSMVLVKMKEIAEAYLGKVGSERNVLIFDLGGGTFDVSILT
IEDGIFEVKSTAGDTHLGGE
DFDNRMVNHFIGEFKRKYKKDISDNKRAVRRLRTACERAKRTLSSSTQASIEIDSLYEGVDFYTSIT
>g1664.t1
MSKGPAVGIDLGTTYSCVGVFQHGKVEIIANDQGNRTTPSYVAFTDSERLIGDAAKNQVAMNPTNTVFDAKRLIGRRFDD
AVVQSDMKHWPFEVVNDNTR
PKVQVEYKGETKSFYPEEVSSMVLVKMKEIAEAYLGKTVNNAVITVPAYFNDSQRQATKDAGTISGLNVLRIINEPTAAA
IAYGLDKKVGAERNVLIFDL
GGGTFDVSILTIEDGIFEVKSTAGDTHLGGEDFDNRMVNHFIAEFKRKYKKDISDNKRAVRRLRTACERAKRTLSSSTQA
SIEIDSLYEGVDFYTSITRA
RFEELNADLFRGTLDPVEKSLRDAKMDKAQVHDIVLVGGSTRIPKIQKLLQDFFNGKELNKSINPDEAVAYGAAVQAAIL
SGDKSENVQDLLLLDVTPLS
LGIETAGGVMTVLIKRNTTIPTKQTQTFTTYSDNQPGVLIQVYEGERAMTKDNNLLGKFELTGIPPAPRGVPQIEVTFDI
DANGIMNVSAADKSTGKENK
ITITNDKGRLSKEDIERMVQDAEKYKAEDDVQRDKVSSKNSLESYAFNMKSTVEDEKLAGKISDEDKQKIMEKCNEVISW
LDKNQTAEKEEYEHQQKELE
KVCNPIVTKLYQGAGGMPGGMPDGMPGGFPGAGGAAPGAGGSSGPTIEEVD
>g1664.t2
MACLAAWQNITLMKGSTLKLSCPLVDGDTNSGEWQSPLGVMFHNHTKGLRDKRYSIEHLSSTMFNVSVSDVTFKDGGVYK
CTQYSPVVTEKVVAVTVVDE
PQGSTGLLRSTTSWPRTDQSATVALTATSLFEASPEATTPGNAMSSMGQHLESMSYQQENERLSRDQGSGSLLIFLVTCL
IIALSVVVGFLAIKLRRAHI
HWKRGEDKPAFPLRNSYRTSHQPRVKYKHGKVEIIANDQGNRTTPSYVAFTDSERLIGDAAKNQVAMNPTNTVFDAKRLI
GRRFDDAVVQSDMKHWPFEV
VNDNTRPKVQVEYKGETKSFYPEEVSSMVLVKMKEIAEAYLGKTVNNAVITVPAYFNDSQRQATKDAGTISGLNVLRIIN
EPTAAAIAYGLDKKVGAERN
VLIFDLGGGTFDVSILTIEDGIFEVKSTAGDTHLGGEDFDNRMVNHFIAEFKRKYKKDISDNKRAVRRLRTACERAKRTL
SSSTQASIEIDSLYEGVDFY
TSITRARFEELNADLFRGTLDPVEKSLRDAKMDKAQVHDIVLVGGSTRIPKIQKLLQDFFNGKELNKSINPDEAVAYGAA
VQAAILSGDKSENVQDLLLL
DVTPLSLGIETAGGVMTVLIKRNTTIPTKQTQTFTTYSDNQPGVLIQVYEGERAMTKDNNLLGKFELTGIPPAPRGVPQI
EVTFDIDANGIMNVSAADKS
TGKENKITITNDKGRLSKEDIERMVQDAEKYKAEDDVQRDKVSSKNSLESYAFNMKSTVEDEKLAGKISDEDKQKIMEKC
NEVISWLDKNQTAEKEEYEH
QQKELEKVCNPIVTKLYQGAGGMPGGMPDGMPGGFPGAGGAAPGAGGSSGPTIEEVD
>g1664.t3
LAAWQNITLMKGSTLKLSCPLVDGDTNSGEWQSPLGVMFHNHTKGLRDKRYSIEHLSSTMFNVSVSDVTFKDGGVYKCTQ
YSPVVTEKVVAVTVVDEPQG
STGLLRSTTSWPRTDQSATVALTATSLFEASPEATTPGNAMSSMGQHLESMSYQQENERLSRDQGSGSLLIFLVTCLIIA
LSVVVGFLAIKLRRAHIHWK
RGEDKPAFPLRNSYRTSHQPRVKYKHGKVEIIANDQGNRTTPSYVAFTDSERLIGDAAKNQVAMNPTNTVFDAKRLIGRR
FDDAVVQSDMKHWPFEVVND
NTRPKVQVEYKGETKSFYPEEVSSMVLVKMKEIAEAYLGKTVNNAVITVPAYFNDSQRQATKDAGTISGLNVLRIINEPT
AAAIAYGLDKKVGAERNVLI
FDLGGGTFDVSILTIEDGIFEVKSTAGDTHLGGEDFDNRMVNHFIAEFKRKYKKDISDNKRAVRRLRTACERAKRTLSSS

TQASIEIDSLYEGVDFYTSI
TRARFEELNADLFRGTLDPVEKSLRDAKMDKAQVHDIVLVGGSTRIPKIQKLLQDFFNGKELNKSINPDEAVAYGAAVQA
AILSGDKSENVQDLLLLDVT
PLSLGIETAGGVMTVLIKRNTTIPTKQTQTFTTYSDNQPGVLIQVYEGERAMTKDNNLLGKFELTGIPPAPRGVPQIEVT
FDIDANGIMNVSAADKSTGK
ENKITITNDKGRLSKEDIERMVQDAEKYKAEDDVQRDKVSSKNSLESYAFNMKSTVEDEKLAGKISDEDKQKIMEKCNEV
ISWLDKNQTAEKEEYEHQQK
ELEKVCNPIVTKLYQGAGGMPGGMPDGMPGGFPGAGGAAPGAGGSSGPTIEEVD
>g1664.t4
MACLAAWQNITLMKGSTLKLSCPLVDGDTNSGEWQSPLGVMFHNHTKGLRDKRYSIEHLSSTMFNVSVSDVTFKDGGVYK
CTQYSPVVTEKVVAVTVVDE
PQGSTGLLRSTTSWPRTDQSATVALTATSLFEASPEATTPGNAMSSMGQHLESMSYQQENERLSRDQGSGSLLIFLVTCL
IIALSVVVGFLAIKLRRAHI
HWKRGEDKPAFPLRNSYRTSHQPRVKYKAGPLAAQSS
>g1665.t1
DEGCRGDPVCESWPCLNGGACHDQFNRFNCSCGPGWEGELCGQETDECSSAPCVHGACEDLLADYRCRCQPGYLGKDCRE
EVDNCLEFSCQNGGLCQEPT
RTCSCPAGFVGKRCQWRFPPVACDAHTECLNGGVCMGGPLGGNCTCKPGYIGPRCETDVDECQSDPCLNGATCLNRLNHF
QCVCVPGYSGRLCEGNKLEL
SEGVPWLVVAVPLTSLCVLLAILATFFLVLTARKKRQSEGTYSPSTQEVAGARLEMGSVLKVPPEERLI
>g1665.t2
MLEIILEYILEDILEYILEYILEYILECETDVDECQSDPCLNGATCLNRLNHFQCVCVPGYSGRLCEGNKLELSEGVPWL
VVAVPLTSLCVLLAILATFF
LVLTARKKRQSEGTYSPSTQEVAGARLEMGSVLKVPPEERLI
>g1666.t1
MCPPSEQGAYDGLPCCFYKEMKVHRVARYAEDYAVSVQYAHMDPDACSSQGCCELSQRIPIIPEDTDCESGAAAADTQGS
LLQANGTAEAEARPGRLQDD
REAGYLSSGQFIGQVHPHEEERRALFQPQCSASRNESGSSTFTSDGGL
>g1667.t1
MLSGSLEGQPAKERSILSVQHHIRQER
>g1668.t1
MHADDDSSETTSGICSSPAFSYTRVKITPVESHSEFVPVKYHKLSKMSDGDYDNESKLMRKAKENPFVPFGIAGLCAIVA
HRLWKMNSRGNTKMSLHLIH
MRVAAQGFVVGSMTLGVIYTMYRDFVVKPRDAQKALEPK
>g1668.t2
MHADDDSSETTSGICSSPAFSYTRVKITPVESHSEFVPVKYHKLSKMSDGDYDNESKLMRKAKENPFVPFAAQGFVVGSM
TLGVIYTMYRDFVVKPRDAQ
KALEPK
>g1668.t3
MHADDDSSETTSGICSSPAFSYTRVKITPVESHSEFVPVKYHKLSKMSDGDYDNESKLMRKAKENPFVPFG
>g1669.t1
MTEYRNQIQAAKQELKVAQKALLSEVGEEVNLQQLLSNPGSFRGRSLQILALQTRVRDLEQQLAASTQRRHPSVEEELLG
PRVLQKTSPQDKNLSHIRTM
EREKREAFERLTAEYDGLLEDHKETKKKLEASRARNTSLTAEMKTLKVQISTLLEKGKHDDELVDTLLKQLSRLQEVLAR
GEQQDAPQSGNTKAPLSNTD
ATPRNTLVQKLMQLVAEKEAKVKELEKEIQQLSLKRGDTQDLPAEGANGKIIISTRDQEMKVKCAEAVHGSQEARREAVT
LEQQLEKARLDPGKTSTRDA
SSRK
>g1669.t2
MTEYRNQIQAAKQELKVAQKALLSEVGEEVNLQQLLSNPGSFRGRSLQILALQTRVRDLEQQLAASTQRRHPSVEEELLG
PRVLQKTSPQDKNLSHIRTM
EREKREAFERLTAEYDGLLEDHKETKKKLEASRARNTSLTAEMKTLKVQISTLLEKGKHDDELVDTLLKQLSRLQEVLAR
GEQQDAPQSGNTKAPLSNTD
ATPRNTLVQKLMQLVAEKEAKVKELEKEIQQLSLKRGDTQDLPAEGANGKIIISTRPVSKHGHKLVSSNPGISVEFRDQE
MKVKCAEAVHGSQEARREAV
TLEQQLEKARLDPGKTSTRDASSRK

>g1669.t3
MTEYRNQIQAAKQELKVAQKALLSEVGEEVNLQQLLSNPGSFRGRSLQILALQTRVRDLEQQLAASTQRRHPSVEEELLG
PRVLQKTSPQDKNLSHIRTM
EREKREAFERLTAEYDGLLEDHKETKKKLEASRARNTSLTAEMKTLKVQISTLLEKGKHDDELVDTLLKQLSRLQEVLAR
GEQQDAPQSGNTKAPLSNTD
ATPRNTLVQKLMQLVAEKEAKVKELEKEIQQLSLKFLNMAINLCLQIQESVWSSADLSAIQSDYKALYNAARVERDRLSE
LVQALQEKDQEMKVKCAEAV
HGSQEARREAVTLEQQLEKARLDPGKTSTRDASSRK
>g1670.t1
MTKHPTPSLCGVARDKAELFRERYTILQQLKTVEALLGSTAKLGEVIVLGMITQLKEGKFFLEDLTGTIQLDMSKAISFF
PHSAYYGNLNFFGGPSTTSV
KVSAKLKQLEDENEDAMFVVLSDVWLDSVEVMEKINTMFS
>g1670.t2
MTKHPTPSLCGVARDKAELFRERYTILQQLKTVEALLGSTAKLGEVIVLGMITQLKEFHNGLYTESCFVLAEGWYEDSVF
HVNAFGFPPTEPSSMTRAYY
GNLNFFGGPSTTSVKVSAKLKQLEDENEDAMFVVLSDVWLDSVEVMEKINTMFS
>g1671.t1
MDERQRSERVAQERSGHTAVVQDNLLFVWGGYVVDDVYWGWNNEVHVFHPKHASWTEPQTNGRPPAPRAAHASTTLGSKG
YVCGGRVMGHCNDALVFQTE
PYPLFRSCVDFICKNISTCKLLGDQVPLLPSKLQHIVHSRMSYYRQPKSNTTLG
>g1671.t2
MDERQRSERVAQERSGHTAVVQDNLLFVWGGYVVDDVYWGWNNEVHVFHPKHASWTEPQTNGRPPAPRAAHASTTLGSKG
YVCGGRVMSPTLGCSSRQGH
CNDALVFQTEPYPLFRSCVDFICKNISTCKLLGDQVPLLPSKLQHIVHSRMSYYRQPKSNTTLG
>g1671.t3
MDERQRSERVAQERSGHTAVVQDNLLFVWGGYVVDDVYWGWNNEVHVFHPKHASWTEPQTNGRPPAPRAAHASTTLGSKG
YVCGGRVMSPTLGCSSRQVL
AFTHCGF
>g1672.t1
MLPLLQVFPVRSRTYGLCSFKRAHSSTELNATTTPSQAFTASESRGQECGSEDVNKNGNSVFSHVRKHAPACSNHEHRGL
GPSRSCVEQVSKTGNDSTFA
NCLNTPVTTPVILYDGSEEELMDTIERDFS
>g1672.t2
MLPLLQVFPVRSRTYGLCSFKRAHSSTELNATTTPSQAFTASESRGQECGSEDVNKNGNSVFSHVRKHAPACSNHEHRVS
KTGNDSTFANCLNTPVTTPV
ILYDGSEEELMDTIERDFS
>g1672.t3
MLPLLQVFPVRTYGLCSFKRAHSSTELNATTTPSQAFTASESRGQECGSEDVNKNGNSVFSHVRKHAPACSNHEHRGLGP
SRSCVEQVSKTGNDSTFANC
LNTPVTTPVILYDGSEEELMDTIERDFS
>g1673.t1
LTNGETFNATVQDVDQVADIATIKITAKHPLPILALGRSSDIRQGEFVVAMGSPFALRNTITSGIVSSAQRGSKELGLSN
SNMDYIQTDAAIDDGEVIGI
NTMKVTAGISFAIPSDRLKVFLDRSAEQKKSRFGESDNRRRYIGVMMLTLTPSIIAELKLRDSSFPDLTHGILIHRVITG
SPANKAGMQPGDVVVEIDGT
RVNTSEEIYQAIRSSNTISMLVQRGQDLLRLQITPEYAE
>g1673.t2
MFLTQDLRQGKQEGVTVVVLRVPVSNGSGFIISSDGLIVTNAHVVANKRGVRVKLTNGETFNATVQDVDQVADIATIKIT
AKHPLPILALGRSSDIRQGE
FVVAMGSPFALRNTITSGIVSSAQRGSKELGLSNSNMDYIQTDAAIDDGEVIGINTMKVTAGISFAIPSDRLKVFLDRSA
EQKKSRFGESDNRRRYIGVM
MLTLTPSIIAELKLRDSSFPDLTHGILIHRVITGSPANKAGMQPGDVVVEIDGTRVNTSEEIYQAIRSSNTISMLVQRGQ
DLLRLQITPEYAE
>g1674.t1
MSPAKMFTHMKERKRQQQQEAGDFGCNTKELRNAGHRVPSQKRPTPTDSDQEEIDHGTCMDAFVRPPERELPSHRFQSKV
AADPSESSRKDAVPFPAPKP

QPHLAEDPLILHSPHVLFPKKPRQDPAPSEQTRPQGHGCKLKQWKIKFTQEGLYVDGIRTSDNIPWHSSIIVDRISKSVL
KTRVGMKTSRSGRMLKPPLE
FWKGGRVIVDASMNVTILEDYETTVYMTVGVTVSPTEKPGGPMGLILLTAVLALCSSWE
>g1674.t2
MSPAKMFTHMKERKRQQQQEAGDFGCNTKELRNAGHRVPSQKRPTPTDSDQEEIDHGTCMDAFVRPPERELPSHRFQSKV
AADPSESSRKDAVPFPAPKP
QPHLAEDPLILHSPHVLFPKKPRQDPAPSEQTRPGHGCKLKQWKIKFTQEGLYVDGIRTSDNIPWHSSIIVDRISKSVLK
TRVGMKTSRSGRMLKPPLEF
WKGGRVIVDASMNVTILEDYETTVYMTVGVTVSPTEKPGGPMGLILLTAVLALCSSWE
>g1674.t3
MSPAKMFTHMKERKRQQQQEAGDFGCNTKELRNAGHRVPSQKRPTPTDSDQEEIDHGTCMDAFVRPPERELPSHRFQSKV
AADPSESSRKDAVPFPAPKP
QPHLAEDPLILHSPHVLFPKKPRQDPAPSEQTRPQGHGCKLKQWKIKFTQEGLYVDGIRTSDNIPWHSSIIVDRISKSVL
KTRVGMKTSRSGRMLKPPLE
FWKGGRVIVDASMNVTILEDYETTVYMTVGVTVSPTEKPGGPMGLILLTAVLALCSSWDVHDPFFAYVHYQKPTTLDNIV
DSQPPKQHGGVVRRTEAPQR
SELTGRPAVDPQPSTLQRKVKPVRRNRKAKTHEPPICPPEKAQPLDASSGSSSPEDEEHVPRRTSKKPPAVREAAPQTGR
NARRQDAPKCRFVDTGSEGR
NAVGDAGRVPKTALPRERNIPATREPAAAHSASSLSDDDGPVSRRKRGVHQSVLDESQSSQSLSMSDSSYTVSRTDKGKV
TARKDKGKVTARKDKGKVAT
RKGKGKVATRKDKVKEMSRPPKPAPVPVCAPAQASRKGDRSTAKRSILQEEDEPWTEAELAKLREAVASFPKHLGCYWVN
VAMMVGTRSPEECQMQHAFQ
GSVKASSKKATAPKKTKESTTDQ
>g1674.t4
LTGRPAVDPQPSTLQRKVKPVRRNRKAKTHEPPICPPEKAQPLDASSGSSSPEDEEHVPRRTSKKPPAVREAAPQTGRNA
RRQDAPKCRFVDTGSEGRNA
VGDAGRVPKTALPRERNIPATREPAAAHSASSLSDDDGPVSRRKRGVHQSVLDESQSSQSLSMSDSSYTVSRTDKGKVTA
RKDKGKVTARKDKGKVATRK
GKGKVATRKDKVKEMSRPPKPAPVPVCAPAQASRKGDRSTAKRSILQEEDEPWTEAELAKLREAVASFPKHLGCYWVNVA
MMVGTRSPEECQMQHAFQGS
VKASSKKATAPKKTKESTTDQ
>g1675.t1
GKKSEDDGIEKENLAILEKIRKNQRQDHLNECHFDKIHG
>g1676.t1
SESPEEESGTGVLGRIGSWFSPWRTQIPRDLKTENATPTGELPPGPDTDAGEAGEEKAESVRAPRESWQREVGTRTRSPN
PERSVLSRDIFSSEKGDATQ
SARKGGPFLSTDWEPAERGPKEEEFLDAREFLEGRRGQDWASCSSGSSSSSGGSSSSSSSRKTVWGDPASENRACDLTRL
SHARVQGVVGKADAVHTQAQ
RQAHNQGGKKLHVYLEEETSVINSENDPCAGQEVVRTRLEKSFHVIAKAKSLDALDSPRNSSQEQWQIRRRGRLSTPAEL
AFTPPSVTDRASPEPEAEEA
ESNRMGRKNTSRRKSRKQSQGDAESSSQENTPTKAQDASGGTLLTGAQAPGVETHLGEAAGNSAPTQSLSGGGKNKTSPT
AIQRQLPQSKQSKGYQGTFS
TGPTDTTWAAVDGEGAMDDEYKLERKTETPESKRRSMKVSKSEVKMFPKKVLVNADPQSGSINGNASQNFQSGLSRRRDP
DKDICETEDFTGKRTLGKLD
EEPKHVVSGGIADKINAFEAQRPGAGKKTFHNPRSADASPVSTISRSLVSTFEEGGSRSKSVERLGDGSWLDVDFPKQRL
RIPDQPPRLSYSSSESNLLD
TAGELGDDDFVEKIKNLCAPFSLPPRKHSHLRPPQPPFAMPAIREDRFEKTFDPDEFKFGLRKKSIRSGPSLLAKLQSNE
TKANLKPARASIVDRCMLLT
SMDTHSRLKERASAHEEMDEDEEEDNTTTTASEKEEKKEETKVRSRLEGSSILSSLCASSARGKRTGLEALPLSGMVSPS
PQTSLSPEASPPPTLPSPTS
PAPLADALDSHRPTALAGSSETQAGVVVVGESAPLFPSFNDIKQPDYLEKYLPREPEKPERGKAGKEQVNTEVSFVVKTL
FPWLMPALCNAFTALLSFS
>g1676.t2
LGRIGSWFSPWRTQIPRDLKTENATPTGELPPGPDTDAGEAGEEKAESVRAPRESWQREVGTRTRSPNPERSVLSRDIFS
SEKGDATQSARKGGPFLSTD
WEPAERGPKEEEFLDAREFLEGRRGQDWASCSSGSSSSSGGSSSSSSSRKTVWGDPASENRACDLTRLSHARVQGVVGKA

DAVHTQAQRQAHNQGGKKLH
VYLEEETSVINSENDPCAGQEVVRTRLEKSFHVIAKAKSLDALDSPRNSSQEQWQIRRRGRLSTPAELAFTPPSVTDRAS
PEPEAEEAESNRMGRKNTSR
RKSRKQSQGDAESSSQENTPTKAQDASGGTLLTGAQAPGVETHLGEAAGNSAPTQSLSGGGKNKTSPTAIQRQLPQSKQS
KGYQGTFSTGPTDTTWAAVD
GEGAMDDEYKLERKTETPESKRRSMKVSKSEVKMFPKKVLVNADPQSGSINGNASQNFQSGLSRRRDPDKDICETEDFTG
KRTLGKLDEEPKHVVSGGIA
DKINAFEAQRPGAGKKTFHNPRSADASPVSTISRSLVSTFEEGGSRSKSVERLGDGSWLDVDFPKQRLRIPDQPPRLSYS
SSESNLLDTAGELGDDDFVE
KIKNLCAPFSLPPRKHSHLRPPQPPFAMPAIREDRFEKTFDPDEFKFGLRKKSIRSGPSLLAKLQSNETKANLKPARASI
VDRCMLLTSMDTHSRLKERA
SAHEEMDEDEEEDNTTTTASEKEEKKEETKVRSRLEGSSILSSLCASSARGKRTGLEALPLSGMVSPSPQTSLSPEASPP
PTLPSPTSPAPLADALDSHR
PTALAGSSETQAGVVVVGESAPLFPSFNDIKQPDYLEKYLPREPEKPERGKAGKEQVNTEVSFVVKTLFPWLMPALCNAF
TALLSFS
>g1676.t3
SESPEEESGTGVLGRIGSWFSPWRTQIPRDLKTENATPTGELPPGPDTDAGEAGEEKAESVRAPRESWQREVGTRTRSPN
PERSVLSRDIFSSEKGDATQ
SARKGGPFLSTDWEPAERGPKEEEFLDAREFLEGRRGQDWASCSSGSSSSSGGSSSSSSSRKTVWGDPASENRACDLTRL
SHARVQGVVGKADAVHTQAQ
RQAHNQGGKKLHVYLEEETSVINSENDPCAGQEVVRTRLEKSFHVIAKAKSLDALDSPRNSSQEQWQIRRRGRLSTPAEL
AFTPPSVTDRASPEPEAEEA
ESNRMGRKNTSRRKSRKQSQGDAESSSQENTPTKAQDASGGTLLTGAQAPGVETHLGEAAGNSAPTQSLSGGGKNKTSPT
AIQRQLPQSKQSKGYQGTFS
TGPTDTTWAAVDGEGAMDDEYKLERKTETPESKRRSMKVSKSEVKMFPKKVLVNADPQSGSINGNASQNFQSGLSRRRDP
DKDICETEDFTGKRTLGKLD
EEPKHVVSGGIADKINAFEAQRPGAGKKTFHNPRSADASPVSTISRSLVSTFEEGGSRSKSVERLGDGSVSKACNAENVP
ENLLPCDFEGRVKAIPTGHS
GQTGCQ
>g1677.t1
WLKLTSDDVKEQIFKLAKKGLTPSQIGVILRDSHGVAQVRFVTGNKILRILKSKGLAPDLPEDLYHLIKKAVAVRKHLER
NRKDRDAKFRLILVESRIHR
LARYYKTKRVLAPNWK
>g1677.t2
LKLTSDDVKEQIFKLAKKGLTPSQIGVILRDSHGVAQVRFVTGNKILRILKSKGLAPDLPEDLYHLIKKAVAVRKHLERN
RKDRDAKFRLILVESRIHRL
ARYYKTKRVLAPNWK
>g1677.t3
LYNPIYRKGLSQSALPYRRSVPTWLKLTSDDVKEQIFKLAKKGLTPSQIGVILRDSHGVAQVRFVTGNKILRILKSKGLA
PDLPEDLYHLIKKAVAVRKH
LERNRKDRDAKFRLILVESRIHRLARYYKTKRVLAPNWK
>g1678.t1
LRITDESGQHVQHIGTTTNPTDSFLFSSLFDGDNGVMVGGSNPLSNFCADIGDNFPGGATAEALLSSLRGDDLCCGLVDD
FVSIASKEDGLFVGNHPSWK
MGFSGDWNIFASGTDTADSSQKSTGKVLKLDLSEEERSPHWSSEEDQNQGDFDSEESRALWESLSKSSDPYNPFFFSACV
STNTNMGKTKQEVRSASDTE
SPSTAKKGEEIAGPPGLNIWVSRSDSESSWGSSDGSCADLDREESERLWEFFSKPLNPYNPMFVTACTASENPPPTHTTP
AANLRQASCRTSSTVVALCS
PQAEHTATPTSLSPWWSSSSEDEEDQLWKSLCQSGDPYHPLNFRACLNSSDTMLMPKPKHRGDLPDQSKIHPKKSTPSSE
EEEEEEEEEEKKEKKKKTVV
LKQTTIPRPVLPEHPQVCHHHLQSTCLPLKRTTGPPDPSAPEQGHATTIRTTTTNSHKQVRFSPVVRVHVMHTWPFARQA
CRKGPWEEMVRDRDRFGRRI
ADVEKAIGYCFTQQHRERIRTNLESSFI
>g1678.t2
LFRFEVHLRITDESGQHVQHIGTTTNPTDSFLFSSLFDGDNGVMVGGSNPLSNFCADIGDNFPGGATAEALLSSLRGDDL
CCGLVDDFVSIASKEDGLFV

GNHPSWKMGFSGDWNIFASGTDTADSSQKSTGKVLKLDLSEEERSPHWSSEEDQNQGDFDSEESRALWESLSKSSDPYNP
FFFSACVSTNTNMGKTKQEV
RSASDTESPSTAKKGEEIAGPPGLNIWVSRSDSESSWGSSDGSCADLDREESERLWEFFSKPLNPYNPMFVTACTASENP
PPTHTTPAANLRQASCRTSS
TVVALCSPQAEHTATPTSLSPWWSSSSEDEEDQLWKSLCQSGDPYHPLNFRACLNSSDTMLMPKPKHRGDLPDQSKIHPK
KSTPSSEEEEEEEEEEEKKE
KKKKTVVLKQTTIPRPVLPEHPQVCHHHLQSTCLPLKRTTGPPDPSAPEQGHATTIRTTTTNSHKQVRFSPVVRVHVMHT
WPFARQACRKGPWEEMVRDR
DRFGRRIADVEKAIGYCFTQQHRERIRTNLESSFI
>g1679.t1
WPPGDPGTPCQGLQHLDGFPGTQKYQPATGTHCPHQAPWHSFKLCAEAAELGCRSAVPPAPLTLRGMEERGGGVPPSDHM
SHL
>g1680.t1
MSVLMYIGNFPQPQKEARKPAAMGWVVFYCSSMEITGNLWPTAPDDKATREQGVSPAELLMGRKIRTTFPILQDNLRPNW
PDEDVIRQADAAAKQRQAYF
YNHRNGLQLRSKIL
>g1680.t2
MCYRGEYMSVLMYIGNFPQPQKEARKPAAMGWVVFYCSSMEITGNLWPTAPDDKATREQGVSPAELLMGRKIRTTFPILQ
DNLRPNWPDEDVIRQADAAA
KQRQAYFYNHRNGLQLRSKIL
>g1680.t3
MYIGNFPQPQKEARKPAAMGWVVFYCSSMEITGNLWPTAPDDKATREQGVSPAELLMGRKIRTTFPILQDNLRPNWPDED
VIRQADAAAKQRQAYFYNHR
NGLQLRSKIL
>g1681.t1
MDGEDDKNSVDIHDNPPAPDNVVREEGDTVYFIYDEEVEVEEKEPEPPPEPVVMVNDKPHKFKDHYCKKPKFCDVCARMI
VLNNKFALRCKNCKTNIHHS
CQSYVEFQKCFGKIPPGFRRAYSSPLYSSDQPDPSNTNRNDPVFDTLRVGVIMANKERKKNDNDKKNMMMMMEEEEEEGQ
QPKENEEGGEGKPEDKKEKG
GDKADEKSKGTFSQSHYYLALYRFKAIEKDDLDFHPGDRIIVLDDSNEEWWRGKMGEKTGYFPTNYLIKVRAAERVYKVT
RSFVGNREMGQITLKKDQIV
VKKGEEKGGYLKVSTGRKLGFFPADLLEEIITVQ
>g1681.t2
EGSYRTMDGEDDKNSVDIHDNPPAPDNVVREEGDTVYFIYDEEVEVEEKEPEPPPEPVVMVNDKPHKFKDHYCKKPKFCD
VCARMIVLNNKFALRCKNCK
TNIHHSCQSYVEFQKCFGKIPPGFRRAYSSPLYSSDQPDPSNTNRNDPVFDTLRVGVIMANKERKKNDNDKKNMMMMMEE
EEEEGQQPKENEEGGEGKPE
DKKEKGGDKADEKSKGTFSQSHYYLALYRFKAIEKDDLDFHPGDRIIVLDDSNEEWWRGKMGEKTGYFPTNYLIKVRAAE
RVYKVTRSFVGNREMGQITL
KKDQIVVKKGEEKGGYLKVSTGRKLGFFPADLLEEIITVQ
>g1682.t1
MQVGRFPTGLCVKGSCPLPRCCPSHQAPPRALQTLVELDVHTLTTICDLYTMPRVAEPVWLTMRSVAPEKQLLCGHFMRE
LALLVEQASKNQLSGI
>g1683.t1
RSALVKLSWLRDLKELQQHSGLPSCSPPVFEMLLSDVHTKLGQIIKLTCRVTGSPAPVVSWLKDGLHLDEDPCHIITAQR
TGTCSLVLDGLTAEDSGQYV
CYATSSVGNASTLARVTVEAPPRFLSRLESMCLMEGEDLQFICSTLTTPLPTVRWWKDGGELTDQSKYRISNDARSGILT
LTIISPTVADIGLYECELWN
QMGCARCKAGLCPAYVPSIDSENVQPQDLSPKAPPFIQVPLESLCVEPGQSALFTAIITGRPAPCVQWYKDEEELSANDN
VEMKQQGARCSLTVLCPEGE
DSGVYTCFAYNDSGHTSCQAQLTVEEGPLEVEEREVELGRRRKLFSVYDIQEEVGRGTFGVVKRVVHRRTGEGFAAKFLP
LRSSTRTRAFQERDLLSRLA
HPRVACLLDFFSTRRTLVLVTEVQSYIQQILEGIGHIHSMNILHLDLKPDNILMVYPPRDEIKICDFGFSQEMDASRHQY
SKFGTPEFVAPEIVHQEPVT
GATDVWSLGVIAFLCLTGRCPFAGESDRATLLKVGEGTLNWETPDVTGRSPEAQDFLRLVLQPEPEYLSFFSRSLTCIGS
VLTLRPIPELLEAPLRETAA

TVQRDPRDNSSTSVSSGSSSEYDEADSWDFFQHASPTEEEEEEEEEEEGYDPLTERARIPMPFIKCHFGSEDEEEEMLGDE
EEEEEDGEMAQSVLERSISK
QSLGSSEMSCQRTPVRERRVSRDSSPSLYLSEGDEGSGSDGSRIPRGSVIRSTFYNSSQQLSPMSARHMSLRDKFQVKKQ
ERGRKPMRRSLSGRLNEPLI
EYVEDETETTRGQRRTSVQPPMQKSCSFDSGVSLSHTNLPPHRRSRSLDECSRRSPGSPKRSLEPSEEEGTQSMKEDFTD
DEVVQKNLLAPPVPCRTSRR
RGSVAPVQGTLRLGGALVSSSLHAGDRRPLMGDRSPLMGDRSPLMGERSPLMGDRSPLIGRPPVEAGNLTGSHSSLAESF
IMEHGDSESSSRLDSCEEPC
PVLSAARFRPGQRYGEQHRPLTVPRSLQFSEVDSKPKSASPLPPPRTRTRSHPNLAASSSSIHRASSGVQPSPSPSLSSL
QAPDRPPRSKDKKGSQGLQR
HASAPALEIKIPQGKSPKLGLLKIFRRQSWTSNSYSQLENPELGPTLGEIMKPDTPTMSLRKKMRASASSLTKLFSKSSS
KEDLAKGGLPVRPNRPDVVQ
LDGGGVLLVWKPVQSIDPAVYCIQYCTDGAEWRALTEEVTDNILVVKDLPRGASYVFRTDSPDGRVIAGKLPLHKTYSFL
SEINR
>g1683.t2
RSALVKLSWLRDLKELQQHSGLPSCSPPVFEMLLSDVHTKLGQIIKLTCRVTGSPAPVVSWLKDGLHLDEDPCHIITAQR
TGTCSLVLDGLTAEDSGQYV
CYATSSVGNASTLARVTVEAPPRFLSRLESMCLMEGEDLQFICSTLTTPLPTVRWWKDGGELTDQSKYRISNDARSGILT
LTIISPTVADIGLYECELWN
QMGCARCKAGLCPAYVPSIDSENVQPQDLSPKAPPFIQVPLESLCVEPGQSALFTAIITGRPAPCVQWYKDEEELSANDN
VEMKQQGARCSLTVLCPEGE
DSGVYTCFAYNDSGHTSCQAQLTVEEGPLEVEEREVELGRRRKLFSVYDIQEEVGRGTFGVVKRVVHRRTGEGFAAKFLP
LRSSTRTRAFQERDLLSRLA
HPRVACLLDFFSTRRTLVLVTEVQSYIQQILEGIGHIHSMNILHLDLKPDNILMVYPPRDEIKICDFGFSQEMDASRHQY
SKFGTPEFVAPEIVHQEPVT
GATDVWSLGVIAFLCLTGRCPFAGESDRATLLKVGEGTLNWETPDVTGRSPEAQDFLRLVLQPEPEYLSFFSRSLTCIGS
VLTLRPIPELLEAPLRETAA
TVQRDPRDNSSTSVSSGSSSEYDEADSWDFFQHASPTEEEEEEEEEEEGYDPLTERARIPMPFIKCHFGSEDEEEEMLGDE
EEEEEDGEMAQSVLERSISK
QSLGSSEMSCQRTPVRERRVSRDSSPSLYLSEGDEGSGSDGSRIPRGSVIRSTFYNSSQQLSPMSARHMSLRDKFQVKKQ
ERGRKPMRRSLSGRLNEPLI
EYVEDETETTRGQRRTSVQPPMQKSCSFDSGVSLSHTNLPPHRRSRSLDECSRRSPGSPKRSLEPSEEEGTQSMKEDFTD
DEVVQKNLLAPPVPCRTSRR
RGSVAPVQGTLRLGGALVSSSLHAGDRRPLMGDRSPLMGDRSPLMGERSPLMGDRSPLIGRPPVEAGNLTGSHSSLAESF
IMEHGDSESSSRLDSCEEPC
PVLSAARFRPGQRYGEQHRPLTVPRSLQFSEVDSKPKSASPLPPPRTRTRSHPNLAASSSSIHRASSGVQPSPSPSLSSL
QAPDRPPRSKDKKGSQGLQR
HASAPALEIKIPQGKSPKLGLLKIFRRQSWTSNSYSQLENPELGPTLGEIMKPDTPTMSLRKKMRASASSLTKLFSKSSS
KEDLAKGGLPVRPNRPDVVQ
LDGGGVLLVWKPVQSIDPAVYCIQYCTDGAEWRALTEEVTDNILVVKDLPRGASYVFRTDSPDGRVIAGKLPLHKTYSFL
SEIN
>g1683.t3
RSALVKLSWLRDLKELQQHSGLPSCSPPVFEMLLSDVHTKLGQIIKLTCRVTGSPAPVVSWLKDGLHLDEDPCHIITAQR
TGTCSLVLDGLTAEDSGQYV
CYATSSVGNASTLARVTVEAPPRFLSRLESMCLMEGEDLQFICSTLTTPLPTVRWWKDGGELTDQSKYRISNDARSGILT
LTIISPTVADIGLYECEMGC
ARCKAGLCPAYVPSIDSENVQPQDLSPKAPPFIQVPLESLCVEPGQSALFTAIITGRPAPCVQWYKDEEELSANDNVEMK
QQGARCSLTVLCPEGEDSGV
YTCFAYNDSGHTSCQAQLTVEEGPLEVEEREVELGRRRKLFSVYDIQEEVGRGTFGVVKRVVHRRTGEGFAAKFLPLRSS
TRTRAFQERDLLSRLAHPRV
ACLLDFFSTRRTLVLVTEVQSYIQQILEGIGHIHSMNILHLDLKPDNILMVYPPRDEIKICDFGFSQEMDASRHQYSKFG
TPEFVAPEIVHQEPVTGATD
VWSLGVIAFLCLTGRCPFAGESDRATLLKVGEGTLNWETPDVTGRSPEAQDFLRLVLQPEPEYLSFFSRSLTCIGSVLTL
RPIPELLEAPLRETAATVQR
DPRDNSSTSVSSGSSSEYDEADSWDFFQHASPTEEEEEEEEEEEGYDPLTERARIPMPFIKCHFGSEDEEEEMLGDEEEEE
EDGEMAQSVLERSISKQSLG

SSEMSCQRTPVRERRVSRDSSPSLYLSEGDEGSGSDGSRIPRGSVIRSTFYNSSQQLSPMSARHMSLRDKFQVKKQERGR
KPMRRSLSGRLNEPLIEYVE
DETETTRGQRRTSVQPPMQKSCSFDSGVSLSHTNLPPHRRSRSLDECSRRSPGSPKRSLEPSEEEGTQSMKEDFTDDEVV
QKNLLAPPVPCRTSRRRGSV
APVQGTLRLGGALVSSSLHAGDRRPLMGDRSPLMGDRSPLMGERSPLMGDRSPLIGRPPVEAGNLTGSHSSLAESFIMEH
GDSESSSRLDSCEEPCPVLS
AARFRPGQRYGEQHRPLTVPRSLQFSEVDSKPKSASPLPPPRTRTRSHPNLAASSSSIHRASSGVQPSPSPSLSSLQAPD
RPPRSKDKKGSQGLQRHASA
PALEIKIPQGKSPKLGLLKIFRRQSWTSNSYSQLENPELGPTLGEIMKPDTPTMSLRKKMRASASSLTKLFSKSSSKEDL
AKGGLPVRPNRPDVVQLDGG
AEWRALTEEVTDNILVVKDLPRGASYVFRTDSPDGRVIAGKLPLHKTYSFLSEIN
>g1684.t1
VLISVLDNNDNAPVFSQPTYDIAISEDTPPDTEVVQVLALDRDEHHRLAYSLHGAVDPGSLRLFRIDPALGSVYTAERLD
HEARAQHILTVMVKDQEFPY
RRGLARVLIEVEDVNDHVPLFTSTLYEGSVYESAAVGSAVVQVTALDKDKGANAELHYAIEGGNAGNAFRIEPVLGIISV
ARDLDLSSIGHYVLTVRVTD
NGSPSLSTTTLVRISVTLSDNAGPKFPQPEYQAEIVENVAVGTSVTTINAVSQSTLTYDIRQGNSQRKFQINQYSGVITT
HKPLDYETQTSYTLTVQATN
MAGMASNATLHIQVVDENDNQPVFQHLSYRGSISEAAQVNSVVLNSDESPLVIKATDADHNQNALLVYQIVEDTAKMFFT
VDSGTGSIRTIANLDHETFS
SFHFHVHVRDSGRPQLTADSPTEVTIEVIDTNDSPPRFTQDAYEAVLLLPTYVGVEALKVSAIDPDQNVPTELTYSLTDG
ILEHFAIESSCGIITVKNNN
FSKERFRFNVKVSDALQVIATDADSEKNSLVRYQIFSDMHNSTDYFHIDSSSGLILTARMLDHEVVQSYDFTVRATDNGF
PPLRSEVSVVVVVNDMNDNP
PVFNQLLYEAYVSELATRGHFVTCVQASDADSSDSDKLVYSILSGNERMNFLIDMKTGIITLSGHRKQRMEPAYSLNVSV
SDGVFTSTAQVHVKVLGANL
HSPLFGLSTYDAELRENAAIGTKVIQVKATDADPGVFGKITYSFVNDVGKDQFSIDGYGFITTVEKLDREDPANKDIILA
VEAQDSGGRASYCTVQVTLL
DENDNAPQFRATEYRASVTSDVAKGFLVTQIQAYDPDNGVNAKVTYSLYSEAHVPVVDILEIDPDNGWMVTKGSFSHMRN
SVLSFFVKAVDSGNPVRHSL
VSVYIHVLAPDALIPSFTQPQYSFTMPEDTPIGASIGMVRLNTPLGHATLSAAFALVNGETGENNKDAVLVVEKDTGIIK
LEKPLDHEVIKAFYFKVTAT
VQQAKLDSVTTVDVELKVLDLNDNKPAFESNSYEATTMEGMPVGTRIIQVHALDPDSGANGQVTYSLGTAIQPAAVERDP
LAGVFSVDSNSGWISTLKQL
DHETNPSYTFTVVAYDLGENLALSATTSVTVVVTDVNDNPPTFLEERYHGSVRESDPPGEVVAVLNTKDDDSSPINRQVS
YHITGE
>g1684.t2
VLISVLDNNDNAPVFSQPTYDIAISEDTPPDTEVVQVLALDRDEHHRLAYSLHGAVDPGSLRLFRIDPALGSVYTAERLD
HEARAQHILTVMVKDQEFPY
RRGLARVLIEVEDVNDHVPLFTSTLYEGSVYESAAVGSAVVQVTALDKDKGANAELHYAIEGGNAGNAFRIEPVLGIISV
ARDLDLSSIGHYVLTVRVTD
NGSPSLSTTTLVRISVTLSDNAGPKFPQPEYQAEIVENVAVGTSVTTINAVSQSTLTYDIRQGNSQRKFQINQYSGVITT
HKPLDYETQTSYTLTVQATN
MAGMASNATLHIQVVDENDNQPVFQHLSYRGSISEAAQVNSVVLNSDESPLVIKATDADHNQNALLVYQIVEDTAKMFFT
VDSGTGSIRTIANLDHETFS
SFHFHVHVRDSGRPQLTADSPTEVTIEVIDTNDSPPRFTQDAYEAVLLLPTYVGVEALKVSAIDPDQNVPTELTYSLTDG
ILEHFAIESSCGIITVKNNN
FSKERFRFNVKVSDVQSYDFTVRATDNGFPPLRSEVSVVVVVNDMNDNPPVFNQLLYEAYVSELATRGHFVTCVQASDAD
SSDSDKLVYSILSGNERMNF
LIDMKTGIITLSGHRKQRMEPAYSLNVSVSDGVFTSTAQVHVKVLGANLHSPLFGLSTYDAELRENAAIGTKVIQVKATD
ADPGVFGKITYSFVNDVGKD
QFSIDGYGFITTVEKLDREDPANKDIILAVEAQDSGGRASYCTVQVTLLDENDNAPQFRATEYRASVTSDVAKGFLVTQI
QAYDPDNGVNAKVTYSLYSE
AHVPVVDILEIDPDNGWMVTKGSFSHMRNSVLSFFVKAVDSGNPVRHSLVSVYIHVLAPDALIPSFTQPQYSFTMPEDTP
IGASIGMVRLNTPLGHATLS
AAFALVNGETGENNKDAVLVVEKDTGIIKLEKPLDHEVIKAFYFKVTATVQQAKLDSVTTVDVELKVLDLNDNKPAFESN

SYEATTMEGMPVGTRIIQVH
ALDPDSGANGQVTYSLGTAIQPAAVERDPLAGVFSVDSNSGWISTLKQLDHETNPSYTFTVVAYDLGENLALSATTSVTV
VVTDVNDNPPTFLEERYHGS
VRESDPPGEVVAVLNTKDDDSSPINRQVSYHITGE
>g1684.t3
VLISVLDNNDNAPVFSQPTYDIAISEDTPPDTEVVQVLALDRDEHHRLAYSLHGAVDPGSLRLFRIDPALGSVYTAERLD
HEARAQHILTVMVKDQEFPY
RRGLARVLIEVEDVNDHVPLFTSTLYEGSVYESAAVGSAVVQVTALDKDKGANAELHYAIEGGNAGNAFRIEPVLGIISV
ARDLDLSSIGHYVLTVRVTD
NGSPSLSTTTLVRISVTLSDNAGPKFPQPEYQAEIVENVAVGTSVTTINAVSQSTLTYDIRQGNSQRKFQINQYSGVITT
HKPLDYETQTSYTLTVQATN
MAGMASNATLHIQVVDENDNQPVFQHLSYRGSISEAAQVNSVVLNSDESPLVIKATDADHNQNALLVYQIVEDTAKMFFT
VDSGTGSIRTIANLDHETFS
SFHFHVHVRDSGRPQLTADSPTEVTIEVIDTNDSPPRFTQDAYEAVLLLPTYVGVEALKVSAIDPDQNVPTELTYSLTDG
ILEHFAIESSCGIITVKNNN
FSKERFRFNVKVSDVFNQLLYEAYVSELATRGHFVTCVQASDADSSDSDKLVYSILSGNERMNFLIDMKTGIITLSGHRK
QRMEPAYSLNVSVSDGVFTS
TAQVHVKVLGANLHSPLFGLSTYDAELRENAAIGTKVIQVKATDADPGVFGKITYSFVNDVGKDQFSIDGYGFITTVEKL
DREDPANKDIILAVEAQDSG
GRASYCTVQVTLLDENDNAPQFRATEYRASVTSDVAKGFLVTQIQAYDPDNGVNAKVTYSLYSEAHVPVVDILEIDPDNG
WMVTKGSFSHMRNSVLSFFV
KAVDSGNPVRHSLVSVYIHVLAPDALIPSFTQPQYSFTMPEDTPIGASIGMVRLNTPLGHATLSAAFALVNGETGENNKD
AVLVVEKDTGIIKLEKPLDH
EVIKAFYFKVTATVQQAKLDSVTTVDVELKVLDLNDNKPAFESNSYEATTMEGMPVGTRIIQVHALDPDSGANGQVTYSL
GTAIQPAAVERDPLAGVFSV
DSNSGWISTLKQLDHETNPSYTFTVVAYDLGENLALSATTSVTVVVTDVNDNPPTFLEERYHGSVRESDPPGEVVAVLNT
KDDDSSPINRQVSYHITGE
>g1685.t1
MSSGPARPEPKPVLWEVNNHGSVHGPVSAPPVKTPKYSGKSDWVAQFELLVDAGGWTVEAKALQLALCLADDALSCLSPG
ERRDYGALVGALQRRFGQCV
KPELLRNKLSSHGRKPGEPLRVLANDIQSLTCRAYAHMPPNVQGELACDQFIRALFAIAASNENSGHCPEPSGGGKAAVG
DRDYRTNKGRGPRSRPADRS
APTNMSVGGVANLDICSEIVLVPMRCARYGGARHSRGRQATPGPEADNSTAYRTAVAGGGHRGGSFGHFHSRGSQTTLPP
EADDVTASRRVVVVGRLGDG
NSCYVPVYMEGVLCSALVDTGLSVTVLRPDVCPEWIQLEPTTCSSARPPGHWHS
>g1686.t1
MLALVPFGSARGGGVIAGHEGDMEEGAVWQLLLSFQDSFAMREDEVGRTHKIDTGDAQAIKCHPWCLPLAHQEARDQAVE
DMLQAGVIEPFDSPWASAVI
LVPKKDNT
>g1687.t1
LNPVTKKDLYPLPRIDESVDMVSLSSWFSTLYLRSRYYQVPLSPETRPKTAYCTGRCLWQFKVLSFGQCNAPATFARLMD
RVLSGIPRQQCLVYLDDILA
HGSSLCFLGLASYYRKFVQGFSCLAAPLHRLLQKDSDFVWTKQCQGEFSRLQRALSEAAVLAPADPSLSFMLDIDASSVG
GEQRYCISRRELLAVVLSIR
HFEYYLCGLPFRGCRCYERKEACERELQAEEENCPSLQQAEGVCRELEIVEGRDWGRPEQDTDFGPVVQWVETQEKPPWE
EVAGLSRVTKELWANFGGAV
GQLWGPLPLHQGVLQQAWKELATGEERWQQFLVAAPMERVAVDVVGPLPRLHRGNRYVLTAIDYFTKWLEAYDIPDQEAE
TVADAQVEGMFSRFGVPETL
HSDQGRNLESRVFGAVCEHLGIHKTCTTLQRPQSDGLMERFHRTMSQQLAILTSQNQRDWDNHLPLVLMSCHSAMQESCS
CHLGEGGWPFTETGWLCTEG
VPPLGPRGVPFLLAVGRTPHSPFSSHQLINLCPLSLCHGEGALAGQGGYRDT
>g1687.t2
LYPLPRIDESVDMVSLSSWFSTLYLRSRYYQVPLSPETRPKTAYCTGRCLWQFKVLSFGQCNAPATFARLMDRVLSGIPR
QQCLVYLDDILAHGSSLCFL
GLASYYRKFVQGFSCLAAPLHRLLQKDSDFVWTKQCQGEFSRLQRALSEAAVLAPADPSLSFMLDIDASSVGGEQRYCIS
RRELLAVVLSIRHFEYYLCG

LPFRGCRCYERKEACERELQAEEENCPSLQQAEGVCRELEIVEGRDWGRPEQDTDFGPVVQWVETQEKPPWEEVAGLSRV
TKELWANFGGAVGQLWGPLP
LHQGVLQQAWKELATGEERWQQFLVAAPMERVAVDVVGPLPRLHRGNRYVLTAIDYFTKWLEAYDIPDQEAETVADAQVE
GMFSRFGVPETLHSDQGRNL
ESRVFGAVCEHLGIHKTCTTLQRPQSDGLMERFHRTMSQQLAILTSQNQRDWDNHLPLVLMSCHSAMQESCSCHLGEGGW
PFTETGWLCTEGVPPLGPRG
VPFLLAVGRTPHSPFSSHQLINLCPLSLCHGEGALAGQGGYRDT
>g1687.t3
LNPVTKKDLYPLPRIDESVDMVSLSSWFSTLYLRSRYYQVPLSPETRPKTAYCTGRCLWQFKVLSFGQCNAPATFARLMD
RVLSGIPRQQCLVYLDDILA
HGSSLCFLGLASYYRKFVQGFSCLAAPLHRLLQKDSDFVWTKQCQGEFSRLQRALSEAAVLAPADPSLSFMLDIDASSVG
GCRCYERKEACERELQAEEE
NCPSLQQAEGVCRELEIVEGRDWGRPEQDTDFGPVVQWVETQEKPPWEEVAGLSRVTKELWANFGGAVGQLWGPLPLHQG
VLQQAWKELATGEERWQQFL
VAAPMERVAVDVVGPLPRLHRGNRYVLTAIDYFTKWLEAYDIPDQEAETVADAQVEGMFSRFGVPETLHSDQGRNLESRV
FGAVCEHLGIHKTCTTLQRP
QSDGLMERFHRTMSQQLAILTSQNQRDWDNHLPLVLMSCHSAMQESCSCHLGEGGWPFTETGWLCTEGVPPLGPRGVPFL
LAVGRTPHSPFSSHQLINLC
PLSLCHGEGALAGQGGYRDT
>g1688.t1
LCEQPCQCPSGLAPQCPRGVPVVLDHCGCCQMCARQLGEHCTETLPCDARRGLQCDYSASFPGDPGECVSQQDLGCEVNG
VTYYDGQAFQPSCDSYCHCQ
GGGVTCVPACPVDARLPMPDCPNPLLYRPLGRCCKEWMCESLDNTVNQDTLAASTPNRLWPDTPGAVGNRFTNQLMPPAL
TCVERSTHWSACSQTCGTGV
STRMSNKNSVCRLEIQTRLCKIRPCQTDQGPHRTLGWGHHGVHCKASYRSPVALRLVHQGCYSTGLHRMLYCGQCTDGCC
CTPHHTHTAQLSFRCLGNRL
LRRHVMVINSCACHRHC
>g1688.t2
PCQCPSGLAPQCPRGVPVVLDHCGCCQMCARQLGEHCTETLPCDARRGLQCDYSASFPGDPGECVSQQDLGCEVNGVTYY
DGQAFQPSCDSYCHCQGGGV
TCVPACPVDARLPMPDCPNPLLYRPLGRCCKEWMCESLDNTVNQDTLAASTPNRLWPDTPGAVGNRFTNQLMPPALTCVE
RSTHWSACSQTCGTGVSTRM
SNKNSVCRLEIQTRLCKIRPCQTDQGPHRTLGWGHHGVHCKASYRSPVALRLVHQGCYSTGLHRMLYCGQCTDGCCCTPH
HTHTAQLSFRCLGNRLLRRH
VMVINSCACHRHC
>g1688.t3
LCEQPCQCPSGLAPQCPRGVPVVLDHCGCCQMCARQLGEHCTETLPCDARRGLQCDYSASFPGDPGECVSQQDLGCEVNG
VTYYDGQAFQPSCDSYCHCQ
GGGVTCVPACPVDARLPMPDCPNPLLYRPLGRCCKEWMCESLDNTVNQDTLAASTPNRLWPDTPGAVGNRFTNQLMPPAL
TCVERSTHWSACSQTCGTGV
STRMSNKNSVCRLEIQTRLCKIRPCQTDQGPHRTLGWGHHGVHCKASYRSPVALRLVHQGCYSTGLHRMLYCGQCTDGCC
CTPHHTHTAQLSFRCLGNRL
LRRHVMFQLGIPKEDPVDSGGVKHAVQPCAVAALMHQEQGHGGP
>g1689.t1
LCSLDGTTVSMGEELETGQCYIAVGTERFRKLPYVELWLSKVQRNKRLGKRAEVDGRRVKSSGDEATEAGAPPANQSRAT
REGRREESSVFPARPVRIHR
NRGQPRVPVRNGPEQTSSLYYKKAGRRRREEVLGAQEVQDDENTHTELPVDQRVAEVVEDEEESHDAQPLNTEKKHPELR
KSDVDNVQEAGWHNGSQTAL
GREQTAAISRVSSTTSLGSRSGPVQRKSGGVEGDGLSVSTKVPSRLDGKWTSN
>g1689.t2
LCSLDGTTVSMGEELETGQCYIAVGTERFRKLPYVELWLSKVDGRRVKSSGDEATEAGAPPANQSRATREGRREESSVFP
ARPVRIHRNRGQPRVPVRNG
PEQTSSLYYKKAGRRRREEVLGAQEVQDDENTHTELPVDQRVAEVVEDEEESHDAQPLNTEKKHPELRKSDVDNVQEAGW
HNGSQTALGREQTAAISRVS
STTSLGSRSGPVQRKSGGVEGDGLSVSTKVPSRLDGKWTSN
>g1689.t3

LCSLDGTTVSMGEELETGQCYIAVGTERFRKLPYVELWLSKVQRFLNIHLLTCVSEPSSTRQVDGRRVKSSGDEATEAGA
PPANQSRATREGRREESSVF
PARPVRIHRNRGQPRVPVRNGPEQTSSLYYKKAGRRRREEVLGAQEVQDDENTHTELPVDQRVAEVVEDEEESHDAQPLN
TEKKHPELRKSDVDNVQEAG
WHNGSQTALGREQTAAISRVSSTTSLGSRSGPVQRKSGGVEGDGLSVSTKVPSRLDGKWTSN
>g1690.t1
LTRVYCVVVEVLCLLLVAVLWGCTNPFLKRGTEGIENVKRTNRVSQVLAEVKFLFLNLQYLVPFLLNQLGSVVYYFTLAT
TDLSLAVPVTNSLTFLLTVL
TGKILGEEFGGKRAVGGLLLTMLGITLCIISSMSHTPGASSDQNNADAAVQ
>g1690.t2
LLVAVLWGCTNPFLKRGTEGIENVKRTNRVSQVLAEVKFLFLNLQYLVPFLLNQLGSVVYYFTLATTDLSLAVPVTNSLT
FLLTVLTGKILGEEFGGKRA
VGGLLLTMLGITLCIISSMSHTPGASSDQNNADAAVQ
>g1690.t3
MVSVVEVLCLLLVAVLWGCTNPFLKRGTEGIENVKRTNRVSQVLAEVKFLFLNLQYLVPFLLNQLGSVVYYFTLATTDLS
LAVPVTNSLTFLLTVLTGKI
LGEEFGGKRAVGGLLLTMLGITLCIISSMSHTPGASSDQNNADAAVQ
>g1691.t1
MGAHDAALREDFNLQHRSIPDGDLLQGTGDTWHLASATDGRHHCENCGFADRGVTSVQPQPGQQQQHGQQVAMRLWVRAH
PYMFALKVA
>g1692.t1
MGPGGNGGGLTQHGPRWTGTHDTVQPPADTTMLNGNALTDKPTANAKASVAANAKANANSVESQYPATRSPKPGAKRGSV
TWAPELESGSGSVSAVVPDT
PLAPPVAAPLAPACNGEVGTLAGGREPVEKRKFSVDDSAQRPRRDSTASPAPPFGISVLECVRDSMVLAWKQPSFAGGAD
ITGYFVDYREVIGGVEGQWH
EANIRAVSDRAYRVCDLKEKRKYQFQVRAANMAGVGIPSLPSDTFLCEEWTIAVPGPPHDLQVTEVRSSSAVLLWKPPVY
QGRDEVNGFYVDIKEAEAAE
EEWRGINEKATNRKFIKIENLKEGEKYVFRVRAQNKAGVGNASEVTEPTLAQTKPGTKEIVVDVDDDGMISLNFECSEKS
ADSKFIWSKNYEEITDTSRI
SVETKGNKAKATFLAPSEEDIGIYSCSVTHTDGASASYDLTEQELRKLLETSHAHKFPSMFFCDFLSVIPLKSELAIELQ
EKGRVRFWLQAEKMSANGKV
DYVFNDNSLSAGEKYKMNFDKNTGVIEMIMDSLTPMDEGTYTFQLQDGKATNQSSLVLIGDVFKALQKESEFQMKEWVRK
QGPHFVEYLGYEVTPECCVI
LKCKVGNIKKDTVAVWQKGGQEIKSDQQLSFTEGVLKLEIAQISKKDAGVYEIILRDDRGKDSSTLNLTDQANSSTPLKI
TSTDEGIRLYTFVSYYNDLL
QVTWHYKDSAISFSDRIKSGVVGEQLWLQIKDPTEKDQGKYAIEFNDGKGGLRRTVELGGQAFDDAIAEFKRLQ
>g1692.t2
MGPGGNGGGLTQHGPRWTGTHDTVQPPADTTMLNGNALTDKPTANAKASVAANAKANANSVESQYPATRSPKPGAKRGSV
TWAPELESGSGSVSAVVPDT
PLAPPVAAPLAPACNGEVGTLAGGREPVEKRKFSVDDSAQRPRRDSTASPAPPFGISVLECVRDSMVLAWKQPSFAGGAD
ITGYFVDYREVIGGVEGQWH
EANIRAVSDRAYRVCDLKEKRKYQFQVRAANMAGVGIPSLPSDTFLCEEWTIAVPGPPHDLQVTEVRSSSAVLLWKPPVY
QGRDEVNGFYVDIKEAEAAE
EEWRGINEKATNRKFIKIENLKEGEKYVFRVRAQNKAGVGNASEVTEPTLAQTKPGTKEIVVDVDDDGMISLNFECSEKS
ADSKFIWSKNYEEITDTSRI
SVETKGNKAKATFLAPSEEDIGIYSCSVTHTDGASASYDLTEQELRKLLETSHAHKFPSMFFCDFLSVIPLKSELAIELQ
EKGRVRFWLQAEKMSANGKV
DYVFNDNSLSAGEKYKMNFDKNTGVIEMIMDSLTPMDEGTYTFQLQDGKATNQSSLVLIGDVFKALQKESEFQMKEWVRK
QGPHFVEYLGYEVTPECCVI
LKCKVGNIKKDTVAVWQKGGQEIKSDQQLSFTEGVLKLEIAQISKKDAGVYEIILRDDRGKDSSTLNLTDQANSSTPLKI
TSTDEGIRLYTFVSYYNDLL
QVTWHYKDSAISFSDRIKSGVVGEQLWLQIKDPTEKDQGKYAIEFNDGKGGLRRTVELGGQDRARVAGGLPDVVTIQEGK
>g1692.t3
MGPGGNGGGLTQHGPRWTGTHDTVQPPADTTMLNGNALTDKPTANAKASVAANAKANANSVESQYPATRSPKPGAKRGSV
TWAPELESGSGSVSAVVPDT
PLAPPVAAPLAPACNGEVGTLAGGREPVEKRKFSVDDSAQRPRRDSTASPAPPFGISVLECVRDSMVLAWKQPSFAGGAD

ITGYFVDYREVIGGVEGQWH
EANIRAVSDRAYRVCDLKEKRKYQFQVRAANMAGVGIPSLPSDTFLCEEWTIAVPGPPHDLQVTEVRSSSAVLLWKPPVY
QGRDEVNGFYVDIKEAEAAE
EEWRGINEKATNRKFIKIENLKEGEKYVFRVRAQNKAGVGNASEVTEPTLAQTKPGTKEIVVDVDDDGMISLNFECSEKS
ADSKFIWSKNYEEITDTSRI
SVETKGNKAKATFLAPSEEDIGIYSCSVTHTDGASASYDLTEQELRKLLETSHAHKFPSMFFCDFLSVIPLKSELAIELQ
EKGRVRFWLQAEKMSANGKV
DYVFNDNSLSAGEKYKMNFDKNTGVIEMIMDSLTPMDEGTYTFQLQDGKATNQSSLVLIGDVFKALQKESEFQMKEWVRK
QGPHFVEYLGYEVTPECCVI
LKCKVGNIKKDTVAVWQKGGQEIKSDQQLSFTEGVLKLEIAQISKKDAGVYEIILRDDRGKDSSTLNLTDQANSSTPLKI
TSTDEGIRLYTFVSYYNDLL
QVTWHYKDSAISFSDRIKSGVVGEQLWLQIKDPTEKDQGKYAIEFNDGKGGLRRTVELGGQAFDDAIAEFKRLQAAAIAE
KNRARVAGGLPDVVTIQEGK
>g1693.t1
FEGTHCEMEVDECISGPCLNQGKCLDQVGRFVCQCPNGFSGDTCQIDIDECSSTPCMNGAKCNDRPNAFECECAEGTALV
SGGPAPGGSLV
>g1693.t2
FEGTHCEMEVDECISGPCLNQGKCLDQVGRFVCQCPNGFSGDTCQIDIDECSSTPCMNGAKCNDRPNAFECECAE
>g1694.t1
MKFADDTTLVGLITQDNETHYRKVNLLTTWCKHNNVLLNVSKTKEIVVDFQRGHTEHSPLTINGAAVERVSSTKFLWVYI
SEYLSWTTNTASLAKKAQQQ
LYFLCKLKREKAPIPILKIFYRDTIESILSSRITEVPGPESPHSAQNLHVLQTNPSSIKSDRRRIKWPAASMTSLWKQFD
DDVDQILEVMAKGDADGKLQ
AMTAVIVSIAAERFGEEEKKEEERTGLAQLMCILRKKIRVLRRVEWHRRRRRERARKRAAFIANPFKFMKDLLGQKRSGK
LVSSQEVIDQHLKQTYSDPA
REQELG
>g1695.t1
LASALALGPPNPMLFNPLNPLNPLASLGLHMKAAAAAAGVVTGGGGLLASPDELYVLLQNLPFTATELEVREMFRGLGVE
AVRILRDGQGRGTGRAMVKF
FSPQDGMEVVKRGGGMMGQRYVDISPATERQWVSLNNASAVKPSQETPEHHRGNAPAGRERARSRSPHRQEWCVYLKGLP
YEAEKRQIQEFFKNLSLVED
SVYIAYGPNGRATGEGFLEFKTEQDYKTALGSHMQYMGSRFIQVHPISRKGMLEKIDAIRKRSGVPGDGKTPEVSKSPRN
CAHITNIPYNVSKKDVRAFL
DGVGIYEEGLKVLTDNHGNGLGQAVFQLRTEEDARKAERLHRQKLNGRDAFVHLVTFEQMKDIERNPPPQNKRGQRNQNQ
QSQQPSQQPNQPSPHQTHIN
PFAGMSGEEFNFLRSTMGNLGSAPFVSPFAAPGNGLVGPPPLPPLGAGLAMTPPLVAGLPGGPILEHPGFRPGAPGGPIL
EPPGFRPGAPGGPILEPPGF
RPGAPGGHTLGPEALRALAPFDGGNRKPAGAPGRVAGANSQARPGGGANNGQQTFGPDGPRNQAAPGNPGNQRPGCPTIV
KLQNMPFTVTVDEIMDFFYG
YQVLPGSVCLQLSEKGLPTGEAMVAFHNHQEASAAVLDLNDRPIGARKVKVTLG
>g1696.t1
MASYFDEHDCEPTNPEEQYRQNALLELARSLMQGLDIDSDAFDLSDWDQRLPPPAAKAAVRTLCVVVISPEQADRGLKCP
VCLLEFEEQETVRQMPCKHL
FHSGCILPWLGKHRHRCEEMCDCPLSCFWNSSVHSQTNSCPLCRLELPTDNPEYEEFKKDKVGPSSTQSGCVSHVEFG
>g1696.t2
MASYFDEHDCEPTNPEEQYRQNALLELARSLMQGLDIDSDAFDLSDWDQRLPPPAAKAAVRTLCVVVISPEQADRGLKCP
VCLLEFEEQETVRQMPCKHL
FHSGCILPWLGKVRSSPHRHNLHTVPSSSSSSSSSPQPHPNDCHNILILQPHHALTTSTTTSHLHNLISPHPHNLSIATA
TNSCPLCRLELPTDNPEYEE
FKKDKVGPSSTQSGCVSHVEFG
>g1696.t3
MASYFDEHDCEPTNPEEQYRQNALLELARSLMQGLDIDSDAFDLSDWDQRLPPPAAKAAVRTLCVVVISPEQADRGLKCP
VCLLEFEEQETVRQMPCKHL
FHSGCILPWLGKTNSCPLCRLELPTDNPEYEEFKKDKVGPSSTQSGCVSHVEFG
>g1697.t1
EDGMSRLQRAEEEVSEVKDIMLDNLQKANERSGKLEDLESRAEDMLVKSKAFEKTTQKVKQKKQWQNRKWKVVLCAFGAA

VVLVILAIIIFYIVQSASGE
>g1698.t1
AWLADPNGGPEGEENIRVLLAEAKRIAALCEDPKERDDILRSISEIVGLTARLVDLRRQGKGDSPEARALAKQIGAALLN
LQSKT
>g1699.t1
VAGLSLRDRVRSSDIRRELGVEPLLLRVERSQLRWFGHLIRMPPGRLPLEVFRARPTGKRPRGRPRTRWRDYISHLAWER
LGVPQEELESVAGEKDVWSS
LLSLLPPRPGPG
>g1700.t1
KDTALTALLLRNDSIAVGGSTLAGSPGGVDEGTTGSATSAQSTASCHIDLRGPRGFIQALPQSISTMHTAAGCTYNVTVY
MGYGVEIQVLRVAVRRLTFG
GRLAL
>g1700.t2
GGSTLAGSPGGVDEGTTGSATSAQSTASCHIDLRGPRGFIQALPQSISTMHTAAGCTYNVTVYMGYGVEIQVLRVAVRRL
TFGGRLAL
>g1700.t3
KDTALTALLLRNDSIAVGGSTLAGSPGGVDEGTTGSATSAQSTASCHIDLRGPRGFIQALPQSISTMHTAAGCTYNVTVY
MGYGVEIQEPSKVFSSGEIL
GLCC
>g1701.t1
MLAFFNRSPRFVGRRQSLIEDARKEREAAAAAAEATDVSEQIVFEEENGKALLNVFFTLKTSKIPALSRSLKVFETFEAK
IHHLETRQCRKPKDQQEALE
YFVRCELHRSDVGTLISSIKRTAEEVRTTKEVKFHWFPKQIAELDKCHHLVTKFDPDLDQDHPGFSDPVYRQRRKMIGDI
AFTYRHGNAIPRVDYTEDEI
GTWREVYVTLRELYNTHACSEHLEAFQLLERHCGYSPDNIPQLEDVSRFLKGRQ
>g1701.t2
LIEDARKEREAAAAAAEATDVSEQIVFEEENGKALLNVFFTLKTSKIPALSRSLKVFETFEAKIHHLETRQCRKPKDQQE
ALEYFVRCELHRSDVGTLIS
SIKRTAEEVRTTKEVKFHWFPKQIAELDKCHHLVTKFDPDLDQDHPGFSDPVYRQRRKMIGDIAFTYRHGNAIPRVDYTE
DEIGTWREVYVTLRELYNTH
ACSEHLEAFQLLERHCGYSPDNIPQLEDVSRFLKGRQ
>g1701.t3
MLAFFNRSPRFVGRRQSLIEDARKEREAAAAAAEATDVSEQIVFEEENGKALLNVFFTLKTSKIPALSRSLKVFETFEAK
IHHLETRQCRKPKDQQEALE
YFVRCELHRSDVGTLISSIKRTAEEVRTTKEVKFHWFPKQIAELDKCHHLVTKFDPDLDQDHPGFSDPVYRQRRKMIGDI
AFTYRHGNAIPRVDYTEDEI
GTWREVYVTLRELYNTHACSEHLEAFQLLERHCGYSPDNIPQLEDVSRFLK
>g1702.t1
MRRQKGVAPAALGGVNLPWTRVLQLHSMSRHESERSISDAALRMVFVEDVELFEIGIVSRRGRGGIKGIEPPTLRLVDDL
LCPPALPTELLPPR
>g1703.t1
METAWLVEFQNVGCSYFPQSRVDCHYTLSSKLPWASNDWIGLFKVGWSSVRDYHTFVWSSAPTDNQEGRDVNCCVQFQAS
YLPKPSSEEYQFVYVDGGGR
VCSCSPAFTFGGPKPLEEMVTLEEGGQGEDGATDMLLVVPRAELLQSRLQECLQERAELLQAQERLLRKKEQEEGRYDRA
RVGWEAERRELKRKVARLQE
ELSRSQDNMVEMEKKQK
>g1703.t2
METAWLVEFQNVGCSYFPQSRVDCHYTLSSKLPWASNDWIGLFKSRVDCHYTLSSKLPWASNDWIGLFKVGWSSVRDYHT
FVWSSAPTDNQEGRDVNCCV
QFQASYLPKPSSEEYQFVYVDGGGRVCSCSPAFTFGGPKPLEEMVTLEEGGQGEDGATDMLLVVPRAELLQSRLQECLQE
RAELLQAQERLLRKKEQEEG
RYDRARVGWEAERRELKRKVARLQEELSRSQDNMVEMEKKQK
>g1703.t3
METAWLVEFQNVGCSYFPQSRVDCHYTLSSKLPWASNDWIGLFKVGWSSVRDYHTFVWSSAPTDNQEGRDVNCCVQFQGT
NFLAL
>g1703.t4

LTAATVLENTGGTLGLVTCVIADVVPPDKLGWVVPSSSEEYQFVYVDGGGRVCSCSPAFTFGGPKPLEEMVTLEEGGQGE
DGATDMLLVVPRAELLQSRL
QECLQERAELLQAQERLLRKKEQEEGRYDRARVGWEAERRELKRKVARLQEELSRSQDNMVEMEKKQK
>g1704.t1
MQPCRFCNLATSQGVDVLHRLGLIAPSSRDTPYEGTSSPSSSSSIYATFPSPQNRPPPGVGVLLDSNSIYENPAGLFFPE
ISHDFSVVVSLSSWRANNAF
LLSVKDGQNRVRFGIQLLPRRVVVYTGDKASVFFTYDWQDGHQHSFALGVRKRSVSFHADCGAVYQWEQTLGRPQPLGSP
RGLLTLGRMNSKAAPFQGRI
CQLDIYPSAQAAAHYCNYLRKQCRLADTFRSSLSPAGLDVGANNPLFEPLIESSSPEAGASHADPNTTSAKAASEQPSLI
RYHTQYSTLRPSVQSRLGEQ
TYLHLGYSLVHTSTPSVHHQSRTPLATTNRLAGLDLTLRTATPATTSMPVQGTRQPQRKVDLRENNTEGDSSPSGPHNPG
ATMSSNTATSRGTQTPHPRL
TDENLEVQHRANKTTLYRHNQLDTSEEHGVDVSYDNEGTEGYDYGYEERDYFYDYEGFLGPKGDPGPEVGAFMNGNLSYG
QEQLLVSQVCAAPCMIKQNQ
AIALQCKGIS
>g1704.t2
MTIAFSCEGVDVLHRLGLIAPSSRDTPYEGTSSPSSSSSIYATFPSPQNRPPPGVGVLLDSNSIYENPAGLFFPEISHDF
SVVVSLSSWRANNAFLLSVK
DGQNRVRFGIQLLPRRVVVYTGDKASVFFTYDWQDGHQHSFALGVRKRSVSFHADCGAVYQWEQTLGRPQPLGSPRGLLT
LGRMNSKAAPFQGRICQLDI
YPSAQAAAHYCNYLRKQCRLADTFRSSLSPAGLDVGANNPLFEPLIESSSPEAGASHADPNTTSAKAASEQPSLIRYHTQ
YSTLRPSVQSRLGEQTYLHL
GYSLVHTSTPSVHHQSRTPLATTNRLAGLDLTLRTATPATTSMPVQGTRQPQRKVDLRENNTEGDSSPSGPHNPGATMSS
NTATSRGTQTPHPRLTDENL
EVQHRANKTTLYRHNQLDTSEEHGVDVSYDNEGTEGYDYGYEERDYFYDYEGFLGPKGDPGPEVGAFMNGNLSYGQEQLL
VSQVCAAPCMIKQNQAIALQ
CKGIS
>g1705.t1
MGINFPKLKLPNPGLDDRIPSYQELEKMEKEEVADRPKWDNKAQYLLTCLGFCVGLGNVWRFPYLCQSHGGGAFMIPFLI
LLVLEGIPLLHLEFAIGQRL
RTGSVGVWRSINPYLAGVGVASLMTSFIVGMYYNTIMAWIMWYLFNSFQDPLPWSQCPLTANGTSVVSECARSSTVDYFW
YRETLNTSTAIDESGGLQWW
IVMSLVAAWTLLYVCCIRGIETTGKAVYITSTLPYLVLTIFLIRGLTLKGSIDGIKFLFTPDVDELMNPTTWLDAGAQVF
YSFSLAFGGLISFSSYNPVH
NNCEQDAVIISIINGFTSVYSATVIYSIIGFRATEKFDDCMERNILELTNAFNYPENIITYTNYKETLDFLNSTSPDIIQ
GLPLQTCDLQKLLSE
>g1706.t1
MAFFWSFAGITCLSSFCLALIFTLPSGSYWLALCDSFAGSIPLLVIAFVELMAITYLYGIDKFNKDIEFMIGHRPNIFWQ
ISWRVVSPLIIFVIFVFYFI
TKVSAPLTYLAWNPSSENFPKLETVFYPDWVYVIIFLVAGVPSLVVPGMALYRILQQRWCQNTTLKMDDLSTISRAVSMH
DKC
>g1706.t2
FNKDIEFMIGHRPNIFWQISWRVVSPLIIFVIFVFYFITKVSAPLTYLAWNPSSENFPKLETVFYPDWVYVIIFLVAGVP
SLVVPGMALYRILQQRWCQN
TTLKMDDLSTISRAVSMHDKC
>g1707.t1
MIIFAFVVRVRDGLPLSASTDFAHNRELQERKQQLRKMSKALSCFPERGTVKGQDLNIYFVSSEGVSYMAVCHRGLSVAK
AFCFLEDLCWAFKACFDSTA
VSLAARPYPFLEFDGTIQKLKQQYNQIGGGPVLEITLAEVQDDLKRHPPKVLTLEELELTNGLANGHIEQGPGSGQNVRL
EPVTPAGILSLVLNIMCASL
NLIRGVHLIEYTFQCHLYVFHSSHKRFKSASLLVVLVLCNSYLFGLRNVWQLLFHTSVACLSTALILSRKVLDRSNDCGV
>g1707.t2
MIIFAFVVRVRDGLPLSASTDFAHNRELQERKQQLRKMSKALSCFPERGTVKGQDLNIYFVSSEGVSYMAVCHRGLSVAK
AFCFLEDLCWAFKACFDSTA
VSLAARPYPFLEFDGTIQKLKQQYNQIGGGPVLEITLAEVQDDLKRHPPKVLTLEELELTNGLANGHIEQGPGSGQNVRL
EPVTPAGILSLVLNIMCASL

NLIRGVHLIEYTFQDDYDSLWSVVAFLMAFVFCLFQCHLYVFHSSHKRFKSASLLVVLVLCNSYLFGLRNVWQLLFHTSV
ACLSTALILSRKVLDRSNDC
GV
>g1707.t3
MPMIIFAFVVRVRDGLPLSASTDFAHNRELQERKQQLRKMSKALSCFPERGTVKGQDLNIYFVSSEGVSYMAVCHRGLSV
AKAFCFLEDLCWAFKACFDS
TAVSLAARPYPFLEFDGTIQKLKQQYNQIGGGPVLEITLAEVQDDLKRHPPKVLTLEELELTNGLANGHIEQGPGSGQNV
RLEPVTPAGILSLVLNIMCA
SLNLIRGVHLIEYTFQDDYDSLWSVVAFLMAFVFCLFQCHLYVFHSSHKRFKSASLLVVLVLCNSYLFGLRNVWQLLFHT
SVACLSTALILSRKVLDRSN
DCGV
>g1708.t1
MGVKDRPQCYFDVELNREPGEKGTGKTTGKELCYKGSTFHRVVKNFMVQGGDFTEDENLLLKHDKAFLLSMANRGKDTNG
SQFFIVHVVFGLVISGFEVI
KKIEGLKTDSASRPYADVRVVDCGQLITKSACDVLEGKRKRASQSADLSPSSQESSSLASSGVASEREADRRTARRRRRH
TRSKRSAKRPKYSAVVLSDQ
GPVDGGYEEEEEEGWNEPGGKRGKPVVRPEEIPPVPENRFLLRRDVPSQEEKPDIPTEQEVLSVPCDLKPTVTKSGRRIK
GRGTMSKSRSASVEERGSSE
TPPHWKEEMKRTQAYQPPSVERWSRGDRWDDRSDSAWSRSQAGSPSQASGRSSTQLPAKKEKKKSKRKKKAKKRKHAKKK
SSKSKPLQAYLSGSEKSGSS
ARQSKLRASLSCSGHGRTFSTSEKEEGEASSESEREVGSKALPRSLGSPKKVIEPEKHKSKKSKRKHKRKRRGETKSVSG
RSKDKAKRSKRKRHKLKETF
HWQPPLEFGEEDDDVESQKEKRAEKHNISGSTKTLVKETPGADHVRNKDHNIPASGNNGSTQTQPFRREEQGHPRPKGSV
HENHPKEKQFDHPSSNRNAQ
DQTSSADDMDICTPEHDTENIVEPLLSKTPESTFETVANSLSKTTQDDNLSSHNQVQPPGLFAVGAAVGTILDLKWKPLK
GMTASAMANLLPVTAKNSRP
VEYQTPRSQGVKMEIKSKSRVRPGSLFDEVRKTARLNQRPRNQESSSEEHSQAESRGESHTPRKSGSVSSHRSRSRGRSR
SYSRSRSRSRSSSYTSRSDS
DYSESSRSRSRSGGRKRWRRRSDSYRSSERRSRSYHSSSRSSSRHRTPSRGSRYS
>g1708.t2
MYPMKGQSCVKATRHAGDPARERCWEKGTGKTTGKELCYKGSTFHRVVKNFMVQGGDFTEDENLLLKHDKAFLLSMANRG
KDTNGSQFFIVHVVFGLVIS
GFEVIKKIEGLKTDSASRPYADVRVVDCGQLITKSACDVLEGKRKRASQSADLSPSSQESSSLASSGVASEREADRRTAR
RRRRHTRSKRSAKRPKYSAV
VLSDQGPVDGGYEEEEEEGWNEPGGKRGKPVVRPEEIPPVPENRFLLRRDVPSQEEKPDIPTEQEVLSVPCDLKPTVTKS
GRRIKGRGTMSKSRSASVEE
RGSSETPPHWKEEMKRTQAYQPPSVERWSRGDRWDDRSDSAWSRSQAGSPSQASGRSSTQLPAKKEKKKSKRKKKAKKRK
HAKKKSSKSKPLQAYLSGSE
KSGSSARQSKLRASLSCSGHGRTFSTSEKEEGEASSESEREVGSKALPRSLGSPKKVIEPEKHKSKKSKRKHKRKRRGET
KSVSGRSKDKAKRSKRKRHK
LKETFHWQPPLEFGEEDDDVESQKEKRAEKHNISGSTKTLVKETPGADHVRNKDHNIPASGNNGSTQTQPFRREEQGHPR
PKGSVHENHPKEKQFDHPSS
NRNAQDQTSSADDMDICTPEHDTENIVEPLLSKTPESTFETVANSLSKTTQDDNLSSHNQVQPPGLFAVGAAVGTILDLK
WKPLKGMTASAMANLLPVTA
KNSRPVEYQTPRSQGVKMEIKSKSRVRPGSLFDEVRKTARLNQRPRNQESSSEEHSQAESRGESHTPRKSGSVSSHRSRS
RGRSRSYSRSRSRSRSSSYT
SRSDSDYSESSRSRSRSGGRKRWRRRSDSYRSSERRSRSYHSSSRSSSRHRTPSRGSRYS
>g1708.t3
MYPMKGQSCVKATRHAGDPARERCWEKGTGKTTGKELCYKGSTFHRVVKNFMVQGGDFTEDENLLLKHDKAFLLSMANRG
KDTNGSQFFIVHVVFGLVIS
GFEVIKKIEGLKTDSASRPYADVRVVDCGQLITKSACDVLEGKRKRASQSADLSPSSQESSSLASSGVASEREADRRTAR
RRRRHTRSKRSAKRPKYSAV
VLSDQGPVDGGYEEEEEEGWNEPGGKRGKPVVRPEEIPPVPENRFLLRRDVPSQEEKPDIPTEQEVLSVPCDLKPTVTKS
GRRIKGRGTMSKSRSASVEE
RGSSETPPHWKEEMKRTQAYQPPSVERWSRGDRWDDRSDSAWSRSQAGSPSQASGRSSTQLPAKKEKKKSKRKKKAKKRK
HAKKKSSKSKPLQAYLSGTS

EKEEGEASSESEREVGSKALPRSLGSPKKVIEPEKHKSKKSKRKHKRKRRGETKSVSGRSKDKAKRSKRKRHKLKETFHW
QPPLEFGEEDDDVESQKEKR
AEKHNISGSTKTLVKETPGADHVRNKDHNIPASGNNGSTQTQPFRREEQGHPRPKGSVHENHPKEKQFDHPSSNRNAQDQ
TSSADDMDICTPEHDTENIV
EPLLSKTPESTFETVANSLSKTTQDDNLSSHNQVQPPGLFAVGAAVGTILDLKWKPLKGMTASAMANLLPVTAKNSRPVE
YQTPRSQGVKMEIKSKSRVR
PGSLFDEVRKTARLNQRPRNQESSSEEHSQAESRGESHTPRKSGSVSSHRSRSRGRSRSYSRSRSRSRSSSYTSRSDSDY
SESSRSRSRSGGRKRWRRRS
DSYRSSERRSRSYHSSSRSSSRHRTPSRGSRYS
>g1709.t1
PIVRPYAGAVGPGFLLVHDNARPHVARGVELMGSKLALRYPDRIPSGMGMSRAALKAEGLAEDNF
>g1709.t2
GVELMGSKLALRYPDRIPSGMGMSRAALKAEGLAEDNF
>g1710.t1
MVANLACSISNNEEGVKLVRMAASQLETLCPQVINAALALAAKPNSKVAQDNMDLFKEQWEKQVCVLTDAVDDITSIDDF
LCVSENHILEDVNKCVIALQ
EKDVDGLDRTAGAIRGRAARVVHVVTSEMDNYEPGVYTEKVLEATKLLTNTVMPRFTEQVEAAVEALSGNPTQPVNDNEF
IDASRLVYDGVRDIRKAVLM
IRTPEELDDSDFETEDDRHSRTSVQTEDDQLIAGQSARAIMAQLPQEQKAKIAEQVASFQEEKSKLDAEVSKWDDSGNDI
IVLAKQMCMIMMEMTDFTRG
KGPLKNTSDVISAAKKIAEAGSRMDKLGRTIADNVRLNPKPKHQRRVIHAE
>g1710.t2
MVANLACSISNNEEGVKLVRMAASQLETLCPQVINAALALAAKPNSKVAQDNMDLFKEQWEKQVCVLTDAVDDITSIDDF
LCVSENHILEDVNKCVIALQ
EKDVDGLDRTAGAIRGRAARVVHVVTSEMDNYEPGVYTEKVLEATKLLTNTVMPRFTEQVEAAVEALSGNPTQPVNDNEF
IDASRLVYDGVRDIRKAVLM
IRVMLVQTPEELDDSDFETEDDRHSRTSVQTEDDQLIAGQSARAIMAQLPQEQKAKIAEQVASFQEEKSKLDAEVSKWDD
SGNDIIVLAKQMCMIMMEMT
DFTRGKGPLKNTSDVISAAKKIAEAGSRMDKLGRTIADNVRLNPKPKHQRRVIHAE
>g1710.t3
MVANLACSISNNEEGVKLVRMAASQLETLCPQVINAALALAAKPNSKVAQDNMDLFKEQWEKQVCVLTDAVDDITSIDDF
LCVSENHILEDVNKCVIALQ
EKDVDGLDRTAGAIRGRAARVVHVVTSEMDNYEPGVYTEKVLEATKLLTNTVMPRFTEQVEAAVEALSGNPTQPVNDNEF
IDASRLVYDGVRDIRKAVLM
IRVIHRGGSHLGVSHVCRLSAHRPQRTPEELDDSDFETEDDRHSRTSVQTEDDQLIAGQSARAIMAQLPQEQKAKIAEQV
ASFQEEKSKLDAEVSKWDDS
GNDIIVLAKQMCMIMMEMTDFTRGKGPLKNTSDVISAAKKIAEAGSRMDKLGRTIADNVRLNPKPKHQRRVIHAE
>g1711.t1
VGRKDRSDALNTAIDKMTRKTRDLRRQLRKAVMDHVSDSFLETNVPLLVLIEAAKNGNEKEVKEYAQVFREHANKLIEIF
SQLFYKIKKVCKD
>g1712.t1
MCPRVRSESGNHTGPSVNLTGSSEDRSFWARWSVDGSIQTIYTKIHLHNVLGSAESDTYNFTLGAIAKPLPPVLSRVECS
SRSCNITWHHSLETHVEVQY
REVPDLREAEQECIPIADSDWQANSSGHWVHDLQPSRRYCFRARAKLPSSLNWSDWSTMVSNRTQEEAPEKELDVWFHRS
TSLPNSLTVVYWKPLNGSEAR
GRVLGYMVHIDDPEENGTQLVSAAVTRVPVPSCVTCPVTVSAYNSKGSSPPARVPTHFRTAHAPQELQAVESKDNVSLYW
RTTDPTSTGYLVEWYLQDQQ
LETLQWMRLSSAKSQAVITELEPNECYDGAVYVFYEDSLGMASFTSVNTVVSGAPSVAKQVGEHGVTLTWTQIPREQRRG
CIRNYTLYVENSKDREDRHT
>g1712.t2
MCPRVRSESGNHTGPSVNLTGSSEDRSFWARWSVDGSIQTIYTKIHLHNVLGSAESDTYNFTLGAIAKPLPPVLSRVECS
SRSCNITWHHSLETHVEVQY
REVPDLREAEQECIPIADSDWQANSSGHWVHDLQPSRRYCFRARAKLPSSLNWSDWSTMVSNRTQEEAPEKELDVWFHRS
TSLPNSLTVVYWKPLNGSEAR
GRVLGYMVHIDDPEENGTQLVSAAVTRVPVPSCVTCPVTVSAYNSKGSSPPARVPTHFRTAHAPQELQAVESKDNVSLYW
RTTDPTSTGYLVEWYLQDQQ

LETLQWMRLSSAKSQAVITELEPNECYDGAVYVFYEDSLGMASFTSVNTVVSVPIGAPSVAKQVGEHGVTLTWTQIPREQ
RRGCIRNYTLYVENSKDRED
RHT
>g1712.t3
VRSESGNHTGPSVNLTGSSEDRSFWARWSVDGSIQTIYTKIHLHNVLGSAESDTYNFTLGAIAKPLPPVLSRVECSSRSC
NITWHHSLETHVEVQYREVP
DLREAEQECIPIADSDWQANSSGHWVHDLQPSRRYCFRARAKLPSSLNWSDWSTMVSNRTQEEAPEKELDVWFHRSTSLP
NSLTVYWKPLNGSEARGRVL
GYMVHIDDPEENGTQLVSAAVTRVPVPSCVTCPVTVSAYNSKGSSPPARVPTHFRTAHAPQELQAVESKDNVSLYWRTTD
PTSTGYLVEWYLQDQQLETL
QWMRLSSAKSQAVITELEPNECYDGAVYVFYEDSLGMASFTSVNTVVSGAPSVAKQVGEHGVTLTWTQIPREQRRGCIRN
YTLYVENSKDREDRHT
>g1713.t1
VWDRERAAASRSLRPLLTLASRLASRPSSWALLLARAVGQPHCYEW
>g1714.t1
LTVAVKCSDAIEDSLRVKVYPSLFSNDKNPDSLDKSIQNLLSALYPPFEATAPTVLSQLFQVIEGRYQGDALRCLLDFLV
PAKHILESVQQAACAQYSDV
IFLCEGWPLCLRDHILIHLAPIHPVVLRPGDFYLQVAPYAHQSARIVVCSLLEEEGLWVEVVEETPVPETCYPCLFTSDW
LQEINQGRHGTPLSQCLLAS
EQGVVRLPWKRVAAPDFVDGPEGVRTRMASAPSSAHSLPTALSLSTPVPYLSTPASVKTSPAFLSPPASPAKQVTPRPPT
DNTQTSVSQRTQQPAAVSVE
TRIRPAKHGIAVSLRLVDTSASGSSRQVKVTDTGPKPVGWVSSNIWDSRCTEVGACPAALSSGITTTSIVEESNNTPASR
DKGEAAFAGVDLGRENHTGV
RAQGARGPADQASCVSGADGEYIDVAQAAMLFGEAKALRKEEQEARAAMQPAPQTQAHMQSFVVQVDPPAQPQWPVELYP
NPPTCNPPHARSGHGALLRP
RVESEPLPSHHSRPVHPQADSHGSPSPRDPSHCARSVRFSEGPCTPCMRRRQTGKVSRAQELRCRYRDSYQAAIQNPVAF
ARDKEKLDALAALEEDSDGS
LCEGRQPPEGARGDPWLSAGRRRRESTESCAEVPRLHVVKCKNATAFGLVSPKINRRRIVNTADIQKIPQPCLARPRPDH
LPLPPPETAIHPLYQGLACL
TGGRDRAGRAVVEVYGDHPGWRSPGAIKELYQMLLYFYTVIRREVRELGLVVVIDARRAPPPPQFFKAMTILQESIDHAI
HCVVLLVDKENNPRRERYHT
GIQAVEACHLTTYLDGTLPYNHRDWLELHQKLHLFVLDLHESSSLLLRAIRKLEGKQKIDTVQEVQQCIVDQRTLMRDVL
EDSRVVGLQREGGAILARLR
RESDLKYPHREDFSDAVDSVTSLYNCVEEQAHVLVQRSNMSLEHLEYLLQLRELEAHFQQIREWFQVEGEHHLLEAERVD
DCKDSVEQTLNSFTSFLLEA
NDRKHHAASLVAEAEQLQGSGLSYPEAQVFGALVQTFRAGLESFLARAEVCCRELQVMVHVCDFCEQATALAGECIEYLD
QSNGPSANTTTTTTMVQDQV
LGSDEPDPSLGPDQGPPTQDPTQDPWSTAGYDSAILKSYQERFHQFSLEQFQEVRSQAGTLRGSRGMRAWNVAWLKCQEA
RQQLQERVQEVDEAYLQRQH
AALSCDCRDMDADVLPAFSDGEEGFLQSTPGPSHPQWEGVISGAVELDKRRPILGRSSSIGAEAHVEPENAYVARGNQGS
KVVPASPHR
>g1714.t2
LLTVAVKCSDAIEDSLRVKVYPSLFSNDKNPDSLDKSIQNLLSALYPPFEATAPTVLSQLFQVIEGRYQGDALRCLLDFL
VPAKHILESVQQAACAQYSD
VIFLCEGWPLCLRDHILIHLAPIHPVVLRPGDFYLQVAPYAHQSARIVVCSLLEEEGLWVEVVEETPVPETCYPCLFTSD
WLQEINQGRHGTPLSQCLLA
SEQGVVRLPWKRVAAPDFVDGPEGVRTRMASAPSSAHSLPTALSLSTPVPYLSTPASVKTSPAFLSPPASPAKQVTPRPP
TDNTQTSVSQRTQQPAAVSV
ETRIRPAKHGIAVSLRLVDTSASGSSRQVKVTDTGPKPVGWVSSNIWDSRCTEVGACPAALSSGITTTSIVEESNNTPAS
RDKGEAAFAGVDLGRENHTG
VRAQGARGPADQASCVSGADGEYIDVAQAAMLFGEAKALRKEEQEARAAMQPAPQTQAHMQSFVVQVDPPAQPQWPVELY
PNPPTCNPPHARSGHGALLR
PRVESEPLPSHHSRPVHPQADSHGSPSPRDPSHCARSVRFSEGPCTPCMRRRQTGKVSRAQELRCRYRDSYQAAIQNPVA
FARDKEKLDALAALEEDSDG
SLCEGRQPPEGARGDPWLSAGRRRRESTESCAEVPRLHVVKCKNATAFGLVSPKINRRRIVNTADIQKIPQPCLARPRPD
HLPLPPPETAIHPLYQGLAC

LTGGRDRAGRAVVEVYGDHPGWRSPGAIKELYQMLLYFYTVIRREVRELGLVVVIDARRAPPPPQFFKAMTILQESIDHA
IHCVVLLVDKENNPRRERYH
TGIQAVEACHLTTYLDGTLPYNHRDWLELHQKLHLFVLDLHESSSLLLRAIRKLEGKQKIDTVQEVQQCIVDQRTLMRDV
LEDSRVVGLQREGGAILARL
RRESDLKYPHREDFSDAVDSVTSLYNCVEEQAHVLVQRSNMSLEHLEYLLQLRELEAHFQQIREWFQVEGEHHLLEAERV
DDCKDSVEQTLNSFTSFLLE
ANDRKHHAASLVAEAEQLQGSGLSYPEAQVFGALVQTFRAGLESFLARAEVCCRELQVMVHVCDFCEQATALAGECIEYL
DQSNGPSANTTTTTTMVQDQ
VLGSDEPDPSLGPDQGPPTQDPTQDPWSTAGYDSAILKSYQERFHQFSLEQFQEVRSQAGTLRGSRGMRAWNVAWLKCQE
ARQQLQERVQEVDEAYLQRQ
HAALSCDCRDMDADVLPAFSDGEEGFLQSTPGPSHPQWEGVISGAVELDKRRPILGRSSSIGAEAHVEPENAYVARGNQG
SKVVPASPHR
>g1714.t3
LFNVKRRAYTAELLTVAVKCSDAIEDSLRVKVYPSLFSNDKNPDSLDKSIQNLLSALYPPFEATAPTVLSQLFQVIEGRY
QGDALRCLLDFLVPAKHILE
SVQQAACAQYSDVIFLCEGWPLCLRDHILIHLAPIHPVVLRPGDFYLQVAPYAHQSARIVVCSLLEEEGLWVEVVEETPV
PETCYPCLFTSDWLQEINQG
RHGTPLSQCLLASEQGVVRLPWKRVAAPDFVDGPEGVRTRMASAPSSAHSLPTALSLSTPVPYLSTPASVKTSPAFLSPP
ASPAKQVTPRPPTDNTQTSV
SQRTQQPAAVSVETRIRPAKHGIAVSLRLVDTSASGSSRQVKVTDTGPKPVGWVSSNIWDSRCTEVGACPAALSSGITTT
SIVEESNNTPASRDKGEAAF
AGVDLGRENHTGVRAQGARGPADQASCVSGADGEYIDVAQAAMLFGEAKALRKEEQEARAAMQPAPQTQAHMQSFVVQVD
PPAQPQWPVELYPNPPTCNP
PHARSGHGALLRPRVESEPLPSHHSRPVHPQADSHGSPSPRDPSHCARSVRFSEGPCTPCMRRRQTGKVSRAQELRCRYR
DSYQAAIQNPVAFARDKEKL
DALAALEEDSDGSLCEGRQPPEGARGDPWLSAGRRRRESTESCAEVPRLHVVKCKNATAFGLVSPKINRRRIVNTADIQK
IPQPCLARPRPDHLPLPPPE
TAIHPLYQGLACLTGGRDRAGRAVVEVYGDHPGWRSPGAIKELYQMLLYFYTVIRREVRELGLVVVIDARRAPPPPQFFK
AMTILQESIDHAIHCVVLLV
DKENNPRRERYHTGIQAVEACHLTTYLDGTLPYNHRDWLELHQKLHLFVLDLHESSSLLLRAIRKLEGKQKIDTVQEVQQ
CIVDQRTLMRDVLEDSRVVG
LQREGGAILARLRRESDLKYPHREDFSDAVDSVTSLYNCVEEQAHVLVQRSNMSLEHLEYLLQLRELEAHFQQIREWFQV
EGEHHLLEAERVDDCKDSVE
QTLNSFTSFLLEANDRKHHAASLVAEAEQLQGSGLSYPEAQVFGALVQTFRAGLESFLARAEVCCRELQVMVHVCDFCEQ
ATALAGECIEYLDQSNGPSA
NTTTTTTMVQDQVLGSDEPDPSLGPDQGPPTQDPTQDPWSTAGYDSAILKSYQERFHQFSLEQFQEVRSQAGTLRGSRGM
RAWNVAWLKCQEARQQLQER
VQEVDEAYLQRQHAALSCDCRDMDADVLPAFSDGEEGFLQSTPGPSHPQWEGVISGAVELDKRRPILGRSSSIGAEAHVE
PENAYVARGNQGSKVVPASP
HR
>g1715.t1
MKKEQYTKILKNNIKQSAEKLDLGHQWTFQHDNDQKLTAKVVKKWLADKNINVLQWPSQSPDLNPIENLWRELKIRVMAR
RPSNLKELELIAKDEWAKIP
VETCKKLQADIQRFLDEATILDPRFKSKMDKDEVWDRIREVAMAANKEAAAADERADQEIQLYRSLPLIPCKDNGTICYV
HQVKMQQDIHPADVPEVTDA
DLMAADELVEWTPLPPPPPPPGTSLLSAAAVPTGQSSHPAMTSGAESGRPILTSELCLWWDPPEDRPLYSQPPATAHAFF
HSRFYRWALYNMWACKLASP
ACGHKLTD
>g1715.t2
GNLITVNGIMKKEQYTKILKNNIKQSAEKLDLGHQWTFQHDNDQKLTAKVVKKWLADKNINVLQWPSQSPDLNPIENLWR
ELKIRVMARRPSNLKELELI
AKDEWAKIPVETCKKLQADIQRFLDEATILDPRFKSKMDKDEVWDRIREVAMAANKEAAAADERADQEIQLYRSLPLIPC
KDNGTICYVHQVKMQQDIHP
ADVPEVTDADLMAADELVEWTPLPPPPPPPGTSLLSAAAVPTGQSSHPAMTSGAESGRPILTSELCLWWDPPEDRPLYSQ
PPATAHAFFHSRFYRWALYN
MWACKLASPACGHKLTD

>g1715.t3
MKKEQYTKILKNNIKQSAEKLDLGHQWTFQHDNDQKLTAKVVKKWLADKNINVLQWPSQSPDLNPIENLWRELKIRVMAR
RPSNLKELELIAKDEWAKIP
VETCKKLQADIQRFLDEATILDPRFKSKMDKDEVWDRIREVAMAANKEAAAADERADQEIQLYRSLPLIPCKDNGTIWWW
SKRDSLPLLSGLPESYLCVQ
ASSTPSERVFSTAGDTINDASAHVCSYVHQVKMQQDIHPADVPEVTDADLMAADELVEWTPLPPPPPPPGTSLLSAAAVP
TGQSSHPAMTSGAESGRPIL
TSELCLWWDPPEDRPLYSQPPATAHAFFHSRFYRWALYNMWACKLASPACGHKLTD
>g1716.t1
MGTEYLECFSCKRKCAAWSQDILDQLDFAYQEHFPAVLTFKLACDRMVIALLNERILGNSATCLRSSLVEQHSRDWMVCS
AVYLSMHRKLLQPGAPLPLV
SLRSMHPMTKKLVMLAAGTAAWVTNLGNEHGQLLMFVLTDSEGTGLKAMAAGLVRRFGEWEGLVVRLDTCHLMRRFARGV
TTGSHQLYGLFMARLSFAMF
EWDPSDVLLLTEAKQAEEGREAHVQLTAKELARHCGAATGAWRRCSAWSRRKWTPLAGRQDTAGVPLIDQTMAEIWWTQR
RHLLCMQDPPGVQMYTKTGE
LSRFTSASGASYRVYLLEGLVRLNDNRGRVAVAASGQSSTMQCYDARLQHDFNQLTQVCLGKTLTENYAQHGQYTVELIG
VEYLYSQTGATLPQDLDQDP
DSAEEDAPEEDAPEESDEGFAEDSVDVRFAVGADGLLRPMQPLPPAATISPPGRGDGGGGGRGKR
>g1716.t2
MGTEYLECFSCKRKCAAWSQDILDQLDFAYQEHFPAVLTFKLACDRMVIALLNERILGNSATCLRSSLVEQHSRDWMVCS
AVYLSMHRKLLQPGAPLPLV
SLRSMHPMTKKLVMLAAGTAAWVTNLGNEHGQLLMFVLTDSEGTGLKAMAAGLVRRFKEAG
>g1716.t3
LVVRLDTCHLMRRFARGVTTGSHQLYGLFMARLSFAMFEWDPSDVLLLTEAKQAEEGREAHVQLTAKELARHCGAATGAW
RRCSAWSRRKWTPLAGRQDT
AGVPLIDQTMAEIWWTQRRHLLCMQDPPGVQMYTKTGELSRFTSASGASYRVYLLEGLVRLNDNRGRVAVAASGQSSTMQ
CYDARLQHDFNQLTQVCLGK
TLTENYAQHGQYTVELIGVEYLYSQTGATLPQDLDQDPDSAEEDAPEEDAPEESDEGFAEDSVDVRFAVGADGLLRPMQP
LPPAATISPPGRGDGGGGGR
GKR
>g1716.t4
MSASALHFQVYLLEGLVRLNDNRGRVAVAASGQSSTMQCYDARLQHDFNQLTQVCLGKTLTENYAQHGQYTVELIGVEYL
YSQTGATLPQDLDQDPDSAE
EDAPEEDAPEESDEGFAEDSVDVRFAVGADGLLRPMQPLPPAATISPPGRGDGGGGGRGKR
>g1716.t5
MGTEYLECFSCKRKCAAWSQDILDQLDFAYQEHFPAVLTFKLACDRMVIALLNERILGNSATCLRSSLVEQHSRDWMVCS
AVYLSMHRKLLQPGAPLPLV
SLRSMHPMTKKLVMLAAGTAAWVTNLGNEHGQLLMFVLTDSEGTGLKAMAAGLVRRFGEWEGLVVRLDTCHLMRRFARGV
TTGSHQLYGLFMARLSFAMF
EWDPSDVLLLTEAKQAEEGREAHVQLTAKELARHCGAATGAWRRCSAWSRRKWTPLAGRQDTAGVPLIDQTMAEIWWTQR
RHLLCMQDPPGVQMYTKTGE
LSRFTSASGASYRVSVKAV
>g1717.t1
ELCTSSTPEFIVDGATRMDMCQGNLGDCWFLSALASLSMHRTLLERVVPPGQGFRNGYDGSFYFRVCTWSHLLHVNQTHV
>g1717.t2
MQELCTSSTPEFIVDGATRMDMCQGNLGDCWFLSALASLSMHRTLLERVVPPGQGFRNGYDGSFYFRVCTWSHLLHVNQT
HV
>g1718.t1
GLEKITTWARMCFKPAKSRSLVLKRGKVMDKFRFSLDSTQIPSVTEKPIRSLGKTFDCTLKDTASIKATHRELEAWLTAV
DKSGLPDWQMKADLAQQLRF
SEHIAETTLRPDIVLFSDSIKQVVLLELTVPWEERMEEANERKWAKYANLVEKCRRRGWRAQCVPIECIY
>g1718.t2
SLVLKRGKVMDKFRFSLDSTQIPSVTEKPIRSLGKTFDCTLKDTASIKATHRELEAWLTAVDKSGLPDWQMKADLAQQLR
FSEHIAETTLRPDIVLFSDS
IKQVVLLELTVPWEERMEEANERKWAKYANLVEKCRRRGWRAQCVPIECIY
>g1718.t3

GLEKITTWARMCFKPAKSRSLVLKRGKVMDKFRFSLDSTQIPSVTEKPIRSLGKTFDCTLKDTASIKATHRELEAWLTAV
DKSGLPDSIKQVVLLELTVP
WEERMEEANERKWAKYANLVEKCRRRGWRAQCVPIECIY
>g1719.t1
MKASERKIFYWEPVSPLLFLVLRLHMEMEDYEDYEDYEPVDCSTAETIRGGTVSYSQGGSKGSVLTYQCSPGYDPFPVSH
RLCDDDGNWSAMISANGRLL
TRAICKEILCPAQLQLDNGDFWPRDQWFPPGTVQSFSCLGGYILSGSANRTCTPSQEWTGTSPICQNNVDACKDPGVPPG
GLRSGSRFQVGDTVSYRCQA
GLDLLGSSQRVCLYSREWTGSAPRCQAYNGFDSPSAVAAVMAASLSGVMDALSPEFQKMEAFGRTIDVEDDSRLHIYILL
DSSGSVAAHYNDSIQAAISL
IRKLDSFDVQIMFHVTSFASTAIDIVTITDTFTSTHAGLVIDSIKKFDPKGKSNIGGNPLAVLARIRSHLGYDNMFVDHT
AESLLDVYVFGVGEVIDRKE
LNNIASHKQNEEHLFILKDIQTLGEVFNSMIDIKTATKCGVAMEETPDDLLLKRWSPTRPWHVTLKTFKAHQLSQIPCYG
SIVSPNWILTAAHCFARSSA
EELSRQIEVKHGSQSELITCEIFIHPNFNMRGLQHRNVSEFYDFDVALVHLNTSIPLSWKSRPICLPCTTAADRAMKKVS
STCRQHRNELLPHNENLASF
INQKSERVETLIHVGEQRQACVSKAAWILKNSTDVTLAELVPERFLCTGGSSTYKNSVSCKGDSGGPLFLRKKMRYFQVG
VLSWGSINLCENTPRAQSRP
RPTLDKDARDFHISLFTIVPWLKEHLSEELEFLVEG
>g1719.t2
MKASERKIFYWEPVSPLLFLVLRLHMEMEDYEDYEDYEPVDCSTAETIRGGTVSYSQGGSKGSVLTYQCSPGYDPFPVSH
RLCDDDGNWSAMISANGRLL
TRAICKEILCPAQLQLDNGDFWPRDQWFPPGTVQSFSCLGGYILSGSANRTCTPSQEWTGTSPICQNNVDACKDPGVPPG
GLRSGSRFQVGDTVSYRCQA
GLDLLGSSQRVCLYSREWTGSAPRCQAYNGFDSPSAVAAVMAASLSGVMDALSPEFQKMEAFGRTIDVEDDSRLHIYILL
DSSGSVAAHYNDSIQAAISL
IRKLDSFDVQIMFHVTSFASTAIDIVTITDTFTSTHAGLVIDSIKKFDPKGKSNIGGNPLAVLARIRSHLGYDNMFVDHT
AESLLDVYVFGVGEVIDRKE
LNNIASHKQNEEHLFILKDIQTLGEVFNSMIDIKTATKCGVAMEETPDDLLLKRWSPTRPWHVTLKTFKAHQLSQIPCYG
SIVSPNWILTAAHCFARSSA
EELSRQIEVKHGSQSELITCEIFIHPNFNMRGLQHRNVSEFYDFDVALVHLNTSIPLSWKSRPICLPCTTAADRAMKKVS
STCRQHRNELLPHNENLASF
INQKSERVETLIHVGEQRQACVSKAAWILKNSTDVTLAELVPERFLCTGGSSTYKNSVSCKGDSGGPLFLRKKMRYFQVG
VLSWGSINLCENTPRAQSRP
RPTLDKDARDFHISLFTIVPWLKEHLSEELEFLVEG
>g1719.t3
MKASERKIFYWEPVSPLLFLVLRLHMEMEDYEDYEDYEPVDCSTAETIRGGTVSYSQGGSKGSVLTYQCSPGYDPFPVSH
RLCDDDGNWSAMISANGRLL
TRAICKEILCPAQLQLDNGDFWPRDQWFPPGTVQSFSCLGGYILSGSANRTCTPSQEWTGTSPICQNNVDACKDPGVPPG
GLRSGSRFQVGDTVSYRCQA
GLDLLGSSQRVCLYSREWTGSAPRCQAYNGFDSPSAVAAVMAASLSGVMDALSPEFQKMEAFGRTIDVEDDSRLHIYILL
DSSGSVAAHYNDSIQAAISL
IRKLDSFDVQIMFHVTSFASTAIDIVTITDTFTSTHAGLVIDSIKKFDPKVHKEKVGTNLYEGLRRVDAMMAQLKENSED
NHFMETQTVIVIITDGKSNI
GGNPLAVLARIRSHLGYDNMFVDHTAESLLDVYVFGVGEVIDRKELNNIASHKQNEEHLFILKDIQTLGEVFNSMIDIKT
ATKCGVAMEETPDDLLLKRW
SPTRPWHVTLKTFKAHQLSQIPCYGSIVSPNWILTAAHCFARSSAEELSRQIEVKHGSQSELITCEIFIHPNFNMRGLQH
RNVSEFYDFDVALVHLNTSI
PLSWKSRPICLPCTTAADRAMKKVSSTCRQHRNELLPHNENLASFINQKSERVETLIHVGEQRQACVSKAAWILKNSTDV
TLAELVPERFLCTGGSSTYK
NSVSCKGDSGGPLFLRKKMRYFQVGVLSWGSINLCENTPRAQSRPRPTLDKDARDFHISLFTIVPWLKEHLSEELEFLVE
G
>g1720.t1
ICGFRGSGCHGLALEVGESVHILEKSEGWYRGFSIRRPNVKVEIH
>g1721.t1
LDFLEKILTFNPMDRLTAEEALAHPYMSDYSFPLDEPIALHPFHIEDEVDDILLMDQSHSHIWDRYHDSQTSEVDWHLHS

THDPDEVQLDPRALSEVTDE
EEVQVDPRKYADGDWEKFLDEPSYDHLSLFPECGHNCNYKAASPSYLDNLIWRDSEVNHYYEPKLIIDLSNWKEQQSKEK
AERKAKSKCEVNGLVKAQIA
HKEAQRDQGLFEKDSEQEKHLTQGNPGPQSQGFDFDSFIAGTIKLSLQPEPFQEAGLLSEVGLLNELNSSVSQLEASRSA
SVPKSISQEKEEKCLVNLAQ
LGGGGSCGGPAETARPLNPWGSFGGGEPAEESSRLINEVCWDIGKEEQKASPYTSYLDRLFSRREDEGAGEIPKSTLSPE
EPSEDKERDACFLGRNDKLV
FNVQLESLALPCFNCSDDLPLKSIQASLAPAAVKCSPQIAHKTYSSIFKHLN
>g1721.t2
LDFLEKILTFNPMDRLTAEEALAHPYMSDYSFPLDEPIALHPFHIEDEVDDILLMDQSHSHIWDRYHDSQTSEVDWHLHS
THDPDEVQLDPRALSEVTDE
EEVQVDPRKYADGDWEKFLDEPSYDHLSLFPECGHNCNYKAASPSYLDNLIWRDSEVNHYYEPKLIIDLSNWKEQQSKEK
AERKAKSKCEVNGLVKAQIA
HKEAQRDQGLFEKDSEQEKHLTQGNPGPQSQGFDFDSFIAGTIKLSLQPEPFQEAGLLSEVGLLNELNSSVSQLEASRSA
SVPKSISQEKEEKCLVNLAQ
LGGGGSCGGPAETARPLNPWGSFGGGEPAEESSRLINEVCWDIGKEEQKASPYTSYLDRLFSRREDEGAGEIPKSTLSPE
EPSEDKERDACFLGRNDKLV
FNVQLESLALPCFNCSDDLPLKSIQASLAPAAVKCSPQIAHKTYSSIFKHLN
>g1721.t3
MDRLTAEEALAHPYMSDYSFPLDEPIALHPFHIEDEVDDILLMDQSHSHIWDRYHDSQTSEVDWHLHSTHDPDEVQLDPR
ALSEVTDEEEVQVDPRKYAD
GDWEKFLDEPSYDHLSLFPECGHNCNYKAASPSYLDNLIWRDSEVNHYYEPKLIIDLSNWKEQQSKEKAERKAKSKCEVN
GLVKAQIAHKEAQRDQGLFE
KDSEQEKHLTQGNPGPQSQGFDFDSFIAGTIKLSLQPEPFQEAGLLSEVGLLNELNSSVSQLEASRSASVPKSISQEKEE
KCLVNLAQLGGGGSCGGPAE
TARPLNPWGSFGGGEPAEESSRLINEVCWDIGKEEQKASPYTSYLDRLFSRREDEGAGEIPKSTLSPEEPSEDKERDACF
LGRNDKLVFNVQLESLALPC
FNCSDDLPLKSIQASLAPAAVKCSPQIAHKTYSSIFKHLN
>g1722.t1
LEGTEVCGCCSLLSPPTANRKSRLQGPRTALQAPAFTGVSHKHTEEQQWVGCCAVLASCLLPTHHTVAAVMETLATLPAW
CLRRTMGAPRERPTTCSQSS
HVADAECQLMKALWKQAGTYVLVRLALALPGRTVHRHH
>g1722.t2
LEGTEVCGCCSLLSPPTANRKSRLQGPRTALQAPAFTGVSHKHTEEQQWVGCCAVLASCLLPTHHTVAAVMETLATLPAW
CLRRTMGAVISQPTAGENIP
RERPTTCSQSSHVADAECQLMKALWKQAGTYVLVRLALALPGRTVHRHH
>g1723.t1
MNSVYTFGLFTVDIFVNAGQVVTGNLAPYFLTVCKPNFTALGCQKALRYISHQEACTGNQGDVLRARKTFPSKEAALSVY
AALYLAMYVTCTVRAKGTRL
AKPVLTLTLMCLAFLTGLNRVAEYRNHWADIMAGFVIGGAIATFLLSFISMQDVWTLWEVRQCRRLIIKYHK
>g1723.t2
MNSVYTFGLFTVDIFVNAGQVVTGNLAPYFLTVCKPNFTALGCQKALRYISHQEACTGNQGDVLRARKTFPSKEAALSVY
AALYLAMYVTCTVRAKGTRL
AKPVLTLTLMCLAFLTGLNRVAEYRNHWADIMAGFVIGGAIATFLWCRCTVRPGKARASLTRTYVPACFHKAFIS
>g1723.t3
LCRMNSVYTFGLFTVDIFVNAGQVVTGNLAPYFLTVCKPNFTALGCQKALRYISHQEACTGNQGDVLRARKTFPSKEAAL
SVYAALYLAMYVTCTVRAKG
TRLAKPVLTLTLMCLAFLTGLNRVAEYRNHWADIMAGFVIGGAIATFLLSFISMQDVWTLWEVRQCRRLIIKYHK
>g1724.t1
RVSSTKFLGVQVSENLFWITNTVSLVKKAKRRLYFLRKLK
>g1725.t1
MTALSSLRTSCLARQDGGQGFALCVTSRQRVQNLSGQNLTDVPVDLSNKTQYLDISDNPLEKLRAAPFQRLSQLCFLKAS
HCGLRRIESDLFAHIPKLKV
LNISHNLLDYIPELSLPLLKVLDLADNQYDRYQLPASFQKMASLSVLHLGSENATQVDFDDFNSLADSSLERLGLGGGVA
WQRYDRGSLAKMKSLQKIIF
KVPFCTGMNIVPCNLMSTLPSLETLDLSNNLLSDSGFWWSVCTYTSVFPKLRRLSLSNNRFVDLAFISQQTHQMNNLESL

DLSFNSIRLEMPCSWPTQIT
ELSLGNNYLGNGVFAYLSKHFQRIDLSKTGITALTPEDVLQFPKLTHLFLSFNSIKVLPMVLSPTLLSLSIDQNALISIS
QENLAGLPRLRTLKAGDNPF
SCTCNSYWFITALNKSLLPDWPLDYACSFPASAAGLPMSEFQTSRMSCETWLQVAVALPVTAVIVAAVCAAFYACDGVWY
TRMLWVWIRVKRRGQRRDNL
LSRVTFRYHAFISYSHQDSVWVESMLVPSLEGAGLTLCVHERDFVPGQWIVDNIINCVEASYKTLFVLSNHFVQSEWCNY
ELFFAQHRALSDQQDSLVFI
LLEPIPTNSLPKKFLKLRSLLMQQTYLEWPKDERKRQVFWANLRSMLQVANHRVLKDIGQELAERASLITDDLAPLLQ
>g1725.t2
MTALSSLRTSCLARQDGGQGFALCVTSRQRVQNLSGQNLTDVPVDLSNKTQYLDISDNPLEKLRAAPFQRLSQLCFLKAS
HCGLRRIESDLFAHIPKLKV
LNISHNLLDYIPELSLPLLKVLDLADNQYDRYQLPASFQKMASLSVLHLGSENATQVDFDDFNSLADSSLERLGLGGGVA
WQRYDRGSLAKMKSLQKIIF
KVPFXMNIVPCNLMSTLPSLETLDLSNNLLSDSGFWWSVCTYTSVFPKLRRLSLSNNRFVDLAFISQQTHQMNNLESLDL
SFNSIRLEMPCSWPTQITEL
SLGNNYLGNGVFAYLSKHFQRIDLSKTGITALTPEDVLQFPKLTHLFLSFNSIKVLPMVLSPTLLSLSIDQNALISISQE
NLAGLPRLRTLKAGDNPFSC
TCNSYWFITALNKSLLPDWPLDYACSFPASAAGLPMSEFQTSRMSCETWLQVAVALPVTAVIVAAVCAAFYACDGVWYTR
MLWVWIRVKRRGQRRDNLLS
RVTFRYHAFISYSHQDSVWVESMLVPSLEGAGLTLCVHERDFVPGQWIVDNIINCVEASYKTLFVLSNHFVQSEWCNYEL
FFAQHRALSDQQDSLVFILL
EPIPTNSLPKKFLKLRSLLMQQTYLEWPKDERKRQVFWANLRSMLQVANHRVLKDIGQELAERASLITDDLAPLLQ
>g1726.t1
MKSHRVLRARAILILGYGVAVRQHHDARATCLYALNVANSFVKCRGSHGFADDVTDAGGGGSTRHHPGDTLGQVGAGGEV
RPRGYKAVTVNAPGGGACAR
RPRVQASGTPAPVVRGTQTTTVVTDSPGEGGSSAESVPP
>g1727.t1
MSSLGTLAFDEYGRPFIIIKDQDKKTRLSGLDALKSHIMAAKAVASTLRTSLGPNGLDKMMVDRDGEVTVTNDGATILGM
MDVDHQIAKLMVELSKSQDD
EIGDGTTGVVVLAGALLEQAELLLDRGIHPIRISDGYDQAAAIAIKQLDKIGETFPFDPNNKEPLIQTAMTTLGSK
>g1727.t2
MSSLGTLAFDEYGRPFIIIKDQDKKTRLSGLDALKSHIMAAKAVASTLRTSLGPNGLDKMMVDRDGEVTVTNDGATILGM
MDVDHQIAKLMVELSKSQDD
EIGDGTTGVVVLAGALLEQAELLLDRGIHPIRISDGYDQAAAIAIKQLDKIGETFPFDPNNKEPLIQTAMTTLGSKV
>g1727.t3
MSSLGTLAFDEYGRPFIIIKDQDKKTRLSGLDALKSHIMAAKAVASTLRTSLGPNGLDKMMVDRDGEVTVTNDGATILGM
MDVDHQIAKLMVELSKSQDD
EIGDGTTGVVAMSLPYTVLAGALLEQAELLLDRGIHPIRISDGYDQAAAIAIKQLDKIGETFPFDPNNKEPLIQTAMTTL
GSK
>g1728.t1
MTYVPGDSFDVLCPNRASEVDAVLHRLGLQDQRRHRAQLAVRTDTKKRGAGLPPHVPDKCTLQYLLTWCLEIRSVPKKAF
LRALVDFAEDSGQKRRLQEL
CSKQGSADYNRFIRDSSLCLLELLTEFSSCRPPLSLLI
>g1728.t2
MPCEKKPRFLVLYGSQKGQAQAIAEGIADEAAEHGLVAEISCLSRHQEHKLERESAPVVFVVSTTGDGEPPDSALKFVKA
IRAKTLPSDHYKHLHYALLG
DTNYANFCNCGKTIDKRLQELGANHFYATGHADDGVGLELVLDPWIEGVWAAIKAAASKMASARDVPLAEKLIDMEKENP
ALTPPLHVELNRLTLADRLT
ADTIEAPSNSDVKLPPAGGLPSPGTPGPXXXXXXXXKPVPGARSDGAGEERAASEPSLTRSQPPLSQSPLNVPALPPAYL
EVTLLPEDAAETVSVLIDFL
LDQA
>g1728.t3
MPCEKKPRFLVLYGSQKGQAQAIAEGIADEAAEHGLVAEISCLSRHQEHKLERESAPVVFVVSTTGDGEPPDSALKFVKA
IRAKTLPSDHYKHLHYALLA
LGDTNYANFCNCGKTIDKRLQELGANHFYATGHADDGVGLELVLDPWIEGVWAAIKAAASKMASARDVPLAEKLIDMEKE
NPALTPPLHVELNRLTLADR

LTADTIEAPSNSDVKLPPAGGLPSPGTPGPXXXXXXXXXKPVPGARSDGAGEERAASEPSLTRSQPPLSQSPLNVPALPPA
YLEVTLLPEDAAETVSVLID
FLLDQA
>g1728.t4
MHTEMFQEVPVINAIQMTQGDSVKKALLLELDISAYPAMTYVPGDSFDVLCPNRASEVDAVLHRLGLQDQRRHRAQLAVR
TDTKKRGAGLPPHVPDKCTL
QYLLTWCLEIRSVPKKAFLRALVDFAEDSGQKRRLQELCSKQGSADYNRFIRDSSLCLLELLTEFSSCRPPLSLLI
>g1728.t5
MPCEKKPRFLVLYGSQKGQAQAIAEGIADEAAEHGLVAEISCLSRHQEHKLERESAPVVFVVSTTGDGEPPDSALKFVKA
IRAKTLPSDHYKHLHYALLG
DTNYANFCNCGKTIDKRLQELGANHFYATGHADDGVGLELVLDPWIEGVWAAIKAAASKMASARDVPLAEKLIDMEKENP
ALTPPLHVELNRLTLADRLT
ADTIEAPSNSDVKLPPAGGLPSPGTPGPXXXXXXXXXKPVPGARSDGAGEERAASEPSLTRSQPPLSQSPLNVPALPPAYL
EVTLLPEDAAETVSVLIDFL
LDQAASEVDAVLHRLGLQDQRRHRAQLAVRTDTKKRGAGLPPHVPDKCTLQYLLTWCLEIRSVPKKAFLRALVDFAEDSG
QKRRLQELCSKQGSADYNRF
IRDSSLCLLELLTEFSSCRPPLSLLI
>g1729.t1
VWQCGGQLEILPCSIVGHVFRNKSPHSFPKGTQVIARNQVRLAEVWMDDYKEIFYRRNQQAGQLAKQVADRLSVARLD
>g1730.t1
YIRLFLVVNIAAFFLLLAVMHLWLPKPRSRRAQRCFYGLLLLDSFILLCLYSSCSHSQC
>g1731.t1
MFRNFNLENRVIREISKEKPLVAPRHKTEVLPPVSSSDAAVETEAIHGKNEPLLSLLKTVYVQSKDPDPAEESPPAAAPE
EEERRPLKFSLLGHQHGLEE
ITDVPKGRLSLVEALKALNNHKTQPSTWTAEKVAQEYSLAPGDTRAVLQFFIPFNVQIIPPKAQGVEQIKAS
>g1731.t2
MFRNFNLENRVIREISKEKPLVAPRHKTEVLPPVSSSDGKNSLPSAVETEAIHGKNEPLLSLLKTVYVQSKDPDPAEESP
PAAAPEEEERRPLKFSLLGH
QHGLEEITDVPKGRLSLVEALKALNNHKTQPSTWTAEKVAQEYSLAPGDTRAVLQFFIPFNVQIIPPKAQGVEQIKAS
>g1731.t3
MFRNFNLENRVIREISKEKPLVAPRHKTEVLPPVSSSDAAVETEAIHGKNEPLLSLLKTVYVQSKDPDPAEESPPAAAPE
EEERRPLKFSLLGHQHGLEE
ITDVPKGRLSLVEALKALNNHKTQPSTWTAEKVAQEYSLAPGDTRAVLQFFIPFNVQIIPPKAQGTSFWRRLLWSVSRFP
LEETLRGSDQENPWSSPYWR
SVWVEEDTEQTETPPEPLVPAAGPTWENEGVKTNPTTAFEINIPGTTSPLNRAVRWSHGADSPRRLVSRTLLLQAELHIG
TTRPGRTGLYSTGLTGLAPR
V
>g1731.t4
LEETLRGSDQENPWSSPYWRSVWVEEDTEQTETPPEPLVPAAGPTWENEGVKTNPTTAFEINIPGTTSPLNRAVRWSHGA
DSPRRLVSRTLLLQAELHIG
TTRPGRTGLYSTGLTGLAPRV
>g1732.t1
MSGQQLEPEALAVSPSAQCPPPPIRSSWESNRLQILTAPTDPLVPSVIVVVVLSVSSADHRDPPRRCLLHDQEKLWSETR
RCAHLDHSRFVPRGVGWAPL
RSA
>g1733.t1
MSGEEHSLSEAEMSPGGSEDCHSLSPTHPRATGGHDSPLNGQSQQLTTLCGAESGGEDGGCRTVPKTEEEDDRFPIGIRE
AVSQVLDGYDWTLVPMPVRV
NSGNKSKPHVKRPMNAFMVWAQAARRKLADQYPHLHNAELSKTLGKLWRLLNEGDKRPFIEEAERLRKQHKKDYPEYKYQ
PRRRKNGKLGPTAEADGQAE
GEASHSQSHYKTLHLEHNGGAGSPLGDLHHHHHHHHHPA
>g1734.t1
LLVSFHANQGLLEGWMPTLLLLRDYNIPAFFTMFSEAELQASLHILHQLEMNILASGPNPFSSHKPEQVQSCPNKPLVYC
NSHFLCYQGLLGSPDG
>g1734.t2
MPTLLLLRDYNIPAFFTMFSEAELQASLHILHQLEMNILASGPNPFSSHKPEQVQSCPNKPLVYCNSHFLCYQGLLGSPD

G
>g1735.t1
RINEEGFRLFLKTYLEVDDLPLDLCQRLFRSFETSEPAPEDGAKEVCLKDVSCYFSLLEDGQPRDKLEFAFKLYDRDGNG
VLDSSVSTAGCWSPSLAFKL
YDRDGNGVLDSSEVDRIIAQMMHAANYLGWDVSELRPVLKDMMVAIDADSSDTVSLEEWVEGGMNNIPLLVLLGLKMTQK
DGQHLWRMKHFNKPVYCNVC
QSMLLGLRKQGLCCTCCKYTVHDRCANRSTDPCTRTYVKSKKETGVPAHDWVLGNCDSGKCDQCQKKIKSYQGLSGKHCV
WCHTMRHDECVAHEPIECTC
GPLRDHILPPWAIYPVIKERPNTVKNGSTASAEDSELNTTPDGQVLQVLSPTHATNARGGRPDAGESSRAAGSRDRARAL
GNRWPRWGKTGEQSDRCLSW
RA
>g1735.t2
RINEEGFRLFLKTYLEVDDLPLDLCQRLFRSFETSEPAPEDGAKEVCLKDVSCYFSLLEDGQPRDKLEFAFKLYDRDGNG
VLDSSVSTAGCWSPSLAFKL
YDRDGNGVLDSSEVDRIIAQMMHAANYLGWDVSELRPVLKDMMVAIDADSSDTVSLEEWVEGGMNNIPLLVLLGLKMTQK
DGQHLWRMKHFNKPVYCNVC
QSMLLGLRKQGLCCTCCKYTVHDRCANRSTDPCTRTYVKSKKETGVPAHDWVLGNCDSGKCDQCQKKIKSYQGLSGKHCV
WCHTMRHDECVAHEPIECTC
GPLRDHILPPWAIYPVIKERPNTVKNGSTASAEDSELNTTPDGQVLQVLSPTHATNARGGRPDAGESSRAAGSRDRARAL
DMQSTLHSLRRDKWRQLSAV
HRHSAAHVC
>g1735.t3
RINEEGFRLFLKTYLEVDDLPLDLCQRLFRSFETSEPAPEDGAKEVCLKDVSCYFSLLEDGQPRDKLEFAFKLYDRDGNG
VLDSSVIAFKLYDRDGNGVL
DSSEVDRIIAQMMHAANYLGWDVSELRPVLKDMMVAIDADSSDTVSLEEWVEGGMNNIPLLVLLGLKMTQKDGQHLWRMK
HFNKPVYCNVCQSMLLGLRK
QGLCCTCCKYTVHDRCANRSTDPCTRTYVKSKKETGVPAHDWVLGNCDSGKCDQCQKKIKSYQGLSGKHCVWCHTMRHDE
CVAHEPIECTCGPLRDHILP
PWAIYPVIKERPNTVKNGSTASAEDSELNTTPDGQVLQVLSPTHATNARGGRPDAGESSRAAGSRDRARALGNRWPRWGK
TGEQSDRCLSWRA
>g1736.t1
MIPRDTRDIKEPSEIFMAGRGSEVGGSSPFSAARLPGRQHRGQEASGGEITSEPSQISPLSNGWRGHMPASQRAQDLCQS
SSASWKSIVSLMMGLGWALG
PATLYTRPRKGLASV
>g1736.t2
MAGRGSEVGGSSPFSAARLPGRQHRGQEASGGEITSEPSQISPLSNGWRGHMPASQRAQDLCQSSSASWKSIVSLMMGLG
WALGPATLYTRPRKGLASV
>g1737.t1
MGVKDPTLVVDGMACLRHWYCCKDWVCGGQWKEYISHLVRWVEAFTSAGIRLVFFFDGVVDEQKRPEWVRRRRRVNGEIS
KVFRHINVHGVQPGRELFCL
PSGLATFTRFALRSLGQEVFCSVQEADYEIAHYAHRYGSMGILGQDSDFLIFNSAPYLSVAKLNLDKLTTVLYRQDSLCQ
TLGLAVNQLPLLACILGNDV
VSEERMQRVRSNAIAAYSEAENPGLIPQSLGLSDPDRKLLERGVRSYLLPGQGPLHGVDTQDVPPGTSCVMERYVSSKIL
KACREKHIAAESFMVYGVVF
EGVVECSNTMEDEEDTELLPQALVFKTCRQRIYGVLLPHAKETSAQIPAVKEWFVFAGNPLKEPEIVYPVPLSTSEDEPS
LESLWFGGGPTVSDPRLARF
LSVFDCQEFLPLYGTIEDDSLLAALCLVTHLVLQPLRFLSCKRAAASQLRYVIPGGRRPVQQLSLEDLDAYLSQAVCGRH
RSHQELRRVQ
>g1737.t2
MGVKDPTLVVDGMACLRHWYCCKDWVCGGQWKEYISHLVRWVEAFTSAGIRLVFFFDGVVDEQKRPEWVRRRRRVNGEIS
KVFRHINVHGVQPGRELFCL
PSGLATFTRFALRSLGQEVFCSVQEADYEIAHYAHRYGSMGILGQDSDFLIFNSAPYLSVAKLNLDKLTTVLYRQDSLCQ
TLGLAVNQLPLLACILGNDV
VSEERMQRVRSNAIAAYSEAENPGLIPQSLGLSDPDRKLLERGVRSYLLPGQGPLHGVDTQDVPPGTSCVMERYVSSKIL
KACREKHIAAESFMVYGVVF
EGVVECSNTMEDEEDTELLPQALVFKTCRQRIYGVLLPHAKEDEPSLESLWFGGGPTVSDPRLARFLSVFDCQEFLPLYG

TIEDDSLLAALCLVTHLVLQ
PLRFLSCKRAAASQLRYVIPGGRRPVQQLSLEDLDAYLSQAVCGRHRSHQELRRVQ
>g1737.t3
MGVKDPTLVVDGMACLRHWYCCKDWVCGGQWKEYISHLVRWVEAFTSAGIRLVFFFDGVVDEQKRPEWVRRRRRVNGEIS
KVFRHINVHGVQPGRELFCL
PSGLATFTRFALRSLGQEVFCSVQEADYEIAHYAHRYGSMGILGQDSDFLIFNSAPYLSVAKLNLDKLTTVLYRQDSLCQ
TLGLAVNQLPLLACILGNDV
VSEERMQRVRSNAIAAYRRSCPYPGGSKGDKVFAVAQLVRSVWGSEAENPGLIPQSLGLSDPDRKLLERGVRSYLLPGQG
PLHGVDTQDVPPGTSCVMER
YVSSKILKACREKHIAAESFMVYGVVFEGVVECSNTMEDEEDTELLPQALVFKTCRQRIYGVLLPHAKETSAQIPAVKEW
FVFAGNPLKEPEIVYPVPLS
TSEDEPSLESLWFGGGPTVSDPRLARFLSVFDCQEFLPLYGTIEDDSLLAALCLVTHLVLQPLRFLSCKRAAASQLRYVI
PGGRRPVQQLSLEDLDAYLS
QAVCGRHRSHQELRRVQ
>g1738.t1
MAAMESGEDFNQKRNTTLQDSQQPSSLALLAATCSRMDTPGEGDSPSDQQSNQQLDLSHGVMTSNGWQVIPVSVQASSGS
TTVSTDSSANSLKNRQVLST
STVGVQGQQQYVVAQPTLQGQQVLTTLSSVMPNIQYQVIPQFQTVDGQQLQFTQAQQDPAVATGQQFQLVSAPNGQQIIA
TTNRATAGGNILTMPGLLQG
AIPIQNLGLGGGLLQNQPQFLANMPMSLNGNITLLPVSTGAGGTGGDGGGDTGLNQLLQQHQPVSSNSSAGYLSGTTTVT
TQTAASYGMTQVQNSNGVAS
SFQHNTVSMGAQPDNRDGQQQQPQQILIQPQQLIQGGSSLQTIQANAAGGQVFTTQEGLQNVQIMPNTGPLILRTVGPNG
QVSWQTIQLQSPMGGQITLA
PVQSLPQLGQAQGATVNTVQIPGLQTINLNTLGSSGLQMHQLQGLPITISNAAGESLQTGGESLDDSTAMDEEDVSPPPQ
GRRNRREACTCPFCKDGEGR
Y
>g1738.t2
MAAMESGEDFNQKRNTTLQDSQQPSSLALLAATCSRMDTPGEGDSPSDQQSNQQLDLSHGVMTSNGWQVIPVSVQASSGS
TTVSTDSSANSLKNRQVLST
STVGVQGQQQYVVAQPTLQGQQVLTTLSSVMPNIQYQVIPQFQTVDGQQLQFTQAQQDPAVATGQQFQLVSAPNGQQIIA
TTNRATAGGNILTMPGLLQG
AIPIQNLGLGGGLLQNQPQFLANMPMSLNGNITLLPVSTGAGGTGGDGGGDTGLNQLLQQHQPVSSNSSAGYLSGTTTVT
TQTAASYGMTQVQNSNGVAS
SFQHNTVSMGAQPDNRDGQQQQPQQILIQPQQLIQGGSSLQTIQANAAGGQVFTTQEGLQNVQIMPNTGPLILRTVGPNG
QVSWQTIQLQSPMGGQITLA
PVQSLPQLGQAQGATVNTVQIPGLQTINLNTLGSSGLQMHQLQGLPITISNAAGESLQTGGESLDDSTAMDEEDVSPPPQ
GRRNRREACTCPFCKDGEG
>g1738.t3
MAAMESGEDFNQKRNTTLQDSQQPSSLALLAATCSRMDTPGEGDSPSDQQSNQQLDLSHGVMTSNGWQVIPVSVQASSGS
TTVSTDSSANSLKNRQVLST
STVGVQGQQQYVVAQPTLQGQQVLTTLSSVMPNIQYQVIPQFQTVDGQQLQFTQAQQDPAVATGQQFQLVSAPNGQQIIA
TTNRATAGGNILTMPGLLQG
AIPIQNLGLGGGLLQNQPQFLANMPMSLNGNITLLPVSTGAGGTGGDGGGDTGLNQLLQQHQPVSSNSSAGYLSGTTTVT
TQTAASYGMTQVQNSNGVAS
SFQHNTVSMGAQPDNRDGQQQQPQQILIQPQQLIQGGSSLQTIQANAAGGQVFTTQEGLQNVQIMPNTGPLILRTVGPNG
QVSWQTIQLQSPMGGQITLA
PVQSLPQLGQAQGATVNTVQIPGLQTINLNTLGSSGLQMHQLQGLPITISNAAGQCVRLGLAFSHGSFS
>g1739.t1
MSAPGLKAAANSKARLIGGVWLSGPKSQLSEGVESRTVNGWPADWSQQSSSLGKRNMVVADDAAFREKSKLLTAMERQKW
LNSYMQKLLVVNTK
>g1739.t2
MSKLPVSTRSSWLMLCILGTSLLTSGSPQPLVQLSPDNSDEVTQEDYDVHLPYFSLRNWGIQLMSAPGLKAAANSKARLI
GGVWLSGPKSQLSEGVESRT
VNGWPADWSQQSSSLGKRNMVVADDAAFREKSKLLTAMERQKWLNSYMQKLLVVNTK
>g1740.t1
DSVVECHATEGGRREKREACLVEDPKVSANRSRAVCHRGNRGLESLKDMLLGLPTVQTGYRFRLQGETGTQHSRTPAEQP

SDGVPLAGKLQASEASRLLL
DTEASLLERREICRQANRMQLQEMIGRLEGLVLIVP
>g1740.t2
MENLYPDRLSYVRLSSAVHTVLCPFAQDSVVECHATEGGRREKREACLVEDPKVSANRSRAVCHRGNRGLESLKDMLLGL
PTVQTGYRFRLQGETGTQHS
RTPAEQPSDGVPLAGKLQASEASRLLLDTEASLLERREICRQANRMQLQEMIGRLEGLVLIVP
>g1741.t1
MTMGKIIFYEDRNFQGRSYETSSDCPDMTSYLSRCHSCRVESGFFMVYDRSNFMGNQYFVKRGEYSDYQRMGMSDCIRSC
RMIPMHRGQFRMRIYERENF
GGQMHEMTDDCDNMMERYRMSECQSCHVMDGHWLMYEQPNYRGRMMYMRPGEYRSMREMGMSGTM
>g1741.t2
MTMGKIIFYEDRNFQGRSYETSSDCPDMTSYLSRCHSCRVESGFFMVYDRSNFMGNQYFVKRGEYSDYQRMGMSDCIRSC
RMIPMHRGQFRMRIYERENF
GGQMHEMTDDCDNMMERYRMSECQSCHVMDGHWLMYEQPNYRGRMMYMRPGEYRSMREMGM
>g1742.t1
MGRITFFEDKNFQGRSYDCSSDCPEFSSHLSRCNSCRVESGCFMVYERTNFMGNQMFLRRGEYPDFQRMSSMTGMMGMTN
MDTVRSCRFIPMHRGQFRMR
IYERENFGGQMHEMMDDCDNMMERYRMSECQSCNVMDGHWLMYEQPNYRGRMMYMRPGEYRSMREMGMSGTMRFNSFRRI
MDMC
>g1743.t1
MTMGKIIFYEDRNFQGRSYETSSDCPDMTSYLSRCHSCRVESGFFMVYDRSNFMGNQYFVKRGEYSDYQ
>g1744.t1
MSMRLVAALEAAVPPPPTGRGSVIIEVTEMLHNASLLIDDIEDNSTLRRGFPVAHSIYGVPSVINSANYVYFLGLEKVLT
LEHPEAVRIFTRQLLELHRG
QGLDIHWRDTYTCPSEQEYRAMVLQKTGGLFGLAVGLMQLFSSWGHNLKPLLDTLGLFFQIRDDYGNLRSREYSENKSFC
EDLTEGKFSFPIIHAIWSRP
ESTQVQNILRQRTDNVDIKRYCVDYLEKVGSFAYTRQTLRTLEEDTCRLIQELGGNPELESLVKHLGKIHREAEAAEAAA
EPASAHPDRLEDLSEKVEAG
GNKPASGQTLHPEPTHPCSDKPAEVSVVGS
>g1744.t2
MGRVWRRRRSFALQTLAEPVGGLEPRPLLIMSMRLVAALEAAVPPPPTGRGSVIIEVTEMLHNASLLIDDIEDNSTLRRG
FPVAHSIYGVPSVINSANYV
YFLGLEKVLTLEHPEAVRIFTRQLLELHRGQGLDIHWRDTYTCPSEQEYRAMVLQKTGGLFGLAVGLMQLFSSWGHNLKP
LLDTLGLFFQIRDDYGNLRS
REYSENKSFCEDLTEGKFSFPIIHAIWSRPESTQVQNILRQRTDNVDIKRYCVDYLEKVGSFAYTRQTLRTLEEDTCRLI
QELGGNPELESLVKHLGKIH
REAEAAEAAAEPASAHPDRLEDLSEKVEAGGNKPASGQTLHPEPTHPCSDKPAEVSVVGS
>g1745.t1
MAVDRRGVGVEVIGVPDLTSNERTVDKLLIHFLRPRNGGGEVQRVLYPLHTPGHALIIYEEPNVEATVNFNTIADTTELK
RLLHRHGLEVIHWDNGWARV
KGSVMMLRPFMVETQKLLKAQTRNLTRPSSSSSPLGSPSPPAAHSKNTHSNSARAKQNPSWSTEAPPYASPSSSPSPTSF
NTSSSSSWAPEGPVDPVDRR
RPTPKPEESSFTSFVTSSDGLQYVQCYKKFAIEDILAKHDVRMDVMALDNDVSRVVVTGWCPPEASIELQHFLSDFGAAL
HVRQIPLGGLDHDAQVGVAK
RIQELKHDFPTVLILQKGDAIHLVGPRRGGKRGAERGKEVKRRSQSLPRSLPYAGGGGGGGGEGEGEPGGGADPTMPASL
GGGRSRPRAGDEGGQDVFRM
AELEALEFGKSDFVLLDEVSMEQFMENLKLRCRSASATSQPGAALQESRRESNRRVKR
>g1745.t2
LTSNERTVDKLLIHFLRPRNGGGEVQRVLYPLHTPGHALIIYEEPNVEATVNFNTIADTTELKRLLHRHGLEVIHWDNGW
ARVKGSVMMLRPFMVETQKL
LKAQTRNLTRPSSSSSPLGSPSPPAAHSKNTHSNSARAKQNPSWSTEAPPYASPSSSPSPTSFNTSSSSSWAPEGPVDPV
DRRRPTPKPEESSFTSFVTS
SDGLQYVQCYKKFAIEDILAKHDVRMDVMALDNDVSRVVVTGWCPPEASIELQHFLSDFGAALHVRQIPLGGLDHDAQVG
VAKRIQELKHDFPTVLILQK
GDAIHLVGPRRGGKRGAERGKEVKRRSQSLPRSLPYAGGGGGGGGEGEGEPGGGADPTMPASLGGGRSRPRAGDEGGQDV
FRMAELEALEFGKSDFVLLD

EVSMEQFMENLKLRCRSASATSQPGAALQESRRESNRRVKR
>g1745.t3
MILFVFTCNKFDHPVEATVNFNTIADTTELKRLLHRHGLEVIHWDNGWARVKGSVMMLRPFMVETQKLLKAQTRNLTRPS
SSSSPLGSPSPPAAHSKNTH
SNSARAKQNPSWSTEAPPYASPSSSPSPTSFNTSSSSSWAPEGPVDPVDRRRPTPKPEESSFTSFVTSSDGLQYVQCYKK
FAIEDILAKHDVRMDVMALD
NDVSRVVVTGWCPPEASIELQHFLSDFGAALHVRQIPLGGLDHDAQVGVAKRIQELKHDFPTVLILQKGDAIHLVGPRRG
GKRGAERGKEVKRRSQSLPR
SLPYAGGGGGGGGEGEGEPGGGADPTMPASLGGGRSRPRAGDEGGQDVFRMAELEALEFGKSDFVLLDEVSMEQFMENLK
LRCRSASATSQPGAALQESR
RESNRRVKR
>g1746.t1
LSLIEEDRAESVEGPQIEDFDSGSQPDSGHTPVALAHREQVALHVDLDDVAMEDPELVESICENAKRYTGLFADSVHELL
PEYRERDIIAKDSLDVYIEH
RLMMEQRGRDPADTRDARNQYPAELMRRFEVYFKTPGTSKPKVVRDVRADSIGHLVSVRGIVTRATEVKPMMAVATYTCD
QCGAETYQPIQSPSFMPLIM
CPSQECVTNKSGGRLYLQTRGSKFVKFQELRIQEHSDQVPVGNIPRSMAVYARGENTRVAQPGDHVAITGVFLPLLRTGF
KQAVQGLLSETYMEAHSITL
MNKTEDDELGSGDLTEEEIHEITADGFYEKLSGSIAPEIYGHEDVKKALLLLLVGGVQQAPKGMKIRGTINICLMGDPGV
AKSQLLCYIDRLAPRSQYTT
GRGSSGVGLTAAVMKDPLTGEMTLEGGALVLADLGVCCIDEFDKMADADRTAIHEVMEQQTISIAKAGIMTSLNARCSIL
AAANPAYGRYNPRKSLEQNI
QLPAALLSRFDLLWLIQDKPDSEADLRLAQHITYVHQHCRQPPTHFTPIDMKLM
>g1746.t2
LSLIEEDRAESVEGPQIEDFDSGSQPDSGHTPVALAHREQVALHVDLDDVAMEDPELVESICENAKRYTGLFADSVHELL
PEYRERDIIAKDSLDVYIEH
RLMMEQRGRDPADTRDARNQYPAELMRRFEVYFKTPGTSKPKVVRDVRADSIGHLVSVRGIVTRATEVKPMMAVATYTCD
QCGAETYQPCAQDPNPPSSV
DVESTRATHAPKATRIPDNPAVFLQIQSPSFMPLIMCPSQECVTNKSGGRLYLQTRGSKFVKFQELRIQEHSDQVPVGNI
PRSMAVYARGENTRVAQPGD
HVAITGVFLPLLRTGFKQAVQGLLSETYMEAHSITLMNKTEDDELGSGDLTEEEIHEITADGFYEKLSGSIAPEIYGHED
VKKALLLLLVGGVQQAPKGM
KIRGTINICLMGDPGVAKSQLLCYIDRLAPRSQYTTGRGSSGVGLTAAVMKDPLTGEMTLEGGALVLADLGVCCIDEFDK
MADADRTAIHEVMEQQTISI
AKAGIMTSLNARCSILAAANPAYGRYNPRKSLEQNIQLPAALLSRFDLLWLIQDKPDSEADLRLAQHITYVHQHCRQPPT
HFTPIDMKLM
>g1746.t3
MAPKDYSAEKEKCKKFLQEFYSEDDNGKKVFKYGAQLVALAHREQVALHVDLDDVAMEDPELVESICENAKRYTGLFADS
VHELLPEYRERDIIAKDSLD
VYIEHRLMMEQRGRDPADTRDARNQYPAELMRRFEVYFKTPGTSKPKVVRDVRADSIGHLVSVRGIVTRATEVKPMMAVA
TYTCDQCGAETYQPIQSPSF
MPLIMCPSQECVTNKSGGRLYLQTRGSKFVKFQELRIQEHSDQVPVGNIPRSMAVYARGENTRVAQPGDHVAITGVFLPL
LRTGFKQAVQGLLSETYMEA
HSITLMNKTEDDELGSGDLTEEEIHEITADGFYEKLSGSIAPEIYGHEDVKKALLLLLVGGVQQAPKGMKIRGTINICLM
GDPGVAKSQLLCYIDRLAPR
SQYTTGRGSSGVGLTAAVMKDPLTGEMTLEGGALVLADLGVCCIDEFDKMADADRTAIHEVMEQQTISIAKAGIMTSLNA
RCSILAAANPAYGRYNPRKS
LEQNIQLPAALLSRFDLLWLIQDKPDSEADLRLAQHITYVHQHCRQPPTHFTPIDMKLM
>g1747.t1
MEKQKLLYQQARLHDRGAAEMVLQTISASKGEMDSMVASTLKLGISILNGGNTTVQQ
>g1747.t2
KPGNDALYMSYATIMAKEKEMEKQKLLYQQARLHDRGAAEMVLQTISASKGEMDSMVASTLKLGISILNGGNTTVQQ
>g1747.t3
LFDSKPGNDALYMSYATIMAKEKEMEKQKLLYQQARLHDRGAAEMVLQTISASKGEMDSMVASTLKLGISILNGGNTTVQ
Q
>g1748.t1

SSVKSEGTPFRGGFRGRGRGRGRGRGRGRGARGHYDHHYGYKAYDVKDGAYGQKFNSTVTYYYDNMSSNDLYTVDHDLLK
DYIKRQIEYYFSLDNLERDF
FLRRKMDQEGFLPIGLIASFHRVQALTTDLSLILEALKDSKEVEIIDLKIRRKEDPEKWPLPGLAMSDAAQTDFSQLINC
PEFVPRQPMEPPTGSSRTSG
GARKQLKTEPDISNLKTMPKGLSASLPDLDSESWIEVKKRPRPSPARPKTPLLQKEKEEMARSPLPLCVSAPSPSAPVNK
EGGVEQDEPEELDFMFDEEM
EQMDGRRNTFTDWSDEDSDCEIDDHDVNKILIVTQTPPYLRKHPGGDRTGQHTSRSKLTTELVKVINDGLFYYEQDLWDD
TCDPEYATIKGQRDTDKMDC
HTYQDGPTANMEDSQEVENFKKVHLISREEFNCLTPEPPVDPNQEVPPGPPKPQQIPTDALANKLFGAPEPSAIARSLPT
TVPDSPNYRSARTPRTPRTP
HRKDSTMTPRFYP
>g1748.t2
SSVKSEGTPFRGGFRGRGRGRGRGRGRGRGARGHYDHHYGYKAYDVKDGAYGQKFNSTVTYYYDNMSSNDLYTVDHDLLK
DYIKRQIEYYFSLDNLERDF
FLRRKMDQEGFLPIGLIASFHRVQALTTDLSLILEALKDSKEVEIIDLKIRRKEDPEKWPLPGLAMSDAAQTDFSQLINC
PEFVPRQPMEPPTGSSRTSG
GARKQLKTEPDISNLKTMPKGLSASLPDLDSESWIEVKKRPRPSPARPKTPLLQKEKEEMARSPLPLCVSAPSPSAPVNK
EGGVEQDEPEELDFMFDEEM
EQMDGRRNTFTDWSDEDSDCEIDDHDVNKILIVTQTPPYLRKHPGGDRTGQHTSRSKLTTELVKVINDGLFYYEQDLWDD
TCDPEYATIKQEVENFKKVH
LISREEFNCLTPEPPVDPNQEVPPGPPKPQQIPTDALANKLFGAPEPSAIARSLPTTVPDSPNYRSARTPRTPRTPHRKD
STMTPRFYP
>g1748.t3
HYDHHYGYKAYDVKDGAYGQKFNSTVTYYYDNMSSNDLYTVDHDLLKDYIKRQIEYYFSLDNLERDFFLRRKMDQEGFLP
IGLIASFHRVQALTTDLSLI
LEALKDSKEVEIIDLKIRRKEDPEKWPLPGLAMSDAAQTDFSQLINCPEFVPRQPMEPPTGSSRTSGGARKQLKTEPDIS
NLKTMPKGLSASLPDLDSES
WIEVKKRPRPSPARPKTPLLQKEKEEMARSPLPLCVSAPSPSAPVNKEGGVEQDEPEELDFMFDEEMEQMDGRRNTFTDW
SDEDSDCEIDDHDVNKILIV
TQTPPYLRKHPGGDRTGQHTSRSKLTTELVKVINDGLFYYEQDLWDDTCDPEYATIKGQRDTDKMDCHTYQDGPTANMED
SQEVENFKKVHLISREEFNC
LTPEPPVDPNQEVPPGPPKPQQIPTDALANKLFGAPEPSAIARSLPTTVPDSPNYRSARTPRTPRTPHRKDSTMTPRFYP
>g1749.t1
LYPYSYHLTLIVALEGHSLLQYMRNTLKRCWLPQLHTRPGRFQTALCCPTFIKLKVKTKRMFARPNHYPKHDERTIPKHN
TWTVGQYCLAVVNTGSTSSI
TRPGVLRLRLPTTIQMRTGNGAQSQIQGRKLLQVASGGNKIQHEFWLTEDQDP
>g1749.t2
MRNTLKRCWLPQLHTRPGRFQTALCCPTFIKLKVKTKRMFARPNHYPKHDERTIPKHNTWTVGQYCLAVVNTGSTSSITR
PGVLRLRLPTTIQMRTGNGA
QSQIQGRKLLQVASGGNKIQHEFWLTEDQDP
>g1749.t3
LYPYSYHLTLIVALEGHSLLQYMRNTLKRCWLPQLHTRPGRFQTALCCPTFIKLKVKTKRMFARPNHYPKHDERTIPKHN
TWTVGQYCLAVVNTGSTSSI
TRPGVLRLRLPTTIQMRTEEESETGSQHARFES
>g1750.t1
MNVMVEKFLDAASIGDLAAVSSLLSRSASLVNQPGTNGWTALMLAARNGHFTVAEALLSHGADRLAVNRSSQTAYDIAKF
WGHTHISHLLVQTDDRSQCI
LLTSDPSQETYFSAEPLDRLSERRSDEAWQQEKQNRPDSVFLLFSNLSPLVRNLEPSTVPGAQTSLCRFSYSLVKELLLK
PESVLVFLGVERRRPSSSSS
SPQNSSEPPAWFAVNSSEDPKVLLELGGEKVFWARRPNRDLLNLSQDEAGILAQARAVLAWHSRYAFCPTCGSSSRSEEA
GYKRTCTNASCRSLQGVHNT
SYPRVDPVVIMLVLHPDGEQCLLGRKKIFPTGMFSCLAGFIEPGESVESAVRREVEEESGVKVGSVRYVACQPWPVPSSL
MIGCLAVALSTHIKVDENEI
EEARWFPRQQVIDALFKGGSPALFLPPQQTIAHQLVKHWIGCTSNL
>g1750.t2
MNVMVEKFLDAASIGDLAAVSSLLSRSASLVNQPGTNGWTALMLAARNGHFTVAEALLSHGADRLAVNRSSQTAYDIAKF

WGHTHISHLLVQTDDRSQCI
LLTSDPSQETYFSAEPLDRLSERRSDEAWQQEKQNRPDSVFLLFSNLSPLVRNLEPSTVPGAQTSLCRFSYSLVKELLLK
PESVLVFLGVERRRPSSSSS
SPQNSSEPPAWFAVNSSEDPKVLLELGGEKVFWARRPNRDLLNLSQDEAGQSK
>g1750.t3
MVEKFLDAASIGDLAAVSSLLSRSASLVNQPGTNGWTALMLAARNGHFTVAEALLSHGADRLAVNRSSQTAYDIAKFWGH
THISHLLVQTDDRSQCILLT
SDPSQETYFSAEPLDRLSERRSDEAWQQEKQNRPDSVFLLFSNLSPLVRNLEPSTVPGAQTSLCRFSYSLVKELLLKPES
VLVFLGVERRRPSSSSSSPQ
NSSEPPAWFAVNSSEDPKVLLELGGEKVFWARRPNRDLLNLSQDEAGILAQARAVLAWHSRYAFCPTCGSSSRSEEAGYK
RTCTNASCRSLQGVHNTSYP
RVDPVVIMLVLHPDGEQCLLGRKKIFPTGMFSCLAGFIEPGESVESAVRREVEEESGVKVGSVRYVACQPWPVPSSLMIG
CLAVALSTHIKVDENEIEEA
RWFPRQQVIDALFKGGSPALFLPPQQTIAHQLVKHWIGCTSNL
>g1750.t4
MGLETQGKGTMGFETQALGTQGGILAQARAVLAWHSRYAFCPTCGSSSRSEEAGYKRTCTNASCRSLQGVHNTSYPRVDP
VVIMLVLHPDGEQCLLGRKK
IFPTGMFSCLAGFIEPGESVESAVRREVEEESGVKVGSVRYVACQPWPVPSSLMIGCLAVALSTHIKVDENEIEEARWFP
RQQVIDALFKGGSPALFLPP
QQTIAHQLVKHWIGCTSNL
>g1751.t1
LWYDSTVNVTVRVHSRLEGVVRYSVPHTFSLSRIVQGPDVVFSGFSSSTLNLSWTPNSWWKVCKVRYTAESGPRTPQEEQ
VEVEPGKEVVYTLQELLPFT
LYRAMVACKPNADNSRSWSFWGSEVKGRTLETAPSEAPEVCYHLERSRGSPGEPPLLRLWLMWTELNVSQTGGRINGYQV
SYNSSTNKLKQAQTTVNVTG
QIALLVVKEESNRVTVKAFNSVGFGPMRTLNTEPHQPALSLPRNLWAFSLFPDHVGFLVQWEMDSVPHTPPTTLGQPAYQ
GASSPAFPVSQFAVEWRIEG
RDSPSGWTRVDNTSAVIQGDLDPEQIYNITVYPVHAPLRGPVRAPFCGPVRYLAVSLQHGALLEVINLRVVDLHKTTSTA
QWLWKKKTSSIRVDRYNAVL
TCKCDSEDQVLLLLPDQSQHTFQNLIPNTEYKHYFMSMVSSPRGSSIGHWLMDKHKQELVRNILTIEDFLVTDLLGDKHP
IKVAAGAQLHLKGSQYMDPH
TAGLGRLLPSQEYVVTAQVVAVSAVYTKNSVLFQSSNSPVATEAFSGETQPLVQHHHHSQHAFLQEEEVAAQLGRFYTPE
VINLCLGEGLDLPGGPPMVC
ESDYVLSSNFTEASPRGRSFFQGIGTEVQ
>g1751.t2
LWYDSTVNVTVRVHSRLEGVVRYSVPHTFSLSRIVQGPDVVFSGFSSSTLNLSWTPNSWWKVCKVRYTAESGPRTPQEEQ
VEVEPGKEVVYTLQELLPFT
LYRAMVACKPNADNSRSWSFWGSEVKGRTLETAPSEAPEVCYHLERSRGSPGEPPLLRLWLMWTELNVSQTGGRINGYQV
SYNSSTNKLKQAQTTVNVTG
QIALLVVKEESNRVTVKAFNSVGFGPMRTLNTEPHQRASSPAFPVSQFAVEWRIEGRDSPSGWTRVDNTSAVIQGDLDPE
QIYNITVYPVHAPLRGPVRA
PFCGPVRYLAVSLQHGALLEVINLRVVDLHKTTSTAQWLWKKKTSSIRVDRYNAVLTCKCDSEDQVLLLLPDQSQHTFQN
LIPNTEYKHYFMSMVSSPRG
SSIGHWLMDKHKQELVRNILTIEDFLVTDLLGDKHPIKVAAGAQLHLKGSQYMDPHTAGLGRLLPSQEYVVTAQVVAVSA
VYTKNSVLFQSSNSPVATEA
FSGETQPLVQHHHHSQHAFLQEEEVAAQLGRFYTPEVINLCLGEGLDLPGGPPMVCESDYVLSSNFTEASPRGRSFFQGI
GTEVQ
>g1751.t3
LWYDSTVNVTVRVHSRLEGVVRYSVPHTFSLSRIVQGPDVVFSGFSSSTLNLSWTPNSWWKVCKVRYTAESGPRTPQEEQ
VEVEPGKEVVYTLQELLPFT
LYRAMVACKPNADNSRSWSFWGSEVKGRTLETAPSEAPEVCYHLERSRGSPGEPPLLRLWLMWTELNVSQTGGRINGYQV
SYNSSTNKLKQAQTTVNVTG
QIALLVVKEESNRVTVKAFNSVGFGPMRTLNTEPHQPALSLPRNLWAFSLFPDHVGFLVQWEMDSVPHTPPTTLGQPAYQ
GASSPAFPVSQFAVEWRIEG
RDSPSGWTRVDNTSAVIQGDLDPEQIYNITVYPVHAPLRGPVRAPFCGPVRYLAVSLQHGALLEVINLRVVDLHKTTSTA
QWLWKKKTSSIRVDRYNAVL

TCKCDSEDQVLLLLPDQSQHTFQNLIPNTEYSLLLMADNNTRATYNFRTPSGAYKHYFMSMVSSPRGSSIGHWLMDKHKQ
ELVRNILTIEDFLVTDLLGD
KHPIKVAAGAQLHLKGSQYMDPHTAGLGRLLPSQEYVVTAQVVAVSAVYTKNSVLFQSSNSPVATEAFSGETQPLVQHHH
HSQHAFLQEEEVAAQLGRFY
TPEVINLCLGEGLDLPGGPPMVCESDYVLSSNFTEASPRGRSFFQGIGTEVQ
>g1752.t1
MTTTFATSQSMQNSNSVSSNTQKVSLSQSADTLVTERDSAMVLNAQTITSETVSRTTTTQITKTVKGGVSETRIEKRIVI
TGDTEIDHDKALAQAIKEAK
EQHPDMSVTKVVVHQETEISPD
>g1752.t2
MQNSNSVSSNTQKVSLSQSADTLVTERDSAMVLNAQTITSETVSRTTTTQITKTVKGGVSETRIEKRIVITGDTEIDHDK
ALAQAIKEAKEQHPDMSVTK
VVVHQETEISPD
>g1752.t3
VSLSQSADTLVTERDSAMVLNAQTITSETVSRTTTTQITKTVKGGVSETRIEKRIVITGDTEIDHDKALAQAIKEAKEQH
PDMSVTKVVVHQETEISPD
>g1753.t1
AESSPVWTDVDHTCRGPPPDHRPYSGDFTASCGREREGDGPPAATAEGAGEQDLSGPRASSRSTTELDLADLNVNDLPCS
PRASHAGVKPAVQPGNTCVC
CALCVPLSVCEGFT
>g1754.t1
LDGLDAKKSAGLGKKMKAISLTMRRKMGKNHHKSFSEEM
>g1754.t2
MPTAFHQLWQITEQLDGLDAKKSAGLGKKMKAISLTMRRKMGKNHHKSFSEEM
>g1755.t1
MFRDTERPFVQPPQAAPGAVPDSLDPRVSDAGGPGGQGAVVGFHPRGPAMDTEMSPKFTSKDEEIDYWKSLSLKYKKSYH
EAQEELQEFQEGSRELEAEL
EAQLGQAEHRLRDLQSENQRLRNEVDNLKVSHVGDLLQESSRPRLLSFQ
>g1756.t1
LNRTLTRTLNRTLPWMGLQIRRPADLTDTRTGRRERQKAVCPFCIPVRLGDGTGVLPRHRKLSARGQHHGGPVARDTDRS
TLWTSSGRHVLVGPQVEQDG
LFSKSFESLSKREKQAVSMVTQTSVSIFSCSTDSAPKVPALIRR
>g1757.t1
MAFYKHGGVEEPKDSECPVCYESLSGSERTLSCGHVFCHDCLVRTLVSITRDGVIRDTIICPVCRHLTFIKKLQDAVGSP
LTKQEDEAKEEQTLEVPLAA
TPGYFTHYPGDLVRSTGTVPLGFDWRRFLRRMCAFQRHVLVSPNLGHNASQIFIISEQGRPMAEDDALSEVSAVVVQQPV
RRRRMKICTTGRWLLFLVSI
FTILALVAATLPWILLA
>g1758.t1
MDTPYIQVDLVKVVELHSTCTEPIQVDLVKVVELHSTCTDGPGEGSTAPVQVDLVKALQNLYRELCLVSSTLDRTGGTGQ
TGAASRTQSLRL
>g1758.t2
MRLNVFLYHCAMDTPYIQVDLVKVVELHSTCTEPIQVDLVKVVELHSTCTDGPGEGSTAPVQVDLVKALQNLYRELCLVS
STLDRTGGTGQTGAASRTQS
LRL
>g1759.t1
MSPVLLLVLLASLVAIASGQSAEDDDVTNTHYSDEEEVTTTDSLLIVSSEAEGDPGDPGFTTIDPSVESENTSIEDVLPG
PENNDTTLEVEDEPLSTIII
LVPVVLVVVIIGMIVCGIAVSRKWNQKATTSGRWSPPPLPGPQSLLRSHAATFRPMFDDDIPSVLELEMEDMDQWVEKQV
PAVL
>g1759.t2
MSPVLLLVLLASLVAIASGQSAEDDDVTNTHYSDEEEVTTTDSLLIVSSEAEGDPGDPGFTTIDPSVESENTSIEDVLPG
PENNDTTLEVEDEPLSTIII
LVPVVLVVVIIGMIVCGIAVSRKWNQKATTSGRWSPPPLPGPQSLLRSHAATFRPMFDDDIPSVLELEMEDMDQWVEKQG
GCLSIAEQYLNTVTEDKKRD
RHFPAEVAIVQFVKE

>g1759.t3
MSPVLLLVLLASLVAIASGQSAEDDDVTNTHYSDEEEVTTTDSLLIVSSEAEGDPGDPGFTTIDPSVESENTSIEDVLPG
PENNDTTLEVEDEPLSTIII
LVPVVLVVVIIGMIVCGIAVSRKWNQKATTSGRWSPPPLPGPQSLLRSHAATFRPMFDDDIPSVLELEMEDMDQWVEKQD
HLRGKLNSLDL
>g1760.t1
MSPKGQKGKAPEKVVHPYSRKAAYLAREEIRLDKKERYLHRFDSELEQIELVNGIKGRQGRLHGARETVIKQTVDRERAM
YESNGFEIPDIINSKHLKTF
REWTGDLKKLPNIKLRQVSNKGLDKVKPDEKAEDGEQDDKHNQEQDAANMSDSD
>g1760.t2
MPKGQKGKAPEKVVHPYSRKAAYLAREEIRLDKKERYLHRFDSELEQIELVNGIKGRQGRLHGARETVIKQTVDRERAMY
ESNGFEIPDIINSKHLKTFR
EWTGDLKKLPNIKLRQVSNKGLDKVKPDEKAEDGEQDDKHNQEQDAANMSDSD
>g1761.t1
LGGEDEQVTYQGSYQGKGSGAGDTAIPTATKHCGVNGDTPQPVGSEDSDAEVCRICHCEGDEEYPLITPCWCTGSLRFVH
QACLNQWIKSSDTRCCELCK
YDFIMETQLKPLNKWERLHMTKAEWRKILCSVVFHLLAISCVSWSVYILIKRTTEDFRLGKEDELWRLALVKYYSAEGVI
EWPFWTKLTVVVMGATSGLF
FMYIQCAAYMQLWRRLKAFNRIITVQNCPEKGLHPPRDPDPGTRLPGDAVEVPVEPSPAPVPGEQTDSDLSVEAAVAPV
>g1762.t1
GPGFLLVHDNARPHVARVCRQFLEDEGIDTIDWPPRSPDLNPIEHLWDIMFQSIRRRQVVPQTVQELSDALVQILEEIPQ
ETIRRLIRSMPRHCQACIQA
RGGNTKY
>g1763.t1
MRMRSLEAIAHLLSLQVEHQTEDLLALTESWFLLLADQPMEMISSICTQPFPELHCSALRIFTAIAPQPWGQRLMIGTPG
FMEFIVDRSTGKTKDAKDAK
FDLVGALVGTSTASNILGSQHYLRLRTYLREGAYYVAAVAVVNTEGAE
>g1763.t2
SFLRRVAGLSLRDRVRSSDIRRELGVEPLLLRVERSQLRWFGHLIRMPPGRLPLEVFRARPTGERFKAVLVRLSQLANLG
ATEMRMRSLEAIAHLLSLQV
EHQTEDLLALTESWFLLLADQPMEMISSICTQPFPELHCSALRIFTAIAPQPWGQRLMIGTPGFMEFIVDRSTGKTKDAK
DAKFDLVGALVGTSTASNIL
GSQHYLRLRTYLREGAYYVAAVAVVNTEGAE
>g1763.t3
MRMRSLEAIAHLLSLQVEHQTEDLLALTESWFLLLADQPMEMISSICTQPFPELHCSALRIFTRLMIGTPGFMEFIVDRS
TGKTKDAKDAKFDLVGALVG
TSTASNILGSQHYLRLRTYLREGAYYVAAVAVVNTEGAE
>g1764.t1
MPVSQQVKPADRDQTPITASSTSGVSRVNMCSECYINQTFVNDDGTTNDHKTSSAPVSPQCDSVRSGIVLDTSTLETEVP
PLPPRFRFRDFLLGEQGFQN
DDR
>g1765.t1
LARGGQACGVLGPEQCPCGSLPSKLLARGCGRFGLLAGDAVDDVTRRTSLDVVSDDVTRRTSLDVVSDDIIRSRSLDVVS
DDIIRSRSLDVVSDDVIRSR
SLDVVFDDITRRTSLDVVFDDLTRRTSVDVVFDDITRRTSLDVVLMTSPGGRLWMLC
>g1766.t1
MVHCTAAMGHYRKSNNLYRLSGQFWCNGVGAQTEHVGVDIHGPFPITDCSNRYVLKAMVFWQLAISVCGYNSSFGDPAEL
HNGQGRNFQSQCYG
>g1766.t2
MVHCTAAMGHYRKSNNLYRLSGQFWCNGVGAQTEHVGVDIHGPFPITDCSNRYVLKAMVFWQLAISVCGYNSSFGDPAEL
HNGQGRNFQSQCYGQFRGAA
YDNRCQGKAFVLGDRVWAFSPSHKKGTPKLAGTGRNIGRYCC
>g1766.t3
MGHYRKSNNLYRLSGQFWCNGVGAQTEHVGVDIHGPFPITDCSNRYVLKAMVFWQLAISVCGYNSSFGDPAELHNGQGRN
FQSQCYG
>g1767.t1

LPFNLSLNLSKISTCFKSSTIVPVPKKAQVTSLNDYRSVTLTSTIMKCFNRLGCVLSPLLYSLYMHDCVSTFSSNPIIKF
ADDTTILGLFTDGDETAYRE
EVNALAVWCQD
>g1767.t2
MHTAPLPFNLSLNLSKISTCFKSSTIVPVPKKAQVTSLNDYRSVTLTSTIMKCFNRLGCVLSPLLYSLYMHDCVSTFSSN
PIIKFADDTTILGLFTDGDE
TAYREEVNALAVWCQD
>g1768.t1
MYNIYNCLLSPECSVGAIADLVFLVDGSWSVGRENFKHIRRFISAMAGAFEIGEDKTRVGIVQYSSDTRTEFPLSRYTRR
GDLMNAISTLPYKGGNTMTG
DAIEYLLNNTFTEAAGARKAFPKVAMIITDGKSQDPVEEHAKRLRNIGVEIFVLGIKGADEDELKEMGSSPHNKHVYNVP
NFDMIQEVQKKIIMEVCSGV
DDQLNVLVSGEEMVEPASNLQVTEIASKSMRVTWDVSVGDVSGYKLTLTPMLPGMKRQEMYMGATQSSINVRDLSPETEY
EISLYALKGLVPSEPVMAME
KTQPIKVSTECSLGVDVQADVVLLVDGSYSIGLQNFAKVRAFLEVLVNSFDVGPNKVQLSLVQYSRDPHTEFALNSHHDI
NAVVRAVRAFPYRGGSTNTG
KAMTYVREKIFISNRGARQNVPRVMVLITDGKSSDSFKDAATKLRNTDVEIFAVGVKDAVRSELEAIANPPSATHVFEVE
DFDAFQRISKELTQSICLRI
EQELLNIKKRSLLPPQNLEFSEVTSRSFRTSWTAATATNVLSYLVRFRKAEDVTGNYISLSVPWETTTAVLPHLFPLTTY
EVNVFAQYDKGDSFPLTGEE
TTLEEQGTVRGLEVTEETTDSFRVSWQAAPGSVIRYHLTYIPLSGAGEMLQTATSGPETTIVLQELFPITTYRVSVSPEY
PSGTGQQKQIDGTTKEVRGS
PRDLRVFDETISTMKLSWRAAPGNVRQYRITYKPADGGDLKETTVKGDTTEALLKSLAPSTEYELFVTASYATGSGDPLL
GTGTTLEGT
>g1768.t2
MYNIYNCLLSPECSVGAIADLVFLVDGSWSVGRENFKHIRRFISAMAGAFEIGEDKTRVGIVQYSSDTRTEFPLSRYTRR
GDLMNAISTLPYKGGNTMTG
DAIEYLLNNTFTEAAGARKAFPKVAMIITDGKSQDPVEEHAKRLRNIGVEIFVLGIKGADEDELKEMGSSPHNKHVYNVP
NFDMIQEVQKKIIMEVCSGV
DDQLNVLVSGEEMVEPASNLQVTEIASKSMRVTWDVSVGDVSGYKLTLTPMLPGMKRQEMYMGATQSSINVRDLSPETEY
EISLYALKGLVPSEPVMAME
KTQPIKVSTECSLGVDVQADVVLLVDGSYSIGLQNFAKVRAFLEVLVNSFDVGPNKVQLSLVQYSRDPHTEFALNSHHDI
NAVVRAVRAFPYRGGSTNTG
KAMTYVREKIFISNRGARQNVPRVMVLITDGKSSDSFKDAATKLRNTDVEIFAVGVKDAVRSELEAIANPPSATHVFEVE
DFDAFQRISKELTQSICLRI
EQELLNIKKRSLLPPQNLEFSEVTSRSFRTSWTAATATNVLSYLVRFRKAEDVTGNYISLSVPWETTTAVLPHLFPLTTY
EVNVFAQYDKGDSFPLTGEE
TTLEEQGTVRGLEVTEETTDSFRVSWQAAPGSVIRYHLTYIPLSGAGEMLQTATSGPETTIVLQELFPITTYRVSVSPEY
PSGTGQQKQIDGTTKEVRGS
PRDLRVFDETISTMKLSWRAAPGNVRQYRITYKPADGGDLKETTVKGDTTEALLKSLAPSTEYELFVTASYATGSGDPLL
GTGTTLEVHACDLM
>g1768.t3
MYNIYNCLLSPECSVGAIADLVFLVDGSWSVGRENFKHIRRFISAMAGAFEIGEDKTRVGIVQYSSDTRTEFPLSRYTRR
GDLMNAISTLPYKGGNTMTG
DAIEYLLNNTFTEAAGARKAFPKVAMIITDGKSQDPVEEHAKRLRNIGVEIFVLGIKGADEDELKEMGSSPHNKHVYNVP
NFDMIQEVQKKIIMEVCSGV
DDQLNVLVSGEEMVEPASNLQVTEIASKSMRVTWDVSVGDVSGYKLTLTPMLPGMKRQEMYMGATQSSINVRDLSPETEY
EISLYALKGLVPSEPVMAME
KTQPIKVSTECSLGVDVQADVVLLVDGSYSIGLQNFAKVRAFLEVLVNSFDVGPNKVQLSLVQYSRDPHTEFALNSHHDI
NAVVRAVRAFPYRGGSTNTG
KAMTYVREKIFISNRGARQNVPRVMVLITDGKSSDSFKDAATKLRNTDVEIFAVGVKDAVRSELEAIANPPSATHVFEVE
DFDAFQRISKELTQSICLRI
EQELLNIKKRSLLPPQNLEFSEVTSRSFRTSWTAATATNVLSYLVRFRKAEDVTGNYISLSVPWETTTAVLPHLFPLTTY
EVNVFAQYDKGDSFPLTGEE
TTLEEQGTVRGLEVTEETTDSFRVSWQAAPGSVIRYHLTYIPLSGAGEMLQTATSGPETTIVLQELFPITTYRVSVSPEY
PSGTGQQKQIDGTTKEVRGS

PRDLRVFDETISTMKLSWRAAPGNVRQYRITYKPADGGDLKETTVKGDTTEALLKSLAPSTEYELFVTASYATGSGDPLL
GTGTTLEARLVNAPGMLLRV
WLWHAEPVHACDLM
>g1769.t1
MCREWERILKAGKAAELGSPKDLVTSDVTDTSFAAHWTAAPGNVRQYRLRWKSMYHDETGEMTVGGDTTDAVLEGLTPET
LYQVSVFAAYGHGEGQPLVG
EETTDG
>g1769.t2
MCREWERILKAGKAAELGSPKDLVTSDVTDTSFAAHWTAAPGNVRQYRLRWKSMYHDETGEMTVGGDTTDAVLEGLTPET
LYQVSVFAAYGHGEGQPLVG
EETTDGTIGLLNI
>g1770.t1
MVNVVQCDDKGDTAVSFFSHVKMASQMPLRARELLEIDICDRGSCSEGDSGVFVNSANSLSDGFSAFQQWRAADAASASN
GLRLSDTSSSPTPVQQVPDL
PGTTTTTTTTDTADSDDPAAHGGMTGGARASIVDCLLVELYDTYSGGKRAYVDSLDSSTEASSSEAFLGRSNTGSNFLQE
LQERHTRRHQMNYLAQK
>g1771.t1
LTGKYPHNHHVINNTLEGNCSSPAWQKTQEPKTFPALLQTQGGYQTFFAGKYLNQYGNAKAGGLGHVPLGWTHWIGLELN
SKYYNYTLSVNGKPTCHGAD
YSQDYLTDVLANASLDFLRYKSSYQPFFMMVSTPAPHSPWTSAPQYQDKYKDTQAPRDPNFNVHGKDKHWLIRQAKTPMS
NSSVKYLDEAFRSRWRTLLS
VDDLVERVVKDLEARKELDNTFIFFTSDNGYHTGQFSLPLDKRQLYEFDIRVPLMVRGPNIKPNQTSPLLVANVDLGPTM
LDIAGYNVNLTEMDGMSFLP
VLEGRANSSSWRTDVLVEYEGEGHNVSDPSCPVLGPGVSECFPDCVCEDSFNNTYACVRSVAPSSNLQYCEFDDNEVRHL
>g1771.t2
MLRASILTGKYPHNHHVINNTLEGNCSSPAWQKTQEPKTFPALLQTQGGYQTFFAGKYLNQYGNAKAGGLGHVPLGWTHW
IGLELNSKYYNYTLSVNGKP
TCHGADYSQDYLTDVLANASLDFLRYKSSYQPFFMMVSTPAPHSPWTSAPQYQDKYKDTQAPRDPNFNVHGKDKHWLIRQ
AKTPMSNSSVKYLDEAFRSR
WRTLLSVDDLVERVVKDLEARKELDNTFIFFTSDNGYHTGQFSLPLDKRQLYEFDIRVPLMVRGPNIKPNQTSPLLVANV
DLGPTMLDIAGYNVNLTEMD
GMSFLPVLEGRANSSSWRTDVLVEYEGEGHNVSDPSCPVLGPGVSECFPDCVCEDSFNNTYACVRSVAPSSNLQYCEFDD
NEVRHL
>g1772.t1
SQQCVFESVVPASRGGFLILRAIGPTRGVSTDSPVTHLDLRTHSVVAPGDSVATLSEAKTGPPSLTLPRYGIKKKEEKEA
EAAAAMEQASEGSLTRPKKA
VPTGCGDEEEEEESIVDTVMKFLPGPLMDMFNKK
>g1773.t1
MGPPKDVSEAVSDRGCIAVSRAVKKCCGECPRGLRSAQRTRSLSSAPSFHQQETGSYSGEGGGYITGPTARFLPPLQLHL
ALRDPASLEPKDLDNLGGEG
TGPPRFQLFPGELAANRLHGACGAGVRALNMNANGIDFLEKDHCASIANESEDNNALHACFYPYSRCCDWQVIAQREKLK
ALKNHMIHFSNKVWYQDDTE
VMNR
>g1774.t1
VGVPNPEGLEKKSMWVDCGIHAREWISPAFCQWFVHEMLQSYNNSDARMTKFLKNVDVYVTPVLNVDGYMFTWGNQS
>g1775.t1
METPSDPTLAFETGQETFPEATVHSTPKKTDGGISPNPPSSLGEDSASYLSSNSRIAGSPARSVLRFGIMQARVTLLDGA
VYTCSIEKRVKGQVLLQKVC
DHLNLLERDYFGLTYNDSDNQKNWLAPSKEMKKQLRGVSWNFYFNVKFYPPDPVRLTEDLTRYFLCLQLRDDIISGRLPC
SIATHTILGSYTVQSELGDH
DPDELGTDYIRELSFAPNQTKEMEEKIVDLHKTY
>g1775.t2
METPSDPTLAFETGQETFPEATVHSTPKKTDGGISPNPPSSLGEDSASYLSSNSRIAGSPARSVLRFGIMQARVTLLDGA
VYTCSIEKRVKGQVLLQKVC
DHLNLLERDYFGLTYNDSDNQKVSPGTSTSMSSFILLILFGSLKTSPDELGTDYIRELSFAPNQTKEMEEKIVDLHKTY
>g1776.t1

MSSKMQSSSGLAQARRSVQQLRVEACMERIKVSKASSDLMHYCREQAKYDPLLMGIPASENPFKEKKPCAIL
>g1776.t2
MQSSSGLAQARRSVQQLRVEACMERIKVSKASSDLMHYCREQAKYDPLLMGIPASENPFKEKKPCAIL
>g1777.t1
MKDREKEFDRRVRENRLDRENDLVRRHKVMKPSQSTPGGGAVEDLLEATQVHPDLSGSPSPQFLLTDQMLLLSRAAWGSL
CLAVVAMAAELTQDQVSWTR
REGEAVSFRCTNTDQCGYNYIYWYQKKEKETFTVILHIHKSNERPLEPPKVSLYPAAGADLKGRRSLLCVARDMHPGVVR
ISWRRKGSGGTENYGEQQEL
RGPSHTASILVVAQEPPLTDQYVCRVEHESGQQEEEMPAET
>g1777.t2
MKDREKEFDRRVRENRLDRENDLVRRHKVMKPSQSTPGGGAVEDLLEATQVHPDLSGSPSPQFLLTDQMLLLSRAAWGSL
CLAVVAMAAELTQDQVSWTR
REGEAVSFRCTNTDQCGYNYIYWYQKKEKETFTVILHIHKSNERPLEPPKVSLYPAAGADLKGRRSLLCVARDMHPGVVR
ISWRRKGSGGTENYGEQQEL
RG
>g1777.t3
MKDREKEFDRRVRENRLDRENDLVRRHKVMKPSQSTPGGGAVEDLLEATQVHPDLSGSPSPQFLLTDQMLLLSRAAWGSL
CLAVVAMAAELTQDQVSWTR
REGEAVSFRCTNTDQCGYNYIYWYQKKEKETFTVILHIHKSNERPLEPPKVSLYPAAGADLKGRRSLLCVARDMHPGVVR
ISWRRKGSGGTENYGEQQEL
RGPSHTASIL
>g1778.t1
VQTLVQQQFEDLECKYSQEKKLYSYISDILTAILSRRLRSGTGLPHKIIMRPNAPV
>g1779.t1
LSRKAKLSIYQSIYVPTLTYGHELWVVTERTRSRIQAAEMSFLRRVAGLSRRDRVRSSDIRRELGVEPLLLRVERSQLRW
FGHLIRMPPGRLPLEVFRAR
PTGKRPRGRPRTRWRDYISHLAWERLGVPQEELESVAGEKDVWSSLLSLLPPRPGPG
>g1780.t1
MAKWSQGRDQTKSGSKTPDGKAKKNQCFLARIGKTGAPYWSQTWEGSSPASVWWPGFDPWGSAGLSPKQQHGGAAALWAH
HLQG
>g1781.t1
METRKRLAKIVLVFVGLFALCWFPNHVLYMYRSFHYQQMDLSLAHLVITLLARVLSFSSSCVNPFALYLLSDSFRRHFNS
QLQCGRAPHPQRQASYLQST
SHIRLTSIKKTTPTAVVAAAVHVTNGGGQRSEVAL
>g1782.t1
MEESPWRKACERGWEEEDEEVGWDGRLRLLNLAWGRRWGGAEEELEALADSRCLLPPPTSSPGHAHLHSPLCSCFGLLGP
RRQTEGRTAKQ
>g1783.t1
AIALLVGAIQAAESRSKENVNATENCVSAVGKVMRFRPECANVPEILPHWLSWLPLNEDKEEAAHTFDFLCDLIESNNPI
VLGPDNSNLPKIFLIIAEGV
ANESIKSEDACSKRLANVVRQVQVRGLFSSLCRDCGVTPHLTEETQDPLQSSEFT
>g1783.t2
LCAEAIALLVGAIQAAESRSKENVNATENCVSAVGKVMRFRPECANVPEILPHWLSWLPLNEDKEEAAHTFDFLCDLIES
NNPIVLGPDNSNLPKIFLII
AEGVANESIKSEDACSKRLANVVRQVQVRGLFSSLCRDCGVTPHLTEETQDPLQSSEFT
>g1784.t1
MALLNWLSSQSDGDKDALMALTGIGVARELFSRLTGQDRVDEFKRECIQSIAEFVRANPRASKAELSAEVNKKVLLFAAR
VKALESTPLL
>g1785.t1
MLMARHSKLQKQILALYRQFLQAGQDKPGFIPRIRDEFRENSRIKKTDVMHIEYLYRRAQRQLEQLRDVNTKQLGSFSKP
KSDS
>g1786.t1
MEVEDQIKRLMDGEWLEVTDLDVHPDQAKPAMLRKTVSYIVCAVILNDKEEVLMVQEAKQECYKQWYLPAGRMEEGEGIA
EAMQREVKEESGFDCQPITL
LLIQEQGPQWIRFIFLAKITGGSLKTPGEADQESLQAAWWDRVSPLQLRGRDILDLIDFGLKYRQDAWHPLTLPVALPCS
HVVQRLVLLFSNAKQQLWVL

LVKAGVLPHHLPTAGAVKTHSLMWASNLVVQDALGSLYYDHDITALGVLGLQHHHHHNHHHHHHSSSSHQQPPGRLQASR
SCDGVCFNMLALLVPDAVQR
DRDGEKLEAVEPSEPPPVENPRYTWHRIQSQELTDSLLRKMHNACTLPIHSLY
>g1786.t2
MLRKTVSYIVCAVILNDKEEVLMVQEAKQECYKQWYLPAGRMEEGEGIAEAMQREVKEESGFDCQPITLLLIQEQGPQWI
RFIFLAKITGGSLKTPGEAD
QESLQAAWWDRVSPLQLRGRDILDLIDFGLKYRQDAWHPLTLPVALPCSHVVQRLVLLFSNAKQQLWVLLVKAGVLPHHL
PTAGAVKTHSLMWASNLVVQ
DALGSLYYDHDITALGVLGLQHHHHHNHHHHHHSSSSHQQPPGRLQASRSCDGVCFNMLALLVPDAVQRDRDGEKLEAVE
PSEPPPVENPRYTWHRIQSQ
ELTDSLLRKMHNACTLPIHSLY
>g1786.t3
MDGEWLEVTDLDVHPDQAKPAMLRKTVSYIVCAVILNDKEEVLMVQEAKQECYKQWYLPAGRMEEGEGIAEAMQREVKEE
SGFDCQPITLLLIQEQGPQW
IRFIFLAKITGGSLKTPGEADQESLQAAWWDRVSPLQLRGRDILDLIDFGLKYRQDAWHPLTLPVALPCSHVVQRLVLLF
SNAKQQLWVLLVKAGVLPHH
LPTAGAVKTHSLMWASNLVVQDALGSLYYDHDITALGVLGLQHHHHHNHHHHHHSSSSHQQPPGRLQASRSCDGVCFNML
ALLVPDAVQRDRDGEKLEAV
EPSEPPPVENPRYTWHRIQSQELTDSLLRKMHNACTLPIHSLY
>g1787.t1
MANVRISERLPSLLDPNLARVAYGSWAVPKLVMELQHPDILTRQRSLAALCDLAHDPEKAYQAIRNGCLEQLKLLLRDDE
PSIRTKTTELLFLLVAHSVG
>g1788.t1
MGTVTSPGLTAGRPDKRKARKTTNRRGATGKMVKRGGAPGVMEEEAHFLEQEVSPAKRWFDGTDQHSSPRRTMTNRTTGA
DPSQGSPEPAPEGSRRKTGR
MRKAPVEWWMLSNAHADTDTQAPSSPPPSSPPPSSPPPSSPPPSPPPPSPPPPSSPPPQPRRQKTHKGGKRQSKQGATLT
SAAPRDTLPVRPPPTPPRAA
AATTTNAGSTPLQRRRMFSVPKTIRHSLATFQDIFTRTAEASPPNAARNAGQPEPWELTPGDPSVCHASSDVLPSAEAPA
GSCSRRPRWPSSTAHTQPNS
RRSGSM
>g1788.t2
MGTVTSPGLTAGRPDKRKARKTTNRRGATGKMVKRGGAPGVMEEEAHFLEQEVSPAKRWFDGTDQHSSPRRTMTNRTTGA
DPSQGSPEPAPEGSRRKTGR
MRKAPVEWWMLSNAHADTDTQAPSSPPPSSPPPSSPPPSSPPPSPPPPSPPPPSSPPPQPRRQKTHKGGKRQSKQGATLT
SAAPRDTLPVRPPPTPPRAA
AATTTNAGSTPLQRRRMFSVPKTIRHSLATFQDIFTRTAEASPPNAARNAGQPEPWELTPGDPSVCHASSDVLPSAEAPA
GSCSRRPRWPSSTAHTQPNS
RRSGSMLTGFQSGPSSHVDLDPNEENEVFPLTTAHCTYWMSLGLHSPPLVPLVLQDEDEANLKEWLSVLWPTPVQEEARQ
TGPGDFEWFSHRDRAIGLQL
DMCSASLCNGKLILGSYMKKPLWVDHCAITVRRLLSAPWNWWNELPANVRTAESLPIFRKRLKTHLFRVHLNPA
>g1788.t3
MGTVTSPGLTAGRPDKRKARKTTNRRGATGKMVKRGGAPGVMEEEAHFLEQEVSPAKRWFDGTDQHSSPRRTMTNRTTGA
DPSQGSPEPAPEGSRRKTGR
MRKAPVEWWMLSNAHADTDTQAPSSPPPSSPPPSSPPPSSPPPSPPPPSPPPPSSPPPQPRRQKTHKGGKRQSKQGATLT
SAAPRDTLPVRPPPTPPRAA
AATTTNAGSTPLQRRRMFSVPKTIRHSLATFQDIFTRTAEASPPNAARNAGQPEPWELTPGDPSVCHASSDVLPSAEAPA
GSCSRRPRWPSSTAHTQPNS
RRSGSMLTGFQSGPSSHVDLDPNEENEVFPLTTAHCTYWMSLGLHSPPLVPLVLQDEDEANLKEWLSVLWPTPVQEEARQ
TGPGDFEWFSHRDRAIGLQL
DMCSASLCNGKLILGSYMKKPLWVDHCAITWWNELPANVRTAESLPIFRKRLKTHLFRVHLNPA
>g1788.t4
MSLGLHSPPLVPLVLQDEDEANLKEWLSVLWPTPVQEEARQTGPGDFEWFSHRDRAIGLQLDMCSASLCNGKLILGSYMK
KPLWVDHCAITVRRLLSAPW
NWWNELPANVRTAESLPIFRKRLKTHLFRVHLNPA
>g1789.t1
MLGDSSCGSEVQPGPLWGLGPHVSRGLAGYVTPYDLMAMWRDGDDESCGLLHAVFALFRVALNGAPDIQGLAGKWGGKAA

RRPFSILSD
>g1789.t2
MTAVCFHCDNALGLGPHVSRGLAGYVTPYDLMAMWRDGDDESCGLLHAVFALFRVALNGAPDIQGLAGKWGGKAARRPFS
ILSD
>g1789.t3
MLGDSSCGSEVQPGPLWGLGPHVSRGLAGYVTPYDLMAMWRDGDDESCGLLHAVFALFRVALNGAPDIQGAS
>g1790.t1
SVLQGHTDYIHCLSVRERAAEVLSGGEDGAYGKWISCLTTDSDWMLCGGGPSLSLWHLRSMSPTSVFPLSGCQRQASFYQ
DMILTVGEGQCVSHCLMGGE
VKAQIPCTPHSINTLAINTNSTEHRWRMSAEVGLGKSAQCSFSLVTGVYLVFRCRCWGDFHAAEYYWLRRGGNVGKSSLL
LRFADNSFSGSYITTIGVDF
KIRTVDIDGERVKLQIWDTAGQERFRTITST
>g1790.t2
DNSLAVGGGDNNIHIMDLENGSFKSVLQGHTDYIHCLSVRERAAEVLSGGEDGAYGKWISCLTTDSDWMLCGGGPSLSLW
HLRSMSPTSVFPLSGCQRQA
SFYQDMILTVGEGQCVSHCLMGGEVKAQIPCTPHSINTLAINTNSTEHRWRMSAEVGLGKSAQCSFSLVTGVYLVFRCRC
WGDFHAAEYYWLRRGGNVGK
SSLLLRFADNSFSGSYITTIGVDFKIRTVDIDGERVKLQIWDTAGQERFRTITST
>g1790.t3
SVLQGHTDYIHCLSVRERAAEVLSGGEDGATCARPQYGKWISCLTTDSDWMLCGGGPSLSLWHLRSMSPTSVFPLSGCQR
QASFYQDMILTVGEGQCVSH
CLMGGEVKAQIPCTPHSINTLAINTNSTEHRWRMSAEVGLGKSAQCSFSLVTGVYLVFRCRCWGDFHAAEYYWLRRGGNV
GKSSLLLRFADNSFSGSYIT
TIGVDFKIRTVDIDGERVKLQIWDTAGQERFRTITST
>g1791.t1
MMGAARVKRRFSAVVDGPVDEEEVMRLAQSAADTEGAG
>g1792.t1
MALNESENSYEFDAPSHVVDLKEFDHADNADHWFEIRTSANDMQLATPLKSDLPFGGASKGNLPKAIVCPTVKADNPEPS
TSADSPPSNIVTSWGGQGTS
QNAARPPTRKATRVPSQNAARTQTKPSAQPRRVSKRKEAAAPVETTGAVVVPPVKKQKRTSAGRAGRRLSGNLRRSCRTP
APTTNSPLPKSSEAQELVRI
KTLQREVAEHRRKNEASYKAALAGDNLPKKLVLSATIPKEFHFSTDSRVKASTSTAGDQVNFQAQLRKHPPSPIKGPKGA
TVPKPFNLSRGNKRKADDAA
AYVPIAEQIAAFQRRTPDRYHLRSRQVQERGPSPVKGEHLKITQPHTPQLMTRQRSRPTQLKSHAELEAEEAEKLHKFRF
KALELNKKILESALNPKKPA
AKEPTRTQGFELQVERRLQQRQVERKAAEEEEEKPHVFHPRPLPSRILGEVVGVPDKKVAHPTVPESPAFALKKRVRMEP
TVEE
>g1792.t2
MALNESENSYEFDAPSHVVDLKEFDHADNADHWFEPSTSADSPPSNIVTSWGGQGTSQNAARPPTRKATRVPSQNAARTQ
TKPSAQPRRVSKRKEAAAPV
ETTGAVVVPPVKKQKRTSAGRAGRRLSGNLRRSCRTPAPTTNSPLPKSSEAQELVRIKTLQREVAEHRRKNEASYKAALA
GDNLPKKLVLSATIPKEFHF
STDSRVKASTSTAGDQVNFQAQLRKHPPSPIKGPKGATVPKPFNLSRGNKRKADDAAAYVPIAEQIAAFQRRTPDRYHLR
SRQVQERGPSPVKGEHLKIT
QPHTPQLMTRQRSRPTQLKSHAELEAEEAEKLHKFRFKALELNKKILESALNPKKPAAKEPTRTQGFELQVERRLQQRQV
ERKAAEEEEEKPHVFHPRPL
PSRILGEVVGVPDKKVAHPTVPESPAFALKKRVRMEPTVEE
>g1792.t3
MALNESENSYEFDAPSHVVDLKEFDHADNADHWFEIRTSANDMQLATPLKSDLPFGGASKGNLPKAIVCPTVKADNPEPS
TSADSPPSNIVTSWGGQGTS
QNAARPPTRKATRVPSQNAARTQTKPSAQPRRVSKRKEAAAPVETTGAVVVPPVKKQKRTSAGRAGRRLSGNLRRSCRTP
APTTNSPLPKSSEAQELVRI
KTLQREVAEHRRKNEASYKAALAGDNLPKKLVLSATIPKEFHFSTDSRVKASTSTAGDQVNFQAQLRKHPPSPIKGPKGA
TVPKPFNLSRGNKRKADDAA
AYVPIAEQIAAFQRRTPDRYHLRSRQVQERGPSPVKGEHLKITQPHTPQLMTRQRSRPTQLKSHAELEAEEAEKLHKFKA
LELNKKILESALNPKKPAAK

EPTRTQGFELQVERRLQQRQVERKAAEEEEEKPHVFHPRPLPSRILGEVVGVPDKKVAHPTVPESPAFALKKRVRMEPTV
EE
>g1793.t1
IRRALLQLLFPEKPFHWARHVAIAVCLLFCVNLLVILVPNIRDIFGIIGATTAPCLIFILPGLFYIRIVPIDQEPMSSRP
KIQAACFTALGFIFMTMSLT
FIGIDWVTGEKRSAGGH
>g1793.t2
IRRALLQLLFPEKPFHWARHVAIAVCLLFCVNLLVILVPNIRDIFGIIVLYSQLSCAGATTAPCLIFILPGLFYIRIVPI
DQEPMSSRPKIQAACFTALG
FIFMTMSLTFIGIDWVTGEKRSAGGH
>g1794.t1
MPIFPVSLQTATSWRGLACETCTCYGDITVCLPPRCPNPRCDYQKGERLRIPGDGCCPECVSTSHHSCQHQGLAYGVKNL
FHSFFVLLFSLVSSPFLLPF
APPQATAAQSVFTLE
>g1794.t2
LISGPLFDLWFHSTATSWRGLACETCTCYGDITVCLPPRCPNPRCDYQKGERLRIPGDGCCPECVSTSHHSCQHQGLAYG
VKNLFHSFFVLLFSLVSSPF
LLPFAPPQATAAQSVFTLE
>g1794.t3
MTVPLETVPWTATSWRGLACETCTCYGDITVCLPPRCPNPRCDYQKGERLRIPGDGCCPECVSTSHHSCQHQGLAYGVKN
LFHSFFVLLFSLVSSPFLLP
FAPPQATAAQSVFTLE
>g1795.t1
LTVTFAAEVHPEVVTAVVTAVVTAVVTEVVTEVVSGAPAGDHRGQAEQWVCPVAEKESNHVHVTTVGCAVKRRGAAWRLG
RCTRATLEKGRAHVKTTTAT
GVVLDHNMTQSLRYVWGNKQDKHGAYLIDRSPEYFEPILNYLRHGQLIVNEGVNIRGVLEEARFFGIEQLGEQLEVALKN
TQPPEDHSPISRKEFVRFLL
ATPTKSELRCQGLNFSGADLSRLDLRYINFKMANLSRCNLTQANLCCSNLERADLSGANLDSGNLQGVKMLCSNAEGASL
RGCNFEDPSGLKANLEGANL
KGVDMEGSQMTGINLRVATLKNAKLKNCNLRGATLAGTDLEVSH
>g1795.t2
LTVTFAAEVHPEVVTAVVTAVVTAVVTEVVTEVVSGAPAGDHRGQADVWGNKQDKHGAYLIDRSPEYFEPILNYLRHGQL
IVNEGVNIRGVLEEARFFGI
EQLGEQLEVALKNTQPPEDHSPISRKEFVRFLLATPTKSELRCQGLNFSGADLSRLDLRYINFKMANLSRCNLTQANLCC
SNLERADLSGANLDSGNLQG
VKMLCSNAEGASLRGCNFEDPSGLKANLEGANLKGVDMEGSQMTGINLRVATLKNAKLKNCNLRGATLAGTDLEVSH
>g1795.t3
LNKHLSLTVTFAAEVHPEVVTAVVTAVVTAVVTEVVTEVVSGAPAGDHRGQAEQWVCPVAEKESNHVHVTTVGCAVKRRG
AAWRLGRCTRATLEKGRAHV
KTTTATGVVLDHNMTQSLRYVWGNKQDKHGAYLIDRSPEYFEPILNYLRHGQLIVNEGVNIRGVLEEARFFGIEQLGEQL
EVALKNTQPPEDHSPISRKE
FVRFLLATPTKSELRCQGLNFSGADLSRLDLRYINFKMANLSRCNLTQANLCCSNLERADLSGANLDSGNLQGVKMLCSN
AEGASLRGCNFEDPSGLKAN
LEGANLKGVDMEGSQMTGINLRVATLKNAKLKNCNLRGATLAGTDLEVSH
>g1796.t1
NTTKIAIARTPTRARRSSVRHSLKLRYSKAELRHSMMQDAVRRASRRSMLKKKSTCGGNSTCKQKPVSSNERITRSMAVN
SPLVGSPALTGKFRGAPAIG
SDLFLYIISRHSGKRKSPDIQDDSPTKKLSPPKKSKPAVRPHMRAFLQTVQKNQMLTMTPMSVGRSSVVKSFIKHTTPLK
IDPK
>g1796.t2
LRYSKAELRHSMMQDAVRRASRRSMLKKKSTCGGNSTCSSNVSEQKPVSSNERITRSMAVNSPLVGSPALTGKFRGAPAI
GSDLFLYIISRHSGKRKSPD
IQDDSPTKKLSPPKKSKPAVRPHMRAFLQTVQKNQMLTMTPMSVGRSSVVKSFIKHTTPLKIDPK
>g1796.t3
LRYSKAELRHSMMQDAVRRASRRSMLKKKSTCGGNSTCKQKPVSSNERITRSMAVNSPLVGSPALTGKFRGAPAIGSDLF
LYIISRHSGKRKSPDIQDDS

PTKKLSPPKKSKPAVRPHMRAFLQTVQKNQMLTMTPMSVGRSSVVKSFIKHTTPLKIDPK
>g1797.t1
ASAVGSGSISFGRNPFGEHEASSHTCSTSPCPAHPALPTMGHLTLTRHGENI
>g1798.t1
MQRREEKGGEEKEEQRMGEDWGRKNKESRDERRGADQQEWTGG
>g1799.t1
MSLWHSSLTPSLPSPLLSPLPRPALTPTQPSPQTSPHPYSAPSPPLLPTSMQPALTALLSPSQPPHQTWTFDFMLGVARR
VCPNLYVVAELFTGSEELDN
VFVTKLGITSLIR
>g1799.t2
MSLWHSSLTPSLPSPLLSPLPRPALTPTQPSPQTSPHPYSAPSPPLLPTSMQPALTALLSPSQPPHQTWTFDTMLSAARA
VRLQFMLGVARRVCPNLYVV
AELFTGSEELDNVFVTKLGITSLIR
>g1800.t1
ANFISKIEIRQRALTQIRPRGFETEGCPSSGCLERAPEFVLARDHQPVMV
>g1801.t1
MILGMVDIARQTVEFLYEENGGIPRDLYLPTIEDIKEEANKFTIDKVRKGLTVVIRCPDSNNTNSTTGGMALPKFAIRGM
LKTFGLHGVVLDVDSVNELV
QVETYLRSEGVLVRYWYPIEMLERPPAGSRRTAANGLVSLDSSNIQIHRELLRCESALARLYCRMALLNIFAPKNPHTFT
RLFHIPAIRDITLEHLQLLS
NQLLAPPLPDGTISSSSILLAQSMLHNLQGQSCSPTDLFYQGNAQPIREWLTVAITRALHQGEESLLELTKQICCFLQNA
PEQFTSEEFPVSESKVSMDV
SFPGAAFVIASCREGQLGCRKE
>g1801.t2
MILGMVDIARQTVEFLYEENGGIPRDLYLPTIEDIKEEANKFTIDKVRKGLTVVIRCPDSNNTNSTTGGMALPKFAIRGM
LKTFGLHGVVLDVDSVNELV
QVETYLRSEGVLVRYWYPIEMLERPPAGSRRTAANGLVSLDSSNIQIHRELLRCESALARLYCRMALLNIFAPKNPHTFT
RLFHIPAIRDITLEHLQLLS
NQLLAPPLPDGTISSSSILLAQSMLHNLQGQSCSPTDLFYQGNAQPIREWLTVAITRALHQGEESLLELTKQICCFLQNA
PEQFTSEEFPVSESKVSMDV
SFPGAAFVIASCREGQLGCRK
>g1801.t3
MGMRRLRCVFQEDHYALLVKAWETKVFPTIRRRFRNEAERKSGLDQIKGALQLGEGSARTGLLGSRRQTVEFLYEENGGI
PRDLYLPTIEDIKEEANKFT
IDKVRKGLTVVIRCPDSNNTNSTTGGMALPKFAIRGMLKTFGLHGVVLDVDSVNELVQVETYLRSEGVLVRYWYPIEMLE
RPPAGSRRTAANGLVSLDSS
NIQIHRELLRCESALARLYCRMALLNIFAPKNPHTFTRLFHIPAIRDITLEHLQLLSNQLLAPPLPDGTISSSSILLAQS
MLHNLQGQSCSPTDLFYQGN
AQPIREWLTVAITRALHQGEESLLELTKQICCFLQNAPEQFTSEEFPVSESKVSMDVSFPGAAFVIASCREGQLGCRKE
>g1802.t1
MVKKGKKEKKVKGAEKTAAKMEKKVSKRSKREEEDLEALIAEFQSLDAKKTQVVETECPPPSPRLNASLSPHPEKDELIL
FGGEFFNGKKTYLYNELYFY
SIKKNSWVKSEIPNPPPPRCAHQVPGAALVIKSEGVTNDSFEELVRAP
>g1803.t1
MPRSTWVALPGAPDDLGPGQTHQTVSGVDSTRGERGEGRTPAAGTERTQTLLSSNCGQLQVSRADRRTVKQGMILIRTND
GHSAPL
>g1804.t1
MHAKATPPQIPDTRRELAELVKRKQELAVCSFRFKLYKFGFVVPETLVNLERQIYAFEGSYLEDTQMYGNIIRGWDRYLT
NQKNSNSKTDRRNRKFKEAE
RLFSKSSVTSVAVSPLKTSNRIRFLLPWPSFSPGPSKATANTVSPSSQQAVCALGGIPDHMNEKREPGSGTESDASPDLQ
NQENEPSQEDTEDGDGTLQD
LKPQKAASSSSTGSHHGNHKKRKNKNRH
>g1804.t2
MAMHAKATPPQIPDTRRELAELVKRKQELAVCSFRFKLYKFGFVVPETLVNLERQIYAFEGSYLEDTQMYGNIIRGWDRY
LTNQKNSNSKTDRRNRKFKE
AERLFSKSSVTSVAVSPLKTSNRIRFLLPWPSFSPGPSKATANTVSPSSQQAVCALGGIPDHMNEKREPGSGTESDASPD

LQNQENEPSQEDTEDGDGTL
QDLKPQKAASSSSTGSHHGNHKKRKNKNRH
>g1804.t3
MHAKATPPQIPDTRRELAELVKRKQELAVCSFRFKLYKFGFVVPETLVNLERQIYAFEGSYLEDTQMYGNIIRGWDRYLT
NQKNSNSKTDRRNRKFKEAE
RLFSKSSVTSVAAVCALGGIPDHMNEKREPGSGTESDASPDLQNQENEPSQEDTEDGDGTLQDLKPQKAASSSSTGSHHG
NHKKRKNKNRH
>g1805.t1
MVAVCNDEKKGFSSQSKRFSLNSPNDNPGPGSYCSFSSAETFSPSFSKKGTTSFPSKAPRLPRHPRRCIPGPDSYNLQSS
PTARHSFGRGPSRVFRAPVA
VPVQEPKFKTPGPNQYDVRGYVLAQRRPDPLMGHYRVDSTVTQNSPMAATAPFRSRSSRFPAQVDHRVPGPGAYDPHQAP
DPVRRPPLPRRHLLVMAMPA
LVVPKGPASPGPAQYDLVDYEGPSRHPASTAAFLSETRRSPQGRRTGQDGPGPGEYASTEGLLLFQYCTMVHGNV
>g1806.t1
SVAVPFQSLSFLSADAQETRLPLQTQQHALRKPERVNR
>g1807.t1
MANLAAAVAAAAANRDPAVDRSLRSVFVGNIPYEATEEQLKDIFSEVGLVVSFRLVYDRETGKPKGYGFCEYQDQETALS
AMRNLNGREFSGRALRVDNA
ASEKNKEELKSLGTGAPVIESPYGDNVRCDEAPESISRAVASLPPEQMFELMKQMKLCVQNSPQEARNMLLQNPQLAYAL
LQAQVVMRIVDPEIALVSED
CRGCVYLSRVL
>g1807.t2
MANLAAAVAAAAANRDPAVDRSLRSVFVGNIPYEATEEQLKDIFSEVGLVVSFRLVYDRETGKPKGYGFCEYQDQETALS
AMRNLNGREFSGRALRVDNA
ASEKNKEELKSKNQPLNQRGLGTGAPVIESPYGDNVRCDEAPESISRAVASLPPEQMFELMKQMKLCVQNSPQEARNMLL
QNPQLAYALLQAQVVMRIVD
PEIALVSEDCRGCVYLSRVL
>g1808.t1
ELLSKKDFPLDVEGSRRLIEDHTQLRKKVLKAPVEELDQEGQRLLQCIRSSDGFSGRNCISGSADFQSLVPKVASLLDKL
HSTRQHLHQSWSSRKLKLDQ
CFQLRLYEQDAEKMFDWVSHNKEVFLQNHTEIGAGYQHAVDLQTQHNHFAMNSMNAYVNINRIMSVGGRLVEGGHYASLQ
IKQISTQLDQDWKSFAAALD
ERSTLLAMSSVFHQKAEQFLSAVESWCKVCSEGGLPSAMQELELAIHRHQSLYEQVTQAYSEVSQDGKALLDVLQRPLSP
GNSESLTATANYSKAVHCIL
DVVHEVLHHQRRLENIWQHRKVRLHQRLQLCVFQQDVQQVMDWIEHHGEVFLSKHTGVGKSLHRARALQKRHDDFEDVAQ
NTYTNADKLLEAAEQLAQTG
ECDPEEIYKAARHLEVTIQDFVRRVEQRKLLLDLSVTFHTHTKE
>g1808.t2
ELLSKKDFPLDVEGSRRLIEDHTQLRKKVLKAPVEELDQEGQRLLQCIRSSDGFSGRNCISGSADFQSLVPKVASLLDKL
HSTRQHLHQSWSSRKLKLDQ
CFQLRLYEQDAEKMFDWVSHNKEVFLQNHTEIGAGYQHAVDLQTQHNHFAMNSMSPPKAWCSLPQKFCYDILGLLDLRLK
DNNAYVNINRIMSVGGRLVE
GGHYASLQIKQISTQLDQDWKSFAAALDERSTLLAMSSVFHQKAEQFLSAVESWCKVCSEGGLPSAMQELELAIHRHQSL
YEQVTQAYSEVSQDGKALLD
VLQRPLSPGNSESLTATANYSKAVHCILDVVHEVLHHQRRLENIWQHRKVRLHQRLQLCVFQQDVQQVMDWIEHHGEVFL
SKHTGVGKSLHRARALQKRH
DDFEDVAQNTYTNADKLLEAAEQLAQTGECDPEEIYKAARHLEVTIQDFVRRVEQRKLLLDLSVTFHTHTKE
>g1808.t3
ELLSKKDFPLDVEGSRRLIEDHTQLRKKVLKAPVEELDQEGQRLLQCIRSSDGFSGRNCISGSADFQSLVPKVASLLDKL
HSTRQHLHQSWSSRKLKLDQ
CFQLRLYEQDAEKMFDWVSHNKEVFLQNHTEIGAGYQHAVDLQTQHNHFAMNSMNAYVNINRIMSVGGRLVEGGHYASLQ
IKQISTQLDQDWKSFAAALD
ERSTLLAMSSVFHQKAEQFLSAVESWCKVCSEGGLPSAMQELELAIHRHQSLYEQVTQAYSEVHCILDVVHEVLHHQRRL
ENIWQHRKVRLHQRLQLCVF
QQDVQQVMDWIEHHGEVFLSKHTGVGKSLHRARALQKRHDDFEDVAQNTYTNADKLLEAAEQLAQTGECDPEEIYKAARH
LEVTIQDFVRRVEQRKLLLD

LSVTFHTHTKE
>g1809.t1
MCNNECDASTEELAHPPELMFDFEGRNPTTFWQSTSWKKYPKPLQVNLTLSWNKTIELTDDIVLTFESGRPEQMVLEKSL
DQGRTWTPYQFYATDCLDAF
TMEPKTVNELTQRTLLDIICTEDYSRGYVWKNDKTVRFEIKDRFALFAGPRLHNMASLYGQLDTTKTLRDFFTITDLRIK
LLKPATGATMVDENNLSRYF
YAISDIKVQGR
>g1810.t1
MEAWRAQFPESEPPCMDLNSQVDIEQELDKCKSSIRHLEKEVNKERFRMIYLQTLLAKERKSYDGQRWGFRKAPQGNAAD
QPAGPESSQALREETTRSAQ
LPESENSFNKPRGGVSPLHQEMETPDIPVGVGSVAALRSNFERIKRANSQSAADGRGLSVAPGGPEKPFYVNVEYHHERG
LVKVNDRDVSDRISTLGCQA
MQMERKRSLPSFPGNLSAAIADIGRSVHRGRSVEGTGCGFSSGSYDDLTARPGRGTPERPGAPECQPYTSVYVGGLMTDR
VAQVVHIRDIHGGEEDGRHL
TWPQRSYSPGSCEDVAGGYTPDCSSNENLTSSEEDFSSGPSTHVSPSPTSCPVYREKSRSPSQQSFESGSPPTPLSMKRL
QQQALASQAVAAHGRRPTRI
GASDGDSTTSSRTSHDNSFQGDL
>g1810.t2
MEAWRAQFPESEPPCMDLNSQVDIEQELDKCKSSIRHLEKEVNKERFRMIYLQTLLAKERKSYDGQRWGFRKAPQGNAAD
QPAGPESSQALREETTRSAQ
LPESENSFNKPRGGVSPLHQEMETPDIPVGVGSVAALRSNFERIKRANSQSAADGRGLSVAPGGPEKPFYVNVEYHHERG
LVKVNDRDVSDRISTLGCQA
MQMERKRSLPSFPGNLSAAIADIGRSVHRGRSVEGTGCGFSSGSYDDLTARPGRGTPERPGAPECQPYTSVYVGGLMTDR
VAQVVHIRDIHGGEEDGRHL
TWPQRSYSPGSCEDVAGGYTPDCSSNENLTSSEEDFSSGPSTHVSPSPTSCPVYREKSRSPSQQSFESGSPPTPLSMKRL
QQQALASQAVAAHGRRPTRI
GASDGDSTTSSRTSHDNSFQGDLVSAVVLLEGPSPWPQAGRPLCPVLPSAFLSERIGIVVVINGIQ
>g1811.t1
MMFWPKLTLRSTFRRGLVTGNKPVIHPLLQWLLQRVPELKKRAYLARYLVKLEVPAEFLQDDVISDTYHQYEELVETFKN
YHKECEQLKTSGFSTAEIRK
DIVVMEEEKDQLIKRVERLRKRVETVPNHQRMLELARQLRVEKEREESLAHQKQEQKNQLFRAEQRLQVSMQKLKDLRQA
SVDADPESLMRRLEEEIKIN
TYIVMEKLPKELESTRRTLHFLQKVASEPSLGPAELQEMEDKIKEVSNEINQLTEKRMMRSDPMDDKLTLFRQQASIIAR
KKEAKAEEIQRAREELAAAE
RELAQRSSQERGDDGEEVIRGDE
>g1811.t2
MMFWPKLTLRSTFRRGLVTGNKPVIHPLLQWLLQRVPELKKRAYLARYLVKLEVPAEFLQDDVISDTYHQYEELVETFKN
YHKECEQLKTSGFSTAEIRK
DIVVMEEEKDQLIKRVERLRKRVETVPNHQRMLELARQLRVEKEREESLAHQKQEQKNQLFRAEQRLQVSMQKLKDLRQA
SVDADPESLMRRLEEEIKIN
TYIVMEKLPKELESTRRTLHFLQKVASEPSLGPAELQEMEDKIKEVSNEINQLTEKRMMRSDPMDDKLTLFRQQVFDLGH
HNDA
>g1811.t3
MFWPKLTLRSTFRRGLVTGNKPVIHPLLQWLLQRVPELKKRAYLARYLVKLEVPAEFLQDDVISDTYHQYEELVETFKNY
HKECEQLKTSGFSTAEIRKD
IVVMEEEKDQLIKRVERLRKRVETVPNHQRMLELARQLRVEKEREESLAHQKQEQKNQLFRAEQRLQVSMQKLKDLRQAS
VDADPESLMRRLEEEIKINT
YIVMEKLPKELESTRRTLHFLQKVASEPSLGPAELQEMEDKIKEVSNEINQLTEKRMMRSDPMDDKLTLFRQQASIIARK
KEAKAEEIQRAREELAAAER
ELAQRSSQERGDDGEEVIRGDE
>g1812.t1
MPASFLNRASSAGPPPPIVIEKLVPRAEEPERPSSSRSSVSFSALSEERLMAAVRLARRDLKKRRQESARSPTASPPPGE
AVHHTGSPDSAQEDAAGVTL
APKTSSPKEKATRSGARSLVQSPQGRCVLAGLGHGRSPPTRDPGLGPSAVSHQPQLSHEIQKVQKELLVYIEKVESGAQK
AEEPLEPEEQQKKEIHCQKQ
AAYSARIIYGLQQQVKDIQDDLEKPCSQKDQSTKKSKAVARLAAAHRSALRAMRLFTQQVSEPPVGKVQSHHKELSQVLR

QLALSAARVKEEDGASTVPA
AALDVLQKLEVRRPSSXALTQPSENGSDLSRGKPKHAAP
>g1813.t1
MSEFLVSLLGERLVNSEKAEVDVQALGCRLSLVGLYFGCSQNAPCKQFNASLCEFYSKFKKSSEHKDKLEVVFISSDQDQ
KHWQDFLQEMQWPALPFKDR
HKKA
>g1814.t1
MCAAKGVAIGIDLGTTYSCVAVFQHGKVEIIANDQGNRTTPSYVAFTDTERLIGDAAKNQVAMNPSNTVFDAKRLIGRKF
DDAVVQADMKHWPFTVTSDT
GKPKIQVDYKGENKSFYPEEVSSMVLVKMKEIAEAYLGQKVSNAVITVPAYFNDSQRQATKDAGVIAGLNVLRIINEPTA
AAIAYGLDKGKSSERNVLIF
DLGGGTFDVSILTIEDGVFEVKSTAGDTHLGGEDFDNRLVNHFVEEFKRKYKKDISKNKRALRRLRTACERAKRTLSSSS
QASIEIDSLYEGTDFYTSIT
RARFEELCSDLFRGTLEPVEKALRDAKMDKGQVHEIVLVGGSTRIPKIQKLLQDFFNGRELNKSINPDEAVAYGAAVQAA
ILSGDTSANVQDLLLLDVAP
LSLGIETAGGVMTPLIKRNTTIPTKQTQSFSTYADNQPGVLIQVFEGERAMTKDNNLLGKFELSGIPPAPRGVPQIEVTF
DIDADGILNVSAQDKSTGKQ
NKITITNDKGRLSKEDIERMVQEADQYKEEDEQQREKIAAKNALESYAFNMKSSLQEDNLKDKVSEADRKKVEEKCEEVI
LWLESNQLAEKEEYQHQLKE
LEKLCNPIISRLYQGGAPTGS
>g1815.t1
MPGKYDPLLGELIKSMLCKRPEERPDVKLILRQPYIKRQIAMFLEATKEKTAKSRKKAVDAAGDNSVSAVSAVSPQQNPK
PQSPPAPPSARPRRRDGAPH
HDAQNGDVSAAPPESHSPNALEASLVSMATISSINIDIQPQQPQPQQQRPAGGEEPAGGEEPAESPARRDRKHKRIPEPS
RGPLPRHPSPRDVSSDGSLV
GGGGGGGRRQHEEERRGSRSPGHLQAVKDPTDKLVVKPPPPPPPPAADDKDDTMELLREAAGGDPLLGLTATLSAVAVGV
PEHSSAEDGRLEGKEEDAST
EHQPSNGQEPQQDASPSPHPASRPGGQAGLTAPHSSTEPCKSQQHRQRGAGHTQAEKLKSRVGSARPLPPPPAEALAGDR
RKRSRVSTEKKRTTTATSSS
SSISPFNGGLLPLPQNERLDGRRSDEEDCSSSTSSTERSEGDGREGKSESTDMQDLVQMMTQTLRMDGREGVNDIMCGST
ALPEFKLNRKYRDTLVLHGK
AWEQTDCSPLNDLPLGAPSGPAKIRRAIECLRTDVVKGLGVKLLDKVLELMEEEDDQKRQVCLREQMGEEKYLTYAVMVR
QLKFFEDVSFKG
>g1815.t2
MPGKYDPLLGELIKSMLCKRPEERPDVKLILRQPYIKRQIAMFLEATKEKTAKSRKKAVDAAGDNSVSAVSAVSPQQNPK
PQSPPAPPSARPRRRDGAPH
HDAQNGDVSAAPPESHSPNALEASLVSMATISSINIDIQPQQPQPQQQRPAGGEEPAGGEEPAESPARRDRKHKRIPEPS
RGPLPRHPSPRDVSSDGSLV
GGGGGGGRRQHEEERRGSRSPGHLQAVKDPTDKLVVKPPPPPPPPAADDKDDTMELLREAAGGDPLLGLTATLSAVAVGV
PEHSSAEDGRLEGKEEDAST
EHQPSNGQEILESTENLLEPFPAITAPEPQQDASPSPHPASRPGGQAGLTAPHSSTEPCKSQQHRQRGAGHTQAEKLKSR
VGSARPLPPPPAEALAGDRR
KRSRVSTEKKRTTTATSSSSSISPFNGGLLPLPQNERLDGRRSDEEDCSSSTSSTERSEGDGREGKSESTDMQDLVQMMT
QTLRMDGREGVNDIMCGSTA
LPEFKLNRKYRDTLVLHGKAWEQTDCSPLNDLPLGAPSGPAKIRRAIECLRTDVVKGLGVKLLDKVLELMEEEDDQKRQV
CLREQMGEEKYLTYAVMVRQ
LKFFEDVSFKG
>g1815.t3
MPGKYDPLLGELIKSMLCKRPEERPDVKLILRQPYIKRQIAMFLEATKEKTAKSRKKAVDAAGDNSVSAVSAVSPQQNPK
PQSPPAPPSARPRRRDGAPH
HDAQNGDVSAAPPESHSPNALEASLVSMATISSINIDIQPQQPQPQQQRPAGGEEPAGGEEPAESPARRDRKHKRIPEPS
RGPLPRHPSPRDVSSDGSLV
GGGGGGGRRQHEEERRGSRSPGHLQAVKDPTDKLVVKPPPPPPPPAADDKDDTMELLREAAGGDPLLGLTATLSAVAVGV
PEHSSAEDGRLEGKEEDAST
EHQPSNGQEILESTENLLEPFPAITAPEPQQDASPSPHPASRPGGQAGLTAPHSSTEPCKSQQHRQRGAGHTQAEKLKSR
VGSARPLPPPPAEALAGDRR

KRSRVSTEKKRTTTATSSSSSISPFNGGLLPLPQERPLSARERRRLRQSQEQAVQPVNAARRASYDVTSYQSKHQDVRAC
RSVSESGEDLSQNERLDGRR
SDEEDCSSSTSSTERSEGDGREGKSESTDMQDLVQMMTQTLRMDGREGVNDIMCGSTALPEFKLNRKYRDTLVLHGKAWE
QTDCSPLNDLPLGAPSGPAK
IRRAIECLRTDVVKGLGVKLLDKVLELMEEEDDQKRQVCLREQMGEEKYLTYAVMVRQLKFFEDVSFKG
>g1816.t1
VPFPRNFQQVCKKMLSRLFRVFVHVYIHHFDSICSMGAEAHINTCYKHYYYFISEFSLIEHSELEPLVREEGAAGAGCWA
GEMGRGAG
>g1817.t1
MVIYSAMADLQVFQHALPSQRGSMAVGQEADSRGGSRPNGTTPVERQRLSDSPAVAVDMEKSQPVNIPDAAKRKKKKRTR
ATDSFTGCFDDVYRLTDEML
GQGAYAKVQGCISLQDGKEYAVKIVEKSAGHSRSRVFREVETLYQCQGSRNILELIQFFEDTSCFYLVFEKLRGGSILTH
IQNRRHFDELEASRVVRDIA
QALDFLHTKGIAHRDLKLENILCEYTDRVSPVKICDFDLGSGVKLSSACTPITTPELTTPCGSAEYMAPEVVEVFTDEAS
FYDKRCDLWSLGVILYILLS
GSPPFTGHCGSDCGWDRGEMCKTCQSHLFESIQGGKYEFPERDWARISEGAKDLISKLLVRDATQRFSAAQVLKHPWVQG
NAPERGLHTPRVLQSVQQQV
CLASWKPPVMCGERMGRVLAGSQVGRNSSTRDLTQFTADAVAFNRQLSHHDEEREDVGAGAPAGPPVVVCSMRLSPPSNS
RLARRRAQSQAVRAVDFLPS
AGGLDPPAP
>g1817.t2
MAVGQEADSRGGSRPNGTTPVERQRLSDSPAVAVDMEKSQPVNIPDAAKRKKKKRTRATDSFTGCFDDVYRLTDEMLGQG
AYAKVQGCISLQDGKEYAVK
IVEKSAGHSRSRVFREVETLYQCQGSRNILELIQFFEDTSCFYLVFEKLRGGSILTHIQNRRHFDELEASRVVRDIAQAL
DFLHTKGIAHRDLKLENILC
EYTDRVSPVKICDFDLGSGVKLSSACTPITTPELTTPCGSAEYMAPEVVEVFTDEASFYDKRCDLWSLGVILYILLSGSP
PFTGHCGSDCGWDRGEMCKT
CQSHLFESIQGGKYEFPERDWARISEGAKDLISKLLVRDATQRFSAAQVLKHPWVQGNAPERGLHTPRVLQSVQQQVCLA
SWKPPVMCGERMGRVLAGSQ
VGRNSSTRDLTQFTADAVAFNRQLSHHDEEREDVGAGAPAGPPVVVCSMRLSPPSNSRLARRRAQSQAVRAVDFLPSAGG
LDPPAP
>g1817.t3
MVIYSAMADLQVFQHALPSQRGSMAVGQEADSRGGSRPNGTTPVERQRLSDSPAVAVDMEKSQPVNIPDAAKRKKKKRTR
ATDSFTGCFDDVYRLTDEML
GQGAYAKVQGCISLQDGKEYAVKIVEKSAGHSRSRVFREVETLYQCQGSRNILELIQFFEDTSCFYLVFEKLRGGSILTH
IQNRRHFDELEASRVVRDIA
QALDFLHTKGIAHRDLKLENILCEYTDRVSPVKICDFDLGSGVKLSSACTPITTPELTTPCGSAEYMAPEVVEVFTDEAS
FYDKRCDLWSLGVILYILLS
GSPPFTGHCGSDCGWDRGEMCKTCQSHLFESIQGGKYEFPERDWARISEGAKDLISKLLVRDATQRFSAAQVLKHPWVQG
NAPERGLHTPRVLQRNSSTR
DLTQFTADAVAFNRQLSHHDEEREDVGAGAPAGPPVVVCSMRLSPPSNSRLARRRAQSQAVRAVDFLPSAGGLDPPAP
>g1818.t1
LAEEHEPETSPQLREREELRRHGAHLLGVLARGGALEPEAMEHIIGGAHLQHTHAEPISFGIDQILNNVEQSCMLGARMH
EQPDYGYGGCVVSAAYGGMN
GGFACSNNGYNNMNHINNNNNNNSCGMASLGGAYNMNMGVSVNGSNVNGAGVIRVPAHRPLSGGHAALQSGGMMSGAINN
LTGLTFPWMESNRRYTKDRF
TGHPYQNRTPPKKKKPRTSFTRLQICELEKRFHRQKYLASAERAALAKALKMTDAQVKTWFQNRRTKWRRQTAEEREAER
QQANRILMQLQQEAFQKTIN
QPVTPDPLCLHNSSLYALQNLQPWTENTAKISSVSACE
>g1819.t1
MSLLRFTATTKSLSSWSFISKSVLTGCSRSASGATNDKQPLRKAKTPQGRLDSTEEKDKDGNQRFPDDVNPDTKEQGGPR
GPEPTRYGDWERKGRCIDF
>g1819.t2
MSLLRFTATTKSLSSWSFISKSVLTASGATNDKQPLRKAKTPQGRLDSTEEKDKDGNQRFPDDVNPDTKEQGGPRGPEPT
RYGDWERKGRCIDF
>g1820.t1

LPSAAPACCVTRWSLPRCKQQGEEEGGSSHFTKEKTPCGETVTSELACPSACPALQQSSQLQSQLLQHCSSQQPLTPEQG
SLTPSRPVPPSQKGSRPTGH
QRYGSPSEAEVPYLTPTVRRTPPG
>g1820.t2
LPSAAPACCVTRWSLPRCKQQGEEEGGSSHFTKEKTPCGETVTSELACPSACPALQQSSQLQSQLLQHCSSQQPLTPEQG
SLTPSRPVPPSQKGSRPTGS
PSEAEVPYLTPTVRRTPPG
>g1821.t1
MFLPEVGNYKRTVKRTDDGHRLCNDLMNCIQERAKIEKAYSQQLTDWSKRWRQLVDKGPQYGTVERAWTALMLEAEKVSE
LHQDVKNGLMNVDFEKVKNW
QKDSYHKQMMGGFKETKESEEGFKKAQKPWAKKLKEVEAAKKNYHAACKEEKLASMREANGKADTSTTADQLKKLTEKVD
KCKQDSQKAREKYEKGLEEL
NNCTPQYMENMEQVFAQTQLFEEKRLSFLREVLLDVKRHLNLTENQSYAAVYSNLECVVTSASPQEDLKWFSNAHGPGMH
MSWPQFEEYSEATHNINKRE
KSRKNQEGVLLTNMSLPTDSPKGGGDHASLSASQEWSDEEQGGPDSGGHSNGAPNPFEEEAGEAGTGVRVRALYDYEGQE
QDELSFKAGEELIKLEDEDE
QGWCKGRLDSGQLGLYPANYVEPV
>g1821.t2
MFLPEVGNYKRTVKRTDDGHRLCNDLMNCIQERAKIEKAYSQQLTDWSKRWRQLVDKGPQYGTVERAWTALMLEAEKVSE
LHQDVKNGLMNVDFEKVKNW
QKDSYHKQMMGGFKETKESEEGFKKAQKPWAKKLKEVEAAKKNYHAACKEEKLASMREANGKADTSTTADQLKKLTEKVD
KCKQDSQKAREKYEKGLEEL
NNCTPQYMENMEQVFAQTQLFEEKRLSFLREVLLDVKRHLNLTENQSYAAVYSNLECVVTSASPQEDLKWFSNAHGPGMH
MSWPQFELPRVLLHIEWIVL
PQEYSEATHNINKREKSRKNQEGVLLTNMSLPTDSPKGGGDHASLSASQEWSDEEQGGPDSGGHSNGAPNPFEEEAGEAG
TGVRVRALYDYEGQEQDELS
FKAGEELIKLEDEDEQGWCKGRLDSGQLGLYPANYVEPV
>g1821.t3
MFLPEVGNYKRTVKRTDDGHRLCNDLMNCIQERAKIEKAYSQQLTDWSKRWRQLVDKGPQYGTVERAWTALMLEAEKVSE
LHQDVKNGLMNVDFEKVKNW
QKDSYHKQMMGGFKETKESEEGFKKAQKPWAKKLKEVRAVFVFLCRICLRTALGAVVEAAKKNYHAACKEEKLASMREAN
GKADTSTTADQLKKLTEKVD
KCKQDSQKAREKYEKGLEELNNCTPQYMENMEQVFAQTQLFEEKRLSFLREVLLDVKRHLNLTENQSYAAVYSNLECVVT
SASPQEDLKWFSNAHGPGMH
MSWPQFEEYSEATHNINKREKSRKNQEGVLLTNMSLPTDSPKGGGDHASLSASQEWSDEEQGGPDSGGHSNGAPNPFEEE
AGEAGTGVRVRALYDYEGQE
QDELSFKAGEELIKLEDEDEQGWCKGRLDSGQLGLYPANYVEPV
>g1822.t1
MRKSSFELSLTNCSDQSQLLFSLWEELQSGTFNVIMEHRQVFAPVDRGQDQPAQQPSFPRVLEETALLSRVLEDAALLSR
VLEDAASRANSTVQRFDRKT
PEMKHETNRLNRRLLGVTHPGCDPPLTTTYGEFNRTASRLLRIKESLQRGRLHVFSQLGVSPSPTVERVLQGSTA
>g1823.t1
QLWSEADSSCSGQPIHLWQFLRELLLKPHSYGRCIRWLNKEKGIFKIEDSAHVARLWGLRKNRPAMNYDKLSRSIRQYYK
KGIIRKPDVSQRLVYQFVHP
V
>g1824.t1
CVSRRVRSPFPHLLRDEDVVSCCLDLVAPSISFRVNGLPVQGMLENFNTDGLMYPAVSFSAGVTVRFLFGGRHGEFRFLP
PPGFAPCSEALLPGGATLKV
EPCQRYTLDLEDGRRELLGPSVALLPPTFTPTPVDISKVRMGASNPQVELPAQLEHIRQRLAENIHELWSMDKIELGWAY
GPTRDESKRFDPCLVEFPKL
PEKEKNQNLQMAQITIKPAPLDLSRTVLCPALETAVGSLAENEHNAWAQELIRQGWTYGAQQDIKARRSPQLVPYSLLED
QSRAAGRDSAREAVCALLAY
GYSLEPLDLEPTALRDPRLLASGEKGRVFRADRRYAVTEGRWYFEFEALTAGDMRVGWARPGCSPDRELGSDGLAYAFDG
STAQWYHQGAEPLGCPWKRG
DVVGCLLDMAQCTMMVSLNGEVLLNHLGSELAAQGFDVEDGLLPAVSVGVNQSGRLNLGRQGATLSCFALCGLQEGYQPF
AVNMAREPALWMSWRQPQFA

SVTPHHRDLTVTRDSGKSDASPSLKVTQRAMTSPHTGGSEMGFYRLSMSVQTAAVLASPAGAALQGTTSPTELWPFVFTG
RKDMEEFEVDSDFEVLMKSA
HGFAGSRDELNHKDQSHDKTSRLKQRRFLLKKSKPGLVSSNSSARLLEDVLVDKDHDSCLVQSSTDPFSVWVGWVTSDFH
QHGPTFDPDRTRTVTVTLGD
DSGKVQESVKRSNCYMVCAGEATGLSQSRRSGGLEIGCLVNTASGLLTFTSSGVDMATFYQVEGSTRLFPAVFIKPTAAN
MFQFNLGCVKSAMPLSAGLF
RSLRSEVAPRCPPRLQVQRL
>g1824.t2
MRYEQPGGYRPAPLDLSRTVLCPALETAVGSLAENEHNAWAQELIRQGWTYGAQQDIKARRSPQLVPYSLLEDQSRAAGR
DSAREAVCALLAYGYSLEPL
DLEPTALRDPRLLASGEKGRVFRADRRYAVTEGRWYFEFEALTAGDMRVGWARPGCSPDRELGSDGLAYAFDGSTAQWYH
QGAEPLGCPWKRGDVVGCLL
DMAQCTMMVSLNGEVLLNHLGSELAAQGFDVEDGLLPAVSVGVNQSGRLNLGRQGATLSCFALCGLQEGYQPFAVNMARE
PALWMSWRQPQFASVTPHHR
DLTVTRDSGKSDASPSLKVTQRAMTSPHTGGSEMGFYRLSMSVQTAAVLASPAGAALQGTTSPTELWPFVFTGRKDMEEF
EVDSDFEVLMKSAHGFAGSR
DELNHKDQSHDKTSRLKQRRFLLKKSKPGLVSSNSSARLLEDVLVDKDHDSCLVQSSTDPFSVWVGWVTSDFHQHGPTFD
PDRTRTVTVTLGDDSGKVQE
SVKRSNCYMVCAGEATGLSQSRRSGGLEIGCLVNTASGLLTFTSSGVDMATFYQVEGSTRLFPAVFIKPTAANMFQFNLG
CVKSAMPLSAGLFRSLRSEV
APRCPPRLQVQRL
>g1824.t3
CVSRRVRSPFPHLLRDEDVVSCCLDLVAPSISFRVNGLPVQGMLENFNTDGLMYPAVSFSAGVTVRFLFGGRHGEFRFLP
PPGFAPCSEALLPGGATLKV
EPCQRYTLDLEDGRRELLGPSVALLPPTFTPTPVDISKVRMGASNPQVELPAQLEHIRQRLAENIHELWSMDKIELGWAY
GPGEVVFAVFGLQTRDESKR
FDPCLVEFPKLPEKEKNQNLQMAQITIKPAPLDLSRTVLCPALETAVGSLAENEHNAWAQELIRQGWTYGAQQDIKARRS
PQLVPYSLLEDQSRAAGRDS
AREAVCALLAYGYSLEPLDLEPTALRDPRLLASGEKGRVFRADRRYAVTEGRWYFEFEALTAGDMRVGWARPGCSPDREL
GSDGLAYAFDGSTAQWYHQG
AEPLGCPWKRGDVVGCLLDMAQCTMMVSLNGEVLLNHLGSELAAQGFDVEDGLLPAVSVGVNQSGRLNLGRQGATLSCFA
LCGLQEGYQPFAVNMAREPA
LWMSWRQPQFASVTPHHRDLTVTRDSGKSDASPSLKVTQRAMTSPHTGGSEMGFYRLSMSVQTAAVLASPAGAALQGTTS
PTELWPFVFTGRKDMEEFEV
DSDFEVLMKSAHGFAGSRDELNHKDQSHDKTSRLKQRRFLLKKSKPGLVSSNSSARLLEDVLVDKDHDSCLVQSSTDPFS
VWVGWVTSDFHQHGPTFDPD
RTRTVTVTLGDDSGKVQESVKRSNCYMVCAGEATGLSQSRRSGGLEIGCLVNTASGLLTFTSSGVDMATFYQVEGSTRLF
PAVFIKPTAANMFQFNLGCV
KSAMPLSAGLFRSLRSEVAPRCPPRLQVQRL
>g1825.t1
QYSRTLRYSTQTVRCLRSSVKPVGEWRLRNGTTLYAVYVAQWSSQRRVSPSSVHSFKVVDGVSSYHLFEGLPTMFPRLAL
SPRLPSKSWQSAVRGFSQTK
FPHTRPHGKALAHRSEFRQARRIVVKLGSAVVTRGDEGGLALGRLASIVEQVAVLQNQGREMMIVTSGAVAFGKQRLRHE
ILLSQSVRQALHTGHNQLKE
MAIPVLEARACAAAGQSGLMALYEAMFTQYSTCTAQILVTNLDFHDEQKRRNLSSTLYELLRMNIVPIINTNDAVVPPPV
PNSDLQGVISIKDNDSLAAR
LAVEMKADLLIALSDVEGLYDSPPGTDNAKLIDIFYPGDQQSITYGSKSRVGMGGMEAKVKAALWALQGGTSVVIANGTH
PKVTGHVITDIVDGKKVGTF
FSEVKPAGE
>g1825.t2
YATCIGYVQYSRTLRYSTQTVRCLRSSVKPVGEWRLRNGTTLYAVYVAQWSSQRRVSPSSVHSFKVVDGVSSYHLFEGLP
TMFPRLALSPRLPSKSWQSA
VRGFSQTKFPHTRPHGKALAHRSEFRQARRIVVKLGSAVVTRGDEGGLALGRLASIVEQVAVLQNQGREMMIVTSGAVAF
GKQRLRHEILLSQSVRQALH
TGHNQLKEMAIPVLEARACAAAGQSGLMALYEAMFTQYSTCTAQILVTNLDFHDEQKRRNLSSTLYELLRMNIVPIINTN
DAVVPPPVPNSDLQGVISIK

DNDSLAARLAVEMKADLLIALSDVEGLYDSPPGTDNAKLIDIFYPGDQQSITYGSKSRVGMGGMEAKVKAALWALQGGTS
VVIANGTHPKVTGHVITDIV
DGKKVGTFFSEVKPAGE
>g1825.t3
QYSRTLRYSTQTVRCLRSSVKPVGEWRLRNGTTLYAVYVAQWSSQRRVSLPTMFPRLALSPRLPSKSWQSAVRGFSQTKF
PHTRPHGKALAHRSEFRQAR
RIVVKLGSAVVTRGDEGGLALGRLASIVEQVAVLQNQGREMMIVTSGAVAFGKQRLRHEILLSQSVRQALHTGHNQLKEM
AIPVLEARACAAAGQSGLMA
LYEAMFTQYSTCTAQILVTNLDFHDEQKRRNLSSTLYELLRMNIVPIINTNDAVVPPPVPNSDLQGVISIKDNDSLAARL
AVEMKADLLIALSDVEGLYD
SPPGTDNAKLIDIFYPGDQQSITYGSKSRVGMGGMEAKVKAALWALQGGTSVVIANGTHPKVTGHVITDIVDGKKVGTFF
SEVKPAGE
>g1826.t1
MWFPVALLTSPGPTVEQQTEMARNGGRVLASLLPEERGEIICRLAELLTEKKEDILNANKKDMELARASGRLSLQLLNRL
SLSTSKLNSLAIGLRQIAVS
SRDSVGRVLRRTRVANNLELEQVTVPIGVLLVIFESRPDCLPQVSALAIASGNALLLKGGKEAAYTNKILHQLTQEALSM
HGIQDAIQLVSTREEVEDLC
RLNKLIDLIIPRGSSELVREIQMAAKGIPVLGHSEGVCHVYIDSEASIDKAIDIVKDSKCDYPSACNAMETLLIHRDLLR
TPMFDQIIDMLRTEEVKIHA
GPKFASYLTFSPSEVKSLRTEYGDLECCIEVVDSMQDAVDHIHKYGSSHTDAIVTENEETAEQFLQQLDSACVFWNASTR
FADGYRFGLGAEVGISTARI
HARGPVGLEGLLTTKWVLRGEGHTAADFSEQGSMKFLHENIPVSHRNLS
>g1826.t2
LPESTSSRAARPTVEQQTEMARNGGRVLASLLPEERGEIICRLAELLTEKKEDILNANKKDMELARASGRLSLQLLNRLS
LSTSKLNSLAIGLRQIAVSS
RDSVGRVLRRTRVANNLELEQVTVPIGVLLVIFESRPDCLPQVSALAIASGNALLLKGGKEAAYTNKILHQLTQEALSMH
GIQDAIQLVSTREEVEDLCR
LNKLIDLIIPRGSSELVREIQMAAKGIPVLGHSEGVCHVYIDSEASIDKAIDIVKDSKCDYPSACNAMETLLIHRDLLRT
PMFDQIIDMLRTEEVKIHAG
PKFASYLTFSPSEVKSLRTEYGDLECCIEVVDSMQDAVDHIHKYGSSHTDAIVTENEETAEQFLQQLDSACVFWNASTRF
ADGYRFGLGAEVGISTARIH
ARGPVGLEGLLTTKWVLRGEGHTAADFSEQGSMKFLHENIPVSHRNLS
>g1827.t1
MELDMAMEPDRKAAVSHWQQQSYLDSGIHSGATTTAPSLSGKGNPEEEEVDNQVLYDWEQGFNQNFSQEQVQDLDGQYAM
TRAQRVRAAMFPETLEEGMQ
IPSTQYDGAHPTNVQRLAEPSQMLKHAVVNLINYQDDAELATRAIPELTKLLNDEDQVVVNKAAVMVHQLSKKEASRHAI
MRSPQMVSAIVRTMQNTNDV
ETARCTAGTLHNLSHHREGLLAIFKSGGIPALVKMLGSPVDSVLFYAITTLHNLLLHQEGAKMAVRLAGGLQKMVALLNK
TNVKFLAITTDCLQILAYGN
QESKVEMLGELIILASGGPQALVNIMRTYTYEKLLWTTSRVLKVLSVCSSNKPAIVEAGGMQALGLHLTDPSQRLVQNCL
WTLRNLSDAATKQEGMEGLL
GTLVQLLGSDDINVVTCAAGILSNLTCNNYKNKMMVCQVGGIEALVRTVLRAGDREDITEPAICALRHLTSRHQDAEMAQ
NAVRLHYGLPVVVKLLHPPS
HWPLIKATVGLIRNLALCPANHAPLREQGAIPRLVQLLVRAHQDTQRRTSMGGTQQQFVEGVRMEEIVEGCTGALHILAR
DVHNRIVIRGLNTIPLFVQL
LYSPIENIQRVAAGVLCELAQDKEAAEAIEAEGATAPLTELLHSRNEGVATYAAAVLFRMSEDKPQDYKKRLSVELTSSL
FRTEPMAWNETGDLGLDISA
QGEPLGYRQEDPSYRSFHAGGYGGDTMGLEPMMEHELAGGHHPGQDYAPVEGLPDLGHAQDLMDGLPPGDSNQLAWFDTD
L
>g1827.t2
DLMELDMAMEPDRKAAVSHWQQQSYLDSGIHSGATTTAPSLSGKGNPEEEEVDNQVLYDWEQGFNQNFSQEQVQDLDGQY
AMTRAQRVRAAMFPETLEEG
MQIPSTQYDGAHPTNVQRLAEPSQMLKHAVVNLINYQDDAELATRAIPELTKLLNDEDQVVVNKAAVMVHQLSKKEASRH
AIMRSPQMVSAIVRTMQNTN
DVETARCTAGTLHNLSHHREGLLAIFKSGGIPALVKMLGSPVDSVLFYAITTLHNLLLHQEGAKMAVRLAGGLQKMVALL
NKTNVKFLAITTDCLQILAY

GNQESKVEMLGELIIILASGGPQALVNIMRTYTYEKLLWTTSRVLKVLSVCSSNKPAIVEAGGMQALGLHLTDPSQRLVQN
CLWTLRNLSDAATKQEGMEG
LLGTLVQLLGSDDINVVTCAAGILSNLTCNNYKNKMMVCQVGGIEALVRTVLRAGDREDITEPAICALRHLTSRHQDAEM
AQNAVRLHYGLPVVVKLLHP
PSHWPLIKATVGLIRNLALCPANHAPLREQGAIPRLVQLLVRAHQDTQRRTSMGGTQQQFVEGVRMEEIVEGCTGALHIL
ARDVHNRIVIRGLNTIPLFV
QLLYSPIENIQRVAAGVLCELAQDKEAAEAIEAEGATAPLTELLHSRNEGVATYAAAVLFRMSEDKPQDYKKRLSVELTS
SLFRTEPMAWNETGDLGLDI
SAQGEPLGYRQEDPSYRSFHAGGYGGDTMGLEPMMEHELAGGHHPGQDYAPVEGLPDLGHAQDLMDGLPPGDSNQLAWFD
TDL
>g1827.t3
MASQADLMELDMAMEPDRKAAVSHWQQQSYLDSGIHSGATTTAPSLSGKGNPEEEEVDNQVLYDWEQGFNQNFSQEQVQD
LDGQYAMTRAQRVRAAMFPE
TLEEGMQIPSTQYDGAHPTNVQRLAEPSQMLKHAVVNLINYQDDAELATRAIPELTKLLNDEDQVVVNKAAVMVHQLSKK
EASRHAIMRSPQMVSAIVRT
MQNTNDVETARCTAGTLHNLSHHREGLLAIFKSGGIPALVKMLGSPVDSVLFYAITTLHNLLLHQEGAKMAVRLAGGLQK
MVALLNKTNVKFLAITTDCL
QILAYGNQESKVEMLGELIIILASGGPQALVNIMRTYTYEKLLWTTSRVLKVLSVCSSNKPAIVEAGGMQALGLHLTDPSQ
RLVQNCLWTLRNLSDAATKQ
EGMEGLLGTLVQLLGSDDINVVTCAAGILSNLTCNNYKNKMMVCQVGGIEALVRTVLRAGDREDITEPAICALRHLTSRH
QDAEMAQNAVRLHYGLPVVV
KLLHPPSHWPLIKATVGLIRNLALCPANHAPLREQGAIPRLVQLLVRAHQDTQRRTSMGGTQQQFVEGVRMEEIVEGCTG
ALHILARDVHNRIVIRGLNT
IPLFVQLLYSPIENIQRVAAGVLCELAQDKEAAEAIEAEGATAPLTELLHSRNEGVATYAAAVLFRMSEDKPQDYKKRLS
VELTSSLFRTEPMAWNETGD
LGLDISAQGEPLGYRQEDPSYRSFHAGGYGGDTMGLEPMMEHELAGGHHPGQDYAPVEGLPDLGHAQDLMDGLPPGDSNQ
LAWFDTDL
>g1828.t1
LSCPQEYYESLLCPLVGPLFTYMLQRLIVKWQLINQRTSANGEEEEEAVCDASQVTQEMLEEQLVRLVTREVLDLLTVSF
ISKKVHEPAGNKDEGEEEEM
MTDLAHTAVSGQPAEDLTELGKCILKHEDIYMNLLTISFVALSWKDTNICHRTASMICWNLLRQVVGGSLLPDAVTWFFI
SVLKGLQLHGQHEVCSATLT
LLAMLIYENLRPRYAELRTVMSQIPDINMEALDQYDHRLMDPKAQKFGDKKRKDHFKKLIAGSVGKALCQQFRKEVHIRN
LPSLFKKPKAEKDLLATEKL
GLEALFSPQRANV
>g1828.t2
LVLSCPQEYYESLLCPLVGPLFTYMLQRLIVKWQLINQRTSANGEEEEEAVCDASQVTQEMLEEQLVRLVTREVLDLLTV
SFISKKVHEPAGNKDEGEEE
EMMTDLAHTAVSGQPAEDLTELGKCILKHEDIYMNLLTISFVALSWKDTNICHRTASMICWNLLRQVVGGSLLPDAVTWF
FISVLKGLQLHGQHEVCSAT
LTLLAMLIYENLRPRYAELRTVMSQIPDINMEALDQYDHRLMDPKAQKFGDKKRKDHFKKLIAGSVGKALCQQFRKEVHI
RNLPSLFKKPKAEKDLLATE
KLGLEALFSPQRANV
>g1828.t3
LFLKQLVLSCPQEYYESLLCPLVGPLFTYMLQRLIVKWQLINQRTSANGEEEEEAVCDASQVTQEMLEEQLVRLVTREVL
DLLTVSFISKKVHEPAGNKD
EGEEEEMMTDLAHTAVSGQPAEDLTELGKCILKHEDIYMNLLTISFVALSWKDTNICHRTASMICWNLLRQVVGGSLLPD
AVTWFFISVLKGLQLHGQHE
VCSATLTLLAMLIYENLRPRYAELRTVMSQIPDINMEALDQYDHRLMDPKAQKFGDKKRKDHFKKLIAGSVGKALCQQFR
KEVHIRNLPSLFKKPKAEKD
LLATEKLGLEALFSPQRANV
>g1829.t1
MSFLRRVAGLSLRDRVRSSDIRRELGVEPLLLRVERSQLRWFGHLIRMPPGRLPLEVFRARPTGKRPRGRPRTRWRDYIS
HLAWERLGFPQEELESVAGE
KDVWNSLLSLLLPRPDPG
>g1830.t1

MWTVGGGGGIKGIEPPTLRFVDDPLCPPALPTEPLPATAIGAATGMVHCDTAVGTPDYISPEVLKSQGGDGYYGRECDWW
SVGVFIFEMLVGDTPFYADS
LVGTYSKIMDHKNSLNFPDDVEISKDAKNIICAFLTDRDVRLGRSGVEEIKRHPFFKNDQWTFDTIRDTVAPVVPELSSD
IDTSNFDEIEDDKGDVETFP
TPKAFVGNQLPFVGFTYFKEDQLLSASNNSLVAHQNMINRNGESAALQNKLHDLEEHLNNEMQVKDELEHKYRAATSRLD
KISKELEEEVSGRKALESSV
RQLEREKALLQHKSMESHRKAESEADRKRCLENE
>g1830.t2
MEDFERVKVIGRGAFGEVQLVRHKASQKVYAMKLLSKFEMIKRSDSAFFWEERDIMAFANSPWVVQLCCAFQDEHHLYMV
MEYMPGGDLVNLTSTYDVPE
KWAKFYTAEVVMALDAIHSMGFIHRDVKPDNMLLDSNGHLKLADFGTCMKMDTVSAAAVVSSSSCFGVKRAMCRHVGGGG
GIKGIEPPTLRFVDDPLCPP
ALPTEPLPATAIGAATGMVHCDTAVGTPDYISPEVLKSQGGDGYYGRECDWWSVGVFIFEMLVGDTPFYADSLVGTYSKI
MDHKNSLNFPDDVEISKDAK
NIICAFLTDRDVRLGRSGVEEIKRHPFFKNDQWTFDTIRDTVAPVVPELSSDIDTSNFDEIEDDKGDVETFPTPKAFVGN
QLPFVGFTYFKEDQLLSASN
NSLVAHQNMINRNGESAALQNKLHDLEEHLNNEMQVKDELEHKYRAATSRLDKISKELEEEVSGRKALESSVRQLEREKA
LLQHKSMESHRKAESEADRK
RCLENE
>g1830.t3
EKVIGRIRDLQMKMEDFERVKVIGRGAFGEVQLVRHKASQKVYAMKLLSKFEMIKRSDSAFFWEERDIMAFANSPWVVQL
CCAFQDEHHLYMVMEYMPGG
DLVNLTSTYDVPEKWAKFYTAEVVMALDAIHSMGFIHRDVKPDNMLLDSNGHLKLADFGTCMKMDTVSAAAVVSSSSCFG
VKRAMCRHVGGGGGIKGIEP
PTLRFVDDPLCPPALPTEPLPATAIGAATGMVHCDTAVGTPDYISPEVLKSQGGDGYYGRECDWWSVGVFIFEMLVGDTP
FYADSLVGTYSKIMDHKNSL
NFPDDVEISKDAKNIICAFLTDRDVRLGRSGVEEIKRHPFFKNDQWTFDTIRDTVAPVVPELSSDIDTSNFDEIEDDKGD
VETFPTPKAFVGNQLPFVGF
TYFKEDQLLSASNNSLVAHQNMINRNGESAALQNKLHDLEEHLNNEMQVKDELEHKYRAATSRLDKISKELEEEVSGRKA
LESSVRQLEREKALLQHKSM
ESHRKAESEADRKRCLENE
>g1830.t4
EKVIGRIRDLQMKMEDFERVKVIGRGAFGEVQLVRHKASQKVYAMKLLSKFEMIKRSDSAFFWEERDIMAFANSPWVVQL
CCAFQDEHHLYMVMEYMPGG
DLVNLTSTYDVPEKWAKFYTAEVVMALDAIHSMGFIHRDVKPDNMLLDSNGHLKLADFGTCMKMDTVSAAAVVSSSSCFG
VKRAMCRHGVFM
>g1831.t1
PQSDFSDLQEPPQGVSEPYPATFIVQSGGSYRRSRLSATKTRYSLNQEFEGPEEFGDYPGDSANDEQPSSTKG
>g1832.t1
LLLCSTGYQSSREDALDQDSSSHGDLFKPEALRAASEKSDSLAQALSLWASGMYKQGSIPSYYLTPQDPSKPFEYFTQPQ
DLSMTGAMDQGSSSGGPSQD
VPRGKEASEAGYGTFDPRSEPSQSQDLTTDNMGFFSQSNEDDSEMAIFYPQTEGSEDLPPQSEAHHHHPPPSEDALEPRA
ASKMSGALDQASPWAFSMFK
QGSGGAGAAAGAGAGPKGKGSSAAGHGTFDPSSRTSQSQGLSNGDDSQMASFYPQGDGSEDLVPQSEFSDLQEPPQGVSE
PYPATFIVQSGRSYKRTRQS
STETQYSLNQEPEEFGVYPGDSANHELPSSTKE
>g1833.t1
LTLCSTGYSSPDDAMEPRAAGKTSGSFNQASPWASSMFRQGSGGAGAGPTGKGSSAAGKGAFDPSSGTAQSQGLSNGDDS
QMAFFYPQGEGSEDLAPQSD
FSDLQEPPQGVSEPYPATFIVQSGGSYRRSRLSATKTRYSLNQEFEGPEEFGDYPGDSANDEQPSSTKG
>g1833.t2
MFRQGSGGAGAGPTGKGSSAAGKGAFDPSSGTAQSQGLSNGDDSQMAFFYPQGEGSEDLAPQSDFSDLQEPPQGVSEPYP
ATFIVQSGGSYRRSRLSATK
TRYSLNQEFEGPEEFGDYPGDSANDEQPSSTKG
>g1834.t1
MESFHNDSKDHYHHHRHHHHHQHVVTTETDSEPDHDGAPGGGGVGSVRALTGCVLFVLIVSTLLGNMLVCAAVIKFRHLR

SKVTNFFVISLAVSDLFVAV
LVMPWKAVSEVAGCWTFGRFCDTWIAFDIMCSTASILNLCIISMDRYWAISSPFRYERKMTQRFAFVMIGVAWTLSILIS
FIPVQLNWHKAAVDNATADN
TEDCNASLNRTYAISSSLISFYIPVIIMVGTYTRIFRIAQTQIRRISSLERAAGHHAQNNNHVESSLKTSFKKETKVLKT
LSIIMGVFVFCWLPFFVLNC
VVPFCDLNKLGDPLCVSDTTFNIFVWFGWANSSLNPVIYAFNADFRKAFSTILGCNKYCSSSTVEAVDFSNELVSYHHDT
TLQKEATVPGGCGGVAQRLP
CIAPRNAEDLEERFDKVSVISDTSRNQGHMLLPAILQFECEAEISLHLMPFSSSGHVECYAIPGQIQD
>g1835.t1
MDHPTGDVPSQPETTNHKEEGSCSSCRLSKGNAETSAAVLSGHCCDNHRNHRSRTGDRSHQGSAFPGDPVDAQRGACPSG
CNAVVIQCVCTATMLDLATG
AEMSPACAYDCKPGSDAVCRGQGRCSSPHSHPGVTAGTCVDGRTASDSAAAAAAAADTSTDRPHETLHTVHSSSLSPRED
TRETETNLADEIEGDSCTDD
RSALTFRGMPAHVEELEFERCNSASQGERSLTEEGSLANGSCLSNPGSGVHLPGDDDGGVLAGPHAEVPFGGFDAGCSMG
GVDGDSDLFSHEGEKSTSLF
PTVVVVERPLLSQSDSQNFRTSPATVKAPPPSSIPNTPTGPSRTTEAPSTAAQQHQEGEEDPTEAEASEAASGLGLSYCG
SPAPAGFPSCSVSCESAPVS
PGSGPEEDPDEADPDDAFADEWEQGQRPGPGVGVPRNTYASSRRQSAPGELAQSEEPSEGETAAEPTPQSRRPGIAEYFG
RATGVLSETHAGPDEADLRC
GKHTPRSVAVTVAGFPRSRPTRSCRGVCLCLFGWVAGVREGSL
>g1836.t1
MNLSLPDQNMHNPSQGCADDDKPELMLPCFRRNTYDEPLASYSNGPIISQLLRKTLHNKRAIDESHYYLGPSAVSSSAIV
ADSSQEDRSSVSSKDSMADV
GSPSFHPSTLASPEVERPMNDHLQAKRARVENIIRVMAGSPNSRLHGDHEKPEMDARESRECREAREVYRENKRKQRLPQ
HQDHNASDEGLENRRLGSSG
GSGSSVDNTKDEECHKLKEQLQSMQKLLRQLQEKFLQVYNQEDSHRDDRDERGGLVEHDNVILSKEPSYLTNGNTFEKRR
ARGTPECKDRTKPADGYLLH
LRETHNLQDALKEELSRVVNECVDKVFKNVSSSGLDVSPLQSLCPSPDLQTSGGTAEKKSQQGCCPPQEPLHPTETACHQ
RSLECHENTDVQSPQDQTEA
LSLVVRKPAMAPLSSVTPTAVKRPYPLHQPAFQFNYSSPLHDSQILEHLLKYGPHTNFSGLSCMAPSLERSSPDAVELPW
DTLSMRSKVTSGHLGHHPRP
SSLGPVTVDNLCLPHVKMECGDLQSMAERNPYLSLNISF
>g1837.t1
LSCPDFLGNEVVTVKTPAFAESVSEGDVRWEKAVGDTVNEDEVVCEIETDKTSVQVPSPAAGVIEELLVPDGGRVEGGTP
LFKLRKG
>g1837.t2
LNAGNEVVTVKTPAFAESVSEGDVRWEKAVGDTVNEDEVVCEIETDKTSVQVPSPAAGVIEELLVPDGGRVEGGTPLFKL
RKG
>g1837.t3
LSCPDFLGNEVVTVKTPAFAESVSEGDVRWEKAVGDTVNEDEVVCEIETDKTSVQVPSPAAGVIEELLVPDGGRVEGGTP
LFKLRKGGPSLDSLTQRKKS
HRVSVVMT
>g1838.t1
LGAVLSGAKAPGRVSHGKVVSGEGPDKERLKNPDGKAKHYQSYLARIGETGAPYWSQTWEGSSPASVWWPGFDPWGSAGL
SPKQQHGGAAALWAHHLQG
>g1839.t1
MMCSIPGADVNATTEVGESAVSSLVFLINESLEASHEDAAQMGRFCVRACELLLAHGTDASRCLVEDGEASLTEASLEHF
DLLLPLTTLLLHRGAAFVCS
RHGAACWSGQALLFQRLRSILQPRHGGVVDQSRASEVLEQAEFVLEAARVNHPSLPLPPGLEPPKLGDDPSSRGLLELHR
RVVEQEASPPALRCLCRAFL
RRHLQPWPLGNKIKALPLPDRLKDFLLPENTQSLKPSWESFRPLHGTQ
>g1840.t1
MGCSAPRGLLLGCGPACGTKRFWTRCTKRFWTRGGRGIRTSERGLAGERGVKGAGYRLDWESRVKLISVPLEKSDPGRRG
SRGGEINVLCTGSPTLQISQ
KNLRPAPWRHSPRPPRANADNPKHKHALPRWTDARPLCDNMGDWNLLGSILEEVHIHSTIVGKIWLTILFIFRMLVLGVA
TEDVWDDEQSEFVCNTEQPG

CKNVCYDQAFPISLIRYWVLQIIFVSSPSLVYMGHALYRLRALDKERHRKKAWLKAELEAGQRIQEDQHRRMERELRRLD
EHKRVRKAPLRGSLLRTYVF
HILTRSVVEVGFIVGQCALYGIGLAPLYKCEREPCPNSVDCFVSRPTEKNIFLVFMLVIAGVSLFLNLLEIFHLGVKKIK
DSLYGYKYGDEDSVCRSK
>g1841.t1
MAKQLEVESQSLGQSSPGLSPELGELDTPNTSKPKEQEPRSQTRSQPSDKRLKSSSEPSKDCEMNRRNRSHREEKESRTK
HISHKLLKSSRSQDAEGHHS
SERAKSPNSRSLDRKQDKAKLASSESNQNSTEPKESHLRDSVQEHRKDKHTDRTSQKKLSSSSKDRRTATTKHHADGNGE
SSRWKGDRADPSGEERRKTE
RRREDEKREKRSEKHRDSDRSRSRDEGRRRVESREVEKSIKDVEQEADSNAPARVEGLKENSPNRKLCFMETLNLTLSPV
KKPQTQKDCVPQGVPRAGGE
EEEDGEIVSRDDDDQPNLEEFLIIDEIEGSMNEMEINHDKEQYTKPSSAIQTSTDEKCSTEKVEEDQNPTHEGEFDPRGS
SPKGSDTVSSTITLEALPQG
DISLPEAIYVLTHSDGSSDGVSCNEPSSSASCVGVSKVSSTTEDMTSPGKRERVQECTPVKGYGSCDRRQRRRSAETDET
SAEPSSSGPLFYDEDSMMLT
LKNLRSIPDIISPLRSPVPPAKRAAVHRHVAAKPPHVKSLQRDFCSTAVVSNSMKPNPNQETKHPDVAQEPQPVPGTSLA
RLPVHSDGVSPSSSDLEEGE
ILSDSNEATTDVSPPKLRSKLTRSVNNIKNKPSPKSTSRAAKQSSEERRLSAKGAGGPVTPPTASPSSGKSRFKKVCPTG
GQTPFGSMEEVMDTLKKVRF
QIRKKYMKLHKVFPKKSFYAMMDHFQQSFLEYVENAIFGEISTQVSELKARLSKLVVSVFRKVMNNGIVNRIFDLQATNL
KQRLWEFVDVQLDYLFMDIQ
VALTTFCKQTVTHSAVNTDQKVANPKNVKIHKKSPLKTSLKLSLSPKQKLVQTIQRRIKPSPVVPCRTGLGRGKDIRITH
MERQNSDPEPNPPVAGFPPR
SIPLTPEKSHRTSTLSHSGTIQDRSDFEILTEQQASSLTFNLVRDTQMGEIFKCLMQGSDLLDASVAAGDATSWSLSTPR
KEFHPGDSLLSITSPSKFLS
PSKFASPGKFVTPTKFVTSPSKLITAWSSISPSKLCSSISPNKLCSPSKLCSPQPKALHMPLNPAMFDESCLLELPSVGG
ANAQRSYSLLADDLAVSLTI
PSPLKSDSHLSFLQPRAVDMMSTPESVISAHFGEDALLDGEDAWEQQDLHLALDTDNSDSSLCSSTASTATAGTGEAAAS
VLFQFKPHLPMQAVVMERSN
DHFIVRIRQSAVEAADATLTDGDDDSLSRTLTEEDRSRGETDPHAPDPRTAERVPKALFLEKSFRKEPSLQTAPPPAPEE
TTPAAPEGPQVQASGKEPPP
RPSDSASSADEMEMSTASEGRLAIVEDGSSGSTPGKRAVDVVSRKRKMHQEKSRAKRARKDEGRAGRRQRKAVSARTSEP
RSSKREKSKDTESPPKTPPS
SLSAKNVVKKKGEVVVAWTRDEDRAILLDLKTKGASRETFSVLSEKLDKSSAQIAERFSQLMKLFKKQGKMDNASP
>g1842.t1
MQFQVLALLLTLACLNLTTAQGSYDDCCLGHSKEIKEKLKHRVVAYTKQEADGGCNILAIVFTFKRGRRFCGNPQEPWVQ
KLKEKIDKENESCCKKVQEY
SSASLHLM
>g1843.t1
NRNAFTVTHVIVPKQCGGPDYCDTENEEELFLIQDQYDLITLGWIHTHPTQTAFLSSVDLHTHCSYQMMLPEAIAIVCSP
KFNEIGYFKLTDRGTEEIST
CKQKSFHPHSKDPPLFT
>g1843.t2
NRNAFTVTHVIVPKQCGGPDYCDTENEEELFLIQDQYDLITLGWIHTHPTQTAFLSSVDLHTHCSYQMMLPEAIAIVCSP
KFNE
>g1844.t1
MDWENMEGQPFSYFTYGACYSEVELDCLTGDYRGCGILCVKTLRTDIVVDVGQSLNPSIDIGQIEGAFLQGLGLYTLEQL
SFSPGGVLHSRGPSQYKIPA
VCDVPLNFNVYLLPDSHNPHAIYSSKGIGEPVLFLGSAVFYAIKDAVAAARAESGLAGPFPLDSPATPERACLACATPFS
QRVPVSQLGPLKPWALNI
>g1844.t2
YISETSTSSVPNTPPSAASYGTDANGMAVKDACLKLYKRLEPIRTKNPEGSWESWISGAFLEKISLSATGFFRGPDCYMD
WENMEGQPFSYFTYGACYSE
VELDCLTGDYRGCGILCVKTLRTDIVVDVGQSLNPSIDIGQIEGAFLQGLGLYTLEQLSFSPGGVLHSRGPSQYKIPAVC
DVPLNFNVYLLPDSHNPHAI
YSSKGIGEPVLFLGSAVFYAIKDAVAAARAESGLAGPFPLDSPATPERACLACATPFSQRVPVSQLGPLKPWALNI

>g1844.t3
MDWENMEGQPFSYFTYGACYSEVELDCLTGDYRTLRTDIVVDVGQSLNPSIDIGQIEGAFLQGLGLYTLEQLSFSPGGVL
HSRGPSQYKIPAVCDVPLNF
NVYLLPDSHNPHAIYSSKGIGEPVLFLGSAVFYAIKDAVAAARAESGLAGPFPLDSPATPERACLACATPFSQRVPVSQL
GPLKPWALNI
>g1845.t1
MRRQQEVTTAELGGVISEHTCYQVCSGSGPQHVSP
>g1846.t1
LDDFKSGRKAYRCPPVGRPEHCRLQTASGSFIAEMQLFVRQLGRKVTGREHGSNKKLAAQSCALSLVRQLYHLGVVEAYT
GITKKKEGEHLEPYDVKLSP
DLEPQLMSLMTELGVIIPPPPEDPAAPLVSLIQGKMASFEPSQRQVGVGVVPWSPPQVNWNPWTSSNIDDGPLAFCTPEQ
ISSDLQSQLGYQHEHNESMN
KASSPCIMGNILSEREALPVKQFEQQIMSALEGNSVVIIRGATGCGKTTQVPQYILDRFITSGRAADCNIVVTQPRRISA
VSVAERVAYERGEEIGSSCG
YSVRFESILPRPHASIMFCTVGVLLRKLETGIRGISHVIVDEIHERDINTDFLMVVLRDVVQTYPDVRVLLMSATIDTSM
FRDYFFNCPIIEVHGRTFPV
QEYYLEDCIQMTHFVPPPNDRKKKDKDEEGGDEDTNCNVLCGPDYTADTRRSMGLMSEKETPFELVEALLKYIETLQVAG
AVLVFLPGWNLIYSMQRHLE
MNPHFGSARYRVLPLHSQIPREEQRRVFDPAPDGVTKVILSTNIAETSITINDVVYVIDSCKQKVKLFTSHNNMTNYATV
WASRTNLEQRKGRAGRVRAG
FCFHLCSRARFDRLEPHMTPEIFRTPLHEVALSIKLLRLGAIGHFLSKAIEPPPLDAVIEAEYTLRELDALDSNDELTPL
GRILARLPIEPRLGKMMIMG
CIFNVGDAVCTISAASCFPEPFINEGRRLGYVHRNFAGSRFSDHVALLSVFQAWDDVRMNGEDAERNFCEHKRLNMSTLR
MTWEAKVQLKDILINSGFPE
ECLLGQMFNAVGPDNGLDVVVSLLTFGSYPNVCFHKEKRKILTTEGRNALIHKTSVNCPFSSQDMTYPSPFFVFGEKIRT
RAISAKGMTLVSPLQLILFG
SKKITSNGDVVELDDWIKLRLPHRVAGAIAALRAALEALVVSVTKDPEYIRQLDPVNQTLLNVTRHVSRPSAAGLNLMTT
SQRYVALCHGGGRDGVVVKG
FLRSAER
>g1846.t2
LDDFKSGRKAYRCPPVGRPEHCRLQTASGSFIAEMQLFVRQLGRKVTGREHGSNKKLAAQSCALSLVRQLYHLGVVEAYT
GITKKKEGEHLEPYDVKLSP
DLEPQLMSLMTELGVIIPPPPEDPAAPLVSLIQGKMASFEPSQRQVGVGVVPWSPPQVNWNPWTSSNIDDGPLAFCTPEQ
ISSDLQSQLGYQHEHNESMN
KASSPCIMGNILSEREALPVKQFEQQIMSALEGNSVVIIRGATGCGKTTQVPQYILDRFITSGRAADCNIVVTQPRRISA
VSVAERVAYERGEEIGSSCG
YSVRFESILPRPHASIMFCTVGVLLRKLETGIRGISHVIVDEIHERDINTDFLMVVLRDVVQTYPDVRVLLMSATIDTSM
FRDYFFNCPIIEVHGRTFPV
QEYYLEDCIQMTHFVPPPNDRKKKDKDEEGGDEDTNCNVLCGPDYTADTRRSMGLMSEKETPFELVEALLKYIETLQVAG
AVLVFLPGWNLIYSMQRHLE
MNPHFGSARYRVLPLHSQIPREEQRRVFDPAPDGVTKVILSTNIAETSITINDVVYVIDSCKQKVKLFTSHNNMTNYATV
WASRTNLEQRKGRAGRVRAG
FCFHLCSRARFDRLEPHMTPEIFRTPLHEVALSIKLLRLGAIGHFLSKAIEPPPLDAVIEAEYTLRELDALDSNDELTPL
GRILARLPIEPRLGKMMIMG
CIFNVGDAVCTISAASCFPEPFINEGRRLGYVHRNFAGSRFSDHVALLSVFQAWDDVRMNGEDAERNFCEHKRLNMSTLR
MTWEAKVQLKDILINSGFPE
ECLLGQMFNAVGPDNGLDVVVSLLTFGSYPNVCFHKEKRKILTTEGRNALIHKTSVNCPFSSQDMTYPSPFFVFGEKIRT
RAISAKGMTLVSPLQLILFG
SKKITSNGDVVELDDWIKLRLPHRVAGAIAALRAALEALVVSVTKDPEYIRQLDPVNQTLLNVTRHVSRPSAAGLNLMTT
SQ
>g1846.t3
LDDFKSGRKAYRCPPVGRPEHCRLQTASGSFIAEMQLFVRQLGRKVTGREHGSNKKLAAQSCALSLVRQLYHLGVVEAYT
GITKKKEGEHLEPYDVKLSP
DLEPQLMSLMTELGVIIPPPPEDPAAPLVSLIQGKMASFEPSQRQVGVGVVPWSPPQVNWNPWTSSNIDDGPLAFCTPEQ
ISSDLQSQLGYQHEHNESMN
KASSPCIMGNILSEREALPVKQFEQQIMSALEGNSVVIIRGATGCGKTTQVPQYILDRFITSGRAADCNIVVTQPRRISA

VSVAERVAYERGEEIGSSCG
YSVRFESILPRPHASIMFCTVGVLLRKLETGIRGISHVIVDEIHERDINTDFLMVVLRDVVQTYPDVRVLLMSATIDTSM
FRDYFFNCPIIEVHGRTFPV
QEYYLEDCIQMTHFVPPPNDRKKKDKDEEGGDEDTNCNVLCGPDYTADTRRSMGLMSEKETPFELVEALLKYIETLQVAG
AVLVFLPGWNLIYSMQRHLE
MNPHFGSARYRVLPLHSQIPREEQRRVFDPAPDGVTKVILSTNIAETSITINDVVYVIDSCKQKVKLFTSHNNMTNYATV
WASRTNLEQRKGRAGRVRAG
FCFHLCSRARFDRLEPHMTPEIFRTPLHEVALSIKLLRLGAIGHFLSKAIEPPPLDAVIEAEYTLRELDALDSNDELTPL
GRILARLPIEPRLGKMMIMG
CIFNVGDAVCTISAASCFPEPFINEGRRLGYVHRNFAGSRFSDHVALLSVFQAWDDVRMNGEDAERNFCEHKRLNMSTLR
MTWEAKVQLKDILINSGFPE
ECLLGQMFNAVGPDNGLDVVVSLLTFGSYPNVCFHKEKRKILTTEGRNALIHKTSVNCPFSSQDMTYPSPFFVFGEKIRT
RAISAKGMTLVSPLQLILFG
SKKITSNGDVVELDDWIKLRLPHRVAGAIAALRAALEALVVSVTKDPEYIRQLDPVNQTLLNVTRHVSRPSAAGLNLMTT
SQRYVALCHGGGRDGVVVKG
FLRSAERVWVQPQMSTNP
>g1847.t1
MAAGKGEKPGSVLQSERVRFIVCFLGVFVCYFYYGILQETITRGEYGHGETKEKFRFAQTLVFIQCIINAIFARILIQFF
EGSRADHTKSWLYGLCSLSY
LGAMVSSNSALQYVNYPTQVLGKSCKPIPVMILGVTILRKKYPLAKYLCVLLIVSGVALFLYKPNKGGATADHLFGFGEI
LLETAESAAVPLRLTVCSAL
FVLKLLSLTLDGLTGVAQDHMRTRFQTSAYHMMLNINLWSTLVLGLVVLWTGEIWEFLSFTERHSSIFYNILLFGLTSAL
GQTFIFMTVVSFGPLTCSII
TTTRKFFTILGSVILFGNVITAMQWVGTLLVFLGLGLDSKFVTRTVNMLATIGKQLRQHPALIPLFIFIGGGVTMSGLYL
ARLGLRNPDVCWDRKNNPEP
WNKMAPTYQYKFYAVNMDYSKLKKNGPDF
>g1847.t2
LIETTMAAGKGEKPGSVLQSERVRFIVCFLGVFVCYFYYGILQETITRGEYGHGETKEKFRFAQTLVFIQCIINAIFARI
LIQFFEGSRADHTKSWLYGL
CSLSYLGAMVSSNSALQYVNYPTQVLGKSCKPIPVMILGVTILRKKYPLAKYLCVLLIVSGVALFLYKPNKGGATADHLF
GFGEILLETAESAAVPLRLT
VCSALFVLKLLSLTLDGLTGVAQDHMRTRFQTSAYHMMLNINLWSTLVLGLVVLWTGEIWEFLSFTERHSSIFYNILLFG
LTSALGQTFIFMTVVSFGPL
TCSIITTTRKFFTILGSVILFGNVITAMQWVGTLLVFLGLGLDSKFVTRTVNMLATIGKQLRQHPALIPLFIFIGGGVTM
SGLYLARLGLRNPDVCWDRK
NNPEPWNKMAPTYQYKFYAVNMDYSKLKKNGPDF
>g1847.t3
MAAGKGEKPGSVLQSERVRFIVCFLGVFVCYFYYGILQETITRGEYGHGETKEKFRFAQTLVFIQCIINAIFARILIQFF
EGSRADHTKSWLYGLCSLSY
LGAMVSSNSALQYVNYPTQVLGKSCKPIPVMILGVTILRKKYPLAKYLCVLLIVSGVALFLYKPNKGGATADHLFGFGEI
LLETAESAAVPLRLTVCSAL
FVLKLLSLTLDGLTGVAQDHMRTRFQTSAYHMMLNINLWSTLVLGLVVLWTGEIWEFLSFTERHSSIFYNILLFGLTSAL
GQTFIFMTVVSFGPLTCSII
TTTRKFFTILGSVILFGNVITAMQWVGTLLVFLVTRTVNMLATIGKQLRQHPALIPLFIFIGGGVTMSGLYLARLGLRNP
DVCWDRKNNPEPWNKMAPTY
QYKFYAVNMDYSKLKKNGPDF
>g1848.t1
LTLLKPSSSPHVYKTYSFQRQPPEGCERVRACDEATSASPGEPVLQASCPDPNKVNFTPHGGSAFCPVSLLKPLLPSMDL
LFRGLTVSPVTGCPGPASPT
TQLGLQ
>g1849.t1
PHLTKKNGRNYIVTDEKRAVEELLDSLELEKSNYHMGLSRVFFRAGTLPRLEEQRDVQTRRNLSLFQAACRGFLARQAFK
KRKIQNLAIRCIQKNIKKNR
GVKGWPWWKLFTTVRPLIEVQLTEEQIRGKDEEIQQLKQRLEKVEKERNELRLNSDRLESRITELSSEVADERNTGESAS
QLLETETSERLRLEKDMKDL
QSKFDSMNKQMESMEMEVMEARLIRASELNGEMDDDDTGGEWRLKYERAIREIEFTKKRLQQELDDKLEVEQQNKRQLER

R
>g1849.t2
PHLTKKNGRNYIVTDEKRVFFRAGTLPRLEEQRDVQTRRNLSLFQAACRGFLARQAFKKRKIQNLAIRCIQKNIKKNRGV
KGWPWWKLFTTVRPLIEVQL
TEEQIRGKDEEIQQLKQRLEKVEKERNELRLNSDRLESRITELSSEVADERNTGESASQLLETETSERLRLEKDMKDLQS
KFDSMNKQMESMEMEVMEAR
LIRASELNGEMDDDDTGGEWRLKYERAIREIEFTKKRLQQELDDKLEVEQQNKRQLERR
>g1849.t3
PHLTKKNGRNYIVTDEKRGVCCDLANRSQNVQATRVEELLDSLELEKSNYHMGLSRVFFRAGTLPRLEEQRDVQTRRNLS
LFQAACRGFLARQAFKKRKI
QNLAIRCIQKNIKKNRGVKGWPWWKLFTTVRPLIEVQLTEEQIRGKDEEIQQLKQRLEKVEKERNELRLNSDRLESRITE
LSSEVADERNTGESASQLLE
TETSERLRLEKDMKDLQSKFDSMNKQMESMEMEVMEARLIRASELNGEMDDDDTGGEWRLKYERAIREIEFTKKRLQQEL
DDKLEVEQQNKRQLERR
>g1850.t1
MRWMSSCCPSQLDRRYSWTLRTDRSGSPQTLLDAVAVQQQRGTCNTQQWASSRRALGAGRRTAQRAGNVANTSGL
>g1851.t1
MSAGSPTLGQPTTGDPGPVPAGDGGADPRPCAGVHCKLCLSEKAPGATHRLDACNCVFCTALLLACSAAVLSGVELVLSG
VELVLSGVELVLSGVELVLS
GAELVLTGVLSFSTFTHISLRHSMLLYRVIIYSIILYSMLLYSIITHSIILHSMLLYRVIIYSIIL
>g1852.t1
MLPSCALCLAQCLQQYVRLAVMEGGGSAVTCPDMACHKTGVLLDSEIASLVTGDQLELYQRLKFERGVQLDPSRAWCPVL
ECQAVCSLSPGTAEGAPTSV
PC
>g1853.t1
SMVTAGLLHLLLTQVVNVSYNVTEREARRAARETAGADATNYSRGFRDNWAEFMTMGDRLKAPAPAPDLV
>g1854.t1
MLSVAGVSSLPDTTLSPLQLMEQRVVLEERAQGFHRAHAQRRVLHSLLDLLTRERLLEWDRLELAQQHSSRRALRRCLLV
WRHYPSLQRAEREKEARREH
LRRRVAEVLPDFLCSPPGPSPSPRPSSFFK
>g1854.t2
MEQRVVLEERAQGFHRAHAQRRVLHSLLDLLTRERLLEWDRLELAQQHSSRRALRRCLLVWRHYPSLQRAEREKEARREH
LRRRVAEVLPDFLCSPPGPS
PSPRPSSFFK
>g1855.t1
EDSLPAHMELLGSFNSLITSVEQLQSGFLLNPDGYSEGELATPPRKLLHTLRQLNPMYEGYLQHDAQEVLQCLLAYIQEA
CDTIKKEQQGGSREEVAADG
LVGPAPAGSEEEEEDGQVNGKRKSDTEAGNAKKKPKSVKSNKPDAEEHADKKPITRSKRRSSREAVANDAAPGDREAKDE
GEEGGEAVACGGEEDGTAEG
ASKASEGKRKKRTRLSWLRPSSKQPSIFSKFRSMGKISSIMAPKSQAEQAGSGRAGSGSGPKRDPTVVDKTAPLPTTGSA
QDQKAAENEDGLDVLERLFQ
GRLVLRTRCLECESFTERREDFQDISVPVQEDQPSGPDDLSEDPKPEQKTLRWAISQFASVERIVGEDKYFCETCHHYAE
AERSLLFDKTPEVVTIHLKC
FSANGLDVDPYAGLSKVNTPLQTPLTLSLEEWCTRAAPPGGCSYQLFAVVMHSGVTISSGHYTAYVRMSELRDVRLQPRG
KGGEWQKEEVKEVKEQEWRG
GGGDGVKQQQQQQQQQQEEEEAQYDDGEVSFGGGVR
>g1855.t2
EDSLPAHMELLGSFNSLITSVEQLQSGFLLNPDGYSEGELATPPRKLLHTLRQLNPMYEGYLQHDAQEVLQCLLAYIQEA
CDTIKKEQQGGSREEVAADG
LVGPAPAGSEEEEEDGQVNGKRKSDTEAGNAKKKPKSVKSNKPDAEEHADKKPITRSKRRSSREAVANDAAPGDREAKDE
GEEGGEAVACGGEEDGTAEG
ASKASEGKRKKRTRLSWLRPSSKQPSIFSKFRSMGKISSIMAPKSQAEQAGSGRAGSGSGPKRDPTVVDKTAPLPTTGSA
QDQKAAENEDGLDVLERLFQ
GRLVLRTRCLECESFTERREDFQDISVPVQEDQPSGPDDLSEVSPDPKPEQKTLRWAISQFASVERIVGEDKYFCETCHH
YAEAERSLLFDKTPEVVTIH
LKCFSANGLDVDPYAGLSKVNTPLQTPLTLSLEEWCTRAAPPGGCSYQLFAVVMHSGVTISSGHYTAYVRMSELRDVRLQ

PRGKGGEWQKEEVKEVKEQE
WRGGGGDGVKQQQQQQQQQQQEEEEAQYDDGEVSFGGGVR
>g1856.t1
RLAYECLSQSIDSAKHSPNLVPTATKPDTVQNESLSYSQYLGMIKSQISCAKDIHNALLECSKKIAGKGQPQGIM
>g1857.t1
DADVRNPFSMLDNMMLNMRSRMDDMHKNFDNLSTDPSSPNSHSFSSSSVTTYSKVGNEPPKVFQASSQTRRAPGGITETR
RALRDSESGLEKMAIGHHIQ
DRGHVVEKKHNKKTGERELSQDFQNMDECMFLSAHAACFRFLQTPAEAQSFDKEWKSKVSEFQPSVPVSRLEAPGVPGAP
AAPRAAHRAALTASEPHRER
RVHIADGRKQSHSDEVKGERP
>g1857.t2
MFSVCDADVRNPFSMLDNMMLNMRSRMDDMHKNFDNLSTDPSSPNSHSFSSSSVTTYSKVGNEPPKVFQASSQTRRAPGG
ITETRRALRDSESGLEKMAI
GHHIQDRGHVVEKKHNKKTGERELSQDFQNMDECMFLSAHAACFRFLQTPAEAQSFDKEWKSKVSEFQPSVPVSRLEAPG
VPGAPAAPRAAHRAALTASE
PHRERRVHIADGRKQSHSDEVKGERP
>g1857.t3
DADVRNPFSMLDNMMLNMRSRMDDMHKNFDNLSTDPSSPNSHSFSSSSVTTYSKVGNEPPKVFQASSQTRRAPGGITETR
RALRDSESGLEKMAIGHHIQ
DRGHVVEKKHNKKTGERELSQDFQNMDESEAQSFDKEWKSKVSEFQPSVPVSRLEAPGVPGAPAAPRAAHRAALTASEPH
RERRVHIADGRKQSHSDEVK
GERP
>g1858.t1
LDRWGRSDEEEEEEQKEEEEKEEEKEKLWLDCVARA
>g1858.t2
LSKVLRGLDRWGRSDEEEEEEQKEEEEKEEEKEKLWLDCVARA
>g1859.t1
VHKIKSTFVDGILSCMAGEVSSCLQGGFTSGFRVVLVGLLVAVVGVVSLTLPLSNQAARHTDSELNIRSF
>g1860.t1
MTEQRPFAAVIREGTEEEEAETAAASPAASRKPKPSDPNPHIQHAFPDPTCKTQKQRGSPSLLPADISQQAVQCKLDSDL
NWMMLIPVGVEGHSAVSLVS
VYDASASNMDNLKPPEKFNLYGDPQC
>g1861.t1
MALPPSCPLICFIKRPWHSGAPLLCCVKTAAAAAAASFTASDIPALVSACRTVEVVAWRPLRPPLHSGVDSRFKMAGNTV
LYIPMEALQRSPEEASKDKE
LIQRLEIVLIHWTRQFKEVLSAQHAVDMGDSSGPLEEITFWKSRCTDLTGISRQLQKPGVRHIQNTLELSKSTYLLPFCR
LAQEIQVGHVFIG
>g1861.t2
MALPPSCPLICFIKRPWHSGAPLLCCVKTAAAAAAASFTASDIPALVSACRTVEVVAWRPLRPPLHSGVDSRFKMAGNTV
LYIPMEALQRSPEEASKDKE
LIQRLEIVLIHWTRQFKEVLSAQHAVDMGDSSGPLEEITFWKSRCTDLTGISRQLQKPGVRHIQNTLELSKSTYLLPFCR
LAQEIQ
>g1861.t3
LICFIKRPWHSGAPLLCCVKTAAAAAAASFTASDIPALVSACRTVEVVAWRPLRPPLHSGVDSRFKMAGNTVLYIPMEAL
QRSPEEASKDKELIQRLEIV
LIHWTRQFKEVLSAQHAVDMGDSSGPLEEITFWKSRCTDLTGISRQLQKPGVRHIQNTLELSKSTYLLPFCRLAQEIQVG
HVFIG
>g1862.t1
MCPGEPACTKLCCSTLIQNLVRLAMFGSAAVISRGAHERGLGPTAPNRTGAAPQRYTRTDIAVTSVSRDRKTSTGSRACW
EAAGAKSYKAGRPKRFNRGP
VFQTDPQTGRVPSEPPELIPRRDVPEFQSV
>g1862.t2
MFGSAAVISRGAHERGLGPTAPNRTGAAPQRYTRTDIAVTSVSRDRKTSTGSRACWEAAGAKSYKAGRPKRFNRGPVFQT
DPQTGRVPSEPPELIPRRDV
PEFQSV
>g1863.t1

RARFGTSLRPADLITLTPVTIAALISLILVTIAALHSLIPVPIAALNSLRPITIAARISLMLVAIAALNSLTPITIAALS
SLISLVIAALISLILVAIAA
LISLILVAIAAVNSLILSP
>g1864.t1
MAYQISVLFIDHGGFENYGSECEGIRITAFLDAGQDNLTPLGRLEKYAFSENLFNREIVARGLLDVLREFSDNENDFISV
METVARMSEDGEPTVRAELM
EQIPNIAMFLHESRPNFPAAFSRYLVPIVVLYLTDPNNQVRKTSQAALLVLLEQGLICKADMETKVCPVLLELTEPSSDD
DYKSEAVAIMCKLVTMLSKD
TVERLLLMRFCELCSDARLFQVRKVCAANFGEFCSIVGQEATEKLLMPKFFDLCSDSLWGIRKACAECFMVVSNCTSPEV
RRTKLSPLFINLISDQSRWV
RQGAFQSLGRFISTFANPSGTGLHFREDGTLLALPRCTLESDSPIASLKCSDTGGCSTDRTIAHSHTPPNRDGRATPSPE
RSLMADLEYAHAFGSHSAPA
ASDTRETPAPVGPNNNNNTNDNNDPTATENDKNQKDMEQADENFNSFYYWRPPLPDISGELEMLTSPTTEQQQQQQQQQL
LQQEVVGEEEEEEEEDEEIME
EFPVSKYSPPKATSDQIKKVLDRLQPHMSDPDVQAQVQVLSAALKVAQLDSPTDSSPTEDQPSGLSEGEGDSDSPPPESD
PAAVQVESQQDPIEEEQTAE
PPPVPESCPIQEQGEMETQTEPQEGQEDSPRGSPVLVRDDSDHSSVESELIESVEEEKEDSAPSPVPEEKPKIQNVIPQQ
LLDQYLSMTDPDRAQTVDTE
IAKHCAFSLPGVALTLGRQNWHCLKDTYETLATDVQWKVRRTLAFSIHELAVILGDQLTAADLVPIFNGFLKDLDEVRIG
VLKHLYDFLKLLHAEKRREY
LYQLQEFMVTDNSRNWRFRYELAEQLILIIELYSHYDVYDYLRQIALTLCSDKVSQVRWISYKLVVEILQKLYASGANDL
GVNFINELIVRFCHCNKWVG
RQAFAFICQAVVEEDCMPMDQFAQHLLPSLLSLSSDSVANVRVLVAKALRQSVMEKAYFKEPGCPYSDELEETVMALQTD
KDRDVRFFATLDPNKALMDT
APL
>g1864.t2
MAYQISVLFIDHVHTGGFENYGSECEGIRITAFLDAGQDNLTPLGRLEKYAFSENLFNREIVARGLLDVLREFSDNENDF
ISVMETVARMSEDGEPTVRA
ELMEQIPNIAMFLHESRPNFPAAFSRYLVPIVVLYLTDPNNQVRKTSQAALLVLLEQGLICKADMETKVCPVLLELTEPS
SDDDYKSEAVAIMCKLVTML
SKDTVERLLLMRFCELCSDARLFQVRKVCAANFGEFCSIVGQEATEKLLMPKFFDLCSDSLWGIRKACAECFMVVSNCTS
PEVRRTKLSPLFINLISDQS
RWVRQGAFQSLGRFISTFANPSGTGLHFREDGTLLALPRCTLESDSPIASLKCSDTGGCSTDRTIAHSHTPPNRDGRATP
SPERSLMADLEYAHAFGSHS
APAASDTRETPAPVGPNNNNNTNDNNDPTATENDKNQKDMEQADENFNSFYYWRPPLPDISGELEMLTSPTTEQQQQQQQ
QQLLQQEVVGEEEEEEEEDEE
IMEEFPVSKYSPPKATSDQIKKVLDRLQPHMSDPDVQAQVQVLSAALKVAQLDSPTDSSPTEDQPSGLSEGEGDSDSPPP
ESDPAAVQVESQQDPIEEEQ
TAEPPPVPESCPIQEQGEMETQTEPQEGQEDSPRGSPVLVRDDSDHSSVESELIESVEEEKEDSAPSPVPEEKPKIQNVI
PQQLLDQYLSMTDPDRAQTV
DTEIAKHCAFSLPGVALTLGRQNWHCLKDTYETLATDVQWKVRRTLAFSIHELAVILGDQLTAADLVPIFNGFLKDLDEV
RIGVLKHLYDFLKLLHAEKR
REYLYQLQEFMVTDNSRNWRFRYELAEQLILIIELYSHYDVYDYLRQIALTLCSDKVSQVRWISYKLVVEILQKLYASGA
NDLGVNFINELIVRFCHCNK
WVGRQAFAFICQAVVEEDCMPMDQFAQHLLPSLLSLSSDSVANVRVLVAKALRQSVMEKAYFKEPGCPYSDELEETVMAL
QTDKDRDVRFFATLDPNKAL
MDTAPL
>g1864.t3
MAYQISVLFIDHGGFENYGSECEGIRITAFLDAGQDNLTPLGRLEKYAFSENLFNREIVARGLLDVLREFSDNENDFISV
METVARMSEDGEPTVRAELM
EQIPNIAMFLHESRPNFPAAFSRYLVPIVVLYLTDPNNQVRKTSQAALLVLLEQGLICKADMETKVCPVLLELTEPSSDD
DYKSEAVAIMCKLVTMLSKD
TVERLLLMRFCELCSDARLFQVRKVCAANFGEFCSIVGQEATEKLLMPKFFDLCSDSLWGIRKACAECFMVVSNCTSPEV
RRTKLSPLFINLISDQSRWV
RQGAFQSLGRFISTFANPSGTGLHFREDGTLLALPRCTLESDSPIASLKCSDTGGCSTDRTIAHSHTPPNRDGRATPSPE
RSLMADLEYAHAFGSHSAPA

ASDTRETPAPVGPNNNNNTNDNNDPTATENDKNQKDMEQADENFNSFYYWRPPLPDISGELEMLTSPTTEQQQQQQQQQL
LQQEVVGEEEEEEEDEEIME
EFPVSKYSPPKATSDQIKKVLDRLQPHMSDPDVQAALKVAQLDSPTDSSPTEDQPSGLSEGEGDSDSPPPESDPAAVQVE
SQQDPIEEEQTAEPPPVPES
CPIQEQGEMETQTEPQEGQEDSPRGSPVLVRDDSDHSSVESELIESVEEEKEDSAPSPVPEEKPKIQNVIPQQLLDQYLS
MTDPDRAQTVDTEIAKHCAF
SLPGVALTLGRQNWHCLKDTYETLATDVQWKVRRTLAFSIHELAVILGDQLTAADLVPIFNGFLKDLDEVRIGVLKHLYD
FLKLLHAEKRREYLYQLQEF
MVTDNSRNWRFRYELAEQLILIIELYSHYDVYDYLRQIALTLCSDKVSQVRWISYKLVVEILQKLYASGANDLGVNFINE
LIVRFCHCNKWVGRQAFAFI
CQAVVEEDCMPMDQFAQHLLPSLLSLSSDSVANVRVLVAKALRQSVMEKAYFKEPGCPYSDELEETVMALQTDKDRDVRF
FATLDPNKALMDTAPL
>g1865.t1
LSIWLALAFPASYAVFVLFQVVCRMVKSIGETLPQKQADTHSDNEHAGAAEERERRELAFSADDEKDLEQRSALGHSCEM
LDQQSVERSLVRRPHRLGEG
RKA
>g1865.t2
LSIWLALAFPASYAVFVLFQVVCRMVKSIGETLPQKQADTHSDNEHAGAAEERERRELAFSADDEKDLEQEMF
>g1866.t1
MDQDEPRYVIDRPTYNIAVFDKDFDKKDRQFPVGEKVKKLFRCSGSRLKSLLYRHLPVLSWLPRYRVKENLLVDVVSGVS
AGTIQVPQGMAFALLASLPP
VNGLYSSFFPLIPYFFMGTAHQMVPGTFAVLSIMVGMVCQRLAPETQFSHYNATLNATVVDTDAMNDVRLGISGTLACLT
AIIQMGLGFMQFGFVAIYLS
ESFIRGFMTAAGLQILISVLKYIFQISVRPFSGPLAVVYTLVDIVGGLPQTNVASLLFAVVSSVVLIASKELGARYRHKL
PFPIPMEIIIVVVATAISGP
LDLPETYHMNIVGEIPLGFPAPILPAVCEWEDMLSTAFSLAIVGYVINLAMGRTLAAKHGYDVDPNQEMLALGCSNFFGS
FFKIHVICCALSVTLAVDNA
GGTSQVGGLLSWAAKLDPRHKSNPGPDILTLCFFSTPQFASLCVMLVVMITMVALGVYLKPLPKSVLGALIAVNLKNTLL
QLSDPYYLWKKSRLDCCVWV
VSFLATFFLSLPYGVAIGVSFSILVASVKGVKIVNYCSPIYFANAEIFRQKVIKKTSLDPGKLILARRKFLKKQQKEKEK
EDKKEKEKESRRRKLSSLVT
MKAQTISQLELQNDFDPKSDNSPTTSYVNFHFNDGGPEEQMPGGPPDSPPALDSQPMPFHTLILDLAGVCFVDLNGVKVL
TKMNSSYEMLGIKLLLASVQ
AQVYQELEEGGAFDGEGGVSRTHLFVTVHDAVLHAQHASARSAAGLSKAGAASPLSV
>g1866.t2
MDQDEPRYVIDRPTYNIAVFDKDFDKKDRQFPVGEKVKKLFRCSGSRLKSLLYRHLPVLSWLPRYRVKENLLVDVVSGVS
AGTIQVPQGMAFALLASLPP
VNGLYSSFFPLIPYFFMGTAHQMVPGTFAVLSIMVGMVCQRLAPETQFSHYNATLNATVVDTDAMNDVRLGISGTLACLT
AIIQMGLGFMQFGFVAIYLS
ESFIRGFMTAAGLQILISVLKYIFQISVRPFSGPLAVVYTLVDIVGGLPQTNVASLLFAVVSSVVLIASKELGARYRHKL
PFPIPMEIIIVVVATAISGP
LDLPETYHMNIVGEIPLGFPAPILPAVCEWEDMLSTAFSLAIVGYVINLAMGRTLAAKHGYDVDPNQEMLALGCSNFFGS
FFKIHVICCALSVTLAVDNA
GGTSQVGGLLSWAAKLDPRHKSNPGPDILTLCFFSTPQFASLCVMLVVMITMVALGVYLKPLPKCNACVCVSLPRLLQSV
LGALIAVNLKNTLLQLSDPY
YLWKKSRLDCCVWVVSFLATFFLSLPYGVAIGVSFSILVASVKGVKIVNYCSPIYFANAEIFRQKVIKKTSLDPGKLILA
RRKFLKKQQKEKEKEDKKEK
EKESRRRKLSSLVTMKAQTISQLELQNDFDPKSDNSPTTSYVNFHFNDGGPEEQMPGGPPDSPPALDSQPMPFHTLILDL
AGVCFVDLNGVKVLTKMNSS
YEMLGIKLLLASVQAQVYQELEEGGAFDGEGGVSRTHLFVTVHDAVLHAQHASARSAAGLSKAGAASPLSV
>g1866.t3
MDQDEPRYVIDRPTYNIAVFDKDFDKKDRQFPVGEKVKKLFRCSGSRLKSLLYRHLPVLSWLPRYRVKENLLVDVVSGVS
AGTIQVPQGMAFALLASLPP
VNGLYSSFFPLIPYFFMGTAHQMVPGTFAVLSIMVGMVCQRLAPETQFSHYNATLNATVVDTDAMNDVRLGISGTLACLT
AIIQMGLGFMQFGFVAIYLS
ESFIRGFMTAAGLQILISVLKYIFQISVRPFSGPLAVVYTLVDIVGGLPQTNVASLLFAVVSSVVLIASKELGARYRHKL

PFPIPMEIIIVVVATAISGP
LDLPETYHMNIVGEIPLGFPAPILPAVCEWEDMLSTAFSLAIVGYVINLAMGRTLAAKHGYDVDPNQEMLALGCSNFFGS
FFKIHVICCALSVTLAVDNA
GGTSQFASLCVMLVVMITMVALGVYLKPLPKSVLGALIAVNLKNTLLQLSDPYYLWKKSRLDCCVWVVSFLATFFLSLPY
GVAIGVSFSILVASVKGVKI
VNYCSPIYFANAEIFRQKVIKKTSLDPGKLILARRKFLKKQQKEKEKEDKKEKEKESRRRKLSSLVTMKAQTISQLELQN
DFDPKSDNSPTTSYVNFHFN
DGGPEEQMPGGPPDSPPALDSQPMPFHTLILDLAGVCFVDLNGVKVLTKMNSSYEMLGIKLLLASVQAQVYQELEEGGAF
DGEGGVSRTHLFVTVHDAVL
HAQHASARSAAGLSKAGAASPLSV
>g1867.t1
MPYLMGCWDMAALPRNMAQWWNELPANVRTAESLPILQKRL
>g1867.t2
LTNQMPYLMGCWDMAALPRNMAQWWNELPANVRTAESLPILQKRL
>g1868.t1
MMAAFAKANLPIPKLTATATDGAPAMIGSVNGLVGLCKADQTFPEFWNFHCIIHREQLVFKSLNLDNVMKPVVEIINYIR
THALNHRQFKNLIAELDKGL
PGDLPLHCTVRWLSKGQVLSRFFELLDAVKLFMEEKDKDYRALGLQVDHGSCFFGRHAVSLGQTEPDSADGQVTSATLIK
QRARYATLVKNLHESFVTRF
RDLQLKRPQITFLVDPLNAEMDCLKAPLVTDEAAAELEMIDL
>g1869.t1
MTSDHRKGGSCAPLPLLLLLLAVSVATAGRGDAEQRNTTLLLTGFTSPRRPSPVPPGEAGHGDQRPMGATGTPPRPLPQT
MLPSNVTAADGLKPPLVDSN
NCRGYFDVMGQFDNTFNCSRGTYIYCCGTCHYRFCCEHQRDRLDQDSCNNYKSPKWATTQISATAPTGNRLAPDFETLQQ
NSSSTAYVIGGVISFTLVVA
VGIRIAFSKVSRQPRHRDMPRALVDILRHQSSPLQGHRGGSTLLSTTATGRSGKTLYTPVLQGKDTR
>g1869.t2
LQSVPHTVALPHLAEKKTDRFLNRGNEDCRVTMTSDHRKGGSCAPLPLLLLLLAVSVATAGRGDAEQRNTTLLLTGFTSP
RRPSPVPPGEAGHGDQRPMG
ATGTPPRPLPQTMLPSNVTAADGLKPPLVDSNNCRGYFDVMGQFDNTFNCSRGTYIYCCGTCHYRFCCEHQRDRLDQDSC
NNYKSPKWATTQISATAPTG
NRLAPDFETLQQNSSSTAYVIGGVISFTLVVAVGIRIAFSKVSRQPRHRDMPRALVDILRHQSSPLQGHRGGSTLLSTTA
TGRSGKTLYTPVLQGKDTR
>g1869.t3
LGRAELRAETLSPGLLHIDLLPCSSSSCSSSTSSSCSDSQPEHASSSAAVCPPVVHRAARLLFTGPARLSPPQKEHRISP
ESSVLLQSVPHTVALPHLAE
KKTDRFLNRGNEDCRVTMTSDHRKGGSCAPLPLLLLLLAVSVATAGRGDAEQRNTTLLLTGFTSPRRPSPVPPGEAGHGD
QRPMGATGTPPRPLPQTMLP
SNVTAADGLKPPLVDSNNCRGYFDVMGQFDNTFNCSRGTYIYCCGTCHYRFCCEHQRDRLDQDSCNNYKSPKWATTQISA
TAPTGNRLAPDFETLQQNSS
STAYVIGGVISFTLVVAVGIRIAFSKVSRQPRHRDMPRALVDILRHQSSPLQGHRGGSTLLSTTATGRSGKTLYTPVLQG
KDTR
>g1870.t1
VFREDGIISGYRDPQSSAKDCLLSSFQLTNETINIWTHFLPTWYFLWRFSILCSTLNFLSDSYTWPLLVYMLLICVYPFT
SSCAHTFSTMSPESRHICYF
FDYGALSLYSLGISTPSSVPSLTGSYMMLVLRCQHALLCLYGQMRCMEGCAISYGSYVMPDSWVNSWLHQHYVPIAVGNT
IFCTSLSCYSR
>g1870.t2
VFREDGIISGYRDPQSSAKDCLLSSFQLTNETINIWTHFLPTWYFLWRFSILCSTLNFLSDSYTWPLLVYMLLICVYPFT
SSCAHTFSTMSPESRHICYF
FDYGALSLYSLGISTPSSVPSLTGSYMMLVLRCQHALLCLYGQMRCMEGGVLMDGWMDGWMDIQVDPHQGCAISYGSYVM
PDSWVNSWLHQHYVPIAVGN
TIFCTSLSCYSR
>g1871.t1
MAEPRKVPFVTISSFNDSPASESKKRRREDEDDSTVVKDGGGGGAAAAGSGGGGGGGGSLFGNVKGGDTEPGDKRPTVRL
NLPLTEPNERGSAEFNYTEL

VQSTLTQVKPKASAVCKGLTPPLDPSDPFADDDRERRELEELAKKFESKYGGAPKKKKRDRLQDLIDIGYGYDDTDPFID
NSEAYDELVPASLSTKHGGF
YINIGTLQFRPASDSEGEEPNAETIHFKKAKDGEERAIKKRRKKPDGINMEDKKPKKNRVTKQGVSALNLHRPEKKKRKK
LMKDSIQLANMLRRKKHVPG
AA
>g1872.t1
ASATGATTFHGRFTPGTFTNQIQAAFREPRLLIVTDPRADHQPLTEASYVNIPSIALCNTDSPLRYVDIAIPCNNKSHLS
VGLMWWMLTREVLRMRGTIS
REHPWDVMPDLYFYRDPEEIEKEEQAAAEKAVGKEEFQAEWTAPTAEFAQTEVADWSEGVAVPSVPIQQFPPATAAAAVA
PAKPAGGEVFAEDWSAQPAT
EDWSAAPTAQAPEWGGATADWS
>g1873.t1
MPVATLLPFTAVPNRKSAGPTSFRLTEKFILLLVFSAFITLCFGAIFFLPDSSKLLSGVFFHSSAVETNTRTGTVSGSGL
SVANDEKILAKIRKDHENAL
TEAKDTLQKRPDEIKQDIKKEKEKILMENLGQGGGEVDRLPSIEYQRPPGATGHEPLDPQTKERRAKVKEVGFVNRA
>g1874.t1
FHGDRLIYFGGYGCKTIQEVRNTSALNFIVEETSWMAVGTTYFRCWGWNNEVVVYDTQTNTWSMPETRGPAPSPRGCHAS
ALLGTKGFICGGLDSKDLDL
YCLDLETWTWTYYDLSSSHVPIGRSMHTLTPTSDHTLLLYGGFGVNEEGVDPSRARSDAWQFDTLRKEWMERADHPHHDK
PRLWHSACRGEDDQVVIFGG
SSNFCILMDSVAVLRSPWQRHCKDVFVFRNQPVSLSRLSEDTMARNQTLLTQQLSWLPHRMLKKIGKRLSYFSRRHPGAP
TWQPEATV
>g1874.t2
FHGDRLIYFGGYGCKTIQEVRNTSALNFIVEETSWMAVGTTYFRCWGWNNEVVVYDTQTNTWSMPETRGPAPSPRGCHAS
ALLGTKGFICGGLDSKDLDL
YCLDLETWTWTYYDLSSSHVPIGRSMHTLTPTSDHTLLLYGGFGVNEEGVDPSRARSDAWQFDTLRKEWMERADHPHHDK
PRLWHSACRGEDDQVAVLRS
PWQRHCKDVFVFRNQPVSLSRLSEDTMARNQTLLTQQLSWLPHRMLKKIGKRLSYFSRRHPGAPTWQPEATV
>g1875.t1
MSRFFATGSDSESEESSSADEITPKTAGPNFRQSLLLSDDEEDTKRVVRSAKDKRFEELTNLIKTIRNAMKIRDMAKCLE
EFEQLCRAFLKSKTIVDKEG
VPPFYIRLLADLEDYLNQLWEDKEGKKKMNKNNAKALSTLRQKIRKYNRDYETEIAAYKENPQESADEEEEKEAEESGSS
SGSSDDDEEGEEAMTAKSFL
KKEVAPEANKFLKGAKGSGDESSSSDDDEDDEDWGSDTVDSGSESSDDGEGKTSSLACRFLKKTQEGEKGLDKKREQRKK
KTKKKERLEEEAEEEAGEEG
EGGWEKVRGGVPLVKVRRLNFNLTCSGLTARRSHEEKKPKMFAKGTEINTTVVVKKLNEILQARGKKGTDRAAQIELLHL
LAGISGEHNLGQGILVKIKF
NIIASLYDYNPNLAAFMKADMWKKCLECIDELLDILFENNNIFIGENIAEDSENLAISETQPFRVRGCILTLVERMDEEF
TKIMQNTDPHSQEYVDNLKD
EGHVCGIIDRLLSYLETKGSTEEVCRIYLRRIMHTYYKFDYKAHRRSLGLQAETKSEQDQEESEGEDSAFIMDRLCKFIY
AKDRTDRIRTCAILCHIYHH
ALHSRWYQARDLMLMSHLQDNIQHADPPVQILYNRTMVQLGICAFRQGMIKDAHNALLDIQSSGRAKELLGQGLLMRNMQ
ERNAEQEKIEKRRQ
>g1875.t2
MSRFFATGSDSESEESSSADEITPKTAGPNFRQSLLLSDDEEDTKRVVRSAKDKRFEELTNLIKTIRNAMKIRDMAKCLE
EFEQLCRAFLKSKTIVDKEG
VPPFYIRLLADLEDYLNQLWEDKEGKKKMNKNNAKALSTLRQKIRKYNRDYETEIAAYKENPQESADEEEEKEAEESGSS
SGSSDDDEEGEEAMTAKSFL
KKEVAPEANKFLKGAKGSGDESSSSDDDEDDEDWGSDTVDSGSESSDDGEGKTSSLACRFLKKTQEGEKGLDKKREQRKK
KTKKKERLEEEAEEEAGEEG
EGGWEKVRGGVPLVKDEDALSQCVCLFGRKKPKMFAKGTEINTTVVVKKLNEILQARGKKGTDRAAQIELLHLLAGISGE
HNLGQGILVKIKFNIIASLY
DYNPNLAAFMKADMWKKCLECIDELLDILFENNNIFIGENIAEDSENLAISETQPFRVRGCILTLVERMDEEFTKIMQNT
DPHSQEYVDNLKDEGHVCGI
IDRLLSYLETKGSTEEVCRIYLRRIMHTYYKFDYKAHRRSLGLQAETKSEQDQEESEGEDSAFIMDRLCKFIYAKDRTDR
IRTCAILCHIYHHALHSRWY

QARDLMLMSHLQDNIQHADPPVQILYNRTMVQLGICAFRQGMIKDAHNALLDIQSSGRAKELLGQGLLMRNMQERNAEQE
KIEKRRQ
>g1875.t3
MSRFFATGSDSESEESSSADEITPKTAGPNFRQSLLLSDDEEDTKRVVRSAKDKRFEELTNLIKTIRNAMKIRDMAKCLE
EFEQLCRAFLKSKTIVDKEG
VPPFYIRLLADLEDYLNQLWEDKEGKKKMNKNNAKALSTLRQKIRKYNRDYETEIAAYKENPQESADEEEEKEAEESGSS
SGSSDDDEEGEEAMTAKSFL
KKEVAPEANKFLKGAKGSGDESSSSDDDEDDEDWGSDTVDSGSESSDDGEGKTSSLACRFLKKTQEGEKGLDKKREQRKK
KTKKKERLEEEAEEEAGEEG
EGGWEKVRGGVPLVKVRRLNFNLTCSGLTARRSHEEKKPKMFAKGTEINTTVVVKKLNEILQARGKKGTDRAAQIELLHL
LAGISGEHNLGQGILVKIKF
NIIASLYDYNPNLAAFMKADMWKKCLECIDELLDILFENNNIFIGENIAEDSENLAISETQPFRVRGCILTLVERMDEEF
TKIMQNTDPHSQEYVDNLKD
EGHVCGIIDRLLSYLETKGSTEEVCRIYLRRIMHTYYKFDYKAHRRSLGLQAETKSEQDQEESEGEDSAFIMDRLCKFIY
AKDRTDRIRTCAILCHIYHH
ALHSRWYQARDLMLMSHLQDNIQHADPPVQVKHQAQILYNRTMVQLGICAFRQGMIKDAHNALLDIQSSGRAKELLGQGL
LMRNMQERNAEQEKIEKRRQ
>g1876.t1
ESEEFIMKLINRPIIVLRGEHGFIGCRKVTGTLDSNRSTYDYFTLEFRDGAYSLQGQVWARTRVRVYPLGKVNGPGCGTV
GPRGSVVYAEVNP
>g1877.t1
LLPDTEYMVSVVCVYDQRESSPAVGVQKTVMDSPGALVFSDVLTNSFTVHWVAPRGPITGYRIRHQMASGGRAKEERLPP
SRSHFTLTGLHPETEYLVHI
YAVNGRRESTPLSGRQKTISDAPTDLKVVESSPTSITIRWDAPAVTVRYYRITHGETGGRGNVQEFTVPGTQSTAQVTNL
KPATNYTITVYAVTARGDSP
ASSTPIYITHRTNSTHVESPSDVEVMGVKASTITVRWSPAQGPIMGYRVTGTPKNGLGPVFSEVVAPDQTEITFSGMMPT
QEYVLSVFALGEDGESTPVV
ESAMTNQTEITFSGMMPTQEYVLSVFALGEDGESTPVVESAMTNVDHPKDLVFSDVDSTSLRISWDAPEGAVTSYRVLYF
SPEEGERELLPAPTGDADSA
VLRDLRPGSEYTVKVIALHDNAASTPLVGTQVT
>g1877.t2
LLPDTEYMVSVVCVYDQRESSPAVGVQKTVMDSPGALVFSDVLTNSFTVHWVAPRGPITGYRIRHQMASGGRAKEERLPP
SRSHFTLTGLHPETEYLVHI
YAVNGRRESTPLSGRQKTISDAPTDLKVVESSPTSITIRWDAPAVTVRYYRITHGETGGRGNVQEFTVPGTQSTAQVTNL
KPATNYTITVYAVTARGDSP
ASSTPIYITHRTNSTHVESPSDVEVMGVKASTITVRWSPAQGPIMGYRVTGTPKNGLGPVFSEVVAPDQTEITFSGMMPT
QEYVLSVFALGEDGESTPVV
ESAMTNQTEITFSGMMPTQEYVLSVFALGEDGESTPVVESAMTNVDHPKDLVFSDVDSTSLRISWDAPEGAVTSYRVLYF
SPEEGERELLPAPTGDADSA
VLRDLRPGSEYTVKVIALHDNAASTPLVGTQVTGEELEIV
>g1877.t3
LLPDTEYMVSVVCVYDQRESSPAVGVQKTVMDSPGALVFSDVLTNSFTVHWVAPRGPITGYRIRHQMASGGRAKEERLPP
SRSHFTLTGLHPETEYLVHI
YAVNGRRESTPLSGRQKTISDAPTDLKVVESSPTSITIRWDAPAVTVRYYRITHGETGGRGNVQEFTVPGTQSTAQVTNL
KPATNYTITVYAVTARGDSP
ASSTPIYITHRTNSTHVESPSDVEVMGVKASTITVRWSPAQGPIMGYRVTGTPKNGLGPVFSEVVAPDQTEITFSGMMPT
QEYVLSVFALGEDGESTPVV
ESAMTNQTEITFSGMMPTQEYVLSVFALGEDGESTPVVESAMTNVDHPKDLVFSDVDSTSLRISWDAPEGAVTSYRVLYF
SPEEGERELLPAPTGDADSA
VLRDLRPGSEYTVKVIALHDNAASTPL
>g1878.t1
SGRYHPDPGPAPVTRFCLNSVDPVEPNAVKGPHGLA
>g1879.t1
LISSSFPPSQEEDFEDMISSADAMESEHGHVFDKVVVNGDLPTAFRELRAALEKASGPDLQWIPAEWTQRATARGSQDHF
VGWI
>g1879.t2

EEDFEDMISSADAMESEHGHVFDKVVVNGDLPTAFRELRAALEKASGPDLQWIPAEWTQRATARGSQDHFVGWI
>g1880.t1
MVRASDQDASWTPPVRGVPGTSNWRSWKALLVRRTSGVPCSVCCHRDPAPDKRSLFNNNDDDDDDDDVKNMHPRTKGTPG
SPCSLS
>g1881.t1
LLTRTGDIVGRWKEHFEELLNPTNTSSVEEAESEDSGEASSIYLAEVSEAVKKLLSGKAPGVDEIRPEMLKALDIVGLSW
LARLFSVAWRAGTVPAEWQT
GVVVPIFKKGDRRVCSNYRGEHSWQPSGPLVSMIPGPGQEVEEHWSTERHTMASPTDAYGTIDFQDSAARVCRAKYVRVA
VDSRAEAVLQLMLNEWQMDR
PKLLISVQGGSENLVLPPKVSQALSKGLVTAALSTEAWILTDGINTGVSRYVGEAVKKFGSHNLRKRNTVGLTPWGMIIN
NNNDLIGRDVYRPYQSLGNP
LSKRACLNNFHSHFLLVDDGTLGKYGCQQGFRRRLEKHIQQQRIHP
>g1881.t2
LLTRTGDIVGRWKEHFEELLNPTNTSSVEEAESEDSGEASSIYLAEVSEAVKKLLSGKAPGVDEIRPEMLKALDIVGLSW
LARLFSVAWRAGTVPAEWQT
GVVVPIFKKGDRRVCSNYRGEHSWQPSGPLVSMIPGPGQEVEEHWSTERHTMASPTDAYGTIDFQDSAARVCRAKYVRVA
VDSRAEAVLQLMLNEWQMDR
PKLLISVQGGSENLVLPPKVSQALSKGLVTAALSTEAWILTDGINTGVSRYVGEAVKKFGSHNLRKRNTVGLTPWGMIIN
NNNDLIGRDVYRPYQSLGNP
LSKRACLNNFHSHFLLVDDGTLGKYGCQQGFRRRLEKHIQQQRIHPQLRTQNSEPRKTFIAK
>g1881.t3
LTRTGDIVGRWKEHFEELLNPTNTSSVEEAESEDSGEASSIYLAEVSEAVKKLLSGKAPGVDEIRPEMLKALDIVGLSWL
ARLFSVAWRAGTVPAEWQTG
VVVPIFKKGDRRVCSNYRGEHSWQPSGPLVSMIPGPGQEVEEHWSTERHTMASPTDAYGTIDFQDSAARVCRAKYVRVAV
DSRAEAVLQLMLNEWQMDRP
KLLISVQGGSENLVLPPKVSQALSKGLVTAALSTEAWILTDGINTGVSRYVGEAVKKFGSHNLRKRNTVGLTPWGMIINN
NNDLIGRDVYRPYQSLGNPL
SKRACLNNFHSHFLLVDDGTLGKYGCQQGFRRRLEKHIQQQRIHPQLRTQNSEPRKTFIAK
>g1882.t1
GEQEEEEEEEEEEEEEEELASASGRERRASVHDKESAQHDEDQPSDEILPAIGGSVEHEHLSAESAPPLSPLIPSEHTPL
DLELQWQDLLAIMEPHALDI
GTPLPSNQDIDGLSGAIQNPLDAAILTNYTSSSNSSNDNQMIYQDVHLMEAALPLGTMSLHHPETHLLPLTPSTELDQQD
GRLNTSGALDNMDINGSLST
SYHRNLLSRDPSGDLNMGLDVENGYTTFDMNPLMQDLTDNFITDDANTGPRSMCTLSPRDPTSFDMNFYQDDFHPVSLTP
HFELSGVSPNLPIIPPSHSL
VDDEEESDYGLPGPLGDLLEDATILDEMSLLDLALEEGFSPEMAARLEEEGYFVPEQAARNVGPHDTNVPNTEEDDDGTS
WSAMAQDNSRILHQESQEKA
DSDSGLSLDCSFSPASPCGSESSCYSSSSTSSSSSSGSSEGSIYSEGNGQKDEEGGVNLDMELEVTIKQEEEEGAVGGYY
SSDVNKIVPSSYQEGKLFNG
LPWQEHIGHDHTYNQAWPGPTSVTGKTSKKQSKYSSGQSRTKPYSRSSSERFAEVELWSRDDRRARSLKIPFSNELIVNL
PVDDFNELLANHRLSEQQLN
LVRDIRRRGKNKMAAQNCRRRKLGALLGLEVDVSGLRRRRAWLRREKQEALKSLQEMERRLEGLYQEVMLSLRDEEGRPL
DASDYALQFGANGSVTVTPR
RQGTLPRADGKAGKKYRDKKCDESK
>g1882.t2
GEQEEEEEEEEEEEEEEELASASGRERRASVHDKESAQHDEDQPSDEILPAIGGSVEHEHLSAESAPPLSPLIPSEHTPL
DLELQWQDLLAIMEPHALDI
GTPLPSNQDIDGLSGAIQNPLDAAILTNYTSSSNSSNDNQMIYQDVHLMEAALPLGTMSLHHPETHLLPLTPSTELDQQD
GRLNTSGALDNMDINGSLST
SYHRNLLSRDPSDDFHPVSLTPHFELSGVSPNLPIIPPSHSLVDDEEESDYGLPGPLGDLLEDATILDEMSLLDLALEEG
FSPEMAARLEEEGYFVPEQA
ARNVGPHDTNVPNTEEDDDGTSWSAMAQDNSRILHQESQEKADSDSGLSLDCSFSPASPCGSESSCYSSSSTSSSSSSGS
SEGSIYSEGNGQKDEEGGVN
LDMELEVTIKQEEEEGAVGGYYSSDVNKIVPSSYQEGKLFNGLPWQEHIGHDHTYNQAWPGPTSVTGKTSKKQSKYSSGQ
SRTKPYSRSSSERFAEVELW
SRDDRRARSLKIPFSNELIVNLPVDDFNELLANHRLSEQQLNLVRDIRRRGKNKMAAQNCRRRKLGALLGLEVDVSGLRR

RRAWLRREKQEALKSLQEME
RRLEGLYQEVMLSLRDEEGRPLDASDYALQFGANGSVTVTPRRQGTLPRADGKAGKKYRDKKCDESK
>g1882.t3
GEQEEEEEEEEEEEEEEEELASASGRERRASVHDKESAQHDEDQPSDEILPAIGGSVEHEHLSAESAPPLSPLIPSEHTPL
DLELQWQDLLAIMEPHALDI
GTPLPSNQDIDGLSGAIQNPLDAAILTNYTSSSNSSNDNQMIYQDVHLMEAALPLGTMSLHHPETHLLPLTPSTELDQQD
GRLNTSGALDNMDINGSLST
SYHRNLLSRDPSDPTSFDMNFYQDDFHPVSLTPHFELSGVSPNLPIIPPSHSLVDDEEESDYGLPGPLGDLLEDATILDE
MSLLDLALEEGFSPEMAARL
EEEGYFVPEQAARNVGPHDTNVPNTEEDDDGTSWSAMAQDNSRILHQESQEKADSDSGLSLDCSFSPASPCGSESSCYSS
SSTSSSSSSGSSEGSIYSEG
NGQKDEEGGVNLDMELEVTIKQEEEEGAVGGYYSSDVNKIVPSSYQEGKLFNGLPWQEHIGHDHTYNQAWPGPTSVTGKT
SKKQSKYSSGQSRTKPYSRS
SSERFAEVELWSRDDRRARSLKIPFSNELIVNLPVDDFNELLANHRLSEQQLNLVRDIRRRGKNKMAAQNCRRRKLGALL
GLEVDVSGLRRRRAWLRREK
QEALKSLQEMERRLEGLYQEVMLSLRDEEGRPLDASDYALQFGANGSVTVTPRRQGTLPRADGKAGKKYRDKKCDESK
>g1883.t1
MRTEHVQNLPRNVSSSSSSSSGSSGSRGGTFPTPQGGDLTPKFWLELKENLQTTHKTRPFTVKNAVLHCERISRRQTEPQ
NSGGAFPPLANRQGQVTPGF
KREDKA
>g1883.t2
LPRNVSSSSSSSSGSSGSRGGTFPTPQGGDLTPKFWLELKENLQTTHKTRPFTVKNAVLHCERISRRQTEPQNSGGAFPP
LANRQGQVTPGFKREDKA
>g1883.t3
MKYVNLVPPGFMRTEHVQNLPRNVSSSSSSSSGSSGSRGGTFPTPQGGDLTPKFWLELKENLQTTHKTRPFTVKNAVLHC
ERISRRQTEPQNSGGAFPPL
ANRQGQVTPGFKREDKA
>g1884.t1
MGMLPGFPPPGMFPFWGPFPGAPPPGAPPANGPAPDTPQGNAEASQAAAGCSPLSANEVRHEKECIIPTHWSPPTTTDPA
AAPPGQPNPGFAFPFAPPPF
PTTPWLPPPPPLPFVSSMPPPPPSLSALSDEELSELEAEGRRGLEARLQCLHNIHTLLDAAMLNIHQYLSTVATLTPPRT
GTSGPARSDPTPPAGATAAE
APTPEESSSSCKNNHMVTRHHCAPGEVHSISRHADSVGGATGFSHPADSTTVATASAAQEPEETEPGRDEEEEEEEEAAG
GGAEPSAAELRRRRLRKLEX
XXXXXXXXXGDDDTVVIASPARQLKGGRRR
>g1884.t2
MGMLPGFPPPGMFPFWGPFPGAPPPGAPPANGPAPDTPQGNAEASQAAAPPGQPNPGFAFPFAPPPFPTTPWLPPPPPLP
FVSSMPPPPPSLSALSDEEL
SELEAEGRRGLEARLQCLHNIHTLLDAAMLNIHQYLSTVATLTPPRTGTSGPARSDPTPPAGATAAEAPTPEESSSSCKN
NHMVTRHHCAPGEVHSISRH
ADSVGGATGFSHPADSTTVATASAAQEPEETEPGRDEEEEEEEEAAGGGAEPSAAELRRRRLRKLEXXXXXXXXXXGDDD
TVVIASPARQLKGGRRR
>g1884.t3
AMGMLPGFPPPGMFPFWGPFPGAPPPGAPPANGPAPDTPQGNAEASQAAAGCSPLSANEVRHEKECIIPTHWSPPTTTDP
AAAPPGQPNPGFAFPFAPPP
FPTTPWLPPPPPLPFVSSMPPPPPSLSALSDEELSELEAEGRRGLEARLQCLHNIHTLLDAAMLNIHQYLSTVATLTPPR
TGTSGPARSDPTPPAGATAA
EAPTPEESSSSCKNNHMVTRHHCAPGEVHSISRHADSVGGATGFSHPADSTTVATASAAQEPEETEPGRDEEEEEEEEAA
GGGAEPSAAELRRRRLRKLE
XXXXXXXXXXGDDDTVVIASPARQLKGGRRR
>g1885.t1
GPSVCGMAVLGTVVILLFASRACYNLTVLALSQNHQVEAFDFDWYNISDQADLRSELGDWGYLAFCAILFIWELLPTTLL
IIVFRVRWPAQEASSSMAIN
NRALPRLYFFDDPQRSEDGSPVPWVHSAHPQSSWYGSETTPLLFASHPPTQNNQHHSLYSTPNN
>g1885.t2
GPSVCGMAVLGTVVILLFASRACYNLTVLALSQNHQVEAFDFDWYNISDQADLRSELGDWGYLAFCAILFIWELLPTTLL

IIVFRSSSMAINNRALPRLY
FFDDPQRSEDGSPVPWVHSAHPQSSWYGSETTPLLFASHPPTQNNQHHSLYSTPNN
>g1885.t3
MAVLGTVVILLFASRACYNLTVLALSQNHQVEAFDFDWYNISDQADLRSELGDWGYLAFCAILFIWELLPTTLLIIVFRV
RWPAQEASSSMAINNRALPR
LYFFDDPQRSEDGSPVPWVHSAHPQSSWYGSETTPLLFASHPPTQNNQHHSLYSTPNN
>g1886.t1
LKTALKSPLKAEADAHASKDLERQKTVTPAKTKGKLFTCQTCGAGLSRTKLLVHQRTHMAAKPVCRTTFAPGELPPHPRT
HSAATHRPSPFPCEQCGKRF
ATRAAVALHQRRHMLIHSAVKPFLCTVGGKSFNQPSILKNHLQAYCYALRRHQQRHHAEAEVEEGGAQKRGRPSCETCGE
ELSSAAGAEGPRVGPHGTEA
FRVRNVRQGLLPE
>g1886.t2
LKTALKSPLKAEADAHASKDLERQKTVTPAKTKGKLFTCQTCGAGLSRTKLLVHQRTHMAAKPVCRTTFAPGELPPHPRT
HSAATHRPSPFPCEQCGKRF
ATRAAVALHQRRHMLIHSAVKPFLCTVGGKSFNQPSILKNHLQLRQVAKSFACDVCGQAYCYALRRHQQRHHAEAEVEEG
GAQKRGRPSCETCGEELSSA
AGAEGPRVGPHGTEAFRVRNVRQGLLPE
>g1886.t3
MALHDPRCAFRSQTEIILHIFMEEVRRLLEETNGISCRENLDPVVKLLVSETVRKLCADEVREAEKTQKTVRRKADAHAS
KDLERQKTVTPAKTKGKLFT
CQTCGAGLSRTKLLVHQRTHMAAKPVCRTTFAPGELPPHPRTHSAATHRPSPFPCEQCGKRFATRAAVALHQRRHMLIHS
AVKPFLCTVGGKSFNQPSIL
KNHLQAYCYALRRHQQRHHAEAEVEEGGAQKRGRPSCETCGEELSSAAGAEGPRVGPHGTEAFRVRNVRQGLLPE
>g1887.t1
MGFRVYTIPLPTTSSLRTRGACQEASPPRLDTDVVVGHGVLILPPLSHCDLKSLAKQPEPCELAEDRPAGSWHGLLLLAD
I
>g1888.t1
MWNEMVKDEEITFTGEESAHTEDCSDCFGAQKKDDSNDKEKKEEEEATAAVHHSIIETWDWGRQP
>g1888.t2
LAARAARGGRDKMPTPSFTLDAQLRFHDKWLKIDLQRAFSPEGLSEMWNEMVKDEEITFTGEESAHTEDCSDCFGAQKKD
DSNDKEKKEEEEATAAVHHS
IIETWDWGRQP
>g1888.t3
MPTPSFTLDAQLRFHDKWLKIDLQRAFSPEGLSEMWNEMVKDEEITFTGEESAHTEDCSDCFGAQKKDDSNDKEKKEEEE
ATAAVHHSIIETWDWGRQP
>g1889.t1
MATGRNDYLCRSVVHLGERSLLLKGSTLAPLGQGGKERAAGIGVRPRTFRLGVRHSNHCSDPQAYVCVFCRAVLSWWVLR
ERLELLGQLGCVLCCCGSVV
LIIHAPKAQPITSRTQLEESLLNPVFLCYSSLVLLLLVVLIVRVAPAHGSSNIMVYVSICSLLGSFTVPSSKAIGLVAEE
AFFGGGSPRSLVLFLGLLGT
LAASVITQFLHINKALERFSCNMFEAIYYVAFTCSVLLASALLFKEWRELTAVDSLAMVCALATVFVGVVLLRVSHEAVL
TWTKGADRQKVE
>g1889.t2
MATGRNDYLCRSVVHLGERSLLLKGSTLAPLGQGGKERVRGIVEHTQSMAVLSWWVLRERLELLGQLGCVLCCCGSVVLI
IHAPKAQPITSRTQLEESLL
NPVFLCYSSLVLLLLVVLIVRVAPAHGSSNIMVYVSICSLLGSFTVPSSKAIGLVAEEAFFGGGSPRSLVLFLGLLGTLA
ASVITQFLHINKALERFSCN
MFEAIYYVAFTCSVLLASALLFKEWRELTAVDSLAMVCALATVFVGVVLLRVSHEAVLTWTKGADRQKVE
>g1890.t1
LCVFLSLQRATEGGATAPRCGSEWRSRRGERRSAEQSAGPRSAECSTWAGHNCSLGQRLQCSVLDVAKLAGSSNLTMVQE
RGKYDFYIGLGLAVSSSIFI
GGSFILKKKGLLRLAKKGSMRAERRGRAQVARQHGGRRSDLQRAASVPTAVGEPAFASTRCPCMNAYLPSMNYASRSKHG
LRRE
>g1890.t2
MELYSNTVDQGYAFCNVSCGDGTWAGHNCSLGQRLQCSVLDVAKLAGSSNLTMVQERGKYDFYIGLGLAVSSSIFIGGSF

ILKKKGLLRLAKKGSMRAER
RGRAQVARQHGGRRSDLQRAASVPTAVGEPAFASTRCPCMNAYLPSMNYASRSKHGLRRE
>g1891.t1
MIRPLSEDRRLQDGVEGWSGGVEEVGQVVLETPELSATPRRLVVASAALGEVITQLLTQPWLPPIQENITMEETQRLFCS
SFPWVRVTEDREQANCLLLP
SAPQQVANSILAELEQVLVSRGQLERALSAGSHILQHYVIYLVALGVAGLVPDQLDASQEHLLVSYKRAYSTTIIKDFM
>g1891.t2
MIRPLSEDRRLQDGVEGWSGGVEEVGQVVLETPELSATPRRLVVASAALGEVITQLLTQENITMEETQRLFCSSFPWVRV
TEDREQANCLLLPSAPQQVA
NSILAELEQVLVSRGQLERALSAGSHILQHYVIYLVALGVAGLVPDQLDASQEHLLVSYKRAYSTTIIKDFM
>g1892.t1
MSPSSAAASPCSSRPRPGSPGEKASFVTQLTSVAKTVLGPMKGPTQDAGAKGKDSPRAGEEKREAAVGKPEASAGGRRGA
QIGAGGSAQAERGAGRTTKH
SS
>g1893.t1
LRSRPQKKTGARVILDPPPLRVVSSEETNQAIAAETPKNEPVANLQPASSRLHLIFLDGVIRGRAVCGRGLLGRAGDSGA
PRLLTAAAVHFRSIKQPLAF
ACRVILTSLPDTHTHTHTHTHTYTHTHTHTYTHTHTHTRTHTHMYTHTHAHTHTQAAERTETQREQRRQEEGLSLAEMGH
MVTSDLLTECPEAAQRGPGR
VLTDRWRGMTPQQHSAVYREREEQRLCKKKQQETERMREASWDLEQMSLARKGEEEEGRERELQRERKIQLDRDNARLAR
EQQAR
>g1893.t2
ETNQAIAAETPKNEPVANLQPASSRLHLIFLDGVIRGRAVCGRGLLGRAGDSGAPRLLTAAAVHFRSIKQPLAFACRVIL
TSLPDTHTHTHTHTHTYTHT
HTHTYTHTHTHTRTHTHMYTHTHAHTHTQAAERTETQREQRRQEEGLSLAEMGHMVTSDLLTECPEAAQRGPGRVLTDRW
RGMTPQQHSAVYREREEQRL
CKKKQQETERMREASWDLEQMSLARKGEEEEGRERELQRERKIQLDRDNARLAREQQAR
>g1893.t3
LRSRPQKKTGARVILDPPPLRVVSSEETNQAIAAETPKNEPVANLQPASSRLHLIFLDGVIRGRAVCGRGLLGRAGDSGA
PRLLTAAAVHFRSIKQPLAF
ACRVILTSLPDTHTHTHTHTHTYTHTHTHTYTHTHTHTRTHTHMYTHTHAHTHTQAAERTETQREQRRQEEGLSLAEMGH
MVTSDLLTECPEAAQRGPGR
VLTDRWRGMTPQQHSAVYREREEQRLCKKKQQETERMREASWDLEQMSLARKGEEEEGRERELQRERKIQLDRDNARLAR
EQQA
>g1894.t1
MLMYEMRGKKQSYDVIDLDPYGSPASFLDAAVQAVRLLCITCTDMAVMAGNNGETCYSKYGAISIKARYCHEMALRIILH
SVDQRAAVYQRYVTPLLSIS
VDFYIRVFLRVHTGQAVVKNSA
>g1894.t2
MRGKKQSYDVIDLDPYGSPASFLDAAVQAVRLLCITCTDMAVMAGNNGETCYSKYGAISIKARYCHEMALRIILHSVDQR
AAVYQRYVTPLLSISVDFYI
RVFLRVHTGQAVVKNSA
>g1895.t1
MDHSQTARLPSIDEPYEKMKNELNKVKAAVERLWYELDGQVLSTETDTLGHVELKLTTVMIPRSQQITDDDERQTEVTQD
GMKAATYDQNVDGLKTEIQA
QRDDIERDRQLVKMDMDEVKKMKESIQRQQQQLDEKLDRVRKEIREREVLNTETDIKKKNLEKSMRRAMMKQQDADWKMV
PQDESQSKWTNYRAHKERRE
LDQRLEKIAQERDALEVMKMKMQLEKAEQATEESGCNAAIADEMKGDMQSTGS
>g1895.t2
MDHSQTARLPSIDEPYEKMKNELNKVKAAVERLWYELDGQVLSTETDTLGHVELKLTTVMIPRSQQITDDDERQTEVTQD
GMKAATYDQNVDGLKTEIQA
QRDDIERDRQLVKMDMDEVKKMKESIQRQQQQLDEKLDRVRKEIREREVLNTETDIKKKNLEKSMRRAMMKQQDADWKMV
PQDESQSKWTNYRAHKERRE
LDQRLEKIAQERDALEVMKMKMQLEKAEQATEESGCNAAIADEMKGDMQSTENS
>g1895.t3
MDHSQTARLPSIDEPYEKMKNELNKVKAAVERLWYELDGQVLSTETDTLGHVELKLTTVMIPRSQQITDDDERQTEVTQD

GMKAATYDQNVDGLKTEIQA
QRDDIERDRQLVKMDMDEVKKMKESIQRQQQQLDEKLDRVRKEIREREVLNTETDIKKKNLEKSMRRAMMKQQDADWKMV
PQDESQSKWTNYRAHKERRE
LDQRLEKIAQERDALEVMKMKMQLEKAEQATEESGCNAAIADEMKGDMQSTGNSSEN
>g1896.t1
LLRRLRSFGVCRTLLRTFYDSVVASAIFYAVVCWRGGSTDRDRSRLNRLIRRASSVLDCPLDSIEEVGERRMLTKLTSIM
DNTSHPLHDSVGSLIMFVLS
VPV
>g1897.t1
LAQVNPGWILSKSTSDIPIESTGYEEVGEISGDPKVSGPSHPPNGTRSKESEEKWQEMCLVYKGLFF
>g1897.t2
LLRWQQQQEEDGEEDEREADPVLDDLYTRRLRQHLHQTSSRRDHDHFLPRFWTPEEEVRVRRILRGSERRPWYRRMLAGK
LVDGRVRVQSASEGMLTLSK
STSDIPIESTGYEEVGEISGDPKVSGPSHPPNGTRSKESEEKWQEMCLVYKGLFF
>g1898.t1
MSRKAIVAAANGGWPALRRVLRCRCQEGRRPPHVSRSSELLAPRDEFTAWLWLLPNHTKVAFIHRRVGLIKIHDTRAVWM
FTQAGAYFRERLTGAVPRAP
FTLQGRGARRGTKPNAAISFTSQPRSYNSQDKLKELRSPGPPCTVSLEIRTIPAARRQRRPSPQSV
>g1899.t1
NGTVALRKCPGYTGLARKYQHMY
>g1900.t1
LSRTHVDWHSVDEVYLYSDATTSKIARTVTQKLGFSKASSSGTRLHRGYVEEASLEDRPPQTTHVVFVVHGIGQKMDQGR
IIKNTGM
>g1900.t2
LKLSRTHVDWHSVDEVYLYSDATTSKIARTVTQKLGFSKASSSGTRLHRGYVEEASLEDRPPQTTHVVFVVHGIGQKMDQ
GRIIKNTGM
>g1901.t1
MMSWMNLDQRRTSSVHGEGGSLDSSAVSSDEIYPDNVQRQVEHVCVRGGLYEVDAKDRECYPVYWNQQDRIPVMRAQWFI
DGTWLPLEEEESDLIEQEHL
ARFRGQQMQDNFESDVVARTVDSKDGECQGVLGIG
>g1902.t1
MSNRHRKDTPQYSVFKNGRLLNTAPSLKGLAADFGADSRAGTGQPGAADTGVVQSDGQQGACWPDMVCFYLGCSFGFEGQ
LREAGVPVRNVEQEPKNVSM
YKTAVPCVPVGVFHCPLVVSMRPVPAGMLTSAVTTTHLNPLAHGAPIHIGDPEPPLAFSHSPGSMFLTDIPDSPLPPSQL
SPLCFLVSQDPLLYSLASQR
AVGKIRQLEKVIGEDPGNRGIQALFVQDELLRSCLALSHSTSVAITTGFPTHYMH
>g1902.t2
LPRSLANDFQAFCSRNPAPLPLLHRSQPGETLCLSLAADVDIRKDTPQYSVFKNGRLLNTAPSLKGLAADFGADSRAGTG
QPGAADTGVVQSDGQQGACW
PDMVCFYLGCSFGFEGQLREAGVPVRNVEQEPKNVSMYKTAVPCVPVGVFHCPLVVSMRPVPAGMLTSAVTTTHLNPLAH
GAPIHIGDPEPPLAFSHSPG
SMFLTDIPDSPLPPSQLSPLCFLVSQDPLLYSLASQRAVGKIRQLEKVIGEDPGNRGIQALFVQDELLRSCLALSHSTSV
AITTGFPTHYMH
>g1902.t3
CQQATVVILPRSLANDFQAFCSRNPAPLPLLHRSQPGETLCLSLAADVDIRKDTPQYSVFKNGRLLNTAPSLKGLAADFG
ADSRAGTGQPGAADTGVVQS
DGQQGACWPDMVCFYLGCSFGFEGQLREAGVPVRNVEQEPKNVSMYKTAVPCVPVGVFHCPLVVSMRPVPAGMLTSAVTT
THLNPLAHGAPIHIGDPEPP
LAFSHSPGSMFLTDIPDSPLPPSQLSPLCFLVSQDPLLYSLASQRAVGKIRQLEKVIGEDPGNRGIQALFVQDELLRSCL
ALSHSTSVAITTGFPTHYMH
>g1903.t1
VDKHRKHLINYIIFLRITPFLPNWFINITSPVINVPLGVFFLGTFLGVAPPSFVAINAGTTLYKLTTAGEAVSWNSLAVL
GVLAVLSILPVCFQKKLQEK
LE
>g1904.t1
LSRGFCVGPFTFLRDPWNWLDFMVISMAYVTEFVDLGNVSALRTFRVLRALKTITVIPGLKTIVGALIQSVKKMVDVMIL

TVFALAVFALVGLQLFMGNL
RHKCIRWPPPTDSLALDGFSNATALNGTMASNGTFNFGEYIENTENHYYLDGSLDALLCGNSSDAGRCPEGYTCMKAGRN
PNYGYTSFDSFGWAFLALFR
LMTQDYWENVFQLILRAAGKTYMLFFVVIIFLGSFYLINLILAVVAMAYDEQNEATQREAREKEEEFQRLLEQLRNQDEQ
LRDQEEPMGGSKATLASKES
LSRHPSFDFADGEGDGGDEAAKDCNGRIIPRLLIRVPTLRQPYSEEGEKDKDEDGRSRGSTLSVVHLELPGLKQRTASAL
TVATIAAMEELEEAQRPCPP
GWYRFADLFLKWDCCEVWVLFKKWVHFVVMDPFVDLAITICIVLNTLFMAMEHYPMTPEFDHMLSVGNLVFTGIFAAEMF
FKLIAMDPYYYFQVGWNIFD
SIIVTLSLVELGLANVQGLSVLRSFRLLRVFKLAKSWPTLNMLIKIIGNSVGALGNLTLVLAIIVFIFAVVGMQLFGKSY
KECVCKISTDCVLPRWHMHD
FFHSFLIVFRILCGEWIETMWDCMEVAGTGMCLIVFMMVMVIGNLVVLNLFLALLLSSFSGDNLSTGDEDGELNNLQIAI
NRISNGAAWIKTLLRRLLTP
LLLRITRQRAEGGPKGGGSVEMNHLDVGGGQGAREDDGISDCLVEGQLDAREDDGMSNCLVEGRRGCLADPEVVLTVPIA
QGESDFENLDDDDEEEEEED
EDDEDDGGQNLHSDLMGVTTMDDGDSSVCSTVDYHPPEPEPEPEEEEEPEPVEPESCFTDSCVRRWACLSVDISQGPGKT
WWNIRRTCFIIVEHDWFETF
IIFMILLSSGALAFEDIYIERRRTIKIILEYADKIFTYVFIIEMLLKWVAYGFKTYFTNAWCWLDFFIVDVSLISLGANL
MGYSDLGPIKSLRTLRALRP
LRALSRFEGMRVVVNALVGAIPSIFNVLLVCLIFWLIFSIMGVNLFAGKFYRCINSTTESLFLMNEVNNHSDCLALQEAT
GDARWVNVKVNYDNVATGYL
SLLQVATFKGWMDIMYAAVDSREVEEQPSYEINLYMYIYFVIFIIFGSFFTLNLFIGVIIDNFNQQKKKISFLICSSNLQ
M
>g1904.t2
YVFTGIYTVEATTKVLSRGFCVGPFTFLRDPWNWLDFMVISMAYVTEFVDLGNVSALRTFRVLRALKTITVIPGLKTIVG
ALIQSVKKMVDVMILTVFAL
AVFALVGLQLFMGNLRHKCIRWPPPTDSLALDGFSNATALNGTMASNGTFNFGEYIENTENHYYLDGSLDALLCGNSSDA
GRCPEGYTCMKAGRNPNYGY
TSFDSFGWAFLALFRLMTQDYWENVFQLILRAAGKTYMLFFVVIIFLGSFYLINLILAVVAMAYDEQNEATQREAREKEE
EFQRLLEQLRNQDEQLRDQE
EPMGGSKATLASKESLSRHPSFDFADGEGDGGDEAAKDCNGRIIPRLLIRVPTLRQPYSEEGEKDKDEDGRSRGSTLSVV
HLELPGLKQRTASALTVATI
AAMEELEEAQRPCPPGWYRFADLFLKWDCCEVWVLFKKWVHFVVMDPFVDLAITICIVLNTLFMAMEHYPMTPEFDHMLS
VGNLVFTGIFAAEMFFKLIA
MDPYYYFQVGWNIFDSIIVTLSLVELGLANVQGLSVLRSFRLLRVFKLAKSWPTLNMLIKIIGNSVGALGNLTLVLAIIV
FIFAVVGMQLFGKSYKECVC
KISTDCVLPRWHMHDFFHSFLIVFRILCGEWIETMWDCMEVAGTGMCLIVFMMVMVIGNLVVLNLFLALLLSSFSGDNLS
TGDEDGELNNLQIAINRISN
GAAWIKTLLRRLLTPLLLRITRQRAEGGPKGGGSVEMNHLDVGGGQGAREDDGISDCLVEGQLDAREDDGMSNCLVEGRR
GCLADPEVVLTVPIAQGESD
FENLDDDDEEEEEEDEDDEDDGGQNLHSDLMGVTTMDDGDSSVCSTVDYHPPEPEPEPEEEEEPEPVEPESCFTDSCVRR
WACLSVDISQGPGKTWWNIR
RTCFIIVEHDWFETFIIFMILLSSGALAFEDIYIERRRTIKIILEYADKIFTYVFIIEMLLKWVAYGFKTYFTNAWCWLD
FFIVDVSLISLGANLMGYSD
LGPIKSLRTLRALRPLRALSRFEGMRVVVNALVGAIPSIFNVLLVCLIFWLIFSIMGVNLFAGKFYRCINSTTESLFLMN
EVNNHSDCLALQEATGDARW
VNVKVNYDNVATGYLSLLQVATFKGWMDIMYAAVDSREVEEQPSYEINLYMYIYFVIFIIFGSFFTLNLFIGVIIDNFNQ
QKKKISFLICSSNLQM
>g1904.t3
QTFVVISKGNTIFRFNADPACYLLSPFSLVRRGAIKILIHSYPSDTIQQHIHPSISFRYRLYKAWYVFTGIYTVEATTKV
LSRGFCVGPFTFLRDPWNWL
DFMVISMAYVTEFVDLGNVSALRTFRVLRALKTITVIPGLKTIVGALIQSVKKMVDVMILTVFALAVFALVGLQLFMGNL
RHKCIRWPPPTDSLALDGFS
NATALNGTMASNGTFNFGEYIENTENHYYLDGSLDALLCGNSSDAGRCPEGYTCMKAGRNPNYGYTSFDSFGWAFLALFR
LMTQDYWENVFQLILRAAGK
TYMLFFVVIIFLGSFYLINLILAVVAMAYDEQNEATQREAREKEEEFQRLLEQLRNQDEQLRDQEEPMGGSKATLASKES

LSRHPSFDFADGEGDGGDEA
AKDCNGRIIPRLLIRVPTLRQPYSEEGEKDKDEDGRSRGSTLSVVHLELPGLKQRTASALTVATIAAMEELEEAQRPCPP
GWYRFADLFLKWDCCEVWVL
FKKWVHFVVMDPFVDLAITICIVLNTLFMAMEHYPMTPEFDHMLSVGNLVFTGIFAAEMFFKLIAMDPYYYFQVGWNIFD
SIIVTLSLVELGLANVQGLS
VLRSFRLLRVFKLAKSWPTLNMLIKIIGNSVGALGNLTLVLAIIVFIFAVVGMQLFGKSYKECVCKISTDCVLPRWHMHD
FFHSFLIVFRILCGEWIETM
WDCMEVAGTGMCLIVFMMVMVIGNLVVLNLFLALLLSSFSGDNLSTGDEDGELNNLQIAINRISNGAAWIKTLLRRLLTP
LLLRITRQRAEGGPKGGGSV
EMNHLDVGGGQGAREDDGISDCLVEGQLDAREDDGMSNCLVEGRRGCLADPEVVLTVPIAQGESDFENLDDDDEEEEEED
EDDEDDGGQNLHSDLMGVTT
MDDGDSSVCSTVDYHPPEPEPEPEEEEEPEPVEPESCFTDSCVRRWACLSVDISQGPGKTWWNIRRTCFIIVEHDWFETF
IIFMILLSSGALAFEDIYIE
RRRTIKIILEYADKIFTYVFIIEMLLKWVAYGFKTYFTNAWCWLDFFIVDVSLISLGANLMGYSDLGPIKSLRTLRALRP
LRALSRFEGMRVVVNALVGA
IPSIFNVLLVCLIFWLIFSIMGVNLFAGKFYRCINSTTESLFLMNEVNNHSDCLALQEATGDARWVNVKVNYDNVATGYL
SLLQVATFKGWMDIMYAAVD
SREVEEQPSYEINLYMYIYFVIFIIFGSFFTLNLFIGVIIDNFNQQKKKISFLICSSNLQM
>g1905.t1
MGVKASIFMIVLSRFASSKGYWKDPYIQYFVRTKPPLSKPLIETHSTDSLLLDGQSLDSDRYCVVGLDLRDVVTLDEKLR
RFQLNPDLRSRCRNSKTLGE
ECRALFGSKRGPAANWVESLRLRQSSGERDGDNPVFIVDTTSCVQQPRS
>g1905.t2
MAARQPFTDSDTADEAVRATCDDATTCKRFASSKGYWKDPYIQYFVRTKPPLSKPLIETHSTDSLLLDGQSLDSDRYCVV
GLDLRDVVTLDEKLRRFQLN
PDLRSRCRNSKTLGEECRALFGSKRGPAANWVESLRLRQSSGERDGDNPVFIVDTTSCVQQPRS
>g1905.t3
MGVKASIFMIVLSRFASSKGYWKDPYIQYFVRTKPPLSKPLIETHSTDSLLLDGQSLDSDRYCVVGLDLRDVVTLDEKLR
RFQLNPE
>g1906.t1
APGSPVLSLSTQYQRDSERVQVLALCPGPKDPQGGGGGGSLLHLLSLSLKELSGPGCADARGRLSEDTLQASTYEVPRAL
QACALGAGEVFAYCEGRNGP
LQRFRLPQLNRLFFELNPLALGSQEATPAVHKHIKIRASSNRRDKRRSATGVASSPPIGRPLSARMQAAFLGQRRASWGR
GRKRRVILWGLATWRCPRAA
PGWPRWAGTACCGSAPPPPWYW
>g1906.t2
VDQGGNVLVTASDRHIFVLDAKPSKTFAVIGFTVAPGSPVLSLSTQYQRDSERVQVLALCPGPKDPQGGGGGGSLLHLLS
LSLKELSGPGCADARGRLSE
DTLQASTYEVPRALQACALGAGEVFAYCEGRNGPLQRFRLPQLNRLFFELNPLALGSQEATPAVHKHIKIRASSNRRDKR
RSATGVASSPPIGRPLSARM
QAAFLGQRRASWGRGRKRRVILWGLATWRCPRAAPGWPRWAGTACCGSAPPPPWYW
>g1906.t3
APGSPVLSLSTQYQRDSERVQVLALCPGPKDPQGGGGGGSLLHLLSLSLKELSGPGCADARGRLSEDTLQASTYEVPRAL
QACALGAGEVFAYCEGRNGP
LQRFRLPQDAGGVPGPTACQLGPGEEAEGHPLGPGHLALSPRGSRLASVGRDGLLRLRATTALVLVIRLDFACVGANTLL
KH
>g1907.t1
MAASVLLGSAESTGPGFSAPGSVLVGNNGGSAPASSWNRSLDRALDEASATGGLNLSGRKLKEFPRSAANHDLTDTTRA
>g1908.t1
MATYVDILKSEFPQIDQEVFNYIKGVLDCGGADFEDDEEVFEAIGGVLQEVSADCKNEDDIRDICLQMFNTLTLPNGHSS
QKQLLLDAPVQLSQMSAECV
TTTNDVQGIWMLKRAQNTTVDAKKLEKAEAKLRAKHDRRNDKDGPKTTSPLVLEEASASQASNKKDTRIDQSGRNRSYDI
RIENFDVSFGERSLLQGAEV
MLAAGRRYGLIGRNGLGKTTLLKMLASRSLRVPAHISILHVEQEVAGDDTVALQSVLESDTVREGLLLQERQLNARIAAG
TADGAESVRLSEIYVQLEEM
EADKAPARASVILAGLGFSAKMQQQTTKEFSGGWRMRLALARALFAKPDLLLLDEPTNMLDVRAILWLENYLQTWQSTIL

VVSHDRNFLNAVVTDIIHLH
SHRLDSYRGDYENFVKTKEDRLKNQQREYEAQLQYREHIQVFIDRFRYNANRAAQVQSKLKLLEKLPELKPLEKETEVTL
RFPDNFEKLSPPILQLDEVE
FYYTAERRLFSGLNVCADLESRICIVGENGAGKSTVLKLLMGELTPINGV
>g1908.t2
MATYVDILKSEFPQIDQEVFNYIKGVLDCGGADFEDDEEVFEAIGGVLQEVSADCKNEDDIRDICLQMFNTLTLPNGHSS
QKQLLLDAPVQLSQMSAECV
TTTNDVQGIWMLKRAQNTTVDAKKLEKAEAKLRAKHDRRNDKDGPKTTSPLVLEEASASQASNKKDTRIDQSGRNRSYDI
RIENFDVSFGERSLLQGAEV
MLAAGRRYGLIGRNGLGKTTLLKMLASRSLRVPAHISILHVEQEVAGDDTVALQSVLESDTVREGLLLQERQLNARIAAG
TADGAESVRLSEIYVQLEEM
EADKAPAREFSGGWRMRLALARALFAKPDLLLLDEPTNMLDVRAILWLENYLQTWQSTILVVSHDRNFLNAVVTDIIHLH
SHRLDSYRGDYENFVKTKED
RLKNQQREYEAQLQYREHIQVFIDRFRYNANRAAQVQSKLKLLEKLPELKPLEKETEVTLRFPDNFEKLSPPILQLDEVE
FYYTAERRLFSGLNVCADLE
SRICIVGENGAGKSTVLKLLMGELTPINGV
>g1908.t3
MATYVDILKSEFPQIDQEVFNYIKGVLDCGGADFEDDEEVFEAIGGVLQEVSADCKNEDDIRDICLQMFNTLTLPNGHSS
QKQLLLDAPVQLSQMSAECV
DAKKLEKAEAKLRAKHDRRNDKDGPKTTSPLVLEEASASQASNKKDTRIDQSGRNRSYDIRIENFDVSFGERSLLQGAEV
MLAAGRRYGLIGRNGLGKTT
LLKMLASRSLRVPAHISILHVEQEVAGDDTVALQSVLESDTVREGLLLQERQLNARIAAGTADGAESVRLSEIYVQLEEM
EADKAPARASVILAGLGFSA
KMQQQTTKEFSGGWRMRLALARALFAKPDLLLLDEPTNMLDVRAILWLENYLQTWQSTILVVSHDRNFLNAVVTDIIHLH
SHRLDSYRGDYENFVKTKED
RLKNQQREYEAQLQYREHIQVFIDRFRYNANRAAQVQSKLKLLEKLPELKPLEKETEVTLRFPDNFEKLSPPILQLDEVE
FYYTAERRLFSGLNVCADLE
SRICIVGENGAGKSTVLKLLMGELTPINGV
>g1909.t1
SVAVLRDCMKDKRLLVFFQERQATLSHSLPLETYLLKPVQRILKYHLLLQELSKHWDHSAQGSEVVEDAIVAMTAVAWYI
NDMKRRQEHAVRLQ
>g1910.t1
MAAMAPALTDAPAEAHHIRFKLAAPSSSLSPGSSENHPNSSNLLLHSSGPARRKASDERALQEQQSQQQQVDTPAQTPSL
GKLQPLVASYLCSDVTPVAS
SKESIALQEILIQQSVLKSHGILPSSLLNGGGDFLLRKKQTFELSGGQLKSLMSTNTNGGSQPMAPVNGLAKKLATMPSS
GCMVAVNGGKPSASASSDPQ
NLSLDSDTTPTIRTLKHHQAHSSRVSLTTDGNSPDPPPAQPPTPPAFGLGGPNEHMNAEEETSHSSLTCPQQPDGERPGS
SQQSSPVLLQPPPPPSSVSK
PPPCSMDTLDSQIRERTLLTSSRQAQIESRLRRLRKRLQVVQAKQVERHVQQQLGGFLGSALSRLAGTGRRQDMSPSTAS
WRTGRQAAVGRDSLGRFLKA
GAMPTELERLCLSGSANLRATESAFDSDVTESSSGGDSDLEEEEVARVDVEQRHVKM
>g1911.t1
MTTVPSSVSMYRGASMYHTGATTVQPDGSTRDAVSRVPRTALSHLDSQKAEKKKKARQLLTDNSSNTTAPPRLDGKLDSL
YTAGWAASSSFSSRR
>g1912.t1
LAGAPGSSVFTVPLARGKNLSVFLLRDCGGTGLAQQLTADGQRGAEDPPRSYSLWFCTPGHVSPRRVRATGKDPPRGRVF
ISSAARGGKDAACPFSPECL
CMLTM
>g1913.t1
MVQRLLYVLTYFIRCSELLESHLLDSVEDEAIVMPGSLITTSLRRGEVEESDYVLITVHKPSGDYLGQGAPAAPSLRGDV
AGCCPPENTLQSSTYLSTGA
EPCTVGKVALARATPMGQEDEEEEADEEDEDEEEEEEEEEEEDSSESQGSRSSVLQAGQGGEVGGLELPIIRAEEDENLSV
PELHSPSTPIAPTTPTPVSP
REKKVTRDMLSAPDCTHPIKMSGARTMGISLEKKPPDKSPSPMATSPVLGVTLATGPMALQLMREEEEQPVNKVTFLIGD
SMSPESDTESRRRKVEEEFK
KHKKHQKDKQQLQPQQLLVQQQQQQQQQQQQQQQQQQLEGFDSTSSSSVISEDQMQLAQARTCALQRVSSKIPQWGCGTG

SADYSHFDEYFSSDHPVETR
TTEDMVSMPPGVSTMDRTHKELLDPSGCDPATWASRGPGTALAEACSGTQLRQSSSSGEGPTTGDSQGSCRCASMEGHSC
CGACSAEQDKGIVSVTAPQD
ASLMKGDQKQVESVLGDWDIPRNESLDSALGDSESEDTEDWQEEVLVPFPGAKQVENYSKPSIANFGRSLFGGYCPTYVP
DFVLHGIPCDEALRQGLMAE
LSHAVQHPVLDEPIAEAVCIIADTDRWTVQVASSQRRATDVNKLGKEVLVSSLVSGLLQSTYQLYKLNLSPNFCVMHLED
RLQEIYFKSKMLAEYLKGQT
RVHVKELGMVLGIESSDLPLLAAVASTHSPYVAQILL
>g1913.t2
QEMVQRLLYVLTYFIRCSELLESHLLDSVEDEAIVMPGSLITTSLRRGEVEESDYVLITVHKPSGDYLGQGAPAAPSLRG
DVAGCCPPENTLQSSTYLST
GAEPCTVGKVALARATPMGQEDEEEEADEEDEDEEEEEEEEEEEDSSESQGSRSSVLQAGQGGEVGGLELPIIRAEEDENL
SVPELHSPSTPIAPTTPTPV
SPREKKVTRDMLSAPDCTHPIKMSGARTMGISLEKKPPDKSPSPMATSPVLGVTLATGPMALQLMREEEEQPVNKVTFLI
GDSMSPESDTESRRRKVEEE
FKKHKKHQKDKQQLQPQQLLVQQQQQQQQQQQQQQQQQQLEGFDSTSSSSVISEDQMQLAQARTCALQRVSSKIPQWGCG
TGSADYSHFDEYFSSDHPVE
TRTTEDMVSMPPGVSTMDRTHKELLDPSGCDPATWASRGPGTALAEACSGTQLRQSSSSGEGPTTGDSQGSCRCASMEGH
SCCGACSAEQDKGIVSVTAP
QDASLMKGDQKQVESVLGDWDIPRNESLDSALGDSESEDTEDWQEEVLVPFPGAKQVENYSKPSIANFGRSLFGGYCPTY
VPDFVLHGIPCDEALRQGLM
AELSHAVQHPVLDEPIAEAVCIIADTDRWTVQVASSQRRATDVNKLGKEVLVSSLVSGLLQSTYQLYKLNLSPNFCVMHL
EDRLQEIYFKSKMLAEYLKG
QTRVHVKELGMVLGIESSDLPLLAAVASTHSPYVAQILL
>g1914.t1
LTWRPKKKKKKGTTRKTKKLPVKGKAKKTAQMSKTPAAPRAARPVPTQQEHSNQTAGESPCRTGQDLDHRWGQSLPEEVL
VSIFQMVVVQDGAIPFLCRV
SRVCRLWNAAAASPSLWRRVSVGYCWVEPGKKQQPRTEERIKETLRGLALDRFSQLREFTLHHWKKNVDYAVEVSAF
>g1914.t2
VISNTAQHDNLTWRPKKKKKKGTTRKTKKLPVKGKAKKTAQMSKTPAAPRAARPVPTQQEHSNQTAGESPCRTGQDLDHR
WGQSLPEEVLVSIFQMVVVQ
DGAIPFLCRVSRVCRLWNAAAASPSLWRRVSVGYCWVEPGKKQQPRTEERIKETLRGLALDRFSQLREFTLHHWKKNVDY
AVEVSAF
>g1914.t3
MSGRGKTGGKARAKAKTRSSRAGLQFPVGRVHRLLRKGNYAHRVGAGAPVYLAAVLEYLTA
>g1914.t4
LTWRPKKKKKKGTTRKTKKLPVKGKAKKTAQMSKTPAAPRAARPVPTQQEHSNQTAGESPCRTGQDLDHRWGQSLPEEVL
VSIFQMVVVQDGAIPFLCRV
SRVCRLWNAAAASPSLWRRVSVGYCWVEPGKKQQPRTEERIKETLRGLALDRFSQLREFTLHHWKKNVDYAVEFPVGRVH
RLLRKGNYAHRVGAGAPVYL
AAVLEYLTA
>g1915.t1
MSPRDLLNRACLWIDAFVPVQVGSVVGGLMMLVGGVASAVMYPGSWQTESSLIAAVFPLIGCSAGFVVAVICRQPWHR
>g1916.t1
LSDLHARPSAEFLPTSKKRLETHFSFMSRDSESALTLHTQTRLPITWKEAYSSGNVNMTTSCSGGKDSCSTVVLAWTFWA
PVSMAMVQHLTVVSE
>g1917.t1
LPVTSRDSKLTEKALLLGQGRDRSTLLLGKGRDRSTLLLGQGRDRSTLLLGKGRDSSALLLGKGRDSSALLLGQGRDSST
DTKH
>g1918.t1
MLCCSALVLLLLSQARGLLSTEEDSLPQEFTLLHVVQGHIGAGNYSYLRLNHDGKIVLVMRSLKGDADLYVSDKTLRPSF
DTYKLQSTTCGQDVVVVPGD
FVRPVGIGIYGHPSYLESEFEMRVFYDQTVALDPFEKSSSEEPDGGNTFPRGSEEDFQEEESIFWTILIGLLKIIVEILF
>g1919.t1
LASCSSLLTVSDPSQGVTLLPYRCFVAHDNAVRAVCFCPASRYMMATGGNDRMVKLWNLKRLYEPISVQRRNLTTEVCWP
LAAPGTFQAQEISYASRGLH

GAYFNDLGYRNARSVFIIPRTGTVWSLSISDWCNSLVACDCLGEVIFALLPEFNCTHPSLRRSVERRFPIYFTDMVRYNS
EEERSQQKAEGGSRGQAEKS
LGQSVTNNNSKPAEEEPASSDEEESSSEGRAGQDHVLKMPPNIETYQEAVKNYYLHFSDSNLQSFKNYKKRAPWKRMSQT
ELKTELDMDKMPLAALHKVR
FNPNMTCHAWVASVGQAGGEGEEGAEAEEADVAVADQ
>g1919.t2
LWDLASCSSLLTVSDPSQGVTLLPYRCFVAHDNAVRAVCFCPASRYMMATGGNDRMVKLWNLKRLYEPISVQRRNLTTEV
CWPLAAPGTFQAQEISYASR
GLHGAYFNDLGYRNARSVFIIPRTGTVWSLSISDWCNSLVACDCLGEVIFALLPEFNCTHPSLRRSVERRFPIYFTDMVR
YNSEEERSQQKAEGGSRGQA
EKSLGQSVTNNNSKPAEEEPASSDEEESSSEGRAGQDHVLKMPPNIETYQEAVKNYYLHFSDSNLQSFKNYKKRAPWKRM
SQTELKTELDMDKMPLAALH
KVRFNPNMTCHAWVASVGQAGGEGEEGAEAEEADVAVADQ
>g1919.t3
LASCSSLLTVSDPSQGVTLLPYRCFVAHDNAVRAVCFCPASRYMMATGGNDRMVKLWNLKRLYEPISVQRRNLTTEVCWP
LAAPGTFQAQEISYASRGLH
GAYFNDLGYRNARSVFIIPRTGTVWSLSISDWCNSLVACDCLGEVIFALLPEFNCTHPSLRRSVERRFPIYFTDMVRYNS
EEERSQQKAEGGSRGQAEKS
LGQSVTNNNSKPAEEEPASSDEEESSSEGRAGQDHVLKMPPNIETYQEAVKNYYLHFSDSNLQSFKNYKKRAPWKRMSQT
ELKTELDMDKMPLAALHKVR
FNPNMTCHAWVASVGQAGVLNLATRVSFKSGERAENSLQH
>g1920.t1
LLQTSLILCSTFPDEAHPSIQNGGTVGTIELKRPVGVHCHGNKAIESEERGDKLLAQKKLCIASAVCLVFMIGEVIGGYL
AHSLAIMTDAAHLLTDLGSM
MVSIFSLWISSRPPTKTMSFGWHRSEILGALVSVMSIWIVTGVLVYLAIERIIRNDYEINGHVMLLTSGCAVIVNIVMAY
ILHHSTTFHGHGEGYHQIDD
NGVNPIAHGHSHALLGGHDNTSVRAAFIHVVGDLLQSVGVMVAAIVIYFRPEYKIADPICTFLFSVFVLGTTFTILKDVF
RILMEGAPKGVEFSSVKEVL
LSLEAVKAAHCLHLWALTLGRSLVSVHLAVDEGADAHMVLREATELLQTKFGFTSVTIQVEPYSEDMDHCARCQDPTT
>g1920.t2
MVPTLTNFVIFLVPSIQNGGTVGTIELKRPVGVHCHGNKAIESEERGDKLLAQKKLCIASAVCLVFMIGEVIGGYLAHSL
AIMTDAAHLLTDLGSMMVSI
FSLWISSRPPTKTMSFGWHRSEILGALVSVMSIWIVTGVLVYLAIERIIRNDYEINGHVMLLTSGCAVIVNIVMAYILHH
STTFHGHGEGYHQIDDNGVN
PIAHGHSHALLGGHDNTSVRAAFIHVVGDLLQSVGVMVAAIVIYFRPEYKIADPICTFLFSVFVLGTTFTILKDVFRILM
EGAPKGVEFSSVKEVLLSLE
AVKAAHCLHLWALTLGRSLVSVHLAVDEGADAHMVLREATELLQTKFGFTSVTIQVEPYSEDMDHCARCQDPTT
>g1920.t3
LQTSLILCSTFPDEAHPSIQNGGTVGTIELKRPVGVHCHGNKAIESEERGDKLLAQKKLCIASAVCLVFMIGEVIGGYLA
HSLAIMTDAAHLLTDLGSMM
VSIFSLWISSRPPTKTMSFGWHRSEILGALVSVMSIWIVTGVLVYLAIERIIRNDYEINGHVMLLTSGCAVIVNIVMAYI
LHHSTTFHGHGEGYHQIDDN
GVNPIAHGHSHALLGGHDNTSVRAAFIHVVGDLLQSVGVMVAAIVIYFRPEYKIADPICTFLFSVFVLGTTFTILKDVFR
ILMEGAPKGVEFSSVKEVLL
SLEAVKAAHCLHLWALTLGRSLVSVHLAVDEGADAHMVLREATELLQTKFGFTSVTIQVEPYSEDMDHCARCQDPTT
>g1921.t1
EISDYDLQELVMKSIGRLTLVRQTFPMPQNATQRCVKHNHRINAAL
>g1922.t1
MLFVDFSSAFNTVIPSKLNTKLGDLGFNTSLRHWITDFLTNRPQFVRSGHSCSSTITLNTGVPQGCVLSPFLYSLFTHDC
RPVHGSNTIVKFADDTTVLG
LISNNDETAYREEVKHLAAWCADNNLLLNTSKTKELIVDFRRERRITHNPIHINGMAVERVSSFKFLGTHITEDLSWSTN
TSSLVKKAHQRLFFLRIL
>g1923.t1
MRTLFCLVTLACFAGAQHQALIDYLERRLLAIEDRISLWHEQTNRYASELRELKQQMVAQLDTLDKEKETLRVSLDGVGA
RVDRVEREMDYMETQNGAQP
CVDVDDKLVEQQVTQVKEKNKARYSKLSDCSEMISSIKAMKILKKLGGPKGMWTKDVGGGSGKVYVLNGTDQGTVYEFSS

VQDFTSSSGVSLARTLSLPS
VWTGTGHLIYDHHLYYVREPLTVVKYDLRNSSLVDSVVFPTEDHVPVYGLTPETVIDLAVDEEGLWVIYATRQNHRHISL
AKMAAKTLDVEQMWDTTCPK
DNAEAAFVICGTLYVVYNTPQPGRSRVQCVFDVNGLVSGEDAPLVYFPKRYGAHSSLKYDPLEKLLYAWDDGYQVMYRLV
MRPKLEV
>g1923.t2
LTETFQRSSEPLSWSTDGPLHFLCTAHSADTTMRTLFCLVTLACFAGAQHQALIDYLERRLLAIEDRISLWHEQTNRYAS
ELRELKQQMVAQLDTLDKEK
ETLRVSLDGVGARVDRVEREMDYMETQNGAQPCVDVDDKLVEQQVTQVKEKNKARYSKLSDCSEMISSIKAMKILKKLGG
PKGMWTKDVGGGSGKVYVLN
GTDQGTVYEFSSVQDFTSSSGVSLARTLSLPSVWTGTGHLIYDHHLYYVREPLTVVKYDLRNSSLVDSVVFPTEDHVPVY
GLTPETVIDLAVDEEGLWVI
YATRQNHRHISLAKMAAKTLDVEQMWDTTCPKDNAEAAFVICGTLYVVYNTPQPGRSRVQCVFDVNGLVSGEDAPLVYFP
KRYGAHSSLKYDPLEKLLYA
WDDGYQVMYRLVMRPKLEV
>g1924.t1
MDFTFRAVTHNAGLIIWRVETQKSGATAMYDIHYWIGSASSQDEQGAAAIYTIQLDEFLGSTPVQHREVQNHESDAFRGY
FKNGMICKKGGVASGMRHTE
TNCYDVERLLHVKGRKRVMATEVEKSWDSFNLGDVFLLDVGKSIIQWNGPESNRQERLKGMMLAKDIRDRERGGRTEIQL
VDGEAEDSSPQAMEALNNIL
GKRTSELKAGAPDEKADQEQKANLILYQVSDEGGNMKVTEVATRPLVQELMNHEDCYILDQGGSKIFVWKGKKANKAEKQ
AAMSRALEFIKVKDYPAHTN
VEAVNDGAESALFKQLFQSWTVKDQTQGLGRTHARGKVAVVSQEKFDATQMHLMPEVAAQQRMVDNGSGEVEVWRIEDLE
LVAVEPEWRGYFYGGDCYLI
LYTYLVNSKQHYILYMWQGRHATQDELAASAFQAVDLDQKYGGEPVQIRVPMGKESKHFMAMFKGKMVIFEGGTSRTAGS
DPEPPVRLFQVRGTDPSSTK
AIEVPALASSLNSNDVFLLKSQSGLFLWCGKGSSGDERDMAKQIASVIGKDSECELVVEGQEMQQQVPDHPPRLFECSNK
TGRFMVEEITQFNQDDLSED
NVMLLDTWDQVFLWVGQGANETERKEAVPTSMEYLRTHPGSRDPDTPVLLIKQGFEPPTFTGWFAAWDPFKWSGGNSYEE
MKKEFADSAPLPVEVQYEEP
QAGQEEEQSYDPHPAGDLLHKQTSELPEGVDPAHKEKYLSDSDFQDIFQLSKDEFVGLPAWKQLKLKKEKGLF
>g1924.t2
MFQSAREEGAEHIWHCWTCSLKETPGDKHSFLLLLLLCLASSELNMDRTVHLTEDRAYSVNMDFTFRAVTHNAGLIIWRV
ETQKSGATAMYDIHYWIGSA
SSQDEQGAAAIYTIQLDEFLGSTPVQHREVQNHESDAFRGYFKNGMICKKGGVASGMRHTETNCYDVERLLHVKGRKRVM
ATEVEKSWDSFNLGDVFLLD
VGKSIIQWNGPESNRQERLKGMMLAKDIRDRERGGRTEIQLVDGEAEDSSPQAMEALNNILGKRTSELKAGAPDEKADQE
QKANLILYQVSDEGGNMKVT
EVATRPLVQELMNHEDCYILDQGGSKIFVWKGKKANKAEKQAAMSRALEFIKVKDYPAHTNVEAVNDGAESALFKQLFQS
WTVKDQTQGLGRTHARGKVA
VVSQEKFDATQMHLMPEVAAQQRMVDNGSGEVEVWRIEDLELVAVEPEWRGYFYGGDCYLILYTYLVNSKQHYILYMWQG
RHATQDELAASAFQAVDLDQ
KYGGEPVQIRVPMGKESKHFMAMFKGKMVIFEGGTSRTAGSDPEPPVRLFQVRGTDPSSTKAIEVPALASSLNSNDVFLL
KSQSGLFLWCGKGSSGDERD
MAKQIASVIGKDSECELVVEGQEMQQQVPDHPPRLFECSNKTGRFMVEEITQFNQDDLSEDNVMLLDTWDQVFLWVGQGA
NETERKEAVPTSMEYLRTHP
GSRDPDTPVLLIKQGFEPPTFTGWFAAWDPFKWSGGNSYEEMKKEFADSAPLPVEVQYEEPQAGQEEEQSYDPHPAGDLL
HKQTSELPEGVDPAHKEKYL
SDSDFQDIFQLSKDEFVGLPAWKQLKLKKEKGLF
>g1924.t3
MDFTFRAVTHNAGLIIWRVETQKSGATAMYDIHYWIGSASSQDEQGAAAIYTIQLDEFLGSTPVQHREVQNHESDAFRGY
FKNGMICKKGGVASGMRHTE
TNCYDVERLLHVKGRKRVMATEVEKSWDSFNLGDVFLLDVGKSIIQWNGPESNRQERLKGMMLAKDIRDRERGGRTEIQL
VDGEAEDSSPQAMEALNNIL
GKRTSELKAGAPDEKADQEQKANLILYQVSDEGGNMKVTEVATRPLVQELMNHEDCYILDQGGSKIFVWKGKKANKAEKQ
AAMSRALVSNKPSAMCDLQE

FIKVKDYPAHTNVEAVNDGAESALFKQLFQSWTVKDQTQGLGRTHARGKVAVVSQEKFDATQMHLMPEVAAQQRMVDNGS
GEVEVWRIEDLELVAVEPEW
RGYFYGGDCYLILYTYLVNSKQHYILYMWQGRHATQDELAASAFQAVDLDQKYGGEPVQIRVPMGKESKHFMAMFKGKMV
IFEGGTSRTAGSDPEPPVRL
FQVRGTDPSSTKAIEVPALASSLNSNDVFLLKSQSGLFLWCGKGSSGDERDMAKQIASVIGKDSECELVVEGQEMQQQVP
DHPPRLFECSNKTGRFMVEE
ITQFNQDDLSEDNVMLLDTWDQVFLWVGQGANETERKEAVPTSMEYLRTHPGSRDPDTPVLLIKQGFEPPTFTGWFAAWD
PFKWSGGNSYEEMKKEFADS
APLPVEVQYEEPQAGQEEEQSYDPHPAGDLLHKQTSELPEGVDPAHKEKYLSDSDFQDIFQLSKDEFVGLPAWKQLKLKK
EKGLF
>g1925.t1
VEFGARMITIDGKQIKLQIWDTAGQESFRSITRSYYRGAAGALLVYDITR
>g1925.t2
AGQESFRSITRSYYRGAAGALLVYDITR
>g1926.t1
MRGLSDKMATSNGGGMEVDGAVIGALIGKQEGRNIEVMNSFELLSHTVDERAHIDKEYYYTKEEQFKQVFKEMEFLGWYT
TGGPPDQSDIHIHKQVCEII
ESPLFLKLNPLTKHTD
>g1926.t2
MRGLSDKMATSNGGGMEVDGAASPSVMASGVTGSVSVALHPLVILNISDHWIRIRSQEGRPMQVIGALIGKQEGRNIEVM
NSFELLSHTVDERAHIDKEY
YYTKEEQFKQVFKEMEFLGWYTTGGPPDQSDIHIHKQVCEIIESPLFLKLNPLTKHTD
>g1926.t3
MRGLSDKMATSNGGGMEVDGAVIGALIGKQEGRNIEVMNSFELLSHTVDERAHIDKEYYYTKEEQFKQVFKEMEFLGWYT
TGGPPDQSDIHIHKQVCEII
ESPLFLKLNPLTKHTDLPVTVFESVIDIINGE
>g1927.t1
LWVLLRQYLGCGLRCLRLRGLLLSARGGVFLSESWLKALTVKCPRLRKLYLLHTDLRGLPSCRLLPPTLQVLELRGCELP
PTFFNQTHASESGGPTEHGG
APSNVVAQQPVQSKAGKRTGTSSGISIERLVLNNVPSFTDQHLQSLTSWERLSRLELRDTFRVTANGLRSCAAKHSGTGM
EGLSKLKFLEIGITGRQGYQ
LQMASLGLGSGWLGLEELSLGGKEVGPGLLCASRLKDLHSLCLWACTLSEMQVLRSCRMIRQLRKLEFLEVFFQPKQAPP
RVEGEGSDQEEEERDSGEAA
AAAGNEEEDGHKRQDGNDPIPRLQRSLAALLPHCMLVFTNCSVQINSD
>g1928.t1
MCSDEDPNEALVFFSIVQGPGRAPVSSSSAAPLSIKDFDIGRPLGKGKFGNVYLAREKKLNAVIALKVLFKSQMEKEGVE
HQLRREIEIQAHLRHPNILR
FYNYFHDRTRVYLILEFAPRGELYKELQKCTRFDDRRTATYMEEISDALMYCHEKKVIHRDIKPENLLLGYRGELKIADF
GWSVHAPSLRRRTMCGTLDY
LPPEMIEGHTHDEKVDLWCIGVLCYECLVGNPPFETASHSDTYKRITKVDLKFPKVVSDGARDLISKLLRHSPLNRLPLQ
SVIDHPWVKANSRRVLPPVC
TPKKP
>g1928.t2
MSTLRTAPTNQEPGPGRAPVSSSSAAPLSIKDFDIGRPLGKGKFGNVYLAREKKLNAVIALKVLFKSQMEKEGVEHQLRR
EIEIQAHLRHPNILRFYNYF
HDRTRVYLILEFAPRGELYKELQKCTRFDDRRTATYMEEISDALMYCHEKKVIHRDIKPENLLLGYRGELKIADFGWSVH
APSLRRRTMCGTLDYLPPEM
IEGHTHDEKVDLWCIGVLCYECLVGNPPFETASHSDTYKRITKVDLKFPKVVSDGARDLISKLLRHSPLNRLPLQSVIDH
PWVKANSRRVLPPVCTPKKP
>g1928.t3
MSTLRTAPTNQEPGRAPVSSSSAAPLSIKDFDIGRPLGKGKFGNVYLAREKKLNAVIALKVLFKSQMEKEGVEHQLRREI
EIQAHLRHPNILRFYNYFHD
RTRVYLILEFAPRGELYKELQKCTRFDDRRTATYMEEISDALMYCHEKKVIHRDIKPENLLLGYRGELKIADFGWSVHAP
SLRRRTMCGTLDYLPPEMIE
GHTHDEKVDLWCIGVLCYECLVGNPPFETASHSDTYKRITKVDLKFPKVVSDGARDLISKLLRHSPLNRLPLQSVIDHPW
VKANSRRVLPPVCTPKKP

>g1929.t1
MAGLPPGDPQLVSMIVNHLKTQGLFDQFRRDCLADVDTKPAYLNLKQRVDNFVSNHLSNHTWSPHLNKNQLRNNIRQLVL
QSGMLEQGVDRIVAQVVDPK
INHTFRPQVERVVREFLSPGSYPDEPPVPDPSADVKPESSALEQVPAAAPPATATTTTAATTTTAAASDAMSILDTITSL
NQGASSWAGPNGEKGRKGLT
SEEPMQVGEKAEQDMAMVDEEDHRQDGLTPEEETEEDKLGLEVQAVDVKTEDSQVELSKEEGEEEGEEVVEEEVRMKEEE
TEGEEGEREGGSQEQADEVK
DKAGGRTGKSPVEKQDDDDLLKSPNQIRQKARERLKEEYSLEDSDLEGLSDITVSSVHTSDLSSFEEDSDEEQPPSESSE
EGELPSDGEGGGDGEAGADK
DGEDDDDKEQKPRRKAYVHKPFLY
>g1929.t2
MAGLPPGDPQLVSMIVNHLKTQGLFDQFRRDCLADVDTKPAYLNLKQRVDNFVSNHLSNHTWSPHLNKNQLRNNIRQLVL
QSGMLEQGVDRIVAQVVDPK
INHTFRPQVERVVREFLSPGSYPDEPPVPDPSADVKPESSALEQVPAAAPPATATTTTAATTTTAAASDAMSILDTITSL
NQGASSWAGPNGEKGRKGLT
SEEPMQVGEKAEQDMAMVDEEDHRQDGLTPEEETEEDKLGLEVQAVDVKTEDSQVELSKEEGEEEGEEVVEEEVRMKEEE
TEGEEGEREGGSQEQADEVK
DKAGGRTGKSPVEKQDDDDLLKSPNQIRQKARERLKEEYSLEDSDLEGLSDITVSSVHTSDLSSFEEDSDEEQPPSESSE
EGELPSDGNLSACGPDPHAF
ALTSSGEGGGDGEAGADKDGEDDDDKEQKPRRKAYVHKPFLY
>g1930.t1
MSPKVKKEAVPAKTEAKSKALKAKKAVLKGVHSLKKKKIRTSPTFRRPKTLRLRRAPKYPRKSAPRRNKLDHYAIIKFPL
TTESAMKKIEDNNTLVFIVD
VKANKHQIKHAVKKLYDIDVAKVNTLIRPDGEKKAYVRLAPDYDALDVANKIGII
>g1930.t2
RDINNKISPLHYTAGNPLVSSFFNMSPKVKKEAVPAKTEAKSKALKAKKAVLKGVHSLKKKKIRTSPTFRRPKTLRLRRA
PKYPRKSAPRRNKLDHYAII
KFPLTTESAMKKIEDNNTLVFIVDVKANKHQIKHAVKKLYDIDVAKVNTLIRPDGEKKAYVRLAPDYDALDVANKIGII
>g1931.t1
MLSSLKKLPMEEQLRRLQEERTCKVCMDKEVNIVFIPCGHLVVCKECAPSLRKCPICRGLVKGTVRTFLS
>g1932.t1
MFRDIATIVAEKCVNPETKRPYTVSLIERAMKDIHYSVKANKSTKQQALEVIRQLKESMEIQRAHMRLRLVLPAKEGKRL
KEKLKPLLKVVESEDFDEQL
EMVCLVDPGCFREIDELIRCETKGRGSLEVLNLKDVEEGDEKLE
>g1933.t1
MDRTLEQTALGLGITGWLCSVFTRCLALWKVSGTVNNVTATLPAYWDGVWLDWDHWDLAHDGSLHCSFYQRLMSLSGNFR
TWRALVVSAIGVGAFAGAIG
AAGIVWFPKRGQVKVTSGVLFVVSGILMLVPIAWTCHHTSQPLEGAEVLRRDWGPALYLGWISFALMVAGGAFLLTRCPR
HAEHAGSEPVSRHPEQEESE
NPLSTINRTAFTNSQYGRRSQPI
>g1934.t1
MHPVRPVVLPWLEPTAGNHGQNHWETQETPDVILIKDEEDINGCTLAEVCEYSADRSTQCGAPSGSLQVDAPGSSHMSSH
NEELRILSVHGRGEGPLAVD
GHDTLFTASELEAL
>g1934.t2
LFSEGSATLRLQISQSLKENEALRTKMKVMRSELFSLRLQTRSNASRAASRFALARANSWKPRTKPLGNGPKPLNDHKVG
GNSPSFTPSKGEASSVTTPA
EQETPDVILIKDEEDINGCTLAEVCEYSADRSTQCGAPSGSLQVDAPGSSHMSSHNEELRILSVHGRGEGPLAVDGHDTL
FTASELEAL
>g1935.t1
WWNELLANVRTAESLPIFRKRLKTHLFRVHLNPT
>g1936.t1
MTDVVSPTALSEVQLRLLCHDDIESVKLLCGDWFPIEYPDSWYQDITSNKKFFSLAATFKGGIVGMIVAEIKGRTKVHKE
DGDILASSFPVDTQVAYILS
LGVVKEFRKHGIEGTQRVNYFPQ
>g1936.t2

MTDVVSPTALSEVQLRLLCHDDIESVKLLCGDWFPIEYPDSWYQDITSNKKFFSLAATFKGGIVGMIVAEIKGRTKVHKE
DGDILASSFPVDTQVAYILS
LGVVKEFRKHGIVYCDKMCSHSAHTTTQGY
>g1936.t3
MTDVVSPTALSEVQLRLLCHDDIESVKLLCGDWFPIEYPDSWYQDITSNKKFFSLAATFKGGIVGMIVAEIKGRTKVHKE
VL
>g1937.t1
MTSIHFVVHPLPGTEDQLNDRLREVSEKLNKYNCNSHPHLSVLEQATLKQCVVGPNHAGFLLEDGRVCRISFAVQPDRLE
LSKPDGSDGSKLNSGSGTGR
SSRPGRTSDPPWFLSGSDTLGRLAGNTL
>g1937.t2
MTSIHFVVHPLPGTEDQLNDSVSGYDLYFDTGDFKLREVSEKLNKYNCNSHPHLSVLEQATLKQCVVGPNHAGFLLEDGR
VCRISFAVQPDRLELSKPDG
SDGSKLNSGSGTGRSSRPGRTSDPPWFLSGSDTLGRLAGNTL
>g1937.t3
MTSIHFVVHPLPGTEDQLNDRLREVSEKLNKYNCNSFAVQPDRLELSKPDGSDGSKLNSGSGTGRSSRPGRTSDPPWFLS
GSDTLGRLAGNTL
>g1938.t1
LALLEKRGQRDFKEIKETKGLTGQKGHQEKRVSWVCEVHRALPAGTDQEETLEWVEPKEKRASMDTLGKMANRAGLAIQV
SREPWAVEVRWGERERREHV
GQLVRWGSWDSLALQGAKATRALADLQVIQEVKDSRVQRVSQVMEVYGDQLDHRGQK
>g1938.t2
LALLEKRGQRDFKEIKETKGLTGQKGHQEKRVSWVCEVHRALPAGTDQEETLEWVEPKEKRASMDTLGKMANRAGLAIQV
SREPWAVEVRWGERERREHV
GQLVRWGSWDSLALQGAKATRALADLQVIQEVKDSRVQRELVVPKGGLGLKEDLERM
>g1938.t3
LALLEKRGQRDFKEIKETKGLTGQKGHQEKRVSWVCEVHRALPAGTDQEETLEWVEPKEKRASMDTLAVEVRWGERERRE
HVGQLVRWGSWDSLALQGAK
ATRALADLQVIQEVKDSRVQRELVVPKGGLGLKEDLERM
>g1939.t1
MNECCLFLKGRHGVPGLTGAKGHKGLQGHLGLRGQDGVHGTPGIPGKAGGPGVQGPVGKSGDVGGRGMVGKPGKPGIRGV
KGKDGLNGLPGTPGVKGIQG
NIGPWGDIGLRGPPGDPGPLGPVGPRGVPGYPGSMGSVGQKGVAGVDGSDRNEGSRGSGGAQRNGCE
>g1939.t2
MNECCLFLKGRHGVPGLTGAKGHKGLQGHLGLRGQDGVHGTPGIPGKAGGPGVQGPVGKSGDVGGRGMVGKPGKPGIRGV
KGKDGLNGLPGTPGVKGEIG
IPGPRAYTGALGIQGNIGPWGDIGLRGPPGDPGPLGPVGPRGVPGYPGSMGSVGQKGVAGVDGSDRNEGSRGSGGAQRNG
CE
>g1939.t3
MNECCLFLKGRHGVPGLTGAKGHKGLQGHLGLRGQDGVHGTPGIPGKAGGPGVQGPVGKSGDVGGRGMVGKPGKPGIRGS
RGVDGPSGKPGPAGIQGNIG
PWGDIGLRGPPGDPGPLGPVGPRGVPGYPGSMGSVGQKGVAGVDGSDRNEGSRGSGGAQRNGCE
>g1940.t1
MPDDDAALGEPIDKEDEASDAPAEEDCSEKPRPKVSEDKEADDKSTNSYSDQNSPISVLSNQETELESRLSDTSDRLTDF
KAPCLGESQKEEERGTPKHK
EEMNSSLEKMRAAYANFLSDSYWTGIGMDLKIGKNTSKANCDSTNGSAKTEFDWHQDALSKTLQQTLSPKPTSKPNLFSS
VHLYRQSNKACGSVFTGASR
FRCKDCSAAYDTLVELTVHMNKSGHYQDNNHGKQSNSSSSSSKSRKRNLHDMEGKEDAQKVLKCMFCSHSFDSLQDLSVH
MIKTKHYQKVPLKEPIPVLT
PKLLPPSKKRAFESARPCSPDSTTGVAGLTDAQRATAISNANNNRYGYQNGASYTWQFETCKSQILKCMECGSSHDTLQQ
LTTHMMVTGHFIKVTNSASK
KGKQLALDPLAIEKMQVLADPATSETEGGKMSPKAASPGEQEKEGQGEGTSEKVEVTETKEDKQESEDQKAGSDTFKYPY
LREEDLEQGSGAGGDILKSL
ANTVASAINKAQTGTPSWSAYPSIHAAYQLSGLIKSTPLSASPPIQLKQSFNHKLRTIAPKGKVYHGVPGAETPEGQHQN
VDIKKEKVDICDGKEIQNIK
FDLLENDDSDCQDDSSSSSKLDADCVNDVNEVIKGKLSPDFDAGKTPSPSASNGHGAAPELLGDSPDTFAINPLSALQSV

LNNHLGKANKPNNARAAKVS
ARTQSIFAEINRNNEKSALRLGNPIRNRPENSFLFVNEDQPIDLTKSKHSKPAGSSLLPASTPVPHKYALSDIADMVKVL
PKATTPKPAVPSRIPSMKLE
TDVRRFEDVSADVYSVHKRKGRQSNWNPRHLLILQAQFASSLFQTSEGKYLLSDLGPQERMHISKFTGLSMTTISHWLAN
VKYQLRKTGGTKFLKNMDTG
HPIFYCNDCASQFRSPPAFISHLESHLGFQIKDMCKLPVEHQTKMEEPELSKALNVRPTEPLGMEEDIDSKFRCKLCCRT
FASNHAVKLHLSKTHSKSPD
NHSQYVEMEKE
>g1941.t1
MYESKHFLLASSPGVFVERVMGGGTVSLALGCLGLLLLYRMLQRLRPGSRVQDSVVVITGASSGLGKECARIFHAAGARL
VLCGRDVVRLQAVALELSAS
AAHSKTPTYAPMTVTFDLADAESLDRAAAEILQCYGQVDVLINNAGISYRGNILDTDLSVQRAVMDTNYFGPIALTQALL
PSMVQRQSGHVVVISSIQGK
IAIPYRSAYAASKHATQAYFDCLRAEVEHHGIAVTVVSPGYVRTNLSLNAVTGDGSKYGVLDKTTAMGRDPADVARAVLE
AVRRRRRKDVLLAGPLPTLAV
YLRNLWPGLFFKIMSTRASKEQRQQDANGGHGGAKGPTQHSDFKR
>g1941.t2
MMYESKHFLLASSPGVFVERVMGGGTVSLALGCLGLLLLYRMLQRLRPGSRVQDSVVVITGASSGLGKECARIFHAAGAR
LVLCGRDVVRLQAVALELSA
SAAHSKTPTYAPMTVTFDLADAESLDRAAAEILQCYGQVDVLINNAGISYRGNILDTDLSVQRAVMDTNYFGPIALTQAL
LPSMVQRQSGHVVVISSIQG
KIAIPYRSAYAASKHATQAYFDCLRAEVEHHGIAVTVVSPGYVRTNLSLNAVTGDGSKYGVLDKTTAMGRDPADVARAVL
EAVRRRRRKDVLLAGPLPTLA
VYLRNLWPGLFFKIMSTRASKEQRQQDANGGHGGAKGPTQHSDFKR
>g1941.t3
MYESKHFLLASSPGVFVERVMGGGTVSLALGCLGLLLLYRMLQRLRPGSRVQDSVVVITGASSGLGKECARIFHAAGARL
VLCGRDVVRLQAVALELSAS
AAHSKTPTYAPMTVTFDLADAESLDRAAAEILQCYGQVDVLINNAGISYRGNILDTDLSVQRAVMDTNYFGPIALTQALL
PSMVQRQSGHVVVISSIQGK
IAIPYRSAYAASKHATQAYFDCLRAEVEHHGIAVTVVSPGYVRTNLSLNAVTGDGSKYGVCSAPNAQQTLMEATTYNVHN
LPLVLDKTTAMGRDPADVAR
AVLEAVRRRRRKDVLLAGPLPTLAVYLRNLWPGLFFKIMSTRASKEQRQQDANGGHGGAKGPTQHSDFKR
>g1942.t1
SLVSPDKGRRTSLTPSGPSSGTALLGPSLMDCNSHDSFQSRGLPDVAEMDSQLACMMSESSVDYIARFNDLAQELAVTEP
SLPPPP
>g1942.t2
MPLVHGFSLRTLESSLVSPDKGRRTSLTPSGPSSGTALLGPSLMDCNSHDSFQSRGLPDVAEMDSQLACMMSESSVDYIA
RFNDLAQELAVTEPSLPPPP
>g1943.t1
MESINMNLEQAQVTPQQMTMSFLPGEEKEVDVEVFAPSKGPLDLYILMDFSFSMKDDLNKLKRLGNQLAALVKGLSNDYT
IGFGKFVDKVVEPQTDMRPR
NLEHPWPDADPPFAFKNVIKLTGKEEFFNSELQKEKISGNLDPPEGGFDAMLQAAVCKDQIGWRNHSTHLLVFSTESAFH
YEADGANVLSGILPRNDEHC
HLDATGKYTKGTQQDYPSVPTLVRLLGEHNIIPIFAVTHHSYTYYEKLHKYFPIAELGLLQDDSSNILSIMEKAFLSIRS
KMSIRAEHLPQAFKAEYLTT
SGKVAQYGAFQVKPGQIGKFKVRLQAHNTVDEQHVCQMKPDDKEGTVRVKPTTFSKALTIETKLLCPSCPCEKSVKVRRV
NSCTRVRATRCNAHGDLVCG
KCQCDNG
>g1944.t1
MLDDIKVQKHHRHHHHHHHHHQEEENEKNEEEEEEEEMEEEAENEEEMEEEEEEEEEENEEELAEEEMEEEEEEEEEENEEL
LAEEEMEEEEEEEEENEEEL
AQEELYPRYLHGAPG
>g1945.t1
MKMCFAFQIEPFASLVNTVRSSVPRLLLNRHAVGPFQKTPLRRVDHVELGDLVESVQRLAQILGWSAEIEDLMRSQGDWC
TSPSVPEAQKHAACSSHEND
SDGLPELCFLKPRQLGL

>g1945.t2
MSKSRPMLEKKASTHPTVPRMTRSSLSQARPGCPGQPQDPRHGQYGKQRRKHTDSGLAQDLSQMSVKGQEVSSLCPVFDD
GNICEASSDSTPQPATLPFV
KECHQARPRIKSSSCSRGGLASVARLMKLGRCKNIVVVAGAGISTASGIPDFRTPGTGLYSNLERYNLPYPEAVFNIDYF
SDHPQAFFSLAKELYPGSHR
PNYIHYFIRMLHHKGLLLRVYTQNIDGLEKMCGVPDDKLVEAHGNFATASCHLCYTPYPAEDAKVAIMSGVVPTCSFCAG
AVKPDVVFFGEDLPQKYFLH
SKDFPKADLLIIMGTSLQIEPFASLVNTVRSSVPRLLLNRHAVGPFQKTPLRRVDHVELGDLVESVQRLAQILGWSAEIE
DLMRSQGDWCTSPSVPEAQK
HAACSSHENDSDGLPELCFLKPRQLGL
>g1945.t3
MSKSRPMLEKKASTHPTVPRMTRSSLSQARPGCPGQPQDPRHGQYGKQRRKHTDSGLAQDLSQMSVKGQEVSSLCPVFDD
GNICEASSDSTPQPATLPFV
KECHQARPRIKSSSCSRGGLASVARLMKLGRCKNIVVVAGAGISTASGIPDFRTPGTGLYSNLERYNLPYPEAVFNIDYF
SDHPQAFFSLAKELYPGSHR
PNYIHYFIRMLHHKGLLLRVYTQNIDGLEKMCGVPDDKLVEAHGNFATASCHLCYTPYPAEDAKVAIMSGVVPTCSFCAG
AVKPDVVFFGEDLPQKYFLH
SKDFPKADLLIIMGTSLQNTSSSILDQL
>g1945.t4
MSKSRPMLEKKASTHPTVPRMTRSSLSQARPGCPGQPQDPRHGQYGKQRRKHTDSGLAQDLSQMSVKGQEVSSLCPVFDD
GNICEASSDSTPQPATLPFV
KECHQARPRIKSSSCSRGGLASVARLMKLGRCKNIVVVAGAGISTASGIPDFRTPGTGLYSNLERYNLPYPEAVFNIDYF
SDHPQAFFSLAKELYPGSHR
PNYIHYFIRMLHHKGLLLRVYTQNIDGLEKMCGVPDDKLVEAHGNFATASCHLCYTPYPAEDAKVAIMSGVVPTCSFCAG
AVKPDVVFFGEDLPQKYFLH
SKDFPKADLLIIMGTSLQRKHFMRQWPGKPAFSQAEPSDRPSSGWAVFCLGQIEPFASLVNTVRSSVPRLLLNRHAVGPF
QKTPLRRVDHVELGDLVESV
QRLAQILGWSAEIEDLMRSQGDWCTSPSVPEAQKHAACSSHENDSDGLPELCFLKPRQLGL
>g1946.t1
KGMIQPMVRKDRTLAPLEESLRQHAHESSGSDTD
>g1947.t1
HFPNKAMPSAGILPWMQGIICNANNPCFRHPTPGESPGVVGNFNDSIISRLFSDSKKILLYSQNDKNLDSFKELAKALQT
LQATTPGRRSDRVEMVTTS
>g1947.t2
MWLCVPHTYVALSYHFSSQPVGSPKGHFPNKAMPSAGILPWMQGIICNANNPCFRHPTPGESPGVVGNFNDSIISRLFSD
SKKILLYSQNDKNLDSFKEL
AKALQTLQATTPGRRSDRVEMVTTS
>g1947.t3
LMVHTADRQRNHTLKVNPSHFPNKAMPSAGILPWMQGIICNANNPCFRHPTPGESPGVVGNFNDSIISRLFSDSKKILLY
SQNDKNLDSFKELAKALQTL
QATTPGRRSDRVEMVTTS
>g1948.t1
MEAEAAAPLSPISAVGGAPLWRLGERRARKPASANIFGGVALWQLQKLFQAAGDQDAEHRAQLVWGHGGEGELAQALIGL
RARGRRRGIRTQRRDSQMGS
RWLKAFHHMR
>g1949.t1
MLQELSNWFWQERLWFPKGLGWADLEDRDGRIYAKGQDLWVALPIALAFLIVRQIFERTVALPLASLLGVKEKERLQVSP
NPTLESFYCNTSKNPTQSAE
KSLCKQTGFSERQVQRWFRRRRNQDRPSVIKKFKEASWRFTFYLLAFIAGLAALIDKPWFYDLKEMWEGFPVLYWYYMIE
LGFYVALLFSVASDVKRKDF
NEQIIHHLAAIVLLSFSWCVNYIRAGTLIMLLHDSSDYFLESAKMFNYAGWRATCNCIFIIFAVIFIITRLVIFPFKIMY
CTWVYPVTIYEPFFGYYFFN
GLMFVLQLLHIFWAVLIIRMAARFLTNNEKVDDERSDKDETDESEEEDDAKKETTKNGAARNGYAAYNNNRCKSE
>g1949.t2
MLQELSNWFWQERLWFPKGLGWADLEDRDGRIYAKGQDLWVALPIALAFLIVRQIFERTVALPLASLLGVKEKERLQVSP
NPTLESFYCNTSKNPTQSAE

KSLCKQTGFSERQVQRWFRRRRNQDRPSVIKKFKEASWRFTFYLLAFIAGLAALIDKPWFYDLKEMWEGFPVLYWYYMIE
LGFYVALLFSVASDVKRKDF
NEQIIHHLAAIVLLSFSWCVNYIRAGTLIMLLHDSSDYFLESAKMFNYAGWRATCNCIFIIFAVIFIITRLVIFPFK
>g1949.t3
MLQELSNWFWQERLWFPKGLGWADLEDRDGRIYAKGQDLWVALPIALAFLIVRQIFERTVALPLASLLGVKEKERLQVSP
NPTLESFYCNTSKNPTQSAE
KSLCKQTGFSERQVQRWFRRRRNQDRPSVIKKFKEASWRFTFYLLAFIAGLAALIDKPWFYDLKEMWEGFPVLTLLSSQY
WYYMIELGFYVALLFSVASD
VKRKDFNEQIIHHLAAIVLLSFSWCVNYIRAGTLIMLLHDSSDYFLESAKMFNYAGWRATCNCIFIIFAVIFIITRLVIF
PFKIMYCTWVYPVTIYEPFF
GYYFFNGLMFVLQLLHIFWAVLIIRMAARFLTNNEKVDDERSDKDETDESEEEDDAKKETTKNGAARNGYAAYNNNRCKS
E
>g1949.t4
MYCTWVYPVTIYEPFFGYYFFNGLMFVLQLLHIFWAVLIIRMAARFLTNNEKVDDERSDKDETDESEEEDDAKKETTKNG
AARNGYAAYNNNRCKSE
>g1950.t1
MSTPGCCHLPGSLCDYSGGGGGGGGETDSSKDSERSDSTSQAQYVAKVTAKDGRPLSTIVKAASLQ
>g1950.t2
MFLSDDRSCATSQAPLPLSSRTGVSWWLPFHPAAMSTPGCCHLPGSLCDYSGGGGGGGGETDSSKDSERSDSTSQAQYVA
KVTAKDGRPLSTIVKAASLQ
>g1950.t3
LSSRTGVSWWLPFHPAAMSTPGCCHLPGSLCDYSGGGGGGGGETDSSKDSERSDSTSQAQYVAKVTAKDGRPLSTIVKAA
SLQ
>g1951.t1
MKNRAVCKELGHIPETHSRRFGFTVDVCCLHTGGEYRLASRQSQGTVSATAPLGCGGTVGAILTCPLEVVKTRLQSSSVT
FYISEVQLSTVNGASVARMS
PPGPIHCLKIGYRNSQLSVSAIDWMKKIRS
>g1951.t2
LIEEKYIRGAAEGFVVRSPGEEACRPLHNTARTTPPSESAETHSRRFGFTVDVCCLHTGGEYRLASRQSQGTVSATAPLG
CGGTVGAILTCPLEVVKTRL
QSSSVTFYISEVQLSTVNGASVARMSPPGPIHCLKIGYRNSQLSVSAIDWMKKIRS
>g1951.t3
MKNRAVCKELGHIPETHSRRFGFTVDVCCLHTGGEYRLASRQSQGTVSATAPLGCGGTVGAILTCPLEVVKTRLQSSSVT
FYISEVQLSTVNGASVARMS
PPGPIHCLK
>g1952.t1
MGVEKERSDTSVIMDEDEFNRSIEPILSKKGKLYVEPPNRDPHDINAHLKVGFDDVIAEPRSTHSFDRVWIGSHAVFELA
KYVFYRVLTTLLAVPMAFLL
GLLFGVISCIHIWYG
>g1952.t2
MGVEKERSDTSVIMDEDEFNRSIEPILSKKGKLYVEPPNRDPHDINAHLKVGFDDVIAEPRSTHSFDRVWIGSHAVFELA
KYVFYRVLTTLLAVPMAFLL
GLLFGVISCIHI
>g1953.t1
MSVIGKRECGTFGNMGLEARDDEGGGTSDRNIMAPKDIMTNSHAKTILNAMNALRKSNTLCDITLRVENTDFPAHRIVLA
ACSDYFCAMFTSELSEKGKS
FVDIQGLTAATMEILLDFVYTETVLVTVENVQELLPAACLLQLKGVKRACCDFLDSQLDPTNCLGIRDFADTHNCLDLMQ
AAELFSQKHFPEVVQHEEFM
LLCQAEVEKLIKCDEIQ
>g1954.t1
MVWEQRTRGVVMLNRVIEKGSIKCAQYWPQREEGDAIFEDTNFRVTLISEDVKVYYTVRQLELENLVAQETREILHFHYT
TWPDFGVPESPASFLNFLFK
VRETGCLNADQGPVVVHCSAGIGRSGTFCLVDTCLVLMSVRKDPSSVRLRDVLLEMRRCRMGLIQTADQLRFSYLAVIEG
AKCIMGDTALQASLPSRS
>g1954.t2
MDNILPSVDHSRILLQLGENDYINASLISMEEAKRNYILTQGPLPNTCGHFWEMVWEQRTRGVVMLNRVIEKGSIKCAQY

WPQREEGDAIFEDTNFRVTL
ISEDVKVYYTVRQLELENLVAQETREILHFHYTTWPDFGVPESPASFLNFLFKVRETGCLNADQGPVVVHCSAGIGRSGT
FCLVDTCLVLMSVRKDPSSV
RLRDVLLEMRRCRMGLIQTADQLRFSYLAVIEGAKCIMGDTALQASLPSRS
>g1955.t1
PRSSCNGSYVCNYEAMGSSSGPGQGVPLPPLLLLPLLLLLLLLGRL
>g1956.t1
MIKELRSSACGNPGVPPKGILNGTQFNVGDKIRYRCVTGYVLDGHSLLTCVTNSAGVSVWDFPVPICRGQPLNRR
>g1956.t2
MIKELRSSACGNPGVPPKGILNGTQFNVGDKIRYRCVTGYVLDGHSLLTCVTNSAGVSVWDFPVPICRETFLIS
>g1956.t3
MIKELRSSACGNPGVPPKGILNGTQFNVGDKIRYRCVTGYVLDGHSLLTCVTNSAGVSVWDFPVPICRATVGHTLSTTDR
HNRQGAVLASALTTSSLHKV
NR
>g1957.t1
MSNPTNVLIIIYMGWVDEKELDSVQDRMYSPKFLALRGSSLFKFSAPPVTTWDWTRAEKSFTVYEIMCKVLKDNDLLDKR
KHCFSVQTETGEDLFYSVEL
DCELLTWEKAFQMATFLEVERIQ
>g1957.t2
LIPPPPTVHMSNPTNVLIIIYMGWVDEKELDSVQDRMYSPKFLALRGSSLFKFSAPPVTTWDWTRAEKSFTVYEIMCKVL
KDNDLLDKRKHCFSVQTETG
EDLFYSVELDCELLTWEKAFQMATFLEVERIQ
>g1958.t1
MDRYNEVKVLLKKWEQSFVQLQQRKPNKEDIDNAPEDTKKLYKEYRSLKQAKENTDSQSDPSGQNGATVEVKANPKDSDS
WGTHLNRSNMPVPSPGLTPR
ERDSLQTSAQYYGAKLKSNLGSLGKERPVSLRKSLTPGRTPLKSWKDRLTEQPNSPGAGAAAAASPLRAPGPALPSLGRV
SSPFNLHGEEQKKQITFIRF
VRAGGPFTGSPAPREPEEPFDAFKPSPQTPLHESPCTGGGNGHSPGLEFPSPRPARTAPAAVATGTPGRTPGVSGRLGVG
VEPPDGADGKTDPRTQRAGG
GPADGDAKSPPGLGGERRGGFRGGPSLLSGLGSVDRGWLERCQVFGEVDGRDRPVSGNTQAPARGQGRERGDEGETRDSG
GRRRRRRSRSWRGWKGKQDG
KESETGRRAREPRRGGEEGGGRGNRKEPEEQSTAAETSDQADEEVPVRATKASKRGRKRQREEEEGDGREAEEDQGAPTK
RKRGPRKKKKRGMRRREVEE
EDPQENLLGEVEEGARAYRAYKTQPAR
>g1958.t2
MDRYNEVKVLLKKWEQSFVQLQQRKPNKEDIDNAPEDTKKLYKEYRSLKQAKENTDSQSDPSGQNGATVEVKANPKDSDS
WGTHLNRSNMPVPSPGLTPR
ERDSLQTSAQYYGAKLKSNLGSLGKERPVSLRKSLTPGRTPLKSWKDRLTEQPNSPGAGAAAAASPLRAPGPALPSLGRV
SSPFNLHGEEQKKQITFIRF
VRAGGPFTGSPAPREPEEPFDAFKPSPQTPLHESPCTGGGNGHSPGLEFPSPRPARTAPAAVATGTPGRTPGVSGRLGVG
VEPPDGADGKTDPRTQRAGG
GPADGDAKSPPGLGGERRGGFRGGPSLLSGLGSVDRGWLERCQVFGEVDGRDRPVSGNTQAPARGQGRERGDEGETRDSG
GRRRRRRSRSWRGWKGKQDG
KESETGRRAREPRRGGEEGGGRGNRKEPEEQSTAAETSDQADEEVPVRATKASKRGRKRQREEEEGDGREAEEDQGAPTK
RKRGPRKKKKRGMRRREVEE
EDPQENLLGEVEEGARAYRAYKTQPARREVDQLALWCGQNNLRPQGL
>g1958.t3
MDRYNEVKVLLKKWEQSFVQLQQRKPNKEDIDNAPEDTKKLYKEYRSLKQAKENTDSQSDPSGQNGATVEVKANPKDSDS
WGTHLNRSNMPVPSPGLTPR
ERDSLQTSAQYYGAKLKSNLGSLGKERPVSLRKSLTPGRTPLKSWKDRLTEQPNSPGAGAAAAASPLRAPGPALPSLGRV
SSPFNLHGEEQKKQITFIRF
VRAGGPFTGSPAPREPEEPFDAFKPSPQTPLHESPCTGGGNGHSPGLEFPSPRPARTAPAAVATGTPGRTPGVSGRLGVG
VEPPDGADGKTDPRTQRAGG
GPADGDAKSPPGLGGERRGGFRGGPSLLSGLGSVDRGWLERCQVFGEVDGRDRPVSGNTQAPARGQGRERGDEGETRDSG
GRRRRRRSRSWRGWKGKQDG
KESETGRRAREPRRGGEEGEATVDANPPTRRGAKAKGGRGNRKEPEEQSTAAETSDQADEEVPVRATKASKRGRKRQREE

EEGDGREAEEDQGAPTKRKR
GPRKKKKRGMRRREVEEEDPQENLLGEVEEGARAYRAYKTQPAR
>g1959.t1
IQPYERVASRGLPHSVAECLDKLVVVKLNGGLGTSMGCKGPKSLISVRNENTFLDLTVQQIEVKN
>g1960.t1
LKSNLTFYYPRRTSSVRGLTVPPETTAKLPEKTEVNPKENNEPIKFSTSKGSHRTWRVDRTMGSHFQRPWWKVLPISVFM
GVFMLWCAFRGEGEIDAILH
KDLYEQLPGLLPDPGPPQEK
>g1961.t1
MVKDKSNGSGTKGSSSDETDAVKRKKDRKTEKKSKKMKVELEGDQQVPEILVKQKKKHKKHKKTHSTEDTSTKEEEEEEE
EQECCCS
>g1962.t1
MDPTMYKEYEHGHNLTVPLNSSLIKEVSSILQEPPGEDLYLISLILSCLYTIFLFPAGFVGNILILVVNLSHGSRLTAPD
LYFVNLAVADLMLVSDSLIE
VFNLKRGYYENAALCTFMALFQQVNMYSSVFFLTWMSIDRFKVLTGSAGRRLPQARLSCCLIWVASSFLTVLPFAIAHAQ
NRGKLHFCFANVSQVLWLEV
MLGFLVPFCVLGVCYWRISRRLLRSRAEPSGPQQWPRRLKALRMIFAAVLVFFLCWLPENVFVCVHLLRGAAEDSTVWQD
YPLAAHVVSLAAFSNSCLNP
LIYSFMGETFREKLRLFLDQNTGWVKQHRATRTENTRFSPAAKMGPTTTRYLQSCNQLSKIIIYPYTQRPL
>g1963.t1
LVNSTITLGPVSPEATGSSATTGSFLYAFLVAVSLVSACFLLYSFIQTCHAQRRIAGLDCLFWAFCGSQFLLVLLSLSLV
ACSTDLDEVALLAAVALSFS
VNVATLCGLLLLVLMACVLTFDPPSQSLLRRPGVSMALVALASVVSSVVLAAVQWTAGGPAHGCTIDPVGDRLSYAAAKL
CLCFIIPCWLLLGILVTGCL
HQWRSRGRFLSGAEEGPVLLTLAAVLFLCQLFYDVALLQVAAPTEAAGNASSASKASKDPLRSAAEAVLFAGSAVSLLLV
LVMHRACRDSLRGALRQLRD
CCRSLGGAQTNRHITGPHVEISDTLPDFHTCSMKETAKESS
>g1964.t1
VNCVTFPLPGEGPEQQLLKPNDWSYCDYFWVRGGSLAIANV
>g1965.t1
MRASKSDNTVSSMEIEDSSNGYAPLIDDNIANVLSGVRRQPARVAGVCLAVLSFLLLVAIIGQAVHHRDVESNQEQNYKT
LTSAQEKLQQSLSDADREKK
QLAAGQTRLQQEYDYISSQKNYLQTNNNILSKERDTLKASQSQQQANTAALTKERDQLKASQSQLQNSNTELAKARDFLQ
KSYDALALKMDGIQKISAAL
EIKKDELQDQNNQLKHNMEQVQSNFTTLLTMITHLKFGQDISAQEKEKLRSSHDIVLEQRDQLNTSLLVLEATSKQMQEG
YTAVVTERDELLAMKANLST
SLDSLLTQNQNLMAERDLLLAKSFLNSILGSGTEVWIDTADDNSEWMWVDGTQMMAS
>g1965.t2
MRASKSDNTVSSMEIEDSSNGYAPLIDDNIANVLSGVRRQPARVAGVCLAVLSFLLLVAIIGQAVHHRDVESNQEQNYKT
LTSAQEKLQQSLSDADREKK
QLAAGQTRLQQEYDYISSQKNYLQTNNNILSKERDTLKASQSQQQANTAALTKERDQLKASQSQLQNSNTELAKARDFLQ
KSYDALALKMDGIQKISAAL
EIKKDELQDQNNQLKHNMEQVQSNFTTLLTMITHLKFGQDISAQEKEKLRSSHDIVLEQRDQLNTSLLVLEATSKQMQEG
YTAVVTERDELLAMKANLST
SLDSLLTQNQNLMAERDLLLAKVKNSTDETGCPSSWLPYQQGCFSCSTTERSWQNSGTQCLQQGNEMSFLNSILGSGTEV
WIDTADDNSEWMWVDGTQMM
AS
>g1965.t3
RIEATLSLTGENSNTATLSRGLVSIPTPARRLSPWATPEKERVQPLYRSLVPEPVLCVEVSPTISSWYRSTSRTSSGSLP
AREVVGPQGCGSSMELFRAV
PGRATWVKARPPDARRRAPLPGLAPVGGPCFPIPGEVA
>g1965.t4
RIEATLSLTGENSNTATLSRGLVSIPTPARRLSPWATPEKERVQPLYRSLVPEPVLCVEVSPTISSWYRSTSRTSSGSLP
AREVVGPQGCGSSMELFRAV
PGRATWVKARPPDARRRAPLPEMRASKSDNTVSSMEIEDSSNGYAPLIDDNIANVLSGVRRQPARVAGVCLAVLSFLLLV
AIIGQAVHHRDVESNQEQNY

KTLTSAQEKLQQSLSDADREKKQLAAGQTRLQQEYDYISSQKNYLQTNNNILSKERDTLKASQSQQQANTAALTKERDQL
KASQSQLQNSNTELAKARDF
LQKSYDALALKMDGIQKISAALEIKKDELQDQNNQLKHNMEQVQSNFTTLLTMITHLKFGQDISAQEKEKLRSSHDIVLE
QRDQLNTSLLVLEATSKQMQ
EGYTAVVTERDELLAMKANLSTSLDSLLTQNQNLMAERDLLLAKSFLNSILGSGTEVWIDTADDNSEWMWVDGTQMMAS
>g1965.t5
RIEATLSLTGENSNTATLSRGLVSIPTPARRLSPWATPEKERVQPLYRSLVPEPVLCVEVSPTISSWYRSTSRTSSGSLP
AREVVGPQGCGSSMELFRAV
PGRATWVKARPPDARRRAPLPGLAPVGGPCFPIPGEQGYSVVAELLGGDGTEPGLKSPKLIVFCFVTGIFY
>g1966.t1
MVIPERERCRSKHLRQGLDLFLGNIKYPDAPRAADVVNALDEKALAAVLLAYGEERHARKISSAIAEARNICPITRTQQL
ASVVA
>g1966.t2
MLQNFDIKSFVSKRPLGRVQPLLGRFSELLSQLEVKPGSVDAVLLDAGCSSMQMDQAQRGFSLSRDGPLDMRMDGDRQVL
AEHPKQMKSKDNDFHVNKWK
RRDKSAVVMVIPERERCRSKHLRQGLDLFLGNIKYPDAPRAADVVNALDEKALAAVLLAYGEERHARKISSAIAEARNIC
PITRTQQLASVVA
>g1966.t3
MLDKGDTSVPERKEVELLDAAFERKAEAERGMVQVMGDGRVQPLLGRFSELLSQLEVKPGSVDAVLLDAGCSSMQMDQAQ
RGFSLSRDGPLDMRMDGDRQ
VLAEHPKQMKSKDNDFHVNKWKRRDKSAVVMVIPERERCRSKHLRQGLDLFLGNIKYPDAPRAADVVNALDEKALAAVLL
AYGEERHARKISSAIAEARN
ICPITRTQQLASVVA
>g1967.t1
RVISQRGELPEQPQKTSHRPPYRPPPGPRRQKSNVPSPTGHCAVCSPGRAGLYLSPGFKAM
>g1968.t1
MTFSIGPQVANETHGHVGADLAALCSEAALQAIRKKMDLIDLEDETIDAEVMNSLAVTMDDFKWALSQSNPSALRETVVE
VPNISWEDIGGLDDVKRELQ
ELVQYPVEHPDKFLKFGMTPSKGVLFYGPPGCGKTLLAKAIANECQANFISIKGPELLTMWFGESEANVREIFDKARQAA
PCVLFFDELDSIAKARGGNV
GDGGGAADRVINQILTEMDGMSSKKNVFIIGATNRPDIIDPAILRPGRLDQLIYIPLPDEKSRINILKANLRKSPISKDV
DLDFLAKMTNGFSGADLTEI
CQRACKLAIRESIENEIRRERERQTNPSAMVSTGPRRSRPLKDHFEEAMRFARRSVSDNDIRKYEMFAQTLQQSRGFGSF
RFPSGTAGGTGPSQGSGGAG
TGPVFNEDNDDDLYG
>g1968.t2
VANETHGHVGADLAALCSEAALQAIRKKMDLIDLEDETIDAEVMNSLAVTMDDFKWALSQSNPSALRETVVEVPNISWED
IGGLDDVKRELQELVQYPVE
HPDKFLKFGMTPSKGVLFYGPPGCGKTLLAKAIANECQANFISIKGPELLTMWFGESEANVREIFDKARQAAPCVLFFDE
LDSIAKARGGNVGDGGGAAD
RVINQILTEMDGMSSKKNVFIIGATNRPDIIDPAILRPGRLDQLIYIPLPDEKSRINILKANLRKSPISKDVDLDFLAKM
TNGFSGADLTEICQRACKLA
IRESIENEIRRERERQTNPSAMVSTGPRRSRPLKDHFEEAMRFARRSVSDNDIRKYEMFAQTLQQSRGFGSFRFPSGTAG
GTGPSQGSGGAGTGPVFNED
NDDDLYG
>g1968.t3
MTFSIGPQVGIQPGPVANETHGHVGADLAALCSEAALQAIRKKMDLIDLEDETIDAEVMNSLAVTMDDFKWALSQSNPSA
LRETVVEVPNISWEDIGGLD
DVKRELQELVQYPVEHPDKFLKFGMTPSKGVLFYGPPGCGKTLLAKAIANECQANFISIKGPELLTMWFGESEANVREIF
DKARQAAPCVLFFDELDSIA
KARGGNVGDGGGAADRVINQILTEMDGMSSKKNVFIIGATNRPDIIDPAILRPGRLDQLIYIPLPDEKSRINILKANLRK
SPISKDVDLDFLAKMTNGFS
GADLTEICQRACKLAIRESIENEIRRERERQTNPSAMVSTGPRRSRPLKDHFEEAMRFARRSVSDNDIRKYEMFAQTLQQ
SRGFGSFRFPSGTAGGTGPS
QGSGGAGTGPVFNEDNDDDLYG
>g1969.t1

DHVTEEEEGELCEKPYVADMSREERGGTLEKNRRTTRPGSTSTVAEEMDQKKVRNKLIKFLMKRPTLQSVKEKGYIRDNV
FGCHLDTLCAQERTSVPRFV
EKCVHAVEKRGLDIDGLYRVSGNLAVIQKLRYKADHEELDLEDGQWEDVHVITGALKLFFRELPEPLFPFSHFDNFIAAI
RSHDYHAKVSYMSDLVKSLP
LPNHDTMKLLFGHLQRVIQHGEDNRMTPQNVAIVFGPTLLRPETESANIAMHMVCQNQIVELILNEYNTIFR
>g1970.t1
LRCLKRRGDDIYTKSWEKEREEGGSARNKQKGGGVIYWTEIGALGFRSAAPSRRETSGXLQPNADTRQKQLAAWCSLALA
YCRHHKLYTLDVMESQESPV
FNNKKIERNLDWLDKNKSRCLVMWRRPEEWGKLIYQWVSKVGMNNSVFTLYELSNGDDTEGEEFHGLEEWMLLRSLQALQ
TEGKAEIISMDDSKGVKFF
>g1970.t2
LGFRSAAPSRRETSGXLQPNADTRQKQLAAWCSLALAYCRHHKLYTLDVMESQESPVFNNKKIERNLDWLDKNKSRCLVM
WRRPEEWGKLIYQWVSKVGM
NNSVFTLYELSNGDDTEGEEFHGLEEWMLLRSLQALQTEGKAEIISMDDSKGVKFF
>g1970.t3
LKRRGDDIYTKSWEKEREEGGSARNKQKGGGVIYWTEIGALGFRSAAPSRRETSGXLQPNADTRQKQLAAWCSLALAYCR
HHKLYTLDVMESQESPVFNN
KKIERNLDWLDKNKSRCLVMWRRPEEWGKLIYQWVSKVGMNNSVFTLYELSNGDDTEGEEFHGLEEWMLLRSLQALQTEG
KAEIISMDDSKGVKFF
>g1971.t1
ALPHHHHHHHHHHHMAPLM
>g1972.t1
MLLWSEARYAELAADAEQQGRRARVRPGEVGCRDFVAKSAFALLRELGIRGRRMKLVTKSMSQAAESAS
>g1973.t1
MVSRRWRTPVVSHAEPDEGSYCGHSIIGALLLPPPRSFVTCTLSYESPLPFLLWSSRPCDSRGSASRSGADGRRLSPSLA
SPASLPAPHPS
>g1974.t1
MSDNVKLFVGNLPLDATHEELNKLFSPHGEVNTCSLLRQYAFITLKGEGAADRAIRHLDGKEYRGRPLVVEESRARPPNS
TKVFVGNLSSTCTADDLHGL
FQSFGRVLDCDKVKARLCSNAGYAFVHMERKEEALLAIEGLNGTMYKGRQLSVELSKAQPLINQLASMDAANLAASMAGG
GPREGLLPRPASSLEHHQSQ
AAILAAAAAAAAGLPIQ
>g1974.t2
LLRYRCQPDIEMSDNVKLFVGNLPLDATHEELNKLFSPHGEVNTCSLLRQYAFITLKGEGAADRAIRHLDGKEYRGRPLV
VEESRARPPNSTKVFVGNLS
STCTADDLHGLFQSFGRVLDCDKVKARLCSNAGYAFVHMERKEEALLAIEGLNGTMYKGRQLSVELSKAQPLINQLASMD
AANLAASMAGGGPREGLLPR
PASSLEHHQSQAAILAAAAAAAAGLPIQ
>g1975.t1
MAQEDPLYDFPEPAPSPHIRRDVHQRAQGSLRSVSVLDRLLLTLSVWLQLSINPATALHILQREPPGAFLVRNSRTSRRN
VLCVRLTDDSIPSFVQQFGI
REQHSTFCLESSAISFPDLPRLIAFYCVSRDVLPFPLELPEAIAKATSHKQLESISHMGIEFWSSQLNYRGPRETPKAKD
HQKKEPGATDESPQMPSQLP
STAASDTLPQPHPANQTLQPSQSHAASSQSETSPTVFQKLCPITTRSPRELNCGSGVGALCFVNPLFIQPQGVLHRRHLF
KRSLKVRVSTETTSSLSPPL
APPPPPPLLPKTRGKCKAKLRMGAQDPQRLPQCQASAEPGAHLPIGIATQIKGDQAQPQDAMMEAQPGAAGPGGQGEDQT
KEQVPEEVAGPEAGDVGDDE
VTAAEGLQDESDYMEPPNSHSQASAPVSPYLSPAPSPMESPSSLLKVSVSPQSSPSLSPYQSPSLSPMLPLSLSPFSSPY
ISPSVEDPYHVPCSTPLGQK
NIAEGDRELGDCEEKENEDEESHKDEGEEEMGLLLEELYASNLNDPECCGSLNSSEGEEEESLSPSPLPQPGVCPLASSS
NPP
>g1976.t1
MVRAGLLALEFYTAQSSSWRQADQNSVFNDKPLKQRRRLLDSKVVLFGATVGVHGGPQAHMQARFVILRVLLEAGEGLVG
LEEVTGADGRPDARITLDRT
KVHTVGKAAIQRFLCKLQATADVAGGRALYEGYSTVTDSGPHNFLRLRDTVLLRKEARKMFVQANTRLNGEGVELVEYEG
SAAGLIRSFTERFQEDAEQL

EADLLELSKRDSLCWCH
>g1976.t2
QDAEDVVYVNWLNMVRAGLLALEFYTAQSSSWRQADQNSVFNDKPLKQRRRLLDSKVVLFGATVGVHGGPQAHMQARFVI
LRVLLEAGEGLVGLEEVTGA
DGRPDARITLDRTKVHTVGKAAIQRFLCKLQATADVAGGRALYEGYSTVTDSGPHNFLRLRDTVLLRKEARKMFVQANTR
LNGEGVELVEYEGSAAGLIR
SFTERFQEDAEQLEADLLELSKRDSLCWCH
>g1977.t1
MVCSLSLLNNNHSVPALCSLRFYLILLILPFFSSPGSLRFYLILLIIRSGRYKMEEDLGTRANRANVSGSEQRRR
>g1978.t1
MLKIDQREITRKLTFPKQPTNPFLEMVKFLLERIAPVHIDSEAI
>g1979.t1
MVLALFRHVLQNKGSLQFEDKWDLMRPIVLKLLRQESVTKQQWFDLFSDVHAVCLWDDKGPAKIHQALKEDILDFIKQAQ
VRVLSHQDDTALLKAYIAEW
RKFFTQCDILPKPFCQLEITLMGKQGSNKKSNVEDSIVRKLMLDTWNESIFSNIKSRLQDSAMKLVHAERLGEAFDSQLV
IGVRESYVNLCSNPDDKLQI
YRDNFEKAYMDSTERFYRTQAPAYLQQNGVQNYMKYADAKLREEEKRALRYLETRRDCNSVQTLMECCVNVLVTSFKETI
LAECPGMIKRNETESEYGRS
ATSRGSSSSGLHQETAPLPLPHFPRNSLHPPLHPIPRHGHGLCRVH
>g1979.t2
MVLALFRHVLQNKGSLQFEDKWDLMRPIVLKLLRQESVTKQQWFDLFSDVHAVCLWDDKGPAKIHQALKEDILDFIKQAQ
VRVLSHQDDTALLKAYIAEW
RKFFTQCDILPKPFCQLEITLMGKQGSNKKSNVEDSIVRKLMLDTWNESIFSNIKSRLQDSAMKLVHAERLGEAFDSQLV
IGVRESYVNLCSNPDDKLQI
YRDNFEKAYMDSTERFYRTQAPAYLQQNGVQNYMKYADAKLREEEKRALRYLETRRDCNSVQTLMECCVNVLVTSFKETI
LAECPGMIKRNETESEYGRS
ATSRGSSSS
>g1979.t3
MVLALFRHVLQNKGSLQFEDKWDLMRPIVLKLLRQESVTKQQWFDLFSDVHAVCLWDDKGPAKIHQALKEDILDFIKQAQ
VRVLSHQDDTALLKAYIAEW
RKFFTQCDILPKPFCQLEITLMGKQGSNKKSNVEDSIVRKLMLDTWNESIFSNIKSRLQDSAMKLVHAERLGEAFDSQLV
IGVRESYVNLCSNPDDKLQI
YRDNFEKAYMDSTERFYRTQAPAYLQQNGVQNYMKYADAKLREEEKRALRYLETRRDCNSVQTLMECCVNVLVTSFKETI
LAECPGMIKRNETESEYGRS
ATSRGSSSSGLHQETAPLPLPHFPRNSLHPPLHPIPRHGH
>g1980.t1
MNDDGDVYTGLPLKEMEEDVTGNLCGSCMANFSLYNYKLKDVFTWQLLKTIAMGQALSLMICGTAVSCQYLAQAGVETPM
FQSFLNYALLFVSYTSYFCC
HKGGENVYQILKTKWWKYLLMGLTDVQANYAVVKAYQYTTLTSIQLLDCFVIPVLMLLSWFLLKTRYRHVHFVAVFVCLV
GVGAMVGADLLAGRDQGS
>g1980.t2
MNDDGDVYTGLPLKEMEEDVTGNLCGSCMANFSLYNYKLKDVFTWQLLKTIAMGQALSLMICGTAVSCQYLAQAGVETPM
FQSFLNYALLFVSYTSYFCC
HKGGENVYQILKTKWWKYLLMGLTDVQANYAVVKAYQYTTLTSIQLLDCFVIPVLMLLSWFLLKTRYRHVHFVAVFVCLV
GVGAMVGADLLAGRDQGSSA
DCYTYRCVNHNIVDSTCRVSLAKPRTFSFVLGLKFKPENFSHTPPPEPLL
>g1981.t1
GDRSGPYCWKNSGQVSRFTSPPRLPTLASFSSCPGFVTGAANRKSPSLAPRPRIHGRCCDTLPRFATGTSSLGV
>g1981.t2
PTLASFSSCPGFVTGAANRKSPSLAPRPRIHGRCCDTLPRFATGTSSLGV
>g1982.t1
MLRCLRVLPIRTTRTFSRCCSSYVSRMRYMNHLKEDEGACLAALDSGDVFLYHALAPLLRKTPQGVFQLPLLPAKGMHLD
RGF
>g1982.t2
MLRCLRVLPIRTTRTFSRCCSSYVSRMRYMNHLKEDEGACLAALDSGDVFLYHALAPLLRKTPQGVFQLPLLPAK
>g1982.t3

MLRCLRVLPIRTTRTFSRCCSSYVSRMRYMNHLKEDEGACLAALDSGDVFLYHALAPLLRKTPQGVFQLPLLPAKGMHLD
RGFR
>g1983.t1
MASVARGFWCSFVHHPHFKSHGGGDDDQTRTAEEEGRSPAFMSGRRGSGVSSPGGGAASPGPVTAGGTPGFGHRGLFEAC
RNGDPERVRKLVTQENVNSR
DTAGRRSTPLHFAA
>g1984.t1
MKPGGVIAYISSASSPESCHSDSSNGSRQSPLGQPDGSIPSRRRGDRPPAVGRAPPAALQKSGRASNTTLKGGITKLNGL
VLLCKVCGDVASGFHYGVHA
CEGCK
>g1984.t2
MESSKVGGVIAYISSASSPESCHSDSSNGSRQSPLGQPDGSIPSRRRGDRPPAVGRAPPAALQKSGRASNTTLKGGITKL
NGLVLLCKVCGDVASGFHYG
VHACEGCK
>g1985.t1
GIGRNWPWASGGSSILAEYGTLHLEFMHLSKLSGNPEFAQKVMNIRKVLEHLDKPHGLYPNYLNPNSGQWGQHHVSVGGL
GDSFYEYLLKAWIMSDKSDE
EAKRMYYDALQAIETNLIRKSSGGLTYIAEWKGGLLEHKMGHLTCFAGGMIALGADGAPGDKTGHQMEQAAEIARTCHES
YARTTLKLGPEAFRFDGGVE
AIATRQNEKYFILRPEVIETYMYMWRFTHDPKYRDWGWEAVQVMWALLLIRFNPEVKVMDG
>g1985.t2
GIGRNWPWASGGSSILAEYGTLHLEFMHLSKLSGNPEFAQKVMNIRKVLEHLDKPHGLYPNYLNPNSGQWGQHHVSVGGL
GDSFYEYLLKAWIMSDKSDE
EAKRMYYDALQAIETNLIRKSSGGLTYIAEWKGGLLEHKMGHLTCFAGGMIALGADGAPGDKTGHQMEQAAEIARTCHES
YARTTLKLGPEAFRFDGGVE
AIATRQNEKYFILRPEVIETYMYMWRFTHDPKYRDWGWEAVQAG
>g1985.t3
MSLVTMSLVTACLTQWDRKELAVGVGGQQYPGRVMNIRKVLEHLDKPHGLYPNYLNPNSGQWGQHHVSVGGLGDSFYEYL
LKAWIMSDKSDEEAKRMYYD
ALQAIETNLIRKSSGGLTYIAEWKGGLLEHKMGHLTCFAGGMIALGADGAPGDKTGHQMEQAAEIARTCHESYARTTLKL
GPEAFRFDGGVEAIATRQNE
KYFILRPEVIETYMYMWRFTHDPKYRDWGWEAVQVMWALLLIRFNPEVKVMDG
>g1986.t1
VPGAPRGLQRRKRGSLTRGHLGRLTVARHSPAGEPGIGQLGLPSSEQVDYDEIWS
>g1987.t1
MTEVTEVAIPSTVDESNGHDPPPAGDEEDGGGRREPETEAGKGLTPSQREVLERCLHALTHAKNDSHTLAALLLITRICP
ASQLDKPTLRRIFEAVGLNL
PARLLITAVRGRDDDAAAATASLPPEELLSLGTALLAALSTDPDMASHPHLLSTVPLLLGLLADGPSGAQGTSGQNPTSE
PPHSKGPKAGDAGDAAGGDG
GGAEGEEAQDSPLDQAMAADCYQILEAVCSLETGPEQLLSRGAVPTLCRAIQHNQVLSQEKGLPLLGCLLSCRSREKTWR
KHPDQLLSLLGRVSTHFRQA
KDQARLDQCAQLLRFLPPPGVATESAALKEVVSAVWAELRPMMQAKLTPRQIGPPLVLSACLLDLCGWELAGPPKFCCLL
VNRACVEVRMGLEEPPGTDL
SPELQHTLTGCYRIMEAAMEQACSQAVALSDGPPQPAITGISLQQSRQVLLVLQEAFSALIYHLQQVDPSRYSDPFVFAT
FRSLCSWLAEETSCLKEEVT
ALLPFLISYAKAHLAGEGPDQSLSHWLSEMSVGEEDGVWAGSQALRYLLPSLCHLSAEDGPRRVLLTLDAPALLVEFVSQ
GWGRLKGRAGVVTPRDPGLE
TACSALLNFTVTEPERVRKDPCFRTLESLLSEALPVLVHKPHLLVLAANFCTLGLMIGRLMPATSGPAESGPRRFFSAAL
RFLRGA
>g1987.t2
MASRYGAMKRAVIGWCVHXRNIGSYMKMTEVTEVAIPSTVDESNGHDPPPAGDEEDGGGRREPETEAGKGLTPSQREVLE
RCLHALTHAKNDSHTLAALL
LITRICPASQLDKPTLRRIFEAVGLNLPARLLITAVRGRDDDAAAATASLPPEELLSLGTALLAALSTDPDMASHPHLLS
TVPLLLGLLADGPSGAQGTS
GQNPTSEPPHSKGPKAGDAGDAAGGDGGGAEGEEAQDSPLDQAMAADCYQILEAVCSLETGPEQLLSRGAVPTLCRAIQH
NQVLSQEKGLPLLGCLLSCR

SREKTWRKHPDQLLSLLGRVSTHFRQAKDQARLDQCAQLLRFLPPPGVATESAALKEVVSAVWAELRPMMQAKLTPRQIG
PPLVLSACLLDLCGWELAGP
PKFCCLLVNRACVEVRMGLEEPPGTDLSPELQHTLTGCYRIMEAAMEQACSQAVALSDGPPQPAITGISLQQSRQVLLVL
QEAFSALIYHLQQVDPSRYS
DPFVFATFRSLCSWLAEETSCLKEEVTALLPFLISYAKAHLAGEGPDQSLSHWLSEMSVGEEDGVWAGSQALRYLLPSLC
HLSAEDGPRRVLLTLDAPAL
LVEFVSQGWGRLKGRAGVVTPRDPGLETACSALLNFTVTEPERVRKDPCFRTLESLLSEALPVLVHKPHLLVLAANFCTL
GLMIGRLMPATSGPAESGPR
RFFSAALRFLRGA
>g1987.t3
MLSCLHHKRTETNMNRLHVSESAAVFRWVIVLRCGVVVASLTVYLLSPRHDLLTFQQRRKMASRYGAMKRAVIGWCVHXR
NIGSYMKMTEVTEVAIPSTV
DESNGHDPPPAGDEEDGGGRREPETEAGKGLTPSQREVLERCLHALTHAKNDSHTLAALLLITRICPASQLDKPTLRRIF
EAVGLNLPARLLITAVRGRD
DDAAAATASLPPEELLSLGTALLAALSTDPDMASHPHLLSTVPLLLGLLADGPSGAQGTSGQNPTSEPPHSKGPKAGDAG
DAAGGDGGGAEGEEAQDSPL
DQAMAADCYQILEAVCSLETGPEQLLSRGAVPTLCRAIQHNQVLSQEKGLPLLGCLLSCRSREKTWRKHPDQLLSLLGRV
STHFRQAKDQARLDQCAQLL
RFLPPPGVATESAALKEVVSAVWAELRPMMQAKLTPRQIGPPLVLSACLLDLCGWELAGPPKFCCLLVNRACVEVRMGLE
EPPGTDLSPELQHTLTGCYR
IMEAAMEQACSQAVALSDGPPQPAITGISLQQSRQVLLVLQEAFSALIYHLQQVDPSRYSDPFVFATFRSLCSWLAEETS
CLKEEVTALLPFLISYAKAH
LAGEGPDQSLSHWLSEMSVGEEDGVWAGSQALRYLLPSLCHLSAEDGPRRVLLTLDAPALLVEFVSQGWGRLKGRAGVVT
PRDPGLETACSALLNFTVTE
PERVRKDPCFRTLESLLSEALPVLVHKPHLLVLAANFCTLGLMIGRLMPATSGPAESGPRRFFSAALRFLRGA
>g1988.t1
MWLQDVFVGLNLSGVLLLTFITLLILDLLKNRDPPNYPPGPLALPLVGNIFNIDPRQPHIYLTKLAAVHGNVFSMRLGRD
RTVFVSGYKMVKEAIVSQAD
IFAERPYSPMATRLYSENVGRN
>g1988.t2
MWLQDVFVGLNLSGVLLLTFITLLILDLLKNRDPPNYPPGPLALPLVGNIFNIDPRQPHIYLTKLAAVHGNVFSMRLGRD
RTVFVSGYKMVKEAIVSQAD
IFAERPYSPMATRLYSENV
>g1989.t1
METLRTNGELTDVTLCVQGVLFPCHRAVLAAASLYF
>g1990.t1
MKTFSKLGYKMRMANDLTMGELEKLLFTVSQEDHSGSASFVCVLLSHGDEGIIYGTDGAVQLQTLTGMFRGDRCKTLVAK
PKLFFIQ
>g1990.t2
CAMKTFSKLGYKMRMANDLTMGELEKLLFTVSQEDHSGSASFVCVLLSHGDEGIIYGTDGAVQLQTLTGMFRGDRCKTLV
AKPKLFFIQ
>g1991.t1
VQEFFGSLTKETGSEMRCIQQTYETIEENIRWVDKHLPLLQAWLDKRTVPSSSPALKCSLGSMAAEEQEKVIFSGDIANV
GANMQVI
>g1991.t2
VQEFFGSLTKETGSEMRCIQQTYETIEENIRWVDKHLPLLQAWLDKRTGRGPAAQPTPHEDL
>g1992.t1
MMTFKVLQDPHVPPGTSGVYRFMSCSCCFRLWTSTCSDEGQPSEGKLDFLFAVFHTTCGSLHYAVGL
>g1993.t1
MYRETRSKESGPEPGSGVACRGPAPFSTQPQDSAPGPPSGAQGPLRRLGVLNGCVVPQPCGPPSPLPYKEVHLGTPFHHA
PPTQETPTQWSHQHPPHLWL
L
>g1994.t1
MKRMHSSCYSPRLIVVVPWVSNGSSSSPPSLTDLTLAGLEMGVLTMKKGEFSRFLLQPRFAYGEMGCPPLIPPAAAILFE
VQLVDYFDSAQVDHFLALEP
NDQNGLPLSVLRGVVNTLRGLGNRSFHQGHYGSAKGRYKQAMAALTTRLNGKEEEEEEEEQQQQEEVKALLLPIYLNLSL

TELRLESPEKALKHARRALD
IDPRNTKALFRCAQAHMDTHEYERAHGVLLAAQAEKPFDCDINSLLMKAAM
>g1994.t2
MKRMGPFGCLALRMEDVMDGGGIRKELVQPGEGPPVPRDASVLIHYSAFLEYADQPYDTTRHLTYPRMMRLGRDLTLAGL
EMGVLTMKKGEFSRFLLQPR
FAYGEMGCPPLIPPAAAILFEVQLVDYFDSAQVDHFLALEPNDQNGLPLSVLRGVVNTLRGLGNRSFHQGHYGSAKGRYK
QAMAALTTRLNGKEEEEEEE
EQQQQEEVKALLLPIYLNLSLTELRLESPEKALKHARRALDIDPRNTKALFRCAQAHMDTHEYERAHGVLLAAQAEKPFD
CDINSLLMKAAM
>g1994.t3
MKRMGPFGCLALRMEDVMDGGGIRKELVQPGEGPPHSSCYSPRLIVVVPWVSNGSSSSPPSLTDLTLAGLEMGVLTMKKG
EFSRFLLQPRFAYGEMGCPP
LIPPAAAILFEVQLVDYFDSAQVDHFLALEPNDQNGLPLSVLRGVVNTLRGLGNRSFHQGHYGSAKGRYKQAMAALTTRL
NGKEEEEEEEEQQQQEEVKA
LLLPIYLNLSLTELRLESPEKALKHARRALDIDPRNTKALFRCAQAHMDTHEYERAHGVLLAAQAEKPFDCDINSLLMKA
AM
>g1995.t1
MSPTSSWFLLLHPDSPLSSSPGSTAVREVQEETGVSSEFVSLLSVRQQHDHPGAFGASDLYLICRLRPLTYRIQFCTEEC
VRCDWLPLAELARTDQTTPI
TSRVARLLLHGLEHGWSAVDLPMEQIPAVYSGRFYQLYHRALPPALTSG
>g1996.t1
MKDWAGSHEVSLPPVRLQLQATAVGEVVIKGLAANRYLAMNRDGRLSAARRATDECYFLERLESNNYNTYRSKKYPEMYV
ALQRSGQYKAGTKTGPGQKA
ILFLPMSAKP
>g1997.t1
MAALKTCLALGVSSRIPAWLCWLYCHAVLLATVFNKSNESSPFTKATASRSIWLSRAPLTGHSSAEHELITFLVFVYLIC
VPIREKGSW
>g1998.t1
LLCWEGRRRSRREQEEEEAMATGEITTLPPTPDDGSGGGFPPANFREPKRLYCKNGGFFLRIGADGRVDGVREKSSPHSK
YCQAAG
>g1999.t1
MAGYLKVLSSLSRSAASLSRNPAVLAPAATCQALQQRNYADKRIKVAQPVVEMDGDEMTRIIWEFIKEKLILSNVDVELK
YYDLGLPYRDQTDDQVTIDS
ALATLKYHVAVKCATITPDEDRVTEFSLKKMWKSPNGTIRNILGGTVFREPIICKNIPRLVPGWTQPITIGRHAFGDQYR
ATDFVIDQPGKFKIIFSPAD
GSQGKEWEVYDFPAGGCGMGMYNTDE
>g1999.t2
MAGYLKVLSSLSRSAASLSRNPAVLAPAATCQALQQRNYADKRIKVAQPVVEMDGDEMTRIIWEFIKEKLILSNVDVELK
YYDLGLPYRDQTDDQVTIDS
ALATLKYHVAVKCATITPDEDRVTEFSLKKMWKSPNGTIRNILGGTVFREPIICKNIPRLVPGWTQPITIGRHAFGDQYR
ATDFVIDQPGKFKIIFSPAD
GSQGKEWEVYDFPAGGCGMGMYNTDEVRTPAQRFISR
>g1999.t3
MAGYLKVLSSLSRSAASLSRNPAVLAPAATCQALQQRNYADKRIKVAQPVVEMDGDEMTRIIWEFIKEKLILSNVDVELK
YYDLGLPYRDQTDDQVTIDS
ALATLKYHVAVKCATITPDEDRVTEFSLKKMWKSPNGTIRNILGGTVFREPIICKNIPRLVPGWTQPITIGRHAFGDQYR
ATDFVIDQPGKFKIIFSPAD
GSQGKEWEVYDFPAGGCGMGMYNTDEK
>g2000.t1
MSVSTLVLTSIKGTPLYMSPELVEEKPYDHTADLWSLGCILYELHTGVPPFYTNSIFHLVQLIVRDTVKWPADTMSPTCM
GFLKGLLTKDPQKRLSWPDL
LHHPFVADGVLVLPGDSVFNPLTVAPSPDVLALKHQQVREKSAGSGKNRLLRKVLAHRDKDQKGKEGEGERAAEDPGSRK
SSPGRTRPNHLTEPEPPREQ
EKVQEAAKMFLNLKKQTSATSKRRGQISRDYEQEFPSVEVGPRFVRTRQNPPPGQLLDGILEDAWQTLHPLRVLSMLLLN
SDHENSLHIGRELGLPHLLF
DLTSVMVDKLNVIKEPWIHDVLSQVLAVLVAYWDRHSEWAQDENRMEELMKPVLKILSKSSSTPSAAVAASVLSMFTHHG

VSVDVDIGTLSEVLKSSSLF
SKHGNGSPSTSPRQEIWCDLWRTIANALETQINPHFCSTKGLHSFLSAMLCVFTKDPSLCLPLFSDSQSECVHTLGLVFH
HNRCSDGAPAGVQEEDLDDL
LDLSCLLLCFPFSLDLSPRTRAKVLDLYDRWGVVTGLMQMIQTRPPALLELPLSLLSRLLLCDPARFVPRLAAASDAAAS
NAAASAAAALPLAQDFFFCP
A
>g2000.t2
MQKGSCFKFLRMTAIFQRTSFARSMSVSTLVLTSIKGTPLYMSPELVEEKPYDHTADLWSLGCILYELHTGVPPFYTNSI
FHLVQLIVRDTVKWPADTMS
PTCMGFLKGLLTKDPQKRLSWPDLLHHPFVADGVLVLPGDSVFNPLTVAPSPDVLALKHQQVREKSAGSGKNRLLRKVLA
HRDKDQKGKEGEGERAAEDP
GSRKSSPGRTRPNHLTEPEPPREQEKVQEAAKMFLNLKKQTSATSKRRGQISRDYEQEFPSVEVGPRFVRTRQNPPPGQL
LDGILEDAWQTLHPLRVLSM
LLLNSDHENSLHIGRELGLPHLLFDLTSVMVDKLNVIKEPWIHDVLSQVLAVLVAYWDRHSEWAQDENRMEELMKPVLKI
LSKSSSTPSAAVAASVLSMF
THHGVSVDVDIGTLSEVLKSSSLFSKHGNGSPSTSPRQEIWCDLWRTIANALETQINPHFCSTKGLHSFLSAMLCVFTKD
PSLCLPLFSDSQSECVHTLG
LVFHHNRCSDGAPAGVQEEDLDDLLDLSCLLLCFPFSLDLSPRTRAKVLDLYDRWGVVTGLMQMIQTRPPALLELPLSLL
SRLLLCDPARFVPRLAAASD
AAASNAAASAAAALPLAQDFFFCPA
>g2000.t3
MSVSTLVLTSIKGTPLYMSPELVEEKPYDHTADLWSLGCILYELHTGVPPFYTNSIFHLVQLIVRDTVKWPADTMSPTCM
GFLKGLLTKDPQKRLSWPDL
LHHPFVADGVLVLPGDSVFNPLTVAPSPDVLALKHQQVREKSAGSGKNRLLRKVLAHRDKDQKGKEGEGERAAEDPGSRK
SSPGRTRPNHLTEPEPPREQ
EKVQEAAKMFLNLKKQTSATSKRRGQISRDYEQEFPSVEVGPRFVRTRQNPPPGQLLDGILEDAWQTLHPLRVLSMLLLN
SDHENSLHIGRELGLPHLLF
DLTSVMVDKLNVIKEPWIHDVLSQVLAVLVAYWDRHSEWAQDENRMEELMKPVLKILSKSSSTPSAAVAASVLSMFTHHG
VSVDVDIGTLSEVLKSSSLF
SKAGERVMVYCLCCYIQSLSQSECVHTLGLVFHHNRCSDGAPAGVQEEDLDDLLDLSCLLLCFPFSLDLSPRTRAKVLDL
YDRWGVVTGLMQMIQTRPPA
LLELPLSLLSRLLLCDPARFVPRLAAASDAAASNAAASAAAALPLAQDFFFCPA
>g2001.t1
MFIRTAFLLCACVAAFLRDVHGVEHEVVFPVRLGVPRNQRGGLASGSGIREERCVVKIRAFQQELVLDLQQDSTFLSPSV
SVQDTVSVSTDVTPELSRCF
YAGYVNKDQYSYAALSLCGGVHGAFGYQGWEYFISPLRNDSANDAESAHSLRRRAAGNNLRGANTTSRCAVDADISPEGA
ESLEKYKHLNAKRNQTESAP
KGISRSKRFASIPRYVETLVVADESMVEFHGDDLKHYLLTLMSVAARLYKHPSIQNSINVVVVKFVMISEADKGPKVSGN
AAMTLRNFCTWQKKMNKNND
KHSDYWDTAILFTRQVISPP
>g2002.t1
MLSPTAGTIGVTKAPGMGSSSSKAEEEAEAEEKEEDGEETEEDIADRLAAELRKEEAEEGDLPLGVDDMLDSGDPVLDLC
YRKFKKLPCRVYKLMHLEKI
YACGNRLRTLPDRLSQLQGLRTLALDFNKMEDVPPAVCQLVNLTCLYMGSNRLVCLPPEVRNLQKLRCLWLESNYFQYFP
KQLYELPHLRTLQIGDNRLK
ALPSDLVRMESLKGLWVYGNRFEEFPRVLLRMEGLEVLDLDRNKISNFPSLRRLPALQLISYDHNPVEGPPRVGQEVTLV
GEGAAEAMQEREDRKERRRR
AAELEAEEQLALAEEEERVIHGILKNSGASPGRVASEAPADGDAPPDGEEEQEAQGEGEGDAQEEEEGSPERAGVMECDS
AGLEYDEEALEYETEELVCE
AEGLDYEGEALEYERVQMDYEYEEEAQGTGTQNGGR
>g2003.t1
MRGLAQNPSNDRSEFIVNEWVNRRVNTLGKKIQLDANYLEGYRRKVRSSAPGAEPFRDAHVTLFVLEFNSTLLLLHVGGK
QTAHSSAEMGGGGQLTEPGE
TTACSGRAGNVYTWDEVQKHCHRNDQWLVINRKVYNVTQWAKRHPGGLRVISHYAGEDATAAVAEDFQALRTRAESLGLF
QAQPVFFCLHLGHILLLEVM
AWMTVWLLGTSWSSTLLCSIILAVAQAQAGWLQHDFGHLSVFKMSSWNHIFHKFIIGHLKGASGNWWNHRHFQHHAKPNV

FSKDPDVNMLHVFVVGESQP
VEDLAWSMSYYLRYFFCYAPFYGLLGSVALISFVRFLESHWFVWVTQMNHLPMNIDHEKQQDWLSMQLNATCNIQQSYFN
DWFSGHLNFQIEHHLFPTMP
RHNYQVLAPLVRALCEKHGIPYQVKTLWQGIVDVVR
>g2003.t2
MRGLAQNPSNDRSEFIVNEWVNRRVNTLGKKIQLDANYLEGYRRKVRSSAPGAEPFRDAHVTLFVLEFNSTLLLLHVGGK
QTAHSSAEMGGGGQLTEPGE
TTACSGRAGNVYTWDEVQKHCHRNDQWLVINRKVYNVTQWAKRHPGGLRVISHYAGEDATEAFVAFHPNPKLVQKFLKPL
LLGELAAAVAEDFQALRTRA
ESLGLFQAQPVFFCLHLGHILLLEVMAWMTVWLLGTSWSSTLLCSIILAVAQAQAGWLQHDFGHLSVFKMSSWNHIFHKF
IIGHLKGASGNWWNHRHFQH
HAKPNVFSKDPDVNMLHVFVVGESQPVEDLAWSMSYYLRYFFCYAPFYGLLGSVALISFVRFLESHWFVWVTQMNHLPMN
IDHEKQQDWLSMQLNATCNI
QQSYFNDWFSGHLNFQIEHHLFPTMPRHNYQVLAPLVRALCEKHGIPYQVKTLWQGIVDVVR
>g2003.t3
MPVVRDMRGLAQNPSNDRSEFIVNEWVNRRVNTLGKKIQLDANYLEGYRRKVRSSAPGAEPFRDAHVTLFVLEFNSTLLL
LHVGGKQTAHSSAEMGGGGQ
LTEPGETTACSGRAGNVYTWDEVQKHCHRNDQWLVINRKVYNVTQWAKRHPGGLRVISHYAGEDATAAVAEDFQALRTRA
ESLGLFQAQPVFFCLHLGHI
LLLEVMAWMTVWLLGTSWSSTLLCSIILAVAQAQAGWLQHDFGHLSVFKMSSWNHIFHKFIIGHLKGASGNWWNHRHFQH
HAKPNVFSKDPDVNMLHVFV
VGESQPVEDLAWSMSYYLRYFFCYAPFYGLLGSVALISFVRFLESHWFVWVTQMNHLPMNIDHEKQQDWLSMQLNATCNI
QQSYFNDWFSGHLNFQIEHH
LFPTMPRHNYQVLAPLVRALCEKHGIPYQVKTLWQGIVDVVR
>g2004.t1
MHQAALLGTAVKDARKYGWKIQDPVSHDWATMAEAVQNHVRSLNWGHRVQLQDKKVKYLNVKGSLVDKHTVRGTAKSGKE
SFLTAKNIVIATGGRPKYPT
HVPGAMEHGITSDDIFWLKEAPGK
>g2005.t1
MLEDVAGSRTFSTASPDSVTSGTRAQCEPALIREEHMVPMANLPILEFCGKCQSGCTVLGCKHRPHLWTLGPHSTLMESV
SHSLSRNMHISGLLKVVL
>g2006.t1
MAQPAQSHLPSQQLQWKRTVIIQHLQRVLQRIPHLGRRSRLFQTDTEPAELGWKEGQILKGLKRLQMRSPNRLSPPASVP
HYKDCMTSNEGIYSLGAKSG
AQAMDPKQAAVKPGTPSFKTEEILASSFHRSLMSSSWKVPQCNLVLAPQRVQITWMLMTRSPQA
>g2006.t2
MAQPAQSHLPSQQLQWKRTVIIQHLQRVLQRIPHLGRRSRLFQTDTEPAELGWKEGQILKGLKRLQMRSPNRLSPPASVP
HYKDCMTSNEGIYSLGAKSG
AQAMDPKQAAVKPGTPSFKTEVPQCNLVLAPQRVQITWMLMTRSPQA
>g2007.t1
MDRPNSNRSGKIRTQIKTQSGSSLNSLGDVFPGSLSCSSSYHCPMNRSLLNNFLIEEGLPIPSHLASSSERLQSLKQQRE
QARRLQIEQQFSSAFGEPVS
EEPESQNAITTIEEKVMLGIEENLHKSQEQEKNNDAKQKSGSSLANWFGFRKSKLPAPSGKKTDLSKVKEEKREQKITSL
LGGKQTKSDKKKERRKSDGK
DWRESHDAVRACIPMACPGAALGDMQGHGDLGPADTKV
>g2007.t2
MIGAKARSQSFGARYMDRPNSNRSGKIRTQIKTQSGSSLNSLGDVFPGSLSCSSSYHCPMNRSLLNNFLIEEGLPIPSHL
ASSSERLQSLKQQREQARRL
QIEQQFSSAFGEPVSEEPESQNAITTIEEKVMLGIEENLHKSQEQEKNNDAKQKSGSSLANWFGFRKSKLPAPSGKKTDL
SKVKEEKREQKITSLLGGKQ
TKSDKKKERRKSDGKDWRESHDAVRACIPMACPGAALGDMQGHGDLGPADTKV
>g2007.t3
MGMIGAKARSQSFGARYMDRPNSNRSGKIRTQIKTQSGSSLNSLGDVFPGSLSCSSSYHCPMNRSLLNNFLIEEGLPIPS
HLASSSERLQSLKQQREQAR
RLQIEQQFSSAFGEPVSEEPESQNAITTIEEKVMLGIEENLHKSQEQEKNNDAKQKSGSSLANWFGFRKSKLPAPSGKKT
DLSKVKEEKREQKITSLLGG

KQTKSDKKKERRKSDGKDWRESHDAVRACIPMACPGAALGDMQGHGDLGPADTKV
>g2008.t1
MGATETGGGGVFSLVQCECDGFIVFNALPVTACKSPVMNIMRYTCLSLLRDVQFTSYGGFKGREVL
>g2009.t1
LKQFFDQNYSHIYYVFFENFVTIEVGLRQKGHKSQREELDSILFIFE
>g2009.t2
MMVRISNSIVACPVILELTSLWCLFTPLIDRAASLENAESSELKQFFDQNYSHIYYVFFENFVTIEVGLRQKGHKSQREE
LDSILFIFE
>g2009.t3
LIDRAASLENAESSELKQFFDQNYSHIYYVFFENFVTIEVGLRQKGHKSQREELDSILFIFE
>g2010.t1
MPVHRDREKKENVAEEMDVEDHEQEASTSEEEDSDTSSVSEEDGDSSEMDEEDCERRRMECLDEMSNLEKQFTDLKDQLY
RERVSQVNSKLSEVEAGVYR
ELCLDSVKNKYECEIQAACQHWESEKLLLFDSVQSELEEKIRRLEEDKHSIDITSVYMLPDLDILEDWTTIRK
>g2010.t2
MPVHRDREKKENVAEEMDVEDHEQEASTSEEEDSDTSSVSEEDGDSSEMDEEDCERRRMECLDEMSNLEKQFTDLKDQLY
RERVSQVNSKLSEVEAGVYR
ELCLDSVKNKYECEIQAACQHWESEKLLLFDSVQSELEEKIRRLEEDKHSIDITSVYMLPDLDILEDWTTIRKVCVCVSR
K
>g2010.t3
MPVHRDREKKENVAEEMDVEDHEQEASTSEEEDSDTSSVSEEDGDSSEMDEEDCERRRMECLDEMSNLEKQFTDLKDQLY
RERVSQVNSKLSEVEAGVYR
ELCLDSVKNKYECEIQAACQHWESEKLLLFDSVQSELEEKIRRLEEDKHSIDITSVYMLPDLDILEDWTTIRKAVATLTP
HNRGKADSESSMFPFRTDRN
RPILNC
>g2011.t1
MMSMNSKQPHFAMHPSLPEHKYTTLHSSSEAIRRACLQTPQ
>g2012.t1
MDCVSGPSEAVAPGAAPSGPPGAVPEGRPVFWAIRYWFSRYTVAALEATIQQNEWKMKQLTKALEQSDKTIESLQAQLRG
YEGSPPGLWGAPGDPAEKQK
LVMMTRSLSDTERQSICSNPEGETLTSKGQSSNFPASPRTSEGEKMMGFTATTFYGTPPKLKPSTPSSAFCSLSLRSPAV
GGGKAGIKPGTSLRKLNFED
PLEKVRSMVSQTENCSPNLPKCPPETSKTPFWGTWQEPKPNAPPSVGPISEDSQMVGDEASAAAQVRPGGSHASSEASMD
AAYHSKVSELDCMMWDSDSS
CSQGSRFSQAASPAADLDTTLVPKPKPAAAPTVSSSASKEENVSVLSFHQNYPPCDPSLGTPDTVSKAGSTGERAAGRGN
AAQCGPSPASSLEGGRGDGL
WSGKACVVQGGDNSLKAGRAIALGSERAGLLQGIAAGLGCSWQGGAPVPSVIEGEGLSQTDDLSFDILFDSSLDGPAAGA
TGSDEPAILPHNGPSPSSTP
GWDQQLVIGQPTKRKSRSPFNTNSPTKLSKFV
>g2012.t2
MLRSQNVDLVQENMNLRAEVSSRSPQKWTACPAPLRPSPRGLHHLARRAPSRRADLSFGPFVIGCDGALPARFFLRSGFS
RYTVAALEATIQQNEWKMKQ
LTKALEQSDKTIESLQAQLRGYEGSPPGLWGAPGDPAEKQKLVMMTRSLSDTERQSICSNPEGETLTSKGQSSNFPASPR
TSEGEKMMGFTATTFYGTPP
KLKPSTPSSAFCSLSLRSPAVGGGKAGIKPGTSLRKLNFEDPLEKVRSMVSQTENCSPNLPKCPPETSKTPFWGTWQEPK
PNAPPSVGPISEDSQMVGDE
ASAAAQVRPGGSHASSEASMDAAYHSKVSELDCMMWDSDSSCSQGSRFSQAASPAADLDTTLVPKPKPAAAPTVSSSASK
EENVSVLSFHQNYPPCDPSL
GTPDTVSKAGSTGERAAGRGNAAQCGPSPASSLEGGRGDGLWSGKACVVQGGDNSLKAGRAIALGSERAGLLQGIAAGLG
CSWQGGAPVPSVIEGEGLSQ
TDDLSFDILFDSSLDGPAAGATGSDEPAILPHNGPSPSSTPGWDQQLVIGQPTKRKSRSPFNTNSPTKLSKFV
>g2013.t1
LPPTIGYLHSLRTFAADENFLTELPRESPAALWGPPALPLGCWSTRSRRSGESAVCPDVPQ
>g2014.t1
NVNVSAPWTCHIMADPLLFEDTSSSQPAMNSQGYYTPGYNMAAPVGGPQADPAGNNLFADPMANAAMMYGSSLANQGKDM
ISRFMSMNKLKYFFAVDTRY

VVKKLMLLLFPYTHQVIWATASPDEIVWFAARAMAFITYILLAGMALGIQQRFSPEVLGLCASTALVWIIIEVLLMLLSL
YLLTVHTDLSTFDLLAYSGY
KYVAMILTVLSGLLFGSDGYFVALAWSSCALMFFIARSLKMKILSSLSSDSMGAGASAKPRLRLYITVATAAFQPFIIYW
LTSHLVR
>g2014.t2
AHSSPPTQSKANVNVSAPWTCHIMADPLLFEDTSSSQPAMNSQGYYTPGYNMAAPVGGPQADPAGNNLFADPMANAAMMY
GSSLANQGKDMISRFMSMNK
LKYFFAVDTRYVVKKLMLLLFPYTHQVIWATASPDEIVWFAARAMAFITYILLAGMALGIQQRFSPEVLGLCASTALVWI
IIEVLLMLLSLYLLTVHTDL
STFDLLAYSGYKYVAMILTVLSGLLFGSDGYFVALAWSSCALMFFIARSLKMKILSSLSSDSMGAGASAKPRLRLYITVA
TAAFQPFIIYWLTSHLVR
>g2014.t3
NVNVSAPWTCHIMAKTRARAPPSADPLLFEDTSSSQPAMNSQGYYTPGYNMAAPVGGPQADPAGNNLFADPMANAAMMYG
SSLANQGKDMISRFMSMNKL
KYFFAVDTRYVVKKLMLLLFPYTHQVIWATASPDEIVWFAARAMAFITYILLAGMALGIQQRFSPEVLGLCASTALVWII
IEVLLMLLSLYLLTVHTDLS
TFDLLAYSGYKYVAMILTVLSGLLFGSDGYFVALAWSSCALMFFIARSLKMKILSSLSSDSMGAGASAKPRLRLYITVAT
AAFQPFIIYWLTSHLVR
>g2015.t1
MTSLLAYTAEEIRRLRRQMEELRKESGHYIELLKAHDINFLEDPTIHWKGKQRCAKVAKVTPTHQLPKGIIVYSNGNVIC
PAGKEPVLEKQPPEAVLVQP
VSDANTGVRVNGVLHVKAPSSAPSLLPGTTVAPLETAAALRVVETSPALPPSVSYITLQIPAVSTTLPQQTPSAVPSQNI
TIAATPVSQLSTQPVVSLAT
VAQAATHSSTAVVADVIGSLVSEDTAMRTVSYTSIPSSAALLRAGAAGSTQTTWTTLHLTGNTVQPVCPSVPTLEGTATN
QRLQQLTVCPVGHKPTAQPI
KIQIRPQVPLQQASIATHIQAQPVQRLPQLHSALLTQTSPQPQCAVPPQPAVVAHPAVMSQPQPSVLQPASVVPHLQSAL
IPQPKPQPLPQPALVPQPQA
TVLPLLQTMQVLQVNPSGATVSGVTTPQNTNNQSVVILQQGNSCSSQSTVREELPNQMSCQHIVIIQTPNQAAPAQQNPQ
QGIQASSAQAAPSAAPQTIT
VNGQVFALQPMNTADKAGMQGNKSTLQLVQPTTAEEPTTNVALNSLGALSSLNKSISQGLPLTIATEPTAMAIKSIPVSV
VGSVTTASKATGPPTKSLIT
TTETSVKLSKSVGSTGSSRSTSSSVTTVSTISAPVCTTVVSDSMAPIRIPGSHTTSTGPSLRNSKVSQACSKPEPQTSPT
SSVSSIAPPPSTVLTFSVAP
DTTPTSAELRKGGVSRSLTQQPDLVPSTPSLPCMADVKQSRGPAREREAQKQRQVSALEKQCMAESTNIDASSRKDYLLP
QPVYTNLDHHETMEHHMTAS
RQTDSPMSAGGGSGRGFSVASMLPQGHGISASSGSFGTFTFTSEQADIVALAMLEQEIPGRRGAGCTVDHGTPATSATTA
WEPPKTSPSSASQERGLAAP
QVKATKPMDAAVAKPPSQGSVRAQVGEGSIIGPTGTRHPAGISYSQAQSHPQVQSQCGTSLSVNNLIRPNSSQQPYPGSP
SLSGQQGSIPSPVGSSAHLS
QSSNGSLPPCSASAQLNDYPPLKSALMRNQAGVGERHVKHVSKRQVQEEAMLTSGKRPKLCPQSAPSLGRMEVKAPDHSQ
MMVGQIPPSSSTIMSRINPD
GAGSLFTSNSFMNTVLRPVDGHCPPQGPPEPNQPVLPHLAQGHPQHTSSQSGQHLGGNPYMKQQQQQQQQQQQQQQQQQQ
QQQQQQQQQQQQQQQQQQQQ
QQQEQHRLYQLQHHLTQSDPAQLHSLHQRALQQQEHQHVQKKRGLVRGNQSGSPAGLQQKQHHMEKPGVQQQQQQQQHSQ
QQQHQQHTQQQSHPHQQQSH
QQSHQQQPQHPQQTHQQQQQQQQHQQQQQLQQQQSSHSRHQQHLQQQIQQQQQQQQQQQQQQHFGARHPEKSCESQAGGP
RGHHAGHLAPQEHLKVGLLP
>g2015.t2
MTSLLAYTAEEIRRLRRQMEELRKESGHYIELLKAHDINFLEDPTIHWKGKQRCAKVAKVTPTHQLPKGIIVYSNGNVIC
PAGKEPVLEKQPPEAVLVQP
VSDANTGVRVNGVLHVKAPSSAPSLLPGTTVAPLETAAALRVVETSPALPPSVSYITLQIPAVSTTLPQQTPSAVPSQNI
TIAATPVSQLSTQPVVSLAT
VAQAATHSSTAVVADVIGSLVSEDTAMRTVSYTSIPSSAALLRAGAAGSTQTTWTTLHLTGNTVQPVCPSVPTLEGTATN
QRLQQLTVCPVGHKPTAQPI
KIQIRPQVPLQQASIATHIQAQPVQRLPQLHSALLTQTSPQPQCAVPPQPAVVAHPAVMSQPQPSVLQPASVVPHLQSAL
IPQPKPQPLPQPALVPQPQA

TVLPLLQTMQVLQVNPSGATVSGVTTPQNTNNQSVVILQQGNSCSSQSTVREELPNQMSCQHIVIIQTPNQAAPAQQNPQ
QGIQASSAQAAPSAAPQTIT
VNGQVFALQPMNTADKAGMQGNKSTLQLVQPTTAEEPTTNVALNSLGALSSLNKSISQGLPLTIATPVCTTVVSDSMAPI
RIPGSHTTSTGPSLRNSKVS
QACSKPEPQTSPTSSVSSIAPPPSTVLTFSVAPDTTPTSAELRKGGVSRSLTQQPDLVPSTPSLPCMADVKQSRGPARER
EAQKQRQVSALEKQCMAEST
NIDASSRKDYLLPQPVYTNLDHHETMEHHMTASRQTDSPMSAGGGSGRGFSVASMLPQGHGISASSGSFGTFTFTSEQAD
IVALAMLEQEIPGRRGAGCT
VDHGTPATSATTAWEPPKTSPSSASQERGLAAPQVKATKPMDAAVAKPPSQGSVRAQVGEGSIIGPTGTRHPAGISYSQA
QSHPQVQSQCGTSLSVNNLI
RPNSSQQPYPGSPSLSGQQGSIPSPVGSSAHLSQSSNGSLPPCSASAQLNDYPPLKSALMRNQAGVGERHVKHVSKRQVQ
EEAMLTSGKRPKLCPQSAPS
LGRMEVKAPDHSQMMVGQIPPSSSTIMSRINPDGAGSLFTSNSFMNTVLRPVDGHCPPQGPPEPNQPVLPHLAQGHPQHT
SSQSGQHLGGNPYMKQQQQQ
QQQQQQQQQQQQQQQQQQQQQQQQQQQQQQQQQQQQQQQQEQHRLYQLQHHLTQSDPAQLHSLHQRALQQQEHQHVQKKRGLVR
GNQSGSPAGLQQKQHHMEKP
GVQQQQQQQQHSQQQQHQQHTQQQSHPHQQQSHQQSHQQQPQHPQQTHQQQQQQQQHQQQQQLQQQQSSHSRHQQHLQQQ
IQQQQQQQQQQQQQQQHFGAR
HPEKSCESQAGGPRGHHAGHLAPQEHLKVGLLP
>g2016.t1
LDVVMEDLGDPVERYHSLSVVLPSGLEKTATVHGSKPVMDLLVTLCARYHLNPSDHTVEVLSHNKNHISFKPNYPIGSLE
AETIVLKAKVAEDKARRPYT
PEVTVRLLVNYRKSHKTVVRVNPRVPLGHLALVISEKCEFQLESTVLLRDSLTQEPLDLTKSLNDLGIRELFAKDMAGKL
LEHFKGPVEGEPHSRNEKKR
RGNNGFLSLFRRKKDKDGRAYAPAPQDLKTLPAVSASSLGLCSASTSTAGMTKKRPAPRPPMIASRSVPDNLSGHRVRGP
QSRPRGGPRGEHRYLPALIP
THARQPTLKGVQVNTPAPSAMEDAFFEGVSCWFNVLSKVSMHECRVLQSPPSWRRMSLVAAVFDSQFPGSIPTVLTAAYL
>g2016.t2
LDVVMEDLGDPVERYHSLSVVLPSGLEKTATVHGRYHLNPSDHTVEVLSHNKNHISFKPNYPIGSLEAETIVLKAKVAED
KARRPYTPEVTVRLLVNYRK
SHKTVVRVNPRVPLGHLALVISEKCEFQLESTVLLRDSLTQEPLDLTKSLNDLGIRELFAKDMAGKLLEHFKGPVEGEPH
SRNEKKRRGNNGFLSLFRRK
KDKDGRAYAPAPQDLKTLPAVSASSLGLCSASTSTAGMTKKRPAPRPPMIASRSVPDNLSGHRVRGPQSRPRGGPRGEHR
YLPALIPTHARQPTLKGVQV
NTPAPSAMEDAFFEGVSCWFNVLSKVSMHECRVLQSPPSWRRMSLVAAVFDSQFPGSIPTVLTAAYL
>g2016.t3
MEDLGDPVERYHSLSVVLPSGLEKTATVHGSKPVMDLLVTLCARYHLNPSDHTVEVLSHNKNHISFKPNYPIGSLEAETI
VLKAKVAEDKARRPYTPEVT
VRLLVNYRKSHKTVVRVNPRVPLGHLALVISEKCEFQLESTVLLRDSLTQEPLDLTKSLNDLGIRELFAKDMAGKLLEHF
KGPVEGEPHSRNEKKRRGNN
GFLSLFRRKKDKDGRAYAPAPQDLKTLPAVSASSLGLCSASTSTAGMTKKRPAPRPPMIASRSVPDNLSGHRVRGPQSRP
RGGPRGEHRYLPALIPTHAR
QPTLKGVQVNTPAPSAMEDAFFEGVSCWFNVLSKVSMHECRVLQSPPSWRRMSLVAAVFDSQFPGSIPTVLTAAYL
>g2017.t1
MGRIFLDHIGGTRLFCCANCDTTLTNRAELISTRFTGATGRAFLFNKVVNLQHSDVQERVMLTGRHMVRDVSCKNCNSKV
GWLYEFATEDSQRYKEGRVI
LERALVRESEGFERTPSDSNS
>g2018.t1
MYLSEPVSAAFKLYCLSVMTLVAATYTVALRYTRTISSGDLYFSTTAVCITEVIKLILSLGMLARESGSPSRLKNSLVDH
VFRSPKELLKLSVPSVVYAV
QNNMAFLALSNLDAAVYQVTYQLKIPCTALCMVLMLHRSLSRMQWFSIFMLCAGVTLIEQNPFVGFVAIAIAVLCSGFAG
VYFEKVLKSSDTSLWVRNIQ
MYISGIAVTLFGVYVTDGGRIAEKGFFFGYTPWVCFVVFLASVGGLYTSVVVKYTDNIMKGFSAAAAIVISTVASVLLFG
LQISKSSSLGGGAGPLPRGC
STASLFIMCKCTGIPLTVTFASGALMVCVSIYLYGLPKQDTSKLPRPGADIPELKQKLITV
>g2018.t2

MYLSEPVSAAFKLYCLSVMTLVAATYTVALRYTRTISSGDLYFSTTAVCITEVIKLILSLGMLARESGSPSRLKNSLVDH
VFRSPKELLKLSVPSVVYAV
QNNMAFLALSNLDAAVYQVTYQLKIPCTALCMVLMLHRSLSRMQWFSIFMLCAGVTLIEQNPFVGFVAIAIAVLCSGFAG
VYFEKVLKSSDTSLWVRNIQ
MYISGIAVTLFGVYVTDGGRIAEKGFFFGYTPWVCFVVFLASVGGLYTSVVVKYTDNIMKGFSAAAAIVISTVASVLLFG
LQITVTFASGALMVCVSIYL
YGLPKQDTSKLPRPGADIPELKQKLITV
>g2018.t3
MCLLQAAYKGDKNKRTTRGKPVSAAFKLYCLSVMTLVAATYTVALRYTRTISSGDLYFSTTAVCITEVIKLILSLGMLAR
ESGSPSRLKNSLVDHVFRSP
KELLKLSVPSVVYAVQNNMAFLALSNLDAAVYQVTYQLKIPCTALCMVLMLHRSLSRMQWFSIFMLCAGVTLIEQNPFVG
FVAIAIAVLCSGFAGVYFEK
VLKSSDTSLWVRNIQMYISGIAVTLFGVYVTDGGRIAEKGFFFGYTPWVCFVVFLASVGGLYTSVVVKYTDNIMKGFSAA
AAIVISTVASVLLFGLQITV
TFASGALMVCVSIYLYGLPKQDTSKLPRPGADIPELKQKLITV
>g2019.t1
LLQSNLDLQPRHLVNFLTKLSHLVASALRELPVRGSPHGVAQARLRLFGGARSVLASGMRLLGIQPVGKM
>g2020.t1
MGEVGMSGGDPGVGVSVFSSTTGESGGAARRGPGATSGPGPVSEGRGWPAWRWGPPDGGPADKG
>g2021.t1
LHPAPSSRQDHACGSEDVTVGVLEDRNPGPSSSGSSVQDVDPGKTDLLAGGSERSSLVPCGGVEDSGEHGAQKRGGVVQD
AGWAARSGTDTDLAVRGNGS
GSRPQLQPLMVETDIDSAGSAPSGHEGLPQGEANRTKHCENRWSGRCHIVETNIDEVDGASALEGSRTHPQALAGETKPR
RKALCGRVTGLLETDLDDIE
STVVGQGSGSNFEGLAGGAKLWLRSYEVMETDIDALEGAAEAPGGSGRVHELLETHIDEPGRGHTGVSQEKGGERSGGVT
LTRRPAGPIAVQSLGEVFET
SLDGSPWDSEDGDVDAVCLELESDGFAYWAEPVRVSSTSPAVSPAETATPGLDYGPVGLDCSSSNPDHLKTLFCVTVDGS
VGVETHSPPPSPSHSPPAHH
GQNLKRRRSVSVQMPSCPSTRTVSHIVKRKDVPYAPAAPKPSLMSTSLPCLDTSSPLRAVQSWTDLQLRRRGLNATLWGR
LQASQSQGSLRVRPAELRLS
PATVFSSDPTFRTTSNSRWSTDSMPESPASFQSNSVSLDTGLWPEDEDSDARGEEGTDRLEETLWEGSQGKQECCCCDRQ
HPGQTRLRKQPSTQSLPYSQ
DELESMIFCLRRFRVVLADIEEQVSEEQALVFSALSDSIREEACDITELRRAVKQEAGNLELQLKELAHHYDENFQMVC
>g2021.t2
LSAGPPSGGPQRQAGHPRPSETGPGPDVAPGPRLAAPPLSPVVEENTETPTPGSPPLMPTSPMTSVSPPSFVRPATHHSQ
RGYSEEEPHGENRGNGSIEA
EPHRGNASLEEEPHGPNRGNGSPEEEPHRGSGSLEEEQYRGSSSLEKEPYTLNNSLEEEPHGRSKQDEGSSPTGPSPPHN
TRLGVLELCSTQTKNSEEES
YQPGPSLHPAPSSRQDHACGSEDVTVGVLEDRNPGPSSSGSSVQDVDPGKTDLLAGGSERSSLVPCGGVEDSGEHGAQKR
GGVVQDAGWAARSGTDTDLA
VRGNGSGSRPQLQPLMVETDIDSAGSAPSGHEGLPQGEANRTKHCENRWSGRCHIVETNIDEVDGASALEGSRTHPQALA
GETKPRRKALCGRVTGLLET
DLDDIESTVVGQGSGSNFEGLAGGAKLWLRSYEVMETDIDALEGAAEAPGGSGRVHELLETHIDEPGRGHTGVSQEKGGE
RSGGVTLTRRPAGPIAVQSL
GEVFETSLDGSPWDSEDGDVDAVCLELESDGFAYWAEPVRVSSTSPAVSPAETATPGLDYGPVGLDCSSSNPDHLKTLFC
VTVDGSVGVETHSPPPSPSH
SPPAHHGQNLKRRRSVSVQMPSCPSTRTVSHIVKRKDVPYAPAAPKPSLMSTSLPCLDTSSPLRAVQSWTDLQLRRRGLN
ATLWGRLQASQSQGSLRVRP
AELRLSPATVFSSDPTFRTTSNSRWSTDSMPESPASFQSNSVSLDTGLWPEDEDSDARGEEGTDRLEETLWEGSQGKQEC
CCCDRQHPGQTRLRKQPSTQ
SLPYSQDELESMIFCLRRFRVVLADIEEQVSEEQALVFSALSDSIREEACDITELRRAVKQEAGNLELQLKELAHHYDEN
FQMVC
>g2021.t3
LSAGPPSGGPQRQAGHPRPSETGPGPDVAPGPRLAAPPLSPVVEENTETPTPGSPPLMPTSPMTSVSPPSFVRPATHHSQ
RGYSEEEPHGENRGNGSIEA
EPHRGNASLEEEPHGPNRGNGSPEEEPHRGSGSLEEEQYRGSSSLEKEPYTLNNSLEEEPHGRSKQDEGSSPTGPSPPHN

TRLGVLELCSTQTKNSEEES
YQPGPSLHPAPSSRQDHACGSEDVTVGVLEDRNPGPSSSGSSVQDVDPGKTDLLAGGSERSSLVPCGGVEDSGEHGAQKR
GGVVQDAGWAARSGTDTDLA
VRGNGSGSRPQLQPLMVETDIDSAGSAPSGHEGLPQGEANRTKHCENRWSGRCHIVETNIDEVDGASALEGSRTHPQALA
GETKPRRKALCGRVTGLLET
DLDDIESTVVGQGSGSNFEGLAGGAKLWLRSYEVMETDIDALEGAAEAPGGSGRVHELLETHIDEPGRGHTGVSQEKGGE
RSGGVTLTRRPAGPIAVQSL
GEVFETSLDGSPWDSEDGDVDAVCLELESDGFAYWAEPVRVSSTSPAVSPAETATPGLDYGPVGLDCSSSNPDHLKTLFC
VTVDGSVGVETHSPPPSPSH
SPPAHHGQNLKRRRSVSVQMPSCPSTRTVSHIVKRKDVPYAPAAPKPSLMSTSLPCLDTSSPLRAVQSWTDLQLRRRGLN
ATLWGRLQASQSQGSLRVRP
AELRLSPATVFSSDPTFRTTSNSRWSTDSMPESPASFQSNSVSLDTGLWPEDEDSDARGEEGTDRLEETLWEGSQGKQEC
CCCDRQHPGQTRLRKQPSTQ
SLPYSQDELESMIFCLRRFRVVLADIEEQVSEEQALVFSALSDSIREEACDITELRRAVKQEAGNLELQLKELAHHYDEN
FQMKMHRLLNEQSLLCSQLR
LLHPPVTTAPPAGGPAVRSVSTQCSLPPVPVAPPPGDPRASADLPPSPEGPGMDSPVMDSPTLGCSATKPDKMDFVGFLQ
RVRC
>g2022.t1
MSEVQATVEFAVELHKFYNVDLFQRGQIRASMKVPSRVPHRVEASLLHPAGSDLAFPASVQEDVISSKTYQILYKNEEVV
VNDVLLFKVMMLLDEKKVEE
SLNEMDFQLVLELYFTDGDYSSEDQSSLQSISSRVLRLHFSLQKGIHQQVNIMFDYFHLSVISMMVHASLVALHQPLISF
PRPVKTTWLNRNAPAQSKDS
VIPNLES
>g2022.t2
MKVPSRVPHRVEASLLHPAGSDLAFPASVQEDVISSKTYQILYKNEEVVVNDVLLFKVMMLLDEKKVEESLNEMDFQLVL
ELYFTDGDYSSEDQSSLQSI
SSRVLRLHFSLQKGIHQQVNIMFDYFHLSVISMMVHASLVALHQPLISFPRPVKTTWLNRNAPAQSKDSVIPNLES
>g2022.t3
MSEVQATVEFAVELHKFYNVDLFYQIRASMKVPSRVPHRVEASLLHPAGSDLAFPASVQEDVISSKTYQILYKNEEVVVN
DVLLFKVMMLLDEKKVEESL
NEMDFQLVLELYFTDGDYSSEDQSSLQSISSRVLRLHFSLQKGIHQQVNIMFDYFHLSVISMMVHASLVALHQPLISFPR
PVKTTWLNRNAPAQSKDSVI
PNLES
>g2023.t1
MSSFLIFARRTDIRMVSLDIPYFADVVLAVNSSMKNTIAIGVDPKEGKVYWSDTTLKKVSRANMDGSEHEEIIATGLMTT
DGLAVDAVGRKIYWTDTGTN
RIEVANMDGSMRKVLIWQNLDSPRAIALFNEMGYMYWTDWGENAKLERSAMDGSDRTVLISNNLGWPNGLAIDKAGSQLL
WADAHTERIEASDLSGLNRR
TLVSPVQHPYGMTLLGPHIYWTDWQSRSIQRADKTTGANAITVCSNLPGLMDIQAVDREKPLGFNKCGRRNGGCTHLCLP
RPNSTSCACPTGVLLKGDGR
SCDDSPETYLLFSNRVSVRRISLDTDDHTDVHVPVPELHNVISLDYDSVDGKLYYTDVTLDVIRRSNLDGTGMETVISQG
LKTTDGLAVDWVARNMYWTD
TGRNTIEVA
>g2023.t2
MSSFLIFARRTDIRMVSLDIPYFADVVLAVNSSMKNTIAIGVDPKEGKVYWSDTTLKKVSRANMDGSEHEEIIATGLMTT
DGLAVDAVGRKIYWTDTGTN
RIEVANMDGSMRKVLIWQNLDSPRAIALFNEMGYMYWTDWGENAKLERSAMDGSDRTVLISNNLGWPNGLAIDKAGSQLL
WADAHTERIEASDLSGLNRR
TLVSPVQHPYGMTLLGPHIYWTDWQSRSIQRADKTTGANAITVCSNLPGLMDIQAVDREKPLGTSPLGTQRRRVPALPVG
QRVAKDTGSTKLRNQRPLWW
ELRFLHEISLMMQRLLVCFCTPRDNLYPFGDIVTLSNLHPPPLSGFNKCGRRNGGCTHLCLPRPNSTSCACPTGVLLKGD
GRSCDDSPETYLLFSNRVSV
RRISLDTDDHTDVHVPVPELHNVISLDYDSVDGKLYYTDVTLDVIRRSNLDGTGMETVISQGLKTTDGLAVDWVARNMYW
TDTGRNTIEVA
>g2023.t3
MSSFLIFARRTDIRMVSLDIPYFADVVLAVNSSMKNTIAIGVDPKEGKVYWSDTTLKKVSRANMDGSEHEEIIATGLMTT

DGLAVDAVGRKIYWTDTGTN
RIEVANMDGSMRKVLIWQNLDSPRAIALFNEMGYMYWTDWGENAKLERSAMDGSDRTVLISNNLGWPNGLAIDKAGSQLL
WADAHTERIEASDLSGLNRR
TLVSPVQHPYGMTLLGPHIYWTDWQSRSIQRADKTTGANAITVCSNLPGLMDIQAVDREKPLGFNKCGRRNGGCTHLCLP
RPNSTSCACPTGVLLKGDGR
SCDDSPETYLLFSNRVSVRRISLDTDDHTDVHVPVPELHNVISLDYDSVDGKLYYTDVTLDVIRRSNLDGTGMETVISQG
LKTTDGLAVDWVARNMYWTD
TGRNTIEVA
>g2024.t1
MDPVVLSYQDSLLRRSDVTLLEGPHWLNDQVIGFAFEYFATERFKNLGETIAFISPEVTQFIKCASCPEELAIFLEPLDL
SSRRLVFLAVNDNSNQTAGG
SHWSLLVYHHAASHFAHYDSQSGGNSLHARRIAGKLEPFLGAGAGGKVRFVEEPCPSQQNSYDCGMYVICNAEALCEGAD
GASSPARLPVQSITPAFITQ
RRRDFCRLIQRLAKD
>g2025.t1
QCLEMTTKRKLIGRLVPCRCFRGEEEVISVLDYSHCSLQQVPKEIFSFERTLEELYLDANQIEELPKW
>g2026.t1
MCHLQKFSPDSSIVGCFSEDEEEEYRGYCRGSTTVGAHWSLLWHAGEEESGRDQLTKLEASSVVRPNLDSLEASVKKEMR
DQDHPGRASFPPPSPSLMSC
DGWGAPSATGSSPQVQGQGFQAVQQHLIPSGYPLFGVSLYRIF
>g2027.t1
EDRDSRFLSSRMAIVAAFRSWSGIINLCKAGNSGIQSLIGLLCIPNMEVRKGLLEVLYEIFRLPLPIVTQDFTEALLSVD
PSRGQDSWRLSDGFVAAEAK
VLLPHRARSRPDLMDNYLALVLSAFITGGLLEGLVEVVTSSEDQLAVRATILLGELLHMANTILPHSHSHHLHCLPTLIN
MAASFDIAQEKRLRASGAVN
HLKRFHEKKKRGLKPYSLYLDHIIRKSVSAHGRRDTHTRAHRDIYAIKDTEEALMTNLRDSHILNHKHNLEWNWLLIATI
LKWPNVNLRNNKDEQMHKFV
RRLLYFYKPSSKLYAGLELDHSRARHLTVVGCQFIEFLMESGE
>g2027.t2
EDRDSRFLSSRMAIVAAFRSWSGIINLCKAGNSGIQSLIGLLCIPNMEVRKGLLEVLYEIFRLPLPIVTQDFTEALLSVD
PSRGQDSWRLSDGFVAAEAK
VLLPHRARSRPDLMDNYLALVLSAFITGGLLEGLVEVVTSSEDQLAVRATILLGELLHMANTILPHSHSHHLHCLPTLIN
MAASFDIAQEKRL
>g2027.t3
VVGAFSPAAAAPRVRCVPREDRDSRFLSSRMAIVAAFRSWSGIINLCKAGNSGIQSLIGLLCIPNMEVRKGLLEVLYEIF
RLPLPIVTQDFTEALLSVDP
SRGQDSWRLSDGFVAAEAKVLLPHRARSRPDLMDNYLALVLSAFITGGLLEGLVEVVTSSEDQLAVRATILLGELLHMAN
TILPHSHSHHLHCLPTLINM
AASFDIAQEKRLRASGAVNHLKRFHEKKKRGLKPYSLYLDHIIRKSVSAHGRRDTHTRAHRDIYAIKDTEEALMTNLRDS
HILNHKHNLEWNWLLIATIL
KWPNVNLRNNKDEQMHKFVRRLLYFYKPSSKLYAGLELDHSRARHLTVVGCQFIEFLMESGE
>g2027.t4
MAASFDIAQEKRLRASGAVNHLKRFHEKKKRGLKPYSLYLDHIIRKSVSAHGRRDTHTRAHRDIYAIKDTEEALMTNLRD
SHILNHKHNLEWNWLLIATI
LKWPNVNLRNNKDEQMHKFVRRLLYFYKPSSKLYAGLELDHSRARHLTVVGCQFIEFLMESGE
>g2028.t1
MGDCPHRDLLVSVEGITVSASPTARNLGVIVDNQLSCTANITAVTRSCRFALYNIRRIRPFLTREATQLLTQALVISRLD
YCNSLLAGLPATAIKPLQRI
QNAAARLVFNRNKFSPVTPLFQDLHWLPVVARIKFKTMLLAYKAVNGTAPTYLQTLVKPHAPARA
>g2029.t1
MELALTQESLSSKEALLSDLEAKLQESTQSCQKKSESVLTCLSVTVSTSCCSRLSLEELRQAVQGLRCSEEKLLSQKSLL
EEKLVSPSNSGPGNGDAPPL
PPVVPPPTLNYEDDAQSVGSMSCHKS
>g2029.t2
MVTMLMSTEQLTTVSLIMPLKHTILQSDSDSAAAEDVKLAIASNFNKRYPTHFSFLMTPEPEEADVEFDASLLEDTENMA
VNEILWNNLTAENLQAL

>g2029.t3
LIMPLKHTILQSDSDSAAAEDVKLAIASNFNKRYPTHFSFLMTPEPEEADVEFDASLLEDTENMAVNEILWNNLTAENLQ
AL
>g2029.t4
MELALTQESLSSKEALLSDLEAKLQESTQSCQKKSESVLTCLSVTVSTSCCSRLSLEELRQAVQGLRCSEEKLLSQKSLL
EEKLVSPSNSGPGNGDAPPL
PPVVPPPTLNYEDDAQSVGSMGV
>g2029.t5
MVTMLMSTEQLTTVSLIMPLKHTILQSDSDSAAAEDVKLAIASNFNKRYPTHFSFLMTPEPEEADVEFDASLLEDTENMA
VNEILWNNLTAENLQALLST
ATMELALTQESLSSKEALLSDLEAKLQESTQSCQKKSESVLTCLSVTVSTSCCSRLSLEELRQAVQGLRCSEEKLLSQKS
LLEEKLVSPSNSGPGNGDAP
PLPPVVPPPTLNYEDDAQSVGSMSCHKS
>g2030.t1
VVGTVRGSMAALGRHGRLGRLARCILHDAPTGLLCDLRRLGRMGRIIGRNFVEVFHHKMQRNELILKDELRLLLHLCQSE
EDVVTASHAIRRYHVENKNF
SFGEFKFGPLFMRLCYEMDLEEMAAATILDKNLRGFFNDVTSFNIAVDMLFMKGRYNDALEVLRDMKSQRVPFNKDTYML
AFAICYKLNTPESFKTCSSL
IEEGYAKGHDISRHAYSFAVALALRQVVLLAMCGAVPEAISVLASAMSPETPPFVKKPEFALEVIELLLLRCEAGPHQRS
VEQMVAALQEGGQVTQQSLD
DMLCHTPAGRRRSLSMGVDRKETHPRLTFCWGDRTEQDVGRLFWNTGVTHFMITGSGVLGWSTMSAEEAPAPTKTVPEDD
GMTWWYRWLCKIAGVIGGIX
VWNCVTVNPLNIAAGVWMVLNAFVLFLCEVPFCCQFIEFANAVAARADKLKPWQKAFFYCGMYVLELADLPSLCPWFCSM
AIFPVFLSFSFTTLFGNGIA
FATGVLYGLASLGKK
>g2030.t2
VVGTVRGSMAALGRHGRLGRLARCILHDAPTGLLCDLRRLGRMGRIIGRNFVEVFHHKMQRNELILKDELRLLLHLCQSE
EDVVTASHAIRRYHVENKNF
SFGEFKFGPLFMRLCYEMDLEEMAAATILDKNLRGFFNDVTSFNIAVDMLFMKGRYNDALEVLRDMKSQRVPFNKDTYML
AFAICYKLNTPESFKTCSSL
IEEGYAKGHDISRHAYSFAVALALRQVVLLAMCGAVPEAISVLASAMSPETPPFVKKPEFALEVIELLLLRCEAGPHQRS
VEQMVAALQEGGQVTQQSLD
DMLCHTPAGRRRSLSMGVDRKETHPRLTFCWGDRTEQDVGRLFWNTGVTHFMITGSGVLGWSTMSAEEAPAPTKTVPEDD
GMTWWYRWLCKIAGVIGGIX
VWNCVTVNPLNIAAGVWMVLNAFVLFLCEVPFCCQFIEFANAVAARADKLKPWQKAFFYCGMAIFPVFLSFSFTTLFGNG
IAFATGVLYGLASLGKK
>g2030.t3
VVGTVRGSMAALGRHGRLGRLARCILHDAPTGLLCDLRRLGRMGRIIGRNFVEVFHHKMQRNELILKDELRLLLHLCQSE
EDVVTASHAIRRYHVENKNF
SFGEFKFGPLFMRLCYEMDLEEMAAATILDKNLRGFFNDVTSFNIAVDMLFMKGRYNDALEVLRDMKSQRVPFNKDTYML
AFAICYKLNTPESFKTCSSL
IEEGYAKGHDISRHAYSFAVALALRQVVLLAMCGAVPEAISVLASAMSPETPPFVKKPEFALEVVTLLLTAFQIELLLLR
CEAGPHQRSVEQMVAALQEG
GQVTQQSLDDMLCHTPAGRRRSLSMGVDRKETHPRLTFCWGDRTEQDVGRLFWNTGVTHFMITGSGVLGWSTMSAEEAPA
PTKTVPEDDGMTWWYRWLCK
IAGVIGGIXVWNCVTVNPLNIAAGVWMVLNAFVLFLCEVPFCCQFIEFANAVAARADKLKPWQKAFFYCGMYVLELADLP
SLCPWFCSMAIFPVFLSFSF
TTLFGNGIAFATGVLYGLASLGKK
>g2031.t1
MAVVSDLVSRHHLHRQTLCPSNGTRGVVSSGSYRNVRESYDRGGRCSGIVSRGEDGKMYKRVKVVKNGLGTHGERCQQHL
TVHLTVSLAKQNSLLHQGIE
VEDTEP
>g2031.t2
MEARGRSATMAVVSDLVSRHHLHRQTLCPSNGTRGVVSSGSYRNVRESYDRGGRCSGIVSRGEDGKMYKRVKVVKNGLGT
HGERCQQHLTVHLTVSLAKQ
NSLLHQGIEVEDTEP

>g2032.t1
ARIRVVSVEGLARDDADQRPVSCSLSLCLVPGKLQLQKSATIRNCSNPVFNEDFFFTDLSEESLAELELRLKVMDRSASG
TLRRGAVRAVVTKPLCQLFP
PEG
>g2033.t1
MVFESVVVDVLNRFLGDYVVNLDCSQLKLGIWGGDAILRNLEIKENALSQLDIPFKVRAGHIGRLELKIPWKNLYTQSVE
ATLDGVYLLVVPTASIKYDA
EKEEKQLQEARQRELQRIEETKQKAAEQDNPKVEKQDTFVEKLVTQVIKNVQVKISNIHVRYEDDVTNPNCPLSFGVSLK
NLSLHTADENWNPCLLDEKA
KLFFKLVQLDSLFAYWNVNSLLFSNHGPDEALLCLQKSIEIKSSLHVSHDFIFRPITADAKLRMNPRSDVDFSSPKVDLV
VNLREVAFELNREQYISILE
LLGSVDLMSRNLPYRKYRPDVHVHSNTHIWWRYVITGVLEVDVKPRLHMWSWQHIQRHRSTVKRYRDLYKLKITSRKPSE
ELLRDLEGPEKTLDIFNITL
ARQQAEMEASKAGLCIYRPGMKVEEEETQGWLSWAWNWGGESPEQPKEVETGGFDRIMTPGEKDKLYTAIGYSETAVNPN
LPKNFEDMKVNFHLEKLSLS
IKEKKDKDEILKLTVETLNSMLTQRPGAQAIKLKAKLSLFEVTGLAGNRPAPTLLSSRKASMATGTPLLSFLFETNPIDQ
SADQRLHVESQPLEIIYDAA
TVNSMSAFFMPPDDLQLDELTNATLMKLEQFRDRTSTGLMYVIETQKVLDLNVNLMASYVIVPQTGFYDATQNILLLDLG
HFKMTSQSKKDLPQLSVGSS
TIEDIMSRAYDSFNIQLSSLQFLYSKPDCDWKKARKQKRSSLHILEPVDLKVIFSRAMVVTDSRMAKFKISAELPLLSLR
ISDDRVRGVLELVDSIPLPK
SRPAPQNTTASKPRQSYTPQLTPRRPPAVADLRSSLLFDSCETISITSLGSEEDLFYDAPTSPIGDKQFFSDMPGPIRSA
DSVRRRGQVKKDPQKNMTEL
LLRFEINEMSVQLCRMAKGREVLVLHLDIEGLGTELKMCTFDMTSNTFLQEICLKCPEYMGMYCLGGYSQCCLYE
>g2033.t2
MVFESVVVDVLNRFLGDYVVNLDCSQLKLGIWGGDAILRNLEIKENALSQLDIPFKVRAGHIGRLELKIPWKNLYTQSVE
ATLDGVYLLVVPTASIKYDA
EKEEKQLQEARQRELQRIEETKQKAAEQDNPKVEKQDTFVEKLVTQVIKNVQVKISNIHVRYEDDVTNPNCPLSFGVSLK
NLSLHTADENWNPCLLDEKA
KLFFKLVQLDSLFAYWNVNSLLFSNHGPDEALLCLQKSIEIKSSLHVSHDFIFRPITADAKLRMNPRSDVDFSSPKVDLV
VNLREVAFELNREQYISILE
LLGSVDLMSRNLPYRKYRPDVHVHSNTHIWWRYVITGVLEVDVKPRLHMWSWQHIQRHRSTVKRYRDLYKLKITSRKPSE
ELLRDLEGPEKTLDIFNITL
ARQQAEMEASKAGLCIYRPGMKVEEEETQGWLSWAWNWGGESPEQPKEVETGGFDRIMTPGEKDKLYTAIGYSETAVNPN
LPKNFEDMKVNFHLEKLSLS
IKEKKDKDEILKLTVETLNSMLTQRPGAQAIKLKAKLSLFEVTGLAGNRPAPTLLSSRKASMATGTPLLSFLFETNPIDQ
SADQRLHVESQPLEIIYDAA
TVNSMSAFFMPPDDLQLDELTNATLMKLEQFRDRTSTGLMYVIETQKVLDLNVNLMASYVIVPQTGFYDATQNILLLDLG
HFKMTSQSKKDLPQLSVGSS
TIEDIMSRAYDSFNIQLSSLQFLYSKPDCDWKKARKQKRSSLHILEPVDLKVIFSRAMVVTDSRMAKFKISAELPLLSLR
ISDDRVRGVLELVDSIPLPK
SRPAPQNTTASKPRQSYTPQLTPRRPPAVADLRSSLLFDSCETISITSLGSEEDLFYDAPTSPIGDKQFFSDMPGPIRSA
DSVRRRGQVKKDPQKNMTEL
LLRFEINEMSVQLCRMAKGREVLVLHLDIEGLGTELKMCTFDMTSNTFLQEICLKCPEYM
>g2033.t3
MVFESVVVDVLNRFLGDYVVNLDCSQLKLGIWGGDAILRNLEIKENALSQLDIPFKVRAGHIGRLELKIPWKNLYTQSVE
ATLDGVYLLVVPTASIKYDA
EKEEKQLQEARQRELQRIEETKQKAAEQDNPKVEKQDTFVEKLVTQVIKNVQVKISNIHVRYEDDVTNPNCPLSFGVSLK
NLSLHTADENWNPCLLDEKA
KLFFKLVQLDSLFAYWNVNSLLFSNHGPDEALLCLQKSIEIKSSLHVSHDFIFRPITADAKLRMNPRSDVDFSSPKVDLV
VNLREVAFELNREQYISILE
LLGSVDLMSRNLPYRKYRPDVHVHSNTHIWWRYVITGVLEVDVKPRLHMWSWQHIQRHRSTVKRYRDLYKLKITSRKPSE
ELLRDLEVGGPSLTPQGSDS
LGVSTGSLTIDVHGGKHSSSSSRIGPEKTLDIFNITLARQQAEMEASKAGLCIYRPGMKVEEEETQGWLSWAWNWGGESP
EQPKEVETGGFDRIMTPGEK
DKLYTAIGYSETAVNPNLPKNFEDMKVNFHLEKLSLSIKEKKDKDEILKLTVETLNSMLTQRPGAQAIKLKAKLSLFEVT

GLAGNRPAPTLLSSRKASMA
TGTPLLSFLFETNPIDQSADQRLHVESQPLEIIYDAATVNSMSAFFMPPDDLQLDELTNATLMKLEQFRDRTSTGLMYVI
ETQKVLDLNVNLMASYVIVP
QTGFYDATQNILLLDLGHFKMTSQSKKDLPQLSVGSSTIEDIMSRAYDSFNIQLSSLQFLYSKPDCDWKKARKQKRSSLH
ILEPVDLKVIFSRAMVVTDS
RMAKFKISAELPLLSLRISDDRVRGVLELVDSIPLPKSRPAPQNTTASKPRQSYTPQLTPRRPPAVADLRSSLLFDSCET
ISITSLGSEEDLFYDAPTSP
IGDKQFFSDMPGPIRSADSVRRRGQVKKDPQKNMTELLLRFEINEMSVQLCRMAKGREVLVLHLDIEGLGTELKMCTFDM
TSNTFLQEICLKCPEYMGMY
CLGGYSQCCLYE
>g2034.t1
MSRLDKRRKGVFGPPMGKKCVIFIDDMNMPALEQFGAQPPVELLRQYFDHGTWYDLKDTSKITLVDLQLIAAMGPPGGGR
NAVTPRFLRHFNICSINTFS
DDTMVRIFSSVVSFYLKNNDFPPEYFTIGNQIVAATLEKAMQNLLPTPAKSHYTFNLRDFSRVVQGCLLLRKESLENKGT
VTRLFVHEVLRVFYDRLVDA
ADRAWLYQLVDHVVKQHFRESFEQVFGHLKKGKQLVEEDIHSLLFGDYMNPDLEEEDRLYAEVPSMERFGQVVELCLEEY
NQTHKNRMDLVIFRYVLEHL
SRISRVLKQPGGNALLVGVGGSGRQSITRLATSMARMAVFQPEISRSYDLNEWRDDLKLLLKNTGVKGQKTVFLLTDAQI
KQEAFLEDVDSVLNTGEVPN
LFAVDEKQEIMEAVRPIAQAGDSHVEFSPLALFAFFVSRCRENLHIVVAFSPIGNAFRNRLRQFPSLINCCTIDWFQPWP
EEALERVADSFLETLELSEH
ERLHVMPICKTFHTSAIQLSQKFLSELGRHNYVTPTSYLELIAAFRQLLTQKRDTVMKAKKRYTNGLDKLAFAESQVGEM
KMELIELQPKLEQAKIDNTK
MMKVIEVESVQVEAKSKLVRVDEEAATLKANAAQALKDECESDLAEAIPALEAALSALDTLKPSDVTIVKSMKNPPSGVK
LVMSAVCVMKDIKPNKMADP
AGTGQKILDYWGPSKKLLGDMNFLRDLRVYDKDNIPVPVMQKIRSEYMTNPDFDPSVVAKASSAAEGLCKWIKAMEVYDR
VAKVVAPKKANLAEAQQSLA
TTMALLDQKRGELKEVEDRLAALQKTFDEKTEEKAQLEFQVDLCARKLERAEKLIGGLGGEKTRWSKAADELQHTYDNLT
GDVLISAGVIAYLGAFTSGF
RQDCAKTWTKLCQIRAWNIAGLPTDSFSVDNGVIVSNSRRWPLMIDPQGQANKWVKNSEKDNNLSVIKLTDADYMRTLEN
CIQFGTPLLVENVREELDPS
LEPLLLKQTFKQGGVDCIRLGESVIEYSHDFRFYITTKLRNPHYLPELATKVSLLNFMITPEGLEDQLLGIMVAKERPEL
EEERNALILQSAANKKQLKE
IEDKILETLQSSEGNILEDESAIQVLDSAKLMSNEISKKQQIAEKTELQIAESREGYRAIAKHSSILFFSIADLANIDPM
YQYSLTWFVHLYVNSIQDSN
KSKILEKRLRYLIDHFTYNLYCNVCRSLFEKDKLLFSFLLCTNLLLAKKEIEYGDLMFLLTGGVGLQNTTPNPDPSWLQD
KSWDEICRASEQPGLHGLKE
AFLSNPDDFKSIYDSKEPYSACLPRPWADRLNGLQKMIISRCLRPDKITPAVSNYVTRNLGKVFVEPPPFDLSKSYLDSS
CCVPLVFVLSPGADPMASLL
KFANDKNMGGGKFQAISLGQGQGPIAAKMIRTSMKEGTWVCLQNCHLAVSWMSTLEKLCEDFSPDTCHPDFRLWLTSYPS
PKWWNELPANVRTAESLPIF
RKRLKTHLFRVHLNPT
>g2034.t2
MSRLDKRRKGVFGPPMGKKCVIFIDDMNMPALEQFGAQPPVELLRQYFDHGTWYDLKDTSKITLVDLQLIAAMGPPGGGR
NAVTPRFLRHFNICSINTFS
DDTMVRIFSSVVSFYLKNNDFPPEYFTIGNQIVAATLEKAMQNLLPTPAKSHYTFNLRDFSRVVQGCLLLRKESLENKGT
VTRLFVHEVLRVFYDRLVDA
ADRAWLYQLVDHVVKQHFRESFEQVFGHLKKGKQLVEEDIHSLLFGDYMNPDLEEEDRLYAEVPSMERFGQVVELCLEEY
NQTHKNRMDLVIFRYVLEHL
SRISRVLKQPGGNALLVGVGGSGRQSITRLATSMARMAVFQPEISRSYDLNEWRDDLKLLLKNTGVKGQKTVFLLTDAQI
KQEAFLEDVDSVLNTGEVPN
LFAVDEKQEIMEAVRPIAQAGDSHVEFSPLALFAFFVSRCRENLHIVVAFSPIGNAFRNRLRQFPSLINCCTIDWFQPWP
EEALERVADSFLETLELSEH
ERLHVMPICKTFHTSAIQLSQKFLSELGRHNYVTPTSYLELIAAFRQLLTQKRDTVMKAKKRYTNGLDKLAFAESQVGEM
KMELIELQPKLEQAKIDNTK
MMKVIEVESVQVEAKSKLVRVDEEAATLKANAAQALKDECESDLAEAIPALEAALSALDTLKPSDVTIVKSMKNPPSGVK

LVMSAVCVMKDIKPNKMADP
AGTGQKILDYWGPSKKLLGDMNFLRDLRVYDKDNIPVPVMQKIRSEYMTNPDFDPSVVAKASSAAEGLCKWIKAMEVYDR
VAKVVAPKKANLAEAQQSLA
TTMALLDQKRGELKEVEDRLAALQKTFDEKTEEKAQLEFQVDLCARKLERAEKLIGGLGGEKTRWSKAADELQHTYDNLT
GDVLISAGVIAYLGAFTSGF
RQDCAKTWTKLCQTLGDPIQIRAWNIAGLPTDSFSVDNGVIVSNSRRWPLMIDPQGQANKWVKNSEKDNNLSVIKLTDAD
YMRTLENCIQFGTPLLVENV
REELDPSLEPLLLKQTFKQGGVDCIRLGESVIEYSHDFRFYITTKLRNPHYLPELATKVSLLNFMITPEGLEDQLLGIMV
AKERPELEEERNALILQSAA
NKKQLKEIEDKILETLQSSEGNILEDESAIQVLDSAKLMSNEISKKQQIAEKTELQIAESREGYRAIAKHSSILFFSIAD
LANIDPMYQYSLTWFVHLYV
NSIQDSNKSKILEKRLRYLIDHFTYNLYCNVCRSLFEKDKLLFSFLLCTNLLLAKKEIEYGDLMFLLTGGVGLQNTTPNP
DPSWLQDKSWDEICRASEQP
GLHGLKEAFLSNPDDFKSIYDSKEPYSACLPRPWADRLNGLQKMIISRCLRPDKITPAVSNYVTRNLGKVFVEPPPFDLS
KSYLDSSCCVPLVFVLSPGA
DPMASLLKFANDKNMGGGKFQAISLGQGQGPIAAKMIRTSMKEGTWVCLQNCHLAVSWMSTLEKLCEDFSPDTCHPDFRL
WLTSYPSPKWWNELPANVRT
AESLPIFRKRLKTHLFRVHLNPT
>g2034.t3
MSRLDKRRKGVFGPPMGKKCVIFIDDMNMPALEQFGAQPPVELLRQYFDHGTWYDLKDTSKITLVDLQLIAAMGPPGGGR
NAVTPRFLRHFNICSINTFS
DDTMVRIFSSVVSFYLKNNDFPPEYFTIGNQIVAATLEKAMQNLLPTPAKSHYTFNLRDFSRVVQGCLLLRKESLENKGT
VTRLFVHEVLRVFYDRLVDA
ADRAWLYQLVDHVVKQHFRESFEQVFGHLKKGKQLVEEDIHSLLFGDYMNPDLEEEDRLYAEVPSMERFGQVVELCLEEY
NQTHKNRMDLVIFRYVLEHL
SRISRVLKQPGGNALLVGVGGSGRQSITRLATSMARMAVFQPEISRSYDLNEWRDDLKLLLKNTGVKGQKTVFLLTDAQI
KQEAFLEDVDSVLNTGEVPN
LFAVDEKQEIMEAVRPIAQAGDSHVEFSPLALFAFFVSRCRENLHIVVAFSPIGNAFRNRLRQFPSLINCCTIDWFQPWP
EEALERVADSFLETLELSEH
ERLHVMPICKTFHTSAIQLSQKFLSELGRHNYVTPTSYLELIAAFRQLLTQKRDTVMKAKKRYTNGLDKLAFAESQVGEM
KMELIELQPKLEQAKIDNTK
MMKVIEVESVQVEAKSKLVRVDEEAATLKANAAQALKDECESDLAEAIPALEAALSALDTLKPSDVTIVKSMKNPPSGVK
LVMSAVCVMKDIKPNKMADP
AGTGQKILDYWGPSKKLLGDMNFLRDLRVYDKDNIPVPVMQKIRSEYMTNPDFDPSVVAKASSAAEGLCKWIKAMEVYDR
VAKVVAPKKANLAEAQQSLA
TTMALLDQKRGELKEVEDRLAALQKTFDEKTEEKAQLEFQVDLCARKLERAEKLIGGLGGEKTRWSKAADELQHTYDNLT
GDVLISAGVIAYLGAFTSGF
RQDCAKTWTKLCQIRAWNIAGLPTDSFSVDNGVIVSNSRRWPLMIDPQGQANKWVKNSEKDNNLSVIKLTDADYMRTLEN
CIQFGTPLLVENVREELDPS
LEPLLLKQTFKQGGVDCIRLGESVIEYSHDFRFYITTKLRNPHYLPELATKVSLLNFMITPEGLEDQLLGIMVAKERPEL
EEERNALILQSAANKKQLKE
IEDKILETLQSSEGNILEDESAIQVLDSAKLMSNEISKKQQIAEKTELQIAESREGYRAIAKHSSILFFSIADLANIDPM
YQYSLTWFVHLYVNSIQDSN
KSKILEKRLRYLIDHFTYNLYCNVCRSLFEKDKLLFSFLLCTNLLLAKKEIEYGDLMFLLTGGVGLQNTTPNPDPSWLQD
KSWDEICRASEQPGLHGLKE
AFLSNPDDFKSIYDSKEPYSACLPRPWADRLNGLQKMIISRCLRPDKITPAVSNYVTRNLGKVFVEPPPFDLSKSYLDSS
CCVPLVFVLSPGADPMASLL
KFANDKNMGGGKFQAISLGQGQGPIAAKMIRTSMKEGTWVCLQNCHLAVSWMSTLEKLCEDFSPDTCHPDFRLWLTSYPS
PKVFLILLFLSCSFGCDTSS
LG
>g2035.t1
VCLQIHRHHLGLGWPDRDCAFGSRPVEVHVDWTADQDYRLPSRDLFASLSQLDGLRFSRLQDLRLDT
>g2036.t1
LNPQNRVEVDSRGSTVENLQRKIRRLEAELEKRPLSPERRSTTTTKEDNKTKEEVVRWEEGKKWQTRMEKLRNGLKEKEG
EVEALSKQLCTLKELYARLE
QENKGLLRKTKGRGVTADQVVGVRGMETEKELEELRRRNGELETQILTIRSQQALPRDAAVDDLTQRNHYLEERLHAMEA

QMSKEPPSRPSSSVTRMAVW
ISTWWLVEDAEAAAEPSDADTDINKEEEQQEEVGGGEDESPKEQENLPWCTQLDASNPIGDSSVSPKSASTEEKDLEGEA
DGEAADTGLGPLGETECGAE
GNPGVVDKPEETNPGQTLVEMEPDEEPCTAQADANPQDEPSSNTVTDIVIAKFAGSSYSRPGSEVFSHGGIRKRETRSDK
RLKTSGRGTGTPSQKDQDVQ
KENLKLSSENLELRLQLAQANVELPGLKREVADLKELCRIMKKEKAEAEKKLSHLRGAGSSGKTVGDLERTVGLMRKVVE
RVQKENDALKRTSAVADPDT
LAALQQQHETLKEKEATERLRVTKSGLEVSNEKLEVELEETKCRLHAALSRAIPEGADSKTWKASVVTRMYENKMKELER
EGEKERAERAELKRKLKEAS
EREEKALRSVHKLEDQVKNVMTADGNATKDAAALRRANEELDQERCALRQKLQELREGSGQASTLPGSAELRNLLKASET
EKKRLQVEVKKLRKDLENFD
PTFFEEIEDLKFNYNVEVKKNILLEEELRKVCGQFGVPANIPSVSIS
>g2036.t2
LNPQNRVEVDSRGSTVENLQRKIRRLEAELEKRPLSPERRSTTTTKEDNKTKEEVVRWEEGKKWQTRMEKLRNGLKEKEG
EVEALSKQLCTLKELYARLE
QENKGLLRKTKGRGVTADQVVGVRGMETEKELEELRRRNGELETQILTIRSQQALPRDAAVDDLTQRNHYLEERLHAMEA
QMSKEPPSRPSSSVTRMAVW
ISTWWLVEDAEAAAEPSDADTDINKEEEQQEEVGGGEDESPKEQENLPWCTQLDASNPIGDSSVSPKSASTEEKDLEGEA
DGEAADTGLGPLGETECGAE
GNPGVVDKPEETNPGQTLVEMEPDEEPCTAQADANPQDEPSSNTVTDIVIAKFAGSSYSRPGSEVFSHGGIRKRETRSDK
RLKTSGRGTGTPSQKDQDVQ
KENLKLSSENLELRLQLAQANVELPGLKREVADLKELCRIMKKEKAEAEKKLSHLRGAGSSGKTVGDLERTVGLMRKVVE
RVQKENDALKRTSAVADPDT
LAALQQQHETLKADYEKMKQEAEEELSARLESKTRGMEKIVTENERLRREVKREKEATERLRVTKSGLEVSNEKLEVELE
ETKCRLHAALSRAIPEGADS
KTWKASVVTRMYENKMKELEREGEKERAERAELKRKLKEASEREEKALRSVHKLEDQVKNVMTADGNATKDAAALRRANE
ELDQERCALRQKLQELREGS
GQASTLPGSAELRNLLKASETEKKRLQVEVKKLRKDLENFDPTFFEEIEDLKFNYNVEVKKNILLEEELRKVCGQFGVPA
NIPSVSIS
>g2036.t3
LNPQNRVEVDSRGSTVENLQRKIRRLEAELEKRPLSPERRSTTTTKEDNKTKEEVVRWEEGKKWQTRMEKLRNGLKEKEG
EVEALSKQLCTLKELYARLE
QENKGLLRKTKGRGVTADQVVGVRGMETEKELEELRRRNGELETQILTIRSQQALPRDAAVDDLTQRNHYLEERLHAMEA
QMSKEPPSRPSSSVTRMAVW
ISTWWLVEAVRVKRWHFGKGTDQENPETHNDAVLLPRDSHPLLGAEDLVWQEQSFADLPQDAEAAAEPSDADTDINKEEE
QQEEVGGGEDESPKEQENLP
WCTQLDASNPIGDSSVSPKSASTEEKDLEGEADGEAADTGLGPLGETECGAEGNPGVVDKPEETNPGQTLVEMEPDEEPC
TAQADANPQDEPSSNTVTDI
VIAKFAGSSYSRPGSEVFSHGGIRKRETRSDKRLKTSGRGTGTPSQKDQDVQKENLKLSSENLELRLQLAQANVELPGLK
REVADLKELCRIMKKEKAEA
EKKLSHLRGAGSSGKTVGDLERTVGLMRKVVERVQKENDALKRTSAVADPDTLAALQQQHETLKEKEATERLRVTKSGLE
VSNEKLEVELEETKCRLHAA
LSRAIPEGADSKTWKASVVTRMYENKMKELEREGEKERAERAELKRKLKEASEREEKALRSVHKLEDQVKNVMTADGNAT
KDAAALRRANEELDQERCAL
RQKLQELREGSGQASTLPGSAELRNLLKASETEKKRLQVEVKKLRKDLENFDPTFFEEIEDLKFNYNVEVKKNILLEEEL
RKVCGQFGVPANIPSVSIS
>g2037.t1
MTDSKAGCLGKTLLRRVPLDDRQVDSQFARCLSTLDLISLGVGATLGAGVYVLCGEVAKEKAGPAIVLCFLIAAVSSILA
GLCYAELGARVPRAGSAYVY
CYVTVGEIWAFIVGWVLILSYIIATASVARAWSLTFDNLVGLAISQWLKSSIPINLPGNIFSEYPDLLAVLLIVLLTALL
SIGVSESALMGKIFTGINVT
VLGFVLASSLVTGDRANWSLTLDHFINATNVTDPELIQKEFGLGGFAPFGVRGILSGAATCFYAFVGFDCIAATAEEALN
PKQSIPISIVASLLCCFVAY
SGVSTALTLMMPYYLLDAQTPLPKAFDFVNQAHGRYLVAVGSLCALSTSLLGCMFPLPRVIHAMASDGLIFRFLSRVWEK
TKTPLRATVISGIAAAPMAL
LFDLDVLVDLMSIGTLLAYTLVALCVIVLRYQPSFPNGGLKTLTLAEAGSDQKIHHEGQEPIEELVEQFTPMQLLRPRTD

IPTNLSGSIVYGTTAVLFLG
VCWDELVMGQYSVVANCAILTLLSFLCLVIIWRQPQTSEISSFKGSGPVSTASSKSPLSQHLKPRSSKTMTSQVDLDM
>g2037.t2
MTDSKAGCLGKTLLRRVPLDDRQVDSQFARCLSTLDLISLGVGATLGAGVYVLCGEVAKEKAGPAIVLCFLIAAVSSILA
GLCYAELGARVPRAGSAYVY
CYVTVGEIWAFIVGWVLILSYIIATASVARAWSLTFDNLVGLAISQWLKSSIPINLPGNIFSEYPDLLAVLLIVLLTALL
SIGVSESALMGKIFTGINVT
VLGFVLASSLVTGDRANWSLTLDHFINATNVTDPELIQKEFGLGGFAPFGVRGILSGAATCFYAFVGFDCIAATAEEALN
PKQSIPISIVASLLCCFVAY
SGVSTALTLMMPYYLLDAQTPLPKAFDFVNQAHGRYLVAVGSLCALSTSLLGCMFPLPRVIHAMASDGLIFRFLSRVWEK
TKTPLRATVISGIAAAPMAL
LFDLDVLVDLMSIGTLLAYTLVALCVIVLRYQPSFPNGGLKTLTLAEAGSDQKIHHEGQEPIEELVEQFTPMQLLRPRTD
IPTNLSGSIVYGTTAVLLVL
VLTLCSVLGVCWDELVMGQYSVVANCAILTLLSFLCLVIIWRQPQTSEISSFKGSGPVSTASSKSPLSQHLKPRSSKTMT
SQVDLDM
>g2037.t3
MTDSKAGCLGKTLLRRVPLDDRQVDSQFARCLSTLDLISLGVGATLGAGVYVLCGEVAKEKAGPAIVLCFLIAAVSSILA
GLCYAELGARVPRAGSAYVY
CYVTVGEIWAFIVGWVLILSYIIGESQTKRATASVARAWSLTFDNLVGLAISQWLKSSIPINLPGNIFSEYPDLLAVLLI
VLLTALLSIGVSESALMGKI
FTGINVTVLGFVLASSLVTGDRANWSLTLDHFINATNVTDPELIQKEFGLGGFAPFGVRGILSGAATCFYAFVGFDCIAA
TAEEALNPKQSIPISIVASL
LCCFVAYSGVSTALTLMMPYYLLDAQTPLPKAFDFVNQAHGRYLVAVGSLCALSTSLLGCMFPLPRVIHAMASDGLIFRF
LSRVWEKTKTPLRATVISGI
AAAPMALLFDLDVLVDLMSIGTLLAYTLVALCVIVLRYQPSFPNGGLKTLTLAEAGSDQKIHHEGQEPIEELVEQFTPMQ
LLRPRTDIPTNLSGSIVYGT
TAVLFLGVCWDELVMGQYSVVANCAILTLLSFLCLVIIWRQPQTSEISSFKGSGPVSTASSKSPLSQHLKPRSSKTMTSQ
VDLDM
>g2038.t1
MEDSSVTPIDPDFEPKSRPRSCTWPLPRPDISAVKPEGADGSESAAGTPPAEEDKHESQQITSEPEKVAAAEGGVVAGVG
GATPRKGSSRRNAWGNQSYA
DLISQAIENSPEKRLTLAQIYDWMVKTVPYFKDKGDSNSSAGWKNSIRHNLSLHNKFLKVHNESTGKSSWWMLNPEGGKT
GKAPRRRAASMDNSSKLLKS
RMRAKQTKKQAGAAALAGAAGSLQGDGPTGSGGGDSPNSSQQFGKWGVNSGSPSSRGSLDDPDMWTSFRPRTSSNASTLS
GRLSPIAPGQ
>g2039.t1
PDVQEVEVSSSSPSPPPSMSLLSPRATQTAGCIRLCEEKHRAKAKKELGLRQERQEQLVVAVANRESEQLKRFEEYMELK
QRKDSQSIRDLMDIQARESI
GRQEKLKEEHRHRMKILNLRLREAEQQRVREAGLERQRQEEGRDRLRNLNSIQEEILQLNQLLEPGPAPDDITAYITRGN
QLCSQVSEVVRTTAEGAFPS
VDDMTVAERALQVMRALVREKREAAAKAQERKRMEEEEVEEKRKQVELQAREEEKKKAAQSAKEKAKKKGLQNSAEDTTL
QWYRGLQDSATQTLSSMQEL
NASKDIQTKKLRMELQKAATTPVSQISSICGSQLRDILEKLDTLLSGRTVVSGGKSVSTAQHAKALEFVSYKMAEKFQKQ
GEEEVASHHEAAFPIAVVAS
GVWERHPRVGELILAHLHKKCPYSVPHYPPMTEGTSLEEYQRILGYRVDDCGVEGQDSFLKRMSGMIRLYAALIQLRWPY
GAKRGPGPHGLNHGWRWLAQ
MLNMEPLADVTATLLFDFLEVCGHALMKQYQGQFWKLIMIIQEEYFP
>g2039.t2
MSLLSPRATQTAGCIRLCEEKHRAKAKKELGLRQERQEQLVVAVANRESEQLKRFEEYMELKQRKDSQSIRDLMDIQARE
SIGRQEKLKEEHRHRMKILN
LRLREAEQQRVREAGLERQRQEEGRDRLRNLNSIQEEILQLNQLLEPGPAPDDITAYITRGNQLCSQVSEVVRTTAEGAF
PSVDDMTVAERALQVMRALV
REKREAAAKAQERKRMEEEEVEEKRKQVELQAREEEKKKAAQSAKEKAKKKGLQNSAEDTTLQWYRGLQDSATQTLSSMQ
ELNASKDIQTKKLRMELQKA
ATTPVSQISSICGSQLRDILEKLDTLLSGRTVVSGGKSVSTAQHAKALEFVSYKMAEKFQKQGEEEVASHHEAAFPIAVV
ASGVWERHPRVGELILAHLH

KKCPYSVPHYPPMTEGTSLEEYQRILGYRVDDCGVEGQDSFLKRMSGMIRLYAALIQLRWPYGAKRGPGPHGLNHGWRWL
AQMLNMEPLADVTATLLFDF
LEVCGHALMKQYQGQFWKLIMIIQEEYFP
>g2039.t3
MSLLSPRATQTAGCIRLCEEKHRAKAKKELGLRQERQEQLVVAVANRESEQLKRFEEYMELKQRKDSQSIRDLMDIQARE
SIGRQEKLKEEHRHRMKILN
LRLREAEQQRVREAGLERQRQEEGRDRLRNLNSIQEEILQLNQLLEPGPAPDDITAYITRGNQLCSQVSEVVRTTAEGAF
PSVDDMTVAERALQVMRALV
REKREAAAKAQERKRMEEEEVEEKRKQVELQAREEEKKKAAQSAKEKAKKKGLQNSAEDTTLQWYRGLQDSATQTLSSMQ
ELNASKDIQTKKLRMELQKA
ATTPVSQISSICGSQLRDILEKLDTLLSGRTVVSGGKSVSTAQHAKALEFVSYKMAEKFQKQGEEEVASHHEAAFPIAVV
ASGVWERHPRVGELILAHLH
KKCPYSVPHYPPMTEGTSLEEYQRILGYRVDDCGVEGQDSFLKRMSGMIRLYAALIQLRWPYGAKRGPGPHGLNHGWRWL
AQMLNMEPLADVTATLLFDF
LEVCGHALMKQYQGQFWKLIMIIQEEYFPRSNSE
>g2040.t1
LELLNNKLTQLDVPSWTFQWFLSFLVSYVIRMWGRWTLELKWESHILAIPRKAQQRLFFRCQLKRLHLSCPILVSLYVES
ILTTSITFPLFKARQQCYAY
CEEHHHVPPLHSDLQVYERS
>g2041.t1
MLEQETHYRQLTQEQLDNFTLDMNAAYARLKGMEDAIDSHVIAEEEARKAHQLWLSVEALGYTLKTSGIDSPTKPLENAA
QAIRESCHDEFAVALAAALP
EESLQRGIYSEESLRSRFNALRSLARRVALIDESHNSLYQYFLSYLQAALLFEEKHEPPPAKITNQDLDPFKILSYASYS
LEQGDLELAAKLVNQLRGEA
RRVVQDWLTEARLTLETKQVVSLLSAYANAVGLGTTQAP
>g2042.t1
MSESNQKTTAKSIPTRRVVINSVDHMPHDYSTTPGGTLFSTTPGGTRIIYDRKFLLECRSSPLAKTPPRGLPDIPGVTSP
PTKDGFKDKAQNGEPLNNNV
IRAPDSKTAGDEAQFEMDI
>g2042.t2
MSESNQKTTAKSIPTRRVVINSVDHMPHDYSTTPGGTLFSTTPGGTRIIYDRKFLLECRSSPLAKTPPRGLPDIPGVTSP
PTKDGFKDKAQNGEPLNNNV
IRAPDSKTAAIDFMSL
>g2043.t1
MPLILLISRVCGIGIIAPCTLGPLCTGPGSLQPLSRLLLLLLLLLLLLLLMNINQWIRNACQVDPCLNGGTCVERGGRVR
CLCLPSYGGDFCQTDVERCE
PGWDKFQGFCYRHYAQRLSWEVAELHCRMTGAHLVSIMSPEEQTFLSSKYEEYQWTGLNDKTIEGDFRWSDGNPLLYDNW
HRGQPDSYFLSGEDCVVMMW
HNGGRWSDVPCNYHLAYTCKKGTSLCGPPPKVKDASIYGRVLQRYRTGAAVRYHCANGFRQRRYPLVSCLPGGMWEEPRI
QCLPIGGRLLEAVGHVYSST
GMKLSAIQGDLEDESTTTEESPQYWDIKF
>g2043.t2
MPLILLISRVCGIGIIAPCTLGPLCTGPGSLQPLSRLLLLLLLLLLLLLLMNINQWIRNACQVDPCLNGGTCVERGGRVR
CLCLPSYGGDFCQTDVERCE
PGWDKFQGFCYRHYAQRLSWEVAELHCRMTGAHLVSIMSPEEQTFLSSKYEEYQWTGLNDKTIEGDFRWSDGNPLLYDNW
HRGQPDSYFLSGEDCVVMMW
HNGGRWSDVPCNYHLAYTCKKGTSEMAAVEYGKCTPLHTMHTNRFEGIIRVLSVKDASIYGRVLQRYRTGAAVRYHCANG
FRQRRYPLVSCLPGGMWEEP
RIQCLPIGGRLLEAVGHVYSSTGMKLSAIQGDLEDESTTTEESPQYWDIKF
>g2043.t3
MPLILLISRVCGIGIIAPCTLGPLCTGPGSLQPLSRLLLLLLLLLLLLLLMNINQWIRNACQVDPCLNGGTCVERGGRVR
CLCLPSYGGDFCQTDVERCE
PGWDKFQGFCYRHYAQRLSWEVAELHCRMTGAHLVSIMSPEEQTFLSSRWKMAAADESPDARKYEEYQWTGLNDKTIEGD
FRWSDGNPLLYDNWHRGQPD
SYFLSGEDCVVMMWHNGGRWSDVPCNYHLAYTCKKGTSLCGPPPKVKDASIYGRVLQRYRTGAAVRYHCANGFRQRRYPL
VSCLPGGMWEEPRIQCLPIG

GRLLEAVGHVYSSTGMKLSAIQGDLEDESTTTEESPQYWDIKF
>g2044.t1
MGQPSAAPSETSLIPVGGATALHHYTVHRSLSSGTSTGEHHPPPRRRVSSCSPVDTMMGSGEEEQEGEEENRGVQAASWR
RMIMPRRSNRDTLPSGDARP
RVTGGS
>g2044.t2
MGQPSAAPSETSLIPVGGATALHHYTVHRSLSSGTSTGEHHPPPRRRVSSCSPVDTMMGSGEEEQEGEEENRGVQAASWR
RMIMPRRSNRDTLPSGDARP
RVTGGSLFRSL
>g2045.t1
MLLVFLLWSQTVQAEHMLGVWSKARVLNEACWSIEPQGRHPVSTASGVWTNGPPERFYGSAEPSSQTHTAVARNFSAKNH
HVCCEWSENRSPTRDTEEQQ
MEDGDPALPRRVVPESGEDHVVRSAV
>g2045.t2
MDHQMVWSSSLQCDLSEPQKQGCSSDYVEQDEQLERPAAGDERRGGERETVQAEHMLGVWSKARVLNEACWSIEPQGRHP
VSTASGVWTNGPPERFYGSA
EPSSQTHTAVARNFSAKNHHVCCEWSENRSPTRDTEEQQMEDGDPALPRRVVPESGEDHVVRSAV
>g2045.t3
MLGVWSKARVLNEACWSIEPQGRHPVSTASGVWTNGPPERFYGSAEPSSQTHTAVARNFSAKNHHVCCEWSENRSPTRDT
EEQQMEDGDPALPRRVVPES
GEDHVVRSAV
>g2046.t1
MEVFLDKEEHLSLSQPGAGQPVCSFMEDICVASAPELDNGGIISPTSECSLKSGNEAKKPSSCCDVLGVFGDHLSLSSAP
LISSSRSLQLLVLFNIVRGA
ALFLGFGEVTLQGGGPDHLVVHVLTRCSGLSLKEE
>g2047.t1
MSRGMTLCWAPHQPRGGAARSSRRSRRRRNQKADAQQWSGTTREGGGADMCLGDDIIGEEREEEEEESRQQRKMKQD
>g2048.t1
LYRRQVRKAPERGPAGRDTLTRYACVSHAAGARASQVLVPQKVNAVLGKNVTLECRVEVGTNLSLTQSSWERRLPTGAVT
VAVYNPLFGISVPPEFSGRL
TFRSPSSRDATILLTDVGFADMGVYTCKVATFPLGNTQASTTVSVLGRQSFQPLVSRM
>g2048.t2
LSGDRAVVKGFALHLISGTCSCEISLPGGCSLVMSTEVDLVLYRRQVRKAPERGPAGRDTLTRYACVSHAAGARASQVLV
PQKVNAVLGKNVTLECRVEV
GTNLSLTQSSWERRLPTGAVTVAVYNPLFGISVPPEFSGRLTFRSPSSRDATILLTDVGFADMGVYTCKVATFPLGNTQA
STTVSVLGRQSFQPLVSRM
>g2048.t3
MSTEVDLVLYRRQVRKAPERGPAGRDTLTRYACVSHAAGARASQVLVPQKVNAVLGKNVTLECRVEVGTNLSLTQSSWER
RLPTGAVTVAVYNPLFGISV
PPEFSGRLTFRSPSSRDATILLTDVGFADMGVYTCKVATFPLGNTQASTTVSVLGRQSFQPLVSRM
>g2049.t1
MMTEEVSKPPEVSGINENSDEFQDVVKNFYSSIEGFHNKIRIVKVEKLDNRLLYSQYKLKKASMYKSGVLNEVERTLYHG
TSEASVKEICLHGFNRSFCG
KN
>g2049.t2
MMTEEVSKPPEVSGINENSDEFQDVVKNFYSSIEGFHNKIRIVKVEKLDNRLLYSQYKLKKASMYKSGVLNEVERTLYHG
TSEASVKEICLHGFNRSFCG
KNDCQQVQDILSCGFAERDDLVAGVPKLFS
>g2050.t1
MTQPEAVVPRRVRCTEYMKKFREPKWETFSKCYEDSLRYRLTRRLMEHSHKPWAWSGWDRVSDSSGGSTPRPSRSKTAPL
SLVPPARAEVRGRDRLSRFE
PPPEPKAAFRSADPEANERDTDPSLAGSGPGAARRRPLAEAPGSSGPSE
>g2050.t2
MNGSKVSKGLKWGNHQLEFTPVSRQIITFAIHNGVYRYKRLIFGVSSAREQYQHEIASALAGIEGVENISDDIVVHAVGR
ATHDQCLQAILKRLQQCGLM
LNPDKCEFNMDHLVFMGILLTDKGIGPTEERVRAIVEARELENVLERQQGEMGPIYYVSRSLSDCERRYSQTEKEALGLV

WAWNMTINEKHAHESEEYVR
FVTVNAIPRALTAREVEEASRTDAELIKVRDRDGEQKGQYKLYADEQSNAEYSNVDVGDKVLVKQDKVDKFTTNFNKTPY
TVVSKNGNWADLEQPVGGLR
GEEDARERPDYLRDADRMTGGDTLLGGDASTVGPVGAVMTQPEAVVPRRVRCTEYMKKFREPKWETFSKCYEDSLRYRLT
RRLMEHSHKPWAWSGWDRVS
DSSGGSTPRPSRSKTAPLSLVPPARAEVRGRDRLSRFEPPPEPKAAFRSADPEANERDTDPSLAGSGPGAARRRPLAEAP
GSSGPSE
>g2050.t3
MTINEKHAHESEEYVRFVTVNAIPRALTAREVEEASRTDAELIKVRDRDGEQKGQYKLYADEQSNAEYSNVDVGDKVLVK
QDKVDKFTTNFNKTPYTVVS
KNGNWADLEQPVGGLRGEEDARERPDYLRDADRMTGGDTLLGGDASTVGPVGAVMTQPEAVVPRRVRCTEYMKKFREPKW
ETFSKCYEDSLRYRLTRRLM
EHSHKPWAWSGWDRVSDSSGGSTPRPSRSKTAPLSLVPPARAEVRGRDRLSRFEPPPEPKAAFRSADPEANERDTDPSLA
GSGPGAARRRPLAEAPGSSG
PSE
>g2050.t4
MNGSKVSKGLKWGNHQLEFTPVSRQIITFAIHNGVYRYKRLIFGVSSAREQYQHEIASALAGIEGVENISDDIVVHAVGR
ATHDQCLQAILKRLQQCGLM
LNPDKCEFNMDHLVFMGILLTDKGIGPTEERVRAIVEARELENVLERQQGEMGPIYYVSRSLSDCERRYSQTEKEALGLV
WAWNMTINEKHAHESEEYVR
FVTVNAIPRALTAREVEEASRTDAELIKVRDRDGEQKGQYKLYADEQSNAEYSNVDVGDKVLVKQDKVDKFTTNFNKTPY
TVVSKNGNWVTVESPEGIQ
>g2051.t1
LLFEDQESGLFSVTLFRKAIDDFKHKARENK
>g2052.t1
NYVFQKRYVKFDGVNLMYFGSEKDIYPKGVIPLAAIQMARPGKDNKFEIVTNHRIFVFRADNE
>g2053.t1
LSGETAILWDRVHSRPPPPWEPSEPWELGWLFPVTPTHDSSQRGAHSSVSIRVSSIIIRCSAAPQDCGPKVQHLMIPGQD
LTPI
>g2053.t2
LSGETAILWDRVHSRPPPPWEPSEPWELGWLFPVTPTHDSSQRGAHSSVSIRCSAAPQDCGPKVQHLMIPGQDLTPI
>g2054.t1
MSGIRDDRMAIWLSWLTALTNFVFTLLGVWLVERVGRRKLTLGSLMGTCVSLVLLAVGFLLSAQHTPPVTFHPSDPALAN
STCVKYQMCEQCMLDPGCGF
CYRDNSSTLFASSCVPVNMVSTERAAWGRQVK
>g2054.t2
MSGIRDDRMAIWLSWLTALTNFVFTLLGVWLVERVGRRKLTLGSLMGTCVSLVLLAVGFLLSAQHTPPVTFHPSDPALAN
STCVKYQMCEQCMLDPGCGF
CYRDNSSTLFASSCVPVNMVSTERAAWGRQVKWWGSGVRAAESH
>g2054.t3
MSGIRDDRMAIWLSWLTALTNFVFTLLGVWLVERVGRRKLTLGSLMVGFLLSAQHTPPVTFHPSDPALANSTCVKYQMCE
QCMLDPGCGFCYRDNSSTLF
ASSCVPVNMVSTERAAWGRQVK
>g2055.t1
KAYINQHWQDVRVGDFIRLSCNEIIPADVLLLHSSDPRGICYIETANLDGETNLKQRQVVTDLPLQGSELTPESFYCRIE
CENPNNDLGRFRGYMEHSNK
VRVGLHNSNLLLRSCTVRNTETVTGIVIYAGHETKAMKNNSGPRYKQSRLERRLNVDVLWCVVLLFAMCLTAAIGGYFHW
HGLWLQRWSDPIYAVAEQVS
PALAGFYVFWTMIIVLQVLIPISLYVSIEIVKLGQIYFIQNDLVLYNEQLDSGIQCRALNITEDLGQIQFLFSDKTGTLT
ENKMVFQCCTIHGVQYPHTE
N
>g2055.t2
KAYINQHWQDVRVGDFIRLSCNEIIPADVLLLHSSDPRGICYIETANLDGETNLKQRQVVTDLPLQGSELTPESFYCRIE
CENPNNDLGRFRGYMEHSNK
VRVGLHNSNLLLRSCTVRNTETVTGIVIYAGHETKAMKNNSGPRYKQSRLERRLNVDVLWCVVLLFAMCLTAAIGGYFHW
HGLWLQRWSDPIYAVAEQVS

PALAGFYVFWTMIIVLQVLIPISLYVSIEIVKLGQIYFIQNDLVLYNEQLDSGIQCRALNITEDLGQIQFLFSDKTGTLT
ENKMVFQCCTIHGVQYPHTE
NG
>g2055.t3
KAYINQHWQDVRVGDFIRLSCNEIIPADVLLLHSSDPRGICYIETANLDGETNLKQRQVVTDLPLQGSELTPESFYCRIE
CENPNNDLGRFRGYMEHSNK
VRVGLHNSNLLLRSCTVRNTETVTGIVIYAGHETKAMKNNSGPRYKQSRLERRLNVDVLWCVVLLFAMCLTAAIGHGLWL
QRWSDPIYAVAEQVSPALAG
FYVFWTMIIVLQVLIPISLYVSIEIVKLGQIYFIQNDLVLYNEQLDSGIQCRALNITEDLGQIQFLFSDKTGTLTENKMV
FQCCTIHGVQYPHTEN
>g2056.t1
FELCEPCFVAGNCLEIAPEAQQYDRVYCGAGVQRDHEDYMRRMLRVGGILVLPLEDKLTKIRRTGYNSWETKKIIAVSFA
PLVLPKHTENCTPEPVPLPS
MFEVRALQDLARISIRHVLTEAPPPRVLPRRRLPVGGQASARPRPAPQQQQLARAVLANRYTFVSRLLPEPLDRGQAYSA
REAXXXXXXXWEAWEEEEEE
EEEEEEEEEEQQTDCRASERLSRESPVNRLREQILGLPLPEPLKMYLLHYREQ
>g2056.t2
FELCEPCFVAGNCLEIAPEAQQYDRVYCGAGVQRDHEDYMRRMLRVGGILVLPLEDKLTKIRRTGYNSWETKKIIAVSFA
PLVLPKHTENCTPEPVPLPS
MFEVRALQDLARISIRHVLTEAPPPRVLPRRRLPVGGQASARPRPAPQQQQLARAVLANRYTFVSRLLPEPLDRGQAYSA
REAXXXXXXXWEAWEEEEEE
EEEEEEEEEEQQTDCRASERLSRVEEEQEEEEEERREAGXXXXXXXXXXXXGGGSPAGVPGEPAQRADTGAAAA
>g2056.t3
FELCEPCFVAGNCLEIAPEAQQYDRVYCGAGVQRDHEDYMRRMLRVGGILVLPLEDKLTKIRRTGYNSWETKKIIAVSFA
PLVLPKHTENCTPEPVPLPS
MFEVRALQDLARISIRHVLTEAPPPRVLPRRRLPVGGQASARPRPAPQQQQLARAVLANRYTFVSRLLPEPLDRGQAYSA
REAXXXXXXXWEAWEEEEEE
EEEEEEEEEEQQTDCRASERLSRESPVNRLREQILGLPLPEPLKMYLLHYREQWKS
>g2057.t1
DSATEEVALKCSCRLKAMIMCQGCGAFCHDDCIGPSKLCVSCLVVR
>g2058.t1
MLSSPNTGLPNPMSSVGPGQPSAPTINTSTPIDPSSMQRAYAALGLPYSCQAPGQGQASAAGQTSGATNAQTQQTLPQQL
RPINAIVVGDAASHSTDSEP
WRGSAQCCRSTSLCGVCSSLFTFLQVPPSSSHAVKTVETCWVCGPAGALEQGTSRKEKSVQGSQCCSK
>g2058.t2
MSSVGPGQPSAPTINTSTPIDPSSMQRAYAALGLPYSCQAPGQGQASAAGQTSGATNAQTQQTLPQQLRPINAIVVGDAA
SHSTDSEPWRGSAQCCRSTS
LCGVCSSLFTFLQVPPSSSHAVKTVETCWVCGPAGALEQGTSRKEKSVQGSQCCSK
>g2059.t1
MALGGTGLSGPTSKPPSLHSDPSLPNTFNTNNQLLSDGSSVASKGNLPTAAPLSATGTRKTWHEHVTQDLRNHLVHKLVQ
AIFPTPDPAALKDRRMENLV
AYARKVEGDMYESANSRDEYYHFLAEKIYKIQKELEEKRRSRLQKQIINQAPMNAQGAQPPGLPPPGTLGPRPQNGPVSI
PNVPNQIMNRMQVPQGINQF
NPMALPNVQMSPAPMGQRALSPMSHAQQINMSSVPAMGMSPSRMPQAQGMMAAHGGGSMVGQTAGQGQFLAQNQFPPGST
TTGALNVTAGPGMGQPQAQA
AVTQQQPQQQQQQQQQQPQQGTNLPMNALAPSLGAQLVSPQTPSRSTP
>g2059.t2
MALGGTGLSGPTSKPPSLHSDPSLPNTFNTNNQLLSDGSSVASKGNLPTAAPLSATGTRKTWHEHVTQDLRNHLVHKLVQ
AIFPTPDPAALKDRRMENLV
AYARKVEGDMYESANSRDEYYHFLAEKIYKIQKELEEKRRSRLQKQIINQAPMNAQGAQPPGLPPPGTLGPRPQNGPVSI
PNVPNQIMNRMQVPQGINQF
NPMALPNVQMSPAPMGQRALSPMSHAQQINMSSVPAAQGMMAAHGGGSMVGQTAGQGQFLAQNQFPPGSTTTGALNVTAG
PGMGQPQAQAAVTQQQPQQQ
QQQQQQQPQQGTNLPMNALAPSLGAQLVSPQTPSRSTP
>g2059.t3
MFRPGCRVQAIFPTPDPAALKDRRMENLVAYARKVEGDMYESANSRDEYYHFLAEKIYKIQKELEEKRRSRLQKQIINQA

PMNAQGAQPPGLPPPGTLGP
RPQNGPVSIPNVPNQIMNRMQVPQGINQFNPMALPNVQMSPAPMGQRALSPMSHAQQINMSSVPAMGMSPSRMPQAQGMM
AAHGGGSMVGQTAGQGQFLA
QNQFPPGSTTTGALNVTAGPGMGQPQAQAAVTQQQPQQQQQQQQQQPQQGTNLPMNALAPSLGAQLVSPQTPSRSTP
>g2059.t4
MALGGTGLSGPTSKPPSLHSDPSLPNTFNTNNQLLSDGSSVASKGNLPTAAPLSATGTRKTWHEHVTQDLRNHLVHKL
>g2060.t1
APTPTTKRAASRPRPPPPDSEVGRPGGPGGSVEGKQVLRRDGRQEAGELIRKWEENLRRDRESERDQEFVTQGDHGTSPW
ELRSETSALRTSGAQMKKGP
WVARETEVKKVLRSPPRSYQGRPKQQNHYSPLSPQSPAAKPARPGRPSSGLQAPRSYLQGSGKADQDSPHHPLQGNSALR
QPPYRRHSSTHAAEQEGASE
DFSRRSLHYAQPPASGQGSGYLPKPQPSFLYTYNSTHSQQHQAQPGAYVHDGAAGGQQSKYRRSSLAGDSEPGDAGQDLS
KTGFSFSIAKLYSLKDLKNK
GRGSPSPTKRNPDSPAQVRKSTG
>g2061.t1
LSASEHFAIEIQTGRDGSKFADLILKKHLDREAQAVHRLVLTAVDGGLPARSGTANIVVRVLDINDNAPSFDKENYRIDL
MENSPIGSLVIKMNATDRDE
GSNSESFYTIRVMENNAPGALLDSLTAYDPDLHENQYLVYFIIEREIANTSMSMLFSINPENGKLYALKTFDYEIEKEFV
FHIEARDSGVPPLSSNVTVH
IIIVDQNDNTPVIVSPWRAHGSVVEEKIPRSTDKGSLVSKVIAIDGDSVLNSRITYQFLQVTDATLFSLDQYNGEIRTMR
MFSYRDPRLQRLVVVAKDNG
EPALSATVTIKLSTVETAVKAYSDMTEVPLEYDIFSDLNLYLVIGLGSVSFLLLITILVTIVLKCQKPKSSKAAAPCRNS
VISERNSTIADSTLVSNDAY
WYSMFLAETRKGKLVVRQPAPKGSRYIVSSLPRSTGLTETSDSAPS
>g2062.t1
CVSSSSHEFTASQHHHLSTMTRLFLPVCLATLVLVSALALKCGKCRPLGEPDRCAGIEFDAITSDGKGKHFFFKDDYMWS
GFHGPALRSSTTFGDIGHVD
AAFRLIRSGNPDGDKVYFFQGEHVSVYHTSLEEGYPKKVTEEFPGVPCHLDAAVECPKPECVTDSVIFFKGSEVYHYDFA
TRTVKTKAWPLLPGCTSAFR
WMDHHYCFHGNNFTRFHPRTGLVVGTYPKDARHYFMNCPNFGHGGTHQLPKCLDIKLDAITSDDAGKTYIFIGSHYTRLS
TDSEGMHAFPMNRMWSQPAG
GVDAVFSYEDKMYLIKGDQVYIYRAGAQYSLIDGYPKTLQEELGIMGSVDAAFACAGQH
>g2062.t2
CVSSSSHEFTASQHHHLSTMTRLFLPVCLATLVLVSALACAGIEFDAITSDGKGKHFFFKDDYMWSGFHGPALRSSTTFG
DIGHVDAAFRLIRSGNPDGD
KVYFFQGEHVSVYHTSLEEGYPKKVTEEFPGVPCHLDAAVECPKPECVTDSVIFFKGSEVYHYDFATRTVKTKAWPLLPG
CTSAFRWMDHHYCFHGNNFT
RFHPRTGLVVGTYPKDARHYFMNCPNFGHGGTHQLPKCLDIKLDAITSDDAGKTYIFIGSHYTRLSTDSEGMHAFPMNRM
WSQPAGGVDAVFSYEDKMYL
IKGDQVYIYRAGAQYSLIDGYPKTLQEELGIMGSVDAAFACAGQH
>g2063.t1
MDHGRTEPVLSEARLQISHVGDVVIKQEILLDSDEDEEVLLGKRATDSAIAFPSCQLKNHKVRLNVPGHNYISHRAKVQE
VTKRHLKMGAGLALKAAVRP
HRPMRKTHHAVSSGAITGHPLAAGSNFHGRTASAFKS
>g2064.t1
MCSPTESPSGANIEVSGGFSCLLINEEGYLQDQSALYPEHTPGSSGSRLGFRGQDQHNGEPPLGRAEDLYGPSESFIQSL
NMGERGGRKHNCNQCSMSFP
DSSSLKAHKQQTHSVGHRHPFSCNQCGKTFTQAHSFKVHQRIHSEQGLHLCNHCGKGFPGIADLKSHKCNQTGDKPYCCT
VCGNKFSRLWNLKLHRRIHT
QEKPHRCSMCDKSFTRADILKVHQRTHTGERPYRCALCGLSFKRLDHLKSHQRKHKSDL
>g2065.t1
VQSDAEQHSSSGRPHAMMPRPREAGRLHA
>g2066.t1
MRRGSDILSAAVSWVSLGKKMSGGLWILGAVAALLSVQLTSSQELSLNNMDFASRLYRAVSSRTDDNVLLSPFSVSAAMA
MLAVGCKDGAQQQLLSALSL
GSLEPQQIPGLFQALQNSITMDGAFPLKQGIAIFPSQDFTLAHTYLDLVQTQFSGKAQTLSYTSQHEAMDSINSWAMSQT

SDRVKDVVGSLNTQTKLLLI
SVAYYQ
>g2066.t2
MRRGSDILSAAVSWVSLGKKMSGGLWILGAVAALLSVQLTSSQELSLNNMDFASRLYRAVSSRTDDNVLLSPFSVSAAMA
MLAVGCKDGAQQQLLSALSL
GSLEPQQIPGLFQALQNSITMDGAFPLKQGIAIFPSQDFTLAHTYLDLVQTQFSGKAQTLSYTSQHEAMDSINSWAMSQT
SDRVKDVVGSLNTQTKLLLI
SVAYYQSKEELFTPVYTSLLALCNIMYSCTFSRSRSFT
>g2067.t1
LDGVLRGGLLPWGLTELAGPSGAGKSQLALQLCLSVQYPTQYGGLGSGALYICTEDSFPIKRLTQLQAEQPCLRTDVPPD
VVSGICFSDNIYVEHAADLG
ALMACMSQRVPVLLARGRVRLLVVDSVAALFRSEFQPDQGLERSHHLMACASTLHKLSSTYQLPILCVNQVTDVMEGDTN
HTGLRPVESKVLPALGMAWA
NQVTVRLMVTRLPGVVSMGTQSSGPRQLDVMFAPHLAMDSCVFGVWREGVRGLPPPSTQDAGS
>g2067.t2
LRLSLGCPVLDGVLRGGLLPWGLTELAGPSGAGKSQLALQLCLSVQYPTQYGGLGSGALYICTEDSFPIKRLTQLQAEQP
CLRTDVPPDVVSGICFSDNI
YVEHAADLGALMACMSQRVPVLLARGRVRLLVVDSVAALFRSEFQPDQGLERSHHLMACASTLHKLSSTYQLPILCVNQV
TDVMEGDTNHTGLRPVESKV
LPALGMAWANQVTVRLMVTRLPGVVSMGTQSSGPRQLDVMFAPHLAMDSCVFGVWREGVRGLPPPSTQDAGS
>g2067.t3
MLYRGECPEWDCGLRLSLGCPVLDGVLRGGLLPWGLTELAGPSGAGKSQLALQLCLSVQYPTQYGGLGSGALYICTEDSF
PIKRLTQLQAEQPCLRTDVP
PDVVSGICFSDNIYVEHAADLGALMACMSQRVPVLLARGRVRLLVVDSVAALFRSEFQPDQGLERSHHLMACASTLHKLS
STYQLPILCVNQVTDVMEGD
TNHTGLRPVESKVLPALGMAWANQVTVRLMVTRLPGVVSMGTQSSGPRQLDVMFAPHLAMDSCVFGVWREGVRGLPPPST
QDAGS
>g2068.t1
MSPRVCKACGCAEIDLDQARGNAVCTSCGSVLEDNIIVSEVQFVEGSGGVTSAVGQFVSSDGVLGP
>g2069.t1
LFLLCVELGWRVEAAGDLGGQPEVLIESECRDRYLWLHVAATGAAPQFEAVDNQGLYSISGQHAVSCGYTISQFRMDGYT
TLRASYYSCYTGNQNDEIFT
FNLNIILMDAEGHWWSQPLSKTCVLPLPLAQREVLCEEHYMEAQKMATSVWQLMFLLENNLAIMSASEAQALGYSLTTSP
NRLVFRSPYQQPHSQLVHVH
EVPVEVIKVVLFFRRKLVVVMNSGSFDGSTLLWDTPRVMQPMVWAGAGFESRGFALGVEGRLVDWDTTVARGWTVTQQGP
LVQVGIPFGSEGGYRQSMVV
NNVYKETYVIFLFYEHIFSTLYDDGSEVETRQRLVTVLDTPLRCRHPFILDQTIHAERVFGVYLGNIPFDVTLAEVTVNG
RRLPVAMTPEYSISRVGHTN
GSHAYVLHVPFDSRVVHRMHLDQGSVQYSMNLNYTLTILPQRLSYHHHTFVSAQLDNAFAPEPRAECTPTGITFSLWKSP
HMEFLWEIGVGADPLTEELA
NLRGYGLYNDTQSITLEVPILSIGYTYQVGETFVAYENEILYNRQLLADEKAIISRDSKFKLTVRCFYPLSTISRLTVDR
IFPTHTPGLGFILGTKGSTV
PPECPFENSPFSRTPQSNAFPKPWPRQQGVSPRSYSPAGRVELPRLTDNPSTVSFDANSPVNHVESVDRFDQTDVGSQEP
KPDRPGVQKLRVSSNVAPRY
RLLNNENTHNLSESVNASSSQKPTDMNDLNHEDEPLSRSALYFQEFLSVNVNQSTTAVTTKLPVSNTTGAVSTRNTTVNS
TEVTRLV
>g2069.t2
LFLLCVELGWRVEAAGDLGGQPEVLIESECRDRYLWLHVAATGAAPQFEAVDNQGLYSISGQHAVSCGYTISQFRMDGYT
TLRASYYSCYTGNQNDEIFT
FNLNIILMDAEGHWWSQPLSKTCVLPLPLAQREVLCEEHYMEAQKMATSVWQLMFLLENNLAIMSASEAQALGYSLTTSP
NRLVFRSPYQQPHSQLVHVH
EVPVEVIKVVLFFRRKLVVVMSDVSLACTVNSGSFDGSTLLWDTPRVMQPMVWAGAGFESRGFALGVEGRLVDWDTTVAR
GWTVTQQGPLVQVGIPFGSE
GGYRQSMVVNNVYKETYVIFLFYEHIFSTLYDDGSEVETRQRLVTVLDTPLRCRHPFILDQTIHAERVFGVYLGNIPFDV
TLAEVTVNGRRLPVAMTPEY
SISRVGHTNGSHAYVLHVPFDSRVVHRMHLDQGSVQYSMNLNYTLTILPQRLSYHHHTFVSAQLDNAFAPEPRAECTPTG

ITFSLWKSPHMEFLWEIGVG
ADPLTEELANLRGYGLYNDTQSITLEVPILSIGYTYQVGETFVAYENEILYNRQLLADEKAIISRDSKFKLTVRCFYPLS
TISRLTVDRIFPTHTPGLGF
ILGTKGSTVPPECPFENSPFSRTPQSNAFPKPWPRQQGVSPRSYSPAGRVELPRLTDNPSTVSFDANSPVNHVESVDRFD
QTDVGSQEPKPDRPGVQKLR
VSSNVAPRYRLLNNENTHNLSESVNASSSQKPTDMNDLNHEDEPLSRSALYFQEFLSVNVNQSTTAVTTKLPVSNTTGAV
STRNTTVNSTEVTRLV
>g2069.t3
LFLLCVELGWRVEAAGDLGGQPEVLIESECRDRYLWLHVAATGAAPQFEAVDNQGLYSISGQHAVSCGYTISQFRMDGYT
TLRASYYSCYTGNQVVVVLP
NDEIFTFNLNIILMDAEGHWWSQPLSKTCVLPLPLAQREVLCEEHYMEAQKMATSVWQLMFLLENNLAIMSASEAQALGY
SLTTSPNRLVFRSPYQQPHS
QLVHVHEVPVEVIKVVLFFRRKLVVVMNSGSFDGSTLLWDTPRVMQPMVWAGAGFESRGFALGVEGRLVDWDTTVARGWT
VTQQGPLVQVGIPFGSEGGY
RQSMVVNNVYKETYVIFLFYEHIFSTLYDDGSEVETRQRLVTVLDTPLRCRHPFILDQTIHAERVFGVYLGNIPFDVTLA
EVTVNGRRLPVAMTPEYSIS
RVGHTNGSHAYVLHVPFDSRVVHRMHLDQGSVQYSMNLNYTLTILPQRLSYHHHTFVSAQLDNAFAPEPRAECTPTGITF
SLWKSPHMEFLWEIGVGADP
LTEELANLRGYGLYNDTQSITLEVPILSIGYTYQVGETFVAYENEILYNRQLLADEKAIISRDSKFKLTVRCFYPLSTIS
RLTVDRIFPTHTPGLGFILG
TKGSTVPPECPFENSPFSRTPQSNAFPKPWPRQQGVSPRSYSPAGRVELPRLTDNPSTVSFDANSPVNHVESVDRFDQTD
VGSQEPKPDRPGVQKLRVSS
NVAPRYRLLNNENTHNLSESVNASSSQKPTDMNDLNHEDEPLSRSALYFQEFLSVNVNQSTTAVTTKLPVSNTTGAVSTR
NTTVNSTEVTRLV
>g2070.t1
LLGSGFHTSIGKESRAQTLQNGKRQIHQLGSQLQLNQHCLDTAFNFFKMVVSKQLTRGRKMAHVIAACLYMVCRTEGTPH
MLLDLSDLLQVRHTLTIRDI
PHLTGNNLKLIV
>g2070.t2
MEPAELNTWEQGVMETPGQFNWTKVWLIERPEKAPLLGSGFHTSIGKESRAQTLQNGKRQIHQLGSQLQLNQHCLDTAFN
FFKMVVSKQLTRGRKMAHVI
AACLYMVCRTEGTPHMLLDLSDLLQVRHTLTIRDIPHLTGNNLKLIV
>g2070.t3
MEPAELNTWEQGVMETPGQFNWTKVWLIERPEKAPLLGSGFHTSIGKESRAQTLQNGKRQIHQLGSQLQLNQHCLDTAFN
FFKMVVSKQLTRGRKMAHVI
AACLYMVCRTEGTPPPDMLLDLSDLLQVRHTLTIRDIPHLTGNNLKLIV
>g2071.t1
MADVFFIVGLPGESQRVPAHKYVLAVGSSVFGAMFYGDLAEGEMEIHIPDVEPAAFLIMLKYMYSDEIELEADTVLATLY
AAKKYIVPALAKACVTFLET
SLEAKNACVLLSQSRLFEEPELTQRCWEVIDAQAELALRSEGFCEIDRPTLEIILQRETLNTSEALVFKAVLEWAAAECG
RQGLVVTAQNRRAVLGKALY
SVRIPAMTLEEFANGPAQSDVLTLEETHKVFLWFTAAKKPWLEFPLAQRTGLSPQRCHRFQSSAYRSNQWRYRGRCDSIQ
FAVDKRIFMAGLGLYGSSGG
KAEYSVKIELKRQGTTLAQNLTKFLSDGSSSTFSVWFEHPVQVEQDTFYTVSAVLDGNELSYFGQEGMTEVQCGKVTFQF
QCSSDSTNGTGVQGGQIPEL
VFFG
>g2071.t2
MFNSEHMADVFFIVGLPGESQRVPAHKYVLAVGSSVFGAMFYGDLAEGEMEIHIPDVEPAAFLIMLKYMYSDEIELEADT
VLATLYAAKKYIVPALAKAC
VTFLETSLEAKNACVLLSQSRLFEEPELTQRCWEVIDAQAELALRSEGFCEIDRPTLEIILQRETLNTSEALVFKAVLEW
AAAECGRQGLVVTAQNRRAV
LGKALYSVRIPAMTLEEFANGPAQSDVLTLEETHKVFLWFTAAKKPWLEFPLAQRTGLSPQRCHRFQSSAYRSNQWRYRG
RCDSIQFAVDKRIFMAGLGL
YGSSGGKAEYSVKIELKRQGTTLAQNLTKFLSDGSSSTFSVWFEHPVQVEQDTFYTVSAVLDGNELSYFGQEGMTEVQCG
KVTFQFQCSSDSTNGTGVQG
GQIPELVFFG

>g2071.t3
LQYVLAVGSSVFGAMFYGDLAEGEMEIHIPDVEPAAFLIMLKYMYSDEIELEADTVLATLYAAKKYIVPALAKACVTFLE
TSLEAKNACVLLSQSRLFEE
PELTQRCWEVIDAQAELALRSEGFCEIDRPTLEIILQRETLNTSEALVFKAVLEWAAAECGRQGLVVTAQNRRAVLGKAL
YSVRIPAMTLEEFANGPAQS
DVLTLEETHKVFLWFTAAKKPWLEFPLAQRTGLSPQRCHRFQSSAYRSNQWRYRGRCDSIQFAVDKRIFMAGLGLYGSSG
GKAEYSVKIELKRQGTTLAQ
NLTKFLSDGSSSTFSVWFEHPVQVEQDTFYTVSAVLDGNELSYFGQEGMTEVQCGKVTFQFQCSSDSTNGTGVQGGQIPE
LVFFG
>g2072.t1
MLEFGEKTHQVSMTALRLLQRMKRDWMHTGRRPSGLCGAALLVAARMHDFRRTVKEVIGVVKVCQATLRKRLTEFEDTPT
SHLTIDEFMRVDLEQECDPP
SFIAGQHKTKIQQLEHELAKKLNEVEGEISGYRDEIETELENSRPKLRGMYSAFAKHTEPPGEEQEDEDEEEEEEEKLTL
SFDQPEGEDLELQVAAQQLT
QDFLCQVMEEVEEEPGPGLPGSLPGCRPCSQSLSSILGIGRSIQDPQEAPEDIQSESGELDLEGIDDQEIEKYILNEREV
ETKTELWMKQNEDYLKEQKE
KEERITKEKEQGTYKVKKSKKRAPIQASTAGEAIEKMLEQKRISTKINYEVLRDLNQGGSGPSSPLPAPSESSSSSTSGR
PRPPRRKKPNRKTNNLAATA
SIMGKRLRPLVSSIPKKKRDTSQVIMPTAPRTPAPSIPSTPLPPETPQSSDTPPGQATPPKLATPPREATPPCEDPPPGE
AVEEEEAEAEPCVSVLQLME
GNEYGCDTEEEEFF
>g2072.t2
MLEFGEKTHQVSMTALRLLQRMKRDWMHTGRRPSGLCGAALLVAARMHDFRRTVKEVIGVVKVCQATLRKRLTEFEDTPT
SHLTIDEFMRVDLEQECDPP
SFIAGQHKTKIQQLEHELAKKLNEVEGEISGYRDEIETELENSRPKLRGMYSAFAKHTEPPGEEQEDEDEEEEEEEKLTL
SFDQPEGEDLELQVAAQQLT
QDFLCQVMEEVEEEPGPGLPGSLPGCRPCSQSLSSILGIGRSIQDPQEAPEDIQSESGELDLEGIDDQEIEKYILNEREV
ETKTELWMKQNEDYLKEQKE
KEERITKEKEQGTYKVKKSKKRAPIQASTAGEAIEKMLEQKRISTKINYEVLRDLNQGGSGPSSPLPAPSESSSSSTSGR
PRPPRRKKPNRKTNNLAATA
SIMGKRLRPLVSSIPKKKRDTSQVIMPTAPRTPAPSIPSTPLPPETPQSSDTPPGQATPPKLATPPREATPPCEDPPPGE
AVEEEEAEAEPCVSVLQLME
GNGAWNTVLLIL
>g2072.t3
LLGWPVLLALGCGSLGVGSNSGSDTWMKLFLLLAVTVASVLLGRLVLAGSHVKKSVALEQQEAGFTALWDPCLYIPRFAH
MLEFGEKTHQVSMTALRLLQ
RMKRDWMHTGRRPSGLCGAALLVAARMHDFRRTVKEVIGVVKVCQATLRKRLTEFEDTPTSHLTIDEFMRVDLEQECDPP
SFIAGQHKTKIQQLEHELAK
KLNEVEGEISGYRDEIETELENSRPKLRGMYSAFAKHTEPPGEEQEDEDEEEEEEEKLTLSFDQPEGEDLELQVAAQQLT
QDFLCQVMEEVEEEPGPGLP
GSLPGCRPCSQSLSSILGIGRSIQDPQEAPEDIQSESGELDLEGIDDQEIEKYILNEREVETKTELWMKQNEDYLKEQKE
KEERITKEKEQGTYKVKKSK
KRAPIQASTAGEAIEKMLEQKRISTKINYEVLRDLNQGGSGPSSPLPAPSESSSSSTSGRPRPPRRKKPNRKTNNLAATA
SIMGKRLRPLVSSIPKKKRD
TSQVIMPTAPRTPAPSIPSTPLPPETPQSSDTPPGQATPPKLATPPREATPPCEDPPPGEAVEEEEAEAEPCVSVLQLME
GNEYGCDTEEEEFF
>g2073.t1
MAKWGEGDPRWIVEERSDATNVNNWHWTERDVTGWSSDKLRELLIGCRVDRDGASCQLTDVTKIEGEASINNRKGKLIYF
YEWVIKASWLGTSPIGMKYK
GTLEVPNLSDENDMDDLDISVSLCKDEPVTPVLQLMKKEGVEKVRKALGEYVTLLKSAFSQGMILPTAGGPSQPPPLPAP
KKVTTSKTIQISNSVPAKTA
PKLPQQASGGTAGVRIPTSSFSLQETFLTSPDELYRTFINQEVFTHSAAQVDGCRGGKFQMLDGNVNGEFAELIPDEKIV
MRWRFKTWPSEHYATISLQL
KNLGEETELSLDCKGVPSGEEERTKEGWKRYYFKAIKQTFGYGASPY
>g2073.t2
MAKWGEGDPRWIVEERSDATNVNNWHWTERDVTGWSSDKLRELLIGCRVDRDGASCQLTDVTKIEGEASINNRKGKLIYF

YEWVIKASWLGTSPIGMKYK
GTLEVPNLSDENDMDDLDISVSLCKDEPVTPVLQLMKKEGVEKVRKALGEYVTLLKSAFSQGMILPTAGGPSQPPPLPAP
KKVTTSKTIQISNSVPAKTA
PKLPQQASGGTAGVRIPTSSFSLQETFLTSPDELYRTFINQEFVQVFTHSAAQVDGCRGGKFQMLDGNVNGEFAELIPDE
KIVMRWRFKTWPSEHYATIS
LQLKNLGEETELSLDCKGVPSGEEERTKEGWKRYYFKAIKQTFGYGASPY
>g2073.t3
MAKWGEGDPRWIVEERSDATNVNNWHWTERDVTGWSSDKLRELLIGCRVDRDGASCQLTDVTKIEGEASINNRKGKLIYF
YEWVIKASWLGTSPIGMKYK
GTLEVPNLSDENDMDDLDVSLLQLACTWLRPLKHLPSISVSLCKDEPVTPVLQLMKKEGVEKVRKALGEYVTLLKSAFSQ
GMILPTAGGPSQPPPLPAPK
KVTTSKTIQISNSVPAKTAPKLPQQASGGTAGVRIPTSSFSLQETFLTSPDELYRTFINQEVFTHSAAQVDGCRGGKFQM
LDGNVNGEFAELIPDEKIVM
RWRFKTWPSEHYATISLQLKNLGEETELSLDCKGVPSGEEERTKEGWKRYYFKAIKQTFGYGASPY
>g2074.t1
VHAEITNHGNRGGARELLRRIVRSPPGWFSEFLEVLRRTEHFYLYNELSVSSGLPGKAKHAVEDSSAIDKGLLETTRESC
VDLYNDEGSDSSMLQDDSER
SFNSSLPDSPGGGADGGAEGGVDSTPQGTDIRLRDYQEEVAAPALEGLNVIVCLPTGSGKTRVAVFITREHLASRRREGR
PGKVVVLVNKVPLVEQHYSS
EFGPFLKHHYKVGRVSGNSQLKISFPEIVRNHDIIICTAQILENHLGSSENPDDERVELSGLVEGVEDMAASPWIIAL
>g2074.t2
VHAEITNHGNRGGARELLRRIVRSPPGWFSEFLEVLRRTEHFYLYNELSVSSGLPGKAKHAVEDSSAIDKGLLETTRESC
VDLYNDEGSDSSMLQDDSER
SFNSSLPGLFNDSPGGGADGGAEGGVDSTPQGTDIRLRDYQEEVAAPALEGLNVIVCLPTGSGKTRVAVFITREHLASRR
REGRPGKVVVLVNKVPLVEQ
HYSSEFGPFLKHHYKVGRVSGNSQLKISFPEIVRNHDIIICTAQILENHLGSSENPDDERVELSGLVEGVEDMAASPWII
AL
>g2074.t3
LRRIVRSPPGWFSEFLEVLRRTEHFYLYNELSVSSGLPGKAKHAVEDSSAIDKGLLETTRESCVDLYNDEGSDSSMLQDD
SERSFNSSLPDSPGGGADGG
AEGGVDSTPQGTDIRLRDYQEEVAAPALEGLNVIVCLPTGSGKTRVAVFITREHLASRRREGRPGKVVVLVNKVPLVEQH
YSSEFGPFLKHHYKVGRVSG
NSQLKISFPEIVRNHDIIICTAQILENHLGSSENPDDERVELSGLVEGVEDMAASPWIIAL
>g2075.t1
LIVIDECHHTQKGGVYNHVMMRYLKQKYRNIQLRKQGKDPMALPQILGLTASPGVGRATKSTKAEEHILKVNFSPCLTHC
TS
>g2076.t1
MFVIHAEGRSVLNLNLIRVSVRPSSWLLHVHDEDPCLTTTGGPWSSITGPTVTPQRRDVCSYRGWSLRLHYMTHSSGDLE
PSQGAPPGEQEIMFSLRRRS
KLRMKRRKMKRREKKRRRRSRRRMRRGAW
>g2077.t1
MADGGLLGRRRFSTCGGTTALHPTGRKLIRNASFGGYNQLAPLSLPGCPGKSRRRPVLLPSGAAGGTGPHCLEPLAHFEC
RCSAPTLHGDVAFDFPFR
>g2077.t2
MADGGLLGRRRFSTCGGTTALHPTGRKLIRNASFGGYNQLAPLSLPGCPGKSRRRPVLLPSGAAGGTGPHCLEPLAHFEC
RCSAPTLHGDVAFDFPFRGL
PPVEESSATRSSHSTHVLLTLQMACGALLSACRTGVASGHLHHAPLLILLLLLLLLFFSLLFIFLLFILSLLLLLRLNMI
SCSPGGAP
>g2077.t3
MADGGLLGRRRFSTCGGTTALHPTGRKLIRNASFGGYNQLAPLSLPGCPGKSRRRPVLLPSGAAGEAIRLSENSLHETNH
YTRLPNGQENSLTLHQTT
>g2078.t1
MNRQLGQKKTRSTGLRTDRQASPWVQSPGRPDALASTPVLLGPDTHTSSMISPPSPSLPPLPPCHPSHAPDGTIAQTEDG
HWLRV
>g2079.t1
ERERERERERERERERQSQSSSFTSTEVQEGAPALSMLQPRPVALQGMRVRRVPLEMPEFDLDHESLQESQENTLMIE

EKAKPKQYYRFWVLPGRWLR
VRYDRLALLALLDRNRRVGENVFAVVLASLVAFLGFLLLLQGFFRDIWVFQFCLVIASCQYSLLKGHNWIIVYSRPVYFC
LCCVLIWVFDLSGRSSSLQP
FSLYGVTFFSGQFLLCARDMLIVLALCFPVIFLLGLLPQVNTFIMSLLEQIDMHVFGGTATTSPLSSLFSLVRSVLVAAL
LYGFCLGAIN
>g2079.t2
ERERERERERERERERERQSQSSSFTSTEVQEGAPALSMLQPRPVALQGMRVRRVPLEMPEFDLDHESLQESQENTLMIE
EKAKPKQYYRFWVLPGRWLR
VRYDRLALLALLDRNRRVGENVFAVVLASLVAFLGFLLLLQGFFRDIWVFQFCLVIASCQYSLLKSVQPDAASPMHGHNW
IIVYSRPVYFCLCCVLIWVF
DLSGRSSSLQPFSLYGVTFFSGQFLLCARDMLIVLALCFPVIFLLGLLPQVNTFIMSLLEQIDMHVFGGTATTSPLSSLF
SLVRSVLVAALLYGFCLGAI
N
>g2080.t1
MLYMPEADTFYLDKMKGEKELLPEIYNGMCNLEKFYTDPKAGDGNSYRLQLWMYTMRLLQYSDAIEHLLTTGQGVILERS
PFSDMVFMEAMFKQGYIRKE
CVAHYNEIKSISICEFLPPHVVIYVDQAAEEVQKKLKQSNKKLFVASTSALAPCGFAALFCSNVNGPVSKRCPGCSCPHL
QNVPLAYLKEIEYSYKKNFL
PQI
>g2080.t2
MKGEKELLPEIYNGMCNLEKFYTDPKAGDGNSYRLQLWMYTMRLLQYSDAIEHLLTTGQGVILERSPFSDMVFMEAMFKQ
GYIRKECVAHYNEIKSISIC
EFLPPHVVIYVDQAAEEVQKKLKQSNKKLFVASTSALAPCGFAALFCSNVNGPVSKRCPGCSCPHLQNVPLAYLKEIEYS
YKKNFLPQI
>g2080.t3
MLYMPEADTFYLDKMKGEKELLPEIYNGMCNLEKFYTDPKAGDGNSYRLQLWMYTMRLLQYSDAIEHLLTTGQGVILERS
PFSDMVFMEAMFKQGYIRKE
CVAHYNEIKSISICEFLPPHVVIYVDQAAEEVQKKLKQSNKNRVITCVAQKLFVASTSALAPCGFAALFCSNVNGPVSKR
CPGCSCPHLQNVPLAYLKEI
EYSYKKNFLPQI
>g2081.t1
VKTQHHEEEKRALSREIIVLNNHLMEAKITVDKLREDNDLYRKDCNLAAQLLQCSKSHHRAHKMSELPLDFQERISSHLE
KLSRGGGGVTAGMCHSPYSD
SVPTSIIAKILEKPEPGSSCPVTRSPSPQVQDADFLSGTGSTDRLNRRIAYKTSDLYCSDTALYCPERWQDGERRQSVDL
NGTRLLQLHAQNSVDSNPDE
EAFHSGSFSHHEAPSPFHAHDEFGTGSLPASSSYSSFSIASDDKGAI
>g2082.t1
LVDPLDESLEAGLSRELLEETGVALPFSEVDHMASCFAPPTSSSFSSSSTPRLITHFYVKKLEEEQIRAMEIAGPTTAME
HGLE
>g2082.t2
LVDPLDESLEAGLSRELLEETGVALPFSEVDHMASCFAPPTSSSFSSSSTPRLITHFYVKKLEEEQIRAMEIAGPTTAME
HGLE
>g2083.t1
DRFPADLELDVFGGDLECDMDAIIRNELMDADCLDLSFESQFPVAPDAERNGGGVPKTSPRSWVPS
>g2084.t1
TPPLLPSALVLPSALVLPEALLLPEASSYPPPSSYPRPSSYLRPSYLRPSYLRPSYLRPPSYLRPPPTLRPRPTRGLLLP
SALLLPEALVLPEALLLPEA
LLMEPKAIPLCSFHLLMMVSM
>g2084.t2
TPPLLPSALVLPSALVLPEALLLPEASSYPPPSSYPRPSSYLRPSYLRPSYLRPSYLRPPSYLRPPPTLRPRPTRGLLLP
SALLLPEALVLPEALLLPEA
LLMEPKAIPLCSFHLLMM
>g2085.t1
PGQPRPHGRTTKKHRRSEQERATPPDPGSPPLHSSNPLISSSSSSSFSSSSPSSSDSDSESERRATVTQVPAVSTSNKRP
VPKRPEPVPPGRPKDCRARS
AGPQGPEAGSASEGQPSEGRKKLYTLVPFGRSEQATAPSHRGLRNLRVQIDLCLLKRVPDTTSNTTTATTNTTTATTTPT

TSTTANHKQPPTPCPSALVK
AKTGRAMRHLHVPGTENRDSKRKRKSENGVSHRDSKRASDPSGQTVSAAAQKPLHRHLATETLHN
>g2085.t2
PGQPRPHGRTTKKHRRSEQERATPPDPGSPPLHSSNPLISSSSSSSFSSSSPSSSDSDSESERRATVTQVPAVSTSNKRP
VPKRPEPVPPGRPKDCRARS
AGPQGPEAGSASEGQPSEGRKKLYTLVPFGRSEQATAPSHRGLRNLRAKTGRAMRHLHVPGTENRDSKRKRKSENGVSHR
DSKRASDPSGQTVSAAAQKP
LHRHLATETLHN
>g2085.t3
PGQPRPHGRTTKKHRRSEQERATPPDPGSPPLHSSNPLISSSSSSSFSSSSPSSSDSDSESERRATVTQVPAVSTSNKRP
VPKRPEPVPPGRPKDCRARS
AGPQGPEAGSASEGQPSEGRKKLYTLVPFGRSEQATAPSHRGLRNLRVQIDLCLLKRVPDTTSNTTTATTNTTTATTTPT
TSTTANHKQPPTPCPSALVK
AKTGRAMRHLHVPGTENRDSKRKRKSENGVSHRDSKRASDPSGQTVSAAAQKPLHRHLATETLHNG
>g2086.t1
MASKRKSTVPCMIPSKSKHMREEIILGCLPDLLPTIPEDSILSISGDGSGHNQHTSPKPPEINRGQKGGMYSCSNCRFES
RDLNYFLDHMHNCHIDFRAQ
PTFYCLNCGVSVVRFEALALHNAKAHRGIMQGSITASLHVNKRDGLTTVEQSLFTDSGEDYRESGLSLTKTPIAKMMKAK
GEHKKIVVSHTVEVRKIDTG
KDVDLNMLTNVPELQNGALSVSGAPAMPRTTVTHAISTTSNQVFHQQTPPLYSSHSSNSFKDLPKVMIPLSSIPTYDAAM
DTSSFLKTTFGKFPYPTKAE
LCYLTVVSEFPEEQIKLWFTAQRLKQGISWSPEEIEEARRKMFNTVFQGQPQQKQPVVPQHASHMVTHRTTVTTQPALLG
QAGPHYPMPNIPFGSLRNRS
TGVITTQANMSTNPKVTRVTYSAPAYPQKFPSVVRTSQVLTKNAHLPALEPTKSNGLELAGGGVCVSSTSSSRSSSSSSC
SSSSSISSYSSCSNGGETGK
RKHMNGNSPTNEVVTCSANISNSEEHGSDGNVHVKMNSIPVEMEGGVATKSKVIVNNTSITDSTRNMHNNNSTIIQHEQS
RFTKPNGDCNNYMNQHTKNC
SISSDCPSTMDNDSVPNLPNHASNSPNESTSTTVITKSCSSLSPLEEGTFTKDFPIKGMSILQQLIKDDSPEQDTCCSEL
KVDPIKINLKKQKMNELEPK
SDLDIFAPLTMNPETFDSPLSQPSFIPPWGTMTPHQVQILRQVFYGTPWPSSQQYEDLSIMTGLPKSEVVRWFSDSRYIH
KNSQRKWLENYQRLPTESEE
PNIRGEANGDASMDPLLPRRKSMEQDPNVHHEGGGAGMNLCQREVWQDAYSPLLGLMGSAEAGERGRPGTSVQPGVLQDP
WLERVKDHPQPMANQSFMEQ
QTDASQAR
>g2087.t1
TGEGCRVVEVVEVEVEVVEVVVVGALCRYALGMEDGTIPDSDITASSAWSDSTEAKHG
>g2088.t1
MFTGGMRESNQDTIELQGLSARGLKHIIDFAYSSEVTLDLDCVHDVLGAAVFLQMVPVVELCEEYLKSAMSVETCLNIGQ
MATSFNLSSLKQSVDAFTFR
HFLQIAAEDDFLHIPLDRLVFFLRSNKLKNCTEIDLFHAAIRWLQHDDARRALASALLGHVRFPLMRSSELVDSVQTVGL
MVEDVLCRRYLLEAFNYQIL
PLRQHEMQSPRTLIRSDVPSLVTFGGTPYTDNDRTVSAKAYYLPDAASRQFKELTEMESGVSHACVAVLDNFVYVVGGQH
LQYRSGEGATDCCFRYDPHL
NQWLRVQAMQEGRIQFQLNVLQGRLYATGGRNRTGSLSSVERYCPKKNEWSNVEPLKRRIWGHAGGCLGDKLYVSGGYGV
SLEDKKTLHCYDPVADQWEF
RAPMNEPRVLHAMIGAKDRLYAMGGRMDHVDRCFDVLAVEFYLPLSDQWTTVSPMRVGQSEAGCCLLNAKIYVVGGYNWH
LNNVTSIVQVYNPDTDEWER
DLHFPESFAGIACTPIILPQTTTQR
>g2089.t1
MIKLCVRRAFRLGGSAWSPRTPAGAPRHVPACLMGTHGRKEPRETLLSSKGAKDFIYRLQPDERTCLLQELQSFESLAAA
QVLLHNGIPFVGFGFLDNAI
MIAAGTQIELSIGVTLGISTMAAAALGNLVSDLAGLGLAGYVEALVSRLGLQAPPLSAQQGDMWQTRLSSHLSSFITCMT
NMELLLNTSYTLHHLHTTCS
SLLLSTPGGDPVVQWR
>g2089.t2
MIKLCVRRAFRLGGSAWSPRTPAGAPRHVPACLMGTHGRKEPRETLLSSKGAKDFIYRLQPDERTCLLQELQSFESLAAA

QANQEASPPTRAQLRSVLLH
NGIPFVGFGFLDNAIMIAAGTQIELSIGVTLGISTMAAAALGNLVSDLAGLGLAGYVEALVSRLGLQAPPLSAQQGDMWQ
TRLSSHLSSFITCMTNMELL
LNTSYTLHHLHTTCSSLLLSTPGGDPVVQWR
>g2089.t3
MIKLCVRRAFRLGGSAWSPRTPAGAPRHVPACLMGTHGRKEPRETLLSSKGAKDFIYRLQPDERTCLLQELQSFESLAAA
QANQEASPPTRAQLRSVLLH
NGIPFVGFGFLDNAIMIAAGTQIELSIGVTLGISTMAAAALGNLVSDLAGLGLAGYVEALVSRLGLQAPPLSAQQGDMWQ
TRLSSHLEETLWSSGDEEEP
GVPSSTDP
>g2090.t1
YKKVRQEIKKRSSDTARLQKKARKGRGDIQPQLDSAMQDVSEKYFLLEETERQALRRALVEERSHFCSFASMLRPVLEEE
VLMLGDMGSLRALNDDLKTL
TMDPHRLPPSSEQVIIDLKGSEVTWSYQTPPSSPSNSLSRKSSMCSGSHSHSPSSHFRFRGSSSSSSSSALPLVPRLSSV
SSHDSGFISHDASCPSISSS
AMSPDGAQDWYRPAPYDLRRIKTTDPSSHLTCDVTTPGEEPARARGGGSVTRSKEEREVHEELSRALARGLHLDLQGPGG
GSLQGSSGYSSQTATPAQTP
SRSDPLQHTPGAPGPVRPPMIPVKTPTVPDRLLCPLDRPPDPHTGPWTNCPAPPPQHPRGAERRPLPGLLEEGEQVEGGG
G
>g2090.t2
YKKVRQEIKKRSSDTARLQKKARKGRGDIQPQLDSAMQDVSEKYFLLEETERQALRRALVEERSHFCSFASMLRPVLEEE
VLMLGDMGSLRALNDDLKTL
TMDPHRLPPSSEQVIIDLKGSEVTWSYQTPPSSPSNSLSRKSSMCSLSSDSRSSGSHSHSPSSHFRFRGSSSSSSSSALP
LVPRLSSVSSHDSGFISHDA
SCPSISSSAMSPDGAQDWYRPAPYDLRRIKTTDPSSHLTCDVTTPGEEPARARGGGSVTRSKEEREVHEELSRALARGLH
LDLQGPGGGSLQGSSGYSSQ
TATPAQTPSRSDPLQHTPGAPGPVRPPMIPVKTPTVPDRLLCPLDRPPDPHTGPWTNCPAPPPQHPRGAERRPLPGLLEE
GEQVEGGGG
>g2090.t3
LVRVNWLLVSSEYKKVRQEIKKRSSDTARLQKKARKGRGDIQPQLDSAMQDVSEKYFLLEETERQALRRALVEERSHFCS
FASMLRPVLEEEVLMLGDMG
SLRALNDDLKTLTMDPHRLPPSSEQVIIDLKGSEVTWSYQTPPSSPSNSLSRKSSMCSGSHSHSPSSHFRFRGSSSSSSS
SALPLVPRLSSVSSHDSGFI
SHDASCPSISSSAMSPDGAQDWYRPAPYDLRRIKTTDPSSHLTCDVTTPGEEPARARGGGSVTRSKEEREVHEELSRALA
RGLHLDLQGPGGGSLQGSSG
YSSQTATPAQTPSRSDPLQHTPGAPGPVRPPMIPVKTPTVPDRLLCPLDRPPDPHTGPWTNCPAPPPQHPRGAERRPLPG
LLEEGEQVEGGGG
>g2091.t1
MYLYVLGVVVFYFLYRWVRELPRVSDKGAKHVYVTGCDSGFGNMLACHLDSLGFSVIAGCFTEKGEEELRKRCSARMTTT
HLNVRSLESIAKVAAMIKDK
VGERGLWAVVNNAGVATPSAPWDWLSFEDYRVMMEVNMDGVVAVTLSVLQLIKKARGRVVNVASVFGRISPTGGAYCVSK
YGVESFNDSLRLNMKPFGVT
VSAIEPGFFRTAVTAYEAHCQSNQKIWAKMPQEVRDDYGHDYLQKVEHIIREVPKMADPDLMKVVHCMEHAVAAVTPRKR
YSPGWDAKLFWLPLSYMPSC
LADYVLLSRAIPPAKAAK
>g2091.t2
MYLYVLGVVVFYFLYRWVRELPRVSDKGAKHVYVTGCDSGFGNMLACHLDSLGFSVIAGCFTEKGEEELRKRCSARMTTT
HLNVRSLESIAKVAAMIKDK
VGERGAFGGLWAVVNNAGVATPSAPWDWLSFEDYRVMMEVNMDGVVAVTLSVLQLIKKARGRVVNVASVFGRISPTGGAY
CVSKYGVESFNDSLRLNMKP
FGVTVSAIEPGFFRTAVTAYEAHCQSNQKIWAKMPQEVRDDYGHDYLQKVEHIIREVPKMADPDLMKVVHCMEHAVAAVT
PRKRYSPGWDAKLFWLPLSY
MPSCLADYVLLSRAIPPAKAAK
>g2092.t1
LSSTAEEVCYDDLGCFSDLPPWGGTTERPVARLPWTPGLIGTRFLLFTQKNKYYQASRGAFALWRRDGGFQSC
>g2093.t1

MKIMKVTNYNKLRATRFIIPGYLEKNDEDWPQDMCRDMLKWDSVNCIAIEWKAGVKTPYAQSANNARVVAAQVAHMINFL
MANYMQTADKFHVVGHSLGA
HAAGQVGTLIPNLGRITGLDPVEPYFQGTLRSVRLDASDATFVDVIHTDALPFTKNKLGLGMAEAMGHIDFYPNGGELMP
GCDKNRGSPSDLDAIWEGNK
AFDGCNHARAYKYYSESTVKAQGFVAYPCPDKESFTA
>g2093.t2
MKIMKVTNYNKLRATRFIIPGYLEKNDEDWPQDMCRDMLKWDSVNCIAIEWKAGVKTPYAQSANNARVVAAQVAHMINFL
MANYMQTADKFHVVGHSLGA
HAAGQVGTLIPNLGRITGLDPVEPYFQGTLRSVRLDASDATFVDVIHTDALPFTKNKLGLGMAEAMGHIDFYPNGGELMP
GCDKNRGSPSDLDAIWEGNK
AFDGCNHARAYKYYSESTVKAQGFVAYPCPDKESFTAVSVSTVCLCDCVFVL
>g2094.t1
MSSPVDFHSQIASIIEVLANAAVAEICKVVDDGYAVVHLEVSQNQKENDFLRRKIRLLELQVAKYRAERMKGPEGIVRFP
GARMLGRPSREALAGPSPQG
RLRGLGRSQKTLPLPRDQDPHQQVVTSTKMEAGEEEPLMIVKVEGAEPSESDHAPDIPDAGSGPTPLAGPEEPRSQSEVS
GSDDLTLLFTSGCSQEPLTG
CDVSVGSQLLPGEQKERHQRRAWPDILPPFTQDLSSRDHSAVAAVMVLDDKGEEFHWSGGGKREEEQGGREEERGREESS
TVGLGFNVAPGPSPPGGPAR
RLPQRPPVTSDPGHSPPHGCGVCGKRFLQEAELQKHAARHRRRRAHACALCGKSFVCRSQLDVHGYVHTGERPFGCPTCP
RRFSHPSNLRRHQKTRRRSD
PGTLAAGSVQCAGQGVKLSLHLQGPGPSLGTPCCRTEPGAAAPGNPSPPGVLWKGQHGGQEDDQQPSCSSQMFEEAPPPR
GSKQEVILIDALQDLTQAGG
DDVALPWIHGGNRPPGDLLLAADGSYGAAF
>g2094.t2
MSSPVDFHSQIASIIEVLANAAVAEICKVVDDGYAVVHLEVSQNQKENDFLRRKIRLLELQVAKYRAERMKGPEGIVRFP
GARMLGRPSREALAGPSPQG
RLRGLGRSQKTLPLPRDQDPHQQVVTSTKMEAGEEEPLMIVKVEGAEPSESDHAPDIPDAGSGPTPLAGPEEPRSQSEVS
GSDDLTLLFTSGCSQEPLTG
CDVSVGSQLLPGEQKERHQRRAWPDILPPFTQDLSSRDHSAVAAVMVLDDKGEEFHWSGGGKREEEQGGREEERGREESS
TVGLGFNVAPGPSPPGGPAR
RLPQRPPVTSDPGHSPPHGCGVCGKRFLQEAELQKHAARHRRRRAHACALCGKSFVCRSQLDVHGYVHTGERPFGCPTCP
RRFSHPSNLRRHQKTRRRSD
PGTLAAGSVQCAGQGVGPGPSLGTPCCRTEPGAAAPGNPSPPGVLWKGQHGGQEDDQQPSCSSQMFEEAPPPRGSKQEVI
LIDALQDLTQAGGDDVALPW
IHGGNRPPGDLLLAADGSYGAAF
>g2095.t1
MPVQPPPTSLAPAQPQGPGLMAQMATTAAGVAVGSAVGHVVGGALTGAFSGGSSSSSPEPARPAYQEAPRPVPGAQGPCH
FEVRQFLDCATTQGDLSLCE
GFSEVLKQCKSSHAMPFIVLDTNISADGLPADFLKKLCSCTAASLGKPEERMNVTVKPGLPMLMAGSSSPCVILSVSAIG
VTDTAEKNREHSANICQFLA
KELALPVD
>g2095.t2
MPVQPPPTSLAPAQPQGPGLMAQMATTAAGVAVGSAVGHVVGGALTGAFSGGSSSSSPEPARPAYQEAPRPVPGAQGPCH
FEVRQFLDCATTQGDLSLCE
GFSEVLKQCKSSHAMPFIVLDTNISADGLPADFLKKLCSCTAASLGKPEERMNVTVKPGLPMLMAGSSSPCVILSVSAIG
VTDTAEKNREHSANICQFLA
KELALPVDR
>g2095.t3
MLRSALIPNKTKVRNHQFRPPPTSLAPAQPQGPGLMAQMATTAAGVAVGSAVGHVVGGALTGAFSGGSSSSSPEPARPAY
QEAPRPVPGAQGPCHFEVRQ
FLDCATTQGDLSLCEGFSEVLKQCKSSHAMPFIVLDTNISADGLPADFLKKLCSCTAASLGKPEERMNVTVKPGLPMLMA
GSSSPCVILSVSAIGVTDTA
EKNREHSANICQFLAKELALPVD
>g2096.t1
DREHLVLKAEEEDEEEEEEEQEQEQEQEQDQEQEQEQKQEQEQK
>g2097.t1

VERVDRITPTLTRLGSPSRVVLLGDGRRAVRITSRELQAWDPDSPLQQLEYSITTPPTHGHLENTVTGGFIQGRFTQQDL
DQQAVVYLLPTDLEITSDHF
QFQLRDPAGNTLPPQRLQLSWSRVQVALSCYRVCETAGSLGVQVVRSGNSADPAYVAIQVEEGTAKVDKDFTHSTASLIQ
FDPGVNVKTWTIYLHDDQLE
ENQESFRITLSNPDNAVLGPRHSVTVEIIDPRGGSCDPEDLKVEGKSSASPPPPPAPTDAAQEEEGPITDIEAELLWDSH
TPPARGDVPRPDDHKYVEEE
VPQDQPLQYPLRPAGGARPLQYTLRPAGGDRLLQYPLRPAGGARLLQYPLRPTGGFRPLQYPLKPAGGAWDGLHRKVGVY
VSSCWL
>g2097.t2
VERVDRITPTLTRLGSPSRVVLLGDGRRAVRITSRELQAWDPDSPLQQLEYSITTPPTHGHLENTVTGGFIQGRFTQQDL
DQQAVVYLLPTDLEITSDHF
QFQLRDPAGNTLPPQRLQLSWSRVQVALSCYRVCETAGSLGVQVVRSGNSADPAYVAIQVTHVP
>g2098.t1
VRLRWTSQLAGQAIPPGDTSLPSVTPLCSPGSSNDKVLENGLLFKELLQTPNFRITVVDDADTVELCGALKNIVAVGAGF
CDGLGCGDNTKAAVIRLGLM
EMIAFSRLFSKDAVSSATFLESCGVADLITTCYGGRNRRVAEAFAKTGKSIEELEREVLNGQKLQGPATSMEVYHILKQK
GLVDKFPLFTAVYQICFEGR
PVGEMISCLQSHPEHL
>g2098.t2
LAGQAIPPGDTSLPSVTPLCSPGSSNDKVLENGLLFKELLQTPNFRITVVDDADTVELCGALKNIVAVGAGFCDGLGCGD
NTKAAVIRLGLMEMIAFSRL
FSKDAVSSATFLESCGVADLITTCYGGRNRRVAEAFAKTGKSIEELEREVLNGQKLQGPATSMEVYHILKQKGLVDKFPL
FTAVYQICFEGRPVGEMISC
LQSHPEHL
>g2099.t1
MNKNIKKGDVSWVKPSEPSFLKKFKSDVGYKEGPDVDTKRQAMPTLDDDSGSDREDELPQVVVLKKGDLSADEVQQIRGG
GASEKEDQPPADGKIVFKKP
AKRSSTDRFQGITASSSKKKKSDTGSKEDKDDKEDKKEDKKEEKKEDKSGRKVKNSLLSFGGDEEEDEEDDEV
>g2099.t2
LLRASGTTMNKNIKKGDVSWVKPSEPSFLKKFKSDVGYKEGPDVDTKRQAMPTLDDDSGSDREDELPQVVVLKKGDLSAD
EVQQIRGGGASEKEDQPPAD
GKIVFKKPAKRSSTDRFQGITASSSKKKKSDTGSKEDKDDKEDKKEDKKEEKKEDKSGRKVKNSLLSFGGDEEEDEEDDE
V
>g2100.t1
MISGDVYALLAGFLGAVASSSAKLSLGADYLKDMCDTGMKRWTTGDHWSSDDGEETPCDWVHIPLRLMCGGLLFTCNAVM
WTFFSKALRHCPSSARATVT
TTASNFVSSAVLGRMIFGETHAALWWVGISLTLSGLLVLHDSTPQIASPDRDKKDN
>g2101.t1
ALNYQSEGRVLQLNRAKFYSNGNCGYILKPACMCEGDFNPHLEDPLPGQMKKQLVLKIISGQQLPKPKDSMLGDRGE
>g2102.t1
LLRPGVNRRLLRPSSSDRRLLLPSENSRSLRPSGGDRRSLRLIEVVGKDDLLCKPLKQGYHFPPVSGAGAQLGSGATDLV
AHLADRSPPCARRVPWYGFL
SAGSI
>g2102.t2
LWPRGGDRRLLRPGVNRRLLRPSSSDRRLLLPSENSRSLRPSGGDRRSLRLIEVVGKDDLLCKPLKQGYHFPPVSGAGAQ
LGSGATDLVAHLADRSPPCA
RRVPWYGFLSAGSI
>g2103.t1
LSPVGDVQPTPAPRSLVAVQPMPPPESAATPESLPPESAASASLEALAPVTAASLETPPPDAAASLESPPPVTAASPECP
GFRLRHRPPEGFHLRHRPHD
GFRLRRQPLEGFPFAADLPRAFGAGLLRDFAFTTGLWEGSPACVAPGQPSGHPPEVPCSTPGNPPPSPLLPPISVLEFMD
VRSRPLRGGFCHVLSATPPS
SVVDSDSQVLRIR
>g2104.t1
MWTWNSSLYFDHCECWEPQGASGSLQWDDLEAVRTTVFGMCCATISLATYTTHHGRDSVSQQKGSGHGPTTHGSSSSSMA
YTAKIGPSILSSNLACLGTE

CVRMMECGADYLHLDVMDGHFVPNLTFGHPMVECLRKSVGPDPFFDLHMMVSRPEQWVKPMSAAGANQYTFHLEATTNPG
NLIKEIREAGMKVGLAIKPG
TTVEELAPWAGQIDMALVMTVEPGFGGQKFMVDMMPKVSWLRSQFPSLDIEVDGGVGPDTIHRCAEVRGPRCATASRSHC
FPFPLLPVPTSPQLTCLCLL
GAPRRREPT
>g2104.t2
MAYTAKIGPSILSSNLACLGTECVRMMECGADYLHLDVMDGHFVPNLTFGHPMVECLRKSVGPDPFFDLHMMVSRPEQWV
KPMSAAGANQYTFHLEATTN
PGNLIKEIREAGMKVGLAIKPGTTVEELAPWAGQIDMALVMTVEPGFGGQKFMVDMMPKVSWLRSQFPSLDIEVDGGVGP
DTIHRCAEVRGPRCATASRS
HCFPFPLLPVPTSPQLTCLCLLGAPRRREPT
>g2104.t3
MWTWNSSLYFDHCECWEPQGASGSLQWDDLEAVRTTVFGMCCATISLATYTTHHGRDSVSQQKGSGHGPTTHGSSSSSMA
YTAKIGPSILSSNLACLGTE
CVRMMECGADYLHLDVMDGHFVPNLTFGHPMVECLRKSVGPDPFFDLHMMVSRPEQWVKPMSAAGANQYTFHLEATTNPG
NLIKEIREAGMKVGLAIKPG
TTVEELAPWAGQIDMALVMTVEPGFGGQKFMVDMMPKVSWLRSQFPSLDIEVDGGVGPDTIHRCAE
>g2105.t1
MLSQDSPAARFMKLLTSKCWAYSPAAMKGKRDVYFGPGFVSYDSYAMAACVDATVVTKSLRCGVHVELQGGVCRGMMALD
PTDQLQKPHRVTVMTECDPE
KFRKLLANSLRQP
>g2106.t1
DEEVVLENPADVPVFVQLLPLALFPSPSVFSGKLADRLPWANLSNINIDTKTLEFRVHKHPGPSKSLAYNLLLQPGETKS
FNVTFTPSSNHSVSSLLIVR
NNLTVMDVVLLEGRGVLEHLRVGGKTQGQGGYLRFKMTEALLRQCTNHTNMTEPTHSLRRTFKVENAGVLPVHITSAQIN
RHVCGGHGFTILDCQEFTIQ
PNSTRELVILELRLVTRGGSEFVFVLNASLPHHMLGPCAETLPRPAWEWNFCLLVSLVMSCMLLLVGVMAYLEAHSRWEM
FRTHLPLETGLFDLRETNSL
THSDDPHDPPLNSRGFYGSGGGASQVGGRHGAEAGGLDRKSRAGQSMVARWTSSSPSANGSASGSAQLANRKAQSGRQQP
IVGLGEDEGQPGTGLPQRRG
LCAGEEEEEEDTGVLEVLDQEEELSGATSGHTSDQPSSLPEQQSELSLKVQAKSKEQKKKKKKKKRKRKEERKEPEEEDEQ
EEEEEAEEEEEEETEGEKKVE
THGDELKDLQDDDASSTTTDTSTTDTSTTDLEASIREQSSPVPDLDYCSNLLPEPDHCIVSEEEKELDPPEWDLPLSTIS
SLEVATLQQISMQTRNTDLF
LKRARLGLCSPPPGLPAPLPMDGYGVTLDATSDETRRAPGTRSAAGRIPDHMQDQEPSSAGYDRSPGAPAMWRPEHPAPI
LTHSASVDNYSSESSGLWTP
LVTRSPPPVSSDHSFSAFGPSNSFNLTRVFRGINEAKKPDPQQSRSEPTEASSSPSSIWDASPVDPLTSWASSCGSPTIP
TTSIFTTSGSPWSTTGPFSS
PIWSTSQDPTLHHCFTTPSNALPTTPETPPSPGELSPTYNPWSMWRPLLARQGSEPWPSTSYDPI
>g2106.t2
DEEVVLENPADVPVFVQLLPLALFPSPSVFSGKLADRLPWANLSNINIDTKTLEFRVHKHPGPSKSLAYNLLLQPGETKS
FNVTFTPSSNHSVSSLLIVR
NNLTVMDVVLLEGRGVLEHLRVGGKTQGQGGYLRFKMTEALLRQCTNHTNMTEPTHSLRRTFKVENAGVLPVHITSAQIN
RHVCGGHGFTILDCQEFTIQ
PNSTRELVILFTPDFSASRVVRELRLVTRGGSEFVFVLNASLPHHMLGPCAETLPRPAWEWNFCLLVSLVMSCMLLLVGV
MAYLEAHSRWEMFRTHLPLE
TGLFDLRETNSLTHSDDPHDPPLNSRGFYGSGGGASQVGGRHGAEAGGLDRKSRAGQSMVARWTSSSPSANGSASGSAQL
ANRKAQSGRQQPIVGLGEDE
GQPGTGLPQRRGLCAGEEEEEEDTGVLEVLDQEEELSGATSGHTSDQPSSLPEQQSELSLKVQAKSKEQKKKKKKKKRKRK
EERKEPEEEDEQEEEEEAEE
EEEETEGEKKVETHGDELKDLQDDDASSTTTDTSTTDTSTTDLEASIREQSSPVPDLDYCSNLLPEPDHCIVSEEEKELD
PPEWDLPLSTISSLEVATLQ
QISMQTRNTDLFLKRARLGLCSPPPGLPAPLPMDGYGVTLDATSDETRRAPGTRSAAGRIPDHMQDQEPSSAGYDRSPGA
PAMWRPEHPAPILTHSASVD
NYSSESSGLWTPLVTRSPPPVSSDHSFSAFGPSNSFNLTRVFRGINEAKKPDPQQSRSEPTEASSSPSSIWDASPVDPLT
SWASSCGSPTIPTTSIFTTS

GSPWSTTGPFSSPIWSTSQDPTLHHCFTTPSNALPTTPETPPSPGELSPTYNPWSMWRPLLARQGSEPWPSTSYDPI
>g2106.t3
MNPSDSDCDSSVSWVLLYNCGSVNWVLLYNCSSDEEVVLENPADVPVFVQLLPLALFPSPSVFSGKLADRLPWANLSNIN
IDTKTLEFRVHKHPGPSKSL
AYNLLLQPGETKSFNVTFTPSSNHSVSSLLIVRNNLTVMDVVLLEGRGVLEHLRVGGKTQGQGGYLRFKMTEALLRQCTN
HTNMTEPTHSLRRTFKVENA
GVLPVHITSAQINRHVCGGHGFTILDCQEFTIQPNSTRELVILFTPDFSASRVVRELRLVTRGGSEFVFVLNASLPHHML
GPCAETLPRPAWEWNFCLLV
SLVMSCMLLLVGVMAYLEAHSRWEMFRTHLPLETGLFDLRETNSLTHSDDPHDPPLNSRGFYGSGGGASQVGGRHGAEAG
GLDRKSRAGQSMVARWTSSS
PSANGSASGSAQLANRKAQSGRQQPIVGLGEDEGQPGTGLPQRRGLCAGEEEEEDTGVLEVLDQEEELSGATSGHTSDQP
SSLPEQQSELSLKVQAKSKE
QKKKKKKKKRKRKEERKEPEEEDEQEEEEEAEEEEEETEGEKKVETHGDELKDLQDDDASSTTTDTSTTDTSTTDLEASI
REQSSPVPDLDYCSNLLPEP
DHCIVSEEEKELDPPEWDLPLSTISSLEVATLQQISMQTRNTDLFLKRARLGLCSPPPGLPAPLPMDGYGVTLDATSDET
RRAPGTRSAAGRIPDHMQDQ
EPSSAGYDRSPGAPAMWRPEHPAPILTHSASVDNYSSESSGLWTPLVTRSPPPVSSDHSFSAFGPSNSFNLTRVFRGINE
AKKPDPQQSRSEPTEASSSP
SSIWDASPVDPLTSWASSCGSPTIPTTSIFTTSGSPWSTTGPFSSPIWSTSQDPTLHHCFTTPSNALPTTPETPPSPGEL
SPTYNPWSMWRPLLARQGSE
PWPSTSYDPI
>g2107.t1
GGAFALGTYTILLLKLYSYKDVNRWCRELSSIKAKTLTRSYSCPSAQHLNGGDQKVCYPGNLTIRDLYYFVFAPTLCYEL
NFPRSPKIRMSFLLRRLFEM
WMIPIIQSSMKPLEDMDLSRMMERLLRLA
>g2107.t2
GGAFALGTYTILLLKLYSYKDVNRWCRELSSIKAKTLTRSYSCPSAQHLNGGDQKVCYPGNLTIRDLYYFVFAPTLCYEL
NFPRSPKIRMSFLLRRLFEM
WMIPIIQSSMKPLEDMDLSRMMERLLRLAVSRATQKPFIKSNLKLQYFFLFIATPQVEHYSAQ
>g2107.t3
GGAFALGTYTILLLKLYSYKDVNRWCRELSSIKAKTLTRSYSCPSAQHLNGGDQKVCYPGNLTIRDLYYFVFAPTLCYEL
NFPRSPKIRMSFLLRRLFEM
WMIPIIQSSMKPLEDMDLSRMMERLLRLAVSRATQKPFIKSNLKLQYFFLFIATPQ
>g2108.t1
MIQKKIFLAFLVISTVSLLLHHGGHLESWTMEAFYLGCPSLCSHPSPGLEPKHTHVAFLKTHKTASSTMQNLLFRFAERH
NLTVALPVQSCGHQFCYPRT
FTAHFVHPHTLPASIITNHMRFSRAELQRFMPTDTMYVTILREPGSMFESLFSYYNQNCQSFKRVPNNSLEAFLEEPWRY
YRPSEKDSMYARNTLTFDLG
GDKDWPPEDVGYAEAFVADVDRLFGLVMIAEHFDESLVLLRHLLSWDLEDVLYLKLNMRTLSSKRSLSPELPSKIREWNA
LDARLYDHFNASLWRQITAM
GPACVAREVRLLRKAQERLVRSCFGGGMPLLRTGAEIKNKDLRPWQPSAKVDIVGYDLPANLSRGASSQVQELCLKLIMP
EVQYTQVLLR
>g2108.t2
VARRSEGSREMIQKKIFLAFLVISTVSLLLHHGGHLESWTMEAFYLGCPSLCSHPSPGLEPKHTHVAFLKTHKTASSTMQ
NLLFRFAERHNLTVALPVQS
CGHQFCYPRTFTAHFVHPHTLPASIITNHMRFSRAELQRFMPTDTMYVTILREPGSMFESLFSYYNQNCQSFKRVPNNSL
EAFLEEPWRYYRPSEKDSMY
ARNTLTFDLGGDKDWPPEDVGYAEAFVADVDRLFGLVMIAEHFDESLVLLRHLLSWDLEDVLYLKLNMRTLSSKRSLSPE
LPSKIREWNALDARLYDHFN
ASLWRQITAMGPACVAREVRLLRKAQERLVRSCFGGGMPLLRTGAEIKNKDLRPWQPSAKVDIVGYDLPANLSRGASSQV
QELCLKLIMPEVQYTQVLLR
>g2109.t1
MEGSDGSEQSGSDQSESQRRRQLDRLDREEAFYQFVNNLSDEDYRLMRDNNLLGTPGEVTEDELLSRLQQIKDGPEPPNS
SPSTETGEATGETPGSSEDS
SNGDTLLDWLNTVRRTGNTTRSGHRGNQSWRAVSQTNPNSGDFRFSLEINVNRSLAEQQAAAAAAAEGGQENPEAPGVPG
EAPGAPGEAP

>g2109.t2
MEGSDGSEQSGSDQSESQRRRQLDRLDREEAFYQFVNNLSDEDYRLMRDNNLLGTPETPGSSEDSSNGDTLLDWLNTVRR
TGNTTRSGHRGNQSWRAVSQ
TNPNSGDFRFSLEINVNRSLAEQQAAAAAAAEGGQENPEAPGVPGEAPGAPGEAP
>g2110.t1
LVTWCVQNNLELNALKTVETVVDFTKNTAPPAPITMCDYPVDAVESFRFLGSIITQDLRAMAEGGDNRRHPHNLQGVLRL
AVEAGAADDGPSPAEPMSEE
RKEWLRGALAEVSKGQLNEVEQMRRCLAVLQKDGMNAKEFEGEEGQRDEEEEEEDERESALEILSELCENLDNARDLMAL
GGLQLCVSQCLGHVQSGVRW
RAAQLLASCAQNMPEVQLHLLAIDALPKLLQLTDSDPHPTVRVKALYAVSCLVREQEAGLQAFASQDGFSVLMRGMQSDD
ERLRTKSAFLLLNLLSAHPE
HKGTVLSMGMVEQLVSVLRSPHSSFHEHVLGALCCLVEDCPQGLKDCRSSSLGLEELLKHRAKDLQGRDECQ
>g2110.t2
LVTWCVQNNLELNALKTVETVVDFTKNTAPPAPITMCDYPVDAVESFRFLGSIITQDLRAMAEGGDNRRHPHNLQGVLRL
AVEAGAADDGPSPAEPMSEE
RKEWLRGALAEVSKGQLNEVEQMRRCLAVLQKDGMNAKEFEGEEGQRDEEEEEEDERESALEILSELCENLDNARDLMAL
GGLQLCVSQCLGHVQSGVRW
RAAQLLASCAQNMPEVQLHLLAIDALPKLLQLTDSDPHPTVRVKALYAVSCLVREQEAGLQAFASQDGFSVLMRGMQSDD
ERLRTKSAFLLLNLLSAHPE
HKGTVLSMGMVEQLVSVLRSPHSSFHEHVLGALCCLVEDCPQGLKDCRSSSLGLEELLKHRAKDLQGRDECQLEEEGDQG
DPTVSGRPALSTRSTH
>g2110.t3
LVTWCVQNNLELNALKTVETVVDFTKNTAPPAPITMCDYPVDAVESFRFLGSIITQDLRAMAEGGDNRRHPHNLQGVLRL
AVEAGAADDGPSPAEPMSEE
RKEWLRGALAEVSKGQLNEVEQMRRCLAVLQKDGMNAKEFEGEEGQRDEEEEEEDERESALEILSELCENLDNARDLMAL
GGLQLCVSQCLGHVQSGVRW
RAAQLLASCAQNMPEVQLHLLAIDALPKLLQLTDSDPHPTVRVKALYAVSCLVREQEAGLQAFASQDGFSVLMRGMQSDD
ERLRTKSAFLLLNLLSAHPE
HKGTVLSMGMVEQLVSVLRSPHSSFHEHVLGALCCLVEDCPQGLKDCRSSSLGLEELLKHRAKDLQGRDECQEELDFCKR
LRATCFQGQQTDDNGMDR
>g2111.t1
LAVEAKKTFSRDKPHVNIGTIGHVDHGKTTLTAAITKVLAEAGGARFKKYEDIDNAPEEKARGITINASHVEYTTANRHY
AHTDCPGHADYVKNMITGTA
QMDGCILVVAATDGQMPQTREHLLLARQIGVEHMVVFINKADAVDDQEMLELVELEIRELLTEFGYDGE
>g2111.t2
LSRRNLAVEAKKTFSRDKPHVNIGTIGHVDHGKTTLTAAITKVLAEAGGARFKKYEDIDNAPEEKARGITINASHVEYTT
ANRHYAHTDCPGHADYVKNM
ITGTAQMDGCILVVAATDGQMPQTREHLLLARQIGVEHMVVFINKADAVDDQEMLELVELEIRELLTEFGYDGE
>g2112.t1
LRTARKLRNHRREQKWHDKQYKKAHLGTALKANPFGGASHAKGIVLEKVGVEAKQPNSAIRKCVRVQLIKNGKKITAFVP
NDGCLNFIEENDEVLVAGFG
RKGHAVGDIPGVRFKVVKVANVSLLALYKGKKERPRS
>g2112.t2
LRTARKLRNHRREQKWHDKQYKKAHLGTALKANPFGGASHAKGIVLEKVGVEAKQPNSAIRKCVRVQLIKNGKKITAFVP
NDGCLNFIEENDEVLVAGFG
RKGHAVGDIPGVRFKVVKVANVSLLALYKGKKERP
>g2112.t3
KCRGLRTARKLRNHRREQKWHDKQYKKAHLGTALKANPFGGASHAKGIVLEKVGVEAKQPNSAIRKCVRVQLIKNGKKIT
AFVPNDGCLNFIEENDEVLV
AGFGRKGHAVGDIPGVRFKVVKVANVSLLALYKGKKERPRS
>g2113.t1
MEDFSGLALPPLFGGHILEAELEPAGVELGPGEDESSVPLESAAGLEADRINGASGCSGAAHHGAGPKKRRHRIPKQEKD
KEPQ
>g2113.t2
MMEDFSGLALPPLFGGHILEAELEPAGVELGPGEDESSVPLESAAGLEADRINGASGCSGAAHHGAGPKKRRHRIPKQEK
DKEPQ

>g2114.t1
SPLLLATGTSNLSPCLIPSTVLSLGQAPSALNSMQAMTLPIAHSAPHSPPLPHSAPLSRAMTPLHLMHQQSGSGGSQVPS
LNPLLMKAAEMYATSTLPLP
RRPVSEMRLGGFQGTSLPRMMALSEPVRVEAMFAHTPAGSEGSAVGGACLLHFLPSDNITLLISEPRDGWHYGQNERTGR
KGWFPYSYTQPHANNMDSHG
SSSLLLSKANSGSAGQLDKLVSAGLPALTPESEEERSLPPPRVSTFRPRPYSMADSHKVSLRPSGAAQGMGEWIGPANRP
SLSMLMQELSSDLASPPPSP
TRTNPFAHVKLRRTVTNDRSAPIIE
>g2114.t2
LSPCLIPSTVLSLGQAPSALNSMQAMTLPIAHSAPHSPPLPHSAPLSRAMTPLHLMHQQSGSGGSQVPSLNPLLMKAAEM
YATSTLPLPRRPVSEMRLGG
FQGTSLPRMMALSEPVRVEAMFAHTPAGSEGSAVGGACLLHFLPSDNITLLISEPRDGWHYGQNERTGRKGWFPYSYTQP
HANNMDSHGSSSLLLSKANS
GSAGQLDKLVSAGLPALTPESEEERSLPPPRVSTFRPRPYSMADSHKVSLRPSGAAQGMGEWIGPANRPSLSMLMQELSS
DLASPPPSPTRTNPFAHVKL
RRTVTNDRSAPIIE
>g2114.t3
SPLLLATGTSNLSPCLIPSTVLSLGQAPSALNSMQAMTLPIAHSAPHSPPLPHSAPLSRAMTPLHLMHQQSGSGGSQVPS
LNPLLMKAAEMYATSTLPLP
RRPVSEMRLGGFQGTSLPRMMALSEPVRVEAMFAHTPAGSEGSAVGGACLLHFLPSDNITLLISEPRDGWHYGQNERTGR
SLLLSKANSGSAGQLDKLVS
AGLPALTPESEEERSLPPPRVSTFRPRPYSMADSHKVSLRPSGAAQGMGEWIGPANRPSLSMLMQELSSDLASPPPSPTR
TNPFAHVKLRRTVTNDRSAP
IIE
>g2115.t1
MPSGPEESLNKPSHWTGRLGRGQRVSGGGEAKPSLTHTERSSICGGMVVSVALCGSYDILSCIQYGSDRARTGLEQGSDR
ARTGIGPG
>g2116.t1
MVRPCNPNEVDVSTPSHPHSLTQGKHSLSATSSFRTPAAYLTSLLYVSRTQQEPLIENSHSGSGHVSKRFHVEISTMQLE
EPPRLEVHVLQQSSTEGLPS
HLNRQLAALPEGCRVFCPRLQGSGRPNMVDSGPLHVSR
>g2117.t1
MSPDSPPIRYRSVHLQLLSLRLVCSRGRSSTWNRVEKRPWMFGFETYLLLEMLPRLEATALQTSCVPTGDRRCCGAAGCR
PPASPQETGGAADSMKTLQH
PARCCGRKTPRCEDLRSPQTGRFSPGRYLQRA
>g2118.t1
LIQNGRLLTLPLVAGDLPALLPDEDGRRLFVALKDNLLSTSLDDITQNPRKLYWPASPDRVQECLMAGKDPETECGNFLR
VLQAYNQTHLYVCGTGAFNP
RCAFIPTSLFLQSEHQSLSYTQTECGKGKCPYDPTQRTASAVVDGELYAGITSDFMSRDSAFFRSLGTRRVIRTEQYDST
WLHGKELTSEPTRTTSPPEG
RGGPTNVFPRHSAHDEASRTLALLTQKTRPSVSVDWPSSPDVFTVALLQNNLKPHLNISPVRLVGSL
>g2118.t2
LIQNGRLLTLPLVAGDLPALLPDEDGRRLFVALKDNLLSTSLDDITQNPRKTECGNFLRVLQAYNQTHLYVCGTGAFNPR
CAFIPTSLFLQSEHQSLSYT
QTECGKGKCPYDPTQRTASAVVDGELYAGITSDFMSRDSAFFRSLGTRRVIRTEQYDSTWLHGKELTSEPTRTTSPPEGR
GGPTNVFPRHSAHDEASRTL
ALLTQKTRPSVSVDWPSSPDVFTVALLQNNLKPHLNISPVRLVGSL
>g2118.t3
LIQNGRLLTLPLVAGDLPALLPDEDGRRLFVALKDNLLSTSLDDITQNPRKLYWPASPDRVQECLMAGKDPETECGNFLR
VLQAYNQTHLYVCGTGAFNP
RCAFIPTSLFLQSEHQSLSYTQTECGKGKCPYDPTQRTASAVVDGELYAGITSDFMSRDSAFFRSLGTRRVIRTEQYDST
WLHGKELTSEPTRTTSPPEG
RGGPTNVFPRHSAHDEASRTLALLTQKTRPSEQRQGGGSGRCTAPRVASTLGRCRDAANASACDDDASLLIGSVQTPSRR
VV
>g2119.t1
MEGEEESVRGEESVASDLSGAIERKKREAKGSCLRIEGQEGYVFKSYSMNLSEVFDTESPAGSSAALVKYFTFPQEEEGV

GDLSPQVDKQSDSEKTKESI
PASPTISDKSLDTDHGEPIMEVEPETDNCDRAAKRIKVEDEEVNEMEEELQEDEGSLLDSSMGPYLPRDDDYSSLFSSYT
STLYDVAMDAVTQSLLSSIR
SNANPRKKSPAWNHFCISPRDSTKAICLYCMKEFSRGKNEKDLSTSCLMRHVRRAHPTVLLQEGTDAPSNHSPSAFNTPS
LIPPSPSSPKNGDLSMIALS
PPSNKTSPSTSSADNQDPSSKEVLPKVEPNPEPGTDAELSLAISTPPFSHSTESGNEDMLDGGLERFPGTPKSSSSRRRS
AVWKHFYLSPADSSKAVCIH
CMNEFSRGKNGKDLGTSCLIRHMWRAHKEVVIEENGQGNHIPPPYTNPPSHLPRSQLQDTLEIKKEFPVPLSPLEVMSDV
AALQHQEDSMEMKGDSDKAL
NLSGQESCLNLSMEVHGDDASDSSPGELPGGTSLSQDHSVFQQNKKIMKRVKSEVWHHFIVSPVDQLKALCRYCPCVISR
GKRGDFGTSCLMRHLMRRHP
DFLKNQKGPDDKDLSPRPYTPLVEPEPDSTKASVDQVTEKKPHTLPVFSKKTSKLWNHFSISPTDPTKVVCLHCSRTISR
GKKTTNLGTSCLFRHMQRFH
GHVLDSNNTISGNVPSAEIKVKQEHTETSSYEENSEKFDENHSVAKKITKLITEMLALDLQPSALVENSGLTRLLEYLQP
QYSAPSSSYFTSTAIPDMYE
RVKDAVLSHLKEAEGGVVHFTTSIWVSSQTREYLTLTAHWASYESNLRPQGQDYHCSALLSVSQIDCDHDMLTIQKQLEY
LWESWVTASGLKFGFTVTDC
SAVRSALEDSDRATVLCFGHTVDLIITEAIKSQRMVQNLLSIARKICERVQRSAKAKEKLAELQKLHELPENQLVQDVPS
KWRTSFSMLERLVEQKKAID
ELSIECNFREMISCDQWEVMLSVCNALKPFEVACQEMSSRTATLGQIIPLIHILNRKVDLLFDETVGIDNMLKSLKEAMV
SRMSATLRDPRYTWATMLDP
RYKTSLFTEDEAEQCKQDLVQELNLSSSTSALNDTPLLPNGRGGEPAAAAIGSSGDNLWSLMDDIRHKIKQEEKPRSSEL
VVLEYLEEDILDQSCDPLDY
WNLKKFQWPDLAKVAARYVGCPPSVVPAESLFSTASASLALNQPRPSLENIEGLLFLKVNLPLIYFQY
>g2120.t1
LWFFQYDHHEFPGVVPRTFLGPLFVSALISPLVVLSSLLDVPKIYTQLMVRGCLGACVVAALWLMQREARKQFGSTVATL
FCLTCASQFHLMFYCTRTLP
NVFALPLVVMACTAWMAQRHRPFIALSAAVIIVFRAELCLLLGLMLMMSLVRRRLSLSELFCCAVPAGILALALSVFVDS
FFWRRLLWPEGQQTSPFLWY
FYSALPRALGCTLPLVPLGLRDPRTRAVLVPSLGFVLLYSLLPHKELRFIIYTFPLLNLVAARGCSYMVEVVGGGCWVVG
AGWRWWVEGAGWWVLGGGGE
RIGPQDFLFCGTELSLALVSFSSWVYGRLLRAVSVHIDPYVAETGVSRFLQQSSTWGYDKREDVRPGDLAMRTYTHILME
ANATSIHLLQDTHEPLVFIP
GYDRVELTPARLPPFRVQMRDKVVILQRKDFAVGDGR
>g2120.t2
MGQRSGAVPRKIRGYSPECPGQTTRLLCTVHISSLKLQVLQLELLFPVSWITVRIPADVRPLTPCVVLCVVLCAVRGCLG
ACVVAALWLMQREARKQFGS
TVATLFCLTCASQFHLMFYCTRTLPNVFALPLVVMACTAWMAQRHRPFIALSAAVIIVFRAELCLLLGLMLMMSLVRRRL
SLSELFCCAVPAGILALALS
VFVDSFFWRRLLWPEGQQTSPFLWYFYSALPRALGCTLPLVPLGLRDPRTRAVLVPSLGFVLLYSLLPHKELRFIIYTFP
LLNLVAARGCSYMVEVVGGG
CWVVGAGWRWWVEGAGWWVLGGGGERIGPQDFLFCGTELSLALVSFSSWVYGRLLRAVSVHIDPYVAETGVSRFLQQSST
WGYDKREDVRPGDLAMRTYT
HILMEANATSIHLLQDTHEPLVFIPGYDRVELTPARLPPFRVQMRDKVVILQRKDFAVGDGR
>g2120.t3
LWFFQYDHHEFPGVVPRTFLGPLFVSALISPLVVLSSLLDVPKIYTQLMVRGCLGACVVAALWLMQREARKQFGSTVATL
FCLTCASQFHLMFYCTRTLP
NVFALPLVVMACTAWMAQRHRPFIALSAAVIIVFRAELCLLLGLMLMMSLVRRRLSLSELFCCAVPAGILALALSVFVDS
FFWRRLLWPEGQQTSPFLWY
FYSALPRALGCTLPLVPLGLRDPRTRAVLVPSLGFVLLYSLLPHKELRFIIYTFPLLNLVAARGCSYMVEVVGGGCWVVG
AGWRWWVEGAGWWVLGGGGE
RIGPQDFLFCGTELSLALVSFSSWVYAVSVHIDPYVAETGVSRFLQQSSTWGYDKREDVRPGDLAMRTYTHILMEANATS
IHLLQDTHEPLVFIPGYDRV
ELTPARLPPFRVQMRDKVVILQRKDFAVGDGR
>g2121.t1
MSVRDGAATMTTLASGAGGGGGGGGAGKAANPQDGRSYLLQIYPRLAANSSACCCLRVQKEATAASVISDAASALGLDSS

RLYVLAEVKECGGEEWILES
GDLPVQRLLLWPRRAQEEHSQSLGFYFLLQESDRDGTIQYVHLPALTKEQEAQRLAARGFLPPPQDDFADLCNLPALNED
GILDNLRTRFHKKKIYTYAG
SILIAINPFTFLPIYNPKYVKMYENHQLGKLEPHIFAIADVAYYAMLRKRVNQCIVISGESGSGKTQSTNFLIHCLTALS
QKGYASGVERTILGAGPVLE
GREVKRLMKLQLDVGWLEFMIVYRKPLRKMDLLHTKYTFK
>g2121.t2
CCVVSEVADAACRGHSSWLRMSVRDGAATMTTLASGAGGGGGGGGAGKAANPQDGRSYLLQIYPRLAANSSACCCLRVQK
EATAASVISDAASALGLDSS
RLYVLAEVKECGGEEWILESGDLPVQRLLLWPRRAQEEHSQSLGFYFLLQESDRDGTIQYVHLPALTKEQEAQRLAARGF
LPPPQDDFADLCNLPALNED
GILDNLRTRFHKKKIYTYAGSILIAINPFTFLPIYNPKYVKMYENHQLGKLEPHIFAIADVAYYAMLRKRVNQCIVISGE
SGSGKTQSTNFLIHCLTALS
QKGYASGVERTILGAGPVLEGREVKRLMKLQLDVGWLEFMIVYRKPLRKMDLLHTKYTFK
>g2121.t3
MEPKQSQGPSQRATFVSLQCSADVYYGCLTQKKGECCVVSEVADAACRGHSSWLRMSVRDGAATMTTLASGAGGGGGGGG
AGKAANPQDGRSYLLQIYPR
LAANSSACCCLRVQKEATAASVISDAASALGLDSSRLYVLAEVKECGGEEWILESGDLPVQRLLLWPRRAQEEHSQSLGF
YFLLQESDRDGTIQYVHLPA
LTKEQEAQRLAARGFLPPPQDDFADLCNLPALNEDGILDNLRTRFHKKKIYTYAGSILIAINPFTFLPIYNPKYVKMYEN
HQLGKLEPHIFAIADVAYYA
MLRKRVNQCIVISGESGSGKTQSTNFLIHCLTALSQKGYASGVERTILGAGPVLEGREVKRLMKLQLDVGWLEFMIVYRK
PLRKMDLLHTKYTFK
>g2122.t1
ANAHQQVEGLLASAQSRETVYYHSTTATAPPRPALHNTARRGQRLEQRIMGQLPVVARSNARFHVLNGPGSVWTTRHLTS
RVDLLRLVSSQLGAQWVGVF
QSLTSASEGEVLAIWRGYASDPDRAQAALQHWVLRDPAASLATLINTLHLHHRPDLLDKIRCVMEDHPEGSANCSQMLIY
AAVSAS
>g2122.t2
ANAHQQVEGLLASAQSRETVYYHSTTATAPPRPALHNTARRGQRLEQRIMGQLPVVARSNARFHVLNGPGSVWTTRHLTS
RVDLLRLVSSQLGAQWVGVF
QSLTSASEGEVLAIWRGYASDPDRAQAALQHWVLRDPAASLATLINTLHLHHRPDLLDKIRCVMEDHPEVARCDSEPVVT
ASPLSHGQGSANCSQMLIYA
AVSAS
>g2123.t1
FRPGEPWKGYPNIDPETDPFVTPGSVINNLSINTVRDTDHLRDRNNGKESPQAHLEQPKPVSPSGAWSSIRASSHSGSLT
STAQSTSARPGESKWSPGGA
GGGSVSNSSLAHELWKVPLPSKGLSVAPPSRPPPGLTGQKPSSGSSAWDSPSLRLGGWGSNESRFTPGSSWGDGGGGSSS
GRTQWLVLKNLTPQIDGSTL
RTLCMQHGPLITFHLNLPHGNAVVCYSSKDEAAKAQKSLHMCVLGNTTILADFASEEEINRFFAQGQSLAASSSGWQAIG
SSQSRMEQSHPFPSRAPEPN
QWNGSDLHSSTLWGGPNYSTSLWGTPTGTEGGRISSPSPISSFLPVDHLAGGGDSM
>g2123.t2
FRPGEPWKGYPNIDPETDPFVTPGSVINNLSINTVRDTDHLRDRNNGKESPQAHLEQPKPVSPSGHSGSLTSTAQSTSAR
PGESKWSPGGAGGGSVSNSS
LAHELWKVPLPSKGLSVAPPSRPPPGLTGQKPSSGSSAWDSPSLRLGGWGSNESRFTPGSSWGDGGGGSSSGRTQWLVLK
NLTPQIDGSTLRTLCMQHGP
LITFHLNLPHGNAVVCYSSKDEAAKAQKSLHMCVLGNTTILADFASEEEINRFFAQGQSLAASSSGWQAIGSSQSRMEQS
HPFPSRAPEPNQWNGSDLHS
STLWGGPNYSTSLWGTPTGTEGGRISSPSPISSFLPVDHLAGGGDSM
>g2123.t3
FRPGEPWKGYPNIDPETDPFVTPGSVINNLSINTVRDTDHLRDRNNGPSSSLNTTMPSNSAWSSIRASSHSGSLTSTAQS
TSARPGESKWSPGGAGGGSV
SNSSLAHELWKVPLPSKGLSVAPPSRPPPGLTGQKPSSGSSAWDSPSLRLGGWGSNESRFTPGSSWGDGGGGSSSGRTQW
LVLKNLTPQIDGSTLRTLCM
QHGPLITFHLNLPHGNAVVCYSSKDEAAKAQKSLHMCVLGNTTILADFASEEEINRFFAQGQSLAASSSGWQAIGSSQSR

MEQSHPFPSRAPEPNQWNGS
DLHSSTLWGGPNYSTSLWGTPTGTEGGRISSPSPISSFLPVDHLAGGGDSM
>g2124.t1
VNYGSKLFVFSYRKRDERLRRVGESRMKEDEEKKKRLEQKISQSDENDTMHVERIAEEKAKKKVAIKRQEQLDQKRRVEE
EARLQKIQQAEEERRQQELV
AKRAAEEEERVRKLAEARRALELRRERERELERERELERERERHKELEMERQAAAERERAEREKALALQCEVESAAREKE
RRELEEKRRLEEEQQRLAAE
KAAREREAAKQKMAATSVALNNVQSSVLKTPVGKGAALNVTMEVE
>g2124.t2
VNYGSKLFVFSYRKRDERLRRVGESRMKEDEEKKKRLEQKISQSDENDTMHVERIAEEKAKKKVAIKRQEQLDQKRRVEE
EARLQKIQQAEEERRQQELV
AKRAAEEEERVRKLAEARRALELRRERERELERERELERERERHKELEMERQAAAERERAEREKALALQCEVESAAREKE
RRELEEKRRLEEEQQRLAAE
KAAREREAAKQKMAATSVALNNVQSSVLNTTMKTGAALNVTMEVESSVLKTPVGKGAALNVTMEVE
>g2124.t3
VNYGSKLFVFSYRKRDERLRRVGESRMKEDEEKKKRLEQKISQSDENDTMHVERIAEEKAKKKVAIKRQEQLDQKRRVEE
EARLQKIQQAGVEPVFDVFL
MKEEERRQQELVAKRAAEEEERVRKLAEARRALELRRERERELERERELERERERHKELEMERQAAAERERAEREKALAL
QCEVESAAREKERRELEEKR
RLEEEQQRLAAEKAAREREAAKQKMAATSVALNNVQSSVLKTPVGKGAALNVTMEVE
>g2125.t1
MNHLSTTTPRLMCITHHVDHVAPRRTPIDPNHCAQPHVTVTSRTPAASQVQHHHGTFIIISFLAFPPEVRLFDYQYPAWT
NVLGYCIGVSSFVCVPTYMV
YHLLSAKGSFKQV
>g2125.t2
MNHLSTTTPRLMCITHHVDHVAPRRTPIDPNHCAQPHVTVTSRTPAASQVQHHHGTFIIISFLAFPPEVRLFDYQYPAWT
NVLGYCIGVSSFVCVPTYMV
YHLLSAKGSFKQ
>g2125.t3
MCITHHVDHVAPRRTPIDPNHCAQPHVTVTSRTPAASQVQHHHGTFIIISFLAFPPEVRLFDYQYPAWTNVLGYCIGVSS
FVCVPTYMVYHLLSAKGSFK
QV
>g2126.t1
DLIKKFLVIDRARRLGNMKNGAEDVKKHRWFKTIEWDSVPQRKLKPPIVPKVSHEGDTSNFDVYPEDDWKKDTPVPQKDL
DLFKNF
>g2126.t2
MAKGGDLIKKFLVIDRARRLGNMKNGAEDVKKHRWFKTIEWDSVPQRKLKPPIVPKVSHEGDTSNFDVYPEDDWKKDTPV
PQKDLDLFKNF
>g2127.t1
MELVRGFMGTKGEKSFMEHGVMVLLASITHTSRTPRHMEKTFPSTEAGAKTQNPLEGHRLEKPRPSPILALNSL
>g2127.t2
MGTKGEKSFMEHGVMVLLASITHTSRTPRHMEKTFPSTEAGAKTQNPLEGHRLEKPRPSPILALNSL
>g2128.t1
MEDPGQAACLHQETNARLSGEHGLMLHADALALQWRAKRLLSSGLLVNVDSVADWASVSGASWKDSASVFIGSGV
>g2129.t1
YTFGPEKVWDRISHVFVFVSSRVDFSPAPEPPSPCLHSCSVSVGEDWRRPCLHTNTAAVVVRAHS
>g2130.t1
PPQRSPPDRPEVKGCVEAPPRHLVAHLIAHVIQGRFHPRQRCRAFTPDTGVCFAGRQDTGTDTLDWPGWSVFC
>g2131.t1
LNRGHRVRQPNLDRGPCVQHPNLNREHRVLNPNLNPGHVLSVWSNIQSAEVHWQTLSLCGDAKGEGVLGEESLPGREPPP
ECFVVHDIHERLAHSRQLKI
YLPKTSERLEFTPVDQPGVTVVYWEKRNHRPSKGSVSGSGSDRRWTLDKVTYADQGTYIQKDFWDKEVSSVKVAVTPRHV
YKKRIPGESLSVSLEGININ
DASLSFSGDNVNITLVRAGAPVGQDLLDYRDRVHLQQGRIEIRDVNTSDVGHYTLSDRWNRVVSITRMDLTDHHDSSGGS
PLLALLLLLGIPAGICCCCR
KKIFKQKASSTLQATAEAQHPPPGGPAGPPPSYHPGPGEPT

>g2131.t2
LNRGHRVRQPNLDRGPCVQHPNLNREHRVLNPNLNPGHVLSVWSNIQSAEVHWQTLSLCGDAKGEGVLECFVVHDIHERL
AHSRQLKIYLPKTSERLEFT
PVDQPGVTVVYWEKRNHRPSKGSVSGSGSDRRWTLDKVTYADQGTYIQKDFWDKEVSSVKVAVTPRHVYKKRIPGESLSV
SLEGININDASLSFSGDNVN
ITLVRAGAPVGQDLLDYRDRVHLQQGRIEIRDVNTSDVGHYTLSDRWNRVVSITRMDLTDHHDSSGGSPLLALLLLLGIP
AGICCCCRKKIFKQKASSTL
QATAEAQHPPPGGPAGPPPSYHPGPGEPT
>g2131.t3
LNRGHRVRQPNLDRGPCVQHPNLNREHRVLNPNLNPGHVLSVWSNIQSAEVHWQTLSLCGDAKGEGVLGEESLPGREPPP
ECFVVHDIHERLAHSRQLKI
YLPKTSERLEFTPVDQPGVTVVYWEKRNHRPSKGSVSGSGSDRRWTLDKVTYADQGTYIQKDFWDKEVSSVKVAVTPRHV
YKKRIPGESLSVSLEGININ
DASLSFSGDNVNITLDLLDYRDRVHLQQGRIEIRDVNTSDVGHYTLSDRWNRVVSITRMDLTDHHDSSGGSPLLALLLLL
GIPAGICCCCRKKIFKQKAS
STLQATAEAQHPPPGGPAGPPPSYHPGPGEPT
>g2132.t1
LNIAELGPDQSLRQSLVSPPKFAFIIPGSSSALVHSPHCPADLQGACSLIQQYPDLRLRQEPNDGATGPQSSVPYHSLLD
CQSVSGLPLQALQSRPDQND
PAKTGASNPDQGYLEMNRYPAVDLSLFGPEPMSNSLLNGLLERHMDELYRQHFTDSMARCNSQLRHSLLHGLVPPPQPGG
QAQASDSLEGSLEAVASEGE
GLKISYLNTATSYFSSPILRISETESAQ
>g2133.t1
MNSNGNKRAPTTATQRLKQDYLRIKKDPVPYICAEPLPSNILEWHYVVRGPEKTPYEGKTPQTASICAVCGWDEGSVNVE
GLILEGLRFVMFGATNGGYY
HGKLIFPREFPFKPPSIYMITPNGRFKCNTRLCLSITDFHPDTWNPAWSVSTILTGLLSFMVEKGPTLGSIETSDYTKRQ
LSAQSLAFNLKDKVFCELFP
DVVD
>g2133.t2
MNSNGNKRAPTTATQRLKQDYLRIKKDPVPYICAEPLPSNILEWHYVVRGPEKTPYEGGYYHGKLIFPREFPFKPPSIYM
ITPNGRFKCNTRLCLSITDF
HPDTWNPAWSVSTILTGLLSFMVEKGPTLGSIETSDYTKRQLSAQSLAFNLKDKVFCELFPDVVD
>g2133.t3
MNSNGNKRAPTTATQRLKQDYLRIKKDPVPYICAEPLPSNILEWHYVVRGPEKTPYEGKTPQTASICAVCGWDEGSVNVE
GLILEGLRFVMFGATNGGYY
HGKLIFPREFPFKPPSIYMITPNGRFKCNTRLCLSITDFHPDTWNPAWSVSTILTGLLSFMVEKGPTLGSIETSDYTKRQ
LSAQSLAFNLKDKVF
>g2134.t1
RICSPVRLYNSRSLTDSDASVESEDSGLRSPGSRTYNTCPRGWGGSLRVKRGSVSTDL
>g2135.t1
MAMSSYMVNSKYVDPKFPPCEEYSQNNYISDQGSDFFSPAQDTDFQQHPGLYTRPNYADQPYSCGTVQDGTAPPRGHVHD
AASRPSPFSSGQDEQCSPVQ
MSGPRTCGQQQNTKSQNGIQAKQPAVVYPWMKKVHVTTVNPDYTGSEPKRSRTAYTRQQVLELEKEFHFNRFS
>g2136.t1
QTTPKKDMAPFERSMEMMPFKQRKCLETRKDEVCSIRSKFPNKLPVIVERYIREKTLPLLNKTKFLVPLELTLGQFLCLL
RYKIDLEASQALFLLVSERC
MSCMSASMGEVYSQHSDQDGFLYITYASQEMFGAPEAAGAPRSRER
>g2136.t2
TPSARARPPQAQQPLRLPPHHPGLKQTTPKKDMAPFERSMEMMPFKQRKCLETRKDEVCSIRSKFPNKLPVIVERYIREK
TLPLLNKTKFLVPLELTLGQ
FLCLLRYKIDLEASQALFLLVSERCMSCMSASMGEVYSQHSDQDGFLYITYASQEMFGAPEAAGAPRSRER
>g2137.t1
MVHSLLQNTALKCAAITYAYVAYYQSFGSRSPVRSVRPEQEKELIRYTQDNHLLSLDQRLFYEKNGFVVIKRLVSEEDID
RFRAAFERICRREVSPPGLV
VMRDVAITSSEFVADQKAVSKIQDFQEDPELFRYCTLPEILRYVECFVGEDIMAMHTMLINKPPDAGKKTSRHPLHQDLH
YFPFRPVDKIVCAWSAMERI

TRQNGCLVVLPGTHTGFLKEHDYPEWEV
>g2137.t2
LKCAAITYAYVAYYQSFGSRSPVRSVRPEQEKELIRYTQDNHLLSLDQRLFYEKNGFVVIKRLVSEEDIDRFRAAFERIC
RREVSPPGLVVMRDVAITSS
EFVADQKAVSKIQDFQEDPELFRYCTLPEILRYVECFVGEDIMAMHTMLINKPPDAGKKTSRHPLHQDLHYFPFRPVDKI
VCAWSAMERITRQNGCLVVL
PGTHTGFLKEHDYPEWEV
>g2138.t1
LSQEGVTLTQHLAAAPLCTPSRAAFLTGRYALRSGLGSHNQVPVLLFLAGSGGLPPTETTFAKRLQKQGYTTGLVGKWHL
GVNCVRRGDHCHHPNNHGFH
YYYGLPFTLFNNCVPGNEIDILGNVELVLRQLSLLLGLGLLTLVSVCVHA
>g2138.t2
MRCAQVEHSIVFLMRRGSGLGSHNQVPVLLFLAGSGGLPPTETTFAKRLQKQGYTTGLVGKWHLGVNCVRRGDHCHHPNN
HGFHYYYGLPFTLFNNCVPG
NEIDILGNVELVLRQLSLLLGLGLLTLVSVCVHA
>g2138.t3
LSQEGVTLTQHLAAAPLCTPSRAAFLTGRYALRSGLGSHNQVPVLLFLAGSGGLPPTETTFAKRLQKQGYTTGLVGKWHL
GVNCVRRGDHCHHPNNHGFH
YYYGLPFTLFNNCVPGNEIDILGNVELVLRQLSLLLGLGLLTL
>g2139.t1
LGVHISDDLSWTDNITAVTKKAQQRLHFLRVLRKNNLDKKLLLAFYHSSIESLLTYCISTWFGSCTEADRARLQRTVRAA
QRIIGCPLPSLSDIYTTSAE
P
>g2140.t1
MSSLVFVVLRPRLLCEHQADSFWTSIHLRSGNPSQSTSLHYMNPYQMNAYAMALKAVGEIIQDYDSDKMFPALGFGAKLP
PDGRVSHEFPLVSEAPAGGG
APSPPGTPGVLPENPRVLRRCSQPPRTTAEINRSPPPPRSAGPHILAGNEMS
>g2140.t2
MSSLVFVVLRPRLLCEHQADSFWTSIHLRSGNPSQSTSLHYMNPYQMNAYAMALKAVGEIIQDYDSDKMFPALGFGAKLP
PDGRVSHEFPLVSEAPAGGG
APSPPGTPGVLPENPRVLRRCSQPPRTTAEINRSPPPPRSAGPHILAGNEMSLDACPSYGRNKSSALLSPL
>g2140.t3
MSSLVFVVLRPRLLCEHQADSFWTSIHLRSGNPSQSTSLHYMNPYQMNAYAMALKAVGEIIQDYDSDKMFPALGFGAKLP
PDGRVSHEFPLVSEAPAGGG
APSPPGTPGVLPENPRVLRRCSQPPRTTAEINRSPPPPRSAGPHILAGNEMSPGERDPAAVCRTLMRTLYALTPLLLFIK
VDACPSYGRNKSSALLSPL
>g2141.t1
MACSRLPERDVQEETFCGHKYSLLIITGPISHRDQAGHIHREIERGIRSWDVNLRSCNLNLYLQEFLTHHIVSLKGPEAS
RWFSWLDVSFFPKVVNMLSE
STNVLDTQVLISPAQEQVHSEVCCLLDGESAHKLLVVAGQSVEESGDLLLHRGLFSPQHLRSILTPQLSDKETSPSFRRR
SSKYSLTLSCPNIGTWREAL
SDHPAVRVNPPEVLPAMESLGEFTSLVTGSLHPSSPFEVLPPPGTVGFLKLSRPCCYVFPAGRGDCAFFAVNGFTMLVDG
GSDPQACFWKLVRHLDRVDA
LLLSHISTSSLAGVNTFLERKVAELELGGSEAKDDLTKRLISPELGVVFFNAPCSLQTNEQPCIDSVLKSTDQATLTIKL
LKKLEIVPQPMFRPPGVPLE
PMTLFQKMGVGQLELYILHPGKTSQEYQALMQSWPDRTETQALPITALASVCALLVWHPACPQEKVVRVLFPGATPQGKL
LEGLEKLRGLAFLQKPTVTT
GDLEVQREHMAAKRTVSQDSGKEVASRVGKEEARDSLVKEKVKVVNGSAVREDAKKGLEKTKARNGNVKPKSGPTEKNVL
KKEGIRKEAKKEEKVVKRED
SGPKRREQVKKEVPPSKLKNENKSKAQKEVKKDTKTGGKKPNKPSGKEIKTGSNLVAKKASVNTELRKTTSQDLKTEGPD
KDHQSDNILDQNHFGSKMST
PEDITEDVLRLRAESVQAVNISEVEHGSEMMECQSGEFEARTPVLEADVSPLTGSDHAEDTAEKPKKTIRVVGFPAPLNL
CSQSDQTDLTPTEYTLLDGA
LKNSPPSRSSVDHPAPASPDEETVGQTSSESRPNSAGHTPYCLSPDDVWCNRDKLSQLQAQIGSSDPTDVVPPLVPSVAA
TEGLQGEAKGSQEGHATFNP
REKHLTSLSLGTFKESGAPDPSPSVTTTTTTHSMPAEAISPQSTEVDESLSMSFEQGSIAVSQREGDDSVHHSLSNGSHC

VETDFRVGMSLQLKKPGRSL
GQGLEMGRPPAPCGIHFESEAHDVDLCLVSPCEFKHFKPADSVPAAGDRHEGAAAVGPQHHGNNNSQRDVSFSESNAPHC
TEDCPSTTADGGLDSDEDES
CSEPTSSLHDHHAHQSLPPDPPPAPLRDGPPAPPQPDSCMPVPQSDPEVHSKAAKVAGMRGKKNAGVLEASHRTGPNGGS
AQTGGKSRGALGLGGGLGRG
YPRTSGTKTAPAKVSSGPEPGKPGGDAELSVHVDLAYIPSGASCATVNGNFFRRVRSSCYVVSGDGPEREELMRQTLDAL
LDGKATWSEPTQVTIIPTFE
SAAMQAWYQQTVERQKELDVVVLGSNSTVAMQDETFPACKIEF
>g2141.t2
MACSRLPERDVQEETFCGHKYSLLIITGPISHRDQAGHIHREIERGIRSWDVNLRSCNLNLYLQEFLTHHIVSLKGPEAS
RWFSWLDVSFFPKVVNMLSE
STNVLDTQVLISPAQEQVHSEVCCLLDGESAHKLLVVAGQSVEESGDLLLHRGLFSPQHLRSILTPQLSDKETSPSFRRR
SSKYSLTLSCPNIGTWREAL
SDHPAVRVNPPEVLPAMESLGEFTSLVTGSLHPSSPFEVLPPPGTVGFLKLSRPCCYVFPAGRGDCAFFAVNGFTMLVDG
GSDPQACFWKLVRHLDRVDA
LLLSHISTSSLAGVNTFLERKVAELELGGSEAKDDLTKRLISPELGVVFFNAPCSLQTNEQPCIDSVLKSTDQATLTIKL
LKKLEIVPQPMFRPPGVPLE
PMTLFQKMGVGQLELYILHPGKTSQEYQALMQSWPDRTETQALPITALASVCALLVWHPACPQEKVVRVLFPGATPQGKL
LEGLEKLRGLAFLQKPTVTT
GDLEVQREHMAAKRTVSQDSGKEVASRVGKEEARDSLVKEKVKVVNGSAVREDAKKGLEKTKARNGNVKPKSGPTEKNVL
KKEGIRKEAKKEEKVVKRED
SGPKRREQVKKEVPPSKLKNENKSKAQKEVKKDTKTGGKKPNKPSGKEIKTGSNLVAKKASVNTELRKTTSQDLKTEGPD
KDHQSDNILDQNHFGSKMST
PEDITEDVLRLRAESVQAVNISEVEHGSEMMECQSGEFEARTPVLEADVSPLTGSDHAEDTAEKPKKTIRVVGFPAPLNL
CSQSDQTDLTPTEYTLLDGA
LKNSPPSRSSVDHPAPASPDEETVGQTSSESRPNSAGHTPYCLSPDDVWCNRDKLSQLQAQIGSSDPTDVVPPLVPSVAA
TEGLQGEAKGSQEGHATFNP
REKHLTSLSLGTFKESGAPDPSPSVTTTTTTHSMPAEAISPQSTEVDESLSMSFEQGSIAVSQREGDDSVHHSLSNGSHC
VETDFRVGMSLQLKKPGRSL
GQGLEMGRPPAPCGIHFESEAHDVDLCLVSPCEFKHFKPADSVPAAGDRHEGAAAVGPQHHGNNNSQRDVSFSESNAPHC
TEDCPSTTADGGLDSDEDES
CSEPTSSLHDHHAHQSLPPDPPPAPLRDGPPAPPQPDSCMPVPQSDPEVHSKAAKVAGMRGKKNAGVLEASHRTGPNGGS
AQTGGKSRGALGLGGGLGRG
YPRTSGTKTAPAKVSSGPGARLHKLGVSVSQAKEPGKPGGDAELSVHVDLAYIPSGASCATVNGNFFRRVRSSCYVVSGD
GPEREELMRQTLDALLDGKA
TWSEPTQVTIIPTFESAAMQAWYQQTVERQKELDVVVLGSNSTVAMQDETFPACKIEF
>g2141.t3
MACSRLPERDVQEETFCGHKYSLLIITGPISHRDQAGHIHREIERGIRSWDVNLRSCNLNLYLQEFLTHHIVSLKGPEAS
RWFSWLDVSFFPKVVNMLSE
STNVLDTQVLISPAQEQVHSEVCCLLDGESAHKLLVVAGQSVEESGDLLLHRGLFSPQHLRSILTPQLSDKETSPSFRRR
SSKYSLTLSCPNIGTWREAL
SDHPAVRVNPPEVLPAMESLGEFTSLVTGSLHPSSPFEVLPPPGTVGFLKLSRPCCYVFPAGRGDCAFFAVNGFTMLVDG
GSDPQACFWKLVRHLDRVDA
LLLSHISTSSLAGVNTFLERKVAELELGGSEAKDDLTKRLISPELGVVFFNAPCSLQTNEQPCIDSVLKSTDQATLTIKL
LKKLEIVPQPMFRPPGVPLE
PMTLFQKMGVGQLELYILHPGKTSQEYQALMQSWPDRTETQALPITALASVCALLVWHPACPQEKVVRVLFPGATPQGKL
LEGLEKLRGLAFLQKPTVTT
GDLEVQREHMAAKRTVSQDSGKEVASRVGKEEARDSLVKEKVKVVNGSAVREDAKKGLEKTKARNGNVKPKSGPTEKNVL
KKEGIRKEAKKEEKVVKRED
SGPKRREQVKKEVPPSKLKNENKSKAQKEVKKDTKTGGKKPNKPSGKEIKTGSNLVAKKASVNTELRKTTSQDLKTEGPD
KDHQSDNILDQNHFGSKMST
PEDITEDVLRLRAESVQAVNISEVEHGSEMMECQSGEFEARTPVLEADVSPLTGSDHAEDTAEKPKKTIRVVGFPAPLNL
CSQSDQTDLTPTEYTLLDGA
LKNSPPSRSSVDHPAPASPDEETVGQTSSESRPNSAGHTPYCLSPDDVWCNRDKLSQLQAQIGSSDPTDVVPPLVPSVAA
TEGLQGEAKGSQEGHATFNP
REKHLTSLSLGTFKESGAPDPSPSVTTTTTTHSMPAEAISPQSTEVDESLSMSFEQGSIAVSQREGDDSVHHSLSNGSHC

VETDFRVGMSLQLKKPGRSL
GQGLEMGRPPAPCGIHFESEAHDVDLCLVSPCEFKHFKPADSVPAAGDRHEGAAAVGPQHHGNNNSQRDVSFSESNAPHC
TEDCPSTTADGGLDSDEDES
CSEPTSSLHDHHAHQSLPPDPPPAPLRDGPPAPPQPDSCMPVPQSDPEVHSKAAKVAGMRGKKNAGVLEASHRTGPNGGS
AQTGGKSRGALGLGGGLGRG
YPRTSGTKTAPAKVSSGPGKPGGDAELSVHVDLAYIPSGASCATVNGNFFRRVRSSCYVVSGDGPEREELMRQTLDALLD
GKATWSEPTQVTIIPTFESA
AMQAWYQQTVERQKELDVVVLGSNSTVAMQDETFPACKIEF
>g2142.t1
MRANKEYSLTEAQQKWRFQSIIMCLPAVLAQRKPATLHALLVPESCTLLSSYGLWQSVGSYFGAELCQVDVQSDGNTDFL
LVGAPLFHHPHENREGQVYV
YKLSNKLELEHVLNVSVPSRGRFGTAISSLADLNGDGLRDVAVGAPTEADNRGAVYIYLGNQHTGIRPTFSQ
>g2142.t2
MCLPAVLAQRKPATLHALLVPESCTLLSSYGLWQSVGSYFGAELCQVDVQSDGNTDFLLVGAPLFHHPHENREGQVYVYK
LSNKLELEHVLNVSVPSRGR
FGTAISSLADLNGDGLRDVAVGAPTEADNRGAVYIYLGNQHTGIRPTFSQ
>g2143.t1
MNTAVGSKHGRCPLLSVFWVFALCQPGSLAFNLDTADPRIYHGNENGFFGHKVLQITSEKDKGILVTAPLQNGSGTVCAY
TKQMESCFTPQEASPGRLFG
LSVAVEPSQPGFTFAAVQFSSEPRKVFDFIDYQNGKAAELLREEPFMQELTNTYYALKFTLENLFENPSVGATPSATKVL
VIITDGDPTDVDDSFQIVNK
YAEKEIIRIVIGGTLVLGSVGSNDWRGSLYEVAEDSHIQDPNMPSSSYMGYSVAAGEKDSVSLYFTGAPRFEHKGMVVVF
RKTEGTWVVVHRVTGNQVPK
IPFGASGQLPYWSADKGADFVWCQKPQSTLGNGNSIQSSIQESNHKELLMAQAVRKAVVSNSLCFSRGEKLGASPGDKTY
FANWVGRSGTQTQAGCSSGF
GLCGAGTIAEGT
>g2143.t2
MNTAVGSKHGRCPLLSVFWVFALCQPGSLAFNLDTADPRIYHGNENGFFGHKVLQITSEKDKGILVTAPLQNGSGTVCAY
TKQMESCFTPQEASPGRLFG
LSVAVEPSQPGFTVCSPNVAHECYDNCYLSGQCYRLNEKLQPMSSFKPRKVFDFIDYQNGKAAELLREEPFMQELTNTYY
ALKFTLENLFENPSVGATPS
ATKVLVIITDGDPTDVDDSFQIVNKYAEKEIIRIVIGGTLVLGSVGSNDWRGSLYEVAEDSHIQDPNMPSSSYMGYSVAA
GEKDSVSLYFTGAPRFEHKG
MVVVFRKTEGTWVVVHRVTGNQVPKIPFGASGQLPYWSADKGADFVWCQKPQSTLGNGNSIQSSIQESNHKELLMAQAVR
KAVVSNSLCFSRGEKLGASP
GDKTYFANWVGRSGTQTQAGCSSGFGLCGAGTIAEGT
>g2143.t3
MNTAVGSKHGRCPLLSVFWVFALCQPGSLAFNLDTADPRIYHGNENGFFGHKVLQITSEKDKGILVTAPLQNGSGTVCAY
TKQMESCFTPQEASPGRLFG
LSVAVEPSQPGFTVCSPNVAHECYDNCYLSGQCYRLNEKLQPMSSFSPAFQEPRKVFDFIDYQNGKAAELLREEPFMQEL
TNTYYALKFTLENLFENPSV
GATPSATKVLVIITDGDPTDVDDSFQIVNKYAEKEIIRIVIGVGNVALDNLYIIASEPKKINTFHINNYNGLPGLLKNFE
EKIFKIEGYSVAAGEKDSVS
LYFTGAPRFEHKGMVVVFRKTEGTWVVVHRVTGNQVPKIPFGASGQLPYWSADKGADFVWCQKPQSTLGNGNSIQSSIQE
SNHKELLMAQAVRKAVVSNS
LCFSRGEKLGASPGDKTYFANWVGRSGTQTQAGCSSGFGLCGAGTIAEGT
>g2144.t1
GEVAGTRKTTPGFRLVEKYAMLVGGVSMHRQDLSGMVMLKDNHVWASGSITKAVQVTRAVCGFSSKVEVECGSAEEAREA
ARAGADVVMLDNLKPRELWA
AARLLKEEFPVLLVEASGGVTPENLSQYFCPHVDVISLGCITQGCPVVDFSLKVQKPRAT
>g2145.t1
MPVYTVNVKWGKEKFDAIELNTEEPPMVFKAQLFALSGVQPERQKVMVKGGTLKGMTLLMMGSAEALPEEPAVRPMFVED
MTEEQLASAMELPCGLTNLG
NTCYMNATVQCLRSVPELKSALKKYSGALRSSGANASQYITAALRDLYETMDKTSSSLPPIILLQFLHMAFPQFAEKGDQ
GQYLQQDANECWLQMVRVLQ
QKLEALEPETPMETESESGAAASTTKKNFIDQYFGVEFETTMKCTEAEDEEPTKAKESQLQLSCFINQEVKYLATGLRLS

KLGRLPAYLTIQMVRFFYKE
KEAVNAKVLKDVKFPLMLDVYELCTSEVQEKMGPVRSKFKVVEDKKLEMAQKKIAAKPGAAKETKFEPFSFPDDLGSNNS
GYYDLQAVLTHQGRSSSSGH
YVGWVKRKE
>g2145.t2
MVFKAQLFALSGVQPERQKVMVKGGTLKGMTLLMMGSAEALPEEPAVRPMFVEDMTEEQLASAMELPCGLTNLGNTCYMN
ATVQCLRSVPELKSALKKYS
GALRSSGANASQYITAALRDLYETMDKTSSSLPPIILLQFLHMAFPQFAEKGDQGQYLQQDANECWLQMVRVLQQKLEAL
EPETPMETESESGAAASTTK
KNFIDQYFGVEFETTMKCTEAEDEEPTKAKESQLQLSCFINQEVKYLATGLRLSKLGRLPAYLTIQMVRFFYKEKEAVNA
KVLKDVKFPLMLDVYELCTS
EVQEKMGPVRSKFKVVEDKKLEMAQKKIAAKPGAAKETKFEPFSFPDDLGSNNSGYYDLQAVLTHQGRSSSSGHYVGWVK
RKE
>g2145.t3
MPVYTVNVKWGKEKFDAIELNTEEPPMVFKAQLFALSGVQPERQKVMVKGGTLKGMTLLMMGSAEALPEEPAVRPMFVED
MTEEQLASAMELPCGLTNLG
NTCYMNATVQCLRSVPELKSALKKYSGALRSSGANASQYITAALRDLYETMDKTSSSLPPIILLQFLHMAFPQFAEKGDQ
GQYLQQDANECWLQMVRVLQ
QKLEALEPETPMEVNMQSVLSSKTESESGAAASTTKKNFIDQYFGVEFETTMKCTEAEDEEPTKAKESQLQLSCFINQEV
KYLATGLRLSKLGRLPAYLT
IQMVRFFYKEKEAVNAKVLKDVKFPLMLDVYELCTSEVQEKMGPVRSKFKVVEDKKLEMAQKKIAAKPGAAKETKFEPFS
FPDDLGSNNSGYYDLQAVLT
HQGRSSSSGHYVGWVKRKE
>g2146.t1
MATMEVEDFMRTDGDSVTVLPCEDGTKPDPECSEVEDLQNSLRQAVHDDTVRPKMQCLMMDPSFSMVTMQGEDSGIEWET
SASRSATPWASEVGAPVELH
APLIRPATPGALPAGRIIMVMDEGSISRRKRSKEKAPSAPSIKRPPQEASDQGDDVVGRPELVGFSLPNDQEQQKEVENS
AGDLEQQVFCLVAEGSEILN
IIVPPMLALVDEEESTAMEDNLSYLEESPVIKSSGITDDNVFDFNDDEPIVTPAAAGLHEAGPVASNPYGANVMDPPGAP
VTRPQARGATSSVDYFEKFT
LIDAQAPGSPAVIKEGQEEQQTEEPEEAKASQHAEEPSAPQSRVDEGGADVGEEDTDGALLDDVFYAGTTDLMTPGSSDV
EECRTPRSPLKQSGSALFGS
QEDILTPIFLPDGPAKIIDPILFEEPKAMAFLYTDLYEEATGSRVQEEDTQSQGSEKSFHSRHSDREARGYLEKYVLKDE
TPAVETTNRDNVQEELDSIR
LMSQGLYDLGDLPANPGKPEINSGEDEITDFFRSSGNSSPCDMNPFNESAQEEDAAQAKIRARLEVIKATQQMFPPSKPR
EKDCSDNRAEQTQSSESYEF
VSEDTDWSGMPEDDLASTLSDESETEHSEEMPVVPPRRKAVSAPKTSLDLTPLRPVAGPDEGHETGSKEQRAEDKETASP
VETADEGDGESEDSSRPQCD
PTADASQAAGSVTEGGDSAGLAAELCWLVSHSAASQGDGTNYKQLLTQGGSTAEVAAHHYHGYPKKDAVRVKGACVRSRH
HFIAMEECLSPDFCNDEKHS
ESFDKPSPEDGELDVFGDLAALPVEVCERRPTCPRCRRPQKVCLCPFLPVHPLEVSTCLYIVQHPAEESRVLRTVPLLAA
CLPPGKCNVIVGRRFSEERH
PDLAAVCRDPRTLIMYPGPGAQNLEELLLADPEARAAGYNVILIDGTWSQAKDLFLRNNLLHLPRQVQLNRTLSSQYVIR
TQPTNICLSTLECAALTLST
LEQKESIQEVLLRPLNALCAFQLQHGAQVHHSKEHLLRTGLYDKPMPKNKRKIKRME
>g2146.t2
MATMEVEDFMRTDGDSVTVLPCEDGTKPDPECSEVEDLQNSLRQAVHDDTVRPKMQCLMMDPSFSMVTMQGEDSGIEWET
SASRSATPWASEVGAPVELH
APLIRPATPGALPAGRIIMVMDEGSISRRKRSKEKAPSAPSIKRPPQEASDQGDDVVGRPELVGFSLPNDQEQQKEVENS
AGDLEQQVFCLVAEGSEILN
IIVPPMLALVDEEESTAMEDNLSYLEESPVIKSSGITDDNVFDFNDDEPIVTPAAAGLHEAGPVASNPYGANVMDPPGAP
VTRPQARGATSSVDYFEKFT
LIDAQAPGSPAVIKEGQEEQQTEEPEEAKASQHAEEPSAPQSRVDEGGADVGEEDTDGALLDDVFYAGTTDLMTPGSSDV
EECRTPRSPLKQSGSALFGS
QEDILTPIFLPDGPAKIIDPILFEEPKAMAFLYTDLYEEATGSRVQEEDTQSQGSEKSFHSRHSDREARGYLEKYVLKDE
TPAVETTNRDNVQEELDSIR

LMSQGLYDLGDLPANPGKPEINSGEDEITDFFRSSGNSSPCDMNPFNESAQEEDAAQAKIRARLEVIKATQQMFPPSKPR
EKDCSDNRAEQTQSSESYEF
VSEDTDWSGMPEDDLASTLSDESETEHSEEMPVVPPRRKAVSAPKTSLDLTPLRPVAGPDEGHETGSKEQRAEDKETASP
VETADEGDGESEDSSRPQCD
PTADASQAAGSVTEGGDSAGLVRRHALSPAHSHHQAAELCWLVSHSAASQGDGTNYKQLLTQGGSTAEVAAHHYHGYPKK
DAVRVKGACVRSRHHFIAME
ECLSPDFCNDEKHSESFDKPSPEDGELDVFGDLAALPVEVCERRPTCPRCRRPQKVCLCPFLPVHPLEVSTCLYIVQHPA
EESRVLRTVPLLAACLPPGK
CNVIVGRRFSEERHPDLAAVCRDPRTLIMYPGPGAQNLEELLLADPEARAAGYNVILIDGTWSQAKDLFLRNNLLHLPRQ
VQLNRTLSSQYVIRTQPTNI
CLSTLECAALTLSTLEQKESIQEVLLRPLNALCAFQLQHGAQVHHSKEHLLRTGLYDKPMPKNKRKIKRME
>g2146.t3
MATMEVEDFMRTDGDSVTVLPCEDGTKPDPECSEVEDLQNSLRQAVHDDTVRPKMQCLMMDPSFSMVTMQGEDSGIEWET
SASRSATPWASEVGAPVELH
APLIRPATPGALPAGRIIMVMDEGSISRRKRSKEKAPSAPSIKRPPQEASDQGDDVVGRPELVGFSLPNDQEQQKEVENS
AGDLEQQVFCLVAEGSEILN
IIVPPMLALVDEEESTAMEDNLSYLEESPVIKSSGITDDNVFDFNDDEPIVTPAAAGLHEAGPVASNPYGANVMDPPGAP
VTRPQARGATSSVDYFEKFT
LIDAQAPGSPAVIKEGQEEQQTEEPEEAKASQHAEEPSAPQSRVDEGGADVGEEDTDGALLDDVFYAGTTDLMTPGSSDV
EECRTPRSPLKQSGSALFGS
QEDILTPIFLPDGPAKIIDPILFEEPKAMAFLYTDLYEEATGSRVQEEDTQSQGSEKSFHSRHSDREARGYLEKYVLKDE
TPAVETTNRDNVQEELDSIR
LMSQGLYDLGDLPANPGKPEINSGEDEITDFFRSSGNSSPCDMNPFNESAQEEDAAQAKIRARLEVIKATQQMFPPSKPR
EKDCSDNRAEQTQSSESYEF
VSEDTDWSGMPEDDLASTLSDESETEHSEEMPVVPPRRKAVSAPKTSLDLTPLRPVAGPDEGHETGSKEQRAEDKETASP
VETADEGDGESEDSSRPQCD
PTADASQAAGSVTEGGDSAGLAAELCWLVSHSAASQGDGTNYKQLLTQGGSTAEVAAHHYHGYPKKDAVRVKGACVRSRH
HFIAMEECLSPDFCNDEKHS
ESFDKPSPEDGELDVFGDLAALPVEVCERRPTCPRCRRPQKVCLCPFLPVHPLEVSTCLYIVQHPAEESRVLRTVPLLAA
CLPPGKCNVIVGRRFSEERH
PDLAAVCRDPRTLIMYPGPGAQNLEELLLADPEARAAGYNVILIDGTWSQAKDLFLRNNLLHLPRQPTNICLSTLECAAL
TLSTLEQKESIQEVLLRPLN
ALCAFQLQHGAQVHHSKEHLLRTGLYDKPMPKNKRKIKRME
>g2147.t1
MMWTFVQDATAVTHEGQRAEDTPWTTATVIQKIGLGHPGVERHVITPVGLTSWRRFITSDQKRRSHAT
>g2148.t1
MTGGQSSSMLVSSRWHPVEMMSVVLFTRLVEQPIFSTRAHVFQIDPSTKKNWVPTSKHAVTVSYFFDSTRNVYRIISLEG
SKAIINSTITPNMTFTKTSQ
KFGQWADSRANTVYGLGFPSENHLLKFAEKFAEFKEAARLAKEQSQDKMELTSTPSQLIVFMFLSITKRLQAAAVRRTVV
SVHQAGNKKESASGELLSPV
TPESINGTDEEDGGETPGTPQHTEPEPQTPTLPFSHSSTSINKHWEAELEALKGNNAKLTAALLESTANVKQWKQQLAAY
QEEAERLHKRVTELECVSGQ
TTVIKSQKADLNQTVEELQAALGAKEEELEKLKAEMEAANEFQLQKESLTQKLQATEDRNQNLEARLTALEQHVESNQLE
RETFRTHLRSLLELLDGKIF
ELSELRDTLAKLIESS
>g2148.t2
MGYVTHYNTQTRPKALTCVLAGAVLDHRTGPPAQEQGELEGCCPGAEVEHPESAERVVRWLLLVLKASRMTGGQSSSMLV
SSRWHPVEMMSVVLFTRLVE
QPIFSTRAHVFQIDPSTKKNWVPTSKHAVTVSYFFDSTRNVYRIISLEGSKAIINSTITPNMTFTKTSQKFGQWADSRAN
TVYGLGFPSENHLLKFAEKF
AEFKEAARLAKEQSQDKMELTSTPSQLIVFMFLSITKRLQAAAVRRTVVSVHQAGNKKESASGELLSPVTPESINGTDEE
DGGETPGTPQHTEPEPQTPT
LPFSHSSTSINKHWEAELEALKGNNAKLTAALLESTANVKQWKQQLAAYQEEAERLHKRVTELECVSGQTTVIKSQKADL
NQTVEELQAALGAKEEELEK
LKAEMEAANEFQLQKESLTQKLQATEDRNQNLEARLTALEQHVESNQLERETFRTHLRSLLELLDGKIFELSELRDTLAK
LIESS

>g2148.t3
MEISAVRSPHNEHEDSMLREQPIFSTRAHVFQIDPSTKKNWVPTSKHAVTVSYFFDSTRNVYRIISLEGSKAIINSTITP
NMTFTKTSQKFGQWADSRAN
TVYGLGFPSENHLLKFAEKFAEFKEAARLAKEQSQDKMELTSTPSQLIVFMFLSITKRLQAAAVRRTVVSVHQAGNKKES
ASGELLSPVTPESINGTDEE
DGGETPGTPQHTEPEPQTPTLPFSHSSTSINKHWEAELEALKGNNAKLTAALLESTANVKQWKQQLAAYQEEAERLHKRV
TELECVSGQTTVIKSQKADL
NQTVEELQAALGAKEEELEKLKAEMEAANEFQLQKESLTQKLQATEDRNQNLEARLTALEQHVESNQLERETFRTHLRSL
LELLDGKIFELSELRDTLAK
LIESS
>g2149.t1
LLTARKKLRKTSVDCTTWRRAGSPMDEVLNSLKRGCFHLRKAELRVLPDPDQDDSQNILAQIRQGVLLRKVRGRPADQNQ
RRPGRGFPPSADALTRSIHE
ALRRIKEASPESESEDEGGPCADWEN
>g2149.t2
LTARKKLRKTSVDCTTWRRAGSPMDEVLNSLKRGCFHLRKAELRVLPDPDQDDSQNILAQIRQGVLLRKVRGRPADQNQR
RPGRGFPPSADALTRSIHEA
LRRIKEASPESESEDEGGPCADWEN
>g2150.t1
MAHSRACTARSMAHLCTHPTAHSSADSTPLSCTHPMAHSSTPQLCTHPMARSTARSVAPSSARSSTHPWPTPLPTPLRPP
HNPFKPSRRHSDCNTHLRML
GRHQLKLNLAKTELLFIPGKDCPHRDLLVSVEGITVSASPTARNLGVIVDNQLSCTANITAVTRSCRFALYNIRRIRPFL
TREATQLLTQALVISRLDYC
NSLLAGLPATAIKPLQWIQNAAARLVFNRNKFSQVTPLFQDLHWLPVVARIKFKTMLLAYKDVNGTAPTYLQTLVKHHTP
ARALRSTTSAGRLVPPSLKG
RMAKCQLFWHCSGGAPCQWQHCRVTPHLPEETQDPLV
>g2150.t2
MAHLCTHPTAHSSADSTPLSCTHPMAHSSTPQLCTHPMARSTARSVAPSSARSSTHPWPTPLPTPLRPPHNPFKPSRRHS
DCNTHLRMLGRHQLKLNLAK
TELLFIPGKDCPHRDLLVSVEGITVSASPTARNLGVIVDNQLSCTANITAVTRSCRFALYNIRRIRPFLTREATQLLTQA
LVISRLDYCNSLLAGLPATA
IKPLQWIQNAAARLVFNRNKFSQVTPLFQDLHWLPVVARIKFKTMLLAYKDVNGTAPTYLQTLVKHHTPARALRSTTSAG
RLVPPSLKGRMAKCQLFWHC
SGGAPCQWQHCRVTPHLPEETQDPLV
>g2151.t1
MTPALFRRIQSATSTANSSSTSAPSYSTSAPSPFAPSSSTSAPAHCVPPSMPKTREYGHSRYMGQPVCSRAEGRSVEDWL
AEKRSTHPPKKVGYLIMLQI
>g2152.t1
ATHGECRVLVRLKSHDRGNGVAVAMPAELSRVITSQGAVVRYLRGGSSEVLFPDGAVSFSPDSGPVWPPTAEPSQGEPDD
ALPPPGRGHWITTAPSGARV
YTVGSTHEHRPISSVLSVRATDPFSKAVMLTRQDRVVCVQDTDGTLTVEHADGTRISSFYQGKATSPTHRHTVFEDVLAS
VLQHIELLPDLWALRSESVT
LMKICARLALPSSPSFPGGRGPGWVSGEPMAEEEETAERVVLVEREGLAGVMVFPERGAARVFLEGGTVATGDADGVYQV
FPASTGRLLVGQDGVCVYNS
GPTPSSLGSQSGSYRLSATDRVTCDITDPEGNHFQ
>g2152.t2
MPAELSRVITSQGAVVRYLRGGSSEVLFPDGAVSFSPDSGPVWPPTAEPSQGEPDDALPPPGRGHWITTAPSGARVYTVG
STHEHRPISSVLSVRATDPF
SKAVMLTRQDRVVCVQDTDGTLTVEHADGTRISSFYQGKATSPTHRHTVFEDVLASVLQHIELLPDLWALRSESVTLMKI
CARLALPSSPSFPGGRGPGW
VSGEPMAEEEETAERVVLVEREGLAGVMVFPERGAARVFLEGGTVATGDADGVYQVFPASTGRLLVGQDGVCVYNSGPTP
SSLGSQSGSYRLSATDRVTC
DITDPEGNHFQ
>g2152.t3
LKSHDRGNGVAVAMPAELSRVITSQGAVVRYLRGGSSEVLFPDGAVSFSPDSGPVWPPTAEPSQGEPDDALPPPGRGHWI
TTAPSGARVYTVGSTHEHRP

ISSVLSVRATDPFSKAVMLTRQDRVVCVQDTDGTLTVEHADGTRISSFYQGKATSPTHRHTVFEDVLASVLQHIELLPDL
WALRSESVTLMKICARLALP
SSPSFPGGRGPGWVSGEPMAEEEETAERVVLVEREGLAGVMVFPERGAARVFLEGGTVATGDADGVYQVFPASTGRLLVG
QDGVCVYNSGPTPSSLGSQS
GSYRLSATDRVTCDITDPEGNHFQ
>g2153.t1
VGPHHPGRPLAHRQAAQPGGDLLWAGAPGFHPWPSKMKMEGPAYLCSVGEMMQSKVGLDGGRKAPKAKKRKIFR
>g2154.t1
MPTGMEKEEELEHHLQRAISAQQVFREKKENMVIPVPEAESNITYYDRLYKGELKAPKQLMHIQPLGLDNEQPDYDMDSE
DETLLNRLNRKMEIKPLQFE
TMVDRLEKASTNQ
>g2154.t2
MSKLSFRARALDAAKPLPVYRNRDLPDLNDCVSINRAVPQMPTGMEKEEELEHHLQRAISAQQVFREKKENMVIPVPEAE
SNITYYDRLYKGELKAPKQL
MHIQPLGLDNEQPDYDMDSEDETLLNRLNRKMEIKPLQFETMVDRLEKASTNQ
>g2155.t1
MTGFLEYIDSGIARGVGGSIPPTVHMSNPTNDFLRSQSQPRPPRPSSLPSPHKPRPNSSCRMPPSSPLARSEPGINPRGG
PVLWAQSQ
>g2155.t2
MRIDVRPLAAGMMMGETRRRATAAALASCIRRRIHQAMVTSLNEDNESVTVEWIENGDTKGKEIDLESIFALNPDVAPEE
EMAPSPETPPPPTPASVKIN
KIAKNRRTIAPSKIEAPSRDNRDSGIARGVGGSIPPTVHMSNPTNDFLRSQSQPRPPRPSSLPSPHKPRPNSSCRMPPSS
PLARSEPGINPRGGPVLWAQ
SQ
>g2155.t3
MRIDVRPLAAGMMMGETRRRATAAALASCIRRRIHQAMVTSLNEDNESVTVEWIENGDTKGKEIDLESIFALNPDVAPEE
EMAPSPETPPPPTPASVKIN
KIAKNRRTIAPSKIEAPSRDNRGEGHV
>g2155.t4
MRIDVRPLAAGMMMGETRRRATAAALASCIRRRIHQAMVTSLNEDNESVTVEWIENGDTKGKEIDLESIFALNPDVAPEE
EMAPSPETPPPPTPASVKIN
KIAKVATVLFVVLLHGARPRSLRSQSQPRPPRPSSLPSPHKPRPNSSCRMPPSSPLARSEPGINPRGGPVLWAQSQ
>g2156.t1
MTRQAVTKMVAVWLLSFLLYGPAIIFWESFRGHSIVPANQCFAEFYFTWYFLLSASTFEFFTPFVSVAFFNLCIYLNIRR
RNKSMTACVEDSSSATAVAA
AAVQQDLRTPRGGGAAWSVFFVKTRKVSSSEPAAISAVIEDDDEDFVPSSGCERGSAQTFVHRKKLSPREGGAALFESRS
PCGAPGRRTQGSRLTRDKKI
AKSLAIIVCIFGVCWAPYTLLMIIRAACSGTCVEHHWYEITFWLLWLNSAINPFLYPLCHSSFRRAFSKILCPKGQSVQP
QIEIQSC
>g2157.t1
MSGGLDVLQMKEEDVLKFLAAGTHLGGTNMDFQMEHYVYKRKSDGVYIINLRRTWEKLLLAARAIVAIENPADICVISSR
NTGQVGLLRPEPC
>g2157.t2
MSGGLDVLQMKEEDVLKFLAAGTHLGGTNMDFQMEHYVYKRKSDGVYIINLRRTWEKLLLAARAIVAIENPADICVISSR
NTGQ
>g2157.t3
MSLHCGDAVMSLPTRGLLPWMISTSVWFLDVVLNGFFSIKWILCLLLSKFSRQPRNTGKAVTMSGGLDVLQMKEEDVLKF
LAAGTHLGGTNMDFQMEHYV
YKRKSDGVYIINLRRTWEKLLLAARAIVAIENPADICVISSRNTGQVGLLRPEPC
>g2158.t1
SGQYNYGSVVGALRSIYQTEGAKALFSGLTATLLRDAPFSGIYVMFYSQAKRALAQEGGGTAYVPLVNFGCGVVAGVLAS
LVTQPADVVKTHIQVSALPC
STSQALRYIYAENGLQGFFRGAVPRSLRRTLMAAMAWTVYEQLMARMGLKS
>g2159.t1
LEVDKKQPALDVLYDVIKSKKHRTWQKIHEPIMLKYLELCVDLRKSHLAKEGLYQYKNICQQVNIKSLEDVVRAYLKLAE
EKTETAKGESQQMVLDIEDL

DNIQTPESVLLSAVSGEDTQDRTDRLLLTPWVKFLWESYRQCLDLLRNNSKVERLYHDIAQQAFKFCLQYTRKAEFRKLC
DNLRMHLSQIQRHHNQSTAI
NLNNPESQSMHLETRLVQLDSAISMELWQEAFKAVEDIHGLFALSKKPPKPQLMANYYNKVSTVFWKSGNALFHACTLHR
LYHLSREMRKNLTQEEMQRM
STRVLLATLSIPITPERTDIARLLDMDGIIVEKHRRLATLLGLQSPPTRQSLINDMVRFNLLQYILPEVKELYNWLEMDF
HPLKLSGRVTKVLDWVRDQA
EKESDLQQYVPHLQSNTILRLLQQVAQIYQSIEFSRLASLVPFVDAFQLERSIVDAARHCDLQVRIDHSSRTLSFGSDLN
YSTKEDSPVGPFLQNMPSEQ
IRNQLTAMSSSLAKAIQIIRPASILQEREEQSQLAIAAYLKNGRKDHQRILARRQTIEERKERLESLNIQREKEELEQRE
AEMQKVRKAEEERLRLEAKE
REKERIMQEHEQIKKKTVRERLEQIKKTELGAKAFKDIDIEVESRTSLGIAIPMTPTWQIKVHRNHICGFTFIYSFSGYE
DLEELDPDFIMAKQVEQLEK
EKKELQERLKNQEKKIDYFERAKRLEEIPLIKKAYEEQRIKDMELWELQEEERISNMKVEREKALEHKKRMSRMMEDKEN
FVSKITAARSFIYEEKLKAF
QERMVEEKKKRLEDRKRQRKEDRRTAYYRQKEEEAQRIHEEQLKK
>g2159.t2
MTNPYTCHGTVICKIREFLEVDKKQPALDVLYDVIKSKKHRTWQKIHEPIMLKYLELCVDLRKSHLAKEGLYQYKNICQQ
VNIKSLEDVVRAYLKLAEEK
TETAKGESQQMVLDIEDLDNIQTPESVLLSAVSGEDTQDRTDRLLLTPWVKFLWESYRQCLDLLRNNSKVERLYHDIAQQ
AFKFCLQYTRKAEFRKLCDN
LRMHLSQIQRHHNQSTAINLNNPESQSMHLETRLVQLDSAISMELWQEAFKAVEDIHGLFALSKKPPKPQLMANYYNKVS
TVFWKSGNALFHACTLHRLY
HLSREMRKNLTQEEMQRMSTRVLLATLSIPITPERTDIARLLDMDGIIVEKHRRLATLLGLQSPPTRQSLINDMVRFNLL
QYILPEVKELYNWLEMDFHP
LKLSGRVTKVLDWVRDQAEKESDLQQYVPHLQSNTILRLLQQVAQIYQSIEFSRLASLVPFVDAFQLERSIVDAARHCDL
QVRIDHSSRTLSFGSDLNYS
TKEDSPVGPFLQNMPSEQIRNQLTAMSSSLAKAIQIIRPASILQEREEQSQLAIAAYLKNGRKDHQRILARRQTIEERKE
RLESLNIQREKEELEQREAE
MQKVRKAEEERLRLEAKEREKERIMQEHEQIKKKTVRERLEQIKKTELGAKAFKDIDIEVESRTSLGIAIPMTPTWQIKV
HRNHICGFTFIYSFSGYEDL
EELDPDFIMAKQVEQLEKEKKELQERLKNQEKKIDYFERAKRLEEIPLIKKAYEEQRIKDMELWELQEEERISNMKVERE
KALEHKKRMSRMMEDKENFV
SKITAARSFIYEEKLKAFQERMVEEKKKRLEDRKRQRKEDRRTAYYRQKEEEAQRIHEEQLKK
>g2159.t3
MTNPYTCHGTVIXFLEVDKKQPALDVLYDVIKSKKHRTWQKIHEPIMLKYLELCVDLRKSHLAKEGLYQYKNICQQVNIK
SLEDVVRAYLKLAEEKTETA
KGESQQMVLDIEDLDNIQTPESVLLSAVSGEDTQDRTDRLLLTPWVKFLWESYRQCLDLLRNNSKVERLYHDIAQQAFKF
CLQYTRKAEFRKLCDNLRMH
LSQIQRHHNQSTAINLNNPESQSMHLETRLVQLDSAISMELWQEAFKAVEDIHGLFALSKKPPKPQLMANYYNKVSTVFW
KSGNALFHACTLHRLYHLSR
EMRKNLTQEEMQRMSTRVLLATLSIPITPERTDIARLLDMDGIIVEKHRRLATLLGLQSPPTRQSLINDMVRFNLLQYIL
PEVKELYNWLEMDFHPLKLS
GRVTKVLDWVRDQAEKESDLQQYVPHLQSNTILRLLQQVAQIYQSIEFSRLASLVPFVDAFQLERSIVDAARHCDLQVRI
DHSSRTLSFGSDLNYSTKED
SPVGPFLQNMPSEQIRNQLTAMSSSLAKAIQIIRPASILQEREEQSQLAIAAYLKNGRKDHQRILARRQTIEERKERLES
LNIQREKEELEQREAEMQKV
RKAEEERLRLEAKEREKERIMQEHEQIKKKTVRERLEQIKKTELGAKAFKDIDIEVESRTSLGIAIPMTPTWQIKVHRNH
ICGFTFIYSFSGYEDLEELD
PDFIMAKQVEQLEKEKKELQERLKNQEKKIDYFERAKRLEEIPLIKKAYEEQRIKDMELWELQEEERISNMKVEREKALE
HKKRMSRMMEDKENFVSKIT
AARSFIYEEKLKAFQERMVEEKKKRLEDRKRQRKEDRRTAYYRQKEEEAQRIHEEQLKK
>g2160.t1
MVAISTPSCLVSSAGPDVVAQFGMETIILYTALMLKKRIIVHHPRIEALLEFTRVLPAFTWHRKDWSILHPNVHLTEVEL
DDLKKCPGYVAGFTNPEVGN
RPDLFDVFVNLPDSTMTVSPAAKEAMAMGKVHKDIGQLIVQSAEDVERTDAQVIKDISVKTKEILANLMSLADECENSKI
TVEGLKRHHFPQATENFLFH

LAAAERLLRI
>g2160.t2
MVAISTPSCLVSSAGPDVVAQFGMETIILYTALMLKKRIIVHHPRIEALLEFTRVLPAFTWHRKDWSILHPNVHLTEVEL
DDLKKCPGYVAGFTNPEVGN
RPDLFDVFVNLPDSTMTVSPAAKEAMAMGKVHKDIGQLIVQSAEDVERTDAQVIKVRDVTFLLADRH
>g2160.t3
MLSVGLIDVVAQFGMETIILYTALMLKKRIIVHHPRIEALLEFTRVLPAFTWHRKDWSILHPNVHLTEVELDDLKKCPGY
VAGFTNPEVGNRPDLFDVFV
NLPDSTMTVSPAAKEAMAMGKVHKDIGQLIVQSAEDVERTDAQVIKDISVKTKEILANLMSLADECENSKITVEGLKRHH
FPQATENFLFHLAAAERLLR
I
>g2161.t1
MDPNLQYWKSEGQAEIKNARSTVASVEKINEKSSVHADSGAVLPFAFRPPGQEDAFETGSFDRRVGLLHDICRASLAFFS
VFTVRSEESDNGIQVFDSNG
NSVGISQAAGDKAVKDTAWSRAALLGTSAAVPNLLVFLLGMTRLFNKNPMLLAPLRHVSTALVFGMMIPVSLSIFPQLGT
IKRETLEQELQAAAVDGQLF
YHRGL
>g2161.t2
MDPNLQYWKSEGQSSVHADSGAVLPFAFRPPGQEDAFETGSFDRRVGLLHDICRASLAFFSVFTVRSEESDNGIQVFDSN
GNSVGISQAAGDKAVKDTAW
SRAALLGTSAAVPNLLVFLLGMTRLFNKNPMLLAPLRHVSTALVFGMMIPVSLSIFPQLGTIKRETLEQELQAAAVDGQL
FYHRGL
>g2161.t3
MLRVGGQEDAFETGSFDRRVGLLHDICRASLAFFSVFTVRSEESDNGIQVFDSNGNSVGISQAAGDKAVKDTAWSRAALL
GTSAAVPNLLVFLLGMTRLF
NKNPMLLAPLRHVSTALVFGMMIPVSLSIFPQLGTIKRETLEQELQAAAVDGQLFYHRGL
>g2162.t1
LCTWALQDCQLRCPPGRLLLYQFAAAVTHPAPAFKGTAVHNGEFKEMSLSDFKGKYLVLFFYPLDFTFVCPTEIIAFSDK
ANEFHDVNCEVVGVSVDSHF
THLAWMNTPRKAGGLGKLHIPLLADLTKQISRDYGVLLEGPGIALRHVPATVT
>g2162.t2
MLMKPVYVVGKTCGWRTPTFRALCTWALQDCQLRCPPGRLLLYQFAAAVTHPAPAFKGTAVHNGEFKEMSLSDFKGKYLV
LFFYPLDFTFVCPTEIIAFS
DKANEFHDVNCEVVGVSVDSHFTHLAWMNTPRKAGGLGKLHIPLLADLTKQISRDYGVLLEGPGIALRHVPATVT
>g2162.t3
MKPVYVVGKTCGWRTPTFRALCTWALQDCQLRCPPGRLLLYQFAAAVTHPAPAFKGTAVHNGEFKEMSLSDFKGKYLVLF
FYPLDFTFVCPTEIIAFSDK
ANEFHDVNCEVVGVSVDSHFTHLAWMNTPRKAGGLGKLHIPLLADLTKQISRDYGVLLEGPGIALRHVPATVT
>g2163.t1
MTVVLCSSRGLFLIDPQGVVKHMSVNDLPVGRSVEETLRLVKAFQFVETHGEVCPASWTPHSPTIKPTPEGSKEYFEKVN
>g2164.t1
QYKKFPQMTSYHRMLLHRVAAYFGMDHNVDQTGKAVIINKTGNTRIPEQRFSEHIKDERNMDFQKKFILKRDDASMEKDD
NQMGSDSAVLTLAGSSFHHC
GAKTEKS
>g2165.t1
MTQCVDTCSQQGGHRGTVTVLLDDNTYAEQKLNAEVKGLPSFFGGSRLATEITLALLDGANDKDAEVQEQVRKSVLTLGK
QQPDRVLAMCQDYLLKHPK
>g2165.t2
LALLDGANDKDAEVQEQVRKSVLTLGKQQPDRVLAMCQDYLLKHPK
>g2166.t1
FNSDRSQLTISWVRVSDYGEYICTARNKIGESTGSIVLHVSEAPAVYVTADEEVVLFGHNVSLSCNATGYPYPQLYWINK
QSRKIVDSTSGRVRVLDGVL
TIEEVAPSDGGLYSCMAVSTSGNASRDIAVHTQPGPSQDVSVLPDATSLVFSLKTQPISGGTPIRSFVLKWRPVSAEKWE
ETVVPVSDPLAITSLQPYSS
YSVCLAAVNTVGRGQCSDTQDVRTQAAREPDSPRLSPVEMKPEGNALSVPVVQLNSGSSPLLHYKIRYRQEKEGAEWKEE
QQPAATAISLSDLSFGEDYN

LEVVAVNANGSSGPTQLSFSTSKQAAGMAKGGVVAIIMVIFLVLLIVVDVTCCYTNRCGLLMFITTKVFGRRVPGLKTME
EGDGTNG
>g2166.t2
PGPLDPARHQFNSDRSQLTISWVRVSDYGEYICTARNKIGESTGSIVLHVSEAPAVYVTADEEVVLFGHNVSLSCNATGY
PYPQLYWINKQSRKIVDSTS
GRVRVLDGVLTIEEVAPSDGGLYSCMAVSTSGNASRDIAVHTQPGPSQDVSVLPDATSLVFSLKTQPISGGTPIRSFVLK
WRPVSAEKWEETVVPVSDPL
AITSLQPYSSYSVCLAAVNTVGRGQCSDTQDVRTQAAREPDSPRLSPVEMKPEGNALSVPVVQLNSGSSPLLHYKIRYRQ
EKEGAEWKEEQQPAATAISL
SDLSFGEDYNLEVVAVNANGSSGPTQLSFSTSKQAAGMAKGGVVAIIMVIFLVLLIVVDVTCCYTNRCGLLMFITTKVFG
RRVPGLKTMEEGDGTNG
>g2166.t3
FNSDRSQLTISWVRVSDYGEYICTARNKIGESTGSIVLHVSEAPAVYVTADEEVVLFGHNVSLSCNATGYPYPQLYWINK
QSRKIVDSTSGRVRVLDGVL
TIEEVAPSDGGLYSCMAVSTSGNASRDIAVHTQPGPSQDVSVLPDATSLVFSLKTQPISGGTPIRSFVLKWRPVSAEKWE
ETVVPVSDPLAITSLQPYSS
YSVCLAAVNTVGRGQCSDTQDVRTQAAREPDSPRLSPVEMKPEGNALSVPVVQLNSGSSPLLHYKIRYRQEKEGAEWKEE
QQPAATAISLSDLSFGEDYN
LEVVAVNANGSSGPTQLSFSTSKQAGMAKGGVVAIIMVIFLVLLIVVDVTCCYTNRCGLLMFITTKVFGRRVPGLKTMEE
GDGTNGDLNLKGLDTPRGSI
QQPQTKEGKLNSEITCDKAPLTKHEKHFGDLPTVDA
>g2166.t4
MAKGGVVAIIMVIFLVLLIVVDVTCCYTNRCGLLMFITTKVFGRRVPGLKTMEEGDGTNGDLNLKGLDTPRGSIQQPQTK
EGKLNSEITCDKAPLTKHEK
HFGDLPTVDA
>g2166.t5
MAKGGVVAIIMVIFLVLLIVVDVTCCYTNRCGLLMFITTKVFGRRVPGLKTMEEGDGTNGDLNLKGLDTPRGSIQQPQTK
EGKLNSEITCDKAPLTKHE
>g2167.t1
LVPARYTDDLVMETKTHQSYFDGNDTAESRWSQIPGAMEYCCSTQESASTSNDILMDIANVSGPAGSPAADCKENNTKKL
DPPVLRLSQTQPFVLPLLNN
SLYSRKQEMDSKELSKTVAESMGLYMNAVREADFGFSQHGGSTSPGKFYAGCGRPLEGNNPCAKLARSPPLGFPQCGVAP
RDCAASGPQQVSSASSQVSS
LSCSPQTSSSVSSHGGGGGGGSCSSSSSSSSSNMVSSTTSPTTCFGPARSSISSPLGQAPSVPPAMGHVQRRNSITCSPV
ESSTVGSPPLISPLNVMKSP
MSSPQSMGSVRSPPPCSSTCNMRSSVSSPTGSNGHVTAGNCNAARSSISSPTAAGGGAMATGSPRNLSFGGFPVSSPASG
LGLVQNDTNSPEAAGLAKDP
DFKNFEFPKVEMVDGEVFNVGLDHMGMVKYIKNEPGTDLRSMCLGSSKCSGNGTAYITQIKSEPNKDEDCMNQPLYNEQP
RSLGLFPASETTYLSLRNNI
DEYSLSGILGPPISSMNGNYEPEGFLNNVLSKGVKQESIDGSYYQENSGMTPSAIVGVNSGGHSFHYQVGAQGTMSFGRN
DVRDQSNPLLNLISPVTALM
ESWKSRPGMAQGAMSARAEGYLGQGCIVDNMSR
>g2168.t1
MFQQQDHLKSQLQEAHPANRQLFQCQECGKQYNTQLGYRRHLVAAHGTAA
>g2169.t1
MTFAWQVLENFSDAPMTPKQILHVIQTKGLKEMSGTAPLACLVTMLHSQVRGDRVKNSIFFKLPGRMSLFTLKKNALQWT
KSPTEADTATEGTGAAAAST
TNPAASATATSTSTGPGLAHTTAPEGAEQESCDSTETTANASGENDGRRPSRDASVDESSSSASCSTEPTPPSQPQTRLS
RAAGQQGRAESQTQHTTRLS
RSRQGKGGVVVRVLETQLKGTGFNTLTASV
>g2169.t2
MTFAWQVLENFSDAPMTPKQILHVIQTKGLKEMSGTAPLACLVTMLHSQVRGDRVKNSIFFKLPGRMSLFTLKKNALQWT
KSPTEADTATEGTGAAAAST
TNPAASATATSTSTGPGLAHTTAPEGAEQESCDSTETTANASGENDGRRPSRDASVDESSSSASCSTEPTPPSQPQTRLS
RAAGQQGRAESQTQHTTRLS
RSRQ

>g2169.t3
MTPKQILHVIQTKGLKEMSGTAPLACLVTMLHSQVRGDRVKNSIFFKLPGRMSLFTLKKNALQWTKSPTEADTATEGTGA
AAASTTNPAASATATSTSTG
PGLAHTTAPEGAEQESCDSTETTANASGENDGRRPSRDASVDESSSSASCSTEPTPPSQPQTRLSRAAGQQGRAESQTQH
TTRLSRSRQGKGGVVVRVLE
TQLKGTGFNTLTASV
>g2170.t1
QSPAAPSTSSSATSQSSPVAIPVTMVPLPATSPVVISAATLSTATLSAVPPATPATPALKKQRPLLPRETVPVVQRAVVW
NPTTKFQTSSQKWHMQKIQR
QQLNQQPAADSTQPQALSPGPGLAQTPPAQTQATATAAPALTPTQPAQSTRYQTRQAVKGDVQIPTASADVAADIAKYTN
KIRRLRIEIEKLQWLHQQEL
SEMKHNLELTMAEMRQSLEQERERLVAEVKKQMEVEKQQAIDETKKKQWCANCRKEAIFYCCWNTSYCDYPCQQAHWPEH
MKSCTQSATAPQQEGEIEPA
VEGSNKSSGPTNNVPVPLRDPAVPPLSDKDCNLEKSSETVSHNPSFQT
>g2170.t2
QSPAAPSTSSSATSQSSPVAIPVTMVPLPATSPVVISAATLSTATLSAVPPATPATPALKKQRPLLPRETVPVVQRAVVW
NPTTKFQTSSQKWHMQKIQR
QQLNQQPAADSTQPQALSPGPGLAQTPPAQTQATATAAPALTPTQPAQSTRYQTRQAVKGDVQIPTASADVAADIAKYTN
KIRRLRIEIEKLQWLHQQEL
SEMKHNLELTMAEMRQSLEQERERLVAEVKKQMEVEKQQAIDETKKKQWCANCRKEAIFYCCWNTSYCDYPCQQAHWPEH
MKSCTQSATAPQQEGEIEPA
VEGSNKSSGPTNNVPVPLRDPAVPPLSDKDCNLEKSSETPEQSQDPLDSCAQAAETENKVEAECHEPDDNGPKEQLEESG
QSPKTSGEVRKTWGFRRTTI
AKRDMPVEATAEPQESRGGAVRRSGRQSKRTDKLEEFLSSTAKRAARRSGPSSIESGDPPSQTPTDAETASEASFDGNAD
TKAAEDKSPSPERRTRSGTR
KPPAGKARGGRLTRSRGGGAATAAKDDGSSENEEDSCEDTAVKGQPQNKSQEDTQEEAKEACLKDEMKASGNSSRAGPEV
DSENQDQAAKQPPKEEKAEE
EKVKEEPNRESEKDTEDEGKEKPPGGFVKRGPIRTYTNKKKASNKSATVAPAPGKQPLAPAMKGAFTARREPNRPNTAQP
ATRNRGSPEGPDDEEDDDEE
DSMTSTSTTSSSSSSDSDEGGYDPNALYCICRQKHNKR
>g2170.t3
QSPAAPSTSSSATSQSSPVAIPVTMVPLPATSPVVISAATLSTATLSAVPPATPATPALKKQRPLLPRETVPVVQRAVVW
NPTTKFQTSSQKWHMQKIQR
QQLNQQPAADSTQPQALSPGPGLAQTPPAQTQATATAAPALTPTQPAQSTRYQTRQAVKGDVQIPTASADVAADIAKYTN
KIRRLRIEIEKLQWLHQQEL
SEMKHNLELTMAEMRQSLEQERERLVAEVKKQMEVEKQQAIDETKKKQWCANCRKEAIFYCCWNTSYCDYPCQQAHWPEH
MKSCTQSEQSQDPLDSCAQA
AETENKVEAECHEPDDNGPKEQLEESGQSPKTSGEVRKTWGFRRTTIAKRDMPVEATAEPQESRGGAVRRSGRQSKRTDK
LEEFLSSTAKRAARRSGPSS
IESGDPPSQTPTDAETASEASFDGNADTKAAEDKSPSPERRTRSGTRKPPAGKARGGRLTRSRGGGAATAAKDDGSSENE
EDSCEDTAVKGQPQNKSQED
TQEEAKEACLKDEMKASGNSSRAGPEVDSENQDQAAKQPPKEEKAEEEKVKEEPNRESEKDTEDEGKEKPPGGFVKRGPI
RTYTNKKKASNKSATVAPAP
GKQPLAPAMKGAFTARREPNRPNTAQPATRNRGSPEGPDDEEDDDEEDSMTSTSTTSSSSSSDSDEGGYDPNALYCICRQ
KHNKR
>g2170.t4
MEENMSPVLSLAPQPEQSQDPLDSCAQAAETENKVEAECHEPDDNGPKEQLEESGQSPKTSGEVRKTWGFRRTTIAKRDM
PVEATAEPQESRGGAVRRSG
RQSKRTDKLEEFLSSTAKRAARRSGPSSIESGDPPSQTPTDAETASEASFDGNADTKAAEDKSPSPERRTRSGTRKPPAG
KARGGRLTRSRGGGAATAAK
DDGSSENEEDSCEDTAVKGQPQNKSQEDTQEEAKEACLKDEMKASGNSSRAGPEVDSENQDQAAKQPPKEEKAEEEKVKE
EPNRESEKDTEDEGKEKPPG
GFVKRGPIRTYTNKKKASNKSATVAPAPGKQPLAPAMKGAFTARREPNRPNTAQPATRNRGSPEGPDDEEDDDEEDSMTS
TSTTSSSSSSDSDEGGYDPN
ALYCICRQKHNKR
>g2171.t1

MTEGFISILTDAGGWNTAKEAYGSFGNNRGKSAFFNDRGNTNRGRFDRGGFSGGGGGGGNSRWVEESRDDEDWSKPTPPN
ERLEHELFSGSNTGINFEKY
DDIPVEATGQNCPHHIESGYCAFAVAGNQR
>g2171.t2
MTEGFISILTDAGGWNTAKEAYGSFGNNRGKSAFFNDRGNTNRGRFDRGGFSGGGGGGGNSRWVEESRDDEDWSKPTPPN
ERLEHELFSGSNTGINFEKY
DDIPVEATGQNCPHHIESAVHA
>g2172.t1
MQYRGAVLSAGFAGLFSFTVLAVAIGTDYWYIINVNNPNYTGSDDLSSHSGLWTTHEGRINQLQVRKVHWYRRTL
>g2173.t1
EPVKPDEGREMANRINAFGYLECSAKTKDGVREVFEMATRAALQVRKRKNSGCLLL
>g2174.t1
LLTQLKEEVVGLFPSPFPRHTTPEKFFIEACDEGADAVLAIDRVSNEMTFTVKREVPPSAVTRPVCGIMGTIRLVAGMYL
IVITKKRNVGSLLGHAVWKM
VEFEVISYKKTVLHLTEAQSQENTTLLSMVSNVLATDGFYFCTDYDLTHTLQRLANTSPDFQEMSLLEREELLPGRGQTR
GSMPFFWSQRPNLKYKPKPL
ISKTANHVNQKGSEKPLEQAFAKMVAGADKEMIQYFMVNAEGKTVAQQTGTFRSNCMDCLDRTNVIQSLLARCSLQSQLQ
>g2174.t2
LLTQLKEEVVGLFPSPFPRHTTPEKFFIEACDEGADAVLAIDRVSNEMTFTVKREVPPSAVTRPVCGIMGTIRLVAGMYL
IVITKKRNVGSLLGHAVWKM
VEFEVISYKKTVLHLTEAQSQENTTLLSMVSNVLATDGFYFCTDYDLTHTLQRLANTSPDFQEMSLLEREELLPGRGQTR
GSMPFFWSQRPNLKYKPKPL
ISKTANHVNQKGSEKPLEQAFAKMVAGADKEMIQYFMVNAEGKTVAQQTGTFRSNCMDCLDRTNVIQSLLARCSLQSQLQ
VSGELHRPTA
>g2174.t3
MYLILTKLSQILRHTTPEKFFIEACDEGADAVLAIDRVSNEMTFTVKREVPPSAVTRPVCGIMGTIRLVAGMYLIVITKK
RNVGSLLGHAVWKMVEFEVI
SYKKTVLHLTEAQSQENTTLLSMVSNVLATDGFYFCTDYDLTHTLQRLANTSPDFQEMSLLEREELLPGRGQTRGSMPFF
WSQRPNLKYKPKPLISKTAN
HVNQKGSEKPLEQAFAKMVAGADKEMIQYFMVNAEGKTVAQQTGTFRSNCMDCLDRTNVIQSLLARCSLQSQLQCLSING
SQRLGVLNVGQRVEEQAQFE
RIYKNGNVRGCLHTVFTV
>g2175.t1
TLIATRISECLADISSWTTAHHIKLNLAKTERWRKDIRGRVAGLPEEGSGGPSRAQRRRRRRRRLMLEKRADRDPRTGSA
GRPGGRVGVEKLLPAALQ
>g2176.t1
VSGLESELLVAQGQVTEVQGALNAAQDELVTLSEELAQLYHHVCLCNNETPNRVMLDYYRQGRGMRGLTAGLKALSADES
RVLLTPRLARRLAAVAASTA
SPGESRSPSQSPSKEPAPGGEGARRDTVVPRLAAPPPRSPGPNTPPTRSPSISASSSSSSSSSPALEPAGELRREPLNIY
NLNAIIRDQVKHLQRAVDRS
LLLSRQRAAARELAPLLDKDKESCLEEILKLKSLLSTKREQVATLRLVLKANKQTAEVALANLKGKYEAEKSMVTNTMTK
LRNELKALKEDAATFSSLRA
MFATRCDEYVTQLDEMQRQLAAAEDEKKTLNSLLRMAIQQKLALTQRLEDLAFDQEQTHRTRGAKPSRTRTGTPKVSPPS
SGLARGSAVTSAPGSLPPSS
LTSPTSAGFDKAHVLTRSPSFVRSAATVLASGLASPTSPGASSKPPSPQQEGARPLGSPPSLETPALPSAWTPHSTALRL
PPSQWTLGLRTLVVDSHSFS
LSVSPTGPSRSLLSREHASDASPASPSPAGSATTITRAPQAGSSPAPPYRSPLLSGRRSAWSATPRSRPLSTLSRPSARY
TPSFSSPYSSFHSPASSYSY
SSAYGSPSYGSSSVHRSSPSSYAHSSSYSPLYPRYYSSHQPRH
>g2176.t2
LESELLVAQGQVTEVQGALNAAQDELVTLSEELAQLYHHVCLCNNETPNRVMLDYYRQGRGMRGLTAGLKALSADESRVL
LTPRLARRLAAVAASTASPG
ESRSPSQSPSKEPAPGGEGARRDTVVPRLAAPPPRSPGPNTPPTRSPSISASSSSSSSSSPALEPAGELRREPLNIYNLN
AIIRDQVKHLQRAVDRSLLL
SRQRAAARELAPLLDKDKESCLEEILKLKSLLSTKREQVATLRLVLKANKQTAEVALANLKGKYEAEKSMVTNTMTKLRN
ELKALKEDAATFSSLRAMFA

TRCDEYVTQLDEMQRQLAAAEDEKKTLNSLLRMAIQQKLALTQRLEDLAFDQEQTHRTRGAKPSRTRTGTPKVSPPSSGL
ARGSAVTSAPGSLPPSSLTS
PTSAGFDKAHVLTRSPSFVRSAATVLASGLASPTSPGASSKPPSPQQEGARPLGSPPSLETPALPSAWTPHSTALRLPPS
QWTLGLRTLVVDSHSFSLSV
SPTGPSRSLLSREHASDASPASPSPAGSATTITRAPQAGSSPAPPYRSPLLSGRRSAWSATPRSRPLSTLSRPSARYTPS
FSSPYSSFHSPASSYSYSSA
YGSPSYGSSSVHRSSPSSYAHSSSYSPLYPRYYSSHQPRH
>g2176.t3
MRGLTAGLKALSADESRVLLTPRLARRLAAVAASTASPGESRSPSQSPSKEPAPGGEGARRDTVVPRLAAPPPRSPGPNT
PPTRSPSISASSSSSSSSP
ALEPAGELRREPLNIYNLNAIIRDQVKHLQRAVDRSLLLSRQRAAARELAPLLDKDKESCLEEILKLKSLLSTKREQVAT
LRLVLKANKQTAEVALANLK
GKYEAEKSMVTNTMTKLRNELKALKEDAATFSSLRAMFATRCDEYVTQLDEMQRQLAAAEDEKKTLNSLLRMAIQQKLAL
TQRLEDLAFDQEQTHRTRGA
KPSRTRTGTPKVSPPSSGLARGSAVTSAPGSLPPSSLTSPTSAGFDKAHVLTRSPSFVRSAATVLASGLASPTSPGASSK
PPSPQQEGARPLGSPPSLET
PALPSAWTPHSTALRLPPSQWTLGLRTLVVDSHSFSLSVSPTGPSRSLLSREHASDASPASPSPAGSATTITRAPQAGSS
PAPPYRSPLLSGRRSAWSAT
PRSRPLSTLSRPSARYTPSFSSPYSSFHSPASSYSYSSAYGSPSYGSSSVHRSSPSSYAHSSSYSPLYPRYYSSHQPRH
>g2177.t1
HSAKTHNVHQIKGSQSEEADTELVLENLEVNTAQPIESDEDHATSFTTTTSTTTTTPKTTTSTTTTLSTMVPTSSTPRIE
RPAPTIVLILDNSDNNSQPL
LHRAGKIMECEGTLQSIEAPQKQHSYGRNEGAWMKDPLAKDSKIYVTNYYYGNNLIEFRNLENFKQGRWSNLYKLPYNWI
GTGHVVYNGAFYYNRAFTKN
IIKYDLRMRYVSAWTLLHDVVYEDTTPWKWRGHSDIDFAVDESGLWVIYPAMDYDYYQHEVIVISRLDPGDLSVKKETTW
RTGLKRNSYGNCFIICGVLY
AVDVYNQKSGEVSYAYDTHTSTEAAPHLPFVNEYAYTTQIDYNPKERVLYAWDNGHQLTYRVTFLDE
>g2177.t2
HSAKTHNVHQIKGSQSEEADTELVLENLEVNTAQPIESDEDHATSFTTTTSTTTTTPKTTTSTTTTLSTMVPTSSTPRIE
RPAPTIVLILDNSDNNSQPL
LHRAGKIMECEGTLQSIEAPQKQHSYGRNEGAWMKDPLAKDSKIYVTNYYYGNNLIEFRNLENFKQVSSDASFESGEGRW
SNLYKLPYNWIGTGHVVYNG
AFYYNRAFTKNIIKYDLRMRYVSAWTLLHDVVYEDTTPWKWRGHSDIDFAVDESGLWVIYPAMDYDYYQHEVIVISRLDP
GDLSVKKETTWRTGLKRNSY
GNCFIICGVLYAVDVYNQKSGEVSYAYDTHTSTEAAPHLPFVNEYAYTTQIDYNPKERVLYAWDNGHQLTYRVTFLDE
>g2178.t1
MNLYRSFGNLLEAYVADGGMYSADVLMLTEYSNAATPASTPASTPDIGINPRSESDDSGFEISISSPADLSPSSAGNPEP
HPMSVPTDGEERSLTPSSTL
TSALRSSVGSSMRSSVSSASFFLPVTDASAAPLTRDTSVSLQHRVEQALLKINPSWRKTRSASTLTRSDAQRRASKKSLS
LPRRTTAVHIPRSESTPAQL
LLRQAGHQRIDRQRRPVSLYHDMGLAEKPIE
>g2179.t1
LTLSSPSYAGYCPQLKYNMGQTYGQLTYKLLTSPEVRRSQRSLLSTGRLQTADIPDRTHEEPDRTADGQHRTPDQAGDEV
VGTTRLHRGEHRNLDTVIRG
YTGVPHKASSSASRGAPTCLVMRSLTRLSPRSAGFVPKRQTYSQSCRGAITKFTRDQNTSLRPQAWGLPAVSCSSPEVQA
RKPNTPLTAVRDEPTELKTP
GAWEPLGSPYLMDERSPHKYFISGFTGYVPKSRFLIGRGYPATTNEALVQFGREAARGLRSSADDPRKLSGALPPVPSVC
PSSRGLLPFYTGHVPGYKFM
YGDTFGKLTHNALGMNKVKRSIGVKS
>g2179.t2
MGQTYGQLTYKLLTSPEVRRSQRSLLSTGRLQTADIPDRTHEEPDRTADGQHRTPDQAGDEVVGTTRLHRGEHRNLDTVI
RGYTGVPHKASSSASRGAPT
CLVMRSLTRLSPRSAGFVPKRQTYSQSCRGAITKFTRDQNTSLRPQAWGLPAVSCSSPEVQARKPNTPLTAVRDEPTELK
TPGAWEPLGSPYLMDERSPH
KYFISGFTGYVPKSRFLIGRGYPATTNEALVQFGREAARGLRSSADDPRKLSGALPPVPSVCPSSRGLLPFYTGHVPGYK
FMYGDTFGKLTHNALGMNKV

KRSIGVKS
>g2179.t3
MESHKYPPKFSKVLVTPDPQYIPGYAGYCPQLKYNMGQTYGQLTYKLLTSPEVRRSQRSLLSTGRLQTADIPDRTHEEPD
RTADGQHRTPDQAGDEVVGT
TRLHRGEHRNLDTVIRGYTGFVPKRQTYSQSCRGAITKFTRDQNTSLRPQAWGLPAVSCSSPEVQARKPNTPLTAVRDEP
TELKTPGAWEPLGSPYLMDE
RSPHKYFISGFTGYVPKSRFLIGRGYPATTNEALVQFGREAARGLRSSADDPRKLSGALPPVPSVCPSSRGLLPFYTGHV
PGYKFMYGDTFGKLTHNALG
MNKVKRSIGVKS
>g2180.t1
MTTTQRHCQPLFQELLLLLQPLSVLPFDLNLLLEPRHRELCSRTSPVPACSALLMTAWPKLQAETPRGHAAGGRPAGSSC
LTDLHLPGLSQVPQSSTQQG
RRGRRDPDPDPGWWIRQPVIVDGVIEEEDRPSAETYSSWSPQYHATGREGVLDGGEATAGPSGRGSADPPVQAEGPAQGG
LRWAKLFGAAPDPPGRPPGA
ARGLGGSQCRRNTRPSQWLQLDRSTVGQLAQSVWAGKFGRAKTE
>g2181.t1
LLRSVSTAMDLIMAHFGSSRDPGEKMRLGNSSSSPTVAGLVLEHLCPAIQNILEDGLRHHNLDVVIGQRRNHSWRVVDAS
TQNGRASHILDNLVSKIHQC
SQLTSSCMKLRAFIMGLLK
>g2182.t1
MGPVDSPYQGGVFFLTIHFPTDYPFKPPKVSLARPTGPKVGGLRYELRPLLVNRTAGSVISKDARAIWFLGIPSN
>g2182.t2
MVDSPYQGGVFFLTIHFPTDYPFKPPKVSLARPTGPKVGGLRYELRPLLVNRTAGSVISKDARAIWFLGIPSN
>g2183.t1
MLGHHVHYVIPYDGDHSLVDSSENYFVSDSVTKQEMDLMLGLLLGFCISWLLLWMDGLLHCAVRAWRASRYYGKIA
>g2183.t2
MDMNALLDRFHNYILPHLRGEDRVCHCNCGRHHVHYVIPYDGDHSLVDSSENYFVSDSVTKQEMDLMLGLLLGFCISWLL
LWMDGLLHCAVRAWRASRYY
GKIA
>g2184.t1
MVVVIICIKTPEPAAVRVALLYPRRCGRSGRARGALSREETLIEAALYRTWPPVLLLPGLGCSNNGTLGGSSRPDLSHRP
QPRYTSRIAPQRPSFKHLFQ
IFVESIAQTGSRSYGGSTGRVWRIWVAGIDVSYPNY
>g2185.t1
MSRKQVVKPARGNKKGDVERKRDERAARRAIAKDRKNRPQDAADDGEEFVSFSNQLQSLGLRLREIPGDGALGDQLEGHS
RGHIRLRQETVQYMKSHRQD
FEPFVEDDIPFEEHLSNLAQPGTFAGNDAIVAFARSQQLKVVIHQLNTPLWEINGVEKQACREMHIAYRYGDHYDSVRRI
GDNSESPAQLRIELHRAFVH
VSGVCFPVFSGRVMAQNLQNAPGQQREFGDGQRTGAGESSPPVREDNVILSTYKHPAVHGEDHRMPL
>g2185.t2
MHPCQRMCGLPELAANTSAAVMSRKQVVKPARGNKKGDVERKRDERAARRAIAKDRKNRPQDAADDGEEFVSFSNQLQSL
GLRLREIPGDGALGDQLEGH
SRGHIRLRQETVQYMKSHRQDFEPFVEDDIPFEEHLSNLAQPGTFAGNDAIVAFARSQQLKVVIHQLNTPLWEINGVEKQ
ACREMHIAYRYGDHYDSVRR
IGDNSESPAQLRIELHRAFVHVSGVCFPVFSGRVMAQNLQNAPGQQREFGDGQRTGAGESSPPVREDNVILSTYKHPAVH
GEDHRMPL
>g2186.t1
MWCRVVLHLLQLLPAAYLGSSGSDGIDHLAEKGQAELLHVRRLANQPRYGECWARALVGVEESCRELTEELQSRVALQFT
HCHLHRSGRKFPACPEGADV
SQCTSNMDAVAFNAFTEFFTHTLSICHFLQSEAWQLRAESTMLSLTEASASVAEQLSSTRLLTEDLLEAQSSALLAQDQI
LLNGQELQHTLRESTQGVRD
LFSQVTDSSREQQVVLSELFNRISFLQSFLLLEAHTLSSGLYNAAGLCTAFLLTCTPRTARV
>g2186.t2
MWCRVVLHLLQLLPAAYLGSSGSDGIDHLAEKGQAELLHVRRLANQPRYGECWARALVGVEESCRELTEELQSRVALQFT
HCHLHRSGRKFPACPEGADV
SQCTSNMDAVAFNAFTEFFTHTLSICHFLQSEAWQLRAESTMLSLTEASASVAEQLSSTRLLTEDLLEAQSSALLAQDQI

LLNGQELQHTLRESTQGVRD
LFSQVTDSSREQQVVLSELFNRISFLQSFLLLEAHTLSSGLYNAAGLCTAFLLTCTPRTARVRFVLLGLVCLNMYLERLV
YQCVMDSSNPAHMHM
>g2186.t3
MWCRVVLHLLQLLPAAYLGSSGSDGIDHLAEKGQAELLHVRRLANQPRYGECWARALVGVEESCRELTEELQSRVALQFT
HCHLHRSGRKFPACPEGADV
SQCTSNMDAVAFNAFTEFFTHTLSICHFLQSEAWQLRAESTMLSLTEASASVAEQLSSTRLLTEDLLEAQSSALLAQDQI
LLNGQELQHTLRESTQGVRD
LFSQVTDSSREQQVVLSELFNRISFLQSFLLLEAHTLSSGLYNAAGLCTAFLLTCTPRTARVRLADGAESWMAEEDQRGH
ECMWMSSDKYTGARL
>g2187.t1
MLFSYSAGCAAEEEPDNRTRGERRHDDQPTGSSEGGDSQMGVEPQVQVSEPEAGPSESRSGPARSGSGDPRPRRSVMRDR
GPLYEDPSLPAGWTRKLKQR
KSGRSAGKYDVYLINSEGKAFRSKVELMAYFQRVGDITSDPNDFDFTVTGRGSSGKARRSTEGVAMKRVVEKTPGTLLVK
RPFDKTESTASTSAAALMVV
TKSRPGRKRKSEQEVAPPQTAPKKRGRKPASASASTPGLPLATTGPGGSSAGSFAAAILAAEAKRRAAKESTSRAIVQET
ALPIKKRKTRETMEEFEKVV
APVATTTPSGLGDGASEQGSAAPSTSELPVSRSEQLPVGRSEQLPVGDDSQPQEPQTPTGQKQNESQKRKDPLKHPSHP
>g2187.t2
MGVEPQVQVSEPEAGPSESRSGPARSGSGDPRPRRSVMRDRGPLYEDPSLPAGWTRKLKQRKSGRSAGKYDVYLINSEGK
AFRSKVELMAYFQRVGDITS
DPNDFDFTVTGRGSSGKARRSTEGVAMKRVVEKTPGTLLVKRPFDKTESTASTSAAALMVVTKSRPGRKRKSEQEVAPPQ
TAPKKRGRKPASASASTPGL
PLATTGPGGSSAGSFAAAILAAEAKRRAAKESTSRAIVQETALPIKKRKTRETMEEFEKVVAPVATTTPSGLGDGASEQG
SAAPSTSELPVSRSEQLPVG
RSEQLPVGDDSQPQEPQTPTGQKQNESQKRKDPLKHPSHP
>g2187.t3
MLFSYSAGCAAEEEPDNRTRGERRHDDQPTGSSEGGDSQMGVEPQVQVSEPEAGPSESRSGPARSGSGDPRPRRSVMRDR
GPLYEDPSLPAGWTRKLKQR
KSGRSAGKYDVYLINSEGKAFRSKVELMAYFQRVGDITSSGKARRSTEGVAMKRVVEKTPGTLLVKRPFDKTESTASTSA
AALMVVTKSRPGRKRKSEQE
VAPPQTAPKKRGRKPASASASTPGLPLATTGPGGSSAGSFAAAILAAEAKRRAAKESTSRAIVQETALPIKKRKTRETME
EFEKVVAPVATTTPSGLGDG
ASEQGSAAPSTSELPVSRSEQLPVGRSEQLPVGDDSQPQEPQTPTGQKQNESQKRKDPLKHPSHP
>g2188.t1
MITSELPVLQDSSNESGVPDAVSLSMSMSEIEDPEVKGKKKRGRPGKQQQKFDKNDLLALKNNALLTHSLSQSATKKPRK
SPTDKAVGVTRGRGKANGVA
QHNGEGGDPVTLFEVVKLGKSAMQSVVDDWIESYKQDRDIALLDLINFFIQCSGCKGTVRIEMFRNMQNAEIIRKMTEEF
DEVAPAFPVLKQLLFCSAGV
STRHSGWQTWP
>g2188.t2
MPGPLWKKFRYNFCEFICVLIRQCQYSIIYDEYMMDTVISLLTGLSDSQVRAFRHTSTLAAMKLMTALVNVALNLSIHQD
NTQRQYEAERNKMAGKRANE
KLELLLQKRKELQENQDEIENMMNSIFKGIFVHRY
>g2188.t3
MITSELPVLQDSSNESGVPDAVSLSMSMSEIEDPEVKGKKKRGRPGKQQQKFDKNDLLALKNNALLTHSLSQSATKKPRK
SPTDKAVGVTRGRGKANGVA
QHNGEGGDPVTLFEVVKLGKSAMQSVVDDWIESYKQDRDIALLDLINFFIQCSGCKGTVRIEMFRNMQNAEIIRKMTEEF
DEDSGDYPLTMPGPLWKKFR
YNFCEFICVLIRQCQYSIIYDEYMMDTVISLLTGLSDSQVRAFRHTSTLAAMKLMTALVNVALNLSIHQDNTQRQYEAER
NKMAGKRANEKLELLLQKRK
ELQENQDEIENMMNSIFKGIFVHRY
>g2188.t4
MITSELPVLQDSSNESGVPDAVSLSMSMSEIEDPEVKGKKKRGRPGKQQQSATKKPRKSPTDKAVGVTRGRGKANGVAQH
NGEGGDPVTLFEVVKLGKSA
MQSVVDDWIESYKQDRDIALLDLINFFIQCSGCKGTVRIEMFRNMQNAEIIRKMTEEFDEVAPAFPVLKQLLFCSAGVST

RHSGWQTWP
>g2188.t5
MPGPLWKKFRYNFCEFICVLIRQCQYSIIYDEYMMDTVISLLTGLSDSQVRAFRHTSTLAAMKLMTALVNVALNLSIHQD
NTQRQYEAERNKMAGKRANE
KLELLLQKRKELQENQDEIENMMNSIFKGIFVHRYR
>g2189.t1
AHVAAFPGKSPPAPSSRCGQTRDADKMCASRPELLWHPPLTQRGAPGSGRPWNDAAAERGVKQEHPGQAYLEDGE
>g2190.t1
MSAELDLSPPEVPEPTLLESLLRYGLFLGAIFQLICILAVIIPSSKGHEQEDTESTDGKAAEQLKKSKGIPPPIRQKLKK
ESKKKR
>g2191.t1
MSSPVERTDMYDHALLGILQHVGNIQDFLQIYFGFLYRKTDFYRLLSCPNDRMGFPPGIAEKMVLKAFKSFERISEQDRD
RRLKELQQREEPKTPPPVVE
QEVSVESEPEQVKAEPNTDAAQLKKAPSEQAEPSSASSSQPDASPQPSASGATQSSPEAKAEAPSTGAPASATSQNQYQA
DPDSYNGATRENYSWSQDYT
DVEIRVFVSKTVVKGRQVSVNLQPSSVRVCVRDGAEEQVLMEGELAHKINTENSLWSLEPGRSVVVCALHLSLSKCGEVW
WNAVLKGEKEIDINQINRER
SMATVDEEEHAVLDRLTFDYHQKLQGKPQSHEM
>g2191.t2
MYDHALLGILQHVGNIQDFLQIYFGFLYRKTDFYRLLSCPNDRMGFPPGIAEKMVLKAFKSFERISEQDRDRRLKELQQR
EEPKTPPPVVEQEVSVESEP
EQVKAEPNTDAAQLKKAPSEQAEPSSASSSQPDASPQPSASGATQSSPEAKAEAPSTGAPASATSQNQYQADPDSYNGAT
RENYSWSQDYTDVEIRVFVS
KTVVKGRQVSVNLQPSSVRVCVRDGAEEQVLMEGELAHKINTENSLWSLEPGRSVVVCALHLSLSKCGEVWWNAVLKGEK
EIDINQINRERSMATVDEEE
HAVLDRLTFDYHQKLQGKPQSHEM
>g2191.t3
MSSPVERTDMYDHALLGILQHVGNIQDFLQIYFGFLYRKTDFYRLLSCPNDRMGFPPGIAEKMVLKAFKSFERISEQDRD
RRLKELQQREEPKTPPPVVE
QEVSVESEPEQVKAEPNTDAAQLKKAPSEQAEPSSASSSQPDASPQPSASGATQSSPEAKAEAPSTGAPASATSQNQYQA
DPDSYNGATRENYSWSQDYT
DVEIRVFVSKTVVKGRQVSVNLQPSSVRVCVRDGAEEQVLMEGELAHKINTENSLWSLEPGRSVVLSLSKCGEVWWNAVL
KGEKEIDINQINRERSMATV
DEEEHAVLDRLTFDYHQKLQGKPQSHEM
>g2192.t1
MAASFSSTPCAMMLRSIVKPGAVWNSLGSRGLHVTAVQCKNRAARIRVGKGDRPVTYEQALHPHDIGHRKGWLSQHPANL
KGEGGAAERALEDVFIRRFI
FGTFHGCLANEIVIKRQGNVITACALMLQKLPPQKFYFLTGYTETLLSHFYKCPVKLEIQTLLNRAVYKYL
>g2193.t1
MRLIHRAAILVGWQTLLSVDDMVEKLVKKLDDMKELNNTYILYTSDNGYHTGQFSLPIDKRQLYEFDIRVPLMVRGPGIA
PKQIVKAPVLNIDLAPTILD
ISGSDLPIMDGQSFLSQMSFVEVYNLTSDASQLENIVKKVDPELLQVMNQRLIKLQSCAGSSCHDNGVCQTAIN
>g2193.t2
MRLIHRAAILVGWQTLLSVDDMVEKLVKKLDDMKELNNTYILYTSDNGYHTGQFSLPIDKRQLYEFDIRVPLMVRGPGIA
PKQIVKAPVLNIDLAPTILD
ISGSDLPIMDGQSFLSQMAPSLRNGTSRACFLVEHTGEGFPESDPACPKLGPGLSSFVEVYNLTSDASQLENIVKKVDPE
LLQVMNQRLIKLQSCAGSSC
HDNGVCQTAIN
>g2193.t3
WQTLLSVDDMVEKLVKKLDDMKELNNTYILYTSDNGYHTGQFSLPIDKRQLYEFDIRVPLMVRGPGIAPKQIVKAPVLNI
DLAPTILDISGSDLPIMDGQ
SFLSQMSFVEVYNLTSDASQLENIVKKVDPELLQVMNQRLIKLQSCAGSSCHDNGVCQTAIN
>g2194.t1
LATSSFPVCSPPRRPARFFSPFISMRSVQSAEQRDRCSSEAGVEPHANESSSRQRWHPLGIEIIDFGLRCLFKEDGGRHA
VGSILREGDSRRCEQLEQLE
QRFTEAGFHVCSFPEVTHAVTASGPRGSPLSVLLGARRVYTRLQDYIKPQSCGAVRQHADTDAELHAVSLSRYAVSLSRY

AAVRQDGSHPF
>g2194.t2
LATSSFPVCSPPRRPARFFSPFISMRSVQSAEQRDRGWVCDGRHRYVTVSTAAGPRTDWESDSLVRTCLCSSEAGVEPHA
NESSSRQRWHPLGIEIIDFG
LRCLFKEDGGRHAVGSILREGDSRRCEQLEQLEQRFTEAGFHVCSFPEVTHAVTASGPRGSPLSVLLGARRVYTRLQDYI
KPQSCGAVRQHADTDAELHA
VSLSRYAVSLSRYAAVRQDGSHPF
>g2194.t3
LATSSFPVCSPPRRPARFFSPFISMRSVQSAEQRDRCSSEAGVEPHANESSSRQRWHPLGIEIIDFGLRCLFKEDGGRHA
VGSILREGDSRRCEQLEQLE
QRFTEAGFHVCSFPEVTHAVTASGPRGSPLSVLLGARRVYTRLQDYIKPQSCGAVRQHADTDAELHAVSLSRYAVSLSRY
AAVRQDGSHPFRRSIEGKPN
RDVTQLQQLDRASRHTLPFTPSAR
>g2195.t1
IITVNAMLFPTSVANSLIAVGTDGLQERDRMVRAAIAIVCELVAITGTPPPLRCSLIVFSVRAALKNPEVVAKRGGLSTI
LKSVIDCRLSRINEALITTI
LHLLNHPQTRQYVRADVELEVRAAALFPLAC
>g2195.t2
IITVNAMLFPTSVANSLIAVGTDGLQERDRMVRAAIAIVCELALKNPEVVAKRGGLSTILKSVIDCRLSRINEALITTIL
HLLNHPQTRQYVRADVELEV
RAAALFPLAC
>g2196.t1
LRGSQRGSQRGSQRGSLRGSQRGSLRGSQRGSQRGSQRGSLRGSQRGSLRGSLRGSPPRGQPSRRPSLK
>g2197.t1
RCERLLLYVFCHELSVGFREPVATSVPNYYKIIKQPMDLTKVKKKLQVRSSQQYRSTQDFVSDVRLVFHNCSKYNEVGGM
SPTAHGHPMHVAPAPL
>g2197.t2
RCERLLLYVFCHELSVGFREPVATSVPNYYKIIKQPMDLTKVKKKLQVRSSQQYRSTQDFVSDVRLVFHNCS
>g2198.t1
MPHTPLIINDCVVECVKSTKFLGVHFTEDLSWSTNTTSLDKKAQQCLHFLRRMRRADLPSSALATFYRGAIESILTSSLS
VWYGSCPVADQKALQRVVRT
AERITRSAQPSIQDLYLSRCLSRATNITKDPQPPNTQTVHPPTIWQALPQHAVWDH
>g2199.t1
MPKAHLRRFKETLWKYYPRAFIASPQNMDLVDLVDRLLENFNLQGSLQRVEALLEEMGQGQLVQYLKELCIQNEVRFELR
KILKSKYGDINENLGTRGEK
MPFDNIFTDLHMATAVENGPNVQHEYRNIGQLGSHHKPEPPTTHKHIFSIDLIEEKCVRALLMIGMAGTGKSMAVQKFIL
DWAEERSHQHISFLFPLPFR
ELKSYEGSKISMLELLNQFYPDTKRLKDICTVDGHVLFLLDGLDEYNHKLDFQTTEVWCDFKEATSFHVLLVNLLRGNLM
YSSLLWVTSRPLRAHGLPSE
AVHQVIEVQGFTDAQKGEYFKKRFTDPLQAARVMAYVNSCKTLHIMCHLPLFCSALSRTLERLFTGQTGAAFGEMPQSIT
PIYTQLLLDLLSQRRFRAPA
RSPTQQREFLLSLGKMAWLMLEKGQVKISRDHWRADGGVDIDEAVVKSGLCTEFTVKQFVMYTDTVYSFIHSTVQEYMAA
LYVFLECKGVFKDPSLKNRL
SLGSKRNRELLKVYSDNLDRTLLCEDGRFNIFLRFLFGMALKSNVELLKPFISSTENWPSVIQDTAALIRKKMSENPRNH
ANRSAILQHCLNELTAEISP
AASN
>g2199.t2
MDLVDLVDRLLENFNLQGSLQRVEALLEEMGQGQLVQYLKELCIQNEVRFELRKILKSKYGDINENLGTRGEKMPFDNIF
TDLHMATAVENGPNVQHEYR
NIGQLGSHHKPEPPTTHKHIFSIDLIEEKCVRALLMIGMAGTGKSMAVQKFILDWAEERSHQHISFLFPLPFRELKSYEG
SKISMLELLNQFYPDTKRLK
DICTVDGHVLFLLDGLDEYNHKLDFQTTEVWCDFKEATSFHVLLVNLLRGNLMYSSLLWVTSRPLRAHGLPSEAVHQVIE
VQGFTDAQKGEYFKKRFTDP
LQAARVMAYVNSCKTLHIMCHLPLFCSALSRTLERLFTGQTGAAFGEMPQSITPIYTQLLLDLLSQRRFRAPARSPTQQR
EFLLSLGKMAWLMLEKGQVK
ISRDHWRADGGVDIDEAVVKSGLCTEFTVKQFVMYTDTVYSFIHSTVQEYMAALYVFLECKGVFKDPSLKNRLSLGSKRN

RELLKVYSDNLDRTLLCEDG
RFNIFLRFLFGMALKSNVELLKPFISSTENWPSVIQDTAALIRKKMSENPRNHANRSAILQHCLNELTAEISPAASN
>g2200.t1
MACPAVVEEVPEKHCWVCFATEREDGSAEWVNPCRCKGCTKWIHQSCLQRWLDEKQKGNNGGPVSCPQCGTEYRIEFPTM
GPLVYSLQHVDTAISRVCPF
AAMGVVVGTVYWSTVTYGAVTVMQVVGHKKGLFLMERADPVFLLMGLPTIPVMLVLGKMIRWEDYILTLWHRYSGQQTRG
RQLHRVGLHRTLSRDHLSVS
RTLCGALIFPSIASLVGRLFFRRLPSSLQRTILGGLAFVLMKGALKVYFKQQQFLIHTNRRILNYPESDGRPGRDGDHER
GEEDMEDSGNE
>g2201.t1
QIIVDPLAFSEERFRPSLEERLESIISGAALMADSSCTRDDRRERIVAECNSVRQALQDLLSEYMGNHTA
>g2202.t1
MDRYDITVQQLHMVSLSCLLLASKFEEREDRVPKLETLNSLGCMSSMNLVLTKQGLLHMELLLLETFQWNLYLPTAAHFI
EYYLSIAVHDGDLRDGWPIG
CLDKTKLYLAKYADYFLEVSLQGKQLSTTHIRAASMHIRALFKHAGTLQNHVFLCFAPSMVAAACLASSRLILHLSPTWP
PRLQHLTAYTWDSLVSCTEK
LMVAHDGDVKEASKQKVPPPNHQQQQQQQQQQQQHQLGPTPNYHTSGQAAPVAQYLHRPMMSYPQQAHPQAVAPGHRSTA
YLNQPTGLPAPIVPQHSHVP
TATTNLEPKASMSNRAYQ
>g2202.t2
MDRYDITVQQLHMVSLSCLLLASKFEEREDRVPKLETLNSLGCMSSMNLVLTKQGLLHMELLLLETFQWNLYLPTAAHFI
EYYLSIAVHDGDLRDGWPIG
CLDKTKLYLAKYADYFLEVSLQGKQLSTTHIRAASMHIRALFKHAGTLQNHVFLCFAPSMVAAACLASSRLILHLSPTWP
PRLQHLTAYTWDSLVSCTEK
LMVAHDGDVKEASKQKVPPPNHQQQQQQQQQQQQHQLGPTPNYHTSGQAAPVAQYLHRPMMSYPQQAHPQAVAPGHRSTA
YLNQPTGLPAPIVPQHSHVP
TATTNLEPKASMSNRAYQVSLNYPCAAPCFDR
>g2202.t3
LPSYKGQSPQLNLRRYFADLIAVVSNRFRLCPAARHLAVYLLDLFMDRYDITVQQLHMVSLSCLLLASKFEEREDRVPKL
ETLNSLGCMSSMNLVLTKQG
LLHMELLLLETFQWNLYLPTAAHFIEYYLSIAVHDGDLRDGWPIGCLDKTKLYLAKYADYFLEVSLQGKQLSTTHIRAAS
MHIRALFKHAGTLQNHVFLC
FAPSMVAAACLASSRLILHLSPTWPPRLQHLTAYTWDSLVSCTEKLMVAHDGDVKEASKQKVPPPNHQQQQQQQQQQQQH
QLGPTPNYHTSGQAAPVAQY
LHRPMMSYPQQAHPQAVAPGHRSTAYLNQPTGLPAPIVPQHSHVPTATTNLEPKASMSNRAYQ
>g2203.t1
MSKVNALLRKANLVVGLGLDSLAVVVERWMRAKKINANSHPTSTHPLHDELSRKRSTKSHMAHPSAISKRRECEQGVGVV
VCKHGGSVEFFPALGGDFEG
AIRDTVVVGPVFPECQYCSYKGPSKRLITKHMNTVHGGQTVNQNMKELHHIKNNPRDSAPKMRNRVTRNTARDMHWLSQD
CLSLPGREFLDKYCSLSNPE
RTLEDTQQFLVRSAVEETDDQKWTKALQSVLSNFPQDINMHPKLENAMMSNSSKDLTVLMVKNKLKVPQNAGSYVKKTTP
PEKLEISPSGSATLDTHCVF
DPNRGQPQLSNDQTHCPGTQNNINEDPSITAKPESAEGGPIQENRENQELRLHQNVEGHSRNNKEIKCDHSCDDGLSASK
EAKAANKRDHRMSSQRKPRG
RRGGRAEKGRPMALEKQLQGLGLKLVLKKDPVKNKQWMSQSPLLPPDGCAAGNHLGPLFSPPAAEMQGQSLVDAPSAESF
IVSLKECAEAPETGLHGTLD
PVAPQSRPQPAEELFSETQGQCCGNGDVPPDGNGKPEGRFPSGEGRAEDAKISAATCGVSERDGRGVDCLTPGRKESSPV
EDVNLLRNNGAIASHAAKPF
IPPP
>g2204.t1
MHSQVRTDLLWPFLVLLLGVPGARPAKCPKQCVCDQIQLTVTCVNKNLTEVPAVDEITVKLDLRGNDIQQLPRGAFKHTP
YLTHLSLQRCNIRAVKEGAF
RSLGRLVFLNLAHNNIEVLYQVPMHTHSI
>g2205.t1
SALNQSMRFLERTASDLPNKVLAALNAIDEAQYLISQNATHLITQETEKYMEAVVGYLHQYVEWVKSSLALEVATCKPFS
NMVDTAHILACSFLLDSM

>g2205.t2
MTLLLYLQSALNQSMRFLERTASDLPNKVLAALNAIDEAQYLISQNATHLITQETEKYMEAVVGYLHQYVEWVKSSLALE
VATCKPFSNMVDTAHILACS
FLLDSM
>g2206.t1
EKFMVRLNKNGGPKNPEKVDRLCALFTDLSSKDLKRDLYIVAQVIRTGRMLLNDSKKGPAHVLYRRPYGCTALAMTDVFH
TITDLKEEKDFVLKIYTVRL
WCQHFLGSSHKELRGAQGSEANMNQVSALFADRDRFAVVVTVVTVVVTVVTVVVTVVWAVVTVVVTVVWAVVTVVVTVVW
AVVTVVVT
>g2206.t2
EKFMVRLNKNGGPKNPEKVDRLCALFTVMDLSSKDLKRDLYIVAQVIRTGRMLLNDSKKGPAHVLYRRPYGCTALAMTDV
FHTITDLKEEKDFVLKIYTV
RLWCQHFLGSSHKELRGAQGSEANMNQVSALFADRDRFAVVVTVVTVVVTVVTVVVTVVWAVVTVVVTVVWAVVTVVVTV
VWAVVTVVVT
>g2207.t1
LRLSCLRRVNDIGILCAELTAYCRSLSAEWLGILKALCCSSNNGNCGFNDLLCNVDVSDLSFHDSLATFVAILIARQCLL
LEDLVRCVAIPSLLNAACSE
QDSEPGARLTCRILLHLFKTPQRNPVPQDGAKSEKSPVGIRSSCDRHLLAASQNSIVVGAVFAVLKAVFMLGDAELRGSG
LSHPASHETASLDVYAKYVL
KSICQQ
>g2207.t2
VNDIGILCAELTAYCRSLSAEWLGILKALCCSSNNGNCGFNDLLCNVDVSDLSFHDSLATFVAILIARQCLLLEDLVRCV
AIPSLLNAACSEQDSEPGAR
LTCRILLHLFKTPQRNPVPQDGAKSEKSPVGIRSSCDRHLLAASQNSIVVGAVFAVLKAVFMLGDAELRGSGLSHPASHE
TASLDVYAKYVLKSICQQ
>g2207.t3
LRLSCLRRVNDIGILCAELTAYCRSLSAEWLGILKALCCSSNNGNCGFNDLLCNVDVSVSDLSFHDSLATFVAILIARQC
LLLEDLVRCVAIPSLLNAAC
SEQDSEPGARLTCRILLHLFKTPQRNPVPQDGAKSEKSPVGIRSSCDRHLLAASQNSIVVGAVFAVLKAVFMLGDAELRG
SGLSHPASHETASLDVYAKY
VLKSICQQ
>g2208.t1
MESVMDHSSWFDLPPSPPPFSPYQGTMEMAPPRGPTFSPLNREQIRKGDPLASQRARLRLSQHPQTPAMLDLQSDFSCSF
APPIIDPPPLTPPAFSPLAS
RSGTNRRTQSIPSAHCRGDGADSNHWLAEGTALDLGPVGPAFTTRHQRSLQPDVHRTSVGSSRFLGDRSKVMKGHPTAVE
REQSGAHSGSQVYSITLIFG
AFPFHLSSDVPHRIGTTSAGGDRGVVEVDTQRRRTRRSRPRSPTLGLETPGDTEIVDPLFIPQVPEGPETLGGIGGCFSR
REDEEEEAEEDAGGETWSPV
FMEYLDELCRDAEFVSTVDFHDGRSVGAFSFSKQR
>g2208.t2
MDHLCWPKVGSILDAEYLEAPEPTGLQHGEGEDPSDARAVDLEAFRSEVSLPGPVGESEPEEDPRPGTGTGPSEFCREHL
DNLEWDDGVRQTTASAVNRY
PFPSPPSLSGLHSLTPLVSPGSPGPTAPGGGARDPSISVPPEQTGGDHPENEVQLQAFLEIGLRVDVTVLPPLDDSNDDE
EEEEEEEEEDASADQVAVRP
EDEALPAVRSEDVQEVGSPSPALLESRPAPTDSTGPFEWRSGARPQPARGAQTSERPAVRPEDRDPHAPPPPPRERATPL
DRGATPTRVYSGPLTRARRR
SESLADREDAEQEEDTRAGERGRGARQEDATEGRPLPSPSGPVSEGRTSLRDDGGKTKRENEWTNFSEEKMLEEDTRASS
PTKRRKPSGGRGGL
>g2208.t3
MESVMDHSSWFDLPPSPPPFSPYQGTMEMAPPRGPTFSPLNREQIRKGDPLASQRARLRLSQHPQTPAMLDLQSDFSCSF
APPIIDPPPLTPPAFSPLAS
RSGTNRRTQSIPSAHCRGDGADSNHWLAEGTALDLGPVGPAFTTRHQRSLQPDVHRTSVGSSRFLGDRSKVMKGHPTAVE
REQSGAHSGSQVYSITLIFG
AFPFHLSSDVPHRIGTTSAGGDRGVVEVDTQRRRTRRSRPRSPTLGLETPGDTEIVDPLFIPQVPEGPETLGGIGGCFSR
REDEEEEAEEDAGGETWSPV
FMEYLDELCRDAEFVSTVDFHDGRSVGAFSFSKQRADMDHLCWPKVGSILDAEYLEAPEPTGLQHGEGEDPSDARAVDLE

AFRSEVSLPGPVGESEPEED
PRPGTGTGPSEFCREHLDNLEWDDGVRQTTASAVNRYPFPSPPSLSGLHSLTPLVSPGSPGPTAPGGGARDPSISVPPEQ
TGGDHPENEVQLQAFLEIGL
RVDVTVLPPLDDSNDDEEEEEEEEEEEDASADQVAVRPEDEALPAVRSEDVQEVGSPSPALLESRPAPTDSTGPFEWRSGA
RPQPARGAQTSERPAVRPED
RDPHAPPPPPRERATPLDRGATPTRVYSGPLTRARRRSESLADREDAEQEEDTRAGERGRGARQEDATEGRPLPSPSGPV
SEGRTSLRDDGGKTKRENEW
TNFSEEKMLEEDTRASSPTKRRKPSGGRGGL
>g2208.t4
MESVMDHSSWFDLPPSPPPFSPYQGTMEMAPPRGPTFSPLNREQIRKGDPLASQRARLRLSQHPQTPAMLDLQSDFSCSF
APPIIDPPPLTPPAFSPLAS
RSGTNRRTQSIPSAHCRGDGADSNHWLAEGTALDLGPVGPAFTTRHQRSLQPDVHRTSVGSSRFLGDRSKVMKGHPTAVE
REQSGAHSGSQVYSITLIFG
AFPFHLSSDVPHRIGTTSAGGDRGVVEVDTQRRRTRRSRPRSPTLGLETPGDTEIVDPLFIPQVPEGPETLGGIGGCFSR
REDEEEEAEEDAGGETWSPV
FMEYLDELCRDAEFVSTVDFHDGRSVGSILDAEYLEAPEPTGLQHGEGEDPSDARAVDLEAFRSEVSLPGPVGESEPEED
PRPGTGTGPSEFCREHLDNL
EWDDGVRQTTASAVNRYPFPSPPSLSGLHSLTPLVSPGSPGPTAPGGGARDPSISVPPEQTGGDHPENEVQLQAFLEIGL
RVDVTVLPPLDDSNDDEEEE
EEEEEEDASADQVAVRPEDEALPAVRSEDVQEVGSPSPALLESRPAPTDSTGPFEWRSGARPQPARGAQTSERPAVRPED
RDPHAPPPPPRERATPLDRG
ATPTRVYSGPLTRARRRSESLADREDAEQEEDTRAGERGRGARQEDATEGRPLPSPSGPVSEGRTSLRDDGGKTKRENEW
TNFSEEKMLEEDTRASSPTK
RRKPSGGRGGL
>g2209.t1
MSHVSWLGFIYHRAEPDTARSVGRTPERSSRQLPTDDHPKPASLLVTSCVIQVTVPRTLGSAVNCTGAEAGEVFLKDMDT
STLLHFY
>g2210.t1
MLRMRTTEDTFKMLHKTVQKGAVRRCIRQKPKKSSLTKFGKGLRNFSDNEGKNRSAAMAHTDVVLTHRRSGAMAPENTTQ
YLMANIYADMSQQLCEPLAQ
RVAYDAPVDVYGESLSPAGVSTALDSIYDDYLAFQQRDFEEVFGL
>g2211.t1
MHHWTGSCRQVTISISVEVHALTLYTLSSHAGDDIQEEGPGKTTIRAENEVEANKLVNNYR
>g2212.t1
LAGTAAPAAPTEPGATKGTVLAGASAPTEPGATKGTVLAGASAPTEPGATKGTVLAGAAAPTEPGATKGTVLAGASAPTE
PGATKGTVLAGASAPTEPGA
TKESSATKRPSTVGTTDLTKSSATKTSTSVTTTESAGQRVPCGVRDVMADACNAIGCCFDDNGCYYGKAVTVQCTKDAHF
IVVVARDATLPNIDLESIAL
LGSVEAAYASYYVESDYPVTKVLREPVFMELRLLDKTDPNLVLTLGRCWTTTTPNPHNLPQWDICVDG
>g2212.t2
LAGTAAPAAPTEPGATKGTVLAGASAPTEPGATKGTVLAGASAPTEPGATKGTVLAGAAAPTEPGATKGTVLAGASAPTE
PGATKGTVLAGASAPTEPGA
TKAAPPESSATKRPSTVGTTDLTKSSATKTSTSVTTTESAGQRVPCGVRDVMADACNAIGCCFDDNGCYYGKAVTVQCTK
DAHFIVVVARDATLPNIDLE
SIALLGSVEAAYASYYVESDYPVTKVLREPVFMELRLLDKTDPNLVLTLGRCWTTTTPNPHNLPQWDICVDG
>g2212.t3
LAGTAAPAAPTEPGATKGTVLAGASAPTEPGATKGTVLAGASAPTEPGATKGTVLAGAAAPTEPGATKGTVLAGASAPTE
PGATKGTVLAGASAPTEPGA
TKESSATKRPSTVGTTDLTKSSATKTSTSVTTTESAVTVQCTKDAHFIVVVARDATLPNIDLESIALLGSVEAAYASYYV
ESDYPVTKVLREPVFMELRL
LDKTDPNLVLTLGRCWTTTTPNPHNLPQWDICVDG
>g2213.t1
MSHKEQVAGDVPPCLTDETTAAGDTQKDHPQTQPGILGRVTGGLFRTTTGVVQATMGGVLWIGGKSLDITKTTVTSLASV
PVMGAGLVKGGVCAVAGGVS
AVGSAVASKVPIGGGKKKDKSD
>g2214.t1

MFYQSVVASVIFFATVSWGAGIRTKDNRLNKLIKKAESVVGSKFVNVEEAKLLAIMDNPSHPLHTTLDKLKSSFSHRLIH
PRCKKERCRK
>g2215.t1
MEDPDMRSQKLDIQVDHGHDNPLEEITLGDHGLTTTMTSDERPAEKPPGPPRTLNLEPGGEAGPKQGEEGEEDEEEEDGG
QVQEKKTKKKAKGMQPSETE
HRDQDRRKQKKPKRKEQEGKGPTSDPQHDRKSPKTGQSKQSSEPRDTETDSGMEFGRAEAADRPRGGEGKRESAAARRKQ
QLSTCE
>g2215.t2
MEDPDMRSQKLDIQVDHGHDNPLEEITLGDHGLTTTMTSDERPAEKPPGPPRTLNLEPGGEAGPKQGEEGEEDEEEEDGG
QVQEKKTKKKAKGMQPSETE
HRDQDRRKQKKPKRKEQEGKGPTSDPQHDRKSPKTGQSKQSSEPRDTETDSGMEFGRAEAADRPRGGEGKRESAAARRKQ
QLST
>g2215.t3
MNYVSVLSPTTSKRVVASVYLSWYPSNRLAFSASGVSEHLAVVSSDRINSLLNHADMEDPDMRSQKLDIQVDHGHDNPLE
EITLGDHGLTTTMTSDERPA
EKPPGPPRTLNLEPGGEAGPKQGEEGEEDEEEEDGGQVQEKKTKKKAKGMQPSETEHRDQDRRKQKKPKRKEQEGKGPTS
DPQHDRKSPKTGQSKQSSEP
RDTETDSGMEFGRAEAADRPRGGEGKRESAAARRKQQLSTCE
>g2216.t1
MPHPLKARVLLXILCSVFNVDIPDPMREVLLGLYIQTVQFCRSSGFNAEQTSVLLSIMKAVHQLNIETSLNNVELCFDSC
SELLLCHSVRVGDNIVVATA
SPAVALLRCVNCFLLPLTQRPPFCVDLFKSHEVTLILQHVNDTYIRNYALYKYIFTPQVRLDLSLSYSGIPEDVLPVESP
TSPDTEKDIGETDPLAEPLG
SQELTENQEIPQNTDPVEAAPGETENQTTFTMG
>g2216.t2
MPHPLKARVLLXILCSVFNVDIPDPMREVLLGLYIQTVQFCRSSGFNAEQTSVLLSIMKAVHQLNIETSLNNVELCFDSC
SELLLCHSVRRPPFCVDLFK
SHEVTLILQHVNDTYIRNYALYKYIFTPQVRLDLSLSYSGIPEDVLPVESPTSPDTEKDIGETDPLAEPLGSQELTENQE
IPQNTDPVEAAPGETENQTT
FTMG
>g2216.t3
MPHPLKARVLLXILCSVFNVDIPDPMREVLLGLYIQTVQFCRSSGFNAEQTSVLLSIMKAVHQLNIETSLNNVELCFDSC
SELLLCHSVRVGDNIVVATA
SPAVALLRCVNCFLLPLTQRPPFCVDLFKSHEVTLILQHVNDTYIRNYALYKYIFTPQVRLDLSLSYSGIPEDVLPVESP
TSPGGQFYSCFLVVINSDTE
KDIGETDPLAEPLGSQELTENQEIPQNTDPVEAAPGETENQTTFTMG
>g2217.t1
MWRGGEKSVTVSGAKSKFEFCAGGSGSGSGGGQDGMPAAGEFGRVNDALSNTNTPLDIGTLTLKTGLVSGPARHFLAARS
DPPSAPSLGARAAGSRPLKP
TVFSRLAALLHFFPPESSFAPNKKQTKGKRGERVRIQSTPVKRRQKGE
>g2218.t1
NGSAANQKAKRPKCPGHRQKAVAVVYDKLCDVISNTAELLRIQLLTDTTVLQVTSMGMTPFFVENISELQLRSIKLVTAV
FARYKKHRQLILEEVFTSLA
RLPTSKRSLRQFRINSSSGDKEPQYIQMVTALVLQLIQCVVQLPSVQHGDDVHNKKEDPDVLITNSYESTMKTAQNFLLV
FLKK
>g2218.t2
NGSAANQKAKRPKCPGHRQKAVAVVYDKLCDVISNTAELLRIQLLTDTTVLQVTSMGMTPFFVENISELQLRSIKLVTAV
FARYKKHRQLILEEVFTSLA
RLPTSKRSLRQFRINSSSGDKEPQYIQMVTALVLQLIQCVVQLPSVQHGDDVHNKKEDPDVLITNSYESTMKTAQNFLLV
FLK
>g2218.t3
MAEYNGSAANQKAKRPKCPGHRQKAVAVVYDKLCDVISNTAELLRIQLLTDTTVLQVTSMGMTPFFVENISELQLRSIKL
VTAVFARYKKHRQLILEEVF
TSLARLPTSKRSLRQFRINSSSGDKEPQYIQMVTALVLQLIQCVVQLPSVQHGDDVHNKKEDPDVLITNSYESTMKTAQN
FLLVFLK
>g2219.t1

MWASSCSQNRVQDPRGRHRLSTVIQPLRQSAGEVVDLTVDEDDLTVVPTT
>g2219.t2
MWASSCSQNRVQDPRGRHRLSTVIQPLRQSAGEVVDLTVDED
>g2220.t1
MSPLVWTIVTTGCVDSNPVSPSATSNRVLFPLLPDARERSAKRFSADGVSNYTSLLLSQEDDMLYVGAREALFALSLSDI
SKTKLQKNMTWGTPVGKREE
CSFKGKNLETDCFNYIKILLRLNSTHLYVCGTYAFSPICAYINTSTFSLERDDSGEVLMEDGRSRCPFNPEYKSTAIIVG
SAWGHVTCMTSCAHVHEACA
DPMCRRPRTAHNVCKSDHIRSCPWMRNGESMSEPLESLLLLQNKMANGELYAGTVSNFQGNEPVIYKSLGPGAALKTENS
LNWLQDPVFVGSAYIEESQP
AGNPVGDDDKIYFFFSEAGKEFDFFDNTIVSRIARVCKVRPASACGRGAS
>g2220.t2
MSPLVWTIVTTGCVDSNPVSPSATSNRVLFPLLPDARERSAKRFSADGVSNYTSLLLSQEDDMLYVGAREALFALSLSDI
SKTKLQKNMTWGTPVGKREE
CSFKGKNLETDCFNYIKILLRLNSTHLYVCGTYAFSPICAYINTSTFSLERDDSGEVLMEDGRSRCPFNPEYKSTAIIVD
GELYAGTVSNFQGNEPVIYK
SLGPGAALKTENSLNWLQDPVFVGSAYIEESQPAGNPVGDDDKIYFFFSEAGKEFDFFDNTIVSRIARVCKVRPASACGR
GAS
>g2220.t3
MLDKICLLQLALVSQSEEIAVCQNARERSAKRFSADGVSNYTSLLLSQEDDMLYVGAREALFALSLSDISKTKLQKNMTW
GTPVGKREECSFKGKNLETD
CFNYIKILLRLNSTHLYVCGTYAFSPICAYINTSTFSLERDDSGEVLMEDGRSRCPFNPEYKSTAIIVGSAWGHVTCMTS
CAHVHEACADPMCRRPRTAH
NVCKSDHIRSCPWMRNGESMSEPLESLLLLQNKMANGELYAGTVSNFQGNEPVIYKSLGPGAALKTENSLNWLQDPVFVG
SAYIEESQPAGNPVGDDDKI
YFFFSEAGKEFDFFDNTIVSRIARVCKVRPASACGRGAS
>g2221.t1
LSHKVWSCPIESGPVQKAVVLSHKVWSCPIESGPVQKAVVLSHKVSTLPRHGRTVHPTVPEGAEQQAQSLFVQEVRYRPA
QSLFVQEVRYRPAQSLFLRE
VRCGPAQKAALRTNLFGCTDIRTVVHLTPCSEVWAESMWASVDFIFPDRRRLRTHVRTMIDVIILSFIVQYQATDPSLAP
VQSTPVYSCIKTYNSDWHVV
GYKYEDYSGDFRQIPNVMVITAHRPPVGRRHASNTGGARYQRERAPDLP
>g2221.t2
MAYWTCRISISDIRDVIQLCSCPKGCGPVQKAVVLSHKVWSCPIESGPVQKAVVLSHKVWSCPIESGPVQKAVVLSHKVS
TLPRHGRTVHPTVPEGAEQQ
AQSLFVQEVRYRPAQSLFVQEVRYRPAQSLFLREVRCGPAQKAALRTNLFGCTDIRTVVHLTPCSEVWAESMWASVDFIF
PDRRRLRTHVRTMIDVIILS
FIVQYQATDPSLAPVQSTPVYSCIKTYNSDWHVVGYKYEDYSGDFRQIPNVMVITAHRPPVGRRHASNTGGARYQRERAP
DLP
>g2221.t3
LSSCSEFSARLEPSRVQASRTKAVVLSHKVWSCPIESGPVQKAVVLSHKVWSCPIESGPVQKAVVLSHKVSTLPRHGRTV
HPTVPEGAEQQAQSLFVQEV
RYRPAQSLFVQEVRYRPAQSLFLREVRCGPAQKAALRTNLFGCTDIRTVVHLTPCSEVWAESMWASVDFIFPDRRRLRTH
VRTMIDVIILSFIVQYQATD
PSLAPVQSTPVYSCIKTYNSDWHVVGYKYEDYSGDFRQIPNVMVITAHRPPVGRRHASNTGGARYQRERAPDLP
>g2222.t1
MQTKPSRKAFTKSCEKLQHYNNGLTETLEAKSSTKPPLDRLLQSELCTTRACLGFSGLVFDLVGTVEVGALSLKGHVLCV
VLFQNQA
>g2223.t1
LHVHMDCVSSQCAAVLRVLDSCRSDMSDLQTSTGASNLHFINSVSKMEELFPTATTCHTLEGFSSTLQDRLGQHIQEIQL
SQRAFRRSIQQRLEQVRDSN
AKLVTSFRLFGDGGNFSPKEVEQLLRLLERTSRRIDALEDSIQADMESLESASLEQDKTSNIFQSLSL
>g2223.t2
MDCVSSQCAAVLRVLDSCRSDMSDLQTSTGASNLHFINSVSKMEELFPTATTCHTLEGFSSTLQDRLGQHIQEIQLSQRA
FRRSIQQRLEQVRDSNAKLV
TSFRLFGDGGNFSPKEVEQLLRLLERTSRRIDALEDSIQADMESLESASLEQDKTSNIFQSLSL

>g2223.t3
LHVHMDCVSSQCAAVLRVLDSCRSDMSDLQTSTGASNLHFINSVSKMEELFPTATTCHTLEGFSSTLQDRLGQHIQEIQL
SQRAFRRSIQQRLEQVRDSN
AKLVTSFRLFGDGGNFSPKEVEQLLRLLERTSRRIDALEDSIQADMESLESASLEQSLSL
>g2224.t1
LYSIDDNIALLNNLNSFFARFEATNNTSNEQHKPTEPPPPRPGSVPVRCQCEEGTLHWQSSGECKATGPDNIPGRVLRDG
AEELKNIVMDIFNTSLRQAA
VPSHFKTSIIIPVPKKPAPSCFNDYSPVALTPIIIKCFECVMSHIKSILPPPTLDPFQFAYQAKRSTEDAISSALHPALT
HLQSHIPLLFTLLTHDCTPT
YSSNHMVKFADDTTLVVVLLTKWCKHNHLLLNVSKTKEIVVDCRRGHTEHSPLTIDDVPVERTHDKKEVNLLTMWCRDNN
LLLNVSKTKEIVVDFRRGHT
EHPPLTIDGAVERVSSTKFQGVHVSEDLSWTTNTASLVKKAQQRLYFLRKLKQVKAPIPIPKSFYWGTIESILSSCITVW
GRSCTEHSRKALQRRVNTVH
SALQCFAAVLLSC
>g2225.t1
LVPQDQRYRVIPPPGGGGAACTCSVPSPRGRRGHKGCRASVSRCVEIKADCRMPASPTWALFPRHHVLLLLPPVQNPGPR
SGM
>g2226.t1
IVTTFIVTTIVIIVIIILALGIVIASRK
>g2227.t1
MLGLTLGFHVLQMDHTWQRVQSMYQWTLENGVLVIIGVYLCVVWTGPRLMKHRGPIDLKGLLLLYNFAMVCLSGYMFQKF
VVLSRLSNYSYLCQPVDYSN
SPLAVRMANVCWWFFFSKVIELSDTVFFILRKKNNQLSFLHIYHHTTMIFNCWAGVKYVPGGQSFLIGLLNSFVHMIMYS
YYGLAGLGPHMQKYLWWKKY
LTTLQL
>g2227.t2
MLGLTLGFHVLQMDHTWQRVQSMYQWTLENGDKRTDPWLLVYSPVPVLVIIGVYLCVVWTGPRLMKHRGPIDLKGLLLLY
NFAMVCLSGYMFQKFVVLSR
LSNYSYLCQPVDYSNSPLAVRMANVCWWFFFSKVIELSDTVFFILRKKNNQLSFLHIYHHTTMIFNCWAGVKYVPGGQSF
LIGLLNSFVHMIMYSYYGLA
GLGPHMQKYLWWKKYLTTLQL
>g2227.t3
MDHTWQRVQSMYQWTLENGVLVIIGVYLCVVWTGPRLMKHRGPIDLKGLLLLYNFAMVCLSGYMFQKFVVLSRLSNYSYL
CQPVDYSNSPLAVRMANVCW
WFFFSKVIELSDTVFFILRKKNNQLSFLHIYHHTTMIFNCWAGVKYVPGGQSFLIGLLNSFVHMIMYSYYGLAGLGPHMQ
KYLWWKKYLTTLQL
>g2228.t1
MSGDDRTLKEGEVSTGVYVRDVLSRGVPKLLSLFTLKARHPPDKRMNPWLLVYSLVSAVDLTLVWVMWTGPRLMERRDHL
KGGLLIMNFWRKRYLPTLQL
EGRPHCPLSPAEHWSALRGCDLHRHDITF
>g2229.t1
VSGVVDGNVIRVLCRLRAIGADSTSPAVTEALWSLANTAVDPERPGDFNQAMMELGARVCTPKAALCGQCPLQSHCHSYR
KVHIKQEVNSERLLGKVGRT
PSTPVDIEECEPWQDDLGVHNFPRKPAKKAPRVERTLTCVALRCGDGGEDEFLLTQRPEKGLLAGMWEFPSLLLEGENSE
VKQQGALCAEVGRILGVRVS
RERLSYVGEVVHIFSHIHQTYVVYSVRVKEEETRREALRWLARPALLEAAVSTGVKKDGKRKKAVQNGKSSTSKKTKLNS
AHGSRQLSLNSFLKPTGRT
>g2229.t2
VSGVVDGNVIRVLCRLRAIGADSTSPAVTEALWSLANTAVDPERPGDFNQAMMELGARVCTPKAALCGQCPLQSHCHSYR
KVHIKQEVNSERLLGKVGRT
PSTPVDIEECEPWQDDLGVHNFPRKPAKKAPRVERTLTCVALRCGDGGEDEFLLTQRPEKGLLAGMWEFPSLLLEGENSE
VKQQGALCAEVGRILGVRVS
RERLSYVGEVVHIFSHIHQTYVVYSVRVKEEETRREALRWLARPALLEAAVSTGVKKILKLYEATGVQTLKDGKRKKAVQ
NGKSSTSKKTKLNSAHGSRQ
LSLNSFLKPTGRT
>g2229.t3

VSGVVDGNVIRVLCRLRAIGADSTSPAVTEALWSLANTAVDPERPGDFNQAMMELGARVCTPKAALCGQCPLQSHCHSYR
KVHIKQEVNSERLLGKVGRT
PSTPVDIEECEPWQDDLGVHNFPRKPAKKAPRVERTLTCVALRCGDGGEDEFLLTQRPEKGLLAGMWEFPSLLLEGENSE
VKQQGALCAEVGRILGVRVV
HIFSHIHQTYVVYSVRVKEEETRREALRWLARPALLEAAVSTGVKKILKLYEATGVQTLKDGKRKKAVQNGKSSTSKKTK
LNSAHGSRQLSLNSFLKPTG
RT
>g2230.t1
MDDMDADLFGPKKKPGSVLPQAKRSPHPGSTKEPPRAETTAGAGGPEGSTVEPTKKPSPAPSAATAQGYRKFDFSDLDDP
LADLLDQLDLEETKSEPKKA
PTKKAPPSPTESPVLKTKEPAKTVKKGEELTFDDDEDDLMDALGFDNDKVIPKKKETVPQKTSSDPPQRARTRLDEILGR
GTPPRLLERPPTGEQKDPPQ
QPMSRQDRDKPTSAKEPLLEDDLGFGSYQPSLGSAPEGRQSRRQSVRFSTEDISSSTLERKSKPSSTATPTTPN
>g2230.t2
MSIVSIILLSSIGFQAEKQITAWSSTSDDFFQKLAEEAEREAGNEEDMDDMDADLFGPKKKPGSVLPQAKRSPHPGSTKE
PPRAETTAGAGGPEGSTVEP
TKKPSPAPSAATAQGYRKFDFSDLDDPLADLLDQLDLEETKSEPKKAPTKKAPPSPTESPVLKTKEPAKTVKKGEELTFD
DDEDDLMDALGFDNDKVIPK
KKETVPQKTSSDPPQRARTRLDEILGRGTPPRLLERPPTGEQKDPPQQPMSRQDRDKPTSAKEPLLEDDLGFGSYQPSLG
SAPEGRQSRRQSVRFSTEDI
SSSTLERKSKPSSTATPTTPN
>g2230.t3
MDDMDADLFGPKKKPGSVLPQAKRSPHPGSTKEPPRAETTAGAGGPEGSTVEPTKKPSPAPSAATAQGYRKFDFSDDPLA
DLLDQLDLEETKSEPKKAPT
KKAPPSPTESPVLKTKEPAKTVKKGEELTFDDDEDDLMDALGFDNDKVIPKKKETVPQKTSSDPPQRARTRLDEILGRGT
PPRLLERPPTGEQKDPPQQP
MSRQDRDKPTSAKEPLLEDDLGFGSYQPSLGSAPEGRQSRRQSVRFSTEDISSSTLERKSKPSSTATPTTPN
>g2231.t1
LSALQSNAVYRLRKTWTLVNREHMAAFDNLCETFPDENCVLANREILLEDGGQPVDDLSPEVSKRCTIPRQMSTSSGTVP
YLGTYLTVLTMLDTALPDTV
QAGLINFEKRRREFEILSQIRLLQAACSQYTLPNHPRIGAWLLGHKLLTDLESYELSRMLEPPVDPCSPGVCSQRSLTKK
LSFFRSSSDVTKKVLPDQIS
MSSSGSSGSEMEDLSTPNSACSIRLQSFSKSCSNVSESSSSPSSSSPPSSSSSSSCSSPESPSLSCSSSRGSQPELCSST
LAATPLEGGALPTPTLASQH
KRSVSMTALPLYNQQVADSCIIRVSVDLGKHNGNLYKSILKHHLEHMSFQDFSLSQLLSQDRELLIPDKANVFYAMSTAA
NFDFVLRRVSENRKTPLRST
ASLGRFAK
>g2231.t2
QECRRLKNFSSVRAILSALQSNAVYRLRKTWTLVNREHMAAFDNLCETFPDENCVLANREILLEDGGQPVDDLSPEVSKR
CTIPRQMSTSSGTVPYLGTY
LTVLTMLDTALPDTVQAGLINFEKRRREFEILSQIRLLQAACSQYTLPNHPRIGAWLLGHKLLTDLESYELSRMLEPPVD
PCSPGVCSQRSLTKKLSFFR
SSSDVTKKVLPDQISMSSSGSSGSEMEDLSTPNSACSIRLQSFSKSCSNVSESSSSPSSSSPPSSSSSSSCSSPESPSLS
CSSSRGSQPELCSSTLAATP
LEGGALPTPTLASQHKRSVSMTALPLYNQQVADSCIIRVSVDLGKHNGNLYKSILKHHLEHMSFQDFSLSQLLSQDRELL
IPDKANVFYAMSTAANFDFV
LRRVSENRKTPLRSTASLGRFAK
>g2231.t3
LSALQSNAVYRLRKTWTLVNREHMAAFDNLCETFPDENCVLANREILLEDGGQPVDDLSPEVSKRCTIPRQMSTSSGTVP
YLGTYLTVLTMLDTALPDTV
QAGLINFEKRRREFEILSQIRLLQAACSQYTLPNHPRIGAWLLGHKLLTDLESYELSRMLEPPVDPCSPGVCSQRSLTKK
LSFFRSSSDVTKKVLPDQIS
MSSSGSSGSEMEDLSTPNSACSIRLQSFSKSCSNVSESSSSPSSSSPPSSSSSSSCSSPESPSLSCSSSRGSQPELCSST
LAATPLEGGALPTPTLASQH
KRSVSMTALPLYNQQVADSCIIRVSVDLGKHNGNLYKSILVELLIPDKANVFYAMSTAANFDFVLRRVSENRKTPLRSTA
SLGRFAK

>g2232.t1
IMAGSNRSGDLRDAQKSIPIGTIAAITTTSLVYMSSVILFGACIEGVVLRDKFGEGVNGNLVIGTLAWPSPWVIVIGSFF
STCGAGLQSLTGAPRLMQAI
AKDSIVPALRIFGHGKANGEPTWSLLLTACICEIGILIASLDAVAPILSM
>g2233.t1
MAISHTAGIKSDIKKVMLDDVFWERVSSSLKILKPIAAAIVKIEVDGAILSNVQCLYAELQTVLPTSLLLKAEETAVVRS
LEKRLEFCMNPVHAAAYMLD
PKYDRGILSGEEISGAYAVITAMSDHLGLDKGKVLGSLAKYRTKQAFGKGMEYGSHASTYLQPPGGRDFVDLRPLHL
>g2234.t1
MPEPSQLAPFDAKEQRLYSELPPDVRAPHPISKAEPSHPAEETHFGRLYPRSRSFGHYPELVTI
>g2235.t1
LVRGPGCVLSITLWRTRLCPQHHSGGPGCVLSITLWRTRLCPQHHSGGPGCVLSITVEDQAVSSASQWRTRLCPQHHTVE
DQAVSSASHCGGPGCVLSIT
VEDQAVSSTV
>g2235.t2
MSERETDRDRGTTGTDLLSCRPGCVLSITLWRTRLCPQHHSGGPGCVLSITLWRTRLCPQHHSGGPGCVLSITVEDQAVS
SASQWRTRLCPQHHTVEDQA
VSSASHCGGPGCVLSITVEDQAVSSTV
>g2236.t1
MLGNKKGLLPEPNLAQLLTSITNPTALQLLLRPYQAGKRG
>g2237.t1
MRGLGCLRAAVAHRAGGLGQLGVGLAKLLRRRFGKNNVILSDIKKPPNNVYHSGPFIYSDILDHKNLREIVVNNNISWLV
HYSAVLSAVGESNVSLARDV
NINGFHNVLDMALEHGLRVFVPSTIGAFGPSSPRDPAPELCVQRPRTIYGVSKVHAELMGEYYHHRYGLDFRCLRYPGII
SADSQPGGGTTDYAVQIFHA
AVKSGHFECNLRSDTRLPMMYI
>g2237.t2
MRVYKQVWRYENAGVGLFEGCSGAQSRRRFGKNNVILSDIKKPPNNVYHSGPFIYSDILDHKNLREIVVNNNISWLVHYS
AVLSAVGESNVSLARDVNIN
GFHNVLDMALEHGLRVFVPSTIGAFGPSSPRDPAPELCVQRPRTIYGVSKVHAELMGEYYHHRYGLDFRCLRYPGIISAD
SQPGGGTTDYAVQIFHAAVK
SGHFECNLRSDTRLPMMYI
>g2238.t1
SLLLVRQEMVVSQKKLGEFCEALKQYLKNVSAQRDCFQ
>g2238.t2
SLLLVRQEMVVSQKKLGEFCEALKQYLKNVSAQRDCF
>g2239.t1
AHRSEVSLYRTLLKLRLLYSPVVTELNEALSNEERNLLSVAYKNVVGARRSSWRVISSIEQKTSADGNEKKIEMVRAYRE
KIEKELEAVCQDVLNLLDNF
LIKNCNETQHESKVFYLKMKGDYYRYLAEVATGE
>g2239.t2
VTELNEALSNEERNLLSVAYKNVVGARRSSWRVISSIEQKTSADGNEKKIEMVRAYREKIEKELEAVCQDVLNLLDNFLI
KNCNETQHESKVFYLKMKGD
YYRYLAEVATGE
>g2240.t1
FFTGSDSIQGTWCKDILQTIMTFTPHNWASHTLSCFPAPLQAFFKQNNVPQESRFNLKKNVEEEYRKWKSMSNENDIITH
FSLQGSPPLFLCLLWKMLLE
TDHINQIGF
>g2240.t2
SDSIQGTWCKDILQTIMTFTPHNWASHTLSCFPAPLQAFFKQNNVPQESRFNLKKNVEEEYRKWKSMSNENDIITHFSLQ
GSPPLFLCLLWKMLLETDHI
NQIGF
>g2240.t3
FFTGSDSIQGTWCKDILQTIMTFTPHNWASHTLSCFPAPLQAFFKQNNVPQESRFNLKKNVEEEYRKWKSMSNENDIITH
FSLQGSPPLFLCLLWKMLLE
TDHINQIGFR

>g2241.t1
AVCRGSHREAVVCVQRCKDMLARLPKETGYLSLMCYNFGVESYNLKTFEESSFWLSQSYDIGKTNTQYCPGPEVQARVLR
LLATVYLEWDCQQFQEKALN
AVSLANKVCMSASGLNLKIRILLRSRASDEAVTVGVNEQLESDAPLEVSLSTVKLLMSADRETLAFDYLKRVCQHFESSP
ELSAASVLHIELLLQRGKEL
LGKQKIEDIITGHYTGKPLTPQCLASLHLLLWDKAGKHFEAKNYIEALQWYNYSLSFYKAGELEPNLAKLQRNRASCLLL
LKQLDKAKEAIKEAERCDPD
SIFTQFSVYKIAVQEKNLEKAVEALTAMGRLAKSPVSREDRLLVCEHAASSLLSLAAQIALENEEQDTAMKALETLCENS
CDEAQVLAALRCLVRLLLST
MEVASDEER
>g2241.t2
AVCRGSHREAVVCVQRCKDMLARLPKETGYLSLMCYNFGVESYNLKTFEESSFWLSQSYDIGKTNTQYCPGPEVQARVLR
LLATVYLEWDCQQFQEKALN
AVSLANKVCMSASGLNLKIRILLRSRASDEAVTVGVNEQLESDAPLEVSLSTVKLLMSADRETLAFDYLKRVCQHFESSP
ELSAASVLHIELLLQRGKEL
LGKQKIEDIITGHYTGKPLTPQCLASLHLLLWDKAGKHFEAKNYIEALQWYNYSLSFYKAGELEPNLAKLQRNRASCLLL
LKQLDKAKEAIKEAERCDPD
SIFTQFSVYKIAVQEKNLEKAVEALTAMGRLAKSPVSREDRLLVCEHAASSLLSLAAQIALEIYTGRDGVEVGRSTQGEM
EVAVGRSTQAEMEVEVGRST
QAEMGWQWADLHRERWRWQWADLHRQRWGGSGQEHRIENEEQDTAMKALETLCENSCDEAQVLAALRCLVRLLLSTMEVA
SDEER
>g2241.t3
AVCRGSHREAVVCVQRCKDMLARLPKETGYLSLMCYNFGVESYNLKTFEESSFWLSQSYDIGKTNTQYCPGPEVQARVLR
LLATVYLEWDCQQFQEKALN
AVSLANKVCMSASGLNLKIRILLRSRASDEAVTVGVNEQLESDAPLEVSLSTVKLLMSADRETLAFDYLKRVCQHFESSP
ELSAASVLHIELLLQRGKEL
LGKQKIEDIITGHYTGKPLTPQCLASLHLLLWDKAGKHFEAKNYIEALQWYNYSLSFYKAGELEPNLAKLQRNRASCLLL
LKQLDKAKEAIKEAERCDPD
SIFTQFSVYKIAVQEKNLEKAVEALTAMGRLAKSPVSREDRLLVCEHAASSLLSLAAQIALENEEQDTAMKALETLCENS
CDEAQVLAALRCLVRLLLST
MEVASDEE
>g2242.t1
DPRFSKILDNLRLQKRGTGGVDTAAVGSTFDISNLDRLGQSE
>g2243.t1
QLTSYPRLREETERIVTTHVRDRESKTKDQVLLLIDIELSYINTNHEDFIGFAKYKTDPTRLTHSKEVRQMPVIRRGWLT
INISIMKGGSKDYWFVLTAE
SLSWYKDEEEKEKKYMLPLDNLNLRDVEKGFMSSKHVFAIFNTEQRNVYKDLRQIELGCDTQEDVDSWKASLLRAGVHPE
KSQADGDDTMTSGDSVSMDP
QLERQVETIRNLVDSYIGIVNKSIRDFMPKTIMHLMINSAKNFIHAELLAYLYEDQNSLMEESPEQAQRRDEMLKMYQAL
KEALVIIGDISTSTISAPLP
PPVNNDWLAKETR
>g2244.t1
MDEAYLRGCQRGDDALCPIFRLGDIVYQAKENFAELAIEGGVIGIQINWDCNLDRLMHHCLPKYSFRRLDEKESNRTLYP
GLNFRFARYHSENGVEMRTL
YKAFGIRFDVMVFGKTTIFIDWLIGTSCYKMEARQNYSERKVESVQDKQQCILSVSFVDESHICLVRRSLKKSLQNVKPI
SVLPRKENTGSHLRAILGMQ
QQDTIPKSTPDTDNGSKPCPQPVPTRPVWCVCGGCSSAPLRQEQLCCRRSSGPCITSSPLFHRLVRSRPLLEAVLLYRDP
LCDPAGSTAALRHCAYEQYI
AWRIGVPPADSHPVIPCCCVEKIRGEFPSQDGNYQGFRPR
>g2244.t2
MDEAYLRGCQRGDDALCPIFRLGDIVYQAKENFAELAIEGGVIGIQINWDCNLDRLMHHCLPKYSFRRLDEKESNRTLYP
GLNFRFARYHSENGVEMRTL
YKAFGIRFDVMVFGKAGKFSFIQLIVYIGSTLSYYALTTIFIDWLIGTSCYKMEARQNYSERKVESVQDKQQCILSVSFV
DESHICLVRRSLKKSLQNVK
PISVLPRKENTGSHLRAILGMQQQDTIPKSTPDTDNGSKPCPQPVPTRPVWCVCGGCSSAPLRQEQLCCRRSSGPCITSS
PLFHRLVRSRPLLEAVLLYR

DPLCDPAGSTAALRHCAYEQYIAWRIGVPPADSHPVIPCCCVEKIRGEFPSQDGNYQGFRPR
>g2244.t3
MDEAYLRGCQRGDDALCPIFRLGDIVYQAKENFAELAIEGGVIGIQINWDCNLDRLMHHCLPKYSFRRLDEKESNRTLYP
GLNFSENGVEMRTLYKAFGI
RFDVMVFGKTTIFIDWLIGTSCYKMEARQNYSERKVESVQDKQQCILSVSFVDESHICLVRRSLKKSLQNVKPISVLPRK
ENTGSHLRAILGMQQQDTIP
KSTPDTDNGSKPCPQPVPTRPVWCVCGGCSSAPLRQEQLCCRRSSGPCITSSPLFHRLVRSRPLLEAVLLYRDPLCDPAG
STAALRHCAYEQYIAWRIGV
PPADSHPVIPCCCVEKIRGEFPSQDGNYQGFRPR
>g2245.t1
MIDVFMKPHGPAPGAESTPRPLFWTHLRIKHRPQAGQTDNRKDRPPLSGPEISGVLNSSSEGKTGVCTRVSMATAPKHRL
SEEIKEDATKSEEDKTFSLC
SQSLLLPQLCLS
>g2246.t1
MSSANASASLVESLDVEVKEVNGNCELQIRWVINVDVSNKHLTGTRIVEGSRGYLCKYSPSFANASLTSLAQLSFEYIVP
VFPGYIDVMVANLPLPPVAN
GLAYKTFSSEIDEDTTLYPRGGKGLSGDTCECSSIPMALFGVMLAGFLILASCVLLFKTNRQRVLASGDFSKVPGSSEPP
VSVLLVYPAECPAFQKTVVA
LAEYLLEHAGCTVAVDLWQQGQIAKLGPMRWVAGQAKTADRVLIVCPQLGKSQDKRPRSLPGELTACKLFSLTEDLDKLC
RSLHNKKQGCLFQRTPHMFY
SKERAMKLQDAIKQLAEHQFSLTAEQEHVPSGVIAV
>g2246.t2
MSSANASASLVESLDVEVKEVNGNCELQIRWVINVDVSNKHLTGTRIVEGSRGYLCKYSPSFANASLTSLAQLSFEYIVP
VFPGYIDVMVANLPLPPVAN
GLAYKTFSSEIDEDTTLYPRGGKGLSGDTCECSSIPMALFGVMLAGFLILASCVLLFKTNRQRVLASGDFSKVPGSSEPP
VSVLLVYPAECPAFQKTVVA
LAEYLLEHAGCTVAVDLWQQGQIAKLGPMRWVAGQAKTADRVLIVCPQPISCHSNHRFLGPSVPASANDLYPLVLNMVAS
RAKNPSELAMFWALQLGKSQ
DKRPRSLPGELTACKLFSLTEDLDKLCRSLHNKKQGCLFQRTPHMFYSKERAMKLQDAIKQLAEHQFSLTAEQEHVPSGV
IAV
>g2246.t3
MSSANASASLVESLDVEVKEVNGNLSNKHLTGTRIVEGSRGYLCKYSPSFANASLTSLAQLSFEYIVPVFPGYIDVMVAN
LPLPPVANGLAYKTFSSEID
EDTTLYPRGGKGLSGDTCECSSIPMALFGVMLAGFLILASCVLLFKTNRQRVLASGDFSKVPGSSEPPVSVLLVYPAECP
AFQKTVVALAEYLLEHAGCT
VAVDLWQQGQIAKLGPMRWVAGQAKTADRVLIVCPQLGKSQDKRPRSLPGELTACKLFSLTEDLDKLCRSLHNKKQGCLF
QRTPHMFYSKERAMKLQDAI
KQLAEHQFSLTAEQEHVPSGVIAV
>g2247.t1
MNTSRVRMLLPWALLARCGPPLTGVQMKRGMRKAGRALSTWRPSLSPGLRQGAALNVVDARNSSSAASTPPANQNTPSYS
YVIVGAGSAGCVLANRLSED
AHESVLLLEAGPKDMLLGSVRLSWKIHMPAALTYNLCDEKYASCLEWASVSFV
>g2248.t1
MDGRVMYWPRGRVWGGSSSLNAMVYIRGHAEDYNRWQREGAQGWDYQHCLPYFRKAQCHELGEDRYRGGSGPLHVSRGRT
NHPLHQAFIEAGQQAGYPFT
QDMNGLQQEGVGWMDMTIHKGKRWSTASAYLRPALRRPNLKTEVCCLTTRVLFEGNRAIGVEYTQNGQKKKVFADKEVIL
SGGAINSPQLLMLSGVGNAD
DLKPLGIPVVQHLPGVGSNLQDHLELYVQQQCSQPITLYKAQKPHHMIKIGLEWLSMFT
>g2248.t2
MDGRVMYWPRGRVWGGSSSLNAMVYIRGHAEDYNRWQREGAQGWDYQHCLPYFRKAQCHELGEDRYRGGSGPLHVSRGRT
NHPLHQAFIEAGQQAGYPFT
QDMNGLQQEGVGWMDMTIHKGRAGKRWSTASAYLRPALRRPNLKTEVCCLTTRVLFEGNRAIGVEYTQNGQKKKVFADKE
VILSGGAINSPQLLMLSGVG
NADDLKPLGIPVVQHLPGVGSNLQDHLELYVQQQCSQPITLYKAQKPHHMIKIGLEWLSMFT
>g2249.t1
WTDLIVGAPNFFDRKAEIGGAVYVYLNPFGHWDDQARPIRLNGTYDSMFGMAVSSIGDLDQDGYGDIAVGAPFDGDGKVF

IYRGSDAGIETKPAQVLDGR
DFDVRRFGYSISGGLDMDKNQYPDLAVGSLNDSVVLFRSRPVIHVVRDISVDPAFIDLEQRNCQGRDGV
>g2250.t1
LGKPAARPLFKVPKPQEPVKPPAQPKPKWQKKQPIPPPPPPPPCQPAPEKLAPVTAHAEETTAQQIQNETIHHTETRKHI
HTKSESTATPLPSGIPVAKA
TEKEPAQPPKKRFVPPLKLPQSPEPAEAPKPRPYARKFKTPLMLAEERFRLQREETEKLKPTPPTSPRADSQAPSNATEA
PSQSTWPTEGRVEEGRAAAA
EETVARDPAPKKPPSHIPSGRPLISPVKKKSPLVSSNITLDNKQSSREHSSLVSGERAQGSSDVFRESQTTAAASESAAK
APPPKALDIKIQSGSNVVSS
SVTEQKQFMKKSSTVKCVSAVQHSVQENINLQSQSEVTVKAEEGKNINVADTQEGRKGTAQPTKIPKVAPSFKVKTFKMP
PEMKDGKCDAVVQKQAVTGG
TQPHLDVGNISQHNETRVIQENSLPTSAAVRNEVEVKIDLKAKQQMSPLVRPKINKSAEPLVKKQTAKTQNEEKQPEPVQ
VISMTEAPASGPQGAGYVAV
SRSHHSSKTEHRQEVVVTESVMQHSIQRQEAVHVHKQKAKVSQQHQQHQQHHQHQQISATTTSQVQALNVKAIKPKGEPR
DTEMTKSTGMLAPKGEVLVS
AESTEMDKCKLMQLLAHIKELQGAPGRIDSGAVRGIVSEVPDWMMGVDEKRNLSEIALHQSKKKLKEMTAYMRNMAEAKL
TFLEAESRAAVEQQGRQTPS
AQTPPPPLAPLLTLKKSDMRATVGGPSPKTPKVTVGLESQREVVEERRTTAQHHAISKTQELTREGVDHRGSPLMSVRSP
SPTFINIESMRRTDSPRYGV
TPSPPPYRAAGTPPPPPRRPYTAQSPLLSRATPSPTLSRSEKVVQLKDYAAKTPRGRTTPSPPPPPPLHHPRPTAEGVLA
EQEESPAFSLMTSYTVSEEE
EQRPGGEAGDDHEGGDSMMTVRDKKSFFEEAQRAEVNKMYIRKDPIEIPERLGADAEEVPLAAMVTEMVKEDLPRVDLSK
LVNRFESPQPQVFPRKDPVV
ITERLGSDPEDVEAEPKIPKTEDVPAFNVRALKDVFETGEHGSQAARELREHIERRESESEPRGHSTDDFGDTAAAHGAS
RCSETRTTTLSECSSSVAAG
GRGSPPSYADVVRGATDILSEAAEEMLRNYQQSWTESQEVVQNVGFTVTEQRSSHIVTRHQQTLATGKVGQRGMRTPYPN
SCVPACVPKIRIPDSELCVS
CRKRVYPMEALIADKQTFHRSCFCCQHCQGKLSLGNYASLHGRMYCKPHFKQLFKAKGNYDEGFGQKPHKELWNAKTPPK
AVTVKSPTPQKRSPDPRTTA
KTPPSSPVVRRAPASEMPDENKTHASKITVVWPPQTDSPKKAFNVDDELKLVKPAWPPQEGAAQKDTEDRRRSRPGRPSG
PALTLKGDNDADKSENGHQQ
ESAGVTEHEGAPVPQLETNAEANATPAQTDIQEPPSADEAERAEEVEDRVDEKDKQVTVKEVQGGRKDVEGQEGMEEAEA
EKVTEHGEHNGAGAPGGIEN
GQQEEAAADDTVKVTAIDGETVEVGPAEAEATNENGNNNNNNNNNNNSSSNDIVPQCSNDPAVDGPGRSELAGDRLLPF
TDSGGEDGGFVPWDQRDEEP
AWMPNHVLRMAQRDDAFVPESAKGSDANLAISVGESGETAAADEGRLPSGGPGESLMGSKVTTASFLEDIFAGLSSTDLL
CDFKCDDFAGEMATSTATTA
PALALEDLLDFSAWGGGDGSGGSLGSSLWAGDHDGSLTAEEQIKRNRYYDDSDN
>g2250.t2
LGKPAARPLFKVPKPQEPVKPPAQPKPKWQKKQPIPPPPPPPPCQPAPEKLAPVTAHAEETTAQQIQNETIHHTETRKHI
HTKSESTATPLPSGIPVAKA
TEKEPAQPPKKRFVPPLKLPQSPEPAEAPKPRPYARKFKTPLMLAEERFRLQREETEKLKPTPPTSPRADSQAPSNATEA
PSQSTWPTEGRVEEGRAAAA
EETVARDPAPKKPPSHIPSGRPLISPVKKKSPLVSSNITLDNKQSSREHSSLVSGERAQGSSDVFRESQTTAAASESAAK
APPPKALDIKIQSGSNVVSS
SVTEQKQFMKKSSTVKCVSAVQHSVQENINLQSQSEVTVKAEEGKNINVADTQEGRKGTAQPTKIPKVAPSFKVKTFKMP
PEMKDGKCDAVVQKQAVTGP
QGAGYVAVSRSHHSSKTEHRQEVVVTESVMQHSIQRQEAVHVHKQKAKGAPGRIDSGAVRGIVSEVPDWMMGVDEKRNLS
EIALHQSKKKLKEMTAYMRN
MAEAKLTFLEAESRAAVEQQGRQTPSAQTPPPPLAPLLTLKKSDMRATVGGPSPKTPKVTVGLESQREVVEERRTTAQHH
AISKTQELTREGVDHRGSPL
MSVRSPSPTFINIESMRRTDSPRYGVTPSPPPYRAAGTPPPPPRRPYTAQSPLLSRATPSPTLSRSEKVVQLKDYAAKTP
RGRTTPSPPPPPPLHHPRPT
AEGVLAEQEESPAFSLMTSYTVSEEEEQRPGGEAGDDHEGGDSMMTVRDKKSFFEEAQRAEVNKMYIRKDPIEIPERLGA
DAEEVPLAAMVTEMVKEDLP
RVDLSKLVNRFESPQPQVFPRKDPVVITERLGSDPEDVEAEPKIPKTEDVPAFNVRALKDVFETGEHGSQAARELREHIE

RRESESEPRGHSTDDFGDTA
AAHGASRCSETRTTTLSECSSSVAAGGRGSPPSYADVVRGATDILSEAAEEMLRNYQQSWTESQEVVQNVGFTVTEQRSS
HIVTRHQQTLATGKVGQRGM
RTPYPNSCVPACVPKIRIPDSELCVSCRKRVYPMEALIADKQTFHRSCFCCQHCQGKLSLGNYASLHGRMYCKPHFKQLF
KAKGNYDEGFGQKPHKELWN
AKTPPKAVTVKSPTPQKRSPDPRTTAKTPPSSPVVRRAPASEMPDENKTHASKITVVWPPQTDSPKKAFNVDDELKLVKP
AWPPQEGAAQKDTEDRRRSR
PGRPSGPALTLKGDNDADKSENGHQQESAGVTEHEGAPVPQLETNAEANATPAQTDIQEPPSADEAERAEEVEDRVDEKD
KQVTVKEVQGGRKDVEGQEG
MEEAEAEKVTEHGEHNGAGAPGGIENGQQEEAAADDTVKVTAIDGETVEVGPAEAEATNENGNNNNNNNNNNNSSSNDI
VPQCSNDPAVDGPGRSELAG
DRLLPFTDSGGEDGGFVPWDQRDEEPAWMPNHVLRMAQRDDAFVPESAKGSDANLAISVGESGETAAADEGRLPSGGPGE
SLMGSKVTTASFLEDIFAGL
SSTDLLCDFKCDDFAGEMATSTATTAPALALEDLLDFSAWGGGDGSGGSLGSSLWAGDHDGSLTAEEQIKRNRYYDDSDN
>g2251.t1
MDDPAAVAPGCRGVYVERTLALIKPDAVHLAEEIEAIILKSGFTVLQTRKLQLSPEQCSDFYAEHYGRLSFPSLTVYMSS
GPIVALALARDNAIAHWKAM
IGPASRARATHPE
>g2251.t2
MDDPAAVAPGCRGVYVERTLALIKPDAVHLAEEIEAIILKSGFTVLQVGTRSLLVATTRKLQLSPEQCSDFYAEHYGRLS
FPSLTVYMSSGPIVALALAR
DNAIAHWKAMIGPASRARATHPE
>g2252.t1
MTEQMTVRGTLKGHTDWVTQIATTPQFPDMILSASRDKSVIMWKLTRDETSYGAPQRALRGHSHFVSDVVISSDGQFALS
GSWDGTLRLWDLTTGLTTRR
FVGHSKDVLSVAFSADNRQIVSGSRDKTVKLWNTLGVCKYTIQDESHSEWVSCVRFSPNSSNPIIVSCGWDKMVKVWNLA
NCKLKTNHIGHTGFLNTVTV
SPDGSLCASGGKDGQAMLWDLNEGKHLYTLDSGDNINALCFSPNRYWLCAATGPSIKIWDLEGKIIVDELRQEVISNNSK
AEPPQCTSLAWSADGQTLFA
GYTDNLIRVWQVTIGTR
>g2252.t2
MTEQMTVRGTLKGHTDWVTQIATTPQFPDMILSASRDKSVIMWKLTRDETSYGAPQRALRGHSHFVSDVVISSDGQFALS
GSWDGTLRLGLTTRRFVGHS
KDVLSVAFSADNRQIVSGSRDKTVKLWNTLGVCKYTIQDESHSEWVSCVRFSPNSSNPIIVSCGWDKMVKVWNLANCKLK
TNHIGHTGFLNTVTVSPDGS
LCASGGKDGQAMLWDLNEGKHLYTLDSGDNINALCFSPNRYWLCAATGPSIKIWDLEGKIIVDELRQEVISNNSKAEPPQ
CTSLAWSADGQTLFAGYTDN
LIRVWQVTIGTR
>g2253.t1
LVGFYPATLAFEFKPNLLPSSVAFHIVRYIEVRRITSLGKELAPVAPYRSSAISRLPPSSCRIVDGEPPESTRDRKALLD
SPLTMENYCEKFQLLLYLEE
LQMLNDIKRYNMPNRDGEYATMVKDQHNKKLLVLEVPGVAENRPSVLVGDRILVCPVGEHLEKYCGYVHAVEKESVKLGF
SSKLRDRFIDGMAFSVEFTV
SRLPMRLQHRATELAKVHKLGVVLFPHGPPPPEAELPSLRLFDRKLEQNPEQYRAVQHIVAGTSNPAPYLVFGPPGTGKT
VTVVEAIKQIEKTKASCRIL
ACAPSNSASDLLCKKILEHVERRKVYRMYASSWDRKRIPSELMDCCNLVGDVYMYPLKEDLMNYTIIVTTLCTAGRLVSG
EIPPGHFSHVFVDEAGHAIE
TECIIPLAGLLQPESGQVVLAGDPKQLGPILRSPLVQQYGMSKSLLERLMDTMPVYQKIVDDSEDEEDEVSGEGLKETFD
PRFVTKLLCNYRSHPAILKI
PNDLFYDGELKVCADEVLRNSYCRWEYLPKM
>g2253.t2
LFASTGDNYEVQVLFQTSLVGFYPATLAFEFKPNLLPSSVAFHIVRYIEVRRITSLGKELAPVAPYRSSAISRLPPSSCR
IVDGEPPESTRDRKALLDSP
LTMENYCEKFQLLLYLEELQMLNDIKRYNMPNRDGEYATMVKDQHNKKLLVLEVPGVAENRPSVLVGDRILVCPVGEHLE
KYCGYVHAVEKESVKLGFSS
KLRDRFIDGMAFSVEFTVSRLPMRLQHRATELAKVHKLGVVLFPHGPPPPEAELPSLRLFDRKLEQNPEQYRAVQHIVAG

TSNPAPYLVFGPPGTGKTVT
VVEAIKQIEKTKASCRILACAPSNSASDLLCKKILEHVERRKVYRMYASSWDRKRIPSELMDCCNLVGDVYMYPLKEDLM
NYTIIVTTLCTAGRLVSGEI
PPGHFSHVFVDEAGHAIETECIIPLAGLLQPESGQVVLAGDPKQLGPILRSPLVQQYGMSKSLLERLMDTMPVYQKIVDD
SEDEEDEVSGEGLKETFDPR
FVTKLLCNYRSHPAILKIPNDLFYDGELKVCADEVLRNSYCRWEYLPKM
>g2253.t3
LVGFYPATLAFEFKPNLLPSSVAFHIVRYIEVRRITSLGKELAPVAPYRSSAISRLPPSSCRIVDGEPPESTRDRKALLD
SPLTMENYCEKFQLLLYLEE
LQMLNDIKRYNMPNRDGEYATMVKDQHNKKLLVLEVPGVAENRPSVLVGDRILVCPVGEHLEKYCGYVHAVEKESVKLGF
SSKLRDRFIDGMAFSVEFTV
SRLPMRLQHRATELAKVHKLGVVLFPHGPPPPEAELPSLRLFDRKLEQNPEQYRAVQHIVAGTSNPAPYLVFGPPGTGKT
VTVVEAIKQIEKTKASCRIL
ACAPSNSASDLLCKKILEHVERRKVYRMYASSWDRKRIPSELMDCCNLVGDVYMYPLKEDLMNYTIIVTTLCTAGRLVSG
EIPPGHFSHVFVDEAGHAIE
TECIIPLAGKSLLERLMDTMPVYQKIVDDSEDEEDEVSGEGLKETFDPRFVTKLLCNYRSHPAILKIPNDLFYDGELKVC
ADEVLRNSYCRWEYLPKM
>g2254.t1
MLAYLPSGYNNHSRIDPTQFLSDKNDVIVSSDQEGTDRKICLWVEDDNKHTVRLHVKNTGTTAVYFTYYTLLNWLEDFTL
EDERKVTRACPLALAPGKTR
TKHMMHA
>g2254.t2
MNDRVKLKDLYNNEFRYRTQFLSDKNDVIVSSDQEGTDRKICLWVEDDNKHTVRLHVKNTGTTAVYFTYYTLLNWLEDFT
LEDERKVTRACPLALAPGKT
RTKHMMHA
>g2254.t3
MNQPVSVHNGHSSPGPSQQASPCFKSETQSQRRLASLLMVKLKKDKTQFLSDKNDVIVSSDQEGTDRKICLWVEDDNKHT
VRLHVKNTGTTAVYFTYYTL
LNWLEDFTLEDERKVTRACPLALAPGKTRTKHMMHA
>g2255.t1
MSFPRRVAGLSLRDRVRSSDIRRELRVEPLLLRVERSQLRWFGHLIRMPPGRLPLEVFRARPTGKRPRGRPRTRWRDYIS
HLAWERLGVPQEELESVAGE
KDVWSSLLSLLPPRPGPG
>g2256.t1
SYQDQGCSAVCAAAALHIPPERSVQTQQAASRDPGPPPLQQHSTAEHRPAHTLITHNRTPPPNGGSSAAEHSSVLKMCGK
RENVVLR
>g2257.t1
MLEHSSELALVQSLYVNSMRCPPSAPGVSVSEEDLPVSQESVCGQRGSLLIVVLVMTSSYDLDFHLAESRLMSSGEAMDE
TRRHKAEDRARSPGPDATRC
NGR
>g2257.t2
MLEHSSELALVQSLYVNSMRCPPSAPGVSVSEEDLPVRLGVQTTPLLWCSSVCRDQESVCGQRGSLLIVVLVMTSSYDLD
FHLAESRLMSSGEAMDETRR
HKAEDRARSPGPDATRCNGR
>g2258.t1
MVGYDPGAQGVVLSPEEVALAGSGSGMITRALISPLDVIKIRFQWNWGVLVKREAHHLGCTQLQIERVSCLRPEGKYWGL
FQASRCILREEGLQAFWKGH
LPAQLLSISFGAVQFASFECLTEAVHKSTPYDSQTPGIHFICGGLSACSATVVCQPLDTLRTRFAAQGEPKVYLSLRHAV
SLMYRTSGPLAFYRGLAPTL
VAVFPYAGLQFFFYRLFGQLLPPKAEGRNSGGNMRSLLCGSAAGVLSKTITYPFDLFKKRLQVGGFEEARVHFGQ
>g2258.t2
MVGYDPGAQGVVLSPEEVALAGSGSGMITRALISPLDVIKIRFQRPQIFHEPNSSGHGGRGLRPATCGVSRSQWNWGVLV
KREAHHLGCTQLQIERVSCL
RPEGKYWGLFQASRCILREEGLQAFWKGHLPAQLLSISFGAVQFASFECLTEAVHKSTPYDSQTPGIHFICGGLSACSAT
VVCQPLDTLRTRFAAQGEPK
VYLSLRHAVSLMYRTSGPLAFYRGLAPTLVAVFPYAGLQFFFYRLFGQLLPPKAEGRNSGGNMRSLLCGSAAGVLSKTIT

YPFDLFKKRLQVGGFEEARV
HFGQ
>g2259.t1
MMQIISDPASNKHSLINMMNRFIAAANNMDETIMVPSLLRDMPLEERAEAAEIETNNNNQPCPNKQRDMYEHYLLLKSIK
NDMEWGLLKREMSSGASFLE
MAVKQEEQKPVTGELPHDDNNSDLEDQFHFHLRGLFGVLSKLTVQADHLTNRYKREIGGGNFMR
>g2260.t1
MPILKKLPGRSKSCHEFPRTTGELILPRLDLDLKDLNDLNHLQDLQDMKDLNPFHEVDLGEDERNGADDPGLIMGEVRGY
HHQQQQQHRTTRDGEVEENE
VNAERHGAGNPKGRPLRGTLERICGVSPLKTLGKLGKGLRISGRSVWGGGGTAQRSPAGANTPPPEKEKRKGLRRSSEEI
MTLLRFTGRRKETRRESLPC
GDLSMDHEAEARRRPSFLKLVSLGKLKRESMSDRGSQEAEDEEEEEEPVVKAREPLSVLEILQLVNNRDLLQADTHIQEL
ERECEVLSGLPNTSNPTLTP
SFPSTPSSLGSSTPSLQDDLLSSNATLDSGRRKAKDVELLYEALQREMWDIVRESLRQPSAGPNLGLVVLVIQQEEHADA
SWALREDKPQREGLQNPSQP
SHRPRRMKQKWRQAVAEATDWSLPNQMDTKAGQLALYLERLRSRMVDDLDAARRNAVSIYPEEFAAFQVYVESYHRAVAK
RLRTITSGPLQITDVYSLLD
WFYNIYNRDVLGNIGASTHINYSHLEPILPQDTVERLEQDCLDIVKDKVTTELIQVLDEEERRWAQTLHIEEYQSNLARS
VIQRLKVDLDRSTTVSQFLG
SRVARCSLIGLADFLYRTFVQRCRQCDPQGSEEIAQRANTSLDRIINQSVRVLSDRLFEHIRPFFDKLVKRKWLNNTVAF
EAIEASIKQSFKKFRRMDPP
PYQTLVGEVHRRVLVEYVRAILRGRVICTSSKMRKRMAFRLQDEAKQLKGLFKDLESGSSWLDSLICHLADIILLEDTPS
IQMEVAMLVKEFPDI
>g2260.t2
MPILKKLPGRSKSCHEFPRTTGELILPRLDLDLKDLNDLNHLQDLQDMKDLNPFHEVDLGEDERNGADDPGLIMGEVRGY
HHQQQQQHRTTRDGEVEENE
VNAERHGAGNPKGRPLRGTLERICGVSPLKTLGKLGKGLRISGRSVWGGGGTAQRSPAGANTPPPEKEKRKGLRRSSEEI
MTLLRFTGRRKETRRESLPC
GDLSMDHEAEARRRPSFLKLVSLGKLKRESMSDRGSQEAEDEEEEEEPVVKAREPLSVLEILQLVNNRDLLQADTHIQEL
ERECEVLSGLPNTSNPTLTP
SFPSTPSSLGSSTPSLQDDLLSSNATLDSGRRKAKDVELLYEALQREMWDIVRESLRQPSAGPNLGLVVLVIQQEEHADA
SWALREDKPQREGLQNPSQP
SHRPRRMKQKWRQAVAEATDWSLPNQMDTKAGQLALYLERLRSRMVDDLDAARRNAVSIYPEEFAAFQVYVESYHRAVAK
RLRTITSGPLQITDVYSLLD
WFYNIYNRDVLGNIGASTHINYSHLEPILPQDTVERLEQDCLDIVKDKVTTELIQVLDEEERRWAQTLHIEEYQSNLARS
VIQRLKVDLDRSTTVSQFLG
SRVARCSLIGLADFLYRTFVQRCRQCDPQGSEEIAQRANTSLDRIINQSVRVLSDRLFEHIRPFFDKLVKRKWLNNTVAF
EAIEASIKQSFKKFRRMDPP
PYQTLVGEVHRRVLVEYVRAILRGRVICTSSKMRKRMAFRLQDEAKQLKGLFKDLESGSSWLDSLICHLADIILLEDTPS
IQMEVAMLVKEFPDIR
>g2260.t3
MPILKKLPGRSKSCHEFPRTTGELILPRLDLDLKDLNDLNHLQDLQDMKDLNPFHEVDLGEDERNGADDPGLIMGEVRGY
HHQQQQQHRTTRDGEVEENE
VNAERHGAGNPKGRPLRGTLERICGVSPLKTLGKLGKGLRISGRSVWGGGGTAQRSPAGANTPPPEKEKRKGLRRSSEEI
MTLLRFTGRRKETRRESLPC
GDLSMDHEAEARRRPSFLKLVSLGKLKRESMSDRGSQEAEDEEEEEEPVVKAREPLSVLEILQLVNNRDLLQADTHIQEL
ERECEVLSGLPNTSNPTLTP
SFPSTPSSLGSSTPSLQDDLLSSNATLDSGRRKAKDVELLYEALQREMWDIVRESLRQPSAGPNLGLVVLVIQQEEHADA
SWALREDKPQREGLQNPSQP
SHRPRRMKQKWRQAVAEATDWSLPNQMDTKAGQLALYLERLRSRMVDDLDAARRNAVSIYPEEFAAFQVYVESYHRAVAK
RLRTITSGPLQITDVYSLLD
WFYNIYNRDVLGNIGASTHINYSHLEPILPQDTVERLEQDCLDIVKDKVTTELIQVLDEEERRWAQTLHIEEYQSNLARS
VIQRLKVDLDRSTTVSQFLG
SRVARCSLIGLADFLYSFQRKVEMFHETQAEFGDRGEGYISRTIALVNCCPPLRTFVQRCRQCDPQGSEEIAQRANTSLD
RIINQSVRVLSDRLFEHIRP
FFDKLVKRKWLNNTVAFEAIEASIKQSFKKFRRMDPPPYQTLVGEVHRRVLVEYVRAILRGRVICTSSKMRKRMAFRLQD

EAKQLKGLFKDLESGSSWLD
SLICHLADIILLEDTPSIQMEVAMLVKEFPDI
>g2261.t1
MADNVLDPGPPSAKRPKLSSPALSVSASDGNEPGHCVVQNWAAATVQNRAATVQIRAAATVQNRTVATVQIRAAARSRTG
PLRSNSDPSNFGSLFDLEHD
LPDELINSSELGLVNGGDLNQIHTSLGGLGAGMGGMGMGGGGGGGGGGQDAATKHKQLSELLRAGAQPQQGAPNNTGAGA
SMALMGGMNATAAGPQGLGP
QGGQQQSGLMQQQQAGMAGGVAGMNRAAMMNAHKANNGQQQHQGLMGGQMMNGSPRMGYPGNGGMSSNSNLLAETLQGQT
GGQQMGPGGPAGIRPQQPGA
LNKMNMMANTVPYGGPYGQPAGQGLPGAGLAPQLQNKAGMANSLAAQFSMDKKTPPGQGMSGM
>g2261.t2
MPWRGVSAVTGSFWLTGPPLVASKTPDAGVFLRKALSRWAVAQKMWSVALCLSRWRVTVSPIGRVPFQQNVHFVCPPLLD
FGSLFDLEHDLPDELINSSE
LGLVNGGDLNQIHTSLGGLGAGMGGMGMGGGGGGGGGGQDAATKHKQLSELLRAGAQPQQGAPNNTGAGASMALMGGMNA
TAAGPQGLGPQGGQQQSGLM
QQQQAGMAGGVAGMNRAAMMNAHKANNGQQQHQGLMGGQMMNGSPRMGYPGNGGMSSNSNLLAETLQGQTGGQQMGPGGP
AGIRPQQPGALNKMNMMANT
VPYGGPYGQPAGQGLPGAGLAPQLQNKAGMANSLAAQFSMDKKTPPGQGMSGM
>g2261.t3
MADNVLDPGPPSAKRPKLSSPALSVSASDGNEPGHCVVQNWAAATVQNRAATVQIRAAATVQNRTVATVQIRAAARSRTG
PLRSNSDPNFGSLFDLEHDL
PDELINSSELGLVNGGDLNQIHTSLGGLGAGMGGMGMGGGGGGGGGGQDAATKHKQLSELLRAGAQPQQGAPNNTGAGAS
MALMGGMNATAAGPQGLGPQ
GGQQQSGLMQQQQAGMAGGVAGMNRAAMMNAHKANNGQQQHQGLMGGQMMNGSPRMGYPGNGGMSSNSNLLAETLQGQTG
GQQMGPGGPAGIRPQQPGAL
NKMNMMANTVPYGGPYGQPAGQGLPGAGLAPQLQNKAGMANSLAAQFSMDKKTPPGQGMSGM
>g2262.t1
MMWRCYREDSRETPDGVLPYSPGKGEQRACLLVSWTRLLDYGWLHTARSPGQSQRRLSGERSALVLGIGIRAIC
>g2263.t1
MRTAEESGTVLHRLIQEQLRFGNRADTHTLLAIQQQALRGSSGSSNSSGGGTGSPRSSLESLTLEEPLQYVHMSTRQEPQ
GQEHRADLLYPENPSACHHL
YQLHGEQLPSYEEAKAHSQYRSAQHPGAGPPAGPWDMKREHARSLSERLMQLSLERNHGPPVPETPMSSSHSYPQLFPTG
PHQGPESRGPPPEYPFCVRI
PGYTLSHSEERGHQYRDPPPPFNSQHRFVSAQPQMSHGAGGVHHATGPRGSDPYITGNPPSMPCGNGSAAQMDALLRENE
RLHQQLEVHVEQTARLQKLE
LEIHRISEAYDTLMKGSAKREALEKTMRNKMEAEIKRLHDFNRDLRERLETATRQRLATEVESTEQKQHIFAKLIEQIAH
HRGGLREKETPPAKRRMPLR
CWGRTVGCDEEQQRERERVERQVQHLRASGEEWARRREALEKALSSAQSRNLTLEEELQRKRAYVSKVERLQSALAQLQA
ACEKRETLELRLRTRLEQEL
KSLRAQQTQSHGAGPADQQLNRTRLQQQLREREERVLALEADITKWEQKYLEESTMRQFAMDAAATAAAQRDTTIINHTP
GSSPNSSFNDELPSQSPRQQ
EMKNRIRALHAQLLEKDAVIQVLQQRSGWESGLLEKHGLRLARSVPASINAVGGSGSQGDLAQGKGKSLWDDRTCEGMLP
SRSSAGPFGRGQDSISQSSY
ISAFSQGTEALMDSRKLAATEARSASVSDLQELKAPLKTFMNISCSDADVVEILI
>g2263.t2
MRTAEESGTVLHRLIQEQLRFGNRADTHTLLAIQQQALRGSSGSSNSSGGGTGSPRSSLESLTLEEPLQYVHMSTRQEPQ
GQEHRADLLYPENPSACHHL
YQLHGEQLPSYEEAKAHSQYRSAQHPGAGPPAGPWDMKREHARSLSERLMQLSLERNHGPPVPETPMSSSHSYPQLFPTG
PHQGPESRGPPPEYPFCVRI
PGYTLSHSEERGHQYRDPPPPFNSQHRFVSAQPQMSHGAGGVHHATGPRGSDPYITGNPPSMPCGNGSAAQMDALLRENE
RLHQQLEVHVEQTARLQKLE
LEIHRISEAYDTLMKGSAKREALEKTMRNKMEAEIKRLHDFNRDLRERLETATRQRLATEVESTEQKQHIFAKLIEQIAH
HRGGLREKETPPAKRRMPLR
CWDEEQQRERERVERQVQHLRASGEEWARRREALEKALSSAQSRNLTLEEELQRKRAYVSKVERLQSALAQLQAACEKRE
TLELRLRTRLEQELKSLRAQ
QTQSHGAGPADQQLNRTRLQQQLREREERVLALEADITKWEQKYLEESTMRQFAMDAAATAAAQRDTTIINHTPGSSPNS

SFNDELPSQSPRQQEMKNRI
RALHAQLLEKDAVIQVLQQRSGWESGLLEKHGLRLARSVPASINAVGGSGSQGDLAQGKGKSLWDDRTCEGMLPSRSSAG
PFGRGQDSISQSSYISAFSQ
GTEALMDSRKLAATEARSASVSDLQELKAPLKTFMNISCSDADVVEILI
>g2263.t3
MRTAEESGTVLHRLIQEQLRFGNRADTHTLLAIQQQALRGSSGSSNSSGGGTGSPRSSLESLTLEEPLQYVHMSTRQEPQ
GQEHRADLLYPENPSACHHL
YQLHGEQLPSYEEAKAHSQYRSAQHPGAGPPAGPWDMKREHARSLSERLMQLSLERNHGPPVPETPMSSSHSYPQLFPTG
PHQGPESRGPPPEYPFCVRI
PGYTLSHSEERGHQYRDPPPPFNSQHRFVSAQPQMSHGAGGVHHATGPRGSDPYITGNPPSMPCGNGSAAQMDALLRENE
RLHQQLEVHVEQTARLQKLE
LEIHRISEAYDTLMKGSAKREALEKTMRNKMEAEIKRLHDFNRDLRVVFLTSIERLETATRQRLATEVESTEQKQHIFAK
LIEQIAHHRGGLREKETPPA
KRRMPLRCWGRTVGCDEEQQRERERVERQVQHLRASGEEWARRREALEKALSSAQSRNLTLEEELQRKRAYVSKVERLQS
ALAQLQAACEKRETLELRLR
TRLEQELKSLRAQQTQSHGAGPADQQLNRTRLQQQLREREERVLALEADITKWEQKYLEESTMRQFAMDAAATAAAQRDT
TIINHTPGSSPNSSFNDELP
SQSPRQQEMKNRIRALHAQLLEKDAVIQVLQQRSGWESGLLEKHGLRLARSVPASINAVGGSGSQGDLAQGKGKSLWDDR
TCEGMLPSRSSAGPFGRGQD
SISQSSYISAFSQGTEALMDSRKLAATEARSASVSDLQELKAPLKTFMNISCSDADVVEILI
>g2264.t1
MADLEDVTLDGRPLNSLRVADLKSALEERGLSKSGPKNALVKRLKGALMLENLQRTSTPHSGLQPNSQIGEEMSQNSFIK
QYLAKQQELLRQRLEREARE
AVDTEEPEDNTSVNNSAPCPAPSQEATPAPVDHHKPLGLADGEGLAGPAHQ
>g2264.t2
MADLEDVTLDGRPLNSLRVADLKSALEERGLSKSGPKNALVKRLKGALMLENLQRTSTPHSGLQPNSQIGEEMSQNSFIK
QYLAKQQELLRQRLEREARE
AVDTEDACSGMEGHNTLNVGRFSHAHQEATPAPVDHHKPLGLADGEGLAGPAHQ
>g2265.t1
MLFFFTEMSWFYYVSPGSSEPQPQLTDTITPTGASSFSSLGGSTQEATHGHPTLEWHPSCSPPGLPALPSPPQCSVPPTL
ETLSCASQSAVPGGGSLPVS
YVTLQEHRCVMSWFLGWSTPQKERFLQDLLGKAVPGKVCTLLDSLNTLQVRDRPPNIFECQLRLWTQWFESWSEEERNHF
LLILEEHDPAFVSQFYQSVA
GTAGRE
>g2265.t2
MSWFYYVSPGSSEPQPQLTDTITPTGASSFSSLGGSTQEATHGHPTLEWHPSCSPPGLPALPSPPQCSVPPTLETLSCAS
QSAVPGGGSLPVSYVTLQEH
RCVMSWFLGWSTPQKERFLQDLLGKAVPGKVCTLLDSLNTLQVRDRPPNIFECQLRLWTQWFESWSEEERNHFLLILEEH
DPAFVSQFYQSVAGTAGRE
>g2266.t1
MGLRGWVCLVTGASRGIGKGIALQLSEAGATVYITGRQERTLRQAASEVKERGGDCLPVVCDSSREEDIRQLFERIKREQ
NGRLDILVNNAYAGVQAIFD
NMGKKFWETDPTIWDSINNTGLRGHYFCSVYASRMMVEQGRGLIVMISSIGGLKYLFNVPYGVGKAACDRLAADMAVELR
RSGVASVSLWPGAVPTELVS
QMLESDKGQDVDPKLKKLWMDGESTEVSGKCIVQLAKDANLMSLTGKVMMTCDVARRYGILDVDGRSVTDFTTLKFLVSQ
VRYLSWLAPIVPSFLRVPRF
MLTLSGGRF
>g2267.t1
MVEGLTEFVCLLQRPGWTQRQDTEPGEGVSTAKGRPPPAPKLGDLVVIKDFRRKAWNSKRWLGPDQVLLTTHTAVKVAER
PSWVHALHCKI
>g2268.t1
LGDDDNETLNNILACQWNFEEEEFLDVSEEAKDFITRLLVKSKSWRMSATASLKHPWLCDTGVHYRLHQKVRAPGVGFIG
MSSRWRSLSF
>g2269.t1
MADITDFETAESGASTTYPIQCSALRKNGHVVMKGRPCKIVEMSTSKTGKHGHAKVNLVGIDIFTNKKYEDVCPSTHNMD
VPNIKRTEWQVGLCSV

>g2269.t2
MADITDFETAESGASTTYPIQCSALRKNGHVVMKGRPCKIVEMSTSKTGKHGHAKVNLVGIDIFTNKKYEDVCPSTHNMD
VPNIKRTEWQVNL
>g2270.t1
DLCVGNDTSSQPSSDSGRPAGDLSKRDSMPTSTSDADVVKVTSTPL
>g2271.t1
MRSRSPLARWTSPLQSEDPHSPDTVISVKRNTVLDPSGPIRYRCPCLCVSVLSRTPQNSARVLRCLHPAREWGLESRHKW
VDNVAHVQITPLTPFARGTP
ACDQTLKLDLTRCQPASPPFRHQGDTRRGELMAFT
>g2271.t2
MRSRSPLARWTSPLQSEDPHSPDTVISVKRNTVLDPSGPIRYRCPCLCVSVLSRTPQNSARVLRCLHPAREWGLESRHKW
VDNVAHVQITPLTPFARGTP
ACDQTLKLDLTRCQPASPPFRHQGDTRRGGP
>g2272.t1
MEEMIEMIYSSHRLAVSVRPRRLASAPGGGGGAALMRRREAFSCRRLRRGCRYPGEWRRGRLIPLNITRQAAFRMKADDG
AWC
>g2272.t2
LKPGDSNPREEVTMEEMIEMIYSSHRLAVSVRPRRLASAPGGGGGAALMRRREAFSCRRLRRGCRYPGEWRRGRLIPLNI
TRQAAFRMKADDGAWC
>g2273.t1
QKDIDGKKDINAALQAERKFFLSHPSYRHLAERMGTAYLQKVLNQETPPGISSSASSSPPPSLLLLDLFSSSSSSFSPPP
PPPPPPLSVHSTQ
>g2273.t2
QKDIDGKKDINAALQAERKFFLSHPSYRHLAERMGTAYLQKVLNQETPPGISSSASSSPPPSLLLLDLFSSSSSSFSPPP
PPPPPPLSVHSTQI
>g2274.t1
CCRNAPQRTRSSDVHQHIDRVHQHIDRVHQHIDHVHDRFGAVHNNHVHDHFGTSDTDHVHEDFGTSAPCTVPLRHRRRAP
DAHLSGTSGDSHGGTLICLY
KSPLRKAKPTARL
>g2275.t1
MEFVLSLFLLCCLSVIAVKCDPGPLIDVVVDRYDIPSVCPREVATEDFVRYHFNGTFFEGGKKFDSSHDRGKAFISQVGL
GRLITGMDRGILGMCVNERR
KITIPPHLAYGSIGT
>g2275.t2
LLSQRIATTAVIAMEFVLSLFLLCCLSVIAVKCDPGPLIDVVVDRYDIPSVCPREVATEDFVRYHFNGTFFEGGKKFDSS
HDRGKAFISQVGLGRLITGM
DRGILGMCVNERRKITIPPHLAYGSIGT
>g2275.t3
MEFVLSLFLLCCLSVIAVKCDPGPLIDVVVDRYDIPSVCPREVATEDFVRYHFNGTFFEGGKKFDSSHDRGKAFISQVGL
GRLITGMDRGILGMCVNERR
KITIPPHLAYGSIGTAQRAVDLRQV
>g2276.t1
MPLDAAYGHAMEQYGAQNWKESVKYLELSLRLHRLLKDSESFCGKNCSAVSRDNDTLFADSTLRIMRHFLLRASCLKKCK
ANFPIFSISYPRRDTLDAFD
KRTPYRYIQYAYYQSNNLEKAVSATHTFLQKNPEDPYLIKNMNYYKTLADVDAFLVDHEERPYESVFLKAVKLYNNEDFS
SSARHMEQATAEFFRTYELC
LAGCEGSYEIVEFKDFYPTLADLYTEALKCKVKCEEDLTPSVGGFFVEKFVATMYHYLQFAYYKLNDVKNAAPCAASYML
FDPDDQVMQQNVAYYRFYRE
QWGLEDHHFLPQPEALKYYNQTTKQKEMLQFALNYLQTDDEDVVSPEEVGTGGPQEPPDAEFEGVGDYEESFVADWWQEP
RTKWDTGEVAD
>g2276.t2
MPLKKNGRSVSLGCLCLVLLIPALVDAQYETYSFKSFPQAEIMPLDAAYGHAMEQYGAQNWKESVKYLELSLRLHRLLKD
SESFCGKNCSAVSRDNDTLF
ADSTLRIMRHFLLRASCLKKCKANFPIFSISYPRRDTLDAFDKRTPYRYIQYAYYQSNNLEKAVSATHTFLQKNPEDPYL
IKNMNYYKTLADVDAFLVDH
EERPYESVFLKAVKLYNNEDFSSSARHMEQATAEFFRTYELCLAGCEGSYEIVEFKDFYPTLADLYTEALKCKVKCEEDL

TPSVGGFFVEKFVATMYHYL
QFAYYKLNDVKNAAPCAASYMLFDPDDQVMQQNVAYYRFYREQWGLEDHHFLPQPEALKYYNQTTKQKEMLQFALNYLQT
DDEDVVSPEEVGTGGPQEPP
DAEFEGVGDYEESFVADWWQEPRTKWDTGEVAD
>g2276.t3
MPLKKNGRSVSLGCLCLVLLIPALVDAQYETYSFKSFPQAEIMPLDAAYGHAMEQYGAQNWKESVKYLELSLRLHRLLKD
SESFCGKNCSAVSRDNDTLF
ADSTLRIMRHFLLRASCLKKCKANFPIFSISYPRRDTLDAFDKRTPYRYIQYAYYQSNNLEKAVSATHTFLQKNPEDPYL
IKNMNYYKTLADVDAFLVDH
EERPYESVFLKAVKLYNNEDFSSSARHMEQATAEFFRTYELCLAGCEGSYEIVEFKDFYPTLADLYTEALKCKVKCEEDL
TPSVGGFFVEKFVATMYHYL
QFAYYKLNDVKNAAPCAASYMLFDPDDQVMQQNVAYYRFYREQWGLEDHHFLPQPLAALRGPFGANSPGMTKKKKSRNSR
EVKPGSAVKEALKYYNQTTK
QKEMLQFALNYLQTDDEDVVSPEEVGTGGPQEPPDAEFEGVGDYEESFVADWWQEPRTKWDTGEVAD
>g2277.t1
GRIEMWVDMFPKEMTAPGPALDISPRKPKKFELRVIVWNTDEVVLEDDDIFTGEKSSDIFVRGWLKGQQEDKQDTDVHYH
SITGEGNFNWRFVYPFDYLM
AEEKIVISKKESMFAWDETEYKIPARLNLQVWDADHFSADDFLGAIELDLNRFPRGAKTPKQCTIEMVTNEAEMPMVSIF
KQKRIKGWWPFVARNEEDEF
ELTVRGLLGSRRRWDVWWCCGAYACD
>g2277.t2
GRIEMWVDMFPKEMTAPGPALDISPRKPKKFELRVIVWNTDEVVLEDDDIFTGEKSSDIFVRGWLKGQQEDKQDTDVHYH
SITGEGNFNWRFVYPFDYLM
AEEKIVISKKESMFAWDETEYKIPARLNLQVWDADHFSADDFLGAIELDLNRFPRGAKTPKQCTIEMVTNEAEMPMVSIF
KQKRIKGWWPFVARNEEDEF
ELT
>g2277.t3
GRIEMWVDMFPKEMTAPGPALDISPRKPKKFELRVIVWNTDEVVLEDDDIFTGEKSSDIFVRGWLKGQQEDKQDTDVHYH
SITGEGNFNWRFVYPFDYLM
AEEKIVISKKESMFAWDETEYKIPARLNLQVWDADHFSADDFLGAIELDLNRFPRGAKTPKQCTIEMVTNEAEMPMVSIF
KQKRIKGWWPFVARNEEDEF
ELTVRGLLGSRR
>g2278.t1
DHRAAAVSADYARGGVEFYDKLLSRNRTLRALMGPKGEEMYAVNGNLFPAHLLQLKHAPYTSRRDGLLSILKDTA
>g2279.t1
MRPYYIPREFSHVIAITAYIPPSADATAACETLHTVVSNLQTSHPHALLLISGDFNHASLSSTLPTFTQYVKCNTRDNKT
LDLLYANIKDAYSSSPLPPL
GRSDHNLVHLLPAYIPM
>g2280.t1
MSETLITSPTFKQYVSCPTREERTLDLMYANIKDAYISSSLPLQGRSDHNLDHLKHCYVPLLKRKPVTTVRSRSEESYEV
WSDPWPMVRPFTDQLQFTYQ
PHLGVEDGIIDLLNRVYSHLDNPANTRDPSYRRSLRGDGEAAEWGLNGGQKRKFKPYIPAVITGNTVRANRNTTATDNTI
PTKMLRCYPNNKPWVTSNLK
ALLNQKKRAFRSGTVQNSNGLHITSSQVRRELKGSNRGKLLDRTASARVC
>g2280.t2
MATQADTVPGFQTVRANRNTTATDNTIPTKMLRCYPNNKPWVTSNLKALLNQKKRAFRSGTVQNSNGLHITSSQVRRELK
GSNRGKLLDRTASARVC
>g2280.t3
MSETLITSPTFKQYVSCPTREERTLDLMYANIKDAYISSSLPLQGRSDHNLDHLKHCYVPLLKRKPVTTVRSRSEESYEV
WSDPWPMVRPFTDQLQFTYQ
PHLGVEDGIIDLLNRVYSHLDNPANTVEAPMASWIVDYLMGRPQFIRLQNCLTGXNMATQADTVPGFQTVRANRNTTATD
NTIPTKMLRCYPNNKPWVTS
NLKALLNQKKRAFRSGTVQNSNGLHITSSQVRRELKGSNRGKLLDRTASARVC
>g2281.t1
LSSLSWANKLDSLTQVSSSDPEGFLAHPVNAYKLMKRLNTEWSQLESLVLQDPSDGFISNISVHRQFFPDEEDEKGAAKA
LMRLQDTYQLDSESFSRGKL

PGSTYSGLHSNVILTVDDCFDMGKTAYNDADYYHAVLWMQQALRQLDTGEDAVASKANVLDYLSYSVYQMGDLPRAIELT
LRLVSIGMHIS
>g2281.t2
LSSLSWANKLDSLTQVSSSDPEGFLAHPVNAYKLMKRLNTEWSQLESLVLQDPSDGFISNISVHRQFFPDEEDEKGAAKA
LMRLQDTYQLDSESFSRGKL
PGSTYSGLHSNVILTVDDCFDMGKTAYNDADYYHAVLWMQQALRQLDTGEDAVASKANVLDYLSYSVYQMGDLPRAIELT
LRLVSIGLI
>g2281.t3
LSSLSWANKLDSLTQVSSSDPEGFLAHPVNAYKLMKRLNTEWSQLESLVLQDPSDGFISNISVHRQFFPDEEDEKGAAKA
LMRLQDTYQLDSESFSRGKL
PGLHSNVILTVDDCFDMGKTAYNDADYYHAVLWMQQALRQLDTGEDAVASKANVLDYLSYSVYQMGDLPRAIELTLRLVS
IGMHIS
>g2282.t1
MQELSQSQQPMSEEPIQLGTYARPKDHLPEREVYEALCRGEGVKMTDKRRSHLFCRYHDGNRNPRLLLKPMLEEDEWDSP
RIVRYLNALSHDEIEKIKQL
AKPNVRSILPPQILHAWLEGEDDPVVARVNQRIEDITGLTVKTAELLQVRSSSASH
>g2282.t2
MQELSQSQQPMSEEPIQLGTYARPKDHLPEREVYEALCRGEGVKMTDKRRSHLFCRYHDGNRNPRLLLKPMLEEDEWDSP
RIVRYLNALSHDEIEKIKQL
AKPNVRSILPPQILHAWLEGEDDPVVARVNQRIEDITGLTVKTAELLQALSMNITKMAPWRASQCHPFGPMQNKT
>g2283.t1
LAGSTKLLVVFVYRPPKASTTFLSELSELLTSVCSMSPATLLLGDFNIHVDSSSCPFAVEFLSLLVCFNIMQHVQGPTHT
KGHTLDLMCSTGTTPSHLQG
LDLAVSDHHAIHFTLPVPLPMDGLVAHYSAALSLSLDSLSPLKTRTASFSCPAPWFTTELCTIKTTDHQLERLYKRGFIK
GLTVHHEAFKDHALALPPPH
TPPSQRVDLSTPHVPLCCLSSFSVDDPQVTKLVTKATASTCSLEPMPTTLVKACLPTLCPIMVDIINMVPSSFKMAAVTP
YSKSQV
>g2283.t2
MSSHELYEPLQSGFRAHHSTETTLIKITNDLLIAADSSHISILILLDVSGACDTVSHTILLNCLSGYLGLTGAALSWFQS
YLSDRKQFVSVRGSNSIPGP
VNQGMPQGSVLGPLLFTIYMLPLGQIIHHHGLSFHCYADDTQLYLSTKPSTQLPPQSLVNCLHSIKTWMSSSLLKLNKTE
LMVVASKALLQKSHIKSLTK
SAFYHLKNISRLRPSLSDSVAGTLIHAFITSRLDCCNGVLSEVPN
>g2283.t3
LAGSTKLLVVFVYRPPKASTTFLSELSELLTSVCSMSPATLLLGDFNIHVDSSSCPFAVEFLSLLVCFNIMQHVQGPTHT
KGHTLDLMCSTGTTPSHLQG
LDLAVSDHHAIHFTLPVPLPMDGLVAHYSAALSLSLDSLSPLKTRTASFSCPAPWFTTELCTIKTTDHQLERLYKRGFIK
GLTVHHEAFKDHALALPPPH
TPPSQRVDLSTPHVPLCCLSSFSVDDPQVTKLVTKATASTCSLEPMPTTLVKACLPTLCPIMVDIINMPGLDPDDLTNYR
PISNLPFLSNILERAVAAQL
HQYMSSHELYEPLQSGFRAHHSTETTLIKITNDLLIAADSSHISILILLDVSGACDTVSHTILLNCLSGYLGLTGAALSW
FQSYLSDRKQFVSVRGSNSI
PGPVNQGMPQGSVLGPLLFTIYMLPLGQIIHHHGLSFHCYADDTQLYLSTKPSTQLPPQSLVNCLHSIKTWMSSSLLKLN
KTELMVVASKALLQKSHIKS
LTKSAFYHLKNISRLRPSLSDSVAGTLIHAFITSRLDCCNGVLSEVPN
>g2283.t4
LVVFVYRPPKASTTFLSELSELLTSVCSMSPATLLLGDFNIHVDSSSCPFAVEFLSLLVCFNIMQHVQGPTHTKGHTLDL
MCSTGTTPSHLQGLDLAVSD
HHAIHFTLPVPLPMDGLVAHYSAALSLSLDSLSPLKTRTASFSCPAPWFTTELCTIKTTDHQLERLYKRGFIKGLTVHHE
AFKDHALALPPPHTPPSQRV
DLSTPHVPLCCLSSFSVDDPQVTKLVTKATASTCSLEPMPTTLVKACLPTLCPIMVDIINMVPSSFKMAAVTPYSKSQV
>g2283.t5
MSSHELYEPLQSGFRAHHSTETTLIKITNDLLIAADSSHISILILLDVSGACDTVSHTILLNCLSGYLGLTGAALSWFQS
YLSDRKQFVSVRGSNSIPGP
VNQGMPQGSVLGPLLFTIYMLPLGQIIHHHGLSFHCYADDTQLYLSTKPSTQLPPQSLVNCLHSIKTWMSSSLLKLNKTE
LMVVASKALLQKSHIKSLTK

SAFYHLKNISRLRPSLSDSVAGTLIHAFITSRLDCCNGVLSEQHITPPPPRPSLASVMSCITYKILLLTYKSLQHLAPQY
TGLLIISRTIWRTFG
>g2284.t1
MGVYLSCNSSLKYFGEKWQVHNELLCLCSIELQMVQLTPRDKVAHNSPIFTLITLFNTTNYCGVIRKLLKFRPCLFLTIP
SRRLPGYHLSLLSPTTNFVS
PFLVSACLSLALFSWPCLSLTIPSRRTSGYHLNLLS
>g2284.t2
MGVYLSCNSSLKYFGEKWQVHNELLCLCSIELQMVQLTPRDKVAHNSPIFTLITLFNTTNYCGVIRKLLKAQQFRPCLFL
TIPSRRLPGYHLSLLSPTTN
FVSPFLVSACLSLALFSWPCLSLTIPSRRTSGYHLNLLS
>g2285.t1
MARWSFLSHHSECGEILNNRSEIPTPSAVLYQLHLKHIAKHLPELDPNSEILLLLGRDILRVHKDNDVTKRIIEYRMRVH
VFDASTRAIGAVAYLKTFQK
DGQIEVGLVMGKARLAPQSEPTIPRLELCAAVLDLIQDELDTKLDVIHFYSDNHLVLRTIFSEITTRRKTTNRGIAFLIH
IARSSKQMNKNSECSKWHRC
KHPRTPEELDQAMQVVLQASQRKLRQKFEEQHMGDLPSERLQRNWIKQCKLSCKHRKGSSGGSSKNNTWETYLPNACRVT
ACGKKLFLCPELGIVSITVE
MIVKFPKNVAKRQKINNKINNEQEGAEDRALGNTTGD
>g2285.t2
MARWSFLSHHSECGEILNNRSEIPTPSAVLYQLHLKHIAKHLPELDPNSEILLLLGRDILRVHKDNDVTKRIIEYRMRVH
VFGNSPSPAVAIYGLRRAIK
EGAQQYDASTRAIGAVAYLKTFQKDGQIEVGLVMGKARLAPQSEPTIPRLELCAAVLDLIQDELDTKLDVIHFYSDNHLV
LRTIFSEITTRRKTTNRGIA
FLIHIARSSKQMNKNSECSKWHRCKHPRTPEELDQAMQVVLQASQRKLRQKFEEQHMGDLPSERLQRNWIKQCKLSCKHR
KGSSGGSSKNNTWETYLPNA
CRVTACGKKLFLCPELGIVSITVEMIVKFPKNVAKRQKINNKINNEQEGAEDRALGNTTGD
>g2285.t3
MARWSFLSHHSECGEILNNRSEIPTPSAVLYQLHLKHIAKHLPELDPNSEILLLLGRDILRVHKVRQQDNDVTKRIIEYR
MRVHVFDASTRAIGAVAYLK
TFQKDGQIEVGLVMGKARLAPQSEPTIPRLELCAAVLDLIQDELDTKLDVIHFYSDNHLVLRTIFSEITTRRKTTNRGIA
FLIHIARSSKQMNKNSECSK
WHRCKHPRTPEELDQAMQVVLQASQRKLRQKFEEQHMGDLPSERLQRNWIKQCKLSCKHRKGSSGGSSKNNTWETYLPNA
CRVTACGKKLFLCPELGIVS
ITVEMIVKFPKNVAKRQKINNKINNEQEGAEDRALGNTTGD
>g2286.t1
LLQVPGLLPGQNRLFTSSSWSLHLQVAKLVTKAMVSMACSLDHMPTTLVKACIRTLCPIMVDIINSCLESGMVPSSFKMA
AVTPILKMPGLDPVDLTAFI
TSRLDYCNGVLFGVCSKALDWLQYVQNSAARVLTHTKPWPSPPPISTFTGFWSSPASPTRSYSSPINPSMPLFISAW
>g2287.t1
VSDSGEGYRVTAQTVLVCCWRSMKEVSMLLGELCQSLPLQYNDAGDAHNGLVTEEQLRTLEHVKCVHSKLVSSDSVGLAD
SHTGRQVNSAIKIGVNFLLF
AETTFRGGGYETTSPPCMYNGRNWNRSRPAGGACVAPGHGKAPLRDCNANASERELQVS
>g2287.t2
VSDSGEGYRVTAQTVLVCCWRSMKEVSMLLGELCQSLPLQYNDAGDAHNGLVTEEQLRTLEHVKCVHSKLVSSDSVGLAD
SHTGRQVNSAIKIGVNFLLF
AETTFREMAVYVPRAAHTARRAAPLHKSFAT
>g2287.t3
VSDSGEGYRVTAQTVLVCCWRSMKEVSMLLGELCQSLPLQYNDAGDAHNGLVTEEQLRTLEHVKCVHSKLVSSDSVGLAD
SHTGRQVNSAIKIGVNFLLF
AETTFRGGGYETTSPPCRPAGGACVAPGHGKAPLRDCNANASERELQVS
>g2287.t4
MAVYVPRAAHTARRAAPLHKSFAT
>g2288.t1
VRDHVWTIIMESVVPSDKGNYTCVVENQYGSINHTYQLDVVERSPHRPILQAGLPANRTAVVGSDVEFECKVFSDPQPHI
QWLKHIEVNGSRVGPDKLPY
VRVLK

>g2289.t1
PGEEDQGGGRGVVSRGPPQEHSCIHHHQLLLVSHHDLPWNHTPGSAYNLPVLQRWPLPPTAHGGGTCMAPTVGL
>g2290.t1
AILDVVINLQFSLIEKLWQTFWRYSPSDTVEGVTITENSGVSEIEERLPRTQLLALCATETVVKWMSTCDYLMYQAVVEI
LIPDVLRPIP
>g2291.t1
LVQLCHKVVDGLCGREPPRIVDYNDTWSLGEWLELTAFLSSLFRKTVGDNCCDEEVLAGLRDVGPGYGEAVLSVLKARRE
EIRQALLQRTHLTSSATLQD
FDWQLKLALSSDKISSLQTPLLNLSLYVRENGVLRPVAIEMSREELHVLISSLEAANK
>g2291.t2
MALCHKVVDGLCGREPPRIVDYNDTWSLGEWLELTAFLSSLFRKTVGDNCCDEEVLAGLRDVGPGYGEAVLSVLKARREE
IRQALLQRTHLTSSATLQDF
DWQLKLALSSDKISSLQTPLLNLSLYVRENGVLRPVAIEMSREELHVLISSLEAANK
>g2291.t3
LVQLCHKVVDGLCGREPPRIVDYNDTWSLGEWLELTAFLSSLFRKTVGDNCCDEEVLAGLRDVGPGYGEAVLSVLKARRE
EIRQALLQRTHLTSSATLQD
FDWQLKTPLLNLSLYVRENGVLRPVAIEMSREELHVLISSLEAANK
>g2292.t1
SQQSLSRTPGPSLVAHRLAAQLGSLWCADSAGDREGDAQPTRVASPSV
>g2293.t1
RAQSGGPGARAPTLPKSAPHIQLSPVKVALGQRLALELTLELPEGAKPTEDAPSFWRLSAEGNEWLLEGQSLAGGIADVS
APVSVSPRLPRTPPPGVDPD
LTPSLTLSVWVYFCAAAGGACMMKAASFCQPLHLLPPPPPPGEEKEGEEGEEEKAVTVAMTHALL
>g2293.t2
MRAQSGGPGARAPTLPKSAPHIQLSPVKVALGQRLALELTLELPEGAKPTEDAPSFWRLSAEGNEWLLEGQSLAGGIADV
SAPVSVSPRLPRTPPPGVDP
DLTPSLTLSVWVYFCAAAGGACMMKAASFCQPLHLLPPPPPPGEEKEGEEGEEEKAVTVAMTHALL
>g2294.t1
MWAGLGYYSRGKRLHEGAQKVVTELRGQMPRTVDSLMKQLPGVGRYTAGAIGSIALGE
>g2294.t2
MLQQTQVVTVIDYYNRWMKEVNKMWAGLGYYSRGKRLHEGAQKVVTELRGQMPRTVDSLMKQLPGVGRYTAGAIGSIALG
E
>g2294.t3
MLQQTQVVTVIDYYNRWMKVVTELRGQMPRTVDSLMKQLPGVGRYTAGAIGSIALGE
>g2295.t1
MATGRNDFLCRSVVHLSERSLLQKDVGRYRMKHLSLMKNVSPALQAPGPPGALHHRPIVCPFKTMGREELQKSHHRHTTN
PYKTFLTEA
>g2296.t1
MVTPSYLMEEDNRDDRSPPLLEATTSTAPVAVSQASEEDLSEPHLDTFFLSRTKNLSLSLVLLLGCYLAILIPAYSDKLA
PLDDDRQLPEDLALLNRSAD
LLLGPCQPKWCLHHLKALSCSAGLLDIPAFVVQGPEEEVEGKERPVPWELPPPLGLRGSEQQAAQALASLPQPGPPAWLQ
REGGCKRCVVVGSGGVLHGS
HLGSHIDQLNAAPVRGFQRDAGSRTTIRLAYPEAAPTSPDEYGDTSMVALVVFKSLDLDWLTSVITKRPLSFWSKLWFWR
GVVEEIPLNAEHFRILNPEI
VHQTGKALHSYTPNQGKLCDRVCLAGFGYDMRHPEARLHYYETLRMDAMKGQVVHDIGSEKLFLRDLVSSGAVTDLTGAL
>g2297.t1
MEEHEKRRAAQKEESGQMGPEGGEAEVNGGMIPANLLQEMLNLGRAENPVGRPRRKRPVKRTSPWASVLMPV
>g2298.t1
LGTVYTEGGLVEGKNIRLGFRHHMDVFKGIPFADIPGRFEKPKRHPGWDGVLKATEFAKRCLQVTLLQTDTRGSEDCLYL
NIWVPHRREISTDLPVMVWL
YGGGFLVGGSMGANFLNNYLYSGQEIADRGDVIVVTVGYRVGTLGFLSSGDASGPGNYGLWDQHAAIAWVHRNIRSFGGD
PDNITLFGESAGAASVSLQT
LSPHNKGLIKRAISQSGVALCPWAVNRNPRLVAEMVARKVNCPTDETMMSCLKMTDAVALTMAGNLAIKSSPESPLLNNL
LLAAAIDGDFLPEDPGHLFN
NTADIDYLAGINDMDGHLFTGMDVPSINRPLVDTKVEDMRALLGAYTKESGKAGQDNAFSQYTSGWGDKPSKETVKRTVV
DIGTDYIFLVPTQAALYLHA

SNARSGRTYSYLFSEPNRLGGGIKPYPSWMGADHTDDLQFVFGKPFSSPLGYWPSHRNLSGYMIAYWTNFAKTGDPNKGL
SVPATWPQFTAAGHQFLEIN
AKMGKNNVRQKMRLRFVHFWTSVLPTLKSVEMYE
>g2298.t2
LGTVYTEGGLVEGKNIRLGFRHHMDVFKGIPFADIPGRFEKPKRHPGWDGVLKATEFAKRCLQVTLLQTDTRGSEDCLYL
NIWVPHRREISTDLPVMVWL
YGGGFLVGGSMGANFLNNYLYSGQEIADRGDVIVVTVGYRVGTLGFLSSGDASGPGNYGLWDQHAAIAWVHRNIRSFGGD
PDNITLFGESAGAASVSLQT
LSPHNKGLIKRAISQSGVALCPWAVNRNPRLVAEMVARKVNCPTDETMMSCLKMTDAVALTMAGNLAIKSSPESPLLNNL
LLAAAIDGDFLPEDPGHLFN
NTADIDYLAGINDMDGHLFTGMDVPSINRPLVDTKVEDMRALLGAYTKESGKAGQDNAFSQYTSGWGDKPSKETVKRTVV
DIGTDYIFLVPTQAALYLHA
SNARSGRTYSYLFSEPNRLGGGIKPYPSWMGADHTDDLQFVFGKPFSSPLGYWPSHRNLSGYMIAYWTNFAKTGDPNKGL
SVPATWPQFTAAGHQFLEIN
AKMGKNNLGTVYTEGGMVEGKNIRLGFRHHMDVFKGIPFAGMPGRFEKPKRHPGWDGILKAYEYRPRCLQLNLIMTDTRG
SEDCLYLNIWVPHGRSVSTN
LPVMVWIYGGAFIWGGSMGANFLDNYLYSGQEIADRGNVIVVTLGYRLGTLGFLSTGDSSLPGNYGLWDQQAAIAWVHRN
IRSFGGDPDNVTVFGESAGS
ASVNFQGLFKRAISQSGVALCPWAVNKNPRSSEGPLVNNLVLAATVDGDFLPDEPANLFHNAADLDYILGVNDMDGHLFT
GLDVPTINSHIVDTPVEDLK
RLLGAYTKEKGLAGMENAFATYSSTWGPKPSQETIKKTVVEIGTDYIFLIPTQVALYLHANATKLGGGRTYSYLFSQPNR
MGGLVLPYPSWMGADHADDL
QYVFGKPFTTPLAYWPRHRDVSRYVIAYWTNFAKTGDPNKGLDVPVTWPLFKTDNHQYLEINSKMDRGYARQKLRMRYVH
FWGSILPNLPLIKYE
>g2298.t3
LGTVYTEGGLVEGKNIRLGFRHHMDVFKGIPFADIPGRFEKPKRHPGWDGVLKATEFAKRCLQVTLLQTDTRGSEDCLYL
NIWVPHRREISTDLPVMVWL
YGGGFLVGGSMGANFLNNYLYSGQEIADRGDVIVVTVGYRVGTLGFLSSGDASGPGNYGLWDQHAAIAWVHRNIRSFGGD
PDNITLFGESAGAASVSLQT
LSPHNKGLIKRAISQSGVALCPWAVNRNPRLVAEMVARKVNCPTDETMMSCLKMTDAVALTMAGNLAIKSSPESPLLNNL
LLAAAIDGDFLPEDPGHLFN
NTADIDYLAGINDMDGHLFTGMDVPSINRPLVDTKVEDMRALLGAYTKESGKAGQDNAFSQYTSGWGDKPSKETVKRTVV
DIGTDYIFLVPTQAALYLHA
SNARSGRTYSYLFSEPNRLGGGIKPYPSWMGADHTDDLQFVFGKPFSSPLGYWPSHRNLSGYMIAYWTNFAKTGDPNKGL
SVPATWPQFTAAGHQFLEIN
AKMGKNNLGTVYTEGGMVEGKNIRLGFRHHMDVFKGIPFAGMPGRFEKPKRHPGWDGILKAYEYRPRCLQLNLIMTDTRG
SEDCLYLNIWVPHGRSVSTN
LPVMVWIYGGAFIWGGSMGANFLDNYLYSGQEIADRGNVIVVTLGYRLGTLGFLSTGDSSLPGNYGLWDQQAAIAWVHRN
IRSFGGDPDNVTVFGESAGS
ASVNFQGLFKRAISQSGVALCPWAVNKNPRRFAEEVALKVNCPTNEKMAACPLVNNLVLAATVDGDFLPDEPANLFHNAA
DLDYILGVNDMDGHLFTGLD
VPTINSHIVDTPVEDLKRLLGAYTKEKGLAGMENAFATYSSTWGPKPSQETIKKTVVEIGTDYIFLIPTQVALYLHANAT
KLGGGRTYSYLFSQPNRMGG
LVLPYPSWMGADHADDLQYVFGKPFTTPLAYWPRHRDVSRYVIAYWTNFAKTGDPNKGLDVPVTWPLFKTDNHQYLEINS
KMDRGYARQKLRMRYVHFWG
SILPNLPLIKYE
>g2298.t4
MVKIGLLVAVGFFLEAASAASLGTVYTEGGMVEGKNIRLGFRHHMDVFKGIPFAGMPGRFEKPKRHPGWDGILKAYEYRP
RCLQLNLIMTDTRGSEDCLY
LNIWVPHGRSVSTNLPVMVWIYGGAFIWGGSMGANFLDNYLYSGQEIADRGNVIVVTLGYRLGTLGFLSTGDSSLPGNYG
LWDQQAAIAWVHRNIRSFGG
DPDNVTVFGESAGSASVNFQGLFKRAISQSGVALCPWAVNKNPRSSEGPLVNNLVLAATVDGDFLPDEPANLFHNAADLD
YILGVNDMDGHLFTGLDVPT
INSHIVDTPVEDLKRLLGAYTKEKGLAGMENAFATYSSTWGPKPSQETIKKTVVEIGTDYIFLIPTQVALYLHANATKLG
GGRTYSYLFSQPNRMGGLVL
PYPSWMGADHADDLQYVFGKPFTTPLAYWPRHRDVSRYVIAYWTNFAKTGDPNKGLDVPVTWPLFKTDNHQYLEINSKMD

RGYARQKLRMRYVHFWGSIL
PNLPLIKYE
>g2299.t1
LDVFKGIPFAAKPGTFEKPKPHPGWTRTLKATRFAKRCLQKSVLQTSTFGSDNCLHLNIWVPHGLHVSENLPVMVWFYGG
GFMVGGSMGANFLANYLYSG
QEIADRGNVIMVSAGYRLGTLGFLSSGDSELPGNYGLWDQHAAIAWVHRNIRSFGGDPDNVTIFGESAGAASVSYQVAVK
VGCPTDDKMVACLKSVSTKQ
LVMAAPLITKGSTDNPVLNTLVLSPVVDGDFVPDDPARLFHNAADIDYLAGVNNMDGHLFTSSDIPAIAKRQNISADDVR
MLLRSYAKDKDQAGLDAAFA
EYTSQWGPSPSQDQMKITAVDIGTDYIFLVPIQRAINLHAATAKSGRTYSYLLSEPSLLVGPGRPYHHWVGTDHADDLQY
VFGKPFTTPKAYGDAHRDLS
GYLISFWTNFARSGLFASAERSRYKASTAHLYWHTLGTPPASVALRH
>g2299.t2
LDVFKGIPFAAKPGTFEKPKPHPGWTRTLKATRFAKRCLQKSVLQTSTFGSDNCLHLNIWVPHGLHVSENLPVMVWFYGG
GFMVGGSMGANFLANYLYSG
QEIADRGNVIMVSAGYRLGTLGFLSSGDSELPGNYGLWDQHAAIAWVHRNIRSFGGDPDNVTIFGESAGAASVSYQVAVK
VGCPTDDKMVACLKSVSTKQ
LVMAAPLITKGSTDNPVLNTLVLSPVVDGDFVPDDPARLFHNAADIDYLAGVNNMDGHLFTSSDIPAIAKRQNISADDVR
MLLRSYAKDKDQAGLDAAFA
EYTSQWGPSPSQDQMKITAVDIGTDYIFLVPIQRAINLHAATAKSGRTYSYLLSEPSLLVGPGRPYHHWVGTDHADDLQY
VFGKPFTTPKAYGDAHRDLS
GYLISFWTNFARSGDPNVGQGKVPATWPQFTSTEQKYLEINSKMDGTYVGQKMRTRFVHLWTDTLQNIPSVKY
>g2299.t3
MEPFWILLAVGLFLGSTSAASLGVVETEGGRVQGTNIRLGDLRSLDVFKGIPFAAKPGTFEKPKPHPGWTRTLKATRFAK
RCLQKSVLQTSTFGSDNCLH
LNIWVPHGLHVSENLPVMVWFYGGGFMVGGSMGANFLANYLYSGQEIADRGNVIMVSAGYRLGTLGFLSSGDSELPGNYG
LWDQHAAIAWVHRNIRSFGG
DPDNVTIFGESAGAASVSYQVAVKVGCPTDDKMVACLKSVSTKQLVMAAPLITKGSTDNPVLNTLVLSPVVDGDFVPDDP
ARLFHNAADIDYLAGVNNMD
GHLFTSSDIPAIAKRQNISADDVRMLLRSYAKDKDQAGLDAAFAEYTSQWGPSPSQDQMKITAVDIGTDYIFLVPIQRAI
NLHAATAKSGRTYSYLLSEP
SLLVGPGRPYHHWVGTDHADDLQYVFGKPFTTPKAYGDAHRDLSGYLISFWTNFARSGLFASAERSRYKASTAHLYWHTL
GTPPASVALRH
>g2300.t1
MKRVQGEPKVGLREAKVLYKKKIERKMQRNSIREVWDAMKTITGSKRSNGTVDGQTALLLQPVSQLPSHLPPPPKPTIPH
PPLHITAEQVLGQLRGLRPG
KPVGPDKVCPRLLKVCATELREPLQYVFNLSLRLGKVPTPWKTSCLIPVPKKTHPSKLNDFRPVALTSHIDEDYGVTAAE
DPETSGPPRTGPATYQEKVG
MEDAITYLLHRTHSHLDKGKGAVRIMFFDFSSAFNTIQPIRLRGKLLQMRVDAHLVSWITDYMTGRLQFPETAVSSQSIE
GDNVEVVTTYNIPRTTLGQ
>g2301.t1
MSSVEEAESEDSGEASSISLAEVSEAVKKLLSGKAPGVEEIRPEMLKALDIVGLSWLARLFSVAWTVPVEWQTRWWFILG
IILLSLPGKVYSRVLERRLQ
PIVEPQIQKEQCGFRPGRGTVDQLFTLAVVLRRISRRSLGEESVRFGNLRIASLLFAHDVVLLASSDLQYALGRFAAECG
SVGMRVSISKSEAMVLCRKM
HSNSMPITEPAFLTSLSSHPESLFLVLPPQHTTA
>g2302.t1
LAVWCEGNNLLLNTSKTRELIVDFRKKNAVPHLPIHINGVTVERVTSFKFLGIHISQDLSWTTNCSNLIKKAHQRLFFLR
ILRKNHLSSDILVNFYRCTI
ESILTNCITV
>g2302.t2
LLLNTSKTRELIVDFRKKNAVPHLPIHINGVTVERVTSFKFLGIHISQDLSWTTNCSNLIKKAHQRLFFLRILRKNHLSS
DILVNFYRCTIESILTNCIT
V
>g2303.t1
MDCSLMDTSQMSNLLNQARLKVLKARDDMIVGFYQLLEPKVTVRCRQQDVEMVQAAIDKDIAIYKDTVKSNIVVQIDQKR

FLSSDM
>g2303.t2
MALSDADVQKQIKHMMAFIEQEANEKAEEIDAKAEEEFNIEKGRLVQTQRVKIMEFYEKKEKQIEQHKKIQMSNLLNQAR
LKVLKARDDMIVGFYQLLEP
KVTVRCRQQDVEMVQAAIDKDIAIYKDTVKSNIVVQIDQKRFLSSDM
>g2303.t3
MALSDADVQKQIKHMMAFIEQEANEKAEEIDAKAEEEFNIEKGRLVQTQRVKIMEFYEKKEKQIEQHKKM
>g2303.t4
MALSDADVQKQIKHMMAFIEQEANEKAEEIDAKAEEEFNIEKGRLVQTQRVKIMEFYEKKEKQIEQHKKIQMSNLLNQAR
LKVLKARDDMIVDLLNEARQ
RLADIARDPARYSTLLEGLILQGFYQLLEPKVTVRCRQQDVEMVQAAIDKDIAIYKDTVKSNIVVQIDQKRFLSSDM
>g2304.t1
MRLETEVEHYSCEQCDPRPLDREVPMLPQPSYAQSGSVYFICLLRDELLLHQGDCVYLMRDSRRTPEGQPIRQSYRLLSH
VNRDKLDIFRIEKLWKNEK
>g2304.t2
HFDCMRLETEVEHYSCEQCDPRPLDREVPMLPQPSYAQSGSVYFICLLRDELLLHQGDCVYLMRDSRRTPEGQPIRQSYR
LLSHVNRDKLDIFRIEKLWK
NEK
>g2304.t3
VWQHFDCMRLETEVEHYSCEQCDPRPLDREVPMLPQPSYAQSGSVYFICLLRDELLLHQGDCVYLMRDSRRTPEGQPIRQ
SYRLLSHVNRDKLDIFRIEK
LWKNEK
>g2305.t1
MQKSVRYNEGHALYLSVVARKEGSKRGVLSKKTTENSRWSDKFFALYQNLLFYFENDQSTRPSGIYLLEGCTCERSPAPK
ASSSVGKEPPDKLQQASYGL
SRSGHQS
>g2306.t1
MCKGLATLPATCLKSAKDIKHKIGFLLQKPESHTVSQKQTNKETKRAVSAVEVERWKTSFTSLMNSEDGRKVFTGFLRSE
FSEENMEFLNACKEFKQLST
AKMAAKGKLIFAQYVEEDSPKQVNLDAATREQTRQNLEASPSASSFDEAQQLIQVLLEKDSYRRFLRSRAMQDLSPTSAG
HSTGTRQQKTAGA
>g2306.t2
MCKGLATLPATCLKSAKDIKHKIGFLLQKPESHTVSQKQTNKETKRAVSAVEVERWKTSFTSLMNSEDGRKVFTGFLRSE
FSEENMEFLNACKEFKQLST
AKMAAKGKLIFAQYVEEDSPKQVTGVVNVNLDAATREQTRQNLEASPSASSFDEAQQLIQVLLEKDSYRRFLRSRAMQDL
SPTSAGHSTGTRQQKTAGA
>g2307.t1
MKVPLGDWAGSLCPGKGGTEIPCRRMRSGSYVKAMGDMEDSEDSEGSPKPSPKSAARRQSYLKATHLSLSEQPAPLLPRK
PRGYPLMREDFLWCPLQSAC
SMQQLSSLPCLKEQSTNRSLDNLDCLVGPAEPPPCHWNSDFKHSFGTLGRGIGAQVCGQVCGRTVPYYEGESQAVDALDL
PAATCFRSRSHSYLRAIQAG
CSQDDDTASVDSDSPPPTSMGCAYSSHPSEYTIRNTSGIKSSSCVQLFVGEAE
>g2307.t2
MKVPLGDWAGSLCPGKGGTEIPCRRMRSGSYVKAMGDMEDSEDSEGSPKPSPKSAARRQSYLKATHLSLSEQPAPLLPRK
PRGYPLMREDFLWCPLQSAC
SMQQLRSVLSRRPSSLPCLKEQSTNRSLDNLDCLVGPAEPPPCHWNSDFKHSFGTLGRGIGAQVCGQVCGRTVPYYEGES
QAVDALDLPAATCFRSRSHS
YLRAIQAGCSQDDDTASVDSDSPPPTSMGCAYSSHPSEYTIRNTSGIKSSSCVQLFVGEAE
>g2307.t3
MKVPLGDWAGSLCPGKGGTEIPCRRMRSGSYVKAMGDMEDSEDSEGSPKPSPKSAARRQSYLKATHLSLSEQPAPLLPRN
SLPCLKEQSTNRSLDNLDCL
VGPAEPPPCHWNSDFKHSFGTLGRGIGAQVCGQVCGRTVPYYEGESQAVDALDLPAATCFRSRSHSYLRAIQAGCSQDDD
TASVDSDSPPPTSMGCAYSS
HPSEYTIRNTSGIKSSSCVQLFVGEAE
>g2308.t1
MSKQRSESPGRIRHLMHSVQKLFAKSQSLEGSAMKGSLNGRAAGGAADGGDEGCRVSRRSKSKDRAKTEGSKQRPRPNTI

ALWSSDDTLDSEAPGPMAVG
YRNPLSMMSLGRAVSDSQAPLRHPVPQGYNTIAASHTLKTSKSSGDLKFLACPGAMQPEGKAEEGRGRPGKEGTLVKRGS
WSTLTLTQARQVLQKGSASV
NKALLKSKSCHQDLAQQFLQVGF
>g2309.t1
MHPCMDGEECKVLPDQKGWSCSTGNKVKTTK
>g2310.t1
MPGISTPRPPPLPAARLALHPTPMSIPAGAEDCQQMFLHPVNVRCLLREYGSLEASPDSITARVVETEGHTVTEEVRRRH
RYLSHLPLTCEFSICELALQ
PPILSKETMDTFADDLEKRKRLRQKKARDEKRRERRIEMEENRKQGKYPEVHIGLENLQQFPAFGSPPKKSSPVLQVDFT
LAPPSPLCSSPVSDGMMFPC
LNGQSASPTVGSVEDDSHCLSFAQMLRDGKARGDPGAKVILKKDMQLAPPVPDSEGESDGSDRVPVPSFQNSFSQAFEEA
LQQMDHGPNSAAQHVVIPG
>g2310.t2
MGQSPATRRSPASSPPRSGSSRGPRSPQSGRAEDCQQMFLHPVNVRCLLREYGSLEASPDSITARVVETEGHTVTEEVRR
RHRYLSHLPLTCEFSICELA
LQPPILSKETMDTFADDLEKRKRLRQKKARDEKRRERRIEMEENRKQGKYPEVHIGLENLQQFPAFGSPPKKSSPVLQVD
FTLAPPSPLCSSPVSDGMMF
PCLNGQSASPTVGSVEDDSHCLSFAQMLRDGKARGDPGAKVILKKDMQLAPPVPDSEGESDGSDRVPVPSFQNSFSQAFE
EALQQMDHGPNSAAQHVVIP
G
>g2310.t3
MPGISTPRPPPLPAARLALHPTPMSIPAGAEDCQQMFLHPVNVRCLLREYGSLEASPDSITARVVETEGHTVTEEVRRRH
RYLSHLPLTCEFSICELALQ
PPILSKETMDTFADDLEKRKRLRQKKARDEKRRERRIEMEENRKQGKYPEVHIGLENLQQFPAFGSPPKKSSPVLQVDFT
LAPPSPLCSSPVSDGMMFPC
LNGQSASPTVGSVEDDSHCLSFAQMLRDGKARGDPGAKVILKKVPDSEGESDGSDRVPVPSFQNSFSQAFEEALQQMDHG
PNSAAQHVVIPG
>g2311.t1
MESITTEAQRTKSAQQQRQTSSAEQEEDIRTRTKRVLVLAQGVRQVRVSSSRKTKVFQVAPTVYIQHHPVSLKHPPCLEL
VIPEDHHHHDA
>g2312.t1
MSPHPIWVSLLLGLLLGFLPMVGTEPEGGAQQLPHPPATPESPPSSSSSSSSSSSASSSSGSNCSDLTVKLEFSTRVVE
HGEQVLGPALESRRPFEGFG
FSRSPRRGCSWRTAARVMFA
>g2313.t1
FDPNPLRRHTFWTLSVGGTFTWLGIYGVNQSVIQRCISCKTKTQARLALYLNLLGLLVILCCAVFCGLIMFSFYTDCDPW
TAGRVSAPDQLMPYFVMEIL
GEFPGLPGLFVACAFSGTLSTVAASINALATVTYEDFVKHCCTNLSKTVTAWTIKGLCVLFGVACTAMAVVASYMGGIVQ
CSSSAGRAKCFSIIIFLTTF
FIFFFFIFLICYLYVLLFLFFAALSIHGMCGGPMLGMFSLGILFPCANSKGAVAGFIAGIAVAFWAGIGSFIYPAPPINS
RPLPLITAGCPWLNSSVGPA
TEAPYLTSLPPRPPLADSWYSLSYLHLSAVGFTVTLTVGLVITSLTGNVHLCRSGAISQLSAAQTPGVNGCCLKAL
>g2313.t2
LSRQSSIVPCSFDPNPLRRHTFWTLSVGGTFTWLGIYGVNQSVIQRCISCKTKTQARLALYLNLLGLLVILCCAVFCGLI
MFSFYTDCDPWTAGRVSAPD
QLMPYFVMEILGEFPGLPGLFVACAFSGTLSTVAASINALATVTYEDFVKHCCTNLSKTVTAWTIKGLCVLFGVACTAMA
VVASYMGGIVQCSSSAGRAK
CFSIIIFLTTFFIFFFFIFLICYLYVLLFLFFAALSIHGMCGGPMLGMFSLGILFPCANSKGAVAGFIAGIAVAFWAGIG
SFIYPAPPINSRPLPLITAG
CPWLNSSVGPATEAPYLTSLPPRPPLADSWYSLSYLHLSAVGFTVTLTVGLVITSLTGNVHLCRSGAISQLSAAQTPGVN
GCCLKAL
>g2313.t3
TPSLSRQSSIVPCSFDPNPLRRHTFWTLSVGGTFTWLGIYGVNQSVIQRCISCKTKTQARLALYLNLLGLLVILCCAVFC
GLIMFSFYTDCDPWTAGRVS
APDQLMPYFVMEILGEFPGLPGLFVACAFSGTLSTVAASINALATVTYEDFVKHCCTNLSKTVTAWTIKGLCVLFGVACT

AMAVVASYMGGIVQCSSSAG
RAKCFSIIIFLTTFFIFFFFIFLICYLYVLLFLFFAALSIHGMCGGPMLGMFSLGILFPCANSKGAVAGFIAGIAVAFWA
GIGSFIYPAPPINSRPLPLI
TAGCPWLNSSVGPATEAPYLTSLPPRPPLADSWYSLSYLHLSAVGFTVTLTVGLVITSLTGNVHLCRSGAISQLSAAQTP
GVNGCCLKAL
>g2314.t1
GNQDGKFSVGFRDGVVRTVVGLDRETQAAYTLVIEAI
>g2315.t1
LGPQECILSGIMSVKGKKVLHMDRNSYYGGESASITPLEDLYKRFNIPGTPPDTMGKGRDWNVDLIPKFLMANGQLVRML
LITEVTRYLDFKVVEGSYVY
KKGSIYKVPSTETEALASSLMGLFEKRRFRKFLAFVSNFDLNDPKTMEGVDPHKTTMKDVYKKFDLGQDVIDFTGHSLGL
YRTDEYVYLDEPCLDTINRI
KLYSESLARYGKSPYLYPLYGLGELPQGFARLSAIYGGIYMLNKPIEDIIMENGKVVGVKSEGEIARCKQLICDPSYMME
RANKVGQVIRVICIMNHPIK
NTADVNSCQIIIPQNQVNRKHDIYVCMVSCAHNVAGKGKYVAIVSTTVETNDAEKELKPALDLLEPIEQKFVSTSDLLAP
TDMGTDSQIFISRSYDATTH
FETTCDDIKDIFKRMTGTDFDFGDIN
>g2315.t2
LGPQECILSGIMSVKGKKVLHMDRNSYYGGESASITPLEDVSLYKRFNIPGTPPDTMGKGRDWNVDLIPKFLMANGQLVR
MLLITEVTRYLDFKVVEGSY
VYKKGSIYKVPSTETEALASSLMGLFEKRRFRKFLAFVSNFDLNDPKTMEGVDPHKTTMKDVYKKFDLGQDVIDFTGHSL
GLYRTDEYVYLDEPCLDTIN
RIKLYSESLARYGKSPYLYPLYGLGELPQGFARLSAIYGGIYMLNKPIEDIIMENGKVVGVKSEGEIARCKQLICDPSYM
MERANKVGQVIRVICIMNHP
IKNTADVNSCQIIIPQNQVNRKHDIYVCMVSCAHNVAGKGKYVAIVSTTVETNDAEKELKPALDLLEPIEQKFVSTSDLL
APTDMGTDSQIFISRSYDAT
THFETTCDDIKDIFKRMTGTDFDFGDIN
>g2315.t3
MGLFEKRRFRKFLAFVSNFDLNDPKTMEGVDPHKTTMKDVYKKFDLGQDVIDFTGHSLGLYRTDEYVYLDEPCLDTINRI
KLYSESLARYGKSPYLYPLY
GLGELPQGFARLSAIYGGIYMLNKPIEDIIMENGKVVGVKSEGEIARCKQLICDPSYMMERANKVGQVIRVICIMNHPIK
NTADVNSCQIIIPQNQVNRK
HDIYVCMVSCAHNVAGKGKYVAIVSTTVETNDAEKELKPALDLLEPIEQKFVSTSDLLAPTDMGTDSQIFISRSYDATTH
FETTCDDIKDIFKRMTGTDF
DFGDIN
>g2315.t4
LGPQECILSGIMSVKGKKVLHMDRNSYYGGESASITPLEDLYKRFNIPGTPPDTMGKGRDWNVDLIPKFLMANGQLVRML
LITEVTRYLDFKVVEGSYVY
KKGSIYKVPSTETEALASSKSLLVCAAML
>g2316.t1
MKRARSVVKCLVFEDAPNGVKAGLAAGMQVVMIPDANLDRGLTQDATLLLASMEDFLPQLFGLPAYD
>g2316.t2
MQDGYCVLELRLMKRARSVVKCLVFEDAPNGVKAGLAAGMQVVMIPDANLDRGLTQDATLLLASMEDFLPQLFGLPAYD
>g2317.t1
MEAALVSWIVDYLSGRPQFVRIQNCVSDRVVSNTGAPQGTVLSPFLFTLYTSDFNYCTESCHLQKFSDDSAIVGCIKQ
>g2318.t1
IQGRDNEIERLNRALSGGRPHDVISLEAQNVSNEKMIAHLNLQIEYLQAANSTLEQKVEAAQQRKQDASSEAADLSVKNQ
ELCQELSHIDHLAKQLEMDK
ELVLQTADQELQEARDQSEGDYEKNRLRDQLVEAQQQRDQLEGLGSFVEQEKARLQEKVEKMTANDRELVLELEALRTKH
GVCRKERSPSRLDAFVRSLE
EERDHYRQEAEHYKRARRPEGPGLS
>g2318.t2
IQGRDNEIERLNRALSGGRPHDVISLEAQNVSNEKMIAHLNLQIEYLQAANSTLEQKVEAAQQRKQDASSEAADLSVKNQ
ELCQELSHIDHLAKQLEMDK
ELVLQTADQELQEARVEIRRQQKTIEDLEDLIQTMRRDQSEGDYEKNRLRDQLVEAQQQRDQLEGLGSFVEQEKARLQEK
VEKMTANDRELVLELEALRT

KHGVCRKERSPSRLDAFVRSLEEERDHYRQEAEHYKRARRPEGPGLS
>g2319.t1
MNGTARQVTRIMKKAEEERTEEETCALLRHQDTVEELCRRQVRSSVLKRKQEEVCDESEELRTKVSQLAETVRQAQHLVV
YTGAGISTVKHVVSQNCDGL
HVRSGLPRHALSELHGNMFIEVCTACVPAREYVRVFDVTERTSLRRHATGRRCGHCGGELRDTIVHFGERGTLEQPLNWR
GAAAAAEKADVILCLGSSLK
VLKKYACLWCMNRPAKRRPKLYIVNLQWTPKDDLAALKINGKCDDVMNLLMEELDIQIPLYNKAEDPIFNLATPLRLEEE
HSHSRALIAAPAGQEDCLPE
AKGPATGKPVQGGWFGRGYSKARKKKKAI
>g2319.t2
MKKAEEERTEEETCALLRHQDTVEELCRRQVRSSVLKRKQEEVCDESEELRTKVSQLAETVRQAQHLVVYTGAGISTVKH
VVSQNCDGLHVRSGLPRHAL
SELHGNMFIEVCTACVPAREYVRVFDVTERTSLRRHATGRRCGHCGGELRDTIVHFGERGTLEQPLNWRGAAAAAEKADV
ILCLGSSLKVLKKYACLWCM
NRPAKRRPKLYIVNLQWTPKDDLAALKINGKCDDVMNLLMEELDIQIPLYNKAEDPIFNLATPLRLEEEHSHSRALIAAP
AGQEDCLPEAKGPATGKPVQ
GGWFGRGYSKARKKKKAI
>g2319.t3
MNGTARQVTRIMKKAEEERTEEETCALLRHQDTVEELCRRQVRSSVLKRKQEEVCDESEELRTKVSQLAETVRQAQHLVV
YTGAGISTVKHVVSQNCDGL
HVRSGLPRHALSELHGNMFIEVGVKAESHGYWHILDIVVLLEARMDDNDMPEVCTACVPAREYVRVFDVTERTSLRRHAT
GRRCGHCGGELRDTIVHFGE
RGTLEQPLNWRGAAAAAEKADVILCLGSSLKVLKKYACLWCMNRPAKRRPKLYIVNLQWTPKDDLAALKINGKCDDVMNL
LMEELDIQIPLYNKAEDPIF
NLATPLRLEEEHSHSRALIAAPAGQEDCLPEAKGPATGKPVQGGWFGRGYSKARKKKKAI
>g2320.t1
MFLFKYTDGITTITITATNNSVTLPTPHHHHHHHPRVQHRLYTITPLQSPTSSHQHHHRHHLNSNHQHLGFQRHDRDRHH
TSTTTTNTLASKNITTHYSN
TPTIITTTTTKNVNTTSATATLTSSTYGGTGACSQHLCPTWGVNTIFGRCVNVVTM
>g2321.t1
MQLHRSVSDCTSGSQSAASFCCPPYENRLLATTRTELSAALGMYSSPYAAAAAASQNYANYFPYSSEPSALYSTLNPQYD
IKDGTGTLHSGITQTAAYYP
YDHSLGQYQYDRYGTVDFNSTARRKNATRETTSTLKTWLYEHRKNPYPTKGEKIMLAIITKMTLTQVSTWFANARRRLKK
ENKMTWSPKNKGGEDRKDDL
EKSDLDCVTK
>g2322.t1
MLFFLSPGIILVQVFDATVDNARVVLQIDNARLAADDFRVKFESELTIRQSVEADIVGLRKLIDDTNMGRMNLESEIEAL
KEELIHLKKNHDNDVMELRS
QISQSGVQVDVDAPKGQDLAQIMAEIRAKYEKMALKSQEELKAWHESQISVVQTEVSQNTEALKGAQVEINELRRTLQTL
EIELESQRSLKLSLEGTLRD
TEMRFNMEIEALNNIIRGLEAELTQLRGNIQNQTQEYEALLNMKMKLEAEIATYRRLLDGGDFKLQDALEDQKVTKKTKV
MTVTQHMVDGKIVSSSTETK
NL
>g2322.t2
VFDATVDNARVVLQIDNARLAADDFRVKFESELTIRQSVEADIVGLRKLIDDTNMGRMNLESEIEALKEELIHLKKNHDN
DVMELRSQISQSGVQVDVDA
PKGQDLAQIMAEIRAKYEKMALKSQEELKAWHESQISVVQTEVSQNTEALKGAQVEINELRRTLQTLEIELESQRSLKLS
LEGTLRDTEMRFNMEIEALN
NIIRGLEAELTQLRGNIQNQTQEYEALLNMKMKLEAEIATYRRLLDGGDFKLQDALEDQKVTKKTKVMTVTQHMVDGKIV
SSSTETKNL
>g2323.t1
MENVMKRNRAHHLVQSQPAGETLRYAYSLSGGARRGPGIRMSRSTSYSGGLDSSIGGSHGYLSLASNAGSFTVGNEKATM
QHLNDRLASYLDTVRSLEEA
KGKLEVQISEFMLKRGPSVEQDYSKYNATITDLRSKIFDKIKENAGLSIAVDNARLATDDFHVKLDYETGMRKAVEADIL
SIRKMLDDTNVIRFHLESNI
ESLKEELITMTKDHRADMAELRAQITQSEVQVDVDAPKGQDLAKIMEEMRAKYERIALKNEEELKAWHESQMMVVQVQVT

ENTTALKDANTLLSDTRRRY
QAMEIELQSVLNLKASLEANLRDVEGRYNMEIERYNEILLRLQDELAQIRSDTRHQSSEYEILLNIKMKLEAEIAEYRRL
LDGEPYHQVEEAKKTVQTQV
VTVTQTLVDGKVVSESKDVKASEKVVSA
>g2324.t1
PLVLGTLWDVTDRDIDRFTAALLESWLAAGPGASLLDHMGPSRQATHLKHLIGAAPVVYGLPVHLK
>g2325.t1
MYVPGSLNDVDHVLVDVGTGYYVEKNVDESKAFFKRKIDFLTKQIEKIQPALQEKHGMKQGSSCGLNPNPNPNPNLSGAH
RQGNTEFCAWEQKRTAVDAT
DHGGMVTTKGVGLNPNAKVWQEIPAPQNGLPESPEDAPWLQTSFSPGEGPEGYSEVAFSEDKGYSTEYSDTSDGVPVLAV
EGVANGMEQPDFGYPLFEPA
AGTTADGELSKEEPLSEEGLRESLRKQLEFCFSRENLCKDLYLMSQMDSDQFVPIWTLACMEDIKVLTTDKELILDVLKA
SPLVEVDGPGEKARPNHKRC
IIILREVPDTTPVQEVEALFKSDSCPKVISVEFAHNSNWYITFQSDTDAQQAYKYLREEVKMFQGKPIMARIKAISTFFA
NNGYRSVDSSVFSQHSQQPF
SSPVYMPQVYSPQQQYPVYPLVTPSWNQSATPYFETPLAPFPNGGFVNSYNSSAPYKGPVAGGRIFNRSRLNVKSHPRPG
TLPSLLPGLSREAGSAPTSP
QPPQTPGAAGGPSQELAVMPGLHIRDLANGDLGLSGRGRRGGARGMRRRREDDRSLRPPPLIEAKVPPPKFDLASSSFPP
LPGCVAPSVAPGAQEESVPE
TRLSDVVRGLRAPPKADAKEEKRESRPPKGPVGVVSCPTPVPAAV
>g2325.t2
MYVPGSLNDVDHVLVDVGTGYYVEKNVDESKAFFKRKIDFLTKQIEKIQPALQEKHGMKQGSSCGLNPNPNPNPNLSGAH
RQGNTEFCAWEQKRTAVDAT
DHGGMVTTKGVGLNPNAKVWQEIPAPQNGLPESPEDAPWLQTSFSPGEGPEGYSEVAFSEDKGYSTEYSDTSDGVPVLAV
EGVANGMEQPDFGYPLFEPA
AGTTADGELSKEEPLSEEGLRESLRKQLEFCFSRENLCKDLYLMSQMDSDQFVPIWTLACMEDIKVLTTDKELILDVLKA
SPLVEVDGPGEKARPNHKRC
IIILREVPDTTPVQEVEALFKSDSCPKVISVEFAHNSNWYITFQSDTDAQQAYKYLREEVKMFQGKPIMARIKAISTFFA
NNGYRSVDSSVFSQHSQQPF
SSPVYMPQVYSPQQQYPVYPLVTPSWNQSATPYFETPLAPFPNGGFVNSYNSSAPYKGPVAGGRIFNRSRLNVKSHPRPG
TLPSLLPGLSREAGSAPTSP
QPPQTPGAAGGPSQELAVMPGLHIRDLANGDLGLSGRGRRGGARGMRRRREDDRSLRPPPLIEAKVPPPKFDLASSSFPP
LPGCVAPSVAPGAQEESVPE
TRLSDVVRGLRAPPK
>g2325.t3
MYVPGSLNDVDHVLVDVGTGYYVEKNVDESKAFFKRKIDFLTKQIEKIQPALQEKHGMKQVLKHFHAAQGALRCCQGRGA
VGGASAAAAYQQGGCKGQAV
RVAARRRQFKALYPWLCCSTRAVVIGSSCGLNPNPNPNPNLSGAHRQGNTEFCAWEQKRTAVDATDHGGMVTTKGVGLNP
NAKVWQEIPAPQNGLPESPE
DAPWLQTSFSPGEGPEGYSEVAFSEDKGYSTEYSDTSDGVPVLAVEGVANGMEQPDFGYPLFEPAAGTTADGELSKEEPL
SEEGLRESLRKQLEFCFSRE
NLCKDLYLMSQMDSDQFVPIWTLACMEDIKVLTTDKELILDVLKASPLVEVDGPGEKARPNHKRCIIILREVPDTTPVQE
VEALFKSDSCPKVISVEFAH
NSNWYITFQSDTDAQQAYKYLREEVKMFQGKPIMARIKAISTFFANNGYRSVDSSVFSQHSQQPFSSPVYMPQVYSPQQQ
YPVYPLVTPSWNQSATPYFE
TPLAPFPNGGFVNSYNSSAPYKGPVAGGRIFNRSRLNVKSHPRPGTLPSLLPGLSREAGSAPTSPQPPQTPGAAGGPSQE
LAVMPGLHIRDLANGDLGLS
GRGRRGGARGMRRRREDDRSLRPPPLIEAKVPPPKFDLASSSFPPLPGCVAPSVAPGAQEESVPETRLSDVVRGLRAPPK
ADAKEEKRESRPPKGPVGVV
SCPTPVPAAV
>g2326.t1
MAARHAGAGPVRPSTNGGPPSDAQHDDMANTEELRLIATYSLRRHPGANGPARCGNDTPAPPPPPADEAVKKKKKKKKGK
SFKSCLGQIFPCVCAPKQDD
AAVGEQASFRHGGGGEEDDDAEEKEREAQTNALAATLMELADESFLPPEIEEDSEDDELEKMAGRLLRQCGDSVDEEYNC
QLVVAQLVGSYSMFSRFMGI
VFRMIGIQTSQPGDQGEKDTKQNIAVTCEVASRLVAMDTQPSRVLDLGARYLRENYPVEAALNEAAEDSDSEDEVD

>g2326.t2
MAARHAGAGPVRPSTNGGPPSDAQHDDMANTEELRLIATYSLRRHPGANGPARCGNDTPAPPPPPADEAVKKKKKKKKGK
SFKSCLGGEEDDDAEEKERE
AQTNALAATLMELADESFLPPEIEEDSEDDELEKMAGRLLRQCGDSVDEEYNCQLVVAQLVGSYSMFSRFMGIVFRMIGI
QTSQPGDQGEKDTKQNIAVT
CEVASRLVAMDTQPSRVLDLGARYLRENYPVEAALNEAAEDSDSEDEVD
>g2327.t1
MDLKDYYLLAALLACLWLDPSIAQELIYPIREELQENVLIGNIPKDLNISHTNAATGASANLVYRLVSKAGDNPLVRVLS
STGEIFTTSNRIDRERLCPG
PSFEDSECSFEIEVVILPNDYFRLIKIKMVVKDTNDNAPMFPSPVINISIPENTLINSRFAIPSATDPDTGPNSVHKYEL
VNGQSAFGLDIVETPEGEKW
PQLIVQQNLDREQKDTYVMKVKVEDGGSPQKSSTAILQVTVTDVNDNRPVFKDSQVEVHIPENSPIGTSVVHLLATDADI
GSNADIRYVFGTQVTPATKR
LFALNTTSGLISVQRPLDREETAIHKLSVLASDGSSSPAKATININVTDVNDNPPNIDLRYIIVPINGTVFLSEKDPINT
KIALITVSDKDTDINGKVIC
FIEKDVPFHLKAVYDNQYLLETSALLDYEGTKEYNFKIVASDSGKPSLNQTALVRVRLEDENDNPPVFTQPVIELAIMEN
NKRDLFLTTLSATDEDNGKN
AEIVYQLGPNASFFDLDRKTGVLTASRVFDREDQDRFLFTVTARDNGTPPLQTQAAVIVTILDENDNNPKFTHNHFQFFV
SENLPKYSTVGVITVTDTDA
GENAAVSLSILNDNENFILDPYSGVIKSNVSFDREQQSSYTFDVRAVDAGRPPCSSAAKVTINVIDVNDNTPVVISPSSN
TSFRLVPLSSIPGSVVAEVF
AVDGDTGMNAELKYSIVSGNNRGLFRIDPVTGNITLEEKPTVTDIGLHRLVVNISDLGYPKSLHTLVLVFLYVNDTAGNA
TLINELIRRTMETPIDRNIG
ESNETYQSGDYLTIMIAFVTGALVVVIVIFVTVLLRCRHASRFKAAQRKKQGAEWMSPNTENKQNKKKKRKKRKSPKSSL
LNFVTIEESKPDDPAHEPIN
GTISIPTELEEQGIGRFDWNATPTTTFKPSSPDLARHYKSASPQSAFHLKADTPVSVKKHHVIQELPLDNTFVGGCDTLS
KRSSTSSDHFSASECSSQGA
FKTKAPMHTRQQVKEHFYWSISSAYNCPVPQC
>g2328.t1
MIYLILISAISGAIVTLIVEFLLVYRRSPEPVARTVQYVKVVPDPALKDYFNSQHVETGQLQPETTASKQQEAVSARHQD
AAVPTTSTTAGPRQQQSSPP
PHSEPLDPSKPETCNFLNAIFLFLFRELRDTPVVRHWLTKKIKVEFEELLQTKTAGRLLEGLSLRDLSLGNSLPVFNTAR
LMKPVQVNADGMPEELNFEV
DLEYNGGFHLAIDVELVFGKSAYLFVKMTRVMGRLRLQFTRTPFSHWSFSFLEDPLIDFEVQSQFEGRPLPQLTSIIVNQ
LKRVIKKKHTLPNYKIRYV
>g2329.t1
NAYDITELKRPLCSSLSQSSSCTTAPLIKSYHGSQEEVHPSGSSCSSGAPYLSIPNHHHHHHQHHHQHHASNANYVADAN
DANYAIGPQCANHGTPINVD
GETSEAEGFADTVLARSHSQVDFKNYVARIVWEADNDGDAYPLDAFHVWCVEGSGSPVGSLSSLGSAASRNGPGDQGDEA
GHGHDRLSRWGPKFQALSDM
YDRPQLELSYRDAVSYMHRSHSQDLLPHPH
>g2330.t1
VVVVLQQLFTLIQNILSKWLDDSEVVEAVCGLFEKSVRTLLHGFGPMVPQLSTMLGQIYCTYPQSSALDLTRQMELLPRF
KDIPPVGAVVLREGKLLVET
LLQAVGGAAPCSLGDHFSEVLFSMNRNCLSLLALWLEESLQDPTFPSSLVSVQHKDAFRQRLLREQTNKRQVKEVVMEFS
LLCRGLQGGGYSTQ
>g2330.t2
VVVVLQQLFTLIQNILSKWLDDSEVVEAVCGLFEKSVRTLLHGFGPMVPQLSTMLGQIYCTYPQSSALDLTRQIFKRKPD
LYLSEHLDVSSLFYCVVLRE
GKLLVETLLQAVGGAAPCSLGDHFSEVLFSMNRNCLSLLALWLEESLQDPTFPSSLVSVQHKDAFRQRLLREQTNKRQVK
EVVMEFSLLCRGLQGGGYST
Q
>g2330.t3
VVVVLQQLFTLIQNILSKWLDDSEVVEAVCGLFEKSVRTLLHGFGPMVPQLSTMLGQIYCTYPQSSALDLTRQIFKRKPD
LYLSEHLDVSSLFYCGVMSL
KFPEAGTVRSASLFFMELLPRFKDIPPVGAVVLREGKLLVETLLQAVGGAAPCSLGDHFSEVLFSMNRNCLSLLALWLEE

SLQDPTFPSSLVSVQHKDAF
RQRLLREQTNKRQVKEVVMEFSLLCRGLQGGGYSTQ
>g2331.t1
LSSGSRDRVILQRDVRNPPSAERRLQGHRQEVCGLKWSPDHQHLASGGNDNKLLVWNSASLLPMQQYSDHLAAVKAIAWS
PHQHGLLASGGGTADRCLRF
WNTLTGQALQSTDTGSQVCNLAWSKHANELVSTHGYSQNQILVWKYPSLTQVAKLTGHSYRVLYLAVSPDGEAIVTGAGD
ETLRFWNVFSKTRCTKESKS
VLNLFTRIR
>g2331.t2
LSSGSRDRVILQRDVRNPPSAERRLQGHRQEVCGLKWSPDHQHLASGGNDNKLLVWNSASLLPMQQYSDHLAAVKAIAWS
PHQHGLLASGGGTADRCLRF
WNTLTGQALQSTDTGSQVCNLAWSKHANELVSTHGYSQNQILVWKYPSLTQVAKLTGHSYRVLYLAVSPDGEAIVTGAGD
ETLRFWNVFSKTRCTKVLTD
TRLQHCLPLFVHVILLESKSVLNLFTRIR
>g2332.t1
LQPHTFASEEAKVANTITHLTSRAHLWGMAPEWEADGLQLDGLIELTSQVNWRLQARCRERRLGQLERRSPIRTRPSETT
VPSTCQHMREAEPMQLLPAP
SMDTSWGCSPTTRPLLILLSGNHHETIQFHILRSPHLPLNLGFPWLCRHNPNIDWSTGSILGWSPSCHQRRPWRSTSTSH
QRQASSVRRPLRLADGIFFI
EKKDKTLWPCIDYLGLNDIMVKNHYLFPLIASAFELLEGATVLRVPGNALWPLQCPAVFQVLVSDVLRDMLDKHIFVYLD
DILIFSKTKEEHVHHVQAVL
QRLLENSLFIKAGKCEFHSRSPSWGTENASKPKKTISTRLN
>g2332.t2
LGHHYHHTIAAVVLLSLFSRLSSSTALLCAESRTDRNRPHTFASEEAKVANTITHLTSRAHLWGMAPEWEADGLQLDGLI
ELTSQVNWRLQARCRERRLG
QLERRSPIRTRPSETTVPSTCQHMREAEPMQLLPAPSMDTSWGCSPTTRPLLILLSGNHHETIQFHILRSPHLPLNLGFP
WLCRHNPNIDWSTGSILGWS
PSCHQRRPWRSTSTSHQRQASSVRRPLRLADGIFFIEKKDKTLWPCIDYLGLNDIMVKNHYLFPLIASAFELLEGATVLR
VPGNALWPLQCPAVFQVLVS
DVLRDMLDKHIFVYLDDILIFSKTKEEHVHHVQAVLQRLLENSLFIKAGKCEFHSRSPSWGTENASKPKKTISTRLN
>g2332.t3
LGHHYHHTIAAVVLLSLFSRLSSSTALPHTFASEEAKVANTITHLTSRAHLWGMAPEWEADGLQLDGLIELTSQVNWRLQ
ARCRERRLGQLERRSPIRTR
PSETTVPSTCQHMREAEPMQLLPAPSMDTSWGCSPTTRPLLILLSGNHHETIQFHILRSPHLPLNLGFPWLCRHNPNIDW
STGSILGWSPSCHQRRPWRS
TSTSHQRQASSVRRPLRLADGIFFIEKKDKTLWPCIDYLGLNDIMVKNHYLFPLIASAFELLEGATVLRVPGNALWPLQC
PAVFQVLVSDVLRDMLDKHI
FVYLDDILIFSKTKEEHVHHVQAVLQRLLENSLFIKAGKCEFHSRSPSWGTENASKPKKTISTRLN
>g2333.t1
MRVSTSKAEAMVLCRKTVDCCLRVGSELLPQVKEFKYLRILFTNEGWLGSATLRRKLISAACIRDLVLSVTIYLIWPGNA
SGSPRRSWKALLGRRTSGVP
CSACCHRDPAPDKRTFNDDDDDDFVINFYLSLVMDRSSSQAAPGPRVYCFSTFFLPKLRGGEGGQAGGHWAVKRWTKAVD
LFLHDLILVPLHLDIHWAMA
VIDLRSQTVKSYDSMGRRHDDICSLLL
>g2333.t2
MRVSTSKAEAMVLCRKTVDCCLRVGSELLPQVKEFKYLRILFTNEGWLGSATLRRKLISAACIRDLVLSVTIYLIWPGNA
SGSPRRSWKALLGRRTSGVP
CSACCHRDPAPDKRTFNDDDDDDFVINFYLSLVMDRSSSQAAPGPRVYCFSTFFLPKLRGGEGGQAGGHWAVKRWTKAVD
LFLHDLILVPLHLDIHWAMA
VIDLRSQTVKSYDSMGRRHDDICSLLLLFLRDEHKVKKGKDLDTSRWTVGSLRASCHMPLFRKVMVWEILNQKLL
>g2333.t3
MRVSTSKAEAMVLCRKTVDCCLRVGSELLPQVKEFKYLRILFTNEGWLGSATLRRKLISAACIRDLVLSVTIYLIWPGNA
SGSPRRSWKALLGRRTSGVP
CSACCHRDPAPDKRTFNDDDDDDFVINFYLSLVMDRSSSQAAPGPRVYCFSTFFLPKLRGGEGGQAGGHWAVKRWTKAVD
LFLHDLILVPLHLDIHWAMA
VIDLRSQTVKSYDSMGRRHDDICSLLLLHSALPFPLPLPLAALQSGLESEGYCS

>g2334.t1
MVSISSRTLTLDNELFRDSGSPYSLSLGEGTGSDGGSSVSCYSPETGELGAMLSSSPGEQEEEDEEEQDMEDDRSSLHIF
PGAGGEEELQAAGPEAGLPQ
FPLRLPSPFSPTLEDIEEFLREKMELVREGLLGPMEDPSPASVCSSSSSSSEDSPSVSEHLSPVLPPPHAAPAAAACRDP
ASTS
>g2335.t1
MLRLRALSAASMGLVLMSRRYHSGPVRFQGRRRLMLAALAGVTGISASTGLLWTRAYADAGPSVRRTEGAEPHGEEFPKD
GESQVEAEKSVEASGGSSGE
EDGAEVEAGSEGKKKKVRSGFRDRKVMEYENRIRAYSTPDKIFRYFATLKVISEHGDAEVYMTPQDFVRSITPNEKQPES
EYQPS
>g2335.t2
MTLVSHFTRRMLRLRALSAASMGLVLMSRRYHSGPVRFQGRRRLMLAALAGVTGISASTGLLWTRAYADAGPSVRRTEGA
EPHGEEFPKDGESQVEAEKS
VEASGGSSGEEDGAEVEAGSEGKKKKVRSGFRDRKVMEYENRIRAYSTPDKIFRYFATLKVISEHGDAEVYMTPQDFVRS
ITPNEKQPESEYQPS
>g2335.t3
MLRLRALSAASMGLVLMSRRYHSGPVRFQGRRRLMLAALAGVTGISASTGLLWTRAYADAGPSVRRTEGAEPHGEEFPKD
GESQVEAEKSVEASGGSSGE
EDGAEVEAGSEGKKKKVRSGFRDRKVMEYENRIRAYSTPDKIFRYFATLKVISEHGDAEVYMTPQDFVRSITPNEKQPE
>g2336.t1
LASLIWRQRCHICESASYESVSVLSTMGCFIAPSLEASKGDPEILRSCDGETPSTTSAGRLPATVPPLLRDKRSIDLHTF
PAISPSESSLARRTAAGGAR
QTEGRREGGGRAAGLD
>g2336.t2
LYRSLASLIWRQRCHICESASYESVSVLSTMGCFIAPSLEASKGDPEILRSCDGETPSTTSAGRLPATVPPLLRDKRSID
LHTFPAISPSESSLARRTAA
GGARQTEGRREGGGRAAGLD
>g2337.t1
MAYSGSNHEHTDLFFSSWDLDSYRQSRYVLCKAIRTAKWSYKDKLESYYKDEFNIELEESNTFPAARLQAPDGIPGRVLQ
ACADQLAEVFADIFNLSLNL
SEIPTCFKSFTIVPVLRPSSVCIPPNPSDDTISMALHTAHLDRRNTYVQILFVNYSSAFNTIVPGKLIIKGCVLNPLLYS
LYTSSSNAIVKFADDTTIVG
LITDGDETADREEVNSLAVWCQDNNLSLNVSKTKELIVDHRKLQRGSHRHSPVDITT
>g2337.t2
MAYSGSNHEHTDLFFSSWDLDSYRQSRYVLCKAIRTAKWSYKDKLESYYKDEFNIELEESNTFPAARLPGCPLMLSVADL
AEVFADIFNLSLNLSEIPTC
FKSFTIVPVLRPSSVCIPPNPSDDTISMALHTAHLDRRNTYVQILFVNYSSAFNTIVPGKLIIKGCVLNPLLYSLYTSSS
NAIVKFADDTTIVGLITDGD
ETADREEVNSLAVWCQDNNLSLNVSKTKELIVDHRKLQRGSHRHSPVDITT
>g2337.t3
MAYSGSNHEHTDLFFSSWDLDSYRQSRYVLCKAIRTAKWSYKDKLESYYKDEFNIELEESNTFPAARLQAPDGIPGRVLQ
ACADQLAEVFADIFNLSLNL
SEIPTCFKSFTIVPVLRPSSVCIPPNPSDDTISMALHTAHLDRRNTYVQILFVNYSSAFNTIVPGKLIIKVND
>g2338.t1
MGLAPHLGTLKAKLATLDPRLATLHPKLATLHPKLATLHPKLATLHHKLATLHHKLATKEVSVSTAVTQVVVQPNLQSIP
GHTVCPYCQQGVITNTKPET
GLLAWAVCGGLAIFG
>g2338.t2
MGLAPHLGTLKAKLATLDPRLATLHPKLATLHPKLATLHPKLATLHHKLATLHHKLATKEVSGNPAAQQVTVTQVVVQPN
LQSIPGHTVCPYCQQGVITN
TKPETGLLAWAVCGGLAIFG
>g2339.t1
MDTSEERPCELLISLYKHVYGPLGATEIFLRIERQQLCTMFIITTQITVPPGIATTSCSLSCAAFHVRGPLLSGRSSPGA
KHWLKCHGRVKDAGSSRSTV
TVMEDVESLSPPPKFV
>g2340.t1

MQNTSSMGRYTPKRLAALSNLGIQYEVPEVLGEPALQLEPHWGLSCNNSGSRGWALRGVPFKVHSGYSYCSYAGWGSRRR
GARARNVACHVAWLTVL
>g2340.t2
MLGMQNTSSMGRYTPKRLAALSNLGIQYEVPEVLGEPALQLEPHWGLSCNNSGSRGWALRGVPFKVHSGYSYCSYAGWGS
RRRGARARNVACHVAWLTVL
>g2340.t3
MGRYTPKRLAALSNLGIQYEVPEVLGEPALQLEPHWGLSCNNSGSRGWALRGVPFKVHSGYSYCSYAGWGSRRRGARARN
VACHVAWLTVL
>g2341.t1
MSIHSDSDLEEELPVYDFLQPRRCGSQTDRAADLVELDSSDTEVASPSSLSAAEFTAADADAVCPGDAVMVVSSDSEEEE
AYVPLALRLKQRQDPLVGTV
GNAKDSHRRPPCHPPWPNQQQQQQLPVQTVAPAAEERSPPTLWPPPTTLTASTASTAVLPKPCLAKSTVAGQSLKTSGGE
VKQRVRGDKASRKMELERQR
AERKALLEAAKALRPEECIKHMVVVVDPGLVQQEGGGALLTSLQELGCSCAIEKQPLPRSVSWLRRAPVGQLGEDISVPE
AHVVIQMTADDFVSLVHNHT
QVGETSALNARADYELIPPLSGTQEQRHGRTGHGPGLSCWVRELQTRNPERILSLAVVDLDKHY
>g2341.t2
MSIHSDSDLEEELPVYDFLQPRRCGSQTDRAADLVELDSSDTEVASPSSLSAAEFTAADADAVCPGDAVMVVSSDSEEEE
AYVPLALRLKQRQDPLVGTV
GNAKDSHRRPPCHPPWPNQQQQQQLPVQTVAPAAEERSPPTLWPPPTTLTASTASTAVLPKPCLAKSTVAGQSLKTSGGE
VKQRVRGDKASRKMELERQR
AERKALLEAAKALRPEECIKHMVVVVDPGLVQQEGGGALLTSLQELGCSCAIEKQPLPRSVSWLRRAPVGQLGEDISVPE
AHVVIQMTADDFVSLVHNHT
QEQRHGRTGHGPGLSCWVRELQTRNPERILSLAVVDLDKHY
>g2341.t3
MSIHSDSDLEEELPVYDFLQPRRCGSQTDRAADLVELDSSDTEVASPSSLSAAEFTAADADAVCPGDAVMVVSSDSEEEE
AYVPLALRLKQRQDPLVGTV
GNAKDSHRRPPCHPPWPNQQQQQQLPVQTVAPAAEERSPPTLWPPPTTLTASTASTAVLPKPCLAKSTVAGQSLKTSGGE
VKQRVRGDKASRKMELERQR
AERKALLEAAKALRPEECIKHMVVVVDPGLVQQEGGGALLTSLQELGCSCAIEKQPLPRSVSWLRRAPLGEDISVPEAHV
VIQMTADDFVSLVHNHTQVG
ETSALNARADYELIPPLSGTQEQRHGRTGHGPGLSCWVRELQTRNPERILSLAVVDLDKHY
>g2342.t1
MAALMSLVLRSRTGNFVHAGNILATQRDLRYHPGAHVGMGTNNTLYALEDGEVHFTKEVYVPPPRSLEATAVICKLPKDV
VLYKTFINVLPTPQEGKFKL
VDMV
>g2342.t2
LGGKSPGRRYGFKKMDGNFVHAGNILATQRDLRYHPGAHVGMGTNNTLYALEDGEVHFTKEVYVPPPRSLEATAVICKLP
KDVVLYKTFINVLPTPQEGK
FKLVDMV
>g2343.t1
MHLANITAGTRWLTLRFMTTVPLTGTPLSTIRGPFVIGKPSDYHLPAAAPRLPSYESVRRKDRRTLVHSMISERFGLRSS
ADCDWEPPPPSYAESLHQPL
DVPSLGLRGLDDEEAFPGQRDQDGHSANADRSATSDHPGAQCLTGQGRDV
>g2344.t1
MPTHSTLSGKRATPAPAAIDTQGDGRSFQEIRQDCLQKKGSTSCHTTLQASILVLCPILTRTSTAGFWLFENVWSSTKMG
GDCWLLAATASLTLHKANLA
RVVPHDQEFDHTYAGIFHFQFWQHNKWLDVVVDDRLPTVRNRLVMLHSASNNEFWSALLEKAYAKLHGSYESLKGGSTME
AMEDFTGGVGEMYDTKSAPN
NLFGIMKKALDRGSMLGCSIDISSSAESEAQTATGLVKGHAYSITGLEEVNYRGRKVQLIRIRNPWGTVEWNGPWSDGSR
EWSQVDQADKDRILKKSDDG
EFWMEFEDFKTNYDKVEICNLTPDSLEDNTPQHWEVNWFEGNWIRGATAGGCRNFIDTFWTNPQFKLMLEDTDDDDDVCS
VVIALMQKNRRKLRKEGMDM
ETIGFAVYEDFFRYNPSKARSRTYINVREVSERFTLPPGKYLLVPTTFQPHHEADFLIRVFSEKKATALEMGSDIDADLP
DPPMPSSPDEETEEEKGLRR
LFDQLAGSDHAISARELQQMLNGVLSRSELKCSSCALRAGSSGVSRRTVENKVDNTGMLEFQEFKVFWEKMKKWIMLFLS

FDTDRSGKMSSYELRIALKA
AGMQLNNKILQLLGLRFADENFDIDFDDYLTCIVRLENMFRTFQALDKNKSGKVNMNIMQPHKKKKSFIQKKAAVTFHLV
HRSQRDPLAADEKAPQHVLL
PVSKVEAEKRQEEQRNFGVFFDDDYDYLQHLKESVPTTEWVASDSSRSARPPVNLRGEGQQQQQQEEDDDDDEEEKEKDP
KSRAIP
>g2344.t2
MPTHSTLSGKRATPAPAAIDTQGDGRSFQEIRQDCLQKKGSTSCHTTLQASILVLCPILTRTSTAGFWLFENVWSSTKMG
GDCWLLAATASLTLHKANLA
RVVPHDQEFDHTYAGIFHFQFWQHNKWLDVVVDDRLPTVRNRLVMLHSASNNEFWSALLEKAYAKLHGSYESLKGGSTME
AMEDFTGGVGEMYDTKSAPN
NLFGIMKKALDRGSMLGCSIDISSSAESEAQTATGLVKGHAYSITGLEEVNYRGRKVQLIRIRNPWGTVEWNGPWSDGSR
EWSQVDQADKDRILKKSDDG
EFWMEFEDFKTNYDKVEICNLTPDSLEDNTPQHWEVNWFEGNWIRGATAGGCRNFIDTFWTNPQFKLMLEDTDDDDDVCS
VVIALMQKNRRKLRKEGMDM
ETIGFAVYEDFFRYNPSKARSRTYINVREVSERFTLPPGKYLLVPTTFQPHHEADFLIRVFSEKKATALEMGSDIDADLP
DPPMPSSPDEETEEEKGLRR
LFDQLAGSDHAISARELQQMLNGVLSRRKEVKFDGLTLNTCHSIINLMDVSLSTVLSDSPVXRLQVDNTGMLEFQEFKVF
WEKMKKWIMLFLSFDTDRSG
KMSSYELRIALKAAGMQLNNKILQLLGLRFADENFDIDFDDYLTCIVRLENMFRTFQALDKNKSGKVNMNIMQPHKKKKS
FIQKKAAVTFHLVHRSQRDP
LAADEKAPQHVLLPVSKVEAEKRQEEQRNFGVFFDDDYDYLQHLKESVPTTEWVASDSSRSARPPVNLRGEGQQQQQQEE
DDDDDEEEKEKDPKSRAIP
>g2344.t3
MPTHSTLSGKRATPAPAAIDTQGDGRSFQEIRQDCLQKKELCDRPRFIVGDANRTDICQGQLGDCWLLAATASLTLHKAN
LARVVPHDQEFDHTYAGIFH
FQFWQHNKWLDVVVDDRLPTVRNRLVMLHSASNNEFWSALLEKAYAKLHGSYESLKGGSTMEAMEDFTGGVGEMYDTKSA
PNNLFGIMKKALDRGSMLGC
SIDISSSAESEAQTATGLVKGHAYSITGLEEVNYRGRKVQLIRIRNPWGTVEWNGPWSDGSREWSQVDQADKDRILKKSD
DGEFWMEFEDFKTNYDKVEI
CNLTPDSLEDNTPQHWEVNWFEGNWIRGATAGGCRNFIDTFWTNPQFKLMLEDTDDDDDVCSVVIALMQKNRRKLRKEGM
DMETIGFAVYEDFFRYNPSK
ARSRTYINVREVSERFTLPPGKYLLVPTTFQPHHEADFLIRVFSEKKATALEMGSDIDADLPDPPMPSSPDEETEEEKGL
RRLFDQLAGSDHAISARELQ
QMLNGVLSRSELKCSSCALRAGSSGVSRRTVENKVDNTGMLEFQEFKVFWEKMKKWIMLFLSFDTDRSGKMSSYELRIAL
KAAGMQLNNKILQLLGLRFA
DENFDIDFDDYLTCIVRLENMFRTFQALDKNKSGKVNMNIMQPHKKKKSFIQKKAAVTFHLVHRSQRDPLAADEKAPQHV
LLPVSKVEAEKRQEEQRNFG
VFFDDDYDYLQHLKESVPTTEWVASDSSRSARPPVNLRGEGQQQQQQEEDDDDDEEEKEKDPKSRAIP
>g2345.t1
LDALQDCIKKVREGPPSLTTVCFYAFHHTERLLNTAEVAADSRLLAAGFDSSAVKLWSLRARKLKARPHRGDVAHVHLAC
DLLEQEEEEEEGSGSEIQTL
RGHSGPVFRTSFLTDSSGLLSCSEDTSIRYWDLGSFTNTALYRGHAYPVWDVDVSPCSLYFASGSHDRTARLWTFSRTYP
LRLYAGHLADVDCVKFHPNS
NYLATGSTDKTVRLWSTQQGASVRLFTGHRGPVLALAFSPDGKYLASAGEDQRVKLWDLASGGLVKDLRGHTDGVSGLSF
SPDSSLLAXXXXXXXXGQLGA
RVGRGAGVPRRDARGRVVGGAGGAVLRGHRQRAQCPVHGLQPPAGHRHRAGEGRTVALWVSTTRDSPVGLLGVVLPLSTA
SQG
>g2345.t2
LDALQDCIKKVREGPPSLTTVCFYAFHHTERLLNTAEVAADSRLLAAGFDSSAVKLWSLRARKLKARPHRGDVAHVHLAC
DLLEQEEEEEEGSGSEIQTL
RGHSGPVFRTSFLTDSSGLLSCSEDTSIRYWDLGSFTNTALYRGHAYPVWDVDVSPCSLYFASGSHDRTARLWTFSRTYP
LRLYAGHLADVDCVKFHPNS
NYLATGSTDKTVRLWSTQQGASVRLFTGHRGPVLALAFSPDGKYLASAGEDQRVKLWDLASGGLVKDLRGHTDGVSGLSF
SPDSSLLAXXXXXXXXGQLGA
RVGRGAGVPRRDARGRVVGGAGGAVLRGHRQRAQCPVHGLQPPAGHRHRAGEGRTVALWQGGSSSEVIILNLLQEVQGLY
I

>g2345.t3
GVEVPAGVLPQNEAALDALQDCIKKVREGPPSLTTVCFYAFHHTERLLNTAEVAADSRLLAAGFDSSAVKLWSLRARKLK
ARPHRGDVAHVHLACDLLEQ
EEEEEEGSGSEIQTLRGHSGPVFRTSFLTDSSGLLSCSEDTSIRYWDLGSFTNTALYRGHAYPVWDVDVSPCSLYFASGS
HDRTARLWTFSRTYPLRLYA
GHLADVDCVKFHPNSNYLATGSTDKTVRLWSTQQGASVRLFTGHRGPVLALAFSPDGKYLASAGEDQRVKLWDLASGGLV
KDLRGHTDGVSGLSFSPDSS
LLAXXXXXXXGQLGARVGRGAGVPRRDARGRVVGGAGGAVLRGHRQRAQCPVHGLQPPAGHRHRAGEGRTVALWVSTTRD
SPVGLLGVVLPLSTASQG
>g2346.t1
VGITLFTVSHRKSLWKHHKYYLHMDGRGNYEFKPITEETVEFGS
>g2347.t1
MGGINFALLICCLSSCWPPCQPLGQVPLRFAAFGDWGGIPVPPYASAHQEAIAAEADWLARTAGLDFVLSLGDHFYFTGY
TFENVFSHKSLLAVPWHLVG
GNHDHRGNISAQMAYSNISRRWNYPALYYELLFTVPHTNVSVTVLMIDTVVLCGNTYDQDQPRGPENLRAAKTQWEWMTS
RLANSK
>g2348.t1
RSGVKSTGLATKPAGISAISCSPE
>g2349.t1
LERLQPQGAESAGPPPAIVERPSADRGVGVGENRRQRRKREAAAAQRRRQGSLWLIQVVGKDPLKL
>g2350.t1
MVVLKLSAVLLAYALVSIQTPSLHAAPSRAAPETESDRVSLVKHSAQKLLDAIVQEFMHTTSAEQPQHAAKGNSVTAEKR
ACNTATCVTHRLADFLSRSG
GTGNRNFVLTDVGAQAFGRRKRHSAL
>g2351.t1
MLPVDSRALWSLSYGQASFAASCLVIVSLVLLLVRQLVKQRRPPGFPPGPSPIPVIGNIFSLATEPHVFLKKQSEVHGQI
FSLDLGGILTVVLNGYDCVR
ECLYHQSEVFADRPSLPLFKKMTKMGGLINHKYGRGWIEHRKLACNSFRYFGSGQRLFERKISEECMFFVDAIDEHKGKP
FNPKHLVTNAVSNVTNLIIF
GQRFTYDDHNFQHMIEIFSENVELAASGWAFLYNAFPWLEYVPFGKHQKLFRNAAEVYRFLQQAIEGFSLGRVPQAPRHY
VDAYLDELEQSAGDPGTSYS
RENLIYSVGELIIAGTETTTNTLRWAMLYMALYPDIQERVHREIDGVLVSGRAASLEDKQRMPYVEAVLHEVLRFCNIVP
LGIFRATSQDANVNGYTIPK
GTMVITNLYSVHFDEKYWSDPGVFSPQRFLDGNGNFVRREAFLPFSLGRRQCLGEQLARMEMFLFFTTMLQRFHLQFPPG
TVPTLTPKLGMTLQPMPYSI
CAVRRQQTRLSSPDTGRQVYSAACQPIKVYTPVSCTEKKLNGSDA
>g2351.t2
MLPVDSRALWSLSYGQASFAASCLVIVSLVLLLVRQLVKQRRPPGFPPGPSPIPVIGNIFSLATEPHVFLKKQSEVHGQI
FSLDLGGILTVVLNGYDCVR
ECLYHQSEVFADRPSLPLFKKMTKMGGLINHKYGRGWIEHRKLACNSFRYFGSGQRLFERKISEECMFFVDAIDEHKGKP
FNPKHLVTNAVSNVTNLIIF
GQRFTYDDHNFQHMIEIFSENVELAASGWAFLYNAFPWLEYVPFGKHQKLFRNAAEVYRFLQQAIEGFSLGRVPQAPRHY
VDAYLDELEQSAGDPGTSYS
RENLIYSVGELIIAGTETTTNTLRWAMLYMALYPDIQERVHREIDGVLVSGRAASLEDKQRMPYVEAVLHEVLRFCNIVP
LGIFRATSQDANVNGYTIPK
GTMVITNLYSVHFDEKYWSDPGVFSPQRFLDGNGNFVRREAFLPFSLGEFTSSAAQTISNS
>g2351.t3
MLPVDSRALWSLSYGQASFAASCLVIVSLVLLLVRQLVKQRRPPGFPPGPSPIPVIGNIFSLATEPHVFLKKQSEVHGQS
EVFADRPSLPLFKKMTKMGG
LINHKYGRGWIEHRKLACNSFRYFGSGQRLFERKISEECMFFVDAIDEHKGKPFNPKHLVTNAVSNVTNLIIFGQRFTYD
DHNFQHMIEIFSENVELAAS
GWAFLYNAFPWLEYVPFGKHQKLFRNAAEVYRFLQQAIEGFSLGRVPQAPRHYVDAYLDELEQSAGDPGTSYSRENLIYS
VGELIIAGTETTTNTLRWAM
LYMALYPDIQERVHREIDGVLVSGRAASLEDKQRMPYVEAVLHEVLRFCNIVPLGIFRATSQDANVNGYTIPKGTMVITN
LYSVHFDEKYWSDPGVFSPQ
RFLDGNGNFVRREAFLPFSLGRRQCLGEQLARMEMFLFFTTMLQRFHLQFPPGTVPTLTPKLGMTLQPMPYSICAVRRQQ

TRLSSPDTGRQVYSAACQPI
KVYTPVSCTEKKLNGSDA
>g2352.t1
MSTTSLDKELQERLREASAIGDIDEVRSLVESGVNVNSQNEINGWTCLHWACKRNHKHIVSYLLNSGADMEILTAKDELA
SQLTSKPEIKKLLGVEVEDL
PEVKETELPIIPNYLSNPPFMYSKLDSKVEVLVAQLQKQNSSGDHEQEDTASSISPTHQPQPPTLSPLADTPNPRFVPSS
SPNATPVPLSQPGPTTGAFI
PLAEQNGVSPGLHNNNHHHHQYSAVNGVL
>g2352.t2
MSTTSLDKELQERLREASAIGDIDEVRTCLHWACKRNHKHIVSYLLNSGADMEILTAKDELASQLTSKPEIKKLLGVEVE
DLPEVKETELPIIPNYLSNP
PFMYSKLDSKVEVLVAQLQKQNSSGDHEQEDTASSISPTHQPQPPTLSPLADTPNPRFVPSSSPNATPVPLSQPGPTTGA
FIPLAEQNGVSPGLHNNNHH
HHQYSAVNGVL
>g2353.t1
MLSAAQLLDELMGRDRNLAPDEKRCNVCRYYLCGFCPAELFTNTRSDLGPCEKIHDENLRKTYEKSSRFMKEGYERDFLR
YLQSLLAEVERRIRRGHARL
ALSQAQQNAGGPGPAGKNDEKSQVLTEKIEDLVLQIEELGSEGRVEEAQGMMKLVEQLKEEREMLSANPSTIESFAAQEK
QMEVCEVCGAFLIVGDAQSR
VDDHLMGKQHMGYAKIKSTVEELKEKLRRRSEDPAEDASPVKKDREAWEREREEREKRRKEEEEKEKEKEREREKEREKE
REREKEKERERDR
>g2353.t2
MLSAAQLLDELMGRDRNLAPDEKRCNVRWDHETVCRYYLCGFCPAELFTNTRSDLGPCEKIHDENLRKTYEKSSRFMKEG
YERDFLRYLQSLLAEVERRI
RRGHARLALSQAQQNAGGPGPAGKNDEKSQVLTEKIEDLVLQIEELGSEGRVEEAQGMMKLVEQLKEEREMLSANPSTIE
SFAAQEKQMEVCEVCGAFLI
VGDAQSRVDDHLMGKQHMGYAKIKSTVEELKEKLRRRSEDPAEDASPVKKDREAWEREREEREKRRKEEEEKEKEKERER
EKEREKEREREKEKERERDR
>g2353.t3
LLDELMGRDRNLAPDEKRCNVCRYYLCGFCPAELFTNTRSDLGPCEKIHDENLRKTYEKSSRFMKEGYERDFLRYLQSLL
AEVERRIRRGHARLALSQAQ
QNAGGPGPAGKNDEKSQVLTEKIEDLVLQIEELGSEGRVEEAQGMMKLVEQLKEEREMLSANPSTIESFAAQEKQMEVCE
VCGAFLIVGDAQSRVDDHLM
GKQHMGYAKIKSTVEELKEKLRRRSEDPAEDASPVKKDREAWEREREEREKRRKEEEEKEKEKEREREKEREKEREREKE
KERERDR
>g2354.t1
MLTFWFLPFLQQLLNLDLRDQNSALLPLSLAMKTPGYIGQPPCGSARLSSSSSPSSLSQHSEFSDAFLPSSHWCQNMPDS
PQPAQQPTSSQWGRSRFWAE
RSLSMVETTSNGSSSSAGGPLGWPYLGAARSASLSTSPTLPSSSSASSLPSAASSSSGSSSSRYKTELCRSFAESSVCKY
GNKCQFAHGLDELRDLNRHP
KYKTEPCRTFHTIGFCPYGIRCHFVHNNEDDLEQPRPRSASSSSSSSSSIPCGLSQPASSRPGRPPLLRQSFSFAGFPST
PHPPLQQALPTLPPPLPPLS
LGSFLRAPGASPPSCADITELLSHALLEMDSAFEASAAQHHHHHPPQQAPSAGPASSSDPRSHFLPSPDSGCSLNGFSPA
ASPSLRQSPGAAAGGFPGPL
GARSLSSTSLSEPEHDGGGSSASSLSGSDGGDGNARRLAVFSQLSVPEDMAGYAL
>g2355.t1
MSEVVFNKALDGYEDDFEKDLDWLISEESRSEDQGPDYDDIEAEIDKELEESEKEGSTKEERANQKAKEKRRRAAARGAG
DDKEEEEEEEDDDDDEERWPS
PMEPLEYDSDRNDSPARGSPTRVSPALAPPPAVAADEQTDEEKKYILDRIQQANRELRDQEAPDATRRRRLHFKEKLVDL
VVPPLEYPHNRDDS
>g2355.t2
MSEVVFNKALDGYEDDFEKDLDWLISEESRSEDQVVFSFVQSLSFCGVLFQGPDYDDIEAEIDKELEESEKEGSTKEERA
NQKAKEKRRRAAARGAGDDK
EEEEEEEDDDDDEERWPSPMEPLEYDSDRNDSPARGSPTRVSPALAPPPAVAADEQTDEEKKYILDRIQQANRELRDQEAP
DATRRRRLHFKEKLVDLVVP
PLEYPHNRDDS

>g2356.t1
MSQGAAGRSPAGRTRRGALVLAGAVAVAGLVANAGYLQRAEMASRVIQVAEPEDDILEKCKGFMSPPVTDIQVLQERKGE
MCTKMEMLIMQTQAEFCRAL
EKVDGGKFKADRWQRKEGKLRTLGRGGGISCVIQDSNVFEKAGVNVSVVFGHLTEEAAKQMKSRGKVLKGKDGKLPFCAM
GVSSVIHPKNPHIPTVHFNY
RYFEIEEDDGSKQWWFGGGTDLTPTYLNKEDAFLFHNTLKEACDKHHPQYYPDFKKWCDRYFYVRHRGETRGIGGIFFDD
LDSPSQEEAFSFIKSCTSTV
VPCYIPLVNKHLNDPFTVEEKNWQQLRRGRYVEFNLVYDRGVKFGLATPGSRVESILMSLPLTAKWEYTHEPAEGSKEAA
MLEVLRTPREWV
>g2356.t2
MSQGAAGRSPAGRTRRGALVLAGAVAVAGLVANAGYLQRAEMASRVIQVAEPEDDILEKCKGFMSPPVTDIQVLQERKGE
MCTKMEMLIMQTQAEFCRAL
EKVDGGKFKADRGGGISCVIQDSNVFEKAGVNVSVVFGHLTEEAAKQMKSRGKVLKGKDGKLPFCAMGVSSVIHPKNPHI
PTVHFNYRYFEIEEDDGSKQ
WWFGGGTDLTPTYLNKEDAFLFHNTLKEACDKHHPQYYPDFKKWCDRYFYVRHRGETRGIGGIFFDDLDSPSQEEAFSFI
KSCTSTVVPCYIPLVNKHLN
DPFTVEEKNWQQLRRGRYVEFNLVYDRGVKFGLATPGSRVESILMSLPLTAKWEYTHEPAEGSKEAAMLEVLRTPREWV
>g2356.t3
MSQGAAGRSPAGRTRRGALVLAGAVAVAGLVANAGYLQRAEMASRVIQVAEPEDDILEKCKGFMSPPVTDIQVLQERKGE
MCTKMEMLIMQTQAEFCRAL
EKVDGGGGISCVIQDSNVFEKAGVNVSVVFGHLTEEAAKQMKSRGKVLKGKDGKLPFCAMGVSSVIHPKNPHIPTVHFNY
RYFEIEEDDGSKQWWFGGGT
DLTPTYLNKEDAFLFHNTLKEACDKHHPQYYPDFKKWCDRYFYVRHRGETRGIGGIFFDDLDSPSQEEAFSFIKSCTSTV
VPCYIPLVNKHLNDPFTVEE
KNWQQLRRGRYVEFNLVYDRGVKFGLATPGSRVESILMSLPLTAKWEYTHEPAEGSKEAAMLEVLRTPREWV
>g2357.t1
LRRANFLLEELKQGNIQRECREEVCTFEEAREAFEDNEKTVSPPRGGMLLMAMKCSVKVCGMLQCFAADEYQQEEQLLEE
EVPKRFWTEYVRESTPAGGM
ETVMGGVHSLYLILPLLLVVLLVAAVAITVWHCHSRKRSERSPGLGHSHYDAGLSVVSMDQWGREYHNGDQSELSAHSSP
AYQSSALASGRASAGDPPPT
YDEAVGQTDVQVETEPPPQYDDIVKTTSVSISQD
>g2357.t2
LRRANFLLEELKQGNIQRECREEVCTFEEAREAFEDNEKTVSPPRGGMLLMKRFWTEYVRESTPAGGMETVMGGVHSLYL
ILPLLLVVLLVAAVAITVWH
CHSRKRSERSPGLGHSHYDAGLSVVSMDQWGREYHNGDQSELSAHSSPAYQSSALASGRASAGDPPPTYDEAVGQTDVQV
ETEPPPQYDDIVKTTSVSIS
QD
>g2357.t3
LRRANFLLEELKQGNIQRECREEVCTFEEAREAFEDNEKTAMKCSVKVCGMLQCFAADEYQQEEQLLEEEVPKRFWTEYV
RESTPAGGMETVMGGVHSLY
LILPLLLVVLLVAAVAITVWHCHSRKRSERSPGLGHSHYDAGLSVVSMDQWGREYHNGDQSELSAHSSPAYQSSALASGR
ASAGDPPPTYDEAVGQTDVQ
VETEPPPQYDDIVKTTSVSISQD
>g2358.t1
MPTVWKQITFSVASVLCIGSVVLLVVALSTERWATGRILCQTGVDIVNASSPELSKFMGHVYYGLFQGGKTKKCGLGDRR
SKIYIFPKLVKTLNSGLHMM
VILFLLVAIGFALVSLSFCVYNARK
>g2358.t2
MPTVWKQITFSVASVLCIGSVVLLVVALSTERWATGRILCQTGVDIVNASSPELSKFMGHVYYGLFQGGKTKKCGLGDRR
SKIYIFPKLVKTLNSGLHMM
VILFLLVAIGFALVSLSFCVYNARKVPYQSIKGHKG
>g2358.t3
MPTVWKQITFSVASVLCIGSVVLLVVALSTERWATGRILCQTGVDIVNASSPELSKFMGHVYYGLFQGGKTKKCGLGDRR
SKIYIFPKLVKTLNSGLHMM
VILFLLVAIGFALVSLSFCVYNARKVPYQSIKGHKGLYLWNVIA
>g2359.t1

MAESTTDTEKKGRDFPRIPKFDGGSKVVDRQRHGPENDTQQTSLAVPFSGHIKDGMRIGKRLVVVGVVEPRPD
>g2359.t2
MAESTTDTEKKGRDFPRIPKFDGGSKVVDRQRHGPENDTQQTSLAVPFSGHIKDGMRIGKRLVVVGVVEPRPDRYNDLSL
FLLLVTW
>g2360.t1
QAEQQTEQQAALREAGLQSPILRLRPLAALIGHLVSDERGGAGPTAVITSIVLRWLLVSALLPTVRKAV
>g2361.t1
PQTEEEPSFDRGLGGLPGGHRGAIAATDAAAHDNSDRLAGVLLHATDGIPC
>g2362.t1
MDAHAAGDCVRPTERLAGATAARGLSSEQGEAAGVMGIMAAIMDLFPVAYVLRCGEWPRCRCCRVARRIYSAFLRWFRNV
FGMDSWNCSIIHAIKSILTR
KSLNSLYGPVLIGEVHKSIAGLQQNLIGYWKLPLAEELL
>g2362.t2
MDAHAAGDCVRPTERLAGATAARGLSSEQGEAAGVMGIMAAIMDLFPVAYVLRCGEWPRCRCCRVARRIYSAFLRWFRNV
>g2363.t1
RGYDRGYDRGDRGYDRGGYDRYDDREYYNNRSYRRRSPSPYYRGTYRSRSRSRSYSPRRY
>g2363.t2
RGYDRGYDRGDRGYDRGGYDRYDDREYYNNRSYRRRSPSPYYRGTYRSRSRSRSYSPRAEV
>g2364.t1
SRPTRIRYMSDLYHLHMFHVSKLLSCSGVLSSNFLGSEPVSRGAALSCEF
>g2365.t1
ICLVGLGLVVFFFSFLLSIFRSKYHGYPYR
>g2365.t2
ICLVGLGLVVFFFSFLLSIFRSKYHGYPY
>g2366.t1
MMSSLGGDKARPPREKTLPAVTHSSQPQRQIQATAEQIRLAQMIYDKNDSDFECKVNQLMEVTGKNQDECMIALHDCNED
VNRAINFLLESTSDTVGHVP
DPRICRRGCALGAASANVQYLNSEPLSPMRALGVAG
>g2366.t2
MSSLGGDKARPPREKTLPAVTHSSQPQRQIQATAEQIRLAQMIYDKNDSDFECKVNQLMEVTGKNQDECMIALHDCNEDV
NRAINFLLESTSDTVGHVPD
PRICRRGCALGAASANVQYLNSEPLSPMRALGVAG
>g2366.t3
MMSSLGGDKARPPREKTLPAVTHSSQPQRQIQATAEQIRLAQMIYDKNDSDFECKVNQLMEVTGKNQDECMIALHDCNED
VNRAINFLLESTSDTVGHVP
DPRICRRGGGRALKDARARVLRTLRCSPHPLELCSV
>g2367.t1
MGGLWNCQSAVKKADSISAYACLQTLHFLALTETLQTTRPLRLPSLSPTPSHTPQDPQGEEELSYRVLLLEHLTRSAFEL
HAVAITDPIKLDVAVINRPP
GPFHDFFDKMDALLSCFPEDGTPLVVLGDFNIQPENSRPARLNRFTPLSSEEVLKLVTSNHATTCPLDPISSSLLQAIST
DILPFITSLVNSSLTSGLVP
TPFKTEWSQTTPQEAYPRHC
>g2368.t1
HRRALRRVFAANRTLMLTRSSFPGVGKYSGHWLGDNAANWNDIKWAIPGMLEFGLFGVPYVSSGGSGRTGRPLARAKRPA
AVVSTTDVQ
>g2369.t1
PILTCTFREVMSASMWSMSKGGEFSELGNGSVAMITNDCGTDAFFPSCLTLNLNQVTGNWAGVFKCEFSEGSVKHAAQLG
LSVALLPDTVEMLVDPLTVD
CSSGLEASVPIKVTAIIANSTEDFKVTWSYRGELQELMDGFHYIFTHPIGCTASPLAHYVNITFINQMCQEKSVRLDVGV
IYAGAPFCEEEVLKNQMWPR
TPAGITVFNRTCGGDRVGYVSRTCVGSAWQDAYYQCISAELSKVLNTADNFKQGFDATQEVAENIFGSLNNGSTKNSGSE
EDLADLSASINILNTMAVAS
GNVALKDALLPNLIGAASNMLNQSWEAVNTSIQNSMSSNYLESVEGLVQNIQANNSKGVDRENVKLSICNSLVEQDCNQN
VFGVAINLNNSQGIIKTMAV
KNLAEKLEKKNKQSKTASLVVSATIQNSSGDSVNIELEFPREHPDYTKVTCVFWNITSHQWSDKGCKLLTGNDENQTICS
CNHLTPFSALMSKTTLILPF

LDEITYLGLGVSICALLLFLIIEALVWSAVVKSNLSHFRHTSLVNISVSLLLADLCFLASAFPDKISETMCLVFTVCKHF
FFLTMFCWMLCLSVMLVHQL
IFVFNPLRKRVFMFLSSIVGYIVPMLIVGCSYVYYRYTGGEYLKKETCWLTYDGLLRGSIHAFLLPVGIVVLTNLFSMCV
VIITLTKTAVPDANKTDKDT
AKSILKVVLFLTPAFGVTWILGFIQLLLGESSDEASVDGTTQPGFVIFIHYAFTILNTFQVRDEVLKLIMAKTSSGDKSE
NNTNTSKN
>g2369.t2
PILTCTFREVMSASMWSMSKGGEFSELGNGSVAMITNDCGTDAFFPSCLTLNLNQVTGNWAGVFKCEFSEGSVKHAAQLG
LSVALLPDTVEMLVDPLTVD
CSSGLEASVPIKVTAIIANSTEDFKVTWSYRGELQELMDGFHYIFTHPIGCTASPLAHYVNITFINQMCQEKSVRLDVGV
IYAGAPFCEEEVLKNQMWPR
TPAGITVFNRTCGGDRVGYVSRTCVGSAWQDAYYQCISAELSKVLNTADNFKQGFDATQEVAENIFGSLNNGSTKNSGSE
EDLADLSASINILNTMAVAS
GNVALKDALLPNLIGAASNMLNQSWEAVNTSIQNSMSSNYLESVEGLVQNIQANNSKGVDRENVKLSICNSLVEQDCNQN
VFGVAINLNNSQGIIKTMAV
KNLAEKLEKKNKQSKTASLVVSATIQNSSGDSVNIELEFPREHPDYTKVTCVFWNITSHQWSDKGCKLLTGNDENQTICS
CNHLTPFSALMSKTTLILPF
LDEITYLGLGVSICALLLFLIIEALVWSAVVKSNLSHFRHTSLVNISVSLLLADLCFLASAFPDKISETMCLVFTVCKHF
FFLTMFCWMLCLSVMLVHQL
IFVFNPLRKRVFMFLSSIVGYIVPMLIVGCSYVYYRYTGGEYLKKETCWLTYDGLLRGSIHAFLLPVGIVVLTNLFSMCV
VIITLTKTAVPDANKTDKDT
AKSILKVVLFLTPAFGVTWILGFIQLLLGESSDEASVDGTTQPGFVIFIHYAFTILNTFQVRDEVLKLIMAKETRVRTTQ
TPRRTEVGVKGVEVTEAVVV
TEEVVVVWVI
>g2369.t3
PILTCTFREVMSASMWSMSKGGEFSELGNGSVAMITNDCGTDAFFPSCLTLNLNQVTGNWAGVFKCEFSEGSVKHAAQLG
LSVALLPDTVEMLVDPLTVD
CSSGLEASVPIKVTAIIANSTEDFKVTWSYRGELQGQLQPVKLMDGFHYIFTHPIGCTASPLAHYVNITFINQMCQEKSV
RLDVGVIYAGAPFCEEEVLK
NQMWPRTPAGITVFNRTCGGDRVGYVSRTCVGSAWQDAYYQCISAELSKVLNTADNFKQGFDATQEVAENIFGSLNNGST
KNSGSEEDLADLSASINILN
TMAVASGNVALKDALLPNLIGAASNMLNQSWEAVNTSIQNSMSSNYLESVEGLVQNIQANNSKGVDRENVKLSICNSLVE
QDCNQNVFGVAINLNNSQGI
IKTMAVKNLAEKLEKKNKQSKTASLVVSATIQNSSGDSVNIELEFPREHPDYTKVTCVFWNITSHQWSDKGCKLLTGNDE
NQTICSCNHLTPFSALMSKT
TLILPFLDEITYLGLGVSICALLLFLIIEALVWSAVVKSNLSHFRHTSLVNISVSLLLADLCFLASAFPDKISETMCLVF
TVCKHFFFLTMFCWMLCLSV
MLVHQLIFVFNPLRKRVFMFLSSIVGYIVPMLIVGCSYVYYRYTGGEYLKKETCWLTYDGLLRGSIHAFLLPVGIVVLTN
LFSMCVVIITLTKTAVPDAN
KTDKDTAKSILKVVLFLTPAFGVTWILGFIQLLLGESSDEASVDGTTQPGFVIFIHYAFTILNTFQVRDEVLKLIMAKTS
SGDKSENNTNTSKN
>g2370.t1
AAGPWVRPTLASGATLDPSCGCYRGNDYGWYTFISRAQLERRSFLKNDDLIITADFNDLTHLLRTEVIPVKPAAHSDDLS
SRGPVREQGPSRQRRAAPGD
PCSPNPCLHHGVCVARHGTATCRCVSGRATFYTGEKCHMMRFQGHVLGALIGGAVGTIVITISILAIVRRRQPGYL
>g2371.t1
MRPTHLLYQVAMLVVANAQQDCFTHPTFREPSNADIKVSCGTKQMAMQMLLCPVYFCGYNESLLALNGEHDKEHCKGTAD
WTVDPPVLRFNFSITEEDIA
VCSNKLTVTEEIGTGVFSDFSSLQFINISGQVKSQDPAMGTVTYREEMMYMFSCRYPLQYLVSNKTQMNVAAASLSINQD
NGSFSSTLRMALFSDKAYSS
SLRIPREGLQLKTRIFVQVAASNLSNRFNVVLDRCYASTAFFSVDSVTHDLFVGCNKDGQTIIAINGMDQNARFSFEAFR
FVKQKTSSTFYLHCSTRLCE
RSSCPTLVENCSTSTAFRRRRSDGGSTRVSDVATVSSGPITTRV
>g2371.t2
MRPTHLLYQVAMLVVANAQQDCFTHPTFREPSNADIKVSCGTKQMAMQMLLCPVYFCGYNESLLALNGEHDKEHCKGTAD
WTVDPPVLRFNFSITEEDIA

VCSNKLTVTEEIGTGVFSDFSSLQFINISGQVKSQDPAMGTVTYREEMMYMFSCRYPLQYLVSNKTQMNVDRGADGAPLS
VAQRFPLSGAGSTPHRAAAS
LSINQDNGSFSSTLRMALFSDKAYSSSLRIPREGLQLKTRIFVQVAASNLSNRFNVVLDRCYASTAFFSVDSVTHDLFVG
CNKDGQTIIAINGMDQNARF
SFEAFRFVKQKTSSTFYLHCSTRLCERSSCPTLVENCSTSTAFRRRRSDGGSTRVSDVATVSSGPITTRV
>g2371.t3
MRPTHLLYQVAMLVVANAQQDCFTHPTFREPSNADIKVSCGTKQMAMQMLLCPVYFCGYNESLLALNGEHDKEHCKGTAD
WTVDPPVLRFNFSITEEDIA
VCSNKLTVTEEIGTGVFSDFSSLQFINISGQVKSQDPAMGTVTYREEMMYMFSCRYPLQYLVSNKTQMNVGADGAPLSVA
QRFPLSGAGSTPHRAAASLS
INQDNGSFSSTLRMALFSDKAYSSSLRIPREGLQLKTRIFVQVAASNLSNRFNVVLDRCYASTAFFSVDSVTHDLFVGCN
KDGQTIIAINGMDQNARFSF
EAFRFVKQKTSSTFYLHCSTRLCERSSCPTLVENCSTSTAFRRRRSDGGSTRVSDVATVSSGPITTRV
>g2372.t1
AVQAAILSGDKSENVQDLLLLDVTPLSLGIETAGGVMTVLIKRNTTIPTKQTQTFTTYSDNQPGVLIQVYEGERAMTKDN
NLLGKFELTGIPPAPRGVPQ
IEVTFDIDANGILNVSAVDKSTGKENKITITNDKGRLSKEDIERMVQEAEKYKAEDDVQRDKVSSKNSLESYAFNMKSTV
EDEKLAGKISDEDKQKILDK
CNEIIGWLDKNQTAEKEEYEHQQQELEKVCNPIITKLYQSAGGMPGGMPGGMPGGMPGGMPGGMPGGFPGAGGAPSGGGS
SGPTIEEVD
>g2373.t1
GEPYLGSAYGIMMCYWDGIVHFIMYLMMISRITDRKSYRTMGLFWAGSLCANMSVFISGIVSGKYGLEIHPAFWINFVFL
LMPVWGTITLFTRAKDRPLI
GGYNAHHVQTMKLIWRPQDLVLVLLLLAAMAFTILRGLVALNSPLEACSIYLKQYEPYLNDPVGYPRVMMLHFLFYGLPL
LGSFVYGLLRPGCTWMPDWT
MFFSGALIQ
>g2373.t2
GEPYLGSAYGIMMCYWDGIVHFIMYLMMISRITDRKSYRTMGLFWAGSLCANMSVFISGIVSGKYGLEIHPAFWINFVFL
LMPVWGTITLFTRAKDRPLI
GGYNDLVLVLLLLAAMAFTILRGLVALNSPLEACSIYLKQYEPYLNDPVGYPRVMMLHFLFYGLPLLGSFVYGLLRPGCT
WMPDWTMFFSGALIQ
>g2373.t3
GEPYLGSAYGIMMCYWDGIVHFIMYLMMISRITDRKSYRTMGLFWAGSLCANMSVFISGIVSAFWINFVFLLMPVWGTIT
LFTRAKDRPLIGGYNAHHVQ
TMKLIWRPQDLVLVLLLLAAMAFTILRGLVALNSPLEACSIYLKQYEPYLNDPVGYPRVMMLHFLFYGLPLLGSFVYGLL
RPGCTWMPDWTMFFSGALIQ
>g2374.t1
MVCLLWFQFGLPKAEKEQYEREQRVEVQQELLKKVVLEMPVYTRTAGGAVRYCDFCQVIKPDRCHHCSTCETCVLKMDHH
CPWVNNCVGFSNYKFFVLFL
GYGSMYCLVISVTVIQYFIKFWTKNLNDSHAKFHILFLFLVASMFFISILCLFSYHLWLIGQNRTTNDPEILELLHLGQQ
LAPNPKGAVHRFPAENHGLR
LGGADSHPNRFTLGCKPSQCLLEVTV
>g2375.t1
FKLSNLYVVILSLLGLIALLLVLCLLSCLYKLHRRSQKNKKLTLIAQQAGKGDGQAFRQGSLVIRVFSFQPEGSGFDGRC
PQPRLYSFST
>g2375.t2
FKLSNLYVVILSLLGLIALLLVLCLLSCLYKLHRRSQKNKKLTLIAQQAGKGDGQAFRQIQSYSPPRTTPQETKGSRTPP
LPSTSPPPLMDQFLS
>g2375.t3
LWTLTPPDQLSSPEDIWQDTPPHGPRSRPLQDHISAACVQLLKRAGVVSDQVGPETRSAWSGDALTPSLSGWLLQAVSCA
PDLFLDLR
>g2375.t4
FKLSNLYVVILSLLGLIALLLVLCLLSCLYKLHRRSQKNKKLTLIAQQAGKGDGQAFRQSLLGGQETRHGGQPCDEQVTP
CPSKEAKRDQLSSPEDIWQD
TPPHGPRSRPLQDHISAACVQLLKRAGVVSDQVGPETRSAWSGDALTPSLSGWLLQAVSCAPDLFLDLR
>g2376.t1

WEQGPWTRCSASCGEAGGQQERSVACVEDDAHGQFSPAEDWKCAHSPRPGSRQLCNAFPCPQWVAMEWSQCTVTCGRGLR
YRVVLCLDHRGQHIGGCRQL
LKPHVKEDCLVPIACHKPRGRSSGIAPATSRLPDNPLHPLSHFLGSFPRIRASSCGAGA
>g2376.t2
MYGFGIVLSHSWEQGPWTRCSASCGEAGGQQERSVACVEDDAHGQFSPAEDWKCAHSPRPGSRQLCNAFPCPQWVAMEWS
QCTVTCGRGLRYRVVLCLDH
RGQHIGGCRQLLKPHVKEDCLVPIACHKPRGRSSGIAPATSRLPDNPLHPLSHFLGSFPRIRASSCGAGA
>g2376.t3
MSSMYGFGIVLSHSWEQGPWTRCSASCGEAGGQQERSVACVEDDAHGQFSPAEDWKCAHSPRPGSRQLCNAFPCPQWVAM
EWSQCTVTCGRGLRYRVVLC
LDHRGQHIGGCRQLLKPHVKEDCLVPIACHKPRGRSSGIAPATSRLPDNPLHPLSHFLGSFPRIRASSCGAGA
>g2377.t1
LGTTRAAGFKTSPGRPLGTTKAAGYKVSPGRPPGSIKSLSRLNKLAYGTCSGAAFPYPLPHKDILCEPSCKDKVAND
>g2378.t1
LENSPKFVETDDFESHPKRQFKTAEIADALMGTETPIDPSDIESLYPKNVKDFQSGFLPPCAEEDAECAAPSQAADGVLL
KKRSETEQVTLDMVHVDTMV
TEEQNTTETTKTHKVNCDERNITGMEDFTVQVLNASQDLMDFLNANGTECSVVLFFTTWCQFSASLAPHFNALPRVFPSM
HFLALDASQHSSLSTRFGTV
AVPNILLFQGVKPMARFNHTDRTLDALTSFIANQTGFEAGPDRNVTDSDRLGPLPGVPVKSIDWLLVFSVLFITGFTFYA
ILRTDSIRWLIPGQEHEHQD
>g2378.t2
MGTETPIDPSDIESLYPKNVKDFQSGFLPPCAEEDAECAAPSQAADGVLLKKRSETEQVTLDMVHVDTMVTEEQNTTETT
KTHKVNCDERNITGMEDFTV
QVLNASQDLMDFLNANGTECSVVLFFTTWCQFSASLAPHFNALPRVFPSMHFLALDASQHSSLSTRFGTVAVPNILLFQG
VKPMARFNHTDRTLDALTSF
IANQTGFEAGPDRNVTDSDRLGPLPGVPVKSIDWLLVFSVLFITGFTFYAILRTDSIRWLIPGQEHEHQD
>g2378.t3
LENSPKFVETDDFESHPKRQFKTAEIADALMGTETPIDPSDIESLYPKNVKDFQSGFLPPCAEEDAECAAPSQAADGVLL
KKRSETEQVTLDMVHVDTMV
TEEQNTTETTKTHKVNCDERNITGMEDFTVQVLNASQVISGFLGSKTLTAVDLLQDLMDFLNANGTECSVVLFFTTWCQF
SASLAPHFNALPRVFPSMHF
LALDASQHSSLSTRFGTVAVPNILLFQGVKPMARFNHTDRTLDALTSFIANQTGFEAGPDRNVTDSDRLGPLPGVPVKSI
DWLLVFSVLFITGFTFYAIL
RTDSIRWLIPGQEHEHQD
>g2379.t1
MYEARGAVGIFTYDLLHNFAETPETLAAMPSVPYDLSSSWCAEGVVSSPINCDDALYSEMYYSDQNAFVRGDAKQGNLTY
NLLIRLDWSAAVDHNILTHL
RAPLVPNTVFKKGRDTTKLPSQGGHDQDDMAVVIQHEGATVLLVSKEGSKVIKRILSPGHCSSCPPEVLKNGLQCGAVTL
SPLHLTATILLTGRHLVAMD
NMNFLHTFEITFPCPSTAPPPIQSTCVAHSHWVWSKVHNMKGESQCWQNLYSDPERVPVTMGLCTSKSSVTLDPSAHTLG
KGAAPIGHTAKDGEGVSGTP
VLGEGILMVQPV
>g2379.t2
MYEARGAVGIFTYDLLHNFAETPETLAAMPSVPYDLSSSWCAEGVVSSPINCDDALYSEMYYSDQNAFVRGDAKQGNLTY
NLLIRLDWSAAVDHNILTHL
RAPLVPNTRILSPGHCSSCPPEVLKNGLQCGAVTLSPLHLTATILLTGRHLVAMDNMNFLHTFEITFPCPSTAPPPIQST
CVAHSHWVWSKVHNMKGESQ
CWQNLYSDPERVPVTMGLCTSKSSVTLDPSAHTLGKGAAPIGHTAKDGEGVSGTPVLGEGILMVQPV
>g2379.t3
ARGAVGIFTYDLLHNFAETPETLAAMPSVPYDLSSSWCAEGVVSSPINCDDALYSEMYYSDQNAFVRGDAKQGNLTYNLL
IRLDWSAAVDHNILTHLRAP
LVPNTVFKKGRDTTKLPSQGGHDQDDMAVVIQHEGATVLLVSKEGSKVIKRILSPGHCSSCPPEVLKNGLQCGAVTLSPL
HLTATILLTGRHLVAMDNMN
FLHTFEITFPCPSTAPPPIQSTCVAHSHWVWSKVHNMKGESQCWQNLYSDPERVPVTMGLCTSKSSVTLDPSAHTLGKGA
APIGHTAKDGEGVSGTPVLG
EGILMVQPV

>g2380.t1
MIARLIRGKSQLLLLEGPDAVEIYK
>g2381.t1
LEQLLPPRHRTSAPRPVDSYLLQDPPTAALIQRCWRTINQLRPWVVVFFTGLAEARDPTRLIPDPRTNAANQHGLRANSL
LFSSSSCRSFQGFGKSCPFT
FPTVREERQALLRSQPEVFSRFLLTLLLCVWRMGGTNTAL
>g2382.t1
FEVIGYGCATCVGNTVPLPDAVVDAIKEGNLVACGVLSGNRHFEGRLSDCVRANYMASPPLVVAYAVAGRVDIDFEKEPL
>g2382.t2
MSCSSSGLLRRFEVIGYGCATCVGNTVPLPDAVVDAIKEGNLVACGVLSGNRHFEGRLSDCVRANYMASPPLVVAYAVAG
RVDIDFEKEPL
>g2383.t1
ETLDEATDRWGIKVERVEIKDVRLPQQMQRAMAAEAEASREARAKVIAAEGEMNASRALKEASLVLAESPPALQLRYLQT
LNSIAAEKNSTIIFPLPMDV
MQHTMSRK
>g2384.t1
LQRDKEELERRFRSELRGLRSRQQGELVALEERLRARHALESLRLQEEQQQEIDDMSESHEASLMEMEKSHNDTLVTLRE
EHARTVKNLKMAHEQQRKSL
EEDFEKLRLSLQDQVDTLTFQNRSLRDRAKRFEEALRRSTDEQIVEALAPYQHIEEDLKSLKEVLEMKNQQIHQQEVKIS
ELEIMAEKNVYLEERVQVLQ
QQNEDLKARIDKNVATSRQLSEENANLQVHVEQESKEKKRLSRTNEELLWRLQTGELSPHMSPSSSPIHQPLSSPASPAR
PHSYHQ
>g2384.t2
LLDVVAFSVARWEHLQRDKEELERRFRSELRGLRSRQQGELVALEERLRARHALESLRLQEEQQQEIDDMSESHEASLME
MEKSHNDTLVTLREEHARTV
KNLKMAHEQQRKSLEEDFEKLRLSLQDQVDTLTFQNRSLRDRAKRFEEALRRSTDEQIVEALAPYQHIEEDLKSLKEVLE
MKNQQIHQQEVKISELEIMA
EKNVYLEERVQVLQQQNEDLKARIDKNVATSRQLSEENANLQVHVEQESKEKKRLSRTNEELLWRLQTGELSPHMSPSSS
PIHQPLSSPASPARPHSYHQ
>g2384.t3
LQRDKEELERRFRSELRGLRSRQQGELVALEERLRARHALESLRLQEEQQQEIDDMSESHEASLMEMEKSHNDTLVTLRE
EHARTVKSIQYFGDITRLKL
EPLISDLKMAHEQQRKSLEEDFEKLRLSLQDQVDTLTFQNRSLRDRAKRFEEALRRSTDEQIVEALAPYQHIEEDLKSLK
EVLEMKNQQIHQQEVKISEL
EIMAEKNVYLEERVQVLQQQNEDLKARIDKNVATSRQLSEENANLQVHVEQESKEKKRLSRTNEELLWRLQTGELSPHMS
PSSSPIHQPLSSPASPARPH
SYHQ
>g2385.t1
MVSPKSPGHGCPRGECVRESERALLQQLRDLCQHQARQVQYLQAQLRKASLGLEVFAITTQHFCQKVC
>g2386.t1
EKMDSQLENEARFCQLMAHSSHDSAIDTDSLEWETEVVEFEKDRVRLPAGGRGRRFHRRKKVMRCGAEVEETRVSLADDM
DLKALGFDIAEGVNDPYLPG
DCGIFVTRVDKGSSADGRLRVNDWLLKVNDVDLTNKDRKQAVKAVLCGGGLVNMVVRRRKSLGGRTVTPVHINLMGHTDS
GISLESGVFVTAVVQGSPAA
RDGSLTVGDRLIA
>g2387.t1
WQGSMATSAPFQNPECIHSTARPDTSCCSWSQQGEEEGEAPGFGPCLQTRHWARVKKGTLGNERDPRRQEVECVQWSGTP
LHKADHRANVLAILTC
>g2388.t1
MLLVSSSLVSSSPVSTNLVFRLDLLGTGTEQVRSRRVGAAVRLFVAVAVLERGGVQVVRLFLMTRNRRYLADV
>g2388.t2
LKHAEMLLVSSSLVSSSPVSTNLVFRLDLLGTGTEQVRSRRVGAAVRLFVAVAVLERGGVQVVRLFLMTRNRRYLADV
>g2389.t1
MAPRGPAPGAGTLGVRDRRGPEGRRSAGLLLVLGPDWLLLSVTKEPAVAGNFPSNLFAFPAVYIVTAQLSMDAMRRPEDE
EWSSVSL
>g2390.t1

EMEGLDLEEPGRPPGGPEGRGHGEESEPSDDEMLSLSSQQSNTSSAAAK
>g2390.t2
MEGLDLEEPGRPPGGPEGRGHGEESEPSDDEMLSLSSQQSNTSSAAAK
>g2391.t1
FGGGMLESRSREEMGKVGSQTSFSGSMEIIEVS
>g2392.t1
MLERCSISWLMGNQLKGRTFTFPPPPEGSPVQRNRLPPRSSQGGGSRGPGFRAPPRLHGNAPPPASLRVSPALSIISVEG
DLKRHLPVSRAVAHRPYVRE
ISPVKESQYEPEKPEEQLNLRGA
>g2392.t2
MLERCSISWLMGNQLKGRTFTFPPPPEGSPVQRNRLPPRSSQGGGSRGPGFRAPPRLHGNAPPPASLRVSPALSIISVEG
DLKRHLPVSRAVAHRPYVRE
ISPVKESQYEPEKPEEQLNLRG
>g2393.t1
MAYERKKARYAELAADAEQQGWRARVRPVEVGCRGFVAKSAFALLTELGIRGRRMKLATKSMSQAAESA
>g2394.t1
MRGGTGNKEVCLSLDQMVASCGVDLSEPAVENENLGAQLRELLNKSAGVFSQHPTDYGHTTTMRFRWTPGCTVEWWACNS
RLICQLGSQHTKDVVQRKQR
FWWAMLEEDTREFMGACPVCSQHKPSHQAPAGLLQSLPVPNRP
>g2394.t2
MRGGTGNKEVCLSLDQMVASCGVDLSEPAVENENLGAQLRELLNKSAGCTVEWWACNSRLICQLGSQHTKDVVQRKQRFW
WAMLEEDTREFMGACPVCSQ
HKPSHQAPAGLLQSLPVPNRP
>g2395.t1
MDLYQLDQTAPQAVFRPCAYQLAEVFADIFNLSLNLSEIPTCFESSTIVSVPKRAQFAYRPKRSTDNGIFMALHTALSHL
DKRNTYVQMLVVDYSSAFNT
IVTGKLIIKLKDLGLSSSLCNWVLDFLVGRPQVARVGNFTSRLKDSF
>g2395.t2
MYDEQIFLPVYPMDLYQLDQTAPQAVFRPCAYQLAEVFADIFNLSLNLSEIPTCFESSTIVSVPKRAQFAYRPKRSTDNG
IFMALHTALSHLDKRNTYVQ
MLVVDYSSAFNTIVTGKLIIKLKDLGLSSSLCNWVLDFLVGRPQVARVGNFTSRLKDSF
>g2396.t1
MGSDSPVLTLAESLFHHCMPEQKRGVISPSDLFLLLVMEVAVDQLSVAVFYEPAEIRTAKEEEEEEILLVWM
>g2397.t1
SDRTTDAPKRLSCPAGAAQLPAQMPFFHRSHLGLTGPVEDTSDLCRLRALVKSCSDIIRYV
>g2398.t1
MHTAFREVTCHGTVPSATQRGSAQFWEPSKECRAFTLRGNKEPRSHQRSAFRHVLLPAYVSIPAVIAVNETYPEWFSMTL
HDSAGPLDRSDSAR
>g2399.t1
MAVGTRLLPKRAPLHLRDLYLHPEGSTVRCWSPAEDHLAHPQRTIWAGFVPDLLDSSYLSLVDAGSSDLLPGPALCFYDS
CRLSHMSLLGPNPTNCIGVT
KKRRDTPAVDAALCSGSYPEFHPGFYPGDALGSPEPSP
>g2400.t1
LAAPSCRQAVNFPVSRAQQSSSRVYIPHLTTTDQCSDNYPSHRTFTPAAPVGPVPPPHTQSSCGDQGGTTMISWWTLGGR
RRALTNSPLSGSLRGSGFLH
GSTPKSSSGSVVIEYQLHMFHGPSRLNTPQDKVHPQ
>g2401.t1
MVLDSEVVLTKMKIFQNFSEKLLVSEDSFSTSSSMSVCEQEVSEPTRTSEENPRLNGGGGEASSPLEEAGSNYLCAMFSD
SQLPRLYKFESEDSGVELPS
GANSPSTPTGSEQSFVVHSRESSCDSCRLNASPMAVPEERVSPGETGESEGKQVTGSPANAGDDNAGEDGDNTLLKSTVT
EEELSFCIEEETDVTLAEDP
VEACCGDVIERTQDSLIQVTGSETRAAVEEVEEEEEEVEEEEEEEEVRPSDQPSVELREGEKEEDLFQPMTSGLRKSSTS
DSLEEYMEECCRLSEV
>g2402.t1
FALSPSGQRLENPTTIEIMVLDQNDNRPSFIKTQFTGSVSEFSVPGTSVMSVWATDADDPSTDNAILSYSIIGQESEPGG
AVTKVMFGINNETGAIYTRD

VGFDREVVKSFRLKLQVGDMSGMGLTSESVALIEVTDINNHAPQFSPSMYHVSGVENRKDYELCRVNTTDRDEPGTGNWE
TKYTVARGNGGGHFVIHTDP
VSNQGVVTVVKPLDFESQPEYTLILTVANRNPLSSKAPSSPVSSATVVVTVVNENEAPRFREDPIQILVPESVVPGTILK
TNLAYDPDQTGLRYDIVRDP
ERWLDVNTLSGDIISRRPFNIRSPHLRNGVYNAVVRATDTGGMATSATLLIMLKETNDYAPQLVPLSGTMCHPCNNPDST
LLLTAQDEDLAPHAEPFTFD
LPDEMALNWTLVPVNKTHARLHSLVELEVGQYAVTVQVMDAGTPTLGSFAQVNVTVCVCDSFSQCRSETAAILGSTVGIS
FIALIIVMAAIALLLLLLLL
AVAINGCTSRRHLQKESALLVGESEEDVRDNVFNYDEQGGGEEDENAYNLDLLNPSDAPHIPASSFCAAPRGKQPLRKDA
PHNLPSPTYPRRPPADPTDI
EDYINDGLEAAGRDPNMPPYDTALIYDHEGDGSLAGSLSSIASGSSDGDQDYDYLNDWGPRFKKLANMYDPR
>g2402.t2
FALSPSGQRLENPTTIEIMVLDQNDNRPSFIKTQFTGSVSEFSVPGTSVMSVWATDADDPSTDNAILSYSIIGQESEPGG
AVTKVMFGINNETGAIYTRD
VGFDREVVKSFRLKLQVGDMSGMGLTSESVALIEVTDINNHAPQFSPSMYHVSGVENRKDYELCRVNTTDRDEPGTGNWE
TKYTVARGNGGGHFVIHTDP
VSNQGVVTVVKPLDFESQPEYTLILTVANRNPLSSKAPSSPVSSATVVVTVVNENEAPRFREDPIQILVPESVVPGTILK
TNLAYDPDQTGLRYDIVRDP
ERWLDVNTLSGDIISRRPFNIRSPHLRNGVYNAVVRATDTGGMATSATLLIMLKETNDYAPQLVPLSGTMCHPCNNPDST
LLLTAQDEDLAPHAEPFTFD
LPDEMALNWTLVPVNKTHARLHSLVELEVGQYAVTVQVMDAGTPTLGSFAQVNVTVCVCDSFSQCRSETAAILGSTVGIS
FIALIIVMAAIALLLSVAIN
GCTSRRHLQKESALLVGESEEDVRDNVFNYDEQGGGEEDENAYNLDLLNPSDAPHIPASSFCAAPRGKQPLRKDAPHNLP
SPTYPRRPPADPTDIEDYIN
DGLEAAGRDPNMPPYDTALIYDHEGDGSLAGSLSSIASGSSDGDQDYDYLNDWGPRFKKLANMYDPR
>g2402.t3
FALSPSGQRLENPTTIEIMVLDQNDNRPSFIKTQFTGSVSEFSVPGTSVMSVWATDADDPSTDNAILSYSIIGQESEPGG
AVTKVMFGINNETGAIYTRD
VGFDREVVKSFRLKLQVGDMSGMGLTSESVALIEVTDINNHAPQFSPSMYHVSGVENRKDYELCRVNTTDRDEPGTGNWE
TKYTVARGNGGGHFVIHTDP
VSNQGVVTVVKPLDFESQPEYTLILTVANRNPLSSKAPSSPVSSATVVVTVVNENEAPRFREDPIQILVPESVVPGTILK
TNLAYDPDQTGLRRPFNIRS
PHLRNGVYNAVVRATDTGGMATSATLLIMLKETNDYAPQLVPLSGTMCHPCNNPDSTLLLTAQDEDLAPHAEPFTFDLPD
EMALNWTLVPVNKTHARLHS
LVELEVGQYAVTVQVMDAGTPTLGSFAQVNVTVCVCDSFSQCRSETAAILGSTVGISFIALIIVMAAIALLLLLLLLAVA
INGCTSRRHLQKESALLVGE
SEEDVRDNVFNYDEQGGGEEDENAYNLDLLNPSDAPHIPASSFCAAPRGKQPLRKDAPHNLPSPTYPRRPPADPTDIEDY
INDGLEAAGRDPNMPPYDTA
LIYDHEGDGSLAGSLSSIASGSSDGDQDYDYLNDWGPRFKKLANMYDPR
>g2403.t1
MTSYEVITKVVVTTHRREWQKGMTDTQREVLYSLMATGRNDFLCRSVVHLGAQDDLRKVTRVAYSMVKQYGMCPGVGQVS
FPQGEEQGAVGRRPFSQGLQ
QQMDHEAKLLIARAYRQTEKLLLDNQDKLTVLANALLDREVVNYEDIEALLGPPPHGPKKMIAPQTWLEAEWDKQDTGEE
PRPPPRGQEDPEPELV
>g2403.t2
MATGRNDFLCRSVVHLGAQDDLRKVTRVAYSMVKQYGMCPGVGQVSFPQGEEQGAVGRRPFSQGLQQQMDHEAKLLIARA
YRQTEKLLLDNQDKLTVLAN
ALLDREVVNYEDIEALLGPPPHGPKKMIAPQTWLEAEWDKQDTGEEPRPPPRGQEDPEPELV
>g2404.t1
MKDCYPFIPTFIQFNLSLVLEGNGPIGNERFKMCSKEWSNPNPGAEGSSMEQGNRRLQQTQAQVDEVVDIMRVNVDKVLE
RDQKLSELDDRADALQAGAS
QFETSAAKLKRKFWWKNCKMWAILIAVIIMIVIIIVIWSYSSG
>g2404.t2
MEQGNRRLQQTQAQVDEVVDIMRVNVDKVLERDQKLSELDDRADALQAGASQFETSAAKLKRKFWWKNCKMWAILIAVII
MIVIIIVIWSYSSG
>g2404.t3

MKDCYPFIPTFIQFNLSLVLEGNGPIGNERFKMCSKEWSNPNPGAEGSSMEQGNRRLQQTQAQVDEVVDIMRVNVDKVLE
RDQKLSELDDRADALQAGAS
QFETSAAKLKRKFWWKNCKMWAILIAVIIMIVIIIV
>g2405.t1
MLIITYIHQGASLQKPTAADFLQPVREKRSAECFPEGCSTESESESESESEEEGLIEVDASYQEALVQLRGSEQENTPKM
RIKCNPIKRDTRTFSITVL
>g2405.t2
MLIITYIHQGASLQKPTAADFLQPVREKRSAECFPEGCSTESESESESESEEEGLIEVDASYEALVQLRGSEQENTPKMR
IKCNPIKRDTRTFSITVL
>g2405.t3
MAVPLLTVMLIITYIHQGASLQKPTAADFLQPVREKRSAECFPEGCSTESESESESESEEEGLIEVDASYQEALVQLRGS
EQENTPKMRIKCNPIKRDTR
TFSITVL
>g2406.t1
MDVVVRRCPFLSRVPQAFFQQARTSLVVYAQRCPVMMDLASKPLAPSLVRSLCSSSSNLPTETVVAASEGSKPDHSSMPA
GHPMPPPGQAAASKCPFLAA
EMGQKNSSVVREVSMELQEDVEEVRTLQKDVSPGHLQQPSLSGPPQSDRGSYGSLMSKLMRQRPKGVSHLLQDNLPR
>g2407.t1
YCDALDRDQQVPVRAVFPRYEEKLSLYECGIVAQISQPLADSDISAYYISTFSFDHALVRSAAWPAGSYSVRCVCVCAIE
DGYPTRA
>g2408.t1
MGCSQRDPSEDASPKSDSELTVKSSESMLRADAHMQWSWGEFPECTRVSKKERSERKELTITPSMSTHFQVILSSEAMEV
DSRDPLDSPSFCSSCSPCGL
VKPQPRTANPEPGRSDDIGATSPSPRGAKWKSSPPARNIGAAVSGHTGSPDVTGVSPDSETCGKTTDSQGKRRDVRKRSQ
HQGPEDIYLDDLTALDPDVA
ARYFPKSGQVSRHWEEVGRRSSSQSPQSMGSAAADSGTECLSDSAGGLDLPDVTLSLCGGVAEDAEIPKERFLEHVITYE
EFSENPAIVDNPNLVVKIGN
RYYNWTLAAPLILCMQAFQQNLPKATEEAWVKEKMPKKSGRWWFWRKSSVKQSVDFKQSSLDSQNPESPSSSLPAPATQM
EESSSDDESKELNSVAPSSE
TAAPPSERVQTGGPAPGLSYRKSLRLSSQQIASLKLREGPNDVTFSITTQYQGTCRCQGTIYLWSWDDNVIISDIDGTIT
KSDVFGQILPQLGKDWTHRG
IAKLYHLVHENGYKFLYCSARAIGMADMTRGYLQWVNDGGAILPRGPLMLAPSSLFSAFHREVIEKKPEKFKIECLTDIR
NLFPAGQQPFYAAFGNRDND
VFAYKQVGVPVCRIFTVNPKGELILEQARGNKTSYSRLSELVEHLFPLRTREQNATFIYPEFSYFCFWRQPIAEVCLEDM
L
>g2408.t2
MNYVGQLLVTVKELYKGINQATLSGSIDVIVVRQPDGTFQCSPFHVRFGKLGVLRSREKVIDIEINGEAVDLHMKLGDNG
EAFFVQETEKCDEAVPSHLV
TSPIPTEESLFVKKKKRRKRKHKADPRREDQQGAEGGDLAGGGRSSGEEQTIPNGRPASVSVKDIVESKSYISGNGLEDP
SEDASPKSDSELTVKSSESM
LRADAHMQWSWGEFPECTRVSKKERSERKELTITPSMSTHFQVILSSEAMEVDSRDPLDSPSFCSSCSPCGLVKPQPRTA
NPEPGRSDDIGATSPSPRGA
KWKSSPPARNIGAAVSGHTGSPDVTGVSPDSETCGKTTDSQGKRRDVRKRSQHQGPEDIYLDDLTALDPDVAARYFPKSG
QVSRHWEEVGRRSSSQSPQS
MGSAAADSGTECLSDSAGGLDLPDVTLSLCGGVAEDAEIPKERFLEHVITYEEFSENPAIVDNPNLVVKIGNRYYNWTLA
APLILCMQAFQQNLPKATEE
AWVKEKMPKKSGRWWFWRKSSVKQSVDFKQSSLDSQNPESPSSSLPAPATQMEESSSDDESKELNSVAPSSETAAPPSER
VQTGGPAPGLSYRKSLRLSS
QQIASLKLREGPNDVTFSITTQYQGTCRCQGTIYLWSWDDNVIISDIDGTITKSDVFGQILPQLGKDWTHRGIAKLYHLV
HENGYKFLYCSARAIGMADM
TRGYLQWVNDGGAILPRGPLMLAPSSLFSAFHREVIEKKPEKFKIECLTDIRNLFPAGQQPFYAAFGNRDNDVFAYKQVG
VPVCRIFTVNPKGELILEQA
RGNKTSYSRLSELVEHLFPLRTREQNATFIYPEFSYFCFWRQPIAEVCLEDML
>g2408.t3
MNYVGQLLVTVKELYKGINQATLSGSIDVIVVRQPDGTFQCSPFHVRFGKLGVLRSREKVIDIEINGEAVDLHMKLGDNG
EAFFVQETEKCDEAVPSHLV

TSPIPTEESLFVKKKKRRKRKHKADPRREDQQGAEGGDLAGGGRSSGEEQTIPNGRPASVSVKDIVESKSYISGNGLEWD
SYPFSDGDWGVRFSFTRRDP
SEDASPKSDSELTVKSSESMLRADAHMQWSWGEFPECTRVSKKERSERKELTITPSMSTHFQVILSSEAMEVDSRDPLDS
PSFCSSCSPCGLVKPQPRTA
NPEPGRSDDIGATSPSPRGAKWKSSPPARNIGAAVSGHTGSPDVTGVSPDSETCGKTTDSQGKRRDVRKRSQHQGPEDIY
LDDLTALDPDVAARYFPKSG
QVSRHWEEVGRRSSSQSPQSMGSAAADSGTECLSDSAGGLDLPDVTLSLCGGVAEDAEIPKERFLEHVITYEEFSENPAI
VDNPNLVVKIGNRYYNWTLA
APLILCMQAFQQNLPKATEEAWVKEKMPKKSGRWWFWRKSSVKQSVDFKQSSLDSQNPESPSSSLPAPATQMEESSSDDE
SKELNSVAPSSETAAPPSER
VQTGGPAPGLSYRKSLRLSSQQIASLKLREGPNDVTFSITTQYQGTCRCQGTIYLWSWDDNVIISDIDGTITKSDVFGQI
LPQLGKDWTHRGIAKLYHLV
HENGYKFLYCSARAIGMADMTRGYLQWVNDGGAILPRGPLMLAPSSLFSAFHREVIEKKPEKFKIECLTDIRNLFPAGQQ
PFYAAFGNRDNDVFAYKQVG
VPVCRIFTVNPKGELILEQARGNKTSYSRLSELVEHLFPLRTREQNATFIYPEFSYFCFWRQPIAEVCLEDML
>g2408.t4
MNYVGQLLVTVKELYKGINQATLSGSIDVIVVRQPDGTFQCSPFHVRFGKLGVLRSREKVIDIEINGEAVDLHMKLGDNG
EAFFVQETEKCDEAVPSHLV
TSPIPTEESLFVKKKKRRKRKHKADPRREDQQGAEGGDLAGGGRSSGEEQTIPNGRPASVSVKDIVESKSYISGNGLEWD
SYPFSDGDWGPTTRQVSLAP
CS
>g2409.t1
MLCGQFKKVGKADCACVSRRGNGIMASRNLYGGPVREAKMFTLVLLVWLASAVQAVKVSPLIEKVSDHKDFKKLLRTRTN
VLVLYTKT
>g2409.t2
LASAVQAVKVSPLIEKVSDHKDFKKLLRTRTNVLVLYTKT
>g2409.t3
LWFHQVWLASAVQAVKVSPLIEKVSDHKDFKKLLRTRTNVLVLYTKT
>g2409.t4
MLCGQFKKVGKADCACVSRRGNGIMASRNLYGGPVREAKMFTLVLLGPYKWQVTVLAQYAEPQMWEGEIVVKREG
>g2410.t1
LGAVLSGAKAPGRVSHGKVVSGEGPDKERFKNPDGKAKYDQRDLARIGETGAPYWSQTWEGSSPASVWWPGFDPWGSAGL
SPKQQHGGAAALWAHHLQG
>g2411.t1
HHNYRDLSSNYMEALWMVSVTFLSIGYGDVVPHTYCGRSICLITGIMIKTAAANVLRETWLIYKHTRVLREKDHTRVRVH
QRKLLLAIHQLRSVRMEKRK
LADQASTLVDLSKMQSLMYDLLSEVQSCRGELDTHVNSLEKHVEELREGFRSLMPLLSNTLSTQNSSIRHLLQERETQAE
NAAAATTGDRAQ
>g2411.t2
HHNYRDLSSNYMEALWMVSVTFLSIGYGDVVPHTYCGRSICLITGIMGAGCTVLVVAVVARKLELTRAEKHVHNFMMDSH
ISKEVPIKTAAANVLRETWL
IYKHTRVLREKDHTRVRVHQRKLLLAIHQLRSVRMEKRKLADQASTLVDLSKMQSLMYDLLSEVQSCRGELDTHVNSLEK
HVEELREGFRSLMPLLSNTL
STQNSSIRHLLQERETQAENAAAATTGDRAQ
>g2411.t3
HHNYRDLSSNYMEALWMVSVTFLSIGYGDVVPHTYCGRSICLITGIMGAGCTVLVVAVVARKLELTRAEKHVHNFMMDSH
ISKEIKTAAANVLRETWLIY
KHTRVLREKDHTRVRVHQRKLLLAIHQLRSVRMEKRKLADQASTLVDLSKMQSLMYDLLSEVQSCRGELDTHVNSLEKHV
EELREGFRSLMPLLSNTLST
QNSSIRHLLQERETQAENAAAATTGDRAQ
>g2412.t1
LLCLSLCSNKIGDTGAAHLATVLGPFALTHGEVVERRRLLLDREQTSSEPSPIERSLAVPSNSSLNTNSSKAMSRGSVRR
RVEPVESPLLETSVQHRNGQ
VLLSGNSALCYLNLSGNQISEQSLPLFLSSLQKQTEGGGLLRLSLQRNRFCPDFEDFVKIQELMALRDPLNKTTASQLEH
EGQGD
>g2412.t2

LLHPTPEEEKRRHKKKRLVQSPNSYFMDVKCPGCYKITTVFSHAQTVVLCVGCSTVLCQPTGGKARLTEGSFRCEMVLRF
EKAENDTLAVVAMAPRCSFR
RKQH
>g2412.t3
LLCLSLCSNKIGDTGAAHLATVLGPFALTHGEVVERRRLLLDREQTSSEPSPIERSLAVPSNSSLNTNSSKAMSRGSVRR
RVEPVESPLLETSVQHRNGQ
VLLSGNSALCYLNLSGNQISEQSLPLFLSSLQKQTEGGGLLRLSLQLAKDLLHPTPEEEKRRHKKKRLVQSPNSYFMDVK
CPGCYKITTVFSHAQTVVLC
VGCSTVLCQPTGGKARLTEGSFRCEMVLRFEKAENDTLAVVAMAPRCSFRRKQH
>g2412.t4
LLCLSLCSNKIGDTGAAHLATVLGPFALTHGEVVERRRLLLDREQTSSEPSPIERSLAVPSNSSLNTNSSKAMSRGSVRR
RDKPSGALNEVEPVESPLLE
TSVQHRNGQVLLSGNSALCYLNLSGNQISEQSLPLFLSSLQKQTEGGGLLRLSLQLAKDLLHPTPEEEKRRHKKKRLVQS
PNSYFMDVKCPGCYKITTVF
SHAQTVVLCVGCSTVLCQPTGGKARLTEGSFRCEMVLRFEKAENDTLAVVAMAPRCSFRRKQH
>g2413.t1
LFCPPSGPSVPQPPVEEKINGILLGFRVRYRELLYDRLRSYALRTITSPSTSWTELTGGSPSVGRPGASVLHVNGGTD
>g2414.t1
FLPKGTWQEVSESTTKVVLDKLLPDTQYTVTVVPIYAEGDGPSLTDNGKTKPLGYARNLQVTDPTTSTLNVRWDPAEGSV
REYIVTWVPTAGGEQEVDQV
SGSTTSMVLKNLEPNTDYTVTVVPVYPEMEGKAQSKNGRTNPMGGVKNLKVVDPTISTLTARWEPAPGNVRDYKVFYSAQ
PGGAEHLETVSGGTTSVILR
GLNSDTVYDVAVVPVYPDVEGVRQAEKGKTTPTDPGYPLYRTGPPCVGSRVLTVGCVTEPLSGVRNLQVTDPTVSSLRVR
WEPAEGDVRQYNVIYAPAAG
GPEAMTQVSGRSTNTVLRGLTPDTEYKVSVVPIYADVEGKRMAENGKTRPLGGVKALQVTDPTTSSLKVRWEPAEGNVRY
YRLFYVPDQGGAEDMEQVSG
GTTNVVLRNLLSDTPYTVTVVPVYPEGEGLRQAETGTTLPRTPPRNLQVFHPTPNSLNTRWEPATGQVQQYRVTYTPLNG
AKPAESEQLFVLQPLKIKRP
GASAIVWDGLKDSVIWSGGGGARCQS
>g2414.t2
FLPKGTWQEVSESTTKVVLDKLLPDTQYTVTVVPIYAEGDGPSLTDNGKTKPLGYARNLQVTDPTTSTLNVRWDPAEGSV
REYIVTWVPTAGGEQEVDQV
SGSTTSMVLKNLEPNTDYTVTVVPVYPEMEGKAQSKNGRTNPMGGVKNLKVVDPTISTLTARWEPAPGNVRDYKVFYSAQ
PGGAEHLETVSGGTTSVILR
GLNSDTVYDVAVVPVYPDVEGVRQAEKGKTKPLSGVRNLQVTDPTVSSLRVRWEPAEGDVRQYNVIYAPAAGGPEAMTQV
SGRSTNTVLRGLTPDTEYKV
SVVPIYADVEGKRMAENGKTRPLGGVKALQVTDPTTSSLKVRWEPAEGNVRYYRLFYVPDQGGAEDMEQVSGGTTNVVLR
NLLSDTPYTVTVVPVYPEGE
GLRQAETGTTLPRTPPRNLQVFHPTPNSLNTRWEPATGQVQQYRVTYTPLNGAKPAESEQLFVLQPLKIKRPGASAIVWD
GLKDSVIWSGGGGARCQS
>g2414.t3
FLPKGTWQEVSESTTKVVLDKLLPDTQYTVTVVPIYAEGDGPSLTDNGKTKPLGYARNLQVTDPTTSTLNVRWDPAEGSV
REYIVTWVPTAGGEQEVDQV
SGSTTSMVLKNLEPNTDYTVTVVPVYPEMEGKAQSKNGRTNPMGGVKNLKVVDPTISTLTARWEPAPGNVRDYKVFYSAQ
PGGAEHLETVSGGTTSVILR
GLNSDTVYDVAVVPVYPDVEGVRQAEKGKTTPTDPGYPLYRTGPPCVGSRVLTVGCVTEPLSGVRNLQVTDPTVSSLRVR
WEPAEGDVRQYNVIYAPAAG
GPEAMTQVSGRSTNTVLRGLTPDTEYKVSVVPIYADVEGKRMAENGKTRPLGGVKALQVTDPTTSSLKVRWEPAEGNVRY
YRLFYVPDQGGAEDMEQVSG
GTTNVVLRNLLSDTPYTVTVVPVYPEGEGLRQAETGTTLPRTPPRNLQVFHPTPNSLNTRWEPATGQVQQYRVTYTPLNG
AKPAESEQLFVLQPLKISED
MNVLKGKADSCVVALDEHRLQRQPADQTRETR
>g2415.t1
MDLVSLNSGGRFCGGSLVSKDRVVSAAHCHNRVEVRLGEHHIRVTGGSEQFISYNINHGIMLIRLSKPATLNQYVQPVAL
PTECAPAGTPGDSNKLRCLD
LTVLSHERTVTIPTLACRPCSACAGFPEGGKDSRPGAVITDHKGPGGDSGGPRWYVMASCRGLVSRGYGCAEREHPWVSA

PWFVSSATGWYTP
>g2415.t2
MDLVSLNSGGRFCGGSLVSKDRVVSAAHCHNRVEVRLGEHHIRVTGGSEQFISYNINHGIMLIRLSKPATLNQYVQPVAL
PTECAPAGTPGDSNKLRCLD
LTVLSHERTVTIPTLACRPCSACAGFPEGGKDSRPGAVITDHKGPGGDSGGPRWYVMASCRGLVSRGYGCAEREHPWVSA
P
>g2415.t3
MDLVSLNSGGRFCGGSLVSKDRVVSAAHCHNRVEVRLGEHHIRVTGGSEQFISYNINHGIMLIRLSKPATLNQYVQPVAL
PTECAPAGTPGDSNKLRCLD
LTVLSHERTGDSGGPRWYVMASCRGLVSRGYGCAEREHPWVSAPWFVSSATGWYTP
>g2416.t1
ETGAGQQQLKPSHAGKVSCPLRVGLTFWHENGKLATTPADDGGGGVSAEVRYCC
>g2417.t1
MSTEMFGKTPMEVAVYQLHNFSISFFSSLLGGDVVSVKLDNSASGASVVAIDNKIEQAMDLVKNHLMYAVREEVEVLKEQ
IKELAEKNNQLERENSLLKN
LASPEQMEKFQLRVPADEMLALDNQSTSGSGHLHHHHHHHHPSQLLTQQQQQQQQQQQQQPCSLSPGSAV
>g2418.t1
MWTAYMGNSFLTHSHEEGYGKRRCAVLTSLILFPEDYFVTRQKVHASPATRGSRALQLKYVLFDETCPALHVEDRRFSDS
LDRPLAITGFDLTEKFLLRK
GTVVEDRPSFRLGSSPLIKPTE
>g2418.t2
MWTAYMGNSFLTHSHEEGYGKRRCAVLTSLILFPEDYFVTRQKVHASPATRGSRALQLKYVLFDFVFSLGPDETCPALHV
EDRRFSDSLDRPLAITGFDL
TEKFLLRKGTVVEDRPSFRLGSSPLIKPTE
>g2419.t1
LDKHRGHSGAWLLSGRIQTTAMDTHRSRLLYLTVLWLTLAEVWSKHVEQTNLFALRACCRRQSHFVYIGQGARRALTESG
QPSERTPSCGESHVCEPTGM
RVDQVLLFEGPREVEVIQECHCETKIPLCVRTPALRTYFFETPYETVVDVGGCSKSKGSPDGFSCVPTKFDSALVETPNK
VNLIRTVVACDLKESCYRVP
YVEYHYEIVAQADGIKEEKLK
>g2419.t2
MDTHRSRLLYLTVLWLTLAEVWSKHVEQTNLFALRACCRRQSHFVYIGQGARRALTESGQPSERTPSCGESHVCEPTGMR
VDQVLLFEGPREVEVIQECH
CETKIPLCVRTPALRTYFFETPYETVVDVGGCSKSKGSPDGFSCVPTKFDSALVETPNKVNLIRTVVACDLKESCYRVPY
VEYHYEIVAQADGIKEEKLK
>g2419.t3
LDKHRGHSGAWLLSGRIQTTAMDTHRSRLLYLTVLWLTRARRALTESGQPSERTPSCGESHVCEPTGMRVDQVLLFEGPR
EVEVIQECHCETKIPLCVRT
PALRTYFFETPYETVVDVGGCSKSKGSPDGFSCVPTKFDSALVETPNKVNLIRTVVACDLKESCYRVPYVEYHYEIVAQA
DGIKEEKLK
>g2420.t1
MMYFNGSNAYAEKSKKNNTKGKLLWKGAQHKPPHRRGAAAFPSAFPKAERVGKRVEPREDNKENRSGSDPPGVQEGAKRR
SVFERAVYDLKHSEKVQDEV
TFGRRVALYELRGEIGSGNFSQVRLGIHDLTNERVAVKILNKARFEKQSQVLHSEISCMQKLAHPNVVRLYEVVETFKRL
YLVMEYASGGELFSHICTRG
RLSDLESKLIFAQVLSAVKHMHDNDIVHRDLKAENVFFATSRCIKVGDFGFSTTCRRGDTLTTPCGSPPYAAPELFRQKA
YAGQSADVWALGILLYFMVS
GALPFTGASLQTLRCSILQGSFVVPAHASQPCHRLLTGILRPAPADRPTVEQMMASDWLRGVEYPQPFPASGLT
>g2420.t2
MMYFNGSNAYAEKSKKNNTKGKLLXKDPDARPLTASAVSASPLLLPPPLSSSRMPGKGAQHKPPHRRGAAAFPSAFPKAE
RVGKRVEPREDNKENRSGSD
PPGVQEGAKRRSVFERAVYDLKHSEKVQDEVTFGRRVALYELRGEIGSGNFSQVRLGIHDLTNERVAVKILNKARFEKQS
QVLHSEISCMQKLAHPNVVR
LYEVVETFKRLYLVMEYASGGELFSHICTRGRLSDLESKLIFAQVLSAVKHMHDNDIVHRDLKAENVFFATSRCIKVGDF
GFSTTCRRGDTLTTPCGSPP
YAAPELFRQKAYAGQSADVWALGILLYFMVSGALPFTGASLQTLRCSILQGSFVVPAHASQPCHRLLTGILRPAPADRPT

VEQMMASDWLRGVEYPQPFP
ASGLT
>g2421.t1
MSSKTSTKDKKSSFGKKLFRRGSVRSVGNFMSRVLRTLSTLSHFVSEVQTEEDKDDGGFTSFKTGSKDLHVDDCDIGGFI
SGDKVPGVSGLKNHGNTCFM
NAVLQCLSNTELFAEYLGLEQYKDDESNEDRPKTNGLHLQRKAPGNRGEVTEQLSGLVRALWTFEYTPQHSREFKNAVSK
SALQYRGNSQHDAQEFLLWL
LDRVHEDLNAVHPDSGPSIRPPIDEDDESLEGPSPPLSAGSFVQELFQAQYRSSLTCPHCQKQSNTFDPFLCISLPIPLP
HTR
>g2421.t2
MSSKTSTKDKKSSFGKKLFRRGSVRSVGNFMSRVLRTLSTLSHFVSEVQTEEDKDDGGFTSFKTGSKDLHVDDCDIGGFI
SGDKVPGVSGLKNHGNTCFM
NAVLQCLSNTELFAEYLGLEQYKDDESNEDRPKTNGLHLQRKAPGNRGEVTEQLSGLVRALWTFEYTPQHSREFKNAVSK
SALQYRGNSQHDAQEFLLWL
LDRVHEDLNAVHPDSGPSIRPPIDEDDESLEGPSPPLSAGSFVQELFQAQYRSSLTCPHCQKQSNTFDPFLCISLPIPLP
HT
>g2422.t1
CGSAPLDHISLNRLSNMRKRYRNGLGPSKEGEAQYSVVHCTGYIKAWPPA
>g2422.t2
CGSAPLDHISLNRLSNMRKRYRNGLGPSKEGEAQYSVVHCTGYIKAWPPAVLALYRLCVEARTSGCYQSLNEAVHRSRGE
GARRFQHAAGASVRAATDGK
QMLCHNKNSCGGGREDDVQE
>g2422.t3
CGSAPLDHISLNRLSNMRKRYRNGLGPSKEGEAQYSVVHCTGYIKAWPPAVLALYRLCVEARTSGCYQSLNEAVHRSR
>g2423.t1
MACLVLTMYCFCFTGAFVASDNSQRFQETCYAFALTPQQVQQISSSMFCLSETSCPQEDHFPPNLCVKVNGKPCTLPGYL
PPTKNGVEPKRPSRPINITS
LVRLSTTVPNTIVVSWTSEIGRVRRPKGSNTALMHTAAARCAPLPLGKMRLTIPCRALTCSHLQCFDATLYIQMNEKKPT
WVCPVCDKKAPYEHLIIDGL
FMEILNSCSDCDEIQFTEDGNWAPMRSKKEVQEVSASYNGVDSDSGRTDASEQKHGSGHDSKKVDVIDLTLDSSSDEELD
DEPPPKRACPSLSPNSPPAT
KG
>g2423.t2
MVMSLRVSELQVLLGYAGRNKHGRKHELLTKAIHLLKAGCSPAVQMKIKELYRRRFPTKMVSPLDLALPTVHSSTSLPTG
LTQLGFDTHGAPSPLLPVSL
LGPKHELGLSHLPSALHPVHPDVKLQRLPFYDMLDELIKPTSLXTSCPQEDHFPPNLCVKVNGKPCTLPGYLPPTKNGVE
PKRPSRPINITSLVRLSTTV
PNTIVVSWTSEIGRVRRPKGSNTALMHTAAARCAPLPLGKMRLTIPCRALTCSHLQCFDATLYIQMNEKKPTWVCPVCDK
KAPYEHLIIDGLFMEILNSC
SDCDEIQFTEDGNWAPMRSKKEVQEVSASYNGVDSDSGRTDASEQKHGSGHDSKKVDVIDLTLDSSSDEELDDEPPPKRA
CPSLSPNSPPATKG
>g2423.t3
MSLRVSELQVLLGYAGRNKHGRKHELLTKAIHLLKAGCSPAVQMKIKELYRRRFPTKMVSPLDLALPTVHSSTSLPTGLT
QLGFDTHGAPSPLLPVSLLG
PKHELGLSHLPSALHPVHPDVKLQRLPFYDMLDELIKPTSLXTSCPQEDHFPPNLCVKVNGKPCTLPGYLPPTKNGVEPK
RPSRPINITSLVRLSTTVPN
TIVVSWTSEIGRVRRPKGSNTALMHTAAARCAPLPLGKMRLTIPCRALTCSHLQCFDATLYIQMNEKKPTWVCPVCDKKA
PYEHLIIDGLFMEILNSCSD
CDEIQFTEDGNWAPMRSKKEVQEVSASYNGVDSDSGRTDASEQKHGSGHDSKKVDVIDLTLDSSSDEELDDEPPPKRACP
SLSPNSPPATKG
>g2424.t1
VSTAGSKESEDRPPSRSQQQPDHPGHPDQPEPLGPSTLPSTVMPALNLTQTLQDSLRKAARHLRRAMVLAHRGAHWACLQ
FACRTAWEQSSSLVDLTARD
PDHPIPLDLLHTTFTPLLVLATDLLMDMMDTLGLWKAFEGDASQEDLESSLGSWVDLRWVQTLVLDTLGLLHAHARWESL
AHYALLYNCYTRGRYTLQVS
PLLVHAQRRLLERVAEFGGPPAPQPHHVRTHSCTETPVHRSMCLVRVPLDVEDTLAAYRLALQNQPHSLRLLQHSRTLLH

LLLAHTHLTAGVQQVGGGGG
LGSPGGQVEICPVVTAGPDPQPPDLTGQDFATPSHLYGLPLGPQHTHTVITAYSASIKFLEAGELRAQALHDLGNLQLYS
GNTRAAHSSWSKAVDCALHG
VGVLDSWEGVSWAGGARSQQDTLKLAGVWGCLQAAVITAKIAQYILTSHIHQRTMCCLLSAHLFKRDLLYGSHSIGEELL
PGVDLFSDPIRVHPATTVAS
LHFLCHWLYSARHYVTLLPMLALFLHFVRSRCRDVQRTVEGALLKVRALTELSMFGQAIKEAVELSRGQGVTLPHGPDIS
RDTQQQTRTFHDNKPILENI
QAVEELVNCRLSAEVRGRYGPVLCRRFSLARVQLVLALCGAAPGPPVRGPCGDTAVSPAEPSASSAGQNRGTVDTDRTGR
GWKEPDRLELGSAREKLSLG
RVKRVLQSNGGPPQAAEGPEYCSSVQQDLEIAIETHLLLSNMHLQRGHAAL
>g2424.t2
VSTAGSKESEDRPPSRSQQQPDHPGHPDQPEPLGPSTLPSTVMPALNLTQTLQDSLRKAARHLRRAMVLAHRGAHWACLQ
FACRTAWEQSSSLVDLTARD
PDHPIPLDLLHTTFTPLLVLATDLLMDMMDTLGLWKAFEGDASQEDLESSLGSWVDLRWVQTLVLDTLGLLHAHARWESL
AHYALLYNCYTRGRYTLQVS
PLLVHAQRRLLERVAEFGGPPAPQPHHVHRSMCLVRVPLDVEDTLAAYRLALQNQPHSLRLLQHSRTLLHLLLAHTHLTA
GVQQVGGGGGLGSPGGQVEI
CPVVTAGPDPQPPDLTGQDFATPSHLYGLPLGPQHTHTVITAYSASIKFLEAGELRAQALHDLGNLQLYSGNTRAAHSSW
SKAVDCALHGVGVLDSWEGV
SWAGGARSQQDTLKLAGVWGCLQAAVITAKIAQYILTSHIHQRTMCCLLSAHLFKRDLLYGSHSIGEELLPGVDLFSDPI
RVHPATTVASLHFLCHWLYS
ARHYVTLLPMLALFLHFVRSRCRDVQRTVEGALLKVRALTELSMFGQAIKEAVELSRGQGVTLPHGPDISRDTQQQTRTF
HDNKPILENIQAVEELVNCR
LSAEVRGRYGPVLCRRFSLARVQLVLALCGAAPGPPVRGPCGDTAVSPAEPSASSAGQNRGTVDTDRTGRGWKEPDRLEL
GSAREKLSLGRVKRVLQSNG
GPPQAAEGPEYCSSVQQDLEIAIETHLLLSNMHLQRGHAAL
>g2424.t3
VSTAGSKESEDRPPSRSQQQPDHPGHPDQPEPLGPSTLPSTVMPALNLTQTLQDSLRKAARHLRRAMVLAHRGAHWACLQ
FACRTAWEQSSSLVDLTARD
PDHPIPLDLLHTTFTPLLVLATDLLMDMMDTLGLWKAFEGDASQEDLESSLGSWVDLRWVQTLVLDTLGLLHAHARWESL
AHYALLYNCYTRGRYTLQVS
PLLVHAQRRLLERVAEFGGPPAPQPHHVRTHSCTETPVHRSMCLVRVPLDVEDTLAAYRLALQNQPHSLRLLQHSRTLLH
LLLAHTHLTAGVQQVGGGGG
LGSPGGQVEICPVVTAGPDPQPPDLTGQDFATPSHLYGLPLGPQHTHTVITAYSASIKFLEAGELRAQALHDLGNLQLYS
GNTRAAHSSWSKAVDCALHG
VGVLDSWEGVSWAGGARSQQDTLKLAGVWGCLQAAVITAKIAQYILTSHIHQRTMCCLLSAHLFKRDLLYGSHSIGEELL
PGVDLFSDPIRVHPATTVAS
LHFLCHWLYSARHYVTLLPMLALFLHFVRALTELSMFGQAIKEAVELSRGQGVTLPHGPDISRDTQQQTRTFHDNKPILE
NIQAVEELVNCRLSAEVRGR
YGPVLCRRFSLARVQLVLALCGAAPGPPVRGPCGDTAVSPAEPSASSAGQNRGTVDTDRTGRGWKEPDRLELGSAREKLS
LGRVKRVLQSNGGPPQAAEG
PEYCSSVQQDLEIAIETHLLLSNMHLQRGHAAL
>g2425.t1
MDCACVGHQRISRNSSKISATPRAPRSPQIWGNLGSELRLRSGRILQVTSCYPAALREMEELEVDILPAAPSWLFKHILT
SCSSKECQGYL
>g2426.t1
MIMVTSICGYRRLKNGLVLLSVTPHLAGGAALHAAVPNRNQHLRKKVCLCFPCGTPADRHPAETNKLRDKPSKGPGIALG
TRGGRCQKQRTDGARGKHGC
RAETTLMEGHSSSPLMTPWSLRANGTGTGTSHGEIPVRYWHLTAETGRRGAERRSETVQKVVEFQRRSVAVGTARLSALR
GVHGNNSSIPAVGMDNQASR
STVSQIHLNLKKKSKFQFPRSLSSSC
>g2426.t2
MVRRGEQKRRTGEENRGAALHAAVPNRNQHLRKKVCLCFPCGTPADRHPAETNKLRDKPSKGPGIALGTRGGRCQKQRTD
GARGKHGCRAETTLMEGHSS
SPLMTPWSLRANGTGTGTSHGEIPVRYWHLTAETGRRGAERRSETVQKVVEFQRRSVAVGTARLSALRGVHGNNSSIPAV
GMDNQASRSTVSQIHLNLKK

KSKFQFPRSLSSSC
>g2426.t3
LNLKEMLHIMFMEQNRKTLQSRNQRTGEQRLKNGLVLLSVTPHLAGGAALHAAVPNRNQHLRKKVCLCFPCGTPADRHPA
ETNKLRDKPSKGPGIALGTR
GGRCQKQRTDGARGKHGCRAETTLMEGHSSSPLMTPWSLRANGTGTGTSHGEIPVRYWHLTAETGRRGAERRSETVQKVV
EFQRRSVAVGTARLSALRGV
HGNNSSIPAVGMDNQASRSTVSQIHLNLKKKSKFQFPRSLSSSC
>g2427.t1
LRNEVKTEEEGGTKALSVEEVQTRIVWYNAQISGNIMRMCADESYTGFIKVQMRLSRPVTVTAAGGRATEGRTNGPSRSL
SAPLLPVTGGPKETAAGQSA
ENRTSFYMPANCVTQLHISSTTTAREVVQGLLKKYKVLDNPRKFALYKETHRDGQVALFQKLPLCECPLLLRLQAGPDHK
QLSFALKENETGEVE
>g2427.t2
LRNEVKTEEEGGTKALSVEEVQTRIVWYNAQISGNIMRMCADESYTGFIKVQMRLSRPVTVTAAGGRATEGRTNGPSRSL
SAPLLPVTGGPKETAAGQSA
ENRTSFYMPANCVTQLHISSTTTAREVVQGLLKKYKVLDNPRKFALYKETHRDGQALFQKLPLCECPLLLRLQAGPDHKQ
LSFALKENETGEVE
>g2427.t3
LRNEVKTEEEGGTKALSVEEVQTRIVWYNAQISGNIMRMCADESYTGFIKVQMRLSRPVTVTAAGGRATEGRTNGPSRSL
SAPLLPVTGGPKETAAGQSA
ENRTSFYMPANCVTQLHISSTTTAREVVQGLLKKYKVLDNPRKFALYKETHRDGQVALFQKLPLCECPLLLRLQAGPDHK
QLSFALKENETGEVEKLFPV
>g2428.t1
NLKIGYFSQHHVDQLDLNVCSVELLLNKFPGRNEEEYRHQLGGYGITGELATRPVASLSGGQKSRVAFAQMTMPCPNFYI
LDEPTNHLDMETIEALAKAL
SKFKGGVVLVSHDERLIRLVCKELWVCEKGAVRRIDGGFDEYRDILQETFKKEGYL
>g2429.t1
LNSIPGPEELGLDGRELESSVVEEVEVLTRGQRENPAWFDWRKNRLTASNVHRIAHSRFVAGTSRAPLASYLSDITGHGS
RVQTKAMKWGVDMEARAVKE
YQVLKSKLLGRDVVVQDSGLFIDPKRPWLAATPDGIVMDRLSGQRLLCLEVKCPFKHRGHTVEEACREDREFCLQIKELP
RQLSVEEELPRQVSGEEELA
SQVSVQEESPSLVSVEEELPSQQTAYQLKQNHSYYSQIQCQLAVTGIQEADLVVFTLKETALVPVTFDPEFWEQTVTKLQ
LFYEEGLLPYLRSMRSDPVV
RPEM
>g2429.t2
LNYGLGDNAVGGDNAQNSQKPLNSIPGPEELGLDGRELESSVVEEVEVLTRGQRENPAWFDWRKNRLTASNVHRIAHSRF
VAGTSRAPLASYLSDITGHG
SRVQTKAMKWGVDMEARAVKEYQVLKSKLLGRDVVVQDSGLFIDPKRPWLAATPDGIVMDRLSGQRLLCLEVKCPFKHRG
HTVEEACREDREFCLQIKEL
PRQLSVEEELPRQVSGEEELASQVSVQEESPSLVSVEEELPSQQTAYQLKQNHSYYSQIQCQLAVTGIQEADLVVFTLKE
TALVPVTFDPEFWEQTVTKL
QLFYEEGLLPYLRSMRSDPVVRPEM
>g2429.t3
MNAISRWFVASTSLVQGGGPDALGQPLLNYGLGDNAVGGDNAQNSQKPLNSIPGPEELGLDGRELESSVVEEVEVLTRGQ
RENPAWFDWRKNRLTASNVH
RIAHSRFVAGTSRAPLASYLSDITGHGSRVQTKAMKWGVDMEARAVKEYQVLKSKLLGRDVVVQDSGLFIDPKRPWLAAT
PDGIVMDRLSGQRLLCLEVK
CPFKHRGHTVEEACREDREFCLQIKELPRQLSVEEELPRQVSGEEELASQVSVQEESPSLVSVEEELPSQQTAYQLKQNH
SYYSQIQCQLAVTGIQEADL
VVFTLKETALVPVTFDPEFWEQTVTKLQLFYEEGLLPYLRSMRSDPVVRPEM
>g2430.t1
MLDHCYSPFKGGPSLDTCLGQTQGLICHLLHGDLDSSRQSRYVLRKAIRTAMWNYKDKLESYYKSSDPRWTWSGLRSLTG
YKGRDPYLQAIRLLNH
>g2430.t2
MLDHCYSPFKGGPSLDTCLGQTQGLICHLLHGDLDSSRQSRYVLRKAIRTAMWNYKDKLESYYKSSDPRWTWSGLRSLTG
YKGRGEGIDNKDPYLQAIRL

LNH
>g2430.t3
MLDHCYSPFKGGPSLDTCLGLICHLLHGDLDSSRQSRYVLRKAIRTAMWNYKDKLESYYKSSDPRWTWSGLRSLTGYKGR
DPYLQAIRLLNH
>g2431.t1
MESCRFLGAIIAQDLKCDLNIRSLTMKGQQRMYFLRQLKKFNLPKTMMVHFYVIESILTSSISISFTLLRRRLRST
>g2431.t2
MSCASLLRSLHHHAHHQKPLPFSLYTNSYTSSHQTVKLLKLMDDTTLIGLISGGDESAYRWETDQLVTWCSQKNLELKAQ
KTVEIVVSLTMKGQQRMYFL
RQLKKFNLPKTMMVHFYVIESILTSSISISFTLLRRRLRST
>g2431.t3
MSCASLLRSLHHHAHHQKPLPFSLYTNSYTSSHQTVKLLKLMDDTTLIGLISGGDESAYRWETDQLVTWCSQKNLELKAQ
KTVEIVVYFIDFFEEPSPTR
PRHPV
>g2432.t1
GTSKTPVSEELPGDKTQNHPTSRKGKRSQKTKHLETQKQAGGGKRARVLPRSSPGTPFPRVPQDPKDRVLALKRRTPLDS
ENQPAGKSRAPRYLLRSRVV
KQSPRRQSPRKPKRLLRKC
>g2432.t2
GTSKTPVSEELPGDKTQNHPTSRKGKRSQKTKHLETQKQAGGGKRARVLPRSSPGTPFPRVPQDPKDRVLALKRRTPLDS
ENQPAGKSRAPRYLLRSRVV
KQSPRRQSPRKPKRLLRKDRSEWLAEPPPESSQFMRRLCSCRDPAALALTPVQKVHPLLLRKPSPSPARELAGLHVNL
>g2433.t1
LASWVVSRFKPTQARADVYWRLRLRIPVEVYGQWVGKPLKERQLLPTIGRCYFYLALENSNATDYISEKLWRNGLGAGAV
PVVMGPSRATYEALAPPDSF
IHVQDFGSVEALANHLRSVASDGEAYAKYFRWQRTHKVEMYRDWRQRLCQICVRYPRLPPHKVYHDLDAWPCNGLISPPV
YTVDRGDWWAELTVTRLGVS
FGVRS
>g2434.t1
MKRPVTWAAAAVGAKPRGSGGSLRAVEPRGASDGWDTGFTCGTCETTGRPKKNDMGRKDEVGGEGRNEVCFFLAAAAALA
VTLRPPGAE
>g2435.t1
LHHDRTIRASWYSTSQWPWDPQSIVVLDKVGAVQKSITVLDIRRLSITDCGHPCTAVDPDGSLVAVSPQRFIDLFKF
>g2435.t2
LAEIDKYSSPGSLHHDRTIRASWYSTSQWPWDPQSIVVLDKVGAVQKSITVLDIRRLSITDCGHPCTAVDPDGSLVAVSP
QRFIDLFKF
>g2436.t1
MAQRKKRLTKRKGRPGRREPEDSDSGADVELVTEDNSCRKLPHFPAATDCTSDVEPDTRQLVRSQNKKKKKSGGFQSMGL
SYPVYKGVMKKGYKVPTPIQ
RKTVPVILDGKDIVAMARTGSGKTAAFLIPMFEKLKGPQAQTGARALILTPTRELALQTMKFTKELGRFTGLKTALILGG
DGMDDQFAALHENPDIIIGT
PGRLMHVIQQMDLKLQSVEYVVFDEADRLFEMGFAEQLQEILRRLPDNRQSLLFSATLPKLLVEFARAGLTEPVLIRLDV
DTKLSDQLKLSFFHLRLDDK
PALLLHLLRNVVKAQEQTVVFVATKHHVEYVKELLTLEGVECAYIYSALDQTARKINIGKFVHRKAMVLIVTDVAARGID
IPLLDNVINYNFPSKAKLFL
HRVGRVARAGRSGVAYSLVCSDEVPFVYDLHLFLGRPLELATPQHTADADGVFGRVPQCVLDDEGAQLRTVHENSLDLSN
IHHVSENAYKQYLKSRPSPS
PESIKRVKNMDLSALAVHPLLGAGMDKMELERLHIVDAIKGYKARSTIFEINSNSKTVACQVMRRKRSRDTKLVEKFSKK
REDLVTETRLLHAHRGPSDR
PPVETLGGEGSEEEEELLQGVFSEVVGGKRRQPAQEDQDSEKPKLKKSKAGGKDDEFFIPYRPKDFDTERGLSLGGEGSM
FDQQAASAVLDLMGDEGGAL
NQHKRIMKWDRKKKRFVSDTGREDQKKKVRLETGQVVSSKNKKNLASAMKSGRKSTRLMTATLKGLQEEEEGEAEEEEEE
GALEEVGLVSGGLC
>g2436.t2
MAQRKKRLTKRKGRPGRREPEDSDSGADVELVTEDNSCRKLPHFPAATDCTSDVEPDTRQLVRSQNKKKKKSGGFQSMGL
SYPVYKGVMKKGYKVPTPIQ

RKTVPVILDGKDIVAMARTGSGKTAAFLIPMFEKLKGPQAQTGARALILTPTRELALQTMKFTKELGRFTGLKTALILGG
DGMDDQFAALHENPDIIIGT
PGRLMHVIQQMDLKLQSVEYVVFDEADRLFEMGFAEQLQEILRRLPDNRQSLLFSATLPKLLVEFARAGLTEPVLIRLDV
DTKLSDQLKLSFFHLRLDDK
PALLLHLLRNVVKAQEQTVVFVATKHHVEYVKELLTLEGVECAYIYSALDQTARKINIGKFVHRKAMVLIVTDVAARGID
IPLLDNVINYNFPSKAKLFL
HRVGRVARAGRSGVAYSLVCSDEVPFVYDLHLFLGRPLELATPQHTADADGVFGRVPQCVLDDEGAQLRTVHENSLDLSN
IHHVSENAYKQYLKSRPSPS
PESIKRVKNMDLSALAVHPLLGAGMDKMELERLHIVDAIKGYKARSTIFEINSNSKTVACQVMRRKRSRDTKLVEKFSKK
REDLVTETRLLHAHRGPSDR
PPVETLGGEGSEEEEELLQGVFSEVVGGKRRQPAQEDQDSEKPKLKKSKAGGKDDEFFIPYRPKDFDTERGLSLGGEGSM
FDQQAASAVLDLMGDEGGAL
NQHKRIMKWDRKKKRFVSDTGREDQKKKVRLETGQVVSSKNKKNLYPVAPLLSHQDFCLSVWDACAAVSQYCDHGASAMK
SGRKSTRLMTATLKGLQEEE
EGEAEEEEEEGALEEVGLVSGGLC
>g2436.t3
MAQRKKRLTKRKGRPGRREPEDSDSGADVELVTEDNSCRKLPHFPAATDCTSDVEPDTRQLVRSQNKKKKKSGGFQSMGL
SYPVYKGVMKKGYKVPTPIQ
RKTVPVILDGKDIVAMARTGSGKTAAFLIPMFEKLKGPQAQTGARALILTPTRELALQTMKFTKELGRFTGLKTALILGG
DGMDDQFAALHENPDIIIGT
PGRLMHVIQQMDLKLQSVEYVVFDEADRLFEMGFAEQLQEILRRLPDNRQSLLFSATLPKLLVEFARAGLTEPVLIRLDV
DTKLSDQLKLSFFHLRLDDK
PALLLHLLRNVVKAQEQTVVFVATKHHVEYVKESVEFLQLTCFSVQLLTLEGVECAYIYSALDQTARKINIGKFVHRKAM
VLIVTDVAARGIDIPLLDNV
INYNFPSKAKLFLHRVGRVARAGRSGVAYSLVCSDEVPFVYDLHLFLGRPLELATPQHTADADGVFGRVPQCVLDDEGAQ
LRTVHENSLDLSNIHHVSEN
AYKQYLKSRPSPSPESIKRVKNMDLSALAVHPLLGAGMDKMELERLHIVDAIKGYKARSTIFEINSNSKTVACQVMRRKR
SRDTKLVEKFSKKREDLVTE
TRLLHAHRGPSDRPPVETLGGEGSEEEEELLQGVFSEVVGGKRRQPAQEDQDSEKPKLKKSKAGGKDDEFFIPYRPKDFD
TERGLSLGGEGSMFDQQAAS
AVLDLMGDEGGALNQHKRIMKWDRKKKRFVSDTGREDQKKKVRLETGQVVSSKNKKNLASAMKSGRKSTRLMTATLKGLQ
EEEEGEAEEEEEEGALEEVG
LVSGGLC
>g2437.t1
LGNHKEDSPFLNNAEAARKNDFYDRNLALFEEELDIRPKVSSFLSRLVNYTNITQGAREHEEEESANLSRRKAPKAPNMG
TLMGVYLPCLQNIFGVILFL
RLTWIVGTAGIAQSLIIVLMCCSCTMLTAISMSAIATNGVVPAGGAYFMISRSLGPEFGGAVGLCFYLGTTFAAAMYILG
AIEIFLKYLLPQAAIFHGGD
SALLNNMRVYGTLCLSLMALVVFVGVKYVNKLASLFLACVIISIVSVYAGALKSISHPPVFTICMLGNRTLVRDLFDVCA
KTVVVGNETVPSQLWRSFCP
QGNVSSAQCDEYFLQNNVTEIPAIPGLASGIISDNMWGDYMRKGEVMERAGLPSRDVPGAVESFGVYVTADIATSFTLLV
GIFFPSATGDLRDAQKSIPV
GTILAITTTSLVYFSSVVLFGACIEGVVLRDKFGDAVNKNLVVGTLSWPSPWVIVIGSFFSTVGAGLQSLTGAPRLLQAI
AKDNIIPFLR
>g2437.t2
LGNHKEDSPFLNNAEAARKNDFYDRNLALFEEELDIRPKVSSFLSRLVNYTNITQGAREHEEEESANLSRRKAPKAPNMG
TLMGVYLPCLQNIFGVILFL
RLTWIVGTAGIAQSLIITMLTAISMSAIATNGVVPAGGAYFMISRSLGPEFGGAVGLCFYLGTTFAAAMYILGAIEIFLK
YLLPQAAIFHGGDSALLNNM
RVYGTLCLSLMALVVFVGVKYVNKLASLFLACVIISIVSVYAGALKSISHPPVFTICMLGNRTLVRDLFDVCAKTVVVGN
ETVPSQLWRSFCPQGNVSSA
QCDEYFLQNNVTEIPAIPGLASGIISDNMWGDYMRKGEVMERAGLPSRDVPGAVESFGVYVTADIATSFTLLVGIFFPSA
TGDLRDAQKSIPVGTILAIT
TTSLVYFSSVVLFGACIEGVVLRDKFGDAVNKNLVVGTLSWPSPWVIVIGSFFSTVGAGLQSLTGAPRLLQAIAKDNIIP
FLR
>g2437.t3

LGNHKEDSPFLNNAEAARKNDFYDRNLALFEEELDIRPKVSSFLSRLVNYTNITQGAREHEEEESANLSRRKAPKAPNMG
TLMGVYLPCLQNIFGVILFL
RLTWIVGTAGIAQSLIIVLMCCSCTMLTAISMSAIATNGVVPAGGAYFMISRSLGPEFGGAVGLCFYLGTTFAAAMYILG
AIEIFLKYLLPQAAIFHGGD
SALLNNMRVYGTLCLSLMALVVFVGVKYVNKLASLFLACVIISIVSVYAGALKSISHPPVFTICMLGNRTLVRDLFDVCA
KTVVVGNETVPSQLWRSFCP
QGNVSSAQCDEYFLQNNVTEIPAIPGLASGIISDNMWGDYMRKGEVMERAGLPSRDVPGAVESFGVYVTADIATSFTLLV
GIFFPSATGDLRDAQKSIPV
GTILAITTTSLVYFSSVVLFGACIEGVVLRDKFGDAVNKNLVVGTLSWPSPWVIVIGSFFSTVGAGLQSLTGAPRLLQAI
AKDNIIPFLRVTITRAGRCK
>g2438.t1
LSPVGSTSSTKNYACNQCGRAYRHASSLLNHKNSHKTGTYFCNSCQKEFPNLMSLKNHRRIHTEPKRYQCPDCGKSFRVS
TALICHRRIHTKEKPFSCQQ
CDKRFSSRSNLRHHMKVHWSGSLLSRGSSSAFLTIPSRPFL
>g2438.t2
LKLILLSYTFSLLNVNLSPVGSTSSTKNYACNQCGRAYRHASSLLNHKNSHKTGTYFCNSCQKEFPNLMSLKNHRRIHTE
PKRYQCPDCGKSFRVSTALI
CHRRIHTKEKPFSCQQCDKRFSSRSNLRHHMKVHWSGSLLSRGSSSAFLTIPSRPFL
>g2439.t1
ISYACLVVLLCVLRASELSGEDVLMALCLVLGWSNVMFFARGFQMLGPYVIMIQKIIFGDLTKFMWLSFIVLIGFSTCLY
LNPLSSHALPPFLSVPLSWL
ALSAAALWMVYMTQDPESLPPYRSFPVTLFSQFELSVGLIDLPVDHTFYTPPIVHVLHCSFSVVSYILLLNLLIAMMSDT
HWRVAQERDELWRTQ
>g2439.t2
ISYACLVVLLCVLRASELSGEDVLMALCLVLGWSNVMFFARGFQMLGPYVIMIQKIIFGDLTKFMWLSFIVLIGFSTSLW
MVYMTQDPESLPPYRSFPVT
LFSQFELSVGLIDLPVDHTFYTPPIVHVLHCSFSVVSYILLLNLLIAMMSDTHWRVAQERDELWRTQ
>g2439.t3
LSAHRSVVSLSKTLNPNLLPGLLSNSSPLLLGTMGWVKRREHIYISYACLVVLLCVLRASELSGEDVLMALCLVLGWSNV
MFFARGFQMLGPYVIMIQKI
IFGDLTKFMWLSFIVLIGFSTCLYLNPLSSHALPPFLSVPLSWLALSAAALWMVYMTQDPESLPPYRSFPVTLFSQFELS
VGLIDLPVDHTFYTPPIVHV
LHCSFSVVSYILLLNLLIAMMSDTHWRVAQERDELWRTQ
>g2440.t1
MKLLFFQECPSGVVNEETFKDIYAQFFPQGEALPGSQPASSVAQMMEPLVLSSGNMCLARVQLPPNASTYAHFLFNAFDT
DHNGSVSFEDFVMGLSILLR
GTIQEKLNWAFNLYDINKDGYITKEEMLDIMKAIYDMMGKCTYPVLKEETPHQHVEIFFQARPPELAIHSTTTCCKMDKN
KDGVVTIDEFVDCCQNDDNI
MRSMHLFENVI
>g2440.t2
MKLLFFQECPSGVVNEETFKDIYAQFFPQGEALPGSQPASSVAQMMEPLVLSSGNMCLARVQLPPNASTYAHFLFNAFDT
DHNGSVSFEDFVMGLSILLR
GTIQEKLNWAFNLYDINKDGYITKEEMLDIMKAIYDMMGKCTYPVLKEETPHQHKMDKNKDGVVTIDEFVDCCQNDDNIM
RSMHLFENVI
>g2440.t3
MKLLFFQECPSGVVNEETFKDIYAQFFPQGASSVAQMMEPLVLSSGNMCLARVQLPPNASTYAHFLFNAFDTDHNGSVSF
EDFVMGLSILLRGTIQEKLN
WAFNLYDINKDGYITKEEMLDIMKAIYDMMGKCTYPVLKEETPHQHVEIFFQARPPELAIHSTTTCCKMDKNKDGVVTID
EFVDCCQNDDNIMRSMHLFE
NVI
>g2441.t1
AGLQYSPDPHTDTVGVGLQYSPDPITDTVGVGLQYSPDPITDTVGVGLQYSPNPHTDTVGVGLQYSPDPITDTVGVRQGC
EEDGGHS
>g2442.t1
MSGRKSPEVYLDLLSQPCRAVHILLSCNKVPFTLRTVALRKGENKTPEFTSLNPMQKVPVLVDNGFALTESVAILKYLVT
KYSLPEHWYPSQPERRAKVD

EYISWHHTNTRPHSAKVFLTEVLFPRMSGSAVDEVRLERALRELKETLDMLESMFLKRQPFLCGDDITLADLLAVCELMQ
>g2442.t2
MSGRKSPEVYLDLLSQPCRAVHILLSCNKVPFTLRTVALRKGEQNHQANSLRGENKTPEFTSLNPMQKVPVLVDNGFALT
ESVAILKYLVTKYSLPEHWY
PSQPERRAKVDEYISWHHTNTRPHSAKVFLTEVLFPRMSGSAVDEVRLERALRELKETLDMLESMFLKRQPFLCGDDITL
ADLLAVCELMQ
>g2442.t3
MSGRKSPEVYLDLLSQPCRAVHILLSCNKVPFTLRTVALRKGENKTPEFTSLNPMQKVPVLVDNGFALTESVAILKYLVT
KYSLPEHWYPSQPERRAKVD
EYISWHHTNTRPHSAKVFLTEVLFPRMSGSAVDEVRLERALRELKETLDMLESMFLKRQPFLCGDDITLADLLAVCELMQ
VRGHLPDTGLPSGLPLPAPR
HK
>g2443.t1
LVLDILTVGGLQKLVKRRGPWGYPPGFFDYIAMDTYSFPAAHASRAVMVAGFLLNHLVLAVPLRILLYLWAFLAGVSRVL
LGRHHLFDVGCGFILGFLHF
HLVESVWLDSDTSADKLIKIWGAFDGKFEKSIVGHKLGISDVSWSSDSNLLVSASDDKTLKIWDNCSGKCLKTLKGHSNY
VFCCNFNPQSNLVVSGSFDE
SVRIWDVKTGKCLKTLPAHSDPVSAVHFNRDGSLIVSSSYDGLCRIWDTASGQCLKTLIDDDNPPVSFVKFSPNGKYILA
ATLDNTLKLWDYSKGKCLKT
YTGHKNEKYCVFANFSVTGGKWIVSGSEDSMVYIWNLQTKEIVQKLQGHTDVVISTACHPTENIIASAALENDKTIKLWK
SDI
>g2443.t2
MATEEKKPETENIKHCSSSGSGSKSNPAKPNYAVKFTLAGHTKAVSSVKFSPNGEWLASSSADKLIKIWGAFDGKFEKSI
VGHKLGISDVSWSSDSNLLV
SASDDKTLKIWDNCSGKCLKTLKGHSNYVFCCNFNPQSNLVVSGSFDESVRIWDVKTGKCLKTLPAHSDPVSAVHFNRDG
SLIVSSSYDGLCRIWDTASG
QCLKTLIDDDNPPVSFVKFSPNGKYILAATLDNTLKLWDYSKGKCLKTYTGHKNEKYCVFANFSVTGGKWIVSGSEDSMV
YIWNLQTKEIVQKLQGHTDV
VISTACHPTENIIASAALENDKTIKLWKSDI
>g2443.t3
MCEHPTLVLDILTVGGLQKLVKRRGPWGYPPGFFDYIAMDTYSFPAAHASRAVMVAGFLLNHLVLAVPLRILLYLWAFLA
GVSRVLLGRHHLFDVGCGFI
LGFLHFHLVESVWLDSDTSADKLIKIWGAFDGKFEKSIVGHKLGISDVSWSSDSNLLVSASDDKTLKIWDNCSGKCLKTL
KGHSNYVFCCNFNPQSNLVV
SGSFDESVRIWDVKTGKCLKTLPAHSDPVSAVHFNRDGSLIVSSSYDGLCRIWDTASGQCLKTLIDDDNPPVSFVKFSPN
GKYILAATLDNTLKLWDYSK
GKCLKTYTGHKNEKYCVFANFSVTGGKWIVSGSEDSMVYIWNLQTKEIVQKLQGHTDVVISTACHPTENIIASAALENDK
TIKLWKSDI
>g2443.t4
LVLDILTVGGLQKLVKRRGPWGYPPGFFDYIAMDTYSFPAAHASRAVMVAGFLLNHLVLAVPLRILLYLWAFLAGVSRVL
LGRHHLFDVGCGFILGFLHF
HLVESVWLDSDTCQTIISISTLSWAPLI
>g2444.t1
FTSTVSAAKSTFYRNKINSSASNPRKQFSMTSSLLNPPSLRPLALLTADDFADHFTKKAVWTDILPFVASLVNSSLSSGL
VPTPFKTAQTAAQEAHPRHC
>g2444.t2
MQSLGFRPEDAAGLCACAAPSPSAAGRMADDRRDPSDWTGGSEPTEDDLDDHKNTTMLCSRLEVSVAETEKRNGKNAMNM
QETFTVYLVETR
>g2444.t3
FTSTVSAAKSTFYRNKINSSASNPRKQFSMTSSLLNPPSLRPLALLTADDFADHFTKKVSSQHPSKLLKPLLKKPTLDTA
DSLGFRPEDAAGLCACAAPS
PSAAGRMADDRRDPSDWTGGSEPTEDDLDDHKNTTMLCSRLEVSVAETEKRNGKNAMNMQETFTVYLVETR
>g2445.t1
HSPALRPSAPAPSLALHSDAKAVSEPGDPGSSAGGRTEDRVAKFRAASEPSFFVKFSPIPDKVARTKSDSQSPPTPVAAA
GTQESNNSNCLDSTLYLTVD
KQNANGSMPPNGSSAVTLSPKESGSDSKGPRKRTGPGVDAHIIPVKRVFISQQPLAFIDNPKPGLSKPAVKRIPRAGTAA

RPESAPCKVTVKQTSVGPAQ
VLALSDSPITQTEGSGTVVKPQALPVKRDDRTVSSSASAALPAAPGSSAADQALLQQIASADPHLIPANSGAHEASVLGD
LKSTMWEEGQLEELQKQAFA
QQIPAEHNQAQADQIAMIGPAGEASSQLALSQEMVDFTGSQPSMDYFPFNDDDMTQDSIVEELVQMEEQMKLKGLQPLFS
TCVDMSLQGQSAGTPASILN
AHQTGPAFFHSAHSSTTPVQTPTPTPTPTPTPTPTPTSEMTLGHSMTRESPCSRMAVVTPVDGAMGRHTPISTPLSNCSSSV
PPSPVECRNPFAFTPINSSM
TGYHDSSVVSSSPVKPMQRPMATHPDKAKLDWINNRYNSNSSGPLSNQSIGILPSYQDLVDDQFRKPHAFAIPGQSFQAQ
SRQDAHFGRLTPISPVQQQQ
QQQQQPPPVTTVTTPTKQESFAVPAPLDSKATTSTGSSTFRCRSVSPAVRQRNFSGNTAAPTSTVTATTTTRAVISPFNA
AITSEMLSILSNSQAAGTVH
SMVQRSQSVPLNIMMQSEMLPVQAQNNTTKITSVLLSKMDSDGDEAVRGLGINNLPSNYTARMNLTQILQTTPTFSGGAT
HQAQLPVSSSPAAFGLQQGY
LAGGPSEQVGFTGDEAQAQPGPSEQEQQQQQQQQQQQQQLQEAAVHTQAQLLLQSAQQQEVAVAAAAAAAEEEQQQLDFN
NTVKDLLGDDGLNPSSQLVG
QVASELNAVASDFSSDIRLTSDLSSSITDLNTLDTNLLFDPNQPQEQYEDSTLEELKNDPLFQQICSDPVNSGFEWLESK
DQPTTVEMLG
>g2446.t1
LLKQKIPASLGEERTSAALRRAFDVWSQATPLTFQEVPAQDNNGVAGGNVSTPAPLADILVLFASGFHGDMSLFDGEGGS
LAHAYYPGPGMGGDTHFDAD
EPWTLDQQDRQDHQNPTGIDLFLVAVHELGHALGLEHSDHPDAVMAPFYSWTPTHNFSLSPDDRAAIQ
>g2446.t2
LLKQKIPASLGEERTSAALRRAFDVWSQATPLTFQEVPAQDNNGVAGGNVSTPAPLADILVLFASGFHGDMSLFDGEGGS
LAHAYYPGPGMGGDTHFDAD
EPWTLDQQDRQDHQNPTGIDLFLVAVHELGHALGLEHSDHPDAVMAPFYSWTPTHNFSLSPDDRAAIQ
>g2446.t3
WPEDAQRGSRRRRYVLTGQRWDTDHLTYSLLKQKIPASLGEERTSAALRRAFDVWSQATPLTFQEVPAQDNNGVAGGNVS
TPAPLADILVLFASGFHGDM
SLFDGEGGSLAHAYYPGPGMGGDTHFDADEPWTLDQQDRQDHQNPTGIDLFLVAVHELGHALGLEHSDHPDAVMAPFYSW
TPTHNFSLSPDDRAAIQ
>g2447.t1
EKLRVGGAQLTELKVRLEYCVGAISQSVPSSTWP
>g2448.t1
VQTLYYSADHKLVDGGLVIEGHSEVFGGEEDHLQFVQKKPPRESTKRQMSCSSSGSLSPSNQAPSAPKPSSRREWKYIAS
EKTTNNTYLCLAVLDGTLCV
IFLHGRSSPQTSPSATPCLLKSLSFEAQPEELEERPLSPMHYARSGLGTAALGGKLIAAGGYNREECLRTVECYDPKEDC
WTFIAPMRTPRARFQMAVLM
GRLYVMGGSNGHSDELSCGETYDPVADEWAQVPELRTNRCNAGVCSLDNKLYVVGGSDPCGQKGLKNCDAFDPVTKAWTN
CAPLNIRRHQAAVCELEGFV
YVIGGAESWNCLNTVERYNPRNNTWTLIAPMNVARRGAGVAVHAGNLFVVGGFDGSHALRCVEVYDPARNEWRMLGSMTS
ARSNAGVALLGELVCAVGGF
DGNDFLNTMEVYNPETDEWSDSTDGFSPSPQ
>g2448.t2
MDVLLSLCVLQVQTLYYSADHKLVDGGLVIEGHSEVFGGEEDHLQFVQKKPPRESTKRQMSCSSSGSLSPSNQAPSAPKP
SSRREWKYIASEKTTNNTYL
CLAVLDGTLCVIFLHGRSSPQTSPSATPCLLKSLSFEAQPEELEERPLSPMHYARSGLGTAALGGKLIAAGGYNREECLR
TVECYDPKEDCWTFIAPMRT
PRARFQMAVLMGRLYVMGGSNGHSDELSCGETYDPVADEWAQVPELRTNRCNAGVCSLDNKLYVVGGSDPCGQKGLKNCD
AFDPVTKAWTNCAPLNIRRH
QAAVCELEGFVYVIGGAESWNCLNTVERYNPRNNTWTLIAPMNVARRGAGVAVHAGNLFVVGGFDGSHALRCVEVYDPAR
NEWRMLGSMTSARSNAGVAL
LGELVCAVGGFDGNDFLNTMEVYNPETDEWSDSTDGFSPSPQ
>g2448.t3
VQTLYYSADHKLVDGGLVIEGHSEVFGGEEDHLQFVQKKPPRESTKRQMSCSSSGSLSPSNQAPSAPKPSSRREWKYIAS
EKTTNNTYLCLAVLDGTLCV
IFLHGRSSPQTSPSATPCLLKSLSFEAQPEELEERPLSPMHYARSGLGTAALGGKLIAAGGYNREECLRTVECYDPKEDC

WTFIAPMRTPRARFQMAVLM
GRLYVMGGSNGHSDELSCGETYDPVADEWAQVPELRTNRCNAGVCSLDNKLYVVGGSDPCGQKGLKNCDAFDPVTKAWTN
CAPLNIRTWTRKHCRRHQAA
VCELEGFVYVIGGAESWNCLNTVERYNPRNNTWTLIAPMNVARRGAGVAVHAGNLFVVGGFDGSHALRCVEVYDPARNEW
RMLGSMTSARSNAGVALLGE
LVCAVGGFDGNDFLNTMEVYNPETDEWSDSTDGFSPSPQ
>g2449.t1
LDPARDPCLKIKCGRHKVCVAEDYKTATCISQRRVSFKDPSLFQSPGPKCVPCPVVHPSPVCGTDGHTYSTKCKLEYQAC
MSGKKISVKCAGMCPCTTLP
EHSSAEKKARLPVTAGSNYTSFVFPWMPGPAK
>g2449.t2
LDPARDPCLKIKCGRHKVCVAEDYKTATCISQRRVSFKDPSLFQSPGPKCVPCPVVHPSPVCGTDGHTYSTKCKLEYQAC
MSGKKISVKCAGMCPCTTLP
EHSSAEKKASEAWRESASLAVNLFTSLLDPL
>g2449.t3
LDPARDPCLKIKCGRHKVCVAEDYKTATCISQRRVSFKDPSLFQSPGPKCVPCPVVHPSPVCGTDGHTYSTKCKLEYQAC
MSGKKISVKCAGMCPCTTLP
EHSSAEKKVTAGSNYTSFVFPWMPGPAK
>g2450.t1
MTVSLTLMDGRTISVPVDSASNSSEVCQMLSQKVQLKDTFGFSVYVALYEKVWALGSGREHVMDAISQCEQEVRRKGGQE
QHAPWRLYFRKEVFTPWHNC
SDDLVSTELIYRQVVRGLKFGEYQCKK
>g2450.t2
MTVSLTLMDGRTISVPVDSASNSSEVCQMLSQKVQLKDTFGFSVYVALYEKVWALGSGREHVMDAISQCEQEVRRKGGQE
QHAPWRLYFRKEVFTPWHNC
SDDLVSTELIYRQVVRGLKFGEYQCKKVNTDP
>g2450.t3
MDGRTISVPVDSASNSSEVCQMLSQKVQLKDTFGFSVYVALYEKVWALGSGREHVMDAISQCEQEVRRKGGQEQHAPWRL
YFRKEVFTPWHNCSDDLVST
ELIYRQVVRGLKFGEYQCKK
>g2451.t1
MLELKRMKKLNEKALVIQRVLRGYKYRRQFLKKKAAALVIQKHWRGHQGRKIYRVVQVGLTRLQARVRSRQMQHQYQKRR
DAAILLQARVRGWLARKEWK
RKRDAVVLLQAHTRGMLARQAVKKKKRDLYLSAKERREEDLAVLERQRRLMEVLKKQKDDDAKVHTDPISDQEMVDNIFN
FLPNMGAGQEGQAPTGFKDL
ENRRMVPEMVDLDELPMEEQDLSDDDDDLDEYSFSKFASMYFQGAATHTHVRHRLAQPLIHHEDEGDVQASLTVWWIILR
FMADLPEPKQRVNPLGDQRG
PGGQSPRVAHALPQDLADRNDRRLSHMVGLDQRVLRGKKDSPAPGETSGGSPRKGSVFTDMLSRNRKASTAPSEVVTGGK
VSEGVPRARKGSMFTDLMSR
SRKASSIPENGIPESPTGSVNTPSIAEEPESNAGLSSAMVTVDTTTLERPMTALEKLHIIVGYAIVRRDLRDEIYCQICK
QLDGNTNRNSYHRGWVLLSL
CLGIFPPSEKFIKYLQSFIRSGPGGYGPYCAERLRRTVLNGVRGEPPSWLELQASRG
>g2451.t2
MLELKRMKKLNEKALVIQRVLRGYKYRRQFLKKKAAALVIQKHWRGHQGRKIYRVVQVGLTRLQARVRSRQMQHQYQKRR
DAAILLQARVRGWLARKEWK
RKRDAVVLLQAHTRGMLARQAVKKKKRDLYLSAKERREEDLAVLERQRRLMEVLKKQKDDDAKVHTDPISDQEMVDNIFN
FLPNMGAGQEGQAPTGFKDL
ENRRMVPEMVDLDELPMEEQDLSDDDDDLDEYSFSKFASMYFQGAATHTHVRHRLAQPLIHHEDEGDVQVYTHLLNPTQA
SLTVWWIILRFMADLPEPKQ
RVNPLGDQRGPGGQSPRVAHALPQDLADRNDRRLSHMVGLDQRVLRGKKDSPAPGETSGGSPRKGSVFTDMLSRNRKAST
APSEVVTGGKVSEGVPRARK
GSMFTDLMSRSRKASSIPENGIPESPTGSVNTPSIAEEPESNAGLSSAMVTVDTTTLERPMTALEKLHIIVGYAIVRRDL
RDEIYCQICKQLDGNTNRNS
YHRGWVLLSLCLGIFPPSEKFIKYLQSFIRSGPGGYGPYCAERLRRTVLNGVRGEPPSWLELQASRG
>g2451.t3
MLELKRMKKLNEKALVIQRVLRGYKYRRQFLKKKAAALVIQKHWRGHQGRKIYRVVGLTRLQARVRSRQMQHQYQKRRDA

AILLQARVRGWLARKEWKRK
RDAVVLLQAHTRGMLARQAVKKKKRDLYLSAKERREEDLAVLERQRRLMEVLKKQKDDDAKVHTDPISDQEMVDNIFNFL
PNMGAGQEGQAPTGFKDLEN
RRMVPEMVDLDELPMEEQDLSDDDDDLDEYSFSKFASMYFQGAATHTHVRHRLAQPLIHHEDEGDVQASLTVWWIILRFM
ADLPEPKQRVNPLGDQRGPG
GQSPRVAHALPQDLADRNDRRLSHMVGLDQRVLRGKKDSPAPGETSGGSPRKGSVFTDMLSRNRKASTAPSEVVTGGKVS
EGVPRARKGSMFTDLMSRSR
KASSIPENGIPESPTGSVNTPSIAEEPESNAGLSSAMVTVDTTTLERPMTALEKLHIIVGYAIVRRDLRDEIYCQICKQL
DGNTNRNSYHRGWVLLSLCL
GIFPPSEKFIKYLQSFIRSGPGGYGPYCAERLRRTVLNGVRGEPPSWLELQASRG
>g2452.t1
MESEDRSADDCRVRGGIYCSYANLREAYHLSLTSYQILHPSALSFRRRQAAGDTWQPVGKVSACPDTARSSSPGGQWVTA
GKTNTRAPRNETPETPEDIG
HCPDGADVDSPLHPPLMLAAFNDTAHAKNRYNSLLLPTTRMHLKEVRLQGNWDPQVFSRRFDLGQPVATVQFLTQNYKD
>g2452.t2
MESEDRSADDCRVRGGIYCSYANLREAYHLSLTSYQILHPSALSFRRRQAAGDTWQPVGKVSACPDTARSSSPGGQWVTA
GKTNTRAPRNETPETPEDIG
HCPDGADVDSPLHPPLMLAAFNDTAHAKNRYNSLLLPTTRMHLKEVRLQELCGVKSQDVDAPAGPSCSQEPAAFEPCIDG
NWDPQVFSRRFDLGQPVATV
QFLTQNYKD
>g2452.t3
MESEDRSADDCRVRGGIYCSYANLREAYHLSLTSYQILHPSALSFRRRQAAGDTWQPVGKVSACPDTARSSSPGGQWVTA
GKTNTRAPRNETPETPEDIG
HCPDGADVDSPLHPPLMLAAFNDTAHAKNRYNSLLLPTTRMHLKEVRLQAPGRGRCMAARSPHPGS
>g2453.t1
ALESKLAACRNFAKDQRARKAFAVDNGNILNGNAGPYAKTQHASYFDKARESVMFPALIPGQGDSELRSSPVNNARKHDP
PLVSGEDLLTGGAAGRLRDC
VGLGSVLTLLERRLCRHASERGGTRRYDGHHRASSRLRTRPGPSGRLRTSTLNLHPPHLAWSLWPPGPSGRLRTSTLNLH
PPHQTWSLWPSENLHPPHQA
WSLWPSENLHPPHQTWSLWPSENLNPPHQAWSLWPSQNLHPPHQACSLWPSENLHPQPPPSAPDMVPLAV
>g2453.t2
ALESKLAACRNFAKDQRARKAFAVDNGNILNGNAGPYAKTQHASYFDKARESVMFPALIPGQGDSELRSSPVNNARKHDP
PLVSGEDLLTGGAAGRLRDC
VGLGSVLTLLERRLCRHASERGGTRRYDGHHRASSRLRTRPGPSGRLRTSTLNLHPPHLAWSLWPSENFHPPHQAWSLWP
PGPSGRLRTSTLNLHPPHQT
WSLWPSENLHPPHQAWSLWPSENLHPPHQTWSLWPSENLNPPHQAWSLWPSQNLHPPHQACSLWPSENLHPQPPPSAPDM
VPLAV
>g2454.t1
MGWVLHQVDQTAAGLLALGLQRGDRLGMWGPNIYEWLLFQFATAKAGIILVSVNPAYQLGEVEFALRKVECNAVVCPTTF
KTQKYVEMLRKICPEIDSST
PGDIRSARLPDLRVVIVTDHRYPGMFHVDDVRQAAGSQHVQQLQDLQKKISFGDPVNIQFTSGTTGAPKGATLSHHNILN
NVYLFGSRVGYTWRPHTRIC
LPVPMYHCFGSVGGGMCMAVHGSSLVFPSAGYDGRANLAASESEK
>g2454.t2
MFRETLPLGWRRCTAAHEYREEQHTSSRLPSPRRRAMRLGLPLLAASVLFVEQGERTNERDPERTPPLHRAVIWTSLSVH
FVEEPLQSGIMFFRVRLQLP
YGLLCGAQRGSQRPLRHTGSLVHRSXHPPSTVETHPPLTLTQTLYSTKKNSGIHVDSRPSIPTLTTSYAHGTSSRSLLHS
TIAETLLQSVERWPDRQALI
FLQEGVRKTFAEFQQDVSLTLTLTLTLTLTLTLTLTLTLTLTLILTLTLTLKGGSSGFSTRAKVDQTAAGLLALGLQR
GDRLGMWGPNIYEWLLFQFA
TAKAGIILVSVNPAYQLGEVEFALRKVECNAVVCPTTFKTQKYVEMLRKICPEIDSSTPGDIRSARLPDLRVVIVTDHRY
PGMFHVDDVRQAAGSQHVQQ
LQDLQKKISFGDPVNIQFTSGTTGAPKGATLSHHNILNNVYLFGSRVGYTWRPHTRICLPVPMYHCFGSVGGGMCMAVHG
SSLVFPSAGYDGRANLAASE
SEK
>g2454.t3

MFRETLPLGWRRCTAAHEYREEQHTSSRLPSPRRRAMRLGLPLLAASVLFVEQGERTNERDPERTPPLHRAVIWTSLSVH
FVEEPLQSGIMFFRVRLQLP
YGLLCGAQRGSQRPLRHTGSLVHRSXHPPSTVETHPPLTLTQTLYSTKKNSGIHVDSRPSIPTLTTSYAHGTSSRSLLHS
TIAETLLQSVERWPDRQALI
FLQEGVRKTFAEFQQDVSLTLTLTLTLTLTLTLTLTLTLTLTLTLILTLTLTLKAVFHIPTGSCVLVEG
>g2454.t4
MFRETLPLGWRRCTAAHEYREEQHTSSRLPSPRRRAMRLGLPLLSANAAPPAGPLCLCVSVPLCLCVSVSLCLCVSLFPF
QSRVRVRLQLPYGLLCGAQR
GSQRPLRHTGSLVHRSXHPPSTVETHPPLTLTQTLYSTKKNSGIHVDSRPSIPTLTTSYAHGTSSRSLLHSTIAETLLQS
VERWPDRQALIFLQEGVRKT
FAEFQQDVSLTLTLTLTLTLTLTLTLTLTLTLTLTLTLILTLTLTLKGGSSGFSTRAKVDQTAAGLLALGLQRGDRLGMWGP
NIYEWLLFQFATAKAGIILV
SVNPAYQLGEVEFALRKVECNAVVCPTTFKTQKYVEMLRKICPEIDSSTPGDIRSARLPDLRVVIVTDHRYPGMFHVDDV
RQAAGSQHVQQLQDLQKKIS
FGDPVNIQFTSGTTGAPKGATLSHHNILNNVYLFGSRVGYTWRPHTRICLPVPMYHCFGSVGGGMCMAVHGSSLVFPSAG
YDGRANLAASESEK
>g2455.t1
MSLDEVLGNRSKVTVVPRGGVGKGSGALQLLLWCTFLYGTPTMFVDMLAHCETTKYDLSSVEGGELGYGMTENSPVTFCG
DPFDTLERRTETVGYIIDHL
EV
>g2455.t2
MSLDEVLGNRSKVTVVPRGGVGKGSGALQLLLWCTFLYGTPTMFVDMLAHCETTKYDLSSVEGGELGYGMTENSPVTFCG
DPFDTLERRTETVGYIIDHL
E
>g2455.t3
MSLDEVLGNRSKVTVVPRGGVGKGSGALQLLLWCTFLYGTPTMFVDMLAHCETTKYDLSSVEGGEGPHNDRHVNEPFRHF
QLGYGMTENSPVTFCGDPFD
TLERRTETVGYIIDHLEV
>g2456.t1
MGPDPQLKWVVSGMVLTQLLACYMVHNLSWKWIFFWAYAFGGCINHSLTLAIHDISHNVAFGNKLAKWNRWFAMWANLPI
GVPYSASFKKYHIDHHRYLG
GDKLDVDIPTDVEGWFFSTPARKILWLFLQPLFYALRPLVVNPKPMGLLEVQNAAVQLAADLLIYYLWGLKPIVYLIAGS
ILCMGLHPISGHFIAEHYMF
LKGHETYSYYGALNMITFNVGYHMEHHDFPSIPGSKLPQVKRIAAEYYDTLPQHTSWTRVLWDFVFDDSIGPYARIKREY
NLVKQG
>g2457.t1
MRTVKRRSRGRRRRRVMRRRRMMRRSDEKEDYEEEKEGDEEECDEKEDYEEEKE
>g2458.t1
MDACVAVLLLALTPLGAAFAPDCEDLVKPFIPEDSTLVFGKWVYVMGSGDPMPYRKALDAMKSSWIELSPTQDSQLVTLR
WGDHC
>g2458.t2
MDACVAVLLLALTPLGAAFAPDCEDLVKPFIPEDSTLVFGKWVYVMGSGDPMPYRKALDAMKSSWIELSPTQDSQLVTLR
NRCIYGEVNGTVSGVATTFR
KNLSNHKGHILHTCPDCLLWTDTFRNGDATGRFIMQFKLCKDKESTE
>g2458.t3
MDACVAVLLLALTPLGAAFAPDCEDLVKPFIPEDSTLVFGKWVYVMGSGDPMPYRKALDAMKSSWIELSPTQDSQLVTLR
WGDHCYNRCIYGEVNGTVSG
VATTFRKNLSNHKGHILHTCPDCLLWTDTFRNGDATGRFIMQFTRTGKIDPKDVDVFKKQVECLNFPENYHSYDGKTELC
KDKESTE
>g2458.t4
MLRCSNRCIYGEVNGTVSGVATTFRKNLSNHKGHILHTCPDCLLWTDTFRNGDATGRFIMQFTRTGKIDPKDVDVFKKQV
ECLNFPENYHSYDGKTELCK
DKESTE
>g2459.t1
MDYFDELDNASGNTGSGMEQPQVEKPTPRNGHRHPARKYYQVSPEAKGFLQGPLSTIMVPTIYAFVFATSLPLNLAAVLM
FLRRKGPRKTAVIYMLNLAC

ADLLFVLLLPLKIAYHYHGNNWVFGAALCRVVTAAFYCNMYCSVLLMTCISVDRFLAVVYPMDALTWRSPRAAYASCTTM
WLLAVGGVAPLLLSEQSLHL
PDLGVTTCHDVQDEGQLRSYYLYFFPVYISLFFFLPLVFTSACYVRIVLALAAANVDNRSRKGRAVVMALAVLVVFVACF
GPSNVLMVVHYAQLARNNNS
SSSSSSSSSSNNNNNKAAAAAADTSYRAYLVATCVGTLSCCLDPLIYYFGSSQYQRKVAALLTCRGPAGRGPGGSSRSQS
RSRSSGSQLTLSTKSTQMES
LQATALGAHYRKLIS
>g2460.t1
MAARNSRRSIKGDEKGGKKSKQQSLKYTGARLHEKGVVLEIEGLQTNQFKNVMFEIAPSEEVGDFEVKAKFMGVEMEKVQ
VHFQDLLQLQYDGVSVMKMF
DKAKVNVNLLIFLLNKKFYGK
>g2461.t1
LDSDPLHTHQRYHSDYSSSSESPSVTSSDPEYRQAKKPEEGPRRYGSQAALVSEQDAYRH
>g2461.t2
PLHTHQRYHSDYSSSSESPSVTSSDPEYRQAKKPEEGPRRYGSQAALVSEQDAYRH
>g2461.t3
MSFRLKDPLHTHQRYHSDYSSSSESPSVTSSDPEYRQAKKPEEGPRRYGSQAALVSEQDAYRH
>g2462.t1
GSFRHFLWQVCKELQSSALSLLLPCPSAAVNRNKGKYILTPCPISYAEEQLLHFLGQLLGIAIRADVPLPLDLLASFWKG
LVGQPLDPEQDLQDADLLTY
NYVKKFESTPLPEPHPFVSERHLISRRRVELH
>g2462.t2
LHLEVAPFVSLKVDRTAAEAFCFLSASGGSFRHFLWQVCKELQSSALSLLLPCPSAAVNRNKGKYILTPCPISYAEEQLL
HFLGQLLGIAIRADVPLPLD
LLASFWKGLVGQPLDPEQDLQDADLLTYNYVKKFESTPLPEPHPFVSERHLISRRRVELH
>g2463.t1
LVVKSLQLSATITITVPEQKSLFLHRRPLRTNEDHSKPSLPASSYGSVLLHGSTSDCTLYCIGTSTPSPTPAMDTLPARL
LPLAMEEFPQPLARACVRRG
SLHCARCGVIEEEPESCCSPVEE
>g2464.t1
MAPEQMVGIVIKSMSDRFLSVVDLLFADMRFAPFSCPERGSWIQGSQEKDLSLMVLESLQPAVCLHKYPRSQIDVNVMVL
ENNGSALAHAITCASLALAD
AGIEMYDLVLGCSIRQDGTSYLIDPTFLEESGSCWGSGENQGSLTVSFLPSLNQISGLQSEGEMAEGTLTAGVRTCIEGC
YKLYPVIQQALVKAVRGAAP
PPSES
>g2465.t1
MDSLLSAAQIREKFIDFFRGYEHQYVHSSAVVPLDDPTLLFANAGMNQFKPIFLNTIDPSHPMARLRRAANTQKCIRAGG
KHNDLDDVGKDVYHHTFFEM
LGSWSFGDYFKVTV
>g2466.t1
LLIGSGCALYPLGWGSEEVRQTCNNSSNLFQLGSCQIGWAYYCTGAGAATAMLLCTWLACFAGKKQKQYPY
>g2467.t1
LKPVFCVCLSTEGQKLFSCHMCNTSFSTKGSLKVHMRLHTGSKPFKCPFCEDRFRTSGHRKTHIQCHYRPNAEVRRSKRN
AAAAASSSSSSSSNSTASTS
SIGGHANHQGQDADGGQGSGAGQHLHLHHHHHHQHHHQQQQQQHQLGAEGLQSVGLLQASGSDPNIYLPGNHVLTGQFDQ
SLLQQGLVGQAILPASMSAV
RPAEPGRQTDGLNLNHSGTRGVMSSQ
>g2467.t2
MCNTSFSTKGSLKVHMRLHTGSKPFKCPFCEDRFRTSGHRKTHIQCHYRPNAEVRRSKRNAAAAASSSSSSSSNSTASTS
SIGGHANHQGQDADGGQGSG
AGQHLHLHHHHHHQHHHQQQQQQHQLGAEGLQSVGLLQASGSDPNIYLPGNHVLTGQFDQSLLQQGLVGQAILPASMSAV
RPAEPGRQTDGLNLNHSGTR
GVMSSQ
>g2468.t1
MDVMQGSVYRLLSEDVTLDPNTAHPRLIISVDGKQVCCGDRHQPVQDTPERFDRVVCVLARQGFTSGHRYWE
>g2469.t1

NADPIHDMLLDVITWVGILLSLVCLLLSLVTFCFFRGLQSDRNTIHKNLCVSLFIAETLFLVGINRADQPPTAVQEAILN
T
>g2470.t1
LNKLREIAAFPRASFISLILQHSLLDEDSMSANMRRLRRQLNFQHYFNDRSENMAALAESRVEGRGVRLVTSTMACY
>g2471.t1
PRAGFKIAFNMLDTDGNQMVDRMEFLVLEEIFRKKKDKNDEDTRRSEQKSLQLYGHQKSHAHN
>g2471.t2
MGFFPEPRAGFKIAFNMLDTDGNQMVDRMEFLVLEEIFRKKKDKNDEDTRRSEQKSLQLYGHQKSHAHN
>g2471.t3
PRAGFKIAFNMLDTDGNQMVDRMEFLVPNILSSCFQKLEEIFRKKKDKNDEDTRRSEQKSLQLYGHQKSHAHN
>g2472.t1
MKEFIMNQEKLAKLQAQVRIGGKGSARRKKKVVHRTASADDKKLQFSLKKLGVNNISGIEEVNMFTNQGTVIHFNNPKVQ
ASLAANTFTITGHAENKQLT
EMLPGILNQLGADSLTSLRRLAETLPKPAGDMPPMVTVEEEDEDDVPVTPGAVTWMFVMHGEGEPVELGPGSGRSLTVST
VDTPNLGEYSCWSGGQKLWS
AYLVHDGSDAEEEESASDSMLSCSAKSYNCVFSCHWTDTDYTMVRLGLGKDCAEGTKNCLWVNSSSQRSEGGFWFELSHS
YPLYMEETSMIEVTAEAMTT
QHFHRVTKKFYLRDIEGKLILSSDTEDD
>g2472.t2
MKEFIMNQEKLAKLQAQVRIGGKGSARRKKKVVHRTASADDKKLQFSLKKLGVNNISGIEEVNMFTNQGTVIHFNNPKVQ
ASLAANTFTITGHAENKQLT
EMLPGILNQLGADSLTSLRRLAETLPKPAGDMPPMVTVEEEDEDDVPVTPGAVTWMFVMHGEGEPVELGPGSGRSLTVST
VDTPNLGEYSCWSGGQKLWS
AYLVHDGSDAEEEESASDSMLSCSAKSYNCVFSCHWTDTDYTMVRLGLGKDCAEGTKNCLWVNSSSQRSEGGFWFELSHS
YPLYMEETSMIEVTAEAMTT
QHFHRVTKKFYLRDIGKSDSIQNY
>g2472.t3
MKEFIMNQEKLAKLQAQVRIGGKGSARRKKKVVHRTASADDKKLQFSLKKLGVNNISGIEEVNMFTNQGTVIHFNNPKVQ
ASLAANTFTITGHAENKQLT
EMLPGILNQLGADSLTSLRRLAETLPKPAGDMPPMVTVEEEDEDDVPDPVTLTCDSVTPGAVTWMFVMHGEGEPVELGPG
SGRSLTVSTVDTPNLGEYSC
WSGGQKLWSAYLVHDGSDAEEEESASDSMLSCSAKSYNCVFSCHWTDTDYTMVRLGLGKDCAEGTKNCLWVNSSSQRSEG
GFWFELSHSYPLYMEETSMI
EVTAEAMTTQHFHRVTKKFYLRDIEGKLILSSDTEDD
>g2473.t1
MASFKPTRIQVVYPKLGEKVTQDTLYWKNYKVHIYGPFSQEPVKTFTRFKDTAYSGRFRSDGQLLVAGCEDSAARLFDVSG
RVAIRMFKGHTKAVHVADFT
SDRYHILTGSDDYTCRLWDIPNSTELTSYAEHTDYIRCGITSKLNRDLFFTGSYDHTLKLFDARVDKSVMTMDHGQPVES
LLLYPSEGLLVSAGVKLYFL
IHSRHVKVYSTTNYKVVHNFDYSASILSLGLAPDDESIVVGMTNGILSIKHKRSPEESKGAVQQRRQPAYRVFVKGKNYV
PKQDDYLVSKPVRQHLAKYD
KLLRKFNVSKALDAAMDQWVRFRKPEIPMAVIKELDRRGTLKNALAGRDEKDLYQLLAFVVS
>g2473.t2
MASFKPTRIQVVYPKLGEKVTQDTLYWKNYKEPVKTFTRFKDTAYSGRFRSDGQLLVAGCEDSAARLFDVSGRVAIRMFKG
HTKAVHVADFTSDRYHILTG
SDDYTCRLWDIPNSTELTSYAEHTDYIRCGITSKLNRDLFFTGSYDHTLKLFDARVDKSVMTMDHGQPVESLLLYPSEGL
LVSAGVKLYFLIHSRHVKVY
STTNYKVVHNFDYSASILSLGLAPDDESIVVGMTNGILSIKHKRSPEESKGAVQQRRQPAYRVFVKGKNYVPKQDDYLVS
KPVRQHLAKYDKLLRKFNVS
KALDAAMDQWVRFRKPEIPMAVIKELDRRGTLKNALAGRDEKDLYQLLAFVVS
>g2473.t3
MASFKPTRIQVVYPKLGEKVTQDTLYWKNYKAPVQIKEFGAITSIDFSPLAPHTYAVTASTREPVKTFTRFKDTAYSGRFR
SDGQLLVAGCEDSAARLFDV
SGRVAIRMFKGHTKAVHVADFTSDRYHILTGSDDYTCRLWDIPNSTELTSYAEHTDYIRCGITSKLNRDLFFTGSYDHTL
KLFDARVDKSVMTMDHGQPV
ESLLLYPSEGLLVSAGVKLYFLIHSRHVKVYSTTNYKVVHNFDYSASILSLGLAPDDESIVVGMTNGILSIKHKRSPEES

KGAVQQRRQPAYRVFVKGKN
YVPKQDDYLVSKPVRQHLAKYDKLLRKFNVSKALDAAMDQWVRFRKPEIPMAVIKELDRRGTLKNALAGRDEKDLYQLLA
FVVS
>g2474.t1
MYIALISLHSLTMVVFHFLYCFEDDWTKCSSFSPPATVILLILLCFEALLFLIFTSVMFGTQVHSICTDETVSHAHTAAL
RGFRALLWAFPLLRFPALSP
PSLLSTQHSDFASGRWPASVARSPLATPEQGKAETYQYVGIERLKGETGKWDKVPCWEAMRAACGGPFSLSWCSPFSGLR
CEKRPLEALDPVPHGEIIEE
DVIEIPLNY
>g2474.t2
MVVFHFLYCFEDDWTKCSSFSPPATVILLILLCFEALLFLIFTSVMFGTQVHSICTDETVSHAHTAALRGFRALLWAFPL
LRFPALSPPSLLSTQHSDFA
SGRWPASVARSPLATPEQGKAETYQYVGIERLKGETGKWDKVPCWEAMRAACGGPFSLSWCSPFSGLRCEKRPLEALDPV
PHGEIIEEDVIEIPLNY
>g2475.t1
MNVSVRSLIEPVGLLRLASLVLSCLSFGLVATAHYNQAYASYWAWCMFTWCFCCFFTLLVLVLEFAALSDKVPVSWEDFT
TAFAMLAALMCLAASTIYPA
FFVCAACPRQIAATITSWLCFLTYGGEVAVTWLRPRGQISGFLSTVPGLLKILETFLACIIFTSLDSAKFDGSPGLQWCV
AVYCMCFICALCIILLTISR
LFSYFPVSFERLVTAYNVLAAMMYLTTMVMWPLYGFQQTTAYSDLWNKVLVVTFMSIFNFVVYTVDCVFSIRMVFWPQES
S
>g2476.t1
GCFGQRMRAWQRWQEAQSTLQRKREAEAKLLWANKPDKLQQAKEDITEVGLPGHRVDGPFGDLG
>g2476.t2
GCFGQRMRAWQRWQEAQSTLQRKREAEAKLLWANKPDKLQQAKEDITEWESRVTQYERDFDRISVTVRKEVLQFEKEKAK
DFKIQIYKYLETMLQSQQRL
IKFWEAFLPEAKAIA
>g2476.t3
MRAWQRWQEAQSTLQRKREAEAKLLWANKPDKLQQAKEDITEWESRVTQYERDFDRISVTVRKEVLQFEKEKAKDFKIQI
YKYLETMLQSQQRLIKFWEA
FLPEAKAIA
>g2477.t1
MNCRFIHPGVNDKGNYSLITKPDLFSPNGAPPGAPHPLLPNNPWGAPPVEELPPPPPPVEPPVESAWERGLRHAKEVLKK
ATIRKEQEPDFEEKRFNVTI
GEDEREFDKENDFFRERNYRIIR
>g2477.t2
MNCRFIHPGVNDKGNYSLITKPDLFSPNGAPPGAPHPLLPNNPWGAPPVEELPPPPPPVEPPVESAWERGLRHAKERRRR
AASQQTALPHGNGRRPQPVL
VFNP
>g2477.t3
MNCRFIHPGVNDKGNYSLITKPDLFSPNGAPPGAPHPLLPNNPWGAPPVEELPPPPPPVEPPVESAWERGLRHAKEVLKK
ATIRKEQEPDFEEKRFNVTI
GEDEREFDKENDFFRERNYRIIRE
>g2478.t1
MHGYNTVNWNDSRGEKTGLLSHNMSHLRCLNTKLSKSDK
>g2479.t1
MSFRASNSSYTLSTPPYRAYSTYAGSGGKGTRISSSTYSGIRSGAPTISSTSAFRVSSGMGGGFGGAAAGAGGGGAGGAG
GSAVIMGNERGHMQNLNDRL
ATYLDTVRNLEKANNELEVKIREALEKGGPQMRDYSKYTPILDDLRRQVLDQTLDNATLVLQIDNARLAADDFNVKFENE
KALRQSVEADIGGLKKVIDD
TNMGRMNIESEIEAVKEELMYLKKNHENDLMEMRNQVSHGGVQVDVDAPKGQDLAQVMEEVRSNYEKMAVKNAQDLKQWH
ENQISEVQVQVSQNTEALQG
AQVEISDLSRQLQTLEIELASQQSLKASLEDTLRSTENRNNMEVEKYNGIIMHLEGELTNLRANIQQQTQDYEVLLNMKM
KLEAEIATYRTLLDGGDFK
>g2479.t2
MSFRASNSSYTLSTPPYRAYSTYAGSGGKGTRISSSTYSGIRSGAPTISSTSAFRVSSGMGGGFGGAAAGAGGGGAGGAG

GSAVIMGNERGHMQNLNDRL
ATYLDTVRNLEKANNELEVKIREALEKGGPQMRDYSKYTPILDDLRRQVLDQTLDNATLVLQIDNARLAADDFNVKFENE
KALRQSVEADIGGLKKVIDD
TNMGRMNIESEIEAVKEELMYLKKNHENDLMEMRNQVSHGGVQVDVDAPKGQDLAQVMEEVRSNYEKMAVKNAQDLKQWH
ENQISEVQVQVSQNTEALQG
AQVEISDLSRQLQTLEIELASQQSLKASLEDTLRSTENRNNMEVEKYNGIIMHLEGELTNLRANIQQQTQDYEVLLNMKM
KLEAEIATYRTLLDGGDFKP
VTSSSGNLARTRSPRWRRQAEQPTTAFRVSCVCCIQSPPTVFQLGSACVCVFNHLASGALWSWRSPAKKKGNKKGKTFSL
TDFLSEDSGGGGGGNAPPPN
YPAKSTSWADETDDLDGDVSTSWHTEEDSFRAPAIDRSILPTAPRSAREPNIDRARLPRSPPYTAFLGNLPYDVSEE
>g2479.t3
MSLFRKMGSDSAILTLAGSSFHHCGAKTEKSCPASAKRPLLESFLLTHSQRLVLFCVPPPQRITMSFRASNSSYTLSTPP
YRAYSTYAGSGGKGTRISSS
TYSGIRSGAPTISSTSAFRVSSGMGGGFGGAAAGAGGGGAGGAGGSAVIMGNERGHMQNLNDRLATYLDTVRNLEKANNE
LEVKIREALEKGGPQMRDYS
KYTPILDDLRRQVLDQTLDNATLVLQIDNARLAADDFNVKFENEKALRQSVEADIGGLKKVIDDTNMGRMNIESEIEAVK
EELMYLKKNHENDLMEMRNQ
VSHGGVQVDVDAPKGQDLAQVMEEVRSNYEKMAVKNAQDLKQWHENQISEVQVQVSQNTEALQGAQVEISDLSRQLQTLE
IELASQQSLKASLEDTLRST
ENRNNMEVEKYNGIIMHLEGELTNLRANIQQQTQDYEVLLNMKMKLEAEIATYRTLLDGGDFKPVTSSSGNLARTRSPRW
RRQAEQPTTAFRVSCVCCIQ
SPPTVFQLGSACVCVFNHLASGALWSWRSPAKKKGNKKGKTFSLTDFLSEDSGGGGGGNAPPPNYPAKSTSWADETDDLD
GDVSTSWHTEEDSFRAPAID
RSILPTAPRSAREPNIDRARLPRSPPYTAFLGNLPYDVSEE
>g2479.t4
MAASAAKKKGNKKGKTFSLTDFLSEDSGGGGGGNAPPPNYPAKSTSWADETDDLDGDVSTSWHTEEDSFRAPAIDRSILP
TAPRSAREPNIDRARLPRSP
PYTAFLGNLPYDVSEE
>g2480.t1
MFKTSYLREVRKDKYERSDVFEEDEGEPEGAGPSAGASAIQGWESLQELNGRFARYINRARVLEQRNAVFRKQLETLQRM
EEASGLQEAFTQQIHVNRQR
IRELSSEHAKLERELREACRTLDEYTSRYRNECEHQEELRCTLEQLNKEADQALLGNLEHQIQNQFLQDDILSTRDRHKK
VSVTIQMFTLAFHCEHQPG
>g2480.t2
MFKTSYLREVRKDKYERSDVFEEDEGEPEGAGPSAGASAIQGWESLQELNGRFARYINRARVLEQRNAVFRKQLETLQRM
EEASGLQEAFTQQIHVNRQR
IRELSSEHAKLERELREACRTLDEYTSRYRNECEHQEELRCTLEQLNKEADQALLGNLEHQIQNQFLQDDILSTRDRHKK
VSVTIQMFTLAFH
>g2481.t1
MWARTPPLSKWWRKEEYGGQLPCALVKNPCTQHINNDHIHGEKKEFVCRWDECSREQKPFKAQYMLVVHMRRHTGEKPHK
CTFEGCAKAYSRLENLKTHL
RSHTGEKPYVCEHESCNKAFSNASDRAKHQNRTHSNEKPYVCKIPGCTKRYTDPSSLRKHVKTVHGPEAHVTKKQRGDAP
PRPHPPKGAGENEGSTKFGG
RDSDGRSEGHGTSRGVEDCLQLKSIKTENSMTYQSSPGGHSSCSSEPSPLSSANNNDSGVEMALHSGGSFGDLSALDDSP
MVESSVPGGGGGGHQAGVGL
QLRKAGGLGGSVTVKLENIKKERLKTVRDACPWANSALQPPQGHRSNGTKLPPMPAIGSLLESSNLTNGLNGSYPGQRII
DPSSCEVTLLNQLNERRDST
TSTMSSIYTLSRRSSGISPCYSSRRSSEASQFGGNRHNNISSADSYDPISTDMSRRSSEASQCGGGGVSGGGGGGGGLPS
LLSLTPAQHYRLKAKYAAAV
GGAPPTPLPNMDRMSLRTRMALYGDPQDAPPYQLHHPPAGTAPRRCSDLAHGSQSMMPHEVPTSLPRRASDPVRRPTLEP
LSLPRVQRYNSMNSMNPINP
PTMQEHYRGLGSQVYTRSDGSLQRHPFAPRPPSISENVAMENMAVDGMMVGGDQPAEDDIVLPDDVVQYLRSQNSVISGQ
SIGQTEYKSNNQTQPYQPGT
THQQQTFYGQRRMAMVNANIPLTGQDFQSCQLSPGGAHQPFQPPSDRMSKNNMPVQWNEVSSGTMDTTAKLNKQPHSIRG
NLAVVQQRQNYGSFQGQGQG
LGTNQQVMPMSQNMAIQGYTHRNNQRMINSSLQQQQRQYNNMNLSEQMSPQQGFGQEVSQNSVPGNTSMRPPFNGMMDQN

NPEILAFRSRTQFEGYPHGN
MAEQQQNLNGLHQQHAGLQNGGRGMLQPRPPMEPKSVGRQHSISSAVMQSRFPQSSDFSSSNANNTSEASPKRPSVSSSE
NPNSAMFYTGQIHMFKPNSE
NFDTPMSPCASQDATNTASAAVANIASPGVNQVTSSTVDSSTGGPGATEHAQIDFDAMLDDGDHSSLMSGTLSPGLLQSL
SQSSSRLTTPRNSVTLASVP
AGISNMAIGDMTSMLTALAEESKFLNMIS
>g2482.t1
TPGHFTPGQYGYGRVQSVAPPAAEIRPGVSPSQRQRRSPVADTFQPAQPRPHGGHRSNSWDASSSSSTSSQYHHHQQQQQ
QQQQHHHHLHGRDHQQPRLP
LPKESRVWAPLYLPGSAYQQPQREPQPSGAQERPYEPLPTTSCPGEHVRFQICDNNVIIALGVSRG
>g2483.t1
MTHLQAGLSPETLEKAKVELKENPDTLHQDIQEVRDMIITRPDIGFLRTEDAFILRFLRARKFNHFEAFRLLAQYFEYRQ
QNLDMFKNLKPTDPGIKQAL
KDGFPGVLSNLDKYGRKILVLFAANWDQSRYTFVDILRAILLSLESMIEDPELQVNGFILIIDWSNFTFKQASRLTPSML
RLAIEGLQCNYIQLN
>g2484.t1
MADSASESDTDGAGSSSATPMSSSASNSGKPSIVISQFRLEELTNRLASLQQENKVLKIELETFKLKCKALQEENRDLRK
ASVTIQARAEQEEEFISNTL
FKKIQALQKEKETLAVNYEKEEEFLTNELSRKLMQLQHEKAELEQHLEQEQEFQVNKLMKKIKKMENDTISK
>g2485.t1
MTGQGRRREEAQTDDTRRREEEQTEDTRRVEEVQTDNTRRRREEQRGEQPEDQKGLHRRGTGAGMRRGERMGERSGDEKR
GEDGREEQGGRRGGGDGERR
VEEEATLV
>g2486.t1
LERIREKLAENIHELWVMNKIQLGWTYGVVRDDNKRQHPCLVEFSKLPEQERSYNLQMSLETLNVSTLSLSPSHRQLHVA
GRGSVVEH
>g2487.t1
MLLNNVWPMFTEHLGLPSVVPTIMGYVDLGPVLEQIMTGTANPGTWYKLERMLNTTLSMLQGTSMWVEDVPVVLMIETLL
QTQVMNMQAQNELIQSLHGP
LVTLTTQLIQSINGSRLNLSVIPERLQQAVERTVKAVQGTNGDHGVRGDLGKLGPPTNETLECHRVLMLWEPVREAAGLD
AETLRAWCRVSLQSVLQRYA
EAQTVYLNFNISVSAVLPSGAASVSAAKITASLRSCYHAFINSSRVSEELIKALCMELSDLQPMGPAAQAQWIQQFEDIQ
LHQSLSSLKMLTDQLLNAAP
FLRPYADAAEQAAAHLLGSIQNGNVSGDIFQEAAYIVLHSLNLTEPALPMVWGNSTALDGASLLRLMKEVVRVIVRTQVF
GDVPEIYRCMERFVATNGTR
LLAEEVAELSAWASEPQGSGMEALARLVPGLQRVFTALLDVLRQMGADLPESTGIFGDLLWNLVHAVQNLAAMEDDHGET
QWGSVTWGHPHNSTQLLERN
HGGKSRRRRESPMVPTREPAVNFMDLFSIDYPALFKAVSIEPSATEVLETAHVFLANPDLRVVMNGVTQDMAMGNDTADQ
ETVVDAVLGVLAYLTDPEVY
AGRWPAMDVLQDVAHLLPEEPSFFRLIKNITESLAMESPAGTPKGPSNCPALFPPNRTATPLCIKPN
>g2487.t2
MLLNNVWPMFTEHLGLPSVVPTIMGYVDLGPVLEQIMTGTANPGTWYKLERMLNTTLSMLQGTSMWVEDVPVVLMIETLL
QTQVMNMQAQNELIQSLHGP
LVTLTTQLIQSINGSRLNLSVIPERLQQAVERTVKAVQTNETLECHRVLMLWEPVREAAGLDAETLRAWCRVSLQSVLQR
YAEAQTVYLNFNISVSAVLP
SGAASVSAAKITASLRSCYHAFINSSRVSEELIKALCMELSDLQPMGPAAQAQWIQQFEDIQLHQSLSSLKMLTDQLLNA
APFLRPYADAAEQAAAHLLG
SIQNGNVSGDIFQEAAYIVLHSLNLTEPALPMVWGNSTALDGASLLRLMKEVVRVIVRTQVFGDVPEIYRCMERFVATNG
TRLLAEEVAELSAWASEPQG
SGMEALARLVPGLQRVFTALLDVLRQMGADLPESTGIFGDLLWNLVHAVQNLAAMEDDHGETQWGSVTWGHPHNSTQLLE
RNHGGKSRRRRESPMVPTRE
PAVNFMDLFSIDYPALFKAVSIEPSATEVLETAHVFLANPDLRVVMNGVTQDMAMGNDTADQETVVDAVLGVLAYLTDPE
VYAGRWPAMDVLQDVAHLLP
EEPSFFRLIKNITESLAMESPAGTPKGPSNCPALFPPNRTATPLCIKPN
>g2487.t3
MLLNNVWPMFTEHLGLPSVVPTIMGYVDLGPVLEQIMTGTANPGTWYKLERMLNTTLSMLQGTSMWVEDVPVVLMIETLL

QTQVMNMQAQNELIQSLHGP
LVTLTTQLIQSINGSRLNLSVIPERLQQAVERTVKAVQGTNGDHGVRGDLGKLGPPTNETLECHRVLMLWEPVREAAGLD
AETLRAWCRVSLQSVLQRYA
EAQTVYLNFNISVSAVLPSGAASVSAAKITASLRSCYHAFINSSRVSEELIKALCMELSDLQPMGPAAQAQWIQQFEDIQ
LHQSLSSLKMLTDQLLNAAP
FLRPYADAAEQAAAHLLGSIQNGNVSGDIFQEAAYIVLHSLNLTEPALPMVWGNSTALDGASLLRLMKEVVRVIVRTQVF
GDVPEIYRCMERFVATNGTR
LLAEEVAELSAWASEPQGSGMEALARLVPGLQRVFTALLDVLRQMGADLPESTGIFGDLLWNLVHAVQNLAAMEDDHGET
QWGSVTWGHPHNSTQLLERN
HGGKSRRRRESPMVPTREPAVNFMDLFSIDYPALFKAVSIEPSATEVLETAHVFLANPDLRVVMNGVTQDMAMGNDTADQ
ETVVDAVLGVLAYLTDPEVY
AGRWPAMDVLQDVAHLLPEEPSFFRLIKNITESLAMESPAVSEVRKQL
>g2488.t1
MIENVLKDVHHLQNTYVMLMTNQSEGGAWMTQVLGSAKEMVMEVMWKAIICQNITNLDQSLAMEWWPLFHMVEEGYSNIS
SNQGDNVTLPMILSEWHMLC
NNTMNLPMFMERMLLGYISDNWMPGNNTYNMAEALLQRVLLIVASLGEDIERSPLWPHVGRYVQMASWIVNYKPDNNTGP
ANCSFDAETMDVYCSADVDW
PQFVQAVANALTSLPTKPEVLIRYLKGTVNLLEHIYGDILRGLITSHIPQLIPEGDALSHYMIKLTDNLLSFVLELTTLK
DSDISNLDPMLPHIFNLLQS
TGLTPLLPLLGSSASINTSTVLDIVLKLGRQNQHLFTFNETDPTLPDLEKLIMNLLSMEGNLTLPLSLSMGHTLLTYSGY
FRPEDLASLQLAIQPYTNQT
SAGFTEAVLSAMELLNKLMEAPGGDPTNIILAYVQQLQRFLISALKLHRIDQVWLSSNQLSAGQVTNLHLVMLDLLQLLS
PEGLQNLTQAGPNATQQLIM
QKLLALLPAKVQLLANDLIQDVMQLVGQLSFCTSADQNCVASLSEMFKFLQQVAEMMLAAKGNVTIQISPTNPFLQTQSS
LNMTTTFFSLLLPLRDARYI
QLTNKTLHFITLIMDTPQPTVAQVQDALKQSNLTLTELEEFTSLFGADDVNSLLVNIVGLIDSQQCFAPQPNVHCAMGLI
GKLSGFLSNVPAFHNESLVF
ALIPEILNNSIVDAEHVIIHDNPHASTVLILNNTLTNVMMSLHQFGLSSPELMQELRVLQDLLKLASIHPYNMLPFSNTN
MSDATYLTKLKIVQWYLKKL
ENITSSSDFSSLFHPIYRMVELEVAIQLASTNFSQFVSDQVMHLKDPIDGAGVNKIGLVIIELVREQMKIILKTCELQND
LAQSFGHPPMCNATTLNALS
YQTGLYLDLIRSWMREPNVTLVFNSMLHWGNSSQNLSTPGNDIHQLLKTVALYLKGEQLAYFSVISNITQSMQMALAVAE
QPGGLQSDRFSEAILAVVGK
VMQSINPEMGPVQQNVLAITADSLKLIVNPNMSYGSARNISLTILRRAETLVMQILPDEVADYVISGIQIIATYFETISM
PDGQDVWNEM
>g2488.t2
MLMTNQSEGGAWMTQVLGSAKEMVMEVMWKAIICQNITNLDQSLAMEWWPLFHMVEEGYSNISSNQGDNVTLPMILSEWH
MLCNNTMNLPMFMERMLLGY
ISDNWMPGNNTYNMAEALLQRVLLIVASLGEDIERSPLWPHVGRYVQMASWIVNYKPDNNTGPANCSFDAETMDVYCSAD
VDWPQFVQAVANALTSLPTK
PEVLIRYLKGTVNLLEHIYGDILRGLITSHIPQLIPEGDALSHYMIKLTDNLLSFVLELTTLKDSDISNLDPMLPHIFNL
LQSTGLTPLLPLLGSSASIN
TSTVLDIVLKLGRQNQHLFTFNETDPTLPDLEKLIMNLLSMEGNLTLPLSLSMGHTLLTYSGYFRPEDLASLQLAIQPYT
NQTSAGFTEAVLSAMELLNK
LMEAPGGDPTNIILAYVQQLQRFLISALKLHRIDQVWLSSNQLSAGQVTNLHLVMLDLLQLLSPEGLQNLTQAGPNATQQ
LIMQKLLALLPAKVQLLAND
LIQDVMQLVGQLSFCTSADQNCVASLSEMFKFLQQVAEMMLAAKGNVTIQISPTNPFLQTQSSLNMTTTFFSLLLPLRDA
RYIQLTNKTLHFITLIMDTP
QPTVAQVQDALKQSNLTLTELEEFTSLFGADDVNSLLVNIVGLIDSQQCFAPQPNVHCAMGLIGKLSGFLSNVPAFHNES
LVFALIPEILNNSIVDAEHV
IIHDNPHASTVLILNNTLTNVMMSLHQFGLSSPELMQELRVLQDLLKLASIHPYNMLPFSNTNMSDATYLTKLKIVQWYL
KKLENITSSSDFSSLFHPIY
RMVELEVAIQLASTNFSQFVSDQVMHLKDPIDGAGVNKIGLVIIELVREQMKIILKTCELQNDLAQSFGHPPMCNATTLN
ALSYQTGLYLDLIRSWMREP
NVTLVFNSMLHWGNSSQNLSTPGNDIHQLLKTVALYLKGEQLAYFSVISNITQSMQMALAVAEQPGGLQSDRFSEAILAV
VGKVMQSINPEMGPVQQNVL

AITADSLKLIVNPNMSYGSARNISLTILRRAETLVMQILPDEVADYVISGIQIIATYFETISMPDGQDVWNEM
>g2488.t3
MIENVLKDVHHLQNTYVMLMTNQSEGGAWMTQVLGSAKEMVMEVEEGYSNISSNQGDNVTLPMILSEWHMLCNNTMNLPM
FMERMLLGYISDNWMPGNNT
YNMAEALLQRVLLIVASLGEDIERSPLWPHVGRYVQMASWIVNYKPDNNTGPANCSFDAETMDVYCSADVDWPQFVQAVA
NALTSLPTKPEVLIRYLKGT
VNLLEHIYGDILRGLITSHIPQLIPEGDALSHYMIKLTDNLLSFVLELTTLKDSDISNLDPMLPHIFNLLQSTGLTPLLP
LLGSSASINTSTVLDIVLKL
GRQNQHLFTFNETDPTLPDLEKLIMNLLSMEGNLTLPLSLSMGHTLLTYSGYFRPEDLASLQLAIQPYTNQTSAGFTEAV
LSAMELLNKLMEAPGGDPTN
IILAYVQQLQRFLISALKLHRIDQVWLSSNQLSAGQVTNLHLVMLDLLQLLSPEGLQNLTQAGPNATQQLIMQKLLALLP
AKVQLLANDLIQDVMQLVGQ
LSFCTSADQNCVASLSEMFKFLQQVAEMMLAAKGNVTIQISPTNPFLQTQSSLNMTTTFFSLLLPLRDARYIQLTNKTLH
FITLIMDTPQPTVAQVQDAL
KQSNLTLTELEEFTSLFGADDVNSLLVNIVGLIDSQQCFAPQPNVHCAMGLIGKLSGFLSNVPAFHNESLVFALIPEILN
NSIVDAEHVIIHDNPHASTV
LILNNTLTNVMMSLHQFGLSSPELMQELRVLQDLLKLASIHPYNMLPFSNTNMSDATYLTKLKIVQWYLKKLENITSSSD
FSSLFHPIYRMVELEVAIQL
ASTNFSQFVSDQVMHLKDPIDGAGVNKIGLVIIELVREQMKIILKTCELQNDLAQSFGHPPMCNATTLNALSYQTGLYLD
LIRSWMREPNVTLVFNSMLH
WGNSSQNLSTPGNDIHQLLKTVALYLKGEQLAYFSVISNITQSMQMALAVAEQPGGLQSDRFSEAILAVVGKVMQSINPE
MGPVQQNVLAITADSLKLIV
NPNMSYGSARNISLTILRRAETLVMQILPDEVADYVISGIQIIATYFETISMPDGQDVWNEM
>g2489.t1
MWSKEVASADMTFVKGLLHSVMGGNMDNHQGPWRSRRSLDTEGPQNVDEDLFVNLGRVVMEGLEVIPDVDAVLQRILRTG
LFSMQTANLAIDTLESK
>g2489.t2
TLLMWSKEVASADMTFVKGLLHSVMGGNMDNHQGPWRSRRSLDTEGPQNVDEDLFVNLGRVVMEGLEVIPDVDAVLQRIL
RTGLFSMQTANLAIDTLESK
>g2490.t1
MTSEDTGLGAVAPTLAGYLGKVNALRSYRLNFQESITLCTNPQCLFPLVSRSLEDVFAELVPAKEQIGGQRKNGHVLRSE
DVASSPPTRLRSDGGEQALG
VTDSPVTAEQPAAMEVDGAEPNLLQDTREKALGGTPRSEGLVPSEAACQGWGAERNGEDVVVPTCSPATPTAGE
>g2490.t2
MTSEDTGLGAVAPTLAGYLGKGQVNALRSYRLNFQESITLCTNPQCLFPLVSRSLEDVFAELVPAKEQIGGQRKNGHVLR
SEDVASSPPTRLRSDGGEQA
LGVTDSPVTAEQPAAMEVDGAEPNLLQDTREKALGGTPRSEGLVPSEAACQGWGAERNGEDVVVPTCSPATPTAGE
>g2491.t1
MGKDPLKPRGRMSSYAYFIQTCREEHKKKHPDASVNFSKFSKKCSERWKTMSSQEKGKFEDKAKLDKVRYEKEMKTYTPC
KMEKKKKIKDPNAPKRPPSA
FFIFCADFRPKIKGETPGLTIGDVAKKLGEMWNNTPIEDKQPYEKKASKLKEKYEKDVAAYRAKTKPGPAAPAKPPAKVE
RKDDDDDDDDDDDDDEDDDD
EDDDDE
>g2491.t2
MGKDPLKPRGRMSSYAYFIQTCREEHKKKHPDASVNFSKFSKKCSERWKEKGKFEDKAKLDKVRYEKEMKTYTPCKMEKK
KKIKDPNAPKRPPSAFFIFC
ADFRPKIKGETPGLTIGDVAKKLGEMWNNTPIEDKQPYEKKASKLKEKYEKDVAAYRAKTKPGPAAPAKPPAKVERKDDD
DDDDDDDDDDEDDDDEDDDD
E
>g2492.t1
MANTSEFGESNPSLQNYATATASAVKLASLGLIICVGLAGNGALSLLVLRDTTLHKAPYYFLLDLCLADVIRSAVCLPFV
MVSIHNGSSWTNSALCCKLI
AFLSILFCFHVAFLLFCVSVTRYMAIAHHRFYSKRMTLCTSVAVICMVWTLSVAMAFPPVFDVGTYKFIREEEQCIFEHR
YLKANDTLGFMLMLAVIVST
THVVYMKMLCFVYDHRKMKPAQLVPAISQNWTFHGPGATGQAAANWIAGFGRGPTPPTLVGIRQASHSGTRRLLVLDEFK
MEKRIGKMYYMITLSFLVLW

APYLTACYVRIFVKGAAVPQVYLTAAVWMTFVQAGANPVISFVFNKELRVRLKACFPWCLSTQTPTEPYCVI
>g2493.t1
MLPTAVLHRQDWFTERRLEKLILSRMTGGLALFDTRTQDSVFLTQWLSGESGWTERVVHQQAVRFPSIPPSSDCPRVEVP
LRKTLNPNLLPGPLN
>g2494.t1
MKLVRLGTGEPVLLLAYLAALTGLCASRSDAPRGVANPFVFDPKAKCDPPCKHSGICIRNNTCFCMRGYEGETCQYAVCP
QGCRNGGSCVAPGICSCPEG
WVGGACHTAVCSQACLSGGKCISPEKCRCRPPYEGPRCEKKRSQYFSLQKVKGR
>g2494.t2
MKLVRLGTGEPVLLLAYLAALTGLCASRSDAPRGVANPFVFDPKAKCDPPCKHSGICIRNNTCFCMRGYEGETCQYAVCP
QGCRNGGSCVAPGICSCPEG
WVGGACHTAYNKTITDFPVDCTNYVAVCSQACLSGGKCISPEKCRCRPPYEGPRCEKKRSQYFSLQKVKGR
>g2495.t1
MLFPRHADFSCGSPVTAVTANCSSEKRFILSSGEHSLGARKVSREISSPVTPTTNTCSEELVLKSVCSGRLFGPIGRRDA
SDQVVPPECSPGGHSLLRNP
YRSVSFHSGGLQQSSLQELVCDHTLEPGWYQFSIFDKPASMPTQCVEVNHCGTQAPVWLSLGEGESLPGPLGLGQLTACT
TWPSVPGGAPDCCLFRIPVT
VRNCGAFYVYLLQPTQGCMAYCAQETEAFSSLTSPGPCGSGQANPEGRCRSEDLTARRPPPPSVPVIWAELSGSVVLLRC
SFQDRSANGSLGFTVSWYRR
SPHDGQPREELKQETTLQTSSTIELDGFNLRLGDKVRIYCSSSSFFLDSPDLLGPTVESQEFFAGIRLRPAMATVPEDGR
DCELSVESTVPLPCDQDPTG
PCTLVLQLRVQTQEANLVLGPDLVLSSCLVELGAQGHCRAGVCSRALVHFTPITDFVKDAGRSTQISVTPIDKKHSFLWN
GYTPEPVQITVEDEPSAYCY
VFTDPHVITFDGRYFTLRASAGQPLEVHARQWECGSVVHAASCVCGVVASDGGDVSRRLGGSGVRVTRSYLGRKMTFSSG
AFVRADVSDWGMSLTVRAPG
SDRNQTAGLCGSFDGQPANDLRLAGSDTKEDLNAFISEWSSSESSCARHGNVELSSIIPTLDVTAEYFVAVELHSDEGNQ
RRTPTPGRLTQHNQGVSSVS
GTHGKGSGLPVTPRDGASSWYGASPASSRPGNRGRRQSYRYISDPPHQRLSTADQEGLAYFFPEDHEPAPHPDSSNSPPT
WPTPSGLTQRQARVRCQRTV
ANSSIALGCGRLLGKAFVSRAVAMCVSDLQLKDDVAWANATLPLLENECERRLVEERRSEGEYQEVVDLLRCPSLCSGNG
QCSDWGCSCFPGFASYDCSS
VSDQIPEITALGEGGLCDVRQGACAVVQVSGVGFKLSHELKCEFVKEKFEHGEFVLDDPRFVQAAFLDSTALECQLPLED
GQVTAGLDPEMSPGRPLARW
QVKVSNDGYSYSNAKVLTVFDGACQSCSSTADVLCTQKERTCNIQSVCYSEGDHHPASPCLTCQPESSRYTWSTAGDNRP
PVIQPLPSRLQSFQGESFLY
QLQAADPDGSSVVFTLASGPGGAALSPSGLLSWEASTGADGSRGRTFQVTVADECGAETQVSAQVFVSPCDCGNGGSCVP
KADAVAGSGEHVCACPEGFA
GGRCEVDVDDCKPNPCRLGRCIDAHNSFSCVCPPGMSGNTCREDVDECASAPCFPGVSCQNTPGSFTCGACPRGYRGDGR
SCALQGSTTSNPKRGPSIHR
KTTVSPSNPHIASDAITSSNGHRSHPAMCAFPCGANMECSLPNTCTCKEGYIGYNCHIAVCRPDCKNQGKCVRPSVCECP
VGYSGLTCEHVSCEPPCQHG
GTCVARNLCTCPYGYLGPHCETTACNPVCLNGGTCVKPAVCACPRGFYGSQCQIVPGTATVHSLKRCTLVVMRRSNKISG
QL
>g2495.t2
MLFPRHADFSCGSPVTAVTANCSSEKRFILSSGEHSLGARKVSREISSPVTPTTNTCSEELVLKSVCSGRLFGPIGRRDA
SDQVVPPECSPGGHSLLRNP
YRSVSFHSGGLQQSSLQELVCDHTLEPGWYQFSIFDKPASMPTQCVEVNHCGTQAPVWLSLGEGESLPGPLGLGQLTACT
TWPSVPGGAPDCCLFRIPVT
VRNCGAFYVYLLQPTQGCMAYCAQETEAFSSLTSPGPCGSGQANPEGRCRSEDLTARRPPPPSVPVIWAELSGSVVLLRC
SFQDRSANGSLGFTVSWYRR
SPHDGQPREELKQETTLQTSSTIELDGFNLRLGDKVRIYCSSSSFFLDSPDLLGPTVESQEFFAGIRLRPAMATVPEDGR
DCELSVESTVPLPCDQDPTG
PCTLVLQLRVQTQEANLVLGPDLVLSSCLVELGAQGHCRAGVCSRALVHFTPITDFVKDAGRSTQISVTPIDKKHSFLWN
GYTPEPVQITVEDEPSAYCY
VFTDPHVITFDGRYFTLRASAGQPLEVHARQWECGSVVHAASCVCGVVASDGGDVSRRLGGSGVRVTRSYLGRKMTFSSG
AFVRADVSDWGMSLTVRAPG

SDRNQTAGLCGSFDGQPANDLRLAGSDTKEDLNAFISEWSSSESSCARHGNVELSSIIPTLDVTAEYFVAVELHSDEGNQ
RRTPTPGRLTQHNQGVSSVS
GTHGKGSGLPVTPRDGASSWYGASPASSRPGNRGRRQSYRYISDPPHQRLSTADQEGLAYFFPEDHEPAPHPDSSNSPPT
WPTPSGLTQRQARVRCQRTV
ANSSIALGCGRLLGKAFVSRAVAMCVSDLQLKDDVAWANATLPLLENECERRLVEERRSEGEYQEVVDLLRCPSLCSGNG
QCSDWGCSCFPGFASYDCSS
VSDQIPEITALGEGGLCDVRQGACAVVQVSGVGFKLSHELKCEFVKEKFEHGEFVLDDPRFVQAAFLDSTALECQLPLED
GQVTAGLDPEMSPGRPLARW
QVKVSNDGYSYSNAKVLTVFDGACQSCSSTADVLCTQKERTCNIQSVCYSEGDHHPASPCLTCQPESSRYTWSTAGDNRP
PVIQPLPSRLQSFQGESFLY
QLQAADPDGSSVVFTLASGPGGAALSPSGLLSWEASTGADGSRGRTFQVTVADECGAETQVSAQVFVSPCDCGNGGSCVP
KADAVAGSGEHVCACPEGFA
GGRCEVDVDDCKPNPCRLGRCIDAHNSFSCVCPPGMSGNTCREDVDECASAPCFPGVSCQNTPGSFTCGACPRGYRGDGR
SCALQGSTTSNPKRGPSIHR
KTTVSPSNPHIASDAITSSNGHRSHPAMCAFPCGANMECSLPNTCTCKEGYIGYNCHIAVCRPDCKNQGKCVRPSVCECP
VGYSGLTCEHVSCEPPCQHG
GTCVARNLCTCPYGYLGPHCETMVCNRHCENGGECISPDVCQCRDGWYGPTCNSAACNPVCLNGGTCVKPAVCACPRGFY
GSQCQIGELESSEFT
>g2495.t3
MGETRPRLAVKSRRLGGSGVRVTRSYLGRKMTFSSGAFVRADVSDWGMSLTVRAPGSDRNQTAGLCGSFDGQPANDLRLA
GSDTKEDLNAFISEWSSSES
SCARHGNVELSSIIPTLDVTAEYFVAVELHSDEGNQRRTPTPGRLTQHNQGVSSVSGTHGKGSGLPVTPRDGASSWYGAS
PASSRPGNRGRRQSYRYISD
PPHQRLSTADQEGLAYFFPEDHEPAPHPDSSNSPPTWPTPSGLTQRQARVRCQRTVANSSIALGCGRLLGKAFVSRAVAM
CVSDLQLKDDVAWANATLPL
LENECERRLVEERRSEGEYQEVVDLLRCPSLCSGNGQCSDWGCSCFPGFASYDCSSVSDQIPEITALGEGGLCDVRQGAC
AVVQVSGVGFKLSHELKCEF
VKEKFEHGEFVLDDPRFVQAAFLDSTALECQLPLEDGQVTAGLDPEMSPGRPLARWQVKVSNDGYSYSNAKVLTVFDGAC
QSCSSTADVLCTQKERTCNI
QSVCYSEGDHHPASPCLTCQPESSRYTWSTAGDNRPPVIQPLPSRLQSFQGESFLYQLQAADPDGSSVVFTLASGPGGAA
LSPSGLLSWEASTGADGSRG
RTFQVTVADECGAETQVSAQVFVSPCDCGNGGSCVPKADAVAGSGEHVCACPEGFAGGRCEVDVDDCKPNPCRLGRCIDA
HNSFSCVCPPGMSVQGSTTS
NPKRGPSIHRKTTVSPSNPHIASDAITSSNGHRSHPAMCAFPCGANMECSLPNTCTCKEGYIGYNCHIAVCRPDCKNQGK
CVRPSVCECPVGYSGLTCEH
VSCEPPCQHGGTCVARNLCTCPYGYLGPHCETTACNPVCLNGGTCVKPAVCACPRGFYGSQCQIVPGTATVHSLKRCTLV
VMRRSNKISGQL
>g2496.t1
MVPVSSDRFERKLLFSMRQNLDAARSQCRISPAESEADSPVVDVWRIQAPDFSPKLYRSLSGRPRGGRFNRRPQALSSGN
TDMTPERDVSKVLHFKRTVA
DISLETHDRREPRRFIKSYKPPDALQAELMFVKLGKFPPLGPYRNPKPHDFRPQDDNLPNMATVTKKDPGDLEFKLSHRD
ICRKYLI
>g2497.t1
LIAWDLQGTYAVVEFANLESVASLREAVAIPVVSNESVVPFKSRLLSQTSLGSGVTPNRQTGPPCKPQATIPINELIQRL
SKEESIDQQVASLTKAYQVT
EENTRLRFLVCSLLKDIAAAYFPECNIRPFGSSVNGFGKLGCDLDMFLDLDGIVGRNVHVVRL
>g2497.t2
GTYAVVEFANLESVASLREAVAIPVVSNESVVPFKSRLLSQTSLGSGVTPNRQTGPPCKPQATIPINELIQRLSKEESID
QQVASLTKAYQVTEENTRLR
FLVCSLLKDIAAAYFPECNIRPFGSSVNGFGKLGCDLDMFLDLDGIVGRNVHVVRL
>g2497.t3
MHGDIKNSFFYESFGTYAVVEFANLESVASLREAVAIPVVSNESVVPFKSRLLSQTSLGSGVTPNRQTGPPCKPQATIPI
NELIQRLSKEESIDQQVASL
TKAYQVTEENTRLRFLVCSLLKDIAAAYFPECNIRPFGSSVNGFGKLGCDLDMFLDLDGIVGRNVHVVRL
>g2498.t1
MLSTEELALWIQHVYRTYAMEDAEERFPEFDPDGDGAVSWEEYSAVTHHGLPALHEAPASSDPEQESIRYLHLKEKRRFE

FADTDGTEGLNLPEFLAFTH
PSEVDHMADFTIEDVLREYDRDGDGFISLTEFIGDVRGNEDSTPSQWEVEETVRFKELYDQDKDGKLNREEQLRWVAPNS
YGSAREEALHLIKEMDHDRD
GKISEAEVLRNQELFMNSEVTDYGRQLNMSHDEL
>g2498.t2
MLSTEELALWIQHVYRTYAMEDAEERFPEFDPDGDGAVSWEEYSAVTHHGLPALHEAPASSDPEQESIRYLHLKEKRRFE
FADTDGTEGLNLPEFLAFTH
PSEVDHMADFTIEDVLREYDRDGDGFISLTEFIGDVRGNEDSTPSQWEVEETVRFKELYDQDKDGKLNREEQLRWVAPNS
YGSAREEALHLIKEMDHDRD
GKISEAEVLRNQELFMNSEVTDYELENVGHLHIQLSGMLRDEVKSMETFREQQKEQKKKRCRDGEEAEQAADKIASAATA
TPKQTEKVSTSDSVVLLGIV
IVSHNM
>g2498.t3
MLSTEELALWIQHVYRTYAMEDAEERFPEFDPDGDGAVSWEEYSAVTHHGLPALHEAPASSDPEQESIRYLHLKEKRRFE
FADTDGTEGLNLPEFLAFTH
PSEVDHMADFTIEDVLREYDRDGDGFISLTEFIGDVRGNEDSTPSQWEVEETVRFKELYDQDKDGKLNREEQLRWVAPNS
YGSAREEALHLIKEMDHDRD
GKISEAEVLRNQELFMNSEVTDYELENVGHLHIQLSGMLRDEVKSMETFREQQKEQKKKSKKSYEQRCRDGEEAEQAADK
IASAATATPKQTEKVSTSDS
VVLLGIVIVSHNM
>g2499.t1
LLNRATFEPGPGGTATIISIASRLAAGFRLSTAVMEVQSWMTDDLLCPAVSESSPGRMNTEEVELLSDSKYRNYVAAVDK
ALKNFEYSSEWADLISALGK
LNKVLQNNAKYQVVPKKLTIGKRLAQCLHPALPSGVHRKALETYEIIFKIIGPKRLAKDLFLYSTTSGHFRSSFHVSPFC
HHSAGLFPLLSNAAMSVKPA
LLGLYETYYLPLGKTLKPGLQGLLTGVLPGLEEGSEYYDRTNTLLEKVAAAVEQSAFYSALWGSILTSPAVRLPGVTFVL
LHLNRKLSMEDQLYVMGSDI
ELMVPAAARLGVEAVSTSVQDSSVLVQRSTLDLILFCFPFHMSQ
>g2499.t2
LLNRATFEPGPGGTATIISIASRLAAGFRLSTAVMEVQSWMTDDLLCPAVSESSPGRMNTEEVELLSDSKYRNYVAAVDK
ALKNFEYSSEWADLISALGK
LNKVLQNNAKYQVVPKKLTIGKRLAQCLHPALPSGVHRKALETYEIIFKIIGPKRLAKDLFLYSTTSGHFRSSFHVSPFC
HHSAGLFPLLSNAAMSVKPA
LLGLYETYYLPLGKTLKPGLQGLLTGVLPGLEEGSEYYDRTNTLLEKVAAAVEQSAFYSALWGSILTSPAVRLPGVTFVL
LHLNRKLSMEDQLYVMGSDI
ELMVEAVSTSVQDSSVLVQRSTLDLILFCFPFHMSQ
>g2499.t3
MNTEEVELLSDSKYRNYVAAVDKALKNFEYSSEWADLISALGKLNKVLQNNAKYQVVPKKLTIGKRLAQCLHPALPSGVH
RKALETYEIIFKIIGPKRLA
KDLFLYSTTSGHFRSSFHVSPFCHHSAGLFPLLSNAAMSVKPALLGLYETYYLPLGKTLKPGLQGLLTGVLPGLEEGSEY
YDRTNTLLEKVAAAVEQSAF
YSALWGSILTSPAVRLPGVTFVLLHLNRKLSMEDQLYVMGSDIELMVPAAARLGVEAVSTSVQDSSVLVQRSTLDLILFC
FPFHMSQ
>g2500.t1
RAPGRLPQEPTQNPAAGSLRDKQPGSRRPKYFPSERRASPRRAGSARCARYVERRSFFQRLTGDNKSEKFFKVFYERMKL
AQQEIKATVTVNTSDLGSKK
KEEEPVDKDAVARKKARHLTLTAPDERDIPVRG
>g2500.t2
RAPGRLPQEPTQNPAAGSLRDKQPGSRRPKYFPSERRASPRRAGSARCARYVERRSFFQRLTGDNKSEKFFKVFYERMKL
AQQEIKATVTVNTSDLGSKK
KEEEPVDKDAVARKKAGEDRSYGLGRAEQVLRPDGLAVASSQPRTAEVSSSKTPHLNCS
>g2501.t1
PLPLQPTSTGHTSTFQPRPSASAAHLHRPHLHVPAPPLCFSC
>g2502.t1
MSVRDFITALTTDGETVECRAEHSVPAILAAQSAAGRSMERQRRVSSADMQEDISPCNKLQDAMTQMMQNKPDKRERGKA
VKQGRGLARGRTRAVEGSPL

MRTTSAENGVR
>g2502.t2
MSVRDFITALTTDGETVECRAEHSVPAILAAQSAAGRSMERQRRVSHATGGLRSADMQEDISPCNKLQDAMTQMMQNKPD
KRERGKAVKQGRGLARGRTR
AVEGSPLMRTTSAENGVR
>g2503.t1
TPLICTLLSSTSALISLPIQRRGLHIFPCTRLLSIQQPAQKKLPCDKREQRKKKKTPDSRRGEMESDALRSEEMRTASRS
ARGGRNAYVPYSWTDTRPDT
HGQDIQSVGSDRHETLRHLVL
>g2504.t1
MEESVAFFVNRSIGVGSGLQGCAAVCEQSRAGPGLGRSESVSLFSAIRCSSLVQYAMANMADITNSQRSLLVSERPNPSI
TMQPFPRSFVRVRARRALGI
RRRGGVRRAPRRWRDADKGRGV
>g2505.t1
GSPDDDGCTVMPAVYSPGSTPPHRLSPQAGSGPQADSLDSEEWAAEAEGEPPAPSSAPCTQLSNGNPSQAVLEFAQYLDS
LFRLDSSSSPPLELSDGESE
SGRGSFLEPEEGPDEGRSTDDKRLLARAVVEPNLVPKPPRTFAGSADPSSGISLSPSFPLSSSGELNDLSPGEGAPLAAH
ALRTSPPCHYPEEHTVSSMV
>g2506.t1
LSPSFFPGSDQAKLPELQPFPSPGGQAVTENEELVWMPGVNDCDLLMYLRAARSMAAFAGMCDGGSTEDGCLAASRDDTT
LNALNTSRTPLLCTERVLRS
GSHDPRWNAPCAQADAETLHESSYDAGKALQRLVKKPVPKLIEKCWSEDEV
>g2506.t2
MFICWAKLPELQPFPSPGGQAVTENEELVWMPGVNDCDLLMYLRAARSMAAFAGMCDGGSTEDGCLAASRDDTTLNALNT
SRTPLLCTERVLRSGSHDPR
WNAPCAQADAETLHESSYDAGKALQRLVKKPVPKLIEKCWSEDEV
>g2506.t3
LSPSFFPGSDQAKLPELQPFPSPGGQAVTENEELVWMPGVNDCDLLMYLRAARSMAAFAGMCDGGSTEDGCLAASRDDTT
LNALNTSRTPLLCTERVLRS
GSHDPRWNAPCAQADAETGRRSACQWPPRASLDGLRRGAHRHALQIAFCLSPPHGDGGASRLHESSYDAGKALQRLVKKP
VPKLIEKCWSEDEV
>g2507.t1
MPIPDLPVLRFPKSPSLSKRQKRAWVIPPINFLENDRGPFPKIMVQIRSDADKEVKIQYMITGPGASEPPVGLFTVDRNS
GHMYVTQPLDREKQDKYMTL
TCFKSMPQIWTSQVLLILISSTEF
>g2508.t1
LAYKNGFALPRPPAVGIGQEPLQSEQLAYNYANELSSETLPLYTDPSSRASVVEFIPAHVALDKKVLRFHGYFQQRVLHS
PDEEYRVRPVVVYYHLEDDS
VCVVEPAVENSGLPQGKLIKRQRLPKDDRGHTYHWKDLNLAVDLAAYGVVYRITDCDAFTREFLESEGILVNDPESMPVD
PYTSHRAKPQRSHISPSDFD
HQRQFLTMDRKVLRFFALWDNTDALHKETRPVSIQYYLVDDTVEIREIHQPNSGRDPLPVLMRRQRLPKTIREASGER
>g2508.t2
LAYKNGFALPRPPAVGIGQEPLQSEQLAYNYANELSSETLPLYTDPSSRASVVEFIPAHVALDKKVLRFHGYFQQRVLHS
PDEEYRVRPVVVYYHLEDDS
VCVVEPAVENSGLPQGKLIKRQRLPKDDRGHTYHWKDLNLAVDLAAYGVVYRITDCDAFTREFLESEGILVNDPESMPVD
PYTSHRAKPQRSHISPSDFD
HQRQFLTMDRKVLRFFALWDNTDALHKETRPVSIQYYLVDDTVEIREIHQPNSGRDPLPVLMRRQRLPKTIREASGQREE
PPPPHCSYNTFTSAIWSLPC
RDGPWASLG
>g2508.t3
MSINARHGLPFLPGNTFRDITKSAYHRSQTLAYKNGFALPRPPAVGIGQEPLQSEQLAYNYANELSSETLPLYTDPSSRA
SVVEFIPAHVALDKKVLRFH
GYFQQRVLHSPDEEYRVRPVVVYYHLEDDSVCVVEPAVENSGLPQGKLIKRQRLPKDDRGHTYHWKDLNLAVDLAAYGVV
YRITDCDAFTREFLESEGIL
VNDPESMPVDPYTSHRAKPQRSHISPSDFDHQRQFLTMDRKVLRFFALWDNTDALHKETRPVSIQYYLVDDTVEIREIHQ
PNSGRDPLPVLMRRQRLPKT

IREASGER
>g2509.t1
LRAAAVNGKQSESLCWSAALSGSGSAGQKDSAIKPAFRATFSSMAAGLAFLLQIIDRLVDWLKTSLPTDLVRIPLEGQHR
LTTSRHN
>g2510.t1
MPLSPHHTCPAYSTLTSPPAKTQDGIALEIQPLKSEEAAESEEKEEVKPVKKVNVTKKEKSVLQGKLTRLAVQIGKAGLI
MSAVTVIILILYFVIDTFGV
QGRTWESTCTPIYIQYFVKFFIIGVTVLVVAVPEGLPLAVTISLAYSVKKMMKDNNLVRHLDACETMGNATAICSDKTGT
LTMNRMTVVQAYIGDTHYKA
VPEPDAIKPETLEIMVNSISINSAYTTKILPPEREGGLPRHVGNKTECSLLGLVLELKRDYQPIRDEIPEEKLYKVYTFN
SSRKSMSTVLKNADGSFRMY
SKGASEIILRKCSHILDSQGKPRVFSPKDRDDMVRRVIEPMACDGLRTICVAYRDFPVEAGEPLWDNENDILNDLTCIVV
VGIEDPVRPEVGPSAER
>g2510.t2
MPLSPHHTCPAYSTLTSPPAKTQDGIALEIQPLKSEEAAESEEKEEVKPVKKVNVTKKEKSVLQGKLTRLAVQIGKAGLI
MSAVTVIILILYFVIDTFGV
QGRTWESTCTPIYIQYFVKFFIIGVTVLVVAVPEGLPLAVTISLAYSVKKMMKDNNLVRHLDACETMGNATAICSDKTGT
LTMNRMTVVQAYIGDTHYKA
VPEPDAIKPETLEIMVNSISINSAYTTKILPPEREGGLPRHVGNKTECSLLGLVLELKRDYQPIRDEIPEEKLYKVYTFN
SSRKSMSTVLKNADGSFRMY
SKGASEIILRKCSHILDSQGKPRVFSPKDRDDMVRRVIEPMACDGLRTICVAYRDFPVEAGEPLWDNENDILNDLTCIVV
VGIEDPVRPEVPDAISKCQR
AGITVRMVTGDNINTARAIATKCGILLPGEDFLCIEGKDFNLQIRNDRGE
>g2510.t3
MPLSPHHTCPAYSTLTSPPAKTQDGIALEIQPLKSEEAAESEEKEEVKPVKKVNVTKKEKSVLQGKLTRLAVQIGKAGLI
MSAVTVIILILYFVIDTFGV
QGRTWESTCTPIYIQYFVKFFIIGVTVLVVAVPEGLPLAVTISLAYSVKKMMKDNNLVRHLDACETMGNATAICSDKTGT
LTMNRMTVVQAYIGDTHYKA
VPEPDAIKPETLEIMVNSISINSAYTTKILPPEREGGLPRHVGNKTECSLLGLVLELKRDYQPIRDEIPEEKLYKVYTFN
SSRKSMSTVLKNADGSFRMY
SKGASEIILRKCSHILDSQGKPRVFSPKDRDDMVRRVIEPMACDGLRTICVAYRDFPVEAGEPLWDNENDILNDLTCIVV
VGIEDPVRPEVPDAISKCQR
AGITVRMVTGDNINTARAIATKCGILLPGEDFLCIEGKDFNLQIRNDRGEVRYAALDALQEGVK
>g2510.t4
MVTGDNINTARAIATKCGILLPGEDFLCIEGKDFNLQIRNDRGE
>g2511.t1
EYSTAVDMWSVGCIFGELLTQKPLFPGKSEIDQINKIFKDLGSPSEKIWPGYGELPAVKKMTFTEYPYNNLRKRFGALLS
DQGFDLMN
>g2511.t2
MVVGSTSPSMSPTRPEAAAAASPPGREYSTAVDMWSVGCIFGELLTQKPLFPGKSEIDQINKIFKDLGSPSEKIWPGYGE
LPAVKKMTFTEYPYNNLRKR
FGALLSDQGFDLMN
>g2511.t3
MSPTRPEAAAAASPPGREYSTAVDMWSVGCIFGELLTQKPLFPGKSEIDQINKIFKDLGSPSEKIWPGYGELPAVKKMTF
TEYPYNNLRKRFGALLSDQG
FDLMN
>g2512.t1
MNPIPTPASCCGDRGAFKDETSRADGVGIPTSRSLVQKWVKSCKGKRSAIVEVRWEHSGAAAPEPLGGDGAASGPESREC
EGADFHSGSWFVPDISPYRQ
AQGWLLPRSVTHLKLAAPLFSTNDSSEGNAHFGLSAISL
>g2513.t1
MMYSSKRKPVLYFKEERRFLSGKITVYKLFGLCMVLVLVSLVLLQLSCSGDMSSSSSSGNMADARSSSQHHPQQQPCPPE
AQAASAVDDPAWGPHKLALI
IPFRERFEELLVFVPFMHAFLNKKKIRHRILVVNQVDHYRFNRASLINVGYMESGNDTDYLAMHDVDLLPLNPALDYSFP
EEGPFHVASPELHPLYHYKT
YVGGILLLTKRHYNMCNGMSNRFWGWGREDDEFYRRLKKAGLQLFRPSGITTGYKTFRHIHDPAWRKRDQKRIASQKQEQ

FKVDPEGGLTNLLYQVESRQ
EVTISGAPCTIVNTKLDCDQKNTPWCL
>g2513.t2
MMYSSKRKPVLYFKEERRFLSGKITVYKLFGLCMVLVLVSLVLLQLSCSGDMSSSSSSGNMADARSSSQHHPQQQPCPPE
AQAASAVDDPAWGPHKLALI
IPFRERFEELLVFVPFMHAFLNKKKIRHRILVVNQVDHYRFNRASLINVGYMESGNDTDYLAMHDVDLLPLNPALDYSFP
EEGPFHVASPELHPLYHYKT
YVGGILLLTKRHYNMCNGMSNRFWGWGREDDEFYRRLKKAGLQLFRPSGITTGYKTFRHIHDPAWRKRDQKRIASQKQEQ
FKVDPEGGLTNLLYQVESRQ
EVTISGAPCTIVNTKLDCDQKNTP
>g2514.t1
LTPTDSHPLIRSPHSDERHKDTAMKPDQNVGCWWCLVGLFLLSLFIVLAWLVQYSLATVRVWRAKRVNQDFGGGSAARHL
PTNQDHRTQTGGLRGFLLRL
RPGRSGRAASEAGAKGLLSTLFSFKSFGEPWQRAWVKALNDQACRHGPLQREATLKRRWRPPDLTRRDRRRLLHSRRRTP
AYTWRRMHPSRRLCTSALAS
LRVGRGHVVSVTVRIRLTPQETQNTFL
>g2514.t2
MLTQPLQREATLKRRWRPPDLTRRDRRRLLHSRRRTPAYTWRRMHPSRRLCTSALASLRVGRGHVVSVTVRIRLTPQETQ
NTFL
>g2514.t3
LTPTDSHPLIRSPHSDERHKDTAMKPDQNVGCWWCLVGLFLLSLFIVLAWLVQYSLATVRVWRAKRVNQDFGGGSAARHL
PTNQDHRTQTGGLRGFLLRL
RPGRSGRAASEAGAKGLLSTLFSFKSFGEPWQRAWVKALNDQACRHGSGKVWVGWYTLYSLVAWALKCQ
>g2515.t1
MINCLLSALGCLATIKLIPAFKDHFISAKLYGIDLNKTSKKQVPESQGVISGTVFLIILFCFIPVPFLGCLVGDQCTGFP
HDEFVQLIGALLAICCMIFL
GFADDVLNLRWRHKLLLPTMASLPLLMVYFTNFGNTVIVVPKPFRVLLGLHLDLGKPRDY
>g2515.t2
LATIKLIPAFKDHFISAKLYGIDLNKTSKKQVPESQGVISGTVFLIILFCFIPVPFLGCLVGDQCTGFPHDEFVQLIGAL
LAICCMIFLGFADDVLNLRW
RHKLLLPTMASLPLLMVYFTNFGNTVIVVPKPFRVLLGLHLDLGKPRDY
>g2515.t3
MSPIPVLPLMINCLLSALGCLATIKLIPAFKDHFISAKLYGIDLNKTSKKQVPESQGVISGTVFLIILFCFIPVPFLGCL
VGDQCTGFPHDEFVQLIGAL
LAICCMIFLGFADDVLNLRWRHKLLLPTMASLPLLMVYFTNFGNTVIVVPKPFRVLLGLHLDLGKPRDY
>g2516.t1
LYYVYMGMLAVFCTNAINILAGINGIESGQALFISGSIILFNLFELSGDYGDDHIFSLYFMLPFFFTTLGLFYHNWYPSS
VFVGDTFCYFAGMTFAVVGI
LGHFSKTMLLFFIPQVVNFIYSLPQLFHIIPCPRHRLPRLNPSTGKLGMSYSKFKRKDLSKLGNLILQVAELLRLLEVHR
GQEEDDDFIECNNMTLINLV
LKLLGPVHERSLTIIMLLIQIKDDDTQLKEAYRICSGMNWMENLCKHMEAHLSASDASEDSDETEGKDGPEELRRHVFFH
CYHTKLKQWGLQVLNSNPNM
GSCSIGYQNRNIIPEIPDNFTCLWEDCE
>g2516.t2
LYYVYMGMLAVFCTNAINILAGINGIESGQALFISGSIILFNLFELSGDYGDDHIFSLYFMLPFFFTTLGLFYHNWYPSS
VFVGDTFCYFAGMTFAVVGI
LGHFSKTMLLFFIPQVVNFIYSLPQLFHIIPCPRHRLPRLNPSTGKLGMSYSKFKRKDLSKLGNLILQVAELLRLLEVHR
GQEEDDDFIECNNMTLINLV
LKLLGPVHERSLTIIMLLIQIKDDDTQLKEAYRICSGMNWMENLCKHMEAHLSASDASEDSDETEGKAEQSCLWRHCGFC
SVDGPEELRRHVFFHCYHTK
LKQWGLQVLNSNPNMGSCSIGYQNRNIIPEIPDNFTCLWEDCE
>g2516.t3
LYYVYMGMLAVFCTNAINILAGINGIESGQALFISGSIILFNLFELSGDYGDDHIFSLYFMLPFFFTTLGLFYHNWYPSS
VFVGDTFCYFAGMTFAVVGI
LGHFSKTMLLFFIPQVVNFIYSLPQLFHIIPCPRHRLPRLNPSTGKLGMSYSKFKRKDLSKLGNLILQVAELLRLLEVHR
GQEEDDDFIECNNMTLINLV

LKLLGPVHERSLTIIMLLIQIKDDDTQLKEAYRICSGMNWMENLCKHMEAHLSASDASEDSDETEDGPEELRRHVFFHCY
HTKLKQWGLQVLNSNPNMGS
CSIGYQNRNIIPEIPDNFTCLWEDCE
>g2517.t1
MALSVRPDTATDLPRGVLPEGGGSSSFKEPVNCARPPRPDRTRPHGRGAAGTITGSSRCGFRLRRPPLISPHRATSPVQT
LPLATTGRTGGAAPEPNIEF
LRPSGEPETGEGRSLASAANLFQPGATFHVLETRFTRTTLHAKASLAAGAHSECVTKCAYGLHIPALRIAFFNAASDQEK
QRVPEFQSSSKQRGSILNML
PTAITSSKDIQENNNIFYA
>g2517.t2
MALSVRPDTATDLPRGVLPEGGGTDTDGWGSSSFKEPVNCARPPRPDRTRPHGRGAAGTITGSSRCGFRLRRPPLISPHR
ATSPVQTLPLATTGRTGGAA
PEPNIEFLRPSGEPETGEGRSLASAANLFQPGATFHVLETRFTRTTLHAKASLAAGAHSECVTKCAYGLHIPALRIAFFN
AASDQEKQRVPEFQSSSKQR
GSILNMLPTAITSSKDIQENNNIFYA
>g2518.t1
PPLRGRERRVEHTYPQSGAFRICVAVNNTISWTEACFDMQVHEEIEELTANSSSPTELNTPTVVVARLASGSNVTWTFSM
GDGATYTTIEPRASHRYADN
GNYTVNVTASNAVSSGWTILPVQVFVFQVLEVKPLECVQERSPVAFQARVSGNTPTPLYQWSFGDGSDDEMHEGDPKVAH
TYHASGNYNLSLHVSSQVNN
ASQAHFISRVCVQPAVANVSLILSKTHFHSAEVIHFHARAQPEFNYSYSWDFDAGKDPVLTHGSGEIMFSYDNPVHFEAQ
MEEGDGVIFSWIVCDRGTSI
LGTHTMFYTFRSVGTFNITVIAENGLGSAQVSILLFVQRELEGLAIVAEEDTGGCASVGGAGVEGAGMEGAGVEGCYFAT
NCVLHLQAVLKEGTNMSFTW
NLIRKEDPSNSAFNLTSKTVEVNFPQPGSLDVFLKAVNLLGQLTVNRTFQFLDPVAGVDLQISDNPIAVNTPMNLTVFAR
GSHLQYLWHVDDDIMPWKEF
SVVHTFTSAGQRKIRVEVFNKVSSHIASRTVQVQEVITDLSFTATNVSELQYVPTAVNVSLQGNILTGSNVTWVWLLQGR
MDTGRKTSVIFLEPQATTIT
LNATNNVSGQVVSREFFVQEKIQGLDLKSSQMYAAVRQPVEFTVSMAAGSDVSLILSISGDYTVTPELNQTYVHRFNKPD
NYMVNLTAHNKVSSMRRNVQ
IQVMEAVQGLTILDCCEAAIPVGVRRTFVANILTGKPVHYLWTFDLHHHRKPTLMGQEVVSYTPEEAGCLTIYLRAFNSL
PSPNITKQIMVQNIIREVTL
DALPVDTFVNKTVTLMASIMPRSSPVQCLWDFGDGTGSLLTNSTTVNCSNLVSWELARVEVNIRVLECEEPEVQVVQAPR
LAIWCAQPTLVEASVDLKGC
VRYGVQYLWQILSTPDCDDQDPDTALHQRMTAVAAKRPLSPRASVYPLPAEVDVRRLQLSIPKMVLAAGNYTLVFSLSYE
GIPLRKAACLQLSVMAARLV
PIIEGGTYRVWSRTQDLQLSAEQSYDPNMDPDNQSLLHYHWECQNTSKGAAHCSSLNFGLGSNGPVLGISGSELEAEVEY
TFRLTVNKEGMVLVQSGHIP
MVYLECVSCKAQSIYEVSQNSYVVLRGTCTNCQGLHHG
>g2518.t2
PPLRGRERRVEHTYPQSGAFRICVAVNNTISWTEACFDMQVHEEIEELTANSSSPTELNTPTVVVARLASGSNVTWTFSM
GDGATYTTIEPRASHRYADN
GNYTVNVTASNAVSSGWTILPVQVFVFQVLEVKPLECVQERSPVAFQARVSGNTPTPLYQWSFGDGSDDEMHEGDPKVAH
TYHASGNYNLSLHVSSQVNN
ASQAHFISRVCVQPAVANVSLILSKTHFHSAEVIHFHARAQPEFNYSYSWDFDAGKDPVLTHGSGEIMFSYDNPVHFEAQ
MEEGDGVIFSWIVCDRGTSI
LGTHTMFYTFRSVGTFNITVIAENGLGSAQVSILLFVQRELEGLAIVAEEDTGGCASVGGAGVEGAGMEGAGVEGCYFAT
NCVLHLQAVLKEGTNMSFTW
NLIRKEDPSNSAFNLTSKTVEVNFPQPGSLDVFLKAVNLLGQLTVNRTFQFLDPVAGVDLQISDNPIAVNTPMNLTVFAR
GSHLQYLWHVDDDIMPWKEF
SVVHTFTSAGQRKIRVEVFNKVSSHIASRTVQVQEVITDLSFTATNVSELQYVPTAVNVSLQGNILTGSNVTWVWLLQGR
MDTGRKTSVIFLEPQATTIT
LNATNNVSGQVVSREFFVQEKIQGLDLKSSQMYAAVRQPVEFTVSMAAGSDVSLILSISGDYTVTPELNQTYVHRFNKPD
NYMVNLTAHNKVSSMRRNVQ
IQVMEAVQGLTILDCCEAAIPVGVRRTFVANILTGKPVHYLWTFDLHHHRKPTLMGQEVSYTPEEAGCLTIYLRAFNSLP
SPNITKQIMVQNIIREVTLD

ALPVDTFVNKTVTLMASIMPRSSPVQCLWDFGDGTGSLLTNSTTVNCSNLVSWELARVEVNIRVLECEEPEVQVVQAPRL
AIWCAQPTLVEASVDLKGCV
RYGVQYLWQILSTPDCDDQDPDTALHQRMTAVAAKRPLSPRASVYPLPAEVDVRRLQLSIPKMVLAAGNYTLVFSLSYEG
IPLRKAACLQLSVMAARLVP
IIEGGTYRVWSRTQDLQLSAEQSYDPNMDPDNQSLLHYHWECQNTSKGAAHCSSLNFGLGSNGPVLGISGSELEAEVEYT
FRLTVNKEGMVLVQSGHIPM
VYLECVSCKAQSIYEVSQNSYVYLRGTCTNCQGLHHG
>g2518.t3
PPLRGRERRVEHTYPQSGAFRICVAVNNTISWTEACFDMQVHEEIEELTANSSSPTELNTPTVVVARLASGSNVTWTFSM
GDGATYTTIEPRASHRYADN
GNYTVNVTASNAVSSGWTILPVQVFVFQVLEVKPLECVQERSPVAFQARVSGNTPTPLYQWSFGDGSDDEMHEGDPKVAH
TYHASGNYNLSLHVSSQVNN
ASQAHFISRVCVQPAVANVSLILSKTHFHSAEVIHFHARAQPEFNYSYSWDFDAGKDPVLTHGSGEIMFSYDNPVHFEAQ
MEEGDGVIFSWIVCDRGTSI
LGTHTMFYTFRSVGTFNITVIAENGLGSAQVSILLFVQRELEGLAIVAEEDTGGCASVGGAGVEGAGMEGAGVEGCYFAT
NCVLHLQAVLKEGTNMSFTW
NLIRKEDPSNSAFNLTSKTVEVNFPQPGSLDVFLKAVNLLGQLTVNRTFQFLDPVAGVDLQISDNPIAVNTPMNLTVFAR
GSHLQYLWHVDDDIMPWKEF
SVVHTFTSAGQRKIRVEVFNKVSSHIASRTVQVQEVITDLSFTATNVSELQYVPTAVNVSLQGNILTGSNVTWVWLLQGR
MDTGRKTSVIFLEPQATTIT
LNATNNVSGQVVSREFFVQEKIQGLDLKSSQMYAAVRQPVEFTVSMAAGSDVSLILSISGDYTVTPELNQTYVHRFNKPD
NYMVNLTAHNKVSSMRRNVQ
IQVMEAVQGLTILDCCEAAIPVGVRRTFVANILTGKPVHYLWTFDLHHHRKPTLMGQEVVSYTPEEAGCLTIYLRAFNSL
PSPNITKQIMVQNIIREVTL
DALPVDTFVNKTVTLMASIMPRSSPVQCLWDFGDGTGSLLTNSTTVNCSNLVSWELARVEVNIRVLECEEPEVQVVQAPR
LAIWCAQPTLVEASVDLKGC
VRYGVQYLWQILSTPDCDDQDPDTALHQRMTAVAAKRPLSPRASVYPLPAEVDVRRLQLSIPKMVLAAGNYTLVFSLSYE
GIPLRKAACLQLSVMAARLV
PIIEGGTYRVWSRTQDLQLSAEQSYDPNMDPDNQSLLHYHWECQNTSKGAAHCSSLNFGLGSNGPVLGISGSELEAEVEY
TFRLTVNKEGMVPQSTTQTV
LVQSGHIPMVYLECVSCKAQSIYEVSQNSYVYLRGTCTNCQGLHHG
>g2519.t1
LFRSSERFLPGRIAIGPARRAPTKPPSEKAETIKVHTSVIFWVSSPVPQRSQAPSPTKRKEDDKTKERGKEKSGATKDGA
EKDRGRDKNRKHRSASTGSS
RSSSSSSSSSGSSSGSSSGSSSSGSSRSGSSSSRSSSSSSSSPSPSRRRHDNRRRSRSKSKSVKKDDRDRDRDRRRRSPT
PKPTKVYLGRLTRNVIKEHI
QEIFSTYGKIKMVDMPMNRLYPHLSKGYAYVEFETSDEAEKALKHMNGGQIDGQEITATAVLTQR
>g2519.t2
LTNRKPLFRSSERFLPGRIAIGPARRAPTKPPSEKAETIKVHTSVIFWVSSPVPQRSQAPSPTKRKEDDKTKERGKEKSG
ATKDGAEKDRGRDKNRKHRS
ASTGSSRSSSSSSSSSGSSSGSSSGSSSSGSSRSGSSSSRSSSSSSSSPSPSRRRHDNRRRSRSKSKSVKKDDRDRDRDR
RRRSPTPKPTKVYLGRLTRN
VIKEHIQEIFSTYGKIKMVDMPMNRLYPHLSKGYAYVEFETSDEAEKALKHMNGGQIDGQEITATAVLTQR
>g2519.t3
MACFLLSIISSSQPDKAAQGSRYRSPLGSKSVKKDDRDRDRDRRRRSPTPKPTKVYLGRLTRNVIKEHIQEIFSTYGKIK
MVDMPMNRLYPHLSKGYAYV
EFETSDEAEKALKHMNGGQIDGQEITATAVLTQR
>g2520.t1
MAMRPGRKRSLLLNNGFLFVSVALVLTSRWARSFEMIVAARLMIGVNSGVSMNVQPMYFGECAPKHLRGAVAFSSAVFTA
FGIFLGQVVGLTELLGAEAL
WPYLLASNALPGILQLLTLPWFPESPRYLLMDRGDKEACILALARLRGGVAPVEEMEEIQQEMQQQQKQQRDSGSVSPWA
LLKDRTRRPQLRTVMAASSA
MMLCGNDSIYFYASYIFREAGVPAEKVQYACLGTGLSEFTASVASNLLIERVGRRWLLVGGYVLMAGFASVFTLALVLQG
AGVAGMSYLSMACVFTYILS
FGIGPAGVTGILPAEIFDQASRPTAYMITGSLMWLNLLLVGMLFPFIVGALGSYCFLPFGAVCVASALFLGRALPETKGR
TLAEITDDFDKLAGTEGGEG

QAKRGPLELGGDCPFTPLEAKVGSIDKEDLLADSQQG
>g2520.t2
MIVAARLMIGVNSGVSMNVQPMYFGECAPKHLRGAVAFSSAVFTAFGIFLGQVVGLTELLGAEALWPYLLASNALPGILQ
LLTLPWFPESPRYLLMDRGD
KEACILALARLRGGVAPVEEMEEIQQEMQQQQKQQRDSGSVSPWALLKDRTRRPQLRTVMAASSAMMLCGNDSIYFYASY
IFREAGVPAEKVQYACLGTG
LSEFTASVASNLLIERVGRRWLLVGGYVLMAGFASVFTLALVLQGAGVAGMSYLSMACVFTYILSFGIGPAGVTGILPAE
IFDQASRPTAYMITGSLMWL
NLLLVGMLFPFIVGALGSYCFLPFGAVCVASALFLGRALPETKGRTLAEITDDFDKLAGTEGGEGQAKRGPLELGGDCPF
TPLEAKVGSIDKEDLLADSQ
QG
>g2521.t1
MGIPWTRTARQADAGELCNTVGLNVKTSERSYEEEAAVDRTLYGIMTCGVIPVCLYVFPVSYFLCAVYASYQSPEREYRD
ARQPGDEVTNNLLLLISAHS
DPPTTNQ
>g2522.t1
MRAQGTIHHPVFIETRHHSCCSSADDVKVPSRLSVSKSLKVPGSVAGSRGGSMLELKETSEEHGEDGEGEKVDKPHVELS
SDEDDLALDDAIEESRAERI
KRSSLQRVQNLKTVFSKEKMDKTRAKTIENLGKTRDKTRANLEKTRQKTKQNLDKTRENLEKTKQKTRLNLEKTRNTIEK
KVGKLSTRMTVNAEQKQKMQ
TSREKVKKSFTPDHVVYARSKTAVYKVPPFTFHVKKIREGAVEAVQGTEMVEVSQEVEAFQEENAEAEEGGVELEMEMMN
GGEEAEEDEDLDEDEEEARQ
DNKDLDSD
>g2522.t2
DDVKVPSRLSVSKSLKVPGSVAGSRGGSMLELKETSEEHGEDGEGEKVDKPHVELSSDEDDLALDDAIEESRAERIKRSS
LQRVQNLKTVFSKEKMDKTR
AKTIENLGKTRDKTRANLEKTRQKTKQNLDKTRENLEKTKQKTRLNLEKTRNTIEKKVGKLSTRMTVNAEQKQKMQTSRE
KVKKSFTPDHVVYARSKTAV
YKVPPFTFHVKKIREGAVEAVQGTEMVEVSQEVEAFQEENAEAEEGGVELEMEMMNGGEEAEEDEDLDEDEEEARQDNKD
LDSD
>g2523.t1
LGVTSYCPHPSDSQSRSGVVRASLACCVATATGVGSIFHGNRDPTEVVLNRIPQPVLARFVRIRPQTWRSGIALRFELYG
CQIT
>g2524.t1
KYMARFGDKMCGFPALVSVLLAGLAAASSSDLFDNQLGDINYCKLQCQLVVKNKSPAKFTLRTLPLACYVHTAKARQ
>g2524.t2
KYMARFGDKMCGFPALVSVLLAGLAAASSSDLFDNQLGDINYCKLQCQLVVKNKSPAKGSFNFLIRILLAACEATRGFSQ
SMRR
>g2525.t1
MSCRRAPFSFFHILLFLLLLLLLILILLLTIIIIILLETQ
>g2526.t1
MKRPTHDVRDEGFFHSNMLTWWTDKTETHTTHSGYVPRDPSLWVRTPGPLTLGTYPGTPHSGFVPLDPGSLTLGMSRGTP
HSGYIPLDPSLWVRPPGPLT
GPLTLGTYPWTPHSGYVPLDPSLWVRTPGPLTLGTYPGTPHSAAAAQTVSGGRDQSQ
>g2526.t2
MLTWWTDKTETHTTHSGYVPRDPSLWVRTPGPLTLGTYPGTPHSGFVPLDPGSLTLGMSRGTPHSGYIPLDPSLWVRPPG
PLTGPLTLGTYPWTPHSGYV
PLDPSLWVRTPGPLTLGTYPGTPHSAAAAQTVSGGRDQSQ
>g2527.t1
ESLICNVCGPSTPEGCLGNTTDSLTCGSAERCAIAYLNFTMVTLKIKGCLPVPFCNMTSGPQSTVLFNSTTVPYTLSTTC
CNTERCNGAASLHVLLSAVI
GAALLALWGGSSL
>g2528.t1
MAAVWTRLLVLGVFVAVAQGLTCRQCPLGVFGRCLFPTEVVCDNATLNCFSGNAQFNATGSLRLHSRGCLDSDLCGRTLT
GSILGAGFSSTFSCCQEDLC
NGVTSVQLSVVAMCSAAVLASLWAS

>g2529.t1
LKKHDNFSEKKSSQSSPCWAEEPALHQQHQYQHHQQTPCLHACPPTERSLPLGYSISHMPLHNNTSTLSITATANTNHTI
RQMRVARHKARGKKSTLRLA
DRNSQNRRVTDYFPIRRSSRKSKAELKFEEKKHIDDLITNGIEEGME
>g2530.t1
MDPGQDLLLAALSESGICPNDFFDVDTQDVSQSSSAHQAISAGALDDGSGVGAAGTEASAPLTTLMIRQKPQPSTTTFVL
NQLNQLPSLGTVVTKQSATN
PIKHTITVTKVVHVASALRGSSGPSSPSVSTAVPLNRDQIQLKDLLRTGGLKKTTTLKGNSLMELMKLKPPSDIAQPVAT
ATATGHLELNNGIKKDFSSK
DATRFWKSPSIKEEEEPEEEEEEELGHAETYAEYMPMKLRIGLRHPDPVVETSSLSSVNPPDVWYRMSIPEEIIDRGWLS
ALQLEAITYAAQQHETFLPN
GDRAAYLIGDGAGVGKGRTIAGIIYENYLLGRKRSLWFSVSNDLKYDAERDLRDIGAKNIQVHSLNK
>g2530.t2
MDPGQDLLLAALSESGICPNDFFDVDTQDVSQSSSAHQAISAGALDDGSGVGAAGTEASAPLTTLMIRQKPQPSTTTFVL
NQLNQLPSLGTVVTKQSATN
PIKHTITVTKVVHVASALRGSSGPSSPSVSTAVPLNRDQLKDLLRTGGLKKTTTLKGNSLMELMKLKPPSDIAQPVATAT
ATGHLELNNGIKKDFSSKDA
TRFWKSPSIKEEEEPEEEEEEELGHAETYAEYMPMKLRIGLRHPDPVVETSSLSSVNPPDVWYRMSIPEEIIDRGWLSAL
QLEAITYAAQQHETFLPNGD
RAAYLIGDGAGVGKGRTIAGIIYENYLLGRKRSLWFSVSNDLKYDAERDLRDIGAKNIQVHSLNK
>g2531.t1
MSFLRRVAGLSLRDRVRSSDIRRELGVEPLLLRVERSQLRWFGHLIRMPPGRLPLEVFRARPTGKRHRGRPRTRWRDYIS
HLAWERLGVPQEELESVAGE
KDVWSSLLSLLPPRPGPG
>g2532.t1
RNSTVTRVIYAFILLLGTIVACIMLSPGVDQQLKRIPGLCEGGAGSLTPGIKGQVNCNMMVGYKAVYRVCFGMSMFFLVF
ALFTINIKSSRDHRAAIHNG
YTKSSAASQQLVLLVDFAHSWNESWVEKMETGNPKGWYAALLVVTVFNYIMSVVAVALFFVFYTKPDDCTINKCFISFNM
LLCIIASLTSVLPKVQESQP
RSGLLQSSIITMYTMYLTWSAMANEPDQKCNPNLLSIFQQMAAPTVAPLEMDNQSVVIIDTEEPVLNTPYLQWWDAKSIM
GLAIFILCILYSR
>g2532.t2
VSSLCGSDSCPVCSCFPHSRNSTVTRVIYAFILLLGTIVACIMLSPGVDQQLKRIPGLCEGGAGSLTPGIKGQVNCNMMV
GYKAVYRVCFGMSMFFLVFA
LFTINIKSSRDHRAAIHNGYTKSSAASQQLVLLVDFAHSWNESWVEKMETGNPKGWYAALLVVTVFNYIMSVVAVALFFV
FYTKPDDCTINKCFISFNML
LCIIASLTSVLPKVQESQPRSGLLQSSIITMYTMYLTWSAMANEPDQKCNPNLLSIFQQMAAPTVAPLEMDNQSVVIIDT
EEPVLNTPYLQWWDAKSIMG
LAIFILCILYSR
>g2532.t3
RNSTVTRVIYAFILLLGTIVACIMLSPGVDQQLKRRIQNATARLVFNRHKFSHVTPLFHDLHWLPVVDRIKFKMMIPGLC
EGGAGSLTPGIKGQVNCNMM
VGYKAVYRVCFGMSMFFLVFALFTINIKSSRDHRAAIHNGYTKSSAASQQLVLLVDFAHSWNESWVEKMETGNPKGWYAA
LLVVTVFNYIMSVVAVALFF
VFYTKPDDCTINKCFISFNMLLCIIASLTSVLPKVQESQPRSGLLQSSIITMYTMYLTWSAMANEPDQKCNPNLLSIFQQ
MAAPTVAPLEMDNQSVVIID
TEEPVLNTPYLQWWDAKSIMGLAIFILCILYSR
>g2533.t1
MAEGGDAEWELSKENIQPLKRGRDISALHQALSQQQEGPSSAVNQQKQYVKWTEQTFPQGGKESNLAMLLERAVTLFTED
KKYHNDTRYVDLWIKFAEIC
PEPLEIYRYMQAQGIGGSQASFYIAWSEEYERHGNSRKADNVYQEGLNNCAEPRDKLQQFRKALQARVSRQVMTGMADDD
SDDERNEPERMSLVDLKHRG
KKKAIVPVNRARAVLGHNSRGLQSQGAPAQSTAKNSCFVVFDESKGPAAAAPEPKWESWAAPPPARAKENTQKAEKWCDV
K
>g2533.t2
MAEGGDAEWELSKENIQPLKRGRDISALHQALSQQQEGPSSAVNQQKQAFECELRIYNGDDPLDVWDRYVKWTEQTFPQG

GKESNLAMLLERAVTLFTED
KKYHNDTRYVDLWIKFAEICPEPLEIYRYMQAQGIGGSQASFYIAWSEEYERHGNSRKADNVYQEGLNNCAEPRDKLQQF
RKALQARVSRQVMTGMADDD
SDDERNEPERMSLVDLKHRGKKKAIVPVNRARAVLGHNSRGLQSQGAPAQSTAKNSCFVVFDESKGPAAAAPEPKWESWA
APPPARAKENTQKAEKWCDV
K
>g2534.t1
MDNRCFCCASKFTLFRKEGHGRPAGGLTGQACALPSSPARVDDSLLNNLNKGEACGLDVNLDLDQGQEQELGMSSKQLEA
EKSRVLEEAMRELRQEQGSQ
EHVLQMAKRLALLKGLDPDKVTMKDIQHPDSDEESEEEAVKRVLKQLSEEAALDEASGYNIPFERSGPKTTNKKAHSKET
GPATRASAAAVKKTAPLDSD
EDDDGGSDEEELPWCCICNEDASIRCHTCDGDLYCDRCFREGHDEDERKEHRASSYSAPKRRRRKRKGQ
>g2534.t2
MGHGRPAGGLTGQACALPSSPARVDDSLLNNLNKGEACGLDVNLDLDQGQEQELGMSSKQLEAEKSRVLEEAMRELRQEQ
GSQEHVLQMAKRLALLKGLD
PDKVTMKDIQHPDSDEESEEEAVKRVLKQLSEEAALDEASGYNIPFERSGPKTTNKKAHSKETGPATRASAAAVKKTAPL
DSDEDDDGGSDEEELPWCCI
CNEDASIRCHTCDGDLYCDRCFREGHDEDERKEHRASSYSAPKRRRRKRKGQ
>g2534.t3
MDNRCFCCASKFTLFRKELGCKNCGRSFCSNCLNFSAVVPRCGNTKQKGHGRPAGGLTGQACALPSSPARVDDSLLNNLN
KGEACGLDVNLDLDQGQEQE
LGMSSKQLEAEKSRVLEEAMRELRQEQGSQEHVLQMAKRLALLKGLDPDKVTMKDIQHPDSDEESEEEAVKRVLKQLSEE
AALDEASGYNIPFERSGPKT
TNKKAHSKETGPATRASAAAVKKTAPLDSDEDDDGGSDEEELPWCCICNEDASIRCHTCDGDLYCDRCFREGHDEDERKE
HRASSYSAPKRRRRKRKGQ
>g2535.t1
SYNMTAAAAAAAGVSSVSSLSPSASQGQRK
>g2536.t1
MPVIINQVEVGIKPATVQMWNPHWIVEYLSGRPQFICLQNCVSDRVGVPVDTVEEYKYLGVYFNNKLDWTRNTEAIYKKG
QSRLFFLRRLRSFNICRTML
RMFYESVVASAIFFAVTCWGSGMRVGDTNRLNRLISRAGDVVGEGLDILTIIADWRMLSRLRSILDNASLFHPGSAEEYL
QQDAPQSDTGNHSCLWPSNY
TTSPSECLPFPPLMWSLSP
>g2537.t1
LVLAHLKSCLPPTLDPHQFAYRKNRSTEEAVSTALHSVLSHLDNNNTYARMLFIDFSSAFNTVIPSKLVTKLTDLGITAP
ICNWLLDFLTNRPQHVRLDH
HCSSTLTRNTEVPQESEQSPFLYSLFTHDCRPGHGSNTFIKFADDTTVIGLIRNNDESAYREEVDRLAVWCEGNN
>g2538.t1
MAESETECDTPGLDTLGSECVIAHSQVDLHYGAETEIMTEEKRGLELEIHGSDLAKIQGLGPELGAVTCVEAIVAETDHD
YIKVEHGEMHCFTGEVRSAN
EALLGEVLLKTDHDHVVKVESDHGGELTVESENGVIIHEAHGLQCNECGEIFGSIADLHQHFEIHKDLNPYICVHCGESF
AVEASLKQHMKIHMKEKPYI
PTGVDMVGKGMMDSFTLKSHQMIHLAEKPHRCSECGKSFAAAITLREHMKMHSEDKPYKCTQCRKSFVRRRHLKKHQELH
ARDKPYTCGQCGKGFATTSN
LKQHQKTHAAAIGDKPHRCTQCSKCFAAAATLREHQRVHSGQKPYKCNQCRKSFVRKRHLKKHQLVHAGGKPYNCSQCGK
GFNHSSSLSRHHKIHLRQNN
MYAAVPSGKGFGYGSPMKRMHQQGDKPYMCHHCEKGFNHSSSLSRHQRVHSDGTCYTCPHCGKRFNHSSSLTRHQRVHMD
EKQQEQQQQQQEQEQQQLLQ
QQQQMQEYSAIPSGKGFSHTITKQRLADGDKPYRCSQCGKGFNHSSSLSRHHRIHIDH
>g2539.t1
MSHPSTLDLDRYSSMSKTHARRSTTTNTKTRSLSRSQPRSGDVPCDFCTTRQERAVKSCLVCQASFCEDHVQTHYEYPAL
MKHKLVKATGQMREKLCAQH
DKLLEAYCRTDNTSVCVLCMMDEHKHHDTVPAGMERTEKQVSLMGTLRGALWGAGQGISAQGPQKQLGSTLHKSQQRIDG
RVKKWQDLRQAVEAVKHSAQ
TALDENERIFTEILRTVERKFIEVREMIESHEKITISRAEILLDRLEEEITLMRKKHNDLEKLSHTDDHIHFLQGWQALS
GPSGYEDLNNVVVAPNYSFE

STKRAIATLKMEVEEATKAETSKISAADAFKLTLDSNTIHPNLHLSDGNKTATMVQEPKNYSSHPDRFDQWQQVLCRESV
SSGRYYWEVDWRGTETDVAV
TYGGIKRKGSGNECSLGWNEKSWSLYCSESKYSFVHNNKSTDLDVPRSSRVGVYLDYRSGVLAFYSVSESMNLLHKIQTS
FTEPIYPAFSVWGFGSSIKL
L
>g2539.t2
MSHPSTLDLDRYSSMSKTHARRSTTTNTKTRSLSRSQPRSGDVPCDFCTTRQERAVKSCLVCQASFCEDHVQTHYEYPAL
MKHKLVKATGQMREKLCAQH
DKLLEAYCRTDNTSVCVLCMMDEHKHHDTVPAGMERTEKQVSLMGTLRGALWGAGQGISAQGPQKQLGSTLHKSQQRIDG
RVKKWQDLRQAVEAVKHSAQ
TALDENERIFTEILRTVERKFIEVREMIESHEKITISRAEILLDRLEEEITLMRKKHNDLEKLSHTDDHIHFLQALSGPS
GYEDLNNVVVAPNYSFESTK
RAIATLKMEVEEATKAETSKISAADAFKLTLDSNTIHPNLHLSDGNKTATMVQEPKNYSSHPDRFDQWQQVLCRESVSSG
RYYWEVDWRGTETDVAVTYG
GIKRKGSGNECSLGWNEKSWSLYCSESKYSFVHNNKSTDLDVPRSSRVGVYLDYRSGVLAFYSVSESMNLLHKIQTSFTE
PIYPAFSVWGFGSSIKLL
>g2540.t1
LHRDNDGVWRWTGEKSDFFDWATGYPRSANCVAYRFETRDWLGYPCFKRYPFLCYENNLVLVTENKTWEEALRHCRSLSD
VSSSCDLVGSQYDNRYVEDH
ALTAVTNEVCSL
>g2541.t1
LYCIRVPGVRCRGCHGYFGTPLDCALAFTHHVPQEYSLVLPEYVPRLHEEIKDFYEYISPRPEEEKMRREVVDRIKEVIH
DLWPSAESEGETDF
>g2541.t2
LDCALAFTHHVPQEYSLVLPEYVPRLHEEIKDFYEYISPRPEEEKMRREVVDRIKEVIHDLWPSAESEGETDF
>g2541.t3
LYCIRVPGVRCRGCHGYFGTPLDCALAFTHHVPQEYSLVLPEYVPRLHEEIKDFYEYISPRPEEEKMRREVVDRIKEVIH
DLWPSAEIPCCRARERPTSR
MSHP
>g2542.t1
SFRSSSRTEESRGAPVFHRTAPRLSAVLSGQRPSDGPLAWVSGPAVNHQFQRRQNPSAARAEDHQLNPNGSLSNQSVRLP
GSRAVCNSTPGLRASLLGNP
RDGSSLLALLPTRMEIPVTPNQRLAWRSEHPACQSNARLVTLDEWRVDARLTSTARLDESSGITVDRVPLGSLDHSPSLT
GLFFLCQSVSERPGGGGVTQ
RDRAVLKEPRGRV
>g2543.t1
MRHAGQVPVRPAVQENKVPIPIAPDAPVSRDPVWIRGEGGRTGQAAPPRPSHRQRGAFRVPPSRHRTRRPGPRRGPRGLR
SRGPPRPLLRPHAPSSRGRE
AFGVMETSPAFGGRGLREVNRPGGSEVFS
>g2544.t1
SQLGRRSRKTKSRSRSPRTRQSPGTRSGSEARGAGPAKQPPPDPATGSAEHSESRLPGIEHAVPGPVVVPAASARGDPLV
PSSDPTPPAAVAAKPSGSWK
PLPPLEVVASVR
>g2544.t2
SPGTRSGSEARGAGPAKQPPPDPATGSAEHSESRLPGIEHAVPGPVVVPAASARGDPLVPSSDPTPPAAVAAKPSGSWKP
LPPLEVVASVR
>g2545.t1
MNPWPNWVTSSPIPWPWLDVAVARLTSAPCWVLYLQVLQEAVWPGGTLPAQPRPERSAAQRLHTKQQCLECLVQLLP
>g2545.t2
MNPWPNWVTSSPIPWPLWLDVAVARLTSAPCWVLYLQVLQEAVWPGGTLPAQPRPERSAAQRLHTKQQCLECLVQLLP
>g2546.t1
WWNELPANVRTAESLPIFRKRLKTHLFRVHLNP
>g2547.t1
LYVIRIAVVTIINITMTSIIIIITAITKIIIINIIIPITKTINIRILSVLGLLKPRQELMNKHRPHKGQERPRWERAGTN
AQRTGQNVRLVEEGHAGWQH
RGGPRDQNNLSNHPSPTPTPSAAVRQETCSCRPHARTSPPTTHVGESSSRHALRRRRLSWRQSA

>g2547.t2
LYVIRIAVVTIINITMTSIIIIITAITKIIIINIIIPITKTINIRILVTSIISVLGLLKPRQELMNKHRPHKGQERPRWE
RAGTNAQRTGQNVRLVEEGH
AGWQHRGGPRDQNNLSNHPSPTPTPSAAVRQETCSCRPHARTSPPTTHVGESSSRHALRRRRLSWRQSA
>g2547.t3
MSQRVRLPTREIPTWKVGLENPQRVAHKAARREEPTAKASPLCGARGVDVSGGQAVTQGWSLGLSRSPSLGLSVTIINIT
MTSIIIIITAITKIIIINII
IPITKTINIRILSVLGLLKPRQELMNKHRPHKGQERPRWERAGTNAQRTGQNVRLVEEGHAGWQHRGGPRDQNNLSNHPS
PTPTPSAAVRQETCSCRPHA
RTSPPTTHVGESSSRHALRRRRLSWRQSA
>g2548.t1
MDCKTKKQANMKLVQARLPFKRLNPEPKDSHNPKRPRTPLCLEPAASDGENETEPSAMSAHNGPALVNGRGPLDGFFNRG
SASPENMCIDLTGEESPIKR
PSKPAAPAPAPARVPTEGKQPNTNKQISTGHCGGSRVPPSKKGSLDCKVASEAESEAEDESEADDDSDLEESVTRLDTTL
DSESEGEEQDESMKANASGP
ENESMVSPSSVSSMSVAESSPEQANNESLNSTSLQEQEERLQLRQEKERQREEAKSAKEKKKEEARKLKEERGRERREKK
EREERDKRDKKEKEDKEKAE
RLKAKEEQRKSKQE
>g2548.t2
MDCKTKKQANMKLVQARLPFKRLNPEPKDSHNPKRPRTPLCLEPAASDGENETEPSAMSAHNGPALVNGRGPLDGFFNRG
SASPENMCIDLTGEESPIKR
PSKPAAPAPAPARVPTEGKQPNTNKQISTGHCGGSRVPPSKKGSLDCKVASEAESEAEDESEADDDSDLEESVTRLDTTL
DSESEGEEQDESMKANASGP
ENESMVSPSSVSSMSVAESSPEQANNESLNSTDPNTTPKVPADEKKLKRRSLKSLQEQEERLQLRQEKERQREEAKSAKE
KKKEEARKLKEERGRERREK
KEREERDKRDKKEKEDKEKAERLKAKEEQRKSKQE
>g2549.t1
VSSTVLSLISLSLISLSCILQIEIRDSKDPRYAVQRFR
>g2549.t2
IEIRDSKDPRYAVQRFR
>g2550.t1
MAVNKVIKYLLFTFNLLFFISGAAILGVSVHAISNRAEYQITDELLPAVNLMICVGAITLALGFLGCCGAIGENRCLLAL
FFLGLLAMLIILLAAGALGA
MARTETSRDLVKAHLEKLGPLSEAPAEVRESFQSLEVAGKCCGFLKGHADWGNSSLQVPDSCNCTDTSRECTELDGRDVY
ATPCLPYLLAWLDRVSDVLM
GVAFTFAAMMVIGMAFSLAIICQISTGSPSVI
>g2551.t1
MDPVQKAVLHHTLGVVPTVKRKHVSCSVCQLRFNSQVLLNDGMKRLKNLSSKMNPV
>g2552.t1
AAPCGGHYSGPSGVLLSPGWPGYYKDSLSCEWVIETEPGRSVKISFDRFQTELSYDFLEVHDGPNLLSPLIGSFNGTQVP
QFLFSSGNFLYLLFTTDNSR
SNSGFKIFYE
>g2552.t2
AAPCGGHYSGPSGVLLSPGWPGYYKDSLSCEWVIETEPGRSVKISFDRFQTELSYDFLEVHDGPNLLSPLIGSFNGTQVP
QFLFSSGNFLYLLFTTDNSR
SNSGFKIFYEVGL
>g2552.t3
LLSPGWPGYYKDSLSCEWVIETEPGRSVKISFDRFQTELSYDFLEVHDGPNLLSPLIGSFNGTQVPQFLFSSGNFLYLLF
TTDNSRSNSGFKIFYE
>g2553.t1
LSACRSVLEQDTEPQLAPRALNLAAHCSYVSPASTTAESTGEQPPSSGTPPTLNMDCSHFCPQAGVYLNLRENVFHHFSL
YVISDCVTMRPRVDTPPSPT
PCKEQPTGARLLAQIIGGIAGIPAACPGKGGTGQSAGC
>g2554.t1
MTRVEECRSLFMPPFNVLPTASGSTGQSETDTAETLVTKRNFSCVELFCSTKKLQPSVRRCAEQAVLTALLHQ
>g2555.t1

MHKTQKAHREFTVTNLPFTALPSVGKSHCCIDITCEDPHHRKLAVCSIICGLSCIGIFSLINSVKAKERTNGGSSAQFKK
KAKYYGIMSIVVWVAILIML
PVLVVFVSFLLTLVD
>g2556.t1
MQFMLLFSRQGKLRLQKWYVPLSDKERKKISRDLVQTILARKPKMCSFLEWRDLKIVYKRYASLYFCCAVEDQDNELITL
EIIHRYVELLDKYFGSVCEL
DIIFNFEKAYFILDEFLLGGEAQETSKKNVLKAIEQADLLQEEAEAPRSVLEEIGLT
>g2556.t2
MHESPQMMLQTASFLWCGSSQMQFMLLFSRQGKLRLQKWYVPLSDKERKKISRDLVQTILARKPKMCSFLEWRDLKIVYK
RYASLYFCCAVEDQDNELIT
LEIIHRYVELLDKYFGSVCELDIIFNFEKAYFILDEFLLGGEAQETSKKNVLKAIEQADLLQEEAEAPRSVLEEIGLT
>g2557.t1
PTPRRHKHSKKSKKKSKKKLKKKKSRAAAEVSSSGESENQGGGPDIKEEPSPRISGEDVQEVLSGELRSEPCVEPQSTAE
VRSVPEETGESTLPELPCQK
EEVEDDVEPQL
>g2558.t1
MMTGLPRRIVKETQRLIAEPVPGIQAEPDEGNARYFHVVITGPEDSPFEGGTFKLELFLPEEYPMAAPKVKTYFIVELRA
TVIDRRVPCHRHSTPGPPVT
SPSSRCTLSLNVTVREMKDGVVSPAETTAG
>g2559.t1
MLCRLPSIHQSGLQPSPYCGSMLGGRVYSDITFPPTKRILGRQPNPAQTAILVTATRWQYYGTPNADVGELQMTWNSSLI
LAEQVNIELWGDREGVALGD
ACFVTLDNLQYTIHGRGEYDLTSSADKELSVQVRTERGLWEGLSSVALRQGSSSGVVRGSSRRGSDGPPRRCCGTRRPSH
SPGQNWMDLLGELP
>g2559.t2
MLCRLPSIHQSGLQPSPYCGSMLGGRVYSDITFPPTKRILGRQPNPAQTAILVTATRWQYYGTPNADVGELQMTWNSSLI
LAEQVNIELWGDREGVALGD
ACFVTLDNLQYTIHGRGEYDLTSSADKELSVQVRTERGLWEGLSSVALRQGSSSGVVRGSSRRGSDGPPRRCCGTRRPSH
SPGQNWMDLLGGTVSQFILV
VRRRKQPRERLPWLSPDDDWAHSREKPGVDRRVPDAVCLGDSAPLGGRLFTSQVRPQRPAGPLQLCRANRDKKRSRHRGG
KNYI
>g2559.t3
MLCRLPSIHQSGLQPSPYCGSMLGGRVYSDITFPPTKRILGRQPNPAQTAILVTATRWQYYGTPNADVGELQMTWNSSLI
LAEQVNIELWGDREGVALGD
ACFVTLDNLQYTIHGRGEYDLTSSADKELSVQVRTERGLWEGLSSVALRQGSSSGVVRGSSRRGSDGPPRRCCGTRRPSH
SPGQNWMDLLGGTVSQFILV
VRRRKQPRERLPWLSPDDDWAHSREKPGVDRRVPDAVCLGDSAPLGGRLFTSQGPQALLVLPLHRSKAHWSVQYLIIVSE
VASEATIQLTWSGARPVLHP
MRLEAPRPWR
>g2560.t1
MTRSRPGSGPPPVVSRQRLRYTYRAGDEWRGSHHPRPPRGQRKGRDIASAGTLSDDERTAPAVEARRPAASHGASGRGFC
VVLCGAFGRRVAHAPAGALR
ALLASLALWLRGPRGMSVLNKPHARRPRMRSRVDFASSPAPGAAVAQCVEQVDRAGRPLTVGSAV
>g2561.t1
MTRVDPDVTDHGSDLDPKRRPNRLFVSGLRRVDEHRTWTTRSVHPLYTLSYCRPKGPPLEPQGRHYQGLMRAPQGSGWLW
TTLHSAWVP
>g2562.t1
MSGCRCALVTSRRQLLLRFHGKLGGGGSVGVEGVDRVGSSTNRRGTHAECSVVHSHPEPCGARMSPW
>g2563.t1
MCLFSSIPPPPPNHLSNPSGGFTAPVVPNANAVLCVPVSPVKKQDGAAAMEMQPLKSAEGGDSDDKEKRKVNVSKKDKSV
LQGKLTKLAVQIGKAGLLMS
SITVIILVLTFVIDTFVVQGLPWLPECTPIYIQYFVKFFIVGVTVLVVAVPEGLPLAVTISLAYSVKKMMKDNNLVRHLD
ACETMGNATAICSDKTGTLT
TNRMTAVQCYIADVHHKVIPDPGALPPKSLDVLMHAISINSAYTSKILVRNLSPPPPPPPPPPHPSPPPRPCVRLADSAS
VSLICAALPRSTQIS
>g2563.t2

MCLFSSIPPPPPNHLSNPSGGFTAPVVPNANAVLCVPVSPVKKQDGAAAMEMQPLKSAEGGDSDDKEKRKVNVSKKDKSV
LQGKLTKLAVQIGKAGLLMS
SITVIILVLTFVIDTFVVQGLPWLPECTPIYIQYFVKFFIVGVTVLVVAVPEGLPLAVTISLAYSVKKMMKDNNLVRHLD
ACETMGNATAICSDKTGTLT
TNRMTAVQCYIADVHHKVIPDPGALPPKSLDVLMHAISINSAYTSKILNAWRLALPWEVTGLPTSGPTSGPRQADLPLGR
RIHASTLASPLSFVGPTSTD
LLRCGFSCSFVSAADTHSRENRTLHHAGGGLWKDMKATMPECVVLWILRSLRATRDTVNFGDLVAARSGDRGTVHTETPL
DSSCHAAAKTG
>g2563.t3
MRLRGSFSGENAWRLALPWEVTGLPTSGPTSGPRQADLPLGRRIHASTLASPLSFVGPTSTDLLRCGFSCSFVSAADTHS
RENRTLHHAGGGLWKDMKAT
MPECVVLWILRSLRATRDTVNFGDLVAARSGDRGTVHTETPLDSSCHAAAKTG
>g2563.t4
MCLFSSIPPPPPNHLSNPSGGFTAPVVPNANAVLCVPVSPVKKQDGAAAMEMQPLKSAEGGDSDDKEKRKVNVSKKDKSV
LQGKLTKLAVQIGKAGLLMS
SITVIILVLTFVIDTFVVQGLPWLPECTPIYIQYFVKFFIVGVTVLVVAVPEGLPLAVTISLAYSVKKMMKDNNLVRHLD
ACETMGNATAICSDKTGTLT
TNRMTAVQCYIADVHHKVIPDPGALPPKSLDVLMHAISINSAYTSKILNAWRLALPWEVTGLPTSGPTSGPRQADLPLGR
RIHASTLASPLSFVGPTSTD
LLRCGFSCSFVSAAAPRRIVIETVIAQTRTRGKTARFIMREAGSGRT
>g2564.t1
KKQQPPPQPRYVSSLSVVIKPSQSPRGRGGKGAGTTGGAPGSNSVDGGGMDAGVSNGARSGRLDPPGGPEAPLFLGPLRD
CSVDEGSDITLSGVIAGTQP
INVSWLHNGQVAHFGKSFCDGKEVRFLVSECLPEDAGIYTCIAENSAGRTSSCASVSVQAPALRFEKPPQHVEVKAGECV
RLSCAFSGSPPIVACWIRNK
LQIVDSGAELWTESGERSSTLVIAAARPQDTGPYTVLVRDRRSSAQHTVTLSVIGRGTPCDHKSEMVMVMEL
>g2564.t2
KKQQPPPQPRYVSSLSVVIKPSQSPRGRGGKGAGTTGGAPGSNSVDGGGMDAGVSNGARSGRLDPPGGPEAPLFLGPLRD
CSVDEGSDITLSGVIAGTQP
INVSWLHNGQVAHFGKSFCDGKEVRFLVSECLPEDAGIYTCIAENSAGRTSSCASVSVQEVIPKKRATSGKAPALRFEKP
PQHVEVKAGECVRLSCAFSG
SPPIVACWIRNKLQIVDSGAELWTESGERSSTLVIAAARPQDTGPYTVLVRDRRSSAQHTVTLSVIGRGTPCDHKSEMVM
VMEL
>g2564.t3
KKQQPPPQPRYVSSLSVVIKPSQSPRGRGGKGAGTTGGAPGSNSVDGGGMDAGVSNGARSGRLDPPGGPEAPLFLGPLRD
CSVDEGSDITLSGVIAGTQP
INVSWLHNGQVAHFGKSFCDGKEVRFLVSECLPEDAGIYTCIAENSAGRTSSCASVSVQEVIPKKRATSAPALRFEKPPQ
HVEVKAGECVRLSCAFSGSP
PIVACWIRNKLQIVDSGAELWTESGERSSTLVIAAARPQDTGPYTVLVRDRRSSAQHTVTLSVIGRGTPCDHKSEMVMVM
EL
>g2565.t1
MHQKDGVNDDDFEPYLSGQTNQSNNYPPMSDPYMPSYYAPSIGFPYSLGEAAWSTAGDPPMPYLTTYGQMSNGEPHFIPD
GVFGQPGALGNTPPFLGQHG
FNFFPGSADFSTWGTSVSQGQSTQQSSVYSNSYGYAPSSLGRAIADGQAGFGSDTQLSKVPVLNSIEQGMTGLKLGTDMV
AAVTKTVGSPLAATAGMSSM
AANAIPQSVSSSAPKPASWAAIARKPAKPQPKVKPKANMGMGCGAAIPPPPIKHNMNIGTWDDKGPLGKAPMAQAMMPPQ
TMVQQPLMAQHQPLLQNPLP
PQHPHQH
>g2566.t1
LAYRRVKGKSPGVVFLPGYGSSMNGQKAEALEEFCRSLGHAYLRFDYTGHGASEGVFAEGTIGTWKKDVLYVLDELVEGP
QIMVGSSIGGWLMLLAAIAR
PEKTAALVGISTAADHLVTTFNALPLETRKEFEEKGIWTVPTKHSEEGEYRFNMEFLREAENHRVLQSPIPVTCPIRLLH
GLKDEDVPWHISMQVAERVL
SGDVDVILRRHAQHRMAEKDDIKLISFSTRSAAMSCSACH
>g2566.t2
LAYRRVKGKSPGVVFLPGYGSSMNGQKAEALEEFCRSLGHAYLRFDYTGHGASEGVFAEGTIGTWKKDVLYVLDELVEGP

QIMVGSSIGGWLMLLAAIAR
PEKTAALVGISTAADHLVTTFNALPLETRKEFEEKGIWTVPTKHSEEGEYRFNMEFLREAENHRVLQSPIPVTCPIRLLH
GLKDEDVPWHISMQVAERVL
SGDVDVILRRHAQHRMAEKDDIKLITALPSGHLGLMLAYILST
>g2566.t3
MAAVALRSCRRGLSHIISNGGVSSLSSTRLEGGPNSIKCIPVVRHKSTVQFATRPDLPKLAYRRVKGKSPGVVFLPGYGS
SMNGQKAEALEEFCRSLGHA
YLRFDYTGHGASEGVFAEGTIGTWKKDVLYVLDELVEGPQIMVGSSIGGWLMLLAAIARPEKTAALVGISTAADHLVTTF
NALPLETRKEFEEKGIWTVP
TKHSEEGEYRFNMEFLREAENHRVLQSPIPVTCPIRLLHGLKDEDVPWHISMQVAERVLSGDVDVILRRHAQHRMAEKDD
IKLISFSTRSAAMSCSACH
>g2567.t1
MANRGPTYGLSREVQEKIDQKYDPDLEQRLVDWIVAQCGGNLERPQAGKHNFQNWLMDGTILCRLINSLYPRGKEPIKKI
PETQMAFKQMEKISNFLQAT
EAYGVITTDIFQTVDLWEGKDLAAVQRTLMALGSVAVTKDDGHYRGDRDWFHRKAQGYRREFTEEQLRQGQSLIGLQMGS
NRGASQSGMTGYGMHRQIM
>g2568.t1
MRVSEMSTSKTLLQDRLCITLFLLLLVVRSVAQEETPTKGCGFPGIPGDPGHNGMPGRDGRDGLRGDKGDRGAVGPLGPK
GKKGENGERGPPGKMGPQGV
LGPIGLKGIKGDFGPPGPRGPKGDIGPLGQEGPQGQVGPRGDRGIQGPLGPPGRPGPKGELGVPGHKGNIGYRGERGGRG
EVGEKGQKGDTLIIPKSAFS
VGLTEQTKLPTPDVPIRFNKIIYNQQNHYNPETGRFTCAVAGAYYFSYHITVYARNVKVALVKNGVKFIHTIDIYQSGED
QAAGGAILNLAVGDKVWLQV
VGGELYNGLFADEDDDTTFSGFLIFGD
>g2569.t1
MLRISPKEWEPETEGHNFTLVHSFWYISGAITLQGVGPHPKALSGRLVSAVWWVFAIVLLVFYFANLSHMQHADNTHATI
KNFEDLSNQDVMSYGTLDGG
SSMTFFKNSNNPVFRRIYQHMERQKSYVANMEEGVARAQKGNYAFIGEAVSLDLAVARHCELMRSEEVVAMRAYAIAASL
GDPLLKNLTVAILQLSESGE
LTYLRDKWWPNSCAGVHESHSTDALGLRNLQGLFLLLGLGLGLGLLIALLELLSKARGLAKDGKPVGTDLWAGCYGYGIY
VYPNSFFRYEGEWNMGRKHG
LGKLLMKDGSYYEGDFVCGEIEGNGLRYWAKTGDSYSGQFDQGELHGFGVLQRARGETYEGQFSYGRREGHGSLTDREGQ
TYLGSFHQDKRHGEGCMQYR
NGDQYEGNWVLDQKQGQGVLRCVDGSLYDVSLFCTASYQLHLSFELQFAIF
>g2569.t2
MLRISPKEWEPETEGHNFTLVHSFWYISGAITLQGVGPHPKALSGRLVSAVWWVFAIVLLVFYFANLSHMQHADNTHATI
KNFEDLSNQDVMSYGTLDGG
SSMTFFKNSNNPVFRRIYQHMERQKSYVANMEEGVARAQKGNYAFIGEAVSLDLAVARHCELMRSEEVVAMRAYAIAASL
GDPLLKNLTVAILQLSESGE
LTYLRDKWWPNSCAGVHESHSTDALGLRNLQGLFLLLGLGLGLGLLIALLELLSKARGLAKDGKPVGTDLWAGCYGNPST
TPGGYGIYVVYPNSFFRYEGE
WNMGRKHGLGKLLMKDGSYYEGDFVCGEIEGNGLRYWAKTGDSYSGQFDQGELHGFGVLQRARGETYEGQFSYGRREGHG
SLTDREGQTYLGSFHQDKRH
GEGCMQYRNGDQYEGNWVLDQKQGQGVLRCVDGSLYDVSLFCTASYQLHLSFELQFAIF
>g2569.t3
ISPKEWEPETEGHNFTLVHSFWYISGAITLQGVGPHPKALSGRLVSAVWWVFAIVLLVFYFANLSHMQHADNTHATIKNF
EDLSNQDVMSYGTLDGGSSM
TFFKNSNNPVFRRIYQHMERQKSYVANMEEGVARAQKGNYAFIGEAVSLDLAVARHCELMRSEEVVAMRAYAIAASLGDP
LLKNLTVAILQLSESGELTY
LRDKWWPNSCAGVHESHSTDALGLRNLQGLFLLLGLGLGLGLLIALLELLSKARGLAKDGKPVGTDLWAGCYGYGIYVYP
NSFFRYEGEWNMGRKHGLGK
LLMKDGSYYEGDFVCGEIEGNGLRYWAKTGDSYSGQFDQGELHGFGVLQRARGETYEGQFSYGRREGHGSLTDREGQTYL
GSFHQDKRHGEGCMQYRNGD
QYEGNWVLDQKQGQGVLRCVDGSLYDVSLFCTASYQLHLSFELQFAIF
>g2570.t1
MASFGWKRKVGERVSKSTVQQFEAEAEKTGRTPGDQDQVDWTHAVKRRREVLLEDCETKSERLKGEGALMAEQGRDNHAA

SSFLPPRDEEGRLSVCSKHK
RFGGLEWATISHALPLDISSQXRRSGETRVYSWIEPPTCRVERGLDGGVKP
>g2570.t2
MASFGWKRKVGERVSKSTVQQFEAEAEKTGRTPGDQDQVDWTHAVKRRREVLLEDCETKSERLKGEGALMAEQGRDNHAA
SSFLPPRDEEGRLSVCSKHK
RFGGLEWATISHALPLDISSQCGRGSSSSVGRAGRPLTVGSVVRSPLPNSLHVEVSPSKTLNPNLLPGRRT
>g2571.t1
MEPGQWKLGPAAAAAQRHEASRREKQEDHERDYQDSIVAVTNKLREVDGPHMKKNLENQIRQWFFECRDATGAFPEYPDE
EDGGSTLIFAEKTPQQLMEE
IAAKEEAEVNKKPKEKDESKDKAKKDKGKAQEEEEEAGLKMLPSAFLPDLEVSSKTYGDVWQNRNESENFTQRHEAELIK
EDKRKVIESELRLQVDELMR
QELANWKLMVDKDKGGKTKASVKKKKASKSGKKKKKEKDLTSDRTFESLHRELVEQGLLKQADDVRLRDFQGDYSYLGTT
LRQNDIEPMPSLSDVRQVMA
LYGVLPLGSQMVHEKAPLIKAILLVGPVGVGKKMLVHAVCQEAGATLFDLSPLNTAGKYPGKTGLAMMLHMVFKVARLMQ
PSVIWIGDAEKMFYKKVPKE
EKELDPKRLKKDLVKALKSIRGEDRVVVMGTTNDPLSADIKLLCKVYNKIILIPRPDYSSRYVLWEQLIRKHGGEVSRAL
DLSSLAKISDGYTPGHMVQV
VRSVLTERRLLQLTGRPLTAAEFVPPLAKFDPVFQDEEEALKNWYAKTPLGKKRVKAATGKEEEEAPSKKKDAKKKAKK
>g2571.t2
RVAELKNEMVEKEFSEYHYMDDVLQDLKLTPADIEIPIPRYFISERAKELQERRQMLSDITSTLEVTAKNKPLRSPAMSM
EEAIKVLQVAERARQGRLRA
RFMRDIQRDKRRQSVAQDQHGADPGAAALCIQKVMEPGQWKLGPAAAAAQRHEASRREKQEDHERDYQDSIVAVTNKLRE
VDGPHMKKNLENQIRQWFFE
CRDATGAFPEYPDEEDGGSTLIFAEKTPQQLMEEIAAKEEAEVNKKPKEKDESKDKAKKDKGKAQEEEEEAGLKMLPSAF
LPDLEVSSKTYGDVWQNRNE
SENFTQRHEAELIKEDKRKVIESELRLQVDELMRQELANWKLMVDKDKGGKTKASVKKKKASKSGKKKKKEKDLTSDRTF
ESLHRELVEQGLLKQADDVR
LRDFQGDYSYLGTTLRQNDIEPMPSLSDVRQVMALYGVLPLGSQMVHEKAPLIKAILLVGPVGVGKKMLVHAVCQEAGAT
LFDLSPLNTAGKYPGKTGLA
MMLHMVFKVARLMQPSVIWIGDAEKMFYKKVPKEEKELDPKRLKKDLVKALKSIRGEDRVVVMGTTNDPLSADIKLLCKV
YNKIILIPRPDYSSRYVLWE
QLIRKHGGEVSRALDLSSLAKISDGYTPGHMVQVVRSVLTERRLLQLTGRPLTAAEFVPPLAKFDPVFQDEEEALKNWYA
KTPLGKKRVKAATGKEEEEA
PSKKKDAKKKAKK
>g2571.t3
RVAELKNEMVEKEFSEYHYMDDVLQDLKLTPADIEIPIPRYFISERAKELQERRQMLSDITSTLEVTAKNKPLRSPAMSM
EEAIKVLQVAERARQGRLRA
RFMRDIQRDKRRQSVAQDQHGADPGAAALCIQKVLSPCTVQRMHSCILPFITVYNSQPNGFGPWVRIPMSAV
>g2571.t4
MEPGQWKLGPAAAAAQRHEASRREKQEDHERDYQDSIVAVTNKLREVDGPHMKKNLENQIRQWFFECRDATGAFPEYPDE
EDGGSTLIFAEKTPQQLMEE
IAAKEEAEVNKKPKEKDESKDKAKKDKGKAQEEEEEAGLKMLPSAFLPDLEVSSKTYGDVWQNRNESENFTQRHEAELIK
EDKRKVIESELRLQVDELMR
QELANWKLMVDKDKGGKTKASVKKKKASKSGKKKKKEKDLTSDRTFESLHRELVEQGLLKQADDVRLRDFQGDYSYLGTT
LRQNDIEPMPSLSDVRQVMA
LYGVLPLGSQMVHEKAPLIKAILLVGPVGVGKKMLVHAVCQEAGATLFDLSPLNTAGKYPGKTGLAMMLHMVFKVVLSEL
LKELMQPSVIWIGDAEKMFY
KKVPKEEKELDPKRLKKDLVKALKSIRGEDRVVVMGTTNDPLSADIKLLCKVYNKIILIPRPDYSSRYVLWEQLIRKHGG
EVSRALDLSSLAKISDGYTP
GHMVQVVRSVLTERRLLQLTGRPLTAAEFVPPLAKFDPVFQDEEEALKNWYAKTPLGKKRVKAATGKEEEEAPSKKKDAK
KKAKK
>g2572.t1
HNIIEIGANWIHGPSEENPVFCLAREYGLLEPEALSPENQAMDIAGHPPWVPSWFTSSGVKLTAEDTAPALGLFAELLDE
AGEFASRGDEPHASMGDFIR
TE
>g2573.t1

MASRVLVQCVRAFARSSARVSLGNLALRTIAVPVSASHRSFSFISNVERTRWLEQSRISNGSVSCRQYGDLPPLTIESIR
ERVMYVLKLYDKVNPEKLQT
TSHFMKDLGLDSLDQVEIIMAMEDEFGFEIPDADGEKLMSPDDVVQYIADKKDVYE
>g2574.t1
LGRLGPQTPTGGLTWPRCQPLALWVWAFTAQLKLSTEMTSVVQSKVRPLITARRSVVRRRETRVQIIHSLTAFFTFCLFC
SPHDAGADRWKSFNLTGSRF
VLGWHLAAPPGPRMPCML
>g2575.t1
MDIRRQLGTTPLNNIKHNLARGWQDVQTSAPYKRTQESLSYAGQVTTAALSTVGVSITRRLAEMRPLGHSISVPAMRPSS
TFKSFEEMVGSVKEKVAGGR
GGSGGNTSGFEKPSSRRNSRDGPF
>g2575.t2
MDIRRQLGTTPLNNIKHNLARGWQDVQTSAPYKRTQESLSYAGQVTTAALSTVGVSITRRLAEMRMLALPSTARPLGHSI
SVPAMRPSSTFKSFEEMVGS
VKEKVAGGRGGSGGNTSGFEKPSSRRNSRDGPF
>g2575.t3
MHISPGTTRNGTVSSDLTEGDIDELNVELTRVEDEIQTLRQVLLAKEKYAMDIRRQLGTTPLNNIKHNLARGWQDVQTSA
PYKRTQESLSYAGQVTTAAL
STVGVSITRRLAEMRPLGHSISVPAMRPSSTFKSFEEMVGSVKEKVAGGRGGSGGNTSGFEKPSSRRNSRDGPF
>g2576.t1
LFSEAMFHLVREKHAELSHSWGFTSHAHHATLAGVVMTRGFTLKGAQVVALGSGTRCLSGGACRDQGWAVKDCHAEVIAR
RALIGFLYCHLELLLGQRDE
AVEQSVFRESAGGGFRLKEGLLFHMFLSSSPCGDARLHCPYETATTHLSGSKPVRRFHCHLRMKTEGGEGTLPVKEQPRA
NQDGGTESPGRPLITMSCTD
KIARWSVLGLQGALLSHLVEPVYLYSLTVGALGHTGHLARTLDRRTARLRHLPSPYHRTPPLLGCVAGSRLRPAGRSPNL
SLNWTLGDGGPEELSTSSGR
GCGGGRPSRLCQRLLFARWLRLQRQLKGEACSQVPSWPRTYFGSKMAAGCYQIARQRFHSALRAAGLGLWPGKPPEPGGL
SV
>g2576.t2
SLTSLLRVLQLFSEAMFHLVREKHAELSHSWGFTSHAHHATLAGVVMTRGFTLKGAQVVALGSGTRCLSGGACRDQGWAV
KDCHAEVIARRALIGFLYCH
LELLLGQRDEAVEQSVFRESAGGGFRLKEGLLFHMFLSSSPCGDARLHCPYETATTHLSGSKPVRRFHCHLRMKTEGGEG
TLPVKEQPRANQDGGTESPG
RPLITMSCTDKIARWSVLGLQGALLSHLVEPVYLYSLTVGALGHTGHLARTLDRRTARLRHLPSPYHRTPPLLGCVAGSR
LRPAGRSPNLSLNWTLGDGG
PEELSTSSGRGCGGGRPSRLCQRLLFARWLRLQRQLKGEACSQVPSWPRTYFGSKMAAGCYQIARQRFHSALRAAGLGLW
PGKPPEPGGLSV
>g2576.t3
LFSEAMFHLVREKHAELSHSWGFTSHAHHATLAGVVMTRGTRCLSGGACRDQGWAVKDCHAEVIARRALIGFLYCHLELL
LGQRDEAVEQSVFRESAGGG
FRLKEGLLFHMFLSSSPCGDARLHCPYETATTHLSGSKPVRRFHCHLRMKTEGGEGTLPVKEQPRANQDGGTESPGRPLI
TMSCTDKIARWSVLGLQGAL
LSHLVEPVYLYSLTVGALGHTGHLARTLDRRTARLRHLPSPYHRTPPLLGCVAGSRLRPAGRSPNLSLNWTLGDGGPEEL
STSSGRGCGGGRPSRLCQRL
LFARWLRLQRQLKGEACSQVPSWPRTYFGSKMAAGCYQIARQRFHSALRAAGLGLWPGKPPEPGGLSV
>g2577.t1
MPGLSCRFYQHRFPEVEDVVMVNVRSIAEMGAYVSLLEYNNIEGMILLSELSRRRIRSINKLIRIGRNECVVVIRVDKEK
GYIDLSKRRVSPEEAIKCED
KFTKSKTVYSILRHVAEVLDYSKDEQLESLFQRTAWVFDEKYKRPGYGAYDVFKQAVSDPAILDGLDLTEEERNVLIDNI
NRRLTPQAVKIRADIEVACY
GYEGIDAVKEALRAGLGCSTEVMPIKINLIAPPRYVMTTTTLERTEGLSVLNQAMAAIKEKIEEKRGVFNIQME
>g2577.t2
MPGLSCRFYQHRFPEVEDVVMVNVRSIAEMGAYVSLLEYNNIEGMILLSELSRRRIRSINKLIRIGRNECVVVIRVDKEK
GYIDLSKRRVSPEEAIKCED
KFTKSKTVYSILRHVAEVLDYSKDEQLESLFQRTAWVFDEKYKRPGYGAYDVFKQAVSDPAILDGLDLTEEERNVLIDNI
NRRLTPQAVKIRAGFHVLAV

PFADIEVACYGYEGIDAVKEALRAGLGCSTEVMPIKINLIAPPRYVMTTTTLERTEGLSVLNQAMAAIKEKIEEKRGVFN
IQME
>g2578.t1
MKARLKGAQTGRNLLKKKADALSMRFRQILRKIIETKTLMGEVMREAAFSLAEAKFAAGDFSTTVIQNVNKAQVKVRAKK
DNVAGVTLPVFEHYQEGGDS
YELTGLARGGEQLSRLKRNYAKAVELLVELASLQTSFVTLDEAIKITNRRVNAIEHGRLLHRVGSLVGLRSYSLPLSLII
PRIERTLTYIITELDERERE
EFYRLKKIQEKKKQLRERTEKEIAARLAALGPIAEPSNMLMEEQDEDMLFE
>g2578.t2
MKARLKGAQTGRNLLKKKADALSMRFRQILRKIIETKTLMGEVMREAAFSLAEAKFAAGDFSTTVIQNVNKAQVKVRAKK
DNVAGVTLPVFEHYQEGGDS
YELTGLARGGEQLSRLKRNYAKAVELLVELASLQTSFVTLDEAIKITNRRVNAIEHGRLLHRVGSLVGLRSYSLPLSLII
PRIERTLTYIITELDERERE
EFYRLKKIQEKKKQLRERTEKEIAARLAALGPIAEPSNMLMEEQDEDMLFE
>g2579.t1
GAAVKAGVQEGDRIIKVNGALVSSMSHQEVVKLIKSGPYVALTLQGPPPSSAALPLEPLSTDLLPNQRTSLGGEAPPPPP
PPLPCGLSSSPSQRITGPKP
HQHAEVQKHASQILQKMLQQEEADLQ
>g2580.t1
NGEGFLDTTLEPPPCGQTVCDNVDVIKHYNHLWNFRKPKTVRYRVINVWDDARRRPTVFDHQISLQHQQLNDAFGPYNIT
WELSVHNVTNSSLRDRLILA
NCDISKVGDDTCDPECNHPLTGYDAGDCTSGHRGRCPEYKQGNGVCDPDCNLENFSYDLGDCCNPNVTDVTKTCFNRSSP
HK
>g2581.t1
MKAAGPDGPDGLEGPSSDFSERPGLTPAAGPERWCGRSSQFMFLLK
>g2582.t1
LSQLCGAWNSDGKEPGCDTGCPAFLHYHHCFDHPLLQIHAVRHQENTSPHMFLLTQKATQVLVQALVISRLDYFNSLTTL
HLLQNAATLFFNPPISSHTT
PLLNTLVAQIRFRLLNAATLFFNPPISSHTTPLLNTLVAQIRFSRRTSRLPLIQSCVRILVQHKVHLKEEPLSH
>g2583.t1
MCDPRMLSKCWGPATSEWIQFFKEAGIPAGLAVTYAVSFVDNRIQKNMLMDLSKEIMMDLGITVIGDIIAILKHAKLVYR
QDMCKMATEAISSGQTSVQA
ELRRTAHTPATRMIANALSRDSPPSTPSRRPDNRLSVTVSNKQASKSGKTVVTQPADEGSGAPAVRRRRVTAEMEGKYIV
NMPKGTTARTRRILAQQARK
GSKRMSVFERLGAESKPDTTNSAGK
>g2583.t2
MCDPRMLSKCWGPATSEWIQFFKEAGIPAGLAVTYAVSFVDNRIQKNMLMDLSKEIMMDLGITVIGDIIAILKHAKLVYR
QDMCKMATEAISSGQTSVQA
ELRRTAHTPATRMIANALSRDSPPSTPSRRPDNRLSVTVSNKQASKSGKTVVTQPADEGSGAPAVRRRRVTAEMEGKYIV
NMPKGTTARTRRILAQQARK
GRGLALLPFHLVDLCLKGSKRMSVFERLGAESKPDTTNSAGK
>g2584.t1
LVGPRYGISHVINARTNLVALLADFSHVNRIEILTEDETNVRLELHVLDVRVGSRSDLSSATLKVNFRESALVFAGLVTL
PITLIMESSDAMNLACLTAG
YYRLLVDSRRSIFNMAHCNSVQGEDAGHDRILEWPYGASLGDSEEEPLPSDGEAYNRHLDHNDNGQRGNARISPYFRNDA
QQPDGADDDEDEDDRGGDPG
GRRSPIPPPLPPSTRHQPQDSPRSAKVSFIFGGAPPRSARGGVMGSYDRLLESPDVPPHRPPYLRHDVSFGSSQPFHYGG
GGGGGGGPHVYSNIGGEECN
EEPLLRDLCYTDTTDDAEDEDDASCEEDSTGGTPVNDDAGNRKRVPNCLNLTAAQAGLAAAAGKTTFLALSGSTDDIIDL
TALPPPEGDDAVAVDDDDDT
LLETLNLAIAAPPPGFR
>g2584.t2
LVGPRYGISHVINARTNLVALLADFSHVNRIEILTEDETNVRLELHVLDVRPITLIMESSDAMNLACLTAGYYRLLVDSR
RSIFNMAHCNSVQGEDAGHD
RILEWPYGASLGDSEEEPLPSDGEAYNRHLDHNDNGQRGNARISPYFRNDAQQPDGADDDEDEDDRGGDPGGRRSPIPPP
LPPSTRHQPQDSPRSAKVSF

IFGGAPPRSARGGVMGSYDRLLESPDVPPHRPPYLRHDVSFGSSQPFHYGGGGGGGGGPHVYSNIGGEECNEEPLLRDLC
YTDTTDDAEDEDDASCEEDS
TGGTPVNDDAGNRKRVPNCLNLTAAQAGLAAAAGKTTFLALSGSTDDIIDLTALPPPEGDDAVAVDDDDDTLLETLNLAI
AAPPPGFR
>g2584.t3
LVGPRYGISHVINARTNLVALLADFSHVNRIEILTEDETNVRLELHVLDVRVGSRSDLSSATLKPITLIMESSDAMNLAC
LTAGYYRLLVDSRRSIFNMA
HCNSVQGEDAGHDRILEWPYGASLGDSEEEPLPSDGEAYNRHLDHNDNGQRGNARISPYFRNDAQQPDGADDDEDEDDRG
GDPGGRRSPIPPPLPPSTRH
QPQDSPRSAKVSFIFGGAPPRSARGGVMGSYDRLLESPDVPPHRPPYLRHDVSFGSSQPFHYGGGGGGGGGPHVYSNIGG
EECNEEPLLRDLCYTDTTDD
AEDEDDASCEEDSTGGTPVNDDAGNRKRVPNCLNLTAAQAGLAAAAGKTTFLALSGSTDDIIDLTALPPPEGDDAVAVDD
DDDTLLETLNLAIAAPPPGF
R
>g2585.t1
SVTYDLSRTPPFIYGLPGDVDHGYGWSVCLAWASLALSTTAGCICTAAPLLSRTPKNSRTSSV
>g2585.t2
SVTYDLSRTPPFIYGLPGDVDHGYGWSVCLAWASLALSTTAGCICTAAPLLSRTPKNSRTSSWSRVLLFLKEPNTTGSLD
PVFSYSAVFLKDPNSSNQSL
HLQRRNTLTKLPISEEDEHLKTVEIVARLS
>g2586.t1
MVLRGRDRPVVDILLSASEGAVKEEKVSKQEKEERRVAELGRPRLGEHARLEVIIEESYEFKNMVDKLVKKTNLALVIGT
NSWREQFRDAITVNSGDGDA
DDDDDKEQDGQVEKRLPSCFDYFMHFLTVFWKLLFAAVPPTDYCNGWACFVVSISSIGLLTAFIGDLASHFGCTVGLKDS
VTAVVFVALGTSVPDTFASK
VAAIQDQYADASIGNVTGSNAVNVFLGIGVAWSIAAMYHQARGGWFRVDPGTLAFSVTLFTVFALVCVAVLLYRRRPEIG
GELGGPRVFKVLTSCLFFSL
WLVYIVLSSLEAYCHISAF
>g2587.t1
MGIAGTPSLLLSCTVLLFIIPSSFETSSAISSVTNSSVTNRTKCGGNTDCMPGVILPIWLPLDPPLGEKLARATVYFLGM
MYMFLGVSIIADRFMASIEV
ITSQERTVTIRAANGEKVVTTVRVWNETVSNLTLMALGSSAPEILLSVVEVCGHNFDAGQLGPNTIVGSAAFNMFVIIGL
CVSVIPDGEHRKVKHLRVFF
VTAAWSIFAYTWLYLILAVISPGVVEVWEGLVTLLFFPVCVGFAYVADRRLLFYRYLHRRYRAGRHKGMIVETEGGPEHL
VDMGGAGRTSLTLGEEGQTG
EESGREMTRILKELKLLRPETDTAGQPLGPRPAPDRAARPDHSSSEVFFEPESYHCPESCGSVALRVVRRGGAARPTTVH
YHTQDGTARSGTDYRATEGA
LLFLPGETEKEISVDVIDDDVFEEDEHFLSAGVAEAVVVRTGGARGAVALPYRTREGTARGGGRDFEDTHGVLEFEDNQL
VYVQT
>g2588.t1
MLALRLKLLLVKEKEYRERKEEDSEREVLALKDELTTLKHFALLVVEEQQCLKEKLEEEGRRVQELTNFTDHAGREISAT
PSRAREEELIALQREAELRN
QSVYSQTQGAMTAKLADKDAQKQQLRQRLANLSRQLDELGGTRAALRRAEEELVELRERVRWAEDGDEGGAGTASLLSEV
DVLRRRVLEMEGKDEELNRM
GDQCRDLDRKLSRETSQCRSLKAEVDKLNSRISQLDKLEEALAKSRQECSTLRGSQDRENSLSNQLCGELDTLRSRVREL
EVAERQLENSEVALKQDLSK
LRALTVVLVEDRKSMSERLRQASEKLGLEKDKQNEQNSLITMTEKLKEEMQQALRSKADLEFRIRDIAKGKDELQSRLHT
EEGKNRELQSKVNTMRRRVQ
VLEDKNEKVTKDNDMDINEPMTHNSNHLDQTDNNKTAELSQEVEMLRRKLLEKEVVESELMKAEEDFESLELRFRNEQER
SKALTQELGEAKRELSRFQQ
AEKQEVNQEHALLRRLQQEQVKSRLLGREVDSLKDKLQKLLGTDESISKVQMDHSTLQRKLAHQEAKNRELAREMEELTD
ELERYRRFSKSLRPGVNGRR
FSDLHQSTKEVQTELADGHPLDYRHRVPFVHDVNLNEQSDDEDLNQNEDQILDISSSMQYNSPSSLNPTNNHVRRYSSNS
ASDTEELGYGNPVKDMVVTQ
TPGQPVHIKVTPDHGLNTATLEISSPTTDNAASYTSTALIPTTSVPHKQRITIIQNAAVSAVKSQSSPTSPDRALSPLTT
GPVSRMVSPNSSRSGTPDHN

NSPVQIVTVSTCSPEPTEIIGQAVFRMSPERQNSWQLRRSNSAQASPSVITTTEDNKIHIHLGSPYVPAPNGTPHSIPKP
GGSYYSLRQEQRTQVLTNGC
HIKSGGKITSSITITPATSPNTHPSNITVSGLYD
>g2588.t2
LLVKEKEYRERKEEDSEREVLALKDELTTLKHFALLVVEEQQCLKEKLEEEGRRVQELTNFTDHAGREISATPSRAREEE
LIALQREAELRNQSVYSQTQ
GAMTAKLADKDAQKQQLRQRLANLSRQLDELGGTRAALRRAEEELVELRERVRWAEDGDEGGAGTASLLSEVDVLRRRVL
EMEGKDEELNRMGDQCRDLD
RKLSRETSQCRSLKAEVDKLNSRISQLDKLEEALAKSRQECSTLRGSQDRENSLSNQLCGELDTLRSRVRELEVAERQLE
NSEVALKQDLSKLRALTVVL
VEDRKSMSERLRQASEKLGLEKDKQNEQNSLITMTEKLKEEMQQALRSKADLEFRIRDIAKGKDELQSRLHTEEGKNREL
QSKVNTMRRRVQVLEDKNEK
VTKDNDMDINEPMTHNSNHLDQTDNNKTAELSQEVEMLRRKLLEKEVVESELMKAEEDFESLELRFRNEQERSKALTQEL
GEAKRELSRFQQAEKQEVNQ
EHALLRRLQQEQVKSRLLGREVDSLKDKLQKLLGTDESISKVQMDHSTLQRKLAHQEAKNRELAREMEELTDELERYRRF
SKSLRPGVNGRRFSDLHQST
KEVQTELADGHPLDYRHRVPFVHDVNLNEQSDDEDLNQNEDQILDISSSMQYNSPSSLNPTNNHVRRYSSNSASDTEELG
YGNPVKDMVVTQTPGQPVHI
KVTPDHGLNTATLEISSPTTDNAASYTSTALIPTTSVPHKQRITIIQNAAVSAVKSQSSPTSPDRALSPLTTGPVSRMVS
PNSSRSGTPDHNNSPVQIVT
VSTCSPEPTEIIGQAVFRMSPERQNSWQLRRSNSAQASPSVITTTEDNKIHIHLGSPYVPAPNGTPHSIPKPGGSYYSLR
QEQRTQVLTNGCHIKSGGKI
TSSITITPATSPNTHPSNITVSGLYD
>g2589.t1
MPVQQHNIMGQPFIELRHRHAENRLRMPPFAPGAMPGNNMDPSMQVQRPPGFPGAQEFRFPSNQGPRMMDPMGAQGGHVQ
LSSSMENLNQQQHMQGNNMP
RQSTLMRSMSQPASNDSHNMATAMMMAAPPAGHSESVAGAPSDAVDEKLDSEESAVKDFEDVEVKDLVDADLENLNLDPE
DGKDLDLETNDLHLEDLFSS
GKFDIIAYTDPDLDDIKKDMFNEELDLSDPMEDNTDSADVQRSSSEKKNSNNSGAETSSTSVSVTTKSETSSEQTQSDVK
QESEGNLGSTSATQEIKTEV
KDGQKSAESTDQQTNGNGTTPNQQGVLSDSTPVLSGLLIKQQPTEQSLNPMADSVNQGVNLMPQPNQVTDAHQHSSGLAM
GQTLSEATAAEASMGGFGTD
PQTGMSQGLEQSTLSQAQQAMLNQALAQQSGQQQRPLLLEEQPLLLQDLLDQERQEQQQQRQMQAMIRQRSSDSFFPSIG
STVDVFADFDAITDPIMKAK
MVALKGINKVMVQNNMGMSPMVMNRFPPGAVPHCPETAPIPPQVVGQDGKLTQVARPNPPNFGPGFVNTAQRAQYEEWLQ
ETQQLLQMQQKFLEEKIGTH
RKSKKALSAKQRTAKKAGRDFPEEDAEQLKHVTEQQGVVQKQLEQIRKQQKEHAELIEEYRVKQQQNNVPPTMGPGISGP
MQGPPGMVPGGPPMVPPTMN
PMMPMQLHPGQPNAPPRMPGGPPPGWHPGAPMPMAGPGMPPMMPPQVPMGNPGQPHPMPMVNMPQRPPQMPPQMPPQMPQ
QMPPQMPQQMPPQMP
>g2589.t2
MQDIPPQMRRSVSVDMAKSMGGPMGMPQHFPPRGMPVQQHNIMGQPFIELRHRHAENRLRMPPFAPGAMPGNNMDPSMQV
QRPPGFPGAQEFRFPSNQGP
RMMDPMGAQGGHVQLSSSMENLNQQQHMQGNNMPRQSTLMRSMSQPASNDSHNMATAMMMAAPPAGHSESVAGAPSDAVD
EKLDSEESAVKDFEDVEVKD
LVDADLENLNLDPEDGKDLDLETNDLHLEDLFSSGKFDIIAYTDPDLDDIKKDMFNEELDLSDPMEDNTDSADVQRSSSE
KKNSNNSGAETSSTSVSVTT
KSETSSEQTQSDVKQESEGNLGSTSATQEIKTEVKDGQKSAESTDQQTNGNGTTPNQQGVLSDSTPVLSGLLIKQQPTEQ
SLNPMADSVNQGVNLMPQPN
QVTDAHQHSSGLAMGQTLSEATAAEASMGGFGTDPQTGMSQGLEQSTLSQAQQAMLNQALAQQSGQQQRPLLLEEQPLLL
QDLLDQERQEQQQQRQMQAM
IRQRSSDSFFPSIGSTVDVFADFDAITDPIMKAKMVALKGINKVMVQNNMGMSPMVMNRFPPGAVPHCPETAPIPPQVVG
QDGKLTQVARPNPPNFGPGF
VNTAQRAQYEEWLQETQQLLQMQQKFLEEKIGTHRKSKKALSAKQRTAKKAGRDFPEEDAEQLKHVTEQQGVVQKQLEQI
RKQQKEHAELIEEYRVKQQQ
NNVPPTMGPGISGPMQGPPGMVPGGPPMVPPTMNPMMPMQLHPGQPNAPPRMPGGPPPGWHPGAPMPMAGPGMPPMMPPQ

VPMGNPGQPHPMPMVNMPQR
PPQMPPQMPPQMPQQMPPQMPQQMPPQMP
>g2589.t3
MGMPQHFPPRGMPVQQHNIMGQPFIELRHRHAENRLRMPPFAPGAMPGNNMDPSMQVQRPPGFPGAQEFRFPSNQGPRMM
DPMGAQGGHVQLSSSMENLN
QQQHMQGNNMPRQSTLMRSMSQPASNDSHNMATAMMMAAPPAGHSESVAGAPSDAVDEKLDSEESAVKDFEDVEVKDLVD
ADLENLNLDPEDGKDLDLET
NDLHLEDLFSSGKFDIIAYTDPDLDDIKKDMFNEELDLSDPMEDNTDSADVQRSSSEKKNSNNSGAETSSTSVSVTTKSE
TSSEQTQSDVKQESEGNLGS
TSATQEIKTEVKDGQKSAESTDQQTNGNGTTPNQQGVLSDSTPVLSGLLIKQQPTEQSLNPMADSVNQGVNLMPQPNQVT
DAHQHSSGLAMGQTLSEATA
AEASMGGFGTDPQTGMSQGLEQSTLSQAQQAMLNQALAQQSGQQQRPLLLEEQPLLLQDLLDQERQEQQQQRQMQAMIRQ
RSSDSFFPSIGSTVDVFADF
DAITDPIMKAKMVALKGINKVMVQNNMGMSPMVMNRFPPGAVPHCPETAPIPPQVVGQDGKLTQVARPNPPNFGPGFVNT
AQRAQYEEWLQETQQLLQMQ
QKFLEEKIGTHRKSKKALSAKQRTAKKAGRDFPEEDAEQLKHVTEQQGVVQKQLEQIRKQQKEHAELIEEYRVKQQQNNV
PPTMGPGISGPMQGPPGMVP
GGPPMVPPTMNPMMPMQLHPGQPNAPPRMPGGPPPGWHPGAPMPMAGPGMPPMMPPQVPMGNPGQPHPMPMVNMPQRPPQ
MPPQMPPQMPQQMPPQMPQQ
MPPQMP
>g2590.t1
MCTEACEEPLVDLCEVLNTDADILGMLGKPSIDSGGSPGLDIRPLADSTPVAPHIQSGRRTPRALHDEPLDGILSPELDK
MDGQLRCLHPPAMQPRPRKS
ILSKLYKIPELEGKDVEDLFTAVLSPTSSQPTPQMPHPQGQGSLPATPGPSAPHPNAGNPMFVRMPMMNGMMGPSQRFPT
NPGGPCIPDNFSPLHRMPFT
DNPRDRKFNQMPREAGPWGSPVQNQGPTPPGSLPEGESEAMSNAQRSTLKWEKEETLGELATVAPVLYTNVNFPNLKEEF
PDWSTRVKQIAKLWRKASSQ
DRAPYVQKARDNRAALRINKVQMSNETVKRHPPQPQPPEVFDPNLPLDTELLFKDPLKPKESEHEQEWKFRQQMRQKSKQ
QAKIEATQKLEQVKNEQLQQ
QQQQQSGSQSEGDGNNSGNQSPASQPSNGSMSPMQPLNPKEGFARPQLPGTPTGGTPEDVFLRPPPPPPSGPSSQPQSPQ
VFSPGSSGSRPSSPWDPYAK
MVGTPRPPNLGTNACRRMPMESGKSPTSLMEQQDRGRPSPAHESFGSPTSMNNDPYAKPPDTPRPAGDMDPFLKPMGPPR
PSQMAQGRPPMGSPGRDAYS
RPMIRNEAYQRMAQNRMILSDPYSRPLLAPIPGSNESGSVGLFKAPMPPPQAQDHFNRPGLHPTDRFSQSQQNDPYAQPP
HTPRPSGGDQSFTSPPRMGQ
HHLQGHPFVQPGPMAHMSRNPYAHAPSTPRPDHYTYDPFAQPPGPSKSAGDPYSQPPGTPRPNMDGNGQSRPFFETFARP
PGTPRPHDNYGQSSTMASPS
SDPYSPVNSRSAGMNQYGHPSHSSQKMSPSHAMDPYAQAPSTPRPTMGDRFPKSPGSQRLPMDMYATNPGQQRPGAGEMF
PQPGTQRPMMNDIYAQPPGT
PRSIMGPDGMNRRQGQMGNHQDPFSPPQGRLQESFPHPGSQTPKHPSLQEDCFTQSPSQRPNQTPVHDPFEQAPMTPRPL
PTERMEMKDQAIAGNGGPGA
QDSGQVPTHLQMPGTEVQSVPLADSEERLRQSGALMTRLGPCPPEHPDLGHRRDLGNQARCSTDLLHPIQDRDQ
>g2590.t2
MCTEACEEPLVDLCEVLNTDADILGMLGKPSIDSGGSPGLDIRPLADSTPVAPHIQSGRRTPRALHDEPLDGILSPELDK
MDGQLRCLHPPAMQPRPRKS
ILSKLYKIPELEGKDVEDLFTAVLSPTSSQPTPQMPHPQGQGSLPATPGPSAPHPNAGNPMFVRMPMMNGMMGPSQRFPT
NPGGPCIPDNFSPLHRMPFT
DNPRDRKFNQMPREAGPWGSPVQNQGPTPPGSLPEGESEAMSNAQRSTLKWEKEETLGELATVAPVLYTNVNFPNLKEEF
PDWSTRVKQIAKLWRKASSQ
DRAPYVQKARDNRAALRINKVQMSNETVKRHPPQPQPPEVFDPNLPLDTELLFKDPLKPKESEHEQEWKFRQQMRQKSKQ
QAKIEATQKLEQVKNEQLQQ
QQQQQSGSQSEGDGNNSGNQSPASQPSNGSMSPMQPLNPKEGFARPQLPGTPTGGTPEDVFLRPPPPPPSGPSSQPQSPQ
VFSPGSSGSRPSSPWDPYAK
MVGTPRPPNLGTNACRRMPMESGKSPTSLMEQQDRGRPSPAHESFGSPTSMNNDPYAKPPDTPRPAGDMDPFLKPMGPPR
PSQMAQGRPPMGSPGRDAYS
RPMIRNEAYQRMAQNRMILSDPYSRPLLAPIPGSNESGSVGLFKAPMPPPQAQDHFNRPGLHPTDRFSQSQQNDPYAQPP

HTPRPSGGDQSFTSPPRMGQ
HHLQGHPFVQPGPMAHMSRNPYAHAPSTPRPDHYTYDPFAQPPGPSKSAGDPYSQPPGTPRPNMDGNGQSRPFFETFARP
PGTPRPHDNYGQSSTMASPS
SDPYSPVNSRSAGMNQYGHPSHSSQKMSPSHAMDPYAQAPSTPRPTMGDRFPKSPGSQRLPMDMYATNPGQQRPGAGEMF
PQPGTQRPMMNDIYAQPPGT
PRSIMGPDGMNRRQGQMGNHQDPFSPPQGRLQESFPHPGSQTPKHPSLQEDCFTQSPSQRPNQTPVHDPFEQAPMTPRPL
PTERMEMKDQAIAGNGGPGA
QDSGQVPTHLQMPGTEVQSVPLADSEERLRQEGLGQPGEMFNRPPPPYPGQGPMRGPMRYPGPFPGDHRGPFPNEGLHFH
QVVLVHMEVKNFSLEVLIKC
RTSHLR
>g2590.t3
LNTDADILGMLGKPSIDSGGSPGLDIRPLADSTPVAPHIQSGRRTPRALHDEPLDGILSPELDKMDGQLRCLHPPAMQPR
PRKSILSKLYKIPELEGKDV
EDLFTAVLSPTSSQPTPQMPHPQGQGSLPATPGPSAPHPNAGNPMFVRMPMMNGMMGPSQRFPTNPGGPCIPDNFSPLHR
MPFTDNPRDRKFNQMPREAG
PWGSPVQNQGPTPPGSLPEGESEAMSNAQRSTLKWEKEETLGELATVAPVLYTNVNFPNLKEEFPDWSTRVKQIAKLWRK
ASSQDRAPYVQKARDNRAAL
RINKVQMSNETVKRHPPQPQPPEVFDPNLPLDTELLFKDPLKPKESEHEQEWKFRQQMRQKSKQQAKIEATQKLEQVKNE
QLQQQQQQQSGSQSEGDGNN
SGNQSPASQPSNGSMSPMQPLNPKEGFARPQLPGTPTGGTPEDVFLRPPPPPPSGPSSQPQSPQVFSPGSSGSRPSSPWD
PYAKMVGTPRPPNLGTNACR
RMPMESGKSPTSLMEQQDRGRPSPAHESFGSPTSMNNDPYAKPPDTPRPAGDMDPFLKPMGPPRPSQMAQGRPPMGSPGR
DAYSRPMIRNEAYQRMAQNR
MILSDPYSRPLLAPIPGSNESGSVGLFKAPMPPPQAQDHFNRPGLHPTDRFSQSQQNDPYAQPPHTPRPSGGDQSFTSPP
RMGQHHLQGHPFVQPGPMAH
MSRNPYAHAPSTPRPDHYTYDPFAQPPGPSKSAGDPYSQPPGTPRPNMDGNGQSRPFFETFARPPGTPRPHDNYGQSSTM
ASPSSDPYSPVNSRSAGMNQ
YGHPSHSSQKMSPSHAMDPYAQAPSTPRPTMGDRFPKSPGSQRLPMDMYATNPGQQRPGAGEMFPQPGTQRPMMNDIYAQ
PPGTPRSIMGPDGMNRRQGQ
MGNHQDPFSPPQGRLQESFPHPGSQTPKHPSLQEDCFTQSPSQRPNQTPVHDPFEQAPMTPRPLPTERMEMKDQAIAGNG
GPGAQDSGQVPTHLQMPGTE
VQSVPLADSLHFHQVVLVHMEVKNFSLEVLIKCRTSHLR
>g2591.t1
MACGAREGTGSQTAQTGPLDAFELSAAAAAALTGRLLAAGGRRPSSSRAALHRYSMFRSRTRGPATAGKNPSGSRAAADA
FVSENIGDTCQGSGRAEPAA
RGTERRPSSAVRSAPSPARRAAAIDLSPVKGSENAGTSNTRTFRLSFPLLQTTNKWFFRLRGVDFPLPFLV
>g2592.t1
MILGAIVEEYAWQFQGISKGVVELFAMSRPSSAGGGSGGLGAGKAGGGKHGGSGSAAAAVAAAAAAGVGSVPGSGSGAPC
SVASLPPANLPGQSSELTAL
FECPVCFDYVLPPILQCQAGHLVCNQCRQKLSCCPTCRGPLTPSIRNLAMEKVASTLPFPCKCSCAVFLRVLFDGLM
>g2592.t2
MSRPSSAGGGSGGLGAGKAGGGKHGGSGSAAAAVAAAAAAGVGSVPGSGSGAPCSVASLPPANLPGQSSELTALFECPVC
FDYVLPPILQCQAGHLVCNQ
CRQKLSCCPTCRGPLTPSIRNLAMEKVASTLPFPCKCSCAVFLRVLFDGLM
>g2593.t1
NLFRPWSSTATLEGKTVLITGANTGIGKETSLDLAKRGARIIMACRDAERGQAAVQEVIDGSQNTNVVYMKLDLSDTKSI
REFAEAVNQGEPKLNILINN
AGVMACPYGKTADGFEMQIGVNHLGHFLLTHLLIDLIKRSAPSRVITVSSMAHAWGAMNLDDLNSEKSYDKKTAYQQSKL
ANVLFTRSLAKKLEGSGVTA
YSLHPGVVQTELWRHLNGPQQAIMKIVSPFTKTSVQGAQTTIYCAVEPTLENESGGYYSDCAPASCSRAAQSDETAKRLW
EMSCQLLSVSWD
>g2593.t2
MQALRNLFRPWSSTATLEGKTVLITGANTGIGKETSLDLAKRGARIIMACRDAERGQAAVQEVIDGSQNTNVVYMKLDLS
DTKSIREFAEAVNQGEPKLN
ILINNAGVMACPYGKTADGFEMQIGVNHLGHFLLTHLLIDLIKRSAPSRVITVSSMAHAWGAMNLDDLNSEKSYDKKTAY
QQSKLANVLFTRSLAKKLEG

SGVTAYSLHPGVVQTELWRHLNGPQQAIMKIVSPFTKTSVQGAQTTIYCAVEPTLENESGGYYSDCAPASCSRAAQSDET
AKRLWEMSCQLLSVSWD
>g2594.t1
MEDDGIAVVGIGCNFPGGEGLENFWKVLVEGRNCSVPIPKERFDVADWYDKDDNKAGKSRAAKAALIEGFNEFDHKFFGI
SDNEVEQMDPQQKQLLQCVY
RALENAGLPMEKASGSRTGVFFGLMNRDYEQSAAHMHPSVINHWTGTGLAMSIAANRVSYIFNLTGPSLAIDCACSSSLV
ALHLACQAIKQGDCEMALCG
GVSCIFEPRVFVALSKAKMISPEGTSKAFSSRADGYGRGEGCGVVLLKPFQKALADHDNIWGIISKTAVNQDGRSVTPIT
KPSMSQQEELLRRIYSKSDL
AMVQYIEAHGTGTPAGDPTEAGSISNAVAKAKPSGSETLRIGSVKSNIGHTESAAGVAGLIKVLLMMKHETIVPSVFYSE
DSGSVDTAALNIKIPMEPER
RSHYKHKYRKVFLVSSILDLEQQLASALNKKIDSTKPDTRVVFVFCGNGVTYRGMCKQLLEEEPKFREKSRVTGGKMLVV
SNLAVAKVLEILPAFSGKVC
IAAFNSPLSCTLSGNSDAIDAIHKRLQTMVTDTKLFLCILEVPAAYHSHMMDPILDNIEKDVNRLSVGDMDCELYSTVTG
DKCLNGDFTTGRQLDRVHFG
NQFKEAVTAVWVPAEVTELSFQLVDLASLSGEDIQTLVHVINTCRHQEVMIWSRVHKLKVGDHVACCYPVGATSKVVIPE
AACYDTDALRFLRETPCLSY
FVLAWAVLKRVLPRAEHQRRPKVNGVLNLHYATLHNKLDYFVCYSSISSFIGNASQCNYSAANSFMDMFCHYRRNNGLAG
QSINWGPLNLGLLLNKDHFQ
RFLETKGMRIMDVMEVHKSLEKCLQINNPQQVVCKFNFSTLSRHVLSQNSSLRERLSALLESEPTANPSAEARLQPLSSL
HENLRTMLSKISNVSMDELD
DDVTLSSLGIDSMLAMTLQNQLFQDTGVTVPLVRILDPNSTLTTLIAILSNGT
>g2594.t2
MEDDGIAVVGIGCNFPGGEGLENFWKVLVEGRNCSVPIPKERFDVADWYDKDDNKAGKSRAAKAALIEGFNEFDHKFFGI
SDNEVEQMDPQQKQLLQCVY
RALENAGLPMEKASGSRTGVFFGLMNRDYEQSAAHMHPSVINHWTGTGLAMSIAANRVSYIFNLTGPSLAIDCACSSSLV
ALHLACQAIKQGDCEMALCG
GVSCIFEPRVFVALSKAKMISPEGTSKAFSSRADGYGRGEGCGVVLLKPFQKALADHDNIWGIISKTAVNQDGRSVTPIT
KPSMSQQEELLRRIYSKSDL
AMVQYIEAHGTGTPAGDPTEAGSISNAVAKAKPSGSETLRIGSVKSNIGHTESAAGVAGLIKVLLMMKHETIVPSVFYSE
DSGSVDTAALNIKIPMEPER
RSHYKHKYRKVFLVSSILDLEQQLASALNKKIDSTKPDTRVVFVFCGNGVTYRGMCKQLLEEEPKFREKSRVTGGKMLVV
SNLAVAKVLEILPAFSGKVC
IAAFNSPLSCTLSGNSDAIDAIHKRLQTMVTDTKLFLCILEVPAAYHSHMMDPILDNIEKDVNRLSVGDMDCELYSTVTG
DKCLNGDFTTGRQLDRVHFG
NQFKEAVTAVWVPGSRVHKLKVGDHVACCYPVGATSKVVIPEAACYDTDALRFLRETPCLSYFVLAWAVLKRVLPRAEHQ
RRPKVNGVLNLHYATLHNKL
DYFVCYSSISSFIGNASQCNYSAANSFMDMFCHYRRNNGLAGQSINWGPLNLGLLLNKDHFQRFLETKGMRIMDVMEVHK
SLEKCLQINNPQQVVCKFNF
STLSRHVLSQNSSLRERLSALLESEPTANPSAEARLQPLSSLHENLRTMLSKISNVSMDELDDDVTLSSLGIDSMLAMTL
QNQLFQDTGVTVPLVRILDP
NSTLTTLIAILSNGT
>g2594.t3
MVPSAGVRGALDHFGEGLENFWKVLVEGRNCSVPIPKERFDVADWYDKDDNKAGKSRAAKAALIEGFNEFDHKFFGISDN
EVEQMDPQQKQLLQCVYRAL
ENAGLPMEKASGSRTGVFFGLMNRDYEQSAAHMHPSVINHWTGTGLAMSIAANRVSYIFNLTGPSLAIDCACSSSLVALH
LACQAIKQGDCEMALCGGVS
CIFEPRVFVALSKAKMISPEGTSKAFSSRADGYGRGEGCGVVLLKPFQKALADHDNIWGIISKTAVNQDGRSVTPITKPS
MSQQEELLRRIYSKSDLAMV
QYIEAHGTGTPAGDPTEAGSISNAVAKAKPSGSETLRIGSVKSNIGHTESAAGVAGLIKVLLMMKHETIVPSVFYSEDSG
SVDTAALNIKIPMEPERRSH
YKHKYRKVFLVSSILDLEQQLASALNKKIDSTKPDTRVVFVFCGNGVTYRGMCKQLLEEEPKFREKSRVTGGKMLVVSNL
AVAKVLEILPAFSGKVCIAA
FNSPLSCTLSGNSDAIDAIHKRLQTMVTDTKLFLCILEVPAAYHSHMMDPILDNIEKDVNRLSVGDMDCELYSTVTGDKC
LNGDFTTGRQLDRVHFGNQF
KEAVTAVWVPAEVTELSFQLVDLASLSGEDIQTLVHVINTCRHQEVMIWSRVHKLKVGDHVACCYPVGATSKVVIPEAAC

YDTDALRFLRETPCLSYFVL
AWAVLKRVLPRAEHQRRPKVNGVLNLHYATLHNKLDYFVCYSSISSFIGNASQCNYSAANSFMDMFCHYRRNNGLAGQSI
NWGPLNLGLLLNKDHFQRFL
ETKGMRIMDVMEVHKSLEKCLQINNPQQVVCKFNFSTLSRHVLSQNSSLRERLSALLESEPTANPSAEARLQPLSSLHEN
LRTMLSKISNVSMDELDDDV
TLSSLGIDSMLAMTLQNQLFQDTGVTVPLVRILDPNSTLTTLIAILSNGT
>g2595.t1
MEGDIAVVGIGCDFPGGEGLDRFWSVLLEGRNCVTDVPEERFDTTAWFHPDGTRPGRTQTTRAALIDGFNEFDHRFFGLA
EAEADCMDPQQKLLLQCTFR
AFEDSGTPMESARLMNRDYETIVNNDPDAVNHYSATGAAMSIAANRISFTFNLTGPSFAVDSACSSSLVALHSACQGIRQ
GDCEMALCGGVSCIIEPRMF
VALSKAKMISPDGTSKPFSSRANGYGRGEGCGMVLLKPLRDALKDLNNIWGIISKTAVNQDGRSVTPITKPSMSQQEELL
RRIYSKSDLAMVHISNAIAK
AKPSGTETLRIGSVKSNIGHTESAAGVAGLIKVLLMMKHETIVPSVFYSEDSSSVDAAALNIKIPMEPEPWRSSGERLAG
VNNFGFGGTNAHAIVKQHKK
LPFTKTCKAMMVGIFTLLRHWGVKPDAILGHSVGAIVSIHNELKASASSQNLFLRLLDVPAAYHSYMMDPILSELILPEK
DHETMLSVPVKLFEVGVHVD
WDQHYRGYEMVVYLRAIAVDVDHFEVCGCFTDEDGHWFSVTV
>g2595.t2
MEGDIAVVGIGCDFPGGEGLDRFWSVLLEGRNCVTDVPEERFDTTAWFHPDGTRPGRTQTTRAALIDGFNEFDHRFFGLA
EAEADCMDPQQKLLLQCTFR
AFEDSGTPMESASGSRTGVYIGLMNRDYETIVNNDPDAVNHYSATGAAMSIAANRISFTFNLTGPSFAVDSACSSSLVAL
HSACQGIRQGDCEMALCGGV
SCIIEPRMFVALSKAKMISPDGTSKPFSSRANGYGRGEGCGMVLLKPLRDALKDLNNIWGIISKTAVNQDGRSVTPITKP
SMSQQEELLRRIYSKSDLAM
VHISNAIAKAKPSGTETLRIGSVKSNIGHTESAAGVAGLIKVLLMMKHETIVPSVFYSEDSSSVDAAALNIKIPMEPEPW
RSSGERLAGVNNFGFGGTNA
HAIVKQHKKLPFTKTCKAMMVGIFTLLRHWGVKPDAILGHSVGAIVSIHNELKASASSQNLFLRLLDVPAAYHSYMMDPI
LSELILPEKDHETMLSVPVK
LFEVGVHVDWDQHYRGYEMVVYLRAIAVDVDHFEVCGCFTDEDGHWFSVTV
>g2595.t3
MEGDIAVVGIGCDFPGGEGLDRFWSVLLEGRNCVTDVPEERFDTTAWFHPDGTRPGRTQTTRAALIDGFNEFDHRFFGLA
EAEADCMDPQQKLLLQCTFR
AFEDSGTPMESARLMNRDYETIVNNDPDAVNHYSATGAAMSIAANRISFTFNLTGPSFAVDSACSSSLVALHSACQGIRQ
GDCEMALCGGVSCIIEPRMF
VALSKAKMISPDGTSKPFSSRANGYGRGEGCGMVLLKPLRDALKDLNNIWGIISKTAVNQDGRSVTPITKPSMSQQEELL
RRIYSKSDLAMVHISNAIAK
AKPSGTETLRIGSVKSNIGHTESAAGVAGLIKVLLMMKHETIVPSVFYSEDSSSVDAAALNIKIPMEPEPWRSSGERLAG
VNNFGFGGTNAHAIVKQHKK
LPFTKTCKAMMVGIFTLLRHWGVKPDAILGHSVGAIVSIHNELKASASSQNLFLRLLDVPAAYHSYMMDPILSELILPEK
DHETMLSVPVKLFEVGVHVD
WDQHYRGWHWFSVTV
>g2596.t1
MMVTRFGMSDKLGVMTYNDLTKQSPETQAAVEREVRVLLKDSYERAKTLLKTYSQEHEKLAGALLRHETLDAKEIRLVLE
GKALDH
>g2596.t2
MTYNDLTKQSPETQAAVEREVRVLLKDSYERAKTLLKTYSQEHEKLAGALLRHETLDAKEIRLVLEGKALDH
>g2597.t1
MSDYVAPRHSAVMERLRRRIELFRRHHNGCENRYENTALERLELERQQTFALHQRCLQTKAKRSNKHRQPPQSIDQAVQR
APGTVGSNSDLGDSGTPGEQ
SRNSTLIAVSEHVNRLLVANII
>g2598.t1
MERKVDQLTEENGNLSVQVREICSQLATSEAEVGRMMESQATIEKELHSKTSYLQTELSQATAQKELQYEQIQILQGKIL
SLEEELCRVSAEEKGENMGP
VMEVAKTEEEIRAKQELVDQMQKEIAEQRQILQQEKHNLQLQVDSISALLKKSQEDAQLKEEQMLQENTRQEAALEQQKS
FYEDQMGRLQEEIQVKQEKM

DILKENTGKVELLNQEIKTLRNQVEMLTFSLNKAEEDIHTKEELLAKQHLDNLTIQETLQEKITVFQEEVDCLRAEVHGL
QEQLECMTTAIRVAEEEIKA
KEQQLAIHQQEKTEQRDMLQKCIAASAEEVEKLKEIQKANDSLFLQSKEEVDHLKAELSTIMSHSAEKDKQLDGLREEVA
LQADVVKMAKEEIQAKEEKL
TSLQMESSHQTDILKCEMHSLEDQVNTITTALSNAKQDLKTQQDMCSRHQQENSQQLEALQEQMCSSKEEIERLIQEIIQ
KEQVLAKVKADTSAHSDGLQ
QEIQSLRSQVHSIADSLRKAEEKLLGQETLLTQQEQESAHQKELLQKKVSASEEEVEKMKEQIKSKEEQLTLLGLNGSEQ
SALLQQQIQGLTSQVESLSS
SLKEAEKEVQAKKEQLTKEQQAGIQQSEVLQKQVVASQEDVKRLNEEIQIKEEQLTTQQKHFQEQVFVSQEDVQRLKQEV
SAKHEEVCTKELLLAKIKEE
GVKQQSLLQEQIHSSLGEIQKVKEALGKSEDLLHQVTEKNEGLQNELSSLLSISADKDKQLNHLREEVTVQIDLYRKANT
DLEAKEHSLAKLQDDISKGR
DDHEHEVQNLKGKIVTINDSLQKAEDMARAKDQLLINSQQESTQQVDLLKQQLQSVNIYLSHIKEAGATSLQQKAALQEL
QATTLIEKETLIQEAAALRS
QLLQADQCQKTLEKELEHQSREKEQMAQAKVALERENKACHKLESALQQELASLRNDKERLMVDITKALEGEKLGKDLQE
QLAAKSEAAEHYKTQMEKAM
SHYNSKKQLLLESQEQVAEMQRSLDVKDHEMKTIATELKLLQLDLDNFKCNEKNLLTRVASLETQLAFADGQLREHNKIR
EDGGRLPGIYFEVADENGVV
QTRGPKDEKPDCSDSLEDNTLEDSLNTTRRPSAPGESSTPLVRSSERLAAKRHALTGASLESLYFTPMNNKNTNRSKRPM
DSSLTSSAELLDSTVKNPPS
SVKRRRTTQVINITMSKKTPGRGGSDHRLPSGDEDETFYSLSSARSQPNLSRGHASRPVSMELFSTPSGKTAATGDQLLS
LPGYRRSTISSTAPPRSECR
VLSPIGCRLL
>g2598.t2
LATSEAEVGRMMESQATIEKELHSKTSYLQTELSQATAQKELQYEQIQILQGKILSLEEELCRVSAEEKGENMGPVMEVA
KTEEEIRAKQELVDQMQKEI
AEQRQILQQEKHNLQLQVDSISALLKKSQEDAQLKEEQMLQENTRQEAALEQQKSFYEDQMGRLQEEIQVKQEKMDILKE
NTGKVELLNQEIKTLRNQVE
MLTFSLNKAEEDIHTKEELLAKQHLDNLTIQETLQEKITVFQEEVDCLRAEVHGLQEQLECMTTAIRVAEEEIKAKEQQL
AIHQQEKTEQRDMLQKCIAA
SAEEVEKLKEIQKANDSLFLQSKEEVDHLKAELSTIMSHSAEKDKQLDGLREEVALQADVVKMAKEEIQAKEEKLTSLQM
ESSHQTDILKCEMHSLEDQV
NTITTALSNAKQDLKTQQDMCSRHQQENSQQLEALQEQMCSSKEEIERLIQEIIQKEQVLAKVKADTSAHSDGLQQEIQS
LRSQVHSIADSLRKAEEKLL
GQETLLTQQEQESAHQKELLQKKVSASEEEVEKMKEQIKSKEEQLTLLGLNGSEQSALLQQQIQGLTSQVESLSSSLKEA
EKEVQAKKEQLTKEQQAGIQ
QSEVLQKQVVASQEDVKRLNEEIQIKEEQLTTQQKHFQEQVFVSQEDVQRLKQEVSAKHEEVCTKELLLAKIKEEGVKQQ
SLLQEQIHSSLGEIQKVKEA
LGKSEDLLHQVTEKNEGLQNELSSLLSISADKDKQLNHLREEVTVQIDLYRKANTDLEAKEHSLAKLQDDISKGRDDHEH
EVQNLKGKIVTINDSLQKAE
DMARAKDQLLINSQQESTQQVDLLKQQLQSVNIYLSHIKEAGATSLQQKAALQELQATTLIEKETLIQEAAALRSQLLQA
DQCQKTLEKELEHQSREKEQ
MAQAKVALERENKACHKLESALQQELASLRNDKERLMVDITKALEGEKLGKDLQEQLAAKSEAAEHYKTQMEKAMSHYNS
KKQLLLESQEQVAEMQRSLD
VKDHEMKTIATELKLLQLDLDNFKCNEKNLLTRVASLETQLAFADGQLREHNKIREDGGRLPGIYFEVADENGVVQTRGP
KDEKPDCSDSLEDNTLEDSL
NTTRRPSAPGESSTPLVRSSERLAAKRHALTGASLESLYFTPMNNKNTNRSKRPMDSSLTSSAELLDSTVKNPPSSVKRR
RTTQVINITMSKKTPGRGGS
DHRLPSGDEDETFYSLSSARSQPNLSRGHASRPVSMELFSTPSGKTAATGDQLLSLPGYRRSTISSTAPPRSECRVLSPI
GCRLL
>g2598.t3
MMESQATIEKELHSKTSYLQTELSQATAQKELQYEQIQILQGKILSLEEELCRVSAEEKGENMGPVMEVAKTEEEIRAKQ
ELVDQMQKEIAEQRQILQQE
KHNLQLQVDSISALLKKSQEDAQLKEEQMLQENTRQEAALEQQKSFYEDQMGRLQEEIQVKQEKMDILKENTGKVELLNQ
EIKTLRNQVEMLTFSLNKAE
EDIHTKEELLAKQHLDNLTIQETLQEKITVFQEEVDCLRAEVHGLQEQLECMTTAIRVAEEEIKAKEQQLAIHQQEKTEQ

RDMLQKCIAASAEEVEKLKE
IQKANDSLFLQSKEEVDHLKAELSTIMSHSAEKDKQLDGLREEVALQADVVKMAKEEIQAKEEKLTSLQMESSHQTDILK
CEMHSLEDQVNTITTALSNA
KQDLKTQQDMCSRHQQENSQQLEALQEQMCSSKEEIERLIQEIIQKEQVLAKVKADTSAHSDGLQQEIQSLRSQVHSIAD
SLRKAEEKLLGQETLLTQQE
QESAHQKELLQKKVSASEEEVEKMKEQIKSKEEQLTLLGLNGSEQSALLQQQIQGLTSQVESLSSSLKEAEKEVQAKKEQ
LTKEQQAGIQQSEVLQKQVV
ASQEDVKRLNEEIQIKEEQLTTQQKHFQEQVFVSQEDVQRLKQEVSAKHEEVCTKELLLAKIKEEGVKQQSLLQEQIHSS
LGEIQKVKEALGKSEDLLHQ
VTEKNEGLQNELSSLLSISADKDKQLNHLREEVTVQIDLYRKANTDLEAKEHSLAKLQDDISKGRDDHEHEVQNLKGKIV
TINDSLQKAEDMARAKDQLL
INSQQESTQQVDLLKQQLQSVNIYLSHIKEAGATSLQQKAALQELQATTLIEKETLIQEAAALRSQLLQADQCQKTLEKE
LEHQSREKEQMAQAKVALER
ENKACHKLESALQQELASLRNDKERLMVDITKALEGEKLGKDLQEQLAAKSEAAEHYKTQMEKAMSHYNSKKQLLLESQE
QVAEMQRSLDVKDHEMKTIA
TELKLLQLDLDNFKCNEKNLLTRVASLETQLAFADGQLREHNKIREDGGRLPGIYFEVADENGVVQTRGPKDEKPDCSDS
LEDNTLEDSLNTTRRPSAPG
ESSTPLVRSSERLAAKRHALTGASLESLYFTPMNNKNTNRSKRPMDSSLTSSAELLDSTVKNPPSSVKRRRTTQVINITM
SKKTPGRGGSDHRLPSGDED
ETFYSLSSARSQPNLSRGHASRPVSMELFSTPSGKTAATGDQLLSLPGYRRSTISSTAPPRSECRVLSPIGCRLL
>g2599.t1
EMPSGGVTVPVLWKLHRETRGRPCTEAPLPPRRKSDFHSAAVRSTASAA
>g2600.t1
LSDQQFRSHANRERVIVKAARHCDGSCPEQDLEKREEEAKIVLTGTVEEIMNMDPVHNMYSCKVRVWRYLKGKTVVNGDI
LLDGGNKVVIGGFGDPGICD
NQVATGDTRIFFLNLATPLTSPAQKNELLLNSSLMRITLRNLEDVEHCVEDRHIQNFQTGRGPVGRVGVTRSA
>g2600.t2
LEKREEEAKIVLTGTVEEIMNMDPVHNMYSCKVRVWRYLKGKTVVNGDILLDGGNKVVIGGFGDPGICDNQVATGDTRIF
FLNLATPLTSPAQKNELLLN
SSLMRITLRNLEDVEHCVEDRHIQNFQTGRGPVGRVGVTRSA
>g2600.t3
LSDQQFRSHANRERVIVKAARHCDGSCPEQDLEKREEEAKIVLTGTVEEIMNMDPVHNMYSCKVRVWRYLKGKTVVNGDI
LLDGGNKVVIGGFGDPGICD
NQVATGDTRIFFLNLATPLTSPAQKNELLLNSSLMRITLRNLEDVEHCVEGLFFNGETCVPGPTLSAATRTHQPPRRGSL
PGRQRGQDCTLGCQRDKTGP
VPELYSVPFSAARL
>g2601.t1
MPIDLVLPLYLSNEEIGYRKTPKPCINPTLTFAPSKGFDRIGPSNALHLKDDDNNNKKAKKKVSFADHRGLSLTRVKTFS
ESQDPIEIPLKVQALFTSAE
AASSSAVVGGDDHHQQLVLDFKQPSADYLLFRRQLEQHGVCLEHCVLRERTLAGTVRVKNLSFEKAVSVRITFDAWKSHN
DVKCAYVMSTYLDTDHDTFS
FEVTLPDALGPDEHIEFAVSYEVRGVVHWDSNQGENYKVVWSSRRDHHQGRRRSSVLGIHFDPYGSPTCSHGLFPDWPSY
AVYENIGPYY
>g2602.t1
MKVFLSILVATAIICTQLWSAVSWFTQFRRMFALCFFVSLVWNWFYLYKIEFAQHQSRLAKSDAVYEQCTGMRKMDWRDS
LTEWYRRTWTLQDDPCQKYY
EDLMVNPILLVPPSKRLRLACMTTRRNTCEWQLAVVPHFATAWRESAGTKAALRVNTLPT
>g2602.t2
VFLSILVATAIICTQLWSAVSWFTQFRRMFALCFFVSLVWNWFYLYKIEFAQHQSRLAKSDAVYEQCTGMRKMDWRDSLT
EWYRRTWTLQDDPCQKYYED
LMVNPILLVPPSKRLRLACMTTRRNTCEWQLAVVPHFATAWRESAGTKAALRVNTLPT
>g2602.t3
MKVFLSILVATAIICTQLWSAVSWFTQFRRMFALCFFVSLVWNWFYLYKMFDSAPQIEFAQHQSRLAKSDAVYEQCTGMR
KMDWRDSLTEWYRRTWTLQD
DPCQKYYEDLMVNPILLVPPSKRLRLACMTTRRNTCEWQLAVVPHFATAWRESAGTKAALRVNTLPT
>g2603.t1

ANDDGDHFPIWGTCQGFQQLSVLTANKNLLTLTNTKAVALPLTLNKAAKSSRLFHSFPEDVMQSLSEENITSNFHSWSLS
LQNFSRSSKLKRFYKILSTN
TDGQVDFISTMEGTVQCDILRYCTLMCPSTSC
>g2603.t2
EPLSNTLTNSLTCKECCWANDDGDHFPIWGTCQGFQQLSVLTANKNLLTLTNTKAVALPLTLNKAAKSSRLFHSFPEDVM
QSLSEENITSNFHSWSLSLQ
NFSRSSKLKRFYKILSTNTDGQVDFISTMEGTVQCDILRYCTLMCPSTSC
>g2604.t1
MSFLRRVAGVSLRDRVRSLDIRRELGVEPLLLRIERSQLRWFGHLIRMPPGRLPLEVFRARPTGKRPRGRPRTRWRDYIS
HLAWERLGVPQEELESVAGE
KDVWSSLLSLLPSRPGPG
>g2605.t1
SPPPPRLIKRKVEVDLLKLCWGDSWKTLKPPKRWRCHRDSSLRNTISPGRRSRGGAAKSREEETSTGWRDSWKLVKPSPA
PPRVGEGQAWEVVSECRFLG
RYPARVVGFSLPVWAGTWKTMIFVLRQQKPWWDREWPSRPPPHPRDKTAQQLALDPQASFDQGDKEVVLKADLPPGQQLL
TLATLSTSDVHSSMSKVDAR
KAAGPDEIPVHVLKACSEHLARVFTDVFNLFLAQAVGPFVFKHNTIIANCFKRLFHT
>g2605.t2
SPPPPRLIKRKVEVDLLKLCWGDSWKTLKPPKRWRCHRDSSLRNTISPGRRSRGGAAKSREEETSTGWRDSWKLVKPSPA
PPRVGEGQAWEVVSECRFLG
RYPARVVGFSLPVWAGTWKTMIFVLRQQKPWWDREWPSRPPPHPRDKTAQQLALDPQLDHLQASFDQGDKEVVLKADLPP
GQQLLTLATLSTSDVHSSMS
KVDARKAAGPDEIPVHVLKACSEHLARVFTDVFNLFLAQAVGPFVFKHNTIIANCFKRLFHT
>g2606.t1
MEGLITGDETAYRYEVQHLAMWWFSDNNQSNPTDWQHAWRMSVTVTTDDTDPATATTKMADVFLPGWSSAWRLAEAPPTS
DRGAEPSNWSDLSWRLGPQS
RWWRPSLASHHHHANVLEKSRLAASLLLTQTLKAEDDDKDEESEEEETEEEETEEEETEEEEETDEEEEEEEEEEMQEGS
MKVLHLRFRELDAVWRSSSW
RAFHSPQEEEEVDSGWRSSWRLQREKEPYCSVQEELLLLRGHGLRGRGLSVAANDGLPDLEEWRKSWRYTGEEEHKETEE
GEEETEEGEEETEEENEETE
ENDETEEEEXXXXXXXMRERERGGDR
>g2606.t2
MEGLITGDETAYRYEVQHLAMWWFSDNNQEVQSNPTDWQHAWRMSVTVTTDDTDPATATTKMADVFLPGWSSAWRLAEAP
PTSDRGAEPSNWSDLSWRLG
PQSRWWRPSLASHHHHANVLEKSRLAASLLLTQTLKAEDDDKDEESEEEETEEEETEEEETEEEEETDEEEEEEEEEEMQ
EGSMKVLHLRFRELDAVWRS
SSWRAFHSPQEEEEVDSGWRSSWRLQREKEPYCSVQEELLLLRGHGLRGRGLSVAANDGLPDLEEWRKSWRYTGEEEHKE
TEEGEEETEEGEEETEEENE
ETEENDETEEEEXXXXXXXMRERERGGDR
>g2606.t3
MVQVKEEVVEEVKVDKEEEEEDEEEETDEDEVKEEEKEEEVKQWWRSWRHTGKVEEVKKEEAEKEVAKVEEAEVKKEKLE
EVKEWVKSWRNTEEEEEDEE
EEETDEEEEVEEEVDDIKVEEEVEEEGDDEEEETDGDEVKEEDKEEEVKQWWRSWRHTGKVEXXXXXXXEEEETDGDEVK
EEDKEEEVKQWWRSWRHTGK
VEEVKKEEAEKEVAKVEEAEVEKEKIEEVKEWGKSWKKTEEETDEEEEVEEEVDDIKVEQEVEKEGDDEEEETDEEEEVE
EEVDDIKVEEEMEEEGDDEE
EETDGDEVKEEDKEEEVKQWWRSWRHTGKVEEVKKEEAEKEVAKVEEAEVEKEKIEEVKEWGKSWRNTEEEEETDEGEEV
EEEVDDIKVEQEEEVEEEGD
DEEEETDGDEVKEEDKEEEVRQWWRSWGYTGKVEEVKKEEAEKEMAKVGEVEVEKKKIEEVKEWGKSWRNTGVEENKEEE
EDTEEDDEEEEETDEEEEVK
EKVEGEVDDIKVEQEEEVEEEGVAEEEETTLDEVKEEDEDEKWRKWWRSWRHTGKVEEVKKQEAEKDVTKVEEAEVEKEK
IEEVNEWGNTGEEEKEDDDD
DDDDDDDDDKEEETDEKGEEDVDDIKVEQDEERIKKEEEEDEEEETDEEEVKQWWRSWRHTGKVEEVKEVEEDIEVEVEK
KKIEEVKDWGKSWRNTGEEE
NEEEEVKEKIEEEVDDIKVEQEEEEEGGDDEEEETTLDEVKEEDEDEKWRKWWRSWRHTGKVEVKKEEAENEVEKVEEAE
VEKEKIDEVKEWGKSWRNTG

EEKEDDDDDDDDDEEEEETEEKEQENVEEDVDDIKVEQDKERIKEEEETDEEEEVKEKVEEEVDDIKVKQEEERIKEELV
KQWWRSWRPTRKVEEVKEEV
EKEKVEEEEEDDDEEEKEDDEEEEEEEEEEVEQEQEKVKEEVVREEDKEELVKQWWRCWRHPRKVEEVEVENEKVEEVKE
WRNTGEEKKEEEDEEEEADE
DEEVKEIVEEVKREQEEQQVKEGEKLKKMKGLEEVKEEDMDDEEEEEVKDEDKEEQEWRRSWRCTENEGTEMKEVGKVTG
EEVEGEWMRSWRNTGGKQEV
EVEEEEEFVDAMEEQEFDDEEEKEAEEIKQWRRTWRKAEGGGDGGNGGQ
>g2606.t4
MEGLITGDETAYRYEVQHLAMWWFSDNNQEVQSNPTDWQHAWRMSVTVTTDDTDPATATTKMADVFLPGWSSAWRLAEAP
PTSDRGAEPSNWSDLSWRLG
PQSRWWRPSLASHHHHANVLEKSRLAASLLLTQTLKAEDDDKDEESEEEETEEEETEEEETEEEEETDEEEEEEEEEEMQ
EGSMKVLHLRFRELDAVWRS
SSWRAFHSPQEEEEVDSGWRSSWRLQREKEPYCSVQEELLLLRGHGLRGRGLSREREEETDEENEETKEEEEKKIEVNQW
WRSWRYTGEKEEVKEKKEVE
KEKEEEEVKEWRRNWRNAEEMVQVKEEVVEEVKVDKEEEEEDEEEETDEDEVKEEEKEEEVKQWWRSWRHTGKVEEVKKE
EAEKEVAKVEEAEVKKEKLE
EVKEWVKSWRNTEEEEEDEEEEETDEEEEVEEEVDDIKVEEEVEEEGDDEEEETDGDEVKEEDKEEEVKQWWRSWRHTGK
VEXXXXXXXEEEETDGDEVK
EEDKEEEVKQWWRSWRHTGKVEEVKKEEAEKEVAKVEEAEVEKEKIEEVKEWGKSWKKTEEETDEEEEVEEEVDDIKVEQ
EVEKEGDDEEEETDEEEEVE
EEVDDIKVEEEMEEEGDDEEEETDGDEVKEEDKEEEVKQWWRSWRHTGKVEEVKKEEAEKEVAKVEEAEVEKEKIEEVKE
WGKSWRNTEEEEETDEGEEV
EEEVDDIKVEQEEEVEEEGDDEEEETDGDEVKEEDKEEEVRQWWRSWGYTGKVEEVKKEEAEKEMAKVGEVEVEKKKIEE
VKEWGKSWRNTGVEENKEEE
EDTEEDDEEEEETDEEEEVKEKVEGEVDDIKVEQEEEVEEEGVAEEEETTLDEVKEEDEDEKWRKWWRSWRHTGKVEEVK
KQEAEKDVTKVEEAEVEKEK
IEEVNEWGNTGEEEKEDDDDDDDDDDDDDKEEETDEKGEEDVDDIKVEQDEERIKKEEEEDEEEETDEEEVKQWWRSWRH
TGKVEEVKEVEEDIEVEVEK
KKIEEVKDWGKSWRNTGEEENEEEEVKEKIEEEVDDIKVEQEEEEEGGDDEEEETTLDEVKEEDEDEKWRKWWRSWRHTG
KVEVKKEEAENEVEKVEEAE
VEKEKIDEVKEWGKSWRNTGEEKEDDDDDDDDDEEEEETEEKEQENVEEDVDDIKVEQDKERIKEEEETDEEEEVKEKVE
EEVDDIKVKQEEERIKEELV
KQWWRSWRPTRKVEEVKEEVEKEKVEEEEEDDDEEEKEDDEEEEEEEEEEVEQEQEKVKEEVVREEDKEELVKQWWRCWR
HPRKVEEVEVENEKVEEVKE
WRNTGEEKKEEEDEEEEADEDEEVKEIVEEVKREQEEQQVKEGEKLKKMKGLEEVKEEDMDDEEEEEVKDEDKEEQEWRR
SWRCTENEGTEMKEVGKVTG
EEVEGEWMRSWRNTGGKQEVEVEVEEEEEFVDAMEEQEFDDEEEKEAEEIKQWRRTWRKAEGGGDGGNGGQ
>g2607.t1
LGLHIPEELSWTAITRAVVKGRHCSAADGKALQRLVNRAQNIMEALFQISVLPQSYQSYQRPWSIPSSPLSLSERKEWTP
DLLRLQGRLGSPVYYNYMCR
KTNLAP
>g2607.t2
LGLHIPEELSWTAITRAVVKGRHCSAADGKALQRLVNRAQNIMEALFQISVLPQSYQSYQRTLCQPWSIPSSPLSLSERK
EWTPDLLRLQGRLGSPVYYN
YMCRKTNLAP
>g2608.t1
MSSFQMSSLEAVLSPSEIKPTTVVSSANVMMVFVGMDWDRERPLSPESTVTTLMSCSGHLPLTHHTIRCGDQDFATASAA
LDAYIADFERWRLAPGGMAR
STGPRLDLQPPGTPSYQPGLPGTLRNRDVLQELLTDTELRFLSLPAASSLHRRADGDEGALTTEELLAIPSDGSLPVTKT
TAYLTDLHYRSLCRCSNSPH
PLSGLSCRSCGQLGGARGQNPAPSPTTLRWREDMDRKCLRSQEVNRLQNSQTAGSVIGANRLSSSHLSGSSQFRGSTYYC
RGTQAPGPAHSVKSSHLQSQ
SHNLRSHPSLNTVGHTQFGHAHQQKPMDEQEMAGRPSGFWEKAGSIPPYAPSWLDQLPGGEEEEQQEEEELLSVSLLTDD
APAVSQEPVSEHQLLLANTG
HTPRMLIN
>g2608.t2

MCVESAEDWTQRTATQVLSVRVEERMDWDRERPLSPESTVTTLMSCSGHLPLTHHTIRCGDQDFATASAALDAYIADFER
WRLAPGGMARSTGPRLDLQP
PGTPSYQPGLPGTLRNRDVLQELLTDTELRFLSLPAASSLHRRADGDEGALTTEELLAIPSDGSLPVTKTTAYLTDLHYR
SLCRCSNSPHPLSGLSCRSC
GQLGGARGQNPAPSPTTLRWREDMDRKCLRSQEVNRLQNSQTAGSVIGANRLSSSHLSGSSQFRGSTYYCRGTQAPGPAH
SVKSSHLQSQSHNLRSHPSL
NTVGHTQFGHAHQQKPMDEQEMAGRPSGFWEKAGSIPPYAPSWLDQLPGGEEEEQQEEEELLSVSLLTDDAPAVSQEPVS
EHQLLLANTGHTPRMLIN
>g2609.t1
MSSAVERTDELVREYLTYRGFSSALRHLDTEIRADRERGLRVDRIVEQLLQLVLTFDLSALRDYWGHLERRLFSRLQEGY
APAVAKLTTSLYRYYVVNAI
QKGNQDKTQEFFQRLGAELQGAVEWRDWFALPFIFAPEQNPTFALYFSRQWADTFLVSLHNFLSALFQCMHILGPALLSF
DAELQQAVMLAEDNQRLRQM
VFALQGSGRGAPQDALGPAESRVQHKLPPYVQHMDRLGESELNFFSTFLPQAKRSSLQTRNSHPGPASSSSSPNQSSLGR
KEPCSASQVSQSHPRQRRHQ
DHEKERKELFSKPLPLTVPLPV
>g2609.t2
MSSAVERTDELVREYLTYRGFSSALRHLDTEIRADRERGLRVDRIVEQLLQLVLTFDLSALRDYWGHLERRLFSRLQEGY
APAVAKLTTSLYRYYVVNAI
QKGNQDKTQEFFQRLGAELQGAVEWRDWFALPFIFAPEQNPTFALYFSRQWADTFLVSLHNFLSALFQCMHILGPALLSF
DAELQQAVMLAEDNQRLRQM
VFALQGSGRGAPQDALGPAESRVQHKLPPYVQHMDRLGESELDLVSSQRAASLVSGGVSSVAAAASPSSRNFFSTFLPQA
KRSSLQTRNSHPGPASSSSS
PNQSSLGRKEPCSASQVSQSHPRQRRHQDHEKERKELFSKPLPLTVPLPV
>g2609.t3
MSSAVERTDELVREYLTYRGFSSALRHLDTEIRADRERGLRVDRIVEQLLQLVLTFDLSALRDYWGHLERRLFSRLQEGY
APAVAKLTTSLYRYYVVNAI
QKGNQDKTQEFFQRLGAELQGAVEWRDWFALPFIFAPEQNPTFALYFSRQWADTFLVSLHNFLSALFQCMHILGVNSLPA
LLSFDAELQQAVMLAEDNQR
LRQMVFALQGSGRGAPQDALGPAESRVQHKLPPYVQHMDRLGESELNFFSTFLPQAKRSSLQTRNSHPGPASSSSSPNQS
SLGRKEPCSASQVSQSHPRQ
RRHQDHEKERKELFSKPLPLTVPLPV
>g2610.t1
MAFPIEVKEKSDDTIADSSSSSTTRGAGLEAEGGGTSSEPPFIKLSQEEYSEHHSSIMHCRDRHRGRVQCKPGGRQRRGP
GRADPRHRRLALGTWNVTSL
AGKEPELVREV
>g2611.t1
MGPLPGHHPDTPRRPDTPNCCSVHKHTQVRAFPSTTCSRIEATLSFTGENSNTAALSRGLVSIPTPALSPWATPEKESSP
SPGVWFQSQCCALR
>g2612.t1
MGDIEAPAPEATEPRPNVLLSILPTKEFALSRKGMLLIAEVLLCFLAFVCFLASTAAAFVTAPLLEFLLLCFLAFVCFLA
STAAAFVTAPLLEFLVALFL
LFAYSTKFNQRFTSFHWPLMDFLRCVSASIIFFVISIISVSKYSDAASKAAGVFGFIATIVFAFDFYIIFNDLADFLKQG
GRASEEPPSRTARTQDDSSD
YDSD
>g2612.t2
MGDIEAPAPEATEPRPNVLLSILPTKEFALSRKGMLLIAEVLLCFLAFVCFLASTAAAFVTAPLLEFLLLCFLAFVCFLA
STAAAFVTAPLLEFLVALFL
LFAYSTKFNQRFTSFHWPLMDFLRCVSASIIFFVISIISVSKYSDAASKAAGVFGFIATIVFAFDFYIIFNDLADFLKQG
GRASEEPPSRTARTQDYDSD
>g2613.t1
LLPPPVSRHQVLKEVTVKAQAAERVKVEVQKVKDKAQAIVDNISADKAIAEEKLEAARPALEEAEAALQVGCSGGSSTAG
GAERRVQRCGWGGAEGPAVR
VGRS
>g2614.t1
MYCSARQEKLRPGDFRASPGLIDVFWMRPGSGCYSCSPGRPHGTQPRGLFYVTLRWGNPERAGRALPRAQRLPVLLSPLT

PPGHTPAATVRVAAKRSVHH
GRETSMWWTDRPPARDTALQNPPGASFLTGGPAPGASPTREARGSTR
>g2615.t1
WRRSEPRDGGGVEDCVVALDGSWSEEPCDRLHHRICEKITEHLEADLKKEVMPVEEEAPSITEFHSSATVLPVGHTARFS
CDASSTPEPTVEWLHDGRPL
ERDGTDHRSEPAAWVERGFLFVRGVSSGVHTVCCMVSNSAGTTKHTAELIVFGKIWIQCVV
>g2615.t2
TCEWVDGTNVTSSWRRSEPRDGGGVEDCVVALDGSWSEEPCDRLHHRICEKITEHLEADLKKEVMPVEEEAPSITEFHSS
ATVLPVGHTARFSCDASSTP
EPTVEWLHDGRPLERDGTDHRSEPAAWVERGFLFVRGVSSGVHTVCCMVSNSAGTTKHTAELIVFGKIWIQCVV
>g2615.t3
DLRVGGWYQRDFKVRLKTQQQLFSESDRVVNTVLKFLFLVVFSWRRSEPRDGGGVEDCVVALDGSWSEEPCDRLHHRICE
KITEHLEADLKKEVMPVEEE
APSITEFHSSATVLPVGHTARFSCDASSTPEPTVEWLHDGRPLERDGTDHRSEPAAWVERGFLFVRGVSSGVHTVCCMVS
NSAGTTKHTAELIVFGKIWI
QCVV
>g2616.t1
PCQRYPLTSLLEGYQCSRHDEHISYGNLGVPVPPFGLQFSSVKGSERVLAVANEEGIVRLYNTERCENPILKGDQMAKLW
DVLSGELLGTFKGHQCSLKS
VAFSKQEKDGFYRQVRQISGAHNKSERNTPSQAKKRRPSVRSMANGLHSQQSVTVVLFQDEHTLISSGAVDGVIKMWDLR
KSYTAHHHEPVPLQTYPYPG
SSMRKLVAVFSGHLNSSFYVKSTISPDDQFLASGSSDHHTYIWKGHSQEVTSMTWSPTDFTKIATCSDDNTVRIWRLDRG
IDGNKSSIGQGNLVGWACPK
TPSTGLKESSPINKITLNVKITSLAFSYLPEDNTMQEVSTPAKTPTAEHMEGLASPRLAVCAPSGANMPPSS
>g2616.t2
PCQRYPLTSLLEGYQCSRHDEHISYGNLGVPVPPFGLQFSSVKGSERVLAVANEEGIVRLYNTERCENPILKGDQMAKLW
DVLSGELLGTFKGHQCSLKS
VAFSKQEKAVFSTGGRDGNIMVWDTRCSKKDGFYRQVRQISGAHNKSERNTPSQAKKRRPSVRSMANGLHSQQSVTVVLF
QDEHTLISSGAVDGVIKMWD
LRKSYTAHHHEPVPLQTYPYPGSSMRKLVAVFSGHLNSSFYVKSTISPDDQFLASGSSDHHTYIWKGHSQEVTSMTWSPT
DFTKIATCSDDNTVRIWRLD
RGIDGNKSSIGQGNLVGWACPKTPSTGLKESSPINKITLNVKITSLAFSYLPEDNTMQEVSTPAKTPTAEHMEGLASPRL
AVCAPSGANMPPSS
>g2616.t3
MYNTLEFVSVPPVPCQRYPLTSLLEGYQCSRHDEHISYGNLGVPVPPFGLQFSSVKGSERVLAVANEEGIVRLYNTERCE
NPILKGDQMAKLWDVLSGEL
LGTFKGHQCSLKSVAFSKQEKDGFYRQVRQISGAHNKSERNTPSQAKKRRPSVRSMANGLHSQQSVTVVLFQDEHTLISS
GAVDGVIKMWDLRKSYTAHH
HEPVPLQTYPYPGSSMRKLVAVFSGHLNSSFYVKSTISPDDQFLASGSSDHHTYIWKGHSQEVTSMTWSPTDFTKIATCS
DDNTVRIWRLDRGIDGNKSS
IGQGNLVGWACPKTPSTGLKESSPINKITLNVKITSLAFSYLPEDNTMQEVSTPAKTPTAEHMEGLASPRLAVCAPSGAN
MPPSS
>g2617.t1
MAGRSRRAWFSVLLGLVVGFTLASRLILPKASELKKASQRRRTSPAGCGLNAGLMKEYSGVLWAPQDNVAPVTQNPGSRS
SNFLFVGVMTAQKYLNNRAV
AAH
>g2618.t1
MAASFSVLTRAEEGQRLEVTGSKKDPGSPTSFNLIHPTTALSSLVSSYKTLLHLQSGGCNLHVDTPRTTRLPGENDTLTK
DAR
>g2619.t1
MAVLHMRNLSIEITLEGFKSTPALLSFEHGYKIPVLENLGATLDQFDTIDFSDNEIRKLDGFPLLKRLKTLLLNNNRICL
LRNPVTNKKHYRLYVISKIP
QIRVLDFQKVKLKERQEAEKMFKGKRGAQLAKDIAKRTRTFTPGAAVQTEKKAGPSQADVEAIKNAIANASSLAEVERLK
GMLQAGQIPGRDLRQGPDDM
VEEEEEEEEESDVVQMAPQEPMGDGGEEDMEDNGEEEEEEEAKGGAEDDGMDEDPPVNGS
>g2619.t2

MAVLHMRNLSIEITLEGFKSTPALLSFEHGYKIPVLENLGATLDQFDTIDFSDNEIRKLDGFPLLKRLKTLLLNNNRIWY
AVAKMCYYNSMVCCRVAERQ
EAEKMFKGKRGAQLAKDIAKRTRTFTPGAAVQTEKKAGPSQADVEAIKNAIANASSLAEVERLKGMLQAGQIPGRDLRQG
PDDMVEEEEEEEEESDVVQM
APQEPMGDGGEEDMEDNGEEEEEEEAKGGAEDDGMDEDPPVNGS
>g2619.t3
MDDVQIIDVNEDGEEVPVVHKSSLENQDMGFFAQAAEDILGQYGWYLVVLTVCLILLIQYLSKRRNSQGNKDSSNETPQA
SLIVRRQEALEASRRRLQED
LDAKAAEFREKQKKAEEAGPSSAVLKPKSDKKLRGSGM
>g2619.t4
MAVLHMRNLSIEITLEGFKSTPALLSFEHGYKIPVLENLGATLDQFDTIDFSDNEIRKLDGFPLLKRLKTLLLNNNRICL
LRNPVTNKKHYRLYVISKIP
QIRVLDFQKVKLKERQEAEKMFKGKRGAQLAKDIAKRTRTFTPGAAVQTEKKAGPSQADVEAIKNAIANASSLAEVERLK
GMLQAGQIPGRDLRQGPDDM
VEEEEEEEEESDVVQMAPQEPMGDGGEEDMEDNGEEEEEEEAKGGAEDDGMDEDPPFGHKMDNGVHFSPANQDLGFFRLA
AEDILARYGWYMVVLTVCLI
LLIQYLSERRARQGNQDSYDETPQDPALIFDHKMDDVQIIDVNEDGEEVPVVHKSSLENQDMGFFAQAAEDILGQYGWYL
VVLTVCLILLIQYLSKRRNS
QGNKDSSNETPQASLIVRRQEALEASRRRLQEDLDAKAAEFREKQKKAEEAGPSSAVLKPKSDKKLRGSGM
>g2619.t5
MDNGVHFSPANQDLGFFRLAAEDILARYGWYMVVLTVCLILLIQYLSERRARQGNQDSYDETPQDPALIFDHKMDDVQII
DVNEDGEEVPVVHKSSLENQ
DMGFFAQAAEDILGQYGWYLVVLTVCLILLIQYLSKRRNSQGNKDSSNETPQASLIVRRQEALEASRRRLQEDLDAKAAE
FREKQKKAEEAGPSSAVLKP
KSDKKLRGSGM
>g2620.t1
DEVFCEMVKSNRLCEKKLFRQFCCRTCLMSG
>g2621.t1
MILGGLTTAGVSSGRRHGIGQLKFQDGTTYTGQFQNGLFHGSGILLFTDGSRYEGEFAQGKFQGTGVFSRYDGMMFEGEF
KSGRVEGYGLLTFPDGAHGV
PRNEGLFKNHKLQKREKCPGVVQRAQASASSARSLAL
>g2622.t1
MSNVNFLHRNRKVVNYSQFNESDAEEDYGGDKPKKVRAPPREPKHKRSKNSQDDSEESDDKLSKSKNDSA
>g2622.t2
MHAISHWPWNRKVVNYSQFNESDAEEDYGGDKPKKVRAPPREPKHKRSKNSQDDSEESDDKLSKSKNDSA
>g2623.t1
MAPRPDRPVCREKHSGNLGAPECPREDSEFNAVRIKLTRRGIVLISGSGIHSGARSQSHCQLPGGPQKKQRASTPAPSPP
NNCAQFKNV
>g2624.t1
KFTCIEVKFHLERQMGYYLIQMYIPSLLIVILSWVSFWINMDAAPARVALGITTVLTMTTQSSGSRASLPKLGWGGRRLT
SSPFTCLTTPWPSTLSHHSM
AQQAVSPLHGPAPCLTTPWPSRLSHHSMAQQPVSPQPLCCACQASQTLIIGIGMIGKYHLSYHITSGSGSPAVHSLPVGV
REQDGPGALPAPAGHVARL
>g2625.t1
MFSLCSEQLRSVLRHAPPQGDVVGDEGGDEGGGEEREEEGDKEGDQERDQEGDEERDRERGEERDEEGDKEGEEEGDQER
DRERDEEGEEEGDEED
>g2626.t1
METCYGPTGYSSTSMVSYVKAIDIWMAVCLLFVFAALLEYAGVNFVSRQQKEFLRLKRRQRRNQKDEELRESRFNFSGYN
ITQCLAMKDVAPTKTPASAA
IPPPPAPKDTDTARKKFVDRAKRIDTISRAAFPLAFLIFNIFYWITYKIIRHEDIHKE
>g2627.t1
MPGSFCVPPTQNPTRRVACVRVPAAVAVWWGGGAVLVGFVVPRETPTTGSTSLTVKSSGFWVLDVEQSSVCPGVMASTNN
STFTHNSTFTNNSTFTHNST
FTNNSTVCELVLVTASRPTVVSSSSVIAEVEKTGRPAPAVCPPGDVCLTQMELSVHVSRLLH
>g2627.t2
LRPVMPGSFCVPPTQNPTRRVACVRVPAAVAVWWGGGAVLVGFVVPRETPTTGSTSLTVKSSGFWVLDVEQSSVCPGVMA

STNNSTFTHNSTFTNNSTFT
HNSTFTNNSTVCELVLVTASRPTVVSSSSVIAEVEKTGRPAPAVCPPGDVCLTQMELSVHVSRLLH
>g2628.t1
PDGVPGWCQRFSLDGGSGVRAVKVIQTSNRPGLIYNIGKWSPPRINVRKGKGRYRDTHIVTFAPRYLLDNRSRHKLAFSQ
REFARGKGTTNPEGYISTLP
GSSVVFHWPRNDYDQLLCVRLMDTPNCTWSGGFEVNKPKSFHVNMRDTLGNCFFLRVEVTLKGATYQICFCDTEQLPPPF
RIDNISEVPVQVWQHGVVDM
RLHTEVKAGAVLDYACDEPTLPPYLVLIAKGAGSSEVTADMNFFREYNKLYYENFIYIAASYTFTSQDAGKRPGTKHQVN
CSELVLDVETKTQRVILKKK
EPGKRSQLWRMTGTGMLCHEGSSPPQSKPAQPRPLDSSLVLDIAGLAAVTDNSFEPLMLRRPDSRRSTTQTWHFSSGMLT
CGLPRLVVQAQGGVGGLYDG
AVVVLGPDSGLMAPGPEQQFINQKMRPGSGVLSVQVLPDGPTRVLQISDFNQRRLARTSPSLEADGSKEDLKKSRRTELE
QELEVLVNLEEGLGVSLINK
LPEELVFTTLAGIDVHFTRTASNEVLELSIQTIQVDNQLLGTTQPVTLCVTPTCSEESAVDTSPTLQVNAVKVPSELMLT
DLFKHLMITARRFTVIIEEK
LLLKLLSFFGYGQMEAELEKLDEHVYERPSEDLSNPKRYYFENLKISLPQVKLSVFTSNKLPPDLKALKSTLGIPLVRFE
DAVINMYPFTRVHPYETQDI
ILNDILKHFREELISQAAQILGSVDFLGNPMGLLNDVTEGMSELIKYGNVGGLIRNVTHGVSNSAAKIVGIRAARQTPEP
LHYMH
>g2628.t2
PDGVPGWCQRFSLDGGSGVRAVKVIQTSNRPGLIYNIGKWSPPRASINVRKGKGRYRDTHIVTFAPRYLLDNRSRHKLAF
SQREFARGKGTTNPEGYIST
LPGSSVVFHWPRNDYDQLLCVRLMDTPNCTWSGGFEVNKPKSFHVNMRDTLGNCFFLRVEVTLKGATYQICFCDTEQLPP
PFRIDNISEVPVQVWQHGVV
DMRLHTEVKAGAVLDYACDEPTLPPYLVLIAKGAGSSEVTADMNFFREYNKLYYENFIYIAASYTFTSQDAGKRPGTKHQ
VNCSELVLDVETKTQRVILK
KKEPGKRSQLWRMTGTGMLCHEGSSPPQSKPAQPRPLDSSLVLDIAGLAAVTDNSFEPLMLRRPDSRRSTTQTWHFSSGM
LTCGLPRLVVQAQGGVGGLY
DGAVVVLGPDSGLMAPGPEQQFINQKMRPGSGVLSVQVLPDGPTRVLQISDFNQRRLARTSPSLEADGSKEDLKKSRRTE
LEQELEVLVNLEEGLGVSLI
NKLPEELVFTTLAGIDVHFTRTASNEVLELSIQTIQVDNQLLGTTQPVTLCVTPTCSEESAVDTSPTLQVNAVKVPSELM
LTDLFKHLMITARRFTVIIE
EKLLLKLLSFFGYGQMEAELEKLDEHVYERPSEDLSNPKRYYFENLKISLPQVKLSVFTSNKLPPDLKALKSTLGIPLVR
FEDAVINMYPFTRVHPYETQ
DIILNDILKHFREELISQAAQILGSVDFLGNPMGLLNDVTEGMSELIKYGNVGGLIRNVTHGVSNSAAKIVGIRAARQTP
EPLHYMH
>g2628.t3
PDGVPGWCQRFSLDGGSGVRAVKVIQTSNRPGLIYNIGKWSPPRINVRKGKGRYRDTHIVTFAPRYLLDNRSRHKLAFSQ
REFARGKGTTNPEGYISTLP
GSSVVFHWPRNDYDQLLCVRLMDTPNCTWSGGFEVNKPKSFHVNMRDTLGNCFFLRVEVTLKGATYQICFCDTEQLPPPF
RIDNISEVPVQVWQHGVVDM
RLHTEVKAGAVLDYACDEPTLPPYLVLIAKGAGSSEVTADMNFFREYNKLYYENFIYIAASYTFTSQDAGKRPGTKHQVN
CSELVLDVETKTQRVILKKK
EPGKRSQLWRMTGTGMLCHEGSSPPQSKPAQPRPLDSSLVLDIAGLAAVTDNSFEPLMLRRPDSRRSTTQTWHFSSGMLT
CGLPRLVVQAQGGVGGLYDG
AVVVLGPDSGLMAPGPEQQFINQKMRPGSGVLSVQVLPDGPTRVLQISDFNQRRLARTSPSLEADGSKEDLKKSRRTELE
QELEVFFLLLATWPCTTSVQ
VLVNLEEGLGVSLINKLPEELVFTTLAGIDVHFTRTASNEVLELSIQTIQVDNQLLGTTQPVTLCVTPTCSEESAVDTSP
TLQVNAVKVPSELMLTDLFK
HLMITARRFTVIIEEKLLLKLLSFFGYGQMEAELEKLDEHVYERPSEDLSNPKRYYFENLKISLPQVKLSVFTSNKLPPD
LKALKSTLGIPLVRFEDAVI
NMYPFTRVHPYETQDIILNDILKHFREELISQAAQILGSVDFLGNPMGLLNDVTEGMSELIKYGNVGGLIRNVTHGVSNS
AAKIVGIRAARQTPEPLHYM
H
>g2629.t1
ILSASKEVVRSKRLTQVLEVVLAFGNFMNKGQRGNAYGFKVSSLNKIADTKSSIDRNITMLHYLIMIFEKNYPDTLHIQQ

DLGSIQEAAKVNMAELEKEV
HNIKSGLRALEA
>g2630.t1
QPGAIGPSGWQLLKILEPPRRVIPHSPGAPQQQPKHI
>g2631.t1
MTPSCISAPSTRTQLPGFSPAPSPGSTPSRIDLHWLLVKSRISYKILLLTNKPIHHLAPQYLTDLLHPYTPSWNLRSSNT
GLLIPPPHQPADLW
>g2632.t1
MADSSGRSPEIQEWSGPYPGQGLGPAEPGMQVSAGSMAQMELMLGCQAPGVGLRGCGGEERLGSQLAYLSYMQPCMGGPA
GSDQHHVSAQQEFPPFLLPT
AACRLPPGGPPGPGLPVAKRTISKTSTEYRLRRERNNIAVRKSRDKARRRILLTQQRAVQLQEENQRLQHRLGQLTQELE
ALRHVLAQRHSMRPAADGGE
SCL
>g2633.t1
MAAPVDNVVLVTGHVRDLQKKVQNGSLQTAKGLSFLDLRYQLLLFYLQDLTHLISLKTEGAKIKASEAMERVVTVRTLSE
SEESGEEGAVPVSERKTAAP
GDRKYIPPKIAPMHYETAGLMTCLPSDGDVTEADRKKERQDRQRRAALRSSVIQELREQYSDTPEEVRDRRDFQTERQSR
DEQHRKNYEESMMVRLNVSK
SERMARKRSLQGMSGQLSSITHFRDITALTGGAARQDGDSSHPKKKPLMKKKTKKRAFKKYK
>g2633.t2
MAAPVDNVVLVTGHVRDLQKKVQNGSLQTAKGLSFLDLRYQLLLFYLQDLTHLISLKTEGAKIKASEAMERVVTVRTLSE
SEESGEEGAVPVSERKTAAP
GDRKYIPPKIAPMHYETAGLMTCLPSDGDVTEADRKKERQDRQRRAALRSSVIQELREQYSDTPEEVRDRRDFQTERQSR
DEQHRKNYEESMMVRLNVSK
SERMARKRSLQGMSGQLSSITHFRDITALTGGAARQDGDSSHPKKKPLMKKKTKKR
>g2633.t3
MAAPVDNVVLVTGHVRDLQKKVQNGSLQTAKGLSFLDLRYQLLLFYLQDLTHLISLKTEGAKIKASEAMERVVTVRTLSE
SEESGEEGAVPVSERKTAAP
GDRKYIPPKIAPMHYETAGLMTCLPSDGDVTEADRKKERQDRQRRAALRSSVIQELREQYSDTPEEVRDRRDFQTERQSR
DEQHRKNYEESMMVRLNVSK
SERMARKRSLQGMSGQLSSITHFRDITALTGGAARQVRAGPPPLLGAGKPNNQTYVVVKLGFHVLAGLDGGVS
>g2634.t1
LRERTPSPVSSANHVTCLLTVNHALLPRPCPCPSMRDVEHGRGEHGRAESRSGYLQAEQRIPISKDIITSSSASAMWFIR
DACGMVCAVITWMLVLYAEF
VVVFVMLLPSKNLTYSLGAVPKGNATKEYIESLQLKPGQVVYKCPKCCSIKPDRAHHCSVCKRCIRKMDHHCPWVNNCVG
ENNQKYFVLFTVRA
>g2634.t2
LRERTPSPVSSANHVTCLLTVNHALLPRPCPCPSMRDVEHGRGEHGRAESRSGYLQAEQRIPISKDIITSSSASAMWFIR
DACGMVCAVITWMLVLYAEF
VVVFVMLLPSKNLTYSLGAVPKGNATKEYIESLQLKPGQVVYKCPKCCSIKPDRAHHCSVCKRCIRKMDHHCPWVNNCVG
ENNQKYFVLFTLEMVGTA
>g2634.t3
LRERTPSPVSSANHVTCLLTVNHALLPRPCPCPSMRDVEHGRGEHGRAESRSGYLQAEQRIPISKDIITSSSASAMWFIR
DACGMVCAVITWMLVLYAEF
VVVFVMLLPSKNLTYSLGAVPKGNATKEYIESLQLKPGQVVYKCPKCCSIKPDRAHHCSVCKRCIRKMDHHCPWVNNCVG
ENNQKYFVLFTGAVTELYRA
GGPGLVARPTALEMVGTA
>g2635.t1
LLARESGIVPGFIGFGSAVTSELGYVPAVHGAEEIDSLVDADFRLVLRKMSKRDTVTKLKAIQEFGSMCQERDAEEVKGV
LPYWPRIYCKISVDHDRRVR
EATQQAFEQLVLKVRRSLAPFLKSLMGHWLMAQCDTYVPAASAACLAFTAAFPPSKQPEALSFCRDEILNVLQDTLLKET
ADTLSDPQSVPEEERETKFI
RMVTSSLLGVKKLLHLLPDGDRNLLEDRLTQLTSTAKFWKYSKHKTPQVRGALSEVVSALCQYTPGLVHREAARASAAVL
LGIDDTDPVVLPPLWEAVLH
VVTAIPAEEDIAVLCEH
>g2635.t2

LARESGIVPGFIGFGSAVTSELGYVPAVHGAEEIDSLVDADFRLVLRKMSKRDTVTKLKAIQEFGSMCQERDAEEVKGVL
PYWPRIYCKISVDHDRRVRE
ATQQAFEQLVLKVRRSLAPFLKSLMGHWLMAQCDTYVPAASAACLAFTAAFPPSKQPEALSFCRDEILNVLQDTLLKETA
DTLSDPQSVPEEERETKFIR
MVTSSLLGVKKLLHLLPDGDRNLLEDRLTQLTSTAKFWKYSKHKTPQVRGALSEVVSALCQYTPGLVHREAARASAAVLL
GIDDTDPVVLPPLWEAVLHV
VTAIPAEEDIAVLCEH
>g2635.t3
LLARESGIVPGFIGFGSAVTSELGYVPAVHGAEEIDSLVDADFRLVLRKMSKRDTVTKLKAIQEFGSMCQERDAEEVKGV
LPYWPRIYCKISVDHDRRVR
EATQQAFEQLVLKVRRSLAPFLKSLMGHWLMAQCDTYVPAASAACLAFTAAFPPSKQPEALSFCRDEILNVLQDTLLKET
ADTLSDPQSVPEEERETKFI
RMVTSSLLGVKKLLHLLPDGDRNLLEDRLTQLTSTAKFWKYSKHKTPQVRGALSEVVSALCQYTPGLVHREAARASAAVL
LGIDDTDPVVLPPLWEAVLH
VVTAIPTGAVFFINWSSLPQALIGPIVCECLEKDVKRSRPDGMTA
>g2636.t1
LGGFNSWSAGFGAALPASYIAILIERKSRRGLLTIYMTNLATETLFRMAVMRGIISPLKHGEVLLFCITASLYMFFFRCK
DGLNGFAFSALKFIAGKEEI
PNHTLVVPSEQGSHAPVESAAAAAVSEPPGRPGPSRTGLLAYIRRLLESVVGYLIQCCLKVPSAFRQMFSKPSRLLSLLY
NKDNFQLGAFLGSFVSIYKG
TSCLLRWLRDVDDELHALIAGFLAGTSMFFYKSTSISMYLFSKLVEAVMEVQNLRPSYWKFLLRLTQGRFALMNRHVLDV
FGTQAAKDFGGFIPKLDPRY
TTTLLPSGGDTTLG
>g2636.t2
MASCSCCCPLRKLLGGFNSWSAGFGAALPASYIAILIERKSRRGLLTIYMTNLATETLFRMAVMRGIISPLKHGEVLLFC
ITASLYMFFFRCKDGLNGFA
FSALKFIAGKEEIPNHTLVVPSEQGSHAPVESAAAAAVSEPPGRPGPSRTGLLAYIRRLLESVVGYLIQCCLKVPSAFRQ
MFSKPSRLLSLLYNKDNFQL
GAFLGSFVSIYKGTSCLLRWLRDVDDELHALIAGFLAGTSMFFYKSTSISMYLFSKLVEAVMEVQNLRPSYWKFLLRLTQ
GRFALMNRHVLDVFGTQAAK
DFGGFIPKLDPRYTTTLLPSGGDTTLG
>g2636.t3
LGGFNSWSAGFGAALPASYIAILIERKSRRGLLTIYMTNLATETLFRMAVMRGIISPLKHGEVLLFCITASLYMFFFRCK
DGLNGFAFSALKFIAGKEEI
PNHTLVVPSEQGSHAPVESAAAAAVSEPPGRPGPSRTGLLAYIRRLLESVAVLPLQGFVRMFSVGYLIQCCLKVPSAFRQ
MFSKPSRLLSLLYNKDNFQL
GAFLGSFVSIYKGTSCLLRWLRDVDDELHALIAGFLAGTSMFFYKSTSISMYLFSKLVEAVMEVQNLRPSYWKFLLRLTQ
GRFALMNRHVLDVFGTQAAK
DFGGFIPKLDPRYTTTLLPSGGDTTLG
>g2637.t1
MTYCGRTCRSLNQVDYSCKINTPVVDGCGCEMGTYMNDQGQCVNSSSCPCYDQDNIINAGETVTKDGVTCTCTNRRFECT
NNVCDEVCSIYGDGHYSTFD
EKKFDFSGLCEYTLIEVNEFQLMDGKYAMVKGTASEFPVQVRKMGLYMVVELKQGIVLMWDQKTSLFIKLSAKYQGQVCG
LCGNYDGNSKNDFTTRSGDK
VVDTLDFGNSWKVAATCPDAELVSNPCSTNPYRAAWSQKQCSIINSLTFQECHSRVDPGPFYQACVTDSCACDTGGDCEC
FCTAVASYAKACTEAAVFCD
YYNYPSGCEWHYKPCGAPCMKTCRNPTGNCSNLITALEGKDHLG
>g2637.t2
METVYSYNMTYCGRTCRSLNQVDYSCKINTPVVDGCGCEMGTYMNDQGQCVNSSSCPCYDQDNIINAGETVTKDGVTCTC
TNRRFECTNNVCDEVCSIYG
DGHYSTFDEKKFDFSGLCEYTLIEVNEFQLMDGKYAMVKGTASEFPVQVRKMGLYMVVELKQGIVLMWDQKTSLFIKLSA
KYQGQVCGLCGNYDGNSKND
FTTRSGDKVVDTLDFGNSWKVAATCPDAELVSNPCSTNPYRAAWSQKQCSIINSLTFQECHSRVDPGPFYQACVTDSCAC
DTGGDCECFCTAVASYAKAC
TEAAVFCDYYNYPSGCEWHYKPCGAPCMKTCRNPTGNCSNLITALEGKDHLG
>g2637.t3

MTYCGRTCRSLNQVDYSCKINTPVVDGCGCEMGTYMNDQGQCVNSSSCPCYDQDNIINAGETVTKDGVTCTCTNRRFECT
NNVCDEVCSIYGDGHYSTFD
EKKFDFSGLCEYTLIEVRKMGLYMVVELKQGIVLMWDQKTSLFIKLSAKYQGQVCGLCGNYDGNSKNDFTTRSGDKVVDT
LDFGNSWKVAATCPDAELVS
NPCSTNPYRAAWSQKQCSIINSLTFQECHSRVDPGPFYQACVTDSCACDTGGDCECFCTAVASYAKACTEAAVFCDYYNY
PSGCEWHYKPCGAPCMKTCR
NPTGNCSNLITALEGKDHLG
>g2638.t1
LSLAGLCAEARPLGVSLYLCHGALAMLNWRRAALGPRWEELLLLVPDTLLLLDTSIKESSTAMVVARVLTLWSAAALDCL
QGERSSRSPSLYQTLQGGSV
LHERLLQHVYSHWEHPLDGVRHQTRALFRNLIALHQHASGSDSARPDPYISTLTHGLLGLEWHMRGKYGSMGCLVELFGA
GHLLGIQPQLPSQLLGLMGD
QNLAPYASDLLERLFVSHKAQLAEEGGAGGRGDAGDGCRMELWHRTWVTPLLQVLCRARLDQTTYILDYFLPKLLRCSPS
SLSHMVQALQDMGPCASGHP
GSRGAVGALMTCLRAARAQGVAPSLEEGLLGGLVPLSLLRQALVHKHDQVRMDALGLLCETHRRTEVLTPQEIELLRHFL
PQNVNCQSPGVRQLTVSLLK
KLLCRVKDSSQLLQKRLTQEKNQEQKDRDTHTLATYKEFLCWMCESLLEVLVPGASFPSCLTSLHLLVLLGQLFTFSTEP
DAFALGSKVTFARAQNVLFC
VASNFLEVKQLASVLLRQLPPSTVGLQDPERLGHVLCAALELSTSTKPYDSVTAAHLLNLLVHQPALPSALLACAQEQGI
PFQLPLQPESQASEALTLEH
NSLAVVLLLLRRLQQEVVSAETSLLQAAASRPLYGRAHCITAVLQHLRPGSLTLTEQWRSVVSELIAVCYRMSDVVSPVV
QSSSPEGLIPMDTDSGGPRH
TTRHQRLRRSEHQAPS
>g2638.t2
LSLAGLCAEARPLGVSLYLCHGALAMLNWRRAALGPRWEELLLLVPDTLLLLDTSIKESSTAMVVARVLTLWSAAALDCL
QGERSSRSPSLYQTLQGGSV
LHERLLQHVYSHWEHPLDGVRHQTRALFRNLIALHQHASGSDSARPDPYISTLTHGLLGLEWHMRGKYGSMGCLVELFGA
GHLLGIQPQLPSQLLGLMGD
QNLAPYASDLLERLFVSHKAQLAEEGGAGGRGDAGDGCRMELWHRTWVTPLLQVLCRARLDQTTYILDYFLPKLLRCSPS
SLSHMVQALQDMGPCASGHP
GSRGAVGALMTCLRAARAQGVAPSLEEGLLGGLVPLSLLRQALVHKHDQVRMDALGLLCETHRRTEVLTPQEIELLRHFL
PQNVNCQSPGVRQLTVSLLK
KLLCRVKDSSQLLQKRLTQEKNQEQKDRDTHTLATYKEFLCWMCESLLEVLVPGASFPSCLTSLHLLVLLGQLFTFSTGT
TRLPLKPXPDAFALGSKVTF
ARAQNVLFCVASNFLEVKQLASVLLRQLPPSTVGLQDPERLGHVLCAALELSTSTKPYDSVTAAHLLNLLVHQPALPSAL
LACAQEQGIPFQLPLQPESQ
ASEALTLEHNSLAVVLLLLRRLQQEVVSAETSLLQAAASRPLYGRAHCITAVLQHLRPGSLTLTEQWRSVVSELIAVCYR
MSDVVSPVVQSSSPEGLIPM
DTDSGGPRHTTRHQRLRRSEHQAPS
>g2638.t3
LKLSLAGLCAEARPLGVSLYLCHGALAMLNWRRAALGPRWEELLLLVPDTLLLLDTSIKESSTAMVVARVLTLWSAAALD
CLQGERSSRSPSLYQTLQGG
SVLHERLLQHVYSHWEHPLDGVRHQTRALFRNLIALHQHASGSDSARPDPYISTLTHGLLGLEWHMRGKYGSMGCLVELF
GAGHLLGIQPQLPSQLLGLM
GDQNLAPYASDLLERLFVSHKAQLAEEGGAGGRGDAGDGCRMELWHRTWVTPLLQVLCRARLDQTTYILDYFLPKLLRCS
PSSLSHMVQALQDMGPCASG
HPGSRGAVGALMTCLRAARAQGVAPSLEEGLLGGLVPLSLLRQALVHKHDQVRMDALGLLCETHRRTEVLTPQEIELLRH
FLPQNVNCQSPGVRQLTVSL
LKKLLCRVKDSSQLLQKRLTQEKNQEQKDRDTHTLATYKEFLCWMCESLLEVLVPGASFPSCLTSLHLLVLLGQLFTFST
EPDAFALGSKVTFARAQNVL
FCVASNFLEVKQLASVLLRQLPPSTVGLQDPERLGHVLCAALELSTSTKPYDSVTAAHLLNLLVHQPALPSALLACAQEQ
GIPFQLPLQPESQASEALTL
EHNSLAVVLLLLRRLQQEVVSAETSLLQAAASRPLYGRAHCITAVLQHLRPGSLTLTEQWRSVVSELIAVCYRMSDVVSP
VVQSSSPEGLIPMDTDSGGP
RHTTRHQRLRRSEHQAPS
>g2639.t1

VHCKLTVHDIHMSFLPLAHMFERVVAGVVLIHGGRIGFFQGDIRLLMDDMKTLRPTVFPVVPRLLNRMFDKIFGMARSPL
KRWLLDFASRRKMAEFKSGV
VRKDSMWDKIIFKKIQ
>g2639.t2
GVVLIHGGRIGFFQGDIRLLMDDMKTLRPTVFPVVPRLLNRMFDKIFGMARSPLKRWLLDFASRRKMAEFKSGVVRKDSM
WDKIIFKKIQ
>g2640.t1
LPPCAARGPVDPWTRGLALQFSGGEELKIKQAVGTFCSNQPFSLELIKTRQKKDSRFNAFIQEAESNRLCRRLQLKDIIP
VEMQRLTKYPLLLDNIIKYT
>g2641.t1
MMPYVNIKFADAECAKKALEQLNGFELAGRPMKVGHVTERTDASTASSFLDNDELERTGIDLGTTGRLQLMARLAE
>g2641.t2
MMPYVNIKFADAECAKKALEQLNGFELAGRPMKVGHVTERTDASTASSFLDNDELERTGIDLGTTGRLQLMARLAEEELER
GVLLEKLLTA
>g2642.t1
SRERKRNASRERHRSRSRSRDRPGRYRARKSPVYVSPRAHVGPRAPARPRKRSKSRSPVKREKSPIRQPIDNLTPEERDA
RTVFCMQLAARIRARDLEDF
FSAVGKVRDVRMISDRNSRRSKGIAYIEFLEASSVPLAIGLTGQRLLGVPIIVQASQAEKNRAAAMANNLQKGSAGPMRL
YVGSLHFNITDDMLRGIFEP
FGRVP
>g2643.t1
TSHARAKGDSADQAAQSASQDSDIARAVARELSPSFHQPEPEASAGTLSDVRSLFSERGSTMAAGDIAATTVETLRRASW
FLCRR
>g2643.t2
TSHARAKGDSADQAAQSASQDSDIARAVARELSPSFHQPGVPRWLLETLQRPPWKLCAGPLGSSVVGDPGSRGAECPLS
>g2644.t1
LRSDKRGTQSDQTVVLAVGLPAGLLVMLCVCALGCYLRRRSLKSPYRELVPALPLFPACTAPVLQVSQGSGPRSGQIPFT
VPPRFEPRGAGGLQGQPGRD
QTQGDPGGQRDILAHRGSLSVRSWYPVGSLLE
>g2644.t2
MPYQTVVLAVGLPAGLLVMLCVCALGCYLRRRSLKSPYRELVPALPLFPACTAPVLQVSQGSGPRSGQIPFTVPPRFEPR
GAGGLQGQPGRDQTQGDPGG
QRDILAHRGSLSVRSWYPVGSLLE
>g2645.t1
LKKLLENEKAYQARKEKEHSKRLAKVREELVKLKSFALMLVDERQLHLEQMDKQGQRVQELGQQLQQREHALGEAGTRAL
EDSQRVVSLEAELREKSSKF
TQLHDEMSAKLVDQEAQDRQLNAKLSGLVHQVEELEENNKTLKKSEDELQELREKISKGECGNSTLITELENLRKRVLEM
EGKDEEITKTESQCKELRKR
LQEEDGKSKILRLEVEKLQKRMVELEKLEGAFSIRKTECTQLNTALEREKCLTKELSDELVALRIRMKEVESSELRLEKS
ELSLKDDLSKLKSLTVALMD
ERKNLMERMKSEEKKNEDLGNMVKTEQRKVTEVTERLIDESKKFLKLKSEMGNKVETLTSENRELSTKIVNEMDKNKDLN
CKVNQLKKRVDGLDQAEKSP
LKDMVKRELGRVPGSALADDNKLMELTFEIEHLKKRLKQLEVVEGDLIKTEDQCDLLEKKFLTEQDKANILSRQIEEMRS
QIARNKAIEKGEEDGSREDL
QRRCKREEAITKDLQADVMALKEKIHELMNKEDQLSQLQVDYSVLQQRFLEEEEKAKNMSIEVIHLTKELDVTKRQSRAL
RPSMNGRRMMDIAMTSTGVQ
TDVLPMDTAEEDCPAVFIRKSVQEENHIMNNFRQKCLKKSSEKSSALERSPSSTSDMSVKKSWIPWMRKKEPSQTGNGIE
KPLHINGEASPTELTMIQKQ
GQPVHIRVTPDHQNSTATLEISSPTGESFFSSSVPLSPNSSHPKSKITIIPTHSASNQRVKSITGIRGPERAKSPVTITT
ISRAKSPDSGRVSSAASGRP
LSPISITTMSTAVVPDTSASPEPQEMTMGRAVFKVTPEKQMVPVPIRKCHTNINGNIITTSEDSKIHIHLGNSAGPKMVV
RPVTESKEMTLSTGTVLRSP
RQMNTATGRANTIKATNSITITPVSSPTRPSQNMMGHEAQTTRTGLTRIPMSKNLKTGKTVLGTLGMSGGVRLESRVESQ
SMRIEVKKSTVNSSALQNGG
KA
>g2646.t1

MALLANQIMDARILSSMNGRVELPQFMRVANQSIVTQASSGPEDTHKNRKYPCPLCGKRFRFNSILSLHMRTHTGEKPFK
CPYCDHRAAQKGNLKIHLRT
HKQGILGKGRGRVREENRLLHELEERAILRDRQMRGSYIGPQQGSLLAQLQKQPPAQNGPAQSPQLSLTPSGAVEGLSHA
SAPAAVASVHDEATQPQPAG
GFRCSFCRGKFRKQQELERHIRILHKPYKCTLCDFAATHEEELIGHVETAHITADTAQGQRAAAGGCDKAKPAGEFPCEV
CGQTFSQAWFLKGHMRKHKD
SFEHCCQICGRRFKEPWFLKNHMKVHLNKLAAKSKLSAEHEMSAKLSSLAQEAPANLYTQYISSIHNRFLSADRAEQQDY
NPILATAGIEVKVREMLGRM
MASSQPALGTEGEERSLLGMNHVRPPLGLSSMEYLQKAMAEADAVNGGAYPGWQVTTTPGLAVEPGFATTKETALQQQQQ
QQQQKCSYLAENGPSGEETK
>g2647.t1
VEAGRYIHGVAVHWYLDSFVPAEISLGTTHHLFPEYYLFGTEACAGWSPVDRGVKLGSWERAEQYAADILQDLNHYVVGW
ADWNLALNPNGGPNWVKNFV
DSPIIVDSKRDVFYKQPSFYSMAHFSKFLLKGSRRVGVSASKETTLEFSAFIRPDGSVVLIILNR
>g2647.t2
VEAGRYIHGVAVHWYLDSFVPAEISLGTTHHLFPEYYLFGTEACAGWSPVDRGVKLGSWERAEQYAADILQDLNHYVVGW
ADWNLALNPNGGPNWVKNFV
DSPIIVDSKRDVFYKQPSFYSMAHFSKFLLKGSRRVGVSASKETTLEFSAFIRPDGSVVLIILNRSALAVQFEVWDHS
>g2648.t1
LPREGALGPRAEPKLGIPRVRFTVRVGAPVRWSFGILRIAQVEKKVLGRGSRRGSARDVL
>g2649.t1
MEAALQISAGIEFQRVVAATLKALSPKVHQLGGDDQASVRGLQIPGWSGGVEWRGG
>g2650.t1
MHRDCPLDCKVYVGNLGNNGNKTELERSFGYYGPLRSVWVARNPPGFAFVEFEDSRDASDAVRELDGRTLCGSRVRVELS
SGERRSRNRGPPPSWNRRPR
DERSRRSPVTRRRPGQSSGGTSPRVLSMPFSTSSNPLPKRPQPTSQS
>g2650.t2
MHRDCPLDCKVYVGNLGNNGNKTELERSFGYYGPLRSVWVARNPPGFAFVEFEDSRDASDAVRELDGRTLCGSRVRVELS
SGERRSRNRGPPPSWNRRPR
DERSRRSPVTRRSVPQSAWLVLCHMTQAWPVVRWHQSTGPEHAVLNLLQPFTKTAAAHFTILTNHRFTFPKVYTTTYQLQ
PSGNQ
>g2651.t1
MSDSTVDAKLESKQQASKDVKESENVAEKYSAMTVSKNSELTMGELSSVLSAVPTHKPVKKSPSLPGDYPGPGRGPGGEV
>g2651.t2
MSDSTVDAKLESKQQASKDVKESENVAEKYSAMTVSKNSELTMGELSSVLSAVPTHKPVKKKPCFQ
>g2652.t1
MAWHLIVHLQNVGPSHLRQYASNPAVMMRSLINMNNPNGMMPRGHLTTINQSHLNAQLGVNMSGPNNVTHTSPSPPTSKS
ATPSPSSSINEDEQDDGHRI
IGEKRPAPVDPSKKPKTPKKKKKKDPNEPQKPVSAYALFFRDTQAAIKGQNPNATFGEVSKIVASMWDGLGEEQKQVYKS
KTEAAKKEYLKALAAYRASL
VSKAAAESAEAQTIRSVQQTLASTNLSPGMVLPSPLSQHSPMPSPGLALQQAMPRAIAPKPLQMRLGGSQMVSPVAVGHQ
NAPSGLPPQMLGQMGAVGAQ
SSAMSQMSPPMPPGQQQHAIQHIQQQQQQQQQQQQQQQQHQMQKHLQHHQMQQQQMHHQHIQQQMQHQHFQHHLQQQLQQ
HHMQEQQQQQQQQQQQQQQQ
QQQQQHMQLQHMQMHQLHQQQIQHIQQQQQQQQQQQQQSQCSPPQHSPGTPHSVSGSTSLGSPQPSSQPQQPHPSQIQAH
AQAMSQVSIY
>g2653.t1
MPSPCGRKGRLNKTGQVVLENKWHKTCVERFSGAHLDVVKERVDILTGLSTGYGTTFHTPSLGDEEFEIPPITPPPEMEH
GLALSEVHSPFQGVQEAAAQ
HRVPLTPQFPPQSLELPSITISRNMMDQDGLAVNNGLPAHVSS
>g2653.t2
MPSPCGRKGRLNKTGQVVLENKWHKTCVERFSGAHLDVVKERVDILTGLSTGYGTTFHTPSLGDEEFEIPPITPPPEMEH
GLALSEVHSPFQGVQEAAAQ
HRVPLTPQFPPQSLELPSITISRNMMDQDGLAVNNGLPAKPPQ
>g2653.t3
MNVKTPVVLENKWHKTCVERFSGAHLDVVKERVDILTGLSTGYGTTFHTPSLGDEEFEIPPITPPPEMEHGLALSEVHSP

FQGVQEAAAQHRVPLTPQFP
PQSLELPSITISRNMMDQDGLAVNNGLPAHVSS
>g2654.t1
SLEVSTFSLPPVEHFCPLWNISAPCGTLLPLWNTSAPCGTFLPLWNTSAPCGTLLPLWNTSAPCGTLLPQTKALLL
>g2655.t1
MRSAVLLLTSSFCLLVAGGSVEDAVERGPVSLKDMFREVEMLMEDTQQMLEEAVDQISTESAKSSLTPLGLPGTFHNYTF
TTHTESNKSTGESHPSHAHV
EISGQWNSLDH
>g2655.t2
MNSPAAVCKMRSAVLLLTSSFCLLVAGGSVEDAVERGPVSLKDMFREVEMLMEDTQQMLEEAVDQISTESAKSSLTPLGL
PGTFHNYTFTTHTESNKSTG
ESHPSHAHVEISGQWNSLDH
>g2655.t3
MRSAVLLLTSSFCLLVAGGSVEDAVERGPVSLKDMFREVEMLMEDTQQMLEEAVDQISTESAKSSLTPLGLPGTFHNYTF
TTHTESNKSTGESHPSHAHV
EISGQWNSLDHLSVQLHLQHKGQNSFVNRTITADQIKGF
>g2656.t1
WWNELPANVRTAESLPIFRKRLKTHLFRVHLNPA
>g2657.t1
MSLIHLVFRPRLALASDNDVTTRAAGPERDHEEEDGQGVALKTKHKKHKKHKSKKSKKRKKKRDRDEKESSSESGVESEG
EAKHRSSRTPAEETGDGDGN
RKAGRHKQHLMGKKKKKRKRRREDGRSSSSSESESDGRGPGSSAARRQEDLPDIIPKQ
>g2657.t2
PRLALASDNDVTTRAAGPERDHEEEDGQGVALKTKHKKHKKHKSKKSKKRKKKRDRDEKESSSESGVESEGEAKHRSSRT
PAEETGDGDGNRKAGRHKQH
LMGKKKKKRKRRREDGRSSSSSESESDGRGPGSSAARRQEDLPDIIPKQ
>g2657.t3
MSLIHLVFRPRLALASDNDVTTRAAGPERDHEEEDGQGVALKTKHKKHKKHKSKKSKKRKKKRDRDEKESSSESGVESEG
EAKHRSRTPAEETGDGDGNR
KAGRHKQHLMGKKKKKRKRRREDGRSSSSSESESDGRGPGSSAARRQEDLPDIIPKQ
>g2658.t1
MFKVALDLEACPLPLPPLHPTSHRRHTNPQPSSRSTEPGQKKTRRGSPPGTRWFTIVDLTNVFFSVPVQT
>g2659.t1
MVDERDGCMVSVLLQRHGNRLRPVACFSAKLDTVVAGFPHCQRAVAAAEKAAMASRDLVGYVMVHKVEPPAFACNAEFGC
DSDLDDDLI
>g2659.t2
MVSVLLQRHGNRLRPVACFSAKLDTVVAGFPHCQRAVAAAEKAAMASRDLVGYVMVHKVEPPAFACNAEFGCDSDLDDDL
I
>g2660.t1
MSNLDLHPLSLPISRLSPAKSNSSITDQLTHHQHGKGDSAYSSFSGGSTVPDYPSPFLQDDLQPSSFNHYSDLKYVKAVY
HPCQVHQSETRSMDQLYHSV
EAISQQHRNSNIGNQRQNGHDRCHADGKVTASPNPPRSHHPPPPPPERLDSFVATRNLENTRGHHQGAKPQPQQLRTHMQ
QMPSDFHLPTMPQDTLKSNQ
MAVPEHGDPNLLYQGWRETQQEPVQSHQLPSYRPPPKHHDAVHFSNSEHIFPVNQSKCGKPQMPASLLNKPPPQGTNQTE
NQKNRQRARSSGGSDGTNKG
GNHFDRHRKRAHSAKESPQSFGPEPNRSSSPGSAGQNTTTSGTQHKGQFYFVTGKQTGTGGRKLSVSSCASEAGSESSTV
VETFSLREKERCHSTMDNFF
RPPQYRPYQSTDKMQVFSPMSQEQDSVFNNEDDNLSSVSNGPSPRTIQPAHSFDSLEQKHLIQCQEIGRHIANHPIFFCG
PEESYTPQGSQRPTKEATTM
RCNDQLNFCKREDLAKKGRRQPLGDVASERINKETTPLLYHLTGANRVAQKTKKDIMNNGKGQETTFNKAGHKDVNTRSK
ERSLQIETVAEVGREVVSNA
CNTLDDSFKKYYKEQLKDAQSKVLRETSFKRKDLQLSWPHRIKEQRPELRPTVIHNFSASQDSETSTETLTPSVASEEME
TGSIKKEVLEEEKDEEGQRE
NGRPLNVAQPQVARIGCRKRLTHQQKKMCFSEPEKLHQLGDSPNHSVCRSLGNELEHFISTECLTTQEPEQHGEHGLVAA
RRKIFETRGRAFSASSLSKT
NLKNLQHKALVEYVERKTGHKVAEPQQPGPQLPPPPRQRHSIGEKPVGWPAKSPASTGGQGFKKKLRPHSAGRILDSSSS

SIRYSQFFSAQPSPSGVIQG
SDQPSLKESQSAQGKSASVGSLLSQTEPTGFFRNRSRSTPHAFQAHGPEDDSSPANAMETESSEQGSVAEEAVVRPAATG
SQQRVRVVAQRGKSMEELGT
LRCSRPPALSKSSEQLDQLWNPPVRPGREKRSLPSLLDDGRRDEMAYLQGLEQARRGMETLGLEKTQPLGGQKQTHRRTP
SKAGSTSQLGADEETRPAAH
AEAMDLSRSASPASSNLSRVRIPSGTPSSHGPVWDEARLATPGSESSDPQSPNGPETRERDIKSTSASPSLLSQARSPIE
SKPGSRDRRSTSGPASSPER
PSDDDPRGASPPPDPDREVTAAPRPPTPPPPPPPGVVDESRGLPLREDASESKSKSAHRGSHPRGAVPLHPSPSDGVP
>g2660.t2
MDSFGLHLERMSNLDLHPLSLPISRLSPAKSNSSITDQLTHHQHGKGDSAYSSFSGGSTVPDYPSPFLQDDLQPSSFNHY
SDLKYVKAVYHPCQVHQSET
RSMDQLYHSVEAISQQHRNSNIGNQRQNGHDRCHADGKVTASPNPPRSHHPPPPPPERLDSFVATRNLENTRGHHQGAKP
QPQQLRTHMQQMPSDFHLPT
MPQDTLKSNQMAVPEHGDPNLLYQGWRETQQEPVQSHQLPSYRPPPKHHDAVHFSNSEHIFPVNQSKCGKPQMPASLLNK
PPPQGTNQTENQKNRQRARS
SGGSDGTNKGGNHFDRHRKRAHSAKESPQSFGPEPNRSSSPGSAGQNTTTSGTQHKGQFYFVTGKQTGTGGRKLSVSSCA
SEAGSESSTVVETFSLREKE
RCHSTMDNFFRPPQYRPYQSTDKMQVFSPMSQEQDSVFNNEDDNLSSVSNGPSPRTIQPAHSFDSLEQKHLIQCQEIGRH
IANHPIFFCGPEESYTPQGS
QRPTKEATTMRCNDQLNFCKREDLAKKGRRQPLGDVASERINKETTPLLYHLTGANRVAQKTKKDIMNNGKGQETTFNKA
GHKDVNTRSKERSLQIETVA
EVGREVVSNACNTLDDSFKKYYKEQLKDAQSKVLRETSFKRKDLQLSWPHRIKEQRPELRPTVIHNFSASQDSETSTETL
TPSVASEEMETGSIKKEVLE
EEKDEEGQRENGRPLNVAQPQVARIGCRKRLTHQQKKMCFSEPEKLHQLGDSPNHSVCRSLGNELEHFISTECLTTQEPE
QHGEHGLVAARRKIFETRGR
AFSASSLSKTNLKNLQHKALVEYVERKTGHKVAEPQQPGPQLPPPPRQRHSIGEKPVGWPAKSPASTGGQGFKKKLRPHS
AGRILDSSSSSIRYSQFFSA
QPSPSGVIQGSDQPSLKESQSAQGKSASVGSLLSQTEPTGFFRNRSRSTPHAFQAHGPEDDSSPANAMETESSEQGSVAE
EAVVRPAATGSQQRVRVVAQ
RGKSMEELGTLRCSRPPALSKSSEQLDQLWNPPVRPGREKRSLPSLLDDGRRDEMAYLQGLEQARRGMETLGLEKTQPLG
GQKQTHRRTPSKAGSTSQLG
ADEETRPAAHAEAMDLSRSASPASSNLSRVRIPSGTPSSHGPVWDEARLATPGSESSDPQSPNGPETRERDIKSTSASPS
LLSQARSPIESKPGSRDRRS
TSGPASSPERPSDDDPRGASPPPDPDREVTAAPRPPTPPPPPPPGVVDESRGLPLREDASESKSKSAHRGSHPRGAVPLH
PSPSDGVP
>g2660.t3
MSNLDLHPLSLPISRLSPAKSNSSITDQLTHHQHGKGDSAYSSFSGGSTVPDYPSPFLQDDLQPSSFNHYSDLKYVKAVY
HPCQVHQSETRSMDQLYHSV
EAISQQHRNSNIGNQRQNGHDRCHADGKVTASPNPPRSHHPPPPPPERLDSFVATRNLENTRGHHQGAKPQPQQLRTHMQ
QMPSDFHLPTMPQDTLKSNQ
MAVPEHGDPNLLYQGWRETQQEPVQSHQLPSYRPPPKHHDAVHFSNSEHIFPVNQSKCGKPQMPASLLNKPPPQGTNQTE
NQKNRQRARSSGGSDGTNKG
GNHFDRHRKRAHSAKESPQSFGPEPNRSSSPGSAGQNTTTSGTQHKGQFYFVTGKQTGTGGRKLSVSSCASEAGSESSTV
VETFSLREKERCHSTMDNFF
RPPQYRPYQSTDKMQVFSPMSQEQDSVFNNEDDNLSSVSNGPSPRTIQPAHSFDSLEQKHLIQCQEIGRHIANHPIFFCG
PEESYTPQGSQRPTKEATTM
RCNDQLNFCKREDLAKKGRRQPLGDVASERINKETTPLLYHLTGANRVAQKTKKDIMNNGKGQETTFNKAGHKDVNTRSK
ERSLQIETVAEVGREVVSNA
CNTLDDSFKKYYKEQLKDAQSKVLRETSFKRKDLQLSWPHRIKEQRPELRPTVIHNFSASQDSETSTETLTPSVASEEME
TGSIKKEVLEEEKDEEGQRE
NGRPLNVAQPQVARIGCRKRLTHQQKKMCFSEPEKLHQLGDSPNHSVCRSLGNELEHFISTECLTTQEPEQHGEHGLVAA
RRKIFETRGRAFSASSLSKT
NLKNLQHKALVEYVERKTGHKVAEPQQPGPQLPPPPRQRHSIGEKPVGWPAKSPASTGGQGFKKKLRPHSAGRILDSSSS
SISSEQGSVAEEAVVRPAAT
GSQQRVRVVAQRGKSMEELGTLRCSRPPALSKSSEQLDQLWNPPVRPGREKRSLPSLLDDGRRDEMAYLQGLEQARRGME
TLGLEKTQPLGGQKQTHRRT

PSKAGSTSQLGADEETRPAAHAEAMDLSRSASPASSNLSRVRIPSGTPSSHGPVWDEARLATPGSESSDPQSPNGPETRE
RDIKSTSASPSLLSQARSPI
ESKPGSRDRRSTSGPASSPERPSDDDPRGASPPPDPDREVTAAPRPPTPPPPPPPGVVDESRGLPLREDASESKSKSAHR
GSHPRGAVPLHPSPSDGVP
>g2661.t1
MSGSEAQTSYTTKEKLGKQQSAMSRYVSSPQPTSSSSSSSSSSSSSSSSSSSHQSLAVPDAGLTQVWRIHREGAVRRNLV
MVMLAKWSH
>g2662.t1
LSDGAESLATSTADTGDELVSGMVVAEGGGKVSKMPSERSGSPSSASGSQSGPSKPPPVLPKSEKALLKAMKLTTRRIKK
EEGQKKPSHKSSSSRKHRAV
KHSTDNPEPIQTEDKPSSNSTIRSEKLSQSVNKEHRQNSEERHHHNKSSRNHTETKAEEAQHPQRDQISDRLEHSHVERS
GYQTGRRDRTESSGLSAEGP
PEVVLTEREGRSSDGHRRPRDKVEHRNYSGDRVISNVPIYKAHVDERPAAERQSKRSQSMDRHVGNHAELRASANISSNM
SEQTDSRNQRIERSIMDELQ
QRGRAKDRAGRGGDAVAKRSHSIDTYPSETSHPSNLSRQSSYNGQQLSRQSSVEHTIVTQSIPMTQRKLLQDPDSGQYFF
VDMPVQVQTKTFYDPETGNY
VQLPVQPPEGAMPQASTVEVLNTPMVLYHGFVPVPMTPITRKSVTQASQREQDEFERRQKERSRQVYANEGRPYLEPVYG
QHDHMLGELLGVEELDCAS
>g2663.t1
MTRVEKRHLGQRMGSMLHHRFPNGFTELLMDETDREVGTLTDRAFRSLCVGDDAVYNDEFSYGFSPFNCHKPLVGEPRRK
TKDSKKQPAHHGDNGGKDTP
PWKRQQQSMTQVSSFLKALSATEKSREGIFNNNNGGEKDSNGESWDKSALRSIQKELSEFSADYHNTLVGGHPGRSGRHP
SGEGSGKKTGKDSAKVKNGK
STIKLRKLNIKNFFLHSEFSPFQMWRDVNRFPFGREEMVTSIPSDKTPKWYDSQLYRELAEAHRIDILNAEDSQPWSQLQ
NNGGECSAGETGS
>g2663.t2
LVGLQATVSSKEETDTHRETQAAGRTDDHGTMTRVEKRHLGQRMGSMLHHRFPNGFTELLMDETDREVGTLTDRAFRSLC
VGDDAVYNDEFSYGFSPFNC
HKPLVGEPRRKTKDSKKQPAHHGDNGGKDTPPWKRQQQSMTQVSSFLKALSATEKSREGIFNNNNGGEKDSNGESWDKSA
LRSIQKELSEFSADYHNTLV
GGHPGRSGRHPSGEGSGKKTGKDSAKVKNGKSTIKLRKLNIKNFFLHSEFSPFQMWRDVNRFPFGREEMVTSIPSDKTPK
WYDSQLYRELAEAHRIDILN
AEDSQPWSQLQNNGGECSAGETGS
>g2664.t1
LAAATASPLVTQKLASPAQGSPVRDSVCGSSPRPVAGQPAPVCPTKHTRPHWSAATLPAFLDAYLRPRMAAVHAEDAASP
LHQYPPVTHLSAGWSVANLR
RRPTGFPVSREATTSTLHSSAPAALALCQLRWLCAFTTITWAIFCRITSLPISHHMDLCQVSGHGN
>g2665.t1
VESELFQSNIQKQGSEKQTKAGTNIKQQGAHLRDTRAENHPQNKTWFSHSVIAATHRTVIPLRLLMKRSGFLAEKMAAPG
PGEYFSVGSHVSCLTCLGQR
LQGEVVAFDYQSKMLTLSILSFPRGKANLNDVILINLAYVSDVDIINDRTETPPPLASLNVSKLANRARTEKEDKLSQAY
AISAGVSLEGQQLFQTIHKT
IKDCKWQEKNIIVMDDVVISPPYQVDNCKGKEGSALSHVRKIVEKHFRDVESQKSMQRSQAQQTQKDSTLSS
>g2665.t2
VESELFQSNIQKQGSEKQTKAGTNIKQQGAHLRDTRAENHPQNKTWFSHSVIAATHRTVIPLRLLMKWRLLDRGSISASG
AMSLASPAWANVCKESGKAN
LNDVILINLAYVSDVDIINDRTETPPPLASLNVSKLANRARTEKEDKLSQAYAISAGVSLEGQQLFQTIHKTIKDCKWQE
KNIIVMDDVVISPPYQVDNC
KGKEGSALSHVRKIVEKHFRDVESQKSMQRSQAQQTQKDSTLSS
>g2665.t3
VESELFQSNIQKQGSEKQTKAGTNIKQQGAHLRDTRAENHPQNKTWFSHSVIAATHRTVIPLRLLMKRSGFLAEKMAAPG
PGEYFSVGSHVSCLTCLGQR
LQGEVVAFDYQSKMLTLKCASSSGKANLNDVILINLAYVSDVDIINDRTETPPPLASLNVSKLANRARTEKEDKLSQAYA
ISAGVSLEGQQLFQTIHKTI
KDCKWQEKNIIVMDDVVISPPYQVDNCKGKEGSALSHVRKIVEKHFRDVESQKSMQRSQAQQTQKDSTLSS
>g2666.t1

FLCGGYVLLLKGYRQRQRYLLNAPDKMGEPPSSTERGCIAPRQAGNLLSAEAGQPALHDPPDYYMLILGRRHADRWVSRR
HNTPHSSMPEPPAQLA
>g2666.t2
GYRQRQRYLLNAPDKMGEPPSSTERGCIAPRQAGNLLSAEAGQPALHDPPDYYMLILGRRHADRWVSRRHNTPHSSMPEP
PAQLA
>g2666.t3
LLKGYRQRQRYLLNAPDKMGEPPSSTERGCIAPRQAGNLLSAEAGQPALHDPPDYYMLILGRRHADRWVSRRHNTPHSSM
PEPPAQLA
>g2667.t1
LDWTDNTHALYKKGQSRLHLLRRLRSFGVCRTLLRTFYDSVVASAIFYAVVCWRGGSTDRDRSRLNRLIRRASSVLDCPL
DSIEEVGERRMLTKLTSIMD
NPSHPLHDSVGSLSSSFRSRLIHPRCKKRGSA
>g2668.t1
MPIENEEKTVEVRGLPEGVDEELLLLYFANKRRSGGGPLVSVEKIGDHAFLELHVVYNAEITVRRPVTKDQRRLLLCGTQ
PNTSMELLELYVENMMEVEV
DEYTLTPLPDRDAVLIHLHQALATDFYSLSSKISKKPLEGSKLTLEQVEQTDSILVENLHPGLSLKTLALHFESSQAANL
KVKDVVMLSESTARVSFVHF
EAVDYVLNHPHKLNDATLTVSPYYAFSQTPACQTQSDPSQSGHSVESKVHVVEVMSETHLVDITIQDSEKLKLFQMSTLP
QDVHQTHPNVNMQIKDDSVY
LTGPDKQSLEDLKITLLDFIAGMAQATLTLDMEQARFLEHDDVREKLLRTFTEHGIAAVYTVLHKVVVVTSLSQDLATQA
CAMLPSQLCAFSVPVKKDYE
CMLYSEEWPAFLQTLGLCSASVSGDGANVDVVTLKDLESEKRSKVTEFLLTPIEQEKFITMEPGMLQYVQMYFHQLLEDM
NQVVIIPVEGKGLCGLRIQG
LAGHCQNALDLLQSIVSSVCTRTLIICETGVARFLVEMEGTSILNEMRAKFQICISLDKVHWAPLQNQDIFDAAWKLMSL
RNFHKVASLTASIPSAITDN
GASDQGSQADEDDDDDDEDEDLYTAGDAGEPENGTALDDTAAGPSSLKSGPEVMGLNEHEQLTLAIQNSLHTTTEEDQLQ
MVLELSKQQGSYASYQMAAP
RPVPRCLSTLLDDTATTDVAHVNVFAGFSSDLIRADIALKKKASLRQVEERMEWKGLAGMSEFHRHCVQVIQRKHAVEIR
VEGDALMVSGFKDFVAEALP
DVRLLLRRVSSCRDESEILSNIQWEREDPDTAAMTPYDPDTVVFIENIWRMKEKEVAVLLHGQPHVIDLVKMEDYNVAAG
SSVTVARTVISD
>g2668.t2
MPIENEEKTVEVRGLPEGVDEELLLLYFANKRRSGGGPLVSVEKIGDHAFLELHVVYNAEITVRRPVTKDQRRLLLCGTQ
PNTSMELLELYVENMMEVEV
DEYTLTPLPDRDAVLIHLHQALATDFYSLSSKISKKPLEGSKLTLEQVEQTDSILVENLHPGLSLKTLALHFESSQAANL
KVKDVVMLSESTARVSFVHF
EGWSSVPYAVDYVLNHPHKLNDATLTVSPYYAFSQTPACQTQSDPSQSGHSVESKVHVVEVMSETHLVDITIQDSEKLKL
FQMSTLPQDVHQTHPNVNMQ
IKDDSVYLTGPDKQSLEDLKITLLDFIAGMAQATLTLDMEQARFLEHDDVREKLLRTFTEHGIAAVYTVLHKVVVVTSLS
QDLATQACAMLPSQLCAFSV
PVKKDYECMLYSEEWPAFLQTLGLCSASVSGDGANVDVVTLKDLESEKRSKVTEFLLTPIEQEKFITMEPGMLQYVQMYF
HQLLEDMNQVVIIPVEGKGL
CGLRIQGLAGHCQNALDLLQSIVSSVCTRTLIICETGVARFLVEMEGTSILNEMRAKFQICISLDKVHWAPLQNQDIFDA
AWKLMSLRNFHKVASLTASI
PSAITDNGASDQGSQADEDDDDDDEDEDLYTAGDAGEPENGTALDDTAAGPSSLKSGPEVMGLNEHEQLTLAIQNSLHTT
TEEDQLQMVLELSKQQGSYA
SYQMAAPRPVPRCLSTLLDDTATTDVAHVNVFAGFSSDLIRADIALKKKASLRQVEERMEWKGLAGMSEFHRHCVQVIQR
KHAVEIRVEGDALMVSGFKD
FVAEALPDVRLLLRRVSSCRDESEILSNIQWEREDPDTAAMTPYDPDTVVFIENIWRMKEKEVAVLLHGQPHVIDLVKME
DYNVAAGSSVTVARTVISD
>g2668.t3
MSNPTNVLSELHVVYNAEITVRRPVTKDQRRLLLCGTQPNTSMELLELYVENMMEVEVDEYTLTPLPDRDAVLIHLHQAL
ATDFYSLSSKISKKPLEGSK
LTLEQVEQTDSILVENLHPGLSLKTLALHFESSQAANLKVKDVVMLSESTARVSFVHFEAVDYVLNHPHKLNDATLTVSP
YYAFSQTPACQTQSDPSQSG
HSVESKVHVVEVMSETHLVDITIQDSEKLKLFQMSTLPQDVHQTHPNVNMQIKDDSVYLTGPDKQSLEDLKITLLDFIAG

MAQATLTLDMEQARFLEHDD
VREKLLRTFTEHGIAAVYTVLHKVVVVTSLSQDLATQACAMLPSQLCAFSVPVKKDYECMLYSEEWPAFLQTLGLCSASV
SGDGANVDVVTLKDLESEKR
SKVTEFLLTPIEQEKFITMEPGMLQYVQMYFHQLLEDMNQVVIIPVEGKGLCGLRIQGLAGHCQNALDLLQSIVSSVCTR
TLIICETGVARFLVEMEGTS
ILNEMRAKFQICISLDKVHWAPLQNQDIFDAAWKLMSLRNFHKVASLTASIPSAITDNGASDQGSQADEDDDDDDEDEDL
YTAGDAGEPENGTALDDTAA
GPSSLKSGPEVMGLNEHEQLTLAIQNSLHTTTEEDQLQMVLELSKQQGSYASYQMAAPRPVPRCLSTLLDDTATTDVAHV
NVFAGFSSDLIRADIALKKK
ASLRQVEERMEWKGLAGMSEFHRHCVQVIQRKHAVEIRVEGDALMVSGFKDFVAEALPDVRLLLRRVSSCRDESEILSNI
QWEREDPDTAAMTPYDPDTV
VFIENIWRMKEKEVAVLLHGQPHVIDLVKMEDYNVAAGSSVTVARTVISD
>g2669.t1
MNNNNNNNNNQSVSADYDYALLELKRPVKQRPMALGVAPHDKPLGRVHFSGYDVEGAPVDGSERVVYRFCAVARESDDLI
YQHCDAQQGATGAGVYVRAR
QQADDGKGKGAWRRKVIGVFSGHRWVELEGSRHTDFNVAVRITPAKYAQICHWIHADPARCEAA
>g2670.t1
MFTTNTGLCLDLRRQPHTCVYPQQLVFSLPSSANPNTAASTRDNRHFGNDEHSSVVPSPPPGEPPTVSLLPRTDGRDGLI
SHGSIRLNQEEAAGVCMNTL
PGACGRDWSQGTDPP
>g2671.t1
MSVSVHENRKSRSSTGSMNISLFHKPSHPDSVLTHLNALRKQCMFTDVTLWAGDRSFPCHRAVLAACSRYFEAMFSGGLR
ESLDSDVNFRNSIHPEVLEL
LLDFAYSSRIIINEENAESLLEAGDMLQFHDIRDAAAEFLEKNLHSSNCLGMMLLSDAHQCKRLYELSWRMCLVHYETVL
ESEEFYSLSKDKLLELILSD
ELEIEDEEVVFNSVMRWLRFDLEGRQHHLPELLRGIRLALLPSECLLEAVAHEELVMADKRSR
>g2671.t2
MSVSVHENRKSRSSTGSMNISLFHKPSHPDSVLTHLNALRKQCMFTDVTLWAGDRSFPCHRAVLAACSRYFEAMFSGGLR
ESLDSDVNFRNSIHPEVLEL
LLDFAYSSRIIINEENAESLLEAGDMLQFHDIRDAAAEFLEKNLHSSNCLGMMLLSDAHQCKRLYELSWRMCLVHYETVL
ESEEFYSLSKDKLLELILSD
ELEIEDEERPPTPISLRRARVVFNSVMRWLRFDLEGRQHHLPELLRGIRLALLPSECLLEAVAHEELVMADKRSR
>g2671.t3
LEVPSEAAMSVSVHENRKSRSSTGSMNISLFHKPSHPDSVLTHLNALRKQCMFTDVTLWAGDRSFPCHRAVLAACSRYFE
AMFSGGLRESLDSDVNFRNS
IHPEVLELLLDFAYSSRIIINEENAESLLEAGDMLQFHDIRDAAAEFLEKNLHSSNCLGMMLLSDAHQCKRLYELSWRMC
LVHYETVLESEEFYSLSKDK
LLELILSDELEIEDEEVVFNSVMRWLRFDLEGRQHHLPELLRGIRLALLPSECLLEAVAHEELVMADKRSR
>g2672.t1
ESLVAMVPPVKTEVQVQLGRMDNQGEAGGVGQRGSPGERGRPGPPGGGGYHSKDAQPMIGPVGPRGERGSPGTPGSSGPS
GNPGSPGRDAVVNYDEIKSF
IRQQVGKIFDERMAYYMSRMPVAVESASPGRPGPPGKDGTPGRQGTPGSPGLPGQLGREGRPGPQGPPGIRGPHGEKGEK
GIGEMGDVGPPGAPGVPGPQ
GYGKMGPTGPVGQQGVPGVSGPPGNPGSKGQDGRCNPGDCRSHTVEYNGKGPNVKGPW
>g2672.t2
ESLVAMVPPVKTEVQVQLGRMDNQGEAGGVGQRGSPGERGRPGPPGGGGYHSKDAQPMIGPVGPRGERGSPGTPGSSGPS
GNPGSPGRDAVVNYDEIKSF
IRQQVGKIFDERMAYYMSRMPVAVESASPGRPGPPGKDGTPGRQGTPGSPGLPGQLGREGRPGPQGPPGIRGPHGEKGEK
GIGEMGDVGPPGAPGPQGYG
KMGPTGPVGQQGVPGVSGPPGNPGSKGQDGRCNPGDCRSHTVEYNGKGPNVKGPW
>g2672.t3
EVQVQLGRMDNQGEAGGVGQRGSPGERGRPGPPGGGGYHSKDAQPMIGPVGPRGERGSPGTPGSSGPSGNPGSPGRDAVV
NYDEIKSFIRQQVGKIFDER
MAYYMSRMPVAVESASPGRPGPPGKDGTPGRQGTPGSPGLPGQLGREGRPGPQGPPGIRGPHGEKGEKGIGEMGDVGPPG
APGVPGPQGYGKMGPTGPVG
QQGVPGVSGPPGNPGSKGQDGRCNPGDCRSHTVEYNGKGPNVKGPW

>g2673.t1
DFTVDCWRWKAELSACVLAPPAGGLGQSSRCHLWVNDSHTTSEVALGCCLTSTWSS
>g2674.t1
VAVAEETAAGAIVVVVVAAEVCVAGAIVVTGEGAAGPVVFAEDGPVDVVETVARDQPVPTGRAAETLTPRQSAGGQSQVG
SRRGPLSPSSRLERLTGLHR
APDVYPTSLIPRIPRPHESSETRQEFQEGLARGTRHLPEKVIGGRKSGDRDAELSHLRGFIFFIC
>g2675.t1
KLLELNNLHSLVSVVSALQSAPIFRLSKTWALISRKDKATFEKLNYLTSKEENYTCMREYIRSLKMVPCIPYLGIYLLDM
IYIDSAYPASGSIIETEQRT
NQMNNLLRVISDLQMSCKYDHLGTLPHIHKYLMSVRYIEELQKFVEDDNFKLSLKIEPGNSSPRIASSKEDLAGLSEVSA
SVRYSRRPTCPDASVGVHVH
TPPPSHHRKSHSLGNNMMCQYGMSESKSATFSTGKARHLLDDSFLESQSPVRTDPHSTSVSNGLSLGSSESSLGEEPSPG
MERGRMYATLGPNWRVPLRS
SPRSCSYVYSPSGASSYHGCAEAGSVTIEGPLRRRTFLKEGRKPRVHAVPYTS
>g2675.t2
KLLELNNLHSLVSVVSALQSAPIFRLSKTWALISRKDKATFEKLNYLTSKEENYTCMREYIRSLKMVPCIPYLGIYLLDM
IYIDSAYPASGSIIETEQRT
NQMNNLLRVISDLQMSCKYDHLGTLPHIHKYLMSVRYIEELQKFVEDDNFKLSLKIEPGNSSPRIASSKEDLAGLSEVSA
SVRYSRRPTCPDASVGVHVH
TPPPSHHRKSHSLGNNMMCQYGMSESKSATFSTGKARHLLDDSFLESQSPVRTDPHSTSVSNGLSLGMSCSKVGPFLSAA
GLPARLTGSSESSLGEEPSP
GMERGRMYATLGPNWRVPLRSSPRSCSYVYSPSGASSYHGCAEAGSVTIEGPLRRRTFLKEGRKPRVHAVPYTS
>g2675.t3
KLLELNNLHSLVSVVSALQSAPIFRLSKTWALISRKDKATFEKLNYLTSKEENYTCMREYIRSLKMVPCIPYLGIYLLDM
IYIDSAYPASGSIIETEQRT
NQMNNLLRVISDLQMSCKYDHLGTLPHIHKYLMSVRYIEELQKFVEDDNFKLSLKIEPGNSSPRIASSKEDLAGLSEVSA
SVRYSRRPTCPDASVGVHVH
TPPPSHHRKSHSLGNNMMCQYGMSESKSATFSTGKARHLLDDSFLESQSPVRTDPHSTSVSNGLSLGSSESSLGEEPSPG
MERGRMYATLGPNWRVPLRS
SPRSCSYVYSPSGASSYHGCAEAGSVTIEGPLRRRTFLKEGRKPR
>g2676.t1
MSPASCHPNLLSSWPLNTLTFCVCVLRPTTGEKTMRYAYPEPAYDRGLKYDKEPDAMQAHMMDQVINNAISYLGTDSLRP
VVQHPGLPVSMADVGPMGNP
LYHQVLPLAHHRLERPRSRNSLPLLPPQPPLPPLFPGHPSSNGQGSLLRHGKQQQQQQHHHLHLLQQQQQLLLR
>g2676.t2
MRYAYPEPAYDRGLKYDKEPDAMQAHMMDQVINNAISYLGTDSLRPVVQHPGLPVSMADVGPMGNPLYHQVLPLAHHRLE
RPRSRNSLPLLPPQPPLPPL
FPGHPSSNGQGSLLRHGKQQQQQQHHHLHLLQQQQQLLLR
>g2677.t1
LSQEKVATFIKRLRAEPRYLLAQNVSTCIDPLEVCLHRQTVQDTVHIFQHSIPTEGKPVTNQKNSGRCWIFSCLNVMRLP
FMKKFNIEEFEFSQSYLFFW
DKVERSYYFLQACVETAQRKEPVDGRLVQFLLSNPTNDGGQWDMLVNLIEKYGVIPKKCFPESHSSEASRRMNDILNHKL
REYCLRLRNMVASNSTEAEL
SDAMDTMIEE
>g2677.t2
LSQEKVATFIKRLRAEPRYLLAQNVSTCIDPLEVCLHRQTVQDTVHIFQHSIPTEGKPVTNQKNSGRCWIFSCLNVMRLP
FMKKFNIEEFEFSQSYLFFW
DKVERSYYFLQACVETAQRKEPVDGRLVQFLLSNPTNDGGQWDMLVNLIEKYGVIPKKCFPESHSSEASRRMNDILNHKL
REYCLRLRNMVASNSTEAEL
SDAMDTMIEEFRQKRELPHF
>g2677.t3
LSQEKVATFIKRLRAEPRYLLAQNVSTCIDPLEVCLHRQTVQDTVHIFQHSIPTEGKPVTNQKNSGRCWIFSCLNVMRLP
FMKKFNIEEFEFSQSYLFFW
DKVERSYYFLQACVETAQRKEPVDGRLVQFLLSNPTNDGGQWDMLVNLIEKYGVIPKKCFPESHSSEASRRMNDILNHKL
REYCLRLRNMVASNSTEAEL
SDAMDTMIEEVL

>g2678.t1
STTPMCCPSRSSILTGKYVHNHHTYTNNENCSSPSWQAHHEPHTFAVHLNNSGYRTAFFGKYLNEYNGSYVPPGWREWVA
LVKNSRFYNYTLCRNGVREK
HGGDYPKVSPGSAHRLTTPPARKSRPLLDVTDTILRMLVFDYLTDLITRDSLSYFRTSKKMFPHRPVLMVMSHAAPHGPE
DSAPQYSTAFPNASQH
>g2678.t2
STTPMCCPSRSSILTGKYVHNHHTYTNNENCSSPSWQAHHEPHTFAVHLNNSGYRTAFFGKYLNEYNGSYVPPGWREWVA
LVKNSRFYNYTLCRNGVREK
HGGDYPKVSPGSAHRLTTPPARKSRPLLDVTDTILRMLVFVFRIEPNATDYLTDLITRDSLSYFRTSKKMFPHRPVLMVM
SHAAPHGPEDSAPQYSTAFP
NASQH
>g2678.t3
STTPMCCPSRSSILTGKYVHNHHTYTNNENCSSPSWQAHHEPHTFAVHLNNSGYRTAFFGKYLNEYNGSYVPPGWREWVA
LVKNSRFYNYTLCRNGVREK
HGGDYPKVSPGSAHRLTTPPARKSRPLLDVTDTILRMLVFVFRIEPNATVAADYLTDLITRDSLSYFRTSKKMFPHRPVL
MVMSHAAPHGPEDSAPQYST
AFPNASQH
>g2679.t1
GGRREVVAGGSLQISNLTEEDSGVYTCVASNGIDNSTIEARAQLTVQGGRWV
>g2680.t1
MPEPSQLAPFDAKEQRLYSEFPPDVRAPHPISKAEPSHPAEETNFGRLYPRSRSFGHYPELMTI
>g2681.t1
MVKEEQEEEMGKRSDKEKEVTGEGMRSEVRWRVKEGGPGGIEEEVEGVQRRGDSSWPFQHLGIWRVAGSISWLPLTRVEG
PLSKRPGHIRNTQHCENNAE
CYLGLASERQKEETKEENLNKEEEK
>g2681.t2
MVKEEQEEEMGKRSDKEKEVTGEGMRSEVRWRVKEGGPGGIEEEVEGVQRSCRPRETEVELDVRGRSADQGDSSWPFQHL
GIWRVAGSISWLPLTRVEGP
LSKRPGHIRNTQHCENNAECYLGLASERQKEETKEENLNKEEEK
>g2681.t3
MVKEEQEEEMGKRSDKEKEVTGEGMRSEVRWRVKEGGPGGIEEEVEGVQRRNTQHCENNAECYLGLASERQKEETKEENL
NKEEEK
>g2682.t1
KDRPRRIRMEDIKKAFPSHSESSIRKRLKLCADFKRTVAQEEDSPWFSIFPIDNEELVYGRWEDSIIWDDQAMARLLAPP
VLTLNPNDENIILEIPDEKE
QRTSSSPSKENKKESALKKSRILLGKTGVIKDEPQQNMSQPEVKDPWNLSNDEFYYPKQQGLRGTFGGNIIQHSIPAVEL
RQPFFPTHMGPMKLRLFHRP
SLKKYSFGALAQPGPHPAQPLLKHIKKKAKMREQERQASGGGDMFFMRTAQDLTGKDGDLILAEYSEEYPPLMMQVGMAT
KIKNYYKRKPGKDPGAPDCK
YGETVYCHTSPFLGSLHPGQLLQVRGHEYIPDVHLSSYSEKSCVVSPATVRSLVLSPPYSEHSNSPQAFENNLFRAPIYL
HKMPETDFLVLRTRQGYFIR
EIVDIVVVGQECPLFEVPGPNSKRANTHIRDFLQVFIYRLFWKSKDRPRRIRMEDIKKAFPSHSESSIRKRLKLCADFKR
TGMDSNWWVLKPDFRLPTEE
EIRAMVSPEQCCSYYSMQVAEQRLKDAGYGEKSFFAPDEENEEDFQMKIDDEVRTAPWNTTRAFIAAMKGKCLLEVTGVA
DPTGCGEGFSYVKVPNKPTQ
QKDDKEPQPAKKTVTGTDADLRRLSLKNAKQLLRKFGVPEEEIKKLSRWEVIDVVRTMSTEQARSGEGPMSKFARGSRFS
VAEHQERYKEECQRIFDLQN
KVLESTEVLSTDTDSSSAEDSDFEEMGKNIENMLQNKKTSSQLSREREEQERKELQRMLMGEESERDHKGRKERRKGLCE
STTGIV
>g2682.t2
KDRPRRIRMEDIKKAFPSHSESSIRKRLKLCADFKRTVAQEEDSPWFSIFPIDNEELVYGRWEDSIIWDDQAMARLLAPP
VLTLNPNDENIILEIPDEKE
QRTSSSPSKENKKESALKKSRILLGKTGVIKDEPQQNMSQPEVKDPWNLSNDEFYYPKQQGLRGTFGGNIIQHSIPAVEL
RQPFFPTHMGPMKLRLFHRP
SLKKYSFGALAQPGPHPAQPLLKHIKKKAKMREQERQASGGGDMFFMRTAQDLTGKDGDLILAEYSEEYPPLMMQVGMAT
KIKNYYKRKPGKDPGAPDCK

YGETVYCHTSPFLGSLHPGQLLQVRGHEYIPDVHLSSYSEKSCVVSPATAFENNLFRAPIYLHKMPETDFLVLRTRQGYF
IREIVDIVVVGQECPLFEVP
GPNSKRANTHIRDFLQVFIYRLFWKSKDRPRRIRMEDIKKAFPSHSESSIRKRLKLCADFKRTGMDSNWWVLKPDFRLPT
EEEIRAMVSPEQCCSYYSMQ
VAEQRLKDAGYGEKSFFAPDEENEEDFQMKIDDEVRTAPWNTTRAFIAAMKGKCLLEVTGVADPTGCGEGFSYVKVPNKP
TQQKDDKEPQPAKKTVTGTD
ADLRRLSLKNAKQLLRKFGVPEEEIKKLSRWEVIDVVRTMSTEQARSGEGPMSKFARGSRFSVAEHQERYKEECQRIFDL
QNKVLESTEVLSTDTDSSSA
EDSDFEEMGKNIENMLQNKKTSSQLSREREEQERKELQRMLMGEESERDHKGRKERRKGLCESTTGIV
>g2682.t3
KDRPRRIRMEDIKKAFPSHSESSIRKRLKLCADFKRTVAQEEDSPWFSIFPIDNEELVYGRWEDSIIWDDQAMARLLAPP
VLTLNPNDENIILEIPDEKE
QRTSSSPSKENKKESALKKSRILLGKTGVIKDEPQQPEVKDPWNLSNDEFYYPKQQGLRGTFGGNIIQHSIPAVELRQPF
FPTHMGPMKLRLFHRPSLKK
YSFGALAQPGPHPAQPLLKHIKKKAKMREQERQASGGGDMFFMRTAQDLTGKDGDLILAEYSEEYPPLMMQVGMATKIKN
YYKRKPGKDPGAPDCKYGET
VYCHTSPFLGSLHPGQLLQVRGHEYIPDVHLSSYSEKSCVVSPATVRSLVLSPPYSEHSNSPQAFENNLFRAPIYLHKMP
ETDFLVLRTRQGYFIREIVD
IVVVGQECPLFEVPGPNSKRANTHIRDFLQVFIYRLFWKSKDRPRRIRMEDIKKAFPSHSESSIRKRLKLCADFKRTGMD
SNWWVLKPDFRLPTEEEIRA
MVSPEQCCSYYSMQVAEQRLKDAGYGEKSFFAPDEENEEDFQMKIDDEVRTAPWNTTRAFIAAMKGKCLLEVTGVADPTG
CGEGFSYVKVPNKPTQQKDD
KEPQPAKKTVTGTDADLRRLSLKNAKQLLRKFGVPEEEIKKLSRWEVIDVVRTMSTEQARSGEGPMSKFARGSRFSVAEH
QERYKEECQRIFDLQNKVLE
STEVLSTDTDSSSAEDSDFEEMGKNIENMLQNKKTSSQLSREREEQERKELQRMLMGEESERDHKGRKERRKGLCESTTG
IV
>g2683.t1
MSTIRRKFGEDYQVVNTNRGTTFSAPVKRKRQRFVEKNGRCNVQHGNLGGETSRYISDLFTTLVDLKWRWNLLIFILTYT
VAWLVMASMWWVIAYIRGDL
SHGGHDASYTPCVANVYNFPSAFLFFIETEATIGYGYRYITEKCPEGIILFLFQSLLGSIVDAFLIGCMFIKMSQPKKRA
ETLMFSQDAVISQRDGKLCL
MFRVGNLRNSHMVSAQIRCKLIKLVLREVARYSAERWSVDRTSNFQASPLTVSPGDL
>g2684.t1
MRSEVAKGFSDFVRQRQPGEKAVEASNTSFNAKEMLLETEIQFIEAAKRNDVQAMKSARGINANVKNVEGLNILHCAAIN
NHCRILEYIIEDLQMQELDK
TDANGDTPLHLAAGNGHLDVVEMLLQSFETRDEINRAKRNALHPVAERGDTSLLRLLLDHKAQTDVQDQQQEAPLHLAVK
NLHIPAIYMLLEAGCNINVT
DKRSQSVMHLAAEQAKVDIVEMLLKAGVDLTLCDKHKKTALGVAARADEVVIADMIIKAERYSAWKAAHPELNESVHSQC
PLTFKLDHRPETKQFRSMVW
HLTHRLLKPGDWKRLAARWDFTKEQVDAIEVQWTGQHSYREHGNRMLLIWHHTVELAGKSPAKELYQGLIYTSNRTAADR
LRMEKESGGKNCNLS
>g2684.t2
MRSEVAKGFSDFVRQRQPGEKAVEASNTSFNAKEMLLETEIQFIEAAKRNDVQAMKSARGINANVKNVEGLNILHCAAIN
NHCRILEYIIEDLQMQELDK
TDANGDTPLHLAAGNGHLDVVEMLLQSFETRDEINRADETALYLATESGHEECAKRNALHPVAERGDTSLLRLLLDHKAQ
TDVQDQQQEAPLHLAVKNLH
IPAIYMLLEAGCNINVTDKRSQSVMHLAAEQAKVDIVEMLLKAGVDLTLCDKHKKTALGVAARADEVVIADMIIKAERYS
AWKAAHPELNESVHSQCPLT
FKLDHRPETKQFRSMVWHLTHRLLKPGDWKRLAARWDFTKEQVDAIEVQWTGQHSYREHGNRMLLIWHHTVELAGKSPAK
ELYQGLIYTSNRTAADRLRM
EKESGGKNCNLS
>g2684.t3
TAGQTQRADEKAPQSFFQRAYTKKLQLQAKGPSRPDKGPGGVGSGFTPRGPDDGKSGGGGPGPAIETRESTRGRPGSSGS
SCVTNVECEGAALPEAGAST
SSSSSPGADCETLTCPVCFKEVRTTDLSVFNTHIDRCLSQTTDTDSESDGDGGPEGPTAREEGAEGADKERQEKSKPEED
LGCFKTSEPLRGTSAARSED

TGNPGANEPPSNHRSFGEVHSSQKASSSRGGHPGMQTRSLGSRPDHGGPSLVCPVCQVPQNTDDLTAFNHHVDLCLNQEV
LHKLGEHSSSSSSSSSVRPP
MTSRKSNVLETEIQFIEAAKRNDVQAMKSARGINANVKNVEGLNILHCAAINNHCRILEYIIEDLQMQELDKTDANGDTP
LHLAAGNGHLDVVEMLLQSF
ETRDEINRAKRNALHPVAERGDTSLLRLLLDHKAQTDVQDQQQEAPLHLAVKNLHIPAIYMLLEAGCNINVTDKRSQSVM
HLAAEQAKVDIVEMLLKAGV
DLTLCDKHKKTALGVAARADEVVIADMIIKAERYSAWKAAHPELNESVHSQCPLTFKLDHRPETKQFRSMVWHLTHRLLK
PGDWKRLAARWDFTKEQVDA
IEVQWTGQHSYREHGNRMLLIWHHTVELAGKSPAKELYQGLIYTSNRTAADRLRMEKESGGKNCNLS
>g2684.t4
TAGQTQRADEKAPQSFFQRAYTKKLQLQAKGPSRPDKGPGGVGSGFTPRGPDDGKSGGGGPGPAIETRESTRGRPGSSGS
SCVTNVECEGAALPEAGAST
SSSSSPGADCETLTCPVCFKEVRTTDLSVFNTHIDRCLSQTTDTDSESDGDGGPEGPTAREEGAEGADKERQEKSKPEED
LGCFKTSEPLRGTSAARSED
TGNPGANEPPSNHRSFGEVHSSQKASSSRGGHPGMQTRSLGSRPDHGGPSLVCPVCQVPQNTDDLTAFNHHVDLCLNQEV
LHKLGEHSSSSSSSSSVRPP
MTSRKSNERDQPMARPNKGKSKRRGPPASPPAKKAKAMGSRNTIDKFFM
>g2685.t1
TNSPVSYLVGSVPTGRLQSDPSIRRLHIWLDTPTRDVAHDYHLASLLAVKYGLPFHVNLGQPTAASKHPPSAQLGHSLPA
TRGQGSSSRAHPITCHVHSI
KVVD
>g2686.t1
LIPPGACNILNSSAIYANNEVSLEEVDIYGFDYDYTLALYSNALDRMIYNTARDFLIEHFKGLLMKIDAFHYIQSGTVYR
GLKPVSDEEVIELYGGTHHV
PLNQDSGFYGKGPKMKQFMDVFSLPEMTLLAVANDYFISNDIEYDPVHLYKDVSEAIGMVHIKGYMYKWIMQDLEKFILS
GEETRAVLQRLVSHGKKLFL
ITNSPFSFVDKGMVYMVGENWREFFDIVIVQADKPHFFNDCVKPFRRLDSKGDLQWDKINSLEKGRIYKQDLMLRHGWRT
GAIVPELELETKVVNTKQYA
LGLTWLQALTGLLERMQTHRDPESQKVLQEWMKEREELRAVTKNLFNPQFGSIFRTCHNPTYFSRRLCRFSDVYMASISC
LLNYDLSYRFYPRRTPLQHE
APLWMDQLCTGCMKTPFLEEMANIR
>g2686.t2
LIPPGACNILNSSAIYANNEVSLEEVDIYGFDYDYTLALYSNALDRMIYNTARDFLIEHFKGLLMKIDAFHYIQSGTVYR
GLKPVSDEEVIELYGGTHHV
PLNQDSGFYGKGPKMKQFMDVFSLPEMTLLAVANDYFISNDIEYDPVHLYKDVSEAIGMVHIKGYMYKWIMQDLEKFILS
GEETRAVLQRLVSHGKKLFL
ITNSPFSFVDKGMVYMVGENWREFFDIVIVQADKPHFFNDCVKRLDSKGDLQWDKINSLEKGRIYKQDLMLRHGWRTGAI
VPELELETKVVNTKQYALGL
TWLQALTGLLERMQTHRDPESQKVLQEWMKEREELRAVTKNLFNPQFGSIFRTCHNPTYFSRRLCRFSDVYMASISCLLN
YDLSYRFYPRRTPLQHEAPL
WMDQLCTGCMKTPFLEEMANIR
>g2686.t3
LIPPGACNILNSSAIYANNEVSLEEVDIYGFDYDYTLALYSNALDRMIYNTARDFLIEHFKYPDGIHKYDYVPDFAARGL
HYDIQKGLLMKIDAFHYIQS
GTVYRGLKPVSDEEVIELYGGTHHVPLNQDSGFYGKGPKMKQFMDVFSLPEMTLLAVANDYFISNDIEYDPVHLYKDVSE
AIGMVHIKGYMYKWIMQDLE
KFILSGEETRAVLQRLVSHGKKLFLITNSPFSFVDKGMVYMVGENWREFFDIVIVQADKPHFFNDCVKPFRRLDSKGDLQ
WDKINSLEKGRIYKQDLMLR
HGWRTGAIVPELELETKVVNTKQYALGLTWLQALTGLLERMQTHRDPESQKVLQEWMKEREELRAVTKNLFNPQFGSIFR
TCHNPTYFSRRLCRFSDVYM
ASISCLLNYDLSYRFYPRRTPLQHEAPLWMDQLCTGCMKTPFLEEMANIR
>g2687.t1
ILNTTLFESWEIVGPYPSWWLFNLLLLLLQALHAYWSCLIVKTACRAVCRGRVWSELPLFELQVSKDDRSDMETSSDEDE
GPPAHQKPHSTAANGTNNQT
HEANGYGPCTEEPH
>g2687.t2

ILNTTLFESWEIVGPYPSWWLFNLLLLLLQALHAYWSCLIVKTACRAVCRGRVSKDDRSDMETSSDEDEGPPAHQKPHST
AANGTNNQTHEANGYGPCTE
EPH
>g2688.t1
MFKTANYPTVDPAPTIMHGTWFSTACGANTPVAVEKSKKKTFNLVKMMSLGNKKGQSKHQQQNNNMPFTIHCQIGKEIKH
ICSNCSRGNDTLDGE
>g2689.t1
METDDREDTQRRCRCHLWRACRAAPGVDDTSLITTVLLQTGGNAGWFARYRQSYKNRVQIQSSFGRKGGLSERSPAWQRG
PPSARVIDFKLSNKPGFQHP
SSPWVSWFWRSPVLPFDDNVCLREPCQNYMKCISVLRFNSSAPFISSPSILFRPIHPIAGLRCRCPAGFTGDYCETEINL
CYSNPCLNGGVCARTEGGFT
CICREDHTGESEGPGAVLMRGT
>g2690.t1
MHHHTPSPCPVSKGLGPPSSLTPTNLPPLWPLPGTTLPAFSPFWFTSEPQDTLAVRGAPALLNCSAHTGAAAAATVHVEW
KKDGTFMTLASDERRKVLAD
GSLLITQVVHSKHNKPDEGTYQCVANIDNLGSISSRAARLSVA
>g2690.t2
MHHHTPSPCPVSKGLGPPSSLTPTNLPPLWPLPGTTLPAFSPFWFTSEPQDTLAVRGAPALLNCSAHTGAAAAATVHVEW
KKDGTFMTLASDERRKVLAD
GSLLITQVVHSKHNKPDEGTYQCVANIDNLGSISSRAARLSVADDSPPGGDYVRGRSVR
>g2691.t1
GAGALRMPASRRAVKVCLWCLCVRYGDYPHLRRNESPYVMLKKNHEQLLGNDKYEGYIVELAAEIAKHVGYHYKLKIVTD
GKYGAKDPEFKMWNGMVGEL
VYGKADVAVAPLTITLVREEVIDFSKPFMSLGISIMIKKPTKSKPGVFSFLDPLAYEIWMCIVFAYIGVSVVLFLVSRFS
PYEWHAEDYEEGGEPQSPTQ
ASASNGAQQGQAASPQNPAQQSPEQNNEFGIFNSLWFSLGAFMQQGCDISPR
>g2691.t2
GAGALRMPASRRAVKESPYVMLKKNHEQLLGNDKYEGYIVELAAEIAKHVGYHYKLKIVTDGKYGAKDPEFKMWNGMVGE
LVYGKADVAVAPLTITLVRE
EVIDFSKPFMSLGISIMIKKPTKSKPGVFSFLDPLAYEIWMCIVFAYIGVSVVLFLVSRFSPYEWHAEDYEEGGEPQSPT
QASASNGAQQGQAASPQNPA
QQSPEQNNEFGIFNSLWFSLGAFMQQGCDISPR
>g2692.t1
MPRLIGNQIRAIMDKKANIRNMSVIAHVDHGKSTLTDSLVSKAGIIASARAGETRFTDTRKDEQERCITIKSTAISLFYE
LDDNDTAFIKQGINGNGFLI
NLIDSPGHVDFSSEVTAALRVTDGALVVVDCVSGKENGQ
>g2692.t2
MDKKANIRNMSVIAHVDHGKSTLTDSLVSKAGIIASARAGETRFTDTRKDEQERCITIKSTAISLFYELDDNDTAFIKQG
INGNGFLINLIDSPGHVDFS
SEVTAALRVTDGALVVVDCVSGKENGQ
>g2692.t3
MPRLIGNQIRAIMDKKANIRNMSVIAHVDHGKSTLTDSLVSKAGIIASARAGETRFTDTRKDEQERCITIKSTAISLFYE
LDDNDTAFIKQGINGNGFLI
NLIDSPGHVDFSSEVTAALRVTDGALVVVDCVS
>g2693.t1
MATPSKTPPGADPKQLERTGTVREIGSQAVWSLSSCKPGFGVDQLRDDNLETYWQSDGSQPHLVNIQFRRKTTVKMLCIY
ADYKSDESYTPSKISVRVGN
NFHNLQEVRQLEMVEPSGWIHISLLDAANSPIRTFMIQVAVLANHQNGRDTHMRQIKIYTPVEESSIGKFPRCTTVDFMM
YRTIR
>g2694.t1
MRSLSIIKERLVFKVGIQGPHTVEEVFEVQLDSLQLQPLQRIQNAAARLVFNRNKFSHVTPLFQDLHWLPVVACIKFKTM
LLAYKAANGTAPTYLQTLVK
PHAPAEHSAQLPQLVNWYRHH
>g2695.t1
MFPAKASSLQNADCMGPLIETLKNSELSSVLSSCEPELQELMRQIDIMMDHKRRDWEAEVRALKLRLQTGEQELRAARSL
GEQRDMEMGLLREQLEDAQG

VRGNLAAHYEEQLQKVHEELSKLKRSYQKLQRKHLKEASEGEKGREELHLKDKAELRPEDSGEVLQLQREVSRLQQALQA
QDHDIRVQQAPRPRPEEELK
EQLSRATEERAVAVSDARKLGAELQRCRQTHAGEVGGVKKEVSRLTGELHQRDLTIAALQGSASAAQRQLSAEMQRAERR
EAELTVLQVKLDSLKTENQR
LSGLLERLDPSQSPKRASACRGSVMEKAMLSLSSLEQENQQLRQEVTQIKSRLQASASPPTQAKSGQSQSTWLGSGGGAG
REKQEKEEVQATKAKLQENT
THHGEEIQRVLARLDELSPGSRQTHRLPDPPSSSSSSSSSSWAIPGRRESGRVRLGHEEGVTEGRHSSSSEEVLDPLEGG
DEETELAAATDAVVSRFLEG
ETLRSEELLLRLETHVQGLREDHARTARKYQPALTSTMTSTMTSTLSPGPSAP
>g2695.t2
MFPAKASSLQNADCMGPLIETLKNSELSSVLSSCEPELQELMRQIDIMMDHKRRDWEAEVRALKLRLQTGEQELRAARSL
GEQRDMEMGLLREQLEDAQG
VRGNLAAHYEEQLQKVHEELSKLKRSYQKLQRKHLKEASEGEKGREELHLKDKAELRPEDSGEVLQLQREVSRLQQALQA
QDHDIRVQQAPRPRPEEELK
EQLSRATEERAVAVSDARKLGAELQRCRQTHAGEVGGVKKEVSRLTGELHQRDLTIAALQGSASAAQRQLSAEMQRAERR
EAELTVKLDSLKTENQRLSG
LLERLDPSQSPKRASACRGSVMEKAMLSLSSLEQENQQLRQEVTQIKSRLQASASPPTQAKSGQSQSTWLGSGGGAGREK
QEKEEVQATKAKLQENTTHH
GEEIQRVLARLDELSPGSRQTHRLPDPPSSSSSSSSSSWAIPGRRESGRVRLGHEEGVTEGRHSSSSEEVLDPLEGGDEE
TELAAATDAVVSRFLEGETL
RSEELLLRLETHVQGLREDHARTARKYQPALTSTMTSTMTSTLSPGPSAP
>g2695.t3
MFPAKASSLQNADCMGPLIETLKNSELSSVLSSCEPELQELMRQIDIMMDHKRRDWEAEVRALKLRLQTGEQELRAARSL
GEQRDMEMGLLREQLEDAQG
VRGNLAAHYEEQLQKVHEELSKLKRSYQKLQRKHLKEASEGEKGREELHLKDKAELRPEDSGEVLQLQREVSRLQQALQA
QDHDIRVQQAPRPRPEEELK
EQLSRATEERAVAVSDARKLGAELQRCRQTHAGEVGGVKKEVSRLTGELHQRDLTIAALQGSASAAQRQLSAEMQRAERR
EAELTVLQVKLDSLKTENQR
LSGLLERLDPSQSPKRASACRGSVMEKAMLSLSSLEQENQQLRQEVTQIKSRLQASASPPTQAKSGQSQSTWLGGGGAGR
EKQEKEEVQATKAKLQENTT
HHGEEIQRVLARLDELSPGSRQTHRLPDPPSSSSSSSSSSWAIPGRRESGRVRLGHEEGVTEGRHSSSSEEVLDPLEGGD
EETELAAATDAVVSRFLEGE
TLRSEELLLRLETHVQGLREDHARTARKYQPALTSTMTSTMTSTLSPGPSAP
>g2696.t1
MGTAKGLTAELAVGRKHARHALLDWRPHKRLCQSWRLSSGPGPPLAPGTFLQDRAPHEKFIPHLLKIQGDTEAWVSSNSR
GSTGGLSRESETA
>g2697.t1
VLIELDSALLDDQPHWYKIQSHHVTSHPLPNSSPYLQRHLYRSLRTHDAEFSDYDCEDGIGVISGMAALHEAVLTGNLEV
VQLLVRRLMRRVSAANSLDF
SGSGPSSAPWPNPMVNVSCVDYSPDFIPHFPFEPDALDEELLRASKGMDCHDNPSRSRSVDLQSDHSPSAQRPAPSTPAL
IR
>g2697.t2
VLIELDSALLDDQPHWYKIQSHHVTSHPLPNSSPYLQRHLYRSLRTHDAEFSDYDCEDGIGVISGMAALHEAVLTGNLEV
VQLLVRRLMRRVSAANSLDF
SGSPDFIPHFPFEPDALDEELLRASKGMDCHDNPSRSRSVDLQSDHSPSAQRPAPSTPALIR
>g2697.t3
VLIELDSALLDDQPHWYKIQSHHVTSHPLPNSSPYLQRHLYRSLRTHDAEFSDYDCEDGIGVISGMAALHEAVLTGNLEV
VQLLVRRLMRRVSAANSLDF
SGSGPSSAPWPNPMVNVSCVDYSPDFIPHFPFEPDALDEELLSTPLNYLSRSTGTAITKKRKRASKGMDCHDNPSRSRSV
DLQSDHSPSAQRPAPSTPAL
IR
>g2698.t1
SYPRLSLSFKLKRNIGYFILQTYMPSTLITILSWVSFWINYDASAARVALGL
>g2699.t1
MFRGITTVLTMTTINTHLRETLPKIPYVKAIDIYLMGCFVFVFLALLEYAFVNYIFFGRGPHLQKKVAEKAAKTNNENKS
RLESNKVQ

>g2700.t1
LFLCCQSASVEAVTELTEFAKNIPGFLHLDLNDQASRALVRRFDAALQPRGCREGGGGEGGVVHNRAGVKIRHLEFPLEA
ESSRSSAAIAAASPQDILME
AVTLLKYGVYEALFTLLASCMNKDGLLVARGGGFITREFLKSLRRPFSDMMEPKFQFATRFNALQLDDSDLALFISAIIC
CGDRPGLVNASLVERLQESI
VQALQLHLEANHRDDAFLFPKLLQKLADLRELVTEHAQLVQDIETTEDTSLHPLLQEIYRDMY
>g2700.t2
LFLCCQSASVEAVTELTEFAKNIPGFLHLDLNDQPGTGCRGMVKGRDELICDVEELTAEGGITVTDKLQASRALVRRFDA
ALQPRGCREGGGGEGGVVHN
RAGVKIRHLEFPLEAESSRSSAAIAAASPQDILMEAVTLLKYGVYEALFTLLASCMNKDGLLVARGGGFITREFLKSLRR
PFSDMMEPKFQFATRFNALQ
LDDSDLALFISAIICCGDRPGLVNASLVERLQESIVQALQLHLEANHRDDAFLFPKLLQKLADLRELVTEHAQLVQDIET
TEDTSLHPLLQEIYRDMY
>g2700.t3
MVKGRDELICDVEELTAEGGITVTDKLQASRALVRRFDAALQPRGCREGGGGEGGVVHNRAGVKIRHLEFPLEAESSRSS
AAIAAASPQDILMEAVTLLK
YGVYEALFTLLASCMNKDGLLVARGGGFITREFLKSLRRPFSDMMEPKFQFATRFNALQLDDSDLALFISAIICCGDRPG
LVNASLVERLQESIVQALQL
HLEANHRDDAFLFPKLLQKLADLRELVTEHAQLVQDIETTEDTSLHPLLQEIYRDMY
>g2701.t1
MMDVLLRDLLPDLDQGISEPLWCDVVLVDGMRHDVLDVLDWIQVWGTGRPVHSMNAFVVQELLTDSSHMRPSIVMHEKEP
RAHCTSIWSNNGSKDLASTW
RSVRPSKDMPPQTITDPPTNRSSWRMLQAAERSPRHLQICHMSSV
>g2702.t1
MDGPLQNTFTCDLCGLSTPFTYFGQKPPNTRAIVLLEECFVTKDPFSPDKERFLVLGSKCSKCGCSACVASDCSLFYTTR
FCMRCVNEHLDQFPTQIQSE
VARRKHAPKTAVP
>g2703.t1
MSDVRLSTASPTLERGVGVGDALPAEPAKPSVCRNLFGNFDRGGFDEDCEATMRTHRRSFRERWHYDPVADRPLPSGGFE
WEAVDSPPEFYVRPAHRRPR
PRGEVDLDAGDRRPEPGEDERPPDREPVQFSRKRRARLLDGFSSGCPAKHSQSDEDGDQPDPLEVPTPTLAEQTPRKPES
QTESNWYEFTSL
>g2703.t2
MSDVRLSTASPTLERGVGVGDALPAEPAKPSVCRNLFGNFDRGGFDEDCEATMRTHRRSFRERWHYDPVADRPLPSGGFE
WEAVDSPPEFYVRPAHRRPR
PRGEVDLDAGDRRPEPGEDERPPDREPVQFSRKRRARLLDGFSSGCPAKHSQSDEDGDQPDPLEVPTPTLAEQTPRKPES
QTESNXTLVDIGG
>g2704.t1
MPGGRAQDGEILRCEDSAYSVRFVPAETARPPGAPPSSPWGPRGRPAGGLSIAVDGPSKAEITFEDRKDGSCGVASVVQE
PEWVSNLVATHEKETGDVRI
CIDPRNLSKALMRPHHPMRTMEGGCRSDVSAASEVFQRAMEQIFAGYACAIIVGDIVIAGKDPDEHEGNLKKVLDLARQL
WSDGRSINCQKAFDRLKTCI
SSPPVVHYYDTETRYVQIEKELLAVVFACHKFDDCIYGKPLVIETDHQPSVTIHNKPFHTIPARLQRMMLHFLVLAVLAV
WSWIFQSDGVRDASSECTGT
TNPAQLKPFWAPRLRQAGLLSRLWQRQQP
>g2704.t2
MPGGRAQDGEILRCEDSAYSVRFVPAETARPPGAPPSSPWGPRGRPAGGLSIAVDGPSKAEITFEDRKDGSCGVASVVQE
PEWVSNLVATHEKETGDVRI
CIDPRNLSKALMRPHHPMRTMEGGCRSDVSAASEVFQRAMEQIFAGYACAIIVGDIVIAGKDPDEHEGNLKKLWSDGRSI
NCQKAFDRLKTCISSPPVVH
YYDTETRYVQIEKELLAVVFACHKFDDCIYGKPLVIETDHQPSVTIHNKPFHTIPARLQRMMLHFLVLAVLAVWSWIFQS
DGVRDASSECTGTTNPAQLK
PFWAPRLRQAGLLSRLWQRQQP
>g2704.t3
MPGGRAQDGEILRCEDSAYSVRFVPAETARPPGAPPSSPWGPRGRPAGGLSIAVDGPSKAEITFEDRKDGSCGVASVVQE
PGPEAIERERSFTDAPPVLG

EDDDVVVPAAMQKKVEQELQRTQTLRVIEPVDAPTEWVSNLVATHEKETGDVRICIDPRNLSKALMRPHHPMRTMEGGCR
SDVSAASEVFQRAMEQIFAG
YACAIIVGDIVIAGKDPDEHEGNLKKVLDLARQLWSDGRSINCQKAFDRLKTCISSPPVVHYYDTETRYVQIEKELLAVV
FACHKFDDCIYGKPLVIETD
HQPSVTIHNKPFHTIPARLQRMMLHFLVLAVLAVWSWIFQSDGVRDASSECTGTTNPAQLKPFWAPRLRQAGLLSRLWQR
QQP
>g2705.t1
MRGRRGRPPKALPTEEEQSQAPVHGLRPRRNLKPRARDSGDEDTESPKRERSRSARGKRRRAAGTSTRGRGRGRGAGYGR
GGRGGRRPAASKTIVYDDHE
SEDDEDDAVSLRSEEEEVVEEEPVSEEEEEEEEEEALKEECEGLDDVLEEDEGDGDDSASHCTESSCRSHTSTPGRRRPR
AHQPRTPILEQKEIPALVLP
ESSEDLLIPKNELLSVASVYEVLRSFSGVLRLSPFRFEDFCAALVGGEQCTLIAETHIALLKAILREEDSSNTSFGPADL
KDSVNSTLYFVDGMTWPEVV
RAYCESDAEYRHVLPWQEGEEFPYGPLRSKVKVLQFLTDQFLTTNMAREELMSEGAVISDDHCRVCHRLGDLLCCETCSA
VYHLECVRLDTVPEDEWQCE
ICAAHRVAGVTDCVSEMQRSRPYIRAEPVGYDRHERKYWFLNRRIIVEEEGEKEQKQVWYYSSRQQLEELMEVLDRQYWE
SDLCAALEEGENKLPGLMRK
KSEKEHGMGQPSFQGC
>g2706.t1
MAFAEIIAARLKLKREEQAARRSAEEAEGAKPAPERASARTETSSPEERPGSGEQAHSGTEAPPAVEEGNDPSPAPATAP
LDPAHVAPASSQENHTPSSE
TSQEDTADAGGKTERSSEEKEAPQSQPAEEQSSGVPGGPEEPDLVDRSSQWSFTSKDGSEDGVKLSGGGTASGGPGAADQ
SLSSNKESSPTPPATSTTTT
TSSTLAKELAGLAELKANASSLFRLGQEGKYRVYHNQYSTNVLALNKHQHRDDHDKRRHLSHKFSLTAAADFKWNGSIHG
SRVLTVSTLRLTIIQLESSV
PGPFMHP
>g2707.t1
MPRHNDQHMRNHWTSDPTKNITTGTITHIPTLPCFQHGGGSWRCFSQGQCTPVPLPSLGTQRIIQGNGTNVGTVISLLCP
DQHRLIGSNMECVMRTNSTQ
WVGETYCRPMSAYGQLGHHVAVLASVVSACIILMLSMAFITCCLHDRFKRKETKRRNHKRDIEEEEWQRYQCVRGRTACS
HHHSRNYNNNNSHRKAGAMG
RGGHDGALVDDKVPCRSCLHPYCDLTAPPSTPLPGESYYPPPPTRDMRLDRDGAGPLQNSRHLQKNPPAPHGPSCQTGRG
ARTSGPAQSLAACSAGGRGA
DAGRQFGGQEHRMPLLSQHASQETYIGNSNKECSIRIITVNIAVATRGRLAELLVHLQQEGKEACLEFCRALQLNAEDCT
LPSRHGLRGPRFFLGCSCLT
ADLAVLCYRRR
>g2707.t2
MPRHNDQHMRNHWTSDPTKNITTGTITHIPTLPCFQHGGGSWRCFSQGQCTPVPLPSLGTQRIIQGNGTNVGTVISLLCP
DQHRLIGSNMECVMRTNSTQ
WVGETYCRPMSAYGQLGHHVAVLASVVSACIILMLSMAFITCCLHDRFKRKETKRRNHKRDIEEEEWQRYQCVRGRTACS
HHHSRNYNNNNSHRKAGAMG
RGGHDGALVDDKVPCRSCLHPYCDLTAPPSTPLPGESYYPPPPTRDMRLDRDGAGPLQNSRHLQKNPPAPHGPSCQTGRG
ARTSGPAQSLAACSAGGRGA
DAGRQFGGQEHRMPLLSQHASQETYIGNSNKECSIRIITVNIAVATRGRLAELLVHLQQEGKEACLEFCRALQLNAEDCT
LPSRHGLRGPRFFLGCSCLT
ADLAVLCYRR
>g2707.t3
MPRHNDQHMRNHWTSDPTKNITTGTITHIPTLPCFQHGGGSWRCFSQGQCTPVPLPSLGTQRIIQGNGTNVGTVISLLCP
DQHRLIGSNMECVMRTNSTQ
WVGETYCRPMSAYGQLGHHVAVLASVVSACIILMLSMAFITCCLHDRFKRKETKRRNHKRDIEEEEWQRYQCVRGRTACS
HHHSRNYNNNNSHRKAGAMG
RGGHDGALVDDKVPCRSCLHPYCDLTAPPSTPLPGESYYPPPPTRDMRLDRDGAGPLQNSRHLQKNPPAPHGPSCQTGRG
ARTSGPAQSLAACSAGGRGA
DAGRQFGGQEHRMPLLSQHASQETYIGNSNKECSIRIITVNIAVATRGRLAELLVHLQQEGKEACLEFCRALQLNAEDCT
LPSRHGLRGPRFFLGCSCLT
ADLAVLCYRRRR

>g2708.t1
MDVLPIKEEGTEEGSWHSQGQSFDTSSVTEISSPLAKDISNKEVDMVSWGRESLEQMQALQRGDLVSGNDIDVLPIKEEC
TEEGTWHNGHQGQSTRAEPL
ASEDFADHYHSESPPLLPPLSDRPEAFPRLPEQQLLHDQTHAELAMKREESNKALACALVSGGTFLRAEEEEEEEEEEEE
ARGHQWAHADCSSDDTGSGD
PGFLYGATRPQYDQSPSLFKNGLPAIRYLSVDLGPPVHSTGKLHLASRLTAARARRRARNLSYKRLQQTHAQGEAHHSGS
GLQPTTQQQQQQQQQQQQQL
QQQHQQQQYMAAAGDMRDVSQGTSAAANHLGFCSNSRGNVALAKARMRALSWRSGGVGDKRFCCTYCDKSFVRFCQLEEH
LRRHTGEKPFSCLQCGRSFT
KQCNLIRHAVVHSGEKPHQCATCGKCFTQRSSLKSHQKTAH
>g2709.t1
MDMLAKTAIVEISKLWDDAFAFVQLEIRQREDEIESLKSKVLMMEKERMELVSKRPPSAPPAAPCFSTPQQPNHLGKPDD
NGPIIVVIQTKSLPNARETA
DTLQNSSTSHERQLELRGSWHDEADEELGVVEPKKEAAESSVVGLKEEAVDDVIQMGDPAQDMGHNSDHGSNHTATEADT
QAAEGAMEDSQQWSSVSIAD
SDDTDDSDCIFRPQQFSESLDKEMKFIQNALDSLDNNQTEAGYIDRIIQTQPGQPMDVQVMGLVPTDKHCPSDQYDLMQI
RALKNCRVREKWFICPFCGK
SFDRSSHLEMHRRIHTGEKPYTCAICGRCFSQRSNLRTHQRTHKEYFPHSLSTELNHQH
>g2709.t2
MLAKTAIVEISKLWDDAFAFVQLEIRQREDEIESLKSKVLMMEKERMELVSKRPPSAPPAAPCFSTPQQPNHLGKPDDNG
PIIVVIQTKSLPNARETADT
LQNSSTSHERQLELRGSWHDEADEELGVVEPKKEAAESSVVGLKEEAVDDVIQMGDPAQDMGHNSDHGSNHTATEADTQA
AEGAMEDSQQWSSVSIADSD
DTDDSDCIFRPQQFSESLDKEMKFIQNALDSLDNNQTEAGYIDRIIQTQPGQPMDVQVMGLVPTDKHCPSDQYDLMQIRA
LKNCRVREKWFICPFCGKSF
DRSSHLEMHRRIHTGEKPYTCAICGRCFSQRSNLRTHQRTHKEYFPHSLSTELNHQH
>g2710.t1
LAQQHQKGVAAHELSAAVCEGDLRPLSVSGPVVCAELDEVFLPLGPLLGHPKTLREFLTHNVSSERRREDVLGPSAVSPR
PRPDDLMFPMFLENMIHTAG
RLASEVQPGPVACV
>g2711.t1
QHYERQQMLVNKLTREMRLEPDSDTTQGTRHQLSRRYAWRSESLSPSCKRSLRSLRRTFRSVVVVSLRVPSSHSRHVNRR
SDSINASLTAFIHGATRLSS
DGVEP
>g2711.t2
QHYERQQMLVNKLTREMRLEPDSDTTQGTRHQLSRRYAWRSESLSPSCKRSLRRCGAVRQIQTDSAENRCVPGGCA
>g2711.t3
MLVNKLTREMRLEPDSDTTQGTRHQLSRRYAWRSESLSPSCKRSLRSLRRTFRSVVVVSLRVPSSHSRHVNRRSDSINAS
LTAFIHGATRLSSDGVEP
>g2712.t1
MPPGRLPLVVFRAHPTVKRPRGRPRTRWRDYISHLAWERLGVPQEELESVAGEKEVWSSLLSLLPPRPHPGYAEEE
>g2713.t1
MEAALSTPHNIQICEVTCDSFRIVWDMSPEDTARATHFFIDLSRKESRDPNRFKHRDVPTKLVAKAVPLPMAVRGHWFLS
PRTEYCVAVQTAVRQPDGDY
LVSEWSQVVEFCTGDYAMEHLQQLLDKATGSTGRLLKFSVFYRNQHPDYFENVRNECGGLMRPSLKDNSGSHGSPINGKL
NGVFLSCNTEFDTGLPPKDS
PYGSLRFQIPAGHLLNPNTNIYFADFYCMYTAYHYVVLVLAPGGSDGDNFCRGRLPRLELSSNPFLTFSAPQMPGEEPLF
CHASDVILEVLYTEPLHLEL
GTVEQISGHHQLMSLTTANAKKDPSCKVCNISVGR
>g2714.t1
LWRYKQHAGQDVLLSRITGTIYDCWPDNVFSMMVQPAVRHQMNWKKMTDTTALPMLTLGSRAQPAGLVEIAVRLCLNQRK
ELCPHVWVPILKPQRPCIRP
PPAPHRLSLDTRCQGCADPLMSFLLDEALDDDMYLSAVDPGKKKRVVFADSKGLSLTDVRVFSEDQDSGRCSPQPVDPPA
SLRRLGAIIGPVLSEEDRYS
CTVSTCCPGTRLQLGFPQPSADFQALRGKLARSTVVLESCAVLTAESSSVLRGTARVKNVSFHKDVRVRITFDSWQSYRD
EPCTYVETRYGGPQTDIFEF

DIHVPKVLDAKKKIEFCLRYSPDGCSQPFWDNNDGQNYGVHICVSSHLCQGENAKQ
>g2714.t2
MVQPAVRHQMNWKKMTDTTALPMLTLGSRAQPAGLVEIAVRLCLNQRKELCPHVWVPILKPQRPCIRPPPAPHRLSLDTR
CQGCADPLMSFLLDEALDDD
MYLSAVDPGKKKRVVFADSKGLSLTDVRVFSEDQDSGRCSPQPVDPPASLRRLGAIIGPVLSEEDRYSCTVSTCCPGTRL
QLGFPQPSADFQALRGKLAR
STVVLESCAVLTAESSSVLRGTARVKNVSFHKDVRVRITFDSWQSYRDEPCTYVETRYGGPQTDIFEFDIHVPKVLDAKK
KIEFCLRYSPDGCSQPFWDN
NDGQNYGVHICVSSHLCQGENAKQ
>g2715.t1
LDDSEEQELSPQLESLLERVACCNWEAESLAGGGGGGGSGGMDGDSCINGIADEGYSGQEDEEQEEEEEEEEVDEGVPRR
RRPVCTLAKVMALCASRLAN
PPLAAEHYQAVCRALFVETLELQTFLTKIRDAKEMLKKIRTDETPEDKSSAELGALKHTDWARLWVQLMKELRQGVKLKK
VGEQPFNPLPTEFSLTPFEM
LMQDIRSRKIQLRKVMVDGDIPTRVKRNAHELILDFIRSRPPLKPVSLPTQEPESLHQRVLAEIKQERKLRPVVPPASRS
FGSMPCLAQCCPCNVKSTSC
IDLSVSDAGLRAPARPRVLLKAPTLAEMEEMNVSEVRRGHGGPRRQ
>g2715.t2
LESLLERVACCNWEAESLAGGGGGGGSGGMDGDSCINGIADEGYSGQEDEEQEEEEEEEEVDEGVPRRRRPVCTLAKVMA
LCASRLANPPLAAEHYQAVC
RALFVETLELQTFLTKIRDAKEMLKKIRTDETPEDKSSAELGALKHTDWARLWVQLMKELRQGVKLKKVGEQPFNPLPTE
FSLTPFEMLMQDIRSRKIQL
RKVMVDGDIPTRVKRNAHELILDFIRSRPPLKPVSLPTQEPESLHQRVLAEIKQERKLRPVVPPASRSFGSMPCLAQCCP
CNVKSTSCIDLSVSDAGLRA
PARPRVLLKAPTLAEMEEMNVSEVRRGHGGPRRQ
>g2715.t3
LERVACCNWEAESLAGGGGGGGSGGMDGDSCINGIADEGYSGQEDEEQEEEEEEEEVDEGVPRRRRPVCTLAKVMALCAS
RLANPPLAAEHYQAVCRALF
VETLELQTFLTKIRDAKEMLKKIRTDETPEDKSSAELGALKHTDWARLWVQLMKELRQGVKLKKVGEQPFNPLPTEFSLT
PFEMLMQDIRSRKIQLRKVM
VDGDIPTRVKRNAHELILDFIRSRPPLKPVSLPTQEPESLHQRVLAEIKQERKLRPVVPPASRSFGSMPCLAQCCPCNVK
STSCIDLSVSDAGLRAPARP
RVLLKAPTLAEMEEMNVSEVRRGHGGPRRQ
>g2716.t1
EPLDQKYSHVSHQALSLMKGCLRMDPCERLTCEQLLEHHYFDSLREKTNGSSREHERPTNKRPRLPRKHLPPGYLPQLTG
SSIFPALDNRKYYNNLRKFN
YHLPNI
>g2716.t2
EPLDQKYSHVSHQALSLMKGCLRMDPCERLTCEQLLEHHYFDSLREKTNGSSREHERPTNKRPRLPRKHLPPGETGEKEK
IYCLSFAKCMFLYLRLTRPD
RRPAHRLLGVLKALIAAATKTDTHRTPLLCKQFITTPVSMTTAACHR
>g2716.t3
EPLDQKYSHVSHQALSLMKGCLRMDPCERLTCEQLLEHHYFDSLREKTNGSSREHERPTNKRPRLPRKHLPPGETGEKEK
IYCLSFAKCMFLYLRLTRPD
RRPAHRLLGVLKALIAAATKTDTHRTPLLCKQFITTPVSMTTAACHRKRTVR
>g2717.t1
LAARFSLLHGRVPHTDGGRGYPSATRPDPPANQTARRHHGAPPIQSWGGAPPTTSRRRRLIGRRVMRHRGAATKVKGSPA
EGCKQSTPARGHCGNMRLLC
GAVRSVVWRGSGTTRLFWGWLNAVFNRVDYERMKTVGPDRAAAEWLLRCGAKVRFLGFERWHHDYNALPTGPLGRYKIQG
IDATESCIMYRGFDHLDGLQ
HLEEVKLIQCVYIEDVCLERLSSTESLQGSLSSLELAACGNVSDRGVLALHRLGNLQRLVLRDLPGVKDHEKTLERLRTA
LPHLDITVDL
>g2717.t2
LHGRVPHTDGGRGYPSATRPDPPANQTARRHHGAPPIQSWGGAPPTTSRRRRLIGRRVMRHRGAATKVKGSPAEGCKQST
PARGHCGNMRLLCGAVRSVV
WRGSGTTRLFWGWLNAVFNRVDYERMKTVGPDRAAAEWLLRCGAKVRFLGFERWHHDYNALPTGPLGRYKIQGIDATESC

IMYRGFDHLDGLQHLEEVKL
IQCVYIEDVCLERLSSTESLQGSLSSLELAACGNVSDRGVLALHRLGNLQRLVLRDLPGVKDHEKTLERLRTALPHLDIT
VDL
>g2718.t1
MRLLVSVVGAAKRKVPNGVTARYLHSSYDVAVVGAGIVGLATARELILRHPNLSFVLLEKEKELSLHQSGHNSGVIHSGI
YYTPGSLKARLCVRGATLAY
EYCDRKGLPYKRCGKLIVAVDQEEVPRLQALYERGLKNNVRDL
>g2718.t2
MMRLLVSVVGAAKRKVPNGVTARYLHSSYDVAVVGAGIVGLATARELILRHPNLSFVLLEKEKELSLHQSGHNSGVIHSG
IYYTPGSLKARLCVRGATLA
YEYCDRKGLPYKRCGKLIVAVDQEEVPRLQALYERGLKNNVRDL
>g2719.t1
MSFLRRVAGLSLRDRVRSSDIRRELGVEPLLLRVERSQLRWFGHLIRMPPGRLPLEVFRARPTGKRPRGRPRTRWRDYIS
HLAWERLGVPQEELESVAGE
KDVWSSLLSLLPPRPGPG
>g2720.t1
MQNLYLFLAQPNCLVHLDLSGTDCTVDLVSSPGLIVSFSSLPCDCVKAGRRFSRRPHSRGALRLSPRRDSPRFLNLQMLS
SITILTAVHVVGYVVSYTRP
GERLGTEVYSK
>g2720.t2
MQNLYLFLAQPNCLVHLDLSGTDCTVDLVSSPGLIVSFSSLPCDCVKAGRRFSRRPHSRGALRLSPRRDSPRFLNLQMLS
SITILTAVHVVGYVVSYTRP
VYSK
>g2720.t3
MQNLYLFLAQPNCLVHLDLSGTDCTVDLVSSPGLIVSFSRDSPRFLNLQMLSSITILTAVHVVGYVVSYTRPGERLGTEV
YSK
>g2721.t1
MTPTPGFNTREQDPIPRAPEPVSQGDPNCKVDLAFLMDGSWSIGKRRFKIQKDFLAEVAQVINLGVAGPMMGVIQYGDDP
VTEISLKSYSNSRDMKAAVE
KVVQKGGLSNVGKALSFINRQYFSDSNGNRGAAPNVAVVLVDGWPTDKVEEASRLARESGINIFFVTIEGPDENEKQNVV
EANFVDK
>g2721.t2
LTPLPLPQLPPPPYPTAILPPPIATLPPPYPTPTIPPPYLHPTSTYRHPTFTLPYLHPSDPPFVSLHPSTAFNRVQPAPA
AERNPSTGPSNPALQQYSHA
APGSALESPRLAPANTGPAGPRPYGNPEPEALFHDEIGRLPPSPVLIGSPERGDRQIHDLLIDSEPSVGKQPPPSLIGAP
CSPPTALSDVSYVAPDAGYT
WGETDAPEGTPPRSSDSEGNRKPVVDGTHTRVEPVDSWRPDTPLYESGFNTREQDPIPRAPEPVSQGDPNCKVDLAFLMD
GSWSIGKRRFKIQKDFLAEV
AQVINLGVAGPMMGVIQYGDDPVTEISLKSYSNSRDMKAAVEKVVQKGGLSNVGKALSFINRQYFSDSNGNRGAAPNVAV
VLVDGWPTDKVEEASRLARE
SGINIFFVTIEGPDENEKQNVVEANFVDK
>g2721.t3
LPQLPPPPYPTAILPPPIATLPPPYPTPTIPPPYLHPTSTYRHPTFTLPYLHPSDPPFVSLHPSTAFNRVQPAPAAERNP
STGPSNPALQQYSHAAPGSA
LESPRLAPANTGPAGPRPYGNPEPEALFHDEIGRLPPSPVLIGSPERGDRQIHDLLIDSEPSVGKQPPPSLIGAPCSPPT
ALSDVSYVAPDAGYTWGETD
APEGTPPRSSDSEGNRKPVVDGTHTRVEPVDSWRPDTPLYESGFNTREQDPIPRAPEPVSQGDPNCKVDLAFLMDGSWSI
GKRRFKIQKDFLAEVAQVIN
LGVAGPMMGVIQYGDDPVTEISLKSYSNSRDMKAAVEKVVQKGGLSNVGKALSFINRQYFSDSNGNRGAAPNVAVVLVDG
WPTDKVEEASRLARESGINI
FFVTIEGPDENEKQNVVEANFVDK
>g2721.t4
LTPLPLPQLPPPPYPTAILPPPIATLPPPYPTPTIPPPYLHPTSTYRHPTFTLPYLHPSDPPFVSLHPSTAFNRVQPAPA
AERNPSTGPSNPALQQYSHA
APGSALESPRLAPANTGPAGPRPYGNPEPEALFHDEIGRLPPSPVLIGSPERGDRQIHDLLIDSEPSVGKQPPPSLIGAP
CSPPTALSDVSYVAPDAGYT

WGETDAPEGTPPLLVEHWSLHLTLLVQLKVCQKVSDMTVGDAEV
>g2722.t1
ACRYGHLQHLEQLLLFGADISSQNATGNTGLHISALHKQTTNDSNESIPLSFELNQLASDEHTFA
>g2722.t2
MWRISRVHVGRCEACRYGHLQHLEQLLLFGADISSQNATGNTGLHISALHKQTTNDSNESIPLSFELNQLASDEHTFA
>g2723.t1
PRQKSAPRARLSTRSRRSDIAGPSSTLGEGDDGDDGDDGDDDDDEDLDYDDDDDDDGDVNLGLGSDLSLNDL
>g2724.t1
LPHGIESTGDTSTQQHREPSTARPDIPTATSRAAHLVPTATPLGTSSALANARADVSSQLSMGGGATVAHESAAVDPLLA
GLVSAFIITAVFITLLLFLK
LRRRDNRPEFRRLQDLPMDDMMEDTPLSMYSY
>g2724.t2
TTTTSHHTEPTTLPANASADLPHGIESTGDTSTQQHREPSTARPDIPTATSRAAHLVPTATPLGTSSALANARADVSSQL
SMGGGATVAHESAAVDPLLA
GLVSAFIITAVFITLLLFLKLRRRDNRPEFRRLQDLPMDDMMEDTPLSMYSY
>g2725.t1
LCGRDHLSFRSYYFPVKTPAQTPQSFGRFSLVLLVRPLAAQPVSWSPPARQRNEQHLAVCTAARPDSGSAARARRGPALL
ALNVIDGDLCEQFNSMDPHK
QKSVAEELDRTPPEVSKKLEDIRTRYAF
>g2725.t2
LCGRDHLSFRSYYFPVKRNEQHLAVCTAARPDSGSAARARRGPALLALNVIDGDLCEQFNSMDPHKQKSVAEELDRTPPE
VSKKLEDIRTRYAF
>g2725.t3
HMRSEFPPLCGRDHLSFRSYYFPVKTPAQTPQSFGRFSLVLLVRPLAAQPVSWSPPARQRNEQHLAVCTAARPDSGSAAR
ARRGPALLALNVIDGDLCEQ
FNSMDPHKQKSVAEELDRTPPEVSKKLEDIRTRYAF
>g2726.t1
MSLFCLGFGDNVDYSFLDVMAKQNKGVARRIYEASDASLQLQGFFEEVSTPLLSEVYLRYPDDVVESLTSHHFSQLFNGS
EIVVAGRMSDSNMDNFLVEV
QAGGHGNDFQVQGHAGVAEWDKIYPEQEYIFGDFSERLWAYLTTQQLLEASELGSPTEKTNASAKALEMSLKYSFVTPLT
SMVVTKPETEEGPGGPLIAD
KLTDGERQSAERSRVDVVGNSKIRPRPRSPPVSRSFQDVDGDPHFMIELPERDDALCFDINESPGTIFNLVRDPVVGFLV
NGQIVAKRQEKGPARGNANT
YFGRLGVVHQALGVRLEVTTQYITLSHDGQQVKLLWSAQASFAAPSVNLKMMNNNSLTVTIRDSIKFRVVKHTKVWRKRH
YQQDYLGFYTLESHRLSQEV
HGLLGQFYHGVEFEVGALRPGEIPEKPDATMYVKGHQLNVTRSWHRDFRKDVQKGVVVPCWFVHSNGTGLIDSSASDYIV
SGLFKTV
>g2726.t2
MIELPERDDALCFDINESPGTIFNLVRDPVVGFLVNGQIVAKRQEKGPARGNANTYFGRLGVVHQALGVRLEVTTQYITL
SHDGQQVKLLWSAQASFAAP
SVNLKMMNNNSLTVTIRDSIKFRVVKHTKVWRKRHYQQDYLGFYTLESHRLSQEVHGLLGQFYHGVEFEVGALRPGEIPE
KPDATMYVKGHQLNVTRSWH
RDFRKDVQKGVVVPCWFVHSNGTGLIDSSASDYIVSGLFKTV
>g2726.t3
MSLFCLGFGDNVDYSFLDVMAKQNKGVARRIYEASDASLQLQGFFEEVSTPLLSEVYLRYPDDVVESLTSHHFSQLFNGS
EIVVAGRMSDSNMDNFLVEV
QAGGHGNDFQVQGHAGVAEWDKIYPEQEYIFGDFSERLWAYLTTQQLLEASELGSPTEKTNASAKALEMSLKYSFVTPLT
SMVVTKPETEEGPGGPLIAD
KLTDGERQSAERSRVDVVGNSKIRPRPRSPPVSRSFQDVYDCPRRGGKDAAVTPLLTSGTCRVTPEHMLPSPADG
>g2727.t1
TVEVHSVTLHSTVTSWFAHTVMTSSALNKANSSQEIFFEVDLPKTAFITNFTMEIEGQVYGGKVTEKEKARKQYDKAVSK
GQTAGLVKASGRKMETFFES
VNVAAKNNVTFILTYEELLQRKFGQYEILTRINPKQLVQNFKIVADIYEPQGITSVDAHGTFLSNELLPLVEKTVTDNKA
HISFSPTLDQQRKCPQCDGS
LIDGDFIIKYDVKRASGLGDAQIVNGFFVHFFAPPDLPRVPKNVVFVIDRSGSMSGTKMHQTREAMLAILQDLHHEDHFG
LIQFDHNVVQWKNSLTKATR

KNVTDAMKYVKKIKDNGSTNINDAVLSAVEMLKRNIRGTTLGQTSVDMIILLTDGMPNSGESHLPRIQQNVQQAIGGNMS
LFCLGFGDNVDYSFLDVMAK
QNNGVARRIYEASDASLQLQVSLCWGVGRI
>g2727.t2
TVEVHSVTLHSTVTSWFAHTVMTSSALNKANSSQEIFFEVDLPKTAFITNFTMEIEGQVYGGKVTEKEKARKQYDKAVSK
GQTAGLVKASGRKMETFFES
VNVAAKNNVTFILTYEELLQRKFGQYEILTRINPKQLVQNFKGITSVDAHGTFLSNELLPLVEKTVTDNKAHISFSPTLD
QQRKCPQCDGSLIDGDFIIK
YDVKRASGLGDAQIVNGFFVHFFAPPDLPRVPKNVVFVIDRSGSMSGTKMHQTREAMLAILQDLHHEDHFGLIQFDHNVV
QWKNSLTKATRKNVTDAMKY
VKKIKDNGSTNINDAVLSAVEMLKRNIRGTTLGQTSVDMIILLTDGMPNSGESHLPRIQQNVQQAIGGNMSLFCLGFGDN
VDYSFLDVMAKQNNGVARRI
YEASDASLQLQVSLCWGVGRI
>g2727.t3
DTEHVGAPRSLQTVEVHSVTLHSTVTSWFAHTVMTSSALNKANSSQEIFFEVDLPKTAFITNFTMEIEGQVYGGKVTEKE
KARKQYDKAVSKGQTAGLVK
ASGRKMETFFESVNVAAKNNVTFILTYEELLQRKFGQYEILTRINPKQLVQNFKIVADIYEPQGITSVDAHGTFLSNELL
PLVEKTVTDNKAHISFSPTL
DQQRKCPQCDGSLIDGDFIIKYDVKRASGLGDAQIVNGFFVHFFAPPDLPRVPKNVVFVIDRSGSMSGTKMHQTREAMLA
ILQDLHHEDHFGLIQFDHNV
VQWKNSLTKATRKNVTDAMKYVKKIKDNGSTNINDAVLSAVEMLKRNIRGTTLGQTSVDMIILLTDGMPNSGESHLPRIQ
QNVQQAIGGNMSLFCLGFGD
NVDYSFLDVMAKQNNGVARRIYEASDASLQLQVSLCWGVGRI
>g2728.t1
DLVVTGVFAFLWLVSSSAWGKGLTDIKFATNPEHLMEACKKGTCGVEAYPSMGRLNASVIFGFLNLILWAGNCWFIYKET
PFHKDPNPAVTMPEGGVPGP
>g2729.t1
MSIHNEDEDADAEEELHKAHKTVSHGAAAATEHATQRANTVWPTDHALRRQPDTPPSMCLGPGHHQRCVWGRLSSATPSA
SPHSSRGFIAPPGLSSSRLA
AEVDPNAESSRSHK
>g2730.t1
KDPVFSCLTTTELQQNLKELKQRNRRVKLLFEIPCTRIIEHSLSKHVVYQIIIMRSGSYDSRHVSIERRYSDFAHFHQRL
MDEFSDELEEVHFPRKLLSG
NFNPEVIAERRLALQDYLAKIYAVHCVRHSCHFPEFFTGLEQRRAHGLLRAGQFKPATEQLQAILGLLEKVAPWQSPTLL
VPTLCALAVGHLDLEQLEEA
FAAAHKALPAVRRYGLRQYKAPLLELLVDVGYRLHRPIAQLQEELRLFRDSERGLVSFGSLKELVVQEYT
>g2730.t2
QNLKELKQRNRRVKLLFEIPCTRIIEHSLSKHVVYQIIIMRSGSYDSRHVSIERRYSDFAHFHQRLMDEFSDELEEVHFP
RKLLSGNFNPEVIAERRLAL
QDYLAKIYAVHCVRHSCHFPEFFTGLEQRRAHGLLRAGQFKPATEQLQAILGLLEKVAPWQSPTLLVPTLCALAVGHLDL
EQLEEAFAAAHKALPAVRRY
GLRQYKAPLLELLVDVGYRLHRPIAQLQEELRLFRDSERGLVSFGSLKELVVQEYT
>g2731.t1
LQILYQSVVASTIFFAAVSWGVGIKTKDANRLNKLIKKAESVVGSKPVTVEEVVEERMLAKLLAIMNNPSHPLHTTLDKL
KCSFSYRLIQLRCENERYRK
SFLPTAIRLISSASVRSTITELD
>g2732.t1
MPENRILHILHGICTGLKVIHDRGYSHRDLKPTNVLLDEDDRPLLMDLGSMNKSQIEVRGSREAMVVQDWAAQRCTISYR
APELFSVESHCIIDARTDIW
SLGCVVYSMMFLQGPFDMVFQKGDSVALAVQNSVIIPQPCSYSEGLQNLLSSMMVSNPQERPAVGWVLDQVQDLRTRSPN
THTNMV
>g2732.t2
LWSVLEKLRDKGSSMPENRILHILHGICTGLKVIHDRGYSHRDLKPTNVLLDEDDRPLLMDLGSMNKSQIEVRGSREAMV
VQDWAAQRCTISYRAPELFS
VESHCIIDARTDIWSLGCVVYSMMFLQGPFDMVFQKGDSVALAVQNSVIIPQPCSYSEGLQNLLSSMMVSNPQERPAVGW
VLDQVQDLRTRSPNTHTNMV

>g2732.t3
LEKLRDKGSSMPENRILHILHGICTGLKVIHDRGYSHRDLKPTNVLLDEDDRPLLMDLGSMNKSQIEVRGSREAMVVQDW
AAQRCTISYRAPELFSVESH
CIIDARTDIWSLGCVVYSMMFLQGPFDMVFQKGDSVALAVQNSVIIPQPCSYSEGLQNLLSSMMVSNPQERPAVGWVLDQ
VQDLRTRSPNTHTNMV
>g2733.t1
WDESSSISSGLSDGSDNLSSEEFNTSPVLNSLPTTPIGSRRNSAVVVTPPQRGLSDCGRASEGRRYKRTGRDGANRRNHG
NPGKEMLRVRNKPLSPSVVP
QTDAGLE
>g2734.t1
MTLNRSLPHCARKGYGVYERAEETAAEETVAEETAAEETAAEETAAEETVAEETAAEETVAEETVAEETAAEETVAEETA
AEETVAEETVAEETAAEETV
AEETAALATVR
>g2735.t1
KQRAELTQQHTEWSRQMTQRHMQQIEDLQAELQTHTQMMALQQDLKQQNQWQVFERQLDESGCLVLELQRENAALREQLK
ERSILEKSETEEKEKEKAET
LKQRDAQLEEEAQRLKEEVEKLRVEMEKLEESQKHWEERKEEETKEEEDELRKKEEHDRRRGEVEEIRRKHRKELQSLAS
EFSSAQTQLQARITALETE
>g2736.t1
MGNTLEEGMNAVIVRHYNHTGKWDLPRTGSACKTLVLLLICVLIVLENLTVLLALWRNKRFHSRMYFLIGNLALSDLLAG
VAYVVNIFTSGHNTFYLSPA
QWLAREGSMFVALSASTFSLLAIAIERHITVFRMQL
>g2737.t1
MWEFMSSRRHSVMVKNVEEGIHRVLTSDYAFLMESTTIEFVTQRNCNLTQIGGLIDSKAYGVGTPMGVYRGCEVRLGFGF
PAGASRAENGCARCIGKVQR
SPLMGTILQGAYVPAPRRSESRHFGRSGWESLSSLRQCVETR
>g2737.t2
MHDGMCWFVFSWSGPAQACIRCVPGVYRGCEVRLGFGFPAGASRAENGCARCIGKVQRSPLMGTILQGAYVPAPRRSESR
HFGRSGWESLSSLRQCVETR
>g2737.t3
MWEFMSSRRHSVMVKNVEEGIHRVLTSDYAFLMESTTIEFVTQRNCNLTQIGGLIDSKAYGVGTPMGAYALTTSWRDWHL
RFFAFVDTQPQTLMLHPSHR
GGGGGILRSDESHACIRCVPGVYRGCEVRLGFGFPAGASRAENGCARCIGKVQRSPLMGTILQGAYVPAPRRSESRHFGR
SGWESLSSLRQCVETR
>g2738.t1
YDVISTTKGPHGEEVSILTTNQTHTAVENLKPESSEPVAFSTESGKDAIWTQFPFKADAYSECNGKQYVKRTWYRKFVGI
QLCNSLRYKIYLSDSLNGKF
YNIGDQTGHGEDHCQFVDSFLDGRTGSQLMADQLPSKSGTSQSALMERSWAPYGSTGETEAVIAM
>g2738.t2
YDVISTTKGPHGEEVSILTTNQTHTAVENLKPESSEPVAFSTESGKDAIWTQFPFKADAYSECNGKQYVKRTWYRKFVGI
QLCNSLRYKIYLSDSLNGKF
YNIGDQTGHGEDHCQFVDSFLDGRTGSQLMADQLPSKSGTSQSALMERSWAPYGSTGG
>g2738.t3
YDVISTTKGPHGEEVSILTTNQTHTAVENLKPESSEPVAFSTESGKDAIWTQFPFKADAYSECNGKQYVKRTWYRKFVGI
QLCNSLRYKIYLSDSLNGKF
YNIGDQTGHGEDHCQFVDSFLDGRTGSQLMADQLPSKSETEAVIAM
>g2739.t1
AGPCAPCGEKVRFSPGATALRDTAVVVEDLDSHLNYTFRVEAHSGVSHFRSHSASASITTELRYTDPPKIIAIQLDENSP
TSLSLSLVLSRKLPPHIGHN
YELMYRQKNVEDRDVTIYTVLVLDKSSVHIKDLTPATTYLVRVQAPGPDGTPGSYSSEYEFQTLALAVPQIQSNSTMVMA
AVVGGAVVLLIMVAVLLLHK
RLEDEFRDQLKPLKTYVDPHMYEDPNIAILKFATEIHPSAVTKQKVIGAGEFGEVFRGVMRSPGRGDAAVAIKTLKAGYT
EKQRQDFLSEASIMGQFSHQ
NIIRLEGVVTKFKHMMIVTEYMENGALDIYLRDHDGEILAFQLVGMLRGIAAGMKYLSDMSYVHRDLAARNVLVNGNLEC
KVSDFGLSRVLEDDPEGTYT
TSGGKIPIRWTAPEAIAYRKFTSASDVWSFGIVMWEVLAFGERPYWDMSNHEVMKAINEAFRLPAPMDCPSAVYQLMLQC

WLHDRSKRPRFTDIVNILDK
LLQSPESLKPIADFDP
>g2739.t2
AGPCAPCGEKVRFSPGATALRDTAVVVEDLDSHLNYTFRVEAHSGVSHFRSHSASASITTELRYTDPPKIIAIQLDENSP
TSLSLSLVLSRKLPPHIGHN
YELMYRQKNVEDRDVTIYTVLVLDKSSVHIKDLTPATTYLVRVQAPGPDGTPGSYSSEYEFQTLALAVPQIQSNSTMVMA
AVVGGAVVLLIMVAVLLLHK
RLEDEFRDQLKPLKTYVDPHMYEDPNIAILKFATEIHPSAVTKQKVIGAGEFGEVFRGVMRSPGRGDAAVAIKTLKAGYT
EKQRQDFLSEASIMGQFSHQ
NIIRLEGVVTKFKHMMIVTEYMENGALDIYLRDHDGEILAFQLVGMLRGIAAGMKYLSDMSYVHRDLAARNVLVNGNLEC
KVSDFGLSRVLEDDPEGTYT
TSGGKIPIRWTAPEAIAYRKFTSASDVWSFGIVMWEVLAFGERPYWDMSNHEVCVTCQTLPCSGGKGTVPWGVQRQLAVF
QQ
>g2739.t3
AGPCAPCGEKVRFSPGATALRDTAVVVEDLDSHLNYTFRVEAHSGVSHFRSHSASASITTELRYTDPPKIIAIQLDENSP
TSLSLSLVLSRKLPPHIGHN
YELMYRQKNVEDRDVTIYTVLVLDKSSVHIKDLTPATTYLVRVQAPGPDGTPGSYSSEYEFQTLALAVPQIQSNSTMVMA
AVVGGAVVLLIMVAVLLLHK
RRMSSGVRRGVEDSYFHSDQLKPLKTYVDPHMYEDPNIAILKFATEIHPSAVTKQKVIGAGEFGEVFRGVMRSPGRGDAA
VAIKTLKAGYTEKQRQDFLS
EASIMGQFSHQNIIRLEGVVTKFKHMMIVTEYMENGALDIYLRDHDGEILAFQLVGMLRGIAAGMKYLSDMSYVHRDLAA
RNVLVNGNLECKVSDFGLSR
VLEDDPEGTYTTSGGKIPIRWTAPEAIAYRKFTSASDVWSFGIVMWEVLAFGERPYWDMSNHEVMKAINEAFRLPAPMDC
PSAVYQLMLQCWLHDRSKRP
RFTDIVNILDKLLQSPESLKPIADFDP
>g2739.t4
MVLGQQSVHVNTWGFSWSPRRGAGAVERAGPFCGYCKEFDGAVPDSSPQVMKAINEAFRLPAPMDCPSAVYQLMLQCWLH
DRSKRPRFTDIVNILDKLLQ
SPESLKPIADFDP
>g2740.t1
MCVPLFVEACAGELISDQRALLDGENAETHMFRLLFEERYFYQMVMCRGGNRGGVFALGLVQGEWKMYVKGLLDREDRSR
YALNVTASDGLFVTRATVDV
VVTDTNDNSPVCDQ
>g2740.t2
MFRLLFEERYFYQMVMCRGGNRGGVFALGLVQGEWKMYVKGLLDREDRSRYALNVTASDGLFVTRATVDVVVTDTNDNSP
VCDQ
>g2740.t3
MCVPLFVEACAGELISDQRGGNRGGVFALGLVQGEWKMYVKGLLDREDRSRYALNVTASDGLFVTRATVDVVVTDTNDNS
PVCDQ
>g2741.t1
LCCWSVDPSSSMSTQLTGHTDPGPEPPSLDTKPRSPASSGEHTTTARP
>g2742.t1
MARRGVPLLPMVFLAMLSHCSTWLTPRISYPQGSPGRPLVHFHSPDVCNTTLLLLSEDGGTLFVGARDAVLSLDVSQKEA
IVVRRKLDWSPSEQDLLQCS
MKGKELMDCPNFVRVLQFLNTTHMYACGTYAFSPRCTYMHTETLLMTSHGSKPEEGRGRCPYDPYQRNTAITVDGELYTA
TVADYRGNRPVISRHLSHVD
LKLDDTLGWLEEPTFISSSFIPSEEKIYFFFSEVGREYDFIDKFIVSRVAQICTVRGHVQSDVGGQRTLQRRWTTFAKAQ
LLCQASSELPYNVVQDMFTL
PPPEGAPADDTMFYGIFTSQWSVNSGQSAVCAFHLRDIKATFSRNYKVLNRDTLRWSTRVQEKIANTGECGLHNASDNAL
RFVKENFLADDSVQPVGRSL
TLVSRDQNYSHLAAQRVQAANHRDYTILFLLTDSGYLHKAVLLEKGAHIIEEVQVFLQPQPIKNMQLSASKGMVFVGTPE
GVVRVPVANCSFYASCAQCV
LARDPFCGWDHVGKACVAISHVPASL
>g2742.t2
MARRGVPLLPMVFLAMLSHCSTWLTPRISYPQGSPGRPLVHFHSPDVCNTTLLLLSEDGGTLFVGARDAVLSLDVSQKEA
IVVRRKLDWSPSEQDLLQCS

MKGKELMDCPNFVRVLQFLNTTHMYACGTYAFSPRCTYMHTETLLMTSHGSKPEEGRGRCPYDPYQRNTAITVDGELYTA
TVADYRGNRPVISRHLSHVD
LKLDDTLGWLEEPTFISSSFIPSEEKIYFFFSEVGREYDFIDKFIVSRVAQICTVRGHVQSDVGGQRTLQRRWTTFAKAQ
LLCQASSELPYNVVQDMFTL
PPPEGAPADDTMFYGIFTSQWSVNSGQSAVCAFHLRDIKATFSRNYKVLNRDTLRWSTRVQEKIANTGECGLHNASDNAL
RFVKENFLADDSVQPVGRSL
TLVSRDQNYSHLAAQRVQAANHRDYTILFLLTGTVANHRDYTILFLLTDSGYLHKAVLLEKGAHIIEEVQVFLQPQPIKN
MQLSASKGMVFVGTPEGVVR
VPVANCSFYASCAQCVLARDPFCGWDHVGKACVAISHVPASL
>g2742.t3
MARRGVPLLPMVFLAMLSHCSTWLTPRISYPQGSPGRPLVHFHSPDVCNTTLLLLSEDGGTLFVGARDAVLSLDVSQKEA
IVVRRKMDCPNFVRVLQFLN
TTHMYACGTYAFSPRCTYMHTETLLMTSHGSKPEEGRGRCPYDPYQRNTAITVDGELYTATVADYRGNRPVISRHLSHVD
LKLDDTLGWLEEPTFISSSF
IPSEEKIYFFFSEVGREYDFIDKFIVSRVAQICTVRGHVQSDVGGQRTLQRRWTTFAKAQLLCQASSELPYNVVQDMFTL
PPPEGAPADDTMFYGIFTSQ
WSVNSGQSAVCAFHLRDIKATFSRNYKVLNRDTLRWSTRVQEKIANTGECGLHNASDNALRFVKENFLADDSVQPVGRSL
TLVSRDQNYSHLAAQRVQAA
NHRDYTILFLLTGTVANHRDYTILFLLTDSGYLHKAVLLEKGAHIIEEVQVFLQPQPIKNMQLSASKGMVFVGTPEGVVR
VPVANCSFYASCAQCVLARD
PFCGWDHVGKACVAISHVPASL
>g2743.t1
PGLSAYFQPFYQPNECGKALCVRPDMMELDELYEFPEFSRDPTMYLALRNLVLAAWHRDCKEVLTPQKCAPHIIVRGLVR
VRCVQEMDRVLHFMTRKGLI
NTGVLAVSQPLLPEQYRLVVVLEARDRIGGRVWDDPSLGLTVGRGAQIVNGCVNNPIALMCEQMGIKMHKLGDKCDLLQE
GGHVTDTAIDKRMEFHFNAI
LDVVSEWRKDKSQNQDAPLGEKVQEVYKNFLQESGIQFSELEEKVLQFHLSNLEYACGSTLDQVLPCSVLVPTLRHALGS
RHTPCPHACPPSHLLPQVSA
RSWDHNEFFAQFSGDHTLLTRGYSSLLLKLAEGLDIRTKCAVQAIDYSGDVIKVTSSKGSQWTAHKVLVTVPLTLLQKNL
IQFYPPLPERKLKALNSLGA
GIIEKIALEFPYRFWDQKVQGADYFGHIPPSPDQRGMFGVFYDLDPQGKQSVLMSVITGDAVPFVREMGEQEVVDECMRV
LRELFKEQEVPEPVNYFVTQ
WSKDVWSQMSYSFVKTGGSGEAYDIIAEDVQGKLFFAGETVTGAYLSGVREASKMAAV
>g2743.t2
PGLSAYFQPFYQPNECGKALCVRPDMMELDELYEFPEFSRDPTMYLALRNLVLAAWHRDCKEVLTPQKCAPHIIVRGLVR
VRCVQEMDRVLHFMTRKGLI
NTGVLAVSQPLLPEQYRLVVVLEARDRIGGRVWDDPSLGLTVGRGAQIVNGCVNNPIALMCEQMGIKMHKLGDKCDLLQE
GGHVTDTAIDKRMEFHFNAI
LDVVSEWRKDKSQNQDAPLGEKVQEVYKNFLQESGIQFSELEEKVLQFHLSNLEYACGSTLDQVSARSWDHNEFFAQFSG
DHTLLTRGYSSLLLKLAEGL
DIRTKCAVQAIDYSGDVIKVTSSKGSQWTAHKVLVTVPLTLLQKNLIQFYPPLPERKLKALNSLGAGIIEKIALEFPYRF
WDQKVQGADYFGHIPPSPDQ
RGMFGVFYDLDPQGKQSVLMSVITGDAVPFVREMGEQEVVDECMRVLRELFKEQEVPEPVNYFVTQWSKDVWSQMSYSFV
KTGGSGEAYDIIAEDVQGKL
FFAGETVTGAYLSGVREASKMAAV
>g2743.t3
PGLSAYFQPFYQPNECGKALCVRPDMMELDELYEFPEFSRDPTMYLALRNLVLAAWHRDCKEVLTPQKCAPHIIVRGLVR
VRCVQEMDRVLHFMTRKGLI
NTGVLAVSQPLLPEQYRLKNVIVIGAGASGLAAARQLQNFGTQVVVLEARDRIGGRVWDDPSLGLTVGRGAQIVNGCVNN
PIALMCEQMGIKMHKLGDKC
DLLQEGGHVTDTAIDKRMEFHFNAILDVVSEWRKDKSQNQDAPLGEKVQEVYKNFLQESGIQFSELEEKVLQFHLSNLEY
ACGSTLDQVLPCSVLVPTLR
HALGSRHTPCPHACPPSHLLPQVSARSWDHNEFFAQFSGDHTLLTRGYSSLLLKLAEGLDIRTKCAVQAIDYSGDVIKVT
SSKGSQWTAHKVLVTVPLTL
LQKNLIQFYPPLPERKLKALNSLGAGIIEKIALEFPYRFWDQKVQGADYFGHIPPSPDQRGMFGVFYDLDPQGKQSVLMS
VITGDAVPFVREMGEQEVVD

ECMRVLRELFKEQEVPEPVNYFVTQWSKDVWSQMSYSFVKTGGSGEAYDIIAEDVQGKLFFAGETVTGAYLSGVREASKM
AAV
>g2744.t1
MIGFVTLTHASISRTFITSRLDYLNSPVRGTQQSPGWAPVCAALRCQVSHPHQDLAAYNPTRIRLHWLPVKSSISFKIFL
ITNKSLHALAPPVPIRSPSP
LYPVSESAALRHTSAH
>g2745.t1
MASLLQPDRVIYLVRGEKKIRSPLSQLYFCRYCSELRSLECVSHEVDSHYCPSCLENMPSAEAKLKKNRCANCFDCPCCM
HTLSTRATNIPAPMPDDPTK
TTMKKAYYLACGFCRWTSRDVGMADKSVASGGWQEPENPHTQRINKLIEYYQQLAQRERQERDRKKLARRRQCMPLAFSE
KYGLGTRLQRQRSGAPLSNL
AGLSVQCVCPTSLKEGEDQKEINIEPAQALDEVEPLPEDCYTRPINLPEVTTLRQRLLQPDFQPPGASQLHPKHKHLLMK
RSLRCRESQVLLTLTNPVEN
LTHVTLVACEDGDPEDLDSTAKVMVPTKELVLAGKDAAADYDELAEPQDFQDDPE
>g2745.t2
MASLLQPDRVIYLVRGEKKIRSPLSQLYFCRYCSELRSLECVSHEVDSHYCPSCLENMPSAEAKLKKNRCANCFDCPCCM
HTLSTRATNIPAPMPDDPTK
TTMKKAYYLACGFCRWTSRDVGMADKSVASGGWQEPENPHTQRINKLIEYYQQLAQRERQERDRKKLARRRQCMPLAFSE
KYGLGTRLQRQRSGAPLSNL
AGLSVQCVCPTSLKEGEDQKEINIEPAQALDEVEPLPEDCYTRPINLPEVTTLRQRLLQPDFQPPGASQLHPKHKHLLMK
RSLRCRKCEHNLSKPEFNPT
SIKFKIQLVAVSYIPEVRIMSIPNLRFMKESQVLLTLTNPVENLTHVTLVACEDGDPEDLDSTAKVMVPTKELVLAGKDA
AADYDELAEPQDFQDDPE
>g2745.t3
MASLLQPDRVIYLVRGEKKIRSPLSQLYFCRYCSELRSLECVSHEVDSHYCPSCLENMPSAEAKLKKNRCANCFDCPCCM
HTLSTRATNIPAPMPDDPTK
TTMKKAYYLACGFCRWTSRDVGMADKSVASGGWQEPENPHTQRINKLIEYYQQLAQRERQERDRKKLARRRQCMPLAFSE
KYGLGTRLQRQRSGAPLSNL
AGLSLKEGEDQKEINIEPAQALDEVEPLPEDCYTRPINLPEVTTLRQRLLQPDFQPPGASQLHPKHKHLLMKRSLRCRES
QVLLTLTNPVENLTHVTLVA
CEDGDPEDLDSTAKVMVPTKELVLAGKDAAADYDELAEPQDFQDDPE
>g2746.t1
DYTHKLVFATERETFEVPVRAIGPRAILDFRDEVRLPVCLVKASTQKTHLVRNIGNADAKFHLHTRRCLAFPEQKPLCPP
HSYAPMLHVQKEIVLLKS
>g2746.t2
DYTHKLVFATERETFEVPVRAIGPRAILDFRDEVRLPVCLVKASTQKTHLVRNIGNADAKFHLHTR
>g2747.t1
LDKVLESSSDERGGQDGSETGYARSTHEPRKRSASGGDAEAEAEAEVKFLNPLIAVGTTEGMEKPLQKGFPQEVYMYVQR
DDFVDGHSTPPGLGARPPSL
K
>g2748.t1
DSKTRSIPQYDRSLEDVLDVRGGIPSPRPRRSLDVEIKVKSPAAAAAAAAAADAAAASQPARLLPMRTFVSRRRLSGCLR
KNRLRPEDDYRR
>g2749.t1
MTMRVVAVVVVLVDGGDRQELCMYSPHEVVPHLNSSLIGRSDRGDIPLMALPPLMSNAGGQYLDLALL
>g2749.t2
MRVVAVVVVLVDGGDRQELCMYSPHEVVPHLNSSLIGRSDRGDIPLMALPPLMSNAGGQYLDLALL
>g2750.t1
MRSKQKGTCLITGGCGYFGYRLARSLHASGAAVVLFDVSPACGELPAGMAFLQGDLRDYAQVERALRGVDCVFHVASYGM
SGREQLDRRRIEEVNVRGTR
NILKACAEVGVPKLVYTSTFNVVFGGQVIENGDESLPYLPLPLHPDHYSRTKSVAEMAVLRANG
>g2751.t1
VDAGFDQHDIPYDFIWLDIEHTDGKRYFTWDPHKFATPKDMLQGLLEKKRKMVAIVDPHIKVDSEYKIHTEIRSQGFYIK
NKDGGDYEGWCWPGNAGYPD
FTKPEMRAWWASMFAYDQYEGSMENLYTWNDMNEPSVFNGPEVTMHKDAVHGPWEHRDVHNLYGFYVVRARPSSSFPSWF
LRMAFFMCLGMRQWPSLRLK

VPFGFDKEAIRCQMATAEGLIQRSGGVERPFVLTRAFFAGSQRYGAVWTGDNAAEWDHLKISIPMCLSLSLVGISLCGAD
VGGFFKTPQSELLVRWYQAG
AYQPFFRAHAHLDTPRREPWLFGPDNTALIREAVRQRYALLPYWYQLFYHAHRTGHPVMRPLWVEYPQDAATFAMDDQFL
LGKDLLVHPVTEEGARGVTA
YLPGKGEVWFDVHTFQKHNGAQDLYIPVTMSS
>g2751.t2
VDAGFDQHDIPYDFIWLDIEHTDGKRYFTWDPHKFATPKDMLQGLLEKKRKMVAIVDPHIKVDSEYKIHTEIRSQGFYIK
NKDGGDYEGWCWPGNAGYPD
FTKPEMRAWWASMFAYDQYEGSMENLYTWNDMNEPSVFNGPEVTMHKDAVHGPWEHRDVHNLYGFYVVRARPSSSFPSWF
LRMAFFMCLGMRQWPSLRLK
QMATAEGLIQRSGGVERPFVLTRAFFAGSQRYGAVWTGDNAAEWDHLKISIPMCLSLSLVGISLCGADVGGFFKTPQSEL
LVRWYQAGAYQPFFRAHAHL
DTPRREPWLFGPDNTALIREAVRQRYALLPYWYQLFYHAHRTGHPVMRPLWVEYPQDAATFAMDDQFLLGKDLLVHPVTE
EGARGVTAYLPGKGEVWFDV
HTFQKHNGAQDLYIPVTMSS
>g2751.t3
VDAGFDQHDIPYDFIWLDIEHTDGKRYFTWDPHKFATPKDMLQGLLEKKRKMVAIVDPHIKVDSEYKIHTEIRSQGFYIK
NKDGGDYEGWCWPGNAGYPD
FTKPEMRAWWASMFAYDQYEGSMENLYTWNDMNEPSVFNGPEVTMHKDAVHGPWEHRDVHNLYGFYVVRARPSSSFPSWF
LRMAFFMCLGMRQWPSLRLK
VPFGFDKEAIRCQMATAEGLIQRSGGVERPFVLTRAFFAGSQRYGAVWTGDNAAEWDHLKISIPMCLSLSLVGISLCGAD
VGGFFKTPQSELLVRWYQAG
AYQPFFRAHAHLDTPRREPWLFGPDNTALIREAVRQRYALLPYWYQLFYHAHRTGHPVMRPLWVEYPQDAATFAMDDQFL
LGKDLLVHPVTEEGARGVTA
YLPGKGEVWFDVHTFQKHNGAQDLYIPVTMSS
>g2752.t1
MTAGLPSAAPLQELPKYLRGYHKCTRDDLHQLAALIYRVKFEDDKSHFANVPKILKELLPQDQLRLFSPEDWKRSIVSLF
NKHSGKSREEAKLSFLKIIY
KWPTFGSAFFEVKQTTDPNYPETLLIAINKHGVSLIDPKSKDILVTHPFTKISNWSSGNTYFHITIGNLVRGSKLLCETS
LGYKMDDLLTSYISQMLTTM
TKQHSSRGNSK
>g2753.t1
CAYVLGANGEPRIFRPRDRDEMVKQVIEPMACEGLRTICIAYRDLPASPEPDWESEAEIVTELTAITVVGIEDPVRPEVP
DAIRKCQRAGITVRMVTGDN
INTARAIAAKCGIIHPGDDFICLEGKDFNRRIRNEKGEIEQERIDKIWPKLRVLARSSPTDKHTLVKGIIDSTVLEQRQV
VAVTGDGTNDGPALKKADVG
FAMVTL
>g2753.t2
VIEPMACEGLRTICIAYRDLPASPEPDWESEAEIVTELTAITVVGIEDPVRPEVPDAIRKCQRAGITVRMVTGDNINTAR
AIAAKCGIIHPGDDFICLEG
KDFNRRIRNEKGEIEQERIDKIWPKLRVLARSSPTDKHTLVKGIIDSTVLEQRQVVAVTGDGTNDGPALKKADVGFAMVT
L
>g2754.t1
MLNPSPISQMFFSISLGFILAWAPYTVVSFLFIFHEDHRYMAPGGFVFPALFAKSSHIYNPFIYFYFNAGFQRELRSLLR
SFCPALVSKRVGARSASD
>g2755.t1
FKLLSQEEGEYFNVPVPPEGEEGNEELRQKFE
>g2756.t1
GVHEPRVASEDYMRLESLVQKVVSPYLGTHGLYSGDGSEAQCCVLEKRLQWCWPKPVDQLSKNPVVRSKSYNIPLLLTPV
AEYDPDAGSVGSGGIRRHSA
WILHGGSSAMELSVCRSASAHPLRSAGLLHGTAATTTLTDLRRAASSSPTSAASSPETVISGGAVESEDSTTSDGIFIDF
TPKESESEGSGQDRQSTV
>g2756.t2
GVHEPRVASEDYMRLESLVQKVVSPYLGTHGLYSGDGSEAQCCVLGILHGGSSAMELSVCRSASAHPLRSAGLLHGTAAT
TTLTDLRRAASSSPTSAASS
PETVISGGAVESEDSTTSDGIFIDFTPKESESEGSGQDRQSTV

>g2756.t3
GVHEPRVASEDYMRLESLVQKVVSPYLGTHGLYSGDGKKRLQWCWPKPVDQLSKNPVVRSKSYNIPLLLTPVAEYDPDAG
SVGSGGIRRHSAWILHGGSS
AMELSVCRSASAHPLRSAGLLHGTAATTTLTDLRRAASSSPTSAASSPETVISGGAVESEDSTTSDGIFIDFTPKESESE
GSGQDRQSTV
>g2757.t1
MLVLKLDSKIGWNWFLIFLPIWIFDAILILMLVAKMAGRCKQALNPRDGHQNLQRTMWYLIALLLKLGFCLALCSRLEKL
MNIWLSVVCIPLWTLLVGAL
LELGHTVFRYRRD
>g2758.t1
EHDKERAGAQPGARPDREDAKEGGGHRHNKPSHGKKEKSGHREAKDSRARSRAAREEAKPGAPRAAHVKKEAKKEKETNN
AEERKKAAAAQGFAAPAEHK
PAPRTLITFDLFKPMDAHQALPVSYADTRVKSHHHGDHSRGVSGARFGADGRTFTPSKGPKAKDPKQAKPAPNSESQLLL
KQPLKPHTPQTQKDFLI
>g2759.t1
MLGMRDMAQETNQSQGPMLCTTGCGFYANPRNNGMCSLCYKDFLQRQNGRGGPPESSSATGSTGESLLSQSQSSSSSSSS
SSSSSSSSSSIALPATIEHA
DTLCGQSSNISTTSTAPSEEDEGSSAAKASPDTDDV
>g2759.t2
MLGMRDMAQETNQSQGPMLCTTGCGFYANPRNNGMCSLCYKDFLQRQNGRGGPPAATETHSTPTAESSSATGSTGESLLS
QSQSSSSSSSSSSSSSSSSS
SIALPATIEHADTLCGQSSNISTTSTAPSEEDEGSSAAKASPDTDDV
>g2759.t3
MLGMRDMAQETNQSQGPMLCTTGCGFYANPRNNGMCSLCYKDFLQRQNGRGGPPGKDALRSASTYSLFNRDARSTGPQWT
FVTVVVLSNRQHRRVVAVTE
PEQQQQQQQQQQQQQQQQQQYSPARHHRARRHAMWTEQLANFVFPHYCNISTTSTAPSEEDEGSSAAKASPDTDDV
>g2760.t1
MYASCDRIAVFLSMHDEVRTEEILQDLFARGKTCFIPKYQTQGSHMDMLRLHSMEDMETLPLTSWNIKQPSDQDLTREEA
LATDLL
>g2761.t1
MALNITAAQEQCRRSAHFGLNRKHAATIGCGRKPSAQSVATPLAGRLQRGSRRQAVGAEHCITVFQWFSERAQAYAVLYE
RVVHRRRRRRHLSESTRVVS
PRNLSFQMKEA
>g2762.t1
LPQSPDPAASWPHSPPPPAPPPPPPPPPPSAAVQEPASGVSSSPPASSPLRREIRSEPEAEEEEEEEKEVLEEDPLPVKQ
EARLFDLDVFISRALKLCGQ
SEDRAEGRSVREGRGSGKEGKQVSINQRVSNS
>g2763.t1
MTRTSIDTHLVFITGLTFMLLQGVSVSSVESEVKPQGFPDDRRDKPGAALELRGTAAPPAETQAKSRPKRCTCYSYKDKE
CVYYCHLDIIWINTPE
>g2763.t2
MTRTSIDTHLVFITGLTFMLLQGVSVSSVESEVKPQGFPDDRRDKPGAALELRGTAAPPAETQAKSRPKRCTCYSYKDKE
CVYYCHLDIIWINTP
>g2764.t1
VRGNIRSVEQDENLRAAIIKVSATRVFRQKYALFTGVGRLTRRGDIRTFQQCGVRPGPGSFLFTGRVHFGEAWLGCAPRY
KDFLQAYLIAKEAQQIPCEL
AVD
>g2765.t1
MGSSRHQNPARLSYAGPKIIRTMTDASEPLHSQLTDTGSHDTPAQPEPEXDERMNTGSTQQPSLQQGARTCSALGVPACS
LVESTAMRDGVMAVSVHPAW
SYASKLTTNHNHNHKHCRTAALLTQHPVATENSQRRQ
>g2765.t2
MTDASEPLHSQLTDTGSHDTPAQPEPEXDERMNTGSTQQPSLQQGARTCSALGVPACSLVESTAMRDGVMAVSVHPAWSY
ASKLTTNHNHNHKHCRTAAL
LTQHPVATENSQRRQ
>g2766.t1

LWSCGRNPAPPAPAILCGSPVDFVESYHFLCTIIAQELNWDLNIRCLTKKGQQRMYFMQQLKKRLECIRTKTSRQENSFF
PAAVGIINKSRDPPHPLPH
>g2767.t1
MGSRPECGPYGLAKVWRSSDEGYKQQSQQQGSTQAALEELENRPRLQPPSLPMKMAAPSQTASELPLEELLALYGYEMPG
RDRCHLAAGLPHITLDKDQV
TKDMYAGDEEQDLAAAAAATTDDDLATSYASNTSDLLRRMQGGDEQTDVGSSEEDCSRTSAPSNEERKDIMVGSMYQAQI
PALSPYTFQGR
>g2768.t1
ISENFFFDLNSEQTKGMLRPHVQTAAITTLARSAIFSITYPSQDVFLVIQLEKVLQQGDIGECAEPYMVFKESDAAKNKE
KLEKLRGQSEQFCQRLGRYR
MPFAWTAIHLMNIVSSAGSLERDTELEVSLS
>g2768.t2
ISENFFFDLNSEQTKGMLRPHVQTAAITTLARSAIFSITYPSQDVFLVIQLEKVLQQGDIGECAEPYMVFKESDAAKNKE
KLEKLRGQSEQFCQRLGRYR
MPFAWTAIHLMNIVSSAGSLERDTELEVSLSGQGARRAPPPGGVVTESQRCGPRPLEWEAVC
>g2769.t1
MQALTYLQRALLVHDLQTLDAAEWESCFNKVRLLPLPRGDVAGYKVLFPLLTKLLDNISPADVGGMEETRMRACTLLSKV
FLQHLSPLLSLPTFAALWLT
ILDFMDKYMHAGSSDLLLEAIPESLKNMLLVMDTAGIFHCSDSRTGYSDLWEITWERIVCFLPNLREDLFKQTVIP
>g2769.t2
MQALTYLQRALLVHDLQTLDAAEWESCFNKVRLLPLPRGDVAGYKVLFPLLTKLLDNISPADVGGMEETRMRACTLLSKV
FLQHLSPLLSLPTFAALWLT
ILDFMDKYMHAGSSDLLVSLEAIPESLKNMLLVMDTAGIFHCSDSRTGYSDLWEITWERIVCFLPNLREDLFKQTVIP
>g2770.t1
LGSGRSDPVLNGGLYSAAYCSSVGGTSPNSPTTVPGSPGFPAGQGAWPDECSICYENAVDTVIYACGHMCLCHTCGLKLK
KMSNACCPICRRQIKDIIKT
YRST
>g2770.t2
MCACSGGLRGGSRRDYNPWRAGLQSARAAAAGPHVCRANQTAGFCGSLSQAAQRSALPASRLPAPGGSITLKVDFGSVFS
DSSQHLTRVVLSSRHVDTPV
LRPPEAHRAPHSRSYPQRDPKKSSRLKRGEAGMSAKVAGRMYFCWCRLSLKGGHALGSGRSDPVLNGGLYSAAYCSSVGG
TSPNSPTTVPGSPGFPAGQG
AWPDECSICYENAVDTVIYACGHMCLCHTCGLKLKKMSNACCPICRRQIKDIIKTYRST
>g2770.t3
MCACSGGLRGGSRRDYNPWRAGLQSARAAAAGPHVCRANQTAGFCGSLSQAAQRSALPASALGSGRSDPVLNGGLYSAAY
CSSVGGTSPNSPTTVPGSPG
FPAGQGAWPDECSICYENAVDTVIYACGHMCLCHTCGLKLKKMSNACCPICRRQIKDIIKTYRST
>g2770.t4
MCACSGGLRGGSRRDYNPWRAGLQSARAAAAGPHVCRANQTAGFCGSLSQAAQRSALPARLAVIVPSARPDHVAADDGLT
FAFLPPTAREL
>g2771.t1
MATGSGWQQYYSPAGQPSSGAGKFSSGSPSATAFQPGIAMEYTHDLHLKMSKKIAQLTKYKPSEDARDSQPELLLPLVNV
QECHVTRQASQSALQRHRGL
NWLPVSLIGAADI
>g2772.t1
MVDCCGGLLTADKTTVPLKSVDVEVTVRDHVATVAATLLYENKEQSPIEAVFVFPLPSDAAVCHFSAKIGQSEVVAKVQE
KQKAREEYDDALSAGQQAFL
LEESEQSPDVFSLSVGSLPPGESASIRLEYVTELAVQADDGLRFCLPAVLNPRYQPRGSDGSGGTVSCGQGTSVLASLVP
YSLSLLAGVCSPRPITKVES
NCSLEPVQYLNQQQTQATVKLASGHKFDRDVELLFYYKDSHLPTAVVEAGQASAKPGSLMADPVVMLSMYPEFPPAVMSS
MTKSGEFLFVIDRSGSMDSP
MSHEGVRQMRIDSAKETLLLLLKSLPIGCYFNIYSFGSSYTHIFPKSVLYSQQTMDEALKVIEDIKADMGGTEILEPL
>g2772.t2
MVDCCGGLLTADKTTVPLKSVDVEVTVRDHVATVAATLLYENKEQSPIEAVFVFPLPSDAAVCHFSAKIGQSEVVAKVQE
KQKAREEYDDALSAGQQAFL
LEESEQSPDVFSLSVGSLPPGESASIRLEYVTELAVQADDGLRFCLPAVLNPRYQPRGSDGSGGTVSCGQGTSVLASLVP

YSLSLLAGVCSPRPITKVES
NCSLEPVQYLNQQQTQATVKLASGHKFDRDVELLFYYKDSHLPTAVVEAGQASAKPGSLMADPVVMLSMYPEFPPAVMSS
MTKSGEFLFVIDRSGSMDSP
MSHEGVRQMRIDSAKETLLLLLKSLPIGCYFNIYSFGSSYTHIF
>g2773.t1
MSKPKGLISGAGGQKDHLLGALLRIKEVIRRRVESSEEDDADVRGMTQRLEDILKDSS
>g2774.t1
MAVSTRLLCGMVVIWGCMLSCSPVDSKRNRGSQGAIPHPDKYNPNESEQQPQPPPQAGSGSRRRQGSSSAPAAEEVLESS
QEALHVTERRYLKRDWCKTQ
PLKQTIHEEGCISRTIINRFCYGQCNSFYIPRHVRREEGAFQSCSFCKPKRFTTMTLTLNCPDQQPSTKKKRIQRVKQCR
CLSIESLD
>g2775.t1
LADDQNFTFSQMDLAALSPLHIDSALFETGLYHNPAAKTPDGSLHGAAAGSPISHDGSAADEADAEVQRLNCSKLIDALD
IQSPAHFKLGVSAGLQSTPY
RTGLDFHEDLNKTPEEDKSDGVALGTPPVPAQAPDGQRPRVADHIQHFNKLTLYSPKGAKAKGGRTPLKFQRTPVRQSVR
RINSLMGDSRRSVPFAGLAG
PAGKAASMESVLPSAPQLHPYRGEEPAPPPNIKGGAAKKPPPVPPKKPSTLARKHKVCALGDVTNRAPAKLGSGASDPAR
QTDASEGGRSRAQQVADRDV
HHYRGSPRNPLNVGRLLSATKPIDL
>g2776.t1
SYFGDSYCSIQAIQDVSTFQLSLQFKTSRRSCLLLLAAGMEDYMFLELHNGKLQVRMDMGAGEVTLTSSLGVQLKNPLEH
RVSVTLQDSKLTMTIDELLP
TSVPVTGADEEELNIDLGVWLGGTGDLDAPFLSNAIPAFRGCLTNVKFESHGFDILNSELKDCRDTEESCSSEFDGDGVA
TSFSSPDSFISLPTWSYSSW
VNRSLEFLMKTTIEDAVLAFHPGRESDFIAVGVAGGYLRVVVDLGEGMQVLNNSLIRLDDDQWHRVILQVNKTSLEITVD
VNSSFLPLNSSQSLDLVGNL
YLGGIQRKMKDVFHDSGILARMESEITSESFIGCFGEIKVNIKRKTLQDALVTKDVHAKCEGEDYDYSNYYYESTTTSPV
QIRYDVVDQSERHCYPTEGM
PEAFRNITKLLDVTTLLVPEGGEAFLDINNLRPTFNLNSVRMWQSHIIFTLKSNPWHGHIEINLNTKDLKTFTLLDVVNK
KIKYLHDGKQKYLDQIQLEV
AVNSDGYLPIKCLRMPHEYTLMVKVIPVNAIPQLGGGDILGSNATIGVQQLAVIAHPRDGDVVYNVTQPLRYGELIMTTS
NGMPKQITSFYQSDLDQGRV
RYVSTDSSDQEKNVIEHIQFDIHLGQVSKWNNTLIVKILPAPVKISHLDPVGVEAEEEWLISHSHLQAIVNGHMANPQSV
KYVVLRPPTLGSLRFRGGEL
SEGDTFTEQDIRNDDLSYMHHVHSSVEDIDQFQFRVSVEDQQSPIYTMPITILADQNADSTAPHLTTESLVVLQGSEDIL
TKRYLWVQSPSSADFVYHVT
QDPKHGRLIRESPPGHPRFDGGIRVFSNEDLELDRLIYKHDGSKTDSDEFHFTASDEANQEMTAQTSVTGIFRITIQSKN
EHMPVRVVDKTFNVVRNGQR
LLTTDVLQFRDDDANFNDTQIIYAHEGILSGQLVSASNPSQPLFRFTQADLRDKHVLFVHRGADRDQFQLQVSDGLHQTT
TLLQLQAGEPYLRLENNTIV
VMDHGSTSTIDTTLLSAGTNLDIRDDGEVAFEVTSPPGDGRIIVSGVEALKFTQEDLKKGVVSYEHNYENLRPKDSFGFT
VRCKGYSQDGTFRIKIFKQG
YRSEPEVVTNEVLVAYEGEHTVIDQDHLKVEQPDILPSDMVFTIKEPPRLGHVVMLVNNSERADSPVLDYIHSFTQNDVN
EGRVLYVSTSQQGSDVFRTE
VSNGFTTVEDLEVALSIVPGMIPLHGHNVTVKEGMGVAITTEILNISHPFYDDANIDLVVDEAPQHGDLRYKDGTVQELT
YFTWEE
>g2776.t2
SYFGDSYCSIQAIQDVSTFQLSLQFKTSRRSCLLLLAAGMEDYMFLELHNGKLQVRMDMGAGEVTLTSSLGVQLKNPLEH
RVSVTLQDSKLTMTIDELLP
TSVPVTGADEEELNIDLGVWLGGTGDLDAPFLSNAIPAFRGCLTNVKFESHGFDILNSELKDCRDTEESCSSEFDGDGVA
TSFSSPDSFISLPTWSYSSW
VNRSLEFLMKTTIEDAVLAFHPGRESDFIAVGVAGGYLRVVVDLGEGMQVLNNSLIRLDDDQWHRVILQVNKTSLEITVD
VNSSFLPLNSSQSLDLVGNL
YLGGIQRKMKDVFHDSGILARMESEITSESFIGCFGEIKVNIKRKTLQDALVTKDVHAKCEGEDYDYSNYYYESTTTSPV
QIRYDVVDQSERHCYPTEGM
PEAFRNITKLLDVTTLLVPEGGEAFLDINNLRPTFNLNSVRMWQSHIIFTLKSNPWHGHIEINLNTKDLKTFTLLDVVNK

KIKYLHDGKQKYLDQIQLEV
AVNSDGYLPIKCLRMPHEYTLMVKVIPVNAIPQLGGGDILVTEKGRTRLSSNLIKTVDTDRRCDEFKITVSSAPSRNIYL
ENDQQPGRPITAFTCTQLID
GLIYFVHKGGDVEDITLDVSDGHSVSQSTTFKMSVTQPHMTVVANTGIVLSQGSNATIGVQQLAVIAHPRDGDVVYNVTQ
PLRYGELIMTTSNGMPKQIT
SFYQSDLDQGRVRYVSTDSSDQEKNVIEHIQFDIHLGQVSKWNNTLIVKILPAPVKISHLDPVGVEAEEEWLISHSHLQA
IVNGHMANPQSVKYVVLRPP
TLGSLRFRGGELSEGDTFTEQDIRNDDLSYMHHVHSSVEDIDQFQFRVSVEDQQSPIYTMPITILADQNADSTAPHLTTE
SLVVLQGSEDILTKRYLWVQ
SPSSADFVYHVTQDPKHGRLIRESPPGHPRFDGGIRVFSNEDLELDRLIYKHDGSKTDSDEFHFTASDEANQEMTAQTSV
TGIFRITIQSKNEHMPVRVV
DKTFNVVRNGQRLLTTDVLQFRDDDANFNDTQIIYAHEGILSGQLVSASNPSQPLFRFTQADLRDKHVLFVHRGADRDQF
QLQVSDGLHQTTTLLQLQAG
EPYLRLENNTIVVMDHGSTSTIDTTLLSAGTNLDIRDDGEVAFEVTSPPGDGRIIVSGVEALKFTQEDLKKGVVSYEHNY
ENLRPKDSFGFTVRCKGYSQ
DGTFRIKIFKQGYRSEPEVVTNEVLVAYEGEHTVIDQDHLKVEQPDILPSDMVFTIKEPPRLGHVVMLVNNSERADSPVL
DYIHSFTQNDVNEGRVLYVS
TSQQGSDVFRTEVSNGFTTVEDLEVALSIVPGMIPLHGHNVTVKEGMGVAITTEILNISHPFYDDANIDLVVDEAPQHGD
LRYKDGTVQELTYFTWEE
>g2776.t3
MQGQTDNNKQVSYFGDSYCSIQAIQDVSTFQLSLQFKTSRRSCLLLLAAGMEDYMFLELHNGKLQVRMDMGAGEVTLTSS
LGVQLKNPLEHRVSVTLQDS
KLTMTIDELLPTSVPVTGADEEELNIDLGVWLGGTGDLDAPFLSNAIPAFRGCLTNVKFESHGFDILNSELKDCRDTEES
CSSEFDGDGVATSFSSPDSF
ISLPTWSYSSWVNRSLEFLMKTTIEDAVLAFHPGRESDFIAVGVAGGYLRVVVDLGEGMQVLNNSLIRLDDDQWHRVILQ
VNKTSLEITVDVNSSFLPLN
SSQSLDLVGNLYLGGIQRKMKDVFHDSGILARMESEITSESFIGCFGEIKVNIKRKTLQDALVTKDVHAKCEGEDYDYSN
YYYESTTTSPVQIRYDVVDQ
SERHCYPTEGMPEAFRNITKLLDVTTLLVPEGGEAFLDINNLRPTFNLNSVRMWQSHIIFTLKSNPWHGHIEINLNTKDL
KTFTLLDVVNKKIKYLHDGK
QKYLDQIQLEVAVNSDGYLPIKCLRMPHEYTLMVKVIPVNAIPQLGGGDILGSNATIGVQQLAVIAHPRDGDVVYNVTQP
LRYGELIMTTSNGMPKQITS
FYQSDLDQGRVRYVSTDSSDQEKNVIEHIQFDIHLGQVSKWNNTLIVKILPAPVKISHLDPVGVEAEEEWLISHSHLQAI
VNGHMANPQSVKYVVLRPPT
LGSLRFRGGELSEGDTFTEQDIRNDDLSYMHHVHSSVEDIDQFQFRVSVEDQQSPIYTMPITILADQNADSTAPHLTTES
LVVLQGSEDILTKRYLWVQS
PSSADFVYHVTQDPKHGRLIRESPPGHPRFDGGIRVFSNEDLELDRLIYKHDGSKTDSDEFHFTASDEANQEMTAQTSVT
GIFRITIQSKNEHMPVRVVD
KTFNVVRNGQRLLTTDVLQFRDDDANFNDTQIIYAHEGILSGQLVSASNPSQPLFRFTQADLRDKHVLFVHRGADRDQFQ
LQVSDGLHQTTTLLQLQAGE
PYLRLENNTIVVMDHGSTSTIDTTLLSAGTNLDIRDDGEVAFEVTSPPGDGRIIVSGVEALKFTQEDLKKGVVSYEHNYE
NLRPKDSFGFTVRCKGYSQD
GTFRIKIFKQGYRSEPEVVTNEVLVAYEGEHTVIDQDHLKVEQPDILPSDMVFTIKEPPRLGHVVMLVNNSERADSPVLD
YIHSFTQNDVNEGRVLYVST
SQQGSDVFRTEVSNGFTTVEDLEVALSIVPGMIPLHGHNVTVKEGMGVAITTEILNISHPFYDDANIDLVVDEAPQHGDL
RYKDGTVQELTYFTWEE
>g2777.t1
VLGAPPLLRGLSRLRRLGIGGATFQALRRGDLDGVTQLEELILNANNLQSYEPGSLAYIWPLGRVLVNLSSPFLTRPGLA
SSLLQDVSYPETRLVLWDLI
LTTNQSVQPFLETTRRRVRSLGFCNLSLSDEGIVSFLQVTTRAPLTSISIEDATLIGEGRWAKADWTEHWTTDEFLLRDG
KLLDTFKFSSLLEMGFLLTY
PRKISLIGCKVRRAVIIDALSKLWIPPTLSR
>g2777.t2
VLGAPPLLRGLSRLRRLGIGGATFQALRRGDLDGVTQLEELILNANNLQSYEPGSLAYIWPLGRVLVNLSSPFLTRPGLA
SSLLQDVSYPETRLVLWDLI
LTTNQSVQPFLETTRRRVRSLGFCNLSLSDEGIVSFLQVTTRAPLTSISIEDATLIGEGRWAKADWTEHWTTDEFLLRDG

KLLDTFKFSSLLEMGFLLTY
PRKISLIGCK
>g2778.t1
GLRRASEASIASQVSGMADSYTATSIANKRSCQTNQSKKFSLLSQLALTSQNKFFLRSPPKSRRKDRVRGPPDSDPGAGL
APPPDTEEEACESLSPTSQF
ESCPSAGLDYAISDLVSLDSQAEVDQGTEGDGTLLRAAVRPANSRLWSSSSFGRDLSLTHLHLHLPRFVFLLVLLILFDG
TGVVVHLWVSLPFSGIIAAR
WWGTKRMDFALYCPDALTAFPTVALPHLFHASYWESTDVVSFLLRQVMRHENSSILELDGKEVSEFTPSKPREKWLRKRT
HVKIRNVTANHRVSDAVFTE
GGQQVLAGRFMYGPLDMVTLTGEKVSLFCTSLCPPYHPSWLRSREVDLHVMTQPPSGEWVHFDMQVTNSSGRVSFPLPES
QRLGVGVYPIKMVVRGDHTF
ADSYLTVLPRGTEFVVFSIDGSFAASVSIMGSDPKVRAGAVDVVRHWQDLGFLIIYVTGRPDMQKQRVVAWLSQHNFPHG
IVSFCDGLVHDPLRHKANFL
KSLTEVRRGSPT
>g2778.t2
SYPSSPVTGPHFRGLRRASEASIASQVSGMADSYTATSIANKRSCQTNQSKKFSLLSQLALTSQNKFFLRSPPKSRRKDR
VRGPPDSDPGAGLAPPPDTE
EEACESLSPTSQFESCPSAGLDYAISDLVSLDSQAEVDQGTEGDGTLLRAAVRPANSRLWSSSSFGRDLSLTHLHLHLPR
FVFLLVLLILFDGTGVVVHL
WVSLPFSGIIAARWWGTKRMDFALYCPDALTAFPTVALPHLFHASYWESTDVVSFLLRQVMRHENSSILELDGKEVSEFT
PSKPREKWLRKRTHVKIRNV
TANHRVSDAVFTEGGQQVLAGRFMYGPLDMVTLTGEKVSLFCTSLCPPYHPSWLRSREVDLHVMTQPPSGEWVHFDMQVT
NSSGRVSFPLPESQRLGVGV
YPIKMVVRGDHTFADSYLTVLPRGTEFVVFSIDGSFAASVSIMGSDPKVRAGAVDVVRHWQDLGFLIIYVTGRPDMQKQR
VVAWLSQHNFPHGIVSFCDG
LVHDPLRHKANFLKSLTEVRRGSPT
>g2778.t3
SYPSSPVTGPHFRGLRRASEASIASQVSGMADSYTATSIANKRSCQTNQSKKFSLLSQLALTSQNKFFLRSPPKSRRKDR
VRGPPDSDPGAGLAPPPDTE
EEACESLSPTSQFESCPSAGLDYAISDLVSLDSQAEVDQGTEGDGTLLRAAVRPANSRLWSSSSFGRDLSLTHLHLHLPR
FVFLLVLLILFDGTGVVVHL
WVSLPFSGIIAARWWGTKRMDFALYCPDALTAFPTVALPHLFHASYWESTDVVSFLLRQVMRHENSSILELDGKEVSEFT
PSKPREKWLRKRTHVKIRNV
TANHRVSDAVFTEGGQQVLAGRFMYGPLDMVTLTGEKVSLFCTSLCPPYHPSWLRSPNSQLIRGQLGLQVDLHVMTQPPS
GEWVHFDMQVTNSSGRVSFP
LPESQRLGVGVYPIKMVVRGDHTFADSYLTVLPRGTEFVVFSIDGSFAASVSIMGSDPKVRAGAVDVVRHWQDLGFLIIY
VTGRPDMQKQRVVAWLSQHN
FPHGIVSFCDGLVHDPLRHKANFLKSLTEVRRGSPT
>g2779.t1
MNDVPSAELRAQLETSLQGLNIQTTCVDHPPVWTVEDMMPYLQDVGGAVTKNLFLKDKKKKTFWLVSVRHDRQVDLGDLS
KRLGLGSGNLRLADEATMLE
NLKVGRGCATALALLFDTDCKVKLLLDRDLLHGGHQAVHFHPMTNTATLGMQPADLLRFLQHTGHDPILHSFD
>g2779.t2
MNDVPSAELRAQLETSLQGLNIQTTCVDHPPVWTVEDMMPYLQDVGGAVTKNLFLKDKKKKTFWLVSVRHDRQWFCVSWF
SEVDLGDLSKRLGLGSGNLR
LADEATMLENLKVGRGCATALALLFDTDCKVKLLLDRDLLHGGHQAVHFHPMTNTATLGMQPADLLRFLQHTGHDPILHS
FD
>g2780.t1
MTSSSPLNPWPHLIGQWAGEKLLNDPSVLSHPYLLLNDPSALSHPNLLLNDPSVLSNPGVSNSFWFRGHIQPN
>g2781.t1
MLPYEATQPRLSRWLPAAKVRPVPVPRTGRYENHTVAVIPVNNLSLAYRTGDMVVLCGGSASAAVNRLMGVAFPARPQVH
LPLPVTKKLSDLLYWRDVKT
TGVVFGAALLLLLSLTACSIVSVASYVALVLLSVTVSFRIYRGILQAIQKSDEGHPFKLYLDQEVALSEDVAHKYSDLVL
ARVNQTIVELRRLFLVDDLV
DSIKFAVLMWILTYVGALFNGLTLLILGLVAVFSFPIIYEKHQVQIDHYVGMVNNQIKDIVGKIQAKVPGAKRKAE
>g2781.t2

MLPYEATQPRLSRWLPAAKVRPVPVPRTGRYENHTVAVIPVNNLSLAYRLAFRTLSDLLYWRDVKTTGVVFGAALLLLLS
LTACSIVSVASYVALVLLSV
TVSFRIYRGILQAIQKSDEGHPFKLYLDQEVALSEDVAHKYSDLVLARVNQTIVELRRLFLVDDLVDSIKFAVLMWILTY
VGALFNGLTLLILGLVAVFS
FPIIYEKHQVQIDHYVGMVNNQIKDIVGKIQAKVPGAKRKAE
>g2781.t3
MLPYEATQPRLSRWLPAAKVRPVPVPRTGRYENHTVAVIPVNNLSLAYRPQVHLPLPVTKKLSDLLYWRDVKTTGVVFGA
ALLLLLSLTACSIVSVASYV
ALVLLSVTVSFRIYRGILQAIQKSDEGHPFKLYLDQEVALSEDVAHKYSDLVLARVNQTIVELRRLFLVDDLVDSIKFAV
LMWILTYVGALFNGLTLLIL
GLVAVFSFPIIYEKHQVQIDHYVGMVNNQIKDIVGKIQAKVPGAKRKAE
>g2782.t1
LVSIGSSYNYGSEDQAEFLCVVSKELHNQSYGTNSEPSEKAK
>g2783.t1
MVLGKLEDFPTSIPFFPGELFEIGPALSENCSRILRKWPARLAQGEVLRECDKKKKKKKKKKKKKKKKKKKRKEDKAEM
>g2784.t1
MPQQHDAVDLRVQVNCGEEAILENSICGSLVVVIQCVSNIHPVEELNPIHLVEELNLIHLVEELNPIHLVEELNLIHLVE
ELNVKCITSSDDMVVGVRYY
ITHSPHRQKQLCIELAFSEH
>g2784.t2
MPQQHDAVDLRVQVNCGEEAILENSICGSLVVVIQCVSNIHPVEELNPIHLVEELNLIHLVEELNPIHLVEELNLIHLVE
ELNVKCITSSDDMVVVLRWL
PGHPGSVLPCSGSERSLYFVFPGRQSS
>g2784.t3
MPQQHDAVDLRVQVNCGEEAILENSICGSLVVVIQCVSNIHPVEELNPIHLVEELNLIHLVEELNPIHLVEELNLIHLVE
ELNVKCITSSDDMVVGVRYY
ITHSPHRQKQLCIELAFMLRWLPGHPGSVLPCSGSERSLYFVFPGRQSS
>g2785.t1
NSIRHNLSLHSRFVRVQNEGTGKSSWWMLNPEGGKSGKSPRRRAASMDNNSKFSKSRGRAAKKKLSLQGGPEGGSESPGS
SYSKWPGSPNSHSNDDFDAW
NSFRPRTSSNASTLSGRLSPFMPEDDLGHEGDVHMVYPGATGSKMASTLPSLTEMAGSLGHSSENVMENLLDNLNLLSPN
NSQVVGGPSTPGSSQSSQSS
PLMQGSPGYPSYSSPSMGPPPQQQQQQQQQQQQQQQQDFRKCMYGGQAGMNALTSRPMQPMAETKPGFGVPSLGGYPCPA
GLLKELLTNDSEQHGDLMPP
TVESVVSPQGGRCMLPSYSSVQHGAKHLARGNGLQHGLTHARPHPHNILNQSPVAPMNGGRLLMSLNHGSGTARLPPLKS
PMQMPYGLVNHLGGVGGGGG
GGGVGGSGIVGGLGGLPGGYCPLNTNGYGRPGPLQPQHHPEKLPSDLDDMSIERFECDVESIIQDTLMDGDSLDFNFEPM
VTQPGFNHSVKTTTHSWVSG
>g2785.t2
MNSIRHNLSLHSRFVRVQNEGTGKSSWWMLNPEGGKSGKSPRRRAASMDNNSKFSKSRGRAAKKKLSLQGGPEGGSESPG
SSYSKWPGSPNSHSNDDFDA
WNSFRPRTSSNASTLSGRLSPFMPEDDLGHEGDVHMVYPGATGSKMASTLPSLTEMAGSLGHSSENVMENLLDNLNLLSP
NNSQVVGGPSTPGSSQSSQS
SPLMQGSPGYPSYSSPSMGPPPQQQQQQQQQQQQQQQQDFRKCMYGGQAGMNALTSRPMQPMAETKPGFGVPSLGGYPCP
AGLLKELLTNDSEQHGDLMP
PTVESVVSPQGGRCMLPSYSSVQHGAKHLARGNGLQHGLTHARPHPHNILNQSPVAPMNGGRLLMSLNHGSGTARLPPLK
SPMQMPYGLVNHLGGVGGGG
GGGGVGGSGIVGGLGGLPGGYCPLNTNGYGRPGPLQPQHHPEKLPSDLDDMSIERFECDVESIIQDTLMDGDSLDFNFEP
MVTQPGFNHSVKTTTHSWVS
G
>g2786.t1
MMVGGYLWGYLADLRGRQRVLVVSLSVNGLFGALASLAPWFWLFLLMRFISGVGVGGSIPVIFSYFSEFLPRLRRGAMIS
SLATFWMAGNILAAGRLPRP
TLPLLATAKLSLPANAGIDVRSPILSPRSLHFCTVVLN
>g2787.t1
MPGQQQVCPKPLSKKVFYLDLPSSRRADALEDDIKLLGGTVEKFFSKDIRYLVSNKREAKYVERLRRELGAPSPVSGQSS

PFPHRGGHRPAGSHKDRLKP
GSPGGYEPGEVSGGESGEGAANRITTPFIKVEDSSRHYRPLYAAMSKMPELNTETAPPCTPFCADKKDQNRNGHSEERGH
VRAKTNRDKNQAGFCECCML
QYDNLSKHVQSETHLAFSKGNEYLVLDRLISTMQCDLALLKPRTKRPKCSVSSTLAVPGPHGREPHKPQVASVTEKGLKP
DRISTAAGSREPLKGLATKP
TPGSVQTSGANGNLRNPDRTSARPLPRHTPHTGGTDSVSDPSARTGPSVQTPALQVELAPSRGSSLDPATLCRHHTEDSQ
RGCSPRTPRPLQEGGGEGVR
DRPSECHQNEPAPREQETRIPACQGVHPHEEWPSPVTTIQRKVRVQKRKRRRVDTGLAVQVRSACDIPDYSLLYLWQLFH
SSDHMDAE
>g2787.t2
MPGQQQVCPKPLSKKVFYLDLPSSRRADALEDDIKLLGGTVEKFFSKDIRYLVSNKREAKYVERLRRELGAPSPVSGQSS
PFPHRGGHRPAGSHKDRLKP
GSPANRITTPFIKVEDSSRHYRPLYAAMSKMPELNTETAPPCTPFCADKKDQNRNGHSEERGHVRAKTNRDKNQAGFCEC
CMLQYDNLSKHVQSETHLAF
SKGNEYLVLDRLISTMQCDLALLKPRTKRPKCSVSSTLAVPGPHGREPHKPQVASVTEKGLKPDRISTAAGSREPLKGLA
TKPTPGSVQTSGANGNLRNP
DRTSARPLPRHTPHTGGTDSVSDPSARTGPSVQTPALQVELAPSRGSSLDPATLCRHHTEDSQRGCSPRTPRPLQEGGGE
GVRDRPSECHQNEPAPREQE
TRIPACQGVHPHEEWPSPVTTIQRKVRVQKRKRRRVDTGLAVQVRSACDIPDYSLLYLWQLFHSSDHMDAE
>g2787.t3
MPGQQQVCPKPLSKKVFYLDLPSSRRADALEDDIKLLGGTVEKFFSKDIRYLVSNKREAKYVERLRRELGAPSPVSGQSS
PFPHRGGHRPAGSHKDRLKP
GSPGGYEPGEVSGGESGEGAANRITTPFIKVEDSSRHYRPLYAAMSKMPELNTETAPPCTPFCADKKDQNRNGHSEERGH
VRAKTNRDKNQAGFCECCML
QYDNLSKHVQSETHLAFSKGNEYLVLDRLISTMQCDLALLKPRTKRPKCSVSSTLAVPGPHGREPHKPQVASVTEKGLKP
DRISTAAGSREPLKGLATKP
TPGSVQTSGANGNLRNPDRTSARPLPRHTPHTGGTDSVSDPSARTGPSVQTPALQEGGGEGVRDRPSECHQNEPAPREQE
TRIPACQGVHPHEEWPSPVT
TIQRKVRVQKRKRRRVDTGLAVQVRSACDIPDYSLLYLWQLFHSSDHMDAE
>g2788.t1
MAVPATVIYFTCYDQLCAALKVRLGEHADKAPLLSGAIARVGSVTVISPLELIRTKLQSQRQSYRELRACISSAVRAGGW
LSLWRGWGPTLLRDVPFSAM
YWYNYEKGKAWMCERSNASEPTFAITFLSGAASGSVMASSKTSSSTLSVMSRIVAENGIPGLFAGFLPRLIKVAPACAIM
ISTYELGKAFFHKQNQETRP
VRFQSGSS
>g2788.t2
MAVPATVIYFTCYDQLCAALKVRLGEHADKAPLLSGAIARVGSVTVISPLELIRTKLQSQRQSYRELRACISSAVRAGGW
LSLWRGWGPTLLRDVPFSAM
YWYNYEKGKAWMCERSNASEPTFAITFLSGAASGSIASMVTLPFDVVKTRRQVELGELQALNLASSKTSSSTLSVMSRIV
AENGIPGLFAGFLPRLIKVA
PACAIMISTYELGKAFFHKQNQETRPVRFQSGSS
>g2789.t1
MASLSLGLHLARKRGSIRSGAVERRNLLTVCRFSVKTLLDRSCFETIDDSSPEFINFVSILEHIFSHRLKGQMTWFGYES
TRTFWDYVKVACSKVPHSCI
RSIESMENVQSSRAKGRAWIRVVLMEKRLSEYISSALREFKTSRRFYEEGAIMLGEEAGLLADTLIGLNTIDFSFCLKGE
GLDGAYPSVIDYTPYLKFTL
NADSISSDEDEMRTVGSSGSESSTPDKMATAATIFTEQSSLVSKCKRLEQKYRMVLEQKSYLEDLVRLREVQLSKTMSNN
SALEQKLADTHSSHSVEKEQ
LEYIILELQDQLTILKNNDLRSRQELTAHLTNQWPSPGTLDASAVALDTLLFRKRTEQWEEKSFQSLEQLSVDMSLSQTS
LDPSHTPSAETRPVGANWPH
HAKEETPSLRGLCGSLTSVASYKSLASLKSSECLASPTTETSTPGLTPS
>g2789.t2
MASLSLGLHLARKRGSIRSGAVERRNLLTVCRFSVKTLLDRSCFETIDDSSPEFINFVSILEHIFSHRLKGQMTWFGYES
TRTFWDYVKVACSKVPHSCI
RSIESMENVQSSRAKGRAWIRVVLMEKRLSEYISSALREFKTSRCGSRFYEEGAIMLGEEAGLLADTLIGLNTIDFSFCL
KGEGLDGAYPSVIDYTPYLK

FTLNADSISSDEDEMRTVGSSGSESSTPDKMATAATIFTEQSSLVSKCKRLEQKYRMVLEQKSYLEDLVRLREVQLSKTM
SNNSALEQKLADTHSSHSVE
KEQLEYIILELQDQLTILKNNDLRSRQELTAHLTNQWPSPGTLDASAVALDTLLFRKRTEQWEEKSFQSLEQLSVDMSLS
QTSLDPSHTPSAETRPVGAN
WPHHAKEETPSLRGLCGSLTSVASYKSLASLKSSECLASPTTETSTPGLTPS
>g2789.t3
MASLSLGLHLARKRGSIRSGAVERRNLLTVCRFSVKTLLDRSCFETIDDSSPEFINFVSILEHIFSHRLKGQMTWFGYES
TRTFWDYVKVACSKVPHSCI
RSIESMENVQSSRAKGRAWIRVVLMEKRLSEYISSALREFKTSRRFYEEGAIMLGEEAGLLADTLIGLNTIDFSFCLKGE
GLDGAYPSVIDYTPYLKFTL
NADSISSDEDEMRTVGSSGSESSTPDKMATAATIFTEQSSLVSKCKRLEQKYRMVLEQKSYLEDLVRLREVQLSKTMSNN
SALEQKLADTHSSHSVEKEQ
LEYIILELQDQLTILKNNDLRSRQELTAHLTNQWPSPGTLDASAVALDTLLFRKRTEQWEE
>g2790.t1
MVCTPLESSLHFPRSVGCTQEPSKPRGTYSTTGTRRFSYGDACVSGQWLTAVILSVCCLLPSCLPAGQTVDYSSVESVVA
RQGDTAILSLLPPSSIT
>g2790.t2
MVCTPLESSLHFPRSVGCTQEPSKPRGTYSTTGTRRFSYGWLTAVILSVCCLLPSCLPAGQTVDYSSVESVVARQGDTAI
LSLLPPSSIT
>g2791.t1
MCLNKLHRKACPFDQTAISTDIEQLPVNTALLQLVGGQVPKAQPVALITSPEDSQHYEEARQCVEELALYLKPLSNTR
>g2791.t2
MPVQAPQWTEFLLCPICTQTFEETVRRPISLGCGHTVCKMCLNKLHRKACPFDQTAISTDIEQLPVNTALLQLVGGQVPK
AQPVALITSPEDSQHYEEAR
QCVEELALYLKPLSNTR
>g2791.t3
MCLNKLHRKACPFDQTAISTDIEQLPVNTALLQLVGGQPVALITSPEDSQHYEEARQCVEELALYLKPLSNTR
>g2792.t1
VAAPGAATRGSFSSYASSGRGSMETGSGGRLSVCLSPTLSKCPDEIEEREEDLGPHRDDARR
>g2793.t1
WSRSDPQLLSRGLCVVETHICTMFKLVVFLLCTAAVPSDSKKDVATGPRLDDHQLYRFSYGAEVLLDRSGGSRRGGTGYR
ITSDVSVHLVWRDPGSKDDQ
LLQLAISNVAVENVAQRSAKDNILRGSTAESLMGKGPLGALTRPYMVHLRNGKVKAFYSYREEPATIKNLKRGLASLLQV
QLKSGKVIEDATERRGSLTL
TQGSPQNDVSGRCTVRYQAAEGRVTRTKLLETCQTAETSFTTHSQVMGVTKEARSVTSFTLSDGFIESAEAEEVHTLVAN
ARRSAATEVTSRQSLRFVKR
EAGPLEVPGKDVAAVVASVDANLAAIGVMAEPVKSQCKGCPTLEEHWQANKNQFEPEGLSKATAPRSFLALIQSIRKATK
EEILKVLKGVSKTALPQVVD
AVTSAQTPASLDAILSFLNFTDAKGLVLQERFLYACGFASHPNEKMLQALLHISKGKIGSSEIKESVVIIMGALVNKLCH
KGGCDSPTVVQVKKMILEGP
ESTEVEAEIQMYLLALKNSLLPQAIPIFTKYAESEVGAFCTISLSALQRYDVKLITEEVKHTVNRIYHQNRRIYEKNVRA
AAADVILSSSPSYEHVKNLL
LSIGHLPHEMNKYMVSKIQDILRFELPASEVVRQAMKDMVSHNYDRFAKVGSSSSYSGFMAKTADVSSTYSLDILYSGSG
IMRRSNMNIYGASSGATLHG
LQVAIEAQGLETLIAATPDEGEQDLESYAGMSALLFDVQLRPVTFFKGYSDLMSKIFSMSGEPMNVVKGLILLTDHSEVI
QLQSGVKVSAEFQGGLAIDI
SGGMEISLWYRESKTSVNN
>g2793.t2
WSRSDPQLLSRGLCVVETHICTMFKLVVFLLCTAAVPSDSKKDVATGPRLDDHQLYRFSYGAEVLLDRSGGSRRGGTGYR
ITSDVSVHLVWRDPGSKDDQ
LLQLAISNVAVENVAQRSAKDNILRGSTAESLMGKGPLGALTRPYMVHLRNGKVKAFYSYREEPATIKNLKRGLASLLQV
QLKSGKVIENDVSGRCTVRY
QAAEGRVTRTKLLETCQTAETSFTTHSQVMGVTKEARSVTSFTLSDGFIESAEAEEVHTLVANARRSAATEVTSRQSLRF
VKREAGPLEVPGKDVAAVVA
SVDANLAAIGVMAEPVKSQCKGCPTLEEHWQANKNQFEPEGLSKATAPRSFLALIQSIRKATKEEILKVLKGVSKTALPQ
VVDAVTSAQTPASLDAILSF

LNFTDAKGLVLQERFLYACGFASHPNEKMLQALLHISKGKIGSSEIKESVVIIMGALVNKLCHKGGCDSPTVVQVKKMIL
EGPESTEVEAEIQMYLLALK
NSLLPQAIPIFTKYAESEVGAFCTISLSALQRYDVKLITEEVKHTVNRIYHQNRRIYEKNVRAAAADVILSSSPSYEHVK
NLLLSIGHLPHEMNKYMVSK
IQDILRFELPASEVVRQAMKDMVSHNYDRFAKVGSSSSYSGFMAKTADVSSTYSLDILYSGSGIMRRSNMNIYGASSGAT
LHGLQVAIEAQGLETLIAAT
PDEGEQDLESYAGMSALLFDVQLRPVTFFKGYSDLMSKIFSMSGEPMNVVKGLILLTDHSEVIQLQSGVKVSAEFQGGLA
IDISGGMEISLWYRESKTSV
NN
>g2793.t3
MLSSISSQHISKGKIGSSEIKESVVIIMGALVNKLCHKGGCDSPTVVQVKKMILEGPESTEVEAEIQMYLLALKNSLLPQ
AIPIFTKYAESEVGAFCTIS
LSALQRYDVKLITEEVKHTVNRIYHQNRRIYEKNVRAAAADVILSSSPSYEHVKNLLLSIGHLPHEMNKYMVSKIQDILR
FELPASEVVRQAMKDMVSHN
YDRFAKVGSSSSYSGFMAKTADVSSTYSLDILYSGSGIMRRSNMNIYGASSGATLHGLQVAIEAQGLETLIAATPDEGEQ
DLESYAGMSALLFDVQLRPV
TFFKGYSDLMSKIFSMSGEPMNVVKGLILLTDHSEVIQLQSGVKVSAEFQGGLAIDISGGMEISLWYRESKTSVNN
>g2793.t4
WSRSDPQLLSRGLCVVETHICTMFKLVVFLLCTAAVPSDSKKDVATGPRLDDHQLYRFSYGAEVLLDRSGGSRRGGTGYR
ITSDVSVHLVWRDPGSKDDQ
LLQLAISNVAVENVAQRSAKDNILRGSTAESLMGKGPLGALTRPYMVHLRNGKVKAFYSYREEPATIKNLKRGLASLLQV
QLKSGKVIEDATERRGSLTL
TQGSPQNDVSGRCTVRYQAAEGRVTRTKLLETCQTAETSFTTHSQVMGVTKEARSVTSFTLSDGFIESAEAEEVHTLVAN
ARRSAATEVTSRQSLRFVKR
EAGPLEVPGKDVAAVVASVDANLAAIGVMAEPVKSQCKGCPTLEEHWQANKNQFEPEGLSKATAPRSFLALIQSIRKATK
EEILKVLKGVSKTALPQVVD
AVTSAQTPASLDAILSFLNFTDAKGLVLQERFLYACGFASHPNEKMLQALLVGAS
>g2794.t1
MQALELHLQREAELNPQASYYNVGLLKYQVSSQDPGRAPLLLSAECQRSGTVTRVSLDYHCCPATAPSTQLAAVQVLLPL
DHTATDLQCQPPATWNAEER
RLLWKLSNLSPTNHSKGQLPLPPLPVFCSFRGIKASEVCCLASFGLCCTFTISLCVQLCCTFTISLRVQLCCTFTISLSV
SSSAARSPSLSLCPALLHIL
KAKRT
>g2794.t2
MQALELHLQREAELNPQASYYNVGLLKYQVSSQDPGRAPLLLSAECQRSGTVTRVSLDYHCCPATAPSTQLAAVQVLLPL
DHTATDLQCQPPATWNAEER
RLLWKLSNLSPTNHSK
>g2794.t3
MQALELHLQREAELNPQASYYNVGLLKYQVSSQDPGRAPLLLSAECQRSGTVTRVSLDYHCCPATAPSTQLAAVQVLLPL
DHTATDLQCQPPATWNAEER
RLLWKLSNLSPTNHSKALLHILKAKRT
>g2795.t1
LCAEKDSIAPPSVTVVEGERSVTLLLAGLEKSTLYALQVLAYTRLGDGPPSGPVLNRTKEDGEGLSLGARSACRV
>g2796.t1
MSYSFPDRLFPTHYNSGPPNCGSKHAAGGWGEYKGILNMPVQGVSGETYPRFVPRRQTRGEPWSPSEPLVLPLLPPKPIP
LWHLDSSDSTDLGQHMGNFY
LKHYSDAFSSVSGLLRDHLDFCPKKCQRSGAMNLGWAADFLSKLKYKKCEYSYLRDHVVAYDNLLRDVIHDIPPALLGGL
LREELQLTGQRLQFSEATTG
GALGHAALPQDTWLVYANGPAFNLLNFQRVSLRSEEAAAPWLDASSKPTSVELKGPVRQISCSTLLTD
>g2796.t2
MSYSFPDRLFPTHYNSGPPNCGSKHAAGGWGEYKGILNMPVQGVSGETYPRFVPRRQTRGEPWSPSEPLVLPLLPPKPIP
LWHLDSSDSTDLGQHMGNFY
LKHYSDAFSSVSGLLRDHLDFCPKKCQRSGAMNLGWAADFLSKLKYKKCEYSYLRDHVVAYDNLLRDVIHDIPPALLGGL
LREELQLTGQRLQFSEATTG
GALGHAALPQDTWLVYANGPAFNLLNFQRVSLRSEEAAAPWLDASSKPTSVELKGP
>g2796.t3

LFPTHYNSGPPNCGSKHAAGGWGEYKGILNMPVQGVSGETYPRFVPRRQTRGEPWSPSEPLVLPLLPPKPIPLWHLDSSD
STDLGQHMGNFYLKHYSDAF
SSVSGLLRDHLDFCPKKCQRSGAMNLGWAADFLSKLKYKKCEYSYLRDHVVAYDNLLRDVIHDIPPALLGGLLREELQLT
GQRLQFSEATTGGALGHAAL
PQDTWLVYANGPAFNLLNFQRVSLRSEEAAAPWLDASSKPTSVELKGPVRQISCSTLLTD
>g2797.t1
MSAGPDVDVWDILQKAPATEYEKIAFQHGITDLRGMLKRLKKMKKEEKKSEAFNRKLDPAYQIEKGNKIKLAIEVANPNA
EVKWLKNGQEIQPTGRYIFE
SVGNMRYLTINHCSLADDAAYACVVGDEKCTTELFVKEPPVTIVKELEDQMVTTGERVELECEVSEEGALVKWEKDGVEL
TRDEAFKYRFKKDGLKHILI
INEASKEDCGHYRVKTNGGESVAELLVQEKDLEVYQSIADLTVKAKDEAFFKCEVSDEK
>g2797.t2
MSAGPDVDVWDILQKAPATEYEKIAFQHGITDLRGMLKRLKKMKKEEKKSEAFNRKLDPAYQIEKGNKIKLAIEVANPNA
EVKWLKNGQEIQPTGRYIFE
SVGNMRYLTINHCSLADDAAYACVVGDEKCTTELFVKEPPVTIVKELEDQMVTTGERVELECEVSEEGALVKWEKDGVEL
TRDEAFKYRFKKDGLKHILI
INEASKEDCGHYRVKTNGGESVAELLVQEKDLEVYQSIADLTVKAKDEAFFKCEVSDEKVKGTWFKNGVEVQPSSRTHIT
HIGRCV
>g2798.t1
EEPGDDFLAVFDSSTDGRRKLASLSKASPDRTTWNILDDQPRVFPLASSARSTGSVDSQPGMKRRDHGLSLAANFTANN
>g2799.t1
YVIMQMLVCWGNYSVLNNDCEKSPLLYFALFTLRCFMQDNLENLL
>g2800.t1
LRENCKYLHPPSHLKTQLEINGRNNLIQQKTAAAMLAQQMQFMIPGTSMQAMGLGPSSSLGYSPYLTPMNHGMGLVSTEM
LPSSQLLVSGSPAVTVQSSN
ASSSPSQKQRSDKLEARGTLMTATTRFHTGLAQREPCLTCSWFAPCGVRVVDGQFKQPRLAPSEADEPLGLAEAAHLWCV
SSSKLTG
>g2800.t2
LRENCKYLHPPSHLKTQLEINGRNNLIQQKTAAAMLAQQMQFMIPGTSMQAMGLGPSSSLGYSPYLTPMNHGMGLVSTEM
LPSSQLLVSGSPAVTVQSSN
ASSSPSQKQRSDKLEREPCLTCSWFAPCGVRVVDGQFKQPRLAPSEADEPLGLAEAAHLWCVSSSKLTG
>g2801.t1
METLEDIYIGSIETDRGTREQVRFYDTRGLRDGMEFPRHYYSFADGFVLVYSIDSKESFKRMEALKKDIDRQRDKKEVTI
VVLGNKLDRDGERKVEHNAA
QNWAKSERVRLWEVSVVDRHTLIEPFVYLASKMTQPQSKSTFPLSRNKNKSS
>g2801.t2
MGKSCKVVVCGLTAVGKTAVLEQLLYANHVAGSEPMETLEDIYIGSIETDRGTREQVRFYDTRGLRDGMEFPRHYYSFAD
GFVLVYSIDSKESFKRMEAL
KKDIDRQRDKKEVTIVVLGNKLDRDGERKVEHNAAQNWAKSERVRLWEVSVVDRHTLIEPFVYLASKMTQPQSKSTFPLS
RNKNKSS
>g2802.t1
MASVDCDDVNSDLDMDMLSDEELEREAEGFKEQGNAFYIKKNYSEAFNYYTKAIDMCPKNPSYYGNRAATLMMLCRYREA
LEDSQQAVRLDDTFMKGHLR
EGKCHLSLGNAMAAKRCFQRVLEMEHDNNQCQQELKNAESILEYEKMAEIGFEKRDFRMVVFCMDRALESAPACHRFKVL
KAECLALLGRYPEAQGVASD
ILRMDSTNGDALYVRGLCLYYEDCIDKAVQFFVHALHMAPDHEKARLACRNAKALKSKKEEGNKAFKEGDYEAAYELYSA
ALTIDPNNIKTNAKLFCNRG
TVGSKLKKLDQAIDDCTKAIKLDESYIKAYLRRAQCYMDTEQYEEAVRDYEKVYHTEKTKGEPPVGLVWFVGLMWLVGFV
GLMWLMGLVELMWLVGFVDL
VGFVDLVGLMWLVGLAGSWGLWGSGAELPLEPGRSIIEHKHFLKNAQLELKKSKRKDYYKVLGVDKNASEDEIKKAYRKR
ALMHHPDRHSGATVEVQKEE
EKKFKEVGEAFTVLSDPKKKARYDSGQDLEEEGVNMGGENLGRTWRRRRRRRRE
>g2802.t2
MASVDCDDVNSDLDMDMLSDEELEREAEGFKEQGNAFYIKKNYSEAFNYYTKAIDMCPKNPSYYGNRAATLMMLCRYREA
LEDSQQAVRLDDTFMKLKNA
ESILEYEKMAEIGFEKRDFRMVVFCMDRALESAPACHRFKVLKAECLALLGRYPEAQGVASDILRMDSTNGDALYVRGLC

LYYEDCIDKAVQFFVHALHM
APDHEKARLACRNAKALKSKKEEGNKAFKEGDYEAAYELYSAALTIDPNNIKTNAKLFCNRGTVGSKLKKLDQAIDDCTK
AIKLDESYIKAYLRRAQCYM
DTEQYEEAVRDYEKVYHTEKTKGEPPVGLVWFVGLMWLVGFVGLMWLMGLVELMWLVGFVDLVGFVDLVGLMWLVGLAGS
WGLWGSGAELPLEPGRSIIE
HKHFLKNAQLELKKSKRKDYYKVLGVDKNASEDEIKKAYRKRALMHHPDRHSGATVEVQKEEEKKFKEVGEAFTVLSDPK
KKARYDSGQDLEEEGVNMGG
ENLGRTWRRRRRRRRE
>g2802.t3
MASVDCDDVNSDLDMDMLSDEELEREAEGFKEQGNAFYIKKNYSEAFNYYTKAIDMCPKNPSYYGNRAATLMMLCRYREA
LEDSQQAVRLDDTFMKGHLR
EGKCHLSLGNAMAAKRCFQRVLEMEHDNNQCQQEVESILEYEKMAEIGFEKRDFRMVVFCMDRALESAPACHRFKVLKAE
CLALLGRYPEAQGVASDILR
MDSTNGDALYVRGLCLYYEDCIDKAVQFFVHALHMAPDHEKARLACRNAKALKSKKEEGNKAFKEGDYEAAYELYSAALT
IDPNNIKTNAKLFCNRGTVG
SKLKKLDQAIDDCTKAIKLDESYIKAYLRRAQCYMDTEQYEEAVRDYEKVYHTEKTKGEPPVGLVWFVGLMWLVGFVGLM
WLMGLVELMWLVGFVDLVGF
VDLVGLMWLVGLAGSWGLWGSGAELPLEPGRSIIEHKHFLKNAQLELKKSKRKDYYKVLGVDKNASEDEIKKAYRKRALM
HHPDRHSGATVEVQKEEEKK
FKEVGEAFTVLSDPKKKARYDSGQDLEEEGVNMGGENLGRTWRRRRRRRRE
>g2803.t1
EVVFWRGGRRQTAQRLTRCQTLGPAATPASPLRAPAGSVLPAESGPIVRTGSCSDRKTDRALFGNTLESVPPEATSCPEI
SSSETALLRESPAGLLEQCL
VVTLRHTAAPQGATGRVSSLVVSVVFHGVSSLVTPITCPHKDSGGEGPYPPPPCLHHHHPTGSEWPLVGKAAVDGGLHPA
GGAASGSTCPHSEVG
>g2804.t1
MDAQRSFPACSKMAAGDTHSSEEVHELAAPLSKALAPHWPQPGRGDQGRGSTRSPGLGLGVGPPPKALEQSPEQGRDRPA
DVEAGGE
>g2805.t1
LEAGSAPLEAGSAPPRRCTAPLNPPASSSSFPPARLWAVRSTTGTRAILPRPPAGQKKSSLMNGGLLVLHQLEWRWRAWP
PAGLRPEVPKPSVRPSGARG
KETSRVSIVMEMIRACQNQKLWRTWPSGRGPLDPEHHSPS
>g2806.t1
GARGGGGGGGAGGLMGFLPEYTGSSDEMEMKQEVVDSFSSDSVQSLDSNSHQGPGKRKRADRKLVSLKRKKLRLMKAMLQE
QRRLSRSMEESSREVRRALQ
QQNFLQIQTLQLQERMMNLLEKMAQPPHPGPSAPWAQGGGKDLGKP
>g2807.t1
MRIMGQPEGVRRRLDLGLNPLADLRHVEVVRGRAGYGFSVSGQGPCLLSGILEGSLADQVGLKQGDRVMAVNGADVSDAP
HETVVQLIGTCNGPLRLAVL
PDGRSPGTPAVGEDDDGGDAGDGRDLYQKVAVFHAVSDDLAHPSVFNPGPASGSKPRPVSEPDMSCWSQHLDSLVRQTQE
ECLVLEDMDSVCTDPEEAAA
TADWSLLNMTMVVGYLSSTELLLHTGESEESCLEAIRQCIRRLGTEQDTHTLVLMKVMFDCVRLCDDTGAVLAAFPAENL
LLGAVCSEDARFFCLVTTAH
VADVRAAPREGGPLRASCHVFFIDPELARHPEHLGIAGRFGFRCTPDPDAAGCLEFPLAPQDVLHFVSVLYGDMGEAVER
IRVRLDLEAQLQAQEGEEQG
AGKQGSTSSTGDSGIGNASPPEERTDRDFPPAEAEAAEAAAGGSHTGVHLPSCPWDYAADEELTPRGRAQTTPSLHCNLV
SHLDEGYPLSESLPGPEALM
GLYGGPPPRLEFQFKPPPPPLPPGKKQNFFGGPLRNSQKWFSKQKWPKSRSGPTDYQAVLANAWPSSVSLGAANANFLAQ
SMSQIPADRYDTADPGVMLP
PKEDWTKRFLEQNTRHGKEGNHKVRY
>g2808.t1
MYEARLCVCVSLTGFEFFEASAKDNINVKQTFERLVDIICEKMSDSLDNVDPAVTGVKQGPQLTEQ
>g2809.t1
MAGYSVASTTPCRPCGPLASSRRLQCPRRFPRGHADNAVNPKTQREEQSSVQEVHDSRFVRPEPGFVHSIKRRVCCSGVS
ESWWVLAQLEAQKATQEFQR
AVEILRAAKETIALAEERLLEEDSRQFDSAWQEMLNHATQRVMEAEQARTRSEVEHKKTAANYNSCIGHMKQLEKKLKRN

INKSRPYFELKA
>g2809.t2
MAGYSVASTTPCRPCGPLASSRRLQCPRRFPRGHADNAVNPKTQREEQSSVQEVHDSRFVRPEPGFVHSIKRRVCCSGVS
ESWWVLAQLEAQKATQEFQR
AVEILRAAKETIALAEERLLEEDSRQFDSAWQEMLNHATQRVMEAEQARTRSEVEHKKTAANYNSCIGHMKQLEKKLKRN
INKS
>g2809.t3
LSSEMAGYSVASTTPCRPCGPLASSRRLQCPRRFPRGHADNAVNPKTQREEQSSVQEVHDSRFVRPEPGFVHSIKRRVCC
SGVSESWWVLAQLEAQKATQ
EFQRAVEILRAAKETIALAEERLLEEDSRQFDSAWQEMLNHATQRVMEAEQARTRSEVEHKKTAANYNSCIGHMKQLEKK
LKRNINKSRPYFELKA
>g2810.t1
MARPGSGVSGLGYPPQNLARVVVWEWLNEHGRWRPYSAAVCHHIENVLKGDARGTVILGQVDGQLTPYIIDLQSMHQFRQ
DTDFLSA
>g2811.t1
MASDKGRTRCDGRVSEEERGFYEMKNRACEYYNRNKVPQQIEDVLNDMFFANPDDIYGYLANFFMKLSAPPRISGLNGRE
IYDANGQLSVQAEVLCTIRN
KQKVQL
>g2811.t2
MNAKKEFLRAEDAGPVAFSCRATAGRTRCDGRVSEEERGFYEMKNRACEYYNRNKVPQQIEDVLNDMFFANPDDIYGYLA
NFFMKLSAPPRISGLNGREI
YDANGQLSVQAEVLCTIRNKQKVQL
>g2811.t3
MASDKGRTRCDGRVSEEERGFYEMKNRACEYYNRNKVPQQIEDVLNDMFFANPDDIYGYLANFFMKLSAPPRISGLNGRE
IYDANGQLSVQAEVLCTIRN
KQK
>g2812.t1
MSRKFFVGGNWKMNGDKKSLGELIQTLNSAKLDANVEVVCGCPAIYLDFVRSKMDAKIGVAAQNCYKVAKGAFTGEISPA
MIKDCGVNWVILGHSERRHV
FGESDELIGQKTAHALENGLCVIACIGEKLDEREAGITEKVVFAQTKVIADNVKDWSKVVLAYEPVWAIGTGKTASPMQA
QEVHDKLREWLKTNVSEAVA
SSVRIIYGGSVTGGTCKELASQKDVDGFLVGGASLKPEFVDIINAKV
>g2812.t2
VHPGDPFKFILSLNDPNAANMSRKFFVGGNWKMNGDKKSLGELIQTLNSAKLDANVEVVCGCPAIYLDFVRSKMDAKIGV
AAQNCYKVAKGAFTGEISPA
MIKDCGVNWVILGHSERRHVFGESDELIGQKTAHALENGLCVIACIGEKLDEREAGITEKVVFAQTKVIADNVKDWSKVV
LAYEPVWAIGTGKTASPMQA
QEVHDKLREWLKTNVSEAVASSVRIIYGGSVTGGTCKELASQKDVDGFLVGGASLKPEFVDIINAKV
>g2813.t1
MREPEREEQPEENMPPKFKRHLNDDEVTGSIRSER
>g2814.t1
MLSAAGREDGRPLFGGAMSARIPHSAKDISDLREVPDNQEVFAHCHTDQSLIVELLEFQSQVEDQDAAKYHFEDIAGSNK
ALKPGTCDVTSVVPLSKSEL
SLSECSSAWLLNGAQCVSKFNEEARNAVTIHLGLFRLPQFSTDVVVTFNDPLSISPDSSSASGTQSTEKWTVQDFHRLLQ
SFTLHDPGLFG
>g2815.t1
MAIPAGRSVVFVTGNAKKLEEVIQILGDRFPYKLISRKIDCLYKLLAGFEDKSAWALCTFAFSAGKEEPVQLFRGITEGR
IVEPRGPRDFGWDPCFQPDG
YEKTYAELPKEVKNTISHRYRALSAMSEHFSQLKDTVPESKKKKLED
>g2815.t2
LVNYGFGMAIPAGRSVVFVTGNAKKLEEVIQILGDRFPYKLISRKIDCLYKLLAGFEDKSAWALCTFAFSAGKEEPVQLF
RGITEGRIVEPRGPRDFGWD
PCFQPDGYEKTYAELPKEVKNTISHRYRALSAMSEHFSQLKDTVPESKKKKLED
>g2815.t3
MAIPAGRSVVFVTGNAKKLEEVIQILGDRFPYKLISRKIDWHWKDCLVLTCLYKLLAGFEDKSAWALCTFAFSAGKEEPV
QLFRGITEGRIVEPRGPRDF

GWDPCFQPDGYEKTYAELPKEVKNTISHRYRALSAMSEHFSQLKDTVPESKKKKLED
>g2816.t1
MNQRNAERRPNLTFQQVDATQTHYEDASYQACLDKGTLDAMASEEGGALARRMLTEVARVLCVGGRYVCVTLAQESVVKL
AVEHFVSLGWAVRLHCLHEA
ESNEDSFALPVFILVCTKFRQPMPAPILEVCLGEDGGPSRSTGVAELLSAVRELQSYSVLRKRLRTSTDASCNPSLTLCH
VKTGLPRYTLTVLDCSPNAR
VPRGNYFGVFIVPQGKESDWQYGSSEGRSQLAASANFKRLVIVAMHRDQLYTDMQAVQAELSQMVMELAPPGMPENQQVP
FLSVGGELGWREVVSRGVSA
LSGEYCVEDVRGEDGQLYRRLVFLSTAGLVQSEGRLVSANAASDQKKKGKKKVKQTAPPAAPTGSLRVDTGFLCCAHHEV
MVAGLTML
>g2816.t2
MYDVGYKKLTNIDISETVVTHMNQRNAERRPNLTFQQVDATQTHYEDASYQACLDKGTLDAMASEEGGALARRMLTEVAR
VLCVGGRYVCVTLAQESVVK
LAVEHFVSLGWAVRLHCLHEAESNEDSFALPVFILVCTKFRQPMPAPILEVCLGEDGGPSRSTGVAELLSAVRELQSYSV
LRKRLRTSTDASCNPSLTLC
HVKTGLPRYTLTVLDCSPNARVPRGNYFGVFIVPQGKESDWQYGSSEGRSQLAASANFKRLVIVAMHRDQLYTDMQAVQA
ELSQMVMELAPPGMPENQQV
PFLSVGGELGWREVVSRGVSALSGEYCVEDVRGEDGQLYRRLVFLSTAGLVQSEGRLVSANAASDQKKKGKKKVKQTAPP
AAPTGSLRVDTGFLCCAHHE
VMVAGLTML
>g2817.t1
ELELMNVYLTAGDQLSLKGVILPNAKGFGIDLGSSCNDVALHFNPRFEDEADGSVLVCNSKADGCWGAEMRESHNPLKQG
EEVKIVLKFNGEMFKVELPG
GQEVQFPNRQGASFISYVCVKGDLKLRSFKICQ
>g2817.t2
MNVYLTAGDQLSLKGVILPNAKGFGIDLGSSCNDVALHFNPRFEDEADGSVLVCNSKADGCWGAEMRESHNPLKQGEEVK
IVLKFNGEMFKVELPGGQEV
QFPNRQGASFISYVCVKGDLKLRSFKICQ
>g2817.t3
ELELMNVYLTAGDQLSLKGVILPNAKGFGIDLGSSCNDVALHFNPRFEDEADGSVLVCNSKADGCWGAEMRESHNPLKQG
EEVKVRYKALASMIVLKFNG
EMFKVELPGGQEVQFPNRQGASFISYVCVKGDLKLRSFKICQ
>g2818.t1
MSASSFVSNRNDFVHVSKGKNTLNGGPCGETPVRPARRAGGYARGACVVASGERRVSVYTLISSSWAPLRCCDRPPENDA
AS
>g2819.t1
QALQDHQAPAATGTPFPVSAPLAPSSISTCAGGGRAGRGLVRLTETNLDQQRLTSNNLGPPKLL
>g2820.t1
MLSCLALVASGLGCGVPSIKPQVSGYNKIVNGQNAVSGSWPWQVSLQ
>g2820.t2
MLLMLSCLALVASGLGCGVPSIKPQVSGYNKIVNGQNAVSGSWPWQVSLQ
>g2821.t1
LAGDKSENVQDLLLLDVTPLSLGIETAGGVMTVLIKRNTTIPTKQTQTFTTYSDNQPGVLIQVYEGERAMTKDNNILGKF
ELTGIPPSPRGIPQIEVTFD
IDANGILNVSAVDKSTGKENKITITNDKGRLTKEDIERMVQEADQYKAEDEAQKEKVTAKNSLESLAFNMKSTVEDEKLR
DKLSPEDKKAIVDKCNEVIS
WLDRNQTAEKDEYEHQQKELEKVCNPVLAQLYQGGDVPGGMPGGAGAGSSSGPTIEEVD
>g2822.t1
MLSRAEASPLRGEPGTARGGRWMFHVQPRVGRMKEILQWNGLIEL
>g2823.t1
MGMFQHSEQRKLSPPWRAPICPARVSFQRFLGKTDVSKEMDEVHAEGRAQNNVHIMSVPQLVRNPAVRWQLVTVIVTMAC
YQLCGLNAIWYYTNGILTEA
GFPSHIIPYITLSTGAVQTLFAIVSRRRGRHLVILPPIKPQFLIFLNIPGSSQPDGAAAQANGAASISFAITCEGRKTAL
ERCEDSNEALTCSLRIPK
>g2823.t2
MGMFQHSEQRKLSPPWRAPICPARVSFQRFLGKTDVSKEMDEVHAEGRAQNNVHIMSVPQLVRNPAVRWQLVTVIVTMAC

YQLCGLNAFTHEQHRCVEDV
MIWYYTNGILTEAGFPSHIIPYITLSTGAVQTLFAIVSRRRGRHLVILPPIKPQFLIFLNIPGSSQPDGAAAQANGAASI
SFAITCEGRKTALERCEDSN
EALTCSLRIPK
>g2823.t3
MGMFQHSEQRKLSPPWRAPICPARVSFQRFLGKTDVSKEMDEVHAEGRAQNNVHIMSVPQLVRNPAVRWQLVTVIVTMAC
YQLCGLNAIWYYTNGILTEA
GFPSHIIPYITLSTGAVQTLFAIVSRRRGRHLVILPPIKPQFLIFLNIPGSSQPDGAAAQANGAASISFAITCEGRKTAL
ERCEDSNEALTCSLRIPNLT
ARPSGFTQPFSCSLPQSYCSPGGFK
>g2824.t1
MRTYAPYITFPVAVVVGAVGYHLEWFIRGTPKPREQKSILELREERKLEEQAGTDGTQVLSLKDKLEFAPRAALNRNRPH
ES
>g2824.t2
MRTYAPYITFPVAVVVGAVGYHLEWFIRGTPKPREQKSILELREERKLEEQAGTDGTQVLSLKDKLELNYGTGMMGSYLG
EMEVTSCMLYVNPKSTVSIS
MAMTSLYLCDLVTRIAAAEDAGSSLLVVAASASILLHGGGLSLIPSYPTMQAGLKCPGADPQGETMERQDPGGDPLPVLP
SKFTPSTASLGCTRVSCKTR
GLCETTQPQSEPTLACMKNWHTAGSHKNLQSPRPGMAAHIS
>g2824.t3
LNYGTGMMGSYLGEMEVTSCMLYVNPKSTVSISMAMTSLYLCDLVTRIAAAEDAGSSLLVVAASASILLHGGGLSLIPSY
PTMQAGLKCPGADPQGETME
RQDPGGDPLPVLPSKFTPSTASLGCTRVSCKTRGLCETTQPQSEPTLACMKNWHTAGSHKNLQSPRPGMAAHIS
>g2824.t4
MRTYAPYITFPVAVVVGAVGYHLEWFIRGTPKPREQKSILELREERKLEEQAGTDGTQVLSLKDKLELNYGTGMMGSYLG
EMEVTSCMLYVNPKSTGLKC
PGADPQGETMERQDPGGDPLPVLPSKFTPSTASLGCTRVSCKTRGLCETTQPQSEPTLACMKNWHTAGSHKNLQSPRPGM
AAHIS
>g2825.t1
GIQLDRFIQVCTQLQSMTQAFRERDQAMTGNVRLSYEDFLSGTVQRLM
>g2826.t1
MGASHSHTAKPKDETQEALTGNAESTDTQNDDDGKNGDVSQFPYVEFTGRDSVTCPTCQGTGRIPRGQENQLVALIPYSD
QRLRPSRTKLYVTISVSICL
LLSSLAVFFLFPRTIDVSYVGVKSAFVSYDKEQRNVFLNITNTLNITNNNYYAISLTNITAQVQFSKTVIGKAKINNSTV
IAPLGKQQIDYTVPTIIVDE
MSYMFDYCTLQSIKVHNIVVMMQVTVTTSYFGHAEQVSQEMYEYVDCGGNTTSLHGHVQVYK
>g2826.t2
MGASHSHTAKPKDETQEALTGNAESTDTQNDDDGKNGDVSQFPYVEFTGRDSVTCPTCQGTGRIPRGQENQLVALIPYSD
QRLRPSRTKLYVTISVSICL
LLSSLAVFFLFPRTIDVSYVGVKSAFVSYDKEQRNVFLNITNTLNITNNNYYAISLTNITAQVQFSKTVIGKAKINNSTV
IAPLGKQQIDYTVPTIIVDE
MSYMLVTVTTSYFGHAEQVSQEMYEYVDCGGNTTSLHGHVQVYK
>g2826.t3
MGASHSHTAKPKDETQEALTGNAESTDTQNDDDGKNGDVSQFPYVEFTGRDSVTCPTCQGTGRIPRGQENQLVALIPYSD
QRLRPSRTKLYVTISVSICL
LLSSLAVFFLFPRTIDVSYVGVKSAFVSYDKEQRNVFLNITNTLNITNNNYYAISLTNITAQVQFSKTVIGKAKINNSTV
IAPLGKQQIDYTVPTIIVDE
MSYMFDYCTLQSIKVHNIVVMMHVNMTVTVTTSYFGHAEQVSQEMYEYVDCGGNTTSLHGHVQVYK
>g2827.t1
MRFCKSVLSVQVLPLENQMRDPKRFPMALNIGMGIVTVLYVTLGTLGYLHFGDSIKGSITLNLPHDAW
>g2828.t1
SRPQWFFGFFLGTGLACVATCGQRAPCCEEEILFCTLNSHKVDMQKLLGGQIGLEDFIFAHIRGETKEVEVTKTEDALGL
TITDNGAGYAFIKVDERMSS
QRRFSINTKGNSLTPFHPDSFLQRIKESSTIDRLKTVCVGDHIEAINDQSIVGCRHYEVAKMLKEQPRGVPFTLRLVGPK
KAFGESRWGTLRRSEPAGLI
>g2829.t1

MVFEVLGHHLLKWIIKSNYQGLPLPCVKSIIRQVLQGLDYLHTKCKIIHTDIKPENILLTVNEPYIKKMAAEATQWQKTG
APPPSGSAVSTAPAPKQVLK
MSKNKKKKLKKKQKKQAELLEKRIQDMEGGAGGEEEEEDDDEETTEDTSSSATVSTSATLQDIASHAPTDGVSEAQQRPE
GKGPRENSGEEELRMEVNEN
GHASSPTGTGGQEESTQEVKRLENHQKQEEEEEKNKKTEEPEENKHDTHTCNGKQEEGEKPEPESLLLSEPVSGEVTVEG
EKEERKEEEMDTQGIRGGSD
EDSQNGSSCSLLVNPLEPLNADKLQVKIADLGNACWVTKHFTDDIQTRQYRSLEVLMGAGYSTPADIWSTACMVFELATG
DYLFEPHSGEEYTRDEDHIA
LIIELLGKVPRKLILAGKYSKEFFNKKGELRHITKLKPWGLLEVLVEKYEWAKEEALSFSTFLLPMLDLVPEKRASAAQC
LSHPWLSS
>g2829.t2
LTSRGPDVCMVFEVLGHHLLKWIIKSNYQGLPLPCVKSIIRQVLQGLDYLHTKCKIIHTDIKPENILLTVNEPYIKKMAA
EATQWQKTGAPPPSGSAVST
APAPKQVLKMSKNKKKKLKKKQKKQAELLEKRIQDMEGGAGGEEEEEDDDEETTEDTSSSATVSTSATLQDIASHAPTDG
VSEAQQRPEGKGPRENSGEE
ELRMEVNENGHASSPTGTGGQEESTQEVKRLENHQKQEEEEEKNKKTEEPEENKHDTHTCNGKQEEGEKPEPESLLLSEP
VSGEVTVEGEKEERKEEEMD
TQGIRGGSDEDSQNGSSCSLLVNPLEPLNADKLQVKIADLGNACWVTKHFTDDIQTRQYRSLEVLMGAGYSTPADIWSTA
CMVFELATGDYLFEPHSGEE
YTRDEDHIALIIELLGKVPRKLILAGKYSKEFFNKKGELRHITKLKPWGLLEVLVEKYEWAKEEALSFSTFLLPMLDLVP
EKRASAAQCLSHPWLSS
>g2829.t3
VCMVFEVLGHHLLKWIIKSNYQGLPLPCVKSIIRQVLQGLDYLHTKCKIIHTDIKPENILLTVNEPYIKKMAAEATQWQK
TGAPPPSGSAVSTAPAPKQV
LKMSKNKKKKLKKKQKKQAELLEKRIQDMEGGAGGEEEEEDDDEETTEDTSSSATVSTSATLQDIASHAPTDGVSEAQQR
PEGKGPRENSGEEELRMEVN
ENGHASSPTGTGGQEESTQEVKRLENHQKQEEEEEKNKKTEEPEENKHDTHTCNGKQEEGEKPEPESLLLSEPVSGEVTV
EGEKEERKEEEMDTQGIRGG
SDEDSQNGSSCSLLVNPLEPLNADKLQVKIADLGNACWVTKHFTDDIQTRQYRSLEVLMGAGYSTPADIWSTACMVFELA
TGDYLFEPHSGEEYTRDEDH
IALIIELLGKVPRKLILAGKYSKEFFNKKGELRHITKLKPWGLLEVLVEKYEWAKEEALSFSTFLLPMLDLVPEKRASAA
QCLSHPWLSS
>g2830.t1
MEDKRKRRSPKVSLPQPPPPPVNPRKLPPLPASKSATFSLGLPQPPSPKNRGKYKRSIGAPGQPKEVLATVAAPPKIARP
HKEKPRAPQPAGPSKVVQSS
PLQHSFLTDVSDVREMEGGLLNLLNDFHSGKLQAFGKVCSFEQLEHVREMQEKLARLHFSLDSHVEELSEDQRKSASDHN
LEHLLCNTKASLGREPGPAQ
VIGPLKHAGRR
>g2830.t2
MEDKRKRRSPKVSLPQPPPPPVNPRKLPPLPASKSATFSLGLPQPPSPKNRGKYKRSIGAPGQPKEVLATVAAPPKIARP
HKEKPRAPQPAGPSKVVQSS
PLQHSFLTDVSDVREMEGGLLNLLNDFHSGKLQAFGKVCSFEQLEHVREMQEKLARLHFSLDSHVEELSEDQRKSASDHN
LEHLLCNLEELSTSMYPFVA
LGPHMAPHRDELELQTKASLGREPGPAQVIGPLKHAGRR
>g2831.t1
MAGGRRGANRTTSYCRSPLSSEPGSLSNGNHSTSSPVTGVRSRTRNGSGAGMSSPPLATQTVVPLKHCKIPELSLDRNVL
FELHLFFCHLVALFVHYVNI
YKTVWWYPPSHPPSHTSLASQSGKLSFPHSIFLVMARFAVLTLTGWSLCRSLIYLFRTYSALSLLFLCYPFGMYIPFFRL
TCDFRRGGPLSPIASIGSKE
VGSVGKGRDYLTVLKDTWKQHTSQLYGVQAMPTHACCLSPDLIRKEVEYLKMDFNWRMKEVLVSSMLSAYYVAFVPVWFV
KSTQYVDKRWSCELFILVSV
STSVILMRHLLPPRYCDLLHKAAAHLGCWQKTLGYVVVKRVWTVEYMWPQGVLVKHNKNVYKAMGHYNVAVPSNVSHYRF
YFFFNKPLRILNMLIILEGA
MIFYQLYSLICSEKWHQTISLALILFSNYYAFFKLLRDRIVLGKAYSYSNSSSDQKVS
>g2832.t1
MPTRRFTPPSTSFPCKIGDGNVTMANPGPLRSRPDTRNVVDVLADHAGELVRTDSPNFLCSVLPSHWRCNKTLPVAFK

>g2833.t1
VDYSFFNRTYEIPSTPSCSAKELAEQQYVHSSRSSFCYENEVALQLVSPDDDDAQNGLRPQTPGAQRPFGSDHPHDN
>g2834.t1
MHFLTVFWKVLFACVPPTEYWNGWACFCVSITVIGLLTAIIGDLASHFGCTVGLRDSVTAVVFVALGTSIP
>g2834.t2
MHFLTVFWKVLFACVPPTEYWNGWACFCVSITVIGLLTAIIGDLASHFGCTVGLRDSVTAVVFVALGTSIPAVHTPGHAW
PRSSAARCRSDDSIQHRSVH
DSLCLPHIRDKTVPI
>g2835.t1
NFKILHKYVALYATHLIKEGSAQAALQLYVQHGAPPNPQNFNIYKRMFLDLLNLTGTDVPEGYRMWADLRNFLLLLCENI
SKSSEANSPSHEEFEQMLLI
AHYCATRCAAEGV
>g2835.t2
LYATHLIKEGSAQAALQLYVQHGAPPNPQNFNIYKRMFLDLLNLTGTDVPEGYRMWADLRNFLLLLCENISKSSEANSPS
HEEFEQMLLIAHYCATRCAA
EGV
>g2836.t1
MALLRSRCRRPEGARVLQPRFFASTASPQVSDSLRVFTLLKKGVPFRTAASHPSSLQQSEVTFSCNSSCSAAFFPDKQGV
SWAKQQRVLYGRPNPSAPPR
RPPEFSATHHQTRNVTQTALLL
>g2837.t1
MAVRVVENSTVKTGQEIRQNRGETLFGGSVQNRMPTAKARRHRVSKARLWTPGGAPAVLVSSRFLMERKCRLQPLGSDGN
HGNQEVEVMSEGALLSSPWE
LSGLHVEVIVLHLIWLKEHQSQDFLRPSHAGPSLLVLSAPPFWSPRPGVHDITPPIFRCHSGVTVASSDLKTFWVAEL
>g2837.t2
MAVRVVENSTVKTGQEIRQNRGETLFGGSVQNRMPTAKARRHRVSKARLWTPGGAPAVLVSSRFLMERKCRLQPLGSDGN
HGNQEVEVMSEGALLSSPWE
LSGLHVEVIVLHLIWLKEHQSQDFLRPSHAVTNVALPLAEPMCGVAFTSVTVASSDLKTFWVAEL
>g2837.t3
MAVRVVENSTVKTGQEIRQNRGETLFGGSVQNRMPTAKARRHRVSKARLWTPGGAPAVLVSSRFLMERKCRLQPLGSDGN
HGNQEVEVMSEGALLSSPWE
LSGLHVEVIVLHLIWLKEHQSQDFLRPSHAGSCVTSGCRAFSSVSAEQNTWFPFQENGPAWPGTASHRPN
>g2838.t1
MLLSYGGGDQMAHVRTLKMKGRDGISMTTRAAEADEELGSRGPGGRDKDLCGAVDRRAGKHTAAIRSAVTDKGAVLNIKH
TGSSGALQEEGHG
>g2839.t1
YILALGPLLFVTVFDVWLERFTTRQAYVINLVSVGVVAFGCTVQQSSFYGYMGMLPKRYTQGVMTGE
>g2840.t1
MAIAVTFTFLTLLFFALHPSPARVMGSSIRSSGPLDPSVLPGAQTVRHAFPAGGRSDVPVKTPGQEPSIKRPPTAVVVES
TKELSEGLPDPTIISDAFSI
STLSSENLSSRPVHSQPTGSRPRTDAASIALSRTSATGKAYTKGPYPPVSSTPSNEVFESAGLEPVKAGRPGNRTGRGPQ
GRNDDWQEAGSVSSPTTLLR
GEPRVSLATVGNEMAPFRSELQATVVRGPAPENADIKPLSGVSQTEEPRLLPLTTTTSISTTPSLEIRTTLPPTLKLTSA
QTVALTASGSLDCGTFPHSD
YSHTRGHHTRGNDYIYIEN
>g2841.t1
VSVRYHIHLFQTWKQTDQRVRLQIPPQSGTKHGHIAVNVSPFSKDTSKETMV
>g2842.t1
MASATSTPATSSSTSRATRSMGRRGTPGDGLSGTSPTRISRIQEKDDLRHLNDRLANYIHRVQELENERSSMLLLLEEKE
DSTSREMGNVRRLYDVELAD
VRKSLDELANERARWQIECGGLKEEHRKLQVRNQKKEADLAHAVSQWRSLEAALRSKDTENTRLLADNKTLTGDVAELQE
QLDSVETVLQETQTQLSSEV
LRRVDLENRMQSLKEQLDLQRNISEQEIQDVRSRHESRIVEVESGRRREFESKLAETMQRLRQDHDLQVRQYKEELERNF
STKVITRTHTRKLENAQQSM
LEKDDVASSTRDELTTTKLRMETLSSQLRQYQKEKGQLEGRVQELERTLDTERGSWHQRLNQKEQELAALRGQLLHQLEE
YESLLDVKLALDMEINAYRK

MLEVEEQRLHLSPSPSQHGAASQTREHASRRVRGKKRKHEGPSGSSPAYKMSTHAASRGSMSVAEVDLEGNYVVLKNDSE
EEQPLGGWVVRRTHPDSGDI
SFHIPPDYVLPGGHTLTIWATGVDAGLEDLILPGHRSWGPITNTRVILLNPEHEEVAERRLVCAQSRGDEERELDFDEEC
VAGSNIQHFRRQDLSKEASC
AVM
>g2842.t2
MASATSTPATSSSTSRATRSMGRRGTPGDGLSGTSPTRISRIQEKDDLRHLNDRLANYIHRVQELENERSSMLLLLEEKE
DSTSREMGNVRRLYDVELAD
VRKSLDELANERARWQIECGGLKEEHRKLQVRNQKKEADLAHAVSQWRSLEAALRSKDTENTRLLADNKTLTGDVAELQE
QLDSVETVLQETQTQLSSEV
LRRVDLENRMQSLKEQLDLQRNISEQEIQDVRSRHESRIVEVESGRRREFESKLAETMQRLRQDHDLQVRQYKEELERNF
STKLENAQQSMLEKDDVASS
TRDELTTTKLRMETLSSQLRQYQKEKGQLEGRVQELERTLDTERGSWHQRLNQKEQELAALRGQLLHQLEEYESLLDVKL
ALDMEINAYRKMLEVEEQRL
HLSPSPSQHGAASQTREHASRRVRGKKRKHEGPSGSSPAYKMSTHAASRGSMSVAEVDLEGNYVVLKNDSEEEQPLGGWV
VRRTHPDSGDISFHIPPDYV
LPGGHTLTIWATGVDAGLEDLILPGHRSWGPITNTRVILLNPEHEEVAERRLVCAQSRGDEERELDFDEECVAGSNIQHF
RRQDLSKEASCAVM
>g2842.t3
MASATSTPATSSSTSRATRSMGRRGTPGDGLSGTSPTRISRIQEKDDLRHLNDRLANYIHRVQELENERSSMLLLLEEKE
DSTSREMGNVRRLYDVELAD
VRKSLDELANERARWQIECGGLKEEHRKLQVRNQKKEADLAHAVSQWRSLEAALRSKDTENTRLLADNKTLTGDVAELQE
QLDSVGALVLDSSAVKFGRE
VETVLQETQTQLSSEVLRRVDLENRMQSLKEQLDLQRNISEQEIQDVRSRHESRIVEVESGRRREFESKLAETMQRLRQD
HDLQVRQYKEELERNFSTKV
ITRTHTRKLENAQQSMLEKDDVASSTRDELTTTKLRMETLSSQLRQYQKEKGQLEGRVQELERTLDTERGSWHQRLNQKE
QELAALRGQLLHQLEEYESL
LDVKLALDMEINAYRKMLEVEEQRLHLSPSPSQHGAASQTREHASRRVRGKKRKHEGPSGSSPAYKMSTHAASRGSMSVA
EVDLEGNYVVLKNDSEEEQP
LGGWVVRRTHPDSGDISFHIPPDYVLPGGHTLTIWATGVDAGLEDLILPGHRSWGPITNTRVILLNPEHEEVAERRLVCA
QSRGDEERELDFDEECVAGS
NIQHFRRQDLSKEASCAVM
>g2843.t1
MSSYADISRTRGRLIICSSSPRRSEPSACMMSEWISWSACSASCGMGMRSRERYVKQYPEDGSLCRLHTEETEKCVVNPE
CSPSSCIVTEWGEWDPCSAT
CGLGMRRRERMVKMPPLDGSMCKAEVLEVDKCMMPDCGEKELQPEGFHWVTSACDSLNAAMVKWRHLSLHKISWVEKPGC
LDCYH
>g2843.t2
MSSYADISRTRGRLIICSSSPRRSEPSACMMSEWISWSACSASCGMGMRSRERYVKQYPEDGSLCRLHTEETEKCVVNPE
CSPSSCIVTEWGEWDPCSAT
CGLGMRRRERMVKMPPLDGSMCKAEVLEVDKCMMPDCGEKELQPEGFHWVTSACDSLNAAMVKWRHLSLHKISWVEKPGC
LDCYHM
>g2843.t3
PSACMMSEWISWSACSASCGMGMRSRERYVKQYPEDGSLCRLHTEETEKCVVNPECSPSSCIVTEWGEWDPCSATCGLGM
RRRERMVKMPPLDGSMCKAE
VLEVDKCMMPDCGEKELQPEGFHWVTSACDSLNAAMVKWRHLSLHKISWVEKPGCLDCYH
>g2844.t1
HGCGNFIRVVQPYNRTHLFMCGSGAYSPVCVYVNRGRRPE
>g2844.t2
MTSVGELITKLHGCGNFIRVVQPYNRTHLFMCGSGAYSPVCVYVNRGRRPE
>g2845.t1
DEWWLTESQAWPWFYLPGLIVLQTCRTQLVEVVAPGNLGLGT
>g2846.t1
GQHAGRKTRVTASKQQQPPPQQQSSGPASPRDPPGVGHDEQDYTKLLEAMDKDLERPEQVVEEPEVLPCLQIKGTKVKLP
RKPKVQKRREEAEARPKVGK
SQGEDPPREETDDNSSITTDTSNTDTEASFREELAVKKNQPLALEKEKEVQAEQSFKPKNKKQANTKEKSHISSSLELPY

VTPLENKRKAFTSKSLSPAL
ASCSPATTKVLPPKPRVPRGKLEELRPSPLVKLLPGGGVGGAAGGGAGRSSSGSSSEGESNVSPPPEWDCVPFNKNANAV
DSLHQISLQTMQADSFLKRP
MRTPPPPPSAATVYLSGGHSSTLVHTPR
>g2846.t2
GQHAGRKTRVTASKQQQPPPQQQSSGPASPRDPPGVGHDEQDYTKLLEAMDKDLERPEQVVEEPEVLPCLQIKGTKVKLP
RKPKVQKRREEAEARPKVGK
SQGEDPPREETDDNSSITTDTSNTDTEASFREELAVKKNQPLALEKEKEVQAEQSFKPKNKKQANTKEKSHISSSLELPY
VTPLENKRKAFTSKSLSPAL
ASCSPATTKVLPPKPRVPRGKLEELRPSPLVKLLPGGGVGGAAGGGAGRSSSGSSSEGESNVSPPPEWDCVPFNKNANAV
DSLHQISLQTMQADSFLKRP
MRTPPPPPSAATVYLSGGHSSTLVHTPRQ
>g2846.t3
GQHAGRKTRVTASKQQQPPPQQQSSGPASPRDPPGVGHDEQDYTKLLEAMDKDLERPEQVVEEPEVLPCLQIKGTKVKLP
RKPKVQKRREEAEARPKVGK
SQGEDPPREETDDNSSITTDTSNTDTEASFREELAVKKNQPLALEKEKEVQAEQSFKPKNKKQANTKEKSHISSSLELPY
VTPLENKRKAFTSKSLSPAL
ASCSPATTKVLPPKPRVPRGKLEELRPSPLVKLLPGGGVGGAAGGGAGRSSSGSSSEGESNVSPPPEWDCVPFNKNANAV
DSLHQISLQTMQADSFLKRP
MRTPPPPPSAATVYLSGGHSSTLVHTPSELGPKVAPTAKGKLIKSVSLPGQNGNPTFAAVAAGYDKSP
>g2847.t1
VSSERRKEKSRDAARCRRGKESEVFYELAQELPMPHSVRSNLDKASIMRLTISYLRMMKLLGSDGPVATATAQEESELVA
QLSGSYLKALEGFLMVLSED
GDMIYLSENVNKCLGLAQFDLTGQSVFDYTHPCDQEELREMLVHRTGSKKPKEPNTERNFFLRMKCTLTSRGRTVNVKSA
TWKVLRCSGHVHVYEAPAEE
TPTGQAEPPVPYLVLICDPIPHPSNIEVPLDTKTFLSRHTLDMKFTYCDERITELMGYDPEHLMNRSVYEYYHALDSDHL
TKTHQNLFAKGQVCTGQYRM
LAKRGGFVWVETQATVIYSNKNSQPQCVVCVNFVLSGIQEEELILSLEQTEDVKPVKEEKKVPLEEEEEEEEGQQQQEEE
EADDDVVDGCQADEAASRAV
TDEEKAPEMDMVQLLTQVPLVSLYDQLKEEPEALTLLAPAAGDTIISLDFSCPDSEIQLLADVPLYNDVMLPSTSERLGR
PLSPLPPSEPPHSVTGEARA
PAPSSQAGSHGQDRSATGPGGSLDLEFPSDMELGSDFDLVEKLFAVDTKPKTPFMTQVHPFLFAVSSSCNLNSGVLVFEQ
AIEDLDLEMLAPYIPMDDDF
QLRALSPDEPVPCEPVEPRASSPLHSYLSSPPSGPGSRTASPPAPGPLEPVATPRAALLLSKRXNPQLDKEESLKMLAAQ
NAQRKRKLGDIPLTEVFGTG
ALLQGTCEQGKKLKVTEVPTVLSHRYILLLPSDLASRLLGSSEGTNPAISLPQLTRYDCEVNAPVQGRQHLLQGEELLRA
LDQVN
>g2847.t2
VSSERRKEKSRDAARCRRGKESEVFYELAQELPMPHSVRSNLDKASIMRLTISYLRMMKLLGSDGPVATATAQEESELVA
QLSGSYLKALEGFLMVLSED
GDMIYLSENVNKCLGLAQFDLTGQSVFDYTHPCDQEELREMLVHRTGSKKPKEPNTERNFFLRMKCTLTSRGRTVNVKSA
TWKVLRCSGHVHVYEAPAEE
TPTGQAEPPVPYLVLICDPIPHPSNIEVPLDTKTFLSRHTLDMKFTYCDERITELMGYDPEHLMNRSVYEYYHALDSDHL
TKTHQNLFAKGQVCTGQYRM
LAKRGGFVWVETQATVIYSNKNSQPQCVVCVNFVLSGIQEEELILSLEQTEDVKPVKEEKKVPLEEEEEEEEGQQQQEEE
EADDDVVDGCQADEAASRAV
TDEEKAPEMDMVQLLTQVPLVSLYDQLKEEPEALTLLAPAAGDTIISLDFSCPDSEIQLLADVPLYNDVMLPSTSERLGR
PLSPLPPSEPPHSVTGEARA
PAPSSQAGSHGQDRSATGPGGSLDLEFPSDMELGSDFDLVEKLFAVDTKPKTPFMTQAIEDLDLEMLAPYIPMDDDFQLR
ALSPDEPVPCEPVEPRASSP
LHSYLSSPPSGPGSRTASPPAPGPLEPVATPRAALLLSKRXNPQLDKEESLKMLAAQNAQRKRKLGDIPLTEVFGTGALL
QGTCEQGKKLKVTEVPTVLS
HRYILLLPSDLASRLLGSSEGTNPAISLPQLTRYDCEVNAPVQGRQHLLQGEELLRALDQVN
>g2847.t3
VSSERRKEKSRDAARCRRGKESEVFYELAQELPMPHSVRSNLDKASIMRLTISYLRMMKLLGSDGPVATATAQEESELVA
QLSGSYLKALEGFLMVLSED

GDMIYLSENVNKCLGLAQFDLTGQSVFDYTHPCDQEELREMLVHRTGSKKPKEPNTERNFFLRMKCTLTSRGRTVNVKSA
TWKVLRCSGHVHVYEAPAEE
TPTGQAEPPVPYLVLICDPIPHPSNIEVPLDTKTFLSRHTLDMKFTYCDERITELMGYDPEHLMNRSVYEYYHALDSDHL
TKTHQNLFAKGQVCTGQYRM
LAKRGGFVWVETQATVIYSNKNSQPQCVVCVNFVLSGIQEEELILSLEQTEDVKPVKEEKKVPLEEEEEEEEGQQQQEEE
EADDDVVDGCQADEAASRAV
TDEEKAPEMDMVQLLTQVPLVSLYDQLKEEPEALTLLAPAAGDTIISLDFSCPDSEIQLLADVPLYNDVMLPSTSERLGR
PLSPLPPSEPPHSVTGEARA
PAPSSQAGSHGQDRSATGPGGSLDLEFPSDMELGSDFDLVEKLFAVDTKPKTPFMTQAIEDLDLEMLAPYIPMDDDFQLR
ALSPDEPVPCEPVEPRASSP
LHSYLSSPPSGPGSRTASPPAPGPLEPVATPRAALLLSKRNPQLDKEESLKMLAAQNAQRKRKLGDIPLTEVFGTGALLQ
GTCEQGKKLKVTEVPTVLSH
RYILLLPSDLASRLLGSSEGTNPAISLPQLTRYDCEVNAPVQGRQHLLQGEELLRALDQVN
>g2848.t1
MKAPSDSSQQWGSYFVDVHKSAGGNGQEEQKLTWCCHSIHKYSTMAIPSVEHIAGLPLDHKFPRFSLDKPSTTGYYPRPP
DSLLGRRGRDTS
>g2849.t1
MQTPPHCLWGPQQRQVLQVVEQSGPGVPAGADGMTDRLGGGRSHSHLASQTTAFEPLMDLSAWGQGPDSNLPPLASLSPD
TPRGERGVVYFGDSMRSDMF
PASSFGRWETVMVVEEMEGEGVPTSAAARAHRAQAQAQEKAREKAKFEVINAYKMSFSDVESWILTFRPYGNRLETRGVM
DPDVQAMWKQAGDSWSHGS
>g2849.t2
MQTPPHCLWGPQQRQVLQVVEQSGPGVPAGADGMTDRLGGGRSHSHLASQTTAFEPLMVVYFGDSMRSDMFPASSFGRWE
TVMVVEEMEGEGVPTSAAAR
AHRAQAQAQEKAREKAKFEVINAYKMSFSDVESWILTFRPYGNRLETRGVMDPDVQAMWKQAGDSWSHGS
>g2850.t1
VEIIYVVVIFIVIAILAIVGIALTLFIRRRIQQIQLFKGPNRILLTLEDLTFIDPQLSKRKITLEDLSESKSAIEDKSTR
SGERSHSVNSIRTTTHETTN
VAVYEVGGDWVWLKKFEDGYFKEVKQSTTKIFTKMKDLRNENVNPFLGFFCDCSMYAVVTEHCSRGSLQDLLGNEDVKLD
WMFKSSLLLDLIKVRGMKYL
HHKDFPHGRLNSRNCVVDGRFVLKITDYGFNELLETQKSPKEERTPEELFWTAPEFLRDPESTRKGTYKGDVYSFAIILQ
EVVVRGPPYCMLGLPPEEII
RKVKKPPPMCRPTVAPDQAPLECIQLMKQCWSELPDRRPAFDEIFNQFKIINKGKKTNIIDSMLRMLEQYSSNLEDLIRE
RTEELEVEKQRTEKLLSEML
PPERTEELEVEKQRTEKLLSEMLPPSVAEALKTGTTVEPEYFDQVTIYFSDIVGFTTISSLSDPIEVVDLLNDLYTLFDA
VLSNHDVYKVETIGDAYMVS
SGLPKRNGNKHAAEIANMSLNILSSVGSFKMRHMPDVPVRIRIGIHS
>g2850.t2
VEIIYVVVIFIVIAILAIVGIALTLFIRRRIQQIQLFKGPNRILLTLEDLTFIDPQLSKRKITLEDLSESKSAIEDKSTR
SGERSHSVNSIRTTTHETTN
VAVYEGDWVWLKKFEDGYFKEVKQSTTKIFTKMKDLRNENVNPFLGFFCDCSMYAVVTEHCSRGSLQDLLGNEDVKLDWM
FKSSLLLDLIKVRGMKYLHH
KDFPHGRLNSRNCVVDGRFVLKITDYGFNELLETQKSPKEERTPEELFWTAPEFLRDPESTRKGTYKGDVYSFAIILQEV
VVRGPPYCMLGLPPEEIIRK
VKKPPPMCRPTVAPDQAPLECIQLMKQCWSELPDRRPAFDEIFNQFKIINKGKKTNIIDSMLRMLEQYSSNLEDLIRERT
EELEVEKQRTEKLLSEMLPP
ERTEELEVEKQRTEKLLSEMLPPSVAEALKTGTTVEPEYFDQVTIYFSDIVGFTTISSLSDPIEVVDLLNDLYTLFDAVL
SNHDVYKVETIGDAYMVSSG
LPKRNGNKHAAEIANMSLNILSSVGSFKMRHMPDVPVRIRIGIHS
>g2850.t3
VEIIYVVVIFIVIAILAIVGIALTLFIRRRIQQIQLFKGPNRILLTLEDLTFIDPQLSKRKITLEDLSESKSAIEDKSTR
SGERSHSVNSIRTTTHETTN
VAVYEVGGDWVWLKKFEDGYFKEVKQSTTKIFTKMKDLRNENVNPFLGFFCDCSMYAVVTEHCSRGSLQDLLGNEDVKLD
WMFKSSLLLDLIKGMKYLHH
KDFPHGRLNSRNCVVDGRFVLKITDYGFNELLETQKSPKEERTPEELFWTAPEFLRDPESTRKGTYKGDVYSFAIILQEV
VVRGPPYCMLGLPPEEIIRK

VKKPPPMCRPTVAPDQAPLECIQLMKQCWSELPDRRPAFDEIFNQFKIINKGKKTNIIDSMLRMLEQYSSNLEDLIRERT
EELEVEKQRTEKLLSEMLPP
ERTEELEVEKQRTEKLLSEMLPPSVAEALKTGTTVEPEYFDQVTIYFSDIVGFTTISSLSDPIEVVDLLNDLYTLFDAVL
SNHDVYKVETIGDAYMVSSG
LPKRNGNKHAAEIANMSLNILSSVGSFKMRHMPDVPVRIRIGIHS
>g2851.t1
YFFPPLEAPRAGSRSGHLLPPSSLIEHCDAEPAQPQQQAALRRLYSKALLSLPEAEAP
>g2852.t1
MADSFNLEDEPLPSLLSGSPGSPGGPVTLGNLTLGSGPGLPVAASTVAKIRAGADNRGNPDEVSYLEGGQLSSNGEKAVF
CLSFKDDMDNADDFIAAHRF
SDMLVKIHMDESQPSARAVEPSKSTALHGRPTELGSDLSTGLLTFTQHRPRNTLGTTSMQTQPLPATTEDDCPDSDEADS
DRFSGSVSSFLANEKLMSVD
SISSDLTDDDVDLNKLPDDELELYFKTLVRPAMQRGRVEGQEIPAAEDFQMPDVRLAATGMDSCPASDEDTEDELEAART
NTSRTQLLLPSTSRQLVGES
NRPSFRPGLEGGSSDDEGPRGRAGPPASGIEQRRSAEGQVINPPVT
>g2852.t2
MADSFNLEDEPLPSLLSGSPGSPGGPVTLGNLTLGSGPGLPVAASTVAKIRAGADNRGNPDEVSYLEGGQLSSNGEKAVF
CLSFKDDMDNADDFIAAHRF
SDMLVKIHMDESQPSARAVEPSKSTALHGRPTELGSDLSTGLLTFTQHRPRNTLGTTSMQTQPLPATTEDDCPDSDEADS
DRFSGSVSSFLANEKLMSVD
SISSDLTDDDVDLNKLPDDELELYFKTLVRPAMQRGRVEGQEIPAAGLSGLPNRTSNTSSAEQERNREQFLDDYDQEDFQ
MPDVRLAATGMDSCPASDED
TEDELEAARTNTSRTQLLLPSTSRQLVGESNRPSFRPGLEGGSSDDEGPRGRAGPPASGIEQRRSAEGQVINPPVT
>g2853.t1
SVNGSKSRATERETIDCNLPEYITPGCKDRPLIPLQPIREEWKPLQCLRVLTVNSWNPPPGNRKMHGDLLYLNVVTVEGR
DLNITSSTRGFYLNQSTAFT
FNPKPALPKILCHSLVELLSQVSPVFRKNFSSLQKKR
>g2854.t1
LPHRVVEVEVDPTCPTVCVEVDLYLANRVVEVEVEVDPTCPTVEVDPTCPTVCVEVDLYLANRVVEVEVEVDLYLADRVL
VLAHPAGRGLLRSHTVQGVV
CLRRYPGVHLDDLIIRN
>g2854.t2
LPHRVVEVEVDPTCPTVCVEVDLYLANRVVEVEVEVDPTCPTVEVDPTCPTVCVEVDLYLANRVVEVEVEVDLYLADRVL
VLAHPAGRGLLRSHTVQGVV
CLRRYPGVHLWIINLKDGVQQCSCVLCSMRVMEVCVCAGGY
>g2855.t1
MADGFLMEVCVDSVESAVNAERGGAGRLELCSSLVEGGLTPSVGLLQVVKQNVKVPIYVMIRPRGGDFLYSEQEVEVMRR
DIQLMKSHGADGLVLGVLTE
DGRVDAELCMELLAFDMVKEPEAALETLVSLGFHRLLTSGCDCSALEGLPVIKTLVKRAKGRIAIMPGGGITERNLQRIL
EGSGAQEFHCSARASRESAM
KF
>g2855.t2
MADGFLMEVCVDSVESAVNAERGGAGRLELCSSLVEGGLTPSVGLLQVVKQNVKVPIYVMIRPRGGDFLYSEQEVEVMRR
DIQLMKSHGADGLVLGVLTE
DGRVDAELCMELLAFDMVKEPEAALETLVSLGFHRLLTSGCDCSALEGLPVIKTLVKRAKGRIAIMPGGGITERNLQRIL
EGSGAQEFHCSARASRESAM
KFRDP
>g2855.t3
MADGFLMEVCVDSVESAVNAERGGAGRLELCSSLVEGGLTPSVGLLQVVKQNVKVPIYVMIRPRGGDFLYSEQEVEVMRR
DIQLMKSHGADGLVLGVLTE
DGRVDAELCMELLAFDMVKEPEAALETLVSLGFHRLLTSGCDCSALEGLPVIKTLVKRAKGRIAIMPGGGITERNLQRIL
EGSGAQEFHCSARASRESAM
KFRWVSTVRY
>g2856.t1
MNHNMTLPEFKFIFYMEWGHRMWGRLVGLAYILPTVYFWRRGYFTGSMKARVVGLCGFVFFQSYDVPRVSQYRLSAHLGS
ALLLYCASLWTGLTLMLPAN

KLADTTRLTQLRRFAKGTGGLVFLTALSGAFVAGLDAGLVYNSFPKMGERWIPDDLLAFSPTLRNLFENPTTVQFDHRIL
AISSLTAITGLYMFSRRVML
PRRARIAISLLAAMAYSQVALGISTLLLYVPTPLAAVHQSGSVALLSLAIWVLAELRKVAK
>g2856.t2
MNHNMTLPEFKFIFYMEWGHRMWGRLVGLAYILPTVYFWRRGYFTGSMKARVVGLCGFVFFQPESYDVPRVSQYRLSAHL
GSALLLYCASLWTGLTLMLP
ANKLADTTRLTQLRRFAKGTGGLVFLTALSGAFVAGLDAGLVYNSFPKMGERWIPDDLLAFSPTLRNLFENPTTVQFDHR
ILAISSLTAITGLYMFSRRV
MLPRRARIAISLLAAMAYSQVALGISTLLLYVPTPLAAVHQSGSVALLSLAIWVLAELRKVAK
>g2856.t3
LVKRGQSTFTAEAGATATPVATTVPNAATNRIVGRWLLGCSGLVVGAVVLGGVTRLTESGLSMVDWHLVREMKPPQTQAE
WEAEFAKYQQFPEFKIMNHN
MTLPEFKFIFYMEWGHRMWGRLVGLAYILPTVYFWRRGYFTGSMKARVVGLCGFVFFQSYDVPRVSQYRLSAHLGSALLL
YCASLWTGLTLMLPANKLAD
TTRLTQLRRFAKGTGGLVFLTALSGAFVAGLDAGLVYNSFPKMGERWIPDDLLAFSPTLRNLFENPTTVQFDHRILAISS
LTAITGLYMFSRRVMLPRRA
RIAISLLAAMAYSQVALGISTLLLYVPTPLAAVHQSGSVALLSLAIWVLAELRKVAK
>g2857.t1
ECPSGVVNEENFKTIYSQFFPQGDSSMYAHFLFEAFDTDKNGSVSFEDFVFGLSIILRGTINDRLNWAFNLYDLNKDGCI
TKEEMLDIMKSIYDMMGKYT
YPNMQDEAPREHVENFFQKMDRNKDGVVTIEEFIESCQKDENIMQSMQLFDNVI
>g2857.t2
LELQECPSGVVNEENFKTIYSQFFPQGDSSMYAHFLFEAFDTDKNGSVSFEDFVFGLSIILRGTINDRLNWAFNLYDLNK
DGCITKEEMLDIMKSIYDMM
GKYTYPNMQDEAPREHVENFFQKMDRNKDGVVTIEEFIESCQKDENIMQSMQLFDNVI
>g2857.t3
LVLSLELQECPSGVVNEENFKTIYSQFFPQGDSSMYAHFLFEAFDTDKNGSVSFEDFVFGLSIILRGTINDRLNWAFNLY
DLNKDGCITKEEMLDIMKSI
YDMMGKYTYPNMQDEAPREHVENFFQKMDRNKDGVVTIEEFIESCQKDENIMQSMQLFDNVI
>g2858.t1
GHTTEVLRLLGSLSPSYQPRHYVIADTDRMIHRIPRSREVLQTWSSSVKSTLIALLYSVPLVFRLRPDVVRSTCR
>g2858.t2
GHTTEVLRLLGSLSPSYQPRHYVIADTDRMSEEKIRAFESAKENADTKSKYTVHRIPRSREVLQTWSSSVKSTLIALLYS
VPLVFRLRPDVVRSTCR
>g2859.t1
AILYAVVCWSCGSSEKNRKRLNKLVRRAGSVLDCTLESIEVVGERRMLTKLTSIMDNPSHPLHETVGHLSSSFSGRLLHP
RCKKECYRRSFIPAAVRLYN
TSL
>g2860.t1
MGYNVIKFTNDTTITTIITNSDNGDIQSRGESPDNNLLLNVGKTKELIVDNKRLRGERHARVSAASDSSGSTSARI
>g2861.t1
MWKTSCLIPVPKTLRPSSPQDYRPVALTSHIIKTLERLILEQLRPMVRPFSDQLQFAYQPHLGVEDKVYSHLDKPASTVR
VSEEYLQQKTPLTEMHHRAT
QEIIPACLKLYNTSL
>g2861.t2
LRPSSPQDYRPVALTSHIIKTLERLILEQLRPMVRPFSDQLQFAYQPHLGVEDKVYSHLDKPASTVRVSEEYLQQKTPLT
EMHHRATQEIIPACLKLYNT
SL
>g2862.t1
LQAHLKKMPDCSEAPFEAESDALMDSWLDVTEKSDAYVDNLQVALELWEKQLALGAEVEGWAGAKLAMFAESHPFHSQQQ
VLDMRDEIQNHEENIERFHK
KSAEIQEMLQSQEPPLELQVVETQLRKRMEQVQELFSDSTDVFEELLAVKKHLTEKIQECAAAVETIQTSVNNVSTSEPQ
ANTLLQDLSEDLDFQEEKAR
VVLKELGLISSVASPQALDPLTQESSSLQDAFSLTRELIQRRRDDQGKDLVTAIRGDRQRFEEFFQDLQLSINECFENPE
SREDVETSLNRLTGFLEARE
SERRLAQLRERLEGGRDQIPPPALEELTSWLEEQQKEVSTFTTHCSNRRKQMQSSLDTFNSLQKQYDVFREWLQNKEKDS

LESDQVSRLLKDLQDHRGSA
ESVGELLGAVRRQGVRADRLLKDGDNLLQRYRNLETRLQEQQAQAHKALEGQREDFHTQAESTQTWIRDLAQGLASPHIQ
GQHQEVKVAAQTVLVSKPDG
DAKIEDLRGRGQSVCEFEDLGESRRKEVQQTVKETEEQWRSVLEKAEESLHRAETQAVVEEKLNDFKTQKESVLSWVRQQ
NQNLRSLGGHMTFEDRLQIP
QNILASKPAGDAKVQGLREQAQPLCEDEALECGARQQVEQTLRDAEAQWAGVLRGAEEAERSALSEDFHLQKNRTLAWVA
ERQRAVDAQGRHAPAPQRRL
AAENVLGSRPEGDRQVNNLRRGGQGLCERLDQEDSRREEVQQAVQEAEERWRLVLTSAKQTLEEAETQVALETEKREGEL
RDFNGHSQEATMWIGALEQK
LTTLAEQANAEDRLHTAQTILSAKQEGDAKMADLQSRVQGLCENEDLEESRRREVQQTLRDGQKRWRLLLESAKQTAEQE
EAQCALEGLLGDIDAQRRST
AAWLEDKQQALATDSHADPEKSIQTAQAVLGSKPEGDGKVDNLRRRGQSVCEREDLKESRRKEVQQTVRETEEQWRSVLE
KAEEVLHRAELQYSLTRELE
AFGGQAGSVGDWVKELQNQADSLGSLVTQGNQEQIESRLKTAQTILGSRSKGESFLSDLSRRAQSLCTREDLEESRRKEV
QQTVKQAQEQWRSILNTTEE
AQRALKAVTERLVSCEFQRCQAQGRLAELQRQSAALPRLFPWPGLGDRRQAAEDARTLLDRTRAQGPVLKALRRQAEEVF
ETTQDPTWRDPTWAGMEECL
PALISELTVSLTAPALV
>g2862.t2
MPDCSEAPFEAESDALMDSWLDVTEKSDAYVDNLQVALELWEKQLALGAEVEGWAGAKLAMFAESHPFHSQQQVLDMRDE
IQNHEENIERFHKKSAEIQE
MLQSQEPPLELQVVETQLRKRMEQVQELFSDSTDVFEELLAVKKHLTEKIQECAAAVETIQTSVNNVSTSEPQANTLLQD
LSEDLDFQEEKARVVLKELG
LISSVASPQALDPLTQESSSLQDAFSLTRELIQRRRDDQGKDLVTAIRGDRQRFEEFFQDLQLSINECFENPESREDVET
SLNRLTGFLEARESERRLAQ
LRERLEGGRDQIPPPALEELTSWLEEQQKEVSTFTTHCSNRRKQMQSSLDTFNSLQKQYDVFREWLQNKEKDSLESDQVS
RLLKDLQDHRGSAESVGELL
GAVRRQGVRADRLLKDGDNLLQRYRNLETRLQEQQAQAHKALEGQREDFHTQAESTQTWIRDLAQGLASPHIQGQHQEVK
VAAQTVLVSKPDGDAKIEDL
RGRGQSVCEFEDLGESRRKEVQQTVKETEEQWRSVLEKAEESLHRAETQAVVEEKLNDFKTQKESVLSWVRQQNQNLRSL
GGHMTFEDRLQIPQNILASK
PAGDAKVQGLREQAQPLCEDEALECGARQQVEQTLRDAEAQWAGVLRGAEEAERSALSEDFHLQKNRTLAWVAERQRAVD
AQGRHAPAPQRRLAAENVLG
SRPEGDRQVNNLRRGGQGLCERLDQEDSRREEVQQAVQEAEERWRLVLTSAKQTLEEAETQVALETEKREGELRDFNGHS
QEATMWIGALEQKLTTLAEQ
ANAEDRLHTAQTILSAKQEGDAKMADLQSRVQGLCENEDLEESRRREVQQTLRDGQKRWRLLLESAKQTAEQEEAQCALE
GLLGDIDAQRRSTAAWLEDK
QQALATDSHADPEKSIQTAQAVLGSKPEGDGKVDNLRRRGQSVCEREDLKESRRKEVQQTVRETEEQWRSVLEKAEEVLH
RAELQYSLTRELEAFGGQAG
SVGDWVKELQNQADSLGSLVTQGNQEQIESRLKTAQTILGSRSKGESFLSDLSRRAQSLCTREDLEESRRKEVQQTVKQA
QEQWRSILNTTEEAQRALKA
VTERLVSCEFQRCQAQGRLAELQRQSAALPRLFPWPGLGDRRQAAEDARTLLDRTRAQGPVLKALRRQAEEVFETTQDPT
WRDPTWAGMEECLPALISEL
TVSLTAPALV
>g2863.t1
MKDLQTVMLRSVQRRTKMKRLQNRLRKSLRNLLIWITKMS
>g2864.t1
MPAVLTNVSVAEVTAAGPLVTAAGIRKHGEELTAEK
>g2865.t1
MPLFASMLLKGAQSDRDKGKICSSPSMLLGSGVKPHCWNESRNIEMLAAACAVGVGCCFAAPIGGVLFSIEVTSTFFAVR
NYWRGFFAATFSAFIFRVLA
VWNRDEETITALFKTRFRLDFPFDLQELPAFAVIG
>g2865.t2
MPLFASMLLKGAQSDRDKGKICSSPSMLLGSGVKPHCWNESRNIEMLAAACAVGVGCCFAAPIGGVLFSIEVTSTFFAVR
NYWRGFFAATFSAFIFRVLA
VWNRDEETITALFKTRFRLDFPFDLQELPAFAVI

>g2866.t1
MFTRTLTTTPTPRCPMERSVRGKARETDRVEPILILIACPQRRTAAAVAKTSVTITPRPEEMASRSPVLGVSESSSSCTA
AVMAAQLSH
>g2866.t2
MERSVRGKARETDRVEPILILIACPQRRTAAAVAKTSVTITPRPEEMASRSPVLGVSESSSSCTAAVMAAQLSH
>g2867.t1
LISRVGEDWIFLILLGLVMALVSWVVDFCIAICLQAQKWMYGGLDSNVFLQYLAWVTYPVVLITFSAGFTQILAPQAVGS
GIPEMKTILRGVVLKEYLTF
KTFVAKVIGLTCALGSGMPLGKEVGQIQCMCYLER
>g2867.t2
LISRVGEDWIFLILLGLVMALVSWVVDFCIAICLQAQKWMYGGLDSNVFLQYLAWVTYPVVLITFSAGFTQILAPQAVGS
GIPEMKTILRGVVLKEYLTF
KTFVAKVIGLTCALGSGMPLGKEGPFVHIASLCAALLSKFMSLFGGIYEVTVSP
>g2867.t3
VCTVRCQKFLISRVGEDWIFLILLGLVMALVSWVVDFCIAICLQAQKWMYGGLDSNVFLQYLAWVTYPVVLITFSAGFTQ
ILAPQAVGSGIPEMKTILRG
VVLKEYLTFKTFVAKVIGLTCALGSGMPLGKEVGQIQCMCYLER
>g2868.t1
MYNYLFVAGGIKGYGDRGKVSDRVFCYNPVDGRWTEVRPLNQARAQLKLVSMDGYLYAIGGECLFTVERYDPRTDRWTTV
APLPKGAFAVAHEATTCNGE
LYVSGGSLFYRLLRYEPRRDEWQECPYNSSRKRSSDMVALKSFIYRFDVNREQGVNVFKYNAIVKMWHDCATRRLGSASP
FRCAVVGNCIYCVNKSQTLQ
FVVEEESAYFVDEPLRAPMEAKGVLFPFVLSLPEKPEAVA
>g2869.t1
MSVANAKHTVLNPVIPYTGPIPGGLHAGEIILVQGTVPPDADRFHIDLSSGCSTKPRSDVALRFDACFEGTASVTCNSLQ
QERWGPEQTLHQLPYKQGAP
FETIILVHEDVFKVAVNGTHLLEYKHRIPLDRVDTFSVSGKSLPYKGSILKGLKPGQHITIKGQVSMYPHSFTVNLRNSG
TENIALHLNPRIKSGMFIRN
SYLGASWGQEERELPFFPFSPGEYFEILMLCQSHQFKLAVNGSHLLEFRHRVQDLSSIDQLEIMGDLELGDVKLW
>g2869.t2
MSVANAKHTVLNPVIPYTGPIPGGLHAGEIILVQGTVPPDADRFHIDLSSGCSTKPRSDVALRFDACFEGTASVTCNSLQ
QERWGPEQTLHQLPYKQGAP
FETIILVHEDVFKVAVNGTHLLEYKHRIPLDRVDTFSVSGKVRVNAIGYIPNSSLPYKGSILKGLKPGQHITIKGQVSMY
PHSFTVNLRNSGTENIALHL
NPRIKSGMFIRNSYLGASWGQEERELPFFPFSPGEYFEILMLCQSHQFKLAVNGSHLLEFRHRVQDLSSIDQLEIMGDLE
LGDVKLW
>g2869.t3
MDIFLDGKSANDPSARQYDYSINEEDGGLVEVIPYTGPIPGGLHAGEIILVQGTVPPDADRFHIDLSSGCSTKPRSDVAL
RFDACFEGTASVTCNSLQQE
RWGPEQTLHQLPYKQGAPFETIILVHEDVFKVAVNGTHLLEYKHRIPLDRVDTFSVSGKSLPYKGSILKGLKPGQHITIK
GQVSMYPHSFTVNLRNSGTE
NIALHLNPRIKSGMFIRNSYLGASWGQEERELPFFPFSPGEYFEILMLCQSHQFKLAVNGSHLLEFRHRVQDLSSIDQLE
IMGDLELGDVKLW
>g2870.t1
MGNSFSNISAFQSLHIVMLGLDSAGKTTVLYRLKFNEFVNTVPTIGFNTEKIKLSNGTAKGISCHFWDVGGQEKLRPLWK
SYSRCTDGIIYVVDSVDVDR
LEEAKTELHKVTKFAENQGTPLLVIANKQDLPKSLPVADIEKQLALHELTPSTTYHVQPACAIIGEGLHEGMDKLYEMIV
KRRKSLKQKKKR
>g2871.t1
PPPKVTSLHLAQKQGGAAIAGESEEEESESGGEGIFRERDEFVVRVEDIRSLKLALQTGREPPPIWRVQKALLQKFSPEI
KDGQRQFCATSNYLGYFGDA
KRRYQRLYVKFLENVSKKDYVRVCSRRPWRRNTPGLRRQPLLRVSSQPPVLTPAEKEEKASLPSQREKTRSREKREAVAK
EEKEEKEKEKGARERMEKER
RVQPPQRKVEKRGADRGKDRGAEKGKERGAERGKERAVERPVRSKQTRSKTEVEPPPKKRKEWQKEAPSSSDSSSEASED
EPVEKEKEVQEENRVLREMF
RSYVEMLVSTALDPDMIQALEDTHDELYLPPMRKIDSMLNEQKRRLLRRVSMSTQHQEVLQAYPQIQVDPLDSGLVRVRL

AGDAYNRKTLNRVKKSQPKP
Q
>g2872.t1
MEELTKEQERLSVVESRILEEEAVFENVTPHLACRASRLGCERADSEQLSLSLRFMGFLETDENDLTPLSPKRKALSFSP
AKWRAAFSEEVAGMAEVSTK
CAERPLTYSDEIAVLTNTRSRDHITPVLRDPYWLPVKHRMNFKILLTTYKALNNLAPSYLCLCLGSADANLLRIPRTKRR
TWGDRACSVAAPSLWNSIPN
HSKNAPTLSSFKKALKTHLFRLAFPS
>g2873.t1
LIGVEYLYSQTSATLPQDFGWDPDSAEESDEACAEDSADLRFTTGPDSLFHLMWPLPPPATTFPPGRLDEEMEVGEEERQ
VREIAAVWHWLSDSDMAVIS
FPPRHKDRLTMGCFKAGPCSNRKMPGVDSAKRSFLAQVQHRLQRQTGWWRPLFWSCAVSIMRAAPLLASGSTAGQLGTRD
YAWIRGNIHNSSDLKETTPL
HLFDVNQRTLLQ
>g2874.t1
MQRFTRGVTTDSHQLYGLFMARLSFAIFEWDTVDVQLLMEAKKAEEGREAHVHLTAKELAHHCRRSTRCVEEMQRLVQEV
LDSFGGVTDIAGVPLIDQAK
MAEIWRRQRRHLLYVQDPPSVQLYTKTGELTRGWGTSASALHFQVYLLEGLVRWNDNRSRAAVASGQSSTMRCYDICMIS
TGSPKSLLAWKLHTAREVHG
YAIGEGLRVRM
>g2874.t2
LVVRLDTWHLMQRFTRGVTTDSHQLYGLFMARLSFAIFEWDTVDVQLLMEAKKAEEGREAHVHLTAKELAHHCRRSTRCV
EEMQRLVQEVLDSFGGVTDI
AGVPLIDQAKMAEIWRRQRRHLLYVQDPPSVQLYTKTGELTRGWGTSASALHFQVYLLEGLVRWNDNRSRAAVASGQSST
MRCYDICMISTGSPKSLLAW
KLHTAREVHGYAIGEGLRVRM
>g2875.t1
VPGPVQEFEDDWENDDLLNDSLVFAMTQEAEPTPLPRTTMSGVTARVDSSPPQGRSLRAPQPGGVPRGGGPRPKPPQRST
FKLEPNPRYPPASTHRSEPL
QAATPAAMRSVTMVKPVTATKPVTTVTPQSSKPLPVAVTRGGGADSTAALNADQSSGDLDCFWDEGGDDQLLFQVCEDIE
NLSNSQPTPAPQGRQPAARG
YARSSSVPGARGDSASCQRGEVQVKGAGVRADQSALSRSHPGSLGPGSFQDHQRTSAGFQPQTVTARPSGNSKSHHATFK
RHLSNPSASSSKVVVSGQVT
AQCSAAEIERKKQEALARKRQRLHRTQNPSVRSARQAADSGASV
>g2876.t1
DWQHGGVRGCFYLEEDTISSLQYRLHLPQRSSVYLSIRPLGLGAHTADKPSPQSWMSVDTALLVTSAGHGKDDCSLVCLT
EAREKDRYVWRGELPAGSYF
LLPFTSGCRLRRRSSRTAPPTKPEPLVYRTDA
>g2877.t1
MGTHMWNSAPARRGRRLSVDGPMAFLGTNPVKFPEIFQKDMSSRLGNGDPSSFWNQYAAAFSSGLAMKTNEISVIQNGGV
PSMSGSLGNGGSSPIGGLTG
SLDKLHSSGPNAALAGLEKMANTENGTHFRFTRFMEDNKEIATN
>g2878.t1
HEAVLEVPQNREFLSTFFSWRLMDRMEHLPPAGSSRWNQTAF
>g2879.t1
VYILICAGGLMMAVGFFGCFGAVRESQCLLVSIVEEVQKFYSSSIADNSNPNYNGTAIASIYHTILHCCGASSPDPALCP
DAEEDTKGCLTAITDFFNEK
LHIIGFIGIGVAGVMVLGMVFSMVLCCAIRNSREII
>g2879.t2
VYILICAGGLMMAVGFFGCFGAVRESQCLLVSLHCCGASSPDPALCPDAEEDTKGCLTAITDFFNEKLHIIGFIGIGVAG
VMVLGMVFSMVLCCAIRNSR
EII
>g2879.t3
VYILICAGGLMMAVGFFGCFGAVRESQCLLVSFFACLLIIFGAEVAAGVFGFLNKEQIVEEVQKFYSSSIADNSNPNYNG
TAIASIYHTILHCCGASSPD
PALCPDAEEDTKGCLTAITDFFNEKLHIIGFIGIGVAGVMVLGMVFSMVLCCAIRNSREII

>g2880.t1
MSQQPIRVDCLVKTVRSEGYFGMYRGAAVNLTLVTPEKAIKLAANDFFRHHLGKDGKGLTVMKEMLAGCGAGMCQVVITT
PMEMLKIQLQDAGRLAAQRK
PCVMLSGKLVAANPMLTRSYNVASTPRPASATQIARQLLHAHGIQGLYRGLGATVMRDVPFSVVYFPLFANLNLLGKPSP
EQPAPFYWAFLSGCLAGSTA
AVAVNPCDVVKTRLQSLSKGANEETYGGVVDCIRKILQKEGPSAFLKGAGCRALVIAPLFGIAQVMYFLGVGEYIVENSP
LRLLSA
>g2880.t2
MSQQPIRVDCLVKTVRSEGYFGMYRGAAVNLTLVTPEKAIKLAANDFFRHHLGKDGKGLTVMKEMLAGCGAGMCQVVITT
PMEMLKIQLQDAGRLGESRF
ACYIKTVPACRVSKVCSDLRFKTTLGRWKHSCTRRGVVLPAQRKPCVMLSGKLVAANPMLTRSYNVASTPRPASATQIAR
QLLHAHGIQGLYRGLGATVM
RDVPFSVVYFPLFANLNLLGKPSPEQPAPFYWAFLSGCLAGSTAAVAVNPCDVVKTRLQSLSKGANEETYGGVVDCIRKI
LQKEGPSAFLKGAGCRALVI
APLFGIAQVMYFLGVGEYIVENSPLRLLSA
>g2881.t1
MDTFVFVGGFLVLMFSPAAPMCLPTDYTLYVEKPECSFCVAINTTICMGFCYSRDSNMQDLVGRRFLLQKGCTYNRVEYR
TAVLPGCPTEGSSLFSYPVA
LGCRCGSCNTAVDECAHRASSNRPTCTKPVTHIYESDFLLPF
>g2882.t1
LSESSNSGLHEDQQGRLLRADNRHQISPLLYSLFTHDCMTPHSSNTTIIDCITDSDGSAYGTEVRALALWCQDNNVLLNI
GKELIVDYRRLQGGRHTAIH
VGGTAVERVSHFRFLWINISVDLRWPHHTGVVMNAARQRLFFLRRLRRLGLDSRILTNFYRAAQRITRTELPTIQDFYSQ
RCRRKAQWILSNPSHPSHIL
FTLLLSGRCFMSGITVIGTPAESYSYGTAFWLFGLSYAITSVVTAEIFVPFFYRLGITSVYEGGLKAVIWTDAVQLLVML
AGFVAVIAKGAVLQGGMARI
WQDAAEGGRLDSFDFDPDPLRRHTFWTILVGGSMIWISVYAINQSQVQRYLSCRSLNHAKM
>g2882.t2
LSESSNSGLHEDQQGRLLRADNRHQISPLLYSLFTHDCMTPHSSNTTIIDCITDSDGSAYGTEVRALALWCQDNNVLLNI
GKELIVDYRRLQGGRHTAIH
VGGTAVERVSHFRFLWINISVDLRWPHHTGVVMNAARQRLFFLRRLRRLGLDSRILTNFYRAAQRITRTELPTIQDFYSQ
RCRRKAQWILSNPSHPSHIL
FTLLLSGRWYRSMRNQQGNTNEEFLLGGRQMHGAPVAMSLTASFMSGITVIGTPAESYSYGTAFWLFGLSYAITSVVTAE
IFVPFFYRLGITSVYEGGLK
AVIWTDAVQLLVMLAGFVAVIAKGAVLQGGMARIWQDAAEGGRLDSFDFDPDPLRRHTFWTILVGGSMIWISVYAINQSQ
VQRYLSCRSLNHAKM
>g2882.t3
LSESSNSGLHEDQQGRLLRADNRHQISPLLYSLFTHDCMTPHSSNTTIIDCITDSDGSAYGTEVRALALWCQDNNVLLNI
GKELIVDYRRLQGGRHTAIH
VGGTAVERVSHFRFLWINISVDLRWPHHTGVVMNAARQRLFFLRRLRRLGLDSRILTNFYRAAQRITRTELPTIQDFYSQ
RCRRKAQWILSNPSHPSHIL
FTLLLSGRWYRSMRNQQSFRGRKGNTNEEFLLGGRQMHGAPVAMSLTASFMSGITVIGTPAESYSYGTAFWLFGLSYAIT
SVVTAEIFVPFFYRLGITSV
YEGGLKAVIWTDAVQLLVMLAGFVAVIAKGAVLQGGMARIWQDAAEGGRLDSFDFDPDPLRRHTFWTILVGGSMIWISVY
AINQSQVQRYLSCRSLNHAK
M
>g2883.t1
AGSYVRDDSVPNLIQLITNSVEMHAYTVQRLYKALLDDISQQPLVQVSSWCIGEYGDLLVSGQCEEEEPIQVTEDEVLDV
LEGLLVSNLSTPVTRGYSLT
AIMKLSTRFSSVNRIKKVVSIYGSSIDVELQQRAVEYNAMFKKYDHMRPALLERMPIMEKTSSNGPTEMVQTNGETEPSA
LETKHPPPVTQPASQVNELL
DLLGGNDVGPVIQTNLPTKQVELLDLLGDLSLTEHELTQMIPGEYAIAASVGQYR
>g2883.t2
AGSYVRDDSVPNLIQLITNSVEMHAYTVQRLYKALLDDISQQPLVQVSSWCIGEYGDLLVSGQCEEEEPIQVTEDEVLDV
LEGLLVSNLSTPVTRGYSLT
AIMKLSTRFSSVNRIKKVVSIYGSSIDVELQQRAVEYNAMFKKYDHMRPALLERMPIMEKTSSNGPTEMVQTNGETEPSA

LETKHPPPVTQPASQVNELL
DLLGGNDVGPVIQTNLPTKQVELLDLLGDLSLT
>g2883.t3
AGSYVRDDSVPNLIQLITNSVEMHAYTVQRLYKALLDDISQQPLVQVSSWCIGEYGDLLVSGQCEEEEPIQVTEDEVLDV
LEGLLVSNLSTPVTRGYSLT
AIMKLSTRFSSVNRIKKVVSIYGSSIDVELQQRAVEYNAMFKKYDHMRPALLERMPIMEKTSSNGPTEMVQTNGETEPSA
LETKHPPPVTQPASQVNELL
DLLGGNDVGPVIQTNLPTKQVELLDLLGDLSLTGT
>g2884.t1
STSPGRPCAGEQGNPGRNVVVEPAALQPLSLPAPQVTCQIRRPCCPDNNIRRSRADAYPFFNPFLFATASPSPRRGGGQP
EIGKRIVRGRDPLRSNRTSQ
PSLLKGGVATRRGVAFRQANERVVLLQGPCLLACLEARGKLKVELGDALYLRGPAETRHCGVYLNRLSAAGASSGWLHGA
AQRQGEGWWSGVDQRPSITV
MESHELEVGSLQEALFIVESGEVYRNPTEVKMSQIVLPCHANHCGELSVGQLLKWMDSTACLSAERHAGCSCITASVDDI
HFEHTIGVGQVVNIIAKVNR
AFTSSMEVGILVTCEDLYIGKQWKVCHAFASFVARRTETGKVQLKQVIPHTQLEQVEYSLAAERRRMRLIHAEIITDLLS
SCAAQL
>g2884.t2
STSPGRPCAGEQGNPGRNVVVEPAALQPLSLPAPQVTCQIRRPCCPDNNIRRSRADAYPFFNPFLFATASPSPRRGGGQP
EIGKRVNVSIVRGRDPLRSN
RTSQPSLLKGGVATRRGVAFRQANERVVLLQGPCLLACLEARGKLKVELGDALYLRGPAETRHCGVYLNRLSAAGASSGW
LHGAAQRQGEGWWSGVDQRP
SITVMESHELEVGSLQEALFIVESGEVYRNPTEVKMSQIVLPCHANHCGELSVGQLLKWMDSTACLSAERHAGCSCITAS
VDDIHFEHTIGVGQVVNIIA
KVNRAFTSSMEVGILVTCEDLYIGKQWKVCHAFASFVARRTETGKVQLKQVIPHTQLEQVEYSLAAERRRMRLIHAEIIT
DLLSSCAAQL
>g2884.t3
LRAPASSGEQSRVPEYSTAQGGRAHGQIVRGRDPLRSNRTSQPSLLKGGVATRRGVAFRQANERVVLLQGPCLLACLEAR
GKLKVELGDALYLRGPAETR
HCGVYLNRLSAAGASSGWLHGAAQRQGEGWWSGVDQRPSITVMESHELEVGSLQEALFIVESGEVYRNPTEVKMSQIVLP
CHANHCGELSVGQLLKWMDS
TACLSAERHAGCSCITASVDDIHFEHTIGVGQVVNIIAKVNRAFTSSMEVGILVTCEDLYIGKQWKVCHAFASFVARRTE
TGKVQLKQVIPHTQLEQVEY
SLAAERRRMRLIHAEIITDLLSSCAAQL
>g2884.t4
STSPGRPCAGEQGNPGRNVVVEPAALQPLSLPAPQVTCQIRRPCCPDNNIRRSRADAYPFFNPFLFATASPSPRRGGGQP
EIGKRVNVSVPASASHLI
>g2885.t1
MKTKMDDLQLFRGDTVLMKGKKRRETVCIVLSDDTCSDEKIRMNRVVRNNLRVRLGDVIGIQPCPDVKYGKRIHVLPIDD
TVEGITGNLFEVYLKPYFLE
AYRPIRKGDIFLVRGGMRAVEFKVVETDPSPYCIVAPDTVIHYEGEPIRREDEEDSLNEVGYDDIGGVRKQLAQIKEMVE
LPLRHPALFKAIGVKPPRGI
LLYGPPGTGKTLIARAVANETGAFFFLINGPEIMSKLAGESESNLRKAFEEAEKNAPAIIFIDELDAIAPKREKTHGEVE
RRIVSQLLTLMDGLKQRAHV
IVMAATNRPNSIDPALRRFGRFDREVDIGIPDATGRLEILQIHTKNMKLADDVDLEQ
>g2885.t2
MGMSAAITDFLDTVDSTNQVNLTWSVLCSSMKTKMDDLQLFRGDTVLMKGKKRRETVCIVLSDDTCSDEKIRMNRVVRNN
LRVRLGDVIGIQPCPDVKYG
KRIHVLPIDDTVEGITGNLFEVYLKPYFLEAYRPIRKGDIFLVRGGMRAVEFKVVETDPSPYCIVAPDTVIHYEGEPIRR
EDEEDSLNEVGYDDIGGVRK
QLAQIKEMVELPLRHPALFKAIGVKPPRGILLYGPPGTGKTLIARAVANETGAFFFLINGPEIMSKLAGESESNLRKAFE
EAEKNAPAIIFIDELDAIAP
KREKTHGEVERRIVSQLLTLMDGLKQRAHVIVMAATNRPNSIDPALRRFGRFDREVDIGIPDATGRLEILQIHTKNMKLA
DDVDLEQ
>g2886.t1
MLWAGCARLLQAHCQARLKDWRQAVTYYTRCINLLGKVCIKAKGVQTQIPSVDLASRRGSKVWTLQRLKGLSFAGRGISF

IQRDQLREALRDLQLSTQIS
PEGVSAGLWLGEVLWRLDRRREAASCWEKSLADGSPAEYGPPSLCSGALPLYLQEPQFSPSLDPTVLRNRVETLSRT
>g2886.t2
MLWAGCARLLQAHCQARLKDWRQAVTYYTRCINLLGKVCIKAKGVQTQIPSVDLASRRGSKVWTLQRLKGLSFAGRGISF
IQRDQLREALRDLQLSTQIS
PEGVSAGLWLGEVLWRLDRRREAASCWEKSLADGSPAEYGPPSLCSGFWNKCNLEAGERTSHWLEYGCTATRTPPLFSFS
FFIRAAGLFHCTYKNPNSAL
PWIPLSFATEWRL
>g2886.t3
MLWAGCARLLQAHCQARLKDWRQAVTYYTRCINLLGKVCIKAKGVQTQIPSVDLASRRGSKVWTLQRLKGLSFAGRGISF
IQRDQLREALRDLQLSTQIS
PEGVSAGLWLGEVLWRLDRRREAASCWEKSLADGSPAEYGPPSLCSGFFIRAAGLFHCTYKNPNSALPWIPLSFATEWRL
>g2887.t1
MNRRLHLSKTKHSKFNESGQLAAFYLFSFVWGCSILTAAEFATNPTFLWEGYPHTHM
>g2887.t2
MGFRKKNKSPPVLSHEFVIQNHADMVSCLAMVILLGLMFEVTAKFAIMFITVQYNVTQALGEIQLRNHEQPSTNVFPCFI
FKKMNRRLHLSKTKHSKFNE
SGQLAAFYLFSFVWGCSILTAAEFATNPTFLWEGYPHTHM
>g2887.t3
MGFRKKNKSPPVLSHEFVIQNHADMVSCLAMVILLGLMFEVTAKFAIMFITVQYNVTQALGEIQLRNHKMNRRLHLSKTK
HSKFNESGQLAAFYLFSFVW
GCSILTAAEFATNPTFLWEGYPHTHM
>g2888.t1
LWSSCVDVPGTEPQSGGWGRVGEEKEKEGEAAAAVVVVVRGRASCGLHFLLAEEEIKTESDVLEGMEVSMRSKVPDPPGS
AERVLVPQKRKVSSPTHSSN
GHSPTDTSPSPVKKKKKPGALGSHNKDQDGRNDFYCWLCHREGQVLCCELCPRVYHAKCLKLPAEPEGDWFCPECETEPF
HKPVSLEQHPDYAEYIFHAM
DLCTLEKVITS
>g2888.t2
LWSSCVDVPGTEPQSGGWGRVGEEKEKEGEAAAAVVVVEVRREGGLPKKKPGFIIPAGVLRVVLAEEEIKTESDVLEGME
VSMRSKVPDPPGSAERVLVP
QKRKVSSPTHSSNGHSPTDTSPSPVKKKKKPGALGSHNKDQDGRNDFYCWLCHREGQVLCCELCPRVYHAKCLKLPAEPE
GDWFCPECETEPFHKPVSLE
QHPDYAEYIFHAMDLCTLEKVITS
>g2888.t3
LWSSCVDVPGTEPQSGGWGRVGEEKEKEGEAAAAVVVVVRGRASCGLHFLLAEEEIKTESDVLEGMEVSMRSKDPPGSAE
RVLVPQKRKVSSPTHSSNGH
SPTDTSPSPVKKKKKPGALGSHNKDQDGRNDFYCWLCHREGQVLCCELCPRVYHAKCLKLPAEPEGDWFCPECETEPFHK
PVSLEQHPDYAEYIFHAMDL
CTLEKVITS
>g2889.t1
LEPVYSFSARSRYQQRRNVFFLTSYTPGEVMDSPPAIPVSNVYNPIPEGHEDHYLEENPELFVWLDVNALEPGFSFSYQQ
GRHDFIQAS
>g2889.t2
LEPVYSFSARSRYQQRRNVFFLTSYTPGEVMDSPPAIPVSNALNDLSSPGSTHTNVGKNSLVYTSGSTLPDGYSGGGPLT
GSQQSTNSKTGGVVYNPIPE
GHEDHYLEENPELFVWLDVNALEPGFSFSYQQGRHDFIQAS
>g2889.t3
EEPVLSDVSDLEPVYSFSARSRYQQRRNVFFLTSYTPGEVMDSPPAIPVSNVYNPIPEGHEDHYLEENPELFVWLDVNAL
EPGFSFSYQQGRHDFIQAS
>g2890.t1
MLPEIPQWCNQLGRYRDQGSTHSFQRSSPEMEPVIDTLLFCRQVRLQVQNVEKPLYRGTFHCFQSIVRQESVCPRFSASA
LHAVCCSLAAGRSWSHHAAD
WLCRQACPGTAHLDGRAGRRVSDTTACLFVLEW
>g2890.t2
MLPEIPQWCNQLGRYRDQGSTHSFQRSSPEMEPVIDTLLFCRQVRLQVQNVEKPLYRGTFHCFQSIVRQESVCPRFSASA

LHAVCCSLAAGRSWSHHAAD
WLCRQACPGTAHLDGRAGRRVSDTTACLFVL
>g2891.t1
MQTSGGRLYSTWGETVLHLGGECTAPEGRLYSTWSETRSSRAVGVFLIGTTANQSEVEPVTRGISDVRIQTQPQFTGLWQ
CAVTTYSKEGVRGFFKGMAL
PVTTISMTSSVVFGTYRNCLQCFSQLRSGAPGGPNTKPEVFLSGMTAGVAQILVMSPGDMVKVRLQCQTESSRGRSDAPK
PKYRGPVHCLLTIVREEGVP
GLYRGALPLMLRDGPSFATYFLTYNTVCEWMTPPGKAGPGEQRASNARAA
>g2891.t2
LINVDIHCPQVRIQTQPQFTGLWQCAVTTYSKEGVRGFFKGMALPVTTISMTSSVVFGTYRNCLQCFSQLRSGAPGGPNT
KPEVFLSGMTAGVAQILVMS
PGDMVKVRLQCQTESSRGRSDAPKPKYRGPVHCLLTIVREEGVPGLYRGALPLMLRDGPSFATYFLTYNTVCEWMTPPGK
AGPGEQRASNARAA
>g2891.t3
MQTSGGRLYSTWGETVLHLGGECTAPEGRLYSTWSETRSVRIQTQPQFTGLWQCAVTTYSKEGVRGFFKGMALPVTTISM
TSSVVFGTYRNCLQCFSQLR
SGAPGGPNTKPEVFLSGMTAGVAQILVMSPGDMVKVRLQCQTESSRGRSDAPKPKYRGPVHCLLTIVREEGVPGLYRGAL
PLMLRDGPSFATYFLTYNTV
CEWMTPPGKAGPGEQRASNARAA
>g2892.t1
LLLTLHYLETSLSSRPNLSLLLIDSISAFYWLDRCGGGESVARQEDKLTKCSELLARLLRGTAGANMATTACAERQGSRQ
EFAVRCISSSSSSSSRGTKA
HKSSSFFVTDGGVVFV
>g2892.t2
LLLTLHYLETSLSSRPNLSLLLIDSISAFYWLDRCGGGESVARQEDKLTKCSELLARLLRDYRITVFATSHAIRRNYKNV
APSSSSCPPSASCSEYEQPY
LCRPWQRLVTHRLLCSRGTAGANMATTACAERQGSRQEFAVRCISSSSSSSSRGTKAHKSSSFFVTDGGVVFV
>g2892.t3
LLLTLHYLETSLSSRPNLSLLLIDSISAFYWLDRCGGGESVARQEDKLTKCSELLARLLRGTAGANMATTACAERQGSRQ
EFAVRCISSSSSSSSRGTKA
HKSSSFFVTDGGVVFVEHHQSLICTHINVWLAGS
>g2893.t1
KLYQSIYNEPFKIPEDDGNDLTLTFFNPDREGWLLKRGGRVKTWKRRWFILTDNCLYYFRYTTDKDPIGIIPLENLSVRA
IHDPSKRHCLELSSCMGQKI
KACKIENRGLVEGKHQAYRLSATNLKEQEGWIASI
>g2894.t1
LDGSDERRCPTGPCGPGQVACLSGGQCLDYRQLCDGVPHCADASDESVDNCGSIGIPPCPGSFSCDNHTCVNTTQVCDGA
PDCPRGEDELVCDKATSPAP
PGHSNTSLPCPGFTCWGGSCVSFNAVCNGVADCPDPSPPVVPGGPSDEQHCRAWGPWGAWGPCSRTCGTGSMSRRRACPP
GDPLRRCRGQDVERQQCFNT
TCAVDGRWLPWVGWSNCSRGCGGVEVRHRGCAPPLYGGVDCAQLPGESSVAMEIKPCPENTTCANLTCPAGLVRHSCAPC
PLSCGHISSGTPCRQDDSCF
SGCWCPEGRVMSPTQHCVAPEECPCEVGGARYWPGQQVKVDCELCVCEGGRPQRCQLNPECSVHCGWSSWSEWGECLGPC
GVQSVQWSFRSPNNPLKHGE
GRTCRGIYRKARRCQTDPCEECEYQGQSHAVGDMWGAGDCQLCQCLPNLGVRCSPYCPHAAAGCPLGFTLVPGVGDMCCY
CEGTGDNATAPQTTVSPLPV
PSTPAGGATPSVPTYPLPPGGECWAPLGVQSLPASSFTASSQQAAHPAGAARLYGWDPHTDLQGWSPEPEEYRDLPQRSP
NDPGGPQQNPYLQIDLLQPY
NITGVLTQGGGAFDAYMSSFYLQFSQNGGQWSTYRQLQSHARPRAKVFRGNHDDRGVAEARLDRMVSARMVRLLPHDFHN
AIYLRLEVMGCGEGHRWLST
PSPPGPVSPGGGCGETELRCGNGRCVPAGPPGVRCDGVDDCGDGSDERGCGTQPPATSAGPHSCPAGQFSCLPPGGCIPA
AQRCDGTPHCAVGEDEWDCH
RLHPSTTPSDRPYSPTPAPLRTPSTAAVTRPAVSPTVSAATHPAEGSPGYRGVCSSALGLEDGRIHYGQMTSSTHRENNP
ADAGRLNIVPNVLIMEPGWS
PLPSDPQPYLQVDFLEPTWVSGVVIQGSQRMLGYLTKYRLAFALHSNLFSDYTEAGGPGSPPKVFEVRMVGRAPVTRWLG
RLVRARYLRIIPLEFRHTFY

LRAEILGCRGDELVTPSGVTMSPPARARCEPGQFACHQGGACVSGSALCDGRPDCRDHSDEMDCGTTTVGSPGLQNLTSP
TGRPGFRGDTTTGAPGLDTS
RPPPGRPGVATT
>g2894.t2
NEFACVGVGQCVPQAWRCDGETDCLDGSDERRCPTGPCGPGQVACLSGGQCLDYRQLCDGVPHCADASDESVDNCGSIGI
PPCPGSFSCDNHTCVNTTQV
CDGAPDCPRGEDELVCDKATSPAPPGHSNTSLPCPGFTCWGGSCVSFNAVCNGVADCPDPSPPVVPGGPSDEQHCRAWGP
WGAWGPCSRTCGTGSMSRRR
ACPPGDPLRRCRGQDVERQQCFNTTCAVDGRWLPWVGWSNCSRGCGGVEVRHRGCAPPLYGGVDCAQLPGESSVAMEIKP
CPENTTCANLTCPAGLVRHS
CAPCPLSCGHISSGTPCRQDDSCFSGCWCPEGRVMSPTQHCVAPEECPCEVGGARYWPGQQVKVDCELCVCEGGRPQRCQ
LNPECSVHCGWSSWSEWGEC
LGPCGVQSVQWSFRSPNNPLKHGEGRTCRGIYRKARRCQTDPCEECEYQGQSHAVGDMWGAGDCQLCQCLPNLGVRCSPY
CPHAAAGCPLGFTLVPGVGD
MCCYCEGTGDNATAPQTTVSPLPVPSTPAGGATPSVPTYPLPPGGECWAPLGVQSLPASSFTASSQQAAHPAGAARLYGW
DPHTDLQGWSPEPEEYRDLP
QRSPNDPGGPQQNPYLQIDLLQPYNITGVLTQGGGAFDAYMSSFYLQFSQNGGQWSTYRQLQSHARPRAKVFRGNHDDRG
VAEARLDRMVSARMVRLLPH
DFHNAIYLRLEVMGCGEGHRWLSTPSPPGPVSPGGGCGETELRCGNGRCVPAGPPGVRCDGVDDCGDGSDERGCGTQPPA
TSAGPHSCPAGQFSCLPPGG
CIPAAQRCDGTPHCAVGEDEWDCHRLHPSTTPSDRPYSPTPAPLRTPSTAAVTRPAVSPTVSAATHPAEGSPGYRGVCSS
ALGLEDGRIHYGQMTSSTHR
ENNPADAGRLNIVPNVLIMEPGWSPLPSDPQPYLQVDFLEPTWVSGVVIQGSQRMLGYLTKYRLAFALHSNLFSDYTEAG
GPGSPPKVFEVRMVGRAPVT
RWLGRLVRARYLRIIPLEFRHTFYLRAEILGCRGDELVTPSGVTMSPPARARCEPGQFACHQGGACVSGSALCDGRPDCR
DHSDEMDCGTTTVGSPGLQN
LTSPTGRPGFRGDTTTGAPGLDTSRPPPGRPGVATT
>g2894.t3
LDGSDERRCPTGPCGPGQVACLSGGQCLDYRQLCDGVPHCADASDESVDNCGSIGIPPCPGSFSCDNHTCVNTTQVCDGA
PDCPRGEDELVCDKATSPAP
PGHSNTSLPCPGFTCWGGSCVSFNAVCNGVADCPDPSPPVVPGGPSDEQHCRAWGPWGAWGPCSRTCGTGSMSRRRACPP
GDPLRRCRGQDVERQQCFNT
TCAVDGRWLPWVGWSNCSRGCGGVEVRHRGCAPPLYGGVDCAQLPGESSVAMEIKPCPENTTCANLTCPAGLVRHSCAPC
PLSCGHISSGTPCRQDDSCF
SGCWCPEGRVMSPTQHCVAPEECPCEVGGARYWPGQQVKVDCELCVCEGGRPQRCQLNPECSVHCGWSSWSEWGECLGPC
GVQSVQWSFRSPNNPLKHGE
GRTCRGIYRKARRCQTDPCEECEYQGQSHAVGDMWGAGDCQLCQCLPNLGVRCSPYCPHAAAGCPLGFTLVPGVGDMCCY
CEGDNATAPQTTVSPLPVPS
TPAGGATPSVPTYPLPPGGECWAPLGVQSLPASSFTASSQQAAHPAGAARLYGWDPHTDLQGWSPEPEEYRDLPQRSPND
PGGPQQNPYLQIDLLQPYNI
TGVLTQGGGAFDAYMSSFYLQFSQNGGQWSTYRQLQSHARPRAKVFRGNHDDRGVAEARLDRMVSARMVRLLPHDFHNAI
YLRLEVMGCGEGHRWLSTPS
PPGPVSPGGGCGETELRCGNGRCVPAGPPGVRCDGVDDCGDGSDERGCGTQPPATSAGPHSCPAGQFSCLPPGGCIPAAQ
RCDGTPHCAVGEDEWDCHRL
HPSTTPSDRPYSPTPAPLRTPSTAAVTRPAVSPTVSAATHPAEGSPGYRGVCSSALGLEDGRIHYGQMTSSTHRENNPAD
AGRLNIVPNVLIMEPGWSPL
PSDPQPYLQVDFLEPTWVSGVVIQGSQRMLGYLTKYRLAFALHSNLFSDYTEAGGPGSPPKVFEVRMVGRAPVTRWLGRL
VRARYLRIIPLEFRHTFYLR
AEILGCRGDELVTPSGVTMSPPARARCEPGQFACHQGGACVSGSALCDGRPDCRDHSDEMDCGTTTVGSPGLQNLTSPTG
RPGFRGDTTTGAPGLDTSRP
PPGRPGVATT
>g2895.t1
ERDMDRKRVEELLEENLVLEMAQKQSMDESLHLGWELEQLSKTPDLTE
>g2896.t1
MGRVETGSVALPAIMRSGAGGPENFQMGSMPQAKQHITSGQMHRGHMPPLTSAQQALTGTINSSMQAVNAAQASLDELDA
LPPLGTDAASQAWRRNKMDE

SKHEIHSQVDAITAGTASMVNLTAGDPADTDYMAVGCAVTTISSNLTEMSKGVKLLAALMEDEGANGQQLLGAAKNLAGA
VSTMLKTAQPANTECFAKKN
RRFNRKILTIPFDPQPRQNLLQAAGNVGQTSGELLGHIGETDADPHFQDMLMQLAKAVANAAAALVLKAKNVAQKTEDSA
QQNRVIAAATQCALSTSQLV
ACTRGATGDEGLLKQVGAAATGVTHALNELLLHIKQYASGARPIGRHGEATDRILDVTENIFSSMGDAGEMVRQARILAQ
ATSDLVNAIKMDAEGETDLE
NSRKLLSAAKLLADATAKMVEAAKGAAAHPDSEEQQQKLREAAEGLRIATNAAAQNAIKKRLVNKLENAAKQAAAAATQT
IAAAQHAASSNRNAASQQQL
VQSCKVVAEQIPQLVQGVRGSQSQPDSPSAQLALIGASQNFLQPGAKMATSAKATVPTIGEQASAMQLSQCAKNLASALA
ELRTASQKAQEACGPLEIDN
ALSVIRGLERDMQEAKASAGAGRLKPLPGETVRTRVLCLEKCSQDLVTSTKSVSSAIAQLLSEATQGHENYT
>g2896.t2
MGRVETGSVALPAIMRSGAGGPENFQMGSMPQAKQHITSGQMHRGHMPPLTSAQQALTGTINSSMQAVNAAQASLDELDA
LPPLGTDAASQAWRRNKMDE
SKHEIHSQVDAITAGTASMVNLTAGDPADTDYMAVGCAVTTISSNLTEMSKGVKLLAALMEDEGANGQQLLGAAKNLAGA
VSTMLKTAQPANTECFAKKN
RRFNRKILTIPFDPQPRQNLLQAAGNVGQTSGELLGHIGETDADPHFQDMLMQLAKAVANAAAALVLKAKNVAQKTEDSA
QQNRVIAAATQCALSTSQLV
ACTRGATGDEGLLKQVGAAATGVTHALNELLLHIKQYASGARPIGRHGEATDRILDVTENIFSSMGDAGEMVRQARILAQ
ATSDLVNAIKMDAEGETDLE
NSRKLLSAAKLLADATAKMVEAAKGAAAHPDSEEQQQKLREAAEGLRIATNAAAQNAIKKRLVNKLENAAKQAAAAATQT
IAAAQHAASSNRNAASQQQL
VQSCKVVAEQIPQLVQGVRGSQSQPDSPSAQLALIGASQNFLQPGAKMATSAKATVPTIGEQASAMQLSQCAKNLASALA
ELRTASQKAQEACGPLEIDN
ALSVIRGLERDMQEAKASAGAGRLKPLPGETLEKCSQDLVTSTKSVSSAIAQLLSEATQGHENYT
>g2896.t3
MQLAKAVANAAAALVLKAKNVAQKTEDSAQQNRVIAAATQCALSTSQLVACTRGATGDEGLLKQVGAAATGVTHALNELL
LHIKQYASGARPIGRHGEAT
DRILDVTENIFSSMGDAGEMVRQARILAQATSDLVNAIKMDAEGETDLENSRKLLSAAKLLADATAKMVEAAKGAAAHPD
SEEQQQKLREAAEGLRIATN
AAAQNAIKKRLVNKLENAAKQAAAAATQTIAAAQHAASSNRNAASQQQLVQSCKVVAEQIPQLVQGVRGSQSQPDSPSAQ
LALIGASQNFLQPGAKMATS
AKATVPTIGEQASAMQLSQCAKNLASALAELRTASQKAQEACGPLEIDNALSVIRGLERDMQEAKASAGAGRLKPLPGET
VRTRVLCLEKCSQDLVTSTK
SVSSAIAQLLSEATQGHENYT
>g2896.t4
MGRVETGSVALPAIMRSGAGGPENFQMGSMPQAKQHITSGQMHRGHMPPLTSAQQALTGTINSSMQAVNAAQASLDELDA
LPPLGTDAASQAWRRNKMDE
SKHEIHSQVDAITAGTASMVNLTAGDPADTDYMAVGCAVTTISSNLTEMSKGVKLLAALMEDEGANGQQLLGAAKNLAGA
VSTMLKTAQPANTECFAKKN
RRFNRKILTIPFDPQPRQNLLQAAGNVGQTSGELLGHIGETDADPHFQFHNSDLT
>g2897.t1
MKFTIILGFISHMVSSHELKVKSEDLKMCPDQPFCVTDKEHCNSLPKSQDPRTQVNLTCYYEPSNSLEGQVSCSWSPASP
WSSLVFTAL
>g2897.t2
MKFTIILGFISHMVSSHELKVKSEDLKMCPDQPFCVTDKEHCNSLPKSQDPRTQVNLTCYYEPSNSLEGQVLYFLGYLLE
RRGVVGTEGKVLSFP
>g2898.t1
LLGWLTLSLAAPTPVPPVLPATAEEEALAEEYISQFYSDVGVTNSTFRSIILTSFNDSLQDMQAFFGLEVTGVLDRDTME
AMKAPRCGVSDISKYGHFAG
RPRWQKSLITYRITRYTPDLRQSLVDSTIAQAFKLYSDVIPLNFQQIYQGTADIMILFKGGCVNLLLVAAHEFGHALGLD
HSRDKSALMFPTYRYVNTNG
YRLPDDDRNGVQALYGSRTPKPKPDPAPVPEPEPDEPEDPDPTSDPSSDPSGEKCSRSLVFDAATSMRNDLYFFKSGYDD
RRNQMDSGYPRYITQDIPGI
GSKVDAAFSNYGYTYFSNGPRQVEYYLPYKRVMRTLLNYGWLNCY
>g2898.t2

LLGWLTLSLAAPTPVPPVLPATAEEEALAEEYISQFYSDVGVTNSTFRSIILTSFNDSLQDMQAFFGLEVTGVLDRDTME
AMKAPRCGVSDISKYGHFAG
RPRWQKSLITYRITRYTPDLRQSLVDSTIAQAFKLYSDVIPLNFQQIYQGTADIMILFKGGCVNLLLVAAHEFGHALGLD
HSRDKSALMFPTYRYVNTNG
YRLPDDDRNGVQALYVPEPEPDEPEDPDPTSDPSSDPSGEKCSRSLVFDAATSMRNDLYFFKSGYDDRRNQMDSGYPRYI
TQDIPGIGSKVDAAFSNYGY
TYFSNGPRQVEYYLPYKRVMRTLLNYGWLNCY
>g2898.t3
LLGWLTLSLAAPTPVPPVLPATAEEEALAEEYISQFYSDVGVTNSTFRSIILTSFNDSLQDMQAFFGLEVTGVLDRDTME
AMKAPRCGVSDISKYGHFAG
RPRWQKSLITYRITRYTPDLRQSLVDSTIAQAFKLYSDVIPLNFQQIYQGTADIMILFKGGCVNLLLVAAHEFGHALGLD
HSRDKSALMFPTYRYVNTNG
YRLPDDDRNGVQALYGSRTPKPKPDPAPVPEPEPDEPEDPDPTSDPSSDPSGEKCSRSLVFDAATSMRNDLYFFKSGSAL
LGHKDVQQENPYSYDDRRNQ
MDSGYPRYITQDIPGIGSKVDAAFSNYGYTYFSNGPRQVEYYLPYKRVMRTLLNYGWLNCY
>g2899.t1
MESLNTFESEMEADGQGHYSLFPALDHIATLSGASETLESEPSSDISEKPNLTWLDAAQMVLEEAGRPMHIKEIKQRIMD
RGLIQSNAKSSLEAVMYREL
PTVFQTQKGSRRFKRIENRNGVFALLGDSDRQQAFSDQSFLVGSPPQTTASSSGSAASLPAFPSPTSSSEPRAKGRRGPR
KNHSEKYRLKYLRLRRAARA
MIFVSEKDILHTCSDGWSLNSY
>g2899.t2
MESLNTFESEMEADGQGHYSLFPALDHIATLSGASETLESEPSSDISEKPNLTWLDAAQMVLEEAGRPMHIKEIKQRIMD
RGLIQSNAKSSLEAVMYRET
QKGSRRFKRIENRNGVFALLGDSDRQQAFSDQSFLVGSPPQTTASSSGSAASLPAFPSPTSSSEPRAKGRRGPRKNHSEK
YRLKYLRLRRAARAMIFVSE
KDILHTCSDGWSLNSY
>g2899.t3
MESLNTFESEMEADGQGHYSLFPALDHIATLSGASETLESEPSSDISEKPNLTWLDAAQMVLEEAGRPMHIKEIKQRIMD
RGLIQSNAKSSLEAVMYREL
PTVFQTQKGSRRFKRIENRNGVFALLGDSDRQQAFSDQSFLVGSPPQTTASSSGSAASLPAFPSPTSSSEPRAKGRRGPR
KNHSEKYRLKYLRLRRAARA
MIF
>g2900.t1
MYRTLLTRTKPWQHVTPSLIDLHWLPVKSRISYKILLHTYKSLHHLAPQHLTDLPHPYTPSRNLRSSDTGLLIPPPAPVG
GPSATELSV
>g2901.t1
TLIATHISECLADISSWTTAHQLKLNLAKTELPFIAGKDCPHRDLLVLVEGITVSASSTARNLGVIVNNQLSCTANITAV
SRSCRFALYNIRRIWPFLTR
EAT
>g2902.t1
METVGQKKEVWRLTLENASTPMAAEDKDREQHMYKDLYLRHKDYLNSLVGHDFEMPPAGVLRLMVNGEIGEWDTLYFSRI
PFCTLNCVQYSSCLCSPLIT
VSAKGFEYDNLYVHFFMELPSNWSTLPFQSLSGVTQTCRTKTIGKDSVALFSYPFSFEAFHLRENESEECLPQWPVLYFK
VLSLDYWQRYRTEGYGYLVF
PATPGNHVVTCQTWRPLQTGTVSGLRRFFIGGSPELEDHSYVRVPGTFKLGGFSQQGAVSNIMEAFQRARRRMQEARESL
PGDLINTSGPLQAESVT
>g2902.t2
METVGQKKEVWRLTLENASTPMAAEDKDREQHMYKDLYLRHKDYLNSLVGHDFEMPPAGVLRLMVNGEIGEWDTLYFSRI
PFCTLNCVQYSSCLCSPLIT
VSAKGFEYDNLYVHFFMELPSNWSTLPFQSLSGVTQTCRTKTIGKDSVALFSYPFSFEAFHLRENESEECLPQWPVLYFK
VLSLDYWQRYRTEGYGYLVF
PATPGLRRFFIGGSPELEDHSYVRVPGTFKLGGFSQQGAVSNIMEAFQRARRRMQEARESLPGDLINTSGPLQAESVT
>g2903.t1
DRRYADLSADQLPSCESLKDTIARALPFWNEEIAPQIKQGKRVLIAAHGNSLRGIVKHLEGMSEEAIMELNLPTGIPILY
ELDKNLKPVKPMQFLGDEET

VRKAMEAVAAQGKAKK
>g2903.t2
TDCVYFGQDRRYADLSADQLPSCESLKDTIARALPFWNEEIAPQIKQGKRVLIAAHGNSLRGIVKHLEGMSEEAIMELNL
PTGIPILYELDKNLKPVKPM
QFLGDEETVRKAMEAVAAQGKAKK
>g2903.t3
CVYFGQDRRYADLSADQLPSCESLKDTIARALPFWNEEIAPQIKQGKRVLIAAHGNSLRGIVKHLEGMSEEAIMELNLPT
GIPILYELDKNLKPVKPMQF
LGDEETVRKAMEAVAAQGKAKK
>g2904.t1
MLGLLHTILLHRSTGKFHYKKEGTYSIGTVGTQDIDCDFIDFTFVRVSSDELDRVISKAVTEFKDALGNTGSSDGMGQIS
LEFYQKKKSRWPFSDECIPW
EVWSIKVNVVHLANEQERQICREKVGEKLGEKVINVVEVINRHEYLPKMPTQSEVDNVFDTSLKDVQPYLYKITYQITDT
LGTSVSTTMRRLIKDTLAL
>g2904.t2
MNCRSEVLELTVEARQVEEAMLGLLHTILLHRSTGKFHYKKEGTYSIGTVGTQDIDCDFIDFTFVRVSSDELDRVISKAV
TEFKDALGNTGSSDGMGQIS
LEFYQKKKSRWPFSDECIPWEVWSIKVNVVHLANEQERQICREKVGEKLGEKVINVVEVINRHEYLPKMPTQSEVDNVFD
TSLKDVQPYLYKITYQITDT
LGTSVSTTMRRLIKDTLAL
>g2905.t1
METLVHYNNPFHGPSVLGVLNEQRLSGQMCDTVLVVGDQRYQAHRSVLAASSEYFQSLFTRTEAEAPNVVHLDFCEADAF
EIVLNYMYSSSLFVDKGSLA
VIQELGYSLGIPFLTNIASTRPHVSYCVSRKRLSFSEEDEADGQTRSVIVCRTRSDRKAAERPPSPRSSTPEASQPPTHH
KPQPVSSKATGTSDDARERK
PVFPYSSILKGNPTLAAMVRPQLTSSVSFSDVEVRHIRSQPGAELGIKDEEPLYHGRSPFQGRPGEPSQAIDRSGPLIKS
LLRRSLSMDSPVPVFSPTLE
LKELHSREQSVVKLASKATGTDTSEHNGGPKAPSPLVLRSQHPGEYDEHAAQGDDGVRVKAEPGSPLPDPLEIIRITIGD
TLPVNLKHFQPNSEQGPRAG
FDSFVKRKGRADNRRYPFKKSKGLNEHYPSENIAVADDIMAEAMAPQNPGLDGNENDAISVNVREPRQGKLFKCWNCLKV
FRSNAGLHRHVNMYHNPEKP
YACEICHKRFHTNFKVWTHCQTQHGVVQNPASSSSSSMLDEKFQKRLIDMVREREIKKALLWKLKRNKQSLQSPKLTKNR
PRTKFTCPYCGKVFLFQSQY
KQHLRTHPDENDAQDASGEGALYPEQDAAGPQTDDRPAAADVYACRLCNVKLSSLSEQGDHERGCRHATVCPYCGLRFSS
TAVKKEHEAHCKYKKLTCLE
CMRTFKSSFSIWRHQVEVHNQNMMTVKDQLNLKPQDADEADEEGSVALGHGRYDHMQTGSSGEEAHYGHASGPPIYDSED
SSSYVPEDLSMGHHGELVVK
EEPMEEAVCESESMDADNNGTAEEAGVWPCEKCGNLFSSHKDLERHQELLCHIKPYICHLCNKAFRTNFRLWSHFQSHMS
SGDDAGAKEVDRRPSSPSPS
PPLTPQTASGHPAPQALAAKPAQTVPVAAAIAEAPSSPEPGSSSECKGKNPEMERQHSHSPLSRSNSVEQSSGTQESDTL
FYHAPTLSALTFKRQYMCKL
CHRTFKTAFSLWSHEQSHGHL
>g2906.t1
MAVCGGHATEARFAERVFDRLSWIQHIVVVVAEERIALMREIVRRFQHARVRVVSGGATRHRSIFNGVRALAACESGAAP
DVVVIHDAVRPFVEEAFLLE
IATAAMENGASGAIRPLVSTVIATTSEGFLDHSLERGKYRASEMPQGFRYRVIFQAYQKV
>g2906.t2
MAVCGGHATEARFAERVFDRLSWIQHIVVVVAEERIALMREIVRRFQHARVRVVSGGATRHRSIFNGVRALAACESGAAP
DVVVIHDAVRPFVEEAFLLE
IATAAMENGASGAIRPLVSTVIATTSEGFLDHSLERGKYRASEMPQGFRYRVIFQAYQKKHLDAC
>g2907.t1
MSSPTGLHFSQADFLARWIPSSSRAGVILNLSPALVDSPGRRSSWGSEPGNPDDSFNSSFAPLPTSTNSSCCVGVDVAAQ
SEGLTGLGEVCRRPGHGTVE
RTGCEQPLNGQNCGPLGHSAVAMDSVEEPGNNKPHDLSLIMKLEQLRQWQHHMQQQLRAHQLEELVQLQEEQQRILGVMS
APQYCGTGLSGPELREVTLQ
VVSPPCEASRSLPSHGPGVPQGSMLGLQEKLESPQEGREDSGHVEDQSEESAAWVFREEQSELGWASSSEHSDHQDPLLP

CDGGTRTGHPHTASHGDLAD
RPIPVGRQKQSFEELLEEQLRLEEQRSNLTQHKQNRQGEEPQGAPPKRPFLRRGQGLSRFKGRPRGPSPPIGTLPGAPPP
SAKPVSRSNPEPATTSQHHK
PVQRKTATLNKENRSPPTAPVVRGDGKAAGVRRQKLLVPQQQQNPVDLEDQRGPGRQHQAHSAGDQNARRSGPLGPPGRG
RMASDPVQHSTGTRQVGPTA
RHDTARVSNGPPTRVPEEKPCPAPPGRRTVASEEEEQGGDGGEYSFEVSFQERMRGWEGELHQEKEALGEFELLEEAAEE
LSFSSNSSFVMKVLHMDRQA
QAAKALHQRRLSSTPIKPPRPPPPKVQPVRSKAGTPPCSEALVPGVGPNPPYNKSSYQDDSMGAGSDATDTDEEDEGDDD
GDSVLTNTDDSTLVEDSDGH
RGDLEFDDDDTWNDLEETAVALSARPLPRTTANRRSPSERTLTRKVAASRGPKTSTAPILTNHELAEPTIPPSKLMARLF
PSLMPKSQNAPVSVAPEPRQ
PDEAPGLQVQSQQLRERLVELELEIERFKKENNALSRLREENERSKEELRREREEFERRREGELARVVKLEEVTKEENRK
LQRDRRLFEQHASAARAIPD
KKEREEIQSLRQQLASLQEELRRKETRWGTTHSRLRQQIDTLNAENAALKEEVNTLERLRLGSWRRSSSPGERRDSATFS
SSSTKGVKFASPLDTRGTSP
LQCGVDAIRGGFRKNSLTGPAGGIKSSLRKPAGPSSSSSSSSSSSAQSPEEVPAARPPDKAPESEPPVPTLEVALTPTLT
QKRVVLQHVEEEEASESTES
NAEVTTHPDGKTEQVLPCGGRLIVFPNGTKKQLSADGLTATVTFFNGDTKQVLADQRVIYYYAEAQTTHTTFPNGMEILQ
FPNHQTEKHFPDGKKEITFP
DQTVKTLFPDGREESVLIDGTVIHVHPYVQNRIEPRRTDLITFTSTCMDDARHLACIMHT
>g2907.t2
MSSPTGLHFSQADFLARWIPSSSRAGVILNLSPALVDSPGRRSSWGSEPGNPDDSFNSSFAPLPTSTNSSCCVGVDVAAQ
SEGLTGLGEVCRRPGHGTVE
RTGCEQPLNGQNCGPLGHSAVAMDSVEEPGNNKPHDLSLIMKLEQLRQWQHHMQQQLRAHQLEELVQLQEEQQRILGVMS
APQYCGTGLSGPELREVTLQ
VVSPPCEASRSLPSHGPGVPQGSMLGLQEKLESPQEGREDSGHVEDQSEESAAWVFREEQSELGWASSSEHSDHQDPLLP
CDGGTRTGHPHTASHGDLAD
RPIPVGRQKQSFEELLEEQLRLEEQRSNLTQHKQNRQGEEPQGAPPKRPFLRRGQGLSRFKGRPRGPSPPIGTLPGAPPP
SAKPVSRSNPEPATTSQHHK
PVQRKTATLNKENRSPPTAPVVRGDGKAAGVRRQKLLVPQQQQNPVDLEDQRGPGRQHQAHSAGDQNARRSGPLGPPGRG
RMASDPVQHSTGTRQVGPTA
RHDTARVSNGPPTRVPEEKPCPAPPGRRTVASEEEEQGGDGGEYSFEVSFQERMRGWEGELHQEKEALGEFELLEEAAEE
LSFSSNSSFVMKVLHMDRQA
QAAKALHQRRLSSTPIKPPRPPPPKVQPVRSKAGTPPCSEALVPGVGPNPPYNKSSYQDDSMGAGSDATDTDEEDEGDDD
GDSVLTNTDDSTLVEDSDGH
RGDLEFDDDDTWNDLEETAVALSARPLPRTTANRRSPSERTLTRKVAASRGPKTSTAPILTNHELAEPTIPPSKLMARLF
PSLMPKSQNAPVSVAPEPRQ
PDEAPGLQVQSQQLRERLVELELEIERFKKENNALSRLREENERSKEELRREREEFERRREGELARVVKLEEVTKEENRK
LQRDRRLFEQHASAARAIPD
KKEREEIQSLRQQLASLQEELRRKETRWGTTHSRLRQQIDTLNAENAALKEEVNTLERLRLGSWRRSSSPGERRDSATFS
SSSTKGVKFASPLDTRGTSP
LQCGVDAIRGGFRKNSLTGPAGGIKSSLRKPAGPSSSSSSSSSSSAQSPEEVPAARPPDKAPESEPPVPTLEVALTPTLT
QKRVVLQHVEEEEASESTES
NAEVTTHPDGKTEQVLPCGGRLIVFPNGTKKQLSADGLTATVTFFNGDTKQVLADQRVIYYYAEAQTTHTTFPNGMEILQ
FPNHQTEKHFPDGKKEITFP
DQTVKTLFPDGREESVLIDGTVIHVHPYVQNRIEPRRTDLITFTSTCMDDARHLACIMHTVFSSLLAMLTCHPPSRLPYT
VAASTT
>g2907.t3
MSSPTGLHFSQADFLARWIPSSSRAGVILNLSPALVDSPGRRSSWGSEPGNPDDSFNSSFAPLPTSTNSSCCVGVDVAAQ
SEGLTGLGEVCRRPGHGTVE
RTGCEQPLNGQNCGPLGHSAVAMDSVEEPGNNKPHDLSLIMKLEQLRQWQHHMQQQLRAHQLEELVQLQEEQQRILGVMS
APQYCGTGLSGPELREVTLQ
VVSPPCEASRSLPSHGPGVPQGSMLGLQEKLESPQEGREDSGHVEDQSEESAAWVFREEQSELGWASSSEHSDHQDPLLP
CDGGTRTGHPHTASHGDLAD
RPIPVGRQKQSFEELLEEQLRLEEQRSNLTQHKQNRQGEEPQGAPPKRPFLRRGQGLSRFKGRPRGPSPPIGTLPGAPPP
SAKPVSRSNPEPATTSQHHK

PVQRKTATLNKENRSPPTAPVVRGDGKAAGVRRQKLLVPQQQQNPVDLEDQRGPGRQHQAHSAGDQNARRSGPLGPPGRG
RMASDPVQHSTGTRQVGPTA
RHDTARVSNGPPTRVPEEKPCPAPPGRRTVASEEEEQGGDGGEYSFEVSFQERMRGWEGELHQEKEALGEFELLEEAAEE
LSFSSNSSFVMKVLHMDRQA
QAAKALHQRRLSSTPIKPPRPPPPKVQPVRSKAGTPPCSEALVPGPNPPYNKSSYQDDSMGAGSDATDTDEEDEGDDDGD
SVLTNTDDSTLVEDSDGHRG
DLEFDDDDTWNDLEETAVALSARPLPRTTANRRSPSERTLTRKVAASRGPKTSTAPILTNHELAEPTIPPSKLMARLFPS
LMPKSQNAPVSVAPEPRQPD
EAPGLQVQSQQLRERLVELELEIERFKKENNALSRLREENERSKEELRREREEFERRREGELARVVKLEEVTKEENRKLQ
RDRRLFEQHASAARAIPDKK
EREEIQSLRQQLASLQEELRRKETRWGTTHSRLRQQIDTLNAENAALKEEVNTLERLRLGSWRRSSSPGERRDSATFSSS
STKGVKFASPLDTRGTSPLQ
CGVDAIRGGFRKNSLTGPAGGIKSSLRKPAGPSSSSSSSSSSSAQSPEEVPAARPPDKAPESEPPVPTLEVALTPTLTQK
RVVLQHVEEEEASESTESNA
EVTTHPDGKTEQVLPCGGRLIVFPNGTKKQLSADGLTATVTFFNGDTKQVLADQRVIYYYAEAQTTHTTFPNGMEILQFP
NHQTEKHFPDGKKEITFPDQ
TVKTLFPDGREESVLIDGTVIHVHPYVQNRIEPRRTDLITFTSTCMDDARHLACIMHT
>g2908.t1
MEKVLCNTHGSICMLKTGVKEGPNKGKSFYVCIDKSGCNFTQVASIPASHCLNHHDSMVELQAISHNPKQPIHRLYYRCV
SGKKAGQKWCGNIPWSEGER
DRRCPLASRQLQPSVAPQDRNPFKASGKSGEESEWRRIQGGAGGGMRGGAEEQGGGRRIGGGGRGSNSDQEEGGENVDSY
RGKALPAGMTIKKRGSDEKG
SCPKGHEEETKTTTKVEMKDKEGDEKKEGKRSVSTTNGEVPSPNSQNSLGDRPQPAPGPVSAAGSKVNSEASSSSRVVEL
PRSTHPPTAEKGNHYRTSAP
KDPREDPQPARSKTTNPSASCYSLRGEEEEEEEDDDDDDDVQIVSVKPSKQTTSPPLAPVQKTLTSFPGFQAASQVKAQH
ADPRGLQNLLKTQLQQKK
>g2908.t2
MLKTGVKEGPNKGKSFYVCIDKSGCNFTQVASIPASHCLNHHDSMVELQAISHNPKQPIHRLYYRCVSGKKAGQKWCGNI
PWSEGERDRRCPLASRQLQP
SVAPQDRNPFKASGKSGEESEWRRIQGGAGGGMRGGAEEQGGGRRIGGGGRGSNSDQEEGGENVDSYRGKALPAGMTIKK
RGSDEKGSCPKGHEEETKTT
TKVEMKDKEGDEKKEGKRSVSTTNGEVPSPNSQNSLGDRPQPAPGPVSAAGSKVNSEASSSSRVVELPRSTHPPTAEKGN
HYRTSAPKDPREDPQPARSK
TTNPSASCYSLRGEEEEEEEDDDDDDDVQIVSVKPSKQTTSPPLAPVQKTLTSFPGFQAASQVKAQHADPRGLQNLLKTQ
LQQKK
>g2908.t3
MEKVLCNTHASHCLNHHDSMVELQAISHNPKQPIHRLYYRCVSGKKAGQKWCGNIPWSEGERDRRCPLASRQLQPSVAPQ
DRNPFKASGKSGEESEWRRI
QGGAGGGMRGGAEEQGGGRRIGGGGRGSNSDQEEGGENVDSYRGKALPAGMTIKKRGSDEKGSCPKGHEEETKTTTKVEM
KDKEGDEKKEGKRSVSTTNG
EVPSPNSQNSLGDRPQPAPGPVSAAGSKVNSEASSSSRVVELPRSTHPPTAEKGNHYRTSAPKDPREDPQPARSKTTNPS
ASCYSLRGEEEEEEEDDDDD
DDVQIVSVKPSKQTTSPPLAPVQKTLTSFPGFQAASQVKAQHADPRGLQNLLKTQLQQKK
>g2909.t1
LLPGPVCHWLSPFIGKGPHISFPSLSLDDSGATAGFPWQRHSRAAMAAAATDASHHITTLTPEDQGRRSAAKLFGRTERE
REREPERVRERGSEGERERG
SAGERERGSEGERGKRQVGEALEKGRVLGGHGSSSECLVCGALFASQEKLHLHAFSHTVEKAFHCSQPHCPKAFSSKYKL
FRHMATHSPQKTHQCSFCEK
MFHRKDHLKNHLQTHDPNKEAFKCEECGKHYNTKLGYKRHVAMHAATAGDLTCKVCLQSYESTPVLLEHLRSHSGKASGG
AKEKKHPCDHCDRRFYTRKD
VRRHMVVHTGRKDFLCQYCAQRFGRKDHLTRHVKKSHSQELLKIKTEPPDMLGLLSSGSPPCSIKEELSPMMCGMGPNKD
PMVGKPFPIGTPFSMGMYNP
HHLQAMSNSGVGHPHPSLMPSSLSASMGMGCHMEPPPGSLHHHPHHHHHHHHHHHHHHSPPLPSHHHHQPPTPQQQPQAQ
PAPKYQLGSTSYLLDKPLKV
EMESFLMDLQSGLPGPLHPAEHHAAASPTKDSLEPSSGLADELCGGDALLSKSPAVIAESLCAANMDFSHLLGFLPLNLP
PYSAPMSTGGLVMGYTSSVA

PSSSSSSSSSSSSSSLHATDPHAMATAAVASTPITSLQLQPQESSIGGLGLGTLHPLAPVFSSSLSATTLPRFHQAFQ
>g2909.t2
LSLDDSGATAGFPWQRHSRAAMAAAATDASHHITTLTPEDQGRRSAAKLFGRTEREREREPERVRERGSEGERERGSAGE
RERGSEGERGKRQVGEALEK
GRVLGGHGSSSECLVCGALFASQEKLHLHAFSHTVEKAFHCSQPHCPKAFSSKYKLFRHMATHSPQKTHQCSFCEKMFHR
KDHLKNHLQTHDPNKEAFKC
EECGKHYNTKLGYKRHVAMHAATAGDLTCKVCLQSYESTPVLLEHLRSHSGKASGGAKEKKHPCDHCDRRFYTRKDVRRH
MVVHTGRKDFLCQYCAQRFG
RKDHLTRHVKKSHSQELLKIKTEPPDMLGLLSSGSPPCSIKEELSPMMCGMGPNKDPMVGKPFPIGTPFSMGMYNPHHLQ
AMSNSGVGHPHPSLMPSSLS
ASMGMGCHMEPPPGSLHHHPHHHHHHHHHHHHHHSPPLPSHHHHQPPTPQQQPQAQPAPKYQLGSTSYLLDKPLKVEMES
FLMDLQSGLPGPLHPAEHHA
AASPTKDSLEPSSGLADELCGGDALLSKSPAVIAESLCAANMDFSHLLGFLPLNLPPYSAPMSTGGLVMGYTSSVAPSSS
SSSSSSSSSSLHATDPHAMA
TAAVASTPITSLQLQPQESSIGGLGLGTLHPLAPVFSSSLSATTLPRFHQAFQ
>g2910.t1
MVNVSVNTQKASRRLGPQTSATPHQHLNTSPTPQHLTNTSPTPQHLTNTSTPHQHLTNTSTPHQHLNNTPTPQQHLNTSP
TPHQHLTNTSPTPQQHLNTS
PPPHHHLNTSPTPHQHLTNTSTTPHQHLTNTSTPHQHLNTSPTPQRGGR
>g2911.t1
MGGLRGGGAQSPRGSAPSRKGTGFNPRTCTAARWANAARYQRAPRGDSAGLLFWIRVSTGAGDRAGRPVDPHPGGPAAWD
RLRSRAGEPEPCRSRITE
>g2912.t1
ATGSQIGMIVNSLTNIGVSLIISFYFSWKLTLVILCFLPFIALSGGFQAKMLTGFAKQDKMAMESAGRISGEALNNIRTI
AGLGKERSFVEIYNAQLDAP
YKAAIQKAQVYGACYGFAQCVVFLANSASYRFGGYLVRQEGLHFSMVFRVISAIVTSGTALGRASSYTPDYAKAKISAAR
FFQLLDRVPQITVYSDKGEK
WDNFRGDVEFIDCKFTYPTRPDMQVLNGLNVSVKPGQTLAFVGSSGCGKSTSVQLLERFYDPDMGRVLIDGHESTRVNVP
FLRSKIGIVSQEPILFDCSI
ADNIRYGDNARDISMNDVMAAATKAQLHDFVMALPEKYNTHVGSQGSQLSRGQKQRIAIARAIIRDPKILLLDEATSALD
TESEKTVQDALDKAREGRTC
IVIAHRLSTIQNSDIIAVMARGFVIEKGSHDQLMAIKGAYYKLVTTGAPIS
>g2912.t2
ATGSQIGMIVNSLTNIGVSLIISFYFSWKLTLVILCFLPFIALSGGFQAKMLTGFAKQDKMAMESAGRISGEALNNIRTI
AGLGKERSFVEIYNAQLDAP
YKAAIQKAQVYGACYGFAQCVVFLANSASYRFGGYLVRQEGLHFSMVFRVISAIVTSGTALGRASSYTPDYAKAKISAAR
FFQLLDRVPQITVYSDKGEK
WDNFRGDVEFIDCKFTYPTRPDMQVLNGLNVSVKPGQTLAFVGSSGCGKSTSVQLLERFYDPDMGRVLIDGHESTRVNVP
FLRSKIGIVSQEPILFDCSI
ADNIRYGDNARDISMNDVMAAATKAQLHDFVMALPEKYNTHVGSQGSQLSRGQKQRIAIARAIIRDPKILLLDEATSALD
TESEKAREGRTCIVIAHRLS
TIQNSDIIAVMARGFVIEKGSHDQLMAIKGAYYKLVTTGAPIS
>g2913.t1
VFKSKYKDDFYPCLLPKSHSTAEDLPFTVPGSGRAKVQVLRSVDRWSAGTCENSILNAYVDTIENSEHYVYIENQFFISC
ADGKSIYNGIGDAIVNRILR
AHSEQKKYRVFVVVPLLPGFEGDINEGGGNAIQAILHFTYRTMCRGEHSILARLKEVEDKWTEYITLCGLRTHTQLAGAP
VTELIYVHSKTLIADDRRYI
IGESPPLQSSANINDRSMLGSRDSELAVLVEDELRVPSTMGGEEYQAGPLTLALRKECFSFGFGRVP
>g2913.t2
VFKSKYKDDFYPCLLPKSHSTAEDLPFTVPGSGRAKVQVLRSVDRWSAGTCENSILNAYVDTIENSEHYVYIENQFFISC
ADGKSIYNGIGDAIVNRILR
AHSEQKKYRVFVVVPLLPGFEGDINEGGGNAIQAILHFTYRTMCRGEHSILARLKEVEDKWTEYITLCGLRTHTQLAGAP
VTELIYVHSKTLIADDRRYI
IGESPPLQSSANINDRSMLGSRDSELAVLVEDELRVPSTMGGEEYQAGPLTLALRKECFRVLLGAASEPSININDDPISDE
FFFLDWNATAKLNATIYEKV
QERQLFVHFCTHRSSDANELISG

>g2913.t3
VFKSKYKDDFYPCLLPKSHSTAEDLPFTVPGSGRAKVQVLRSVDRWSAGTCENSILNAYVDTIENSEHYVYIENQFFISC
ADGKSIYNGIGDAIVNRILR
AHSEQKKYRVFVVVPLLPGFEGDINEGGGNAIQAILHFTYRTMCRGEHSILARLKEVEDKWTEYITLCGLRTHTQLAGAP
VTELIYVHSKTLIADDRRYI
IGESPPLQSSANINDRSMLGSRDSELAVLVEDELRVPSTMGGEEYQAGPLTLALRKECFR
>g2914.t1
DDTSPCFPAVAPSPTRTQPAVAMETISVKLLTAVGPKGGRRAEPTSSVSRSRGPHTQEHLASLQWFSALGLSEEQAVCFQ
EADSSLRGGPGAHLKSCCVD
A
>g2914.t2
VAPSPTRTQPAVAMETISVKLLTAVGPKGGRRAEPTSSVSRSRGPHTQEHLASLQWFSALGLSEEQAVCFQEADSSLRGG
PGAHLKSCCVDA
>g2915.t1
PCAPGPGAGHSLSGSWEVPGGAAGQPSRSQRHLQASCSQMPSPTPARS
>g2915.t2
PCAPGPGAGHSLSGQPSRSQRHLQASCSQMPSPTPARS
>g2916.t1
MFRGPCGLVPQGLLSAEQAKRKPLFVDEGPHSGARPRLSAPEVQEILVGSSLDHDTIMQNPFTGESRLADRELRFTPLTT
TSSCLSCCSPRVSPAQSVTQ
VALCSSGRRGPRECSDVLG
>g2916.t2
MFRGPCGLVPQGLLSAEQAKRKPLFVDEGPHSGARAPEVQEILVGSSLDHDTIMQNPFTGESRLADRELRFTPLTTTSSC
LSCCSPRVSPAQSVTQVALC
SSGRRGPRECSDVLG
>g2917.t1
LSSSWRRARSLQGRQKPSLQDFEPASKQASSPSSHTVGPEPRAESPENPHPNPTHPEETANYLRCPGERQAETCSVQRAG
GGRPQRRGEGCRASPVLI
>g2918.t1
DIRQLRLKQDGFIREISDLQETVEWKDKKIGALERQKEYTDAIRVERDELRDEVVKLKDILKKHGIVLGPDLNVNGDIVD
PGTDGSSQPGSQPGPDAQTP
PTEGNSSSMLGEKPL
>g2918.t2
DIRQLRLKQDGFIREISDLQETVEWKDKKIGALERQKEYTDAIRVERDELRDEVVKLKDILKKHGIVLGPDLNVNGDIVD
PGTDGSSQPGSQPGPDAQTP
PTEGNSSSMLAQGGSAELAVATETVQVGGLLQPREEGMENKHIIADVCEALVAEVMSVVARTVHGGLATKRDDIQSNSQT
VSDVEESPQREADVVDGLKD
DEPEGSLTIAPITQPPDESLTIAPITQPPDESLTIAPITQPPDESLTIAPITQPPDESLTIAPITEPPVESLTIAPIAES
HPEPGNVDDDENEELENEEL
SSRPQVKGAAGKKKKKKRKGKKKKPAVVSEERQKEDEKEDGVPENVNENDTRKNDSKSGTKEDGSFMGSEIASSSPLKIL
KGSQMDQLQENQDEENVLEP
GIVEPEVCGSVTNAKSNLDVSDPVLNSESNNSTDCLDMDGMSLKGYNMNLTRAMFRSLINLHIQWKTKVIKSVESRNGKQ
SQRNLLRFILFPMRNTLIED
QTSQRSENISLTRAMVRNVMGLHIQWKTKKINSAQRKKGNPNHLK
>g2918.t3
DIRQLRLKQDGFIREISDLQETVEWKDKKIGALERQKEYTDAIRVERDELRDEVVKLKDILKKHGIVLGPDLNVNGDIVD
PGTDGSSQPGSQPGPDAQTP
PTEGNSSSMLAQGGSAELAVATETVQVGGLLQPREEGMENKHIIADVCEALVAEVMSVVARTVHGGLATKRDDIQSNSQT
VSDVEESPQREADVVDGLKD
DEPEGSLTIAPITQPPDESLTIAPITQPPDESLTIAPITQPPDESLTIAPITQPPDESLTIAPITEPPVESLTIAPIAES
HPEPGNVDDDENEELENEEL
SSRPQVKGAAGKKKKKKRKGKKKKPAVVSEERQKEDEKEDGVPENVNENDTRKNDSKSGTKEDGSFMGSEIASSSPLKIL
KGSQMDQLQENQDEENVLEP
GIVEPEVCGSVTNAKSNLDVSDPVLNSESNNSTDCLDMDGMSLKGYNMNLTRAMFRSLINLHIQWKTKVIKSVESRNGKQ
SQRNLLRFILFPMRNTLIED
QTSQRSENISLTRAMDGMSLKGYNMNLTRAMFRSLINLHVQWRTKMIKPVESRNGQQSQRNLLRFILFPMRNTLIEDQTS

QRSENISLTKAMLDDEEEEE
EDEIDEGHSFDFDEMDLEASITITSITTTSEEAEKATGVTSQDANRDLGQCEASERSIQKVSEVCPAGEPPGNTEETATV
ECLVVDSSEPKNPTSSTEPQ
STTGKEQGASEAADKGIDQLHNVVESSGVADEQRNTLNEGEGERRGCSGGVGFEMGKEALVSSIDEGQEVLKGGDQLRVG
NTEDQVSSQTALSSTKKDSK
NVPKGKGKGKGKGKEDCKMS
>g2918.t4
MENKHIIADVCEALVAEVMSVVARTVHGGLATKRDDIQSNSQTVSDVEESPQREADVVDGLKDDEPEGSLTIAPITQPPD
ESLTIAPITQPPDESLTIAP
ITQPPDESLTIAPITQPPDESLTIAPITEPPVESLTIAPIAESHPEPGNVDDDENEELENEELSSRPQVKGAAGKKKKKK
RKGKKKKPAVVSEERQKEDE
KEDGVPENVNENDTRKNDSKSGTKEDGSFMGSEIASSSPLKILKGSQMDQLQENQDEENVLEPGIVEPEVCGSVTNAKSN
LDVSDPVLNSESNNSTDCLD
MDGMSLKGYNMNLTRAMFRSLINLHIQWKTKVIKSVESRNGKQSQRNLLRFILFPMRNTLIEDQTSQRSENISLTRAMDG
MSLKGYNMNLTRAMFRSLIN
LHVQWRTKMIKPVESRNGQQSQRNLLRFILFPMRNTLIEDQTSQRSENISLTKAMLDDEEEEEEDEIDEGHSFDFDEMDL
EASITITSITTTSEEAEKAT
GVTSQDANRDLGQCEASERSIQKVSEVCPAGEPPGNTEETATVECLVVDSSEPKNPTSSTEPQSTTGKEQGASEAADKGI
DQLHNVVESSGVADEQRNTL
NEGEGERRGCSGGVGFEMGKEALVSSIDEGQEVLKGGDQLRVGNTEDQVSSQTALSSTKKDSKNVPKGKGKGKGKGKEDC
KMS
>g2919.t1
MRPLISQFDPCLPKPSSYSLSLRRGGVEKVVPQSPKAQERDILKEVFANDLSSPTGIIASCRKPIRGAAGRPIQPSDFWN
DENLLLSPKSLSTTTSSTST
YTETRHRSSNLALSPPSSSIASSASRLVSAAPPAVQTKAVRRGFSVWLKLFFLALLVSFLFFVYQTMEVEVLSPFSSSET
EQTSTKA
>g2919.t2
MRPLISQFDPCLPKPSSYSLSLRRGGKECSDTHVAEGKQQKITPFLSDLSPVRRHGCVALLSKQSYSDKVEKVVPQSPKA
QERDILKEVFANDLSSPTGI
IASCRKPIRGAAGRPIQPSDFWNDENLLLSPKSLSTTTSSTSTYTETRHRSSNLALSPPSSSIASSASRLVSAAPPAVQT
KAVRRGFSVWLKLFFLALLV
SFLFFVYQTMEVEVLSPFSSSETEQTSTKA
>g2919.t3
MRPLISQFDPCLPKPSSYSLSLRRGGGWRPLVACVVFSWKVEKVVPQSPKAQERDILKEVFANDLSSPTGIIASCRKPIR
GAAGRPIQPSDFWNDENLLL
SPKSLSTTTSSTSTYTETRHRSSNLALSPPSSSIASSASRLVSAAPPAVQTKAVRRGFSVWLKLFFLALLVSFLFFVYQT
MEVEVLSPFSSSETEQTSTK
A
>g2920.t1
MKRSPRHQGNLQHGSPRRGASRLPFNAAWSWNSSGRSLCVHARASADFPLNTRESSVQSVEGMPSFLRAPDDQIGMSGGV
ASFVCQALGDPRPRITWMKK
GK
>g2921.t1
MCVCAQRVDYKAMAASPLFERYCEMAVQLQRVELLSLTREEKLAFFINTYNALVIHGYLRLGAPTNMWQRYRFFNYVSYL
IGGEVFTLQDIENGVLRGNR
KGMAQLLKPFSKTDPRLQMALPDAEPLIHFALNCGAKGCPPIKTYTPQDIDGQLRTAAEAFLENDDGCVVDSGKQEVRLS
QIFKWYKADFGGTDEKLLNW
VLEHMGDSAKKSNLQGVLTAGKTKVSYLPYDWSTNSTH
>g2921.t2
RVDYKAMAASPLFERYCEMAVQLQRVELLSLTREEKLAFFINTYNALVIHGYLRLGAPTNMWQRYRFFNYVSYLIGGEVF
TLQDIENGVLRGNRKGMAQL
LKPFSKTDPRLQMALPDAEPLIHFALNCGAKGCPPIKTYTPQDIDGQLRTAAEAFLENDDGCVVDSGKQEVRLSQIFKWY
KADFGGTDEKLLNWVLEHMG
DSAKKSNLQGVLTAGKTKVSYLPYDWSTNSTH
>g2921.t3
MPFLSAVFSAPKKNKKSGKRCPDWPGRVDYKAMAASPLFERYCEMAVQLQRVELLSLTREEKLAFFINTYNALVIHGYLR

LGAPTNMWQRYRFFNYVSYL
IGGEVFTLQDIENGVLRGNRKGMAQLLKPFSKTDPRLQMALPDAEPLIHFALNCGAKGCPPIKTYTPQDIDGQLRTAAEA
FLENDDGCVVDSGKQEVRLS
QIFKWYKADFGGTDEKLLNWVLEHMGDSAKKSNLQGVLTAGKTKVSYLPYDWSTNSTH
>g2922.t1
LWLHNTVLRVFTEDTPKSLFSHTGANSCFAGRRRGKVFAKCSLKAMHHTPASSRSASVSCELGGFKVGADSN
>g2923.t1
LLGLCNNFAYVVMLSAAHDILTKQQLDNSTTPVVLLADILPSLVIKLSATFYIHQVPYGFRVLFCVSATVASFLLVSFST
TMSMSILGVVFASMGSGLGE
LSFLSLTAFFSRDVLEGWGSGTGGAGVAGALLYSALTQAGLSPQVTLLTMLVVPAAMLLSYFFLLAFPPMFPQWRRSREA
GPVGPVGPAGRGGVSEERRR
LMDEIEEEEEEEEYEQQEKGPRAYLQLCLSA
>g2923.t2
LLGLCNNFAYVVMLSAAHDILTKQQLDNSTTPTPHSLTVDFEVRNSSNSTRYDCNPVSTAVVLLADILPSLVIKLSATFY
IHQVPYGFRVLFCVSATVAS
FLLVSFSTTMSMSILGVVFASMGSGLGELSFLSLTAFFSRDVLEGWGSGTGGAGVAGALLYSALTQAGLSPQVTLLTMLV
VPAAMLLSYFFLLAFPPMFP
QWRRSREAGPVGPVGPAGRGGVSEERRRLMDEIEEEEEEEEYEQQEKGPRAYLQLCLSA
>g2923.t3
MLSAAHDILTKQQLDNSTTPTPHSLTVDFEVRNSSNSTRYDCNPVSTAVVLLADILPSLVIKLSATFYIHQVPYGFRVLF
CVSATVASFLLVSFSTTMSM
SILGVVFASMGSGLGELSFLSLTAFFSRDVLEGWGSGTGGAGVAGALLYSALTQAGLSPQVTLLTMLVVPAAMLLSYFFL
LAFPPMFPQWRRSREAGPVG
PVGPAGRGGVSEERRRLMDEIEEEEEEEEYEQQEKGPRAYLQLCLSA
>g2924.t1
MLKPGEQSGSAFLKVDPSYLHHWQQLFPQTPQTQLKTAGSLAALCPPERVSVPVESLRASRLNNHLISSTNCSSTSSTSS
SSSSSSAALTPSSTSCSSGS
SVTSSLAGLSQLPVPQQIALFGQALVPACSGAGDLVVVVPPDTQGLHPGGGLLPGPPSCGVVSSSVRSSSSSDPKTLSSS
CSSSSTCRGRLTSEELDYYL
YGQQRMEIIPLNNHSGELSNRKEKHVPLERLYY
>g2925.t1
TEPPTVSRVVGVAGGLPLSWAADANASSGYLVEWRNASCLRRCPVGWTEAGAGSTNVTMATEPFQDGLLYDFSLYGLSPN
ASVLLQRWKGYPKETGPFTS
VPNLMANQVKSDIQLTWGDIPPADRGGFLLGYCVYLNNGSQLTLIGTSHLLPAPRGHSGQSNLSDPDSRSYWVRNKLVSS
YKFTVKAYNSAGEDAGSTTS
ITMEAYNWLLLEILASLGTMTLFLFIITCVCYRKRKWVKKAFYPDIPEPKLAGDWSRTQGTLDVKPHANSMVHIVDEPEW
DSSKEKLVVIPEEDEDEERA
VRDDPVDTDEPMSIRYYNQVVDERPIRPRYPDSSESSASSLDSARTDVTYTGIQTS
>g2925.t2
MSDTEPPTVSRVVGVAGGLPLSWAADANASSGYLVEWRNASCLRRCPVGWTEAGAGSTNVTMATEPFQDGLLYDFSLYGL
SPNASVLLQRWKGYPKETGP
FTSVPNLMANQVKSDIQLTWGDIPPADRGGFLLGYCVYLNNGSQLTLIGTSHLLPAPRGHSGQSNLSDPDSRSYWVRNKL
VSSYKFTVKAYNSAGEDAGS
TTSITMEAYNWLLLEILASLGTMTLFLFIITCVCYRKRKWVKKAFYPDIPEPKLAGDWSRTQGTLDVKPHANSMVHIVDE
PEWDSSKEKLVVIPEEDEDE
ERAVRDDPVDTDEPMSIRYYNQVVDERPIRPRYPDSSESSASSLDSARTDVTYTGIQTS
>g2926.t1
MTSARYVDFLNGHFLPWCKRKPGGSRDIMMSMQVNSVSHTAKGTIDWLLCKSWCSPPSPPDHNPIEILRNILKRNEGGCS
IPQNSSSGRQYQHRQMK
>g2927.t1
LCRKTGRLKFPLRREQSTADRSAAHPSIYSSACFTRHRPLSSSPPPTRDRPPSLTYKLAQCHSATSDSSLGITAKDDDLF
QDHRRHADTLKTKKPRFWLS
FLRVFFPWLCAFGCAEPLSGTLARSTTDVSINTETRLSAGEHGREDRLSVRFSLLIGQSVNGIPETLTAVYRRAERPGGP
DGGRAQRQASERLTSGERAL
GCQIAVSLRWRHKRDPSLAALSTTAVTDRNAREPFFAFGLRNRQETRRTPPATAPGHRRQAYRTRCGPGLSPF
>g2927.t2

LCRKTGRLKFPLRREQSTADRSAAHPSIYSSACFTRHRPLSSSPPPTRDRPPSLTYKLAQCHSATSDSSLNSLGDNNTNR
GEYGDGDRLSFLRVFFPWLC
AFGCAEPLSGTLARSTTDVSINTETRLSAGEHGREDRLSVRFSLLIGQSVNGIPETLTAVYRRAERPGGPDGGRAQRQAS
ERLTSGERALGCQIAVSLRW
RHKRDPSLAALSTTAVTDRNAREPFFAFGLRNRQETRRTPPATAPGHRRQAYRTRCGPGLSPF
>g2927.t3
LCRKTGRLKFPLRREQSTADRSAAHPSIYSSACFTRHRPLSSSPPPTRDRPPSLTYKLAQCHSATSDSSLGITAKDDDLF
QDHRRHADTLKTKKPRFWVK
RRSDVRPLDRSVSGGVEPVGAIPFLTLSRLLKLSFLRVFFPWLCAFGCAEPLSGTLARSTTDVSINTETRLSAGEHGRED
RLSVRFSLLIGQSVNGIPET
LTAVYRRAERPGGPDGGRAQRQASERLTSGERALGCQIAVSLRWRHKRDPSLAALSTTAVTDRNAREPFFAFGLRNRQET
RRTPPATAPGHRRQAYRTRC
GPGLSPF
>g2928.t1
MGSVTSAFTRTRNALVKVGSEPDHHDAARGCSAPEADRAKKKSARSNVTSSQTGRQSARQQSLSEVLGPPDGDGPKPTPK
SSHCKSQ
>g2929.t1
MVEMHVQAQEQHLQVDMAEVAKPDLTAALRDVRLQYENLATKNLQESEDWYKSKFSDLTESAARTSEALRAARQEANDHR
RQMQALTCEVDSLKGTNESL
ERQMRETEENFSMETSGYQDSIARLEDDIHNMKDEMARHLREYQDLLNVKMALDIEIATYRKLLEGEESR
>g2929.t2
MVEMHVQAQEQHLQVDMAEVAKPDLTAALRDVRLQYENLATKNLQESEDWYKSKFSDLTESAARTSEALRAARQEANDHR
RQMQALTCEVDSLKGTNESL
ERQMRETEENFSMETSGYQDSIARLEDDIHNMKDEMARHLREYQDLLNVKMALDIEIATYRKLLEGEESRWSTSQHRTMK
NLIKGIGFYAVKDPNSRSNK
RQTTKQEDNCCFKCLYVYKIP
>g2929.t3
MVEMHVQAQEQHLQVDMAEVAKPDLTAALRDVRLQYENLATKNLQESEDWYKSKFSDLTESAARTSEALRAARQEANDHR
RQMQALTCEVDSLKGTNESL
ERQMRETEENFSMETSGYQDSIARLEDDIHNMKDEMARHLREYQDLLNVKMALDIEIATYRKLLEGEESRPHVESTTTKK
VLIKTIETRDGQVFNLK
>g2930.t1
SCHTNRVFIDSEELASALHFKKHIEVLHYLLQLWPVLSPGQPWTPTTAASTPTPHTSLFLTTTTAVVPDPPGVPPPLRRR
RERPQVLNLSEVEMAGVLRP
RPGRLDSPSNRYAGDWSRCGESYFGEPEITVLPGPPRGREEADYDDVPSEDTEDGGEEEEQQEKETKVEEVKEEEDVQEK
PEGGEQHGEGGSEKESENGK
EVALEEEKEDEKEEERDREEKEEENEEEQEEELVNSDSCGCSQPLENKMEEEVATMEMEEEEEEEQQQQEEEELEKEEEE
EEEEDRLHNHILPPHLPKEH
AYQAYVKIQDISPVLSNRVNLRDLQESIDTLIGNLERELNKNKLNIGY
>g2930.t2
SCHTNRVFIDSEELASALHFKKHIEVLHYLLQLWPGQPWTPTTAASTPTPHTSLFLTTTTAVVPDPPGVPPPLRRRRERP
QVLNLSEVEMAGVLRPRPGR
LDSPSNRYAGDWSRCGESYFGEPEITVLPGPPRGREEADYDDVPSEDTEDGGEEEEQQEKETKVEEVKEEEDVQEKPEGG
EQHGEGGSEKESENGKEVAL
EEEKEDEKEEERDREEKEEENEEEQEEELVNSDSCGCSQPLENKMEEEVATMEMEEEEEEEQQQQEEEELEKEEEEEEEE
DRLHNHILPPHLPKEHAYQA
YVKIQDISPVLSNRVNLRDLQESIDTLIGNLERELNKNKLNIGY
>g2931.t1
GASGQSSPGTPKSPGSLTKAAVEANKVKKVAVVRSSPKSPGSLKSRSPAPLAAAAPLPDLKNVRSKIGSTDNIKHQPGGG
KVQILDQKMDVSNVQSKCGS
KVNLKHVPGGGNVQILDKKMDVTNVQARCGSKANLKHTPGGGKVQIIEKKLDLSNVQSRCGSKDNIKHVAGGGNRGLEGQ
REEPPAREQKGSCALGWDCC
PLMTSPLTGSQTDGQMDRQRQKDIDGQIQRPSDR
>g2931.t2
GASGQSSPGTPKSPGSLTKAAVEANKVKKVAVVRSSPKSPGSLKSRSPAPLAAAAPLPDLKNVRSKIGSTDNIKHQPGGG
KVQILDQKMDVSNVQSKCGS

KVNLKHVPGGGNVQILDKKMDVTNVQARCGSKANLKHTPGGGKRGLEGQREEPPAREQKGSCALGWDCCPLMTSPLTGSQ
TDGQMDRQRQKDIDGQIQRP
SDR
>g2931.t3
GASGQSSPGTPKSPGSLTKAAVEANKVKKVAVVRSSPKSPGSLKSRSPAPLAAAAPLPDLKNVRSKIGSTDNIKHQPGGG
KVQILDQKMDVSNVQSKCGS
KVNLKHVPGGGNVQILDKKMDVTNVQARCGSKANLKHTPGGGKVQIIEKKLDLSNVQSRCGSKDNIKHVAGGGNRVGMIC
VSLSEQRGLEGQREEPPARE
QKGSCALGWDCCPLMTSPLTGSQTDGQMDRQRQKDIDGQIQRPSDR
>g2932.t1
DNSQPSPFTNNPADTKAKHFLPLLSVLAACVRESPACPAVTEYQMEPTECGAGGRPRAPGGPRRAV
>g2933.t1
MTHLPSLTCPSPPAPKVKTHFFSSDESEEEEDHRKKFKIKIKPLPSDCPVAAPSVDQLKASIGNIALSPSPM
>g2934.t1
MHLTTMKERXKRLLSHCLWRGVNEVIGHNASVQQATAREKHGNVTDHDGRANRRPARVAATWETLAEHPDAGRQTRAVFV
VSSTGRRASRPAASQPR
>g2935.t1
MKTKFINGVSSSPSMSLGLLVTESALHVQPESEEHCKEVMSRPDQPDSETKRKAFLWCREFLHGAWRSVSEEDFHITIIR
GGLSNKLFLCELPGSLDSVG
DEPRSVLLRLYGAILQANKDRLAWQCASLSGTELASWRTASMQRISWRAAKKRDARSDRICITAGEAGLVSFAPLRERLC
NNDRLGPNKCPRRGAEAMVL
ESVMFAILAERELGPKLYGIFPQGRLEQYVPSRKLETCELQDPNISAEVAQKMAAFHAMRMPFNKEPKWLFGTMEKYLNQ
IMRMAFTRESHLRLFNRLVS
YNLPQEMDALKSLLESTNSPVMFCHNDCQEGNILLLNTSRAQKLMLIDFEYSSYNYR
>g2935.t2
MKTKFINGVSSSPSMSLGLLVTESALHVQPESEEHCKEVMSRPDQPDSETKRKAFLWCREFLHGAWRSVSEEDFHITIIR
GGLSNKLFLCELPGSLDSVG
DEPRSVLLRLYGAILQANKDRLAWQCASLSGTELASWRTASMQRISWRAAKKRDARSDRICITAGEAGLVSFAPLRERLC
NNDRLGPNKCPRRGAEAMVL
ESVMFAILAERELGPKLYGIFPQGRLEQYVPSRKLETCELQDPNISAEVAQKMAAFHAMRMPFNKEPKWLFGTMEKYLNQ
IMRMAFTRESHLRLFNRLVS
YNLPQEMDALKSLLESTNSPVMFCHNDCQEGNILLLNTSRAQKLMLIDFEYSSYNY
>g2935.t3
MSLGLLVTESALHVQPESEEHCKEVMSRPDQPDSETKRKAFLWCREFLHGAWRSVSEEDFHITIIRGGLSNKLFLCELPG
SLDSVGDEPRSVLLRLYGAI
LQANKDRLAWQCASLSGTELASWRTASMQRISWRAAKKRDARSDRICITAGEAGLVSFAPLRERLCNNDRLGPNKCPRRG
AEAMVLESVMFAILAERELG
PKLYGIFPQGRLEQYVPSRKLETCELQDPNISAEVAQKMAAFHAMRMPFNKEPKWLFGTMEKYLNQIMRMAFTRESHLRL
FNRLVSYNLPQEMDALKSLL
ESTNSPVMFCHNDCQEGNILLLNTSRAQKLMLIDFEYSSYNYR
>g2936.t1
LTDGDNTATGATCGRRTVTPIHTHNTKHQDSSKGVDTPPRPEGGGCQLIPDPTAHHKMDADMDAARDCKG
>g2937.t1
LQQELQAMKQQQELVEKERRLEQQHQQQQQQQLQQQQQQQLQQQQSQQEREQERHRREQHLSSLSLRGKDRSRESAVAST
EVKQKLQEFLLSKSAKDPIC
SGATHSLIHHPKLWYTSSHHTSLDQSGSPLGGASPTCQYTLPSPVESKGDFPLRKTGL
>g2937.t2
LQQELQAMKQQQELVEKERRLEQQHQQQQQQQLQQQQQQQLQQQQSQQEREQERHRREQHLSSLSLRGKDRSRESAVAST
EVKQKLQEFLLSKSAKDPIC
SGATHSLIHHPKLWYTSSHHTSLDQSGSPLGGASPTCQYTLPSPVESKGDFPLRKT
>g2938.t1
DGPPRLDRPSSPAGLDPDPPSTGSPLLSPDSSSHDAVLSAPAVSPADSESLSPDELELLAKLEEQNRFFSRQKTQIMISP
ESAGLAPRQHKAQLELSTVQ
EHVALKASLLMSHTGLYSDRAPLRAVENHNGGKGNAPGVRRAETKLKKKKKKHVKHSGSHDNR
>g2939.t1
MFLYDNSIDERNKNMDGFAGGNFAANQCRNLLAHPTSLAPSTTYTSSEVPASGIGEPVKQCSPCSATQSSPNASLPYGYF

GNGYYPCRMSHSSVKTCAQP
SGYGDKYMDTSISGEEFPSRAKEYAFYQGYSSGPYQASYLDVPVVPALSAPSEPRHHESLLPMEPYQPWAIANSWNGQVY
CAKDQPQSNALWKSSLQGGL
LFINR
>g2940.t1
DHGPHHGTQQGHHGNLTPEAPHHPPVRGHGNNNNNQGKDKHAAAGEVSSSPEQMERMLHSLKDRLEDLQKRNTSNTYSNS
LKDLLQRKITTLEQQLQHRT
AALSSPDRYDDHGHHDGGGGSSHPDDIGRHDGDHSDDTHPNDHRRDDEDEGGGGGREDGSHGDGVHREERGGDRDDHDDD
DHHRDGEGDGGDHGDRDEGH
HDGSQNGNEADSRGFLPRTGYQTSGSRSHFHVNKLEDMLSQLHLAGSSRKNVKSPNAFQIGFPIRTNYMYGRLKKSLLHE
IFALTLCLWIKAGVGPGLGT
PFSYSAPGQANELVLIEWGNNPMELLIDDKSVSLPLSLTDGKWHHVCVTWSTRDGQWEVFQDGVQRGSGKNLSAWHPIKP
GGIFILGQEQDTMGGRFDAT
QSFVGEISDLQMWSHVLTPGDIYSLASCGSHLSGDVIAWSEAEVELHGGATKYPFDPCH
>g2941.t1
CPHLLSGDPSRLFSKRFVYDINAPEYTVSHALLSIPTSLCISNEVHDCSGSTLTLCSGPQHRGQLPRRCERLRAREGPAC
LRLSSLTPYGGKQMK
>g2941.t2
LRCPHLLSGDPSRLFSKRFVYDINAPEYTVSHALLSIPTSLCISNEVHDCSGSTLTLCSGPQHRGQLPRRCERLRAREGP
ACLRLSSLTPYGGKQMK
>g2941.t3
CSGSTLTLCSGPQHRGQLPRRCERLRAREGPACLRLSSLTPYGGKQMK
>g2942.t1
MVSKCNCVVWLPLSLAFVLAVAGDSEVILKRPKREWIIAPKGLKENFDHTIKQYIAKIRSDKEYESVVTYELLGIGASKS
PAGRFTVDPLTGLVKVNSIL
DREEIANYTLRGTAKFSNGQRAETDVELHIIVLDENDCPPVFQVNQIGSVNESSAAGTFVMRVIATDADEEGLHSKIYYS
IEGSSSSMFTIDSSTGEVMV
RQSSLDREYEGRVQENTVNVEVLRVRAIDLDLKHSDNWLSVFSFQSGNEAGYFSISTDSTTNEGVIMIRKPLDFEEVKVV
NLALSVSNKAAYNFGSSHME
VTQKTYPIKINVVNQKEGPHFHPSVKVVTISEDRSTISINKVITNYAARDSDTLQTATNVWYAKYEDAASWLIIDERTAD
IKLNKLPDRESKFLINGTYY
AKIICITKDLPTKTATGTIAIQVKDFNDNCPELTVTNQTVCRGDTAVYVTAVDRDEYPNSAPFDFRVISENKQEWITEQL
NQTTTILRNKADLWPGYYKV
ALKIMDQQGHSCESAQVLNMLVCTCLEGTKTCVSRGTHTAVFGASGVLLFLLGLLLLLREYIVGCWACRNVR
>g2942.t2
MVSKCNCVVWLPLSLAFVLAVAGDSEVILKRPKREWIIAPKGLKENFDHTIKQYIAKIRSDKEYESVVTYELLGIGASKS
PAGRFTVDPLTGLVKVNSIL
DREEIANYTLRGTAKFSNGQRAETDVELHIIVLDENDCPPVFQVNQIGSVNESSAAGTFVMRVIATDADEEGLHSKIYYS
IEGSSSSMFTIDSSTGEVMV
RQSSLDREYEGRVQENTVNVEVLRVRAIDLDLKHSDNWLSVFSFQSGNEAGYFSISTDSTTNEGVIMIRKPLDFEEVKVV
NLALSVSNKAAYNFGSSHME
VTQKTYPIKINVVNQKEGPHFHPSVKVVTISEDRSTISINKVITNYAARDSDTLQTATNVWYAKYEDAASWLIIDERTAD
IKLNKLPDRESKFLINGTYY
AKIICITKDLPTKTATGTIAIQVKDFNDNCPELTVTNQTVCRGDTAVYVTAVDRDEYPNSAPFDFRVISENKQEWITEQL
NQTTTILRNKADLWPGYYKV
ALKIMDQQGHSCESAQVLNMLVCTCLEGTKTCVSRGTHTAVFGASGVLLFLLGLLLLLLLPLLLLFCLCGGPAAMGNFKA
IPFDAKEQLIAYHTEGQGED
KAVPLLSIPTEVDHGHSVVDGVTSGGSRFVGGGKYTAGL
>g2942.t3
MVSKCNCVVWLPLSLAIRSDKEYESVVTYELLGIGASKSPAGRFTVDPLTGLVKVNSILDREEIANYTLRGTAKFSNGQR
AETDVELHIIVLDENDCPPV
FQVNQIGSVNESSAAGTFVMRVIATDADEEGLHSKIYYSIEGSSSSMFTIDSSTGEVMVRQSSLDREYEGRVQENTVNVE
VLRVRAIDLDLKHSDNWLSV
FSFQSGNEAGYFSISTDSTTNEGVIMIRKPLDFEEVKVVNLALSVSNKAAYNFGSSHMEVTQKTYPIKINVVNQKEGPHF
HPSVKVVTISEDRSTISINK
VITNYAARDSDTLQTATNVWYAKYEDAASWLIIDERTADIKLNKLPDRESKFLINGTYYAKIICITKDLPTKTATGTIAI

QVKDFNDNCPELTVTNQTVC
RGDTAVYVTAVDRDEYPNSAPFDFRVISENKQEWITEQLNQTTTILRNKADLWPGYYKVALKIMDQQGHSCESAQVLNML
VCTCLEGTKTCVSRGTHTAV
FGASGVLLFLLGLLLLLLLPLLLLFCLCGGPAAMGNFKAIPFDAKEQLIAYHTEGQGEDKAVPLLSIPTEVDHGHSVVDG
VTSGGSRFVGGGKYTAGL
>g2943.t1
MEIGHSDAQDIWKGMDKQHCLHLLYLFSVCHVLILVQPNCAFDVTYDRLFRALDALRQKVLPLIRAAIKDTQVSKEWKLN
CRPCPPRLLFVFQMNGALKV
PTGTDSAGTADKPKKHSPRRRLQHALEDQIYRIFRKSRVLTNQSSNCLFSVPANQPFVYVVPGPDEDPLGALLGHLRSSC
ALREQDPNSSLTGPRRYQQI
RRSARQPHTTVDYSGVSGQLVDCSLKEFLWQHIDLVLTKKGFDDSVGRNPQPSHFELPTYSKWVQVACKLHQVLIHNTEE
DSADYALKVQSQLKVLEGFL
DADAKFSENRCQKALPLAHSAYQSNLPHNYTTTVHKNQLAQALRVYSQHARGVAFQRYALQLHEDCYKFWSNGHQLCEER
SLTDQHCVHKFHLLPQPGEK
PDMDHNPPVLHHNSRGRSTSACNCGRKQAPREDPFDIKAANSEFYLMVEDKCCLKLERIDFPVFQPSTPDPAPAPASACQ
DTARPGSDSSAPSEGEKLKD
PTPAAAAVSGSAAHSHTPGDSTGLSLALSLGQSTDSLGAYGEADGSEAQVSHKRPSLVDRQPSTVEYLPGMLHTGCPKGL
LPKFSSWSLVKLGPAKSYNC
HTGLEQPGFLPGSSFLLPWDLVIRSRSSEEDAGLSEPLDGGGSSWPAPNKSLVGKRGSTGGLGRGRRRDDVARAFVGFEY
EDGRGRRFLSAGPDKIVKVL
GPGGAKEPATRVLNTDMPLYVPSPAQGRGLKPHYAQLMRLFIVVPDAPLEITLNPQVQPGPLPCPVFHPERTELALPSDG
LWVLRFPYSYMTERGPCYPP
KENQPLSAYKVLRG
>g2943.t2
MQTGVLKESFINTLADKHIFSLFGGLEEKNSENGSLIQAVYSQENRVLYLVLTSVYDNRQLLRACESLSMEIGHSDAQDI
WKGMDKQHCLHLLYLFSVCH
VLILVQPNCAFDVTYDRLFRALDALRQKVLPLIRAAIKDTQVSKEWKLNCRPCPPRLLFVFQMNGALKVPTGTDSAGTAD
KPKKHSPRRRLQHALEDQIY
RIFRKSRVLTNQSSNCLFSVPANQPFVYVVPGPDEDPLGALLGHLRSSCALREQDPNSSLTGPRRYQQIRRSARQPHTTV
DYSGVSGQLVDCSLKEFLWQ
HIDLVLTKKGFDDSVGRNPQPSHFELPTYSKWVQVACKLHQVLIHNTEEDSADYALKVQSQLKVLEGFLDADAKFSENRC
QKALPLAHSAYQSNLPHNYT
TTVHKNQLAQALRVYSQHARGVAFQRYALQLHEDCYKFWSNGHQLCEERSLTDQHCVHKFHLLPQPGEKPDMDHNPPVLH
HNSRGRSTSACNCGRKQAPR
EDPFDIKAANSEFYLMVEDKCCLKLERIDFPVFQPSTPDPAPAPASACQDTARPGSDSSAPSEGEKLKDPTPAAAAVSGS
AAHSHTPGDSTGLSLALSLG
QSTDSLGAYGEADGSEAQVSHKRPSLVDRQPSTVEYLPGMLHTGCPKGLLPKFSSWSLVKLGPAKSYNCHTGLEQPGFLP
GSSFLLPWDLVIRSRSSEED
AGLSEPLDGGGSSWPAPNKSLVGKRGSTGGLGRGRRRDDVARAFVGFEYEDGRGRRFLSAGPDKIVKVLGPGGAKEPATR
VLNTDMPLYVPSPAQGRGLK
PHYAQLMRLFIVVPDAPLEITLNPQVQPGPLPCPVFHPERTELALPSDGLWVLRFPYSYMTERGPCYPPKENQPLSAYKV
LRG
>g2944.t1
MAETAKDPPHSSFSSSSSSSCLHVLGNVSYLLLSVTTSCRPRRPYTSCIAASLPREDRTSRHSPSTSSSETRSVKGRIGS
GSLSWRGGLISRRKSSKNLR
KVFSSVADPAISSHSWTDKRTLKPANIGLEYFE
>g2945.t1
MEVSWRAILMDHSWQQPQGGAVSASACRQLKQQEREEGMRLKEEQQQEWWAAQMGISPAEAELAAGPKRTPPHSIPPHPF
SPFIPRLKAWPTKPDWVQVG
GRFPETDPKMDPETDTDDVTEERLMERLTGHLRVASTGP
>g2945.t2
MEVSWRAILMDHSWREGSPRLHILCWFWFEQPQGGAVSASACRQLKQQEREEGMRLKEEQQQEWWAAQMGISPAEAELAA
GPKRTPPHSIPPHPFSPFIP
RLKAWPTKPDWVQVGGRFPETDPKMDPETDTDDVTEERLMERLTGHLRVASTGP
>g2945.t3
MEVSWRAILMDHSWQQPQGGAVSASACRQLKQQEREEGMRLKEEQQQEWWAAQMGISPAEAELAAGPKRTPPHSIPPHPF

SPFIPRLKAWPTKPDWVQVG
GRFPETDPKMDPETDTDDVTEERLMERLTGHLRVASTGPCTTT
>g2946.t1
LKREIVSGVTGTLFSWLLVKGGGVCSSQNFRGLWSSRTSPFLADGVTTLYHSLHLRFMTRAPERIDDGALKWLIERISQM
WNNVT
>g2947.t1
MSNIEGADSVRVTSDSPQHLDPSLGAEPQEKTSEDQGISAALLGVKQELKEMLLEVFDGAFAPSCGDKKACSAESFDFSS
SIDTRASAADIHPIKAQLQL
LMGRADDLQNQLAHAQDQLEREHLSTLVPTFLSSSKPLFTYLESRARSRLSQQPAGPLPSTSSSTASLLELSQRLCERLE
QLVLNYAQQGLLSVDESQSD
SLSHFYIGQCVIDGLKVSVFRYHSATPFLSGGGAQPGLYKRMRWNVERLSDDRLHPHQQQQQQQEEEPEERQLGTGGDTE
YFFMCYEDIPEGVSREEGEG
LAPGDLATRTWSIGRWVQTVPDPDIEDIYDWVLCEVPRARYLKLVSLGSEEPSVCHATDCLLGVLLLQQTGGKDDIPFSE
>g2947.t2
PSCVRHGKRSGRTCRAWDTAVCTRPAFHSAPRDEGTVRDDTKDKREKQRLHLGQERCCRALERPGLSKKTGVGLNYLALC
LSFMVAYTRLLWAWPGHNRR
SSPVWGGRNLTRADADHTLQKPRCSLPPLVKHRNQSAGTRPDVSRAEPQEKTSEDQGISAALLGVKQELKEMLLEVFDGA
FAPSCGDKKACSAESFDFSS
SIDTRASAADIHPIKAQLQLLMGRADDLQNQLAHAQDQLEREHLSTLVPTFLSSSKPLFTYLESRARSRLSQQPAGPLPS
TSSSTASLLELSQRLCERLE
QLVLNYAQQGLLSVDESQSDSLSHFYIGQCVIDGLKVSVFRYHSATPFLSGGGAQPGLYKRMRWNVERLSDDRLHPHQQQ
QQQQEEEPEERQLGTGGDTE
YFFMCYEDIPEGVSREEGEGLAPGDLATRTWSIGRWVQTVPDPDIEDIYDWVLCEVPRARYLKLVSLGSEEPSVCHATDC
LLGVLLLQQTGGKDDIPFSE
>g2947.t3
MSNIEGADSVRVTSDSPQHLDPSLGAEPQEKTSEDQGISAALLGVKQELKEMLLEVFDGAFAPSCGDKKACSAESFDFSS
SIDTRASAADIHPIKAQLQL
LMGRADDLQNQLAHAQDQLEREHLSTLVPTFLSSSKPLFTYLESRARSRLSQQPAGPLPSTSSSTASRLCERLEQLVLNY
AQQGLLSVDESQSDSLSHFY
IGQCVIDGLKVSVFRYHSATPFLSGGGAQPGLYKRMRWNVERLSDDRLHPHQQQQQQQEEEPEERQLGTGGDTEYFFMCY
EDIPEGVSREEGEGLAPGDL
ATRTWSIGRWVQTVPDPDIEDIYDWVLCEVPRARYLKLVSLGSEEPSVCHATDCLLGVLLLQQTGGKDDIPFSE
>g2948.t1
MESHHCLGRNHMGEQPKMELINEGRAGAGSHSSHEEPAGERSAMKRLSSMWSSFRGRKNDKLKDVPESPGETGPPAPQDR
EQRYIRGQYFFEYLVVVSLK
KASDDSYEPQITYQFPKKDGMVRFQREEEEKMLKAITLFCFPEGINWAPMTEYQSETFSFVLTEVDGSRRNGYCRRLLPK
GNKACPPEAYCIISRLACFG
LFSKIFDEVEKRRQISMAMIYPFMQKLRESPFPAPGHSVEIKSFIPESGTEVRLRPKPYRD
>g2948.t2
MGEQPKMELINEGRAGAGSHSSHEEPAGERSAMKRLSSMWSSFRGRKNDKLKDVPESPGETGPPAPQDREQRYIRGQYFF
EYLVVVSLKKASDDSYEPQI
TYQFPKKDGMVRFQREEEEKMLKAITLFCFPEGINWAPMTEYQSETFSFVLTEVDGSRRNGYCRRLLPKGNKACPPEAYC
IISRLACFGLFSKIFDEVEK
RRQISMAMIYPFMQKLRESPFPAPGHSVEIKSFIPESGTEVRLRPKPYRD
>g2948.t3
EQPKMELINEGRAGAGSHSSHEEPAGERSAMKRLSSMWSSFRGRKNDKLKDVPESPGETGPPAPQDREQRYIRGQYFFEY
LVVVSLKKASDDSYEPQITY
QFPKKDGMVRFQREEEEKMLKAITLFCFPEGINWAPMTEYQSETFSFVLTEVDGSRRNGYCRRLLPKGNKACPPEAYCII
SRLACFGLFSKIFDEVEKRR
QISMAMIYPFMQKLRESPFPAPGHSVEIKSFIPESGTEVRLRPKPYRD
>g2949.t1
MSSSKTTAVSLHSSAKQQNGNGDVRELSDVWKALSQSKVALRHIENRLEATPGTGVLLESVMDPKRYASRKLSRRG
>g2949.t2
MSSSKTTAVSLHSSAKQQNGNGDVRELSDVWKALSQSKVALRHIENRLEATPGTGVLLESVMDPKRYASRKLSRRGRGAD
DAGGSSKRRGHGNHSPEKSS
SRSPLRNTTLDSNVRKNNVEFREPLASFREATPPPLQPSQLEACSTQPESGPSLTPTQSPSDPSLSQLVYQRDTRDRQTD

RDLDSTHSSALEGTEVRYLN
DQSALDATMPRPVQAALATEGED
>g2949.t3
MNVEQVFLAPAAIAGRGADDAGGSSKRRGHGNHSPEKSSSRSPLRNTTLDSNVRKNNVEFREPLASFREATPPPLQPSQL
EACSTQPESGPSLTPTQSPS
DPSLSQLVYQRDTRDRQTDRDLDSTHSSALEGTEVRYLNDQSALDATMPRPVQAALATEGED
>g2949.t4
MSSSKTTAVSLHSSAKQQNGNGDVRELSDVWKALSQSKVALRHIENRLEATPGTGVLLESVMDPKRYASRKLSRRAGRGA
DDAGGSSKRRGHGNHSPEKS
SSRSPLRNTTLDSNVRKNNVEFREPLASFREATPPPLQPSQLEACSTQPESGPSLTPTQSPSDPSLSQLVYQRDTRDRQT
DRDLDSTHSSALEGTEVRYL
NDQSALDATMPRPVQAALATEGED
>g2950.t1
AYKTIEDEDLRFPLIYGEGKKARVLATIGVTRGLGDHDLKVHDSNIYIKPFLSCCPEVWFPGVKVKVYNLALYKHGPDDV
LVMGTDGLWDVLSNQEVAEA
VTSFLANCDPDDSHRYTMAAQELVMRARGVLRDRGWRITNERLGSGDDISVFVVPLMYGNQQL
>g2950.t2
MGVPLKVWFPGVKVKVYNLALYKHGPDDVLVMGTDGLWDVLSNQEVAEAVTSFLANCDPDDSHRYTMAAQELVMRARGVL
RDRGWRITNERLGSGDDISV
FVVPLMYGNQQL
>g2950.t3
AYKTIEDEDLRFPLIYGEGKKARVLATIGVTRGLGDHDLKVHDSNIYIKPFLSCCPEVKVYNLALYKHGPDDVLVMGTDG
LWDVLSNQEVAEAVTSFLAN
CDPDDSHRYTMAAQELVMRARGVLRDRGWRITNERLGSGDDISVFVVPLMYGNQQL
>g2950.t4
AYKTIEDEDLRFPLIYGEGKKARVLATIGVTRGLGDHDLKVHDSNIYIKPFLSCCPEVFSSAPYAPLSFPQALLSAFSSS
PPRVLSSSSSSPPCPLILAL
LSSRSS
>g2951.t1
MVSKLTSLQQELLSALLDSGVTKDVLIQALDDMDPSPATGYGVKLENTLHMSPPSSKMNGNDSDSKPVFHTLTNGLSKSK
LSGDEGSEDGDDFDTPPILK
ELQSLNTEEAAEQRAEVDRMLA
>g2952.t1
PELLDDPASLSVDCDKWSFGTTVWEIFNNGETPLKGWDLEMVAMVTEQYHACYKLDVGGDIPGKNPKRHSGSDAWSASCS
GEIGEVEPTDSSHHLCVKSV
CWRRQ
>g2953.t1
ALAAGRPLKPRDNEGQG
>g2954.t1
MLMPPKRETVAATNPTPVKMMKIGTDTQDFACDPLEDKYRFSECPSYSPFAHQESEPAGFEEEDGSPAIRTDTITLLMKI
EQLQAQLKYERRCRILAERE
LRELKEMNGLMLQMRHTAHELRATLDQVRQGTSDAISSQGLAEEEISLATDPDGEMRIPYHIAEVDTRASPAEDSNFLYL
SESLRVPRNLYERISEITDF
RKYTSALLMMLFDRETLATHSLQGRRTNVTGDEGQKPQLPPEILGSII
>g2954.t2
MLMPPKRETVAATNPTPVKMMKIGTDTQDFACDPLEDKYRFSECPSYSPFAHQESEPAGFEEEDGSPAIRTDTITLLMKI
EQLQAQLKYERRCRILAERE
LRELKEMNGLMLQMRHTAHELRATLDQVRQGTSDAISSQGLAEEEISLATDPDGEMRIPYHIAEVDTRASPAEDSNFLYL
SESLRVPRNLYERISEITDF
RKYTSALLMMLFDRETLATHSLQGRRTNVTGDEGQKPQLPPEILGSIIG
>g2954.t3
MLMPPKRETVAATNPTPVKMMKIGTDTQDFACDPLEDKYRFSECPSYSPFAHQESEPAGFEEEDGSPAIRTDTITLLMKI
EQLQAQLKYERRCRILAERE
LRELKEMNGLMLQMRHTAHELRATLDQVRQGTSDAISSQGLAEEEISLATDPDGEMRIPYHIAEEDSNFLYLSESLRVPR
NLYERISEITDFRKYTSALL
MMLFDRETLATHSLQGRRTNVTGDEGQKPQLPPEILGSII

>g2955.t1
MASTFFIKKSGNSAFPKKGHSLATSKRKANSEEDLGGKSKGRAKKPNSRHNEEISSDSETEGTAVQNEREEDEFDETPQE
KKLRLAKLYLDQLRDEGFNF
APHLSLQLEQKGKLQRSVAKDLLPPDASEIRLLRGHQLPVTCLVISTDEKFIFSASKDGSIIKWDVETGKKLHNIKGGRR
GTEARHEGHTTQVLCMAISS
DGKYLVSASVTEAPAPGALRKQKTCVTQSGDTVLWLPRSHG
>g2955.t2
MASTFFIKKSGNSAFPKKGHSLATSKRKANSEEDLGGKSKGRAKKPNSRHNEEISSDSETEGTAVQNEREEDEFDETPQE
KKLRLAKLYLDQLRDEGHVH
GGPSLYSQLLPPDASEIRLLRGHQLPVTCLVISTDEKFIFSASKDGSIIKWDVETGKKLHNIKGGRRGTEARHEGHTTQV
LCMAISSDGKYLVSASVTEA
PAPGALRKQKTCVTQSGDTVLWLPRSHG
>g2955.t3
MASTFFIKKSGNSAFPKKGHSLATSKRKANSEEDLGGKSKGRAKKPNSRHNEEISSDSETEGTAVQNEREEDEFDETPQE
KKLRLAKLYLDQLRDEGFNF
APHLSLQLEQKGKLQRSVAKDLLPPDASEIRLLRGHQLPVTCLVISTDEKFIFSASKDGSIIKWDVETGKKLHNIKGGRR
GTEARHEGHTTQVLCMAISS
DGKYL
>g2956.t1
MREYKVVVLGSGGVGKSALTVQFVTGTFIEKYDPTIEDFYRKEIEVDSSPSVLEILDTAGTEQFASMRDLYIRNGQGFIL
VYSLVNQQSFQDIKPMRDQI
IRVK
>g2956.t2
MREYKVVVLGSGGVGKSALTVQFVTGTFIEKYDPTIEDFYRKEIEVDSSPSVLEILDTAGTEQFASMRDLYIRNGQGFIL
VYSLVNQQSFQDIKPMRDQI
IRVKR
>g2957.t1
LAEAGWTEEQQQEEEEEEVAGGGRGAPGQKEHKEEQKQEQVRRRGGGERPLLPRRDPGGEEQIQKGATRPAGPHPPLVLR
PATRTVRPGPSHRTPVMGEW
CHLRNDSIDL
>g2957.t2
LAEAGWTEEQQQEEEEEEVAGGGRGAPGQKEHKEEQKQEQVRRRGGGERPLLPRRDPGGEEQIQKGATRPAGPHPPLVLR
PATRTVRPGPSHRTP
>g2957.t3
MHAVKKQASCCLNPLRLGWGDFIDVCVCPVSALAEAGWTEEQQQEEEEEEVAGGGRGAPGQKEHKEEQKQEQVRRRGGGE
RPLLPRRDPGGEEQIQKGAT
RPAGPHPPLVLRPATRTVRPGPSHRTPVMGEWCHLRNDSIDL
>g2958.t1
GVVQPTLSVLPPSRVELQQGSATLLCMASGGFPSDWKLGWKVGGSSRSLGVSDSLGVLGKDGHYSWSSSLTLPADQWRKA
GSVSCEASKNSQTPVTQTLN
PGQCSE
>g2959.t1
MAAMQVEAVLVSWIVDYLSGRPQFVRLQNCVSDRVVSNTGAPQGTVLSPFLFTLYTSDFNYCTESCHLQKFSDDSAIVGC
IKQGVPVDTVEEYKYLGVCF
NNKLDWTRNTEAIYKKGQSRLFFLRRLRSFNICRTLMRMFYESVVASAIFFAVTCWGSGMRVGDTNRLNRLIRRAGDVVG
EELDTLTTVAERRMLSRL
>g2959.t2
MAAMQVEAVLVSWIVDYLSGRPQFVRLQNCVSDRVVSNTGAPQGTVLSPFLFTLYTSDFNYCTESCHLQKFSDDSAIVGC
IKQGVPVDTVEEYKYLGVCF
NNKLDWTRNTEAIYKKGQSRLFFLRRLRSFNICRTLMRMFYESVVASAIFFAVTCWGSGMRVGDTNRLNRLIRRAGDVVG
EELDTLTTVAERRMLSRLRS
ILDNASHPLFSTLAQRRSTFSRRLL
>g2960.t1
LRVHADCFTGSDAVDAVLSYLMQNVVFCASEVSRLKAARLCQALMEAKVFEPVGTKLFRRDKEVVFEDVSCSLYRFLDCK
QLSSSASKDCDAENVIPGEL
ESKKKKTSRQNELRTIANPLAVGPSDRRVERLLRSINLQPSLPTAPEAKLPSSSFLSKAVVEDLWKQQTLLQLLQIVELP

MLDRILTSPARAPTQARPAA
PPGLGQQDLIISNTCLDRELHHML
>g2960.t2
LWQNIIRALQAHVDVRSRRKHLRVHADCFTGSDAVDAVLSYLMQNVVFCASEVSRLKAARLCQALMEAKVFEPVGTKLFR
RDKEVVFEDVSCSLYRFLDC
KQLSSSASKDCDAENVIPGELESKKKKTSRQNELRTIANPLAVGPSDRRVERLLRSINLQPSLPTAPEAKLPSSSFLSKA
VVEDLWKQQTLLQLLQIVEL
PMLDRILTSPARAPTQARPAAPPGLGQQDLIISNTCLDRELHHML
>g2961.t1
MFLSFLFVSTNHVAGGRDLLCAVSPQKFCSNTVCLHRAHVNFIRTVNVPTPARRESCASQHAPGLTQTRSVSRDPGIHTP
CPASGRDPGGRAPAPVPTDG
HLYRALRTL
>g2961.t2
LNERFMFLSFLFVSTNHVAGGRDLLCAVSPQKFCSNTVCLHRAHVNFIRTVNVPTPARRESCASQHAPGLTQTRSVSRDP
GIHTPCPASGRDPGGRAPAP
VPTDGHLYRALRTL
>g2961.t3
LCAVSPQKFCSNTVCLHRAHVNFIRTVNVPTPARRESCASQHAPGLTQTRSVSRDPGIHTPCPASGRDPGGRAPAPVPTD
GHLYRALRTL
>g2962.t1
FCSSESERRHLAPRAAASTLRPSEKDHRKVRPLTRSPLRGRARWSGDTPDP
>g2963.t1
MYPCHILMCSPPLRRQWMGRDRPALHSSLIQDENFHRLLFSQQQQQQQQQQQHHHHQQQQPRVPLDESRHYNQASAPPR
MLHPAAHLPQQSPIMVDLHD
QMHQGSVPISYTITTVTTHGFPIHAGPPLAGCNTQQLPACSVMFSGQLSLLCCLPPPVSLKRKSLNVRSIHLSKARHAGR
SVSPFRAD
>g2964.t1
MFNKSFGAPFGGGTGGFGTASTFGQQNAGFGTTGGFGGSAFGQTATTGGLFGATQNKPGGLFGSSTFSQPVTSSTTSGFG
FGAAGGTSTS
>g2964.t2
MSVERYVAVCFPLRHAHIATIRHTGAAIAAAWTFCSLNSFTRVLILVGWNHDYDLDAGFGTTGGFGGSAFGQTATTGGLF
GATQNKPGGLFGSSTFSQPV
TSSTTSGFGFGAAGGTSTS
>g2965.t1
MRGKLPNVTPALMDLEVRDRDGEQKEQHKLYADEQRNAEYSNVGVGDKVLVKQDKVDKFTTNFNKTPCTVVSENGNWVTV
ESPEGIQYSWNTTSVKKYNT
DQNGAEKVKDDSTESYPEQHQAMNSSGCPAMPAEHNSSPTPLLKPQRQRKPPERLKDFVIS
>g2966.t1
MLQGPYGGFDKDKPLLRLPFTDFAVGTVLLALTGLLACLAFSLLYHFEESTYTHCHVPNYLPSISASISLVPERYIWRFC
IGLHSAPRYLVAMAYFSFYR
GRFSRRWPELLLSGMALLFSVAENTGLLLLTYVASTETYSVHKNGFIAFIASSLLHMLITCRLWQVIKRHCVHPEEVTSY
RWKIRLFMFNVSCCISAAYF
FRRHNKYCEQGIYTLFALCEYMVVLSNMAFHMTAFWDFGSKEVMVATRPEDKRY
>g2966.t2
MLQGPYGGFDKDKPLLRLPFTDFAVGTVLLALTGLLACLAFSLLYHFEESTYTHCHVPNYLPSISASISLVPERYIWRFC
IGLHSAPRYLVAMAYFSFYR
GRFSRRWPELLLSGMALLFSVAENTGLLLLTYVASTETYSQYTLPQLPCVHKNGFIAFIASSLLHMLITCRLWQVIKRHC
VHPEEVTSYRWKIRLFMFNV
SCCISAAYFFRRHNKYCEQGIYTLFALCEYMVVLSNMAFHMTAFWDFGSKEVMVATRPEDKRY
>g2966.t3
MGMRMLQGPYGGFDKDKPLLRLPFTDFAVGTVLLALTGLLACLAFSLLYHFEESTYTHCHVPNYLPSISASISLVPERYI
WRFCIGLHSAPRYLVAMAYF
SFYRGRFSRRWPELLLSGMALLFSVAENTGLLLLTYVASTETYSVHKNGFIAFIASSLLHMLITCRLWQVIKRHCVHPEE
VTSYRWKIRLFMFNVSCCIS
AAYFFRRHNKYCEQGIYTLFALCEYMVVLSNMAFHMTAFWDFGSKEVMVATRPEDKRY
>g2967.t1

LQPIFSGSKTHLNRFEYWSASWPLIGQPVVCTFKPGLVCTFKLSIRKAHRGL
>g2968.t1
TTPASTSISTFTTTTATGGHLHHQSRTGGQDDLLPLPVTSMGSDPHSGHQPLAPAWDSPSQRGLLEGPEDFQDAVSSHGG
RGGGGGGRGEGRSARTTTPS
SATASHAGAGVPNGFHFTLGVNAASGSGPRGTGGLRDSNEEDWC
>g2969.t1
MTTSLFKPKPLDTAGRAKKPMIPGRVAPSLSPDTAPKPSPYGAARHQSRAAFAIKEAHQENLPPCDGDGIQT
>g2970.t1
LGHRPAISRKLPPGSVPPDLAGLQQANQGTTPDRLPCRPRAGSCYRNGPGRSNRYHNYSARAIMGLRVGEEVRLRER
>g2971.t1
MDAPPEIILDESGRKGSVGSEGQGTAVLLPGRFEVHQELFQVQQERPVASIDRTTWSSRGESPAGV
>g2972.t1
MTTARTENPIILGLSNQNGQMRGSVKPAGVPGGGGGVQQQPSQMKTSCTINNGSSQPTPTANTVIKPGDDWKKNLKLPPK
DMRMKTSDVTATKGNEFEDY
CLKRELLMGIFEMGWEKPSPIQEESIPIALSGRDILARAKNGTGKSGAYLIPLLERIDLKRDCIQAVGIVPTRELALQVS
QICIQVGKHMGGVKVMATTG
GTNLRDDIMRLDETVHVIIATPGRILDLIKKGVAKVNQVQMIVLDE
>g2972.t2
MRGSVKPAGVPGGGGGVQQQPSQMKTSCTINNGSSQPTPTANTVIKPGDDWKKNLKLPPKDMRMKTSDVTATKGNEFEDY
CLKRELLMGIFEMGWEKPSP
IQEESIPIALSGRDILARAKNGTGKSGAYLIPLLERIDLKRDCIQAVGIVPTRELALQVSQICIQVGKHMGGVKVMATTG
GTNLRDDIMRLDETVHVIIA
TPGRILDLIKKGVAKVNQVQMIVLDE
>g2973.t1
VYSVGKEEAASESVKFVVPKDLSEGHVNNKQESYKFRECLIKMLVKRRSSKPSPNQSQTGHNTGLLRSLLTHLQCGPLLA
GLGDADRMLRAVLVSNLVL
>g2973.t2
KRPKRSSAFPQVLFDPNPNPTFVYSVGKEEAASESVKFVVPKDLSEGHVNNKQESYKFRECLIKMLVKRRSSKPSPNQSQ
TGHNTGLLRSLLTHLQCGPL
LAGLGDADRMLRAVLVSNLVL
>g2973.t3
MCKPGFCLADCCLWLPQVYSVGKEEAASESVKFVVPKDLSEGHVNNKQESYKFRECLIKMLVKRRSSKPSPNQSQTGHNT
GLLRSLLTHLQCGPLLAGLG
DADRMLRAVLVSNLVL
>g2974.t1
FSSEEEGPAPKQDNRKYMNQPHLRFEITSDDGFSVKADSIEGTTACFSFIERRGVAWRAVIDGVLEARAGFQLRQLPLGV
VSGPRMLGVLHDATIFLLEQ
LEGAGSCRRHRFRFHRCDATEEELPLNPSGCARAEVCT
>g2974.t2
FSSEEEGPAPKQDNRKYMNQPHLRFEITSDDGFSVKADSIEVAWRAVIDGVLEARAGFQLRQLPLGVVSGPRMLGVLHDA
TIFLLEQLEGAGSCRRHRFR
FHRCDATEEELPLNPSGCARAEVCT
>g2974.t3
FSSEEEGPAPKQDNRKYMNQPHLRFEITSDDGFSVKADSIEGTTACFSFIERRGGKTLRRHWRPGHMGVSRVDTGSVAWR
AVIDGVLEARAGFQLRQLPL
GVVSGPRMLGVLHDATIFLLEQLEGAGSCRRHRFRFHRCDATEEELPLNPSGCARAEVCT
>g2975.t1
MLDFYDEQAVFPESFWPQSEPAQPMTFNPRGRLLFQYPVSQSQPAPYFVGGPMGGMDSMAGSEPSQQAPPPAPLTPASAP
PTSSCSAGPSPSSQGSETSF
DCTHCGKSLRSRKNYSKHMFIHSGQKPHQCSICWRSFSLRDYLLKHMVVHTGVRAFQCSVCGKRFTQKSSLNVHMRTHRA
ERTFQCSVCHRAFTHRTLLE
RHALQHAHHNPQGPGQGQGPGP
>g2975.t2
MERYSEEEELLEERGERGRDGERDARGAGHSRKQRQPLRLQVQWGGEREGLPWLPLSGMRRCTCFFLPFFFSSPAPLLLS
TPLTALAHIAAFTPPHSPAA
ASSSSYRAGAPMDCCLHAAGPRPALGHHRTRRVCRPVCNPLLCVCGGQVMGGEGVVVKDEGVQDGSVYALQDRRPDGEDE

GNQDSMTSFGQCSAARCYKG
SLENVEGWKQKLPRCLLVFCNTTQQRYTAGPHVTDSEPQDALAGRASVTCTERKGKPTALILLSRHVFLFPIPLDMWDSF
RTAPVLPIPLDMWDSFRTAS
VLPIPLSLFSSGALPPV
>g2975.t3
MERYSEEEELLEERGERGRDGERDARGAGHSRKQRQPLRLQVQWGGEREGLPWLPLSGMRRCTCFFLPFFFSSPAPLLLS
TPLTALAHIAAFTPPHSPAA
ASSSSYRAGAPMDCCLHAAGPRPALGHHRTRRVCRPVCNPLLCVCGGQVMGGEGVVVKDEGVQDGSVYALQDRRPDGEDE
GNQDSMTSFGQCSAARCYKG
SLENVEGWKQKLPRCLLVFCNTTQQRYTAGPHVTDSEPQDALAGRASVTCTERKGKPTALILLSRHVFLFPIPLDMWDSF
RTAPVLPIPLDMWDSFRTAS
VLPIPLSLFSSGALPPDFYDEQAVFPESFWPQSEPAQPMTFNPRGRLLFQYPVSQSQPAPYFVGGPMGGMDSMAGSEPSQ
QAPPPAPLTPASAPPTSSCS
AGPSPSSQGSETSFDCTHCGKSLRSRKNYSKHMFIHSGQKPHQCSICWRSFSLRDYLLKHMVVHTGVRAFQCSVCGKRFT
QKSSLNVHMRTHRAERTFQC
SVCHRAFTHRTLLERHALQHAHHNPQGPGQGQGPGP
>g2975.t4
MLDFYDEQAVFPESFWPQSEPAQPMTFNPRGRVNKPLTPPPTSQSINNQLLFQYPVSQSQPAPYFVGGPMGGMDSMAGSE
PSQQAPPPAPLTPASAPPTS
SCSAGPSPSSQGSETSFDCTHCGKSLRSRKNYSKHMFIHSGQKPHQCSICWRSFSLRDYLLKHMVVHTGVRAFQCSVCGK
RFTQKSSLNVHMRTHRAERT
FQCSVCHRAFTHRTLLERHALQHAHHNPQGPGQGQGPGP
>g2976.t1
QRKSSLGRLNARLHRGRRRAQIIHSPSFRESGEVRGDEPLCSPVRTRPLSSLFG
>g2977.t1
MTTIVHDTAEAVCLCEEYNLYLKPTAKMTISVALPQLKLPGKSISNWEVMERLKAMVAPEQLSALRISKSTMDFIRFEGE
VENKGLVKSLLSRLDGKSIK
LSGFTDILKVRALENKMDFPTRHDWDSFFRDAKDMNETLPGERPDTIHLEGLPCRWFSQKDSQHPDRPSEEVLATIFQGF
GKVRNVDIPMLDPYREEMMD
KNFNTFSFGGHLYFEAYVQYQEYHGFTKAMDTLRGMKLMLKGDDGKAMACNIKVTFDTSKHLSSTAVRQRSQERLKLQEL
ERQREEQKRREKEDEDRRKE
EERRQKEQEEEERERRREERVRKREQKAREREEKRSLKRVKRRQDEEQRRLQLKIATEERRLLLAQRNLESIRLI
>g2978.t1
MCALTVEMSSVPLQYQIFIPMVNKVMLKHRVNHQRYDILICRIVKGYTLAEEEENPLVFQHRQLRGNQGDTLASGPVEPG
PMKKLHVSTTALQKAWGAAR
KVSKDDWLEWLRRLSVVLLKESSSPALRSCWSLAQTYIPLARDLFNAAFLSCWSELSEDQQDELIRSIELALTSQDIAEV
TQTLLNLAEFMEHSDKGPLP
LRDDNGIVLLGERAAKCRAYAKALHYKELEFQKGPSPLILEALISALQSAAKHPFNCLIPWSHHAHFTGKPGVYQQFPPG
SLARGNVARVCQGNARC
>g2978.t2
MCALTVEMSSVPLQYQIFIPMVNKVMLKHRVNHQRYDILICRIVKGYTLAEEEENPLVFQHRQLRGNQGDTLASGPVEPG
PMKKLHVSTTALQKAWGAAR
KVSKDDWLEWLRRLSVVLLKESSSPALRSCWSLAQTYIPLARDLFNAAFLSCWSELSEDQQDELIRSIELALTSQDIAEV
TQTLLNLAEFMEHSDKGPLP
LRDDNGIVLLGERAAKCRAYAKALHYKELEFQKGPSPLILEALISALQSAAKHPFNCLIPWSHHAHFTGKPGVYQQFPPG
SLARGNVARVCQGNARTDSH
KEE
>g2978.t3
MCALTVEMSSVPLQYQIFIPMVNKVMLKHRVNHQRYDILICRIVKGYTLAEEEENPLVFQHRQLRGNQGDTLASGPVEPG
PMKKLHVSTTALQKAWGAAR
KVSKDDWLEWLRRLSVVLLKESSSPALRSCWSLAQTYIPLARDLFNAAFLSCWSELSEDQQDELIRSIELALTSQDIAEV
TQTLLNLAEFMEHSDKGPLP
LRDDNGIVLLGERAAKCRAYAKALHYKELEFQKGPSPLILEALIRTDSHKEE
>g2979.t1
MQKMATAKMTLVALGVALLLLAGARAQNPKKKSFPKAQKGSCCDEVRSLKVQVANLTSILEELGRKQEADLMAVVRQVME
LDKLNRQQESRVTEAESKYS

EINNRVEILQLQGAQSITQTSSDVMYDCASLYGKNYRISGEYRLPKDDFLRTPELTVFCDMETDGGGWTVIQRRRVGLTS
FDRDWKQYKNGFGTIRGDFW
LGNEHIFRLTRQPSTLRIEMEDWAGETRYAQYGSFSLSNELNSYKLFLTNYSGNAGDSLRYHNNTNFSTKDKDNDKCVDD
CAALRKGGYWYNCCTDSNLN
GMFYRRGLHTPTTDGISWYGWHGSVYSMKRVEMKIRPHSFQP
>g2979.t2
MTLVALGVALLLLAGARAQNPKKKSFPKAQKGSCCDEVRSLKVQVANLTSILEELGRKQEADLMAVVRQVMELDKLNRQQ
ESRVTEAESKYSEINNRVEI
LQLQGAQSITQTSSDVMYDCASLYGKNYRISGEYRLPKDDFLRTPELTVFCDMETDGGGWTVIQRRRVGLTSFDRDWKQY
KNGFGTIRGDFWLGNEHIFR
LTRQPSTLRIEMEDWAGETRYAQYGSFSLSNELNSYKLFLTNYSGNAGDSLRYHNNTNFSTKDKDNDKCVDDCAALRKGG
YWYNCCTDSNLNGMFYRRGL
HTPTTDGISWYGWHGSVYSMKRVEMKIRPHSFQP
>g2979.t3
MATAKMTLVALGVALLLLAGARAQNPKKKSFPKAQKGSCCDEVRSLKVQVANLTSILEELGRKQEADLMAVVRQVMELDK
LNRQQESRVTEAESKYSEIN
NRVEILQLQGAQSITQTSSDVMYDCASLYGKNYRISGEYRLPKDDFLRTPELTVFCDMETDGGGWTVIQRRRVGLTSFDR
DWKQYKNGFGTIRGDFWLGN
EHIFRLTRQPSTLRIEMEDWAGETRYAQYGSFSLSNELNSYKLFLTNYSGNAGDSLRYHNNTNFSTKDKDNDKCVDDCAA
LRKGGYWYNCCTDSNLNGMF
YRRGLHTPTTDGISWYGWHGSVYSMKRVEMKIRPHSFQP
>g2980.t1
MSTSGTLTSYYVDSLILPESEELSAPRYPSGPGGLQQSRQSASINDHNDIGTCTFSTKASVFSPSWSHVQAPFPGTVYHH
HYGPHQGSVSGDSDARYMQS
WLLEPMSGSLPMTGLQASHNYGIKPEGLAARADGALPGSHTALLLSDYANGTVATTPVEKDALPLQGGDIKGEGDDKPGL
DPNNPVSNWLHASATRKKRC
PYTKHQILELEKEFLFNTYLTRERRYEVARQLNLTERQVKIWFQNRRMKMKKFNKDGRPKDE
>g2981.t1
EVVEYMVQHFKPQLFGDRKPVYDGKKNIYTVLALPIGSEKVDFEVTIPGEGKDRIFKVSIRWLAKVSWRLLQETLVSGRL
QVPLDSVQALDVAMRHLASM
RYTPVGRSFFSPPEGYYHPLGGGREVWFGFHQSVRPAMWKMMLNIDVSATAFYKAQPVIEFMCEVLDIRNIDEQPKTLTD
SQRVRFTKEIKGGLLSSGKN
FSINLDHLTHLQIPRTSLT
>g2981.t2
MVQHFKPQLFGDRKPVYDGKKNIYTVLALPIGSEKVDFEVTIPGEGKDRIFKVSIRWLAKVSWRLLQETLVSGRLQVPLD
SVQALDVAMRHLASMRYTPV
GRSFFSPPEGYYHPLGGGREVWFGFHQSVRPAMWKMMLNIDVSATAFYKAQPVIEFMCEVLDIRNIDEQPKTLTDSQRVR
FTKEIKGGLLSSGKNFSINL
DHLTHLQIPRTSLT
>g2981.t3
EVVEYMVQHFKPQLFGDRKPVYDGKKNIYTVLALPIGSEKVDFEVTIPGEGKDRIFKVSIRWLAKVSWRLLQETLVSGRL
QVPLDSVQALDVAMRHLASM
RYTPVGRSFFSPPEGYYHPLGGGREVWFGFHQSVRPAMWKMMLNIDVSATAFYKAQPVIEFMCEVLDIRNIDEQPKTLTD
SQRVRFTKEIKDSKNLTNIR
PSHTPADNQGPH
>g2982.t1
VIMGAVSGMLLRVASPIHPDIVMVIAFPGDILMRMLKMLILPLIISSLITGLAGLDAKSSGRLGSRAMVYYMSTTVIAAI
LGVILVILIHPGDPKLKENL
GEGIKNDDVSSLDAAFDLIRNLFPENLVQACFQQIQTVTKKVEVIVEEAVNATTVDGLVANVTKEPQFTVKKSLQFKSGM
NVLGLIGFFIAFGICMGKMG
ERARLMLEFFNILNEIVMKIVIMIMWYSPFGIACLICGKIISIDDLEVVARQLGMYMITVIVGLIIHGAIFLPGIYFAIV
RKNPFTFFMGIFQAWITALG
TASSAGTLPVTFRCLEENLGIDKRVTRFVLPVGATINMDGTALYEAVAAIFIAQMNGIYLDPGQIITVSLTATLASVGAA
SIPSAGLVTMLLILTAVGLP
TQDISLLVAVDWLLDRFRTSVNVMGDSYGAAIVYHLSKAELDELDANTAKSDDIEMSKTQSSYYDDMKNHHENNSNQCVL
SATATTSANNAVVVDECKVP

LMLPDIETSM
>g2982.t2
MGAVSGMLLRVASPIHPDIVMVIAFPGDILMRMLKMLILPLIISSLITGLAGLDAKSSGRLGSRAMVYYMSTTVIAAILG
VILVILIHPGDPKLKENLGE
GIKNDDVSSLDAAFDLIRNLFPENLVQACFQQIQTVTKKVEVIVEEAVNATTVDGLVANVTKEPQFTVKKSLQFKSGMNV
LGLIGFFIAFGICMGKMGER
ARLMLEFFNILNEIVMKIVIMIMWYSPFGIACLICGKIISIDDLEVVARQLGMYMITVIVGLIIHGAIFLPGIYFAIVRK
NPFTFFMGIFQAWITALGTA
SSAGTLPVTFRCLEENLGIDKRVTRFVLPVGATINMDGTALYEAVAAIFIAQMNGIYLDPGQIITVSLTATLASVGAASI
PSAGLVTMLLILTAVGLPTQ
DISLLVAVDWLLDRFRTSVNVMGDSYGAAIVYHLSKAELDELDANTAKSDDIEMSKTQSSYYDDMKNHHENNSNQCVLSA
TATTSANNAVVVDECKVPLM
LPDIETSM
>g2982.t3
VIMGAVSGMLLRVASPIHPDIVMVIAFPGDILMRMLKMLILPLIISSLITGLAGLDAKSSGRLGSRAMVYYMSTTVIAAI
LGVILVILIHPGDPKLKENL
GEGIKNDDVSSLDAAFDLIRNLFPENLVQACFQQIQTVTKKVEVIVEEAVNATTVDGLVANVTKEPQFTVKKSLQFKSGM
NVLGLIGFFIAFGICMGKMG
ERARLMLEFFNILNEIVMKIVIMIMWYSPFGIACLICGKIISIDDLEVVARQLGMYMITVIVGLIIHGAIFLPGIYFAIV
RKNPFTFFMGIFQAWITALG
TASSAGTLPVTFRCLEENLGIDKRVTRFVLPVGATINMDGTALYEAVAAIFIAQMNGIYLDPGQIITVSLTATLASVGAA
SIPSAGLVTMLLILTAVGLP
TQDISLLVAVDWLLDRFRTSVNVMGDSYGAAIVYHLSKAELDELDANTAKSDDIEMSKTQSSYYDDMKNHHENNSNQCVL
SATATTSANNAVVVDECKAH
GVSAGVDIALRSRAPAKRPAMY
>g2983.t1
EYHAPGLSQETVAQWSRLADLLEGAGARVRRVSLPHTQHSIVCYHVLCCAEVASNMARFDGLEYGHRSAVDSSTEAMYAT
SRHEGFNDVVRGRILCGNFF
LLQQCVATLFSPGQVKKQRVE
>g2984.t1
MAAAYRVVVSSVSCYNSVVVDRRVHKHAVHYCAGPCGALSQGLDCAVAHRGTCSELLVAPDPGPIDRNCSTHTYSHNITT
ATGGTTYQVSAGHGNNLSMD
TSGSLERAGSIGNVSIGEENPPWRVKKSPSGGGSMGNLASATSLKDITGEAINLASGKLKEFSFDKLRVSTSSQVTFRKG
RKVRPDSFSRRSTDLDIIYG
HFSSSVTTNGFATNSLVNAAARASNDENLPPFNTAKGVTVEEVKVKGGTGSLSATTKVGGGSTGSLSSACSGSLDQGLNT
VASLYLNTLGEENLIARLLE
KTRAEGGIGGAGGEDIRACLDILLKCSEDLKKCTDIIKQCIRRKGPGGGSGSDDEGANPESIYRAVMTRLSSYLKRLPLE
LEGIGGLGRLAGGQGGQGAL
SSGHSELAELVNSLHSFQQGPFSPIFGHDQPPRYEDVVQSPPLPKSPSIPPSAPTPSSCSASYSSTSSSNSSSSSSPSRA
ESVNAMQLQSSKPTQSPLRN
SPIPLSTHSPLATPMEALYIEEDADVXTPYRNDDIDKLLLDLENLSQSMNHQREKEPPLPVKTRKMMGEGGMGKPASTAP
PQPTSNLQSKAPILKPASHL
FTNGSTSTSTQGLIPVPLQGDSHGEGCCREEEDGALLLRILESIESFAQELVESGAGSTGSSERSSGKEREVMRLLQDTL
ATAGRARTPPQNTQMAALSS
VPPVNQSESKLYLFFWHNLPPPHPHPHPSPSLCPYPRLSPRFKLRNQSWWLKCLHLHLERYLLLRSSCMLQLPALLSLNP
QPPLL
>g2984.t2
MAAAYRVVVSSVSCYNSVVVDRRVHKHAVHYCAGPCGALSQGLDCAVAHRGTCSELLVAPDPGPIDRNCSTHTYSHNITT
ATGGTTYQVSAGHGNNLSMD
TSGSLERAGSIGNVSIGEENPPWRVKKSPSGGGSMGNLASATSLKDITGEAINLASGKLKEFSFDKLRVSTSSQVTFRKG
RKVRPDSFSRRSTDLDIIYG
HFSSSVTTNGFATNSLVNAAARASNDENLPPFNTAKGVTVEEVKVKGGTGSLSATTKVGGGSTGSLSSACSGSLDQGLNT
VASLYLNTLGEENLIARLLE
KTRAEGGIGGAGGEDIRACLDILLKCSEDLKKCTDIIKQCIRRKGPGGGSGSDDEGANPESIYRAVMTRLSSYLKRLPLE
LEGIGGLGRLAGGQGGQGAL
SSGHSELAELVNSLHSFQQGPFSPIFGHDQPPRYEDVVQSPPLPKSPSIPPSAPTPSSCSASYSSTSSSNSSSSSSPSRA

ESVNAMQLQSSKPTQSPLRN
SPIPLSTHSPLATPMEALYIEEDADVXTPYRNDDIDKLLLDLENLSQSMNHQREKEPPLPVKTRKMMGEGGMGKPASTAP
PQPTSNLQSKAPILKPASHL
FTNGSTSTSTQGLIPVPLQGDSHGEGCCREEEDGALLLRILESIESFAQELVESGAGSTGSSERSSGKEREVMRLLQDTL
ATAGRARTPPQNTQMAALSS
VPPVNQSESVSAVPPKHSHLNPPAAPPAPVLHTPVPYTPAPSEALSAAVPEIVSLLLAQSAATTPTPTSIPIPLPLPEAE
PKVQAEESELVAQMSPPSPG
TLPAPPVVLHAPTPSIAQLESSATVVVVTPIAGRDDDVTICNTAAVRDTGSTLLIQQTPEVIRVSELPMM
>g2984.t3
MAAAYRVVVSSVSCYNSVVVDRRVHKHAVHYCAGPCGALSQGLDCAVAHRGTCSELLVAPDPGPIDRNCSTHTYSHNITT
ATGGTTYQVSAGHGNNLSMD
TSGSLERAGSIGNVSIGEENPPWRVKKSPSGGGSMGNLASATSLKDITGEAINLASGKLKEFSFDKLRVSTSSQVTFRKG
RKVRPDSFSRRSTDLDIIYG
HFSSSVTTNGFATNSLVNAAARASNDENLPPFNTAKGVTVEEVKVKGGTGSLSATTKVGGGSTGSLSSACSGSLDQGLNT
VASLYLNTLGEENLIARLLE
KTRAEGGIGGAGGEDIRACLDILLKCSEDLKKCTDIIKQCIRRKGPGGGSGSDDEGANPESIYRAVMTRLSSYLKRLPLE
LEGIGGLGRLAGGQGGQGAL
SSGHSELAELVNSLHSFQQGPFSPIFGHDQPPRYEDVVQSPPLPKSPSIPPSAPTPSSCSASYSSTSSSNSSSSSSPSRA
ESVNAMQLQSSKPTQSPLRN
SPIPLSTHSPLATPMEALYIEEDADVSSKATPGTPYRNDDIDKLLLDLENLSQSMNHQREKEPPLPVKTRKMMGEGGMGK
PASTAPPQPTSNLQSKAPIL
KPASHLFTNGSTSTSTQGLIPVPLQGDSHGEGCCREEEDGALLLRILESIESFAQELVESGAGSTGSSERSSGKEREVMR
LLQDTLATAGRARTPPQNTQ
MAALSSVPPVNQSESKLYLFFWHNLPPPHPHPHPSPSLCPYPRLSPRFKLRNQSWWLKCLHLHLERYLLLRSSCMLQLPA
LLSLNPQPPLL
>g2985.t1
EDGDRDALRSERRTAEKRLRTAKNLGVTLSQLSFATNISAMTRSCRFNTRRTCLFLTQKAT
>g2986.t1
LPACAIRSLKLIQKVAARLFFNPCKFCHITPLLHTLAYHWLLVAAHWWSSWNPEGPLLQKSLRRFPERGPLVPQRGTFPA
RRVTLISFAPLAHNRA
>g2987.t1
LGLGLDVCRTFPPRHPIPESIAEDMPEEALWGSSSSSLSVGESSVGEWLQRLGLERYEKGLLHNGWDDLEFLSDITEEDL
EEAGVLDPAHKHILLESLRQ
QNQ
>g2988.t1
HRTLYIGVHVPLGRKSHRRHRHHGHRHRKRSKERDSSTDDGHESPSHTDTPAQRVQFLLGTEDGDEEHIPHALFTELDEI
CLREGEDAEWKETARYVAIV
M
>g2988.t2
HRTLYIGVHVPLGRKSHRRHRHHGHRHRKRSKERDSSTDDGHESPSHTDTPAQRVQFLLGTEDGDEEHIPHALFTELDEI
CLREGEDAEWKETA
>g2989.t1
MKWANCDGTPGKCECKLLVDNVIPKCFLMKAEVYRAKKNLTTRTIGGKPMETAFLDNDGIYDPDCERNGVFKAKQCNNTE
ECWCVNSAGVRRTDKGDTNI
VCDKLVET
>g2990.t1
MDCETAGWKCHPRAECLQVNVNVTCVCRTGYQGNGLECEDIDECLSGLHGCHPNAPCINTLGSYDCVCLAGYVGDGLQCQ
DIDECQRDNGGCHADATCTN
YEGGRGCGCRTGYSGNGFQCTDNDECSDSRICHWDATCTNNPGSYMCTCNAGYKGNGNYLCLDIDECSETPVVCSSSFGY
KGCKNLPGSYQCTCDAGYQS
NSNTCEDIDECANNTCSQFADCKNTIGSYRCTCFSGFVGNGQSCVDNNECNGNNNCHPSAVCINIVGSYQCSCLTGFLGD
GQNCQDVDECLTAGICHTSA
VCVNTPGTYSCNCGRGYLYNNSRCQDEDECASGLCSPHATCTNVPGSFNCECIPGYNGDGLICVDEDECLLARQCHTNAV
CVNRPGTYNCSCAAGYSGDG
VVRCTDVNECLVDNGGCSNMASCVNVQGSFSCLCSTGFTLVNETLCLDVDECEENRVACSANERCLNTRGSYECRCRAGF
YRPGSQVSCTDVDECQSAPC

HANATCLNTEGSHECACNRGYSGNGTHCGDVDECLAEGACHRRALCGDGFYCQDVDECLSNATCPPVSACINSPGAHVCS
CLNGSVAIDGLCVLPAARCD
PACHAHGLCHASPSGYQCVCDLGFIGDGLTCSDIDECQLENVCPRNETECVNVPGSFACVCKAGFTLNDTDCVDVNECET
QEHECSDFARCENSIGSHLC
LCLSGFAGDGITCLDIDECQGDSAGCHPVAACTNTAGSFRCTCPPGTEGSGFDCRDVDECSQNSTLLHNCSVHAVCGNTD
GSYLCRCQDGYHGDGFDCED
VDECRSGDACDSDMSCHNTPGSYACVCVLGLVYEQGTCVSLGDCLNASHACHPKALCYPDQSSFYCHCHKGYYGDGTDCQ
DVDECDEGSAESPWDACPPF
SSCVNGDGSYVCECWDGYRSTGKRCQDVDECASGNFTCPDNSTCDNVDGGYRCPCTPGFSLSNDSLCSDVDECSLGLDDC
PRVSDCFNTMGSFFCDCWAG
YHGNDTGAWCADIDECLGNSTCPEHSTCNNTDGHFMCTCNSGFLSVEDLCVDVDECNTTGRAPPCANGVCMNTPGSYYCE
CKMGYWSNDTECVDVDECSD
LFNSSVECQPFATCVNTPGSYSCLCDEGFTLVLTNGTSWTNGTIWTNETIWTNETAWTNETTWTNETTWTNVTTSTIGTV
WTNESSWATRTECQDIDECE
GNPCPEYSTCDNVEGSFLCLCNKGFTPDSFSYLSSNSSENGSNVYLLNGCEDIDECALDSICRPDQVCVNLPGGYACSCR
KGYHEGQGACDDTDECGGAP
CHPSARCWNTEGSFSCHCLPGHRGNGTWCEDVDECTALSRPCHPMARCHNTLGSFWCQCEPGYAALGPHCVDLDECLQRN
GPCHRGAACSNRVGGFECRC
ARGWDPTADEGHGKRGCVDRDECARPPACSGAALCTNVPGSFLCSCPSNDTECEHMAPKESELFPFGPEVGDHLVEVDSE
DGNSPYITPPLGLPFMGKLY
NRVF
>g2990.t2
MDCETAGWKCHPRAECLQVNVNVTCVCRTGYQGNGLECEDIDECLSGLHGCHPNAPCINTLGSYDCVCLAGYVGDGLQCQ
DIDECQRDNGGCHADATCTN
YEGGRGCGCRTGYSGNGFQCTDNDECSDSRICHWDATCTNNPGSYMCTCNAGYKGNGNYLCLDIDECSETPVVCSSSFGY
KGCKNLPGSYQCTCDAGYQS
NSNTCEDIDECANNTCSQFADCKNTIGSYRCTCFSGFVGNGQSCVDNNECNGNNNCHPSAVCINIVGSYQCSCLTGFLGD
GQNCQDVDECLTAGICHTSA
VCVNTPGTYSCNCGRGYLYNNSRCQDEDECASGLCSPHATCTNVPGSFNCECIPGYNGDGLICVDEDECLLARQCHTNAV
CVNRPGTYNCSCAAGYSGDG
VVRCTDVNECLVDNGGCSNMASCVNVQGSFSCLCSTGFTLVNETLCLDVDECEENRVACSANERCLNTRGSYECRCRAGF
YRPGSQVSCTDVDECQSAPC
HANATCLNTEGSHECACNRGYSGNGTHCGDVDECLAEGACHRRALCDVDECLSNATCPPVSACINSPGAHVCSCLNGSVA
IDGLCVLPAARCDPACHAHG
LCHASPSGYQCVCDLGFIGDGLTCSDIDECQLENVCPRNETECVNVPGSFACVCKAGFTLNDTDCVDVNECETQEHECSD
FARCENSIGSHLCLCLSGFA
GDGITCLDIDECQGDSAGCHPVAACTNTAGSFRCTCPPGTEGSGFDCRDVDECSQNSTLLHNCSVHAVCGNTDGSYLCRC
QDGYHGDGFDCEDVDECRSG
DACDSDMSCHNTPGSYACVCVLGLVYEQGTCVSLGDCLNASHACHPKALCYPDQSSFYCHCHKGYYGDGTDCQDVDECDE
GSAESPWDACPPFSSCVNGD
GSYVCECWDGYRSTGKRCQDVDECASGNFTCPDNSTCDNVDGGYRCPCTPGFSLSNDSLCSDVDECSLGLDDCPRVSDCF
NTMGSFFCDCWAGYHGNDTG
AWCADIDECLGNSTCPEHSTCNNTDGHFMCTCNSGFLSVEDLCVDVDECNTTGRAPPCANGVCMNTPGSYYCECKMGYWS
NDTECVDVDECSDLFNSSVE
CQPFATCVNTPGSYSCLCDEGFTLVLTNGTSWTNGTIWTNETIWTNETAWTNETTWTNETTWTNVTTSTIGTVWTNESSW
ATRTECQDIDECEGNPCPEY
STCDNVEGSFLCLCNKGFTPDSFSYLSSNSSENGSNVYLLNGCEDIDECALDSICRPDQVCVNLPGGYACSCRKGYHEGQ
GACDDTDECGGAPCHPSARC
WNTEGSFSCHCLPGHRGNGTWCEDVDECTALSRPCHPMARCHNTLGSFWCQCEPGYAALGPHCVDLDECLQRNGPCHRGA
ACSNRVGGFECRCARGWDPT
ADEGHGKRGCVDRDECARPPACSGAALCTNVPGSFLCSCPSNDTECEHMAPKESELFPFGPEVGDHLVEVDSEDGNSPYI
TPPLGLPFMGKLYNRVF
>g2991.t1
MNCFVECHHRKRAAMAVSQASWKRQESGEAASRGHVRNADDPRLMDLQLLAAMKVREYVLKEQKLW
>g2991.t2
MNCFVECHHRKRAAMAVSQASWKRQESGEAASRGHVRNADDPRLMDLQLLAAMKVREYVLKEQKLWMLLCASVLN

>g2992.t1
MRDYKLTVHQACKLDAYPSPRVEDLFASTGRGQSITVKWNIPRGVFKAVSALKSAAYGHLPAPLHP
>g2992.t2
MRDYKLTVHQACKLDAYPSPRVEDLFASTGRGRTLRNITAPEMIDEPRRCVCHTRPDTIVSDNGATFTGKLISGVVQESD
IRHVGTDSFHPASNGPAEWA
VQN
>g2992.t3
MLKNKAEPWVSVGSPQYVALAGICARRTYAGESTPSPGALEVDMAYSSQKACKLDAYPSPRVEDLFASTGRGQSITVKWN
IPRGVFKAVSALKSAAYGHL
PAPLHP
>g2993.t1
LSSGAEDRCGRPPHHGGATPGADPRVFPANERAHALEVVRPLHPSGSGSGSGRGRSAGRRHRRDSGTRYALQFRLSQGAL
GAPGRPLGGAWSAAAAKRHV
GDIYEASSDVLPSLAHSSILFQALLSVETQTPGSDISLSENLQNVARALPLWQVLRDLVQMKGSELDAGRSYSCYDVFIT
SPASDALRSSVVHRVGMRRV
TSSRPAASRMKPSVSVVFRELL
>g2993.t2
LSSGAEDRCGRPPHHGGATPGADPRVFPANERAHALEVVRPLHPSGSGSGSGRGRSAGRRHRRDSGTRYALQFRLSQGAL
GAPGRPLGGAWSAAAAKRHV
GDIYEASSDVLPSLAHSSILFQALLSVETQTPGSDISLSENLQNVARALPLWQVLRDLVQMKGVSQLPPGSELDAGRSYS
CYDVFITSPASDALRSSVVH
RVGMRRVTSSRPAASRMKPSVSVVFRELL
>g2994.t1
MTPCIPPNPPSEEMEPRGDSGSLFVDWWQERGRQVRVPAVGESTAGGVRACTTGQRLRRWERRRVVAQHNAAASPGLPPP
PRYLEDVIGPGARRRRKIKS
PAELQLQYKVSVLR
>g2995.t1
LSAGLKRTICVSEFTQEALNALRLLLDSDQILAPRRLAQTAAATWECHFPGPFVRYHVVNFKHSLAAWRRGGARPIKETP
GSGEDVRRARQADCVLGAWA
AEKSVIIKLQLCLFFPRRGRLEDFLRALTSRPAE
>g2996.t1
MWMTSVVALSLLALLASAEDRKLSSHATMLADNSAHLAFSLYHNMAKDKGTGDNILISPVVVASSLGVVALGAKDSTASQ
VKTVLRADSLKDEHLHSGLS
ELMSEVSDAKTRNTTWRISNRLYGPSSVVFADAFVKNSKKHYNFDHSKINLKDKKSAVNSINEWAAKSTDGKLPEVTKDV
PNADGAMLVNAMFFKPHWHE
KFFEQMVDNRGFLVTRSLSVAVKMMHRTGLYEFHEDTENRLFALNMPLGRNQASMIFIMPYHLEPLERLEKLLTQEQVDT
WVSKMEKRAVAISMPKVSME
VSHNLQKHLADLGLTQAVDKTKADLSNISGKKDLYLSNVFHASALELDIQGNPFDTSIFGSEKLRNPKLFYIDHPFIFLV
KDSKTGAILYIGRMVKPNGD
KMRDEL
>g2996.t2
MTSVVALSLLALLASAEDRKLSSHATMLADNSAHLAFSLYHNMAKDKGTGDNILISPVVVASSLGVVALGAKDSTASQVK
TVLRADSLKDEHLHSGLSEL
MSEVSDAKTRNTTWRISNRLYGPSSVVFADAFVKNSKKHYNFDHSKINLKDKKSAVNSINEWAAKSTDGKLPEVTKDVPN
ADGAMLVNAMFFKPHWHEKF
FEQMVDNRGFLVTRSLSVAVKMMHRTGLYEFHEDTENRLFALNMPLGRNQASMIFIMPYHLEPLERLEKLLTQEQVDTWV
SKMEKRAVAISMPKVSMEVS
HNLQKHLADLGLTQAVDKTKADLSNISGKKDLYLSNVFHASALELDIQGNPFDTSIFGSEKLRNPKLFYIDHPFIFLVKD
SKTGAILYIGRMVKPNGDKM
RDEL
>g2997.t1
MWFFILAVWPVVYISNYIKGQRETVSLGYGVENTTCSRYLPGPGMSEQHPPVGIQTHSLGPSESLGSFPKLPNARKWVGN
SLVLMRSSSGGLVSERHINN
TQRRGTTAEDTQSPKLPSELVKLVFTGLGSEILQSDRFLPKVKISIIFGCALQRALWLSTE
>g2998.t1
MVLTPGELDEARLGEGGYSRAHSGTLHCQSYKLFLVSVIYSKAKLASACGGIVYFLSYVPYMYVAIREEVAHDKITAFEK

CIASLMSTTAFGLGSKYFAL
YEVSGVGIQWRTIRQSPVEGDDFNLGLSMMMLIVDACLYGVLTWYIEAVHPGMYGLPRPWYFPLQKSYWLGSGRVETWDW
PWCGGGASRLSVMEEDQACA
MEHRRSDKDNSLQNNNLPEDEATANPFALVSEISEILFSVATVVIH
>g2998.t2
MVLTPGELDEARLGEGEYHLLQLRSDRLFNFLVSVIYSKAKLASACGGIVYFLSYVPYMYVAIREEVAHDKITAFEKCIA
SLMSTTAFGLGSKYFALYEV
SGVGIQWRTIRQSPVEGDDFNLGLSMMMLIVDACLYGVLTWYIEAVHPGMYGLPRPWYFPLQKSYWLGSGRVETWDWPWC
GGGASRLSVMEEDQACAMEH
RRSDKDNSLQNNNLPEDEATANPFALVSEISEILFSVATVVIH
>g2998.t3
MVLTPGELDEARLGEGGYSRAHSGTLHCQSYKLFLVSVIYSKAKLASACGGIVYFLSYVPYMYVAIREEVAHDKITAFEK
CIASLMSTTAFGLGSKYFAL
YEVSGVGIQWRTIRQSPVEGDDFNLGLSMMMLIVDACLYGVLTWYIEAVHPGMYGLPRPWYFPLQKSYWLGSGRVETWDW
PWCGGGASRLSVMEEDQACA
MEHRRATAALQSPSSRFDVIGEISRCSSGIAVDKDNSLQNNNLPEDEATANPFALVSEISEILFSVATVVIH
>g2999.t1
LLRRLRSFGVCRTLLRTFYDSVVASAIFYAVVCWRGGSTDRDRSRLNRLIRRASSVLDCPLDSIEEVGERRMLTKLTSIM
DNTSHPLHDSVGSLSSSFSS
RLIHPRCKKERFRRSFIPAAVRLYNTCST
>g3000.t1
MSSGNMMSKITFYEERNFQGRSYDCSSDCADMSSYLSRCHSCRVERGCFMVYDRTNFMGNQYFMRRGEYADYMSMMGMSD
CIRSCRMIPMHRGNYRMRIY
ERENFGGQMHEMMDDCDNVMDRYRMSNCMSCNVMDGHWLMYEQPQYRGRMMYMRPGEYRNFMHQGMNPMRFMSMRRINDS
YY
>g3001.t1
MGPCEMPNHKLLPNAALDDRMGYMAAYCCTGLDQNRLGFLASPTAGPTQPIYLPARSGVFLMLPVPAASVVSRCSAQLEQ
CGLNQNRPPPGRCGPTEWAQ
GSQQPADPMGILTLVCIGVVKLYLGSGDRV
>g3002.t1
EKPFKCEFDGCDRRFANSSDRKKHMHVHTSDKPYICKVCDKSYTHPSSLRKHMKVIKVFITP
>g3003.t1
MDELVHDLVSALEESSEQAARGFGDGGDHALAVGCLLKRQARKRRGRKRRSDNPHPPWEAGHLSEGSESSGEEHKVGVSV
GASVPGNRVYSFCVVFFIMD
YRACAGGVSGANSHARDNSDSDEQLGPKRRTPLLADTGRGKRPLWQDDLGLLGSAEGT
>g3004.t1
MMQMIQMMQMMQMIQEAFTELLHNKDAERRCLNWFTAEFEQNDTEQEASPPAPESNQPDSGLTWPESDAYNQPKSMRGRR
ACCVGADRTRDLGIQSSNRS
MVQNGDRPGTFLDGFPEISLSGKQI
>g3004.t2
MQMIQMMQMMQMIQEAFTELLHNKDAERRCLNWFTAEFEQNDTEQEASPPAPESNQPDSGLTWPESDAYNQPKSMRGRRA
CCVGADRTRDLGIQSSNRSM
VQNGDRPGTFLDGFPEISLSGKQI
>g3005.t1
MCEWNKSSVGLWGSPYSSNTASFPAPHGQGNKSSPAETTDRPKDGRGPGQRPGDARGGPWRPAEACRASDQVLRLQSVYH
VPSSRCRGAGPCVLLQTRLL
HIADARFDF
>g3006.t1
GAAVDKSFRMPVFHTKTIESILEPVAQQISHLVIMHEEGEVDGKAIPDLTVPVAAVQAAVSNLVR
>g3006.t2
GAAVDKSFRMPVFHTKTIESILEPVAQQISHLVIMHEEGEVDGKAIPDLTVPVAAVQAAVSNLVRREASAMFA
>g3007.t1
MLRSEFPALVPGAQGSVAQLFMDVSLYMLGSDSTVDHMVAAFFGRLFPLAYRRLLGGGAAGSEECLRGAWKDSAAFGPYP
KLMMTRLSRSLLATRVFLQA
LNLGIEVVNTTNHLRPGRDCGRSLLRLWYCPHCQALLGAPACGGFCQAVMQGCLGGAAEVQPHWRIYVDGLGKLAGAMRG
EQDMEAVVLRLPSVIKLALK

HAVSARVRLAAVVSFSSVH
>g3008.t1
LGKCFLANPVKKNYHAASEDCIAKGGTLGTPVTGHQNDRLYEYVLESIGSEEHVWLGINDMVTEGHWVDQSGQSVRFKNW
ETDITLQPDGGRGQNCAIMS
TSANGKWFDENCRAEKVSVCEFNIV
>g3009.t1
MKSQATVSWIVNVPAKKEAHLAFVNLSRPLKCSDRHSNIRVQLLGSLEEMYSRREDEDAEMQQVVPGSFYLNMSNCMPEK
GQLSVVTEITLRKSRNLLLT
IILSVVSVLLVLAALVLTVVCVVIRKKKKEMNRNQVSIYNPAGTVFRPGESHLPGSDEDEYHVYASIEDTLVYTHLLKKE
LEVEGDDDKADTYRPFTGPT
EPPRPLSSMNPEVGTYRPFPGTPNVGPPVPQANRPPSRDQTMVHNGLYDPDGQSDLGVSEDDVSGPPALGARMEPEGSD
>g3010.t1
MIRSLLSACLFSTTGRIHHAMVTALNEENESVTVEWLENGDTKGKEIDLESVFALNPHFAPEEDVPLSPETPPSSTSTAA
KATKLPKSRRTIVPPKTENP
MRENRAASVGTTRARPSQQVQASEPPPPAINPQPTQQQTLLQQQMARRKSNCVKEVEKLQEKREKRRLQQQELREKKAQE
VDATLPNYEIMCMIRDFRAS
LDYRPLTTTDLIEEHRICVCVRARPLNKKELTTKDLDVITIPSKDVVMVHEPKQKVDLTRYLENQTFRFDYAFDDSTTNE
MVYRFTARPLVETIFERGMA
TCFAYGQTGSGKTHTMGGDFSGKNQDCSKGIYALAAREVFVMLGKPNYKKLDLQVFDLLNRKAKLRVLEDGKQQVQVVGL
QEREVKCTEDVLRLIEVGNS
CRTSGQTSANAHSSRSHAVFQIILRRRGKMHGKFSLIDLAGNERGADTSSADRQTRLEGAEINKSLLALKECIRALGRNK
PHTPFRASKLTQVLRDSFIG
ENSRTCMIATISPGMASCENTLNTLRYANRVKEFGISPSDIPFSQSGGGGGRSELSPTYEYGDLANRTKELSLDGEGLMD
SPLGDHHAQLEVLEAQWGVG
SSPQRDDLKLLCEQNEEEVSPQLFSFHEAVSQLVEMEEQVLEDHRAVFGESIRWLEDEKVLLEMTEEVDYDVDTYVTQLE
QILDQKIDILSELRDKVKSF
RSALQEEEIASKKIPSMTKKCRAL
>g3010.t2
MIRSLLSACLFSTTGRIHHAMVTALNEENESVTVEWLENGDTKGKEIDLESVFALNPHFAPEEDVPLSPETPPSSTSTAA
KATKLPKSRRTIVPPKTENP
MRENRAASVGTTRARPSQQVQASEPPPPAINPQPTQQQTLLQQQMARRKSNCVKEVEKLQEKREKRRLQQQELREKKAQE
VDATLPNYEIMCMIRDFRAS
LDYRPLTTTDLIEEHRICVCVRARPLNKKELTTKDLDVITIPSKDVVMVHEPKQKVDLTRYLENQTFRFDYAFDDSTTNE
MVYRFTARPLVETIFERGMA
TCFAYGQTGSGKTHTMGGDFSGKNQDCSKGIYALAAREVFVMLGKPNYKKLDLQVFDLLNRKAKLRVLEDGKQQVQVVGL
QEREVKCTEDVLRLIEVGNS
CRTSGQTSANAHSSRSHAVFQIILRRRGKMHGKFSLIDLAGNERGADTSSADRQTRLEGAEINKSLLALKECIRALGRNK
PHTPFRASKLTQVLRDSFIG
ENSRTCMIATISPGMASCENTLNTLRYANRVKEFGISPSDIPFSQSGGGGGRSELSPTYEYGDLANRTKELSLDGEGLMD
SPLGDHHAQLEVLEAQWGVG
SSPQRDDLKLLCEQNEEEVSPQLFSFHEAVSQLVEMEEQVLEDHRAVFGESIRWLEDEKVLLEMTEEVDYDVDTYVTQLE
QILDQKIDILSELRGDEGHD
ERIVLRHRDTDKVKSFRSALQEEEIASKKIPSMTKKCRAL
>g3010.t3
MIRSLLSACLFSTTGRIHHAMVTALNEENESVTVEWLENGDTKGKEIDLESVFALNPHFAPEEDVPLSPETPPSSTSTAA
KATKLPKSRRTIVPPKTENP
MRENRAASVGTTRARPSQQVQASEPPPPAINPQPTQQQTLLQQQMARRKSNCVKEVEKLQEKREKRRLQQQELREKKAQE
VDATLPNYEIMCMIRDFRAS
LDYRPLTTTDLVXLTTKDLDVITIPSKDVVMVHEPKQKVDLTRYLENQTFRFDYAFDDSTTNEMVYRFTARPLVETIFER
GMATCFAYGQTGSGKTHTMG
GDFSGKNQDCSKGIYALAAREVFVMLGKPNYKKLDLQVFDLLNRKAKLRVLEDGKQQVQVVGLQEREVKCTEDVLRLIEV
GNSCRTSGQTSANAHSSRSH
AVFQIILRRRGKMHGKFSLIDLAGNERGADTSSADRQTRLEGAEINKSLLALKECIRALGRNKPHTPFRASKLTQVLRDS
FIGENSRTCMIATISPGMAS
CENTLNTLRYANRVKEFGISPSDIPFSQSGGGGGRSELSPTYEYGDLANRTKELSLDGEGLMDSPLGDHHAQLEVLEAQW
GVGSSPQRDDLKLLCEQNEE

EVSPQLFSFHEAVSQLVEMEEQVLEDHRAVFGESIRWLEDEKVLLEMTEEVDYDVDTYVTQLEQILDQKIDILSELRDKV
KSFRSALQEEEIASKKIPSM
TKKCRAL
>g3011.t1
MLDNMPSPQVLQPVVKLKVDELYLNWLSDPSTQSVLKNYLTLIKSGQPIDLGHVDAGDRRLLAFGVADLRWSGIKKRVYL
KTAKEFLNTNTILEGLAKYS
INIMLMRPAAAGPALTPDPDIDSPASLVLALETSELGQWDEVWQGIRRSDCSPQMSLIVRIHGIRSL
>g3012.t1
MEVVSSELLPKPAKTGLGVASSPAKHSFSDLLAGLRPGEILRCGHREGLALRWTRRYRHRGKTPRSGPLARAPSAHHLTS
EPEVWWRRHFDMRTVEGGGM
EGIEPPTLRSVDDPPCPPALPY
>g3013.t1
SQPGSALPSAITSQPPAQPAILSPLRE
>g3014.t1
MGCGLRKLEDPEDCSPGKIYSTLKRPQVETKTDTVFEYVLLDFSLEDMQLHGSKTPLGTPWAFRSPPSRPCGLQLRPPIP
GLLCHVVCRLWSSVASFTGP
LLVQALLAEHGELGAWGRQHIKGRIMSETSS
>g3014.t2
MGCGLRKLEDPEDCSPGKIYSTLKRPQVETKTDTVFEYVLLDFSLEDMQLHGSKTPLGTPWAFRSPPSRPCGLQLRLWSS
VASFTGPLLVQALLAEHGEL
GAWGRQHIKGRIMSETSS
>g3015.t1
IHTDSDEGEGTIKYTISGEGAGTIFIIDELTGDIHATERLDREEKAFYTLRAQARDRLSNDPLEPESEFVIKVQDINDSE
PKFLEGPYIGSVAELSPIGT
YGPETYFMGVIPATRIV
>g3016.t1
MAPAFRLSLKAKASDNMSHMMVDFSQEREMLQGLSFLPVPATPEIQTAECQVCDNTVSVAWTLQEPDSKIDHYILEHRRT
NHDGPPRIREEYPWMVVEGI
REMEHTLTGLRFDTRYMTFRVKACNKAVAGEFSEPVTLETHAFLFKLDAASAHQNLKVEDLSVEWDSCGGKVQEIRKDKN
RTNQSPMHSPARASLMSPKR
VPTARAGRDRFTAESYTVL
>g3016.t2
MAPAFRLSLKAKASDNMSHMMVDFSQEREMLQGLSFLPVPATPEIQTAECQVCDNTVSVAWTLQEPDSKIDHYILEHRRT
NHDGPPRIREEYPWMVVEGI
REMEHTLTGLRFDTRYMTFRVKACNKAVAGEFSEPVTLETHAFLFKLDAASAHQNLKVEDLSVEWDSCGGKVQEIRKDKN
RTNQSPMHSPARSDKTHWSC
SPELLH
>g3016.t3
MAPAFRLSLKAKASDNMSHMMVDFSQEREMLQGLSFLPVPATPEIQTAECQVCDNTVSVAWTLQEPDSKIDHYILEHRRT
NHDGPPRIREEYPWMVVEGI
REMEHTLTGLRFDTRYMTFRVKACNKAVAGEFSEPVTLETHAFLFKLDAASAHQNLKVEDLSVEWDSCGGKVQEIRKDKN
RTNQSPMHSPARASLMSPKR
VPTARAGRDRFTAESYTVLGEWRRCNGSTTQIRVLTIDARLYHARPLEHFVVLSACVYILGHAFYLEM
>g3017.t1
MVNSTRVRSIYESRCTPGPPGPPGPPAPLFGRDPSEAPLDPREEPGLYLRSSPRRPAFRWPFAG
>g3017.t2
MVNSTRVRSIYESRCTPGPPGPPGPPAPLFGRDPSEAPLDPREEPGLYLRSSPRRPAFRWPFAGVGCQRG
>g3018.t1
MTEPASKKQGFKKCRSATFSIDGFSFTIGKTQVSLSWSEDH
>g3019.t1
MRAQERFALPLDGYVTMPHGGDLLVATQGFHTQVTVTQANGITANHLCVRLLEDAPKPLTCHLDDRLVPAAGGCLWHLFP
TTLLPGGLPTHLDDSRWEID
GSFHLHLSLLISRRRRVVDMA
>g3019.t2
MPHGGDLLVATQGFHTQVTVTQANGITANHLCVRLLEDAPKPLTCHLDDRLVPAAGGCLWHLFPTTLLPGGLPTHLDDSR
WEIDGSFHLHLSLLISRRRR

VVDMA
>g3019.t3
MRAQERFALPLDGYVTMPHGGDLLVATQGFHTQVTVTQANGITANHLCVRLLEDAPKPLTCHLDDRLVPAAGGCLVTASL
SAGRSSGLGRSIVSGSRHRW
HLFPTTLLPGGLPTHLDDSRWEIDGSFHLHLSLLISRRRRVVDMA
>g3020.t1
DSTLQRGETLGEFISDGGLGRTPWRLAMMILIQ
>g3021.t1
MSAERLQHTYNQQGYISGLPVLSPAELSDARQAFSALEDEFGTEHTQYGLHNVHLQYQWVMGLAKHPRLLEAIQAIIGPD
VLLLDSRFICKYPVCGADGG
VPDTLLPYVAWHQDMRYWGIDGGPVLSVWLALDDSLQDNGALKVVPGTHQSGMLPHQLAKRSGNMLSVNQEIPEDLVEGD
KAVLCPLLAGQMSDPDRPRK
FNAVVLASGTDQFNHFSS
>g3021.t2
FVHVPEPNASSSSDVHARPAPPASSDGSGFRPPGGSPSPPSSSSCFSTFNRPGAGPPDVGTHYPPGGVQDLKEQLEALRC
QTQIYEAEFETEHKDHKNTL
QENRRLRRKREEMRQQVALLQEQLKVYEDDFRKERSDKQVLQRLLEKKNSPVKDPVLVHRCNNEKHPAELSDARQAFSAL
EDEFGTEHTQYGLHNVHLQY
QWVMGLAKHPRLLEAIQAIIGPDVLLLDSRFICKYPVCGADGGVPDTLLPYVAWHQDMRYWGIDGGPVLSVWLALDDSLQ
DNGALKVVPGTHQSGMLPHQ
LAKRSGNMLSVNQEIPEDLVEGDKAVLCPLLAGQMSDPDRPRKFNAVVLASGTDQFNHFSS
>g3021.t3
FVHVPEPNASSSSDVHARPAPPASSDGSGFRPPGGSPSPPSSSSCFSTFNRPGAGPPDVGTHYPPGGVQDLKEQLEALRC
QTQIYEAEFETEHKDHKNTL
QENRRLRRKREEMRQQVALLQEQLKVYEDDFRKERSDKQVLQRLLEKKNSPVKDPVLVHRCNNEKHPAELHIYPALALEP
TIQEQLKPTEMSAERLQHTY
NQQGYISGLPVLSPAELSDARQAFSALEDEFGTEHTQYGLHNVHLQYQWVMGLAKHPRLLEAIQAIIGPDVLLLDSRFIC
KYPVCGADGGVPDTLLPYVA
WHQDMRYWGIDGGPVLSVWLALDDSLQDNGALKVVPGTHQSGMLPHQLAKRSGNMLSVNQEIPEDLVEGDKAVLCPLLAG
QMSDPDRPRKFNAVVLASGT
DQFNHFSS
>g3021.t4
FVHVPEPNASSSSDVHARPAPPASSDGSGFRPPGGSPSPPSSSSCFSTFNRPGAGPPDVGTHYPPGGVQDLKEQLEALRC
QTQIYEAEFETEHKDHKNTL
QENRRLRRKREEMRQQVALLQEQLKVYEDDFRKERSDKQVLQRLLEKKNSPVKDPVLVHRCNNEKHPAGHRSNHLRPLKK
KQS
>g3022.t1
MLLRVVVWLNWAGWCGAAVLLRWWRLGVAELIRLGGAVGVCLESISSSWLRSHGLQRPGRPGNTDHRDQGDPETRTRETR
ETRTKETRASRKQRRSESRE
YGDQKRPGTSGDQRDQTSRLLHSRK
>g3023.t1
MLASYPRHLLADCRVCVCRGAAEDCSYVSLFRYLNLNNKTSPQVHVRPVKNWTTPSVVRVNFVVYGIMEVLWSNEFLRWN
PARFCGINQLEVAREMMWLP
DILIEEDASDSGSTIHSRNLLLTANGNVVASSMRRLEATCQMNLYRFPMDVQRCNITVGSFSSDGTGIIVSQLHSGNVLT
TLAKAHMSTKGEWEMAGITV
TPTTLDYDGEKQHRVVYRVTLKRRPMLYIMNCILPLFYFLVLDMGTFFISEARGEKLSLKVTLLLSISVLLLILQDILPS
TTDQLPLIALYSIVIFSLMA
VSLMEAMLVSFLIDMDHSTAKPDPTPSCVTMDTEVKAPQPEHSRGAENPEEEQREKEKAGQPDHFLLKLILKEVMSVRQE
VLSETTPGDRKLGFYGRLAA
KIDVTFFWVYLICNGGFLIYLFYIWMVV
>g3023.t2
MLASYPRHLLADCRVCVCRGAAEDCSYVSLFRYLNLNNKTSPQVHVRPVKNWTTPSVVRVNFVVYGIMEVLWSNEFLSAS
DSGSTIHSRNLLLTANGNVV
ASSMRRLEATCQMNLYRFPMDVQRCNITVGSFSSDGTGIIVSQLHSGNVLTTLAKAHMSTKGEWEMAGITVTPTTLDYDG
EKQHRVVYRVTLKRRPMLYI
MNCILPLFYFLVLDMGTFFISEARGEKLSLKVTLLLSISVLLLILQDILPSTTDQLPLIALYSIVIFSLMAVSLMEAMLV

SFLIDMDHSTAKPDPTPSCV
TMDTEVKAPQPEHSRGAENPEEEQREKEKAGQPDHFLLKLILKEVMSVRQEVLSETTPGDRKLGFYGRLAAKIDVTFFWV
YLICNGGFLIYLFYIWMVV
>g3023.t3
MLASYPRHLLADCRVCVCRGAAEDCSYVSLFRYLNLNNKTSPQVHVRPVKNWTTPSVVRVNFVVYGIMEVVGVTASDSGS
TIHSRNLLLTANGNVVASSM
RRLEATCQMNLYRFPMDVQRCNITVGSFSSDGTGIIVSQLHSGNVLTTLAKAHMSTKGEWEMAGITVTPTTLDYDGEKQH
RVVYRVTLKRRPMLYIMNCI
LPLFYFLVLDMGTFFISEARGEKLSLKVTLLLSISVLLLILQDILPSTTDQLPLIALYSIVIFSLMAVSLMEAMLVSFLI
DMDHSTAKPDPTPSCVTMDT
EVKAPQPEHSRGAENPEEEQREKEKAGQPDHFLLKLILKEVMSVRQEVLSETTPGDRKLGFYGRLAAKIDVTFFWVYLIC
NGGFLIYLFYIWMVV
>g3024.t1
TRAGRSCLAALGFSMTEMGLVRVETQQHHAAVHGGRKIQELFLGDIHTEWTQRTHHRPSGYQQPLQNAMHHVHPCPVCAL
VYRSTQQRPPVLPRTPAASL
ALAGRWVSRRCEARPSVLFLTRDFTFHPDRHAWEGVYRHYSDPGCSEATFTLAASGHYARGNPSARLPGATEMAFKVTRV
KVTPWDESSVRLLNGTRPGH
CGPSGRWEAGVERDVTPTAGCSALGIKLPHKEYELLKTEMDPWKHPLLFTGERPTDGLSPDRPLRRPTSFQPPMVLCGGG
GKASAAHRSGGSGGAGSHSQ
QVQFAASGSERLAQLGLLALGAAVCSWFWV
>g3025.t1
MDDQTRMLTGLAGLGGLSQADVGDPDAVRKSLGQPQQDIGDILQQIMAITDESLDEAQARSTCVKGPTIAVIAM
>g3026.t1
MAPRDFVLCVIVTGDLLNEDSETSHLVRSRNGFPHETQPAQQHPPLTRAHAGDTCLTTPAASGAHNQRGVHASSFISLFT
IPNDASVCFVAWPSAALHSG
AVLPNPVSEPRRVMQQPARIQEEREHWQRGASLWEVLSAGEERSPGSGGPRRTP
>g3026.t2
MAPRDFVLCVIVTGDLLNEDSETSHLVHPPLTRAHAGDTCLTTPAASGAHNQRGVHASSFISLFTIPNDASVCFVAWPSA
ALHSGAVLPNPVSEPRRVMQ
QPARIQEEREHWQRGASLWEVLSAGEERSPGSGGPRRTP
>g3027.t1
LFLWCEVLVRMDRSLMPEGRDWKSLCPGTSSLPPLDWPLPSQFGQYELKIEVHPKAHHRAHYETEGSRGAVKATSGGHPI
VKKFYTHIVGLLEPEMNPTV
AALCLRTRTYVLEPYLAYVD
>g3027.t2
MDRSLMPEGRDWKSLCPGTSSLPPLDWPLPSQFGQYELKIEVHPKAHHRAHYETEGSRGAVKATSGGHPIVKKFYTHIVG
LLEPEMNPTVAALCLRTRTY
VLEPYLAYVD
>g3027.t3
LFSWLTAEGKKLFLWCEVLVRMDRSLMPEGRDWKSLCPGTSSLPPLDWPLPSQFGQYELKIEVHPKAHHRAHYETEGSRG
AVKATSGGHPIVKKFYTHIV
GLLEPEMNPTVAALCLRTRTYVLEPYLAYVD
>g3028.t1
IEEEEFDKYNIPSKTDSDKYKVIRTFSFLKNRMTSTRNKSKGKGKDREAKERQVNGHQFATGSCLGPTVCLICEKPASSR
DLLHCS
>g3028.t2
IEEEEFDKYNIPSKTDSDKYKVIRTFSFLKNRMTSTRNKSKGKGKDREAKERQVNGHQFATGSCLGPTVCLICEKPASSR
DLLHCSS
>g3029.t1
LSLRDRVRSSDIRRELGVEPLLLRVERSQLRWFGHLIRMPPGRLPLEVFRARPTVCCHRDPAPDKRSLFNNNDDDDDDDD
DECGNANCVVLHVPEDTMDA
AGPLDFPKLKPFLNCQPAAQLSQCDATRSQVASELIIRWTSLGLHNPIRTPGLRGVLRSAGTDPRLAVAVVRAAPAERGV
RTHVNEGPERVWTVSSVDRR
GGRTSRTPLRAGRGQCVRGEAAPSCYTDHSAFLVR
>g3029.t2
LSLRDRVRSSDIRRELGVEPLLLRVERSQLRWFGHLIRMPPGRLPLEVFRARPTVCCHRDPAPDKRSLFNNNDDDDDDDD

DECGNANCVVLHVPEDTMDA
AGPLDFPKPAAQLSQCDATRSQVASELIIRWTSLGLHNPIRTPGLRGVLRSAGTDPRLAVAVVRAAPAERGVRTHVNEGP
ERVWTVSSVDRRGGRTSRTP
LRAGRGQCVRGEAAPSCYTDHSAFLVR
>g3029.t3
SIYVPTLTYGHELWVVTERTRSRIQAAEMSFLRRVARLSLRDRVRSSDIRRELGVEPLLLRVERSQLRWFGHLIRMPPGR
LPLEVFRARPTVCCHRDPAP
DKRSLFNNNDDDDDDDDDECGNANCVVLHVPEDTMDAAGPLDFPKLKPFLNCQPAAQLSQCDATRSQVASELIIRWTSLG
LHNPIRTPGLRGVLRSAGTD
PRLAVAVVRAAPAERGVRTHVNEGPERVWTVSSVDRRGGRTSRTPLRAGRGQCVRGEAAPSCYTDHSAFLVR
>g3030.t1
LHSSEVPVNGEVPVNGEVPVKGEVPVNGEVPVNGEVPVNGSLMQSEAGQPLGHPGRRCCWC
>g3031.t1
TVANYVQWRSVLSRVTALSRRFLYRYLDYSRVTTGTTSLMSQVDKCVGYIKNLLILPTGRLFIDTHFQEDKKHMPYARQE
KSIG
>g3031.t2
TVANYVQWRSVLSRVTALSRRFLYRYLDYSRVDKCVGYIKNLLILPTGRLFIDTHFQEDKKHMPYARQEKSIG
>g3031.t3
TVANYVQWRSVLSRVTALSRRFLYRYLDYSRVSMTHQLRLVTRSNLELLPWCPVTTGTTSLMSQVDKCVGYIKNLLILPT
GRLFIDTHFQEDKKHMPYAR
QEKSIG
>g3032.t1
LSLRLQPLKTKPADPALSGLAPPTPRPDCTRGLWVSSRLPSAAPFSCNRGSSGVSLPAPRRAFQHPGLLRLSNHTAKFHM
GALFKNEEDIQKNAEIRRAP
SSA
>g3033.t1
MKKHKRARAGPAESASSAAGSRCPQSRPHAGFIGARGRSSASQTLTREQSGVNGARRSYEDPRGPSPSDGRARAPHRPFG
TFWKKKRLFLGSVFGPQHGD
ALTLRTTPGPGYLNGDGLVGENNTLNSALNLRKVTMSESAATSSPAKRSARRKPASHPKYSEMVRKAMESENGRSGCSRQ
WIQKYIKKNFTVGDNSDLQT
KITLKKMVTNGDLLHIKGIGASGSFALPRVKKDTSKPKVVKPKMPKFKASKQKVSPQKKSPQKKQVRKKPLAKSPKSVAK
KDKASKPLKSKTVKKIQQKV
TKPKPKPKPKATPKTKPKAKPKSKASKKTPAKRVSKKK
>g3034.t1
GKVLRYFPTERGQQVSQGPITLTLTTQKTTPTHVERMISLQYRDQSLKRTVVHLQYNSWPELGLPDSKGSLLGFIQEVQG
HFQHQRPLHTPIIVHCSSGV
GRTGAFCLLYAALQELEAGNGIPDLPLLVKKMRQQRKNMLQEK
>g3035.t1
MGEIKCNLFSSTSFRPFELLPLQARDYSGPPDPGPLPLYHFLSGRGAAEGTATVIAGRFERTSSNEKALCEIIYTGPFWF
ALWKPTSEAHCARLRPSAAG
EHPVSPSTSGVRFLQCDLHQL
>g3035.t2
MGEIKCNLFSSTSFRARDYSGPPDPGPLPLYHFLSGRGAAEGTATVIAGRFERTSSNEKALCEIIYTGPFWFALWKPTSE
AHCARLRPSAAGEHPVSPST
SGVRFLQCDLHQL
>g3036.t1
MVLCRKTVDCSLRVGSELLPQVKEFKYLGILFTNEGKMEREMDRRIGAASAVMQALYRTVVVKRELSQKAKLSISQPIYI
PTLTYGHELWVVTEKTRSRI
>g3037.t1
LSVYSGGILFKDSCRVGHNEVIGVCYTGPDAHGLGRDHWNEMLAYPRKPITHWHALGEVETPSAHTPHPPSHSAQGAVHL
AATGGALQS
>g3037.t2
LSVYSGGILFKDSCRVGHNEVIGVCYTGPDAHGLGRDHWNEMLAYPRKPITHWHALGEWPGRASSFESQGSCPSPKPPTT
P
>g3037.t3
VGHNEVIGVCYTGPDAHGLGRDHWNEMLAYPRKPITHWHALGEVETPSAHTPHPPSHSAQGAVHLAATGGALQS

>g3038.t1
MAAQVATLNTSPPSELKKPDREPKEESVPGEKQTDKKPSGLDGGSPGRGDLQDGADGGNAGGGGEPEMKNGNGNPPRPNN
NQNDSIGPDGNNHPGMVHHH
GSAFPPPSYGYSQHYGRAPFHQHGGQQSPGMAAAAGPAVQSSNMIDPYQPNSHDHGFSSHQFNNYSPFPNRTPYPGQGYG
MNSPRNTQAPTAGGQPAKQQ
PAGGPTAMAGSYNNQRYNMGNPQPTSTPTLNKLLTSPSSTRGYPNYPPGDYSGPEGAAKGPADMGSSGGGGGPYGGSNPA
WPQRTHHPPPMSPGSAGPAL
GRNQPPGPMDPMGKMRGQPYGAGSPYSQQPQQGPPTGPQQGPGYPGQAYGPQGPQRYPVGMQGRAPAGMGGMPYGPQMGS
YGQQGPGGYGPQSQAQYYGQ
PGQAPHPGQQQTPYAQPPSGQQGGQTPYPGQPHPAPSSAAHSQGAPPYQQPHMPPQGQGPQ
>g3039.t1
MLEWISLKVLGAARLLTCIMDYCSRSFMVYFRAIVVALVPLYGQILDLLAKVSQAQPMPFLTGSPLPTDLMEFLGPSWSS
LQPKGLSLARLSETLDPSNL
TTPSTKKHCDDVGTALQRAQDLDSDCSPSFQIFKNVRRKAMESPFSLKRQHGKQEEVFHTQVRSAATFKNVATHLKEMIE
WCGSQKRKREKQYFSFLHLK
CRSLRGKLRRLKGEACWVLSPMDPLPVSCHSPSALWRKTHPKTRFQPLRLRVRATRARVKAGAQRKPQPSCLLCLDKVF
>g3039.t2
MLEWISLKVLGAARLLTCIMDYCSRSFMVYFRAIVVALVPLYGQILDLLAKVSQAQPMPFLTGSPLPTDLMEFLGPSWSS
LQPKGLSLARLSETLDPSNL
TTPSTKKHCDDVGTALQRAQDLDSDCSPSFQIFKNVRRKAMESPFSLKRQHGKQEEVFHTQVRSAATFKNVATHLKEMIE
WCGSQKRKREKQYFSFLHLK
CRRLECLESAGYSLRGKLRRLKGEACWVLSPMDPLPVSCHSPSALWRKTHPKTRFQPLRLRVRATRARVKAGAQRKPQPS
CLLCLDKVF
>g3039.t3
MLEWISLKVLGAARLLTCIMDYCSRSFMVYFRAIVVALVPLYGQILDLLAKVSQAQPMPFLTGSPLPTDLMEFLGPSWSS
LQPKGLSLARLSETLDPSNL
TTPSTKKHCDDKAMESPFSLKRQHGKQEEVFHTQVRSAATFKNVATHLKEMIEWCGSQKRKREKQYFSFLHLKCRSLRGK
LRRLKGEACWVLSPMDPLPV
SCHSPSALWRKTHPKTRFQPLRLRVRATRARVKAGAQRKPQPSCLLCLDKVF
>g3040.t1
LIFVVDSNDRERVNEAREELMRMLAEDELRDAVLLVFANKQDLPNAMNAAEITDKLGLHSLRHRSWYIQATCATSGDGLY
EGLDWLANQLKNKK
>g3040.t2
LVEGLIFVVDSNDRERVNEAREELMRMLAEDELRDAVLLVFANKQDLPNAMNAAEITDKLGLHSLRHRSWYIQATCATSG
DGLYEGLDWLANQLKNKK
>g3041.t1
MASSRDGETRRGGRPEPVDESEGGSTTSSYNSDCSADLEPEWLDNVQKNGELFYLELSEDEEEVALARAAHGNAAGLSTN
HVRFSDKEAEIITEADRKQC
AGSPARAEPKLKRLARLLRRKRPSQRRGGKAGVKDG
>g3042.t1
PAATGGSVGGDSGHGSSQTDAYEELFHLPSAVDLEPLDTDPEEAAHKVIDLSQYSPTFFGPEHAQVLSSHLLHSSLPGLT
GTEQMSLMALADTIATTSTD
LQDSQSRKGGETLDDCGLRFLLAVRLHTFLSTSLPPAHRAQLLRQGLSTCHYAWAFHSEAEEELLNMLPALQRGEPVWAE
LRSMGLGWWLRSTNKLRRCM
EKVGDPGLQDSMEESWSQHNAKMTDFFSNNFSEDRWRKAALKNAFSLLGKQRFQHSAAFFLLAGSLKDAVEVCVEKMQDL
QLALVISRLYESEFGATSTY
KRILRRHILGQDKQASGSLFLTH
>g3042.t2
PAATGGSVGGDSGHGSSQTDAYEELFHLPSAVDLEPLDTDPEEAAHKVIDLSQYSPTFFGPEHAQVLSSHLLHSSLPGLT
GTEQMSLMALADTIATTSTD
LQDSQSRKGGETLDDCGLRFLLAVRLHTFLSTSLPPAHRAQLLRQGLSTCHYAWAFHSEAEEELLNMLPALQRGEPVWAE
LRSMGLGWWLRSTNKLRRCM
EKVGDPGLQDSMEESWSQHNAKMTDFFSNNFSEDRWRKAALKNAFSLLGKQRFQHSAAFFLLAGSLKDAVEVCVEKMQDL
QLALVISRLYESEFGATSTY
KRILRRHILGQDKQASGSLLKSPVITGALCASQIEAHRDPFLCSMAHWVLEDYSSALDTLLQQPAADPRSPGITSET
>g3042.t3

PAATGGSVGGDSGHGSSQTDAYEELFHLPSAVDLEPLDTDPEEAAHKVIDLSQYSPTFFGPEHAQVLSSHLLHSSLPGLT
GTEQMSLMALADTIATTSTD
LQDSQSRKGGETLDDCGLRFLLAVRLHTFLSTSLPPAHRAQLLRQGLSTCHYAWAFHSEAEEELLNMLPALQRGEPVWAE
LRSMGLGWWLRSTNKLRRCM
EKVGDPGLQDSMEESWSQHNAKMTDFFSNNFSEDRWRKAALKNAFSLLGKQRFQHSAAFFLLAGSLKDAVEVCVEKMQDL
QLALVISRLYESEFGATSTY
KRILRRHILGQDKQASGSLMAHWVLEDYSSALDTLLQQPAADPRSPGITSET
>g3043.t1
VWVDAAAQIFFSLGPGFGVLLALSSYNPFTNNCYRDAIVTSLVNCLTSLVSGFVIFTVLGHMAEMRKVDVEDVAKDQGPS
LLFITYPEAIANMMGSTFFA
IIFFVMMITLGLDSTFGGLEAIITAVLDEYPDTFSHRRELFVLLLVIVCFLGSLSTLTHGGAYVVKLLEEFGVGSSIIAV
GFLEAIAVSWFYGINRFSND
IKAMLGSAPGMFWKVCWVVISPAFLAYIIVSSMVKPPPLTLFDYKYPDWSIKVGYMVGLSSFMWIPIYMVYKLVWTPGSL
KQVCRT
>g3043.t2
AAAQIFFSLGPGFGVLLALSSYNPFTNNCYRDAIVTSLVNCLTSLVSGFVIFTVLGHMAEMRKVDVEDVAKDQGPSLLFI
TYPEAIANMMGSTFFAIIFF
VMMITLGLDSTFGGLEAIITAVLDEYPDTFSHRRELFVLLLVIVCFLGSLSTLTHGGAYVVKLLEEFGVGSSIIAVGFLE
AIAVSWFYGINRFSNDIKAM
LGSAPGMFWKVCWVVISPAFLAYIIVSSMVKPPPLTLFDYKYPDWSIKVGYMVGLSSFMWIPIYMVYKLVWTPGSLKQVC
RT
>g3044.t1
MACVQQGRTAQETQLLPNVVATEQQSLVVVKKLLAIAVSSITYLRGLFPEKAYGTRHVEEQKVMILREERSCPAANQIVQ
CNNKNSSAVASMVHGNSKKA
SILLVRKLYTLMQNLGPLPDNVCLNMKLSYYDEGSQQRATSSLVTYGVEICSLVFMSPPMVVS
>g3044.t2
MACVQQGRTAQETQLLPNVVATEQQSLVVVKKLLAIAVSSITYLRGLFPEKAYGTRHVEEQKVMILREERSCPAANQIVQ
CNNKNSSAVASMVHGNSKKA
SILLVRKLYTLMQNLGPLPDNVCLNMKLSYYDE
>g3044.t3
MACVQQGRTAQETQLLPNVVATEQQSLVVVKKLLAIAVSSITYLRGLFPEKAYGTRHVEEQKVMILREERSCPAANQIVQ
CNNKNSSAVASMVHGNSKKA
SILLVRKLYTLMQNLGPLPDNVCLNMKLSYYDEVFMSPPMVVS
>g3045.t1
MYLLDIIQCHLISCCQDSQEKNSSPIKSEEAKLKAKYPGLSHKPGGADFLMKRLQKGAQPKYFDSGDYNMAKAKMKNKQL
PVAGPDKNLVTGDHIPTPQD
LPQRKSSLVTSKLAG
>g3045.t2
MSSENLDSDTQIDYEDEKQDSQEKNSSPIKSEEAKLKAKYPGLSHKPGGADFLMKRLQKGAQPKYFDSGDYNMAKAKMKN
KQLPVAGPDKNLVTGDHIPT
PQDLPQRKSSLVTSKLAG
>g3046.t1
MFSVTGNMSLAALVLAVLTIVLATSPVGANEDCLWYVDKNGTWHNGFDCPLITFCCGNCNRRYCCLDAFKMITEREQKRC
MLFQFRCCCCLHCSAR
>g3047.t1
PERWTNIPLLVKIFKLIINELSSVVEANATRANAADWSQDSTGMWEEQEGEEGADDEDEEEDEGLAGQLLSDLIASNKYD
DEYYEDDEEDDPDALKDPLY
QVDLQAYLTDFLTQFAQQPCYSMFSGHLNDPERRALQSIGL
>g3047.t2
MIHRSSGLPDPERWTNIPLLVKIFKLIINELSSVVEANATRANAADWSQDSTGMWEEQEGEEGADDEDEEEDEGLAGQLL
SDLIASNKYDDEYYEDDEED
DPDALKDPLYQVDLQAYLTDFLTQFAQQPCYSMFSGHLNDPERRALQSIGL
>g3048.t1
LDCGGGVREQHGPEGLARDVTADHRSHRKTGGPRPGKRFSVHTSFSNKPTVGLLDSTLLPGSDSTAGRKTADPGHARGQT
SFHANAPHLRVVPPIGRGPL
PSGGTAEGSWIDATGENNTLTSAPVTRCLCGPGPDRSITHGETPNGARIWKPAGPLDVAQQGDTATANGIISPYSAARFR

LESTEGISFGGAIVVSWRPF
PGTRHMAPSVRPRRGGRVLGE
>g3048.t2
LDCGGGVREQHGPEGLARDVTADHRSHRKTGGPRPGKRFSVHTSFSNKPTVGLLDSTLLPGSDSTAGRKTADPGHARGQT
SFHANAPHLRVVPPIGRGPL
PSGGTAEGSWIDATGENNTLTSAPVTRCLCGPGPDRSITHGETPNGARIWKPAGPLDVAQQGDTATANGIISPYSAARFR
LESTEGISFGGAIVVSWRPF
PGTRHMAPS
>g3048.t3
LDCGGGVREQHGPEGLARDVTADHRSHRKTGGPRPGKRFSVHTSFSNKPTVGLLDSTLLPGSDSTAGRKTADPGHARGQT
SFHANAPHLRVVPPIGRGPL
PSGAAGPLDVAQQGDTATANGIISPYSAARFRLESTEGISFGGAIVVSWRPFPGTRHMAPSVRPRRGGRVLGE
>g3049.t1
DLASVLTPEIMAPILANAEVQQRLTPYLPSGESLPQSSEELHHTISSPQFQQAMSMFSSALASGQLGPLMNQFGLPAEAV
DAANKGGECFVHILGNLPYV
EAFAKAMETENKSTEDGDAKDKKDDDEDMSLD
>g3049.t2
DLASVLTPEIMAPILANAEVQQRLTPYLPSGESLPQSSEELHHTISSPQFQQAMSMFSSALASGQLGPLMNQFGLPAEAV
DAANKGGEYVEAFAKAMETE
NKSTEDGDAKDKKDDDEDMSLD
>g3049.t3
DLASVLTPEIMAPILANAEVQQRLTPYLPSGESLPQSSEELHHTISSPQFQQAMSMFSSALASGQLGPLMNQFGLPAEAV
DAANKGGECFVHILGNLPCA
HMYVEAFAKAMETENKSTEDGDAKDKKDDDEDMSLD
>g3050.t1
MEVSNKKTPASTKLETSAHRLDLPRLATKRPSPRWHPPPPPLARGSAYSSLPNSSRTSVRCEVTICVQPRPRTRNISGSP
GPKAPKKVHFIRSMRQYDTR
GSRIMLICAKRSLYAAFSVLPYGDSFELSDAKLDTQRQRLSSVVATDLIPSLEGLELGAYGKTVSYARFLYPTNALVRPR
SSGLPEGCAAVPTPQSRFRG
NGQRNLTAPARLNSSVAQEPCTEEMLEYDPSWLSDPQWPCGRHKRVLIFASYVTTVIEYVKPSDLKKDMNETFKEKFPHI
KLTLSKIRSLKREMRAMGEE
GSLQPVTIAMAFVYFEKLVLQGRLNKQNRKLVSAACVLLAAKISSDLRKPEVRQLIDKLEERFRINRRELIPLEFQVLVA
LEMGLYLPESKVMPHYRRLV
KQG
>g3050.t2
MEVSNKKTPASTKLETSAHRLDLPRLATKRPSPRWHPPPPPLARGSAYSSLPNSSRTSVRCEVTICVQPRPRTRNISGSP
GPKAPKKVHFIRSMRQYDTR
GSRIMLICAKRSLYAAFSVLPYGDSFELSDAKLDTQRQRLSSVVATDLIPSLEGLELGAYGKTVSYARFLYPTNALVRPR
SSGLPEGCAAVPTPQSRFRG
NGQRNLTAPARLNSSVAQEPCTEEMLEYDPSWLSDPQWPCGRHKRVLIFASYVTTVIEYVKPSDLKKDMNETFKEKFPHI
KLTLSKIRSLKREMRAMGEE
GSLQPVTIAMAFVYFEKLVLQGRLNKQNRKLVSAACVLLAAKISSDLRKPEVRQLIDRFAVRLLCLSVASLMRKLEERFR
INRRELIPLEFQVLVALEMG
LYLPESKVMPHYRRLVKQG
>g3051.t1
MAAKSDGAPVSLRNESSPPECPSSLDQRSPQCRAAEKRYSLLDCCWTLCALLVFFSDGASDLWLAADYYLRENYWWFALT
LVFVIVPSVVVQVLSFKWFA
YDYAETTESGTAAAAVVAASGTENNFSTKDSDQRGAGRAAAVGVLPATGTGGGARGCCRVFMWIFQAIIHIFQLAQVWRY
VHALYLGVQSRWRGDHERHH
FYWSMMFQSADISMLRLLESFLKSAPQLVLQLSIMVQARQVLPLQGLSASASLISLSWMIASYQKVLRDSRDDKLPMTYK
AVIVQILWHLFTIGARTMAF
ALFASVFQLYFGIFIVAHWCIMTFWIIQGETDFCMSKWEEIIYNMMVGIVYIFCWFSVREGRTRCRMLIYSLTVFAENVA
LTTLWYLYRGQQTSDFYAVV
LVCVVASSYALGTFFMCVYYCLLHPDGPISGWGYIVEKERLDDEPLTSPASPLPPDVVGSPPRTLPRTKDREQGVGGEAD
VFQVRATRGAQASAPRLTPR
TEGPVIRIDLPRKKYPAWDAHFIDRRLRKTILVLEGAAPVTPRIQYRCLGMAKEVMEYETTV

>g3051.t2
LRNESSPPECPSSLDQRSPQCRAAEKRYSLLDCCWTLCALLVFFSDGASDLWLAADYYLRENYWWFALTLVFVIVPSVVV
QVLSFKWFAYDYAETTESGT
AAAAVVAASGTENNFSTKDSDQRGAGRAAAVGVLPATGTGGGARGCCRVFMWIFQAIIHIFQLAQVWRYVHALYLGVQSR
WRGDHERHHFYWSMMFQSAD
ISMLRLLESFLKSAPQLVLQLSIMVQARQVLPLQGLSASASLISLSWMIASYQKVLRDSRDDKLPMTYKAVIVQILWHLF
TIGARTMAFALFASVFQLYF
GIFIVAHWCIMTFWIIQGETDFCMSKWEEIIYNMMVGIVYIFCWFSVREGRTRCRMLIYSLTVFAENVALTTLWYLYRGQ
QTSDFYAVVLVCVVASSYAL
GTFFMCVYYCLLHPDGPISGWGYIVEKERLDDEPLTSPASPLPPDVVGSPPRTLPRTKDREQGVGGEADVFQVRATRGAQ
ASAPRLTPRTEGPVIRIDLP
RKKYPAWDAHFIDRRLRKTILVLEGAAPVTPRIQYRCLGMAKEVMEYETTV
>g3052.t1
MTILCGSGTEHCVPRRPLLYILLDSVVDKTEAFSFREEASFPLNGPIREAPHALRSIPDTEGWAPAFAVCRTSLAE
>g3053.t1
LDGRFLPGVRHGGRVQGSLCHFLRVPSIDPATRGEFQQQNCEHCGRVSAHLQPQARQNHSGSGGRMRPPRYRSDVFAGI
>g3054.t1
LNKEPRFKRDFESGLPTRKTSDSGSGETVLYVLDTSTVVCIAFAAFVIGAMLTGALWFIYSNTGGSHAPLACREPFVLPC
SDGLIHHHKLCPVGLIHLAE
>g3054.t2
LNKEPRFKRDFESAVLYVLDTSTVVCIAFAAFVIGAMLTGALWFIYSNTGGSHAPLACREPFVLPCSDGLIHHHKLCPVG
LIHLAE
>g3054.t3
LNKEPRFKRDFESGLPTRKTSDSGSGETVLYVLDTSTVVCIAFAAFVIGAMLTGALWFIYSNTGVQDNRRTQQKERDVNI
MDQSRSKVGADPATVLTVLL
LEFPSRCRVYGGHPQEVT
>g3055.t1
LEHVQTVRFNNYALTNNKQRTLCQDGEPKTPGRSCSRNPTQDFYCQAGHSLGEDRSSEQGWTSERSPGRFGQALWLQTLH
SYLSSLACYVEKARGREHSL
RYTSGPPHHQEHRATLTAPNGKTKQLRCIV
>g3056.t1
MDLCSAAMQNSGPAEDLKTTVMQLRETVLQQKETIMNQKETIRELNSKLTRCESQSLPESGPVGRRPGSKNTMGDQYSRG
NNTIQANSLKDLLQNKIDDM
EKQVLSRVNTLEETKPGPRNDTDQRNKVESTLTSLHHRITDLEKGKEHRHMEKFQLTFPLRTNYMYAKTKRSLPEMYSFT
VCLWIKSNASPGVGTPFSYA
VTDQANEVVLIESGDNPMEFLINDKVAKLPFLINDGKWHHLCITWTTRDGVWEAFQDGVKRGTGENLAPYHPIKPHGVLV
LGQEQDTLGGGFDATQAFVG
ELANLNFWDRKLSIGDIYNLATCNSKAQTGNVFSWTEANIEIFGGATKWTFEPCRSLN
>g3056.t2
MDGFSWKLFVISCLIVEGSSQDFGQTQFICTSVPKDMDLCSAAMQNSGPAEDLKTTVMQLRETVLQQKETIMNQKETIRE
LNSKLTRCESQSLPESGPVG
RRPGSKNTMGDQYSRGNNTIQANSLKDLLQNKIDDMEKQVLSRVNTLEETKPGPRNDTDQRNKVESTLTSLHHRITDLEK
GKEHRHMEKFQLTFPLRTNY
MYAKTKRSLPEMYSFTVCLWIKSNASPGVGTPFSYAVTDQANEVVLIESGDNPMEFLINDKVAKLPFLINDGKWHHLCIT
WTTRDGVWEAFQDGVKRGTG
ENLAPYHPIKPHGVLVLGQEQDTLGGGFDATQAFVGELANLNFWDRKLSIGDIYNLATCNSKAQTGNVFSWTEANIEIFG
GATKWTFEPCRSLN
>g3056.t3
MDLCSAAMQNSGPAEDLKTTVMQLRETVLQQKETIMNQKETIRELNSKLTRCESQSLPESGPVGRRPGSKNTMGDVSRGP
TDTLAQLGHTLQTLKQRLEN
LEQYSRGNNTIQANSLKDLLQNKIDDMEKQVLSRVNTLEETKPGPRNDTDQRNKVESTLTSLHHRITDLEKGKEHRHMEK
FQLTFPLRTNYMYAKTKRSL
PEMYSFTVCLWIKSNASPGVGTPFSYAVTDQANEVVLIESGDNPMEFLINDKVAKLPFLINDGKWHHLCITWTTRDGVWE
AFQDGVKRGTGENLAPYHPI
KPHGVLVLGQEQDTLGGGFDATQAFVGELANLNFWDRKLSIGDIYNLATCNSKAQTGNVFSWTEANIEIFGGATKWTFEP
CRSLN

>g3057.t1
WPVRRHGSVDRWSRDPERKAPPTPDFSANRTAPLVVAERYVTSPAVVVVVGLLLLLRAGMAI
>g3058.t1
MQRLCSLGEDGGAIPSEVVNVKLLVSSELLLDILLAHTVGHMQAKKRYLILFSTGACVILLFCFGGIQIPLPRRGPNRRD
GRSFGDDHVGSQWRRSSGTL
QPLDPWDQVETEDYNGHISPQQKRDSISGVYIGRQCRMDTCFDFSKCKSNGFKVYVYPQQKVEKMSESYQNILSSIEGSR
FYTSDPGQACLFVLSLDTLD
RDQLSPQYIHNLKAKVQSLPLWNGGKNHIIFNLYSGTWPDYTEDLGFDIGHAMLAKASISTENFRPNFDVSIPLFSKDHP
RTGGERGYMKQNSIPPYRKY
MLVFKGKRYLTGIGSDTRNALYHVHNSEDVVLLTTCKHGKDWQKHKDARCDRDNAEYDK
>g3059.t1
MGKHEEEEEEEEEEEEEEEEEEEEEEEEEEEEEEEEEEEEEEEEEEEEEEEEEEEEEEEEEEEEEEEEEEEEEEEEELS
VFILSISFVVLRHALDYTDL
HSKR
>g3060.t1
MTLHYQFLLNQFFQRPAKSQIRYVVFTTKHHDGFTNWPSPTSWNWNSVDVGPHRDVVGELAVAIRKRNLHFGLYHSLFEW
FNPLFLADQASGFKTQDFVF
EKALPELVQLVNAYKPDLIWSDGDWSAPDTYWNSTEFLAWLYNDSPVKDVVVTNDRWGAGCYCKHGGYYNCADRYSPTEV
PTHKWEKCQTIDTLSWGYRR
YMKATELLTLPSILKVVDGRSGENYNLSDWKWQL
>g3060.t2
MRKNYPPGFQYADFAQQFHAEFFDANSWAEILEASGAKYVVFTTKHHDGFTNWPSPTSWNWNSVDVGPHRDVVGELAVAI
RKRNLHFGLYHSLFEWFNPL
FLADQASGFKTQDFVFEKALPELVQLVNAYKPDLIWSDGDWSAPDTYWNSTEFLAWLYNDSPVKDVVVTNDRWGAGCYCK
HGGYYNCADRYSPTEVPTHK
WEKCQTIDTLSWGYRRYMKATELLTLPSILKVVDGRSGENYNLSDWKWQL
>g3060.t3
GLKYEEEIEFMRKNYPPGFQYADFAQQFHAEFFDANSWAEILEASGAKYVVFTTKHHDGFTNWPSPTSWNWNSVDVGPHR
DVVGELAVAIRKRNLHFGLY
HSLFEWFNPLFLADQASGFKTQDFVFEKALPELVQLVNAYKPDLIWSDGDWSAPDTYWNSTEFLAWLYNDSPVKDVVVTN
DRWGAGCYCKHGGYYNCADR
YSPTEVPTHKWEKCQTIDTLSWGYRRYMKATELLTLPSILKVVDGRSGENYNLSDWKWQL
>g3061.t1
MKTLERLFLNLLKPQVHHAQDPLQFAYRNNVGVEDAILYLLHRAHSHLDKGRGTVRILFLDFSSAFNTIQPLILQDKLLR
MQVDPCGARRTICSSTPQKP
RSWSWTLGSPDHVHSRLYFLRRLLSFNIASKLLRMFYQTVVASVLFYAVVCWGGSATKKNSTRLDRLIRRAGSVVGLKLD
SVVTVAEKGTLKKLLAVMDD
ASNPLHTVISAQRSQLSNRLLLPKCKTNRFKDSFVPRAIKLYNSALGGRGRSSRRPH
>g3062.t1
ILVKLCSFVSPAEELAQKDDLQLLFSAITSWCPPHNLPWRRSAGEVLATISRHGLSVNVVKYIHEKECLATCVQNMQQSD
DLSPLEIVEMFAGLSCFLKD
SSHLSQTLLDDFRMCQGYAFLCDLMLRLEQAKEDESKDALKDLVNLVTCLTTYGVTELKPAGLTTGAPFLLPGFVLPLPS
GKGNTVRNLQAFSVLQNAFL
RAKTSRLARMVLDAIANIYAAEPANYFILEAQHTLSQFAERVAKLPEAQVKYFELLEFVVFNLNYVPCKELFSVSVLLKS
STSHACSSTAVRMLLKLARH
DAVFSDVLREVGLLEVLVNLLHKYTALLKDPAVQQNTATQHNPQNNEHGRCECETTEEDGMSSSRRRDYPKWMSSGDSRS
NSVVEEQKQLAWLVMETLTV
LLQGSNINAGLFREFGGARCVHNIIRYRQCREQALLIIQQLVVSPSGDDDMGTLLGLMTSAPCTEMELKADILRALLTVL
RESHRTRTVFRKVGGFVYVT
SLLVAMERSLCQPPRQGWELANQNQVFELLLTIFCTLTAAMRYEPANSHFFRTEIQYEKLADAVRLLGCFSDTKKLGPTS
VFPSNAQPFQRLLEEDVPPL
GGAGEGVCPTLKHCSKLFIYLYKMATDSFDRHGYYGTSGPPAQVKAVTELKVHHVHPQAGPPQPSSDVVVIHPGAVLAIL
DLLPSVSSDSQPEHALDLQL
AVSNLLQVLVHSERNQQRAFERLASQALLPMALREFLRLGNPLNCGAWDKKLLKQYRVHKPSSLSYDAEMRSSMTTSMEG
FGPDSVFTTPGEDSGQYRIS
RSLVRSSEDSAVPLTRVKCLVSMTTPHDIRLHGSAVTPAFVEFDTSLEGFGCLFLPSLAPHNAPTNTTSNSALSEGAVPS

GRGTGERVFPPASGLTYSTW
FCVERFSAAPHLHPLRLLTVVRRASASEQHYVCLSITLSPSDRSLSVSTKEELLHSYSDESREEGSFYELLPCCARVRCG
DQILEGQWHHLVVVMTDESR
EEGSFYELLPCCARVRCGDQILEGQWHHLVVVMSKGMLKNSMASLYLDGQLLNTVKLHYVHSGPGGSASANPPVVSTVYG
YIGTPPAQRLVSSLLWRLGP
SHFLEEVVPAAGVAAIYELGPNYVGSFQAIYIPCKDSKTEGVTPSLVSLVPEEKVSFSLYAVSVSSLTVAKIRKVYNKLD
SKAIAKQLSVSSHDYATPVK
LIHNAAGHLNGPARTIGAAVIGYLGVRAFMPKPVATNLQYIGGAAAILGLVAMASDVEGLYAAVKALVCVVKSNPLASKE
MERIKGYQLLAMLLKRKRWL
LNSHILHLTFSLVGTVDSGHETSIIPNSTAFQDLLCDFEVWLHAPYELHLSLFEHFIELLTESSEAVKNAKLLREFQLIP
RLLLTLRDTSLSQPTVGAIG
QVLSLLLQGFPHPYDLLRFGQFISSTLPTFAVCEKFVVMEISNEEKIDGGNDEDFGGLLSANLILLRNRLLDILLRLLFT
SKEKATINPQACEELVRTLG
FDWLLMFLEEHVHSSTVTAALWILVVLLSHHNILHRFKEGLCGGGWLDHTDSVLTNKIGTVLGFNVGRSAGGRSTLREIN
RDACHFPGFPVLQALLPKHT
NVPEIYFLLMALFLQQPVTEMPDSLQFDLDSIWTFIFGVPASSGAALVGSLHNVCTEAAFLLLAMLRSMLNLPWQSEEGS
WLREYPITLMQFFRYMYHNV
PDLTPMWHSSEFLCSLAASVFPFNIRPYSEMVSDLDDETGSPTEEFKGFAGDSGMNRSQSEYCNVGSKTYLTNHPAKKYV
FDFMRVLIMDNLCTTPATKQ
TPVLDLLLEASPERSTRTQQKEFQSFILDGVMDHLLAADVLLGEDSSLAVSSGGSYQTLVHNIFYFSQRVVDKLWQGVFN
KDSKLVVDFIVQLIGQSKRR
SQGVSLDSVYHSLNRTVLFQLCRPHKTVAQQVALLDALRVLTVNRNLVLGPGNHDQEFIACLAHCFICLHAGSVEGFGLE
AEARMTTWHVMMPAENQSDA
SSNQEASEGRQLLLKAVARVWTELMHSKRSMLEDIFKVSLPSNDRGHVDVSTARPALEELALRSWQNHLAQEKKLIGRGE
AVTPASQSKLSRVSSGFGLS
KLTGVRRNKKDPSLNKNQLSAQVHTHKQA
>g3062.t2
ILVKLCSFVSPAEELAQKDDLQLLFSAITSWCPPHNLPWRRSAGEVLATISRHGLSVNVVKYIHEKECLATCVQNMQQSD
DLSPLEIVEMFAGLSCFLKD
SSHLSQTLLDDFRMCQGYAFLCDLMLRLEQAKEDESKDALKDLVNLVTCLTTYGVTELKPAGLTTGAPFLLPGFVLPLPS
GKGNTVRNLQAFSVLQNAFL
RAKTSRLARMVLDAIANIYAAEPANYFILEAQHTLSQFAERVAKLPEAQVKYFELLEFVVFNLNYVPCKELFSVSVLLKS
STSHACSSTAVRMLLKLARH
DAVFSDVLREVGLLEVLVNLLHKYTALLKDPAVQQNTATQHNPQNNEHGRCECETTEEDGMSSSRRRDYPKWMSSGDSRS
NSVVEEQKQLAWLVMETLTV
LLQGSNINAGLFREFGGARCVHNIIRYRQCREQALLIIQQLVVSPSGDDDMGTLLGLMTSAPCTEMELKADILRALLTVL
RESHRTRTVFRKVGGFVYVT
SLLVAMERSLCQPPRQGWELANQNQVFELLLTIFCTLTAAMRYEPANSHFFRTEIQYEKLADAVRLLGCFSDTKKLGPTS
VFPSNAQPFQRLLEEDVPPL
GGAGEGVCPTLKHCSKLFIYLYKMATDSFDRHGYYGTSGPPAQVKAVTELKVHHVHPQAGPPQPSSDVVVIHPGAVLAIL
DLLPSVSSDSQPEHALDLQL
AVSNLLQVLVHSERNQQRAFERLASQALLPMALREFLRLGNPLNCGAWDKKLLKQYRVHKPSSLSYDAEMRSSMTTSMEG
FGPDSVFTTPGEDSGQYRIS
RSLVRSSEDSAVPLTRVKCLVSMTTPHDIRLHGSAVTPAFVEFDTSLEGFGCLFLPSLAPHNAPTNTTSNSALSEGAVPS
GRGTGERVFPPASGLTYSTW
FCVERFSAAPHLHPLRLLTVVRRASASEQHYVCLSITLSPSDRSLSVSTKEELLHSYSDESREEGSFYELLPCCARVRCG
DQILEGQWHHLVVVMTDESR
EEGSFYELLPCCARVRCGDQILEGQWHHLVVVMSKGMLKNSMASLYLDGQLLNTVKLHYVHSGPGGSASANPPVVSTVYG
YIGTPPAQRLVSSLLWRLGP
SHFLEEVVPAAGVAAIYELGPNYVGSFQAIYIPCKDSKTEGVTPSLVSLVPEEKVSFSLYAVSVSSLTVAKIRKVYNKLD
SKAIAKQLSVSSHDYATPVK
LIHNAAGHLNGPARTIGAAVIGYLGVRAFMPKPVATNLQYIGGAAAILGLVAMASDVEGLYAAVKALVCVVKSNPLASKE
MERIKGYQLLAMLLKRKRWL
LNSHILHLTFSLVGTVDSGHETSIIPNSTAFQDLLCDFEVWLHAPYELHLSLFEHFIELLTESSEAVKNAKLLREFQLIP
RLLLTLRDTSLSQPTVGAIG
QVLSLLLQGFPHPYDLLRFGQFISSTLPTFAVCEKFVVMEISNEEKIDGGNDEDFGGLLSANLILLRNRLLDILLRLLFT

SKEKATINPQACEELVRTLG
FDWLLMFLEEHVHSSTVTAALWILVVLLSHHNILHRFKEGLCGGGWLDHTDSVLTNKIGTVLGFNVGRSAGGRSTLREIN
RDACHFPGFPVLQALLPKHT
NVPEIYFLLMALFLQQPVTEMPDSLQFDLDSIWTFIFGVPASSGAALVGSLHNVCTEAAFLLLAMLRSMLNLPWQSEEGS
WLREYPITLMQFFRYMYHNV
PDLTPMWHSSEFLCSLAASVFPFNIRPYSEMVSDLDDETGSPTEEFKGFAGDSGMNRSQSEYCNVGSKTYLTNHPAKKYV
FDFMRVLIMDNLCTTPATKQ
TPVLDLLLEASPERSTRTQQKEFQSFILDGVMDHLLAADVLLGEDSSLAVSSGGSYQTLVHNIFYFSQRVVDKLWQGVFN
KDSKLVVDFIVQLIGQSKRR
SQGVSLDSVYHSLNRTVLFQLCRPHKTVAQQVALLDALRVLTVNRNLVLGPGNHDQEFIACLAHCFICLHAGSSVEGFGL
EAEARMTTWHVMMPAENQSD
ASSNQEASEGRQLLLKAVARVWTELMHSKRSMLEDIFKVSLPSNDRGHVDVSTARPALEELALRSWQNHLAQEKKLIGRG
EAVTPASQSKLSRVSSGFGL
SKLTGVRRNKKDPSLNKNQLSAQVHTHKQA
>g3062.t3
ILVKLCSFVSPAEELAQKDDLQLLFSAITSWCPPHNLPWRRSAGEVLATISRHGLSVNVVKYIHEKECLATCVQNMQQSD
DLSPLEIVEMFAGLSCFLKD
SSHLSQTLLDDFRMCQGYAFLCDLMLRLEQAKEDESKDALKDLVNLVTCLTTYGVTELKPAGLTTGAPFLLPGFVLPLPS
GKGNTVRNLQAFSVLQNAFL
RAKTSRLARMVLDAIANIYAAEPANYFILEAQHTLSQFAERVAKLPEAQVKYFELLEFVVFNLNYVPCKELFSVSVLLKS
STSHACSSTAVRMLLKLARH
DAVFSDVLREVGLLEVLVNLLHKYTALLKDPAVQQNTATQHNPQNNEHGRCECETTEEDGMSSSRRRDYPKWMSSGDSRS
NSVVEEQKQLAWLVMETLTV
LLQGSNINAGLFREFGGARCVHNIIRYRQCREQALLIIQQLVVSPSGDDDMGTLLGLMTSAPCTEMELKADILRALLTVL
RESHRTRTVFRKVGGFVYVT
SLLVAMERSLCQPPRQGWELANQNQVFELLLTIFCTLTAAMRYEPANSHFFRTEIQYEKLADAVRLLGCFSDTKKLGPTS
VFPSNAQPFQRLLEEDVPPL
GGAGEGVCPTLKHCSKLFIYLYKMATDSFDRHGYYGTSGPPAQVKAVTELKVHHVHPQAGPPQPSSDVVVIHPGAVLAIL
DLLPSVSSDSQPEHALDLQL
AVSNLLQVLVHSERNQQRAFERLASQALLPMALREFLRLGNPLNCGAWDKKLLKQYRVHKPSSLSYDAEMRSSMTTSMEG
FGPDSVFTTPGEDSGQYRIS
RSLVRSSEDSAVPLTRVKCLVSMTTPHDIRLHGSAVTPAFVEFDTSLEGFGCLFLPSLAPHNAPTNTTSNSALSEGAVPS
GRGTGERVFPPASGLTYSTW
FCVERFSAAPHLHPLRLLTVVRRASASEQHYVCLSITLSPSDRSLSVSTKEELLHSYSDESREEGSFYELLPCCARVRCG
DQILEGQWHHLVVVMTDESR
EEGSFYELLPCCARVRCGDQILEGQWHHLVVVMSKGMLKNSMASLYLDGQLLNTVKLHYVHSGPGGSASANPPVVSTVYG
YIGTPPAQRLVSSLLWRLGP
SHFLEEVVPAAGVAAIYELGPNYVGSFQAIYIPCKDSKTEGVTPSLVSLVPEEKVSFSLYAVSVSSLTVAKIRKVYNKLD
SKAIAKQLSVSSHDYATPVK
LIHNAAGHLNGPARTIGAAVIGYLGVRAFMPKPVATNLQYIGGAAAILGLVAMASDVEGLYAAVKALVCVVKSNPLASKE
MERIKGYQLLAMLLKRKRWL
LNSHILHLTFSLVGTVDSGHETSIIPNSTAFQDLLCDFEVWLHAPYELHLSLFEHFIELLTESSEAVKNAKLLREFQLIP
RLLLTLRDTSLSQPTVGAIG
QVLSLLLQGFPHPYDLLRFGQFISSTLPTFAVCEKFVVMEISNEEKIDGGNDEDFGGLLSANLILLRNRLLDILLRLLFT
SKEKATINPQACEELVRTLG
FDWLLMFLEEHVHSSTVTAALWILVVLLSHHNILHRFKEGLCGGGWLDHTDSVLTNKIGTVLGFNVGRSAGGRSTLREIN
RDACHFPGFPVLQALLPKHT
NVPEIYFLLMALFLQQPVTEMPDSLQFDLDSIWTFIFGVPASSGAALVGSLHNVCTEAAFLLLAMLRSMLNLPWQSEEGS
WLREYPITLMQFFRYMYHNV
PDLTPMWHSSEFLCSLAASVFPFNIRPYSEMVSDLDDETGSPTEEFKGFAGDSGMNRSQSEYCNVGSKTYLTNHPAKKYV
FDFMRVLIMDNLCTTPATKQ
TPVLDLLLEASPERSTRTQQKEFQSFILDGVMDHLLAADVLLGEDSSLAVSSGGSYQTLVHNIFYFSQRVVDKLWQGVFN
KDSKLVVDFIVQLIGQSKRR
SQGVSLDSVYHSLNRTVLFQLCRPHKTVAQQVALLDALRVLTVNRNLVLGPGNHDQEFIACLAHCFICLHAGSVEGFGLE
AEARMTTWHVMMPAENQSDA
SSNQEASEGRQLLLKAVARVWTELMHSKRSMLEDIFKVSLPSNDRGHVDVSTARPALEELALRSWQNHLAQEKKLIGRGE

AVTPASQSKLSR
>g3063.t1
MIRQEQGEQERHSDWLDFIEQRQAEATLRRHFYTHWLKSEAERSANVRNRYRSNSTLKSGAAFKRSEHRCSVQYRLNSVQ
YRLNSGATGT
>g3063.t2
QGEQERHSDWLDFIEQRQAEATLRRHFYTHWLKSEAERSANVRNRYRSNSTLKSGAAFKRSEHRCSVQYRLNSVQYRLNS
GATGT
>g3063.t3
MIRQEQGEQERHSDWLDFIEQRQAEATLRRHFYTHWLKSEAERSANVRNRYRSNSTLKSGAAFKRSEHRCSVQYRLNSVQ
YRLNSGATG
>g3064.t1
HHCRRCGRCFCDKCCSRKLALPRMCFVDPVRQCGACGLLSQRESDFYDKQIKVLTG
>g3065.t1
MDTMQELIPFVKEMLLSKESSRGMLKVYLVGTAFAVVGTVIGLVETVCHPFSGGHLDDAEVMFMLEEPRTHVDPPVKRRG
RKEEQQEEVQGGTYFETDKS
PATLSKTHGPQDHRIAANRPHAS
>g3066.t1
MHPVVAGSGATASRRSEAAHHNQEQADKVGGSFQLPNHGPATPKAGTNGPSLGVSQNLKKARLLRSGVWNEPNVNQRVSP
PSAELNGQGGALQNGNREGS
PHAGESTLLASLLQSFSSRLQSVALSQHASKPASECSPPPKAPATEKEPLPVYGTASSRLKGLMRKNKLQNHSNTPYSRR
GHNQDRPPESPRSAHSATPP
SAPVSTETVSCAERLKAVANMVKIRSSPAPSPKPSVACSQLALLLSSEAHLQQYSREHALKAQLSGRSASERLAAMATQQ
LGQGKRPPSAGEAPPVAQDG
LNSLTTQNGKTSTNAATATLPRVAHTCPQSPSLLHSHSQSQTSPPPVAQGVSHAPSQPPREKRGFDSRPMRPPQTCSSLL
LLLLNNHNNQKQLTKNGHLE
ESCGLLPPSGSSSVTSDSECSTQERSVAKDSSDAESTYSSCSPIDLSLRGRVNAHTAGYKVNTTTTASSSSSSSLSSSSA
VFSSTPAAFSPPPSAHCSSV
TFSSPTAVPSASTSFSSPSSSYNTSSLDKLTETLINKWKPEPSWSQGPKSKEPEVIPDLKSHSKVTLMQLLLERRNNDNK
GNRCAANEDLPLDVTMATMS
RGPADELVSWDETRTKSPLDKLGATAQSRCSLSRDPGSSLSLHSSPSPHIQSSPLDLCKSKSFPAEKASEPAFSASKLLQ
NLAQCGTASPSPPMPLSKVQ
GQDVEVGKPLVLLERLNAPIHRNTTTPLSERPSGSGTPFSRSEASPPSSQIENLLERRTVLQLLLGTGSAAATVHRKDRP
VAGGAPRTVEWPGMSFEKSP
GTSVICDSSNGPTIDIKVKTETEEEVAQSPATSVDSAVRKRRGGPDKNSPTPDPPHDFKSEPRPAEVIAKYGLLSQLLKQ
QNATYYSSAAMQTETQPRQV
KEEQRDYQGPSPKKRRPFPDLTDSLQSIRSPRAVDSVDGGNPHPSAFQDDPPQQRRPKEEEALSGSPPSEYLARESRGFN
VLKQLLLSDNCLKELAQQPR
GAPSPSVLQTNGKANGSILSQPTHNNHNFLHSPWHPHSSLSSGLPGNLRPLPTPPACDIPLRSPWGRNVSHLSPWQVPPK
RDAPTLVKQEPESPSRWAGQ
EEEAEGCDSNPDSPRLTRSNPILYYMLQKGSAQLRKEARDQTEGNPSVVRVKEEPISDVHAYEHKLSSAPHSPSHNEKHS
HKSQRLSQLSE
>g3066.t2
MTHGEEPGPETHKDSAVLTYLEGLLMHPVVAGSGATASRRSEAAHHNQEQADKVGGSFQLPNHGPATPKAGTNGPSLGVS
QNLKKARLLRSGVWNEPNVN
QRVSPPSAELNGQGGALQNGNREGSPHAGESTLLASLLQSFSSRLQSVALSQHASKPASECSPPPKAPATEKEPLPVYGT
ASSRLKGLMRKNKLQNHSNT
PYSRRGHNQDRPPESPRSAHSATPPSAPVSTETVSCAERLKAVANMVKIRSSPAPSPKPSVACSQLALLLSSEAHLQQYS
REHALKAQLSGRSASERLAA
MATQQLGQGKRPPSAGEAPPVAQDGLNSLTTQNGKTSTNAATATLPRVAHTCPQSPSLLHSHSQSQTSPPPVAQGVSHAP
SQPPREKRGFDSRPMRPPQT
CSSLLLLLLNNHNNQKQLTKNGHLEESCGLLPPSGSSSVTSDSECSTQERSVAKDSSDAESTYSSCSPIDLSLRGRVNAH
TAGYKVNTTTTASSSSSSSL
SSSSAVFSSTPAAFSPPPSAHCSSVTFSSPTAVPSASTSFSSPSSSYNTSSLDKLTETLINKWKPEPSWSQGPKSKEPEV
IPDLKSHSKVTLMQLLLERR
NNDNKGNRCAANEDLPLDVTMATMSRGPADELVSWDETRTKSPLDKLGATAQSRCSLSRDPGSSLSLHSSPSPHIQSSPL
DLCKSKSFPAEKASEPAFSA

SKLLQNLAQCGTASPSPPMPLSKVQGQDVEVGKPLVLLERLNAPIHRNTTTPLSERPSGSGTPFSRSEASPPSSQIENLL
ERRTVLQLLLGTGSAAATVH
RKDRPVAGGAPRTVEWPGMSFEKSPGTSVICDSSNGPTIDIKVKTETEEEVAQSPATSVDSAVRKRRGGPDKNSPTPDPP
HDFKSEPRPAEVIAKYGLLS
QLLKQQNATYYSSAAMQTETQPRQVKEEQRDYQGPSPKKRRPFPDLTDSLQSIRSPRAVDSVDGGNPHPSAFQDDPPQQR
RPKEEEALSGSPPSEYLARE
SRGFNVLKQLLLSDNCLKELAQQPRGAPSPSVLQTNGKANGSILSQPTHNNHNFLHSPWHPHSSLSSGLPGNLRPLPTPP
ACDIPLRSPWGRNVSHLSPW
QVPPKRDAPTLVKQEPESPSRWAGQEEEAEGCDSNPDSPRLTRSNPILYYMLQKGSAQLRKEARDQTEGNPSVVRVKEEP
ISDVHAYEHKLSSAPHSPSH
NEKHSHKSQRLSQLSE
>g3067.t1
MVVSVWRDPQEDVAGGPPNGPNPAAQPAREQQQAASAAPHTPQTPSQPGPPSTPGAAGDKGSQNSGQSQQHIECVVCGDK
SSGKHYGQFTCEGCKSFFKR
SVRRNLTYTCRANRNCPIDQHHRNQCQYCRLKKCLKVGMRREAVQRGRMPPTQPNPGQYALTNGDPLNGHCYLSGYISLL
LRAEPYPTSRYGSQCMQPNN
IMGIENICELAARLLFSAVEWARNIPFFPDLQITDQVSLLRLTWSELFVLNAAQCSMPLHVAPLLAAAGLHASPMSADRV
VAFMDHIRIFQEQVEKLKAL
HVDSAEYSCIKAIVLFTSGYHEQQEK
>g3067.t2
MVVSVWRDPQEDVAGGPPNGPNPAAQPAREQQQAASAAPHTPQTPSQPGPPSTPGAAGDKGSQNSGQSQQHIECVVCGDK
SSGKHYGQFTCEGCKSFFKR
SVRRNLTYTCRANRNCPIDQHHRNQCQYCRLKKCLKVGMRREAVQRGRMPPTQPNPGQYALTNGDPLNGHCYLSGYISLL
LRAEPYPTSRYGSQCMQPNN
IMGIENICELAARLLFSAVEWARNIPFFPDLQITDQVSLLRLTWSELFVLNAAQCSMPLHVAPLLAAAGLHASPMSADRV
VAFMDHIRIFQEQVEKLKAL
HVDSAEYSCIKAIVLFTSGDTSLSPWPDDDVGQLMHL
>g3067.t3
MAMVVSVWRDPQEDVAGGPPNGPNPAAQPAREQQQAASAAPHTPQTPSQPGPPSTPGAAGDKGSQNSGQSQQHIECVVCG
DKSSGKHYGQFTCEGCKSFF
KRSVRRNLTYTCRANRNCPIDQHHRNQCQYCRLKKCLKVGMRREAVQRGRMPPTQPNPGQYALTNGDPLNGHCYLSGYIS
LLLRAEPYPTSRYGSQCMQP
NNIMGIENICELAARLLFSAVEWARNIPFFPDLQITDQVSLLRLTWSELFVLNAAQCSMPLHVAPLLAAAGLHASPMSAD
RVVAFMDHIRIFQEQVEKLK
ALHVDSAEYSCIKAIVLFTSGYHEQQEK
>g3068.t1
MCRRWNSHLQECRRTHFSRSSGRRDDMLLCMMESWIGESVGSRLIQPSHPAMSKSLKKKSHWTNKVHESVLCRNTEGVLG
FELKGGAENGQFPVIGEVTS
GKVVCQTSGRLSQDELLLEVNETPVAGLTTRDVHAVVRHCKDPVRLKCVRQG
>g3069.t1
LYGHQQVSCRDNNHSIDLQGANQLVAQEAVRKPSTLRPPPARTGEPTASKS
>g3070.t1
LFRGDRDRDANTTTRGDNGVYGVGTTHGDRTTYGDRTTYRDRTTHGVGTTHGVGTTHGDRTTYGDRTTYRDRTTHGVSTT
HGDRTTHGDRTTYRDRTTHG
VSTTHGDRTTHGVGTTHRDRTTHGVGTTHGVGTTHRDRTTHGVGTTHRDRTTHRDRTTHGVRT
>g3071.t1
MASDGSSSGSSGSIPDTQQIAQQRLRIIAGHLQKPLDTSDGDIRALECGAPDHQPGAATTKTVPRRRQEVMKWNGWGYSD
SRFHFNKKGQAEFTGK
>g3071.t2
MASDGSSSGSSGSIPDTQQIAQQRLRIIAGHLQKPLDTSDGDIRALECGAPDHQPGAATTKTVPRRSVILYGKKPQEFDI
ESWPAKRSLQTRQHTAIVGP
QEVMKWNGWGYSDSRFHFNKKGQAEFTGK
>g3072.t1
MTRRLMFPGLSLLLPFYDPLCAFVLGRSWSPHVVYLYQGHGVLGLLTDAGAASLKAGRCHCGFPNKVL
>g3073.t1
MMSQALKATFSGFTKEQQRLGIPKDPRQWTDSHVAEWLTWTVNEFSLKNVDYHKFRMNGASLCALGKERFLDLAPDFVGD

ILWEHLEMLQKGYGIEHAQC
VPPSEYSEPGFITESYQTLHPISSEDLISLKYESEYPSVILRDATLNSLQNEYFSVKQEVVSPDNMCVGRLSRGKLGGQD
SFESIDSLESCDRLTHSWSS
QSSFSSLQRVPSYDSFESEDYPSALHGHKPKGTFKDYVRERSDLNKDKPVIPAAALAGYT
>g3073.t2
MMSQALKATFSGFTKEQQRLGIPKDPRQWTDSHVAEWLTWTVNEFSLKNVDYHKFRMNGASLCALGKERFLDLAPDFVGD
ILWEHLEMLQKEYSEPGFIT
ESYQTLHPISSEDLISLKYESEYPSVILRDATLNSLQNEYFSVKQEVVSPDNMCVGRLSRGKLGGQDSFESIDSLESCDR
LTHSWSSQSSFSSLQRVPSY
DSFESEDYPSALHGHKPKGTFKDYVRERSDLNKDKPVIPAAALAGYT
>g3074.t1
PQPHVQPHAPREEGPPAGEGRRCDFPVQTALKGTVEYRLEAGALKIPASYYGREAAFPRSSVWAAGEGACRALVHTQAYS
RIREGISSHGETLKP
>g3075.t1
MSMSHSSPNYDVINDKPSGLVFQNKKDRKILELNPWVASGDNWSRSEVQSDLYVQVVIYDHKTRRITLTPKPLR
>g3075.t2
MSHSSPNYDVINDKPSGLVFQNKKDRKILELNPWVASGDNWSRSEVQSDLYVQVVIYDHKTRRITLTPKPLR
>g3076.t1
CEESRLLDPPMVESGWLPDRPGKPKVVVRVNWERLVETPVREAFNSHLRKNFSCIPGEVGDIESEWVMFKTSIAEAAARS
CGRKAIGACCGGNLRTCWWT
PAVKEAVKLKKEAFRAWLAQGSPEAADRYREARRAAASAVNEAKTRVWEEFGEAMEK
>g3076.t2
CEESRLLDPPMVESGWLPDRPGKPKVVVRVNWERLVETPVREAFNSHLRKNFSCIPGEVGDIESEWVMFKTSIAEAAARS
CGRKAIGACCGGNLRTCWWT
PAVKEAVKLKKEAFRAWLAQGSPEAAD
>g3077.t1
MWEGLQPRKVINKVHDADALGGVINGSSLPSLFTSSLTDRVSTAIHPRQQGEGESPKPRHAPSKLTPREALSLGGEPELT
SPQPEDRGSGAADS
>g3078.t1
VCCTGPPILWLRGRELGFTAEGERLTGGELARSMARLRRLTLSLLTGMNRRGNAVG
>g3079.t1
RQAAVTFLIGMPCRSPRLKDLLSYTSAPLGALVHAECSGEATPGFSPRLASFISAISPVEKQAPR
>g3080.t1
MAPLSLCHLRPLKLLSSLRLQMMTKPSEQPSSSSASSSRQHQSHPEETEEELREHQGLSSRGLVIKQEPLDPQEQEERER
QTEELLFRQVGGAQEVVDWG
GLWRWWEGEKEVEGGAERGVVVEDT
>g3081.t1
MPHLLPRLVPVSRCKKYVIQRISEADPSMNVSSVDLGGRPNEQKDPQVKEVVEQLLKIADELNQNAELQ
>g3081.t2
MSLTWRSLCVPGRYVIQRISEADPSMNVSSVDLGGRPNEQKDPQVKEVVEQLLKIADELNQNAELQRYAAHVAPHREEDP
RPPPHTT
>g3082.t1
MHHEISKDSLCPISSRPARSSPPPPWLSESLRAHRTELRTAERKWKKSGCATDLSHYQHLLSDFTSTVSAAKSTFYRNKI
NSSASNPRKLFSMFSSLLNP
PSPPPPSSLTADDFADHFTKKVTDISLSLTP
>g3083.t1
VQHEVLCSAKNSYEVLEFLGRGTFGQVVKCWKRGTNDVVAVKILKNHPSYARQGQIEVGILSRLSSENAEEHNVVRALEC
FQHRRHTCLVFEMLEQNLYD
FLKQNKFNPLPLKIIRPILQQVATALKKLKCLGLIHADLKPENIMLVDPTRQPYRVKVIDFGSASHVSKAVCSTYLQSRY
YRAPEIILGLPFSEAIDMWS
LGCVMAELFLGWPLYPGALEYDQHAPLPCCRHHRHVVDANLSSIPRAAKEQGAVPRATNADVHNGCCPISPQAELKVLHM
CTFEHSECLLGRAVKVKVSG
TKGPACNARPRTPAGPGTDVRGADADHVPVQHAAVSRRNNVFLSGSGSPLILVRYISQTQGVPGERLLNKGTKTSRFFRK
ESDSPYASWRLKTTDEHEKE
TGLKSKEARKYIFSCLDDIAHVNLVMSPDPSDMHAEKADRREFVSLLKAMLLVDAEDRTAPSDVLSHPFLAMTHMLDYPH
SN

>g3083.t2
VQHEVLCSAKNSYEVLEFLGRGTFGQVVKCWKRGTNDVVAVKILKNHPSYARQGQIEVGILSRLSSENAEEHNVVRALEC
FQHRRHTCLVFEMLEQNLYD
FLKQNKFNPLPLKIIRPILQQVATALKKLKCLGLIHADLKPENIMLVDPTRQPYRVKVIDFGSASHVSKAVCSTYLQSRY
YRAPEIILGLPFSEAIDMWS
LGCVMAELFLGWPLYPGALEYDQHGIQHAPLPCCRHHRHVVDANLSSIPRAAKEQGAVPRATNADVHNGCCPISPQAELK
VLHMCTFEHSECLLGRAVKV
KVSGTKGPACNARPRTPAGPGTDVRGADADHVPVQHAAVSRRNNVFLSGSGSPLILVRYISQTQGVPGERLLNKGTKTSR
FFRKESDSPYASWRLKTTDE
HEKETGLKSKEARKYIFSCLDDIAHVNLVMSPDPSDMHAEKADRREFVSLLKAMLLVDAEDRTAPSDVLSHPFLAMTHML
DYPHSN
>g3083.t3
VQHEVLCSAKNSYEVLEFLGRGTFGQVVKCWKRGTNDVVAVKILKNHPSYARQGQIEVGILSRLSSENAEEHNVVRALEC
FQHRRHTCLVFEMLEQNLYD
FLKQNKFNPLPLKIIRPILQQVATALKKLKCLGLIHADLKPENIMLVDPTRQPYRVKVIDFGSASHVSKAVCSTYLQSRY
YRAPEIILGLPFSEAIDMWS
LGCVMAELFLGWPLYPGALEYDQHAPLPCCRHHRHVVDANLSSIPRAAKEQGAVPRATNADVHNGCCPISPQAELKVLHM
CTFEHSECLLGRAVKVKVSG
TKGPACNARPRTPAGPGTDVRGADADHVPVQHAAVSRRNNVRYISQTQGVPGERLLNKGTKTSRFFRKESDSPYASWRLK
TTDEHEKETGLKSKEARKYI
FSCLDDIAHVNLVMSPDPSDMHAEKADRREFVSLLKAMLLVDAEDRTAPSDVLSHPFLAMTHMLDYPHSN
>g3084.t1
IDVDLNDFQWAPVNHTSKSSTTTSYQSSSNVASSSAALAYEAADPFPPPPTEQELPDYCPACSLHSQGVDQRSSEQKEYY
FKHKSMAELKRLYKHIHPDV
RKNLEADFLSQLTEEERARFETEGERVEGEVQQTCYLFENDGGSPDRGSSPEGDYLEWEEILKGEVQSMRWMFENKPLDT
IKDDTPGGGEDNNIAQQEII
AGKDVRYTAWMFENQPMGTRGTETPETTGQSEKVKELARGDVRTATWLFETQPLDYLNKIYQEEDEQVIEVVIDKDISGG
DVKTARYLFETQHLDSLGKT
ETIDESHFLNLKSELEEIKGEVKTTTCMFETQPMCVIRGDSGEMLEITKIRREETEKGDVKTSRWMFETQALDMIHQDPS
KVKLICGVSMEEDVQGGVNK
GKWLFETKTLDAIKEEDWECSKREREEIIGADVRQHCRVFETQPMDTLKDNANARPLCAEEIVGGDVRSAKYLFETVPME
NLKDLLEVGKLQKMVASEEE
KGDVRHQKWMFESQPLSNIREEKREITRTVNMEALDKGDVTNYKERFETMDLSKCEGTQRIQVEGVTSGSVRSNKVLFES
TPMYAMQDSAGHYHEVKTVR
REEVVKGDVRSCRWMFETRPIDEFDESIDKFQIIKGISKQEIQSGDVKTAKWLFETQALDAIKYEHEERQAKEDMEIEKG
DVKTCRWLFETQPMDILYEK
EEKSETDVQQVQKGDVKTCTWLFETQTLDNIRDHAETESETILKTCTINQEDIQGKDVRLARFLFETENLDSLSGEDSGS
FRRVTEIDIDSGDVSRMKYI
FENRSSDIMTSTSDETMQRLKTKQAEDIQKGNVNNCTWMFENQAIDTIKEDETRESRTVTDVQGGNVDKGRFIFETYSLD
QIQDENTETGISKLTSVLSK
EIQKGDVKNYTMMFETQPLYAIRDKDGHYHEVTTVTKEEVMRGDVKGARWQFETKPLDSIRDSEEVYVIKAVTEEGINKG
DVSSARWRFETQPLDEIAED
MKVRSKTVEDVQGGDVKSNKQRFEADETSPKYVRTVSVSEIQKGDVRSATWMFETRTIDKIHSEAAEYNGMETVTKEDVI
KGDVTQSVWLFEKQPLDQIR
EKGDEDDMERVVTKEVIPQADVKSTTWLFETTPVHEFNESSTERTAVTGKSIKETLEELYCQKMVDSQGILIEADEIGDV
RMAKFKLMNQDTPEIQKEEV
IRGDLNNIMMNLLNRRESTEKGITVDVEERGNIDTTVRQLFSQEKAANIEKEAIIRGDIQEAINNLLKQEGSSKRGLLIQ
EDEKGDVRMTIYSLLNKTES
DSVEREDVIQGNVSKTLHRLMSNPDADQARKIKVGDTERGNVSFYSTCIESGALDYLRQLRCESDDAPEKSEKERIVGGD
VEETKLILRRNHQQVGRTVA
EDDIVPGDVHSTVQVFMSEPNVSYRNLEKQDIVKGDLWAAMDSLTQAANQRVVMEKEEVVKGDLTLTLRSLQEAQHQAKD
VEKPDIIRGDIRGALQSLEK
SATTKTEATVEDLVPGDIKGTLRSLEEAKQAVKEVEKEEIVKGDIYAAMQNLHEVSSERKLYQHQASEQGDVKGTIQLLL
EPSTSTRTQRRRSIEGDVKT
SIKTLYEGQEMTQAEKEEVIKGDVQGAIKCLMQKKQYSSPKRMSSKRVKVAVKAPPHTREVEHTCAHTCAHAAKSASVTV
AATPAVTNLPQSVQTQRHIQ

KHGESRSVKSEAVIQEGSGHSQRMSTREESVTLPQPQAQIVPATLVSVQNKQTPNSPQPDGKSAVDTKVKQGTEQSVSTK
QTSVSTKQTSVSTKRTQETR
TVKHVHAAVVERQAVSTTSSVTSSSAQGLNVSASSERVSTSQRQSGITENKALTQKQDIKSGRGEYRNLDNKGKGLIKKK
KPEINFPPPPSSPPPPSESE
FSL
>g3084.t2
QQYEKTIEDATPSKQVKIDVDLNDFQWAPVNHTSKSSTTTSYQSSSNVASSSAALAYEAADPFPPPPTEQELPDYCPACS
LHSQGVDQRSSEQKEYYFKH
KSMAELKRLYKHIHPDVRKNLEADFLSQLTEEERARFETEGERVEGEVQQTCYLFENDGGSPDRGSSPEGDYLEWEEILK
GEVQSMRWMFENKPLDTIKD
DTPGGGEDNNIAQQEIIAGKDVRYTAWMFENQPMGTRGTETPETTGQSEKVKELARGDVRTATWLFETQPLDYLNKIYQE
EDEQVIEVVIDKDISGGDVK
TARYLFETQHLDSLGKTETIDESHFLNLKSELEEIKGEVKTTTCMFETQPMCVIRGDSGEMLEITKIRREETEKGDVKTS
RWMFETQALDMIHQDPSKVK
LICGVSMEEDVQGGVNKGKWLFETKTLDAIKEEDWECSKREREEIIGADVRQHCRVFETQPMDTLKDNANARPLCAEEIV
GGDVRSAKYLFETVPMENLK
DLLEVGKLQKMVASEEEKGDVRHQKWMFESQPLSNIREEKREITRTVNMEALDKGDVTNYKERFETMDLSKCEGTQRIQV
EGVTSGSVRSNKVLFESTPM
YAMQDSAGHYHEVKTVRREEVVKGDVRSCRWMFETRPIDEFDESIDKFQIIKGISKQEIQSGDVKTAKWLFETQALDAIK
YEHEERQAKEDMEIEKGDVK
TCRWLFETQPMDILYEKEEKSETDVQQVQKGDVKTCTWLFETQTLDNIRDHAETESETILKTCTINQEDIQGKDVRLARF
LFETENLDSLSGEDSGSFRR
VTEIDIDSGDVSRMKYIFENRSSDIMTSTSDETMQRLKTKQAEDIQKGNVNNCTWMFENQAIDTIKEDETRESRTVTDVQ
GGNVDKGRFIFETYSLDQIQ
DENTETGISKLTSVLSKEIQKGDVKNYTMMFETQPLYAIRDKDGHYHEVTTVTKEEVMRGDVKGARWQFETKPLDSIRDS
EEVYVIKAVTEEGINKGDVS
SARWRFETQPLDEIAEDMKVRSKTVEDVQGGDVKSNKQRFEADETSPKYVRTVSVSEIQKGDVRSATWMFETRTIDKIHS
EAAEYNGMETVTKEDVIKGD
VTQSVWLFEKQPLDQIREKGDEDDMERVVTKEVIPQADVKSTTWLFETTPVHEFNESSTERTAVTGKSIKETLEELYCQK
MVDSQGILIEADEIGDVRMA
KFKLMNQDTPEIQKEEVIRGDLNNIMMNLLNRRESTEKGITVDVEERGNIDTTVRQLFSQEKAANIEKEAIIRGDIQEAI
NNLLKQEGSSKRGLLIQEDE
KGDVRMTIYSLLNKTESDSVEREDVIQGNVSKTLHRLMSNPDADQARKIKVGDTERGNVSFYSTCIESGALDYLRQLRCE
SDDAPEKSEKERIVGGDVEE
TKLILRRNHQQVGRTVAEDDIVPGDVHSTVQVFMSEPNVSYRNLEKQDIVKGDLWAAMDSLTQAANQRVVMEKEEVVKGD
LTLTLRSLQEAQHQAKDVEK
PDIIRGDIRGALQSLEKSATTKTEATVEDLVPGDIKGTLRSLEEAKQAVKEVEKEEIVKGDIYAAMQNLHEVSSERKLYQ
HQASEQGDVKGTIQLLLEPS
TSTRTQRRRSIEGDVKTSIKTLYEGQEMTQAEKEEVIKGDVQGAIKCLMQKKQYSSPKRMSSKRVKVAVKAPPHTREVEH
TCAHTCAHAAKSASVTVAAT
PAVTNLPQSVQTQRHIQKHGESRSVKSEAVIQEGSGHSQRMSTREESVTLPQPQAQIVPATLVSVQNKQTPNSPQPDGKS
AVDTKVKQGTEQSVSTKQTS
VSTKQTSVSTKRTQETRTVKHVHAAVVERQAVSTTSSVTSSSAQGLNVSASSERVSTSQRQSGITENKALTQKQDIKSGR
GEYRNLDNKGKGLIKKKKPE
INFPPPPSSPPPPSESEFSL
>g3085.t1
MLKGLSRVIKAGFGLRLGQDGVGVRVFYSSPSQKVNGVARLYQQLEVEVAFANPLPETLRNCIFTAEGSGLLLDPVEERL
PDLKPNRGIRVVLSFFPYRL
GQKTLAVDFDCSTFKNVKASITINVTQ
>g3085.t2
SQSHSEDDDDEGPVRAWPSQYSLHNTEDSRVIKAGFGLRLGQDGVGVRVFYSSPSQKVNGVARLYQQLEVEVAFANPLPE
TLRNCIFTAEGSGLLLDPVE
ERLPDLKPNRGIRVVLSFFPYRLGQKTLAVDFDCSTFKNVKASITINVTQ
>g3085.t3
SQSHSEDDDDEGPVRAWPSQYSLHNTEDRVFYSSPSQKVNGVARLYQQLEVEVAFANPLPETLRNCIFTAEGSGLLLDPV
EERLPDLKPNRGIRVVLSFF

PYRLGQKTLAVDFDCSTFKNVKASITINVTQ
>g3086.t1
MPPRCRWLELKQAGNECFKTGQYGEASERYSQAIKELEKAGKKSPDDLSILYSNRAASYLKDGNCSECVKDCNMSLDLVP
FGVKSLLRRAAAYEAMERYR
QAYVDYKTALLVDCNIPAAHDGTNRMTKVLTEMDGLRWREKLPAIPVVPMEVRERLAQQARQSANASQQQHNGTGNVPGT
QSAHM
>g3086.t2
LNPPPRGPWNLLETASPSPSASTTPEDSVGRWLELKQAGNECFKTGQYGEASERYSQAIKELEKAGKKSPDDLSILYSNR
AASYLKDGNCSECVKDCNMS
LDLVPFGVKSLLRRAAAYEAMERYRQAYVDYKTALLVDCNIPAAHDGTNRMTKVLTEMDGLRWREKLPAIPVVPMEVRER
LAQQARQSANASQQQHNGTG
NVPGTQSAHM
>g3086.t3
MPPRCRWLELKQAGNECFKTGQYGEASERYSQAIKELEKAGKKSPDDLSILYSNRAASYLKDGNCSECVKDCNMSLDLVP
FGVKSLLRRAAAYEAMERYR
QAYVDYKTALLVDCNIPAAHDGTNRMTKVLTEMDGLRWREKLPAIPVVPMEVRERLAQQARQSANASQQQHNGTGNVP
>g3087.t1
RFQPHLGVEDGVIYLLSKAYSHLDKPASTVRVMFFDFSGAFNTLRPGEKMAAMQVEAALVSWIVDYLSGRPQFVRIQNCV
SDKVVSNTGAPQGTVLSPFL
FTLYTSDFNYCTESCHLQKFSDDSTIVGCIKQ
>g3087.t2
FQPHLGVEDGVIYLLSKAYSHLDKPASTVRVMFFDFSGAFNTLRPGEKMAAMQVEAALVSWIVDYLSGRPQFVRIQNCVS
DKVVSNTGAPQGTVLSPFLF
TLYTSDFNYCTESCHLQKFSDDSTIVGCIKQ
>g3088.t1
LFTDSTLRVQDVLKEFHSGDHQGQAKFGEFIDAAGFSLFLKTYLEEDDFPEDLCQRLFRYFQHFEHNEAESQNGE
>g3088.t2
LVHIELLLTTWKNYVLFTDSTLRVQDVLKEFHSGDHQGQAKFGEFIDAAGFSLFLKTYLEEDDFPEDLCQRLFRYFQHFE
HNEAESQNGE
>g3088.t3
LLTTWKNYVLFTDSTLRVQDVLKEFHSGDHQGQAKFGEFIDAAGFSLFLKTYLEEDDFPEDLCQRLFRYFQHFEHNEAES
QNGE
>g3089.t1
MALVSGISLDPEAAIGVTKKLPPKWVDGVDEIQYEITRVRQKMKDLALLHDKHMNRPTLDDSTEEEHAIEITTQEITQRA
VRGMQARCGHCTEQEERLLR
NVVSSLAQSLQELSTGFRHTQSSYLKRMKNREERSKHFFDSGPLLEEDAELALFDRGFTDDQLVLVETNTVMVEEREKEI
RQVVQSISDLNEIFRDLAGM
VVEQVCFKFIVTC
>g3089.t2
MALVSGISLDPEAAIGVTKKLPPKWVDGVDEIQYEITRVRQKMKDLALLHDKHMNRPTLDDSTEEEHAIEITTQEITQRA
VRGMQARCGHCTEQEERLLR
NVVSSLAQSLQELSTGFRHTQSSYLKRMKNREERSKHFFDSGPLLEEDAELALFDRGFTDDQLVLVETNTVMVEEREKEI
RQVVQSISDLNEIFRDLAGM
VVEQ
>g3089.t3
MALVSGISLDPEAAIGVTKKLPPKWVDGVDEIQYEITRVRQKMKDLALLHDKHMNRPTLDDSTEEEHAIEITTQEITQMF
HRCQRAVRGMQARCGHCTEQ
EERLLRNVVSSLAQSLQELSTGFRHTQSSYLKRMKNREERSKHFFDSGPLLEEDAELALFDRGFTDDQLVLVETNTVMVE
EREKEIRQVVQSISDLNEIF
RDLAGMVVEQVCFKFIVTC
>g3090.t1
MAKRPGTDDGMLVSPTILRDFSVGVNAGDGNMDCIDGAAELSLEEILVLYSQPINEEQAWAVCYQCCRTLARTRRGRNAK
SADASSSVGDGERRIDGPGD
VRIQRDGTVLLQYQDTTGHRPNEASQHSGHIVALSAF
>g3090.t2
MAKRPGTDDGMLVSPTILRDFSVGVNAGDGNMDCIDGAAELSLEEILVLYSQPINEEQAWAVCYQCCRTLARTRRGRNAK

SADASSSVGDGHRPNEASQH
SGHIVALSAF
>g3091.t1
DLRGNSFQCNCETKWLMVWLKNTNATASDVLCSGPADMKGKRLNDLPLPPGQCIST
>g3091.t2
LLSTPSPTPRDLRGNSFQCNCETKWLMVWLKNTNATASDVLCSGPADMKGKRLNDLPLPPGQCIST
>g3092.t1
DIAQRYRISKYPTLKLFRNGMMMKREYRGQRSVAAIADFIRQQKVDPIKEVLSLEEVNKVDRSKRNIIGYFERKESDSYH
TFEKVANILRDDCVFFAAFG
EVSQVERISGDNVIFKPVGETVPDMVFLGSLPNFDLSYAWVQDKCVPLVREITFENGEELTEEGIPFLILFHVKEDTDSL
DKFQQEVARQLISEKGTINF
LHADCEKFRHPLLHIQKTPAECPVIAIDSFRHMYVFPEFKDLM
>g3093.t1
SEVAILYNDRSVLENHHVSAAYRLMAEDEMNIFTNLNKEDWRELRALVIEMVMSTDMSCHFQQIKTIRNHLPQSHEMDKV
KVLSLMLHAADISHPAKSWS
LHHTWTNNLMEEFFRQGDKEVQLGLPFSPLCDRKATMIGQSQIGFIDFIVEPTFSILVDATEKVMLPLIDEERRARESGH
RRSSLTGSGSVTVESVQRHS
SGRHGNSEDGQTDFSLAAVDLPSLRLRLSGLISSNKERWKELSTQGAGPEEDVIGDA
>g3093.t2
SEVAILYNDRSVLENHHVSAAYRLMAEDEMNIFTNLNKEDWRELRALVIEMVMSTDMSCHFQQIKTIRNHLPQSHEMDKV
KVLSLMLHAADISHPAKSWS
LHHTWTNNLMEEFFRQGDKEVQLGLPFSPLCDRKATMIGQSQIGFIDFIVEPTFSILVDATEKVMLPLIDEERRARESGH
RRSSLTGSGSVTVESVQRHS
SGRHGNSEDGQTDFSLAAVDLPSLRLRLSGLISSNKERWKELSTQAKKEPESSPRKGEPSDSTGREEACSTSPDGQHSPH
VKLNSDAVWAGWAHQDEEKL
SVGQVELKWFTFIQMEVAKCSSLASCLHSTPRRRTVCCYLRLASCKRWGSQLLEVKWKSEV
>g3093.t3
SEVAILYNDRSVLENHHVSAAYRLMAEDEMNIFTNLNKEDWRELRALVIEMVMSTDMSCHFQQIKTIRNHLPQSHEMDKV
KVLSLMLHAADISHPAKSWS
LHHTWTNNLMEEFFRQGDKEVQLGLPFSPLCDRKATMIGQSQIGFIDFIVEPTFSILVDATEKVMLPLIDEERRARESGH
RRSSLTGSGSVTVESVQRHS
SGRHGNSEDGQTDFSLAAVDLPSLRLRLSGLISSNKERWKELSTQAKKEPESSPRKGEPSDSTGREEACSTSPDGQHSPH
VAAPLENTTWNGVVKLNSDA
VWAGWAHQDEEKLSVGQVELKWFTFIQMEVAKCSSLASCLHSTPRRRTVCCYLRLASCKRWGSQLLEVKWKSEV
>g3094.t1
MDELESDWLPRDQNPNDTRQLFKTGRL
>g3095.t1
FSLWDLLVLPTEQHPFLKIAKPLSSLNPLILAAKEAMKGNR
>g3096.t1
MSGLNASLGYFISTVAFGVSSRVLLSRWPRLDFLLELAASFVLAACLLEVQTIVEVGQWAGGLGPDVCVTILFVVLLTHG
VACGSASGNPTLTLVRFLTL
EATPVPTVLAVAGQFVGAHVALLAALHYWGLELTDMHMIKNLMATSCSTSLQVSVLQGFFTECVCALAFHLAHLSLRRRS
ALIRVPLVAVLLTFLSHAAS
GYTSAYMNPSLAYSLTFHCPGFTLTEYATVYWLGPLTGMSMAVFLYMGHIPRVFSKNLLYSQKTRFRVPKGNSSGEQEDK
KKK
>g3097.t1
LSADGSRAILGESLSHEDTSSWSGSLSSPPTPFKAFTEGDEQPYSLSESLGKLVCQQLQQGNVVRVGSHRSRRRDELQRR
DAKIVTPRARSAYIPLPIVS
ETCPVLSSGGEEYKHTLFGSSGFHTLSPSQPIPPPQRRTVLSAIVRITDSLEAGAVGKKRSQGWRERPPVTAEAWR
>g3097.t2
MLDCDGAASGWILFFPKKEKLLSQGLSADGSRAILGESLSHEDTSSWSGSLSSPPTPFKAFTEGDEQPYSLSESLGKLVC
QQLQQGNVVRVGSHRSRRRD
ELQRRDAKIVTPRARSAYIPLPIVSETCPVLSSGGEEYKHTLFGSSGFHTLSPSQPIPPPQRRTVLSAIVRITDSLEAGAV
GKKRSQGWRERPPVTAEAWR
>g3097.t3
LSADGSRAILGESLSHEDTSSWSGSLSSPPTPFKAFTEGDEQPYSLSESLGKLVCQQLQQGNVVRVGSHRSRRRIVSETC

PVLSSGEEYKHTLFGSSGFH
TLSPSQPIPPPQRRTVLSAIVRITDSLEAGAVGKKRSQGWRERPPVTAEAWR
>g3098.t1
MSGFAEFVPPPECPVFEPSWDDFSDPLGFINKIRPIAEKTGICKIRPPEDWQPPFACDVHNFRFTPRVQRLNELEALTRV
RLNFLDQIAKFWELQGSKIR
FPHVERKVLDLYRLSKVTHP
>g3098.t2
LGFINKIRPIAEKTGICKIRPPEDWQPPFACDVHNFRFTPRVQRLNELEALTRVRLNFLDQIAKFWELQGSKIRFPHVER
KVLDLYRLSKVTHP
>g3098.t3
MSGFAEFVPPPECPVFEPSWDDFSDPLGFINKIRPIAEKTGICKIRPPEDWQPPFACDVHNFRFTPRVQRLNELEALTRV
RLNFLDQIAKFWELQGSKIR
FPHVERKVLDLYRLSK
>g3099.t1
LVPLPRVPHLPPRVHNYPGLQLPGSQGRISPKRHHIPSNTYKQHIRWHRRPDTPGSSNVRCGVDSAGLVVFQFCWVLQSL
FKLSKADFYSWVRSTGNVC
>g3100.t1
MLSGLMVHLPVYKGGGEYSHITLLFCCDFPDVGRAVYAPEHITERVVQLFRSKTEFTFLASIQQKASTSGVIFSIHESEH
R
>g3101.t1
MFRIAAFFALLVCACSAESCTDPVITPSAYTTNDAVISSESVFIVELSLACANGAQSVALYADVNGRQFPVTRGQDVGKY
QVSWSLPHKQASSGTYQVKF
FDEESYSSLRKAQRNNEDVDAIQPLFSVNVDHRGAWNGPWVSTEVLAALIGILVYYLAFSAKSTIQA
>g3101.t2
ESCTDPVITPSAYTTNDAVISSESVFIVELSLACANGAQSVALYADVNGRQFPVTRGQDVGKYQVSWSLPHKQASSGTYQ
VKFFDEESYSSLRKAQRNNE
DVDAIQPLFSVNVDHRGAWNGPWVSTEVLAALIGILVYYLAFSAKSTIQA
>g3101.t3
MFRIAAFFALLVCACSAESCTDPVITPSAYTTNDAVISSESVFIVELSLACANGAQSVALYADVNGRQFPVTRGQDVGKY
QVKFFDEESYSSLRKAQRNN
EDVDAIQPLFSVNVDHRGAWNGPWVSTEVLAALIGILVYYLAFSAKSTIQA
>g3102.t1
NQAMVSHSAVSVRILDVNDNPPELATPYEASICEDAKPGQASPLECTP
>g3103.t1
MSFLRRVAGLSLRDRVRSSDIRRELGVETLLLRVERSQLRWFGHLIRMPPGRLPLEVFRARPTGKRPRGRPRTRWRDYIS
HLAWECLGVPQEELESVAGE
KDVWSSLLSLLPPRPGPV
>g3104.t1
MKYGLPLVPKPTGSRQSFPPDYKLFTGSAGVSIPNKPTRSRLGGPDPLDYISMYRNIGSPPQDIQEHWHYVSFGLSDLYG
DNRVHEFTGSDGPSGFGFEL
TFRLKREVGETAPPTWPAELMQGLARYVFQSGKTAHV
>g3104.t2
MKYGLPLVPKPTGSRQSFPPDYKLFTGSAGVSIPNKPTRSRLGGPDPLDYISMYRNIGSPPQDIQEHWHYVSFGLSDLYG
DNRVHEFTGSDGPSGFGFEL
TFRLKREVGETAPPTWPAELMQGLARYVFQS
>g3105.t1
MPAAYKGECREEDDPMPFLVPTEKERMDAINKPYDIKRSCWVRDERAAFVAGEIQSEDGDKVTVRTTKSTTVTVKKDDIQ
QMNPPKFFQASDMANLTFLN
EASVMENLRSRYVNMRIYVRLRLRPAAAGKGWRRWGLFAVNLVMMCRTDNRFALQRLFCVTINPYQWLPIYGAKVAQIYK
GKKRNEVPPHLFSVSDNAYH
DMMIG
>g3105.t2
MPAAYKGECREEDDPMPFLVPTEKERMDAINKPYDIKRSCWVRDERAAFVAGEIQSEDGDKVTVRTTKSTTVTVKKDDIQ
QMNPPKFFQASDMANLTFLN
EASVMENLRSRYVNMRIYVRLRLRPAAAGKGWRRWGLFAVNLVMMCRTDNRFALQRNNTAFCLFCVTINPYQWLPIYGAK
VAQIYKGKKRNEVPPHLFSV

SDNAYHDMMIG
>g3106.t1
LQRDNTHSLINFLTSPGHHSHRLLKFQIHFNLTIRIRSILQFILMVFHRSLHHQLQRLKCKNL
>g3106.t2
RDNTHSLINFLTSPGHHSHRLLKFQIHFNLTIRIRSILQFILMVFHRSLHHQLQRLKCKNL
>g3107.t1
MRALGIAVTLGLLLVSARALSLPLLLALSTTSSSRPPCDSPEVEAAAMEAQDFLNAQHTHGYKYVLNRIDDVRVINTSKG
EELYIMEVELLETNCHVLDP
KPLANCTVRPKRLTAVEGDCDVVLKKEGGVLSVTAFKCKTEESTEDLCLGCPTLLPLNHTSGLAFVHQSLGKINNKLNRT
FIVTEVGRMSQQLVSLGYKF
VAEYVIAEAECGAGDCVPLDNASTNVPEVNSTAASLLPVIHATHGLNHHKLTHIHDPEFTGLLSAESVESTESAEVMLVV
AESSQTSVPADAPPTIVDAV
VEVEIPPKDLPDEVMPAVEPTLGVDLPAGDDATKAVHKDSSAFQTRAVMMYPKMSCRDLAVHYRELAAEN
>g3107.t2
MRALGIAVTLGLLLVSARALSLPLLLALSTTSSSRPPCDSPEVEAAAMEAQDFLNAQHTHGYKYVLNRIDDVRVINTSKG
EELYIMEVELLETNCHVLDP
KPLANCTVRPKRLTAVEGDCDVVLKKEGGVLSVTAFKCKTEESTEDLCLGCPTLLPLNHTSGLAFVHQSLGKINNKLNRT
FIVTEVGRMSQQLVSLGYKF
VAEYVIAEAECGAGDCVPLDNASTNVPEVNSTAASLLPVIHATHGLNHHKLTHIHDPEFTGLLSAESVESTESAEVMLVV
AESSQTSVPADAPPTIVDAV
VEVEIPPKDLPDEVMPAVEPTLGVDLPAGDDATKAVVHKDSSAFQTRAVMMYPKMSCRDLAVHYRELAAEN
>g3107.t3
MRALGIAVTLGLLLVSARALSLPLLLALSTTSSSRPPCDSPEVEAAAMEAQDFLNAQHTHGYKYVLNRIDDVRVINTSKG
EELYIMEVELLETNCHVLDP
KPLANCTVRPKRLTAVEGDCDVVLKKEGGVLSVTAFKCKTEESTEDLCLGCPTLLPLNHTSGLAFVHQSLGKINNKLNRT
FIVTEVGRMSQQLVSLGYKF
VAEYVIAEAECGAGDCVPLDNASTNVPEVNSTAASLLPVIHATHGLNHHKLTHIHDPEFTGLLSAESVESTESAEVMLVV
AESSQTSVPADAPPTIVDAV
VEVEIPPKDLPDEVMPAVEPTLGVDLPAGDDATKAHKDSSAFQTRAVMMYPKMSCRDLAVHYRELAAEN
>g3108.t1
MARQRGFRGTLSSAKDACSPCGGCGRGAAAAERDVVSSPRGGRASNAEDPQCAPATSPVKSACRREEQQAGTDTSRNTLQ
TKNKGQRQVLWAVEDLKQAV
KKSVGLLLHQPQPPAAPGRGGAGEGARTDVCSTESRRRLAIRRPASARGKPPPPRVQSRRRRMERPASERVPARSARACR
GAGCPLNAGRTPESPTKFLS
GFCRAYGSLIPLQKCDIRRSLRWPEMDVRERYDGSARGPMGSDSAVLTLAGSSFHHCGAKTEKS
>g3109.t1
MIPQPADGCGRAAVLPQIRTSVWLSEWDAERAAGKQMPLRSALSVVLEADSYREHSPQGGADGNDDSRPLNAHQRTSRWV
GSAAVPRRRYPPHVRAQAYI
TGWSGEVSTPRTAAEHCSYYTMPVVLRPWMRLAALVLCALVCLSRVADAYPPKPESPGTDAAPEDWAKYHTALRHYVNLI
TRQRDTGALWEQRLAFLRLK
DKVQT
>g3109.t2
MWWCALRRALSVVLEADSYREHSPQGGADGNDDSRPLNAHQRTSRWVGSAAVPRRRYPPHVRAQAYITGWSGEVSTPRTA
AEHCSYYTMPVVLRPWMRLA
ALVLCALVCLSRVADAYPPKPESPGTDAAPEDWAKYHTALRHYVNLITRQRDTGALWEQRLAFLRLKDKVQT
>g3109.t3
MIPQPADGCGRAAVLPQIRTSVWLSEWDAERAAGKQMPLRSALSVVLEADSYRDFWAVFREARRPCDAVIGDGRQSSAEM
IPVHYNSEHSPQGGADGNDD
SRPLNAHQRTSRWVGSAAVPRRRYPPHVRAQAYITGWSGEVSTPRTAAEHCSYYTMPVVLRPWMRLAALVLCALVCLSRV
ADAYPPKPESPGTDAAPEDW
AKYHTALRHYVNLITRQRDTGALWEQRLAFLRLKDKVQT
>g3110.t1
MTDSGKDKALFLRQSLCSRSGGGGAPVVRAVPNSGSHPTLPPSALQGNKKSIAKHNSFVPKDQLISLLLPCGGKQCVKKN
QQRQMRRSAEISPVASRSTP
SLCPAAGTGSQSCRERERVKPHAPCSSIPLNYLSWSTGSSITKKEEKVGWRNHNSVLAPQWWNELPANVRTAESLPIFQK
RLKTHLFRVHLNPA

>g3110.t2
LMTDSGKDKALFLRQSLCSRSGGGGAPVVRAVPNSGSHPTLPPSALQGNKKSIAKHNSFVPKDQLISLLLPCGGKQCVKK
NQQRQMRRSAEISPVASRST
PSLCPAAGTGSQSCRERERVKPHAPCSSIPLNYLSWSTGSSITKKEEKVGWRNHNSVLAPQWWNELPANVRTAESLPIFQ
KRLKTHLFRVHLNPA
>g3110.t3
MTDSGKDKALFLRQSLCRSGGGGAPVVRAVPNSGSHPTLPPSALQGNKKSIAKHNSFVPKDQLISLLLPCGGKQCVKKNQ
QRQMRRSAEISPVASRSTPS
LCPAAGTGSQSCRERERVKPHAPCSSIPLNYLSWSTGSSITKKEEKVGWRNHNSVLAPQWWNELPANVRTAESLPIFQKR
LKTHLFRVHLNPA
>g3111.t1
MDMEVDIITTPKGFHCNLCNTDMPNKPSLDEHVKGRKHQTLTGVRATRAAQEARSVFVSGFPKDTSQSQITEHFQRFGEV
ANVIIDKDKGVYAIVQFSEV
DSQLRALDETLHEMDGQRLRVKPREKKGFKLINKKKTDFRNLQEVLERLKPALAQAESVNEQMQAVVERFQLSENKRKAR
ELLVQLIQEVFTEFFPDSQI
LPFGSSVNTFGIHSCDLDLFLDLENTKTFQARARASTEPVAEASSDDGRSEDSILSDIDLSTASPAELLELVAAILKKCV
PGVHKVQVVASARLPVVKFH
YHQLDLQGDISLNNRPLVYTIRYWARQKQLAGNVGGAGPLLNNYALTLLVIFFLQNSDPPVLPTVDQLKDMACEEEECVI
EGWDCTFPSQAIAVPPSRNE
QDLCVLLASFFSFYATFDFAGSVISLREGRALPVTSFLGRGGGEEEEDATEAERPGPHRNPAPKLGPLNVLDPFELSHNV
AGNLSERSQQNLQRECQEAE
KYCRSLQYLRKSAKGKPWGLVRLFTPQGAGPNRKNPDGPVDKELSIDVPFKLALLPESFQHQLQRAGDGFRPLWFQRVCA
AAAQVFQDVLKCSPGPFTEP
TLPGRDAGRADTAPGSCESMDATSEELNASGDSGVAGQGSPRGSKRPLSSESSDASTSPQEKKPRLGGGPRPGSPCWTWT
QTHAVWAGRRKVRRELMMKE
AADGRPEGSSSALEVQVTDHIREKEPAVKVPLVFRVQAKLVGGTQSTKVQLKVHPPSEGRETFQDFFHFLEVFLPKMVDT
LMEKSM
>g3111.t2
MDMEVDIITTPKGFHCNLCNTDMPNKPSLDEHVKGRKHQTLTGVRATRAAQEARSVFVSGFPKDTSQSQITEHFQRFGEV
ANVIIDKDKGVYAIVQFSEV
DSQLRALDETLHEMDGQRLRVKPREKKGFKLINKKKTDFRNLQEVLERLKPALAQAESVNEQMQAVVERFQLSENKRKAR
ELLVQLIQEVFTEFFPDSQI
LPFGSSVNTFGIHSCDLDLFLDLENTKTFQARARASTEPVAEASSDDGRSEDSILSDIDLSTASPAELLELVAAILKKCV
PGVHKVQVVASARLPVVKFH
YHQLDLQGDISLNNRPLVYTIRYWARQKQLAGNVGGAGPLLNNYALTLLVIFFLQNSDPPVLPTVDQLKDMACEEEECVI
EGWDCTFPSQAIAVPPSRNE
QDLCVLLASFFSFYATFDFAGSVISLREGRALPVTSFLGRGGGEEEEDATEAERPGPHRNPAPKLGPLNVLDPFELSHNV
AGNLSERSQQNLQRECQEAE
KYCRSLQYLRKSAKGKPWGLVRLFTPQGAGPNRKNPDGPVDKELSIDVPFKLALLPESFQHQLQRAGDGFRPLWFQRVCA
AAAQVFQDVLKCSPGPFTEP
TLPGRDAGRADTAPGSCESMDATSEELNASGDSGVAGQGSPRGSKRPLSSESSDASTSPQEKKPRLGGGPRPGSPCWTWT
QTHAVWAGRRKVRRELMMKE
AADGRPEGSSSALEVQVTDHIREKEPAVKVPLVFRVQAKLVGGTQSTKVQLKVHPPSEGRETFQDFFHFLEVFLPKMVDT
LMEKRSSSEAQVMALLEVQT
HRSVGEHLARLISDPRGFMAAQADDKSLERLQRDALCTMITPLASERLS
>g3111.t3
MDMEVDIITTPKGFHCNLCNTDMPNKPSLDEHVKGRKHQTLTGVRATRAAQEARSVFVSGFPKDTSQSQITEHFQRFGEV
ANVIIDKDKGVYAIVQFSEV
DSQLRALDETLHEMDGQRLRVKPREKKGFKLINKKKTDFRNLQEVLERLKPALAQAESVNEQMQAVVERFQLSENKRKAR
ELLVQLIQEVFTEFFPDSQI
LPFGSSVNTFGIHSCDLDLFLDLENTKTFQARARASTEPVAEASSDDGRSEDSILSDIDLSTASPAELLELVAAILKKCV
PGVHKVQVVASARLPVVKFH
YHQLDLQGDISLNNRPLVYTIRYWARQKQLAGRTPCVCPGVCRHNTSGLIYCLLIVDKVVDKTTGNVGGAGPLLNNYALT
LLVIFFLQNSDPPVLPTVDQ
LKDMACEEEECVIEGWDCTFPSQAIAVPPSRNEQDLCVLLASFFSFYATFDFAGSVISLREGRALPVTSFLGRGGGEEEE
DATEAERPGPHRNPAPKLGP

LNVLDPFELSHNVAGNLSERSQQNLQRECQEAEKYCRSLQYLRKSAKGKPWGLVRLFTPQGAGPNRKNPDGPVDKELSID
VPFKLALLPESFQHQLQRAG
DGFRPLWFQRVCAAAAQVFQDVLKCSPGPFTEPTLPGRDAGRADTAPGSCESMDATSEELNASGDSGVAGQGSPRGSKRP
LSSESSDASTSPQEKKPRLG
GGPRPGSPCWTWTQTHAVWAGRRKVRRELMMKEAADGRPEGSSSALEVQVTDHIREKEPAVKVPLVFRVQAKLVGGTQST
KVQLKVHPPSEGRETFQDFF
HFLEVFLPKMVDTLMEKSM
>g3112.t1
LLAEAAAAVEPVAAVEPVAAVTRPAARGRAGKRAAVETVEVPAEAPVEPANKGEEVEEKDKDDDDDEEKVQPKRGRLSKK
TKSTPKDQDSLANVGDGKTS
KSVNATPAPTPVLKATRGRKAAVAAAAAPVAPAAKRKASRGQSKAEEEDLSEVSQPVKRTRKGTKAL
>g3113.t1
MHVQSSKRVLVATEKNVFASVNTRTGDLAWRHVDSTGPEGNIDSLLLHGQDAVLMVGDGRLLRSWDINVGGLNWEMVLDS
GSFQAASFVGHLDSVRYVAV
LKKAAISLHYLYNGHQKWLENLPESESVQYQAVYSEGDGEVYALGVVPHSHISIVGFSVEDGEVLKQVTVEAPWLSDLGA
NCVVVGQGVLVCVDVATMSL
YTLDLHLESQMTQIPLQALQLELTAGFQPVLTSTQPNAAHSPLSEFFLQLGPEHHLLLQLKDGKVATLRDFKPSLLVSFA
TTGEKTVAAVMATKNTT
>g3114.t1
WDLVCGDYWKVPLQHICFMTGWILGYILLGTLCDWAGRRRGFLMSVSLSSVLGVALCISNSPVVFLLLRLAQGAMLAGVL
LASYIS
>g3114.t2
WDLVCGDYWKVPLQHICFMTGWILGYILLGTLCDWAGRRRGFLMSVSLSSVLGVALCISNSPVVFLLLRLAQGAMLAGVL
LASYISSK
>g3115.t1
MCKMGTVENEPAQSCLSTIRVKRVLLVDTLEDRINSLLDVLLSRDVLNRDDREEVLCQLGPRARVRKVLDILECKEYNQI
KFKHKEVLRRRSESMLYYNT
RHGEKILFSQQYVNLLLVNGHQGLDIKRHEVLAYGRKRLSLQQKSTEHRRITPAQLFSVVNGIRPVKKILVTGVAGIGKT
ILVQKLLLDFGNNKDHLGFD
FIIHMTFRDLNLVNKPVNLRELVLRKNRHLAKELDNIMANDDKLLIIFDGFDEFRHYRRCDVDAFVCEPDDFGEVEEVFT
SLLLGELLPNTSIIVTSRPT
AISHIPVGCIDRFVLIAGFSLDEIQDFFHRYFQDKGLAERLFTVVRENELMLTLCYIPAFCYIICCILKESKDLAGDNPR
TMTDIYVQYLVALLRTHTQE
RAKASGGDQGTCTGQQLSDGVLKLGRLAYLKLMDQQTLFYESDPDVAPLEGCPLASTFLDKMVAQDPGCTEDVYSFAHLT
VQEFFAAVYCAMSDQPLPDV
LQCDSSPTEETVKGHLDLFSRFLSGILSERNSKLLARHVGLSCDDGKAEAYRQRMVHNLRSLCENGAHILNSLHCLFEQQ
NISLGQDVQAETLRLNVSDE
SLSPMDYNTIKYFLKATAGNLSELDLTGTDVSHEPLRDLMPHLLKCESLXIGWADIGDEELLVLSNAIRRKKVLKELWME
GNRASCNGLLCLSDLTPNPL
RKV
>g3115.t2
MGTVENEPAQSCLSTIRVKRVLLVDTLEDRINSLLDVLLSRDVLNRDDREEVLCQLGPRARVRKVLDILECKEYNQIKFK
HKEVLRRRSESMLYYNTRHG
EKILFSQQYVNLLLVNGHQGLDIKRHEVLAYGRKRLSLQQKSTEHRRITPAQLFSVVNGIRPVKKILVTGVAGIGKTILV
QKLLLDFGNNKDHLGFDFII
HMTFRDLNLVNKPVNLRELVLRKNRHLAKELDNIMANDDKLLIIFDGFDEFRHYRRCDVDAFVCEPDDFGEVEEVFTSLL
LGELLPNTSIIVTSRPTAIS
HIPVGCIDRFVLIAGFSLDEIQDFFHRYFQDKGLAERLFTVVRENELMLTLCYIPAFCYIICCILKESKDLAGDNPRTMT
DIYVQYLVALLRTHTQERAK
ASGGDQGTCTGQQLSDGVLKLGRLAYLKLMDQQTLFYESDPDVAPLEGCPLASTFLDKMVAQDPGCTEDVYSFAHLTVQE
FFAAVYCAMSDQPLPDVLQC
DSSPTEETVKGHLDLFSRFLSGILSERNSKLLARHVGLSCDDGKAEAYRQRMVHNLRSLCENGAHILNSLHCLFEQQNIS
LGQDVQAETLRLNVSDESLS
PMDYNTIKYFLKATAGNLSELDLTGTDVSHEPLRDLMPHLLKCESLXIGWADIGDEELLVLSNAIRRKKVLKELWMEGNR
ASCNGLLCLSDLTPNPLRKV
>g3115.t3

MCKMGTVENEPAQSCLSTIRVKRVLLVDTLEDRINSLLDVLLSRDVLNRDDREEVLCQLGPRARVRKVLDILEXYNQIKF
KHKEVLRRRSESMLYYNTRH
GEKILFSQQYVNLLLVNGHQGLDIKRHEVLAYGRKRLSLQQKSTEHRRITPAQLFSVVNGIRPVKKILVTGVAGIGKTIL
VQKLLLDFGNNKDHLGFDFI
IHMTFRDLNLVNKPVNLRELVLRKNRHLAKELDNIMANDDKLLIIFDGFDEFRHYRRCDVDAFVCEPDDFGEVEEVFTSL
LLGELLPNTSIIVTSRPTAI
SHIPVGCIDRFVLIAGFSLDEIQDFFHRYFQDKGLAERLFTVVRENELMLTLCYIPAFCYIICCILKESKDLAGDNPRTM
TDIYVQYLVALLRTHTQERA
KASGGDQGTCTGQQLSDGVLKLGRLAYLKLMDQQTLFYESDPDVAPLEGCPLASTFLDKMVAQDPGCTEDVYSFAHLTVQ
EFFAAVYCAMSDQPLPDVLQ
CDSSPTEETVKGHLDLFSRFLSGILSERNSKLLARHVGLSCDDGKAEAYRQRMVHNLRSLCENGAHILNSLHCLFEQQNI
SLGQDVQAETLRLNVSDESL
SPMDYNTIKYFLKATAGNLSELDLTGTDVSHEPLRDLMPHLLKCESLXIGWADIGDEELLVLSNAIRRKKVLKELWMEGN
RASCNGLLCLSDLTPNPLRK
V
>g3116.t1
HLSKNPEMMELNMGGCAGFTPGPLGEMLQLFSKLEQLNISWCEFTSDHVKAVVGNLPSSMTDLNLSGYRENLAQEDLEVL
VERCPNIVTLDISDSVFLRT
TCIPTLEQLPHLKHLSLSRCYQIYAAALGDVGERFTSLRLLDAFGLVPDARVFSKTYEMKHVAVNTRAISVIARPTPVLA
DVHSMWDRRCRLRFHY
>g3116.t2
HLSKNPEMMELNMGGCAGFTPGPLGEMLQLFSKLEQLNISWCEFTSDHVKAVVGNLPSSMTDLNLSGYRENLAQEDLEVL
VERCPNIVTLDISDSVFLRT
TCIPTLEQLPHLKHLSLSRCYQIYAAALGYVHLLRSLARKAHHRSALHNYELRDVGERFTSLRLLDAFGLVPDARVFSKT
YEMKHVAVNTRAISVIARPT
PVLADVHSMWDRRCRLRFHY
>g3116.t3
MLQLFSKLEQLNISWCEFTSDHVKAVVGNLPSSMTDLNLSGYRENLAQEDLEVLVERCPNIVTLDISDSVFLRTTCIPTL
EQLPHLKHLSLSRCYQIYAA
ALGDVGERFTSLRLLDAFGLVPDARVFSKTYEMKHVAVNTRAISVIARPTPVLADVHSMWDRRCRLRFHY
>g3117.t1
IRLHLEGTGINPTPVDLHEQQLSLEQHHQAHRITMLESRRGASVRPMHAQQAHLGCPRLLRCIGVVPXTGTQDESFSEPS
LLPVRSLNEIFIGESLSSRV
KYKSFNPHLTFSLHRASYYEISVDDGPWEKQKSSGLSICTGTGSKAWKLTSLCSCPVTEEYNQSLVFSPEDRRLFFSIRE
PIVNRVFSSIRQRGFANKCW
DACMVVDGGTSFEFNDGAIATISMSEEDQLRTVLL
>g3117.t2
IRLHLEGTGINPTPVDLHEQQLSLEQHHQAHRITMLESRRGASVRPMHAQQAHLGCPRLLRCIGVVPXTGTQDESFSEPS
LLPVRSLNEIFIGESLSSRV
KYKSFNPHLTFSLHRASYYEISVDDGPWEKQKSSGLSICTGTGSKAWKLTSLCSCPVTEEYNQSLVFSPEDRRLFFSIRE
PIVNRVFSSIRQRGFANKVC
VRSRCWDACMVVDGGTSFEFNDGAIATISMSEEDQLRTVLL
>g3117.t3
IRLHLEGTGINPTPVDLHEQQLSLEQHHQAHRITMLESRRETGTQDESFSEPSLLPVRSLNEIFIGESLSSRVKYKSFNP
HLTFSLHRASYYEISVDDGP
WEKQKSSGLSICTGTGSKAWKLTSLCSCPVTEEYNQSLVFSPEDRRLFFSIREPIVNRVFSSIRQRGFANKCWDACMVVD
GGTSFEFNDGAIATISMSEE
DQLRTVLL
>g3118.t1
MASSSTFSQVIFNDIPKTYLMSTPVTCCYTLAVGFQPTTRDWVGIFKVECYLPKEESDFYQFCYVDGAGHVRGASTAFSF
QHPVENMDCSMETDLLVVTT
QDEVERNARQTEELREAVEQLRAQAWVLEKALEEKQREVDDLKEQNKEKDRKASDLTEELNRARRQSESTALSLKQKEQE
AERLMEQISIQTQHHLDRDQ
HNATEKETPEINTATGAEAGEKYEQAVIQIQQLKVELEQQRLHHTQSADIAELRTKIRGEFKEELIRLKDVIQLLEVDLK
HSERERETLSAELNQLKHLA
LDVDTLKAENRGLRENLSQQEAQASRPDQDYKAQCSTLRGQLEHTQTLLAGQSAECKDKSRCAEEAERELLNTKEQLHHK

DMENDLIKQDSSQLKLRLAE
FQVVIAEKDAEIAEIHDIIALRENRLEIQNQAKEQLERRQQEIITEKDAEIAKSIGVVALRENRLKIQNQEKEALLRENQ
RLRANMEELCRTRSQEQNAL
PKATGKGGGDLDTKGK
>g3118.t2
MRLSVQKVLRTSGFSTDGEDMASSSTFSQVIFNDIPKTYLMSTPVTCCYTLAVGFQPTTRDWVGIFKVECYLPKEESDFY
QFCYVDGAGHVRGASTAFSF
QHPVENMDCSMETDLLVVTTQDEVERNARQTEELREAVEQLRAQAWVLEKALEEKQREVDDLKEQNKEKDRKASDLTEEL
NRARRQSESTALSLKQKEQE
AERLMEQISIQTQHHLDRDQHNATEKETPEINTATGAEAGEKYEQAVIQIQQLKVELEQQRLHHTQSADIAELRTKIRGE
FKEELIRLKDVIQLLEVDLK
HSERERETLSAELNQLKHLALDVDTLKAENRGLRENLSQQEAQASRPDQDYKAQCSTLRGQLEHTQTLLAGQSAECKDKS
RCAEEAERELLNTKEQLHHK
DMENDLIKQDSSQLKLRLAEFQVVIAEKDAEIAEIHDIIALRENRLEIQNQAKEQLERRQQEIITEKDAEIAKSIGVVAL
RENRLKIQNQEKEALLRENQ
RLRANMEELCRTRSQEQNALPKATGKGGGDLDTKGK
>g3118.t3
LSVQKVLRTSGFSTDGEDMASSSTFSQVIFNDIPKTYLMSTPVTCCYTLAVGFQPTTRDWVGIFKVECYLPKEESDFYQF
CYVDGAGHVRGASTAFSFQH
PVENMDCSMETDLLVVTTQDEVERNARQTEELREAVEQLRAQAWVLEKALEEKQREVDDLKEQNKEKDRKASDLTEELNR
ARRQSESTALSLKQKEQEAE
RLMEQISIQTQHHLDRDQHNATEKETPEINTATGAEAGEKYEQAVIQIQQLKVELEQQRLHHTQSADIAELRTKIRGEFK
EELIRLKDVIQLLEVDLKHS
ERERETLSAELNQLKHLALDVDTLKAENRGLRENLSQQEAQASRPDQDYKAQCSTLRGQLEHTQTLLAGQSAECKDKSRC
AEEAERELLNTKEQLHHKDM
ENDLIKQDSSQLKLRLAEFQVVIAEKDAEIAEIHDIIALRENRLEIQNQAKEQLERRQQEIITEKDAEIAKSIGVVALRE
NRLKIQNQEKEALLRENQRL
RANMEELCRTRSQEQNALPKATGKGGGDLDTKGK
>g3119.t1
MADVVEYEEYEEYEEYEEEISTEQ
>g3120.t1
MRAYHTYSPPPTLTAHLYHSRPPSREESGAGTRSRLAGWSCPRGGKEVLQSVELILTPLVRYGEELKARARC
>g3121.t1
MDTTSIHLITQCRTDDVLDVTHGGLQRERPPRFRAACGRDGAPRLTAAPLGPAAHAPPAAEGSLLSLVMCSPEKPTAHAA
AVK
>g3122.t1
MPEVKSIFREVLPKQGQLSMEDVPTLVLCKPKLLPLKSVTLEKLEKMQLEAQEAIKQQELASKQTQ
>g3123.t1
GEPALICEKHRVPVADLPILVFYGKCQSSSTVPGSEHRAH
>g3124.t1
MSRLPHAPGGFKYVAGVGKVYPSQLEDQNTTLKHGEREYTAAGPDMIARGSVDLESLVDCEAQWGIEQMSGRAEQATGEE
GEVGRMGDGLSDEECSRHEA
HKTDGEDFKTEEPLLEFPLGRERSESTEAVSETVEAVASENIVRSSIWYDEVALQEDIVSYDDDDVANKMSTTHDSPNVQ
PNFEVQIRDPDEMKEMSGQM
EAEEDEELPLEALLQMPSLEPSPSQPSIPQQALPLAGRPIGGINDGRMSGYSQTTWWLSPKPSAVDLPVKVELVSSNARR
ATKPQNRPAEANPFWGTHLA
RGGPRSARRATKPAAPLTSWAPATDTGGGDDSPV
>g3125.t1
LEAGSEGGNLLEAGGGGATLWTQGMQLGAASLERLQTREAESLGPPADKGQALTAPSDRGAGVGRTATRERGAGVGRTTP
SLRPSGGNRRLLWPSGGNGG
LLRPIQVVGKDNPP
>g3126.t1
SQNPQAEGHPSRVSLKGWVTPDRTAEQVLLAVGEVAVASDRVAVPSDRVAVPSARVEVPSDRVAVASDRVAVPSARVALP
SARAAEPGIVLEVCVSLVEP
QRTFAEKRPRCEQWIDEGQHRVLLGSSRRERTLLWNPTAGRLKEPGRKRRIMLEERVLVQAEMETMQSSVGVQPSAGAPV
DLRMEPRTGVLTGVLVGLDA

GQREQRLQQELENLKKQQELQKQRLFADFQKQHEVLTRQHEVQLQEHLKQQQEVLAAKRQEELEQRRQQEQQEEQEKLQH
QLLLLRHKDKTKESAIASSE
VKLKLQEFLLNKKEPAVNHWVIISPPVSVAGAQLGSGAVGLVARLADRGPPCARWVPQNGLASAGLF
>g3126.t2
VAVASDRVAVPSDRVAVPSARVEVPSDRVAVASDRVAVPSARVALPSARAAEPGIVLEVCVSLVEPQRTFAEKRPRCEQW
IDEGQHRVLLGSSRRERTLL
WNPTAGRLKEPGRKRRIMLEERVLVQAEMETMQSSVGVQPSAGAPVDLRMEPRTGVLTGVLVGLDAGQREQRLQQELENL
KKQQELQKQRLFADFQKQHE
VLTRQHEVQLQEHLKQQQEVLAAKRQEELEQRRQQEQQEEQEKLQHQLLLLRHKDKTKESAIASSEVKLKLQEFLLNKKE
PAVNHWVIISPPVSVAGAQL
GSGAVGLVARLADRGPPCARWVPQNGLASAGLF
>g3126.t3
LLALWMEESQNPQAEGHPSRVSLKGWVTPDRTAEQVLLAVGEVAVASDRVAVPSDRVAVPSARVEVPSDRVAVASDRVAV
PSARVALPSARAAEPGIVLE
VCVSLVEPQRTFAEKRPRCEQWIDEGQHRVLLGSSRRERTLLWNPTAGRLKEPGRKRRIMLEERVLVQAEMETMQSSVGV
QPSAGAPVDLRMEPRTGVLT
GVLVGLDAGQREQRLQQELENLKKQQELQKQRLFADFQKQHEVLTRQHEVQLQEHLKQQQEVLAAKRQEELEQRRQQEQQ
EEQEKLQHQLLLLRHKDKTK
ESAIASSEVKLKLQEFLLNKKEPAVNHWVIISPPVSVAGAQLGSGAVGLVARLADRGPPCARWVPQNGLASAGLF
>g3127.t1
MNEYNNVTGITKEHVFEMTMMWCVWLQGLVKLLSSRDSSVLLGSLMALSSLAESPEGRERIGELPLVEDLLVILQEYDSL
SKRMAAELLRLLVPVLGVRR
QLRDLEGLPVLLGLLHGRHLRLLWSSAWILVQLCQDQDTRAEGTYVSDRSSVETLSSANAAGRIQRQHCSEELTPQEQKD
NALALQSACCAALTELVLDD
SNAYHLVQENGIYVVARLILPQDPAAKAASLQCYAFRALRFIFSVERNRGLFRRLFPTELFEMFIDVGHYVRDLTAYEAL
QAAVARYTEEELQALREGIE
SSNQNCPPIRVINGYAVLDHLGTGAFGSVFKVLSLCSSIYSYVYLFILVRKQCGQNLLALKEVDLHNPAFGKDKRSRDSN
VERIVSELTIIKEQVQLYMK
EKLDVQILVMTHPNIVKYYKTFLEGDRLYIVMELIEGVTLADLFSALKEKQQKLTEQRVWNIFVQMCLALRYLHKEKRIV
HRDLTPNNIMLGDRDKVTIT
DFGLAKQKQESSKLTSVVGTILYSCPEIVKCEPYGEKADIWALGCILYQMTTLQTPFYSTNMLSLATKIVEAVYEPVEDA
TFSERVTGMIKWCLSPDPDQ
RPDVVEVSASISDILMSLMDSFYASHHALQRRADRDRKRAQKYFLERHKREMGCMISNPPQGPEESTMKTQTPAPSSTAC
GLDHSQQDSGQGDESEEDVD
VGSKSNNGFWKKPGASAGISISQRRLRELDDPIQRFLVLLHKIVFITQLPPAPHSSSSHKRRLVERFKKSLFHQDSSALG
LKVTLSKVLQGSSEPVELGS
ASSDWWQQSLSIKAPLYSPQSQDMMDEGTFRDSVSYEQMQAVIEEVLEESRYYEGTGIRVSHGKVVSGEGPDKERFKNPD
GKAKHYQSYLARIGETGAPY
WSQTWEGSSPASVWWPGFDPWGSAGLSPKQQHGGAAALWAHHLQG
>g3127.t2
MTPPDMFQKLSTLEGQRDWVIRSEAHRGLVKLLSSRDSSVLLGSLMALSSLAESPEGRERIGELPLVEDLLVILQEYDSL
SKRMAAELLRLLVPVLGVRR
QLRDLEGLPVLLGLLHGRHLRLLWSSAWILVQLCQDQDTRAEGTYVSDRSSVETLSSANAAGRIQRQHCSEELTPQEQKD
NALALQSACCAALTELVLDD
SNAYHLVQENGIYVVARLILPQDPAAKAASLQCYAFRALRFIFSVERNRGLFRRLFPTELFEMFIDVGHYVRDLTAYEAL
QAAVARYTEEELQALREGIE
SSNQNCPPIRVINGYAVLDHLGTGAFGSVFKVLSLCSSIYSYVYLFILVRKQCGQNLLALKEVDLHNPAFGKDKRSRDSN
VERIVSELTIIKEQVQLYMK
EKLDVQILVMTHPNIVKYYKTFLEGDRLYIVMELIEGVTLADLFSALKEKQQKLTEQRVWNIFVQMCLALRYLHKEKRIV
HRDLTPNNIMLGDRDKVTIT
DFGLAKQKQESSKLTSVVGTILYSCPEIVKCEPYGEKADIWALGCILYQMTTLQTPFYSTNMLSLATKIVEAVYEPVEDA
TFSERVTGMIKWCLSPDPDQ
RPDVVEVSASISDILMSLMDSFYASHHALQRRADRDRKRAQKYFLERHKREMGCMISNPPQGPEESTMKTQTPAPSSTAC
GLDHSQQDSGQGDESEEDVD
VGSKSNNGFWKKPGASAGISISQRRLRELDDPIQRFLVLLHKIVFITQLPPAPHSSSSHKRRLVERFKKSLFHQDSSALG
LKVTLSKVLQGSSEPVELGS

ASSDWWQQSLSIKAPLYSPQSQDMMDEGTFRDSVSYEQMQAVIEEVLEESRYYEGTGIRVSHGKVVSGEGPDKERFKNPD
GKAKHYQSYLARIGETGAPY
WSQTWEGSSPASVWWPGFDPWGSAGLSPKQQHGGAAALWAHHLQG
>g3127.t3
MFQKLSTLEGQRDWVIRSEAHRGLVKLLSSRDSSVLLGSLMALSSLAESPEGRERIGELPLVEDLLVILQEYDSLSKRMA
AELLRLLVPVLGVRRQLRDL
EGLPVLLGLLHGRHLRLLWSSAWILVQLCQDQDTRAEGTYVSDRSSVETLSSANAAGRIQRQHCSEELTPQEQKDNALAL
QSACCAALTELVLDDSNAYH
LVQENGIYVVARLILPQDPAAKAASLQCYAFRALRFIFSVERNRGLFRRLFPTELFEMFIDVGHYVRDLTAYEALQAAVA
RYTEEELQALREGIESSNQN
CPPIRVINGYAVLDHLGTGAFGSVFKVLSLCSSIYSYVYLFILVRKQCGQNLLALKEVDLHNPAFGKDKRSRDSNVERIV
SELTIIKEQVQLYMKEKLDV
QILVMTHPNIVKYYKTFLEGDRLYIVMELIEGVTLADLFSALKEKQQKLTEQRVWNIFVQMCLALRYLHKEKRIVHRDLT
PNNIMLGDRDKVTITDFGLA
KQKQESSKLTSVVGTILYSCPEIVKCEPYGEKADIWALGCILYQMTTLQTPFYSTNMLSLATKIVEAVYEPVEDATFSER
VTGMIKWCLSPDPDQRPDVV
EVSASISDILMSLMDSFYASHHALQRRADRDRKRAQKYFLERHKREMGCMISNPPQGPEESTMKTQTPAPSSTACGLDHS
QQDSGQGDESEEDVDVGSKS
NNGFWKKPGASAGISISQRRLRELDDPIQRFLVLLHKIVFITQLPPAPHSSSSHKRRLVERFKKSLFHQDSSALGLKVTL
SKVLQGSSEPVELGSASSDW
WQQSLSIKAPLYSPQSQDMMDEGTFRDSVSYEQMQAVIEEVLEESRYYEGTGIRVSHGKVVSGEGPDKERFKNPDGKAKH
YQSYLARIGETGAPYWSQTW
EGSSPASVWWPGFDPWGSAGLSPKQQHGGAAALWAHHLQG
>g3128.t1
MGDGSVNSYEEVDANTTGRDYSNDVVARAQWYKDNGY
>g3129.t1
GQFDNTFNCSRGTYIYCCGTCHYRFCCEHQRDRLDQDSCNNHRPPVGRRSRRWSRLGPRGSRGPLKEAEVEDGDEGPTGA
AETPPKPLPQIMLPSNMTAA
DALKPPLGAAQVAPPPRRQVDVDVCRGYFDVMGQFDNTFNCSRGTYIYCCGTCHYRFCCEHQRDRLDQD
>g3130.t1
MCQHCGVWLRGPSGCLAQSDVAMLNVVYVKAVNPLKHSRLCDQSGELRLGGLVDASHPPSSPPPAAGTSQRIYPTHQRST
PASPILPLPLDTESSLSLTL
VPCPLAHEAQPIAPHVHGTPIPTSLPNPAISSWVPAEDPHKSTLPLPVSLPLSSTMMEPGTVSALTDECLLQPSRTCLGC
FIETTDHNSIHETPHDPTSN
PDPEPGLSVRIGDVGREDFSDISNISIQCLSHAGEAGSHYGEQLLSDQLLNFPLPKAPGEGKRGDGDKITDCDDPEDDAT
TKNLYEGLLLDKVNGEEVLL
ANAGQDWGYFESFISESKMELLDLCSKNELSVNLFSEEDVDNLFDDEDDDSTLSSDVCSLKIRYESFQDNMREKTNVLQE
ETQFNFFPSVLANCGKKEEG
VLVPKRGAEKHPPKSDELILVAEQVEKAGDCSSGSPLDGSQGSPMSTPKVNYLLDFNSTEESGEYSDDSSCTGSSSDTLQ
EGKIKKGHSRRFLSPSNPLN
YGLRSKRKVRYSDDYLYDVDSIESEKNTEKKEKAPSGVTKEETTPMDIAGVTEAPTCPLTLDSNAIAQAVTAKSRSQERE
EREARLGGNATGRIRKFKSE
ARLRSMKMKAAEGKEGRSVTNVMDACAAVAQIEGPTAGLGEASSNTVTTHFSDTSATNSTPEEKFPFVSPTCSPDKATPS
EEVEAGVSVIPGGYLQTLLD
GTDSSGGAAISYFPQQPSRQQYPLGLSLEEKQFTSMQLAQSCVLSPPSESELQQSPQNCPNFPQMWHPQLCPSHSQSFGP
ETNETPILPNNCPAALPLND
SLPVSNYNQLSSEADRLLYEKSYLAEAGLQPGADMQGCQSAALDGQVQYQRGSLCTDNGRLISYDSVGSLSASSSNYSSL
SLKSCEREGEEEGRDNFLAH
CSPKVVIQQSVDALTPLRESSDLLDISNFTPDKFRHSSLSELSPPETPNLSPQVVWREIKMAGKAGEYQEGNDSTLDNGR
EVKWNCDVIPQQEHSGPYAA
EDGQFALQHFSGEGSLGLDKKALVSTDFDEHTTEMNGMKSTKTKKKGSYKQTPAGQGPKKSRAPKATKTEKVKVPKQNSR
STKKIKAMLEGKAAKNQAGG
CGTGLTDSSSTGDWSGTGWSENNSLVGDDQREFEEPSNILSNIVSGMAEVQRFMMASIEPLWDPMAEACITPEANSLNLK
TLKILAGTEADLKKKGALLT
GAGRGRKAGGKGGKNQAKFNPSHPLFPQLALGCNMFDKPNFINPGPAHKKLYRHKTSAKFPRIETLKGKRAERDPNKDIA
LMASFETLR

>g3130.t2
LRLGGLVDASHPPSSPPPAAGTSQRIYPTHQRSTPASPILPLPLDTESSLSLTLVPCPLAHEAQPIAPHVHGTPIPTSLP
NPAISSWVPAEDPHKSTLPL
PVSLPLSSTMMEPGTVSALTDECLLQPSRTCLGCFIETTDHNSIHETPHDPTSNPDPEPGLSVRIGDVGREDFSDISNIS
IQCLSHAGEAGSHYGEQLLS
DQLLNFPLPKAPGEGKRGDGDKITDCDDPEDDATTKNLYEGLLLDKVNGEEVLLANAGQDWGYFESFISESKMELLDLCS
KNELSVNLFSEEDVDNLFDD
EDDDSTLSSDVCSLKIRYESFQDNMREKTNVLQEETQFNFFPSVLANCGKKEEGVLVPKRGAEKHPPKSDELILVAEQVE
KAGDCSSGSPLDGSQGSPMS
TPKVNYLLDFNSTEESGEYSDDSSCTGSSSDTLQEGKIKKGHSRRFLSPSNPLNYGLRSKRKVRYSDDYLYDVDSIESEK
NTEKKEKAPSGVTKEETTPM
DIAGVTEAPTCPLTLDSNAIAQAVTAKSRSQEREEREARLGGNATGRIRKFKSEARLRSMKMKAAEGKEGRSVTNVMDAC
AAVAQIEGPTAGLGEASSNT
VTTHFSDTSATNSTPEEKFPFVSPTCSPDKATPSEEVEAGVSVIPGGYLQTLLDGTDSSGGAAISYFPQQPSRQQYPLGL
SLEEKQFTSMQLAQSCVLSP
PSESELQQSPQNCPNFPQMWHPQLCPSHSQSFGPETNETPILPNNCPAALPLNDSLPVSNYNQLSSEADRLLYEKSYLAE
AGLQPGADMQGCQSAALDGQ
VQYQRGSLCTDNGRLISYDSVGSLSASSSNYSSLSLKSCEREGEEEGRDNFLAHCSPKVVIQQSVDALTPLRESSDLLDI
SNFTPDKFRHSSLSELSPPE
TPNLSPQVVWREIKMAGKAGEYQEGNDSTLDNGREVKWNCDVIPQQEHSGPYAAEDGQFALQHFSGEGSLGLDKKALVST
DFDEHTTEMNGMKSTKTKKK
GSYKQTPAGQGPKKSRAPKATKTEKVKVPKQNSRSTKKIKAMLEGKAAKNQAGGCGTGLTDSSSTGDWSGTGWSENNSLV
GDDQREFEEPSNILSNIVSG
MAEVQRFMMASIEPLWDPMAEACITPEANSLNLKTLKILAGTEADLKKKGALLTGAGRGRKAGGKGGKNQAKFNPSHPLF
PQLALGCNMFDKPNFINPGP
AHKKLYRHKTSAKFPRIETLKGKRAERDPNKDIALMASFETLR
>g3131.t1
MATPQTPTLTRSVLDLTNDTPGPGLKETHRPAVIRSLAEAFSALGGLIQKIRRPGRK
>g3132.t1
MESSRVWTRALVCAVLLSVVYGADLYNSESEEDLSTRALRDFYPKGPNLTNEKQLLGALHDVLEKLQTRKMPLWEKKFGQ
VPTCDVGEQCAIRKGARIGK
MCDCPRGSFCNFFLLKCL
>g3133.t1
MTITNNLSTISSPSPLSYFLWVSGNRTATHSMARPRFNYCLLCQLYVGLMLVSAGLCMVQSNECPQLCVCEVRPWFTPQS
TYREAITVDCNDLRLTRIPG
NLSSDTQVLLLQSNYIARSSEELEQLFNLTELDLSQNNLSKIRDVGLANMSQLTTLHLEENQITEMPDYCLQDLSNLQEL
YINHNQINTISAYAFSGLHN
LLRLHLNSNKLKAIDSQWFDSTANLEILMIGENPVLGIMDFNFKPLGNLRSLVLAGMDLTDIPGNAFVGLDNLESLSFYD
NKLVRVPQRALQKLPNLKFL
DLNKNPVHKIQAGDFKNMLRLKELGINNMAELVSIDRYALDNLPELTKLEATNNPKFSFINKQAFHDVPALESLMLNNNA
LNALYQSTVEYLPNLREISI
HSNPLRCDCVIQWMSSNKTSIRFMEPLSMFCAMPTELRGQQVREALQKDSVEHCLPMISHDSFPNHLNLDVGMTVDLDCR
AMSQPEPEIYWVTPLGNKVA
MDTLSDKYNLNSEGTLRISHIQVEDSGRYTCVAQNSEGADTRVTALRVNGTLLDSTQLMKIYIKQTESHSILVSWKVNSN
VMTSNLKWSSATMKIDNPHI
TYTARVPVDVHEYNLTHLQPATEYEVCLTVSNIHQQMQKSCVNGTTKQATFAVELSDQGTNIALAVVMGTIFTIVSLASL
SMYVAKRWKRKNYHHSLKKY
MQKTSSIPLNELYPPLINLWEADSEKEKEAVPETKPSQVDTTRSYYMW
>g3134.t1
MFKGLGTWLGLENAANPPRSADPDPDPDQDRSGPEEEPGHVVQQQNQVNQQEEDPTRTRGSSSPRD
>g3135.t1
MACRTKLSLNLLVLVRWLLYRLPDGRSTNRLWLGWLGFRDDPLGLPSAALAVEFLAGWQLGPGDALGCPDHPLQSLAVEG
SAVPGRVAASQILSMTGEKK
RGADRNRGGEQTAGGGNTSYSARVPRVEKKTLAARNRRRGTAPGRCSLEIVRLKSMLLRLLPVRWTMKTLMVFDFDHTVV
DDNSDTWVIRCLPDQTLPKA
LKDSYQKGQWTDYMGRVMSYIGDQAVSPDDIRVVMETIPFTQGMVDLLAFISAHKNAVDCVVISDSNGIFIDWILGAAGL

RSAVDRVLTNPAGFDERGYL
KVQRHHSHDCARCPVNLCKRTALELYLKERSVGGVDYHRTIYVGDGGNDLCPASCLRGGDVVMPRRGFTLEGLLAKPLGS
SLKARVVPWSSGTEILEELR
AHMLPPGP
>g3135.t2
LMACRTKLSLNLLVLVRWLLYRLPDGRSTNRLWLGWLGFRDDPLGLPSAALAVEFLAGWQLGPGDALGCPDHPLQSLAVE
GSAVPGRVAASQILSMTGEK
KRGADRNRGGEQTAGGGNTSYSARVPRVEKKTLAARNRRRGTAPGRCSLEIVRLKSMLLRLLPVRWTMKTLMVFDFDHTV
VDDNSDTWVIRCLPDQTLPK
ALKDSYQKGQWTDYMGRVMSYIGDQAVSPDDIRVVMETIPFTQGMVDLLAFISAHKNAVDCVVISDSNGIFIDWILGAAG
LRSAVDRVLTNPAGFDERGY
LKVQRHHSHDCARCPVNLCKRTALELYLKERSVGGVDYHRTIYVGDGGNDLCPASCLRGGDVVMPRRGFTLEGLLAKPLG
SSLKARVVPWSSGTEILEEL
RAHMLPPGP
>g3135.t3
MACRTKLSLNLLVLVRWLLYRLPDGRSTNRLWLGWLGFRDDPLGLPSAALAVEFLAGWQLGPGDALGCPDHPLQSLAVEG
SAVPGRVAASQILSMTGEKK
RGADRNRGGEQTAGGGNTSYSARVPRVEKKTLAARNRRRGTAPGRCSLEIVRLKSMLLHQPSASPSQDGFILLSWTMKTL
MVFDFDHTVVDDNSDTWVIR
CLPDQTLPKALKDSYQKGQWTDYMGRVMSYIGDQAVSPDDIRVVMETIPFTQGMVDLLAFISAHKNAVDCVVISDSNGIF
IDWILGAAGLRSAVDRVLTN
PAGFDERGYLKVQRHHSHDCARCPVNLCKRTALELYLKERSVGGVDYHRTIYVGDGGNDLCPASCLRGGDVVMPRRGFTL
EGLLAKPLGSSLKARVVPWS
SGTEILEELRAHMLPPGP
>g3136.t1
MSLSLEKVPALWKTSCVVPVPKTPHPKEPNHFRPVALTSHLMKTMERIILRHLRPL
>g3137.t1
MMGRQCHSAQAHLYRAKEDSQESENSLYHSGSSKDPEGSVAGAGPGAGQEYAPASPNSSSESLNQRGALEMQAAELQHYQ
VEMQHSLPPQGSQTSPPPPP
PPLPPYSQKFGTLGLSRKDSLTKAQLYGTLLN
>g3138.t1
MMRGKTVIVTGANCGIGKAVAGELVKLQARVIMACRDQESAEQAARDIKKEAGPNQGEVIVKHMDLASLSSVRTFCAEIS
KEESKIDVLINNAGIYQCPY
TTTVDGFEMQFGVNHLGHFLLTNLLLDRLKAAAAGRVVVVTSKLYKNGHINFEDLNSEHHYNKAFCYSQSKLANLLFCME
LSRRLGVTSGVTANAVSPGI
VRTRLGRHIHIPLLARPLFYLATLTFFRSALQGAQTLLYLACSPQVEGVSGKYFADCAEDALLDKATDAQAARRLWDLSE
RMVGLRS
>g3139.t1
MGSDSAVLTLAGSSFHHCGAKTEKSLDFASRPFPPFSGGGTSRPAERVPVLENILPGPSAHNSRPVTLEDYRPVALTSHI
TKVMERLVLAHLRPLVSPSQ
GPLQFAYQPHVGVDDAIIYLLQKTHSSLDCPNTSVRVVFFDFSSAFNTIQPRLPNWRTCFLFRCLPHHMG
>g3139.t2
LSLFLKMGSDSAVLTLAGSSFHHCGAKTEKSLDFASRPFPPFSGGGTSRPAERVPVLENILPGPSAHNSRPVTLEDYRPV
ALTSHITKVMERLVLAHLRP
LVSPSQGPLQFAYQPHVGVDDAIIYLLQKTHSSLDCPNTSVRVVFFDFSSAFNTIQPRLPNWRTCFLFRCLPHHMG
>g3140.t1
MSSSAFKGEGHVKPEATVEDSQITGKVPITRSCLGSSRMWELRKNTRELHEQVSSAEEISPPPPQKVLLVSRP
>g3140.t2
FKGEGHVKPEATVEDSQITGKVPITRSCLGSSRMWELRKNTRELHEQVSSAEEISPPPPQKVLLVSRP
>g3141.t1
MSSQLQVFSPPSISSSAFCRVKKLKVESNVWDVSTTEGYGSMAGPSAYSFTPAMAVPPFAPSLVFPPAAPGSRGQVVVRA
ADSTGSAPRGSG
>g3142.t1
RCPLVLPTSVRQADGQDVRAACATTFRMPRE
>g3143.t1
RGHAVHGALPTLSTHTPPLGTVVSEADTVMSFVNLVEQERDLLQPRPYWKEFRFDLTPLPQGETVTAAEFRIYKTLTMGQ

RANRTLHISVYEIQRDNRHR
EPELVLLDIQSVPAGQEGWLAFDVTSASNRWLLHPRSNLGIRLYVETEEDRSLSAGWIGLVGRRGPRSKQPFMVTFFRAS
QIPCRPPRALKPNPRKKKHK
YDLPVPSVHDHSHTNSGRQACKKHELYVSFSDLGWKDWVLAPTGYSAYYCDGECLYPLGSCMNATNHALIQQVVHLLKPE
VPKACCAPTKLSPISVLFYD
EHNNVILKKHRNMVVKTCGCL
>g3143.t2
LPTLSTHTPPLGTVVSEADTVMSFVNLVEQERDLLQPRPYWKEFRFDLTPLPQGETVTAAEFRIYKTLTMGQRANRTLHI
SVYEIQRDNRHREPELVLLD
IQSVPAGQEGWLAFDVTSASNRWLLHPRSNLGIRLYVETEEDRSLSAGWIGLVGRRGPRSKQPFMVTFFRASQIPCRPPR
ALKPNPRKKKHKYDLPVPSV
HDHSHTNSGRQACKKHELYVSFSDLGWKDWVLAPTGYSAYYCDGECLYPLGSCMNATNHALIQQVVHLLKPEVPKACCAP
TKLSPISVLFYDEHNNVILK
KHRNMVVKTCGCL
>g3143.t3
RGHAVHGALPTLSTHTPPLGTVVSEADTVMSFVNLEHVSSVRSSSLNDLFVTDSIRVEQERDLLQPRPYWKEFRFDLTPL
PQGETVTAAEFRIYKTLTMG
QRANRTLHISVYEIQRDNRHREPELVLLDIQSVPAGQEGWLAFDVTSASNRWLLHPRSNLGIRLYVETEEDRSLSAGWIG
LVGRRGPRSKQPFMVTFFRA
SQIPCRPPRALKPNPRKKKHKYDLPVPSVHDHSHTNSGRQACKKHELYVSFSDLGWKDWVLAPTGYSAYYCDGECLYPLG
SCMNATNHALIQQVVHLLKP
EVPKACCAPTKLSPISVLFYDEHNNVILKKHRNMVVKTCGCL
>g3144.t1
MGPSISDSRIGCGESGRCAPLGNYACNEDLSTRVYLGHLGRQVTVTMSEMFNRASRQDAKLTFLSCQPSFSSENGTDGAI
DELTLSKIGQTDVCLTGHSI
IFSGPAKDSRLPLLSRWRLKYRSVQASK
>g3144.t2
QGLFPITLATRRVEHRAAPMGPSISDSRIGCGESGRCAPLGNYACNEDLSTRVYLGHLGRQVTVTMSEMFNRASRQDAKL
TFLSCQPSFSSENGTDGAID
ELTLSKIGQTDVCLTGHSIIFSGPAKDSRLPLLSRWRLKYRSVQASK
>g3145.t1
MPGSQSRSTTVLKLVGIRNPAAMLSGS
>g3146.t1
VGSEPASSSFHVARGDTSSEDERGPRRRNVPPFEAHRRRTSDEDDENEGLAFAVGAKADEKRGFSMNKCIVGALVLLCLG
SLFLSGEFCSPLRSAVANTR
FVASGPRHPSIAQPSDAVSFSATRTTGGCSWMTLLN
>g3146.t2
VGSEPASSSFHVARGDTSSEDERGPRRRNVPPFEAHRRRTSDEDDENEGLAFAVGAKADEKRGFSMNKCIVGALVLLCLG
SLFLSGEFCSPLRSAVANTR
FVASGPRHPSIAQPSDAVSFSATRTTGGCSW
>g3147.t1
MEQELCYGNGSCVRTVYPTAIRTILYLCLGSVVVLTVLGNLLVIVAIAHFKQLHTPTNYLTLSLAMSDLLLGVLVMVPSM
VYSVETCWYLGEMFCKLFSS
SEVLLCTASILNLSFISIDRYYAVCHPLLYPRKITVHTALIMILVTWCVSAVVGFGMVFLKLNILGMEEFYYNNVACEGG
CIFFQTGVSSTVSSILSFYI
PGVIMVGIYLKIYMVAQRQVRSMGLQNTSSSKVDKHQRKATKTLAIIMGVFLSFWMPLFIINPTNPW
>g3147.t2
SMEQELCYGNGSCVRTVYPTAIRTILYLCLGSVVVLTVLGNLLVIVAIAHFKQLHTPTNYLTLSLAMSDLLLGVLVMVPS
MVYSVETCWYLGEMFCKLFS
SSEVLLCTASILNLSFISIDRYYAVCHPLLYPRKITVHTALIMILVTWCVSAVVGFGMVFLKLNILGMEEFYYNNVACEG
GCIFFQTGVSSTVSSILSFY
IPGVIMVGIYLKIYMVAQRQVRSMGLQNTSSSKVDKHQRKATKTLAIIMGVFLSFWMPLFIINPTNPW
>g3148.t1
MRDVEIAVLRGVLTPGEETEWQHNSSTTA
>g3149.t1
LDVSQRQGVEDLVSAAPCDFDHAELFTLLQRLTLRHRMDDSYSCLGRGGFDEEVKNRLVIRAGGTLRRPHKVTLL

>g3150.t1
YWAAGQPGAVLWSCDGGWGLGLEAENKANEDMRGASWLEEEDQRKRRRSRKNNNKNKTIKEQRRRRKRKRALGLNQT
>g3151.t1
VVQSRRPCPTILTRQGHRISSKGKFGGALNRAPDLSALHGLVFGAPLPHQYHALMEQMDNLYSYRNAMVASEEAKEELNT
QRQVLLSAEHVKREKEMDAN
KQQLTEIHSQLGNLQELCEGKPACVKGAFKSLMAPFVFSERMSVSTLGKRAFRDMKPEEGAGEPSGPQPRRPRTSS
>g3152.t1
MSRRRCCKCDCKIPLYGLPKNDELKKQWLRFIFTTVPQEYSASLSLCSLHFTSEQSRLGTQTLDSPQMVPVLTVFRGHTE
GFCFPSGKRLCVKEEVGATI
TKVKVEPFDPSYDPGDHIVKEEPDAPRHAQCFVEPAVVHKWKKDQRVLLQQLQGGKVAACGGMQSDSPGHSAKFGSYSLM
HMESNTIIDLQLVQSNEVGG
SYHMEKEGLKRSLDLLEDTGLDVDYIVTERHPQIQKYLRERKVTHYYNVWHFEKVYKRLRNKRLLKDVGKLSNSQTSSLE
AFHSLSLRFAPKNVVFPFIG
LLCRLYLAIMHCNENAGRKQAETSSGQAAFKVKSRKGRWKSTSNYLDALLELVFNAVFQDPAPFVA
>g3152.t2
MRLPAVKRDTFRRHAQCFVEPAVVHKWKKDQRVLLQQLQGGKVAACGGMQSDSPGHSAKFGSYSLMHMESNTIIDLQLVQ
SNEVGGSYHMEKEGLKRSLD
LLEDTGLDVDYIVTERHPQIQKYLRERKVTHYYNVWHFEKVYKRLRNKRLLKDVGKLSNSQTSSLEAFHSLSLRFAPKNV
VFPFIGLLCRLYLAIMHCNE
NAGRKQAETSSGQAAFKVKSRKGRWKSTSNYLDALLELVFNAVFQDPAPFVA
>g3152.t3
MSRRRCCKCDCKIPLYGLPKNDELKKQWLRFIFTTVPQEYSASLSLCSLHFTSEQSRLGTQTLDSPQMVPVLTVFRGHTE
GFCENQRNTGPETCSGFQDL
PLSSTPTEAPGFPSGKRLCVKEEVGATITKVKVEPFDPSYDPGDHIVKEEPDAPRHAQCFVEPAVVHKWKKDQRVLLQQL
QGGKVAACGGMQSDSPGHSA
KFGSYSLMHMESNTIIDLQLVQSNEVGGSYHMEKEGLKRSLDLLEDTGLDVDYIVTERHPQIQKYLRERKVTHYYNVWHF
EKVYKRLRNKRLLKDVGKLS
NSQTSSLEAFHSLSLRFAPKNVVFPFIGLLCRLYLAIMHCNENAGRKQAETSSGQAAFKVKSRKGRWKSTSNYLDALLEL
VFNAVFQDPAPFVA
>g3153.t1
MHFDNRGLRGLASRDPNYSRALAKSAKKEAGAGFHAVNTSSLSACQLAETELVILSCDASDEEQLKRAGLS
>g3154.t1
MGPLPPTAELCQRLTSSIIFPCGLEQPALMCHHGNAPHCISLGPTWPTRVQRPPRKVWVIPTGQACERN
>g3155.t1
MTTTPYVRVAPPPGDKQLAGAATRRPEPEQEKQSDHTVKIAGVTAGVLLFIIIFLGVILLMKK
>g3155.t2
MHDWTWFGGRGLGRPEPEQEKQSDHTVKIAGVTAGVLLFIIIFLGVILLMKK
>g3156.t1
MDPCKVGAVSEWPQPCSRVTSHPGRLTLCCSVPQSTLKSSPTITGCQLPTIAPAKGVKPVLDRFTIKWFMDVCKRGRGLQ
YLVD
>g3156.t2
MDPCKVGAVSEWPQPCSRRLTLCCSVPQSTLKSSPTITGCQLPTIAPAKGVKPVLDRFTIKWFMDVCKRGRGLQYLVD
>g3156.t3
LENQVFVKAEKCDFHVPKVSFLGYIVEEVSVQMDPCKVGAVSEWPQPCSRVTSHPGRLTLCCSVPQSTLKSSPTITGCQL
PTIAPAKGVKPVLDRFTIKW
FMDVCKRGRGLQYLVD
>g3157.t1
EQAQILKKEGMKRVLVLGSGYVSGPLVEYLTRDPGTQVTVASAILQQAEELAGRYPNTIPVMLNISQDGEEGRLDCLVKD
HHLVVSLLPYSFHPLVARHC
IRRKINMVTASYLSPEMKSLQQSAVDAGITIVNEMGLDPGIDHMLAMECIDQAKMDGCTVESYSSFCGGLPAPECSDNPL
RYKFSWSPSGVLMNTVSQAV
YIKDHQVVTVPAGGALMESATPMDFLPGFNLEAFPNRDSTQYAEPYGIQDAHTLIRGTLRFKGFSDAMSGFVKLGLINTE
PAPILQTSSRVTWRELLCTQ
MGLSSSSMTQEGFEEAVYQRTGESDFRMNTLRGLGMLSNDSVPRAPTVLSALAKHLEVRLSFDKGERDMIVLRNDVGLRH
PTGELETKHVSLVVYGEHNG
FSAMAKTVGYPAAIAARMILDGEILTKGLVVPMTRDVYGPALRRLEDEGLRIICKSTLQE

>g3157.t2
EQAQILKKEGMKRVLVLGSGYVSGPLVEYLTRDPGTQVTVASAILQQAEELAGRYPNTIPVMLNISQDGEEGRLDCLVKD
HHLVVSLLPYSFHPLVARHC
IRRKINMVTASYLSPEMKSLQQSAVDAGITIVNEMGLDPGIDHMLAMECIDQAKMDGCTVESYSSFCGGLPAPECSDNPL
RYKFSWSPSGVLMNTVSQAV
YIKDHQVVTVPAGGALMESATPMDFLPGFNLEAFPNRDSTQYAEPYGIQDAHTLIRGTLRFKGFSDAMSGFVKLGLINTE
PAPILQTSSRVTWRELLCTQ
MGLSSSSMTQEGFEEAVYQRTGESDFRMNTLRGVSGSCVFCLVKPLERHILQRWSLAAAGVLEPALYSRNYFERLSKPRL
GMLSNDSVPRAPTVLSALAK
HLEVRLSFDKGERDMIVLRNDVGLRHPTGELETKHVSLVVYGEHNGFSAMAKTVGYPAAIAARMILDGEILTKGLVVPMT
RDVYGPALRRLEDEGLRIIC
KSTLQE
>g3157.t3
EQAQILKKEGMKRVLVLGSGYVSGPLVEYLTRDPGTQVTVASAILQQAEELAGRYPNTIPVMLNISQDGEEGRLDCLVKD
HHLVVSFHPLVARHCIRRKI
NMVTASYLSPEMKSLQQSAVDAGITIVNEMGLDPGIDHMLAMECIDQAKMDGCTVESYSSFCGGLPAPECSDNPLRYKFS
WSPSGVLMNTVSQAVYIKDH
QVVTVPAGGALMESATPMDFLPGFNLEAFPNRDSTQYAEPYGIQDAHTLIRGTLRFKGFSDAMSGFVKLGLINTEPAPIL
QTSSRVTWRELLCTQMGLSS
SSMTQEGFEEAVYQRTGESDFRMNTLRGLGMLSNDSVPRAPTVLSALAKHLEVRLSFDKGERDMIVLRNDVGLRHPTGEL
ETKHVSLVVYGEHNGFSAMA
KTVGYPAAIAARMILDGEILTKGLVVPMTRDVYGPALRRLEDEGLRIICKSTLQE
>g3158.t1
LDIDPSLRNWILDFLTNRPQSVRIDGHTSSTLTLNTGVPQGCMLSPLLYSLFSHDCTHGSTTIVKFADDTTVISLISNND
ESAYREQVQCLAVWCADNNL
SLNAMKTKEIIVDYRKSRGGTHTPLHINGTAVERVTSFKLVTHLGVHISENLSWSLNTSTLIKKAHQHLFFLRRLKKAQL
SPQILVNFYRSTIESTLTNC
VTVWYGDAPLVPHSPPSRLSRGSGACV
>g3159.t1
LAAVLRDVGSSNAVTVGVGVACMAFLYAVKQVNERCKARMPVPVPGEIVVVIVATALSSGLGLSERHGVAVVGRIPTGLL
PPALPHFHLLPRLLVDSLAI
AVVGFSMAISMAKTFALKHGYSVDGNQVSHRHGSRGSHLRGEELLALGLCNFVSSFFQTFAISCSMSRSLVQESTGGKTQ
>g3159.t2
SLAAVLRDVGSSNAVTVGVGVACMAFLYAVKQVNERCKARMPVPVPGEIVVVIVATALSSGLGLSERHGVAVVGRIPTGL
LPPALPHFHLLPRLLVDSLA
IAVVGFSMAISMAKTFALKHGYSVDGNQVSHRHGSRGSHLRGEELLALGLCNFVSSFFQTFAISCSMSRSLVQESTGGKT
Q
>g3159.t3
LAAVLRDVGSSNAVTVGVGVACMAFLYAVKQVNERCKARMPVPVPGEIVVVIVATALSSGLGLSERHGVAVVGRIPTGLL
PPALPHFHLLPRLLVDSLAI
AVVGFSMAISMAKTFALKHGYSVDGNQELLALGLCNFVSSFFQTFAISCSMSRSLVQESTGGKTQ
>g3160.t1
FRISERDLTEIQLHTVDSINDLHRMHPEHRGVRPPLLPAHNPPPSNGNLCFQGVTDVNQAGQPISAKWQKHLGDVWWTGP
SRVHAAACGVVMFLLLTLVV
AVYFLVQQGGAIRMLTEAVREKQDVSMEISKLVQELQALRHNLTALRDRI
>g3161.t1
MLCFMAVISEGDISEETGQDTLDEGDASGSKGEEQAGEGSRVSELSAPSDAESGVGGEVGREDEDTDSMDGSGLFSLTED
GGPKREGGRRERARASDGGR
RAARKRNRPALVPNRSSTTDEDDDDDEEDREEEEDDDDEEEDEEDAQAMEVWLGSDVSDLRGPTWRAVPSLRSREIGRDS
QQFVRRVCGARGLVQRLELQ
GRLERHTGCVNTLHFNPSGTRLASGSDDLRVIIWDWAVRRAVLEFDSGHKSNVFQAKFLPHSGDSTLAMCARDGQIRVAE
LSATQRCKSTKRVAQHKGAA
HKLALEPDSPCSFLSAGEDAVVFGIDLRLDRPANKLVVVKEGDKKVGLYTIFVNPAKTQQFAVGGRDQYI
>g3161.t2
MAESDGKSSAANGEGSRVSELSAPSDAESGVGGEVGREDEDTDSMDGSGLFSLTEDGGPKREGGRRERARASDGGRRAAR
KRNRPALVPNRSSTTDEDDD

DDEEDREEEEEDDDDEEEDEEDAQAMEVWLGSDVSDLRGPTWRAVPSLRSREIGRDSQQFVRRVCGARGLVQRLELQGRLE
RHTGCVNTLHFNPSGTRLAS
GSDDLRVIIWDWAVRRAVLEFDSGHKSNVFQAKFLPHSGDSTLAMCARDGQIRVAELSATQRCKSTKRVAQHKGAAHKLA
LEPDSPCSFLSAGEDAVVFG
IDLRLDRPANKLVVVKEGDKKVGLYTIFVNPAKTQQFAVGGRDQYI
>g3161.t3
MAVISEGDISEETGQDTLDEGDASGSKGEEQAGEGSRVSELSAPSDAESGVGGEVGREDEDTDSMDGSGLFSLTEDGGPK
REGGRRERARASDGGRRAAR
KRNRPALVPNRSSTTDEDDDDDEEDREEEEDDDDEEEDEEDAQAMEVWLGSDVSDLRGPTWRAVPSLRSREIGRDSQQFV
RRVCGARGLVQRLELQGRLE
RHTGCVNTLHFNPSGTRLASGSDDLRVIIWDWAVRRAVLEFDSGHKSNVFQAKFLPHSGDSTLAMCARDGQIRVAELSAT
QRCKSTKRVAQHKGAAHKLA
LEPDSPCSFLSAGEDAVVFGIDLRLDRPANKLVVVKEGDKKVGLYTIFVNPAKTQQFAVGGRDQYI
>g3162.t1
MEYWRQCALWLINCKVLPVNHRVTAETAQVFDLAQTLRDGVLLCQLLNNLKPHSINLKEINLRPQMSQFMCLKNIRTFLS
SCAEVFGMKRCDLFEAFDLF
DVRDFGK
>g3162.t2
MEYWRQCALWLINCKVLPVNHRVTAETAQVFDLAQTLRDGVLLCQLLNNLKPHSINLKEINLRPQMSQFMCLKNIRTFLS
SCAEVFGMKRCDLFEAFDLF
DVRDFGKESL
>g3163.t1
CVDIMELSEQQDMRKFHYHTLKLYCALCALGNTRVAHALCGHLDQSQLLYIIDNKYLSGMLREGFYSVLISIHLETAREA
RLMMNNEFIIPVTPETRSIR
LFSDASKRHLPPGVGLSTSLKPRLNFAPPCFVGSKREQHLYSPQIPLDALKEKAISMLTEAVRAGGHHIRDPVGGSVQYQ
FVPILRLISTLLTMGVLGSG
DVHKV
>g3164.t1
MSVMFYNPSSALLQRVAGGQVHATQAVTVLEASGRVGGRVETYRHSNDDWYAELGAMSIPSF
>g3165.t1
MESIPAGRDSSSSPTSKQELSYPSSNHLKPNQVSETVLYGIPIVSLVIDGQERLCLAQISNTLLKNYSYNEIHNRRVALG
ITCVQCTPVQLEILRRAGAM
PISSRRCGMITKREAERLCKSFLGAHSPPKLPENFAFDVSHECAWGSRGSFIPARYNSSRAKCIKCSYCNMYFSPNKFIF
HSHRTPESKYTQPDAANFNS
WRRHLKLTDKNSADEVAHAWEDVKAMFNGGSRKRTLPMSGPDPGSSLKSHGPRLSPSSPEIPPKIIRSEDNRGSMNITTT
RSYPVIPVPSKGFGMLQKIP
PPIFPHPYGFPAFGLCQKKEDSLLGEQAKHNLSGVLWPGTKDSAYHPFPMFWPTAGSLPMPPYHQSQHKPPSELLCPPRP
NDMDLSEHSDRSTNTSKDSM
IDSERCSSTQSTRNEEDKSGDEARTMEGMTLVPRKINYVSAFRPVIKDADSIAKLYGTRGSYNSSRKGYLSPDFLSESSS
YRSASPCVDSDGEPDVDVET
NNKSPEDEDSRGPSSLCLRGLPGQSLAHGLPTSDAESKTASHGDIQRMSGHVAQSSEKDVQNKPLPENHVATSYAEVYTP
ERDELQRNSPYHFRPANYQR
GLLTSNGKEATARAPSTQKDQETQAKEELQKQLLEQVELRKKLEREFQHLK
>g3165.t2
MESIPAGRDSSSSPTSKQELSYPSSNHLKPNQVSETVLYGIPIVSLVIDGQERLCLAQISNTLLKNYSYNEIHNRRVALG
ITCVQCTPVQLEILRRAGAM
PISSRRCGMITKREAERLCKSFLGAHSPPKLPENFAFDVSHECAWGSRGSFIPARYNSSRAKCIKCSYCNMYFSPNKFIF
HSHRTPESKYTQPDAANFNS
WRRHLKLTDKNSADEVAHAWEDVKAMFNGGSRKRTLPMSGPDPGSSLKSHGPRLSPSSPEIPPKIIRSEDNRGSMNITTT
RSYPVIPVPSKGFGMLQKIP
PPIFPHPYGFPAFGLCQKKEDSLLGEQAKHNLSGVLWPGTKDSAYHPFPMFWPTAGSLPMPPYHQSQHKPPSELLCPPRP
NDMDLSEHSDRSTNTSKDSM
IDSERCSSTQSTRNEEDKSGDEARTMEGMTLVPRKINYVSAFRPVIKDADSIAKLYGTRGSYNSSRKGYLSPDFLSESSS
YRSASPCVDSDGEPDVDVET
NNKSPEDEDSRGPSSLCLRGLPGQSLAHGLPTSDAESKTASHGDIQRMSGHVAQSSEKDVQNKPLPENHVATSYAEVYTP
ERDELQRNSPYHFRPANYQR

GLLTSNDEGSSKEEPSSTVEEVESKSFTEQHSEESDEGKEATARAPSTQKDQETQAKEELQKQLLEQVELRKKLEREFQH
LK
>g3166.t1
MSRGIGGKNNADISRERTRFARVNTGWKPRFTLFPSLSTSKMPGVTVKDVNQQEFVRALAAFLKKSGKLKVPDWVDLVKL
AKHKELGPSDDNWFYVRAAS
TVRHLYLRGGAGVGSMTKIYGGRKRNGVCPAHFSVGSKNVARKVLQALELLKMVEKDPNG
>g3166.t2
MSRGIGGKNNADISRERTRFARVNTGWKPRFTLFPSLSTSKMPGVTVKDVNQQEFVRALAAFLKKSGKLKVPDWVDLVKL
AKHKELGPSDDNWFYVRAAS
TVRHLYLRGGAGVGSMTKIYGGRKRNGVCPAHFSVGSKNVARKVLQALELLKMVEKDPNGGRRVTSQGTRDLDRIAGQGQ
GPPMTQQNRSPDGGASVP
>g3166.t3
MPGVTVKDVNQQEFVRALAAFLKKSGKLKVPDWVDLVKLAKHKELGPSDDNWFYVRAASTVRHLYLRGGAGVGSMTKIYG
GRKRNGVCPAHFSVGSKNVA
RKVLQALELLKMVEKDPNGGRRVTSQGTRDLDRIAGQGQGPPMTQQNRSPDGGASVP
>g3167.t1
MGLQLSFILLWFVSDAVFGQLSYSVSEEANRGTAVGNLAKDLNLNLQELESRMFQVVIGSKRKYFEVNLKTGVLFVNERM
DREELCPEEPKCTVSVEAVI
NNPLKLFRIEVDILDANDNAPLFTEPLQMLHIAESTLPGVKFALSEATDADVGKNGVNTYKLSPNEYFTLAVHKAEAAAS
AELVLQKGTTVIAVNATDAD
EGVNSEIIYSLRSKDQDHVLDIFQIDSATGVITVKGNVDFEVKKAFEIRVEARDKGQPPMTAHCKVLVEVIDVNDNAPEI
TVTSLLDGVKEDAQAGTAIA
LVSVLDKDGGRNDRESASQHNVTITANDEGSPRLSSQSFISVHVTDVNDNAPHFSEPSVRIYVKENCPVGATIESISAID
ADVDSGVPPLSSNVTVNVFI
LDENDNSPGILPPYSEHGSVNSENIPYSAEAGYFVAKIRAVDADSGYNALLSYHMSEPKGTNLFRIGTSTGEIRTKRRMS
DNDLKTHPLVVLVSDNGEPS
LTATVSIDVVVVENTGDIQTQFRHVPLKEDSFSALNLYLLIAIASVSVIFLLSLISLIAVKCHRTDNSFS
>g3168.t1
LGGSCSVGRVGGQSGSSTNRRVSGSIPLIPPPPTGHMLNPKNVLYDIATMQLCANKLDKKDFFGKSDPFLVFYRSNEDGT
YVSVG
>g3169.t1
PPPRCKDIDASACSGDQAFQEVRVQQAVVEFILNHTEQIFNHGTGLCKAKEGLSFACGEKYATLPLSGGHCGPMKLMSLE
EAQARSLGPGHPVHRERQRE
NSLPDASTATLYHTVLDISDTKRKFSGKSKKWKSIFNLGRSGNESKGKLSRNGSVFIRGPNTDDDRTGSPPGAANGALSP
DAKSRTLGSDSLYDLREEDQ
RWDFKGKTTPTGAAAGGSSPATNQREAAGSSSSSSSSSTPQKTLPEQLKVFKGDDLAGFKPTSPKNRRMLYSGSAHHHHS
ASKPSFPGSLFPLESSSPRH
QRKAVNISEPFAVSVPLRVSAVISSNSTPCGAHVKDKTGAGTAAGLRPAPGEGSSNSSSSSSSLLQLETDGRGKQAGDAL
EQATSDLTTADPNDRVSQEA
ADGKGLSKLERSGEQFTSSPGKLDEGSSPTVRELWSDLHHELKITEPELDLLDKTFKPIFCPEGSYEISSLRKSSTQGSS
EVLKTKSGRGQSQRSSSSPT
LSVFHGGPKAAAVILTDHPSSGPNRVRKQASESDILHPFYQNMDFAVGDKKLNPFPCAPSKDEKMSKPHRALMVKSTSLG
TSTDFKQWQDLSGNKSLDFT
QANLVEKPVDDAGDGRFSGGASKGAVEIETYENHKPSEEVLVETTSTKIPSEAGQEPLAEVKKVATSSKCLVDRPRTSSE
GGQNPLAQIPRAGISSIRLE
DRPKTLVFYHLDEEEACAGHSEELDLVETLDELSSGSSQWVTSPLHTPEIEDLFRNNLLLAPPSSTLSSAEGVGGGTSLH
SLNTNKPSPAGNGTPSGTPQ
VSQGQRNAEAPEEPKPPRFQLLLPRTNGTGLNAAPPQTGKGASAIKPKSSSGNGNPPPQRFRRQMSQEACPPPSLRRDEV
AASKHARPCSLNLDLVGRRC
IRDISNQQQPQGRNPQEPPAFQRGPPPPRPVDNGPAEALDLELFLSNSHAPLRRNSAPVSVSSVRTAFMIKTSQAKAVPV
IPPKVQYTQVPHSRRDADVH
GAAREQENQNPRRTGAQE
>g3169.t2
PPPRCKDIDASACSGDQAFQEVRVQQAVVEFILNHTEQIFNHGTGLCKAKEGLSFACGEKYATLPLSGGHCGPMKLMSLE
EAQARSLGPGHPVHRERQRE
NSLPDASTATLYHTVLDISDTKRKFSGKSKKWKSIFNLGRSGNESKGKLSRNGSVFIRGPNTGTIPTLSTGLNHNSTYGQ

SLVKIKPIITAQKAVEFVAH
QPSRLDVTWNDDDRTGSPPGAANGALSPDAKSRTLGSDSLYDLREEDQRWDFKGKTTPTGAAAGGSSPATNQREAAGSSS
SSSSSSTPQKTLPEQLKVFK
GDDLAGFKPTSPKNRRMLYSGSAHHHHSASKPSFPGSLFPLESSSPRHQRKAVNISEPFAVSVPLRVSAVISSNSTPCGA
HVKDKTGAGTAAGLRPAPGE
GSSNSSSSSSSLLQLETDGRGKQAGDALEQATSDLTTADPNDRVSQEAADGKGLSKLERSGEQFTSSPGKLDEGSSPTVR
ELWSDLHHELKITEPELDLL
DKTFKPIFCPEGSYEISSLRKSSTQGSSEVLKTKSGRGQSQRSSSSPTLSVFHGGPKAAAVILTDHPSSGPNRVRKQASE
SDILHPFYQNMDFAVGDKKL
NPFPCAPSKDEKMSKPHRALMVKSTSLGTSTDFKQWQDLSGNKSLDFTQANLVEKPVDDAGDGRFSGGASKGAVEIETYE
NHKPSEEVLVETTSTKIPSE
AGQEPLAEVKKVATSSKCLVDRPRTSSEGGQNPLAQIPRAGISSIRLEDRPKTLVFYHLDEEEACAGHSEELDLVETLDE
LSSGSSQWVTSPLHTPEIED
LFRNNLLLAPPSSTLSSAEGVGGGTSLHSLNTNKPSPAGNGTPSGTPQVSQGQRNAEAPEEPKPPRFQLLLPRTNGTGLN
AAPPQTGKGASAIKPKSSSG
NGNPPPQRFRRQMSQEACPPPSLRRDEVAASKHARPCSLNLDLVGRRCIRDISNQQQPQGRNPQEPPAFQRGPPPPRPVD
NGPAEALDLELFLSNSHAPL
RRNSAPVSVSSVRTAFMIKTSQAKAVPVIPPKVQYTQVPHSRRDADVHGAAREQENQNPRRTGAQE
>g3170.t1
ETARQVGGLREMLVDVNWTVGAVNHTVSSDISIHHLKIQDMQIQISNITEDTTSLWVTHVHNEAQLRSEMEILNTITEDL
RLKDWEHSMFLKNLTILEGA
HSLFYS
>g3171.t1
SVGGPENFNLGSMPSAQQQITTGQMHRGHMPPLKDVLSEDDGGSQGVGLPAARRVGWIPFVTSPFAPH
>g3172.t1
MEEEVVVVVVVVVEEEVVRW
>g3173.t1
MECMVKVVEVMEVVVVLVLVEEVVVEVEEEVVVVVEMVVVVVVVVVELEVVVVVVEEVVMEEVVVVVEVVVVMVVVVVVV
MVEVVGVVLVVVEEVVLVVV
GGW
>g3174.t1
MRTREALSQLPPRLRWPSSTLAFVFVWPSSVLAFVFVWPWSAPLERGCVRALVTCSDQGFMLAQIPEYFLYKY
>g3175.t1
PYLPPFASSLETLHTLLASKKRLSEMAFRLLPTDTLTDLTPSPGPDHQYSLGSSGEEASEPVDPDRRRAAMAQHKVAEVR
KLVAKHCAASPAYQMLKARF
LSCFTVPALLATITPDQQQGVLKTVKRARKRLSEKRERDHRETVKDFHRDLVVLGKDYEESSRSTCEDLLSWLGEMDQSL
ETLMDGIECQQQVSLQELND
AWKRVKETFVLKRGRIRELGVNLTGYEIQRTQQIAVLLKKYCTLLERISFLSPSDVYRLLDTEAMMINNALLANHRSIAR
LRLHLLEEDLQKESVLRLRW
QDRSASWVTGRVHQEVERIREQFWRSEGHNKLCLKNRDASSSTSGTSIAQHYNRHYVLWCHEALCSLALSEEEVQDIVSS
QILSLIGPCQRQAEERLAIL
DRDVDVVARHAGILSRTVWHVLRGATLLWETHSQAVEHREQRLEEELRLLRCQQEEEIQRKEAHLDVLLDRLRQESSEEA
LRSSLDTALAYLDDIPNRRV
TSESLSHECHDSREILDITEGRCTEME
>g3175.t2
PYLPPFASSLETLHTLLASKKRLSEMAFRLLPTDTLTDLTPSPGPDHQYSLGSSGEEASEPVDPDRRRAAMAQHKVAEVR
KLVAKHCAASPAYQMLKARF
LSCFTVPALLATITPDQQQGVLKTVKRARKRLSEKRERDHRETVKDFHRDLVVLGKDYEESSRSTCEDLLSWLGEMDQSL
ETLMDGIECQQQELNDAWKR
VKETFVLKRGRIRELGVNLTGYEIQRTQQIAVLLKKYCTLLERISFLSPSDVYRLLDTEAMMINNALLANHRSIARLRLH
LLEEDLQKESVLRLRWQDRS
ASWVTGRVHQEVERIREQFWRSEGHNKLCLKNRDASSSTSGTSIAQHYNRHYVLWCHEALCSLALSEEEVQDIVSSQILS
LIGPCQRQAEERLAILDRDV
DVVARHAGILSRTVWHVLRGATLLWETHSQAVEHREQRLEEELRLLRCQQEEEIQRKEAHLDVLLDRLRQESSEEALRSS
LDTALAYLDDIPNRRVTSES
LSHECHDSREILDITEGRCTEME

>g3175.t3
MAESRVIPSGRLYRQMFDAQRLSEKRERDHRETVKDFHRDLVVLGKDYEESSRSTCEDLLSWLGEMDQSLETLMDGIECQ
QQVSLQELNDAWKRVKETFV
LKRGRIRELGVNLTGYEIQRTQQIAVLLKKYCTLLERISFLSPSDVYRLLDTEAMMINNALLANHRSIARLRLHLLEEDL
QKESVLRLRWQDRSASWVTG
RVHQEVERIREQFWRSEGHNKLCLKNRDASSSTSGTSIAQHYNRHYVLWCHEALCSLALSEEEVQDIVSSQILSLIGPCQ
RQAEERLAILDRDVDVVARH
AGILSRTVWHVLRGATLLWETHSQAVEHREQRLEEELRLLRCQQEEEIQRKEAHLDVLLDRLRQESSEEALRSSLDTALA
YLDDIPNRRVTSESLSHECH
DSREILDITEGRCTEME
>g3175.t4
PYLPPFASSLETLHTLLASKKRLSEMAFRLLPTDTLTDLTPSPGPDHQYSLGSSGEEASEPVDPDRRRAAMAQHKVAEVR
KLVAKHCAASPAYQMLKARF
LSCFTVPALLATITPDQQQGVLKTVKRARKNSPLLQEGDGRPATHFSGITTHRPLETKNTSQDSSQSGPGQTPQNQARPN
C
>g3176.t1
MPAATVDHSQRICEVWANNLDEELKRIRHVIRKYNYIAMDTEFPGVVARPIGEFRSNADYQYQLLRCNVDLLKIIQLGLT
FMNEEGEYPPETSTWQFNFK
EDMYAQDSIELLTTSGIQFKKHEDEGIDTLYFAELLMTSGVVLCEGVKWLSFHSGYDFGYLIKLLSNANLPEEEVDFFEI
LRLYFPVIYDVKYLMKSCKN
LKGGLQEVAEQLDLERVGPQHQAGSDSLLTGMAFFKMREMFFEDHIDDAKYCGHLYGLGSGSA
>g3176.t2
MSPCLSSFCGSASPFLCRQAVSEEGASVLVHCSDGWDRTAQVCSVACVLLEPYYRTLKGLMVLLEVRKTQKVSATNSPTT
FETQVLIEREWVSFGHKFSH
RCNHLDGDSKEVSPVMDQFLECVWQLTEQFPCAFEFNERLLIAVHHHMYACQYGDFIGNNQRERLELGLQNKTHSIWKHL
WDSRCDYINPLYRTDNCQSQ
GVLRPSTAPYCFKFWSGLYNRFDRGMHPRQSVEDYLMAVREETLQLEEQLASHKEANTPQDYTGGFLAGSPCQQRSGTMP
AATVDHSQRICEVWANNLDE
ELKRIRHVIRKYNYIAMDTEFPGVVARPIGEFRSNADYQYQLLRCNVDLLKIIQLGLTFMNEEGEYPPETSTWQFNFKED
MYAQDSIELLTTSGIQFKKH
EDEGIDTLYFAELLMTSGVVLCEGVKWLSFHSGYDFGYLIKLLSNANLPEEEVDFFEILRLYFPVIYDVKYLMKSCKNLK
GGLQEVAEQLDLERVGPQHQ
AGSDSLLTGMAFFKMREMFFEDHIDDAKYCGHLYGLGSGSA
>g3176.t3
MSPCLSSFCGSASPFLCRQAVSEEGASVLVHCSDGWDRTAQVCSVACVLLEPYYRTLKGLMVLIEREWVSFGHKFSHRCN
HLDGDSKEVSPVMDQFLECV
WQLTEQFPCAFEFNERLLIAVHHHMYACQYGDFIGNNQRERLELGLQNKTHSIWKHLWDSRCDYINPLYRTDNCQSQGVL
RPSTAPYCFKFWSGLYNRFD
RGMHPRQSVEDYLMAVREETLQLEEQLASHKEANTPQDYTGGFLAGSPCQQRSGTMPAATVDHSQRICEVWANNLDEELK
RIRHVIRKYNYIAMDTEFPG
VVARPIGEFRSNADYQYQLLRCNVDLLKIIQLGLTFMNEEGEYPPETSTWQFNFKEDMYAQDSIELLTTSGIQFKKHEDE
GIDTLYFAELLMTSGVVLCE
GVKWLSFHSGYDFGYLIKLLSNANLPEEEVDFFEILRLYFPVIYDVKYLMKSCKNLKGGLQEVAEQLDLERVGPQHQAGS
DSLLTGMAFFKMREMFFEDH
IDDAKYCGHLYGLGSGSA
>g3176.t4
MSPCLSSFCGSASPFLCRQAVSEEGASVLVHCSDGWDRTAQVCSVACVLLEPYYRTLKGLMVLLEVRKTQKVSATNSPTT
FETQVLIEREWVSFGHKFSH
RCNHLDGDSKEVSPVMDQFLECVWQLTEQFPCAFEFNERLLIAVHHHMYACQYGDFIGNNQRERLELGLQNKTHSIWKHL
WDSRCDYINPLYRTDNCQSQ
GVLRPSTAPYCFKFWSGLYNRFDRGMHPRQSVEDYLMAVREETLQLEEQLASHKEVRGHR
>g3177.t1
MCRCAVTVGAEGVQERAQHTALRNASDQGQRNVTVNLWVVNESWLFSLLWCTGVSSVTTNSCHRFQRMEQPDWTLGPTYY
TVIWITLDLLSFLIISGLVI
YQGVAEQHRCGPLERCVNNGGAACNACDTERAVDLLRQYQANLTTPQEQALNASVGKVSAIFGSQLFHALLGIKGRQQRG
KGQRVDLE

>g3177.t2
MCRCAVTVGAEGVQERAQHTALRNASDQGQRNVTVNLWVVNESWLFSLLWCTGVSSVTTNSCHRFQRMEQPDWTLGPTYY
TVIWITLDLLSFLIISGLVI
YQDVRVAAVQPGERSCRAVWTQGRFNHFSFVHTSRVREGAVYLILFGVAEQHRCGPLERCVNNGGAACNACDTERAVDLL
RQYQANLTTPQEQALNASVG
KVSAIFGSQLFHALLGIKGRQQRGKGQRVDLE
>g3177.t3
MASSILLFWRELGQRNVTVNLWVVNESWLFSLLWCTGVSSVTTNSCHRFQRMEQPDWTLGPTYYTVIWITLDLLSFLIIS
GLVIYQGVAEQHRCGPLERC
VNNGGAACNACDTERAVDLLRQYQANLTTPQEQALNASVGKVSAIFGSQLFHALLGIKGRQQRGKGQRVDLE
>g3178.t1
MQHLVMRLVFFLPKAHGMLTGFLEAQTTIPSPAHADGQEEKKILFYHPSEVEKNEKIRNVGLCEAIVQFTRTFSPTKPAK
SLHTQKNRQFFFEPEDNFWI
VMIVRNPMVEKPNKDGKPPTVEYQEEEVLDNVYGAVLRQCYSMYKLFNGTFGRAMEAGGVHLLVQKLEKFFYR
>g3178.t2
MQHLVMRLVFFLPKAHGMLTGFLEAQTTIPSPAHADGQEEKKILFYHPSEVEKNEKIRNVGLCEAIVQFTRTFSPTKPAK
SLHTQKNRQFFFEPEDNFWI
VMIVRNPMVEKPNKDGKPPTVEYQEEEVLLFNGTFGRAMEAGGVHLLVQKLEKFFYR
>g3179.t1
ECCGTARHLLLILLPRFFLQRFVPGARTLTEAETKTFVSAADDDNDGRIGVEGQ
>g3179.t2
FFLQRFVPGARTLTEAETKTFVSAADDDNDGRIGVEGQ
>g3180.t1
GTYFLISRSLGPELGGSIGLVFAFANAVAVSMHTVGFAETVQSLMHESDVVMVDRLNDIRIIGVITVTCLLAISLAGMEW
ESKAQIVFFIVIMVSFINYI
IGTIIPATPEKQAKGFFSYKADIFAANFVPDWRGADGSFFGMFSIFFPSATGILAGANISGDLKDPTMAIPKGTLLAIFW
TTISYLIIAATIGACVVRDA
SGLLNDTVAGSSGNDSCAGLACSYGWDFGGCMRNNSCVHGIS
>g3180.t2
GTYFLISRSLGPELGGSIGLVFAFANAVAVSMHTVGFAETVQSLMHESDVVMVDRLNDIRIIGVITVTCLLAISLAGMEW
ESKAQIVFFIVIMVSFINYI
IGTIIPATPEKQAKGFFSYKDAHMITNLLPSLTADIFAANFVPDWRGADGSFFGMFSIFFPSATGILAGANISGDLKDPT
MAIPKGTLLAIFWTTISYLI
IAATIGACVVRDASGLLNDTVAGSSGNDSCAGLACSYGWDFGGCMRNNSCVHGIS
>g3180.t3
GTYFLISRSLGPELGGSIGLVFAFANAVAVSMHTVGFAETVQSLMHESDVVMVDRLNDIRIIGVITVTCLLAISLAGMEW
ESKAQIVFFIVIMVSFINYI
IGTIIPATPEKQAKGFFSYKDGSFFGMFSIFFPSATGILAGANISGDLKDPTMAIPKGTLLAIFWTTISYLIIAATIGAC
VVRDASGLLNDTVAGSSGND
SCAGLACSYGWDFGGCMRNNSCVHGIS
>g3181.t1
WGVEVTGHLQTRYSQHQGWFNLASAAADLHAVYFWMIPACFHLHRDLGLKMVWVAVLGDWLNLLLKWVLFGERPYWWVYE
TTFYGEGPVPVLKQFPITCE
TGPGSPSGHTMGATGVWYAMVTTLVSMAAEKGCPPLLHRFLQLVLWMLVALFILLVSVSRVYMAAHFPQQVIGGVIAGVL
VARSVSRMQWIYSASLRQYL
YTTLFLTFFVVGVYLVLKAMGVDVMWTLEKAQKRCARAEWVHLGSSPFASLMRNMGTLLGLGLGLHSPLYTDARRRGRGV
AFKVGCVGVSMALLWLLDGY
RVSSHWHALFYALSFGKNALALLIPTALVPGALGLLFPGGRAEKDL
>g3181.t2
MDLFHSWGVEVTGHLQTRYSQHQGWFNLASAAADLHAVYFWMIPACFHLHRDLGLKMVWVAVLGDWLNLLLKWVLFGERP
YWWVYETTFYGEGPVPVLKQ
FPITCETGPGSPSGHTMGATGVWYAMVTTLVSMAAEKGCPPLLHRFLQLVLWMLVALFILLVSVSRVYMAAHFPQQVIGG
VIAGVLVARSVSRMQWIYSA
SLRQYLYTTLFLTFFVVGVYLVLKAMGVDVMWTLEKAQKRCARAEWVHLGSSPFASLMRNMGTLLGLGLGLHSPLYTDAR
RRGRGVAFKVGCVGVSMALL
WLLDGYRVSSHWHALFYALSFGKNALALLIPTALVPGALGLLFPGGRAEKDL

>g3182.t1
MSKQLTVEEWRPCHPITISPLTLHPAHFQRTVTWTQLHLSVVLLTWTQLHHPVVLVTWTQLHLPVVLVTWTQLHLSVVLV
TWTQLHLSVVLVAWNISKSA
VQPRQSALRHTGALANR
>g3182.t2
MSKQLTVEEWRPCHPITISPLTLHPAHFQRTVPVSWTQLHHPVVLVTWTQLHLSVVLVTWTQLHLSVVLLTWTQLHHPVV
LVTWTQLHLPVVLVTWTQLH
LSVVLVTWTQLHLSVVLVAWNISKSAVQPRQSALRHTGALANR
>g3183.t1
LVLLVVVVVVVGHKLQELGRKVTSIREPPIVKARFGPTPVTAKAGACKTFRMSLSTKLTLDKVDVKGKRVIMRVDFNVPM
QGKQITNNQRIKAAVPTIKH
CLDNGAKAVVLMSHLGRPDGVPMPDKYSLEPVVAELKALLGKDVTFLKDCVGSEVEAACTAPAPGSVILLENLRFHVAEE
GKGKDAAGNKTKASQAEIDS
FRASLSKLGDVYVNDAFGTAHRAH
>g3183.t2
SEASHCPGLVLLVVVVVVVGHKLQELGRKVTSIREPPIVKARFGPTPVTAKAGACKTFRMSLSTKLTLDKVDVKGKRVIM
RVDFNVPMQGKQITNNQRIK
AAVPTIKHCLDNGAKAVVLMSHLGRPDGVPMPDKYSLEPVVAELKALLGKDVTFLKDCVGSEVEAACTAPAPGSVILLEN
LRFHVAEEGKGKDAAGNKTK
ASQAEIDSFRASLSKLGDVYVNDAFGTAHRAH
>g3183.t3
SEASHCPGLVLLVVVVVVVGHKLQELGRKVTSIREPPIVKARFGPTPVTAKAGACKTFRMSLSTKLTLDKVDVKGKRVIM
RVDFNVPMQGKQITNNQRIK
AAVPTIKHCLDNGAKAVVLMSHLGRPDGVPMPDKYSLEPVVAELKALLGKDVTFLKDCVGSEVEAACTAPAPGSVILLEN
LRFHVAEEGKGKDAAGNKTK
ASQAEIDSFRASLSKLGDVYVNDAFGTAHRAHR
>g3184.t1
TSEQQVSNHSPEHEALLRPQAAEEEEEEDEEDEEGTVGRAAHS
>g3185.t1
MPGSSQPPLFSSYPWKNPKLLPRQLSADADATATKTTALCRCSMSKRGKAGRLGVISAGYCFIMLLINVLPGLWELGAAL
RSGSITALVSSIHTLSPSSR
TDGEKL
>g3186.t1
LPRSTAALDLLRKTRSRGRVGSRKRRAGPSRSGRKLEKRLRGVMRTARRASNVEVPSFLRQLAKETEKMVTFFFKGGPRE
RDAGEDGDPDGEDAAPSPYL
DRPVLEKQVAEGGCAGGLSYAVASMQGWRTQMEDAHTCMAQLKGELEAWSYFAVFDGHAGSTVAQYCSRNLLDHILATVK
TPSIVSMMLGGAALL
>g3186.t2
LPRSTAALDLLRKTRSRGRVGSRKRRAGPSRSGRKLEKRLRGVMRTARRASNVEVPSFLRQLAKETEKMVTFFFKGGPRE
RDAGEDGDPDGEDAAPSPYL
DRPVLEKQVAEGGCAGGLSYAVASMQGWRTQMEDAHTCMAQLKGELEAWSYFAVFDGHAGSTVAQYCSRNLLDHILAT
>g3187.t1
VFSYFHAQYTFFHQGFGLLKGLEPTMETMATQLAQLSADCTVKRKDLENQHLLVQQRMAGARQRWSPLVEDEHAAEHWIL
ENHNKLLNSATDEEAEHCSD
DRVPEQTEEHCSDGRVPEQTEQHYSYGHVPEQTEDSTDCSGSSRTSEHKALCQQLLPIESDRSIPDTMGSKETRAPDTMD
SKETRTPDTMNSKETRTPDT
METKETSKPGDSAEGGNSNQTHVSIDEGMSSSASKKDCMLVLHEAHLMISRPLASWHGE
>g3187.t2
VFSYFHAQYTFFHQGFGLLKGLEPTMETMATQLAQLSADCTVKRKDLENQHLLVQQRMAGARQRWSPLVEDEHATDVEVE
DLSLHMNVQGDEPNDLELKA
EHWILENHNKLLNSATDEEAEHCSDDRVPEQTEEHCSDGRVPEQTEQHYSYGHVPEQTEDSTDCSGSSRTSEHKALCQQL
LPIESDRSIPDTMGSKETRA
PDTMDSKETRTPDTMNSKETRTPDTMETKETSKPGDSAEGGNSNQTHVSIDEGMSSSASKKDCMLVLHEAHLMISRPLAS
WHGE
>g3188.t1
MSESLYEKFLAPEEPFPLLSQRANLSDVGTLDVSDFGCQLSSCHRTDPLRRFHSNRWNLTSCGTSVASSECSEELFSSVS

VGDQDDCYSLLDDQELTSLD
LFPEGSVCSDVSSSISTYWDWSDSEFEWQLPGSDIASGSDVLSDIIPSVPSSPCLFSKRKAKPHPHPHRNLDELPWSAMT
NDEQVEYIEYLSRKVSTEMG
LREQLDIIKIIDPCAQISPTDSEFIIELNCLTDEKLKQ
>g3188.t2
MSESLYEKFLAPEEPFPLLSQRANLSDVGTLDVSDFGCQLSSCHRTDPLRRFHSNRWNLTSCGTSVASSECSEELFSSVS
VGDQDDCYSLLDDQELTSLD
LFPEGSVCSDVSSSISTYWDWSDSEFDLKHVSTTVPSQLPGSDIASGSDVLSDIIPSVPSSPCLFSKRKAKPHPHPHRNL
DELPWSAMTNDEQVEYIEYL
SRKVSTEMGLREQLDIIKIIDPCAQISPTDSEFIIELNCLTDEKLKQ
>g3189.t1
IDILKYIIYGLAGGFFVLGVLLLVEGFFTTGAIRDLYGEFKITACGRCLTAFLMFLAYLFFLVWIGVTAFTGLPVFMYFN
IWSMCQNASLVDGANLCLDL
RQFGVVPISEERKVCTGSEKFFKMCESNELDMTFHLFVCALAGAGAAAIAMVHFLMALAANWGYLKDASRMQKYEDIKSK
EEQELHDIHSTRSKERLNAY
T
>g3189.t2
IDILKYIIYGLAGGFFVLGVLLLVEGFFTTGAIRDLYGEFKITACGRCLTAFLMFLAYLFFLVWIGVTAFTGLPVFMYFN
IWSMCQNASLVDGANLCLDL
RQFGKRGRVVPISEERKVCTGSEKFFKMCESNELDMTFHLFVCALAGAGAAAIAMVHFLMALAANWGYLKDASRMQKYED
IKSKEEQELHDIHSTRSKER
LNAYT
>g3190.t1
LKLNEGYQSRSQAVGMWQVLVDERILVHVKLELNFHDKDTQFYRFLESEFSQDPMKPTEKDSEKAFEKDSERDSEDELQE
GLSLLSQMGPDALLTMILRK
>g3190.t2
LLKLNEGYQSRSQAVGMWQVLVDERILVHVKLELNFHDKDTQFYRFLESEFSQDPMKPTEKDSEKAFEKDSERDSEDELQ
EGLSLLSQMGPDALLTMILR
K
>g3190.t3
LKLNEGYQSRSQAVGMWQVLVDERILVHVKLELNFHDKDTQFYRFLESEFSQDPMKPTEKDSEKAFEKDSERDSEDELQE
GLSLLSQMGPDALLTMILRK
LS
>g3191.t1
MSKDELILPKDFPQLKNDTFLRAARGEQTEHVPVWCMRQAGRYLPEFREFRAGKDFFDTCRSPEGCCELTLQAMGMEVQM
VPGKGPTFPDPLKDPEDLQR
LKTKVDVKKELDYVFKAITLTRHKIEGKVPLIGFTGAP
>g3191.t2
MSKDELILPKDFPQLKNDTFLRAARGEQTEHVPVWCMRQAGRYLPEFREFRAGKDFFDTCRSPEGCCELTLQPLRRFDFD
ASIIFSDILVVPQAMGMEVQ
MVPGKGPTFPDPLKDPEDLQRLKTKVDVKKELDYVFKAITLTRHKIEGKVPLIGFTGAP
>g3191.t3
MSKDELILPKDFPQLKNDTFLRAARGEQTEHVPVWCMRQAGRYLPEFREFRAGKDFFDTCRSPEGCCELTLQAMGMEVQM
VPGKGPTFPDPLKDPEDLQR
LKTKVDVKKELDYVFKAITLTRHKIEGK
>g3192.t1
MAYIEKRNYIHRDLRAANVLVSESLLCKIADFGLARVIEDDQYTAREGAKFPIKWTAPEAINYGSFTIKSDMWSFGVLLY
EIITLGKIPYPGMNKSEVIS
SIQHGYRMPQPHNCPAELYQIMMSCWKDKPDDRPTFDYMQSVLGDFYTATEAQYQPQL
>g3192.t2
IAEGMAYIEKRNYIHRDLRAANVLVSESLLCKIADFGLARVIEDDQYTAREGAKFPIKWTAPEAINYGSFTIKSDMWSFG
VLLYEIITLGKIPYPGMNKS
EVISSIQHGYRMPQPHNCPAELYQIMMSCWKDKPDDRPTFDYMQSVLGDFYTATEAQYQPQL
>g3192.t3
LQIAEGMAYIEKRNYIHRDLRAANVLVSESLLCKIADFGLARVIEDDQYTAREGAKFPIKWTAPEAINYGSFTIKSDMWS
FGVLLYEIITLGKIPYPGMN

KSEVISSIQHGYRMPQPHNCPAELYQIMMSCWKDKPDDRPTFDYMQSVLGDFYTATEAQYQPQL
>g3193.t1
LLGMVAHMMYTTAFQLTVSLGPEDWKPQTWDYSWSYM
>g3194.t1
KPLLWTPFIQRPLHKRKGGQVRFSNDQTIELEKKFETQKYLSPPERKRLAKMLQLSERQ
>g3194.t2
KPLLWTPFIQRPLHKRKGGQVRFSNDQTIELEKKFETQKYLSPPERKRLAKMLQLSERQVCSVPV
>g3195.t1
LVNRVQGDREVRRVHVVDEEPEGPQASRAPRGQQATMDPQAHPVRGGLKDPRGQSASPDQRAHLDLQEKMVCLDTLASVE
RQ
>g3195.t2
MSLQHLRVHVVDEEPEGPQASRAPRGQQATMDPQAHPVRGGLKDPRGQSASPDQRAHLDLQEKMVCLDTLASVERQ
>g3195.t3
LVNRVQGDREVRRVHVVDEEPEGPQASRAPRGQQATMDPQAHPVRGGLKDPRGQSASPDQRAHLDLQEKMVCLDTLASVE
R
>g3196.t1
MSTNLSFYPGEDKEREPIVVVGPVDISKSPVSLTFSACEGKVCLNLNKYVKLKVVVLGVAVLVMEVVVMVLVVVMEVEVV
VMEAAVVVVVMEVEMVMVVV
MEVVVVMEEVVVMEEVVVVMEVMVVMEVVVQVVVVVEVVVGMVMEVVVGRRWWW
>g3197.t1
NSKEEALNTALGGDQLHLQDSGLQELSRAVIAELHHMPGNDACADCGAPEPTWLSTNLGILTCIECSGIHRELGVHYSRI
QSLTLDVLSTSELLLAVSIG
NTRFNDIMEAGLPNDSVKPLPQSDMNARKEYIIAKYVERRFVTRREDADPCRAYDAIRGRSLTALLQLYAEGVDLAKPLK
LPDGQVRRHRCSRGASSCCF
REQQ
>g3197.t2
WVSVLQNSKEEALNTALGGDQLHLQDSGLQELSRAVIAELHHMPGNDACADCGAPEPTWLSTNLGILTCIECSGIHRELG
VHYSRIQSLTLDVLSTSELL
LAVSIGNTRFNDIMEAGLPNDSVKPLPQSDMNARKEYIIAKYVERRFVTRREDADPCRAYDAIRGRSLTALLQLYAEGVD
LAKPLKLPDGQVRRHRCSRG
ASSCCFREQQ
>g3197.t3
NSKEEALNTALGGDQLHLQDSGLQELSRAVIAELHHMPGNDACADCGAPEPTWLSTNLGILTCIECSGIHRELGVHYSRI
QSLTLDVLSTSELLLAVSIG
NTRFNDIMEAGLPNDSVKPLPQSDMNARKEYIIAKYVERRFVTRREDADPCRAYDAIRGRSLTALLQLYAEGVDLAKPLK
LPDGQ
>g3198.t1
LVRLQNCVSDRVVSNTGAPQGMVLSRPFLFTLYTSDFNYCTESCHLQKFSDDSAIVGCIKEGDEGEYRTVVSNFVSWCEL
NHLQLNGTKTKELIVDLRRT
KSLVCPVFIQGVPVDTVEEYKYLGVYFNNKLDWTRNTEAIYKKGRLFFLRRLRSFNICRTLLRMFYESVVASAIFFAVSC
WGSGMRVGDINRLNRLFRRA
GDVVGEELDTLMIVAERRMLSRLRSILDNASHPLFSTLAQRRSTFSRRLLSPRCTTERHRKSFLPVAIKLYNTSL
>g3199.t1
QQMELARQQQEQIAKQQQQLIQQQHKINMLQQQIQVNMPYVMIPAFHPSAQALQVPSDPQLSLPLQPIPCKP
>g3199.t2
QQMELARQQQEQIAKQQQQLIQQQHKINMLQQQIQVNMPYVMIPAFHPSAQALQVPSDPQLSLPLQPIPCKPA
>g3199.t3
IAKQQQQLIQQQHKINMLQQQIQVNMPYVMIPAFHPSAQALQVPSDPQLSLPLQPIPCKPA
>g3200.t1
MSGLDHAFRCCEILNIPDLPVNTPPVMNGVDVNGLSECVGGGVSLLVRDSGGCSALHLAAQSGHTPLVSYILHQVVRGRL
LYVAALGAGKTPADQAQAEG
DYELSSYLSTQQLLTPTPVTHEDLETAV
>g3200.t2
MSGLDHAFRCCEILNIPDLPVNTPPVMNGVDVNGLSECVGGGVSLLVRDSGGCSALHLAAQSGHTPLVSYILHQGSKVLL
DLRDREKGDTALHKAASGKQ
HAVCRLLVEAGASLKRTNFLGKTPADQAQAEGDYELSSYLSTQQLLTPTPVTHEDLETAV

>g3200.t3
CCEILNIPDLPVNTPPVMNGVDVNGLSECVGGGVSLLVRDSGGCSALHLAAQSGHTPLVSYILHQVVRGRLLYVAALGAG
KTPADQAQAEGDYELSSYLS
TQQLLTPTPVTHEDLETAV
>g3201.t1
MAASLLRRDKRATAAHLKADLNQTDNVALILQLQELLDTVLNPETPATDTEALDWCKCLIAGGDGFEEFCKTVRSYDNAA
LCGLVWTANFVAYRCRTCGI
SPCMSLCAECFNNGDHTGHDFNMFRSQAGGACDCGDGNVMRESGHRLKTGENVPSVPRDLLLMSEMVLPRFIISVIQYLR
EGYTEPDAGSERDLQKVLQQ
LEPQISFLEELTKMGGAMRTVLTKILTNQQMFKDLSMGQEDNPYAKQNYEKYLSALKNSGLVSAEEKALPPSAELVAGAE
GAAGSLGSSGAAAAGSPDES
GKEEDQDGGQSVGQRKRVKLSSATKDSSIMDSLKHKCFLEELLFWTIKYEFPQKMVTFLLNMLPDQDYKITFTKTFVQHY
AFIMKTLMKSHESDTMSNRI
VHISVQLFSNEELARRVTEECQLLDVMVTVLLYMMESCLVKSELQ
>g3201.t2
MAASLLRRDKRATAAHLKADLNQTDNVALILQLQELLDTVLNPETPATDTEALDWCKCLIAGGDGFEEFCKTVRSYDNAA
LCGLVWTANFVAYRCRTCGI
SPCMSLCAECFNNGDHTGHDFNMFRSQAGGACDCGDGNVMRESGHRLKTGENVPSVPRDLLLMSEMVLPRFIISVIQYLR
EGYTEPGEITDAGSERDLQK
VLQQLEPQISFLEELTKMGGAMRTVLTKILTNQQMFKDLSMGQEDNPYAKQNYEKYLSALKNSGLVSAEEKALPPSAELV
AGAEGAAGSLGSSGAAAAGS
PDESGKEEDQDGGQSVGQRKRVKLSSATKDSSIMDSLKHKCFLEELLFWTIKYEFPQKMVTFLLNMLPDQDYKITFTKTF
VQHYAFIMKTLMKSHESDTM
SNRIVHISVQLFSNEELARRVTEECQLLDVMVTVLLYMMESCLVKSELQ
>g3202.t1
FLRTRTCPEYLEGRSAPRPQRPQWAPASLRIDAPVNPGAVRDAAPAAWNARSVAGCQ
>g3203.t1
ARSMTGLSGEVFGNHDNHSNNSNNNSNNNNNNNNNHSLFPTSRLSVEDCCAFSRTP
>g3204.t1
DCLVP
>g3205.t1
LFCWTPFFVVHTTRAVCETCDIPASLMNIVTWLGYVNSALNPIIYTIFNTEFRKFFKKFLHGCCCHRFSFFGG
>g3206.t1
MDSPAKDSLVHPGFYRSEIQKTTWDVPERYEALKAIGSGAYGTVCSALDKQTKEKVAIKKLYRPFQSLIHAKRAYRELRL
LRHIENDNTFLEYQPLQQAR
QTYALSEEAIYSAHLTYRLLRQGVRGAYRS
>g3207.t1
MISEGNFLGVGEREPGGGCGGPGRSVDQSSLHSEGFNETESHQRPGSGRPPQLPTIGPNETERSVPVWFGFRPFPPGYLS
HVTRAVPIPRGGVHLQPHCL
GCPTWLQQSGGAE
>g3207.t2
MISEGNFLGVGEREPGGGCGGPGRSVDQSSLHSEGFNETESHQRPGSGRPPQLPTIGPNETERSVPVWFSFAARVWFGFR
PFPPGYLSHVTRAVPIPRGG
VHLQPHCLGCPTWLQQSGGAE
>g3207.t3
MISEGNFLGVGEREPGGGCGGPGRSVDQSSLHSEGFNETESHQRPGSGRPPQLPTIGPNETERSVPPFPPGYLSHVTRAV
PIPRGGVHLQPHCLGCPTWL
QQSGGAE
>g3208.t1
LPFFREREKRHTAQRLMDASEQAHEGILLHDLLQPAGWMYREGLEYDSLRHIKNGSYGDVVCIQDRRTGFQCAAKRVAKR
HFSSEEVSTWSALDSPHVVR
FLGAVTEGPNVVLFMDLKPGCLAQVLKERVRLPEDLALHYMRQVLWALEHLHQRRVLHLDVKADNVLLSADGRNTFLCDF
GLSERSDENGKSTRTHRGPA
FPGTETHMAPEVAGGSQRCSKADVWSSCCMLLHMLNGCPPWVRYYAPPLCLKISREPPPLWEVPSHCNHFTARLLRAGLR
KDPRRRASARQLRRKTTKAL
RAVGGLHPSSLHAACPLSTEAPPTQAPMCWISPWRVAAVLQDSSEYEEVVSGLDSEEEMVGEEEEEKEEDRGPHPRSLRV

LGWGLDCDPEVDIYMGEEEF
WEKRGVLTQRDRNYEGDWEEEEEGEGEEEVEETWESSPSPGLFCALREHFPLLRSGLQRGPASWGSEQELKHLREDVAPD
NPVGNLSPEPRDYPPSCLSC
TDSSQISDQDSDHWSDDLSSGVFSSYNSQTDGQLEWLLSANQQSSHCFEGVDIWIEDVRGACLRIRERRRVKVGHVAIGI
SDQISVKAFTLETLDRKLVS
FQQEVQESGLWLRCVPAPDRCPRWYWRINDGKLDLRGAL
>g3208.t2
KVTRADSRFLLPFFREREKRHTAQRLMDASEQAHEGILLHDLLQPAGWMYREGLEYDSLRHIKNGSYGDVVCIQDRRTGF
QCAAKRVAKRHFSSEEVSTW
SALDSPHVVRFLGAVTEGPNVVLFMDLKPGCLAQVLKERVRLPEDLALHYMRQVLWALEHLHQRRVLHLDVKADNVLLSA
DGRNTFLCDFGLSERSDENG
KSTRTHRGPAFPGTETHMAPEVAGGSQRCSKADVWSSCCMLLHMLNGCPPWVRYYAPPLCLKISREPPPLWEVPSHCNHF
TARLLRAGLRKDPRRRASAR
QLRRKTTKALRAVGGLHPSSLHAACPLSTEAPPTQAPMCWISPWRVAAVLQDSSEYEEVVSGLDSEEEMVGEEEEEKEED
RGPHPRSLRVLGWGLDCDPE
VDIYMGEEEFWEKRGVLTQRDRNYEGDWEEEEEGEGEEEVEETWESSPSPGLFCALREHFPLLRSGLQRGPASWGSEQEL
KHLREDVAPDNPVGNLSPEP
RDYPPSCLSCTDSSQISDQDSDHWSDDLSSGVFSSYNSQTDGQLEWLLSANQQSSHCFEGVDIWIEDVRGACLRIRERRR
VKVGHVAIGISDQISVKAFT
LETLDRKLVSFQQEVQESGLWLRCVPAPDRCPRWYWRINDGKLDLRGAL
>g3208.t3
LPFFREREKRHTAQRLMDASEQAHEGILLHDVRQHPPSRALERERRQAGVLRYKRLLQPAGWMYREGLEYDSLRHIKNGS
YGDVVCIQDRRTGFQCAAKR
VAKRHFSSEEVSTWSALDSPHVVRFLGAVTEGPNVVLFMDLKPGCLAQVLKERVRLPEDLALHYMRQVLWALEHLHQRRV
LHLDVKADNVLLSADGRNTF
LCDFGLSERSDENGKSTRTHRGPAFPGTETHMAPEVAGGSQRCSKADVWSSCCMLLHMLNGCPPWVRYYAPPLCLKISRE
PPPLWEVPSHCNHFTARLLR
AGLRKDPRRRASARQLRRKTTKALRAVGGLHPSSLHAACPLSTEAPPTQAPMCWISPWRVAAVLQDSSEYEEVVSGLDSE
EEMVGEEEEEKEEDRGPHPR
SLRVLGWGLDCDPEVDIYMGEEEFWEKRGVLTQRDRNYEGDWEEEEEGEGEEEVEETWESSPSPGLFCALREHFPLLRSG
LQRGPASWGSEQELKHLRED
VAPDNPVGNLSPEPRDYPPSCLSCTDSSQISDQDSDHWSDDLSSGVFSSYNSQTDGQLEWLLSANQQSSHCFEGVDIWIE
DVRGACLRIRERRRVKVGHV
AIGISDQISVKAFTLETLDRKLVSFQQEVQESGLWLRCVPAPDRCPRWYWRINDGKLDLRGAL
>g3209.t1
MNDVESRHAHKCETASAVGRKGPLRTEHRRVVLWLLAVSLRFVIHYAGICRRYWIVTFPAEFNLDLGLIRMTEELRDLAA
ELGFKEHFKLIDISTM
>g3209.t2
MGSSTLGKAAPFDLLLEECIQAFDENGELKNSHLPRTLLLMHRWYLTSTELAGKLLMIYPFPSSTRPPATFQGYNCCG
>g3209.t3
MGSSTLGKAAPFDLLLEECIQAFDENGELKNSHLPRTLLLMHRWYLTSTELAGKLLMIYPFPSSTRPPATFQGYNCCASA
VGRKGPLRTEHRRVVLWLLA
VSLRFVIHYAGICRRYWIVTFPAEFNLDLGLIRMTEELRDLAAELGFKEHFKLIDISTM
>g3209.t4
MKRCDASRGLSKTPTMGSSTLGKAAPFDLLLEECIQAFDENGELKNSHLPRTLLLMHRWYLTSTELAGKLLMIYPFPSST
RPPATFQGYNCCASAVGRKG
PLRTEHRRVVLWLLAVSLRFVIHYAGICRRYWIVTFPAEFNLDLGLIRMTEELRDLAAELGFKEHFKLIDISTM
>g3210.t1
LLFDHLEPMELAEHLTFLEFKSIRRISFTDYQSYVTHGCLQDNPTLERSIALFNGVSQWVQLMVLSKLTPQHRAEVITKY
INVAQKLLQLQNFNTLMALV
GGLSHSSISRLKETHSHLAPEVTKVGTLTFRPLNGCVQSLD
>g3210.t2
LFDHLEPMELAEHLTFLEFKSIRRISFTDYQSYVTHGCLQDNPTLERSIALFNGVSQWVQLMVLSKLTPQHRAEVITKYI
NVAQKLLQLQNFNTLMALVG
GLSHSSISRLKETHSHLAPEVTKVGTLTFRPLNGCVQSLD
>g3210.t3

LLFDHLEPMELAEHLTFLEFKSIRRISFTDYQSYVTHGCLQDNPTLERSIALFNGVSQWVQLMVLSKLTPQHRAEVITKY
INVAQVGAAGPSGSTQQLVT
FKLLQLQNFNTLMALVGGLSHSSISRLKETHSHLAPEVTKVGTLTFRPLNGCVQSLD
>g3211.t1
MERALTLLEPKVSPECSGPLGMEGGIISNQQITASSTHRALFGLQKWYPYFARLNKKGL
>g3212.t1
PPQQTANITVQIPPKTEPQDPAPVPAAKASEEAATPGPSHPGLAKVQLIVDRVTKLEQEVRCFDGKKNDKRYLLLEEMLT
KELLALDSVDPEGRADVRQA
RRDGVRRVQTILEELEQLDERPRVPPSESDSLPQKDEPSMMVTGNMEASREIS
>g3213.t1
MELFQAKDHYILQSGDRALWCSRQDGTMSVRPDLPLGLVLIRQKALVGSLPGNHKVYKITKIAVIPLSDEEPQELELELC
KKHHFGIDRPEKLAPSPDDS
KFLMKTLTQIKSNVAVPIKKKVMSHDARL
>g3213.t2
MELFQAKDHYILQSGDRALWCSRQDGTMSVRPATDLLLAWNPVCLGLVEGLIGKIQLHADLPLGLVLIRQKALVGSLPGN
HKVYKITKIAVIPLSDEEPQ
ELELELCKKHHFGIDRPEKLAPSPDDSKFLMKTLTQIKSNVAVPIKKKVMSHDARL
>g3213.t3
LSTQCPGIMELFQAKDHYILQSGDRALWCSRQDGTMSVRPDLPLGLVLIRQKALVGSLPGNHKVYKITKIAVIPLSDEEP
QELELELCKKHHFGIDRPEK
LAPSPDDSKFLMKTLTQIKSNVAVPIKKKVMSHDARL
>g3214.t1
LVVGQIDKLTSDFDFDLEPDDWTVATASSTSSSERGLGEAFRLDLLNPDVLSDSWEFCSYLEATGAAAAATAALGSGEHP
>g3214.t2
LEVDFGLLLVTLVVGQIDKLTSDFDFDLEPDDWTVATASSTSSSERGLGEAFRLDLLNPDVLSDSWEFCSYLEATGAAAA
ATAALGSGEHP
>g3215.t1
LVESSGSGAALVQNKETAGRLGLRSQTSQPTTRPQNGGTRASWSGETAHSHCQNQAGRSGSSEGDGGLLITPHTPAANMR
SNRDNAGAEVKRSSSKTLDK
DPRETWKSARDDKRQSEDLDVTGGGGISQSGPKLCKREPVTTTTPPRPSPVPSSACRPSPVPGDALLNARVSVMLRRIRG
ELGVSAPRRADSGSRETEVR
SPPESLPRTARLETSPVVVPGKRRLMKKGKLMPRIGSDQENPPPGPEDITQEQPLHRDPSKAERSPSTTPDNGVCSPARA
SVTGERVPIVLDGNPSERRD
QRGGGLSNTQRGGKKRKAKSSMGTENLDQLLAVSQQEEEVCKSLGELEVTLRQAQKALQATYTKVQRLLLLKKQFMTQVN
VLRAKRIEILQGMQ
>g3215.t2
MKMLQKKHSRKSKTRQCQPVTKADSAGQLVESSGSGAALVQNKETAGRLGLRSQTSQPTTRPQNGGTRASWSGETAHSHC
QNQAGRSGSSEGDGGLLITP
HTPAANMRSNRDNAGAEVKRSSSKTLDKDPRETWKSARDDKRQSEDLDVTGGGGISQSGPKLCKREPVTTTTPPRPSPVP
SSACRPSPVPGDALLNARVS
VMLRRIRGELGVSAPRRADSGSRETEVRSPPESLPRTARLETSPVVVPGKRRLMKKGKLMPRIGSDQENPPPGPEDITQE
QPLHRDPSKAERSPSTTPDN
GVCSPARASVTGERVPIVLDGNPSERRDQRGGGLSNTQRGGKKRKAKSSMGTENLDQLLAVSQQEEEVCKSLGELEVTLR
QAQKALQATYTKVQRLLLLK
KQFMTQVNVLRAKRIEILQGMQ
>g3215.t3
LVESSGSGAALVQNKETAGRLGLRSQTSQPTTRPQNGGTRASWSGETAHSHCQNQAGRSGSSEGDGGLLITPHTPAANMR
SNRDNAGAEVKRSSSKTLDK
DPRETWKSARDDKRQSEDLDVTGGGGISQSGPKLCKREPVTTTTPPRPSPVPSSACRPSPVPGDALLNARVSVMLRRIRG
ELGVSAPRRADSGSRETEVR
SPPESLPRTARLETSPVVVPGKRRLMKKGKLMPRIGSDQENPPPGPEDITQEQPLHRDPSKAERSPSTTPDNGVCSPARA
SVTGERVPIVLDGNPSERRD
QRGGGLSNTQRGGKKRKAKSSMGTENLDQLLAVSQQEEEVCKSLGELEVTLRQAQKALQATYTKVQRLLLLKKQVNVLRA
KRIEILQGMQ
>g3216.t1
MVAPGQIKPRNCRRIVVLGAPKVGKTNIVRRFLGDEFDERYAPTAEDFHRKMYHIRGKPYLIDILDAAREREFPAKRRLS

ILTGDIFLLAFSLDDRDSLK
EVCNLRNEIISGKTTLMKLKDTPRVPIVICGNKVDLAVGRVVSRLEISEILGEDTAYFETSAKAGSGLEDVFRALVTLGG
LPSEVIPSQHQTISLHTYQS
LFSHQRSARKLSSECAVDAPCAALCPLARRPSFNSDLKLVLGSSSSKNKPKKCQIQ
>g3217.t1
HWQAFCPAADLHKVFGAFADVQNLLLSGHLILDDRGLRCSWDGLALLDSLLPTLLLALERHAGPRGQTGGPRGQTGGPRG
QTGGP
>g3217.t2
MHWQAFCPAADLHKVFGAFADVQNLLLSGHLILDDRGLRCSWDGLALLDSLLPTLLLALERHAGPRGQTGGPRGQTGGPR
GQTGGP
>g3218.t1
IYFHDQEKLLSRQGQQGWNRLFDLLQAELAVRPADVHVNVKLVELYSKDGRLDEAVKHCLSSEKKGQLRKSLEWYSVVVH
TLQEYLAQPGVSNNEKLCRR
LKGELLLAHCNLLRLKLSVSGVQPALDALRSFDEAMRSISASAGGPADELCEVFGEMRAHLYLHTGTLLLKMAQEHEQNW
RAVTDLATLCFLLSYQAPRP
KPKLTKGDQLAPQLLELMASDRQSQAGHMLLNLSSDTQTFITEVVEAFGNRIGQNSLFELLFGAQASAQTSFIANDDLCS
INTQVPELSQLAKWDDGSIM
LHGGDLQHLSWLGLQWTLLAQRPTLRDWLKQLFPRLNLETSKLDTNAPESISLLDLEVFVCGVVFCCHCQLQETAKISCV
GQQPLSEPRCLPLPLLRSLS
SDRQREWWDAIYSLINKQALPGTSAKLRMAVQHGLSTLRAGEKHGLQPALVISWAQHLSHMGMEVNSYYDQKEYIGRSVH
YWKSALPLLDKIKNRRSIPE
PLDPLFMHFPNRDIQISIVKGYEEEAMIGFASLLDIEGKTEEAIATLETINNVSSSWHLAQASLSFKLLKLSEEAGSGVE
ETQDRCIMFLRKFRTYLSKI
YNSDDLEQNEVHDLKHNSSGNLGSPHHRMYGDSYVDGHQEPYTPVPSFQGPQLP
>g3218.t2
IYFHDQEKLLSRQGQQGWNRLFDLLQAELAVRPADVHVNVKLVELYSKDGRLDEAVKHCLSSEKKGQLRKSLEWYSVVVH
TLQEYLAQPGVSNNEKLCRR
LKGELLLAHCNLLRLKLSVSGVQPALDALRSFDEAMRSISASAGGPADELCEVFGEMRAHLYLHTGTLLLKMAQEHEQNW
RAVTDLATLCFLLSYQAPRP
KPKLTKGDQLAPQLLELMASDRQSQAGHMLLNLSSDTQTFITEVVEAFGNRIGQNSLFELLFGAQASAQTSFIANDDLCS
INTQVPELSQLAKWDDGSIM
LHGGDLQHLSWLGLQWTLLAQRPTLRDWLKQLFPRLNLETSKLDTNAPESISLLDLEVFVCGVVFCCHCQLQETAKISCV
GQQPLSEPRCLPLPLLRSLS
SDRQREWWDAIYSLINKQALPGTSAKLRMAVQHGLSTLRAGEKHGLQPALVISWAQHLSHMGMEVNSYYDQKEYIGRSVH
YWKSALPLLDKIKNRRSIPE
PLDPLFMHFPNRDIQISIVKGYEEEAMIGFASLLDIEGKTEEAIATLETINNVSSSWHLAQASLSFKLLKKFRTYLSKIY
NSDDLEQNEVHDLKHNSSGN
LGSPHHRMYGDSYVDGHQEPYTPVPSFQGPQLP
>g3218.t3
IYFHDQEKLLSRQGQQGWNRLFDLLQAELAVRPADVHVNVKLVELYSKDGRLDEAVKHCLSSEKKGQLRKSLEWYSVVVH
TLQEYLAQPGVSNNEKLCRR
LKGELLLAHCNLLRLKLSVSGVQPALDALRSFDEAMRSISASAGGPADELCEVFGEMRAHLYLHTGTLLLKMAQEHEQNW
RAVTDLATLCFLLSYQAPRP
KPKLTKGDQLAPQLLELMASDRQSQAGHMLLNLSSDTQTFITEVVEAFGNRIGQNSLFELLFGAQASAQTSFIANDDLCS
INTQVPELSQLAKWDDGSIM
LHGGDLQHLSWLGLQWTLLAQRPTLRDWLKQLFPRLNLETSKLDTNAPESISLLDLEVFVCGVVFCCHCQLQETAKISCV
GQQPLSEPRCLPLPLLRSLS
SDRQREWWDAIYSLINKQALPGTSAKLRMAVQHGLSTLRAGEKHGLQPALVISWAQHLSHMGMEVNSYYDQKEYIGRSVH
YWKSALPLLDKIKNRRSIPE
PLDPLFMHFPNRDIQISIVKGYEEEAMIGFASLLDIEGKTEEAIATLETINNVSSSWHLAQASLSFKLLKLSEEAGSGVE
ETQDRCIMFLRKFRTYLSKI
YNSDDLEQLPVSMEEVMDLLNEVNQQLEEIGENTEEKEEKGGARRAPIRSTPESAAHVSRINFTTASPNKSTVSPSKIHL
NEVHDLKHNSSGNLGSPHHR
MYGDSYVDGHQEPYTPVPSFQGPQLP
>g3219.t1
PDVQHGPSVWKPVCSFKVRSPEELAPGRDCCLDVLY

>g3220.t1
MWSYPSAEVMQYCRSSHSLLGSEGRSRGEKRRDGETEWEMSGGWDSGRRGGGRCMEEGERWLQVQRMPSPLRRPGRGGAE
RDGITPPDG
>g3221.t1
MRIPSNPNEVCVEDMYQNNILWSSILRTFSDSVVASAIFYAVVCWRGGSTDRDRSRLNRLIRRASSVLDCPLDSIEEVGD
RRMLTKLTSIMDNTSHPLHD
SVGSLSSSFSSRLIHPWCKKERFRRSFIPAAVRLYNTCST
>g3221.t2
TFSDSVVASAIFYAVVCWRGGSTDRDRSRLNRLIRRASSVLDCPLDSIEEVGDRRMLTKLTSIMDNTSHPLHDSVGSLSS
SFSSRLIHPWCKKERFRRSF
IPAAVRLYNTCST
>g3222.t1
SLKNVQDLSGFRVADDRLHWAVKYLVAPKHADGSDAEVS
>g3223.t1
MSLYHTRLQHLGVPPPSLGISNHPPVVYLYGGENSGMVPALSFLSARQQEPPQKPPYSYIALIAMAIKNAPERRATLSGI
YKFIMDRFPFYHDNKQGWQN
SIRHNLSLNDCFIKVPREKGRPGKGSYWTLDTKCLDMFENGNYRRRKRRLKSRQQQESDSPSGTKRTRVSGGQSSTRQAF
TEAPGSEDSYTTPAFSKIQH
RGNVQNRRRALAEHPPGDSNRYHGQPGSVAHQNRNPRRAWKIFQSLHPHAIRKRRMQDPFSVDERMILQKALKCIRMLSL
FV
>g3223.t2
MSLYHTRLQHLGVPPPSLGISNHPPVVYLYGGENSGMVPALSFLSARQQEPPQKPPYSYIALIAMAIKNAPERRATLSGI
YKFIMDRFPFYHDNKQGWQN
SIRHNLSLNDCFIKVPREKGRPGKGSYWTLDTKCLDMFENGNYRRRKRRLKSRQQQESDSPSGTKRTRVSGGQSSTRQAF
TEAPGSEDSYTTPAFSKIQH
RGNVQNRRRALAEHPPGDSNRYHGQPGSVAHQNRNPRRAWKIFQSLHPHAIRKRRMQDPFSVDERMKFRHQV
>g3224.t1
LAESWFRAKLSRQRSYSSALSYEDSEPELSRSLGVHALIDNMVSFISGDVGLAPAFREPDEGMSTSPQATVLAMEQQQSR
AELRLEALHQIVVLISGMEE
KGSQQGPVPGRPGAAFQSSSLLTSVRLQFLAGCFGLGTVGTGGLKGENVQLHHYQDGVKAAKRSLQMDIQTAVHKIYQQL
SVTLERALQANKHHIEAQQR
LLLVTVFALSVRYQPVDVSLAISSGLLNVLSQLCGTETMLGQPLQLLHKPGVSQLSTALKVASTRLLQILAISTGTYADK
LGPKVVQSLLDLLCSQLKNL
LSQAGVSLLSSGKDQEDTKQDDGADAEKKDFRALLRKQHTAELHLGDFLVFLRRVVSSKAIQSKMASPKWTEVLLNIAAQ
KCCSGVPLVGNLRTRLLALH
VLEAVLPACEANVEDDQMTQVVERLFSLLSDCMWEAPVAQAKHTIQIKERVQELKLQGDAEEEDENLPIQEVSFDPEKAQ
CCVVENGQGLTHGSGGKGYG
LASTGISSGCYQWKFYIVKENRGNEGTCVGVSRWPVHDFNHRTTSDMWLYRAYSGNLYHNGEQTLTLSSFTQGDFITCVL
DMEARTISFGKNGEEPKLAF
EDVDATELYPCVMFYSSNPGEKVKICDMQMRGTPRDLLPGDPVCSPVATVLAEATTQLIRILHRADHWTHRINKIMVERL
HTIKACFREAGSGGGGGQRL
KKSRSVQS
>g3224.t2
LYSSPDSLAESWFRAKLSRQRSYSSALSYEDSEPELSRSLGVHALIDNMVSFISGDVGLAPAFREPDEGMSTSPQATVLA
MEQQQSRAELRLEALHQIVV
LISGMEEKGSQQGPVPGRPGAAFQSSSLLTSVRLQFLAGCFGLGTVGTGGLKGENVQLHHYQDGVKAAKRSLQMDIQTAV
HKIYQQLSVTLERALQANKH
HIEAQQRLLLVTVFALSVRYQPVDVSLAISSGLLNVLSQLCGTETMLGQPLQLLHKPGVSQLSTALKVASTRLLQILAIS
TGTYADKLGPKVVQSLLDLL
CSQLKNLLSQAGVSLLSSGKDQEDTKQDDGADAEKKDFRALLRKQHTAELHLGDFLVFLRRVVSSKAIQSKMASPKWTEV
LLNIAAQKCCSGVPLVGNLR
TRLLALHVLEAVLPACEANVEDDQMTQVVERLFSLLSDCMWEAPVAQAKHTIQIKERVQELKLQGDAEEEDENLPIQEVS
FDPEKAQCCVVENGQGLTHG
SGGKGYGLASTGISSGCYQWKFYIVKENRGNEGTCVGVSRWPVHDFNHRTTSDMWLYRAYSGNLYHNGEQTLTLSSFTQG
DFITCVLDMEARTISFGKNG
EEPKLAFEDVDATELYPCVMFYSSNPGEKVKICDMQMRGTPRDLLPGDPVCSPVATVLAEATTQLIRILHRADHWTHRIN

KIMVERLHTIKACFREAGSG
GGGGQRLKKSRSVQS
>g3225.t1
AVAAVCSALKARTAAFHSVGPYGSGVKSESAGWLGSLVIFCAFLRQQFHLMQLGPRLPEEQATGWRQRLFFLLRRFGLDS
RILTNFYRCTIESILAGNIT
AWYGSCTALDRKALQRVMAPQCIIKTELPTIQVLYSQCYKRKAQWIISDPSHPSHSREFFVGLSKRTNQKGAEILADAFK
RSKGAEVQTYNLYVCAHIER
GSLIRSPYI
>g3225.t2
GSGVKSESAGWLGSLVIFCAFLRQQFHLMQLGPRLPEEQATGWRQRLFFLLRRFGLDSRILTNFYRCTIESILAGNITAW
YGSCTALDRKALQRVMAPQC
IIKTELPTIQVLYSQCYKRKAQWIISDPSHPSHSREFFVGLSKRTNQKGAEILADAFKRSKGAEVQTYNLYVCAHIERGS
LIRSPYI
>g3225.t3
AVAAVCSALKARTAAFHSVGPYGSGVKSESAGWLGSLVIFCAFLRQQFHLMQLGPRLPEEQATGWRQRLFFLLRRFGLDS
RILTNFYRCTIESILAGNIT
AWYGSCTALDRKALQRVMAPQCIIKTELPTIQVLYSQCYKRKAQWIISDPSHPSHSREFFVGLSKRTNQKGAEILADAFK
RSKVGRARNTSNGRRPGGIL
IRCPNHLNWLLSTRRSSGSTPSSLRMSELLTLSLRLSPATLRRKLISAACIHDLVLSVTTQSS
>g3226.t1
NVSTTESSHMFVSLLAEGSSIRYDSSMQVDSGYNTTSAGTASLIDGLSSVSNGKESFSSNLVDEPFPFPRQTKVKVNAPV
GRLNVVAELTF
>g3227.t1
LFFKSRGILNEEGFILFVRKNAIIVLIPKFGLEGTVFFDGKGKTGPKLVFDEEGPTLMVEQHTFKIFDKVKVTISLDDSN
IQHQKIRMSLVEPEIPGVSV
PVPVPAPVPEPLAKKAKLDR
>g3227.t2
MGAGLFFKSRGILNEEGFILFVRKNAIIVLIPKFGLEGTVFFDGKGKTGPKLVFDEEGPTLMVEQHTFKIFDKVKVTISL
DDSNIQHQKIRMSLVEPEIP
GVSVPVPVPAPVPEPLAKKAKLDR
>g3227.t3
LFFKSRGILNEEGFILFVRKNAIIVLIPKFGLEGTVFFDGKGKTGPKLVFDEEGPTLMVEQHTFKIFDKVKVTISLDDSN
IQHQKIRMSLVEPEYQYQHQ
YQSHWPRRPNWTAESLTKLQ
>g3228.t1
MTSASTKVGEIFSAAGAAFTKLGDLTMQLHPVADTSPAGAQVKTPVKRKPPSDGGSVVPPEATRKAVKKAGPGGTSQVLS
GPAASARRAPPSAQPTVKKQ
KGAGGPISRSSAALQWFDSYLNGSRWHLLAPISELRDRI
>g3228.t2
MSGDFLVTTQTGSQVGEIFSAAGAAFTKLGDLTMQLHPVADTSPAGAQVKTPVKRKPPSDGGSVVPPEATRKAVKKAGPG
GTSQVLSGPAASARRAPPSA
QPTVKKQKGAGGPISRSSAALQWFDSYLNGSRWHLLAPISELRDRI
>g3228.t3
MTSASTKVGEIFSAAGAAFTKLGDLTMQLHPVADTSPAGAQVKTPVKRKPPSDGGSVVPPEATRKAVKKAGPGGTSQVLS
GGPISRSSAALQWFDSYLNG
SRWHLLAPISELRDRI
>g3229.t1
MSCLVCGPKLATCGLVLSLWGVIMLGLLGVFFRTHSAVLIEDVPLAEEDLRDPNPPQKIYGLYNQVAYNCFIAAAIYLVV
GVFSICQVKLNKRKEYLVQ
>g3229.t2
MSCLVCGPKLATCGLVLSLWGVIMLGLLGVFFRTHSAVLIEDVPLAEEDLRDPNPPQKIYGLYNQVAYNCFIAAAIYLVV
GVFSICQVKLNKRKSKHGKL
ELDVQSSSSGGQAMKSANMEATPSSI
>g3230.t1
PGDDPAGEEAGEDGKGVKRKREEEGTQGTGNKRMDDGVASMLADFIACPPDDEDCTSSSSLPCLD
>g3231.t1

MSSSFPKSESRTSLLKSSTGGPSSHLLEPAPESHRSHHHHHHRHHRPAAPPNSSHAEESFWSAECDVSARRPLCHHSAQG
HRDTATARDRNRIPSLSDND
DTAPFFKRTDHKELAESNGRHDGRSSSPSSFSPAPPSSQSYRRSRRSSTASSTIGLHLRAVLNSLFGQ
>g3231.t2
LSARTRAALPHSQVVKERGKPAATADRGALQSHRSHHHHHHRHHRPAAPPNSSHAEESFWSAECDVSARRPLCHHSAQGH
RDTATARDRNRIPSLSDNDD
TAPFFKRTDHKELAESNGRHDGRSSSPSSFSPAPPSSQSYRRSRRSSTASSTIGLHLRAVLNSLFGQ
>g3231.t3
LSARTRAALPHSQVVKERGKPAATADRGALQSHRSHHHHHHRHHRPAAPPNSSHAEESFWSAECDVSARRPLCHHSAQGH
RDTATARDRNRIPSLSDNDD
TAPFFKRTDHKELAESNGRHDGRSSSPSSFSPAPPSSQSYRRSRRSSTASSTIGLHLRAVLNSLFGQTPSHHPSRLALLP
NASSGCWSYDLVMLC
>g3232.t1
MEWSLGRVRELVADGMQSIRDCESSAVGTAACLLLLFLWYCYRVGREHGSSPLRGRYGAEPGHLRAGVVSSSEAMGGVKA
QQCLLDEQNGLAYCQSADCF
RCTATSEGLNQRLYHSLQEYAKRYTWSGMGRVHKGVRDQGRYLSSRPTIQRPEVFFLPDLPSAPFFSRDAQKHDVELLER
SFPALLAEFGNVYNQPPARS
GSSLPPGWKANSTPHGQWWTYYLVNQGTPLALNARRCPRAWRVLGQLRTFIANNVFGNACFSVLTPGALITEHYGPTNVR
LRCHLGLRVPSACELVVGGE
PQCWSEGSCLLFDDSFLHRAFHEGGAEDGPRVVFMVDLWHPNVAAAERQALDYIFTPGR
>g3233.t1
MSSYLTNSKLYSDKNGNLLKAGAIMRFEKMADTLEMVANQGADALYTGKIAENLIHDLQNEGATLTMEDLASYKARVSDA
WSVSLGEYQIHLAPPPSGGV
LLGLILNIMKGYGLNPESLVGEERALTYHRFVEACKFANGLRKYLRDPQFNSGAVAEKITEESFADHLRGLISSDQSQDA
QYYNMTPYRDSFGTTHVSVL
DEHGTAVAVTSSINHMFVVSYHTSVWKKHELG
>g3233.t2
LARGGFPIPPVQARFIPYLAGNINCSLRKLYSDKNGNLLKAGAIMRFEKMADTLEMVANQGADALYTGKIAENLIHDLQN
EGATLTMEDLASYKARVSDA
WSVSLGEYQIHLAPPPSGGVLLGLILNIMKGYGLNPESLVGEERALTYHRFVEACKFANGLRKYLRDPQFNSGAVAEKIT
EESFADHLRGLISSDQSQDA
QYYNMTPYRDSFGTTHVSVLDEHGTAVAVTSSINHMFVVSYHTSVWKKHELG
>g3233.t3
LRGYELAHRLYGKLPWANLFQPTIQLARGGFPIPPVQARFIPYLAGNINCSLRKLYSDKNGNLLKAGAIMRFEKMADTLE
MVANQGADALYTGKIAENLI
HDLQNEGATLTMEDLASYKARVSDAWSVSLGEYQIHLAPPPSGGVLLGLILNIMKGYGLNPESLVGEERALTYHRFVEAC
KFANGLRKYLRDPQFNSGAV
AEKITEESFADHLRGLISSDQSQDAQYYNMTPYRDSFGTTHVSVLDEHGTAVAVTSSINHMFVVSYHTSVWKKHELG
>g3234.t1
VLQLGSDILPQYKQETPKTPPHIILHYCLFKTTWDWVILILTFYTAIMVPYNVSFKTKQNNVTWLVVDSIVDVIFLVDIV
LNFHTTFVGPAGEVISDPKL
IRMNYLKTWFVIDLLSCLPYDVINAFENVDEVTRHALAPQDCRLDEAAGSLREAPFSPP
>g3235.t1
MVAGSALMEPSVKSGANQVADVVFVIEGTANLGPYFDSLRKNYILPAIEYFNGGPPAETDFGGDYGGTQYGLVVFNTVDC
APESYAQCHAPTSSAFEFVS
WIDSI
>g3235.t2
MEPSVKSGANQVADVVFVIEGTANLGPYFDSLRKNYILPAIEYFNGGPPAETDFGGDYGGTQYGLVVFNTVDCAPESYAQ
CHAPTSSAFEFVSWIDSI
>g3236.t1
PGGRGADWAPGGRQSGLLMFIVCVDGRSEQSCLVCRAQLFVFCRVVLWRRGPHSHDPNEFEFSGSQLWVI
>g3236.t2
LFVFCRVVLWRRGPHSHDPNEFEFSGSQLWVI
>g3237.t1
VAPSVEQPEIQPQMDYDHHMETQKNNSNNNINTNGADNSDSPTAVETMKNTEESRENTEAHVATPKDVTALLTPPLEEPT
SHSSNSRPSPPSPSHHPPSS

PPNSPLSSDPPPSSDRHGYRFRCSRCSLAFPTHEKLQLHWQYHAMRAATECPLCARRCRSQEALQRHMQNTHAQPDGANP
QAAHHLLSHAAQTQQEHDVR
LSPQVSEEADEEEDDDMEAEALIMEKKDVKTEERKMAGKEAQVGVSGAQETILDLEKHHQAESGLEPTPSFYMKKGSNPT
MDRYLDPSRPYKCTICSESF
TQKTILLVHFNSVSHLHRARRALQESGPGQANPETPRGPDPRPYRCRLCGVGYSQSSTLDIHLRSVLHQTRARASQNTAL
QVPAFGSATPAPVPSTTAQT
AVTSEGTSKSLPLTKKADLVTPSISSLISSAAEAQLALHNQAESQLAKRSAELLSSRNQLLLMQQQQLAQAQAQLQQHTT
LLQSQLFNPALLQHLQLGPE
SLLKQHFSIAPDNLLSLQQQLLLPFYLSGDMKLNPELVLKSLELNQADSCITALMEQLNPKATRESPPHIEEKPTQRQSP
TSAQPPPNESVESEGKVKTS
SGGSVVTQTEQEYIVPGLKEEKKEAAIPVIKAEKQKDETNQVDCSPHKRLGFDCPPPRVPYAAVNGEPLRTLLQSYGYEL
ALQYIQSRQRHQLKINHMVP
EGHECIEERQAYLDRRMKDYIEFKAGKVEGCDGTPKAEAVNGDRIDGSLEKKKEKEQEILSKEEKERCLDCGKMFSDALV
LKTHQEYIHRQLFPTAVLEK
FSKEYRLQYDQLYPLAQTKSEETSDVAPPTPKPDSVPEAALEPVEQRPASVSEPKAKTLAPLTDFPPTTPVNSPPPSPQP
QASPKHEGPLPSPSPPATSK
TPSQGMGLPNLPLQLPQFPLSSLPLPPMPFPMDLPLLPSVVMQSMALQSQPWLDSGVTSDMAKLYQSQLSPALLSQQPEL
SPALLAQQSQMSHLLLAQPS
HHSPVLQQPQGTSTALDQQQGKRPRTRISEEQLGVLRQHFDINSLPGDEDVIKMSSQCGLPHKVIKHWFRNTLFKERQRD
KDSPYNFNNPPTTVLDEPQE
AAAQMQYLTEAASSVSPGLPSSHSPQPQRADPHRGEPSRGRRSSRTRFTERQLETLQSVFEATPYPREEEYDRLSALLSL
PNRVIVVWFQNARQRARKNQ
EKGEEEDGTEGRARAEDAPRRSRGGYKNDTDEDCGEEGHGDRQNENSMDLAYEYYTHADSPVDANSPSRRAESKHELLNG
KSNTASKKQDEPAAAQDEKM
QAPVELQMEIPEADKRNQNKACTREMPTPKPQPKPELSSEKPQHTSGSCSEKMVASIQASSQFSQDNTIPSSAPAIDRPA
PSPLNPPSECETDRACMPDT
TLGRSTQIHTQNPLQHQTPTQFQCSFCPMSLPSPQLWQEHQTRHLLAAQSQVQLIHPGFSDRTMPYMMIHPNQTLMASQL
LASAMSQMHPNPAHPMLPQM
NGLHLNNTLSDHLSNSLSGLPHSSLSSAKQSSKASTDVSCEAPGGGREAEEEQRRDKRQRTTITPEQLEVLYQRYSLDSN
PTRGVLESIARDVGLKRRVV
QVWFQNTRARERKGQFRSTTTGTSFSLGLNHLRCLFCRALFKVKSALDAHIRSRHWAEAERAGYNLSASNGSASQIGMSM
SSLLDDAGPSASSGHSPSPG
FAMSTKDRHARPLMSSHCSAIEPNSPEDEGAYEEEEEYPCDEGSSVAEQGSLSPDGIGGPSGDWGETLSQRQYHHQQQQR
QRTQMSHFQVLQLRDFYRSH
RTPNRQECEALGQDLGLPHRVVQVWFQNARAKEKRARSMSSDSADREQSELSAGAGERDRA
>g3238.t1
IARSAPDIEDILEDTFISKLAAAVKKMTAEYATSHNVTISHIINCCKAIRLLSEKKCDIDTNPNPQIYEIYSTISILLVS
DEPLTVHHDFGTIYQIRLKP
EEMGNAEFTSEPDGPSVKLPPYSGLKSHLQQYSKVITQLGFFHKLPHQTNETIVENVCSFSLSHGLTPINLGNLSEMIQI
FIPNNASGLRNETVEMKDST
TVLTSFNVTDTNATIVFNVQPSANVSLLLLLAAGSPPNATNASNSIILNQTVGYRWMVTPDMLEETLGVWFLKAVLFNAS
DDERLDVQFLSFITKCMFWD
MVREEWSIEGCQVGLLSTPVLTQCLCNHLTLFGNSFFVMPNALDLSETASLFQTITDNYVVLALLCAFFGLYLVTVLWAW
YSDRRALVRRKITVLEDNHP
CASYNYLLSVQTGHRKNAGTSSNLRNLRLQHDSSGGHPSWYINKVMVQDMQTRQVCQFLCDCWLSSDRGDGTTKKVFNAA
KNNEIASFRNIFQNRTSTGF
RDEHIWVSIVDPPWRSPFTRAQRVSCCMSLLLCTMAINIAFWNIPADPNSPVLFSFGSLHITWQEIMVGVQSGLLMFPIN
ILIITIFRSIKPRLSIGEKK
KKSEQEAPQPPVTIPSILQETEEVVMSLCRSPKNNVCCLEERLESTYQLPLALDKIHQVLLLMQGEGESTPHWVFCSRYL
LLCLAHLLLCLEKLDVKNFH
SPADSQQLFDITNQLLRKAEMVYTCHKAAWYTINILLSRKKKKISAGCSLPWWFVFIAWFLLLSISGISTFFTLLYGLKY
GKEKSIQWVISLGLSLFQSI
FILQPLKVIGVAVFFALLLRPVAVEETEEVETVIFEQRDRCRRYTGRQGD
>g3238.t2
IARSAPDIEDILEDTFISKLAAAVKKMTAEYATSHNVTISHIINCCKAIRLLSEKKCDIDTNPNPQIYEIYSTISILLVS
DEPLTVHHDFGTIYQIRLKP

EEMGNAEFTSEPDGPSVKLPPYSGLKSHLQQYSKVITQLGFFHKLPHQTNETIVENVCSFSLSHGLTPINLGNLSEMIQI
FIPNNASGLRNETVEMKDST
TVLTSFNVTDTNATIVFNVQPSANVSLLLLLAAGSPPNATNASNSIILNQTVGYRWMVTPDMLEETLGVWFLKAVLFNAS
DDERLDVQFLSFITKCMFWD
MVREEWSIEGCQVGLLSTPVLTQCLCNHLTLFGNSFFVMPNALDLSETASLFQTITDNYVVLALLCAFFGLYLVTVLWAW
YSDRRALVRRKITVLEDNHP
CASYNYLLSVQTGHRKNAGTSSNRGAVDMFLLSTPFPLGELRNLRLQHDSSGGHPSWYINKVMVQDMQTRQVCQFLCDCW
LSSDRGDGTTKKVFNAAKNN
EIASFRNIFQNRTSTGFRDEHIWVSIVDPPWRSPFTRAQRVSCCMSLLLCTMAINIAFWNIPADPNSPVLFSFGSLHITW
QEIMVGVQSGLLMFPINILI
ITIFRSIKPRLSIGEKKKKSEQEAPQPPVTIPSILQETEEVVMSLCRSPKNNVCCLEERLESTYQLPLALDKIHQVLLLM
QGEGESTPHWVFCSRYLLLC
LAHLLLCLEKLDVKNFHSPADSQQLFDITNQLLRKAEMVYTCHKAAWYTINILLSRKKKKISAGCSLPWWFVFIAWFLLL
SISGISTFFTLLYGLKYGKE
KSIQWVISLGLSLFQSIFILQPLKVIGVAVFFALLLRPVAVEETEEVETVIFEQRDRCRRYTGRQGD
>g3238.t3
IARSAPDIEDILEESDQQLFYMETNALTNIQRDTFISKLAAAVKKMTAEYATSHNVTISHIINCCKAIRLLSEKKCDIDT
NPNPQIYEIYSTISILLVSD
EPLTVHHDFGTIYQIRLKPEEMGNAEFTSEPDGPSVKLPPYSGLKSHLQQYSKVITQLGFFHKLPHQTNETIVENVCSFS
LSHGLTPINLGNLSEMIQIF
IPNNASGLRNETVEMKDSTTVLTSFNVTDTNATIVFNVQPSANVSLLLLLAAGSPPNATNASNSIILNQTVGYRWMVTPD
MLEETLGVWFLKAVLFNASD
DERLDVQFLSFITKCMFWDMVREEWSIEGCQVGLLSTPVLTQCLCNHLTLFGNSFFVMPNALDLSETASLFQTITDNYVV
LALLCAFFGLYLVTVLWAWY
SDRRALVRRKITVLEDNHPCASYNYLLSVQTGHRKNAGTSSNLRNLRLQHDSSGGHPSWYINKVMVQDMQTRQVCQFLCD
CWLSSDRGDGTTKKVFNAAK
NNEIASFRNIFQNRTSTGFRDEHIWVSIVDPPWRSPFTRAQRVSCCMSLLLCTMAINIAFWNIPADPNSPVLFSFGSLHI
TWQEIMVGVQSGLLMFPINI
LIITIFRSIKPRLSIGEKKKKSEQEAPQPPVTIPSILQETEEVVMSLCRSPKNNVCCLEERLESTYQLPLALDKIHQVLL
LMQGEGESTPHWVFCSRYLL
LCLAHLLLCLEKLDVKNFHSPADSQQLFDITNQLLRKAEMVYTCHKAAWYTINILLSRKKKKISAGCSLPWWFVFIAWFL
LLSISGISTFFTLLYGLKYG
KEKSIQWVISLGLSLFQSIFILQPLKVIGVAVFFALLLRPVAVEETEEVETVIFEQRDRCRRYTGRQGD
>g3239.t1
MLGGGFKAERLKVNLRLVINRLKLLEKKKTELAQKARKEIADYLSAGKDERARIRVEHIIREDYLVEAMEILELHCDLLL
ARFGLIQSMKELDPGLQESV
STLIWAAPRLQELDPGLQESVSTLIWAAPRLQSEVAELKIVSDQLCAKYSKEYGKLCRTNQIGTVNDRLMHKLSVEAPPK
ILVERYLIEIAKNYNVPYEP
DAMVRPEVSPGEEANLIDVDSDNKPRGGGGGGGGGGGGGFTAPGMDFPHSSAFNYPSKGAEPYSSPLGTYVDHFPVGGGN
PPQLPSCPPTYESVIAPLPR
ASKQLYDNNTLPELPSVPDTLPTTLGDRHTTDDIDFDDLSRRFEELKKKT
>g3239.t2
MLGGGFKAERLKVNLRLVINRLKLLEKKKTELAQKARKEIADYLSAGKDERARIRVEHIIREDYLVEAMEILELHCDLLL
ARFGLIQSMKELDPGLQESV
STLIWAAPRLQELDPGLQESVSTLIWAAPRLQSEVAELKIVSDQLCAKYSKEYGKLCRTNQIGTVNDRLMHKLSVEAPPK
ILVERYLIEIAKNYNVPYEP
DAMVRPEVSPGEEANLIDVDSDNKPRGGGGGGGGGGGGGFTAPGMDFPHSSAFNYPSKGAVIAPLPRASKQLYDNNTLPE
LPSVPDTLPTTLGDRHTTDD
IDFDDLSRRFEELKKKT
>g3239.t3
MLGGGFKAERLKVNLRLVINRLKLLEKKKTELAQKARKEIADYLSAGKDERARIRVEHIIREDYLVEAMEILELHCDLLL
ARFGLIQSMKELDPGLQESV
STLIWAAPRLQELDPGLQESVSTLIWAAPRLQSEVAELKIVSDQLCAKYSKEYGKLCRTNQIGTVNDRLMHKLSVEAPPK
ILVERYLIEIAKNYNVPYEP
DAMVRPEVSPGEEANLIDVDSDNKPRGGGGGGGGGGGGGFTAPGMDFPHSSAFNYPSKGAEPYSSPLGTYVDHFPVGGGN
PPQLPSCPPTYESIDDLPVT

PSVPSQVIAPLPRASKQLYDNNTLPELPSVPDTLPTTLGDRHTTDDIDFDDLSRRFEELKKKT
>g3240.t1
MSRQLSQLLTPDLPAGANTQFGGCPQPGGSLSGGHLSSMAGLKSLLQHPVKGDQRVKTTSEVKADKDRPSDQDEDSLGTC
AMRNSTGINSSNSSGGGGGN
SAGGGGGGGGGGGGGGGGSGGGGGGGFNQILGSLLWDRSLPADGGLFQLQYMDLEEFLTDNGMGSIPNNNSSNNSSSTAQ
VPSQTSPSAVPNQSSQCPPS
SPPPCSSASSTSSSPSLIGLEVAQPQGMLGGSECLHGSQTSMNDSCESPCSSSSSSCPPLLTPTGSGPDGMGMFDMDPSD
MALSSIPGQDNFDPRRHRFS
EDELKPQPMIKKARKMLVPEDLKDEKYWSRRCKNNEAAKRSRDARRLKENQISVRAAYLERENAALRQEVAEMRKELGRC
RNILSKYENHLADQ
>g3240.t2
MSRQLSQLLTPDLPAGANTQFGGCPQPGGSLSGGHLSSMAGLKSLLQHPVKGDQRVKTTSEVKADKDRPSDQDEDSLGTC
AMRNSTGINSSNSSGGGGGN
SAGGGGGGGGGGGGGGGGSGGGGGGGFNQILGSLLWDRSLPADGGLFQLQYMDLEEFLTDNGMGSIPNNNSSNNSSSTAQ
VPSQTSPSAVPNQSSQCPPS
SPPPCSSASSTSSSPSLIGLEVAQPQGMLGGSECLHGFSAHALIIYTTGGSQTSMNDSCESPCSSSSSSCPPLLTPTGSG
PDGMGMFDMDPSDMALSSIP
GQDNFDPRRHRFSEDELKPQPMIKKARKMLVPEDLKDEKYWSRRCKNNEAAKRSRDARRLKENQISVRAAYLERENAALR
QEVAEMRKELGRCRNILSKY
ENHLADQ
>g3240.t3
MSRQLSQLLTPDLPAGANTQFGGCPQPGGSLSGGHLSSMAGLKSLLQHPVKGDQRVKTTSEVKADKDRPSDQDEDSLGTC
AMRNSTGINSSNSSGGGGGN
SAGGGGGGGGGGGGGGGGSGGGGGGGFNQILGSLLWDRSLPADGGLFQLQYMDLEEFLTDNGMGSIPNNNSSNNSSSTAQ
VPSQTSPSAVPNQSSQCPPS
SPPPCSSASSTSSSPSLIGLEVAQPQGMLGGSECLHAFLPPTSTSPVELLINTMVTKHFSVLWWSPKCQQQTMSPKL
>g3241.t1
MRLKKKKPGTLLLKIRRGSEHGGGGYELVSTGGAKVQVMRSLKSVKNFTCPMCPNAMFSCQAGLLVHRALKHPQKALSPQ
ERLQCSACGKQSHSPLAAFV
HRATHRAKGSFSCRRCSACFWNAALLDRHKAFCCRRAKGFQQGSSISLKPSKMAAQRGNPEDCSGSSFQPEVYGY
>g3242.t1
AGSEPAREASYAECSLRERLLMRNVATTNQAKTNVDRNVTKCLLYPPWTLGPLNVPVKSATDNQLSDKRHAVVGSKHYQS
GANMVYHGRYVSERAPLAVA
HGEGADGGSSARKARTHSDPVWAALSSASLVPRRRHLNPVAVGDGDRGPQPQVPKRSVCAAPCSSSEDSGAENRPAGIQK
QAKSKSLRHMRPKPASKETA
APKRDIARGASSSGLARRYVTDPRPQSVPGPAAITNESPGDGLPQTSVPNDVFITCTPSSPVTVTDSDDSLSCSDSPLPL
AERLRLKYLV
>g3243.t1
MDCDIEAASAKIIAEWDWKKEESAFGDEQDSLESNSLGSSCDSICSSPELSSTSSGLQDYQDLSYGFIRRSKDPCSTQRQ
TKHKMSTKRRMKASEREKMR
MRSLAEALHQLRDYLPPDYSKSGQPLTKIQTLKYTIEYINQLEDILNHA
>g3244.t1
LTERIRDQCFCSLQHKKHGSVNITVTDEPDTLFQKVSVLVKGHDRSVLDSYEFFATMAAKELGITLGKVFEPPKDIERLT
LLKSIHIFKKHRVQYEMRTH
YRCLELFRITGSTAQVYLEYIQRNLPEGVAMEVSKIAMVKVPEHILEPMWEEPPTDNSPTTDLKG
>g3244.t2
LTERIRDQCFCSLQHKKHGSVNITVTDEPDTLFQKVSVLVKGHDRSVLDSYEFFATMAAKELGITLGKVFEPPKDIERLT
LLKSIHIFKKHRVQYEMRTH
YRCLELFRITGSTAQVYLEYIQRNLPEGVAMEVSKLLWE
>g3244.t3
MAISWWYKLMVLRSSIHTASSLASASSPITVTDEPDTLFQKVSVLVKGHDRSVLDSYEFFATMAAKELGITLGKVFEPPK
DIERLTLLKSIHIFKKHRVQ
YEMRTHYRCLELFRITGSTAQVYLEYIQRNLPEGVAMEVSKIAMVKVPEHILEPMWEEPPTDNSPTTDLKG
>g3245.t1
MFAAVEHGPVLCSDSNILCLSWKGRVPKSEKEKPVCRKRYYEEGWLATGNGRGVVGVTFTSSHCRRDRTTPQRINFNLRG
HNSEVSGGV

>g3246.t1
MAAGGGCLCRRSAPSPAVPVQPPHFFPRQRRRAASQELFRAAVSVLAKVQNGYIIFTMRQQVDKSPETPTYQPLQ
>g3247.t1
MEGEAGLMDPEQSDTERTGERMAEDEFACAGMLRDSLTSQRLSVEKMFRVSFGIGADDVPHNDGQMWLKLRGASHNVAAA
KIGSHGCLLISGLTEQVVQA
YSLITDLLERFQGAQDQCTETGSRALGKSLDASRAFNCLVRKWEDRHTVELLALPEAVKQSLLTLVKESGLYSSVDSVEL
SPVRIDHQTGQGPQGPMTGG
YARPGWEDSVGTVAFNSGRHLGRDRARDPHGHEPDHRRVAPYLTAEPAVPAVVRPSTGLAGPPSGPPPSDALVPPEYTAV
ATGGRGGDRGDGDEQRLEEE
QVVMLEAVKGKEKEQEEEILQDEEVVLSVGSRKEFRLLLKFFTAMGYTEQVVRQVLARTGPREASQILDLVQQEQDRSDK
NDQSPVINWQKMGGQTQRNC
PTETEVGEGWMGEEELNGGRSTGRLPGGRQPSGAALGRIGGSHAGLLYPTAKRGGPQTGGAMGVSREPSRDGGDGEQVEE
DFVLAVLKRAAASCGYTEEK
VEEVYSCLPDLSTHQLILRLQREAGMLGAPREEGDEAPVLEREEEGAGAAEGEWGGASGHPAAHAGPNEKKPSMAPRPRE
PLPVPWVSTAAAPPE
>g3247.t2
MEGEAGLMDPEQSDTERTGERMAEDEFACAGMLRDSLTSQRLSVEKMFRVSFGIGADDVPHNDGQMWLKLRGASHNVAAA
KIGSHGCLLISGLTEQVVQA
YSLITDLLERFQGAQDQCTETGSRALGKSLDASRAFNCLVRKWEDRHTVELLALPEAVKQSLLTLVKESGLYSSVDSVEL
SPVRIDHQTGQGPQGPMTGG
YARPGWEDSVGTVAFNSGRHLGRDRARDPHGHEPDHRRVAPYLTAEPAVPAVVRPSTGLAGPPSGPPPSDALVPPEYTAV
ATGGRGGDRGDGDEQRLEEE
QVVMLEAVKGKEKEQEEEILQDEEVVLSVGSRKEFRLLLKFFTAMGYTEQVVRQVLARTGPREASQILDLVQQEQDRSDK
NDQSPVINWQKMGGQTQRNC
PTETEVGEGWMGEEELNGGRSTGRLPGGRQPSGAALGRIGGSHAGLLYPTAKRGGPQTGGAMGVSREPSRDGGDGEQVEE
DFVLAVLKRAAASCGYTEEK
VEEVYSCLPDLSTHQLILRLQREAGMLGAPREEGDEAPVLEREEEGAGAAEGEWGGASGHPAAHAGPNEKKPSMAPRPRE
PLPVPWVSTAAAPPEPHGPY
IYPQAYAQPPGHLSTTGQNTAP
>g3248.t1
RPGDTVVMERRRRHGDSSWTNVMSNVTPQRLRDGPGS
>g3249.t1
DEESEEDDADIISRDALKRQSQLIIDSKTKKKPRKKKK
>g3249.t2
FYTRIEGKLPDYNIRIQLPEEQRQNPFNEDEESEEDDADIISRDALKRQSQLIIDSKTKKKPRKKKK
>g3250.t1
TIVTSSLPSTVASHMMYPGGHTVMYATPTSSLTDAGLTLLNTFSQSGPAPNHEPGPQVFLTPLPPVTQIPVSAVQLHSMV
IGQQAAGSSTLTELQVVNMD
ATPPTKED
>g3250.t2
TIVTSSLPSTVASHMMYPGGHTVMYATPTSSLTDAGLTLLNTFSQSGPAPNHEPGPQVFLTPLPPVTQIPVSAVQLHSVS
THSKYTHCKYSASTCSMVIG
QQAAGSSTLTELQVVNMDATPPTKED
>g3251.t1
MSFHEAPDVVALGLLLRSYNVVKIQLQPQPQSFCCFTGCTNVALLPLLATQALFIRTYVQNLFHGQQDICGTRTGPEDHG
AIVVYPKFNCGPV
>g3252.t1
MAVAPAASRKRLLKDEDMTKIEFETSEEVDVTPTFDTMGLREDLLRGVYAYGFEKPSAIQQRAIKQIIKGRDVIAQSQSG
TGKTATFCVSVLQCLDIQVR
ETQALILAPTRELAGQIQKVLLALGDYMNVQCHACIGGTNVGEDIRKLDYGQHVVAGTPGRVFDMIRRRSLRTRAIKMLV
LDEADEMLNKGFKEQIYDVY
RYLPPATQVCLISATLPHEILEMTNKFMTDPIRILVKRDELTLEGIKQFFVAVEREEWKFDTLCDLYDTLTITQAVIFCN
TKRK
>g3252.t2
MAVAPAASRKRLLKDEDMTKIEFETSEEVDVTPTFDTMGLREDLLRGVYAYGRPGIRGHRGRNVASGFEKPSAIQQRAIK
QIIKGRDVIAQSQSGTGKTA

TFCVSVLQCLDIQVRETQALILAPTRELAGQIQKVLLALGDYMNVQCHACIGGTNVGEDIRKLDYGQHVVAGTPGRVFDM
IRRRSLRTRAIKMLVLDEAD
EMLNKGFKEQIYDVYRYLPPATQVCLISATLPHEILEMTNKFMTDPIRILVKRDELTLEGIKQFFVAVEREEWKFDTLCD
LYDTLTITQAVIFCNTKRK
>g3252.t3
MAVAPAASRKRLLKDEDMTKIEFETSEEVDVTPTFDTMGLREDLLRGVYAYGFEKPSAIQQRAIKQIIKGRDVIAQSQSG
TGKTATFCVSVLQCLDIQVR
ETQALILAPTRELAGQIQKVLLALGDYMNVQCHACIGGTNVGEDIRKLDYGQHVVAGTPGRVFGQSCSPSGLPETFSGAP
VLSYWSSR
>g3252.t4
MIRRRSLRTRAIKMLVLDEADEMLNKGFKEQIYDVYRYLPPATQVCLISATLPHEILEMTNKFMTDPIRILVKRDELTLE
GIKQFFVAVEREEWKFDTLC
DLYDTLTITQAVIFCNTKRK
>g3253.t1
HDSGLHRSTEPTVVEVSQHSSTNHHMDGRRNCRHTPP
>g3253.t2
HDSGLHRSTEPTVVEVSQHSSTNHHMDGRRNCRHTPPQKACLIHLGAIRKPQPKQEE
>g3254.t1
MSEHDEGDDPLLDSPPLLSRDLKSVLVTSVLQLEEIDVGLYRGKNYWIPLSGRLFGGQIIGQALVAAAESVSDNLYAHSL
HCYFVRPGDPDVPVLYKVDR
VRDGRSFCVRSVKALQRGEPILVLQASFHMDQPSSLRHQFAMPEVPPPEDLLSLEDLIRFVLGRPDLEQKVKQGFNKVLA
NEVPIEVRPVNPLEFSREVT
TEPKRMFWVRARGHI
>g3254.t2
DPDVPVLYKVDRVRDGRSFCVRSVKALQRGEPILVLQASFHMDQPSSLRHQFAMPEVPPPEDLLSLEDLIRFVLGRPDLE
QKVKQGFNKVLANEVPIEVR
PVNPLEFSREVTTEPKRMFWVRARGHI
>g3254.t3
MDQPSSLRHQFAMPEVPPPEDLLSLEDLIRFVLGRPDLEQKVKQGFNKVLANEVPIEVRPVNPLEFSREVTTEPKRMFWV
RARGHI
>g3255.t1
LGVRRAESNGVDDEEGGGEHDIETPHGVLHVTMRGVPKGNRPVILTYHDIGLNHKSCFNTLFNYEDMQEITQHFAVVHVD
APGQQEGAPPFPTGYQYPTM
EQLAEMLPSVMTQLKVNSVIGIGVGAGAYILSRFA
>g3255.t2
LGVRRAESNGVDDEEGGGEHDIETPHGVLHVTMRGVPKGNRPEHDIETPHGVLHVTMRGVPKGNRPVILTYHDIGLNHKS
CFNTLFNYEDMQEITQHFAV
VHVDAPGQQEGAPPFPTGYQYPTMEQLAEMLPSVMTQLKVNSVIGIGVGAGAYILSRFA
>g3256.t1
LKRRKAFAAMKAARIKKMLAEKITRKVTRKLIYKRAEAYHREYRTLYRREVRQARTARKVGNFYVPAEPKLAFVIRIRGI
NGVSPKVRKVLQLLRLRQIF
NGVFVRLNKASINMLRIAEPYIAWGYPNLKSVRELIYKRGFGKINKQRIAVTDNSLIEKTLGKYGIICMEDLIHEIFTTG
KNFKVANNFQWPFKLSSPRG
GMNKKTTHFVEGGDAGNREHQINRMIQRMN
>g3256.t2
MLLLINVASSAAAPWTRKVTRKLIYKRAEAYHREYRTLYRREVRQARTARKVGNFYVPAEPKLAFVIRIRGINGVSPKVR
KVLQLLRLRQIFNGVFVRLN
KASINMLRIAEPYIAWGYPNLKSVRELIYKRGFGKINKQRIAVTDNSLIEKTLGKYGIICMEDLIHEIFTTGKNFKVANN
FQWPFKLSSPRGGMNKKTTH
FVEGGDAGNREHQINRMIQRMN
>g3256.t3
MEATVLRIGELMDGKVKRTRKVTRKLIYKRAEAYHREYRTLYRREVRQARTARKVGNFYVPAEPKLAFVIRIRGINGVSP
KVRKVLQLLRLRQIFNGVFV
RLNKASINMLRIAEPYIAWGYPNLKSVRELIYKRGFGKINKQRIAVTDNSLIEKTLGKYGIICMEDLIHEIFTTGKNFKV
ANNFQWPFKLSSPRGGMNKK
TTHFVEGGDAGNREHQINRMIQRMN

>g3257.t1
MNPLIRQPRKKKDRLQMKPIRAASDTEYGSLSVRVSGYDMTLVEHYSQYIHNLCHRLGIRVTESYGLPTKSTEVLLMQEQ
GTKMYVDALLKTHERIVQIS
SMTATLCPTFMDAVLQNQPEGVQLSVKE
>g3258.t1
LSLVEGLTHLLECRSVSGACRAMHGLENSLLTPSGYYHLLPFFPSG
>g3259.t1
MEIYYHNNEEHDTNSDNIEMACGGTRSWVEVLPPGRFLRAWFFTCRAEPRLVSGNGEVSGDSSGECNFGDPLSQEAGGLG
APRQPDDPPPKKKT
>g3260.t1
MLPVLLLVCVAGGTRAYRPDQDHPPQQGVRAGPGSCPAPCHCEEDSIYVMVDCSELGLSSVPENLSPFTAYL
>g3261.t1
MMERTKWSDYAASPTKAMVAPPTAVFLQPKWSVNMLTTGEQRKIIPIARAPTQAGGKEQQEEEGWR
>g3262.t1
DSTKDKTNLIVYIICGVVAIMALVGIFTKLGLEKAHRPQRENMSSSAAEMERWLEDIRMAVDLAEQISDPQCDLLSSSLA
DTVANVCQPMSTMNTLPATA
VQDWGYGWLALRCLLAC
>g3262.t2
AIMALVGIFTKLGLEKAHRPQRENMSSSAAEMERWLEDIRMAVDLAEQISDPQCDLLSSSLADTVANVCQPMSTMNTLPA
TAVQDWGYGWLALRCLLAC
>g3262.t3
DSTKDKTNLIVYIICGVVAIMALVGIFTKLGLEKAHRPQRENMSSSAAEMERWLEDIRMAVDLAEQISDPQCDLLSSSLA
DSPGVLPRGWIALNLKGRIC
FPLPFVSVQPL
>g3263.t1
LPELEIVSLLSEGQPNYTLRADSVFGYDHDDWLHTPLLPQEVVLGLTHQQIEETFKYF
>g3264.t1
LPYSVSVLSPVEIDSFRILLYALIFLLSVAGNLLIIVVLALNKRMRTVTNCFLLSLALSDLMMAVFCMPFTLIPNILEDF
IFGAAMCKIVAYFMVGRAA
>g3264.t2
LSPVEIDSFRILLYALIFLLSVAGNLLIIVVLALNKRMRTVTNCFLLSLALSDLMMAVFCMPFTLIPNILEDFIFGAAMC
KIVAYFMVGRAA
>g3265.t1
AGYHMVIVKDHLCNVSEITRLVHMYVPDATIESSAGAELSYILPKESTSRFELLFAELEMNREELGIASYGASVTTMEEV
FLRTPRGKPTPAGPG
>g3265.t2
AGYHMVIVKDHLCNVSEITRLVHMYVPDATIESSAGAELSYILPKESTSRFELLFAELEMNREELGIASYGASVTTMEEV
FLS
>g3265.t3
AGYHMVIVKDHLCNVSEITRLVHMYVPDATIESSAGAELSYILPKESTSRFELLFAELEMNREELGIASYGASVTTMEEV
FL
>g3266.t1
MGHKSLDKIITLQKNPANIRNLCILAHVDHGGKEYLLNLIDSPGHVDFSSEVSTAVRLCDGAIVIVDAVEGVCPQTQAVL
RQAWLENIRPVLVINKMDRL
ITELKLTSMEAFSHLQKILGQTSSLINAPVNRGRDGPSSAFAGKREPEK
>g3266.t2
MGHKSLDKIITLQKNPANIRNLCILAHVDHGGKEYLLNLIDSPGHVDFSSEVSTAVRLCDGAIVIVDAVEGVCPQTQAVL
RQAWLENIRPVLVINKMDRL
ITELKLTSMEAFSHLQKILGQVNAVTGALFTSKVLEERAEKEEQKEKERDPSGSQAEQTPAADQVYDWSAGLEETDDSHL
YFSPDQGNVVFASAIDGWGF
SVQQFAHIYSQKMGIREEVLRKTLWGDFFLNAKAKKIMKGAQ
>g3266.t3
LFTSKVLEERAEKEEQKEKERDPSGSQAEQTPAADQVYDWSAGLEETDDSHLYFSPDQGNVVFASAIDGWGFSVQQFAHI
YSQKMGIREEVLRKTLWGDF
FLNAKAKKIMKGAQ
>g3266.t4

MAASQRTAAGSVVLDYGRSQYRAPRVARGFPMFSLGALFTSKVLEERAEKEEQKEKERDPSGSQAEQTPAADQVYDWSAG
LEETDDSHLYFSPDQGNVVF
ASAIDGWGFSVQQFAHIYSQKMGIREEVLRKTLWGDFFLNAKAKKIMKGAQ
>g3266.t5
LDVGVQTQAVLRQAWLENIRPVLVINKMDRLITELKLTSMEAFSHLQKILGQTSSLINAPVNRGRDGPSSAFAGKREPEK
>g3267.t1
MKPKPEMDNNKTRMDGTTTFKTDFIPHEVTRRPGRQPAEYRPNSGKIDLGTTYTRDFNPYEITQPFVPLRPREMTHSMEK
PKMLVAAAPRESFKPANVVQ
RSGVPLESFTSNRLAFVPHVVEPRRQRPPETYKPSGQPLESITTNRQDFQGLAAELPQSCKPQTNEYSSMAASSLPLQGS
TENKDQFQKWPVSLPQFHKK
EVYVGPTEQMDLSTTTAEAFVKHQIQPFVFAKPQLPPKRSTVPLE
>g3267.t2
LTEYSEKFPVHTGLYNPPNSMKPKPEMDNNKTRMDGTTTFKTDFIPHEVTRRPGRQPAEYRPNSGKIDLGTTYTRDFNPY
EITQPFVPLRPREMTHSMEK
PKMLVAAAPRESFKPANVVQRSGVPLESFTSNRLAFVPHVVEPRRQRPPETYKPSGQPLESITTNRQDFQGLAAELPQSC
KPQTNEYSSMAASSLPLQGS
TENKDQFQKWPVSLPQFHKKEVYVGPTEQMDLSTTTAEAFVKHQIQPFVFAKPQLPPKRSTVPLE
>g3267.t3
MDNNKTRMDGTTTFKTDFIPHEVTRRPGRQPAEYRPNSGKIDLGTTYTRDFNPYEITQPFVPLRPREMTHSMEKPKMLVA
AAPRESFKPANVVQRSGVPL
ESFTSNRLAFVPHVVEPRRQRPPETYKPSGQPLESITTNRQDFQGLAAELPQSCKPQTNEYSSMAASSLPLQGSTENKDQ
FQKWPVSLPQFHKKEVYVGP
TEQMDLSTTTAEAFVKHQIQPFVFAKPQLPPKRSTVPLE
>g3268.t1
NTVETVISFDQGAKWQALRTPQNSRCDRQTPANRCRLHIHASYSTTNKMNVPMLPLSQPNAVGLILAHGSMGDPESVLSP
DVYVSDDGGYTWLLALRGPH
QYAILDSGGLLVAVEHSNSPIRQIKFSTDEGQCWHVHNFTDDPLYFSGMDSEPGSRSMNVSLWGYRTVISKWEVITIDFG
KLLDRDCGDEDYVQWLAHSA
DLSEPSSGCMLGYMEHYQRLRKDSMCWNGRGYAVTKQPTPCPCTKDDYH
>g3269.t1
MSGDEMIFDPNMTKKKKKKKKPFMLEEEGGDGVAGEEAKEVEAKEVEPEGGDDKEVDFDEDEGRKKEPSDDLDDLNFLNQ
KKKKKKTKKFLENEVEEGIK
ELKIEGEQPEPMEEDDLDLMLPAKKKKSKKVDFDEGLETLEKEDVLDDDEGKNNDGISFSSSTGPAWAGTERDYTYDELL
NRVFNIMREKNPDMVAGEKR
KFVMKPPQVVRVGTKKTSFVNFTDICKLLHRQPKHLLAFLLAELGTSGSIDGNNQLVIKGRFQQKQIENVLRRYIKEYVT
CHTCRSPDTILQKDTRLYFL
QCETCHSRCSVASIKTGFQAVTGKRAQLRAKAN
>g3269.t2
MSGDEMIFDPNMTKKKKKKKKPFMLEEEGGDGVAGEEAKEVEAKEVEPEGGDDKEVDFDEDEGRKKEPSDDLDDLNFLNQ
KKKKKKTKKFLENEVEEGIK
ELKIEGEQPEPMEEDDLDLMLPAKKKKSKKVDFDEGLETLEKEDVLDDDEGKNNDGISFSSSTGPAWAGTERDYTYDELL
NRVFNIMREKNPDMVAGEKR
KFVMKPPQVVRVGTKKTSFVNFTDICKLGSIDGNNQLVIKGRFQQKQIENVLRRYIKEYVTCHTCRSPDTILQKDTRLYF
LQCETCHSRCSVASIKTGFQ
AVTGKRAQLRAKAN
>g3270.t1
MGKPLSRPGCFQKNSCCRAKHGVGEGGGVRGGGGSIVEGGYGDGYVPQRSIYDTMCLNQQIDSQHHHHHHHMGGSMREAA
AEGSFNYSASSSLRVSRPAG
DMFDQQPRSLTPNPSFVRRLDERAIYDSLKLGVQEADGVDCHSPGRFKRSASPSASSFSAPGISSSSSKKHHHQQQQQLQ
QQQQQQQQQQQQQQQQQQQH
HHHPHHHHGNSGKSREGRHSWKAVTPPNHPNHMDCLELTSEDMLERGGGYLNPAAYPPTGSSSLASPLISPFTGSPHSSG
YHTPHFFSPAKPRPAQTQSL
RCSPPHVTQPRHYSQTQSLRLSPTHELTHSPYRAPLVQLSARDLQEDMRERAWSGRADREREREWERERERMERGWAER
EWEKERERGRLERERARVRE
RRDTSVSSFGTFGYGRSGPLCSDRDSVFLESDQVHDTTPLLPPQQSLSPSPNAAPRMGTGAVGRNRAGSKVGPETMSASV
RTSNSGGGVETKTKTEHSPQ

SGFNAVSGSNTEKMNPDLNIARHKDEKKTTAIDGGNPESLPCEKAVKKVDKSKIGSIVPDIDFGAQKLSTPKPNSRTRPG
TATGLRPGLVSPKLGSGLGE
DPAAPPDGMESTWPRRVIVRKRTVRQGGALHNLPILPPLPSVLSALEKRFPHHLPRLAHHSENPLTNPALIGVGVGRCSL
KEPSHLELGLCASVVSRASL
KEQNLEQWRATKEHIDLRMHSRNTESREESAAARGEKKASGVERDELKTKEVKAGNQGDRPGERENSGQRKCEQSYGVSK
VEDVITKQVEPRGVDAQVEI
PESVKRERVTKDIQLALEVKANEVEDSTRRALPSPEQPKPKVKEATEVAAKPEVRIDLEGEWMEERDIGEEAGELESWDV
VLEMVNTIWEDGWAGGRGAG
AAEEGDDTGSVSGSLQRWPLLRPPAGFGGSHPPSSAASELSLAELEKRARELDSDLEHLDLSQPHRDPHGLMLPKPQKER
TDMYQTHPGPQREKCAFLTG
VGSRSQVRLELAPAIIEKVITEVDTSWPAKTAAGVPTIGISSTKEDSSPDSNLTLESDSSGVFLSLSNQSQDEMGSDSDQ
PMSGSDLGSSSTSFDRDGEE
AGVKEWGREESAELDWCYPSLLDPSSHEHAGGDGSEGEEGGEHENKDRRDTEKESVSVKTSIQMFSSVSLPTSPSSKQQI
KQFLNPNLPMRSSGLDCGDL
DPFVQSDSFVYLAVSTSSGTQADPAETTDRPPHGTAPAIPTKHPVAPRAHEGPSPAQKDTVPGTHLVPQRPEEGDFLSTD
SFVYLAAPACLLLGPEGTAP
YSARDSDSDSSGSGGADVSVLGCGSLAGDSDWDSDASDSDPHRPSSRAHGAGG
>g3270.t2
MGKPLSRPGCFQKNSCCRAKHGVGEGGGVRGGGGSIVEGGYGDGYVPQRSIYDTMCLNQQIDSQHHHHHHHMGGSMREAA
AEGSFNYSASSSLRVSRPAG
DMFDQQPRSLTPNPSFVRRLDERAIYDSLKLGVQEADGVDCHSPGRFKRSASPSASSFSAPGISSSSSKKHHHQQQQQLQ
QQQQQQQQQQQQQQQQQQQH
HHHPHHHHGNSGKSREGRHSWKAVTPPNHPNHMDCLELTSEDMLERGGGYLNPAAYPPTGSSSLASPLISPFTGSPHSSG
YHTPHFFSPAKPRPAQTQSL
RCSPPHVTQPRHYSQTQSLRLSPTHELTHSPYRAPLVQLSARDLQEDMRERAWSGRADREREREWEREREREMERGWAER
EWEKERERGRLERERARVRE
RRDTSVSSFGTFGYGRSGPLCSDRDSVFLESDQVHDTTPLLPPQQSLSPSPNAAPRMGTGAVGRNRAGSKVETKTKTEHS
PQSGFNAVSGSNTEKMNPDL
NIARHKDEKKTTAIDGGNPESLPCEKAVKKVDKSKIGSIVPDIDFGAQKLSTPKPNSRTRPGTATGLRPGLVSPKLGSGL
GEDPAAPPDGMESTWPRRVI
VRKRTVRQGGALHNLPILPPLPSVLSALEKRFPHHLPRLAHHSENPLTNPALIGVGVGRCSLKEPSHLELGLCASVVSRA
SLKEQNLEQWRATKEHIDLR
MHSRNTESREESAAARGEKKASGVERDELKTKEVKAGNQGDRPGERENSGQRKCEQSYGVSKVEDVITKQVEPRGVDAQV
EIPESVKRERVTKDIQLALE
VKANEVEDSTRRALPSPEQPKPKVKEATEVAAKPEVRIDLEGEWMEERDIGEEAGELESWDVVLEMVNTIWEDGWAGGRG
AGAAEEGDDTGSVSGSLQRW
PLLRPPAGFGGSHPPSSAASELSLAELEKRARELDSDLEHLDLSQPHRDPHGLMLPKPQKERTDMYQTHPGPQREKCAFL
TGVGSRSQVRLELAPAIIEK
VITEVDTSWPAKTAAGVPTIGISSTKEDSSPDSNLTLESDSSGVFLSLSNQSQDEMGSDSDQPMSGSDLGSSSTSFDRDG
EEAGVKEWGREESAELDWCY
PSLLDPSSHEHAGGDGSEGEEGGEHENKDRRDTEKESVSVKTSIQMFSSVSLPTSPSSKQQIKQFLNPNLPMRSSGLDCG
DLDPFVQSDSFVYLAVSTSS
GTQADPAETTDRPPHGTAPAIPTKHPVAPRAHEGPSPAQKDTVPGTHLVPQRPEEGDFLSTDSFVYLAAPACLLLGPEGT
APYSARDSDSDSSGSGGADV
SVLGCGSLAGDSDWDSDASDSDPHRPSSRAHGAGG
>g3271.t1
LKTIDDHIGHADLGLKPRPVSSPRVGGPRFRISALFLLLLLLLPPKATLALWACVRACPSSCTCTQERSCSVLCDRLAMA
ELPKEFPCEASSINVDKNRI
KFLSERAFGTLPSLRSLSLDHNNISFITPGAFKGLPNLVDLKMAHNEYISYLHTRTFTGLKKLVRVDVSDCNLFNIPDRI
FLEQTALREVLCFKNNFRRI
PGAFRDMVNLTHIYLEKNKIEAVAYNSLLGLSNLRFLNLEENRINVIHDQSFQDLQRLEYFYLNDNLLSVLPQETFRGLT
HLKMLNLGGNLLTNVSKTWF
SDLVELEVLYLDRNRLVAIEEGSFENLTSLMTLHLNSNNLTALPFPVFQPVYFLGRLYLFKNPWECGCSLEWLKEWMENY
KLVRNIPCASPSSVAGLDLS
EVVFIQANGTCVDPPELNATATPSAAPSDTAKRFNSLISRLLQLEQQEMMGNVTEGFRNDTLRDPEDALLSAGAQRQAVR
WGLPHVHLLVAWFLLGFGSH

SEIVFHVFGT
>g3272.t1
MALASVLGSDCADFSFDCHKPYAFGSGLADEPTSIENLSFALKGSLDSADQDEVERKIEFLHGTTTLAFKFDHGVIVAVD
SRATAGAYIASQTVKKVIEI
NPYLLGTMAGGAADCSFWERLLARQCRIYELRNKERISVAAASKLLANMVYQYKGMGLSMGTMVCGWDKRGPGKC
>g3273.t1
MALEDFCSFRRNDHLWTPTSLLWPSQDPPGIPQHPHSGRGGLEKGNRGDGPPLRFVLPPTVSPHRLLPSVRRPFPLAHGT
TTLGFVFQGGVIAAADTRAS
AGGLVMCPMADKIIPVHSHLVVTSSGSGADCKLWARALEREVRLYRLRHGHHLSVGGTAKLLALMLHPFKGTEVCVALTF
CGWDQGQNGCKHDGGDMGSA
EALSQKSSSSSSSPSCSASSSSSTSPGPSSPSTSSSSPYTASSTASVGPKVIYVCSDGARLQGELFSVGSGSPYAYGILD
GGLRWTLSQEEAASLAREAV
FRATHRDAYSGNNVDVVYITENGWSRREREDLKEEYHRRKEEIGKIVEGRCRKN
>g3274.t1
MASGSTGSRVSCGRDLNCVPEVADTLAAVAKLGFDFLCMPLFHPRFRREYELAPAKARPGAQTRSDLLLCGRDWNTLIVG
KLSPWIDADAEITTERRNSE
AALVQELNFAAYLGLPAFMIPLKGAHSANLARVLVNHIQTGHHTSNFWIRVPLIAPEDTHDELVENEQSKAAPDDTSVDE
KTWIWWHSFRTLCDYNKRIC
VAIEVGADMPSEAVIDKWLGEPIKAAILPTSIFLTNKKGFPVLSKAHQRIIFSLFKLEAQFIFTGTSRHSEKDFRSYLQY
LEYLNQNRPAPNAYELFAKG
YEDYLQSPLQPLMDNLESQTYEVFEKDPIKYSQYQQAVYKCLLDRVPEEQKDTNVQ
>g3274.t2
MASGSTGSRVSCGRDLNCVPEVADTLAAVAKLGFDFLCMPLFHPRFRREYELAPAKARPGAQTRSDLLLCGRDWNTLIVG
KLSPWIDADAEITTERRNSE
AALVQELNFAAYLGLPAFMIPLKGAHSANLARVLVNHIQTGHHTSNFWIRVPLIAPEDTHDELVENEQSKAAPDDTSVDE
KTWIWWHSFRTLCDYNKRIC
VAIEVGADMPSEAVIDKWLGEPIKAAILPTSIFLTNKKGFPVLSKAHQRIIFSLFKLEAQFIFTGTSRHSEKDFRSYLQY
LEYLNQNRPAPNAYELFAKG
YEDYLQSPLQPLMDNLESQTYEVFEKDPIKYSQYQQAVYKCLLDRVPEEQKDTNV
>g3274.t3
MASGSTGSRVSCGRDLNCVPEVADTLAAVAKLGFDFLCMPLFHPRFRREYELAPAKARPGAQTRSDLLLCGRDWNTLIVG
KLSPWIDADAEITTERRNSE
AALVQELNFAAYLGLPAFMIPLKGAHSANLARVLVNHIQTGHHTSNFWIRVPLIAPEDTHDELVENEQSKAAPDDTSVDE
KTWIWWHSFRTLCDYNKRIC
VAIEVGADMPSEAVIDKWLGEPIKAAILPTSIFLTNKKGFPVLSKAHQRIIFSLFKLEAQFIFTGTSRHSEKDFRSYLQY
LEYLNQNRPAPNAYELFAKG
YEDYLQSPLQPLMDNLESQTYEVFEKDPIKYSQYQQAVYKCLLDRVPEEQKDTNVQELVSPLRCQNGEEM
>g3275.t1
MPSALAVFACRPNSHAFQERHVYLDEPVKIGRSVARCRPAQNNATFDCKVLSRNHALVWFDHKTGKRNSRLWVHTVVMTL
EVSSYQTLRLIEYK
>g3276.t1
MALQAGPEELGEQVVEELEDLEDQDASSISPAEEITLPVGPGGDDESEPLLLPWDRFSAWLHCICVVGFDLELGQAVEVI
YPHHSKLSEKEKTSICYLSF
PDSNSGQVLFLRLPMFQ
>g3276.t2
MALQAGPEELGEQVVEELEDLEDQDASSISPAEEITLPVGPGGDDESEPLLLPWDRFSAWLHCICVVGFDLELGQAVEKT
SICYLSFPDSNSGQVLFLRL
PMFQ
>g3276.t3
MALQAGPEELGEQVVEELEDLEDQDASSISPAEEITLPVGPGGDDESEPLLLPWDRFSAWLHCICVVGFDLELGQAVEVI
YPHHSKLSEKEKTSICYLSF
PDSNSGCLGDTQFCFRFRQGSNRKSS
>g3277.t1
MASPQPGSPPVVDQYTPPSTESEEGQHNNREVTKEQLTKKRGRGRPPKTKPSFKCSACQEAFRSPSALRSHKLSAHGKVH
PHAQEHACSQCAKTFSSKAQ
LSKHERSHAAQRPFQCPDCHKAYKTPTELRNHSRSHSGEKPFVCTECGKAFMQAICLRIHMTQHSGERPFSCPQCSKSYP

MLSKLKVHMRSHTGEKPYFC
AECGKSFADPSVFRKHRRNHQGHRPYVCEECGKSYTELKDLKNHERSHTGEKPYLCSDCGKAFSRSSSLACHQRIHSQSK
PYQCEQCGKGFTQLSSYQSH
LRTHSGEKPFLCPQCGKMFSDPSSFRRHQRAHLGFKPYPCDKCAKRFRQPADLAVHERVHSGERPYKCQSCDKAFVASWD
LRRHMLVHTGLRPFSCTECD
KSFAERSSLNKHRRVHSGERPFKCAKCFKSFVVSSSLRKHERTH
>g3278.t1
MPSLIEHMDSHLQDDEDRKYKCDECGRGYRHAGSLANHKKTHEVGSFQCQICGKENSNALALKSHLRSHTSQKKYACMDC
GKAFRLASQLATHERVHRSK
HVKEQSFRMVEDEYAEAEHQDEMQEDDQHLLFEQSATSCGLSLENSPAEDEMGPLDGAEMSDDPGNRPFKCDLCDKSYIH
YRSLTNHKKTHQTGMFECTV
CFKLFNNMAALYSHQRTHRGRSGAEPAARDRDSPYASTPLSQFSPQNQDTSVNFCHLCQVLFSTDEEFQEHIQMHNSSSA
PYSGHADMHDNHNVSYENSI
HSPESNFYASPVNNIPSVSSSIDNHGDYDHSEQILNNGQMFTDCSSGRTPTAAAPQVAEENEDAAEAEGGSDSGERRFKC
EVCGKRYRHSGSLINHRRSH
QVGIYQCSICRKSYPHLAALKSHLRLHKAQPSSFSLNSEGDWLSSEPLTLESHHAGFLPQEDDDDDDAGDGDPALAGDLE
ASGDHGDAPSYHSQFSQGFP
QGRPVHLPQDEHMVQRHQCADCGETFANIAGIKAHACPLHLRGHQAGYSGDMSFGPSNGECGTRRPDDGMDFSGVNGNHH
DQSYFSEEDFHEHAGSERLS
GGGGEDADEEDEEEEEEEEEDDDGDLYQCSVCGNRYTSMRALRSHLRGHTQAHGTPASSGPSSMSSHEEMKDDESGEMMV
MICSTCGESFAKRQDLIAHQ
LSHNAPRAEAEVGDPHTLNHHHHHHHDDDNHHRGHGADAKEEPQSSICGRCGLFCTSYH
>g3279.t1
MFPHHREGITVYRLAEGGLRRATCDNSRGPCNISTAQQATSGDRGTAVMSVTIKAYLLGKDDVHKEIRRFAVDEGVSTSF
EYLTNKVVDVFSNLRNIGFQ
MYYRDEDSDMIAFSSDDELMMALTFVQDNTFRLFIKEKKEHRRDFPLHAFPGGVPPFTFPPPPVPGAPHAPPPPHHGPPP
LHPNVTCDGCQGPVAGTRFK
CTVCPDYDLCSTCQAMGLHREHPLLPIWHPLANLMEWFPRGKLMRKMRHCMWNGAQQPTKPGAPGGQSTSGAQSTSGAQS
TSAPGTSTSPPSSDTSSSSS
GSSQTNAEFLKNIGEGVAAMLSPLGIDVDIDVEHDGIRTKVTPPATPAQSGAPASPTVQVDGATAVPDGPGPRSGEMEVD
EPNSLGRTPCDKGTKDTEEE
EWTHLTPKEVDPSTGELQSLIATPSEGQEGPTGLREAALYPHLPEEADPRLVESLSQMLSMGFSDEGGWLTRLLQTKACD
IGAVLDTIHYSGPPRK
>g3279.t2
LRRWLSEHVERRGETRELFLATPTTRHIKHVYDQQFRLPTMCRTDAQNSQNSRKRKCSGCSAKTSRTANTRSASSGDANA
RGDANTSGDANSSDASASSA
SQPALPAALTETINVFDSVLAGEEDVVDLTCEGTEPVATVVDLTNNDSVVVSNISLDHCGSSRPEGREATGESYVVSSDE
EEEDLPTLPNTALLSSLQAS
SQARATPGIISCPVCMDSYIEIVETRRLVVSTKCGHIFCSQCIRDSLARSHTCPTCRKKLTPRQEGITVYRLAEGGLRRA
TCDNSRGPCNISTAQQATSG
DRGTAVMSVTIKAYLLGKDDVHKEIRRFAVDEGVSTSFEYLTNKVVDVFSNLRNIGFQMYYRDEDSDMIAFSSDDELMMA
LTFVQDNTFRLFIKEKKEHR
RDFPLHAFPGGVPPFTFPPPPVPGAPHAPPPPHHGPPPLHPNVTCDGCQGPVAGTRFKCTVCPDYDLCSTCQAMGLHREH
PLLPIWHPLANLMEWFPRGK
LMRKMRHCMWNGAQQPTKPGAPGGQSTSGAQSTSGAQSTSAPGTSTSPPSSDTSSSSSGSSQTNAEFLKNIGEGVAAMLS
PLGIDVDIDVEHDGIRTKVT
PPATPAQSGAPASPTVQVDGATAVPDGPGPRSGEMEVDEPNSLGRTPCDKGTKDTEEEEWTHLTPKEVDPSTGELQSLIA
TPSEGQEGPTGLREAALYPH
LPEEADPRLVESLSQMLSMGFSDEGGWLTRLLQTKACDIGAVLDTIHYSGPPRK
>g3279.t3
MFSMGLQSPSTNQSGSGSSRPGMDVDGSGVMSSTNSRKRKCSGCSAKTSRTANTRSASSGDANARGDANTSGDANSSDAS
ASSASQPALPAALTETINVF
DSVLAGEEDVVDLTCEGTEPVATVVDLTNNDSVVVSNISLDHCGSSRPEGREATGESYVVSSDEEEEDLPTLPNTALLSS
LQASSQARATPGIISCPVCM
DSYIEIVETRRLVVSTKCGHIFCSQCIRDSLARSHTCPTCRKKLTPRQEGITVYRLAEGGLRRATCDNSRGPCNISTAQQ
ATSGDRGTAVMSVTIKAYLL

GKDDVHKEIRRFAVDEGVSTSFEYLTNKVVDVFSNLRNIGFQMYYRDEDSDMIAFSSDDELMMALTFVQDNTFRLFIKEK
KEHRRDFPLHAFPGGVPPFT
FPPPPVPGAPHAPPPPHHGPPPLHPNVTCDGCQGPVAGTRFKCTVCPDYDLCSTCQAMGLHREHPLLPIWHPLANLMEWF
PRGKLMRKMRHCMWNGAQQP
TKPGAPGGQSTSGAQSTSGAQSTSAPGTSTSPPSSDTSSSSSGSSQTNAEFLKNIGEGVAAMLSPLGIDVDIDVEHDGIR
TKVTPPATPAQSGAPASPTV
QVDGATAVPDGPGPRSGEMEVDEPNSLGRTPCDKGTKDTEEEEWTHLTPKEVDPSTGELQSLIATPSEGQEGPTGLREAA
LYPHLPEEADPRLVESLSQM
LSMGFSDEGGWLTRLLQTKACDIGAVLDTIHYSGPPRK
>g3279.t4
LRRWLSEHVERRGETRELFLATPTTRHIKHVYDQQFRLPTMCRTDAQNSQNSRKRKCSGCSAKTSRTANTRSASSGDANA
RGDANTSGDANSSDASASSA
SQPALPAALTETINVFDSVLAGEEDVVDLTCEGTEPVATVVDLTNNDSVVVSNISLDHCGSSRPEGREATGESYVVSSDE
EEEDLPTLPNTALLSSLQAS
SQARATPGIISCPVCMDSYIEIVETRRLVVSTKCGHIFCSQCIRDSLARSHTCPTCRKKLTPRHLHKAIDNCSLLVLKLA
AYT
>g3280.t1
LTRGTSSTDYNQADPGLTRGTSSTDYNQADPGFAIQTVAKDDNKAMPGFTMRTNTTESNQTKPVVRNTGSDRVYRHNKPC
SFGPSAAPPISRWTQFLPVS
RPQEEGVEDDDDKEEGETHLVSNTESKPLGLLGSLATPCSVSNIREPGHTSEHPKALSSPILHQPCPSLPVNSLFNTCDD
FEDFNFDDMF
>g3281.t1
MVQEFHVLRCFSCQTFQVQQEFGRGTGADCRCHVQKLNTKRGEMELEQVTWSLREKDMEAGQSLGGGARIVGGHKVRRSD
KESRWDKYLLNMPQGGEEQG
EEPEDTLHLSISRLHGEGNGSWVGPCLKQAQKLPSAAKASSLSLVLVLLDLSAAFDTVNHQTLLSTTARLVFNQHKFSHV
TPLFHDLHWLPVVTRIKFKT
MVLAYNAVNGAAPTYL
>g3281.t2
MCGEKQSVIKEFGRGTGADCRCHVQKLNTKRGEMELEQVTWSLREKDMEAGQSLGGGARIVGGHKVRRSDKESRWDKYLL
NMPQGGEEQGEEPEDTLHLS
ISRLHGEGNGSWVGPCLKQAQKLPSAAKASSLSLVLVLLDLSAAFDTVNHQTLLSTTARLVFNQHKFSHVTPLFHDLHWL
PVVTRIKFKTMVLAYNAVNG
AAPTYL
>g3281.t3
MVQEFHVLRCFSCQTFQVQQEFGRGTGADCRCHVQKLNTKRGEMELEQVTWSLREKDMEAGQSLGGGARIVGGHKAEESR
WDKYLLNMPQGGEEQGEEPE
DTLHLSISRLHGEGNGSWVGPCLKQAQKLPSAAKASSLSLVLVLLDLSAAFDTVNHQTLLSTTARLVFNQHKFSHVTPLF
HDLHWLPVVTRIKFKTMVLA
YNAVNGAAPTYL
>g3282.t1
MASVNSSDWSALCDKFTTALNLTEVESHKDPENDPFRSKYKAREILREIYSSLKNSEATETDGESSGERGEEQAEDGEKD
GQRFIGDSPAGLRAAKLAAV
EYFLGVNHVETEELSAGQEHLMNCTKILDKWSTSPVNVSLFIHVREGIPPTDMTEYFSTEENHLTHQERTKRFELAYTHT
KYYLAQVYKYL
>g3283.t1
KRRCGGLNCDGAAAEAGNALESARRAERELNKAMGEVEGLFTKVAEAKSRAEEAKGQAQAALDKAMATKNKVERSNNDLR
DLIKQIREFLTQEGADPDSI
EAVANRVLELSIPASPQQIRHLAEEIKERVRSLSNVDAILEQTQDDVRKAEQLLLDAKRARHSAEGVKTTTEAVKQALLD
ARTAQTSAEKAIARAKSDIG
DTETRLAQVTTPVLLAQIESETSDSEKDLRESMERLGTLGQQIDALKTKRVSNNMAAARAEETATMARDKANEAKQILDG
QLTDKFQEVQQRVDTKAKAV
QEAKQRAESLRDQAKELLADAHNKLLRLAELEKDYEENQRRLEGKARQLDGLEDKMKTILSDINKQIQIYNTCQKSAGFV
TKQSASHFTGFATPPDRPTC
GPTRRTPARRVFQPQGALPQRASVCSDSVPGELSGPIAKIAGPGRKRHTSDPEPCSECRDGQPLSAKCLKMASGSGETAG
RRGGGPSQHQRGGERAGERG
GAELSSSSSSSTSKKKQKDRANQESREAKRAAAAAAGGAIAEAKKGWCCVSILATALMETSDTLEAERA

>g3283.t2
KRRCGGLNCDGAAAEAGNALESARRAERELNKAMGEVEGLFTKVAEAKSRAEEAKGQAQAALDKAMATKNKVERSNNDLR
DLIKQIREFLTQEGADPDSI
EAVANRVLELSIPASPQQIRHLAEEIKERVRSLSNVDAILEQTQDDVRKAEQLLLDAKRARHSAEGVKTTTEAVKQALLD
ARTAQTSAEKAIARAKSDIG
DTETRLAQIESETSDSEKDLRESMERLGTLGQQIDALKTKRVSNNMAAARAEETATMARDKANEAKQILDGQLTDKFQEV
QQRVDTKAKAVQEAKQRAES
LRDQAKELLADAHNKLLRLAELEKDYEENQRRLEGKARQLDGLEDKMKTILSDINKQIQIYNTCQKSAGFVTKQSASHFT
GFATPPDRPTCGPTRRTPAR
RVFQPQGALPQRASVCSDSVPGELSGPIAKIAGPGRKRHTSDPEPCSECRDGQPLSAKCLKMASGSGETAGRRGGGPSQH
QRGGERAGERGGAELSSSSS
SSTSKKKQKDRANQESREAKRAAAAAAGGAIAEAKKGWCCVSILATALMETSDTLEAERA
>g3283.t3
KRRCGGLNCDGAAAEAGNALESARRAERELNKAMGEVEGLFTKVAEAKSRAEEAKGQAQAALDKAMATKNKVERSNNDLR
DLIKQIREFLTQEGADPDSI
EAVANRVLELSIPASPQQIRHLAEEIKERVRSLSNVDAILEQTQDDVRKAEQLLLDAKRARHSAEGVKTTTEAVKQALLD
ARTAQTSAEKAIARAKSDIG
DTETRLAQVTTPVLLAQVRAQIESETSDSEKDLRESMERLGTLGQQIDALKTKRVSNNMAAARAEETATMARDKANEAKQ
ILDGQLTDKFQEVQQRVDTK
AKAVQEAKQRAESLRDQAKELLADAHNKLLRLAELEKDYEENQRRLEGKARQLDGLEDKMKTILSDINKQIQIYNTCQKS
AGFVTKQSASHFTGFATPPD
RPTCGPTRRTPARRVFQPQGALPQRASVCSDSVPGELSGPIAKIAGPGRKRHTSDPEPCSECRDGQPLSAKCLKMASGSG
ETAGRRGGGPSQHQRGGERA
GERGGAELSSSSSSSTSKKKQKDRANQESREAKRAAAAAAGGAIAEAKKGWCCVSILATALMETSDTLEAERA
>g3284.t1
GRTHGEFRLTCGARWSGVGRCGCSGSDPERAWLGRSESQPGLMKDCWGTRHMEDSFAALLG
>g3284.t2
SLPGSGRTHGEFRLTCGARWSGVGRCGCSGSDPERAWLGRSESQPGLMKDCWGTRHMEDSFAALLG
>g3285.t1
MTGLVGASHAVEKRSASQEKIWASRGGRVPKASWVTSVGRSVPKVKGPTKSLLMSRRGTELRTAPARLAKGST
>g3286.t1
MARRTHLNPKVQLHSSKVFSGVKQQTLEDVAQSIQEKQYKRIVVMAGAGISTPSGIPDFRSPGSGLYDNLQQYDLPYAEA
IFEIAYFHHNPRPFFALAKE
LYPGNFQPNLTHYFVRLLQDKGIPPERLVEAHGTFATATCTVCRRTYEGQDLRVSGNPI
>g3286.t2
MARRTHLNPKVQLHSSKVFSGVKQQTLEDVAQSIQEKQYKRIVVMAGAGISTPSGIPDFRSPGSGLYDNLQQYDLPYAEA
IFEIAYFHHNPRPFFALAKE
LYPGNFQPNLTHYFVRLLQDKGQLLRMYTQNIDGLERCIPPERLVEAHGTFATATCTVCRRTYEGQDLRVSGNPI
>g3287.t1
MFLFSDPKDAITFLEKTREKVKSSEEAVILCKTTIGSLKLEINDLPATKKIIEEVEEILNNLPGVTSVHGRFYDLSSKYY
RIIGNHASYYKDALRYLGCV
DIKDLPETEKQERAFTLGLAGLLGEGVYNFGELLMHPVLESLRSTDKQWLIDTLYAFNGGNVEKFQSFKSAWGQQPDLAT
NESKLMQKIQLLCVMEMTFT
RPANNRQLTFQEIGQSAKIPINEVELLVMKALSVGLIKGNIDEVDQKVQMTWVQPRVLDVQQIKGMKERLDLWCGDVKNM
AMLVEQQAHDILT
>g3287.t2
MFLFSDPKDAITFLEKTREKVKSSEEAVILCKTTIGSLKLEINDLPATKKIIEEVEEILNNLPGVTSVHGRFYDLSSKYY
RIIGNHASYYKDALRYLGCV
DIKDLPETEKQERAFTLGLAGLLGEGVYNFGELLMHPVLESLRSTDKQWLIDTLYAFNGGNVEKFQSFKSAWGQQMTFTR
PANNRQLTFQEIGQSAKIPI
NEVELLVMKALSVGLIKGNIDEVDQKVQMTWVQPRVLDVQQIKGMKERLDLWCGDVKNMAMLVEQQAHDILT
>g3287.t3
MSLNKPHVVQVKSSEEAVILCKTTIGSLKLEINDLPATKKIIEEVEEILNNLPGVTSVHGRFYDLSSKYYRIIGNHASYY
KDALRYLGCVDIKDLPETEK
QERAFTLGLAGLLGEGVYNFGELLMHPVLESLRSTDKQWLIDTLYAFNGGNVEKFQSFKSAWGQQPDLATNESKLMQKIQ
LLCVMEMTFTRPANNRQLTF

QEIGQSAKIPINEVELLVMKALSVGLIKGNIDEVDQKVQMTWVQPRVLDVQQIKGMKERLDLWCGDVKNMAMLVEQQAHD
ILT
>g3288.t1
CCLLRLVTVGLFRKYLSFLELPVSWLWPSALCCVTGPGGYAKAKAAMFCHVTQLLWFRRLYVFFSRYMFVNTFQVFPRGP
RNLKIY
>g3289.t1
MYPKHQRVDDLVWCGEIFELKAELNNEKKEKRKEAVKKVIAAMTVGKDVSSLFPDVVNCMQTDNLELKKLVYLYLMNYAK
SQPDMAIMAVNSFVKASHRL
MFTVSPRPVDGL
>g3289.t2
LRAPSAFPHCRLMYPKHQRVDDLVWCGEIFELKAELNNEKKEKRKEAVKKVIAAMTVGKDVSSLFPDVVNCMQTDNLELK
KLVYLYLMNYAKSQPDMAIM
AVNSFVKASHRLMFTVSPRPVDGL
>g3289.t3
MYPKHQRVDDLVWCGEIFELKAELNNEKKEKRKEAVKKVIAAMTVGKDVSSLFPDVVNCMQTDNLELKKLVYLYLMNYAK
SQPDMAIMAVNSFVKASHRL
MFT
>g3290.t1
MSSELNVPMGSPTPAASEHLQADGGGMDYRDWVRRSYLELVSSNHHSVQALSWRKLYLSRAKLKASSRTSALLSGFAMVS
AMQGLNGACP
>g3291.t1
MSVFHFLHSDWYYARSPLLKPHLSVDIISHLYELNEVQFDVASRGHDLDASWPTFARRTLGMPNSPGHTWRAPSRSSSIN
SLASTYSQGLLSELNESLAS
CGEDTSAMESLRLELDQSELSQQRLLSRVQQLGEEAGELRGVVAELQRQLDVSLAAQEDQLSLQAALKALEGREETLSRE
LEALRSGTQAREAGQNLLQD
KLSAAEGKNMELLAKLDGVLNEKGQQATSHFDSAQKIHELLDRLKEAESGKMEAVSDMEDNRRQVERLEEELKIKDLALK
DIEVKLGELVTSTSEEKAKL
EARVLEQCNTTDTLAENLSLRDKEASNFQRQLEALQKLLEEKERQVVEVKQRAQYDEETARQSTIFLKESLEGEVNALKE
LLKIKETELTSISKTLQRFE
SEKDSLSRERHSLTVSNSELETNVKEHTKKIEDYKTQCTNLMELNEKLLSTVKRNEELKKEMAANRVAMEAELVSLRASD
KQLRSQLNDAMVTVDEKEKR
LREDNCSLDESLQRAAMAAQLSETTSKRLEQENQALREDRATVSAALSSMQAELKSFNGQIGDLEKSLGMSRKSEAALQE
QVKAKEAQLESKESDLKELQ
TKVKMKENREKELKKAKAKAEAALARQSEVIERLSTEKQAVEESQLERSSLHARENQEVGERLVLVEGQLEVSMKDVSRL
QAEVLDLRVQLQRAGGEHLK
CQTKLEVMEGQRAEFMSLTEQLQAQTEALNQSHVAELLGCKHKEEALMEEQVRQAAAHAELSTSFAAAQQELAQLKAENE
RLVVENGETREGVHRANTEM
AELGMTICRLTAEKEEARERWTREAAKIEELQDGATREAERLEACLSALRQENTSLREGMIQVENLPRAVVELQEQLGKA
EGQVQTLQRASREEMDAVRF
QISSESMSHQSQLKSVNVQLTTVKAQLHSELETVSRLKGALSEQEAVNEQHAHLIMEKDSHITKCEATMRHNELEIQQLR
DSVSRTEEALFALRTVSEEL
KESLETTEAEKQSDYQKMSAEIDDLSRTKSTLEERLIELLKDKDALWQKSDALEFEQKLRAEERWWLVDKEATQCLDCRG
HFTWYLRRHHCR
>g3291.t2
MSVFHFLHSDWYYARSPLLKPHLSVDIISHLYELNEVQFDVASRGHDLDASWPTFARRTLGMPNSPGHTWRAPSRSSSIN
SLASTYSQGLLSELNESLAS
CGEDTSAMESLRLELDQSELSQQRLLSRVQQLGEEAGELRGVVAELQRQLDVSLAAQEDQLSLQAALKALEGREETLSRE
LEALRSGTQAREAGQNLLQD
KLSAAEGKNMELLAKLDGVLNEKGQQATSHFDSAQKIHELLDRLKEAESGKMEAVSDMEDNRRQVERLEEELKIKDLALK
DIEVKLGELVTSTSEEKAKL
EARVLEQCNTTDTLAENLSLRDKEASNFQRQLEALQKLLEEKERQVVEVKQRAQYDEETARQSTIFLKESLEGEVNALKE
LLKIKETELTSISKTLQRFE
SEKDSLSRERHSLTVSNSELETNVKEHTKKIEDYKTQCTNLMELNEKLLSTVKRNEELKKEMAANRVAMEAELVSLRASD
KQLRSQLNDAMVTVDEKEKR
LREDNCSLDESLQRAAMAAQLSETTSKRLEQENQALREDRATVSAALSSMQAELKSFNGQIGDLEKSLGMSRKSEAALQE
QVKAKEAQLESKESDLKELQ

TKVKMKENREKELKKAKAKAEAALARQSEVIERLSTEKQAVEESQLERSSLHARENQEVGERLVLVEGQLEVSMKDVSRL
QAEVLDLRVQLQRAGGEHLK
CQTKLEVMEGQRAEFMSLTEQLQAQTEALNQSHVAELLGCKHKEEALMEEQVRQAAAHAELSTSFAAAQQELAQLKAENE
RLVVENGETREGVHRANTEM
AELGMTICRLTAEKEEARERWTREAAKIEELQDGATREAERLEACLSALRQENTSLREGMIQVENLPRAVVELQEQLGKA
EGQVQTLQRASREEMDAVRF
QISSESMSHQSQLKSVNVQLTTVKAQLHSELETVSRLKGALSEQEAVNEQHAHLIMEKDSHITKCEATMRHNELEIQQLR
DSVSRTEEALFALRTVSEEL
KESLETTEAEKQSDYQKMSAEIDDLSRTKSTLEERLIELLKDKDALWQKSDALEFEQKLRAEERWWLVDKEATQCLDCRG
HFTWYLRRHHC
>g3291.t3
LSVDIISHLYELNEVQFDVASRGHDLDASWPTFARRTLGMPNSPGHTWRAPSRSSSINSLASTYSQGLLSELNESLASCG
EDTSAMESLRLELDQSELSQ
QRLLSRVQQLGEEAGELRGVVAELQRQLDVSLAAQEDQLSLQAALKALEGREETLSRELEALRSGTQAREAGQNLLQDKL
SAAEGKNMELLAKLDGVLNE
KGQQATSHFDSAQKIHELLDRLKEAESGKMEAVSDMEDNRRQVERLEEELKIKDLALKDIEVKLGELVTSTSEEKAKLEA
RVLEQCNTTDTLAENLSLRD
KEASNFQRQLEALQKLLEEKERQVVEVKQRAQYDEETARQSTIFLKESLEGEVNALKELLKIKETELTSISKTLQRFESE
KDSLSRERHSLTVSNSELET
NVKEHTKKIEDYKTQCTNLMELNEKLLSTVKRNEELKKEMAANRVAMEAELVSLRASDKQLRSQLNDAMVTVDEKEKRLR
EDNCSLDESLQRAAMAAQLS
ETTSKRLEQENQALREDRATVSAALSSMQAELKSFNGQIGDLEKSLGMSRKSEAALQEQVKAKEAQLESKESDLKELQTK
VKMKENREKELKKAKAKAEA
ALARQSEVIERLSTEKQAVEESQLERSSLHARENQEVGERLVLVEGQLEVSMKDVSRLQAEVLDLRVQLQRAGGEHLKCQ
TKLEVMEGQRAEFMSLTEQL
QAQTEALNQSHVAELLGCKHKEEALMEEQVRQAAAHAELSTSFAAAQQELAQLKAENERLVVENGETREGVHRANTEMAE
LGMTICRLTAEKEEARERWT
REAAKIEELQDGATREAERLEACLSALRQENTSLREGMIQVENLPRAVVELQEQLGKAEGQVQTLQRASREEMDAVRFQI
SSESMSHQSQLKSVNVQLTT
VKAQLHSELETVSRLKGALSEQEAVNEQHAHLIMEKDSHITKCEATMRHNELEIQQLRDSVSRTEEALFALRTVSEELKE
SLETTEAEKQSDYQKMSAEI
DDLSRTKSTLEERLIELLKDKDALWQKSDALEFEQKLRAEERWWLVDKEATQCLDCRGHFTWYLRRHHCR
>g3292.t1
MTYKMVTEMEWKCCHGYSGEDCNVGPVGGSGTQGSTTRPRPRPGQGGAGNGYGQGGTGTGQGGGDSGRGDSDKMRQLEER
IQSLTKDLHNVQSTLRGMND
RSQEESGRRQGPGGGGAGGSGGTSPGTGGGGRNPADAAQPEIKEMINSIQTKLDQLDNRTQDHDKTLVNINNHLVNGKGK
ELEGGLSGGGVDGGKGNSLK
EEILSELERRVSLSCSFCQEGVEDLRNQQQQDRERIRALEKQLAALDTRRSQGCCDGINDLRSRVDDAERKITTTAENGG
RGGGGPGDTLVTEDKLDGRL
RDLERRINSSVRQTEKSCSFVENEVKDLRTVFLDRFDDQGFRIADVELDVGLVKDLVTEHDKRLARLENTTSLIGRKVQE
CGCSHDGGEGRVGGGGASNG
WGGRGDGGGNRVTGGGGIGGGGIGGEGGGDGGDGGGRGGFGGSGGAGGRGSGGAGGRGSTGGTTGGTIGGGRGGTGGLTG
TNGTTEKSLEWRVIANEDQI
HRFSTTLKDLSVSGDSLLDKVVDLSHDVRQIKALTGDRGEHFNRIVTEIEMLGHGCGETCSRVEDELRKLKNYTRSALDR
FQIDIKTLQGSGASDQGPCS
QVCSNLQEEVGKLREDMEKCTGQCKNGLDTSTDGGSGGSGGTGLSGGGLGGEKPLDGHSVIGGTLNNNHLKRLQGELSEV
LLTFSSINDTLKGLEHTTHK
HGSVITDLGNTKDKIISELDKIQQEVNEHIEDSRDRLEGMNKDVRRFESTLVVEMGDCRRSGDGLEKRLSKLEGVCSRMD
GVSDSLQKIKEGLNKHVSGL
WSCVSGLNSTLITHGGILDDIQNEQLDAVHGKIKNLNSSVNHILSEFQILLQQDLTGG
>g3292.t2
MTYKMVTEMEWKCCHGYSGEDCNVGPVGGSGTQGSTTRPRPRPGQGGAGNGYGQGGTGTGQGGGDSGRGDSDKMRQLEER
IQSLTKDLHNVQSTLRGMND
RSQEESGRRQGPGGGGAGGSGGTSPGTGGGGRNPADAAQPEIKEMINSIQTKLDQLDNRTQDHDKTLVNINNHLVNGKGK
ELEGGLSGGGVDGGKGNSLK
EEILSELERRVSLSCSFCQEGVEDLRNQQQQDRERIRALEKQLAALDTRRSQGCCDGINDLRSRVDDAERKITTTAENGG

RGGGGPGDTLVTEDKLDGRL
RDLERRINSSVRQTEKSCSFVENEVKDLRTVFLDRFDDQGFRIADVELDVGLVKDLVTEHDKRLARLENTTSLIGRKVQE
CGCSHDGGEGRVGGGGASNG
WGGRGDGGGNRVTGGGGIGGGGIGGEGGGDGGDGGGRGGFGGSGGAGGRGSGGAGGRGSTGGTTGGTIGGGRGGTGGLTG
TNGTTEKSLEWRVIANEDQI
HRFSTTLKDLSVSGDSLLDKVVDLSHDVRQIKALTGDRGEHFNRIVTEIEMLGHGCGETCSRVEDELRKLKNYTRSALDR
FQIDIKTLQGSGASDQGPCS
QVCSNLQEEVGKLREDMEKCTGQYGGSGGSGGTGLSGGGLGGEKPLDGHSVIGGTLNNNHLKRLQGELSEVLLTFSSIND
TLKGLEHTTHKHGSVITDLG
NTKDKIISELDKIQQEVNEHIEDSRDRLEGMNKDVRRFESTLVVEMGDCRRSGDGLEKRLSKLEGVCSRMDGVSDSLQKI
KEGLNKHVSGLWSCVSGLNS
TLITHGGILDDIQNEQLDAVHGKIKNLNSSVNHILSEFQILLQQDLTGG
>g3292.t3
MTYKMVTEMEWKCCHGYSGEDCNVGPVGGSGTQGSTTRPRPRPGQGGAGNGYGQGGTGTGQGGGDSGRGDSDKMRQLEER
IQSLTKDLHNVQSTLRGMND
RSQEESGRRQGPGGGGAGGSGGTSPGTGGGGRNPADAAQPEIKEMINSIQTKLDQLDNRTQDHDKTLVNINNHLVNGKGK
ELEGGLSGGGVDGGKGNSLK
EEILSELERRVSLSCSFCQEGVEDLRNQQQQDRERIRALEKQLAALDTRRSQGCCDGINDLRSRVDDAERKITTTAENGG
RGGGGPGDTLVTEDKLDGRL
RDLERRINSSVRQTEKSCSFVENEVKDLRTVFLDRFDDQGFRIADVELDVGLVKDLVTEHDKRLARLENTTSLIGRKVQE
CGCSHDGGEGRVGGGGASNG
WGGRGDGGGNRVTGGGGIGGGGIGGEGGGDGGDGGGRGGFGGSGGAGGRGSGGAGGRGSTGGTTGGTIGGGRGGTGGLTG
TNGTTEKSLEWRVIANEDQI
HRFSTTLKDLSVSGDSLLDKVVDLSHDVRQIKALTGDRGEHFNRIVTEIEMLGHGCGETCSRVEDELRKLKNYTRSALDR
FQIDIKTLQGSGASDQGPCS
QVCSNLQEEVGKLREDMEKCTGQCKNGLDTSTDGGSGGSGGTGLSGGGLGGEKPLDGHSVIGGTLNNNHLKRLQGELSEV
LLTFSSINDTLKGLEHTTHK
HGSVITDLGNTKDKIISELDKIQQEVNEHIEDSRDRLEGMNKDVRRFESTLVVEMGDCRRSGDGLEKRLSKLEGVCSRMD
GVSDSLQKIKEGLNKHVSGL
WSCVSGLNSTLITHGGILDDIQNEQLDAVHGKIKNLNSSVNHILSEFQILLQQDLTGPPGPTGPPGVKGSVGPPGPMGPL
GPPGRAGDYGPRGSPGKERE
SADGGRIKQRDGWVKMGG
>g3293.t1
GPRGPMGPGGPTEPFTPGGPVGPGGPAVDETRVSKTQQVSIGGGLEVPQRETSCCVRLHVD
>g3294.t1
MASPGVCQRIMEKRNRKEGFGSLANPKRFMNQDFQRLKQYCTEQQLRYIDNMFPPNQNSIGRVSLSTSDMARIEWLRPGS
HILQQVMPAEQSFRDYCGIF
HFRFWRFGSWVDVVIDDKLPTINGKLIFVHSNTPNEFWPALLEKAYAKVCGSYANMNAGTPAEALVDFTGGVHICYDLAN
ALPDLWSTMYRAAQSKSLMG
CGTPQGETSANTVATNGLVEGHAYTITGVKQVVSNGQPVPLIRLFNPWGQGEWLGDWSDKSPLWQTIGAEDRLMCLDVAD
NGEFWMAMKDFCRFFSDLDI
CCLCPDFLDGAESCHWKPLLYEGRWVAGTTAGGCLNNPEMFWTNPQYRFKVEGLNSGCERGDNMLVSLMQKPEKGSRKLK
EILFIGYSIFKRLDIVFFQY
VASNGKFPAAFFASNLAVAQTKKHVNSREVMELFLLPPGEYLIVPSTFKPNETASFLLTILSKSETHAQ
>g3294.t2
MASPGVCQRIMEKRNRKEGFGSLANPKRFMNQDFQRLKQYCTEQQLRYIDNMFPPNQNSIGRVSLSTSDMARIEWLRPGS
HILQQVMPAEQSFRDYCGIF
HFRFWRFGSWVDVVIDDKLPTINGKLIFVHSNTPNEFWPALLEKAYAKVCGSYANMNAGTPAEALVDFTGGVHICYDLAN
ALPDLWSTMYRAAQSKSLMG
CGTPQGETSANTVATNGLVEGHAYTITGVKQVVSNGQPVPLIRLFNPWGQGEWLGDWSDKSPLWQTIGAEDRLMCLDVAD
NGEFWMAMKDFCRFFSDLDI
CCLCPDFLDGAESCHWKPLLYEGRWVAGTTAGGCLNNPEMFWTNPQYRFKVEGLNSGCERGDNMLVSLMQKPEKGSRKLK
EILFIGYSIFKYVASNGKFP
AAFFASNLAVAQTKKHVNSREVMELFLLPPGEYLIVPSTFKPNETASFLLTILSKSETHAQ
>g3295.t1
LNCDFNTAWLSGDNNAGDQARSRRDQRVGAERVRLGSTHRDSATRNHKGTERRGREAESEGPSRHTQTMQVTKKATERMN

LPEDSDFRSSP
>g3296.t1
MMLPRTLVISLRGAEVDVAQGSGACALRIRAPAVVRLEGALHWGETTEIEMRAESTVQSILSVRCSACRGFGGSRRRRHA
GGNRSKPSSLMAAFSLLRQV
EPGAAVISQAAEAFGKNMQIFQDEAKWRHKRDLAASELLRLEDVELMADLSGCPPPARPAACLRRSPLDDYRSISGLCNN
RRNPTWGAANTALLRWLPAQ
YEDGAGQPKGWNPGRLYNGFPLPPVEAVSKHIIKSSFKRKEDSYSQLMIEWGQFIDHDMTFTPQSTGCAADRGVDCLQTC
HNVHPCFPIQGLLRDLAGPS
GRLAVHDRFRDGGGRPYLPPVDAGPSACFQDPRTPRGQRVECFAAGDSRVNEGLSLIALHTLWLREHNRIADALKGLNSH
WSKETVYQETRKIIGALHQI
ITMRDYIPKIIGRDSFDRYIGPYVGYDPAVNPSASNVFATAAFRFGHATISPVVRRLNESFQTHERFPPLRLHRTFFSPW
RIVKEGGIEPVLRGLVGTAA
TSVNTDELMAEELTEKLVVLNIPWKLDLAALNLQRGRDHALAGGLVEDHLKDSRNGPLFSCLIAKQMKSIRDGDR
>g3296.t2
MMLPRTLVISLRGAEVDVAQGSGACALRIRAPAVVRLEGALHWGETTEIEMRAESTVQSILSVRCSACRGFGGSRRRRHA
GGNRSKPSSLMAAFSLLRQV
EPGAAVISQAAEAFGKNMQIFQDEAKWRHKRDLAASELLRLEDVELMADLSGCPPPARPAACLRRSPLDDYRSISGLCNN
RRNPTWGAANTALLRWLPAQ
YEDGAGQPKGWNPGRLYNGFPLPPVEAVSKHIIKSSFKRKEDSYSQLMIEWGQFIDHDMTFTPQSTGCAADRGVDCLQTC
HNVHPCFPIQGLLRDLAGPS
GRLAVHDRFRDGGGRPYLPPVDAGPSACFQDPRTPRGQRVECFAAGDSRVNEGLSLIALHTLWLREHNRIADALKGLNSH
WSKETVYQETRKIIGALHQI
ITMRDYIPKIIGRDSFDRYIGPYVGYDPAVNPSASNVFATAAFRFGHATISPVVRRLNESFQTHERFPPLRLHRTFFSPW
RIVKEGGIEPVLRGLVGTAA
TSVNTDELMAEELTEKLVVLNIPWKLDLAALNLQRGRDHALAGYNDWREFCGLKRLKTKEDFQGVVKDKVVVDKILKVYK
HPDNIDVWLGGLVEDHLKDS
RNGPLFSCLIAKQMKSIRDGDR
>g3296.t3
MLPRTLVISLRGAEVDVAQGSGACALRIRAPAVVRLEGALHWGETTEIEMRAESTVQSILSVRCSACRGFGGSRRRRHAG
GNRSKPSSLMAAFSLLRQVE
PGAAVISQAAEAFGKNMQIFQDEAKWRHKRDLAASELLRLEDVELMADLSGCPPPARPAACLRRSPLDDYRSISGLCNNR
RNPTWGAANTALLRWLPAQY
EDGAGQPKGWNPGRLYNGFPLPPVEAVSKHIIKSSFKRKEDSYSQLMIEWGQFIDHDMTFTPQSTGCAADRGVDCLQTCH
NVHPCFPIQGLLRDLAGPSG
RLAVHDRFRDGGGRPYLPPVDAGPSACFQDPRTPRGQRVECFAAGDSRVNEGLSLIALHTLWLREHNRIADALKGLNSHW
SKETVYQETRKIIGALHQII
TMRDYIPKIIGRDSFDRYIGPYVGYDPAVNPSASNVFATAAFRFGHATISPVVRRLNESFQTHERFPPLRLHRTFFSPWR
IVKEGGIEPVLRGLVGTAAT
SVNTDELMAEELTEKLVVLNIPWKLDLAALNLQRGRDHALAGGLVEDHLKDSRNGPLFSCLIAKQMKSIRDGDR
>g3297.t1
LISWTGRKIDPVGVDYILQKLGFHHARTTIPKWLQRGVMDPLDKVLSVLIKKLGTALQDEKEKKGRDKDDH
>g3298.t1
QFSRYFKELPAVALRSGKTAGRRMYLTRSQGDTTVSLVEELRKTLCSLWQGNQTAFSPDSLFYAIWKIMPSFRGYQQQDA
HEFMRYLLDHLHRELQGNQN
GVPTSASGPGLVRPASTAGKCCINGITTVVTSIFGGILQNEVNCLICGTESRKFDPFLDLSLDIPSQFRQKKSKDQEPGP
TCTLSDCLRSFTDLEELDDA
ELYMCLKCIKRQKSTKKFWIQKLPKVLCLHLKRFHWTAFLRNKVDTYVEFPLHSLDMRPFLLEVRMSDPELIHQPRRDRV
AGDQSAGSSLRLVGSGHYTA
YGCHEGRWYHFNDSTVTVSSEDTVRKAKAYILFYVERPPKGACANAVAVDQTAPSAGEPVKGEDASPCGVTAGEADQADG
TVGGEEEEEEEEQEQEQEQE
QEQEQEQEGLAGGRAGRASPDPAATDSVSQALPSDAGQ
>g3298.t2
QFSRYFKELPAVALRSGKTAGRRMYLTRSQGDTTVSLVEELRKTLCSLWQGNQTAFSPDSLFYAIWKIMPSFRGYQQQDA
HEFMRYLLDHLHRELQGNQN
GVPTSASGPGLVRPASTAGKCCINGITTVVTSIFGGILQNEVNCLICGTESRKFDPFLDLSLDIPSQFRQKKSKDQEPGP
TCTLSDCLRSFTDLEELDDA

ELYMCLKCIKRQKSTKKFWIQKLPKVLCLHLKRFHWTAFLRNKVDTYVEFPLHSLDMRPFLLEVRMSDPELIHQPRRDRV
AGDQSAGSVGSGHYTAYGCH
EGRWYHFNDSTVTVSSEDTVRKAKAYILFYVERPPKGACANAVAVDQTAPSAGEPVKGEDASPCGVTAGEADQADGTVGG
EEEEEEEEQEQEQEQEQEQE
QEQEGLAGGRAGRASPDPAATDSVSQALPSDAGQ
>g3298.t3
LPAVALRSGKTAGRRMYLTRSQGDTTVSLVEELRKTLCSLWQGNQTAFSPDSLFYAIWKIMPSFRGYQQQDAHEFMRYLL
DHLHRELQGNQNGVPTSASG
PGLVRPASTAGKCCINGITTVVTSIFGGILQNEVNCLICGTESRKFDPFLDLSLDIPSQFRQKKSKDQEPGPTCTLSDCL
RSFTDLEELDDAELYMCLKC
IKRQKSTKKFWIQKLPKVLCLHLKRFHWTAFLRNKVDTYVEFPLHSLDMRPFLLEVRMSDPELIHQPRRDRVAGDQSAGS
SLRLVGSGHYTAYGCHEGRW
YHFNDSTVTVSSEDTVRKAKAYILFYVERPPKGACANAVAVDQTAPSAGEPVKGEDASPCGVTAGEADQADGTVGGEEEE
EEEEQEQEQEQEQEQEQEQE
GLAGGRAGRASPDPAATDSVSQALPSDAGQ
>g3299.t1
VSRFDRPSLKERALLSVSTVIRLYRDRLGHRFEGKRRAGMCRGPSAEFRASHNNGVLQAD
>g3300.t1
FSLETNQNLLRGLLGQTGSSSQTNQNTVAYIKLKISGDLSPERSINLFHCLNELNDRSLVEEIQQYLTSGNLSRESLSPA
QWSALVFILQSSDEELDVFD
LKKYSASEDGLLRLLPVVKASKTCL
>g3300.t2
VDKAIQSENGHLDLFLRFLLGFSLETNQNLLRGLLGQTGSSSQTNQNTVAYIKLKISGDLSPERSINLFHCLNELNDRSL
VEEIQQYLTSGNLSRESLSP
AQWSALVFILQSSDEELDVFDLKKYSASEDGLLRLLPVVKASKTCL
>g3301.t1
MTQTEAVISSPSSQHSWIQNDCLEEHPRNQQGRQPYLLTVGRQKNQMDIFITMDKHLIPGQANRSLWA
>g3302.t1
MRSFILTSLSNVPSTSQLLKRREVCVPLRIEQLSAELDAWRTCMYTNSSHSDVQLSCFTGHRIQGPVFLLEDGESAISLN
DALMWAKVNPFSPLGTGVRI
VPF
>g3302.t2
MVRASDQDASWAPPVRGVPDTSNCQLLKRREVCVPLRIEQLSAELDAWRTCMYTNSSHSDVQLSCFTGHRIQGPVFLLED
GESAISLNDALMWAKVNPFS
PLGTGVRIVPF
>g3303.t1
MPPGRLPLEVFRTRPTGKRPRGRPRTRWRDYISHLAWERLGVPQEELESVAGEKDVWSSLLSLLPPRPGPG
>g3304.t1
MNASDSPGTYARILFVDFSSAFNTILLALLQDKLSQFHVPNSTCSSSNHQSVKLLKFADNTTLFGLISGGDESA
>g3305.t1
PTGYMETTLSYSTIEDLQLLTWDNAPKYCVQLSFPGGTVLLQGTASALHEWSEVCCPASRRMKKGPSHD
>g3305.t2
LGIVTAREASHSYRRHLCLNKHMCVNTGAVTRHWPTGYMETTLSYSTIEDLQLLTWDNAPKYCVQLSFPGGTVLLQGTAS
ALHEWSEVCCPASRRMKKGP
SHD
>g3306.t1
MTFREQRRQTLWASQATSWSPCHRFPEQQNQESGSRDRPSAPPRRGEATSSFVLVARWVGGTHGSAAPAVAPAHG
>g3306.t2
MGRRNGAPLSAPRSPSAPPRRGEATSSFVLVARWVGGTHGSAAPAVAPAHG
>g3307.t1
VGVHSVALTQSAKVLERVREAGPVDAGSRSSLSRRG
>g3308.t1
MVRSESYGPLSLSVPQRSEPVCLWSSRSLADVLTVSPVSSSAAAFPRSPSTQTTVGVGGEQEGVVGSPRTTEARSSHEAP
GLGKTLERTLRLNVSPTAKD
AH
>g3309.t1

MAPTQYRTLLRFIHCSLLISAALCDAEAEEDEHAKHSYTVQMFNDAVPTAPHFVMFYAPWCGHCQRLQPAWNELADKYNS
MESPPVYVVKVDCVQDTKFC
SKDHGVRGYPTLKLFRPEQESVKYQGPRDLQSLETWLLKALQADPEEPESEPEPPAAPEPKQGMYELTVGNFKAHVAKGK
GDTGGCVLLFP
>g3310.t1
VCAAGESRNSVTGQFRVEGITLPDQFTDCTR
>g3311.t1
MKVLLVFAAFALLQHVSPGSARSLPPPTASNTQVKLLQEMYYFARQANATVPGRLRKAMVPVIDDCENCTDSIQNTTNVT
FELQELFIILMDCINKEVIG
L
>g3311.t2
MKVLLVFAAFALLQHVSPGSARSLPPPTASNTQVKLLQEMYYFARQANATVPGRLRKAMVPVIDDCEKNTFCKIQMTLSE
LPANDKINVLCDSLMLYNNH
THMNCTDSIQNTTNVTFELQELFIILMDCINKEVIGL
>g3312.t1
VFLSDDEPESGSRHGNVGHGDPITYDILRAHEGHGGHLERPACTLEDLCRQKLIALQK
>g3312.t2
HEGHGGHLERPACTLEDLCRQKLIALQK
>g3313.t1
KPWRVEAAGTTTTTTTSTTTTTASTTTTTTTTTTITTTTTTTTTTTITITTTITTTTAFSPSVSHHYGALLLPSFLCWGR
GGTLVTAREQNSKALTAALK
G
>g3314.t1
MPRSFLVDSLILREANDKGNENSPPLFPYAVHSSHHALPGSCHSRKAGLLCFCPLCMAASQLHPSPPALPLLKASFPAFG
SQYCHSALSRQHSSANGVNL
GHGPGIYQATYSMPDPRQFHCISIESNNAKLQSSKRMRTAFTSTQLLELEREFTSNMYLSRLRRIEIATYLNLSEKQVKI
WFQNRRVKHKKEGKGNGHRA
GAHNCKCSSARCSEEEEEDSPVSPSSSSEKDDVDLSVSP
>g3315.t1
MWQVQPLLGLPASRSIGLLTLNVEVHQQNVNYDAVLPHQNPTKVCGSPQEARPYLEQHPVTRSENRRGQQQCDANFPCFI
RPPGSTEDALQRRTQCCSG
>g3316.t1
MSGIALSRLAQERKAWRKDHPFGFVAVPTKNPDGTMNLMNWECAIPGKKGTPWEGGLFKLRMLFKDDYPSSPPKCKFEPP
LFHPNVYPSGTVCLSILEED
KDWRPAITIKQVSPRPGPHTASS
>g3316.t2
PVVLQPRSSKRSPSRRKSCVIPSPTIMSGIALSRLAQERKAWRKDHPFGFVAVPTKNPDGTMNLMNWECAIPGKKGTPWE
GGLFKLRMLFKDDYPSSPPK
CKFEPPLFHPNVYPSGTVCLSILEEDKDWRPAITIKQVSPRPGPHTASS
>g3316.t3
PRSSSSPVVLQPRSSKRSPSRRKSCVIPSPTIMSGIALSRLAQERKAWRKDHPFGFVAVPTKNPDGTMNLMNWECAIPGK
KGTPWEGGLFKLRMLFKDDY
PSSPPKCKFEPPLFHPNVYPSGTVCLSILEEDKDWRPAITIKQVSPRPGPHTASS
>g3317.t1
PPGPSSLCVDLRPPAPSLLLKSGGGFQHDIRGERTLFLCVAMETVDNVGVDGGLGWGRGGSLSSAGPAVRTSDPTHALGD
GGPSHGNPGRKRGQGQILWS
GGEMANGLTLNAYEGKEQEEQEQEEQQEQEEQQEQTSLQGQC
>g3317.t2
TQRCSRTPTGDTGHQTQFLCVAMETVDNVGVDGGLGWGRGGSLSSAGPAVRTSDPTHALGDGGPSHGNPGRKRGQGQILW
SGGEMANGLTLNAYEGKEQE
EQEQEEQQEQEEQQEQTSLQGQC
>g3317.t3
DTGHQTQFLCVAMETVDNVGVDGGLGWGRGGSLSSAGPAVRTSDPTHALGDGGPSHGNPGRKRGQGQILWSGGEMANGLT
LNAYEGKEQEEQEQEEQQEQ
EEQQEQTSLQGQC
>g3318.t1

LKGFQEEDSRRSPPSPQEGEPCDVTIVPSYHRTIVPSYRCGPLEVSSYLEYQGLQVPRAKGRGPCSELCQTSPLSQDLVV
KSPHFVFQEDIVDLGGRLCG
G
>g3319.t1
MGGRHSRSFSKEELSEVSTSHGRRSVSVRDDQNGSNGGGLSSAAERIRKQTTVRFGYSTLSLAMRGLDDPPMELWELREL
EKLNLSMNCLRSLPPPLGAL
DNLVVLNLWGNNLSSLPPEIGMLKKLRVLFAYRNRLSEVPEELGSCTSLEVLSLADNQITTLPESLASMRNLTKLNLSNN
RMAHIPTCVYSMKGLVFLHL
ACNRLETIADQIQDLVNLKILIVEGNSLHSLPKTLCFLESLELLNVDFNDLQSVPVEMYLMSGLRKLACHPLDKGLHIIH
NPLVKPIQEVLQGGLSALYN
YLKPS
>g3320.t1
GPGVQMAHLEIQDLRVQRVNLVISGRKENLDSXARLECQVALERRDQGVNPAPKDLQGTKDLMESQDLRGHPVGRESQDP
SESRDL
>g3320.t2
GPGVQMAHLEIQDLRVQRARLECQVALERRDQGVNPAPKDLQGTKDLMESQDLRGHPVGRESQDPSESRDL
>g3320.t3
GPGVQMAHLEIQDLRVQRVNLVISGRKENLDSXARPAAQEREAFQENLARLECQVALERRDQGVNPAPKDLQGTKDLMES
QDLRGHPVGRESQDPSESRD
L
>g3321.t1
VCAAIMALVFDLKALVDMMSIGTLFAYTLVAVCILILRYQADLTEDSGSSKPESFSLMSMLSPPSQATPRTSTNVSRITI
SIVFIAIAVSLLVSRAGASL
RGHEWWSLLVFTVLLALLGFLVAAVWRQPQNSAEAAFMVPFLPVLPILSIFVNVYLMIQLGADTWARYAVWMAVDEEGET
PVFESTAAELGNVTRPRLPE
LSIISPGLDTKASAPKPAPQVERQGSGDEASPEKVKVYVRVRPLSKEERERAEEQGCVTIQNEDTLLLKAPKDSQTMKSA
ERGIAQSIHKFSFSKIFGPE
TTQQEFFEDTMKNMVKDVLHGENRLLYTYGVTNSGKTYTIQGVGREPGLLPRALVSTFRKLQGRLYAAMDLKPAMYQDVK
RLEPSEVRAEGQRRDSLLKE
DESVCGRRGGTSTAYDSGIAGLSSTINTQLEDSDNVCLDPDSLSHSGGEGLEEGVQYSVWVSYYEIYNEFLYDLLEAPPT
ATPRKRATLKLSDDKQGNPY
VKELTWIQVRSAEEAWRVLRAGRRNQSFASTHLNQNSSRSHSIFSLRLLHIHPEAEASKAAHISELTVCDLAGSERCKDQ
RNGERMKEANNINTSLHTLG
RCIAALRHNQNNRSRPPQVVPFRDSKLTRVLQGYFCGRGTSSMVVNINPCAGIYDETLQALKFSAIATQ
>g3321.t2
VCAAIMALVFDLKALVDMMSIGTLFAYTLVAVCILILRYQADLTEDSGSSKPESFSLMSMLSPPSQATPRTSTNVSRITI
SIVFIAIAVSLLVSRAGASL
RGHEWWSLLVFTVLLALLGFLVAAVWRQPQNSAEAAFMVPFLPVLPILSIFVNVYLMIQLGADTWARYAVWMAVDEEGET
PVFESTAAELGNVTRPRLPE
LSIISPGLDTKASAPKPAPQVERQGSGDEASPEKVKVYVRVRPLSKEERERAEEQGCVTIQNEDTLLLKAPKDSQTMKSA
ERGIAQSIHKFSFSKIFGPE
TTQQEFFEDTMKNMVKDVLHGENRLLYTYGVTNSGKTYTIQGVGREPGLLPRALVSTFRKLQGRLYAAMDLKPAMYQDVK
RLEPSEVRAEGQRRDSLLKE
DESVCGRRGGTSTAYDSGIAGLSSTINTQLEDSDNVCLDPDSLSHSGGEGLEEGVQYSVWVSYYEIYNEFLYDLLEAPPT
ATPRKRATLKLSDDKQGNPY
VKELTWIQVRSAEEAWRVLRAGRRNQSFASTHLNQNSSRSHSIFSLRLLHIHPEAEASKAAHISELTVCDLAGSERCKDQ
RNGERMKEANNINTSLHTLG
RCIAALRHNQNNRSRPPQVVPFRDSKLTRVLQGYFCGRGTSSMVVNINPCAGIYDETLQALKFSAIATQVRTSPAR
>g3321.t3
MALVFDLKALVDMMSIGTLFAYTLVAVCILILRYQADLTEDSGSSKPESFSLMSMLSPPSQATPRTSTNVSRITISIVFI
AIAVSLLVSRAGASLRGHEW
WSLLVFTVLLALLGFLVAAVWRQPQNSAEAAFMVPFLPVLPILSIFVNVYLMIQLGADTWARYAVWMAVDEEGETPVFES
TAAELGNVTRPRLPELSIIS
PGLDTKASAPKPAPQVERQGSGDEASPEKVKVYVRVRPLSKEERERAEEQGCVTIQNEDTLLLKAPKDSQTMKSAERGIA
QSIHKFSFSKIFGPETTQQE
FFEDTMKNMVKDVLHGENRLLYTYGVTNSGKTYTIQGVGREPGLLPRALVSTFRKLQGRLYAAMDLKPAMYQDVKRLEPS

EVRAEGQRRDSLLKEDESVC
GRRGGTSTAYDSGIAGLSSTINTQLEDSDNVCLDPDSLSHSGGEGLEEGVQYSVWVSYYEIYNEFLYDLLEAPPTATPRK
RATLKLSDDKQGNPYVKELT
WIQVRSAEEAWRVLRAGRRNQSFASTHLNQNSSRSHSIFSLRLLHIHPEAEASKAAHISELTVCDLAGSERCKDQRNGER
MKEANNINTSLHTLGRCIAA
LRHNQNNRSRPPQVVPFRDSKLTRVLQGYFCGRGTSSMVVNINPCAGIYDETLQALKFSAIATQ
>g3322.t1
MLVCAAVFLVGAVLLTHTDARYTADWKSLDARPLPSWYDEAKFGIFVHWGVFSVPGFGSEWFWWHWQGQQPPDPKCVRYM
TENYPPGFSYPEFAPDFHAQ
FFNPEDWADIFQASGAKSLHYGLYNSLYEWFNPLYLSDKKNGFKTQEFVLRKLLPELYNMVVRYRPELIWSDGDWEAPDT
YWNSTEFLAWLYNDSPVKDT
IVTNDRWGSGCYCKHGGYYNCADKYTPGELPKHKWEKCMSVDLQSWGYRRNMKIVELMDLPGIIEDLVRTVALGGNYLLN
VGPTPDGMIPPLFEERLRGA
GVWLEVNGEAIYGSSPWRVQRQNKTDTLWFTSKGPTVYAFMTTKPTRSILTLYEPKTSEHTKVTLLGNPTPLKWSPIQPS
AGLIVLLPELPAVPGKAWTL
RLDGVL
>g3322.t2
MLVCAAVFLVGAVLLTHTDARYTADWKSLDARPLPSWYDEAKFGIFVHWGVFSVPGFGSEWFWWHWQGQQPPDPKCVRYM
TENYPPGFSYPEFAPDFHAQ
FFNPEDWADIFQASGAKSLHYGLYNSLYEWFNPLYLSDKKNGFKTQEFVLRKLLPELYNMVVRPELIWSDGDWEAPDTYW
NSTEFLAWLYNDSPVKDTIV
TNDRWGSGCYCKHGGYYNCADKYTPGELPKHKWEKCMSVDLQSWGYRRNMKIVELMDLPGIIEDLVRTVALGGNYLLNVG
PTPDGMIPPLFEERLRGAGV
WLEVNGEAIYGSSPWRVQRQNKTDTLWFTSKGPTVYAFMTTKPTRSILTLYEPKTSEHTKVTLLGNPTPLKWSPIQPSAG
LIVLLPELPAVPGKAWTLRL
DGVL
>g3323.t1
LLRPVTRGDLLGLASGPDARPVSPGHGAEQTHPPGSPADKMAAKTGDGPRANVRTNALNSGTYTAKNNSVYAVIFGWPPN
QRLQLSLPKTSDATNVTLLD
YPKMPLQWKPTGGNGLVIAMPPMAASAGNAWTLKLEGVA
>g3323.t2
PDARPVSPGHGAEQTHPPGSPADKMAAKTGDGPRANVRTNALNSGTYTAKNNSVYAVIFGWPPNQRLQLSLPKTSDATNV
TLLDYPKMPLQWKPTGGNGL
VIAMPPMAASAGNAWTLKLEGVA
>g3324.t1
PPLAGHITEDFRQRVPFSVFTTNPCRIQYCSQEIVIIREDLVNKMCRNCLRMPTNSLDIPNHFVKTILSQGHLTPLPLYV
SPVFWAYDYTLRVYPVPDVV
IFADKYDPFSVTNADCLCINPGSFPRSGFTFKVYYPSNRTVEDSCGVCVDGVLYLFAGHHARGNINRIFCLNLRAPHLIW
EEMKDLKGLPPSCKDKLGCW
VQKNRLIFFGGYGYAPQGQHRGTFEYDESSSLVWNNPSRGWNNHIHILDLETSTWSQPITKGNTPSPRAAHACATLGNRG
YVFGGRYKNFRLNDLYYIEM
DTWEWHEMSVPQGGPSGRSWHSFTPVSDDYVFLFGGFTTERETL
>g3324.t2
PPLAGHITEDFRQRVPFSVFTTNPCRIQYCSQEIVIIREDLVNKMCRNCLRMPTNSLDIPNHFVKTILSQGHLTPLPLYV
SPVFWAYDYTLRVYPVPDVV
IFADKYDPFSVTNADCLCINPGSFPRSGFTFKVYYPSNRTVEDRTKRITGGNLFTSMSGSCGVCVDGVLYLFAGHHARGN
INRIFCLNLRAPHLIWEEMK
DLKGLPPSCKDKLGCWVQKNRLIFFGGYGYAPQGQHRGTFEYDESSSLVWNNPSRGWNNHIHILDLETSTWSQPITKGNT
PSPRAAHACATLGNRGYVFG
GRYKNFRLNDLYYIEMDTWEWHEMSVPQGGPSGRSWHSFTPVSDDYVFLFGGFTTERETL
>g3324.t3
PPLAGHITEDFRQRVPFSVFTTNPCRIQYCSQEIVIIREDLVNKMCRNCLRMPTNSLDIPNHFVKTILSQGHLTPLPLYV
SPVFWAYDYTLRVYPVPDVV
IFADKYDPFSVTNADCLCINPVRTL
>g3324.t4
MDEEMGEDLPAREEEDDDESEGDEEFDWIVENDDDEDDDEEEEDDDPLVTEESETFEVDTPPERSGHIAVVDRNCMYVWG

GYKNAVNHGFFDLYLPRNVL
WEYNMETGVCCGVCVDGVLYLFAGHHARGNINRIFCLNLRAPHLIWEEMKDLKGLPPSCKDKLGCWVQKNRLIFFGGYGY
APQGQHRGTFEYDESSSLVW
NNPSRGWNNHIHILDLETSTWSQPITKGNTPSPRAAHACATLGNRGYVFGGRYKNFRLNDLYYIEMDTWEWHEMSVPQGG
PSGRSWHSFTPVSDDYVFLF
GGFTTERETL
>g3325.t1
LWTWLEELQKELLDDVYAESVEAVQELIKRFGQQQQTTLQATVNVIKEGEDLIQQLR
>g3325.t2
MTASFDVLRSASAAFFVPNNEYTFYKFKAPNPRLLAGYLWTWLEELQKELLDDVYAESVEAVQELIKRFGQQQQTTLQAT
VNVIKEGEDLIQQLR
>g3326.t1
LTSSQAARIHTFLHMFLHTFLMFPHTFLMFSHTFPHTFPHTFLQTFLQTFPHMFLHTFLHTFLQRNMTMLAGAAQTRPLL
RSGARAPTPRDPAPPGCQQS
PRCSPSSREPCRPIKLSITSQPVQDESQLPGRRCLACLQHSSRRSVMPTLEPNRAVLVVYQPAWTAGAGPLLTINTDLEN
VAIKVNLVGHSSG
>g3326.t2
LTSSQAARIHTFLHMFLHTFLMFPHTFLMFSHTFPHTFPHTFLQTFLQTFPHMFLHTFLHTFLQRNMTMLAGAAQTRPLL
RSGARAPTPRDPAPPGCQQS
PRCSPSSREPCRPIKLSITSQPVQDESQLPGRRCLACLQHSSRRSVMPTLEPNRAVLVVYQPAWTAGAGPLLTINTDLEN
VAIKVNLVGHSSG
>g3326.t3
MFLHTFLMFPHTFLMFSHTFPHTFPHTFLQTFLQTFPHMFLHTFLHTFLQRNMTMLAGAAQTRPLLRSGARAPTPRDPAP
PGCQQSPRCSPSSREPCRPI
KLSITSQPVQDESQLPGRRCLACLQHSSRRSVMPTLEPNRAVLVVYQPAWTAGAGPLLTINTDLENVAIKVNLVGHSSG
>g3327.t1
MVSFQKGDSLGLRLAGGNAVGIFIAGVQEGSPAEEKGLRAGDQIVKVNNVDFRNIFREEAVLFLLKIPKGQDITILAQRR
HDVFSVVLVSGRSDSFFIRT
HFEYEREGPQSLAFGCGEILKVVDTLYDGKLGNWLAIRVGKEKQLLEKGVIPNKIRAEQMAAVLSPPKDTVVADRAEFWR
LRGQQDTRKTDPLRRSQPEP
SARDANTPPPTYEKVVLREAGFRRPVVLFGPIADAATERLAKEMPDQFVVAKTEPKDAGCLRSSGVVRLNTIRQIIEQNK
HALLDVTPKAVNTLNYTQWY
PIVIFLSPDSKQGVKLLRQRAMPHSGRSARKLYVQALRLKKTCSHLFTGTIHLNSANDAWYSALKESIKQQQMQGVWVSE
GKLERIETELDCPDVELVSY
LSGMSTDYLSVDGQQASDVAAHQGTCGHSQPDAELLNHCPRPPLTHVSAMSQVLSQEVSKHVKHAILWTTCKTCKPFFLE
F
>g3327.t2
MDNVPHSFAVQSLRNCSKEARITVKRARTIVTHQHPHRSSGGEDYSKADTDDGWRHDVVHSHRVESTGCGRSEDRRDKIR
GGSRERGGSPGAEDPSKGGG
GSGGGGRPNGHCQTCDTWGKPGGDGKGAGETLHLSGKYECDAGEVGEKYLSLARSSPLREHPGVTGELEPLEKPVSVVLV
KEAPYEEYGLRLGSQIFIKQ
MTATGLAAKDGNLQEGDIILKINGTVTENLSLNEAGKLIEKSRGKLQLSVQRDHQQLLVRIPVLIDSDSEHEDVSEMESY
QDYSSLEDRRSHHSDQSSHS
SNDRYSSRENLIPVTKATAQRVKKPEDLRLPSERKADPSPTEDVGAQKRRKFMGLKPTPRVTVALLQHHMSSAWKHSLSR
PNTVMVSFQKGDSLGLRLAG
GNAVGIFIAGVQEGSPAEEKGLRAGDQIVKVNNVDFRNIFREEAVLFLLKIPKGQDITILAQRRHDVFSVVLVSGRSDSF
FIRTHFEYEREGPQSLAFGC
GEILKVVDTLYDGKLGNWLAIRVGKEKQLLEKGVIPNKIRAEQMAAVLSPPKDTVVADRAEFWRLRGQQDTRKTDPLRRS
QPEPSARDANTPPPTYEKVV
LREAGFRRPVVLFGPIADAATERLAKEMPDQFVVAKTEPKDAGCLRSSGVVRLNTIRQIIEQNKHALLDVTPKAVNTLNY
TQWYPIVIFLSPDSKQGVKL
LRQRAMPHSGRSARKLYVQALRLKKTCSHLFTGTIHLNSANDAWYSALKESIKQQQMQGVWVSEGKLERIETELDCPDVE
LVSYLSGMSTDYLSVDGQQA
SDVAAHQGTCGHSQPDAELLNHCPRPPLTHVSAMSQVLSQEVSKHVKHAILWTTCKTCKPFFLEF
>g3327.t3
MGFGLAVSGGQDNPNMDNNDTSIVICDVLQGSPADGLLLVIQVNSIPMDNVPHSFAVQSLRNCSKEARITVKRARTIVTH

QHPHRSSGGEDYSKADTDDG
WRHDVVHSHRVESTGCGRSEDRRDKIRGGSRERGGSPGAEDPSKGGGGSGGGGRPNGHCQTCDTWGKPGGDGKGAGETLH
LSGKYECDAGEVGEKYLSLA
RSSPLREHPGVTGELEPLEKPVSVVLVKEAPYEEYGLRLGSQIFIKQMTATGLAAKDGNLQEGDIILKINGTVTENLSLN
EAGKLIEKSRGKLQLSVQRD
HQQLLVRIPVLIDSDSEHEDVSEMESYQDYSSLEDRRSHHSDQSSHSSNDRYSSRENLIPVTKATAQRVKKPEDLRLPSE
RKADPSPTEDVGAQKRRKFM
GLKPTPRVTVALLQHHMSSAWKHSLSRPNTVMVSFQKGDSLGLRLAGGNAVGIFIAGVQEGSPAEEKGLRAGDQIVKVNN
VDFRNIFREEAVLFLLKIPK
GQDITILAQRRHDVFSVVLVSGRSDSFFIRTHFEYEREGPQSLAFGCGEILKVVDTLYDGKLGNWLAIRVGKEKQLLEKG
VIPNKIRAEQMAAVLSPPKD
TVVADRAEFWRLRGQQDTRKTDPLRRSQPEPSARDANTPPPTYEKVVLREAGFRRPVVLFGPIADAATERLAKEMPDQFV
VAKTEPKDAGCLRSSGVVRL
NTIRQIIEQNKHALLDVTPKAVNTLNYTQWYPIVIFLSPDSKQGVKLLRQRAMPHSGRSARKLYVQALRLKKTCSHLFTG
TIHLNSANDAWYSALKESIK
QQQMQGVWVSEGKLERIETELDCPDVELVSYLSGMSTDYLSVDGQQASDVAAHQGTCGHSQPDAELLNHCPRPPLTHVSA
MSQVLSQEVSKHVKHAILWT
TCKTCKPFFLEF
>g3327.t4
MRCVSQDPQMGFGLAVSGGQDNPNMDNNDTSIVICDVLQGSPADGLLLVIQVNSIPMDNVPHSFAVQSLRNCSKEARITV
KRARTIVTHQHPHRSSGGED
YSKADTDDGWRHDVVHSHRVESTGCGRSEDRRDKIRGGSRERGGSPGAEDPSKGGGGSGGGGRPNGHCQTCDTWGKPGGD
GKGAGETLHLSGKYECDAGE
VGEKYLSLARSSPLREHPGVTGELEPLEKPVSVVLVKEAPYEEYGLRLGSQIFIKQMTATGLAAKDGNLQEGDIILKING
TVTENLSLNEAGKLIEKSRG
KLQLSVQRDHQQLLVRIPVLIDSDSEHEDVSEMESYQDYSSLEDRRSHHSDQSSHSSNDRYSSRENLIPVTKATAQRVKK
PEDLRLPSERKADPSPTEDV
GAQKRRKFMGLKPTPRVTVALLQHHMSSAWKHSLSRYSAEF
>g3328.t1
ERHGGLLQVRTRPAVTLIPPARSVALGAPSMPMGEQNHTQDNPKMFSSQSEEWFHQMQHEQGGQRAECGWWCSEGTEETA
AQRREHTEERAA
>g3328.t2
ERHGGLLQVRTRPAVTLIPPARSVALGAPSMPMGEQNHTQSEEWFHQMQHEQGGQRAECGWWCSEGTEETAAQRREHTEE
RAA
>g3329.t1
MSAAVVLDLSHLRDVSVQEEKVRYLGRGYVLVLRLATGFSYPVTQSATMGGRSDVEAVGALLKRFLGLEELQKRRQQEED
DAFVEEEEEEEDHSSDSQDE
PLTAQ
>g3329.t2
VLDLSHLRDVSVQEEKVRYLGRGYVLVLRLATGFSYPVTQSATMGGRSDVEAVGALLKRFLGLEELQKRRQQEEDDAFVE
EEEEEEDHSSDSQDEPLTAQ
>g3330.t1
MLGEGAESKRWLSAPGHTVERLFLGHVTRVAQAVGEAWPHLNVVMWDDMLRGMSRDTLKASGLVGLVQPMLWDYNPGLNV
EETVSLLEKYCGAGLADLWA
ASAFKGSTSVHTSVTNTQRHVDNHLQWLKVAAAVSPGVRLQGIALTGWQRYDHMSVLCELMPVALPSLASCLQTLIRGHF
GPEVQATVTERLGISSVEVE
TLDRTAVADSRFAGRRLAGLIVELSQLLNSEEVRHFESNMFVRGWFSPYHRQRRAVNPLICMQIQSQATTLLTMLEETAT
ATREEMQRLYPEATAQEWME
QHVSPVAAPLRRITEEIGASAPL
>g3330.t2
MLGEGAESKRWLSAPGHTVERLFLGHVTRVAQAVGEAWPHLNVVMWDDMLRGMSRDTLKASGLVGLVQPMLWDYNPGLNV
EETVSLLEKYCGAGLADLWA
ASAFKGSTSVHTSVTNTQRHVDNHLQWLKVAAAVSPGVRLQGIALTGWQRYDHMSVLCELMPVALPSLASCLQTLIRGHF
GPEVQATVTERLGISSVEVE
TLDRTAVADSRFAGRRLAGLIVELSQLLNSEEVRHFESNMFVRGWFSPYHRQRRAVNPLICMQIQSQATTLLTMLEETAT
ATREEMQRLYPEATAQEWME

QHVSPVAAPLRRITEEIGACLSEMIXXXXXXXXXSETPPPPPPTGIIIDVSEDDFRTLAFF
>g3331.t1
MSPGSGYQFGILVLYGEQQSEYGSRYVVASQGFQGCVSAAIECVSPKGNPDARQRAAFLGLLAARRHSCLNWFQRHDM
>g3332.t1
MRSREERNATTEHSLCFQEPRPAGSRPVQNKPLDSIGVETSGVTERSPRTNVLNEDVGYGLSMARWWASLILKFFGVISI
FAEFSRYGRAHLLGTGVLEI
KSVDVGVVAIRALSSNYYLAINKKGDLYGAVSPPQTETSTASATQANNRG
>g3333.t1
VSCGAPKAPANGGVLTSDYSVGTRVSYFCSDGYRLSSKELTSAVCQPDGTWSNHNKIPRCSGTVAAARGGPAGA
>g3334.t1
MQPLLSSGPHLWNSLSEDLRVNIMIRAALTNIQTKWRNYRHRFVPWIALNLHRNQRSLRHVTKCVQDKPIHDEVVSESLL
KLFRTLLERDRAAEGHGPKE
TLRSVLTQRADARGTCEGRRVAENDDVIFQRLGFCVHRRPSSVPQAGLGVFVSRGFVPEGTVVAMYPGTVYEPYEPVFFQ
SVRNPFVFRCLDGVLVDGND
RGISKLVYKSCSRRDMLGPHRLSDFSWLTADPQNPLAVGQYVNNCTDSPANVCYQEYDVPREFPLELCQYLPNVKYSHEI
QRPLRCVILVSLRDISQDEE
LFSNYYTIVDS
>g3334.t2
MIRAALTNIQTKWRNYRHRFVPWIALNLHRNQRSLRHVTKCVQDKPIHDEVVSESLLKLFRTLLERDRAAEGHGPKETLR
SVLTQRADARGTCEGRRVAE
NDDVIFQRLGFCVHRRPSSVPQAGLGVFVSRGFVPEGTVVAMYPGTVYEPYEPVFFQSVRNPFVFRCLDGVLVDGNDRGI
SKLVYKSCSRRDMLGPHRLS
DFSWLTADPQNPLAVGQYVNNCTDSPANVCYQEYDVPREFPLELCQYLPNVKYSHEIQRPLRCVILVSLRDISQDEELFS
NYYTIVDS
>g3335.t1
MLQEVVLESTVSSYSPRELTGSTEYRVTLQAIAAGRHSPLLTTTFTTNVCPPVGRLYPHPRDCAQVLLNGESSSGLHTIY
LTGDRSQPIQVYCDMTTDQG
GWLVFLRRQNGKLEFFRNWRNYTAGFGNMEDEFWLGAITNSAHYELRVDLRDNGETAYAQYDNFTIAEPRTRYKIYVGNY
SGTAGDSVIYHQGRPFSTFD
HDNDAAITNCALSYKGAFWYKNCHRANLMGKYGDTSHSKGINWYHWKGHEHSIQFAEMKMRPVNFSNFESRRKRS
>g3335.t2
LTGSTEYRVTLQAIAAGRHSPLLTTTFTTNVCPPVGRLYPHPRDCAQVLLNGESSSGLHTIYLTGDRSQPIQVYCDMTTD
QGGWLVFLRRQNGKLEFFRN
WRNYTAGFGNMEDEFWLGAITNSAHYELRVDLRDNGETAYAQYDNFTIAEPRTRYKIYVGNYSGTAGDSVIYHQGRPFST
FDHDNDAAITNCALSYKGAF
WYKNCHRANLMGKYGDTSHSKGINWYHWKGHEHSIQFAEMKMRPVNFSNFESRRKRS
>g3335.t3
MLQEVVLESTVSSYSPRELTGSTEYRVTLQAIAAGRHSPLLTTTFTTNVCPPVGRLYPHPRDCAQVLLNGESSSGLHTIY
LTGDRSQPIQVYCDMTTDQG
GWLVFLRRQNGKLEFFRNWRNYTAGFGNMEDEFWLGLTNLHKITNSAHYELRVDLRDNGETAYAQYDNFTIAEPRTRYKI
YVGNYSGTAGDSVIYHQGRP
FSTFDHDNDAAITNCALSYKGAFWYKNCHRANLMGKYGDTSHSKGINWYHWKGHEHSIQFAEMKMRPVNFSNFESRRKRS
>g3336.t1
MLKKPIVIRAKNHTLIGKRKTNNFLFTTGFGDNISWRTLDDGKKEAEASGLPMMVIIHKTWCGACKALKPKFAESKEISE
LAHNFIMINLEDEEEPKDEA
FSPDGGYIPRILFLDPTGIVHPEITNKNGNPSYKYFYSNADHVAAGMKEAQEKLTGDAYRKSHVGDEL
>g3336.t2
LIGKRKTNNFLFTTGFGDNISWRTLDDGKKEAEASGLPMMVIIHKTWCGACKALKPKFAESKEISELAHNFIMINLEDEE
EPKDEAFSPDGGYIPRILFL
DPTGIVHPEITNKNGNPSYKYFYSNADHVAAGMKEAQEKLTGDAYRKSHVGDEL
>g3336.t3
MLKKPIVIRAKNHTLIGKRKTNNFLFTTGFGDNISWRTLDDGKKEAEASGLPMMVIIHKTWCGACKALKPKFAESKEISE
LAHNFIMINLEDEEEPKDEA
FSPDGGYIPRILFLVAAGMKEAQEKLTGDAYRKSHVGDEL
>g3337.t1
MNQEKLAKLQAQVRIGGKGTPRRKKKIVHRTATAEDKKLQSSLKKLVVNNIAGIEEVNMIKDDGMVIHFNNPKVQASLSA

NTFAVTGHSETTPLTELLPG
ILTQLGSDSLSNLRQLARRFPGPRQALDTQERQQGDDEDENVPDLVQNFDEASKGEANC
>g3337.t2
MFFFLVVMNQEKLAKLQAQVRIGGKGTPRRKKKIVHRTATAEDKKLQSSLKKLVVNNIAGIEEVNMIKDDGMVIHFNNPK
VQASLSANTFAVTGHSETTP
LTELLPGILTQLGSDSLSNLRQLARRFPGPRQALDTQERQQGDDEDENVPDLVQNFDEASKGEANC
>g3337.t3
MNQEKLAKLQAQVRIGGKGTPRRKKKIVHRTATAEDKKLQSSLKKLVVNNIAGIEEVNMIKDDGMVIHFNNPKVQASLSA
NTFAVTGHSETTPLTELLPG
ILTQLGSDSLSNLRQLARRFPGPRQALDTQERQQGDDEDENVPGLRSDVH
>g3338.t1
MRSRGRYVKLLGRWTEQLHLATTLLSGRWILILLRGARRDIKEFCHLLKTVKVDVDSSGKKCKERMMKVLWDGPFALPSS
SEHGLVSQGSSTNQSCFLEK
EYQSKLELTEAFQEANMPEMYQQILPFLTL
>g3339.t1
MELDNSSLELYYTNNTAEFPENTTTSRWDEATLLGLQIFISALLALITLATVLSNAFVIATIYLTRKLHTPANFLIGSLA
VTDLLVSILVMPISIVYTVS
KTWALGQILCDIWLSSDITFCTASILHLCVIALDRYWAITRALEYSKRRTMRRAAVMVAVVWVISISISLPPLFWRQAKA
HEELTECMVNTDQISYTLYS
TFGAFYVPTVLLVILYGRIYVAARSRILKTPASRAKRFTTAQLIQTSAGSSLCSINSASIQEAHLHSATGVGGASPLFTN
SVKVKLADNVLERKRLCAAR
ERKATKTLGIILGAFIVCWLPFFVGTLVTAICKECWFDPLLFDVFTWLGYLNSLINPVIYTVFNDEFKQAFQKLMKFKRC
S
>g3340.t1
IRETAFVYAITAAGVT
>g3341.t1
RATRGQHRAGNAGPNRTRGDETLEDFSGRPPSLLDVRGQRTCRELYLHHNLLAALIQSVLVRGSERQSENDFPLTFHLQL
RPGAAPEPLKRRPAKRPRIC
CPVVALISPLPCGPLESYQHYDRRRACGV
>g3341.t2
ISDRATRGQHRAGNAGPNRTRGDETLEDFSGRPPSLLDVRGQRTCRELYLHHNLLAALIQSVLVRGSERQSENDFPLTFH
LQLRPGAAPEPLKRRPAKRP
RICCPVVALISPLPCGPLESYQHYDRRRACGV
>g3341.t3
MTWRTLRGGTRSGSVAYVLLTACFRSQIAPLEGSTEQGTRDRTERAVTRRWRTSAGPSDPWSSSLRPSGRPPSLLDVRGQ
RTCRELYLHHNLLAALIQSV
LVRGSERQSENDFPLTFHLQLRPGAAPEPLKRRPAKRPRICCPVVALISPLPCGPLESYQHYDRRRACGV
>g3342.t1
SPAASLPVRGVHLDTSQCAGHNKWSKVRHIKGPKDEARGRMFMKFGMMIRIAVKDGGPNPDFNAPLANILEQCRSNNMPK
ASIDTALKNAEKNKAGTQQT
YEARGPGGCVFLIEVLTDNNTRTHQDLKHLLHKKGGMLADGARHMFDRKGVVVVEAGGLSTEDALEMAIEAGAEDVLETE
EEERPRLQFICSQTELSSVR
TWLENRGGIRVVSSGLEFVSHTPTLLADSQLEVASVLLEALNDHCDVLRVWDNIEAER
>g3342.t2
GLRGLLASGPLDRLASSPAASLPVRGVHLDTSQCAGHNKWSKVRHIKGPKDEARGRMFMKFGMMIRIAVKDGGPNPDFNA
PLANILEQCRSNNMPKASID
TALKNAEKNKAGTQQTYEARGPGGCVFLIEVLTDNNTRTHQDLKHLLHKKGGMLADGARHMFDRKGVVVVEAGGLSTEDA
LEMAIEAGAEDVLETEEEER
PRLQFICSQTELSSVRTWLENRGGIRVVSSGLEFVSHTPTLLADSQLEVASVLLEALNDHCDVLRVWDNIEAER
>g3342.t3
CAGHNKWSKVRHIKGPKDEARGRMFMKFGMMIRIAVKDGGPNPDFNAPLANILEQCRSNNMPKASIDTALKNAEKNKAGT
QQTYEARGPGGCVFLIEVLT
DNNTRTHQDLKHLLHKKGGMLADGARHMFDRKGVVVVEAGGLSTEDALEMAIEAGAEDVLETEEEERPRLQFICSQTELS
SVRTWLENRGGIRVVSSGLE
FVSHTPTLLADSQLEVASVLLEALNDHCDVLRVWDNIEAER
>g3343.t1

MSSPAALKREGGALMSPQSLDLLGPSKVKERTQKVTEKVTQVLSLESDVLPKYKLQLPETTWWILLHYSPFKAFWDWIIL
LLVLYTAVFTPYSAAFLLDE
HGDRACGYTCDPLNVVDVMVDVLFIIDIVINFRTSYVNHNNEVVTGPGRVAKHYITGWFAIDLFAAIPFDLLIFRSGSDE
MATLIGLLKTARLLRLVRVA
RKLDRYSEYGAAVLFLLMCTFALIAHWLACLWYAIGFLERPYTETGWLDNLAQQLGKTYNDSDASSGPSIKDKYVTALYF
TFSSLTSVGFGNVSPNTNSE
KIFSICVMVIGSLMYASIFGNVSAIIQRLYSGTTRYHTQMLRVKDFIRFHQIPGGLCLRLEEYFQHAWSYTNGIDMNAVL
KGFPECLQADICLHLNRSLL
QNCKAFHGGSQACLRALAMRFKMVHAPPGDTLIHYGDVLDSLFFISRGSIQVVRDNVVVAILEKCDIFGEPIHLHDDPGK
SSSDVHAITYCDLHRILRED
LLEVLDIYPDFANNFWKNLEITLDLRCNEDKVLTVVCPQTDQGQHPEDSSDDWEYQQQPRQRGYSLDCRIRPDGIDHEDS
FPLHSIAPRHSAPQAGSGSS
LSPSAEDLREAIPRGTRLHPSEEVRLDYASSADRLPPPVSRTAGREQALEIASSLRPGMFYYWPDRQSLQFSSRPRSARC
TNPYHHTAGETDLGSRLDVL
QSQLNRLETRMTADITVILQILQGPTAPPIPPAYSTVSSTDPPIPSGPCAVIHSPFKGVPSTGVHSTGMSSTSTGDLCPF
TEVPSIGVPNLYTGPPGPST
RVSSSSTGAPSPSTDPPSPSTGPPSPTTGDLSQTSGVHSPSTGPPSPSTGDPSTSSGIHSASTAPTSPSTGVPSPSTAPP
SPSTGVPSPSTGFPSTCTGA
PDSLSSVYGPRTPALPTMYTSSPILVESLTSTQPGERPNRRTDPNRESTCQGTDPTRKPTSQGTNQSARWNLWPRFNPFP
ICPLIPLLHQSPGLNHKLQK
SHQQKFHLLLNQLACPINTTSGLCLRSIIHMDNSTRDYNDAASKKSTYRKSTSKNASGAQSIPPVAYASLLQQSIWTTAI
SIHAHSHPSRTHRSTRDSNE
ATSQKSTYRKSTSKNASGAQSVPPVAYASLLQQSIWTTGTAISVHAHSTTPYYTRAHNCAADYNNKAPDDCYNGGTGHRE
GTGHREGTGHREGYTTLGAT
YKSSNG
>g3343.t2
MSSPAALKREGGALMSPQSLDLLGPSKVKERTQKVTEKVTQVLSLESDVLPKYKLQLPETTWWILLHYSPFKAFWDWIIL
LLVLYTAVFTPYSAAFLLDE
HGDRACGYTCDPLNVVDVMVDVLFIIDIVINFRTSYVNHNNEVVTGPGRVAKHYITGWFAIDLFAAIPFDLLIFRSGSDE
MATLIGLLKTARLLRLVRVA
RKLDRYSEYGAAVLFLLMCTFALIAHWLACLWYAIGFLERPYTETGWLDNLAQQLGKTYNDSDASSGPSIKDKYVTALYF
TFSSLTSVGFGNVSPNTNSE
KIFSICVMVIGSLMYASIFGNVSAIIQRLYSGTTRYHTQMLRVKDFIRFHQIPGGLCLRLEEYFQHAWSYTNGIDMNAVL
KGFPECLQADICLHLNRSLL
QNCKAFHGGSQACLRALAMRFKMVHAPPGDTLIHYGDVLDSLFFISRGSIQVVRDNVVVAILEKCDIFGEPIHLHDDPGK
SSSDVHAITYCDLHRILRED
LLEVLDIYPDFANNFWKNLEITLDLRCNEDKVLTVVCPQTDQGQHPEDSSDDWEYQQQPRQRGYSLDCRIRPDGIDHEDS
FPLHSIAPRHSAPQAGSGSS
LSPSAEDLREAIPRGTRLHPSEEVRLDYASSADRLPPPVSRTAGREQALEIASSLRPGMFYYWPDRQSLQFSSRPRSARC
TNPYHHTAGETDLGSRLDVL
QSQLNRLETRMTADITVILQILQGPTAPPIPPAYSTVSSTDPPIPSGPCAVIHSPFKGVPSTGVHSTGMSSTSTGDLCPF
TEVPSIGVPNLYTGPPGPST
RVSSSSTGAPSPSTDPPSPSTGPPSPTTGDLSQTSGVHSPSTGPPSPSTGDPSTSSGIHSASTAPTSPSTGVPSPSTAPP
SPSTGVPSPSTGFPSTCTGA
PDSLSSVYGPRTPALPTMYTSSPILVESLTSTQPGERPNRRTDPNRESTCQGTDPTRKPTSQGTNQSARWNLWPRFNPFP
ICPLIPLLHQSPGLNHKLQK
SHQQKFHLLLNQLACPINTTSGLCLRSIIHMDNSRTHRSTRDSNEATSQKSTYRKSTSKNASGAQSVPPVAYASLLQQSI
WTTGTAISVHAHSTTPYYTR
AHNCAADYNNKAPDDCYNGGTGHREGTGHREGTGHREGYTTLGATYKSSNG
>g3343.t3
MSSPAALKREGGALMSPQSLDLLGPSKVKERTQKVTEKVTQVLSLESDVLPKYKLQLPETTWWILLHYSPFKAFWDWIIL
LLVLYTAVFTPYSAAFLLDE
HGDRACGYTCDPLNVVDVMVDVLFIIDIVINFRTSYVNHNNEVVTGPGRVAKHYITGWFAIDLFAAIPFDLLIFRSGSDE
MATLIGLLKTARLLRLVRVA
RKLDRYSEYGAAVLFLLMCTFALIAHWLACLWYAIGFLERPYTETGWLDNLAQQLGKTYNDSDASSGPSIKDKYVTALYF
TFSSLTSVGFGNVSPNTNSE

KIFSICVMVIGSLMYASIFGNVSAIIQRLYSGTTRYHTQMLRVKDFIRFHQIPGGLCLRLEEYFQHAWSYTNGIDMNAVL
KGFPECLQADICLHLNRSLL
QNCKAFHGGSQACLRALAMRFKMVHAPPGDTLIHYGDVLDSLFFISRGSIQVVRDNVVVAILEKCDIFGEPIHLHDDPGK
SSSDVHAITYCDLHRILRED
LLEVLDIYPDFANNFWKNLEITLDLRCNEDKVLTVVCPQTDQGQHPEDSSDDWEYQQQPRQRGYSLDCRIRPDGIDHEDS
FPLHSIAPRHSAPQAGSGSS
LSPSAEDLREAIPRGTRLHPSEEVRLDYASSADRLPPPVSRTAGREQALEIASSLRPGMFYYWPDRQSLQFSSRPRSARC
TNPYHHTAGETDLGSRLDVL
QSQLNRLETRMTADITVILQILQGPTAPPIPPAYSTVSSTDPPIPSGPCAVIHSPFKGVPSTGVHSTGMSSTSTGDLCPF
TEVPSIGVPNLYTGPPGPST
RVSSSSTGAPSPSTDPPSPSTGPPSPTTGDLSQTSGVHSPSTGPPSPSTGDPSTSSGIHSASTAPTSPSTGVPSPSTAPP
SPSTGVPSPSTGFPSTCTGA
PDSLSSVYGPRTPALPTMYTSSPILVESLTSTQPGERPNRRTDPNRESTCQGTDPTRKPTSQGTNQSARWNLWPRFNPFP
ICPLIPLLHQSPGLNHKLQK
SHQQKFHLLLNQLACPINTTSGLCLRSIIHMDNSPYYTGTRDYNEAASKKSTYRESASKNASSAQSIQPVACASVLQ
>g3344.t1
LFTFGVIADIQYADIDDGFNYTRTRRRYYRSSLQLLRNARDSWSRAAVKPSFILQLGDIIDGFNKQYDSSDQALDTVMRE
LSTSPAEVHHVWGNHEFYNF
SRDTLMKSKLNSTPHCDSGSGGPLTGDIYAYQFNPAPKFRFVVLDAYDVSILGRTVSSVQYNNALNILKEHNSNEDLNHP
PAEEGIKRKFVKFNGGFSRD
QLNWLDGVLSLSDENEERVIIVSK
>g3344.t2
LFTFGVIADIQYADIDDGFNYTRTRRRYYRSSLQLLRNARDSWSRAAVKPSFILQLGDIIDGFNKQYDSSDQALDTVMRE
LSTSPAEVHHVWGNHEFYNF
SRDTLMKSKLNSTPHCDSGSGGPLTGDIYAYQFNPAPKFRFVVLDAYDVSILGRTVSSVQYNNALNILKEHNSNEDLNHP
PAEEGIKRKFVKFNGGFSRD
QLNWLDGVLSLSDENEERVIIV
>g3344.t3
MESCSENPPLFTFGVIADIQYADIDDGFNYTRTRRRYYRSSLQLLRNARDSWSRAAVKPSFILQLGDIIDGFNKQYDSSD
QALDTVMRELSTSPAEVHHV
WGNHEFYNFSRDTLMKSKLNSTPHCDSGSGGPLTGDIYAYQFNPAPKFRFVVLDAYDVSILGRTVSSVQYNNALNILKEH
NSNEDLNHPPAEEGIKRKFV
KFNGGFSRDQLNWLDGVLSLSDENEERVIIVSK
>g3345.t1
RGMPFLWALGTPRLCISTIPFSFYHHHRHHHHHQHHHHHHHHHHHHHHHHHHHHQHHHHNTTNTTIMKHQHPTKTTTGTTI
E
>g3346.t1
MKGHKCDVCSREFTLSANLKRHMLIHNSIRPFQCHVCFKSFIQKQTLKTHMIVCGKSFNRMYNLLGHMHLHAGSKPFKCP
YCTSKFNLKGNLSRHMKVKH
GMDVSPEGQGEA
>g3347.t1
GEDEEEEEEEEEEEEEKEAEKISGKSKQEMGRIKGFCKPGNEGEDSRSRRSGGKRDEGRSLIAHGGGSGSRPHPVADVAL
QDAASRALLLDSELQA
>g3348.t1
ACGVDFEIKAYIANAIDDADEVIEKKCVFFLCFRIVTSRAVAKDTCRLMIRKIQFAPDNTNPGPKVEVPKPLQLSASLGK
ENYYHGDPISIKVKINNDSS
KVVKKIKISVEQSTAVVLYSSDTYVKPVCSEEYGETINANATFEKEFSLTPLLANNSEKRGIAVDGRLKDGDTNLASTTL
SQGGDKEMQGIIISYKVKVT
ACLSSGG
>g3348.t2
ACGVDFEIKAYIANAIDDADEVIEKKDTCRLMIRKIQFAPDNTNPGPKVEVPKPLQLSASLGKENYYHGDPISIKVKINN
DSSKVVKKIKISVEQSTAVV
LYSSDTYVKPVCSEEYGETINANATFEKEFSLTPLLANNSEKRGIAVDGRLKDGDTNLASTTLSQGGDKEMQGIIISYKV
KVTACLSSGG
>g3348.t3
ACGVDFEIKAYIANAIDDADEVIEKKCVFFLCFRIVTSRAVAKDTCRLMIRKIQFAPDNTNPGPKVEVPKPLQLSASLGK

ENYYHGDPISIKVKINNDSS
KVVKKIKISVEQSTAVVLYSSDTYVKPVCSEEYGETINANATFEKEFSLTPLLANNSEKRGIAVDGRLKDGDTNLASTTL
SQGGDKEMQGIIISYKVKVT
ACLSSGGDVVAELPLILMSPKPAEMYRNTLMEVKLSN
>g3349.t1
MPCVQTQIGILPIDSLSSEFFNTDLNARLVMDISDQKDQLSASSLPSINTLVASGYVGEFDAYSCKVTSAAPGPSASFSP
SPSPVAGGSSPQQNLKLDDF
QVYGCYPGSFAFGYLDETLSSCGSDYYNSPVYAAASPSTPSYQPPATPIWDSFSPYSSAPSSVAAEKAVLAHQLSFFTFN
PPTEEISPLGQRQDQVTDDP
FSLACQQHASPLCCPPISPEHRGLESPRSMEGNMTSPQTCSPVAGDGRCAVCGDNASCQHYGVRTFVRTDSLKGRRGRLP
SKPKTVSEASPSTPPVNIIA
SLVRAHLDSNPTLGKLDHSKYQETVVSLSEKEDSGDIQQFYDLLSGSMDVIKKWAETIPGFTDFCNEDQELLLESAFVEL
FILRLAYRSSPEKSKLIFCN
GVVLHRMQCVRSFGDWIDSIMDFSQNLHRMNLDVSSFACLAALVIITDRHGLKEPKRVEDFQNHLITCLREHMSGNGSYN
IRTQPNYLSKLLGKLPELRT
LCTQGLQRIFYLKLEDLVPPPPIVEKIFMDTLPF
>g3349.t2
MPCVQTQIGILPIDSLSSEFFNTDLNARLVMDISDQKDQLSASSLPSINTLVASGYVGEFDAYSCKVTSAAPGPSASFSP
SPSPVAGGSSPQQNLKLDDF
QVYGCYPGSFAFGYLDETLSSCGSDYYNSPVYAAASPSTPSYQPPATPIWDSFSPYSSAPSSVAAEKAVLAHQLSFFTFN
PPTEEISPLGQRQDQVTDDP
FSLACQQHASPLCCPPISPEHRGLESPRSMEGNMTSPQTCSPVAGDGRCAVCGDNASCQHYGVRTFVRTDSLKGRRGRLP
SKPKTVSEASPSTPPVNIIA
SLVRAHLDSNPTLGKLDHSKETVVSLSEKEDSGDIQQFYDLLSGSMDVIKKWAETIPGFTDFCNEDQELLLESAFVELFI
LRLAYRSSPEKSKLIFCNGV
VLHRMQCVRSFGDWIDSIMDFSQNLHRMNLDVSSFACLAALVIITDRHGLKEPKRVEDFQNHLITCLREHMSGNGSYNIR
TQPNYLSKLLGKLPELRTLC
TQGLQRIFYLKLEDLVPPPPIVEKIFMDTLPF
>g3349.t3
MPCVQTQIGILPIDSLSSEFFNTDLNARLVMDISDQKDQLSASSLPSINTLVASGYVGEFDAYSCKVTSAAPGPSASFSP
SPSPVAGGSSPQQNLKLDDF
QVYGCYPGSFAFGYLDETLSSCGSDYYNSPVYAAASPSTPSYQPPATPIWDSFSPYSSAPSSVAAEKAVLAHQLSFFTFN
PPTEEISPLGQRQDQVTDDP
FSLACQQHASPLCCPPISPEHRGLESPRSMEGNMTSPQTCSPVAGDGRCAVCGDNASCQHYGVRTCEGCKGFFKRTVQKN
AKYVCLATKDCPVDKRRRNR
CQFCRFQKCLSVGMVKEVVRTDSLKGRRGRLPSKPKTVSEASPSTPPVNIIASLVRAHLDSNPTLGKLDHSKYQETVVSL
SEKEDSGDIQQFYDLLSGSM
DVIKKWAETIPGFTDFCNEDQELLLESAFVELFILRLAYRSSPEKSKLIFCNGVVLHRMQCVRSFGDWIDSIMDFSQNLH
RMNLDVSSFACLAALVIITD
RHGLKEPKRVEDFQNHLITCLREHMSGNGSYNIRTQPNYLSKLLGKLPELRTLCTQGLQRIFYLKLEDLVPPPPIVEKIF
MDTLPF
>g3350.t1
PRLKNVDRSSAQHLEVTVGDLTVIITDFKEKAKPPPATATSADLHSGSGSDNTERRGSRATSPRGESSSVNGDAH
>g3351.t1
MRCYLHALLICIFLAVFASLYVLNQLASSLDAKDEWTHSKSQGVPHSGRHDTGERLNAKTSKDERMHLAVVACGPRLEET
LVMLKSAVLFSTKLLHFHIF
AEDHLHKSFKEALDSWPAAVQLKFSSTTYPITFPSDNAAEWKKLFKPCASQRLFLPLILKDVDSVLYVDTDILFLHPVDR
IWALLSRFDGSQLAAMAPEH
EEPRIGWYNRFARHPYYGATGVNSGVMLMNMSRIREALFKNDMTSVPLRWGEMLMPLLQKYKLNITWGDQDLLNIIFHHN
PEGLYVFPCQWNYRPDHCIY
GSNCQQAEQEGVFILHGNRGVYHDDKQPAFRAVFRAIQK
>g3351.t2
LNQLASSLDAKDEWTHSKSQGVPHSGRHDTGERLNAKTSKDERMHLAVVACGPRLEETLVMLKSAVLFSTKLLHFHIFAE
DHLHKSFKEALDSWPAAVQL
KFSSTTYPITFPSDNAAEWKKLFKPCASQRLFLPLILKDVDSVLYVDTDILFLHPVDRIWALLSRFDGSQLAAMAPEHEE
PRIGWYNRFARHPYYGATGV

NSGVMLMNMSRIREALFKNDMTSVPLRWGEMLMPLLQKYKLNITWGDQDLLNIIFHHNPEGLYVFPCQWNYRPDHCIYGS
NCQQAEQEGVFILHGNRGVY
HDDKQPAFRAVFRAIQK
>g3351.t3
MRCYLHALLICIFLAVFASLYVLNQLASSLDAKDEWTHSKSQGVPHSGRHDTGERLNAKTSKDERMHLAVVACGPRLEET
LVMLKSAVLFSTKLLHFHIF
AEDHLHKSFKEALKFSSTTYPITFPSDNAAEWKKLFKPCASQRLFLPLILKDVDSVLYVDTDILFLHPVDRIWALLSRFD
GSQLAAMAPEHEEPRIGWYN
RFARHPYYGATGVNSGVMLMNMSRIREALFKNDMTSVPLRWGEMLMPLLQKYKLNITWGDQDLLNIIFHHNPEGLYVFPC
QWNYRPDHCIYGSNCQQAEQ
EGVFILHGNRGVYHDDKQPAFRAVFRAIQK
>g3352.t1
TALARTAAMPLLEEPTVPQDHPPTLPVVIIGNGPSGICLSYLLSGYKPYLENGAVHPNPILYRKLQEAKQLPITEQDLEY
LSEGLEGRSGNPVAVLFDTL
LHPEADFGYEFPPVLQWRQDKQQHIPHLVLGSATPGGAWHAMEGSMLTISLGIWMELPGVNYRDLTNGKRRGVTNDRATP
EEISSYYRNYVKLMGLQKNF
IDNTYVTSVQKLCRGHKTEAVEDQSSEQGIGGVGNGENGELEGNGVQCEDSGGGGKLWEVRGYQRLQKDTHVPFCLFAEN
VVLATGASDFPARLGVEGEG
LPFVLHSISDLGLAVSQGQLDSSSDPVLIVGAGLSAADAVLCACSANIPVLHVFRKNIDNPDLIFKQLPKTLYPEYHKVY
NMMCSQSHTTGVAVLSAANG
PQTGSIASSMCAKICSRPLHAVSNMATNGGSNISLDYTSFPEHCVVSFQPDRKCILQGNNSLKAFKISMALVLIGTNPNL
CFLKGQGQYLGINPTKPISC
KQNPIDIHPYSFECAKEPGLFAMGPLVGDNFVRFLKGGALGIASCLIKRLKKNEKLIDGGGGEII
>g3352.t2
MSTVMLVTALARTAAMPLLEEPTVPQDHPPTLPVVIIGNGPSGICLSYLLSGYKPYLENGAVHPNPILYRKLQEAKQLPI
TEQDLEYLSEGLEGRSGNPV
AVLFDTLLHPEADFGYEFPPVLQWRQDKQQHIPHLVLGSATPGGAWHAMEGSMLTISLGIWMELPGVNYRDLTNGKRRGV
TNDRATPEEISSYYRNYVKL
MGLQKNFIDNTYVTSVQKLCRGHKTEAVEDQSSEQGIGGVGNGENGELEGNGVQCEDSGGGGKLWEVRGYQRLQKDTHVP
FCLFAENVVLATGASDFPAR
LGVEGEGLPFVLHSISDLGLAVSQGQLDSSSDPVLIVGAGLSAADAVLCACSANIPVLHVFRKNIDNPDLIFKQLPKTLY
PEYHKVYNMMCSQSHTTGVA
VLSAANGPQTGSIASSMCAKICSRPLHAVSNMATNGGSNISLDYTSFPEHCVVSFQPDRKCILQGNNSLKAFKISMALVL
IGTNPNLCFLKGQGQYLGIN
PTKPISCKQNPIDIHPYSFECAKEPGLFAMGPLVGDNFVRFLKGGALGIASCLIKRLKKNEKLIDGGGGEII
>g3353.t1
AISNKDQHSISYTLSRAQTVVVEYTQDSNTDMFQVGRSTESPIDFVVTDTLAGGEGHPDAPSVQSTISRFACRIACQRSP
PHTARVYAAGFDSSKNIFLG
EKAAKWRTSDGQMDGLTTNGVLVMHPRHGFTQDSKQGVWREISVCGNVFTLRETRSAQQRGKM
>g3353.t2
LSRAQTVVVEYTQDSNTDMFQVGRSTESPIDFVVTDTLAGGEGHPDAPSVQSTISRFACRIACQRSPPHTARVYAAGFDS
SKNIFLGEKAAKWRTSDGQM
DGLTTNGVLVMHPRHGFTQDSKQGVWREISVCGNVFTLRETRSAQQRGKM
>g3353.t3
AQTVVVEYTQDSNTDMFQVGRSTESPIDFVVTDTLAGGEGHPDAPSVQSTISRFACRIACQRSPPHTARVYAAGFDSSKN
IFLGEKAAKWRTSDGQMDGL
TTNGVLVMHPRHGFTQDSKQGVWREISVCGNVFTLRETRSAQQRGKM
>g3354.t1
LTGQHTDCTRRREGVGARVLTPNLKVSCIAFFTFGAPFPPCCLGSPGGHKMCLTETRAKLHVVFHRLSAYHIVLVSEPTK
LHPASWVCTL
>g3355.t1
MAVALMQIAMDWLSGEKTSLALVGDGAQRVFPFNPRVPLSPCTLQALKLCQLSSSVSLLEHVCYLNSWSPPDYYLYSTPG
PRLLLIYKVLHHLKLLSHAA
QKST
>g3355.t2
MAVALMQIAMDWLSGEKTSLALVGDGAQRVFPFNPRVPLSPCTLQALKLCQLSSSVSLLEHVCYLNSWSPPDYYLYSTPG

PRLLLIYKLLLGC
>g3355.t3
MDWLSGEKTSLALVGDGAQRVFPFNPRVPLSPCTLQALKLCQLSSSVSLLEHVCYLNSWSPPDYYLYSTPGPRLLLIYKV
LHHLKLLSHAAQKST
>g3356.t1
IPSPHSSEDHIPRAQKGKREKKRDEEKRGDAFHSSPTEEREAESSSSVRPAVSCRPLHINDSAAVPRLTPFLMGLIGLVR
CLLPGGMCSGPRSLRDRGRE
SYPAVETRRVVAEELISSVRSHRL
>g3356.t2
AQKGKREKKRDEEKRGDAFHSSPTEEREAESSSSVRPAVSCRPLHINDSAAVPRLTPFLMGLIGLVRCLLPGGMCSGPRS
LRDRGRESYPAVETRRVVAE
ELISSVRSHRL
>g3356.t3
IPSPHSSEDHIPRAQKGKREKKRDEEKRGDAFHSSPTEEREAESSSSVRPAVSCRPLHINDSAAVPRLTPFLMGLIGLVR
CLLPGGMCSGPRSLRDRGRE
SYPVITPLTSRRKKEARKASHSRLHELYLLTVTEVSLRGPALLKGGLSRVKKRLYYKCLSSRLPGCAGQNARPLRLPYIE
NISLPLESGRVTKALFLGPH
DRRQAESDHAVETRRVVAEELISSVRSHRL
>g3357.t1
MDDANINMGDPNDSGYPTSPTSEAPEQPAAAANRRLSLRQSPRRGRRSQGEPSPPAGRKGTSSGSLISNLKLLINSESPT
DSVFSKMVRDCCDNEDFLLE
RQVVEVRDEKVVINVSGLMYETQLSTLHRFPDSVLGDPMKRIKHFDPIKNEYFFDRNRPSFDGILYFYQSGGKLKRPANV
PLDVFAAEIVFYELGNEAME
QFREDEGFVTEQEVLLPTNELHRQFWLLFEYPESSSAAKSVALVSVMVIVISIFIFCLETLPEFRDDRDFIPTVTQLING
TTVHSMAYPPQKGLISYVTD
PFFVVETICIIWFCFELCVRFVVCPSKSAFFNNIMNVIDIVSITPYFVTLGTELAATPDEDINSGQNASLAILRIIRLVR
VFRIFKLSRHSKGLQILGQT
LKASMRELGLLIFFLFIGVILFSSAIYFAEVDEPQTAFVSIPDGFWWAVVTMTTVGYGDMCPITMGGKMVGTLCAIAGVL
TIALPVPVIVSNFNYFYHRE
TEQEEKQIIDAATEATQKSMAAANNKYGSTPSLSKSNGTWQNEKNGVQ
>g3358.t1
LLVWRSEERGAVEELDSGTRKILRLLAGEEHGNTADDSRQSQGHSGRVCVVDSPPLEIRFNIRVNKESPGYSYTSMNGNP
GQRSPAGIVNTALAGVPCLP
PCEGGP
>g3358.t2
LVWRSEERGAVEELDSGTRKILRLLAGEEHGNTADDSRQSQGHSGRVCVVDSPPLEIRFNIRVNKESPGYSYTSMNGNPG
QRSPAGIVNTALAGVPCLPP
CEGGP
>g3359.t1
MISCDGSDFTSSSSLSKKCSPLRRELAHSEGKVVKSVFVPNIGWASQLTSGEVWVQFNDGSQLVVQAGVSCIAYTSPDGH
TIRYKENEKLPEHVKEKLHC
LSTILGLLANPAVHRLHQPH
>g3360.t1
MRGQTSTQKQRQRAHAHLTHWLPTWLPMWLSTPTAPRGATLVEALNVRGTREMVQGGCTFKDFQTFPPYANQWVGPCVEK
AFSEVRAKYCWQTPFIXAEE
Q
>g3361.t1
MTHVMEVRSGPAATQWFTVVDLANAFFSIPVTPGERGTLGPTYCRDIVSPQRYNEVLKLRLESLELSPGPDQVWTLIECS
TLMVRLPETQKQVGPGQVLL
LYLIMTFVRTFYLLHYSAQAAKLALTEACRDG
>g3362.t1
SFIPKFINHLFRCKPISMVGAEQLLLDTHSLKTDLLDLPSIGSQVLRKAPASYTKIVVKGMTRAEMILKPSSTITTTTIT
TITTITTTPPRP
>g3363.t1
AALQCTSDQVKYDPERLPPENSSFLERSFVCRFRCLLDNSSGFLALKFQGRLKYLHGQSVMTEDGSRVQSQLALFSIAVP
VQNPSILEIRTKTLIFQTKH

QLDFTPMGIDNRGKVVLGYSEIELCMRGSGYQFIHAADMMFCADNHIRMIKTGESGLNVFRLLSKSSGWVWVQANAKLVY
KGGRPDFIIARQRALVNAEG
EEHLRQRRLQLPFNFTTGEALLYEVGPTLDVTLMQSDRAFNSSMSGDSGSANDQQEAVGGLLGCFLNQDKNVYIQDAEPQ
LPVDQVFMESRALVNVPSDP
WPSLGLRADNNVAIKEEDPSVVAMMDALEDLAQDGELVTALAGMDVDPNELIEWENTLKKLSQEENGDDSNRTKYELESL
LSNDIFAYIDNVLFKEIAEA
NLTTSQSSCFSAVNNNPGNLLGQVAHYSGSGPISEMMMFQSPNDEANTHSPAKGLGPAVVSQDPRNQPASATGQGLAPQT
PAMFNSTQKLSHYGPPVPEA
RLPAIPQLLTDFFNPSVNLPDLSLPTLAGNNRRPFEPCVQTPISHYQGVAGNALSNQMLSTQTLSNQTLSNQTPSNQTPS
NQMLSTQTLSNQTLSNQMPT
NQTGSNLTLSNQTLSTQSLQSCPLIGMPSAPMAANGHLLHGSIQQPAIHVAPNVVAPVPPQLPRNDFSLPVNPSANSTLF
TGDCLVQGGAPFQTHSNHRV
PQWQQSLQPQQQSLPHASMAQNAHALPTGSHSQVLESQRLAGLWAQNYNGVNHPPSHGGLADPLRTTPSSCMLDKPLRAP
PATHTLSRPHPLTNGNLASA
NGTGFVPATMPPCQWGQGPGVPMAGAAAIGQDNAAAGAPPRQLLPGNISSSTADDMHAMQHYLDCNKHTQINMLSAKTED
RDLTMSVFFPSTTPRGSPKQ
MLSLPAEDNDLLAIPPLVDGNMYFSDQSQLNCCNF
>g3364.t1
MAEWLCSESGPASYLDMDESYRPVKLRKVVLGFPCSPNQNDFKFDLSLNYKMANIIQTYSDQKPALVFCSTRKGVQQSAT
VLAKDARFIMSIEHKQR
>g3364.t2
MAEWLCSESGPASYLDMDESYRPVKLRKVVLGFPCSPNQNDFKFDLSLNYKMANIIQTYSDQKPALVVGGIFAILVYVGS
D
>g3365.t1
MLGVGYHHAGVDVSDRKMVEEAFTRGDVPVLFTTSTLAMGVNLPAHLVVVKSTTHYVSGSCDEYSEADMLQMIGRAGRPQ
FDTSATAVIMTKTQTKDKYM
KLMNGMEIIESSLHSHLVEHLNAEIVLQTISDVSMALDWIRSTFLYIRALRNPKHYGFSPDLDQCGIEAKLQELCLKNLY
SLSSMGLISMDEDINIKPTE
AGRLMARYCVAFDTMKQFNSVIGHESLAELVGTVELVSKSREFSDVQLRVSDKRTLNTLNKDKNRVTVSLIQAQLGSIPI
QEFGLSQDTAKIFRNGTRIS
KCLSEFLIQRSKAGLSAQLSSLTLAKCFRAKLWEDSPYVSKQLEKIGLTLSTAMVNAGLTTFGKIEQTHPRELELVSTFR
PQTPFFVFCTTETESEPKPN
LTQGKEFLFLEMLIVNRHPPFGNQIKESVVNLPKYEVSLEQLPRYSSATAEMVVTVKIRNLEQLLSRRSPPDHHHHHTTL
VM
>g3365.t2
MGCLVGDLVMLGVGYHHAGVDVSDRKMVEEAFTRGDVPVLFTTSTLAMGVNLPAHLVVVKSTTHYVSGSCDEYSEADMLQ
MIGRAGRPQFDTSATAVIMT
KTQTKDKYMKLMNGMEIIESSLHSHLVEHLNAEIVLQTISDVSMALDWIRSTFLYIRALRNPKHYGFSPDLDQCGIEAKL
QELCLKNLYSLSSMGLISMD
EDINIKPTEAGRLMARYCVAFDTMKQFNSVIGHESLAELVGTVELVSKSREFSDVQLRVSDKRTLNTLNKDKNRVTVSLI
QAQLGSIPIQEFGLSQDTAK
IFRNGTRISKCLSEFLIQRSKAGLSAQLSSLTLAKCFRAKLWEDSPYVSKQLEKIGLTLSTAMVNAGLTTFGKIEQTHPR
ELELVSTFRPQTPFFVFCTT
ETESEPKPNLTQGKEFLFLEMLIVNRHPPFGNQIKESVVNLPKYEVSLEQLPRYSSATAEMVVTVKIRNLEQLLSRRSPP
DHHHHHTTLVM
>g3365.t3
MLGVGYHHAGVDVSDRKMVEEAFTRGDVPVLFTTSTLAMGVNLPAHLVVVKSTTHYVSGSCDEYSEADMLQMIGRAGRPQ
FDTSATAVIMTKTQTKDKYM
KLMNGMEIIESSHLVEHLNAEIVLQTISDVSMALDWIRSTFLYIRALRNPKHYGFSPDLDQCGIEAKLQELCLKNLYSLS
SMGLISMDEDINIKPTEAGR
LMARYCVAFDTMKQFNSVIGHESLAELVGTVELVSKSREFSDVQLRVSDKRTLNTLNKDKNRVTVSLIQAQLGSIPIQEF
GLSQDTAKIFRNGTRISKCL
SEFLIQRSKAGLSAQLSSLTLAKCFRAKLWEDSPYVSKQLEKIGLTLSTAMVNAGLTTFGKIEQTHPRELELVSTFRPQT
PFFVFCTTETESEPKPNLTQ
GKEFLFLEMLIVNRHPPFGNQIKESVVNLPKYEVSLEQLPRYSSATAEMVVTVKIRNLEQLLSRRSPPDHHHHHTTLVM
>g3366.t1

MPHPKCEWHHLFFFIVLFGKGHSVKNDATELLEGHLFDDLAEVPTNESLNRAQNGGRLLESPREGPVSALEQNQVGCREL
RSTKYISDGQCTSLNPVKEL
VCAGECLPAHLLPNWIGSSHTGRYWSRREAQEWRCVIDRTRTQRIRLQCQDGSSRTYRIMVVTSCKCKRYSRQQNQSDLH
RSKEQAAPSEASDPVSHAAK
RRKDQETTIGPPENRAGR
>g3366.t2
MPHPKCEWHHLFFFIVLFGKGHSVKNDATELLEGHLFDDLAEVPTNESLNRAQNGGRLLESPREEQNQVGCRELRSTKYI
SDGQCTSLNPVKELVCAGEC
LPAHLLPNWIGSSHTGRYWSRREAQEWRCVIDRTRTQRIRLQCQDGSSRTYRIMVVTSCKCKRYSRQQNQSDLHRSKEQA
APSEASDPVSHAAKRRKDQE
TTIGPPENRAGR
>g3367.t1
SLVFLSSAFSSWHYAFNHHTDLTMSDIEDDNEISFAVKLLGRVEVLRPDGMQILQEAMQRLKTPDRDSLGDSVDKMQKKN
KVHLFLSSSGIDILEKKTKY
MLYTCPISSVSFCGVLPAAPKVFGFVAKHPAADMYHCYLFQSQKFSHILVSVIGDAFRVVKKEQNVRGQRDLIVEALRHK
NKVLQRENAELKQRGSTLAL
ISPVDTETNDTNDEDADDDSLNGRQEDIAQFPYVEFTGRDSITCPTCQGTGRIPSEQVNELVALIPYSDQRLQPQRTKLY
VVLSAALCLLVSALVAFFLF
PRSVVVVDDGIRMVTVQFDRTGTRVLMNMTSTLNFTNSNFFSVLVKSLSCQVLYMKMVVGTQQLDNVITIQPLSMKQ
>g3367.t2
MSDIEDDNEISFAVKLLGRVEVLRPDGMQILQEAMQRLKTPDRDSLGDSVDKMQKKNKVHLFLSSSGIDILEKKTKYMLY
TCPISSVSFCGVLPAAPKVF
GFVAKHPAADMYHCYLFQSQKFSHILVSVIGDAFRVVKKEQNVRGQRDLIVEALRHKNKVLQRENAELKQRGSTLALISP
VDTETNDTNDEDADDDSLNG
RQEDIAQFPYVEFTGRDSITCPTCQGTGRIPSEQVNELVALIPYSDQRLQPQRTKLYVVLSAALCLLVSALVAFFLFPRS
VVVVDDGIRMVTVQFDRTGT
RVLMNMTSTLNFTNSNFFSVLVKSLSCQVLYMKMVVGTQQLDNVITIQPLSMKQ
>g3367.t3
LTMSDIEDDNEISFAVKLLGRVEVLRPDGMQILQEAMQRLKTPDRDSLGDSVDKMQKKNKVHLFLSSSGIDILEKKTKYM
LYTCPISSVSFCGVLPAAPK
VFGFVAKHPAADMYHCYLFQSQKFSHILVSVIGDAFRVVKKEQNVRGQRDLIVEALRHKNKVLQRENAELKQRGSTLALI
SPVDTETNDTNDEDADDDSL
NGRQEDIAQFPYVEFTGRDSITCPTCQGTGRIPSEQVNELVALIPYSDQRLQPQRTKLYVVLSAALCLLVSALVAFFLFP
RSVVVVDDGIRMVTVQFDRT
GTRVLMNMTSTLNFTNSNFFSVLVKSLSCQVLYMKMVVGTQQLDNVITIQPLSMKQ
>g3368.t1
MFNYTRAFLGAKDLFPYDESKEKFGKPTKRKGFSEGLWEIENNPTVTHEAYEAEEKESLSDGKQEAGAAEKAEAEGSSDE
DEGALVIDEKNERGGAKRKA
GESTEVRWPRETGTTSQASPKRLKDTGGEGDSKADDKKSNAEAKANNVAGPLAAALSSQSECKAESKSDAPVEEKLTAEK
HFRYLGKTWDYRGTYIASEA
PLLVQNVALIDPTDRKPTPIEWKFTEEGETVRVSSRTGRIIPKPIAERDDGIVPQQWKDGPKDTSPDDTLEKTYVPSLKT
LEEEVMEKLGIQEDRRPRKS
YWY
>g3368.t2
LPEGAVKSPSNKYQVFFFGTHETITSVMFNYTRAFLGAKDLFPYDESKEKFGKPTKRKGFSEGLWEIENNPTVTHEAYEA
EEKESLSDGKQEAGAAEKAE
AEGSSDEDEGALVIDEKNERGGAKRKAGESTEVRWPRETGTTSQASPKRLKDTGGEGDSKADDKKSNAEAKANNVAGPLA
AALSSQSECKAESKSDAPVE
EKLTAEKHFRYLGKTWDYRGTYIASEAPLLVQNVALIDPTDRKPTPIEWKFTEEGETVRVSSRTGRIIPKPIAERDDGIV
PQQWKDGPKDTSPDDTLEKT
YVPSLKTLEEEVMEKLGIQEDRRPRKSYWY
>g3368.t3
LPEGAVKSPSNKYQVFFFGTHETAFLGAKDLFPYDESKEKFGKPTKRKGFSEGLWEIENNPTVTHEAYEAEEKESLSDGK
QEAGAAEKAEAEGSSDEDEG
ALVIDEKNERGGAKRKAGESTEVRWPRETGTTSQASPKRLKDTGGEGDSKADDKKSNAEAKANNVAGPLAAALSSQSECK
AESKSDAPVEEKLTAEKHFR

YLGKTWDYRGTYIASEAPLLVQNVALIDPTDRKPTPIEWKFTEEGETVRVSSRTGRIIPKPIAERDDGIVPQQWKDGPKD
TSPDDTLEKTYVPSLKTLEE
EVMEKLGIQEDRRPRKSYWY
>g3369.t1
MSVKETNRPLIPYTLTKSVEPKASKPSIPAKGLLDSSASPSAIKAAAKSAALQLARQIAAEDHDNEEDVAPENYFSLGES
TAPIPVAAASPDLESPAPTV
ALPYVVPRVDDDGQSDAPLDFGSSHEGPGAWSGQPPEYPQPLDAAAASGYYNQPYYQGANPQQADEDDDEDGPGSSEMFDE
EAFMRLQGKRNRGKEEVKFL
EIKGDDQLIGSQQWMTKSVSEEKQERKSFSKVETTKKAHHAACKEEKLAASRENTSKLENNNNPDAQKKLQDKVEKCQQE
MEKTKERYEKSLDELDKLTP
QYMENMEQVFEQWQQFEDKRIGFFRELLLEVKQHLDLSTNP
>g3370.t1
MSCQTTVLYLPPMFGDNCHTFAFGNHVGGFLSCGFSRRPALRAPGKCRSALCST
>g3370.t2
MFGDNCHTFAFGNHVGGFLSCGFSRRPALRAPGKCRSALCST
>g3371.t1
MAAGLHRALGLSLLVLLVDVKLSTGSF
>g3372.t1
MDWDVFQTAANNNTNDYTEYVTGYISKYIDDVALTVTVRTSPNQKPWVNGVVCTALKAQTAAFHSGDPCEKTRWMLDFLT
SRPQAVWIGNATSSTLTLST
GTPQGCVLSPLLYSLFTHSARPHIAPTPSLRLLMTEQSITAWYGSCTALDRKALQRVVRDLYSQRCRRKALRIIGHNPSH
PNRRLFTLLPSGR
>g3372.t2
MDWDVFQTAANNNTNDYTEYVTGYISKYIDDVALTVTVRTSPNQKPWVNGVVCTALKAQTAAFHSGDPCEKTRWMLDFLT
SRPQAVWIGNATSSTLTLST
GTPQGCVLSPLLYSLFTHSARPHIAPTPSLRLLMTEQSILADSITAWYGSCTALDRKALQRVVRDLYSQRCRRKALRIIG
HNPSHPNRRLFTLLPSGR
>g3373.t1
MSGVLKHNTLTLGMLCQGGVHGQGRSSTLNFNLYNNIKHEPPAGHWLPLPPPPANATPQQGAMLDNDQEGGTASSQWALY
SLGRRDVPPDNIMKKNVHGL
NPSHQPHNTMIVTSSSASSATPPHRGLGQQAYESGAINKGGQYQQGMVMAISPSGGGGQYQALPLALPSPSQPYSSGGMP
MSMTPSGNQPQYASHQPSLP
ATNPQYHGNHGNHGNQGMVHGSQVGMVTHTNPRPQSARCLLQTKGQKSMDGFQEQLCVRIEKNPGLGFSISGGISGQGNP
FKPSDMGSS
>g3373.t2
SGVLKHNTLTLGMLCQGGVHGQGRSSTLNFNLYNNIKHEPPAGHWLPLPPPPANATPQQGAMLDNDQEGGTASSQWALYS
LGRRDVPPDNIMKKNVHGLN
PSHQPHNTMIVTSSSASSATPPHRGLGQQAYESGAINKGGQYQQGMVMAISPSGGGGQYQALPLALPSPSQPYSSGGMPM
SMTPSGNQPQYASHQPSLPA
TNPQYHGNHGNHGNQGMVHGSQVGMVTHTNPRPQSARCLLQTKGQKSMDGFQEQLCVRIEKNPGLGFSISGGISGQGNPF
KPSDMGSS
>g3374.t1
MAVSVHTYGWLLRAALVACSRAGAGNPNTELGPDATAAGHGVSDLEGIPVVALNWHDVKAPFLVALWILVAGTAKMGRFG
PCVFTDLPSVSALSVPLGRG
VRDMACEA
>g3375.t1
MADQNHIGIERDNCNQPLSGGPRGFSERDGKETTKGRRLGKELRREDLLFLLSMLEGELQARDEVITVLKAQITDPALLE
GHYAFRGPDNVLRALQRDSL
QTQQEQLQDVYEKPMAELGRLEEAHRRTSRRMLRQLVEVERSHSGALSRLEEQEEMHRSFIQKSDDLTTLLEQDRERFKP
LANESKWVSNATLPPKQSDA
DVTYSEDVSSRCTRGPALCAEVVRLSRLTYRQSLLCNLGVGLDPPLGPLP
>g3376.t1
LDLSHCLLGTRLYVRLLLYTRSNLRCGRELSHLRPSSEPLFDLSRPSAFVIHGYRPTGAPPVWIDHLVRLLAERDLNVI
VVDWNRGAANLNYFTAVANT
WHAAHNLTAFIANMQEEGASLSAVHLIGVSLGAHLAGFVGANLKGKIGRITARALT
>g3376.t2

LDLSHCLLGTRLYVRLLLYTRSNLRCGRELSHLRPSSEPLFDLSRPSAFVIHGYRPTGAPPVWIDHLVRLLAEREDLNVI
VVDWNRGAANLNYFTAVANT
WHAAHNLTAFIANMQEEGASLSAVHLIGVSLGAHLAGFVGANLKGKIGRITAKITKEWLRNNSVNILDWPSQSPDLNTIE
HLGRDLKMAVHQRSPSNLTE
LERICKEEWQRIPKSRWQQPPNV
>g3377.t1
TTEEELFSNTHESPAFVEFLEFLGEKIRLHDFKGFRGGLDVTHGQTGSESVYSHFRNKEVMFHVSTKLPYTEGDAQQLQR
KRHIGNDIVAIVFQEESTPF
VPDMIASNFLHAYVVVQALNACSDNVLYKVSVTARDDVPFFGPPLPEPAIFTKGPEFHEFLFTKLINAEYACCKAEKFAK
LEERTRSALLETLYEELHIN
SQAMMGIGGEDDKLENGTERTRSALLETLYEELHINSQAMMGIGGEDDKLENGTVAGGGFFESFK
>g3377.t2
TTEEELFSNTHESPAFVEFLEFLGEKIRLHDFKGFRGGLDVTHGQTGSESVYSHFRNKEVMFHVSTKLPYTEGDAQQLQR
KRHIGNDIVAIVFQEESTPF
VPDMIASNFLHAYVVVQALNACSDNVLYKVSVTARDDVPFFGPPLPEPAIFTKGPEFHEFLFTKLINAEYACCKAEKFAK
LEERTRSALLETLYEELHIN
SQAMMGIGGEDDKLENGTERTRSALLETLYEELHINSQAMMGIGGEDDKLENGTVAGGGFFESFKKLQGGNSNRIHCWTL
PRASLHPDSALLVVFVLLVG
AFHFGVRKAVSVTGLRAD
>g3377.t3
TTEEELFSNTHESPAFVEFLEFLGEKIRLHDFKGFRGGLDVTHGQTGSESVYSHFRNKEVMFHVSTKLPYTEGDAQQLQR
KRHIGNDIVAIVFQEESTPF
VPDMIASNFLHAYVVVQALNACSDNVLYKVSVTARDDVPFFGPPLPEPAIFTKGPEFHEFLFTKLINAEYACCKAEKFAK
LEERTRSALLETLYEELHIN
SQAMMGIGGEDDKLENGTERTRSALLETLYEELHINSQAMMGIGGEDDKLENGTVAGGGFFESFKHCDC
>g3378.t1
MWSHILRAETGRKQFSILCAPKGGSRQPRLLSCPAHVCNKEQQKATGPPRRSLSTTRVQPNVFTPERHSTLYFMMEARAR
PALCGGPLSWRRVNPGVNAA
PRLASGVESDPRAALWCGGRVMRGGHLCLSLLEIAGAKFSVGRL
>g3379.t1
MDLKVGRCGLFLVLLSGLARCSAAFVFWTALLELSYHDDQLNKTVTRFCECGVYGRNSLIEGASGRLVMPFQDPMGCGQP
PIYSRNGSKSPWIALVKRGN
CTFSEKIKEAKEEGAAAIVVYNVEGTGNSTMHMSHPEATDLVAIMIGNVVGVEIARLVAGGTAVDMLIDPGSLHGPWMDT
YWLYLLSIAFFIVTAASIGY
FVFVSAHRLHNLRSQRRSERRLKTDAKKAIRQLEVRTVKRGDPETGADAQTCAVCIESYKAGDVITVLTCGHIFHKACIE
PWLLEKRTCPMCKCDILKAL
GVESEEDKEEMRASAYPPPDVTVVTVSGGDPMYEVPLTGGQNLDPQEYQHRYVNGAFEGDSQDRRG
>g3380.t1
NIAEALVSKGLATIIRYRQDDDQRSSHYDELLAAEARFIFMDDNAPAYIGGNIREWLLETGVPQMEWPALSSDLNPIENL
WDQLSRRVEACNSVPQNLND
LRYALREEWDAMPQQTIGQLVNSMRHESL
>g3380.t2
LVSKGLATIIRYRQDDDQRSSHYDELLAAEARFIFMDDNAPAYIGGNIREWLLETGVPQMEWPALSSDLNPIENLWDQLS
RRVEACNSVPQNLNDLRYAL
REEWDAMPQQTIGQLVNSMRHESL
>g3381.t1
SAIFLLRSMQREECFTPIRANFCAPLNKIN
>g3382.t1
MHLPPKLRRLYGPGGKYLHDHGDKFDRERLILDQACDSLKISQAPQVSISRSKSYRENASLSRGEGDQSPGEGSEDGSLG
LVELGPLKFEEDPGKEEETC
RDTAIALSPKDEERESLNAGDGDGRDGEDSVCLSAGSDSEEGMLKRKQRRYRTTFTSYQLEELERAFQKTHYPDVFTRSV
RAPGRWAPDDDGYDI
>g3383.t1
MLKVISAGLQFGNISFTKEKNHDQASMPDDTAAQKMCHLLGISVVEFTRAILTPRIKVGREYVQKAQTKEQADFAVEALA
KATYERLFRWLVHRINRALD
RRQRQGASFIGILDIAGFEIFQLNSFEQLCINYTNEKLQQLFNHTMFILEQEEYQREGIEWNFIDFGLDLQPCIDLIERP

AHPPGVLALLDEECWFPRAT
DRSFVDKLSGEQGSHPKFFKPRQPRQEADFAIIHYAGKVDYKANDWLVKNMDPLNDNVASLLHQSSDHFISELWRE
>g3383.t2
MLKVISAGLQFGNISFTKEKNHDQASMPDDTAAQKMCHLLGISVVEFTRAILTPRIKVGREYVQKAQTKEQADFAVEALA
KATYERLFRWLVHRINRALD
RRQRQGASFIGILDIAGFEIFQLNSFEQLCINYTNEKLQQLFNHTMFILEQEEYQREGIEWNFIDFGLDLQPCIDLIERP
AHPPGVLALLDEECWFPRAT
DRSFVDKLSGEQGSHPKFFKPRQPRQEADFAIIHYAGKVDYKANDWLVKNMDPLNDNVASLLHQSSDHFISELWRE
>g3384.t1
RSPLVYVLLDAGFGRDPVYGDSDSLLGDGPGDITDVXXXXXXXXXAPCRKRRFRALAAASDTATSDPATSDPAAKRAPPET
SARHRRRNVPSRQYSGGFAL
AGAVRPGPGV
>g3384.t2
RSPLVYVLLDAGFGRDPVYGDSDSLLGDGPGDITDVXXXXXXXXXAPCRKRRFRALAAASDTATSDPATSDPAAKRAPPET
SARHRRRNVPRLFISVGVAP
SSPQSRQYSGGFALAGAVRPGPGV
>g3384.t3
RSPLVYVLLDAGFGRDPVYGDSDSLLGDGPGDITDVXXXXXXXXXAPCRKRRFRALAAASDTATSDPATSDPAAKRAPPET
SARHRRRNVPRLFISVGVAP
SSPQSRQYSGGFALAGALEEEVGDQGDGTADPTVSGRPALSTPLYPLSYCRPVKKVYM
>g3385.t1
MSPNETLFLESTNKIYKDDISSSSFVNFQIWDFPGQVDFFDPTFDYEMIFRGTGALIFVIDAQDDYVEALGRLHLTVSRA
YRVNPDINFEVFIHKVDGLS
DDHKIETQRDVHQRANDDLADASLEKLHLSFYLTSIYDHSIFEAFSKVVQKLIPQLPTLENLLNIFISNSGIEKAFLFDV
VSKIYIATDSSPVDMQSYEL
CCDMIDVVIDVSCIYGLKEDGSGSAYDKESMAIIKLNNTTVLYLKEVTKFLALVCILREESFERKGLIDYNFHCFRKAIH
EVFEVGVATQRSTGPQPAAT
PCKTVALNGTPRNTV
>g3385.t2
MFLSFRRLLFFSFQVVFHKMSPNETLFLESTNKIYKDDISSSSFVNFQIWDFPGQVDFFDPTFDYEMIFRGTGALIFVID
AQDDYVEALGRLHLTVSRAY
RVNPDINFEVFIHKVDGLSDDHKIETQRDVHQRANDDLADASLEKLHLSFYLTSIYDHSIFEAFSKVVQKLIPQLPTLEN
LLNIFISNSGIEKAFLFDVV
SKIYIATDSSPVDMQSYELCCDMIDVVIDVSCIYGLKEDGSGSAYDKESMAIIKLNNTTVLYLKEVTKFLALVCILREES
FERKGLIDYNFHCFRKAIHE
VFEVGVATQRSTGPQPAATPCKTVALNGTPRNTV
>g3385.t3
MSPNETLFLESTNKIYKDDISSSSFVNFQIWDFPGQVDFFDPTFDYEMIFRGTGALIFVIDAQDDYVEALGRLHLTVSRA
YRVNPDINFEVFIHKVDGLS
DDHKIETQRDVHQRANDDLADASLEKLHLSFYLTSIYDHSIFEAFSKVVQKLIPQLPTLENLLNIFISNSGIEKAFLFDV
VSKIYIATDSSPVDMQSYEL
CCDMIDVVIDVSCIYGSVSSCLLLPLEIPHFLKEDGSGSAYDKESMAIIKLNNTTVLYLKEVTKFLALVCILREESFERK
GLIDYNFHCFRKAIHEVFEV
GVATQRSTGPQPAATPCKTVALNGTPRNTV
>g3386.t1
MATATSRYYSEDGRATKRQKTDGMATGYEDPHKTLPSVVVHIRGLVDGVTEADLVEALQEFGTISYVVVMPKKRQALVEY
EDMNGSSTAVTYAADNQVYI
AGHPAFINYSTSQKISRPGDSDDSRTVNNVLLLTIMNPIYPITSDVLYTICNNCGPVQRIVIFRKNGVQAMPTRLNVFKN
DQDTWDYTNPNLGGP
>g3386.t2
MATATSRYYSEDGRATKRQKTDGMATGYEDPHKTLPSVVVHIRGLVDGVTEADLVEALQEFGTISYVVVMPKKRQALVEY
EDMNGSSTAVTYAADNQVYI
AGHPAFINYSTSQKISRPGDSDDSRTVNNVLLLTIMNPIYPITSDVLYTICNNCGPVQRIVIFRKNGVQAMCGCRLEFPS
QRYGTRYRCNGFLNSNITFD
SVQSAQRAKASLNGADIYSGCCTLKIEYAKPTRLNVFKNDQDTWDYTNPNLGGP
>g3386.t3

MATATSRYYSEDGRATKRQKTDGMATGYEDPHKTLPSVVVHIRGLVDGVTEADLVEALQEFGTISYVVVMPKKRQALVEY
EDMNGSSTAVTYAADNQVYI
AGHPAFINYSTSQKISRPGDSDDSRTVNNVLLLTIMNPIYPITSDVLYTICNNCGPVQRIVIFRKNGVQAMPTRLNVFKN
DQDTWDYTNPNLGGPGK
>g3387.t1
VATLFFEDKNEEDEQHSCAVYSHLTNTLLPQPCDAKHEWICELHRARRNQGSKPATIRYSGYLLFPCQELNKPYWYTEQS
EPWVFYRGAEYLLARQLFDW
KAVSLACQMMGAQLLSIHSKEELRFIQQRMAKVSVGATDWWIGLSINLPREEVSWSDKTELDLQNWAEGASYGGPVTHKL
CVSMSSASALGAQSVQEYHT
FNSLQSSQSVAGVSRPGSLVGFGSETGRDSETCFLLHLPDRPEEGKSWPDAHSICSSFQGSLVAIEDPIQQAYITMLLQG
SAVGVWIGQNFDDTEWWSNG
RPVGYTNWSPKNSPAEDEWLSAAVASGEPLCTVLSNHHNLHLAGKWYDDRCSETGYGFVCQKPQDPSKPPTHTYHHPIPD
NVDYKSRSYKVLPGNMSWYK
AQQACMESGSELASITDAYHQAFLTVLVNRLAAPHWIG
>g3387.t2
VATLFFEDKNEEDEQHSCAVYSHLTNTLLPQPCDAKHEWICELHRARRNQGSKPATIRYSGYLLFPCQELNKPYWYTEQS
EPWVFYRGAEYLLARQLFDW
KAVSLACQMMGAQLLSIHSKEELRFIQQRMAKVSVGATDWWIGLSINLPREEVSWSDKTELDLQNWAEGASYGGPVTHKL
CVSMSSASGKWSVGECSSLH
GYVSLGAQSVQEYHTFNSLQSSQSVAGVSRPGSLVGFGSETGRDSETCFLLHLPDRPEEGKSWPDAHSICSSFQGSLVAI
EDPIQQAYITMLLQGSAVGV
WIGQNFDDTEWWSNGRPVGYTNWSPKNSPAEDEWLSAAVASGEPLCTVLSNHHNLHLAGKWYDDRCSETGYGFVCQKPQD
PSKPPTHTYHHPIPDNVDYK
SRSYKVLPGNMSWYKAQQACMESGSELASITDAYHQAFLTVLVNRLAAPHWIG
>g3387.t3
VATLFFEDKNEEDEQHSCAVYSHLTNTLLPQPCDAKHEWICELHRGQELNKPYWYTEQSEPWVFYRGAEYLLARQLFDWK
AVSLACQMMGAQLLSIHSKE
ELRFIQQRMAKVSVGATDWWIGLSINLPREEVSWSDKTELDLQNWAEGASYGGPVTHKLCVSMSSASALGAQSVQEYHTF
NSLQSSQSVAGVSRPGSLVG
FGSETGRDSETCFLLHLPDRPEEGKSWPDAHSICSSFQGSLVAIEDPIQQAYITMLLQGSAVGVWIGQNFDDTEWWSNGR
PVGYTNWSPKNSPAEDEWLS
AAVASGEPLCTVLSNHHNLHLAGKWYDDRCSETGYGFVCQKPQDPSKPPTHTYHHPIPDNVDYKSRSYKVLPGNMSWYKA
QQACMESGSELASITDAYHQ
AFLTVLVNRLAAPHWIG
>g3388.t1
MRKHSTSLRVRLPVFVVVTQLVIVVLFAFFVTYDDQSDAKLQGNETVAMDNALYKDYPFFTDIQVMIFVGFGCLLAFFRQ
YGFGGLVFNFLTATFAIQWA
ILVQGYFQFSHNGKIHLGVINLINAEFACAVVLISFGAVLGKTSPLQLLVMALLEIPVFAFTEWAVLTYLKINDAGGCIL
IHLFACYFGLGVTFVLYRPR
LNQGHAKESSSYQSDILSAMGTLFLWVFWPSFNSALALKGDDQHRAVLHTFMALSASTLTAFALSAMLNRTGKLSMADIQ
NVTLAGGVTVGASVDMMISP
AAAYALGMLGCTACMLGYRYLSPFMARHLRIQDQCGIHNLHGLTGLISCTAGICAILAASEEVYGPSLYETFAHRAPMEG
DPKLLELQEQIPGLQPGLGR
TAREQALYQVAALLSTVVVVMADDFEIEAMLEAPFRKGEMCDRGGIELLQFKNENG
>g3388.t2
MRKHSTSLRVRLPVFVVVTQLVIVVLFAFFVTYDDQSDAKLQGNETVAMDNALYKDYPFFTDIQVMIFVGFGCLLAFFRQ
YGFGGLVFNFLTATFAIQWA
ILVQGYFQFSHNGKIHLGVINLINAEFACAVVLISFGAVLGKTSPLQLLVMALLEIPVFAFTEWAVLTYLKINDAGGCIL
IHLFACYFGLGVTFVLYRPR
LNQGHAKESSSYQSDILSAMGTLFLWVFWPSFNSALALKGDDQHRAVLHTFMALSASTLTAFALSAMLNRTGKLSMADIQ
NVTLAGGVTVGASVDMMISP
AAAYALGMLGCTACMLGYRYLSPFMARHLRIQDQCGIHNLHGLTGLISCTAGICAILAASEEVYGPSLYETFAHRAPMEG
DPKLLELQEQIPGLQPGLGR
TAREQALYQPHLSVRRANARTPSLVSAVMADDFEIEAMLEAPFRKGEMCDRGGIELLQFKNENG
>g3389.t1
MPSASTGSVVVTDDLNYWGGRRIKSKDGAKSEPVFEPATGRVLCQMVPCGAEEVDQAVECAQAAYLKWRKMAGIERSRIM

LEAARIIRERRDNIAKLEVI
NNGKTIAEAVYDIDAAWQCIEYYAGLAPTLSGDYTITLT
>g3390.t1
MSTMFADTILIVFISVCTALLAEGITWVLVYRTEKYKRLKAEVEKQSKKLEKKKETITESAGRQQKKKIERQEEKLKNNN
RDLSMVRMKSMFAIGFCFTA
LMGMFNSIFDGRVVAKLPFTPISYIQGLSHRNLLGEDFTDCSFIFLYILCTMSIRQLLFLVGKLTLQFFFQNIQKMLGLA
PSRAATKQAGGFLGPPPQAA
KFS
>g3390.t2
MSTMFADTILIVFISVCTALLAEGITWVLVYRTEKYKRLKAEVEKQSKKLEKKKETITESAGRQQKKKIERQEEKLKNNN
RDLSMVRMKSMFAIGFCFTA
LMGMFNSIFDGRVVAKLPFTPISYIQGLSHRNLLGEDFTDCSFIFLYILCTMSIRQLTLQFFFQNIQKMLGLAPSRAATK
QAGGFLGPPPQAAKFS
>g3391.t1
MIMIIVIICIIFIVIFVIITIVIIIIIIKEERIQPAASTSSKLSIAAFTQQMGQCGMGAQSLSSVCSKIMELLGQNKIDH
HQRQVVILSLDSFYRVLTPE
QKARALKGQFNFDHPGIYSPGWGGVRDPAVGRF
>g3392.t1
VKFQYTFETVTGFLLPYAIIVTSYMLILRRLKQTKFRRTVRSEKLILGIVVTFGVFWLPYHVINMIQVGAEWFDPASPTR
GRLVHIWKSSRAVTSALAFI
SSCANPVLYTFAGKSYIKRDGFAFMARLFEGTSLDQSVGKKSLSGNEGNRLNGL
>g3393.t1
FAGYQCDLMAAVPKQARELPSNKAGTPA
>g3394.t1
MDGLETPYEIEVPIDFIDCSLNLQAPFWGNPWHSHASLETLSIQCILDLLWGPILLPVKQALIDLFLLLSLFPWQKEEAL
VSSGQNSLSFIHLHLVLAIL
QFSIHNLWTLPTYMYVKLLKLLDKKVIWPPNYLGDHGYVTVSEQEVLRFFNYQTCSDKARSFIR
>g3394.t2
MDGLETPYEIEVPIDFIDCSLNLQAPFWGNPWHSHASLETLSIQCILDLLWGPILLPVKQALIDLFLLLSLFPWQKEEAL
VSSGQNSLSFIHLHLVLAIL
QFSIHNLWTLPTYMYVKLLKLLDKKVIWPPNYLGDHGYVTVSEQEVLRFFNYQTCSDKARSFISHLEEVIGSDANDPQPG
PRYFCRLCSLEANLPNMINH
IIGRKHRQKYLPPPFHQEDHYLGPEEEGRGQYQRRKPPHQEDYMEEDHDRPEFMDSDYHRGYPEDPMGGGQHRDYPLPRA
EGRPMRGFVEEMPHSESYER
DASPYRRPRPESDPLKQFYSEELRRERARANEAAATAVATDGRSYPSRDPQETGGGGGGGGGDNNSNRTVYAYMSAGSER
QNPGGRPRGPRYPPEAPGRG
SADYPRQGDRFDDEDRRGAPDESDGDEYCHRAADYPEAQRWDYTDGHHSHQGRPDQDRRGNPEPRRNADHQPLDRDYLFE
MVGALRDKTAAPPREAGSPY
QKVPEGVRGMSEIPEPFRRFLQRNASSQDIRKRKSRFSDATQEEMEGSKRMFMQSDGFAVSEEPRASRQSREWPEAHGAQ
RLDPYAESNKNIEIENGEEA
[truncated: 531,772 more chars]
